# Supplementary material for: RLP/K enrichment sequencing; a novel method to identify receptor‐like protein (RLP) and receptor‐like kinase (RLK) genes
Source: New Phytol. 2020 May 22;227(4):1264–76. doi: 10.1111/nph.16608 (PMC7383770; doi:10.1111/nph.16608)
Supplement: Supplementary file 1 — Notes S1 fasta file of the 977 RLP RLK genes from DM genome used for generating the RLP/KSeq enrichment library. Notes S2 fasta file of the 977 RLP RLK genes from M6 genome used for generating the RLP/KSeq enrichment library. Notes S3 Eighteen additional known RLP and RLK genes from Solanaceae species. Notes S4 The 2x bait library used in this study. Notes S5 Java script for calling the informative SNPs. [file NPH-227-1264-s001.pdf]

***New Phytologist* Supporting Information**

Article title: RLP/K enrichment sequencing; a novel method to identify receptor-like protein (*RLP*) and receptor-like kinase (*RLK*) genes

Authors: Xiao Lin, Miles Armstrong, Katie Baker, Doret Wouters, Richard G.F. Visser, Pieter J. Wolters, Ingo Hein and Vivianne G.A.A. Vleeshouwers

Article acceptance date: 27 March 2020

The following Supporting Information is available for this article:

Notes S1 Fasta file of the 977 RLP RLK genes from DM genome used for generating the RLP/KSeq enrichment library

Notes S2 Fasta file of the 977 RLP RLK genes from M6 genome used for generating the RLP/KSeq enrichment library

Notes S3 18 additional known RLP and RLK genes from Solanaceae species

Notes S4 The 2x bait library used in this study

Notes S5 Java script for calling the informative SNPs

Notes S1 Fasta file of the 977 RLP RLK genes from DM genome used for generating the RLP/KSeq enrichment library

>PGSC0003DMG400009417 C-LecRK

AATTAACTACTCTGTCTGAACAACACTTTCTTCTTCAAAGAAATTCAACTAACCATTGGAGACTCCAAATG
TGAACTCTTGACCTTTCTCTCTCTAAAAAAAACCCTATCCACCCACCCCATATCCACAATGCCCAACCCCAC
ACTCAAAATCATTCTAGGTTTTGCTATACAGTGAGAACAAGTGATGATACTGATGGCTGTTAAACCTCTG
TTCCCTTTGTTTCTACTTACTTGTTTTCTGCTTCACCTCTGTGCTTCCACTTCTGTAAGTAAAGAAAAGATT
ATAGTACCTTTTATTTAGAGAAAAGGGTGTCTGAATATGTTATCAGAATTCATGTTAGTGTGTGAATTTG
GAGAAAGTTTTGATTTTTATTTTTTTGAATTTTTTTCAGGTCCCTAATAAGACACTTGATTCACAAATAGTT
TCATCTTCTGGGAATCAAAGTCATGATGATGGTAATGTTTTGGAATTAATTAGATTATTGTTCTGTCTATT
TCCTTGGAACTGATAGATTGGCTTGTGTTGATTAGATTAGGGCTCTTTTTAGAATCAGTAATGGAGTTGC
ATCTATAATCATCTTTTCAACATAATGTTCAACTACTATATGTTACTTTGTGTATAATATGTTGGGATTTCT
TACTGGTGCTTCTTATGTAAATTGTAATGTGTGCATATCTATGTGTCATTTGCTAATTTAAACTAGCATAG
TGAACTTTTGAGTTTGGAGAAAATAAAGGCTGGCGAATCCAATCTTGTTTATCAAGTTGCTATAGGTTTA
AGGTCTACAACTTGGGGTATTTTGTGCCAAACTATTATTCATAAATCAAGATTTTGAGAACTACGTCACTT
CCCAGCATCATTAAGCTCAGGCCATAAAAGACTGTCTCGCAACCAGTTATCCCTCGAAAAAATAAAAATA
GAAGATGAACTTGCAACTATTTATTAATCAGCTTCCTACTTCTAAAGAAAATTAATTGACCAGCCTCTCGA
TTCCGGTTTACAAAGTTATGGATTCCCATTCCTCCAAGACAGTTGTTCTTGGAATATTCTTAGAACAATAT
TCTATTTGACTGATCGATTATCCTTCTAAGAAATCCTGGTGGTCATAGTCACCTGATTGAATTTAATTTATT
CGGTCGACAAAGGAGCAGAAGTAGATTCTGCTCATTTCATGCATAAAGGCATGCTTAGGTTGCTCTTAA
AGTGAAACTTTGGTTCTATTTGAGGGAGATAATATATATGCTTTTATGGTGGATTAATGATGGCCTTGAT
GAATAATGTTGTCCAGTTCCTTTCCTATTGAGGAACTCCTCCCTTGGAGTTTCTTTTGTTTTGATGATATGC
ATTTCACCATTTTTTTTCCTAAAAAAACTGCACTAGATGTCGGGAGGACTCATGAGGTGATGCATAGAGC
TCACCTGGTAGTTGTCTAAGAGGAGGAGGTGGCCAGATGAAGCATGTGACACTTACAATTTCACCAGTT
AGAGGTTTTGCTATTACATTAGGCAATGGAATGACGAGAATAACAGCGTGTTTTATGTTGATCAGCATGT
AGAGCAGCAGTAATTGCGCTAGTATTCTAGCTTGATAGTACTACATCTTTCATTTGAGATAGCACATCATT
TTCTTTTGAAATGGGCATAGACTGAAAGCAATAGGGAAAATAATGATGATCCAACTTTATCCTACTTTTG
CCCCCTGCTATTCTTGAATATTGATGGTTTTTACAGGATATTTGCAGTCATGTGGAATGTGATTCCTACAT
ACTCTATATGCCTCTTTAGTAAGACCTGTGCTTTCAAGTCTCAAGTTATCCCTGCAACTTATGGTGTTAGA

CATACTGGTACACGTGTAGAAATGTCATGCCAAGAACCATCTAGCTGTTCATTTAAGTTTCCACAATTAC
GAAGAACCTTTCTTTTCCATTCGCTCTGTAGGAAGAAGAACAAAGTTCTAGTTTTTATTGAACTCGCATG
GGTTTTAAAAAAGACGTATATTTGTTGGTACATCAGTGAATAAGGCTGTGGATCATCTCAGAGAAAACT
GACTAATTGAGTGAACACAATTAGGAGTCTTGGATGGAACAATTATGGAGAATGTTCATCTGTCTAAAG
GGGATAAGATGGGAGAAACCAGGGAGCATTAAAGGAGTCCTGAGCAGTTGGAATAGAGATGGCAATG
CTTCTGACAAGGAGAAGAGATGGAAACTTGTTCCAGCATGTATTTGGTGGACAATCTGGAAAGAGAGA
AACAACCGGAGTTTTGAAAATTGGCAGAATAGCTTACAGAAGATCAAGATGGACTGCTTAGCTCTTTTCT
ATTTTTGGTGTAAACAAGATATATTAACTAGGACAGAAGACATCTTTGATGTGCTAGATTGTTTATAGGC
AGCAGTTTAGACAATGACTATTTTATTCTGTAAGTTGTAACACTTTTGAAGGGGAACTACACTTTTGTTGT
AGTATACCTGTGGATATATAGACTAAAGTTACCAATTTTTTTTTAAAAAAACAATTAGGAGTCCAAGCTG
AACGAAAAGAGAAGAAAACACTGGAGCTTAATTTTTTTTTTAAAGATTCAGTGTTCTCTTCTCTTTTCGTT
CAGCTTGGACAAGCTTAGTTTAATGCAAGTTTGGCTTTGTTTGTCAGAACTTGGTACTTTCATTTATCCAT
CATACTTGAATCTAGCACACCTTCCCTCTCAACAAAGGAAAAGTGAAAACTGAGAACACACTGTCGATGT
CAAACCTCCACTAATCTGAGTGATATCACACAATCCATAACACGGAGGCCAAGAAACGACTACTCCTATC
AGGTTCATAAGACACAGGGGTAGAGGGCAATGACCTGGTGTCAACATCGTTCTTGGGTCTTCTTATTTC
GTAAGCTTAGTTTGAGATGGCAGTGGAGGCCAATTGTGGTGAACCGTGAATCTTTCAATCTTCAAATCTT
GGAGACCTTATCTGAAATGAATTCTAATTTTGAAGCTGATCCCTCAATTGTAACCTTTGTAAAAAAAAAA
AGTAAATAAATCCATTAATGAAGATAAAGATACTTTTAGGGTGAAAAGTCATCTTCAGTTGTGGTATTAG
AAATAACTAGTTCAGAATACCCGAGCTTTATTGTCCATTATGAGGTATTTATTAGATATAAACAAGTTCAT
GATACCTGAGTTTTACCTACCTAGATATAAACAACTATTCCTACCTAGCACTTTCCAGTCCCCGCAATCTT
ACTAGTATTATTTACAACTGAAGTCGATGATCAAACTTAAGGAAACAACAGAAAGAAAAAACATTAAAA
CTACAAATATAGAAAGATGGTGAAAAGAATCAACAAATGTGTACAAGAGTTGGAGATCATGTGGCTAA
ATGGATGGGGTTAGTGCAGGGATGGCAAGTTTGAGCCACTTTTATAACATTAGGAACACATATAAACTG
ATAGAATAACATTAGGGGTATAGATGAACTGATAGTATAATGGAGGGTAAAATTAAACCTTTTCAATAG
TACAGGAAGTGAGGGACAAATATGACCCTTTTCCTAATTTCTTTTCTTCTTCTTAGATAAATGAAGAAGTT
AGATCAAGTATTATTAAAACACTAGTGCAACTGAAACTTATTTGGACCTCTCAACGCGTGGTTTTCTTAGT
GTATGTTTTTTATTTTTAACTGGTCAAGTTATTATGTCTCATCTCTTTGGCACTGTTGTTTATTGTTTCGCAA
GCAGGAAGATGTGATCCTGGTTGGTTTAGCAGCCCAAATGGGAAAAAGTGTTTCAAATATAATGCGACC
TCTTTGCAATGGAATGAATCAGAGACTTACTGCAATGGTTTTGGAGGAAACCTGGCTGCCATAGCATCTT

CAGAAGAACTGAACTTTGTCCAAAAGCTCTGTGGCAATGATAGTAACGGATGCTGGGTTGGCGGAAGG

AGCATCAATACCACAGCTGGGTTGGGTTGGAAATGGTCTGATAATGCTTCTAGCTGGAATGAGAGTATT

GCTCCTAAGACAACTTTGGACTCAAGTTGCAAAAATTTCTCATGCTATGGCTTTAAGTCGGTGGATTTGT

GTACATCGATAACCAATGGCGCTATTATAGCTGAGAGATGCAATGTAACTCATGCTTCTATATGCACACT

TGATGCTGGTAAGTGATTGCAGACCAATTTATTTCTAATTGTTTCTTTCACCCAGTGTAATTTTGCAAAGT

AGGAGCAAAACCCTTTCCCTGATATAGTGTGGTTATACTATGTGATTAAAAGCTTCATAGTCCCCTTTTGT

TTTTTCCATGAGAAGTGCATTTTAGCTAACCATTATACAGGCCTACATTGTTATCTGGATAAATCTATTAC

ATTCCTTAATTGGTTTTTAAATAATCTTCTAAATTTTACATTAGGGAGCAAGTGTCGCCATATGCGTTGCC

ACAGGGAGTATCTCATTATTCTTGCAGTTGTGAGTGGGTTGATCCTTTGCACTACTTTGGCTATTGTGATA

TGGCTTCTTGTATTTCGGCGAAGTAAAAGACGCAAAAGATCATCGCGTGCATCATTAGCATTAGTACCCC

CGTCTTGGAAAATTTTCAATCGTGATGAAGTAAAGTCCATGACAAAGAATTTCAGTGAAGCAAATCGTCT

CCTTGGGGACGCCAAAACAGGTGGTACCTATAGTGGGCTTTTACCTGATGGTTCAAGGGTGGCTGTGAA

AAGATTGAAGAGGTGCGGTATTCAGAGGAAGAAAGAGTTCTACTCTGAGATTGGAAGGGTTGCAAGAC

TTCATCATTCAAATCTGGTATCTATCAAAGGATGTTGCTATCATCATGGTGACCGCTATATTCTGTACGAG

TTTATTATCAATGGACCCCTGGATAGGTGGCTACACCATATTCCTAGAGGAGGTAGGAGTTTGGACTGG

AGCATGAGAATGAAGGTCGCCACCACCCTTGCTCAAGGGATTGCGTAAGTAGTGTTCCAACATTATCTTC

ATGTGCATTGTAGATTAGGAATAAATATTTTAACTTATCCTTGAGGTGGATTGATTTTAGTCAGAAATGT

GAGTTTATTCTTTAAATTTTATCATACTTCCCTTCTTTGTCATGAGTATGAACTCATATATACATGTTTGGG

TTGATGTCCAATGAAGATGTTATCGTGGAAGTCAAACAAGGGTGAAAATAGATGATAAGAATGCATTAT

CTATATTGTTAAGAAAAAAAGGTGTGTTATCTGTCTTGCAATATTCCTTTTCGTATTTTCTTTTTTCTTCCCT

AGATTTTTCATTCAGAAGAAAGTCAAATACTGCACATCTTGACCAAGATACTTCTTGTTAACATATAATGC

AATTAATAAATGAAAACCTTTTTAGTTTACAACATTCGTTCCAGTACTAAGGAGAATTCACTTCCTAGGGA

TCTACCTATCTGCTTTTCCCACTCCAGCTAGATCTTACCATTGCCAAACGTGTCTTTGCAGCACTGGCCTTC

TGGCTTCTCGTGTTTCCTTTCAGGTTTGGGGTGGATCTTTCCTTTCTCAATTATCACATACTCGGTTAGGTC

GCCCGTGTGTGTGTCTTTCAATCTAAGTGTTTGAGCAGGAACTCTCTTCATGAATCTCACTAAAATATTGT

AGGAAAGCTATGATTGATTTTCTATTCGGGGATTCATTATTAACTATTATGACTCTATTTATTGGTGACAG

AATTGTTGGACTTTTATCTAGGGAATATTTCCTTGACATGGAAATTATGGCAAATAAATTTTTCAGGGGTT

TGTGGGACTACTCACCACCTAACCCCCAACAACAAATATACATGGAAGAAATATTGAAGTGTACAAAAA

GAAGCCAATATATTATTCCAATTATTCACGATGATAGAGTGAAATAATTAGCATAAGATAGACCAAACCT

TAGGCTTATCTACATCACCAGATTTAATATATGCAATGTATTATGTATGGTCCCTGGTAGTTTAACTTTTC
ATGGACTTCTTTGTTAAGAGGTATTATTTTCCATAATTTATTCAGTGTAAGTTGAAAAATATAGGCCACTT
GAGTAAGCTGGGTTCATTTCATAATGTACATACTTTTAAAGATTTCAATAGTTTGCAAGTAGGTCTTTGTA
ATTTTTATATGCTTCAAGGAATTCAGACAGAAGTGGGTGAGAAAAGATGGAATACTTAAGGAAATGGAT
ACCAGCGACATCTTAGTAAGAAAATAAAAACTGCATATTACTGTCCTGTTGTCTTCATGAGTTTTTGTTCT
GAGTGTCTTGCCTTGTTCTAGGGCGGATGTCTATGGAAAGTGAAGTCATAGAAAATAGGATGTCTATGG
AGGTCACTGTTATAAGCGAAGCAACCTAGATGCCTAATATATCAATGGATAGATACTTTAAGACCAGTCA
TGCCTATGTCATATTTGTTAAAAAAGGGAAAATAAACAGAGACAATTCAGGGTCTATTACTAGTTTATTA
CCTTTGTAATTTATGGAATTGAAGGATCTAGCAAGTATGTTAATCAACTGCATAAGGTGAAAATGACTGG
TCTGGTTGTATTTTTAACCATGAAAGGTGGTGATAGTTGTTAAAACTCTGTTTTGTGTCACTGTGTCAGCT
AGTGCACAAGAGGATGTGCGAGTGGCCAAAGCATGACATTGTTTGTTTGCTATCTCTATGATGGCAGTT
TCTTTGCTTATATTGTTAGTGCTTACTTTCATCAAATGTGACAGGTTTCTTCATGACAAAATGAAGCCACA
AGTTGTGCATAGGGATATCCGTGCAAGCAATGTTCTTCTTGACGAAGACTTTGGCGCCCATCTAATGGG
AGTTGGTCTGTCTAAGTTTGTACCATGGGAAATTATGCATGAGAGGAGAGTCATGGCAGGTGGTACTCA
TGGGTATCTTGCTCCGGAGTTTGTTTATAGAAATGAGCTTACTACAAAGAGTGACGTCTACAGCTTTGGA
GTCCTCTTGCTTGAAATAATTAGTGGACGAAGACCAGCACAGGCAGTTGATTCTGTTGGATGGCAAAGT
ATATTTGAATGGGCCACGCCTCTGGTGCAGGCTCATCGATATGTTGAACTCTTGGATCCTCTCATATCATC
TTCTTCCTCTTCTCAGATTCCTGAAGTTGGAGTCATCCAAAAAGTTGTTGATCTTGTTTATTCTTGTACACA
ACATGTACCATCAATGCGTCCGAGGATGTCACATGTCGTCCATCAGCTACAACAATTAGCTCAGCCCCCT
ATTGTAAAGTAAGTGGATAAGAATTATAGTTCAGCATTTCTTTTGACTGAAACCAGCCTGCAATCTTTTAT
CCTAGTTGCAAGGGCTAATTGTATTAGGCATTATTTTTGAACTTGACATGCAAGGCCTTGAGGCTTGAAC
CAGTCCATGATGGTTGAGATGTAATACAACAACTAATCTGTATCACCAAAGCCAGTATGCTTTCATCTGC
TGCTGCTTTGCATTGTTTACTTGTAGCACCTGTATTTCTAAAATTTGTTTCTGTTTCCTGTGTTAAAATATC
ACTATGCATAACAACCAATTCAATAAATGTCCATGAAATCTTG

>PGSC0003DMG400000179 G-LecRK

CAAATATTCTCTGAGCTTTTCAATGTTTCCTTAAAAACCAAAATCTTTTTTTCTTTAGCTAAGCAAAATTCA
TCACCATGATTCCACAGAAAGCAATTTTCATTTTACTTTTCATCACAATTTCTATGGCATTTGAGGTCAGTT
CAGAAACAGCAAAATCAAGAATGCAAAATGTAAACATGACCCATGTAGCTGAACTCACTAAAGAGTCTT
TTTCAGCTGAAAGAAAGTTGGGATCTTTAGAAGGGAGCATAAAGTTTGAATCTTTGAACAAATCTAGAG

TTTTACTAAGTGGGAACTTCACTGTTTTGAGTGAAAACAAGACATTTGAAGTGGGGTTTTTCAAGACTAA
TGATGAGAGTAAATGGTATTTAGGGATTTGGTTTGCTTCAATTCCTACCCCTACTTATGTTTGGGTTGCTA
ATCGTGAAAAACCCATCAAGAATCCATTTTTAGCTACTTTGGAGATTAGTGAGGATGGGAGATTAGTTTT
GAAGGAAGATTCAAGAACCATTGTATGGGAAACAACCAATTTGGAGAAAGCTAGTGATGTGAAGCTTTT
GGACCAAGGGAATTTGGTTCTTGTTTCTAATGAAGGTGTTTTGGTATGGGAAAGTTTTGATTTTCCTACA
GATACATGGCTTCCTGGGATGAACTTGACTGCAACAAAATGGCTGACATCTTGGAGAAGTACTAATGAT
CCATCACCTGGAAGGTACTCTTTAAGGCTCCAACCACATAGTTATGGTGAGATAGTTCTTTTTTATAATGG
TACTTATCCATATTGGTCCACTGGGAAATGGAGTGAAAATGCATTTGTTGATGTACCAGAAATGACTGTT
CCTTACATTTACAAGTTCAATTTTGAATCACCTTTTACCCCTATGGCATCATTTGGATACTCAGCGGTGAC
ATTAGAGAATGGAATGCCTCCTCCTTTAACTAGATTCATGGTTGATTTCACTGGACAGGTTAAACAGTTC
ACTTGGATCCAACAGGCGCAAAGTTGGAACATGTTTTGGTCACAGCCAGAGAATATGTGTAGTACTTAC
GGTCTTTGTGGGAATTTGGGGTTCTGTAATAGTAAGTCATTGAATCCATGTAAGTGTTTACCAGGGTTCA
CCCCTTTGGATGGTGATTCATGGGATGCAGGGGATTTTTCGGGTGGCTGTCACCTCGAGAGTAATGAAG
TTTGTGGTGAGAATGATAGGTTTGAAGAGGTTGGAATGGTTAAATATGATGGAGCAAGGGTAGTTTCA
GTTTCTGGAACTAGAAATGAATGTGAGAGAGAGTGTTTAGGTAATTGTTCTTGTATTGGTTTGTATCACA
ATGAGAAGACTAATTTGTGCAAGAGTTTATATGGGTCATTGCTCAATCTTAGGAATCTTACTTCCGATGG
CACTGTTGAGGATAAGCTATATGTTAGAGTTCAAGGAGGAGGGAATACTCGAAAGAATCAGATTCAAG
TCAGATTACTTGTGATCGAAATGATCTGTGGATTTGTGGTAATTCTGTTAGTTGGAATTGGGACTTTCTT
GGTAGTTAGGAGGAGAAGAGTAAGGAGGAAGAAAAAAGATGAGGAAGATGTATTTCCTATAATGAAT
TTGAAGGTATTTTCATACAAAGAGCTTAATGCTGCAACAAAGGGATTCTCGGAGAAGCTTGGTCATGGT
GGTTTTGGAACTGTGTTCCTAGGGGAGCTATCGGATTCTTCACTCGTAGCTGTGAAGAGACTTGAAAGA
CCTGGTGGTGGTGAAAAGGAATTTCGAGCTGAGGTATGCACAATAGGGAACATTCAGCATGTTAATTTA
GTGAGATTGAGAGGATTTTGCACTGAAAATTCTCATAGGCTTCTGGTTTATGAGTACATGTCAAAGGGTC
CTCTTAGCGCATATTTAAGGCGAGATGGTCAGAATCTAAGTTGGGATGTTAGATTCCGAATTGCTGTAG
GGACAGCAAGAGGTATTGCTTATTTACATGAAGAGTGTCGCAATTGTATAATACATTGCGACATTAAACC
AGAGAATATTCTGCTACATGAAGATTTTTCAGCAAAGGTTTCTGATTTTGGACTTGCAAAGCTCCTTGGT
AGGGACTTTAGTAGAGTGTTGGCAACCATGAGGGGTACCTGGGGCTATGTTGCACCCGAGTGGATCTCT
GGATTAGCGATCACAACGAAAGCTGATGTATATAGCTATGGCATGACATTATTGGAACTGATAGGTGGT
CGACGCAATGTAGAGTCACCACCTTCAGCTAAAGGCGAAGAGGGTGGAACAGAGGAGAAGTGGTTTTT

CCCACCGTGGGCAGCACGTCAAATAGTCGAAGGAAACATTGCTGCAGTTATGGATGAAAGACTACATG
GAACGTACAACCTCACGGAGGCAGAACGTGTGGGATTGATAGCTATTTGGTGCATTCAGGACGATGAA
TCAACGAGGCCTTCCATGGGAATGGTGGTGAAAATGTTGGAAGGAGTAGTCGAGGTGACAACGCCTCA
ACCTCCGAAGCTGCTCCAAGCATTAGTATCTGGTGAATCATTCCATGGGGTAGGAATTGATTCAAGTAAC
GGGACATCACGAGACGGAGGTTTCAGCTCGGGTTATAATCCTCAACGTTCAAGATATTCAAAAGACTCT
CAAGCTTCAAGTTAGGTCTGCTTCTCAACTAGTATGCAAGTTGATTTTTCCCAGCTTCACTGAATGTAGAG
TTCTTTTTTATTGTCTTCAAAATCCCACTGAGAATTAGTAGCAAAATGCTCATCATGGTATTTTGTAAAAT
GTATCATGAATATAGAAAATCTTGTTTACTTTGCTTAAAAATTGTCACTTTTTTTTTTTTTGTAAATTCCAA
AGAACAAAAGCAAAACAAAACATACATTTCACTTTTGAAGGAATGTATGTTTGGATCAGAGGGTCGGGG
CAGAGCTAGTAAATTACACTATTGATATATGGTTAAAATATTTTTTACGTATATATAGTAGACATTGAACC
CTTTAACTTCTTCGTGTGTTTACTTCTTCATATTTTGAACTCTTTAATGAAAATCCTGACTTGCATAGGGTT
ATAGGAGAGGGCTTCCTAACCTTAGAATGAGACGGTAGTTGAAAATATCAAAAGTTATAATAACGGAAT
TATTTAATGGATATATTTTTATTGGTAGATATTTTGTATAGTGAACTTAAAATGAGATATAAGATATAGGT
GTATAATATAAAGCATTAAAACATGTGTTTGGATTATACCAACCAAACATTATGCAAAATTTTATACTGA
GATTATTTTTGTTACTATGGTAAACCAAATGACTATAATAAGAATATACCCTCTAACCTAATTTAATGGGA
GAGTTAATTTTATTTTTTATAAATGAGTGTAACGTTTGTACCTGCCCAATTATAACCACACTTTACTTTCCT
AAGTTTAATTTACATAACTGTCTTGACTACAACAATAACAACTAACCACTTGTCAATAGTTTCCTCTTTTGT
AGCTAAATAATATAACAGAAATTGAAACAAGAAAGACCATATACTCACACAAACAAGCAAACAGAAAAA
GGAGAAATGTTTTCCTAACTTGTCAACTTTAGTAACAAAATTTTCCCAAAATTAATTTTTTCAAAAGAGTG
TAAAGATTGAGGGGGTTAAAGAAAATGGTCAAAGGAGAGCAGAGGTAGACAACTCTGCACTCTCACCA
AAGAAGTTTGTTACCTTGGAAATGGAGACACCTGGCAGTTCAGGTGGGGAGGTGCCGATATATGGGGG
CTCCTCGTCTATGGAGCCGACTGAGTTACACTCGGCTCCATCTACCATGCTGGCAGATTATCCGCCAGTA
CAAGGCTTTCAAGATTTGAAGAACTTGGTTAGCATGGAATCTGGGAAGTTATGGACTATTGCAGGTCCC
ATAGCCTTTAATATTCTGTGTAATTATGGGATTAATTCTTTCACAAGTATATTTGTTGGACATATTGGTGA
TGTAGAACTCTCTGCCGTTGCCATATCTTTATCGGTGATAGCAAATTTCTCATTCGGATTCTTGGTTAGGT
TCTTTTTTTGTTCTTACTAATTCTCAAAGTTCTGTTCTTCTTTATATTATGTTATATGGGCATTGAGTATTCA
TATAGCCGACCCCAACTTGCTTGAAATAGCGGGATAGTTGTTGTTATTGTTGTGGTTGTTTGTAAAATGT
AATACAACGAAGATATGTTGGATTTTGTCACTCTTTGATGAGACTTATGACTATAGATCACAAATAGTTT
ATTATGAAACAGAACTAGGATTTTGATTGTCAAATGATATAAAGTCTCAAAAAGGTTAGAATAAGCATTC

TTTTAGGTTATTGGTTATGTTAGTTTTCTAATTCTTCATCTTTTTTTTGATGGATTATCAGTTAGGCATGGG

TAGTGCACTTGAGACACTTTGTGGACAAGCATTTGGTGCAGGCCAAATAGAATTGTTAGGAGTTTACTT

GCAGAGGTCATGGATAATACTTGTTGCTTCTTGCTTCTGCATAATGCCACTCTACATCTTTTCCACGCCGA

TACTGAAGTTATTAGGCCAAAGAGATGACATAGCAGAGTTAGCTGGAAAATTTTCGATACAAATCATAC

CTCAAATGTTCTCCCTTGCTATCAACTTCCCGACCCAAAAGTTCTTGCAAGCACAAAGCAATGTCGCGATC

CTGGCATGGGTTGGGTTCATGGCTTTAGCCATGCATATCGGGGTGCTCTTTCTTTTTATCAAAGTTTTCCG

ATGGGGCGTTACTGGTGCTGCTGCAGCCTATGATGTTTCTGCTTGGGCTATTGCTCTGGCTCAAGTGGTT

TATATTGTTGGCTGGTGCAAAGATAGTTGGAAGGGGTTGTCTTGGTTGGCCTTAAAGGAACTTTGGCCC

TTCGTAAAGCTTTCTGTTGCATCAGCAGTGATGATTTGCTTGGAGATCTGGTATTTTATGACTATAATTGT

TCTAACTGGTCATCTTGAGGACCCCGTCATCGCTGTTGGATCTCTTTCTATCTGGTACAATCTTGCAAATA

TGCACTTAAAATTTATAATGTGTTCATAATTGTTCTGTTTTTCAAGCTTAGATTCTTTCTTCTTCATTGTTTC

AGTATGAACCTTAATGGATGGGAAGGCATGTTGTTCATTGGAATTAATGCAGCAATAAGGTATACTACT

ACTCTACTAGTTATAGTAGGACATTGTCAGTGCACAAAAGTTAAACTCTAACGAATAGTGTAACTGCTAA

CGGTTATTTTCATTTGTTGATATTTCAGTGTTCGCGTTTCCAACGAGCTTGGATCAGGCCATCCTAGGGCT

GCAAAGTATTCCGTCTTCGTCACAGTAGCTGAATCTCTCATGATTGGGATATTCTGCATGGTACTAATCAT

ATTAACCAAGGACCATTTTGCATTGCTTTTCACAAGCAGTGAGAAAATGCAAAAAGCTGTTTCTAAGTTG

GCATATCTTCTTGGTGTTACAATGCTGCTTAATAGCGTTCAGCCAGTTATATCAGGTACCAAAATATGTGT

GTTTCTTACTCAAACAATTCTCCACACACACATCAGTTGATGTGCCAATCAAATAGTAACTATTTGGTACA

AATCTATGTTGCTTGGACTCTTCAAAAAATGTCAATGGGTGTGTGTCGGATTCTCGATTAGTAGAGTATT

GTTGAAGAACCCGCACAATCAAAAGTGAAGAGTCCGCGCAACTTTAGGTACAAACTCAAAGAATAAATT

AGAATGTTTCTAATATTGAAGGTGTTGCTGTTGGAGGAGGATGGCAAGCACTAGTGGCATACATTAATT

TGGCTTGCTACTATGTCATTGGCCTCCCACTTGGGTTCCTCTTGGGCTATAAAACAAGTTTGGGAGTTCA

GGTAACAACATAAATCTTCTTGTATTAATTTAGCTCCGGTTTGACCATAGATTAGTAGTTTAAAAACAAA

GGTATACTAAATTGTTCTTTTACTTGATCTCAACTAGACATCTTTGCCCTCCAATTCTGAGTCTGCACAAGT

AGATACGTAAACTTATATAAAGTTGAACAAGTAGACACATGTGTCCTATATGACATAATGAAAATCAGTT

TGGGTGTGTCTCACGTGAATTGACACGTAGGACACATTTATCTACTTGTTCAACTTTATACAAGTTTAAGT

GTCTACTTGTGCATACCGAAAGTTGTAGGGCATATATGTCAACTGAAGTCAAGTTAAAGAACGTTTATGT

ATAATTTTGAACATTGTTTGTTCATGAAATTTGGTCAGTTTTCTAAAAATATTCAATTGAGCTAGTTTTTG

GATGAAATTTCACGCCTACTCACAAAACAAGTAAAATGCATATCCAAACATAACTTCATATTCCAAAAAC

TATTGTTCAACACAACTTCAAAAACTCACTTTTTCAAGTTTCAACTAAATCTATGGTCAAAACTCAAACTCA
AACGTTAGCTTAGTTTTAAGTTTAAATTTTAGCTGGAAACTTTGATATAACGATAAAGTTATTTCGAATAA
ACTATTGATATTTGTATCCGGATAAATTGTCTACATTACACTAATTGTCTCTTCATTTTTTTTTTCTCTTTTCT
GTAGGGAATATGGATGGGCATGATTTTTGGAACTTTTTTGCAAACAATAATTCTTTGTGTTATCGTATATA
AAACCAATTGGAACGAAGAGG
>PGSC0003DMG400000915_ G-LecRK
ATGGAGTTCATCTTATTCTTTCTCATTTTATCTTTGTCCAGTATTCCTGTCTTTGGTTTTTTATCAAAAGAAT
TTGTCTATCCCAACTTTACTGCTTCCAATTTCCAATTTGTCGATAGCACCGGAAGTTTCTTGGTTTCTCGCA
ATGGAACATTCAAAGCTTCTATCTTCAATCCCGGTCCTGAACAAGTTAATTTCTATTTGTGTGTTATCCAT
GCTGAATCCAATACCATTATCTGGTCTGCAAATGGTGATGCACCTATTTCAACTTCAGGAATTATGAGGT
TGACCAAGAAGGGTATTGGTATTACTGAAAAAGATGGCAGTTTTAAGTGGTCAACTCCACCATTAAAGT
TAGCAGTATACGCGATGCAGCTAACTGAGACTGGAAATCTCCTTCTCCTTGATCAGTTTAATGGGACACT
TTGGCAAAGCTTTGATCATCCGACAGATACAATTGTAATTGGACAGAAGTTGCGTGTTGGCATGATGCTT
TCAAGTGCAATAGCAGGCGATGACTTGTCAAAGGGTCATTACAGGCTTTCTTTGTCTGCTTCCGATGCTA
TGTTACAGTGGCAAGGTCTGACATACTGGAAATTGTCCATGGAAACCAAAGCTTATACTAATTCAAATTA
TGCAGTGGAATACATGTCTGTCAATCAAACAGGTCTTTATCTTTTTGGTCAAGGTGGTTCTGTAGTTGTG
ATAATGGTGAACTTGTTACAATCCACTTTTAGAATTGCCAAATTGGATGGTTCAGGTCAGTTGATCGTTA
TTAGTTTTGTTGGCTCTGACATCAAACGAGATTTTGCTGGGCCAGTGGATGGGTGTAGAGTACCTTATGT
TTGCGGAGGGCTTGGTGTATGCACCTCTGATGTATTATCAGATAATCCTAAATGTTCCTGTCCTGCTAACT
TCAATCTTGGATCACATGATTCAAGCAGCTGTGTGCCTAGTGATAGTTCCTATTCTTTGCCAGTTTCTTGT
AATTCCACCAACCACAGTAATCTGTCGAATTCTTCCTCCATGTCGTACATAGGACTTGGCTTTGGTATGGA
CTACTTCACTACTGATTTTACCAAGCCCTTCAGGGATGGGGTAAATTTGTCCCTTTGTCAAAATTTCTGCT
CAGTAGACTGTTCATGCTTGGGGATATTTTATGCAAACTCGTCTGGTTCTTGTTACAAACTTGAAGATGA
AATAGGGTCAATTATGTTAAGGACTAGTAGAAATGATGATCTGTTGGGATTTGTTAAGACCCTGGTTGG
AGTTTCAACAACCTTAGGAAACAATGATAATTTTGATCAGGAAGCTGCAGACTTTCCTTTAGTTGCAACA
GTGATCTTACCTTTCACAGGAATCTTCCTACTCATGGCGCTGGGTTTCATTATGTGGAGAAGATCTAGAC
CCCAAAGAATGGGAAAGATAGAGTCGAAAAAAAGCCAACCCAGTTCTCCTTCTTCAGAGGATCTGGATG
CCTTCTCCATCCCAGGATTACCTGTACGGTTTGAGTACAAACAGCTTAAGGCTGCAACTGATAATTTCAA
GACTCAGATCGGCACAGGAGGCTTTGGTGCTGTATATAAGGGTGTGCTCCCTGACAACACTCTGGTTGC

AGTAAAGAAGATAATAAATTTAGGCATTCAAGGGCAGAGAGACTTTTGTACTGAGATTGCAATCATTGG

CAATATTCACCACATAAACTTGGTCAAGTTGAAAGGTTTCTGTGCTCAAGAGAGACAACGCCTATTGGTT

TATGAATATATGAACCGTGGATCGCTTGATCGCACTCTTTTTGGTCATGTACCTGTATTGGAATGGCAAG

AACGGGTTGAAATAGCTATTGGTTCTGCCCGCGGACTTGCATACTTGCACAGTGGCTGTGAACAAAAGA

TTGTTCACTGTGATGTGAAGCCAGAGAACATTCTCTTGCACGATAATTTTCAGGTGAAAATATCTGATTTT

GGCCTCTCCAAGCTGCTGAATCGTGAAGAATCCAGTCTATTCACAACAATGAGGGGGACTCGTGGCTAC

CTTGCTCCTGAATGGCTCACAAGTTCTGCAATCTCAGACAAAACGGATGTGTATAGCTTTGGAATGGTAC

TCCTTGAAATTGTAAGTGGAAGAAAAAACTGCTCTTTAAGAACACAAAGTCACAGCCTTGATGATAGTG

CTACTGGAGATCACTCATCATCTTCATCTGCACAGGGACTTGTTTATTTCCCTTTATTTGCATTAGAGATG

CATGAGCAAGGAAGGTACCTTGAACTTGCTGACCCAAAACTTGAAGGGCGGGTCAGTGGTGGAGATAT

TGAGAAGTTTGTGCGGATAGCATTGTGCTGTGTCCATGAGGAGCCATGTCTCAGGCCAACTATGGTTAG

TGTTGTTAGCATGTTAGAGGGAGAGATACCACCGACTGCGCCAAGAATGGAATCTCTAAACTTTTTACG

CTTCTATGGGCGACGGTTTTCAGAGGCATCAAACATGGAAGAAACTGGAGGCCAGATAGATGCCATGTT

ATATCCGCAGGCAAATACTTCTCATACAACCTCTCGGAGCGTCTCTAATGCTTACTTCTCTTATATTTCGTC

CCAACAGATCTCTGGTCCAAGATAA

>PGSC0003DMG400001059 G-LecRK

AAAAATTTCATACTAGAACAATAGTTCTTTAATGGCATGCCTAAAAATCCTACTATTTTTCCTCTTCATGAA

CATTTCTTTTGCCCAAAAAAACACAATTTTTCAAGGCCAACACCTTAGAGTTGGAGAAAAATTGGAATCA

GCAAACAAGGAATTCAGGCTTGAGTTTTTTAGCCTTGATGCCAATAAAACACAATATATTGGCATATTCT

ACAATTTACCCTCTAACATGACCTTATTCCCCGGTGATGATCGACCCGTTTGGGTTGCAAATCGCGATATT

CCGATACAATATGCATCAGGTAACACTCTCACATTGGATGATGATGGAAAGTTGAAAATTTTTTATGGAG

GGGACAGTTTTGTTATGTTAAGTTCTTATAATGTAACAACAAGAAATGCAAGTGCTACACTATTTGACAA

TGGCAATTTTGTGTTGGTGGAATTGAATACTAATGGTTCAGAGAATAAAACATTGTGGCAAAGTTTTGAT

TATCCTACTGATACACTATTACCTGGGATGAAACTTGGGAGAAATGTTAAAACAGGACAGATATGGTCA

CTGACTTCTTGGATAAGCAAAGATGTACCTGCCTCGGGGTCATTTACGATTGGTATAGACACGAATGAC

ATTGACCAATTGATAATATGGTGGATGGGAAGTGTGTTTTGGACGAGTGGACGCTGGGGAAATGAGAC

GTTTGGCAATGTATCGCGAGTATCACACTATGAGTATGTTAACTTGAGTTTTGTTTCGACAGAGGATGAG

AAATATGTGACTTATTCGGTCAGTGAGACTCGAACTTTGTCTAGATATGTAATGGATACATTTGGTTTTAT

TAAGGAAAGAGGAGCAGCAGGACCTTTTGGGGTGTGTTTTTACAAGCCTAGTGCTGGTTGTGTGACACA

AGAATCGATTGGGTGCTCGATCAGAAACGATTCATGGTTCAAGAGGAGGCCAAATAGTGTCTCTGGTAA
TAGATTTAGGTTTGCAAACAACAACATGAGTTTATTTGATTGCAAGAAACAGTGTGAGAAAAACTGTTCT
TGTGCTGCTTTTGCCTCTATCACAGCTACTGGTACTGGTTGTGATATTTGGAGTAACGTATCGATTTTGAA
TTCACTTGAATCAGATGTTTTCACTCTTGATGATGAAAGAGGTATATTCATATTTACCTCATTGTCTTTTTT
ATGTTGTTGTCGTGTGACCAGGAGGTCGCGGGCTCCAGCCTCTTGCAGATATGCAGGGTAAGGCTGCGT
ATAATAGACTCTTGTAGTCCAAGGAAGTGCATTGTTTCTATGTCTAGCAAGTAAAGTTAGTAAAGCGGAC
TCTAACGATGAATTGTGCAATGTGTATTCATAGATTTGGTGCCAATAAGAAATCGGAACAGCACAGATG
TACCAAGCACAGTCTCACTTCCTCCAGATTTAGCACCAGAACCATCTCCTAATTCAACGTCAGGATCGTCT
AAAGGAACTTGTATGTATTGTGTCTAAATCTTGCTCTTCAACTATTGTATATGATTGAAGGTGAAAACCTT
TTTTTGATTGTTTCCTCCATATTATCCATTTAAGAAAGAACTATACATTTTAGGCACGTATTCTTTTGTAGT
ATCATCATTACACAATCTAGTCCTTGTTTCGAGTATATCCAGAGAAAGGTCATTTAGATCATCTGTGTTGA
TGTTGTTACTGCTCCTTTCCTGAATGTTCTGCACTGTTTTCCTTTTTACTTGTCATGTTCTCTTCACTGTCGT
TTTTCCATTTCCATAACTACTTTTATTTGTTGCTCTTGAGCCGAGGGTCCTTTAGAAACAGTCTCTCTACCT
CCATGTGGTAGGGGTAAGGTCTGCGCACACTCTACCCTCTCCAGACTCCACTTGTGGAATTTCACTCGTT
ATGTTGTTTTGTTGTTGATGCAGCAACAAAATGGTGGATATGGCTTATAGCTATAATCGGTTTGACACTA
TTCGTAGGACTTAGCTCCCTATGCTACTTTCTACACGGAAAAGGTGAGCACTCGAAGAATTTAGCTAGAA
GCTTGCACCAGTAAAACTCAACTATGTTGCTCGGACTCTGCTAAAATGCTGCCACAGTACCCCTGTCGGA
TCCTCAAAAAAAAAAACTCACTACTTTTGAAGGATCTGACACACACCAAACAGCATTTTTTAAAAGTCCG
AGCAATATAGAAATGCTCTATACTATGGGTTCTGATCCCACAACTTCCTCTTTCACTATCAGGGAAAGCA
AAAGCAACAGCTTTGCTATTGCTTAATCAAACAGCAAATATATCTAAAAAGAGAAAAATAGACAAGAAA
ATGAGCCATGAAGTGCAGTTATACAGCCTCGAGAGCCTAGCAATCGCCACAGACAATTTTTCACCAGGA
AATAAGCTTGGGGAAGGTGGATTCGGACCAGTTTACAAGGTAAACATGAAACCTTTTATATGAATATGA
TTCACGTAAGCATAAATACTTAAACGAGTCATTATTTTTCAGGGAGAAATGGCTGATGGGCAAGAAGTA
GCTATAAAAAGACTTTCGACTAGTTCAGGACAAGGCCTATTGGAGTTCAAGAATGAAATTCTATTGATTG
CAAAACTTCAACATACTAACCTTGTTAGGCTTCTAGGATACTGTACTCTACGCGAAGAACGGATTATAGT
TTATGAATACATGCACAACAAAAGCCTAGACTTCTTCCTCTTTGGTAAATATCATGTTTTTCATCTTCAACA
ATTAGAACAACTTTGTCAAGAAATTTCATTTTTTACAATGATATGAACAGATAGTAACAAAAAGGAGCTA
CTAAATTGGGACACACGATTCAGAATCATCGAAGGGATTGCTCAGGGTATACTGTATCTGCATAAATATT
CAAGGTTGAAAGTGATTCATAGAGATTTAAAAGCAAGCAACATCTTACTTGACGCGGAAATGAATCCAA

AGATATCAGACTTTGGCATGGCGAGGATATTTGGAACACAAGAATCTGAAGCAAACACTAAAAGAATTG
TTGGAACACAGTAAGTTAATAAGCAATGTTTTCATATACAAGTTTTTATACAATATTACTTGAAGTTCTGA
ATACATTTTTCGGATACTACTTCAGTGGCTATATGTCTCCAGAGTATGCCTTGAGAGGCATTGTTTCAACG
AAAACGGATGTATTCAGCTTTGGAGTTTTACTGTTAGAAATTGTTAGTGGCAAGAAAAACAACAGCTGC
TATGATTCCGAACATCCACTAAACCTCATAGGACTGGTAAGTTTCTAGACTATATTATGAACATATCTCGC
GAAATGGAGAAACATATAACTATTAGTACTATGTTTTTAGGCATGGGAATTGTGGAGAGATGAGAGAG
CTTTGGAGCTAATAGATGCAACACTAATTGAATCATGCTCGCGCGATGAAGTAATGAGATGCATTCATGT
AGGACTTCTTTGTGTGCAAGATTATGCAAAAGATAGGCCTTCAATGTCAAATGTTGTTTCAATGCTTATG
AATGATACGAAACAACCACCACCACCACCAGAACGACCAGGATTTTTCATCGAGAGAGGGGATCAACGT
GCAGAGATCTCCGAAGAAGTAGTCAGATACTCGATAAACGGGCTATCAATATCAGAGTTGAGAGCAAG
ATAAGATACAAGATGGTCAATTGGGAAGTAACTTATTTAACATTCTTTTGATGAAGTGTAAAAAGTCACC
AACTTCAAGTACATACTTATAGGACTAATTTCGAGTCCTCTTTCTTTGATACTATTACATTATGATGCATTT
TGTGAGTAGAATATTTGAGACGGATCCAGAATTTAAGTCAAAGATGGTAAACGAGATAAGGCAAGGCG
AAGTTATGGCAATGGAGACTTCACCATTACCTTCCCCCTCCCGCCTGATAGCAACGTCACCAGTTACATTT
AAAATGTTAGAGAATACACTTGACACTCATCGATTATATAAGTCGAAAGAGAATATCAGAGGAGTTTCT
GAACTATCAATATTCGTATATTAGAAAAAAGAGGTAACTGAATGTACATATATTTTCTCATTCTGAGCTTT
ACATGTTCCACAAATGGGCTGTGAATTAGTTTACACTCAATTAACTACAAAAATAAAGAACAATTTTGGT
TTTCACCTAAACTCACTACAATTCTGATGATTTAGCTTCATCAATATTGTTATCTTTATCGCTAACAAAAAC
TAAACGATCACCATCTCTGATCAACTCTATTTCGTCGATTTCAGCTCCATCTTTGCTTACAACTTTGGCCTG
AGAGATACCATATCTACTAGAACCAATTTGAAGTAGCTCTTTAAAACTCTGTGGGAGTAAAATGAGTTTT
CCCCCGTTGTCTCCTTTCTCAGGGCAACACACTGTCACTCGGGGAGCATAAGTCTTGGTTGTCATGACAC
TTACATTACTGTCGTTTGCAGATAAAAGCACGTCGTGCTCATTGGTCTGCTTTGCTGACATTATCCCGAAT
AATGAGTTGTGGAAGTTATTAGACCTACGTCTAGGACGTGAACGCCCTAATGATCCGTCTAGTCCTAGA
AATGAGACTCCATGGGATGCAGGGGTGATTGTTGGTTCACTTTTGAACCTCCCAAGAAATCTAACCCCTC
GTTCCTCGGGGATAGGATCAACGGATCGAGTTCTCATCATTACCCTGGATTCAAAGAGTTCTTTGATGTC
TTCATGTCCTTGTTGCTCAGCAAGGTCCCGGGGGGTCCAATTATGTTCGTCTGGTTGATCAACGTTAGCC
CCTCGATCCAAGAGATACTTTACTATTTCTATGTTACCTTCACAAACAGCAACATGGAGAGCTGATGAAC
CATTGATCTTGGGAAGCGTGACATCCCCGCCATAACGGACAATCTCCTTGAGCAAACTCAAGTTGTTTTG
TTCAACAGCAACGCAGGCGAAATGTCCCACATCACCAGCTGATAGTTTAGCACCGTTGTTAACGAGTAA

CTGGATAACTGACTCGTGCTTCCCCATGATAGCCTCCCACAATGGCACATTGCCTTCTGAATCTGTGTATA

CACAAGAAACAAAATCAAATTCTTATATCTGGCTGTGTCGGTGGACATGATAGGAAAAGGAGACAACTG

ATAGAGTGTGAAACTCGCAAAAAAAACGATAGTTGAGGTGTGTTTTTGACTATAAACTCTCTAATCTTTG

GACAAAAACAAACTTTTGGCAACAACAGAATTTGAAAAAAGAAATGGAGCATACCTCTACTATTGGGAT

CAGCCCCGAAATCGAGCAGAAGAACCACACAGTTCTCAATGCCTTTAGACGCAGCGATA

>PGSC0003DMG400002017 G-LecRK

ATGAATGCGATTTGCGTAGCAACAGATACTATATCAATGAACCAACAAGTTGTAGATGGTGAAACATTA

GTTTCAGCAGGAGGTATCTTTGAGCTTGGGTTCTTCAGTCCTAATGGATCCCAAGGGCGATATGTCGGG

ATATGGTACAAGGGATTACCAGTTGAAAAAGTTGCATGGGTTGCAAACCGCGAAAGAGCATTAATTGG

ATCATATGGAGTTCTTATGATTAATGCAAGTGATGGAAATTTAGTCCTTGTGGATGATAAAAATATGTCT

GTCTGGTCTACAGAAATTAATCTGGTATCGAACAATAACACAATTGCTGTACTGACTGATCAGGGGAATC

TTGTTCTCCGCGATAAGAACGAGTTGAATGAATCTGTACTGTTATGGGAGAGCTTCAATCATCCATCCGA

TACTCTCTTGCCTCGTATGAAGATTGGGCTGAATACTAGAACAGGGGAGCAAATAGTCGTCACGTCGTG

GAAAGATGAGAAAAATCCATCACTGGGAAGTTTTAGCACTGCTGTGGTGGATCCTCATGAACTTTTACA

AGTACTTATTTTGAATGGCTCATCGAAGCATTTCAGATCGGGGCAGTGGAACCGGAGAAGTTTCATAGG

AGTGCCTAGCATGACTAAAGACTTCCATAATGGCTTTCAACTACTTATGGATAATAATGGGGAGGAACTT

TATTTTACTTATGCTATACTCAATTACTCTGCTCCAACATTGATATACATTGATTCCTACGGAGCGATAATT

CAAATGGATTGGGATGCAGAAGAGAATAAATGGTTAAAGGTGCAGGTAGTACCTAATGGTCAATGTGA

CTTGTATAACACATGTGGTCCTTTTGGAAGTTGCAAGGATAATGATTCACCAATATGCAAATGTTTGACG

GGCTATGAACCAAAGTCTATCGGTGAATGGAACGAGGGGAATTGAACTAGTGGTTGTGTCCGGAGAGC

ACAATTCCAGTGTGAGGAGACGATCACTAGTACGAATTCTGATGATGAGAGAAAGAGAGGGGAAAAGA

ATTCTGATCACTTGTTTGGCATATGCTTATGTGGATGGAATAGGCTGCATGATTTGGGGAAAAGAACTTA

TTGACCTCCAAGAGTTTGCCCGTGGTGGAGAGCAACTATACATTCGCCTCGCACATTCCCAACTTGGTAG

ATACAGATACACTAATTCTATGTTGCTCGGACACTCCAAAAATGTTGTTGCACCCGTATAGGATCCTCCA

AAAAACACACTATTATTGGAGGATCCTACATGCACACAACAATACTTTTGAAGAGACTGAGCAACATAG

CACCAATTTTCTCTATAAACATCCCATTTTATTACTACTCGAACGAAACTTCACTTCATTTTGGATTTGTTTT

AGTAGTTTGCCTATGTTATTTGTCTCTAATCATTTGTTGGATTCACATTCTCCTTTTATGACTGTAGTGTTC

GGGCCAGCTTTAGTGCGCCTGAACTATTCTCAGAGACAGAGCTAGGATTTTGAGTTTATGGATTTTGGAT

TCTAGAAAAAACAAATTACTGGGTCTTGAATAAATTATTTATACTTATTAAATGGATTCTTAAACACAAAA

AATATATGGTCTAGCCCAAAGCTACTGGATTCTACAGACTCTGTAGACAGGCTTGTAGCTCCGCCTTTTC
CATCGAGTACTTGCTACCTCTCACCAATAGGGATACTGAGTAATTCTGCTCACTAAGGCATAAGTAGATG
TGAAAAAACACCTAGTATGGTTTTGCTTCCATCGGAATCTGAACTTGAGACCTCATGTTTTAGTTTTTTTC
TTATCGAGTTGCCTTTTGTCATGCAGATAAGAGGAGGATGCTAACGGGAGCCCATATTGCAATTATTGTC
TCAGCGACATTCGGCCTGATTCTTATTGTTTGCACTATATATGTATTGAAGATCAGGAAAAAAGGTAAAA
AAAAAAAAAAAACAGTCAATTTTTACAGTTGTTAGAGGCACAACTTTTCGCTATCTTTTGGAAAGACTTT
AATTTTTACTAGATTTTATCAGGCATTGGGAGTTTAGTCCCTTTTCGCATAGCTTCAAAACACTCTTCAGC
AACAAATTTGCTTCATAACAGTTTTACTCAAGGTAGAAGTACAGAATTACCAGTTTTCAGCTTCAAGTGC
ATAGAAGCTGCTACTAAGAACTTTTGCTATCGAAATAAACTTGGAGAGGGGGGATTTGGACCTGTATAC
AAGGTATATATATATATATCACTCGTGTGTGCTTTAATCATCGAAAAAGTAGAGCAAATTTCACATGGTT
TATACTCATTTGCTTATTAGTTGAAGTCATGTGTGATTCATATATAGGGGGTATTAGCAAATGGACAAGA
GGTTGCTATTAAACGACTTTCGAGGAGCTCAGGACAAGGCATTGAAGAGTTCAAGAATGAGGTTACACT
GATTTCTGGACTTCAACACAGAAACTTAGTCAAACTGATGGGATTTTGCGTTCACGGGGAGGAAAAGTT
GCTGATCTATGAATACATGTCCAACAGAAGCTTAGACGTTTTCATATTCGGTGAGCTAATTAACAAGAGT
ACTTAAACAGTACTTTTCTTGATGAATTATGATTAAATTACTCTTTGTGCTTGGCAGGAGGATCAAGATCT
AATGATAAGGCGGCATTGAGTTGGAATCAACGATATAGTATCGTTGAAGGGATAGCTAGAGGTCTTCTT
TATCTGCATCGTGATTCACGACTAAAAATCATTCACAGAGATCTTAAGACAAGCAATATTCTGTTAGACG
AAGATATGAATCCCAAAATCTCAGATTTTGGAATGGCTAGGATTTTCGGAGGGAATCAAACTATAGCAA
ATACCAACAGGATTGCCGGTACCTAGTAAGTATTAATTGTTCATATATAGCAGTGAAATTCATCATAACT
AAATTGTATATTTGTGTATCAATGCAGTGGTTACATGTCCCCTGAATATGCAATACAAGGATTTTTTTCCG
AGAAGTCGGATGTCTTCAGTTTTGGTGTCATATTGTTAGAGATTATCAGCAGTAAAAGAAACAACAGCT
ATTGTCACTCTGAAGACTCTCTCAACCTCCCGGACCAGGTGAAAAAAGAGAAAAACTGAAAGAATTTCTC
ATTTTCTCATATACAGTCAAACCTCTTTATAATAACGTAATTTCTTGTCTAGCTGCTATAGCAAAAATGTA
GTATAAAGAACATATAAATATAACATAATATGGAAGTTCGAGAAAAACTAGGCCGAGGATGATTGTTAT
CGAGAGGTCTGAATGGACTTGAATATTCTTCTATCCTATACTACTACTATTTTCACATGACAAAACTAATA
TCAATGGATTTTATAGGCATGGCAACTGTGGAATGAAGGCAAGATACTCGAGTTTCCGGATCCAAAGGT
GTTTGGTTCACTTGAAACACCAACACAAGTTGTTAGATGCATCATTGTGGGATTGCTTTGTGTGCAAGAA
CATGCAAGTTACAGGCCAAATACAGCTACAATTGTTTCCATGCTCTGCAGTGACACACTTCTTCCTCCGCC
TAAGCGGCCGGCATATAGCAGCAGAGGCTCTGATGATCCAAGCATTTCACCTTGCTCCAGAAATTCCATT

TCTATTTCAAATGTTGAAGGACGTTAA

>PGSC0003DMG400003313 G-LecRK

TTCAAAATTACATATTTCAAAACTTCTGTATGAATTAGGATTTCATACTATAACATGAATATAAAATTTTCA

ATTCTTGTTTTACCTTTACTTGTTGTTTCATCTTATTGTTCAACAATCTTTAGTTTATCATCTGAAGGTTCAC

TTTCTGCTCCACAAGATTTAATTTCGTCTCGAAATGGAAAATTCACTGCTGGTTTCTACTCTGTTGGTGAT

AATGCATATATCTTTGCTATATGGTTTACTAAGTCATTGGCTGATGGAACCAACACAATTGTTTGGATGG

CTAATCGAAATCAACCGATAAACGGACAAAAATCACATCTGTCGCTGTTTAAATCAGGCAACCTTGTACT

GATTGACGCGAATCAGATCAATGTTTGGGAATCTGGTACACAATCATCAACTTCCTCTGTTGAGTTAAGA

TTGTTGGATAATGGAAATCTTGTTCTTGTAACTTATGAAGGTCAAAGATTATGGCAAAGCTTTGATTCAC

CAACTGATACACTTCTTCCTGAACAACCACTTACGAAAACCTCAAAGCTTGTTTCGCGTAGAAGCTTGACT

AATTTCTCCTCTGGATTCTATCAGTTACATTTTAATGAAGATAATGTCCTGCATCTTGTCTTTGATGGAATA

GAGATGACAAGTGTGTTTTGGCCAAGCCCGTGGTTAATCGTTTGGGATGCAGGTTAGTAGAGACAGATT

CAGAATTTAAATTTGATTCGTTAAATCCTTAGTTTGTTGTCACTCAACTCACATATATTTTTGCATGAAAAA

TACTAGTTTTAGTTGAACTCATTATATGTTTCATTTGAGTGTATTGGTTAGTAAAGATGGATTCAGGATTT

AAATTTAGTTGGTTCAATCTTTAGTACAACTCATTTTTTTTCGGTTTAACTGAACTCACTGCTGATCATGTC

TGCAGGTCGATCCACTTACAATGACAGCAAAACAGCAGTTCTTGATCGTTTTGGGAATTTTACATCGAGT

GATCGATTTGGATTCCAATCTGCTGATTATGGTGTGGAATTGCAAAGAAGATTCAGCCTTGACGTAGAT

GGTAACCTTCGATTGTACAGCCTAGATAAGCTGAGCAACATTTGGAAAGTTTCTTGGCAGTTATTTCCAG

CAGCCTGCAGGATTCATGGAGTTTGTGGATTGAACAGTTTGTGTTCCTATGATCCTGTCTCTGGCAGAAA

ATGTTCTTGTGTACCAGGATACAGGATGAAAAATCCTAAAGATTGGTCCTATGGTTGTGAACCAGAATTC

AGTATCTCTTGCAACGATACAAGCTCGTTGGACTTCATTCCACTTCGCCATGTTGAGTTTTACGGGTATGA

TATTGCATATTTTCGCAATAAAACTTTGCAAGAATGTAAAGATTTGTGCTTGAAACACTGTGATTGTAAA

GGTTTTGAATACAAGTATGTTGAGGGTAATGGCACCTATGGTTGTTACCCGAAAACACTTCTGTTCAATG

GCTATGTTCAAGCTAGTTGGCCTGATTTTGTTTACCTTAAAGTGCCTAAAGCACAACGTGCCAGGCAGGA

GCAGTATGAAGGAAACCTTCAATGTGACGATAAAAAGGTGATGTTGGATAGAGCTTATAGAAGGAAAG

AACATGGTGGATGGATGAAGCCATTCATTTGGTTAGTTGTTGTAGCTGGAGTCTTTGAGATTCTCTGTGT

TCTCACTTACTTTATTAAAACGAGAAAAAGCTCGAATGAAACCACACAAGGCTACCTTCATCTTTCGACA

AGATTCAAGAAATTCACCTATGCTGAGCTTAAAAAGGCCACTTCCAATTTCAGTGAAGAGATAGGTCGA

GGAGGCGGTGGTATTGTGTACAAAGGGAAGTTGTCTGATGACAGAGTTGCAGCTATAAAGTCTCTCTAT

GGAGCCAGCTATCAAGGAGAAGCAGAATTTTTAGCTGAAGTGAGCACTATAGGCAACGTTAATCACATG
AATTTGATCGAAATTTGGGGATATTGTGCTGAGGGAAAGCACAGGCTTTTAGTTTATGAGTACATGGAG
TGTGGTTCTTTGTCAGATAATTTGAATGCCAACAATCTTGATTGGGAAAAAAGGTTTGAAATCGCGTTGG
GAACGGCTAAAGGACTTGCATACTTGCATGAAGAATGCTTAGAATGGGTTTTGCATTGTGATGTAAAGC
CTCAGAACATACTTTTAGATTCCAATTACAAGCCAAAAGTTGCTGACTTTGGGCTTTCAAAGATATTTAAC
AGAGGTGGCTTGGATCATTCAAACTTTTCCACGATAAGGGGGACGAGAGGATACATGGCTCCAGAATG
GGTATTCAAGATGCCTATTACATCCAAAGTCGATGTCTATAGTTATGGCATTGTTTTATTAGAGATGATCA
CAGGTAAGAGTCCAGAAGTTTGTGCTGATGGTGACTCGGGGGATGATGATTCTATGGGACTAGGATTG
TTGGTCACTTGGATTAGAGAAAAAATGCGCGAAAACAGTGAAAGAAAATCATGGATTCAACAAATTGTG
GATCCTGCATTGAATGGTAAATTCGATTTGGAAAAAATGGAGATCTTATTAGAGTTAGCTTTGCAATGTT
CGGAAGAAGATAGAGATGCAAGACCTACCATGTGTGAGGTGGTGGATAAAATGCTTCATCCAGAAAAT
TTCGAGTTAAAATTAGACATGTTAATGTAGTTTCTCAAGATGTAAAATCTTGCCAAAGCTGATATAGTAC
AAGCTTATATGTAATGTCTCTTGAGTAGCTAGTACTTTTGAATTCCAAAATGTAAATCCTTATAGAATGAC
ATAGTTTCTTAACAATATTGTGCATTATAGCTACTGTTATGGTCTTGTGCATCAGTGCTAGATGGAAAGT
GTGCAAGAGTACAT

>PGSC0003DMG400003982 G-LecRK

GAAAAGATAAAATCTATGGCTGCTTTGCTATGGTTATTAATCTTATCTGCATTTCATGCAGTGACACTAGC
ACAACAAAGACAGTTTAATATAACTCTTGGCTCTTCTCTAACACCTACTGCCAATTCATCATGGTTTTCAC
CGTCCAGGCGCTTTGCCTTTGGGTTTTATGAACAAACCAATGGCTATGCTGTTGGAATCTTAATTGTCGG
TATGCCTAAGAAGACAGCAGTGTGGACAGCAAATAGGAACAGTCCTGTTGTTCCGAGCAACGCTGTCTT
GCTCTTAACTAATGATGGAAGGCTCATTGTACAAGTAGGAGGACAAGAAATCACTGTCGTAAATCTCTCT
GGTCAAGTCATTGCTTCAGCTTCCATGCTGGATACTGGCAACTTTGTGCTCTATAATTCTGATCACAGTAT
CATATGGCAAAGTTTTGACAATCCAACAAACACCCTTTTACCAGGTCAGCATATATCAGCTGGACAAGAG
CTGTTCTCGAGTGCTTCAGTAGCAGATGATTCATACGGTATTTTCCGTCTCAAAATGCAAGATGATGGGA
ACCTCGTTCAGTACCCTGTCAATACACCAGATACTGCCCCATATGCTTACTACTCAACACGCACTTCTAGA
GTAGGGAATACTGTTACACTGAATCTTGATGATGACGGCCTCCTCTACTTGCACAATTCCACTAAGAGGC
TAAGAAATCTAACCAGAGGAGGCTACCCTAGAGAAAGGACCATCATCTATATGATGAAAATTGATGCTG
ATGGTATTTTTCGAGTCTATTCACATTCTCTGAACCAACAGAACAGCTCCGTGATATGGTCATCAACAAAT
GATGGCTGTAATCCAATAGGCCTTTGTGGGCTTAATGGGTTTTGCACCAATATAGATGATCAAGCTAAAT

GTTTGTGTCTTCCGGGATTTGATTTTGTAATGCCAGGAAATTGGAGCGCAGGCTGTGAAAGGAATTTCA

CCGCAGAAACCTGTCGGTTAAAGGAGAGCACTTCCAAATACTATGCTATGAGAACAGTTGAAAACACAG

GATGGGAAGATAGTACTTATGTTGTTTTGGCCGGAACGACCAAGGAAGATTGAGACCAAGCCTGTCTAC

AGGATTGTAACTGTGAGGCTGCTCTGTTCAAAGACAGAGAGTGTAGAAAGCAAAGGCTGCCTCTGAGG

TATGGAAGGAGAGAACTGGATAATTCTAATCTAGTATTGGTCAAGGTGGGCACTAATTTCATACCCAAC

AAAGGGGTGCCTAATCAGACGATTGAAGAAACAAAAGGCAAAAAGCTAAGGATAGACATCCTAATTGC

CAGCATTACATTGGCTGTCTTTGCTTTGTTGGTGTTAGGCATCTCTGGATTTCTCATTCACAGAATTCATG

TCTGGACTTACAGAAAAATCCAGGATAGTAGAAGTGTTCAGTTATGTGAGGATGTTGCTCCACGAGCAT

TTTCCTATGCAGAACTAGAGCAGGCAACCAGTGGCTTCAAAGAAGCGTTAGGGAGGGGGGGATTTGGA

ACAGTATTTAAAGGGATATTGGCAGAAGGTCAGAAAGTGATAGCTGTTAAGAGACTAGACAAAGAATT

GGTAGAAGGGGAAACAGAGTTCCAAACTGAAATAAAAATCATTGGAAGGACACATCACCGAAATCTAG

TCCGTCTCCTTGGTTATTGCCTGGAGGGATCAAGAAGGCTTCTGGTGTATGAGTACATGACCAATGGGT

CACTTGCGGACATACTTTTTACACCAGAAAAACAGCCCATGTGGGAGGAAAGGTGTGGCTTTGCTAGAG

ATATTGCAAGGGGCCTCCTATATTTGCATGACGAGTGTGACACACAAATCATCCATTGTGATATAAAGCC

TCAGAACATACTCATGGATGATCAGTACTGTGCAAAAATATCTGACTTTGGAATGGCCAAGCTCTTAAAG

AAAGACCAAAACAAGAACTTACACAGGGATAAGAGGGACCAGAGGCTATGTTGCACCAGAATGGCACC

GAAATCTGCCAGTCACTGTGAAAGCAGACGTTTACAGTTTTGGTGTTGTCCTATTGGACTTGATTTGCAG

GCGGAAGTTTGTTGATATGAGCCTTGATGAAAATGAGGCAATTCTTGAATTCTGGGTTTACAATTGCTTT

GATGCAGGAGAGCTAGATAAACTAGTCAGGGACGAGGAGGTTGATAGAAGACAATTCGAGAGAATGG

TGAAATAAGCATTGGTGCATTCAAGAGGAACCATCACTTCGCCCTTCAATGAAGAAAGTTCTACTGATGC

TGGAAGGAACAGTAGAAATTCCAGTGCCTCCTAGTCCTTCTTCTTTTCTAAGTGCCATATAGTTTTAAACT

TCTTCACTTTTTGTTTCAAGTATCCCAACTTTCCCATAGAGAGCAGAGTACTTTATTATAATTGTATCAATT

TTCTACATCATTCTCCACCTCTTCTAGCCATCAGTTATCTTCTGCTGTGGTGTATCTCAAGGCTTATGTTTC

CATTTTGAGATTTGTCCATATCAACACTTCTCTTCAAATTTACTTGTAGTATTTGAATTGTAAATACTAATT

T

>PGSC0003DMG400003999 G-LecRK

ATGGCTGCTTTGCTATGGTTATTAATATTATCTGCATTTCATGCAGTAGCACTAGCACAACAAAGGCAGT

TCAATATAACTCTTGGCTCTTCTCTAACACCTACTACCAATTCATCATGGTTTTCGCCGTCCAGGCGCTTTG

CCTTTGGGTTTTATGAACAAAACAATGGCTATGCTGTTGGAATCGTAATTGTCGGTATGCCTAAGAAGAC

AGCAGTGTGGACAGCAAATAGGAACAGTCCTGTTGTTCCGAGCAACGCTGTCTTGCTCTTAACTAGTGA

TGGAAGGCTCATTGTACAAGTAGGAGGCCAAGAAATCACTGTCGTAAATCTCTCTGGTCAAGTCATTGC

TTCAGCTTCCATGCTGGATACTGGCAACTTTGTGCTCTATAATTCTGATCACAGTATAATATGGCAAAGTT

TTGACAATCCAACAAACACACTTTTACCTGGTCAGCATATATCAGCTGGACAAGAGCTGTTCTCGAGTGC

CTCGGAAGCAGATGATTCTTTCGGGATTTTCCGTCTCAAAATGCAAGATGATGGGTACCTTGTTCAGTAT

CCTGTCAATACACCAGATACTGCCCCATATGCTTACTACGCATCCTACATCCGTGGAGTAAGGAATAATT

TTTCACTGAATCTTGATGATGACGGCCTCCTCTACTTGCTCAATTCCAACAATAGTCTAAAAAATCTAACC

AGAGGAGGCTACCGTAGAGAAAGGACCATCATCTATATGATGAAAATTGATGCTGATGGTATTTTTCGA

GTTTATTCACATTCTCTGAACCAACAGAACAGCTCCGTGATATGGCCATCAACAGATGATAGGTGTGATC

CAAAAGGCCTTTGTGGGCTCAATGGTTTTTGCACCAATATAGATGTTCAAGCTAAATGTATGTGTCTTCC

GGGATTTGACTTTGTAATGCCAGGAAATTGGAGCGCAGGCTGTGAAAGGAATTTCCCCACAGAAACCTG

TCAGTTAAAGGAGAACACTTCCAAATACTATGACATGAGAACAGTTGAAAACACAGTATGGGAAGATAG

TACTTATGTTGTTTTGGCCGGAACGACCAAGGAAGATTGTGAACAAGCCTGTCTACAGGATTGTAACTGT

GAGGCTGCTCTGTTCAAAGACAGAGAATGTAGAAAGCAAAAGCTGCCTCTGAGGTATGGAAGGAGAGA

CTTGGGTAATTCTAATCTAGTATTGGTCAAGGTGGGCACTAGTTTCATATCCAAAGAAGGGGTGCCTAAT

CAGACGATTGAAGAAACAAAAGGCAAAAAGCTAAGGATAGACATCCTAATTGCCGCCATTACATTGGCT

GTTTTTGCTTTGTTGGTGTTAGGCATCTCCGGATTCCTCATTCACAGAAATCATGTCTGGACTTACAGAAA

AATCCAGGAGAGTAGAAGTGTTCAGTTATGTGAGGATGTTGCTCCACGAGCATTTTCCTATGCAGAACT

AGAGCAGGCAACCAGTGGTTTCAAAGAAGCGTTAGGGAGGGGAGCATTTGGAACAGTATTTGCAGGA

ATTTTGGCAGAAGACCAGAAAGTGATAGCTGTTAAGAGACTAGACAAAGAATTAGTAGAAGGGGAAAC

AGAGTTCCAAACTGAAATAAAAATCATTGGAAAGACACATCACCGAAATCTAGTCCGCCTCCTTGGTTAT

TGCCTGGACGGATCAAGAAGGCTTTTGGTGTATGAGTACATGACCAATGGGTCACTTGCGGACGCACTT

TTTACACCAGAAAAACAGCCAACGTGGGAGGAAAGGTGTGGCTTCGCTCGAGATATTGCAAGGGGCCT

CCTATATTTGCATGACGAGTGTGAAACACAAATCATCCATTGTGATATTAAGCCTCAGAACATACTCATG

AATGATCAGTTCTGTGCAAAAATATGTGACTTTGGAATGGCCAAGCTCTTAAAGAAAGACCAAACAAGA

ACCTACACAGGGATAAGAGGGACCAGAGGCTATGTTGCACCAGAATGGCACCGAAATCTGCCAGTGAC

AGTGAAAGCAGACGTTTACAGTTTTGGTGTCGTCCTATTGGAATTGATCTGTAGGCGGAAGTGTGTAGA

TTGGAGCCTTGATGAAAATGAGGCAATTCTTGAATACTGGGTCTACAATTGCTTTGATGCAGGAGAGCT

AGATAAACTAGTAGGGGACGAGGAGGTTGATAGAAGACAATTCGAGAGAATGGTGAAAATAAGCATCT

GGTGCATTCAAGAGGAACCATCACTTCGCCCTTCAATGAAGAAAGTTCTACTGATGCTGGAAGGGACAG

TAGAAATTCCAGTGCCTCCTAGTCCTACTTCTTTTTTAAGTGCCATATAA

>PGSC0003DMG400004000 G-LecRK

ATGCCTAACAAGACAGCAGTGTGGACAGCAAACAGGAACAGTCCTGTTGTCCCGAGCAACGCTGTCTTG

CTCTTAAATAATGATGGAAGGCTCATTGTGCAAGTAGGAAGCCAGGAAAACTCTGTCATCAATCCCTCTC

GAGCCATTGCTTCAGCTTCCATGCTGGACACGGGAAACTTTGTGCTCTATGATTCTGATCGCAATGTCAT

TTGGCAAAGTTTTGACAATCCAACAAACACACTTTTACCTGGTCAGCATATATCAGCTGGACAAGAGCTG

TTCTCGAGTGCCTCGGAAGCAGATGATTCTTTCGGGATTTTCCGTCTCAAAATGCAAGATGATGGGTACC

TTGTTCAGTATCCTGTCAATACACCATATACTGCCCCATATGCTTACTACGAATCCCACATCAGTGGAGTA

GGGAATAATGTTTCACTGAATCTTGATGATGACGGCCTCCTCTACTTGCTCAATTCCAACAATAGTCTAAA

AAATCTAACCAGAGGAGGCTACCGTAGAGAAAGGACCATCATCTATATGATGAAAATTGATGCTGATGG

TATTTTTCGAGTTTATTCACATTCTCTGAACCAACAGAACAGCTCACCGATTTGGTCATCAACAGATGATA

GGTGTGATCCAAAAGGCCTTTGTGGGCTCAATGGTTTTTGCACCAATATAGATGCTCAAGCTAAATGTAT

GTGTCTTCCGGGATTTGACTTTGTAATGCCAGGAAATTGGAGCGCAGGCTGTGAAAGGAATTTCCCCAC

AGAAACCTGTCAGTTAAAGGAGAACACTTCCAAATACTATGACATGAGAACAGTTGAAAACACAGCATG

GGAAGATAGTTCTTATGTTATTTTTGCCGTAACGACCAAGGGAGATTGTGAACAAGCCTGTCTACAGGA

TTGTAACTGTGAGGCTGCTCTGTTCAAAGACAGAGAATGTAGAAAGCAAANNNNNNNNNNNNNNNN

NNNNNNNNNNNNNNNNNNNNNNNNNNNNNNNNNNNNNNNNNNNNNNNNNNNNNNNNNNNNNNNNNNNN

NNNNNNNNNNNNNNNNNNNNNNNNNNNNNNNNNNNNNNNNNNNNNNNNNNNNNNNNNNNNNNNNNNNN

NNNNNNNNNNNNNNNNNNNNNNNNNNNNNNNNNNNNNNNNNNNNNNNNNNNNNNNNNNNNNNNNNNNN

NNNNNNNNNNNNNNNNNNNNNNNNNNNNNNNNNNNNNNNNNNNNNNNNNNNNNNNNNNNNNNNNNNNN

NNNNNNNNNNNNNNNNNNNNNNNNNNNNNNNNNNNNNNNNNNNNNNNNNNNNNNNNNNNNNNNNNNNN

NNNNNNNNNNNNNNNNNNNNNNNNNNNNNNNNNNNNNNNNNNNNNNNNNNNNNNNNNNNNNNNNNNNN

NNNNNNNNNNNNNNNNNNNNNNNNNNNNNNNNNNNNNNNNNNNNNNNNNNNNNNNNNNNNNNNNNNNN

NNNNNNNNNNNNNNNNNNNNNNNNNNNNNNNNNNNNNNNNNNNNNNNNNNNNNNNNNNNNNNNNNNNN

NNNNNNNNNNNNNNNNNNNNNNNNNNNNNNNNNNNNNNNNNNNNNNNNNNNNNNNNNNNNNNNNNNNN

NNNNNNNNNNNNNNNNNNNNNNNNNNNNNNNNNNNNNNNNNNNNNNNNNNNNNNNNNNNNNNNNNNNN

NNNNNNNNNNNNNNNNNNNNNNNNNNNNNNNNNNNNNNNNNNNNNNNNNNNNNNNNNNNNNNNNNNNN

NNNNNNNNNNNNNNNNNNNNNNNNNNNNNNNNNNNNNNNNNNNNNNNNNNNNNNNNNNNNNNNNNNNN

NNNNNNNNNNNNNNNNNNNNNNNNNNNNNNNNNNNNNNNNNNNNNNNNNNNNNNNNNNNNNNNN
NNNNNNNNNNNNNNNNNNNNNNNNNNNNNNNNNNNNNNNNNNNNNNNNNNNNNNNNNNNNNNNN
NNNNNNNNNNNNNNNNNNNNNNNNNNNNNNNNNNNNNNNNNNNNNNNNNNNNNNNNNNNNNNNN
NNNNNNNNNNNNNNNNNNNNNNNNNNNNNNNNNNNNNNNNNNNNNNNNNNNNNNNNNNNNNNNN
NNNNNNNNNNNNNNNNNNNNNNNNNNNNNNNNNNNNNNNNNNNNNNNNNNNNNNNNNNNNNNNN
NNNNNNNNNNNNNNNNCAGAAAAATCCAGGAGAGTAGAAGTGTTCAGTTATGTGAGGATGTTGCTCC
ACGAGCATTTTCCTATGCAGAACTAGAGCAGGCAACCAGTGGCTTCAAAGAAGCGTTAGGGAGGGGAG
CATTTGGAACAGTATTTAAAGGGATTTTGGCAGAAGGCCAGAAAGTGATAGCTGTTAAGAGACTAGAC
AAAGAATTGGTAGAAGGGGAAACAGAGTTCCAAACTGAAATAAAAATCATTGGAAAGACACATCACCG
AAATCTAGTCCGCCTCCTTGGTTATTGCCTGGACGGATCAAGAAGGCTTCTGGTGTATGAGTACATGACC
AATGGGTCACTCGCGGACGTACTTTTCACACCAGAAAAACAGCCAACGTGGGAGGAAAGGTGTGGCTT
TGCTCGAGATATTGCAAGGGGCCTCCTATATTTGCATGACGAGTGTGAAAACACAAATCATTCATTGTGA
TATTAAGCCTCAGAACATACTCATGAATGATCAGTTCTGTAGCAAAAATATCTGACTTTGGAATGGCCAA
GCTCTTAAAGAAAGACCAAACAAGAACCTACACAGGGATAAGAGGGACCAGAGGCTATGTTGCACCAG
AATGGCACCGAAATCTTCCAGTCTCTGTGAAAGCAGACGTTTACAGTTTTGGTGTCGTCCTATTGGAATT
GATTTGTAGGCGGAAGTGTGTTGATATGAACCTCGATGAAAATGAGGCAATTCTTGAATACTGGGTTTA
CAATTGCTTTGATGCAGGAGAGCTAGATAAACTAGTCAGGGACGAGGAGGTTGATAGAAGACAATTCG
AGAGAATGGTGAAAATAAGCATCTGGTGCATTCAAGAGGAACCATCACTTCGCCCTTCAATGAAGAAAG
TTCTACTGATGATGGAAGGAACAGTAGAAATTCCAGTGCCTCCTAGTCCTTCTTCTTTTCTAAGTGAGATA
TAG
>PGSC0003DMG400004928 G-LecRK
GGATCATTCACTATGGAAATTGGAATGGTATCAACCAGCAGAAAGCAAATGAAAAATTTTCTGCTTATGT
TGCACATATTGTTATGTGGTTTTGTATGCTCAATTTCTAGTGCAAAAGATACCATCACTACAACTGACTTT
CTAATAGAGGGTAAGACCATTGTTTCATGTGATGAAAGCTTTGAATTGGGATTTTTTTCCCCAGGTAGTT
TCGCGAATAATTGGTATGTTGGAATATGGTACAAGCACGACAACGTGCCTGATAAGTCTGTTGTATGGG
TTGCCAATAGAGAAACTCCACTCAACAATACATCAGGTGTTATGCTGAAAATCGTTGATCCAGGACGACT
TGCTCTTGTTACTTCTGCTAATACGAGCATATGGTCTACTAATTCGTTGAGACCTTTGGCTGTGAAGAATC
CAATTGCGCAGTTGTTGAATTCAGGGAATCTTATCATAAGAGATGCAAATGATACTAAGCCTGAGAACTT
CCTGTGGCAGAGTTTTGATTATCCTACTAATACGCTCTTACCAGGCATGAAACTTGGTAAGAACTTTGTG

ACTGGACAGGAATTTTACCTGTCCTCATGGAAGAATGAATATGATCCGGCTCCAGGGAAATATACGTAT
CATTGTGATCCTACTGGATATCCTCAGAATGTCATGAGGAAAGGTAAAGTTAAGGTGTTCAGTGGCGGA
CCATGGAATGGACTCCGCTGGAGTGGTGTACCAGGATTGACAAAAAATACTATTTATACATTTAAGCTG
GATTTTGATGAAAAGAAAGCAATTTACAGTTACGCGCTCGTGGATAAATCTGTTTTATCGAAATTAACCT
TGAACAGTCATGGTATGCTTAAGCGTTGGACGTGGGATGAGAAGATAAAGGAATGGCATGTTTACCTG
GCTTCACCAGCAGATGCCTGTGAAAATTATGGAGCATGTGGTGCCTATGGTAGTTGCAACATCATATTTT
CTCCTGTTTGTCGTTGCTTAGACAAGTTTGTGCCTAAAGATCAGAAAAATTGGTCAGTGACAAAGTGGTC
CGGTGGATGTGTTAGAAGGACACCCCTTAATTGCCAGAATGGAGATGGATTTCTCAAGTACTCTGATAT
AAAGCTACCTGATACACAGTATTCACGGTTTGATGCGAGAATGACACTCCGAGAATGCGAGAAAGTTTG
CCTAAAGAATTGTTCTTGTATGGCTTACTCAAATCTTGATATACGCAATGGTGGGAGTGGCTGTTTGCTT
TGGTATGGCGACTTAATTGACATTCGAGAATTACCTGGAGGACAGGACATTTACATTAGGATAGCTAAC
TCAGAGTTAGGTAAAATTTATGCAACAAATTCATTACTATTTGATAAATATGAAGCATTAGCTGAATGAA
GTAAACTTCACAATTATTAGCTTTAGTTGTGTTTTGCTTATCTATTTGTTAAAAAGTTCACATAGCATATAT
TTAGTTTATTTTACCCTCCATTTTTTTTAGGCTCGAAGAAGAAAATGAAAGCACTTGTGCTGAGTTTATCA
CTGTTGAGCGGAGTGTCTGTCATTGGCCTGATCATATGGCTGTATATTTTGATAAAGACGAGGAAGAAG
AAGAGGAAGATGATTCTGAAAGATGATCTAGAACTTCCACTGTTTTCCCTATCAACAGTAACTAAAGCGA
CCTCTAATTTCTCAGACAAGAACATGCTCGGAGAAGGTGGATTTGGATCTGTTTACAAGGTAGCATAACT
TGTAGGTTTATTATATGTACTATAATTGACAGTAAACAGATAAGTAACACTTTGAATTATCACTGTCAAG
ACATCCTTAGCTTCAATATGAGTCAGCTGTATGTTATGATATTGTAGGGAATCCTGGAGGGAGGACAAG
AAGTAGCTATGAAGAGGCTCTCAAAATCTTCCTCGCAAGGAGTCAACGAGTTCAAGAATGAAGTTAATT
GTATTGCCAAACTTCAGCATCGAAACCTTGTGAAGCTTATAGGTTGCTGCGTTGAAGGAGAAGAGAAAA
TTCTGGTCTACGAATACATGCCCAATAGAAGTCTTGATTTCATCATATTTGGTATGCTTACTCTTGCTTAAC
ACATAAAACCTCAAGTACTGCTCATAGTATAGCATTGAGCTTGCAGCTTTCACCATGATTTTGGAAAAGG
TTGTTAATTTCAAATGGAAGTAGATTCTGTTTAACATACTAAAAGCAGTTAGAGCATCAGTTACTATACTA
ATTGTTGGAAGATCTGTTAACAGATGAAGACAAGAGCTCGATACTCGACTGGCCTAAGCGCTTCAACAT
CATCAATGGAATTGCTAGAGGACTTTTGTATCTCCATCAAGATTCTCGGCTAAGAATTATCCATAGAGAC
CTCAAAGCTAGTAACATTTTGCTAGACGCTGACATGAACCCCAAGATCTCGGATTTTGGCATAGCTAGAA
GTGTGATAGGGAATGAGACGGGTGCTAATACACATCATGTGGTTGGGACACAGTAAGTGATTTCACACT
ACGACCTTTTAGCATTTATCTCATGTACTACTCATATAGTATCATCTGTGTAACACTATTTTCTGTTTGATA

TGTGATGCAGTGGCTACATGTCCCCAGAGTATCTAATACATGGTGTTTTCTCGATAAAATCAGATGTATT
TAGCTTTGGCGTGTTGGTTCTAGAGATAATCAGTGGTCGAAGAAACAGAGGATTTTTCCATGAAAGTCA
CAGTATCAACCTTCTCGGACATGTAAGTTTTGTATGTTAAAAAGTACTTACATATTCAGATTTCAGACCAC
TCGTAAGGTAATTACAAGTGATAGTGTAACTAAACTGATAGTGTAAAAAGTCTTACTAAGTGTCGGTGT
ATATAACTTAAATCTATTAAACACGCTTCACTTGCTTGTACTGATCTGTTATTAGCAGGTTTGGAAACTTT
ATAAAGAAGGTAGACCATTGGAGCTGATTGATGTGCATATTACAGATTCATGTTATTTTACTGAATTACT
ACGGATAATTCATGTCGCTCTATTATGTGTACAACATAGTCCAGAAGACAGGCCAGACATGTCTGAAGT
GGTTGTGATGTTGGCTAATGATGCCATTTTGCCTGAAGCTAAGGAACCTGGTTTTTACACAGAAAGCAA
ATTCCCTGATTCTGAATATTCTACAAGCATGTATTCGAAAAACGAAATCACTATTACACAGTTAGATCCCC
GGTAAATTAAATTTCATATTCTTGTAGATCTTTAAATGTCTAGAAGCTTGAATAACAGAGGAGAACATTT
TATGAGTTGTATCATATTAGGAAAGAATTTACCTAACTCTTGAATTAAAAAAAGACAGCCTGGAGCACTA
AGCTCTCGACATTCATGGATTCAGGTCAGAATTGGACCACACGGGTCTATTGTACGACAGTTTTACAGGC
ACTTGAGTATTAGTTACAAATTGCCATTGTTGCGTATGTTACATGGTTTTGTTATACTTACAGATACGTAT
CCACTCTGGGTGTGTGTAAGAATCTAATATGAATACTACAGTTTTTTTTGAAATTTTAATCTACTCTCCA
>PGSC0003DMG400004930 G-LecRK
ATTAAAAGGCTTAGGCATCAAACACATAGATGGAATCTAAATACTTCATACTTTTCTTACTATCTTTCTAC
TCTTTTATACCGAAGATTAGTTCTACTACGACAAATGTAATCAATACTAATCAGTTCATAACAGATGGTGA
CACTATTGTTTCATCTGGTGGAACCTTTGAGCTGGGATTTTTCAGCCCCAATGGTTCCACAACGCGATATA
TTGGGATATGGTACAAGCAAATTCTTCCTTATGTCCAAACAATTGTATGGGTTGCAAACAGAGAGAAAC
CACTCACCAATACATCTTCAGTCGTTTTGAAGGTCAACAAGCCGGGAATACTTGCTCTACTTAATGACAA
GAATGAAACTATATGGTCCACGAACACCTCACGATCAGTCCAAAATCCAGTTGCAGTGCTGTTGGATTCT
GGTAATCTTGTCCTAAAAGATGCAAATGATGACAATCCAGAAAATTTCCTATGGCAGAGTTTCAATTTTC
CAACTGATACTCACTTGCCTGATATGAAACTCGGAAAGAATTTTAAGACTGGTATTGAAGTTTACCTTTTA
GCATGGAAGAACGATAATGATCCAACTCCAGGGGAATACAGTCTCCACATTGATCCTACTGGATATCCA
CAAGGCCTCATTAGACATGGGGCAAGGGTATCAGCTCGAGCAGGACCATGGAATGGTTTACGGTGGAG
TGGAGCACCTGCACCACTACAAACACAAACCAGCATATATACTTTTCAATTTGTTTTCAATGAAGAGGAG
GTTTACTATAGTTTTTCTCTCATTAACAACTCACTGTTAACAAGGTTAGTCCTGACTAACAATGGTTATATA
CAACGTTTGACGTGGGTGGATAGGACAAAGAGTTGGCATCTTTACCTCAATATTCCTCTGGATACTTGTG
ATACATATAGCTTATGTGGTGCATATGGGAGCTGTGTCATAGATAGTTCTCCTGTTTGTGGATGTTTGGA

CAAGTTTGAACCTAAATATCCACAAAATTGGCAAACGGGAGACTGGTCACAAGGGTGTGTTAGGAAGA
CACCTATTGATTGCAACAAGGAACATGGATTTCTAAAATATTCTGGAATCAAGTTGCCAGAAACTAACAA
CTCTCAGTACAATAAGACCATGACACTCGAAGGATGTAGGCAAGTATGCTCAACGAACTGTTCTTGCAC
AGCTTATTCGAGCCTAGATATAAGCAATGGAGATAAAGGTTGCTTGTTTTGGTCTGGGGAGTTGATTGA
TATCAGAGAGCTCTCTGGAAGAGGACAAGACATTTACATACGAATGGATTCTTCAGACATAGGTAAGAA
CTTATGTTAACGAAATTTCTAATGATGTGTTTTTGAACTTTTAAATTTTTTAACATATGTAGACCATATTAT
ATATGCAAAAGTAGTGGCCTATATCAATTCGAAGAAGCTAATTTAGAAAATGGTTGTAGTATCTCAGGC
AAGTTCAAACAGAAAGAAGACAGGGACAGTACTCGCGGTGAGTTTCTCGTTGTTGGTGGCAGTGATTCT
GCTAGGCCTGATTCTGTTCATGTACATACGTAAAAAGAAGAAACTAAAACTCAAAGAAGATTTTGAGCT
GCCACAGTTTCAATTGTCGTTAATAACAAGAGCCACAAATAACTTTTCGGTCAACAACCAGATTGGAGAA
GGTGGATATGGACCTGTTTATAAGGTATAAACTCAATTTTTTTTTGGAATAGTGACCAGGATAGAGATGA
CTTTCATTCTGCAATACTCGCATCTTTGTAAAATATATTTCCTTCTGATATACTTTTTCCATTTCAAGGGAG
TACTGGAAGAGGGACAAGAAATTGCTGTGAAGAGACTGTCAAGGACTTCCATGCAAGGAATTGATGAA
TTCAAGAATGAAGTTACCTATATTGCCAAGCTTCAGCATAGAAATCTTGTGAGACTTCTGGGTTGCTGCA
TTCAAGGGGAAGAAAAAATGTTGATCTATGAATACATGCCTAATAAAAGCCTGGACTCATACATATTTG
GTTAGGATTTCTAACCTACTATAAAAGCAAAAATGAGTAATGAACCATGTTTTCAAATTAAAGCTGCTAA
AAAAAAAGAAGTTTTTTATTTTTTTTTGTAGATCAAACAAAGAAAAAGTTACTTGATTGGTCAAGGCGTT
TCGACATCATTAATGGAATTGCTCGTGGCTTATTGTATCTCCATCAAGATTCTAGACTACGTATTATCCAT
AGAGACCTTAAAGCAAGCAATGTTTTGTTAGATACAGAAATGAATCCAAAGATATCAGACTTTGGGATG
GCTCGAAGTGTTGCAGGAAATGATATGGGAGCAAAAACATGCCATGTAGTTGGGACACAGTAAGAATC
GATCATACAGAAGTACTCTCATATGAAATCCCCCTTCTTAAATTTCTCATGGTGTAGTTCTTTTTTTGGAAC
AGCGGTTACATGTCCCCTGAATATGCAGTAGATGGAATCTTCTCAGTAAAATCAGATGTGTTTAGCTTTG
GCGTATTGGTGTTAGAGATTGTGAGTTGCAAGAAAAATAGAGGATTTGTCCATGAAGATCACAACCTTA
ACCTTCTCGGTCACGTAAGCACTCTATACATTCTTGTCTATATTACATATAGAATTTGTCATGATCTAAATT
ACTCAAACAGGCATGGAAGCTTTACAAGGAAGATAGGTCCTTGGAACTAATTGATGAGAAGCTAGCTGA
ATCTTGTCATATTTCTCAAGTTTTGAGGTCAATTCAAGTGGGACTATTATGTGTGCAACAATGTCCTGAAG
ATAGACCAAACATGTCTTCTGTGGTTCAAATGCTAGGTAATGAGAGTTTACTGGCAAAAGCTAAAGAAC
CAGGATTTTTCATGGAAAGAACGCTACATGATTCAGATAATTCAGGAATGCAGACAGGAAGTTCGAAAA
ATGAAATAACAATGACAATGTTAGATCCTCGGTAGAGTACTGTGAATGAGGCAACTACTGTTGATAAGC

ATGAGTAACAAATTATTTTAGAGGGCGTGATAACGACATTGAAGCGCCTGAGAAGCGCTCAAGCATCAC
CTCAGGGATTAAGCGTGCCTTTGAGTATTTAGAAAAATGGTTCATAATTGCCATGCTATTATAAGTATCT
ATTCAACTTGAATTTTCATTTGTATCTTATCTTAGAAAGAAATGTAAATTCTAGTAAGCAAAAATGTCCAA
ATTATATAGCCTATGATAATACATAAAATCGAATCAAACCGACAGATGCACGAGCTCTCCAATATCTGCA
TGGAAGAGTCCAATATGTCGTTGAAATTAAAGGTCACAAACTTTGGATTTCCATATTTGAGTAAAGAGA
AGAAGATAGGGAAAAAGGCCATAAGCCCACATTGACCGTGCACGGCCTTTCATATCCATCTCCGCCCAT
AATAAGGGAAAGTAAACTAATCGGGTATGAATAGATAAGAAAGTGAGATTTGAATCCAACCCGGCC
>PGSC0003DMG400005044 G-LecRK
ATGTCACCAGAGTATGCAATGGGAGGACTATTTTCTGAGAAATCTGATGTCTACAGCTTTGGCGTATTGC
TACTAGAGATTGTCAGTGGAAAGAGAAACAGCGGATTTTATGACCACGAAAGGCATCTCAACCTTCTCA
GTTATGTAAGTGCAAAATCAGCAGGATCTCTTGCCACAAGTTATTCACACAAACTTAGAGTATTCATTTC
AAAATGATTTCAGGCGTGGAAGCTACGGACTGAAAGCAACGGATTAGACTTAATGGATAAATCAGTTTT
GGACTCAGATTCCTCTGCAACAGTGCTGAGATGCTTACATATTGGCCTGCTTTGTGTCCAGGATCATGCT
GCTGATAGGCCATCAATGCCGTCTATCGTTCTTATGCTAAGTAGTGAGATGGATCTTCCTCAACCAAAGC
AACCTACATTTATATTTCAAAGATGGTTGAATTCTAATACCCAATCGCAAATCAGCAAAACTCAGTCTGTT
AATGATATTACTGTCTCTGTTGCAGAAGGACGATAA
>PGSC0003DMG400005689 G-LecRK
AAAAAAACCTGGTCAACAAAAGAAATCAAAGCCAACAGCATGCTTGTCTCTGTTTTGGTCATTCTATTTT
GACTGTAAACATTCATGTGTGGATTAGAGAGCTTCTCTCATATGGCTACCTACAGAATTCTGATTGCTTTC
ATCCTCGTGTTATCCTGTCACTTTTTGCGTGCAGATTTCAACTCAATATTGAAGGGAGAAAGTTTAGCTGA
TTACCAACAGCTGGTCTCAGCAAATGGCTTCTTCAAAATGCAATTTTTCAGTCCAGGCAAGTCAAGGAAC
CGCTACTTGGGTATATTTTCCACTCGGCCTAGTCTTGATTTGTACTCTGATGTTAATGGAGGAGGAGATG
AAAAGGCTTTGTGGATAGCTAACAGAGACGATCCGATAACCGATACATCTGGAAGTCTTATGATAGCTC
CTGATGGCAGATTAATAATTTCTCATAAAGAAGGTAATGTTACTTTGTTCAGTGCTACACCAACAACAGC
CACTAACTTAACTGCTATACTACTCGATAATGGGAATTTTGTGCTGAGGGAACTGAATACCAATAGCTCT
GTAAATCGAACGCTATGGCAAAGCTTTGATTATCCAACTGATACACTACTGCCTGGAATGAAGCTTGGG
ATTAACTTGCGAACGGGGCATAAATGGTCATTGACTTCATGGGTAAATGATCAAGCACCTGCTTCAGGG
TCTTTTACCTTTGGTCTTGACCCTAATGGTACAAATCAGCTGATGATCTTGTGAATGGGCAAGGTCTATTG
GAAGAGTGGCCCGTGGCCTACTGGCCATCTCGCTCTTAAATATGTCTCAAATGAGGATGAGAAATACTTT

ATGTATACTGTTGAATTTGAATATCAAAGATACTCTGTGAGTCCATTTGGAATAATTCAGGATACTTTTAA
AGGAAATGCAGTGTTTGGTAATTGTAGTAATGAGACTCCCCATGCTGGCTGCGTAAAGCAGGTATTGCC
CCAGTGCAGGGCAGCTAAAAAGTACTGGTTTGAGCTTAGACAAGTTTACATGTTTGGAGACAGCGTTAA
ATTTGATGAGAACTATACCTTGAGCCTCTCAGATTGCAAGGCCAAGTGTGTGAATGATTGCTCGTGTGTT
GCCTATGCCTCAGTCGATAGTGACACCGGAATTGGCTGTCAAATTTGGGGGAATGACACGAGTTTTGTG
ACAGCACAAAACAGTCTAGCCAGAGATGTTTTCTTTCTTACAAGAAAAGGTAATTCTTGAGTACAAAGTT
ATTCAATAGAGTGAAAGATCAAAAGACATGTTAATAATGTTGCATCTCATATTATGATTACTTTATCCAGC
TCAAGATAAATTGTTTTGTGAGGGCATCACACTAATGTCACCAATCTGCATTACTCTCATTTGCATGTCAT
TCCAAAAATTACTAGTACTAGAACAAGAAATTTTCTCCGATCGCAATGCTGCAGGATTTGAGTTAATAAG
AGTGTCATCTTCATTTCAAAAATACACTCGAGCAAATGAACAACCATACCTTACAGAATCTTGTTTTGGA
GGCTATTAAATTATTATTTTTTGGTATTTCTAGAGTACCGACAATTATGATAGCATCATAGTGTATTCCCA
AGGTAAATATGTGGAATTTTGGTCATAAACACTGACTTACAATTTTGACAGATAGGAAGCGGAAATGGT
GGATATGGTTGACAATAGCAGTGTCCCTAATGGTATTTGCGTTTATTTGCTCCTTATTCTGTCTGATACGA
AGAAAACTCAGAGCAAGAGGTGAGCAAAACATCAGCTCTAGCATTAGTCTAAGGTGACACTTATTTGTC
TTTGAAATCTTCAATTTTGCATTTGTCACCTAGGCAAAGTCAGACAAATGGAGAAAATTTTATACGAGCT
TGAAGATCCTAAGACCATTTCTGGCCAGTACAATACTAACAAGACTGCTAGACTAAGGAAGAAGTTCAG
GCATGATAGACACATATTCAGCCTAGAGACTATGAATATGGCCACAAACAATTTCTCGTCTTCTAATAAG
TTGGGACAGGGCGGTTATGGACCTGTTTATAAGGTAAAAGTTTAGAAGCTTTTTGTCTTAAGCTGATTTT
TAGATTTGCAAGTAGCAAAAATTTCAGGGGATGTTGCTCGACGGACAAGAGATAGCCATCAAGAGACTT
TCAAGAAGTTCAGGGCAAGGATTGGTTGAGTTTCAAAACGAAATTGTGCTGATTGCTAAACTCCAGCAC
ACTAATCTTGTTAGGCTTTTAGGATGTTGCATTGAAGGGGAAGAGAAGATATTAGTTTATGAATACATG
ATGAACAAAAGCTTAGACTTCTTCCTATTTGGTATGGATTTCCAATTTCATGCCTAAATTGCTTAATTAATC
ATATTTGATAACTAATACCCCTTCCCATTTTGATTGAATAGATCCTAGCAGAAAAGATTCACTAAAATGGA
ATACGCGTTTGAACATTATTGAAGGAGTTGCTCAGGGACTACTTTATCTCCATAAGTATTCGAGGTTGAG
GGTGATTCATAGAGATCTAAAAGCCAGCAATGTCTTGCTTGATGACAATATGAACCCCAAAATCTCAGAT
TTTGGTTTGGCCAGGATATTTGGAATGCAAGAATTTGAAGCAAACACGGAGCGAATTGTTGGTACCTAG
TAAGTCCTTAATAGATATAATTATCTAACAATGGTGATTCACTGTGTCCTTTTTATTATGTGCTAAGTTGCT
ACCTTTTCAGTGGTTATATGTCTCCAGAATATGCCATGAATGGCATTGTGTCCATGAAGACAGATGTATT
CAGTTTTGGAGTTCTAGTACTCGAGATTGAGTGGCAAAAGAAACAACAGCTGCTATCATTTAGAACGCC

CACTAAACCTCATCGGATATGTAAGTTCCTCTCGTAAAATATCAATATAGCATGATCAAGAGGCGGATGG
AGTGTTTATTCAATGAGTTCAACTAAACCCAATACTTTAGAAACGAAACATAAATATACAACATCAACAT
ACCCATTGGAATCCCACAAGCCACAAGTTGGGTCTGGGAGGGCGGTGTGCACACAGACTTACCTACCTA
GTAAAGGCTAGGCTGTTTTCAATAGACCCTCGACTCAAGACACGAAACATTGATATACATGTGAAAATCA
CTTGAATTATAAAATTCTGAAGTATAATGAGTCTAGTTCTAAAAACTTTAAAGTTCAACCTGTCAACTTTA
CATTGTTTTGGATCCACCCTGGCATGATCACTTAGTTGAACAAGGAAATTTCAAACTTGATATGCTCAATT
TGTCTCAGGCATGGGAATTGTGGAAAGCAGGCAGCGTCCTTGAAGAACTAACAGATCCTGTATTGACAA
ATGAATCAACACCAACGAATGAAGTGATGAGATGCATTCATGTAGGATTACTCTGTGTTCAAGTCAATCC
TATGGACAGGCCATCCATGTCAAATGTGGTTCTGATGCTCACAAATGATAGCCTACAACTATCCGTCCCC
AAGCAACCAGCATTTTTCATTGAGACAGCTATGACTGAGACTGAAACTCGCGAAGAAGTTGGACATTGT
TCCACAAATGGGCTATCAATTTCAGATATGGTAGCGAGATGACTTTCATCATTGCACCCCTCAAAATTGT
AAAAAATGGTAATATGCAATCTCCCTTGTGATACAAAATAAAAACCACTGCTTGTCTCCTTCATCCAGATT
TTACTTTTTCTAGCGTCGATATTAAGTATGAGTTGTTGACAAAATAAAGTGAGTAAGCTTGTTAGAGCCT
TTCAAGATTATACTGTGTCTAAACAATTTTTATACATTTGTTTTGAATGCAAATGAGGATTCAGAGTATCA
ATTGTAACTGAAATCAGAACTCATAAATGTAGTAATTGTTACTAGTTTTGCAAAGATGATTAGTATCTGT
ACATTAGAAAATCAAATGGATGTTTCCCACGTTCCTTTGTTGCTTG
>PGSC0003DMG400007073 G-LecRK
CCAATCTCAAGAAGCAAATGTATCATCTAACAAACAAGCTAAAACAACAAACTAAACATGTCTTCTTCCT
GGCCTACTACACTTCTTGCCTCTCTCTTTCTTTTCTCCCAAATATTTTCTTGCATTGCTCAAGTTCCAGCAGA
AAACACCTTTAAATTCGTCAATGATGGCGAATTAGGAGATTATGTTGTAGAATACAGAGCAAATTATCGC
GTTATTAGTATTTTCAATAACCCATTTCAGCTCTGTTTCTACAACACAACTCCTAATGCATGGACATTAGC
ATTGCGAATGGGCACTGTCCGTTCTGAATCACTCATGCGTTGGGTGTGGGAAGCAAATAGGGGAAACCC
CGTTAAAGAAAATGCGACATTCACGTTTGGAACTAACGGAAATCTCGTATTGGCGGATGCTGACGGTCG
AATCGCTTGGCAGACCAACACGGCCAATAAGGGGGTCACCGGGTTCAAATTGTTACCTAACGGTAACAT
GGTGCTACATGACTCCAAGGGTAAATTCGTCTGGCAGAGTTTCAACTATCCCACTGACACATTACTAGTG
GGCCAAACTCTCCGATTGTCCGGCCCGAATAAGCTTGTGAGCCGGGCCTCGGTGAAAAAGAATGCAAAC
GGGCCTTATAGCTTGGTGGTGCAGCCGAGATTGTTCGCTGTCTACAAGGGAAACAAGGTAGACGTGGA
ATTAGCTTGGTTCGATTATGGGAATAACACGTTGGAATCGATAAAATTGAATAGTGGGAATCAAAGATT
GAAATTGGATTATAGATTTGCAAAATCAACACAGAGAAGTTCACATGTGATGGCATTTACTAAATACAAC

ACAACATTGACATATCTTAGACTTGAAATTGATGGAAATCTAAAGGCCTACACTTTTGTTAGAGATGAAG
AAGCAGATGAATTCCGTTGGAAAGTAACTTACAGTAGGATTTGAAACGTCTAAATCTGTATACACATTAT
CCTTTACGAGCAAATTTCACGGGTTATATTATTATTGTTGTTGTTATATTGTTGTAAACGTAGCTCTATAA
GATAATGATTAGTGTGTCCAGTACAATAAGTGGGTCCACAGCTAGTAGTCCAATAAATGAGTACTTCCTA
TTTAATGTTAATTATATTTTTCTTTTTCCCTTTTTGAAAAAATATTATGTTTTGGTTGAATTAAGA
>PGSC0003DMG400007455 G-LecRK
ATTTTTTAGAGGAATGAGGTGGAAAAAGGAAAAAAAAGAAATTGGGTCCTTTGTGATTTCTTGCATTGG
CAGTAACCAACGTCCAAAGGCCAAATCTCATCGATTGTGACATTGTAACAAAATATACATAATTCATCAA
TCTTCATAAACCTATCATCATAATCATAATCAATACAACATTATTGTAAACAAAACAAATATTCCTTCTTGT
ATATACACACAATATTAAAAAATTATAAAGTTTTTTACATTCTTGATTGTGTACGTGGGAAATTGGGCAG
CAAATTTGCAATTTTTTATCCTGTATCTAATGCTTTGTGGATGTTCTCACAATTTGATTCACCTTTTTGGAG
TGGGGTTTCCTTAGAAAGGGTAAATTCTACATAATCTTGAATTTCTGTATCTTTTTCTTGATTTGGGTATCT
GGATATTCGAATTATTCTGTTTGGTGTTGTGGGTGTTTCTTGAAACTGCATTTGATGATCTGGGGTACTTT
TATTTTGGTCATAGTTGCTGGTGATATCATTATTTGGCTGCTTTGTTTAGAAAGATTGAATCTTTTTTACTC
TGATAAGGTTCTGCAGTGTGTGGAGGGATTGTTCTTATGAATTATAGCTAAAAAGGAGATATTGTTCTGC
TTTAGACCTGTCACTAAATTCTGTGTCCTGTGGCAACATTTTGATTTCGCGTAAAATCTGATATGTTTAAG
GTTGAGGTTGAGGTTCTTGTTGTGTTGAAAATCCTAGCTGTTTAACTTGGGAGTGGTGTGTGTGTATACT
ATGAGTGAATTGGTTGGTGAGTTTGGATAAATTTTGGAATAAGAGGAAGTAATTTACTGCATATTAGGG
ATCTTAGTTTTATGTCAGCAAGTCTGCATTGTATAAGAAATGTTGTAAATGTAGCTAGCTTTGGTGGTGG
TGAGATGTTAAAACTTTAATCATCCACCTCCAAGTTATAGAATTTGTAGTTCCATTATCTGTGACTTCTCTC
CTGGCTCGTTACTCTCTTTCGACAAATGTAAATATAGTCTGTCCTAGCCTCACTACCAATCTTATAGACTTA
TAATGGATTCTTCACCATCTCATGAAGAATCTGCTCCTCCAACTGATGTGGCACCTCCAACGTTTAGTCCT
CCGCCTACACCTTCTAGTCCATCTGATAGCTCACCACCACCTCCATCTTCTAGTCCACCTCCTAGTTCACCA
CCACCTGCATCTTCTAGTCCACCTCCTAGTTCACCTCCACGTGAATCAACTAGTCCACCACCTAGATCACC
ACCACCTCAATCTTCTAGTCCACCACCAGCCTCACCTCCACCTGAACCAACTCCTCCTTCTCCTGTCAGTTC
ACCTCCACAAACTTCATCACCTCCCCCTGCATTGTCACCTCCGCCACACGTACAGAATCCAGAACCTCCTC
CAGCACCACAATCACATGGAAATTCATCCCCACCACAATCTCCTGAACCTAAAAAAGGTTCATCATCATCT
TCACCACCTTCACCACCTTCATCCAACTCACCTGGGGATGGAGGTGGAGGTGGAAATTCACCACCATCCC
CAACATCTAAGTCATCAGATAATTCGCCTCCTTCACCAGCCTCAGGCCCTCCTAAAGATTCGCCACCTTCA

CCAGCTGCATTATCTCCAAGTAACTCACCATCTTCTGATTCACCCTCAAACTCATCAACACCAACATCTGT
AATACAATGGTCACCACCACCTCCATCCTCTAATGGTGCACAATCTTCTCTTAATCCTCCTTCATCACAAGT
TCCTAGTAACCATAGTTCCGGAAACCGTTCAACAGGAAGTCCAAAGCCTGCTGGTGGCAACAGCAATGG
TACTGCAGAGACAGTTTCCATTGGCGTTGTAATTGTTCTTTTTTTGGTTGGTATCATCGGAGCAGTTGCAT
GGTGCATACGGAAGCGAAAGAAGAAGAATTCTGTCCATTGTAGTGGTTATGTCAAGCCAATCTCTGTGT
GCTCCTCTCCAAAATCAGGTAAATTTTCAGAAACTATAAAATCATTCCTTTTAGTTTGTTATTCAGTGCTCA
AGATGCTTGCAAGAAAAATCAATGTAATATTTGTAGTTGAGACCTTTCAAGTGAAATCTGAGAAGATGT
GAAAGTTGTGTACAGAGTCTGCTTTATTGAAGGTCAATGAATCAACACCTGAGAGAAATGGAACGAGCT
CTAATTTCCTCAATTCTCCAGCAGACCCTGGCGGATTCAACAATGCAAAGACATGGTTTACTTATCAAGA
ACTTGTTGAGGCCACTAATGATTTTTCAGCGAATAATGAATTAGGGAAGGGTGGATTTGGTTCTGTATAC
AAAGGCTATCTAGCAGATGGAAGATATGTCGCGGTAAAGCAGCTAAATATTGGTGGGAGCCAGGGCGA
ACGGGAATTTAGAGCTGAAGTTGAAATCATTAGCCGGATACACCATCGTCATTTGGTTTCACTTGTAGGT
TATTGCATTTCAGAGAACAAAAGGCTCCTCGTTTATGACTATGTCTCCAATAATACCCTTTACTTCCATCTT
CATGGTAAGCATAACATTTGTAGATTTAATTATTTCATTTGTTATGTTTGGTCACTAAGTGAGCTGTTGAT
ACTTGCAAGTTACAGCACAAGGGAGGCCTGTCATGAATTGGCCAACACGTGTTAAAATTGCTGTTGGCG
CAGCTCGTGGAATAGCTTATCTGCATGAAGATTGTAATACTGTTCTCTTCCTCAATTCTTATGAGTTATCC
ATGTTTTATTCACATGATATCTTTCATTGGTTGAACAAACGTCCCCCTTATAGTTAAAAGAGAATGTACAG
TTAAAAACTTTACCACAACTTGGAATTAGTTTTAATGATCCCTAAAGCTGTGGCGGGATCATGGATGGCA
ATCTAAGCTCCACACCTTCTTCCTACTCTTCCTAAAAGCGTACATTAACGAAAGGAAAATAAAAAAGAGA
GAGTAAACCAAACATGTAGTAATAATTTTCAGCTCATAGAAGTAGAAGACCTTCCAGTTGGCATTTCCTT
TTTGTAATGTAATGATATTACTTATGCAGTTTAGAATTGAGTTCTAAGGTCCTTTTTCATGCCATAAATTAC
CTCGTTATATAACTTTTTGGCATGAGTGTAAATTATCATATCTGCATTAAGATTGTAATTAGTGTTCTGTTC
CTTAATCCTTTTATGTTATCCATGTTTATTTTTATTTCAGTTCGTTTGTATGGCTGAAAAAAATGTCTCCTCT
TTCCTGCCCCAAAAGTTAGAGAGAAAGCAGGAGGTCAAAAATGTTACCATATGACGAATCGGATTGTGA
TATGAGATTGTGTGTTCCTATCATATCACTTACATTCTTTTTATTGATCCCTACTCCACTAGCTGAACAGCT
GTGGTAAATTAAATGATGCAATCTGAGCTACTTCCAGAACCAACTTTGTCCTATTATCCCTACAAGAGGA
CTTGAAGTAAAAAGAGAAAATGGAACGTATAAATAGTTTAAGCTCGATGGAAGTAGAAAGATCTTAGTA
TTGGATTTCCCTTTGGTAATGTTATAATATAACTTCCGCAATTTAGAATTGTGTCCTCTGTTTTTATGCATC
TCGCAAATTATCTCGATTATATGACCTTTTGGCAGTATAAATAATGAATTCTAATCACTTCTCTAATTAGTA

ACTTAAAAGATGCTTTTGTAGCTGAATGTACCAAATGAGGCTCAAAATCAAACTAAGACTGAACAAAGC
ATGATTGGTTCCATTGAGAGAACAGAAAAAATTGTATGACTCAGTTCTTTATACATTCTTGTATTGCACCA
TGTAGTTACATTCCCGAGAAAGAAATATTTTAATCCATGAGCAGGCATAGTCGCCTTGAACATGAGTATA
TCTTCCACACTGAAGTGAGCGCATCAGGCGCTGCATTTGTAGTGCATTATATGCTTTTCGATTGCCAATAT
TCACTCTACGACAAGCATTTGAATAAATCTTCACTGATCGAGCCAGGTTGAAAAGTGAGAGAAACATGT
TCAACTGCTCTTGTGAAGCATATAGTGAAAAAGCAAGTGACAAACTGGTTTCTCCATATAACTAAGCTGA
ATGTGTTAATCATCACTGACTTATTATGTTATACTGTTGAATAAGTAATGAAAAGTTAAGAAAAGTAGAC
CAGGCGACGCGATTGTGATTTTCTATGTTCATTTCCTTCATTTTTGTTACTTGAGCTGCCTTTAATTATATA
GTTAAAGTATGATACTCCTTTTTTATTTGGTTCTTAATTAATTCTTTTGGTGTGGATGACAGGTTATCCTCG
TATTATTCACAGGGACATTAAGTCATCAAACATTCTTTTAGATGATAACTTTGAGGCCCATGTATGTCTAA
GTCTTCGAACTCCTATATCCACATTCTTTGTCTCGCGAGTTGTCTTTTACAATTAATTTACTTTCTACAGGT
TGCTGATTTTGGACTAGCTAAATTAGCTCAGGATGCAGAAAGTCATATCACAACACGTGTCGTGGGAAC
ATTTGGGTATGTTTCAGTGTCAACTTTTATATTAAGTGGACAGCATAAGATAAATATTTTATTCCATCTTTT
TCAATTTCACATATTTATTCTAATTCCTTAGATCTGACTCAGATACATGGCTCCTGAATATGCATCAACTG
GCAAGCTGACTGAAAAATCTGATGTATATTCCTTTGGCGTGGTACTTCTGGAGCTAATTACTGGAAGGA
AGTCAGTGGATACATCTCAGCCTTCAGGGCAAGAGAATCTAGTTGAGTGGGTATGTTGTAGCCAATCTA
TTATTTGTCTTTCTCGAAATCCATATTTTCATCAGGTCTCCTTTATTACCTCTTTGTTAGAAATTTTGACATT
AATATCCAACCTAAGGCAATGAAAGTGCATATCAGCATGCAGAGAGAACTTAATTTTTAGTCCTTTTAGC
TAGGATAACCTACAAAATTTCATTTGAGTCAGGATTTGGTTGTTCCTCCGTCTTTTCCCATACTGCACAAG
AACTTGGTAGCTGATATGTGAAGAAACCGCCATTTTGCTACTGTACAGTAGTAGGCCTCTAGTTGGTCTG
CTAGTGAAGTTCCTCTTCTTTATTTTTCCCTTTTTTACCATGAAATGATGCCTTATCCTGGGAAGAATGTGG
TTGCAATGCTGTAAGCCTGAAACATTGTGTCTGCATTTTTTTTTAAATTTTACAACAATCAAACAGCTTGA
TCAAGTTATCCGCTTTAATTTCATTGATCCTTTAATGTTAGAAAACTGAAAAACCATTAACAGTAGCAGCC
GAAAACTTGTATCTTTTATCATGCTGAATGTGTGAATTCCATTTACTATAGTTATTTTATTTTCGAACTCTT
TCGTTTTCTTGTCGTCAGTGCCTAGTAGCAGTAGCCAGTTTACAAAGCTTTTAATGCCTTGTATTCTGGTA
CTAGTTTCCACTACTAAATTTGAACCAGGCTTAAAAATAACTACTCCATTATATTGTACTGTAACTTGATTT
TTTTGTTGTCCAGGCACGACCTTTGCTTAGTCGAGCACTTCAAAAGGAGGAATTTGATCTGTTGGCAGAT
CCGTGCCTTGAAAAAAACTACGTTGGCATGGAGATGTTCCGAATGATTGAGGCTGCTGCATCTTGCGTG
CGTCACTCATCTGCAAAGAGACCAGCAATGGGACAGGTAACTCTTGTTCTGTTATATTGAAAACCTATGC

AAAAGACACCTTCAAGACTCTTCTTGTGAAGGGCATTTACAACATTCTGAATTCTGATCATGGTTATGCTT
CGTAGATCATGAGAGCTTTCGATGGTATGGCTATACAAGATCTGTCAAACGGGATGAAAGTTGGTGAAA
GTGCAATACATAGCGCTGCACTACAATCTGCAGAAATAAGTTGGTTTAGGAAAATGGCATTCGGTAAGC
AAGATTTTAGTTCGGATTTTTTCAGCGAAAGTAATCAGAATAATAGAGAGTCAAATGAACATCCTTAGAG
GATTTTATAGAACAAAAGGGCTTATTGAAGCTATGTATGCATCTGTTGATACATCAAAGGTACCTTATTA
GCTTAGAGATGCCTTGGTTTAGCTTTCATATGTATAAGTTCCCTTTTGGCTTTGAGTAATTTTC
>PGSC0003DMG400008493 G-LecRK
TAAAAAGGTGTATATAGCTAGCGTCCGGCATGCTTTTATTTCAACTCTAATGGCTTTTGATTTTCAATCAC
AACAAGAAATCATGTACGTATAGATTATTATCTAAATTTTCATGTTTTTCCCATTTGGCTAAGTAGATTTTA
TAGTAGTTGATTTTTTTTTCCTATTTCCTTTTCATTAATTTGCAGAAATATTAGGTGCTACAAAGTTAAAAT
TTTGAATATTTGAGCAATGGGTTGGAATTGCTTTGGAGTTTTGCAATCATACAAAAACAATAGAGAGACT
CAAATCCAAGGTAAGTACACTCATAACTCTTATTTCTGTTTTCTCAATTACATGCTTAAAACAAATCGTTCT
TATAAAATTTAGAACCCAGAAAATTTAAAGATTTGACGCTTCTGCTCTACTTTTTGTAGTCCATCCAAATT
TGAGAAGTACTTCACATAATTTTCATTTTCTTTTTGTGTTTTTCATTGTGTAGAGTTCACGACCAACAATGT
GAGGGTTTTTTCCTATAACTCATTGAGATCAGCAACAGGTCACTTCCATCCATCTAACAAAATAGGAGGT
GGTGGTTTTGGAGTTGTGTATAAGGTAATCATCTCGTTTATGGTGTCATGCCTTTTAATGGTCCTTGTGG
ATGCTAGTTATTAAGTAGTGAGTTTTAACTTATATACGTTCAAAGTTTAAAAGAATCTTTTCACAGTCAAG
TCACTTAAAAGATAAATATAATAATTTATCCATTCGAAATGGGATTAGCTAGTAAGTAGATGCATTTAAG
GGTGTAGCCTAATGATTAATAAAGTGGGATGAGACCAATGAGATTTTTAGGTTCAAATCTCAACGGAGG
CCCAAAAAGGGTGTAGCCTAGATAGACACCTTCCAATGTAACTAAGCTTTGGTGGGTAGAGTACCGATG
GAATAGTCGAGTTACATGTAATCTCATACGAACACCACCATCATAAAAAAAGAAAGTAAAATAAGATAG
ATTACCTGCTACATTAATAATATATATATGTTAAATCTTTTATGTATTTCCACTCTCTGTACTTGTGCAACTT
TGTTCTTTAAATGTAGGGAGTTTTGAGAGATGGAACTTGTGTTGCCATTAAATGTCTCTCTGCTGAATCA
AAACAAGGGACTAAAGAATTCTTAACAGAGATTAATATGATTTCAAATACCCAACATCCAAATCTTGTTC
AGCTGATTGGTTGTTGTGTTGAGAGTGGCAATAGAATGTTGATATATGAATACTTGAAGAATAACAGCC
TTGCCAGTGCTTTACTAGGTAATGTTCTCACAGATCAGTTTTAAAAAAAAACTTATTTTGAACCAAATGAA
CATGACATAGTTGAAGTTAGGAATGTTAAATGAGCGGATTGGACTGAATCTGGACGAGTTAAAATTGTT
ATTTCAGGTTCTAATGGTAAACGCGTTGCCCTGAATTGGCCTCAGAGAGTTGCTATATGTTTAGGTACAG
CATCTGGTTTAGCATTTCTTCATGAAGAAGCCTCACCACCGATTGTTCATCGAGATATTAAGGCTAGTAAT

ATCCTTATAGATGAAAACCTTCATCCTAAAATTGGAGATTTTGGTCTGGCTAAGCTTTTTCCTGAAAATGT

CACTCATCTAAGTACGCGAGTAGCAGGAACAATGTGAGTAGTTACATTGAATTTCAGTTTCATAACATTC

GATATTCCTCTCCTGTCTCGTCTCAGTATGGTATTCTGATGTGCAGAGGTTATCTTGCTCCGGAGTATGCC

TTGTTTGGACAACTCACAAAAAAGGCTGATGTTTATAGTTTTGGAGTTCTTGTACTTGAAATCATAAGTG

GAAGAAGCAGTAGTAAATCAGCATTTGGAGTGGAGCTCTTGGTTCTGGTGGAATGGGTAGGATTATACT

TTCAGCTTTATGATCTTCAAGATTCATCAGTAATCAGCATTTTGAAAGTTTACGCGTTGCAGGTATGGAA

GCTAAGAGAAGAAGGACAGCTTCTGGAAATCATTGATCCAGAGCTAACAGAGTATCCAGAAACTGAATT

ACTGCGGTTCATCAAAGTTGCTCTCTTTTGCACACAAGCAGCACCTACCCAAAGACCTAATATGAAACAA

GTGATTGAGATGTTGTCTAAGGAAGTCAACCTCAACGAGAAGTCGTTAACTGAACCAGGAGTTTATAGA

CCACATAGCTCGAAACGATCAAGTTATGGTAGTCTGCAGAAATCATCCTCCGCGGGGAAGAATGGGGT

GCAATCTGTAAATCCTTTTGTAACATCAACAAAGTTTGAGAGCTTTCAGAGTGTCACCCAAATGCTTCCTA

GATAATGTTAACTGGTGTTCTTATTCTTCATACTTGTATAATGTCGTTATTCTAACGTGGGCTATCGAGGA

GTAAATGTATTGTCTCTCATTAGACCTAGCTTTTTTCATACATTTTCTCATGTAATGTAAAGTTGGCAGTA

GCTGATAAGGGAGAAAGAGAGACAATCACACAGAATGAGCTACTATGTAACTCCAAATTTCAGATATGA

AGATGAAGTTTACAGCAGAGACTATGAACACAGTAAAGGATAGAAACTAGGAGAAACTTTTCTCTATGC

ACATATTAGCTCCTAAAACTAGAAATGAAGGTAAAAACTTCCTGATAGGAGTTATTCTCAAACTCATAAA

CATATAAAGGACGACTGCTTTACAAGATCAACACAGTAGCATTTTCATTGTGCAGCAGAGAATAAACCTT

CAAGGTGCAGCAGTAGTAGCTTGAGGCTTTGCAGAGGAAGCAACAGAAGTTTCTATTCCTGTCGTTGCA

TCTTTTCCTGGTTCCGAAGATGGTTTTGCAGAAGGCTTCATATGGTCAGGGGGGTTGTGTTTGAGTTTCA

CAAGTATTTGACGCATTCTAGAGAGTTCAGTTGGCCTGCCAGGTTTGGGTCCTTGGATGACATTGATGAA

TGGCTGCTTCTCCTTATCCTTCTTTCCTTTCTTTTCTATGGTCGGGACCTCCTTTGGGATAAGCTCTCCAATT

GTCCTCCAATAAGCATTATTAGCTTCAGTATGGAACTTCTCTTGGTTGGCCAAGAATAACTGGAAAAAAG

AAAAAGATGACACATGAACTGCAACACATTGATTGACTAAGGAGGAACTTGCCGTACTGTGGAACTATG

TTTAGAGATTTGACAAACCATAAAACCAAAGACATAGTTCTGTGCAGATTAGCTAATAAAACTCACCAAA

GCACAAATACTCAGCATAGATTGTACTAAAGGTTATCAGCACATTTTGATCTCTTGTGGAAACCTTCCACT

TTCAATTTTTTTAATTTACACCTCTTACTTAGTGTTAACTCTAAATCATATGAAATGTAAACAGATAAATGT

AGAAACTGGTTTTCTCTGTGTGCCTGCCTTGGTTGAGTCGAAAGTACCAATACAATGACTTTTAAAAAAC

AAAAGTAACTTAAAAAAGAGAGTAAAAAGTACCATGATTAAAAATTGGATAGTACTTTTACCAAGTTAA

CTAGGTTAATCTCCCTCTCTCAACACTCATATCCTATACCGAAGATTTGAAAGCAACGATTAGGAAGAGA

AGGTAACTAGATGCACGACACATCAGACCAGTGATAATTTTCCTTCCAGCTAAAGCAACTGTTTGGAAAA
CAAACACCCAGCACAAGCAAATTTATGCCAAACTAGTTAACAAACCATGACTTTAAGCAAAAATGCCTCG
GAAAGATTTCTAATTTATATAGCTATAGGTCTCACCTTCTCCTTCTCCCGGTTAGCAGCTATGTTGTTCTCA
CATCTAAGTTTCCATTTCCTATAGTACTCTGCTTTGTATTCCTCTGCTTCTTTAATTATTTCCCTAACAATTT
CTTTCTCCCTTTTCTCCTTCTCCTCCAGCCGTATGGCGTTCAAT
>PGSC0003DMG400008508 G-LecRK
TTTAAATTTATTTTTAGCAATCACTCATTCTACCAGCAAATTGTGCAGCCATAGAAATGAAGAATAGCAGT
TCAATTCTGATACTTGTGCATTTTTTATGCTTTAGCCTCATCACTAATCTTTCCCTAGGAGGGGACACCATT
TCTGTGAATGAATCTGTTTCTTCAGGCCAAACAATTATCTCTTCAGGTGGGAACTTTGAGCTTGGTTTCTT
CAGACCAGGCGACTCTCGCTCCTACTACATAGGCATATGGTACAAGAAACTATACCCTCAAGCAGTAGTA
TGGGTAGCAAATAGGGATAAACCACTTGATAGTGCTGATGCTAACTTGATAATTAGTCAAGGTAACCTG
GTGCTTCTCGATAGACTTCAAAACTCAATTTGGTCTGCACTTACTGAGAACATCAATCCAAATATTTCAGT
TGCTGCAGTTCTTCGTGATGATGGGAATTTTATTTTGAGTGATGTGTCAAAGGCATCAATGCCCTTACTA
CTTTGGCAAAGTTTTGATCACCCAACACACACTTTTTTGCCTGGTGCTAAGATTGGATATGATAAACGGA
CACAAAGAAAACAGGTTTTGGTTTCATGGAAGAATTCGAGTGATCCGGCACCAGGAATGTATTCTCTAG
AAATGGACCCAAAAAATGCTCAATATGTTATAAAATGGAATAGGACTACAGAGTATTGGGCAAGTGGTT
CATGGGATGGCCAAAGATTCGGCTTAGTGCCTGAGATGAGCTTAAACTACATATATAATTACAGCTATAT
CGACAATGAGAATGAGTCATATTTTACATACTCCCTTTACAATAGTACAATCACATCAAGATTGATAATG
GATGTCTCGGGACAAATTAAGCAACTGTCATGGTTGGATGGTAGCATTGATTGGAATTTATTCTGGACTC
AACCAAGAGAATCTTGTCAGGTTTATGCTATATGTGGGGCATTTGGAGTTTGTGATGAAGCTAATGCAA
CCTGCAATTGTTTGAGTGGTTTCAAGCAAAGATCAGATGCAGAATGGAATTCAAATGATTATTCCAGTG
GCTGTGTAACAGACGAAAAGGTGCAGTGTGATGCAATTACTGAAGACAAAGATAGTTTATGGATAACTT
CAATTGTGAGAGTACCTGCTTCTCATAATACTAATATCACAGTTGGGACAGCCTCACAGTGTAGATCTGC
ATGTTTCAACGATTGCTCTTGCACTGCTTATACTTATGATGGTTCCGGTACCTGTTCCATCTGGACAGGGG
ATCTGTTCAATCTGGAACAACTCAGCACAACTGAGTCAAAAAGGACAATATTTGTCAAACGCGGCTCACC
TGAAGGTTAGAATAGCATATTAACATAGCTTGATTCTTTATTTCATATAAGTACTATGTTATGGATAGAAT
TCCTTATTAACCATTAGCACTTAACAACAATGATTCTCCTTTCAGCTCAAACTAAAGCTAAGAAGTCGATG
AAGCTAAAAGCAATCCTTTCGTCAATATCAGTTTTGATGTTTCTACTCATAGGCAGCATTAGCTACATTTA
CTATAAAAGAAGAATTGCAAAAAGAGCAGGTAGTTGCTGAATGAATCTTACTCTGAAAGATATTTTCTCT

TATAGCATTTTGAACTTACTTTCAAAGGTCATTCTCAATCAGATAGAAGTAAAGGTATTCAAGGGGCACA

TAAATCTCACTGGCACAAAGCTGAAGGAGAAGCAAAAGTATTAATGAATGAAAACAGTGATGAAGCGA

TTGATGTTCCATATTTTCACTTGGAGACTATTTTGGAAGCTACAGACAACTTCTCAAATGCGAATAAGCTC

GGACAAGGAGGATTCGGTCCTGTTTACAAGGTTCAAACTTCATTCCTACATCTATATGGACTGGCATGGC

CATATTGACCTATAGTAATGAGATGAATTTTTTTTGCAGGGTATCTTTCCGGGCGAAAAAGAAATTGCAG

TAAAAACGTTATCCAGTCAATCAGGACAAGGCATAGATGAATTTAAGAATGAAGTGACTCTAATTGCAA

AGCTTCAACATCGAAATCTGGTGAGGCTATTGGGTTATTGCATCAATGCAACGGAACAAATATTACTTTA

TGAATATATGCCGAATAAAAGTTTAGATACATTCATATTTGGTGAGTTAGCCTTGTTAAGTTCTTTATTCT

CAAAGTCATACATTTCTTATAATGATCTAATTTTATTGACGTACCAAACTTTGTCATGGTAACGATGCAAG

ATGGAACACTTTGTCAATTATTGGATTGGAAGAAACGTTATGACATCATACTGGGCATTGCACGGGGTC

TATCTTATCTTCACCATGATTCACGACTAAGGATCATTCATAGAGATTTAAAAACCAGTAACATTTTATTA

GATGAGGAGATGAACCCGAAAATTTCAGATTTTGGCTTGGCAAGGATTGTTGAAGGAAAAGTAACAGA

AGCAAATACAAAGAAAGTAGTGGGGACCTAGTAAGTATTATATCTCTTCCTGTCATTAATTTCTTACAAA

AGGAGCTTTGGATAAGATCTTATACTCAAATGTTTTGTTCACCTGATGAAATCTAGCGGCTATATGTCTCC

TGAATATGCACTAGATGGATTGTTTTCCATCAAATCAGACGTATTCAGTTTTGGAGTAGTCGTACTTGAG

ATAATCAGTGGAAGAAGGAATACAGGATTTTATCAGTCAGAAGAAGCCTTAAACTTGTTGGGCTATGTA

AGTGAAGAAACTTCCCATGACTTAATTATTATATCTTCTTCTTAGCTTTGTTTCTCTGATAGAAAAATTATC

ACAGGCATGGAAATTGTGGACAGAAAAAACTGAGATACAATTAATAGAGAAGTCACTACTTGAATCATG

CAACAAAAGTGAAGCATTGAAGTGCATAAATATTGCACTCCTGTGTGTACAAGAAGATCCAAATCATCG

TCCTAATATGTCAGATGTCATTTTAATGCTGGGAGGGGAAGGTACTAACCTTCCAACACCTAATAGACCA

GCTTTTGTAATAAGGACACATGCTTCTAGTACATCTTCTTCTTCCTCCGATAAGAAATACATCGTATCCAA

CAATCAGGTGACGATTACAGTTGAAGAAGGACGTTAGCAGGAATAGTGAAGGCATTTAATTCGTATGG

AAAGGGCTAGGGGTGGCACTAGCTAGGACTTAAAGATGAGCCATATTATACTTTTTTATCCTTGTTAACC

TCTACTTTGGTCCTAAAAGAGTCCAGGGATAATTGTGTGTCGGTACTTGGCCAAATGTCGTCTTCTCTATA

ATAGTTAGGACAAAAGGTGGAAATGTTGGGAAAACTTTCAAGTGTTTGTACTCAAAACATATACAATTTT

GCTTTAGAATGTAGTAGGTCTTAGCAATCAAGTACAAGAGTAATTTGATTATTCCTGATGCCTTCAAATG

TACAAGCAATGTAATTTTTCTTTCTTATTCAATATCTTATTGGC

>PGSC0003DMG400008523 G-LecRK

CACACCCAAAAAATCAACTTGTTTGACTACTTTCATGGACTCTGAAGAGAAAAGTATACCTCCGTCAACA

CTTATTTTCTCTTATTGTTTCGTTCATCATCTTCTTTACCATGGAAATGAAGAACAATCATTCGTTTCTAGT
ACTTTTTTTGTGCTTTAGTCTCGGTACCAATCTTTCTATCGGAGAAGACACCATTTCTATGAATGAATCTG
TTTCTTCAGGGCAAACAATTATCTCTTCAAGTGGGACCTTTGAGCTTGGTTTCTTCACACCAGGTAACTCT
TTTAAATATTATCTAGGCATATGGTACAAGAATATTTCATCGCAAACTGTAGTTTGGGTAGCAAATAGGG
AGACACCAGTTAGTGATGCTGCTCACTTGACAATTATTCAAGGCAACCTAGTGCTTCTCGATAAATTTCA
AAGCTTAGTTTGGTCAACAAATATTTCAAGGAGTGTACCTCCAAAAAATTTAGTCATAGCAGTTCTTTGT
GACGATGGAAATTTGATTTTGAGTGATTTGTCATCAAACTCATCAATACCGTTACTACTTTGGCAAAGTTT
TGACTACCCAACACATGCATTTTTACCTGGTGGTAAGGTTGGATATGACAAACGCACACAAAGAAAACA
GGTTCTGATTTCATGGAAGGATTTGAACGATCCGTCACCTGGATTGTTTTCCATGGAATTAGACCCACGG
CGTGCCCAACTTGTTATAAAATGGAATAGGACAACACAGTATTGGGCAAGTAGTTCATGGAATGGCCGG
ACGTTCAGCTCAGTACCTGGGATGAGGTTAAACTACATATTCAATTACAGCTATATCGACAATGAGAATG
AGTCATATTTTACATATTCCCTTTATAATAGTGAAATCCCATCAAAATTTATCATGGATGTCTCAGGACAA
ATTAAGCAACTCTTATGGTCGACTAGTTTGGATGATTGGTATCCATTCTGGGCTCAACCAACGGAACAAT
GTGATGTTTACGCCAATTGTGGGGCATTTGGAGTTTGCAACAATGTTAATTCATCCTGCAATTGCTTGAG
CGGTTTCAAAGCAAGATCTGATGCAGAATGGAATTCAAATAATTATTCCAGTGGCTGTGTAAGAGTAAG
AGACCAAGAGGTGCAGTGTAACGGAATTACTGAAGACAAGGATAGCTTTTGGATGAATTCAATAGTGTC
ATTACCTGCTTCTCAAGATACTAATATCACAGTTGCGGAAGCCTCACAGTGTAGATCTGCATGTTTCAAC
AATTGCTCTTGCACTGCTTATACCTATGATGTTTCTGGTGCATGTTCATTTTGGACCGGGGATCTGTTCAA
TCTACAACAACTCAGCAAAACTGAAACAGAAAAAACTATCTTTGTCAAAAGTGGCTCACCTGAAGGTTA
GAATAACATAAAATAACTTAGCTTAATAAGTATCGAGGTCCACTTTTGCATTATGTTTTTCGAGAAAGAT
GTTAAACATATCTGACTTGTTTTTGGCTCTCTACTTTCAGATCAAACTAAGGCTAAGAAATCGATGAAGCT
GGTAGTTCTACTTTCATCCACAACAGCTTTTATCGTTCTATTCATAGGAAGCTTTAGTTACATTTACTATAG
AAGAAGAAGAAGAAGAATGACAAAAGGAACAGGTATTTACTGAATGAATCTCACTCTGGAAAATATTTC
GTTCGTACCTTTTTTTAACTTAATTTGCTTTCTCAATCTTATTGAGGTAATGAGGTCATTCTGTTTTTCTTAT
TCCTTTTCCCTAAATGATACTCAAGGGACACAAATATCTCACTTGGACAAGATTGGAGGAGGAGAAATC
ATTGATGTTCCATACTTTTGTTTGGAGACCATTTTGGTAGCTACAGATAACTTCTCGAATGCAAATAAGCT
CGGACAAGGAGGATTTGGTCCTGTTTACAAGGTTAAAATATAATTATTGCACCTCTGTGTACTGGTATGG
CTTAATTGACCAATAGTAATGAGATGAACAACTTTTAACAGGGTATCTTTCCAGGCGGAAAAGAAATTGC
AGTGAAAAGGTTATCTAGTCATTCAGGACAAGGCATAGATGAATTTAAGAATGAAGTGACTTTAATTGC

AAAACTTCAACACAGAAATCTGGTCAGGCTTTTGGGCTATTGCATCAACGGAAAGGAACAAATTTTACT
GTATGAATATATGCCAAATAAAAGTTTAGATACATTCATATTTGGTAAGTTTGCCAAGGTATGTTCTTTAT
TCTCAAGCACCTATACTATTGACAAGATTTAATCTTTGTCATGATATTTATGCAAGATGGAGAACTTTGCA
AATTATTGGATTGGAAGAAGCGCTATGACATCATATTGGGTATTGGACGAGGTCTTGCTTATCTCCACCA
TGATTCACGACTAAGGATCATTCATAGAGATTTAAAAACCAGCAACATTTTACTAGATGAGGATATGAAT
CCCAAGATTGCAGACTTTGGCTTGGCGAGGATTGTTGAAGGAAGAAGAACAGAAGCAAAGACAGAGAA
AATAGTTGGGACCTAGTAAGTATAACATCCTTCCTATTGATCATTTTTCCCTTTTGCTTTTGTAAAATACAT
CTAACACGTTTTCGACATGGTGTAATCAAGTGGCTATATGTCTCCTGAATATGCATCAGATGGTCTGTTTT
CCCTCAAGTCAGATGTATTCAGTTTCGGAGTAGTCATACTTGAGATGATCAGTGGACGAAAGAACACAG
GATTTTACCAATCAGAAGAAGCATTAAACTTGTTAGGTTATGTAAGTGCAGAAACTCAGACCTACATCAT
TTTTGTCCTTTTTGCAAGTTTTGATATCTAACTGGAAGTTATCACAGGCATGGAGAATGTGGATAGAAGA
AAGGGCAATGCAATTAACAGAGAAGTCATTACTAGAATCATGCAATAGAAGTGAAGTGATGAAGTGTA
TCAATGTTGCACTCTTGTGCGTGCAAGAAGATTCAAATGATCGTCCTAAAATGTCAGATGTCATTGTAAT
GTTGGTAGGAGAAGGTACGAGTCTTCAGAGACCTAATAGACCAGCTTTTGTAATAAGAACACACGCGTC
TAGCAGACCATCTTCTCCCTCAAAAGTCTCTAACAATCAAGTGACAATTACAGAAGAAGGAGGACGGTA
GTAATGGTGTGAGATCATCTTAATTTTCATTTATATATGATACAATTGTAATGTTTTCCTGCAGACTTCTAA
AGCACGAGCAGAAAAATAATCTATATTTTGTTAGTATCTTGATTGTAATTTGTAGCCATGACATTGTTATC
CTTACACATATATTACATGCACTTTTC
>PGSC0003DMG400008524 G-LecRK
GTGAAGTTTCTCCACTTGCCATAATCTTGTCTCCAACCCCATTAAGAGAAAAAAAAAATTAAACAACAGA
GCAAGAAAAGTTGGAGCTCAAGAAGGTGAATGATACTCAAAGAAGTGAAATAAAGGAAGAAAAATTGT
TTGGATTTTCTAAAATCCAATTTAATTTAAGGTAAATTAGTTTTAGGTACTTTTAAACTTTATTTTGGAGTA
AATATAATGTGTATGTTATTCATTTGTTTTTGTGGCTTTTATGGTTCTTGAGTGATTGATTTTTGGATTTT
CATCTTGGTTGTTGAATGTTATTTGCTTTTTGTGTGTGTGTGTGTGTGGATTTCATGGTTCTTGAGTTTAA
TCTGATTCAATAATGAATTCTTGAAAGTTAGTATAGTTTTTCATATTGAATTTACATGTGGAAACTACTTTT
GATGTTGACTTTTATGTTATAAGCATTCTAGGTGGAAAAGTTATTTAAAAATTGTTATATATAACTTAAAT
CCTTTTGGTTTTTGTCATTTCAGGCATTTATGTTTGTTATTGGGTATATGTGTTATCAATTACTACAATTAC
CTAAAAAGAAAAGTTGTGTCTTACTACTCTCAACATCATCTCGTTGTCTAGCAAGATATGTCTAGTTCTCC
CTCTACTTACCTATCCATTAGGAAAGTGGAACTTTCTTGACCTTCGAAAAGGTATAAAATGGTTTTATAAA

TATGGTATGGTATATGGACTAATTTTTTAGGTGCTACTTGCTTGCTTCAAGCACAATACTTTGATTTCTTTT
TAAAGTTTTAGACAGAGGTGGAGCCAAGATTTAAAATTTATGAGTTTTAGATTCTTTTTATGTTACTGGG
TTCTAAATTGATAAAATGTATAAATTCAAAAGATTTTAAAGACAAATACAAGATTTAAACTAAAACTACT
GGATTCAGACTGAACCTGTAGCTGATATTCTAGCTCCGCCCCTAGACTTTTTTATTAGCATCCTCAAATAC
TAAAGTGGTCTTATTGCCCCTTCTTTTGTCACACTTTTTAGCTAGTTTTCTCAAAATGCATGAAAAGGAAG
AGATGCTCTATTTCATCTTTTAAGTTCAATTCTTGAGGCTATATCTTGAATTCATTTTATTTTTTTTAAATAA
ATAAAATTGAGGGCCTAAGAAAGAGATTACAATGTGGGGAATCGAATACTCACACAGGCAGACAATCA
ACTGAGTTACGAGATTCTCCTTTATCTTGATTTCACATGATTTGCTATATTTTGTTGTGATTCAAGTTGTCT
ATCTATTTTAGCTTTAGGTTATCAGTTCTGTTCCTATTTCAGGCTAATTGCAAATGATTTGAGGCTTTGAA
GGTTTACGAGGATGTTGGACTTAAACAAAATAGAACCTGAAGAAGGAAAGCTAGACTTGGATCTTCGTC
GCGTTAACTATGATCTTGATGACAAATCTCATCTTAGATATGGTGATTGGGTCACTTTCAAGAGCCATTC
ATCTAAAAAAGTTAGAATCTCTCGACCTGAATCTCCGAGAGGTGAAAATTCAGATAGACAACCTCAGGA
TGCAGATTATCCCTCACTTAGCTATGAACTCGAAAGTGAGATTCTATCCAGGTTTCCTAGGTGGGAGTAT
TGGAAACTTAGCCTCGTAAACAAGAGATGTTCAATGCTTCTAAAGAGTGGTGAGATATTTGAGATTCGT
AAAGAGAATGGATTTAAAGAACCTTCGGTATATATGTTAGCAAGTGGGGAGACTAATTGGTGGACGTTT
GATAGAGAGTTTAATTCCCGGAGGAAAATCCCTGATTTACCATCAGATGTGTGCTTCACATTTGGCGATA
AGGAATCGCTATGTGCAGGCACACATTTACTTGTTTCGGGTAGGGAAATCGATGGTCTTGTTATATGGA
GGTTCGAATTAGCAACGAATTGTTGGTACAAAGGTCCTTCTATGGTTAATCCAAGATGTTTATTTGCATC
AGCTACTTGTGGCACCTCTGCTTTTGTTGCTGGTGGTGTTGGTATTATGGCGAACAGTGAGGTCTATGAC
ACAGCTGAGAAATACAACCCCGATAGCAGATCGTGGGACCCACTACCGAGGATGAAGAGGAAGAGGA
AGCTTTGTTCAGGATGTTACATGGACAAGAAATTCTACGTGATCGGGGGGAGGAATGAGAACGGAGAG
CTTACCTGTGGAGAATTCTTCGATGAGGGTAAGAACAAGTGGGAGCTGATTCCGGACATGTTGAAGGAT
GATCCAGTCCTAACATGCCACTCACCACCCCTCATTGCTGTCGTGAACAATGAGCTGTACTCACTCGAGG
CATCTTCAAACCAGCTGAAGCTTTACCTGAAAAAAACCAACACTTGGAAAATGTTAGGACAAGTGCCAG
TTAGAGCTGATTCAAACAGAGGATGGGGCATTGCTTTCAAGTCATTGGGAAATGAACTTCTTGTAATCG
GAGCTGCTTCATCGTCAGCCTCTTATTCGGGTAATTCCATGGCTATATATACTTGCTGTCCAGATCCTGAT
GCAATTGAGTTGCAGTGGCAACCTCTTGACAGTGGTCGAAATCGACTTAGTAGCTTTATCTTAAACTGTT
CTGTTATGGTAGCTTGAATAATGTCATTTATCTAGCAAACAACTTGTGTATTATGATCAAAATATATAAGT
GGCATATGGCCACTGGTTTATGATATTTCTTGCATATATTCACTAATGTTTGCTCTGCTATGTTTGATGTA

ATTTGCATTTATTGTTGCATTATTATGATTTATATTGAGGTGTTGTTCTAA

>PGSC0003DMG400010240 G-LecRK

AAAACAATGTCTTCTTTGCTTGTAATAACACTTTGTTCCTTATTCATAGTAACATTAGCCAATGTTCCTCCT

TCCAAAACCTTCAAGTACATAAATGAAGGCTCATTTGGCGAATACTCCGTTGAATATTTTGCAGATTATC

GTGTGTTGGACATTGTCAACTTCCCTTTCACCCTTTGTTTCTTTAATTCTACTCCAAACACCTTCATACTTG

GGCTACGAATGGGTCATCGTAGATCTGAGTCCATTATGCGATGGGTCTGGGAGGCTAATCGTGGTAAGC

CTGTCCGCGAAAACGCCACTCTAACGTTTGGTACTAATGGGAATTTGGTACTAACTGATGTCGATGGCAC

GGTAGCTTGGCATACCAATACTGCCAACAAAGGTGTAACAGGGCTAAAACTTTTGCCTAATGGCAACTT

GGTTTTACATGACAAAAATGATAGATTTGTTTGGCAAAGTTTTGATCACCCAACGGATACACTTTTGGTG

GGCCAAAGTTTAAGGGCTAAAGGCCCATCTAAGCTTGTTAGTAGAAAGTCCAGTAAAGAACCTGTTGAT

GGGCCTTATAGTTTTGTCCTAGAAGGGAGACATTGGGCCATGTATTATATAAGTGTAAATTCACAAAAG

CCTTTACCATATTTCAAATCAGATGATTTTGGTAATGGGAAAGGCTCTTTAGCCAATTTTGATTTCTATTG

TGAGCCAGAATATGGTGAAAATTATGCTTTTGAGCTAGGATTTAGGATTAACATGACCAATTCTCCTAGC

TCTGGTACTTCTATATTTACTAGGCCCAAATACAATAGTACATACACTATGTTTAGAGTTGATATGGATGG

TAATTTAAGAATGTACACTTATGAACCAAATGTTGATTGGGGAGCATGGGAAGTCACATATCGACTTCTC

GATAGGGACAACGATGGAAAAAGTGAATGCAAGCTACCGAGGAGGTGTGGATCATTAGGCGTTTGCG

AGGATAGCCAGTGTGTGGCGTGTCCGAGCCCTCGAGGCTTGATTGGGTGGAGTAAGGGATGTGCACCA

CCAAAGTTGCCAGTATGTAGGGGAGGATCAAATGTGGACTATTATAAGGTGGAAGGAGTTGAACATTTT

ACTAGTGATTATAATGAAGGAGTTGGGCCTATGAAAATAAGTGAATGCAAAAATAGGTGCAACAAAGA

TTGCAAATGTGTGGGCATTTTCTACAAACAAGAGTCATCAAAGTGCTTGGTTGTACCTGAGCTAGGGGC

TTTGGTTAAAGTGGAAAATTCCTCCCATGTTGGCTACATCAAGATCTCAAAGTAGATGAAGTTATTACTG

ATTTATATTTATATCAAGCAAGCATGTTATATTTTTTAAATTTCAGTAATAATAATGTAACTTTTGTCAAAA

TATAATAAATAATAAATGCAC

>PGSC0003DMG400011251 G-LecRK

ATGTTAATTTCTCCAAAAAGGAAATTTAATTTGGGATTTTTTAGTCCAAATGTTTCTAATAATCAAAGGTT

TGTTGGTATATGGTATGTTGATGGTCCAAAAAATTCATTTGTTTGGGTTGCAAACAGAGATAAGCCAATT

TTTGATAAAAATGGTGTTTTTACTATTGAGAAAAATGGGAATTTAGTGGTGAAAAATGGTCGTGGCGAT

TTAATGTGGACGAGTAATGTTGCTGCTATGAATAATAATTGTACAGCACATCTTTCTGATTATGGAAATC

TTATGATGTTTAATGGCAATAACAGGGAGTTGTGGCAGAGTTTTCACCATCCTACAGATACATTTTTACC

AGAAATGAGATTTTATTTGGATGAAGTTTTGAGGTCATGGACTAGTGAAAGTGATCCTTCACCTGGACG
GTACGTAATCTTGTATCGTGTGATCATCTTATCTGAAATCTTTTATTTACTTAATTTTGTTTTGTACATGCC
CCCTCACGTGTGGATGAATTTTTTATATAATGTGTGGTGGTGAGACTCTCTGATAGCATGTTGCGTTTGT
GACATCTCATTTAAAAGCTTAATTGAGAATGTGCTTTTTTATTTTACGTAATTTTGTTTTGAACCCCTCACG
TGTTTGACTCGTGGAAAGTTGAAAGACAGAAGGGTTGTTGGTCAGTCACGGACTATTTGATTTCATTATG
ACGATTCAATGTTAATGACCTCATTCAATATTTTTTTTTTTTGATCCTTCACTATCGTGGGCTGGATGGATA
GCTAGTAGTTGTGAGTAACAGTCCCCTTTTTATAGATTAAAGGTGATGATTTTTTGATATTGGGTGGCAG
TGAGACTCAAAACCTAAGAACTCAGCTTGCCCTGATATGGTAATGCATTTGTGCCATCTCATCCAAAAGA
TTAAGTTGAAAATATGTTTTTCTTCACTTAATTTTATTTTTAGCATGCCCCCTCACATGTTTCTTGAATCTTT
GATATTGGGTGGTAGTGAGACTTGAAACCTAAGAACTCTACTTGCACTGATATGGTAATGCATTTGTGAC
ATCTTATCGAAAAGATTAAGTTGAAAACATGTGTTTCATAACTTAGTTTTGTTTTTTAACACGACCTCTCAT
GTGCTTCTTGAATCCTTGATATTGGATGGCAGTGAGATTCGAACCTAAGAACTATGTCTGTTCGGATACA
ATGTTGCATTTGTGACATCCCAAAGTCAAAGAGCATATTTTTATTTACTTAATTACGTTTCCAACAGGTAC
TCATTAGGAGTAAATACTCGCGGATCACCACAAATTGTGATCTTTGATGGAGAGAAGAGACGTTGGCGG
AGTGGATATTGGGATGGACGCATATTCACAGGCGTTACAGATATGAAGCCCGAGTATCTACATGGTTTC
AAGCTTTACAATGAAGGAGATAAGCTATACTTCACTTACACTGTATCAGATCCTTCTGATTTGGTGAGGT
TCCATATTAGTCCAACCGGATACGAGCTGGAACAAAGGTGGGATAAAGATAACGAAAACTGGAGTATA
ATACAGTCTCATCCATCAGGTGATTGTGACTTGTATAACTTATGTGGGAATTTCGCGAAATGTGATATCA
CATATTTGAAAAAATGCATTTGTTTAGTTGGATTTGTACCGAAAGATTTGGGGCAGTGGAATGCGAGGA
ACTGGTCAGAAGGGTGTGTTAGGAGGAAAGAAGTGGAATGCAGAGGAAATAACAGTGTCTTGAAGAG
TGGTAGTGGAAAGAAGGACGGATTCTTTGAGATTGAGAAAATCAAGTTGCCGGATTTTGCTGATACTGC
ATATTTACAAAATATCGATGAGTGTCGAAGCATGTGCCTTGAAAATTGTTCATGTACTGCTTATGCTTTTG
TTAGCGGAATCAATTGCATGATGTGGAGTGGAGATTTAGTCGATATGCAGCAGTTTCAGGAAGGCGGA
AACACTCTCTATGTTCGCCTTGCTGATTCTGAATTTGGTAAAAATGTGTACTATTTTTTCTGTTGTTATTCA
TATAATCATTTTAGATGTTCTTATTATAAACTTTCTTGAAACAGCTGGAAGCAACAAGACAGTCAAAATTG
TGGTAATATCCGTTATGGTAGCCGGGGCTTTTCTTGTATGTATGGTAGTTTTTCTACTATGCAAGTACAAA
GCCAAAACGCGAGGTAAGATTGCAGAAAAGCTGGTGTTTTACGACAGTCATTTATTGTTATGCAATATA
CCTATATCTTCTTCAATGTCCCATTTTCAGTGTCCAATAAGATCAACCAAATGGAGACAAGCGTTCCAACT
AGGAGCGGAGAGTTTTCTATGAACACATCAGGAGCAGGAGACTTGAGTATTGAAGGGCATCAAGGAAG

TGGTTCAGAACTTATATTCTTCAGTTTCAGCGGTGTAGCAGCAGCTACCGATAACTTTTCTAACGAAAAC
AAGCTGGGACAAGGTGGATTTGGCCCTGTCTACAAGGTTGGTTTCCATTCAATTGACAAGCGTGTCTTAC
TTTTCTAGAGAAGTATGACATTATTGTGTTAAACTAACGTCGATGTTTGTATATTTAGGGAAAACTACTAT
GTGGCGTAGAAATTGCAGTGAAGAGACTTTCAAGAAAGTCAGGACAAGGTGTGGAGGAATTCAAGAAT
GAGATCAAGCTAATCGCCAAATTACAACATAGAAATCTTGTTAGACTTATGGGATGCTGCATTGAAGGA
GAAGAAAAGATGCTTTTTTACGAGTACATGGCCAACAGAAGTTTAGATTCATTTCTATTTGGTTAGTACT
CTAGATATGCTACAAATTATTTCATCGATAGAGAGATCGATCACTTACAATTATTTTGGTTGATAAATTTT
TGACAGATCCTGTTAAGCAAGCTCAATTAGACTGGAGGAAACGCTTCAACATTATTGAAGGGATTGCAC
GAGGGCTATTGTATCTCCATAGAGATTCGCGACTCAGAATAATCCATAGGGATTTAAAGGCTAGTAACA
TTTTGCTAGATGAAGAGATGAACCCGAAAATTTCAGACTTTGGTATGGCTAGGATATTTGGTGGAAATG
AAAATGAAGCAAACACAAATAGAGTAGTTGGGACATAGTGAGTATCATTTGATTTTTATACCTTTAAGAG
TGAGTACCTTTAACTTGCTCAAAGAACTAACTCCATCAACGCGTTTCTTTTTTTGTTTTTACTTAAATCAGC
GGATATATGGCTCCTGAATATGCAATGGAAGGTTTGTTCTCCGGAAAATCTGATGTTTATAGCTTTGGTG
TACTATTACTGGAGATCATCTGTGGACGGAGGAACACAAGCTATCGTTCAAATGAACACTCTGGCATTAT
TGGTTATGTATGTCCAATAACTACTTCTCGATATCTGTTGTGAAGTTAAATCTTTTAACAACAATTCTACGT
TTTTTCTTTTTAGGCATGGCAGAAGTGGGATGAAGGTACACCAATGGACTTAGTAGACCGTTCTATTTGG
GACGAGTGTCAATATGATGAAGCATTGAGATGTATACAGTTAGCACTAATTTGTGTACAAGACATGGCA
GTTCACAGACCGAGCATATCATCCATCGTGTTAATGTTGGAGACGGACAATATACGATTGCCCTTGCCTA
GGCAACCTACATATACCTCAATGAGAAAACATGAAGATGCAGAAACATGGAATGAGAAGCAGGATTTTT
CTGCAAACAATGTCACAATTAGTGTTATAGTTGGTAGATGA
>PGSC0003DMG400013383 G-LecRK
AGACTCCATAGCTGTAAAGCTTGCAAACGATGTCGCTCCATCCTTCTTCTTCATCCTCACTTCTCTTCTTCC
TTCTCTTCTTCTTCTTGAACCCCTTTTTCTCTTCTTCACAAAAAATCCTCTCTTCATTCAATTCAAAATCCCCT
CCTTGGAATCCAACCCAAAACCAAATCCTACTTTCACCCAACTCCACTTTCGCTGCTGGGTTTCTTCAATCT
TCCCAAAATTCCTTCAATTTCTCCATTTGGTACTACAAAATCCCTGTCAAAACCATTGTTTGGTCAGCTAAC
CCAAATTCCCCACTTAATTCCTCAGCTACCCTTTTCATTTCTTCCTCCGGTGAACTCAAGCTAACCCCATCT
ACATCTTCCTCAGCTCCAAATTTATGGCCTTCAATTATTCGAAACACCTCCTCTGTTCTTTTCCTACAAGAA
GATGGAAGCTTGGTTTATGGAAACTGGAACAGTTTTTTGAACCCAACTGATACTTATCTTCCGACCCAGA
ACATCACCGGAACAAATTTAACTTCCGCTAATGGAAAATTCCAGTTTGATGGATCGAATTCGAATACCCT

TTTCTTCAATGGAAACGATAGTTATTTCACTTTTTCCCAAAATGCCCTTCAGAGGTTGGAGGAAACAGGG
GAAGTTACTCAAGTTAATGGAAAATTTGTTTCGTCGGATTTCGGGGAGAAAGGGAAACTGAGAAGGAT
GAAATTGGATGAAGATGGGAATATGAGAATCTATAGTTTTGATCTTAGTTCAAGTTTAGCTAAAAATTG
GACAATTGTTTGGCAAGCAGTTAATCAATTATGTACAATACATGGTACTTGTGGTACAAACTCTATATGC
TTGTATGATACTTCTACAACTCAAACTTCTTGTGTTTGTCCACCTGGTTTTAGGAAAGACACTAGTAAATC
TTGTGTGAGGAAAATTCCATTGATGACGAAGGATTCGAAGGCTAGTAAGTATTTACCTCTTGATTTTGTT
AGTTTTACTGGTGTGGGTAACCAAACAGACCTTAAAGCTTTGAGCTTTTCGAGTTGTGAAAAAAATTGTT
CAGATAAGAATGATTGTTTAGGTTTTTTGTTTAAGTATGATGGGACTGGATATTGTGTCCTTGTTTTGGA
GAAATTGTTGTATGGATATTGGTCACCAGGTACTGAGTTTGTTATGTATTTGAGAGTTGATAGTAGAGA
GAACGATATATCGAATTTCATAGGCATGACTAGTTTGATGGAGACTTCATGTCCAGTGAGAATTAGCCTT
CCATTTCCACCAGAAGAGTCTAAGACAACCACTAGAAATATTGTGATCATTTCGACGATTTTTGCAGCTG
AGTTGATTAGTGGAGTGTTCTTCTTTTGGGCATTTCTCAAGAAGTATATTAAGTATAGGGATATGGCTAG
GACTTTTGGTCTTGAAGTCATGCCTGCGATTGGACCAAAAAGGTTTAGCTTTTCTGAGATCAAGAATGCA
ACTAATGATTTCACTGATAAAATTGGGAGGGGTGGATTTGGAGATGTTTACAAAGGGAAGTTGAGTGAT
GGACGTGTTGTTGCTGTGAAGTGCTTGAAGAATGTCAAAGGTGGTGATGCTGAGTTCTGGGCTGAGGT
CACGATCATAGCACGTATGCACCATTTGAATTTGGTGAGGTTGTGGGGGTTTTGTGCTGAGAAGGGTAG
AAGGATACTTGTGTATGAGTATGTACCTAATGGTTCATTGGGTGAATTCCTCTTCCAAAAGTCTCTGATCC
AGTCACCAGATGGACAGAAACCTATACTCGATTGGAATATAAGGTACAGGATTGCGCTTGGTGTGGCAC
GAGCAATTGCCTATCTACACGAAGAGTGTTTGGAATGGGTATTGCATTGTGATATCAAGCCTGAAAATA
TTCTCTTAGGCGATGATTTCTGCCCAAAGGTGTCTGATTTTGGGCTAGCTAAGCTAAAGAAGAAAGAGG
AGATGATGACCATGTCAAGATTTCGAGGCACGCCAGGATATGTAGCACCAGAATGGACCAAGGCTGAT
CCAATTACTCCAAAAGCCGATGTGTACAGCTTCGGATTGGTGCTGTTGGAGATAGTTTCAGGTACAAGG
AACTTTGAGCATCATAACTCAAAGGTGGAAAGTGATCAATGGTTTTTCCCTGCTTGGGCATTTGATAAGG
TGTTTAAGGATATGAATGTTGATGATATTTTGGACCCTCAAATCAAACAATCGTATGATTCAAGGGCACA
CTTTGACTTAGTGAACAGGATGGTGAAGACAGCAATGTGGTGCATCCAAGATCGGCCAGATGCACGAC
CATCAATGGGGAAAGTGGCTAAGATGTTGGAAGGGACGGTGGAGATCATCGAGCCAAAGAAGCCTACC
ATTTTCTTCTTATCAGAGGAATAATATATGGCTCAATCCTCTTCATAATTTCCTCAAAATGCTGCCTCAACA
AGTTGCAGTAAGCATGTATTCCATGTTGTTTTGTATTGTAATAAAAGAAAGTAGATAGGAATTTGGATCA
TGTTGTGTTTTGTCTGTTTTCTTTTTCTTTTCTTTTTTCCAGTTTTGTAATCTCTCTTGTGTTGTAGTTGTAAA

GTACATTTTGATCAATGAAGAAGCAAATGGTTGTTACATAGAAG

>PGSC0003DMG400013882 G-LecRK

TTCATTTTGGTCCAGAGTTTAAAGTTTGAGGAACCTAAACAGAGAAGATTACCTTGTTAGAAAGCATCAG
TTATGTGTCAGAAGAAAGAGTTGTTGATAGTAATTGTTTCATTTCTTGTCCCATTTTGTAGTTCCATAGAT
ACCATTTCCTTTAACCAATCTCTCAAAGATGGTGATTTGTTGATCTCTTCAGGGAAATCTTTTGCTTTGGG
TTTCTTTGGGAATTCCCCTGATAAACGTTACGTCGGAATTTGGTACAACAATGTTCCTGAACTAACTGTTG
TTTGGGTTGCCAACAGAGACAATCCTGTCAATGGCACATCTGGGATTCTCACCATCGATTCAACTGGAAA
TCTTGTTGTACATCTCGATGCGGAAACAAAAACTGTAGCCTGGAGAACAAATGTTTCTTCAGCTACAAAA
CGAGCAGACTCGTATACTGCTAAGCTGGAGGATTCAGGGAATTTTATATTGTTTCAAGACTCCAAAATG
GATGTTACTGAGTGGCAGAGCTTTGATTATCCTACCAATACTTTGCTTCCTTCAATGAAATATGGAATAG
ACAAGAGGACGGGTTTGAACCGGTTTCTAACATCCTGGAAATCGTTGAACGATCCAGGCACTGGAGAGT
ATCGTTACACAATGGAGCTCAATGGGACACCTCAAGTTTTCTTGTACAAGAACTCTAGCAGGATTTGGCG
GACAGGATCCTGGACGGGTCATGGTTGGAGTGGTGTACCAGAAATGAGCCCACGATTCATCTTCAGCCT
TAGTTATGTGGACAATGATACCGAGGTTTCCTTGACATATGGCATACATGACAGTTCAATAATCTCAAGA
ATGGTCCTGAATGAATCCGGGATCGTGAACCGGTTAACCTGGCAAGAGAGTGAGCAAAAGTGGGTGCA
ATTTTGGTCTGCCCCTAAAGACCCTTGTGACAATTATGAACATTGTGGAGCATTTAGCAATTGCAATCTGT
TCAATTTGGCTGAATTTGAATGCAGTTGTCTTCCAGGGTACGAGCCAAAGCTAAGCCGACAATGGTTCTT
GAGAGATGGCTCTCATGGATGTTTGAGGAAGAAAAATGAAGAGGTGTGCAACAATGGTGAGGGATTTG
TCACATTAAGTCATGTAAAGGTCCCCGACACAGGTGCAACTAGGATGAACAAGAGCATGGGATTGAAG
GAATGTGAAGAGTTGTGTCTGAAGAATTGCTCCTGCACAGCCTATGCAAGTGCTAATATTAGTGCTGGA
GGAAGTGGATGCATCACTTGGTATGGTGAGTTGATAGACATAAAGCAATTTACAGATGGGGCCCAAGA
TTTATATATCAGAGTCTCTGCATCTGATTTAGGTAAGGAGTTAACTTAATATATTATGTGAGGATTTAGGT
AATTCAATATTATGTGGAGAAGAATGTGCAAGTTGATCATTTTTGGTTAGTAGTAGTACACAGTATCTTA
CCCTGGTGTTTACTGGAAGTTCTCATTTTTTGTTTGTTCAGCGCAATTCTCGAAGAATTCAAGAGGCCATA
ATAGGAAAAGAATGATAGGGATTCTAGCAGGGTCTGCTGCAGCAATAATTCTTGCACTCTCTTTGGCAT
GTTGTTTGGTGATCAATATAAGGAGAAATGGTAAGGATATTATTCTACATCATACTTTAGTCGAATATTTT
CAGTATCTGTACAGCAGCTATAATGGTGAAGCAACTATAGCAACCTAGACCTTCTACTACCTTTCTATATC
GTGCAGCAAAAACAATAACATTACTGCAAATTTCATTTGATCCTAATCGGTGTTTGCTGATGTCACAGAT
AATGAGAGAAGTGAAAGTATAGCATCCTATGACGGCATGGATGAAAGTAAACATACAGAAATCTCTATC

TTTGATTTAACCACAATAACTAATGCCACTGATAAGTTCTCTGATGCTAACAAACTTGGTGAAGGAGGAT
TTGGTAGCGTATACAAGGTAACAACTCCCAAACATGATTGAATTCCTAACAATCAACATTATCTTTATAG
GAGGCTGCAAATAGATTTAATAATACTTGTTAAACCAAGATTTTCTCCCTAAAAATAAGAAGAAGAAGCT
ATGTTTACCAAATAATAATCTGGAATCAATATCAGGGTCACCTGACAGATGGACAAGTAATAGCTGTCAA
AAGACTTTCAGTAACTTCAGGACAAGGGACCGAGGAGTTCAAGAATGAAGTCACACTAATTGCAAGACT
TCAGCACAGAAATTTGGTGAGGCTTTTAGGATGTTGCGTTCAACGAGGAGAGAAGATGTTGGTTTATGA
ATATATGCCAAACAAATCCTTGGACAGTTTTATTTTTGGTAAGGAACATCTCCAGAACCGGAATTTCTTCC
ATTGACCTCCAGTCTCTACATGCTTTTACATGTTTACTGATGATTAATGATTACTGCTTTAACGTGTGTGT
GTGTGTCCCTAAAAGATTTCTTATGCACAAATTTTTAAACTGTGATCCAGATAAAACAAAAGGGTCTTTG
TTGGATTGGGGAAAACGGTTTGAAATCATACATGGGATTGCACGAGGAATGTTATACCTTCACCAAGAC
TCTAGACTAAGAATTATTCATAGGGATCTAAAAGCAAGCAATGTTTTACTCGATGCCTCCATGCAACCAA
AAATATCAGACTTTGGAATGGCCAGAATATTTGGAGGTGACCAAATGGAAGCAAACACAAATCGAGTTG
TTGGAACATAGTAAGTATGTCAAGTTGTGTATAAACATAAATGAGTTATGTTAGATATGTGCATGTGATC
TTTAAAATGATAAATCTTGACATTGCTTATCTATTGCAGTGGTTATATGTCACCTGAATATGCAATGGTAG
GGCACTTTTCAGCAAAGTCTGATGTTTTCAGTTTCGGGGTTTTGTGTTTGGAAATTATCACTGGCAGAAA
GAACAACAGTCATAAGGATCAGGAGAAATCTCGACATTTAGTTGGATATGTAAGTACAAACATATATAT
GCTGTTAAAATCGAGAAATTGCATTTATTATCTTACAGTTGCCTTAGACATAAGCTGTGTCATGGATGGT
AGTACTGCAAATAATAATTTGGAAACAATTCCCTTTCAGGTTGTTAAAAGTGCTGAATAGATCGAGATTT
TGTATTTCAGAATTTCAATTTTTGGTGTTATAGTCGCTTCTCTTATAGAGAAGAAATTGTCTGAAATTTTG
ATTGTGATGCATTGTGGTGTTTCTATTTCAGGTTTGGGATCTTTGGAAGGATGAAAAGGCTTTAGATGTT
GTTGATCCATTGTTAGGTGGCTCATATGAAGCTTGTGAAGTTTTGAGATGCATCCACATTGGCCTTTTGT
GTGTACAACCGTTTACAGATGACAGACCGACTATGTCAGAAGTGGTCTTCATGCTGTGCAATGAAACAA
ATCTTCCTTTCCCTAAACAACCTAGCCTTATATTCAGATCAGAGAATCATAGTTCAGTTACACACTCTTCAT
CAGCAAGCATAGCAACATCATCTGTCAACGATATGTCTATTTCAACAGTTCATGGTCGCTAGAGCTTATG
CTAACTATTCAAGAACTATTTTGAAAGAAATACAGAATTCCTTTTTCTGTACTTTGCTTTCATAGTATTAGA
GAGATTTATTGGTGGGTTATACACAACATTTAACATGCTAGTCCATTCTTGATTCAAACTAGTGGGGTTG
GGCTTTAATAGCACTGGAACCAAAACTGACTTCTGATTCCCCATGGCCCATGTTACAGACTTAAAGGTAG
GTGGGTGGCAAAGGATTGGTAGGAAAGAAGCATAAACACAGAGGGTTTCTATTTCATCTGTGAATTAA
ATGGTGCTCTTTGTCAGCTGTTTTTGATAATTGCCCCACTTCATGTTTTCTTTAACAGTCTTCTCTGTGTTC

ATCAATGCTCTAAAAGCAACAAAGGCTAATCCTGCTAGCCACACACAACTTTTCAATTTTTTTATTGATTT
GCAGGATAGTAATTTGGTTGTGGAGTTTTGTCAATAAATGAGATATTTAACTATTATTAGTTGGTTATGT
TTTTCCTCAAAAAGTTATGGATAAGAAAATACAAACATGTAAGTTATCTTATGTAAGATTTTAACTTTTGT
TTCATAATTAGATCATGAGATAGACATGTATCAAATACAACAATAATATTATGATCATGAGTAAGAGCAC
TGTTGTCTCAATCTTTCTGTGATGAGTATCTGTAAAAGTAATATCAACTAGTCTGTTGAAAATCTTTTAGC
ATTAAATGCATTGAATCATTGAAGACTTATATCATACTTTGGGAAGTAATGAGCACTAGATACATTTGAT
ACTCAGATAACAGAAATACATATACACGTCAGACAACTAACGAACATAAGGTGCGGTAATTGTCACCTC
GTTGATCGAAGATGATGCTGTTTCTGTAGTAATCAAGTCTGAGTTACGGGTAATGAAGGGCTTCTTCGG
GGAGGGAACCGTTGCTTCAATACTTAGCATGGACAGAATTGTTGACATGGTTGGTCTGTCTGCTGCGTT
GAGTTGCAAACACAATAGTCCTACTAGAATGCAGCGTAATGCTTCCTCACTGGGACAAGAATCAACTATT
GATGGATCTACCAGCTCAAAGGCCTTACCATCATTCCACAACTCCCAGGCCTGAAGCAGCAGTTCA
>PGSC0003DMG400013885 G-LecRK
GGGATGTGTACCAAAATACCATCTTCATTCCTTCCCTCTTCCTATCAAACAAGAGAACAAGTACACCAACA
AATGTCTCAAACACCAACACTGCAGCAAAACAAAATACTCTTTTCTTAAATTCCATAATGAGAAACACCA
AAGATTACTACTTTCACCAATTCTTGTTCTTGATTTTGATTTCCCAATTTCTTCATCCAATTCTTGCTATACC
CACTGATACAATCACCCCAACACAGCCTTTAACCAAAGATCAAACCTTAGTATCTTCCGATCAACACTTTG
AATTAGGCTTTTTCTCTCCCGGCGGTGCAAATTCAGACAAATGGTATATCGGTATTTGGTACAAAGAGAT
TCAAGACAGGACTATTGTCTGGGTTGCAAACAGAGCTAAACCCCTTTCAGCTTCATCAACATCAGTCCTA
AAAATCACAGAAATTGGTACCCTTCTTCTCGTCGATGGCCAAACTGGAAATCCCGTCTGGTCTTCCGATC
AAACTCCGGCGACCAACGTAGTAGCCCAACTCCTTGACTCCGGGAACTTTGTCATTCGACCGGAAAACG
ATGACAGAGAACAGAGCTACCTGTGGCAGAGCTTTGATTATCCAACAAACACTTTATTACCTGGGATGA
AACTTGGGTGGGATTCAAAATCCGGGATGAATAGAAACATAACATCATGGAAATCAGCAATCGATCCAG
CTCCCGGAGATTACACCTTCAAGATCAACACCAGTGGGTTCCCGGAAGTTTACTTAACAAATAAACAACA
AATTATCTACAGGAGCGGCGCTTGGAATGGAATTAGATTCAGTGGGGTACCGGAAATGAAGGCAAGTG
AGATTATCTCATTCGAATTTCAGTTCAAATCAGATGAAATTACTTACATGTTCAAATTACACAACAAAACT
TTATACTCAAGATTGTCTGTGAGTCATACTGGTTTTCTTGAAAGATTCACTTGGATCCCAACAAGCAACAT
ATGGAATCGATTTTGGTATGCTCCAAAAGATCAATGTGACGGATACACAGAGTGTGGGGTATCTGGAAT
TTGTGACACAAATATTTCACCAATCTGCAAATGTATGGTGGGATTCAAGCCTAAAAATCAAGTGGCCTGG
GAATTGCGAGATGGGTCTAATGGTTGTGTTCGTTTTCATAAATTGGATTGCAAAACTGATAAATTTAACA

TATTGAGGAACATGAAGTTGCCAGATACGACGAATTCGTTTGTGGATACAAATATGAATTTGGATGAAT
GTGAAGCTATGTGTATGAAGAATTGTTCTTGCACGGCCTATACAAATTCGAATATTAGCGGAAGTGGGT
CGGGTTGTGTGATTTGGAGCACCGAACTTGTTGACATGCGGCAGTATGCGGCGGCGGAAGGTGGCCAA
GTTCTCTACGTTAGGGTGGCTACTTCTGATGCAGGTATCATCCTATTTTTCTACTTCTTTTTTCGCTTAGTA
CAATGATAACGAATTAACGATTAATAATCGATAAAAAATAGTTGTATTTAAATCGTATTAAAAAGAATCT
GTACAATTAATTTTAAGTAATTTGGGGTTGGAATTTGATTGTTAGATAATCCACTTCTGTGTTAGGATCAA
ATTTTGGGGATGATTTGAGTCGACTCGGCTGCACGAGTGGAGTTTGTTGGAATTTTTTATTTACTTGTTA
GGTTGGATGATATTTCAACACAGAGAAGTAGAGTAAAATAGAAAGGGTGATGAAATTTTTGGAAAATAT
TTTTGTGGAAGTTTGGAGAGTATTTCCATGATTGGAAATATGCAATTTAATACTTTTTATCTCAATTTATG
TGCTACACTTTTTAGTTTGTCTACAAAGGAATGATGCATTTTTTTTGGTAAGTAAACATTTTATTTGTCAA
GTAAACGTTACAAAACAACATTTTTATATTTAGTACTAACAATTTATTATCTTTAATTAGATGATTTATAGT
CACACAAACATCTATGACTTGTTTAGACCACACATTTCAAAAATCACTCTTTCTTAAATTTTATATCAAATC
AAATTACGTCACATAAATTGAGACAAATAAAGTAGTATAATGTTTTGCACATTTTTGTCGGAGAATTCAA
GTTTGATGCAAAGTTAATTTATTTAATTCATTCGGCGAACTTGCTGTTCTTATGTTCTCTGAATTATTCCAC
TATATAAACTGTATATATTGTTTTGTGTTTGAATTTATTTCCATAAATTCAAAAGTCGAGGACAAGTTGAA
AAATCTGTATGTCTCAAAATTGTTACTCATTTTTGCATGTCATCCGATCCAAATATCCTTGAATTATATTAT
TCTTTTGTCATTTTAATAAGAAGCATATTACACATTATAATAATTTTAATTTACTCTTTTCTGATTGAGTTCC
AAAGTTGGCAAACTTAGCCCTTGATCAAAGATAAACCATGTGCATGCAGTTAGTTTTGCCTCTAATAGAT
GAAATTATAAAAATATATTTTACTTTTTTGAATTCTTCTTTTCTATTTTATTTTTTTGGGTATTGCTATTTTTT
TAAAATTCCCCCCCCAAACGTCATGCAGAAATTAAATTAGAGTATTTATTTTGAAAATAAATACTCTATTC
CCTTTAAACAACACGTATAGTTGTCTTTTAATATCCTTGGTCCCCTTTTTAAATTCCCTTTCACGTTACCTAC
TTTCGATCTAATATTTTATATAATCAACTAGATTTCAAAATAAAAATAAAAATTCATAATTGCTAGTTGAA
AAAACAAGAAGTTAGTATAATAATTATTGAGTGCATTTAATCCCGAAAAGGACTTATAAACAACCTACTT
TTCAAAGTTATTCTGCTAATGAGTTTGCCAATATATTCTTTGTGGTATGCACGTTTTCAATATAATTTTTCT
TTTTCTATGCTTTGTGTATTTTACTTTGAAAAATCGAAAAGTATAAAAAAACCTAACCATATCTACCAATC
CCCATCAACAATTTGCCTTCAATAGGGACTTAATTTTATTTATTTACTATATTTGGACTTTCTTTTTCTCATT
TGATTTGATAGGGATGATTAGTATTTGGAAAATATGTTACACAGTCAAGTATCTCAAGGCTCTAAAAGTA
ATCAGCCACCTAATTCCACCTTCTTTCCAATCACCTATTCATAAATTTGAACAAACATTATAGATTTATTGT
CACAATTCAGCTCGTAATTAACGTTCAGATTGAAATATTAATAATAAAAGGTAACAAAGTAATCAAAATG

TGCATTTGCTTTTTCTTTTGTACTTTCCTCTTTAAATCTGCATGCTTAAGAGGAAACTTTTAATTATGGTGG
GAAATAAAGTTTCCAAATAAAGCTTATTTAGCTCTAAATTATTTTCAATTAATTGGTCCCACAATAAATGG
GTCCCACAAAGAGATACTCCTTTGCAAATCAAAACGGTGACGAAGCAATTTCCTGGTCGGTGTAATGGG
CCCTTACATGGCCGTCGATCTTGACTTTTATTGGGCCATCCCGGCCCAGTTGTTTTCCCTTTTTTCTCCTTTT
TAGAGAAAAAAAGCATATGTTTCCTAATGTAATACAAGAAAATGATTGGACACCAATTCCTTTGACACGT
TTGGGAAAAGGTCATATACACGTTTGAAAAGTAAGGTTTGGTGGGGTAAATTAATTCAAATATTACAAA
GGGATTAAGAAGTATATATTAAGCTAACTATTTTTTTTTACCAAGAATATGTCAAGGATGATATTAATAG
TTTTTTTTGGACACAACTTTGTGAAGTAGGTGAATTAGATCATGTTTAGTGACACCATTGGACTGTAGTCT
TTTCAATTCAAACTTTTCTATTGTCACCGAAAATGATTTTGAACAACACTATAGGTTTTCATACATAAATA
GACATTCAAATTTGGCCTAAGTAAACACCTTAACTTTAGTATACATACCTAGACACCTCAAATTATTTCCT
CCGTGTCAATTGAACTCTCCAACTTACAAAATGATAATCTAGACACCTTCAAAATTAATGTGTCAAGTCAG
CATGGGTGTCCATGAGACATAGTGGGACGAGTTGGAGGCGTCTAGATATGCTCTTTTGAAGTTGGAGTG
CTTCCTTTGTCAGCTGAGGTCAAGTTTGAGTGTTTCTTTATGTATTATGCTAGTAAAAAAATGATGATCTTC
TTTTTGCAGCACTAAACTCTTGAATAAAATTAAGCATGATTAATTAACTTGAAAAACTTTTAATTTCAGTG
CAAATTGGAGGTGTGGGATCTGGAAATAGCTCTGGCAAGACAAAAATAGTTGCAATAGCTGCTGGGGT
TACAGTTGGTATAGCTCTTGTGCTATTTGGACTAACCCTTTGCTTCTTATCAAAGAGAAGAAAACATCAA
GGTTCAATCCGAACTAAATCCGTAAACAGAGGTATTATATGAATCTATCATGATAAATAAGATTGTCTAA
TATAAAATGCTTCATTTTCTAATGAATGTTTCTTATGATCCTAAACCAGGCACTAGCGAACGAAGTCAAG
AACTTCTGATGAATGCAACTATTATTCCAAGTAAAAGAGAATTTTCTGGTGAAACTTCCACGGAAGAGTT
TGAATTGCCATTATTTGATTTCAGCACCCTAGCTACGGCTACAGAAAATTTTTCTGATGCGACTAAGTTGG
GCCAAGGTGGATTTGGTTGTGTTTACAAGGTAAAATCTCAGTTATATGTAAACCATAGTGGTATAATAAA
GAAGAAAAAATGATCGACGAACTCAATAATATAATCTTTTGATCAGGCAATGCTGGTTGGTCAAGAAGT
AGCAGTCAAAAGGCTCTCGAAGAATTCCGGACAAGGAGTAGAGGAATTTAAAAATGAGCTAAGATTAA
TTGCCAGGCTTCAACACAGAAATCTAGTCCGGCTTCTTGGCTGTTGTGTTGAAATGGAAGAGAAGATGC
TGATCTATGAATACCTGGAGAATAAAAGTTTAGATTCAATTTTATTCAGTAAGTCTTTTTCGTTGTATCGT
CAAGAGAGTAGTGTTTCACATCATTTACTTACATGCTTATATAATTTGTGAGTCACATTTGAATATGCTCC
TCAGATAAACAGAAAAGCTCGCTGCTCGATTGGCAAAAGCGGTTCAATATTATTTGTGGCATTGCTCGG
GGGCTTCTATACCTTCACCAAGATTCAAGATTTAGGATTATCCACAGAGACCTTAAAGCAAGCAATATTC
TGCTTGATAAGGATTTGACCCCCAAAATATCAGATTTTGGCATGGCAAGAATATTTGGCGGAGATGAGA

CTGAAGGAAATACAAAAAGAGTAGTTGGAACCTAGTAAGCACTCAACATAATTTCTTCACATAATTCATA
GAACCTTTAAAGCTGTTACTAAATCTGTTTTTTTGTTTTGATGTAGTGGTTACATGTCTCCGGAATATGCA
ATGGATGGCCTCTTCTCTGTTAAGTCCGATGTTTTCAGCTTTGGGGTTTTGGTGTTAGAAATAGTAACCG
GGAAGAAGAACAGGGGATTCTATTATCAAAACAACCAACTCAACCTTCTTGGACATGTAAGTCCACAGA
TCATATGTTAGCTTCATTATTGGTAATACATTCATTATTTGATTCATTTCTGATAAACAAAGTAATCGATTA
TGCAGGCGTGGAGACAATGGAAAGAAGGGAGCGGCTCAGAGCTACTAGATCCATCTTTTGGAGAATCA
TTTTCTCCGTCTGAAGTAATGAGATGCATACAGGTTGGACTGTTGTGTGTCCAGGAACAAGCGGAAGAC
AGACCAAACATGGCCACTGTGGTTTTGATGTTAGGCAGTGAAAACGCGTCACTACCTCAGCCTAAAAAC
CCAGGATTTTGCCTTGGAAGAAGACCCGTTGATTCTGATTCATATTCGACTAATTATGAAGAAACTTGCA
CTGTCAATCAAGTTACAGTTACCATGATAGATCCCCGGTAGTTGATGGTGACGCCCTCAGAAAGTGTATA
TTACTACAAGAGGTTCACAATTTTCTCTTGTATACTTTCTCAGTTTTTGCTTTTTGGGTAAGTTTTGTGTAA
GATAAAGAGTATAATTAGGTCTTTTTTTCTCATTACATACAATTGTGGGTTCAAAAATCTCTTTTTTCATCA
TCAATGTAATAGTCCAATCATTCCTTTTCCATATCCTTAATTGAAATCCATTGTAAATTACTGTGC
>PGSC0003DMG400013886 G-LecRK
GCCTCATCAGAACCTTCTTCTTTCTTTTTGTTTTCTATACCCTTTACCATTCTCCTTTGTAAAATTCCCAATTT
TCTCCATTCTTGAATTGTGGCATCATCATTTCCATTTCCAAATTCTTGAATGGCCACTGGCTTTAGATTTCC
CCCCTTTTGGTTCTTATTTCTTTTCATTCTTTCTTGTTTCTCCTCATTCTGTTTAGGTGGTGACAGAGTTACG
CGAGGAGAGATTTATAAAGATGGTGATAACATCACTTCTAAAGGAGGGGATTTTGTGTTGGGGTTCTTT
AGTCCTGCAAATACCAGTAAAAGGTATCTTGGTATATGGTATGTTGACGTACCAGTAAAGACATATATTT
GGGTTGCCAACAGGAACAATCCTGTTCATGATAAAAATGGTACCTTTTCAATTAATGAAAATGGGAATTT
GGTGGTTAAAGATGGACATGGGGATTTACTATGGTCGTCTAATGTTTCTGTTAAAACCACAAATTCTACG
GCTTGTCTGAGAGACGAGGGCAATCTTGTCATCCTTAATAATGACAGGAATGCTACGAGGTTAAATTCT
GAATTGTGGGAGAGCTTCTCGGATCCTACAGATACATTTTTGCCTGGAATGGAAGTTCTCATAGAAAGA
CAAGGCCAAGAGCAAAAAGTTTTCCGTTCTTGGACAAATGAGAGTGACCCTTCACCTGGGAGGTACTCA
ATGGGAGTTGATCCTCGTGGAACACCTCAGATTGTTATTTGGGATGGACCGAATAGACGATGGAGGAG
TGGACATTTTGATGGAGCGGAATTCATTGGTGTTCCAGATGTGATTAGAACTACTTTTTTCTCTGGCTTCA
GAATTCAAAACGAAGGAGACAATAAATTGTTACTCACTTACAGTGCCTCAAATACATCTTCTTTTGTGAG
GTTTCAGATTACTGTGACTGGAAATGAGCTGCAACAGAGGTGGAACGAAGACCAGGGTGAATGGAACA
CGTTGCAATCTAGGCCTGTAGGTGGTTGTGACTTGTATAACTTCTGTGGGAATTTTGCAGAATGTGACAA

AGATGTATGCCAATGTTTAAAAGGTTTTGTACCACGTGTTCAGGAGCAGTGGCATGCTGGGAACCGAAC
CGAAGGATGTGTTAGGAAGACAGAATTGGAATGCAGGAGAAATAGCAGTGTTTCAAGGAATGATAGTT
CAAAAGATGATGGATTCTCGACTATTCGGAGAGTTAAATTACCTGACCATGCTAATGTTTCAGAAATAAC
CATAGACGAGTGCAAAATCAGGTGCCTGAATGATTGTTCCTGCAATGCTTATGCTTATGTCAGAGGAATC
AATTGTATGATGTGGCGTAATGATTTAGTTGATATAGAGCATTTTCAGGAAGGTGGGAACACTCTTTATG
TTCGTCTTCATCCTTCAGATATCGGTAAGCACAATACTTTCTGCTGAATTCATAAATCTCTTACCAGTTTTC
TTCTCATTCGTGCAATTATACAAATCTAAAACCAAAGTAGTTTGTCTTTTAGTACAAAGATCAATTTCTTGT
TTGTCTTTACTCTTTATTTGTAGCAAGCTCAAACAACAAGCAATATATCTTTTTCTTCCTTGTGTTTGAAGA
ATGATTAGCCTTTCTAATTTCTTGATTTCTCCTTTGAATATCTTTTGAAACAGGTAAAAAAAAGAAGACGA
TCATAATTGTTGTAATATCAATTCTAGCAGCTCTGGCTTTGGTTGTTATGGTGGCCATTTGGCTAGTATGC
AAGTACAGGGCCAGAAAACGAGGTAAATTTTGATGCAGTAACATTTCTGACTTGTTAGTTGCATTTATTA
TATGTTCCATATTGTACTTATACCTTTGACTTGCAATTTTCAGAATCCAAGAGGACCAGTGAAATTCCCAA
AAACCATTTAGTTAGGAGTGGAGAGTTCTCCACGGAATATTCTGGACCAGGCGACATCAGTGCTGAAGG
GCATCAAGGAAATGGTTCAGAATTAGCATTTTTCAGCTTCAGCATGGTGGCCACAGCTACTGACGACTTC
TCTCTTGCAAACAAGCTTGGGCAAGGGGGATTTGGCCCCGTTTATAAGGTAAATTTTCAATCTTGATAGA
TTATTCTGTTCCTTTAGGAAGTATATGGCCATGTTTTGGACTAAAGTTGATGTTTTTCAAATCTTTAGGGA
AAGCTACCTTGTGGTCAAGAAGTTGCTGTAAAAAGGCTTTCACAAAAGTCTGGACAAGGCGATGAGGA
GTTCAAGAATGAGATCACTCTAATAGCAAAATTGCAACACAGAAATCTTGTTAGACTTTTGGGTTGCTGT
GTTGAAGGAGAAGAAAAAATGCTGATTTACGAGTACATGCCCAACAAAAGCTTAGATACATTTCTATTT
GGTAAGTTCAGGAAATTCTATCAAAATAATTTCTGCTGCTCCCACAGATGTACTATAGCAACACATCTCAT
TGAGATAGACACTAACAACTGTATTGGCTGTTAAACTTTGACAGATACTGTTAGGAAATCCCAGTTAGAC
TGGAGAAAACGCTTCAACATTATCGAAGGAATTGCCCGAGGATTACTGTATCTCCATAGAGATTCAAGG
CTTAGAATAATCCATAGGGATCTAAAGGCTAGTAACATTCTGTTGGATGAAGAGATGACCCCAAAAATTT
CAGACTTTGGCATGGCCAGAATATTTGGAGGAAACCAAAATGAAGCAAATACAAATAGGGTCGTAGGA
ACATAGTAAGTATCATTTGATTTTAAATCTTTTAAGAGACTAATTTCAAAATTTCTTGTTCCTGGAGCTAA
ATCCATTTCATTTTGTACCTTGTTGCTAAACAACAGTGGATATATGGCTCCTGAGTATGCTATGGAGGGC
CTGTTCTCTGGCAAGTCTGATGTCTACAGCTTCGGCATAATATTGCTTGAAATTATATGTGGACGTAGAA
ATACAAGCTTTCGGACAGATGAGCATTCAGGCATCATTGGATATGTAAGTCTGACTAATTTCTCCACCTA
TTTCAATCATTTTAGTCAAGAACAAGCTGTCTAAATAATGGGGCTAATTTCTTGTGTGATGAGCTATGAC

GGAGTGGTCATCTTTCTCATATTTTTCTGGTGGAAAATATTTCAGGCATGGGAGAAGTGGGAAGAAGGT
AGACCAATGGACCTGGTGGATCGTTCAATTTGGGATGGTTGTCAACACAATGAAGCATTGAGATGTATA
CACTTGGCATTGCTTTGTGTTCAAGATTTGGCGGCTCACAGACCAAACATGTCTTCTGTGGTTTTGATGCT
GGAAACAGACAATGTAAGGTTGCCCTTGCCTAGGCAACCTACCTATACTTCAATGAGAAGATCTGTGGA
TGAAGATATATGGCATGGGAATCAGGATGTAACATCTTCCAACAATGTCACAGTTAGTGTACTAATAGG
TAGATGATTGGTTAATGCTTGTTCCTTCTAACATACCTGCTTCATTTAAGAGAATGGGATCAAGTAGTGT
AACTATGCCAAGTTATACTTGGAACTATGTTCATAATTTTTTGTTAGCTAGGTACACTTTAGCACATCATG
TGTATTGTAATTAGAAGGTGGTTCAGCTCTCAAAGCTGAAAGAGAGATATGTCACAACCTTGTTCAATTA
GTATGGCTGAAGTTTTTTTCTTTACTGTTTGTAGATGGAGATGCTCCACCAATCGAGCAAACCCCACTTGT
CATATCTAGTTTTTTTTTTTTTATGACAAAGGAAACCCGCAGCCGCTACCCTTTGGGTGCGCACAGGTAAA
ACCCCTGCTCCTATGCAATAGCTCGCAAACCACATAAATCAAGCATTTCAATCGGTCCGTATATATCACTG
TATAATCAAGGAACAGAATTACATGCTTGTTATCTCCTATCACTTCAAGAAACCATTTTGCAGTTCTTCTA
AAGCTTGAGAACTAGTATTTTGTCCATTGAAGCACAAGCAATAGTTATTACCAGGTTCTGCATAACTTGA
ACTATCTGTAGGGAGATCAGCTTGTGTTCCCTTTGAGACAGAAGGGGAAATGGAATTATTTACTATGTA
ATTAATCAGTGTCAATTTAGTGGTGGTTGACTTGTTGCATGTAACTCAAATATGGAAG
>PGSC0003DMG400013887 G-LecRK
AAAAATTTCACAAGTTACACGCTTTATATTTTGGTTAAGCACGCACGCCACGTAGTTAATGTGGTGCGTA
AAAGTCACACCTTTGGAAAGGTTTGGTATGTAAAGAGATTCCCATGGCTGGGGAGTGTGTAGAAGTGAT
TTGGCTAAGAGTTCACAGATTAACTAGGTATTAGGCCTATTTTCTACAGTAAACTGTTTTTCCCGTAAACA
TTTTGCAGAGATGGTTGGTGTGTCATTGAGTCAATACCCGTAGGAAAAGGACAATCCATTAAATTCACAT
CAGAAATTGTCAACATGATACAAAATCCTGCCCTTATATTCTCAGAGCTAAACATATCAGCCCAAGTAATT
TTTCAGTATTGCAGGTATCAAATGCACAAGAAGTACTCAGAAACATAGTAAGCTGTAGACGTAGAAGCA
AAATGGCCACTTAACAAATCATTTGAGACATGGGTCCAGCAATTAATTGACACCCAATTGAAAAGTAAAC
GGATGAGAACATAAAAAAGATAAGTATCCAAGAACCTTATTTGACTAAAGGAAAAGAAAGGTCATCCTT
GAGGTTCTCAGCTCTTACACTTCAAATGATAAGACTGTACCTTTTGGAAGTAAAGAAGTTAGAATGAGCA
TATCTATGCTTTTTCATCTTTTACTCTATTGTTTTATGTAGTTTTTTCCAGTGCCAATACTTCAGACACCAT
TAGCAGTAGTGAGCCTGTGAGAGACTCAGAAACTGTATTTTCTAGAGGCAAAAGATTTAAACTGGGTTT
TTTTAGCCCCAGGAATTCTGCAAAACGTTATGTAGGGATTATGTTTAACCTACCATCACCAACACCAACT
GTAGTATGGGTAGCTAACAGAGATGAGCCTTTACACGATTCTAGTGGAATACTCACAATATCAGAAGAT

AGCAATCTTGTCATGTTGAATGGACAGAAGGAGATAATTTGGTCATCCAGTATTTCAAACTCTATGAAGA

ATTCCACAGCACAACTCTTGGACACTGGAAACTTAGTCTTGAAAGACAGCTCAAATGGGAGAGTTCTAT

GGGAAAGCTTTCAATATCCTACGGATTCTCTCTTACAGCTCATGAAAATGGGCACTGATAAGCGTACTAA

CACAACAACTCTCCTGAACTCATGGGGGAGTCCTGATGATCCATCCGTTGGGAGCTTTTCAGCTGGAATT

CAACATCGATACATTCCTCAGTTTCTTATTTGGAAAAACAGTTCTCCTTACTGGCGGGGCGGTCCATGGA

ATAAACAGATATACATAGGATTACCAGAAATGAATTCTTTCTATCTCTATGGTATTGACCTAGTAATTGAT

AATGCTGGCACAGCATACCAAACTTATTCAGACCCAAATCAGTCTAGGATACTCTATTATTCCTTGAATTC

AACAGGGTCTTACCAGGAGAAGGTTTGGGATCAAAGTAAGAAGGATTGGGTGGTAACATGGGCAAATC

CCCGAACAGAGTGTGATTTTTATGCCAAGTGTGGGACATATGGAAGCTGTAATCCAATGAATTCTCCAAT

ATGCAGTTGTATACAAGGTTTCAAACCAAAAAATGATGGAGAATGGAAGAAGGGAGATTGGTCTGGTG

GATGCATCAGAAGTACTGCACTAGACTGTGAAAGGAACAAAACTGATATTGAGAAGGACAAGCAGGAC

GGGTTTCTGAAGCTGCAGACAATGGGAGTGCCAGATTTTGTAATTTCGGTACCTTCTGCAAAAAAAGAC

TGTGAGAGTGACTGTTTGAGTAACTGTTCCTGCACTGCATATTCATACTACAGAGGCATAGGTTGTATGC

ATTGGAATAGAAGCTTAGTTGATATTCAAGAATACTCCAGGGATGGGGCGGCTAGTTTGTTCATTCGCCT

TGCCTACTCTGAACTTGGTAACATACTTCTTTACAACCTTGAGCTTTCAATTGTTTAGCTTGTTTTTTTTTT

TTGGAACTACAAGGAGTTGAAATTTAAAGTCTTCTTGTCTTTGAAGAATATAACCAAGACTCCCAATAAA

ATCTATAAAACCATCGAATTAGGCTCAAATTCTCAATTAATGTATTGCTTAGCTCTTTTCTATTTTTGGTGT

AAACAAAGATATATTAACTAGGACAGAAGACATCTTTAATGTGCTAGATTGTTTATAGGCAGCAGTTTAG

ATTAAGACTGTTTTATTCTGTAAGTTGTACTCCCTCTGTCCCAATTTATGTGACTTACTTTCCTTTTTGGTCA

GTCCCAAAAAGAATGACACATTTCTATATTAAGTAATAATTTAACTATAAAATGTTTATTTTACCCTTAAT

GAAATGATTTACAGCCACACAAATTTCTATCATTCATTTTGGACCACAAGTTTTAAAAGTCTTCCTTTCTTT

CTTAAACTTCGTGCCGAGTCAAAGTACCTCACATAAATTGGGACGGAGGGAGTAATACTTTTGAAGGGG

AACTGCACATTTTTTGTAGTGCACCTGTGTATATATAGACTAAAGTTACCAGTTTCAAAAAAAATAAAAAT

CAATTAGTTCTATCACTTCCATGGAAAGAAAGATATGCTCAAGAACAGTCTATGGCTCATACATGTTTGC

AGCTGCAAATGACAAGAAAGACATTCCTGTAGCAGCTATTGCAACCACAGTCTCAATAGGCTCAATAAC

AGTTATCTTGTGTGGATATCTTTTCTGGAAATTGTTGGCTAAGCACAGAGGTAATTAATCAAATAAGATT

TTTGAGAAGACAAACTAGAACTTCTCTGTCTGCAAATGGTTTGATTTCTGAATGTCCACTCTGAACCAGA

AAGGAAGAGGAAAAATGAAGCACTCTTAAGAGAAGCATCTCCAAAATTTTACCAAGAAGGCATGAGTA

AAGATGACATCAATCAAGTCAAAATTGAAGACATCACCTTGTACAGATTTGACATGTTAGCGAATGCCAC

TGACAGTTTTCACCCAGCTAGCAAGCTGGGGCAAGGAGGCTTTGGTCCAGTCTACAAAGTAATGTTTCTT
GATGCATCTCTGCAGTGTTCTGACAAATGAACACAATATAAAATAAATTAGATCAATTATCTGCAGGGAC
AATTGCCAGATGGACAAGAAATTGCAGTAAAAAGGCTTTCACAGTCTTCTGGTCAGGGGCTACAGGAGT
TTATGAATGAGGTCGTTGTGATTTCTAGACTACAACATCGTAATCTTGTTAGACTCCTTGGCTGTTGCACA
GAGAGAGGGGAAAAGATGCTGATTTATGATTTCATGCCAAATAGAAGCTTAGATGCATATCTTTTTGGT
CAGTGCAGAATTTTCTTTCATTGGTTTTGCCTGTCCTTCAACTTAAGTACTTCCAGAATCAAAAGAAAGCT
GTATTTCTTTAAGGAAACAAAATCCATCATACATTTCTCGGTGATACCTTTTCAGGTTCACACCAGGAAAA
GTTCCTTGATTGGAGTAAACGAGCCATCATTATTGAAGGAACTGGTCGAGGCCTCCTTTACCTTCACAGA
GACTCAAGACTACGAATTATTCATAGAGATCTGAAGGCAAGCAACATCCTGTTGGATGAATACCTAAAC
CCAAAAATTTCGGATTTTGGCATGGCGAGGATTTTTGGAGGCAATCAAGATCAGGCCAGCACTATAAGA
GTTGTTGGCACATAGTATGTTTGCACTGCTTCCTCCATTTTTTCATGGATGAACTGCCATCATTAGTCATT
GAGTTTCTGTGTAATATCTTATGCAGTGGTTACATGGCCCCTGAATATGCAATGTACGGAAGATTCTCAG
AAAAATCAGATGTCTACAGCTTCGGTGTGTTGATCTTGGAAATTGTCAGTGGAAGGAAGAACTCTAGTT
TTTATGATGATGAAGATGAACTGACTCTACTTGCATATGTAAGTTCATTCTTGCAACTATATATAAAGCTT
TAATGTTCTGCTAGCTCATCAAACCAAAAAAATAGAGGAGAAAAATTATGAAACTTCTGAAAATAACATT
TTTGCTGTTTGACAATGTCTTTGTTCATTAGGCATGGAAGTTGTGGAATGAAAACAATATCATAAAATTG
ATAGACCCCAAAATATTTGATTCAAGCTTTGAAAAACAGATGGTGAGATGTGGACATATTGGATTATTAT
GTGTTCAAGAATATGCAGAAGATAGGCCAAATGTCTCCACAGTTCTGTCAATGCTCACTAGTGATATTGC
TGAACTACCTACTCCTAAACAACCTGCATTTACCGGAGGACATGCTTCACCACAGCAAGTATCTTCTAAA
AGCCAAGGGTCCGTGAATGCTGATACCATTACTATATTGGAACCACGATAGGATACAGAGTTTTCAACA
GGCAGGCAAAGATCATTACTTTCCTTAAAATGGTAACAGTAGCATAGTATTTCTTGAATGTGCATAGGGT
AAAACACGCTCCTTTGCAATAACTTGCAAAACACACAAAAGAGGTAAAAGTGCACTAGCTATGTGATGA
ATTAATTACAGAGTGAGCATGGAGAACATGAAACAGTTGATCAATTTTTAACTTTGCATTATGAAAATCC
TTAGAAAGAAAGTAGACAAAAGGAAAAAAAGAATCGCAAATCCCCAATTAACAAGTTCAAATAATGGC
AATCAAAGACTTGAAAAATCATACCTACTGTACTGATACACGAAACGGCGTCGGCGAAAAATAGCCACT
CGTTTTACCACTGTTTTTCTTAGAAAATTACAGAGAAGTGAATTGGTGAATTAATCAATGTTGAGGAAAA
GTTAACTCTGCACTATTTTAGGTCATTCACACAAGTTATTTGGGTGTTGGTTAAAGACTATAATTGTTTCA
TTTTTGTTTTAATTTTGCATAAACTAATGTGAAACTTACCTAAATCTCACTAGTTTAGGAGTTAATTACTTA
TATATATTTTAGTTTGTAATATTACGTGTGTTACCCCCAATTTTGGTGCACTAAGAAACATCCAGATACAT

GTATTTCTGAATACATGGGCCAAAACTAGGTGTAAGTTGTTCTAGATACATTGTATCCAAGTAGATTCAC

ATGTATTTCGTATACATAAAAAAATTGGTTCGCCTCCTTCGCCTCTCTCCCATATCGCTCACCACTCTCCAA

TGTATTAGGGATATATAATAAATCTCGCTCACCTCTCTCCCTATTTTAGTCTATCCAGAA

>PGSC0003DMG400015476 G-LecRK

TCCAAGTATTGTTTTGGGGGGTTGGGGCTGGGGAAAACTGCTAGACTAAGATGAAAATTTTACAATGTT

AACATTTCTCTCTCTGTTCCATTTGACAATCATTATCCATTCTTGTTCTTCATTGAGGTAAAAAACTTTTATT

TATTTATTTATTTATTCCCATATTATATAATGCAAGTAACATTGTTCATGGAAATATCGTAGGGACATTGA

AGAATCGTTGAGAAGAGCAGTTAACGGGAGATAGATAGATATTGAGGGAATTTTTATCATTTGTTAAAA

ATATGGATTCATCTCCTGGAACTGCTTCCTCCAATGATACTGATTCTAAAGAAACCCCTTCTTCAAATGGA

ACTCCGCCTTCATCTTCAGATTCAAAAAATGATTCTCCTTCGTCTTCGTCTTCGTCTTCGTCTTCATCTCCAC

CACCAAAAAATGAATCTCCACCTTCATCACCACCACCACAATCTCCTCCTCCATCTCCTAATGCATCACCAC

CTCCAAAAAATGATTCTCCTCCGCCTTCTTCACCACCACCATCCTCTTCTGAATCTAATTCATCACCATCAA

AAGATAATAATAATAATAATCAATCCCCAAATCCTTCTTCTAATACACCTTCACAATCACCACCACCATCAT

CCACGTATAATACTCCATCAACCCCAAATAGTCCTAAAAATCCTCCATCATTTCTATTATTTTCTCCACCAC

CGCCTGCTTCTTCACGTGAATCGCCTGCACCTCCTCATGCACATTTGTCTCCTCCTCCACGCTCAGGATCT

AATCAACCATCACCTGCAAACGATGATTCCTCAAGTACTGTTGGTATAGTAGCTGGAATTGCTGTCGGAG

GCATCGTCATAATTGCATTAATCATTCTATGTGTATGGTGTAGTAAAAGGAAGAAGAAACAGAGATACT

ATATGGCTCCTGGACCTAAAGGTATGTTACTAATTACTAATAAAGTGTTGTTTAAAGCATAGTATTACTTT

TTAATTTTCGATTAAATATGTTTTTGATTAGGTGGTGATCCATACTACAATAACAACAATCAATGGAATAG

ACGTCAGTCGATGGATCATGTAATGAAGGTACCACAAAGTGAAATGGGGACTCAGAGTGGTTGGGCTG

GAGCTACTCCACATCAGCAGGGAGCTAGTTCAAGCGGCGAATTTGGCTCAGGTTATTCAGGCCAAGCAG

TGCCATTGCCTTCTCAATCACCTAACATGGGACTTGGTGGTTTCTCCCAGAGCCAATTCAGTTATGAGGA

GCTAGCTAAGGCCACAGATGGATTTTCTCAGGCCAATCTGTTAGGACAAGGTGGTTTCGGATACGTGCA

TAAAGGTGTTTTGAATGATGGAAGAGTGGTTGCCATCAAGAGTTTGAAAGCAGGAAGTGGACAAGGAG

AACGAGAATTTCAAGCTGAGGTTGAGATCATTAGCAGAGTTCATCATCGTCATCTTGTGTCCCTCGTTGG

ATATTGCATTGCTAATGGACAACGCATGTTGGTCTATGAATATGTTGACAACAAAACCTTGGAGTTTCAC

CTTCATGGTACATTCTTTCCTATTCAATACTCACTACTTCTTCTACGATCATGTTAATTTTATTGTGAAATAA

GAGAATCCTTTTCCTAGTTGATTCCCAAATTTCGATTCAATATATTTCTTAATTCTAGTTTTGATAAGGGTG

AAATCAAATTCATTATTGCAGGAAAAGGTCAACCTGTCATGGATTGGGAGACCAGACTTAAAATTGCTTT

GGGATCAGCTAAAGGACTTGCTTATCTCCATGAAGATTGTATGAACATTCTAATCTACACTTTCACCTCTC
TCTGTCTCTCTTTATCTCTCTCTCTCATTTCTTGATATATACATGCAGGCCAATATAGAATTATCCACCGCG
ACATCAAGGCTGCAAATATTCTACTAGACCTCAATTATGAAGCCTTGGTAATTAAATGATCAGCCACATT
TGATATGTCAATGAAGATATGAAGAGCAAGTTTTATGTTAAAACCTCGCGTTTTTGTTTAAGAAAGGTTT
ATTAACCATCACAATGCCTTTTAGATTTAAAGGAAAATTTTGGTTTATTAGGTGGCAGATTTTGGATTGG
CTAAGCTTACTTCGGATAACAACACTCATGTATCAACTCGTGTTATGGGAACATTCGGGTGAGTCAAGCA
TAGCTACTTAGTCGTATAAAACTTGTATTCAACAATCAGAACCAATTATGTATTTTAACATACTTCAGATG
TCTCTAAGCTAGAAAGTTACTTGCAGGTACTTAGCTCCTGAATATGCATCAAGTGGCAAACTAACAGAGA
AGTCAGATGTGTTTTCATATGGAGTAATGCTGTTGGAGCTCATAACCGCAAAAAAACCTGTTGATCCTAG
CAATATGATGGAGGACAGTTTAGTAGATTGGGTTTGTTCTAGATCCTCAGTTCACATTTGATTGCGCCAA
GATGTGCTTAGACTTTTAACTTTTGTTTGAGGTATTGATCTAATATATGAATGTATACAGGCTAGGCCCCT
TCTTACAAGAGCACTGGAAGAGGAAAAATATGATGGATTGGTAGACCCACGTCTTGAAGGGAACTTTG
ATACAGATGAGCTACATCGCATGATAGGTTGTGCTGCTTCTAGCATTCGCCACTCTGCTAAAAGACGTCC
CAAAATGAGTCAGGTATGATTACCCTCTCTAGAAGATAACAAATACTGCATATTTACATTATGATTTAACA
TGTTGTAGCATATTTTGGTGGTTTGCTTATATGGACTCGAGAAATTCATATCTTTACATGATATCAGAGTA
AGGTTCATCTCCGTTTGGACAAAATGACTTGTTTCACTTATATAAATATTATGTATTATGAACAGATTGTA
CGTGCATTGGAAGGCAATTCATCGTTGGAGGACTTGAACGATAATCCAAAACCAAGCAAGGTTCCTAGC
TTTGCCGCGTCAAATGGGGCACCCACTCAGAGCTATGATACTGGTATGTATAATGCTGACATGATGAAG
TTCAGGAAAATGATTATGCCAACCCAAGAATTCAGCAGCAGTCCAAGTGAGTAATGAATCAACCCTTCTT
CCTCCTCCTCAAGCAACGATTCAACACGCGGGCAAGAGTAAAAAAAGAATGTACCTAAAGCTTGAGATA
TTTTTTCAAAACATATTCTTGGCTGGAACTTATAACAGAGATTTGGGATTACAAAAATTAATGTTTATGAT
GAACATTCACTACTCATATCTTGATTTGAATAACAAAGATTATAGGATCAGAATACAATTATACTTTTTTT
GTTTCAATATGTACATACAATTTATTCCTACAAAAATGTTGAAGATCTTTATGTTTAAC
>PGSC0003DMG400017034 G-LecRK
GCTAACCACAAAACCACTACACTTCAATGTAATCCTTCTGTCTCTTAATTAATAGTACCGTTTATGTACTAT
TTGATGAATGTAAAAACATTTAAAAAGACTCACTTGATAGGTAAGTCACTATTTTAAGGAGTGGGGGGG
ACACTACTAATGTTGTAACATTATAATCATGCCAAACTTTTTATTTTCTTTCATCTTGTCTATACATTCCTAA
CAACAACAAAGTCCTGGAGCAAAAGCCTATTGATTCACCAGTTTGCTAACCTACTTTGTCGGCTATGTGC
ACTCTGTAACCAGAATAGAGCTCTGGACTTAAAACCACCCACTTTTTGAAGTTTGGAAGGCGGCGCCTAA

ACTAGTGAGTAACATTCCACACATGAAAATTTATGAGCTTTTTTATCTCATTTGCTGGTTGGCTCATGGAT
ACTGCACCTCTGATATTCACATTGGTTATCAGGTAAGTCTTGCTATACCAACATCATATTGCAAAGGATTC
ATAGGCAGGGCTTTTCTAATGGAAACTGATCACAACCAAATTGGACCAAATTTTAGAGGTGGAATCAGT
GTTGAAGCCAATAATGATAAATACACATGTTCACTTGATGTTTTTCTGGGAGATGTCAAGGTTTGGAGTT
CTGGTCATTTGTCACAATTCTACACAATAGACAAATGTACACTTGAGCTAACTCAGTATGGAGACTTATT
GTTGAAAGGGGGTCTAGATGACAGAATTGGATGGAAAGCTGGAACTTCAGGACAAGGTGTCAAGGTA
ATTAATTGTCAATCTTTAGATATAAGTTGTATATGCAAACAAAAGGGAAATAACATTTCTTAGTATATGTT
TTTTGAGGTAAAACTATAAAAAGAACAAATTATTATGTGCAACAATGCTAATATATGACTTCTTCTGCAG
AGACTGAACTTACTTGGGACAGGTAATTTGGTGTTAGTTGATGCTATGAACTTGATTAAATGGCAAAGTT
TCAATTTTCCAACTAATATTATGCTTTGGGGTCAGAGACTAAGCTCAAGAACTCGGTTAACGTCTTTCCCT
AGCAACTCTAGTTTGTCATATTCTTTTGAGATTCAGTATGACAAGATTGCATTGTACTTATACTCTGGGAA
ATCGAAGTACTCGTATTGGGAATATACACCTTTGGAATTAGATGGCCTGAATATCACATATGTTGAGTTA
ACTTCTAACGCGCTGGAGATATTCAACAACGAAAATCGTAGAATTGGAAGGATAACATCAGACAAGCCA
GAGCCCTTGAGATTTTTAGCATTGGGAAATAGCACAGGGAATTTGGGATTCTACTATTACTCATCTGACA
AAGGAAAGTTTGAAGCTTCATATCAAGCACTAAACACTAGCTGTGATCTTCCGTTGGCATGTAAACCTTA
CGGTATATGTACATTTTCAGAGAAGTGTTCTTGCATAAGACTAATAAAAAGAGGAGATGGATTGCTTTCT
GATTGCACTGAAAATGTAAGAATTTGTGGAAGAAATGATTCCCAAATGCTAGAATTACAAGGCGTTACG
AGTGTATTAAGAAGCAATCCTTATAAGATCAATGTGACCAAAGAAATATGTGCAAATTTGTGCTTGGATA
ACTGTACATGTGTTGCAGCATTACATTTTTCTTATGAGAATGATCCCGCGAAACCTGGAGAATGCTATGT
GTATGGACTAGCTAGAGGAGTTAAACAAATCGAAAGAGATGGAAGATTGAGTTATATGGTGAAGGTAC
CAAAGGGAACTGATCAAGGTCATGATAAGCACTCTCATTGGAAGAAATGGATTCCAATTGTTGTAGGAG
TGGTTGATGGAGTTGTATTATTACTTGTTTTAGGAGGAGTTGGATACTATGTAATCCGAAAGAGAAGAA
AGTCGTGCGTAGATACTGGTCACAGCAATTGATATTGTATAGATAAAATTTTGATTTCAAGATAACATTT
TCTACATCGAGATAGCAATTAACCGATTCTCATGAAAAATCAAGGATGAAGCTCGAGTGACAGATACTA
AACTTGTAGCCAAAAACTTAAATTTTTGAACTTGGCTTGGCTTGATGAATTGCCTGAGATTGAGTAGAAT
TCGAAGAAATAGAAGTGTCAAAGGTATGCAAGCAATCTTTTCAGGGTTAAAATGTTGTTACTTCACAAG
AGATTTTTCGATTCCTAAACTGACATGTTGAATTTGTTGATGTCTAAGGTTAATTAGAGAGAAACACTCTA
CGATATTCTATACTTCAAAAATTGACTTACAAAATGCGCGGCCAAATGTCC
>PGSC0003DMG400017106 G-LecRK

CGAAGTATTAAAAATCACAACAAACACATTATACTTACATCCCTATATAATCTCCTTTAACAATGGCTCCT

AATTATGTGATGATAGTTGCTCTGATAACATTTATTTGCTTTTCACTATCTTTTGGTGATGATGCTATGTTA

CCAACGTCACACAACAATCTCATAGACAAACCTAAGTACTGCAATAAAAGTTGTGGAAATGTCAACATCC

CTTTCCCATTTGGTATGACCCCAGATTGCGCTCTACGCAATCAGTTCCTTGTCACTTGTAATTACTCCTTTC

ATCCTCCCCAGCCTTTTTTAGGAAACAGCAGTATTGAAATCACAAGCATATCTCTCTCTGGGCAACTTAAG

GTCTTACATTTCATATCCAGAGATTGTTATCGTGATGGGATAAGCACGAATAGAACCAAGCCATATATAG

TATTACCATCGTTATTAACTGTGGACAACACTGCGAATAGCTTCATCGCCGTTGGCTGTGACACGTATGC

AATTGTCCAGGGTTATTATACCAAGTTTGGAACTGAAATCGAGTATCTCCGCTACATAACAGGATGCATG

TCCATGTGCTATAGTATTGATGAAGCTGATGATGATACATGTTCTGGGGTCGGTTGTTGCAAGACATCCA

TCCCTAAGGGAACATCGAATGTCAATATAACACTAAGCAGCTATAATAATTATAATGATACTAATATGAC

AAATGACCCTAGCTGCAGCTATGCTTTTGTGGTCGATCAAGCTAATTTTAACTTTTCTAAGAGTTACCTCA

GCAGTTTACAGAACACGAAGAAGCTTCCTCTTGTGCTAGATTGGGCAGTTGGTGAAGACAAATGTGAAA

TAGCTAAGAGAAACTCGACTGCATATGCATGTAAGAGTAACAACAGCTATTGCCATGATGATTCAAATG

GTTATCGATGTTCCTGCATGCAAGGATATGATGGGAATCCATACCTGAGTTGTCAAGGTATGTATATGTT

TGAGAGTTTCCTAATATGATTTTAATTATATCAAATCTATCATTTTGGAAATGAATGTTTACGTTATCATAC

GTTTGTACTCTACGAGTCTGCAGATATCGACGAATGTATAGATCGGAAACCAGATTATAGATGTGCCAA

GGATGCCATTTGCCAGAATACAGAAGGGAGTTATACATGCATATGCCCTCCAGATTTCAGTGGTAATGG

TACAGTATGCAATCGTGACAATCCTCAGAGAATAACTCAAAATATTTATTTTGTTATAGGTACCTCTCTTA

ACTTCTTTCAAATCGATAACAATTTTATTTTCACGAACTATATACATCGACCTAAAATGTCTAATAAAGTTT

AAATATTTTTATACAGGTGCTACAGCAGGGGTTGCTTTTGCGATTATTATTGTTTTTGGCTGGTCATACAC

TGCATTCCAAAGGAGAAAGATGTCAAAGATGAAGAAGAAGTTTTTCCAGGAAAATGGTGGATTAGTTCT

ACAACAGCGACTTACAAGGGAAGAGGGATCAAGTCATAATAATACAATAAAGATTTTCACAGCGGAAG

AGCTAGAAAAGGCAACTAATGGCTTTGACAAAGATAGAGTTGTTGGTCACGGAGGTTTTGGAACTGTGT

ACAAAGGATATCTGAAAGATAATTGCATCGTTGCCATAAAAAATCTAAGGTAATTGATCGCAATCAGATC

GAACAATTCATAAATGAAGTCCTTGTCCTTTCCCAAATCAACCATAGAAATGTGGTTAAGCTCTTAGGTT

GTTGCCTCGAGACGGAAGTTCCACTATTGGTGTATGAATTTATCAGCAATGGAACACTTTCTGAACACTT

ACATGATAAATTTAAGGCGTCAACTCTTTCTTTGGATATTCGTCTGAAAGTTGCTGCAGAAACTGCTGGA

GTACTTTCCTACTTGCACTCTGCAGCTTACCCTCCTATCATCCATAGAGATATAAAATCAGTCAACATTCTT

TTAGACAAAAACTACACAGCCAAAGTGTCTGATTTTGGAGCATCCAGGTTGGTTCCTGCTGATCAAACAG

AGTTATCTACATTGGTTCAAGGAACTCTAGGATATCTAGATCCTGAATACTTGCAAACAAATCAACTTAA

TGAAAAAAGTGATGTATACAGTTTCGGAGTAGTGCTTATAGAGCTCCTAACCGGTAGAAAGGCTTTATG

TTTTGAAAGACCAGAAGAAGAGAGATGTCTAGCTCAATATTTCATATCTTCAGTGGAAAAGGGTCATTT

GTTCGATATTCTAGACAGCAACATAGTCTGTGATGAGGTAAATGCAGGGCAATTGAAAAATGTTGCTGT

GCTTGCCCAAAGGTGCTTAATTCTTAAAGGGGATGATCGACCTACTATGAAGGAAGTTGCAGCAGAATT

GGAAGCTGGATCAAAATTGAAGCACTCGTGGGCTCAAACTGATCAGCAGTCCGAGGAGATTAAGTCCTT

ACTGCCTGGATTTGGATATGAATATTGTGAACACAGTATCCATATTGATAGTGTAACGAGCCATGTAACT

TTACCATTTCCTGGAGGAAGGTGATCAATCATGCATGTTAATTTTGCTCAGAAGCACGAGCTCAAAACGG

CCACGGCTTCAACATGAATTTCTTTTTTCTCTTGGGCATGTTAGAAATATGAACTAAGTTTGATAAGTTGC

ATCTCTTTTATTTGTCCAGACATAATAAATTTGAGTAACAAATTAATGTCAAGTGTAGTTATTTATTATGTA

TCCGAGTAAAAATCAACACATTTTGAGAGAATCTATGTATTATTTTATGC

>PGSC0003DMG400017277 G-LecRK

ATGATTGCAGAGCAGAGAGTGTATCTTCCTATCCTTCTCTTCATTTGTCTCCACTCCTTTTCATCCCGGTTT

GCTTTCTCGCAACTTTTCTGGAACTATAATGTTAGTGTTTTGAATTCTACTACTGGACTTTCCAATTCTTGG

ATCAATTTGCCATATGTTAGAGTTTACAATACCTCCGAGCCTTCTGAAATGACGCCTATTATTCGGAGCA

GAAAGGATAGCCCTCAGTTTCTCTTTGCCTTCTATTGCCCCCTTCTAACTAACGGTACCACAGAATGCCTT

CTTGGTATCCTTTTGTATCACAACAGATTCAACGGGGTTTCAAGTGTTCCCCAGTTAGTTTGGTCTGCTAA

CAGGAACCATCCAGTGAAAGTCAATGCAACGTTGCAACTAGGCCAAAATGGCAACTTGGTCTTGACAGA

CTCTGATGGCACTCTTGTTTGGTCCACTAATACTACTGGAAAATCTGTTTCTGGCTTAAACATGACAGAAA

TGGGAAATCTTGTGCTCTTTGATAAGAGAAAACAAGCAATTTGGCAGTCTTTTGATCATCCAACAGATTG

TTTGCTTCCGGGGCAGAAGTTGGTTTCTGGTAGGAAGCTGATAGCAAGCATTTCAGGATCCAATCAGTC

CTTTTCTTTTACTGTTCTTAATGGACGCCTGGTGTCTTCCATAGATACCAACCCACCTCAGTATTATATCGC

TTCAAGTGGGGATTATAGTCCTCTTTACGATTTTGACGGTCGAACCTTTACTGCTCTACAAGACCCTGATA

CATCCCAATTCATAAAGCTTGGGCCTGATGGACATTTAAGGTTATACCAGTCGGATGCATTTGATTGGAA

ACAGTTAGATGAGGTCATGAATTCATATCTGGGGAACTGTGGGTACCCAATGGCGTGTGGAAGATATA

GCATTTGTACAAATGATGGGCAATGTAATTGTCCGGTAGAAGGAAACTTCTTCAGGCCAATAAATAGGA

ACCCGGATCTCGGGTGTTCACAGCTAACATCTATTTCTTGCAACTCTTCACATTATCATAGTCTTATAGAG

CTCAGCGACACCACATATTTTGCATTTGAGATCAACTTTTATGCAAGTTCAAACATGTGGTTTGAGGGAA

CAAAGATGGAAAATTGCAAAGCGGCCTGTTTGAGTAACTGTTCCTGCAAAGCTGCTGTTTGGTCTAAAA

CTCTAAGGAAAAACTGCTTATTACTGAATGAAGTATTATCTCTTAAGGACAACTGGTCTGGTAGTGACAA
GACAAAAGTTTTTCTCAAGGTGCAGAATTTCGCAAAGTCTCAGTATCAGCCGCCAATCGTTTCTCAAAGA
AAGCAATCAAGACCTCTGAAAGTGATTGTAGCATCTGCTCTTGCTGCTTTAGTGGGAATAATTTTAAGTA
TTAGTGCATGGTTTGTTCTTTTCAAAAAGAGGACACTGTCTGTCAAGGCTGGGGATTTTCTGGATTTAGC
ACCAATCTTACCGGGAATCCTAACTCGATTCTCTTACAATGAGTTGAAAATAATTACACAAGATTTCAGCA
GAAAGCTCGGGGAAGGAGGATTTGGCTCAGTATATGAAGGAACACTGAGTAATGGCAACAAAATAGCT
GTGAAGCGTCTGAATGGTTTAGGTCAAGTAAAGGATTCATTCTTAACAGAGGTAAAGATAGTTGGTAGC
ATTCACCATGTCAATTTGGTAAAACTCATTGGCTTTTGTGCTGAAAAAGATCACTGGGTTCTGATCTATGA
GTACATGGTAAATGGATCATTGGATAGGTGGATTTCTCATGAAAAGCAAGAAAATGGGCTTACATGGCT
TACGAGGCAAAGGATAATATCAGATATTGCAAAAGGATTAGCGTATCTTCATGATGAATGCAATCATAA
GATAATTCATTTGGACATCAAACCACACAACATCCTCTTAGATCAAAATTTCAATGCTAAGATATCTGATT
TTGGGTTGTCGAAACTAATTGAGAAAGACAAAAGCAAAGTTGTTACTAGAATGAGAGGAACACCAGGG
TATTTAGCTCCTGAATGGTTGAGGTCTGTAATAACCGAGAAAGTAGATGTATATGCCTTTGGAATTGTGC
TCTTGGAAGTTCTTTGTGGGCGAAAGAATTTGGATTGGTCGCAGGCTGACAAAGATAATGTCCATTTGCT
TAGTGTCTTTAAGAGAAAAGCAGAACAAGATCAGCTCATTGATATGGTTGACAAAAACAACGAGGATAT
GCAGATCCACAGGGAAGCTGTGATGAAAATGATGAGCCTCGCAGCATGGTGTGTGCAAGGTGATTTCA
ACAAGAGGCCTTCCATGTCATTGGTGGTTAAGGTACTGGAAGGTTTGGTGTCTGTTGAAACCAACTTGG
ATTTCAATTTCACAAACCTAACTGAGGCTGGGGCAGGCAACCAACAGATGGAAGTCCCTATCAGTTCAA
TACTGCCTTCAGTTTTATCTGGACCAAGGTAAACAATATGCTTGAGAATTATGACAATATTTCCAATTCTA
GTGTAACTTCATAAATGTAATATTCAGTAACTTTGCATTAGTT
>PGSC0003DMG400017278 G-LecRK
AATTTATTCAAACTAAATAACTTATTTTGCTAAAAGTTACTGCAGTAACTAAAAGCAAACTTCAACAAAGC
ATTTCAGCAGACAATTCGAGTCAAGGTTTATTTTCTCTTACTGTTCTCAATGGAAGCTGGGCCACATACAT
AGATTCTGATCCACCTCAGTATTACTACACTTCAGAAAATCCCAATAGTCCTTATTGGAGTTTTGATGGTC
ATACCCTTACTGCTTTACAATATCCTCCTACATCGATAGCTCAATACATACAGCTTGGGCATGATGGACAT
TTACGCGTATACCAATTGGACAACCTTGAACAGAATTTGGTTTCTGGTCAGAAGCTGACAGCAAGCGTTT
CAGCAGACAATTCGAGTCAAGGTTTATTTTCTCTTACTGTTCTCAATGGAAGCTGGGCCACATACATAGA
TTCTGATCCACCTCAGTATTACTACACTTCAGAAAATCCCAATAGTCCTTATTGGAGTTTTGATGGTCATA
CCCTTACTGCTTTACAATATCCTCCTACATCGATAGCTCAATACATACAGCTTGGGCATGATGGACATTTA

CGCGTATACCAATTGGACAACCTTGAAAAAAATATGCTCGGCCTTAATTGGAAAGAGGTAGGTGACATT
TTGACACTCTCTGACTGTAGGTACCCAATGGTGTGTGGGAGATACAGCATTTGTGTAGATGGAGGACAA
TGTACTTGTCCAGTTGAAGTTAACTTCTTCAAGCCATTTTCTGAGAGGATGCCTAATCTCGGATGCACTGA
GCTGACGCCCATTTCTTGTGACTCTTTGCAGTATCATAGTCTTGTAGAGCTAAAGAATACTGACTATTTTT
CATTTCGGTTCAATGATGAACTAAACTCGAGCGTATTTTGGCTTAAAGGGAAAAAGATTGAAGATTGCC
AAAGGGCATGTCTGAGTAACTGTTCTTGCAAAGCTGCTGTTTATGATCCGTATCTGAATGTGAGTCTACT
ACGAGGGAACTGTTTGTTACTGAATGAAGTCTTCTCTCTCATAGACAGTGCAGTAGGAAGCGGCACGAC
AGTTTTTCTTAAGGTGCAGAATTCCTCAAATGCACCAGTCATTTCCCCTAGACAGAAATTGCCTTTCAAAG
TTATCATAGGATGTACTTTGATTGGGATAATTTTGATTCTTTCAGTTTGTTCTGCTCTTTCCAAAAAGAGA
TCGGAGAAACGCAAAGCTGGTGATTTTCTGGATCTAGAACCAATCTTACCGGGAATCCTAATTCGATTCT
CTTACAATGAGCTGAAAATAATTACACAAGATTTCAGCATAAAGCTCGGGAAAGGAGGATTTGGCTCTG
TATATGAAGGAACACTGAGAAATGGCACCAAAATAGCTGTGAAGCATCTGGATGGAGTAGGTCAAGTA
AAGGAATCATTCTTAACAGAAGTAAAGACAGTCGGTGGCATTCACCATATCAATCTGGTAAAACTCCTTG
GATATTGTGCCGAAAAAAGCCACAGGCTTCTAATCTATGAGTACATGGTGAATGGATCGTTGGATAGGT
GGATCACGCAAGAAAATGGGCTTACATGGAACACAAGGCAGAGGATAATATCAGATATTGCGAAAGGA
TTAGCTTATCTACATGAGGATTGCAGCCATAAGATAATTCATTTGGACATTAAACCACAAAACATCCTTAT
AGATCAAAATTTCAATGCTAAGATATCTGATTTTGGTCTGTCGAAGCTAATTGAGAAAGACAAAAGCAA
AGTTGTGACTAGAATGAGAGGAACGCCGGGGTATATAGCCCCTGAATGGTTGAGCTCAATAATCACTGA
AAAAGTTGATGTGTATGCTTTTGGAATTGTGCTCTTGGAAACTCTCTGTGGACGAAAGAATTTGGACTGG
TCCCAAACTGATGAAGAAGATGTCCATTTGCTAACTGTTTTTACGAGAAAAGCAGAACAAGAGCAGCTC
ACAGATATGGTTGACAGAAACGAGGATATGCAGCTCCACAGAGAAGCAGTGACAGAAATGATGAGCCT
TGCTGCGTGGTGTCTACAGGGCGATTTCTCCAAGAGGCCTTCCATGTCATTGGTGGTTAAGGCACTCGA
AGGTTTGGTGACTGTTGAAACCAACTTAGATTATAATTTCAGAAATGTACCTAAGGTTGGGGCAGGCAA
CCAACCGAGGGAAGCCGCTATCAGCTCAAAATTGCCATCAGTTTTATCGGGACCGAGGTAAACAGTATG
TCTAATTCTAGCACAAATTCTTACGGTGTATTAGATTACAATTCATGAATGTAATATTCAGTAACTTTGCA
TTAGATTTTTTGAGTTTCATGTAATTATCATGTAGAAGCTCCTCAATTCATATGTGACAGGAGATATAATA
TTGCATTAGCTTTTCCTCATTTTTTGTAAGGGAAGTATCAAGACTCGAGAACAGCATGAATCGGATATCG
AGGGCCAGATGCTCTGCC

>PGSC0003DMG400017282 G-LecRK

GAAAAAACTACTTGAATAAAAGGATGAATTTTAAGATTGATACTGATCAATCAAACAGAGCCTTCTTTCT
GAAAAAAATAGTACAATGGTGCTTAATTATAGTGCCATTCTTTTCATGGCCAGTCTCCTCTTCATTTCTTC
ACAGCATCTAATTAGTTGTCAACCTCCTGAAACATTTATCACCAATTCCTCCATTTCCTGGATCAATAGCC
GCGCCCGCTCCTATGTAGTGAATTCCACAGATGGAGCCACACTGACACCCATCCTATCGAGAGAAAGGA
ACGAGACTCTGTTTGTCTGTGGTTTCTTATGTGATTACAATGGTACTGCTTGCGTTTTTGGCGTCCTTTTAT
CCCCAAAGATCAGTAACAACAATTCTTATCTATATTACGCGAGATTAGTTTGGTCAGCAAATCGGAATAA
TCCAGTTAGAGCTAATGCAACCTTGCAACTTAGGCAAGATGGAGGTTTATTCTTGATGGACTCAGATGG
CACTTTGATTTGGAGTACAACCACAAGCACAAGAGGAAAGGTTGTTACGGGATTCAAATTGACAGTACT
GGGAAATATTGTTCTCTTTGACAAAAGTAACGACATCATTTGGCAGTCTTTTGATCATCCTACAGACACTT
TGGTTCCTGGACAAATGACAAATGCTGGACAAATGCTGAGATCCAGTGTATCAGCAACAAATTGGAGTG
AAGGTTTGTTCACATTTGAAGTTCAACTATTTGGCTTTTTAGCATACATTAAGTCAAATCCACGTCAACTC
TATGTCTCAAGACTTGGTGGGGATAATGGTTCCCCGTTTCCTGGACTAATCTTTGATTCGAAAATAATTAT
TTTCAACGGGACATTAATCTTAAATTTTGCATTTTCAAATACTAGATTCATGAGGTTTGGGAATGATGGG
CATTTAAGGGTGTATCACTGGAGAGGATCTTGGTTAGAGGAGGCTGATCTATTGACAGACTACATTGGT
GAGTGTGGGTATCCAACCGTGTGTGGGAATTACTCTGTTTGTGCAAATGGGCAGTGCACTTGTCCTCAA
ACCGCGGTTGATCAGACTAACTTTCTCCAGCAAATAAATTACAGGCAGCCAAACCAGGGGTGTGTCCTT
ATAACACCTATCTCTTGTGAACATTCCCAATATCATATTTTGATGGAGCTTAAGTACATAGCTTATATTCCC
CTCTATTTGCAATATGGTACCAACAAAACAGATTTGGAGAATTGCAAAACGTCTTGCTTAAGCAATTGTT
CTTGCAAAGCAGCTCAATTTCAATTTAGTGGTCCTAATAACACAATGGGTCACTGCGCATTGTTGAATGA
TGTGTTTTCGCTCACATACGATTATGGAGCACCCAATAAGACCACTCTTTTCATCAAGGTGCAGAGCTCTT
TTAACTTACAAGCCTCTCCATCACTTGTCTCTTCAGAGAAGAAATCAAAGCGTGTCTCAGCAATTGTAGG
ATCCACCATCGGAGCTTCATTTGGTTTGCTTCTCATGGTTTTAACTTGCTTTGCCTATATTTTCAGAAGGA
GAAAAGGGCTCGAGGAAGATGAGGAGGAGTTCCTTGATCAAATACCAGGAATGCCTACTAGATTTTCCT
ACGAGGAACTAACTGTGATGACAGAAAATTTCAGTGAGAAACTTGGGGAAGGGGGATTTGGTTCTGTA
TTTGAAGGAACAATGAGTGATGGCACCAAAATAGCGGTGAAGCGTCTGCAAGGTTTTGATAATGTGAA
GAAATCATTCTTAGCTGAGGTAGCAACAATCGGTAGCATTCAGCATGTCAATTTGGTAAAACTTGTTGGA
TTTTGTGCTGAGAAATCACACAGACTTCTGGTTTATGAATATATGGCCAATGGCTCTTTAGATAGGTGGA
TTTTTCACGGAAAGCAGGAACGATCTCTCACATGGGATATCAGGAAAAAGATCATATCAGACGTCGCCA
AGGGCCTAGCTTACCTACATGAAGACTGTAACAACAAAATTATTCATTTGGATATTAAACCTCAGAACAT

TCTTCTTGATCATAATCTCAATGCAAAAGTATCAGATTTTGGGTTATCAAAGCTTGTGGGGAAAGATGAA
AGTAAGATAGTTACAACAATGAGAGGAACACCAGGCTATTTAGCACCTGAATGGTTACATGAGGTCATT
ACTGAGAAAGTGGATGTTTATAGCTTTGGGGTTGTGATTTTGGAAATCATCTGCGGCCGGAAGAATTTG
GATCGTCATCAAGATGAGGATGATATGCATTTGCTTAGTTTGTTCATGAGAAAAGCAGAGGAGAGACAA
CTCTTGGAAATGGTGGACAAGAACAGTGAAGACATGCAGCTACATAGAAAAGAGGCTGTGGAAATGAT
GAAGATTGCAGCCTGGTGTTTACAAAGTGATTATACTAAGAGGCCTTCCATGTCGTTGGTAGTTAAGGT
GTTTCAGGGTTTAGTGGCTGCTGAAACCGACTTGCATTACAGCTTCACATTTCCAACAATGACGAGAAGA
GTTGCCGGAACTAATCAAGAAAGGGAATCTGTGGTTGGTATCAGTTTGCCACTTCCATCACAGTTGTCAG
GACCTAGGTAAATTCTTAGTAGTATTTATCATCAAGACTGAATAGCTAATACGAAAAGCCATTTTGTGTTT
TTCATAATTCCTTATTATATCAATACTATTTCAGAAACATAATTTAGAAAAGTAGAACACACTGTCTACTA
GAAAAATGTTAGTTTTCGTGCAGTCGAATTAACTTATTGAGTATTGATAAATGGATTGCCTCCTCCGTATT
GATGAATTAAATTTGATTGCGC
>PGSC0003DMG400017284 G-LecRK
AAAAAAGGCACAACAGAAAGAACTCATATATTTTAGAAAAATATGTTTGTAGAAAAGAGACTGTATTTT
CCTATCCTTCTCTTCATTTACCTTCTCTTTTGTTCGTCTCGATTTACTATATCACAATCTTTACAGGACAACA
AGATCAGTCTTTCAAATTCTACAGCAGGGCTTGCCGGGCCTCTGTTGTTCGTCTCGATTTGCTATATCGCA
ACCTTTACAGGACTACAACATCACTCTTTCGAATTCTACTGCAGGGCTTGCCGGGCTTTCCTCTTTCTGGA
TCAACAGGCCCTCTCTTCTTGTTAATTCCACCGAGGACTTCTTTGGTTTCGTGACACCCATACTTCAGCGA
AAAAATGCTGGCTCACGATTCCTCTGTGGCTTCTACTGCAATTACAATGCCACAGAATGCCTTCTTGGTAT
CCTTTTGTACCACAACGAGAACAATATGATAGATAAACCCCAGTTAGTTTGGTCTGCTAACAGGAACCAT
CCAGTGAAATTCAATGCAACCTTGCAACTAGGCCATGATGGCAACTTGGTCTTGACAGACTCCGATGGC
ACTCTTGTTTGGTCCACTGATACAATTGGGAAATCTGTTTCTGGCTTAAACTTGACAGAAATGGGAAATC
TTGTTCTCTTTGATAAGAGAAAGCGCACAATTTGGCAGTCTTTTGATCATCCTACAGACTCTTTGCTTCCA
GGGCAGAATTTGGTTTCCGGGCAGAAGCTCATAGCAAGCGTTTCAGCAACCAATCGGAGTCAAGGTTTG
TTTTCTCTTACTGTTCTCAATGGAAGCTGGGCTGCTTACACAGATACTGTTCCGCCTCAATACTACTACATT
TCATCCTATGCTGATAGTTCTTATTACAGTTTTGATGGTCAAACCTTTACTGCTTCACACAATCCTACTAAC
TCGACAGCTCAATTCATGAAGATTGGGCCTGATGGACATTTAAAGGTATACCAATGGTCTGTAGTTGATT
GGAAAGAAGTATCTGACATTTTGTTGCCAAATGTAGAAAACTGTGGGTACCCAATGGTATGTGGAAGTT
ATAGCATTTGTACAAATAACGGGCAATGTACTTGTCCGCAACAGGAAAACTTCTTCAGACCATTTTCTGA

GAGGAAACCAGATCTTGGATGTTCACAGCTGACTTCCATTTACTGCAACTCTTTGCAGTATCATAGTTTCA

TAGAGCTCAAGAATACTACATATTTTTCATTTAACATTGATCAGGAACTAAATTCGAGCAAATTGTGGCTT

GGGCAGAAAAAGTTGGAAGATTGCAAAAGGGCCTGTCTGAGTAACTGTTCTTGCAAAGCTGCTGTTTTT

GAATATAATTGGAATGGGGCTCGAAGAGGGAACTGTTTGTTACTGAATGAAGTTTTCTCTCTCACAGAC

AGCGAAGAAGGAAGAGGCAAGACAGTATTTATTAAGGTGCAAAATACCTCAGAGGCGCAGACTCGGTC

TCTAATCATTCCTGGAGGAAAGAAATCAAGACCTTTCAAAGTGATAATAGGATCTACTCTTGCAGCTTTC

TTCGGGATAATTTTAAGCATAGCTACTTGCTTTGTTATTTTCAGAAAGAGGGCACATGAGTCCAGAAAGG

CTGCGGATATTTTGGATTTAGCACCAATCTTACCGGGAATTCTAACTCGATTCTCTTACAATGAACTGAAA

ATAACTACAGATGATTTCAGTAGAAAGCTCGGGGAAGGAGGATTTGGCTGTGTTTATGAAGGAACACT

GAGAAATGGAACTAAAATAGCTGTGAAGAATCTGGATGGTGTAGGTCAAGTAAAGGAATCATTCTTAAC

AGAAGTAAAGGCAGTCGGTGGCATTCACCATATCAATCTTGTAAAACTCATTGGATTTTGTGCCGAAAA

AAGCCACAGGCTTCTAATCTATGAGTACATGGTGAATGGATCGCTGGATAGGTGGATTACGCATGAAAA

TCAAGAAAATGGGCTTACATGGAACACAAGGCAGAGGATAATATCAGACATCGCCAAAGGGTTAGCAT

ATCTACATGAGGATTGCAGCCATAAGATAATTCATTTGGACATCAAACCACAAAACATCCTTCTAGATCA

ATATCTCAATGCTAAGATATCAGATTTTGGGTTGTCGAAGCTAATTGAGAAAGACAAAAGCAAAGTTGT

GACTAGAATGAGAGGAACACGGGGTTATTTAGCTCCTGAATGGTTGAGCTCGGTAATCACTGAGAAAG

TTGATGTATATGCTTTTGGAATTGTCCTCTTGGAAATTCTCTGTGGGCGAAAAAATTTGGATTGGTCCCA

AGCTGATGAAGAAGATGTACATTTGCTAAGTGTCTTTAGGCGAAAAGCGGAACAAGAGAAGCTCATCG

ATATGGTTGACAAAAATGAGGATATGCAGCTCCACAGGGAAGCAGTGACAGAAATGATGAGCCTTGCT

GCATGGTGTCTACAGGGCGATTTTTCCAAGAGGCCTTCCATGTCATTGGTGGTTAAGGCATTGGAAGGT

TTGGTGACTGTTGAAACCAACTTGAATTATGATTTCACACATGTACCTGAGGTTGGGGCAGGCAACCAA

GAGAGGGAAGTCATTATCAGTTCAAAATTTCCTTCAATTTTATCGGGACCAAGGTAAACAATATGCTTAA

GATTTTGTCTTTGAAGGAACCAACATATGGTTGCGTAATGTCAAATATCGCAACATTTTCAATTCTAGCAT

AACTTTTTTACCGTGTATGAGATTATAATTCATGAAATGTAATGTTCAGTAACTTCTATATTAAATTTTTGA

GCTTCATGTTACTATCATGTAGAGGCTTCTCAATCCATGTGCGAAAATAAGTATAATATTGTACGTGGTG

GTGCAGCTACTGAATTCAAACCAGATGAACTGTTCTTGGATCTTAA

>PGSC0003DMG400017286 G-LecRK

AATCAGGCAGATGTTATCTTCCCATTGGTTAGTTGAGAAATGTGATTTGGAAAACATTTCCTTCTGACTTG

ACGCGTAAATGATTCGCAAAATTGGGTATCACTGAACTCCACACATCTCAAAAGTATTCTTAGCCACAAC

ATTATGAACTACTGGAAGATGCTAACCTACATGTAAAATACAGTTTGGATATTCTGTATGCTCATTTCTTT
CTTCAACTATACACCACAAGATTCCATCTATTTTATTGGTTTTGATTAGGAAACAATGTTTGCAGAACAGA
GACTGTGTTTTCCTATCCTTCTCTTCATTTATCTCCTCTTATGTTCATCTCAATTTGTTATATCACAATCTTCT
CGGGAATTCAGCATCAGTCCTTCGAATTCTACTGCAGGGCTTGCCGGGCTTGCCGGGCTTGCCGGGCTT
TCCTCTTTATGGATCAACAGGCCGTCTCTTAGGGTTAATTCCACGGCTGACGGCTTCTTTACTTTGACAAC
ACCCATACTTCAGCGGGGAAATGCTGGCCCACGATTCCTCTGTGGCTTCTACTGCATTTACAATGCTACA
GAATGCCTTCTTGGTATCCTTTTGTACCACAACAAATATGACGAGCCGAATGGTGTGATAGATAAACCCC
AGTTAATTTGGTCTGCTAACAGGAACCATCCAGTGAAAGTCAATGCAACCTTGGAACTAGGACAAGATG
GCAACTTGGTCTTGACAGACTCTGATGGCACTCTTGTTTGGTCCACTCATACAACTGGCAAATTTGTTTCA
GGCGTAAACTTAACAGATATGGGAAATCTTGTGCTCTTTGATAAGAGAAAGCGCGCAATTTGGCAGTCT
TTTGATCATCCTACAGACTCTTTGCTTCCAGGGCAGAATTTGGTTTCTGGGCAGAAGCTCATAGCAAGCG
TTTCAGCAACCAATCGGAGTCAAGGTTTGCTTGCTCTTACTGTTCTCAATGGAAGCTGGGCCGCTTACAC
GGATACTGATCCGCCTCAATATTACTACATCTCTTACTATCTTGAGAGCCCTTATTACAGTTTTGATGGTC
GGACCTTTAATGCTTTACAATCTCTTGATAAATCAGCAGCTCAATTCATGAAGATAGGGCCTGATGGACA
TTTAAAGGTATACCGATGGTTTATAGCTGATTGGAACGAAGTATCTGACATTTTCTCGCAATATGTAGAA
AACTGTGGGTACCCAATGGTATGTGGAAGTTACAGCATTTGTACAAATAACGGGCAATGTACTTGTCCG
CTACAGGAGAACTTCTTCAGGTCATTTTCTGAGAGGAAACCAGATCTTGGATGTTCACAGCTAACTTCCA
TTTACTGCAACTCTTCGCAGTATCATAGTTTCGTAGAGCTCAAGAATACTACATATTTTTCATTTAACATTG
ATCAGGAACTAAATTCGAGCAGATTATGGCTTGGGCAGGCAAAGTTGGAAGATTGCAAAAGGGCCTGC
CTGAGTAACTGTTCTTGCAAAGCTGCTGTTTTTGAATATGGTTGGAATGGGACTCACAGAGGGAACTGTT
TGTTACTGAATGAAGTTTTCTCTCTCAAAGACAGCGAAGAAGGAAGAGGCAATACAGTATTTCTTAAGG
TGCAGAATTCCTCAAAGCCGCAGACTCAGTCTCTAATCATTCCTGGAGGAAAGAAATCAAGACCTTCCAA
AGTGATAATAGGATCTACTCTTGCAGCTTTCTTTGGGATAATTTTAAGCATAGCTACTTGCTTTGTTATTTT
CAGAAACAGGACCCGTGAGTCCAGAAAGGCTGCGGATATTTTGGATCTAGCACCAATCTTACCGGGAAT
CCTAACTCGATTCTCTTACAATGAGCTGAAAATAATTACAGATGATTTCAGTATAAAGCTCGGGGAAGGA
GGATTTGGCTGTGTTTATGAAGGAACACTGAGAAATGGCACCAAAATAGCTGTGAAGAATCTGGATGG
TGTAGGTCAAGTACAGGAATCATTCTTAACAGAAGTAAAGGCAGTCGGTGGCATTCACCATATCAATCT
GGTAAAACTCATTGGATTTTGTGCCGAAAAAAGCCACAGGCTTCTAATCTATGAGTACATGGTGAATGG
ATCGCTGGATAGGTGGATTACGCATGAAAATCAAGAAAATGGGCTTACATGGAACACAAGGCAGAGGA

TAATATCAGATATCGCCAAAGCGTTAGCATATCTACATGAGGATTGTAGCCATAAGATAATTCATTTGGA
CATCAAACCACAAAACATCCTTCTAGATCAATATCTCAATGCTAAGATATCAGATTTTGGGTTGTCGAAG
CTAATTGAGAAAGACAAAAGCAAAGTTGTGACTAGAATGAGAGGAACACGGGGTTATTTAGCTCCTGA
ATGGTTGAGCTCGGTAATCACTGAGAAAGTTGATGTTTATGCTTTTGGAATTGTCCTTCTGGAAATTCTCT
GTGGGCGAAAGAATTTGGATTGGTCTCAACCTGATGAAGAAGATGTCCATTTGCTGAGTGTATTTAGGA
GAAAAGCGGAACAAGAGCAGCTCATTGATATGGTTGACAAAAATGAGGATATGCAGCTCCACAGGGAA
GCAGTGACAGAAATGATGAGCCTCGCTGCATGGTGTCTACAGGGCGATTTTTCCAAGAGGCCTTCCATG
TCATTGGTGGTTAAGGCGTTGGAAGGTTTGGTGACTGTTGAAACCAACTTGAATTATGATTTCACACATG
TACCTGAGGTTGGAGCAGGCAACCAAGAGAGGGAAGTCATTAGCAGTTCAAAATTTCCTTCAATCTTAT
CGGGACCAAGGTAAACAATATGCTTAAGATTTTGTCATTGAAGGAACCAACATATGGTTGCGTAATATC
AAATATTGCAACATTTCCGATTCTAGCATAACTTTTTATAATTCATGAAATGTAATGTTCAGTAACTTCTAT
ATTGAATTTTTGAGCTTCATGTTACTATCATGTAGAGGCTTCTCAATTCATGTGCGAAA
>PGSC0003DMG400017315 G-LecRK
CAACAATGTTTGCAGAAAAGAGACTGTATTTTCCTATCCTTCTCTTCATTTACCTTCTCTTTTGTTCGTCTC
GATTTACTATATCACAATCTTTACAGGACAACAAGATCAGTCTTTCAAATTCTACAGCAGGGCTTGCCGG
GCTTTCCTCTTTCTGGATAAACAGGCCATCTCTTATTGTTAATTCCACCACCGATGGCTTCTTTGGTTCAAC
AACACCCATACTTCAGCGGGGAAATGCTGGCCCACGATTCATCTGTGGATTCTACTGCAGGTACAATACC
ACAGAATGCCTTCTTGGTATCCTTTTGTACCACAACAAATACAACGAGGAGAACGGTATGATAGATAAAC
CCCAGTTAGTTTGGTCTGCTAACAGGAACCATCCAGTGAAAGTCAATGCAACCTTGCAACTAGGCCATG
ATGGCAACTTGGTCTTGACGGACTCTGATGGCACTCTTGTTTGGTCCACTAATACTACTGGAAAATCTGT
TTCTGGCTTAAACTTAACAGAAATGGGAAATCTTGTGCTCTTTGATAAGAGAAAGCGCGCAATTTGGCA
GTCTTTTGATCATCCTACGGATTCTTTGCTTCCAGGGCAGAATTTGGTTTCCGGACGGAAGCTCATAGCA
AGCGTTTCAGCAACCAATCATAGTCAAGGTTTGCTTGCTCTTAGTGTTCTCAATGGAAGCTGGGCTGCTT
ACACGGATACTGTTCCGCCTCAATACTACTACACTTCATCCTATGCTGATAGTTCTTATTACAGTTTTGATG
GCCAAAGCTTTACTGCTTTACACTATCCTACTAAATCAAAAGCTCAATTCATGAAGATTGGGCCTGATGG
ACATTTAAAGGTATACAAATGGTCTGTAGTTGATTGGAACGAAGTATCTGACATTTTGTCGCCATATGTG
GATAACTGTGGGTACCCAATGGTATGTGGAAGTTATAGCATTTGTACAAATAACGGGCAATGTACTTGT
CCGCCACACGAGAACTTCTTCAGACCATTTTCTGAGAGGAAACCAGATCTTGGATGTTCACAGCTGACTT
CCATTTACTGCAACTCCTCGCATTATCATAGTTTTGTAGAGCTCAAGAATACTACATATTTTGTATTTCAGC

TCAATCTTGAACTAAGTTCGAAGATATTTTGGCTTGAGGGGAAAAAGTTGGAAGATTGCAAAAGGGCTT
GTCTGAGAAACTGTTCTTGCAAAGCTGCTGTTTTTGTATATGATTGGGAAGGGGCTCAAAGAGGGAACT
GTTTGTTACTGAATGAAGTTTTCTCTCTCACAGACAGCGAAGAAGGAAGAGGCGAGACAGTATTTCTTA
AGGTGCAGAATACCTCAAGGGCGCAGACTCAGTCTCTAATTATTCCTGGAGGAAAGAAATCAAGACCTT
TGAAAGTGATAATAGGATCTACTCTTGCAGCTTTCTTTGGGATAATTTTAAGCATAGCTACTTGCTTTGTT
ATTTTCAGAAAAAGGACACATGAGTCCAGAAAGGCTGCGGATATTTTGGATCTAGCACCAATCTTACCG
GGAATCCTAACTCGATTCTCTTACAATGAGCTGAAAATAATTACAGATGATTTCAGTAGAAAGCTCGGG
GAAGGAGGATTTGGCTGTGTTTATGAAGGAAGACTGAGAAATGGCACTAAAGTAGCTGTGAAGCATCT
GGATGGTGTAGGTCAAGTTAAGGAATCATTCTTAACAGAAGTAAAGGCAGTCGGTGGCATTCACCATAT
CAATCTGGTAAAACTCATTGGATTTTGTGCCGAAAAAAGCCACAGACTTCTAATCTACGAGTTCATGGTG
AATGGATCGCTGGATAGGTGGATTACGCATGAAAATCAAGAAAATGGGCTTACATGGAACACAAGGCA
GAGGATAATATCAGATATCGCCAAAGGGTTAGCGTATCTACATGAGGATTGTAGCCATAAGATAATTCA
TTTGGACATCAAACCGCAAAACATCCTTCTAGATCAGTATTTCAATGCTAAGATATCAGATTTTGGGTTGT
CGAAGCTAATTGAGAAAGACAAAAGCAAAGTTGTGACAAGAATGAGAGGAACACGGGGTTATTTAGCC
CCTGAATGGTTAAGCTCAGTAATTACTGAGAAAGTTGATGTGTATGCTTTTGGAATTGTGCTCTTGGAAA
TTCTCTGTGGGCGAAAGAATTTGGATTGGTCCCAAGCTGATGAAGAAGATGTCCATTTGCTAAGTGTCTT
TAGGCGAAAAGCGGAACAAGAGCAGCTCATAGATATGGTTGACAAAAACAATGAAGATATGCAGCTCC
ACAGGAAAGCAGTGACAGAAATGATGAGCCTTGCTGCATGGTGTCTACAGGGCGATTTTTCCAAGAGG
CCTTCCATGTCATTGGTGGTTAAGGCATTGGAAGGTTTGGTGACTGTTGAAACAAACTTGAATTATGATT
TCACACATGTACCAGAGGTTGGGGCAGGCAACCAACAGAGGGAAGTCATTATCAGTTCAAAATTTCCTT
TAATTTTATCGGGACCAAGGTAAACAATATGCTTAAGATTTTGGATTTGAAAGAACCAAATATGGTTGCT
TAATGTCAAATATTGCAACATTTTCAATTCTAGCATAACTTTTTTATGGTGTATGAGATTA
>PGSC0003DMG400017318 G-LecRK
ATAAAATTATGACTTATATTTTCTTTCCTCGTGAAAATTTCTTCAGGCCATCTTCTGAGAGGAAACCAGAT
CTTGGATGTTCACAGCTGACTTCCATTTACTGCCCCTCTTTGCAGTACCATAGTTTCATAGAGCTCAAGGA
TACCGCATATTTTTCATTTCAGTATAATCATGAACCAAGTTCGATCATATTTTGGCTTGAGGAGAAAAAGT
TGGAAGATTGCAAAAGGGCCTGTCTGAGAAACTGTTCATGCAAAGCTGCTGTTTTTGAATATAATTTGTA
TGGGAATCGAAGAGGGAGCTGTTTGTTACTGAATGAAGTCTTCTCTCTCATAGACAACAACGGACAAGG
AAGAGGCAAGACAGTATTTCTTACGGTGCAGAATCAGTCTTCAATCATTCCTGGAAGAAAGAATCAAG

ACCTTTCCAAGTGATTATAGGATCTACTCTTGCAGCTTTCTTCGGGATAATTTTAAGCATAGCTACTTGCT
TTGTTATCTTCAAAAAGAGGATACATGAGTCCAGAAAGGCTGCGGGTTTTCTTGATCTGGAACCAATCTT
ACCAGGAATTCTAACTCGATTCTCTTACAATGAGCTGAAAGTAATTACAAAAGATTTCAGTAGTAAGCTC
GGGGAAGGAGGATTTGGATCTGTTTATGAAGGAACACTGAGAAATGGCACCAAAATAGCTGTGAAGCA
TCTGGATGGTCTAGGTCAAGTAAAGGAATCATTCTTAACAGAAGTTAAGGCAGTCGGTGGCATTCACCA
TATCAATCTGGTAAAACTCATTGGATTTTGTGCAGAAAAAAGCCACAGGTTTCTTATCTATGAGTACATG
GTAAATGGATCATTGGATAGGTGGATTACGCATGAGAATCAAGAGAATGGGCTTACATGGAACACGAG
GCAGAGGATAATATCAGATATCGCCAAAGGCTTAGCGTATCTACATGAGGATTGCAGCCATAAGATAAT
TCCTTTGGACATCAAACCACAAAACATCCCTCTAGATCAATATCTCAATGCTAAGATATCAGATTTTGGGT
TGTCGAAGCTAATTGAGAAAGACAAAAGCAAAGTTGTGACTAGAATGAGAGGAACACGGGGTTATTTA
GCCCCTGAATGGTTGAGCTCAGTAATCATTGAGAAAGTTGATGTTTATGCTTTTGGAATTGTCCTCTTGG
AAATTCTCTGTGTGCGAAAGAATTTGGATTGGACCCAAGCTGATGAAGAAGATGTCCATTTGCTAAGTG
TATTTAGGAGAAAAGCTGAACAAGAGCAGCTCATGGATATGGTTGACAAAAACAACGAAGATATGCAG
CTCCACAGGGAAGCAGTGACAGAAATGATGAGCCTAGCTGCATGGTGTCTACAGGGTGATTTTTCCAAG
AGGCCTTCCATGTCATTGGTGGTTAAGTCATTGGAAGGTTTGGTGACTGTTGAAACCAACTTGGATTATG
ATTTCACACACGTACCTGAGGTCTGGGCAGGAAACCAACAGAGGGAAGCCATTATCAGTTCAAAAATTC
CTTCAATTTTATCGGGACGAAGGTAAACAATATGCTTAAGGTTTTGTCTTTTTAGGAACCAATTTGTGGTT
GCATAATGTCAAATATTGCAACATTTCCAATTCTAGCATAACTTAAGGTTTATGAGATTATAATTCATGAA
CTGTAATGTTCAGTAACTTCTATATTAAATTTTTGAGCTTCA

>PGSC0003DMG400017327 G-LecRK

AGAGACGCGTTCCCAAAAGAAAGGCATTATTAGAACACAGGAGGAGTTGCTAGCAAAGCAGATTTCAA
ACATTTTCTTTTGTTAGCTTTAGTAAGAAATGATTGCAGAGCAGAGATTGTATTTTCATCATGTCCTGGTC
ATCATTTATTTTCAGTTTTTTTCATTCAGGTTTGCTTCATCCCAGATTTCATGGTACCTAAATGGCAGTTAT
TTGAATTCTACTGCTGGACTTTCCACTTCATGGACCAATAGGCCATTTTGGTTTGATGCACAATCCACATA
TGGATTATCTCTCTTGACACCTATCCTTCTGAGTGGAATAAATGGAAATAAATACCACTTCTGTGGCTTCT
ACTGCAATAGCCAAAGTGTTGAATGCCTTCTCGGGATCTTCTTTGCATTCCCGGACAGCTCCTCTACTGAC
AACAAATTCATGGATCCCCAGTTGGTTTGGTCTGCTAATCGAGACCGTCCAGTGAATGCCAATGCAACCT
TGCAACTAGGAAAAGATGGCAACTTGGTCTTGGAAGACTCTGATGGCACTTTTGTTTGGTCCACTAATAC
TACTGGAAAATCTGTTTCTGGCTTAAACATGACAGAAACGGGGAATCTCGTGCTCTTTGACAAAGCCAAT

CGCACAGTTTGGCAGTCGTTCGATCATCCTACAGATTCTTTGCTCCCGGGGCAGAGCTTGGTTTCTGGGA
GGAAGCTCATAGCAAGCGTCTCAGCAACCAATTGGAGTCAAAGTTTGCTTTCTTTAACTATTATCAATGG
AAGCTGGGCCACTTACATCGAATCTGATCCACCTCAGTTTTACTATGCTTCAAGTAATCCGGATACTAGTA
CTTATTTCAGTTTTGATGGTCAAACACTTACTGTTCAGCAAGGCCCTCCTAAATCACCAGCTCAATTCATG
AAGCTTGGGCCTGATGGACATTTATGGGTATACCAATGGGACGAGAATGCATTAAATTGGAAAGTCATA
TCTGATGTTTTGTTTAATGTACATGCAGAGAACTGTGGGTACCCAACGGTGTGTGGAAGATATGGCATTT
GTACAAGTAATAGGAAATGTAGATGTCCGCCAGAGCGAAATTTCTTCAGGCCATTAGATGAGAGGAATA
TAGATGATGGATGTTTACAGCTGACTTCCATTTACTGCAACTCTTCGCAGTATCATAGTTATGTGGAGCTC
AAGAATACCACATATTTTGCATTTGAGTTCAGTGATGACCGAATTTCGGGTATATTACCGTTTGAGGGGA
AAAAGTTGGAAGATTGCAAAGGGGCTTGTCTGAGAAACTGTTCCTGCAAAGCTGTTGTCTTCAGATATG
ATTCGGATGGGGCTACAACTGGGAGCTGTTTATTACTGAATGAGGTCTTTTCTCTCGTAGACACCGACGA
CGGAATGGACAAGAGAGTATTTCTTAAGGTGCAGAATTCCTCAAACACTCAGAATCAGTCTAAAACCATT
TCTGGAGGAAAAAAATCAAGACCTCACATAGTGATAATAGGATCTACTCTTGCAGCTTTCTTTGGGATAA
TGTTAAGTATCACAGCTTTCTTTGTTATCTTTAAAAAGAGGACAAAAGATTCCAGAAAGGATGGGGGTTT
TCTGGATCTCGAACCAATCTTTCCGGGAATGCTAACTCGGTTCTCTTACAATGAGCTAAGAATAATAACT
GAAGATTTCAGCAGAAAGCTCGGGGAAGGAGGATTTGGCTCAGTATATGAAGGAACACTGAGTAATGG
CACCAAAATAGCTGTAAAGCGTCTGGATGGTCTAGGTCATGTGATGGAATCATTCTTAACAGAAGTAAA
GATAGTCGGTGGCATTCATCACATTAATCTGGTAAAACTCATTGGATTTTGTGCTGAAAAGACCCAAAGA
CTTCTGATCTATGAACACATGGTAAATGGATCACTAGATAGGTGGATTTATCATAAAAATGAAGAAAAT
GGGCTTAGATGGCATACAAGGCAAAGGATTATAACAGATATTGCCAAAGGATTAGCTTATCTTCATGAC
GAATGCAGCCAGAAGATAATTCATTTGGACATCAAACCACAAAATATCCTTCTAGATAAAAATTTCAATG
CTAAGATATCTGATTTTGGGCTATCGAAACTGATCGATAAAGACGAAAGCAAAGTTGTAACTAGGATGA
GAGGAACACCAGGGTATTTGGCTCCTGAATGGTTGAGGTCAGTTATCACTGAGAAAGTAGACGTGTATG
CCTTTGGAATTGTGCTCTTGGAGCTGTTGTGTGGTCGAAAGAATTTGGATCGGTCCCAAGCTGATGAGG
ATGTCCATTTGCTAAGTGTTTTCGAAAGAAAATCGGAACAACAGCAGCTTATGGATATTGTTGACAAAAA
CAATGAGGATATGCAGATCCACAAAGAAGCAGTGACAGAAATGATGAGCATTGCTGCGTGGTGTCTAC
AGGGAGATTTCACCAAGAGGCCTTCCATGTCTTTGGTGGTCAAGGCATTGGAAGGTTTGGTGTCTGTTG
AAACAAACTTGGATTATGATTTCACAAGCCTACCCGAGGTTGAGGATGACAACCAACAGAGGGAAGAC
ACTATCAGTTTAATATTGCCTTCAATTTTATCTGGTCCAAGGTGAACAACTAGCCTAAGAGGTTGTAAGTT

TTTTTTTCCTTTCCCAAGTCTATGTCTTTCTTTTGTGAAAATGTATTTCCATGCTTCATCACCAGATCCATCT
TTCTCTTGTACATAAATTTGGCTAACAGGTAAACTATGTCATAATGTAAAAGGTAAACTTAATTAGCTTTG
ATAGATAACCAATTAACGTCTCGTGGCAGAGATGGATAGCATTTTCTTTCTAATTTGCATCTTTGTGGTCA
ATACATCTTCATATGCAGCACATTTTATATTTCTAAGAGTAAGGGGTCTACTTATTCTGTGTTATGTACAG
AGAAAACTGCAG

>PGSC0003DMG400017330 G-LecRK

AAACAACCCAACATCTTAAATTTAAGTTCAATCATTTTTCATCTCAATGATGAACTAAAGGCCCTTTGCCT
TCTTTGGAAAACCAAAAAATTGGTCTTTATGGCATTAAGCCAGTTTTTACCTTTCTTCTTCTTCATTCTTTCT
TGTTCTTTTCATTTGATAACTAGCCAACCTTATGATTATCCATCTGCAAATCTTTCTACAACTTGGATCAAC
AGTGTTTCTGCAGACCATTCAGTGAATTTCACTGATGGCTCAACAATAAGAGCCATACTACTCAGAGGAA
CTTTTGGTCCAAAATATGCTTGTGGTTTCTACTGTAATGGAAATTGTGATACTTACCTCTTTGCCATATTCA
TTGTGCAAACCAACAGTGCTTCCCGAATTACTCTTCCCGCGATTGGGTTCCCACAAGTTGTTTGGTCTGCT
AACAGGAATAAACCTGTTAGGATCAATTCTACTTTGGAACTTACAGCACAAGGAGATTTGGTGCTTAGA
GATGCTGATGGTACTTTAGCTTGGTCAACTAACACTACTGGGAAGTCTATTGCTGGCTTAAACTTGACCG
ATGAGGGGAATCTTGTTCTGTTTGATTCGAAGAATGCAACTGTTTGGCAATCGTTTCATCACCCAACTGA
TGCTTTGGTTCCAGGGCAGAGGTTAGTACCAGGGATGAAGCTAACAGCAAGTGTTTCAACAACAAATTG
GACTGAGAGAGGTTTGTTTTCTCTCTCTTTTACCGATGATGGTTTTGTTGCTTTGGTAGAGTCAAATCCTC
CCCAAACATACTTTGACGTGACTATTGGAAGATTGAATGCAAGTAGAGGCTCAAATTATGTTGTGTATTT
GAATGGTAGTCTAGCTTTACTCTCAAACTCTAGTAATTCCAGCAATTCACAGACACTGGTTTCATTTAGTC
TAGCGACCTCAGCTCAATATATGAGACTGGAGTCTGATGGACACTTGAAAGTGTATGAGTGGCAAAGTG
GGTGGACTGAGATGGATGATCTCCTCACAGGTTTTAACGGCGAGTGTTATTACCCCATGGCCTGTGGAA
GATATGGCATTTGCTCAAGGGGGCAGTGCAGTTGTCCCAAATCGAGCTCTAATTCAACGACCTATTTCAG
ACAGATAGATGATAGGCAGGGTAATCTGGGTTGCTCTGAGGTCACAAGGCTGACATGTAATGCTTCAAG
TAACCACAGATTTTTGGATCTTCAAGATGTGGACTATTTCACGTTTACGACAGATATCAGAAACACTAGT
ATGAATACCTGTAAAGATGCATGTTTGAGGAACTGTTCCTGTAAAGCTGCTTTGTTTCGCAGTGGTTTAA
ACTCATCCACGGGAGACTGCTACCTACCATCCGAGATCTTTTCACTAGCGAATAATGAGAAGGACAAGA
CAAGGTATGATTCCCATGCATTTATAAAAGTACAGGTTGAACCTGAAATAGCTGCTGCTAAAGAGAAAA
GGCGTGTTAATGGTGCTATACTGGGCTCTGTTATAGGAGTTTCCATCTTAGGGATCATAGTTGGAATTGC
AGTTTTCATATTCTGGAAGAAGAAGAGAAAGGCCAATGAAGATGAGGAGGATTATCTAGATCACGTACC

AGGAATGCCGACTAGATTTTCATACGATGATCTAAAGGCTGCAACGGAGAACTTCACCAAGAAGCTTGG
TCAAGGAGGATTTGGGTCAGTTTTTGAAGGGTGCTTAGCAGATGGCACAAAGATTGCAGTGAAATGTCT
TGATGGAATAGGACAAGTCAAAGCGTCATTCTTAGCTGAGGTTGAAACTATTGGCAGCATACATCATGT
AAACTTGGTGCAATTGATTGGCTTCTGCGCTGAGAAATCTCATAGGCTTTTAGTATACGAGTGCATGAGC
AACGGATCACTAGAAAAATGGATCTACCATGGGAAACAGGAGCAAACTCTAGATTGGAACTGCAGGAG
GAAGATTATTCAAGACATAGCCAAAGGATTAGCCTACCTTCACGAAGAATGCAGGCAAAAGATTCTGCA
TTTGGATATTAAGCCCCCAAACATACTATTGGATGAGAAGTACAACGCTAAACTTGCTGACTTTGGGCTT
TCAAAGCTAATTGATCGGAATCAGAGCCAAGTTATGACTCAGATGAGAGGCACTCCTGGTTATTTGGCT
CCTGAATGGCTTAGTGGAGTCATAACAGAGAAGGTAGATGTCTACAGCTTTGGCATTGTGCTCTTGGAA
ATTTTGAGTGGAAGAAGACATTTTGAAGCATCGGAGTCTGAAGACCAACAGGTAATGGTGAACTTATTC
AGGAGAAAGGCAGAGGAAGGGAAGTTGGTGGATCTTATTGATAAGCACAGTGAAGATATGCAGTTTTA
CAAAGAAGAAGTAGTAAAGGCGATGCAGATTGCTGCCTGGTGTTTACAAAGCGATTTCACGAAGAGGC
CATCAATGTCGATGGTGGTCAAGGCCATGGAGGGTGTTATAGATGTTGACAAGGATCTAGACTACAGCT
TTAGACCACAAACTGTTTCCGCAATACCATATATCAGTTTTGCAGATTCAACTCCTTTATTGCCTTCGATCC
TATCAGGTCCAAGGTGAGGAGGAAGTTCCTTGGAATTTTTGTTAATTTCTTTTTTTTATGCTATTGCAGCT
TGCTCGCTGCTGTGTAAATTACTTCTCCAATGTTCACAAAGTTATGATTGTTTTCTTCAATTTATT
>PGSC0003DMG400017331 G-LecRK
TTTTAACTCATCTTCCTCTAGTGTTCTTAATGGCTTTATGCAAGATTATTCCTTTTGCCTTCATCCTTCTTTG
TTGTTCTTGTTATCTGATATCGGGCCAACGTTTCGATTACCCCACTGCAAATCTTTCTACAACTTGGATCA
ACAGTGTTTCTGCACCCCATTCAGTGGATTTCACTGATGGCTCAAGAGTAAGGGCTATACTACTCAGAGG
AACTTTTGGTCCAAAATATGCTTGTGGTTTCTACTGTAATGGCAATTGTGATACTTACCTCTTTGCCATTTT
CATTGTGCAAACTAATAGCGCGTCCCAAATTACTTCTCCCGCGATTGGATTCCCACAAGTTGTTTGGTCTG
CTAACCGGAACAATCCGGTTAAGATCAATTCAACTTTGCAGCTTACAGCACAAGGAGACTTGGTGCTCA
GAAATGCTGATGGTACTTTGGCTTGGTCAACTAACACTGCTGGCAAATCTGTTGCTGGTTTAAGCTTGAC
CGATGAAGGGAATCTTGTTCTGTTTGATTTGAAGAATGCAACTGTTTGGCAATCGTTTGATCACCCGACA
GATGCTTTAGTTCCAGGGCAGAAGTTAGTATCCGGGATGAAACTAACAGCAAGTGTTTCAACAACAAAC
TGGACCAAAGGAGGTTTGTTTTCCCTCTCTGCTATCGATAATGGTTTGGTTGCTTTCATAGAGTCAAATCC
TCCCCAAACATACTTTGACGCGACTATTGGTGGATTGGATCCTAGTGGAGGATCCAATTATGTTAAGTAT
TTGGATGGAAGCTTAACTTTCTTCACAAATTCGAGTGGTACAACCGAGTTGGTTCTGGTTTCTATTACTCC

AGCATCCTCTGCTCAGTATATGAAACTCGAGTCCAACGGACACTTGAAAGTGTACGAGTGGAAAAATCG

ATGGGGAGAGGTGGATGATCTCTTGACAGGCTTTCGTGGTGTGTGCAATTACCCCACAGTCTGTGGAAG

ATATGGCATTTGCACAATGGGACAATGTAGTTGTCCCATAAGCTCTAATTCAACTACCTATTTCAGACCG

ATAAACGTTAGGCTGCCTAATCTTGGTTGCTCTGAGGCCAAAAAGCTAACTTGTAACAACTTAAAGAAAC

ACAGACTTTTGGAAGTTGAAGATGTGGACTATTTCGCATTTACTGCAGATATTAGTAACACAGATGTGAA

TACTTGTAAACGCGCATGTTTGAATAAATGTTCCTGTAAAGCTGCATTCTTTCGTAGTGGTTTAAATTCTT

CCAGGGCCATAGGGGAATGCTACTTACCAACTGAGATCTTTTCACTAATGAATAATGAGAAGGACAAGA

CAAGGTATGACTCTGTCGCGTTTATAAAAGTACAGGTTTAAGCTAAACCAGCTGCTGCAATAGGGAAAA

AATCGTGTTTTATGTACAAAAATGAATCTATGGGAGGACATGGATGGAAACTTAGCAAATTATGCACGG

GATCTTGAATAAGTTCATGCTAGACATGAACTATATGAAGGTATTATTACATAAATAAATGGTTGGATAA

GCCAAATTAGTGTTAAGAGTTCTCTGTATTCCTTCATTTCTCTTCTTCTCTGTATTACACTTTCATTTCTGTT

TGTTATCTGTAATTTCAATTCAATAGACAATAATAAAGACA

>PGSC0003DMG400017946 G-LecRK

AGCTATGCAAAATTTCCACGCGACATATCAACATAGTAGCAAATAGATTATGAAGCTTCATATTCTGAAC

ATCTCCATGAATCCATCTGCAAGTGAAATCAAATTCCATCTGCTATCTCATTTTAATGGAGGCCATAAACA

TACTTTACTTCTTGTCATCCATATGGATTCTGTCTCGTGTGAGTGCTCAACATACCATACCTGCAAATCAA

CCTCTAACAGATGGAAGCACCATTATTTCATCAGATGGCAAGTTTAAGTTGGAATTTTTTTCCCCTGGGA

CATCCGGGAAACGCTACTTAGGAATATTGTTTAATAAAGTTACTGTACAGACAGTGGTATGGGTTGCTA

ATAGAGACAATCCACTCAATGACACATCCAGTGCGCTAAATCTTACCAGACAAGGAATTCTTACTCTTCT

AAATGGTTCTGGCAGAATCATTTGGTCCTCCAATTCCACTAGACATGGGAAAAATCCAGTAGCACAACTC

CTTGATTCAGGCAATCTTGTTGTTCGAGATGCAGCCGAGGATAAGCCTCAGAACTACCTGTGGCAAAGT

TTTGACAATCCAGGTGACCCAGCTTTACCTGGAATGAAGGTTGGGATTAATCTGAAAACTGGTTTTCATC

GTTCCCTTTCGTCATGGAAGAGCACAAATGATCCTTCCAGGGGTCAGTTTACCTGGACATTCGATACTGG

TGGATTCGTACAAACATTCATTATGAATGGTTCCATTGAACTCTACAGAGCTAGACAATGGAATGGTCGA

GTCTTTCCTAATGCACCATCTAGTGACACTTCGTGGAATGGTTATAACTATACATACTTATCTGATCCTAA

TGAAATATCATTCATGTATGAACTTACAGACAGCTCTATCATTGCAAGGGTAGTGATGCAACTAAATGGA

TTTCTACAGCTTTCAGAATGGAATAATCAGACCCAAAATTGGGACGATGTTGTTAGTGCACCAGCTGATA

ACTGTGACATTTATGGCCAGTGTCATTCATATGGTTTGTGCAACAGCGGCAATTCCCCAATCTGCAGATG

TTTGGATAAATTTGAACCCAAAGATCCAACAGAATGGGCAAGGGGAAATTGGTCAGGTGGCTGTGTTA

GAAAGACAGCAATATTGAATTGCCAAAAGGAAGTTAAATTCTTCAAGTATCAAGGCATCAAGTTGCCAG
ACACTCGTTTTTCCTGGTACAGTGGAGGAGTGACTCTTAATGCATGCGAGGAATTGTGCTTGAGAAACT
GCTCGTGTATGGCTTACGCAAATCCAGACATAACAGGGACAAATGAAGACTGCTTACTTTGGTTTGATG
AGCTGATGGATATCAGAGAGTTTGGTGCTACTGGGCAAGATATCTATGTAAAGTTGGACTCTTCTGAGT
TAGGTATAAATCTGTTTCTTTACTTTTTCATGTAAGGCTGTTAAGTATGCTACATTAGATTTTGTGAGCAA
GTTGAGGTTTCTCTACATTATTTGAAAAACAAAACAATATCTTTTTTCCTTCTTATGAATTTATTTCTGTCA
TCAGGTAACTCCAGTACAGGGAAAGTAAAGAAACTGAGAATCAGCTTGCCACTGGCAGCATTAGGTCTG
TTCCTAGTACTATGCTTAATCATGTACATAAGGCAAAAGAAGAAGAATCAGCAATATTTTAGAAAAGGTA
AACCTCAGAGGTTTTGCTGCTTTTGAATTTGTGGTGCGTTTTTTCTGGAGTTACATTTGGTAAATATCATA
ATTTTTCTCTTTACGTCCAACTAAGTCCTTATTTTAACAGGAAGAAGACCAAGAATCTCTGAAATGTTGTG
CACTAAAGAAAGGAAAGATAAAGAGCTAGATTTACCGTTGTTTGATTTTGAAACTATATCTCATACTACC
AATAACTTTTCATTGAACAACAAGCTTGGAGAGGGAGGTTTTGGACCTGTTTACAAGGTAACGATTTTAA
CTTCAGCTCTTTCTGTTTCATCCAAAAGAACTGCCTAAAATGGAATTTCCGAACCTTTAAAAGGAAACACA
TGTTTCAGTAAGAAAATTGATATGTCATTCTCCTATTTCATCTAAACAGCAATGTCAAAATCTCGTTTATT
GTTTGTCAGTAAAATATTCTGGATAAGCTGATTCAACTAATTTCCTGGATATCTAGGGTATGCTGGAAGA
TGGGCAAGAAATTGCAGTAAAGAGACTTTCAAGATACTCAGCACAGGGAACTGATGAGTTCAAGAATG
AGGTTATCTTCATTGCTAAACTCCAGCATCGGAATCTTGTGAAGCTTCTTGGTTGCTGTATCCAAGCAGA
GAAAAGATGTTGGTTTATGAGTACATGCCAAATAATAGCTTGGATTGGTTCCTTTTTGGTTGGTTCTCAC
TTCTCTCAACTCTTAATACATTTGGTTATCTGTAGTAATAGTTCAATCAGCTGCAGATGGATTTGGCCATC
ATCAGATGGAGTTGGTTATCACTTTTCTGAACTCTTGATTTTTGCAGATACAGATAGGAGGTCACTGCTT
GATTGGCCTAAGTGCTTCCATATTATTAATGGAATTGCTCGCGGACTTCTCTATCTTCATCAAGACTCAAG
ATTGCGGATAATCCACAGAGATCTTAAACCTAGCAACGTTTTGCTAGACATTGACATGAACCCGAAGATA
TCTGACTTTGGCATGGCAAGAAGTTTTGGAGGAAATGAGACTGCAGCCATGACAAAAACAGTGGTTGG
GACATAGTAAGAATCTTTAAACCGATTAAGTATTTGCAGTCTACTAAAAAGCTACTTACTTTGTTTATTTG
ACATTATCCTACAGTGGGTACATGTCCCCGGAGTATGCAGAAAAAGGAGAATTTTCAGTGAAATCAGAT
GTATATAGCTTTGGAGTTCTAGTACTGGAGATTTTAAGCAGGAAGAGAAATAGAGGGTTCTTGCATCCA
GACCATGACCATAATCTTCTAGGACATGTAGGTGATCTTCTTTTCTATCTTATATGTTAAGTAAAACTAAT
AGCATCTAACTGCATGATAGTAAATGTTCAGGTGTGGATCCTTTTCAAAGAAGGCAGGGTTATGGAAGT
AATTGATACACAACTAAGGCAGTCATGCAATCAGTCTGAAGTTCAAAGATCAGTTCATGTAGGTCTATTA

TGTGTGCAGCAGTGTCCAGAAGATAGGCCGAATATGGCATCAGTTGTGCTCATGTTGGGTAGTGATGTC
GCACTACCCTTGCCTAAAGAGCCAGGCTTTTTCAATGGCAGGAACCGATTCACTGAAGCTGACAGTTCAT
CTAGCAAGCATGGCGAAACTTCAGTGAATGAATTAAGCATCACACAGTTGGATGCCAGATAGGAGATAA
AATTTCAGGTCATGGATGTTTTCTGTATGCTATATTAACTTAATTGTTGCAGTCATATAGAACAAGAACTA
CTGGTTGTTCAACTAAATAGTTTTACTCAAGAATCTGTGGCATGGTATTAAGCATGACAACTTGACACTC
AGTATGCTGTATATAAAGATAGGAGCAGCAGAGTTGTGAGAAGAGCTAAAAGCATTCTCTACATACAAA
AGCACCTAGTACATTTGCTTATGAAAGAAGAACAACTTATTCTGTAAAAGACTTTCAGCCTCCCTTGGGG
AAGTAAGAACAATTTTTCCTTTGTTGGCTTGTATGAGTCCACGACTAGTCAGGAGACCAGGTCCCTTGAC
TGTAGTAATAACAGGAAGTAATGTAAATGCTCAAAGTAAAGAACATAGATGTTTACCTGGAAACTTCCTT
GCTCAAGAGAGGAAAAGCTACGACCTGTCACACGAGATTTCTCAAAACTCCAATAACTTCAAGTAACTTG
TGAGTACTGACTCTAGTAATCACAAGGATTAAGATATTTATCCTACAAACCTTTAAACACCTCTATTACAA
AACACAAAGCAATAACCCTATTGTCCACTTTATCAACTACCTCTAAGTAGAATAGACTTCAACAAACACCA
CTCAGCTAACTCAAGCCAAAAACTTAACTAGCACTAATATGGAATGGCATGATCTATCTTCCCGTATAAC
ATAGGAATAGATTCTTAATAAAAGACCATAAGACACTAGACTCAAATAACAACTTCAGACTCTGCAAGAC
TAAATTGGGTCCCTCATTACGTGTCAGAGTAGTTCTTTCTTTAAAACGGGTTTTGCTTTTGAATAATCCTA
ATTGCAAAAGCTAGGTTGTTTCTTGTCAACAAACAACTTAGCAGCATGTCATTGGTCGAGGACTGTTGTC
CTTTCTGTGCCTCTACTGCAGTGTCCACCAACCACTGCAGTTTCTACAGCTTTAGAACTGCACTTCATTGA
CTTGTCCTTTACGACAAAAACATAGAGGACTAGGTCCCTTTACTAGATCCTATTTCTTTATAGCTTGTTAA
ATCATCAAAATCCCAAAATACAACATTTCCCTCCTTTTTGATGATGATAAATTATTGCCCATGTCTACATAT
CTTTTGCTTGCACATTCCCCCTATCAACATGCGTCCCTTGTCGGAAAGACCTGATCCCTTGATCTGAACAT
GCTTACATGTCATCAGTGTATCTTCCTCCTTTTGACATTATAGAAAAGTAAGAACAGTAAATCAAGCAAA
ATCACATGCATTCTGGGTAAATTCATGGCCACTAGACAATACTAATATGAACAAACAGGCAGAAAGAAA
GGAACGAGAAACAAACATTCTTGGTTTTCCAACTCCGCCTTCTCACTTTTCCATTGGTCAAACCAAAATTG
AGTAGCAAACTTTGGTTGATAAGCGGCCAACTCCGCCTTCTCCACCGTAGAAAAAATCTATAATTCCAAC
TATCTTATACACCTTATCAATGAACTCTTAAGGATCCTCATCAACCTTGGAAACATGGAACTTTGAAGGAT
TCAACCTAGTTAAGTCCCTTATCCTTGTCACTGCCGTACCCTCATTTGGATTCATGGGAGCAACAACTTCT
CCAATTTATCGGGTGGTCATAACTTGAGTCAACACTTGAAAAACGGCTCAAAATTCAGCATGAGTCACTT
GCTTGGCCAAAGGATCAACCAGAACTTGAGGAACCTCGGGCTCCATATTTTTACATGTATTCCTTTTCAC
AAAAACTCTTCGAGGGGCCATATCCTGAAAAGCACCGAAC

>PGSC0003DMG400017949 G-LecRK

TATATATCAGCTATTTCATCAATAATCCCAAAGGAGAATCGTGCAATTTCCTTTAGTAAAACAAGGATGA

TGGAAAGCAGACATGTTCATTTGTTATTATTATCTTACATGTTTTGTGTTTTGATTCTTACTAGAGCTGCA

GATAATATACGTAGAGATGAACCTTTAAGAGATGGAGAAACGATTGTTTCGTCAGGTGGAAATTTTGAG

TTGGGATTTTTCTCCCCAACTCCAACATCGAGTAAACGATACCTGGGAATATGGTTCAACAAAGTATCTG

TACAGACAGTGGTATGGGTTGCTAATAGAGACACTCCACTGAATGATAAAAATGGTATGCTCAATTTTAC

AACACAAGGAAATCTTACTCTTGTAAATGGTTCTGGCAGTGTTGTTTGGTCTTCCAATACCACAAGAGGA

GGCGTGCAAAATCCAATAGCACAGCTTCTAGATTCAGGCAATCTTGTTGTTAGAGAAGATGCAAGTGAG

AATTCGAATTATGTATGGCAGAGTTTTGACTATCCTGGTGACACAGCTTTGCCTGGAGTTAAGATTGGGA

TCGATCTTAAGACTGGTTTTCGTCGTTTCCTCTGGTCATGGAAGAGCAGAAATGACCCTTCTAATGGTCA

ATATAGTTTCATATTTGATCCTAATGGATTCCCACAGCCATTTCTCATGAACGGTTCCATCCAACGCTTCA

GGGGCGGAGCATGGAATGGTCAAAGCTTTGCTAATTCACCATCTCTACTACCAAGTCCTTCATATAAATA

CATATTTGCATCCGATCCAGACCAAGTATACTTTACTTATGAGCTCATTGACACTTCTGCTATTGCAAGGG

TAGTGATGCAACTACATGGGTTTCTAGAGCTTTCGACATGGAATAATCTAACTCAGAATTGGGATAACTT

TGGTAGCGCACCAGCAGACAACTGTGACATTTATTCCCAATGTAACGCATATGGTTTGTGCAACAGTGG

CAATTCTCCAATCTGCAGTTGTTTGGATAAATTTGAACCCAAAGATCCAACAGAATGGGTAAGGGGAAA

TTGGTCAGGTGGCTGTGTTAGAAAGACACCAGTATTGAATTGCCAAAAGGAAGTTAAATTCTTAAAGTA

TTCAGGGATCAAGTTGCCAGACACTCGATTTTCCTGGTACGGTCGAGGAGTAACTCTTAATTCATGTGAG

GAATTGTGCTTGAGAAACTGTTCATGTGTGGCCTATGCAAAACTAGACACAACAGGGACAAATGAAGAC

TGCTTGCTTTGGTTTGATGAGCTGATGGACATCAGAGAGTTTGGTTCAAGTGGGCAAGATATCTATATAA

AGTTGGACTCCTCTGAGACAGAGATAAGTACAGGTTGGTATGCTTCTTTACATTAGTCTTGAGTTTTCTA

GACCAAATTGAACTGTCTATTAAAAATTTGAAAAGACTAAGATGAATTTATATGAATCATCAGGAAACTC

CAGTAAAGAGATATTAAAGAAATTGAGAATCAGCTTGCCATTGGCAGCATTAAGTCTTCTCTCAGTGCTA

TGCTTGATCTTGTACATTAGGCATACGAAGAACAAGAAGAAGAAAGAGGAGGAGAATCAGAATCAACA

ACGTTTTAGTGAAGGTAAACCGGATGTTTTGCTACTTTGAAATTTGAAATAAGTGGCTTGAATTGATGGT

AAATCCTAGAAATTCCTCTTAACATCCAATTAAGTCCTTATTTTGACAGGAAGCTCTGAAATGTTGTACAT

TAATAAGAGCAAAAAATGATGATCTAGATTTACCATTGTTTGATTTTGCCACTATCTTGGAGGCTACCAAT

AACCTTTCTTTGAGCAACAAGCTTGGAGAGGGTGGTTTTGGACCTGTTTACAAGGTAACAATGTTAACTA

CAGCTCTTTCTTTTTCTCTAAACAAACTGCTTATCTTCAATATATTATATATGCTGGAACATTGACAAACTT

TCGGAACCTTTAAAAGGAAACAAGTTCCCGATTAGAAAATATGTCTCCGATTCTCCAGAAATGTCATTAA
AAGTTAATTCCACTTCAAACAAGCTATCTAAAAAAGTTTACTCAGAATTTTTCAATCGAAAAACTAGAGA
AGCTAATTGCCTGAGGATATGTAGGGTGCGCTGAAAGATGGACAAGAAATAGCAGTAAAGAGACTTTC
AAGATACTCAGCACAGGGAACGGATGAGTTCAAGAATGAGGTTATCTTCATTGCTAAACTCCAACATCG
GAATCTTGTGAAGCTTCTTGGTTGTTGTATACAAGCAGAAGAAAAAATGTTGGTTTATGAGTACATGCCT
AACAATAGCTTGGATTGGTTCCTTTTTGGTTGGTTCTCACTTCTCTCAACTCTTAATACATTTGGTTATCTG
TAGTAATAGTTCAATCAGCTGCAGATGGATTTGGTTATCACTTCTCTGAACTTTTGATTTTTGCACATAAA
GATAGGAGGTCACTGCTTGATTGGCCCAAGTGCTTCCATATTATAAATGGAATTGCTCGAGGACTTCTCT
ATCTTCATCAAGACTCAAGATTGCGAATAATCCACAGAGATCTTAAACCTAGCAATGTTTTGCTAGACAT
TGATATGAACCCGAAGATATCTGACTTTGGCATGGCAAGAAGTTTCGGAGGAAATGAGACTGGAGCCA
TGACAACAAGAGTGGTTGGAACATAGTAAAGATCTTTAAACCAAAGAGCTACTTACACTCTACTAAAAA
GCTAACTTTCTTTATTTGTGATTTAAAAAAAAGAAGAGATACTTACTTTCTTTGTTTGACGTTATCCTACAG
CGGCTACATGTCTCCGGAGTATGCAGAAGAAGGGAAATTCTCAGTAAAATCAGATGTTTTTAGCTTTGG
AGTTGTAATTCTGGAGATTTTAAGCGGGACGAGAAATAGAGGGTTCATTCATCCAGACCATCACCATAA
TCTTCTAGGACATGTAGGAACTCATCTTATACTTACAGCGTAAAAATCATGTTAGTTAAAATTATAGCATC
TAACTGCATGATGTGAAATGTATGTTCAGGTGTGGATCCACTTCAAAGAAGGCAGGGTTATGGAAGTAA
TAAATACACATCTAAAGGAGTTATGCAACAACCTATGTGAAGTGCAAAGATCAGTCCATGTAGGTCTATT
GTGTGTGCAGCAGTGTCCAGAAGATAGGCCTAGCATGTCATCAGTTGTGCTGATGTTGTCTAGCGATGT
TCCATTGCCCTCACCTAAAGAGCCTGGTTTTTTTACTAGCAGAAGCCGATTCGGTGAAGTTAATAGTTCAT
CTAGCAAGCTTGGCGAACATTCTGGAAATCAATTAAGCATCACACTATTGGATGCTAGATAGGATATATA
TATATATATTTTCATTGTTTGCTTGTAGAGGCTAGTGTCACGGACTTAGAGTCTCACTAATGTTCCGTGCG
GTACTCGACAACTTACACAGGAATCTAATTACAGTGGCGGGACATGACACTAGGCTTTATTTACTTTATT
CTTTACTTCACAGAGCTCAACACAGCTTCCAGTTGTACAACCAAACAACTTAAAGTGAATTTGTCAAATGT
ACAAGTGCTTTCTCAGTCTAATAATTCTTCATAATCTATATTGTCCTCACTCCTCAGATTGTGAAATGAGTT
GAGACAATTTGTCATAATTGTACAAAGGGCATTGGACAGCTCTTTTTCAAAACAAGTACAGTTGATAGTA
TTTTAATTAACAACTTGACGGTGATCAACAGGTTGGTCAATC

>PGSC0003DMG400018452 G-LecRK

CTTCAACCATGTTAACAGTTAGAACTTGTCCTTCTTTAATTTCTCCCCCATTGATATCATGGCTCTCTATTTC
TTACTTGTGTTGTTTATCTTTGGATCCGCTAGAGCTCAATCAAATCCACCTAGTACAATAAGATTAGGATC

TTCTCTTCATCCCACTGGACAATCTGTAGCATGGTACTCAGCTTCAGGCCATTTCGCCTTCGGCTTTTATCC

ACAAGGAAGTGGTTACAAGGTGGGAATTTGGTTAGTGGGAGGCTCCAATAACACTATTGTGTGGACCG

TGTCTCGTGATGATCCAGAAATATCTGCTGATTCTACCTTAGCTTTCATTGATGGAATTGTAGTTCTGAAA

ACAAATAACAGTGAGGATAAGACTGTTGCTAGTTCGTCAGAATCTGCTTCTTATGCCAACTTGCTTGATA

ACGGTAACTTTGTTCTTTACAATCAAGATCATGAAACTATCTATGAGAGCTTCAGTTTTCCTACTGATACA

ATTTTAGGTGGCCAGAAATTAACAAGTGGGGGTAAATTGGTTTCTAGTTTCTCCTCCACAAATCACTCCT

CAGGGAGATTTCGCCTCTGGATGCAGGATGATGGGAACCTTGTTGCATACCCCAGAAACTTGTTTCGGC

CAGTAGATGCCTATTGGTCTTCACAAATGTACTGTGATGGTTGCAGAAATCTCCTGCTTCTTAACAGCACT

GGCATCTTATTTCTGATCAATGACACTGATTCTTCAGTACTTCGACAATTATACACTCCACTTGTACAGAA

TAACAGAGCTATTTATCGGGCCACTCTAGATTATGATGGGAATTTTCGACTCTATTCTCACGAATTCAACT

CCAATGGGAATTCTAAAATGGTAATGGAGTGGGAGGCAATTGATGATATTTGTCTTGTGAAGGGTTTCT

GTGGCCTTAATGGCTATTGTGTTGGAAGTAATCACAATGCATCATCTTGTATGTGTCTACCAGGGTCATA

CTTGAGAGAGAATGATCCAAGCTTTGGTGATTGTCAAAGGAACTTCACCAGAGGAAAGTGCATTAATGG

GAAAGAAGATGTAAGTGTCTACAAGATTACTACCACAACCAATTTGACCTGGGAGGATCCTCCTTACTTT

GTTACATCACTTCTAGGAAAAGAAGATTGTGGTAAATCTTGTTTGGAGGACTGTGATTGTGATGCTGCTC

TTTTCGATGAAACTGGCCAGTGTATGAAACACAAGCTTCCTCTGAGGTATGTTAAAGGTGCTCCTGAAG

GATCTCGCACTGCTTACTTCAAAGTCAGCAATCTAGCAGTGCAAAATCCAACAACGGATTCAGTGAAGCC

ACCATGGGTGGTGATCTTGGTTTTATCGATAAGTTTCGTTATGTATTCAGGTACTGCTCTTGCATTTTCAG

GCTTTTATGTCTTCAAATTCCGGATAATAAAGTACAGGAAGCTACTTCAGACAGGAACCACAGGCTTAAC

AAAAGATTTCATACTCAAAACTTGCTCTTACAAGGAGCTAAAACAGGCAACGGATGGTTTCAAAGAGGA

ATTAGGAAAGGGTGCTTTTGGAGCAGTTTATAAAGGGAGCTTTGACAAAGGTAAAAACCTTGTGGCAGT

GAAGAGGCTAGAGAAGGTAGTGGAAGAAGGTGAAAGGGAGTTCAGAGCAGAAATGAAGGTCATTGGT

AGAACTCGACACAGAAACTTGATTCGTTTGCTAGGATATTGTGCTGAAGGATCAAAAAGAGCTCTTGTTT

ATGAATATATGAGCAATGGTTGCCTAGCAAATCTGTTGTTCCGAGGAGCTACTCGGCCGGATTGGAATC

TAAGAGTAAATATTGCACTAGATGTGGCCAGGGGAATCCTCTACCTCCATGAAGAATGTGAAGCTCCAA

TTATCCACTGTGATATAAAGCCACAAAACATCTTACTCGACGAGTTGTTGACAGCTAAAATCTCAGATTTT

GGTCTGGCCAAGCTGCTAATGCCTGATCAAACAAGAACATTCACTGGTGTAAGAGGGACTAGAGGGTA

TTTAGCACCAGAGTGGCAAACAAATGCCCCCATCTCTGTCAAGGTTGATGTCTTCAGCTATGGTATTGTA

TTGCTAGAAATCATTTGCTGCAGAAGAAACATTGAAGTCTGTGTGACTAAAATAGAAGAAATCCAGCTTT

CTATATGGGCATATAGTTGCCTTGTTGAAGGTGAAGTAGACAAGCTTGTTGGAAGTGAAGAAGTAGACA
AGAAAACCTTGGAAGGAATGATCTCTGTGGCGATTTGGTGCATTCAGGACGAACCAGCTCTTCGTCCTTC
CATGAAAAAAGTATTACACATGTTGCAGGGAATTATTGATATTCCAATTCCTCCATGTCCAAGTCCCAGT
AGTTAATTTCAGTAGTTGTAAGTGGTTTGGTTTATTATATTGTTGTTTTCTTCAATAACATCTTTTAATGGA
AATTTTAGTGAGGATATATAGTTAGTTCAACATACATGAGTTCAGATTATGTTTTATTTTAATGAGATCAT
TTGCAGGCCCTATGGGTGTAATCTTGAAAGATATTACTCAAAAATGAGAATGTTTTGAAGTGTCAGAGT
ATCAAAATACTCAACTATTACACTAGGAGATTATCAAAATTATGCTTTCCATCATATGTTGGGAGTTGCG
AAAATTTAGACATATATACAAGTATATGTGGGAATATTTAAGGTGTTGAGAGCCACTAGATTTGGAGCTT
CTAATTAAGGTGGATAACACTTCAATATAGTAACATGATTGCTGATATTATGCAGGGAAAGGGTATTCCT
TGATAGTATTGTGGTTAAAGGCTTAAAGCAAGGAAGACTCTTTATCAAACCAATGGTATTATATATTGCT
GTAGAAAAGGAAATTAAATAGTGGATGCAATGGCTAACTGGAGCTTAAAGGGGCATACATCTATCAGTT
CAATGATCTACTACAAGAAGCTAAAGCTGCATACAACAAATTGAACAAAATTCAGGTGCTTAGTCAAAG
GTGCAGAGTATGCAACAAGATTCTTGTAAGAACTTCATGCATCAAGACATGATAAACCACTTCATATTTT
GTATGTTCTTATAAAATCAGATAGAAAAAGTAGTCGAAAGATGATTTTATTAATTCAGTAGTCA
>PGSC0003DMG400018457 G-LecRK
GGAATACTACTGCCATTTAAAATTGGAAATGTCCAAGTTAGTATACAAAAGAATACTTAGCAAAGAGTAT
ATCTATATTCTCATCCTTGTGTCATGGCTAACTTAACATTTACTTTCATGTTGTTGATGCTTCCATTTGTGTT
TGCAGCTATTCAAGTTGGAGCTCAGCAACAGCCCTCCAACATAACCTTAGGTTCTTTTCTTTATCCGAACC
GAAATCCAAGTTATTGGCCATCTTCTTCAGGCCTTTTCTCATTTGGTTTCTACCAAAAAGGGGATGGATTT
CAAGTTGGCATATGGCTACCTGATAAAACTGTTGTTTGGACTGCAAATAGAGATGATCCTCCACTTCATT
TGGATGCATATATCGAGTTCACAAAGGGAGGCAAACTTATACTATGGACAAAACAGAACAAAAAAATCA
TTGCAGAATCTCCACAGCCAGTAACTTCAGCATCCATGCTTGATTCTGGTAACTTTGTTATCTATAACAAA
ACAATTGTTGTTTGGGAAAGTTTTGATTATCCAACAGACACCATCCTGGCCGGTCAGCGTCTAACATTGG
GCCAGAGCCTTGTCTCTAGTGTTACTGAAGTTGATCATTCAAGTGGAAGATTTTATCTTAGTATGCAGGC
TGATGGGAACCTTGTTGCCTACCCAACCAATTATCTTAATGGACCTGAGGCTTCCTATTGGGGTTTTATGC
TACAAAGCAGAGGTGAATTTGGTGAGGTTAGCTCAGTTTTCATAGGTCTTACTCCAACAGGCCAACTCTT
TCGGAATGCTTCTAATGGTTTTCGTTTCCAAGTCAATACCATAGGGAATACCTCTAATCAATCATCAACCA
ACAAAGCAGCAGCAATTTATCGCGCAACTCTTGATCCTGATGGGATTTTTAGGCTTTATACACATAACTTT
GACAACTCAAGCTTGGAAATCAATTGGTCAGCGCTGGAAAACCAATGCAAAGCCAGGGGATTCTGTGG

TTTAAACAGTTATTGTACTGCAAAAAATGGCAGCAGCATAGGCGATTGTTCTTGTTTTCCTGGATTTTCAT
ATGTCAACCGTGCAATGAAATTCCAAGGCTGCTATCGGGGATTCGTTTATGAAGAGTTTTGTGGAAGCA
GCAGAGACTCAACATTGGCCTACAATGTCACGGCGATTCAACACTTGAAAGTTGGAGGATATCCATTTTC
TCAGGTTTCAATGGTTGAGCAGGACTGTAGAAATTCTTGCTTGGATGATTGTACTTGTTGGGCTGCTCTA
TACATGAATGGAGTTTGCAGCAAATTTAAGCTTCCATTAATTGATTCGACAATTGAACAAAATGATGAGT
CCATCAAGAGCTTCGTCAAGCAGAGCTATAACATCTCCCAAATGGCAGGTCACTCTACTCCCTATCCTGG
AAAAACAGGGAATAGAAATGAAAGTCGGAAAGAGATAATATTGATTCTATCCCTGGTTCTTGGTTCTGT
AGCATTTCTGTTCACTGTTTTTGCAATTTTCAGTTATATTTTCTACAGAAGCAGAGCGGACGAATACCAAA
AGCTATTGGAAAATCCATATTTGGGGCCAAATGAGGAGTTCACTCTTCGATCATTTTCTTACAGTGAGCT
TGAGAAAGCCACTGAACATTTCAAGGAAGAGATAAGACATGGTTCATTCGGGAACGTCTATAAAGGAAT
TCTATCAGAAGGTAACAGAACAGTTTCTGTAAAACGACTGGAGAAAATCGGGGATGAAGGAGAAAGGG
AATTTAAAGCAGAAATGACTGCGATTGGGCAAGCTCGTCACAAAAACTTGGTTCATTTGCTTGGTTTTTG
CTTGGAGGGATCAGAAAAAATTCTTGTGTATGAGTACATGAGCAACGGGTCACTCGCAGATATCATATT
CAATTCAGAAACAAGACCTTCTTGGGAGCAAAGGATGAGACTTGCACTGGACATATCAAGGGGAATACT
TTATCTGCATGAAAAGTGTGAGACTTGCATAATTCACTGCAACATAAAGCCACACAATATCCTAGTAGAT
GATTCGTGGACGGCTAAAATCTCTGAATTTGGGCTTGCAAAGTTTCTGGTGCCTGATCAAGTAGGAAAC
CAACTTCAGCTTAAAGGAACAAGAGGATATTCAGCACCAGAGTTGCAGAATAGTGCATTGATCTCCGAT
AAGGTAGATGTTTATAGCTACGGAGTAATGCTCTTGGAGATCATATGCTGTAGAAGTAATATGGATGTC
AACGTCTCAACCGAGGATGAAATCTTTCTTCCTAATTGGGTACACAAATGCTTTGTCGAGAATGATATCA
GAAAGCTAGTGGGAGATAAGGAAGTAGATCATGCGAAGTCCTTGGAGAGGATGGTGAAGGTTGGATT
GTTATGCATTCACGATAATCCTGATCTGCGCCCTTCTATGAGGAATGTAGTTCTCATGTTAGAAGGTACT
ATGGATATACCATTTTTGGCATGGTATTAAGTCTTTTACACTTCGTTTGGCTGGTTGCTACATCTCATTCA
AAATGTATCATATTATATTGTATTGTGTTGTGTACTTGCATTGTATTGTACTGTTTTCTTTTTATGAATACA
AC

>PGSC0003DMG400019062 G-LecRK

ATGGTTCTGTGTGGTATTATTTATATAATTTATCTGAAAAGATCGGCAAGAAGTAAAGGTAACAATGTAG
AAATTTCATCTGCTTTTTCTCAACTACTCCAACTTTTGGATATCGTAATCACTTCAATTGTTTTGATTATAG
AAGCCAGAGATATTGTTTTGGGAATGCCTATGGAGTACTTGCCTCATAGAGATAGTTTTGATGAAGATTT
AATCACTGAAGATGATAAGAAACGGATTGATGTCCCGTTTTTCAGCTTGAATAGCATACTAGTTGCTACT

GAAAACTTCTCAAATGCAAGTAAGCTCGGGAGAGGTGGATTTGGACCTGTTTATAAGGTTTTGCAACTTT
ATTTTTCTTTCCTTCTTCTGTTCTAAGTTGTTTTACCATTCAGATTTTTTTCTTTCGTTTTCATTATATAACAG
TCCGGAGATATATTTATGCAGGGTAAGTTTCTCGGAGGTACAGATATGGCAGTTAAAAGGTTGTCAAGT
AATTCTGGACAAGGTGTTGAAGAATTTAAAACTGAAGTAATGTTGATTGCCAAACTGCAGCACAGAAAT
TTAGTTAGGCTTCTAGGCTATTGTGTTGAGAGAAATGAAAAGATTTTGTTATATGAATATATGCCCAACA
AAAGCTTGGATACGTTTCTATTCGGTTAGTATACTTCCCTCCCTACCATGAAACTCGTTGTAGTAATAGTT
TTATGATTGATTCTCTCTGAAGTTATCTTACAAACCTTGAAATTAGATCATGCATTCTGTCGATTACTGGA
CTGGAGGATTCGTTTTGAAATTATATTGGGTATTGCTCGAGGGTTACTTTATCTTCACCAAGACTCAAGG
CTAAGGATCATTCATAGAGATCTAAAGACGAGCAACATATTGTTAGATGAAGAAATGAATGCCAAAATA
TCAGATTTTGGCTTGGCAAGGATTGTTGAAGGCAAAAATACAGAAGCCAATACTAACAAAGTCGTTGGA
ACATAGTAAGTCTCTTAGTTCTGCTTCACCTTTGATTTGGTGGGAAGAAGATAGATATATACACACCTAA
TGTATTGGTTTACACAAACTAGATGCTAAAATAACCTATTCTGGAACACTAAAAACAGTGGCTATATGTC
TCCGGAATATGCATTGGAGGGACTCTTTTCAATCAAGTCTGATGTATTTGCCTTCGGAGTAGTCCTACTC
GAGATCGTAAGTGGGAGGAGAAACATGGAATTTTTCGAAGATGTAAACCTCATTGGTCATGTAAGTACC
AAAATTTCCCTCATTAATATACATCTCATTCTACTTAAAGCTTTCATTGACTCAAAATTAGCATTTCACAGG
TATGGAGACTGTGGATGAAAGACAGTGCATTAGACATGATGGATCAAACCATAGTTGAGTCGTGCGAT
GAAAGTGAAGTGCTAAAGTGCGTTAATGTGGCACTCCTATGTGTTCAAGAAGATCCAGCTGAGCGTCCT
GTAATGTCAAATGTTGTTTTTATGCTTGGAGGTGAAAGCATAACACTACCAAGACCAAATCAACCAGCTT
TTATAGCACGAAGAAATAGTGCTGGTGCAAGTACATCTTCTTCGTCTTCTAAGCTATACAACACTTCCAAC
AATGGGTTGACAATAACTCAACGAGTAGGCCGATGA
>PGSC0003DMG400019237 G-LecRK
GCCAAGTGTAATTCAAGAAGTGGACAACTTTGTCAAAATTCAAAATTGTCCAAAATCTTGCAAGCCCCAC
TTTGTCTTCCAAGTAATTCAGTCACAAAAATGAAGTCTTGGTCATATTGACATGTTATCACATCACCAACA
CCCTTTTAATATTTTTAAAATTTTTTTTGCCTTATTTTGTGTAACCAAGAAATGAAAAAGGGCTAGTAGCT
ATGGAGAAATTTCTACCCTTTTTGCTTCTTTGCCTTTTAATTTTTCTTCGAAAAGTTAATGGACAAGGAGA
TATGATAACTACCACTAAGTTTATCAAAGATGGTGAAACTATTGTTTCTTCTGATGGAACATTTGAGTTG
GGGTTTTTCAGTCCTGGAAATACTTCTACAAATCGTTATGTTGGAATTTGGTACAAGAAAATTTCTGTTAT
TACTCCTGTTTGGGTTGCTAATAGGTTGGTTCCACTTACTAATAAATCCGGGGTTTTGAAAGTTATTCAAT
CGGGCGGTGTTGCTCTTCAAAATGTTACAAATTCAACAATTTGGTCGACAAATTCATCGAGATTCGTGCA

AAACCCTGTTGCCCAGTTGCTGGACACGGGGAATTTTGTGTTGAGGGATGCTAATGATCCGAACCCAGA
GAATTTCCTTTGGCAGAGTTTTGATTACCCTACTGATACGCTGATTGCGAATATGAAACTTGGAAGAGAT
TTGATTACTGGGTTTGAAAGGTATCTTAGTTCATGGAAAAGCAGTGATGATCCTGCTCCAGGGGATTATA
CTTATCATTGTGATCCAACGGGTTATCCACAGGACGTTATGCGAAAAGGGGCTGATGTGATTTATAGAG
CAGGGCCATGGAATGGTTTAAGGTGGAGTGGTGCACCTAATATGGTGAACAATTCAATCACTTCGTTTG
GTTTGGTAATGAACAATAAGGAAATTTATTATAAATATGAACTCGTGAACAAATCTGTAGTTTCAGCTCT
AGTAGTAAAGCCTAATGGGAACACTATGCGTTTGATTTGGATCGCGAAGACTCAGGGTTGGGTTAACTA
CCATTCTGCTGATGCAGATGACTGTGACACTTACAAATTATGTGGTGCATATGGGACTTGTAACATTTTG
AGTGATCCTGTGTGTCATTGTTTAGATAAATTCGAGCCAAAGCATCAGGATGATTGGAATAGGGCAGAT
TGGACTAGTGGTTGTGTCCGGAAAACCCCATTGAATTGTACTGGAGATGGATTTATTATGTATTCTGGTG
TTAAACTGCCTGATACACGAACTTCTTGGTTCAATGAGACCATGAGTTTAGATGAATGCAGGGCGGTTTG
CTTGAGAAATTGTTCGTGTATGGGTTATACTAATCTGGATATACGCAATGGAGGAAGCGGGTGCTTGAT
ATGGATTGAGGAGCTGATTGACATCAGACAGCTATCTCAATCAGGACAGGATATCTACATTAGGATGTC
TGCTTCAGAAATAGGTAATATGCAATTTACTGAGGTTTTCTTTATTACACAATGCTTGTTATTAAGAATGA
TGTATTGGATAGTTCAATTCTGTAAGGCTGATTAACGAACGAAGAGTAGTGCATTTACAGGGAAATTAG
GTGTAAACATCCTCAAGCAATGGATCCTTTTGTTGAGAATAAGACTTATATGACCATCTTGTTCTACAATA
AAATAATTTGCACAGGATTTAAAAAGTTTATCGGAAATTCTAATTCAAACAACTACCTGAAGAAACTTTT
GGATGGAAAATGTTGCAGGTTCTGCTGGATCAAGTTCAAAGGGAGATAAATCTGTTATACTTGCAGTAG
CTTTGCCACTATTGTTTGCGTTGATTCTTCTAGGTTTAGGTGTTGGCCTGATTCTTTATAAACGGAGAAGG
AGAGAGGATCCAGTGGTTATGACAAGGGGTAAGTGAACATAACAAGTTTTTATCTGTTGAAAAGCCGG
AAGTAATTAATGGTTATTAGTTCTGTATTCCATTAGTATCTCTTTTCAAGCTCATCGTTTGAACAAAGTCTG
CTTGGTTGTTCAAATTGCAGGGAGATTTAGTGGTCACAACAACAAGAACGATAACACTAATCAAAGTCA
CCATGAAGATTTTGAGCTACCACTTCTTGATTTGCTTACATTAATCAATGCTACTGACAACTTTTCAATTGC
GAACAAGATTGGAGAGGGTGGCTTTGGACTAGTTTACAAGGTAGAAACATTTGATCCTTGAGAGCACAT
TGAAACCTTAAATCGATTTAACTTTTTGGTTGAAATGAAAGTAAGTTCTACTTTTACAGGGTGTGTTAGA
AGGGGGGCAAGAAGTAGCAGTGAAGCGGCTATCAGAAACATCAAAGCAAGGAATTCACGAGTTCAAG
AATGAAGTTAACTGCATTGCTAAACTTCAGCATCGAAATCTTGTAAAGCTTCTGGGATGCTGCGTTCAAG
GAGAAGAGAAGATGTTGGTCTACGAATACTTGCAAAACAAGAGCCTTGATATATATATATTTGGTTAGT
CTTTCACATCCAGTGTACATCCATTTCTTTCAAGTGAACTTCTGATGTGATTTCACTGAGCTTAGTTTCATG

TTTAGGAGAAATTATAATAAGTTGAAAACATTTGGCAGATGAAGAAAGGAGCGCATTACTTGACTGGCC
AAAGCGCTTCAACATTATCAATGGAATTGCTCGTGGACTGATGTACCTCCATCAAGATTCAAGACTTAGA
ATCATCCACAGAGACTTGAAAGCGAGCAATGTCTTACTTGATACTGAAATGAACCCGAAGATATCAGATT
TTGGGATGGCTAGAAGTTTTGGAGGAGATGAGACTGGAGCCAATACACGCCGTGTTGTTGGAACATAG
TAAGATTTAATCTTTAATTTTACATGTAGTTGTTACATTCACATGTGCATTTCCTGCATTATATTGTTACTT
GTCCGCGTAGCTTGGGATTTTCAAAAGCAAAAAAGAAGATATTTAACTTGCCTTGTTCTACCCCGCTCTG
TCCTGCATAAGAGCTCCTCTACCCTGCCCTATATAAGATTAAACCCACCCTATCCCATTGCCATCTCTAATT
ATATAGTCCTTTGTTATCGTTGTTGCAGTGGTTACATGTCCCCGGAATATGCAGTCGATGGGATATTCTC
AGTGAAATCAGATGTCTTTAGCTTTGGAGTATTAGTACTAGAGATTGTGAGCGGAAAGAAGAACAGAC
GATTCGTTCATCCAGACCACCACCTCAACCTTCTTGGACATGTAAATACTATGTCCCAACTCCTATGTGTA
AAAGGACTTTACTCAAAGTATCTGTTAATGAGTTCAAGAGTAGTATATAAACTATTGACATTGTCAATGT
ATATAACTTAAATCGTTTGTTAACGGATGAGTCATTATATGCAGACATATATGCTACATAAAGAAGGAAG
ATCATTGGAGCTAGTTGATCCAAAACTGGTGGACTCATGTAATATATCAGAAGTACTAAGATCAATCCAT
GTAGGTTTATTATGTGTTCAACAAAATCCAGAGGACAGGCCAAGTATGTCAACTGTGATTATGATGCTG
AGCAATGAAGGCATTTTGCCATCAGCTAAACATCCTGGCTTCTTTACTGAAAGGAAAATTGGTGATGTTG
ATCAATTTTCTTGGAGTACACAAACACCTAGTTCTATAAATGAAGTTACCATCACATTGTTGGATGCTCGA
TAGGAAGAATGTCGAGTGATTTCTGAGATGATCACTCAACTAATAGTTACTATATCAGAAAGTCACTTTC
TTTGTTAAAAGATCATTGGAATGTGATTTTCTGAGATAATTACTACTACTTAAGTAACCATCTGAGAACTC
AACCAGAATTGTATTAAAATCTTCTCCTCTTTTCTATTTTATTTATTTAGTTAGAAATTTTTGTATTTCAACA
ACATACACAGTGTGATCCCACTTGTATTCTTTTGTAGTAACATATTTGTCTACATTGTACGTTAG
>PGSC0003DMG400019238 G-LecRK
ATGAATGCAATACAAAGGAATGATTTTTACTTGTTATTGTTATTGTACTTGTATTCGATCAACCATACTTTT
GGTGCTACAGATAGTACTATAACAGCTACTCAGTTTCTGAAAGATGGCGATAAAATTGCTTCACATAGTG
GAAGCTTTGAAATGGGGTTCTTCAGTCCAGGTGATTCAGAGAATCGCTATGTGGGTATATGGTACAAGA
ACATATCTGTTAGGACTGTGGTGTGGGTAGCTAACAGAGAAGCTCCTTTGACCGGTGAATCAGGTATCT
TGAAAGTCATTGAACAAGGAATTCTTGTCCTTCTCAATGGTACTAACAATGTTGTGTGGTCAACTAATAC
CTCTAGATCTGTGCAGAATCCTGTTGCACAGTTGCTGGATTCGGGGAATCTTGTTTTAAAACAAGCTGAT
GATGATAACTCGGGAAATTTCCTCTGGCAGAGTTTTGATCACCCAAGTGATACACTATTACCAAGCATGA
AGCTCGGTTGGAACTTTGTAACTGGCCGAGAGGTGTACTTGTCATCATGGAAGAACGAGGAGGATCCA

GCTCCCGGTGATTATACATATCATTGTGATCCTTCTGGATATCCACAGAACATCCTGAAGAAGGGTTCAG
ATGTTGTGTATCGGTCTGGACCGTGGAATGGTCTTCATTTTAGTGGTGCATTAAGCTCAAGAGACAGTCC
TTTGTATACATTTGGGATATTCTCAAGTAAGACGGAGGTGTATTTTGGATTTTATCTCACATCTTCGGTTA
TCACAAGGTTGACACTAAGTCAGAATGGTGCATTACAGCGTTGGACTTGGGGTGACCGTGCACAGGATT
GGATCCCTTACCTCTCAATACCCACAGACAACTGCGATGTTTACAAACTGTGTGGCGCATATGGTAGCTG
CAACAGTCAAAATTCTCCTGTATGTGGATGCTTAGACAAATTTGTGCCAAAACATAATGAAGATTGGCAA
AAGGCAGACTGGTCAACTGGCTGTGTTAGGAGGACTGAACTAAACTGCCTCCAGGGAGATGTATTTTTG
AAGTATTCGCATATTAAATTGCCAGACACACGGAATTCCTGGTCCAATGTGACTATGACACTAGAAGAAT
GCAAGACTATATGCTCTAGAAATTGCTCATGTATGGCTTACTCAAATATTGACATACGCAATGGAGGAA
GTGGATGCTTATTGTGGTTCAAGGATCTGCTTGACATTCGACAGCTATCTAAAGAGGGACAAGATATCT
ATATAAGAATAGCTGCCTCTGAATTAGGTATGTTTGTTACTGAAACAGTTAACAATGATTTGTCATTTGTA
ACTTGAAAATAAAAACCTAAATTTATGGTTGCTGATACTTGGGGTTAAGCAGAGGTAAACTTAAATTTCA
ACTGTGCTTATGCTTATAAGTATCTAGTACTAGAACGAAATTTCAAAGTCTTGCGTGCTGTATATATATAT
ACTAGTTTCTGAGCACATGCTTCGCACGTGTGTTTCATGTGAATTTTTATAGATACGTTAGAATGACTAAA
ATTTCATGAAAATTTATATATGTAAATTGTAATGAACAATAAAATGCAACAATTACAAAATAAGTATAAA
GACTTACGACAATAAAACATTCATAGAAAACTAAATTAAGAAGTCTAATAGAGAAAAAAAAAAAAAAGAC
AAAAAGAAACAGATTTAAGAAAAAATACAAGGTAGACATTATTCTTATAAGACTGATACATATATAGAT
GGAAAAATAAGTACAAAATTAGTACATAACTCTCCGAGTTCTTATCGATTCCTTCCATATCCTATCAAAAT
TCAAGAGTCTTCTTCATCTACTTTAACTATAGAATAAAAATAATAATAGCCTAATAATCTTGGTAGGGATT
TCATGAGATAAAGAAGTTGAATTTATATAGATAGAATAGAAGGAATATCAAAAGATATCTTAAAGTGCT
AGTTTAAATATTTAGAGGTTATGAATATGAAAAGATGAGAGTTATGAATATTAAGAGTATAAAAGTTGA
ATAAGTAATTAGAAAATAATAGTAAATAAATGAAGAATATGAAAAAGAAAATTAAAGTTAGCAAACCAT
GTAGAAAGAACAACTAAAAGTTCTTATCATGTAATTAAGCATCAATGAATTTAAATTTGTGAAGCTAGAG
TCATTCTTTAATAATAATCAAGAGGACATTATCAATGTGTGCGTCTCCATTTTGCAGATTTGCACCCTTCA
AATTATTAAATTTCGTTAGTTATCATAAAGGATAATACTTTATCCACATAAATTATATTAAAGGAAAAAGT
TATGAGAAGAGGAAAAGTATAGCAATTTAGGTATAGACTTTTTTTAATTTTTTCTTTTATATATATAGATT
ATAGATGATAAATATTAATAAATTAGATATTTAATTAGGAATTAATCTTAGGAAGTCTAAAAGTCATTAA
TATTTTAATTAATTTTTTTTTAAAAAAAAATTATAATTATTATATACATATAGATTATAGATGATAAATATTA
ATAAATTAGATATTTAATTAGGAATTAATCTTAGGAAGTCTAAAAGTCATTTAACATTTTAATTAAACTTA

TACAATCAAATATTTAAATATTTTATAACAATTTAATTAATAGAAGGGGTAAAACCGTAATTCAACTTTCA
ACTTTTTTTGTTCATGCTTATAGTAATATATGATATATATATATATATATATGTATAGCTAGGAACTAAAAC
TTTAGTAGAATAAAAGGCTACTACTTAGATTGAGGTGTTGAATTTTAAAGCTAAATCTATAAGCATAATG
AGTTATTGCATCTGTCAGCTAAAAATCTAAGACGAAATAAAAGAGGAATTTAGTTGGAAAAACTCATTG
AGAACTAAAAATATGATTTGCAGAATACCTCTACTAAATGTCAACAAATTAAAAATGACGTACCGTACTT
CAAAGTCCAGCAGAATGTTGTAACTCAATATCAGTTATCAACTCCCCCAAGTGATCAACAGGTAGAGACA
GTCAAATTTATTGATTACTTTGTTGGAATGGACCTTGCAGATAGTCTGGAGAAATCAGATGGTGAAAAA
GGAAGAGCACTATGCTGGATTCTTCCATTATCCGTTGGTTTAGTTCTAGTAATCCTTAGCATATTGCTGAT
ATGCCACAGAAGAAGGAAGAAGGCTTTAAAGCTTACAACGAAAGGTAAGCTGGATAGGTCACTTTTTTG
CCTGATCGTTGAGCAAAATAGAGGAAGTGAAATTCAATTAGAAGTGAAGACGGTCTTTGCTGCTTTAAC
ACAATTCAATCAATTTCCTTTTAGCTGCAGAAAAATCGAGATACAGCGGAAGTATCAAGCAGGATTACAA
CAGTGGTAGTTGCACTGAAGAATTTGAGATACCACTGTTTGACTTATCTACCATAACTAAGGCTACCGAT
AACTTTTCAGTAAACAGAAAGATTGGAGAGGGGGGCTTTGGTCCTGTTTATAAGGTAAAAACACTTGTT
TCATGGGCAATTTTGTACAAGAAAAATACCAGCTTATCACACAAAGTCTAGATGACTGACAGAAGCTTCC
TGGTCCTCCAGAGCAAAGCCTTCATCTTTAGTAGGCAAAATGTAATGAATTAAGTATATGCTGGAGCGTT
TTTTGACCTAAACTATACTTAAACTGATTCTCATAATTTCTCCAGTCCTTTTTCGTGGGAAGCAGCTTACTT
TGTACCTTATACTTTCAATTTAGATATTATTTATTGTTTTTGAGAAAAAAGATATAAAATATATCGGAAAC
AATCTCTCTACCTTCAAAAGGTAGTGGTAAGGTTGTGTACACACCAGTTACCACCCTCCCCAGACCCCAC
TTGCGGGATTACACCAAGTATATTGTTGTTGTACGTTATGTAAAATATTTAACTTGCAGATTTTGATGTTT
AGGGAATACTTGAAAAAGGACGAGAAATAGCTGTCAAGCGGCTGTCTAAAACATCCACGCAAGGAGAA
GATGAGTTCAAGAACGAAGTGGTATATATTGCCAAACTTCAGCATAGAAATCTTGTGAAGATTCTTGGA
TGTTGCATTGAAGGAGAAGAAAAAATGCTGATATATGAATACTTGTCAAACGGAAGTCTTGATTCATTTA
TATTTGGTAACAAATTCCCATACCAATATTGTGTATTCTGAATTATCTTCTCCCAGTTTCGAATCTCCCATC
AAATAAGAAAAAAAAAAAACATGTAAGGAGTGAACTAAAACCTCAGCTTTATTAGAGCCTAATAATAATG
TACACAGTAGCCCACATTGATCTTTACGTTCAATACGTGAATGGACGGATTTAGTTTGATGTTTTAGAACT
ACTTTCTATCTATAGCTTAGTGAACCAACAAAATTTGGCAGGTGGCAGACAAAGCAGGATATTAGACTG
GTCTGAGCGATTTCACATCATCAATGGGATTGCTCGGGGACTTGTGTATCTGCATCAAGATTCTCAACTG
CGGATAATTCATAGAGACCTGAAAGCTAATAACATCTTGCTAGACAATGATATGAATGCAAAGATATCA
GACTTTGGTATAGCAAGATGCCATGAAGAGGATGGTAATGAAGCCATGACAAACCGAGTTATTGGAAC

ATAGTAAGAATCTATTACTGGGTAGTAGGAAATGCTTGAGATAGTTATGTTTTCCAGCATTAGTAACTAT
ATTTTGTGTACATTTTCTGCAGCGGATACCTCTCACCAGAATATGCGCTATATGGGCAATACTCTGTAAAA
TCAGATGTGTATAGCTTTGGTATCTTAGTACTGGAAATTGTGAGTGGAAAAAGCAACAGACGATTCGCT
CCTTCAAACCTTAACCGTAGTCTAATCGGGCATGTAAGATTTTTATGTATTTTTCTTATTTATACATTGGTG
TTAGCAACTAACAAGTATCTTGCAAGGCTAGTGATGCTTATAAGCTTGTAAAATCTACAGGCATGGGAA
CTCAACAAGGAAGGTAGGTCAATAGAACTACTTGATGAACGCGTAGGTGATTCTTGTTCTACACCTCAA
GAAGTGGTACGATCAATTGGTGTTGGTCTATTATGTGTCCAAGAACGTCCAGATGATCGTCCAAGTATGT
CTTCCGTAGTTCTAATGTTAAACAATGAAGGTACGCTGCCTCAGGCTAAACTACTAGCTTTTTACATGGA
AGGAGATGCATCAGATACTGAATTACTTTCTACCCAATCACATATTACAACGGATGACACCCCGTTAGAA
ATTAGATGA
>PGSC0003DMG400019241 G-LecRK
TTGATTTTTCACATTTTCCCAAATATTCCAAAAGGACTAAACAATTTCCATGAAACGCTTTAGCTTTTTCTA
TTTCTGTTCCTCTAATATAGTGTCATTTCTATTCATATTTTCCACTGCTTTAGACACTATATCGAACGATAG
CCCCATCAAAGATGGACATACCATAGTTTCAGCTGGTGGAAACTTTGAGCTTGGATTTTTCAGCCCTGGA
AATTCCAAGAATCGGTATATCGGTATATGGTACAACAATTTACCTAAAGGCAGAGAAGTAGTGTGGGTT
GCCAATAGAGTGAACCCACTGAATGAAACTTCTGGCATATTAACAGTCAGTAGCAAAGGGATTGTACTA
CTCAATGGAAACCAAGATGTGATTTGGTCATCAAATTCATCGAAAAGTTTGATAAAACCAGTGGCTCAAC
TTTTGGACGCTGGAAATCTTGTCTTAAAAGATGACAGTTTGGTAAATCAAAAAGACTATGCTTGGCAGA
GTTTTGATTATCCAGACAGTACTTTGTTACCTGGCATGAAATTAGGCCTTAACTTGGTCACGGGCAAGTA
TTGGACTATGTCGTCGTGGAAGAGCAGTGATGATCCTTCTCCAGGAGAATATTTGGATCGCCTCGACAC
CAGTGGTTATCCTCAATTCTTTGTGTGGGAAGGTCCTGCAATAAAGTTTAGCTCAGGAATATGGAATGG
ACATTTATTTGTTGGAGGGCCAAATTTGAAACCAAATCCTTATTATACATTTGAGTTTGTGAACAATGACA
AGGAGATCTACTATAAATATGAGCTTATCAATACTTCTATTCCTACCAGGTTGGTCTTGAATCCAGCAGG
CCTGCTTCAACGACTCTTATGGATCGAACGGAACCAGAACTGGTTCCTTTACTCCACTGGACAGATGGAT
AATTGTGACCGTTATGCCTTGTGTGGTCAATTTGCGCAGTGCAACATCAATGACTCACCTCCATGTGATT
GTTTGAGAGGATTTCAGCCTAAAAATCAACAAGGATGGGATGCAGCAGATTGGTCTAGTGGGTGTGTA
CGAAGAACTCCGTTGACATGTGGGACAAGTGACAGGTTTCTGAAGTATTCTAGTGTGAAATTGCCAGAT
ACACGCCATTCTTGGTTTGACAAAAGCATTGGCCTCGAGGAATGCCAAAGGCTGTGTCTGAAAAACTGT
TCGTGTACAGCATATTCCAATCTTGATGTTAAAAATGGCGGGAGTGGATGCTTACTTTGGTTTAATGAAC

TCGTTGATATCAGAGAATATGCTGAACTTGATCAAGATCTCTATGTAAGGATGGCTGCATCAGAATTAG
GCAAGTCTCCTTATGCTAAGATGAATCATACCACAGGGAATCTTAGTAACTCAATTGATTGATTGACTAT
TTGAACTTCCACCTTGTTAACGAGGGTTGGGTTCCCAACCTCGTAATCCCCTTTCCCATTTCCCATTTAGCC
TTCACCTACCCCCAACCCCATTTCAAAAATAAGAATCATACCGGAAGGTTTTCACAAGTAATAATGAATTT
TCACAGGTTCAGGCTATATGGGAAACACAAGAACATCAGTCATTGCAATTATATTAACAGTTTCAGCAAT
CATCCTTGTTGGTTTTCTATTTTGGTTTGCTATGCAGAGAAAGAAAGGGGAAAGAGGTTAGAAAATTTGT
CCGTCGCGTTGAAAGTATAGTTATTTCATTCACCTCAATCTCTTTACAAAATAAAGTTCAATTTTACAGGA
GTAGGAGAAGGAGAAGGAAAGGAAGATATGGAATCACCTTTGTTTGATGTGATGACTGTTTCTGCTGC
AACTAATAATTTCTCATCTGCTAATATCATTGGAGAGGGTGGTTTTGGATCTGTTTATCGGGTAACTAGA
ATTATATTCTCTGTGTATCTTAGTCAAATATATGAATGATTCTCTTGTGACTTAGACTTCGCGTGGATTAT
GTGACAGGGAAAACTATCAACAGGACCAGAAATAGCAGTAAAGAAGCTCTCTAAACATTCCGGACAAG
GTTTTGAAGAGTTGAAGAATGAAGTTGTTCTGATTTCCAAACTTCAGCATAGAAATCTAGTTAGGCTTCT
AGGCTGTTGTCTTGAAGGGGAAGAAAGGATGCTAATTTATGAATATATGCCAAACAATAGCTTGGACTT
CTTCATCTTTGGTTCGATTCTCTTGACGCATTTCATCAAAAGATTCATCTTATCATATCAGCATTGTTCTGC
TAATGCAAAATGAACTGATGTTTCTGCAGATGAATGCAGGAAGAAACAACTTCCCTGGGAAAATCGTTT
TCGTATTGCAATGGGAATATCAAGGGGCATTCTTTATCTTCACCAGGACTCCAGATTAAGGATCATACAC
AGAGATCTAAAGACCAGCAACATTTTACTGGACAGTGAATTGAATCCCAAAATTTCTGACTTTGGACTGG
CTAGGATTATTGGTGGTGACCAAAACGAAGCAAGAACAAAGCGAGTAATAGGGACATAGTAAGTTCAA
TTAGATAACTCATTGATGCTTTCATCTTTTCCTCTGTTAGAGTTCCACATTGGTTGGGGAATTGACTGATT
TATATGGACTTGGGCAATTCTCCCTCATAAACTAGATTTGAGTTGAGTTAGACTCAAGTGTCATATCCTTC
CATGGTATCAGAGCCAGGTAAATCCCGATTGGGGCTCCTGGTCCATGTTGGTGGTTTGGTTATATGAAA
TTGGACAATCTTTCCCCTTATGAGCTAGCTTTTGGGGTTGAGTTAGGCATGTGTCAAATCTTTACAACTTC
CATAAAATCATTTAACCCATATTAAACATTATATATGTCTTTGTGCAGTGGCTATATGTCTCCAGAATACG
CAGTTGATGGGAAATTTTCAGTGAAATCAGATGTCTTCAGTCTTGGTGTTCTTCTACTAGAAATAGTCAG
CGGAAGAAAGAACAGAACATTTCATCATCCAGATCACCATCATAGTCTTATAGGACATGTAAGCAAGTT
ACAAATCCAGACATTTGCTTTTGTCATTTCATACTACATTTGTATTTCCTACATGTCATTGAAAACTATACT
TGATTTTGCTATAGGCTTGGTTATTATGGAACGAAGGAAAGGCTTTGGAACTAATCGATGATTGTTTAAA
AGAATCGTTTGTGGAATCCCAAGTACTAAGATGTGTTCATGTGGCACTGTTATGTGTTCAACGACTAACA
GATGAACGACCAACAATGTCATCAGTGGTTTTCATGTTGAGCCATGAAGAAGTGGCATTACCTCAACCA

AAGGAGCCTGGTTTCTTCATAGAGAGAAGTATAGCTGAAACAGATCATTCAAATGAGAAAAGATGCATC
AGTGACAATGTTTTGACATTAACAATTCTTCAACCAAGATAGCAATGGCGGAACCAGAAATTCAAGAAC
CTTCTATTGTATAAAGGTGATTCAACAACTAATGTATATACAATACAGAAAATCTGTTCATCCCTATATGT
ACAGTAAAATATTTTGACGAAGGGGCTTCAGTTGAAACCCCTCTAGACCATGCAGCTCCGCCCCTGATTG
TTATTGGACGAGCATTTACTAACATGCTTACATAGAACAATTGCGTATATCTTTCTGATATTTGATTAAGA
CTCACGAGCCATCGGGACCATTGTGTCAAGTTGTGCAAATTGTCAGAACATGATGGAAATGGACATGAA
AGTTGTTACACAGTATAATGACTTATAGTAGATCTGAATGGCTAAATGATAGCCACTGACTTTGTTGAGC
AATTATTTTTCCTCATGACTACAAATCAATATCATGTCCTGGGGGAGTGTTTCTTTGCAATCACATTCATC
ATATCTTTTTCCACATCTCTATCACCAAACTCGAGTTTTGTGCAACTTTTTCCTGGTTTTTCTACCCAGTGTT
CAGTACCAGCTTTGGGGGCTTTTTTTGAAAAATGCTAAACAGGGTAGAGCAAGGTGGGTCGATATCTTA
GCAGGTCAAGGCTTGCCTCACCTTAACCCGCCCTATTGACATCCCTACCTACTTCAAGGTAAGCCAGCTTC
AACAAGACGCCCATAAATCCCAGAATGCCTACCTTCCTAGTTCCTGGAACTATGTAAATCTTTGCATTTGA
TTCTCATTTGAACAGCATACAGATTTATATTCAGAGGTAGTTACAATAAGTAGAACAAGGAAATGTTCTC
GAAAACACTCACAAAATAAATAATTCAAAAAAATGAAGTCATAGCCAACAGAAAAAAAGGCCGTTCAAT
CAAGCCAAAAAACAAATCCCTTGATGGTTTATACTCCTAAGGCATAAAGCAGTGCAATGGAAGCTCCTAC
AGTGATTGTCCAGAGATATATGGTTTGAGACAAAGAATTAGCACCACTGCATTGGTCATTAGAACCACT
GAAGCATCCAGGTCTACTCACCTTAGGTTCTACTCCCCCTGTGGAAAAGCTACTCTTTGGTTTGCCATTGC
TGAAGAGTACACACCAGAAATAGGGGCCACCACCACCGGAGCCACTCACAGCAGCACCAACCTCAGTGT
GATTCTTGCTATAGAGAATATCCAAACTCTTGGTATTTCTTATAAGAATATCATTGAATGCTTCAGAAGGT
TCGGCATACTTGGATTGACATGCGAGAAATCTTCCAGTTATTTGAGCAAGCGATGACGCCTGCACACCAC
AGTTAGGGGCAAATTCTTGGGCGAATTCAGATTCAGCAGGTTTCTTTCCATCTGGCCCTCCAACTACTTTA
CAATCACCTCCATATGCTTTTATATATTGCAGAGCCAAGCATGCCAAGCCAGGGTTACTGTATAAGGAGG
ATAATTTATTCGCAGTTCTATTACTATTAAGGGCAGATACCAGCTCATCAGCAGGATTATCAGTTACCTTG
GCTGCAGTTTATCAAGCCAATATATGTGTTAGAGGTTGATCATAAAGGCAATAATAGTGAATCAAGATC
GAGAAGCTATCTGCTTGAAGGAATACACATTGGGAATGACAAGCGTAAATTACATCTAAACCCAAGAGG
CTTGAGCACATGTCAGTATGCTTTCGGTAATTGGAAGTACACGCAATTATCTAAGACTGACTAAAGGAA
GAAGAGCCAGAGAAGGAGAGTAAACATCATTTCGGCTGTGACTATAATTCTAGCACACTCATGGAAAGA
TTATCAACAACCACCCTATTATCAAGAGATCTCTAACAAGAAAGATACAAAATTAGGGCTCCAACATAGT
ATTTGAAGGCATAAACTAAAGCATCTTGAGCACTGTCCTTCCATTAGTGGGCTAGAGTCCCACATTGGTT

GGGAAAAGACTGGTGGCTTGTTTATATTAACTTAGACAACCCTCCCCTAAAAGCTAACTTTTGAGGTTGA
GTTAGGTCCAGATATCATATCTTTACCTGGGCGGCAAAGGGTGTTGGAATGGGTTAGAGTCCTCATTGG
TCTCCTTATATGGACTTAGACAACCCTTTCCTCATGAGATGCCTTTTGTGGCAAGGGGTGTTGGAATGGG
TTAGAGTCCACATTGGTCTCCTTAGATGGACTTAGGCAATCCTCCCCTCATGAGATGGCTTTTGTGGTTG
AATTAGGTCAGGTGTCATATCTTTACGGGTATCTTCTCATGTTACCACAAATTACAAAGACCGAAAAACC
AATTTGAGCAGCTTCCCCTAACTATTCACACTATTCACAAAATCAGAATTTGCAGATTGACTAAATAGTTT
CTAGTCAGTGCTATAAATGCAACATGCCAACAAAATCAGAATTTGCAGATTAACTAAAGAGTTTCTTGTC
AATGCCATAAATGCAG

>PGSC0003DMG400019275 G-LecRK

CTTTTTGATGTAAAAATCATGAAGGCATTACTTAGGGAAAGTTTCTACTTATTCTTGTTCCTGTGTTTGTA
TTCAATCCATCAAAGCTTTGGTGCTACAGATACAATAACTACAACTCATTTTCTAAAAGATGGTGATGAT
GATATTACTTCACCTGGTGGAATATTTGAAATGGGGTTCTTCAGTCCAGATAATTCGGAAAATCGCTATG
TCGGTATGTGGTACAAGAACGTATCTGATAGGACTGTGGTGTGGGTTGCCAATAGAGAAGCTCCTTTGA
GGAGTGGATCAGGTATCTTGAAAATAATTAAGTCTCGACTTGTCCTTGTTGATGGAACTAACAATGTTGT
GTGGTCAACTAATACCTCTAGATTGGTTCAGAATCCTGTCGCGCAGTTGCTAGATTCTGGAAATCTTGTT
GTTAAAGAGGCTGGTGATGATAGCTCGGGGAATTTGATATGGCAGAGTTTTGATCACCCAACGGATACG
CTATTAGCAGGCATGAAGCTTGGGAGGAATTTTGTAACCGGAAGAGAGGTGTACTTGTCATCATGGAA
GAATGAGGAGGATCCAGCTCCCGGTGATTATACATTCCATTGTGATCCTACTGGTTACCCACAGACTATC
GTGAAGAAGGGCTCAGATGTTGTGTATAGCTCTGGACCGTGGAATGGTCGATATTTCAGTGGAACACAA
AACTCGAGACTAGGTACTTTCTATACATATGGAGTATATTCAAGTAAGACAGAGGTGTATTTTTTGTATA
AGCTCACATCTTCAGTTCTTGTGAGGTTGACAGTAAGTCATAATGGAATTCTACAGATTTGGACTTTGGA
TGACAGAGAACAGGGTTGGGTGCCTTTGATCTTAGTACCAGCAGATAAATGTGAGGTTTATAATTCATG
TGGCGCATATGGTAGCTGCAACAGTCAAGATTCTCCAGTATGTAGATGCTTAGATAAATTTGTGCCCAAC
AATAGTGATGCTTGGAAGAGGGAAGATTGGTCAGGTGGCTGTGTTCGGAAAACTAAACTAAATTGCCTT
CATAGAGATGTATTTTTGAAGTATTCGCATATTAAATTGCCAGACACACGGAATTCCTGGTCCAATGTGA
CTATGACACTAGAAGAATGCAAGGATATCTGCTCAAAAAATTGCTCTTGTATGGCTTACTCAAATCCTGA
CATACGAGGAAGTGGATGCTTATTGTGGTTCGGGGATCTGCTTGACATTCGGAAGGGAACCAATGGACT
GCAAGAGATCTACATAAGAATGGCTTCTTCTGAATCAGGTAGGCTTGTTGCTACTTAAAGATTTTTGATA
GATTACCTAAAGATAGTAAAGGAATTTGGGGTTGCAAGCACTTTGAATAAAGCAGACTGTATTTTTGTG

CCTATGCTTGTAAACATCTAGTAGTGTCAACTGCCAGCTCCTACCATATATAACCAGGAACTAACAAATTA
TAGTAGAACGGAAAGCGACTCCTTTAGAGTGAAGTTGGTCTGAACTAATAAGCTGAATCTACAAGCATA
TGACTGGTTAGACCCTGTTCAATTTTTTAGAACTAATTGGTAAGCACTTAGTTTTCTATAGAACAAGCACT
TGATATGCAGTACTCCAAAGGCTTGCAGAACCCTTAATAACTCAGAATAAGAAATCATCTCTCCCATCCG
CTTGCAGAACCTTTAATAACTCAGAATAAGAAATCATCTCTCGAATGGATTGGAGAAAGATTGAAATTCA
TTATTTGTCTCAGTTTAAATGGTTACTTGCAGATGATCAGGAGGAATCGGATGGAAAGAAAGGAAGAGT
ACTTTTCTGGATTCTGCCATTATCAGTTGGTCTGATTCTGGTATTCCTTAGTCTGCTAATCTACCATAGAA
GAAGGAAGAAGGCTTTAGAGCTAAAAAACAAAGGTAAGCTGGATCCCCTTTTATTTTTCTTGACGTCTAA
GCAAAATAGAGGAATAGACATTTAAATATTAGTGAAAAGAACTTCATAGTTGTCTGATATTCACAACTTA
AACAACATTCACTTCATTTTCTATTTCTTAGAAGCAAAATTACATCATTTGGTTTTGGCAGGAAGGACAGG
ATGTGGGGGCAATTGCGCTGAAGAATTTGAAATACCACTGTTTGACTTATCTACCATAGCAAACGCTACC
AATAACTTTTCAATTGACAGACAGATTGGAGAGGGAGGCTATGGACCTGTTTACAAGGTATACACACAC
TTAAACCCTTGTCCTTCAGAGTAAAGCTTAATCTTCAGACACAATTTAGGAGTTGTATGATCAAAGGTGT
AATGACAAAAACCTTTGCTTTGTGAAACTAAAAGGAGACAGATACAATAGCTAAGGGAAGAAAGGTGG
GGATATGGGGGATGTCCTTCTCAGAATGTGTCCTAGAACCACTATCTTAGCTTTTCCAATAAGTGTTGTC
GACAATTAGCTCAAGTTAGTCAGTCTAGAGTCTTTAGAGGGGATATCCTTCTGGTGCGTGAACTTGTACA
TTTCTAAACATTCTCCGGATGTATTTTTATGAGTTAAATTATCTTTCTCTTCTGCATCTCAAGCCTTTATAGT
GAGCCGACATTTTGATCAACTTAGATATTTAAAATATTAACATGCAGATATTGTGATGTTTAGGGCATAC
TTGAAGGAGGGGAAGTAGCTGTCAAGCGGTTATCTAGAACTTCCACACAAGGAGAAAATGAGTTCAAA
AATGAAGTTGTATATATTGCCAAACTTCAGCACAGAAATCTGGTGAAGATTCTTGGCTGCTGCATTGAAG
GGGAAGAAAAAATGTTGATCTATGAGTATTTGCCGAATGGGAGTCTTGATTCATTTATATTTGGTACAAC
TTCTCAATCCAATATTAAGAATTTGAATAATAATTGTACTCTCGTATTATTTTTCCATCGACAAAGAACTG
ATTTTGCTTTAGGCTTTAATAGTACCTTCCACTATACCTACTTAGAATGTTAATAACCCAACATTATTTGGC
AGATGACGCACAAAGCAAGGTATTAGACTGGCCTAAGCGATTTCACATTATCAATGGTATAGCTCGGGG
ACTTATGTATCTGCATCAAGATTCTCAATTGAGAATAATCCACAGAGACCTGAAAGCTAACAATATTTTG
TTAGACAAAGACATGAATCCAAAGATATCAGATTTCGGGATAGCAAAAATATGTGAAGAGAATGACATT
GGAGCCAAGACAAACCGAGTTGTTGGAACATAGTAAGAACCTTCAACTGGGTAGTTAGGAATTTGAACC
TAAGACGAGTTTTGAGATATTAAGCATGTTTTCCAGCACTAGTAATTCTACATTTTGTTTACATTTTTATGC
AGTGGGTACCTCTCACCGGAATATGCATTGCACGGAAGATACTCAGTAAAATCAGATGTATTTAGCTTTG

GTATCTTAATATTGGAAATTGTGAGTGGGAAAAGCAACAGAAGATTCTCTCATCCAGATCACAACCTTAA
TCTACTTGGACATGTAAGATTTGATTAGTTTTATTTTACTTATCCTTTGGTGTTATGTTATCTCCTAGTAAC
AGAGATCTGCAAATCTAGTCAAATGCTAATAAATCTTGTAATCTACAGGCATGGAAACTCTATAAAGAAG
GTAGGTCAACAGAACTACTTGATGAATACCTAGGTGATTCTTGTTCAACAACCGAAGTGGAACGATCAA
TCTGTGTTGGTCTATTATGTGTCCAACAAAGTCCAGAAGACCGTCCAAATATGTCTTCTGCAGTTATGAT
GTTAAGCAATGAAGGTGAATTGCCACAGGCTAAACGGCCAGGATTTTACATAGAAAGAGATGCACCAG
ATGGTGAATTATATGCACAGAATACAGTGAGTGAGACCCCCATCACAATATTAGCTGCCCGATAAGAAA
ATATTTGTCCACTCGCTTCCACAACAAGCTTGCAGTTTTAATAACCTTTTCTTTAATTCTTTGTAGAAATGT
GGTGAAAATTTTGTGTATGGCTTTGCCTTATTGGTGTATTAACTATGTCAAACTGACTCAAACATATGTAA
AGTGCTGATTCGCTGTTGCTTCTCAATACAAGGAAGAGGTTAGCCAACAAATCAAGTTCAAGAACATTA
GCCTCAAACCCAAACACAAGCTCCATAGAAAAAGATGGTGTTACATCAGTCCATAAAACTGTTCTTAACC
TGGCGATATGATCTACGGATTTTAATCTATCGCAAAACTGTTTTAGTTTTAGTACTTTACTAATGGATTTT
GTCACAACTTTCAATAAATCAAATTCAGGTTTTAAACTTTGATGAGACATTTCCTCAATCAAACTAGTATC
ATTTTAGTCATTACTTGTACTTAATTTCCTTTCTACTGTCATTACTATAATAACAACAACATGATCTGGAAA
GGGTAGAATGTATGCATACCTTACCCTTACCTCGAGATAAAGAGGTTGCTTCTGATAGACCCTTGTCTCA
AGGATAAACAAAATAACGACACTGAAGGAGCCATGGACTGTCATAGTATAATCTATAGCAAATCACAAT
TACTCCATGTCCAATCATTTAGAAGGGGAACGAGGAAGCAATAAGTTTCGGTACTTAACCCCTGTTCTCC
AATGTGCACAGATTATATATAACGTTAATTGGAACAATAGCATTTCGTAATTGATGAAAGAAAAATATTC
GTACCACGAGTTAGCCGCCGGTAGCGTTGGCCGATTCGGAGCACTGAATTTTGATTGGATTCGCCTGAT
TACCGAGCACTGAAAAAGGAAATGAAGGTACAACTTCAATTATACAACTTTACACCAATTTATGAATCAA
AGATGAACTTCCTCATAGATACAGATGAATGCATATCGGCAGTAGAATTAGATGAAAATGAGAGAGGAT
TTGACAAAGCATATCATCCTAAAATACACTATAATTATACAATCTTAACACAACACATGAATAATTAAATG
ATAGAAAAATTAATAATTTCAATTTTTTGTGATATTCTCAAGCAAAGGGATCACAGTGCACATTTCAAGT
AATTAAAGTTTAAATATGCGTAGTTTAAATATCACAAACTTGCTTGGACATAAAATTATTACGATTTCTAA
CAAGATATTAAAATATTATCTAATTTTTTCATGCTCAAAAGTCAAGAACTATTGACCTCCTATAATATTTCT
TAATTCCACATCAGCCGCCGTTTAGTCTTAGTTATCCAGTCAAATCTATGAAGACCCCATTATATTCGAAC
TGCAGCAACATTTTCCTCATGTTTGATACTGCTCAGAACATATATGCCTTTTACTAAATTATTCGTAAAATA
TACTATTCAAAAGTCCACCAAAAGCAGCCATTAACTTTTTTAAAAGCAGCAGGACTTGAAGCAGCCTCAA
CTCTCATTAAAAAGCTAAGACTTTTCATAGTCATAGACCTTTCACTCCTTTCAAATAATACTCTTTCATATG

TTAGAAAGCTCAAGTGATGGCTTTTTAACACTAGTTTGTTCAGTTTCTTCTTGTAAGATTCACCGTCTGC

AAACAAGTCAAAGTGTCATCACTATGCTACATTTGACATTGAAAAAAATTGGATCTCAAAGCATGAAGG

CATTACTAAGGGAAAGTTTTTACTTATCTTTGCTCCTGTGTTTGTGTTTCATCCACCGAAGCTTTGGCGCT

ACAGATACAATAACTACAACTCATTTTCTGAAAGATGGTGATGATAATGTTGCTTCAACTGGTGGAATAT

TCGAAATGGGGTTCTTCAGTCCAGGTAATTCGAAAAATCGCTATGTGGGTATGTGGTATAAGAACATAT

CTGTTAGGACAGTGGTGTGGGTTGCCAACAGAGAAGCTCCTTTGACTGGTGGATCAGGTATCTTGAAAA

TAATCAAGCCAGGAATTCTTGTCCTTGTCAATGGTACTAACCATGTTGTGTGGTCAACTAATACCACTAG

ATCTGTGCAGAATCCTGTAGCGCAGTTGCTAGATTCTGGAAATCTTATTGTCAAAGAAGCTGGTGATGAT

AGCCCGGGGAATTTGATATGGCAGAGCTTTGATCACCCAACGGATACGCTATTAGCAGGCATGAAGCTT

GGTAGGAACTTTGTAACTGGCAGAGAGGTGTACTTGTCATCATGGAAGAATGAGGAGGATCCAGCTCC

TGGTGATTCTACATATCACTGCGATCCTTCTGGTTACCCGCAGAATATCCTGAAGAAGGGGTCGGATGTA

GTATACCGCTCTGGACCATGGAATGGTCGCTCTTTCAGTGGGAACCGAAACTCAAGAGAAGGTCCTTCC

TATACATTTGGAGTATATTCAAGTAAGACAGAGGTGTATTTTGGATATAACCTCACAACTTCAATTATCGT

GAGGTTGACATTAAGTCACAATGGAGTCCTACAGGTTTGGACTTGGGGTGATGGAGACCAAGGTTGGA

TCCCTTTCCTCTTAATACCCGCTGATAATTGTGATATGTATAAGTTGTGTGGCGCATATGGCAGCTGCAAC

AGTCAAGATTCTCCAGAATGTGGATGCTTAGACAAATTTGTGCCCAACAATAGTGATGCTTGGAAGAGG

AAAGACTGGTCAGGTGGCTGTGTTCGGAGAACTGAACTAAATTGCCTCCAAGAAGATGTATTTTTGAAG

TATTCGCATATCAAATTGCCAGACACACGGAATTCCTGGTCCAATGTGACTATGACACTAGAAGAGTGCA

GGAATACCTGCTCTAAAAATTGTTCTTGTATGGCTTACTCCAATTCTGACATACTCAATGAAGGAAGTGG

ATGCTTACTGTGGTTCAAGGATCTGCTTGACATTCGGCAGGGACCCAACGGAGGGCAAGATATCTACAT

TAGAATGGCTTCTTCTGAATCAGGTTGGCTTGTTGCAACTTAATATTTTTTGATAGATTGCCTGAGGATA

GTAAAGGAGTTTAAGGTTGCAGGCACTTTAATAAAGCAGACTGTAGTTTAGTGCCTATGCTTACTATGAT

ACTATCTACTGCCAATTCCTACCATATTTAGCCCGTAACTACCAAATAATAGCAGTACAGAAAGCTACTAT

TTTTTAGTGAAGTTGGGATGAATTTATAAGCCAAATCTGCAAGCATGATGACTATAGAACAAGCATTTGA

TATGACTTGCATTACACAAGGAATTGAAGAACCTTGACAACTCAACCTCAACAAACTAAAAAGGATGTG

CTGTACTCCAAAGGCTTGCAGAGCCTTTTATAACTCAAAATAAAAATCATCTCCAATGGATAGGATAATT

GCAATTTATTATTTATGTTTGTTGAATTGATACTTGCAGATAGTCTGGAGCAATCAGATGGAAAGAAAAG

GAAAGTACTTTTCTGGATTCTACCATTATCAGTTTGTTTGATTCTGGTATTCCTTACTCTGCTGGTCTACCA

TAGAAGAAGGAAGAAGGCTTTAGAGCTCAAAAACAAAGGTAAGCTGAATACCCCTTAATTTTTTCTTGA

CATCTAAAGTCTAAGCAAAATAGAGGAAAAGACATTTAAATTATACTGAAAAGAACTTTCACTTCATTCG
CTTTCTCTTATAGGCAAAATTATGTCATTTGGTTTTGGCAGGAAGGTCAGGATGCAGTGGAAATTACAAG
ATGAATTACAACCGTGGCAATTGCACTGAAGAATTTGAAATACCACTGTTCGACTTATCTACCATAGCAA
AGGCTACCAATAACTTTTCAATTGACAGACAGATTGGAGAGGGGGGCTTTGGACCTGTTTACAAGGTAT
AAACACTTAAGACCTGGGTCTTCAGAGTAAAGCTTCATCTTTAAATACAATATAGGAGTTGTATGATCAA
AGGTATAAGCCTTTGCTTTGTGAAACCAAAAGGAGACCTATACAATAGCTAAGGGAAGAAAGGTTGGG
ATATGGGGGATGTCCTTCTCAGAATGTATCCAAGAACCACTAGTCTAGCTTTTCAATAAGTGTTGTCCCA
AGTTAGTCAATATAGAGCCTTTAGCATTTTCAAGAAAGTGCTGCAAGAGTAACCCATCACCCTTTGGGTG
GAACTGCAGATTAAAGTGTTGAGGATATAGTTTGTTTAAGAAGAGAGTCATTTGTTCTTTGTAGTCATTT
ATTTGTTCATCTTTCCATGGTGACAAGTGGCAACTTGTTCCCGCATCAAAAGGGCGAATATTTCTTATAGA
CTTTAGTTTTAATTTTAAAGAAGATTATCATAGGGCTAAACTGAAAATATAATGAACTTATAATAGAGAT
TTGGCACATTTGACCTAAATTTGTTAACGGAGATTTTGATCAACTTAGGTATTGAAAATATTAACATGCA
GAGATTGTGATGTTTAGGGCATACTTGAAGGACAGGAAATAGCCGTCAAGCGGTTGTCTAAAACTTCTA
CACAAGGAGAAAAAGAGTTCAAAAATGAAGTTTTATATATTGCTAAACTTCAGCAAAGGAATCTGGTGA
AGATTCTTGGCTGTTGCATTGAAGGGGAAGAAAAAATGTTGATTTATGAGTACTTGCCAAATGGGAGTC
TTGATTCATTTATATTTGGTACAACTTCTCAATCCAATATTATGCATTTGAATAATAGTTGCATTTCAGTGA
CTTGATGCCTATAGGCATACACTGATTTTCTTCCAACTTCAAAATCCCCATAAAGCAGGAAAAAAAATGT
AAGGAGAGAACTAAGACCCCAGTTGTTTAAGAGCCTAATAATTACATACTTGGTAGTTCAAACTTCTCGT
ATTATGTTTCCATCAGCAAGGAACTGATTTTGTTTTTGGTTTTAATACTACATTCCACTAAACCTATTTAGT
GTGTTAATGACCCAACATTATTTGGCAGATGACATACAAAGCAAGGTATTAGACTGGCCTAAACGATTTC
ACATTATCAATGGCATAGCTCGGGGACTTATGTATCTGCATCAAGATTCTCAATTGAGGATAATCCACAG
AGATCTGAAAGCTAACAATATCTTATTAGACAAGGACATGAATCCAAAGATATCAGACTTTGGCATTGCA
AAAATATGTGATGGGAATGACATTGGGGACAAGACAAACCAAGTTGTTGGAACACAGTAAGAACCTTA
TACTTGATAGCTAGAAATTTGAACCTAAGAAGAGTTTTGAGATATTAGGTACGTTTTCCAGCATTAGTAA
TTCTATTTTTTGTTTACATTTCTATGCAGTGGGTACCTCTCACCGGAATATGCATTGCACGGAAGATACTC
AGTAAAATCAGATGTATTTAGCTTTGGTATCTTAATATTGGAAATTGTGAGTGGGAAAAGCAACAGAAG
ATTCTCTCATCCAGACCACAACCTTAATCTACTTGGACTTGTAAGATTTGATGAGTTTCATTTTACTTATCC
TTTGGTCTTATGTTATCTTCTAGTTACAGATATCTGCAAATCTAGTCAGCATTAATAAATTGTGTAATCTAC
AGGCATGGAAACTCTATAAAGAAGGCAGGTCAACAGAACTACTTGATGAATACCTAGGGAATTCTTGTT

CAACACCCGAAGTGGAACGATCAATCTGTGTTGGTCTATTATGTGTCCAACAATGTCCAGAAGATCGTCC

AAGTATGTCTTTTGTGGTTATGATGTTAAACAATAAAGGTGTATTGCCACAGGCTAAACGGCCAGGTTTT

TACATTGAAACAGATGCACCGGATGGTGAACTCTCGTCTAGCCATACTGAGACCCCCATCACAATATTGG

TAGCCCGATAAGAAAATATTGTTTCACTTGCTTTCAACAAGCTTGCAGTTGTAAAAAATCTTTTCTTTTATT

CTTTGTAGAAATGTTGTAAAAAAATATTTGGTATATCAGCTATTTCGAAC

>PGSC0003DMG400019276 G-LecRK

AAAAAATGAAGTCTTGGTAATCTCTGAGACAATCTTACTGAAAAGAATACCACAAGAGATGGAATATGT

TAAATTCTTTTTAACAATTTTGAAGAAGAATGCAATGCAACAAAAGGTAAATATAACTATCATGGATATA

ACAATATTTCAATTTATTCTACTTCTCTTGTATTTTGTGTTTCTTCAAAATGTTAGTGGTCAAGGAGATACC

ATAACTACAACTCAATTCATTAAACATGGTGATACTATTGTTTCGTCTCATGGAACGTTCGAGTTAGGATT

CTTCAGTCCTGGAGATACTTCTACAAATCACTATGTTGGAATATGGTACAAGAAAACCTCTGCTACTACTC

CTGTTTGGGTTGCCAATAGACTAGCTCCTGTTGCTAATAAGTATGGGGTGTTGAAAGTTATTCAGCCAGG

TCTGATTGTACTTCTGAATGATACTAATGCAACTGTTTGGTCCACAAACTCATCGAAATCTGTACAGAATC

CTGTTGCTCAGTTGTTGGACACAGGGAATTTTGTGGTGAGGGATGCTAATGATCCGAATCCAGAGAATT

TCCTCTGGCAAAGTTTTGATTATCCTAGTGATACGTTGTTGGAAGGTATGAAACTTGGGACGGACTTGGT

GACTGGATTAGAAAGGTATCTTACTTCTTGGAGAAGTAGTGATGATCCTGCTCCGGGGGATTATACTTAT

CATTGTGATCCTACGGGTTATCCACAAAACCTTATGCGAAAAGGGGCTAATGTGACGTTTAGAGCAGGG

CCGTGGAATGGTATCAGGTGGAGTGGTGCACCAAATCTGGTAAACAACTCTATCATTTCTTTTGGCATGG

TTATGAATAGTAGGGAAATTTACTACAAGTATGAGATGGTGAACAAATCCGTTATTTCAACATTTGTTTT

AGAGCCTTATGGGAAAGCTATGCGTATCATTTGGATTGGAAAGGTTCAGGGATGGGTAAATTACCACTC

TGCAGTTGTGGATGACTGTGACACCTACAAATTATGTGGTGCATATGGGACTTGCAACATTTTGAGTGAT

CCTTTTTGTCAATGTTTAGATAATTCGAGCCAAAACATCCTGATAATTGGGAAAGGTCTGACTGGTCTAG

TGGTTGTGTCCGGAAGATCCCACTGAATTGCACAGGAGATGGATTTATTAAGTATTCTGGTGTTAAACTG

CCTGATACGCGAAATTCTTGGTTCAATGAGACAATGACACTAGATGAATGCAGGGTAGTTTGCTTGAGA

AATTGTTCGTGTACGGGTTATACTAATCTGGATATACGCAAGGAGGAAGGGGGTGCTTGCTATGGATTT

GGGAGCTGGTTGACATCAGACAGCTACCTGCATCAGGACAGGATATCTACATTAGGATGTCTGCTTCAG

ACATAGGTAATTAATAATTAATATGCGATTTCATAAATCATAACCATGTCAATTGTACTTCAATGCCAGAA

ATTCAACTCATATGAGGGACAAAGGTAATCCAAGTCCACATTATGGGAATAAGACTTCAAGGACCGTCC

TGTTTTCCTCGACATAAATACAACAAATATAAAAAGAGAAAAAACTTCTAGCATTACTAGAAACAACTAT

TGATAATATTTCGATGGGAAATGTTGCAGGTTCTGCTGGATCAAAAGGAAAGATAGCTTTCATACTTGCA
ATAGCTTTGCCACTATTGGTTGCATTGATTCTGCTTGGTTTAGGTGTAGGCCTGATTCTTTGTAAACGAAA
AAGGAGAAAGGATCCGGTGGTTACAACAACAGGTAAGTGGAAGTACCAATTCTGTCTATCGTAAAACCA
GAAGCAACTAGTGGTCATTAGTTCTGTATACCACTAGTATTTCATTTCAAGCTCCTTATTTGAGCAAGGTC
TACTTGTATGTTCAAATTGCAGGGAGATTAGGTGGTCACAGCAACAAGAATGATAACAGTAATCAAATT
CACGAGGAAAATTTTGAGCTTCCACTTTTTGACTTATTAACGTTAACCAATGCTACCAACAACTTTTCATTT
GCAAACAAGATTGGAGAGGGTGGCTTTGGACAAGTTTACAAGGTACAAACATTCCTCAGACCCCAAGTA
ATGATACTCGAGACTTAATTTCTTTGCTTTTCTTGAAAGGAATATTAACTCTTGGAGTCAGTACATACAGC
AGTGTTTGCTAGTATGATGAGCGGAATGAGTGTTAAGTTGAGAGCCTTGTTTCAAGACCGATAATGTTTT
ATAAAATAAAGGTTTCATATTCAAAGAAAAGGAACATCAGCTGGAAAAGATTACCAACCATGAGATAAG
CTGTGGAATGAAATGTGCAAGTAGATGCTGCCTGAACAAGAACTTGATTGCACTAGTAAGGAAAAAAAT
GACGATTCACTCCATCAAATTTCTAACGGGACTGCTACTTTTACAGGGTGTATTAGAAGGTGGACAAGA
AGTAGCAGTGAAGCGGCTTTCAGAAACATCAGAGCAAGGACTTCACGAGTTCAAGAATGAAGTTAAGT
GCATCGCAAAACTTCAGCATCGGAATCTTGTAAAGCTTCTAGGATGCTGCATTCAAGGAGAAGAGAAGA
TGCTGGTGTATGAGTACTTGCCAAACAAAAGCCTGGAATTATATATATTTGGTTAGTCTTTCACATCTACT
GAACGGCCATTTCTTTCAATGCAATCAATTGTGATTTCACGAAGGTTGGTTTCATTGTTATAGAAACCTTT
AGTAATAAGTTGAAAACATTTGACAGATGAAGAAAGGAGTGCATTACTTGATTGGCCTAAGCGCTTCAA
TATTATCAATGGAATTGCTCGAGGACTGATGTATCTTCATCAAGATTCTAGGCTCAGAATCATCCACAGA
GACTTGAAAGCTAGCAATGTCTTACTTGATATTGAAATGAATCCAAAAATATCCGATTTTGGGATGGCTA
GAAGTTTCAGAGGAGATGAGACAGGAGACAATACAAGCCGTGTTGTTGGAACATAGTAAGATTTAATA
TTTGATTATATATGTAGTCATGAACACATGGTGCATTATATAATCCTGTAGTTTCTTTATTGCAGTGGTTA
CATGTCCCCGGAATATGCAGTCGAGGGGATATTCTCAGTGAAATCAGATGTCTTTAGCTTTGGGGTATTA
GTACTAGAGATTGTGAGTGGAAAGAAGAACCGACGATTTGTTCATCCAGACCACCACTTCAACCTTCTTG
GACATGTAAGTACAGTAACTCCTAACGGTAAAAAGACTCAGAAAAAGTGTTTGTTAATGCATTTAAGTTT
TATGTGCCGACAGTAAATTACATGTAAATCTCTATTATAAGAGCTTGTTAACTAACATACTATCACAAGTT
AAACTACAATGCTAGAGTATAAAGTCTTTGTACTGTTAGTGTGTATAAGTTAAATCCTTTGTTGATGGAG
GGATCGTTTTATGCAGGCATGGATGCTTCATAATGAAGAACGGACGTTGGAACTAGTAGATCCATATCT
CGTAAATTCATACTATATATCAGAAGTACTAAGATCAGTCCATGTGGGATTGTTATGTGTTCAACAGAAT
CCAGAGGACAGGCCAAGCATGTCAACTGTGATTATGATGCTGAGCAATGAAGGCATTTTACCACTGCCT

AAACATCCCGGTTTCTTCACTGAAAGGAAAGTTGAAGGTCATGATCAATTATCTTGGAGTACACAAACAC
CAAGTTCTATAAACGAAGTTACCATCACGCTGTTGAATGCTCGATAGAAGCATATCATGGCCATAGTCAG
TGGCAAAGCCAGGATTTTCACTAAGGGTTTCAAAATATGAAAGAAGTAAACACACAAAGTCAAGACGAT
TCAACAACTATTATATATACATAAACAATTAATTGTAGACCTTTATATATATAGTGTAATTTTTTGGCGAA
AGGGTTCGGATGAACCCCTTGCATCAACTTGGCTCCATCCTTGTCGATTGTGAGTGTCATTCATTTCTCAG
AATTGTATTAAAATCCTCCCTTTTTTTCTTTAAGAAATTAGAAAGCAAAATCTGCAGGAATTGTATTGAAA
TATGTCTAAATTTTAAGGCTTTGAATTCAATTTCAG
>PGSC0003DMG400019278 G-LecRK
ATGAAAGGGAAACATTTTTTATCTTCTTGCTCCATTTTTCTTTCCATCTTGCTAATTTCCACAGCATTAGAC
ACAATCACAACAGAAAAACCAATTAGAGATGGTGACACAATTGTTTCAGCTGGAGGGGTTTACGAGCTT
GGATTTTTCAGCCCTGGAAATTCGAAGAATCGCTACGTTGGCATATGGTTCAAGAAGATAGCAACTAGA
ACTGTTGTCTGGGTTGCCAACAGAAACATTCCACTGAATGACAATTCAGGAGTGTTATTACTCAATCCCA
ATGGAATTCTTGTACTTGTTGACAATTCCAATGCCTCAATTTGGTCATCAAACTCATCAAGATTGTTAATG
AATCCAAAAGCACGGCTCCTGGATTCCGGTAACCTTGTTGTCGGTGATGGAAATAATAGTGAACGAGAA
GTTAATTTCGTGTGGCAGAGTTTTGATTATCCAGGAGATACTTTATTACCTGGGATGAAGCTTGGACGTA
ATCTGGTCACGGGCATGGATTGGTACATAGAGTCATGGAAGAGCACTGATGATCCTGCTGCGGGTGAA
TATATAGAACGTCTTGATTCTCATGGATACCCTCAATTTTTCGTGTGGAAAAATTCGTCTATAGTATATAG
CACAGGGCCATGGAATGGTATCGCATTTAGTAGTAGTCCTAAAAATCAACCAGCTATATATTATGCTTTC
GAGTTTGTTATTAAACAGAAGGAGATTTACTTTAAATACGAGCTTAATGAGTCCCTGCCCACCAGGGTG
GTGATCAATCAGGCTGGAATGGTAGAACACCTAACATGGATCGAGCGTAATCACAGATGGATAGTCTAC
GTATCAGCACAATCTGATAATTGTGATCGTTTTGCTTTATGTGGTCCTTATGCAAGTTGCAACATCAATAA
CTCTCCTCCATGTGACTGCTTGCGAGGTTTCGAGCCTAGGTATTGTTAAAAATATGCCTTCACACGACTCC
TCTGTCTGTTCCAATACCATGTTAATTCATTAATTATGTCTTCACAGGTATCCTGAACAATGGTATGCAGT
GGACTGGTCTAATGGTTGTATAAGGAAAACTTCTTTGTCTTGTAACCAAGATGGTTTTCTTAAATTTAGG
AATATCAAGATGCCGGATTCTAGACACTCCTGGTATAATGTAAGCATTAGTCTTGAAGAATGCAAGAAA
ATGTGCTTGGCTGATTGCAATTGTACAGCCTACTCAAATCTTGATATAAGAAATGGCGGAAGTGGATGC
TTACTATGGTTCGGTGAACTCATTGATATTAGAGAGTACAACAAAAATGAGCAATACCTGTTTGTGAGA
GTTGCTGCTTCAGAATTAGGTAATGACCCTACTCTATACCATTTTTGTGTTCTTTTGCACAATATAAGCAT
GACACCTTAGAGTTTAAGGAACTTTACTATCTGATGTCACTGATAGTAACATATGATAGTCGCGGAAGCA

ATTCATATGTCAATTGAAGACTGCTTATGGACTTTTTATTAAACAGATTCAGTCAGGAATCGGAGGGGAA
AGAGGTCAGCCCTGATTGCGGTCATTTCAGCACTAGTAGCAACTTCTATCCTCATCTTTCTAGCTTGGTTT
ACCTTCCAAAGAAAGAACAAAAAAACAGATAAACATACTGGAGGTATTAGAAAATTGTTAACTGTGAAT
GCTTATTTACCTCCATCTAGCTAACATTGTTCGACTTTATCAAATGATTGAGGAAAAGTTCAATTATATCA
TGTCTGTGATTGGAAAATATATGTTGACAGGTTCAGAAGTTGGAAACAAGGACCTCGAGTTGCCATTGT
TTGATTTAGTTACTGTTACTACTGCCACTGAAAATTTCTCTTCTGCGAATGTGATTGGTGAGGGTGGCTTT
GGACAGGTTTACAAGGTAAATCAACTTTGATCTTCAACTAAAAGCTACAAGCATATTTGACCCTAGAGAG
AAAATCGCAGGGTATTCTACCAGATGGACTAGAGATAGCAGTAAAGAAGCTATCAACGTATTCTGGACA
AGGCGTTCAAGAGTTAAAAAATGAAATTGCTCTCATTTCCAAGCTGCAACATAGGAACCTTGTCAAGCTT
TTGGGTTGCTGCCTTGAAGGAGAAGAAAAGATGCTAATCTATGAGTTTATGCCCAACTCTAGCTTGGACT
GTTTCATTTTTGGTCTGCTTTTCGTGATTCATTAACTAAATATTACTACTTGATAAGTCCTCAATATCTCTG
TTATCACTCAAGAATTGGTTTTACATTGTGCAGATCCAAGCAGAAAAGCTTCACTTGCATGGAAGAATCG
CTTTGAAATTTCTGTGGGAATATCTCGAGGTCTTCTTTACCTTCACCAGGACTCAAGATTTAGAATTATTC
ATAGAGACCTCAAGACCAGCAACATTTTACTAGATGGAGACATGAATGCCAAAATTGCTGATTTTGGCCT
TGCCAAAATTTTTGGCGGAGAACAAGTGGAAGGAAATACTAAGAGAGTGATAGGGACATAGTAAGTTC
TACAAATCTTTATGATAGACATTCAATCAGTACTTGAACTTAAATTTGATGAGTTGATGCAGTGGATATAT
GTCACCTGAGTATGCTGTTGATGGGAAATATTCAGTAAAATCCGATGTATTTAGTATCGGCGTTATCATT
CTAGAGCTAGTCAGTGGCAGAAGGAACAGGAAATTTCATCATTTGGAACATTATCACAATCTTTTGGGA
CATGTAAGCACATACAACACTAAACTTCGTTCCCTTAACTCACTTTTTCTATAGTGAATGCATTTGTATAA
ATGTGTTCTGTTTGTAATTTAGGCATGGTTACTTTGGACTGAAGACAAAGCGTTGGAACTGATGGACGA
ATGTTTGAAAGAATCATTTTCAGAATCTCAAGTGTTGAGATGCATCCAGGTTGGCTTGTTGTGCGCCCAA
AAACACCCTGAGCATAGGCCTACAATGGCATCAGTAGTTTTCTGGTTGGGAAATGAAGGCCTGGTTCTT
CCTCAACCAAAGCAGCCTGGATTTTTTATAGAAAGGAATTCAATGGATTCAACAGAATCAGCTCAATTTA
TCAGTTAGCTCGTTGTAATCGGATAAGGGCATTGTGAATTCAAGTTCAATCAACACATATAATATCTAAT
TAATGAGAAGAAAATCTGAAAGTGTGTACACACATTTCAGCACACGCTTTGTAGCACAATTAATCCGGAT
TCACAGGGGAAAGTTCGACTTTTTGGGAGAAACTAAAGTCTCCTCATCAAAAGTGACTCCATTCAAGAA
GTTGAAGATTCTTCTTGTTTTTTCTCTCAGTTGAATATTAGATGGTGACTTTACAACTTTTTAAAATAAATG
CATTAATATTAATAGAAAATAAAAAAGACATCAGTTCCTTTTTTCTTAGTTCTGCCATTACTAGTCCAAGA
TGTCACATCGTCATTAACACTAAATCATTTTAGGAAGACTTTAGGCAATTTACTCCAATACATTTCATGTG

TTTACACCAAGAAAATAGATGCATGCCATATGTTTGTATGAATAGTTCTATAGCGTGCAATTGCAAACAC
CAGGTTAATGGTGGAAATGGAAAGTCAAAGGCATACAACTTTTGACAAGTTCAAACTTTGACATCTAATT
CTTGGCAGCAGTTGTGATTGGCCTTTCATTCAAAAAAGTTCTTTTTTTCATTTTATAGTTGTGATTGGCCTT
TCATTCAAAAAAGTTCTTTTTTTCATTTTATTTATATAAAACATTTTTGGACATGGTTGTTAATTCTAAACT
GAGAAAAAAAAAAGTTATCAGTATCTTTTCTGTGTCAAGGCTTTTTTGAACTTGTCAATCAAAAACATGAG
TCAGAGTCTATCAAAAGGGCTACTGTTTTTATTCCTTTGCTCACATTTTTTCTTCATCTTACTAATTTCCGC
GGCATTAGACACGATCACAACAGATGAATCCATTAGAGATGGTGACACAATTGTTTCAGCTGGTGGGAT
TTATGAGCTTGGATTTTTCAGTCCTGGAAATCCGAAGAATCGTTATGTTGGCATATGGTACAAGAAGATA
TCACCTAGAACTGTTGTCTGGGTTGCAAACAGAGATATTCCACTTAATGACACATCAGGAGTGTTAACAC
TCAAACCCAATGGGATTCTTGTACTTGTTGATAATTCCAATACCTCAATTTGGTCATCAAACTCATCAAGA
TCATTAAAGGATCCAAAAGCACGGATTCTGGATTCCGGGAACCTTGTTGTCAATGATGGAAATGAAAGA
GACCTGAAAATTAATTTCGCGTGGCAGAGTTTTGATTATCCAGGAAATACTTTTATACCTGGCATGAAAC
TTGGACGTAATTTGGTCACGGGCATGGATTGGTACATGTCATCATGGAAGAGCATTGATGACCCTTCTCC
TGGTGAATATATAAACCGTCTTGAATCTCATGGATACCCGCAATTGTTTGTGTGGAAAAATTCAACTATA
GTGTCTAGCTCAGGGATATGGAAAGGTAATGCATTTACTGTTAGTGCAAATAGTAAACCAAATACACAT
TACACTTCCGAGTTTATTATTAATCAGCAGCAAATTTACTACCAATTCAAGCTTAAGAACGAGTCACTGCC
CAGCAGGATGGTGCTTAACCCGGAAGGGCTGATAGAACACATAACATGGATTGAGAGCAGTCAAAGCT
GGTTTCTGTACTCAACAGTACAGATTGATAGCTGTGGTCGATTTGCTTTATGCGGTCCTTATGCAAGTTG
CAGCATCAATAACTCCCCTCCATGTGACTGCTTGCAAGGTTTCAATCCTAGGTATTGTTAAAATGAGTGA
TCATCAAACCTTAAACTATTAGAGATAACACACTTGATTACTTAGTTATGATTTTACACTCCCCTTCATGTG
TGTGCTTGATTCTATTTCTTGGGCCAAGCACATAGATATTATATTGATTACTTAATTATGACTTCACAGGG
TTCCTCAACAGTCTGCAGCAGATTGGGCTACTGGTTGTGTTAGGAGAACTTCTTTGGATTGTAAACAAGA
CGGTTTTCTTAAATTTACGGGCATCAAGATGCCTGATTCTAGAAACTCCTGGTTTAATGAGAGCATTAAC
CTTGAAGAATGTGAGAAATTATGCTTAGCTGATTGCAACTGTACAGCCTACTCAAATCTTGATGTTAGAA
ATGGCGGAAGTGGATGCTTACTATGGTTCGGAGATCTCATTGATATTCGAGAGTTGAGCCAAAATGAAC
AAAATCTGTTTGTGAGAGTTGCTGCTTCAGAAATAGGTAATGACCCTACTCTGCACCATTTTCATTTTGTT
GTTCAATATAAATACACAGATCTTTGAAACTGACGAATCCCTGGTCAACAAATTACTGATAACCAAGTTT
GCAGGCTTCATAACAAAATAAGACGTATATGTAAATTGAAGACTGCTTATGAACTAATTATTGAACATAT
TCAGACAGGAAGCGGAGGAGAAAGATGTCAGTCCTGATTGGTGTCATTTCAGCAGTGGTAGCAACATTT

ATCCTCAGCTTTTTAGCTTGGTTTTCCTTCCAAAGAAAGAAAAGAAGAATAGGTAAATATAGTTGAGTCA
CTCTTGTCTACCAACTTTATAAGAAAATTGTCAAGTCTAAATGCTGTGCTGGCTAGGTGAATTTTCTATCT
AGCTGACATTGTTTGCCTTTACCAAATTCTCATTGATATCATGTTTGTGGTGGGCGAATGTATATTGACAG
GTCCAGCTGTTGAAAATGAGGACATGGAGCTTCCATTGTTTGATTTAGTTACTGTTACTAGTGCCACTGG
GAACTTTTCTGCTAAGAATGTGATCGGGAAGGGTGGATTTGGACCGGTTTACAAGGTAAATCAACTTTG
ATCCTCAACGTCTAACTTGAAATGGCATCTTTGACCCTTGACATAAATTTCAGGGTATCCTACCAAATGGA
CAAGAGATAGCAGTAAAGAGGCTATCAAAGCATTCTGGACAAGGCTTTCAAGAGTTAAAAAATGAATTC
GTTCTCATTTCCAAGCTGCAACACAGGAACCTTGTTAAGCTTTTGGGTTGCTGCCTTGAAAGAGAAGAAA
GGATGCTAATCTATGAGTTTATGCCCAATGCTAGCTTGGACTATTTCATTTTTGGTCTGCTTTTCATGATC
CATTAATAAATAGTACTTGATATAAGTCCTCAATATCTCTGCATCACTCAATGATTAGTTCTACATTGTGC
AGATCCAAGCAGAAAAACTTCACTTTCATGGAAGAACCGCTTTGAAATTGCTATAGGAATATCTCGAGG
TCTTCTTTACCTTCACCAGGACTCAAGATTAAGAATTATTCACAGAGATCTCAAGACCAGCAACATTCTAT
TAGATACTGACATGAATGCCAAAATTTCTGACTTCGGCCTTGCCAAAATATTTGGTGGAGATCAAGTGGA
AGGAGAAACTAAGAGTATAGTAGGGACATTGTAAGTTTCTACACATCTTTAAATCGTATAATCTTTATTA
TATTAGATCAGTACATGAGCATAAACTTATTGAATTGATGTAGTGGATATATGTCCCCGGAGTATATTGT
TGATGGGAAGTATTCAGTAAAATCAGATGTATACAGCATCGGCGTAATCATTCTTGAAATAGTTAGTGG
CAGAAGGAACAGGAAATTTCATCATTTGGAACATCTTCACAATCTTTTGGGACATGTAAGCATATACATT
ACACAACTTCATTCCCTTAATTCGCTCTTAGCTTTCTATTGCGAATACATTTGTATAAATGTGTTCTGTTTG
TAATATAGGCATGGTTACTTTGGACTGAAGGCAACGCGTTGGAACTGATGGATGAATGTTTGAAAGAAT
CATTTTCAGAATCTCAAGTGTTGAGATGCATCCAGGTCGGTTTGTTATGCGTCCAAAAACTCCCAGAGGA
TAGACCTATAATGGCATCAGTGGTTTCTTGGTTGGGAAATGAAGGTCTGGTTCTTCCTCAACCAAAGCAT
CCAGGTTTTTTCACAGAGAGGAATCCAATGGAATCTACTGATGAAGAATGTCTAAGTAACAACGCGACG
TTAACAGTTCTTGAACCAAGATAGACAAATAATCACTATTGGATGTTTTGAAATGTATTTACTTTGTCTTA
TTTGTCAAACAGTACTAGTGTATATATGATAGAATATACTAAAAGAGATAGTTTAATGCCAGTGTTCATA
TCAAGGCAGGGTAAATAATTGATATACACTTCTTGTCTTTGATAATTGAGCAATCAATGGACTAATTATT
GGCAAAATTTGTAAGCACAAGTAGTTAGAGGTATCTATATCATGCATTACTTTTTTTGGGGACAATGAAT
ATATTCATCAAACATAGTTGCTATACATGATACATAAGCCCTATTCTATATGAAGTATCTTTTTTTGTTTCC
CACTCGGTGTCTGGATCCCACATTGGAGCTCTGGCTAAATTTGGATTGCGTATAGCAGGA
>PGSC0003DMG400021804 G-LecRK

TGAATGTCTGCGACTTCCCTGTTGATGACCCATTTTGGCCGTCTCCATTACCAAACTACTAGTCTTTGATT
GTGTTGCAAAGAGCTCCAAAAATGGAAGAAATTCGCTAATAAATAAGCTGCATTTTATTTTTCCAACCAT
GAAGCTGCTACAAACAACTATTCATTTCTTGTTCGTTTTCTACTTATTTTCCATTTTCCAAATTTCATTAGGT
GATACCATAACAACAACTCAATTTCTCAAAGATGGTGAACCCAGTATCACTTCATCCGGTGGAAGTTTTC
AGATGGGGTTTTTCAGTCCAGGTAATTCCAAAAATCGTTATATAGGTATATGGTATAGCAACATATCTGT
AACAACCGTTGTTTGGGTTGCCAATAGAGAAGCTCCTCTAGGAACTAATTCAGGTACCCTAAAAGTTATC
AAACCAGGACTACTTGTTATAGTGAATGATAGTAACCACATCATATGGTCTACCAATACCTCGAGATCTG
TGCAAAACCCTGTTGCAAAGTTGCTTGATTCTGGAAATCTTGTTGTTATTGATGCTGGTCATGGTGTTGG
TGATGATATTAAAATTGGGGATTTTCTCTGGCAGAGTTTTGATTACCCAACTGATACCCTTTTGCCCGGG
ATGAAAATTGGGTGGAATTTTGTAACTGGGAAGGAACTGTATTTGTCGTCGTGGAAGAACGAGGAGGA
TCCAGCTCCGGGTGATTATACGTACCATTGTGATCCTTCTGGTTACTTGCAGAACATTTTGAAGAAAGGT
TCAAAGGAGGTATACAGATCTGGGCCATGGAATGGCTTAAGGTTTAGCGGAGCAACAAGTTCAAGACA
AAGTCCATTTTATACATTTGGTGTGATTTCAACTAAAACTGAGGTGTATTTTAGTTATCACCTTCTAGCTTC
GGTTATTACAAGGTTTTGTTTGAATCCAAATGGTGCGTTACAACGATGGACTTGGGGCGATAGGAATAA
AGGTTGGGCACTTTACCTTTCATTACCAACAGATAATTGTGATACTTATAAGTTGTGTGGAGGATATGGT
AGTTGTAGTAGTTTGAATTCTCCGGTATGTGGATGTTTAGAAAAGTTCGAGCCTAAACATGTTGAAGATT
GGGGGAAGGCAGATTGGTCAAGTGGTTGTGTTAGGAGGATTGAACTGAATTGCATCAAGGGAGATGG
ATTTTTGAAGTATTCGAAACTTAAATTACCAGACACGCGGAATTCATGGTTTAATGTGACTATGAACCTT
GAAGAATGCCGGAAAGTTTGCTTGAGAAATTGCTCTTGTATGGCATACTCAAATCTTGACATACGCAATG
GAGGAACCGGTTGCTTACTGTGGTCTGAAGATCTGCTTGATATTCGACAGCTAGCCAACGAAGGACAAG
ATATCTACATTAGAATGGCTGCCTCTGAATTAGGTAAGTTTATTGCTGAAAATATAAAATAAATAGTTTT
GAGATTGATTTCGTAAAGACGGTAAATTTATGAGTTGCAGGAGCCTGGGATTAAAAAAAAAAGTGAATT
TTCTTGTAGACACTGCCAATGACTACTATGAAATCCATAAATGAAAGTAGTTAATGGTAGGAGTACCGTT
ATCCTTTTTGTAGTGATGAATCTTTTATTTGTTCCTAAGAAAAGGAATTTGTCCATTTTTGGGGGTCTCAA
AGGTGCGTGGAAACGCATATGAACTCTTGAATGGGAAAAGGATAAAACTCCACTTCCTTCTGAATGGGT
AGTCAATCCTATGTGTCGATAGGAGCAGTTGACAATTGAATCCGATTTTGACCATTATTTCACATCAAAC
ATGTTGTACTATAACGACAAAGTAAAACTCACAGATTTAACAGAACAAAATACTATGAAATTAGTACAGC
TACGTCTCTAGATTAAGAAAAAAAAATGAGTTAGAGTTGAGCTATGCTGAACTGGTGACCACTGACCATG
ATGTCTAGACAAATAAAAGAGCTTTAATGTAGTCATGTCTAATAAGCTGACGGAGAAAGTCGAACTGAT

TACCTATATTTGGGTTACATACTTGCAGCTAGTCAGGTGAAATCAAATGGACACAAACGAAAATTACTCT
CCTGGATTATCCCGTTATCGGCTGGAGTGATTTTGGTAATCCTAAGCCTAGTGATCTGGATTAGAAGAAG
GAAGATAGCTTCAGAGAAAAAGAAAGGTAACCTCGACAAGTCATTATGTTCGGAATATAATTAGTAGAT
GAATTCAAATTCAAAGGAAAATCTTTCTTGTCTAGTATTTGCCAAACTGAATTTGGTATATCGGTCTTTTG
ACAGGATGCTGGGGGAACAATGGCAATTACAAGATGGATTACCTCAACGGAAACCTCAGTGAAGAATA
TGAGTTACCACTGTTCGACTTATCTACAATAGCTAAATCTACGAACAACTTTTCAGGGATGAGCAAAATT
GGAGAAGGTGGATATGGACCTGTTTACAAGGTAATCACTTATAGCATTTGCACCTGTTTACATGAAATAA
GCAATTCACAGCCCACATAATTGAGCATAGAGAGATTCGGGAAGACTGACAGTAAGGAATGATTTATCG
ACTTTATAGTTTTGGAAGAGTTTTATAAACATTAGCTAAACTTGATTGAATTTATAATAGTTGCTCAATTC
TTCATAATTTGAGCTTCTACCTTTTGGTGGAAGTATAATTTGATTTTCATGTTCAGTGTTGAAAAAACTTA
ACATGTAGATATATGATGTTTAGGGCGTGCTTGAACACGGACAGGAAATAGCTGTGAAGCGGTTGTCTA
GGACTTCCACACAAGGACAAGAGGAGTTCATGAATGAAGTGATGTATATTGTGAAACTTCAGCATAGAA
ATCTGGTGAAGATTCTCGGATGTTGCATCGAAGGAGAAGAAAAAATGTTGATCTATGAGTACATGCCAA
ATGGAAGTCTTGATTCATTTATATTTGGTAAGGATTTCTCAAAAAAAAAGAAAAAAATTGTGCTTAATAG
TTACATACATCTCAGTTGATAGATGCCCTGTAGGAGTACCCAAATAATCCTCTTAGCCCAAAGTTATGTTT
TATTGTTACTTTTCCGATTTATTTCGATGTTTAAATCCCACTCTCAGTTTTATCTGATTTGTGTGAATTAATG
TTCCAACTATGAAACTTTTAACAGATGACACACGAAGCACGGTATTAGACTGGTCTAAGCGCTTTCACAT
AATCAATGGGATAGCTCGGGGACTTATGTATCTGCATCAAGATTCTCAGTTGAGGATCATTCACAGGGA
CCTGAAAGCTAACAACATTTTACTAGACAATGACATGAATCCAAAGATATCAGACTTCGGTATAGCAAGA
AGTTGTGAAGACGACAAGTTTGGAGCCAAGACACACCGAGTAGTTGGAACATAGTAAGAATCTCAAGA
TGATATTTTACTGAATCATTCAAAACTGGAATAGCAGAAGAGCCTTGACATTATCATAGTATGTTAAAAT
CAAAAAATAAAATCAAAAAATTATCATAGTATGTTTACCCAGTATTAGTAACGCTAATTTCTGTTTACGTT
TTATGCAGTGGCTACCTCTCACCGGAATATGCTGTACATGGTGTCTACTCGGTGAAGTCAGATGTATTTA
GCTTTGGTGTCTTAGTTCTTGAAATTGTGAGTGGGAAAGGGAACAGAAAATTCTCTCATCCAGACCATAA
CCTTAACCTCCTCGGACATGTAAGGGTTGTGTTTTTCATTGTCCATCAGTAAATTATCTCGTAAACCTGGT
CATGCTAACACGATAGTAAAATCTACAGGCATGGACACTCTATAAAGAAGGTAGATCAATGGAATTACT
CGGGGATTTCCCCATTGATGTTCATTCTACACCTGAAGTTATACGATCAATCCATGTTGGTCTATTGTGTG
TTCATCATCGTCCAGAAGATCGTCCAAGTATGTCTTCAGTGGTTATGATGTTAAACAATGAAGGTGTACT
GCCACCTGCTAAACAGCCAGCTTTCTTCGTAGAAACAAATACACCAGATAATGAATTCTCTTCTAGTCAAT

ATGCACATAGTACAGTGAATCAGATCACCATCACAACATTAGAAGCCAGATAGTAAATTCTTCATTGCAA
ATAGCAATCTATTATACCCTTCATTCTTTTCTTCTTTGTAACAACGTAGCAATCTTATCGTAATAGTGTACC
CATCATATCAAAATCTTGGATATGCCTCTGCACGCGATCCTTCTCTGCCAGCCATAAACAGCTAATCATAG
CAATGCAGGGGGTTAAATAGGGAAAAGATGTAAAAATCACAAAACAACTTTCAGGATCGGTATATATTC
TCTCTCTTGTATGTGTTATTTTGCTTTTGTTCATATATAGTTTAGTATTGATTTATATGTATGTGTTTATCTA
TTAATTAAGAAGATGGAAGAATGTGTAGATCCAACACTAAAAGGATCATTTTGTAAGATATATAGACAT
GTTATTCAAAG
>PGSC0003DMG400021993 G-LecRK
AAAAGTATGAAAATTTTATGATGATGAATTGATGGAGGACAAAAAAGTTTATTCCTTAGCCCTTTTACTT
GTGATTTTATTGATCACACTCATCGTTATCGCACGTATAACGTTGAAATATTCGGAGACATTTTTCCTCAT
TTTCGGAGCTGATGTTGCGTTGATAGTTGGTGTTTTTGCTGGTGTTTTGATCCGAAGGAAGTTCAACAAC
AGGAGGTTATTGCTAGAAAATCAGTTAGACTCAGATGGACGCGAGCTGAGGATCGAGTATAGTTTCCTA
AGGAAAGTAGCTGGTGTTCCAACAAGATTTAAGCTGAAAGAGCTCGAAGAAGCAACCGATAACTTTGG
ATCGTTGGTAGGACGTGGATCATCAGCTTGTGTTTTCAAAGGTGTGCTTAGTGATGGTACATCAGTAGCT
GTTAAAAGGATAGATGGAGAGGAGCGTGGCGATAAGGAATTCAAATCAGAAGTTGCAGCTATTGCTAG
TGTTCAACATGTTAATCTTGTACGTTTACTTGGATATTGTAGTGTTCCTCCTTCAGGACCTCGATTTTTGGT
TTATGAGTATATTGTTAATGGATCACTTGATAATTGGATTTTTCGAAAAAGGGGAATTCGAGGGTGTTTG
TCGTTGGATTTGAGGTGTAGAGTTGGTCTAGATGTGGCTAAGGCACTTTCTTATTTGCATCATGATTGTA
GATCGTGTATTTTACATCTCGATGTCAAGCCTGAGAATATACTTTTAGACGAGAATCATCGTGCTATTCTA
GCTGATTTCGGGTTGTCTAAGTTGATGGGGAAAGATGAGAGTAGAGTTGTTACAACGATTAGAGGTACT
CGGGGGTATTTAGCCCCCGAGTGGCTCCTGGAGAACGGGATTTCTGAGAAAAGTGATGTTTATAGCTAC
GGAATGGTGCTATTGGAGATGATTGGAGGGAGGAGAAATATTACTTTAGCTGAAAATGGGAAGAGTAC
CAATTCAAAGAGTAAGTTCAGCTATTTTCCTAAGATTGTGAGTGAGAAATTGGAGCAGGGGAAGATCAT
GGAAGTCCTGGACGAGAGGCTCGTCCATGAGGCAGCTACAGGAGGTGTAGCTGTAGAAATGCAAGTG
AAAAGATTGGCGTGTGTGGCGTTGTCTTGCATCCAAGAACGGCCTAGTCTTAGGCCAACAATGGCACGA
GTCGTTGAAATGTTGGAAGGTCGTGGGCCAGTAGAAGCGCCTAGACAAACGACAATGTTGATCCTTGAT
CTATTAGACGAGGGTGTGGATCATCATCCTGGGATACCTAGGGTTGCTGCAGCCATGGCAAGATCTAGT
GATACTAATTCGCTTCGCTCTTATACAATGTCTATTCTCTCAGGGAGGTAGTACCTACATAATGCATAAGG
GATTTCGTCTGCTTTCTGTCAAATTTTTCAATATCAATAGGTTGATTGTTTCTCGAGTGTTGTATCCTGAAT

CCGAAAGAGTGTACTTGAATTCTCCCCCTAACAAAAAAAAGTGTATGAATTTGCTTTTGTTTACGCCAAA
ATATA
>PGSC0003DMG400022391 G-LecRK
AAAGATAACATTCAAAGCATAACGAAAAGAGAAAGAAGTGTGAGCAAATAGTATTGCAGGCTGCAGCT
ATAGGTATTCTCCATTTTAGTTTATACAGAACTAATTCACATGCAAATTGGCAGAAGAAAAACTTCATAA
GCAATGAAATTAAGCACAAGAGAGATACTTCTGTTTCTTTCTATATCAACTGCTTCAGGTATCATTAGCAC
CAATAAATTCCTGCGAGATTCAGAAACTTTAGTCTCCAATGACAAAAGATTCATATTCGGGTTCTTTAGTC
CTGAAAATTCGACGAATCGTTATGTTGGTGTTATGTTTAATGTCCAACCACCAACTGTTGTATGGGTTGC
CAACAGAGAGAAACCTTTACAGGATTCTAGTGGAAGAGTGACAATATCTGATGATGGAAATCTTGTAAT
CTTGAATTCACAGAATAGGAGTATATGGTCATCAAATATTTCACCAGCTGTGAGAAATTCCACAGCGCAG
ATCTTGGATACTGGAAACTTAGTTTTGAACGATAGCTTCAATGGGAGGGTTCTCTGGGAAAGTTTTCGG
GATCCTTCAGATTGCTTCTTGCAGACCATGAAAATTGGCGTTGATGTAAGTACTAACACGACAAATCTGC
TGAAATCATGGATAAGTCCTTCAGATCCATCTGTTGGGAGTTTCTCAGTTGGTATTCAACCTGAAACAGT
TCCCCAGATTTCCATATGGAAGAATGGGAAACCTCATTGGCGTAGTGGTCCATGGAATAAACAGGTTTT
CATTGGGGTACCAGACATGACTTCATTCTATCTCAATGGATTTGATCTAGTTAATGACAACAAGGGCACC
GTGTACCTTACCTATTTATATGCAAATCAGATTGAGCTGACGTTTTTCACCTTGAACTCAACAGGGTTTTT
GCAGCAGAAATATATGGATCCTAGTAAGAATGATTGGGAAGTAACATGGGAATTTCCTGCAACTGAGTG
TGATTTTTATGGAAAATGTGGACCTTTTGGAAGCTGTGATCCTACAAGCTCACCAATCTGTTCTTGTTTAG
AGGGATTTAGGCCGACAAGTGAAGAGGAATGGGGAAAAGGAAACTGGACTCGTGGATGCAACAGAAA
GTCCATGTTAGAGAGCGAAAGAAACAGTTCTAACCTTGAGCAAGGGAAGCAAGATTGGTTTCTGAAGCT
GCAGTCAATGAAAGTGCCGGATTCTGCTATTTGGGTACCTTTTGCAGATGAAGATTGTGTTAACGGTTGC
TTGAGGAATACTTCATGCATAGCTTATTCATACTACACAGGCATAGGATGCATGCATTGGGAAGGAAGC
TTACTTGATGTTCAGAAATTCTCCATCGGTGGGGCAGATTTATTCCTCCGCCTTTCATACTCTGAGCGTGG
TAAGACCTGCATTTCAGAACTATAATATAAGCTATAAAACTCTGCTTGCCAATGACATATGTATAATGGA
CACTTATTTTGTAATGCAGATCAAAAGAGAGAATATAAAGTAGTCATTGCCATCATAGTCCCAGTAGGCT
CAATAATTCTTGCCATTTTCGGATACATTTCCTGCAAATATGTAGCTAAGCGCAGAGGTAACAAGCTTAT
CTGAAACATTAGAAAATCCATAGTAGCTTTAATCATACGAATCACTTTATCTCTAATGTCCTCTTGAATCA
GGATGGAAGAGAATGAGTAAGATCTTATCAAGTGAATCATCGCCAAACTATTACAAGGAAGACAAGATT
ACAGAGGACATCAATCAAGCTAAATTGGAAGAACTGCTTGTATACAACTTTGATATCTTAGCAAACGCAA

CTGAGAATTTTCATCTGTCCAGCAAGCTTGGACAGGGAGGTTTTGGTCCAGTTTACAAAGTAAGGATTTG
TTTGATTGAGTGTCTTACAATTGTTTTTAAGAAAACATAACCAAGTATATGATATAATTGCAGGGGAAAT
TGCCAGATGGACAAGAGATTGCTGTGAAAAGGCTTTCACAGTCTTCTGGTCAGGGGCTGCAGGAGTTCA
TGAATGAGGTTGTGGTGATTTCAAAACTTCAACATCGTAATCTTGTTAGACTTTTTGGGTGCTGCATAGA
AAGAGGGGAAAAGATTCTGGTTTATGAATACATGCCAAAAAGAAGCTTGGATGCCTATCTCTTTGGTTC
GTTATGATTAGTTTTCTTTTATTTTCCATAGTCTAACTAAAACAAACTTTTCTTTAATTTAAACTTGTCAAAT
TCGCGATGTATTTTCAGGGTCACAACAACAAGAGGAAGAGTTCCTGGATTGGAGTAAACGTGTGATCAT
TATTGAGGGAATTGGTCGAGGCCTTCTTTACCTTCACAGGGATTCAAGACTAAGGATTATTCATAGGGAT
TTAAAGGCCAGCAACATTTTGTTGGATGAATACCTGAACCCCAAAATTTCAGATTTTGGGATGGCAAGG
ATTTTTGCAGGCAACCAAGATCAGGCCAACACAAGCAGAGTAGTTGGAACCTAGTAAGTTCTTGTATCTT
TCCCTTTTATCTTTTCATGAATGAAAAGAACACAAAAAGTAAACATTCAAAATTATAACATGATGCAGTG
GTTACATGGCACCTGAATATGCAATGGAAGGAAGATTCTCAGAAAAATCAGATGTTTATAGCTTTGGAG
TATTGTTATTGGAAATTATAAGTGGAAGGAGGAACACTAGCTTTCACCAAGATGATGGTGCATTAAGCC
TGCTAGCATTGGTAAGTTCTTATGAAAATTAAGCTCCAATTCATATGAAATGTCAACTTTTCATAGTTCAA
GTTTCCAATGTTTTGTCAACGTGCTTGCTCTTCAGGCGTGGAAATGTTGGATTGGAAACAAGATTGTGGA
ATTGGTTGATCCCAAGATAACTGACCTGCACCTTGAAAAAGAAATGGTGAGATGTGTACAAGTTGGATT
ATTATGTGTACAAGAATATGCAGAAGACAGACCAAATGTCTCCACAATTTTGTCTATGCTCACCAAGATG
TTTATAGCTTTGGAGTATTGTTATTGGAAATTATAAGTGGAAGGAGGAACACTAGCTTTCACCAAGATGA
TGGTGCATTAAGCCTGCTAGCATTGGTAAGTTCTTATGAAAATTAAGCTCCAATTCATATGAAATGTCAA
CTTTTCATAGTTCAAGTTTCCAATGTTTTGTCAACGTGCTTGCTCTTCAGGCGTGGAAATGTTGGATTGGA
AACAAGATTGTGGAATTGGTTGATCCCAAGATAACTGACCTGCACCTTGAAAAAGAAATGGTGAGATGT
GTACAAGTTGGATTATTATGTGTACAAGAATATGCAGAAGACAGACCAAATGTCTCCACAGTTTTGTCTA
TGCTCACCAGGGAAATTGATGATTTACCAAGTCCTAAACAACCTGCATTTACAACAAGACCGAGCTTTTC
CAAAAAATGCACTTCTAAATCTCAAGGCTCCATTAACAATGTTACCGTTACTATTATGGAAGGACGATAA
GGCATATACGATTTTTATTTAGGCATGATAGTATTCTTTTCTCTGTAATTATTACTGTTTCTGGTTCTTATT
ATAATGGTTCCACTCAATATTACCTCAGTACATTAAGAATCCAAGAGAAAAGTGTTAACCCTGCAAGTCA
CTTATGCTTGATAACAGAACCCATATCCAAGCAAGAAGAAGCAAAGCTAGACTTCAAACTCAGAGTTGT
CAACAAGTAGCCTAAACAGGTGTTTGCAGAAATTTAGAGAATCCTATCCACGGATTGAGATCATTTAGA
GTTAGCTTTGTATTACAGCTGACTCTGAAGATGAACAACTTTTCTAGACAAAGGCATTCTTACAGCGTGG

TTGGAGGGTCACCAAATCAACAGGGAAGCCTGTAGGAAAGGGGAACATGCATCCAATTCACATCAGAA
ATTGCTGAAATGATGTGAAATCTTGCACCACTTTTGTTCTTGGAACTCAAGTCATGTCAGCCCAAGAATA
TTTGCAGTATCACAGGTAACAAAGGACAAGAAACCTTCTGAGATAGCATGTAAATTTTTCAAAATTCAGA
CAAAGATTACTCCTTTCCTTAGAATTGTTAATAAACCTATTATTCTGCTGATGCCTTTACTAAAT
>PGSC0003DMG400022426 G-LecRK
GAAATATCGTAACTAATAGAGTAGCATTTACATATAATCTTAGTCCAGGTAGGGGTGAATAGGATGGAA
TGTCACTTATGGAACTTGTTTTTTTGAAAGTCATTTTCCTGATTTTCCGAGAAACTTGTTATCCTAGTCAAT
TGAAAACAAAAGAGCTGGAATCATATTGAATAATTGGTCCCAGTTTGAGGAAAATCCTTACGGTAGTCT
ATCTAAGCACCACAGATTATTCTGAAGACTTGTAAATTCAAATCTGAATACCAATATTCCATAGCCATTGT
TTTTATGAGCTAGCAACATGAAGTCTACTAGTGTCTTCTTGTTTGTATTCTTGCTGTATTTGAGTCACTTCA
AATGTGATTCTAGGGACATCATAACTTCAAGGAACATATTAAAAAACAAAAGGAAAACTCTTGTGTCAG
CTGGACAGATATTCGAGCTGGGACTCTTCAACACACGATCTAAAGACGGAAAACTTAGAAATTATGTTG
GCATATGGTACCGGGTGAGTCCCAAAACTATTGTGTGGGTTGCCAATAGGGACAGACCAATTCCCGTAT
CAGATGAAATTTTGGCTATTGGCATTGATGTGGATGGTAATCTCAAAGTGTTGGACTCAAAGAAGAACT
CTTACTTTTCAACAGGACTCGTTAGTGCATCTTCTTCTAAGCGTACAGCAAATCTCTATGACTCAGGAAAT
TTGGTTTTAATTGATGAATTGTCAGGCAAGAGATTGTGGCAAAGTTTTGACAACCCCACTGACACCTTCC
TTCCTGGTATGAACATGGACAGTGCATTGAAGTTGACTGCTTGGAGGGTTACCCAAGAGGATCCCAGCA
CTGGAGATTACACGTTTCGGCAAAATACAGCAACCAAATTTGAGTACAACATACTATCCCAAAAAAGGG
GCCTTCACTGGAAAGCCTCTGTTTCTGCTAAGCCCTTTAGTTCCAGGGAACTGCCCTCTCTTGTGGGATCA
ATGTTAACCAATTTCAGTTTCAATACAGAACAACAATATACCCCGAAATTGAACAAGTCACCGACTGATG
ATGTTTCATCTGTGCCAGACTTTAATTACACAAGGCTACTACTGAATTCCTCAGGGGAAATACAATTATAT
GGCTGGAGTAACGAGAGTACAGGGTGGTTACAAATATGGTCAGAACCACAAGATCCATGCGATGTGAA
TGAAAGTTGTGGGAAATTTGGAATTTGCAACAGTGGGTTTATGTCTCGGTGCAAGTGTTTGCCAGGATT
TGACCCTGATTCTCCAGATGAATGGACGGTTGGGACTTGTACTGATGGTTGTTCAAGAAAGTCAGTTCGT
GGTTGCAACAAGAAGTCAGAATTTGACACATTCTTAAACTTTTCAACTATCAATGGGAAACTTTGTTTGC
ATACCTAACAAGGTGTGGATCACCACCGACAAATCCTTTTAGTTGTATCCTCCATTTTATTCACCTACGCA
AGTTTCAATTTCGTTTGGTTCAGAGCATGTAGGACAAAGAGGATGGAGTGGAGAGAAGGCAACATCCTT
GAATCTCAAAGTCCTGATTATTTCTGTCAGTGTAGCTGCTGCTGGAACTATAACCATAACCATTTGCTATG
TAATCTACCATAGAAAAAAGGTGGCAAGAAGGAAAGGTAACAAATAGCACAAGCTTTTATATAAATGAT

ATATGTATTGTGAATTTGGTGCTAATTTTGAACTCACTTTATGTAATGAGAAGCCAAGAAAATAAGTTTG
GGGAATGGGATAGAGTACTTGTCCGAGTGTGGAGGTTCATCTAAATATTTGGTAACCGAAGATGATAA
GAAACGGATCGATATCCCATTTTTCAACTTGGAAAGCATACTAGTCGCTACGGACAACTTCTCAGATGCA
AACAGGCTTGGTCAAGGTGGATTTGGCCCTGTTTACAAGGTGGTGGTGATTCTCACATGTACCATTTTTA
AAATCATTTTTCTAACTACAGCTAATATTGTACGTACAGGGTAAGTTCCCTCAAGGACAAGAAATGGCAA
TTAAGAGGTTGTCAAGTCATTCTAGTCAAGGTGCAGAAGAATTTAAAAACGAAGTAATGTTAATTGCCA
AACTCCAACACAGAAATCTAGTCAGACTTTTGGGCTACTGCATCGAGGAGAACGAAAAGATCTTATTATA
TGAATATATGCCCAACAAAAGCTTAGACACCCATATATTTAGTGAGTCTACTTACTTTTCTTCTCAGAACT
CCATTATAGAATCTCTCCCGTTGGTTCAGTGAACCTCTAAATTTCATTATACATACAGATAGATACAAGGG
TCAATATTTATTTAACTAAACCATTGTTCAACTAAAACTTTGCTTTGTTGGGGTCATTATTGTTACATTGGA
AAACAGAAAACAAGGGAATTTCATTTTGGTGTGGAAAGATCTTTGTATTAGCAAAAGCTTAGGAATTAG
AATGCTTAATTTCTGTAATTGGTGAAAAGTAAGATCTAACTGTGATTTAGTTGTTCTTTGAGTTCACTCTA
CACAAATACCTCGGGTACTACTAGAAGGGGATTAAGTTTCTTAAAGACACAATGTAAATTCAACTTTGAC
TTGATGGACTTGTTCCGGATAGTTCTCTTGAGACATTTATCAAACATATTGGCTGTGTTTGGTATGAAGG
AAAATGTTTTCCTAGAAAAACAAATAGATTTTTTATTTATTTTCTCATGTTTGGTTGGTGAGGAGAAAAGA
TTTTTAGTGTCTGGTTTTTGAATCAAAAATATTTTTGAGAAATATCTTTTATTACTAGAGTAGAAAATAATT
TTTGAAATTGAAAATATTTTTAAAAANNNNNNNNNNNNNNNNNNNNNNNNNNNNNNNNNNNNNNNNNNNN
NNNNNNNNNNNNNNNNNNNNNNNNNNNNNNNNNNNNNNNNNNNNNNNNNNNNNNNNNNNNNNNNNNNNNN
NNNNNNNNNNNNNNNNNNNNNNNNNNNNNNNNNNNNNNNNNNNNNNNNNNNNNNNNNNNNNNNNNNNNNN
NNNNNNNNNNNNNNNNNNNNNNNNNNNNNNNNNNNNNNNNNNNNNNNNNNNNNNNNNNNNNNNNNNNNNN
NNNNNNNNNNNNNNNNNNNNNNNNNNNNNNNNNNNNNNNNNNNNNNNNNNNNNNNNNNNNNNNNNNNNNN
NNNNNNNNNNNNNNNNNNNNNNNNNNNNNNNNNNNNNNNNNNNNNNNNNNNNNNNNNNNNNNNNNNNNNN
NNNNNNNNNNNNNNNNNNNNNNNNNNNNNNNNNNNNNNNNNNNNNNNNNNNNNNNNNNNNNNNNNNNNNN
NNNNNNNNNNNNNNNNNNNNNNNNNNNNNNNNNNNNNNNNNNNNNNNNNNNNNNNNNNNNNNNNNNNNNN
NNNNNNNNNNNNNNNNNNNNNNNNNNNNNNNNNNNNNNNNNNNNNNNNNNNNNNNNNNNNNNNNNNNNNN
NNNNNNNNNNNNNNNNNNNNNNNNNNNNNNNNNNNNNNNNNNNNNNNNNNNNNNNNNNNNNNNNNNNNNN
NNNNNNNNNNNNNNNNNNNNNNNNNNNNNNNNNNNNNNNNNNNNNNNNNNNNNNNNNNNNNNNNNNNNNN
NNNNNNNNNNNNNNNNNNNNNNNNNNNNNNNNNNNNNNNNNNNNNNNNNNNNNNNNNNNNNNNNNNNNNN
NNNNNNNNNNNNNNNNNNNNNNNNNNNNNNNNNNNNNNNNNNNNNNNNNNNNNNNNNNNNNNNNNNNNNN

NNNNNNNNNNNNNNNNNNNNNNNNNNNNNNNNNNNNNNNNNNNNNNNNNNNNNNNNNNNNNNNNNNNNNN
NNNNNNNNNNNNNNNNNNNNNNNNNNNNNNNNNNNNNNNNNNNNNNNNNNNNNNNNNNNNNNNNNNNNNN
NNNNNNNNNNNNNNNNNNNNNNNNNNNNNNNNNNNNNNNNNNNNNNNNNNNNNNNNNNNNNNNNNNNNNN
NNNNNNNNNNNNNNNNNNNNNNNNNNNNNNNNNNNNNNNNNNNNNNNNNNNNNNNNNNNNNNNNNNNNNN
NNNNNNNNNNNNNNNNNNNNNNNNNNNNNNNNNNNNNNNNNNNNNNNNNNNNNNNNNNNNNNNNNNNNNN
NNNNNNNNNNNNNNNNNNNNNNNNNNNNNNNNNNNNNNNNNNNNNNNNNNNNNNNNNNNNNNNNNNNNNN
NNNNNNNNNNNNNNNNNNNNNNNNNNNNNNNNNNNNNNNNNNNNNNNNNNNNNNNNNNNNNNNNNNNNNN
NNNNNNNNNNNNNNNNNNNNNNNNNNNNNNNNNNNNNNNNNNNNNNNNNNNNNNNNNNNNNNNNNNNNNN
NNNNNNNNNNNNNNNNNNNNNNNNNNNNNNNNNNNNNNNNNNNNNNNNNNNNNNNNNNNNNNNNNNNNNN
NNNNNNNNNNNNNNNNNNNNNNNNNNNNNNNNNNNNNNNNNNNNNNNNNNNNNNNNNNNNNNNNNNNNNN
NNNNNNNNNNNNNNNNNNNNNNNNNNNNNNNNNNNNNNNNNNNNNNNNNNNNNNNNNNNNNNNNNNNNNN
NNNNNNNNNNNNNNNNNNNNNNNNNNNNNNNNNNNNNNNNNNNNNNNNNNNNNNNNNNNNNNNNNNNNNN
NNNNNNNNNNNNNNNNNNNNNNNNNNNNNNNNNNNNNNNNNNNNNNNNNNNNNNNNNNNNNNNNNNNNNN
NNNNNNNNNNNNNNNNNNNNNNNNNNNNNNNNNNNNNNNNNNNNNNNNNNNNNNNNAGGGGGGATGGGTG
GGTCGGGGGAGAGTTTTGGAAATTGTTTTCCATAAATTTGAAGGAAAGTCATTTTCCGAAGTGTTGATTT
GAAAAGCATTAAATCCAACCAAACATGAGAAAATTGGAAACACACCCATTGTAAGTAAACATATTTGATT
TCTAAAAAACTGCTATATATGTAATGTCCCTTCAATTGTTCTTTTGATCATCAATTGGTTTCGTTTTTGCTA
ATGTTGTGATTTTCTACTTGTTCTGCAAGATCATAGTGTACGCCCATTGTTGAATTGGAATATCCGCTTTG
AAATCATATTGGGCATTGCTCGTGGACTTCTTTATCTACACCACGACTCCAGATTACGTATCATTCATAGA
GATCTAAAAACGAGCAATATTCTATTAGACGAAGAGATGAATGCCAAAATATCAGATTTCGGCTTGGCC
AGGAATGTTGAAGGCAAAAGTATTGATGCTAAGACGCAAAAAGTTGCCGGAACTTAGTAAGTATATATA
TATATATATGCTTTCAGTTTAGTTTTCTGCATAATATAATCGCCAAAAAACTTATGAAATTACAAGTATAT
CGGAAATTGTGCAATCACCTGTTTATAGAGAGCAGCCTTGTTTCATGAACTAGCCCCTCTCAAATCCCCA
CTTGATCAGGAATTCTTCATTGACAGTGTCACCGGAGCTAGATGGATCTTAATTACCGGCGTGATATTAT
TGACTTTTTAGTCAAAATCATTCTTAAAATATAGAGAAAATACATTAAAAAAAACCCTGAATTTGTCCGCT
AAATTTACTTTAGTACTTTTAGCTTTATAAATAATCATTTACTTCTAAATCTCATTTCAAGTGAATTAAAAT
CCACCCAAAAAGCTAACGTCGCAAATAAAAAAAAAGAAAAGGCTAGTGAAAACGTAAAAAATCAAAAT
AAATACTCTCTCTGTCCCTAATTACTTGTCCACTTTTCCTTTTATAGTTATTTTTAATTATTTGTCCATTTTGA
CAAATCAAGAAATGACAATTTTTTTTACCTATTATACCCTCAATTAATTACTTTGAAAAAAGTAGAACTTTT

TGAAAATCTTAAATTTTAATTCATCTACTTCATAATTAATAAGGGTAACATGATAAACTTACTATGTCAAT
AATTATTTTCTTTAATAGGTGTGTCAATTCAAAAGTGGACAAGTAATTAGGGACAGAGGAAGTAAACAA
ATAGGCCTTTTTTTTCTTTCCAACCAATAAGGTTTCATCTTCCTCACACCCCACCACCCCACCACCTTTCTCC
ACCATAACCCCACCCTTACGCCTCGAACCCCCCACCCGCACCTCTCTTTCCCCTATTTCGAACCCTGCCGCC
ACCCACACTTGCCTCACCCCTTTCTTCTTCTTCTTCGAGGTCCCGTCCCTGCCACGTTCTTTCTTCTTTTTCA
AAACACTCCACACATTCCTTCATTTCAAATTATTCTTGCTTATTCTTAGAAATGATGAATAGGAAAAAAAA
TGAAAAAACCAACAACTTATTATTGCTTCAAGATCTTACTTTTGGAAGGGTTGTTGATTGTGATGAACTT
GAAGTTTTTTTAATAAAAATAATATAATTTCTTCATCTTAAATTATTTTTTTTAATTTGTACTCCTATTATTT
ATTTATTTTGATAATGTGCTTTAAAAATTGAATGTTTTCTAGTCTTGGATCCATAAAGTTTGATTTTATTTT
TATAATCTTAGATTAATTCTCAATTTTTTTTCTATTTTTTACCTTTCTAAAATTGTATAAATAAAAAGAAACA
AAAAAGAATTAAAAAAACTCAAAATTTAAATAAATTTCAAGGTGGCGCTGATGTGACAATGATGTGGCA
TTGATTTGGCAATGAAATGGAGCATGTGTAATACACTTGCCATTGTGAGACTGATATTGTAATATTAGAT
TTGAAAAAAAATCACTTCAAAAATGTTAAGGGGTATTTAAACGTCCATGTAGTTTAAGTGCTAAAGTAAG
TTGTGCTGCCAAGTTTAAGGGGTTTTTTTATGTGTTTTCTCTAAAATATACCCTAAACTTAAACTATATCC
TTAAAATATTATTCTTATCAAGAAAACATTTTTCAAGTATTTAAAAGTGAATAATTTTCATCCTTCTACTTG
AATTTATGAGAAATCTAACAAATCCCACAAAAATCAAGTTATTCCTTCATAATTATTATTCTTAGATACGC
CAAAAATACTATCGTGAACTTTTATGGTAATTAAGTTTAGCATTATGCAAAATCTTTAAATTTGACAGTTT
TTTTCATTTATTTTTCATGTAATTTCATATTTAAAATATTATTAGTTGGTATAAGTTTCTTCTCAATTGAATA
TACTAGAAGTGACGTTTGTCGGAAACTTCCCCTTCTAATAATGTAGAATGAAACTCAAATGCGAGACATG
ATCAATATTTTGATTACTTCAATATCAGATTGACCCAAATATTTATTGATCTAAATACAATTTGACTTTGAA
AAAGGTAAAGGTAATTAAGCTTCAGCTTATTTCTGTTATTAAATGAGAAAAAGAGCTGATCATGTATCCT
CACACCTTCCAAACATTTTTTGTTTCAGTAAGCAATGGTGTCTAAGATTCTTAATATTTATTTTCGAAACTT
CTTATAGCAGGTAATGAAATTATGCAAGAAAACTTTTGTTGATAGTCACATGAATTGCTCATGTTTTGTA
GGATCCCTTAGTTTTTTGACTATATCTAACATAAGGATGAAAATTGTTAACTTTCAAATATTTAGAGGATG
TTTTTTTGCCAAAAAAGATATTTTAAGGATGTAGTTTAAGTTTGGGGTATAGCATAAGGATGTTTTTGAC
CAAAAACTTTGATATTGTTTCCCGGTAGTATTTTTCCCCAATTGAGTTGTGCTATCATGGGCCGAGCCTCT
TTTACCTGAAAATTATGCGGTGAATAGAGGTTAAATGTTAATTATTATGAATATATATTTAATTTTGTATA
TTATTCACCTAACTAAAAAGAAAATTTGCAAATCCTCGCCACAGGCGTTTACTGATCATGTACTCATTCAT
CTACTCAAGGCCGTTTTTATCTCATATACATGGTGGTATACGTTATCTTGACTCATACATGATGGGATACA

CTATCTAAGATAATTAATCCTGGGATAACTTGTTCGTTTAATTGGATCAATTCTTGTCTTGTGATATACAG
CGGCTATCTTGCACCAGAATATGCATTGGAAGGACTTTTTTCAATCAAGTCTGATGTTTTTGCTTTTGGAG
TGGTTGTACTGGAAATCATTAGTGGAAACAAAAACTTGGATGTTCTTGAAGACACAAACCTCTTGGGTC
ATGTAAGTACCTCTCTATTTAGAACATGTAATCTTAAGACATAAACGAAATGTGAAGATTGACATATAAA
GTAGGCCTTCTGTCCCAATTTATATGAATATTGGGCATCGTTGATAACTCGAAATTAAATATAATTACAG
GCTTGGAAACTATGGATGGAAAACAAGGCATTGGACATAATGGACCAAAGTCTAGTGGATACATTCAAT
GAAATTGAAGTAGTAAAGTGCATAAACATAGCACTTTTATGTGTGCAAGAAGATCCAGGTGATAGGCCA
ATTATGTCAAATGTGATAATTATGCTTGGAAGTGATAGCATGCCTCTTCCAAGACCCAATCAACCAGCTT
ATGTCACAAGGAAAAATTACACAATAGGAGCAAGCACATCATCCTCCTCTTCTTATAATTATAATAATTAC
TATAATTATGATTCATTCACTGGCTCCAAAAATGAGTTGACCATCACTGATATGCAAGGCCGATAAATAT
TATATAGTAGTAGTAAGTTTTGCACGATATGGACATGTTAAATACATATTCTAAGGTTAGTGTGAATGAC
AATATTTATGTAGTAGGCGGATCTACGTAGAGGAGAGGGAATGCAAGGGCACCCGATCGAACACTCGA
GAAGAACTTCATGTATAAATATCTCGAGTATATATGTGTATGTACTCCATTAAAATCTATGTGTGTTAGCA
TATATATATTGAATGTAAATATAGTAATTTGTATATTATGATAAATATAGGGCTAATAGGTTAGGATTAG
CAGTACTATTCTCTTGTATGGAGCACACAATTGAAGCAGACATGTTGATGCTCAGCTTTGTGCTTATAGT
AATTCTTGGATAATACTAATATTTGTACTGGTAATAAAATCCTTGTTAATCCTCTAAATGTTGCATTAGTTT
TC
>PGSC0003DMG400022787 G-LecRK
CACACTTCGACTAATTCATAGAATACGTATCACCTCTCACCAGCAACAAAATCTTGATTTGTGGAAAAAA
AAAAAACCAAAGTGATGATCACTAGTCTACAATATTTGCTTGTTCTTCATTTTTCTTACTTAGGACTTTGCT
TATCAATATGTAACATTAATAGCACTCAATATCTAAAAGATCCAGCAACTTTATGGTGTCCTGATAACATG
TTCAAGTTGGGATTTTTCAGTCCTGGAAATACTACGAATCGTTACGTTGCTATTTGGTACAACTTCTCAGA
AACAACTGTTGTTTGGGTAGCAAATAGAGATAAAGTACCATTGAAAGATTCATTTGGTGTTTTCAAGATT
TCTGAGGATGGAAATCTTGTTGTGATGAATGGAATGAAAGATGTTATTTGGACGTCGAACATTTCATCTA
CTTCACACGTTGTTACGAGTTCTGTTGCTCAGTTACGAGGAGGGAACCTTGTTTTACTTGACAACTCGAA
AAATAGGAACATAATATGGCAAAGTTACCAACATCCTAGTGATACATTTTTGCCTAAAATGAAAATACCT
ATTAACTCTGTTACAACAGGGGAGAAAACAGGGCTAAGATCTTGGAAAAGTCCTTTGGATCCAAGTTTT
GGTGATTTTTCGACTAGCATTACCGCGATTCTTCCTCAGCTTTTGATTTGGAAAGGGAAGAATCTATACT
GGCGAAGTGGTCAATGGAATGGTCAGGTTTTTATTGGAATAAAGGGTATGCATAGAGTGCACACTGGT

GGATACAATGTTGTTGATGATGAAGAGGGCACTGTCTATCTTACGGGTTTTGTTGAACGAAAAAAACCT
ACAAGGTTTGTCTTGGACTCGAGTGGGAACTTGGTACAATCATACTGGGATGGGATTGAGAGGATTTGG
AAGTTTCCGTGGTCAGCTATCGAAAATGATTGTGATGTTTATGGAACTTGTGGTCCATTTGGGAGCTGCA
ATTCATTGTATTCGCCAATTTGTTCGTGTTTAAGAGGGTTCGAGCCAAAGAATATCGATGAATGGGAAAA
GGAGAATTGGACTAGTGGTTGTGTGAGGAGGAAAAGTTTGCAATGTGATCAAGAAAACAACAAAACAA
GTACAAGAGATGGATTTTTGAAGATGGGATACATGAAAGTGCCTGATTTCGCTGAATGGTTACCTCCTA
AGCTTGAAGATGAATGCAGAAGCCAATGCTTGAGTAATTGTTCTTGCTTAGCTTATGCTTTCGACACAGG
AATAGGCTACATGTCGTGGAGCGGATACTTGATTGACATTCAACAGTTTCAAGCCTCTGGCACGACACTT
CACATCCGTGTGGCACATTCTGAACTCGGTATTGATTCATTGTTTCTTGATTTGCCACTGTTCCATTAATGT
TTCCAGTAGTCTTACATGATTTGGTTTCGTCTGCAGATCATCACGGAGAGATTAGACTAATCGCAACTCT
AGTGATTGTATCGTCTTTTGTTGTTTGCGTTGGTGTGTATCTTATATGGTTTTGGATGTCAAGGCAAAGA
GGTAAAATATGGTTTCACTTTAAAAGGAACAATCATTTAGATGGAAAGAAGATCTGTTTGGGAGAAATT
ATTAGTAATGTTGGTTTGGATGAGTTGCCAATTTTCAAGTTTGAAGTGCTTGCAATGTCAACAAATCAGT
TTCATGACAACAATAAGCTTGGACAAGGTGGTTTTGGTCCGGTATACAAGGTAAACCTGTTTTTTTCTTC
GCGTGAACCAATATGTTCCAACTCCGGTTCTTGTAAATGCAACTGTCACGTAGTATCGTTTACCTGTTTAT
TGTTCGTAGGGAGTATTAGCAGACGGGGTGGAAATAGCTGTCAAGAGGCTTTCGACTGTCTCAGGACA
AGGGATGGAAGAGTTCATGAATGAAGTGTTGTTGATATCTAGAGTCCAACATAGAAACCTTGTTAGATT
ATTGGGATGTTGCATAGAAAAAGAAGAGAAGATGTTGATCTATGAGTATTTGCCAAATAAAAGCTTGGA
TGTTTTTCTCTTTGGTTAGTCCTAAAAGAAACTCTTACTCTGCGATATATAGTCAGACCTTTTTATAACAGT
CATTCTCTATAATAACATTCCACTATAACAACCAAGTTTCCTTTGGAACCGATTTTAATGTTATGTTTTTTA
TAATAGCATTCACTATAGCAAGCCAAAAAATCATCCAGACAAACGACATTGTTATAGAGAGGTCTGACT
GTATGTTCATTATAGCTTTCCTCTTTTATCTCATGCAAAAGCTTAAGTTGTTATAGAAAGAAATTGTCTTTC
GATGAATGTTTAGATCAAACGCATAAAGGGATGTTGGATTTGAGCAAACGATTGCACATTATCGAAGGG
ATAGGGCGAGGACTACTATATCTACATAGAGATTCAAGAATAAAGATAATCCATAGAGATCTAAAGCCA
AGTAATATCTTGTTAGATAATGATTTCAATCCAAAGATTTCAGACTTTGGCATGGCTAGGATTTTTAAATG
CAATCAAGATCAAGCAGAGACAAGGAAAGTTGCAGGAACATAGTAAGTATATATATAGCCATTCAATAT
GCTAAGTTTTCTTCTTGTCTAAAAGTTCCCACATTAAGCTAATTCGTTGATTTATCGTTGAATTTGCAATGC
CTTAGTGGCTATATGGCTCCAGAGTACGCGATCAAAGGGATGTTTTCTGAGAAATCGGATGTTTTTAGCT
TTGGAGTAGTGTTGTTGGAAATAATGAGTGGGCAAAAGGTTGCAAGTTTTTGGAATGAGGAGATCTCAT

TAAGCCTTCTTGGATATGTAAGTTCCTTTAAAACTCTCTTGTACTTAATTGTGAGAAGCAAAAATATATTG
ATAAAGAAGTTCATTAGAATCTAACTTTAGAACTTGATATTGAGTGTTATAGCCTTGTGAAATAAGGACA
TTTTGATAGATCGAATTGAATGTTATTAACGCTCAAAACTTTAGTACAACCTTTTCTGATCCCATTGATGG
GAAGACCCATATTATACCAACATCTCTTTAGGTTCTTCGATCATCTAGAGGTGTATATTTCCATTCTGCTT
GGATCCAGTATCATTAAGCTTCAAATCCTGAGTGCGTCTCATTGTTAATTATTTAATAAATCTAAATACCA
CTAAAACAATTTATTTATTTTTCAGGCATGGGAACTATGAAAAGAGAATGATTTATCAAATTTTATTGATC
AAGTGATATGGGATCCCAACTTTGAAATTGTGTTGACAAAATGCATACAAATAGGTTTGTTGTGTGTTCA
AGAATTTGCTAGAGATAGGCCAACAGTTTCAAATGTTCTTTCCATGTTTTCAAGTGAAGTTATAAGTCTTC
CAACACCATTGAAGCCTGCTTTTGCTAATTTTTATGATGATAATGGAGTTGGTTCTGTAAATTATGTCACT
TTGACAAACCTTGATGCTAGATAATTAATATGAAATATTGTGAAT
>PGSC0003DMG400024258 G-LecRK
ATGGTGTCATCGACTCAATTCAGGAGGTTTACATATACTGAACTTAAGAAAGCAACAAAAAACTTCAAG
GTTGAGCTAGGAAGAGGAGGTTCAGGAGTTGTTTATAAAGGAGTTTTAGCAGACGGAAGAGCGGTAGC
AGTCAAGAAACTTTCAAATGAGTTTCAAGAAGAATTTTGGGCAGAGATGACTACCATAGGAAGAATCAA
CCATATGCATTTGGTGAGGATGTGGGGATTTTGTTCTGAAGGTAGACGTCAGCTTTTGGTCTATGAATAC
GTCGAGAACTCATCACTAGACAGGCATCTTTCCAGGGCTGATATTCTTGAATGGAAACAAAGGTTTGCA
GTGGCACTAGGGACAGCCAAAGGTCTGGCATATCTTCATCATGAATGTCTTGAATGGGTGATTCATTGT
GATGTTAAGCCTGAAAATATACTTCTCGATAGTGAATTCGAGCCCAAGATTGCAGATTTTGGACTTGCAA
AGCTGTCCCAAAGAGGCGGCCCTGGTTCAGATTTCTCCAAGATTCGCGGTACTAAAGGCTATATGGCTC
CTGAATGGGCTTTGAACCAACCTATAACAGCAAAAGTTGATGTTTATGCCTTTGGGATTGTAATACTAGA
AATGATCAAAGGAAGTAGGCTGTCAAGTTGGGTGGTGGATGATAGTGAATGTGATCATGAACAAGATT
CACAGCTCGGAAAATTTGTCAGGATGGTGAAGAGAAAGATTCAAAGTGGAGAAGATTCTTGGGTGGAG
AAACTTGTCGATCCACGACTAGAAGGCAAATTTGGCAAGAACCAGGCAGTGACACTGATTGAAATAGGC
CTTTCTTGTGTAGAGCAAGACAGAAACAAAAGGCCTACAATGGCCTCAGTAGTCCAAACATTGCTGGAT
TGTGAAGATGAAACAACACAAATAACATAG
>PGSC0003DMG400024259 G-LecRK
ATGAAAAGTTTGAAACTTTACTATCTTTTTCCATTTTTAGTTTTTTCATTTCTTGTTTCTTCTTTCCCTTTGAT
AACATCATCAAGAATTTTGCTTAAAGGTGATTCTTTTTCAGTTAAAGATGATTCACATTTCATTACTTCTCC
ACAAAAAACATTCACTTGTGGTTTTTATCCTATAGAAAGTACTAGTAATGCTTATTATTTTGGAATTTGGT

ATACAAATTCAAGAGATAAAACTGTTGTTTGGAATGCTAAACAAGATAGGCCTGTTAATCTCAAAGGGT
CAAAGTTGACATTAAGAAAGAATGGAGCTTTAGTTTTAACAGATGTTGATGATACAATTGTTTGGCAAAC
AAACACAACATCATTTGATGTTGAAAAGGCTGAGGTTCTTGAAACAGGAAACTTAGTATTGAAGAATCC
TAAAGGTGATGTTTTGTGGCAGAGTTTTGATTTGCCTACTGATACTTTATTGCCTGATCAAAGATTTACCA
AGAATCATAGATTAGTTCCTCCTTTGAGGAAAGGGAGTTTATCACCTGGATACTTTAGTTTGTACTTTGAT
AATGATAATGTATTGAGAATGATTTATAATGGTCCTTTAGTTTCGAGTATATACTGGCCTAATCGCGAAA
GGAGTGCGCCCCAGAATGGTAGAACTAGTCAGGTGGATACTGTAGTTGCACATTTTGATGGAATGGGT
AGATTTTTCTCAAGTGATATGTTGCAATTCAATGTTTCTGATATGGGGCTTGGGATATTGAGAAGATTGA
CTATAGATACTGATGGTAATTTGAGGATGTATAGTTTGGATAACTTGACAGGGTTGTGGAAGATGTCGT
GGCAAGCTATTGCACAACCTTGTGCTGTGCATGGAATGTGTGGAAGATTTTCGATATGTACTTATGTATC
GAAGCCTAAATGTACATGTCCTCCTGGATATGAGATTGTTAATGGAAGTGATTGGAGTAGAGGTTGTAG
GCCAATGTTTAGATCAAGTTTGGAGTCTAAACAACATGTAAAGTTTGTGGAGATTCCTAATGCTGATTAT
TGGGGTTTTGATCTCAATGCCACTACTCCTTTGTCATTTGAATCTTGTAGGGCATTGTGCTTGGACGATCC
ACGTTGTCGTGCATTTGTTTATAGGTTAACTGGTGTGGGATCATGCTATACTAAAGGCATCCTTTTTAATG
GATACAGGTCTCCTAGTTTCCCTGGAAGTGTATTCTTGAAACTCCCTATGAATCTAAGTGCATCAGAATC
AGGTCATTTGATGCTTGAAGGTACTGATGCAAAATGTGGATTGAGTCCTGAAAACTCTCTTTTTGGTTCT
CTTTCTATGTATGTATTGGATTTTAAGAAGGTGAAGTGGATTTATCTTTACGTGTTTTGTTTCACCCTTGG
TGGAATAGAGATTCTGTTCTTCGTGTTAGGCTGGTGGGCGTTGTTTAGTAAACATGGGATTCCTGCTTCC
ATTGAGTATGGATATAAAATGGTGTCATCGACTCAATTCAGGAGGTTTACGTATACTGAACTTAAGAAA
GCAACAAAAAACTTCAAGGTTGAGCTAGGAAGAGGAGGTTCAGGAGCTGTTTATAAAGGAGTTTTAGC
AGACGGAAGAGCTGTAGCAGTCAAGAAACTTGCAAATGAGTTACAAGAAGAATTTTGGGCAGAGATGA
CTACCATAGGAAGAATCAACCATATGCATTTGGTGAGGATGTGGGGATTTTGTTCTGAAGGTAGACGTC
AGCTTTTGGTCTATGAATACGTCGAGAACTCATCACTAGACAGGCATCTTTCCAGGGCTGATATTCTTGA
ATGGAAACAAAGGTTTGCAGTGGCACTAGGGACAGCCAAAGGTCTGGCATATCTTCATCATGAATGTCT
TGAATGGGTGATTCATTGTGATGTTAAGCCTGAAAATATACTTCTCGATAGTGAATTCGAGCCCAAGATT
GCAGATTTTGGACTTGCAAAGCTGTCCCAAAGAGGCGGCCCTGGTTCAGATTTCTCCAAGATTCGCGGT
ACTAAAGGCTACATGGCTCCTGAATGGGCTTTGAACCAACCTATAACAGCAAAAGTTGATGTTTATGCCT
TTGGGATTGTAATACTAGAAATGATCAAAGGAAGTAGGCTGTCAAGTTGGGTGGTGGATGATAGTGAA
TGTGATCATGAACAAGATTCACAGCTCGGAAAATTTGTCAGGATGGTGAAGAGAAAGATTCAAAGTGG

AGAAGATTCTTGGGTGGAGAAACTTGTCGATCAACGACTAGAAGGCAAATTTAGCAAGAACCAGGCAG
TGACACTGATTGAAATAGGCATTTCTTGTGTTGAGCAGGACAGAAACAAAAGGCCTACAATGGCCTCAG
TAGTCCAAACATTGCTGGATTGTGAAGATGAAACAACACAAATAACATAG
>PGSC0003DMG400024260 G-LecRK
ATGAAAAGTTTGAAACTTTACTATCTTTTTCCCTTTTTAGTTTTCTCATTTCTTGTTTTGTCTTTCCCTTTGAT
AACATCATCAAGAATTTTGCTTAAAGGTGATTCTTTTTCAGTTAAAGATGATTCACATTTCATTACTTCTCC
ACAAAAAACATTCACTTGTGGTTTTTATCCTATAGAAAGTACTAGTAATGCTTATTATTTTGGAATTTGGT
ATACAAATTCAAGAGATAAAACTGTTGTTTGGAATGCTAAACAAGATAGGCCTGTTAATCTCAAAGGGT
CAAAGTTGACATTAAGAAAGAATGGAGCTTTAGTTTTAACAGATGTTGATGATACAATTGTTTGGCAAAC
AAACACAACATCATTTGATGTTGAAAAGGCTGAGGTTCTTGAAACAGGAAACTTAGTATTGAAGAATCC
TAAAGGTGATGTTTTGTGGCAGAGTTTTGATTTGCCTACTGATACTTTGTTGCCTTATCAAAGATTTACCA
AGAATCATAGATTAGTTCCTCCTTTGAGGAAAGGGAGTTTATCACCTGGATACTTTAGTTTGTACTTTGAT
AGTGATAATGTATTGAGAATGATTTATGATGGTCCTTTAGTTTCGAGTATATACTGGCCTAGTCGCGATA
GGGATGTGTATGGGAATGGTAGAACTAGTCAGAATAGTAGTAGATTTGCTTATTTTGATGGAATGGGTA
GATTTTTCTCAAGTGATATGTTGCAATTCAATGTTTCTGATATGGGGTTTGGGATATTGAGAAGATTGAC
TATAGATACTGATGGGAATTTGAGGATGTATAGTTTGGATAACTCGACAGGGTTATGGAAGATGTCGTG
GCAAGCTATTGCACAACCTTGTATTGTGCATGGAATATGTGGAAGATTTTCGATATGTACTTATGTATCG
AAGCCTAAATGTACATGTCCTCCTGGATATGAGATAGTTGATGGAAGTGATTGGAGTAGAGGTTGTAGG
CCAATGTTTAGATCAAGAAGTTTGGAGTCTAGACATGTCAAGTTTGTGGAAGTTCCTAATGTTGATTATT
GGGGTTTTGATCTCAATGCCACTACTCCTTTGTCATTTGAATCTTGTAGGGAATTGTGCTTGGGTGATCCA
CGTTGTCGTGCATTTGTCTATAGGCGTACTGGGGAGGGATCATGCTATACTAAAGGCATCCTTTTCAATG
GATACAGGTCTCCTGGTTTCCCTGGAAGTGTGTTTTTGAAACTCCCTATGAATCTAAGTGCATCAGAATC
AGGTCATTTGATGCTTGAAGGTACTGATGTAAAATGTGGATTGAGTCCCCAAAACGTTCTTTTTGGTTCT
CCTTCTATGTATGTATTCGATTTTAAGAAGGTGAAGTGGATTTATCTTTACCTGTTTTGTTCCACCCTTGGT
GGAATAGAGATTCTGTTCTTCGTGTTAGGCTGGTGGGCGTTGTTTAGTAAACATGGGATTCCTGCTTCCA
TTGAGGATGGATATAAAATGGTGTCATCGACTCAATTCAGGAGGTTTACGTATACTGAACTTAAGAAAG
CAACGAAAAACTTCAAGGTTGAGCTAGGAAGAGGAGGTTCAGGAGCTGTTTATAAAGGAGTTTTAGCA
GACGGAAGAGCGGTAGCAGTCAAGAAACTTTCAAATGAGTTTCAAGAAGAATTTTGGGCAGAGATGAC
TACCATAGGAAGAATCAACCATATGCATTTGGTGAGGATGTGGGGATTTTGTTCTGAAGGTAGACGTCA

GCTTTTGGTCTATGAATATGTCGAGAACTCATCACTTGACAGGCATCTTTCCAGGGCTGATATTCTTGGAT
GGAAACAAAGGTTTGCAGTGGCACTAGGGACAGCCAAAGGTCTGGCATATCTTCATCATGAATGTCTTG
AATGGGTGATTCATTGTGATGTTAAGCCTGAAAATATACTTCTCGATAGTGAATTCGAGCCCAAGATTGC
AGATTTTGGACTTGCAAAGCTCTCCCAAAGAGGTGGCCCTGGTTCAGATTTCACAAAGATTCGCGGTACT
AAAGGCTACATGGCTCCTGAATGGGCTTTGAACCAACCTATAACAGCAAAAGTTGATGTTTATGCCGTTG
GGATTGTAATACTAGAAATGATCAAAGGAAGTAGGCTGTCAAGTTGGGTGGTGGATGATAGTGAATGT
GATGACGAACAAGATTCACAGCTCGGAAAATTTGTCAGGATGGTGAAGAGGAAGATCCAAAGTGGAGA
AGATTCTTGGGTGGAGAAACTTGTCGATCCACGACTAGAAGGTAAATTTAAGCAAGAACCAGGCAGTG
ACACTGATTGA
>PGSC0003DMG400024426 G-LecRK
CATTGTTTGGGAGAACGGCAGGGTTTAAACGACGTGTTTTGACATAAAGTGTGATCATCAAGACTTTAT
AAAAATGTCTTCATCATCTAAGGATGATCAATCATCCAATACACCATCATCGGATTCTTCATCATCTTCTTC
GAATTCTCCACCGTCTGGCTCATCCTCAAATTCTTCGCCATCCCAAGACAATTCATCTTCATCCAATGATTC
AAAATCCCAATCTCCTCCTCAAAGTAATTCATCATCTTCTCCGCCATCAGGGGATAATAATAACTCGTCTC
CTTCCGGTGGTAACAACAACAATAATGACAATAACAACGACAACAAGGACAACAACAACAATAATGGTA
ACAACAACAACAACAATGATGGTAACAACAACAACAATGGTAACAATGATAACGACAACAATAACAATA
AGAATGACAATAACAACAATAATGGTGGGAACAATAACAACAACAACAACAATGGTGGTAACAATAAC
AATAACAACAACAACAATGGTGGTAACAACAATAATGGTAACAAATCTTCACCACCAGGAAAATCGTCA
AATTCAAATGGAGGTTCATCACCTCCTCCACCCCCACCACCACCACCTCCTCCACCACCTCCTCCTTCTCCT
TTTTCTCCCAACTCCGGGTTTCTTTCTCCTCCAAAGCATAAGCCACCACCAAGCAAGTCCTTAGATAATAA
TAATAACAACAAGCCTAATGATAACGGGACAGCCATCATTGTAGGAGTTGCTGCTGCAGGAGGTCTATT
ACTTCTTGTCATGTTAGTTTTCCTTATATCATGTTGTAAACGCAAAAGGAGAAGGCCACGCGACCAAATG
GGCTACTACAGGGACAATTCTCATGGAGGCAATAGTATGTTCTTTCTTCTCTTAACTTATACTGTATGTTT
TATGTTGAGCATACTGTTTTCAAATAGAATTAGCTTCAAATGCAGTAGCTATGAAGAGAACTGGTAAATG
TGCAGAGTACTGAATATGTTTAGGTTTAAATAGTGTTAGTATTGAGAATGTATATATGGTGCAGGTACTG
ACTACTATAACAGTGGACCATATGGGAATTGGCACAACAACAACAATATTCAATCAACGGATCATATGG
TGAAAATGCCTAATCCTCAAGCACCGAGTACTAACGTAAGTTCAGAACTGAGTTGGCCAATAGCACCTCC
ACCACCACCACCAATGATGAGTAGCAGTGAATTAAGCTCAGCTGCTTTCTCTGGTCCGCAACAACCTCCA
TTGCCACCCCCACACCCATCAATGGCTCTTGGTTTTAACCAAAGTAGTTTTACATACGATGACTTATCTAC

CGCAACTGGAGGATTTGTTAAGTCTAATCTATTGGGACAAGGTGGTTTTGGCTTTGTACATAAAGGGGT
CTTGCCTAATGGTAAGGATATAGCTGTCAAAAGTCTAAAAGCTAATAGTGGACAAGGTGAACGAGAATT
TCAAGCTGAAGTTGAAATTATTAGTCGTGTACATCATCGACATCTTGTCTCTTTAGTCGGTTATTGCATTG
CTGGATCTCAAAGGTTGTTGGTATATGAGTTTGTTCCTAATGGCACTCTTGAGTATCACCTTCATGGTATT
AACTTAATTTCCTTTTTTTTATATCTAAATACTCATTCTACATATGTCGTATTTAACTCATCGATCCCTTATT
TAATTAGGAACTGGTCGCCCTGTTATGGACTTCCCTACTAGGCTTAAAATTGCATTAGGATCTGCCAAAG
GCTTCGCGTACCTTCATGAAGATTGTAAGTTCATATATACTATTCTCATTTAAGCACTTGTCTATATATTCT
CATAAAAAATTAAACTTCTATTTCATATATAAATTACAGGCCACCCTCGTATTATTCACAGAGACATTAAA
GCTGCTAATATTTTACTGGACCATAACTGTGAAGCTAAGGTATTTGGTAAAAATAGTTGAAACATTGATT
CCTCATTCAGATTTCTAACAAATTTTATTATTACTCCCTTTGTAAGGTTGCTGATTTTGGATTAGCTAAACT
TTCCAACGACACTAATACACATGTTTCTACGCGTATCATGGGCACTTTTGGGTGAGTTTAGTTTGATAATT
CTTCAAATATCCAATGGATTTGCATTAATCGAAAGAGTATATTTATTTGTGTAGGTACTTAGCACCAGAAT
ACGCTTCAAGTGGCAAGCTAACTGAAAAGTCTGACGTCTATTCTTATGGTGTTATGCTGTTGGAACTTAT
AACTGGGCGTCGTCCTATAGACATCAGCAGTGATGATGATACTTTAGTGGAATGGGTATACCATGATGA
TTACTTTACATCTTCAAAATTAATTCAGTAGGACATCGATCAGATTATATGTATATATATGTGCAGGCTAG
GCCAATTCTGATTCGTGCAACAGAGGGTGGAAACTACGATGAATTAATAGATCCACGTTTGGAAGGAAA
TTTTGATGCCCAAGAGATGCTATGTATGGTAGCTTGTGCTGCTGCATCCATCAGACATTCAGCAAGAAGA
CGTCCCAAAATGAGTCAGGTTAATTTCTTCTTAATATTCGTCAAAAGCTTTTCCTAATTCACAATTTTAAAA
AGTATAGTGGTCCAGCGATTGACATATATATATGTTGTAGATTGTACGTGCTTTAGAAGGTGATGTGTCT
CTGGATGATTTAAATGAGGGAATGAAGAAGTCCCCGAGTACTGCAATGTTTGGTTCTAGTGAAAGTTCT
GAATACGATGGAGGTTCATATGACCTTAAGAAATTCAAGAAATCTGGAATGTCGAGTCAAGAATTCACA
AGCAGTGAACATGGTACCGGTGAATTTGTCCATTCCGGTGGTGAATCTCAAGAACATGGGCAGAAAAG
GCACACCCCTTAAGGAAAATGGATCATACCTTCAACTTCTGATGGATCTATAAGTAATAATTACACACATT
TGTTGGGCTTACAAGGATGCTTTAGGCATTAAAAACTAGTTTTATGGGGGAAATATAATTATGTATTTAA
ACAACAATTGTGAAGCTTTATTAGAGATTTGAATGCGGGTTGTAAAGAGTATCACATCCTCCTAACCATT
CTGTGGTTGGCTTTGGTTGTCAGTTGAATCATTTAGTTTTGTTCGACTTTGGAAGTTGGGATATATACTAA
TTCTGAATTGTTTTCAATATATTTCGTCAATGTATCTGCTAATTACTAATCTTTTCTTTGTTTA
>PGSC0003DMG400024998 G-LecRK
GAAGAATTAGCATCAAAAATTTGAATCCATGAAATTTCTAGCTTTTTTATTCATTTACTTCAATTTTCTTTC

AATTGTACTAGCATTAGACACCATAACTATTGATCAACCTATTAAAGATGGTGACACAATTGTTTCATCA
GGTGGTGTTTATGAACTTGGATTTTTCAGGCCTGGAAATTCGACGAATCGATATGTTGGTATATGGTACA
AGAAAATATCAACTGGAACTGTTGTTTGGGTTGCTAATAGAAATAATCCGTTGAGTGATAGTTCAGGTG
TGTTGATGATCAATCCTGATGGAATCTTAGTACTTGTTAATAGTACTAATGTGACGATTTGGTCAACGAA
TTCATCAACAATCTTGAAGAATCCAATAGCAAGATTGTTAGATTCAGGTAATACTTGTTATGTTTCGTTTC
TGGAGAGTGAAACTACATGAACTTTGACCAACATTTAACATGTATTTTTTACCATACTAGAAGAATTGCA
AGTACTTTTCGTATACTTTTTAAAAATCTGTAAATTTTAATTTTGAAATATTGAGTTAATGTAATCTAATCT
GATTTCGTAATCTTTTCATCAAGGAAGGAAATGAAAATAGAATTGAGATTTTCACCTGGGAGAGTTTTGA
TTATCCAGGGGATACTCTGTTGCCTGGAATGAAGCTGTGTCCAGAAATACTTGTCATGTTTAGTTTCTCG
AGAGTTAAACTGAATGAACTTTGATCAACATTTTAACATGTTTATTTTTAACCATACTGATATGAGAAGAA
CTGCAATTTGTAGTACTTTTCGTATAATTTTTACTACTGTTGTTTCTTTTACTTTGGTTATCCTTTATATTTGT
ACTGTAACTACTTTTCTTTCTTCAATATTTTCATCTTGAAGTTCAATTATTGCTTTTCTTTCAAAGACGAGG
GTTTATCAGAAACAAACTCTGTGTATGTTGTTGTCGTAGTAGTAGTACTTTTAGTATAGTTTTTTAACTAT
CTTAATTTTAATTTGAAAATACTGAGTTAATCTAATGTAATCTGATATCAGGTAATCTTGTCATCAGGGAA
GAAAATGAAAACAGGCCTGAGTTTTTCACCTGGCAGAGTTTCGATTATCCAGGGGATACTCTGTTGCCTG
GAATGAAGCTCGGGCGGAATTTGGTAACAGGCCTGGATTGGTACTCGTCGTCGTGGAAGAGTCCTGAT
GATCCTGCTATAGGTGAATTTGTTGAAAGGATGGATGTTCAGGGGTATCCACAGTTATTTGTATGGAAA
GGTTCATCAATTACATTTAGTTCAGGTCCCTGGAATGGTTTAGCATTTAGTAGCAGTCCTAGTTTGCAACC
GAATACGTATTTTACGTTCGGTTTTGTACTGAATCAGGAGGAAGTTTATTACAAATATGATCTTAAGAAT
GGATCACTGCTCACCAGGGTGGTACTAACCCCTGGAGGATTGATAAATCACTATACCTGGATCGATCGT
ACGCAGAGTTGGTTCCTTTATTTGACAGCACAGTTTGATAACTGTGATCGTTTCGCGTTGTGTGGTCCTTA
TGCAAGATGTGTCATTAACAACTCGCCCCCGTGTGACTGTCTGAGGGGTTTCGAGCCAAAGTATCCACA
GGAATGGGATGCAGCTGACTGGTCTAGTGGATGTGTTAGGAGGACTCCGTTGGCGTGTCAGCAAGATG
GATTTAGGAAGTTTACGGGGATTAAAGTGCCTGATACGCAGAAATCATGGTTTAACGAGAGCATTGGAC
TTGAGGAATGCGGAAAGTTGTGCTTGGCTGATTGTAACTGTACAGCCTACTCAAATATGGATGTTCGAG
ATGGTGGAAGTGGATGCTTACTCTGGTTCGGAGACCTCATCGATATCAGAGAACTTAGTCCAAATCAGC
AGGACCTGTTTGTGAGAGTGGCTGCTTCAGAAGTAGGTAATAACTACTTACTGTTTTTCGTTTCTCTAACT
TGATCTTGTTGAACGTGGCGAAAAACATGTATGTAAAATTTCAGCAAGTATGTATCTAGATAGTCAAAA
GTCTCGATAGTTAGCTCGTGGAGTCTTCAGTCAATAGAAAAAACATCTATGAATGTGTGTTGGTGCATTG

TTCTCTCTCACTCAAAGACGGAACGTTGCCTGTTTCTTATGGCCTACAATTTTTGTTGGGAACTCATGAAT
GTGAATCATATTGTTATGACAATGTTGGAATTTTCTGATTGATTATGTACGAACTTATCGACAGACAAGA
AACGAAAGAAAAAGAAGTCAAGGCTGATTGCAATCGTATCAGCAGTAGCCGCGACTTGTATCCTCAGCC
TTCTAGCTTGGTGTGCATTATTCCACAGAAGAAAAAAGAGTAAAGGTAAACAACTATTGTTCATCGTTAT
CAAAAGATCGTGTTGCTAACGTTCTATTTGTATCAGGCTAATGTATACATTTATACCAATTGATTGCTCAT
TTTACAGGTAGGGAAGTTGGAGCAGACGATATGGAGTTACCATTGTTCGATTTAGTAACTGTTGCTAAC
GCCACTAAGAACTTCTCTTCTGCTAATATTATTGGAGAGGGTGGCTTTGGACCTGTTTATAAGGTACAAT
AACTATCCCATCGTCGAATTTTAGCTAAAAAAAGGCATCTTTAACACTTTGTTTTGAAAAAAAAATTCAGG
GGAAACTACGAAATGGACCTGAGATAGCGGTGAAGAGGCTTTCGGAATACTCAGGACAAGGTCTTCAA
GAGTTGAAAAATGAACTGATACTGATATCCAAACTACAACACAGGAATCTTGTCAAGCTTTTAGGTTGTT
GCCTTGAAGGAGTAGAGAGGATGCTGATATATGAGTATATGCCAAACAATAGCTTGGACTATTTCATTTT
TGGTCCGGTTCTCTCGACTAGTCACCTTTATACTATGTTGCTCGTATCCTCCAAAAAATGATGTTGCACCA
GTGTCGGATCCTCAAAAGATGCACTACTTCTGGAGGATCCAACATGGGCGCGGAGGCATTTTTGGAGGA
CTTGAGCAACCTACTAATAAATGAATGGATGAGGATTCACTGACATTTTACATATTGCAGATCCAAGCAG
AAAAGAATCACTTTCTTGGAGAAATCGCTATGAAATCGCGATGGGAATATCTCGAGGCCTTCTCTATCTT
CATCAGGACTCAAGATTAAGAATTATACACAGGGACCTGAAGGCCAGCAACATTTTGTTGGATACTGAC
TTGAATCCAAGGATTTCTGACTTTGGTCTAGCTAAGATATTTGGTGCAGACCAAATGGAAGGAAAAACA
AAGCGAGTAATCGGAACATAGTAAGTCTTATAAACAACCTCTATGATAGATGTTTTTGTTTGTTACAATC
AACAAGTTGATCAACTTAAATTTGATGCATTGTGTAGCGGATACATGTCTCCGGAGTATGCTGTCGATGG
GAAATATTCAGTGAAATCTGATGTCTTCAGCCTTGGCGTGCTTTTACTTGAAATTGTCAGTGGCAGAAAG
AACAGGAAGTTTCATCATTTGAGTCATCATCATAATCTTTTGGGACATGTAAGTACATATAGCACTAAATT
TTTACCCTTTAGCCTCTTTTTTTACTAAACACTCGACCCCGTCTGAGCTTACTCACATCACCAGTCCCAAGC
TCCCATAAAGGAAGAGGGTTGCAGCCAGTATAAAACTAGTCAGTTCATGTCGAATCCTCACAATACAGT
CGGAACTGAGGCATTGTAGTAGTTGTTATAGCCTGTTTTCCATTAAAAAAACTTGACCTGCCTGAGTTTA
CTCATATTACCGGTCCCAAACCCCCATAAAGGAAGAGGATCGCAGTAGGCTAGAAGCCATTATAAAACT
AGTCAATTCATGACGAATCCTCATAATACAGTTGGGGCCGAGGTGTTGTAGTAGTAGTTGTTATAGCCTT
ACATTTCCATTCCCAAGCCCCTACAGAAGTAAGAGGGTCACAATAGGCTAGCAGCCAAGCATAAAACTA
GTCAATTCATGACGAATCCTCATAATGCAGACGGGACTGAGGCGTTGTACTAGTCCACATAGCCTTTCTT
TTACTAATGCAAGTGTATAATTCATGATGACTGAGGCATTGTAGTAGTCACCGTTGTAGCTTCTTTTTACT

AAATGCATTTGTATAACTGTGTTATCATAAAAAATATTTCAGGCATGGTTACTATTGAATGAAGGCAAAG
CATTGGAACTAATGGATGAATGTTTAAAAGACTCATATGTTGAATCACAAGTGTTGAGATGCATTCAAGT
AAGTTTATTGTGTGTACAAAAACTCCCTGAAGACAGGCCTACAATGGCTTCAGTAGTTTTCTGGTTAAGC
AATGATGGTGTGGAATTGCCTCAACCAAAGCAGCCTGGTTTCTTTATAGAGAGGGATTCAACTAATCAAT
CAAATGAATCAACTGATGAAAGATGTGTTACTGACAATGAACTTTCACTAACAATTCTTGAGGCAAGATA
GACAAAATATGAACTTGCAGTCGTGTGACCAGGAGGTCAAGGGTTCAAGTCATGACAATAGCCTCTTGC
GG
>PGSC0003DMG400025558 G-LecRK
AAAAAAAACTCAAATCTATGGCTGTTTTGCTATGGTTATTAATCTTATCTGCATTTCATGCAGTAACACTA
GCACAACAAAGGCAGTTTAATATAACTCTTGGCTCTTCTCTAACACCTACTACCAATTCATCATGGTTTTC
ACCATCCAGGCGCTTTGCCTTTGGGTTTTATGAACAAACCAATGGCTATGCTGTTGGAATCTACATCGTC
GGTATGCCTAACAAGACAGCAGTGTGGACAGCAAATAGGAACAGTCCTGTTGTCCCGAGTAACACTGTC
TTGCTCTTAACTAATGATGGAAGGCTCATTGTACAAGTAGGAAGCCAGGAAAACTCTGTCATCAATTCCT
CTCGAGCCATTGCTTCAGCTTCCCTGATGGACACGGGAAACTTTGTGCTCTATGATTCTGATCGCAATGT
CATTTGGCAAAGTTTTGACAATCCAACAAACACACTTTTACCTGGTCAGCATATATCAGCTGGACAAGAG
CTGTTCTCGAGTGCCTCGGAAGCAGATGACTCATTAGGGATTTTCCGTCTTTCCGTCTCATAATGCAAGTT
GATGGGTACCTTGTTCAGTATCCTGTCAATACACCAGATACTGCCCCATATGCTTACTACGCATCCTACAT
CCGTGGAGTAAGGAATAATTTTTCACTGAATCTTGATGATGACGGCCTCCTCTACTTGCTCAATTCCAACA
ATAGTCTAAAAAATCTAACCAGAGGAGGCTGCCGTAGAGAAAGGACCATCATCTATATGATGAAAATTG
ATGCTGATGGTATTTTTCGAGTTTATTCACATTCTCTGAACCAACAAAACAGCTCACCGATTTGGTCATCA
ACAGATGATAGGTGTGATCCAAAAGGCCTTTGTGGGCTTAATGGATTTTGCACCAATATAGATGCTCAA
GCTAAATGTATGTGTCTTCCTGGATTTAATTTTGTAATGCCAGGAAATTGGAGCGCTGGCTGTGAAAGG
AATTTCACCACAGAAACCTGTCAGTTAAAGGAGAACACTTCCAAATACTATGACATGAGAACAGTTGAA
AACACAGCATGGGAAGATAGTTCTTATGTTATTTTTGCCGTAACGACCAAGGGAGATTGTGAACAAGCC
TGTCTACAGGATTGTAACTGTGAGGCTGCTTCGTTCAAAGACAAAGTGTGTAGAAAGCAAAGGCTGCCT
CTGAGGTATGGAAGAAAAGACCTGGGTAATTCTAATCTTGCATTGGTCAAGGTGGGCATTAATGTGTTA
GACAACAAAGAGTTGCCTGATCAGAAGATTGAAGAAACAAAAGGCAAAAAGCTAAGGATAGACATCCT
AATTGCCAGCATTACATTGACTGTTTTTGCTTTGTTGGTTGATGTTATTTTAGTTTTGATGATTTGACAAAC
AAAGGGATCCTATAATGGTACCTGATACCTTGTGACAATTTGTTGAAAATGCCAGCTGGAAAAAGTATA

GAAAGTTGGTGAGGTTGGTGCACGGCTTCCAAGCATGCCAGAATCCTCAGCCAATAGCATGGCTTCCTT
GTGTGTGTCTATTAGATAAAAGGCTTTAATCAATAGACACATTTTTAGTTTTTGCATTCCTGAAATTCTCA
ACCACACAAGCAAAAATCACTCCTATCAAGAAGTATTATCCTCAAGAGCAACAGGGACCTGATAGAGCT
GCTTGCTGCTTAGTTGTCTTGAGATTATATCTTCCGTGCTTATATTGTAAATCTATTCCTAATCTATAAAGG
AATTAGATCGTTGCTTTGTTCATCTAAAAGTCTAAGCCAGTTAAGTGTAACTGGCTTAGTGGGCAGTGTT
GTATCTACTTAGTCAAATTCTAAGTCATCTAGAGTTAGGTGGTTTAGTGGGCAGTGTTGTATCTGCTTAG
TTGAAGTCTAAGTTATCTAGAGTTAGGTAGCTTAGTGGGTAGTGTTGTATCTGCTTAGTTGTTTTCTTTGT
AGTAGAGTTACTACATAGAGGGGGAAGGGATTTAGAGGTAAATTCCTAGGTTGCAACTGCCTGTAATCT
AAACTGTTGCTCCGTTTGAGTGAAATGGAGTTGGATTGAAAGCCTGTGAGGACAGGTCGTGGTTTTCAC
CGCCCTTGAACAAGGGGATTTCCACGTAAACTACTTGTGTTGTTTTCTTTATTGTTTACGCCTCATTTATTG
TTCATACTGAGTCAAGGGACCTGGTCCGTTGACTGATAGTGGACGCATACATCTTATCATTGGTGTTAGG
CATCTCTGGATTTCTCATTCACAGAATTCATGTCTGGAGTCTGGACTTACAGAAAAATCCAGGAGAGTAG
AAGTGTTCAGTTGAGCGAGGATGTTGCTCCACGAGCATTTTCCTATGCAGAACTAGAGCAGGCAACCAG
TGGTTTCAAGGCGTTAGGGAGGGGGGCATTTGGAACAGTATTTAAAGGGATTTTGGCAGAAGGCCAGA
AAGTGATAGCTGTAAAGAGACTAGACAAAGAATTGGTAGAAGGGGAAACAGAGTTCCAAACTGAAATA
AAAATCATTGGAAGGAAACATCACCGAAATCTAGTCCGCCTCATTGGTTATTGCCTGGACGGATCAAGA
AGGCTTCTGGTGTATGAGTATATGACAAATGGGTCACTTGCGGGCATACTTTTTACACCAGAAAAACAG
CCCATGTGGGAGGAAAGGTGTGGCTTTGCTCGAGATATTGCAAGGGGCCTCCTATATTTGCATGACGAG
TGTGAAACACAAATCATCCATTGTGATATTAAGCCTCAGAACATACTCATGGATGATCAGTTCTGTGCAA
AAATATCTGACTTTGGAATGGCCAAGCTCTTAAAGAAAGACCAAACAAGAACCTACACAGGGATAAGAG
GGACCAGAGGCTATATTTTGTGCTAAGTGACTAGCAACCGTCGTTGTAGTATTTGAATTGTAAATACTAA
TTTAGGAGAGTTACAATTTTCTCCATAGTGTAAGCGAATCTGCAACAACCACAAC
>PGSC0003DMG400026154 G-LecRK
GTCTCACTGAAATGAAATAGAGGGCAAGAGCTACCTATTTGAATTTATGTTCTTCATTCAAATCATCAAC
AATTCCATAAAACAATGCAATTAAGTAATTTTCTGTTGTTGTTGTTGTCTGCAGCATTGATGATTTTACAT
CGATGTGATGCAAGGAGGAGCACCATTGATGGAAGCAGTAAATTAGTTGACAATGGCGAAACCCTCGT
ATCTGCTGGAGAAAATTTCGAAATGGGATTCTTTTCTGATGATGCCGGGTTAAACAAATATGTAGGCATA
TGGTATTACAAATTAAGTCCTAGAACTGTTGTATGGGTTGCTAATTGGAATAATTCGATTCAGGGTAAAA
GAATAATGAATGAGGATAATTCGGTTGTCGTTGAAGATGGTAATCTCAAAGTTATAAGTAATGGGTATA

CTTATTTCTCCACACAATTAGGTAGTGGTTCTAACAGGAAAGTAGAGTTGTTGGATACAGGGAATTTAGT
TTTAGTTGATGAAGGAGCTGAAATGTGGCAGAGTTTTCGCAATCCAACCGATACATTCCTACCAGGTATG
AAAATGGATAGCAGTTTGAATTTGACTGATTCAAAATATGAGAACTACATATTCCGATTAGATCAAGCAA
GTGACAAGGAATATGTTATAGTACTTCCAAAGCAGGGCAAAATTCTCTGGAAAGGGTCTGCGGAATCAG
GAAAGTTAAGTTTCAGTGAGATGCCTGGTTATGTTGCCTACTTGTTATCCAACTCCACTAATAATTCATTG
GAATCGATAGGAACATTTAACAAGTATAGGCTCTTAATGAACTCCTCGGGGGAAATACAATTCTACGGT
TGGGATAAGGAGATCAGTGGATGGTCTTTGAGGTGGTCAGCGCCAAATGATAAGTGTGATTGGTATAA
GTATTGTGGGAAATTTAGCATTTGTAACAGCAAACGTGAGCCAGTATGCAAATGTTTGCCCGGGTATAA
GCTCAATCCTCCAGATAATTCAAAAGCAGGAGAATTTTCAGGTGGTTGTTCAAGTATGTCAGTTAGCTCT
TGCAATGAAGACAATGTAGAAGTGCTCGACACGTTCTTGGATTTGAGGTCGATGAAATTCAAGAGTCCA
GACAGGATATTCAGTAATAATAGTACCCGTGAGGACTGCAGGAGAATTTGTCTAGGCAACTGTAAGTGC
CAGGCCTATACTTACCATGATTCTGTATGCCGGATTTGGGTCACTAGTCTCATGTATCTCCAGGAGAACT
ATGCTGGTGGCTTCAACATCTCTGTTCGTGTCAGCATTTCCGATATTGGTAATTTCTTTTTCGATTTAACTT
ACGTACACAGACAGTTTAAGCTAATGTGTGACCACAAATAGTACAGTAATTTGATTTTTGTGCATTGCAG
AAGCAACAAGGAGAAATTGCAAGCCGTGTGGCATAAACCTAATACCCTATCCATTAAGCAGTGAACCAA
ATTGTGGTGACCCTTTGTATTATAGTTTCTCATGTGATGATTTAGTTGGTCAGGTAAGCTTCCTCACATCG
AATGGCAAGTATACTGTCGTTAACTTTGATAAAGATAACAAAACATTTGTTATAGAAGCAGCTCATAAAG
AATCTGTTGGTACTTGTGATGACAAAGGACCGGTGACAGGAATTTCTTGGTTCAACCAGTCATCACCTTT
TAAGGTGATAAACTGGTGTTACAATCCCACAGAGAATCTTAATTCAGGACCTTTTAGTCGAGGGAAAGA
TTTAATATTGATTAGTTGGAAACCACCATTGGAGCCATTTTGCAAGACTTCAGAAGATTGCAATGATTGG
CCAAATTCTAGTTGCAATATGACAAAACAAGGAGAAAGAAGATGTATATGCCAAACTGATTACAAATGG
AATGGCTTGATTTTAAATTGTTCCTCGAGTTTAGGTATATGGATCAATGTTACTTACTACAAATGATTTTC
TTGCCAATAGTTCAATGATACCTTTTCCTATGTTCTCCATTTGACCTTTTATATGACCAACACAAATTTCAT
TTCTTAAATTTGCTTCAGAACTTGGAACACAAGGATCATTCATTGCGAAGCTGGCATCCTCACGGAATCA
GAGAACCCTTGTTATTTCCATCTCTGTAGTTCTTGGAGTTATAACTTTGTGCAGCATTAGTTATATAATTTA
TCAGAACACTAGAGTGACAAGAAGTAGA

>PGSC0003DMG400026155 G-LecRK

ATCTGATTGAACGAAAAAACCAAGTCTTACCATGACTATCAACATGATCTTAATTGATGCTGTGCATCTTC
TGTTATCAAATTTGGTGCAACCATGCAACTGCAACATTATTCGATTCGTCTACTTTTTGTGATCCTAACAT

GTTTATCTATCAATATTCATCAGTCTCTTGGAGTTGAAATGATCTCAGTAAATCACTCTGTTTCTGGAAAC

CAAAGTATAGTCTCTTCGGGCGAAAACTTTGAATTAGGATTCTTTTTCCTGGTAGTTCGAAAAATTACTA

TCTTGGTATATGGTATAAGAATGTAATGCCACAAACAATAGTATGGGTAGCAAACAGGGAGAAGCCACT

TTCTGCTACTGATATGAATTCTGTTGAGTTGAAAGTTTTGGATGGTAATTTAGTGCTTATTACTGAATCCA

AAAACTTGTTTTGGTCCACAAATATCAGCAACACAATTAGTTCAAATACACTAATGGCAATTCTTTCTGAT

GATGGTAATTTGATTTTGAGTGATGGATCAAATTCAACTACACCACTTTGGCAAAGCTTCGATAACCCGA

CTAATACATGGTTGCCGAATGCTAAGGTGAAGTATGACAAGCGTACAAATACAACAAAGATTCTTACTTC

TTGGAAGAACTCAGAGGATCCATCTCCGGGGATTTTTTCCGTGGAAATGGATCAGAGTAATAAGCAGTT

CTTGATAAAGTGGAACAGGACTGAGATGTACTCAGCTACTGGATCTTGGAATGGGCGTATTTTCAACAT

GATGCCTGAGATGAGTTTGAATTCTGATAGATACAGTTTTTCATATGTTGATAATGAAAATGAGAGCTAT

TTTACCTATTCCCTCCGTAACTCTTCCAAAATCAGATTAACACTAGATGTCTCTGGGCAAATCAGGCATCT

GATTTGGGCCGAAAATTTAAAGGAATGGCAAATTTTTACATCTCAACCAAGACAACCCTGTGAGGTGTA

TGCTAGTTGTGGTGCCTTTAGCATTTGCAATAAGGAGTCCGCGACCTTCTGTAATTGCTTGACTGGTTTC

ACACCTAGATCTGATACAGAATGGGACTTGAATGATCATTCTGGTGGCTGTGTTAGGAAGGAAAGCTTG

CAGTGTGGTGATGGTAAAATGAAGGGCGGTTTTTCGGAGAATCCCAAAGTGACATTGCCCGGTTATTCT

CTAACTGTGCCGGCTGCTAGTTCTGAGGAATGCCAGTCGACTTGCTTGAGTAACTGTTCTTCATGTAATG

CATATGCTTATGATAATAATGTGTGTTCAATTTGGAATGAAGTTGTGAATCTGAAGCAACTCTCACCAGG

AGATGGTAGTGGAAGTGTGATTTATACCAGACTTGCTGTCTCTGATGAAGCACGCGGTGAAGGTAAGTT

TAAAGTTAGGAGTTGTATTTGATAGTGTTTGCCATTTCTATGTATGCAATGCTCATCTCCAAATAGCTACC

GATAGAGCTTTCTATGGATGTCTGTTTTTTCTTGTCTGATGTTTCAGTTCTTTACCTCATGTCTTTCAACAG

TACGAGTCTATATATTCTCTAAATCTTAGTGCTCATAAGAATTAACTAGGCAATTATAAGTTTACGCAAAC

ACCTTCTATGTTTTAAACAAACATCAGATCGCCACTGACCAAATTTAAGTTTACGCAACATCCTCTACGTT

ATAAACACACATAAGATAGTCACTGACCAAATGTATGGTTTTCATGATTTGATTAGTTACAACTAGCCAA

AATGAGTTGAAGACCTTTCGCCAGTAAACATTACCAGTTCGCTGAGTTCCCACATCAGCTCTTTAAGGAT

GGGATGGTCTTTTCATATGGTTTTGGACAGTTCTTACCTCATAAACTAGCTTTTATAGTTGAGTTAGCCTC

AAGGTCCATTTCTTATCAAGTTATTAGAATCAGACCCATCCCAAAATAGTCTATTTCTTGTCACAATTTCCT

TATGTTTCTTCTTTCTGTCATCAGATGCAAATTCAGGGAAATTGTCCCTGAAGGTTAAAGTCATCATATCT

GGCGTGGTAGCCGTTGCAGTTCTACTTCTATGTAGCTTTAGCTACATTTATCATAGGAGAATAATGTCAA

AGAAAACAGGTACTTGTGTTGCTATTACAATCTCTGGAGTAGTTGTTTTACCACTTCCAGAAAAATTTCTC

CTTCCATTTTTTTTTTCCGAATCAGATCAAGAGAGATATCTCAAAAGGCTGCTCTTTTTTTTATTTTTTTATT
TTTTATCTTAGCAAGCCAACAAAGCACAGGAGGCAATCCAGTTCCACATTGGTTAAACAGAGAAAGAGA
AGCACAAAACCTGATTAACGAAAATGATAAGCAAAGCATTGCCGTTCCATTCTTTAGTTTGGAAAACATA
CTAGCAGCCACAGATCATTTCTCAGATGTAAATAAACTTGGACAAGGAGGTTTTGGTCCTGTTTATAAGG
TTAGCATTAATAATTCATTTTAACATGAGTTCAACTTTATGTAGTCATTTCATAGCATCCTAGTTTACACAT
TGAAAATCTTTCAGGGTATATTTTCAGATGGACAAGAAATTGCTGTGAAAAGGCTATCAACCCAATCTAG
ACAGGGCATTGAAGAATTCAGGAACGAAGTCATACTGATCTCGAAACTTCAACATAGAAATCTAGTCAG
ACTTTTAGGCTACTGTATCACAGGGTATGAACAGATTCTACTTTACGAGTATATGTCAAACAAAAGTCTA
GACACCTTCATATTCGGTTAGTATATATATCTGAACTTATCTTATTTCTTAATCTCTCTATTTACTTGATCAA
TATTGTTAAATTTTCTCGTTGTTTACCAAAATAAGATCCAACACTTAGCAAGTCATTGAAATGGAGAAAG
CGATTTGAGATCATATTAGGAATTTCTCGAGGGCTTCTCTATCTTCATGAAGACTCAAGGCTCAGAATCA
TTCATAGGGATCTGAAAACAAGTAACATTCTTTTGGATCAACAAATGAACCCAAAAATATCAGACTTTGG
CTTGGCAAGGATAGTTGAAGAACAAACAACAGAAGCAAGTACCAAAAAAGTGGTTGGAACTTAGTAAG
TAATACAAACATGACAATGTTATGAACTTTGCTTAATTCCATCTCACATCTGACTCATGATAATTCCATCAT
TAATTTAGCGGATATATGTCTCCTGAATATGCATTGGAAGGAGTATTCTCAATTAAGTCAGACGTATTCA
GCTTAGGAGTAGTTATACTGGAGATAGTAACCGGAAGGAGAAATACAGGATTTTATCAATCTAAAGAAG
CATCTAACCTTTTGGTCCATGTGAGTGCCTAGAGTACATGTTAATTTTTTCCAATATGTATGTTGGACTTC
ATATTCATTATTCTAAATCATGATATCACAGGCATGGAATTACTGGAAAGAAAAAAGAGCACTACATTTG
CTGGATCATTCATTGCTCGAATCATGCAACCCGAAAGAAGCAATGACGTGCATAAACGTTGGGCTCTTAT
GTGTGCAAGAAGATCCAGGTGATCGTCCTTCCATGTCAAATGTTGTAATGATGCTTCGTAGTGATAGTAC
GTCTCTTCCAAAGCCTAATCAACCTGCTTTTGTGTCAAGGAGAAATGTGTCTAATTCAACTTCTGTTTCAT
CTGGTACACCACCTAGCTTCACCAAAATTGAGTTGACAATCACAGTTGAAGAAGGCCGATGATGTGATG
AATTCATTAGTTTTTCAAATGAATTTAAACGCGAAGTTTATTTATTTTTTCACCTTAGATTAAAAAACAAAA
TTACAGGAATACATCATCTTTTTCTTTTTCTCAATTTTTTTGTTGTTTAGGCAGTAGAAGACTTAGGACCAG
TACTGCTGATATTTATAGTGAAAACTGAAAGGTATATATGCTTAATATTGTGTACAACATAGAATATATT
GTGTTGATAAAAAAAAGTTCATTTTCTGTTTCATTTTGGTCTCTTCTACCTTCTTAACTTGTCTTAAAAGGC
CATTCAGTAAGAATGGAAGAACAAAAAAG
>PGSC0003DMG400026156 G-LecRK
TTTAAACAGGAGCAAATTACAAGGAGGTGTACTAGGAAAAATAATGGAGTTGGATTGTGTTGTAGCTAT

TTTAATTGTATTTTGTTGTTCTTTCGTTACGCTTGTGGCGAGTGAAGAAAGTATCAAGATGGGCAAATCA
ATCACTGGTAATCAGAAAATGATTTCAGGTGGTGGAAGTTTTGCCTTAGGCTTCTTCACTCCGCGTAATT
CTACATTTACCTATCTTGGCATATGGTACAACACGATCCCCGAACAAACAGTCATTTGGATTGCTAATAG
AGAATCTCGAATTCCTCAGAATTCTACTGTTGTTTTCACTATTGGTGATGATGGGAATTTAGTGATTTTTG
ACGTAAAAGATCAACTTATTTGGTCATCAAATGTTTCATCCATAACTTCAAATTCTACTGTAGGAGCACTA
CTAGATAATGGAAATCTAGTGCTCATACATGGTGAGTCTGATATATTGTGGCAGAGCTTTGATCATCCTA
CCGATACGTTTATGCCTGATATGAAACTTGGTTATAATAGAAAAACAGGACAACGGACTCTGATCAATTC
GTGGACAAGTAATGAAGATCCTCGGCCTGGAAATTTCTCGTTTGGGATAAATCCTGAAGGGCGGACGC
GCTTTTATATATTGAAGCAGAACAGTATTTATTTTAGGTTTGATGATAGTACTGAATACAGTGGAGGGAA
TATCGGAGGAATTGCTTGGTATATTTCTGTTGTTTCGGGAAATGATGGAGTTTTTGTTACGTATGGTTAT
ACCAAAGGGTTGGTAACATTAAGGATTGTTTTGCATCCTAATGGTTACATTCAGTTGCTGGTATGGCGTC
AAAATGCTAATAAGTGGATAGTTAAACTTCAGGCACCAGAATCTCTTTGTGAACGTTACGCGGAGTGTG
GTCCATTTGGCAGCTGTGATGTTGGATCAAGTGGATTATGCAGATGCTTAACTGGATTTGAACCAAGATT
TTCGACAGATTGGGAGAACGGGAAATGGAATGATGGTTGTGTTAGAAAAGTAGCATTGAGTTGTGATG
GCGGAGACATATTCTTGAAACATGAAGAGGGTATGAAGTTTCCGGATCATTCTATCTCCTTAGGACATAT
GAGCATTAAGGACTGTGAAACGCGGTGCATTAGGAACTGTTCTTGTTCAGCTTATGCTTATTCGAATAGC
ACTTGTTTAATTTGGTTTGGAGATTTACTGGACCTTTCCCACAACTTTTCTGGTGGCAGAGTTCTCAATGT
TCGAGTTCGTGGCTCCGAGCCAGGTATTTTCACTTCTATCCTTACTAATTCTACTAACAAACGATTCTCTAC
ACGTCTTTGGTCAAGAAAACTAAATTTATTTGCTTTTTGTCTTCTGGATGCAGTTACTCATGGTGGATCTA
GGAATTTGGCTCATAGACACAAAGTTTTCGTTGCGAGTATTGTTTCTGCAATATCTATCATCTTGGTTCTT
ATAAGTATTTTTTTCTTTATCTTCGAAAGAAAGCACTTGAGAAGACAAGGTATGTTTAGTGTACGTCTTAA
TTGTAAATACAAATACTGAATTCGAATTTCGTATGGGTTACACTGATTTGCTTTACTGATTGCTGTTTGAA
ATTAGGCTGGAAGAATGGAACTAAAGCTTTCATTAGGTCCGTGTCTGGTTTATATTCGGTTGAAAAAAG
CGATATGAAGTTGCTGCAGTACAACCTTCAGAAAATAAGAGAGGCTACTAACAACTTTCATGGAGAACA
CAAACTTGGAGAAGGTGGATTTGGTCCTGTGTTTAAGGTGCTAAAACTAAATTCATTGTTGATATTTTAC
TGCTGCTACTTTGAGAGAGACGTCAACGTCTCATTTTCGTTTCATGACTATGGTGGATGTGGATTTACAT
ATTCTGTTTCAGGGATTTTTAACTGAATTCGGAGACGTAGCCATCAAAAGGCTAAGCAAGAGGTCATCA
CAAGGACTGGAGGAGTTTATGAATGAACTGAAGCTAATCGCGAAACTGCAGCATAAGAATCTTGTCAGC
CTCTTAGGATGCTGTGTTGAAGGGGAGGAGAAAATACTAATCTATGAGTATATGTCCAATTGCAGCTTG

GACAAATTTCTGTTTGGTTCGTCTTCTACTCCGTTTTAAGACTTCACATAACTTAAATTTTTACTCCTCTCA
ATTCCCTTTGGTTAATGAGATGTTCAAATGTCTTTTTAGACCCTTCGTTGAAGGTTACACTCGATTGGGTT
ACACGTTTGGGGATAATAGAAGGAATTGCTCAAGGAATGCTTTACCTGCATAAGTACTCGAGATTAAAA
GTCATCCACAGGGACTTAAAAGCAAGCAACATTCTTCTAGATCAAGAGATGATCCCAAAAATATCAGACT
TTGGAATGGCAAGGATTTTCGGGACTGATCAAACACAGGCCAATACAAATCGAGTCGTTGGTACATAGT
AAGTAATAACCCCTTATGATTTGTGGTTTCCAAGCTTTTATGTCATTTGTGCATATCTAATGGATACATATT
TATACAAATATGGCATAGTGGCTATATGTCTCCAGAATACGTAGTATATGGCCAATTTTCAGAGAAATCA
GATGTTTTCAGCTTCGGAGTATTGCTGTTAGAAATCCTGAGTGGTGAACGTAACTCAGACTTTTTAATGA
CTGAAATTTCCGCGTCCCTCTTAGGATGGGTAAGAACACTCAAGACTTTAATTAATTCTGCTTTCTTTCAC
TTTGTTTTCATGTTTTTTATTTATGGTGCAACTCATTTGAACTTTTTTAAGGCATGGAATAAGTGGAAAGA
AGGAAAAAATCTTGGAGTTGATTGATCCGTCCATAAGAGAAACTTGCGACAATAACAAAGCAACAAGATG
TATTATAGTTGCCCTTCTTTGTGTTCAAGAAATCCCTGTTGATCGACCAACAATGTCTGATGTATCTTTCAT
GTTAAGCAACGAAACAACTCCTATCCCTGAACCAAAAGAACCTGCTTTTCGTAGCAGCTGGCAATCTCAG
CAGTTAAGTGATGTATCCATAAATGAGATGACGATTACCTTGCCAGCACCGCGATGAAATGATTTAAGTT
CTCCTACTTCTTGTGGTAAATTTTTCTTGTTAGCATAGAGCTGTCATAATTTTTTTTTGTAGAACTTGCATA
AGTGTAGCTCTGGAAGAAATATAGACTAAATTTCTCTGTTTAATTGATGTTTTCATGTTTTGTTTTTTTTCT
GTTCCTACAGTGTATCAAGTTTGTGAAAATGTTGTTTTGAACATTCACTTGGCTT
>PGSC0003DMG400026183 G-LecRK
AAAACAATGGAGTTGGATAGTTTCTTACCTCTTTTACTGTTAATTTTCTCATGTTCTTGCTTACCCGTTCTT
CCTTGCCAAAATTGTGTCAAAGTTGGCAAATCAATCACTGGGAATCAGAAATTGGTTTCAGCTGATAGA
AGTTTTGCCTTAGGATTTTTCACTCCATCAAATTCAACGTACACCTATCTTGGCTTATGGTACAACATAATC
CCTCAACAAACAGTCGTTTGGGTTGCCAACAGAGAATCTCCAATCCCACAGAATGCTTCACCAGTTTTCA
CTATCGGCGATGATGGAAATTTAGTGATTTTCGATGAAAAAGGCAAACTCATCTGGTCATCAAATGTCTC
ATCAACAGCACCGGAATCATTATTAACTTCAAATTCTACTATCGGAATATTGTTAGATAACGGGAACCTT
GTTCTTCGACACTACGGTGAGTCCACTTATTTGTGGCAGAGCTTCCAGTATCCTACAGATACGCTATTAC
GCCATATGAGATTTGGTTTCAATAAAATTAGTGGCTTACGAAATGTGATCACTTCATGGACAAGTAATGA
CGATGCTCGCCCTGGAAATTTCTCTTTTGGAATAGACCCTACTGAAGGGGTTATGCGTTTTGTCATTTGG
AACCGAAACGGTATTTATTATAGATTTGACGATAGTTGCAGAGCGTATATACTAGCAACAGTGTGGTGTT
TCACTGTTGTTACTGAAAATGATAATGTATATCTTAATTTAGCTTACTACAGAATCTTTCTGAGATTAAGA

ATCGTTCTGAATCCGAGCGGGCACCTTCAGGTGATGGTGTGGAATCGGAATGGAAGTAACAGATGGAA

TGTTGAATTTGAAGCACCACAATCTAAGTGTGACCTTTACGCACAGTGTGGTCCGTTTGGTATCTGTGGG

ATCAGATCAAGTGGATTATGCAGTTGTTTAGACGGATTTGAACCTAAATTTTCGACAGATTGGGCAAATG

AGAAATGGATTGGAGGTTGTGTGAGAAAAATAGCGTTGGGGTGTGATGGTGGAGATGGATTTTTTAAG

CACGAGAATATGAAATTGCCAGATCATGCTGTCTCTTTAGGAAATATGAGCACCGAAGAGTGTGAAACT

CAGTGCATTAAGAACTGCTCCTGTTCAGCTTATGCTTGTACGAAAAACGCCTGTTTTATATGGCATGGAG

ATTTACTGGATCTTGGGCATGACACTACTAACGGCAGAGCTCTCTATGTTCGAGTTCACGTGTTAGAGCT

AGGTATGTTTACTTGTATCATTGCTGGTTCTGTTAACAACAAGTGAAAGACTAGTATAATTTTCGTGTTGT

GGATGCAGTTACTGATAATCTATCTGGGAATTCAGCTCGAAGATACAAAATTCTCACTGTGAAAATTGTT

TCTGCAATTTTTGCAATCTTGCTTCTTGTTAGTACCTCTGCTTTTATCTTCAAAAGAAAGCGCTTGAAAAG

ACGAGGTGAATCTAGCATTTCTAGACTGTAATGATTTCAGATTTCATATTGTTTACAGTGCTTTGCCTTAC

TGATTGTTGTTTGAAACTAGATAAGATGGACGGAGATTCTGCTTTAGTTAGCTCCATGCCTAGTGGATCT

TTAGTTGGGAAAGATGATATGAAGTTGGTACAGTACAGCCTGCAGAATATAAGAGACGCAACAAACAA

CTTCCATGAAAGTAACAAACTTGGAGAAGGTGGTTTTGGCCCCGTGTTTAAGGTGCAAAATTCAATATTT

TGATGTTCGTATTTAATTTCATGAATATAGAGAATTGTGGCTTTAAACATTCTGTTTCAGGGATCTTTGGC

TGAATTTGGAGACGTAGCCATCAAAAGGTTAAGCAGGAGGTCATCACAAGGACTGGAGGAGTTCATGA

ATGAGTTGAAGCTAATAGCGAATTTGCAGCACAAAAATCTAGTCAGCCTCTTAGGATGTTGCATTGAAG

GGGAGGAGAAGATATTGATCTATGAGTATATGCCCAATTGCAGCCTGGACAAATTTATTTCTGGTTATAC

TCATTAGTATTATTTTCACGAGAATCATAACTTTGTTTTCCTCATTTCTGTTGGCTAATTGGATATTCATTTT

TTTCTTTTAGACCCTTCGTTGAAGGTTACACTCGATTGGGATACACGTTTTGGGATAATAGAAGGAATTG

CTCAAGGAATACTTTACCTGCATAAGTACTCGAGATTAAAAGTCATCCACAGGGACTTGAAGGTAAGCA

ACATTCTGCTAGATCAAGAGATGATCCCAAAAATATCAGATTTTGGAATGGCAAGGATTTTCGGGACTG

ATCAAACACAGGCCAGTACAAAACGAGTGGTTGGTACATAGTAAGTATAGTAGCCGCTTCTGTAACCAG

TTTTTATGTTACTACCCCTTGTGTATAGCTACTTAAGACTGATTTACGCCAATATGGCATAGTGGCTATAT

GTCTCCAGAATACGTAGTATATGGCCAATTCTCGGAGAAATCAGATGTATTCAGCTTTGGAGTATTGCTG

TTGGAAATCTTGACTGGTGAACGAAACTCAGACTTCTTCATGACTGAAGTTTGTGTGTCTCTATTAGGAT

GGGTAAGTACATTCGCGCATTTGAAAACTAAGTCCAAGTTTGTGTCAATTTCTTTTAACATTTCACATCAT

TTGGCATGTTAGGCATGGAAAATGTGGAAAGAAGGAAGAATGTTGGAGCTAATAGATCCATCAATAAG

GGCGACCTGCGACAGTAACAAAACAACACGGTCTGTTCTGGTTGCTCTTCTTTGCGTTCAAGAAATTCCT

ACTGATCGACCAACAATGTCTGATATAGTTGTCATGTTAAGCAACGAAACAACTCCCATCCCTGAGCCAA
AAGAACCGGCTTTTCGCAGCAGCTGGCGACATCAAAAGTTAAACGATGTTTCCATAAATGAGTTGACAT
TTACTTTGCCAGTACCTAGATGAGAGGATTTAGCTTTCCATATTTCTTATGGAATTAATCTTATTTTGAAA
AGAACCATCTCAAAAATGTTTAGCACTGTAAATAAAAAATCAAAAACCAATTAAATTACTCCCTCTGTCCC
AGTTTATATC
>PGSC0003DMG400026184 G-LecRK
ATGGCGAAGAAAACAGGTTTGTGTTGCTATTACAGCCGTTGCAATTCTACTTCTTTTTTTGCTCTATGAA
TCAGATAAAGAGAGATATCTCAAAAGGCTGCTCTTGTTTTCTGATCTTAGCAAGCCAACAAAGCACAGG
AGGCAATCCAGTGCCACATTGGTTCAACAGAGAAAAAGACGCACAAAACCTGATTAACGAAAATGATAA
GCAAAACATCGCCGTTCCATTCTTTTGTTTGGAAAACATATTAGCAGCCACAGATGATTTCTCAGATGTA
AATAAGCTTGGACAAGGAGGTTTTGGTCCTGTTTATAAGGTTAGCACTATTTTTTCACTTCAATAGTTCCG
CTTTATGTAGTCATTTCATAGCATCCTAGTTTACACATTGAAAATCTTTCAGGGTATATTTTCAGATGGAC
AAGAAATTGCTGTGAAAAGGCTGTCAACCCAATCTAGACAGGGCATTGACGAATTCAGGAACGAAGTC
ATACTGATCTCGAAACTTCAACATAGAAATCTAGTCAGACTTTTAGGCTACTGTATCACAGGGTATGAAC
AGATTCTACTTTACAAGTATATGTCAAACAAAAGTCTAGACTCCTTCATATTCGGTTAGTATATATATCTG
AACATATCTTATTTCCTAATCTCTCTATTTACTTGATCAATGTTGTTAAATTTTCTCATTGTTTACCAAAACA
AGATCCAACACTTAGCAAGTCATTGAAATGGAAAAAGCGATTTGAGATCATATTAGGAATTTCTCGAGG
GCTTCTCTATCTTCATGAAGACTCAAGGCTCAGAATCATTCATAGAGATCTGAAAACAAGTAACATTCTTT
TGGATCAACAAATGAACCCAAAAATATCAGACTTTGGCTTGGCAAGGATAGTTGAAGAGCAAACGACA
GAAACTAGTAATTAA
>PGSC0003DMG400026185 G-LecRK
ATGATTTCAGTAAATCACTCTGTTTCTGGAAATCAAAGTATCGTCTCTTCAGGCGAAAGCTTTGAATTAG
GATTCTTTAAACCTGGTAGTTCGAAAAATTACTATCTTGGTATATGGTATAAGAATGTAATTACACAAAC
AATAGTATGGGTAGCAAACAGGGAGAAGCCACTTTCTGCTACTGATATGAATTCTGTTGAGTTGAAAGT
TTTGGATGGTAATTTAGTGCTTATTACTGAATCCAAAAACTTGTTTTGGTCAACAAATATCAGCAACACAA
TTAGTTCAAATACACTAGTGGCAATTCTTTCTGATGATGGTAATTTGATGTTGAGTGATGGATCAAATTC
AACTACACCACTTTGGCAAAGCTTTGATAACCCGACTGATACATGGTTACCGAATGCTAAGGTGAAGTAT
GACAAGCGTACAAATACAACGAAGATACTTACTTCTTGGAAGAACTCAGAGGATCCTTCTCCGGGGATT
TTTTCCGTGGAGATGGATCAGAGTAATAAGCAGTTCTTGATAAAGTGGAACAGGACTGAGCTGTACTCA

GCTTCTGGATCTTGGAATGGGCGTATTCTCAGCTTGATGCCTGAGATGAGTTTGAATTCTGATAGATACA
GCTTTACATATGTTGATAATGAAAATGAGAGCTATTTTATGTATTCCCTTCGTAACTCTTCCAAAATTAGA
TTAACACTAGATGTCTCTGGGCAAATCAGGCATCTGATTTGGGCCGAAAATTTAATGGAATGGCAAATTT
TTACATCTCAACCAAGACAACCGTGCGAGGTGTATGCTAGTTGTGGTGCCTTTAGCATTTGCAATAAGGA
GTCCACGACCTTCTGTAATTGCTTGACTGGTTTCACACCTAAATCCAATACAGAATGGGACTTGAATGAT
CATTCCGGTGGCTGTGTAAGGAAGGAAAGCTTGCAGTGTGGTGATGGTAAAAAGGGCAGGTTTTTGAT
GAATCCCAACGTGACATTGCCCGAATATTCCATATCTGTTCCGGCTGCTAGTGCCGAGGAATGCCATTCT
ACTTGCTTGAGTAACTGTTCCTCATGTAATGCATATGCTTATGATAATAATGTGTGTTCAATTTGGAATGA
AGTTGTGAATCTGAAGCAACTCTCACCAGGAGATGGTAGTGGAAGTGTGATTTATACCAGACTTGCTGT
CTCTGACGAAGCATACGGTGAAGGTAAGTTTAAAGTTAGGAGTTGTACTTGATAGTGTTTGTCATTTCTA
TGTATGCAATGCTCATCTCCAAATAGTTACTGACAGCTTTCTATGGATGTTTGTTTTTTCTTGTCTAATGTT
TCTTCAGTTCTTTACCTCATGGACTTCAACATTACGAGTTCTCTAAATCTTAGTGTTCACAAGAATTAAATA
GGCAATTATAACTTCACATAAACAGACCTTACGTTTTAAACAAACATCAGATAGTCACTGACTAAATATA
AGTTTACGCAACACCCTCTATGTTATAAACACACATAAGATAGTCACTGACCGAATGCATGGTTTTCATG
TTTTCATTAGTTACAACTAGCCAATATGAGTTGAAGACCTTTAGCCAGTAAACTATTACCAGTTTGTTGAG
TTCCCACATCGGCTCTTTAAGGATGGGATGGTCTCTTCACATGGTTTCGGACAGTCCTTGACTCATAAACT
AGATTTTATAGTCGAGTTAGCCTCAAGGGTCCATGTCTTATCACATTATTAGAATCAGACCCATCCCGATT
CTTTGTTTACCCGATATTCTGCCCCATAATATTGTTCACGCTCCAGTTGGCAGGCCTAGGCATGAGCGAT
GTTGAGTTCCCACATAGTCTCTTAAGGGATGAGATGTTTTTTTTATATGGTCTCGGACAATCATCATATCA
TGAAGCTAGCTTTTAGGGACAAAAGTTTATTTCTTGTCACAGTTTTCTTATGCTTCTTCTTTTCTGTCATCA
GATGTAAAATCAGGGAAATTTTCCCTGAAGCTTAAAGTTATCATATCTGGCGTGGTAGCCGTTGCAATTC
TACTTCTATGTAGCTTTAGCTACATTTATTGTAGTAGAATGATGGCGAAGAAAACAGGTTTGTGTTGCTA
TTACAGCCGTTGCAATTCTACTTCTTTTTTTTGCTCTATGAATCAGATAAAGAGAGATATCTCAAAAGGCT
GCTCTTGTTTTCTGATCTTAGCAAGCCAACAAAGCACAGGAGGCAATCCCGTGCCACATTGGTTCAACAG
AGAAAAAGAAGCACAAAACCTGATGAACGAAAATGATAAGCAAAACTTCGCCGTTCCATTCTTTTGTTTC
GAAAACATATTAGCAGCCACAGATGATTTCTCAGATGTAAATAAGCTTGGGCATGGAGGTTTTGGTACT
GTTTATAAGGTTAGCACTATTTATTCACTTCAATAGTTCCGCTTTATGTAGTCATTTCATAGCATCCTAGTT
TACACATCGAAAATCTTTTAGGGTATATTTTCAGATGGACAGAAATTGCTGTGAAAAGGACTAATCAACC
CAATCTAGACAGGGCATTGAAGAATTCAGGAACGAAGTCATACTGATCTCAAAACTTACTAACATAGAA

ATACTAGTCAGACTTTTAGGCTACTGTATCACACGGGTATGAACAGATTCTAACTTTACGAGTATATGTC
AAACAAAAGTACTAGACACCTTCATATTCGGTTAGTATATATATCTGAATTTCTTAATCACTCTATTTACTT
GATCAATGTTGCTAAATTTTCTCCATGGTCACCAAAACAAGATCCAACACTTAGCAAGTCATTGAAATGG
AAAAAGCGATTAGAGATCATATTAGGAATTTCTCGAGGGCTTCTCTATCTTCATGAAGACTCAAGGCTCA
GAATCATTCATAGAGATCTGAAAACAAGTAACATTCTTTTGGATCAAGAAATGAACCCAAAAATCTCAGA
CTTTGGCTTGGCAAGGATAGTTGAAGAACAAACAACAGAAGCAAATACCAATAAAGTGGTTGGAACTTA
GTAAGTAATACAAACATGTTAATGTTATGAACTTTGCTTAATTGCATCGCACATCTGACTCGTGATTATTC
CATCATTATTTGGTTGATTTAGCGGATATATGTCTCCTGAATATGCATTGGAAGGAGTATTCTCGATTAA
GTCAGACGTATTCAGCTTAGGAGTAGTTATACTGGAGATAGTAACTGGAAGGAGAAACACAGGATTTTA
TCAATCTAAAGAAGCATCTAATCTTTTGGTCCATGTGAGTGCCTAGAGTACATGTTAATTTTTTCCAATAT
ATATGTACGTTGAACTTCATATTCATTATTCTAAATCATGATATCACAGGCATGGAATTACTGGAAAGAA
AAAAGAGCACTACATTTGCTGGATCATTCATTGCTCGAATCATGCAACCCGAAAGAAGCAATGACGTGC
ATAAACGTTGGGCTCTTATGTGTGCAAGAAGATCCAGGTGATCGTCCTACAATGTCAAATGTTGTGATG
ATGCTTCATAGTGAAAGTACGTCTCTTCCAAAGCCTAATCAACCGGCTTTTGTGTCAAGGAGAAATGTTT
CTAATTCATCTTCAGTTTCATCTGGTAAACCACCTAGCTTCACCAAAATTGAGTTGACAATCACAGTGGAA
GAAGGCCGATAATGTGATGAATTCATTAGTTTTTACGCCAATTTTCTCCGCATGAAATTATTTTTTCACCTT
AAGAGAAGATAAAATACATCAAATCAACTTGATAGCAAAACTTTTTATCAGTTTAACTACATTGGTG
>PGSC0003DMG400026810 G-LecRK
CAAATTGGAAGAGACATTTTTCAAAGTGTCTGAAACCTTTTTCAGACCACTCCCTTTTACTCCCAGAAAAA
AATTGCTTTGTTGATTTTCCGTCAGTCCCTTCTCTTGCTTTCCAGGGAAAGATAAAGAGTTGAAAATCTAC
AATGTGAGGCACTTAAATTTTGCCTCTTTACTTTACTGGTGAGTCATATCTCTTGTTAATTTAAGCTCACCA
CTCAAAACTTCCCCAAGATACTAACTTTCTGTTTCACTCTAGCTTTTTCACTGAGATATGTTGATTTTAAAG
CTTTTTCACTTGTGGGGTTCTCATTTTCTTCAAATCTTGAAATACCCATTTAAGGATTTCAACTTTTGGAGT
TATGTTAGACCGAGTACTTTTGCTCTACATTATCCTTAAAATTGGGACGTAATTGTAAAGATCCATTCTCT
CTTGCATGTGCACTTAGATTTGTTCTCTTATCTTTCCTTAAAAATATTTTTTTCTCTTACCTAAATTGCTCTTT
TTTTGCTTAGAAAAATTGAGATATATATGTGTGTATGTGGACTCATGGTTTCTTTGCAACGCGTATTTTGT
CAAAGGCATGTTTGTTTCCAAGTCTATGCGTCTATATTGACTTTTAAATAGTTAATTTTTGGGGTATTAAA
CTACTCCCTTATTAGGTTTTCAACTTTGGATGCTTGCAAAACATTTTTTAGTGTCACTTTAAAGTATAGAAA
AGTGGTTGAATATGCATAAGGACCTAAATAGGGTCCCATTTGTAGTTTTGAACCAGGATTTCAAACAAA

AGCTGTTTTCTTAAGCCATTTTAAATTAATTTTCTTTGTGTACTGTTTATTTTATGGTCCATCTGTAGGTCAT

ATTTTTTTTAGTTCAATCATAAGGTGTGGATCTTTTGTTTTCTCACATGGCAAGTTTCTTATAGGGAAGCA

TTTGCTGTTTCAAGAAAGTGTCTGCAGGCAATATTCTTCCAATTGATGTGGTTTCATTCTCAAAAAAAATA

TTCTTCCAATTGATGTGGTTTGGATGTCGTTTCTGAGGTTCAATGCTTCCCTTCTCTTTCAATCATTTTCCC

ACCTTTTGTTTGATCTTGGTGTAACCTTGAATCAATTGGTAGTTTGAGAGTTTTACCTTATAGTTGATAGT

GATATTTCAATTCCTTTACAAAAGAACAGTTTCTTGTTCATTGTAAATCAGTGGAATGACTGAACATATTT

TTTGTTGATATGCAGAAGCTGGAACCAGCTTTCTGTCTTTTCATCTGCTTCCGCAAGTAGTGACAGATATG

GATAGAGGAGACAGAATAATTGCCTGTTATTCTAATTTGCTGCTTTCTGTCTCTATCGGGTTCTTGCTTTT

TTCAGTTGTCAGTTCACAAATTCCTTTGGGTTCCAAACTAACAGTAGAAGAGAACAATTATTGGTTCTCAT

CCAATAAGGACTATGCAATTGGATTCTTGAACTTCTCCGACCAGTATAGCTTTGGTGTCCGTTTCAATGCA

ATTTATATACCTAGCAGTGAGCAAGCAGCCATTTGGACTGCAGGGTGGAATGTTAAAGTTAGTAGCAAA

GCATATTTTGAGCTTTCCCTAACTGGGGAAATGATATTGTTTGATCCAGCAAAGGGAAAAATTGTTTGGC

AAAGCAAAACTGGCAACACATCTGTTGAATCAGCTGTTCTTCTTGATGATGGCAACTTTGTCCTGTTAAA

TAGGAATAAGAGTGCTGTTTGGCAGAGTTTTGAAAGTCCATCTGACACAATGCTGTCTGGCCAGAGTTT

ATCTGTTGGCCAGTCTCTTCGAGCTTCTAGCAGGGGTTCTTTAACAAGTTATTACAGTCTTCATATGAATG

TTTCTGGTGAGATGCAGCTTAGGTGGGAGACTAGTATCATTTACTGGACTGTTGGGGGTCCTAAATCTG

CTGTACGAGCCATTCTTGGCTCTGACGGAATCCTCCAACTACTTGATCAGCAATCGCAAGCTGTTTGGTC

AGTCTATGGTGAGGACCACAATGATTCTGATGTGAAGTTTCGGTTCCTCAGACTAGATTCTGATGGTAAT

CTTCGGATATACTCATGGGAAAATAATGCAACATCATGGAGAACAGTTTGGCAAGCTATAATTAATCAGT

GTGATGTCTTCGCAACCTGTGCTACCAATGGTATTTGCACCTTGAATGCATCTGACTCTTATGTTTGTTGG

TGTCCATTTAGATCTACAAGAGACTCAAACTCTGAATGTCTAATTCCATACAAACCAAGTTGTGAATCCG

GTTCATCCATGATCCTTCATGAGCATATGTATTTGTACGGAATATACCCACCAAACAACACAGTTGTGCA

AACCAATTTGCAGCAGTGTAGAACTCTGTGTCAGAAAGATCCATCCTGCCATGCTGCATCCTTTATCAAC

AATGGCACTCCACAATGCCACATGATGAACTCCCGGTATGTTGGTGGTCAATCAGATCCTTCTTTGGGTT

CCGTTACTTTTGTCAAAACTTGTTCAGACCCAATTGCTGTTTTACCTCCGCCTGTACCTGCTCCGAGAAAG

GTATCACAGAAAATTTGTGTTGCTTGTCTGTTAGAAGTTGCTGCTGCGTCAATCGTTGTGTTTGTCATGCT

TCAATTCAGCATTGGAGTTTACCTTTTTAGGAGAAGGAAACATATGATGCAGAAATCTGCTTTACCCCAT

ATAGTGCCTAATGCTACTGGCTGCATCGTGTTTTCTTACTCTGAAATCAAAGACCTGACTGATAATTTTAA

GCAACAAATTGGGCAGAATGTGTTCAAAGGTCTACTTCCAGATAATAGGCTTGTTGCAGTTAAAGATCTC

AATGCCTCTATTGATGAAAGGAGGTTCCGGGCTGCAGTTCTAAAAATAGGAAGCATATACCACAAAAAT
CTTTTGAGGCTGGATGGTTATTGCTGTGAGTCCGGCCGTAGGTTATTGGTTTATGAGCTTGCCAAATATG
GTTCTGTTGACAAATGTTTAGAAGAACCTAGAATGTGCAAGAGATTGACGTGGAGAAAAAGAATGGAT
ATATGTTTGTCAGTGGCAAGGGCCATATCTTACTTGCATACAGGATGTAGGGAGTTTATTAGTCATGGAA
ATCTTAAATGTGAAAATGTTGTCTTAGATGATGAATTAGAGGCCAAAGTTAGTGAATTTGGACTGAGGA
CTATTCAAGCTGAGGCTTCCAGTAGCGGGGGGTTAGCAGAGACAGATGTAAGAGATTTTGGCAAAGTG
ATGGTGGTATTAATCACTGGATGCCAAAATGCGGATGAAGCTTGTTATTGGTCCTATGAAAAATGGGTG
AAAGCTGAAAAGGAAATGGCCATGGATCAGCGAATTGTGGTTGGCACCGATTCAGAAGAGTTAGAACG
AGCATTAAGAATTGCATTTTGGTGCTTACAAGGTGATGAACGTATGAGGCCATCAATGGGAGAGGTAAT
CAAGGTACTGGAGGGAACTCTGACTGTCGATCCTCCACCACCTCCTTTTGCTCATAATCAACAGTGGCCA
CCTGATGATGAATCACCATCTGAATCATATTCAGCACTGTAGAAGTTCTAATTCGTATATTTCATTCCCAT
TAAGTCATCTGTTTCTGTACAGAACTTACCAGGCACCTTCGACTTGGCATGATGTAGAATGCCTAGTTTTT
CCAGTATGGCATGCTTAATCTGAAATTCAAGACGAGCATCCCACTCTGCCTCTTCTGAAACTGTAACCTTG
GTATGTAACCCTCCAAGTACAGTTTTGTGTTAGAAAATCCTGATTCAGAAATTCAGGTTTCTTTTTTAAGT
TTTTTATTCTGTTCATGTTAATTATCACTGCTTATCACATGTGTTTACATTTGATATTTTCTTGGAGGTGACT
AAACTTCCTTAACTCTGCCCAAAATGTCTCCTTGGCTTGTGCTATCAGTTGGCACTGGCTTTTTTACTAGG
CAGTGGTCCAAGAGATCGTGGATGAGGTTTGAACCTGGACATCTCTATTCTTAGGATTACTCTGACCATT
AAGTCGATCCTAACTTAAAATGGAAATGGAAGTTTGTGTCTCTTTTTCTTTACAGAGGTGGTTTCCATGCT
ATTTGCACCTCGACCATTTCACCAGGTACTTGGTATCTCCACCAACCTAGGTATCAGGTAACTCTATCTCC
AAAGCTTAGACAAATGAAAAGAAATCACCTAGCATTTTGAATTTCGTTGATAGGTCACACTTGTGTTTTA
TATATGTTCTGTTTTGATTTTGGCTTGCTGCACCTTTGTTGCCTGGTAAGTAGGGTCATTTTCGGTGAACA
TCCATGATCAGTATTTCAGCAATGAACACAATTCATTATTTTTATTAGTGAAAGAGTTG
>PGSC0003DMG400027233 G-LecRK
CACAACCAAAAAGTTATGTTATGACTTTTGTGAGTTTGTCTTAAATCTAAATTTACAAACTCGAAAAAGTT
ATTGTAAGTAGCAGAGTGGCCTTGCTAGTACAATCCACTTATATGCTAGGTAGCAGAGTAGATTGCTACC
GCGATCAACTGAAATTCAGCTCTCTATTGCTCCAAAGAAAACATTTTGACATGTGTAGTCTTGCTGTCAG
GATCCACATAGAGATATCTTGGTTATCGTGACCAATTGAGATTCAAACACTTTGTTGCTCCAGTGAACAA
AAAAACATTTTCATAGAGTAATCTTGCTGTCAGAATCTACTGAGATATCTTGGTTATCGTGACCAATTGAT
ATTCAACACTTTTTTGCTCCTGTTCCATTTCATATAGTAAGTCTTGCTACCAAGATCAGCTGAGATTTAGCT

TTTTGTTGCTCCGATTCATTTTCTTGTTTCTATTAACAAAAAAATATTTTGATAGAGTAATCTTGTTGTCAG
GATCCACTGAGATATCTTGGTTATTGTGACCAATTGATATTCAACACTTTGTTGTTGCTCCAATTCCATTTC
ATATAGTAAGCCTTGCTACCAAGATCAATTGAGATTCAGCTTTTTGTTGCTCCGATTCGTTTTCTGTTCTCC
TTTTTCAAGTAAGTAAAGTAGGTTGACTCGCTACTTTACCTTCTGTGTATAATGAGACGGAACAGAGTAA
AAAATGCAACTTATAAGTATGGTATGCAAAAAGATTCTTGATTTCAAGAACTGATTTTTTTTTTCAAGTTT
TCATTGTTGTTAAGAGCATGGGATCATCTTTTTTGTTATTCATTGTTACCCTTTTGTTATCTTGTTTTCTTGT
ACACTCTGGACCTCTTTCTCTTCAACCATTAACTCCAAATTTCACTGCTTCAAATTTCAAGTTCATTGATAC
TTCTGGATCATTCTTGTCATCACCAAATGGAACTTTTAAAGCAGCAATAACAAACACAAAATCTCAAGAA
AGATCCTATTACTTTGTCATTGTTCACTCGGAATCACATGTAGTTGTTTGGTCAGCAAATCGCGATATGCC
TGTATCTGATTCAGGTGAGTTGCACCTTTCTGTCGATGGCCTTGCTCTTTTTGATGATTCAGGTCACACCG
TTTGGTCTGCGAAGCGTAGTAGTACTAGTAGTTCTGTTACTTCAATGCAGCTTCTTGAATCAGGAAACTT
GGTGTTGGTTGATGCTTTTAATAATACTGTGTGGGAAAGTTTTGATTCCCCGACAGATACAATCGTTGTA
GGCCAACGATTGCCTGTTGGGAAATCGTTGGTTAGTTCAGTGAACGAAGATGAAATTGCTAAAGGCGAT
TATAAACTTGTGGTTGTAGAGAATGATGCAATGTTACAATGGAATGGAATGACTTATTGGAAGTTAAGT
ATGGAACCTAAGGCTTTTACTGATGCATATACTCTGGTGGAGTATATGATGATCAGTAGCAACGGGTTG
TTCCTTGTAGGGGGCGAATGGGACGGAGAGAGTGATTCAGGTTATTTTGGATGAGGTGAAAGATCCTGA
CTTTAGGATTGCGAAATTGGAAGAGAATGGACATTTCGGTGTCAAACGATTCAGTAACGGAAATTGGAT
GTCTGAATTTGATAGTCCTATTGATAGTTGTCGTGTTGCTTTCACTTGCAAGAAGCTTGGAGTTTGTGAT
GAGGGGAGCTGTTCTTGCCCACCGGGATTTCGTGTTAGTTCTGAGGTGAATGGTAGTTGTGCTCCAGTT
GATAGAAATCTGGTAATGCCAGTTTCTTGTAATGCATCTTTGAATATGAATGTAACTGAATTGGGAAATC
GTGTTTCGTACTTGAGGCTAGAGAATGGTATGGACTATTTTGCTAATGATTTTATCGAACCTGTGAAGCG
TGGTGTTAATGTATCTGCTTGTCAAGATCTTTGTTCTAAGAATTGTTCTTGCTTGAGTGTTTTTCATGATCA
GTCGTCGGGGTCATGTTATATGATTGAAAATTTTCTTGGTTCGATTTTGAGGGGCTCGGATTCTGGTAAC
GGAAGAGGTAGATTGGGATACGTTAAGGTCATTTCTGAACCGTCATCATTTGATCCAAATGACAACTCA
AGTGACAAGAGGTCAAGATTACCCGTTGTTGCTTTGGTTTTACTTCCTTCTTCTGGGCTTTTCCTGATCAT
TGTGATGATGGCTGGGATTATGTGGTTGATGAGAAGAAAAAGATTGATGCAGATTTCAGGGAAAGAGT
TTGGACGGACGGATTCTTCTTTGTTTGCTGAGCTTGATAACATTTCCATACTGGGGTTGCCTGTGAAATTT
GATCATGAGGAGATTCGGGTAGCTACGGAGTGTTTTAGAAATCAAATTGGTACTGGTGGATTTGGAACA
GTATATAAAGGTACACTTTCTGATGGAGCTGTTGTTGCTGTCAAGAAGATGAATGCTCTCGGGGCTCAC

GGAAATAGGGAGTTTTGCACTGAGATCGCGATTATTGGGAGAGTTCACCATGTTAATTTGGTCAGTTTG
AAAGGATTTTGTGCACATAGGGGAGAAAGGTTTCTGGTGTATGAATATATGAATCGAGGCTCATTGGAT
CGAACCCTTTTTGGACTTGGACCTGCCTTAGATTGGCATACCAGATATGAAATCGCGCTTGGAACAGCAC
GTGGGCTTGCTTACCTGCATGGTGGTTGTGAGCAGAAGATCATACATTGTGATGTGAAGCCAGAGAACA
TACTCCTCCATGACAACCTGCAAGTAAAAATATCAGATTTCGGGCTTTCCAAGCTTCTTAATTCTGAGCAG
TCTAGTTGGTTTACCACGATGAGAGGAACTCGAGGCTACTTAGCTCCTGAATGGCTGACTAGCTCAGCC
ATAACGGAGAAATCAGATGTCTACAGCTACGGAATGGTGCTACTAGAAATAGTACGAGGGAAGAAGAA
CTCCTCGTTCCAACCACCAAATGACACTACTTCTCAAAGCGAAAGTAGTGAAAGGAACAGGCTTTCTCCA
TCGTCTCTTGCCTCAGCAAATCAGCCAATCTACTTCCCATTATTCGCGTTGGAAATGCATGAACAGAAAA
AGTACCTGGAGCTAGTGGATCCTCGTGTTCTGGGGAGTGTCAAAAGTGAGGAAGTCGAGAAGCTAGTA
CGTGTGGCTTTGTGTTGTTTGCATGAAGAGCCAACTCTGAGACCAACAATGGCCAATGTTGTTGGCATGT
TAGAAGGTGTGTTGCCTTTGGCTACGCCACAGGTTCAATCACTCAATTTTCTACGATTTTATGGTCGTAGA
TTCACTGAAGCATCAACTATCGATGGGGATCAAGAGGTTAATGTGTTTGAGTTACATCAACAAAGCAGG
AACCGTAGCAGTACTACTAGTAGCTCTTACAATTCCTTCTCTTATATGTCTTCACAGCAAGTCTCAGGTCC
AAGATAGCACTAGTAAATCAAACGTTGCTCAAACACTACAAAAATGTTGTTACATTCGTGTCTGGTCCTC
CAAAAGTGCATAGCTTTGGGAAGATCCAACCTACTTTTGACATTATTTTTGGAGAGTCTAAACAACACGG
AGTAAATCTGCTTTTTTAGTTCAATATGTTATGCTACTGTTTGAAAACTTTAGTTGTAATATGATGTTTGTA
CCTCTTCTGTGAGGCAGTAGAAAGAGTATAAATATAAGTTGTAATTTATTCAATTTC
>PGSC0003DMG400028461 G-LecRK
AAATACAACATCAGCTTCAAATCATCTTTACCCTATCCAGACAAGTAGTTAAGCAGATGGGAATAGATAC
ATTTCGATGATCGTCAAGTCATTGATATAAATTAGAGATTTCACAGATGAACTATTCGTATGTATAAGAA
TTCTTTTCCCTTGAAATATGTTTTTTGGTCAATGAACATTTTAGTTTTTTTGTCTAAACAGAATCATGGCTT
TACCCTCTGTCAAACATTTGTTGAAGCTTTTGATCATTGTGCTCCTTGAGAACCTTACTTTGGCTCAATTTC
CTGATTATCACACAGTGAAAATACCCACAACGTGGATAAACAATAATGTATCGTCTATCAATGTTACAAA
GCCCTTGACATGGCCTTTTTACTCATTCAATCAATCATCTGGACCTTTCATGCGAATTCTGATGCTCAAAG
AGATAGGAAAGGACATGGGATATGCTTGTGGTTTTGCTTCAAATGGAGAGGATGATTCCTTTGTGTTTTC
CATTGGAATTATACGTCTTCGTCAGGACTTTCTAGGAGCTCAGCGCTGGACTGACCTTGAGCTGGTCTGG
TTTGCAAACAGAAACCATCCAATTCATGAAAATGGAACGCTGCAATTGTTGCAAGATGGAGATTTAGTG
TTAAAAGATGTGGATGGAACTCTTGTATGGTCTACAGGCACCGCGAATAAGTTTGTTTCGGGCATTAAG

ATGATGGATACTGGGAACCTTGTGCTTCATGACATGTATAATCAGACTGTCTGGCAGTCTTTTGATCATC

CAACAGATGCATTGCTTCCAGGCCAGAAATTAAGGTCTGGTCAAAGACTTGCGGCTAGAGTTTCTTCGTC

CAATTGGAGTGAAGGTAACTACTATATCTCTGTAACCAATCAAGGCTTGTTTGCATTTTACATATCAGAC

AAGCCGCAGATGTATTTTAAGTTTTTGGTCAGTGGAGAAAGGGATAGCTTTGATGAAAGTTATGTCAAA

GCAGTTAATGGTACTCTTGCCTTGTACATATCTTCAACCGAGCCGAATGAGCCAAATGCTGTCTTCTCAA

GACCTTCAAGGATGAAATATTTGAGATATGATTATGATGGACATCTTCGAGCCTACACCGAGGGTAGTG

ATCAGACAAACGATCTTTTGGCAGATTTCATTGGGCTTTGCGATTACCCCACAGCTTGTGGCAGTTTGGA

GCTTTGCTCAAATGGAATTTGTTCTTGTCCAGGTCCATTAATAAAAAGTAATGATCGACAAAACAACGGA

TGTGTGGAAGTTAGTCCGGTGGAATGTGATGGACAACGCTCCCACAGAATGGACCGTGCTTCGGATGTC

TATTACTTTAACTATGTCGACACTGATGCAGCAGCTCTGAGAGGAACAGATGAGGAAAGTTGCCAAGAG

TTGTGCTTGAAGAACTGTTCTTGTAAAGTAGTTCTATTCCGGTATTTCACTAACTTTTCAAGTGGTGACTG

CTATTTACCCTCTCCTGTTCTGTCACTTATAATTGATGGAAAGGGAAGAAGTGTCTATGAATCATCTGCAT

TCATCAAATTACCAAATGATGCAGAAAAAGGTAAGTCTACAGCTACAAGAAGAACTGGCATTATAGCTG

GATTAACTGGTGGAACAGTTCTTCTGATAGCATTAACTGTTGGCATCTTGATTGCTTTCAACAGAAAACA

CACATTACAAGAAAATAATGATGATTACTCAGGGGAGATATCTGGTCTTGTAAGATTTTCTTATGAACAG

CTGAAGATGGCAACTTGGAATTTCCAGAAAAAGCTTGGTCAGGGAGGTTTTGGCTCAGTTTATGAAGGA

GTTATTCGAGATGGTCAGAAAGTAGCAGTGAAGGTTCTTGATGGTTTTGGCCAAGGAAAGAGGGAATT

CTTGGCAGAGGTCCAGACCATAGGAAGCATCCATCATGTTAATCTTGTTAGACTAATTGGAGTTTGTGCT

GAGAAAGAGCACACAATATTAGTTTATGATTTCGTGAGTAATGGATCATTGGATAAATGGATTTTTGGC

ACATCCTCTACTCAATTTACCCTTGATTGGAAAATAAGAAGAAAAATCATCCATGATATAGCAAAAGGAT

TGGCTTATCTTCATGAAGAATGTATGCAAAGAATAGTCCACTTAGATGTTAAGCCGCAGAACATTCTGCT

GGACGAGAATCTTTGTGCAAAAGTATCTGATTTTGGCTTGGCCAAGTTGGTGGATAAAGATCAGAGCCA

CATAGTGACCAGAATTAGGGGAACCCCTGGATACTTGGCTCCTGAATGGTGTAGTGCATTCATTACAGA

AAAAGCAGATGTCTACAGCTTTGGAATTGTTGCAATGGAGATCCTCTGTGGACGCAAGAACGTGGACTA

TTCACACTCACTGGAACATCCACATTTGCTTAGTTTATTTATGGAGAAAGCCGAAAACAATCAGCTTATC

GATATGATGGGAAACTACAGCGATGATCCACAATGCAATACATCAGAAGTCATCCGTATGATGAAGCTT

GCTGTCTGGTGCTTACAGAATGATTTTACTCTGAGGCCTTCCATGTCAATGGTGGTTAAAGTAATCGAGG

GGACAATGGACATTGAATCTCAGTTAGACTATAGTATTCCAAATTCACAAACAATTGCAGCAATCAAAAG

AGTGGCAGACACCACGACTAACTTGTTTCCTTCAATTCTTTCAGGACCTAGGTAAGCATTTCTAAGCATG

ATGAAGAGTCAATAGAGAAAAGAAAATCCCCCCTCCTTTATCTTATTTCTTGTCTGATCCCATGATCATCA
TTGTTGTATAGCTATTTATTGGTTTCATTTGTGTTGTTTTTCTGTGACTCAGTAAGATACTTCAATTAATTA
AGAACCATGGCGTGATTTCCA

>PGSC0003DMG400028617 G-LecRK

GTCACAGTAAAAGTTCTACAAACAATATCATCGAAACTTGGGAACAGATACTGGAATGTCAACCGAGCC
TCTTGTACGCAGGAGAGGGATTTTAACATGACGGATCCTAATTATGATGAAGTTTTCAGCAATGTAAAAT
GTAATTGTTCATTCAATAATGGCTCAGTTTGCCATGTTGTAACAATGTAAGCTCTGCTTTGATTTTTAAAA
CTTCACATTTTACTCTTAATCAGAAAGTTTTACAGTTAAACAGATGCCATCTTCTGCATCATGTAATGAGA
ATTTCTTACACTTTTTTTGTTGATACAGCCAATTGAAGCGTCTTAATATGACAGGAATTCTGCCTCCAGAA
TTTGCAAATCTTACTCATCTGCAAGAATTGTGAGTATCAGTTATAGATTTTTCTCCCCTTTCCGATTACAGA
AACTTTTATTCAATTGTTTTTATAAAATCGTAACAGTAGTAGCAAGTTAACTATTTGTTGCTTTTATACCCT
TCGAGGTGGCCTAGTGGTCTGGGCTTGGGAATTCCATGTCGGAGGTCTCAAGTTCAAAACCCCTTGTCA
AACGTAAACAAGGGGTTTGCCTTCTGGGTCGAGCTCGTCACACCAGGCTTGCCTAGTGCGGGTTACCTC
TCCTGTGTGCATAAGAGTGGGGGTTTTACCTGTGGCTTAATCAATTTTTGCATTTCCAGAGATCTGACTC
GTAATTACTTAAGTGGATCGATTCCCTCAAGCTATGGCCAGCTTCGCGTCACTAATTTGTAAGTTCTTTTC
TGTTTAATCTTTTCAATGAAGAATTTTTGACATTTTGTTCAAACTCAGGTCACTCTTGGGTAACCGTATCA
GTGGGCCGATTCCGAAGGAGTTAGGTGACATTTTCACACTGGAAGAACTGTAAGTTGAAGATATTGCAT
ACTTCCTTTAATTTTCTACATATGTCACATTCATGAATTCCATATTTTCAGAGTTTTGGAAAATAATCTACT
TGAAGGACCTCTTCCTCCGAACCTTGGTAGCTTGAACCGCCTCAGAAGAATGTAAGACATAGATTCAAC
GTCTAATATCTACTTATTGTTACATCTATTCCAAATTATTTGGCACTTTTCAATTCCCAGTTGTCAAACTAA
ATTGCATTTCTTTCACCGTTTTTATTTGGAAAGAACTGTCAAGTTCATGAGGTTAATGAAGTATATCCTAC
CCATGATAGAACGATTTTACTCACTAATGTATTGGTACCTAAAATAGTGGTGTCAACGAACCACTCAACA
AGAGCAAAATACGCTCAAATTTATTTGTGCAAAAGAAGAAGAAGTTCAGAAAATTCGTAAGGAATAATC
TGAGGAAATAGATTTATTTATAGCCATAGAAGTACTGTTTCTGACAGTTTGCAACCTTCCAGAATACCTTA
AGAGAAGTACTATTTCGGAAAGTTTCCTTAAGATTGTTGTTTATTTGTAATAATAGGATACTTAGAACAA
TACTTTGCAGAACTTCGTAAGAAACATCACTTCATATTCCCTTCATTTCTCATTTATTATTATTATTATTATA
CCCAACACTTTTAATATGAAGAAAGAGTTAATCGAGTCTAATTTAGCTCCAAAAGTTGGTCAAATTGTAT
CAAACATAAGCAAAAAGTGTCGATCAAATTGGGATGAACAGTGTGATTTTTCAGTTATCATGGTTAATTG
ATGGATCATTTTGCAGGTTGCTTTCTGCCAATAATCTCAATGGAACTATACCTGAGAACTTCAGCAACCTT

AAGAACATGAGAGATTTGTAAGCAAGAAAATGTTATAAGAACATTTGACTTTTGTGCTTCAATCTCTTAA
CTCTTTCGTCTGTTTGCAGTAGGATCGATGGGACTAGCATATCTGGAACCATACCAGATTTTATTGGCAA
TTGGACCGAAATGGATAGATTGTAAGTATGCATCTTGTAGTTGTAAATGTTGATGGAATATTCTTCTCCC
GGAAGGATTCTAAACTTTAAAAAAAGCAAAAAATTGGTATCATACATTGTATTTAATAGCAAATATCATG
AAACTTTTTGCAGAGACATTCAAGGAACGTCAATGGAGGGACCTATTCCTCCTACAATATCCCAGTTGAA
AAACATGACACAGTTGTAAGTACACACGTCTCTCGTACTACCTGCTATAGCGGAAGTTCTCATGTGCATT
GTTGTTTTCAGGACAATATCTGATTTGAGAGGAAAACAAATGCAATTCCCAAATCTTCAAGGCTTGAGGA
ATATGACAAGGCTGTGAGTAGTTGGAAATTTAGCTATTACATCTCTTCATTTTAATTTCGTATTCTCATGG
TTCTTTAGTTTGACTCAGGACTTTGAGAAATTGCTCAATTTTTGGTCCGATTCCGAGATATGTAGGTGACA
TGCCTTTGAAACTTTTGTAAGTTTGTCATCTCCTCTTTCGATTTTTTTTTTTGTCTATAATACAAACCTGGA
ATTACCCTATATTGTATAAAAGGAAAATCTTCTACATACCAATGCATTCGTCGTATTTGCATGATGGAGCA
AAGATTTTCACTAATTGTTTGGATACCACAGTGGTTTGCTTTGCTGTCTAGTTTGATGCTAAACCTGCCTG
CTTCTGATAACAGTTATTTAATTACTTGCAGGGACCTGAGTAACAATATGTTGAATGATACAATCCCAGA
TACATTTGAGCAGTTGAATTTTGATAACATGTAGGTACCTTGCAAGTGAAACTTTTACAAGTTGAAGCAT
AAATCTTGTACATGTTTGTTTTATTTCTTTTCTCTCGAAGTTTTCATTCGGTTATAACAAGCAAAAGTTTTA
TCTGCAATGTAGGTTGCTTGGTAACAACACATTAAGCGGAGCAATTCCCAGCTGGATGTTTAGTAAAAG
AGAGAACATGTTGGTATTTGCTCAATTTATTCCGTTTTCCTCATCTTAGTAGTAAACTTTGGTTATTATTAT
AGTTAGCCACACTTCAAAGTTAACAGTTTTTTTCCCATAGCTTTTTGTTTACTGCAAGGACACATTTACAA
GAATTTGATGGCGTCGACAACTACAATTTTTTGCAGTGATTTATCATACAACAATTTCACCCATACAACTA
CTCCAGGATGCCAGTCCTCTACCATGTGAGCATTCATTTATTATTGCTTTTTTTATTAGTGAGAATTTCGGT
CCTTTAACTACATAACTTACTAACATTCTTTCTTTAATAGAAACTTAGCCGCCAGCTATTCAGATACAATG
AACACGTTCAAAAACATGTAAGTCCTCCTAAATGTTGTCTTATGCATCAGGTGCTAGTTTGTTTGATGCCT
TCTATCTATTGTCGGCTTTATCTATTCGAAATTGTAGGGATGCCTGGTGTTCCATGAAACCCCTCATTTGT
CCCACAGAAGCGAAATGTAAGAGTTACATCCTATTAACCTTATTTTTTTTATACCTGAAAATTAATAAATG
AAGTAGCAGTTTGTCAAAGGACCTCAGGGGTTATGTCTGGATCGTTTACCACTGAATCGACTCTTGGAGT
GGCAGACTGTAGAGTTTGAAGTACAAAAGCATAGCCTTGGAGTTGAGAGATAATTTCACAACCCTTCCT
CATGTTATGGTCTTTATGTCAATTGCAGATACTTCGCTATTCATAAACTGTGGAGGAGGTAGAACTACAT
TTGAGGGAAATGATTATGAGGAGGACAAGATCAGTTCGGGTCCATCATATTTTTCTTCGTCCTCCGATAA
ATGGGCATTCAGCACTTCAGGAGTGTACGTGGGTCGAGTAAATGCTAGCTATATTGCAACAAATACATT

TTCACTAGATGTGTCCGGTCCTGACTTTTACAACACAGCACGCCTTGCCCCAAATTCTCTCAAATATTATG

GGCTTTGTCTACGAGGAGGTAGCTACAGAGTGCGCCTTCACTTTGCTGAAATAATGTTTTCTAATGACTC

AACATATAGCAGCCTTGGAAGGCGGATATTTGATGTAGCAATACAAGTAAGTTTGAGGCCTAACTTCTTT

TGTAAATAATAAATATGTAATCTAAAATGTTGATTTATTCAGTTCAGATATGATTTGGTCACTTGATGTAG

ATATACTGAGTTCTGATATGATTGGCAACTTTCTGATTTAATATTGAATGTTGGAGATAATTTTGATTTCT

TACACAGGGGAGAGTAGTTTTGAAGGATTTCAACATCATGGAAAAAGCTAATGGTGTTGGAAAAGGTC

ATACCGAGGACTATGATAATATTACTGTTAGTAGCACTCTGGAGATTCACTTGTATTGGACCGGGAAGG

GGACTAACGCCATTCCAGAAAGAGGTGTATATGGACCTCTGATTTCAGCTATCACAGTGACACCAAGTC

CGTTTATCTCTCTCTGCCTATAGACTTTACACTTTTCTTCCCTCCCACAATCTTATCCCATTTTCTTCTTGCA

GACTTTAACACAGGCAACGGGTTACCTGTCGGAGCAGTTATTGGCATTGTACTTGCTTCAATTGTGGTGG

TTCTGATAGTGTTATTTGCTCTGTGGAAGAAGGGCATTTTTGGAGGGAAAAATAATGAAGAAGAATTAG

GTAAACATACCTCAGAATTAATTAATCACTCTCTATATTTGATATGGTGATACTTTTGTTGTGATAATCAG

AATTATTTACGAGATTTCTAGCTGCGACATACAAATAATTATCTGCCTGTAATTGACCAGCATTGAGATG

ATTGTTCTGTTGGTGGATCGACATCATGACATATCATAGATAGTTAGCACACAACTAAATGCTCATGACT

GCTTAAACTATATATCTTCGTCATACAGATGTTTTAGAAGATCTATATATACTGACGTTTGCAATATTCTTC

TCGTTTACAGAACTCAGAGCCCTTGATTTACAAACAGGTCATTTTAGACTTAGACAAATTAAAGCTGCTA

CAAATAACTTTGATCCTGCCAACAAAATTGGTGAAGGAGGGTTTGGACCAGTTTACAAGGTATCAAATC

TCTATCTCTCTCTACCCATACTGATTTTGCGCAAGGATAGCTTGTGGTTCTGTGGTGATATTTCCTACTGT

GCGTAACGTTTATAGGAAAATAGCTGATATAATCCATATCCTACGTTCTTCCTTCCGTGTGTGATATGATC

GTTGTGAATTAATCTTGCTATCTGTAGGGTGTACTCTCGGATGGTGCAGTGATAGCTGTTAAGCAGCTGT

CTTCCAAGTCGAAGCAAGGAAATCGTGAATTTGTCAATGAGATAGGCATGATTTCAGCTCTACAACACCC

AAACCTTGTCAAGCTTTACGGTTGTTGTATAGAAGGGAACCAGTTATTAGTGATATACGAATACATGGA

AAATAACTGTCTTGCTCGAGCTCTCTTTGGTAATGCTTTGCAATAACATTTTGAAAATTATTAATCATGTA

ATGATAATTTTATGCTTATTAGTCCTCGTATTCTCCTTTCTTAGGCCGTGACGACCAGAGGTTGAACCTAG

ACTGGGCAACCAGAAAAAGGATATGCTCAGGAATAGCAAAAGGGTTAGCTTACCTTCATGAAGAATCA

AGGTTGAAAATCGTACACCGAGATATCAAGTGTACCAATGTGCTTCTTGATAAGGATCTTAACGCTAAG

ATTTCCGATTTTGGATTAGCTAAATTAGATGAAGAAGAGAACACTCATATTAGCACCCGAATTGCTGGAA

CAGTGTGAGCCTTCTTATCATAACTATCTCTGTACTATCATTTGAATTTGTTCGTCATGGTCAGTTTGAAG

TATCTTACATTTCTTTTTCCGTTTTTGAAGAGGTTATATGGCTCCCGAGTATGCAACGAGAGGCTACTTGA

CAGATAAAGCTGATGTTTATAGCTTCGGAGTCGTTGCATTGGAGATTGTCAGTGGGAAAAGCAACACAA
ACTACAGGCCAAAAGAGGAATTTGTTTACCTTCTTGATTGGGTAAACTATCTTTCTACTAATATCTGCAAC
ATTATATCTTTAGTTTAAGACCACAAGATGCAAATGTCTTTGTTACTTTCTTAAACTCTGTACGGAGGTAT
CTTTGCATATTTAGTTTTTCTTTTCTCTAGCTAATTTGATGTTTGCTATGTATGTGAATGTTTGTTTTAGGCT
TACGTCCTACAAGAACAGGGAAATCTGTTAGAACTAGTGGATTCACATCTCGGTTCCAATTACTCCAAAA
AAGAGGCAATGCAGATGATAAATATTTCTCTTTTGTGCACAAATCTCTCTCCTACTCTCCGACCATCCATG
TCTTCTGTGGTGAGTATGCTCGAAGGGAAACTCCCCGTGCAAGCACCAATAATCAAGCTGACTACATCA
ACCAACGAAATGAGATTTAAATCGTTTGAAAAAGTTTCACATGACAGCCAAACAACATACTCTCAGGAGT
CTCAAGGACAAGCACCTTGGAATGATTCGTCAGGGTCAGGGTCTCTGCCTGCTAAAGATGAAAAGTTTA
GTTCCTCCAGTAGACTTCTTCCAGATCTTTATGATATAAACATAGATTGATTTTGATTTTGATTTTCACACA
GGATCAAATATTTATTTTCTTGTGTATACTACTTTGTAA
>PGSC0003DMG400029520 G-LecRK
CCCCACCCCACCCCCTCCTTTGAATTTTCTGAAAATTCCACATAAACACGGTTCCTTAATGTAACAAAGAA
CCTTAAAATTTGTAAGGACAAGTTATAATTTTCTATTGTTTTATACTTTGTACAAAGTCCTCTTGAGTTTTG
TAATGGACTAATTATTTTTGTGTACCCGCTATGACCAAACCCAGAAAAATTCTTGAGAACTTTTTGAAACC
CTTTTTCTCAAGTTCCAACAAAGGTAAGCAAAAATTCTTGTGAGGGTACCCTTAATCTTGATTTTTTTTTT
GTTGTTCTAATGGTGATTTTGGGTTTTTTTCTTGTTGTGAATTGACTAATCAAAAAAAGGGAAGAATGAC
GACTTAGAGAAAATTGCAGCACAAGAACAGAAGCAATTCTCTTTTGGAGTTCTTGTTTCTGCTACCAAAG
ATTTTCACCCAGATAACAAGCTTGGTGAAGGTGGATTTGGGCCTGTATTCAAGGTAAAAAATAAAAACA
AACTGAAAATCAACAAAATTGAGTCTTTCTTGTGATTTGGGTGTTAACAAAAACTCAAGCTTTCTTGAAA
AGATTGAGTCGTTTTGTGATTTGGGTGTTAACTGAAACTCAAACTTTCTTGAAAAAATTGAGTCTTTTTGT
GATTCTTGAAGAAAATGAATCTTTTTGTGATTTGGGTATGAACAGAAACTCAAGCTTTCTTGCAAAAATT
GAGTCTTTTTGTGATTTGGGTATGAACTGAAACTAAGGCTTTCTTGAAAAAATTGAGTCTTTTTTTGATTT
AGGTATTGAACAGAAACTCAAGATTTCTTGAAAAATTGTGTCTTCGTTCTTTATAGGGGAAATTGAGTGA
TGGGAGGCAAATAGCTGTGAAGAAGCTTTCACATAGTTCAAGGCAAGGGATGAAGGAATTCAAGAATG
AAGCTAAGTTGTTGGCTCGTGTACAACACAGAAATGTGGTGAATTTGTTAGGGTACTGTATACATGGGG
TAGAGAAGTTGCTTGTCTATGAGTATGTTGCTAATGAGAGCCTTGACAAAATCCTCTTTAGTAAGTTTTAT
TACTACTAGTTTTTATCTTCTTAGCTCTACTAGATTTTGTATGTGCTTTTAAAATGAAATGAAATGACATTC
ATTGAGAGGCGTAGCCAGGATTTTTAGTTTATGTGTTCTAGGTCATAATTGTGTTTGTTTACTGAGTTCTG

GATAGATTAGTCGTACTTATTAGATGAATTTTTTAATGCAATAACAAGGTTTGAGCCAAAGTTACCAGGA
TCTGCAGAACTAGTAGACACCTGTGGCTCCGCCCTTGTCTTCACTATCCAAAGATTAAAACAAATTGAAC
TGCAGATGGATGCTCTTGTCAGTCTTTTTAGGAGTGAGATACTGGAAATAGCCTTGGGATAAGCATTTTC
ATTTTGCTATTGGGTCCAGAAATTTGTTCTTTCAGTTACGTTGTCAGTAATCTAGTTCAATGATCTCTGTGT
CAGTCCGTGAAACTACTAGAGACCGTCATATTGTTCTGTATCCGTGTGTTTTACTTGCTTAACAAACTCTA
AGCTCAAATGTTCCTTGAACTCCAGTGATGGACCATAATGTTAAAATTAAATTTTCTCTTGACATCCAGCA
GCTAACTTGTGGCTGTTCTTTCTGTCGTCTTTCTTCTTTTTTTGATAACTTTGGTGTTTGGGCCAGCATGTG
TGCACCTCGACTATTTCATGGAATACTTTTATCCTCCTATCAATACATGTATCATGTAAATCTGTTCACCAA
GGGTCAGACAGATGAAAAGAAACTACATAGCTTGTTTTTTTGCCTTTGCTAGGATTCGTACCCCACCCAT
TGTGTTCATTCCAGCTTCATCAACCTTAGGCGGCCGCAGTATGTTATTGAAACTTCTTCTGTCGGAAACAG
CCTCCCATTCCCCACTAAGGTACATCATACACTCCCTAGACACCACGCGTGGGATTACACTGGGTATGTT
GTAGTAGTATTTGTAGATATTGAAACTCCTTGTACTTCTCAAGAATTCTGTAACCCTGAAACATCATTCGA
CGACATTTGAGGACCTTAACTTCCTCGTGTAAAACTTATCAGTAGAAGCAATTTGTGATTCCAAGCCCGA
ATATAATATAATTCTTCAAGCAAGAAATTTTTCTCATAGTGGTCTTAGTAAAACCAGACTCTTTACTTTAT
GAATCTTTTTAACCTTTCCGAGTTTTGAGCAAGAAGAAAGATGTTTAGTAGTTTTAGAAGCTAGTACTGA
CTCTTATATTCTTGTATTAGAATCTGCTAACAGAGATGCACTCAGCTGGAAGCAACGGCATGACATAATT
GTTGGCATTGCTAGAGGGTTGCTATATCTTCATGAAGGTTCACATACTTGCATTATCCACCGTGACATAA
AAGCTAGCAACATCCTACTTGATGACAAATGGGTGCCCAAGATTGCTGATTTTGGAATGGCTAGACTCTA
CCCTGAAGATAAAACACATATTAATACCCGTGTAGCTGGTACCAAGTATGATTCTATCGAACCTTATAAA
CTGTGTTTAGTAATGACTATGTAGAAATTTCATTGAATAATATATTGCACAAGTACACTCACTTGATATTG
CTAACAACTCTCTTTGATAATTTAACTTGGAAGCCAGTGGCTATATGGCGCCAGAGTACGTTATGTATGG
ACATCTATCTGTTAAGGCTGATGTATTCAGTTTCGGAGTTGTAGTTCTGGAGCTCATAAGTGGCCAGAAG
AATTCAACCTTTAACAGAGATCCTGATTCTCGAAGCCTTCTTGAATGGGTAAGAACTTGATTTCTCTGCGC
TTTTCAGTCTTGATATTCCTTGAAATTTCTGAATTACAAAAATAAGCACCGGTCAATGCCTTCGTTCCTGTT
GAGTATAATAGGAGAGTCCTCGGAATCAATTCGAGCTTCCTATAATCATGTAACAGAAAAAACAATATA
GCCATATGGTAGAATTTTTTAACTACTTGAAAAGTAAGATCGAACTACTTTTGTCTATGTTATAGTGCAAA
ACATAATCAAATCGGTAGGTGGAGGGAGAAGGACTTTACATTTAAGGTATAGGGATACTCTTGATGGTG
TGAGGCCTTTTGGAGAAAACTTTTGTGGCTGTGACCCTAAGTGGATAGTAGCACACCATGTAAAGGTAT
TTTCTAGGCCGACTTAGCCTATAAGCAGATAGTAGAGCCAATTATTTGACGGGTTGAGTATGGAGATGA

AGGCATTGAGTGAGTTTGCTGCTAGTCGTGGTAAATGCACAAGAAAAACACTATCAGTCTTGGTGATTG
ACCACACGGTACTAGGTCCGGGGAGTGTGTCGTTCTCGGGTGAATCGTGATTCGAGAGAATATTTGTGG
AGATGCGAACAAAGTCTCATATTGGTAGTCGAAGTAGAAAAATAGCTATATATAAGGTGTTGGGATGGT
CTTACGGTGTGTGGGGTAAAAACGTGCATGCTTGGCCCGAAACAACAGTATTAAACCATGTTTAAAGTG
TCTTTGAGCGGGGTTAGCCTAACTTTGTACCAGATGGTGATCATGATTAAATGTTGTTATGGTTGTAGGC
GTACAGGCTTTACAAGAAGGGTCGGAGCTGGGAGATTATGGACCCTGCACTGGCACAATCGGCTATCCC
CGATGAGATAGCAATGTACATACAAATTGGCTTATTATGCATTCAAGCGGATCCACCACTACGTCCAACC
ATGCAACAGGTCGTGGTGATGCTATCAAAGAATCCTGGATCTCTTGATAGAGAACCAACTAGACCTGGA
TATCCCGGTTCAAGAATTAGATCTCACAGACCAGGTGGTGGTACGTCTCATACAGCTGGAACCTCGGGT
GCATCCAATCCTTCTACATTCAGCTCCTCGTCTATTAGCAACACTGTTAGTGCAACTGCAAGTTCATCCAC
ACCTACAAGACGGAGATCGGACCCTCATGGAAAACGTCCTGTGAGAGATTAGGCGCGTTGTTGTTGGG
AGATTTTTTCTTATTCTTTTTGTGTATACATTCAATTTTTGTAGCATGTAAATTGGTTACAAATAGAGATCA
ATGAGTATGATGAATCTTAGTGACAGTTAACATGTAAAAGAAGTGTATGCAGTTACTCTCAGGATATAA
AACTACGTGGCACAATATATAAATGTGTCCTTTAATTTGGCCTCGTTTTATAATTATACCCTCTAACTTTAG
GTGTGCACAAGTAAACAACTTAACTTTTATAAAATTGAAGAAGTAGACATGAGTTTTACGTGGCATGATA
TACATTGGACACCATGTAGGATGAATATTGTCACGTCAGACATGTGTGACTATTTGTTTAATTTTATGCAA
ATTTAAGTGTTTACTTGTGCACACACAAAGTTGGAGAACATAAATGTAAGTTGAGGTCAAATTAGAAGG
CATATTTATA

>PGSC0003DMG400030209 G-LecRK

TTTTGATATTTTCTTTCAATTCAAATTAATAAACCAAAACGCCCAATTCTTGGCCAAAAATGAAAATTCAA
CTCAGAAGGAGGCCAAAGTAGAAAAGTAAAGTCAAAGTCGACACTTCTTCTTCCCACCTTCCCCTGTTTT
CTCCACCTCCACAACAATGGCACACAACTCATAAATCCACACCAAATACCTCATTCTTCTTCACCCATTTCC
TCCAAAAATGAGACTACTATTCATACTTTTCACCATTCTTATCATCACCATAGAAGCTGCAGATATTCTTCC
TAATTCTACCCTTTCAGCTTCCAACCCAAACTCCAAATGGTCATCTCCAAACAACACCTTCTCTCTTTCCTT
CCTACAGCTCGACCCCACAAACCAGAGTTCCTATTTTGCTGCTATTTCTTACAACAATATACCCATATGGA
AAGCCGGCGCCGATACCGTTAACGGTGGAGCAGTTGATATCTCTGGTGAACTACGGTTCCTCTCTAACG
GTGACCTTCAGCTCATAAGTGGGTCTTCTGGTTCTGTTGTTTGGAGTTCAGGAACTGTAAATCGTGGTGT
TTCTTCAGCTTCTCTTGATGATAATGGAAATTTTCGTCTCAAAAACGGTACGGTATCGAACATTTGGTCTA
CTTTTGATAACCCAACTGATACTATTGTTCCTGGACAGAATTTCACTAATAATCATGTTTTGAGATCTGGG

TTGTATTCTTTTAGCCTTATGAATAATGGGAACTTGTCACTTTTGTGGAATGGTTCTATAGTGTATTATAA
TTCTGGGTTGAATTCTTCTGTAAATGTTAACTTGTCTTCGCCTAGTTTGGGAATGCAGCCTATTGGGATTC
TTTCACTTTCTGATCCTAGTCTTTTGAACCCTGTGAATGTTGTTTATAGTAGTGATTATGCTGATGAAGGG
AACATTTTAAGGTTTTTCAAGCTGGATAATGATGGAAATCTTAGGATTTATAGTTCTACACAGGGTAGTG
GGACTCAAAATGTGAGATGGGCTGCTTTAACTGATCAGTGTCAGGTTTTTGGTTATTGTGGGAATTTCG
GGGTTTGTAGTTATAATGAAACTGATCCAACTTGTGGATGTCCTTCGCAGAATTTTGAGCTTACTGATCC
GAATGATAGTCGAAAAGGGTGTAGGAGGATAGTTGAACTTAGTAATTGTCCTTCCAATGCAACTATGTT
GCAATTGGATAATGCTAAGTTCTTGACTTATCCTCCGGAATTATCTGAGCAGATTTTTTCTGCTGGTATTT
CAGCATGTAGGTTTAACTGTCTTGTTAATGGTGCTTGTGTTGCTTCGACTTCTTTAGCAGATGGCACGGG
GATGTGTTATATTAAACAGCCAAATTTTGTTAGTGGCTATCAGGCGCCGACCCTTCCTAGCACTTCATTTC
TTAAGATATGTGGGCCTGCAATGCCTAATCCTACTGCAGTTTCAGACACTGTTCAGGAGAAGAATGGAG
GGAGGGTTCCTGGCTGGGTTGTCGCAGTTGTGGTTGTGGCTAGTGTTTTGGGTTTGATCCTGTTGGAGG
GTGGTTTGTGGTGTTGGTGCTTTAGGAACAGTTCTAAATTTGCATCACTGTCATCTCAATATGCACTTCTT
GAATATGCCTCTGGTGCCCCTGTGCAGTTCACATTCAATCAGCTTCAGCGGGCAACCAAGGGGTTTAAG
GAGAAGCTTGGTGCAGGAGGATTTGGGGCTGTTTATCGGGCAGTTCTGGCTAATAGGGCTGTTGCTGC
AGTAAAGCAGCTGGAAGGAATTGAGCAAGGAGAGAAGCAGTTCAGAATGGAGGTTGCTACTATTAGTA
GCACACACCATTTGAATTTGGTGAGATTAATTGGGTTTTGTTCTGAAGGGCGCCACAGGCTACTGGTCTA
TGAGTTCATGAAAAATGGTTCTCTTGATAAGTTCCTCTTTTCAGAAGATCATTCATCGGGAAGGCTTTTGA
ACTGGGAGCAACGGTATAATATTGCTTTGGGGACGGCAAGAGGTATCACATACCTTCACGAGGAATGCC
GAGATTGTATAGTTCATTGTGACATAAAGCCTGAGAACATACTATTGGATGAGAACTACGTTGCCAAAG
TATCAGATTTTGGCCTCGCCAAGCTCATTAACCCGAAGGACCACAGACATAGAACCTTGACAAGTCATGT
GAGGGGTACTAGAGGATACTTGGCTCCCGAGTGGCTGGCCAATCTTCCTATAACTTCCAAATCTGATGT
GTACAGTTATGGTATGGTATTACTGGAAATAGTGAGTGGTAAAAGGAACTTCGAAGTCTCTGAGGAGAC
TAACCAGAAGAAGTGCTCTTTGTGGGCATATGAAGAATTTGAAAGGGGTAATATGGAGGCCATTATGG
ACAAAAAGCTTTCCAATCAGGAGATGGATATGGAACAAGTTATGCGAGCAATTCAGGTGAGCTTCTGGT
GCATTCAAGAGCAACCATCTCAAAGGCCAACGATGGGCAAGGTGGTTCAGATGTTAGAAGGCGTTTTTG
AGATTGATCGGCCACCAGCACCTAAGGCTACTGAAGGGTCCTTTGCTGGAACCAGCTTAAACACCAGTA
GCACAAGCGGTCTCTCCACCTTTGCAGCCTCGGCCCCAGCTCCCTCTTCATCATCATCATTCCAAACGGCA
GGATTCCAGTCTTCAGCTTCTGCAAAGAATGTCGATAGGCAATCATCATCACTTCTACACTCCGAAATTA

AGTGAGTCTAAACCATACACTTTAGATTCCCTACAAGAACTGATGGGAGGTTTAGTATCTCAAAGATCAT
TGTTTGACTTGTGTCTTACAATTCATATTGTATAATAGATAGGGTTTGCATATATATTGATATGTTTCATAT
TAGAAAATACCACTCACTTATTTTCCATATCCTATTATTCACCAGAATTCGACGACTCTGTCCTGTCCTGTT
TGTGGTGCTTGTCCCATTTCTTTTGATAATGAAGCTAGGGGTGTGCATCAGTCTGGTTCGATTTTATGTAT
TATTAGTTCGATTTATCGATTTTCGGGTTGGGGATGATTTGGTCAGGGGGTTGATGTGAACTAGAAACA
TGGCTATAAAATAAT

>PGSC0003DMG400030225 G-LecRK

ATGTCTAGTAAAGCTGGTCATAATGCACCTGTATTTGAGAAGTACCAGCAGAAAGCAAAGAACTTTATG
TGTTCATGTCTTGCAAAGGGTGACAGAAATACCCAGAAAACTCCTGGAGGTCTCATCTATAGGCAAAGA
TGGAACAATATGTATTTTGTTACTAGTGCTGCATTCCTTGGCACTACTTATTCTGACTATTTGGCCTCTGCT
GGTAAATACCTCAAGTGTTCTTCTGGTTCTGTTTCTCCGAATGAGCTTCTCTCATTCTCTAAGTCTCATGTA
GAAATTTTAACTTTGATAGAGTGCTACAAGCCTGTATTATGACGATTCATCATAAATTAAAGCATGGAAC
TAGCAATGTGTTCTTGGTGAACTAATCATCATGTTTGATGTTGTTCTAGGTGGACTACATTCTTGGTGACA
ACCCGAGAGCAACAAGTTACATGGTGGGATATGGAAACAATAACCCAAGACAGGTACACCACAGAGCC
TCCTCCATTGTCTCGTTTAAGGTTAATCCTAGTTTTGTTATCTTTCGGGCAGGCTATCCTACCTGTTATAGT
AGAAAGGCGAGTGATCCTAATCTCCTTACTGGTGCTCTTATTGGTGGACCTGATGCCTATGACAACTTTA
CTGATCAAAGAGATAACTATGAGCAAACTGAGCCTGCCACTTACAACAATGCTCCGTTGATCGGTGTGTT
GACAAGACTTCATGCTGGTCACAACGGTTACAATCATCTCCTCCCAGGTACATTTTAGTGAATTAACATCT
TTATATGAGTATTTTGAGGATTCATCATAAAATGACTAGTTTTAGCTAGCCATCCACCTACTACAACTTGC
TTGGGATTGAGACGTAGTAGTTGTTGTAGTACTAAATTATTCGTATAACTCATTTAGTCTCAAGTTCTAAC
CTGTAATGGAGAAAATCCACTCAATGACACATCCAGTGCGCTAAATTTTACCAGACAAGGAATTCTTACT
CTTCTAAATGGTTTTGGCAGAGTAATTTGGTCCTCCGATTCCACTAGACATGGGCAAAATCAAGTAGCAC
AACTCCTTGATTCAGGCAATCTTGTTGTTCGAGATGCAGCCAAGGATAAGCCTCAGAACTACCTGTGGCA
AAGTTTTGACAATCCAGGTGACACAACTTTACCTGGAATGAAGGTTGGGATTAATCTGAAAACTAGTTTT
CATCGTTCCCTTTCATCATGGAAGAGCACAAATGATCCTTCCATAGGTGAGTTTACCTGGACATTCGATA
CTGGTGGATTCCTGCAAACATTCATTATGAATGGTTTCATTGAACTCTACAGAGTTAGACCATGGAATGT
CCGAGTCTTTCCTAATGCACCATCTCATGACATATCTTGGAATGGTTATAACTATACATACTTATCTAATCC
TAATGAAATATTATTAATGTATGAACTGACATGTAGCTCTATCATTGCAAGGGTAGTGATGCAACTAAAT
AGATAA

>PGSC0003DMG400030553 G-LecRK

AAAAAATGTCTCCTCTCTTTGTTTTTCTCTTTGTACTCTCTTTTTTCTCTCTAAAAAATTTAGCAAAAGCTAA
CAATTTCACAAGCCAAAATAGTTCAATTATTGGTAACACAATTGGTGGAAACAAATGGATTTCAATTGCC
TCAACAATAGTTTCTTCAAGTGGTAATTTTGAAATGGGATTCTTCAAACCAGGTAACAATTCCAATTATTA
CATAGGTATATGGTATAAAAAAATAAGTAAACAAACTATAATTTGGGTTGCAAATAGAGAAACACCAAT
TTCCATATATGAAATGGATTTTGCACAATTCAAGATTGATAATGGTAATTTAGTACTCATAAATGGTACTA
AACACATAATTTGGTCCACAAATATCAATTTTACAACAAATTTCAACAATTCACAAGTTGTAGCAACACTA
TTTGATGATGGCAATCTAGTATTAAGTAATCAGGATATTACCAATTCGACGAATTATCTATGGACAAGTT
TTGATCATCCCTCTCATACGTTCATGCCTGGTTCAAAATTCGGTTACAACAAACATACAAGATTTAAACAG
GTACTTACTTCATGGAAAAACGTGAACGATCCATCACCAGGACCCTTTACACATGAAGTTTACATGGAAA
ATAAGTATATTGGACAAGGTGTTAATATGTGGAACCATAGTGTTGTTTATTGGAATAGTGGACCATGGA
CAGGTAACAATTTTACAGGGGTACCATATCAACCAAATCCTATGTTTAATTATACATATGTTAATAATGAT
GATGAGGTTTATTATATGTATAATTTCTTTAACCCTTCATTAATATCAAATTTTATTATGGATGTTAATGGA
CAAACAAAACAAGTATTATGGATGGATAGTACTAATGATTGGAATGTGTTTTACACTGATCCAAAACAA
GTTTGTGATGTTTATTCTTATTGTGGAGGTTTTGGTATTTGTAATGAGGTAAACTCTACTTCAACATGTGA
TTGTTTGAATGGTTTTAAGCCTAAGTTTGAGAAAGATTGGAAATTGAGTAGTTTTTCAAGTGGATGTATG
AGGAAAACAAGTTTGAATTGTGGTGATTTTGGTGAAAAGGATAGGTTTTGGATGTATAAGAATATGAGA
TTACCTACAAATAATGAGAGTTTGGGAGTTGGAAATGAAGTTGAGTGTGAAAGTGGTTGTTTGGAGGAT
TGTGATTGTGTTGGTTATGCTTATGGTAGTGGAAATATTGGGTGTTTGGTATGGAAAAGGGAGATGTTG
AATTTGCAACAACTTTCTCAAGATAATGTTAATGGAAGCACAATTTATGTTAGGCTTGCAGCTTCTGAGTT
TTCAAGTAACCAAGGTAATGACTTGCAATTTCTCCTATTTTTTTGAATTTTAGATGCATGATGTACTTTATT
TTGTTTTTTTGGTTCTCGTTAGGTGTCTGATATCTGTTTTGGGATCTGACTAAGATGTATTCGCACCACTTT
TAGGGATGGGGTGGGGGAAGGGGAAGCTTTTCCTACCGAAAACGACTTCAATCTCAAAACTTGAACTA
GAGACTAGAGACATCTAGTTGAGGATAGATGAATACTTAACAGTTACCACTCCACCACAACCTTTAGTAA
TACATATCTACAGTTACCATTCCACCACAACCTTTATACATATATATATACTTCAAATTGTTACCTTTGTAG
GTAGAGTGGATTTACCTAGTTTATAAAGTTTCTTTTGAGTCCAAATTTGAATTTCATTAATTACTTCTCCTC
TTTTTTTTCTCTATTATCAAGAAAATGAAAACCAAAAACACATATTTTCTTTGTTTAACGATCCAGTATCTG
AAATTTACTGGCTTGATTAATTTAGATTTACATCGGGGTAAGCCTAATTAAGGTGTTAAATCTCTTTCTAT
TAATTCATTATTGAATTCGAGACGTCTGGTTAAAAATGGAGGCCATCCCCTCACAATTAGTGACACATTA

AAACATAACATTTTTTTTCTTTCTCTCTTTTGTTATTTGTCTATTTATGAACTTGTCATTAATCTTTTCATTTT
ACTTGGTAATAATGTGTTCTTTGCTCTCTTCATTAACAGATCAAAAGCAAACTTCAACAAAGCTGAAAATT
GCAATTCCTATTGGTGTAATTGCTGCACTTCTTATTCTATCATGCTTTACCTACATATACTATAGAAAAAG
AAGAAACTCAAAAGTCAAAGGTAACAACAAATGTCTCTTTTTTTTAATGACATTTTTCTCCTTTTTATTAAT
AAAAATATACAAAATTTAGTATTATCAAGACTCTAAACTCATACATTTTTTTAATTTTTAAATATAGAGAG
TACTACAAAATTTCACAGACAGAATACAAAAGGAGAAGGACTAGAATTGATAGATATTCAAGATGATGA
TATTGAGGTTCCATTCTTTAGTTTTGAAAGTATATTAGTAGCAACAGATGACTTCTCTGAACAAAATAAGC
TTGGTCAAGGGGGATTTGGTCCTGTTTACAAGGTATTTATGCAAATATCATTTTGTAATTTATTTTTATAT
GTGGCGCACAGTTTGAAATTCGATTTGTATAGGGATAGAGGTACAAACAGGTTTGACAGAACCCAATAA
TTTTGGTTCAGACCTTATATTTTTGTTTAAAAGATCGATGAATTAGTATAAATATTTTATCCAGATCCCAAT
AAGCTCTCTTTTCAAAAATCTGAAATCTATACACTCAAAATTCTAGTTTGCGGCATGTACTACTTTTCTTAT
TAGTTGTCATGTCTTTTTTACTGCCACATTTCCCTTTTTCAAACTACTTTGAAATGCTTTTCTTGAGTCTGAG
CGTCTATCAGAAACAATTTCTCTATTTCCATGAGGTAGGAGTAAGGTCTATGTACCATCTACCCTCTCTAT
ACTCCACTTATGAAATTACACTACTGAATATGTTAGTAGTATTATATTGTTTTTGCTGTGGTGGCCGAACC
TGTGACGTGCACCTAACCAACATAATCACTTTTTAAATAAATATATTGTATCACAGTATTTATTTTTAAAA
ATAAATAATTTTTTTGTAGGGAATATTTTCTGGAGGAAGAGAAATTGCATTAAAAAGGTTATCAAGTCAT
TCTGGACAAGGCATAAATGAATTCAAGAATGAAGTAATGTTGATTGCAAGGCTTCAACATAGAAATCTT
GTTAGACTTTTGGGTTATTGCATTCAATCAAGTGAAAAGATTTTACTCTATGAGTATATGGCAAACAAAA
GTTTAGACACCTTCATATTTGGTTAGTATTTTTATTTTTTAACTAATATCTTTATACTGTCGATGTATATAA
GTTAAATTTATTTGATTGTTTTTCTTGCATAAGATCGAAAACGAAGCGTATTATTGGATTGGAGAAAAAG
ATTTGAGATTATAGAGGGAATTGCTAAAGGACTTCTTTATCTACATCATGATTCAAGATTAAGGATCATT
CATAGAGATCTCAAAACAAGTAACATATTATTAGATGAAGAATTGAATCCAAAAATATCAGATTTTGGAT
TAGCAAGAGTTGTTGAAGGGAAAATTACACAAGCAAATACAAATAAAGTGGTTGGAACCTAGTAAGTA
CTCATAACTCCCTATTGTGATGTGACCCTTCTCCAGAATCTGCTAATGTAGGATGTTTTGTGCATCCGAGT
TCAGACTGTCCTTTTTAAAATAATTTGCCAATTGTGTTGATTTTCTTGTTCATTAATATGTTTTTGTTGATAC
TATTAATGCAGTGGTTATATGGCTCCGGAGTATGCAATAGACGGATTATTCTCAATTAAATCAGATGTAT
TTAGTTTTGGAATAGTTATACTCGAGATCATCAGTGGAAGAAGAAATACTGGATTTTTTAATCAAGAAGA
AGCGTCAAATCTCTTAGGCCTTGTGAGTGAATAATTACTTAAATTCTTGCTTTTTTTTTGCAACACTGTCAC
GGAGTTTCAAACTTCATAGGTTAAACTCCAGTACATTGCCATGGAATTTCACCTATGAAATTTGAATCGA

TCTCCATGACATTTATTTTTCAATTACATGACTGAAAAGTGACTAGTCCGCGCTATTTATTACAAAACTTTA
TGTTAACATAAATATGTGAACTATAATTTAGGCATGGAGATTGTGGACAGAAGATAAGTCAATGGACTT
AGTGGATCAATCACTACATAAATCATGCAACAAAGAGGAAGCAATTAAATTGATAAATATTGGGCTTTTA
TGTGTACAAGAAGATCCAAAAGATAGGCCCAATACATCAAATATAATAATGATGCTTGGTAGTGAAAAT
TCCAATATTATTTCACTTCCAAGACCAAATCAACCAGCTTTTATGACAAGAAAATGTGGTAATAATGACAC
ATCTTCTTCTTCTAATGCTAAGTCTGATGGTGTCTCCAACAATCAGCTTACAGTCACCATTGAAATGGGTC
GATAACGCACTACTAGAAATCAGTCAATTTTTCATCATGAACATGTTCATTGAAAAATCTATCGAAAATAT
TTCTGATAGAACGTCTGTTGAAAATTGACTGATTTAAGACTGAGATGCCAACAATTTTGACATAGCAATA
TTGTAGTTTTATCTTTACTTATTCAATGTATATATTCAACTTAAGTATGTACTTTAGAAACATCTTTAGTAA
TTAAATTATGTATTTTT
>PGSC0003DMG400031860 G-LecRK
CTTTGATAGTACAAACAATTTCTGGTTAGACCAAGAAATATAGTGAGCCATTGTGTATATGAAATCATTT
GTTACTTCCTATTGTCTAAACCAAGGCCTTCTGATTTCATACAAGATGAGAATATCTCTTAATCTTCTTGCA
TTCCTCCTCCTAGTCCTTCATCATCAGTCAAATGGCCAAAATAATCCAAATATTAGCATAGGTTCTACTTTC
ACTGCTGGAGAAAATGTTTCTTTGTTATCCTTGTCTGGGGACTTTGCCTTCGGATTTTATCCTGTTTCAGA
TACTCTTTTCCTAGTAGGAATTTGGTTCAACAAAATCCAGGAAACAACACTTGTCTGGTCAGCAAATCGC
GACAATCCAGCTGAAATCAGATCAACTATTAGTCTAACAAATGGTGGTCAGCTTGTGCTCAACTATGCTA
ATGGCACAGTTCAACAGATTTACAATGAGTCTGCAAGGTTGGGAACCATGCAAGATGATGGAAATCTTG
TGTTGAGAGACTCAAGTTCAAATAAAGTTTGGGAAAGCTTTAAATCTCCCACTGATACACTTTTGCCAGG
ACAGACTTTATCATCAGCAGGAAAGCTCTATTCTAATTCTATTTCAGATGGGAATTCCAAGTACTCTAAA
GGGAATTTCATGCTAGAAATGCAACTTGATGGAAAATTGGTACTATCTGCTTATCATTTTGGGGATACAG
GATATTGGCTGAGTAGTTTAGATGATGAAGGAAACCGTGAAGTGAATTTAGTTTTCGACCAGAGGAATG
CATCTTTGTACCTTGTGAAAGATAACAATAACACAATTTTTTCTTTTCCATTGAATGTGCCAACCCCAGTT
GAAGACTACTACCACCGTGCAACTATAGACAGTTTTGGCAACTTCCAGCAGTATGCTTATCATAAGACTA
ATGGTAGCAATTGGATTAGAGTTTTGAAGATCCCTTCAGAGCCATGTATGGTTAATGCTGTTTGTGGGGC
TTATGGATTCTGCAGCTCAAATGATAATGAAACTGTAAATTGTGACTGTCTTCCGGGGTATGTCTTCTTG
GATCCAAGTAATCCTACCAAAGGTTGTCATCCAGAGACAATGATCAACTTCTGTGCAGGTCATCTATCTA
GCGGAAATTTCACAATAGAGGCTATTCAGAATAGTGACATGCCTTACAATGAGGTTGGGGATTATGAAA
TTTATCGTGATGTTGACGAGGAGGAATGCAAGAAAGTTATCATGGAAGATTGTTATGCCATGGCTGCTT

CTCTAATCAATAGGGAATGTCACAAGAGGCGTACTCCTATACTCAACGCCCGTAGAATTTCTATGACCAC

AGGTAGTGTTTCCTTCATCAAGGTGCCTATTAAGTCAGTTAAAGATGACATATCAACAAAAAAGAAATCC

AACACCAGAGCCCATCTCACTGCTGGCCTTATAACTACCAGTTCACTTACTTTCCTATTTGGAGCCTTGGC

TTTTTACTATCATCCTGCTCCTTGGAGACTTTTAAAACGAGAGTGGAATCCAAATTCATCAAGAATAGGT

ATCAATTTCCGCGAGTTCACATACAAAGAACTGCATGAAATTACAAATGGCTTCAGCAAGCAACTTGGAA

AAGGAGCTTCTGCAAAAGTTTACTATGGTAATCTAAAGTTAAAGGATGTTCAAGTAGAGATTGCAGTGA

AGCTGATGAAAGACGTTGCAGAGCCAAGTGAGAACGTATTCATGACAGAGCTAAAAATCATAGGCAGA

ACTCATCACAAGAATTTGGTAAAACTATTAGGATTTTGCATTGAGGATAATCATTTCATTTTGGTTTACGA

GTTGATGAAGAACGGAGCTTTATCAGATTTTTTGTTCAAGGAAGAAATACTGCCTACTTGGAGTCACAG

AACTGAAATGGCATCAGGAATTGCAAGAGGACTGCTTTACTTACATGAAGAATGTGATTCTCCAATCATC

CACTGTGACATAAAGCCTCAAAATGTACTTCTTGACAGCAAATACAATGCCAAGATTTCAGATTTTGGGC

TATCGAAACTCTTGAAGAAGGATCAAACAAGAACAGATACTTGTGCAAGAGGGACAGTGGGATATTTA

GCACCAGAATGGCTAAAGAATGCTCCAATAACACCCAAAGTTGACATTTTTAGTTTTGGTGTAATGTTAC

TAGAGATCACATGTGGTAGAAGGCATATTGAGCTTAGCAGGGTAGAGGAAGAGAGCGAAGACGATGA

AGGTGATGATTTATTGCATGTAAATTGGGTTTCAGGCTGTGTGAGAAGTGGTAGAATTGACAAACTTGC

AAGGAGTGATCCTGAAATATTGAATGATATTAAAAGACTTGAACGATTCGTCAAGGCGGGATTGTGGTG

TGTCCATCCTGATCCAACTGTTAGACCTTCAATGAAAATAGTGATGCAAATGCTTGAAGGCATAACTGAG

GTTGGAGTTCCTCCAATGTTTTATCACTAAATGATTGTTTAAATCATCAACAGGGAGGAGCATTGGAACA

CGACGAAAAACTGAAAGGAAAAGGCCTCCTTGGCATTGCAAACTGGTAGTGAAAAATCCTGGAGTACTT

GTGATATTGTAAAATTAGTCTCTGTCTCAGAATTTGATTGTTAAGTTTTTTCTTTTTTGTTTGAAGTCCTGA

ATTTAAATTATTACTTTTGCTAGTTTATAATCTGTGCTTCAGTTTCTCCCTTGTCA

>PGSC0003DMG400031861 G-LecRK

GAAAAGAATTCCACACATTCCAGTGACCTCATAATCATAGCCTTCTGCATTATATTATGCAAGTCTTTGAA

TCATGGCCTTTTGACTAAAAGATTTTAATTCTCCATTTGTATCTAAGTTATAATATCTCAAAAAGAATAAA

GTATTTTCCATAAGATTCCAACAATGGTTTCTTGGAAAATCCTGTTATGGTTTGTCTCAATTTATCTTGCAT

TTATTAGCTCTTTCTGTCAAGAAACTACAAATATTGTCCCTCTTGGTTCCAATATCACAGCTGGAACTGAA

TCTTCACACTGGCTTTCATCCTCTGGAGATTTTGCTTTTGGTTTCTACCATCTTAACAGTACTGGACTATTC

CTTGTTGGAATATGGTTTGATAAAATCCCCGATAACACTCTCGTTTGGTCAGCCAATCGTGATGATCCAG

TTCATGCTGGATCAAAGGTTAATCTAACGTTGAGTGGACGCCTCGTGTTGAAAGATACTAATGGGAGAG

AGTTTGTTTTGTACAATGGCACAGGCACTAGTCATGATACTATGCAAGATGATGGAAATTTTGTCCTGAA
AAATTCATCCTCAGGTGTCTTGTGGGAAAGCTTTGATTTCCCAACAGATACAATTTTGCCTGGTCAATATC
TTGATATGGGGCAGGCGCTGTTTTCCAGTGCCAATGGGACAGTAGATTACTCGACGGGGAAGTATAGA
CTACTGATGCAGACTGATGGGAATGTGGTCTTGTCTGCATATAGAACTGCTGATATTGGCTACTGGAATT
CTATAACTGAAAACAATAAGAATGTAAGACTGGTCTTCGACAACACCAGTGATACTTTGCTCATTACAAA
TGGAAGCTCTATCATATCCAACATGACATTGACAGAGAAGTTGCCTGATTCAGTTCGAGATTATTACCAC
AGAGCTATGATCACTGATAAAGGAGATTTCCAACAGCTCTTCCACAGAAAAGTAAATGGGAGCGGTTGG
AATGTTGCACTGCAAGTTATCGTGAAGCCTTGTATTGTGAATAACATTTGTGGTGTATATGGCTTTTGCC
AATCACCAGACAATAAGGAAGCCAATTGTAGTTGCTTGCCAGGCTATTCTCCAAGGGATCAGTACACTCC
ATCTAAAGGATGTTATCCGAATGAGATGAAAGATTTTTGTGATCCGAATTCTTCCCTTTCGGATGTCTATG
TTGAAAGGATCTCTAATGCTGATTTCCCTAATAGGATGTATGCTGAACTAGAAAGGGTATTTGATACTAC
AGAGGAGCTTTGCAGGCAGGAAGTGTTGAATGATTGTCTTTGTGAGGCAGCTGTGTTTAATGATTCAAC
ATGTTTCAAGAAGAGGATGCCTATACAGAATGCCAGAAGTATTAATCCTGATACAAATAACATGGTTGC
ATTTCTTAAAATTAGCAACTCTTCGATAACTCAAAACAAAAAGCACTCTATTTCAAAGGGTGTTTTGGTAG
CAGGTGTGACAGTATGTTCAGTACTCGCTCCGCTTTTTGCAGCAATTGCTATATATTATCACCCTTTTGTG
AAGAAACATAGAGATGCCAAAACCCCTCCAAAAAGAAAGGCAATTGAGATGAATTTAAGGGCATTTTTG
TTCCAGCAGCTGCACGAAGCCACCAACGGTTTCAAGAATAAATTAGGCCAAGGAGCTTCTGGTGCTGTT
TATAGCGGAATTCTCAAGTTAGAGGATGAAGAAGTTGAAGTTGCTGTGAAGAAATTAGGAAATGGAAT
TGAACAAGGTGATGATAAAGAATTTTTGGCAGAAGTAAGGGTGATAGGCCTAACACATCACAAAAATCT
GGTTCATTTGATAGGCTTCTGCAATGAAAAAAGCAACAGGCTTGTTGTTTATGAATTAATGAAAAATGG
GGCCGTGTCAAATATTGTCTTTCGCGATGGACAAAAACCAAGCTGGAAGCTAAGAAGTGACATAGTACT
TGACATTGCCAGAGGCCTGTTATACTTGCATGAAGAGTGTGAGAACCAGATCATTCATTGCGACATTAA
GCCACAGAATATCCTTCTTGACAAGAACTACTCTGCAAAAATTGCTGATTTTGGTCTTGCAAAGCTTCTTA
TGAAAGACCAGACGCGAACAAACACGAATTTTAGAGGAACCATGGGTTATATGGCACCAGAATGGCTA
AAAAATGTGCCCGTGACAACCAAAGTTGACATCTACAGTTTTGGTGTTTTGCTGCTTGAGATTATTTTCTG
CCAAAGGCACATGGACTTGAACCCAATCGGCCAAGAAAATGAAGAACCCGAGTTGATCTTAGTCGATTG
GGTTCTTCATTGTGTGAGAAACGAGAAGCTGAGAGCTGTTGCTAGTCTTGATGAAGAAATTATGTTACA
TTTTCACAATTTTGAGAGAATGACTATGGTTGGCCTATGGTGCTTGTGTCCTGAGCCGAATCTTCGACCA
TCTGCAGCAAAGCTTGTTCAGATGTTGGAAGGAACTATAGAACTAGGTGTTCCTCCTATGGTTGATACAA

CTATATGAGCCATTTTCTGTATTATCTTGTAATTTTTCCAACAATTAAGTGTCATTCTGTTTTATTAATCATT
AGACCTGGTTAAATTTCATTGTTTTTTCTCATAGTTCTCTGGTGCAAAAGTTAGTGTTT
>PGSC0003DMG400032782 G-LecRK
TCTCTCTCTCTCTCTCTCTCTCTCTCTTATCTCATATCAGCAAAAAGCCACACAAAACCTGCGTTTGCACCA
AACAAATGCAATTTTGTTACCTCTTCTGTTCATCACTCTTCTTGCATACGTGCTGTCTTCCTGTGTAGACTG
TCTTCCTCTCTGGTTGTTTCATTCAAATGAAATTTTTCAGAGCTTTGACTTGAATGAATTCTTATTGCTTCTT
CTGCTCTTGTGGGTCCTTGTTCACTTTCACGTTTTTTGATTCTTGGACCATAATTTCATACCCCATGTGAAT
GTGATAGACAATGTCATTTATGAGTTCCCTGCCTTCTTTCTTATCATAATTCTCATTCAACTTTTCCCCTTTT
GAATCTGAGGTTTTGGTGGATATTTTCCAGATTGTTCTTGTTCCAAATTGGGTAATGGAGTCTTGGATTTC
CCTTTATTTAGTAATGATCTTCCTATTTCTGCCTGAGACTTGTATAGCTAGTGTTCAGAATAAAGGCAGAC
TGAATCTGGGGTTTCAAGGCTCTCAAATGACTTGGATAGACAACGATGGATTAATTCTTGTTTCCAATAG
CTCCAAATTTGCATTTGGATTTAACCCTACTAATGATGTTACCTTGTTCCTTCTTGTTGTCATCCATGTAAG
TAGCTCTACCATCGTTTGGTCAGCTAACAGAGATTCCCCTGTTCGAAATAATGATAATTTTGTTTTTGATG
ATACTGGAAATGCCAATTTGCAAAGTGGGAAATCAACAATTTGGTCTACGAATACGGCTGATAAAGGAG
TTTCCGCCATGGAATTGAAGGATTCGGGGAATCTAATTTTGGTTGGGAAAGATGGTAGCGTTATTTGGG
AAAGCTTCACACATCCTGTTGATACTCTTCTGTCAGGCCAGAATTTCACTCAAGGAATGAAGCTTGTAAG
CACTCCTAACAACAATAATTTGAGTTATTCACTTGAATTTAAGTCTGGGGATATGGTTCTTTCTGCAAGTT
TTCAGCCTCCACAGCCTTATTGGGCTATGGGGAAAGATGATAGAAGGACTATCAATCAAGTTGGAGGAG
GTGTCACTTCAGCAATTCTTGATGGAAATGCTTGGAAAATTTATGGTGAGAAGAGAGTGTTGCTCTGGC
AGTTCATTTTCCCTGATGATAAATATCCAAATGCGACTCGGTTGGCTGTTCTTGGAGAAGATGGTTCCAT
TACTTTTAGCATTCTTCAAGATGAATCGAAGCTTGACTCGGGGACAAGAATTCCACAAGATGAATGCAG
CAGACCAGACTCTTGTGATCCATATTTCATTTGTTATAGTGGCAACAAGTGTCAGTGCCCATCAGCCCTTC
CATCTTGCAAACCTGAGACTGCTTCGTTTTGCAATAAAGATGTGGAGCTTGTAGATGCTGGGGATAGTCT
TGGTTATTTTGCACTTGGTTTTGTTTCTCCATCTGCTAAAACTGATTTAAATGGTTGCAAAGCTTCTTGCGT
TGGCAATTGCTCATGTGCTGCTATGTTCTTTGATAGCACTTCAGGGAATTGTTTTATGTTTGATCAAATAG
GAAGTTTACAAGGTTCGGTTAATGGAGCTGGTTTCAAGTCATACATTAAAGTATCAGCTAGCCAGGGGA
ATGGGGATAGCGGTGGTGGAGGTGGAGGTGGGAAGGGACGATTACCTATTGTCTTTGGTATCGTCATT
TCTTCGGCTATTGTCATTCTTGGCCTAATTTATGGAGGTATTAGATATCAGAGGAGAAAGAACAACAAGA
TGCCTGATTCTGCAAAGGGGAGTTCAGAAGAAGACAATTTCTTGGAGGGCCTATCAGGGATGCCGATTC

GTTTTAGTTACAAAGAACTTCAGAATGCAACTAATAACTTCTCAATAAAACTTGGGCAAGGAGGGTTTG

GTTCAGTTTACCAAGGGGTCCTCCCAGATGGGACTAGACTTGCTGTGAAGAAATTGGAAGGCATTGGCC

AGGGAAAGAAAGAATTTCGAGCAGAAGTTAGTATTATAGGCAGCATCCATCATCTTCATTTGGTGAGAC

TGAGAGGTTTTTGTGCTGAAGGAACACACAGGCTTCTGGCTTATGAGTACATGGGAAATGGATCTTTAG

AGAAATGGCTTTTCAAGAAGAACAAGGAATTCTTGCTAGACTGGGATACACGATTTAATATTGCACTTG

GCACAGCAAAAGGCCTAGCTTACCTCCACGAGGACTGTGATGTAAAAATTGTGCATTGCGATATCAAAC

CTGAAAATGTACTTCTTGATGATCATTTCCTCGCCAAGGTCTCAGATTTTGGCCTCGCTAAGCTGATGACC

AGAGAGCAGAGCCATGTTTTCACAACCATGAGGGGTACAAGAGGATATCTCGCACCAGAATGGATCAC

AAATTATGCCATATCAGAGAAAAGTGACGTTTTTAGTTATGGAATGGTTTTGCTTGAGATAATTGGAGGT

AGGAAGAATTATGATCCTTCACAAAGTTCAGAGAAATCCCATTTTCCCTCATACGCCTTTAGGATGATGG

AAGAGGGGAAATTGGAAGACCTTATCGATAGGAACTTGAAAGTTGAAGAAGAGGATGAAAGGGTCTCC

ATAGCAATTAAAGTTGCTCTGTGGTGTATACAGGATGACATGTCTCTCAGGCCATCCATGGCTAAGGTTG

TGCAAATGCTGGAAGGGATCTCCCATGTCCCTTCACCGCCAACTGCTTCTCAAATGGGGTCACGACTTTT

TTCAAGTTATTTGAAATCACTAAGTGGCGAGGGTACTTCATCCGGCACCTCTGCACCATCAGACTGCAAC

AGCGATGCTTATCTTTCGGCTGTTCGGCTCTCAGGTCCAAGATAGCATTTGCAAGAAGCATAAACATAGT

CGTTCATGTAAGGGCTGTTGTAGATGATTGACAAACATGTGCAATGGTTTCATACCTTTTAGTTTTTCTGT

ATATTAAAATTATCTAGGAAATTTTTTTGCCATATTAGCCATGACAAAGGTTGGAGTTGATTGAGAATGT

TTTATTTGTATATATATCAATGCTATAAATGAGTTGAAAATAAACAAAGAAAATGCATTTGGTAACACTC

ATCCTGGATTTCAAGTACAGCATGTGTG

>PGSC0003DMG400034859 G-LecRK

ATGTACCTGGGATTATGGTACAAAGATGTAGGTCCCGGTACCGTTGTATGGGTTGGCAATAGAAGAAAT

CCAGTTAATTGTGGTGCATTGCTAGGACTAGACACAGAAGGAGACATTTTCCTCCAAGATATACTGAGA

ACAACAGTATGGATTCATAAGCCCAACAGTACAGTTCCCTTACTAGTACTGAAGCTCCTTGATTCAGGTA

ATCTTGTGTTTAGAGATTCTAGCAATCTGACGGCTGGAGAGTACCTGTGGCAAAGCTTTGACCACCCAGT

TGACACATTGTTACCAAGAATGAAGCTTGGCTGGGACAAGAAAACTGGCATCGACCGATCCATAAGGTC

CTGGAGGACAGAAGGTGATCCAGCTCCAGGTGATTATTTGTTCAGGTTAGACTCAGGTGACTCAGGTCA

GTTACCCCAACTAGTTCTTGAGAAAAACCAGCGAATACATTCAAGATGGGGTCCTTGGGATGGACAGAA

ATTCAGTGGCGGTTATGCCCTCATGGATAACCATGCATTCAGACCAATATTTCATTCCGATACAGATGCG

TTTTTTTTTCACATTCGAAGCCAAGAACGATTGGAGTTTGAAATTTTCACTGAATCCAGACGGAAAAATA

CAGTTCTTGAGGTGGAATAATGCCTACCAATTACTGGGAGGAAGTGAATACACTGAATAAGGACACATG
TGACCAGTATAGAACTTGTGGGCCTTATGGAGTTTGTAGTGATGGAGATCTCCCCTGCGGGTGTCCTGA
TGGTTTCACAGCTGCATCACCAGATGAATGCGGCATGATGAATTTTACGGAGGGATGTAGGAGAAACAC
CTCACTTAACTACACCAATAAAGACGTCTTTGTGGAGAATACTGGACTGAAGTTGCCTGATAATGCTACT
TACTGGGGAATGTTGTACCACCAGGAGTGTGAACAGAAGTGCTTAAATGAAAGATCGTGTATGGCTTAT
ACTAACATCAATATCAACGGAAATGGAAGCAAATGTGTCGTTTGGTTGGGAAATTTACTTGATATAAGA
CATTCACAGATAGCTGGGAACAACATTTTTATACGGATGGCACCTGAAAAACTAGGTAAAAATGATTGC
TTGCTTTAACTGATTTAATTCCTAGATTATATTATTAATATGGTGCAAAACAATGTGTGCAAGAATGACAT
TTGAAAGGTGAAGTAGTTAATTGTTGTGTAGGTTTGATTATTTACACAGAAACATCAATTAATAGAATTC
CCATATTAGTGCTTGATTAAGTGGTTCATCGCAAATTGATGTTTGAAGCATTAGATTGAATGTTGTCACAT
AATTGCTACACAATCGAGCATAAGATGAAACAACCCTGCATGCATCTAGTTATTCTTATTACAAAATCTTT
AACATAATGAGCAAATACCTCTAGAATTCCTATAAAGTTTATGTCAAATCTGAAGCCTAATTTTATGAGTA
AGAAACAACTAACACTTTTAACTTTCTTATGGACAGATGCACCTGTTTCCGACAGCAGATGCAAAAATAA
ATGGACAGTTTTTGCTTCTTTAATCTCAGAGCTGGCACTACTTATTATTTTGCTTATAGCTGTTGTGGTTCT
ATGTCAAAAAATGAGGACAGAGGTGCTAGGTAAAAAGAATCACCTTGCGCTGCTATTATATTATTTTCAT
AGTTCTTCATTCCAGACTAAGCTTAGTTTCACTAGAACACTATTATTTAGCAAACACAAGACCTAAGTAAG
AAAGCTAGCGTAATACTCATATTAAGGTATGATTCACATCCATTATTTTCTAATTAATTTGCTTTCTCATAG
CAGATGAACAAGAAATCAATCAGCCGACAAATTCTCAAGGAGCTTTGCTGCAAGGCACTAATGTCATTG
CATATGATTCCAGTGATCTAGTTGCAGCCACTAACAATTTTTCGCTGGCTAATAAGATAGGGCATGGTGG
CTTTGGCAATGTTTATAAGGTTATTATTTCTTTTCGACAAGCATGAAAATGCATATTTACAGTAACTCAGA
CAACAAAATTAATACCTAAATGATACATCTGTAGGGTGTACTAGAGAATGGAGTAGAAATAGCAGTGAA
GAAGCAGGAAGTGACTTCATGTCAAGGATTTACTGAGTTCGAGAATGAAGTCAAGTTAATAGCAAAGCT
ACAGCATCGTAACCTAACCAAATTGTTGGGATACTGCATTAATGGAACAGAAAAATTCCTAGTCTATGAG
TTCATGGCGAATAATAGCCTAGACAAGGTCATATTTGGTATGACATCTTAAATAACATTCTTCTTTTTTAC
TATTGTCTGTGTTGTTTTGATATTGGAAAAGCCACAAAAGACAGAAATTTAGGAGTATCCGAGTGAAATT
GAGTAACTGTTTTCTGTACATGTCTAGATCCTGCAAGAAGGGGTACAGTAACATGGCCAATGCGCTTTAA
CATTATCAAAGGAATCGCAAAAGGATTAGTTTACATGCATCATGACTCAAGGTTGACAATAATCCACCGG
GACTTAAAAGCTAGCAATGTCTTGCTAGACAGAGAGATGACACCCAAGATATCTGACTTAGGGCTGTCA
AGGGTATTCGAAGATGATGTTGAAGAAAAGACTCAGCATTTAATTGGAACACGGTTAGTATATTATCAT

TAAGACTACATGTTTATGTATATTTTAGAGTTTTTACAGATGGTTTTATGTTGTGGTTGTAGTGGTAATTA
TATGCCTCCTGAGTACATGAAAAATGGACATTATTCAACAAAACAGACGTTTTTAGTTTTGGAATCCTGG
CATTGGAGATGTGAGTGGCCAAAAGAATTATGATTATCGGCACCCAGTCTATGATATTGGTCTAGTAGA
CTATGTAAGTACATCATCGATCCCCTGGTTATCGTCATTACTTCACTTAAATTGGTTACATAGAAGTTTCA
CCTTGTGCTAAAACAATTTACATGATTGATGATCGATCTATTGTTGGTCAGGCTTGGAGAATTTGGAGTA
AGGGGAATGCCATAGAACTACTGGATCCAATGATTGAAAAACCAGGTGATGAGAATGAAGTTTTAGGA
TGCATCCTTGTTGGGCTCTTGTGTTGTCAACGCCGTGCCCAAGACAGAACTTCAATGATTCAGGTGGTTT
CCTTACTGGAGGAGAATGAAATGTCAAGGTTAAATTTTGTGCCTCAGGAACCTTAA
>PGSC0003DMG400035841 G-LecRK
GGAATTCTTACTCTTCTAAATGGTTCTGGCAGAGTAATTTGGTCCTCCGATTCCACTAGACATGGGCAAA
ATCCAGTAGCACAACTCCTGGATTCAGGCATTCTTGTTGTTCGAGATGCAGCCGAGGATAAGACTCAGA
ACTACATGTGGCAAAGTTTTGACAATCCAGGTGACACAACTTTACCTGGAACGAAGGTTGGGATTAATC
TAAAAACTAGTTTTCATCGTTCCATTTCGTCATGGAAGAGCACAAATGATCCTTCCAGAGGTGAGTTTAC
CTGGACATTCGATACTGGTGGATTCCTGCAAACATTCATTATGAATAGTTCCATTGAACCCTACAGAGCT
GGACCATGGAATGTCCGAGTCTTTCCTAATGCACCATCTCGTGACACATCTTGGAATGGTTATAACTATA
CATACTGA
>PGSC0003DMG400042814 G-LecRK
ATGGAGATCAAAAACAATTATTTTCTCCTCTTTTCTTTGATGTACCTATGCTTCTCTCTCAAAACCCATCTC
TCCATTGAAGCTGCTGACACCATTTCTGCAAATCAATCCCTTTCTGGAGACCAAACAATTATCTCTTCAGG
TGGAAAATTTAAACTTGGTTTCTTCCAACCAGGTAATTCTTCCAACTACTACATAGGCATGTGGTACGATA
AAGTGGTTGAACAAACTGCTGTTTGGGTTGCAAACAGGGAAAAACCAGTTCTTGATAAGAGCACTGCAG
AGTTAAAGATCTTAGATGGTAATTTAGTACTGGTGGATGAATCTCAAACTCCAATTTGGTCCACAAATAT
CAGTTCCAGCAACTCGAGTTCTGTTGTAGCAGTCCTCCGAGATGATGGTAATTTGATCTTGACAGATGGG
TCTAATTCAACACCACCACTTTGGCAAAGTTTTAATAACCCCACCAATACTTGGTTGCCTGGTTCTAAACT
TTCTTACAACAAAGCCACCAGAACAAAGCAACTTCTTACTTCATGGAAGAGTGCAGACGATCCTACACCT
GGGCTGTATTCACTTGAACTCGACCCAAATGAGAAGCAGTATATTATAAGGTTCAATAGAAGTGAGCAA
TATTGGAATACTGGACCATGGAATAATCGAATTTTCAGAGATGTGCCTGAGATGAGGACTAACTATATTT
ACAATTTTAGCTATGAGGACAATCAGAATGAAAGCTATTTTACATATTCCCTTTATGATGATTCTATCATA
TCAAGATTCATTATGGATGGCTCTGGACAGATTAAGCAACTCACATGGTTGAATACTTCGAATCAGTGGA

ATCTGTTCTGGTCTCAGCCAAGACAACAATGTGAAGTTTATGCATTTTGCGGGCCATTTGCTACCTGTCA
GGAAACCAATCCCTTCTGTAATTGTTTGGATGGTTTCAAGCATAGTTCAGAGACTGATTGGAATCAAAAT
GATTTTTCTGGAGGATGTGAGAGGCAAACGAAGTCGCAATGTGGCAATGGTAAGGGGGAGAAAGATG
ACTTTTGGATGCATCCCCAGATGAAAGTACCTGAAAATGCTCAAAATATTTCTGCTGGAAGTGCTGAAGA
ATGCAGATCTACCTGTTTGAATAATTGCACTTGCACTGCTTATACTTATGAAAGTTCATGCTCAATTTGGA
ATGGTGAACTCTTGAATATGCAGCAACTCCCACAAAATGATGGCAGGGGTGAGTTGATCTATGTCAGGG
TAGCTGCATCTGACATCCCAAAATCAAAGAGTAAAAAGGGAATTCCAATTGGTGTTTCTGTGGGGTCTG
CTGCTGCCGTACTGATCCTTCTGGGCATTCTTTTTGTTGTATTCCGGAGAAGGCGTAGGCATATTGGAAG
TGGGAAAATAGTGGAGGGTTCATTGGTTGCATTTGATTACAAAGACTTGCAACATGCGACTAAAAATTT
CTCAGAGAAGTTGGGTGGTGGAGGCTTTGGTTCTGTTTTCAAAGGGAAACTATCTGATTCATCTGTTATT
GCTGTTAAAAGGCTCGATAGCATCAGCCAGGGAGAGAAGCAATTTCGATCAGAAGTCAGCACAATCGG
GACAATCCAACATGTTAATCTTGTACGCCTTCGTGGATTTTGCTCTGAAGGTAACAAGAAACTGCTGGTT
TATGATTACATGGAAAATGGATCCTTGGATTCACATATTTTTACCGAAAAGCAGTCAGATGTTATGGATT
GGAAAACGAGGTACCAGGTGGCACTAGGGACAGCTAGAGGCTTGACTTATCTCCATGAGAAGTGCAGG
GACTGCATCATACATTGTGACATAAAGCCTGAAAACATCCTTCTTGATGCACAATTATGCCCCAAAGTAG
CAGATTTTGGCCTGGCAAAGCTTGTTGGGCGCGATTTTAGCAGAGTGCTTACTACTATGAGAGGAACAA
GAGGTTATCTTGCACCAGAATGGATATCAGGGGTGGCCATTACAGCCAAAGCCGATGTTTATAGCTACG
GCATGATGCTTTTGGAAATTGTATCAGGGAAGCGAAACTCGGAATACTCTCACGATGGGAAAGTGAAAT
TCTTTCCTAGTTGGGCTGCACGTGTAGTAGTTGATGAAGGCGATATCCTTAGCCTATTGGACAATAGGCT
GGACAGAGCTGCTGATGCTGAGGAGGTGTCAAAAATATGTAAAGTTGCATATTGGTGCATCCAAGATG
ATGAATTCCAAAGACCCTCAATGGGCCAGGTTGTTCAGATTCTTGAAGGAGTTTTGGATGTGAATTTGCC
TCCTCTCCCACGCTCTCTTCAAGTTTATGCAGACAATGAGGAGCACATCGTTTTCTTCACCGAGTCATCCT
CGAGCCAGACTAGTTCACAAGCTCAGAGCAAGACTTCAAGTACAACATCTCAATCTAAAAGCATCACAG
AGTCTACGAATTCCAGGTCTTGA

>PGSC0003DMG400042886 G-LecRK

ATGGACTTTGTTAACTCAATAACTCCTTTGAAACTAACTCAGTTTTTCATTATTTCCATCTTTTTCTCAATTT
TTTTTCAAATTAAAGTTGAATCTGAGACACCCAACCAGTTTTTCAAAGGTTTTAAAGCAAACCCAGATCCC
CAAATTTCGAATTTTCAGCCTCTTCTTACTGATTCTACTGGAAATTACTCACTGAGTTTTCTTCGAGTTGAA
GAAAATCAACTCACTCTTGCTATTATCCATGTTAAATCTTTCGAGTCGATATGGGTTGCTAATTTGACCCG

TTTAGCTAAATGGGCTGACCCGACTGAGCTCTACTTCAATGGCAGCCTTGTTCTGTCTGATTCACGTTCAG
GGGTATTTTGGTCAACTCACACTGATGGAGACCGTGTATGGCTTTCAAATACATCAAATTTGCAAGTTCA
GAAGGTTGATAATGGAGTGACGTCACCTTCTGTTTTATGGCAAAGTTTTGATTTTCCCTCAAATACCCTTG
TGGAAAATCAAAATTTCACTAGTGCCATGACTTTAGTTTCGTCAAATGGGCTTTATTCCATGAGATTGGG
CTTTGATTTTTTCGGGTTGTATTTTAAAGGTGAATCGGGTTCGGATTCGGGTCAGATCTACTGGAGGCAC
AAGGCATTAGAAGCTAAAGCTGATGTGATGGAAGATCAAGGACATATTTATGTTGTATTAAAGTCAAAT
GGATTTTTGGGAATGTACCAAAATGAGTCAGTTCCAGTTGACATTGAATCATTTAACAGCTTTCAACAAC
CCGTATCCGGCGTCCGTCGGGTACGGGTCGAACCGGATGGTAACTTAAAAGGGTATTTTTGGGACGGG
TCGAGTTGGGTATTGGACTATCAAGCGATAAAGGAGACATGTGAATTGCCTAGCCCCTGTGGGGCGTAC
GGGCTCTGTCATCCAGGCAAGAGTTGCTCTTGCCTGGATAACAGCACGGACTACAGTATTGGCGGGTGT
GCCCCGCCGAATAGTCAGGAGTCCCGCGATTTTTGCGGGGCCTCCTATGATCTTGATCACAAGAAGTAC
AAGGGGTTATCAAGAAATGGTGTTGAATTGCCTAACACTGAGTTAATGGACTATCAACATATGGTTTCTT
TTCAAGAATGTCAAAGTGCATGTGAAGGAAATTGTACATGTTGGGGTGTGGTGTACACTAACACATCTG
GATTTTGCTACATACTAAATTACCCCATACAAAGTTTAGTAGGGGTGAGGGATGAATCCAAGATGGGGT
TTTTCAAAATGAGGGAGGGTGTAGGAAAAGATAAGATGGGGGTGGGGTTTGGGGTGAGGGTAGGGTT
ATTATGTGGGGCCATATTGGTATTAGGTGGGGTCATAGGTTTAGGGTATTATAGATATATAAAAAGAAA
AAGAGAAAGAGGAGTTAGTGGTTATGTTGATGATGATGGAGTAGTGGTTGGACCATATAAAGATTTGG
GAAATGCAAGTTTTAGGTCAATTGAACTAATTGAAAGATGA
>PGSC0003DMG400044943 G-LecRK
ATGGCTTCCATGCTTTCTTTGTTTACTATCTGTCATCTTTTTATTTCCATCGCCCTGTATGTATGTTACGCTG
CTGCTACTGACTCCTTGTTTGCATATCAAATCCTCACCAACTTGAACCCTTTAGTTTCATCCAACCATAATT
TCGTAATTGGATTTTTTGGTTATTATTCATCGACTCTACCTCTATGCAAGACGTACCTGGGATTGGGGTAC
AGGAGTGTAGATTCCAGGGCTATAGTATGGGTAGGTAATGAATTGAATCCACTTGGATGTTTTACAGCG
CTAGCACTAACCAAAGAAGGATTTTATGTCAAAGATATAGTGCTGGGAACAACGGTATGGATTCATAAG
CCCAACAGAACAGTTCCGTCACCAGTTCTGAAGCTCCTTGATTCAGGTAATCTTGTGTTTGGAGATTCTA
GCAATCTGACGGCTGGAGAGTATCTGTGGCAAAGCTTTGACCACCCTCTTCACACATTGTTACCAGAAAT
GAAGCTTGGCTGGGACAAGAAAACTGGCATCGACCGATCCATGAGGTCCTGGAGGACAGAAGGTGATC
CAGCTCCAGGTGATTATTTGTTAAGGTTAGACTTAGGTGACTCAGGTCAGTTACCCCAACTAGTTCTTGA
GAAAAACCAGCGAATACAATCAAGATGGGGTCCTTGGGATGGACAGAAATTCAGTGGCGGTTATGCCC

TCATGGATAACCATGCATTCAGACCAATATTTCATTCCGATACAGATGCGATTTTTTTCACATTCGAAGCC
AAGGAGGATTCAAGTTTGATACTTTCACTGAATCCAGATGGGAAACTACAATTCTTGAGATGGAATAAT
AATGCTACCAATTTCTGGGATGAAGTGAAGACACTCAATATGGCCATATGTGACCAGTATAGCACATGT
GGGCCTTATGGAGTTTGTACCGATGGAGATTTCCCCTGTGAATGTCCTGATGGATTCACAGCTGCATCAC
CAGAAGAATGGGACAAGATGAATTTCACGGAAGGATGTAGGAGAAACACCTCACTTAACTATACAGAT
AAAAACGTGTTTGTCAAGAACACTGAACTAAAGTTGCCTGATAAAGCTACTTACTGGGGAATGTTGTAC
CCTCAGGACTGTGAACAGAAGTGCTTAAACAAAAGATCGTGTATGGCTTATACTAACATCAATATCAACG
GAAATGGAAGCAAATGTGTCGTTTGGTTGGGCGATTTACTTGATATGAGACGTTCACGGATAGCTGGGA
ACGACATTTTTATAAGGATGGCAAATGGAAAACCAGGTAAAAATGATTGCTTACTTGTGATATGATTGCT
TGCTTATGACACGACAACTAATTCAATGCCTAAATTATTTTTAGTATAGTGCAAAGTTATGTGTGCATGAA
TGACATTTGAATGGTGAAGTAGTTAATTGTTGGTGCATAAACATCAATAAATAAAATTTCCATATTAGTG
CTTGATTAAGTGATTCCTGCAGATTTGTCTTTTTTTTAAAACAAAAAGTATCTATAACTTGATTCTTACATG
AACCACTCATCCCTCATCTCTATCAGAGGGAAACAGTTCTATAACTTTTCAAAATGCATATGTATCTCCGT
TATATAGAAACGTTAGATGTGTTTCAAGCATTTTACTGAATGTAGTCACATAACTGCTACACAATCTAGC
AAAAATTGACACTAACCTGCATGCATCTAGTTGCTCTCATAGCAAAATCTTTAACTTTGTATCAACAGAAT
ATTATGAGCAAGAAAACAACTAACACCTTTTACTTTCTTATGAACAGATGCACCTGTTTCCCATGGGAAA
AGAAAATTGAATGAGGGATCGATAGCAGTGATTTCTGTATTAGTTCTGGAAGTACTTATCTCGATTTCAT
ATATTGTGTATACACATCGAGCAAAGAGTACAGAGGTGCTAGGTAAAATGAATCACCTCTCGCTGCTTAT
ATATTATTTTCGTAGTTATTTTTTTCAGCCCAACCTTAGTTTCACTCAAACACCATTCATGAGCAAAACCTC
CTTTTAACTCTTGAGACAACAAGAATGAACAGAGACATTGTTCAAAAGCATCTTGTTCTGCCAATACCCTT
TAGTTTGTAATGAAAAACAAAATTTACTTAGAGCACACATGACCCTGTAACCTAGCTCGCGGTTCGCTTC
CATTATTGTCTGATATATTTGTTTTTGAAAAAGAATATTTTCATCTGAGCAGATGAACCAGAAATCAATCA
GCCAGAAAATTCTGTAGGAGCTTTGCTGCAGGGAACAGATGTCATTGCATATGATTCCAGTGATCTAGC
CGCAGCCACTGACAATTTCTCGCTGTCTAATAAGATAGGGCATGGTGGCTTTGGCAATGTTTATAAGGTT
AGTATTTCTTTTCAACAAGCATGAGCATGCATATTTACAGTAACTCAGACAACACAATTAATACCCAAATG
ATACATAAATCTGTAGGGTGTACTAGAGAATGGAGTAGAAATAGCAGTGAAGAAGCAAGACGTGGCTT
TGCGTCAAGGAGTTGAGGAGTTCGAGAATGAAGTCAAATTAATTGCAAACCTACAACATCGTAATCTAA
CCAAGTTGTTGGGATACTGCATTCATGGAATAGAAAAATTCGTAGTCTATGAGTTCATGGCGAATAAAA
GCCTAGACAAGGTCATATTTGGTAGGTCATCTTAAATAACAACTTTGTTTTACTATTATATGTGTTAATAT

GATATTGGACAAGCCATAAGAGACAGAAATTTTAGGCCTATCGGAGTGAAATTAATAGGGTAACTGTTT
TCTCTACATGTCCAGATGCTGCAAGAAGGGCAACAGTAACATGGCCAACGCGCTTAAACATTATCAAAG
GAATCGCAAGAGGACTAGTTTACATGCATCATGACTCGAGGTTGACAGTAATCCACCGGGACCTAAAAG
CTAGCAATGTCTTGCTAGACAGCGAGATGACACCCAAGATATCTGACTTTGGGTTGGCAAGGGAATTTG
AAGACGATGTTGAAGTCAAGACTCACCGTGTAGCTGGAACATAGTAAGTAGACTAGCACTCATTATCAT
TAACACTACATGTTTATGCATATTTTACAGATAGTCTTATGTTGTGGCTGTAGTGGCTATATGTCTCCTGA
ATACATGCAAGCTGGACATTATTCAACAAAATCAGATGTGTTCAGTTTTGGAATTTTGACATTGGAGATT
GTGAGTGGCCAAAGGAATTCGATTTATCGGCACCCAACCTATGATATTGGTCTAGTAGGCTATGTAAGT
ATATCGATCATCCCCTGATTAAATATGGTCACTACTTGTACACCTAAGTTTCACCTTGTGCTAAAACAATT
TACATGCATGATTGATCTTTTGTTGGTCAGGCTTGGAAAATTTGGAATGAAGGGAATGCCATAGAACTA
CTGGATCCACTGATAGATAAACCAGATGATTTGGATGAAGTTTTAGGATGCATCCATGTTGGGATCTTGT
GTTGTCAACGGCGATCCCAAGACAGACCTTCAATGGTTCAGGTGGTTTCCTTACTGGAGGAGAATGAAA
TGTTAAAGTTTAATTGTGTGCCCCGGGAACCTTACTTCTATAAGGAGACGAGTCAGAGTAGCAGGAGTC
GCGAAAGTATGAATGGGCTAAGTATTACTGAATTCACCGGAAGATCATGA
>PGSC0003DMG400045997 G-LecRK
ATGACTTCTTTAGTTATGCTTCTTGTTGGTCTCTTATTGTTTCCCCTTTCAGCCATTGCTCAATCTTACCAAA
ATGTTAGTTTGGGTTCATCACTCACTACAAGTGATGTTACTAGCTTTTGGTCATCCCCATCTGATGAATTC
GCTTTGGGCTTCCAAAAAATTGGAAATGAATCAAGGTACTTACTAGCCATATGGTTCAACAAAATCACAG
ACAAAACCATAGTTTGGTCAGCCAATAGGAATAATCTTGCCTTAGATGGATCCAAAGTTCAGCTCTCCGC
GGATGGAAGACTAGTCCTCACTGATCCAAATGGTCAAGAGATGTGGGCTCGTGGCATGGCGAATGCTC
AATTGGCCTATGGAGCCATGCTTGATAATGGAAACTTTGTGCTAGCAACTAGTAGTTCAGACACTCTATG
GCAGAGTTTTAATGAGCCCACCGATACGATTTTGCCTGGTCAAGTACTTAACCAAGATAACAGTCTTGTT
TCAAGTTTCTCTGACACGAATGTATCGAGTGGGAGGTTTGAGTTCATACTTCAAACTGATGGAAATCTAG
TGCTTTACACAGTCAATTATCCAGCTGAGGCAACAAATGCTGCATATTGGTCACCTATGTCAGTTGGCAG
TGGTTATCAGGTGATCTTCAATCAATCAGGCTTTATTTTTCTTCAAGCAAAGAATGGAACTTTGATAAATT
CGATATCTTCTAATGTGGAAAATTCAAGAAGCCAATCAATGTATCATCGAGCGATTCTTGAATATGATGG
AGTTTTTAGGCACTATGTCCATCCGAAATCTTCTGGTAGGGAACCGATGGCATGGTCTAGTTTGTATAAC
ATTCCTGATAACATCTGTTTGTCTATCAGGCAAAGTACAGGAGGGGGTGCCTGTGGTTTCAACAGCCTAT
GCTCAATAGGAACTGATCAGAGGCCGAGATGTGATTGTCCACTCGGATATATCTTGGATGATCCGAATG

ACAAATTAGGCAGCTGCAGACAAAATTTCTCTGAGCAAAACTGTAACCATGAATCGCGAGAAGTTGAGT
CTTTCACATTCCACGAAATGTTGGATACTAACTGGCCTGACTCTGATTATGAGTCTCACCGCGATGTTTCA
GAGGATTGGTGCAGACAGAATTGCTTGAATGATTGTTTTTGTGATGTTGCCATTTACAGTGACGACAAC
GTCTGTTGGAAGAAAAGGTATCCTCTTTCAAATGGGAGAGTAGGTCCAACGATCGGAGGGAAGGCTTT
GATAAAAATAAGGAAAGACAACTCCACAGTGGGAACACCAAATGTTGAAATAAGAAAAAAGAAAAATC
AGTCTACTCTGATCATTTCTGGTTCTGTGTTGTTGGCCAGTTCAGTTTTCATGAATTTGTTCTTAATTCTTTT
GGCCCTATTGTACATTTTTAAGTTTATAGGAAAAAAACGAAAGAGGACTGCACCGTATTCAGCTGTTCCA
GGAGTGAACTTAAGAAGTTTTAGCTACAAAGAGCTAGAACAAGCTACAAATGGATTCAAGGAAGAATT
AGGAACTGGAGCTTTCTCAACAGTTTACAAAGCGGTTCTTGATGATGAAAATGGGAAAGTCGTTGCTGT
CAAAAAACTCCGAAACATGGTAACAGAAGGAGAAGGAGAAGAAGTATTTGAAGCTGAAGTGAATTCAA
TCAGCAGGACTAATCACAAGAACTTGGTCCAACTCCTTGGATTCTGCAACGAAGGCCAACACCGCCTTTT
GGTATATGAACACATGAAAACCGGCTCAATAGCACACTTGCTATTCAAAGATTCTAGGCTTAGTTGGTCC
AAAAGGGTACAAGTTGCCATCGACACTGCCAAAGGACTTTGTTATTTGCATGAAGAGTGCAGTACCCAA
ATTATACATTGTGACATCAAACCCCAAAATGTGCTTTTGGATGAAAATTTGACAGCAAAAATAGCAGATT
TTGGTATGGCCAAGCTACTGAAAAAACATCAGACTCAAACAACCACTAGGATACGTGGAACCAAAGGGT
ACGTAGCCCCAGACTGGTTCAGAAGCATGCCTGTAACTGTGAAGGTTGATGTGTACAGCTTCGGAGTCT
TGCTCTTGGAGTTGATTTGCTGCAGAAAAAACTATGAGCAGGATGTGGCAAACGAGAACGAAATGATA
CTACTGGAGTGGGCTTATGATTGCTATAAAAGAAATAAGTTGCATTTACTTGTAGGCGATGATGAAGAG
GCATTAGAAGATATCAAGAGATTCGAGAAGTTTTTGTTGGTTGCAATTTGGTGCATTCAAGAAAATCTAG
CATCAAGGCCTAACATGAAGAAAGTCATGTTAATGCTTGAAGGTTCTGTTGAAGTATCAATTCCTCCTGA
CCCTTTTTCGTTTGTCGATTCTATTTAG
>PGSC0003DMG400046324 G-LecRK
ATGGTTGTTAATTATAGTGCCACTCTTTTCATGCTCAGTCTCCTCATTCTTTCTTCACAGCATCTGATTAGT
TGTCAACCTTCTGAAACTTTTATCACCAATTCCTCCATTTCCTGGATCAATAACCGCGCCCGCTCCTATGTA
GTGAATTCCACAGATGGAGCCACACTGACACCCATCCTATCGAGAGAAGGGAATGAGGCTCTGTTTGTC
TGTGGTTTCTTATGTGATTACAATGGCACTGCTTGCGTTTTTGGCGTCCTTATATTACCAAATACCTATGA
TTCTTATCTATACTACCCGAGATTAGTATGGTCAGCAAATCGGAACAATCCAGTTAGAACCAATGCAACC
TTGCAACTTAGGCAAGATGGAGGCTTGTTGTTGATGGACTCAGATGGCACTTTGATTTGGAATACAAGC
ACAAGCGGAAAGGTTTCTGTGGGATTCAACTTGACAGTAATGGGAAATCTTGTCCTCTTTGACAAAAGT

AACGACATCATTTGGCAGTCTTTTGATCATCCTACAGACACTTTGGTTCCGGGACAAATAATGGTCCCTG
GACAAAAGCTGATATCCAGAATATCAGCAACAGACTGGAATGAAGGTATGCTCACACTTGATTTTAAAT
ATTCTGGCCTTTCTGCTTACATTGAGTCAAATCCACGCCAACTCTATGTCTTGAGACTTGGTGTGGAAAAA
CCTTTCCAGTTTCCTGGACTAGGCTTTTCTTTGAAAGGTTATGGTTCCAGAGGGATGTTAACCATAGGTTT
CGAATTTTCAAATGCTCGATTCATGAGATTAGGGGTTGATGGGCATTTAAGGGTGTATCACTGGAGAGG
ATCTTGGTCAGAGGAGGCTGATCTATTTACATACTACATTGGTGAGTGCGGGTATCCAACCATGTGTGG
AAATTACTCTGTATGTGGAAATGGGCAATGTACTTGTCCTCAAACCGCGGTTGGATTTGGTCAGACTAAC
TTTCTCCAGCAAATCAATTACAGGCAGCCAAACCAGGGGTGTGTCCTTGTCACACCTATCTCTTGTGAAC
ATTCCCAATATCATATTCTTATGGAGCTTGAGGACATAGCTTATATTCCCCTTTATTTTCAGTACGGTACCA
ACAAAAACAGATTTGGAAAATTGCAAAAGGTCCTGCTTAAGTAACTGTTCTTGCAAAGCAGCTCAATTTCA
ATTTAGTGGTCCTGGTAACACAATAGGTGACTGCGCATTGTTGAATGATGTGTTTTCGCTCACAGACAAT
TATGGAGCACCCAATAAGACCACTCTTTTCATCAAGATGCAGAACTCTTCTTACCTACAAGCCTCTCCACC
ACTTATCTCTTCAGAGAAGAAATCAAAGCGTGTCCCAACAATAGTAGGATCCACCATCGGAGCTTCATTT
GGTTTGCTTCTCATGGTTTTAACTTGCTTTGCCTATATTTTCAGAAGGAGAAAAGGACTCGAGGAAGACG
AGGAGGAGTTCCTTGATCAAATACCAGGAATGCCTACTAGATTTTCCTACGAGGAACTCACTGTGATGA
CAGAAAATTTCAGCGAGACACTTGGGGAAGGGGGATTTGGTTCTGTATTTGAAGGAACAATGAGTGAT
GGCACCAAAATAGCGGTGAAGCGTATGCAAGGATTCGGTAATGTGAAGAAATCATTCTTAGCTGAGGT
AGCAACAATCGGTAGCATTCAGCATGTCAATTTGGTAAAACTTGTTGGATTTTGTGCTGAGAAATCACAC
AGACTTCTGGTTTATGAATTCATGGCCAATGTCTCTTTAGATAGGTGGATTTTTCACGGAACAGGGGAAA
AATCCCTCACATGGGGTGTCAGGAAAAAAATCATATCAGACGTCGCCAAGGGCCTAGCTTACCTACATG
AAGATTGTAACAACAAAATTATTCATTTGGATATTAAACCTCAGAACATTCTTCTTGATCATAATCTCAAT
GCAAAAGTATCAGATTTTGGGTTATCAAAGCTTGTGGGGAAAGATGAAAGTAATATAGTTACAGCAATG
AGAGGAACACCAGGCTATTTAGCACCTGAATGGTTGCATGAGGTCATTACTGAGAAAGTGGATGTTTAT
AGCTTTGGGGTTGTGATCTTGGAAATCATCTGCGGCCGGAAGAATTTGGATCGTTATCAAGATGAGGAT
GATATGCATTTGCTTAGTTTGTTCATGAGAAAGGCAGTGGAGAGACAACTCTTGGAAATGGTGGACAAG
AAGAGTGAAGACATGCAGCTACATAGAAAAGAGGCTGTGGAAATGATGAAGATTGCAGCCTGGTGTTT
ACAAAGTGATTATACTAAGAGGCCTTCCATGTCGTTGGTAGTTAAGGTGTTGCAGGGTTTAGTCGCTGCT
GAAACCGATTTGGATTACAGCTTCACATTTCCAACAATGACGAGAAAAGTTGCCGGAGCTATTCAAGAA
AGGGAAGCTGTGGTTGGTATCAGTTTACCATTTCCATCACAGTTGTCAGGACCTAGGTAA

>PGSC0003DMG401004946 G-LecRK

ATGGGATTTTTTCCCCCGACTAGTTTCACGAATAATTGGTATGTCGGAATATGGTACAGGCACAATGTAC
CTGATAAGTCTGTGGTATGGGTTGCCAATCGAGCAAATCCATTCAACAACAAATCAGGTGTTAGGTTGA
AAATCATCGATACAGGACAACTTGCTCTTCTCACTGCTGACAATAAGAGCATATGGTCTACTAATTCGTC
AAGGAGTTTGGCTGTGAAGAATACAGTTGCACAGTTGTTGAACTCGGGGAATCTTGTCGTAAGAGATGT
AAATGATGCTAGGCCTGAGAACTTCCTGTGGCAGAGTTTTGATTATCCTACTGATACACTGTTACCAGGC
ATGAAAATGGGTAAGAACTTTGTGACTGGACAAGAATTTTACCTGTCCTCATGGAAGAAGGAATCTGAT
CCAGCTCCAGGAGAATATACATACCGTTGTGATCCTACTGGATATCCTCAGAATGTCGTGAGGAAAGGT
AAAGTTAAGGTGTTCAATACCGGACCATGGAATGGAGTCTTCTGGAGTGGTGTACCAGGAAGTATACAA
AATACTATTTCCACATCTAAGCTGGATTTTGATGAAAAGAAAGCATTTTACAGCTTCACGCTTCTGGCTTC
TGTTATGACCAAATTAACCATAAAAAGTAGCGGAGTGCTTCAAATTTCAACGTGGGTTGAGAATAGACA
GGAATGGCAAGATTATGTTTCTTCATCGGGCGATACTTGTAACAACTATGGAACATGTGGTGCATATGG
TAGTTGCAACAACGTGCACACTCCTGTGTGTAGCTGCTTAGACACGTTTGTGCCTAAAGATCCAAGAAAT
TGGGCAATGAAAAATTGGTCCAGCGGGTGTGTTAGAAGGAAACCCCTTAATTGCCAGAATGGAGATGG
GTTTCTCAAGTACTCTAGTATAAAGCTACCAGATACACAGTATTCATGGTTTGATGTGAGTATGACACTC
CACAAGTGCAAGCAAGCTTGCTTGAGGAATTGCTCGTGTATGGCTTACTCAAATCTTGATATACGCAATG
ATGGGAATGGCTGTTTGCTTTGGTATGGCGACTTAATTGACATTCGAGAATTACCTGGAGGACAGGACA
TTTACATAAGGGTTGCTACCTCAGAGTTAGGTAACATTTGTGAATTTGATCAAAGTTGGAGTATTAGCTG
ATTGATTTAAACTTCCCAATTCTCAGATTTAGTTGTATTTTACTTAGATATCTGTTAAAAGTTCACACAGTA
TATGTTCACTCTTCCATGTCTATTTAGTTTATTTCACCCCTCCATGTTTTGTTCAGGCTCGAAGAAAACAAA
ACTACTCGTGTTAAGTCTGTTACTGTTGATTGGAGTGACTGTGACTGGCCTGACCATAGGGCTGTATATT
TGGAAGAAGAAGAAGAGGAAGATGAATTTGAAAGATGACCTAGACCTCCCACTGTTTACCTTATCAACT
CTAACTAAAGCATCCTCTAATTTCTCAGTCGAGAACAAGATTGGAGAAGGTGGATTTGGATCTGTTTACA
AGGTAACATCTTAAGTTTATGAATCATTCTTTCAGTAAACAATATCTACTATTATTGACAGTAAACAGATG
ACACTTACTGAATCACTAAACCAGTTATATGTTGATATTATAGGGAATCCTGGAAGGTGGACACGAAGT
AGCTATAAAGAGGCTATCCAAGTCTTCCTCGCAAGGAGTTAACGAGTTCAAGAATGAAGTTATTTGCATT
GCCAAACTTCAGCATCGAAATCTTGTGAAGCTTATAGGTTGCTGCATTGCAGGAGGAGAAAAAATGTTG
GTCTATGAATACATGTGCAACAGAAGTCTAGATTTCTTCATATTTGGTCTGGTTACTCTCTCTTAACTCGA
AGAACCTCTATTAATACAAATAGTAAAGTTGATAGTTTAATCACATTATCAGTGTTCTAACGGAGGACAT

GTTAACAGATGAAAAGAGGAGCTTGTTACTCGACTGGCCTAAGCGCTTCGACATCATCAATGGAATTGC
TAGAGGACTTCTGTATCTCCATCAAGATTCTCGGCTAAGAATTATCCACAGAGACCTCAAAGCTAGTAAC
ATTTTGCTAGATGCTGACATGAACCCCAAGATCTCAGATTTTGGCATAGCTAGAAGCGTTGTAGGGAAT
GAGACTGGTGCTAATACACACCATGTGGTTGGGACACAGTAAGAAATTTAATACTACTCATTTTTTAGCA
TTTATATTATGTATATAATATCCTTTACTTAACACTATTTTTGTTTAATGTGTAATGCAGTGGCTACATGTC
CCCAGAGTATGTAGTCCATGGTGTTTTCTCAGTGAAATCAGATGTATTTAGCTTTGGTGTGTTAGTACTA
GAGATAATCAGTGGTCGGAGAAACAGAGGATTTGCCCGTGAAAGTCACAGTATCAACCTACTCGGACAT
GTAAGTCTTGTTTGTAATTAAAGACGAGTTTAAATTTTGTGCACTAACAGAGTAAAATAATCCTTAAACA
ACGAGGTCACTTATAAGGTAGTTAATTGATAACACCTCATAAAAATGATAGTTAAACTGCATTGAACTTA
AATCCATCAGGCACATCAACCGCTACATGTTTCTTGATTATGTACTGATTGTGTTATTTATAGGTATGGAA
ACTTTACAAAGAAGGTAGACCATTGGAGCTAATTGATGGGGATCTAATGGATTCGAGTTATATTTCTGA
ATTGCTACGCCTTATCCATGTCGCTCTGTTATGTGTACAACAATGTCCAGAAGACAGGCCAGACATGGCT
ACAGTAATCTTGATGTTGACTAATGATGCTATTTTGCCTCAAGCTAAGGAACCTGGTTTCTTCACAGAAA
GCAAAGTCACTTCTGGATGTTCTACAAGCATGAGTTCAACAAACGAAATTACCGTCACACAGTTAGAACC
CCGGTAAATTACATTACATGTTCATTCAGATCGTTCAATGTCAACAAGCTTGAATTACAGTGTTTGTAAAG
CCATAATGGCCTATGTTATATGGTATCTGTTATTAGCAAGTCTTCAATATGGGTGTGTATCAGTATTTAGT
GTGGATACCTTTAGTTTTTATTAATTCTAATGTATTTTAAAGTATTCGAACAAAGTACCTATTCAGCATGT
GTACATCAAAATAAATTAAAGATCCATATAAAATAAATCAACACAATGAGAGTTCACTACTTCACAGTAA
CAGAAGAAGCTATTGTTGTTGGTGTTTAGAAGCTCCAGTCCCATAGTACTCCATTATTTACAGAGTATCA
GTTATGTTGTTCCCATTCTCCGAGTTTACACGATCAAACATCAGGCAGTCACTGCACCAACAATTAGAAT
GATAGCAGAATGGTTTGATTAAAGTAATGAAATTGCCAAGACTAGAGTGATTGGAAGGATTGCAATTCT
AATTTCTTACTTTCTTTCTAGGAAATGTACAAAAGATATTATCACTGCAACTGACTTTATAAATGGAAACA
ACTAAAATTGAAAGACATAAAACGCATTCACTCAATGCGATTTATGAGTAAAATTCTGTTTCTTCTTTTTT
CTTGTTGAAATTACTAGATTAACCCTTCTGTTTAATTTTATTTGTACGCTATTTTAAAAATATATTTATTTAT
TTCATTTATTACTTTTATAATATCAAGAAAAGATAAATATTTTATCTTTTTAAAAAAATGTCATGTCGTTGA
AAACTTGAAAGACCCTTCTTAGATACCTAAATAAAAAGAGCTGAGAATGAAAGGCATAAAACACATTCA
CTGGATTCGATTTATGAGTAAGATTCCGTTTCTTCTTTTTTATTACTGAAATTACCATATTACCCCTTCTGTT
TAATTTTATTTGTCCACTATTTTTAAAATATATTTATTTCTTTCATTTATTACTTTTATAATATCAAGAAAAG
ATAAATATTTTATCTTTAAAAAAAATGTCATGTCGTTGAAAACTTGAAAACTCGTTCTCAGATGCCTAAAT

AGAAAGAACTGAGAATGAAAGACATAAAACGCATTCAGCGAATGCGATTTATGAGTAAAATTTTGTGTC
TTCTTATTTATTGTTGAAATTACTATATTACCTCTTCCGATTAATTTTATTTGTCCACTATTTTTAAAATATA
TTTATTTATTTCATTTATTACTTTTATAATATCAAGAAAAGATAAATATTTTATCTTTTAAAAAAAATGTCAT
GTCATTGAACACTTGAAAACCCCTTCTCAGATACCTAAATAAAAAGAGTTGAGAATGAAATGTATAAAAT
GCATTCAGCGAATGCGATTTATGAGTACAAATCTATTTTTTATTTTTTATTGTTGGAATTACTATATTATCT
CTTCCATATTTCATTTATTACTTTTATAATATCAAGAAAATATAAATATTTTTTCTTTTAAAAGAAAAGAAA
TGTTATGTCGTTGAAAACTTGAAAACCTCTTCTCCAGATACCTAAATAGAAAGAGCTGAGAATGAAAGGT
ATAAAATGAATTCACTGAATGCGATTTATGAGTAAAATTCTCTTTCTTCTTTTTTATTGTTGAACTTACTAT
ATTACCCTTCCGTTTAATTTTATTTGTAAACAATTTTTAAAATATATTTATTTATTTCATTTATTACTTTTATA
ATATCAAGAAAAGATAAAAATATTTTATCTTTAAAAAAAGACACGTTGTTGAAAACTTGAAAAACCCTTC
TCAGATACCTAAATAGAAAGAGTTGAGAATGAAAGACATAAAACGCATTCAGCGAATGCAATTTATGAA
TAAAATTATGTGTCTTCTTATTTATTGTTGAAATTACTGTGTTACCCCTTCCGTTTAATTTTATTTGTCCACT
ATTTTTAAAATATATTTATTTATTACTTTTATTTAGGTAGATGAGAAGGGTTTATTACCTAGTGCAAAAGA
TATTATCACGGCAACTGACTTTCTAGCAGATGGAAAGACCATTACTTCATCTGATGGAAGCTGTGAGATG
GCTAGTTTCACGAATAATTGGTATGTTGATGAAAAGAGAGCATTTTACAGCTTCACGCTTTTATTCCTAAC
TGTTAGATTAACAGATCCTTGGTTATGACTTATGACTATCACATTAACCAACTTGATAAATAGGAGTTTTT
GTCCACTTGAAACCACAAGTGTGAGACCCGGTCTCAATTTGAGACCCGTGCCGACCCCGACAGCCGACG
CGAGATCCGACCCTGACCTTGACCCTAACATCTGACCCAAAACCCGACCTCAACCCTAACACTCTGTTGA
CACCAAATTTTGACCCTCCTTTTTTCTAATTTATTTCGTTGAACTTCTAAATTGGTAACAAGTCGAATACTT
TATAATTCACTATTATTTTCCATTTATCACTATTTCTATCGTAATGATCTATATTACTTTAACTTTTATGATA
TTTATGTTTATCATCTTTTTATTATCATTTTCACTATTTCAAAATTATTATTTTTTATCAATGTTACTATTAGT
ACTTTATTGTCGTCTTCTTTAAAATAGATTGTCGTTATTTTATTTTAAGTTTCTTACTTACTTAACTATTTTA
CGTAATATATATTATATTAGTTATTAAATTAAAAGCTCAAATGATTTAATTTAAAGAAGGAAAAATGCCAT
GAAAGCCTATCCATTTTCAGCCCTTTTTACAATCTTAAAATATGCAACAATTTGGCTATCCAAAATGCAGC
CCAATAGGCCCATACGTTTCAGCCAAGTTCACTAAAAAACCCAAATTCGCAGCCCTAATCCAAGACTCAC
CAGCAGCAGCGCTAAGCATCCCTTTCTCCTCTCTTCCCTCTTCTAAGCAAAGTTCCAATATAGTGCAGCCA
TTGACAGTTTTCATCCTTACGTTTTGAGCTTAATTTTGTCCTTGTTGAGGCAGCCAACGAATGCTTTCTTGT
TTCATGCTTCAAAACGCAGCAGCTGCTTTTCAAATTTCAAACAAAAATCTCACTATTTCGGACGATTATAT
GCAGCAATATCACTGCCATATTTGACCAATTTCAAACGGATACTTTCCCGCTCAAGCATGCCTTAACCCTA

ATCTTCACCTATAAATACATCCTTATATATTTACAAAGAAAAAAAAAAAGGTTTTCAGTCACCTTCTTCCCCT
CTAACTTTACTAGTCTTTACAACTTTAACAAAAAGGTTTTAATCTCTCTTTGCTCCTGTTGGACGACAACAA
AGGCATTTCAAAGTTGTTCATTACGAGAGTATAGATTCAAGGCCGATAACAATCCCAGTTCACTGCCCCA
CAATCGGTAATCTCGACCGCTTTACTTTGTTATATTTTACTCTTTTATCCAGCTCGCCAGCGTGTTTGGTAT
TAGTTTCTATATGAAAAATGACAAATGATTAAAGGCTGGGCACCGAACCGGAATGGGACCATCGAACCG
GTTTGATCGGGTTGTTGATCGGTACCGGGATGAACCGGACAGGAACTACCGGGATGGAGGCTCAGTTC
CATCCCGTCCCACTATATACCGGGATAGAACCGGGATGGACCGAAATGGATCGGAACGGGATGGGATA
A
>PGSC0003DMG401005047 G-LecRK
TTAACACCAAAAAACACATTTTTCATCTATGTTCTTGCTTCTACTTTCTTCTAATTTCACAAATCCTCGCAAT
AGCAGCAACAACAACAGATAATACGATAACGATTACAAATCCCCTTACGATATCGAAAACTTTAGTCTCT
CAACAAAAAAAATTCGAATTAGGATTTTTCACTCCTGGTGGTCCGAATTCAGACAAATGGTACGTCGGA
ATATGGTACAAAGAAATCAAAGAAACGACGATAGTTTGGGTTGCGAACAGAGAAAACCCCGTCACCAA
TTCATCGTCTCCCCCTGTGTTAAAAATAACACAAGATGGACGTCTTGTTATTGATAATAGAGATGGAAAT
TATACGTTGTCATTAAATTCCGCGAATAATAATACTACATTTATCGCTAAGTTATTGGATTCAGGTAATTT
CGTCGTTCTAACAGAGAAAGACGAAATTATGGTGTGGCAGAGTTTTGATTATCCAACGGATACTCTGTTA
CCCGGGATGAAACTTGGGTGGGACTCGAAAACGGGGTTTAATCGGAATATAACGTCATGGAAATCGCC
ATTTGATCCTTCTCCGGGTAATTATACGTTTAAACTTGATGTTAATGGTTTGCCTGAAGCTTATTTGACAA
ACAGAGATACAATTTTCTATAGAAGTGGACCTTGGAATGGTGTTGGATTTAGTGGTGTACCAGAAATGA
AGCCAACAGATATTATAGTTTTCGAGTTTCAGATGAATAAGGATGAAGTTTACTACACTTTTGAGGTATG
TTCGTCTCTTCACGTATGAGTCTGATTCTTTTTTAGTAATGACAGGTAGCGTTATGTTGAGTCTCATGCTA
GTGGTGGGATCGAGGCAATCTTCACTTCATGAACAAGTGTCTATTTCTTATCATGGTATCAGAGTTAGAC
TTGTCTGTTCAATTCTTCGTTTATATGATGTTAGTGGGGTTAGGGGACTCTTTTGTCTTCTTATACGGTCTT
GAGTAATCTTCACCTTATGAGGTAGTTTTTGGGGTTGAGTTATGTCTAACGTCTATTTTTTACATGACAGA
TTTATCTATGCTCTGATACCATGTTAATTTTGTAAAAACGTGTTTTTACTTACTTAATTATGTTTTTGTTAG
GTGCTTGACAAGAAAATATGTTCGAGATTGTTAGTGAAGCATAACGGTTTTTTAGAGAGGTATACTTGG
ATTCCGACTAGTAACATTTGGAACAAATTCTGGTACGCCCCGAAGGACCAATGTGATTTATACGAAGAG
TGTGGTGTTTCGGGAATTTGTAACGCGAATTTATCGCCGGTATGCAAATGTCTAGTAGGGTACAAGCCT
AAAAATCAAGTGGCATGGGATTTAAGAGATGGATCAGATGGATGTATTAGATATCATGATTTGGATTGT

GAAACTGATGTTTTTAACATATTGAAGAACATGAAATTGCCACAAAGTTCAAGTTCATTTGTTGATACTA
AGATGAATTTGGAAGAATGTGAAAAAATGTGTAGGTACAACTGTTCATGCACGGCGTACACCACCGCAA
ACGTTACGGGTTCGGGTTCGGGTTGTGTCATTTGGACAAAGGAATTAGTCGATATGCGCCAATATTCGG
CAGCAGAAGGTGGACAATTTCTCTACGTTAGAGCTGCTTCTTCTGACGCAGGTACATTTTTCATTTCAATC
TGATGTTCGATATCTGTTTGAATTGTTATAGAAAATACGTTTTTTGTGGTAAAATAGTACTCCATTTCAAA
ATAATGAAAAGGTAATTTAATTTGCTTCTATAAAACTTGAATCTGAAACCTGTGGTTATTATGAAAAGAG
TGCTTAATATCCTAGCGCTATTCTTAATTTAGTAGGAGAGAGTTGAGTCGAACTCAATTTAATGGAGTGG
TTGAATGTTGGTAGAGATATATATGAAATTTTAATGTATTTGTTGGGTTGAATAATAATAATAATAATTG
GTCTCATTTCATGAAGTTTGAACTACATATTTTTAAATGATAAAAAAATGGTCGTCTAGGCAATTTTTTAC
CCCTTTATTTTAGAAATTTGAAGTAATATCATGGATTGGACACGTGGAGATTCGCCACGTCACACTAGGT
TGTTAGCACGCCCTTGTTGAGATTTTACAAAACTCTGAAACGATTGTGTAGATGATGAAGTTCAATATTTT
ATTTTAGGATATTCTGGAACAATTTTGTAGGTGATAAAGTTCGATATTTCTTTTTTAGGAGATTCTAGAAC
GATTGTATAGATAATGGAGTTCGGTATTTCATTTTTAGGAGATTCTAAAATGATTGTGTAGATGATAGAG
TTCGATATTTCGTAATGAAAAAAAAATGCTTGTTTAAAATATCTTGTTAGTATATATTTAATGAGAGTAAT
TAATAGAAAAGTAGTTGGTGCTATACTTTGAAAAAACAAGAGGTTAGTACATATTATCATTACAATAAGC
AAATTATAATTAATTAAAGTCCATTGACTCTTGACAAAGACTTGAAAACAGCCAACTATATATTCGAATAT
ATATTATCTTGCATTATATTTTATTTGATATTGAGAGAGACAAAACATATTCTTTGTGGTAGGCACGTTTT
AATTTTCACTTAATTTTCCTTTTTCTTTTTTTGAATTATTATGCATTGAAAAATTAAAGAAAAAAATGTAAA
CCAAAACATATGAAAAATACCCTCAAAAGTCCTAAATTAGTTAATTATATGGTTTATCTACTATTAGGTGT
TTGAATTTTTTTTTCCTTTTTGATAAGATTGGGATGGTTAATTAGTTAGAAAATAATGCACCTACATCTCA
AGCTTCTAGAATTAATTATTTTCCATTCAAAAGAAAATTCTACTAATAATACAAAGTTTTTATTCTCCCTTT
GGTAAGTTAACTAAAACTGAGTTTCCTTTTTCTTTGTTATCTTATTCTTTCCTATGTATAAGCATGAGATCA
TATTTCAACTAGAATTTAAGTTAAGTTATATATACAAATAATATGGAAAAAAAATGCTAATAGTAACAGA
CATATGAATTAAAGGATAAATAAAAACACTCGATAATACACTAATAAAAAAGGTGAGTATAAAAGGTAC
ATTTCTAACTTTATTAATTAGTTTGATGTGCACTTACAGCCTTACATGTTCACGTAAACTTAGTAGTTTTTA
TCCAGATTCTATAAATATATATATTAATAAATTCACTAAATATTTAGAGGGTCAGTAACCGATTTACATGA
ATTCAGTAGCTTTTATCCAAACCATCGTTTTCCTTCGGGTGAAGCTATAATAAGTCTGTAGGCAGGTTCG
ATAGAACTTAGTAGCTTTGACCTATAGTCTATATTTTTGCTTAAAAATCCACTAATCATGTATAAATTATTT
ATCAATAACTCAGCAAGCTATTTTTTCTAGAATTTATAATTCATAAACTTAAAATTTTAGCTCAGATTCTTA

TTATAAATATTCATAAACTTCAAATCTTGGATCCGATTCGTTATAAGTTAATGCTTACCTAAAAAAAGAAG
GTGAGAAACGGGTTTAAAATTAAAACTTGGTCATAAAATGAACAAAGCGTGTGTAACCTTTTTAAATTTT
GTATTTTGTATTGCTAACGCAAATTGTTGCGTGACATAATGAAAAATACATTAATTTTTTGCTGCTTTTATT
GTTATATTTTCATGAGGATATCATATCTTTTTTTAATTATTATTTATTTTTTAAAATTTTGACAATAAATGGA
AAAACTAAAATTAGTGGACAATTGGTCCTAAAAAGTTAGGTGTAAGTTCACACAAAAGATTCTTTTTGAA
ATGGATGAGAAGAAATCATTTTTTTGAACCCACTTTATATTCCAAAAATAGTCACTTGGCCGATTATTTTG
ACTTTGATTAATTTCAAATTTACATATAGTATGAGTTCTACTTTTAATTTTATTTTAACTTTGATTAATTTCA
AATTTACATATAGTATGAGTTTTACTTTTAATTTTTCATAACGTTTACTTTCATATTATAAACATAAAAAAT
AAATACTTTTTGACAAGGACAGTCTTTGGGTCATTTGAATGGTGTACAAGTTACATTAAAATTATAATATT
TAAAAATAACTAATGTATAATATTATTTAGTATATATGTATTTGACTTTACATTGGATAAATTAGTATCAA
CACTTATTTTCAATTTTAACCTTAGATTTTCTAAAGGACCTTGGAAGAAGAGTCTCAAGAAAGGGCATTTT
GGTCATTTTTCGTATTTTATCTAGAGATTATTATCTGCATTAAAGGTTGTATAAAAATAATATCACAATTA
CGGTGTAACGACTAACCCCAAAATCAAACTTTCAATCACACGGAATAATATAATCTCACATTTCATCTCGG
GATTGTTATCTATTATTCCAAATATCAAACGACTATAGGAATTACTAAGTTATGTAATGTGCACTAATGTT
TTTGGATTCATGACAAAGGAAACTTGACCTTCCGTTCATTTGTATTTTTCGAGCAATTCACACCTAGTTCA
AGTTACTCAATCGCGATAAAAAATAATTTTGTGACTTTACTGTTAAAAAAAAATACAACCTTTGATGGAA
GACTCATTTTTATGTGTTAATTTTCAGCAAAAAGTGGAAATGTTGGATCTGAAGATGGTTCTGGCAAAAC
AAAAAGAATTGCTATGGCTACTGGAATTACAGCTGGTGTTGTTCTTGTTCTAATTGGAGTTGTAAGCATT
TGCATTTTATCAAAGAGAAAAAAATTATTGGAAGGTCCAATAAGAAACAAAGCAGAACAAAGAGGTAT
GTTATTATAAGCCTATAATTTCGATTTCAGTATGGTATTCGATATTTACTTAAGGCATAATACATAAAGAA
TGCACATCTATAGACACCACGACTCCTTTACTGTGTCTTATGGACACCTGACGTTGTTGTGACACATAAAT
TTTGGAGATGTCTAGATGATCATTTTGTAAGTTGGAATGTACGTTCACTAGACATGATGGTGACGAGTTG
AGGTGTCTAACTATACATTACTCAAAATTAGAGGGTTCAGTTGTCAGTTGAGGTCAAGTTAAGGTGTCTA
TCTATATATTATACAGTAACCATCTCATAATTCATATTTCTTACACTATTTTTGAACCAGGCTCTATTGAAA
GAAGTCAAGATCTTCTAGTGAACACAGGCATTATTCCAAGTAAAAGAGAAATTTCTAGTGAAACAGTCG
CGGACGAGTTTGAATTGCCATTATTTGATCTCAGTACCTTAGCTATGGCTACTGAAGATTTTTCTGATGCA
AACAAGCTAGGCCAGGGCGGTTTTGGTTGTGTTTACAAGGTAAAGCTCATCTTGACGTTAAAAAAGCAA
AAACTTATGTTGCTCGGATTGGTCAAAAATGTCATCATATCCATGTCAGATCCTCCAAAAATACATTACTT
TGGAAGATGTAACACGGGTGTAGTGACATTTTTCGAGGATCATAGCAATACATCAAAAGTAGTACTTTTT

TTCATTTACAAGTTAAAGCTGATCATATATCAGTAAAAAGAATATATGCTGCTCAGACAGGACAAAAATG
TCGTTGCATGCACTCGTGTCAGATCCTCCAAAAATGCATCACTTTGGAGGATTGATCTAACAAGGGTGCA
ATGACATTTTTTGAGTATCTGAGCAATAATGAAAAACTAGTCCTTTTTTTTTTGCATTTACAATGTAAAACT
CAACTAGATGTGAAAAAAACCTATGTTGTTCGGATTCTACAAAAATGTCATTGCACCCCTGATCTGACAC
GGGTGTTACAACATTTTTCAAGGATATGAGCAACATATGAAAAACTAGTTTTTGCCTTTACAAGGTAGAC
GTGAAAACAACCTATGTTGTTCGTATCCTACAAAAATATCATTACACCCTTGTCGGATCTCCAAGAAAAT
GCTTCATTTTTGGAGGATCTGACATGAGTGCAGTGACATTTTTCAAGATTCGAGCAACATTGGGAAAGA
CGGACTCTGTTTTATTGGAAAATGTGAAGACATTCAACAAAAGAACATCTTCTTTTTTTTTATTATCAGG
GAATAATAGATGAAGGTCAAGAAATAGCAGTGAAAAGGCTGTCAAAGAATTCAGGCCAAGGAGTAGA
GGAATTCAAGAATGAGCTAAGATTAATTGCTAGACTTCAACACAGAAACCTCGTTCGCCTTCTTGGATGT
TGCGTTGAGATGGAAGAGAAAATGTTGATATACGAATACATGGAAAATAAAAGCTTGGATTCAATCTTA
TTCAGTAAGTCGTTCTTATGATCAGTAACTATGTTTATGTGTGCTATTGGACTTCAAAATTAACAAATATA
CGCGTCTCAGATAAGCAGAAAAGCTCGCTGCTAGATTGGCAAAGACGATTCAACATTATCTGTGGGATT
GCTCGGGGGCTTCTGTATCTTCATCAAGATTCAAGATTTCGGATTATCCACCGAGACCTCAAAGCAAGCA
ACATTCTCCTTGACAAGGAAATGATCCCGAAAATATCAGATTTTGGCATGGCACGGATTTTTGGAGGCG
ATGAGACCGAAGGAAATACAAAGAGAGTTGTTGGAACCTAGTAAGTAGAATCACATTATTCCTTATAAA
TCATTCATAAAAAACACTTCGAAAAGTGTTGGTAAATCGACGTTTTTCTCTCTTAATGTAGCGGTTACAT
GTCTCCTGAATACGCGATGGATGGTCTATTCTCTGTTAAATCAGACGTTTTCAGCTTCGGAGTTTTAGTGT
TGGAAATAGTAACAGGGAAGAAGAACAGAGGATTTTATTTTCAGAACAATGAAAGAAACCTTCTTGGCC
ATGTAAGTACACCAAAATTATCACAACTTAGTCATCATTTCTAACTAAGTCGGAACAGAATTTTGAGTTTA
TGCGTTCTGAATTCTAAAAAAATATAGCTAATTAGTAGGTTCTCGATAAATTATTTATGCATGTTAAGTAG
ATTTCATAACATAACTACAAGGTTTTTACTAAAGTTGCTACTGAGTTCTGTCGAACCTGTAACTTTCTCTCT
AGTTCCGCCCCTAAATGTATAATCCGCTCTGCAGGCGTGGAAACTATGGAGAGAAGGAGGAGCCTCAG
AACTACTCGATTCATCAGTTGGTGAATCATTTTCTCCGTGTGAAGTAATCAGATGCATACAGGTCGGGTT
ATTGTGTGTTCAGGAACAAGCAGAAGATAGACCGAACATGGCAACCGTTGTATTGATGTTAGGAAGTG
AAACCGCAACAATGCCTCAGCCTAAACACCCTGGTTTTTGCCTCGGAAGGAGACCTGTTGATGAACACTC
GGAAACTATTTACGAAGAAACTTTCACTGTGAATCAAGTTACGATTACCATGCTTGATCCCCGGTAGAGT
TAAACGTTCAGACAGATCATAGGCTTGCCCGCTTCTACGTGACAGAATAGAAATAAAGGCAAATTCCACT
GATGGGAGTGTGAACATAGAGATTTCACGACGAATGAATGCAATACATGGATGATGGATCCCTGCTGG

CAAAAATGTATAGATAGTACATTAGTGTTTTTTCTCCACCCGACCATATCAGGAGGATCAATAGTGTTTTA
ACTTACCCTCCTATAGTACATTAGTACTACGAGTGGTTAGCAATTTTCTCTTTGTACACTTACCTTGATTTT
TTTTACAAAAATAATGTTTTTTTTTGGGTAGTTAATTATAGAA

>PGSC0003DMG401009323 G-LecRK

GCTGCCAAAAATGAAATCTTTAGCCAATTCCATCTTCATCTTAATATTGACCTTATTCTGCAGCCATACAG
GTATCATTACCCTTCCATCTGTGGCTGCAATAACCTCAATGAAGCCTGGTGATGAACTCAATCACACTCA
GGTTCTTGATTCTGAAGATGGAACATTCAAGTTAGGATTTTTTTCTATCTCACAAACAAATAACTCTTATC
TTGGTATATGGTATGCAGGTGATCCACAAGATAAGAAGCTATGGATAGCCAATCCAAATACGCCTTTATT
GAACAATTCAGGATTGCTCACTATAGATACTACAGGAACATTGAAAATCACTAGTGGAGGGAAAACAGT
TGTGAATATTACCCCTCCTTTATTGACAGGAAGTTTAATAGCTAGGCTTCAAGATTCTGGAAATCTTGTGC
TTCAGGATGAAACTGAGAACAGGACTTTATGGCAAAGCTTCGATCATCCTACCGATACGCTTTTTCCAGG
TATGAAGCTTGGTTATAACCTTACAACAAAGCAGAATTGGACTTTAACTTCTTGGTTGAGTAGTTATATTC
CGGCTTCGGGGGCTTTTACTTTAAGTTTGTAGTCCATTCAAGATGCATTTCAGCTGGTTATACGTCGAAG
GGGCGAGGTTTATTGGACTAGTGGCGCTTGGAGGAATCAAAGTTTTCCATTCTTAACTGCATTGCATGAT
TCTTCTAACAGATATCAGTATAATCTCAACTTGGTGTCTGAAAAAGATGGTGTGTTCTTCCAATTTGATGC
GCCAGATGGAAGTTTCCCAAGTCTTGTGTTGACTTTTAATGGGGCTATTCTTGGTGGTGGTGAAGACAG
TCGTATATACGCCCTTTATAATGAGTTTTGTTATGGTTATGAAAGTCACGATGGTTGTGTCTCTAATCAGT
TGCCTGAGTGCCGAAAGGATGGGGATAAATTTGAGCTAAAGAGTGCATACTTCACTACTGGTAGTGCTA
CTTCTAATTATGATGATAATGCAAACATTAGTCTTGGTGATTGTATGAAGAATTGCTGGGAGCACTGCAG
TTGTGTTGGCTTCATCACCAGCAGCAATGGAATAGGCTGCATAATTTGGAATGGAAATGGGGAGTTTCG
AGTTGATGAGAGTGGTAGTGCAGTGCAACAATATGTTCTGGTTTCATCAAAAGGTAAATCCTTATATTAG
GAATACACCTTGTTTATGAACTAATCTATTTTGAATAGATCAAGAGTCACCTGTATAACACTCAGCAACCT
ATGTGTGCAGGAAAACAAAAGAATTGGATATGGATAGTAATTGTTGTAGCTATTGTTGTCCCTATGCTG
ATATCTGGTTTCATCTGCTACATTATAGTGAGAAGGCGCAAACTACAAGGTAAATCTCTGAAAAAGTGAA
AAATGATTAACTAGTAAGCTTTTTTATTAATTATGTTAAGCATGTGTAAATATCTACAAACTTTTCTTGCAC
AGCTGAGAAAAGAAGGGAGGAAGAGTATATACGTGAGTTGACAGCATCCGATAGCTTCAACGACACCA
ATATGAAAGAAAAGGATGGAAGGGAAGTCCAAGATTTGAAGATATTTAGCTTTGGATTTGTATTGGCAG
CCACAAACAATTTCTCAAGTGAAAACAAGCTTGGAGAGGGTGGTTTTGGACCTGTTTATAAGGTATAATT
ACATCATATGCCTTCGGAAAACTTCAGTGCCATCATTGCATTAACTGAATTACCTGTAAAATTTTATGACA

GGGAAAATTTCCAGATGGGAGAGAGGTGGCAGTCAAGAGACTTTCACGAACATCAGGTCAAGGGCTTG
TAGAGTTCAAGAATGAGCTTATACTCATTGCAAAAGTTCAGCACACAAATCTAGTTCGAGTTCTAGGATG
CTGTATTCACGAAGATGAGAAAATATTGATATATGAGTATATGCCTAACAAGAGCTTGGACTTCTTCCTA
TTCGGTTCGTTACCATTTCAAAACAAAGAATTAATAGCTCAAATAAGTTCTGAAAATTTTGACCTGAAAT
GCTTTCTGTCTTTGACAGATCCTGAAAGAAAGAAGCTACTGGATTGGCAGAAACGTTATGAAATTATCG
AAGGAATTGCTCAAGGATTGCTCTATCTTCACAAATACTCAAGAATGAGAGTGATACATAGAGATCTTAA
AGCCAGTAATGTGCTATTGGATGAGAACATGAATCCTAAGATAGCTGATTTTGGTATGGCAAGAATTTTC
AAACAGAACGAGACGGAGGCAGTGACTGCCAGGGTTGTTGGAACATAGTGAGTAGAACTTATTTCATG
ATATAAGTTTAATTCATTTAGACATTTTCATATTGACTAAACGGGTAATGTTTCCTGTCCTCTATACAGTG
GTTACATGGCTCCCGAGTTCGCAATGGAAGGAGCATTCTCAATCAAGTCTGATGTCTTCAGTTTTGGAAT
CTTAATGCTGGAAATTGTGAGTGGTAGGAGAACTACTAGCTTACAACAATTCGATCGTCCACTCAATTTT
ATAGGTTATGTAAGTTTTCCTTGACACCCCTTTTTCCTTTTAAGTACTATAATACATAAAGTTTGAGAATAC
TGATCATTTTGGTTTAGGCATGGGAACTATGGAAAGAAAAGTGTGCACTGGAATTGAAGGATCCAGCTC
TTGGAGATTTATGTGACACTAAACTGCTGTTACGAGTTATTCATGTCGGACTATTATGTGTACAAGAAGG
AGCAACTGATAGGCCAACAATGTCTGATGTTATCTCACTGCTAGGTAATGAAAGTATGCCACTGCCTACG
CCAAAACAACCAGCATTTTTCACAGGAAGAAATGAAGCAGAATCAAATTCATCGGGTATCAAAGCAGAA
CAATGTTCGGTTAATGATTGTTCCATCACTGTCATTGAAGCAAGATAGCTTAGAGACATTATCTTGCTGTA
CTCTGTTGTGCAACACTCAGTAGCTATACATGAAAAGCATGTTTCCTTATGGTATAGATTAGTCAATTTGT
ACTGTTAGTACTTGTATAGCTGTCTTTAGCTGTGTTTGTAACAATACATAAATTTCTTTCCTACAAAGACC
AGAATTATCAATAACTTTTTTTTTTTTTTGCATTTTCTCACTTAAATGTAGTAACATTTATTAGTATAACAAC
CTTAAGATTGTAAGATTCTCTTTATTTATTAGCTCCTGTATATTTTTAAAATTAGGAGACTCATCAATGATG
ATATTTTTTTGTGGTCCACATAAATGGATAACTTATCTGAGGATCATAATTCATAAGGATGACTGTAAACT
AATCTAGTCCAAACAACTTACTCGACGAGTTAAGGACATTCTTAATCTTGATTCAACAATGCATCAATGA
AGTTCACAGTGTATTTCAACAAGTAGATATGATATCTTTTTGAATTATATTAGCAAGCAGCAAAATGAAA
TAGAGAAATATGAGGAAGAAAAAAGAAATTAGAAAAGTCAAAGAAATAACAGAAACAAAATGCAAATT
GATTTCACATTTCAACAAGAAAATTAAAGATTGTCTCTTTCAACAAATTTTCTTACAAGTAAGGTAATATT
TCTACTTAGAAAAATCTTCAAAATTTCTGGCTGGAAAATGTTTAATATAATTGTCTATTCTCTAATGCATTA
TTGGCTTTGAACAGTAAACAACCTAAAAAGGAATAATCTATTATTATCTGCAGCAACATTTCTTTCTTTCT
GGCTGCCAAAATGAAATCTTTAGCAAATTACATTTGCTTCCTCATCTTGACATTATTCTGCAGCCAAACAT

GTATCATTAGCCTTCCATCTGTAGCTGCAATAACCTCAATGAAGCCTGGTGATGTACTAAATCACTCACA
GGTACTTGATTCAGAAGGTGGAAGATTCAAGTTGGGATTTTTCTCTATCCCACAAACAAATAAAACTTAT
CTTGGAATATGGTATGCAGGTGATCCAGTAGAGAAAAAATTATGGATAGCCAATCCAAACACACCTATA
TTGAACAGCTCAGGATTGCTCACATTAGATTCTGCAGGAACATTGAAAATCACCAGTGGAGGGAAGACA
GTTGTGAATATTACCACTCCTTTATTGACAGGAAGTTTAATAGCTAGGCTTCAAGATTCTGGAAATTTGG
TGGTTCAGGATGAGACTCGGAACCGGACTTTATGGCAAAGCTTTGATCATCCTACCAGTTGTCTTTTGCC
TGGTATGAAGCTTGGTTATAACCTTACCACAAGGCAGAATTGGACTCTAACTTCTTGGTTGGTTAGTAGT
ACAATTCCGGCCTCAGGGGCTTTTACTTTAAGTTTGGAGGCAATTCAAGATGCATTTCAGTTGGTTGTTA
GACGAAGGGGTGAGGTTTACTGGACTAGTGGGGCTTGGAACAATCAAGGTTTTCCATTCTTACCTTCATT
CCGTGATTCTGCTACTACTTATCAGTACAATCTCAACTTGGTATCCGGTACTGATGGGATGTTCTTCCAAT
TTGAGGCTACAAAGGGAAGTTTCCCAAGTCTTGAGTTGTTTTCAGATGGAGCTATTGCTGCTGGAGACG
GTAGTATATACACCCGTTATAATAAGTTCTGTTATGGTTATGGAGGTGATGATGGTTGTGTCTCTAGTCA
GTTGCCTGAGTGTCGAAAGAACAGTGATAAGTTTGAGCAAAAGAGAGGAGACTTTATAGATTTATCTGG
TACTACTACTAGCAATTATGACAATGCAAGCATTAGTCTTGGTGATTGTATGCAGAAGTGCTGGGAGCA
CTGCAGTTGTGTTGGATTCACCAGTCTTAACAGCAATGGGACAGGCTGCCGAATTTGGAATGGAAAGAG
GGATTTTCGAGTCGATGAGAGTGGTAATGCAGTTCAAAGATATGTTCTGATTTCACCAAAATCATCTAAA
GGTTAGTCTTTAATTATGTCATAAATACACCTTCTTATGAACTAATTTTTTGGTCGAACAAACACATTTTAT
TGCAAACACCCAAAAACAGAATACAGTATATCCTAAGTAAAAAGATACAACTGACCACAGCTAACCAAA
TTTAGAAACATCCCCTACTCTTCCTCCCTTCTATACAGTTTTCTATTTGTAAAGTTCTTGTTGGTGTTTTTTT
AAAAATGGGGCAGCACAAAAAGATTGAAAATGTCTCTCAATATTTTATTAATCATAATAGGGCTTTAAAT
AGCCAACTTGATAACATAATCTGCAGAAAATCTGCATAACAAGAATTTAAAAAGACTAAATAATCTCAAC
AACTAAAACAACCTAACAAATATTTAAAATTCAAATTCATCCTAAAACTAGGTAGCTTATTATTTTAAAAA
ATGACTGCAGTAACTAAAAGCAAACTCAACAGTTCTTCTATCCATTCTCCGTCTTTACAGTTTTTCAATTTT
CAATATATAGACTGTATTAGTGAAGAATCAACTGTATAATTTTCATCGTGTTACGTGTGCAGGAAAAACT
TGGATATGGATAGTATTATCTATAGTTATCACAATGCTAATATGTGGTTTGATTTGCTTGATCAAAACAA
GAATACAAAAACTACAACGTAAGTCTCTGAAAAGTGATTTACTAACTAGTATGTTGTTTATCAGTTATTTT
AAGCATGTATGTAAATCTACATACTTCTCTTGCATAGGTGAGAAAAGAAAGAAGGAGGAGCATATACGT
GAGATGAACGCAGCAGACAGCTTCAACAACACAAATCTGAAAGAAGAAGATGAAAGGGGAGTACAAG
ATTTGAAGCCTTAAGATCAATGAAGCTCTTGATACCACTGTTGGTGTTTTTTAAAAGTGGGGCAGCACAA

AAAGATTGAAAATGTCTCTCAATATTTTATTAATCATAATAGGGCTTTAAATAGCCAACTTGATAACATAA
TCTGCAGAAAATCTGCATAACAAGAATTTAAAAAGACTAAATAATCTCAACAACTAAAACAACCTAACAA
ATATTTAAAATTCAAATTCATCCTAAAACTAGGTAGCTTATTATTTTAAAAAATGACTGCAGTAACTAAAA
GCAAACTCAACAGTTCTTCTATCCATTCTCCGTCTTTACAGTTTTTCAATTTTCAATATATAGACTGTATTA
GTGAAGAATCAACTGTATAATTTTCATCGTGTTACGTGTGCAGGAAAAACTTGGATATGGATAGTATTAT
CTATAGTTATCACAATGCTAATATGTGGTTTGATTTGCTTGATCAAAACAAGAATACAAAAACTACAACG
TAAGTCTCTGAAAAGTGATTTACTAACTAGTATGTTGTTTATCAGTTATTTTAAGCATGTATGTAAATCTA
CATACTTCTCTTGCATAGGTGAGAAAAGAAAGAAGGAGGAGCATATACGTGAGATGAACGCAGCAGAC
AGCTTCAACAACACAAATCTGAAAGAAGAAGATGAAAGGGGAGTACAAGATTTGAAGATATTTAGCTTT
GGATTGATATTGGCAGCCACAAACAACTTCTCGAGTGATAACAAGCTCGGAGAGGGTGGTTTTGGACCT
GTTTATAAGGTACATTTTCAGTATGGTATATCATATATGTACCCCAAAAACTTCTGTAGTACTCTCATTAT
ATGAAATAATAATCTGTAAAAATGTATGACAGGGACAATTTCCTGATGGGAGAGAGGTGGCAATCAAG
AGACTTTCAAGAACATCAGGTCAAGGGCTTGCGGAGTTCAAGAATGAGCTTATACTAATTGCCAAAGTT
CAGCACAGAAATCTAGTTCGTGTTCTAGGATGTTGTATTCACGGAGATGAGAAAATGTTGATATATGAG
TACATGCCTAACAAGAGCTTGGACATCTTCCTTTTCGGTTTGTAACCATTTCAAAATAAGATCTAATCTTT
CAAATAACTGTCGAAATGTTTGATGAAATGTTTTCAGTCATTGACAGATCCTGAAACAAAGAAGCTACTG
GATTGGCAGAAGCGCTTTGAGATTATAGAAGGAATTGCTCAAGGATTACTCTATCTTCACAAATACTCAA
GAATGAGAGTGATACATAGAGATCTAAAGGCTAGTAATGTGCTCTTGGATGAAAACATGAATCCTAAGA
TAGCTGATTTTGGTCTGGCAAGAATTTTTAAACAGAATGAGACGGAGGCAGTGACTCGAAGGGTAGTTG
GAACATAGTGAGTTGAGCTTATTTCTTGATATTGACCTAGAGAACATGTATACATATTCATACTGACATA
GGTGGAAAATGTTTTCTATACACAGTGGTTACATGGCTCCCGAGTTCGCCATGGAAGGAGCTTTCTCAAT
CAAGTCTGATGTCTTCAGCTTTGGAGTCTTAATGCTGGAAATTTTGAGTGGTAGGAGAAATGCTAGCTTA
CAACAATTCAATCGTCCGCTCAACTTAATAGGTTATGTAAGTGTTTCTTGACATCCTTTTTATATTTGAAGT
ACTAAATGTACAAAGTTTGAGATAATTGATCATTCTGGTTTAGGCATGGGAACTATGGAAAGAAGGGTG
TGGACTAGAACTGAAGGATCCAGCTCTTGAAGATCTTTATGACACTGAACAATTTTTACGAGTAATTCAT
GTCGGACTACTATGTGTACAAGAAGGAGCAACAGATAGGCCAACAATGTCTGATGTTATCTCAATGCTT
GGTAATGGAAGTATGTCATTGCCAATTGCAAAACAACCTGCATTTTTCACAGGAAGAGATGAAGCTGAA
TCATATTCGTCATCAAACAAAACAGAACAATGTTCAATTAATGATTGTTCCATTACTGTCATTGAAGCAAG
ATAAGATTAGAGATGTTATTTTGTTGTAATAAAGATTGCAGTAGATCACCAATGTTTTAATGACCTCTAA

AGTAACTTGAAGGCTCTTTTTTGTTAAGGTTTAAAAGCTAATTTTTTCTGCTTCATACATATTAGAACTCG
G
>PGSC0003DMG401013876 G-LecRK
GAAAAACTTGAGACTTTAAGAAAGGGGACGAGAGGAGTACACATTTTTGTCCATAATTTCCAAAGCAGC
TGACGACAACCCTTTTAACCCTTTGTCACGTAATTCCTACTCAAGCAAATCTTTCGTTGGGCTGTCTAATTC
TTGTGCCTCTTCGTAGTTTAACCTTAGGCCCAACATCTTAGCCAGCCTCAGATAATTGGTCCATAAGCATT
ATCTGGGCTATCCATGTTCACAACATTATTAGTACTTATTTTTTAGTGGAATGATTGCACTACTGCAGTTT
GATGTCATTTTCATCAATTGGATATTTTCCTTGCGTAGAATTTTCAAATATTTTGACTAAGGTGTTTGATTA
GTTGCGTAACTTTTGGTTTGGTAGAATTCTCAAAGACTAATTTTATTTTTTCTGTTTCTCTGAGATGATAAC
ATTGATATACTTATGGATACTTGAATGTTTGTTTGTGTTAATTCGTCTATTCCCTCGACGTGTCAAAACTTT
TTAAAAACAGGTATTTGGGAATGAAGATATTGTTGGTTGATTTAGTTCTCTTGCTTTCTTGTTTTCATTTA
GTTAGGTGCAATAATACGCGTTCAGACAACTTATTGCCTGGACAAACCCTTTCCTTTTCTGCAGCAGATA
CTTTAGTGTCCAAACAAGGGAAGTTTGAACTCGGTTTCTTCTGTCCAGGTAATTGTGAAAAGCTTTTTATT
GGTATATGGTATACAAACATAAAGCCCAAGACCGTGGTTTGGATAGCAACTAGGGACAACCCCATCCGC
CCTCCGTTCAATAGTTCTCATCTAGAGCTGTCTGATGGAAATCTAGGAATCTTTAATGAACGTAAACAAA
GAGTTTGGGACTCTAATTTATCCACAGCAGATGTCAATAGGGCAGTACTTCTTGATTCTGGAAATTTCAT
TTTGACAAATGGCATTGAAATACAGTGGCAGAGCTTTGATTATCCTACAGATACATGGCTGCCTGGTGC
GAGGATTGGATTTGATAAGTCCAAGAACACACTCCAGAAACTTACTTCTTGTATGGATGCAAATGATAC
GGCTTCAGGACACTTCTCTTTGGAATTAGACCCTAATCAAAATGGTGAGTTAGTCATGCTACGTAACCTC
GTGGTAATGTGGATCTGGGTGCCTTTGAATCTCGTGGTATCCAACTTCAGTTATAATCCAACTGAACATA
GTGCATTTATTACATACAATGTCTTTGGTGAATCTGTCATTTCTCGAATTGTCCTAGATCATAGCAGGTTC
ATGAGGGAACTCTTCTGGTCTAATGAGCATCAGAATTGGATGGTTGTGCGGTCTGTACCTGTTGATATGT
GTGACGCAACGAATATATGTGGTGCTTTTGGAATTTGTGATATAAATACCAGTCCAATCTGTGGATGTTT
ACAAGGTTATGAACCCAAGTTTCCATTAGGTTGGGATACTAATGATTATTCTGGTGGGTGTGTGAGAAT
AACTCCGCTGCAGTGTGGCAGCAATAACGATGGTTTTGTTCGTATGCAAAATGTTGAACTTCCTGCATCA
TCTGAATCAGTGCAAGTGGGGAATGATCAGATCTGTGAATATATATGTTCATCCAATTGCTCATGCAATG
CTTATACTTACAGCTATAGTGGAGTGTGCTTATTATGGAATGATGATCTTATCAATCTGAGAAGATTATCT
GATAATTCACGTCAAGCAGTGTTAAGCATAAAAATGTTTGAGCTTTCAAACAAAGGTTAGGTGCTTCTAA
ACTGCTTCTTGTTCTAATTGTGCTTACTTGAAACTGACATTATGTTCATTTTCTCCTCTCCCCATCTATCTTA

TTGCTCAGGCAATAAAAAGTCATCGTTTGTAGTGCTAGTAGCATCCATCACTTCAGCAATTTTTGTCTGTG
GTACATGCTGTTACTTTCTATGGAGACGAAAGCTCAAGAAAAAAGGTACTTCTTTATATTTCCAATAGCT
ATGCCAGCGCTTTATGAATGTTAATGTGGCCTACATTGGTTGAGGCCATGAGGGTATAGGATCTTGTCTC
CTTATATTGTCTTGGCTATCCTCACCTCATGAACTAGCTTTCAATCGAGATTGACATTTTCTTAACAATGAA
CATATGTGGGATGCCAATAAACGAACCTAGTCCTTCTTGATTCATTATTAAACTGTGCAGTCTTCGTGTGA
TATTCTAGTTCTTACTAGTAGTAGCAGGTTATCTGACTTAACTGTGTTCTCGCTGTCACACTCACTACAATC
ATGGATTAGATTATAAGTTAAAATTACCAATGTACAGCTTCATCTATAGACTTCATGTTTCTATTGCCTTTT
CAGGGATTTTGAGAAAGATGAAGTTCAGGGAAATGTTACTTTCTGACTCAGCAACTAACATGTCAAAAC
CTGGAACTTCCATTGGAAAGGGACAAGAAAAGAATGGTGACATTGAACTGAAGTTCTTTGAACTACATG
ACTTGAAAGCCGCGACAGACAATTTTCCCCCTGATAATAAGCTTGGAGAAGGAGGTTTTGGTCCTGTTTT
TAAGGTATTGTTACTACAACCTGAATACCTGCAACACAAATATGCGTTCACACCTAAAGAAAATGGAAAG
AAATCCCTCCGGAAATAGCCAGATAGTATGTATCCTGATTTTAGTTAATATCTTTGCTAGCTGTACTTATG
TCTTGGAGGTGTGATAAATGTTTTTTTGCAATTTCTAGGGTCAATTGCCAGATGGGCAGCAAATAGCTGT
GAAAAGGCTATCAACTCAATCAAGGCAAGGCATAAGTGAATTCAAAACTGAGGCCCTTCTCATTGCAAA
ACTCCAACACAGAAACCTTGTTAGGTTCCTTGGCTGTTGTGTAGAAGATGAGGAGAAAATGTTGGTATA
TGAATATATGCCAAATAAAAGTCTAGACTATTTCATATTCGGTATATATGATAATTTACTACCATCTGTTTT
TGTGTTAACCCTTTCCTCCTGTCAAATGTGTGCTTACTGGATAAATTTGATATGTACAACTTAGACGAGAG
CAGGAGGTCATTGCTTGATTGGAAAAAGCGGCATGAGATCATTATTGGTATAGCTAGAGGGATACTCTA
TCTGCATCAGGACTCAAGATTAAGAGTAATCCACCGTGATCTGAAAGCCAGCAACATTCTTTTAGATGAA
GACATGAACCCCAAGATATCAGATTTTGGTACAGCAAGAATATTTAGCGGAAACCAAGATGAAGCAAAT
ACTCTTCGAATAGTTGGCACATAGTAAGCAAGCAAATACTGAAAGTTCTTTTCCCATACTACTTTGCCTAC
TCATTATAGCTCCTTTTCTATGCAGTGGCTATATGTCACCTGAATATGCTCTAGCTGGTCTCTTCTCAGTAA
AATCTGATGTCTTTAGCTTTGGTGTCATATTATTGGAGATCATTAGTGGAAAAAAGAACAGAATCTCTTA
TAATAGTGATTCCCCTCCAAATTTGATACGACAGGTAAGTTGTAAAATGCAAATGTTCGAATCTTTCTTTG
ATTAAGAATCAAATTTATCCATTTCAATGAACTACTGATTCAGGCCTGGGAGCTTTGGAATGATGGCAAG
GCCTTCGAGCTGATAGATCCAACGATAGTTGATTCTTGCCCCAGTGAGGAAGCACTACGCTGTATTCAAG
TGGGACTATTGTGTTTGGAAGTGAATGCAGGGGACAGACCAACCATGTCATCAGTTCTATTCATGCTAA
GTAATGAAGCAACGATTCCCTCCCCAAAGCAGCCATTGATTACCCCTAACTCTGACTCGAGTACAACAGA
AACAACACCATCTTCGATCAATGAGGTGACTATTACTGCACTTGATGGTCGTTAGTCAGCTGATGACTGA

TGATTCAGCCAATCCGTTCAAGTGTGGGAGGGTTATTGACCCATCCATTTACTAGCTCAACGAATTGGGT
TGATATGTTGTCCAAATTGATTCGGGAGAGATTGTCAAAATAATTTTCAGAGGACATTTTTTTATTTGATA
TGTTATATAAATAGTTACAATAAAGAAAACAATAATTTTAGGTATTAAAAATTCATAAAAGAACAAATAA
AATAATTAAACCTTACTAAGAATTGAACGAATTGAGTTACAACTCACATTGTAGTCCTGTTTCAGTCAAAT
TTAATCCACTCAAATTCGACTCAACCCGTCTATTTGAGACCTTTGATCT

>PGSC0003DMG401019239 G-LecRK

ATGAGCGGTAATGGCTGGGGAAGGGGGCAAGTACATCCTTTTGTTCTCAGTTCGTGGGAATTGCTTCCA
TCACTAACCAGTTCAGCTCGGCTAGTGGGCTTTTGTGTTCGAACCTCGAATCCGGACCTGTTCGACAAGA
CCAGATCCATCAACAAACCTAGGTCCAATATTTGCGTCTACGAGCTAGCCATCAACAGACGGACATCCTT
TTACCGCAGTCTTCCCCTATTGGGCATAGACTTTTCTACGTTAGGCTCCTGTAAGGGCGGGTATGAGAAG
GGTCCCCCTCTCCCTTGTCAAAGCTTAGTTATCCGTGAATGAGAACTCGTTTTGCTTGTTGGCTGGCTTAT
GATGGTTTCCTGTCCCAATTCAACATATTCCTTTTTTTGCTCCTGCTTGGTAGCAGGCTTGTGTGCGAGCT
TGGTCCGGTTTCGATTCCTAAAGCCCACGGAGAAGGGTGTGGAGCCTCGCTTTCCATGCTGCTCCTAGTT
CGTGGGAATTGCTTCCATCAAATAGCCGTACCATTAACTGATTAAATTAAAAATAGCTACATTGGGGTAA
AATGCTTTCTAACAAATGCGACTTCATATCCAAAAAGATTCAAACCCAAGACTTTTAATTAAGAATGAAG
GAGTCACATCACCACAACTGTGTCAGTGATTTGATGTTGTTGTTGTAAGCAAATAAGATAGAACATGGCC
CCAACATCCATGTGTGGCTGTGATTAAATTCAACTATTCATTATAAACAAATAATCTCAAAACCAAAGAAT
ACGATGAGTATTAATCAAACCAGCCAAGCTAAGGTGTGTTCAAATAGGACTTGGGTCAAGTGTCATCAA
TATGCTGCATGAACTGGTCATAAGAGTCGGAAGGCAGACTTGCTAAGCACTTCGACATAGAGGAAAGA
CAACACAACGTCTATTGGATCACAAAGCATGAAGGAATTGCTAAGGGAGAGTTTTCACTTATTCTTGCTC
ATGTATTTGTATTTTGTTCAACGCAGCATTGGCGCTACAGATACCATAAGTACAACCCACTTTCTGAAAG
ATGGTGATGCCAATATTACTTCACCTGATGGAACCTTTGAAATGGGGTTTTTCAGTTCAGGTAGTTCCAA
AAATCGCTACGTGGGTATGTGGTACAAAAGCTTATCTGTTAGGACAGTGGTGTGGGTTGCCAACAGAGA
AGCTCCCTTGACCAGTGGATCAGGTATCCTTAAGGTTATTGAGCCAGGACGTCTCGTCCTTCTCAATGAC
ACTAACAAAGTTGTATGGTCAACTAATACCTCTACATCTGTGCAGAATCCCATTGCACAGTTGCTGGATT
CTGGGAATCTTGTCGTGAAACAAGCTGGTGATGATAATTTCCTTTGGCAGAGTTTTGATCACCCAACTGA
TACACTATTACCAGGCATGAAGCTTGGTTGGAACTTTTTAACTCACAGAGAGGTGTACTTGTCATCGTGG
AAGACCCAAGAGGATCCAGCTCCAGGTGATTATACATATCACTGTGATCCTTCTGGATATCCACAGAACA
TCCTGAAGAAGGGGTCAAATGTTGTCTATCGATCAGGGCCGTGGAATGGTCTTCATTTTAGTGGAACAA

GAAACCCAAGAGGCAGTCATTTATACAAATATGAGATATTCTCAAGTAAGACACAGGTTTATTTTGGATA
TAAGCTCATATCTTCAGTTATCACAAGGTTGATACTTAATCAGAATGGTGCCCTAAAACTTTGGACTTGG
GGTGATCATGTATCTGATTGGATCCCTCAACTCTCAATACCCGCAGATAACTGTGATGCTTACAAACTGT
GTGGCGCATACGGGACTTGCAACAGTCAAGATTTTACTGTATGTAGATGCTTAGACAGATTTGTGCCCA
ACAATAGTGAAGCTTGGAAGAAGAGAAACTGGTCAGGTGGCTGTGTTCGGAAAACAGAACTAAATTGC
CTTCAAGGACATAGATTTTTGAAGTATTCACATATCAAATTGCCAGACACACGGAATTCCTGGTCCGATG
TGACTATGACACTTGAAGAATGCAAGAATACCTGCTCTAAAAATTGCTCTTGTATGGCTTATGCAAATCC
TGACATACGCAATGGAGGAAGTGGATGCTTGTTGTGGTTCGAGGATCTGCTTGACATTAAGGTGGTTTC
CGATGAAGGTCAAGATATCTACATAAGAATGGCTGCTTCTGAACCAGGTAGGTTTTGTTGTCACTTAAAG
ATTTTTGATGGATTACCTGAAAATAGTAAAAGAATGTATGGTTGCAGTCACTTCTTTGGAACCAATTGAT
AGGTATTTAGTTTTCTGTAGAACAAGAAGTTGAGATGCATGCGGCTTGAATCAAAGAAAAAATCTTCTCA
AAATTAAAGAGATTTATAGTAACATTCAAAAATTAAAAATGACTTGCATTATATTGAATTCTAGAACCCTT
ACAACTCAATATCAACAAATTCAAAATGATGTGCAGTACTCCAAAGGCTTACAGAATCTTTTTTAATTCAA
TATAAAATTATCTCCCTCGTGGATTGGACAGAAAGTGAAATTGATTATTTTTGCGGAATGGATACTTGCA
GATAGTCTGGAGAAATCAGATGGAAAGAAAAGAAAAGTACTTTTCTGGATTCTGCCATTATCAGTTGGT
CTGATTCTGGTAATTCTTAGCATGTTGATCTTCCATAGAAGAAGGAAGAAGGCTTTACAGCTCAAAAGG
AAAGGTAAAATAGGATAAATCTCATTTTTTATTGACATCTTAGCAAAATCAAGGAACATAATTCAAAGAA
AAGTGAAGAACTTTAATATTTGGTCTGGATCACAGCTTAAACAAAATTCACTTCGTTTGCAGGCAGGTAG
GTCCGGACGCAATGGCAACTACAAGATGGATCATAGTGGTAATTGTGAGGAAGAATTTGAGATACCAA
TGTTTGACTTATCTACCATAATGAAGGCTACCAATAACTTTTCAATAGACAGAAAGATTGGAGAGGGGG
GCTTTGGACCGGTTTACAAGGTATAACTGGTGCACAGGCAAAAACTTCCTGATCCTATAGAGTAAAGTTT
CATCTTTAGATTCAGTATAGAAGTTGTATGATGAAATGCATAATGAAATAAAGCTTTGGCTTGTGAAACC
GAAGGAGACATACACAATAGCTAAGGAAGAACAAGGATACGGTGGAACACAAACTGATTGTCTCTTTCT
AATTGGAACACAATTGACTCTGTGTACTTCCTACATCAAGTAAGGTAATGAAAATATTAACATGCAGAAA
TTGTGATGTTTAGGGCATACTTGAAGGACGGGAAATAGCTGTCAAACGGCTGTCCAGAACTTCCAAACA
AGGAGAAGGTGAGTTCAAGAATGAAGTTTTATATGTTGCAAGACTTCAGCACAGGAATCTGGTGAAGA
TTCTTGGTTGCTGCAGTGACGGGGAAGAAAAGATGTTGATCTATGAGTACTTGCCGAATGGGAGTCTTG
ATTCATTTATATTTGGTACTATTCTCCATCCAATATTGTGCAAATTAAATAAATAATTGTGTTTCAGTGATG
ATGCCTATAGTAATACTTCATTAATCTTTTTTATTTTGAAGTAAAAGTAACTAGTAGTACTTTGGTAATTTT

GTTCCAATTATGAAAGCCCCATAAAGCAGGAAAAATATTGATAGACCAAACCTGTTGTGGGAGTATTTA
ATACTACTTTCTCCTATAACCAATTAGTCAGTTAATGACCCAACAAAAAAAATTGGCAGATGACACACAA
AGCAAGGCATTAGACTGGCCTAAGCGATTTCACATTATCAATGGTATAGCTCGGGGACTTATGTATCTGC
ATCAAGATTCTCAATTGAGAATAATTCACAGAGACCTGAAAGCTAACAACATTCTGCTAGACAAGGACAT
GAATCCAAAGATATTAGACTTCGGCTTGGCAAAAATATGTGAAGAGGATGACATTGGAGCCATGACAAA
CCGAGTAGTTGGAACATAGTAAGAACCTTCCACTGGATAGTATTATTTTGATAAGATTTACTGGATAATA
ATTTGAACCAAAGAAGAATCTTCAGTTAGTAAAGGCATGTTTTCCAGCATTACTAATTATAATGTTGCGC
ACATTTTAATGCAGTGGGTACCTCTCACCGGAATATGCATTGCACGGAAAATACTCAGTAAAATCAGATG
TATTTAGCTTTGGTATTTTAGTATTGGAAATTGTCAGTGGGAAAAGCAACAGAAAATTCTCTCATCCAGA
TCATAACCTTAATCTACTCGGGCATGTAAGATTTTACTTGTTTTATTTCTTATTTTTTTGATAACCGAGGTG
TTGGAGCCAACATACTTGAGCCAAGCGTCTATCAAAAATAGTCTCTCTACCAACGAGGTAGTGGTAAGG
CCTACATACACACTACCCTCCCCAAACTCCACTTGTGGGATTATACTAGTTATGTTGTTGTTGTCGTGGTG
CCGAGGCCAGCTTGCGCACACCTCAACTTATTCCATGGGATACCTGCAAACTCCCACCACCAATAGGTAC
CGGGTAACTCTGTCCAACAAGGTTAGGACTGATGGGAAGAAATTGCCTAGTGTTTTTTGTCTCTGCTGGA
ATTTGAAACTGATCATCGACCACTAGGTCACACCCTTGGGTGCAGTTGTTTAATTTTATTTATTCCTTGGT
GTATGTTATATACTAGTAACAAATATCTTGTAAAACGACCCCTGCTAATAAGCTTGCAATATCTACAGGC
ATGGAAACTTTATATACAAGGTAGGTCAATAGAACTACTTGATGAATGCCTAGGTGATTCTTGTTCAACA
TCTGAAGTGGTACGATCAATTAGTGTTGGTTTATTATGTGTCCAACAATGTCCTGAAGATCGTCTAAGTA
TGTCTTCCGCAGTTCTGATGTTAAACAATGAAGGTGTGTTGCCGTTGGCTAAACAACCAGGTTTTTACAT
AGAAGGAAATGCACCGGGTGGTGATTTCACTCCTAGCCAATCTGCACAGATTACAGTGAGTGACACCCC
CATCACAATACTAGCTGCTCGATAGAAGATATTGGTTCACTTGTTTGCAACAAGCTTGCAGTTGCAATGA
TCTTTTCTTAATTCTCTATAGTAATGTGGTGAAAAAAATGTGTGCATGATTGTGCTTTTTTAGGTGTATCA
ACTATGTCAAACATATGTAGGAAGCTTGCTGACTGGCTGTTACTTTGATTTGCGCAGTATATCTGAT
>PGSC0003DMG401019242 G-LecRK
ATGTTACTAACTTCTGCAGCACTAGACACAATCACTACAGATAAATCCATTAGAGATGGTGACACAATTG
TTTCAGCTGGAGGGGTTTATGAGCTTGGATTTTTCAGCCCTGGAAATTCGAAGAATCATTACGTTGGCAT
ATGGTACAAGAAGATATCACCTACAACTGTTGTGTGGGTTGCAAACAGAGATATTCCACTTAATGACACT
TCAGGAATGTTAACACTTAATCCCAATGAAATTCTTGTACTTGTTGATAAATCCAATGTATCAATTTGGTC
ATCAAACTCATCAAGATCGTTAAAGAATCCAAAAGCATGGCTCCTGGATTCCGCGAACCTTGTTGTTAGT

GATGGAAATGATAGAGAACAGGGAATTAATTTGGCGTGGCAGAGTTTTGATTATCCAGGAAATACTTTA
TTACCTGGAATGAAGGTAGGAATAGATTTGATTACGGGCATGGATAGGTATGTAACGTCATGGAAGAG
CACGGATGATCCTACTCCTGGTGAATATGTAGATCGTGTTGATTCACATGGATACCCTCAATTGTTCTTGA
TGAGAAATTCATCTGTAGTGTTTTGCTCAGGGCCATGGACTGGTGCTGCGTTTTCTAGTAGTCCTAGTAA
TAAACCAACTTTGTATTATACGTTCGAGTTTGTTGTCAATCAGAAGGAAATTTACTTTAAATACGAGCTTA
AGAATGACTCTATGCCCACCAGGGTGGTGCTCAACCGGATGGGGTGATAGAACACTTAATATGGATTGA
GCATACTCAGAGCTGGTTTCTCTACTTGACAGCACAACTGGATAATTGTGATCGTTTTGATTTATGCGGA
CCTTATTCAAGTTGCAACATCAATAACTCCCCTCCATGTGACTGTTTGAAAGGTTTTGAGCCTAGGTTTGT
ATTTGTTAAACTTTGTGATCATCTCGCGTTAAGCTATTAGAGAGAGTGCACTTTTGAATTTTGATTACTAA
GTTATGTTTTCACACACCCCTCATGTGTGTGCTTGATTCTTTTTCATGGACCAAACACGTGTAATTATTTTT
TTGCCTTCACAGGTATCCTCAAGATTCTGCAGCAGACTGGTCTAGTGGCTGCATAAGGAGAACTTCTTTA
AATTGTACCCATGATGGTTTTCTTAAATTTACGGGTATCAAGGTGCCGGATTCTAGAAACTCCTGGTATG
ATGAGAGAATGAACCTTGAAGATTGCGAGAAAATGTGCTTGGCTGATTGCAATTGTACAGCCTACTCAG
ATCTTGATGTTAGAAATGGCGGAAGTGGATGCTTACTATGGTTTGGAGAACTCATAGATATACGAGAGT
TCAGCCAAAAAGAGCAAAATCTGTATGTGAGAGTAGCTGCTTCAGAATTAGGTAATGACCCTACTCTGC
ATAAATGCACTAACTCTTTAGAATTGAAGGAACTCTGCTACCTAATTTACTTACATGATGGACATTAAAGT
TGATTAGGTGCTTTAGTAGTTGGTAATATGGCGATTTCATTGTGAAGGATTTCATTTTCGGTGGTACTGG
AACGGAAGTAGCTTGCTGCAAAGAGCATTTGAATAATTTCATATCTTACTCTACGACTATATGGATACTA
GGACATAACCGGTTAGTAGGATTGCTAAGGGATGAGACACTTCATTCCCTTAAAAGGCTTAAAAACATG
CTAGACCTCATGACAGATCAAGGCTGTTGACAGCGGGTGATAATCATATGCATTCTGTCCTTAGTAGTGC
CGCTTGTAATCAGTGACACTGTACTTTAATATGTGTCATCTTTCAAAGAAGATCTCCAATTTAACAATGCA
GCTTGTAATCAATTAAACATTGACTACTTTTTCAGTAATCTTGTTGATTGAATGCCCTGGTCTACAAGTTA
CAAAGTCTGCAGGGCTTCATAACAAAATAAGAAGTAATACAATCAATGCAATTAACTACTGATATGGCTA
ACTCATATGTAAATTGAATACTGATTATGAACTAATATAGAACATGTTCAGACAGGACGCGGAGGAGAA
AGAGGTCAGTCCTGATTGGTGTCATTTCAGCAGTGGTAGCAACGCTTATCCTCAGCTTTTTAGCTTGGTT
TTACTTCCAAAGAAGGAAAAGAAGAAGAATAGGTAAATATGCCTGAGGTACTCTTGTCTATCTGGCTGA
CATTGTTCGCCTTGACCAAATTCTCATTGATAGCATGTTTGTGGCAGGCGAATGTATATTGACAGGTTCA
AAAGTTGAAAATGAGGACATGGAGCTTCCATTGTTTGATTTAGTTACTGTTACTAGCGCCACTGGAAACT
TCTCTTCTGCTAATGTGATTGGGGAAGGCGGATTTGGACCGGTTTACAAGGTAAATCAACTTTGATCTTC

AACTTCTAGCTGGAAGTGGCATCTTTAACCCTTGATAGAAAATTGCAGGGTATCCTACCAAATGGACAA
GCAATAGCAGTAAAGAGGCTATCGGAGTATTCTGGACAAGGCATTCAAGAGTTAAAAAATGAAATCGTT
CTCATTTCCAAGTTGCAACATAGGAATCTTGTCAAGCTATTGGGTTGCTGCCTTGAAGGAGAAGAACGG
ATGCTAATCTATGAGTTCATGCCCAACGCTAGCTTGGACTATTTCATTTTTGGTCTGCTTTTTCGTGATTTA
TTGCCTATACTAGTACTTGACAAGCCCTCAATATCTCTGATCACTCAATGATTAGTTCTACATTGTGCAGA
TCCAAGCAGAAAAGCTTCACTTGGATGGAAGAATCGTTTTGAAATTGCTATGGGAATATCTCGTGGTCTT
CTTTACCTTCACCAGGATTCAAGATTAAGAATTATTCACCGAGATCTCAAGACCAGCAACATTTTATTAGA
TAGTGACATGAATGCCAAAATTTCTGACTTCGGCCTTGCCAAAATTTTTGGTGGAGATCAAGTGGAAGG
AGAAACTAAGAGTATAGTAGGGACATTGTAAGATTCTACACATCTTTAAATCGTATAATCTTTATTATATT
AGATCAGTACATGAGCATAAACTTATTGAATTGATGTAGTGGATATATGTCCCCGGAGTATATTGTTGAT
GGGAAGTATTCAGTAAAATCAGATGTATACAGCATCGGCGTAATCATTCTTGAAATAGTTAGTGGCAGA
AAGAACAGGAAATTTCGTCATTTGGATCATCATCACAATCTTTTGGGACATGTAAGCACAAAACTTCACT
CCCTTAATTCACTCTTAGCTTCCTATTGTGAATACATTTGTATAAATGTGTTGTTCTGTTTGTAATTTAGGC
ATGGTTACTCTGGAGTGAAGGCAACGCGTTGGAACTGATAGACGAAAGTATCAAAGAATCCTTTTCAGA
ATCTCAAGTGTTGAGATGCATCCAGGTTGGTTTGTTATGCGTCCAAAAACTCCCCGAGGATAGGCCTACA
ATGGCATCAGTAGTTTTCTGGTTAGGCAATGAAGGTCTAGTTCTTCCTCAACCAAAGCAACCTGGTTTTTT
CATAGAGAGGAATTCAATAGAATCAACAGAGTCATCAACTGATGAAGTATATGTAAGTAGCAGCGTGTC
GATAACAGTTCTAGAGCCAAGATAA
>PGSC0003DMG401028902 G-LecRK
TTTTGGCCATTTTGTGTCAATCCTCTAAATAGAAATTCCCCAGTCAGCAAAGATGCAATCTTGGAGTTTGG
CACCACAGGAAATCTCTTGTTGATGGATGGAGACACCACAGTGTGGGCATCAAACACCTCGGAAGCAG
GCGTTGAGTTGGCTGTCATGTCTGAAAATGGCAACTTCATTCTCTACAGCACCAATCTAAGTACCATTGC
ATGGCAAAGCTTTTCACATCCATCTGATACTCTCTTGCCTGGTCAATCCTTAACAGTGTCACTAGAATTAA
CATCATCAAAATTACCTTCTCATGGTGGTTACTACACTTTAAAAATGTTGCAGCAGCCTACTTCACTAAGT
CTTGCATTGACCTATAATGTCCCCAACTACTACGATTCATCGCCTGAATTTTACAGCAATTTTTCTTATTGG
TCAGGACCTGAAATATCAAATGTGACTGGAGATGTTACTGCAGTATTGGATAAAGCAGGAAGCTTTGGT
ATAGTTTATGGCTCATCTTCAGATGGAGCAGTTTATGTATATAAAAATGATGGAGATTATGGAGGACTAT
CATCGGCTGTAAATCAAACGAATTCCAATGTTCCATCTATTCTTCGTCGATTAACCCTTGAGGTTAATGGA
AATTTACGTTTGTATCGATGGGATAATGATGTGAATGGATCAAGGCAATGGGTTCCAGAGTGGGCAGCT

GTGTCTAATCCATGTGATATTTCTGGAGTTTGTGGCAATGGGATATGTAATTTAGACAGAAGCAAAACA
AATGCTTCTTGCACATGTTTGCCAGGGACTTCTACGGAGGGAGATGATGTTTCATGTTCGGGAAATCCTT
CAGTGACAGGGAAATGTGGACCTCAGCATGAAAATATGATGTCCCAGTTCAAGATTTCTACGGTTCAGA
AAACGAATTATTATTTCTCAGAATCATCTGTTATAGGAAATTATAGCGATAAAGGGTCACTGTCCAAATG
TGGTGATGCTTGCTTATCAAACTGTGGTTGTGTTGCATCTGTTTATGGACTTAGTGAGGAAAAGGCTTAT
TGTTGGTTACTCAGGAGCTTGGAATTTGGTGGATTTGAGGATCCTGTTTCGACCTTATTTGTGAAGGTTG
AGGCCAATGCCTCGGTATCTGAAAAACCTGGGAATTCATCAGATGAGTCGAAGAGTAGGCACGATAAG
GTTTTGGTACTTCCTATAGTCCTGAGTATGACTGTTCTCATTTTGCTTCTCTGTTGTTTATTGTATATCAAT
ATCCATAGGAGAAGATCTATGAGAAGGGCACTTGAGGGATCTTTGCTGTTGTCTGGAGCTCCAATTAGC
TTCAGCTACCGCGACCTGCAATGTTGGACTAACAATTTTTCCGAGTTACTTGGAACAGGTAAAGATATAG
AATTTTTATTACTTCTATGATACTAACCTCTCAACTATAGAAATAACAAGCACAGAAAACTGTTCTCTTTCA
GATAAGGTAAGAATATGATCAGTAACATTGCTGCTTTGACAGGTGGATTTGGAAGTGTGTATAAAGGAA
GTTTAAAGGATGCAACATTGATTGCAGTAAAGAAACTTGATAAAGTTTCACCTCATGGGGAGAAGGAAT
TTATAACAGAGGTCAAAACCATTGGCTCTATGCATCATCTGAACTTGGTTCGTCTATGCGGTTACTGTTCT
GAGGGAACCCAACGGTAAGTTTTAACTTTTTTTCATTGTAATTTGTGATGTATAGATTGGGAAGTACGTA
GAATTGTAGCATTACAGTCAAGCACTAGCACAACTTTCACTAAAATTTTCACTCTTTGGCAGGCTACTAGT
GTATGAGTACATGAAAAATAGCTCATTAGACAAGTGGATATTTCATTCATTTAGTACAAGAAATAGACTA
CTGGATTGGGCGTCACGTTTTCGCATAGCAGTTGGCACTGCACAAGGGATAGCCTATTTTCATGAGCAAT
GTAGGAACAGAATTATACATTGTGATATCAAGCCAGAAAACATACTTCTGGATGAGAATTTCTGCCCTAA
AGTATCTGATTTTGGACTAGCTAAGTTAATGGGAAGAGAGCACTCTCATGTTGTCACAATGATCCGAGG
AACGAGAGGTTATTTAGCTCCCGAGTGGGTCAGCAATCGACCTATCACTGTAAAAGCTGATGTTTACAG
CTATGGCATGCTTCTTCTAGAGATCATTGGTGGTCGGAGAAATCTTGATATGACTTGTGATGCAGATGAC
TTCTTTTATCCTGGATGGGCTTACAAGGTACACACGCAATCCTCTAATTGTATTCATAGTGTTCCCTCCTTT
CTGGTACATGTTCTCTTCACTATCTTTCTCCTTTTTTTCAGGAGATGACTAGTGGAACACCCGTAAAAGTT
GTAGATAGGCGACTTAGAGGAGCAGTAGAGGAAAAAGAGGTAACGAGAGCACTGATGGTCGCCTTCT
GGTGTATTCAGGATGAAGTGTCAAACAGGCCTTCCATGGGAGAAGTGGTGAAGATGTTGGAAGGATCA
GTTGACATGAATATGCCACCAATGCCACAGACAGTTTTGGAGTTAATAGAGGAAGGCTTAGATCAAGTA
TACAAGGCAATGAAGAGGGAACTCAATCAGTACAGTTCTTTCTCCATTGCTACTCATCCATCATCTAATG
CTACATGCAGTCACTCCAGTATGTCACCTAGATAGTTGAAGGTGAAATCGAACACAGTTTTTCCTAGGAT

TAAGTAATCGTATCATCATTGATTATTCATTCCCCGTCTCCCAAAAGAAGTCCCGGAAAAAGAATAAGAA
GGGAACACAACCACTTTTGTATCATGTGTTGCTACAACACCATACACGTTGCAAGAACTCACTGTCTTGTT
TTATTGTTTAATTTCACTCAATTCCTCTGGTCTCTAAGATATCTCCACCATTTCCTCTGTATAATTCGTCACT
ATCTCTTTTTCGGTTTCTTTTATA
>PGSC0003DMG401029444 G-LecRK
GGTAATAGGAAGAGTATTAGAAAACCAAAAAAGATGACCACTAGCAGCAGAAATGTGCATCATTTTGTT
CATCTAATTCTTGTTATTCTTCATTGTTTTAATACAGGATTTTGCACAGAAGTAGATAGCATTACCAGCAC
TCTATCTCTGAGGGATCCTGGGATTTTATCATCTCCAGGAGGCGTCTTGAAGTTGGGATTTTTCAGTCCTC
TAAACAGCTCTAACAGGTATGTTGGTATTTGGTACAATTTTTCTGAAACGATTGTCATTTGGGTGGCTAA
TAGGGACAAACCTTTAAGAGATTCTTCTGGGGTTGTCAAAATATCTGGTGATGGAAATATCTTAGTTATG
AACGGGGAGGAGGAGATTCTTTGGTCATCAAATGTTTCAACCTCTCAGGTAAACTCAATTGCCCTTCTCC
AAGATTCTGGGAATTTTGTTCTTGTAGATCATCTGAACAATGGGAGCACAATATGGCAGAGTTTTGAACA
TCCTTCTGATTCAATTGTCCCTAAAATGAGATTAAGTGAAAACACAAGGACAGGGGAGAGGGTAGAAGT
AAAATCTTGGAGAAGCCCTTGGGATCCTAATTTCGGAAACTTTTCTTTAGGAATGAATTCTGGATTCATTC
CTCAGGTGTATATCTGGAAAGGTAGTCATCCCTATTGGCGAAGTGGTCAATGGAATGGCCAGATCTTTA
TCGGAGTGCAGGGTATGTATTCTGTGTCGTCTGATGGATTCAATGTAGTGAACGATCGCGAAGGTACTG
TATACCTTACTGGCCCTGGTGATTTTGATTTCTTAACGAAATTTGTCTTGGATTGGAAAGGGAATTTGGTT
CAATCATATTGGGATGTGAATGAGACAAACTGGAAGATAATATGGTCAGCTCCCAATAACGATTGTGAA
GTTTATGGAACATGTGGTCCATTTGGGAGCTGCAATTTGGAGTCGCCGATCTGTTCTTGTCTGAAAGGTT
TTGAGCCAAAGCACAGGGAAGAATGGGAAAAGGGGAATTGGACTAGTGGTTGTGTTAGGAGGAAAGC
TTTGCAATGTGAAGTAAGGAATAACTCAGGGGATTCAAGTAAAGAAGACGGATTTCTAAAGATAGGGT
CAATAAAATTGCCTGATTTTGCAGAAAGGTCATCGACTAGAGAAGACCAATGTAAAAGCCAATGCTTGG
GATATTGTTCCTGCATAGCGTATGCATATGACTCAGGTACTGGCTGTATGTCATGGAGTAACAACTTGAT
TGATATTCAGCAGTTCCAAAGCTCGGGGAAAGATCTCTATATTCGCGTGGCACATTCAGAGCTTGGTATG
TATAGTGGTGTTGAATAGAAGACTCATTATCCACCCAGAATAGCTTAATCAGTCATTTGAGATATCATGG
ACTTATTGAATTCCATATATATACATTAAGTAGCTTACTTCTGTAGAGAACTGATTTTTGTTCACCTTTTGT
GATTGCTCTACATTACAGATCATCATAAAGACATAAAGAAAATTGTAATTCCAGTAATCCTTGGTTTTCTT
ACACTCTGTGTTTGTCTGTTCCTTTGCTGTACATGGATGGCCAGACTTAGAGGTAAAACAAGCTATAAAG
TTTTAGTATTATACATTTAGAAGCACTTTGTTTGCTATAGTAGTTAATTTGTATCCCTGCTGATATTAGGA

GTAAAAAGGAAGAAGATAAATTTACTTGGTGACAGAAGTGCAGTTCATATGGAAGAGTTACCAGTCTTC
AGCCTCGATACGCTTGCAAATGCAACATCCCAATTCCATGAGGATAAGAAGCTTGGTCAGGGCGGTTTT
GGTCCAGTTTACATGGTACAATTTTCTTCTCTCTCGATCACTTTTCAAATTTCAGTTGCTGAAATTGCTGAT
TACTTGTAGGGAAAATTGGAAGACGGGAAAGAAATAGCAGTCAAGAGGCTTTCAAAAGCCTCGGGACA
AGGGCTGGAGGAGTTCATGAATGAAGTGTTGGTGATCTCGAAAGTCCAACATAGAAACCTTGTTAGACT
TTTGGGATGTTGCGTTGATAAAGAGGAGAAGATGTTGATTTATGAATATATGCCCAAGAAAAGCTTGGA
TGTGTTTCTCTTTGGTTAGTCCTTGCTTTTAATAAGCATTGTACTTCTCATAGCAGTTCTTTGTTACTCCAC
ATCTATGTTGTTCGGACTCTTCAATAATGTCGATGGATGCTTGTTAGATCCTCCAAAAGTACTGCACTTTT
GGAGGATCCGACACGAGAACATCAACAATTTTGGGGAGTCTGAGCAACATTGCTCCACATTTCTTCTGA
AGCAAAGTTAATCCCTTCTCCATTCATATTTGACAAACATCTAGATGAAGGCCACCGAGGCATTTTGGAT
TGGAGAAAACGTTCCACTATAATCGAAGGGGTTGGTCGAGGACTCCTTTATCTTCACAGAGATTCAAGA
TTGAAGATAATTCATAGAGATCTGAAGCCAAGTAACATTTTGCTCGATAATGACTTCAATCCAAAGATTT
CAGATTTTGGCATGGCTAGGATTTTCGGATCTGACCAAGATCAAGCAGACACAAGGAGAGTAGTTGGTA
CTTAGTAAGTGGACATTGATCTATATCTACCATAGCTTTACACAGTTTTCTCCTGTAAAAAACCATATCAT
ACATACTTACTAACTAATCATTTTTTACACGAATGTGCTGACATTTTTACAGTGGATACATGGCCCCTGAA
TATGCAATGAAAGGAAGATTCTCTGAGAAATCGGACGTTTTCAGCTTTGGAGTTCTAGTGTTAGAGATC
ATCAGTGGCCGAAAAAGTACAAGTTCTTGGAATGAGACATCCTCTTTTAGCCTTTTCGGATATGTAAGAT
TCCCTTTATATGCTACTATTCAAATACTAAAAATGCTCATACTGCAAAAACTATACAGTCAAAACATTTCT
ACATCGACCTCGTTTGTCCAGATATCTTTTGGTTGCTATAGTGAAATGCTATTATAGAGAACATCCAAAGT
AAATTTCACCATTATAGTAACGTGTTGTTATAGTTCGATTGTGTGTACCTTATATTTTTACATTATGATGGT
AGAATTACTTTTGCTGTCACTGTGTTTTATGCATCAATAAGTTTGATGATCAATTTTTCACTGTTTTCAGGC
ATGGATGTTATGGAAAGAACAAGATTTATCAACTTTTATCGATCCATTCATATTGAACACGAGCTCGGAA
ATGGAGATCAGAAAATGCATACAGATTGGTTTACTATGTGTTCAAGAATTTGCTGAAGACAGACCAAGT
ATTTCATCTGTTCTTGCTATGCTTACCAGTGAAACTACAAGTCTTCCTGCACCATCACAACCTGCTTTTACT
GAAAGACATGATTGTATCTTCAAAATGTGCAATGTAACTAATTGTACTTTGAACAATATCAGTATCACAA
ACATAACTGGTAGATGACATGAGTATATGAAATTATTATTTTTAAGATATGCAAAATTATCCATTTGTCTC
AATTTGTATGATATCTTTGTCATTCATTTTTCGATTACATGTTAAATATTCGTGTTGAATCAAATATCATAA
TCCACAAGTGTAATATGTGTTTGCCAATCACAGAATATATTATGGGATGTATTTGGAATAAAATGAAAAG
GCATTTAGGTTTAATGTCAATTTGTGCAACAATACATTTTTCATTACACTATGTGCATTATTACCACTTATA

GTTGACAAGCATGTTGACGTTGAGATGCTAGCTTATTGTCAAGGTCCCCACTCAATTCTCACACTAACGT
GGCTTTTGGATTTTCACTCATTTTACTTTTTCGAAATAGTTTAAATATATCAATCATTATATTTCTGGTCTA
AGGTTGAATTAAGCTTAGATGTCATATCTTTACATCAATACTCATTTTATTTTATTTTCTTCTTCTTTTTTCA
ATGAGCATTTAATAATTTAAGGAAAAGAAATGTGTTATTTCAACAAGTTAAAACTTGTCAATTCACAATG
TTATTTCAGGAACATACTACACAATGATAGTGATGGGAGAAAAGACTTATATTGTCAGCGTATAGAAGT
TAAACTCTACATTTTACATTATTCGTTTTTAAGTGTCAATTTTTGCATTGGCCTAAAAGTCTCGGATAGAG
AATGATATGTGGTTTGTGATATTTCTATAGGGTCAACAAATTAAAAATCCTATTTGGCTGCTTGTGATATT
AGACTTTGACAACATATTGTGCTTTTGTTCATATAGTGTGGTAATAAGAAAAGTCTTAGAAAACCAAAAA
GATGAGCAACAGCAGCAGCAGAAGAAGAAATTTGCAATATTTTGTTCATATAATTCTTGTTATTCTTCGT
TTTATCGATACAGGATTATGCAGTGAAGTGGATAACATTACCAGCATTCAATCTCTGAGAGATCCTGGAA
TTTTATCGTCTCCAGGAGGCGTATTCAAGTTAGGATTTTTCAGTCCTCAAAACAGTACTAATAGGTATGTT
GGTATTTGGTACAATTTTTCTGTAACAACTGTCATTTGGGTGGCTAATAGGGACAAACCTTTAAGAGATT
CTTCTGGTGTTGTAAAGATATCTCGTGATGGAAATGTCGTTATAACGAATGGAGAGGAGGAGATTCTTT
GGTCATCAAATGTTTCAACTTCTCAGGTAAACTCAATTGGCCTTCTCCAAGATTCTGGGAACTTTGTTCTC
GTTGATCATCGGGACAATATGAGCACTATATGGCAGAGTTTTGAACATCCTTCTGATTCAACTATTCCTA
GAATGAGAATAAGTGAAAATACAAGGACAGGGGAGATGGTAGAAGCAAGATCTTGGAGAAGTCCTTC
GGATCCTAATATCGGAGACTTTTCTTTAAGAATGAACTCTGGAGTCATTCCTCAAGTGTATATATGGAAA
GGAAACCGTCCCTATTGGCGAACTGGTCAATGGAATGGACAGATCTTTATAGGAGTGCAGAATATGTAT
GCTGTGGTATCTGATGGATTTAATGTAGTGAATGATCGCGAAGGTACTGTATATTTTACTGGACCTATTC
GTGATAATTTTTTAAGGATATTAGTCTTGGATTGGAGAGGGAACTTGGTTCAATCATATTGGAATGTGAC
TGAGACAAACTGGAAGATAATATGGTCAGCTCCCAGTAACAATTGTGAAGTTTATGGAACGTGTGGTCC
ATTTGGGAGCTGCAATCATTTGGAGTCGCCGGTCTGTTCTTGTCTGAAAGGTTTCGAGCCAAAGCATAG
GGAAGAATGGGAAAAGGGGAATTGGACTAGTGGTTGTGTTAGGAGGAGTGCTTTGCAATGTGAAGTTA
AGAATAACACCGCGAATTCAAGTAAAGAAGACGGATTTCTAAAGATGGAGTTGATGAAATTGCCTGATT
TTGCTGAAAGGTCATCTACTTCAGAAGACTTATGTAGAAGCCAATGCTTGGGGAATTGTTCCTGCATAGG
GTATGCATTTGATTCAGGTATCGGCTGTATGTCGTGGAGTGAAATGATTGACATTCAGCAGTTCCAAAGC
TCGGGGAAAGATCTCTATATTCACGTGGCAAATTCAGAGCTTGGTATGTATTCTGGTACTGAATAAAAAA
CTCATTGTCAGTCATTTCAGATAGGCGTAATACATAAATGTGCCCTTTTACTTGGCCTCAACTCACATCTA
TGCTCTCATGAACACTCAAAGTTAGATGGCATGGATGTCAGCAAAGGCCAAATCAAAAGACACATTTAT

GTATTATGCCTTTGAGATATGTTCGACTTAATTGAATTCCATGTATTCTGTAAAGAACTGATATTTGTTCA
CCTTTAGTGTTTTCTGCAGATCATGGTAAAGACATAAAGAAAATCGTAATTCCAGTAATTGTTGGTTCTCT
TACACTCTGTGTTTGTCTGTTCCTTTGCTATACAATGGTGATCAGACGTAGAGGTAAACATGCTAATAAG
TTTTTGTATTACACATTAAGAAGCACTTTGTTCGCTATAGTTAACTTGTATTCCTGCTGATATTAGGAGTG
AAAAGAGAGGAGGTAGCTTTACTTGGTAACAAAAGTCCAGTTAATATGGAAGAATTACCAGTCTTCAGC
CTCGATACGATTGCAAATGCAACATCCCAATTCAATGAGGATAATAAGCTTGGTCAGGGCGGTTTTGGT
CCAGTTTACAAGGTACGAGTTTCATCTCTCTCGATCACTTCCCATAATTCAAGTTACTGATATTGGTGATT
CCTTGTAGGGGAAATTGGAAGATGGGAAAGAAATAGCTGTCAAGAGGCTCTCAAAAGCCTCGAAACAA
GGGCTGGAGGAGTTCATGAATGAAGTGTTGGTGATCTCGAAAGTCCAACATAGAAACCTTGTTAGACTC
TGTGGATGCTGTGTCGATAAAGAGGAGAAGATGCTGATTTATGAATATATGCCAAAGAAAAGCTTAGAT
GTGTTTCTCTTTGGTTAGTCCTTGTTACTTCTCATCTGCATTACTCCACATCTATGTTGCTCGGACTCTTCA
ATAATGTTGATGGATGCTTGCTAGATCCCCCAAAAGTACTGCATTTTTGGAGGATCCAACATGAGTACAT
CAGCATTCTGAGCAACATTACTCCACATTTCTTCTGATCCTTTTGTTTAAATTCTCCATTCATATTTGACAA
ACATCTAGATGAAGCACATCGAGACATTTTGGATTGGACAAAACGTTCCATTATAATCGAAGGAGTTGG
TCGAGGACTCCTTTATCTACACAGAGATTCAAGATTGAAGATAATTCATAGAGATCTAAAGCCAAGTAAC
ATCTTGCTCGATAATAACTTCAATCCAAAGATTTCAGATTTTGGCATGGCTAGGATTTTCGGATCTGATCA
AGATCAAGCAGACACAATGAGAGTAGTTGGTACTTAGTAAGTGATCATTTATCTATATACAATACCATAA
CTTTGCATAATTTAACTACTCGAATCGTACTGACATATTTACAGTGGATACATGGCACCCGAATATGCAAT
GGAAGGAAGATTCTCAGAGAAATCCGATGTTTTCAGCTTTGGAGTTCTAGTCTTAGAGATCATAAGTGG
TCGAAAAAGTACAAATTCTTGGACTGAGACATCCTCTTTGAGCCTTTTGGGATATGTAAGACTCCTCTTAT
AGTTGCTATAGCGAAATGTTTGTTATAAAGAACGTATAATATAACATAACATGAAGAATCAGTTCCAAAA
AAGAACTTGGCTGTTATATAATGAAATGTTATTATAGAGGACGACTATTATAAAGAGGTCTGACCGACT
ATATCATGTACCTTATATTTCAGGCATGGAAGTTATGGAAAGAACAAGATTTATCAACTTTTATCGATCC
GTTTATATTGAATCCGAGCTCGGAAATAGAGATCAGAAAATGCATACAGATTGGTTTACTATGTGTACAA
GAATTTGCTGAAGACAGGCCAAGTATTTCATCTGTTCTTGTTATGCTTACTAGTGAAACAACAAGTCTTCC
AGCACCATCACAACCTGCTTTTACTGAAAGAAGACACTTCAGAATGTGCAATGAAAATAGAGAAACTAA
GTTTACTTTGAACAAAATGAGTATCACAAACCTTACTGGTAGATAACATGAGCATATGAAATTACTTTTTA
AAGATTTGCACAATTGTTATAGTATGATCCATTCGTCTCAATTTGTATGATACCTTTTGTTTCAAAATGTTT
GACAATATTGGGTCCGTGTAAAGCACAGAATATTTGTTATTAGTTACAAA

>PGSC0003DMG402003983 G-LecRK

AAAAAAGAGAAGAAAAGCTAAAATCTATGGCTGCTTTGCTATGGTTATTAATATTATCTGCATTTCATGC

AGTAGCACTAGCACAACAAAGGCAGTTCAATATAACTCTTGGCTCTTCTCTAACACCTACTACCAATTCAT

CATGGTTTTCGCTGTCCAGGCGCTTTGCCTTTGGGTTTTATGAACAAAACAATGGCTATGCTGTTGGAAT

CTTAATTGTCGGTATGCCTAAGAAGACAGCAGTGTGGACAGCAAATAGGAACAGTCCTGTTGTTCCGAG

CAACGCTGTCTTGCTCTTAACTAATGATGGAAGGCTCATTGTACAAGTAGGAGGACAAGAAATCACTGT

CGTAAATCTCTCTGGTCAAGTCATTGCTTCAGCTTCCATGCTGGATACTGGCAACTTTGTGCTCTATAATT

CTGATCACAGTATCATATGGCAAAGTTTTGACAATCCAACAAACACCCTTTTACCTGGTCAGCATATATCA

GCTGGACAAGAGCTGTTCTCGAGTGCCTCGGAAGCAGATGATTCTTTCGGGATTTTCCGTCTCAAAATGC

AAGATGATGGGAACCTCGTTCAGTACCCTGTCAATACACCTCATTAGGGATTTTCCGTCTCAAAATGCAA

GATGATGGGAACCTCGTTCAGTACCCTGTCAATACACCAGATAGTGCCTCATATTCTTACTACTCAACAG

GCACTTATGGAGCAGGGAATAATGTTACACTGAATCTTGATGATGACGGCCTCCTCTACTTACTCAATTC

CACTAAAAGTCTAAGAAATCTAACTCAAGGAGGCAACCGTAGAGAAAGGACCATTATCTATATGCTGAA

AATTGATGCTGATGGTATACTTAGAATTTATTCAAATTCTCTGAACCAACAGAACAGCTCCGTGATATGG

TCATCAACAAATGATAGGTGTAATCCAAAAGGCCTTTGTGGGCTTAATGGTTTTTGCACCAACATAGATG

ACCAAGCTAAATGTTTGTGTCTTCCGGGATTTGATTTTGTAATGCCAGGAAATTGGAGCGCAGGCTGTG

AAAGGAATTTCACCGCAGAAACCTGTCGGTTAAAGGAGAGCACTTCCAAATACTATGCTATGAGAACAG

TTGAAAACACAGGATGGGAAGATAGTACTTATGTTGTTTTGGCCGGAACGACCAAGGAAGATTGTGAA

CAAGCCTGTCTACAGGATTGTAACTGTGAGGCTGCTCTGTTCAAAGACAGAGAGTGTAGAAAGCAAAG

GCTGCCTCTGAGGTATGGAAGGAGAGACTTGGGTAATTCTAATTTAGTATTGGTCAAGGTGGGCACTAA

TTTCATACCCAACGAAGGGGTGCTTTATCAGATGATTGAAGAAACAAAAGGCAAAAAGCTAAGGATAG

ACATCCTAATTGCCGGCATTACATTGGCTGTTTTTGCTTTGTTGGTGTTAGGCATCTCTGGATTCCTCATTC

ACAGAAATCATGTCTGGACTTACAGAAAAATCCAGGAGAGTAGAAGTGTTCAGTTATGTGAGGATGTTG

CTCCACGAGCATTTTCCTATGCAGAACTAGAGCAGGCAACAAGTGGTTTCAAAGAAGCGTTAGGGAGG

GGAGCATTTGGAACAGTATTTAAAGGAATTTTGGCAGAAGACCAGAAAGTGATAGCTGTTAAAAGACTA

GACAAAGAATTAGTAGAAGGGGAAACAGAGTTCCAAACTGAAATAAAAATCATTGGAAGGACACATCA

CCGAAATCTCGTCCACCTCCTTGGTTATTGCCTGGAGGGATCAAGAAGGCTCCTGGTGTATGAGTACATG

ACCAATGGGTCACTTGCGGACATACTTTTCACAACAGAAAAACAGCCCACGTGGGAGGAAAGGTGTGG

CATCACTCGAGATATTGCAAGGGGCCTCCTATATTTGCATGACGAGTGTGACACACAAATCATCCATTGT

GATATAAAGCCTCAGAACATACTCATGGATGATCAATACTGTGCAAAAATATCTGACTTTGGAATGGCCA
AGCTTTTAAAGAAAGACCAAACAAGAACCTACACAGGGATAAGAGGGACCAGAGGCTATGTTGCACCA
GAATGGCACCGGAAACTGCCAGTGACAGTGAAAGCAGACGTTTACAGTTTTGGTGTCCTCCTATTGGAA
TTGATCTGTAGGCGGAAGTGTGTTGATATGAGCCTTGATGAAAATGAGTCAATTCTTGAATACTGGGTCT
ACGATTGCTTTGCTGCAGGAGAGCTAGATAAACTTGTCGGGGACGAGGAGGTTGATAGAAGACAATTC
GAGAGAATGGTGAAAATAAGCATCTGGTGCATTCAAGACGAACCATCACTTCGCCCTTCAATGAAGAAA
GTTCTACTGATGCTGGAAGGGACAGTAGAAATTCCAGTGCCTCCTAGTCCTACTTCTTTTTTAAGTGCCAT
ATAGTTTTGAACTTCTTCACTTTATGGTTTTTGTACTTCAAATTTCAAGTATTCCAACTTCCCCATAGAGAG
CTGACCACTTTATCATCCATACTTGAGCTAAATAAAATATAGTTCTATCAAATTTCTACATCATTC
>PGSC0003DMG402004946 G-LecRK
ATTTTAAGGCCTTCAGAACTAGCTAGATTTACACTGAAAAGGTCAGGTTACCTCCAGATATCTAAACCTG
CATAGATTAGGTAGCATGTAATAAAGCAGTTCTTAATGGAAAGTTTTGTGCTTATATTGCAGATATTGTT
ATGTGGTTTTGTATGCTCAATTTCTAGTGCGAAAGATATCATCACTGCAACTGACTTTCTAACAGATGGA
AAGACCATTACTTCATCTGATGGAAGTTTTGAGATGGGATTTTTTTCCCCGGCTAGTTTCACGAATAATTG
GTATGTTGGAATATGGTATAAGCACGATGTACCTGATAAGTCTGTGGTATGGGTTGCCAATAGAGAAAA
TCCATTCAACAACACATCAGGTGTTATGTTGAAAATCATCGATACAGGACAACTTGCTCTTCTCACTCCTG
CTAATACAATCATGTGGTCTACTAATTCATCGAGGCCTTTGGCTGTGAAGAATACAGTTGCACAGTTGTT
GAACTCGGGGAATCTTGTCATAAGAGATGCAAATGATACCAAGCCTGAGAAGTTCCTGTGGCAGAGTTT
TGATTATCCTACTGATACACTCTTACCAGGCATGAAGATGGGTAAGAACTTTGTAACTGGACAAGAATTT
TACCTGTCCTCATGGAAAAACGAATATGATCCAGCTCCAGGGGAATATACGTATCATTGTGATCCGACTG
GATATCCTCAGGATGTCATGAGGAAAGGTAAAGTTAAGGTGTACAGTACTGGACCATGGAATGGACTC
CGCTGGAGTGGTGTACCAGGACTGACAAAAAATACTATTTATACATTTAAACTGGATCTTGATGAAAAG
AGAGCATTTTACAGCTTCGCGCTTTTAGGTTCTGTTATGACCAAATTAACCATGAACAATAACGGCGTGC
TTCAGCGTTCAATGTGGGCTGAGAATAGACAGGAATGGCATGTTTACATTTCTTCACCGGAAGATACTTG
TGACAATTATGGAACATGTGGTTCATATGGTAGTTGCAACAACATACTCACTCCTGTGTGTAATTGCTTA
GACAAGTTTGTGCCTAAAGATCCAAGAAATTGGGCAATGACAAATTGGTCCGGCGGGTGTATTAGAAG
GAAACCGCTTAATTGCCAGAATGGAGATGGATTTCTCAAGTACTCTGGTATAAAGCTACCAGATACACA
GTATTCATGGTTTGATAGGAGTATGACACTCCACGAGTGCAAGCACGCTTGCTTGAGGAATTGCTCGTG
TTTGGCTTACTCAAATCTTGATATACGCAATGGTGGGAGTGGCTGTTTGCTTTGGTGTGGCGACTTAATT

GACATTCGAGAATTACCTGGAGGACAGGACATTTACATAAGGGTTGCTACCTCAGAGTTAGGTAACATT

TGTGAATTTGATCAAAGTTGGAGTATTAGCTGAATGATTTAAACTTCCCAATTCTCAGATTTAGTTGTATT

CTACTTAGATATCTGTTAAAAGTTCACACAGTATATGTTCACTCTTCCTTGTCTATTTAGTTTATTTCACCC

CTCCATGTTTTGTTCAGGCTCGAAGAAAACAAAACTACTCGTGTTGAGTCTGTTACTGTTGATTGGAGTG

ACTGTGACTGGCCTGACCATTGGGCTGTATATTTGGAAAAAGAAGAAGAAGAGGAAGATGAATTTGAA

AGATGACCTAGACCTCCCACTGTTTACCTTATCAACTCTAACTAAAGCATCCTCTAATTTCTCAGTCGAGA

ACAAGATTGGAGAAGGTGGATTTGGATCTGTTTATAAGGTAACATCTTAAGTTTATGAATCATTCTTTCA

GTAAATAATATCTACTATTATTGACAGTAAACAGATGACACTTACTGAATCACTAAGCCAGATATATGTT

GATATTATAGGGAATCCTGGAAGGTGGACACGAAGTAGCTATAAAGAGGCTATCCAAGTCTTCCTCGCA

AGGAGTTAACGAGTTCAAGAATGAAGTTATTTGCATTGCCAAACTTCAGCATCGAAATCTTGTGAAGCTT

ATAGGTTGCTGCATTGCAGGAGGAGAAAAAATGTTGGTCTATGAATATATGTGCAACAGAAGTCTAGAT

CTCTTCATATTTGGTCTGGTTACTCTCTCTTAACTCGAATAACCTCTATTAGTACAAATAGTAAAGTTGATA

GTTTAATCACATTATCAGTGTTCTAACAGAGGACCTGTTAACAGATGAAAAGAGGAGCTTGTTACTCAAC

TGGCCTAAGCGCTTCGGCATCATCAATGGAATTGCTAGAGGACTTCTGTATCTCCATCAAGATTCTCGGC

TAAGAATTATCCACAGAGACCTCAAAGCTAGTAACATTTTGCTAGATGCTGACATGAACCCCAAGATCTC

AGATTTTGGCATAGCTAGAAGCGTTGTAGGGAATGAGACTGGTGCTAATACACACCATGTGGTTGGGAC

ACAGTAAGAAATTTAATACTACTCACTTGTTAGCATTTATATTATGTATAATAACCTTTATTTTACACTACT

TTCTGTTTAATATGTGATGCAGTGGTTACATGTCCCCGGAGTATGTAGTCCATGGTGTTTTCTCAGTAAA

ATCAGATGTATTTAGCTTTGGCGTATTAGTGCTAGAGATAATCAGTGGTCGAAGAAACAGAGGATTTGC

CCGTGAAAGTCACAGTATCAACCTACTCGGACATGTAAGTCTTGTTTGTAATTAAAGACGAGTTTAAATT

TTGTGCACTATATGGTAGCTACAGATATTTCTCTATAATAATTAATTGATAACACCTCGTACAAATGATAG

TTAAACTGCATTGAACTTAAATCCTTCAGGCACATCAACTGGTGCATGTTTCTTGATTATGTACTGATTGT

GTTATTGATAGGTATGGAAACTTTATAAAGAAGGTAGACCATTAGAGCTAATTGATGGCCATCTAATGG

ATTCGCGTTATATTTCTGAATTGCTACGCTTAATCCATGTCGCTCTGTTATGTGTACAACAATGTCCAGAA

GACAGGCCAGATATGCCTACAGTGATCTTGATGTTGACTAATGATGCTATTTTGCCTCAAGCTAAGGAAC

CTGGTTTCTTCACAGAAAGCAAAGTCACGTCTGAATGTTCTATAAGCATGTGTTCAACAAACGAAATTAC

CGTCACACAGTTAGAACCCCGGTAAATTACATTACATGTTCATTCAGATCGTTCAATGTCAACAAGCTTG

AATTACAGTATTTTGTGCTGTAAAGCCATAATTGCCTATGGTATCTGTTATTAGTAATTATGGGTGTGTTG

TAGTTTTTATTAATTCTAATGTATTTTGAAGAATTCGTACAAAGTACCTATTCAACATGAGTACATCAAAA

TAAACGAAAGAT

>PGSC0003DMG402005047 G-LecRK

CAATGGTTATCACATGAAAAGAACCATAGCAATTGTGACAATCTGCATTGGTGCAATACTCATCGCGCTC

TCGTTTGCTTGTTGTTTGGTAATAAGGAAAAGGAGAAGAGGTAAAGGGTACCCCTTAATTTCTTCGTGTG

TTTACTACTTTATTGAATCCCCTTAGTGAAATCTTGGCTCCGCGACTTACAATAGTGAGTTACTTCATGTA

CAGATAAGGAGGACCAATTCACCAGTTTGAACACTTTGACAAGGAATTTAGCATCCTATGAGAACTCTTC

AAGAGGTAATGAGATGGATGGAAGTGAACACGTCGATGTGTTAATCTTCGATCTAAGCACTATCATTTC

TTCCACTGATGATTTCTCTGATGCTAACAAACTCGGAGAAGGAGGCTTTGGTAGCGTTTACAAGGTACCT

CCCGATATTTTACCAATTTCTTACTCATTTGTTGAGCCAATTTTACTTGACAACAAGCTTACATTTTGATTT

CAGGGCCAGCTAAACAATGGGCAAGAGATAGCGGTCAAAAGACTTTCGAAAAATTCAGGGCAAGGAGT

GGAGGAATTCAAGAATGAAGTAACGTTAATCGCGAGAGTACAACACAGGAATCTTGTGAGGCTTTTAG

GATGTTGCATACAAAGAGGAGAGAAAATGTTGATCTATGAATACTTGCCAAACAAAGGCTTGGACAGTT

TCATCTTTGGTATATACATTTGCATATTGTGTTAAAATTTTAAAAAATAGGCATGTTACTGATGGATTGCT

TATGTATGAATTTCGAATCGGTATTGCAGATAAAACTAAAGGGTCGCAGTTAGATTGGAGAAAACGATT

TGAAATCATCGTTGGAATAGCACGAGGATTATCATACCTTCACCACGACTCACGAGTAAGAATCATACAC

AGGGATCTAAAAGCCAGCAATGTTTTACTAGATGCCTCAATGCACCCGAAAATATCAGATTTCGGAACT

GCTAGGATATTTGGTGGTGACCAAATTGAAGCTAACACGAATCGAGTCGTTGGAACATAGTAAGCATCA

CTTTTATGTATGAACATATGTGTATTTTTCTCAAAGGCACTTAAACTTGTATAAAGTTGAACAAGTAGACA

CACTCGTCCTACATGATGATTCGCATAAGATACACTCGAACTGATTTGTATTATGCCATGTAAGACGCAT

ATTATGTCATGTAGAACATGTGTGTCTACTTGTTCACACCTCATAGATGTCAGTTGAGGCTAAGTTAAAT

GACACGACGTTTATGTATTATGTCATATTAAAATTGTGGATGTTGACATAGCTATTGGTTCATTTCAGTGG

TTATATGTCACCAGAATATGCAATGGAAGGGCATTTTTCAGTGAAATCTGATGTTTTCAGTTTTGGTGTTC

TGTTGTTGGAGATCATTACTGGCAGGAAGAACACTACACATTATCAGGATCATTCATTAAATTTAGTTGG

AAATGTAAGACATCTCTTCATCAAGAACTCACCTTTAGTTAAGAAGATGTATCTTGTTTTGCTCTAGCCCT

TATACCATTGCTCTAGGGAAGGATATGTGGAAATGTTCATCTCTTCTTTTTTTTCTTATACGGTGGAGTCA

GGGCTAGCTTCTGCATACTTTGACTATTACAATGTACCTGCTACCCTCCACCAACATAGGGATGGAAACA

ATGGCGGGAGCTACATGCATTGGAAGGGTATTCAAATTGAATCCCCTTCTTTAGGAAGCTATGATAGAA

ACTATTGGGTGTGTCTGAACAAAGTCCCACATGAAAAGTAAAAAAAAACTACTTTAAGATGTCGGATAA

TCTTAATGTTATGAAGTCTTCTGGAAAAAAACTATGCGGGTTTGGTCCAAGGGAACAATATCATGCCATG

TTCAGAGTATCTTATTGGATTATTTTAGCCTAACAACTGATATCAGAGCCAGTGGTTTGGCAGGACAATT
ATTGAGATGGTAGAGTGATGGAGTGGGGTCCGACTTGGTGCCTTTGACCATCTATGGGCCGATTTACTA
TCTTTACCTGTAGCCTTAGAGATGCTTACACAAAAAGAAGCTAGTGGCCGTTCGTGACCATAAATACTCT
ATTGATATGGTGGCACGCAGTGGAATCTCCAGATAAGCCTATAAAGTGATAGGTCATGTGGAACTCAAT
TTAAGGAGAAGACTGTTGAGTGTACGTAAACAAAGTCTCACATGAAAAAATAAAAAAAATATAACTTAT
AAGGTATTGGATACTCTTAATATTGTGAGGCCTTTTGAGAAAACCGTTGGGGTTTTGTCCGCAAACTGAC
AATATCACATCATGTTAAAAGTATCTTTGAGTTATTTTAGCCCAATAGAAACACATTCTTTCGTCAAACAT
ATATGAAATTTTGGACCCCCTTAACCCCATACACGACATTTTTGTATTTTCCTTGAATCCCCTAATTGAAAA
TTTTGATTCCGCCACTGACCAGAAATGTTAACTTTGCTCTCCCTTTTTTCAGGTATGGGATTCATGGAATG
ATGACAAAGCTATAGATGTTGTTGATCCCTCATTAGGGGATTGGTATGAAAGTAGTGAAGTTTTGAGAT
GCATCCAAATTGGACTATTATGTGTACAATCATATGCAAATGAAAGACCAATGATGTCTCAAGTTGTCTT
CATGCTTTGCAATGAAACAAAATTGTCTAATCCTGGACAACCTGGTTTTGTGTTCAGATCAAGAAATTCTT
CTTCACTTCCTTATTCATCATCAGCTAGTATTGGAAATTCTGTAAATGACATTTCTATTACTGCACAACATG
CTCGCTAAGTGTGTGTCAGATCCTTCAAAAGTAGTACCATATATTTTTGTGGATCTGACATGGGTTCATCA
ACATTTTTGAAGAGCACTGAGCCATATGGTCATTAGGTAATTTATACTTGATCTATTTATTGATTTAGTTT
AAGATTTTTTACATTTATCAATGATGTATTTTAACGTGTTATAACAGGTAACGTATCTTTT
>PGSC0003DMG402007101 G-LecRK
CAAGAAGCAAATGTATCATAACAAACAAGCTAAAACAACAAACTAAACATGTCTCCTTCATGGCTTACTA
CACTTCTTGCCTCTCTATTTCTTTTCTCCCAAATATTTTCTTGCCTTGCCCAAGTTCCACTTGAAAACACCTT
CAAATTCGTCAATGAAGGCGAATTAGGACCTTATGTTGTAGAATACAGAGTAGACTATCGCGTTATTAGT
ATTTTCAATAACCCATTTCAGCTTTGTTTCTACAACACAACTCCTAATGCATGGACATTAGCATTGCGAAT
GGGCACTGTCCGTTCTGAATCACTCATGCGTTGGGTGTGGGAAGCAAATAGGGGAAACCCCGTTAAAG
AAAACGCGACATTCACGTTTGGAACTAACGGAAATCTCGTATTGGCGGATGCTGACGGTCGAATTGCTT
GGCAGACCAACACGGCCAATAAGGGGGTGACAGGGTTCAAGTTGTTACCTAATGGTAACATGGTGCTA
CATGACTCTAAGGGTAAATTCGTCTGGCAGAGTTTCAACTATCCCACTGACACATTACTAGTGGGCCAAA
CTCTCCGATTGTCCGGTCCGAATAAGCTCGTGAGCCGGGCCTCGGTGAAAAAGAACGTAAACGGGCCTT
ATAGCTTGGTGGTGCAGCCAAAATTGTTCGCTATCTACAATAGGACTAAGTTAGGCGTGGAATTAGCTT
GGTTCGATTTTGGAAACAGTACGTTGGAATCAGTAAAATTGAATAGTGGAAATCAAAGATTGAAATTGG
ATTATAGATTGGCAAAATCAACAAAGAGAAGTTCACATGTGATGGCATTTACTAAATATAACACAACATT

AACATATCTTAGACTTGAAATTGATGGAAATTTAAAGGCTTACACTTTTGTTAGAGATGAAGAAGCAGAT

GAATTCCGTTGGAAGGTAACTTACCAAATGCTTTGAGAAACTTTTTAAAATAAAAAATAAAATAATATAT

ATTAAAAACGTAACTTTATAAGATAAGGATTTCATTTCCAGTAAAAATAAGTGGAGAAAATGAGTGGGT

CCACAGCTATTATTTTCCTTTTTTTTTTTGGTTGAAGGGTGTATTTTATTATTATGATTTCTAGTTTTTTGTT

TTCTGTTAAAGGATGTATTTAATTATGTTTTCTACTAGCTATGACAAATGTGAA

>PGSC0003DMG402009323 G-LecRK

ATGAAGCTTGGTTATAACCTCACCACAAAGCAGAATTGGACTTTAACTTCTTGGTTGAGTTTTAGTGTTCC

TGCCTCAGGGGCTTTTACTTTAAGTTTGGAGGTTTTTGAAAATGCATTTCAGTTGGTTATTCGTCGAAGG

GGTAAGGTTTATTGGACTAGTGGGGCTTGGAACAATCAAGGCTTTCCATTCTTACCTTTATTCCATGATTC

TGCTACTATTTATCAGTATAATCTCAATTTGGTATCCGATAAAGATGGGATGTTCCTCCAATTTGATGCAA

CAGAGGGAAGCTTTCCAAGTCTTGCATTGAATTCAGACGGGGATATTCTTGGTGGATACCGGTTTACAT

ACACCCTTTTTAAAGACTTCTGTTATGGTTATGAAAGCGAGGATGGTTGTTTCTCTAATCAGTTGCCTGAG

TGTCGAAAGGATGGGGATAAATTTGAGCAAAAGAGTGGAGACTTTATACATACATCTAGTATTACTACT

GAGTTTTATGACAATGCAAGCATTAGTATTGTTGATTGTATGCATAAGTGCTGGGAGCACTGCAGTTGTG

TTGGCTTCACCACCGCCAGCAGCAATGGGACAGGCTGCCGTATTTGGAATGGAAATGGGGATTTTCGAG

TCTATGAGAGTGGTAATGCAGTGCAAAGATATGTTCTAGTTTCACCAAAATCATCTAAAGGAAAGTCTTT

ATATCAGGAATACTCATTTTTATGTACTCAATTGTCAACTGTATAACTCTCAGCATGCTATGGGTGCAGGA

AAAACTTGGATATGGATAGTAATTAGTGTAGCTGTTTTTATCGCGTTGCTAGTATCTGGTTTCATCTGCTA

CATTATAATGAGAAGACGCAAACTACAAGGTAAATCACCAAACTGTGGTTTTAGAAGCTAGTAGGTTGT

TTAGTCAATTATTTTAAGCATGAATGTATATCTACATACTCTTCTTACATAGATGAGAAAAGAAAGGAGG

AAGCATATATACGCGAGTTGACAGCAGTAGACAGCTTCAACACCGCAAATCTAAAAGAAGAAGATGGA

AGGGAAGTACAAGATTTGAAGATATTTAGCTTTGGATTCATATTGGAAGCCACAAACAACTTCTCAAGT

GAAAAGATGCTCGGAGAGGGTGGTTTTGGGCCTGTTTATAAGGTATATTTGCACTATGTTATATGTAGCT

CAGACATTTGCTTACTGCTCTCACTACATAACGAATCAACTATAAAAATGGATGGCAGGGAAAATTTCCA

GATGGGCGAGAGGTGGCAGTCAAGAGACTTTCAAGAACCTCGGGACAAGGGCTTGTGGAGTTCAAGA

ATGAACTCATACTAATTGCCAAAGTTCAGCATACAAATCTAGTTTGAGTTCTAGGATGTTGTATTCACGG

AGATGAGAAAATGTTGATATATGAGTACATGCCTAACAAGAGCTTGAACTTCTTCCTCTTTGGTCTGTTG

CTATTCCAAAAAAAAAAAAAAGACTAATTTTTCAGGTAATTTTTTAAAATTTTGATGTGAAATGCTTTCTCT

TTAACAGATCCTGCAAGAAAGGAGCTACTGGATTGGCAGAAACGATTTGCAATTATCGAAGGAATTGCT

CAAGGATTGCTCTATCTTCACAAATACTCAAGAATGAGAGTGATACATAGAGATCTAAAAGCCAGTAAT
ATGCTTTTGGATGAAAACATGAAATTTTGGTCTGGCAAGAATTTTCAAACAGAACGAGACGGAGGGAGT
GACTGGCAGGGTTGTTGGAACATAGTGAGTTGAGCCTAGTCATTTCTTCGATATAAATCTATGGAACAT
GTGTACTTATTCATACTTACTGAGTAAAAAATGTTTTCTATCCTCTGCACAGTGGTTACATGGCTCCCGAG
TTCGCCATGGAAGGTACTTTCTCAATCAAGTCTGATGTCTTCAGCTTTGGAGTCTTAATACTGGAAATTGT
GAGTGGTAGGAGAAATACTAGCTTACAACAAATTGATCGTCCACTTAACTTAATAGGTTATGTAAGTTTT
CTTTAAACCCTTCTTTCTTTAATGTACTAAAGTTCACAAAGTTCAACAATACTGATCATTTTGGTTCAGGCA
TGGGAACTATGGAAAGAAGGGTGTGGACTAGAACTGAAGGATCCAGCTCTTGAAGATCTTTGTGACAC
TGAACAATTTTTACGAGTTATTCATGTCGGACTATTATGTGTACAAGAAGGTGCAGCAGATAGGCCAAC
AATGTCTGATGTTATCTCAATGCTTGGTAATGGAAGTATGTCACTGCCAATTGCAAAACAACCTGCATTTT
TCACAGGAAGAGATGAAGCTGAATCATATTCGTCATCAAACAAAACAGAACAATGTTCAATTAATGATT
GTTCCATTACTGTCATTGAAGCAAGATGA
>PGSC0003DMG402013876 G-LecRK
TTAGTCCTCTATCAGATTTCCCCCAAAGTTTCATTGCCCATCACCTAGGAACTTTCTTTATTCTCCTCTTTCG
TTTTTACGTAGTATGAAGATAATATTGGTTCTCTTGCTTTCTTACTTTCATTTAGTTAGCTGCAACAGTACT
CGTTCAGATACCTTGTTGCCTGGCCAGTCCTTTTCCTTTTCTGCAGAAGATACTTTAGTATCCAAACAAGG
GAAGTTTGAACTCGGTTTCTTCTGTCCAGGTAATTGTGAAAAGCTTTTTATTGGTATATGGTATACAAACA
TAAAGCCCAAAACTGTGGTTTGGATAGCAAATAGGGACAACCCTATTCGCCCTCCGTTCAATAATTCTCA
TCTAGAGCTGTCTGATGGAAATCTACAACTCTTGGATGCACGTAAACAAAGAGTTTGGAGTTCTAGCTTA
TCCACACCTTCAGCATATGCCAATAAAGCAGTACTTCTTGACTCTGGAAATCTTGTTTTGACAAATGGCAT
TGACAGACAGTGGCAGAGCTTTAATTATCCTACAGATACGTGGCTGCCTGGTGCGATGATTGGATTTAA
TAAGTCCAAGAACACACTCCAGAAACTTACTTCTTGGAGGGATCTGAATGATCCAGCTTCAGGAAGCTA
CTCTTTGCAATTAGGCCAGAATCAAAATGGTGAGTTAGTTGTTCAACATAATTTCCAGGAAGAATGGAG
GTCTGGGCCTTGGAATGAAGGAGGGTTTGCTATTCTAGCACAACAAAATTATTATAAGGATTTATTCGAC
TTCAGTTATGATCCAACTGAATATAGTAAATATATTACTTACAATGTCTTTGATGAATCTGACATTTCTCG
AATTGTCTTAGAATTTAATGGGTCGATGAAGCAATGGTTCTGGTCTAAAGATCATCAGAGTTGGCAGTTA
GTGTGGTCTCGATCTACTGATAATTGTGACCGAATAAATTTCTGTGGTGCTTTTGCAATTTGTGACATGA
ATGCCGGTCCACCCTGTGAATGCTTAAAAGGTTATGAAAAGAAGTTTAAATTAGATTGGGATAGTAGAA
ACTATTCAGGTGGGTGTGTGAGAAAAAATCCGCTGCAGTGTGGCAGCAATAACAAAAATTTTGTCCATA

TGCCAAATGTAAAACTTCCTGTGTCATCTGAATCAATGAAAGTAGGAAATGATCAGATCTGTGAATATAT
ATGTTCATCCAATTGCTCATGCAATGCTTATGCTTACAGCAATAGTGGAGAGTGCTTATTATGGTATAGC
GATTTGATTGATCTGTCAAGATTACCTAATAATTCAAGTCAAGTAGAGTTCAACATTAAGCTTTTTGAGC
GCTCGAACAAAGGTTAGGTGCTTCCAAGCTGCATCTTATTTCTAATGTGCTCCTTTTCAACCAAAAATATG
TTATTTTCCTCCTTTCTTGATCTTCCTTAATGTTTAGGAAAGAAGAAACTACTACTGGTAGCACTAGTAGT
GTCCATCACTTCTGCAAGTTTTGTCTGTGGTACATGCTGTTACTTTCTATGGAGAATAAAACTCAAGGAA
AAAGGTACATTCTAATTCTTGCTAGTAGCAGTTGAGTAACTTCAGTTTCACTTCTATTTGGCATGACTGAA
AGAGGTCAATCAAAATGTAACCTTTGATTTAATTAGTCATGGAAAGGAAATTATATTCTCTGAATCATGC
TTAGATAAAAGTTAAAATTGCCCATGTCCAGCTTCTTCTAAACTCATCAATAAACTTCCTGTTTATATTGAT
TTACATCAGGGATTTTGGGAAGGAAAAAGTTCAGGGAAATGTTACTTTCTAAATCAGCAACTAACTTATC
AAAACCTGGAACTTCCAGTAAAATGGGACAAGAAAAGAAACTTTCCATCGAGCTGAAGTTCTTTGAATT
ACGTGAGTTGAAAGCTGTGACAGACAATTTTTCACCTGACAATAAGCTTGGAGAAGGAGGTTTTGGGCC
TGTTTTTAAGGTATTGTTACTACATAAGAAAATATATTGACAACCTGAATAACTGCAACACTAAATTCTAC
TCAGGCCCAAAGAAAATGGGAAAAAGTCCCTCTTGAGGTGAACAGGTAGCGTAATGGTTTTAACTAAAA
TCTTTTCCATACTTGTGTTTTAATGGTGTGATTAATTGCATTGCAATTTCTAGGGTCAATTGCCAGATGGG
CAGAAAATAGCTGTGAAAAGGCTATCAACTCAATCACAGCAAGGAATTAATGAATTCAAAACTGAGGTC
CTTCTCATTGCTAAACTTCAACACAGAAATCTTGTTAGGTTTCTTGGCTGCTGTGAAGAAGACGAGGAGA
AAATGTTGATCTACGAATATATGCCAAACAAAAGTCTAGACTATTTCATATTTGGTATGATAACTTCTTAT
CTGATGTTATGTTAACTCTTTCCTCCTAAGTAGTGCTTACTGGATAAATTTGATATGAACAACTTAGACGA
GAGCAGGAAGTCGTTGCTTGATTGGAGAAGGCGGTATGAAATCATTATTGGAATTGCTAGAGGGATAC
TCTATCTTCATCAGGACTCAAGATTAAGAATAATACACCGTGATCTGAAAGCCAGCAACATTCTTTTAGA
TGAAGATATGAACCCCAAGATATCAGATTTTGGTACTGCTAGAATATTTAGTGGAAACCAGAATGAAGC
TAAGACTCTTCGAGTTGTTGGAACATAGTAAGCACGAAAATACCAAAAGGGATTTCTCTGTCGCATCCTT
ACCATTTATCAATACTTTGCGCACTCATTATTGCCCTTTCCCTTTGCAGTGCCTATATGTCACCTGAATATG
CTCGAGCTGGTCTCTTCTCAGTAAAATCTGATGTCTTTAGCTTTGGTGTTATATTATTGGAGGTCATAAGT
GGCAAAAGGAACATGGTCTCTTATAATAGTGATTCCCCTCCTAATTTGATACGACGGGTGAGTTTTAAGA
TACAAACATTTGAAACTAACTTTTGATTTAATAATCAAACTTATTTGCTAAATGAACTGCTGCTTCAGGCC
TGGGAGTTGTGGAATGATGGTAAGGCCTTTGAGCTGGTAGATCCATCAATAGTTGATTCTTGTCCCAGT
GAGGAAGCATTACGCTGCATTCTAGTAGGACTATTGTGTTTGCAACTCAACGCAGCAGACAGACCAACC

ATGTCAACAATTCTGTCCATGCTAAGTATTGAAGCAACGGTTCCCTCCCCGAAGAAGCCCTTCATTACCC
GTAACTCAGACTTGATTACTACAGAAACAGCATCATCTTCGATCAACGAGGTGACAATTACCGCACCTTA
TGTTCGTTAGTTGTCTGACGTGTATATGTATTTCTGTTATCTGAGTATCAAATGTATCTAGTGCTCATTACT
TCCCAAAGTATGATATAAGTCTTCAATGATTCAATGCATTTAATGCTAAAAGATTTTCAACAGACTAGTTG
ATATTACTTTTACAGATACTCATCACAGAAAGATTGAGACAACAGTGCTCTTACTCATGATCATAATATTA
TTGTTGTATTTGATACATGTCTATCTCATGATCTAATTATGAAACAAAAGTTAAAATCTTACATAAGATAA
CTTACATGTTTGTATTTTCTTATCCATAACTTTTTGAGGAAAAACATAACCAACTAATAATAGTTAAATATC
TCATTTATTGACAAAACTCCACAACCAAATTACTATCCTGCAAATCAATAAAAAAATTGAAAAGTTGTGT
GTGGCTAGCAGGATTAGCCTTTGTTGCTTTTAGAGCATTGATGAACACAGAGAAGACTGTTAAAGAAAA
CATGAAGTGGGGCAATTATCAAAAACAGCTGACAAAGAGCACCATTTAATTCACAGATGAAATAGAAAC
CCTCTGTGTTTATGCTTCTTTCCTACCAATCCTTTGCCACCCACCTACCTTTAAGTCTGTAACATGGGCCAT
GGGGAATCAGAAGTCAGTTTTGGTTCCAGTGCTATTAAAGCCCAACCCCACTAGTTTGAATCAAGAATG
GACTAGCATGTTAAATGTTGTGTATAACCCACCAATAAATCTCTCTAATACTATGAAAGCAAAGTACAGA
AAAAGGAATTCTGTATTTCTTTCAAAATAGTTCTTGAATAGTTAGCATAAGCTCTAGCGACCATGAACTG
TTGAAATAGACATATCGTTGACAGATGATGTTGCTATGCTTGCTGATGAAGAGTGTGTAACTGAACTATG
ATTCTCTGATCTGAATATAAGGCTAGGTTGTTTAGGGAAAGGAAGATTTGTTTCATTGCACAGCATGAAG
ACCACTTCTGACATAGTCGGTCTGTCATCTGTAAACGGTTGTACACACAAAAGGCCAATGTGGATGCATC
TCAAAACTTCACAAGCTTCATATGAGCCACCTAACAATGGATCAACAACATCTAAAGCCTTTTCATCCTTC
CAAAGATCCCAAACCTGAAATAGAAACACCACAATGCATCACAATCAAAATTTCAGACAATTTCTTCTCT
ATAAGAGAAGCGACTATAACACCAAAAATTGAAATTCTGAAATACAAAATCTCGATCTATTCAGCACTTT
TAACAACCTGAAAGGGAATTGTTTCCAAATTATTATTTGCAGTACTACCATCC
>PGSC0003DMG402014514 G-LecRK
TTCATGCATTTCTCACTTCACTCCATAACTCCTATGCATCCACACTCACCGTTCCCGTCGCCGTCGTCGGAA
GCCAAAAATGGTATCACTTCAAGAGCCATTCCTAAACCGCCGCCAACAGTCATTAATTTTACACATTCACC
ACTGCCGCCTTCGCCGTCATCACCGAACGCGTCGTTAAAGCCAATAATTAAACCTATTGATGATAAAATC
GCTTTACATCCACCGCCACGGCGGCCGATATATACTCATCCGTTACCGAAGCTTCTTCCGAAACTTACGTT
ACCACCGCCTCCGGTAACGGCGTTAATGTTTCCTGTGAATGGTTCTTCTGCACCAGTTTCTAGAGATGAA
ACTTCGTCGATTAAAGTTCAATCGCCGTTGTCAAGTGCATCAATCGGATCTGTAATTGGAATTTCTGTAG
GCGTTACCGGAGCTTTCTTCATTGTTGCAATTATACTCTTCGTGTGTTGCAGGAATAAGCTTAAATGTGG

AAAATTACGTGATCGTGAATCTCCACTGCCAAAAGGTAAAGCTTTTTCAGTTTGATACAGTAGTTGAATC
AGTAATATATACAGTAACAACTAAAGCTGCTTTTTCAGGGGAAATGTGTTTCACTTAACCAGAAAATATT
TTTCTTTGCTCATGAAGCTTTCAATGTTCTGTTTATCTTCAGTAATTGATTACCTTGCTAGCTTGATATTTTG
CAAAGAAATGACGTTTCAGGGTTAAGTACTTCACTTCGCCTGAATTTTATTTCGCCAAAGATGATTTCGAT
ACAACAACGTACCCAGTATAATATCCCACAAGTGGGGTTTAGGGAATGCGATGTATATGCAGCCTTACC
TTGCTTGTGAAGATAGAGAGACTTTTTCCACTAGATACTCGGTTCGAGTAATCATTTCAAATATCAAGTAT
TTAAGCAAATACAGTAAAAAATTAGGTGCCCGGGGTGACCCAGTGGTTTGTACTTGGAACTTCCATGTTC
GAGGTCTCAAGTTTGAAATCCCTTGCAAGCAAAAGCAAGGGCTTTGTCACACCAGGGTTGCTTAATGCA
AGTTACCTCTCCTGTGTGGTTTGCGAGCTATTGCATAGGAGTAAGAGTTTTACCCTGTGAGCGCTCAAAG
GGTAGCTTCTGTGAATTTCCCTTGCCTTTAAGAAAAAAAAATTAAGAAGAGAACAAGTAGCTACATCAAA
TAACACAATAATTGAAGCAAAGGAAATAATAGGTACTGCTAAAGTCGAAGAAAAAAATTAGTAAGAGG
GTAATAATATAACAATACTAATAAGGGAATTAGACAACGCTCAACTACTACTAACCTTCTAACTTAATCAT
TGATCTTCATATCTTTCTATCTAAGATCATGTCTGCGATAAGCTGTAGGTGAGCCATGCCTTCTCAAATCA
CCTCTCCCAAAGACTTCTTCGGCCTGCCCACCTCTACGGCTCTACCTATGCTTATACCCACTACAACCAAC
CTCTCACACCCAGTGAGGTACGCAAGATTTTTTGTAAGGCATGTCGGTATTTAAAAATAAATAATTGATA
TAGCAACATTATATTAATATCTTGTACAGGAGCAGACCCCTTCATTTGCTGTAATTTTTTGCCTTGCATCTT
CTTGATGCCTTCTAATGAAATCTCCTTACTTCATAAAAAAAAAAATTATAAAAATTATTTAAAGCATACTA
ATAAAAATAAATCAAGTAAACTACTACAGCTTATTATTGTGTCCTATTCTATTTTGGACAAGTAATCAAAT
TTCTATAATAGGATATAAATTGTATTTTAGTTCAATTGGTTTATCTTATCCTGATTTTGAATAGGAATTGAA
GGTTGGAGGTTCAAATCCCTATAACACTTACTTTTATCTAAAAAGGCCCCAAAATATAACTCAAATTTGG
GCTTGTTGAGGGTCGAACTCGGCCACACTTTAGAGAATGACTACACCATCTGCACGCAGTAAACCAATA
AGCCAAGGAAGCTGTTCAAGTAGTGTCATTAATATTTTTTCTACTTATATTTGAGTTTTCAGATTTTTAGA
TATAATTATATAATACTAAAAATTTTCATCGAAGCAGTGTCGGTTGACACTCCTTGACATAGGGTGGGTC
CGCCCTTGCTCGCACCTCATCGCTGCGGCGTACATTTTCTTTTCACATACCCGAATCATCTCAGCCTCGCT
CCCTCACTACCATGGAGGCCACTCCTGCCTTTTTCTTGAATAACTTTGTTCTTGATCCTATTTCTCCTATAT
GCCCACACATCCATCCCAACATCCTCATTTCAGCTACTCTCATTTTTTGGACGTGCGAGTTCTTGATTGGTC
AACACTCTGCCCCTTACTACATAACTAGTCTACCACCACTCCATAACACTTACCTTTTAAGGATTGGTGGC
ACATTCTTATCATACAAGACAAAAGGTGCTAGCTTCCATTTCTTCCACCCCGCTTCACGAGGATGTGTAAC
TTCATAGTCAATCTCCCCATTTCCTTGAATTATTGACCCTAGATACTTGAAACTTTCTTTCTTGGGGGTGAC

TGGTGTATTAATCCTTACTTCTACATCTTCCATGTGGATATGTCATGAACTTAAACTCTAAGTATTCAGTCT
TGGTCCTACTCAATCCGAACCCCTTAGACTCCTGGGTCTGTCTCCAAACCTTTAATATATTGTTGACTCTGT
TTTATGCCTCGTTAATCAAAACTATGTCCTCTGCAAATAGTATACACCATGGCACCTCTCCTTGGATATGC
CACGTCAGTTGATCCATCACCAAAGCAAAAAGAAATAGATTAAGAGTTTATCCTGATGCAACCTCATCAT
GATTGGGAAGAGACCTGAGTTTCCTCCTATTGTCCTGACTCGTGTCTTGGTACTATCATACATGTCCTTAC
TTTCCCTAATATAAGCCTCAGGTACACCTTTAGCCTCCAAACATCACCAAAGAACCCTCTAATGACGTTGT
TGTAAGCTTTTTCTACGTCAATGAACATCATATGTAAGTCCTCTTTCTCTCCCTAAACTGCTCGATTAATCG
CCTAACGAGATGTATGGCTTCTGTAGTTGAAAGCCCTACGATAAGTCCAAATTGGTTCTCGCTGATAGAT
ACACCCCTCCTCAACCTCATCTCCACCACTCTCTCCCAAACTTTCATAGTGTGGGTCAACTAACAACTTGAT
ATCCCTATAACTGGTACAATCCTGAATATCACCCTTGTTCTTGTACAACAAGATCAAAGATGATTTAATTA
CTATTTAAATTTATCATTATTTTGTTTTATGATTTAGTAGGAGTGGTGACTTTATCTCTAAATTCAACTATT
GAAAATTAGTGATCCAAATTGCTATTTTTTCCTCGTACAATTTAATAGTAACTTCTTTAAAAATTTACTAGT
AATGAAATTAGTGACTTATGTTTCTAAATTCATTGTGACAGACAATTATCATTTCTTAAATTTTATACTTCT
TATGTTCATTTTATATGATTGTGTTTGAATGTGTATGGACTACGGAGTTTTAAGAAGAAAAAGGGACTTT
TTGAGACATAATCGAGTATATGTCACATACCAAAAGTATCTATAGGATCTTATGCTTTTAAACATGCCAT
GCCATTCTGGAAATGTGACAGTTTGGACATTAATCTCTTAAATGTGAAACTTTCTCCGTCTGACCAGATAT
AATGCTTAAATCTTGTTTTAAGAGGCTCCTTGTTCAGAGCTTGTTTTCATTGATTGCAATTTGCATTTAGA
ATGGGGTCCAAATTGTTGATATTATGAAATTTAGCAGTTTAATCTATCAACATAAGTTCAAAAAAGCTGT
TTACTAACCAGTATGAGTTCAACTTGGTATATTCATTTGCTAATTGTTTCGGTCGATTCATCAGATGACTT
GTACCATGTTCTGCGTCAAAGTTTTAGACAGCAGAATGTAGCAAATGGTTCTCCTGTTTCCCCAAAGCAA
GTGACACCATTCCGCCCACTTCACTTCTTTGACAACAATGCTCATTCCAATTCCCTTAATTCAGAAGCTGG
CAGTCATTTTTCACCAAAAGACCACATAGTGGATTTAGCTGTTTCGGGTGGCAATTTCACATATGAAGAG
CTGTGGTTAGCAACTAGTGGATTCTCTACATCCAACCTTCTAGGTGAAGGTGGATTTGGGTATGTACACA
AAGGAGTCCTCCCTACTGGAAGAGAAATTGCAGTTAAACAGTTGAAGGTTGGTAGCCATCAAGGGGAG
CGTGAATTTCAAGCAGAAGTAGAGACTATTAGCCGAGTGCATCATAAACATCTTGTGTCTTTGGTTGGAT
ACTGCATGAATGGAACAAAGAGATTGCTTGTTTACGAATTTGTTTCCAACAGGACCTTAGAGTATCATTT
ACATGGTATGTTCTTTCTCTTTTTCCTTAATTCTGGTTGTTGGTCGAATCCTGTATCAATAAGATTTTGTGA
CCAGCTTAAGGCATTTTTTGAATTATGCTAATATTTGTACAGCAGAGAGTTGATAATTAAGGTTAAAATA
AATCTACCTTAACGGGGGCTTCCTTAATGCAGCTTAGTTTCACTGTATTGATTTTGAAAAGCATGTAAAAT

ACACCTCACGAGTTATGAGTTCCATGTTAAATGGACAATCTTTAAGGGGGTACTTAACCAAGCCTTTTAA
GTAAGATTCCTCGTGGTATATGCCTATACCACAAAAAGGTGGCCTAGAATTTCATCAAACCATGGTTTTT
AACATGAGAAGGTAAAAGAAGAGCTTGCAAATACATGCTACTTCTGTAACCCAAGCCCTTTAATCTTGCA
TGGTAAAGCACTGCTATTTCTATGCTTTTGGAAAGACCAGCATTTAATATCTTCTGACTTCTGCTGTCCCA
GGCACTTCATTTGCAATTTTCCACATACTTTTCTATACCCTTATGGTGTATTATTTTAACACACTTCATGTG
AGGTACATTTGATGGAGGATCTTTCATAAGTACCACATTTTAGATGTGTGATTTGTTTCATCATCTGTTTT
AAGGTTTGTATAAAGAATGTTCCTATCCACCCTGATGCATGTTTTCTTTTGAACTTTTGTTTTACAGAAAT
GCTCTTGTATCAGTTTATACTTCTCTGATCACTTCTAAATATCCGATTGAATAGAGTTTCCATAAGTTCCTC
TGATATAAAGGTCTTTCTACCAAGATGCTATTTAGAACAGATTGTATGATATGCAGGGGAGGCACAATC
CACGATGAAATGGGCATCTAGAATGAAAATTGCTATAGGGTCAGCCAAAGGATTGGCTTATCTGCATGA
AGACTGTGAGTGCGCTTCATCTCTGTCTTGGCTTCCTATGTTTGTTCTTAATTTGCATGTTCGTCCATTAGT
TGACTAGAGGTGTCCAAAGGGGTAAATTGAATATTAAGTGGCCTGGATGCTTTTTAACAATGTTGAACA
TGTTTCCACAGGGAACTCTTGTTAGTGTGTTTGTAGATTGTGACAAGTTGGGTAACATAATGGTTTTCAT
CACATATCTATCTTCACTTTTTCTCCTGAAAAGGATGTAAATCTAATTTTCTCCCTCCCAATAGATACCATT
GATCCTCACTTCTATAATACCATTTCTGAATCTCCTCGAGATTTGAGAGCTGAATACCTCGGTTCTAGGGT
CTGCTAATGGATAGCAGAAGGCCATAATAATTCCTAAATTGTGAAAGATATAACATGAATATGCATCAG
GCAAACTGTACTTTCCCCATATAATTTTATATGCCATGTTCTTTCCTGTCAAATATGAAATCAAATAGTTTT
AGTATATTTTCTTCCACTACTACCAATTTAATATAGATGTCAACGTATCTTTGCGCTCTGTAGGACAATTTA
ATTGTGGTTGCTTGAATTAGCTTGAATAGAATAAATATCACTTCTTTTGATGTAGCAACAACAGGTAATC
GTTCTCTGAAGATACTAACTTGTGGCTGCAGGTAACCCCACTATCATTCATCGTGATATCAAGGCAGCTA
ATATTCTTCTTGATTCTAACTTTGAAGCTAAGGTAACTTTGTGTTTCGCTTTTTGGTATTGCCACTATATTT
TCTGTTCTTTTTTTTGAGAACTGTTTTTTTATTTTTTTTGAGAACTATTTTCTGTTCTATCTTTACTATCAAAG
AAAGGTCGGGCTGTTTTCTATTATCGACAACTGGCTTCTGAGTACACCTAGAATCCACTGTTGATGTAAA
TAGTTTTTTGTTCTTAACAATAAAATCCATATGATGAGATTAATTTGAACCCAAATGAGCATCTACTCAGG
TGCAAATGTTAACTGCATTATTGATTCTGCTTTTTGTTTCTTTAATTTTCATGTGACAACTAAGAACATATT
TCCTCGCAAGTTATTTAACATGAATGGGAACTTTGATTTGTATCCAGGTTGCGGACTTTGGACTTGCCAA
GTTCTTATCTGATTCAAACCATCATGTCAGTCATATCTCCACTAGAGTGGTGGGAACTTTTGGGTGAGTTT
TATTGATCCTGTCTCTGCTACCATGAATTAGATATCAGAAGGAAAAAAATTGACTAGATCAAGCAAAATT
ACTATATACAGGTCACCATACTGAGTGGACCAGTCTCTTCTACTTTTATCAAGTAAAAAGTGGTGCTTCCC

TCGTTCTAGCGTTAATAATATTCATTTTTTTGGGCTAACCTCATACGTAGCATAGGCTATGCCCTTCCCAT
GTTAACTTTGTTTCATCCAACTTGCAAACTGATATCTATATCAGATACCTATAGAAAATCGAGCTAGAATG
GGACTCTCAAATCTGGGGATTTTAAAGACATTGATGACTTGCATGACCTCGATTTTAGGTCAACCTGCTC
ATAATGCAGTGATTCCTCTCAGAGTTATAACCGAAGTTTTCCTGCTAGTCGGAGGATGAGAAGAGCGCA
TGGGATGTTACTGCTGTTTTCCCGTGTCTGATAGTGTTGGGTTTCTTAGTCCTTGATTTTCCCTTACCAACA
CTGATTGGGGAAAGAGGGGGTTTGGGGGGTTAAGGAGAGGGGCTTGGATTTTGGAAACTGTAGAACT
TAAGCTACTTCATGATTAGTAATGATTTCTGGATTACACAGCTTCAGGTAGATGGACCACGTAAAAACTA
ACAGGGTAAAACCTACATTTCCTGATCACAATTTCTTCATGTATTTGATAAAGCTGACGTCTTGCAGGTAT
CTGGCCCCAGAGTATGCACAGAGTGGAAAGACCTCAGATAAGTCCGACATCTTTTCTTTTGGAGTCATGC
TCCTGGAGTTAATTACAGGGCGTCCACCTATAATCTCAACTGAATCCTCCGCATGCAGCAGTTTGGTCAT
ATGGGTATGTTAACTGTTCCACTTTCTTGACCGCTATTCAGCATTCTTAAGTGCCTAAAACTAATGTTCAA
CTAGATGCATGAGTAAAACGTTTGTGTGCAACCTTTATAACTGGTTAAATAGCATAATGCTTAAATTTACT
TTCTGTCCAACTACACTAATCAAATCCTCAATATGGAATAACATTCATCTTACTATAATCTTATCCTTAATT
AGCTATGTATGCACTGTATTTAATCTGAGGCAGAACATGCAAGCACAGCTTATGCAATTCTCTGAGGTGG
CCTTTCTATTTATAAGAGATCTTGATCCTCCGGAAGGTTTATCATTTGTGGTTTTAGAAGTACTGGGTGGT
TTTATCCTTTATTGCTTTTCTTTTTTGCGAGTGATAAATGGTTGAGAAATTTTATCACGGCAGTAATCAATA
TCACGGAAACAACCTTTCTACCTCCACGAGGTAGTGGTAAAGTCTGCGTATACTCTACCCTCCTCAGACC
CCACTTGTGGGACCTCACTGGGTATGTTGTTGTTGTTGTTTGTATTAATCAATCACTTAAAAAAAGAACCA
TGGAAAAGCATTGGCTTAAAATCAGAATTTGGCAGACCACCGTCCTTGTACAAAAGAAATGTGCAAATC
AAAAGGATCTGGTGTTCTTGAAAAGTTCATCTCTGTCAAGCCAAAGGCATAAAAATTCACGTCCATCTTA
CAAATTTTTAAAAGAAATTTGTCATCCTTGTACCAAATGATTTGGCTTTTGGACACCCGTTTTGATGATGG
AACATATCTCAAGTTTTCTGTTGCATCTTTTACAACTATTAGTTTGTCCAAAGTTTCAGTCTTAAAGCATGG
AGTTCACCATGAATTTGATATCATACTCTATCAAATTTTCCCAAAATAAGGCTCCCTTTTTAGGAAATCTT
GGAGATACGGGTTCCTGGAATCATTTTGTTGATCTCAAAGATTATTACTGTTGGTTCTACTTAAGTCCCAC
AATTGATCAGAATTAATTACATAAGTTAATTCGAAATTGAGATCAGAAAGACGGATTTCAGAGTTAGCA
GCTTCTTGCATGCTGCCTACTCACAGACAGAATTAGTTATGTCACTTTAGTTAATCAGAATTAACTTTACA
CCTCTTCCGGGGCATTTTTTTTCATAACCTCGTCATCAAATACAATATTTTGTATTAGGAATCATTACCGTA
GTTTCTGGTTTTCATATTCTTGGTTTTTGATTAAGTACTTTTTGAGTGTCGTTTATTAACTGGATGTACACA
AATTTCTTGATCAACATATTGGTTTCTTGTAGCAAAATAACAAAATATTTTGGGAACTAGAAGCATCTCTA

GGCCGATGCTTAAATGCATTTTCAAAGATATGGATATCATTGTTGTCAACCCTCTTCTTATAGTATACCAC
TTGTACATAGCATAAGAAGACTCTTTCTGATACACAGGCAAGGCCTTTGCTTAGAAGTGCATTGGATGAT
GGAAAATTGGACGCCCTAGTAGATCCATGTTTGGGACAAAATTATAACAGTGAAGAGATGGCTAACATG
ATTGCTTGTGCTGCTGCTTGTGTGCGTCATTCATCACGGAGAAGACCGCGGATGAGTCAAGTAATAACA
TCTTCTCTTAATCTTTTTCTGTTAGTGCTGGCGGATTTCTTTAAAGGGGTTTATAAACCAAAACCTTCCTAC
TTATTGTTCAGGTAGTTCGTGCTTTAGAAGGCGATGCCTTTATCCTGGACCTTGATGAGGAAAATAGGCC
AGGACAGAGTACCATTTGTGACTTTGATTCTGATGGAAGCAACTATTTTGAGAAGTTCAAGAAAATCCA
GCGGAGCACTCTGAAATCAAAAGAAGAATGGTAATACCACCGTGCACCGGGGGCTGTCATGGAAGTAC
ACCAACTGAAAATGGAGTTAGATGAACGAAGCTCTTCTCCCCTTTTTATCTTTCTTTTCACTGATGTAGAT
GATAATTCGAGGGTGAAATATATGATTGCAACCATCAAAGTTGTCTCACTTAGTGAGAGGATTGATAGT
TTGTAGTTACTTGAAAGTGCAAAAAGAGAAGAAACAGGAATTGGCATGAGATATTCAGTATATCACCAA
GTAAAAATCTGTGTATAAAGATGCATGTTCAGATGTACATATAGATTTCTTCCCCTCTCTCTTTTCTTATTC
CTTTTTGC
>PGSC0003DMG402019242 G-LecRK
ATGAAAGGCCTATTTTCATTATGCATTTGCTGCCAATTTCTCTTCATTTTACTAACTTCTGCAGCATTAGAC
ACAATCACTACAGATAAATCCATTAGAGATGGTGACACAATTGTTTCAGCTGGAGGGATTTATGAGCTT
GGATTTTTCAGCCCTGGAAATTCGAAGAATCGTTACGTTGGCATATGGTACAAGAAAATATCAACTGGA
ACTGTTGTTTGGGTTGCCAACAGAAACATTCCACTGAATGACAATTCAGGAGTGTTAACACTCAATCCCA
ATGGAATTCTTGTACTTGTTGATAAATCGAATGTCTCAATTTGGTCATCAAACTCATCAAGATCGTTAAAG
AATCCAAAAGCACAGCTCCTGGATTCCGCAAACCTTGTTGTCAGTGATGGAAATGATAGAGGCCTGGAA
AATAATTTCGCGTGGCAGAGTTTTGATTATCCAGGAAATACTTTGTTACCTGGCATGAGGATTGGAAAA
GATTTGGTCACGGGAATGGATTGGCATGTAACGTCATGGAAGAGCATGGATGATCCAACTCCTGGTGA
ATATGTAGATGGTCTTGATTCTCATGGATATCCACAATTGTTTGTGTGGAAAAATTCATCTGTAGTATTTA
GCTCAGGGCCATGGAATGGTATTGCATTCAGTGGTAGTCCTAATAATAAACCAAATACATATTACAGTTT
CGAGTTTGTTATTAATCAACAGGAAATTTACTACACATATACAATTAGGAATGACTCCATTCCCACCAGG
GTGGTGCTCAATCCGTCTGGTGTGCTAGAACACCTAATATGGATAGAGCGCAGTCAGAGCTGGTTTCTC
TACTTGACAGCACAATTTGATAATTGTGATCGTTTTAGTTTATGTGGACCTTATTCAAGTTGCAACATCAA
TAACTCCCCTCCATGTGACTGTTTGAAAGGTTTTGAGCCTAGGTTTGTATTTGTTACACTATCTGATCATC
TCACGTTAAGCTGTCAGAGAAAGCACACATTGGACTTTTGATTACTTACTTATGTTTTCACACGCCCCTCA

TGTGTGTGCTTGATTCTTTTTTTGTTACCTAATTATGTCTTCACAGGTATCCTCAAGATTCTGCAACAGAAT
GGTCTAGTGGTTGCGTTAGGAGAACTTCTTTGGATTGTACCCATGATGGTTTTCTTAAATTTACGGGTAT
CAAGATGCCTGATTCTAGAAACTCCTGGTATAATGAGAGCATGAACCTTGAAAATTGCGAGAAAATGTG
CTTGGCTGATTGCAATTGTACAGCCTACTCAGATCTTGATGTTAGAAATGGCGGAAGTGGATGCTTACTG
TGGTTTGGAGAACTCATTGATATACGAGAGTTCAGCCAAAATGAACAAAACCTGTTTGTGAGAGTAGCT
GCTTCAGAATTAGGTAATGTCCCCACTCTGCACCATTCTCTCGTCTTAACAATATAAATACACAACTCTTA
GAATTGAAGGAACTCTACTATCTTATTTACTTGATAGACATTAAAATTGTTTATCAGCCATTAAATGGCTG
CACATCTACAATAGTTTCCCCTTAAGGAGGGAGCTGATAATCTAGTACGAACGTATCCATCTAGTAGTGC
TGTACTTGCATTTGCCTCTCAGATCATCCACTAGTCCCCTTTGTTGCAGACTTTATTTTGTATAGAGTTTGA
CACTAGCAGCTATATTCAGAATGAATAACCATATAGGAGAGCAAAGATTGTTGCGTAACAGTTTCCTCCA
AGGGATTTTAGGAAAGCTGCCTCTCAGCTTCTGATGTGCTAGACCTTATGGAAGATCAAGGATGTTAAC
AGCGGGGGATGGTCGTACACACTCTATCCTTTAATAGTTACTGATTAGCAAGTCTGCGGGGTTTCATAGA
AAAATAAGAAGTAATACAATCAATGCAATTCATTACTGATATGGCCAACTCATATGTAAATTGAACATAT
TCAGACAGGAAGGGGAGGAGAAAGAGGTCAATCCTGATTGGTGTCATTTCAGCAGCAGTAGCAACATT
TATCCTCAGCCTTTTAGCTTGGTTTTACTTCCGAAGAAGGAAAAGAAGAAGAGGTATATATATACTTGAG
ATACTCTTGTCTTTCTGGCTGACATTGTTGGCCTTTTACCAATTCTCATTGATATCATGTTTGAGGCGGGC
GAATGTATATTGACAGGTTTAGAAGTTGAAAATGAGGAAATGGAGCTTCCATTGTTTGATTTAGTAACT
GTTACTAGTGCCACTGAGAACTTCTCTTCTGCTAATGTGATTGGAGAGGGAGGTTTTGGACCCGTTTACA
AGGTAAATCAACTTTGATCCTCAATGTCTAGCAAGAAGTGGCATCTTTGACTCTTGATAGAAAATTGCAG
GGTATCCTACCAAATGGACAAGAGATTGCAGTAAAGAGGCTATCAAAGCATTCTGGACAAGGCTTTCAA
GAGTTAAAAAATGAAATCGTTCTCATTTCCAAGCTGCAACATAGAAACCTTGTCAAGCTATTGGGTTGCT
GCCTTGAAGGAGAAGAAAGGATGCTAATCTATGAGTTCATGCCCAACGCTAGCTTGGACTATTTCATTTT
TGGTCTGATTTTCATGATTCATTAACTAAATACTACTTGATATGTCCTCAATATCTCTGCATCACTCAACGA
TTAGTTCCACATTGTGCAGATACAAACAGAAAAGCTTCACTAGCATGGAAGAATCGCTTTGAAATTGCTA
TGGGAATATCTCGAGGTCTTCTTTACCTTCACCAGGATTCAAGATTACGAATTATTCACAGAGATCTCAA
GACCAGCAACATTTTATTAGATAGTGACATGAATGCAAAGATTTCTGACTTCGGCCTTGCCAAAATTTTT
GGTGGAGATCAAGTGGAAGGAAAAACTAAGAGAGTAATAGGGACATAGTAAGTTTCTACACATCTTCA
AATCATAGAATCTTTATTACATTCAATCAGTACATGAGCATAAATATATTGAGTTGATGCAGTGGATATA
TGTCCCCGGAATATGCTGTTGATGGGAAATATTCAGTAAAATCAGATGTATTCAGCATTGGCGTAATCAT

TCTTGAAATAGTCAGTGGCAGAAAGAACAGGAAATTTCGTCATTTGGAGCATCATCACAATCTTTTGGG
ACATGTAAGCACAAAACTTCATTCCCTTAATTCCCTCTCAACTTCCTATTGTAAATACATTTGTATAAATGT
GTTGTTCTGTTTGTAATCTAGGCATGGTTACTTTGGAGTGAAGGCAACGCGATGGGACTGATAGATGAA
TGTTTGAAAGAATCATTTTCAGAATCTCAAGTGTTGAGATGCATCCAAGTTGGTTTGTTATGCGTCCAAA
AACTCCCCGAGGATAGGCCTACCATGGCATCAGTAGTTTTCTGGTTAGGCAATGAAGGCCTGGTTCTTCC
TCAACCAAAGAAACCTGGTTTTTTTATTGAGAGGAATTCAACGGAATCAGCAGAGTCATCTGATGAAGG
ATATCTAAGTAACAACGTATCGATAACAGTCCTAGAGCCAAGATAGACATAAATTCCAACAATTGTAACT
TTTGAAATGTATCCACTTTGTCCTGTTTTTGTCAACAGTATTAGTGTATATATGGACTATATCATGAGGTG
CATTGAGGAAGATAGTCTAGTATTTCTGTTTTTCATTGACATGTGTATTCTTCGATACTATAATAAACGTA
ACGAATTGGCCTATTCTGAATAACTTGTAAAAATCATGTCTCCGCTGCTGACCATCAAAAT
>PGSC0003DMG402026153 G-LecRK
CAGAGATATTGTTTTGGGAATGCCTATGGAGTACTTGCCTCATAGAGATAGTTTTGATGAAGATTTAATC
ACTGAAGATGATAAGAAACGGATTGATGTCCCGTTTTTCAGCTTGAATAGCATACTAGTTGCTACTGAAA
ACTTCTCAAATGCAAGTAAGCTCGGGAGAGGTGGATTTGGACCTGTTTATAAGGTTTTGCAACTTTATTT
TTCTTTCCTTCTTCTGTTCTAAGTTGTTTTACCATTCAGATTTTTTTCTTTCGTTTTCATTATATAACAGTCCG
GAGATATATTTATGCAGGGTAAGTTTCTCGGAGGTACAGATATGGCAGTTAAAAGGTTGTCAAGTAATT
CTGGACAAGGTGTTGAAGAATTTAAAACTGAAGTAATGTTGATTGCCAAACTGCAGCACAGAAATTTAG
TTAGGCTTCTAGGCTATTGTGTTGAGAGAAATGAAAAGATTTTGTTATATGAATATATGCCCAACAAAAG
CTTGGATACGTTTCTATTCGGTTAGTATACTTCCCTCCCTACCATGAAACTCGTTGTAGTAATAGTTTTATG
ATTGATTCTCTCTGAAGTTATCTTACAAACCTTGAAATTAGATCATGCATTCTGTCGATTACTGGACTGGA
GGATTCGTTTTGAAATTATATTGGGTATTGCTCGAGGGCTACTTTATCTTCACCAAGACTCGAGGCTAAG
GATCATTCATAGAGATCTAAAGACGAGCAACATATTGTTAGATGAAGAAATGAATGCCAAAATATCAGA
TTTTGGCTTGGCAAGGATTGTTGAAGGCAAAAATACAGAAGCCAATACTAACAAAGTCGTTGGAACATA
GTAAGTCTCTTAGTTCTGCTTCACCTTTGATTTGGTGGGAAGAAGATAGATATATACACACCTAATGTATT
GGTTTACACAAACTAGATGCTAAAATAACCTATTCTGGAACACTAAAAACAGTGGCTATATGTCTCCGGA
ATATGCATTGGAGGGACTCTTTTCAATCAAGTCTGATGTATTTGCCTTCGGAGTAGTCCTACTCGAGATC
GTAAGTGGGAGGAGAAACATGGAATTTTTCGAAGATGTAAACCTCATTGGTCATGTAAGTACCAAAATT
TCCCTCATTAATATACATCTCATTCTACTTAAAGCTTTCATTGACTCAAAATTAGCATTTCACAGGTATGGA
GACTGTGGATGAAAGACAGTGCATTAGACATGATGGATCAAACCATAGTTGAGTCGTGCGATGAAAGT

GAAGTGCTAAAGTGCGTTAATGTGGCACTCCTATGTGTTCAAGAAGATCCAGCTGAGCGTCCTGTAATG
TCAAATGTTGTTTTTATGCTTGGAGGTGAAAGCATAACACTACCAAGACCAAATCAACCAGCTTTTATAG
CACGAAGAAATAGTGCTGGTGCAAGTACATCTTCTTCGTCTTCTAAGCTATACAACACTTCCAACAATGG
GTTGACAATAACTCAACGAGTAGGCCGATGAAATCAATATACATATAAGTTAAATGCATAACGCACACA
AGCCAATAATGTGCCGGTATATCTTTACTTCAATTTCAGTTTGAATTTCTGTTAAATTATTTTGGCACGCA
AAATAACCGATTGTGTTGTAGTCTCGGA
>PGSC0003DMG402029906 G-LecRK
CAAAGAAGTACAAAACACTCATGAAGTAGAAATAATTGGGGACTAAGACTATTTCCCACCGTGATCAAA
GTAACAGTCTTATTTTCAAAATTATCACTTCCACTTAATTATGAAATGTTTTCATCTTTTATGGATAGTGGT
GTATAAAAGATAAAACTCACATTTATATACCATAGTTTTTGATATGTGAAAATGACATACTTCATAAAATA
TCTTCTTGTTCTTGTTCTTGTTCTTCTCATCTTTCCCCTGCATATTTTAGCACAAACTATACCATTAGGCAGA
TCTCTCATAGCAAACGATGAAACAACACCATGGCTTTCTCAATCTGGAGATTTTGCATTTGGATTCAAGA
AAATTCAAAGTCAAAATCAGTTCTTGCTTTGCATATACTATGCCAAGATTAAAGATGCCACCATAGTTTG
GTATGCAAATGGTGGTAACCTCGTGCCAGGAGGCTCTATCGCGGAGTTAAATCCTCAAAAGGGACTAAT
TCTCCGCGATCCTAAAGGGAAAATGATCATTTGGAGCACTGGTCGGATTGGTTCTAATGTTGCTTATGCT
GTCATGAATGATACTGGAAACTTTGTTCTTGTTGGAGTTGATTCTTCAGTTTTATGGGAAAGTTTTCGATA
TCCGACTGATACCCTTATGCCTACTCAAATACTAGAGATTAACAACAAGCTTGTTGCTCGAAAATCGGAG
TCTTTCTTTGTTCCTGGTAGATTTTATCTTCGTATGTTGAGTGATGGAAATTTGGTTCTTGTTACTCAATCC
AAGCCAACGAATTTCGATTATGATGCTGAGTATTACAACAGTCATACTTCTGATTCAGGAGATGAAGCCA
ATTCTGGTTATCGATTGGTATTCGATGAGTTTGGTTCAGTTTACATTTTAAAAAGGAACAATCAAAGACTT
GTGTTGACACCTCCCAATGTTCCTTCAATTTCAGAAAATTATCACAGGCTAAGTCTTGATTTTGATGGTGT
TTTAACTCATTATTATCATCCTAAGAGTACGGGGGATCAGAAGTGGAGTACTCTTTGGTCTTTGCCTGAT
AACATATGTCTAGCAATTCTTGAAGATGTTGGAAGTGGAGTTTGTGGATTCAATAATGTATGCAATCTAG
GTGAAAATCAAAGACCATATTGTGAGTGTCCAAAAGGGTATTCGTTGATCGATCCAAATAATAAGTATG
GAAGCTGCAAACCAAAATTTGTTCCAAGTTGTGATGAATTTGGACAGGGAAATCCTGAAGAATTATATG
ATTTTGATGTGGTGACAGATGTTGATTGGCCGTTATCAGATTTCGAGAGGATTTATCCTTCTGCTGAAGA
AGAATGCAAGAAGGCTTGTTTAGAAGATTGTTTCTGTGCTGTTACTATTTATAGAAGCAATAGTTGTTGG
AAGAAGAAACTTCCATTGTCAAATGGGAGAGTAGATACCTCTTTGAATGTAAAAGCTTTCCTTAAAATAA
GGAAAATTTGAACATAAGAGCTTCTTAAATTAGATATAATGTATGTCTGCTATAGGTTCATGGTTGAATT

CGTCGATCCAACTTGCAATTTTACACAATTCAATAGTTTGGATTTGTAATAATTAATGGACCATATTGTAT
AACAAACTTTGATTAGCAAATAGTGAAATATTTCATACATTTCAAGAAACATATTGCACTGAGATACAGA
TACAGGTTATTGATGTACTCTTTAGGTCCAAGCAAATGAATAATGGAAAAAAAAAATGGTTGATCTAAAT
AGAATCGACAAACGAAAAAGGGTCAGGAGGAATTGATACTTCAACAGAACCTTCAAGCATTAACATGAC
TTTCTTCATGTTAGGCCTTGATGCTAGATTTTCTTGAATGCACCAAATTGCAACCAACAAAAACTTCTCGA
ATCTCTTGATATCTTCTAATGCCTCTTCATCATCGCCTACAAGTAAATGCAACTTATTTCTTTTATAGCAAT
CATAAGCCCACTCCAGTAGTATCATTTCGTTCTCGTTTGCCACATCCTGCTCATAGTTTTTTCTGCAGCAAA
TCAACTCCAAGAGCAAGACTCCGAAGCTGTACACATCAACCTTCACAGTTACAGGCATGCTTCTGAACCA
GTCTGGGGCTACGTACCCTTTGGTTCCACGTATCCTAGTGGTTGTTTGAGTCTGATGTTTTTTCAGTAGCT
TGGCCATACCAAAATCTGCTATTTTTGCTGTCAAATTTTCATCCAAAAGCACATTTTGGGGTTTGATGTCA
CAATGTATAATTTGGGTACTGCACTCTTCATGCAAATAACAAAGTCCTTTGGCAGTGTCGATGGCAACTT
GTACCCTTTTGGACCAACTAAGCCTAGAATCTTTGAATAGCAAGTGTGCTATTGAGCCGGTTTTCATGTG
TTCATATACCAAAAGGCGGTGTTGGCCTTCGTTGCAGAATCCAAGGAGTTGGACCAAGTTCTTGTGATTA
GTCCTGCTGATTGAATTCACTTCAGCTTCAAATACTTCTTCTCCTTCTCCTTCTGTTACCATGTTTCGGAGT
TTTTTGACAGCAACGACTTTCCCATTTTCATCATCAAGAACCGCTTTGTAAACTGTTGAGAAAGCTCCAGT
TCCTAATTCTTCCTTGAATCCATTTGTAGCTTGTTCTAGCTCTTTGTAGCTAAAACTTCTTAAGTTCACTCCT
GGAACAGCTGAATACGGTGCAGTCCTCTTTCGTTTTTTTCCTATAAACTTAAAAATGTACAATAGGGCCA
AAAGAATTAAGAACAAATTCATGAAAACTGAACTGGCCAACAACACAGAACCAGAAATGATCAGAGTA
GACTGATTTTTCTTTTTTCTTATTTCAACATTTGGTGTTCCCACTGTGGAGTTGTCTTTCCTTATTTTTATCA
AAGCCTTCCCTCCGATCGTTGGACCTACTCTCCCATTTGAAAGAGGATACCTTTTCTTCCAACAGACGTTG
TCGTCACTGTAAATGGCAACATCACAAAAACAATCATTCAAGCAATTCTGTCTGCACCAATCCTCTGAAA
CATCGCGGTGAGACTCATAATCAGAGTCAGGCCAGTTAGTATCCAACATTTCGTGGAATGTGAAAGACT
CAACTTCTCGCGATTCATGGTTACAGTTTTGCTCAGAGAAATTTTGTCTGCAGCTGCCTAATTTGTCATTC
GGATCATCCAAGATATATCCGAGTGGACAATCACATCTCGGCCTCTGATCAGTTCCTATTGAGCATAGGC
TGTTGAAACCACAGGCACCCCCTCCTGTACTTTGCCTGATAGACAAACAGATGTTATCAGGAATGTTATA
CAAACTAGACCATGCCATCGGTTCCCTACCAGAAGATTTCGGATGGACATAGTGCCTAAAAACTCCATCA
TATTCAAGAATCGCTCGATGATACATTGATTGGCTTCTTGAATTTTCCACATTAGAAGATATCGAATTTAT
CAAAGTTCCATTCTTTGCTTGAAGAAAAATAAAGCCTGATTGATTGAAGATCACCTGATAACCACTGCCA
ACTGACATAGGTGACCAATATGCAGCATTTGTTGCCTCAGCTGGATAATTGACTGTGTAAAGCACTAGAT

TTCCATCAGTTTGAAGTATGAACTCAAACCTCCCACTCGATACATTCGTGTCAGAGAAACTTGAAACAAG
ACTGTTATCTTGGTTAAGTACTTGACCAGGCAAAATCGTATCGGTGGGCTCATTAAAACTCTGCCATAGA
GTGTCTGAACTACTAGTTGCTAGCACAAAGTTTCCATTATCAAGCATGGCTCCATAGGCCAATTGAGCAT
TCGCCATGCCACGAGCCCACATCTCTTGACCATTTGGATCAGTGAGGACTAGTCTTCCATCCGCGGAGAG
CTGAACTTTGGATCCATCTAAGGCAAGATTATTCCTATTGGCTGACCAAACTATGGTTTTGTCTGTGATTT
TGTTGAACCATATGGCTAGTAAGTACCTTGATTCATTTCCAATTTTTTGGAAGCCCAAAGCGAATTCATCA
GATGGGGATGACCAAAAGCTAGTAACATCACTTGTAGTGAGTGATGAACCCAAACTAACATTTTGGTAA
GATTGAGCAATGGCTGAAAGGGGAAACAATAAGAGACCAACAAGAAGCATAACTAAAGAAGTCATGTA
AGAAAGAGGCAAAGTTATAAATGGATATTATGTACACC

>PGSC0003DMG403026153 G-LecRK

GTTTACTTTTTCATACCATAAAAAATGTTACTGACTCAAGTAGTTGCTGGAAAAGTTTAAGTTATGCAAAT
GAGTAATTTTCTCTTGTTGTTGTTTGCAGTGTTGGTGATTATATGTGGATGTGATGCAAGGAGCACGATC
GATGGAGGGAGTGAGTTAATAGACAACGGGGAAACACTGGTATCTGCTGGAAAAAAATTCGAAATGG
GATTCTTTTGTGATGATGCCGGGTTAAACTGGTATGTAGGCATATGGTATAACAAGAAAGGTCCTCGAA
CTGTTGTATGGGTTGCTAATTGGAATGATTCGATTCAGAATTCAACAAAAAATAAGGAGAATGTTTCCAT
GGTCGTTGAAGATGGTAAGCTCAAAGTTTTGAATATTAGTGGACACATTTATTTCTCCTCAGAAGTAGAT
GGTAACTCTGGCGTTAAAGCAGAGTTGTTGGATACAGGGAATTTGGTTTTATTTGATGAATCAGGAACT
AAAATGTGGCAGAGTTTTCGGAATCCAACCGATACATTTCTACCAGGCATGAAAATGGATAGTAATTTG
AAGTTGATTGATTCAAAAAATGGCAACTACAAATTCCAATTAGATCAAACAAACAACAAAAAATATGTCA
TAGTTGATGAGAATGGAATGCATAAAATTCACTGGCAAGGGTCTGTAGGAGGGTTTGCGAAATCAACT
GCTTATGAATTCTTCAGATTCAATGATATGCCCTCTTTTGTAACCTTCTTTTTATCCAATAATTCAGTTGAA
GCGAGAAGAACATTATTCAATAATTCAGTGGAAGCGACAAGAACATTATTCAATAATAATCGTAGTTTTA
CTCCGATTGATTTTTCAAGGTTAGCCAATCATAGTAGCAAAGATTCTATTTTCGAAGGTACTAGACTCCTG
ATGAACTCCTCTGGAGAAATACAATTCTACTTATGGGATAACCAGAGTGGATGGTCCTTGTTATGGTCTG
CCCCAAAAGATGAATGCGACGAGTTTAGAAAATGTGGGACATTTGAGATTTGTAACAGCAATGGAAGTT
CAGTGTGCAGCTGTTTGCCTGGGTTTCAGAGTGATCCTCCAGAAGATAATGGAGACGGGGAATATTCAG
GTGGATGTTCGAGAATATCAGATAACTCATGCAAAGAAGATACATTTGTGCATCTGAGCTCAATGAAAT
TTGGAAATTCAAATTGGACAGATAAAAAGATTAATACCAGTGAGAACTGCATGAGTCATTGTTTAAGCA
GCTGCTCATGCAAAGCATATACATATTATAACAATTCTGGATGCTGGTTTTGGGGGTCGAATCTCAATAA

TCTCCAGGAGAACTACCCTGGTGGCTTCAACCTCTCTGTCCGCGTGCCCCCTACAGAGGCGTATGTATGT
GAAGGGGAGGGGGTTCACATGCACCCATCCTCCCCTTCCTAACCCCTATATAATACTACTATTTTTCTAAT
TATCTTTTTAAATTTGTATTGGTGAACCCAAGGTTAAAAAGAATACTCTAGTATATTGATAAGTAAGTACA
AATAGGGGGTGCATAACTTATAGATTTGTGGTTCAAATTTTTCTATCCACATTAACATTTTTAATTTTTAAT
TAATATAACAAAATGTACCACTTTTCATAGATCTTTTTAATAATTTATAAATAAAAAGAGGTTCAAATACC
CATATTTTTAGGTTGCATTAGCTATGTCT
>PGSC0003DMG400000578 L-LecRK
CAATCATTAAGGCATTAAAATTTACATTCCATCTGAATTACTATATTTTGTTTACTGAAACTGAAATAGGA
CAAAAAATTGCATTATGCAAGTGAGGACTTTAAGTTCTCTTGTGTTTGGAAGAAAGTGAAATGAAGTTAT
CTGTAGACCACATCCTTATGTACAACCTTGACAATTGATAATTTGCTACTCAGAAAATTCAAATTCCAAAA
AAATTTATCACTAGCCAAGAATTAGCTCATCATGGATTACTTCTTCTGGTTTTTCTTCTCGTTTGCCATACT
AGTTAATAGTAATTTAGGTTCGTTCGCGGACGGACCTTCAATTAACGTGACGAAGCATATCTCTTTTCGA
GATTTCAGTTCTAGTAATCCCAGATTGAAGCAAGATCTTACACTTCTCGGTAGTGTTATCGTCTCGGATG
AAAAAAAATCCGTTCAAATTCCTGATCCTGAGCGAGAAGGTGATGATCTAAAGCATCTAGCAGGACGAG
CTATATATTCTTCACCTATCCGTTTCTTTGATCCTCGGACTCAAACACCGGCTTCTTTTGAAACGACTTTCT
CGTTTCAATTTGAAGTGAAGTCCAATTCAAGCAATGAGACGTCAGATCAGGGGAAATCTGTTGGTGGTA
GTGGCCTTACTTTTATAATCGTTCCGGATGAATTGACTGTTGGCCGTGCTGGTCCGTGGCTTGGGATGCT
GAACGATTTGTGCGATGAGGATTATAAAACAGTAGCTATTGAGTTTGATACTCGGCAGAACCCTGAATT
TGGTGACCCGAATGATAATCACTTGGGCATTAATTTGGGTAGCATAGTATCAACTGCAGCGATTAATGCT
TCGGATGCCGGAGTTCAATTGAATGACGGATCAATTCACAGAGTTTGGATATCTTATGATGGGCGAAAG
GGTTTTATTGAAATTCGTCTTGCACCTGATGGCAGAGGATATCCTTCTAAACCAGTTTACTCTGGTTTACT
TGATCTTTCACCTTACTTGAATGAGTATATGTTTGTTGGATTTTCAGCTGCTACTGGTAACCATACACAAA
TCCACAACATCTTGTCATGGAATTTCACCTCAATTAGTCAAGCTTCCCTTCGAATTCCTTCGACAGAGACG
TGCCAGAACAAAATCATGCTTCAAAATAGCACACAATCTGAAATTGATCATCGGAAAACGCCTAATAGTT
TCTTTATTTTCCTTGCTGTTGTCATTCTTCTGGTAATCGTTCTTATTAACCTCTATTTCAGTAGCTACAAGCG
AGATGACAATTCCGACGAAGCATTCACTCTGCCCGAGAAAAAGCAGAGACCGCGGCCACCAAACAAGG
CACGTCGCTTCACAATAGCTGAGATCTCCATCGCAACAAGGAATTTCAGCGAGTTACAAATATTAGGCA
GTGATGAGAAGAGCATTACGTATAAGGCCACGATCCTGAACGGGTGCAACGTTGTTGTAAAACGATTTT
TAACTCAATTTTTCAACACACACGGGTTTGAAAAGCGTCAATTTCACAAGGAAATCAAGGCTATCACCAG

AATTCGTCACCCGAATTTGGTCCCGATTAGAGGATGGTGCTATGACAACCAAGAAACAATAGTTGTGTA
CGATTTCATCCCCAATGGAAGCCTCGATAAATGGCTGTTCGGCGTTGGAGTTTTGCCTTGGACGAGACGT
TTTAAGGTCGTTAAAGATTTAGCAGATTCTCTTGTCTACCTTCATTCAAAGCAACTCGCTCACAAAAACGT
TAAAAGTAGCAGCGTGTTTCTTGACGTGAGCTTCAGAGCAGTAGTGGGTGATTTCGGGTTTGTGCTTAC
TTCAGCCGGGTCAACCCGGTTTGAGGCCATGGTGAGTCAGACAGCTGATGTGTTCGAATTCGGAGTAGT
CGTGCTAGAGATCATTGCGGGTCGGAGCAGAAAGTCCAACCCGGGAGAACGGGATTTGTTGGATCTTG
CATGGGCAATGCATGAGGTACAACAGAAGGAAACTCTAGTGGATCGTAGAATGGGCGCGGTAGTGAAC
CTGGAGCAGGCGATTCGGGCTTTGGATATCGGGTTGCTCTGCACGTTGAACGAGAACAAAGGAAGGCC
TACGATGGAAGAAGTTGTGGAGTTCCTAAATATGGAGAAACCGATACCGGAGTTGCCATCGGGTCGAC
CCGTTTGTTTGTTCCCGTACAGCAGCACCACAGGCTTATGCAGTGGGTATACTTGTACAGCATTCAAATG
AAGTTACGTGTTGCTTGACTAATTGGTTCTTCGGCGTTCCACGTAAGCACCAATTATTCTTATCTATTATA
CTACAAAATAATGACAGGGAAGCACATGTATATGCTATGTGACGTTATTTTCATGACATAGACAACTATT
GTCTGGTCTATGGTCGGAGTTTAATATCACAAGAACTCCTAAAAAGATTTGAAATTATTGTAAATTATGT
ACTGCAATTCAAATGTTCAATATATAAAATTGTATGTAATCTGTTTATTCCTCCA
>PGSC0003DMG400000789 L-LecRK
ATGCGTTTTTGTTTGCAAAATCTCACAAGGGTCACTCTGTTCTTCATCTTCACCATAAATTCACCTTCCCTT
TCAGCTCCTTTCTTGCCTATAAACAATGTAACTCTTTATGGCGATGCTTCATTCACCGCAAAATCAATCAC
CCTCACCCAAGAACGCAACTGTTCATCATCATCATCAACACCTCCCATTTCTGGCATTGGAAGAGCTTTCT
ACACATACCCAGTTCGTTTTCTTGATTCTTTAACCAATAACACTGCTTCTTTCTTGTGTACTTTCTCTTTTAC
TATACTTCCAACCCCTTCTTGCCCTTTTGGTGATGGCATGGGGTTTTTGGTCACTTCTGATGTTGATTCTTT
GAGCATCTCTGATGGGTATATGGGTCTTCCGAATCCCGACTCGGAAGATTCCTTCTTGGCTGTGGAATTC
AATGCCAATGATAACCGTATTGGTGTTGATACTAAAGAAATTAGGTCTTTGGCTTCTACTAATGTTGATTC
AGCTGGGATTGATTTGAAAAGTGGGAAAGAAATGGCGGGTCGGATTGAGTATAAAGATTCAGAGAAAA
TGATCAGAGTTTGGATTGGGTATGAACTGCAAATTAGGCCTCCTAATCCTGTTCTTTCTACCAGAATTGAT
GTTTCCAATCAGTTGAAGGAGTTTATGAGGATTGGTTTCACTGCTAAAGGGTCTGCAGTTTATAGCATTA
GTCGTTGGCGATTTAGAACGTTTGGATTGATTTCGTCTCCAATATCATCGTCTTGGGATCAATCCGATGA
AGGAAACTGTTTGATGTGTTTCCCTGAAGAGGAAATTGGTGGGCATATTTCTGATTCTCATCATAGTAGT
AGTAGTAGTGGTAGTAAAAGTTTACTGAAATTGACTTATGGAGGGTTAGCTGCAATTGTCACACTTGTTG
GTTGTGCTTTGTCTGTTGTGTTTGTTCTTGCGTTGAGAAGGAAAAAACGCGATAGAGTAGGGGAGACCA

ATGAACGGCAAATGTGTAGATTACAAGGAAATAGAGTGCCTCAAAGATTGTCATTATCTGAAATAAAAT
CAGCTACAGAGTGTTTTAATCATGAAAGGATAATCGGAGAAGGAGCATCTGCTGTTGTGTATGAAGGG
GAAATTCCTTCTAGGGGATCTGTGGCTGTTAAGAGATTTGTCCAAGGGAGTAGATTGGGTCCTTCACAT
ATTCCTTTTAATACTGAATTTGCTTCTATGGTTGGCTGTTTAAGACACAAGAATTTGATTCAGCTTCAAGG
GTGGTGTTGTGAGAGGAATGAATTGGTGCTAGTGTATGAATTCATGCCTAATGGTAGCCTTGACAAAAT
CCTCCACGAGCGATCGCATTTGACTAAGTTTCTGACATGGGAGAGAAGATTGAACATAGTTATTGGTGT
GTCATCTGCTCTTATGTATCTTCATGAAGAGTGTGAGAATCATATAATCCACAGAGATGTGAAGAGTTGC
AATATAATGCTTGATGCTGAGTTCAATGCCAAGCTTGGAGATTTCGGTTTAGCAGAGGTGTTTGATAATT
CTAAGACAAGGGATGCTACTGTACCAGCTGGAACAATGGGATATTTTGCACCTGAGTATGTCTATACTG
GTATTCCAACTGTTAAAACAGATGTGTATAGCTTTGGTGTTGTGGTACTGGAAGTGGCATCAGGTAGAA
AGCCTATCGACGAAGGTGGTGGTTTGATTACTGATTGGGTGTGGGACTTGTGGGAGAAAGGGAGGATA
ACTGAGGCTGCTGATCCTAAACTAATGGGGCGGTTTCAGAAGAATGAAATGGATAGAATGCTCATTGTG
GGACTTTCTTGTGTGCATCCCGATCATGAGAAGAGACCGCGAATGAGAGATGTTTTCCGTATGCTTAAA
GATGAAGCTCCACTTCTCATTTTACCTCCAATGAAGCCCACTGTGAGAATTCAATCTGTTTTACCAGAGAG
CTGTGAAGAAATCATGAATTGGGCTGCAAGAATGGAGGATACACCATGGTCCACTCCCAGAACTCATTT
TAGCAAGAACTAG
>PGSC0003DMG400001773 L-LecRK
ATGGAGATTTACTCATTACTAGTTGTATTGCTCTGTTTCTTGGCTGCTTTAGCTGCTGCCACTGAGTTTGA
TTCTGGGACTTTAACTCTTAGTAGTTTGAAGCTTTTAGGTGATGCCCATTTGGGGAATAATACTATAAAG
TTGACTCGTGACCTTGCTGTTCCAAATTCCGGTGCCGGAAAAGTTTTGTATTCTAAACCAGTAAAGTTCC
GGCAGCCGGGTCTTGATTTTCCGGCAAGTTTCTCTACGTTTTTCTCGTTTTCTGTTAGTAATTTGAATCCTT
CATCGATTGGAGGTGGATTGGCTTTTGTTATCACACCGGATGATGAGTCGGTTGGTGATTCCGGTGGGT
ATTTGGGGATTTTGGATGCAGAAGGAGGGCAAAATGGGAATTTTGGAGTTGAATTTGATACACTTATGG
ATGTTGAATTTAAAGATATTAATGGAAATCATGTGGGGTTAGATTTAAATTCAATGATTTCAACTCAGGT
TGGTGATCTTGATTCTATTGGTGTTGATCTTAAAAGTGGTGATTTGGTTAATTCTTGGATTGATTATTTTG
GTTCTACAAAAAAATTGAATCTTTATGTTTCATATTCTAATCTTAAGCCAAAAGAACCATTTTTATCTGTTA
CAATTGATATTTCTGAATATGTAAATGATTTCATGTTTGTGGGGTTTTCTGGTTCAACACAAGGGAGTAC
AGAGATTCATAGTATTGAATATTGGAGTTTCACTTCATCATTTGATACAAATCCTAAATCTCCCCCTGAAG
CGGTGGCGGAGCAGCCTCCGCCCCCGCCAACTGCTAGTTTGATGAACCCCACGGCGGATGTAACTACAT

NNNNNNNNNNNNNNNNNNNNNNNNNNNNNNNNNNNNNNNNNNNNNNNNNNNNNNNNNNNN
NNNNNNNNNNNNNNNNNNNNNNNNNNNNNNNNNNNNNNNNNNNNNNNNNNNNNNNNNNNN
NNNNNNNNNNNNNNNNNNNNNNNNNNNNNNNNNNNNNNNNNNNNNNNNNNNNNNNNNNNN
NNNNNNNNNNNNNNNNNNNNNNNNNNNNNNNNNNNNNNNNNNNNNNNNNNNNNNNNNNNN
NNNNNNNNNNNNNNNNNNNNNNNNNNNNNNNNNNNNNNNNNNNNNNNNNNNNNNNNNNNN
NNNNNNNNNNNNNNNNNNNNNNNNNNNNNNNNNNNNNNNNNNNNNNNNNNNNNNNNNNNN
NNNNNNNNNNNNNNNNNNNNNNNNNNNNNNNNNNNNNNNNCCACCGGCTAGTTTGATGAC
CCCCACGGCGGATGTAACTACATCCGCCACATCGCCCCCTCCGGAGCAACAACAGATTGCTCCGGCGGA
CGCTAATGGTACTGCTAATGCAGTACAGAGAAACAGCAGCAAATGTCATAATAGTTTTTGTAAGCAGGG
TGCTGGTGCTGTTGTTGGGGTTGTTACTGCTGGAGCATTCTTTCTAGCTTTTGCTACATTGGTACTTATTT
GGTTGTATTCGAAAAAATTCAAGAATGTGAAGACTTCAGAGACTATGTTGCCTTCTAATATTATCAAAAT
GCCAAAGGAATTTAGCTATAAGGAGCTTAAAATCGCGACGAAAGGGTTTGATTCGACTAGGATTATAGG
GCATGGTGCATTTGGTACTGTCTATAAAGGGATTTTGTCTGAGACTGGTGATATTGTTGCGGTAAAGAG
GTGTAGTCATAATGGACAAGGGAAAGCTGAGTTCTTTTCTGAATTGTCGATAATCGGAACACTTAGACA
TAGAAATCTTGTTAGGCTACAAGGATGGTGTCATGAGAAAGGTGAAATCTTGTTAGTTTATGATTTGATG
CCAAATGGTAGTCTTGACAAGGCGTTATTCGAGTCCAGAATGATCCTCTCATGGCAACACAGGCAAAAA
ATCTTGTTAGGTGTTGCTTCTGCATTAGCATATTTACATCAAGAATGCGAAAACCAGGTGATACATAGGG
ATATCAAGAGTAGTAACATTATGTTGGATGAAGGGTTCAATGCAAAGTTAGGTGATTTTGGACTAGCAC
GACAAATCGAACACGACAAGTCCCCCGATGCAACGGTAGCAGCCGGTACAATGGGGTATTTGGCACCA
GAATACTTGTTGACAGGTAGAGCAACTGAGAAAACTGATGTGTTTAGTTATGGGGCAGTGGTTCTTGAA
GTGGCAAGTGGGAGGAGGCCAATTGAGAAAGAAACAACAAGTGGTGTTGGATTAAACAGCAATTTGGT
TGAATGGGTGTGGGGATTACACAAAGATGGGAGTCTACTATCCGCAGCTGATTCAAAACTGAATCTCGA
ATTTGATGAAAAAGAAATGACAAGAGTGTTGTTAGTTGGTTTGGCTTGTTCACATCCTGACCCTATAGCT
AGGCCAACAATGAGGGGGGTGGTACAAATGTTAGTAGGTGAGGCTGAAGTACCAATTGTACCAAGGGC
TAAACCATCAATGAGTTTTAGCACATCTCATTTGTTAATGACATTGCAAGATAGTGTTTCTGACTTGAATG
GTATGATCACACTTTCAACTTCTTCATCAGAAAACAGTTTCAATGGTGTTGGTGGACATGATGGCATGGA
CCTAGTTTAG
>PGSC0003DMG400004396 L-LecRK
AATATTTTGGGCAACGAAAAACTTGAGTCTGCACTTGAAAATATAAAAACATTATAGAGTAGTGTAGCA

ACATACATTTGTTTAACTATGGCTGTTTTTGCATCTGTATCATACTTTGTTTTGATAATCCCCTTTGTCACTT

CATTGTCTTTCAATTTTGATAGTTTTAATCCCAATGATCAGAATATAACATATGAGGCAGATGCTTATCCA

AACGGTGTAATTCAGCTCACCAAAAACGAGCAGGGTAGTGGTTTAAACGATAGCATAGGCCGAGCTAC

ATATTCTAAAACACTCTATCTTTGGGACAAGGCTTCTGGGAATATCACCGATTTCACTACTCACTTCTCCTT

TGGAATCAATTCACTGGGAAAAAACTTATATGCTGATGGTCTTGCCTTCTTCCTCGCTCCTGCAGGTTCAA

GAATTCCTGATAAGAAGTCAGCCGCAGGAGGCGGCCTTGGCCTTGCAATTGATAGTCGACAAAATACAT

CGAAAAATCATCCTTTTGTTGCTGTTGAGTTTGACACCTTTAAGAACTATTATGATCCACCAGGTGATCAT

GTAGGTGTCAACATCAACTCCATGGTATCTGTTGTTAATGTGACTTGGTTTAGTAGCATTCCAAATGGTA

AGAGAACTGATGCCTGGATTACTTATAATTCAACCTCAAAAAATCTTAGTGTTGTCTTCACCGGTTTCCAA

CCACAAGGGAATACCACAGTCACTTTCCTGCAGACTCTATCTTACAATATTGATCTGAGGGACTACTTGC

CAGAATGGGTCACTTTTGGCTTCTCAGGTGCAACAGGATCCCTCTTTGCATTACAAACCATTTATTCTTGG

AATTTTACTTCTTCTTTAAAATATAATGACAACATAACAGATCCATTTGTGCCCCTACCAAGCCCTGTGCC

ACATCCAGATGTATCCCTACCAACCCCAGATCCAGATCCAGATCCAGATCCTTCAAGCAAAAAACTGTCA

GGACTAGTAATTGGATTGATTTCTGGCGGTTGTGTTTCGGTAGCACTATCTGTTTTGATATTGTTTGCATT

TTGGAGAAAGAGGAAGGTCAGAGAATATGAAGATGAAGATGAAGATGATATTATTGACAGTTCCATGA

CTAATGAATTTGAAAGAAGCACAGGACCAAAGAAGTTCTTGTACAGTGAGTTGGTTAGATGTACAAATT

ACTTTTCGCGGGAAGAGATGCTTGGGCAGGGTGGGTTCGGAGGTGTTTATAAAGGATATCTCAGTGAA

TCCAACTCCTATATTGCTGTTAAGAGGGTTTCAAGGGAGTCAAAGCAAGGAATAAAAGAGTATGCATCA

GAAGTCAGGATCATCAGCCGGTTAAGACATAAACATTTGGTGCAACTCATTGGTTGGTGCCATCAAAAA

AGAGAACTTCTACTTGTCTATGAGTTTATGCCTAATGGAAGCTTAGATTACGATCTCTTCAAGGGAAGAA

GCCATTTGACATGGTCAATAAGATTCAAGATTGCTCAAGGCTTGGCCTCAGCGTTGCTTTATTTACACGA

TGAATGGGAACAATGTGTGGTGCATAGGGACATAAAGTCGAGCAACATTATGTTGGATTCCAATTTCAA

CGCTAAACTTGGGGATTTTGGTTTGGCTAGGCTAGTTGACCATGATAAGGGATCCCAAACAACAGTTTT

GGCAGGCACGATGGGTTACATGGCTCCTGAATGTCTCACCACTGGCAAAGCTAGCAAGGAAACAGACG

TCTATAGCTTTGGTGTTGTCGCATTAGAAATAGCTTGTGGAAGGAAACCTATTGATCCTAAAGCTGAGGA

GCATCAAGTAAATATTGTCGATTGGGTTTGGAGACTTTACGGGATGGGAAATATTCGTGAAGCAGTTGA

TCCTAGACTCTCATCAGAGATCAATGAAGAGGAGGTGGAGCACTTGCTAATTGTTGGTTTATGGTGTGC

TCATCCAGATAACAATTGCAGGCCTTCTATTAGGCAAGCAACTCAGGTTCTTAATTTTGAAGCTCCGTTGC

CCACACTCCCTCCAAACATGCCTGTACCGACATATTGCAGTCTGCCACAATATGGACCAACTACTTCATTT

GCATCACCATACGACTCTAATGGTCCTGGGATATCTGAAATACACTCTTCAGGGACTAGAGACTACACTG

GTTCTTCAAATAACACAGCAGCTTCTGCAGCATCTTCGCCTTCCGCATCACTTTTGTACACACGTTGATAT

TGTCTTATAAATGAACAGGTCTTTTATTTCCCTGCATTCAATCTTTCAATTGCAGTGTCCAACTTTTGGTCT

ATGTATTCTTTATTATTTCTACTTTTA

>PGSC0003DMG400004397 L-LecRK

ATGGCTATTTTTGCATCTTTATCTGCTCTCACATACCTTGTTTTGATAATCCCCTTTGTCACATCACTGTCCT

TCAATTTTGATAGTTTTAATCCCAATGATCAGAATGTAACATATGAGGCAGATGCTTATCCAGCAAATGG

TGTAATTCAGCTCACCAAAAACCAGCGCGATAGTGGTGCAAACTATAGCATAGGCCGAGCCACATATTC

AAAAACACTCCATCTTTGGGACAAGGCCTCTGGGAATGTCACAGATTTCACTACACACTTCTCCTTTAGC

ATAAATTCACAGGGCATGAACATATATGCTGATGGAATTGCCTTTTTCCTCGCCCCTGCAGATTCAAGAA

TTACTGATAACTCAACCGCAGGAGGCGGCCTTGGCCTTGCAAATTATTATAGTCGACAAAATACATCAAG

AAATCAATCTTTTGTTGCTGTCGAGTTTGACACCTTTAAGAACATTGATGATCCACCAGGTGATCATGTA

GGCATCGACATCAACTACATGGTATCTATTGTTAATGTGACCTGGTTTAGTAATATTCCGAATGGTACGA

GAACTGATGCCTGGATTACTTATAATTCAACCTCAAAAAATCTTAGTGTTGTCTTCACTGGTTTTCAACCA

CAAGAGAATACCACAGTCACTGTCCTGCAGACCCTATCTTACAATCTTGATCTGAGGGAATATTTGCCAG

AATGGGTCACTTTTGGCTTCTCAGGTGCAACAGGAACCTACTTTGCAATACAAACCATATATTCTTGGAA

TTTCACTTCTTCTTTAAACTATAATGAAAACATAACAGATCCAGATGTACCCCAACCAAGCCCTGTGCCAG

AAGATACTCCAAGCAAAAATAAGTCAGGACTAGTGATTGGATTGATTTCTGGTGGTTGTGTTTCAGTAG

CAGTATCTGTTTTTATATTGTTTGCATTTTGGAGAAAGAGGAAGGTCAGAGAAGATGAAGATGAAGATA

TAATTGATGGTTCCATGACTAATGAATTTGAAAGAAACACAGGACCAAAGAAGTTTTTGTATGGTGAGT

TGGCTAGATGTACAAATAACTTTTCGCGGGAAGAGATGCTTGGGCAGGGTGGGTTTGGAGGTGTTTATA

AAGGATATCTCAGGGAATATGACTCCTATATTGCTGTTAAGAGGATTTCAAGGGAGTCAAAGCAAGGAA

TAAAAGAGTATGCATCAGAAGTCAGGATTATCAGCCGGTTAAGACATAAACATTTGGTGCAACTCGTTG

GTTCGTGCCATCAAAAAAGAGAACTTCTACTTGTCTATGAGTTTATGCCTAATGGAAGCTTAGACCACCA

TCTTTTCAAGGGAAAAAGCCATTTGACATGGCCAATAAGATTCAAGATTGCTCAAGGCTTGGCCTCAGC

GTTGTTTTATCTACGCGAAGAATGGGAACAATGTGTGGTGCATAGGGACATAAAGTCGAGCAATATTAT

GTTGGATTCCAATTTCAATGCTAAACTTGGGGATTTTGGTTTAGCTAGGCTAGTTGACCATGATAAGGGA

TCCCAAACAACAGTTTTGGCAGGCACAATGGGTTACATGGCTCCTGAATGTGTCACCACTGGCAAAGCT

AACAAGGAAACCGACGTCTATAGCTTTGGTATTGTCGTGTTAGAAATAGGTTGTGGAAGGAAAATGGG

CTTGGAGACTTTACGGGATGGGAAATCTTAGTGAAGCAGTTGATCCTAGACTCTCATCAGAGTTCAATG
AAGAGGAGGTGGAGCACTTGCTAATTGTTGGCTTATGGTGTGCTCATCCAGATAACAATTGCAGGCCTT
CCATTAGGCAAGCAATTCAGGTGCTTAATTTTGAAGCTCCATTGCCCACACTCCCTCCAAACATGCCTGTG
CCGACATATTGCAGTTATGGATCAACTACTTCATTTGCATCACCATACGACTCCAATGGTCCTGGGATATC
TGGAATACAGTCTTCAGGGACTAGAGACTACACTGGTTCTTCAAATAACACAGCAGCTTCTGCAGCATCT
TCGCCTTCCGCATCACTTCTGTACACACGTTGA
>PGSC0003DMG400004401 L-LecRK
GTTGTTTTTGCCTCTCTCAAATACTTCTTTCTTATCCCTTTAGTCACTTCACTCTCCTTCAATTTTGATACTTT
TACACCCAATGATCACAATGTAACCTATGAGAGAGATGCTTCTCCACAAAATGGTGCAATTCTACTCACC
ACAAACCTCCTCAATCGCAATATCAATGCTAGTATAGGTCGAGCTATTATTCAAAACCACTCCATCTATGG
GACAAGGCCTCTCGAAATCTCACTGATTTCACTACACATTTCTCCTTTAGCATTAATTCACAGGGAAGAGC
TGAATATGGTGATGGTTTTGCCTTCTTCCTTGCCCCTGCAGGTTCAACAATTCCTTATAATACAACAAGAG
GTGGAAGCCTTGGCCTTACAAGTAATAATGAGCGACTAAATTCATCCAGTAATCATTTTGTTGCTGTGGA
GTTTGACACCTATCAGAACCTGTATGACCCAAAGGGTGATCATGTAGGTGTCGATATCAACTCTATGCAA
TCTGTTGTTAATGTGACTTGGTTTAGTAGCATTCCAAATGGTACGAAAACTGATGCCTGGATTACTTATA
ATTCTAACTCAAAAAATCTTACTGTTGTCTTCACTGGTTTTCAACTCCAGTCTCCAAGGGAATAATATCAC
AGTCACTGTCCTGCAGAACCTATCTTACAATCTTGATCTGAGGGAATATTTACCAGAATGGGTCACTTTT
GGCTTCACTGGTGGAGGAGGAAATAGCTTTGCATTTCAAAGAATCTATTCTTGGAATTTTACTTCTTCTTT
AGATAATATGACAGATACAGGGGTAACCTTATCAAACACCAAGCCAAAGGATGCACCAATAAAAAGTAA
GTTAGGACTTGTGGTTGGATTGATTTCTGGTGGTTGTGTTTTGGTTGCAGTATGTGCTTTGGTGTTGTTT
GCATTTTGGAGAAAGAGTAAGGTCAGAGAAGATGATCATGTCTTTGATGGTTCCATGACTAATGAATTT
CAAAAAAGTGCAGGACCAAAGAAGTTCTGGTACAGTGAGTTGGCTAGATGTACAAATAACTTTTCACAG
GAAGAGATGCTTGGGTGGGGGGGATTCGGAGGTGTTTATAAAGGATATCTCAGTGAATCCAACTCTTAT
ATTGCTGTTAAGAGGGTTTCAAGGGAATCACAGCAAGGAATAAAAGAGTATGTATCAGAAGTCAGGAT
CGTCAGCAGGTTAAGACATAAACATTTGGTGCAACTCATTGGTTGGTGCCATCAAAAAAGAGAACTTCT
ACTTGTCTATGAGTTTATGCCTAATGGGAGCTTAGACCACCATCTTTTCAAGGGAAAAAGCCATTTGACA
TGGCCAATAAGATTCAAGATTGCTCAAGGCTTGGCCTCGGCGTTGCTATATCTACATGAAGAATGGGAA
CAATGTGTGGTGCATAGGGATATAAAGTCTAGCAATATTATGTTGGATTGTAATTTCAATGCCAAGCTTG
GGGATTTTGGGTTAGCTAGGCTAGTTGACCATGATAAGGGATCCCAAACAACAGTTTTGGCAGGCACAA

TGGGTTACATGGCTCCTGAATGTGTCACCACTGGCAAAGCTAACAAGGAAACAGACGTCTATAGCTTTG
GTATTGTCGTGTTAGAAATAGGTTGTGGAAGGAAACCTATTGATCCTAAAGCAGAGGAGCATCAAGTAA
ACATTGTCGATTGGGTTTGGAGACTTTATGGGATGAGAAATATTCTTGAAGCAGTTGATCCGAAACTATC
ATCAGAGTTCAATGAAGAGGAGATGGAGCATTTGCTAATTGTTGGCTTATGGTGTGCTCATCCAGATAG
TAATTGCAGGCCTTCTATTAGGCAAGCAGTTCAAGTGCTTAATTTTGAAGCTCCATTACCCACACTCCCTC
GGAACATGCCTGTGCCCACATATTGCAGCTTCTCACACCATTTACCAACTATTTCACTTTCATCAACACAT
GAAACCAACGGTACAGAGATCGCCCTTGCCCAAATCCAGCATTCAGTAAACGAAAACAACATTGATGCT
TCATTAAAAAGAACATTTTCTTCAGCATCTTCATCTTCAACATCAGTTTTGTATCCAAGTTAATATTGCCCT
GTTAAGATCCTGCATTTTGTCTTCCATTAGCAGTGTCCATCTTTTCCTTTCTTACTTTTTGTTGTTTCTCTTCT
AATGAAAGAGGATTGAATGGATTACATTTTCTGTAATTTTAACTGTTCTATCAAAC
>PGSC0003DMG400008822 L-LecRK
ATGTTCTTCAATTTTTTCATACTAGTAGCTCTCTTTTTAGTTGATTTTTCACCAGCTTCTTGTGAAGTTGATG
CATTCATTTTCAATGGATTCCAATCAGCTAATCTTAGCTTGGATGGCATAGCACAGTTCACATCTACTGAT
CTTTTGTTGTTAACAAATTCAGGAACACAAAATCAGGGCCATGCTTTCTATCCAAATCCAATTCATTTCAA
GAACTCACCTAATGGTTCTGTATTTTCTTTCTCCACTACTTTCGTGTTCGCGATAAGGTCTGATTATGGAA
ATTTGAGTGGTCATGGACTGGTTTTCGTTATCGCGCCACATAAAGGACTTCAGGGGTCTTTGGCAAATCA
CTATCTTGGTCTTTTTAACTCTAATAATAATGGGAATGGATTAAACCATGTCATTGGAGTTGAGCTCGATA
CAATCTATAGCGAGGATTTTGGTGATATCAATGACAATCATGTTGGAATTGATATAAATGGATTGAGGTC
TGTAGCAATTCACACAGCAGGTTATTTTGATGATACTGGTTTTTTTCATAACTTGACTTTAATTAGTGGCA
AAGAAATGCAAGTTTGGATTGATTACGATGGGAGGACTAAGCAAATGAATGTAACAGTAGCTCAGTTAC
ATATGGAAAAACCAGTTAGACCACTTTTGGCTTTGAAACATGATCTTTCGTCGATTCTTGATCAGACTATG
TATGTTGGTTTTTCATCCTCAACAGGTTCAGTCCCAACACATCATTATATCTTGGGATGGAGCTTCAAGAC
AAATGGGAAAGCTCAAGAACTCTCGCAACTTCCTGAGCTTCCTCGTCTTGGGAGTAAAGGGACATCAAG
ATTTGTAACGATTGGTTTGCCTATAATCTCGTTGGTTTCAGTTGTTGCAGCTGTGTTAGCGGTGGTTTATT
ATGTAAGAAGGAAGAAGTATGAAGAAATTCATGAAGATTGGGAACGCGAGTATAGACTACAAAGGTTT
AAGTATAAAGAATTGTACATTGCTACTAAGGGATTCAGAGAAAAGGAGGTATTGGGAGCTGGAGGATT
TGGTAAAGTTTATAAAGGAGTGATGCCTATCACTAAACTTGAGATAGCTGTAAAGAAGATATCTCATGA
ATCAAGACAAGGTATGAAGGAATTTATTTCGGAGATTGTAAGCATTGGTCGTATGCAACACAGGAATGT
AGTACAACTATTGGGTTATTGCAGGCGAAAAGGGGAGTTGATTTTGATATATGAATACATGTCTAATGG

AAGTCTAGACAAGTATTTATACGATCAACCAAGATACACCCTCGATTGGAACCAAAGATTCAGAGTCATT
AGAGGTGTAGCCTCTGGACTTTTTTTCCTACACGAAGAATGTGATCACGTAGTGATTCATAGAGATGTTA
AGGCCAGTAACGTCTTGTTAGATGGTGAACTAAATGGCAAGTTAGGAGATTTTGGACTCGCGAGGCTAT
ATGGTCATGGGACCGATCCTCAGTCTACTCGTGTTGTTGGTACTCTTGGTTACCTTGCACCAGAGCATAC
TAGAACTGGCAGGGCAACACCTAGTAGTGATGTATTTTCATTTGGGGCATTTCTGCTTGAAGTTGCATGT
GGTAGGAGGCCGATACAGCCAAGACAAGACGGTGATGATCTGATTTTGGTCGATTGGGTGTTCTCGTGC
TGGAATAGGGGTAATATACTTGATGCTGCTGATCCAAACATAGGCATTGATTTTGTTCCAGGACAAGTG
GAGTTGGTCTTAAAGTTAGGCTTGTTCTGTTCTCATTCAGATCCTTCGTGTAGGCCAACCATGCGACAAA
TCTTGTTGTTCTTGGATGGTGTTGTGGCCTTACCAGAGTTATCAGAACTCGGTGTTTCATCAGCTAACCTA
ACATTTGAACATCGCGGAGGTTTTGATGATTTTGTCAAGTCGTATCCAACTTCTTTGGGCTATGCATATTC
CGGCTCTCCATCTGTAACAGACTCCTTTCTCTCTGGTGGCCGATGA
>PGSC0003DMG400008897 L-LecRK
ACTAGATTTGACTATATTGTGTGATTATTTTTCATATATACATATTGAAGTTTGGCTGGCCTCAAGAAAAT
GACATTACAGAGTATACATTGTAGACTTGAACATTGGGAGATAAATAGTCATATTTGTGATTGTAGAAA
GTTAACCCCGCGAACATTATTATCAGTTCTATCCCTATAAGAGTTTTGAGCAGACATGGAGAAATTGTTTT
TAGCCATGATTTTTCAGTTTCTTGTTTTACTTTTTCATGCTCCATTAGGCTATGCAGCTGCAGAAGATCTCG
ATTTCACCTACAACGGGTTCCACTCAACAAACTTGAGTCGTGATGGAATAGCAGAGTTCACATCAGATG
GCTTGTTAAGGCTAACTAATGCTTCACAATTGCAGGAGGGTCATGCTTTTTATCCTAGACCTATCAATTTC
ATGAATTTAGCATCTAATGGTTCAGATTTCTCTTTCTCAACCACATTTGTCTTTGCCATAGTGCCTGAGTAC
TCGATTTTAGCTGGTCATGGTATGTCTTTTGTGATTGCACCAGCTAGAAGGCTTCCAGAAGCACTTCCAA
GCTCTTATCTTGGCTTGTTTAATGATTCTAGCAATGGAAATGCCACCAGCCATGTTGTTGCGGTGGAGTT
TGACACGCTACAGAACCGTGAATTTGATGATATTGACAGCAACCATGTAGGCATTGACATCAATGGCCT
AAAATCTGTAATAGCCAAGCCGGCCGGTTATTATACCAGTAATGAGAAATCCTTCAATAACTTAACTCTT
GCTAGTGGCCAGCCAATGCAAGTTTGGGTGGAATATGATTCTGTGGATAAGCATATGAATGTCACAATG
GCTCCACTGCACGTTGCAAAACCAGACCGTCCTCTCCTGTCCTTAGTCTACGATCTTTCATCTGTCATGGA
TGAAAACGTGTCTATAGGCTTCTCAGCATCCACCGGTGCAGTTGTTTCAACACATTATATCCTTGGATGG
AGCTTTAAAATGAATGGAGTGGCTCAAGGGCTTGATCTCTCTCAACTTCCTAAGCTTCCTCGAGTAGTAC
CTAAGAAAGAATCCAAGCTTTTATTGCTTGGTTTGCCCGTGATTTCTTCAGTTTCATTTGTGATTGTAGTC
CTGGTAATAATTTATTACATAGGAAAGAAGATGAAGTTTGCTGAATTGCAAGAAGAGTGGGAGCTTGAT

TATGGACCACACAGGTTCAAGTACAAGGATTTGTACACTGCCACAAAGGGCTTTAGCAGCAAGGATTTG
TTGGGATCCGGGGGATTTGGCAGAGTCTACAGAGGGGTATTGCCCATATCCAACATTCAGATAGCAGTC
AAGAGGGTCTCTCATGAATCAAGACAAGGGATGAGGGAATTTGTTTCAGAAATTGTTAGTATTGGCCGG
CTACGCCACAGGAATTTAGTACCACTTTTGGGTTATTGCAGGCGTAAAGGAGAGTTACTTCTGGTTTACG
AATGCATGCCTAACGGAAGCCTAGACAAGTATTTGTTTGACAAACCAAGCTGCACTCTTGATTGGAACCA
AAGATTTCGAGTCATCAAAGGCGTAGCATCAGGACTAGTCTATCTTCATGAAGAGTGGGAACAAGTAGT
GATTCATAGAGATATAAAAGCGAGTAACGTGTTGTTAGATGTTGAATTAAATGGAAGATTAGGAGATTT
TGGGCTAGCAAGATTGTATGATCATGGGACAGATCCTCAAACTACGCATATCGTTGGAACTCTCGGATA
CCTTGCCCCAGAGCATACAAGAACAGGCAAAGCCACAACTAGCACTGATGTGTATTCATTTGGGGCATT
TTTACTTGAGGTGGCTTGTGGGAGAAGGCCAATAGATCAAAGGGTATCGGACGAGGACCTCGTTTTGGT
TGACTATGTATTCTCTTGTTGGACTAGAGGTGATATTGTTGAAGCCATTGATCCAAACTTGGGCAATAAC
TATGTAAAAGAAGAGGTTGAGTTGGTACTAAAACTTGGACTATTGTGCTCTCAGACAGAACCAGCAGCT
AGGCCAAATATGAGGCAAGTGTTGATGTACTTGGAAAATGCCCTGCCTTTGCCAGAGTTATCACTGATG
CAAACTTCAAATATTAGCTTAACTTTTGCAGGCTTTGATCATTTTGCAATGTCATGGTCTTCTTCTGTTACT
GATTCCCTTCTCTCCGGTGGTCGATGACTCGGGCGAAATTTTAATATTTTCCTAGTAAAAATAAAACTTGT
TATATAAGCTATATTTCTTCCACCTAAGGTTGTATACTTGGTTCATTTAGGCATCTTGGAG
>PGSC0003DMG400010466 L-LecRK
CGTGTTTCCTTTGGATTCTCTGCTTCATTCACACCCCTCTTCTTCACCCCCATAATCCTCACTCTTCTCCCCC
TGTTTCCAGCTTTCTTCAAAAAATTTCACTATTTTCTAAGCTAAAAAAAAAGCTAAAAATCAAATCTTTGT
GCAATTTGATCTTCATTGTGCAATGGGGTTTTGCTAAAATCTAGCAATGAAGTCAATTCTTGAAGAACTTT
CCAGCAACATTCCTGTAAAAGTCATCCTGTTGTAGTGTTGTTCTCTCTTGTCTACTTTCTTGCATTTTCACT
GTTTCTAAGCTCAAAAAATAGCTAAAAATCAAATCTTTGTGCAATTTGATTTTCATTGTGTAATGGGGTTT
TGCTAAATCAATTCTTGAAGAAATTTCCTTCTGTAAAAGGCATCATGTTGTAGTGTTGTTCTCTCCTTAATC
CTCACTCTACTGTGTCAGTTTTCTACTTTTTTGCATTTTCACTGTTTCTAAGCCCAAAAAAATAGCCTACCC
TTTTTGTATCTTCACCAAATTCTTGTGATTTGGTGTGAAAAATCTTACCTTTTATACAATTTGATCTTCATTC
TCTGATGGGTTTTGCTAGAATCTAGCAATGACATCAAATCTTGAAAAAAAATTCAGGGACTTTCTTGTAT
GGTTCATCTTATTGTTCTGTTTCTGTAGTATTAGTTCAACATTTGGTAAAACTGATTTCGATTTTGAAAATT
TAACACTGAGTAGTTTAAAGCTTCTTGGTGATGCACACTTGAGTAACAACACTGTAAGGTTGACAGGTG
ATCTTGCTGTACCAAATTCCGGTGCCGGAAAAGTTCTGTATTCAAAACCAGTAAGATTCCGGCAACCGG

GGTTTGATTTTCCGGCGAGTTTCTCGACATTCTTTACATTTTCAGTTACTAATTTGAACCCATCGTCAATTG
GTGGTGGTCTTGCTTTTGTGCTTACGCCGGGTGATGAGTTAGTAGGTGATGCTGGAGGGTATATGGGAA
TCTTGGATGCTAAAGGGACACAAATTGGTACAATTGCTGTGGAATTTGACACCCTTATGGATGTTGAATT
CAAAGATATTAATGGAAATCATGTTGGTTTGGATCTAAATTCAATGATTTCAACTGAAATTGGTAATTTG
GATTCCATTGATATTGATCTCAAGAGTGGTGATTTGATTAATTGTTGGATTGAATATTCTGGTTCTAATGG
AGAGATGGATATATTTGTGTCATATACTAATCTCAAGCCAAAAGAATCATTTTTATCAGTTAACATCAATC
TTGCTGAGTATGTAAATGATTTCATGTTTGTGGGGTTTTCTGGTTCAACTCAAGGGAGTACTGAGATTCA
TAATATTGTGTGGTGGAGTTTCAGTTCATCATTTGATGCAACTCCAAACTCGGGGGAGGTGGTGGCTCC
ACCACCACCGCCAACGACTAGTTTGATGAACCCAACGGCAGATTCTGTCGCGTTGCCACCACCTTCTATG
GCTCCATCAGAGTCTAATAGTAGTACACCAGGTGTATTACAAGAAAAGAGAAGTAGGAAATGTCATAGC
AATTTTTGTAAACAAGGTCCTGGAGCTGTTGTTGGTGTGGTGACTGCTGGTGCATTTTTTCTTGCATTTGC
TACATTGGTACTTATTTGGTTATACTCCAAAAGGTTCAAGAGAGTTAAAAATTCTGAGATTGTGGCATCT
GATATCATCAAAATGCCTAAGGAGTTCAGCTATAAAGAGCTTAAATTGGCCACGAAAGGTTTCATTCCAA
CGAGAATTATAGGCCGTGGTGCATTTGGGACTGTTTACAAGGGCATTTTATCGGATACTGGTGGCATTG
TGGCAGTCAAGAGATGTAGTCATAATGGACAAGGGAAAGCAGAGTTCTTATCTGAATTATCTATAATTG
GAACCCTTAGGCATAGAAATCTTGTTAGGCTTCAAGGATGGTGCCATGAGAAAGGTGAAATTTTGTTGG
TTTACGATCTTATGTCTAATGGGAGTCTTGATAAGGTGTTATTTGAATCAAGAATGGTTCTTTCATGGTCA
CATAGGCGAAAAATTTTGATAGGTGTTGCTTCTTCCTTGGCATATTTGCATCAAGAATGCGAAAACCAGG
TGATTCATAGGGACATTAAGAGTAGTAACATCATGTTGGATGAAGGGTTCAATGCAAGATTAGGAGATT
TTGGATTAGCAAGGCAAGTTGAGCATGACAAGTCCCCCGATGCAACGGTAGCAGCTGGGACAATGGGC
TACTTGGCTCCAGAGTACTTGTTAACCGGAAGGGCAACCGAAAAAACTGATGTTTTCAGCTATGGAGCA
GTGGTTCTTGAAGTGGCTTGTGGAAGGAGGCCAATTGAGAGGGAAACAACTAAAGTTGAGAAAGTTAG
AGTGAATAGCAACTTGGTTGAATGGGTATGGGGGGTGCATAGAGAAGGGAATTTACTAAATGCAGCTG
ATTCAAGACTTTGTGGTGAGTTTGATGAACAAGAAATGAGAAGGGTTCTAATGATTGGGCTAGCTTGTT
CACATCCCGACCCTACGGCTAGACCAACAATGAGAGGTGTAGTTCAAATGCTGGAAGGTGAATCTGAAA
TCCCAATTGTCCCAAGAACTAGACCTTCTATGAGTTTTAGCACATCACATCTTCTAATGAGTTTGCAAGAT
AGTGTTTCTGACTTAAATGGTTTGATCACACTTTCCCCTTCATCATCCGAAAGTAGCTTCACTGGTGGTCG
CAATGGCAATGGCATGGAGTTGGTCTAAATGCAAGACTAAAAAAGACTTGACTTTTCGAGTTAATGCAC
CCATAAGAGCCCAACAATCCCACATATGGGGCCACCATCAAGGCGGAACAGACACAATAACTACTCTGA

TTGGGCGTGGACAAGGAAAAAGTGGCATCTTTTTTGTTTTGTTCATAAATTCATTAATTTTATTCATAATA
TATATGTACCAAATAAGCTTTAAGATTTAGTAATGTAATTTTCTCCTTTTCCTTTCATATACTATAGTCCATT
GAGTGCAAGATTAGAGATATTTAGTGGGGAAAATAAA

>PGSC0003DMG400011071 L-LecRK

AAAAAAAAAAAAAATTCTCACTATCCATCACCAAATCTTGAAAAAAAAATTCTTGTGTATCGTCTTTTTATAA
GGCAAATCAACTTCGATTCTTGAATAATCCACATCACTTCTGCTTCTATATATCTTACTTTAAAATGTTCAA
TTTTCTCACATTCCTCTGCTTTACCATTTCCTTCTCAATTCTTGCTTCATCTCAACTTGATGAATTCATTTTCA
CAAAATTCAATCAACCAAACAACAACATAACCTTAAGTGGAGTAGCAAAAATAAGCCAAAATGGGTTTA
TTCAATTAACCAATGACACAAGTAGACTAATAGGCCATGCTTTTTACTCTTCACCTTTCCATTTCAAGCCTA
CTAATGCCTCTACCTTCTCTTTCTCAACGTGTTTCGCTTTGGCCATAGTCCCCGAGTATCCAAAACTCGGG
GGCCATGGCCTAGCGTTCACAATTTCTCCGTCAAAAGACTTTAGTACCGCTCTTCCGAGCCAGTACTTAG
GTCTGCTCAATGCTAGTGATATTGGTAACTTCTCTAACCATATTTTTGCTGTTGAATTCGACACGGTACGT
GATTTTGAGTTTGGTGATATTGATGATAACCATGTTGGTGTTAATATTAACAGCTTAGAATCAAATAAAT
CAGCTGCAGCTGGCTATTTTATTAACGATCAAAATGTGAAACAAGATTTGAATCTTAAAAGTGGGAAAG
TCATTCTTGTTTGGGTAGAATATGATTCTGTTACAAAATTAGTTAATGTAACTCTTTCACCAAATTCTTTAA
AACCAAAAATTCCTCTTTTATCTTATAATTTTGATGTCTCTCCAATTTTTAAAGAAAATATGTATGTTGGCT
TCTCTGCTTCTACTGGTTTGCTTGCTAGTTCACATTACATTTTGGGTTGGAGTTTTAAGTTAAATGGAGAA
GCAAAATCACTTGACTTGGATTCTCTACCATCACTTCCTGGACCTAAGAAGAAGCATACAAGACTAATTA
TTGTCATCTCGATTATAGTGTTTGTGTTCGCGTTAATTTCAATCTTGATTGCTTTTTATACAATACGGAGAT
TCAAGAATGCTGATGTGATTGAGCAGTGGGAGCTTGAGATTGGTCCTCATAGATATTCGTATCATGAAC
TCAAGAAAGGTACTAGAGGTTTTAAGGAGAGTGAACTACTCGGGCGAGGTGGATTTGGGAAAGTTTAC
AAAGGTGTTTTGACAAATTCAAAAGCAGAAATTGCTGTGAAACGAATTTCTCATGAATCTAAACAAGGG
TTGAGGGAATTTGTGTCTGAGATTGCGAGCATTGGAAGACTCCGTCATAGGAATTTGGTTCAATTGTTA
GGATGGTGTAGACGTCGTGGTGATTTGTTGCTTGTTTATGAGTTTATGCCTAATGGAAGTTTGGATAACT
TCTTGTTTGATGAGCCAAAAATGGTGTTGAAATGGGAACAAAGGTTCAAGATTATCAAAGGGGTTGCTT
CTGGTTTACTTTACTTACATGAAGGCTATGAACAAGTCGTGCTACATCGAGACATTAAGGCTAGTAATGT
GTTACTAGATGGTGAATTAAATGGTAAGTTAGGGGATTTTGGACTTGCTAGATTGTATGAGCATGGGTC
TAATCCGTCCACGACTAAAGTTGTAGGTACATTAGGGTACCTTGCACCAGAATTACCAAGAACAGGACG
AGCCACAACAAAGACTGATGTGTTTGCCTTTGGTGCTTTGTTACTCGAGGTGGTTTGTGGACGTAGGCC

AATTGAACACAAAGCAGGACCCGAGGAGTTTGTTCTCGTGGACTTGGTGTGGGATAAATGGAGAGAAG
GTAAAATACTTGACATTGTGGACTATAGATTGAAAGGCGAATACAATGAGAGTGAGGTTTTGATGGTGT
TAAAATTGGGACTAATGTGCTCAAATAACAAGCCATTGGCGCGACCAAGTATTAGGCAAGTTGTAAGAT
ACTTTGAAGGTGAAATTGGGATGCCTGAGGCTCCAACAACACAAGGTTCTGATGATCTTGAAGGATTTG
GATTCGATGAAAGTGAACGTTGGAATTCCTTAGCATCGACTGGTATTATGATCAAGTCAAGTTCATTTAC
ATGTTTGTCTAATGGAGATGAAAATAGTACTAATTATGTTTCCTTTTCTACTTCACCACTTCCACTTCTATA
TAGTGGTGAATGTACCTAGATACTAATATGAACTAGCATTGCAAACTTTTCTATATTTTCCTGATGAACAC
TAATGTACATTGTTTTTTCCTTTTCTTTGTGTTACCTCTTTGTAGTAGTTTTTTTCCCACAGAAAACTTAGGA
AGCTTCCCGGTATTTCAGTGAGAACCTGTTTTCCACCATGTGTTTTCCCAGTGTGCTGGTTCGATGGGAA
ATGGGTGGAAAAATCATGTTTTATAATACAAATTTTTCATTGATTCGCGATGGAA
>PGSC0003DMG400018184 L-LecRK
TAGGAATCAAAATAATTAGTCAAAGGATGATCAGAACTTAGTCATCTTCATCATTTTCAGAGAAACAGCA
CTCATCTCTAATTCCCTTCAAAGTACCATGTAATTAACACATAATTTCCCCGTTTCCTTCACAAAATTCTCTT
CTTCTTCTTCATGCAAACCCCAACAATATGCCACCATTATCACTGTTCTTCATTCTCATCAACCTTACAGTC
TTCCCATCCATTTCCGCCGTCGAATTTCTCTTCAACTCCTTTACTGCTAACACCACCCCTGCCCTCAACCTC
ATCGAGGATGCCCGCCTTGAGCCACCAGTTATCCGCCTCACAAATGACTCCAACCAATTTTCCCTTGGCC
GAGCTTTTTACCCATCTCAAATTCCTGTAAAATCCGCTTCAAATTCCACTTCAATTTCGTCCTCTTTCTCTAC
CCAGTTCATCTTCTCTGTACTCCCTGATGATTCCTCTAGTCCCGGATTCGGAATTGCTTTTGTTCTCTCTGC
TTCAACTTCCCCACCTAATGCCCTCTCCAGTCAATACTTTGGACTTTTCTCTAACGCCACCGTTCACACGGT
GGCCCCACTTCTCGCCGTCGAGTTTGATACGGGTCGTAACCCGGAATTCAATGACCCAGATAGAAATCAT
ATCGGGATTGATCTTAACAGCATTGAATCTATTGTTACCCAAACGGCTGGGTATTATAGTTCTTCTGGTA
ATGATAGTGATTCTTTTGTGCCTTTAAATTTGCGGTCTGGGCAAAATATACATGCTTGGATTGAGTTTAAC
GGACCTGAATTCGAGATTAATGTTACTATTGCTCCGGCGGGTATGTCACGGCCTGTTAGAACTTTGTTGA
GTTATAGAAACCCCATAATTGCGAATTACACGTCTGCCCAGATGTATATGGGGTTCTCTGCTTCCAAGAC
TCAATGGGTTGAGGCGCAAAGACTTCTAGCTTGGAGTTTCAGTGATTCTGGAGTTGCAAGGGATATTAA
TACTACGAATTTGCCGGTTTTTCAGCTGGAAAATTCCGGGTCTTCGCTATCTCCCGGTGCTATTGCTGGG
ATTGTTATTGGTTCTGTGGTTGCTGTGTTGGGTTGTTTATTTATATTTTATTGGTTTTGGTGGCGTAAGAA
GGAAGAAGATGATGTTATCGAAGATTGGGAACTTGAGTACTGGCCTCATAGATTTTCATATGAAGAACT
TAGCCAAGCTACAAAAGGGTTTTCTAAAGATGAGCTACTTGGGGCTGGTGGTTTCGGTAAAGTATACAA

GGGAACATTTGCTAATAGCACAGAAGTGGCAGTGAAATGTGTGAACCATGACTCAAGACAAGGAATTA
GGGAATTCATGGCTGAGATATCTACTATTGGAAGGCTTCAACATAAGAATTTAGTACAAATGAGAGGGT
GGTGTAGGAAGGGGAATGAACTTATGATTGTGTATGATTACATGCCTAATGGGAGTTTGAATAAATGGA
TATTCGATAAGCCGGAGAAGGTTATGAATTGGGTAGACCGGAGGAGGGTTCTAGCTGATGTTGCAGAG
GGGTTGAATTATTTGCATCATGGTTGGGAACAAGTAGTTGTACATAGGGATATTAAATCTAGCAATGTTT
TGTTAGATAGTGAAATGAGAGGGAGATTGGGTGATTTTGGGCTAGCTAAGTTGTATACTCATGGTGGTG
TCCCTAATACTACTAGGGTAGTAGGTACATTAGGGTACTTGGCACCTGAAGTGGTGACAAGGGCTAACC
CAACTGCTGCTAGTGATGTTTATAGTTTTGGGGTGGTGGTTTTGGAGGTGGCATGTGGGCGAAGGCCG
ATTGACTTTGGGATTGTGTTGGAGGAAGAGGAAGTGTTGATTGATTGGGTTAGACAAAAGTACAGGGA
AGGGCGATTATGTGAGGCAGCAGCAGATAAAAGGATTAAGGGGCAGTGTTCAGAGGAAGAAATGGAA
GCTATGTTGAAACTAGGACTTACATGTTGTCACCCTGATCCTCTCCGTCGACCTACTATGGGAGAGGTTG
TTGCTGTATTGCTTGGTGAGAATGTGGATGCAACGCCAAATGAGTTGATCGTTGAATTAGCACCTAGTG
AAAGGAACACGGGAGATAGAAGCGACTGGTCAACTGAGGAATCAGAACCACTTTCGGCTGTGTAGATA
TTGTACTTTTGTTTCCTTTATTGTTGAATCTTTTTCATCCCTTGAAATTATTGTTTAAAGGTTTGTTCTTTGT
ATTGTTCTTACATGCTTTTGAACTCTAAACTTAGCATTAGTTGCAAATAGTTATGGATATGCAATTACTCA
AGATATCAAAACTGCTATGTCTTTGCGCTACCTTAAAGGGTCAACATCCATGGCAATTAGCAAACGTCTA
TACGCATTAAGATTCGCCTCGGTGGAATAACG
>PGSC0003DMG400018677 L-LecRK
ATTTTACACTACTCCAACATGCTTCAAAATAGCTTCTTTTATTGACCCTTCCTCTTATTAAAAGCCCTTACAT
CTCTATACTTCTTTTCACTACACAATATGGAATTTTTGACACCAAAAATCTTCATATTTTTCTCTTGTTTTCA
ACTCATATCACAAGCCAAAATCATAACATTTGACCAACAATATGGTGACCCTTTTGATCATACATATGTCC
CCCTTCTTGAAATCAAACATCCTGCACAAATAAGCAATCAAGCACTTCAAATCACCCCTGACACAGCTTCC
TCTGCTTACCAAATGTTCAATAATTCAGGCAGAATCCTATTAAAACGGCCTTTCAAACTATGGGTAGACA
ATGATATTTCAAGGACAGCGTCATTCAACACATCATTCCTAGTAAACATATATAGACCGGATAACCAAAC
ACCAGCTGAAGGTCTAGCTTTCTTGATATGTCCAAATTTTGATTTGCCACTAAATAGCCAGGGCCAGTAT
TTGGGCCTAACGAATGCTACAACAGATGGTGAATTATCCAACAAAATAATCGCGATTGAGCTAGATACA
TTCAAACAAGATTTCGATATTGATGATAACCATATTGGGATTGATATAAACAGTGTTAAATCCGTTAAGT
CGGAACCTTTAACACCACATGGAATTGAACTAGCTCCAATAGGTGCTAGATTTTACAATATATGGATACA
CTATGATGGATTCAAGAAAGTACTTGATGTGTACATTATTGAACAAATGACGAAAAATGGGACAACACC

AACAAGGCCAAAAGTACCAATACTAACACACAATGTTGATTTGAGAGAATTTGTAAAGCAAGAATCATA
TTTTGGATTTTCAGCTTCAACAGGACATTTCAATCAGTTGAATTGTGTGTTGAGATGGAATTTAACAGTTG
AGTATTTTCAAGAAAAGAATGATCAAGAAAAGGTACTAATAATAAGTCTAAGTGTTGGTGTATCAGTATT
GGTTGTTTTATTGATTTTGTCAGGGTATTTTGGGTATTTTTTTTATAAGAAAAAAAGGGATGATAGGTCA
CAATCTAATATATTAGGGGCATTAAAGAGTTTACCTGGAATGCCTAGGGATTTTGAGTTTAAAGAATTGA
AAAAAGCTACTAATAATTTTGATGAAAAGAATAAACTTGGTGAAGGTGGATTTGGAGTTGTGTACAAAG
GGTATTTAGTTGGTGAAAATTTGGAAATTGCTGTGAAGTGGTTTTCTAGGGAAAGTATCAAGGGTCAAG
ATGATTTCTTGGCTGAGTTGACAATTATCAACCGTTTAAGGCATAAACATCTTGTCAAATTACTTGGTAAG
TATTTTGAACTCTTTACTTTTTATGCTTTTTTTTGAGTATTTGTTCTTTGTTCAAGCTCAGATAAAGGAGGA
ATGTTATGATCTTAAGATTTTGATATATATGCTTATTGAGTTTAAGCGTAATTGGTTGATAGATTCGTCAT
GTCATTGGAATTTTATTCATGTTCTATGTATAGGTAGCAAAATCTACTAAGATTCTACTGAAAAAAGAGA
CAGGTTTTACACATAATCATTTCTTCGTTTTGAGGACACTTTTTATTTATTGAGTCGTGTTTCTTATCCTTCC
TCGACATAGTAACCGCTTTCATATTTTTTATAACTTTCAAGTACAAGTGCATGTTCATCCATCTTCATAGTC
ATTTCTTTGTTCCTTTAGTCGTTTTGATAGGACTTTTTTTATTGAGTCGTATTTCTTCTCCTTCTTCAACATA
GTGATTTGTTTCATGTTTTTTTTTCAAATTTCATAACTTTCGACCACAAGTGCATGTTCATCCATCTTCGTTT
TGAACGAAAGCTTAAATTAAATTTTATTAATTATGGTACCAAGAATATTTTCTTGTGTTCTAAGGAGTTCC
ATTTTGACTCATGTTAGGCCCATCTCCATCTTGAAAATGGTCCCAATAATTATATCCAACTACTACAAGAT
AAGTGAAAAAAGTTAAAATCTTTGATGCAACTTGCAAGTGTCAAAAATGGTGTGCTCATGTACCTTTTAC
AATAGTTTGAAAGTGATAAACATATACTTTTTTGTCTCAAAATATTTATTTATATATTTTTATACGCCTTTT
AAGAAAACATTAATTAGTAAAGATATTTAATTATTTCCCCCTTATTTATATTTTAAAACATAATCTCTATTT
ATATGTCATATTTGAGGTATCTGTAAATTTCAAAAATAATTACTAACTACTAAATATAAAATGAGGTAGG
GGTGATTAATTTTAACTTGAATTTTTAAAATATAATCCAAAGTTTCTTGGAGGACCTACTAACTAATAATT
TTAGTGCCCTCTTGATGAAAAATCCAAAGTAATAAATATTAAATTCTTAGCTGGAAGACAGAAGAGCACT
GATTCCATTTCCTTTGTCTTTTTGGTTTTCTATACCACCAATTTCAAACATAGGGTCCCACACTCTTTTGTTC
TTTGTTAGCTTCGGTATAAATATCGAGTAATTCTAATCATTTAAATTAATTATATGAAAAAAATTGTCTAG
TATTTTTGTTTCGATTAAATTTGAATCATTGTTTGAACAAACTTTTTTTTGTAGGGAAGCTTCCTGTCTTTG
AAAATCACTTTCATTCAAATGTCACCTATTATGATAAGTTTGGTACTTTCTAATATTAATATAATATAATTG
GACAATTGCTTCAAAATGCACTCAACTCCAATTAAGACTAAGATTGTAACCCTACAACATGTCATCATAAT
TGAGAGAGGTCCATATATTCCAAAATTAGGTCCTACAAAAAAGTTGATTGGAACACATACAACCCAACTT

TTTTTGCCTTGTAAAGATTTGATATCCTCAAAATAGTATTGATAAGATATATTTATAGATTATTATTATTGT

AGTAACAATTTAGCTTATATATGCATTAACAATTTTAACTGCATCTGTGTGAGCTCGCTCAAATTGTCTGT

CCTTAATCCGAATAAAGAAGGAGGGTTGTTGAGGATCACTCAGAAACACCATTCTCTATCCCATAAGAG

TAGGAGTAAGGCTGCATCAATTCATAGATCATTATTATTGTAGTCATAATTTGGCCTATATGTATATCTAC

TAATAACAATTTCAACTCTGTCATCAGTGTGATCTCGCTCACATTGTCGGTCTCAAGATTGAATAAAGAA

GGAGAGTTGTCGAGGGTCAACCGAAAACGGTCTCTCTGCCTTATAAAGATAGGAATAAGGATATTTACA

TCTTACCTTTGTCAGACGTACCCCACTTGTAGGATTTCACTGAATTTGTTGTTATTGTTGTTGCACTAACAA

TTTCAAGTGATCTAAAGTAATTTTTGAACCTCCTTTACCTTCTAATGAGAACGTAACCTAAATGTTGCATG

TAGTTATCATTTAGGTGACCTGCAGGTAATGGTATAAAAGTTCTTTTACGCTTTCAGTGTATATATATGGT

AATCACATGCATATTATTGTGACAGGATGGAGCCATAAGCATGGTAAGCTACTACTTGTATATGAATACA

TGCCAAATGGTAGCCTGGACAAACATCTCTTCTCATCAGGGCAAGAAAAAGAGCCACTCAGCTGGAGTG

TGAGGTACAACATTGTATCAGGAGTCGCGTCAGCTCTGCACTATCTTCACAACGAATACGAGCAGAAGG

TAGTCCATCGCGATCTCAAGGCAAACAACATCATGCTCGACTCAAACTTCAATGCACGTTTAGGTGATTT

TGGATTAGCACGAGCAATTGACAACGAAAAGACCTCATATGCTGATGAAGCTGAAGGTGTACTTGGCAC

GATGGGATACATCGCGCCAGAATGCTTCCACACAGGAAAAGCAACTCAACATTCTGATGTCTATGCATTC

GGAGCTGTGTTGTTGGAGCTAATATGTTGCCAAAGGCCTGGAACTAAAGTTAATGGCTTTCAACTCTTTC

TTGATTGGGTTTGGTTCTTGCATCGCGATGGGAGAATCCTTGAAGCTGTTGACAAGAGGCTCGAGGATC

AATACGTGGTTGAAGAAGCAGAGAGATTGTTACTACTTGCTCTGGCTTGCTCCCATCCAATTGCCAATGA

AAGGCCTAAAACACAAACTATAGTTCAAGTTATATCAGGATCAGTACCAGCACCACAAGTACCACCATTC

AAACCATCATTTGTATGGCCTTCTATGGTTCCAATTGACATAGATTCATCGAGCATCGTCGATACAATATC

CATCACAACTCCTCAGTACAGTTCAGAGAACAACAGTATTGAATATCAAAGCAAGTAGAGATAGCAATA

GCTTGATTTAAGGCCACTAAGATATTTTTTTGCTTATTGTGTAATGACAAGAATAGCAGCTCATGTATAT

ATTTCTCAGCCTTTTATGTTAAATTCCATCCTATACATTGTTCCTTCTGCGGTTTGACTGTTTGATTTGTTGT

ATTAAAAATGACATGTATTGCATAATTTCTAAAAAGAAGTTGTTCG

>PGSC0003DMG400020326 L-LecRK

CAACAATAGAACAAATTAGCATATTTTCTCTCAATCAAGAAAATATTACCAATGTTGTTAAATTTTGTCAT

ACTAGTGTCTCTCTTCTTTGTACCAACTTCTTGTGAAGTTGATGGATTCATTTACAATGGATTCAAATCAA

GTAATATTAGCTTAGATGGCATTGCTAATATTACATCCAATGGTCTTTTGTTGTTAACCGATTCCAAAACA

CAAGATCAAGGCCATGCTTTCTATCCAAATCCAATTCATTTCAAGAATTCACCTAATGGTAATGCATTTTC

ATTCTCTACAACCTTTGTGTTCGCGATAACGTCTGATTATCCACCATTGGATGGTCATGGACTAGTTTTTG
TTATCGCGCCACAGAGAGGAATTCAGAAGGCTTTGGCAAACCACTATCTTGGCCTTTTTAACTCAAGTAA
TAATGGGGATAAATCAAACCATGTTGTTGGAGTTGAGTTTGATACAATATATAGCGCGGATTTTGGTGA
TATGAATGACAATCATGTTGGAATTGATATAAATGGATTGAGGTCTGTAGCAAATCACACAGCAGGTTA
TTTTGATGATACTGGTTTGTTCAACAACTTTACTCTGATTAGTGGCAAGGCAATGCAAGTTTGGGTGGAT
TATGATGGCAGAACTAAGCTAATCAATGTGACAGTAGCTCCTTTACATATGGAAAAACCAGTTAGTCCAC
TTTTGGCTTTGAAATATGATCTTTCGCCGATTCTTAATCAGATTATGTATGTTGGTTTTTCATCATCAACTG
GTTCAGTCCCAACACATCATTATATCTTGGGATGGAGCTTTAAGGCAAATGGGAAAGCTCAAGAACTTTC
TCAACTTCCTAGTCTTCCTCGTGTTGGGCGCAAAGGGACTTCAAGATTTGTAACGATTGGTTTACCGGTG
ATCTCTTTGGTTTCAATTGTTGTAGCAACCTTAGTGGTGGTTTACTATGTAAGAAGGAAGAAGTATGAAG
AAGTTCTTGAAGATTGGGAACGTGAGTATAGACCGCAAAGGTTCAGTTATAAAGATTTGTACACTGCTA
CTAAAGGATTCAGAGAAAAGGAGCTGTTGGGAGCTGGAGGATTTGGTAAAGTTTATAAAGGAGTGATG
CCTGTAACCAAACTTGAGATAGCTGTAAAGAAGATATCTCATGAATCAAGACAAGGGATGAAGGAATTT
GTTTCGGAGATTGTAAGTATAGGCCGTATACAACATAGGAATGTAGTACCACTTTTGGGCTATTGTAGG
CGAAAAGGGGAATTACTTTTGGTTTATGAATTCATGTCTAATGGAAGTCTAGACAAGTATTTATACGACC
AACCAAGACTCACCCTCGATTGGAACCAAAGATTCAGAGTCATTCGAGGTGTAGCATCAGGACTATTTTT
CCTACATGAAGAATGTGACCATGTAGTGATTCATAGGGATATTAAGGCTAGTAATGTCTTGTTGGATGG
TGAACTAAATGGCAGGCTAGGAGACTTTGGACTCGCGAGGCTATATGGTCATGGGACCGATCCTCAATC
AACTCGTGTCGTTGGTACTCTTGGTTATCTTGCACCAGAGCATACTAGAACTGGCAGGGCAACACCTAGT
AGTGATGTATTTTCCTTTGGGGCTTTTCTGCTTGAAGTTGCCTGTGGTAGGAGGCCGATAGAACCAAGAC
AAGCCGGTGATGATCTGATTTTAGTCGATTGGGTGTTTTCGTGCTGGAATAGAGGTAATATTCTTGAGG
CTGTTGATCCAAACTTAGGCAATGATTTTGTTCCAGGGCAAGTGGAGTTGGTTTTAAGTCTAGGCTTGTT
CTGCTCTCATTCAGAGCCCTCATTTAGGCCAACCATGCGACAAAACGTGTTGTTCTTAGATGGTGTTGTG
GCCTTGCCAGAGTTATCAGCCCTCGGTGTTTCATCTGCTGGTCTAACATTTGACCATCGCGGAGGTTTTG
ATGATTTTATCAAGTCGTATCCAGCTTCTTCTGTTAATGCACATTCCCCCGCTTCATCTGTAGCAGAATCAC
TCCTTTCTGATCCCCGATGATTCATGGTGAGTTGGACATAACTTCTTTGGTGGAATATTGATATACTGATC
CTGGTAAATATAACATGATTTTAGTGATTATGTACTTCTGATAAATTATTGTAATTCATACACCTGAAACA
TATTGTGACATGTCTTAAGATATCAGCCATGGTATCCTGATCTTATTGTTTACTTCCAATGCTAAATAAAT
ACTAATCAGAGATTCAGCAATGGC

>PGSC0003DMG400020327 L-LecRK

AAAAAATGGATTAAAAAGATCATTTTTTTGTCATATCAAGAAATGTATAAACAGTTGAACAAATTAGCTT

TATATGGTCACAAATCACAATATTATCTCTCAATCAAGAAAATATTGGCAATGTTGTTCAATCTTGTCATA

CTAGTAGCTCTCTTCTTAGTTGGTATTGCACCAGCTTCTTGTGAAGTTGATGCATTCATTTACAATGGATT

CCAATCAAGGAATCTTAGCTTGGATGGCATAGCAGAGTTTACGCCCGATGGCCTTTTGCTGTTAACAGAT

TCTGAAACACAAGATCAGGGCCATGCTTTCTATCCAAATCCAATTCATTTCAAGAATTCAACTAATGGTAC

TGCAGTTTCATTCTCCACAACATTTGTGTTTGCCATAAGGTCTGATTATGGAATTTTGAGTGGTCATGGAC

TGGTTTTTGTTATCGCGCCACAGAGAAGAATTCAAGGAGCTTTGGCATACCACTATCTTGGCCTTTTTAAC

TCAACTAATAATGGGGATAGATCAAACCATGTTGTTGGAGTTGAGCTCGATACAATCTACAGCGAAGAA

TTTGGTGATATCAATGCCAATCATGTTGGAATTGATATAAATGGATTAAGGTCTGTAGCAGTTGAAACAG

CAGGTTATTTTGATAATACTGGCTTGTTTCATAATTTGACTTTGATAAGTGGTCAGCCAATGCAAGTTTGG

GTGGATTATGATGGCAGCACTACGCAAATTAATGTAACAGTAGCTCCATTACATGTGGAAAAACCAGTT

AATCCACTTTTGTCTTTGAAAACTGATCTTTCGCCGATTCTTGATCAGACCATGTATGTTGGTTTTTCATCA

TCAATTGGTTCAGTCCCAACACATCATTATATCTTGGGATGGAGCTTCAAGATAAATGGGAAAGCTCAAG

AACTCTCTCAACTTCCTAATCTTCCTCGTCTTGGACGCAAAGGGACATCAAGATTTGTAAAGATTGGTTTG

CCATTAATCTCTTTGGTTTTACTTGTTGTAGCAACCTCAATGGTGGTTTATTATGTAAGAAGGAAGAAGTA

TGAAGAACTTCTTGAAGATTGGGAACGCGAGTACAGACCACAAAGGTTCATGTACAAAGATTTGTACAC

TGCCACTAAAGGATTCAGAGAAAAGGAGCTGTTGGGAGCTGGAGGATTCGGAAAAGTTTACAAAGGAG

TGATGCCTATCACCAATCTTGAGATAGCTGTAAAGAAGATATCTCATGAGTCAAGACAAGGGATGAAGG

AATTTGTTTCGGAGATTGTGATCATAGGTCGTCTACAACACCGGAATGTAGTACCACTTTTGGGTTATTG

CAGGCGGAAAGGAGAGTTGCTTTTGGTTTATGAATACATGTCTGGTGGAAGTCTAGACAAGTATTTGTA

CAACAAACCAAGATTCACCCTTGATTGGAACCAAAGATTCAGAGTTATTAGAGGTGTAGCATCAGGACT

ATTTTTCCTACACGAAGAATGTGACCATGTAGTGGTTCATAGGGACATTAAGGCCAGTAATGTCTTGTTG

GATGGTGAACTAAATGGTAGGCTAGGAGACTTTGGACTTGCGAGGCTATATAGTCATGGTACCGATCCT

CAGTCTACTCGTGTCGTTGGTACTCTTGGTTACCTTGCACCAGAGCATACTAGAACTGGCAGGGCAACGC

CTAGTAGTGATGTATTTTCCTTTGGGGCTTTTCTGCTTGAAGTTGCCTGTGGTAGGAGGCCAATAGAGCC

AAGACAAGACGATGATGATCTGATTTTAGTCGATTGGGTGTTCTCATGTTGGAATAGGGGTAATATTCTT

GAGGCTGTTGATCCAAACATAGGGATTGATTTTATTCCAGGGAAAGTGGAGTTGGTCTTAAAGCTAGGC

TTGTTCTGCTCTCATTCAGAGCCCTCGTATAGGCCAACCATGCGACAAATTTTGTTGTTCTTAGATGGTGT

TGTGGCCTTACCAGAGTTATCATCACTCGGTATTTCATCAGCTGGCCTAACGTTTGACCATCACGGAGGT
TTTGATGATTTTGTCACGTCCTATTCATCTTCGATGAATGCACATTCCCGCTCTCCATCTGTAACAGACTCC
TTTCTCTCTGGTGGCCGATGATTCTTACATTAAATATGTTAGTAACTTTAACTTGTAAGTTGTAATCATGG
TTAGCACCTCATCTTAGTTTTAATTGAATCTCTATACTGCTGCACCTCAAATAAGATGGAAATGCA
>PGSC0003DMG400020328 L-LecRK
AAAAAAAATTAGCCATGTTTTTCAAGATTGTGTCCTTGATTGTGTCTGCTCTTTTTGTTGTTACAAATTCTG
AAGACTTTGGATTCATTTACAATGGGTTCAATAGAGCAAATTTGAGTCTTGATGGCATAGCACAATTTAC
ATCAAATGGCTTATTAGAGTTAACAAATACTTCTAGGCTACAAAAGGGGCATGCTTTTTATCCTACACCA
ATTAATTTCAAGAACTTACAAAATGGTTCAAATTTCTCTTTTCCTACCACTTTTGTCTTTGCTATTGTGCCTT
CTGTTTTACCTGGTCATGGTATGGCTTTTGTAATTGCACCTGTTGGTGGCCTAGTAGATGCACTTCCAAGC
CCATTTTTAGGCCTGTTCAATGATAACACTACTGGGAGAGTCAGTAACCATGTTTTCGCGGTCGAGTTTG
ATACTTTACAGAACAGAGAATTCAATGATATTGATGGTAACCATGTTGGAATTGATATCAATGGATTGAA
ATCCGTTGAATCAAAGACAGCAGGTTATTATTATGGTGTTAAATTCAATAACATGACTCTTGCTAGTGGT
CAGCCAATGCAGGGTTGGGTGGACTATGATGGTGTGGCTAAGCAAATTAATGTAACTCTGGCTCCAATG
AATGTAGCAAAACCAAATGTACCACTTTTGTCATTGTTGTATGATCTTTCACCAATCTTGAATGAAACCAT
GCATATCGGTTTCTCTGGATCGACTGGCTCGGTTGTTTCAACACAATATGTTCTAGGATGGAGCTTTGAA
ATGAATGGAATAGCTCAAGGTCTTGATCTTGCTAGACTTCCTAAGCTTCCTCGAGTTGGACCGAAGAAAC
AATCTAAACTTTTGTTGGTTGCTTTGCCAATGATATCAGCAGTTGTTGTAGTAATAGCCTTTTCTGTGTTG
ATTTACTATGTCGGAAGGAAGAGGAAGTTTGCGGAATTGCTTGAAGATTGGGAGCTTGAATATGGACCT
CATCGGTTCAAATACAAAGATCTTTATATCGCGACAAAGGGATTTGCTAACAAAAAGTTGTTAGGTTGTG
GCGGTTTTGGCAGAGTTTACAAAGGAGTATTACCAACTTCTTCAATTGAGGTAGCAGTTAAGAAGGTGT
CTCATGACTCAAAACAAGGATTGAGGGAATTTGTAGCAGAAATTGTTAGTATTGGTCGTCTACGCCACA
GGAATTTAGTGCAACTTTTGGGATATTGCAGGCGTAAAGGAGAGTTGCTTTTGGTTTACGAATACATGC
CTAATGGAAGTCTTGATAAGTTCCTATATGACAAACCAATATGTGCCCTCAGTTGGAACCAAAGATTTCG
AGTCATCAAAGGCGTAGCATCAGCACTAGTCTATCTACATGAAGAATGGGAACAAGTTGTAATACACAG
AGATGTGAAGGCTAGTAATGTGTTGTTAGACAGTGAATTGAACGCGAAATTAGGAGATTTTGGACTAGC
AAGATTGTATGATCACGGGACCGATCCACTCACTACACATGTAGTTGGAACTGTAGGTTACCTTGCCCCT
GAGCAGACAAGAACAGGCAAAGCCACAACCATTAGTGATGTATATGCTTTTGGTGCATTTTTGCTTGAA
GTGGCTTGTGGGAGAAGGCCAATAGATCCACGAGTATCGGATGAGGACATCGTGTTGGTTGATTACGT

ATTTTCTTGTTGGAGTAGAGGTGATATTCTTCAATCCATTGATCAAAATTTGGGGAATGAATATGTTAAA
GAGGAAGTTGAATTGGTACTAAAACTCGGACTTGTTTGCTCTCAGACAGAACCAACAAGGAGGCGAAG
TATGAGGCAAGTTTTAATTTACTTGGAAGATATCGTGCCTCCGCCATTGCCAGAGTTGTCATTGTTACAA
AGTTCTGCCCATTATTTTTCATTTCCAGGTTTTGATCATCACTTGTCTATGTCTTCATCATTTTCTCCAGAGA
AAGTACTTTCATATCCCTCCACCGATTCGAACTCTCTTCCATTCACTGGTCCATGATTAGAACTCTTTCTGT
ATTGCCTTGATGAATTGTGATTCCAATCCCAACAGCAACAACAACTGTACTTCAATTCCAAACAAGTTCGT
GTTAACTATGTGAATTCTTACTGTCTATATCGTTTCAATTAAGCATGTAATGTTTGTTACGATACAAATAA
TGGACTAATGTACACTTAGAATGATCCGGTTTCTTTTGATCTATAATGTTGTCGACATGTATTGCTA
>PGSC0003DMG400023671 L-LecRK
AAAAAACATAGTTTTTTGTTATATGTTGATATGTATATTCTAGGCAAGATTAGTTATTTAACTAACTTTGTT
CATCTTTTGATCATATTCATATTTTTGGTTCTTAGAGTGGATTGTATGAGTTTCAGCTACCAAAAATTCACC
AAGCAAAACGAAAAAGATTTCATTACTACTAACGATTCGTACATAGCCGTTGATGCTATCCAAGTAACTC
GCGATGTTCGTGGTGCTTCCATCAGTAATTTGTCTGGAAGGATATGGTATTCACATCCTATGAATCTATG
GAACAGAAGGAAAAACAGAACAGCCTCGTTTAATTCAACGTTTGTGATCAATATCAAACCTGAGAGTGA
TCCATGGGGTGAAGGATTGGCTTTCATATTGACTAAAGAAAGTGGTTCTCACTCAATCCCGGATAACAGT
TATGGACAATGGCTTGGGATTGTGAATGAAAGTACTAATGGATCTTCCTCAAACAACATATTTGCAGTTG
AATTCGATACAAGGAAAAGTTACCTTGATGATCTTGATGATAATCATGTTGGCATTGACATAAACAGCAT
CAATTCTGTCAAGCAAGTCTCGTTGATTGATCGTGGTGTTAACCTTTCACTAGCTGTTGATGTTATAGCAA
GTGTCCGGTATGATGGAGAATCCAAGATGTTGAAGGTTTATACATTCATGAGCAATCAAACCGGAGACA
ATGAGAGAAATCCCATCATTTCCATGCCTATTGATCTTTCTAGTTATCTCCCCGAGGATGTTTTCGTAGGA
TTTTCAGCCTCAACTGGGATATATAATCAGCTGAACTGCATAAAGGCATGGAATTTTACAAGCACAGATA
TTGGGAACAGTAATGGAGTTAGCCTGTTATGGTTGTGGATCTTGATACCAGTAATGTCAGTTCTTGTTAT
GTTCTTAGGTGGGGTTTTCGTATATTTCAGCTGGTGGAGAAAAAAGAAGAGGAGGCAAGTGTTAGACC
AATCTGAGAATATAGAGATTCAGATTCAGAATTCAGCTACTGCACCTCAGAGATTCCATCTGAAGGACTT
GAAACGAGCAACGGGGAACTTTGATCCGAAGAACATTCTTGGAAGAGGAGGATGTGGAGTAGTCCTCA
AAGGGATGTTAGATCAAAAAGAGGTGGCTGTGAAGAGATTCTTTAATGATTCAAGTCAAGGGGCAAAA
GATCTCATAGCAGAAGTCACAACTATAGGCAATCTGCACCATAAAAACCTTGTCAAATTGATAGGATGGT
GCTATGAGAGCAATGAACTCCTTGTAGTCTATGAGTTCATGCCAAATGGGAGCTTAGACAAGTTGATATT
TTGCGAAGAAAACGGAAAAGGTTTGAGTCTGAATTGGGAAATAAGGTATGGTATCATCTGTGGGACTG

CTCAAGCACTTGATTATCTGCACAATGGATGTCAAAAAAGAGTACTTCACAGAGACATAAAAGCCAGCA
ACATCATGCTTGACTCAGAACTCAATGCTCAGCTAGGGGATTTCGGATTAGCTAGGACAGTTCAAGTGA
ATGGAAAGACTCACCATTCAACAAAAGAGATTGCTGGTACTATAGGCTATATGGCACCAGAGAGTTTCC
ATATAGGCCGAGCCACAGTTGAAACAGACGTCTTTGCATTTGGAGTGCTAGTTCTTGAAGTTGCTTGTGG
TCGAAAACCTGGAAATCGATATGAGGAGAATAATTATACTAATAAAGTCATTGAATATGTATGGGACTT
GTACAAGATTGGAAGGATCATTGATGCCATAGATGTCAGATTGGATAGAGACTTCAATGAAGAACAAGC
CGAATGTGTGTTAATATTAGGATTGGCTTGTTGCCATCCAAATCCTTACGAAAGGCCAAGCATGAAAACT
GCTTTACAGATTCTTACAGGGGAATCAGTTCTACCAAACATCCCAACTGAGAAACCTGCATTTGTATGGC
CAGCCAGAGCTCCATCGTCCAACGAAGCTGCTAGTGGCTCTCTCCTGGATGGCCAACTTACACAGATTAC
AGTACTCAGTGGCAGATGACTGCAGAGTATGAGGAACCTCAGCAAGGTTAGGTAACTCTTTAGGAGGC
AAAAGATACCTGTAAATTCACATATGTAAAGGCATGAATGTCTCTTTATTTTTTTCTTTCTATGTATGTGG
TGGGGAGTGGGAGATATCTTCTTCCGAGTGTTCGTTTTTCTGCATTATAAATAATTTTGTATTTAGTCAAT
GTCATAATCAACTTTCCAAGTAAAAACTGAAAACAAAAGCAGCAAGCATTGTAACAAAGGCAGCTAGCT
ACATGAGTTACTCCTACAGAACATAAATGTTGTACCAAAACCACAAAGAAGTTGTGAAAATGAAAATTTC
ACGATCCATCATCATCTTCATGTTCTATCTCGAAGAAACTCTTTCGTACTCCATCAATAACAGTTTCTCTGG
ATTGTTCATGTCTGCTATGTCCATCGTT

>PGSC0003DMG400026646 L-LecRK

GCACAAAAAAACTTAACAATTTGTACTTCCAACAACTCCATGGACAATCCCTATAGCCTAGTTATTCTTGC
ATTGATCTTTTTCCCTATTCTTGTGAGAGCTCAATCTTTTGATTTTGCTTACAATGGATTCAATAACTCAAA
TATCATTAGAGATGGAGTTGCAATAATCAATCCAAGTGGTGCACTTAAGCTCACTAATAGATCACAAAAT
GTTATAGGTCATGCGTTCCACCCCAATCCTGTACCAATTTTCAACTCCAGTACTAAAAATGTTACTTCATTT
AGTACATACTTTGTCTTCGCCATTGTCCCTCTTGAGAAAACCTCTGGTGGCTTTGGCTTTGCTTTCACCTTA
TCTCCATCCCCGGGTTTTCCAGGTGCTCAGGGTGATCACTTTCTTGGAGTCCTCAACAACACGAACAATA
GAAAGGATACTAACCACATCTTTATGGTTGAATTCGACACTGTGGATGGTCATGATGAAGGTGTGGATA
CAGATGGAAATCACATTGGGATTAACATCAATAGCATGATATCTATCGCGTCACAACCAGCCAATTACTA
TGTTAATGGTACTTCTAGAACAGAGGAGGTACATCTACAAAACGGAGAATCAATTCAGGCTTGGATCGA
TTATGATGGAATGAGCAAAGTGGTAAATGTAACTATATCTCCCAGAACAGTTCCAAAGCCAATTCGACCT
CTGATCAGTGAGGTCGTAGATTTATCTCCTGTGTTGAACCAAACTATGTATGCTGGTTTCTCAGCAGCCA
CGGGAGATAAAGCGAGCTCTCATTACATCTTGGGTTGGAGTTTTCGGTTGAATGGAGCTGCTGATCCTTT

AGATCTTGATAACCTACCTGTTGCCCCACCTGAAGTGGTAAGACCCTCCAAGAATTCCCATCTCAAAAGG
GCCTTGATAGGAACATTCTGCTCCGTCGTTATCCTGCTCCTTGGGGCATTTTATATCTATAGAAGGATGA
GGCAGCATGAAGTTCTAGAGGACTGGGAGCTGGATTGTCCTCACAGGTTCCGATTTAGAGATCTTTACA
AGGCAACTAAGGGATTCAAGGAAAGTGAACTAATTGGAGTTGGAGGATTTGGTGCTGTTTACAAGGGT
GTTTTGCCTACTAATGGAGCCGAGGTTGCAGTAAAGAAGATATCGAGCAATTCTCTTCAAGGAATGAGA
GAATTTGCAGCGGAGATCGAAAGCTTAGGCAGGTTAAGGCACAAACACTTGATCAACCTACAAGGCTG
GTGCAAGAACAAGAATGATCTTCTCCTAGTGTATGACTTTGTCCCAAATGGAAGTCTTGATTCATTACTTT
ACAGGCAGAAAAGGGACATTGTTCTAACATGGGAACAGAGATTTAACATCATCAAAGGGATTGCTGCA
GGGCTTCTTTACTTGCATGAAGAATGGGAACAAGTGGTAATACATCGCGATGTGAAGAGCAGTAACGTC
CTAATTGATGGTGAAATGAATGGTCGATTAGGGGATTTTGGGCTAGCAAGATTATACGACCATGGCAAG
AATTCACACACAACAAATGTTGTTGGCACAATAGGGTACATTGCACCAGAATTGACACGGACAGGGAAG
GCCTCAACGAGCACGGATGTGTATGCATATGGTGTACTACTTCTTGAAGTAGCTAGTGGAAGACCTCCT
ATTGTCTATGAACCAGGGAAAGGAGCTTTAGTACTAGCTGATTGGGTGATAGAATGTTTACAACTAGGT
AATGTTCTTGATGCAGTTGATCAAAGGTTGAATTCAGTCTATGTAGATAAAGAGGTTCAAATGATTTTGA
GACTTGGGCTTGTTTGTTCACACCCAAGACCAGAAGCTAGGCCAACAATGAGACAAATAATGAAGTATC
TCACTGGAGATGAATCACTTCCAGTTTTTGAGCAGCAGTTGAGCTGTGTTGGATCTGATAGGGTTGATG
AAATCACAGCCAAGTTCTTGGAAGTGTTTTCTACTGATACAATCAATATATCACGTCGTTCGCTATCCGTC
GGACAGATATCATCTAGTTCACTCCATTCTGGTAGATGAAAACTCTGTATAAAGGACATACACTCTTCTTT
TGTTTTGTTTAACCACTTTGTTGGGATTGATTAAGTAGCTTCTATTTACCCAAATCCTGTTTTTCTTCCTCAT
AATTCTGCAATTTAGATAGGTCATTTATTTCTAGGCTCATTCAGCATTCACAAGTTAACATAATCTCACTTC
TATGTCTGCAACATCACTTATCACATCCTATATTTTTGTTTATGTACAAAC
>PGSC0003DMG400026647 L-LecRK
TAATTGTCTCTTTAAATTGAATATAAATATTGCACAAAAAACTTAACAATTTGTACTTCCAACGACTCAAT
GGACAATTCCTATAGCCTAGTTATTCTTGCATTGATCTTTTGCCCTATTCTTGTGAGAGCTCAATCTTTTGA
TTTTGCTTACAATGGATTCAATAACTCAAATATCATTAGAGATGGAATTGCAATTATCAATCCAAATGGT
GCACTTAAGCTCACTAATAGATCACAAAATGTTATAGGTCATGCTTTCCACCCCAATCCTGTACCAATTTT
CAACTCCAGTACTAAAAATGTTACTTCATTTAGTACATACTTCGTCTTCGCCATTGTCTCTCTAGAGAAAA
CCTCTGGTGGCTTTGGATTTGCTTTCACCTTATCTCCATCCCCGGGTTTTCCGGGTGCTAAGGGAGCTCAT
TTTCTTGGAGTCGTCAACAGTAGTAACGATGGCAATGATACTAACCACATCTTTATGGTTGAATTTGACA

CTGTGGATGGTCATGACGAAGGTGTGGACACAGGTGGAAATCAAATTGGGATTAACAGAAATGGCATG
AGGCATATCACGTCAGAATCAGCTGATTACTATAATAATGCTACTTCTGAAACAAAACCAGTAGATCTAC
AAAACAGAGAATCAATTCAGGCTTGGATCGATTATGATGGAATGAGCAAAGTGGTAAATGTAACTATAT
CTCCCATAAAAGTTCCAAAGCCAATTATACCTCTGATCAGTCGGGATGTAGATTTATCTCCTGTGTTGAAA
GAGACTACGTATGCTGGTTTCTCAGCAGCCACGGGAGATAAAGCGAGCTCTCATTACATCTTGGGTTGG
AGTTTTCGGTTGAATGGAGCTGCTGATCCTTTAGATCTTGAAAAGCTACCAGTTGCCCCACCTGAAGTGG
TAACATCCTCCAAGAATTCCCATCTCAAAAGGGCCTTGATCGGAACATTTTGCTCCGTGGTTATCCTGCTC
CTTGGAGCATTTTATATCTATAGAAGGATGAGGCAGCATGAAGTTCTAGAGGACTGGGAGCTGGATTGT
CCTCACAGGTTCCGATTTAGAGATCTTTACAAGGCAACTAAGGGATTCAAGGAAAGTGAACTAATTGGA
GTTGGAGGATTTGGTGCTGTTTACAAGGGTGTTTTGCCTACTAATGGAGCCGAGGTTGCAGTGAAGAAG
ATATCGAGCAATTCTCTCCAAGGAATAAGAGAATTTGCAGCGGAGATCGAAAGCTTAGGCAGGTTAAG
GCACAAACACTTGATCAACCTACAAGGCTGGTGCAAGAACAAGAATGATCTTCTCCTAGTGTATGACTTT
GTCCCAAATGGAAGTCTTGATTCATTACTTTACAGGCAGAAAAGGGACATTGTTCTAACATGGGAACAG
AGATTTAACATCATCAAAGGGATTGCTGCAGGGCTTCTTTACTTGCATGAAGAATGGGAACAAGTGGTA
ATACATCGCGATGTGAAGAGCAGTAACGTCCTAATTGATGGTGAAATGAATGGTCGATTAGGGGATTTT
GGGCTAGCAAGATTATACGACCATGGCAAGAATTCACACACAACAAATGTTGTTGGCACAATAGGGTAC
ATTGCACCAGAATTGACACGGACAGGGAAGGCCTCAACGAGCACGGATGTGTATGCATATGGTGTACT
ACTTCTTGAAGTAGCTAGTGGAAGACCTCCTATTGTCTATGAACCAGGGAAAGGAGCTTTAGTACTAGCT
GATTGGGTGATAGAATGTTTACAACTAGGTAATATTCTTGATGCAGTTGATCAAAGGTTGAATTCAGTCT
ATGTAGATAAAGAGGTTCAAATGATTTTGAGACTTGGGCTTGTTTGTTCACACCCAAGACCAGAAGCTA
GGCCAACAATGAGACAAGTAATGAAGTACCTCAATGGAGATGAATCACTTCCAGTTTTTGAGCAGCAGT
TGAGCTGTATTGGATCTGGTAGGGTTGATGAAATCACAGCCAAGTTCTTGGAAGTATTTTCTACTGATAC
AATCAATATATCACATCGTTCGCTATCCGTCGGACAGACATCATCTAGTTCACTCCATTCTGGTAGATGAA
AACTCAGTAAAAAGGACATACACTCTTCTTTTGTTTTGTTTAACCACTTTGTTGGGATTAATTAAGTAGCT
TAGAATAATATATAACTAGGAAAAAGGTAAGTCTCAAGTCAACATTTTCACTTAGCTGGAGTGCAAAAA
CCATGTAATTTAGAGAAGAAAAGTTATAAAATTAGTCTAGAAAGTAAATTTGTAATTATAAAAAAAAAA
AAAATCTATGTAGAATATTCGTTATAAAATCTATTTGTAATAAAAATGGTGACATGATTCAGA
>PGSC0003DMG400027140 L-LecRK
CTTTAAACCCCACTCTTTCACTTTCCCAACTCTTTCTTCATTCCTTTGTAATCACACAGACAAATCACATGTG

GCAAATCAAAGTAGAACTCTACAAAAAGAATGTCTAGATTCAGCTATAAATCATCACCCCACACAATTCT
TCAACTCTACAAACAAATTTCAATCTAACTATGCCATGGCCAAACAAATTACACCAACCCTTTTCTTGATTC
TCTTTATTTTGCTGTCAAGAACAGTTTTTTCTGATGAATTTGTGTATAATGGCTTTAATGGGGTTGAAGCT
AGTAATCTTACTGTAAATGGGATTGCTCAGATTTTAAAGAGTGGCGCTTTAAAGCTTACAAATGAAACAT
CTAGAGTTGTTGGTCATGCTTTTTACAAAAATCCAATTAAATTCAAGAATTCCCAATCTGGTAAAGTTTCT
TCCTTTTCTACTGCTTTTGCTTTTGGGATTGTTCCTGAATATGCTAAGTTAGGTGGGCATGGCTTTGCTTTT
ACAATTTCAAGGACTAAAGAGATGAAAGGGGCTCTTCCTAGTCAGTATTTAGGTCTTTTAAGTTCAAGTA
ATATTGGGAATTTTTCAAATCATGTATTTGCTGTTGAATTTGATACTGTTCAAGATTTTGAATTTGGTGAT
ATTAGTGATAATCATGTTGGTATTGATGTGAATGATTTGAAGTCTAATGCTTCAGTAAATGCTAGTTACTT
CTCTGAAGGAAGTTTTAGTAAACAAAAGCTTTTTCTTCAGAGTGGAAAGACAATTCAGGCTTGGATTGAC
TATGATTCAAGTAGAAATGTGTTGAATGTTACACTTTCACTTTCTTCAACAAAACCAGCCTTTTCAATTTTG
TCTTTCCCTTTGGACCTTTCTCTAGTTTTTGAGGAATTCATGTATGTTGGATTTTCTGCTTCAACTGGTTTG
CTAGCTAGCTCACATTATATATTTGGTTGGAGTTTCAAGATGAATGGGAAAGCTCAATCTTTGGATCTTG
ATTCATTGCCTGCTCTCCCTAAGCCTAAGAAGGATCAAACAGTCTTGATTGTAGCTACCACGATATCGGC
TGTTGTGTTTCTTGTATTCGGACTCGTTTTAGCCTTGTATGTCATTTGGAAAATCAAGAACATAGATGTGA
TTGAACCTTGGGAACTTGAAATAGGTCCACATAGGTTTTCTTACAAAGAGCTAAAGAAGGCTACTAGGG
GTTTTAGAGATAAAGAGTTGCTCGGGTTTGGTGGATTTGGTCGAGTTTATAAAGGAACACTGCCTAAGA
CGAATACCGTTGTTGCTGTGAAGCGAATACATCATGAAGCAAAACAGGGTCTTCAAGAATTTGTGTCTG
AAATCGCGACTATTGGTCGCCTTAGACATAGAAATTTGGTTCAGCTCTTAGGATGGTGTCGGCGAAGAG
GTGATTTGTTACTTGTTTATGACTTTATGCCTAATGGAAGCTTAGACAAATACATTTATGATGAACCAAGA
TTTACTTTAACTTGGGATCAGAGGTTCAAGGTTATTAAAGGTGTGGCTTCTGGTCTGTTGTATTTACATG
AAGAATGGGAACAAACTGTGATCCATAGAGATATTAAAGCAGGGAATGTGTTGTTAGATTCCGATATGA
ATGGACGACTTGGAGATTTCGGACTTGCTAAGTTATACGAGCATGGAGCAAATCCTAGTACAACAAGAG
TAGTCGGTACGTTGGGTTACTTAGCTCCAGAATTAACCAAGATCGGAAAGCCTACACCAAGTTCAGATG
TTTTTGCCTTTGGTGCATTGCTGCTAGAAGTTGCTTGTGGGAGGAGGCCAGTTGAGGCAAAGGCATTGC
CTGAGGAGTTAATCCTAGTGGATTGGGTATGGGATAAGTGGAAGGAAGGTGCCATTCTTGAAGTGGTT
GACTCGAGATTAAATGGCGAGTATGATGAGATGGAGGCCATAATGGTGCTTAAATTAGGACTCATGTGT
TCAAATAATACACCATCTAAGAGACCTAGCACGAGACTAGTGGTGAGATACTTGGAGGGGGAGGTGAC
ACTCCCAGAGACGCTGGCAGCTCCCGATGAATATGATGGAAAGAAGGGTGGTACTAGCGGTATGGAGT

TAGAGGACTTAATAGATTCCTATCCGTCTTCGTCTTATTTTGAGAAAGTAAGTACATGGTCATCAGCTTAT
GACGGTGAAGGCGATATTGATATTGAAGCTAATTCAGTAACGCCATTAACGGATTCTGGCAGAGAGGA
CAACAGATAGCCATGCAAAATGGAGGGACCATTCTACTTGCCCTCCCAAACTCCCTCCATATTTTAGTACT
TTTTATGGTTTGATTTTTGAACTTAAATGTAAATTATGGCTTTTGAAAAGAAAATGATATTAGTATCAACC
ATTTCTATGGTTTTGAGAATATTGGTGTTAGTAAAATGTGAATATATTTTTGCAATCAAGTCAATTTTGAA
TCTTGATGAAGCTTG

>PGSC0003DMG400029735 L-LecRK

GAATGATATTTCCCCATTTTCTCTGCTTCCCATAATTCTTTATCCATGTGAATTAGCTCTGCTTCTTTCTTAG
AATGAACCAATATTATCCAAAAATTCTTCTTTCTTACCTGTTATTAACCCTATTTTCTTGTAATCAATCAGTT
GCAGAAACTGATTTTGTCCTCAATGGCTTTACACAATCAGATGTGTCATTTTTTGGGAATGTTACAATCGA
ATCTAGAGTTCTTACTCTTTCAAATGACTCAACTTTTTCAATTGGTAGAGCTCTGTACCCTTCAAAGATTGT
CACAAAAGAAGCCAATTCATCTAAAGTTCTTCCCTTTTCAACATCTTTCATTTTTGCAATGGCTCCTTACAG
AGACAGGCTGCCAGGACATGGTATAGTTTTCTTGTTTGTGCCACACACAGGTATTGATGGTACTACTTCT
GCACAGAATTTAGGTTTCTTGAATTTCACAAATAATGGGAATCCTGATAATCATGTTTTTGGGGTTGAGT
TTGATGTTTTCAAGAATCAAGAATTCAATGACATAAATGAGAATCATGTTGGAATTGATGTTAATTCTCTT
GCATCAGAGTTTGCTCATGAAGCAGGATATTGGCCTGATGAGAAAAATAAGTATAATAGTGATGGTAGC
CTAAATGAGGAGTCTTTAGAGACTTTAAAGTTGAATAATGGAAGAAATTATCAAGTTTGGATTGATTATG
CTGATTTCAACATTAGTGTAACTATGGCACCAGTTGGTGTGAAAAGGCCTAAGCAGCCTTTGTTGGATTT
TCACCTAAATCTTTCCCAGGTTTTTGAGGAAGAGATGTATGTGGGGTTTACAGCAGCCACTGGAGAACTT
GCTCAAAGTCACAAGATTTTAGCTTGGAGTTTTAGTAACTCGAATTTTTCGATAGGTGATGGTTTGATCA
CACAAGGGCTGCCTTCGTTTGAGCTTCCTGAGGATCCAGTTTATCGATCGAAGGGATTCATTGCAGGTAT
GACAGTGTCACTCTTGTTTCTTGTTGTGGTCATTGCTGTAGCTTCATGGCTTTTGATTAAGAGAAATAGG
AGAATGAAAAAGGAAAGAGAGGATATGGAAGATTGGGAATTGGAATATTGGCCACATAGGATTACTTA
TCAAGAAATTGATGCTGCAACAAAGGGTTTTGCTGATGAAAATGTGATTGGAATTGGAGGTAATGGGA
AGGTTTATAAAGGGGTTTTGGCTGGGGGTTCAGAGGTTGCAGTAAAGCGAATTTCTCACCAAAACAGTG
AAGGGGCAAGGCAATTCTTGGCTGAGATTTCGAGTCTTGGTAGGCTAAAGCAAAGAAATTTGGTGTCAT
TAAGAGGGTGGTGCAAGAAAGATAGAGGCAGCCTGATTGTGGTTTATGATTATATGGAAAATGGGAGC
TTGGATAAAAGGCTGTTTGAATCTAATGAGAGAAGCATGCTGAGTTTTGAAGACAGAATTAGGATTTTG
AAAGACGTGGCATCAGGGGTTCTATACTTGCATGAGGGATGGGAGTCAAAAGTGTTGCATAGGGACAT

TAAGGCTAGCAATGTTTTACTTGACAGGGACATGAATGCAAGGCTAGGTGATTTTGGTCTAGCTAGAAT
GCATGATCATAGTCAAGTGGCTAGCACGACTCGAGTTGTTGGCACGGTGGGATACTTGGCGCCAGAGTT
TGCCAAGACTGGCCGTGCCTCCACACAAACTGATGTGTTTGGATATGGAGTGCTAATTATGGAGGTGAT
GTGTGGAAGGAGGCCTATAGAGGAGGAAGGCAAGCCACCTTTAGTGGATTGGCTGTGGGAACTAATG
AGACGAGGCGAATTGATTAACGCCTTTGATAGTCGATTAAGGATTAATCAAGATTTCAATGAAGAGGAA
GCTTTAAGGGTGTTGCAACTAGGCATGATATGCGCGAGCCTAGACGCCAAAGCTAGGCCAAGCATGAG
ACAAGTAGTGAAATTCTTTGACAGGAATAGTGATATTGATGAATATGAAACTGAGGATATGGATGCTTA
CCTTCTAGAGAGTTTGAGATCTAATACCATGTTGTCCAATTTTTCCTTGAGTTTAAGCCATGGTTCACATC
CAACATTTGAAGAAATTAGAGAAGGTTTGTCTTCTTCCATGTCAATTTCTTGGACAAATTCTCTGGTGGAT
GGTAGATGAGTTTAACATTTGGTTGGTTTATGTCCATCACTCTGATTTAACTTATGTTACCTTCCAAGGCC
TAGCTTTGATTGAATTTGCATTTGGCAATGCTGCTTCATGTACAAATTTATATGATGGAGTAGGAAAAAG
GATTTGGTTATGACTTGTGAGATATATACATGTATTCATTTCTTTTTTTGTACCTTGATGCTAGTTACAGTG
GAAGAGTTTGTTCTCTTTTTTTTTTTTTAACTATAGAATTTGTTCTTTATCTTTTTCATTTTACACGAGATAA
G

>PGSC0003DMG400030144 L-LecRK

GTATATATAAAATTTATTTTTTAGCAAAGCACTAAACAGAGTGTTTGGTGTTTATTGTATATATATGCAGT
TGCATGTCTAACAATGTGATTAACATATTGTCCAACTATGGTTGTTTTTGTCTCTTTATCTTCTCTCAAATA
CTTGCTTCTTATCATAATTATCCCTTTAGTCACTTCACTCTCCTTCAATTTTGATACTTTTACACCCAATGAT
CACAATGTAACCTATGAGAGAGATGCTTCTCCAGAAAATGGTGCAATTCTACTCACCAAAAACCTCCTCA
ATCGCGATATTAATGCTAGTATAGGCCGAGCTATATATTCAAAACCACTGCATCTATGGGACAAGGCCTC
TCGAAATCTCACTGATTTCACTACACATTTCTCCTTTAGCATTAATTCACAGGGAATAACTAAATATGGTG
ATGGTTTTGCCTTCTTCCTTGCCCCTGCAGGTTCAACAATTCCTTATAATACAACAAGAGGTGGAAGTCTT
GGCCTTACAAGTAATAATGAGCGACTAAATTCATCGAGCAATCATTTTGTTGCTGTGGAGTTTGACACCT
ATCAGAACCCCTATGACCCAAAGGGTGATCATGTAGGTGTCGATATTAACTCTATGCAATCTGTTGTTAA
TGTGACTTGGTTTAGTAGCATTCCAAATGGTACGAAAACTGATGCCTGGATTAGTTATAATTCTAACTCA
AAAAATCTTACTGTTGTCTTCACTGGTTTTCAACTCCAAGGGAATAATATCACAGTCACTGTCCTGCAGAA
CCTATCTTACAATCTTGATCTGAGGGAATATTTGCCAGAATGGGTCACTTTTGGCTTCACTGGTGGAGCG
GGAAGGAGATTTGCATTTCAAAGAATCTATTCTTGGAAATTTACTTCTTCTTTAGATAATATGACAGATAC
AGGGGTAACCTTATCAAACACCTTGCCAAAGGATGCACCAACAAAAACTAAGTTAGGACTTGTGGTTGG

ATTGATTTCTGGTGGTTGTGTTTTGGTTGCAGTATGTGCTTTGGTGTTATTTGCATTTTGGAGAAAGAGT
AAGGTCAGAGAAGATGATTATGTCTTTGATGGTTCCATGACTAATGAATTTCAAAAAAGTGCAGGACCA
AAGAAGTTCTTGTACAGTGAGTTGGCTAGATGTACAAATAACTTTTCACAGGAAGAGATGCTTGGGCGG
GGTGGATTCGGAGGTGTTTATAAAGGATATCTCAGTGAATCCAACTCTTATATTGCTGTTAAGAGGGTTT
CAAGGGAATCACAGCAAGGAATAAAAGAGTATGTATCAGAAGTCAGGATCGTCAGCCGGTTAAGACAT
AAACATTTGGTGCAACTCATTGGTTGGTGCCATCAAAAAAGAGAACTTCTACTTGTCTATGAGTTTATGC
CTAATGGAAGCTTAGATCACCATATTTTTAGTGGAAAAAGCCATTTGGCATGGCCACTAAGATTCAAGAT
TGCTCAAGGCTTGGCCTCGGCGTTGCTATATCTACACGAAGAATGGGAACAATGTGTGGTGCATAGGGA
TATAAAGTCTAGCAATATTATGTTGGATTCTAATTTCAATGCCAAGCTTGGGGATTTTGGGTTAGCTAGG
CTAGTTGACCATGATAAGGGATCCCAAACAACAGTTTTGGCAGGCACAATGGGTTACATGGCTCCTGAA
TGTGTCACCACTGGCAAAGCTAACAAGGAAACAGACGTCTATAGCTTTGGTATTGTCGTGTTAGAAATA
GGTTGTGGAAGGAAACCTATTGATCCTAAAGCAGAGGAGCATCGAGTAAACATTGTCGATTGGGTTTG
GAGACTTTATGGGATGAGAAGTATTCTTGAAGCAGTTGATCCGAAACTATCATCAGAGTTCAATGAAGA
GGAGATGGAGCATTTGCTAGTTGTTGGCTTATGGTGTGCTCATCCAGATAATAATTGCAGGCCTTCTATT
AGGCAAGCAGTTCAGGTGCTTAATTTTGAAGCTCCATTACCCACGCTCCCTCGGAGCATGCCTGTGCCCA
CATATTGCAGCTTCTCACGCCATTTATCAACTATTTCAGTTTCATCAACACATGAAACCAGCGGTACAGAG
ATCGCCCTTGCCCAAATCCAGCATTCAGTAAACGGAAACACCACTGATGCTTCAATAAAGAGAACATCTT
CTTCAGCATCTTCATCTTCAACATCAGTTTTGTATCCAAGTTAATATTGCCCTGTTATAACCCTGCATTTTG
TCTTCCATTAGCAGTGTCCATCTTTTCCTTTCTTACTTTTTGTTGTTTCTCTTCTAATGAAGAGGATTCTTTG
GAT

>PGSC0003DMG400047213 L-LecRK

ATGTTGCTATTTTTAGTAATTTTTGTTATCCCTTCTTTATATTCATTGAACTTCAATCTCACCAATATCAATC
CTTCTTATGTTAATCTCTCTATCAATGTCACTGGAGACGCCTACGTAACGAAACAGGGCATACAAGTTAC
GCCTAATGAACGTAACATTACATTAAGTAGGAAAACAGGACGCGCCATGTACATTGAGACACTGCAGTT
ATGGGACAAAGCTACAGGGGAAATGACAGATTTTACTACACATTTCTCTTTTGTTATTGATTCAAATGGT
AACGATCGTTTTGCTGATGGACTAGCCTTTTTCTTAGCTCCTGTTGGTTCGAGTATTCCAGTTGGTTCGTT
TGGCAGTGGCCTTGGTCTTGTTGATGCTGAGACAGAAAACATGTCATCACATGAGTCATTTGTTGCTGTA
GAGTTCGATACACATGTGAATAACTGGGACCCCTCAGACATACATGTAGGCATCAATATAAATTCTATGG
AATCTGTTGCTACAACAATATGGAAAAATGACATTAAACTTGGGAAGAAAAATGATGCTTGGATTAGTT

ACAATGCTAATTCCAAGGTTCTTGAAGTTGTTTTCACAGGATTTGAGAAAAAATATAATCGAGACAAACT
AAGCTACCCGGTTGATCTGAGGGATTATTTGCCTGAGAATGTTAGTTTTGGCTTCTCAGCATCAACTGGA
CAATTGTTTCAGAAAAACAATGTCAAGTCTTGGGATTTCAATTCAAGTTTGAGTTTTGATGCTATCAAAGT
TCCACAACAGCCTAGTGCAAGTCCTCCTATACAAGCTCAAGATCCAAGAAATCATCCTATTCAAGGTCAA
AATCCAAATGATCCTCCCATTACTCATGTTCAAGAACCCAAGGGTCCTCCTCAAGAAGTACTCAGCACCT
CGGGAAAAGGAAGCAAGGGACTACTAGTCGGATCAAGCATAGGTTTACCTATTCTGATTCTTGGTTTGA
TAACTGCCAGTTGCTTTTTGTGGAAGAAAAAGAAGAAAGGAGATAAAAAATTAGACCATGTCTTCATTG
ATCTAGATATGGATGATGAATTTGAAAAGGGTACCGGTCCTAAGAAGTTCTCGTATGGTGAATTAGCTC
GTGCAACAAACAACTTTGCTGAGGGACAGAAGCTTGGAGAAGGGGGATTTGGTGATGTTTATAAAGGT
TTATTGAAGGAATGTAACTCATATGTTGCTGTTAAGAGAGTGTCAAAAGGGTCTAAACAAGGGATAATG
GAGTATGCATCAGAAGTGAAGATCATCAGTCGATTAAGGCATAGAAATTTGGTTCAACTTATCGGTTGG
TGCCACGAGAAAGAGAAGCTTCATCTTGTTTATGAATTGATGCCCAATGAAAGCCTAGATAAACATCTTT
TCAAAGATAAGTCATTGTTGGTCTGGGAAATAAGGTGGAAAATTGCTCAAGCAATTGCCTCAGCTTTGCT
GTATCTACACGAAGAGTGGGAACAATGCGTAGTCCATAGGGACATAAAAGCAAGTAATGTCATGTTGG
ATTCAAACTTCACCGCTAAATTAGGTGATTTTGGGTTGGCTAGGCTTATTGACCATGACAAAGGATCTCA
AACAACAATGTTAGCAGGGACCGTGGGGTATATGGCACCGGAATGTATCATGAATGGAAAAGCTAGCA
AGGAGTCAGATGTATACAGCTTTGGAATAGTAGCATTGGAAATAGCAAGTGGAAGAAGATCAATAAAT
ATCAAAGCACCAGAAGATGAAGTGAGATTGGTTGAATGGGTGTGGAGCCTCTATGGAACGGGAGAGCT
AGTCGAAGCAACTGATCCAAGATTGAATAAAATGTTTAATGAGAAAGAAATGGAACGTTTAATGGTCAT
CGGTCTATGGTGTGCTCATCCAGACAACAAACTCAGGCCATCAATAAGGCAAGCAATCCATGTTCTAAAT
TCTGAAGCCCAATTACCAAATCTTCCATCAAGAATGCCAGTTGCAACATATTCACCCCCTCCATTGAATAT
GTTTTCGTCTCCATTTTCAAATACCTATGAAGTTAATAAGCAGGAGCAGACTATGACTTACACTATTTCCT
CAAGTGTCTATACTTCGTCAGCTGCTTCATCGACAAAATCACTTTTGTAA
>PGSC0003DMG401009415 L-LecRK
CTCCATTTTTCACTCATTGATCAATTATTTTATTTGCATTTACAAAAAAGTAAGACAAAGCCAACGTGCTTC
AAAGTTTCTGCTCATTGATTCTTTCTTCTTCCCCTGTTTTCTTGCCCATCACCAAATCTACTTCCTCTGTAGC
CACCACCATCTCTGAAGGGACTCACTGCTGATGAAAAATGGGATTGTTAACACCAAAAACACTCACCATT
TTCATCATTCTCTTCTCTTTACTACACCTCAAACTTCAAGCTCAAAAAAATGGAACATTACAACCTAGAATAT
GCATCTTTTGATCAAACTTTCTTTGACATCTTTGAAGTTGAAAGTCCTGCAACAATCAGTAACTTTGCTCTC

CAAGTAACCCCTGATTCTGCTTCTTCAGATTTCAATCTCTACAATAACTCAGGAAGAATCCTCTTTAAGCG
ACCATTTAAGCTCTGGGATGGTGATGTTTTAGACAATAAAACAAGGGTTGCATCTTTCAACTCTTCTTTTC
TTGTCAACATTTACAGACCAAAGAATGAAACAGCTGCTGAAGGGTTAACATTCTTGATTTCACCAGATTT
GGAGAAGCCCGATAATAGCCAGGGCCAGTATTTAGGGTTAACAAATGCTACTACAGATGGGAAATCTA
GTAACAAAGTTGTTGCTGTTGAGCTTGATACTTCTAAACAAAGCTTTGACCCTGATGGTAACCATATAGG
TATTGATGTTCATAGTGTTAGATCTGTAAAGGTTGAATCTTTAACACCCCTTGGTATAGAACTTGCTCCTA
TGGGTGCAAGATTCTATAATGTTTGGGTACAATATGATGGAATCAAGAAAGTTCTTGATGTTTATATTGT
TGAACAAGCACAGAAAAATGGGTCTACCCCACCTATACCAAAAACCCCCATTTTAACATATAATCTTGAT
TTGAAAGAACATGTAAATCAAGAATCATATTTTGGGTTTTCTGCATCAACTGGGACTAACTACCAACTCA
ACTGTGTATTGAGATGGAACTTAACAGTTGAGTACTTCCCAGAAAAGAAAAGTCCTTGGTTGAAGATTG
TTTTAGGTGTTGGGATTCCTGTAGTGGTGCTTTTGATTCTTGCCGCGGCGTATTTAGGGTATTGTTATCAT
AAGAAAAGGATTGATAGGTCACAGTCTAACATATTGGGTGCACTCAAGAGCTTGCCTGGAACACCACAA
GAGTTTCAGTTTAAGGCTTTGAAGAAAGCTACAAACAATTTTGATGAAAAGAATAAGCTTGGGCAAGGA
GGATATGGAGTTGTTTACAGAGGATTTTTAGCTGGTGAGGAGAACAAAGATATTGCTGTCAAATGGTTT
TCTAGGGAAAGCATCAAAGGGCAAGATGATTTCTTGGCTGAGCTTACTATTATCAATCGTCTTCGACATA
AACATCTTGTCAAATTGCTTGGTAAGTGTTCTTAGTTTTTCTTCAAACATAGATTTTGTGCATTTGTTTGAG
TAGAGTATTAGAGTTATATTATTGTGACTGTTTTGTTTATGATCTTCAATAACTGTTAGGGAAATGGTACT
GGTAACTGGTAAAGTTGCTGCAATGTGATCGAAAGTTCATAGATTCGGGGTGTGGATATAGTCTCTTAG
GCTGCGTAAAGTACACCCTTGTGGTCTAATCCTTCTCAGTATAGCTACAAATTTGAGTCTCAAATAAGGC
CTTATCTCCAATATTTCAATTGAAAAGTAAACTATACAAATAGGGTAAAGATTAGTTTTTATTTTTGGGTA
TGTACATAAAATGTGTGAATACCTTCAAGGCAAAGGAAGTTGTAAGTGTAGAGTAAATTTTGCTCAAAA
TTAGTTGCATTTTCTTGAATTTCCCTTATAGAATTCTTTCCCCACCCCTCCTGCCATAATTAAGCAATGAGA
CAAGGATAATGGCGAGAGGATCCAAATTTAGCAATCAGAAACAGAAATTGTCCTCTATGTTTTTTGATTC
TCAATGAAAGAGAGAATTCATTACCAGGATTCATTGATGTGACTTGAAAGAATGGAACCAAAAACCTTG
TAAAAGTTACTGACATAATTTAAAGCCAAAGATTGGTGGGACATCAAATTTAATACTCCAATTTGTTTGT
CATTTTGTCTTTCTTTTTTTTGGGTATAAAAAAGAAGGGAAAAGGTTCAAATATGCCATCGAACTTTTAGA
AAAGGCTCATTTATGCCATCCGTTAAAAGTTTGACTCATTTATGTCATTTCTGTTAAAGAAAATGCTCATC
CATGCCATTTTTTTTAACTCCGGTTTTGCAAAACCATTTTTTACACACCAAACATGGTTTAACACTTTTCTA
TTGGTGTTTTTGGATCAAATGTACATTTTTTGCTTGTTTTTCTTTAAAAAACACCAAGAACACATGAAGAA

CATAAACAACTCCAAAATTTTCAACGTCTTCAATTCTTCATGAATCACCTCAAATTTCAAGAGTAGTTTCG

CTCCGAGCTAGATACCAAAACTCAATTTCTTTTGAGCTCGAATTCAACCTAACTCAACAAGACCCCAATTG

AAATTTCTTCAAAGTTTCAAAACTTGAACATTCTTTAAATGGTAGATTCTCCAAAACTTCAAATCATTTGA

AATTTTGAACATAAACTCAAAAAACACTAGTCAACAAAGATCTAATGTTAAAAACAAGAATCAAAGCAA

ATTTTGAAATTTTGGGACTGATTTTCGTTTTTTGGAATATATATTTTTAAATATGGGTGAATTTTTTGAACT

CTACAAATGATTTTAAAATTTTTATTTTTTTAAAAAAAATAATTAATGGTGTAGCCGAAATTTGAGATGAT

TTCGTGAAGAATTGAAGACGTTGAAAATTTTTGGAGTTGTTTATGTTCTTCATGTGTTCTTAGGCGTTTTT

AAAGTAAAAACAAAGCAAAAAATAGTACATTTGATCCAAAACAACCAATAGAAAAGTGTTAAACCATGT

TGGTGTGTAAAAAATGGTTTTGCAAAACCGGCGTTAAAAAAAAATGGCATGGATGAGCTTTTCTTTAAC

AGCAATGGCATAAATGAGCCAAACTTTTAACGGATGGCATAAATGAGCCTTTTCTAAAAGTTCGATGGC

ATATTTGAGCCTTTTCCCAAAAAAGAATGGCTTTTACTATTTTATTTCAACTCCTTCTTTTAAACTTTACAAC

TAATATGTTTGAGACTATAAGATTGAAAAATCTTTTGATATACATATGTTTAAATTAAGACCATAAAAATC

TAAAATCTTCTTTATTATCATAAACTCTGCAAGTCAAAAACAAGACAAACATATTGAAACGGAAGGAGTT

TATGTAATATTATTATGTTGTTCGGACTCTCCAAAAATGTTGTTGCACATGTATTGAATTCTTAAAAAACA

ATACTACTTTTGAAGGATCCGATCGGTATCCATCACATATTTAGAGAGAGCGAAGCAACACTAATACAAT

AGACTCATTAACCGCGTGAGCACATGCCCTTGTCTTTTTATGCTTTTCTTTTTCTAGTAACTAGTAATTGAT

CTTATTCAACTAATTTATGTAATTTTAACAAGGGACTAGTCTTTCTATTCTTACATTTAGTATGCTAGGAAT

TTTGACATGGGACTAGTTTTTCTAGTCTGACATTTAGCGTGCTAGGAATGTACTAGGTTGGACCATCTTCT

GACAAGCAAATTTCCCCTTCTGAAGAATCAAAGTCTTCTTGAACATTTAATAACTTGGTACTTCTTTTTGTC

TTCAATCTCCTTTTCCTTTCTTAGAATTCTCAAGTCATCCCAAGGGTTTGTCAAAGTTTCTTTTACCCTGACT

AACTAGATTTGCGTAGCATAGGGCCCAACAAAGTGAGAAACGCTATATATCAAGACCATTTTTTAATGCT

TGAAGTCGAGATCTCTAGTTAAGGAATCACACTCCCCGTTAGGCGATAGTAAATGTTTTAATAGTTGATT

CATACCATTTGGTAAGTAAGAGTTTAAGCCAAAAATATCAAGGAAGTAAGAAAAACTAATGGAATGCCT

TAAAGTTTTGACCAAGAACCAACTTGTTACTATGGTCCCTCCAATTTAGAAAAACAATGCACTATCTTGAA

GCCCTTCATTTTGACTTGCTTAAAAGAAGATGATCATCCTTTGCTGACCATGGAATAAGCAGCCATTCGTC

AAAGTTCAAAACAATTTTTTATGTCAAAATTTTGGGAAGTTTCCTTCTTGTCCTAACATCCCTTTCATTCAT

TGTCAACGATTAAGACAAGTTTTGGATGTTAGTTGAATGATGGTAAATTAAATCCAATGAAAAGGACAT

TAATTCTGTATTTTAGTATACATTCATCTTCAAACACCTAATTTTTGTGTCACCTACTCTTGTCATCTGACCC

TACAAAGAGATAAGGTTTGTAAAATACAAAAAAAAGTTTACCATTTGGGTATTCTGCAACTTCTGTGTCT

AGCTGTTTTTACTGATAAAAAACAAACATATGCATCTTACAAAATTACATGTTATGGTGACAGGATGGTG
TCACAAGAATGGAAAGTTACTGCTTGTATATGAATACATGCCTAATGGCAGTCTAGATATGCACCTCTTT
GCGGGACCAGATAAAGAGTCGTTGAGCTGGAACGTCCGCTACAAGATTGTTCAAGGTGTTGCCTCAGCA
TTGCACTATCTGCACAATGAGTATGAGCAGAGGGTGGTCCATCGCGACCTGAAGGCGAGTAACATCATG
CTTGACTCAAAGTTCAATGCGCGCCTTGGGGACTTTGGCCTTGCACGGGCACTTGACAACGAAAGGACC
TCGTATGCTGAGGCAGAAGGAGTGCTTGGCACAATGGGATACATTGCACCAGAGTGTTTCCACACAGG
GAAAGCCACACAGCAATCTGATGTATACGCGTTTGGGGCAGTGTTGTTGGAAGTTGTATGTGGCCAGAG
ACCTGGAACCAAGATTAATGGCTTTCAATTCTTTGTTGATTGGGTTTGGTACTTGCACCGCGATGGCCGT
ATCTTGGAAGCTGTTGATCCAAGGCTTGGAGGTGACTATGTTGCTGATGAAGCAAAGAAACTTCTACTG
CTTGGTTTGGCTTGTGCACATCCTATAGCTATTGACAGGCCTAAAACACAGGCAATAGTTCAGATTATAT
CAGGGTCAGCACCAGCACCAGAAGTTCCACCATTCAAGCCAGCATTTGTGTGGCCATCAATGATGCCAA
TTGACATAGACTCAAGTGTTATGGACACAACATCCATTACTACTTCTCACTTCAATTCAGGATGGAGTCTT
GACTATCAAAGCAGGGAGACCCCAACATATGCAGACCACTCTTTGGTGTAGTTACTTTGATGGTAAACA
ACTATTACCAAAAGGAAAAATGATTTTTTTGGCCTTCTATTTTTTAAAAAATTTCCTGTAATTATGTTTTCT
TGGGAGGAGGTTAAAAATTTCCTTCAGTTTTTTATTTTTTGTTTGATGTTTTTTTCCCTTATCAATTTGTTAT
TGGGGCATGATGAAAAGATCAGAGCAACCCGTGTACATTCATATAACTCAGTAGTGTTACATTATGTCAT
ATTTACTGTTTTCCTGTCTTTGTCAACCAAAATTATGTAAAAGACACTCATGTTGAGCCAAATTGGAGGA
GACAGAAAATGAATAGTGGCGAAGTATCTTTGTTGCCTCAATTGTACTACTACATACAACTTAAAGAATT
CCAAGATCTCAAATTGTAATGAAAGATAAGAACTGATCAATTCTTATAAAAGCAATAGATAACAGAAGA
ACACAATACAAAGATGGATCATAGATGCTTTGTTGATATGCATAAATGCTCTGCCATTTTATAGGCTATT
GTAGTATTAGATCGTTGACAATATATAGTAGATGAAATCCCACTAAGATTTATCTACTGTCAAGATCATTT
CTGTAAATCAATATAACGGAGAACCTGAGATGCTTGCCAAAGTTTTACAAGTTAGTGCGCATAAGATCA
AGAGTTTCCCCCGCCTTTAACTTTGTTGATAAACGGAGCTTCCTATAATCATTGAAAGTGAAAGGAACAT
AGATACGGGGGTGTTCAGCATCCACCAGCTCAGGTGGTGGTTCCACTGCTGTGTCCCTTGGTCCCAAGA
GGAATGAAGCAATAGACACCCGTACTTTTGCTTCCTTGCATATCACTCTATGCTTGACAGTGTGTAATCTT
CCATTGCTCCATACCTTCACATAGGCCATAGCAAAAAGCAAGTATCATTGTCTAGTTCTTTTTTCGGAATC
TTAGAAGGGTTAAGGGAGTAGGAATTATCTAACTTCCTGCTAAACAATCCCCGCCAGAGGCTAGCATAA
GATTGTTGGAACACGTCAAATCTTGAAAATGAACTAGGTAGAGTAGCAGCATAGTTACCGTGCCCATGT
CTCCAAGATTCACCAGGAGGGTGTTTGGCCAAGGATCAACTGCTACAAATTCACCAGACTTCTTCATGAC

CTCAAGACCGCCAACACTTTCATCATCCTGTAGAATAGTAAGGAATCCTGAGTCTGTGTGTGTCTGAACC
CCAGAGGAACCTACAGCTTCTGGGGTGAAGTGGTACTTGTTAATCCTAAACTGACAAGGCCAATCCTCA
AAGGAAACATTTGGCAGTCCCAACCCTTCACTCATCTTTCGCAGAATGTCCATCATTAGTCCATTAGTTGC
TGCAGCATACGTCTTGATTGTCTCTCTGTTCACAGTAGGAATCAAACTGCTTTTAAGCATTCTAGTAAAGC
AGTCTTTT
>PGSC0003DMG402009415 L-LecRK
TGCAACTTCTGTGTCTAGCTGTTTTTACTGATAAAAAACAAACATATGCATCTTACAAAATTACATGTTAT
GGTGACAGGATGGTGTCACAAGAATGGAAAGTTACTGCTTGTATATGAATACATGCCTAATGGCAGTCT
AGATATGCACCTCTTTGCGGGACCAGATAAAGAGCCGTTGAGCTGGAACCTCCGCTACAAGATTGTGCA
AGGTGTTGCCTCAGCATTGCACTATCTGCACAATGAGTATGAGCAGAGGGTGGTCCATCGCGACCTGAA
GGCGAGTAACATCATGCTTGACTCAAAGTTCAATGCGCGCCTTGGGGACTTTGGCCTTGCACGGGCACT
TGACAACGAAAGGACCTCGTATGCTGAGGCAGAAGGAGTGCTTGGCACAATGGGATACATTGCACCAG
AGTGTTTCCACACAGGGAAAGCCACACAGCAATCTGATGTATACGCGTTTGGGGCAGTGTTGTTGGAAG
TTGTATGTGGCAAGAGACCTGGAACCAAGATTAATGGCTTTCAATTCTTTGTTGATTGGGTTTGGTACTT
GCACCGCGATGGCCGTATCTTGGAAGCTGTTGATCCAAGGCTTGGAGGTGACTATGTTGCTGATGAAGC
AAAGAAACTTCTACTGCTTGGTTTGGCTTGTGCACATCCTATAGCTATTGACAGGCCTAAAACACAGGCA
ATAGTTCAGATTATATCAGGGTCAGCACCAGCACCAGAAGTTCCACCATTCAAGCCAGCATTTGTGTGGC
CATCAATGATGCCAATTGACATAGACTCAAGTGTTATGGACACAACATCCATTACTACTTCTCACTTCAAT
TCAGGATGGAGTCTTGACTATCAAAGCAGGGAGACCCCAACATATGCAGACCACTCTTTGGTGTAGTTA
CTTTGATGGTAAACAACTATTACCAAAAGGAAAAATGATTTTTTTGGCCTTCTATTT
>PGSC0003DMG400000295 LRR-RLKs
TTTTTACCAAATGTTCATAGAAACTTAGAAAAAATCACCAAAAAAAATGTCCAAAATTATTTTTTCACTTTT
CATTTTTCTGCTTTTCATCATTTCTCATGGAAAATCCCAAGAATCTCCAAATCAAGAAAAGGCCATTTTACT
CCAACTAAAACAACACTGGTTTACTTCACCAAATGTCACAAAATGGATTTCATCATCAAATCATTGTTCTT
GGGATGGAATAATTTGTACCCAAAATTTAGTTTCTGGTATTCAAATCCCTTTTGGAAATATCTCAAAACCA
ATCCCAAATTTCATTTGTGATCTTAAAAATCTCACCTTTCTTGATTTGAACCATAATTTCATCCCTGGAAAC
TTCCCTGATATCTACAATTGTTCAAATCTTGAATTCTTAGACCTTTCTTATAACTACATGGCTGGTAATCTC
CCTGATGAGATTAACCGTCTTTCGAGTAATCTAAAATACCTAAACTTAACTTCCAACAACTTCAATGGTGA
TATCCCTAAAGGAATTGGTGGATTGAGTCAGCTCAAAGTACTTGACCTGAGGGGGAATTTGTTTAATGG

ATCTTTTCCTGAAGAGATTGGTGAATTGTTGAATCTTGAAGCACTTATGTTGAGCTCGAATGGTTTTGCTC

CACAGGCTATACCATCACGATTCACCAAGTTGAAAAATTTGAAAATTTTTTGGATGACAGAAGCACATTT

GATTGGAAATATCCCGGAATATATTGGTAACATGACAAGTCTTGAATTTTTGGATTTGTCGAACAATGGA

TTGAGTGGTAGTATCCCTGATGGTTTGTTTCAGCTCAAGAACTTGAGTATTGTGTATTTATTTACTAACAA

ATTGTCAGGGGAAATACCACAGTCAGTTTCATCAATGAATTTAGATGTTGTTGATTTGTGTAACAACAGT

TTGACAGGAAAAATACCAGAAGATTTCGGAAAGTTGACGAAACTGAGTGGATTGGCTTTGTTTTTCAAT

CAATTATCAGGGGAAATACCTTTGAGTATAGGCAAATTATCATCATTGGTATCTGTTAAGTTGTTTGGGA

ACAAGTTATCAGGTGAAATTCCACCTGATTTTGGTCGATTTTCGAAACTTTTTGACTTCCAAGTTTCAGAA

AATCAACTTGTTGGAAAGCTACCTGAGGGTATTTGCAATAACAAGGCATTAGCTAGGATGGTTGCTTTTG

GCAACAATCTAACAGGTGAACTGCCAAGTTCACTTGGAAGTTGTGACAGTCTGAGGTATCTTCGAGTCG

AAAATAACCGACTTTCTGGTGAGGTTCCTGATGGATTATGGACAGGTAAGAGTTTGTCAATGGTTTTGAT

GAAGAATAACTTGTTAACTGGTCAGCTTCCACATAGAGTGGCATCAAATTTGTCACAAGTTGATATCAGC

AATAACAAGTTTTCGGGGGAATTACCAGCTGGTATGGGTACTTGGTACAATCTAAGTGAGTTCAAGGCA

AGTAATAATCTCTTAAGTGGTCAAATCCCTCAAGAATTGACTCTTCTTCCAGGGATAACTAAACTTTTTCT

TGATGGTAATCTACTATCTGGAAATTTTCCATCGAATATATCGTCGTGGAAGACTCTTGTCACATTAAATA

GCAGAAAAAATCAGCTTTCAGGTCCTATACCTTCTGCACTTGGCCTTTTACCAAATCTCATTGATTTGGAC

TTGTCAAGTAACCAATTTTCAGGTGTTATTCCAACTGAATTAGGTAACTTGAGGTTTACTTCACTTAACCT

GTCATCCAATCGCCTGTCGGGTGAAATCCCATCTCAGTTGGAAAATGCAGGTTTTGGTAAGAGCTTCTTG

GATAATCCTGGTCTTTGTGCAATTAATCCATCAGTAGAAGTCGCGTCTTGCAAGAGGGAAACTAAGTCA

GATAAGTTTCCTGTTGGACTTGTTGCTGCTCTTGCTAGTGTAGCAGCAGTTACTTTCCTTGTGGCAGTTCT

GTATGGTCTGTTTGTGTTGAGAAGTCATAGAAAAAGAAAACAAGAATCAGTTTTGACATGGAAACAAAC

ATCATTTCACAAATTAGACTTCACGGAATCAGACATTGTATCCAATCTGACCGAAAACAACATAATTGGA

AGTGGAGGATCGGGACAGGTCTACCTCGTGCCTTTAAGCCGATCAGGTGACTATGTTGCTGTCAAGAGG

ATTTGGAGAAACCAAAGGTTGGATCACAAGCATGAGAAACAGTTTCTTGCTGAAGTTCAGATATTAGGC

ACGATTCGACACACCAATATAGTGAAACTCCTCTGCTGCATCTTCAGTGAAGAGTCGAAACTTCTTGTCT

ACGAATATATGGAGAACAGGAGCTTGGATATATGGCTTCACTCAAAGAATAGGTTGAACAATGCCTCAA

GATCAACACCACATCTGGTCTTGGAATGGCCTAGGAGGCTGCAAATCGCGATAGGAGCTGCCCGTGGTA

TTTGCTACATGCACCATGACTGCTCACCACCTATTATTCATCGCGATGTGAAGTCAAGCAACATCCTATTG

GATTCCCAATTCAATGCAAAAATTGCAGATTTTGGCCTTGCCAGGATGTTGCTCAAGCCTGGAGATAACA

CAGTGACAGCAGTAGCTGGCTCTTTCGGATACATTGCACCAGGTATAAGGCCATCAACTTATCTATAACA
TCATTATAAGTTTAGAAGTTGGTTTAATCTCTTACATGGAAAAAAGTTTCCTATGCTACACAATTCAATAA
CATATCAAGGGGTCGTTTTGTTCACGTACTAGCTATGCATGAGTTGTAACACGGGTTGTCTATGTAGAGA
TTGTTATGCAAGTAGTTATTCTTAGTACACTAATTCTCGCGTAACTCATATAAATGCTAATATATTCATGAT
GGTATTTCAGAGTATGCACGAAAAACTAGAGTGACAGAGAAAATTGATGTGTATAGTTTTGGAGTCATA
CTATTGGAACTGGTGACTGGAAAAGAAGCCAATCTTGGAGATGAAGATTCATGTCTTGCAGACTGGGCA
TGGCGTCACCTTCAAAAAGGGAAACCGATGGCTGATGCATTAGATGAGGACATCAAAGAAACGCGATA
CTTGGAAGAAATCTTCATTGTGTTTAAGCTTGGAATCTTTTGCACCAGCACATTTCCTTCATCAAGGCCAA
CAATGAAGGAAGCTCTGCAAATCTTGATCCAATGTAGCAACAGTTCACCGACATCTGGAGAAAAGAAAA
ATGAAACAGAACAGGATGTTTTGCCACTCCTCAAGAACTCAAGGAGTGACAGGATTGAAGAAAATGAT
GATGTTGGTTTAACATCACTTGTTTGATGGCATTACAATATGATTAGAGTGACGGAGAGCCTTGACGTAA
CTGGTAAAGTTGTTGTGGAAATAGTCTCTTGCAGAAATGCAGGGTAAGGTTATGTACAATAGATTCTTGT
GGTCCGGTCCTTCCCAGGACCTTGCTCATAGTGAAACTTCGTGCACCGGGATGCCTTTTTTTAACAATAT
GATTAGAGTAAGAAATAGCAAAGAATAAGACCAATAGATAATGTATCAGGTAGTGAAAACTAGCATTGT
ACATACACTGTATTGGATCCATAGTGTTGCTTTGCATTGTTTTGCTACATAAATTTTGCTTTTCCAATGTTT
TCTTTACTCATAAAATAACTGTCAAAAAGGTACATTAAAACCATAAACTATTGAATGGAGGATAGATTTC
CTAAAAATCCAGAAAAAAATAGAAACAAGTTTGAACTACCTCAGTACTTTAACTTTTGGCAACCATACAAC
AACATATCCGATGTAATCCCATGAGTCCCAAGCTTTTGGCAACAATACAACAACATATCTGATTTAATCCC
ACAAGTGGCTCTGGGGAGGGTAGAGTGTACGCAAATCCTACCCATATCTCGTAGAGATAGAGAGGTTG
TTTCCGATAGTAACTTTTTGGCAACAATAAATCTTGTAAAAGATCACACAAAAATGTGGCTACTAGAAGA
ACTTTATTTGTATCCTAGCTTTATTTCTGGCTAGTGAGGAAAAAATTTAAGTTTACACTTGGGATAAAGAT
GATCCTTAGAGGCACCAGGAGTGAAAAGGTCAGCATATTGATGTTTAGCTGAAGAGAGATGTGCTCATT
TGATACATTGAACTATGGCTCACTTCAAAGCTCACTCTTGCATGATGCGGTGCACAAGGAAGCAAGAAA
TATTTCAATCATCTCGGAGAAGAAAGTAAGAAGAGGGTTCTCTTTAGCAAGAAGAGGGGTGATTGTAAA
CGTACAAACTTTGACTAGGCACTTTTGGTTGTGTCACTCTGACCATGATCATGATTCCTCGAGATCTGTGT
ATTGGGAATGCGAGGCAGCCAAAAGTGCAGCTCCAACACCTGAACCATCATTAGAAAGCTCAATGACTA
TGCTGTCCGCAGTGTCTCCCAGTAACTCTTTCATAGTTTCCTCCAAGCAATTTCTGAACTTGGTGTAACGC
TCAAACAATGCACCGTCCACAGCTATGACAGACCTCTGGTTCTCTCCGTCCTTCAAAGTGTCTTTTCCCAA
TTTCTTGAGGATGCCCACGATTCCTGCTGCAGAAAGACGAGCTCCACGAGAGGTGATAATGTCGCATAG

CTCCACAACTATTTTCCTCATTTTCAAGGTAGAATTAGGTACCTGTGAAAAAAATGTATATATAGCAAATA
GGATGAGAAAGACGTTCTCCACTTAATTAAGCCTAGCCAACTTAGTGATGTTCAGCCAGGAGCTAGGAA
TTTGAGTTTATGGGTTCCGGATAAATTATTTACACATATTAAGTGGGGTTTTCAGTACAAATACAAAGCT
ACTGGTTTTTTGTCCTATCATATATAGGACAAAAAAGATGAGAACTTCAAAGACTAGTAATGTTTCACTCT
AAGTCAAATGTTTGATCTTAATACCTCTAAGATATCCTTCAGTTTATTGCCAACCACCTTGAGATCAGCCG
ACTCGTCGTGATGCATAGCAGCCATATCCGGAGTCCTGCATGTGTGGCAGTAAATAACCTTTTTCACAAT
ATTTGACAATTCAAAGCTTACATATGCTTAATAAACGTACACGAATCTAGTCCAACAAGAAGCATAACTT
ACAGTGTAAGAGATTAGTGCAATTACATAAACCTTAAGTTGCACTAATTGCATGGAGAAGAATAAGGTT
AAAATTGATGAATGCAAATAGGGCCTGCCAAAAAATAAATTACTGCCGTAATAAATGTGTTGGTCAAAG
CAATACCCTACCTCAATATGAAAGGAATTTTCAGTTTGGATGGGACATAATCACCGAATAATGAAGCTTC
TTTAGCCATTCTACACAATACTCTACGCAAAATTTCTCCAAGATACATCCCGGAGATCATCTTTTCATAAA
TCTGTGCAGTTTCCGAAAGTTCAACAACAGATGTAAGATAGAAAATGTAGAAAGGAAGGGGATTGTCTT
AGTTACCTGCTCCCCGGGGTTTAAACTCTCAACATCAAGATTTTGGTCATATTCTGTTACTGGAAGATGTG
ATGAGAGGAAATTACCCCATTCCATGTTGATAACCTTTAAAGACCAGTCAAAATTACGTTTAAGTAAACA
AACGTGTATGAATCTACCAAGGAAAATATTTTTAGGAAATGAATTAGGCACATATCAAGTCATGAAGGT
GTTATGAAATGTCATAAGCATCTACCATCTCTCCGGATTTCGGCAACAGACCATGCCATTTGGGAATCGC
ATGAGCCCGTTCAACATATGCTGCATTGGTTCCAGTACCCAAAATTACTGCAGCAATGACATCAGGATTA
TTGTACCGACCTCCTGCTAACGTTCCAACAGTATCATTGACCTAAACAGCCAAGTCAACTAAGAAATTCA
GGCTCAGGAATTGCTTAAGATTAAAAATCAGAAATATCACACATACAAAAACCTAATACATGAAAATCAT
CAGCTTACTTATATTTTATCGAATTTGATTGGGAAAAAAACTTACTAAAGCAGCCACACGCACATCAAGG
CCGGCCCTTTCCATTGCTTTTGCTAATTCTCCCACCACATCTTGGCCAACCTATGTTAGATTCCATGAGGA
GTAGTTCAGAATTGAAATACAATAAAACGTTACAAAAGGAACTCTTTATAACGTACTTTAAATCAAATCG
GTGAAAATGCAATGAAACAATGATCAACTTTGCTTGTATAAAGTTGTATGCATTAAATCCTCAACAATGC
CAACCTATGTTAGGCCATGAGGAGTAGTTCATCATTGAAATACAACATAACGTTGGAAAAGGAAATCCT
ACAACGTATTTTAAATCAACTCAGTGAAAAAGCAATGAAATATACGTTGTCGGACTCTCCAATAATATTG
CCGCACCTGTGTCAGATCCTTCAAAATGTACTGCTTTTGAAGGATCCTACAAGCACCTGGCCAGTGGCGA
AATTTTTGAAGAGTCTGAGCAACATAGATGGTATAATCATCAACGTTGCATGCATAAAGTTGTATGCATT
AAATCATCATCAATGCAAAACCATACATTGAAATGAATGGACTAAATCTAAGGTTGATTGTAGGCATGAT
CAATCGGAAGTGAAAATCAGAAACTCTGTATGACTAACCACGTCTTCTATGGAGAAGCCCTTTGTCCATT

TAATAAGAGTTCCTGATGCAATTGACAATTGTTTCACAGGGAAAGAGAAGGTAAAGCCTAATTCCCTTTG
TCTACCAGGTGGAAGATGAAAATCATCACCTTCTGTAGCTACAAATTCTGCAAGTGCCGTGGCAATAAA
ATCAAATAACACCTGCAATTAACAGTTTGAAGTTTAATAATGTGAGTACAACCTTGATTGTATTAAAAAA
GTTGCAGGATGCATGGCTGATACAGCTTAAGACTTACTTCAGATGATGATCCAGCCATCACATTCTGTGG
AATTGAAACTTCTTTAACTTCATGTTTAACTATACGCTTTTCGTTCCCACCCAACTGCACACGCATCACACG
AAAGTTTGTGCCGCCTAGATCCAATGCATAAAACAGACCTTCTTCGTCCCTAAGATGAAGGAATAAAAGT
AATACCAGTCGTTAAACTGAAGATACAAACATACATGGAAAACATACCCCACCCCTAATGCACTACAGA
GGTCTATTGGCAGCAGCTTTTGAAAATTTGTCGATACATGTGAATTTTTATACCCAATTTTAGTAAGTTTA
TCACAAGAACATGGAAATGTTACCAAGTCCCTACCTCCGCCTACTACTATAGGTATGCCATTTTTAAACTT
TAGAGAATGTCAAACTGATTCATATGTCATATATGCTTTTCTTATGGATCTAACTTCATTATGTTGAACAC
AGAGATGTCTGTGTAGACAGCACAGTAACAAATAATGAGCAGACAGAAGAAGATGGAACTGAAAAATT
TACACACATTATAAGTGTTAATGAATGGAACTAGCAATGTAAAATTCAAAAAAGGTGGTCAAGATACAA
AACAGTAGAAGAGAATATAGAATAAACATCAAACTCAGGATCCATAGATAACAAGTGACCTGATCACTT
GTCAGTTAGAAACTTCTTAGTGGACTCCAAGTTCAACAAATACTTCAAATAATAGAGTTTCATTTTTGCTT
TTTGTGATTTATAACGATGCTCAAGGTAATCACCTTAAGAACATCAAAAGAATCATAATTTAGGACTAAA
AAATAATAATTAAAAACAATTTGAAAAGTACTTAATCAATTATCCAAACATTACGAAAGTGAAATCAATC
TCCATGATCACAGCTTTATTCAAAAAAGAGAAATTCTTGACACCCAAATTAGCAAAAATCAGTTTGTGCA
CATATACACACACAAATACTCATCCAATGTGGAGGCTATCAACTTAGCTAATAAGCATCTTAAGTTTGCC
AATGAATCGTTAGTAAAACACACAATCTGATATAACTCTCCACCCCATAAGCTAAAATCAAGAGACAACA
CTACCAAGATCACAGCTTTATTGAAAAAAGAGAAATTCTTGACACCCAAATGAGCAAAAATCAGTTTATA
CACACAAAAATACTCGCCCAGTCGGATGGTTATCAACTTAGGTAATAACCATCTTGAGTATGTCGATGAA
TCAATTGTAAAAACACAAAAAAGAACTGACATAACTCCAACCCAGAAGCTAAAATAAAGAGACCACCTC
CAACCGAGGTGGAGCTAGAATTTGATGTTTATTGGTTCTGAACTTGTTGAGAAAAAACTATTAGGTTCAT
CCGAACCTGTATCTAATATGTAGCTCCACCCCTGCCTCCAAAATCACAACTTTACTAAAAAAGAGACAAA
TTCTTGAACACCCAAATAAGCAAAAGACAGTTTATGCACATTCTTAAACACCCAAATTAGCAAAAAACAG
TTTATCCACATTCATAAACACCCAAATAAGCATAAAAACAGTTTATCCACATTCTCAAACACCCATATAAG
CCAAAAAAAAAACAGTTTATGCACATTCTTGAACACCCAAATAAGCCAAAAACAGTTTATCCACACTCTT
AAACACCCAAATAAGTAAAAAACAGCTTTTCTTAAACACCCAAATAAGGCACTCAAGGGTGTGGCCTAG
TAGTCAACAACGTGGATTGAGCACCATGAAGTCTCAGGCTCAATTCCCAGCTCCTATCTATTCTAGCCTT

GGTGGACTCAGTGGCGAATTTATGTGTAAAATTCGGGTCACCCGAACCCGTGCTCCTCCGACTAAACTTG
ATATATTACTTGTATAATTATTAAAGAACATATTAAATAAACACTTGTTGAACCCGTTCTTAAAGTTGCTT
GATGGTTTACTGGTTTGAGAGCTAGATTTTAACACCAAGGTCCTGAGTTCAATCCCAGCCAGCCACAATT
CACTTCTAATTTTTTTAGTAACTAATAACACTAGTTTTTACAAAAAAAAACGAAATTCTAAAGCAATGGTG
CACCCGTTTTCTTGAAATCCTGGATTCGCCTCTGTGGACTGAATACTGATACTTATTGTTGGTGTGAGATG
CCAAGATTTAGTTGAGGTGCGCGCAAGTTGATCCGGATATCAAAACAAAGAACACCCAAATAAGCAAAA
AAAAAACAGTCTATACACATTCTTGAACACCCAAATAAGCAAAAATCAGTTTCTTCACACACACAAAACA
CAAATACTCACCCAGTAGGAAGGTTATCAACATAGCTAATAAGCATCTTGAGCTTACTCCCACCTTCAGA
AGCAAGTCCAGCATGCATCTCTACAGTCATAGCATCAGCTACCTGCTTAAGTTTCCCCATTGGAGTTGCA
CACTTCTCCTCAAAATCCTTCAATAAAGCTTCAACTTTTCCCCACTCCCCAGAATTCTTCATCCTGCGCTTC
ATTAACAAAACTGCCACCCCACATACTACAGCAGCAGTACACACAACTGATACACCTACAACCAATTTTC
CCATATTTTCTTGAAAAAAAATCAAACTTTTTGTGTATATTTGAGTGAATATACTGTAAAGATTGAAACTT
TTTTGGTTGATTTTAAATGGGGTTTGTTAAAGATTCAGAATTTGATGTGTGGAGTGACAGAGAAAGAAG
AAAAATGGGAGTTGCAGAGGATATTTTATT
>PGSC0003DMG400000358 LRR-RLKs
TTTTTACCAAATGTTCATAGAAACTTAGAAAAAATCACCAAAAAAAAATGTCCAAAATTATTTTTTCACTTTT
CATTTTTCTGCTTTTCATCATTTCTCATGGAAAATCCCAAGAATCTCCAAATCAAGAAAAGGCCATTTTACT
CCAACTAAAACAACACTGGTTTACTTCACCAAATGTCACAAAATGGATTTCATCATCAAATCATTGTTCTT
GGGATGGAATAATTTGTACCCAAAATTTAGTTTCTGGTATTCAAATCCCTTTTGGAAATATCTCAAAACCA
ATCCCAAATTTCATTTGTGATCTTAAAAATCTCACCTTTCTTGATTTGAACCATAATTTCATCCCTGGAAAC
TTCCCTGATATCTACAATTGTTCAAATCTTGAATTCTTAGACCTTTCTTATAACTACATGGCTGGTAATCTC
CCTGATGAGATTAACCGTCTTTCGAGTAATCTAAAATACCTAAACTTAACTTCCAACAACTTCAATGGTGA
TATCCCTAAAGGAATTGGTGGATTGAGTCAGCTCAAAGTACTTGACCTGAGGGGGAATTTGTTTAATGG
ATCTTTTCCTGAAGAGATTGGTGAATTGTTGAATCTTGAAGCACTTATGTTGAGCTCGAATGGTTTTGCTC
CACAGGCTATACCATCACGATTCACCAAGTTGAAAAATTTGAAAATTTTTTGGATGACAGAAGCACATTT
GATTGGAAATATCCCGGAATATATTGGTAACATGACAAGTCTTGAATTTTTGGATTTGTCGAACAATGGA
TTGAGTGGTAGTATCCCTGATGGTTTGTTTCAGCTCAAGAACTTGAGTATTGTGTATTTATTTACTAACAA
ATTGTCAGGGGAAATACCACAGTCAGTTTCATCAATGAATTTAGATGTTGTTGATTTGTGTAACAACAGT
TTGACAGGAAAAATACCAGAAGATTTCGGAAAGTTGACGAAACTGAGTGGATTGGCTTTGTTTTTCAAT

CAATTATCAGGGGAAATACCTTTGAGTATAGGCAAATTATCATCATTGGTATCTGTTAAGTTGTTTGGGA
ACAAGTTATCAGGTGAAATTCCACCTGATTTTGGTCGATTTTCGAAACTTTTTGACTTCCAAGTTTCAGAA
AATCAACTTGTTGGAAAGCTACCTGAGGGTATTTGCAATAACAAGGCATTAGCTAGGATGGTTGCTTTTG
GCAACAATCTAACAGGTGAACTGCCAAGTTCACTTGGAAGTTGTGACAGTCTGAGGTATCTTCGAGTCG
AAAATAACCGACTTTCTGGTGAGGTTCCTGATGGATTATGGACAGGTAAGAGTTTGTCAATGGTTTTGAT
GAAGAATAACTTGTTAACTGGTCAGCTTCCACATAGAGTGGCATCAAATTTGTCACAAGTTGATATCAGC
AATAACAAGTTTTCGGGGGAATTACCAGCTGGTATGGGTACTTGGTACAATCTAAGTGAGTTCAAGGCA
AGTAATAATCTCTTAAGTGGTCAAATCCCTCAAGAATTGACTCTTCTTCCAGGGATAACTAAACTTTTTCT
TGATGGTAATCTACTATCTGGAAATTTTCCATCGAATATATCGTCGTGGAAGACTCTTGTCACATTAAATA
GCAGAAAAAATCAGCTTTCAGGTCCTATACCTTCTGCACTTGGCCTTTTACCAAATCTCATTGATTTGGAC
TTGTCAAGTAACCAATTTTCAGGTGTTATTCCAACTGAATTAGGTAACTTGAGGTTTACTTCACTTAACCT
GTCATCCAATCGCCTGTCGGGTGAAATCCCATCTCAGTTGGAAAATGCAGGTTTTGGTAAGAGCTTCTTG
GATAATCCTGGTCTTTGTGCAATTAATCCATCAGTAGAAGTCGCGTCTTGCAAGAGGGAAACTAAGTCA
GATAAGTTTCCTGTTGGACTTGTTGCTGCTCTTGCTAGTGTAGCAGCAGTTACTTTCCTTGTGGCAGTTCT
GTATGGTCTGTTTGTGTTGAGAAGTCATAGAAAAAGAAAACAAGAATCAGTTTTGACATGGAAACAAAC
ATCATTTCACAAATTAGACTTCACGGAATCAGACATTGTATCCAATCTGACCGAAAACAACATAATTGGA
AGTGGAGGATCGGGACAGGTCTACCTCGTGCCTTTAAGCCGATCAGGTGACTATGTTGCTGTCAAGAGG
ATTTGGAGAAACCAAAGGTTGGATCACAAGCATGAGAAACAGTTTCTTGCTGAAGTTCAGATATTAGGC
ACGATTCGACACACCAATATAGTGAAACTCCTCTGCTGCATCTTCAGTGAAGAGTCGAAACTTCTTGTCT
ACGAATATATGGAGAACAGGAGCTTGGATATATGGCTTCACTCAAAGAATAGGTTGAACAATGCCTCAA
GATCAACACCACATCTGGTCTTGGAATGGCCTAGGAGGCTGCAAATCGCGATAGGAGCTGCCCGTGGTA
TTTGCTACATGCACCATGACTGCTCACCACCTATTATTCATCGCGATGTGAAGTCAAGCAACATCCTATTG
GATTCCCAATTCAATGCAAAAATTGCAGATTTTGGCCTTGCCAGGATGTTGCTCAAGCCTGGAGATAACA
CAGTGACAGCAGTAGCTGGCTCTTTCGGATACATTGCACCAGGTATAAGGCCATCAACTTATCTATAACA
TCATTATAAGTTTAGAAGTTGGTTTAATCTCTTACATGGAAAAAAGTTTCCTATGCTACACAATTCAATAA
CATATCAAGGGGTCGTTTTGTTCACGTACTAGCTATGCATGAGTTGTAACACGGGTTGTCTATGTAGAGA
TTGTTATGCAAGTAGTTATTCTTAGTACACTAATTCTCGCGTAACTCATATAAATGCTAATATATTCATGAT
GGTATTTCAGAGTATGCACGAAAAACTAGAGTGACAGAGAAAATTGATGTGTATAGTTTTGGAGTCATA
CTATTGGAACTGGTGACTGGAAAAGAAGCCAATCTTGGAGATGAAGATTCATGTCTTGCAGACTGGGCA

TGGCGTCACCTTCAAAAAGGGAAACCGATGGCTGATGCATTAGATGAGGACATCAAAGAAACGCGATA
CTTGGAAGAAATCTTCATTGTGTTTAAGCTTGGAATCTTTTGCACCAGCACATTTCCTTCATCAAGGCCAA
CAATGAAGGAAGCTCTGCAAATCTTGATCCAATGTAGCAACAGTTCACCGACATCTGGAGAAAAGAAAA
ATGAAACAGAACAGGATGTTTTGCCACTCCTCAAGAACTCAAGGAGTGACAGGATTGAAGAAAATGAT
GATGTTGGTTTAACATCACTTGTTTGATGGCATTACAATATGATTAGAGTGACGGAGAGCCTTGACGTAA
CTGGTAAAGTTGTTGTGGAAATAGTCTCTTGCAGAAATGCAGGGTAAGGTTATGTACAATAGATTCTTGT
GGTCCGGTCCTTCCCAGGACCTTGCTCATAGTGAAACTTCGTGCACCGGGATGCCTTTTTTTAACAATAT
GATTAGAGTAAGAAATAGCAAAGAATAAGACCAATAGATAATGTATCAGGTAGTGAAAACTAGCATTGT
ACATACACTGTATTGGATCCATAGTGTTGCTTTGCATTGTTTTGCTACATAAATTTTGCTTTTCCAATGTTT
TCTTTACTCATAAAATAACTGTCAAAAAGGTACATTAAAACCATAAACTATTGAATGGAGGATAGATTTC
CTAAAAATCCAGAAAAAATAGAAACAAGTTTGAACTACCTCAGTACTTTAACTTTTGGCAACCATACAAC
AACATATCCGATGTAATCCCATGAGTCCCAAGCTTTTGGCAACAATACAACAACATATCTGATTTAATCCC
ACAAGTGGCTCTGGGGAGGGTAGAGTGTACGCAAATCCTACCCATATCTCGTAGAGATAGAGAGGTTG
TTTCCGATAGTAACTTTTTGGCAACAATAAATCTTGTAAAAGATCACACAAAAATGTGGCTACTAGAAGA
ACTTTATTTGTATCCTAGCTTTATTTCTGGCTAGTGAGGAAAAAATTTAAGTTTACACTTGGGATAAAGAT
GATCCTTAGAGGCACCAGGAGTGAAAAGGTCAGCATATTGATGTTTAGCTGAAGAGAGATGTGCTCATT
TGATACATTGAACTATGGCTCACTTCAAAGCTCACTCTTGCATGATGCGGTGCACAAGGAAGCAAGAAA
TATTTCAATCATCTCGGAGAAGAAAGTAAGAAGAGGGTTCTCTTTAGCAAGAAGAGGGGTGATTGTAAA
CGTACAAACTTTGACTAGGCACTTTTGGTTGTGTCACTCTGACCATGATCATGATTCCTCGAGATCTGTGT
ATTGGGAATGCGAGGCAGCCAAAAGTGCAGCTCCAACACCTGAACCATCATTAGAAAGCTCAATGACTA
TGCTGTCCGCAGTGTCTCCCAGTAACTCTTTCATAGTTTCCTCCAAGCAATTTCTGAACTTGGTGTAACGC
TCAAACAATGCACCGTCCACAGCTATGACAGACCTCTGGTTCTCTCCGTCCTTCAAAGTGTCTTTTCCCAA
TTTCTTGAGGATGCCCACGATTCCTGCTGCAGAAAGACGAGCTCCACGAGAGGTGATAATGTCGCATAG
CTCCACAACTATTTTCCTCATTTTCAAGGTAGAATTAGGTACCTGTGAAAAAAATGTATATATA
>PGSC0003DMG400000728 LRR-RLKs
CTTCTCTCTTACCTATCAAACTGAAGAAACTGAAAGAAAAAGAGTTGGTAGAGAGAAAACAGTATAGGG
ATTAGCATTTAGAACTCTTCTTTGCTTTATCAAGAACAAAGAATCAAAGCAAAAGCTTAGTTGATTCACTC
AAAAAAGGTAATCCCTTTAATTTTCTGACACAGAATGTATATTCCCTTTTTTATTTCAAGAATCACTATGAT
ATAGAAGGGGTATAACAAATTAGAGATGCCCATTTATATATATTTGAGAAATGATGAGAATTTTCGAAG

TTAAATGAAGGTTGAGCAAGAATGGCGAACCAACAGGGCAGAGTGGTTTTGTTTTGTTTTGTGTTTTTCT
TGCTGTTTGACTCTACATTTCAGCAGAGGGTTAGTTCTTCAGCTGAGAAATTTGCTTTGCTTCAACTGAGA
TCTTCTTTAGGGTTAAGAGCCAAAGAATGGCCTATAAAGGGTAACCCTTGTTTCAACTGGGCTGGGATTC
GTTGCAAGAATGGTCGAGTTACTGAGATCAACATATCTGGTTTTAAACGTACTCGAGCTGGGGGTCAAA
CCCCTCAATTCTCAGTTGATGCTCTCCAAAATTTGACTCTTTTGGAATCTTTTAATGCTTCCAATTTTGTAC
TTCCTGGTTCTATTCCTGAATGGTTTGGTCTTAGGCTTGTGTCTCTTCGGGTACTTGATCTCAGGTCATGT
TCCCTCATTGGTCCTATTCTTCCTTCCCTTGGCAATTTAACTAGTTTAGTTACTCTCAATTTGTCCAACAAT
GGTCTCACTGGACAAGTGCCTCGAAGTTTCGGTCAATTGTCACGCCTATCAAGTCTTGATCTTTCGCATA
ATAAGCTTCTTGGGGTAATTCCTGATACATTTGTTTCTCTTAAGAACCTTACCTTGCTTGATATGTCTTCAA
ATTTCTTCAATGGGGCTATTCCTTCAGATATTGGGACTTTGCTTCAGTTGAAATCCTTGAATCTGTCTGAC
AACAGCTTTTCTACTTCGATACCCACCCAACTCGGTAACCTTTCTAATTTGGTTGATCTTAACCTCAGCTTC
AATAGTCTAAGTGGAGTGGTCCCTGAATTAGGAGGATTACGGAACTTGAAGAGTATGGTAGTTGGGAA
TAATAGGCTGTCTGGATCGTTGCCTAATGCTTTGTGGTCGATGCCTGGGTTGCAATTCCTAGATGTCTCT
GCGAATAACCTCACTGGTATTCTGCCAAATGTTAGCTCCGTTGTCAATGCCACTGGTGCAGTGTTTAATCT
TTCTCATAATACGTTTTATGGAAATTTGCCGTCTTTAAACAGAAGCTTCAGTTTTCTAGATCTATCAGGAA
ACTATTTTGAAGGCAAACTCCCAAATTATGCACAACGCAACGCGTCAATTAGTAGCAATTGCCTCCAAAA
TGTGACAAGTCAAAGGAATAGGAGCGAATGTGCTTCTTTTTATAGCGGACGTGGCTTGTTGTTTGATAAT
TTTGGAGAGCCAAATGCCACAGAGCCTCTTCCAGCTTCTAAATCCAACAGAAAGAGTCACAGAAATAAA
ATCATATTGGCGGCTGTTCTGGGTAGCGTTGGATTACTTGCACTTGTCTTTATCTGTATATTGCTGCTGAT
TTTTTGCACCCGCAAACGGGGTGCCACGAATCAAAGAGCCACTGAGGTTGGGCCCGGTCCTGCCAGTTC
AAGTCCACCACCTCCTGCACCTGGAGTATCATTGAATTTTTCAAGTTTAGGAGATGCATTTACCTACCAGC
AGATTCTTCAAGCCACAGGTGAGTTCAATGATGCAAATCTTATGAAGCATGGTCATTCAGGTGATCTCTT
CCGTGGTACCCTAGAAGGCGGGACCCTTATAGTTGTCAAAAGAATAGATGTGCAGTCTGCAAGGAATG
AAGCATACTTGTCAGAATTGGACTTCTTCAGCAAGGTTTCTCACTCAAGATTAGTACCTTTTATGGGGCA
CTGTTTGGAGAATGAGAATGAGAAGTTCGTGGTGTACAAGTATATGCCAAATGGAGATCTTTCAAGTTC
TTTGTTTAGAAAAAACAATTCAGATGATGATAGTTTGCAGTCATTGGATTGGATAACAAGACTGAAAATT
GCTATTGGAGCTGCTGAGGGTTTGTCTTATCTACATCATGAATGTAACCCGCCTCTAGTGCACAGGTATC
AAGGACTTGTCATATTACATTTATCTTACTTCCATGTGCCACCACTTTTATGGTTAATCTGAGAGAACTTG
AATAGCAATGCTGGACTTATGTGATTTACTATTAGATTTCCATGTTCGCATGTGCACTTATATTTCACTATC

ATGATTACAAGTTTTCATTTAAACATGTTTATCATATATCTTGGAAGAGTTGCAGGCTACCAGCTACTCTT
TCTATGTATATTCATTGGTTATACATAATTGGTGACTCTTGGGCTAGACTCAAGGTAATAAAGGATATGA
CTTGATGACTTGGAGAGCGCCCTTTTTGGTTTGTACACGTTGGAGATACTAGGAAAAATGCTCTGAATGA
AAGCTATCAAATATCAGTGTATTTCGTGTCTCTGAATTAGGCGGGACAAGATTCCCATAGAAGGCTCTCA
AATTTTCAGCCAAAAATTCAGTGCAGCGCAGACACTCTGCACGTTAAAGGTGCTTGCAACTTTATGGATT
GTGCCCATGTCTGGTCGACTGAAACTCATCTAAAGCCTATTTATAAATGATAAATATTAGATGTCTGCTTC
GCTCTTAGCTTCTCGAGATTTATAAAAGGAGGCAATTTATGGTTGATGGTTTTCTGATTTGCTATCAGTTG
GAGATCTGTAATGTTTATTGCCTCGTGAGACTATTATTATACAGAGTTCATCACATAAGGAATAGCCTCC
ACAATATCTTGTACCTGGAGCTTGACTCTATCAACTTGAGGGCTTCACTTACTATTTTTATGAGCTACACC
TGCATGTAATAAAATTAAGAGCTTAGTAAACCTAAAACCTGTCCTTTTAGGGCTTAGCAATGTAAAGTCA
AGAGCCATGCACTTCTAAAATTGCTGTAAGCTCAGCTTCTATAGTTCCATGCATGGCTGCGATATCTCTAA
TCTAGGCAGTTTATATTGTGTTAGATTCGGTATGAGGGATGTGGCCTTAGAAGATCTTGATGACATTATA
TTTCTATTGTGCAGAGATATTGTGAATGCAGAAATAGAAATTTTCTCAAACTAAGAACAGTAATGTTTTTT
TAAAAATTGTTTTCTTTTCTTATTATTGAACCTTCTCCATACATCAATCCTTCTTTTTTTGATAACCGTGGTG
CCCGGGCTAGCTTGTGCACACCTCGACACAATCCCATCAATCTTAATCAACTAAAGGTAGTCCTCATATG
ATACTCCTTATTGTACCTTTTATCTCCATGGAACCTAAGCCTGTCAGTGAACTATCTTATTTCTCTAAAACT
TTTCATCTGCATGTTTTGGGTTCAACCATTATTCTTTCACCGGGCAACCCTTAGGGATTTGGGTTAGTGGC
AAGAGCACAACTCATTATGTGTGGGTTAAGATCACGTTATCAGTTTGAATCCTACCATAGACTAAAGCTT
GGTATTTAAGTGGAGAAAGGTAGAAGGCTGGGACCCATTATCCACCGAGTTTTGGATCGTGCGCCAATA
GAGCTGGGAGGATTTCTCAGTTACAAACAAAAGATCCTTTCATGGGGAATCCAATAGACTTTCTTGAACC
GAATATCCATTTCAGAAGGAGTTTCCAAAGGAGATGCTTCTCTAAATGTCCGTATAGTTTTTCTGCTGCA
GTTCACCGTGAAATTCTCAATGGTTTAATTGCATGAATTAGTTTCTTGGATTCAATCTTACCTCCACCCCCC
ACCCCCAAAAAAGAACTTTATTTCTGTCAGAAATTTTTTAGTGCATCATGTAGTGACATCTTCTTTTTCCTT
TGCCTACTATTCTCGCAGAGATGTTCAAGCAAGCAGCATACTTCTTGATGATAAGTTTGAAGTACGGCTA
GGGAGTCTAAATGAGGCCTGTGCACAAGAAGGAGAGAGCCATCAAAACCGAATTTCCAGGCTGCTCAG
ATTTCCACAGTAAGTAGTTGTTGTTGGTAAATTCTATGTTCTAACCATCCTTTTCTTTTTGAAGGCTAACTA
CTATTACTACTAGGTTGTTATGTAATGGTCAATTTTTGTCTCCCATGATGGATCTTAAAATCAGGCAGTTT
TATTGTTAATATATCTGTCTCTCCAATTTGATGTTATAGAAACTACTTTGGCTTGGTTTCTAAATTGGATTT
GAGTTCCACAGTTTTGCTGCCAGCTCAAGTCTTTCTGGTGTATATTTATTAGCTCTGATATAAATGAGAAT

CCTAACGTTCATGCAGCAACCTTACTCTTAACCTTGTTATCTGTTCTTATACAAAAGGGCTTTAGCTTTGTT
TCTAAATTATGACAACTTGTAATATAGGCCCTGACTAGTTTAGTCCTGATGATAGTTATCATGCAGCTGCA
TATTATCTTATTTCTTCTCAGTATTCAAGTTACAAGGAACACCGTAAAGAACAGTCAGTCTAGTCTGAAAG
ACCATCACCCACTTGTTAATTACTTGTTCCATCCCTCAGTAAATTATTCCTTTTCTACGTTATATGAATCTTC
TTGCCTTGATTTACCTATGTCTAGCGGGTGTCACATGGGTGTTGTAGTTTAAGTAGAAATTAGCAAAAAA
TTGTATGTACCCTTACTGTATACTTAAAACTCATGTATGGGATATGCATGCGTAGCTGAATCCATGTAACA
TAGTCCTTTGATGTACATGTTTTCACTTAAGGAGAATTTGTTGGTTTTGCTATTTGGTAGGGAATGCATAG
TTCTCATTCTTTTAGCTTTCCATCCTGTGCTGAAGTAAGTTTAACTATTTCTTACTCTCATTGTCTAGCTTTA
ACTATTACCTATTCTCGTTATGCCTACACATTGAATGCAGGAAATTATTAAATTTTGTATTGTGGGGTAAA
TACTAAATACTTCTGGAACGACCTAGATATAATGGCATGTGAAAAATTGGTTTGAAGTCCCTATGTTAGA
TGCCAAAGATTTGAGGCCTTCTATTCTATTATTTTAATCTTCATGGCTTCTGTTAAAACTTTCCAGGTTCTC
TTATTTTAGAAATTGAACTTACGACCACTGATCATGATTATATTCAATCATAATCCAGAAAGCTTTAGAAG
TTTTTGTTTTATAGTGAAAACTTTTCCATATGTATTTCCTGTCTTATGAATGGTTATCACTGTTTGTCCAGC
TTTTCGTCGAAGCTTGTTGCATGATCTCAAAAGTTTTGTTATATTCTTTCAACCAGTACTGTTTTATAGTTT
ATACTGCTTGTTGACATAGTTCTCCCTTTCTTATTTGCAGGACGTCGGAACAAGGTGCATCAGGTATGTG
CAACTTTATTTAGTTCTACTTTCCTTAAATAGAGTTTACTTGGATATTCTTAATATTTATTAAATTGGTGCA
AGTAACTATTTAAAATTATTTGCGTCACATTTCCGGTATTGTTTGTTGGTGGGTAAAATCTCCTTAAATTG
ATCTAGGCAACAAATCAAAACTGTAGATTCTCATTAGAAGTAAGAATAAGTGAAACTATTAGGTGGAAA
TTTACGATATAAAGCTGATTCCAAAGAGCGTAAAGGGCTGGAGATTTTGATGGCGATGTTTTGCTTTACT
TTTTGTGGGAGTATGCTTTGTTGCTTGAACTATCACTAGGGAATACTTCAAAGTCTACTTATCTCGTCCTA
ATATTCTTTCTTACCCGAATGGACAGGTACACCTAGTGCCACTTGTGCGTATGATGTCTACTGTTTTGGAA
AGGTTTTGCTCGAGCTAGTAACTGGCAAGCTAGGTATCAGTGCATCCAATGACGCCAGCATGAAGGAGT
GGTTGGACGGTACGCTTAAGTACATTAGTATATATGATAAAGAACTTGTCACAAACATTGTGGATCCTTC
CCTAATCATAGACGAAGATTTATTAGAGGAAGTATGGGCGATGGCTATTGTTGCCAGATCCTGCCTCAAT
CCCAAACCGTCAAGGAGACCCTTAATGAGATACATCCTTAAAGCTTTGGAAAACCCTTTGAAGGTAGTTA
GGGAAGAACACACTAGCTCAGCAAGACTTAGAGCAACTTCATCGAGAAGTTCATGGAACGCTGCACTGT
TTGGCAGCTGGCGTAGCTCCTCAGATGTAGCAGCCGTACCTGCTGCAGCTTCAGCACATAAGTTGGAAG
GAACAAGTAGTTTGAAGCAATCCGGAACAACAGGCTCTCAAGGAAGTGGTCCTAATGGGGACAACAAC
GGTCATTCTTCATCAACTAGACGACAGTCCAAAGAGATTTTCCCTGAGCCATTGGAGGAGCACGATGTA

GAGAGAGGACCACAGGCGGAGTAGAAGGGAGGGTGATTGTCATTAATTCTTTGTTAATAATGCAGTAG
GATCTGCATTAAGGTTGCATTTTTGTTTGGGGACACCTTGTACATTCTCCGTTTCCGGAATGCTTGAGTTT
GCAAGCCATTATCTTGCCCTGGTCTTGAAAATGGAGATGCATAGTTTACCTGGGGACAGCAAACATCAA
ATTGTTTATTGGGGTTTTGGGATTTTAAGTTCTTTTTCTTTTTCTGGGGATAGAGTTGGAAAACAGGATTT
AGTAGTGGTGGTGGTTCTTTTTGTTAATTTTGTTTTTGGGGGGTTTATGTTTGCTTCCAAATTCCAATTCTT
TTCTTATAATTCTTCTCTTTTACTAGTCTCTCCATGGAATGTAAATTATACACAATCTTTCATTTTCCAATTG
GAGGAGAGGAAAAAAGAAAAAAAAAGAGTATCCC
>PGSC0003DMG400000996 LRR-RLKs
GGCTGTTGTCTCTTTTTTGCCTTAAACTTGAACTGCTTCTAAAATTTTCTACTCCACATATTGCTGCAGGCA
TGAAGTCTTTTAATGTTTTCCTTTGTGTTATGTTTTCTTGCTTATTATGTGTTCTTTCTGTAGATGCATCTTC
CAGGCTTGCTGGAAATGAAACGGATCGGTTAGCCCTCCTCAGCATCAAAGCACAGATAACATATGATCC
TTCTGGTGTTACGAATTCTTGGAATGATTCCTTCCATCATTGTAGTTGGCAAGGCGTTACATGTAGTGCTC
GACATCAAAGGGTGACTATGCTGGATTTAAGCTCTAAGCAGGTTGTAGGAACATTAGTTCCTCAGATAG
GAAATATGAGTTTTCTTAGAGAACTAATTCTTCAAAATAACACCTTTAACAGTCAGATACCACGCGAAAT
CGGAAGGCTATTCAGGTTGAAGAATTTAGTGCTTAAGGACAACTCATTTACTGGTGATATTCCAGTGGA
ACTATCAAATTGCTCTAGACTCATTTATCTTGATTTGGATGGTAATTCACTGACTGGGAAAATCCCCGTGG
AGCTTAGTTTGTCTTTGAGAAATCTTCAGGTACTTTTTCTTAGATCAAACAATTTAACAGGTGAACTACCA
TATTCTTTGGGTAATCTTTCATCCCTTATTGCCTTAGCAGCAATAGAGAATAGATTAGAAGGAAGTATTCC
GTATTCGTTAGGCCAATTGACTAACTTGAGTTATATCTCTCTTGGTGGTAATATGCTTTCTGGTTCAATTC
CCCTTTCAGTTTTTAACTTATCCTCTCTGTATCATCTTGCTGCACCAGTTAACCAACTTAAAGGAACTCTTC
CCACTGATATTGGATCCACTCTTCCGAGTCTTCGCATAGTTTTTTTGTTCTCAAACTTATTAAGTGGTGTGC
TTCCAAGTTCCATTTCTAACTTAACCAATCTTGAAATTCTCTCTTTATCAAGAAACCAGTTGTCCGGGAAA
ATCCCTTCTCTGGAAAAGCTCCGTAATCTTCAAGGACTAGCTATGCATTTCAATAATCTTGGAACCGGAA
GAGAAGATGACATGGATTTCTTTTCTTCATTGGTTAACATTACCAGTTTTAAAGAACTGAGTTTGAGTGT
GAATAATATAGCAGGCCAGCTGCCTAAGAATATTGGCAACTTAACCAATTTCCGGTCTATTGGTTTTGCT
AGGAACAAACTATTCGGACGAATACCTGATGGATTTGTTGATCTCAGCAACATGGAAGTGGTAAGTTTG
GAGTATAACCAATTGACTGAAGAAATTCCAGCTAGTCTTGGTAAACTTCAAAAGCTCAAGTATTTCTATG
TTAACGGAAACAAACTCTCGGGGGAAATCCCTTCCTCTATTGGGAACATAACATCTTTATATGGACTTAA
TCTAGCTCAAAACAACTTAGAAGGGACTATTCCTTCAGTTCTTGGAAACTGTCAGTTGTTGCAAATGTTG

TATCTTTCTCGAAACAGACTCAGTGGGACGATACCAAAAGAGGTTCTTAGTATCTCATCCTTGTCTATCCA
ACTAGACCTGTCTGGAAACCAGTTAAGTGGATCACTTCCCCTTGAAGTAGGAAGTTTAGTCAATCTCGGG
TATCTTGATATATCCGAGAACAAGTTATCTGGAAAACTTCCGAGCACTTTAAGCAGTTGCATCAAGTTGG
AAAACCTCTATGTTCAGGAAAACATGTTTGAAGGTGTGATCCCATCATCTTTAAGTTCTTTGAGAGGGAT
GGAATATTTGGACTTGTCGCGTAATAATTTCTCTGGTCTTATTCCAAGGTACTTTGAAACTTTCATATCAT
TGAAAAGCTTAAACTTGTCATTTAACAATTTTGAGGGTGAAGTACCTCGAGAAGGAGTTTTCAGTAATGC
AAGTGCAGCAATAGTCAACGGAAACAGAAATCTTTGTGGAGGTTCTTCCGCGCTAAAACTACCACAATG
CAACTTTCCAACATCAAAGAAGGGAAGATTAATGTCATCAACCTTGAAGATAGCTATATCTATTGCTAGT
GCATTATTTGGAGTAGCACTTGTTCTGGTGCTCCTAATTTTGTGTTTCTTAAAGAGGAAACGTAGCCCTTC
ACTCGATTTATCAGATGATTCGTTCTTGAAAATCTCCTACGGGGAACTTCTGAAAGCAACTAATGGATTC
TCCTCAGATTATTTAATTGGTAAGGGTGGTTTTGGTTCTGTATACAAGGGAATTCTTGGTCCAGATGAAA
AAACTGTCGCGATCAAAGCACTCGATTTGCAGCATAAAGGAGCTTTGAAAAGCTTCATTGCAGAATGTG
AAGTCCTAAAGAACCTCAGGCATAGAAATCTTGTCAAGTTAGTAACTGCATGCTCAGGTACTGACTTTCA
AGGCAATGATTTTAAGGCCCTCATTTACGAGTTCATGGTTAATGGTAGCCTAGATGATTGGCTACATTCA
TTTTCTAATGATGGAAGTCTGCACGTTCGATATCTAGATCTTTATCAGAGAGTAAACATTGCATCAGACA
TTGCTTTCGCGCTAGATTATCTTCATCATGGAACCCAAACACCAGTTGTTCATTGTGATTTGAAGCCAAGC
AATATCCTTCTAGACAAGGACATGACTGCCCGTGTTGGCGATTTTGGTTTATCAAGATTCCTCCAAGAAA
CATCTCAAAGAGAAACTAGCACTATTGGTATCAAAGGATCAGTTGGCTATGCAGCTCCAGGTAAACATC
TCTTTCCCTTTTTTAGCAACTGTCACATTCCAACTTTCCGCCTGACATGTTTAAGATCATACTATGTTTAAG
ATCGTAATATTCAAAAGTCTTACATACTTTCTTAAACTTCGTGGCAAGTCCAAACCAGACAAACATAAAAC
GGAGTGATTTTGATATGGACTAATGAATTTTTTTCTGCAGAATATGGAATGGTAAGTGAAGTATCAACTT
ATGGTGACATATACAGCTATGGAATCATCATACTGGAGATGTTAACAGGAAAAAAACCGACAGACGAC
GCCTTTTCAAATGGATTGAACCTTCACAACTATGCTAAAATGGCTTATTCTACTGGTAGAGTCATGGAAA
TTGTTGATCAAATGGTTTACCACAACTTGCAAGAAATGAAAACCAAAGATTACATTGAAGAGTGTTCAAT
TTGTATGTGTAAAATTGGGATTGCTTGCACTATAGATTCACCAAAAGAAAGAATGAGAATTAGTGATGTT
ATTAAAGAGTTGCAATTAGTCAAAGAAACTTTATCTTAATGTGATAGAACACTATGATATTAGAAGTAAA
TCATAAAACATTATCTATTTTGTTCTATATATTGTGAGTTGGTAAAATTTC
>PGSC0003DMG400001185 LRR-RLKs
TCAATGGTAGTTCCTCTTGTCTCCTTGTTTTACAACTTTTTCCTCTTTCTCATACTTTCGTCCTCCAACGTAA

CTGCTCAACCATCTAGCACAGACACAGACTTTTCATGCTCGTTTAACTCTTCCTTTTCTTGTGATACTTTTA

TTTCATATCGTGCCCGTCCACCAAACTATTTAGATGTCGGAAGTATATCTGATCTTCTTGAAGTGAGTCGT

TTAAGTATAGCTACGGCCACAGGTCTAGCCTCAGAGGATACCGAGCTATTCCCTGACCAGCTTTTACTTG

CACCCGTTAAATGCTACTGCAATAGTTCTCATTATTTCTCCAATGTCACCTATCAGATCAAGAAAGGCGAC

AACTTTTATTCAGTTTCAATAGGAGCTTTTGAGAACCTTACTAATTATCACGTGGTGCAAGATATGAATCC

AACACTAGATCCAACCAACTTGACAATTGGCGCAGAAGCTGTTTTTTCTTTGTTTTGTAAATGCCCCACAC

ATTCTGATCTCGAAAAGGGACTTCAGTACCTGGTCACTTATGTTTGGCAGCCTTGGGATGATGTGTTGCC

TGTAAGCAATATGTTTGGAGCATCTGCAGCTGACATTCTGGCTGCCAACAATTACAGAAATTTCACTGCT

GCAATATATTCTCCTGTCTTGATACCAATAAAACTTCCTATTATTCTGCAATCTTACCCCTCTTCTGCAAGC

AGTAGAAAATCCAAACATGGATGGATAGTAATTACTGTTTTAAGTATTATGGGGTTGCTTGCAGTTTTTT

CATTTTGCTTGATGGTATACATGCGTCATCTAGACGAGAAGAGGAGGGCTAACTTAGCCCATAACTCTTC

TACTCTTGAAACTTCTGAGCTCTTTCATACCAAGAAAGCCTCAGAAGGTGAAATTACGGACCATAAGAAC

ATTCAAGATAAGCTTCTTCCTGGAGTTTCTAGCTACATTGGAAAGCCAATAATATATGACCTGAAGATCA

TCATGGAGGCAACTGTCGACCTCAGTGAGAGATACAGGATAGGAGGATCAGTGTACAAGGTTACAATG

AACGACCAAGTTGTAGCAGTCAAGAAAACAAAACAAGCCTCGGAGGAAATGACAATACTTCAAAAACT

ACATCACGCAAATCTAGTGAAACTAATGGGCGTTTCATCAGACGATCGTGGGAATTCCTTTGTAGTTTAT

GAATATGCTGAAAATGGCTCACTGGATCAATGGTTGTTTCCCGGATTATCATCATCTTCTTCTGCCTCTGT

CTTAGTGGCATCACTTGGTTGGAGTCGAAGATTACACATAGCCTTGGATGTTGCAAATGCTCTGCAATAC

CTGCATGAACATACTCAACCTAGTATAGTTCACGGGGACATCCGAACGTGTAGCATACTTCTTGATTCAA

GGTTTAAAGCCAAAATTGCAGGTTTCTCTACAGCCAGACATGCCACCAACTCAATGATCCTGAAAGTTGA

TGTGTTTGCTTTTGGGATTGTGCTGTTGGAGTTGCTTTCAGGGAAGAAAGCAATGGAATCAAAAGATTA

TAATGATGAGACTCTGATCATCTGCAAAGAAATTAGTGGAATCTTGGAAGTTGAAGAGAACAGAAAGG

AGAAGTTTAGAAAATGGATGGATCCAAAGTTGAGCTTTTACCCTATTGATGATGCTCTAAACTTGGCTGC

CTTGGCGACAGCATGCACATCGAAGCAATCAGCAGAGAGGCCTAAGATGACAGATATTGTCTTCAACCT

CTGTTTTCTTGCTCAATCATCTTTTGAAATGTATGGACCATCTTGGACTTCAGGTGAAGCTGAGGAGATT

GTCCAGATTGTTAGTCCAGTAATAGCACGTTGAGTTAAAGATGGAGCGTAAATATAGTTCAAGAACTTTT

GTCAGTTCGTCTCAAATTTGTAACATATTCTGGATTATAAAGCCACAGGAACAATAATCAAGCTGATCTC

CAGAATATATTGCAAATTCGAGTTATCTTGTCCAGCTTTTCCCTGTATTTCTCTTGCTGCTAAGTTAGTTGC

TAGAGTATATTCTTGTATTGGATGTTTTTCTCAACTTTGAAATAAGTGGAGTGCAATTTGACCTGTATTTC

TTTCGTCCTCTCAAAAGAAAAACAGAAAAAATTAAGTTTCTGAAAAGACACTGCAAACTCGTTCTTATTTA
TATTTATATATTTAAGATCCAGACGAGTACATGTTTA
>PGSC0003DMG400001301 LRR-RLKs
TAATTTCTTAGTAGCTAGCTTCTAAAGGCTCCACTCATACTTGTAAAATAACAGACTGGGTTTTCATTTGT
CAAGTCAAATGCCTCAACAGTCTTCATCTTCCTTCTCCTTGCTATTTCCTACCTATTTCTCTTGCGCATATTA
TCAAACACCTTGTGCCAATTACCCAAAACTGCTGTTGGATCCCTAAACCCCCCAGCTAACGATGGCTCGT
TTCAGATTTCTCCTTTTTCTTCTGTTGATTCTGATTTCTCTTGTTCTTCCGTGCCTTGATGCTACTGTAGTTG
AAGATGATCTCAAGTGTTTAGAAGGATTTAAGAAGTCTTTGGAAGATCCAGATGGGAATCTGAATTCAT
GGAACTTTAAAAATACTACTGTCGGAGCTATCTGTAAGTTTACGGGTGTTAATTGCTGGAATGACAACG
AAAATCGCATCATCAGTCTCTCTCTTTCGAATATAAACCTCGGAGGGAAGGTTACAGAGCCTGTACAGTA
TTGTGCCAGCTTGACAACCCTTGATCTCTCTGGTAATCGCTTTTCTGGTCCAATTCCTTCTCAAATTTGTAC
ATGGCTACCTTTTTTGGTAACCCTAGACTTGTCCGGTAACGATTATTCCGGCCCTATCCCTGCTGATCTTG
CTAAATGCATCTTTTTGAATAAATTATCGCTCAGTGATAACAAGCTTACTGGAAATATTCCCCCTGAATTT
TCTAGTTTTGGTAGGCTTAAAAGTTTTTCTGTGGCAAATAATCAACTCTCCGGTAGAATCCCGGCAGCCTT
CGATTCTTCTAACTTTAATTTTGAAGGAAATAGTCTCTGTGGTGGACCTTTGGGGAAATGTGGAGGACTA
AGTAAGAAAAGCTTAGCTATTATTATTGCAGCAGGAGTCTTTGGTGCTGCTGCTTCTATGTTGTTGGCTT
TTGGTGCGTGGTATTGGTTTTTTACCAAGTCTGGAAAGAGGAAGAGAGGGTATGGAGTTGGGAGAGAT
GACTCTGATAGTTGGGCTGATAAGCTGAGGGCTCACAAGCTCACTCAGGTTATGTTGTTCCAGAAACCG
CTTGTCAAGGTTAAATTAGCTGATTTGTTGATTGCCACAAATGGTTTCAGGACGGACAATGTTATCAACT
CAACTAGAATGGGTACTACTTATAATGCTGTGTTACGTGATGGTTCTGCACTTGCCATTAAGCGGCTTAA
TACTTGCAAACTGAGCGAGAAGCACTTTCGTGATGAGATGTATAGGCTAGGACAACTTAGGCATCCTAA
TTTGGTGCCACTGTTGGGGTTTTGTGTTGTGGAAGAGGAGAAGCTTTTGGTTTATAAACACCTGTCAAAT
GGTACTTTGCACTCATTCTTGAATGGGAATGCAAGTGAACTGGATTGGCCTACTCGGTTTAGAATTGGTT
TCGGTGCTGCTAGGGGCCTTGCTTGGTTACATCATGGTGTCCACCCACCTATCTTGCACCAAAACATATG
CTCTAATGTTATTTTCCTCGATGAAGACTTTGATGCTAGAGTAATGGATTTTGGGCTGGCAAGGTTGATG
ACATCAGATGCAAAAGAGTCTAGTTATGTGAATGGGGAGTTGGGTGAATTTGGCTATGTAGCTCCAGAG
TACTCTAGCACAATGGTGCCTTCACTGAAAGGGGATGCTTATAGTTTTGGGGTAGTGCTTTTGGAGTTG
GCTACTGGGCAAAAACCTCTTGAAGTTACTGCTGGTGAAGAGGGATTCAAGGGTAATTTGGTAGACTGG
GTGAATCAGCTCTCTGCTTCAGGTCGCATTAAAGATGCAATTGACCAAAACATGCGTGGGAAGGGGAAT

GATGAAGAGATTGTGCAATTCCTAAAAGTTGCTTGTAATTCTGTGGTTTCTCGGCCCAATGATAGGTGGT
CTATGTATCAGGTTTATGAAGCACTTCAGAGCATGGCTGAAAAACAGGGTTTCTCCGAACAGTATGACG
AATTCCCGTTACTATTTGGCAAAGAAGGTGCTACTAGAAGCCCTGTTTGAAGCCGGAATTGCGAAATTAT
TACTAAATGTTCCACTCCAAATGAACATGATGCATCCTGTTGTAAGTTTTCAGTTCCTATTGGTGTAAGTG
GCATGGCACTGTTTTGATTGGCTCAGAAGTACACCCTTTAAATGTTTCTGTATTATATTTGCAAAATGCAC
AAATCATTCTTAAGTATTTGCTTGCTTGATATTTGTAGTATAGGAAGCAGCTGTTAATTATTGTTCATGTT
TGTGATTACCATTGCCTATAGCATTAGATGTTATGTATTCAAAGGATTCTCAACTGAACGTCTCATTTACT
GGAAACTGGTCTATATTACTATTTACTGCCTGTCTAGCTCTTGTTTGGAAGCCAATGGGGCCG
>PGSC0003DMG400002012 LRR-RLKs
TGGAAGTAGTTAGTCAAAATTGTGTCTAAATGAAAGGTTTTTGGTGGTTTACTGTTTCTTCATTTTTATAA
GGAAAGTTGGTTTGACTAAAATAACAACCCCTATTGACTTGCTTATGGTATAGGGAAATGTAGGGTCTCT
TGCTTTTGATTTTGATGTATTGGTTATTGGTGAGTGAATTTAATGGTGGCAATTTGCATTTTGGGACATTA
TTGGTGCTGCTGTTGGACTTCAATGAATTTAGTGGCTGGTGTTGTTGCTGCTTGTTCTTTTATCTAGGTTT
ATATACTCTCTTATTTCACTTACATAATGATTAGTGATGATTGTAGTTTGTGTTTAAAAATTTTGCTAATTC
ATTTTCTGGGATTCTTGAAATGATTGTGCAATCTTTAGTCTTTCTACTTGGTGGGGTTTGTCTACTTTCTTG
GCCCATTCTGAATTATTAGCTTGTCAAAACGCTGTCTTTTTTCCTTTTTAGTTCAGTATTATATGTAACTCT
CTGTTAGTGTCACACGGGTTTTTCATTGGGAGGAAATGCAATCTACCATTTGTTTAGTGCTGCTATTTTTA
GTTGAGTTAGCTAAAGGAAGCTTAGATTTGGATGCTCTTTTGGAGTTTAAGAAGGGAGTTTTGAAAGAC
CCTTCTGGGAAAGTACTTTCTTCATGGGATTCTAAGTCATTGGGACCAAATGGTTGCCCCCAAAACTGGT
TCGGTATCGGTTGCAGTGATGGCCATGTCACTTCAATTGAACTAAATGATGTAGGTCTGATTGGAGTTTT
GGATTTTGCAGCTATTAGTGGCCTAAAAATGCTGCAGAATTTGTCTGTTGCAAACAACCAGTTAAGTGG
GAAAATTACTGAGGAAGTTGGGTTGATTATGTCATTAGAATTTTTGGATCTTTCCAAGAACATGTTTAGG
GGTTCTATACCCTCTAAGCTGACTAGTTTAAAGAACTTAGTATCTCTTAATCTTTCTTTAAATAGTCTCGAT
GGAATGGTTCCCACTGGGTTTGCTAGTCTTGAGAAATTGAAGTATCTGGATTTGCACTCTAATGCCTTCT
CAATTGATATTATGCTTCTTCTGGCCTCATTGGGGGATGTAGAGTATGTAGACCTCAGTAGCAACAAGTT
TGTTGGATCACTTGACCTGCAAGTAGGAAATTCGAGCTTTGTCTCGTCAATTCAGTATCTGAACATTAGT
CATAATAACCTGGATGGAGAGCTATTTCCTCACGATGGAATGCCATATTTTGACAGTTTAGAGGCGTTTG
ATGCAAGTGACAATCAGCTTACTGGTACTATTCCGTCCTTCAATTTTGTAGTCTCCCTTCGAATTCTTAGG
CTCGGGAACAATCAGTTGTCTGGATCACTCCCCGAAGCTCTTCTAGAGGATAGCTCAATGATTTTATCAG

AATTGGACCTAAGCCAGAATCAGCTTGCAGGTGTGTTGATCCTTTCTTCTTTAGTTAATTTGTTTGGTTGA
AGTCAATCTAAGTAATCTATGGTTAATTACTACTCCCTTTGTCCCAATTCATGTGACACTTTTTATTTTTTG
AGAGTCAAACAATTTAAGTTTGACCGGAAATTTGCGCGTGGAATCTTCAACTTTTTTGAAATGAAATTTA
TATATTTGTAAACTACGTAAAAAGTACTATAAGTCACAATAATTGGCAATTCAAAATAATTAAAAGATAT
ATGATAAATTTACGGTCAAAGATAGAATTGTTTGAATCTCGAAATCCGAAAAGTGCCACATAAATTGGG
ACGGAGGGAGTACTATCTATGATCTGAGGTTGTTGTCAGTTAGATAACAAATGAATAATATTTAGATTGC
TATCTGCTGGAAAGCATGCTTGTTTTGGTTGCAATCAAATGTAATGTATAGAACACAATTCCTGAGCACT
TCGGAATTGTGTTCTAGAGGAATGCTGTACAATTCTCTGCTTATGTGTAATTATCATACACATTGTTCCCA
ATTCTTCCCTAGGTTGGAGATATTTCATACTTAGACTCAACTCTAAGGCAATTATTGCTGTACTTGCATTTT
AATTAGTGGAATGTTCCAGTGTCTTTTTGATTGTTTTTTTTTGCTGCATTATTAATTTCATCTCTCCTCGAG
CCGAGGGTCTATTGGAAATAGCCTCCCTACCCTCACAAGGTAGGGGTAAGGTTTGCATACATGCTACCCT
CCCCAGACTCCACCTATGGGATTATACTGGATTTGTTGTGGTTGTTGTTGTTTGCTGCGTTATAAACAAGC
TGCATAAAGATATTTTTCATGTGGTTGGAGTTCTGGTGTATTTTAAGTCTTTCGTTGTTCTTTTAATTATTA
TATTTAGTTGTTCTTACCACAGGCCTAATCCAAGCTACTTCCTTTCCAAACTATTTCATGAAGCCATTTGCT
ATTTTGGCAGGTCCCATTGGAGGTATAAGTGCTGTGAATCTCAAGCTTCTAAATTTATCATATAACCAGC
TCTCAGGCCCCTTGCCTTTCAAGGTTGGGCGCTGTGCCATCATAGACCTGAGCAACAATCGGCTAACTGG
AAATGTGTCCCGGATCCAAGGTTGGGGAAATTATGTTGAAGTTATTGTGTTAAGCTCAAATGCATTGAC
AGGAACCTTCCCCAACCAAACTTCCCAGTTCTTGAGGCTTACTTCGTTAAAGATTTCAAACAACTCACTTG
AAGGTGTGTTACCAACTACGTTAGGTACATACCTCGAACTTAAAACAATTGATCTTAGCATTAACCAACT
TAGTGGTACTCTCCTTCCCAGCCTTTTCAACTCTACCAAATTGACTGATATTAACGTTTCTTTCAACAAATT
TACCGGTAGTGTACCTATCATGGCATTTAACTCAGAGAATCTAAGCTTGATTTCTCTAGATGTTTCACATA
ATGCATTAGCTGGTCCGTTACCTCCGGGGCTGGACAAGTTCCTGGACATGGTAAACCTGGATCTCTCTGA
TAACAAATTTGAAGGTGGCCTTCCTAATGATCTCTCAGACAAATTGGAATTTTTTAATGTGGCTAATAAC
AATTTTTCTGGACCTGTGCCACAAAATTTATGGAGGTTTCCTGACTCCTCATTCCATCCGGGAAATCCCTT
GCTTGTACTTCCAAAGCAAGCCAAAGCTCCTTCAGAGGGAGATTCAACTTTAAGCTTGAGAAGTCATGG
TTCTCGCATGAAATCTACCATCAGGGCTGCCCTCATTGCTGGGCTGATCTGTGGTGTATCTGTTATAGCTT
TGTTGACATTAATAATCTACCGTAAGGCCCATCAGCGAGATGGCGGGAAGGACGATATGAAAGGAACC
AAGGAAAAGAAAGGTATGGAACTATTCTTCTTCTAATAAGACAGGTGATCAGATCAACTGATACTAATG
CCTTTTCATTTCCACAGGTTTGTCTTTATCTGATATAGAATGTGGACATGACACTAGAGAACACAGCGTG

CCAGTATCAACAGTTCAAAATGAGCCATTATCTTCACCTATATCTGTCATGTCGTCCGCCAATCTCTCTCCT
TCTAAGGTTCAAGATCAATCCAAAAGCCCAAACTCTCTCAGGGTTTCTTCTCCTGATAAACTGGCTGGAG
ATTTGCATCTACTAGATAACGCCTTGAAGGTCACTGCTGAAGAATTATCGTGTGCTCCTGCCGAAGCTGT
GGGCAGAAGTTGTCATGGAACATTGTATAAAGCTACTCTTGGTTCAGACCAAGTACTTGCTGTCAAATG
GCTTAAGGAAGGGATAGTGAAAGGCAAGAAGGAATTTGCTAGAGAAGCTAAGAAACTGGGGAGTATT
AGGCATCCTAATTTAGTTTCTCTACTGGGTTACTACTGGGGTCCCAAGGAGCATGAGCGGCTTCTCATAT
CAAATTACACCGATGCACCATGTTTAGCTCTTTATCTTCTTCGTAAAGGTCAGTCTCCCCTTTTCTATCTAT
AGAGTATATCACCTGCATCTATTTCTGTAGCTTTTCTGATTTTTGTATGGTGCTGAAATTGTGATCCAGTTT
TACCAAAGAAATTAGTAGTACTCCTTCAATGAAATCAAACATAATGGACCGTGAATGCGCACAACCTTGA
ATGTGTATATTGTTATTGTAGTTGTCAAGATTGCTCATACCTGATATGCTGAAGTTTAATCACCATCTTGC
CACTTGATTTCCATTCCGGTGCCTCAAGTGTACTTTACTTCCAAAAGATATTCTAGCTTGCTGGTTGAATA
TGTATCTGAGACTATTAATACACATTTCACATAGCAACTTTCATGAAATTTATAAATGGAGCTATCTTCCA
AAATCTGAACTTTTCAATTCATATATATTAGCAATGTTAGAATTACATATAGCAGAAAAGATACACACCTG
ATTTTTGCTTTCATGGTAATACTAAAAGGTAAAACAGTTCCACCTTCGCTAATTATGCCTTGATGGTGATA
GTGGGAGAGTCAAAAGTCGAAAAGAGGATTCCTCTCTTGTTTCTCTCATTCAAAACTGTGCATGAGAATT
TTATCTCACGTGGCTCATTTTGTATTGATTACTTTGGTTGGGCTCGATTGATGTCATTTTGATAAGGGCAT
CCCGGTGCACTAATTAGAGACTGCTACTTTGAATTCCGCTTATTGCATATAGCACTGTACTTTGTTTTCTA
GTTTTGAGTGATGATATGGATGCCGGTTTCATTCTTTGATGTATGCAGATGCAGAACCCTGTAAGCTACA
TCCTTTATCTCTCGATGATCGACTCAAGATCTCTGTAGATGTTGCTCGTTGTCTAAACTACCTTCACCATGA
AAGTGCCATACCTCATGGCAACCTGAAATCCACAAATGTACTTATAGACACGTCTACCGTGAATGCCCTA
CTAACTGACTATAGTCTTCATCGGTTGATGACATCTGCTGGAACAGCTGAACAGGTTCTAAATGCCAGTG
TACTCGGTTATCGTCCACCTGAATTCGCGAGTACAAGTAAACCTTGCCCGTCATTGAAAAGTGACGTCTA
TGCTTTTGGTGTTATCTTGTTGGAACTTTTGACTGGAAGAAGTTCTGCAGAAATTGTTCCTGGGAACTCA
GAAGTGCTGGATTTAACCGAATGGGCGAGATTATTGGCCTTTCAAGACCGTTCAATTGAGTGTTTCGATC
CATTCTTATTGGGAAAGCAAAGCAACAACGAAGACATGCATACGATTCTTGATAGCATGCTTCAGGTTG
CACTAAGGTGCATTTTGCCAGCTGATGAAAGGCCTGATATGAAATTTGTTTTCGAGCAACTTTGCTCGAT
AGCGCAATAACACAAGTTGAGGGGAGGGGTAAATTTGAATACTATGTAGTTGTAGATTTGTATCTTAAA
AGTTGATATGTCATTCATGTTCCATTTTTAATTCAATTGTTTTAGAAACAAATTCATTTGTGAATTAGCACA
TTAAGTGATGAATGAAGTTGTTGTATTCTTCACATTTATTGACAAGAAATAAAGTTTTCTTGTAT

>PGSC0003DMG400002689 LRR-RLKs

TACACAAATTCAACTCTTTTGTTCCTTAAATTCAACTTTGTGTTTTTTTGTTCTCTTCCTAACTTCCCGCGCT

TTATGCCATAAACCCAAAAAAATGAACCGTTTTTTCTCCTTCATTCTCCTCCTCACACTTCTCATCTCCGCC

GTGACCGCCGCCGTAACCGCCACCGGCGACGGCAACGACACCGCCGCACTCCTCCTCTTTCAGTCCCAA

ACTGACATCCACGGCACTCTCCTTCACAATTGGACACTTCCGGTGAACTCCACCACCGCCTGCACAGCTC

AATGGCTTGGTGTAAAATGCATAAACAACCGTGTCTCCGCCGTAATTCTCCCGTCGTTCAATCTCCGGGG

ACCCATTACTGCTCTCTCTTCTTTACCCTTACTCCGCCTTCTCGACCTCCGTAACAATCGCCTTAACGGAAC

CCTCACACCCATTACCCAATGTACTAATCTTAAGCTCATTTACCTCTCCGGCAACGATTTCTCCGGCGAAA

TTCCGCCGGAAATTTCTTCTCTCCGTCGTCTCCTCCGCCTTGACGTTTCAAATAACAACCTTGAAGGTTCA

ATTCCGACCCAGATTGCGAATTTGACCCGATTACTCACACTTCGTTTACAGAACAATGAACTTTCTGGAAC

AATCCCAAAATCCCTAAATTCTCTTCAAAATCTTAAAGAATTGAACTTTTCGAATAATGAGCTTTATGGGT

CTGTTCCAAATGGTTTATATAATCATTTTGGTGAAAATAGCTTTGATGGAAATGAGGGTTTATGTGGAAT

TGGAAATTTACCTCAATGTTCATATACAGGGGAAATAGTTCCTTCAAACCCTAGCTCTTTGCCTACAACCT

CCACTGCAACAATCGAAGAACCCCACGAAAAATCGCGAAAAGGGTTGAGTAGAGGAGGGGTAGTAGC

AATAGTGATGGTGAATGTGGTGGCATTGTTAGTTGTAGTGTCATTTATGGTAGCATATTACTGTGGGAA

GTATTCAAGAACTCAGAGTTTTTCAATGTCAGGAAGTGAGTGTGGGAAGAGGAGGAGTAGTTATTCGA

GCGAAAAACGAGTTTACGCTAATAACAATGGTGGTGGTGGTGGTGATAGTGATGGTACAACAGCTACT

GATAGAAGTAAACTCGTGTTTTTCGATAGGAGGAAGCCATTTGAGCTTGAGGAATTGTTAAGAGCATCA

GCTGAGATGCTTGGTAAAGGGTGTTTAGGTACTGTTTATAAGGCAGTTCTTGATGATGGGATTACAGTT

GCTGTAAAAAGATTGAAAGATGCAAATCCATGTCCAAGAAAGGAGTTTGAACAGTATATGGATGTTATT

GGAAAGCTTAAGCATCCAAATATGGTGAAACTTAGAGCTTACTATTATGCCAAGGAAGAAAAATTGCTT

GTTTATGACTACTTGCCTAATGGGAGTTTGCATTCACTTCTTCATGGTAAACATTAGTAAATGTTCATGTC

AAAGATTAAAGATTGTTTTGTAATTCAAGATTGTCTCTTTTGGGGGTTGTTAAGGTCTATATTGCTCGGA

CTCTTCAAAATGTCGATGGGGTGTGTCGGATCCTCCAAAAATAGTGCATTTTTGGAGAATTCTGACGTTG

TAATTCAAGATTGTCTTTTTTGGGGGTTGGTAAGGATTATATTCCTTGGACTTTTCAAAAATATCGATGGA

TGTGTGTCAGATTCTCCAAAAGTCTCTAGAAATAGTGCATTTTTGGAGAATCTTACACGGGTGTGACGTG

TTTTGTAATTCAAGATTGTCTTTTTTGGGGTTGGTAAGGGTTATATTGCTCGGACTTCTCATAAATGTTGG

CAGGTACGTGTCGGATCCTCTAGAAATCTCCAGGGATAGTGTATCTTTGGAGGATCTAACGCGGGTATG

GCAACATTTTTGGAGAGTCCGAGCAAGTTGTAGGTAAGATACTAATATTGATGGGATTTTTTTTTCTTGA

TTCTAATAGGAAACAGAGGACCAGGTAGAATTCCATTGGATTGGACAACAAGGATTAGCTTGGTGCTAG
GGGCAGCTAGAGGGCTTGCTCACATTCATGAAGAGTATGCAGATTCAAGAATCCCACATGGGAATGTG
AAATCATCTAATGTGTTGCTTGACAAAAATGGTGTGGCTTGTATATCTGATTTTGGGTTGTCATTGCTTTT
GAATCCTGTTCATGCCATAGCTAGATTAGGAGGATACAAAGCGCCCGAACAATCTGAAATCAAGAGGCT
ATCTCAAAAATCTGATGTTTATAGCTTTGGTGTACTGTTATTGGAAGTGCTCACTGGTAAAGCTCCCTCAG
AGTACCCTTCACCGACTAGGCCTCACGATGAGGAGGAAGAATTGCCAGTGGATTTGCCTAAATGGGTTC
GATCAGTAGTAAGGGACGAGTGGACTGCTGAAGTGTTTGATCAAGAACTGTTGAGGTACAAAAACATT
GAGGAGGAATTGGTGTCAATGCTACATGTAGCCATGGCATGTGTAGTGCCACTACCCGAAAAAAGGCCT
ACTATGGTTGAAGTTGTTAAGTTGATTGAGGAGATTAGAATTGAACAATCTCCATTAGGTGAAGATTAT
GACGAATCGCGCAATTCTCTTTCCCCCTCCCTAGCCACCACTGAAGATGGACTCCCCGGTTACTAATTTTC
GATCATCGTGGCATTGATGTTTCATAGTCCTAGTAGTATATGTTCAAATTTGCAGCATCTTACTGCAAATT
GGGAAGTTCAAAAGCTATGGACTTATGTTTAGTAGTTATTTGATTGCTTTGTGTTATTCCAAATTACTGCA
CTTTTGAAAAATCTGATGATCCAACAAGCATATAAGAAGTGCAGCAATCAGTCCATTATGTTTTTTTTTAA
TGTAATGTAATGTAATGTTAATTCTTTGTATTTAATTCATTTTCAAGATTTGTGTTCTAAGGCAAAAC
>PGSC0003DMG400002870 LRR-RLKs
TTTTTTTTATAATATTAAAAATCTCATTTTGCATTGTGGGTAGTTCTTTTCTTGGAATTCTTGAATTAGTAA
AAGCAAGAAAAAATGAAGGCTATTTTGGGTTATTTGTACTTATTCAACTTCTTGGCTTATTATGCTCATGCT
ATGCTTACTCCAGCTGGTGTCAATTATGAAGGTTAGTTCTTGTGATGAAATATTGGGCTTTTGTGCTTTGA
TTATTGTCTTATTTTGTTGTTGTTATTGTTAATTTTTTTGTATGTTATGTATTTGTTATCCATTTTGCATTTTT
TTCGTTGTTATTACTGGGACGGAGCTAGCAAGGGCTGAGGTGCCTTCCACGGAAAGTTATATCGTTTATA
CATGGTTAAAATTATTTTTATGTATACATAGTAGATGTTAAATCCCTTTGGTTTCTTTGTCTGGTTACTTTT
TATATTTCGAACTCCTTAGTGAAAATTCTGACTACACCAGTCGTTGTTACAATTTTTTTTTTAAAAGCTAAG
GGTTTATTGAAAACAACCTCTTTGAGGTAGGATAAGGTCTGTGTACACTCTACTCTACCCTCTTCAAATCA
TGCTTGTAGAGTCACACTGAATATGTTGTTGCGGATTTTGAAATTAAAAGCTCAAGATTTTTTTTTTCTTT
GGCAGTGCAAGCTTTAATGGAGATAAAGAAGAATTTAGATGATCCTCATAATGTTTTAAACTGGGATGG
AGATGCTGTTGATCCTTGTAGCTGGAATATGGTGACTTGTTCATATGATAAATTTGTCACTAGCCTGTAA
GTAAAACAATTTTCACTTATTCTAAAAATAGTACACCTAACAAGATTTCTTAATATAGTTGAATTATTGAT
TATGCAGGGAAAGTCCCAGCCAAAATTTGTCTGGCAAAATATCACCATATATCCACAACTTGACACACCT
TGAACTTATGTGAGTACTTACTTAACCAAAGTAAATAAAAAACGTAATACATAAACGTGTCCTTTAACTT

GGTGTTATCTATCATCTATGCCTCCAATATGGATGTGCAGAAGTAGGCATACACGTCCTATGTGAAAATT

CTCATCCTACGTGTATTATGCTACGTAGGATGTGAGTGTGCCTACTTGTGCACCCCCAAAGTTGGAGGAT

AGATGTCAGGTGACACCAAGTTAAATGACATATTTATGTATTAGGCCTGAATAAGATATCAAGACATTTG

AGTTTTGATTCTTATTCTTAGATGTTAATATGAGTGTCCTGTTTTTACAGACTTCTACAGAGCAATAATATA

TCAGGATCAATTCCTATGGAGCTTGGAATGCTTAAAAAACTCAAGACAATTGATCTTTCTGATAACAAGT

TAACCGGCGAAATCCCGTCATCTTTAGCTCAGCTGAAAGGCCTTCAATACTTGTAAGCAAAGAGAATTCT

GAGATCTTATACAAACAAGTTTTTTTGGGGTTAAAAACTAATGATGTTTGTTCTTGATTTTTGATGATTAT

AGGAGATTAAACAACAACAGTCTAACTGGAGCTATTCCTTTGGACTTGGCCAATATGACTCAGCTCTCAC

TTATGTAATTTTCTAATACCATTTTCATGTTATGTTATGTTATATGTTCTTTATAACAACATTTTGCTATAGC

AGCTAAAAAATATCCAGACAAATGACGTACGTAGTTATAGAGAGGTTTGACTGTATTTTTCACTTTGGAC

ATATATGTATTATGGAGTTGTGTGTTTTGCATGTTTAGGGACTTGTCTTTCAACAATCTCAGTGGTCCTGT

ACCAAGGCTTCTAGCCAAAACATTCAAGTAAGGTTCTTGAAGAATTCTCTGTGTATCACTTTTTCCTTAAT

ATCAGTAAATGAAACCAGTTTTTTTGTATTTATTTGTCTTTTTTTTAAACCTTATTGACACAGTATTTTGGG

AAATCCAATGATATGTGCAACTGGAAAAGAGTCAGAGTGCAATGGAACAACACCAATGCCCCTCTCCTT

CTCTTCAAGCAATCCACAGAGTAAGAACTAACCTTATTTTAACTGCCAAATGATAGTTTTTTTGTTTTTTTT

ATGACCGTATTGTCTGAGCTAGCTTTTGTACACCTTAACTAATTCCACGAGATACCTACCACCTCCCACCA

GCAACATGTACCAGGTAACTCTCTCCACCAAGGTTAGGATGGAGGGGAGAATCACCTGAGACCTCATGG

CTCTTAACCACTTCATTGACCAGTAGGCCACACCCTTGGGTGCACAACCAAATGATAAATAAGCGCCTCT

CTACCTTCACAAAGGTAGGGGTAAGGCTACATACACACCACCCTCTCCAGACCCCATATATGGGATTACA

CTGGGTATGTTGTTGTTGTTATTGTCGGTCGATATAATGATTATGGTGCTTTTACTAGTCATACAGATTTA

TGAAGTAAATTTGCTTCACTGGTTTAATTTTCTCTGCTGATAGATTCAATATTTATAGTTCATTCCCAGCTA

AAAATATTCTGTGTTATCTCAGATGTTCAGCCTTCTGGAAAACCGAAAACTCACAAGGCTGCGTTAGCCT

TTGGAACAAGTCTAGGATGCATCTTCCTACTAATCGTTGGGTTTGGATTCTTTCTGTGGTGGAGACAAAA

GCACAATAAGCAAATTTTCTTTGACAGTAATGGTGAGTATTGCTTGATCAGAACATTTTGAAGTTACGGT

CTAGCTCATCGATCCTCTCTTATAAACGTTACTGTCATAAATTGCAGAACATCATATTGAAGAAGTGTGTT

TAGGCAACTTAAGGAGGTTTCAATTCAAGGAACTTCAGTCTGCCACAAACAATTTCAGCAGCAAGAACA

TACTAGGGAAAGGTGGTTTTGGTAATGTTTATAAAGGTCGTCTTAGTGATGGGATTATTGTAGCTGTGA

AAAGGCTCAAAGATGGAAATGCAGTTGGAGGCAATCAACAATTTCAGACTGAAGTCGCGTTGATCAGTC

TGGCAGTTCATCGGAATCTCCTCAGGTTGTATGGATTTTGTATGACACCAACTGAAAGGTTGCTGGTGTA

CCCTTACATGTCTAATGGAAGTGTAGCTTCGCGTCTCAAAGGTAAACATATCTAAAAACCTGTCTCCTTTG
GCCTATAATTACATTATCCTTCTTATTTGCTCAGCTCATAACGTATCATTTCTTGCAGCCAAACCAACCTTG
GATTGGGGTACAAGAAAAGGAATAGCTTTGGGATCTGCAAGGGGTTTATTATATCTACACGAACAATGT
GATCCAAAGATCATTCATAGGGATGTGAAAGCTGCAAATATATTGCTTGATGATTACTGTGAAGCTGTTG
TTGGAGATTTCGGATTAGCAAAGCTTTTGGATCATCACGATTCACATGTCACAACCGCGGTTAGAGGAA
CTGTAGGACATATAGCTCCGGAATATCTCTCCACAGGACAGTCCTCTGATAAGACGGACGTCTTTGGTTT
TGGTATTCTCTTGCTAGAGTTAATCACTGGACAAAGAGCTCTGGAATTTGGTAAAGCAGCAAACCAAAA
AGGTGCCATGCTTGATTGGGTAAGTACAACAAATCCCTATAATTATAGTTACATTTCTCTTTTTCAGCATG
CTTTGACATGACTTCTTGAGCCGAGGGTCTATCACAAACAACCTCTCTACATCCCAAGGTAGAGGTAAGG
TCTGCGTACACTCTACCCTCACCAGACCTTATTAGTGGTATTACACTGGATATGTTGTTGTTGTATATCTG
TAAGGTGTGCATTAACACTATCTAAAATATGAACTCTATAAGGTATTAAGAGATGCAATGAACGAAGTA
CGAACCATCTTTAAATCCTGGATCCGTCCTTCTGTGTTTTTTTACCTGCAGGTAAGAAAGATTCAACAAGA
GAAGAAGCTAGACATGTTGGTGGACAAAGACATGAAAAACGAGTACGATACGATAGAGTTAGAGGAA
ATGGTTCAAGTAGCATTATTGTGCACTCAGTATCATCCAAGTCATAGACCAAAAATGTCTGAAGTTGTAA
GAATGCTTGAAGGAGATGGACTAGCAGAGAAATGGGAAGCATCTCAAAGAGCAGAACCAACAAGATG
CAGCAGAGCTAATGAATTTTCTTCTTCAGAAAGATACTCTGATCTTACTGATGATTCTTCATTACTTGTAC
AAGCAATGGAACTATCTGGTCCAAGATGATAATTGAATTAAGACACAATGTCACTTTTGCTAATGGGAG
GTGACAAGTCCCTCGTGGAATAAGTCGAAGTGCGTGAAAGCTGGCTCAGACATCACATACATAAAAAAA
AAACATGTGTCATATTGAGCTTTGTTTATAAGTTTATACAGAAGCTGCAAATAGAATATCATACATTCTGA
TTGTAAAGGATAATATAATAACTTTGTAAGGCTGAGAGTTGTTTTTCCTTTTTTGGTTCTTGTTTGTTTGA
GTGGAAATGTTTATGGGATGTTATAATTTGCTTATGTGTATATATAATTAGAAATAGAAC
>PGSC0003DMG400002902 LRR-RLKs
AGAAAAGAAACAGAGACGCATTTGCAGATTGTTTCTTTAAGGGGAAGGTGGACATGTCTAGTAAACAT
GGCCGCCGTTCCCCTAATCCTTCATCTCCTTCCTCAAGCATCGTCTCCGAAAAAACACCATCTTTTCCTTAT
TCATATCTCCATTTTCTTCCTCATTTCTCTATCTAATCCACAATTTTCTTTCTCCATTTCCGAGGACGATGCC
TTGATCAAATTCAAAGAATCTTTAAAAAACACAACATCTTTAGATTCATCTTGGCTTAAAGGTACTAGTCC
ATGTGACAAAAACAAGAAATGGGATCGTATTCAATGCGAAGGGAACGTGGTTGAAGGCCTCCTTTTAG
GTGAAGCTGGCTTATCAGGAGAACTAGATATTGATCCTTTGATTGCACTTCCAGGTCTCCGAGTTCTTGA
AATCGCGAATAATTCATTTTCAGGCACAATCCCTGAATTCTTCTTGCTTGGTGCACTTAAATCTATATATAT

AGATGGAAACCAATTCTCAGGTGAGATTCCTAAAGATTTCTTTGCCAAAATGGGGTCTCTTAAGAAGATT
TGGTTTGCACGAAACAAATTCTCCGGTTCAATTCCTGAATCTTTAACCAACTTGAAGTACTTGATGGAACT
TCGTCTTGAAAGTAATGCATTTTCAGGACGCGTTCCATCTTTCTCACAAGTGAGTTTAACATCTATTGATC
TTTCGAATAACAAATTACAAGGCGAAATCCCTCAGGGGATGTCAAAATTTGGTGCTGATCCTTTTAAAGG
AAATGCAGACTTATGTGGACAACAATTAGGGAAAGAATGTAATAAGGGAATCAATAAGACTCAAGGTG
CACCAATGTCAAAATTGAAATGGATCATTCTTGGTATAGTAGTTAGTCTTTTATTGGTGACCATTCTTTTT
CGCGTCAAGCGCAAGGAAGATCATTTCGACAAGCTTGGGAAAGAGAATCTTGATGAGGGGTTGCACGT
TGGTAGCTCGATTAGGAAGAGCATGAGCATTCATAGCAAAGGAGGTGATTCTGGTCGTGGATCAACAC
GAAGGGGAGGGTCACAAGGTGGTAAGGCTATGGGAGATCTCGTGTTGGTGAACAACGAAAATGGTAC
ATTCGGATTGCCTGATTTGATGAAGGCAGCTGCTGAGGTACTTGGAAACGGAGTGTTAGGATCAGCTTA
CAAGGCAAAAATGGGAAATGGAATGTCTGTTGTGGTTAAGAGATTGAGAGAGATGAATAAAATGAATA
GAGATGTATTTGATGCAGAAATGAGAAAACTTGGAAAGTTAAGACACAAAAATTTACTTCAATTATTGG
CATATCATTACAGAAAGGAAGAGAAATTGATGGTATCTGAATATGTTCCCAAAGGCAGCCTATTATATCT
GTTACATGGTATGACAATTTCTTTCATCTACAGTCAAACCTCTCTATAACAACATCGTTTGTCTAGATAAG
TTTTTGGCTGCTATAGCGAAATGTTGTTATCTAGAACATAAGTTTCATATCTCAACTGTTACTCCCTTTGTA
TTGAAACATACCTCGATGATGAGTTGGCCTACACACACCTGTTGTTGATTGAGAACAGATATTTGACCAA
CTCAACATCGAGGTGTGTTTTGATACAAATGGAGTGAATAGTTAAGGTAGGTTAAGAGAAAAATGGATA
GTTAAAGTATGAAACTCGTGAAAAAAGGATAGTTTAGGTGTGTTTTTGACCATTATGGTAAAAATCTATT
CCGAAGAAAACTTGGTCACTATTATAGTGAAATGTTATTATAGACAATGATTGTTATAGAGAGGTATGAC
TGTATATCAAATCATTAAGGTCTTCACTTCTTCTTGACATTTGTATGAGTAGATTTTATCTGATAATGTGAT
CCGATCTATACTTACCTGTCCCTTCCAAGACATCCGGTCACAATTGGGACGATACAAAGAAGATTAGCAT
GATCCCACAAGTTTTAGGATTCACAGTTTACAGAAGGATTGCTTATGACTGTTAGTACATTCATATACATT
TTGTTCTTCTATGTCAACTATCCTAATTTTGAAATTTCGTGCATACTTTCAGGTGATCGAGGGATATCACA
CGGTGAGCTAAACTGGCCTACTCGTCTAAGGATCATCCAGGGAGTTGCTAGTGGAATGTGTTATCTTCAT
TCCGAGTTTGCATCGTATGCAGTGCCTCATGGAAACCTCAAGTCTAGCAACATTCTACTCAATGAAAAAT
ATGAGCCACTTCTATCAGATTACGCCTTCTATCCACTGATCAACAACACGCAAATTGTTCAATCCCTTTTC
GCGTATAAAGCACCAGAAGCCATACAACATCAACATCTCACTCCAAAATGTGACATATATTGTCTTGGAA
TCATAATACTTGAAATCCTTACAGGGAAATTCCCTTCTCAGTATCTCAACAATCAAAAAGGTGGAACGGA
CGTTGTTCAATGGGTGCAATCAGCTATAGCTGACCAAAGAGAATCTGAATTGATTGATCCAGAGATAGC

GTCTGCAACAGACTCCGTTGAACAAATGGTGAAACTCCTTCATGTAGGAGCTGCTTGTACTATAAGCGAT
CCGGATAAGAGGATTGATATGAAGGAAGCATTAAGGAGGATAGAAGAGATAAGTTTAATATGATGAAC
ATAAACCTTCCAAAAGGTAATAGGTTAGTAGATTCGTGTGAAGCTTTAGTTTCGTTACCTGCCAGAGCGA
TCCTTAGCCTCCTTTGATTCCGTTTTATCTCAGTTAAACTCCACAACGTCTCTCGACTCGTTCAAATGAATG
TTGATGCAGGAAACAAATGTCATTTTCTTCAAATACTGGAGCTGAGGTTCATTTGCCAAACAAAACAGAG
TTTACTGTATATAAAACACCAAAATGAGACTTCTAAATTACCAAAAACATAAAGTTGATTACTAATTCTAC
ATGTTTTGTTTTATTGTATAGAATTTGTTTCTTTTTTTGTGTGTGTGAGTTAAAGAGGGACATGGATCCTC
AAGGCAATGATCAATTTAGACATAGATTTTGAAGACAACCTTCCGGCTTGATCTCCTCCTTTCTGCTTATG
TTAGCTCACGTTGTCACACAATCTAATCTAACGCGCTCCCATCAAGAGATCGTTTGATGATTTAGCAAAC
AAGATGATCTAGCTTTGATTTGTCAAAAAACCTATGTTGTTCGGACTCTCCAGAAGGGTTTTCGTGCACTT
GTTAGATCCTCCCAAAATGCACTACTTTTGAAAGATTTGACATGCATCCTATGACATTTG
>PGSC0003DMG400003002 LRR-RLKs
ATGTTTGACACAACTGTATGTGATGAAACAGTGACCAGAGATGCATTGCTCAAGTTTCTTGCAGAGGTAT
CTAACAATAATAGTACAATTCTTGGTACTAATTCGGGCTGGAACTCGACATCTGATCCTTGCAAGGACAA
GTGGCTTGGTGTTACATGTAACGCGAAGACTCTCTCCGTGGAGAAAATCATTCTTGATGGTTATGATTTT
TCGTCTGGAACGTTTGATGTTACCACGATTTGTAATGTGGAGCCTATCGCGCACACCCTTTTCTTCCTGAG
TCTGAAAAATGGCCTACTTCAGGGGAAAAACTTTGACAGTATAGATAAATGTAAGCATCTAAGACATCTT
GTTCTTGGTGGAAACCGGTTCTCAGGTAGCATTCCACAATCATTTTCAAGATTGAATAACTTGAAAAGAC
TTGACATATCCAACAATAGATTATCAGGTGTTCTCCCTGATTTATCTAGGATTTCGGGCTTGATTGAATTC
GAGGCTCAGAATAACCAGCTATCAGGAGAGATACCTGAATTTGATTTCTCAAACTTTGTTGTATTTAATG
TCTCTTTCAACAATTTCAGTGGATCGATTCCGCCTGAGGGTGATAGTTTCCCTGTTAGTAGATACTTGGAA
AATCCTCTGTTGTGTGGCTCCCCGTTGGCTGGAAAATGCTCATCACTGTCCGAAGATTCAGAATCAGCTG
ATCATGACAAGCCAAAGAGCAGCTTGTCAAGAGATGAAATTCTCATGTTTTCAGGTTATTTCCTCATAGG
CCTGGCTCTGGTTCTTCTTATCCTACTATGCGTCTATAGAAAACGGAAGCAGAAGCAGCAGAAGAAGAA
AGAATCAAGAATCGAGCTGATGAAAAAGGTTGCTACAGTTGATCATGACAGTACTACTACATCAATGGA
TCAATCAAAGAAAGGCGCGAGCAATTCAGATATCTCATCAGCTGTCATTTCTTCCGATGACAGCAACACC
GCAGTTTCACAGTCACTCGTGGTTCTTACGAGTCCTGAGGCGAATAACGGATTGAGATTTGAGGATTTG
CTTAAGGCTCCAGCAGAGTTGCTTGGAAGAGGGAAACATGGTAGTATGTACAAAGTGATGTGTGACAA
TCCCAAAATGACACTGGCTGTTAAGAGGATAAAAGATTGGTCAATAACTGGTAGTGAATTCAAGAAGAG

AATGCAGAAACTAGACAAAATCAGGCATCCAAATGTGTTGCCTGCTGTTGCCTTTTATTCTTCTAGACAA
GAGAAGCTTTTGGTCTATGAGTACCAAGACAATGGCAGTCTTCTCCTACTTCTACAAGGTAATTCACTCT
ACTAGTCTATTCTTCTGTGTATTGAATTGTGCGACCATTTCATCTAGGCATATAAACAGACACTCGAACTT
GGCCTCAACTGGCAAGTAAGCACTCAACTTTGAGAGAGTGCACATCTAGACACCTCAAATTGGTCTCAA
CTGACGAGTTGAGGTGTCTAGATGTGCATACTCAAAATTGGAGTGTTTACTTGCAAGATGATGCCAAAA
TTTGAGCGTCTGTTGATGTTTTATCAATTATGACTTTCATTTAAGAACGTAAGTTGCATGAAGGTCTGATT
CTTTTTCATCAGCTAAGCATGTGAACATTCTTATTCATAATAGGTGCATAAAGGTTAGATATCAATATGTT
CTATTACCATATGAAATTGTGAGACGTGCTCATCTAAAGACTTAAAAACTGTTAAAAAGCGCGAACTTTT
GCTTACTTAACGATGTTGGATCAATGAGTAGTATTAAGGCTTTTAGGCATGTGAAAGACTTTATTTAAGG
TACTTATTGTCCCCTCATGATTTAAACATGTCAAAATTCTCTTTTAGAACCTCAGTTAGAAACCTCTGTTTG
CCTTATTATCTTCAACACTGGTGTATGAATTTGCTTTTCAGGAATCCAGACAGGCCAAAGATTCCATTGGA
GTAGCAGACTTTCTATAGCAGCAGGTATCGCCGATGCTTTAGCTTTCATGCATCAGGAGCTTCAACATGA
CAGAATTGCACATGGAAACTTGAAATCATCAAACATTTTCTTGACCAACAACATGGAGCCAATGATCGGC
GAATATGGCCTTATGAGCATTGCAGAAATCAGTACTACTACAGCAAGCAATTCCCATTATGTCTTCCCTAT
AACTGATGAAAATGTAGGAGCAATCTTTAAAGAAGATGTTTATGCATTTGGTGTCATTCTTTTGGAGTTG
CTAACAGGGAAAGTGCAAAACAATGGAATAGAACTTGCAAGTTGGGTTGTTTCTGTGCTAAGAGAAGA
ATGGACAGTTGAAGTGTTTGACAGAACTTTGATACAAGAAGGTGCTAGTGAAGAAAGGATGGTTAATTT
GTTACAAGTTGCTGTTAAATGTGTGAACCATTCTCATGAAGCTAGGCCTAGTATAAACCAAGTTGCTCTC
ATGGTTACCACTATACGAGACGAGGATGACAGATCCGTCGATGTTTCTGCAAGTACTAGTACTATCATCT
GA
>PGSC0003DMG400003175 LRR-RLKs
ATGGGTGGTAGATGGAATACTTATGGATTCAAGTTATCATATCTTGCACTTCTGATTATTCTTTATCACGT
TCACGAATGTAGCTCACTCAATTCTGAAGGTAATTATATTCTATGTTTGGACTGTCCTACTGGTTTTGGTG
TTTATGTCTTAGTTGTATCTAGTTTGTTATGTCCATTTGAATATTGGATTGGTCTATTTTACAAAACAAGG
ATTGGCATTGTTAGGACTACGATCTAAAGTGGATTGTGATCCATATGGAATTCTTGCAAACTGGAATCTT
GATCATTGTGACCCATGTATGTGGTTTGGAGTACAGTGTCTGGCAGGCAAAGTGCAAATGTTGTAAGTC
CCTTGTTACATACGATCATATCTGTTAATGAGTATTCAATTCCATAAAACTTACTTTATAATTGTAATGTTT
TGTTTCTTTTTTAGTTAAGAACAAATCTGTTAGATTTATTTTAATGACAACAGATGTGCAAAAGATGAACT
CATATGTTTTGACTTTTGAGCTATCTCTCATGTTGTTAATTATTTTCTTTTTCTTCTCTGTTTAGGGATCTTC

ATGGGAATTCTTTGGAAGGCACATTGGCACCAGAGCTTGGGAATCTCACTCACTTAAGATCAATGTAAG

TTCTTACCTACGACAAAATGTTTTTTGCTTTTTTAGTTCATAGGTGCAAATTTGTGGGGAATGAAAGAGA

GATTGTTGCTTGTCATGGTTGAAAATTGATTAAAACTTAGTATTGGTTGAAAATAGCCTGGCAGCTTAAA

AATACCTCCTTCTTTTGATTTCTTTTTTAACATGCAATTTTTTATAAAAAAAATTGATTTTCCTCTTCTTCTTT

TGGTTTTGTGCTTAAAAAAACTCCCTAAATAATCTTGTCTTGTTTGGAAGCTCTTTTGAAAGAGCTTGCCT

CTGGACAATGATAGGATTTGAAAGGAGTGTTTCTAAGGATCGTTCTTCCGTTACAATATTCCATAAATCA

TAGGTTTCAAGATATGAATTCATTTTCACTGACCAAATCTGATAGTTTTTGGTAGTGAAAATTTATGGGAC

AATGAAAGAGAGATTGTTACTTGTCATGGTTGAAAATTGGTTTAAAATAGCCTTGCGCATTGAAAATAA

GCATAGGTTGAAAATAACCCCTAATGAGTTCTAAAAAATGGATTGAAAATAGTCCTGGTGGGTTAAAAA

TAGCCTTGACAAGTTAAAAATGGGTTGACAATAGTTCTTTAGAAGAACTCACAGATCCCATAAGATCAAT

GGGGCTTTGGATACTACTGTTGCTATTTTTTTAAAGATGGTGCAGCTAAATAATGTTGAAAATGTCTCTC

AATTTTTATTAGGCATAAGAGGGGTTTAATAGACAACTTGAAAACATAACTTGCAGAAAATCTGCATAAC

AAAAAGTAGTAGATTAGTAAATTGGGTGGAATAGACTAAAGATAATTAGTTCCATGTCTAATAATCATA

GTTTTCATCATCTTGCATGTTGTATCTTCTTGTGTGTTGTGTATGACTCCTTTTTTAATCTTAACAATTTAAA

CATTTTTTTTCTCTATGATTGACTCACAGTGTGCTGTCAGAAAACAATTTTTTTGGTGCCATTCCAAAAGA

ATTTGGATGACTTCGAAGGTTGGAAGTGCTTGACTTGACAGATAATAATTTGAGTGGGAGAATTCCAGC

AGAAATAGGAGACCTGCATGCGCTAAGAAGCTTGTGAGTTCATAATTTAATTTGTGTGCATGTGAAGAT

GTGTGCTGCCTATATTAGTTTATATATGTGAGTATTCATGAGGAAGAACCATTAAAAAGATTCTTTTGAA

CCGTGTCTTATACCTTTTCACAGGTTGATTCGTAACAACAACTTGGAAGGAAAAATTCCATTTGAAATTG

GGAAGCTTCGTTTGCTCTCCAAATTGCAATTTGACGAAGCACTCACCCCTGCTGCTGGGGGAACATGCTG

TTTAAATAGAAAATTTGCACACTGGTAAGGTTGTTTTGGTTTTATTGGACCTTTTATATCTGTGTTCTGGT

TCTTGTTACATTTATGTAACCAAATATCAACATGCAAAGATGTTTGAAATGTATGAAATTTGTATTTAAGC

CATTTCAGGCTGCCATTATTTTCAGAAATGGTCATAACTCCTGTCATAAGGTTTTCTTGGTGGGAGTGAG

GGGGTGGGTGAACACAAGTTGATAAAGTTGAGTATTAATGACAACAAGGAATATTGTGGTTTATGTGA

ATTCAAATAAAGCCTGGGAGATAGGTGATTTCATAGATGTGGCGATGCAATGACTTGTGCTGCTAATGC

TTGAAATTTACTGTCTAGTTTGACATCTAATACTAATACTTGACCATACATCAACTCAAACGAAAAAGCTT

TGTAAACTACAAGGAGGTGATTTTCCAGTCATTGCATCCATCCAAGGCTGGGGTTGAGGATGAGGGATG

CAATATATTGGAGATCTTGGGATTGATCCTGGAATCAAGAGAGCATCAAGTTATCATATTGGTTCTAATT

TATTTTAATTTTTTATCAAGATACTCTTCTCAGATAGCTTTAACTCTTACATAGAATACTGACTATCAAACA

TAATAGCATGGTTACTTTCAATAGCACTTACCTTCTTAATCAGATATTTTCATTTGCCTGCTTCTCTCTGGT
AATTTGATGTCTGTTTCTGGCAGCATTTGTCATGGCAGTTTGATACCATTAAAGACCGTAGACTCCTTTAT
CAGACCACTTAAAGGGACACTTGTGCGTTATTTCACTCTGTAAGTAACTAAATGGTTTTCAATCACCATGT
GTTTCTACTTACATTTTATCACTCTAGAAGCGTCTTTGTGTCAATAAAGGTTCTCCAGACAACTTGGAAGT
GGCTCATTGGATAATCAGATAGGGTATTCCTCTGACAACCTACCAAGTGAGTGAAAAAGTTTTACCCCAC
ATCATTAGCAGGAATTATGTTGAAAGTGCACAAAAGATTGTTTTCACTCTTCTTTTTTAGCAGGTTCATCT
AGGCCTCACATCATCCATACTATTTAGAACCAGGCGAATATCGCACGCCACAAGTTAGTAGAACAATCTA
GCAACCTTGCTGCTGTTTCTGCCAATGCTGGGAAACATTTGGGTCCTATTGTTTCAGTGCCAAGCACCAG
AAGTAGTGGGTCATTTCATGCTGTGAAAAATACCTAAGGAACACCTCCTACACCCTTACCTGCCTCGCAA
CGACAGCCTGAGTCTCAATCCAAACCAAATCCAATGGGACAGTCAAGTAGTGTTGTTAAATAGCCAACT
AATAGACAAGCTCCACCTGCCCAAAAATCTGGTGATACATGGAAGTATTTAGTTGGAGGTTTTGGGTTTT
TTCTGCTCATTGTTGTTGTAGCCGTGTTCTTAGTTTACAGAAGCAGAGCTGCAAAACCAATAGGCCCTTG
GAAGACTGGGTTGAGTGGACAGCTGCAAAAAGCATTTGTCATCGGTACTTCAATTGAAATTTGTGACAA
GAATTACTGTTAATATTCTGTCCACATTCTGATATTCAATGACAAAATCACTGTTGGTTGATAATAGTCCT
AAATCACAGCCATAAAAGAAGGTATAATCCTGATGTTGATTGCAAAAAATTGTTTCTCATTGCAGGGGTC
CCTAAGCTTAACAGGTCTGAGCTTGAAACAGCATGTGAGGATTTTAGCAATATAATTAGTAGTCATGATG
CATATATAATCTACAAGGGAACTCTTTCTAGTGGAGTAGAGATTGTTGTTGCCTCGACTACTCTAACTTCT
CTCGAAGATTGGTCTGAGAGTTCAGAAATAGCCTTTAGGAAGAAGGTTTGTTGTTTCCATTTACATCAAA
AATAAAATATTATCTCTCCATATATCTCACAGTCTTACACTTCACATAATGACATTGTTGTTATCTCTTCAT
CCTTTTCGGTTGTAAAAGACATGACATTTTGTGATTGTGTTATTGAACAGATTGATACACTTTCAAGAGTG
AACCACAAAAATTTTGTTAATCTTATTGGATACTGTAAGGAGGATGAACCTTTCACTAGGATGATGGTCT
TTGACTATGCTCCAAATGGAACTCTTTCTGTGCATCTACACGGTAAGATTCTTTGTTGTCCACAATTCAGC
TTTAATTACTCACAATGATGGCTGGAGTTAGTTCAGAATAACACTTCTTATTATTTTATTTTAAAAAAAAG
ATGTTTAGAATGACTTAAAATATAAGTTTAATTGTGTCAATTGAAGAAACCGATCCTCTTGAATGGTCTG
CAAGGATGAGAGCCATAATGGGGACAGCATATTGTCTTCAGTACATGCATGATCTAAATCCTCCAGTGC
CCCATTCCAGCTTAGATTCAAATGCCATATTTTTGACTGACGATTATGCTGCTAAGGTCTGCTCCATCTTCT
TCAATATGTATATACAATTTATCTACCATATTGAACTAAAAACCATTTTTCATTTGTATTTCATATTGAAGT
AGAATTTTTTATATTTTCACAGATTACAGAGATTGGTTTCTGGTCAGAACTAATAGCCAAGTCTAAGGAC
TCTAGGGATGATTTAGAAAATTCTGAGCTGTCAGCTCTTGCTGATCTTGAAACAAATATCTATAGTTTTG

GGATTCTGTTACTAGAAATAATTTCTGGAAAGTCACCTTATTCAGAAGAACAAGGATCTCTTCTGAACTG
GGTAAATCTCCTTCATATGTTTCCCTTATTTTCTTATACTTGATCGTTACAATGCGTTCCCTAGTCTGCATG
CATTTTCTTGTGTGAATGTCTTTTCTTTCAGACTTATACCAATGCAAAAGCATATTGATAACTATTATTTTA
TTTAGGCGGAGCAATACCTTAAAGATAAGATGAATGTGAGCTCCTTGGTTGATCCAACACTCAAGTCCTT
CAAAAATAATGAGCTTATGGCTATCTATGTGGTAATTGAAGAATGCCTTCAAGAAAATATAAGGAAGAG
ACCAACGATAACTGAAGTCATTGCAAAATTAAGGGAAGCGATTGATATATCGCCTGATGCTGCAGTTCC
AAGGCTTTCTCCCCTCTGGTGGGCTGAACTCGAGCTCTTATCTTCTGAGGCAGCTTAG
>PGSC0003DMG400003210 LRR-RLKs
TCGCAGTATGGATCTAAAAACGTGCTGTATTATATTAAAAAAAAAAAACTGACAGAGACTGACTCCCTCA
TTAAGAATTATTGAACAACCAGCCAAAGCAAAGCATTAAATATTCTCCTATTTTGGTTGTTTCCTAAGTCA
TTCATTACCAAATACCACACTAATTAAGCATATGATTAGCTGAGGACACATATTATGAAACAACATTCTCT
GGCGCCTCAGCCCAAAAATTAGTTCTTGAACTAGCAGCTCAGTGTTTTAAGAGGTCAGAGATGTTCTTGT
TTCTGGTTAATTGCCTTTTCTTCTTTGCCAATTTATTTGCAGTCTTTTCGACCTCATCGCCTCAAGATTCTGT
TCATCTACTTGGGTTTCACAGCAGTCTTCCAGAGCTCTCTCAACAATTGCTCCCTTGGAATCAGTCTGTCT
CACACTGCCGATGGAAAGGTGTTACCTGCTATTCAGACATAACTTCTCATGTCAAATCATTGATTCTAACA
GACTTATTGCTACCTGGAACCTTGGACAAGGCCTTTCCTAATCTCTGCCGTCTTCCTCGCTTGGTTTCTCTT
GATCTCAGTGGCAACCATTTCACTGGTGGCATCCCTGATATGCTTGCTAACTGCAGCCAACTTGACACGA
TTCTACTCAATGAAAACAGATTTTCAGGATCCATCCCACCAGAGATTTTCAAATCAAGCAAACTCATAAA
ACTTGATTTGGGTTTAAACCAGCTCACTGGCACTATTCCCTCTGAGGTAAGCCTTTCTACAAACCTTCAAC
ATCTCGGGCTGTGGAACAATTTCCTTAGTGGAAATATTCCAAAAGAGCTATTTGATTTGCCAAACTTGAC
ACATCTCCACCTCTACACAAACGAGCTGACAGGACCTCTACCCGACTTCCCATTCTCGTGCTCCCTTTCCG
AGTTCTTTATTTATGAGAACCGGTTCTCAGGTTCTTTGCCAATTACCCTAGGCAATTGTCACAACCTTACTT
CATTCTCTGCATCATCTGCTCATCTTGGAGGAGTTATTTCACCAGAGGTTTTTAGGGACCTTTCAAATCTT
GAATTTCTCTATTTGGATGAGAACAACTTTGAAGGGGGAATTCCTGAGACCTTATGGGATGGGAGCTTA
CAAGAGTTAGCTCTTTCCTTGAATAAATTTAATGGTAGTATATCCGAAAAGATTGGTGGTTCTCTTCAGAT
AAATTACATTGATTTGTCAGTTAACAAATTAACTGGTCAGCTTCCAAGATCAGTTGGACGTCTGAAGAAT
CTGAACAAGCTTTTTCTGTATGATAACATGCTTAGTGGTTCATTACCAGCAGAACTTGGGAACTGCACTT
CTCTTGTTGCGATAAGTCTCGCCACTAACTTCATCGGTGGGGAAATTCCTCAAGAGCTCTGCAACCTTCA
CTCCCTAATAAAATTTCAAGTGTACGGAAATCAAATTCAGGGCCAAATTCCAGAATGCATTGGCAGAATA

AGTGGACTGGAGGAGCTGGATCTTAGTGAGAACCGATTGATCGGCAAGATACCACCTGGAATCACAAA
TATGACGAAACTTGTGTTACTCTCTTTGGCTCATAATAATCTTACAGGGGAGGTACCACCTAATATTGGA
AAAAACAATTTTCCTGGGTTATTTAAGGTTGATTTAGGTTATAATAACTTCAGTGGACCAATTCCTTCTGA
ACTATGTAATGGCAACAGGCTTGGAGTCCTGGTTCTTGAAAACAACAGTTTTAATGGAAGCTTCCCAACA
TATATAGCAAAATGTGAATCCCTCTATAGGGTAAAAGTCCCCAACAACAATCTGCAGGGGAGTATTCCT
GACTACATTGAAAAGAATGAGAACATCTCTTACTTGAATGTTCGGGGAAATATGCTTGCGGGAAGGATT
CCAGCAGCATTTGGTTATTGGACCAATCTTTCAACGATTGATCTTTCAGAGAATATGTTTTCTGGTTCCAT
ACCTGCCCAAATTGGGAAGCTGCAAAATCTTGTAAGACTGAACATTTCTTCAAACAGACTGACAGGAAA
AATTCCTCTTCAGTTAAGTTACTCTGCGAAACTGGAAGAGTTGGATCTCAGCAACAACAATCTTTCGGGA
AGAATTCCGAAAGAAATTGCATCATCTTTAGTACTGACAAATCTTCTACTCCAGGACAACAAACTTTCTG
GTACTCTTCCAGACACTTTCTCCTCCTCACAAAAGCTTGTAAAGTTGCAGCTCGGGGACAACTTGCTTGA
AGGCTCAATTCCATGCAGTCTGAGTAAACTTAGGGAACCTAATGTTGCACTGAATCTGAGCATGAATAA
GTTTAGTGGTCAAATTCCTAAGTGTCTGAGCAATCTAGACAATTTAGAAATCCTTGATATATCAAGCAAC
AACTTGTCTGGAGCAATACCATCAGAAATGGACAAGATGAGGTCCCTTTCGTTTCTCAACATATCATTTA
ATAACTTTTCAGGGAAGGTACCCATTTCATGGGGAAAACTATTAAGTTCACATCCAGGAACTTCCCAGGG
AAATCCAGGACTCTGCTTATCAGACACTGAAAGTAGCAGCTGCAAGCATGTGAAAAAATCACAAAGAAA
CTGGAAGACCTTGGCTGGTGTAATTAGTGGATGTGTGTTTTCCGTGGCAGTCATAGCTGCGGCAATATA
CTTGCTAGTGACTCGGATTCAGCACCCTTCCCTGCTGAATAAGCATCGGCTCGTCAAGTATCATGCAAAA
ATTGAAGATCTGCCAGATCGTATAAATTTTGAGGACATTGTACGTGCAACAGAAGGCTGGAGCGAAAAA
TATGTAATTGGAAGAGGCAAGCATGGAACTGTTTATAAGATGGAATCTGCGAAATCCAAAAAGCTTTGG
GCTGTCAAGAAGGTTGACTTAGCTCAGAGAGCATTCAGCGATGAAATGAGAAGCCTAAATTCTGTCAGA
CACCGCAATTTGGTAAGATTGGGAGGACACTGCACCAGACATGGATATGGCTTCATTCTAACAGAGTTT
ATCCCTGGAGGAACTCTTCATGATGTCCTTCACCAGAGAAAACCACCTGTAGTCCTGGACTGGGAGCCCC
GCCATCGTATTGCTCTTGGTGTTGCTCAAGGTCTGTCTTACCTTCATCATGATTCTGTCCCTCAAATCATTC
ACAGAGATCTCAAATCAGATAATGTAATGCTGGATACTGAGATGGAGCCAAAGATTGGAGACTTCGGG
ATTGCTAAAACAGTATCAGACTCTGATGAAAACTCGACAAACTCCAAAATTGTTGGGACACTAGGATAC
ATTGCTCCAGGTTAGCCACACGTTACTATGCTAGTCTTCAGTAATAATTTAGTTGGCAGATTACTTTTGAT
GATTTAATAAATTTTATTATTTTCTCCCCCGGTCCCCCCTCCTTCTCCTTGTCTGTCCATCAATTCAGAGAAT
GCGTACTCAGTTCAATTGACAGAGAAAAGTGATGTCTATAGCTATGGAGTTCTTCTACTGGAGCTCTTCT

GCAGAAAAATGCCAGTAGACCCCAGCTTTGAAAAAGGATTAGACATTGTTTCCTGGGTGAGGAAAAACT
TGCACAGAAGCGACAACAATTTCCTTCACTTGCTTGATGAAGAAATTAGTTTTTGGTACATAGAAGAACA
ATGGAAGGCCCTGAAAATGGTGTATCTAGCGCTTCAATGTGCTGAACTCGAGGCAAGCACCAGGCCTGC
AATGAGGGATGTAGTAAGGTCTCTTGTCGAGTTAAATCATAGATGTAAAAGTGAAGAAATGAAACTTTG
AATAGTGAAAGCCATAATCAACTTTTCCATATTCCAACGTCTTTTTTTTTCTTTGCTTACATAAAAATTGAA
CCATTTCTCGATTTGTGTGTGTCATCCTTGCATTCAAGGGCTAAATTCCAACATAACCTGATCGTGGGTGT
TTCTGTTTAACTTTGTGACATCCACTGCAACATTATAGATTAAGAAAATGCATGACGTTTCAAATCAAATT
TTGGTAAAAGCGGTTTTCGCATTCTAGCCATAGCTCTTTTCATAATGGATCACTGTCCTTAATCAACTTTT
GCTTGTTACTTTCTCCAGCTGCTCCAAAATCTGAACAGTTTTGAG
>PGSC0003DMG400003211 LRR-RLKs
TAAAAATGATGTTTGCGGGATACATAGAGTTGTGCACGGGATACATTAGGTTTAATACATCCCACGTAG
CGGGATACATAGCGTTGTGCACGGGATACATAGCGTTGTGCACGGGATACATAGGTAAAATAAGGGAT
TTTTGAAATTTTTGAAATAAGTAGGGATAAATGGATATTAAGGTAAGTAAAGGAGTGTAGTTAAGTAAT
TTGTCCTAAAATAATCTCCTATATTCATTGTTCCCTCGGTTTCACACGTATCTTGAAAAAACAGGCTGACC
TCAACTTAAAACCTAAATAATAGTCTCAGGTTCCAGCCCAAAAATTAGTTTTTGAACTAGCTCCTCATTGG
ATGCAGCTTCTTTAAAAGAGTTCAGAAATGTTCTTGTTTGATCTTGTTCACTGTTTTATCTTCTTTGCCAGC
ATTGTAGCGGTTTTTTCGGAATCATTGCCTCAAGATTCTGTTCATCTACTTGGGTTTCGAAGCAATCTTCC
AGATCCCTCTCAACAATTGCTCCCTTGGAATCAATCTGTCTCACACTGTAAATGGAAAGGTGTTACCTGCT
ACTCAGACAAAACTTCACAAGTCAAATCATTGTATTTCAGAGACTTCCTTCTATCTGGAATCTTGGACAAG
GCCTTTCCAAATCTTTGCCATCTTCCTCGTTTGGTCTCCCTTGATCTCAGTGGCAACCATCTCACTGGTGGC
ATCCCTGCTATGCTTGCTAACTGCAGCCAACTTGACACGATTTTCCTCAACGACAACAGGTTTTCAGGATC
CATCCCACCAGAGATTTTCAAATCCAAAAGGCTTGTATACCTTGAGTTGGGTTACAATCAGCTCAATGGC
ACCATTCCTTCTGAGGTGGGCCTTTCGACAAGCCTTCAATATCTTGGGCTGTGGAACAATTTCCTTAGTG
GAAATGTTCCAAAAGAGCTATTTGGTTTGCCAAACTTGACAGATCTCTACATCCACACAAATAATCTAAC
GGGACCTCTCCCGGACTTCCCATCCTCATGCTCACTTTCCCAGCTTCTTATTCATCAGAATCGCTTCTCAGG
TTCTTTGCCAATTTCCCTAGGTAATTGTCACAACCTTTCTGCATTCTATGCAACATCTGCTCATCTTGGAGG
AGTTATTTCCCCAGAGGTTTTTAGGGGCCTTTCAAATCTCGAATTACTCTCTTTAGATGACAACAACTTTG
AAGGGGAAATTCCTGAGACCTTATGGAATGGAAAATTACAAGAGTTGGTTCTTTCCATAAATAATTTTAA
TGGAAGTATATCAGAAAAGATTGGTGGTTGCCATCAGATGACTTACATTGATTTGTCAGCTAACAAATTA

AGTGGTCAGATTCCCAAGTCAGTTGGACGTCTGAAGAATTTGTACAAGCTTCTTCTGTATGATAACATGT

TTAGTGGTTCATTGCCAGCAGAAGTTGGGAACTGCACTTCTCTTGCTGAGATAAGTCTTGTCAGTAACTT

CATCAGTGGGGAAATTCCTTCAGAAATTTGCAGCCTTCAAAACCTAGAAACATTTAATGCATTCAAAAAT

AAAATTCAGGGCCAAATTCCAGAATGCATTGGAAGAATAAGCGGGCTGCAGGAGCTAGCTCTTTATGA

GAACCGATTGACCGGCAAGTTACCGCTTGGAATTACAAATATGACGAAACTTTCATTTCTGTCGTTGGCT

CATAATAACCTTACGGGGGAGGTACCACCTGATCTTGGAAAAGATAATTTTCCTGGCTTAGTTAAGGTTG

ATTTAGGTTATAATAACTTCAGTGGACAGATTCCTTCTAAACTGTGTAATGGCAACAGGCTTGCAGTCCT

GGCTCTTGAAAACAACAGGTTTAATGGCAGCTTCCCAACCTATCTAGCAAACTGCAAATCTCTCTATAGG

GTAAAACTTCCCAACAACAATCTGCAGGGGAGTATTCCAGACGACATTGAAAAGAATGAGAATATTTCT

TACTTGGATGTTAGACAGAATATGCTTGTAGGAAGGATTCCAGCTGCATTTGGTTACTGGACCAATCTTT

CAATGATTGATCTTTCAGAAAATATGTTTAATGGCTCCATACCCACAGAACTTGGAAAACTTCAGAATCTT

GTAAAACTGAGCATTTCTTCAAACAGACTGACCGGACAAATTCCCTTTCAGTTGAGTAACTCTGAAAAAC

TGGAGGAGTTGGATCTCAGCAACAACAGTCTTTCAGGAAGAATTCCTAAAGAAATTGCATCATCTTCAGT

ATTGACAAATCTTCTACTCCAGGACAACAAACTTTCTGGTGCTCTTCCAAGCACTTTCTCCTCCTCACAAAT

GCTTGTAAAGCTGCAGCTGGGAAACAACTTGCTCGAAGGCCCAATTCCATGCAGTCTGAGTAAGCTTAT

GCAACCTGGTTTTTCACTAAATCTGAGCATGAATAAGTTTACTGGTGAAATTCCTAGGTGCCTAGGCAAT

CTTGACAAATTGGAGGTCCTTGATATATCAAGCAACAACCTGTCTGGTGCAATACCATCAGAAATGGAA

AAGATGAGTTCCCTTTCGTTTCTCAACATATCATTTAATAGCTTGTCAGGGAAGGTACCCAATACATGGG

AAAAACTATTAAGTTCACGTCCAGGATCTGCCTTGGGAAATCCAGGACTTTGCTTAATAGACACGGAAG

GTAGCAACTGCAAGCATGTGAAAAAATCACAAGTAAAATGGAAGACTTTGGCTGGTGTAATTAGTGGAT

GTGTGCTTTCCATGGCAATCACAGTTGCTGCAATGTACTTACTAGTGACTCGGATTTGGCACCCATCCCT

GCTGAATAAGCATCGGCTCGTCAAGTGTCAATCAGGAATTGAAGATCTACCTGATGGTATAACTTTTGA

GGACATTGTACATGCAACAGAAGGCTGGAGTGAAAACTATGTTATTGGAAGAGGCACGCATGGAACTG

TTTATAAGATGGAATCTGCCAAATCCAATAAACTTTGGGCTGTCAAGAAGGTAGACTTAGCTCAGAGAG

CATTCAATGATGAAATGAGAAGCCTAAATTCTGTCAGACACCGCAATTTGGTAAGATTGGGAGGATATT

GCATGAAACATGGATATGGCTTCATTCTAACAGAATTTATACCTGGAGGGACTCTTCATGATGTCCTTCA

CCAGAGAAAACCACCTGTAGTCTTGGACTGGAAGTCCCGCCATCGTATTGCTCTTGGTATTGCTCAAGGT

CTATCATACCTTCACCACGATTCAGTGCCTCAAATCATTCACAGAGATCTCAAATCAGATAATGTAATGCT

GGATTCCGAGATGGTGCCAAAGATTGGCGATTTCGGGATTGCTAAAATGGTATCAGATGAAGAAAATTC

AACGAACTCCAATATTGTTGGGACTCTAGGATACATTGCTCCAGGTTAGCCACACGTTACTATGCTAATT

TTCAGTATAATTCAGTTGGCAGATTACTTTGATGAACTAATAACTTTTACTACTTTCTTCCCTGGCCCCCTT

TCTCTTTTTGTCACTCCATCTATTCAGAGAACGCATACTCAGTTCAACTGACAGAGAAAAGTGATGTCTAT

AGCTACGGAATTCTTCTTTTAGAGCTTTTCTGCAGAAAAATGCCAGTAGATCCCAGCTTTGAAGAAGGAT

TAGACATTGTTTTCTGGGTGAGGAAAAACTTGCAGAGAAGCAACAATTTCCTTAGTTTTCTTGATGAAGA

AATTAGGCTCTGGAATGTAGAAGAACAATGGAAGGCCCTGAAACTAGTGGATTTGGCGCTTCAATGTGC

TCAACTCGAGGCAAGCACCAGGCCTGCAATGAGGGATGTAGTAAGGTCTCTTGTCGAGTTATGAAGAG

AGGAATGCAAGATGATTTGAAGGCAGGTGCAGTTCAATCCAGAAGATCAAGAGCTCCTGCATTTCTCTT

CTTTATTTTTGGCGTAAAGAACAGTTAGTTGATGATGTTGAATCATTACTAGACTCTATGGCCTCTTTGTA

AGTTTTGTTTGAGGCTGTATCTGTAGATATGATATACCAGGTCTTTATTAGGGTGCTGATGAATATAATA

TGTTACCAAATTCAAAAAGAGAATAGATGTAAAGATAAGTAATGAAACTTTTAGTAGTGAAACCTGTGA

TCAACTTTCCAAATTCCAACATCTCATTTTTTTCTTTTTTTGCTTACATCAAAAAATTGGACAAAGAAAATA

CATGATGTTTCAGTTTAACAAAATCGTGATCTTGGGTTTTTCAGTTTAACTTTGTGACATCCAATGTAGCA

ACATAGACTAAGAAAATGCATGATGTTTCAAATCAATTTTTGGTAAAAGCAATTTTCACCTTCTAGCCATA

GCTCTTTTCATCATGGATCACTGTCCGTAATCAGTTTTTGCTTATTACTTTCTCCAGCTTCTCCAAAATCTG

AACAGTTTTCAGGTATCACAATTAAAATGAGAGTGTGAAGGAACAAGGCTTTGCTACAAGCTAGTCCTT

ATCTTATGGACTATAACATTCCGGATTATAAAAGAAATCAAAAGAACAACCCCCACCCAAAAAGATAAG

CTTTTGTTGAATTTGGTCGTACTAATGATGGTTCCTGCGAAAGAGTGCAGAAGACCTCCGCCTTACCGGT

CTTTCTTTGTGTTCCAAACTTGCAGTAGCAGAATTATTTGCAGAAAATATAGATATGATGAATAGAAATG

GCTTGTGGACAATCATTGTATTTCAAACAAATACAGATATTCAAATAAGGTAGATATAACTCAGATATGA

GTTACCAACAGGACTTCTAACAAGACTTAACAGACGAATGGCAAATACATAGGAATATCAGATCAGGAG

AGTAAAATAATAGTCCTTGAAGAATTGATCAGAATTCAGTAGAAAGGAAATATGAAAGAAGTGCAAGA

ATAAACTGGAAGAAATACTCTGCAGGTGACAAATAGATGAAAGACATACCTCAGTTACGAAAACTGCAA

CACCCGAGCACAATACGATGCGCACCTAAATCATCCAAGTAGGAGAAGCCCAACTTAAATGTCCCAGAT

TCCTTTTTCGTATTTTTTATGTAGTTGACTTGGATGTGCATTACAAGACAACTGTCACAAGATTTTGAGAA

TTTTTCATGTTAAAATATCCACCAGAATTTGCATTGGATATATTCACATGGCACCACATATGCTGACAAAT

CTCAGACTTCTAATAATAGAGATTCCATTCTGAGACAGATCCACTAAATTCCCAAGTTTCTGCTCTTAGCA

AGTGCAGCACCATCACATATGATGATAAGTCTAGGACCTCTAGTAATAAAGCTTCCCTTCTGAGCCAAAT

CCACTAAATTCCCAAGCTTCTGCTCTTAGCAAGTGCACTTTTGTGCAAGACGATTAGAGACAAACTCCAG

GAATTTGGTGCTTGCTCTAATACCAAAGTTACAAGCAGCTCAAAAGAGAAAATATAAAACACTAAACCA
CATTAAAGAAAAGCTAGGAAGCTAAAACCAAAATTGAAGAAGAAAAAGTTCCACACTGTTTCTCTTAAC
AGAACAGGTGAAATTTCTCATAAAGAGCTGATCATATTCTATATCCAATCACTTTACTGAAGTTAGAGTA
CAGATAAATGTTTGAAAACCCTAGTGTCCAAAGATAGGGACAATAGAAATATGAAGCTGTAAAATGCTC
CAAAAAAAAATAATTAAATTACTGAGAGGATTTCCAAAATAGCCTCGAGGTAAAATATTTATACAAGGTT
AAACTTGTCTGCTTCCTCAGTCCAATAAAGCAGAGTTCAGGAGTTTTACTTTGAAGAGACCGAACAAATT
TAGAGAACTACTCCATTACTGGCTATGTAACCTATGGCTAACTAGATTAAATTAGAAGCTCAATGTGCAT
AGACGTGGTGTTCTTGGGTTATACTTAAGACCAGCTAACTTGAAGAAGAATTCATTAGTATACATGGTTA
CGGACTGAGGCACAGTGCAACAGGATAATATTTTCAACTTAAGTAAGAAATTACCATTAGAAAGATAGG
ATGTCCTTGATCACATCATACAGAATTAGATACCTAATGGAGCTTGCATGTCTGCTTAATTAACACTAAAC
AATGTTAACTATTGCATATCCTCGCCACTTGAATATTTTCATGCAACATATTTTTGTCATTTGGATGTATAT
ATGTTGAATGAAATTAAGGAACCTAAAAGTCAGTTCACCAGACAATCTATAATCAGTTATGTACCACATC
TTTTTACCTAATACATTTTTTGTCAATCAACACCACTATTTATACCCTATACTACATATCAAAGGGACAAGA
TGAAGTTTGCAAGGATCAATGCCTAGTTTATACTCAGCATACAAATAAAGCTCATATAAGATACAAAACC
AACTAAACCTCTTTTCATCTCCCAAATATAGTTTGAGCAATAAAATAGAGAGATGGAAAAGTATTATTTG
ATCGCGAAATTATGAACCAGAATAGATTGTGGCAGCAACACCAGACCTCAGTCACAGTCATACTAAGTC
ATCTACTTTGTTGTTTTTCACATACTTTTCTGCTTCAATTTTAATCAAAAGCAGAATGCTGATAGCCTGATA
TGCATTCAACTACAGATGTATGGATCCTTTTTTTTTTTTGATGACAAGGGAAACCCGCAGGTGCGCACAG
GGTAAAACTCTCGCTCCTATGCAATAGCTCGCAAACCACATAGGAGAGGTAACCCGTGGGTGCGCACAG
GGTAAAACTCTCGCTCTTATGCAATAGCTCGCAAACCACATAAGAGAGATAACCCGCACTAGGCAAGGC
TGGTGCGACGAGCTCGACCCAGAGACAAACCCCTTGCTTTCGCTGGCAAGGGGTTTCGAACTTGAGACC
TCCAACATGGAAGTGCCAAGCCCAAACCACTGGGCCACCCCGAAAGGTCAGATGTATGGATCCTTTTCA
CCTAGATATTCAACATCAAGGCATCTCAGAATTCTCAAAATCAATTTACTCCTGGCAACTTCTAATTTTGA
AATCCCTAAGATCATAAAGGGGCTGTGCCCAAGCATTAGAAAACTAGCATTGCCTTCGAAGGTCAAATT
ATCGATTTAATCAATGGTACAGAGGGCTTTTAGAAGCGGAGAAATTGGGAACAGCATTGTACCTGTTAA
TTGTTGATGGAGTCATCGGAATCAAAAGTAACTCAAATTAACGAAGACAAATAAATAAAAATCTCATTTC
TCAACTCAAACTAAATTGGATGGGCATAAATTTGTGGAACCCATTAAGCTTTTTTCTCCAACTTGC
>PGSC0003DMG400003280 LRR-RLKs
GGAAATTCTGTCTCTCTCTGGAGAGCGGCTCATTGTTTCTCCACAAAAAATGGCCGCTGTCCTCTTCCAGC

TCCACCACCTTATTTTCTTATTTGTTTTTTTCCTATCTCATCCAACTCCTTGTGTTTCCATTGGAGATGATCA
AGTCCTTCTTGAATTTAAAGAATTGTTATTAAATACCTCACTTCTTGATTCTTCTTGGAAAAAAGGAACAA
ATCCTTGTGATAATAATAACAAATGGTTTGGTGTACAATGTGATAATAAAAATGTTATTCAAGCCCTACTT
CTTGGTGGGGTTGGCCTTTCAGGGAATCTTGATGTTGATGTACTAATTAGCCTTCAAGGCCTTAGAGTTG
TTAATCTTGCAAACAACTCTTTCTCAGGAACAATTCCTGATTTTTTTAGGCTTGGTGCTCTCAAGTCTTTAT
TCATTAATGGAAACCAGTTTTCCGGAGACATCCCTGGAGATTTCTTCTCCAAAATGGGATCTCTCAGGAA
AATATGGTTTTCGCGGAACAAGTTTTCAGGGAAAATCCCTGATTCTTTAGCAGGTTTAAAATATCTTTTAG
AACTCCATTTGGAGAATAATGAATTCACAGGACCTATTCCAATTTTGTCACAGGCTAATCTAGCAACTAT
AAACTTCTCAAACAATAAATTACAAGGTTTAATTCCGCAAAGTTTGTCTAAGTTTGGTTCGAACCCCTTTC
AAGGAAATCCTGATTTGTGTGGAAATCAAATAGGGAGGGAGTGTAAGGCTGTAGCCTCTGGTGAAAAG
TCTGAAGGTTCTGGAAGTACAAAATGGATAATTATAGGCTTAGTGGTTGTTTTGCTATTGGTGGCTATAT
TATTTAAGTCAAAGCGTAAGGATGATCAATTTGAAAAGCTTGAAAAAGAGAGCCTTGATGAGGCTGTGA
AGGTGCACCTTAACAAGAGAAGCATGAGCACGCGCACCTCTATGCGTTCATCAAGGAAAGGACGCTCAA
GAAGTGGCAGCGATATGGGTGATCTAGTTGTGGTAAATGATGAAAAGGGTATATTTGGGATGCCTGAT
TTGATGAAGGCAGCAGCTGAAGTCCTTGGTAATGGAGGATTGGGGTCAGCTTACAAGGCGGTATTGGG
GAATGGAGTGTCCGTCGTGGTGAAGAGGTTGAGGGAAACCAATAAATTTAACAAGGAAAGTTTTGATG
CAGAGATCAGACGACTCGCTAGGATAAGGCACAGGAACATATTGCAACCATTAGCATACCATTACAGGA
AAGAGGAGAAGTTGGTGGTGTCTGAATACATTCCCAAAGGCAGCCTGTTATACCTATTACATGGTATCG
ATATTTCTTCTTTGCCTCTCATGTCTCATCTTCAATGGTAAAAGAGTCCATGAAAACAGCCTATATATTATT
GTGTACAACTTAATGCTAGCTTCTATTTCATGTGCATAAGTAGAGACTATCTCATTATGGGATATCATTTA
CGTTTATCAAATGGTCTGTTAATCATTCCCTTGAAATGACTCTTTTAAAGTTTTGGGATTACATAATTCATA
AAAATGATCTTAGATAGCCCGAAACATTTCCATTCTTGTAATTGAGGTAATATTCACATTATGTATTTAGC
ATGCCAATTTGGGATCTGCTTCTATCAATATTATAAACTCTTTTTATCCAAAATTTTCAGGTGATCGGGGG
ATAGCACATGCTCAGCTAAATTGGACTATTCGCGTTAAAATCATCCTGGGAGTTGCCAGTGGAATGAAA
TTTCTTCATTCAGAGTTTGCATCATATGATGTACCTCATGGGAACCTTAAGTCTAGCAACATTCTTCTTTCT
GCAAATAATGAACCACTTCTCACAGATTATGCGTTTTATCCACTACTCAACGATTCACAAGCTGTCCAATC
TCTTTTTGCTTATAAATCCCCAGAAGCCATACTAAACCAACAAGTCACTCCAAAAAGTGATGTATATTGTC
TTGGAATCATAATACTTGAAATCCTAACTGGGAAATTCCCATCACAATATCTCAGCAACCAAAAGTGTGG
AACTGACGTTGCACAATGGGCGTGGTCAGCAATTGAAGAGAATAGAGTATCAGAATTGATTGCTCCAGA

GATAGAAACAGAAAAGGATTCCCTTGAAATGATGGAGAAGTTCCTTCATATAGGAGCTGCATGCACTGA
GAGTGATCATGATAATAGAATCGACATGAAGGAAGCCATAAGGAGGATAGAAGAGATAACAAATTTAA
TGTAGTAATTGGTTAGATGATCAGGAGGTTTCAATTTATTTTAGGGAGGAGGGAAAAAAAGGTACATAA
ACCTGTTTGAGTTCTCCAACTAATGATCCACGCACGAGCTCTTCCCAGTAGAATGTGGGCATTTTACGTTC
CCTGAGATGAATGCTGTTTTCATTTGATTTGACTTGCCTGTATCGAACTGGTGTGACATGGGCGTGGATA
CTTTGTGAGGATTCTTCAACATGCACTTAATGTTAGTAGGAAACTTAAAGTACCCATACGAAGAATTACA
CAAACTTGTATATTGGATACATTAACATTATGCATTTTCAATTTTTTCTCTCTATAGTTCATTTTTTTCAGAT
AACCAATGCAGATACTCG
>PGSC0003DMG400003429 LRR-RLKs
AAGCAACACGTCCACACTTCCAAACCACAAAAAAATAAAAAATAAAATGTCTTCTAGTCATTTTCTTTCAT
TCCTTTTCCTTTCTCTTCTAATTTCCGGGATCTTTTCGGACCTTAACGCGGATAGAGCCGCTCTACTCCACC
TGAGTGCGGCCATCAAAGGCCGCACTCTTAGGTGGAACACAACCAATTCAACCCCATGTTCATGGGAAG
GTGTCGAATGTGATACTACAATCAACCGTGTAATTGAACTCCGTCTCCCCGGATATGGTCTATCCGGTGA
GATGCCCTTAAATTCCATCGGAAACTTAACTGAACTCCGTACTCTCAGCCTTCGGAGTAACTCTTTATCCG
GTCTTCTTCCTTCTGACATAGGATCATGTACAGAGCTACGTATACTTAATTTGGAAAACAACGATTTCTCT
GGTTCAATTCCAACGACTTTTTTCAATCTGAACAATCTTATACGCGTGAGTTTTTCAGGGAACAGGATCTC
CGGTGAGATATCGGACGCGTTTAACAATCTGACTAGGATGAGAACTCTGTATTTGGAGAATAACAACTT
TTCTGGCTCACTTCCTGATTTGAAAAACCTCTCACAGCTCAATGAATTCAATGTGTCTTTCAATAGATTAA
CCGGTTCGATCCCTTCGAGTTTAGGTCGATTCTTAGCTTCTTCATTTCTTGGAAATTCACTCTGTGGCTCTG
CTTTCTCTCTTAGTCCTTGTCCTGAAAACAATAGCCTCACCAGTCAATCTGATAAACTTTCCGGCGGAGCA
ATAGCTGGAATAGTAATTGGCTCGATTATCGGATTGTTCATACTACTATTAGTTTTGTTTATGTTAGTGAG
ATCAATTTATAGGAGTAGAAAAAGCTTCCGTCAAGTGAATGTGTCTCCTACGCCGAATCAAGTACTTTCA
AGTCCTCATGATTCAATAGTGACAGAAAATCATGATATCGAAAATGTTTTTTCGGAGAGGAAAGTTTGTG
AAGATAGGACAAAAGGGATGGTGTTTTTTGGGGAAAGTTTTGAGTTGTTTAGTTTGGAGGATTTATTGA
TGGCGTCTGCGGAGGTTTTGGGGAAAGGGGCAACTGGAACTACTTATAAAGCATATTTGGATAGAGAT
GTTGAAGTTGTTGTGAAGAGGTTGAGAAATGTTTGTGTTTTAGAGGAGGAGTTTAGGGGAAAAGTGGA
GCAATTGGGAGGAATTAGTCATGGGAATTTGGTGCCTCTTAGAGCATATTATTATGGAAGGGATGAAAA
GCTTGTTGTTTATGATTCTATGCCTACAAGTTTGTATGCTGTTTTGCATGGTAAGTATCTTATATCTAATAA
ACTTTAGCTTATTGTTGTTTTAATAATAATATTTGCTACTTGCTTCTTTTAGGTAGTCTCTGGTTTTGGTTA

GGGAGTAAATCAAGTTAATTAAAATTCTTAGCTTAGATTATATTAGCTTCTAGTTAATTGTTTTTTAGCTTT
AGCTATGAAAACTGAAATTATGTGGAAGAGATGGTATGAAAACAGGGGAAAGGAAGAAGATCATTTGG
TAGTTCTCTTTTACATTAGTTAATTGTCATCAGATCCACTGTCGCTACTCGCTATAGGCTTAGCAGAATTTA
CAAAGAGTGTTAAAATGTAAAGTCACTAGTAATTGCCTCTAAAAATATATTTAGCAGCAATTAAGCTATT
GTCGTAAATTAATAGTGGCTAAAGATAACTTTTGATGTTGTGTATATTGATGTTCACATCACAACTAATG
AAGGACCCATGTAATATTATTAGAGAGAACAAGAACTTTCTGTGTAGAGGGAAAGCCTCCTTAATTAGT
ATTTAGTTGAATAGGCCTTCTATCAACTCACATTCTATTAAATGTTACGGAAACGAACTATAGAATTTTAC
TAGTATTAATTATTTCGTATTCTAACCAATTACTATTACTATGAGTTAGTTTTAACCTTTCTGATTTTGTTAG
GCGAAGGAAAAGAAGCATTGACATGGGTAATAAGGAGCAGAATTGCATTAGGAGTTGCCAATGGTATC
GAATATCTCCATTCACTAGGCCTTAAAGTCACTCATGGCAACATCAAGTCATCTAACATCCTCCTCACACA
CTACTACGATGCATATCTATCCGAATTTGGCATAACACAGTTGATTTCTTCAACATCAAACTCCAAAATGA
CTGGATACTACGCGCCTGAGGTGACAGATATTCGTAATGTTTCCCAGAAAGCCGATGTCTATAGCTTTGG
CACGATTCTGTTGGAACTCCTAACGGGTAAAAATCCTAGTAGTGTCATCAACGACGAGGGAATTGACCT
GCCTAAATGGGTGAAATGTATAGTTGAAGAGAGGGGGACAACACAAGTGTTTGATCCTGAGTTGATTA
GATTTCAGAATTGTGATGAAGAGCAAATGGTTTCATTGCTGCATCTTGCTATTTCTTGTACATCCCACCAC
CCCGAAAGGCGGCCGCCTATGGCTGACACTACCAGGAGGATCAAAGAAATTGTTATGTGAGTTTTGTTA
ATGTTTGATCTTCCCCCCTCATTCTTGTCCAAATGAAGGGGCGAATTCATACCTTCAAAATTGGGCACGTG
AATATGTGATCTCTCTATAAAACTAGATATTTTATGTATATATATTTTGAATTAATATAATATTATTTATTG
CCTC
>PGSC0003DMG400003551 LRR-RLKs
GTTAAAAAGTGCTGGTCCTTGTTTTGAAAAGTGCTCATTTGCACATTCTCCACTTCCTCCCTCACTCTTCTT
CTCTGTGTCACTCTGTGCCTCTGTGCAAAGTGAGGGGGCCTCTGCTATGGCCATTAACACTCATCTTCTCC
ACTTTATCATGCACCCATTTTAACCTTCATTATAAACCAAGCCAACTCCTTTTTCTTATGTTCTTGATTTCTT
CTTCTTCTTCTTTCATGGCTGCTTATCTACTTCTGTTTCTTGTTTTTCTCTCTACATTATGCTCTGCTCAGAG
AAACTCACAAACCCTTTTGGAAGTTCAAGCGCTTACTTCTTTTAAACTTAGTATTCATGATCCACTCGGTG
CACTCACTGACTGGGATTCTTCTTCCCCTTTTGCTCCCTGTGATTGGCGAGGTGTTTTCTGCGTCAATGGC
CGGGTCGGTGAACTTCGTCTCCCTCATTTGCAACTCAGTGGGCCACTCACTACCCAAATTGGTAACCTGC
GCATGCTGCGCAAGTTAAGCCTCCGGTCCAACTTCTTTAATGGTACTGTTCCAGCTTCGTTATCCAAATGT
ACCCTTTTGCATTCTGTTTTTTTACAGGGTAACGCATTTTCCGGTAATCTTCCGCCGGAAATATTTAACCTT

ACTGATTTACAAATCTTTAAAGTTGCCGGAAATCAGTTGTCTGGTGAAATCCCCGGAGAGCTTCCTCAGA
GTCTACGATACTTTGATCTTTCGTCAAATTTGTTTTCAGGAGATATTCCGAGGAAATTTTCTGACAGGTCT
CAGTTACTTCTGATTAATCTTTCTTATAATCGTTTTTCCGGCGAAATTCCGGCGAGTCTCGGCCGGCTTCA
ACAGCTTCAGTACCTTTGGCTTGCGTACAACAACTTGGTAGGAACTTTGCCCTCTGCAATTGCTAACTGTT
CATCGTTGGTTCACTTGAGTGCTGAAGGAAATGCTATAGGGGGTCTTATTCCGGCAGCCATTGTTGCTTT
ACCGAAGCTTCAGGTGATATCTTTATCACATAATAATCTCTCTGGTTCCTTACCAGCTTCATTGTTCTGCAA
TGTTTCCATTTATCCTCCCTCCCTTAGGATTGTTCAGTTGGGTTTCAATGCATTCACGAATATTGTCAAGCA
AGAGTCTTCTAAATGTTTTAGTTCTTTGCAAATTTTGGATCTTCAACACAACCAAATACATGGTGAATTTC
CTTTGATTTTGACGAACATTTCTGCATTGACGTCGCTGGATGTGTCATGGAATTTGTTCTCTGGAAAAATC
CCAAGTGCCATTGGGAACTTGTGGAGATTGGAGGAACTGAGAATGACAAATAACTCATTTGAAGGTGCT
CTTCCATTTGAGATTACGAACTGCAGTGACTTGAAAGTTCTTGATCTTGAAGGGAATCGAATGACGGGT
GACATTCCAATGTTTTTAGGTGATCTTAGAAGCTTGAAGACATTGTCACTGGGAAGAAATCAGTTTTCGG
GTTCCATTCCTTCTAGTTTTAGAAATTTGAGTAATCTCGAAAATCTCAACTTGGGAGGAAATGGTCTCAAT
GGAAGCTTGCCCGAGGAGGTAATGGGTTTGAGCAATTTAAGCACACTGAATCTCAGTGGAAACAAGTTT
TCTGGAAGTATGCCAGTTGATATTGGGAATCTTCAGCAGCTCTCGGTTCTGAATCTGAGTAAAAATGGCT
TTTCAGGCACTATTCCTTCTAGCATTGGGACTTTGTATAAGCTAACGGTTGTTGATTTGAGTGGACAGAA
TTTCTCAGGTGAAATACCATTTGATCTTGCTGGTTTGCCTAATCTACAGGTTATAGCTTTGCAAGAAAACA
AGTTGTCTGGTAATGTTCCTGAAGGTTTCAGTAGCTTATTGGGTATGCAATATTTGAATCTTTCTTCCAAT
TCCTTTTCTGGCCATATACCATCAACATTTGGGTTCTTGACTTCATTACTTGTACTTTCTTTGTCTAACAACC
ACATAAATGGTTCGATTCCTCCCGACTTGGGCAACTGCTCTGCTCTGGAGAATTTGAATCTTCACTCAAAT
TCATTGAGTGGCCAAATACCTGCTGATCTCGGACGTCTTTCCCACTTGAGTGTGCTAGATTTGGGTAGAA
ACAACTTAACAGGTGAAGTCCCAGTAGATATTTCCAATTGTTCATCCTTGACATCACTCGTGCTCGACTTG
AACCATCTTTCAGGGAACATACCAGAGTCACTATCCAGGTTATCAAACCTAACTGTCCTTGACCTCTCTAC
TAACAACTTCACTGGAGAAATCCCAGCTAATCTTGCCATGCTCTCTAGCTTGGTGAGTTTCAATGTGTCAA
ACAATAATTTGGGTGGCCAGATTCCGGAGATGTTGGGCTCCCGTTTTAACAACTCATTGAATTATGCTGG
CAACCAAGGTTTGTGTGGGGAGCCATTGGAAAGAAGATGTGATACATCTGGTGATGGTGGGAATAAAT
TGATTATGTTCATTGCTGTGGCTGCCAGTGGAGCTCTTCTCCTTTTATCATGCTGCTGCTTATATACTTACA
ACTTCTTGAGGTGGCGCAGGAAGCTCAAAGAGAAAGCAGCTGGAGAAAAGAAGCATAGTCCTGCAAG
GGCTAGTTCAAGGACCAGTGGTGGTCGTGGCAGTGGCGAGAATGGTGGACCCAAACTTGTTATGTTCA

ATAACAAAATCACACTCGCTGAAACAATTGAAGCAACTAGAGAATTTGACGAGGAACATGTGCTAAGTA
GAACACATTATGGAGTGGTCTACAAGGCCTGTTACAATGATGGAATGGTGCTCTCAATTCGTAGACTTTC
AAATGGATCACTAGGTGAGAACATGTTCAGAAAAGAGGCAGAATCACTTGGCAGGGTGAAGCACAGGA
ACCTAACAGTTTTGCGTGGGTACTATGCTGGCCCGCCTAACCTCAGACTACTTGTCCATGATTACATGCCT
AACGGGAACCTTGCAACACTGCTTCAAGAAGCATCCCACCAAGATGGTCATGTCCTCAACTGGCCAATG
CGCCACCTCATTGCACTTGGCATTGCTCGTGGCCTGGCTTTCCTTCACTCATCCCCAATGGTCCACGGAGA
TGTTAAGCCGCAGAATGTCCTATTCGATGCAGACTTTGAAGCCCATCTTTCAGAATTTGGCCTAGGCAAG
CTTGTAGTAGCCACACCAACCGAGCCCTCCACATCAACTTCAGTTGGTACCCTAGGCTATATATCTCCAG
AAGTAGCATTAACCGGAGAAACCACAAGAGAATCAGATGCTTATAGCTTTGGCATTGTTTTGCTGGAAC
TACTAACAGGAAAAAGACCCTTAATGTTCACACAAGATGAGGACATAGTGAAATGGGTGAAGAGACAA
TTGCAAAGAGGGCAAATTTCCGAACTATTAGAGCCAGGATTGCTCGAATTGGACCCTGAATCATCAGAA
TGGGAAGAGTTCTTGCTAGGAATTAAAGTAGGCTTGCTATGCACAGCACCTGACCCACTTGATCGTCCCA
CCATGGCCGACATTGTGTTCATGCTCGAAGGCTGTCGTGTGGGACCTGATATCGCGTCTTCAGCTGATCC
CACATGCCAACCTTCACCCGCCTGAATCCCCCGAGGTTCATTCTAATTCTTTTCCCTTTTTTATTATTAGCT
CTGTATAAGTTGTGAGAGTAAATTTGCAATGCAATGCAACGCAACAGTAGCTAGCTAGCAACTTAGTCG
TTCAATTTCATGCTTTTTTTTTCTTGTAGATTTTTTTAAGGTAAAAGAGAAACAATATTTCTCTTTTATTGAT
TCTTGTTTTATGCTATTATTCAAACACCTCACTCCCCATGGTGCCGGGATTTTTCTCCTTTGGCGTGGGGA
CCAAAATGTAGTGCTATCAATAAGTCAAAGAAAGCTAA
>PGSC0003DMG400003714 LRR-RLKs
GCTCAACACATGGCGTTAGTCTTTGTCAGTGCTGCTTCTTCTTCTCCTTCTGAAACTGAAACTGAAACTTT
TTTTTTTTTCAGCTCATACGCTCCTCTCTTCCCCACTTCCATAACAAACTCCATTAACACCCACCATTCTTGA
TTTTTCCCCCATTTGATCTTCTTCTTCTTCTCTCTATCATTACTCACCACCACTCTTTCTCATGTCAACGGCTA
CTTTTCTTCTCTCTCCGCCATTGGTGGTGGTGGCACTTACATTGCTATTTTCCTGTGTTTTTTCCATGGCCG
AAAACGTTACAGAGACGGAAATGGCAGCCTTAATGTCGTTCAAACGGAATTTGGAAGACCCACTTGGG
GTATTAGACGGTTGGGATTTTTCTACCTCATTAGCTCCGTGTGATTGGCGTGGGGTTCTGTGTGATGCCG
GCAGAGTTCGTGAACTCCGCCTACCTGGGTTGCAGCTGAGTGGACGGCTAACTGACCAGGTTGGTAATT
TACGCCAGCTACGGAGGTTAAGCCTACATTCTAACAACTTTAATGGCTCTGTCCCACGCTCTTTAGCTCAA
TGTGCTTTGCTACGCGCTGTTTACTTCCATTACAACTCTTTTTCCGGTGAACTCCCGCCGGCTATCTCGAA
CCTCACTAATCTTCAAGTTTTAAACTTTGCTCACAACTTCCTTTCCGGTCATGTCTCTGGTAATGTTCCAGC

AAGTCTCCGGTTTCTCGATCTTTCTTCTAATCTCCTCTCCGGTGGCATTCCGGGTAACTTTTCAGTTGGTTC
TCAGCTCGAACTGTTAAATTTGTCGTTTAACCGGTTCTCCGGTGAAATTCCGGCGAGTATCGGAGCTTTA
CAGAAGTTAGAGTATCTCTGGCTTGATTCTAACCAACTTTACGGCACTATACCTTCAGCAATCTCTAACAT
TTCCACATTAATACACTTGAGCACTAGTGATAATCATCTGCAGGGATTAATCCCGGCAACAATCGGTTCA
CTTTCCAGTTTACAGGTAATCTCATTGTCGCAAAATCAACTATCTGGTGTGGTCCCTGCATCATTTTTCTGT
AATGGGTCAGTAAATGCTCATGCTATTAGGATCATTGAGCTGGGTTTCAATGCATTCACGGGTTTAACTA
AACCTGGGAATGCAACATGTTTGAGTGCTTTAGAGGTCTTGACCCTTCACGGGAATCACATTAATGGTGT
TTTTCCTGAATGGTTGACGAATTTTTCGTCGTTGAAGGTTCTTGATATCTCCGGGAATGCAATTTCTGGAA
CTCTGCCAGATGATATAGGGAATTTAAGGTTTCTTGAGGAGCTTAGACTGGGTAACAACACATTAACTG
GTGATATTCCTGCTAGTATTGTGAACTTTGGTTCATTGGGGGTACTTGATTTAGGTGGAAATCGGTTTTC
TGGATTGATTCCACAGTTCTTGGGTAAATTGACAGGGTTGAGAATGCTGTCTCTTAATGGGAATCGCTTT
TCTGGTTCGATTCCGGATAGTTTGGGGAGTCTTTATCAGCTGGAGTTTTTGGATTTGAGCATGAATGATT
TGAATGGGAGTTTGCCTCAAAACCTTATGTTGCTTAGCAATTTGACTAGTTTGAATTTGTCCAGCAACTTG
TTTTATGGTGAAATTCCAAGAGGAATTGGAAGCTTGCATGGGTTAGAGGTTTTAAATGTTAGCAATTGT
GGGTTCTCAGGGAACATTCCGACTAGTATCGGAAGTTTGTTGAGGCTGACAACTCTTGACGTGAGCAAG
CAAAACCTATCTGGTGAATTACCCTTTGACATTTTCGGGTTGCCAAGTCTACGAGTTGTGGCTTTACAAG
AGAACATGTTAACTGGGGATGGTCTGGAAGGTTTTAGCAGTTTATCTGGTCTGGAATATTTGAATCTGTC
ATCTAATGCCTTTTATGGACAAGTTCCGAAGACATATGGTTTTCTTACATCCTTGAAAGTTCTTTCTATGTC
TAAAAGTGGTATTAATGGCTCAGTTCCAGCAGAGTTGGGTAACTGTTCCGGCCTTCAAGTGCTTGAGCTT
CGAGGAAATAAGTTAACAGGTCAAATTCCGAAGGATTTCTCACATTTATCTCATCTAAGGAAGCTTGATT
TGGGCCACAATGGATTAACCGGGGAAATCCCTGAAAACATCTCTAATTGTTTATCTTTGGCTACTCTCTTG
TTGGATTCTAACCATATCTCAGGTCACATACCAGAGTCGTTGACGAAATTATCAAACTTGGAAATGCTGG
ATCTCTCTTCAAATAATTTGAATGGATCAATTCCTTCAAGTCTGTCCCTGATTTCCAGTCTGAAGTATTTGA
ATATTTCCCATAATCATCTCGAGGGTGAGATTCCAGAGGCATTGGGTTCTCGGTTCAAGGACCCTTCTTT
GTTTGCTGCGAATAATGCTTTGTGTGGAAAGCCATTGAAGGAGTGCAATGACGGTAAAAGGAAAAGAA
AGAAGTTGATCTTATTCATTGTTTTGGCTGCTGTAGGGGCCTTTTTCGTGGCAGTGTGTTGTTGTGGGTA
CATCTACAGCCTTATACTTTGGCACAAAAAGTTAAGAGGTTCAGCTGAAGGGAAGAAGCGAAGCCCAG
GCCGTGCGAGTTCAGGGGGAGAAGGGGGCCGTGGAAGTGGAGAGAATGGAGGTCCAAAGCTTGTTAT
GTTTAACAACAAGATCACTTATGCAGAGACTTTAGAAGCGACACGACAATTTGATGAGGAAAATGTGTT

GAGCCGTGGAAAGTATGGCCTAGTATTCAAAGCCACTTTTGCAGATGGAATGGTCTTGGCTATCCGCCG
CCTTCCTGATACATCAATCGAAGTGAACACATTCCGCAAGGAAGCTGAATCCCTCGGCAAGGTGAAACA
TCGCAATCTGACTGTTGTTCGTGGATACTATGCAGGACCACCACCTGATGTTCGATTTGTTGTATACGATT
ACATGCCTAATGGAAATCTTGCCACACTTTTACAAGAGGCATCGCATCAGGATGGCCATGTGCTCAATTG
GCCAATGCGCCACCTGATTGCTCTTGGCATTGCTCGTGGCTTAACTTACCTTCATTCAGTTTCATTGATCC
ATGGAGATGTAAAGCCACAAAATGTATTGTTTGATGCAGATTTTGAAGCTCATCTTTCGGATTTTGGTTT
AGATAAACTAAGCCTAGTAGCAACTCCAGCAGAGACTTCAACATCTTCCACCTCAGTTGGAACTCTAGGC
TACATAGCACCTGAAGTGGCATTAACTGGACAACCCACAAAAGAAGGTGATGTATACAGTTTTGGGATT
GTATTGTTAGAGATACTAACAGGAAGAAAGCCAGTAATGTTCAATGGGGACGAGGACATTGTCAAGTG
GGTGAAGAGACAACTACAAAGAGGCCAAATTTCAGAATTGCTCGAGCCGGGGTTGCTTGAACTGGATC
CTGAATCATCAGAATGGGAAGAGTTCTTGTTAGGAATCAAAGTTGGTTTGCTTTGCACCATGCCTGATCC
TCTTGAAAGACCATCGATGACCGATATTGTATTCATGCTCGAAGGCTGCAGAGTTGGTCCTGACATTCCC
TCTTCAGCTGATCCTACAACTCTTCCTTCACCAATGTGATATGAAAATTCAATTCTTTGCAATGAAATCAAT
TTGCAAGAAAATGTCCTTCATTTTTCTCAGTTCAATATTGTTCATGCATTTCTTCAATGTATTGCTACTCCA
TTCATTATCATTTTGTTTGTACATTGTTCATTCAAGAGTTGGCTGGTTTTGTGCA
>PGSC0003DMG400003790 LRR-RLKs
TTGAAAACCAAATATTACAGATGACAACATTTCATACTACCTTAGTTTACATCTTGTATGTGCTACTTCTTT
TTTCACTTCCATTAAGTATCACATCTTCAGCAAGAACAGAAGCTGAATCTCTTGTCAAATGGAAGAGAAA
TTTACCTTCTACTTCTTTCTTGGACACATGGTCCATTTCGAATCTCGAAAACTTGTGCAATTGGACATATAT
TGTGTGTAATGATGGTGGAACAATTTCTGAGATCAATCTTTCTGATGCAGCCCTCTCAGGCACACTTGAT
CACCTTGATTTCACTTCATTTCCGAGTCTCGTGAATTTCAATCTTAATGGAAATAACTTCAGTGGATCGAT
ACCTTCGAGTATTGGCAATGCCTCATTGCTCACATTCTTGGACCTGAGTAACAATATCTTATCAGGTGTCA
TACCTGAAGAGATTGGAAAATTGAATCAACTTGAGTATCTGAGTTTTTATAACAACAATATCGAAGGTGT
GATGCCGTATCAGATTAGCAATCTGCAGAAGGTAATGCACTTGGATCTTGGATCAAATTTCTTAGAAACT
CCTGATTGGTTGAAGATGAGAAATATGCCTGTGTTGACGTATCTAAGCTTTGGTTATAATGAACTGAGGT
TAGAATTCCCTGAATTCGTACTTCGTTGTCATAATCTAACATATCTTGATTTATCGATAAATCATTTCAATG
GTTCGATTCCTGAAACAGTATTTACCAACTTGATCAACCTTGAAAGACTTAATCTTTCATCTAATTCATTTC
AAGGTTCATTGTCACCAAATTTTACTAAGTTGTCTAAGTTAAAAGAACTTCAGCTAGGTGTTAACATGTTT
TCTGGTTTAATTCCTGATGAAATTGGCTTGATTACTAGTCTTGAAGTTCTTGTACTTTTCAACAATTCATTT

GAAGGAAAGATTCCATCTTCTATAGGTAGACTCATAAATCTTCAAAAGTTGGATCTTCGAAAGAACGATT
TGAATTCAACTATTCCTTCTGAGCTTGGCTTTTGTACTAAACTCACTCTCTTAGCTCTAGCAGAAAATGAC
CTGCAAGGGTCATTGCCTTTATCATTCTCCTCTCTTGCAAAGTTATCTGATTTGGGGTTGTCTGATAATAG
TCTTTCTGGTGAAATCTCATCGAATTTCATCACCAATTGGACTGAGTTGACATCTTTGCAGCTTCAAAACA
ATATGTTTACAGGGAAAATCCCACCCGAAACCAGTCAATTGACAAACCTTGTATATCTTTACCTCTATCAT
AACAATTTCACTGGTTCCATTCCCTATCAGATTGGGAACTTGCAAAACTTGTTGGATTTAGACTTTTCTGA
TAACCAGCTTTCAGGCATAATACCTCCAACCATTGGAAATCTGACCAATCTAAAGATGTTGCAACTTTTTC
GCAACAATCTCAGTGGAACCATTCCTCCTGAGATTGGAAAATTGATATCCCTTGAAACCATCGACATCAA
TACCAACCGACTAAGTGGTGAGCTGCCGGACAGCATTTCTGACCTCAGTGAGCTGAAGTTTCTTTCTGTA
TATACTAATGATTTCTCAGGCAGTGTCCCTAAGGACTTTGGAAAGAACAGTCCTCAATTGTCAAGTGCCA
GCTTTGCAAATAACAGCTTCACTGGTGAACTACCTGCTGGATTGTGTAGCCCGAATCTTGAGGAGTTGAC
GATAAATGGGAACAAATTCAGTGGGAAGTTACCAGATTGCTTGAAGAACTGCACACTTCTAAGACGAGT
ACGGCTTGAAGGCAACAATTTATCTGGTAATCTTGCAGATGCATTTGGAGTGCACCCGAAACTTGTTTTC
CTTTCTCTTAGTGACAACCAACTTTCAGGTGAACTCTCACCTGACTGGGGGAAATGTGAAAATCTCACAA
GTCTAAGAATGGATGGAAACAAATTTTCTGGTGTAATCCCAAGTGAGCTAGGGAACCTGAGAGCGTTGC
GCGTGTTAGCTTTGGAAGGAAATGAATTGACTGGTGAAATTCCCTCTGAACTGGGAAGGTTAGACCTGC
TCTATAATCTCAGCTTGAGTAAAAACAATCTTACTGGAGGCATCCCTCAGTCTGTTGGCAATTTAACTAA
GCTCCAGTATCTTGATTTGTCGACAAACAAGTTAAGTGGTAACACACCAGTAGATCTTGGGAAGTGTGA
GAGCCTGTTGAGCTTGAATCTTGGCAACAACTCATTATCAGGTGGCATTCCATCTGACCTTGGTAATTTG
ATGCAGTTGAGTATTCTTTTGGACTTGAGTGGCAATTCACTAACGGGAACCATTCCACAAAACTTGGCCA
AACTCACCTCATTAATGCATCTCAACCTCTCACATAACAACCTCTCAGGTAGAATTCCTCCAGCATTATCTC
AGATGATCAGTCTTCAGGAGATGGATTTTTCCTACAATGAGTTTTCTGGACCAATCCCAACCGATGGAGT
ATTTCAAAGAGCACCCGCTAGATCTTTTCTTGGAAACTCTGGTTTGTGTGGAAATGTAGAGGGATTATCC
TCATGTAATTTGGATACTCCCAATGACAAGTCCAGAAATAATAATCAAAAGATCCTCATTGGGGTACTTG
TACCGGTGGTCAGCCTCATACTTTTGGCAATCCTGTTTGTTGCATGTCTCGTGTCTCGAAGGAAGGCTAA
GCAATATGATGAGGAGATCAAAGCTAGCCAGATACATGAAAATACAGAATCTCTGATTTGGGAAAGAG
AAGGGAAGTTTACATTTGGTGACATAGTGAAAGCTACTGAAGATTTCAGTGAAAAGAACTGCATTGGAA
GAGGAGGCTTTGGAAGTGTCTATAAAGCTGTTTTGCCCTCTGGGCAGATTGTTGCAGTCAAAAGACTCA
ACATGTCAGACTCAAGTGATATTCCATTGACTAATCGTCGAAGCTTTGAGAATGAGATTAGAACTTTGAC

AGAGGTGAGACACAGGAATATAATTAAGCTCTTTGGTTACTGTTCCAAGAATGGATGCATGTACTTGGT
TTATGAGTACATAGAAAGAGGTAGCCTGGGGAAAGTTCTATATGACAATGAGATGGGAATGGAACTTG
GATGGGGTACAAGAGTGAAAATTGTGCAAGGAATAGCTCATGCACTCGCTTACTTGCATCATGACTGCT
CTCCGCCCATTGTTCACCGTGATGTATCATTGAATAACATTTTGCTTGAATCAGAATTCGAGCCACGACTC
TCTGACTTTGGCACAGCAAAGTTGTTAGCTTCAGACTCATCAAATTGGACCACAGTTGCTGGTTCTTATG
GCTATATGGCACCAGGTATGTTACATACTCATTACCTAATGCATCCATATTTCTGTTGATTAAGTAGTATA
TTCATTGGAAGGATACTTCAATATTATTGTGTCTTTCTTCTTGTGCAGAGCTTGCACTTACCATGCGTGTT
ACAGAAAAGTGTGATGTTTATAGTTTTGGAGTCGTAGCGATGGAGACTATGATGGGAAGGCATCCAGG
GGAGCTTTTAACTTCATTATCAGCATCAACAACATTGTTTCCAGAAATACTTTTGAAGGATGTTCTTGACC
AAAGACTTCCACCTCCCACAGGCCACTTGGCAGAGGCAGTGGTTTTTGTGATCACGATTGCCTTGGCATG
TACACGTACCACTCCTGAATCGCGACCAACCATGCGTTCTGTAGCACAAGAATTGTCTGTTCAGACTTTG
CCTTACCTTCCACAGCCATTGGGGACAATAGAAATGAGCAAACTAACAAGTTTTCAGAAATAATGAGAA
GATAGAGCCTCATGTACAGGTGTGAAATGTACAGGCTGTAGAGTTTTAGTGTTGTAATGCAACTTCCTCA
AGATCTAGAATCTATATAGTATCAAGGATGAAACCTTGTATCTATTCAAATTACTATGTTTTGGTTGCACT
TTTCCCTAGAAAGAAAGAAATAATATCATCCTGTTAATCATGAATGTTACAAAGTTGGTATATTAATTTG
GACAAGTATGAATGTTACAAAGTTGCACAACCTTATCCTAGTTTTCACTTTATTGCTAGGCAAAACACTA
GCAAAAAGATACAATGTGTTCACTTGTAATAAAAATAGCAACCTAACAAAAATTTGGCAAAGAATACAT
CACACAGAAACCAGGCATGGTACTTAGCATTCCACTCCACACTGCTTGAGTATAGGTCCCTTCTGCTTTG
GTAGGAATGGGTCTATTCGTACCCAAACGAGTGAGAAGATTGAAGCAAGAAGGATTGACCAGAGGACC
ACAATGGTGGGAGTCCTGTTCTGCCTGCCCATCAATCCCTTTAGGAAAGGATAGAGATGCACAATAACC
CAAAAGGCAAAGAATAACTTTCCGAAGAGAGGACCCCATGAACCATAACCATTGTTGATGGCATCCGAA
ACACCAGCTACAACACCAACCATATTCAAGATGATCAGAGTGGTTGGTGGGATGAGAAGTGTGGTCCAT
TTGAAAAGGTAAAGCTCCCCAAATTCAGCGTCATCAGCTGCTTTTGATGTTACAGTGAAGTTTGTGTCTA
CACCAGCAAGAACTTTAAGAAGGCCTTGGAAAACTGCAAACAAATGTGCTGAAACACCACCGATGACCC
AAAATTGCTCGTTACGCCACCAGGCCTCAATGCTTACTCCACTCCATCGCAGCTCGAGTACACTTGTGGC
TATGATGGAGATAAAGAGAGCCATGAACCAAATGCTTGCAAAGTTGTTAAGCTACAAAACAAACATGGA
TTTTGATTTATAAAATCTAAATCAAATCTTTATATTACTGAACCAAATGACTTCAGTTCACTTACTGTGGG
GACGATAAATTTTCCAGTGAGAAGGCATACAGCTGGAAGGGTGCAGTAAGCTAGCAGAGCAATGGAAG
TGAATGGGTAAACAATAGTGTTAATGTATGCTAGCCTTTCCAACCACTTGAGTTTTCCACCCCAGGCATA

CCAAAGGGGACAATGACGACTCATGAAGATTTCGACAGAACCAAGAGCCCATCTCAGGACTTGGTGCA
ATCTATCTGATAGATTGATTGGTGCTGATCCCTTAAAAGCGGCTCTCTTCGGACAGCAATAAACCGATCT
CCATCCTCTGCAATGCATCTTGAATCCAGTCAAAATGTCCTCTGTGACAGAACCATAAATCCATCCAA
>PGSC0003DMG400003918 LRR-RLKs
TCTATCTGGGAATCTTTTAGTAGGAGAAATACCACAAAACCTGTACAACTTGTCAAACCTTGTATATCTTG
ACTTACATCATAACCAACTGAATGGAAGTATTCCATCAACCATTGGAAATTTGTCTAACTTGCACTTTTTG
GATTTATCACAAAATCTGCTATCTGGGTCAATCCCTGTTGCTCTTGGGGATTTGCAAAATTTAACTCATTT
TAATGTATCTTACAACCTTTTATCTGGTGCTATTCCCTCAATTGAGAGCATCAAGAAGTTTGGACCCTCTG
CCTTTTTTCATAATAGTGGTCTTTGTGGTGATCCTTTAGAAGTTTCATGCTCTGCCAGTGGCACTACTCCT
GCAAAAGGGAAACCAAAATTGAGTGTCTCAGCTATTGTTGCTATTGTCGCTGCTTCAATAATCCTTACAG
GCGTTTGTCTGATAACCATCATCAATATGAAGGCTCGACGGAGAAGGAGGAGAGAAGATGAAACATTC
GTTGTAGAGAGCACGCCACTGGCTTCAACAGATTCAAATGTCATTATAGGAAAGTTGGTTCTTTTCAGCA
AGACTTTGCCTTCAAAATATGAGGACTGGGAGGCTGGCACAAAAGCCTTGCTAGACAAGGAGTCTTTGA
TTGGTGGGGGGACAATTGGATCAGTTTACAGAACTTCCTTTGAAGGTGGTGTATCAATTGCAGTGAAAA
AGCTCGAGACATTGGGGAGAATCAGAAATCAAGATGAATTTGAGCATGAAGTTGGCCGGTTAGGGACC
CTTCAGCACCCCAATCTTGTTGCTTTACAAGGGTACTACTGGTCCTCTAGCATGCAGTTAATATTGTCGGA
GTTTGTTCCAAATGGAAACCTATACGACAATTTACATGGACTTAGTTATCCTGGAACTAGCACTGGTGCT
GGTAACCCAGAATTGAATTGGCCAAGAAGGTTTCAGATAGCAGTTGGAACAGCTCGAGCACTCACATAT
CTTCACCATGATTGCAAACCTCCAGTCCTTCATCTTAATGTCAAGTCCACTAATATACTCCTGGATACAAA
CTATGAAGCCAAATTGACTGATTATGGCCTGAGTAGGTTCCTTCCCCTGCTGGATAACTATGGACTAACC
AAGTTTCACAATGCAGTTGGTTATGTTGCACCAGAGTTGGCGCAAAGTCTGAGACTAAGTGATAAATGT
GACGTTTACAGTTTTGGGGTGATTCTGCTGGAGTTGGTCACTGGGAGGAAACCAGTAGAGAGCCCAGT
AGCAAATGAAGTGGTGGTCTTGTGTGAATATGTTAGAGGATTGATAGAAAGGGGTGCTGCTTCAGACT
GTTTCGATAGAAGTTTGAGGGGCTTTGCAGAAAATGAGCTGATTCAGGTAATGAAGTTAGGCTTGATTT
GTACTTCTGAAATATCCTCAAGGAGACCTAGCATGGCTGAGGTAGTGCAGGTTCTTGAGTCCATCAGGA
ATGGCTTGGGATCATAGTAAAGAATCAGTAGAGCCATAAGACTATTTGAAACAGCAGCTGTTCATAGAA
TGAGAAGAAAGCAGTCTAGCTGTTGTGCTAGACTCACTTCTCATGAGATTTGATGAGGGAATCAGAAAG
GGTCTCAAATAAGAGAGACGCGCAGACCAATTAATAGTATCAGTCATATTCTTGAATTTGGAATCAGAG
TGATTAGGAGGTGACAGCTTTTGGTACAAAGAGAAGATTCTTTAGTATTTGATTTGTGATTCTTTTAATCA

ATTGGTCATTGTAAGTTTACAATAAAGCAATTCTTTTCCTGTAAGATGCAAAGGTATTTTGATGTCTCATA
TGCAGCTTGGCAACTGCTTTTTATAACCATTCAATATTGCTCTTCATTTACTGTTAAGAATTACCTTTGACA
TGCATATATCAACAACCTTTGAAGGGATGAAAGAAATCGATTCATTTCTCGAATTACATATCTAGTGCTA
ACACAAGAATGAATGTGAACAAATAATGAGTCCATACATCTACCTGTAACTATACAACAATGAGATAATA
AGAAAATATCAAGATTTAAGACCGACCTTATTGACACAAAGAATTTGGAAATGGATTTCATTTGAAGCCA
TTTCTACATGCTATATGTTTTTCCTTGCATCAACTTTGACTGCTAATTTAGCTATCTTCAAAATCCATAAACT
GGGAGGAGGAATGCATTTGTGAAAATGACAGTTGGAGAGGACCAAGAACACACTTCTGGAATCATCTG
CATACAATCTGGCTTTTTTTTGTTTGTTTTCTCTTCATGTAGTTCTCAGTGCTGCTGCATTCTGATCTCATTA
TCCGAACATTGATAACTTATGGATCCATTAAATCACATAACACGCGACCCTTAAAATCTCTGCATAAGTTT
TGGACCTTTGGTTACGGTTTACTGTAAGGGATCATATCATTAGCAACTTAACTCAGAGACTTACGTAACT
CCAAAACTTTCTGGATACGTTGCATAACTGTGGAAATGTCCTTCTCACTTAATGTCGAAAAACATAACCT
GAACCACCCAGGTTCAACACAGTGGCAGGAAGAACCAGGAGTTGCATTGATCTTAGCTACATTTAGTAG
ATTATCCCAAAGCTCAATCTCACCCTTCTCATTATACGACCGAATTAACCTGCTCATGTCAGCCCAACAAT
AGAAACCCCCACTACTTTTGGTGCACTCAATTCCCAGCTGCTTCAATCCACTGACAAATAGAGAAGACAT
CTTTCTTAGCCTCTCTCTGTTCTTTTTGATAAATTGTTGTACAAACTTTGCATCTGATAGCATCTGGATGAT
TAAATGCTGTGTTGGGGCTGAAATGGATGAGAATCTTGTCAACTTTTTCGCAGCAGCTAGAACATTTTCA
TTGCAAGAATATATGACCCCCACCCTGAAACCAGGAAGAGAAAGGTCCTTAGAGAGACCATACACAATG
TGTACCCTGCTCCTGTCAAAATCTTCAGAATCAATTATCTCTGCTATGCTGACAAATTCTTCACGTCCATAA
GTTGACCCTGCCAAGATTTCATTGGATATTACATGGATGTTCTTCTCAGTTGCAAAGTCTAAGAGGTTAT
AAAGTGTCTCCCTAGAGAAAATATTTCCCACAGGATTTGAGGGGTTAGAAATGATAATCCCTCGTACTTT
AAGGCCACGTTTCTTAGCTTGGTTAAAAGCTCGGTCAAGAGCGTCAATGCTCAAGTTAAAGTTGTCTGCA
CTGCGGCAAGGAACAGGTATAATCTCCACTCCAGTTCTCCATTTGACATCCCTATCAAGACTGCAAACGC
ATTGAGCTCATTTTATACACTATATACGTAAAGATGACCAAAAGATTGAAATTTATTTGGGAAGGAGAAT
AAAACTCACTCAGGATAATATGGTGAGGGAACAAGAAAAGCATTTCCAGGATCAGCTAGGCAGAAGCT
CAGTATCTCAAGCGCAGGAGCGGCACCACCAGTTAGAACCATCTGCGAGGGGCTGAAGGACACTGATC
TCTCCAGAGCTTGAGACATAAATTCTCCCACAGCCTGCATACAGGTGAGGAAACATGAAAATCAATTCAA
AGAGATACCATAAATAATGTAGAATCACACAAAACACCTATTGTTTTTATGAATCGATCTTCATAGTGGC
TCAAGGATGATTTCATATATTGCTGTTATACAATTCTCAAACCCACAATTTTAAAAGGTAAATCCATCAGA
TCATCTCCCACCCTAGGATACCCGATGACAAAGGTAGCCACTCATATTTCAAAGAGGCCAAAATTGCCTT

CCATCTTCAACAGATACTGCAAATCTAGCAATAGATCCTTCAACAGGTTACACAATGATGAGTATCGAGG
TGAGTATTTGTCAAGGAAATGATAGATCACCTCTCAGAACAACTGAGTCCATTATTCACTATTAACTTGCT
TATAACGAAAACCATGCAAAGTAAAGCAGGTTAAACTATTCTCTTATGTGCTCAAACTAGATTTTTTATCA
TATCACACTGCAAGGGATCCTATTGTTCCGGTCAGGACAAACATAACTCAGAAAATCATCAAATGCTGAA
ACAGCATAAATGACTGAGCTAGATCAACAATAATTATGAATTTCCAACTCAAGTTTTACCAATGTAACGC
TCAACTAGGAGAAGATATTTACCACAAAAAAACACCCCCATCCCTCTGGATACTGTTTCAATGCTTTGGC
TGGTCAAACACATTTTCAGGTTTCAAAATTTTCCTATCATTTCTCTTCTTTTGTATTTTAACCTCCTTTATTA
GACAATCATGGGCTCCAAAAGCTCAAGAAAAAAATCAAAACATTCAGAACCGTGTTTATTTTTCAAAGTG
TTGCTTAGCGTCGATAGCGTAGAAGTTAGAGCATTGACGAACTTTTCAGAAGTTAATTGGCACTGTAAA
ATGAACGTGAAGCTTATTCAATGCTTTTCCTTATGTTTGTTGCACTTTATAGACTTAGAAAGCTAAAGACG
GGACATACTAAAACCTTGTATACAATAGGTAATTTTGGGATTGTCAAATGCCATAACCAATCCCGCATCC
AGGGGTTCACATTTCAAATTAAACCAGCTATAAAGCACACTGGACTAGCGGATGGGTGATTGATATGGA
GTAACTGTTACACAAGCATATGAACATAACATTTTTGAAATTATATGGACATGACATGAGCCCATCCTAG
GTTACAACTATAAACTAATACCACCCAAATAGGTATCTCGTTGATTTAATCCTATAGTCTAAACTCAAAGT
AGCAACTTTAAATTATACATACAAACTTTTTCAAGTAAATTTTTTGTTCAATTTCCTTCTCTAGCCTGTAAA
AACTATAACCATATGAACAACTACTAGTACTTTATTAGTTCAAGAAGAACATATTATATCCAATACATGTC
AACATTATATCAACAAACAAGTCACTAGTAGTGTGCAAGGAAAAATCAAAAAGCTTACCACTTTCAACTC
CAACAACCCATCAAATGGCTGATAAGTAGCTATTCCGGTTATACTCGAATCTTGAGTCATCATCCATCTTG
ACACATTTTCTGCAAGCCATTCTTGAACTAAATCCAATGACAACTATAACCATACAAATAGAGACAAAAT
TCAAAATCATTGAAACAACAAACAAAAGTAGATTCCCCAACAAGAAATGTACTGCATTTGGTACATTTCT
CTAGAAAATGATTCAATATATCCAAACATTCAAAATTATTAGTACCTTGTTTTCTGCTAATCCTAGCTGAA
TAACACCATCTGGATTACTTGACTCATCATAAGGGTCTTCAGAAGCTCTTTTCAATCCAACATAGTAAGG
GCTACTATCAATTTGTTTTGAAATAATCGAATTAGCTCTAGCAGATACCTGAGATTGCCCAGTAGTTCCCT
TCGGTGACAGTAAAACACGTGAGTGAACCCTTTGTAACCCAGACCCAGAAGACACTTCCGGCAAATGGG
TCGACGACGTTGACCTCGCCGGAGCCGTAGATTCGCCGTTATTGTCACCACCGGAGGAACGATTTCGTTT
TAGGTAGAGTTGCCAAAAATAGAAAAGAGCACAGGGTATAACTGAACCTAAGAAAAGACCACCCCGAC
CTTGAACTACACCTTGTAAGGGTACTATCACCCTCATCGCCGTCGTGGCTCCTCCTCCGTCTCTGCCGCCG
GCGCCGCCAGTTGAAATGGTTGTTGTTGTTGTTCTAGTTGGTGAACGGTCTCGTGACCTTGTCATAACTG
TGACATAGTGGGGGCAGAGAGAAAGACGTCACCTTGTCGGAGTATGACGTCAGTCAGCTATCTGACCG

GCGAGACGGTGGACGGAGAGTACGAAGGGAGGTGAGTGATGGAGAAGAGATTTGGAGAAAATGGTG
GCAAATAGTTGGTCGAATTATTATTTATCTTTTTGTCCGCCTAAAGTTGTTTATTTT
>PGSC0003DMG400003945 LRR-RLKs
ATGAGAAAATTAGGTACATTTGTTCATTGTGTTGTTCTTGTTTGGTGTTTTTGTTTGCTTGAATTGGAATT
GGTATCTCCCATCACTGAAAAGGAGATCCTGCTTCAATTCAAGGGTAACATTTCTAGTGACCCTTTTAATA
GTTTAGCTTCTTGGGATCCTAATGGAACACCTTGCTTAGACTTTAGTGGTGTTTTCTGCAATTCTGTTGGA
AATGTGGTGAAGATTGTGTTGTGGAATACTAGTCTTGGTGGGGTTCTCTCCCCTGCCTTGGCAGAACTTA
AGTCTTTGCGCATCCTTACCTTGTTTGGGAACCAATTCACTGGCAATATTCCTGCAGAGTATGGGGGGAT
TGATTCATTGTGGAAGATTAACTTCAGCTCTAATGCCTTTTCTGGTTCAATTCCTGAGTTTCTTGGGGATT
TATCAAATATAAGGTTCCTTGATTTGTCAAGAAATGTGTATAGTGGTGAAATTCCTGTGTCTTTGTTTATG
AATTGTAACAAAACAAGGTTCATTTCCTTGTCACATAACAATCTTTCAGGTCCAATTCCAGCATCAATTGG
AAACTGTCAGAGTCTTGAAGGAATAGACTTGTCTTTTAATGGTCTTAGTGGAAGTTTACCTTCACAAATTT
GTGACATTCCAGGATTAGTGTACTTGTCTGTGAGGAGCAATGCACTATCAGGACTTGTTCAAGAACAGG
TTTCTCCATGTCAAAGGTTGGAGCTTTTGGATCTTGGTAGCAATGACTTCTCTGGTTTGGCTCCTTTTGGG
GTTCTTGAGCTGGTGAATCTTACATATTTCAATGTTTCAAGTAATGGTTTTGAAGGGGAGATTGTTAACT
CTGGGACTTGTAGTCAGCGACTGGAAGTGTTGGATGTTTCAAGGAATATTTTTTTTGGTGAAATTCCCTT
GAGCATCTCGAAATGCAGCGCGTTGAAGTACTTGGACATGGCATATAACAGAATTAATGGAAGTATCCC
TATTGAGCTTGCAGATTTGAATAGCCTTTCCGTTATGCGATTGGGTGATAATTTGTTAGGTGGGACAATC
CCAACGGAGCTTGGTAGTATCGAATGGCTTGCAGTTTTGGACCTCCATAATCTCACCCTATTTGGTGAAA
TCCCAAATGAGATAAGCAACTGCAAGCTTCTTCTTCAACTGTGAGTATATCACCATCTTAGTTTAATTTGT
TTTATTTCATCATTGCTCTGATCTGGAATTGAACCGTCTAATATTTGCTGCAGGGATCTATCTGGGAATCT
TTTAGTAGGAGAAATACCACAAAACCTGTACAACTTGTCAAACCTTGTATATCTTGACTTACATCATAACC
AACTGAATGGAAGTATTCCATCAACCATTGGAAATTTGTCTAACTTGCACTTTTTGGATTTATCACAAAAT
CTGCTATCTGGGTCAATCCCTGTTGCTCTTGGGGATTTGCAAAATTTAACTCATTTTAATGTATCTTACAA
CCTTTTATCTGGTGCTATTCCCTCAATTGAGAGCATCAAGAAGTTTGGACCCTCTGCCTTTTTTCATAATA
GTGGTCTTTGTGGTGATCCTTTAGAAGTTTCATGCTCTGCCAGTGGCACTACTCCTGCAAAAGGGAAACC
AAAATTGAGTGTCTCAGCTATTGTTGCTATTGTCGCTGCTTCAATAATCCTTACAGGCGTTTGTCTGATAA
CCATCATCAATATGAAGGCTCGACGGAGAAGGAGGAGAGAAGATGAAACATTCGTTGTAGAGAGCACG
CCACTGGCTTCAACAGATTCAAATGTCATTATAGGAAAGTTGGTTCTTTTCAGCAAGACTTTGCCTTCAAA

ATATGAGGACTGGGAGGCTGGCACAAAAGCCTTGCTAGACAAGGAGTCTTTGATTGGTGGGGGGACAA
TTGGATCAGTTTACAGAACTTCCTTTGAAGGTGGTGTATCAATTGCAGTGAAAAAGCTCGAGACATTGG
GGAGAATCAGAAATCAAGATGAATTTGAGCATGAAGTTGGCCGGTTAGGGACCCTTCAGCACCCCAATC
TTGTTGCTTTACAAGGGTACTACTGGTCCTCTAGCATGCAGTTAATATTGTCGGAGTTTGTTCCAAATGG
AAACCTATACGACAATTTACATGGACTTAGTTATCCTGGAACTAGCACTGGTGCTGGTAACCCAGAATTG
AATTGGCCAAGAAGGTTTCAGATAGCAGTTGGAACAGCTCGAGCACTCACATATCTTCACCATGATTGC
AAACCTCCAGTCCTTCATCTTAATGTCAAGTCCACTAATATACTCCTGGATACAAACTATGAAGCCAAATT
GACTGATTATGGCCTGAGTAGGTTCCTTCCCCTGCTGGATAACTATGGACTAACCAAGTTTCACAATGCA
GTTGGTTATGTTGCACCAGAGTTGGCGCAAAGTCTGAGACTAAGTGATAAATGTGACGTTTACAGTTTT
GGGGTGATTCTGCTGGAGTTGGTCACTGGGAGGAAACCAGTAGAGAGCCCAGTAGCAAATGAAGTGGT
GGTCTTGTGTGAATATGTTAGAGGATTGATAGAAAGGGGTGCTGCTTCAGACTGTTTCGATAGAAGTTT
GAGGGGCTTTGCAGAAAATGAGCTGATTCAGGTAATGAAGTTAGGCTTGATTTGTACTTCTGAAATATC
CTCAAGGAGACCTAGCATGGCTGAGGTAGTGCAGGTTCTTGAGTCCATCAGGAATGGCTTGGGATCATA
G

>PGSC0003DMG400003958 LRR-RLKs

TATTTTCTAACTAACTCTCTTTTCTCCTTTGACAATTCACACCAATAAACTCCCCATTTTTCTTTCTCTCTACT
GTGAAGCACCATTGAAATCCAATGCAAAAGCTCACTAGTACTTCATAAAAAACTCAACTTTTCACTCAAA
TCTGAAAGTTCACAAAGTTCAGAGAAATGGGGGTTAATTATTTCCTCTTAACTTTCATTTTTGCTCTGTTT
GTTCTTGAATTTTCTTGCATAGAACCTGTTTGTGGAAATGCTGAGGTGAGAGCTTTGATGGAGATTAAAT
CAGCTTTGGACCCAGAAAGCAAGAAACTTTCTTCATGGACAAGTGATGGTGATCCATGTAGTGGTTCTTT
TCTTGGTGTGATTTGTAATGAACATCATAAAGTAACAAATATTACTCTGACAGATAAAGGACTTACTGGA
AAGTTGTCACCAGCAATTGCTGAGCTGAAGTGTTTGTCTGGTCTTTACTTGCATTATAATTCTTTAACAGG
GGAAATACCAAAGGAACTTGGGAATTTGACTGAGTTGAATGATCTTTATCTCAATGTGAACAATCTTTCT
GGGATTATACCACCAGAACTTAGAAACATGGCAAGTCTACAAGGTTTGTAGCCTTTGTAAACACCTTTCT
GTTTAATTTTGAGGGAGAATGTTTCTCTTTTTTTCGTAGCTCCCACCTTTTTGCTGCCTTTGTGACTCGATC
TGTCGTGATTTCTAAAAACAGTTGTATTAAATGATTTGTCATTTTCGAAGTTTTAAGATAAAATAATTATC
CTTTTCTCATTCTACTTTTAATAGTAATTGTTCTTGAAGATCCTGTTTTGGTGACTCGAACCGACAACCTTA
AAGTTGGAGGTAGAGAGTGCTTACAACTTGAGCAACCCCTGCTATCAGGGAAATTGCTTGCTGTTTCATT
TTACTGTTTTTTGTGTGTGCTGCTGAATAATGAGTCTACATGAATTTAGGTAAACAGAGTAGACAGGTTG

CAAAATTTCTTAGAGATCTATGCAGATTTTTTGGTTCTGTAAAGATAGGACTTTGTGGGGATTGATGGTT
TACTCGTTTTTTTGAGTTCCTTCATCCATAAATTAAACAGTGCAAGTTTGAGAGAAAATAAAGTGTTACTA
GTATATATATGAATTGTTGAATTTCCTAACATAAGGAAAGCTTTAAGTACAACAGTAGAAGTGGCTCAAA
ATTGCTTTAAGTAGCAGATTCGAATTCCAAGTATCCCTTGTTAGAATTCCTACTTCCGCCACTGTTGCACC
CACACCCCCTTTTCTGGTCTTTATTCTTTCTTGTTAATTATATTTTTGGTCTGAACAGCATACTGAAATTTCT
TTTCCAAATAAGGTGATGAAAGAAATGCAAGAACTTCTTTTTTTCCCCTTTCTACAATGATATGTGCTCTG
AGATGATTTGTGCATTTTGTGGTTCCACAGTGCTGGACTTGAGTTGTAACCAGTTGACAGGGAGCATACC
AACAGAGATTGGGTTCTTGAAGAAGTTGAGTGTTCTTGAATTGCAACGGAACAGGCTGACAGATAAAAT
TCCAGAGAACTTAGGAGTCCTCGGGACGTTGAAAAAGCTATATTTGGGATTCAATCAGCTATCTGGTCC
GATTCCATTAAAATTAGCTGCTGCTCCCCAGTTGGAGGTTCTAGAAGTACAAAATAACACCCTCTCTGGA
GTTATTCCTCCAGGTTAGTTGCCCTTTTTGAGTTCTCAACATTTTATAAATCCTTTTTGCCTATCTTGAAGTT
ACATCTTTTGAATGTGTTTTGATTTTAAACCTCTTTTTCTTCTTTTTATCTGGACTGTTTGTAGCTCTAAGGA
GATTAAATGAAAAAATTCAATTATGAAAGCAATCCTGGTTTATGTGGTACTGGATTTACTTCATTGAGAGT
TTGCACTGCCTGGGATCATGTCAATGTGAATCAAGTTGGTTCTGATGTACCTGATTCCAATAATAACGGT
GTCCCTATAGATGTCCCAGGAGCTGCTCATATTAGTCCGTTGCCTTGTAATCAAACTCATTGTTCAAGATC
ATCAAAATTTCCTCAAGCAATTATCGTTGCTTCTGTGATTACAGTCACTGTAACTTTGGTAGTAGCTGTAG
TTTTCGGTATATTTAGACGAAGAAGGCTAAAACAAAGAGTAGGGAACACAAGTGATGCATCTGATGATA
GGCTTAGCACTGATCAGGCAAAGGAAATATACAAGAGAAGCCCTTCTCCACTACTTACTGTTGAGTACTC
AAATCGTTGGGACCCTATGACCCCTGAAAAGAGTTGCAATAGCATGTTCAATGAGTTCTTAAGTGGATTC
AAGTTCAATTTGGAAGAGGTTGAGTCTGCAACTCAGCATTTTACAGAGACAAATCTATTGGGACGGAGC
AATTTCTCTGCCGTCTATAAAGGAATTTTAAAAGATGGATCTACGGTAGCTGTAAAAAGCATCAGCGTGA
CAAGCTGTAAGTCGGAGGAGACGGAGTTCATGGAAGGATTGAGCTTATTAACCTCCCTGAAGCATGAA
AACCTTGTCAAGCTAAGAGGTTTCTGTTGTTCAAAGGGAAGAGGCGAGTGCTTTTTAATCTATGATTTGG
CCTCTAAAGGGAACCTTTCTCAGTATCTTGATGTTGAAGTAAACAGCAACCATGTTCTTGACTGGTCCAA
AAGAGTTTCGATCATCAAGGGGATTGCAAAAGGTTGGTCTTCTTTGCTTTTGTTCATCATTACATCTTCAG
CATTCTCTACAAATTTCTCAGAATATTTTAAAGTTTGGAAATATTATGGTTGTATAATATTTTCCCCATAAT
GTGGAGTCTTCTAAGAGTTCCATTGCATAGGCAGTTTATTACCACTAAAGTGCTTCTCACAGGAGTCAGG
AACAGGAAGTGACTGTAGATTCATTTGCATGGTAATTAGTGGCAGAGTCAGGAGGGGTTCAAAGTATG
AAGAAGTAAACACACAAAGAAGCCGAAGGGGTTCAACATTTACTATATATACATAAAAAATAATTTTAA

CCATGTATAAACAACGCAATTTTCTGCCGACCACCTCGGCCTACCTAGCTCCACCCCTGTGGTTAATATAT
TATCAAATTACTCCTCCATCCCAAAATAAGTGTCGCTTTAGTTCATTTCACGCCCATTAAGAAAAGTAATA
CATGCAAGGCATAGTTTACTAAGTCACCCCTAGTTATTAGATGAAGGAGGAGTAGTTCTTTAATATAAAG
GTATTGTTGGAAACAAGGAGTACTATTTTGTCTTGACTTCTAAATAGACAATTATTTTGGGATAATTTTTT
ATGCTAAAATGACACTTATTTGGGACGGAGGGAGTAGTATGAATACAGAGAGAGATTGGACTTATTGCT
TTAATTGAGAATTAAAGTTCCCTTGCCATCAAGGAAAGCAGTAGGACATGTCTCAAGAACCTCATTAATA
TGTGCATTTAATAGTTCTCCTATGTTCACTATTTCGAAACCAGTGCCTTTGATTTGATTAAAGATGAAATA
GCACAGGTGCACCATCACTGCATTAACTTCAGTTATTCATATTAGAACAACAGGCAACAGATATTTGGGC
TAAGTTTTAGTATAGCTAAGGTCTCTAATTAATAAAATCTTTAATACACTATGATGGCGAATGATTTCAAC
TTACCAACTATATTGTTTTTGTGAGAATCAGGGAAAACTTACCCATGTTTTTTGCGTTCTATCTTAATATGA
TTCCTTCATATTTCCTTTTGACTGATACCAATCGTTATGCATCTTTCTAACAGGACTAGGATATCTGCACAG
CACCGAACCAGACAAACCTTCCATGGTTCATCGCAACATATCTGTGGAAAAAGTCCTCCTTGATCAACAG
TTCACTCCACTAATACTGGACTGTGGCCTACTAAAGCTACTTGCTGATGATGTTGTTTATTCAGCACTTAA
GGTCAGTGCTGCACTAGGATATATGGCTCCTGAATATATTACAACTGGACGATTCACAGAAAAGAGCGA
TGTATATGCATTTGGAGTAATTATCCTTCAAGTACTCTCTGGTAAAGGACTTCTGGACTGCTCAATGAGA
CTTGCTGCTGAATCTTTTAACTTTGAGAACTTCATTGACCCTAATCTCAAGGGAGCATTCTCCATAAGTGA
AGCAACTATGCTAACTAAACTTGCAGTAAGTTGCACCCTTGAGGATCCAGATAGCAGGCCAAGTATGGT
TTTAGTAAATGAAGAATTGAACAGGTCTAGTTGTGGTTGAAAAAGGAAAAATTGAAGTCCTTTTTGTTCG
CGTTGAAATTTGTTGATGATTATTAACCATAGGGAACACGTGCCATAGCGATAGAGCTGCAGTTTTCTAT
CTATTATGCAATATCACTTGTTTCTGTATATAATGAGGCAGATTGAACAAATTCTTTTGATGGTTTGAAGG
TGAAACTGGGACGCCCGTTGGCATCCTTGACTGTTGTAGCTCTCAGAAGAGATAAAGAGGAAGCTAATG
AACTTGTACCATTATTTGATCTTTTCACTGTATTTTAATGTCTTTTTCAGGGCCAATATTCTGATAAATGCA
TTAGTCTTTGTATCGAGTGATCCAAATTCAATACAGTGCCTATGGCATATGGGTACAGACAAA
>PGSC0003DMG400004146 LRR-RLKs
CAAACTATGAAAAAAAATTCATCTGTTTTGCTTGTTACTTTTGAGTGTTTTTTTCTTTGTTTCATCAAATAAG
GATGAAGTTTCTATATTGTTAGCAATAAAGTCTAGTCTTGTTGATCCAATGAATCATCTCAAAGATTGGA
ACTTTTCAAACAATGAAAGTTCTATTCATTGTAAATGGAATGGTGTGTATTGCAACTCAAAAAGCTATGT
AGAGAAGCTTGATTTGTCGAACATGAATCTTAGTGGCAGAGTTTCAGATCAAATTCAAGGACTTCAGAG
TCTATCTTTACTTAATCTTTGTTGCAATGACTTTTCCACTTCATTGCCAAAGTCATTAGCTAATCTGACATC

TTTGAAGAGCATTGATGTGAGCCAGAACAACTTTGTTGGGAACTTCCCTGATGGTATTGGAATGTCGAA
TCCTGGTTTGAAATATGTCAATGTTTCAAGTAATAACTTCGAGGGGGTTCTCCCTGAGGATCTTGGCAAT
GCTACGTTGCTCGAAGTCATGGATTTTCGAGGGAGTTTCTTTGAAGGTTCAATCCCTGGATGTTACAAGA
ATTTGAAGAATCTGAAGTTTCTTGGCTTATCAGGGAATAATCTTTCCGGAGATATCCCTCGTGAGCTTGG
TGAATTGAAGGCTATGGAGACTATGATTCTTGGATATAATCAGTTTGAAGGTTCAATACCAGCTGAGTTT
GGTAATATGAGTAGCCTTAAGTATCTTGACTTGGCTGTTGGCACTTTAAGTGGACAAATTCCAGCTGAGT
TAGGTAAGTTGAAGAACCTCACTACAGTGTACTTGTATCAAAACAGTTTTGAAGGTAAGATTCCACCTGA
AATTGGAAATATAACATCTTTAGTTTACTTGGATCTTTCTGATAATAAGATCACTGGGGAGATTCCGAAT
GAATTAGCAGATTTGAAGAATTTGCAGCTGCTAAATCTCATGTGCAATAACTTAACGGGTCCTATTCCTA
CCAAACTTGGTGAGTTAGAAAATCTTGAGATACTTGAACTATGGAAAAATTCTTTAAATGGCTCTTTGCC
AATGAATCTTGGGAAAAAATCTCCCTTGCAATGGTTAGACGTTTCTTCGAATTTCCTGACAGGTGAGATC
CCTCCTGGCTTGTGTGATTCTGGCAATCTAACAAAGCTCATTCTGTTTAATAATTCCTTCTCAGGATCAAT
CCCATTAGGCCTGTCTAACTGTTCTTCACTCGTTCGAGTTAGAATCCAAAATAATCTTCTATCAGGAATGA
TTCCGGTTGGGTTTGGAACACTTCCTATGCTCCAAAGATTGGAATTGGCTAAAAACAATCTCACTGGTGA
AATTCCTGTGGATTTCACTCTCTCCACCACTCTTTCTTTTATCGATGTCTCCTCGAACCACCTTGAATCATCT
TTGCCTTCTAGCATTTTGTCCATTCCTAGCCTGCAAACCTTCATAGTTTCCAATAACAATCTTAAAGGCAAC
ATTCCTGACCAATTCCAAGATTGTCCTTCCCTTTCTTTGCTTGATCTTTCAAGCAACCATTTCTCGGGGAAA
ATTCCTCAGAGTATTGCTTCATGTGAAAAGCTGGTGAAGCTTAATCTAAGAAACAACCAATTCAGTGGTG
AAATTCCAACTCACATTGCCACATTGCCAACTTTATCTATTCTTGATCTATCATACAACTCGTTAGTTGGAA
AAATACCAATGGACTTTGGTAGCTCTCCAGCTCTAGAAATGCTTAATCTTTCCTACAACAAGCTTGAGGG
TCCAGTTCCAAGAAATGGGATACTCATGACTATTAATCCCAATGATCTGATAGGAAATGCTGGTCTTTGT
GGTGGCATACTTCCACCGTGTTCTCAAAGTCTCACCATAACTTCAAATGCGAGAAAGAATCGTGTCAATC
ACATAATCGTGGGATTCATTGTTGGAATCTCAGTTATCTTAGCTGTTGGGATCATGGTTCTAGCAGGGAG
ATGGATGTACAATAGATGGTACTTGTGCAACAGCTTTTTCAAGGAATTTCGGTTCAACAAGAACAACTCA
GAATGGCCTTGGAGGCTTGTAGCATTCCAGAGGCTTAACTTCACAAGTACTGATATTCTGGCTTGCCTAA
AGGAGTCAAATGTGATAGGCATTGGTGGCAATGGAATTGTCTACAAGGCTGAGGTTCTGAGGCCACATT
CTGTTGTAGCAGTTAAAAAGTTGTGGAGATCAGATGGAGACATTGAAGCAGGAGATGATCTCGTTGCA
GAGGTTGATCTTTTAGGGAAGCTTCGTCACAGGAACATAGTTAGGTTACTAGGCTATCTTCACAATGAG
ACTGATATCATGATGTTATCTGAATACATGCCTAATGGAAATCTTGGAGCTGCTTTACATGGAAAAGAAG

ATGGAAAAATGCTCGTAGATTGGTTGTCGAGGTACAACGTTGCCCTTGGTATTGCTCATGGACTCGCTTA
TCTTCACCATGATTGTCATCCACCAGTCATACATCGCGATGTCAAGTCAAACAACATTCTCCTAGATTCAG
ATTTTGAGGCAAGAATTGCAGATTTTGGCTTGGCAAGGATGATGCTTCACAAGAATGAGACTGTTTCTAT
GATAGCAGGATCATATGGCTATATAGCACCAGGTTAGTTACATTTCCTGATAATGCCACTCACAAGAAAA
TTCATTTCATTTTATATAGTCTCATTTTCCTTTCTCTGTTGTTTTTGGCAGAATATGGATACACATTGAAGG
TTGATGAGAAGAGTGACATATACAGCTATGGGGTGGTGCTTTTGGAGCTTGTAACAGGAAAAATGCCAT
TAGACCCTTTGTTCGGAGAGTCGATTGACATTGTAGAATGGGTTAGAAGGAAAGTAAAGAACAAGGCA
TCAGAAGAAGCATTAGATGCTGATGTAGCTGGCCAATGTAAACATGTCCATGAAGAGATGCTTCTTGTC
CTAAAAATTGCACTTCTTTGCACTGCCAAGCTTCCCAAAGAAAGACCATCAATGAGAGACATTATAACCA
TGCTTGGAGAGGCAAAACCAAGAAGGAAAAGTATTTGCCAGAATTGGGGTTATAGTACTAGTGCTAAT
AAAGACAAGTTAATCTTTGCTCATTCACCAGTTATAGGCCTTCTATAAGTACCAAAACCTTATTTTTCCACT
TCAATATCTAGTATATCTTCATTTTCATTAGTTCAAATGTAAA
>PGSC0003DMG400004667 LRR-RLKs
ATCAAAAGCTGTCACTTAGCTTACTGATGAAAAACTCATAAACCCAAAATTTTCAACAAACAATCCCAAA
AAAAAAAAAAAAACTTCAAAATTTTGAAGAATCATAGCAAAAAAAAGGTAATCTTTGCCAAAAAAATTC
AAGAACAATAGCAAAAAAAAAAAAAAACTCCATTGTTGATTGTTCCTAGATCTCTAAAACCTCCATTAATG
GCACCCCACAAGCTTTTTTTTCTTGTTAACTTCTTCTTCACCTTCATTTCATTCACATTTTCCGATGACAGCA
CCGTCATGTCAAATTTTCTGAAGGGTCTGTCTAAACCACCATCTGGTTGGGATGCTTCAAAATCTTACTGT
GATTGGAGTAAAGTTAATTGTGATAAATCGAGTAATACTGTTGTTTCCATTAATCTAGATTCTCAAGGTCT
TTCTGGGGTTTTGCCTTCTGACCTTAATCAACTGTCAAATCTCAAAACTCTTTCAGTTCAGAGGAATTCAC
TTTCTGGGGTTCTTCCATCTTTCGCAAACATGTCGAATTTAGCTGAGATTTACTTGAATGACAATGGATTC
ACATCTGTTCCTCAAGATTTCCTTTTGGGTCTTACTAGTTTGCAGATTTTTAGTATTAGTGAGAATGGGAA
GTTGAGTCCTTGGCAAATTCCTAGTTATTTGATTGATAGTAACAATTTGGTTAATGTTTATGCTAGTCATG
CTAGTTTGTTTGGTGTTATACCTGATTTTCTTGGTAAATTCCCTAATCTTCAGGATTTGAGATTGTCTTATA
ATAATTTTACTGGTTCTTTGCCTGCTAGTTTTGGGGATTCTGAGATTAAGAATTTATGGCTGAATAATCAG
GTTAAAGGGTTATCAGGAACTATTGATGTGATTTCAAGTATGTTTCAGTTGTCACAAGTGTGGTTACATG
CTAATTCTTTTACTGGTCCTATACCCAATTTGTCGAAATGCGAAAATATATTTGATTTGCAGTTAAGGGAT
AATGATTTTACTGGTGTTATACCTGATTCAGTGATGAAACTTCCTAACTTGTTGAATATATCTTTGAAAAA
CAATAAGTTGCAGGGTCCAATGCCAGAGTTCAGAAAGGAGGTTAAGGTGGAGATTGAGGGTACTAATA

GTTTTTGTTTGGATACTCCTAGACCTTGTAATTCACAGGTTATGACGCTTTTAGATGTGGCGGGTGGTTTT
GGATATCCAGGGTTTTTGGCTGATTCTTGGAAAGGAAATGATGCTTGTAATGGATGGTCACATGTAAGT
TGTGATGGATCGAAAAAGAATGTGGATGTTGTAACACTTGGTAAGCTACGTTTATCGGGTTTTATATCTC
CTGCTTTTGCTAAATTGACTAGTTTGAGGAATTTGTTCTTGAATGATAACAATCTTACCGGATCTATACCG
GAAAGTTTGGCAGCGTTGCCTATGCTTCTAACTCTTGATGTTTCTAATAACAATTTATCTGGTCCTGTACC
TGCGTTTCGATCAAATGTGAAACTTATTAATGGTGGTAACGTGTTTCTTGGAAAGAATATAAGTGATGGA
CGGGGTAGTGGTGGATCACCGGGGTCTGGGTCAAGTAGTGATGGTGGAAATTCATCTGGTGGTTCGAA
GGGATCCTCAGTTCAACCTGGAGTGGTTGCTGGTGTTGTTATATCTGTCGTTATATTTATCCTTGTTCTGT
TGTATGTGTCTTACAAATGTTACATAAGGAGACGGCATAAGAGATTTGGAAGGGTTCAGAATCCGGAAA
GAAGCAATGATATGGTTAAGCCTGGTGTAGTGATAGGTAATGGATATGCAGGAGTCCCTAGTGAATTAC
AGAGCCAGAGCAGTGGGGATCATAGTGAGATGCCGATATTTGAAGGTGGAAATGTTGCCATTTCCTTTC
AGGTGCTTCGACAAGTGACAAACAACTTCAGCGAAGAAAATATCTTGGGTAGAGGAGGATTTGGAGTC
GTTTATAAGGGTGAATTACATGATGGGACTAAGATTGCTGTGAAGAGGATGGAATCTGGCGCGATGGG
TACTAAAGGAATGAATGAGTTCCAAGCTGAAATCGCGGTCCTTACCAAAGTTAGGCATAGGCATCTGGT
CGCTCTTCTTGGTTCCTGTGTTAACGGCAATGAAAGACTTTTGGTGTATGAGTACATGCCACAAGGAACT
TTAAGTCAGCATTTGTTTGAATGGCAAGAACACGGGTGCCCAATTTTGACCTGGAAGCAAAGGGTCACA
ATCGCATTAGATGTTGCAAGGGGAGTCGAGTATCTCCACAGCTTGGCACAGACAAGTTTTATCCATAGG
GATTTGAAGCCCTCTAACATACTTCTTGGGGATGACATGAGAGCCAAGGTTGCAGATTTTGGGTTGGTT
AAGAATGCCCCCGATGGGAAATATTCTGTTGAGACACGGTTGGCTGGAACTTTTGGCTATCTGGCGCCT
GAATATGCTGGTAAGTCTGTCTTTCCTCTGCTGATCTTTAATCCTTTTAGTTGTAAAACTGGTAGAAAACT
AAAACAATATCCTATCATTAGCTTTCATGTTCTTTTTAATCTTCACTATATTAGAGATAAATGGAGAGGTG
TTAGCTTCATTGAAGTTCATTTCCATGTTCAAATGCTAGTTTGTTAATCTCGATCTGATTCGTGGATTGAT
ACCGCAAACGTTGTCCAGTAAATTAACTAATGTTAGCAAAAAAGAGTACGATTTGAAAGACAAACATAA
GGTAGAAATCTAGAAGCCCACTCATCCGGTTTTCAAATTGAAATTCGGATGTTGGTACAGCGTGTGTGA
CTGATCCCATTGTATCTAAATAGAAATTTGGCTTTCGGAATATCACTCTTCTTCAAGACATTAAGTTCAAC
ATATCTAGATTTAGTGTTGGCTGTCAATAGTTTTCTGCTCGTAACATGTTTAATTTCCTTTAGAGATCTTGT
TCTGGCCTATTTCTTGCATGTCTTAGCAAGGTTGTCTGTGATCATTACAAAATTTGGATTAAACGTGACTG
CTCCTCTTTATTTACAGCTACTGGACGAGTCACGACCAAAGTAGATGTGTATGCGTTTGGAGTTGTTCTA
ATGGAGATCATCACTGGTAGAAAAGCATTAGATGAGACCTTGCCAGATGAGAGGTCTCATTTGGTCACC

TGGCTTCGCAGGGTCCTTGTCAACAAAGACAACCTCCGAAAGGCTATTGACCCAACTCTTGATCCTGACG
AGGAAACATATGAGAGCATTTGTAAGGTTGCCGAGTTAGCAGGCCATTGCACTGCACGAGAACCATTCC
AGCGCCCTGACATGGGACATGCAGTCAATGTCCTTGCCCCCCTCGTAGAACTGTGGAAGCCTACAAGAA
ATGAAGACGAAGACAGTGGCATCGACCTCCAAATGAGCCTTCCTCAAATTCTTCAGAGATGGCAAGCTG
ATGAAGGAACTTCAAGAATGTTCGATGACATCTCCTTCAGTCAAACACATTCAAGTATACCCTCAAAACC
TTCTGGATTTGCAGATACTTTCAATTCAACAGATTGCAGATGACATTAAGGCAACAATCAAGAAAGGCAC
ACAATTCATTTGGTTAATTGGTTTCGTCTTAACAACGCGTTTTACTTCGTCTTTTCACAACCCTTTTGGTGA
TAGATCATTAGACCAAAGAAGCTAAGGTGGATAGCAGAGTTTATTTGTGGTTTTGTTAGAAGAGAAAAA
TTGTATTAAATTCTTTTAATCTTACATAGTTGATGTAAATAGATTTGAGTTCTTGTGTTAAACATGCAAAAT
ATACTCTTCTTATTAAGTTTTTTACTACAAGATTTCATTATTTGTGCCACTAAGTGTGTA
>PGSC0003DMG400004963 LRR-RLKs
CTTTTATTTCCTTCCTCTCTAAGAAAAAAACAATTTAATACAAATGACTATTCAAAAAATTATCACCCTTGT
GTTATTTTTTCTTACTTTGACGTCCACTTTTGTTCAAGGTGATCAATCTCAGTTCTTTGTACTCATGAAGAA
ATTTGTCACAGGGAGTTCTTTGTCTAATTGGGATATTGGAAAACCAATTTGTCAATACAAAGGTGTCGGT
TGTGATGAAAGGGGAGACGTTATCAAAATTAACATATCGGCCTGGTATTTATCAGGCCAATTTCCAAGT
GATGTGTGTAGTTATTTTCCAAGATTGAAAAGTCTTCATATTGGTCATAATAATTTCCAAGGTGGTTTTCC
TAAGTACCTAACAAATTGTTCTTTTTTGGAAGAATTGAACATGACTAAAACATCACTTACAGGACAAATTC
CTGATTTGTCACCAATACAATCTTTGAAACTACTTGATCTTTCATGTAATCAATTGACAGGGGATTTTCCTT
TGTCAATTATTAATCTCACCAACTTAGTTATCCTGAATTTCAATGAAAATCGCCATTTCAATCCGTGGCGA
TTACCAGAGGACATTTCCAGGCTTATAAATCTCAAGTGGATGATTTTAACAGCATGTAATATGCATGGGA
CAATTCCAGTGTCAATAAGTAACATGACTTCTCTTGTTGATCTTGAATTGAGCGCGAATCGCCTTGTTGGT
AAGGTGCCTAGAGAACTTGGGAAGTTGAAGAATTTGAAACTCCTTGAGCTTTTTTACAACTTACTTGATG
GTGAAATCCCCGCGGAGCTAGGGAATTTGACAGAACTTGTGGACTTAGACATGTCCGCGAATAATTTCA
CAGGGAGAATTCCAGAGTCTATAAGCCGGCTTCCTAAGCTGCAAGTGTTGCAACTTTACCATAACGCGCT
ATCAGGAGAGTTTCCAGCAGCACTTGCTAATTCAACAACTTTAACTATCCTGTCTCTTTATGACAATCTGT
TTACAGGAGAAGTTCCACAACACTTTGGTCTTTCATCAGCTTTGTTGGCATTGGACTTGTCAGAAAATCG
ATTTTCTGGAAAGCTACCGCCTTTTCTGTGTAGTGGAGGTAAATTGAGTTATATTCTTTTACTTCAAAACA
TGTTCTCAGGTGAACTGCCTGATGGATATGTGAAATGTCAGTCTGTTCTTCGCTTTCGAGTGAATTACAA
TCAGTTGGAGGGAAGAATACCACAAGAGCTTTTTACTCTTCCACATGTTTCAATCATTGATTTGAGCTATA

ATCATTTTAGTGGTCCAATTCCAACAACAATTGGAAGTGCTAGGAATTTATCAGAATTGTTTATGCAAAG
TAACAAGCTTTCAGGTTTGCTTCCTTATGAAATATCTACATCTTCTAATCTTGTGAAGCTTGATCTTAGCAA
TAACCTTTTGTATGGTCCAATTCCTTCTGAAATTGGTGGTTTAAAAAGCCTCAATTTGTTACTCTTGCAAG
GTAACAAGTTCAATTCTTCAATCCCCGAGTCACTTTCTTCGCTTAAATATCTCAACTATCTTGATCTGTCTA
ACAATCTTTTGATAGGAAAGATCCCTGAGAGCCTAGGTGAATTGTTACCAAACTCTATGAACTTGTCAAA
CAACTTACTTTCCGGTGCTATACCTCTTTTGTTTATAAAAGGAGGTGTTTTGGAAAGTTTTTTAGGCAATC
CGGGACTTTGTGTCCCTACCTCTTTGAACTCATCAAACACAAGTTTTCAAACATGTTCACATAGCTATAAC
CATAAGAAGAGGAACAACATTGTTTGGGTTATTGGGACATCGGTGGGGATAGTCATTGTTGGATTAGTC
TTGTTTATCAAACGATGGTTTGGTAACAAAAAGGAGGTGATGGAACAGGATGATCATTCATTGTCATCA
TCATTTTTCTCCTTTGATGTTAAGAGTTTCCATCGATTAAGTTTTGATCAACGTGAGATCTTTGAAGCTAT
GGTTGAGAAGAATATTGTTGGATATGGAGGGTCAGGGGCTGTCTATAAGATCGAGTTGAGTAATGGAG
GAGTAGTTGCTGCAAAGAAGCTGTGGAGTCACAAACATAAGCATTCTGTTTCTGAGGATAAATTGGTTT
TGGATAAGGAACTTAAGACAGAAGTTGAGACTCTTGGTAACATAAGGCACAAAAACATTGTGAAATTGT
ACTGTTATTTCTCAAGTTTGGATTGCAGCTTGTTGGTTTATGAATACATGCCTAATGGAAACCTTTGGCAT
GCTCTTCATGGTGGGAAGTTCGTGTTGGATTGGCCTATTCGTCATCAAATTGCACTCGGAATAGCTCAAG
GATTGGCCTATCTTCACCATGATCTTATGCCACCGATTATTCATAGAGATATCAAATCCACCAACATCCTC
CTGGATATTGATTATCAACCAAAAGTCGCGGATTTTGGTATAGCCAAAGTGTTGCAAGCCAGAGGAGGC
AAAGATTCCAGCACCACGGTCATCGCAGGAACTTATGGTTACTTGGCACCAGGTATAATTTTCCCAATTC
ATAAGTGTTACTTCAATCTTTGAGCTTGATTGAGTAGATTAAATTAAATCCTTTATGTGCAAATATCATCC
CATAGTTGAAGTGTTCATATTGGCCTAATGTTACTCTAAATTGGCCATTTAATATAGTGAAAAATTTAGAA
CATTGCCTAACGTTAAGAGTACATTTGACCCTTTTTCGTTCTTAATTATGGCTTTTTATGTTGTAAAAAACA
TCACATATGCTCAAGTTTCTTGATTGAACTTTCATTTAATACATGTTTAAGTGTTGAAATTAGGCTAATGTT
ACTCAACTTTGGTGTAACAGAGTATGCATATTCTTCAAAGGCAACTACAAAGTGTGATGTGTATAGTTTT
GGAGTTGTTCTAATGGAATTGATTACTGGAAAGAAGCCAGTGGAGCCAGAATTTGGAGACAACAAGAA
CATTGTTTATTGGGTATCAACAAAAGTGGAGACTAAAGAAGGTGCATTTGAAGTATTGGATAAAAAAGT
CTCAGATTCTTTCAAAGAGGACATGATTAAGGTTCTACGTATAGCTATACGTTGTACGTATAGTACACCA
ACACTTCGTCCTACCATGAATGAAGTTGTTCAGTTGCTAATCGAAGCGGATCCTTGCAAGTTCAATTGTT
GCAACATGTCAAATAAGAAGAAGAATGACACAGAAGAAGTGATCAATAAGCCACCAAAGAACATATGA
GTTGTAAAAAATTTAGTGTATCAAACTAGTATACAAGGTATATAGAATATGTGTACTGAGATATTACACC

CTCAAAAGAATCAGTCTTTATGTTTTCTAATAAATGGTACTACTTTTTATTAGTATATATATAGATTCTCCA
ATTAGTGTTACCTTGCTTTTTTATATTTTTGGCCTGTTGTAAGTATTTGGGAATTTTATGAA
>PGSC0003DMG400004966 LRR-RLKs
GCGCTCTTCATACTCGTCTCCTTCACACGTTTTTCTGAAGGCGGAAGAAAAAAACTTCTCTTCGTCAACTT
CTTCGTCAGCTATGGCGGCGCTATTCAATTGCCGGCCGGAACTTATCCACTTCTTCTCTATTTTCTCCTTCT
TCCTTCTCTTCCCCGTCGCTTTTTCCGACGAGCTTCAAACACTTTTATCCATCAAATCCTCTTTATCAAACCC
CACCACCAATGTATTCCAAAATTGGGAACCCAATACTCCTCTCTGTAAGTTCACTGGCATCACATGTAATT
CCGATGGGTCAGTCAAAGAAATCGAGCTTTCGAGTAAAAAAATTTCTGGGTTTGTTCCTTTTGATAAAAT
ATGTTCACTTAATTCATTGGAAAAGCTCTCTCTGGGTTACAATTCACTTTCTGGGGAAGTTACGGATGATT
TGAACAAGTGTGTTAGCTTGAATTACTTGGATGTTGGTAATAATGAATTTACTGGGTATTTCCCTTATGTA
TCTTCACTCAGTGAGTTAACCCATTTTTATGCAAATAACAGTGGGTTTACAGGAAAATTTCCATGGAATTC
TTTTGCGAATATGAGTAATTTGATTGTGCTTAGCCTTGGTGATAATCTGTTTGATCGGACACCGTTTCCTG
AAGTGATTTTGAAGCTTAATAAATTGAATTGGCTCTACTTATCGAGTTGTGAGCTTGAAGGGGAAATTCC
TGAAGAAATTGGAAATCTTACAGAGCTGATTGATTTAGAGCTGTCGATGAATCACCTTACTGGTGAAATT
CCTTCTGGAATTACGAAGCTGAAGAAATTATGGCAGCTTGAGTTGTATGAAAACCAGCTTACAGGGAAG
TTGCCGGTTGGATTTGGGAATTTGACAAGTCTTGAATATTTTGATGCTTCGGCTAATAATCTGTACGGTG
ATCTGTCGGAAATTCGAAAGTTGAATCAATTGGTAAGTTTGCAGTTATTGCAGAACCAATTTTCAGGGGA
AGTACCAGCAGAATTGGGAGAGTTCAAAAAGCTGGTGAACATTTCTTTGTACACTAACAAACTCACTGG
TCAGCTTCCGCAGAAGTTGGGTTCTTGGGCGAATTTTGATTTCATTGATGTATCAGAGAATAGTTTCACT
GGTCCAATTCCACCGGACATGTGTAAGATGGGGACGATGAGAGGATTATTGATACTTCAAAACAATTTC
ACTGGCGGAATCCCTGAAAGTTATGCTAATTGTACAACAATGACGCGGATTCGAGTGAGCAAAAACTCA
CTCTCCGGTGTGATTCCTGCAGGAATTTGGGGGCTACCAAAGCTCGAAATCCTTGATGTTGCAATGAATG
AGTTTGAAGGTACTATTACTTCTGATATTGGAAATGCAAAATCTTTAGGTGAAATTGATGCGGCTAATAA
CAGATTCTCTGGTGAATTGCCTTTTGATATTTCGAATGCTTCTTCCTTAGTGAAAATTGATTTTAGTAACA
ATCAGTTTTCCGGTGAAATTCCGGGAACAATTGGGGAGCTTAAGAAGATTGGTAATCTTAATTTACAGA
ACAACAAGTTTTCTGGTTCAATACCAGATTCTTTAGGCTCTTGTGTTTCACTAAGCGACATTAACATGGCT
AACAATTTGCTTAGCGGCTCTATTCCTGTTTCTCTAGGATCATTACCGACTTTAACTTCATTAAACTTGTCG
GAGAATCAGCTTTCAGGGAAAATTCCGACGAGTTTATCAAATCTGAAGTTAAATCTTCTTGATTTTTCCAA
CAATCAGTTGACTGGAGCAATACCAAACTCCCTATCAATTGATGCTTATAAGGGTAGCTTTGCTGGGAAT

AATGGTCTTTGTAGCCAAAACATTAAGAACTTCCGTAGATGTTATGGTGAATCTGGAAAGCCTAGAGAA
TGGTACACTCTGTTGATATGTTTATTGGTGGCTGTGATTGTGGTGCTCGTTTCATTTGCGGGTTATTTGTT
CTTGAAGAAGAAAAGTCATAAGGAACATGAGAGGTCTTTGAAGCAAAATTCTTGGAATACAAAGTCATT
CCATATATTGACCTTCACAGAGGATGATATTCTTGATGGAATCAAGCACGACAATTTGATTGGAAAAGGT
GGTTCTGGTAGTGTATACAGAGTGCAGCTTTCAGATGGAACAGATTTCGCGGTGAAACATATTTGGACT
TCTGATTCAGGAAACAGGAAAATATCAGGAACAACATCACCAATGTTGGGAAAACCAGGGAAGAAATT
GAAAGAATTTGAGGCTGAGGTTGAAACACTGAGCTCAATTCGACATGTGAATGTGGTGAAATTGTACTG
TAGCATAACGAGTGATGACTCGAGCTTGTTGGTGTATGAATATATGCCAAATGGAAGTTTGTGGGATCG
ATTGCATACTTGCAAAAAGATGTCACTTGATTGGGAGACGAGGTACGAGATAGCTCTTGGTGCAGCCAA
AGGACTGGAGTATTTACACCATGGTTGTGATAAGCCAGTGATTCATAGAGATGTTAAATCAAGTAACAT
CTTGTTGGATGAGTTTTGCAAGCCAAGAATCGCTGACTTTGGTCTTGCTAGGATTGCTCAAGCTGATTCA
ACCAAAGATACAACTCATGTCATTGCTGGAACACATGGATATATTGCTCCTGGTAAGAAGCTCATTATAT
TGTAGTTGATTTTATACGTAGAATTTGAGTATATCTACTTAACACTAACCATGTATTCATTTTTCACCATCG
GTAAAATTGACTTACGAAATAGGCAACTCCCCATATCAAGCAATATGGTATCTTGAAAGATATAGTGAAA
ACTTGCTATAAACAGTTGGATCACTATGTTGCTCGGATCCTTCAAAAATGTCACTGCACCTGTGTTGGTTC
CCTCAAAAATAGCGCATTCTTGGAGGATTTGACATGAGTGTGACAACATTTTTGAAGGATCCGAGCAAC
AATTGAATTGCACTAATAAGAGTGTGAAACAATATTAGTGTTAATTAACTTAGATCCTATCTTGATTATCT
ATTCATTTGGTCATAGTGAGTGTCTCATTTCTGGCATCCTTTTTGAACTTATTTGAATTGAATTGGTGTTGC
AGAATATGGATACACACACAAAGTGAACGAGAAAAGTGATGTATATAGTTTCGGAGTTGTATTGATGGA
GCTTATATCTGGGAAAAGGCCAATAGAATCCGAATACGGAGAGAATGGCAACATAGTTACATGGGTGA
GCAGCAAATTGAAGAGCAAGGAAAGTGTATTAAGCATAGTGGATTCAAGCATTCTTGAAGCTTTCAAGG
AAGATGCAATCAAAGTCTTGAGGATTGCTATTGTATGCACATCTAGGCTTCCCACATTAAGGCCAACAAT
GAGAAATGTAGTCAAAATGCTGGAAAAAGCAGAACCTTGCAGATTGGTTGGGATAATTGTAAGCAAAG
ATGATGGTAGTATCAAGACAGAACAATTGAAGGATCACACCAAGATGTAATTAGATCCCCCGTCTTTGCT
CGTTTCACTCTTCAACCTGATCTCTGTCCTCCATGGAACGTCGAATCTTGTAGCAGTATGTAAAGTTTTGA
AGATGGCCTCTTGGCCTTGTAACATATAGTAGAAGCTAGATCCATACTAGTATGTGGAATTAGATTGAAT
TTAGAGATGCATGGCATAAGTGCTATAAGATGTAATACTTGTATGTGCTAAATAAAAATGGTTTAGTTAT
TCAAATCCCTTTTCACCCTGCA
>PGSC0003DMG400004972 LRR-RLKs

GTTCATGCTCTAAATAACTTCAGGGAAGCTATTTATGAAGATCCACATCTGGTGTTCTCAAATTGGAATG
CCTTAGATTCAGATCCTTGTGGATGGTCTGGAATTTTTTGCTCAACGGCACGAGATCATGTCATAAAAAT
GTGAGAGAAGTGCCTTCTGCATATGGCGTTTTCGTTTTCTTTCTATCTGTTTGTTGGATTGATTTTTCTCAA
TTGGATGCAACTTATTTCATTACTTTTTGATACATGTTAGTAGCACAACTGAGCCTTTACTAACGAGTTTA
CAACGTCTCCTGTCTATGTAATTTTGCAGTAACATTTCTGGTGCTTCTTTGAAGGGTTTTCTTTCGCCGAA
CTTGCATTTGATTTCTTATTTGCAAGAACTGTAAGCTGTTCCTTCCATGAAAAACTTATACTTGTAACTCCG
GTGCATAAAAGATTTGGTAAAAGCTTAATGCTATGTATTGGTGATATGCAGGATATTGCATGGAAATGT
ACTGATTGGTACAATACCGAAGGAGATTGGCTTGTTGAAAAATTTAAAGGTCTTGGATTTGGGTTCTAAC
CAGCTCGCAGGACCAATTCCTCTCGAGCTTGGCAATTTGTCAAACATTATTAAGATGTTAGTGCAATTTG
AGTGTTCGTTTTGTATGTGTACTTTCGTTTTTGACTTGCATGAAGCTTCCTTATCTCATCTAGTTTTGTTAT
GTCTCTGCAGAAACCTTCAATCCAATGGATTAACAGGTAAATTGCCCTCTGAGCTTGGTAAATTGATATA
CCTTCAGGAACTTCGCCTGGACAGAAATAAGCTCAAAGGACCACTGCCTTCCAGTAATGGCTCAAATTTT
ACATCTAGTGCGTATGGGATGTAAGTCGAATATGGACAGTGCTATTTCTTCAATTCTATGCGTTTTTAATC
TTTGGATTTGCTGTTATGTAGGTTTTACATTATCATGTTGGTAGAAGATTTAATGTTGCAAATGCTTTTGA
GACGTGGTTCATTTGATTTTTCAATCTTGTACACTATGAAGTTGCCTTTTTTCGTCTTTTTGGGGGAGGGG
GTGGATATCTGAAGCTTGAAATCTTTTATTTAAGCCGTTAACTATACCCTTACTAAATGATTCTCTCTAAA
ATGTAATGTTTCCAGGACTTACGAGCATTATACATTCAAAACTTTTGGAATATTTGAAGCTTGATTAAATT
TATATATCTTTTTAATCTTCTAAGGAGCCCATAGTATCTCAATTAGCATAAGATTTGCTGCAACATTTTTGG
CTGCAGTTTGTCTGGACATTTACAGAGGCTTAAAATTCAAAGATTATGGCTAATGTTTAATCTTTTTGTCG
TCTTGATTGATAGTGCTGGACTTCTGTTGACATGTATGTCTGTTGTACTTGAACTTAATTTAATACATTTGT
TGTTCTCCTAGAAATTTCACACAATTACTGAGTGTAATGAAATCTTAAAGGGTTCAACATGTATATTCAAA
AGTCATCACCGACGTTATCGGCAATATTCTGTATAGTTTTCTGGAAAAATCAGTAAAAAGCCTCCTATGC
TTGCTTGAACTTGTAATTGCTCTACATAGATCCCTTTAAGTTTTATCTCACAGTTGGTTTCTTAGTAATCGT
CTTTTATTTTTGTTTAGGCATGCCTCTGGTGCCAAACCTACTGGTCTTTGTCGTTTATCTCAACTGAGAGTT
GCTGATTTCTCCTTCAACTTCTTCTTTGGTAGCATACCGAAGTGTCTGGAGTACCTTTCAAAGTATAGTTC
TTAATTCCAATCCACAAGAATTTTTTATACTCTTTATATCAATTTCTGAGTGCCTATTATTTTGTTCTTTCTA
TAGAACTAGCTTTCAAGGAAATTGCCTTCAAGCAAAAGATCCCAGGCAGCGTTCTACTGCTTTATGTGGT
ATGTGTAATCTGCTAGACAACTCTTACACTTTAGCATAATCATTAGCCAAGCCTTTGAATCTTTTATGCAT
ACATTAGTGTGCGAAAATGCAAGCTGGTCATGCTTACTTTTGATTGTATATCCTAAAGAGGCTTCTGCGA

GTAAGTTCGCCCATTTCACTAACAGTCGCAGTTGAACAGATCAACTTTAAAATAATTGAATTGATTGTTGT
AACTTTTGCATTATAGGAGGCACTCCACCTTTCAAAAGCAATCCAGCACAGAGCAATAATAAGCACCGG
ACTGTTGAAGGAAGAGCCAAACATAAGTCTGCTTCAAAACCTACCTGGCTTTTTGTGTTAGAAGTGGTAA
CTGGAGTTATTGCGGGTTCTCTCTTCCTTGTGGCTATTTTGGCTGCCATTCAGAAGTGTAAGAACGAATC
TTCTATTATCATCCCTTGGAAGAAATCAGCAAGTATGAAAGACCATTTGAAAGACCATATGGCAGTTTAT
GTAGGTGAGTACCATGCAATTTCAGTTGGGCGATTACAGTTAAGAATTGCCTTTAAACATTTGTATTACT
TGATTCTTTATTGACTTAGGGTGTGTTAGGTATGAAGGAAAAATGGTTTCCTTGGAAAATGTTTTCCAGA
AAATAAGTGGATTTCTTACTTATTTTCTTGTGTTTGGTATGTAAGTAAAAAACACCACCCTAATAAGATTT
GTATATAATCTAGGCAAATACTATGGGAGGTGGGAGTAGGGGTCCCGGGTGGTGGGGATGGGGGTGA
GCTGAGGTGTGTTGGAGGGTGGGGAGGACACAATTGATGTGAAATACCACTTGTGGAACTTGTTTTCCC
TACCTCCATACCGAACACGCCCTTAATTCTTTCTCATATAGGATCTGATTCTTCAATTTAGTACTACTAATG
TTTCTGTTACTTCAGTTATCTTTACTATTATTGTTGTTAATACATAAATATGCCCTTTAAGCCACATAGGAC
TCATGTGCCTACTTGTTTAACTTTATACAAGTTTAAGTGTCTGCTTGTGCACATCCAAAGTTAAATGACAT
AAATGTGAAATGAGGCCAAATTAAAAGGCACATTACTGTATTATGCCTTTTTCTTTTCTATGATGTTTTCA
CAATAGCTTCCTTACTCTTGTAATTTTAAAAACCTGTTTTGGAAAACATTTTTCTTGAACCGAGGGTCTATC
GGAAACAACTTCTCTACTTCCACAAAGGCAAGTTTAAGGTCTGCATACACCTCACCCTCCCTAGACCCGA
CTTGTGAGCTCACACAGTGTATAGGATCACAAGTTCAGAAGCATTAATTATAATTCACATTTCTATTAGTA
ACAAATGTTTCTTTGTCAGATACTGAGATGCTAAAGGACGTTGTAAGATATAGCAGACAAGAACTTGAG
GTAGCTTGTGAAGATTTCAGCAACATTATTGGATCCTCACCAGACAGCTTGGTCTATAAAGGAACTGTGA
AAGGCGGACCTGAATTAGCTGTAATTTCCATTTGTGTCAAAGAAGAGCAGTGGACCACCTATCTAGAGC
TTTATTTTCAGAAAGAGGTGAAGCCTGTTCTCTCTTTAAAATACATGGCTACTGCTGAGCTCTCGTGCGCA
ATAAAAATTTCATCTTTGCTGCAATGCTCTAACTTGTATGGCTTACAAACTCAGGTGGCAGAATTGGCCA
GAATAAATCACGAGAACACTGCAAAACTTCTAGGTTACTGCAGAGAGAGCAGCCCCTTCACAAGGATGT
TGGTGTTTGAGTATGCATCTAACGGGACTTTATATGAACACCTCCATTGTGAGTGGAAAAAACTATTTTA
CACATTTGCTTACTGTATGTTTTAGAGGCTGAAATCCGAACTCTTTGAACTTATCACAGATGGAGAAGGA
TGCCAACTTTCTTGGACGCGGCGAATGAAAATAGTTATTGGCATCGCTAAAGGACTGAAATATCTCCATT
CAGAACTTGACCCGCCATTCACTATATCAGAACTGAATTCAAATGCTGTATATCTCACAGAAGATTTTTCT
CCCAAGGTAAGCAAAAGATTTCTGTATCACTGCTGCAAAACCAATTCGCTGTTAAACTTTCTGGTTAATCT
TACTGTCAATTGTGCTCATCCATAAGACAATTTTAGTTTACTCTCCACGGTTTCTGCTCATTTAATCTTTGA

ACTCGCCGAGTTTCCTTACAAACAATTTGGGCTTACATTTTTCCATTTTTGTTTCCTTCAAGCTCAACCCTT
TGTGCTTTTTAACCCGAGTAATAAAAGGCTGATTTTTCATACCCTTTTCTCTCAAAGTTTAATTTATCTGGT
GCAGCTTGTTGATTTTGAAAGTTGGAAAACAACATTTGCCAGATCAGAAAAGAATTCCGGTGCTATCAG
TAGTGAAGGAGCAATGTGTGTCCTTCCAAATTCTCTACAGAGCCGTCATCTTGACATGCAGGGTAACATC
TATGCATTTGGAGTACTACTGCTCGAAATAATCAGCGGAAGACCTCCATATTGCAAAGACAAAGGGTGC
TTAGTAGATTGGGTAAGCATTTCTTTTCATGAAGTTTTTGTATAGGCATAGTACATAAATATACACTCAAA
CTTGGCCTCAGATGGCAAATAAGTACTCCAACTTTGAGAGTGCACGTCTAGACGAGTTGAGTTGTTTAG
ATGTGCACTCTCAAAGTTGGGAGTGCTTACTTGCTAGCTTAGGCCAAGTTTGAGTGTCTGCTTGTGTATT
ATGCCTTTTGTATAATGATAACTCTCTCTCTGTGGAACCTGGACTCGTGCTTTATGGAATCTGAAGTTGAC
TTTGATATGGCAGGCAAAAGAATTCCTTGAATTACCAGAGGTATTGCCTTATGTTGTAGATCCCGAGTTG
AAACATTTCAGATACGAAGACCTCAAAGTGATTTCTGAAGTGGTTAATCTCTGTATCCACCCAAGTTCGA
GTAGCAGGACTTCGATGAGAGATTTGTGTGCTATGCTGGAGAGCGACATTGACACATCCATAACTGCAG
AGCTCAAGGCATCGTCTTTAGCATGGGCTGAGCTTGCACTCTCATCCTAA
>PGSC0003DMG400005103 LRR-RLKs
CTCATGGCCACCCTCTTGAGAAAAAATTCTCCACTTTCTCTCTCGTGGATTTCATGTTCCCACAGAAACCA
AACATCAAACATAGCCATATCAAACTTTCTTCTCTATTCTTCTTACTAGCGAAAAAAAAAAAAAATGTCAGC
CAGTGCCAACTTTTTCCGGCCATGCCCAATTTTATTGCTCTGTTTTTTCATATTCTTGATTTCTCCTTCTCAT
CAACAAGATGAGTTACAACTCCTTATGCAATTCAAATCCACTCTGAAAACAACACAAATTTCACATCTTTT
TGACACGTGGACACCTCAGAATAACATTTGCAACTTCACTGGAGTTTTCTGTGATTCTGACAGTAAATTG
GTTAAAGAAATCATTCTTTCTGAACAGAATCTATCTGGGGTTGTCTCTTTTGATTCACTATGCTCTCTGAA
ATCACTAGAAAAAATTTCTCTTGGTACCAACTACTTGTATGGTAGAGTCAGTGACCATTTGAAAAACTGT
ACAGAGTTGCAGTATTTGGATTTGGGTAACAATTCTTTTTCAGGGGAAGTTCCAAATTTGTCTTCTTTAAG
CCAATTGGAGTTTTTGAATCTCAATAGAAGTGGATTCTCTGGTTCATTTCCATGGAGTTCACTTGGAAATC
TTACTAGTCTAACTTTCTTGAGTTTGGGTGACAATTCATTTAATAAAAGTCCATTCCCTCTTGAAATCTTGA
ATCTTGATAAGTTATATTGGGTTTACCTTACCAATAGTAGTATTGAAGGCCAAATTCCAGAAGGTATTGG
AAATCTTACTCTGCTTGAAAATCTTGAGCTTTCATATAATGACTTGTCAGGCAAAATCCCAGATGGAATA
ATCAAACTTACTAAGCTTAAACAGCTTGAGATTTATTCTAATGGACTAACAGGAAAGTTCCCTGTGGGGT
TTGGGAATCTCAGTAGCCTTGTCAATTTTGATGCTTCAAGTAACAATCTTCAAGGTGATCTTTCAGAGCTC
AAGTCTTTATCTCTTCTTGAATCTTTGCAGCTGTTTGAAAACCATTTTTCTGGTGAGATTCCTGTTGAATTT

GGAAATTTCAAGTTTACAGAACTGTCATTGTACAGAAACATGTTTTCTGGTTCTCTTCCTCAAAATATTGG

ATCATGGGCAGAACTTCAGTACATTGATGTTTCTGAGAATATGTTCACTGGTTCAATACCACCTGATATG

TGCAAGAAAGGGAGTATGACTGATCTTTTGCTTCTCCAGAACAACTTCACTGGTGGGATACCTTCGAACT

ATGCTACTTGTTTGTCGTTGCAGCGTTTACGGGTTAGCAACAATTCACTTTCAGGAGTAGTCCCTAGTGG

AATTTGGAGTTTGCCAGATTTGGAAATCATTGATCTCACATTGAATCTATTTGAAGGCCCAGTGACATCC

AATATTGGTGAAGCAAAGTCTTTAGCACAGTTGTTCCTAGCCTACAACCGGTTCAATGGTCAATTACCAC

AGACAATATCAGAAGTTTCTTCATTAGTAGCCATTAATCTGAGCGCGAATCAGCTCTCTGGTGATATTCC

AGCAGCAATAGGTGAACTTAAGAAGCTTAATACTCTTCATTTAGAGTATAATTTGTTTTCTGGAAGTCTTC

CAGATTCTATTGGATCATGTGTTTCTCTCTGTGAAATTAATCTTGCTGGTAATTCACTTTCTGGTGCAATTC

CCGAAAGTCTTGGCTCTTTGCGTAGCTTGAACTCTCTCAATCTATCTGATAACACGCTCTCAGGTCAAATT

CCAGCGACCCTTTCGAGTTTAAGATTAAGCCTTCTTGATTTGTCAAACAATAGGTTGAGTGGTAGCATAC

CGGATTCTTTATCAATAAAAGCTTTTAGTAATAGCTTTTTGGGAAATCCAGATCTTTGTAGTGAGAATTTT

GGTAGCCTCAGGCCATGTTCATCAGATCCTCACACATCTAGAGACCACAGGACAGTCATGTTGTGCTTGA

TAGCTGGGGTAGTTGTTCTTGTTCTGTCACTTACGTGTTTCGTGTATGTGAAGTTTAAGCACAATAATCA

GAACACTCCCGTAAAGAGACTTGATTCTTGGGATATCAAACAGTTTCATGTATTGAGCTTTAGTGAAGAT

CAAGTCTTGAAGGCATTGAAGCAAGAAAATTTGATTGGAAGAGGTGGTTCAGGGAATGTGTACAGATT

AGTCTTGAATTGTGGAAAACAGTTGGCTGTGAAACACATTGTTAAATCCGATTCTGGTGACCAAAAAAG

TTACCGGGACAGCTCAGCCATACTGGTGAAGGAGAATAGAAGATCAAAGGAGTATGATGCTGAGGTGA

CCACGTTAAGTTCCATTAGGCATGTCAATGTTGTCAAATTGTACTGTAGCATCACAAGTGAGGACTCAAA

TATGCTGGTTTATGAATACTTAACTAATGGAAGCTTATGGGACCGATTGCACACGTCTCAGAAAGTCAAG

ATGGATTGGTTGGTGAGATATGACATTGCATTAGGTGCTGCTCAAGGGCTTGAGTATCTGCATCACGGA

TATGACAGCCCCGTGATGCACCGGGATGTCAAGTCTAGCAACATCTTGTTGGATGAACAGATGAAGCCA

AAAATTGCTGATTTTGGATTAGCCAAAGTTTTGCATGTTAATGGTACTAAGGACTCTTCTCAGGTTGTAG

CTGGGACGCATGGCTACATTGCTCCTGGTAAATATTTTTCTCTTCCCTTTTCCATTTCATAGTCACTTAAAA

ATTATTTGGCAAATTCTAAATTTTATTCTTTCTTCATTGTTTGTCACAACAGAGTATGCTTACACAACCAAG

GTGACCGAGAAGAGTGATGTTTATAGCTTCGGGGTGGTGCTAATGGAATTGGTGACTGGAAAGAAGCC

AGTGGATGCAGAGTTCGGGGAGAACAGTGACATAGTCCAATGGGTTTGTAGCAAGATAAGAAACAACA

CTAGCATGATCGATTTGGTGGATTCAAGCATTTTCGAGGGGTTCAAAGAAGATGCTGTTGAGGTTCTTA

AAATTGCAGTTCATTGCACATCAAGGACGCCAGCATTAAGACCTTCCATGAGGATGGTAGTTCATATGCT

AGAAGAAGCCGAGCCTTGCAAGCTGACAAATGTAGTCGTAAATTCACCGAATGAGGATGGCAGGAAAC
AAGGACTTGCTAACTAATGGCAAGTCATGATGTTGAACTATCTCGTTGAGAGCAATGGAATTTTGCAGA
ATATTGAACTATGTTGTAAGTTCACAAAGAATGCTTAGTGTACATATGACACTTTTGAAGAAATAGTGTA
TTTCACAGCTGCATTAATAAATCCAACTAGCAAGTAATGTATTTCACATCCAAATTACTTATACAAACTGA
GCAGTTGATCTACCCTACAACCAATTTCATTTTTCT
>PGSC0003DMG400005254 LRR-RLKs
AAAGATTTAAAAGGTAATTGGCACCTTGCTTTTTAGTGGCCTACAATGGAAAGAATGACTTTATTGCCAA
AAGGACTTTAGGCAAAGTATGCAATAAATCCTCTTCCCACTCACATTGAAAATCTGTCTTCAGTAGTTCAA
TTAGTATCTGTCATAATTCTTGCCTACTGGATTGATCCATTTTCAATATGAAGAGGTAGTAAATCAAATGA
AGTTTCCCTTTCATTGAACAACACAACTCTCAGATCCCTTTCATGCATATCATATAAAAGTAAGTTAACTT
GTGTGCCATATATTTTAGAACTAAAAGAGTCAGTAAGGCAAAATCTTGGCTTTGTCGCAATAACCAAATG
AATTTTTTCTATTCTGAGATAGAGTTCTTCCTTTTTCGTTACTTCTTTAGCTGATACTATTTCTGGATTGTAT
AGTTTTCTTGCATTGTGTTAAAGCTTTTTCATTGTATTTGTTAATGGTTTCTTATAGCAATCTGATTTTATCT
CTCTTTCACAGTGGCTAATGAGCTCTGATTTTGTCTAATGCTCTTCAAAAATAGTCAAGGAGAGAGGGAA
GAAGTGAAGGACTGCTGTAACTAGGATCTTGCTTTTGCATTTTTGTCAGCCAGATCAGTGAAAACATAAT
GAAGCTTCAGCCTTTACTGGCAGCTATTGCTTTTCTTATTCTGCTGCTATCAGTATTTCTTCATGTTATAGC
TGATCTTGATTCCGATAGACAGGCGCTACTCGACTTTGCAGAATCTGTCCCTCATATACGAAAACTCAAC
TGGAATTTGGCTCTCCCTATTTGCAAATCTTGGGCTGGTATAGCTTGTAACAAAGATGGAACGAGAGTCA
TTGCAATCCATCTCCCTGCTGTAGGACTTTTTGGTCCTATCCCAGCTAATAGTATAGGCAAGCTAGATGCT
CTTAAAGTCCTTAGTCTAAGAGCCAATTATCTTAATGGAAGTGTTCCTTCTGATCTCCTTTCGATACCGTCT
CTCCAATCTGTCTACCTTCAGCATAATAGCTTCTCTGGTGATATTCCTGTTTCTTTATCTCCTCGTCTTGGT
GTACTAGATCTCTCCTTTAACTCTTTTACTGGAGAAATTCCCTCCACAATCAAGAACTTGACGCGATTATC
TGTGTTGAACCTTCAATTCAATTCATTGACAGGAGAAATCCCCAGCCTTGACACCCTCAGGTTAACTAAC
CTGAATTTGAGCTACAATATGCTAAATGGCTCCGTTCCAGACCCCCTCCAGAAGTTTCCACTTACATCATT
CGCAGGAAACTCTCATTTATGTGGTACACCTCTAAATAGCTGTTCCTCGACCCCGTCACCTTCTCCAGCAG
CTGATGGCTCTGCAATCCCTGAAAAACAGAAGGCTGTCCACTCTAAGAAACTAAGTACCGGAATCATCAT
TGCCATTGTGGTAGTGGTTTCTTTAGTAATGTTTCTCCTAGTTTTAGTAATCTCCTTCTGCTGCTTGAAAAA
GAAAGTGAGTCATAGCACTAGCATAATTAAAGAGAAGGTGGCTAATGGGGGAAGAAGTGAAAAACCC
GAGGACTTTGGAAGTGGTGTGCCAGATGCTGAGAAAAACAAGTTGGTGTTTTTCGAAGGTTGCTCTTAC

AGCTTCAATCTGGAGGATCTTCTTAGAGCTTCTGCTGAGGTTCTTGGAAAAGGGAGTTATGGTACAGCTT
ACAAAGCTGTCTTGGATGAGGCAACAATTGTAGTGGTGAAACGGCTGAGAGAAGTAGGGGTTGCCAAA
AAGGAGTTTGAACAGCATATGGAGATAGTTGGAAGGGCTGGGAGGCACCCTAATATCGTTCCACTCCG
CGCATATTATTATTCCAAGGATGAGAAACTTCTTGTTAATGAATACATGCCTGCTGGTAGCTTGTCAGCG
GCCTTGCACGGTATAAATTGCTAACCTCTATCTAACGTATGTTGCTCAGACTTTCCAAAATGTTGCTGCAC
CCGTGTTGGATCCTCCAAACATGTACCTGTCCACATTTTTGAAGAGTCCGAGCAGCACAACCTCTAAACT
TTTTTCTTGCACATTAATTGCTTTGATCATCTGTAATGATTCATATTTTTACGCAATCTTGATAATGAATGA
CAGGTAATCGAGGTATTGGAAGAACTCCACTAGATTGGGATTCAAGATTGAAGATATCTCAGGGAGCA
GCAAAAGGAATTGCCCACATCCACACTGAAGGTGGTGTGAAATTTACTCATGGAAACATCAAGTCCTCC
AATGTACTCCTCACAAGAGACCTAGATGGATGCATTTCTGATTTTGGCTTAACACCTCTGATGAACTACAT
ATCATACAAATATAGATGTGCTGGTTACCGCGCACCAGAAGTTATTGAAACACGGAAAGGGACACAAAA
ATCCGATGTTTATAGCTTTGGAGTGCTGCTCCTTGAGATGCTAACAGGTAAATCCCCTCTCCCATTATCAG
GGCAGGATGAGGTAGTTGATTTGCCTAGGTGGGTCCGGTCCGTTGTAAGAGAGGAATGGACAGCTGAA
GTTTTCGACGTAGAGCTATTGAAGTACCAGAATATTGAAGAAGAGATGGTGCAGATGCTTCAGATTGGA
CTAGCTTGTGTAGCAAAGGTGCCTGATATGAGACCTGCTATGGGAGAAGTGGTTAGAATGATTGAAGA
AATTCGACAACCAGAAGGTGAAACGAGACCTTCCTCGGAGGACAGCAGGTCCAAGGACTCTAATGCAC
AAACACCTGAATGAAAAATGCAGTTTTAGTCATTCAGCTTTCAATTCTTGATCATTTTAAGAATGCAATTT
ATCTCCATTGCCAACTTTTGCATGTTCTTGGACATACAAGTAGTGTTATTTTTATTTTATTTTTTTATCTTGA
TGTTATATCCTATTCTAGAAACTTATCAGCTCAACATAAATGAGAACCTTTGTTTTTCTCTATTTTTAAATC
TTTG
>PGSC0003DMG400005485 LRR-RLKs
AATAAAGATAAAAAATAAACCTAAAACTAACCTACACTACCCCATTAACCCCTTATACTACTACCCACTCC
ACTCACAACCCCACAGTCCCACATTAATCCATCACTCCCCTCAACCACACACACGTACCACACACTACTAA
ACATCAACAACACACCCCTTCAACCCATATACACAGAGTCACAAAAAACGAAGAAGGACGACACTCAAG
AAAACAAAAAGAAAAACTTTCCATTTTATTTCATTATCCTCTTAAAAATTCACCAACACATTCACATTCCTC
TTCCTTTCTCATTATTATTTTCATATAAACACCATACATATACCACCACACACACCTACAAAATTTGTGGGT
ATTCTAATTGAGCTTCAGTTAAGATTTTGCGGTATTCAACTTTACCTTATCTTTCTATTTATTTATTTATGGA
ATTTGATGTAATTTTATGACAAGTTCTTTTTATATTTTGTTTCATTCATGAATTTGATTGGGATGATTAATT
TGTTAGTTGTTATATCTCTATTTCATGAATAAGGTAGCTTTAATNNNNNNNNNNNNNNNNNNNNNNN

NNNNNNNNNNNNNNNNNNNNNNNNNNNNNNNNNNNNNNNNNNNNNNNNNNNNNNNNNNNNNNNNNNNNNN
NNNNNNNNNNNNNNNNNNNNNNNNNNNNNNNNNNNNNNNNNNNNNNNNNNNNNNNNNNNNNNNNNNNNNN
NNNNNNNNNNNNNNNNNNNNNNNNNNNNNNNNNNNNNNNNNNNNNNNNNNNNNNNNNNNNNNNNNNNNNN
NNNNNNNNNNNNNNNNNNNNNNNNNNNNNNNNNNNNNNNNNNNNNNNNNNNNNNNNNNNNNNNNNNNNNN
NNNNNNNNNNNNNNNNNNNNNNNNNNAAATATATGACCCGTTTCAAAGGTCGGTTTCAATTAAGAATGTG
GTTAAACATCTTTCTTTGGAACACTTAAAAAAAACTCAAGGATGCAGTGACGCACCTTGTCCAAATTGTT
TTAATAATTATTTTTTGTTAATTCAAACCAAGAGGTGTAAATTGAAATTATTAAGTGTTATGAAATCGAGA
GGGGAAGACAGTTATGTCTCTAAATGTCAAGATCGAGAATGCAGTTATGCATCCGGTTTAAGACTAATA
AAAATTCAACAATTAATAAAATAAGTGAACCATGGCCTTAGACACAAATTATCCAAAAATAGTAAAATAA
TGTATAAGTCAATAAAAGCGACCGTGCTAGAACCACGGGACTCGAGGGGTGCCTAATACCTTCCCCTCG
GTCAACAGAATTCCTTACCCGAATTTCTGGTTCGCAGACTAATATAAAGAGTCATTTCCTTTTGATAAGG
GATTAAACAAAAAGGTGACTTGGAACACCATAACTCAATTCCAAGTGGCGACTCTGAAAAAATAAAATA
ATCCCTAAATCAATAACGTCACTTAAATTGGAAAAAACCCTTCCGCCGCCTTGCGCAAAAAGGGGTGTGA
CACTAACAACGATAAATCTTAGGAATAATAGCTTCCATGGTGAAGTCCCTATGGAAATTGGCAACCTCTT
GCAACTGCAACATCTTAATCTCACTTGGAATTCATTTACTGGAACCATTCCTGCTAATCTGAGTTACTGTA
AAGAACTTAGATCACTTGCTCTAGAATATAATAGTCTGGTTGGTAAAGTTTTACCTGATCAGTTCAGTTCA
CTTTCAAAGTTGAATTATTTAGGCCTTGGAAGCAACAATCTAACAGGAGGTATCCCATCTTGGATAGGAA
ATTTTTCGACTCTTCGAGGTCTTTCCCTTGCAATCAACAATCTACAAGGACCAATACCTCGAGATATTGGA
CGTTTGTCAAACTTGCAAATATTCCAAGTTTATGGAAATCAGTTGAATGGTACAATTCCTCAATCTCTTTT
CAATATATCCTCTGTTTACTATTTCTCTGTTACTCAAAACTTGTTGTATGGAGAGCTTCCATCAGATATAG
GCCTTACTCTTCCGAATCTTGTGGTGTTTGCTGGTGCTGTGAACGATTTCACTGGACCAATTCCTGTTTCA
TTATCAAATGCAACAAAACTTGGTGTACTTGAATTGTCTCAAAATAAGCTCACCGGAAATGTCCCTACGA
GTTTAGGACAATTGCAAGGGTTGTATAGGATGAACTTTGAGATCAATAGTCTTGGAAGAAACACAAGTG
GGGATTTGAGGTTTCTTGATTTCCTAGTTAACTGCACAAGTTTGCAAGTTCTTAGCTTTGAGGACAACATT
TTGGGAGGTGAATTGCCTAAAACAATCGGTAACCTTTCCACGAGGCTAGAAATATTTGCTCTTGGTTATA
ATATGATAGTTGGTTCTCTTCCTACAGGACTAGAAAATCTTGTTAATTTGACCCTTCTATCACTGGATAAT
AACTACTTGAGAGGCAGTGTTCCTGAGTCTTTAGGTAAGCTTCGACGCCTGCAAGGACTGCTGTTGAAT
GGAAACAAGTTATCAGGAAGGATTCCATCTTCTATTGGTAACTTAACATCTTTGTCTACTTTACACATTGA
GGACAATGAATTAGAAGGAAACATACCTCCGGAGCTCGGGCAGTGCATCCGCTTATCAAGGCTAAACCT

TACAGGAAATAATCTTGTTGGTTCCATACCAAAGGAGCTTGCAGGTCTTTCTTCTCTTTCGATTTCCTTAG
CCTTAGCGAACAATTCGTTGACCGGTTCCTTGCCAGCTGAATTTGGAAAGTTGATAAATCTCAAGGAAAT
GGATATTTCACATAACAAATTATCAGGTGAAATTCCAAGTACTCTAAGTAGCTGTGTCAGCTTAGAGCGC
TTCATAGCGAATAACAATCTGTTTCGAGGAGAAATTCCTGAATCCTTGCAAGGTTTAAGAGGCTTAGAA
GAAATTGATTTGTCACACAATAACATCTCAGGAGGAATACCTGAGTTTCTTGGGAAACTTCCATATCTTA
GGAAGCTTGATCTTTCATTCAACGAACTTGAAGGCGAAGTACCAACTGAAGGGATCTTTGCAAATGAAA
CCGCCGTCTCAATCTCAAGGAATGATGAACTATGTGGAGGTCCTCCAAACTACAATTTTCCTACATGCCC
TAAACAAAAAGATGCATCATCAAAGAAACACATTAGTTCAAGGATAAAAGTAGCAATTATTATTTCGGTT
GCATTTCTATTTTTACTTTTGTGTTCTTTTGCAGCTTGCTATATAGTAATTAGGAAGTCGAGAAAAAGAGA
TCTTACTGGACGGTCCTCAAGAGAAAGGCAGTCTGATCATTTTGATGATGAAGAACCAACTTTATTTAAG
GATCCAATTTTGACAGCAAAGATAACTTACCAAGATATATTCAAGTCAACCAATGGATTTTCTGAGGATA
ATCTAGTCGGTACAGGAAGTTTTGGTTCTGTTTACAGAGGAAAGTTTCAGGTTTTTGATAAAGTAATGGC
AGTGAAAGTATTGAACCTTCAACAAAGAGGTGCTTTGAAAAGCTTTTCGGATGAATGTAGAGCTTTGAA
AAGCATAAGGCATCGAAATCTACTTAAGATAATAGCTGTTTGTTCAAGCATCGATTACCAAGGTAATGAT
TTCAAATGCATAGTCTTTGAGTTCATGGAAAATGGAAGCTTAGATGACTGGCTGCACTCGAAAGGTGAT
GAGAAATACCTCAATATCATCCAAAGACTAAACATCGCAATAGATGCTGCTTCAGCACTTGATTATCTCC
ACAACAATTGCCAAGTATCAATTGTTCACTGTGATTTAAAACCGAGCAACATACTCCTTGATGAAGAGAT
GACTGCCCATGTTGGTGACTTTGGATTGGCAAAATTTCTCTTCAAATCATCATGGAACAAACATACATCT
ATTGCACTAAAGGGTTCTATTGGTTATATCCCACCAGGTATGTCATTAATCCATGTTGATTTCTCTTGCAT
ATCTATTTCCCTTATCAGGGGTGCTAACATGTAAGATGTTTGTGTACCTCAATCTCATATACTCCCTCCGTT
TCAATTTGTTTGTGTGGTTTTGACTTGACACAAAGTTCAAGAAAAATAAAGAATACTTTCGAATCTTGTCG
TCTTAAACTAAAGATACATAGAATGTACGAACATGCCCTTTAATCTATTGAAATTTAAAGAGTTGTCAAA
AAGCAAAGAGACACTCTTTTATAAACGGACTATAAAGGAAAGTAAGACAAATGAATTGAAACGAAGGG
AGCTTATCTTTCTGATGCTTAAGGTGTCGAATGAACAAATCAATAACTAAGCATTCATATGAGGTTGTCA
TTTTCATGTGAGTGAACATATGTTCAGTGTCCCAACATAATTCATCATTATCTTGCAGAATATGGATCAGG
TGTAAATGTGTCCACTCTAGGAGATGTTTATAGCTTTGGTATCATGTTGCTAGAGTTGTTTACCGGTAGA
AGGCCAACCGATGAGATCTTCAAAGATGGCCTGAATATTCACCAATATGTCAAATCCCATCTGCCTAGAC
GTGTCACGGAGATTGCTGATGCATCATTGCTTTTAGCATATGAAGAACACAATATATATGAGGACAATG
CAAGTGATTTGGAAGAAAAGGCAATACTTCAAGATGATGAGTATATATCCAAGCTAAACACCAGCACTA

TTATACAAGAATGCTTGATCTCAATCATGAAAATAGGACTTTTATGTAGTAGCTCATCGCCAAGAGATCG
AATGCCAATAAGCATAGCTTTGAAAGAGATTCACACAATCAAGAACTTATTTCTAGAATCTAAGAGGATA
AATAATAGTATAGATAGATCAGATGGTAATAAGCTAATCAAATTATCATCTTCAGAATTTTATTGATGAA
GGCAAATATACATAGAATTACAACTTTGAAATAGATATTATTTACAATTAGTACTATAGATAGCCTCACA
AAATGTGAATTGTAACCAAGAAATTAAGGGATATGATTGAGTTTTTTTTTTTTTTTTGAGTAATGGATGG
AGTTCCTTTTTCCATCCTATCCCATTCACC
>PGSC0003DMG400005896 LRR-RLKs
GAGAAATCAAAGGACAGAAGGAGACAGAGAAAAAAAGGTGTCATGAATTTGTACTTGTCTTTAGGGTT
TGATCCACAACTCTTTGCTAAACTCATCTCTCTTTCTTACAAAATTCACTCACATTATTAAAAAAAGGTACC
ATTTTTCCAACAATTTATAGTTATTATGTACTTGCATCTTTATATGTTACTGTTATGTACTTTGTTCATTTGG
GGTTCACAATTCTTGAGAAAAAAGTACTATGTAATTTTATGATAATCTTGAATTTTCATCATGTGTGTATA
TAAGTATTGTGATTTCAGAGGTGTTGTTATATACTTTTGTACATTAGGTGTATTTCTTGTAAATGTTGTTTC
TGGTGTAACAAATTTGAATGATTTCAAGATTTTGAGTGATTTTAGAAATGGATTAGAGAATCCTGAACTA
TTGAAATGGCCTTCCAAGGGGAATGATCCTTGTGGTCCTCCTGCATGGCCTCATGTGTTCTGTTCTAGTG
ACAGAGTTACCCAAATTCAGGTTCAGAGTTTGGGTTTAAAGGGTCCTTTACCTCAGAACTTGAATCAACT
TGATAAGCTTCAAAATCTTGGCCTTCAGGGGAATGGTTTTAGTGGGAAATTACCTACTTTCAATGGATTA
TCTGATTTGAAATATGCATACTTGGACAACAATGAATTCGATACGATTCCTGGTGATTTCTTTAATGGTCT
TAGCAGTGTTCAAGTTTTGGCTTTGGATTATAATCCGTTTAATGAGAGTGGATGGTCTATTCCGATTGAG
TTGCAAGATTCAGCTCAGTTGACGAATTTTTCTTGTGTGCAATGCAATATAGTTGGACCTGTGCCTGATTT
TTTTGGGAAATTGCCTTCACTAAGTGCGTTGAAGCTGTCGTATAATCGTTTGACCGGTGAGATACCAGAC
AGTTTTCGTGATTCAATGCTTCGGATTTTGTGGTTGAATAATCAAGATAGTCCTGGAATGACGGGTCCAA
TCGACTTAATTGGTACTATGGATCAGCTTATGTTGTTATGGCTTCAAGGCAACAGTTTTAGTGGACCAAT
TCCTGATACAATTGATGATTTAAATGACTTGAAGGAGCTGAATTTTAACGGAAATCAACTCGTTGGTCTG
ATTCCGCAAGGTTTGGCAAATCTAGACTTGAGTGTGTTAGACTTGAACAATAATAAGCTCATGGGTCCTA
TCCCAAAGTTTAGAGCTGCGAATGCTACATATTCCTCAAATTCTTTCTGTCAGCCAACTCCTGGTGTTCCT
TGTGTTCCTCAAGTTAATGCACTTTTAGATCTTCTTTGCGGTTGGAATTATCCAGCCAACCTTGCTCCTGA
ATGGAGTGGCAATGATCCATGTACAGGTCCTTGGTTGGGAATCAGTTGCAATCCTAAAGGCCAGATTAC
GATTGTTAATCTACAGAACAAGAACCTTACGGGTACGCTTAGCCCTTCACTTGCAAACTTGGATTCACTG
CTTGAAGTTCACTTAAAAAGAAATAGTTTGCATGGTCGAGTTCCTGCAAACTTAACAGAGCTCAGATCCT

TGAGATTGTTGGACTTAAGTGGGAACAATTTTGATCCACCATTACCGAAATTCCGGGATAGTGTGAAAG
TCATTACAGACGGAAATGCTCATCTTGTTGCTAATGTAACTGCGGCAGCTCCTCCTCTGAGTATTAGCCC
ATTTCCTCCATTGAGCCACAGTCCTAAATCAAGCAAGGAGTTACCATCGAAAAGCCCATTTCCCGGTGAT
AATCAGCCAACATTACCAGATACACCGCCCTCCCCAGAGAAAAGTTCAGGTTCAGACTCAAAGTCTCCCA
GTGGAACCAAAGGGCAGACCACATCAGAGAACCATGATAAGACCATGATCATTGTAGTTGTGAGTGCA
GCTTCTTTCGTTTTCACTCTTCTTGCCGTTGTTTTGTATTTCAAGAGCAAGAGAAAACGAGAGAAGGATTC
AGGTACTATTGTTATCCACCCCAAAGAGCCATTCGATCAAGATAACATTGTTAAGATCACTATCTTAGAA
GATCCCATGATGTACAGCTTACAGAGTGGTACTACAACAACTACTACAACTAGTGGTGGAACAAAAGGT
AGTCGAGTGATTGAGATTGGAAACCTTGTCATTTCAACCCAGGACCTTCGGAGGGTAACCGATAATTTT
GCACCAGAGAATGAGCTTGGTCGTGGTGGCTTTGGAGTTGTTTACAAAGGCGTAATTGAAGATGGAATT
CAAATTGCAGTGAAGAGAATGGAATCTGCCATTATCAACAGCAAGGCATTGGATGAATTTCAAGCAGAA
ATAGCTGTGCTTTCTAAGGTTCGACATCGCCATTTAGTTTCACTTTTGGGTTATTCCATTGAAGGAAATGA
GAGGCTTTTGGTATATGAATATATGTCAAAGGGCGCTTTGAGTAGACATCTTTTTCGCTGGAAGATCCTG
AACTTAGAGCCTTTATCGTGGACAAAGAGACTCAATATTGCTCTAGATGTCGCTCGAGGGATGGAGTAC
CTTCATAATCTGGCACACCAGAGCTTCATACATCGCGATCTCAAATCATCAAACATTCTTCTAGATGATGA
TTTTAGAGCAAAAGTTTCTGATTTTGGATTAGTGAAACTCGCTCCTGACAAAGAAAGGTCCGTTGCAACA
AGGCTTGCAGGAACATTTGGATACCTTGCTCCCGAGTATGCTGGTATGTGTGCATTTTTTTCCATTGGTAT
CTCTTTCTTAGACACTCGTTTCGACTGTTCTGGTAAATATTATCATTTAAGCATCAATTGTATTTTTTACGC
TCGGGTAAGCTGTTACATGAACACATCTCTACCTGAACGAAACCAATACTCTTTTAGTCCCTCTATTTTAA
GAAATGCATCAGTTCTATAATGAGAATCCAGACTGCTAAATTGCGCTTCTGACCTTCCAATCCCTTCCTTC
TCCAGCCAAAATAGAACAGAGTAACCAATGGAGCATTTACGTTTTTCCTTTTTGTTGTCGGTGTGCGCTA
GCTACTAAATTGATATACCTCGGACATTGATGTATCAATCTCGCATCATTCAGTGATATTCTCATTCTGTTC
ACTATCTTTGTTAAACAATTCAAGTAGAAGGTTTCACATCCTCTCTCCATATGATCATTCAGAGCATAGTT
ATCATCTTGACTCCACAAAAAGAAACAGAAACTCTATCCCTTTAAAGATACTTTTCTCTTGAGGCATGGTA
CTGAAATATGTTTGCGTAAAGCTGTTCAACAAGCATGTCATACCTGTTAATTTTGGTGTAGAAACTATTG
CTTACTTTATCTTTATGTTACCCGTATATTTTAGTTCTCAGTCTTGTTGAACGAATTACCTAGTCACCTTTCA
TATATCCCAACTCACATTTTACCTCTCCAGTGACCGGAAAAGTCACAACTAAAATAGATGTCTTCAGCTTT
GGGGTGGTGTTGATGGAACTTGTGACTGGACTAACTGCCCTTGATGAGCATCGGTCCGAGGAAACTCG
ATATCTAGTTGAGTGGTTCTGGCAGATAAAATCGAATAAAGAAAACCTCCTTGCTTCCGTTGATCCAGCT

CTTGACGTGAAGGAAGATATCCACAAGAGCATTTGCACTATGGCTGAATTAGCTGGACATTGCACTGCG
AGAGACCCCAATCACAGGCCTGACATGTCACACGTTGTGAATGTGCTTGGCCAACTAGTTGAGTCTTGG
AAACCCGTAGAAGAGTCGGATGACTATTCAGGAATTGACTACAGTCTACCTCTTCCAGAAATGTTGAAG
GACTGGCAGGATGAAGACACTGGAGACTTCACCAGTACTAGTCAAGATAGCAGAGGAAGTATTCCTGC
TAAGCCAAATGGATTTGCGGATTCTTTTACATCCAACGATGCTCGATAGGTGAAGTTGGTTTTGGCTACC
AAAGTCCATTGCTTCCTTACTATTAGTTTTGCTAGCATTTTCGACATGAATTTTGCTTTTGGCTCGTGAAAA
TAATCTATGGAGGCGTTGCGTCCAGTCTATATATGGTGAATATACATATACCGGGTTGTCATGAATGATT
GATCAAGAGATCAAATTGGTGTCGTCCAGATACAGAGCAAAAAACAGAAAAAACTTTTCAATTTAATGT
TGCTTACACAGTTATTTGTTTTGAATGTTGCTTGTAAAAGTTATATTTATAATAGGTTGGTCCAATTTGCT
GTATAAGTTATTTACATGACACTCTTGAGAATCTTATGCATGAATTTGAGAGTGATGCTCAAATTAATAAT
GCAAAAAAAG
>PGSC0003DMG400006065 LRR-RLKs
ATGGAGAAGCACATTTTCTTATTGATTCTTCTCTTTCTAGTTCAATTTTCTGTATCATTTGCTAACTCAAAT
GTGACAGACAAAGAAGCTTTACTAGCTTTCCAAAATCTTATTACAAGTCCTACTCATTTTTTGGCCAACAA
TTGGACCAACAATACTTCTTTTTGCTCTTGGTTTGGTGTCAGTTGCAGTCCAAAAAGGCAAAGGGTTGTA
GCCTTGGATCTTTCTAGTTTGAATCTTCGAGGTACAATTTCCCCGTCTTTGGCTAATTTGTCCTTTCTCAGA
GAGCTTAATCTTGGGAACAACAACTTTCATGGCGCCATCCCTTATGGCATTGGCCACTTGCCTCGCTTGC
GAGTGATTGATATTCAAAACAATCAGCTCCAAGGAAGTATTCCAACAAGCCTATTTCAACACCAGAAAGT
TCAAAAGATTTCATTGGCTTTCAATAAACGCAGTGGTGAAATGTGGAATGGTTCATGGTATGTACCAGA
ACTCAAGGTCTTGTATCTCACCAACAATAGTCTCACAGGCATAATCCCGCCTTCTATTGGAAATTCCACAA
AGTTGAGGAACTTCAGTTTGTACGGTAATAGAATCAATGGCAACATTCCGAAGGAGATTGGTAATCTTA
GCCAACTTGCAGAGTTGTCCTTGGGAGATAATCAATTAACAGGTTCCATTCCTGCAACACTGTTTAATAT
CTCGTCGCTACTTATTGCATCTCTGAAAATCAATAACCTTTCTGGTCCTCTCTTGCTTGATGAAGGGAATA
TTGTGTCAAATCTGAAGCATCTAAGTATATCTAAGAACCAAATTTCTGGTATCATTCCTTCCAATATTTGC
CAACTCAAAGAGCTCAAAGTGTTGTCCATATCTTTCAACAAGATAACTGGAGATATACCCAAAAATATTG
GTTGTTTAGCTAAGCTCGAGGTGTTCTATATCGGAGGTAACGCACTAAGTGGGACTTTTCCTGCTTCATT
GGGAAATATATCTACTCTTGAATATCTGGATTGTCCAAACAATTGCATAGGGGGGCAAGTTCCTCCAGA
ATTAGAGAAGCTGTCAAATTTGAGGCAATTAAACCTTGGCCAGAATTATAATATTATTGGTCAAATTCCG
AAGGCTATTTTTAACATGTCCTCTTTGGAAATGATTGCTTTCAATCTCAACAACCTCTCGGGTAGAATTCC

AGCCACTACAGGTCTTCATCTTCCGAACCTTGAAAGACTTTACTTGTCTAAAAATCATCTCGAAGGGGAA
ATTCCTCCGCACATCACAAATGCTTCCAAACTTAAGACATTGGAGCTAAATAGTAACTTTTTCAGTGGCA
GTATTCCAACTAATTTGGGAAATCTTCGTGAGCTGCAACTGCTGCACCTAGATCATAATCAACTTACCAAT
GAACCAAGAGAGCATGAGTTGCTATTCTTCAGTTCTTTGGCGAACTGTAGGATGTTGCGAGATCTAGAT
ATGAGTACCAATATGTTGAATGGTACTCTGCTCAATACTATCGGAAATCTTTCATCTACTATTGAATTCTT
TGCAATAATAGATGCATACATCAATGGCCCCATCCCCACAGGTATAGGCAACATGAGCGGTCTAATATCT
CTAAGCCTTCATGGAAACAACTTGGTGGGAGGTTTCCCTTCTGAGATTGGTAAGCTTAAACAACTACAAG
GACTATATCTAAATAACAATAAATTGCAGGGACATATTCCAGAGGTGGTATGTCGTTTATCTAATTTGGT
TCAATTAGCTCTAGATGATAATGAGCTCTTTGGGTTAATTCCAGCATGTATAGGAAATTTTAGCATGCTA
CAATATCTTTCATTGAGTTATAACAATTTTGCATCAAAGCTTCCTTTGAGTCTTTGGAAGATGAGTGGCCT
TCTCCACCTAGACGTGTCACAAAATTCTATAGAGGGAGAAGTTCCACTGGATATTGGAGAACTGAAAGC
AATTGTAGATCTATATCTTTCTAGTAACCGCCTTTCGGGCATGATACCAAACAGTTTGGGGGAACTCCAA
ACCCTGGAGTCTCTTGACCTGTCAAACAATTCATTTTCAGGCCAAATTCCATTATCCTTTGCCAACTTGAT
AAGCTTAGAATTCATGGATTTGTCTTTAAATGCCTTGGCAGGTACTATTCCTAAGTCTTTGGAAAAACTCT
TGTACCTTAAAGCCATCAATGTTTCATTTAATGAATTAGAAGGTGTAATACCTAGTGGTGGTGTGTTTGC
AAATTCCACTTTGCAATCATTTCTTGGTAACAAAGGCCTATGTGGAATGCACATATTGGATGTTCCTGCTT
GTGCTGTCACTAATCTTGGACATCAATCAAAGTTTAAGAAGCTTGTGCTAAAAATTGCTATTCCAGTGGT
TACTTCATCCTTTCTAATATCCTTGTTTGCTTCAATTTGGATGATGAAACGACAAAAGAAGGGAAATTCCA
AAGACGTGGAAAAGGTACCGGAGGTCAAGACACATCAATTAGTATCTTATCACGAGATTCAAAGAGCTA
CAAATTATTTTGATGGATCAAACTTAATTGCTGTGGGAGGTTCTGGCTCTGTGTACAAAGGTACATTATC
TAGCGGAGTTGTGGTTGCAATAAAGGTTCTAGATTTGCAAAATGAGGAAGTATGCAAGAGGTTTGATAC
TGAATGTGAAGTGATGAGAAATGTTAGGCACAGAAATCTTGTATCGGTGATTACTACTTGCTCTAGCGA
ATACATTAGAGCTTTTGTCCTGCAGTATATGCCAAACGGGAGTCTTGAGAATTGGTTGTACAAAGAAGA
TTGCCACTTGAACCTTCTTCAAAGGGTTTCCATAATGTTTGATGTGGCTGTGGCTGTTGAATATATGCATC
ACAGACATCACACTCATATTGTTCATTGCGATCTAAAGCCAGCCAACGTTCTTTTGGATGAAGAAATGGT
GGCACATGTTGGTGATTTTGGAATTTCCAAAATCTTAGCTGTAAGCAAGTCCATGGCATCTACCGAGACA
TTGGGCACTCTCGGATATATTGCACCAGGTATACTAGTCTCTTTCTTTTTTACTACACAAATTATAACTAA
GCCCTTCCAAAATCTAAACAAAAAGCAAGTGTTTGTTTGTTTTGTTGTTATATTGGATGAATAATTTTTTTT
CATCTTTTGTTCTTTTAAGAATATGGCTTGGAAGGAATAGTGTCCACTAGTGGCGATGTCTACAGCTATG

GTATCATGCTAATGGAGGTTTTGGCAACAAGAAGACCAACTGATGCAGAGATATTCAACGAAAATCTTG
GCTTGAGGGAGTGGATAAGGCGAGCATTTCCAAAAACTATCATAGAAGTTGTGGATGCCAATCTTTTTC
CTGAGGAAGAACAAATCACTTCTAAAAGTGAAATCTGCATAATTTCCTTGATAGAATTGGCTTTAGATTG
CGCAAAGGAAAGGCCAGAGTCAAGGTTAACCATGAAGGATGTAGTCAAGAGGCTTAACAAAATCAAGA
ACACATTTTTGGAAATATGA
>PGSC0003DMG400006280 LRR-RLKs
CCAAAGCCAAAAATTACTATCATATTTTATCAGTATCAATGGAATCTTTCAAAATACTTGTTGCATCTTCTT
CTTCTTCTTACCTTTGATGTTTTCCGAAGTATTCGAACTTCAATCACTAGTTATCTAAATGGCTACTTCTAA
CACAAGTCTCTTCTTTTTCGCGTATTTCTTACTCGTGTTCCTCATTACTCCATCTCAATCGCGTAACGTGTCT
CTGAGACGACAGGCTAAAACTCTAGTTTCATTGAAACAAGCATTTGTAGTATCCTCTGTTCCTAGTACTCT
GTCTACTTGGAACATGTCAAATTATATGTCTATATGTTGTTGGACCGGTATCACCTGTGATGATACCAAAT
CAGTAACTACCATTGATATATCCAATCTAAACATTTCTGGCTCTTTATCACCTGATATTCATGAGCTCACTA
GACTTCGCGTCCTGAATATTTCTAACAACTTGTTAGGTGGGAACTTGAGCTGGGAGTATCGAAAGTTTAA
CGTACTTCAAGTGTTGGATGCTTATAACAACAATTTCACTGGTCCACTCCCCTTGGGAGTTACTCAACTTC
TGCAGCTGAAGTATTTGAATTTCGGGGGTAACTACTTTTCAGGGAAAATTCCTTTGAGTTATGGTAGTTT
TAATCAGCTTGAGTTCCTGTCTCTTGCTGGGAACGACTTGCACGGTCCTATACCAAGGGAGCTGGGCAA
CGTTACGAGCCTCAGGTGGTTACAGTTGGGTTATTATAATCAATTTGATGAGGGGATTCCGCCAGAGTT
GGGAAAACTTGTTAATTTGGTTCATCTAGATCTTTCTAGCTGTAACTTGACGGGTTCGATTCCAGCAGAA
TTGGGCAATCTTAATATGTTGGATACTCTTTTCTTGCAAAAAAATCAACTTACTGGTGTATTTCCTCCTCA
GCTAGGGAATTTGACAAGGTTAAAATCTCTTGATATATCCGTCAATGAACTCACAGGAGAGATACCAGT
TGACTTGTCAGGACTCAAGGAGCTCACATTGTTGAACCTCTTTATCAACAATTTGCACGGTGAGATTCCT
GGATGTATCGCGGAGCTGCCAAAGTTGGAAATGTTGAATCTTTGGAGGAATAATTTCACTGGATCGATT
CCTTCTAAGCTTGGGATGAACGGTAAACTAGTTGAAATTGATCTTTCTAGTAATAGACTCACTGGCTTGA
TACCAAAATCTCTATGCTTTGGGAGGAATTTGAAAATTTTGATTCTGCTTGATAATTTTCTGTTTGGACCT
TTACCTGATGATTTTGGGCAGTGTCGAACCTTGTCCAGAGTCAGAATGGGACAGAATTACTTGAGTGGA
TCAATACCAACAGGGTTTCTTTATTTGCCTGAGTTGTCACTGGTGGAACTGCAGAACAACTACATCAGTG
GACAACTCTCGAACGAGAAAACCTCAGCGTCTTCTAAACTTGAAGGGCTGAACCTGTCGAACAATCGCT
TGTCTGGTGCACTTCCCAGTGCTATTGGAAATTATTCAGGGCTGAAGAATCTTGTGTTAACTGGAAATGG
TTTCTCAGGAGATATCCCTTCTGATATTGGCAGACTAAAAAGCATCTTAAAGCTGGACCTGAGTAGGAAC

AACTTCTCTGGCACAATCCCCCCTCAGATTGGTAACTGTCTTTCCTTAACTTACTTGGATTTGAGCCAAAA
TCAACTTTCTGGTCCTATCCCAGTTCAAATTGCTCAAATTCACATCTTAAATTACATCAATATTTCCTGGAA
TCACTTCAACGATAGCCTTCCCGCGGAGATTGGCTCGATGAAGAGCTTAACTTCAGCAGATTTTTCCCAC
AATAACTTATCTGGATCAATACCTGAAACAGGACAATATTTATATTTCAACTCAACTTCCTTCATTGGCAA
CCCTTATCTCTCTGGATCCGACTCGACTCCTAGCAACATTACATCCAACTCACCGTCAAAACTTGGAGACG
GAAGTGACAACAGAACTAAGGTTCCAACAATATACAAGTTCATATTTGCATTTGGGCTCTTATTCTGCTC
CCTCATTTTTGTTGTCTTAGCAATAATCAAGACAAGAAAGGGGAGTAAGAATTCAAATTTGTGGAAGCT
GACAGCATTTCAGAAGCTTGAATTCGGAAGTGAAGACGTCTTGCAGTGCTTGAAAGACAACAACGTCAT
AGGGAGAGGTGGAGCAGGGATAGTGTATAAAGGAACTATGCCAAATGGTGATCATGTCGCGGTGAAG
AAGTTGGGAATAAGCAAAGGCTCACATGATAACGGCCTATCTGCTGAACTTAAAACATTAGGAAAGATC
AGGCATAGGTACATTGTGAGACTGCTCGCGTTTTGTTCAAACAAGGAAATCAACTTGCTAGTTTATGAGT
ACATGCTAAATGGAAGCTTAGGTGAAGTGCTTCATGGGAAGAACGGCGGGCAACTCCAATGGGACACT
AGGCTAAAAATTGCCATAGAAGCTGCCAAGGGCCTTTCTTATTTGCACCATGATTGCTCCCCTATGATAA
TCCACCGCGATGTCAAGTCCAACAATATATTGTTGAACTCTGAACTTGAAGCTCATGTTGCAGATTTTGG
TTTAGCCAAGTACTTCCATAATAATGGTACCTCTGAGTGCATGTCTGCAATTGCAGGGTCTTATGGTTAC
ATTGCTCCAGGTAACTAGTTTGTTCATACATTAGTTGATTTAATTTCTTCTCGACTGGCCTATTATGCACTC
GGACCAATCTACATCCTACTAATAATGGTCTTTACATGTTATCTTTTTGTATAGAATATGCATACACGCTG
AAAATTGATGAGAAAAGCGATGTGTATAGCTTTGGAGTGGTGTTGTTGGAGCTTATAACAGGACGAAG
GCCAGTTGGTAATTTTGGAGAAGAAGGAATGGACATTGTACAATGGGCGAAAACGGAGACAAATTGGA
GCAAAGAAGAGGTGGTGAAAATCTTGGATGAGAGGCTAAAAAATGTTGCAATTGTAGAAGCTATGCAA
GTATTTTTTGTAGCAATGCTTTGTGTTGAAGAGTACAGCATTGAGAGGCCTACAATGAGGGAAGTAGTC
CAAATGCTTTCTCAAGCTAAACAACCAAATACTTTCCAAATCCAATGATCTAATTGTGGCTCTACTAATTC
TTATAGTATATCTTTTTTTGTTTCTTGTGTGCTTGGGAAGACCCCTTTTGTTAGCTTTGCAAACAAATTGCA
ATTATGTTAGATCTTAGGGCATGATATCTAACTATCCTCTCAACTTGGAATAGTGTATTGGATGTGTAGA
ACTAGTATTAGTATCCGCGTGATGTGTGGCGAATATCAAAAGAAATTGATTACATAAAATGAATCTCTCT
GTTTC
>PGSC0003DMG400006364 LRR-RLKs
ATGAATCTTGCAAATTATGGGATTAGAGGTAATCTTCACCATCTAAATTTCACTTCTTTTCCTCATCTTCAA
TTGATTAACTTTTCCAGTAACTCACTCAATGGTACACTTCCAAAAAACATGTTCAATCTTTCAAGATTAAG

TTTCCTATATTTGAGTTACAATCAATTTGATGGAGTGATTCCACATGAAATAGGGCTATTGAAGAATCTC
AAGCAGTTAGACTTGTCGATAAATCGTTTTATTGGCTTTATCCCTTCATCTATTGGTAACTTGATTAAACTT
GAGTTGTTATTTTTATCAGGTAATGAACTCTATGGCCCTGTCCCTTCAAGTTTTGGTAACTTAAAATCCCT
TGTGCATTTGTGTTTGCTTAGAAATAAACTCAATGGATCACTTCCAAAAGAGTTTGAAAATCTTACACATA
TGCAAACTTTTCAAGTAGCAGAGAACAATTTTTCTGGTCATTTGCCACAAAATGTGTGCCTTGGTGGATC
ACTTGCTAAATTCATAGCATATGACAATAAATTTATTGGTCATGTCCCTAAAACCTTGAAAAATTGCTCTA
CGCTATCAAGAGTTAGGCTTGATGGTAACCAATTGTCTAGCAATATATCAGAAGCTTTTGGTGTCTATCC
AAGTCTAGTTTACATGGATTTGAGTCACAATAAACTATATGGCGAGTTATCGTCACAATGGGGCTTTTCT
CATAATCTAACAAGTTTGAAGATCTCCAAGAACAACTTGTTTGGTGCCATACCAGTTGAAATAGGAAATT
TGATCAAACTAGAAATGCTTTATCTCTCTTCAAATCACTTGACCGGCGAAATTCCTAAAAGTTTAGAGAG
GCTAACTCTCTTGCTGGAACTTGATTTACATGAGAATAGAATTTCAGGAGAAATACCTATAGAAGTGGG
GAAGTTGTCTAAACTTACAAAACTTGACTTAGGTGCGAATAATATGAGTGGAACGATTCCAGGAGAAAT
AGGAGATTGTAGGCAGCTTTGGTACTTGAATTTAAGCAAGAACATGCTGAACACAACTATTCCTTCTAAT
TTAGGTAACTTGCACTCTTTAGCCTATCTTGATCTCAGTTATAACATGCTCAGTGGTGAGATACCGTGGCA
CATAGGGAGTTTGAGAAGTTTGGAAAGAATGAATCTTTCGCGTAATAACTTGTCTGGTTCAATCCCGCCT
AGTTTCAATGAACGCGTTAGTTTGAGGTCAATTGATATATCTTGCAATCAATTGGTTGGTCCCCTTCCCAA
GATCGTGGCATTTCAAAATGCATCGCGTGAAGAATTAAGAGATAACAAGGATTTATGCAGCAATGATCA
TACTGGTATGAGACCTTGCTCTTCATTGAGGAAAAAAGAGAGAACAAGTAGAAAGCTTATACTGACCTT
AACTCTGTCTCTAATTTTGGTTGCCTTGCTTCTACTTGTGATTACTTGTATACTTTTTCGAATGAAAAGGAA
AAGAAATAGAAGTATTCAGCTTAGAGAACCATCCAGAAATTCTTTTTCAGTTAGGGGCTTTGATGGGAA
AATAGCACATGAAAACATAATTGCAGCAACAGAAAACTTTGATAGCAAATATTGCATTGGGAAGGGAG
GACATGGAAGTGTCTATAAGGTTGAGTTACCATGTGGTCAAGTTGTTGCAGTGAAAAAAGTTCATGCTT
TAGAAGATGAAGAATCTGATGACAACTTGATCAAAAGCTTTTCAAAAGAGATACAAGCTCTAGTAAACA
TCCGCCATCGGAATATTGTGAAGCTTTATGGGTTCTGTTCACACGCGCGCCATTCATTCTTGGTTTATGAG
TTGTTGGAAGGAGGAAACTTGTCACAAAACCTTAGCAATGAGGGTAGAGCAAGAGATTTAAATTGGCTC
AAGAGGGTTGATATTGTCAAGGGGATAGCAAACGCGTTGTGTTACTTGCATCACGCGTGTTCACCTCCT
ATAGTTCATAGAGACATATCAAGTAACAATGTTCTCTTAGATGATGAAGGCAATCCCCATGTTTCTGATTT
TGGTACTGCTAAACTCTTAAGGCCTAACTCCACCAATTGGACATCGTTTGCTGGAACTCTTGGCTATGTA
GCTCCAGGTATTTAGTCTTCTTTACTTTCTAAACTTCGTGTTAAGTCAAAATCAGACAAACAAATTGAAAC

AGTAACTAGGAATTCATTACTTCAAAATCGTTCACTCTTTCACACAATTTTCTAATTTGTTTGTAGAGCTTG
CTTACACAATGAAGGTAAATGAGAAGAGTGATGTTTACAGCTTCGGGGTGCTATCACTAGAAGTTATTC
TAGGTCATCATCCAGGTGATATTATTCAAGCTATATCATCATTTTCGCCAGCATCAGGAGTGAATGGAAC
GCTGTTAAAGGAATTGATTGACAAACGGACACTAGCTCCTGGAAAACAAGAAGCAGAAGAATTGATCA
AGATCACAAAACTAGCATTCGCGTGTCTTCATCAGAGTCCTCTAGCTCGGCCAAGTATGAAGCAAGTTTG
TGCATCTTTATCAAAAGAAAATTGGCCTTCAAAAGGCTTGTTCTCCACTGTTACATTAGGCCAGTTGCTTG
AACCAACACTACTAAGATGTTGA
>PGSC0003DMG400006502 LRR-RLKs
CAGCACTTAGCCATGGCCTATTATCTTGACAAGATCTTTCTTCTCCTATTTCTCTTCACACTTAGTTCTTCCT
CAAAATCAGATAATATGACAGATATTGAAGCACTCTTTGCTATCAAGAATGAAATACTTGATCCCTTTGA
ATCTTTGATTTCATGGAATGAGTCTGTTCCTTTATGTCAATGGCAAGGTGTGGTATGTGGAACGCGAAGT
CAACGAGTCACTGAACTAAACCTTCTTGACCATAAGCTGGGTGGTGTGTTATCACCATTTGTTGGAAATT
TGAGCTTCCTTGTACGACTAGACATCGAAAACAATACCATTTCTGGCAATATACCTCCTGAATTAGGCCG
TTTGACAAGATTAAGCCATCTATATTTGCAAAACAATTCATTACATGGTGAAATTCCTGTCAATCTATCAT
TTTGTAGTAACCTCGTTGAGTTATGGGCACACAAGAACAATCTTGTAGGTGTGCTTCCGAAGGAGCTTG
GATTTCTTCCCAAACTCAAATATTTTGATTTCAGTTACAACAAGCTCATTGGTGAAATCCCGAAATCTTAT
GGTAACTTTTCAGGTCTCTTGGAAATGTACCTTTTGTATAATGATCTTGTGGGTAAAATCCCTGATGAGTT
AGGGAAACTGAAAAGCTTGGAAATATTTGACGCAGACTTCAATAGGTTATCAGGTAGAATTCCGAGCTC
ACTATTCAATCTTTCCTCCTTGAAAGTCATTGATGTGTCAGATAACCAATTCGAAGGTACTCTGCCTCGGG
ATTTAGGGATCAACCTTCCTAACCTGGAGTGTCTCAGCATTAGTGAGAATCAATTCAGTGGATCGCTTCC
TTATTCACTTTTCAACCTCTCAGGACTCGCGTACCTTCTTGTTGGCAAAAATAACTTAGGAGGACGTGTTC
CTAGGTTTGATAAGTTGCATAATTTGTATATTTTAAGCATGGAAAATAATCAGTTTGGGAGTGACAAATC
TGATAATGAGTTGACCCTCATGTCCTCCTTAACAAATGCAACTAACCTGAACTGGCTGTATCTGCAAAAC
AACGCGTTTGGAGGGACATTTCATGAGTTTTTCAGCAACCTTTCAAGTAATCTGGTATATGCAGACTTGT
CAAGGAATCGGTTTTCAGGACATATCCCGATAGACATTGGGAAATTCATCAACTTGGAAGAGCTTAGGC
TGAGCGGAAACCAACTCACAGGTATCATCCCTCCGACTATTGGGAAGCTTTATAAGCTACGATATTTATA
TCTGTCCCAAAACAAAGTCTCAGGTAGTATACCATCTTCCATTGCAAATCTGAGCCTTTTAACAGAGTTAA
ATCTTGATCATAACAATCTTAGAGGTAGAATACCTTTAAGTGTTGGAAAATGTCAAAATTTGTTGTATCTT
AACCTGAACCGAAACTATTTAATCGGTTCAATACCCAAAGAAATTTGTCTCATCACTTCCTTAGTTGTTCT

AAACCTTTCTAGTAACAACTTGACTGGCGCCCTTCCCAAAGAAATCGGTAGCCTGAAAAATCTCTTATCA
TTAGATGTCTCTTTGAATAATCTCACTGGTGAAATTCCTAGTACACTTGAGAGTTGTGTGGCTATTGTTAA
TTTAACAATGAAAAGGAATTTATTTCGCGGGATCATTCCTTCTACTTTCAATTCACTAAAGAGTCTTGAAG
TTTTAGATCTTTCACAAAATAAGCTATCAGGTATGATTCCAAAGTACTTGGAAGGATTTGCCTTGCATTTC
TTAAACTTATCGTTTAATGATTTTGAAGGTGGCCTTCCACAACGAGGCATCTTTGAGAACGCGAGTGTAG
ATTCTTTCGTTGGAAATCCTAGAATCTGTGGTGGTGTACCTGGCTTGAAACTTTCCAATTGCAATTTCAGT
CATTCAAAGAAAATAAACTTTAAGCTTGTTATTTTAGTAATATTAGGAATTTTGGGGCTAGTTGTTATGGT
TTTTGCTTTTTTCTTCTATCGATTCAGAAGGCCCAAAAGAACATTTCCTTCATTAGATAACAATCTAAACCA
ACTCATAGCTATGTCCTATCAAAGTATTCTCAAAGTTACCAACGGGTTTTCTGCGAGCAACTTGATTGGT
GTTGGGAGTCATGGATATGTTTACAAGGGAATATTAGAAACGGATGGTAAACACGTTGCTATAAAAGTG
TTGAATCTTTTACAATATGGAGCTATCAAAAGTTTCATAGCAGAATGTGAGGCCTTGAGAAATATTAGAC
ATCGGAATTTAGTTAAGCTACTTACTGCATGTTCTGGTGTTGATTATCGTGGCAACGAATTCAAGGCATT
GGTTTACGAGTTTATGGCCAATGGAAGTTTGGAGGACTGGTTGCATCCAGATAATTCAAGACCAAATGT
TCAGCCAAGAAGGTTGGGATTTCTACAAAGGCTGAATATTGCTATTGATGTTGCTAGTGCAATTCATTAT
CTTCATAATGATTGTCAAATATCAATAGTTCATTGTGATCTCAAACCAAGTAATATACTCCTTGACAATGA
ATTGGTTGCCCATGTTGGGGATTTTGGATTGGCAAGATTCCTATATCTTACTGATGAAACCACTTGTAGA
ATTCAGACAAACTCTTCAACTTTTAAAGGATCTATTGGCTACATAGCTCCAGGTAAATTTCACTCTATATA
AATGCCTCTAAAGACTTTAGTGACTCTGGATGCAATGACATTAACTCAGTTGCCGCTATATTTTCATTGCA
GAGTATGGTATGGGAAGCGAGGCATCAACGCAAGGTGATGTGTATAGTTTTGGTATCGTCTTACTTGAA
ATGTTAACCGGGAAAAGGCCAACGGATGACATGTTTGGCGGTGATTTAAGCCTTCATGATTTTGGTAGG
ACTGTCATGCCTGATGGAGCACTTGAAATAGTAGATCCACTTCTTAATTTGGAAGAGGAAGAGATAAGC
AGAGAGAGGTCTCAAATTCCAAGATTCATGAGAAGGCAAAAAATGGTAGAGGGTTTAATCTCCTTATTT
GGAGTTGGTATTGATTGTTCAATGTATGATTCAAGTAAAAGAAAAAACATGAAGGAGGTTGTTAGAGA
GTTGTGTTCTATAAGAGATTCTCTTGTTGGTTGTACAATATGAAACACTTTTCTTGTTTTGTTTTTGTCTTG
TAAGGTTCCAATAATATTTAATGATGAATCATTTG

>PGSC0003DMG400006563 LRR-RLKs

AGTAAACATACAGAGCTAAGGAGATCAAGTGTCATTACATTTCTGTATATTTAGGTCTCTGAAATGCATA
GCATAGTTATTTAAAATTTATGTTTACTTGTGAGTAAGATTTTGGAACTTGCGTTGTATTTTTAAAGGTGT
GAAATTATGAACCAATGCAGGTATTCTTTAAAGCCAATAATTGAAAAGTTGGATAAACATATGTTTTTTG

CTCATGGAGCTGTTAATTGTATAACGCCTGGCCATTTCTTTCATGATTACGGTGGTGTCTTGTGAGGACC
TTGATTGTTCCAACATATACCTGCATTTACCTCTAAGCATTAGGCATATAAGGAAGAAGATGCCTAACTCT
TTTTTTGTCAGCTAGGATTCAAACTCTAGATTTCATGGTTCTATATTCATCGATCACTAGACCACACCCTTG
GGTTTGTATCCCTTTTCGACTTTCAGCTTGTGCATTTCTTCATCTGTTGTATTCCCTTTCTCTCTTTTGATAA
TGTTTGACCCTTTATTTGCCTTGTGGTTTCTCTTTCCTATTCAGTCTATCAGGAAAATGGTGTGAGGGTTC
AATAAGTTCACTAAACATCTGAAGACTAAGATGCTAATATTGATAGTGATCAGTTCAATTTCCACCTTTAA
CTTTTCTAAATTGCATCGATCCTAGCAGAGCTCTTATTCTATTTTTACTTTTCTTTACATGTTAGATGATGA
TGGAGAGAAAGGGTATGAAGGTATAACAGATATAATGAATTCATGATACATGTTGTGAAAAGACAACTT
ACCAAAAAGTTATATATGAGAAAGGAATAAACCGAAACATATTTTGGACTCCCACATTATGTTTGAATTT
TTGAAAGACAGATTATGGAGTATTTTATCATGATAGAACATGTTGGTAGAAATAAACATGAAGTTAGGT
CCTCATCTTGCACACTTATTGAAGATCAAATGCTGCTTCTGGTATTGCAGAAGTGTCAGAAATTGCTCTTT
GCAGGGATCCATTCCTGATTTCAGCAGAATTGCAAACCTAAGCTATCTGTAAGTGCGAGTTTGTTATTGT
TTTGTTCTTCTTCTTTTGTTAAGCTTTATCTGTATATTTTTACTTCAAGATTGTTCAACTAGTAGATCTCTGA
AAATGTCTAGCTTCTGCTTGCTATACAGTGATCTCAGCTGGAATCAACTTTCTGGATCTATACCACAGAAT
AAGCTTTCCAACAATATGACAACTATGTAAGCCTTTTGATTTGGCCTCGATAGTTTCTTCATATAGTTATA
TAAATTGACAGATGTTGGCTTTTGTTTCCACAGCATTTGAACTGGATTGCCTGGGTTCCTTTTACTTTTTA
GCATATTCTAATGTATATTTCAAATGCATGATGCAGTATTCTATCACATAACCGTCTTAATGGATCTATAC
CCAAGAACTTTTCTCTCCTTCCTTCCCTGCAGAAACTGTGAGAATCTGGACTTCGTCTTGCAATATCTACT
ATTTCACTGAAGGAAATGTACCATTAACGAATTGGTGGGACAAATTTGTCACAGGTCTCTTGAGAACAA
CTTTCTGAATGGTTCCATTTCCACTGACATTTGGCAAAACAAGATCTTTAATACAACTTCCAGACTTGTGA
TGTAAGATGTGAAGCAGAAAAATAACAGATCTTATTTCAATAGAAGTATTTACTTCCAGAGTCAAACCTT
TGAATGAGGATTTCTTTCTAACAACTCTTTCATTGATTTTGCAAACCTAGTGATCTGCGGAATAATTCAGT
TTCAAACATTTCGGGTGCTTTTGAACCTCCTGTAAATGTCACACTAAGGTATGATTTCTTCTTTCAAATTCA
AAAATAGAACAATTTGAATGTCGGTTTATGTCCTCATGGGCCACATAAATAGGACAAACAAATGGGTAA
AGAAACTAGAATAACAGAAGCAATATGCCATACCAAAAAGGGAAAGTACGTCGATTTTCCTCGTGTGTG
TGACAACAATTACTTAAATTACTTGAAGAAGCACAAATGCATGGAATCACTTCTGTTATGTTTTATCCACA
CACACCGATACACATGCTCTTAGCAAATTCATGTATGGTGCTTTATAAAATGTAAACAAAATGGAAAGAA
CAAGGAGTTGAGAGAACCTTTGTGGTTTTCAGTTTTTTCATTGTTTGAAAAATTTAAGTATGCTTAAAAA
GGAGGTATAGATAGAAAAGGGAATTCTGAAATTCTTAGTCACATATTTTCATCTAGTTGCAGTGGAAAA

GGATGAAATGATGAAGGGCCCTATAATATTGGTTATGGTCCTGATCAGCGTAAATCTTGAAAAAGTACA
AAGTTTTACCCAATAATATAAATGAGAAGTGTGTGTGTGGGGTGGGGGGGGGTGGGGGGTAAAAAGA
TATAAATAGAGGGAAACTATGAGCATAAAAGGAGTTTTTGTCTTTTGTTGAGTCTCTCATTTATTCGTTGA
AAGGAGGCCTGAACAAATGGAGAGGAAGGAAGTTTCTGTAAAATTTGAGTCCACTACATGCCTTATGAC
ACATCTAATCTAAGTTGCTCGGACTCTTCACTTTTGGTGCCGCACCCGTGTCGACAGGATATGGGTGTGG
GATTCGTGTCCGATTTTGGGTATTTGGACCAAAATTGATGGAGAAAGTCTGGACAGTTTTAATGATTTTT
ATAATCAATACAAAAGCTAAGGTGAAATTGAAGAAAATGGAATACCTTTTATATAGAAATTTCTATGTCA
CTCATTTTCCTTTGATCTCCTTCCAAGATTCTCTTCAAGTTCTCCACATAATATCTCATAATATAGGTTTTAT
AACTCTTTTAGATATTTCAATTATATTTAGGCGAATCCCTGCACCCGTATCCATTCATGGATCCATATCCCT
GAATCTTAAAAGTTATATCATATCATGAAGGATCGGACCTTTAGATCCGCACCCGTATCGGACACCCACA
CCCTAGTCCAAGCAACTTAGATTCTAATAGTTTGTGAAACTGAGGGAGTTTTTCTGCAGTATATCACATC
AAGACAAGTGATATAAACTGGTATCATTAAATTCTTGAAGAAGGAAAACATCACATTTAGGAAAGTGGA
GAATGGAAATTGTGCCAAACAAAAAGAGTTATTGGTCTAAAATTACTTGTACATCTTTGTTACTGTCTCT
GGAGTTCATGTTTTCAGTTATGCACTCATTCTTGTTGTCCAATTATTCATAAATATCTGATTTTGACAGTGA
ATTGAGGTCATCTTTCAAAAATTACTTACTGATCTCTTTCTATCTCATGAGGTCAATTCATCTTGAATTCTT
AATTGAAAATCTGCTCAATGTTTCTCAAAAGAAACATGTCAATAAAAATGTTTTAGATTTTGTACTATGTT
AAAATTCATTTATTATTTTCATAGTCTTCCCCTAACTTTAGCAGACGATTTCCAGGTTCCAAAGCAATCCCA
TCTGCAGCAGTACAAGTATCAGAAACATTGGTAAATACTGTGGCCCTGACTTTGGAGCAGATGACGACG
AACCTAGTAATTCAACAAATCCAAGTGGTGTTTGTCCTATTCATGCTTGTCCAACTGACAACTATTATGAA
TATGTTCCTGCATCACCTAAACCATGCTTTTGTGCCTCACCTCTTAGAATTGGGTACAGGTTGAAAAGTCC
TAGTATTTCTTATTTTGATCCGTACAAGCAACTATTCGAGTCTTATGTGACTAGTTCCCTTCAGTTGGATCT
TTACCAATTGTGGATCGATTCATATTTTTGGGAAAAAGGACCTCGTCTGAGGATGTATTTGAAGCTTTTC
CCGGTGGTTGGGGACAATACATTTAATGAAAGTGAGATTCTTCGTATCAGTGAGATTTTTGCCTCTTGGG
AATTCCAAGGAAGTCACCTATTTGGACCATATGAGCTCCTGAACTTCACACTCCTCGGACCTTATTCACAC
TGTAAGTATTCACAGCTTAAATAGATTCTAATTCAACTTGTAACAACTTAGGCTGTGTTACTACAATTGCA
TGACAATTGAATCATTGACATGCAGCATGTGGATAAGATTGAAATATTTGACTATCATTGCCACTTCTAG
CCAACAAGTATAAGGATCTTAATTCATATAATCAAATAGTTTAGATAAGCAACAAAAGACTATGATCCAT
GATCTTCTAAGGACTTCATATATATTGGGTTGAAAAAGACAATGAAGTCTTTGTTTATATCCAAAGTTTTT
TTAAGACTTCAGATATTTTACGTTGAGGAACCTTGAAGGATCTCACTAACTTGATTAAAAGGTTGAAACT

TCAAGGGGTTCAAACTTAGATAGTTTGGTCCAGTCTCTCTTCAAGAGCTTCAATTCTGGAGGTCTCAGCC

CTTACAGTTTTTGTTGGGATGAAAGTGGATAAAAAAAAATGGACCTTAGGCCTCCCAAAAAGCTAGCTC

ATGAGGAGGGGATTGTCCAAAATCATATAAGGAGAATAACCCTTGTCACTCCAACACCTTGCTGCACTCC

CAAGAATTGACATTGGAGCGTGGACAATATAATAAGGGGGGGTTCAACACCGGAAACAATAAATTGGG

ATCAGTGTGGATCTGAGACCATGTGAGAAATAAAGAAGAAAAATATTACTCCCTCTATCCAGTTTATATG

ACTTACTTTTCTTTTTAGTCAGTCCCAAAAAGAATAACACATTTCTATATCAAGTAATAATTTATTAAAATG

TCAATTTTACCTTAATGAAATGATTTATAGCCACACAAATATCTATCACTTTTTTTTAGACCACAAGTTTTA

AAAGTCTTCCTTTCTTTCTTAAACTCCGTGCCAAGTCAAACTAACTCATATAAAATGGGATGGAGGGAGT

ATTAAAATTATGTATTCTTTTATTACATTGAGCCCTATTTATAGATTCTACATTACAATTCATTTCCAAGTA

GGACAATATACACAATCCTATTCGAACACTCCCCCCTTAAGCTGGTGCATACAAGTCAGATGTACCGAGT

TTGTTAAGATGTAATTAATATGAGGATCAGTGAGGGACTTGGTGAAAATATTTGCAAACTCATCACTCGA

CTTCACAAATTTTGTAACAATATCTCTTGGGATCATCTTTTCTCTGACAAAGTACCAGTCAATCTCAATGTA

CTTAGTCTTCTCATGAAATACCACCGATTTGATGTGATATCAAGGGCTGCTTGGTTATCACAGACAAGAT

AATGGCTCAATTGTTTGATTTCCTTTGTACAAGCTTTGATTTCTTATATTATCTGCAGTGAATCCTGAAATG

AAAGGTAAAAGACAAAGCAAAGGCGTTGTGATAGCTTGCATTGTAGCAGCAGGTGCTTTTGCTGCCTTT

GTTTCCTCAATTGCCACTATATTAATCACAAGACGACGTGCTAAGTATCAAAAGATCTTGTCAAGAAAAC

GTTTGTGTAAGTCCTCTTATTAGATTAGCTATAATTTTACTTTATCAAGTCTGCTATTTTTAATTCTTTCTGC

AAAATTGATGACTATGGGTTTTTGTTTGCAGCTTCAAGCCTCTCTATAAAAGTAGATGGCGTTAAAAGTT

TCACCTTCAGAGAAATGACTTCGGCCACTAACAACTTTGATACCTCAACTCAAGTTGGTGAAGGAGGATA

TGGTTCCGTCTTTAGGGGAATTTTAGCTGACAAAACAGTTGTTGCTATCAAGCGAGCAAAAGTAGGATC

CCTGCAGGGGCAAAAGGAATTCTTGACCGAAATAGAACTATTATCAAGGTTACATCACCGTAATCTGGT

TGTGTTGCTTGGATACTGTGACGAAGAAGGAGAGCAGGTATGACAAAAGCTGAAAACCTTTTCAATTCC

AACTGTTTTAGTTACTAATAATTCACACCATTCATCATTTTTAATCATAGTTAGTCATATTTTTTTAGTGTA

GCATCATAAACCTTCTTGTATTCTTTCTCACTTCAGTTGCTAATGATCAGACTGAATGTGTAGCAGACTAA

ATGCTTTCTTGAGCTACTGAAGTCTTCTTGTTTAGCACAAAGAGTGATATAGTCGAGCAATTATGAGTCTT

CTGTATACATTTGCAGATGCTGATTTATGAGTTCATGCCCAATGGAACTTTGCGAGACTGGCTTTCTGGT

AAGAACTGTTTCCTGGTTGTTTCTAGCTTTCATATGATTAATCCTCCTCAATTTCTTTCTTTTAGTTTAGTAG

TGAAGATCCAATGCGGAGTTGCAATGTTTATAGATGTTCCTTGCACATGTTGTGTCATTAGTAGTACTAT

GGTGAATTGATAATACAGGCTTCAGTATTTTATTGTGTTTGTGGAGAAACCCTTGTCAAGGTGGTGATCG

ATCTAAAAGTTGCATAATGGAAAAGATATTGCAGGAAATCTAATAGTCAGAGAAACATAGATTCCATCTT
CTTTTTCCTTATCAGAACAAGAAAAGTTGTTAGTTTCTTTATGCGGCTGTGATAAACTTAAAATTGACATC
GGTCAAGAAACTTTTGTATTGCTATCTAAGTAGGTGATTTCCATCATTTGACAGCCAAATGTAAAAAAAA
TTTGAAGTTTGGAGCAAGGTTGGGGATCGCACTGGGGGCATCTAAGGGCATCCTTTACCTCCATACTGA
AGCTGATCCACCCATATTCCATAGGGATATTAAAGCCAGCAACATTCTTTTAGACTCTAAACTCACTGCCA
AAGTTGCTGATTTTGGATTGTCTCGGCTTGCACCTGTCCAGGATGATGAAGGTCTTCTGCCTAATCATGT
GTCAACAATGGTGAAGGGGACACCTGTAAGTCTGTCTATGCTCTAGATTTAAGGAAATAATTGTGCAAT
GATCTTACTGATTTTCTTATGTATATGCACAGGGTTACCTTGATCCCGAATATTTCTTGACCCGTAAAATG
ACAGATAAAAGTGATGTCTACAGCCTTGGGGTTGTATTTCTGGAGATCTTAACTGGCATGCATCCTATCT
CACATGGAAAAAACATTGTCCGTGAGGTATTTCTGTCGGCTGAACACCTATTGGAATACAAAAACAGTG
AATGTTTGTCTAGCATTTTAGCAATAAAAGCATAGGTGTATGTATATAACCTTTAACTGTCCATGACAGG
TAAAAATGGCTCATAAATCGGGAGTAATGTTCTCCATTATGGATAAGAACATGGGATCTTACCCCTCCGA
ATGTGCGGAGAAGATGGTGGAGTTGGCCCTGAAGTGTTGCCAGGACAAACCAGAAGACAGACCCTCAA
TGCTGGAGGTGGTTAGGACGCTAGAAACCATACTCCAAATGATGCCATATACCGACACCGATCCTTTGG
ATAATAAAGCCAGTTTCAGTGAAACTACATCATCAGCTTCATTTTCTAATACTAGAAGTGGAGACCTATTT
ATATCATCATCCAATGTATCAGGTGGTGATCTTATTAGTGGTGTTACTCTTAATATTACACCTCGTTAACA
TTAGCTTGTCTTCCACTGCATAAACATTGACTTGTACACTATGACTAGTAGGAAGTCTATACGACTTGTCT
TTTATGTTGTATACGAGTGTACAATAATACCATCTTACTGCTTCTAGATTTATGTATATTCCTCAAGAATCT
GTTGTGACAGACAACCCTTGTATATGTATAGTACATTATACATGAAGTGCCTTATCACTGTCCATAGAAA
TGCTACATGTAGTTGGTTATCATCCTTGCA
>PGSC0003DMG400006923 LRR-RLKs
TTTTGAGGACTGTGAAGTAAAAAATATTGATGAGAAGCCCATCTCTTTCTTCTGCAAGAACCGAACCCAA
CGGAAAGGTCACCATTAATGGCGGAAACCTCATGAAACTCGCGACACTACCTATCTTCTTCTCATTCAAT
GCATGTAGCATTAAATTCCTTCTTCTGTCTATAGCAACCCAACTTCTCCATTTTTCTACATTATATATACTAC
TCTACTTTACTCTTCTCCACTTTTGTGCTTCTTTTAATTTCACCTCTTAAGCCGTACAAGACCCATTTATTAG
TGCTTCATTATAGAGTTCTCTGTTCTTTTTGCTTCTTTCTCTTGACCCCCAGAGTGGGTTTTTGTTCTATTTG
TTTTCTCTTCTTCCAAAATATCTCTGCCAGGAAAAAGCAAAACATTCCTTCAATGTGCAGTTTAGTTTGTTT
GTTTGTTTTTTCTTGATGGGTCTTTGTCAAGATTTGTGTTTGGTTCAGAATTAGTTTAATTTTCACCCAAAA
ATATTCTATCCATTTGGAGTTTTCCGCAGATTTAGACATAAAAAAATGCTTGACGTCTGCAAGAATTGAA

GCTCAAATTGTTCCCTTCAGTTTATTCATAAATTTCTGTTGAGTATTTAGAGGACTGTGTTAAATGGGGTG
CCTAAAGATATTTGTCATAGTTGTATTCATGTGGGCTTTATTGATCCCAAATTCTCATCAATTAGGGTCCT
ATGAAACTCAAATTCTTCTTCAGTTACGGAGGCACTTGGAATATCCAGTGCAGTTGGATGTTTTGGAGAA
TTACAATGGGGATTTTTGCAGTTTGGGTTCAACATTGAATATGAGCATTATTTGCGAGAATAACTCTGTT
ACTGAGCTCAAAATCAAGGGAGATAAGCTAGTAAAGGTCAATGAATTCCATGGAGTTGCAGTTCCAAAC
AATACCTTATCTGAACGTTTCTCCATTGATTCATTTGTTACCACTTTGACAAGGTTAAGCAGTTTAAAAGT
TCTCACTTTGGTTTCTTTGGGCATTTGGGGACCTCTCCCTGTTAAAATTCATCGGTTGTCTTCTCTAGAAGT
TCTGGATATGAGTTCAAATTTTCTTTTTGGTAAGGTTCCATCTGAGATGTCTACAATGGTGAAGCTTCATA
CTTTGACATTTGATGGCAATTTTTTCAATGAGAGTCTCCCTGAGTGGTTTGATTTGTTACCTAATATCACT
ATTTTAAGCATGAAGAATAATAGGTTGAAGGGTAAATTTCCTCATTCAATTTCTAAAATCACAGGCCTAA
CTGATATTATTCTGTCACATAATGCTCTTTCTGGTGAATTACCTGATTTAAGTGCTTTGTCCAATTTGCATT
TGTTGGATTTAAGAGAAAATCATTTGGATTCTGAACTGCCACTGTTGCCACAAGGATTGACCACTATTCT
TCTTAGTAGTAATGCTTTCTCTGGTGAGGTCCCAGAAGAATTTGGCAAACTAAATCTACTTCAACACCTTG
ATCTGTCAAATAATGCTCTTACCGGAACGCCGCCTGCTGATTTGTTCTCTTTGCCAAGTATTAGTTACTTG
AATTTAGCGTTTAACGTTCTGAGTGGTTCACTTCCTGAACATCTCAACTGTGGGAGTGAACTTGGTTTTGT
TGATATTTCTGATAATAGACTGCTTGGTATGCTTCCTTCTTGCTTGAACGCCAGTTCGGATAAGCGAATTG
TAAAGGTCAGTGGAAACTGCTTGTTGGATACACAATATCAGCACTCTGAGTCCTACTGCAAACAAGCTA
GTTTGGCAAAGAAACAAACCACTGGAAAAGAGATAGCAATATTGGTAGGTGTTGTTGGGGGAATTGTA
ATACTTGTGGTGTTTTTGGCTGTTGTTCTTCTCATCTTTTGTAGAAGACAACATGCACGTCACGATGTGGA
CCGGTACATGTTTCCCAAAGTTGTGCAAGATAATGGGCAACCCAATATTTCTGCTGAGCTCCTGGCAAAT
GCTAGTAAGTTGTAGTTTGATTTCATTTTCAGTGTCAACTGTAAATTTTTTTCCGGGATTTGATAAGAGTT
TCTTTAACTTATTTTACCTCAGGAATCATTTCTCAAACAACAGCACTAGGATCACAAGGTGCCCCATCATA
TCGGGTGTTCTCCATGGAAGAGTTGGAAGAAGCGACAGAAATTTTTGATAAATCAGCATTACTCGGTGA
AGGCTCCATTGGAAAAGTACTGCTCTGAAACTATTTATGACTCCCAAATAATTGTAGGAAGAGGACTTA
GCGTATTGTTTCCTTGCATATATCCATTCAAAATTCATTTTATATTTTGTCGGAAGAATAATATTTTTGTTG
CTTATTTAGCTTTTGTCCATATGATGCCATATTATTCCAGAGGTCTTGATCTCCGTTATCAATTGAAGCAA
ATCAAATTAGTTTTGTAGGTAGGTTTCATTGAATAACTCGTATCAGACAATTAAACAGATGCACAAGTCG
TCAATATAGGCAGTTGATTAAACAGTTTTTTGCCTTTATGATTGATTGTCTGTGTTCATGAATGCTATATG
CATCTTGACTTTGCAATTTAATACCATATAATGGTGGTCACAGTTAAAAGTAATGTTCGTTTTGATTTGGT

ATCTACTGAACTTTGACAATTAATATCAATAAAGTCAGTGCGCTTGATTCGCTTATTTAAAGTGCATATGC
AGTGAGTTGATATCTCTTCAAGCCTTCTGTCCCAGGATTTTTTCTTTCTTGGGATCAAGTTGGACAATTTTT
ATTTAACTAACACCCTTCTATGTCATTCTCTCTATCTCAGATTTACAAGGGAAAATTAGAGAATGGAACCT
ATGTTGCTGTAAGGGAATTGACTGTTCATAGACGATGCACTAGTTGGAATTTCAAACTTCGAATGGATTT
GCTGTCAAAGTTTCGCCATCCCCATTTAGTTAGCCTTCTGGGTCACTGCATTGATGATGGGGTGCAAGAT
GACTCAACAGTCCACAGACTTTTTCTGGTTTATGAATTTATTCCTTGTGGAAACTTCCGTGCTCGTCTTTCA
GGTCAGTAGCGTAAATATTTTAGTACCAACAGTTGCATAGACATATACTTGATTGAATATGCAAATGCTG
CAGGTATATTTCTGGTTATGCTGATTGCTAATTTTCAGATAACTATTTAGTATGATAGTTTTGGTGAATCT
GTCTTTTTTTGAATATTGTGCGGGGGAAGGGGGTTAAGGATGAGAAACACATGAATTTATTGCGTCTTTT
GTTATTTATTCTCTAGTAAGTACAAATACAGAGTATTGTTATCTTCTGGATAGTTTCTTTCCGTCTATTTGT
CCAAGCTTGCGATCTGAAATTTTACAACAAAGTGAAAGACAATTATTGTAGTTTGATGAAGCAATCTGTG
TTAGCGTTTCATTGATTTGTCGAGATATACTTGATCGTATGTTCTTTGCATGGTCTGTTAGCCGACACTTG
CACTGCAAAATGTTTTGACCATCTTTTTCTTTGTGTTGTTCATTTTATTTATCCCGATATATCCAATGAACC
ATAGGGAATACATGTTGACCTTGTGTAAATCATCATTTTCACTTGCAGAAACTACTCCAGGAAAAGTCCT
CAACTGGTCAGACAGGTTGGCGGTTCTGATTGGTGTTGCCAAGGCTGTGCACTTTCTCCACACGGGTGT
AATCCCTCCTTCATTCGGCAATAGCTTGAAGACAGATAGTATATTGCTTGATGAGCATCAGATTGCAAAA
CTTAGTGATTATGGAATGTCAATCCTTATAGAAGAAAGTGAAAAAGTTGAGGTATGTTGAAATGATATT
CATATGATGAAGGGTAGGGGTAAGGTCTGCGTACACCCCACACCCTCCCCTGACTGTTATTACACTGGG
TTGTTGATGTTTTAGGGTAATATCTGATACTGATGATATTCTTTTTCAGGCAAAAGGAGATGGCCACAAC
GCCTGGTATGTAACTCTTTTGCCCAAATAGAGTCATACATCAGTCTGTTTGCAACTTCTATTCCTAGAAAA
ACTGATAGATTTTTTCTTAGTGCCTCTTCTGACTATGGCATCCCTTAGAGATAGATAAAAATGCTTGGCAA
TGACAGTTCAAGAATGTTGAGCAGAGACATATTCTCAACATTAAAATTTTGTGCATTTCTTCTCATGCAG
GAACACGAGGAAGAAAGAGGATGATGTGTATAACTTTGGTTTCATATTACTGGAGTCGCTGGTGGGTCC
TTTTTTTAGTGGAAAAGGAGAAACATTTTTGCTCAATGAAATGGTATGGAACTACTAATTTTGATTTTTGA
TATGCAACTCCTTTCTCATTGGCAGTAGCACAGAATGCTCTTCAGCATTGTTTCTACTGTGGATCACAAAC
TTTCGTCCCGTTTGTAGTTAAGGAGTTTTGAAATGAATGTCTGTGACACTTACTATGACTTGGAAGGTAA
GCAGTAAAGTCTTTTTTTTTCCGATGACAGACATCCTTTGGTAGCCAAGATGGTAGGCGTAAAATTGTGG
ATCCTGCTGTGCTAACCACTAGCTCCCAAGAATCTTTGTCAATTGTAATATCACTCACCAACAAATGCATC
TCTCCCGAGTCACAGAGTCGCCCCTCATTTGAGGATGTCCTCTGGAACCTACAATATGCAGCTCAAGTCC

AGGCTACAGCCGATACAGATCAAAGATCTGATGCAACTTCATTGTCATGAGGTATTCAAGAAATTCAACT
GCATCTCATCAGCAATACTTGCTCATGCCGTTGTCTCAATTGGGATGCAGGCTGAATGCATGATGTTTGT
GAAGCACAAAATGGTTGCAAACCTCAGCCTCATGTTCAATCTTCTAGTCCTCGTTATCATTTTCTTTTGTAT
AAAAATGTAAATTTGTCGATTCTTTGGATTTTAGAATTTAGAAGTGGGTGATGTTGCAAAACAGAGACTA
GTCTTCACATGAATTTGTATTACCCTCCTTCAGAAAGGATAGAAATGAAAGTGACAGAAGGCTTAGTAG
CCGTGCAACCTCAAAAATTGCCCTTCATGCTTATGTGTAAGATAAGCATACATGTATATCAACATCAGAC
TAATAACTGATCTTCATTGCCTTGGTACTTGCATTGATCCAGATATGAGAAAATCAAAGAGACTAGAGTC
CCTCCAATTCTTCCTAAAC
>PGSC0003DMG400006942 LRR-RLKs
GGTGCTTACTCAGGGGAGTCCACTCCATTAACAGGACTAACACTAAATTAAACTTTACAAATTCTCTAAA
TCCATAATTCCAAGGTTCCACTTCATCATTCAAAGTCAAATGTCATCTTCCATTCTTTTGCTTTTTTCATACA
CTTATATTTCTCATGTAAACAATTCATCAAACACCTTCTATGCATCCCTACTCCCTAAAGCTCAAAGATGA
AACTTTTCAGATTTTTCAATCTTCCCCTTATAGTATTCATCTTATTGATTCTTGTATTTCAACCCTTTCTTCAT
TGTACTGCTATTGCTGGTGCTGTAGCTGAAGATGATATAAAGTGTTTAAAAGGAGTCAAGAACTCCTTAA
CAGATCCAAAAGGGAACTTGAATTCATGGAATTTTGCTAATTCTACAGTGGGGTTTATATGTAAATTTGT
TGGTGCTTCTTGCTGGAATGACCGTGAAAATCGTCTCATAAATCTTGAACTTCGGGATATGAATCTTGGA
GGTAATGTTCCAGATTCGTTAAAGTATTGTCGAAGCTTGCAAACTCTTGATCTTTCTGGTAATCGGATTTC
TGGTTCAATTCCTTCTGATATTTGTACTTGGCTACCTTTCTTGGTAACCCTTGATTTGTCGAACAATGAATT
TACCGGTTCTATTCCGTCTGATCTTGTTAGTTGTTCCTATTTGAATAAATTGATGCTTAATGATAATAAGCT
TTCTGGGAATATACCACCTCAGTTTTCTAGTTTGGGGAGGCTTAAGATATTTTCTGTGGCAAACAATGAT
CTTTCTGGTAGGATTCCGGAAGCTTTTGATTCGGTTGATTCGTTTGATTTTGGAGGAAATGATGGGCTTT
GTGGTGGTCCGTTGGGGAAATGTAGGAGGCTTAGTAAGAAAAGTTTAGCTATTATTATTGCTGCTGGTG
TGTTTGGTGCTGCTGCTTCTCTGTTGTTGGGTTTTGGGGCTTGGTATTGGTATTTTACGAAGGCAGGGAA
AAGGAGGAAGATGGGGTATGGATTAGGGAGAGTTGACTCGGAAAGGTGGGCGGATAAGTTGAGGGC
TCATAGGCTTACTCAGGTTACATTGTTTAAGAAACCGCTTGTGAAGGTTAAGTTGGCAGATTTGTTGGCT
GCCACGAACAATTTTAGTACGTCGAGTGTGATAAACTCGACTAGGACAGGGACTACATTTAGAGCTGTT
CTACGTGATGGTTCTGCGCTTTCTATTAAACGGCTTAAAGCTTGCAAGCTGAGTGAGAAGTTGTTTCGAA
TGGAGATGAATGGACTAGGACAAGTTAGGCATCCTAATTTGGTGCCACTTTTGGGCTTTTGTGTTGTTGA
GGAGGAAAAACTTTTGGTTTATAAGCACCTGTCAAATGGTACTTTGTATTCATTATTGAAAGGGAGTGCA

AGTGTGTTGGATTGGCCTACTAGGTTTAGGATTGGCTTGGGTGCTGCAAGGGGCCTTGCTTGGCTTCAT

CATGGTTGTCAGCCACCAATATTGCACCAAAACATATGTTCTAACGTTATTTTCCTTGATGAGGATTTTGA

TTCTAGAATAATGGATTTTGGGTTGGCAAGGCTAGTGACACCTCCAGATGCAAAAGAGACTAGTTTTGT

GAATGGGGAGTTGGGTGAATTTGGCTATGTTGCTCCGGAGTACTCAAGCACAATGGTGGCTTCACTGAA

AGGGGATGCTTACAGCTTTGGGGTTGTGCTTTTGGAGTTGGCTACTGGGCAAAGACCTCTAGAAATCAC

TGCTGCTGATGAAGGTTTTAAGGGAAATTTGGTGGACTGGGTAAATCAGCTCTCTGTTTCTGGTCGGATT

AAAGATGCAATTGATAAGCATATTTGTAGAAAGGGCCATGATGAAGAGATTGTAAAATTCCTCAAAATT

GCTTGCAATTGTTTAATTTCTCGGCCCAAGGAGAGGTGGTCTATGTATCAGGTATATGAAGCACTGAAG

AGCATGGCTGAGAAACATGGTTTCTCTGAACATTATGATGAATTTCCCTTACTATTTAACAAACAAGAGA

CCAGCAGTCCCATTTGATACATGAATGGGAAAGCACTATGAGATGTTTCACATCTGGTGAACACAAATG

CATTATGGTATGTTCTTCACTTCCCTTAGGTGCAAGTGGAATCAGGCACGATACATATGGTTCTGTGAAG

TACTACCACAGATATTGCTGTATTATACTGTGAAGTGTCAAAACATTCTGAGGTGTTCTGCTCGAAATTTA

TGTTACAGATAGCTAATATTATTGGTATCTTAGGTTTATGTTTCTCTTCTGCTCATACTTATGCATTAAACA

AGACAGTAAGCAAAAGCTGTCACAGTTTATTTGAAAGCTATAATACCTACTAATTTAACTGTTTATAACT

GACCTGGGCATTGGCCTTTTTAGGGTCGAGATATATTCAGTATGTCCCGTACAGTCATTTATGGTTTGTA

AGATCAAATCACCCTAGGTTTCCCTAATGGATTGGTTCGGTAGAATATCTGCCTCCACACTGATATTAATT

CATCTAATCGGAATGCTATCAAGCAGAGTTATTAGTAATTACCCCTTCTGATTAATTTTGGATGATGTTGA

TCAGTAATTGTAGGTCCTTTTGCAAAAATTGTGTCTGATTGGGAATAATTTTCACTAAAATGGAAGTTGA

ATGGGCTTTCTGAAAGTTATGTGCCCTCTTAAATTTGATTTGGCAGCTGAAATTTTCATTCACTTGATTCT

AATCTGCTTCTTTACAATCTCAGAAACATAATGCACAATTATAGTTTCCAAGTAGGAAAAAAAAATTCTTG

TTGACAGTTGAAACTCTTTGCAATAGAAGTTATCAAGCCTCTTACTTTTTCCCTCAAGCTCAAGCTCGGAT

GCGTGTTTCCATATGCTCTCCTGCTTCCCTGAAAAGTCATTTTGTAATATTTCAAAACAACTACTTGTAGTC

TAATAATAGTAAATGAAGGGAAAAGGTCTGTTAGCAAGTTATCTCATATCTGTC

>PGSC0003DMG400007234 LRR-RLKs

CTCATCTTTACATACCTTATTTTCCTCAACCAATTTTTTCTTACAATCTCTTAACCATGAACCATTTCTTTCTC

ACTACAATTTTCTTCTTTCTCATACAATTTTCACTTGTTTTCACTACTTCAAATTTTCCTCTTCAACTCATTTC

ACTTTTATCCTTAAAATCTTCATTTCAAGATCCAAACAATACATTTCAAGATTGGAATCCTACATCAACTTT

CTCAAATTTTGGTTCACAGCCCTTTTGGTGTTCATGGTCAGGCATCAAATGTGACACAAAAACAAGTCAC

ATTACATCACTTAATCTCTCAAAGAGGAACCTTTCTGGTAAAATACCTCAAAATATTGCATCCTTGATACA

CTTACATCACTTAAATTTAAGTGGAAATTCATTTGATGGACCTTTACCATCCATCATTTTTGAATTTCCATT
TCTTAGAACACTTGATATTAGTCATAATTATTTCAATTCAGCATTTCCTCTTGACGGGCTAACGAATCTCA
AGTCCCTAGTACACTTGAATGCCTATAGCAACAGCTTTACAGGCGTGTTGCCTGTAGAGCTTGTTGGGCT
ACAAAATCTTGAGTATTTGAATCTTGGTGGCAGTTACTTCACTGGTGAAATTCCGGCAAGTTATGGGAAT
TTCAACAAGTTGAAATTCCTACATTTAGCCGGAAATTCACTGAGTGGGAAAATTCCAGAGGAATTGAGA
TTGTTGAACCAACTCGAACATCTTGAAATTGGATACAACAATTACACTGGAAATGTTCCTCCTGGATTTTC
CTCGTTATCGAATTTAGCTTATCTTGACATCTCACAAGCTAATCTTTCAGGTGAAATTCCAATTCAGCTTG
GAAATTTAACAAAGATTGAATCTCTATTTCTTTTCAAGAACCATTTTATCGGTACAATTCCCTCGAGTTTTT
CCAAACTCACATTGCTGAAATCACTGGATTTATCGGATAATCGTCTCTCAGGCAACATTCCTTTTGGATTT
TCAGAGTTGAAAGAGCTTATCATGTTGTATTTGATGAACAATAACTTATCAGGTGAAATCCCTGAAGGG
ATTGGTGAGTTACCAAATCTCGAATTGTTAGCTCTTTGGAACAATTCACTCACTGGAATTTTACCGCGAAT
GTTAGGATCAAATGCTAAGTTACAAAAACTTGATGTGTCTTCAAATAATTTGACTGGTCCAATTCCCCAA
AATCTCTGCCTCAGCAACAACTTAGTTAAACTCATTCTGTTTTCGAACGAGTTTACTGGTGAGATTCCTTC
ATCCTTAACTAACTGCAACGCTTTATCCAGGTTGAGAATCCAAGACAACAAACTCAATGGCACGATTCCA
TCAGGATTCGGTTTTTTGCCTGATTTCACTTACATGGACATCAGCAAGAACATATTTTCGGGGCAAATTCC
AAAAGATTTTGGAAATGCCCCGAAAATGATGTACTTGAACATTTCTGAGAATTTTTTCGGTAGTAACTTG
CCTGATAATATCTGGAACGCCCCGAATTTACAGATATTCTCAGCAAGTAACAGTGGCCTTGTTGGAAATC
TCCCTGATTTTAAAGGATGTCAAAATCTCTATAAAATCGAACTCGAAGGAAACAATCTCAATGGTAGTAT
TCCATGGGACATTGAACATTGTGAAAAGCTTATTTCTCTAAATCTTCGACGAAATTCATTTACAGGAATCA
TTCCTTGGGAAATATCTGGTATTCCTTCGATAACAGACGTTGATTTGTCACATAATTTTCTCACAGGAACA
ATCCCTTCCAATTTCGAAAAGTCTAGCACGTTGGAACATTTCAACGTGTCGTATAATCAGCTCACTGGTCC
ATTGCCTTCTTCAGGGTCTATGTTCTCAACGTTCCATCCTTCGTCTTTCGTAGGAAACGAAGGTCTTTGTG
GTACTATCATACATAATCCGTGCAGGACGGATGAACTCAAAGATGGAAAGACAGATTTTTCGAACCATT
CAAAGAAAACAGATGGTGCATTGATAGTATGGATTGCGTCTGCAGCATTTGGAGTAGTAATTATCATTCT
TATTGTGGCAATTCGATGTTTTCATTCAAATTACAATCGAAGATTTCCATGTGACAAGGAAATTGGACCG
TGGAAGTTAACAGCATTCAAAAGGTTGAATTTCACAGCAGATGATGTACTCGAAAGCATAGCAATCACA
GATAAGATCATCGGTATGGGGTCTGCAGGTACTTAACGGATCTATATTGTTAGTTATAATGAGTTCAGTT
GAACCCGCAATTTTTCATTAAGTATATGTGGTGGAAAAATCATACAAAATTGGTGTACATATATATTTTTT
TTAAAATGCTAACATCACCTAGACAAGTTGGCAATGTGGACTTTTATCAATATAACTCTCAAATGGAAAA

GTATAAGATAGACTAGATCTTACCTTAAAATTGTTACATAAATATTTAGTTTTGAGTGCACTTTCAAATAA

CTTCGATCCTTAAAGCCATTAAGATTATATTCTAGATCTGCCTCTATCTATAGATATAATGTACAATTTCCA

CCTTGAACTTAGAATGGGGTCCGCGCGTAGTGGATGATCTGTAGTGTCAGCAAAGTCGAAATGTACAAA

GTCTCTTAAGCAATAGTAGAATAAATATTACATTTCAAAATCATGTTTTCAAATAAGTTGGAATCTTGAAT

ACTAAGATTATATTCTAGATTCGCCTCTGTCTGTGGGTATAATGTACCATTTCTAGGTGAAACTCAAAATA

TGGTCCGCAACTAGTGGATGATTTATAGTGTTAGTGAAGTCGAACATATATAGAGAAATGTGCAAAATC

TCTTTGACATTAGTAATTTTAAAAGAAGCTTGAATCTTGAATACTAAGATTATATTCTAGATTTGTCTTTGT

CTATAGATATAATGTACAATTTCAAGTGAAACTCAAAATGGGGTCCGCAACTAGTAGATGATCTATAGTG

TTAGTGAAGTCGAACATAGAGAAATCATGTGCAAAAATCTCTTAAAAGTACATGAGTAACTTCCAAAATA

GTTTGAAAGCTTGAATACTAAGGTTATATTCTGAATCCACCTTTGTGTGTGCGTGTCTATATATAGGTACA

GTGTACAAGGCGGAAATGCCAGGTGGCGATATCATAGCGGTGAAGAAGCTATGGGCGGGGAAGCAAC

ATAAGGAGACGACGAGGAAACGACGTGGCGTGTTGGCAGAGGTGGATGTGTTAGGCAATGTAAGACA

TAGGAACATTGTGAGACTACTAGGTTGTTGTAGTAACAATGAATGTACTATGTTGTTGTATGAGTACATG

CCTAATGGTAGCCTTGATGACTTGTTACATGACAAGAACAAAGATGCAAATTTGGTTGGTGATTGGTTTA

CTAGGTACAAAATTGCACTTGGTGTTGCACAAGGGATTTGTTATCTTCATCATGATTGTGACCCTGTCATT

GTTCATCGTGACCTAAAACCTAGCAACATTCTTCTTGATGGTGAAATGGAAGCTAGGGTTGCTGATTTTG

GGGTTGCTAAATTGATTGAATGCGACGAATCGATGTCCGTCATTGCTGGATCTTATGGCTACATTGCACC

CGGTAAGTAATTTCGAATCTCCCTTAATTTTTTAAACTATTTTCTAAGGTTGATTTAGCTGTCAAGAGGTT

ACAATTTAACTGACAACGTGCACGAATTTGAAATCTAATAGTTTCATGTTGTGTTATATGTATGTTTGTAG

TCTTTATAGGGGTCCATACTCCGTATTAGTGGCGTTTCTATAAAACCTGTCACGAATTATTATAAATCGTG

ATGATTGAGCTAGTTGTCCGTAACATTATTACTTACGATGGGTAGTGGGAACCCCTAAAATATTACTTTT

AGTGACAACTTTTAAGGTGGCCATGAAAAAAACATCACCGAAAATCACATTTTTCATAGTCGATTTTTTA

AAAAAATTATCTTAATTTTAATTTCATATTGTTTTTCCAACTAACACTTTAAACATTATTATTATCTTTAACA

CTCAAAAAGTTCATCAAAATGTTTCCGATTTGCCAATATTAGTAACAATAATGATTATATACCCAGTTAAA

TTGTTTTCTATAAATTTATCAATATTAAGCTTGATATTTATTAAAAAGATAGACATATGAATCTTTATTATA

TAACTTTTTGAAAGATATTTTTCATTAACCAACCAAATATTAAAACACAATTTCATAAAAATATTTCCCCAC

AAAAGACTTGTGTGAACAAAAAATCGGATTTATTCAAGCTTATTATTTTAATAAATAAATAAAATAATTA

AGGTTTGTTTTGTGCTTTTTTTATTTTTTTTACGTTGTGTTATGTTTATCCAAAAATATTTCGTCATCGATCG

TGTCGGTTTAGTCCGTTGTGTAGGGTGACGAAAAGGGTCATGACTTGCGTGCTTAACGTGGTATCATGT

CATATTGTATTTTTGGTGAACTTCATCATTTTGTTTGGTCCTTTTCAATGTGTCCACTTTCTTTGTGAATCCT

CTTCCTTGGACGGACTTCAGAAATTCAGTAATTTTTGTACGGTTATATATTTGTATTGAGAAATTCAGTTT

AAAGTGTGTTGTTGGTTCAGTGATGATAGAAAAGGCCTAATTTGAGCTCTTTCAAGGGGGGGGGGGGA

ATATTTCCGTTGATCTTGTATAAGGGTGACATGGCACCTAGAGGCGAACTCACATGTATAGGTGGGGTG

CACGTGCACCAGTTAACTTTGGAAAAAATCATGAACATATGTATATTTATTTTGAGAAACTGACAACAAC

TAGATATAATTAACAGTGTTCCCATGAAAAGTATAGAATGACACTTGGAACAGGCCAATGCATCCGCCCT

CGCGACCTAGGTTCGAGCCTAGTCAAAACCACATTTTATAGTTTTTATTATAAGTGATTTCTAGATAAGG

GATTCAAGTTTAGAATCATTCAGTAGAACAAACCCATGATGCACCCAAAGTCTTCAAATCTTAGATTCGC

CTCTGATGGCACCACCATTACCATTTTTATTTTTGCTCTTTTTCTCCGTGATTTACTACTTATAATTGTTTTA

ACCTTTTAGGGACCATGTCCACTATTGCGCGTGAATTATTCCCTTGGCTCTTGAGGCTTTTGATTTTCACTT

TATGTTATTTCTTTGGGCCCACAAAGATTTTCCCAAAGTATAATATATGAATCTTCATGATTATAAAACCC

TTTAATTTATTAGTCCTAGTACTATATTCTTTATTAAACATATAAATTCAAAAGTCAAATACTTTGGGCTAT

AAAAGCCAAAATGTTATGATAACTCGATTTCTTGTGTGCAAGATAGTTGTGATCTCACTTAATTATCTTAC

TTGCTAGTGCTACTTGTAAAATAAAATCGAATCAAATATAATCCTATCAAATATGTTTAGGATTTAATCTT

TTTTACACTATATATAATAGTAAATAATATAAGATTCAGATCACTAATCTAACTTTGTACATTTATATATTT

TTATGATGCAGAATATGCGTATACGTTGCAAGTGGACGAAAAGAGTGACATTTATAGCTATGGAGTGGT

GCTAATGGAAATTTTGTCCGGAAAAAGATCGGTAGATTCGGAATTTGGCGATGGAAATAGCATTGTGGA

TTGGGTAAGGTCCAAGATGAAGATTAAAAATGGTATAAATGATGTTTTAGATAAAAATATTGGTGCTTC

GTGTCCTCGAGTGAGGGAGGAAATGATGTTGATGCTTAGGGTGGCGTTGCTATGTACTAGCCGGAACC

CGGCTGACCGGCCTTCCATGAGGGATGTTGTGTCCATGTTACAAGAGGCCAAGCCCAAGAGGAAGTTG

CCCGGAAGTGGTGGTGACAATTCAATTGCTCTTGTTTGTTTGGCACAAAAGGCCAATGTGGAGTGTTAA

TATAATTGGAGATTTGGGGTTTCTTTTCTTTAGTCTTTTTTTTTTTTCCAATTGTGGGGGATTGGTATAATC

GTATAGGATAAGTACTCAATGTTTCCTTTTAAAACTTTAATTAGTGGAATTTTAGGTATTATGATTTCTGA

G

>PGSC0003DMG400007517 LRR-RLKs

GGCATGTTGTTTTTGCTCTCACTTTTTTACCACCAAAAACCTCTGTTTTTTTATTTGTTTTTTTGAAAGAGTC

TGACATAAAGCTGTTTGAAACTTTGAGTGAGTGAGTGAGTGAGTGAGTGCAAGTTTTCTGTAAAATGAA

GCAGCAAAGCATGAGGAATTGTACCACTTTCCAATGATTTGTGTTTTTATTTGTGAAAAAACCAAGTCCC

AAATTCTTCTTTTATAAAAAAAGATCTCATTTTTACTCTTTGTTTAGCTGTTTTCTTGTTTAAGACAAATGG

AAACAAGAAGTGTTTTTGGTTTTGTGGTGTTGTTGAGTTTCTTGGTTTACTCTAATGCTATGCTTACTCCT
GGTGGTGTTAATTATGAAGGTTAGTTCTTGTTATTTTTAATAAAGATTTAAACTTTGAGACATTATTGGAA
TGAGATTGATCATATTAGAAAATATCAAGAATTTTAAAGTATGATTTACTCCTGTTGGTGTTAATTATGAA
GGTTGTTTCTTGTGGTTTTTCTCATAAAGATTGAAACTTCATGACTTTTTTGTTGAAAAAATCATATGGGG
TGTTTGAAATTTTTGAAAGGAATGAGATTTTGATCAAATTTTAGAAATATCAAGAATTTTGAAATATGCTT
ACCCTTGTTGGAGTTGATTATGAAGGTTAGTTCTTGTGGTTTTTCTCATGAAGATTGAAACTTTATGACTT
TTTATTTGAAAAAATCATATGGGGTGATTGAATTTTTTGAAAGGAATGAGATTTTGATCAAATTTTAGAA
ATATCAAGAATTTTGAAATATGCTTACCCTTGTTGGAGTTGATTATGAAAGGTTTAGTTCCTTGTGGTTTT
TTCTTCATTGAAGATTGAAACTTTATGACTTTTTATTTGAAAAAATCATATGGGGTGATTGAATTTTTTGA
AAGGAATGAGATTTTGATCAAATTTTAGAAATATTAAGAATTTTGAAATATGTTTACCCCTGTTGGAGTT
GATTATGAAGGCTAGTTCTCATAAAGATTGAAACTTTGATACCTTTTTGTGAAAAATTCATAAGGGGTTT
CTGATTTTATTTTTTTGAAAGGAATGAGATTTGAACAAATTTAAGATATTTCAAGAATTTCATCTATTTTAA
CTTTCTTGTTATGTTGATTTTGGACAGTGCAAGCTTTAATGGAGATCAAGAACTCATTACATGATCCACTT
GGTGTTTTGAATTGGGATGAAAGTTCTGTTGATCCATGTAGCTGGAATATGATCACTTGTTCAAATGATA
AATTTGTCACTGGCTTGTAAGTATACCCGATTGTCCGGTATCTGCATTGGAGCCCGACTGACAAAACCTT
TCTATTCCTAATACTTGAACCTGAGACCTCTAGTTAAGGATGAAAGTGCTTACAAGTTTTCTTGAATTGTG
CAGGGCTTGTTCAAGCCAAAGTTTGTCTGGGAAAATATCACCATACATCCATAACTTGACTCACTTGGAT
CTACTGTGAGTACTTTATATGAAGAAAAAATAATGAAATTTCAAATGATTGATTATTTGAATCAAGAATA
TAGAAAATAGTAGTACTAATATTACTTTTTAACTTTTTTTTTTAAACAGGCTTCTGCAGAGCAATAATATA
ACAGGACCAATTCCAACTGAACTTGGAATGCTTCAGAAACTTAATACAATTGATCTTTCTGATAATCAACT
CACTGGGAAAATCCCTTCATCTTTAGCTCAATTAAAAAGCCTTCAATACTTGTAAGCAAAGAAAATTTTAA
AGATTTTGATATAAAAATGAGTTCTTGAATTATAAAAAGTTAATGATTATGGTTTTTTTTTCTTTTTCAATT
GTGTTATAGGAGACTAAACAACAATAGTCTTAGTGGAGCTGTTCCTTTGGAATTGTCTAATATGACTCAA
CTTACTCTTCTGTAAGTTCCTTAGCCTCATTTAGTTTTGTTTATTTTCTCATACTTGAAGGGGAGCCTCGGA
GCAACAGTAAGCTTGTCTCCTTGTTATCTATATAGGTCACGCGTTCGAGTCAGGGGTAGGCTGCCTATAG
TATACCCTTTGGGGTGCGGCCTTTCCCCAGACCTTGTGTGAACGTGGAGTGCTTTGTGCACCTTTTTCCTA
ATTGCTTTCTGTTTTCATGAGTATATTTTGATGGGATTTTTGTTGTTTTTTTCTAGAGACTTGTCTTTTAATA
ATTTGAGTGGTCCTGTGCCAAGGCTTCTAGCTAAAACATTCAAGTAAGTTTCTTGAACAATTCTTTGAATT
TCATTTTTTTTCCCTTTGCTAAAGATTAGAGAAAATAAATCATTTTTTCTCTCATTGATGCAGCCTTTTGGG

AAATCCTATTATATGTGCAACTGGAAAAGAACCAGAATGCAATGGAACAACAACATCCATGCCTTTGCCC
TTCACTTTGAACAACTCACAAAGTAAGAATCTTGAATCATCCTCCTAAGTTTTTTTTCGAGCCAGCTTGTG
TGCCCCTCGACTATTCCACAAATCTTGGTTGTTGTCAAGTCTCCTGCTTTCAACGTCGTTATGATTAACTAC
AACTCTATTATAAGGATTCATCATGAAGTCTTGGATAGTTTCTGTGTTGTACTATTCACAACTGAGAAGTC
TCTTGTTATATCTCAGATGCTCAGCCTTTTGGAAGATCTAAAACTCACAAAATTGCCTTAGCCTTTGGGAC
AAGTCTTGGATGTATCTGCCTACTAATTATTGGGTTTGGAATATTTCTATGGTGGAGACAAAAGCACAAC
AAGCAAATTTTCTTTGACATCAATGGTGAGTATGATATGCAGCTAAAACCTATATTGCTCGAATCTCGAG
ATACTAGTAATGTCTTGCTTTAACCAAGAGGATCATGCTTGTTGATAATTTCAGAACAACATCATGAACA
AGTGTGTTTGGGAAACTTGAGGAGGTTCCAATTTAGGGAACTTCAAGTTGCCACAAACAACTTCAGCAA
CAAGAAGATACTGGGAAAAGGCGGTTTTGGAAACGTGTACAAAGGTTGTCTTAGTGATGGCACGATTG
TTGCTGTAAAAAGGCTCAAAGATGGAAATGCAATCGGAGGAGACAAACAATTCCAAACAGAAGTTGAA
ATGATCAGCTTGGCAGTGCATCGCAATCTCCTACGTTTGTATGGGTTCTCCATGACACCAACTGAAAGGC
TTCTGGTTTACCCTTACATGTCTAATGGAAGTGTTGCTTCTCGTCTCAAAGGTAAAAAGAAACAACCTTA
GAGAACTAAAGATTCTGCAAGTATTCTTAAATATGGTGGGATGGTTGAGATACCGTCATCCTTAACCATA
GTAGGCTTATCTGTGTGAACCGGAATAGTCGAACTAGTGGACTTCAGATACCTGATGGTAGATTTCTCTA
TTTCTGTTGTTTCTTGCAGCCAAGCCACCATTGGACTGGACTACAAGAAAAAGAATTGCATTAGGAGCTG
CAAGAGGTTTACTATATCTACATGAACAGTGTGATCCAAAGATTATCCACAGGGATGTGAAGGCAGCAA
ACATATTGCTCGACGATGACTGTGAGGCTGTTGTGGGAGATTTTGGATTGGCAAAGCTATTGGATCATC
GTGATTCACATGTCACAACTGCAGTTAGGGGAACTGTAGGACATATAGCTCCTGAGTATCTCTCTACAGG
ACAGTCCTCTGATAAAACAGATGTTTTCGGGTTTGGAATTCTCCTGCTAGAGTTGATATCTGGCCAGAGA
GCTCTAGAATTTGGTAAAGCAGCAAATCAGAAAGGCATTATGCTTGATTGGGTAAGTATTACACATTCCA
AGATGTGACAAAACCCTCCGTTTCAATTTTTCTTACTTTTCGTTTTAGTCTGTTTAGAAAAAAAATGCCTAC
GACCATTCTATATCTACTAAGTATGTTTTTTTATTTCAACATTCTATTTTACCTTCAAAGGGTATTTGGTGA
CAAGATTCAAAAGTCGTATTAACATACTGAAACTTTGTGCTCGATCAAACTAAGACAAACAAAATGAAAC
GGAGAGAGTATCTCGATTCTTGTTGGGGTTGATAATATCAATGTGCATATTTGTTGCAGGTAAAGAAGA
TTCACCTGGAGAAAAAGCTTGATTTGCTGGTGAGCAAGGACTTAAAGGACAAATATGATCGGATCGAGC
TTGAGGAAATGGTACAAGTAGCTCTAATATGCACTCAGTATCACCCTAGTCATAGACCAAAAATATCAGA
AGTGGTAAGAATGCTCGAAGGAGACGGACTTGCAGAGAAATGGGAAGCTTCTCAAAGAGCAGAATCTA
CGCGCTGCAGAGCCAATGAGTTCTCCTCTTCAGAAAGATATTCTGATCTTACTGATGATTCTTCATTGCTT

GTTCAAGCAATGGAATTATCAGGACCAAGGTGATGTAGATTATACAAGATAGTCAATTAGATGAAACAA
AAAAGGGCATCTGAGATAGAATGCTGAAGTAATATATGTATGTTATTGAGCTTGTTTTTTATTTCTTCTTT
AGATAACTGAGAAGTCCTCGAGGGTCAATAGTGCACGGTTCATAACTCAGTGGATCATAGACACCCCCC
TTTACTCTTCTCCACTTATATACCAGACGTGGTAGGGTTCGATTGAGCTTGTATATATGATCATATGGAAG
CTGCATAAAGGAATGGGATATGTTCAGACTGTCTAGGAAAAAGATTTATGAGCAACTGACCTCCCCAAC
CACACCCACAACCCCTACTTTTTTTTCCTCTTCCTGTTTTGAGTGATAATTTTTCTGGGATGTGGAATTGTA
ACTTTGAGCTTGTAAGAATAATATTGTTGAAATTCAATTTCAGCTTATAAATATTGTATCATAATTCTCATT
GAAAAATATTGTTCAAAGGGATTATCTTTTCCATTCAAAA
>PGSC0003DMG400007634 LRR-RLKs
TATATATTCATTTTTATCAATTTGGCATCTTCTTCACACCTCCAATGTTGACCAAACTTTCCAAAATCAACT
CATAATTCTAACCCCCACCTTTTTTTTTCATGGAGTTTTCCCATTTTCCGACAGGCATCTTCGCCGGAAAAC
AGTCCCTTTATCTCATTTTACTACTAAACTTCATACCCATTTTCGTAACTTCACAATCTCCGGCCACTACTG
AACGTGACACTCTACTGAAGATTAAGCGTCAATGGGGTAACCCATTAGCACTTGACTCATGGAACTCTAC
TTCTTCTCCTTGTAGCTGGCCGGAGATTGAGTGTGATGATGGGAAGGTTACCGGAATAATAATTCAAGA
AAAAGATATTACTGTTGAAATTCCAAGTTCTATTTGTGAACTTAAAAATCTTACTTTTCTTAATCTCCGGTT
GAATTATCTTCCCGGGAAATTCCCAACTTTTCTTTACAAATGTTCCAATTTGCAGCATTTGGATCTATCTCA
GAATTACTTTGTGGGTTCCATTCCGGAGGATATTTACCGGCTTGGGAAACTCAAGTATCTTAATCTGGGT
GGTAATAATTTCACCGGAGATATCCCGCCGTCAGTCGGGAATTTGACGGAGCTGGAGACCTTGTGTATG
AATTTGAACTTGTTTGATGGGAGTTTCCCGGCGGAAATCGGCAACTTAGCCAATCTTGAAAGTTTGGGG
TTGGAGTTTAATGAGTTTTCTCCGATGGCACTACCGCCGGAGTTTGGGAAGCTGAAGAAGATAAAGTAT
ATTTGGATGAGAGATACGAAATTGATCGGTGAAATCCCTGAAAGTTTCGGGGATTTTGAAAATCTTGAG
CTTATTGATTTTGCTTACAACAATCTGGAAGGAAAAATCCCTTCTGGGTTGTTTCTGTTGAAGAATTTGAC
GATGATGTACTTGTACGGTAATCGGCTTTCAGGTCGAATCCCTGAAACTTTTGATTCATCAAAGTTGATG
GAACTTGATGTTTCCAACAACTATTTAACTGGAACAATCCCAGAAAGTTTTGGAGGGTTTAAACATTTGG
AAATTATGAATCTGTTTGGTAACCAATTGTATGGAGCAATTCCAGAGAGCATAGCCAAGATTCCATCTTT
GAAGGTATTCAAAGTGTTTAGGAATAAACTGAATGGAAGCTTACCATCTGAAATGGGACTTCATTCGAA
ACTCGAATCTTTTGAAGTTTCACTGAATTCCTTCACTGGAAACTTGCCAGAGCATTTGTGTGCTGGAGGA
ACTCTGTTTGGTGCTGTAGCTTATGCTAACAATTTGTCTGGTGAAATACCAAAATCACTCGGAACTTGTTC
TAGTCTACGTTCGATTCAGCTCTACAAGAATCAGCTTTCGGGTGAGATTCCATCTGGGGTTTGGACTTTG

GTTGACATGACAAGTTTGTTGTTGAGTGATAACTCATTTTCCGGGGAGCTACCAAGTAAAATTGCATTCA
ATTTTACGCGGTTAGAGATTAGTAACAACAGGTTTTCTGGTGAAATTCCTGTGGGGATATCTTCTTGGAG
GAGTTTAGTGGTGCTTTTGGCAAGTAACAATTCATTTTCAGGTCGGATTCCAGTGGAATTGACTAGTCTT
TCTCAGATAACTCAGCTAGAGCTTGATGGTAATTCGTTATCTGGAGAACTTCCAGCCGATATAATATCAT
GGAAGTCGTTGACAATATTGGATCTTGCCAGGAACAAACTCTCTGGCAAGATTCCAGCAGTTATAGGTTT
AATCCCTGATCTTGTTGCTCTAGATTTGTCTCAGAATCAATTTTCAGGTCCAATTCCACCTCAACTAGGTG
TAAAAAGGATCACTAGCCTTAACGTATCATCGAATCAACTTACCGGGAATATCCCTGATGCATTTGCTAA
CCTAGCCTTTGAAAACAGTTTCTTGAACAATCCCAGTCTTTGTACCACCAACTCACTTCCATATCTTCCAAG
TTGTAATAATGCGAAAGTCGCCAATTCCAAAAGATTGTCCCATAGAGTCCTAGCCCTAATTCTAGTCCTT
GCATTCGCTGTTTTCCTATTTAGTGTTGTATCGACCTTGTTCATGGTTAGAGACTACAGGAGGAAGAAGC
ATAAGCGCGATGTTGCAAGCTGGAAGTTGACTTCATTCCAGAGGTTGGATTTCACAGAGGCGAACATTT
TGTCAAGTTTGACGGAAAATAACATGATTGGAAGTGGTGGATCGGGTAAGGTGTACAGAATTTCCATTG
GCAGACCAAATGAGTATGTTGCTGTGAAGATGATTTGGAGTGATAGGAAAGTAGACTACATTCTGGAA
AGGGAATTCTTGGCTGAGGTTCAGATATTGGGATCAATTAGACATTCCAATATAGTGAAGTTATTGTGTT
GTATATCAAGTGAGGATTCAAAGTTGCTCGTTTACGAGTACATGGTAAATCACAGTCTTGATGGATGGCT
TCATGGGAAGAAGAGAGTTTCCTTGAGTAATAAAGTCATGGATTGGCCAAAAAGATTGGAGGTTGCCAT
TGGAGCTGCTCAAGGACTTTGTTATATGCACCATGACTGCACTCCTCCGATCATTCATCGCGATGTAAAA
TCAAGCAATATCTTGTTGGATTCTGATTTCACAGCCAAAATAGCTGATTTCGGGTTAGCCAAGATTTTAG
AGAAGAAAGGAGAGCTTAACACCATGTCTGCTGTTGCAGGTTCCTTTGGTTACATAGCTCCAGGTAAAA
GCAAAATCCAATCTTGAACTTCGTATTACCTCAATTTCGTCAAAAGAATGACTAACTAATAATCTACTGTT
TGGTTGCAGAGTATGCTTATACAACGAAAGTGAACGAGAAGATTGATATCTATAGCTTCGGGGTGGTGC
TATTGGAGCTAGTGACAGGAAGACAACCAAATTTTGGGGACGAGCATACAAGCCTGGCAGAATGGGCA
TGGAAGCAGCACGGAGAAGGGAACACTGCCATCGATAATATGTTAGATACAGATATCAAAGAAACATG
TTACTTGGAAGAAATGAAGACTGTGTTCAGACTCGGGCTTATATGCACGAGCAACTTACCTGCAAACAG
GCCTTCCATGAAAGAGATTCTGCAGATACTTCATCGATGCAAGTCCTTTAGGTATTCTGGAGGAAAATCA
CCAGATACAGAGTATGATGTTGCTCCTCTCCTTAGCGGGAACAACAGCGAAAAATACATTGCTAGTTACA
AACGTATTAATTCCAATAAGGTAATAGATGATAGTAGTGATGATGGTCTAATAATTTCCACTGTGTAATA
TTGGAGATGCCAAAGTAAACTGAGTTGTTGTAAAATGAGTGCCACTTAAGTAACGTACAAGTTTTGAGG
ATGTTGTTAGAATAGAAAACCCCTATAAGAACGTTGCTTTTCTAGTGTACTCTTGAATTTGTTCACTGCTT

GTAGATTGAGTGAAATGCATATTTAACTTTTGCTAGTTATGTAGATTGGAAAAG

>PGSC0003DMG400007709 LRR-RLKs

CATCAATCCAATGCAATCCCTTCAGTCCAATCACTCAATAGAAAGTTAACTTTGTAAAACCCAATTGCTTA
CTTCGTTTCTTTTCACATACATAATAAACAAAGGGATTCTCTTTTAAAAAAACTGACCACATCTCTGTATCT
GTGAGTTTTTGGCCTATGTTTTGGCTTTTTCTGTTGTGTTTGTATCTTTGTGGTATATATTACTTAGTTAAT
AATAGAGGGAGAGGATGTTGATCTTGAATGGATATGGTGGAGAGTTTCATTGAAGGAGAAAAAATAGCT
CAAACATAGAATTTGTTGGGATTAGTTCATATTCCAATATTATTAGATCTCAAGGTTACCACCTTAGTCTT
TTTTCTTTATCAAGAAGATTCCCTTTTTATTCATTCATTCTAATTATAGATGGATGCCCATTTCTCATAATTG
TGTAAAGATAGCAACTTTGGTGTTTTGAAGAAATATTGGAAGCTGAAAAGAGCAGGATTTGTGGTTGCT
ATTGAGTGAAAACAGCATCTATCTTTCTCTCTCTTTTTTTTTTAGCAAATTGGTTCTGTTCTGATTCTGAGA
GTGACCCTTTATGGATGAAGAAGTGGTTTTGATGTAAAGAATGTATTCTTGTTGTCTTAATGTTTTATGG
GGAGGTTTTGTTAGGCAAGTAATCCATTTTCTGACAAGGTAAATTGTAGAAAAGTAGAGAATGGACAGA
AAGTGGTGTATGTTCTTGTATGTGGTTTTAACATTGATGGAGAGTTGTTATGCTTCTCTTTCCCCTACTGG
TATCAACTATGAAGGTAAACTTCTTTCCTACATTTCCTTTCTCTCTTGGGGGTGAAAATCATCTTCATCTCA
CATAATTATATGCAGTTGTTGCCTTGATTGAAATTAAGAAGGCCTTACATGATCCTTACAATGTTCTTGAG
AATTGGGATGTCACTTCTGTTGACCCTTGCAGTTGGAGAATGGTTACATGTTCAAATGATGGATATGTTT
CTTCTCTGTAAGTAGTTGAGCTTCAGTAATTTTCACTCTACATAATCATAACACCTAAAGTAAACTCTTTTT
TCTTCCCTTCATCAGTGGACTACCTAGTCAAAGTTTATCTGGAACCTTGTCACCTGGAATTGGAAACCTCA
CTAAATTGGAATCAATGTAAGTTCTTCACTGTATTTTTCTCTTAGTACTTCATTTGATTATATGTTAGCTAA
ATAGTAATACTATTCCCAATATCGTGTAGATTGCTGCAAAACAATGCTATTTATGGTCCTATTCCTGATGT
GGTTGGTAATTTAGAAAAGCTTCAGACACTTGATCTCTCCAACAACAAGTTTGATGGCGAAATACCTGCT
TCTTTCGGAGATCTCAATAATTTGAATTATTTGTGAGTTCAGGAAATTTTTCTTATTTTTCACTGTTTCTTAT
CAAACTACACTTGAATCTTATTGTGTATCTTCGCTGTTGAGCAAGACAGACGATTAAACAACAATAGCCT
CACAGGAACCATTCCTCAATCTCTCTCAAACATTGGAGGCCTCGCTCTCGTGTACGTCTCCATACCCTCCT
TTACCATCGAAATTTGTTATGCTTATCCTTGAAAGACCGGTCTAAATTACTCTAATGTTCTGTTTCTTCCTC
GTACATTTTACTGATTTAGCTGATCTTGATTGTAGGGATGTTTCTTTTAATAATCTAAGTGGTCCATTGCC
CAAAATATCTGCAAGAGCTTTCAAGTGAGTCACCTTTTCTTCTTTCAATACATAATTGCTGCAGTTGAAGT
AGTAATTCTCAATATTAATGAAATTGACTGGTTCATGACTCTGTATATATACTTCTCATTAACAATAATTAT
GTAGTGCTTCTAGGATAATGGAAAGTATTTATTTTTGATAGATTAGTGAACCTTATTATATTGAATTTAAG

TTTCTCATCATGATTTTCAGAGTTATTGGAAATCCTTTGATTTGTGGCCAAAGTTCGGGGAACAATTGTTC
AGCAGTCTATCCAGAACCGCTGTCCTTCCCGCCAGATAGTTTAGGAGGTCTGCATAACATCACTCACTTT
CATTCACCTTCCGTTCCTTTCATTTAAAAATGGAAAATAGTAACAGACACAGGAGGTTTTGCTATCTCTAT
TGTTGAAATTGTATTGTCTGTTTTCTTCAATTTCATAGATCAAAGAGCAGGAAGTAAAAACCATCATGTG
GCTGTTGCTTTTGGAGCAAGTTTTGGTGCTGCTTTCTTGGTTTTAGTAGTCATAGCACTGGTTCTTTGGTG
GCGCTACCGACATAATCAGCAGATCTTCTTTGATGTCAATGGTTAGCTGATTTTCTTTTGCTTTTTATCCAT
TTAAGATAATTCAATTTCTTTGATTTTTTGTTTTTTCTATTTCTGATTCATTTGGTTATGTACATATTTTCTCG
AATGTATTAAACTCCGTAGATTTGTCATTATTTGATTATTTTTTTTCAAGTAAGGGTGTTTCAGAGTTTGG
TGGCTTGTGGTTTAACTGGTTATCTTAGCTTCTCAATATACATCTTTCATTTAATCTGACATAGAAAAGGT
TTACATTGACATTTGATTGTAAATCTAGTAGTCTAGTATTATGAAGGACCCAAATTTGCAAGTAGTAGAT
ATCTAACAGCAGTTCTACATTTACATCGAGAATATGTTGAATAAAATGCTATAATCCACATCTTGGACTA
GATAACTTCCCTGCTTTAGACCAGCTTAATTCATATCCTGACCAACCTTGTCAGGTGAATCAATTGTTATT
TGCTCCCCCTCCCATTGAGAAGAAGTTTTCCCTGACAAACTTATAGGGTGGTTGCATATTGTGGACAAAT
TTTGGGGACTTGTCACTACTCATTCTTTCTCATCCCTTTCCTTGAGCATTAGTATTTTTCGTCCGTCTGTCAC
CTGCTATTTCTACAGCTGTATCTGAGCAATGATTTCTCAAATGCAGAACAATATGATCCAGAGGTATGCT
TGGGTCACTTGAAAAGGTATGTCTTTAAAGAACTGCGAACTGCAACTGATCATTTCAGCTCAAAAAATAT
TTTGGGTAGCGGAGGATTTGGAGTTGTGTACAAGGGACGCTTAAATAATGGAACTGTCGTGGCTGTCAA
AAGATTAAAGGACTACAATGCTGTTGGTGGTGAAATCCAATTTCAGACAGAAGTTGAGTTGATTAGTTT
GGCTGTCCATCGAAATCTTCTCCGTCTTTGGGGTTTTTGTTCAACTGAAAGTGAACGGCTTCTTGTTTACC
CCTATATGCCAAATGGAAGTGTAGCAGCACGATTAAAAGGTCAGCCTCTTGGCCCGCATGTTTTGTTAGT
AATAATTGCTATGCCTCCTAGCTTCAGATCATTCCTCCAACATATATGTTCTGTGAAGTTTGACCTTTAAG
GAAGGTGTTTGTTAACTAATTTCAACTGTGGTTACCTTTCTTTTACATTTTTAGGTGCCTGGTCACACCTTT
CTAACTCATTATGGGAAGTTAACTAGAAAATACTCTCTTAAAGCTGCACAATTTTCTATTACAGAAAGTG
AGGCTTAACAGACTAACGTATTATCTTACAAGAACAAATCTTGTGAAGAAATGGACATTGGATCTAATTC
AAACTTAAAAGTTAGCTCATGAGGTAAGTATTGCTCAAAGACCACAACCCATTCCCTCAACCTATGTGGG
ACTTCTTAAAGGTAGATGAGCTTAGCTGATCTTTAATTCCATACGCTGTAAAGTGTAAATCAGTTTCTTTT
CTCCGATATGAGAGTGTCACTAGGGCTTGAATACCAACAAACAAAAATTGGATTCTTTTCCCTTAAGCTG
GGACTTGATAATATTGCTTGTTTAAGGACTCCCCTCTTTGCCTTCTCTCTATCCAAGTTAAAACTTGTTAAT
TTTAGAGAGGTTATCCTCTTCTCATTCTCTCAGTTTTATGCAATTTGGAATAATGAAGGAAGAAAGTGAA

TTAAAATTAAAATTTCACCATTGCACATGATAAAGCTTTTGAAAAGAGAAAGAAGTTTATTCATCTTATAC
AACAATTTGCTTCTAAAAAGCATTATGAGACTTGTCATAATTTATGGCTTAAGGAGATTAAAGTGGGTTT
GAACCATTCACGTAGTCCATATTTTACCCATATTTATGTAGATGGATTCCAGTTTATCTTCCTTTAACCCTG
AAAAGAGCATATGCATCCCGAGCTTTTTACGTTACGCTTCAATAGAACGAATTATCTTTACTTCAATAATA
GATATTAGGCCTTTCAGCAGGTTAGTTCACGAATTCTCCTATTTCAGTAAGAGACGTGTTTGGGCCTGCT
TTGAAGACAGCCAGTAACTACTAGCACAAGATCCAGTATTTGAAAAGGTTCCATACCCATAAGGGTTCCA
AGCTTCAACCCACTGCTCGAGCAACTTGCTATCTATTTTAACTCAATCCACCCAAATCCGGTCCAATGCAC
CCATTTGCCACCACTATAACTGATACTATCTTTTATTTTTTAGTACAAGCTTTTAATGTTTGACCCATCCTAT
TTTGTGTCCCTTATCATATTTCTTCTTCTTGAAGTATATGGTAGGCCATCATCAGCGACCACAGTGATGCA
GTTTATTCTGGCGAACATTGCCTTCCATTAACCATGTCTTTTGTGGTTCCTAAATTTGCAGATCACATCCAT
GGCAGGCCAGTTTTGGACTGGTCAAGGCGGAAAGGAATAGCAGTAGGTACAGCAAGAGGGCTAGTAT
ATTTGCATGAACAGTGTGACCCCAAAATTATCCATCGTGATGTCAAAGCAGCGAACATTCTGTTGGATGA
GGAATTTGAAGCAGTTGTTGGAGATTTTGGGTTAGCAAAGCTCTTGGATCACCGGGATTCTCATGTAAC
GACTGCTGTCAGGGGCACAGTTGGTCACATTGCTCCAGAATATTTGTCAACGGGTCAATCGTCTGAAAA
GACTGATGTGTTTGGGTTTGGAATCTTGCTGCTTGAGCTAATTACAGGTCAGAAGGCTGTGGACTTTGG
ACGAGGGGTGAACCAGAAAGGTGTTATGCTTGACTGGGTAAAGAAACTTCATGCAGAGAAAAAGCTGA
ACCTTATGGTGGACAAAGATTTGAAGAACAACTTTGACCGGATTGAACTTGAAGAGATGGTTCAAGTTG
CTCTTCTGTGTACCCAATTCATTCCAACTTATCGGCCAAAGATGTCGGAGGTATTAAGGATGTTGGAAGG
AGATGGATTAGCTGAAAAATGGGAGGCTTCACAAAAAGTTGAGACACCTAGGTACAGAACATCTGAAA
ATACACCGAAGAGATATTCTGATTATATAGAAGAATCATCACTAGTAGTCGAAGCAATGGAGCTTTCAG
GACCTAGATGACAACATCTGATTCTATCCTTGAGTTGAAATATGAGAAATAGCTGTTGGAATGCCTGTAT
TATTAATCTGTAAAAAAGAACACAAAGCTCATGTATAATTCGATCAACACCTAACTTCATACTTCTCTCCC
TCTCAATTTTGATAAAATGTGCCATTCATTTTTTGAAGATGGGGGCACAAGTCAGCCATCCTCATGTACA
GTGGTCTGATTGTCATGGTGGATGAATCATGAATAAGGTTTTCGGTTTTCTTCTTTTGTTCATGTACTGAA
ATGTTTAGTCTCGTCATAGTGAAGGGAAAAATGAAAATATGAAGAAAGGTAGTAGTAGGTAGGGGTGT
CAAAAATGAGCCGTTTAATCGGATCATAATTTTATGGGTTGAGCTCAACAT
>PGSC0003DMG400007862 LRR-RLKs
TTTTGTTATTTGTGGGTGATTGAGTCTTTTTCTTGCATTTTTATGCTTGAGGAAAACACTGACATTGATGTT
TTGTTTTATTTGGTATTTGCAGTTTTGGGAACTTTTTTGTGTTGGCTATGCAGAATTGTTTTCCAAAGAAA

GATTGAATTGAATTGAAGAGAAAAGATTGTATTTTTGGGGCAATGGATGGGAAGAGATTTGTTGTATGC
TGTTTGAATTTGGAGATTTTGGTGTGGATTCTTTTAGCTTTTGCTGTTCAGCTTTCAGATGAAGCTAACCC
TGGAGATGGTAAGGAACGACAGTCGCCAAGTCTTCGTAGGCGTTTGGACATGAATTGAGTGATATACG
AAAAAAAAAAGATTAGTATTTGATGTTCAGTTGAAAAATAGTAGTATTTAGAAACTGAAGTTGTGTTTCG
ACATGCATTTTAGTTTTTAAAAAGAGTTGTAAAACAATGTTTTGAAAAGAGGGATAACAAATTCATGTCC
AAACGGGTAAGTAATTTCCTGTGTAAATGCTCATAAGGGAATTCCAATTTCATGTTTTGTGCAATGCTGA
TTATGTGAGAATATGTAATTATCTAAGCGAGTTGTTGATTTGAGCTCAAAAGTGAATGCATTTGCTTATT
GTCCTTGTATATTTGAACTAGTAGGTGGTCTTCTTGCTTCTTATAAAGTTTGTACCTTTTGTTTAAAAAAAA
TTGAGGATAGGGTAAATGGTCGAGGGATTACAAGGTGGAGAATCGAACTCTCATCAATAAGGTAAAAG
TTCTAGTAGCCAACCAGTTCAGCTACTAAGATTTCCCTATAAAGTTTGTACCTTCACTTGCATTGTCCTGA
CATTTTATTGGGTTTGTAAAGTTGAGGACTAACAGCTTTTGCGCCTATTCTGGTTAATTGCCTCTCAGGGA
AGTTCGTAGTTAGCCTTTGTTAATTGATTGGGTTCTAAATTTCCACATGGTTTGTTTCTGTAATTGATTGG
ATTCTAAATATTTATATGGTTTCATGGATCAGTTTCTGCACTTTCTTCTTTATGTTTGGAATTTCTTAACAAT
TCTCCTTTTGTATTGGTTAAGTTGCTGCTATTAATAACTTATATGCTTCTCTGGGATCGCCATCCCTTCCGG
GCTGGGGTGCTGTTGGTGGAGACCCATGTAATGGACTTTGGCAAGGGGTCGAATGTGACAATGGAAAC
ATACTGACAATGTAAGGACTTACCATTTACTACATTGCGTAGATTTTTGTGGAACTGAAATGCTAATCCTA
CTTATCATGTTGTAGTCGACTCAATGCTGCTAACTTGGGAGGTGAACTTGGGGATAACTTAGGCTCATTT
TCTTCTCTCAAAATAATGTAAGCTTCTCTCATATCTCCTAACTTAAAAGTTTGCTTGTTTGTATGCATGTGT
TTTTCATAGACATAATACGTGAACACACTCAAACTTGGCCTCAGTTCGCAAGTAAACACTCCAACTTAGA
GAGTGGACATCTAGACACCTCAACTGGAAACTAAACACTTCAACTGGTCCCCACTGCATCTCGTGGTTTG
GAGGTGTTTAGATGATCATTTTGTTAGTCGGAGTGTTGAACTGACACGAGTTGAGGTGTCTGGTGCATA
CTCAAAGTTGGAGTACTTACTTGCCAGTTGAGGCCAAGTTTACGTGTCTGTTATGTATTATGCCTTTTTCC
TATTATTAAATGTTTCCATTTAGTGGAGTCCACTGTAATGATATGTGAATACTAATTGTGGTCATCTCCTG
TGGCAGAGATCTGAGCAACAACCATATTGGCGGAAGCATTCCGTCTAATCTGCCAGTTACCTTGCGGAA
TATGTAAGTTCTACCATTTGACTTATGAAGGATCCCCAGAAACACGAGAAAAAGAAAGAGGACATATTG
ATAAACCTAGCTTTACTTATTGAGTAAAACAGTTGATATTCTTTACCTTGGACAGTTTTCTTTCAGCTAAC
GCATTCACTGGAAGTATCCCTACTTCTTTGTCTTCCCTGAGTCAACTTTCAGCTATGTAAGTATCACTTTTA
GTGTAGATAAAAAGTTGCTTACTTCATAAAGAATCAAACTCAGGTATTTCGCTGTGTGTCTGCAGGTCTC
TGAATGCCAACCATTTGACTGGAGAAATTCCTGATTCCTTTCAAGGCCTTACCGCTTTGGTCAATCTGTAT

GAAAGCAAACAAAAGCTTTTACATACTTCCATTTAGTTTTTTTCATATGAATAACTGGTTTGGGTGTTTAT
CTTTCAACAGTGATTTGTCAAGTAACAATATAAGTGGATCATTGCCACCATCTGTAAGAAATTTGTCTTCC
TTGACCACTCTGTGAGTAATTCAACATTATACAGTTTGTTTATAACTTCATAGTTTGATCAATATGATAAA
ACTAACTTCTTGATTTCCAGATATTTGCAGAATAATCAGCTTTCTGGAACTCTTGATGTTTTGCAGGATCT
TCCCCTTGCAAATTTGTACTCTTCCAAACTAGATTTTACTCGAATATAATTCAGTTCACTAGAATGATAGA
ATCTTACTAACATCTCTTCACACTCCCTGTGTCCTTTATAGGAATGTGGAAAACAACTTGTTTTCTGGGCC
CATACCTCAAAAGTTGTTCTCTATTCCCAATTTCAAGTAAGTACATTCATTTTATGCTTTCATATACTCGTTT
CATTTCTGTTTATGTTGAGTCAACATCTTGTATATTAAAATGTAATCTTGGTCACATTTAATATCAATGTAT
CCCTCGTCGTTTGTTGGAGATATCATTTGACATGTAACATATCCCACAAAAAAGATATATAATCATAGAA
GACAATAGACATGTATGTCGAAACCGCGGAAACAAATATCTTTTCAAGTTCAATGTTACTTAATACTGCT
CCTTATTTTCCAATAATGCCTCACTTTTGTACTATGTACCAGTGGTTATAACCAAACATATAACTTTTCTAG
CTCCCAATATCCCCCTTTAAAGCTTGAGAACTTGTATCGTCTGTGCTATGCTTCATACACACAAGTAAGCC
TTTCAGAAACTGCTTGTTTACAAGTTCCTGAAATCTTACTTAGTAAAAAATGGATGTGCTTGTATGCTATG
TTGCTCGGACTCGTATTGGATCCTCCCAAAGTAATCCGACACTGGTGCAACAACATATTTGGAAAGTCCG
AGCAACATAGCTTGTATGCTGCAAGGTCATTTTGTACAAAATGACCAGAAATATAGGCTTAATGTCTGAC
ATGGATATGAATCCTAAACTTGCCCTTAGCATAAATGAAAGCTCAATGAGTAAAAGTAAGAATAAAACA
AATAAGAAATTGACCTGAAAAAAGAAAAAAAGAATCCTGACAAGCATGTCATTTAAATACATGAATTCT
ATCATATGGCCAGTCCCTCAAAACTTGGAAGTAAACAACTCATATAGACACAATAAATAGACAACAGAT
ATAGGTATAGATTTACAAACCGTAGTACAATATGCATATGAAAACATACAAACAGTTAAGTTATGATATT
CATGACCTGTAAAATACACAAAATTGCAAGTTATTTAGTTTAGCAAGCTGGCTCTTGGTCTAAACTTGAT
CATAATTTAAGTTCACATTCCATGGTGGCTATACTCTAGTTAGGTCACCCACCTTAATCATATATCGAGCC
CCTAACTTTCAGGAGGCAAAAATAACATGGAAGTCAGGCAGTCTTATTTATTAGCTGACTTAAATGGTAC
AAGTTGGTAGATATATTTGATATATGCAAAGATCAATGTATCAGTTGTTGGATCATTTTGTTACCATCTGA
TACATGCCTTGTCGATTATGATCAGTGATGGTTTGTACTGTCTTAGTTACCGGTTTTGTTTTTCGTGCAGA
AAAGATGGAAATCTTTTCAACAGTAGTATTGCTCCTTTACCTCCACCCACATCATCAACAGCACCTCCCAT
TTCTGGGTCTCCAACTTCAGCACAGACGCCATCTTCTCAAAGCAAACCTAAAACACAGGCCGATGGACCA
TCAGCATCTGCAGAATCAAGTTCAACAGGAAAAAAAAAGTCTTTATCTGCTAAAAGGGTGGTCTGGATA
TCAATTGCAGCAGTTTTGTCATTTATCATATTGGTTTTAGCAATCCTTCTCTGTCTGCCAAAGTGCCTCAG
GAAATGGCACGAGACTCAAAGAACGCCCAAACACCATGAAATAGCCCCTTATATGGGTGCTAGAGAGA

ATCTTGGAGATAGTGGTTCATTAGTCCAACCTGGCCATGATATAGAAAAAGGTGCTACAAAATACTCAAT
ATCTATGTTATTTTATGATATCTTTTATCACTGTTTCTTAAAGTTCAACTTTTCCCCCTTAGGGTTCGTTTGG
TAGGATGTATTAGAGAAATTAATACATGTATTAGTGATTGTATTATTTAATCCTTTGTTTGATAGGATTTT
CAGCATATGTATAACTAATACTATTTAATATTATAGGGTGTATAACTTATACATAACCAACCATGGTATTA
GTTAAACAATGGTTATTAATACATGGATAAGTATGGTTAAAGACAGAACTGTCCTTAATATATCAAACTA
AACCGTGGATAAGAAACTATATCTAGCATAACTAACCCCAACATAATTAATCCCAACATTACTCATCTCG
GCACTACTAATACACCCTATTTCAATACTATTATACGTGCTACCAAACGACCCCTTAGTTACTTGAAAAAA
AAAAGATACTGGGTCTGTTGTGTTGATGCACTTGAGCCAAGGGTCTTTCGGAAATAGCCTCTCTACCATC
ACGAGGTAATGGTAAGGTCTGTATACACTCTACCCTCCCCAGAACCTACTTAGTGGGATTTGACTAGGTA
TGTTGTTGTATTGGAAGTCTGTTGTGCAGCATTTAGAACTGAAAAGTTAATGAATCTCAAAGTTTAACCT
TCTACTCTTTAGTCTTTAGTGATAAGTGGTTTTCTTATGTGTTAAACAGCTCCCCTAGTTGTGACGCCAAA
AGAAAAGCAACCAAGAAGACCGGTTCCCGTTCCGAAGCCACAAGATGAGCAGCAGGTAAATGTGCAAA
CCATGAGTGCAGTGCCAAAGAAGGATGATAGTGAGATAAACCTTGGCAGATTGGATATTGATTTGATGC
CACCACCTCCGCCTCCTCCTCCGCCGACTCTTCCGCAAGAGAGAGTTATTGTCAAGCCGATTGCGCCTGC
TGAAGATACAGCAACGAGGCTTCCAACCAGGCCACTTCCACTAACTTCTGTGAAATCTTACACAATTGCA
TCACTTCAGCAATACACTAATAGCTTCTCTCAAGATAATCTGTTGGGATTAGGAATGCTGGGTAGTGTAT
ACAGGGTGGAGCTTCCTAATGGAAAGGTACAACTTTCAATCGGCATGAATTTATTATGATGTACCAATTT
ATTAACTGTAGTTTCCTACTTCCACTAGATTGTCGAGAGTCTTGACTTACTGCTTTTTCTCTCCTTTTATTTC
TCCATCATGTTTGGTATTCTGGAATGCTAAATTATCAGCAAGTAGCATCAAAATTGCCTCTTTATCTCTAG
TATAAATTGTTAGGTTGCTTAGATTTATCAAGAATGGTTGTGATACAAGCACAATTATATGCAAAAAGGC
CTTTTTTAATGGAAAATTCACCTTCATATATCCTTAATTAGAGCACTCCTTAGCAAATCTATTAAATGAATT
CTACCTATTTTATCTGATATAATGTTTCTTGAATTACTTTAGCTAGTTGAGACCTGCTTAAGGACAAAAGT
TGTAATTGCAATATTTTCCCTCTAGGTTTCTTCTAGTTCTTTTCTTTTCCTCGTTCCAAATTAATCTTCAACT
AGTATTTTTTTTCCAGTAACCAACATCATCGATGAAATCTTGTCCTAGAAGTGTATTTCAAATTTGTTAAA
ATGAAAAACAGCCTTTTATACTATTTTCTGCCTTGACTTGTCTCACTTTATAAGAGGAACCGCCAGATACT
GCTGCATCTTTTTTCTGGTCATGATTTTAACTATTAAAGGTAAGTATATGCATGCTGTTTCATTTATATAAA
TTACTTGGGGATGTATATGCATTTCTCTGGCATTCAAATCTTACCGCTCTTCCTTCACTTGCACTATGCACC
ACATCATTCAAGTGGTTGCAACATTTCACTTCTGACACATGCTCCTTTTGGATGCTTCTGCAGTTGCTTGC
TGTCAAAAAGCTGGACAAAAGAGTTTGCGATCAACAAAAGGATGATGAGTTTCTTGACCTAGTAAACAA

TATTGACAGAATTCGTCATGCTAATGTTGTTGAGCTGATGGGCTATTGTGCAGAGCATGGTCAAAGGCTT
CTGGTCTATGAGTATTGCTGTAACGGGACACTACAAGATGCACTGCATTGCGACGATGAATTCAAGAGA
CAACTTTCATGGAATACTCGCATGAGGATGGCTCTTGGAGCTGCAAGAGCGTTAGAGTACGTCAAATAA
CATTACATACATTTAATTATGCACATCATGTTTGTGCACACTCGCGTTCATGCGTGTGTGTATAAGTGTTT
TGGGACCTATAATAAGTGTTTCTGCACACACTATATGATAAATCAGTGCTATTAAATACTGAACAATAAA
ATGGTTCTGGTATAGTGATAAATTCAATAACAAGCTCATTTTAGTCCTCAACTTTCTTGTGTTGTGTATAT
TACATGATAAAAAGCCACACAAACGACCTGGAATGGTCTTAACTATATGCAAAATGTTAGATGTAGCTGT
CTTTTGCTTCCTCTCCTTTCTTTTCTTCTGCATGTCCAAGAAAAAGATGAACAAAATTAAATTAACTGGTTT
GCACTGCTATGCTGTAAAAGGGAAGCCCGATGCACAAGGCATCCCGCATTAGCAGTCTGGGGAAGGTC
CATACACCAAGGGGTGTGATGTAGACAGCCTATTCCAATGCAAGCAGTAGTGGCTGCTTCACGGCTCGA
ACCGGTGACCTTATAGGTCACACAGAGACAATTTCACAATTGCTAAGCAGTCTGATTCTCATTTTTTAATT
AGATTTGCATGAAAAAGTGAGGTAGACTGCTAGAGTATGTTTAAATTAATAATCATTCTTGTTTGATGAA
ATGCAGGTATCTCCATGAAGTCTGCGAGCCACCTATTATTCATAGAAACTTCAAGTCTGCCAATCTTCTGC
TTGATGAGGAGTTGGCTGTACATGTTTCAGATTGTGGTTTGGCTCCACTTATATCATCTGGTGCTGTGAG
TCAGGTAACTTGTGACCCCAATGTTATGGAGTAGAAAGAATTGAAATGCTTCCTTGGATTATTAGTTTCC
TCTATGAGCTGCAAGTGGAAAGAAAAGTGAATTTCTAGTGTTTGATAGAAGTGGACATGGGAGCTGAA
TATATAATTTAAAAAAGGAACAGTAGTGATGGTCAAAGCGTTTTCAACAAGATGTTGGAATAAGGAGCA
GTAAGAATGAATTCCCGTAAATGTGTAAAGTATATTCTTTGGGAAATGGTTGGTTCACACCCTGTAGGAT
AGGGTGAAGTGTTGTCGAAGGCGCGCTTAAAGCACGCTTGAGCCCTGAAGCGAGGCTCAAAATGTGTT
TAGCATTTCACATCGCTTAATGTGAGATTAGGGGTCTATAGGAAACAACCTTTCTACAGTTGAGGTAGG
GGTAAGGTCTGCGTACACTCTACCCTTCCCAGACTCCACTGCGTATGTTATTGTAAGTAAACACAAATGG
TGAAGGTGCTCTTCTCATGATGTGTTTTATATGAACAGAAAATTGCTCTAGGATGCATGTGGATCATCTA
TCTTGCTGTTATTTGTTGTTCTTTTGATAAAGTAGTCTTGCTTAGTCTTGGGTTTGATTACTGTTACGTTCA
ATTAAGGTATGTTAAAAACTATATCAAGTTGACATTACACTGATTTTTCAGTTGTCGGGACAACTTCTCAC
AACATATGGCTACGGTGCTCCCGAATTTGAGTCAGGGATTTACACTTCCCAAAGTGATGTTTACAGTTTT
GGTGTGGTGATGCTGGAACTAATAACAGGCAGAATGTCATATGACCGGTAAAAAAGCCTTCTGCATCCT
TTTGAGCTAATATAGGTGTGCCTCATTTCTAAGTTAACACACCTGCATATCATATTCAGGACACGGAGTC
GAGGAGAGCAGTTCTTGGTCAGGTGGGCAATCCCTCAGCTACACGATATTGATCTCTTGACAAGGATGG
TTGATCCTGCTCTCGATGGAAAATACCCCATTAAATCACTATCACACTTTGCTGACATAATTTCTCGCTGT

GTTCAGGTGAGTTATTCTGTTGTTCCTTCATGTGAAACACATAAATGCTTACTAATATACATTGTCTAATC
GCTGAGTGGACCGAATAGTCTTAGATTGTTCGTCATCTCAATCGGTTGGAAGGATATTTCTAGGATCTAG
TTGTGGAATAGGTTTGCCTTCAAATGTCACTTAACTTTTAAACTCTTATACAGCATGAGCGAGAATTCAG
GCCACCAATGTCAGAAGTTGTTCAGGACCTCATACAAATGATAAGAAGAGAGTCTCACAAAAGATCTGA
TGGTGAATGATTTGAGTTAGACGCGCATACTGGGAATTCAGCCGGTGAGAAACTAAGATTATTTCTTCTT
CGAAGGAGCTGAGATACTCGGAAAAAGAAAGGCGGTTTGCAGGCCAAGCACCGTGCTAAAGACAACTT
CAAACTGAAGCAAAATGCACCTCAAAGAAGCGCGATTCATGAAAATACCAACACATGCAGGCGACTTAA
TGACCTGTAAAAACAGCTTGCTCATCTGATTCTTGTCAAGCTGCATCATTTTAATGTTCATTTAGGCTTAC
ATGTGAGCACCAATGCATATAATTCATTGATCTTGACAAAATGGTATATTTTCTTGAAGTTGTATATCTTT
ACTTGCTGCAACATGTACAAACTTCATTTGGTTGTACAAGGATACCTTCATTCTTTCTCATGTACTTGTGG
CACATGCCAAAGTTATTTACTACTAGTCATTACCTAAGGAGTTGGAGACCTACAACCACTCAACCATGTA
ACAATATGTAAA
>PGSC0003DMG400007948 LRR-RLKs
TTAATAATTAATCCTAAAATAACTTATTTCGACCGTGAAACATTATGGCCATGGCTGACACAGAAACAG
AGAGAAAAATAGCCAGACTAAATATTGGAAGTCCCCACTTTTCTTTTTTCTTTCTCAAAAATCTTCACGTG
AATCTGACTCTGCCTTTTGCCATATCTTCTTAGACAGCTGCTTCTCTCTTCACTCTTCCACCTCTACACATAT
TCCACAACAATTCAATCTGGAAAAAATTTCTTAAACAAGATGAAAATACAACAATTCTTACTACTTACCAT
TCTTCTGTTTTTATCTGTTTCTTTTCATTCCACTGTTTCGTTGATTCCTTCTGATGCATCAGCATTGTTAGCA
TTCAAATATAAAGCTGATTTGGACAACAAACTTGCATTTTCTGCTAATACAAGTTCCAGATTCTGCAAATG
GAAAGGGATACAATGCTCGGAGAAGAAAGTGATTCGTATTGTTATCGAAAGTTTTAGCTTACGAGGTAC
GTTTCCAGCTAATACATTGTCTATGCTTGATCAACTCAGAGTATTGAGTTTACAGAACAACTCACTTACCG
GTCCGATTCCGGACCTTTCTCCACTTATTAATCTCAAAGTTCTTTTCCTGGACCATAATTTGTTCACCGGTT
CAATACCAGCTTCCATTTTCACTCTTCATAGGCTTAAAACCCTTGATCTCTCCTATAATAATCTCACCGGTT
CAATACCCGTTGCAATCAACGGGTTGAACCGGTTGTACTATCTCCGGCTTGACTCGAACCGGATAAACG
GATCAATCCCACCGCTGAACCAATCTACCCTCCATATCTTCAACATTTCTCACAATGCCCTTTCTGGTCCTA
TTCCGGTAACGAAAACGTTATCCCGGTTTAAAACGGCGTCGTTTTCAGACAATAAAGGACTTTGTGGTGA
GATCGTACACAAAGAATGCCGTCCAATACAACCGTTTTTCAGTCCATCCACCGCTGCCTCCACTAAAATTA
CGCCGCCGCCGTCTAAAACTCCGGCAGAACTCGGCCAAAACGAAGAGTTACGACAGGGTAGCCCGTTA
AACCGTAAAGAAAACAAATCACATAAAAGATCCTTACTCATTATTGGAGTTTCAACAGCTTGTTTAGTCCT

TTTATGTTCAGTCATACTATTAGCATTAGCGTCGAAGAAGCACAGGAACTCGAAGAAATTGGGAGAAAC
TAAGAAATCCGTATTCGATCCAAGTGTTAGCGGCAATGCAGAAGCAGTAATAAGAATTGAAGAAGACA
ACAATGAACTGGAAGAGAAGGTGAAGAGAGTACAACAAGGAATGCAGCAAGTGATGGGTAAAAGTGG
GAGCTTAGTATTCTGTGCAGGTGAGGTGCATGTGTATACCCTGGAGCAGCTGATGAGAGCTTCTGCTGA
GCTTTTAGGTAGAGGAACAATGGGGACTACTTACAAAGCAGTGCTTGATAACCGTCTGATTGTATGTGT
TAAGAGATTGGACGGTGGGAGATTGGCGGGTACAAGTCAAGAAGAGTTTGAGCAGCATATGGAATCA
GTGGGCGGGCTTAGGCACCCGAATTTGGTTCCTTTTCGGGCTTATTTTCAGGCCCGACAAGAAAGGCTTT
TGGTTTATGATTATCAACCCAATGGCAGCCTGTCTTCTCTTATTCATGGTACGTTCTTCTCCGTATATCTTG
TACTTATTATTTATTAAGATGCGCATAAACTATACGAAAAATATTATTAATGCAATTATTTTGACGAGAAG
TAAAATATAATAAAATGAACGGAGTATTTTGAAGGTGTTGTCCAATCAAATAGGGTCCATACACCATACC
CTATTGTTACAGTTTCTCAACCCCAATGTTCTCCCAAACACAGAGAATCTTGCAGCTGACCCTGATGACAT
TTTTGGACACATATTTCTGTCATCGTAAAATACTTTTATATTTGTTTAGTGTCCTAATCATAGATTATAGTA
TTATTATTGAAGCTAGTTCTGTCTGCATTAAGAATGTTATTGTTTCACGTGTGGTTGCGTAACGTGAAGTT
AATAAGAGTACGGTGGTTAGCATTAATAATTCATAAAGTAATATAAATAATAGCTTTATTGCATATACTG
AATTTTTTGGTCAATCTGACGTGGCATATGAAATAAAGTTTGACTGGTCAACCTGCCACGTGTACAGGTT
CCAAGTCATCAAGAGCAAAGCCACTTCACTGGACTTCATGTCTGAAAATAGCAGAGGATGTAACTCAAG
GACTTTCCTACATCCACCAAGCATGGAGGCTTGTCCATGGCAATCTCAAGTCATCTAACGTCCTCCTCGG
CTCCGATTTCGAGGCGTGCATAACCGACTACTGTCTCTCCATCCTAGCTGTCCCCTCAGACGATGACAATC
CTGATTCTGTAGCTTATCAGGCTCCAGAAATTCGGAAGCTGAATCACAACAACCACCACCACCACCGCCA
AGCTAGTGCCAAGGCTGATGTCTATTCGTTTGGTGTTCTTTTGCTTGAACTTCTAACGGGTAAGCATCCTT
CAGAACATCCATATCTAATGCCAGATGATATGTTACATTGGGTGAAGTCTACTAGAGAGGATCATGATG
GTAGCATTGGAGAAGATAGTAAGTTGGAGATGCTTCTTGAAGTTGCTATGGCTTGTAGAGTGAGCTCAC
CAGAGCAAAGACCAACAATGTGGCAAGTGTTAAAGATGATACAAGAGATTAAAGAAGCTGTTGTAATG
GAAGATAGTAATGAAATGGACCTTCTCACTGGCCCTCCTAAATCCTAATTCTTATTTAAATCTTAGTTATT
ATAAAATTGAATTTGTATTATATTCACTACATGAGACTTTTCAATATTAGTAATTGCAAAAGAAAGGAAC
AAAAGGGGAAAGACTTATACTTTTGTTACATGACCCTCATTCACCCTATTATATTGGATAACTATGTCTCA
CTTCCAAACAAATCGGTATGTTTTATGCTAGTTGTCAGCCTACTACAACCCTCCTCCTTTATCTGGTCTCGG
GATCAACATGTTTGTTGTTGGCAGTGTTTTTTCTCAATAACATATTTCAATTTGAC
>PGSC0003DMG400008146 LRR-RLKs

AGTTGTTCAAGTTTGTATCTTTCTTCTATTTGTACTTAAAACTGCAATTTTTATCACTTCTGCAACTTATTTG
TTGTTTTTGGTACATGGGGTTCATTCTTGAGTAGTAAAGATTGCATCTTTATTGTTCTGTTTCATTTTCAAG
ATTCTTGAAATGGGTCATTTCCTTTAAGTTGTTCAAGTTTGTATCTTTTGTTTTCTAAACCCTTCTGTTTCTA
CTCTAGTTGTCTCTACAAGGTTTCTTGGTTTTTGGGTTCTAGCATTTTGTTTCTTTATGGTTTCTTGATTGTT
CCATTTGGGCTTTTTTCTTTTTGGGGAAAAAAGGGAAGAAATTGAAGATTTTCTTGAAAAAAACAACTTA
AAAATGGTAACTTTGAGGTTGTTCACTGTTTTGCTGGTGGGGTTGTTGTTAAGGTCTCAATTTGGGATTG
TAAAGGCTCTTCATGAACAAGCCATTTTGGAAGCAATTGGCAAAGAGCTAGCAATACCTGGTTGGGATC
TGAATAGCACTGATTTCTGTTCTTGGCATAGCATTAGTTGCAGCAGCTCAAACAGTTCAATGGTGGAAAG
GCTGAATCTTTCGGGTTTTCGATTACAAGGTAATGTGACTCTAATATCTGAGCTCAAAGGATTGAAATGG
CTGGACCTTTCTAATAACAATTTCCAAGGTTCAATCCCACAAGCATTTGGGAATTTGTCTGAGCTTCAGTT
TCTTGATTTGTCTTTTAACATGTTTAGAAACTCTATTCCTGGTGAATTAGGTAAGCTCAAGAACCTTAGAG
CATTGAATCTTTCAAACAATATGCTCACTGGTTCAATACCTGATGAGCTTGAAGGGATGGAGAATTTGCT
TTATTTCCAATTATTTACCAATAAATTGAGTGGTTTTATTCCAATGTGGATTGGGAATTTGACTAATCTTA
GAGTGTTTGCAGCCTATGAGAATGAGTTTAGTGGTGATATTCCAGTTAACTTAGGCTTACATTCTGAGCT
TTTGTTGTTAAATCTTCATTCAAATCAGCTTGAAGGGACAATTCCTGAGAGTATTTTTACTATGGAAAAGC
TTGAATTTTTAGTTCTGACTAATAATAAGTTGACTGGCACTATTCCTGATTCAATTGTGAATTGCAAAGGC
CTTTCAAGCATTAGAATTGGGAATAACAAGCTGATAGGTGGTATCCCTAAAGGAATTGGAAATATAAGT
AGTCTTGCTTATTTTGAAGCAGATAATAATACACTTTCCGGGGAAATCGTCTCAGGATTTGCGAAATGCT
CTAATCTCACTCTTCTTAATTTAGCTTCGAATGGATTTTCCGGAACTATCACTCCAGAGTTTGGTGAGCTC
GATAATCTACAGGAGTTGATTGTTCCGGGGAATAATCTCTATGGAGAGATTCCAACTTCAGTTCTGAGGT
GCAAGAATCTCAACAAGCTTGATTTAAGTAACAACAAGTTCAATGGAACCATTCCAGGAGATATATGCA
ACACAACCAAATTGCAGTTCTTGTTGTTGGGACAGAATTCCTTGAAAGGGGAAATACCCCGTGAGATAG
GGAACTGCGTAAAACTGCTTGAGTTGCAAATGGGGAGTAACTATCTTACTGGAAGCATCCCTTCTGAGA
TTGGTCATATGAAGAACTTACAGATTTCATTGAATTTGAGTCATAATCATCTCCATGGACAGTTGCCTAA
GGATCTTGGAAAACTTGACAAGTTGGTTTCTTTAGATGTATCTAATAATCAGCTGTCGGGGAATATTCCG
TTGGAATTGAAAGGCATGTTGAGCTTGATAGAGGTTAACTTTTCGAGCAATCAATTCACAGGACCAATA
CCTGCTTTTGCTCCATTTGAGAAGAGCTTAAATTCAAGCTTTCTTGGAAACAAAGGGCTCTGTGGTGAGC
CTTTGAGTAGTGATTGTGGATATGATTTTGAACACAACGGTAATCATCACCGAGTTTCCTACCGGCTCAT
CTTGGCTGTTGTTGGTTCTGGTTTGGCAATTTTTACAGTTGTCACAGTGGTTGTTTTACTATACATGATGA

GGGAGAAACAAGAGAAAACCACCATGGAAGATGGAAATACCACTGATGAAACTTGTAGCAAACCAGTG
ATCATAGCAGGGAATGTTTTCGATGAAAATCTCAAGCAAGCAATAGATTTTGATGCAGTTGTAAAGGCA
GTCAGGAAAGATACAAATAAGATTAGTACTGGAACTTTCAGTGATGTGTATAGAGCAGACATGCCTTCC
GGGATGATTTTGTATGTTAAGAGCCTAAAGTCGATGGACAAGACTATAGTTCATCACCAGAGCAAGATG
ATCAGAGAGCTTGAAAAGCTAAGTAAACTCTGTCATGATAATCTGACGAGGCCTCTCGGGTTTGCAATCT
ATGAGGAAGTTGTTCTACTCTTACATCAATACTATCCCAATGGGACATTGGCTCAGTTTCTTCATGAGTTT
AGCCAGAAACCTGAGTACGAACCTGACTGGCCTACAAGACTTTCCATTGCCATTGGCGTTGCAGAAGGG
TTAGCGTTCCTTCATCATGTGGCCATAATCCATCTTGATGTTTCTTCAGGGAATGTGTTTCTTGATTCTAAA
TTCACCCCTTTGGTTGCTGAAGTTGAAATATCCCGACTTCTTGATCCATCTAGAGGGACTGCAAGTATTA
GTGCTGTTGCTGGCTCATTTGGATACATTCCCCCAGGTATATTCTATGGCTTGAAAGACTTGGCTAATCAT
TTTACTACCTCGTGTTTCAATTTGTTTGTCTTACTTTCCTTTTTAGTCGGTTTAAAAAAAATGTCTTTCCTTT
CTTTGTAACTCTTTAATTTTAACTTTACACCTGACATTATTAAGACCATGGACATTTTGTATATTCTATCTG
CCAGACAAACAAATTGAAACGGAGGGAGCATTTTCTTTTTGTGGAAAATATCTGGTGTCTGACTAGTTCC
TTTTTCTTCAACAGAGTATGCATATACAATGCAAGTAACAGCTCCCGGAAATGTTTATAGCTATGGCGTT
GTTTTGCTCGAGATCCTTACCACCCGACTACCCGTTGATGAAGCTTTTGGTGAAGGAGTTGATCTGGTCA
GGTGGGTACATGGTGCTTCTGCGAGAGGAGAAACACCAGAGCAGATACTTGATGCAAGACTTAGCACC
ATTTCTTTTGCTTGGAGGAAAGAAATGCTTGCAACTCTAAAAGTCGCATTGATGTGCACCGATACCATTC
CAGCAAAACGACCAAGAATGAAGAAGGTGATAGAAATGCTTCAAGAGGTTACACAGAGTTGAATCAAA
ACTCGTATCATCCAAAAGTCTCTGCTTAATCTCAGACCTGCAGATCATGAAAACCCCTGTAGAAATACAA
AACGGTATGAATTCACTGTTCTTAGCGTAGACTAGTGGTCTAAAAATAGTTAAACTATGTATATTGTCTG
AGGTTGCTGTTCTTCTCTGATGATCAGCTCCGAGGTTGTAATTTTGATTTTCATATTGGAGCGAGTAAGG
AGGTTATATGGTAAGGGTTAAAACCAGAAACAGTTCTTGATATGTACCATTCTGTACAAGGCCTTCAATC
ATATATACTTCTTTTTTCTTGTCTATGTGTAAATGGAGCTTTGGCATTTCATATGCAACAGTAAAATCTTTA
TGTATATATAATATACAGTAGATGTTGAACCCTCTTGGTG

>PGSC0003DMG400008296 LRR-RLKs

TTATGTACTCCTAGGTTTCGAGTAGGAGACGAAATTCTTGTTGTCTTTTCTTGTGTGGGGGTACATACAA
CTTGTTCTTTAACACGGAGCATCCTAGATTAGAGGCATGCTTGGTATTTTGTAACAAATTCATAGAATGC
AAGTCATTTTCCTTGGTGTGAATGGATGACTAGTTGACTGCCACTGAAAGCAGTGATTCACTGTACGATT
CACTAAGAAATGTGTATAAAGGCTTCATTGTCAGCATAAGTTTTTTCAGTGAGTGAAGCTTCTTTCTTTTG

AAACAATGATGTCAAAGACAGTTGTATATGCATTAGCCATATTTTCTATTACCTTTCTCATACCTCTGTCAT
CTGGGCAACATCCAAGCTTTGAAGTTGAAGTTGCTGCTTTGAAAGCTTTCAAGAGCTCAATCTCTGACGA
CCCTTTCGGTGCACTTGTGGATTGGACGGATGCAAATCACCATTGCAACTGGTCTGGAATCATATGTGAT
CCTTCTTCAAGCCATGTCATCAACATTTCGCTTATTGAGACGCAGCTCAAAGGCGAAATTTCTCCGTTTCT
AGGAAACCTCTCCAAACTCCAGGTTCTTGATCTAACTTTGAATTCATTTACTGGAAATATCCCACCCCAGC
TGGGGTATTGCACAGACCTGGTTGAGCTCGTTTTTTACCAAAACTCTCTTTTTGGTGAAATTCCTGCCGA
GCTGGGAAACCTAAAAAAACTGCAGTATATAGATTTTGGAAATAACTTTCTAAATGGGAGTATACCTGAT
AGTATATGCAACTGCACTGAATTGTCGTTAGTAGGCTTCATCAACAACAATCTCACAGGCAAATTACCGT
CTGAAATTGGTAAGTTGGCCAATCTTCAGTTGTTTGTAGCTTATACAAACAATTTGGTTGGTTCTATTCCT
ACCTCCATTGGAATGCTTACAGCTTTGCAAACCCTTGATCTGAGCGAAAACCAGTTATCTGGACCTATAC
CATCAGCAATTGGCAACTTGTCAAGTTTAGGAATTCTTCAGTTACATCTCAACTCTCTTTCTGGGAAAATC
CCATCTGAACTTGGCCTCTGTATCAATCTTTTTACCTTGAACATGTATACTAATCAATTTACTGGAAGCAT
ACCTCCTGAGCTTGGAAATTTAGAAAACTTGCAAGTGCTCAGATTGCATAACAATAAGTTGAACTCCAGT
ATACCTGCCTCAATATTCCACCTGAAGTCATTAACTCATTTAGGACTCTCACAGAATGAACTAACTGGTAA
TATTCCTCCCGAGTTGGGATCTTTAACGTCACTAGAAGTGCTTACCCTTCACTCAAATAGGTTATCGGGG
GAGATCCCATCAACTATAACAAACCTGGCAAACTTGACATATTTGTCTTTGGGCTTTAATTTATTGACTGG
ATCACTTCCATCGGAATTTGGGTTACTCTATAATCTGAAAAATCTAACTGCAAATGATAACCTCCTGGAA
GGATCTATTCCATCGAGCATAACAAATTGTTCTCATCTTCTTGTTTTAACCCTCGCTTATAATAGAATAGC
GGGGAAAATACCAAATGGGTTGGGGCAATTGTCCAATCTTACATTTTTGTCTTTGGGATCAAACAAAAT
GATGGGGGAGATTCCTGATGATCTCTTCAACTGTTCAATGCTTGAAATTCTAGATCTGAGTGATAACAAT
TTCAGCGGGAAACTCAAATCAATGATTGGCAGACTCTCTAAACTTCGAGTTTTAAGAGCTCGTACTAATT
CATTTCTTGGGCCAATCCCACCAGAGATTGGTAAACTGAGTCAACTCGTGGATTTAGTGCTTCACGAAAA
CAGTTTCTCAGGTGCGATACCACCAGAAATTTCAATGCTTTCAAACCTTCAGGGTCTTTTGCTATCTGACA
ACAAGCTGGAAGGTGAACTTCCTGTGCAGCTTTTTGAGCTCAAACAGCTCAATGAACTCCGGCTGAAGA
ACAATAATTTCTTTGGTCCACTACCGCAACATATATCCAAACTAGAATCACTGTCGCTCATGGATCTGAGT
GGAAATAAGCTTAATGGCACAATCCCAGAGAGCATGGCGAGCCTCCGCAGATTGATGACCGTAGATCTT
TCACACAACCTTCTTACCGGAACTATTCCCAGAGCAGTACTTGCCAGCATGAGAAGTATGCAACTTTACT
TGAACGTTTCCAGCAATTTGTTGCACGGAGAGATCCCAGATGAGATAGGCGTGTTAGAAATGGTTCAAG
AGATTGACATGTCAAATAATAATCTGTCAGGCAGCATTCCCAGATCCCTAGAACGCTGCAAAAACTTATT

TTCACTGGACCTATCAGGAAACATGCTCTCTGGTCCTGCTCCAGGTGAAATTTTCACCAAATTAAGAGAG
CTTGTGTTCTTGAACCTCTCAAGGAACAGATTAGAAGGTAGTCTTCCTGAAATAGCAGGATTATCACATC
TTCGCTCTCTTGATGTTTCACAAAACAAGTTCAATGGAATCATTCCCGAGAGATTTGCCAATATGACTGCA
TTGAAATATCTCAACCTTTCTTTCAACCAACTTGAAGGTCACATTCCAAAGGGGGGTGTATTCAACAATAT
AAGGTCGGAGGATTTACTGGGAAATCCATCTCTATGTGGAACAAAGTTTCTCAGTCCATGCAACATAAA
AAGAAACCGAACAAGTTCTCATGGTTTTTCCAAGAAAACCTGGATCATACTTGCAGCACTTGGATCTGTT
TTCAGTCTCATTCTTCTTGTCCTGGGAATAATTTTATTTCATCGGTACATGAAGAAGAAGAAAAAAGTGA
ATGACGCGGAGGATACAAATCCAAAGTATACCGCAGCATTGAGCCTCCAGAGATTTTATCAAAAGGATT
TGGAACATGCTACCAATAATTTCAGTCCGGAAAACATTATTGGAGCCAGCAGTTTAAGTACTGTGTACAA
AGGTACACTGGAAGATGGGAAGATTGTAGCAGTTAAGAAGCTGAATCACCAGTTCTCAGCAGAATCTG
GTAAATGCTTTGATAGGGAAGTCAAGACTCTGAGCCAACTCAGACACAGGAACCTAGTTAAGGTGCTAG
GTTACGCTTGGGAAAGCAAGAAGCTAAGGGCTTTAGTTTTAGAATACATGGAGAATGGGAACTTGGAC
AACATGATTTATGGTCAAGTGGAGGATGACTGGACGTTGTCCAATAGGATTGATATTTTAGTTTCAGTTG
CAAGTGGACTGTCATACCTGCATTCAGGCTATGATTTTCCAATAGTGCACTGCGACATGAAGCCTTCAAA
TATTCTTCTGGATAAAAATATGGAAGCACATGTGAGTGACTTTGGGACAGCTAGGATGTTGGGTATTCA
TCTCCAGGATGGGAGCAGCATATCATCAGCATCCGCATTTGAAGGAACTATTGGTTACATGGCGCCAGG
TAATAATTCTTACAACTTCTTGACCCACACTTTACTAACTAGATTATGTGTTACAATAGCATGAATACAAG
ATGTCTACAATGTAAACGAGCTTATATGGGTACCACAAATAATATTTTGTCAATGGTGAATGTTTCAAAT
ACATTCCATTTTAGCATCATTCAGTAGTAACATCACTTCTGTCAATCACAATATGCAGCACAGCAGATTTT
TTTTTCCTTTCAATCACAAAATACTGATTTAACTTTTATGTGGAAGAATAGACAAGCAAGTAAATGATGCA
ATGTTTAGAACATATAATCTGTAAAGTATTATTTTAAATGATAATGTCAGTTTTAATCTATCCAAAAATCT
AGCGCCAGCTTTTACATGCTCCTAATTTAATTTGAGACCTCACTTACATGAAATGTTATCTATGCAACAGA
GTTTGCATATATGAGGAAAGTGACCACAAAAGTAGATGTATTCAGCTTTGGTGTCATTGTGATGGAGAT
CATTACAAAAAGAAGGCCAACAAGTCTTACCGGAGCAGATGAATTACCAATGACTTTGCATCAAATTGTT
CAGAATGCCCTTGCGAATGGCATAAACAAGCTAGTTCAGATTGTGGATCCTAATCTAGCTTCATATGTCT
CCAAGAAACAGGATGTACTAGAGGGACTTCTTAACTTGGCTTTGTCCTGCACCTCTGCTGATCCTGAAGA
TAGACCTGACATGGAACAAGTTCTGTCTTCTCTTTCAAAGCTAAGTAAGATGGACTGCATGCCTTCACAT
TTGGTAAAAGATTAAATTTGATGTAGAAGAAGCTGGAATATCATCACTCTTAAAGTGAACCACCACCAA
GTTTCTCATGGGGCTAATGGAACTAGTTCATAACTTAAAATGTATTTACTATTTAAATAAATTTTCCGTAT

TTCAAGCCATAAATGATCTCTCGAGTATTCCTGCTTTACTAGAGAGATATAATGGAACTGTCCCGTCAGA
TTATTTTGAAGTTGACATGCATCATGAAAATGAACAA
>PGSC0003DMG400008403 LRR-RLKs
AGTGACCATATGTATGAAAAATCAAATTAGTTATTTGCTGATCTGAAGCAAATTTTTGAACAATGGCAGC
CACTCGGCTGTTACTCTGCTTTATCATCTTCTCGTCTGGCATCGATTTCATATATACTGTTACAGATCCCAG
AGATGGTAAGAACTTTGTATAAAATTGTCGGATCCAGTATTTAAACTTTATGAGTTTTACTATTTGTTGCA
GTTTTTGTGAACTTTTACTTATAAATTTATGTTCTGTGTTGAAAGTACTGAGTTCAGATAATAATGTTGTT
ATGAATAAGTGTGAACTTGGTTGATTGCAGTAACGATACTTCGATCACTGAAAGATCAGTGGGAAAATA
CGCCGCCTAGTTGGCAGAAATCGGATGATCCTTGTGGAACATCTTGGGAAGGTGTAACCTGCAACAACT
CAAGGGTAACTGCATTGTGAGTATGCTTTTTCTCTTCCAATATCTAGGGATCGTTTGGTTTACGTTCTAGT
TATACGATGACTAATAACACATGAATTGTTATGCATGATTGTATAATTTAAGTGCATTTCCGCCTTTAGAT
GCTCTTATCCACGTATTACTCGTACATGTATTGTTGTTCCACATTCTATCTGTGAAAAATAATATATAGATT
CTCGTCTAAGTTATACAAGGTTTTAGTTTCCAATATTAAACTTCGCATGACTAGTGCAGAGTTTAATATCG
GAAACAAAACATCATGTTAGTTATATGGAAACTATATTATCTTACAAGTTTTAAGATTGAAAACCAAATG
TAGTATAAGTAATACGAAAATTTATACATAAAGTTAATTTTTTAGGAAAAAATACATAATTAGACATCCTT
CGCTATCATATATATATCATTACCTATACTTTCAAAACGTATCTTACCACTAATTAAGAGTTTCCTAACATA
TATTGAGGAAGATGCACTTAGATACATGTATTTTTGCTACCAGATACAGTAAAATAGGGAGAGAGGAGA
GCGAAATGGGGAGGGAGGCGAGCGAGATATGAGAGTGGTGGGTGAGATGGGAGAGACGCGAGAGA
CAAACGAGATCTGTTAAGTATCCTAGATACATGTGAATTCACTTGGATACAATATATCTAGAACAATTAC
ACCTAATTTTTACCCTATGTATCCCGAGATGCATGTTCTGGATGTATGTGGACGCAACAAATGGTAAGAT
ACATAATATTGCAAACTAGAGTGTATCAAAGTAATTAGCTCCTAAATTAGTGGGATTTATGTAAGTTTTTC
ACCTTTTTATACTTCAAACCAAGCCACATCTTAGTTTTTGAAATTGTTGTTTCACCAACGAAGCAGTTGTTA
ACAATTGATTGTGTTTATTGTTTCTTTCAGGGGATTATCAACAATGGGACTGAGAGGAAAATTAAGCGGT
GACATTGGAGGGCTCACTGAATTAATTTCCTTGTGAGTTTGGTTTTTAAATCCAATAAACTTCTGTTGATT
ATTAGCAATAAGGTTGTATTTGTCACCATAATTTGCTCACTTCAGTTGCTAAACTTGTATTGCAGAGACCT
GTCGTTCAACCGAGGACTTACAGGTTCTCTTTCTCCGCGGATAGGAGATCTGCAGAAACTAAACATATTG
TAAGACTACGAAAACTTGATCATCTTGATTGTATAGTGTATACCTGCTATGTACATAAAATTGACATTTAT
AATATTTTCGTCATAGAATGATCATTTGATCAGCTTTCTTCCTCGTAATTTGTATCATGATTCAATGACATA
CATAACTTACATGTACGTGAATATATCTATTTACTTTTCAGAATCCTTGCGGGCTGTAGCTTCAGTGGTAG

TATACCGATGGAGTTGGGAAGACTTGCTGAGTTATCATTCTTGTAAGAATTTCTCAGCCATATTAACTTGT
GATCATTTTAGGTGTATCGTCAACTCATTTAGTTCCATGTCAATTTCTTTGACAGGGCACTTAACTCAAAT
AATTTCACCGGTGAGATTCCACAAACTCTTGGCAATCTGTCAAAACTGTACTGGCTTGATCTAGCAGATA
ACCAGTTAACAGGACCAATTCCTGTTTCAACCTCCTCAAGTTCGGGGTTAGACCTTCTAAAAAAAGCAAA
ACACTTGTGAGTAAACTTTATATGTCCATATTACATGCTGTTCTTAAGTTGGGATATATACCTCTTAAGGT
CTGACTTCTACGTTATGTCATTATCGCAGCCATTTTAACAAAAACCAACTTTCAGGCTCCATTCCGGACAT
TCTTTTTAGTGCTGACATGGTGCTAATACATGTGTAAGTCAAGAAACTAAAGGGTGATTCACGCTATTCT
TCCTGCCCTCCTTCTCACTTTGTGAACTTCTGATATGGCAGACTGTTTGATGGAAATCAACTTAGCGGGA
GCATTCCATTTACACTAGGACTTGTTCAGACACTTGAGGTTCTGTGAGTAGAACTGACCGTCTTATTTATC
CTATCTTTCATGCTACTTCACCTTCTGTTTTGGATTATATGTTTGTACTCTTTTATCTGTTGCTGGTATTCAT
GTCACCTAACATATACAGTCGGCTCGATAGAAATGCCTTAAATGGTAGCGTCCCATCAAACCTCAACAAT
CTCACAAGCGTTGTAGAGTTGTAAGTATACTTTTTCCCATCATTGCTCACTCAATTTTCTGCTTCAAATCAA
TAACTTGTAGATTGAAGTTGTACTTTTGTGCACTTATTTCTGTTTTCAGGAACTTAGCTCACAACGAACTG
TCCGGTCCACTACCAGATTTAACTGGAATGAATTCCCTTAATTATCTGTAAGGAAAATATCCAGAATCCT
GATTTTCCCTAGTTGTTTCTCAATGTTGAAGTGATACATCATATTTTGTTTCTCTTCCAGGGACTTGAGCA
ATAATTCCTTCCATAAATCGGAGGCGCCTATATGGTTCTCGACTTTAGAGTCTCTCACCACTCTGTGAGTA
TTAATCAGTTATGAATGCACCTATTAGTTTCCCAAAAAGTTTGATGAATCTAAGTAGGAAGAACTGATGT
AATGCTCAGAGTCATTGAGTATGGCTCACTCCATGGATCAGTGCCTCAGAAACTTTTCGCTTTACCTCAAT
TACAACAAGTGTGAGTATCGCGATCCTAACTGAATATTCCATGTGTATTACCTGCAATGAAGAGGTTGTA
ATGATCGTAATAACTTATGCAGGAAACTGAGGAACAACGCATTGAATGATACGTTGAACATGGGTGGCA
TCAGTGGCAGGCAACTCACACTTGTTGATTTACAAAACAACGAGATCTCCTCAATAACACTTGGCTCTGG
TTACAAAAATACCTTGATGTGAGATGGCTCATTTCTCCTTTTTCGTCAATTAGATTATGTTTTGTCTCTTTC
TTTCTCCTTGATTGCTTTGTGGTTGTCGTCAGACTGATTGGAAACCCTGTATGTGACACGGCCCTTGGAA
ACACAAACTACTGCCAGCTTCAGCAGCAATCTGCAAAGCCTTACTCAACCAGCCTAGCTAACTGTGGGAG
CAAATCATGTCCTGCTGATCAAAAAGTTAGCCCACAAAGTTGTGATTGTGCTTATCCGTACCAGGGAACA
TTCTACTTTAGAGGACCCTCTTTTAGGGAGTTGTCCAATGATAATACGTTCCATTCACTCGAAATGAGCCT
GTGGGTGAAGTTGGATCTCACGCCCGGTTCAGTTTCTCTGCAGAACCCCTTCTTCAACATTGATGATTATC
TTCAGGTGCAGTTAGAATTATTTCCACCAACAGGAAAGTATTTTAATAGATCAGAGGTTGAGAGGATTG
GATTCTCCTTGAGTAACCAAACTTATAAGCCGCCTCACGAGTTTGGACCGTACTATTTTATAGCATCTCCT

TACACTTTCCAAGGTATAATTACTGAGTTTCTTTACAACTGTAACTTCTAGACCAATATACTGACTTAATAC
TAAAACTGTTTCAGCCGAACGTGGAGAAACTTCTATTAGCTCAAGACAAGTTATAGGAATAGCTACCGG
GTGTACCATCCTGGTCCTCTTGCTTGTTGCTCTTGCAATATATGCTATTCAACAAAAGAAACTTGCTGAAA
GAGCTATTGGATTGAGTAGACCATTTGGTAATGTCATCTCTTATAGAAAAAAACAGATGAGTTATAACTA
TATATACATTCAAGCCTCTCTATAACAGTCATCCTATATGATAACATTTCACTATAACGACCAAGTTTTCTT
TAGAACTGATTTTTCATGTTATGTTGAATTATATGTTCTCTTTAACAATATTTCGCTATAGCAGCCAAAAA
AATATCAGAACAAACGACGCCTTTATAAAGAGGTTTGACTGTATGTCCAGAATGCAAGTAGGATTGAAC
TCAAATGTCTGATTACTTTGACCTTCCTTGTCCAGCATCATGGGCTCCAAGTGGCAACGACAGTGAAGGT
GCACCACAACTAAAGGGAGCAAGATGGTTTTCTTATGATGAACTTAAGAAATGCACGGGTAACTTCTCG
GAGAGAAATGAGATTGGTTCCGGTGGCTATGGGAAGGTTTGCTATGAAAATCCATTGTTTTAGAATAAA
TTAAAGAGGATAAACTCTTCAGCTGCTTTTCTTATTCGCGTGTGTTCTGTTTCCTAGGTCTACAGAGGAAC
CCTCGCGAATGGGCAAGTAATTGCTATCAAAAGGGCTCAACATGGTTCCATGCAAGGTGGACAAGAGTT
CAAGACGGAAATTGAGTTGCTTTCACGGGTTCATCACAAGAACCTAGTTGGTCTGGTTGGATTCTGTTTT
GAGCAAGGCGAACAAGTACTGGTATACGAGTATATGCCAAATGGTTCACTCAGGGAGAATCTGTCAGG
TAAGTTACATCCTCCTATACATTAAAGGCTACTTCCTATTTATAGTAATTTACACTAAAAAGTTCCTAATAT
TTCCATTTCTCTGTAGAAGTTAATTTCCACTATGCTCTACAGGGAAAACGAGCATTTATCTAGATTGGAAG
AGGAGACTTCGTGTTGCGCTTGGTTCAGCCAGAGGATTAGCTTATCTGCATGAGCTTGCCAATCCTCCTA
TAATTCACAGAGATGTGAAATCAACTAACATTTTGTTGGATCAGAACTTAACAGCAAAGGTCGGGGATT
TTGGCTTGTCCAAACTTGTATCAGATAGCTCAAAGGGTCATGTTTCTACTCAAGTTAAAGGCACATTGGT
CAGTACCACATCCTGCACATTTTCGCTTTCTATTTCAAGAAAAAGATTGAATTTTGTTGAACTCGTGAAGC
ATGAAATTGAATTTAAGACTATTGACTACATTGCAGGGTTATCTAGATCCTGAGTACTATATGACTCAAC
AACTAACCGAGAAGAGCGATGTGTATAGCTTTGGTGTTGTAATGCTAGAACTCATAACTGCAAAACAAC
CAATAGAGAAGGGGAAATACGTTGTTCGAGAGATGAGAACAGCAATCAATAAGAACGACGAGGAGCA
TTATGGCTTGAGCAATATGATCGATCCAGTCATTAGAAACATGCCAAATCTAATTGGTTTTACAAGGTTT
GTGGACGTGGCGATGCAATGTGTTGAAGAGGCTGCTGCTGATCGTCCAACGATGAGTGAAGTGGTAAA
AATGCTTGAAAGTATTCTGCAAAATGATGGTCTGGAAACAAATTCTACGTCTGCATCATCATCGATAACA
GATTTTGGGACAGCAATAGCTGCTTCTAGACATCCTTATAATAAAGAGGCTCTACAGAGAAAGGAGATA
AATGATACTCATGCTTTTGATTACAGTGGTGGATACACTCTTCCAACAAATGTGGAACCAAAATAGTGAC
AACCAAAAAAAAGGACTTGTCTCTACTGTCTCTCATTCTTTAATTTATTCTCTATTAAAAAAATTGGAAAA

TTGGTGAAAGGGATAAGAGAATTTAGTTGTTGTATTGTCCCTATATTGGCTACTTTTCGCGAAAACAATC
ACGTATATTATCAAGATCGAAGTTCAGATTTTCTATTGCCAAGGCTTACAGTCTAGTAGTATCAGTGAGA
GTCTCCTATACACTAAGTGTGAGTTCGATTTTTACTGTCCCCTATCCTCAAATGTAATAGAAAAGAGTTAT
AAGGAAAAAAAAGATTTTGTACATCAAGGCAAAGATTTTGTATATCAAGGCTTACAGTCTAGTGGTACA
CGTAAGAGGTCTTCCATACACTAAGTGTGAGTTCGATTTTTTACTATCTCCTTTCCCGTTCCATGATTCTAC
AATGTAATAGAAAAAAATAAAAAATTTAAAAGTTCAATTTTC
>PGSC0003DMG400008581 LRR-RLKs
ATGAATTTATTAACCATTTTCATACTCTTCTTGTTCTACTATTACAGTTTTTTTGCTAACGGGGTGTTTGCG
AAAGTTGATTTGAATGATGAGGTGTCTATTCTACTTTCTATTAAAGAAAGTCTCGTTGATCCATTGGATCA
TCTTCGTGATTGGACAGTGCCCAATAATGCAGCTGCAGGGAACAATAGAAGTATTATTGTTCCCTGCAGC
TGGACGGGAGTTGAATGCAACTCCCATGGGGCTGTCGAGAAGCTTGATCTGTCCCACATGAATCTCACT
GGCACAGTGTCTAATGATATCCAAAAGTTGAAGAGTTTGACTTCTCTCAATCTATGCTGCAATGAGTTTT
CATCGCCATTGCCGAAAAGTTTGTCAAATCTAACTGCATTGAGAAGCATTGATGTGAGCCAGAACTATTT
TGTCTATGACTTCCCTGTAGGTCTTGGAATGTCAGAAGCGCTTATGTATTTGAATGCTTCGAGTAATAACT
TCTCCGGTTATCTTCCTGAGGATATTGGAAATGCTACATTGCTCGAGACATTGGATTTTCGAGGGAATTT
CTTCGAAGGTTCAATTCCTAAATCTTATAGGAATTTGGGGAAATTGAAGTTTTTAGGTCTTTCAGGGAAC
AATTTGACTGGCTACATTCCGGGAGAGCTGGGGCAGCTTTCGTCGCTTGAGACTGTAGTACTGGGATAC
AATCTTTTTGAAGGTGGAATTCCAGCTGAATTTGGAAATCTGACAAATCTCAAGTATCTTGATCTTGCTAT
TGGAAATCTTGGAGGTTCAATTCCTTCTGAGCTAGGAAAGCTGAAGCTGCTTGATACGATTTTCTTGTAC
AAGAACAAGTTTGAAGGCAAGATTCCGCCAGAGATTGGCAATATGACTTCATTGCAGTTGCTTGATCTG
TCTGATAACATGCTTACGGGTGAAATTCCAGCTGAGATTGCTGAACTTAAGAATTTGCAGCTTTTGAATA
TAATGTCGAACAAATTATCAGGTTCAGTTCCCCCTGGGATTGGAGGTTTGACTCAGCTGGAAGTAGTTG
AGCTGTGGAATAACAGCTTATCAGGTCCTTTGCCAAGTGATCTTGGAAGAAATTCGCCATTGCAATGGG
TGGATATTTCATCCAACTCATTTACCGGTCCAATTCCAGCAGGATTATGCGCCAAAGGTAACCTCACTAA
GCTTATCATGTTTAACAATGCTTTCTCTGGTCCAATTCCAACTGGTTTATCAACCTGCACGTCCCTCGTTCG
TGTAAGAATGCAGAACAATCTTCTTTCGGGGACGATCCCAGCAGGTTTTGGTAAACTTGGGAAACTACA
GAGGCTGGAACTGGCAAACAATAGTCTGACAGGACAAATCCCAAGTGATCTTGCTTCTTCTACCTCTCTT
TCTTTTATTGATTTCTCTAGAAACCACATTCAGTCTTCTATTCCTTCATTCATTCTTGCAATTCCAACTCTTC
AAAATTTCATTGCCTCCGACAATAAGATGACAGGCGAAATCCCGGATCAATTCCAGGATTGTCCTTCTCT

TACTGTTCTGGATCTATCCACAAACCATTTCACTGGAGATCTTCCAGCTAGCATTGCTTCCTGTGAGAAGT
TAGTAACTTTGAACCTTCGAAACAACCAATTAAACGGTCCAATTCCAAGAGCTATTTCCATGATGCCCAC
ATTAGCTATTCTAGATCTATCCAATAATTCTTTAACTGGTGGGATACCTGAGAACTTTGGCAATTCACCAG
CACTAGAAATGCTGAATGTTTCTCATAACAAACTGGAGGGTCCTGTTCCGGAAAATGGCATGTTGAGAA
CAATCAATCCAGATGACCTGATTGGAAATGCCGGTCTATGTGGTGGAGTGCTTCCTCCATGTTCTCATAA
TGCAGCATACACATCAAAGCAGAAGAGCTTGCACGCGAAGCACATCATCACAGGATGGCTAACTGGTGT
AGCAGCTCTTCTGCTTTTTTTGACAGCAGGTCTTGTAGCTAGATCTCTATACAAGAGATGGCATGAAAAT
GGGAGCTGCTTTGAACCAAGCTTTGAGATGAGCCGTGGTGAATGGCCATGGAGGTTGATGGCATTCCA
GAGGCTAGGATTCACTAGTAATGACATCTTGGCTTGCCTTAAAGAGTCAAATGTCATTGGAATGGGAGC
AACAGGGGTTGTATACAAAGCTGAAATGCAACGCGAGAATATGGTTGTTGCAGTAAAGAAGCTGTGGA
AATCTGGAACTGATATTGAGATGGGGCACAGTGATGATCTCGTAGGTGAGGTGAATGTTCTTGGAAAAT
TGAGGCATCGAAATATTGTCAGGCTTCTGGGGTTCCTTCACAACAAACGTGATGCAATGATACTATACGA
GTATATGCAAAATGGTAGCCTTGGAGAGGTTTTACATGGTAAACAAGCAGCAGGGAGACTGCTTGTTGA
TTGGGTCACTCGATATAACATAGCCCTTGGAGTGGCACAAGGCCTTGCTTATCTCCATCATTACTGCCATC
CACCAGTTATTCACCGGGATGTCAAGTCAAATAACATACTTCTTGATGCAAATCTAGAGGCAAGAATTGC
AGATTTTGGTTTGGCAAGAACGATGCTCAAGAAGAATGAAACAGTTTCCATGGTTGCTGGATCTTATGG
ATACATAGCCCCCGGTAAGTCCTTCAAATTTTTCATCAATGTTTACTCTAGAGTGATTTTTGTTACTAATAT
TTAACAATCATTTATCTGGATTTTGACTTTGCAGAGTACGGATACACGTTGAAAGTGGATGAGAAGAGT
GATATTTACAGCTATGGAGTTGTTCTCATGGAGCTCCTAACGGGTAAACGACCTCTAGATCCTGAATTTG
GGGAATCTGTTGACATTGTAGAGTGGTTTCGGATGAAGATCCGAGACAACAAGTCTTTAGAAGAAGCAC
TTGACCCACATGTAGGAGCAACACAACATGTTCAAGAAGAAATGTTGTTAGTTTTAAGGATAGCAATTCT
ATGTATAGCCAAACTCCCCAAGGACAGACCATCCATGAGGGATGTTTTGACGATGCTCGAAGAGGCAAA
GCCTCGGAGAAAAAGCAGTAGCAACAGTGGAGATAGCCATGATGCTACTACTAACAAAGATAAGCCAA
TATTCAGTACATCACCTGTAAATGGTCTTTTGTAA
>PGSC0003DMG400009068 LRR-RLKs
CGTGATTAAGAGTGGTTAATTTCCTTTCCCTTTTTCGTAATAGTAATGTGTGTCTTCCTTTAGTGAAGAGC
TTCAAAAAAATGGCGGAAATGGTGGAAAATGCTCTTCTGGTGCTCCCTGCCTTACTGGCACCCACTTCCC
CTCTTCTTCTAAATCCAAGCAAATTGTTAAAGAATTGATTTTGTGTATTACTGTTAACTTAACTTCTTCATA
TCATAGTAGTAGTAGTTCCACTTTTGTCTCCACGTGCTTTTCCCACTCTTTTCGCTTCGTTGTTAACCAAAT

ACATACATATACCCCATTTCTTTATTTGCTTTCTTCTTCTTCAAATTAACTGCCTTTCAATCATTTATTGTCTC
TCACCCACTCCCCCCCTTCTTCTCATTTAATCATTGTCTCTCTCTAGCTACTTACTTAATTCTGCATTTACTTC
CCATCATTTTCCTCTTCATCTACTCCTTCTTTTCAGTTTACTCTACCAAGTGTTTGATTTTTTGCCTCACTGG
TACTTGCTAGTATACTATACAGTTTCTGAATTTTGTGGGGTTTTCCGTTTTCTCCATTTTGATGTGATTTGG
CTATCTGGGTATTGCTATTCAGGTTCATTTCAATGTTCTTTTTGTTATTGCCGACAGTTTTCAAATTATGCA
TACTTTGCTTTAAGGTCAAGAAAAGTGTTAAAAATTCAATCTTTTTATGTGTATGTGCTTGGAAATAGGT
GAAGAACGTGAAATGCCTGTTTGTTCCTTTTTCTTTTTCTTTTTTTTTTCATTTTTTGGCAGCCCCAAAAGTT
GACGGAAGAAATTTGTAGCTGGTTCTTGTTTTTACTTTAGCTATTCTGTTTATTGAAACCTAACTTTTACTT
AACTTGCGTGGTTGACCCAAAAACAAAATGTGTTCTGAGTGTCTACGTAACTTTAGCTGCCCTATAAATT
GTATATTTATGGTTAAGAAACGGTTCTCTAGTTTAAATGGTAATTATTTATAGTGAATAATGAACATCATA
CCGTTTCAATGTCTGATTCCATTTTTATTTTTTTTTGAAATTCTTGTAGAGATGAAGGGTGCTGTTACTAGA
AGCTGATGCCTTTGTAGTTTAAGCTCAGATGAGGAGAAGGATGGCTACAAAGTTCCTGTTTTTTTCAGTT
TTATTCTGCACAGCTCTGTTCTGGCTAGCAAGTAGTGAACCATATGAAGACAAAGAAGCGCTTCTTGATT
TCCTTAATAATGTTAATCATACCCGAAACCTGAATTGGGATGAGAGAACTTCTGCTTGCAGTTCTTGGAC
TGGAGTCACCTGTAATCATGACAAATCAAGAATTATAGCAATTAGGTTGCCTGGAGTTGGATTCCGTGG
TTCGATTCCTGGAAACACTCTTAGCCGCTTGTCTGATCTTCAGATCTTGAGTTTAAGGTCTAATAGTTTTA
GTGGTAGTTTACCTACTGATTTTGCTAAACTTGGAAATCTTACTTCTATTTATCTTCAATCCAATAACTTTC
AAGGTCCATTGCCTGCTGATTTTTCTGCTTGGAAAAGCCTTTCGGTTTTGAATTTGTCAAATAATGATTTT
AGTGGGAGTATACCTTCTTCAATATCGAACTTGACTCATTTGACTGCTCTGGTTCTTGCTAATAACTCGCT
TTCTGGCAGTATTCCTGATCTCAATCTCCCTAGTTTGCAAATATTAGATCTATCCAACAATAACTTCACTG
GAAGCATCCCCAATTCTCTTCAAAGATTTCCTGGTTCAGCTTTTGCTGGTAACCAGCTTTCTCCTGCAAAT
TTTTCACCTTCATTTCCTCCAGTTCCTCCTCCAAGTGTCCCACCAAAAAAGAAATCGTTTAAACTCCGTGAA
CCTGCAATACTTGGGATTGTAATTGGAGGGTGTGTTTTAGGTTTCTTGGTAGTAGCTGCAGTGCTGATTA
TGTGCTTTTCGAAGAAAGAGGGTAAAAGTGGGGCCACAGAGAAATCAATCAAGAAAGAAGATATAGTC
AGGAAGGGAGTTTCCAGCAGTCAGCATGGAGTAGGGAATCTCGCATTCTTTGAGGGTTGTAATCTTGCA
TTTGACCTTGAAGATCTGTTGAGAGCTTCAGCGGAAGTGCTTGGAAAGGGAACATTTGGCACTACATAT
AAGGCAGCTTTGGAGGATTCTACCACAGTTGTGGTGAAGAGATTGAAGGAGAGTGTGGGAAGAAAAG
ATTTTGAGCAACAGATGGAGGTTGTTGGCAATATCAGGCACGAGAATGTGGCTCCACTGAGGGCATACT
ACTATTCCAAGGATGAGAAGCTCATGGTATATGATTTCTACAGCCAAGGGAGTGCATCTTTGATGCTGCA

TGGTATGTTGAGCACCCAACTTCTACTACTCTTGCAGTTTTATTTTTGAACCGAACCATAGAGAACTGATT
ATCAGGACCAAAAAGATAATGCAGATCATTTTTAGCTATGATGTGTCTTGTGCAGTAGTATTTGAAATTG
CTTGTAGGCTAATTCCAACGTAACCAATTACCTTTGAATTAATAGAGTATCTGCTGTTGTTGTCCTCTTGA
TAGTTTTTTTAATCACCAATAGTTTATAGGACAGCATAAGTGTCTCGAAGCCATAACTACATGCCACTTTT
AGGCCATGTCTTAGTGCAACTTCTGTTTTAATTTGTCAAAATTCAAGAACATCATGTTGCTCGCGAACTTT
GCTTTGGTGTTACAGTCAACTTTTATCATTATTAGGTGGATAGTCTCTTAACTGCACCTTCAAGTATTCCT
ATCATTATATCTGATGCACTCTGTAATTGCAGCAAAGAGGTCTGCTGATCGAGTTCCTCTGGACTGGGAA
ACCCGACTACGCATTGCCATTGGTGCAGCTAGAGGCATTGCTCAAATACATGGACAGTCTGGGGGAAG
GCTTGTCCACGGGAACATAAAATCCTCAAACATATTCCTCAACTCCCAAGGTTTCGGTTGTATTTCTGATC
TTGGCTTGGCAACTATAATGGGTCCAATTGCTACACCAATCGTGAGAGCTGCTGGTTATCAACCTCCAGA
AGTTACAGATTCAAGAAAAGTATCTCAAACCACTGATGTCTACAGCTTTGGCGTCCTAATACTTGAACTT
CTCACTGGAAAGTCACCTACACATGCCACAGGTACTAGCGACATTGTCCACTTAGTCAGGTGGGTACATT
CTGTCGTTCGTGAGGAGTGGACTGCTGAAGTATTCGATGTGGAGCTTTTGAGGTATCCAAATATAGAAG
AAGAAATGGTAGAGATGTTACAAATAGGTTTGACTTGTGTATCCAGAATGCCAGAGCAGAGACCAAAA
ATGACAGAAGTAGTAAAGATGGTAGAGGGTGTTAGGAGAGTGAATACGGGGACTCGAACATCCACTGA
AGCTTCTACACCGAACCTGACACCACCGATGACTGAAATAGGGTCGTCTTCCATTACACACTGATATTTCT
TTGTTATTTAGTGATTAGTTACTGTTCTATATGCTCAATTGTTTCATTGTTATAAGTTTTTTCCTCAGACTTC
TGTCAAAACATTATTTCAGCTACAAAATTGAATAGAAATATTTACTTGATC
>PGSC0003DMG400009180 LRR-RLKs
CCAATATTAATCAAAATAAAAATAGGAAAGTTCTGACAAAGCCTTTCTTCTGTTCCCGAGCTGAAGCTTG
GGGTTTTGTGTTTTGTGGTGCCCGGCACTGGTGGTGAAAGAAAACTCCGACGGCTGTGACTGAGGTGG
AAGAGGAGAAAACGACGCCGTGAAGATGAGGTTGCTGCTGCTGCTGCCGATACTCATCGGCATTTTTCT
GTATTGTGCTAATTATGCCATTGCCGACACAGATCCAAATGACGGTCAGCTATTGCTTTTAATCATTCTCA
TTTATTGTTCCTATCTGTTTGTCTGTATGCTTACTGATCATTAATGGAGGTTGATTGATTTGGAAAGTCAA
TGAGTAATCTACCTGACAATGAACTCGGCAACCGTCGCAAATTTGTTTGATATTTTCCTTACCATATATAT
GTAAAAATGCGACAAGTTCCTTTCTGATCAGGGCATGAATCATATAGAGGGAGCTCTCTTGGATTCTTAT
ATATTATGTATGTATTGGCCATGTCGGTGGACTTTTAGTACTGTTGAATCTTTGCATTATTACTTCCATTCA
TTCTGAGAACTGACATATTGAAACTTCGTCGGACATATTTGACTGAGTTACCATTTTCAGCCAAGTTGCTC
CAGAAGATTTTGGCTTTTCTTCTTTCTGCTGCTTGGAAGCGGGACAGGGTAGTGGAGGTGGGCCGCCAA

AACAAGTGAGGTTAAAAATGATCAAGGGCATTATAACCTAAAAGACTCTTCTCCCAGTGATAATACACAT
ATAGGTTCATGGCTTGATCAACGCACTTGTTGCGTTTCACCATGTTTATTGATGTCTTGTAGATATACCAC
TCGTTAACAGTTTGATTACTCTTCAAGCTATTATTAATGAAAAGAAACTTTTTGTTTGAACAGTATAAGAA
TATGTTGACCTGTAAGTATGCCTAGCTGATTTTTTTGGTGGCGCTTTGATTTTCAGCATCTGCTCTGAGAG
TTCTGTATAGCAGCCTAAATTCACCTGGACAGCTAACAAAATGGAATGCCAATGGTGGTGATCCTTGTG
GAGAGTCCTGGACAGGTATTACTTGCTCGGGCAATAGAGTTACTGAAATGTGAGTTCCTCTCTTCCTTAC
TGTTGTAAGTAATTATTGTTGATGTTTTCCTTCTTTATCTTCTTCATCATGTTTCAATTCTTGGAATTGTTGA
CTTGGTGATTTCTGATGCTTTCTTCCCTTTTCATTTTCCTCCAGCAAGATATCAGGTCTTGGACTCTCTGGT
TCACTGGGCTACCAATTAGCAAGTCTTACATCGGTGACTAATTTGTAAGATAAATGGCCTTCAATAATGT
CCTCATAAATGTTTCTTCGATCTATATCTATATCAAATTCTTACACACTTCTAATTTTGCAGTGACATAAGT
AATAACAATTTGGGAAACCAGTTACCTTATCAGCTTCCTCCAAATGTGCAAAGATTGTATGTCTCGTAGTT
AGTATATTTTCATCATTCCAGATGCTATTTTTCCACCTATCTTTCTTAAACCTTGCCTTGCAATGCAGAAAC
CTTGCTGCTAATGGTTTTAATGGTGCCCTTCCATATTCCGTTTCGCAGATGACTTCCCTCCGATACTTGTAT
GTAAAATCTTTGAATTTGACTGCAATTTGCTTTCCAGCAATAAGCTTTTGGTTTAAGGTTGAGCTTTTCAA
TGACTTTAATATTTTTATCTGGCTGTAGAAATGTCAGTCATAATCAAATTCAAGGACAAGTGACTGTCGC
GTTCGACTCTCTGTCTTCTCTCAATACATTGTAAGCCGTATCTTCTCTCATGCCATTGCTTGTGTGTTTTAA
TATTACAAAGTTGTGGTCATAACTCATATATGAGGTGTTGTAGGTAAAACATGTCATTCTTACTTCATTAG
TATAAACTTTTCTTCTCCCATCCGAGTTGTTGACTTGTTGCTTTGGTAAAGAAAGTCAATATCATGAAAGA
CAGTATCTGATCCTTGTTGTAATCTGTAAACTTATGTGGTTGTCCCAGTTAAGTCAACTAAGAAGCTGTAT
ACATTTCGAGATATTCTTAAGTCAAGTCTTAAAAACCTCTAAATGACTAGTTATCATTCTCTGTCTCATAGT
TTTTGATTGATTGATCTGTATGCTTCAGGGATTTCTCATTCAATGCTATGACGGGTGATCTTCCTCAAAGC
TTCAAGGCACTGACTAGTATGAATAAAATGTGAGCTAATAAATTTTCTGCTTCGTCCTTGTAGACAATTG
GTTGAGTCCTTCAGTGGTGAAGTTTACATGTTGAAGTATGCAGGTATTTGCAGAACAACCAGTTTACAG
GAACTATTGATATCTTAGCCAATCTTCCTCTTGATGATTTGTGAGTTTTCAGTCTATGCTTCTTTTTAGATG
TTATCCCTGATGTTTTATCTTATTTATATAGACTCGATTGAAAGCATGGTTGAAAGTCATGTGTATTCCTTT
ATGCTTTAGGAATGTTGAGAACAACCGTTTTACTGGCTGGGTTCCTGATCACTTGAAAGGGATCACGAA
GTAAGTATATCATGTTAGCGTAATAAATTTTGAAACTTATGTCAGAAGTATTTTGAATTTATTCAGGGTTT
GTGACATTTCTGTTACAGATCAAATGGTAACTCATGGAACTCAGGATCTGCACCCCCTCCTCCACCTGGC
ACACCCCCTGCAAGCAGGCCGCACCACAAATCTGGAGGCAATAATAGCCCTACTGATGGTGCAAGCAGT

GGTGATGGTGGTGGTAAATCGGGGATAAGGGGTGGAGCCATTGCAGGCATAGTGATATCTGTTCTAGT
TGTTGGGGCAATTGTAGCATTCTTTGTTATCAAGAAGAGATTGAAGCGGTCATCGACGGACATTGAGAG
ACATGATAATCAGCCATTTGCTCCTCTTGCTCCCCCCCAGGAAGTACATGGTACTTCTTTTCATAATTTCTT
GGTTTTATCAGTTAAGATGCCTTTTAGACCCTTTCATGGCCTCTGTGATTGTTATTTAACTGTGGAACATA
TCAATCAACCACACTATCATCAACCTCATTCCTGTATCCTGCTTAAAAAGGTGTAAAATAGACCTGCTGCA
TAAAATGGAAATTTTATGAAAGTGAATTTGGAGAATCAATCTTAACCTTTGTGCTTTATCACTGCCTGTG
GTGTATAGCTAATAGTGGATCAACACAGCTACTGTCTTCGCTATAAAAGAAAAACACCAAAGTAAAAAC
AAACTTTATATCGAAATGTAAAACCGCTAAGGCTGATAGGTCCTATCTTCCATGGAAGCAAAACTTCTTG
CTTACTGAAAAGTTCCAAGAACTTGATTGTAGAAATCTGAACTGATGTTTAGACTCTGTATTGCCTGTTG
AAGCTGATAGTGGGATAAAGCGAACTTCAGCTTTTTTGATAATGAAGAAAACTTCAGCTTTTTGATCTCT
TTCAATGGATCTGGTTCTATGCTTGTTATAAGTTAGCTCCCCTTGATGGTTTTCCTTCCTATAAAACTACTA
TTCATAAATCTTATTGACCTTTATAGAACAACTATTGCTTCAAAATGCAACTCTCAGCTAAGTGAAAGTTT
CTCCCATTCTAAACCATTTGTGCTCACCACCAAATTCACCTCTTTGCAAATTCTAATTCTTTGCTTTTCTCAA
CTCCATCACTCGTGGTGCATAGTTATGCCTTTTAAGCCTCATCTCACTTCCGTATCTCATTAATCCGAACCT
GAGTATGCATTTGCCTGTTTGTAACACTAACAGTCTTGTGCTTCTTCTCTGTCTGGATTTTCTGTTCATTCA
TTGTCCTTGTTTTTTACTGCAGAATTGAAAACTAATGAAGCTTCCTCTGCGCCAATAGTGAAAGTATTTGA
AGCTCCTGCAGTAGTAAATCTAAGACCTCCACCTATTGAACGCCACAAGTCATTTGACGAAGTTGACATA
GCAGCAAAACCAATTGTCCCACCCAAGAAAGTTAATACAGCTAAAATAGATGCAAGACAATATTCAATC
GCAGACCTACAGATGGCGACTGATAGCTTCAATGTTGATAACCTTATTGGCGAGGGATCATTTGGGCGT
GTTTATAGGGCTCAGTTTGATGATGGAAAGGTATGATTTCTCTCTTATGACTAACTGACTATCTATGTGA
AGAAAATGGCTCTCTCTAATTGGATAAAAAATTCATATCATGAATTTTTTATGTGCTGCGAATGAATGTTT
AAGCAAGATAGTTGAAATGACAGATAAATGTTCAATATGAAAAAGTACCTATCTAAAGAAAATGAAAGA
AAGAAGTTTGATCATTAAAAATTAGTTGAAAAATTATGTTTGTTCAAAACTCAGTTCGGAGCTACTATCA
TCTTGTCCTGTGTAACTGAACCATCCTCGTAAGTTCCTTTCTAATTTTTGAAATGCCATAGGTTCTTGCAGT
CAAGAAAATCAACTCTTCTGCACTCCAGAATCCTGAAGATTTTCTCGATATTGTTTCTGAGATATCACGGT
TGCACCATCCAAATGTTACTGAGCTGGTTGGATATTGTTCAGAGCACGGGCAGCATCTGCTGGTTTATGA
ATTCCACAAAAATGGTTCTCTGCATGATTTCCTGCATCTGTTAGATGAGGAAAGCAAGCCTCTAACATGG
AATAGCCGAGTTAAGATTGCACTAGGCACAGCAAGAGCACTAGAGTGAGTTTCCATGATCTACAAAGAT
ATTTGATGCCATTTTATAATGTTCACGGAGCTCATAACAAGGTTTTATATTTTCCTTGTACCATTGGTTGA

AATACTAGCTCTGTCATATATTTTCTGGTATGTGTAGTCAAACAATAATGAAGTTTCTTTTCCTGGGGGGG
GGGGGGGGGGAAAAAAAGAGTTTTGGAANNNNNNNNNNNNNNNNNNNNNNNNNNNNNNNNNN
NNNNNNNNNNNNNNNNNNNNNNNNNNNNNNNNNNNNNNNNNNNNNNNNNNNNNNNNNNNN
NNNNNNNNNNNNNNNNNNNNNNNNNNNNNNNNNNNNNNNNNNNNNNNNNNNNNNNNNNNN
NNNNNNNNNNNNNNNNNNNNNNNNNNNNNNNNNNNNNNNNNNNNNNNNNNNNNNNNNNNN
NNNNNNNNNNNNNNNNNNNNNNNNNNNNNNNNNNNNNNNNNNNNNNNNNNNNNNNNNNNN
NNNNNNNNNNNNNNNNNNNNNNNNNNNNNNNNNNNNNNNNNNNNNNNNNNNNNNNNNNNN
NNNNNNNNNNNNNNNNNNNNNNNNNNNNNNNNNNNNNNNNNNNNNNNNNNNNNNNNNNNN
NNNNNNNNNNNNNNNNNNNNNNNNNNNNNNNNNNNNNNNNNNNNNNNNNNNNNNNNNNNN
NNNNNNNNNNNNNNNNNNNNNNNNNNNNNNNNNNNNNNNNNNNNNNNNNNNNNNNNNNNN
NNNNNNNNNNNNNNNNNNNNNNNNNNNNNNNNNNNNNNNNNNNNNNNNNNNNNNNAGGTTT
TTTTTCGGGGGGGGGGGGGGGGATAGAATGAAGCTTATGGAATAGTTACTTTCTTCCTGAAGAGGAAGT
GGAGAGTTATATATTGTGAACTCCAATACTAAGCACTTAGTAATTAAAGCTGTGTTCCTATGTTTAAATCA
GACTTAATCATTTATTATGAAGGCCATTAAATAATTCAGATTTCTGGAGCATTGATGATCAGTAACATGC
AAGAAGATGATACATTATCTGAACATTAAAAGAAAAGAAAATATGTGTTGCGAGCCATTGTTTATCCACC
CTGTTTAATATACTGATCTTTCCATCTTCTTCTTCTCAAGTTTCTAGAGTAGTAGCTAAAATAAGCATCCTT
TGCAGGTATCTGCACGAAGTTTGTTCACCATCTTTGGTTCACAAGAATATCAAGTCTGCAAATATTGTACT
TGATGCAGAACTCAATCCTCATCTATCAGACTGTGGATTGGCAAGTCTAATTGCTGATGCAGATCAGGTA
AGCTATAGCTCCTAGCTTTAGGATTTCCTTTGCTAAGTCTCATTTGTTGGATGTTGCTATATCTTCTAATGT
ATGAATGTGGGTTCTGATTGACTAATGCATATGCATAAGGGTGCTCATGCACATGCCTATATGCCTGTAC
TTGCATCCTCTTTGCCTATTCATCTGTCCTTGTAGAAAAGAAACCAGAAATTTGATTCAGATCTTCGATAT
AAGTCATCCACAGTATCATAACTATAGAAGGCGTATACTCTTTACCATGAACTATCAAATATCAAAAAAG
GAATTTTGTTGCCATGTTAAAAATTAGATTGACTCAGAAGCTGAGAAGTATCTACTAGTTTTTCGCAAGG
GGTCTTATTTCCTCTTTTTCAGCTAGTATAGATTTTCATCATAAAGCCAGTTTCCTGAGATATTCACATGAC
TATCAATCTCTTGTCGTAGGCATTGAACCATAATACAGGATCGGGATATGGTGCCCCCGAAGTTGCTATG
TCCGGTCTCTGTACCATAAAAAGTGATGTGTACAGCTTTGGAGTGGTTATGTTGGAACTTCTTACCGGAC
GAAAACCTTTTGATAGGTAATGTTATTTAGCATCCCATGATATTATGATGTAAGTTTCTTAAAGAAACAGT
ACAAATTTGAAGTTTCAGAAAATTTTCTGATGATATTCTCTGTTCTCTTAATGCAGCGCAAGAACAAGATC
TGAACAATCTCTAGTTAGATGGGCAACACCTCAGCTGCACGATATTGATGCCCTAGCTAAGATGGTTGAT

CCAGCACTCGAGGGGCTCTATCCTGTTAAATCTCTATCACGCTTTGCTGATGTAATAGCTCTCTGTGTCCA
GGTAATATTTCGAACTGGAAGTGAATGATTAGCTTTCAGAAATCTGCTACTTAGTTTTCATTAATCTAGTT
CCTTAATTATCTCAATGGACCTCTCATTTTTTTTTCTTTGTGGCAGCCTGAGCCCGAGTTCAGGCCACCTAT
GTCGGAAGTGGTTGAAGCATTAGTTCGGCTAGTGCAACGAGCAAACATGAGCAAGAGGACATTTGGTG
CTGATCAGGGTACTTCAAGGGGTGAAACAGATGGCCCGGACTATGAACCATAGTTTCCATGTGACGTTG
AGTACTCAACATGTTGCCTATTCTAACACGTGGAGGTGTTTTTTTTTTGTTTCTATTCATGAGAAATGAC
TTCGAGCCTGTTGTTTTGCAGACTTGGAGTGCATTGTACCAAAACGAGACGCTAAAATCTGTTCATAGTG
CATAATTTATTTTCAGTTCTTTTCCTCTTCCTTCTAATTTGTTCTTACCAGCGGGATTAAAGAAATAATATT
ATTTCATATTTTCATATGGTGTCACATTACAAACTATCATCTGAACAAACATGTATGTACTAGTGCCTTATT
CCAACA
>PGSC0003DMG400009181 LRR-RLKs
GGAGGAATTGAGGAGAGGAGAATAGTATGGTAAAGTCTCTCTCTCTCTCTCTCCCACGTATATTACAC
GCTTTCAAATATCCATAGAATAAACAAAGTCCACCAGCCATCTATCTATCTAGCCGTCCCAAAAAGTAAA
CGCTTTATTTCAAAAACTCATACCTCACCTTTTTGCTACATAAAAATTGCACCTTGTCAACATCAACTACAC
TCTCTCTCTTCTCTTCATGGGGTTAGACTAAAGTCTGCACGATCAACAGGACAAAGAATTGCCAAAAAAA
TACCCACATGGTTGATTGGAGTATCTTGGCATCTTCAACTGTCTTTCTTCTTCATCTTTAGTTGTCTAAAAA
TAATAATGATCATGGATAATAAAGACAGCCGCCACCAATCTGGTCTGACATATCATCAATTTCGAGCAGC
CATGCTTCTTACTTCCTTTCATTCTTCTTCTTCTTTACCCCACCATAAATCTTTCTATCCAATAATATTGATA
GTTGTTCTACTTCTTTCTTCTATTGCTACCATCTGTCATGCTTCCTGCAATCAGCTTGATCGTGACTCTTTGT
TATCATTCTCTGTTGGTATCTCTTCACCTTCTCCTTTGAATTGGTCCTCTTCCGAGGATTGCTGCACCTTGT
GGGAAGGTGTTGGTTGTGATGATAATGGCCGAGTAACCGCTCTCTGGCTTCCCTCAAGAAGCCTTTTTG
GAAACATAACCCCTGCTATTGCAAATTTGAGCAAACTCTCTCAACTCAGCCTGTCAAATAATCGATTTTTT
GGTCCCCTCCCAGATGGATTTTTCAAGTCATTTAGTAGCTTGCAGATCATTGACTTGAGTTATAACCGTTT
ATCAGGACGATTGCCGCTATCAGATAGATTGCCATCACCCATCAAGACAGTAAATCTCTCTAGCAACCAC
TTCAATGGGACAATTCTATCATCATTTCTCGAGCCAGCAATTAATTTAGAGAGTTTCGATATTAGCAACAA
TAGCTTCTCTGGTCCAATACCCTCCTTTATTTGCAGCTACTCAGCCGCTGTCAGAGTTCTTGATTTCACTTC
CAATGACTTCAGGGGCCAGATGCCCCAAGGGTTTGGGAGTTGCTCCAGTTTGGTTACTTTGAGAGCAGG
ATTCAACCATCTTTCGGGATTCATTCCTGATGATATTTATAGTGTGTCAACACTGCAAGAAATCTCTTTAC
CTGGCAATAAATTTTCTGGACCCATCCCAGAAAGCATTGTCAACCTTGTCAACCTCAGAATCCTCGCACTC

TATGGCAATGAATTGACAGGTTTGATCCCTCAAGATATTGGAAGGCTCTCCAGATTGGAACAGTTGCTCC
TCCATATAAACAATCTAAATGGCACTGTGCCGCCATCGCTGATGACTTGCACCCGTCTCACTGTGCTCAAT
TTAAGGGTCAACTTCTTGGAAGGTGAACTCTCAGCTCTTGATTTCTCCAACCTTAGTCGACTTGGCATAAT
CGACCTTGGAAATAACTTCTTCACTGGAAGCATTCCACAAAGCCTTTTTTCATGCAGGTCATTAACTGCAA
TCCGTCTGGCTACTAACTATCTGACAGGAGATATCTTGCCTGGCGTAACGTCTTTACAAGCTCTGTCCTTC
CTCTCGGTTTCCAATAACAGCCTAACCAATTTTGCAGGGGCAATAGAAGTCCTTAAGGGTTGTAAGAATC
TTACCACACTAATCCTCACCAAAAACTTTTATAATGAAACGTTGCCTGATAATGGGAACTTGATTGGGTCT
GAAGATTTTCAAAATCTCCAAATTCTGGGTTTAGGTGGTTGTAATTTCACCGGACAGATACCCACATGGC
TGGTTAAACTTGGGAGGGTAGAGGTTCTAGACCTTTCTATGAATCAAATCACAGGCAAAATTCCAGGTT
GGTTGGGGACTTTACAGAATCTATTTTACCTGGATTTGTCTCAGAATTTTCTATATGGAGGCTTCCCAGTT
GAACTTACTCAACTGCAAAGACTCGCATCACAAGAGGCTGCTGATCAGGTCGACAGAGGTGCTTTAGAA
TTGCCTGTGTTTGTTCAGCCAAACAATGCCTCAAATCAGCAATATAATCTACTGTCAAACCTGCCACCAGC
TATTTACCTTGGAAACAACAACCTTGATGGCAATATTCCAACTGAGATTGGCCAGCTGAAGTACATTCAT
GTTCTTGATCTGAGCAAGAACAACTTTACAGGAAACATTCCAGAAACAATATCCAACCTCACTAATCTGG
AGAAACTAGACCTCTCTGCCAACAACCTCTCAGGTGAAATTCCTTCTTCACTCAAGGGTCTTCATTTTTTG
TCTTCTTTCAGTGTTGCTCACAACAATCTTGAGGGACCTATTCCAACAGGAGGTCAGTTTGATACATTTCC
TATCACTAGTTTCTTAGGTAATCCAGGACTGTGTGGTCAAATCCTGCAGCACCCTTGCCCTGATCGATCA
GGAACTACACAACCTTCTGCAGTTAGAAAAACCGCAAAAAGGAAAATCTTAATTGGACTCATCCTAGGA
ATCTCCTTCGGCATTGCCTTCACAGTCATCATCATAGCATTCTGGATATTCTCCAAGAGGAGGATCCTTCC
AAGAGGTGACGCCGAGAAAAATGACTTGGAAATTGTGTCCTACAACTCCACTTCTGGATTGTCTGCTGA
GATTGGAAAGGATAACAGCATGCTTGTTATGTTTCCAACCAATAAGGATCAGATTAATGATCTCACAATT
TTTGACATTCTGAGAGCCACGAATAACTTCAACCAAGCAAACATAGTAGGTTGTGGAGGCTTTGGTCTG
GTCTATAAGGCAACTTTAGCAGATGGAACTATGTTGGCTGTTAAGAAACTTTCAGGAGATACGGGTTTA
ATTGAAAGAGAATTTAAAGCAGAGGTGGAAGTTTTATCCACTGCCCAACATGAGAATTTGGTCTCTCTTC
AAGGTTACTGTGTGCATGATGGCTGTAGGTTGCTGATCTATTCCTATATGCAAAACGGAAGTCTGGACTA
CTGGTTGCACGAGAAAACTGATGGAGCATCCCTACTTGATTGGCCAACTCGACTGAAGATTGCACAGGG
GGCAAGTTGTGGGCTGGCTTACATGCATCAGATATGTGAACCACATATTGTTCATCGTGACATAAAGTCC
AGTAACATCCTCCTTGATGAAAAGTTTAAAGCGCATGTGGCAGATTTCGGATTGTCCAGAATGATTCTTC
CTTACCAAACTCATGTCACCACTGAACTGGTTGGTACCCTCGGCTACATCCCACCAGAGTATAGTCAATC

ATGGATAGCCACTTTGAGAGGAGATGTCTATAGTTTTGGAGTTGTGATGTTAGAACTCCTGGCTGGCAG
AAGACCTGTGGACATGAGCAAACCAAAGATGTCAAGGGAATTGGTTGTATGGGTGCACCTAATGAGAA
ATGAGGGTAAACAAGAAGAAATATTTGATCCTATACTGAGAGACAAGGGTTTCGAAGAAGATATGCTTC
AAGTGCTTGATGTTGCCTGTATGTGTGTCAGCCAGAATCCTTTCAAAAGGCCAACTATAGCAGAAGTGG
TTGAGTGGCTCAACAGAGTAGTATCCAACGAGGGAGCACCTAAATAAGGTGGTGGTACACATAAATTTG
ATGTAGATGTGAGTGTCTGTATAGGTGAACTTTTGCTTTTTACACGTAGGAAGTTAACATATAGGAATAA
ATGTATAAAGTGAACAATTTCCTCAATAAATTACATTTACGATTTTGCGTCATGGCTAACATTGGATGACA
GGTAATG
>PGSC0003DMG400009196 LRR-RLKs
GGTTATGCCTCAAATTGATGGTGGTTTGATGTGGTTAGGCAAAGGAAGGATGATGACGGAGGTGGAGG
ATACCGGCCGGTCACCAGTTACGTTTGCATGTTCTTCATCATCATCATCATCATCAAGAAGAAGATCATAC
AATGCATTGTGTCTCATTTTCCTTGTTGTTGTTGTATCCTTGATCACATTTGTATCGGCTCAACCGGACGAT
GACGACACCGGCCCAGATGATCATCATTATCTTCCCGATGCAAAGAGCTCTTCGGAGGCCCTCTTCAACT
TTAAAAGCTCACTATCTACATCTTCAGGTAAAGGAAAAGATGTTCTCGGGTCTTGGGTTCCATCGACAAG
TCCCTGTACCGGGAACAATGCCAATTGGTTGGGAGTAATTTGCTTCGAAGGGGATGTATGGGGTTTGCA
ACTAGAAAACTTGGACCTCTCAGGTGCAATTGATATAGATTCATTGCTTCCTTTGCATTTCCTCCGGACCC
TAAGCTTTATGAACAACAACTTCAAGGGGGGAATGCCTGATTGGAACAAACTTGGTGCCCTCAAGTCAT
TGTACTTGTCCAATAATAACTTCTCTGGTCAGATCCCCGATGATGCCTTCAAGGGTATGACTTATCTCAAG
AAACTTTATTTGGCCAACAACCAATTCACTGGTAACATTCCCACCTCCCTCGCTACCTCCTGCCCCAGGCT
CTTCGAGTTGACCCTCGAAAACAACAAATTCACTGGCTCTATACCTGATTTCCGCCGTGGCCTTCTCAAAC
TCCTCAATGTATCCAACAACCAACTCGAGGGTCCAATCCCTCCTTCTCTTTCTTTAATGGATCCAACAACA
TTCGCAGGTATCATTGGTTAATTTAATTATGTACCGATCTAGCCTTTTTAATTTATTATTATTATACATATA
CATGACAGGGAACAAAGGCATGTGTGGGAAGCCTTTGGAATCAGTTTGCAACTCCCCAATTCCAGAAGC
AAATACTACTACTCCGCCTAACAGTCTCAACTCTACCATCAGCACCCAATTATCTGGTGATATAAATAAGA
AGTCTCCCTCTCTCCTGAGTAGAGTGATGTTGATCGTTGCAGTTTGTTTGGTGGTCTTATGTCTCGTGATT
GTGTTAATTCTTATCATTCGCCGCCGCAGCAGCAGCCAAAATAATTCACAACTCACCAGCCGGGCTGTTG
AGTCAAATAATATTGATGGGGATCAAAACATGACCTTTTCATCATCAGCTCTTCCTGATATGACGATGAG
CGGCAATCCAACTTATGCAAGACACGATAACAATAACAAGGCAGTAGAGGCACCACCAGCAGCAGCAG
CACCAGGCGCAACGGTGGTGGGTAAGTTGTCCTTCGTTAGAGATGACAGGACAAGATTTGATCTACAG

GACTTGCTAAGAGCATCCGCGGAGGTGCTTGGCAGTGGGAACCTGGGGTCCTCTTACAAGGCACTACTT
ATGGATGGCCAAGCCGTTGTGGTGAAGAGGTTTAAGCAAATGAACCATGTCGCTAAAGAAGACTTCCAC
GAGCACATGAGGAGGTTGGGCAGACTAAGCCACCCGAACCTACTCCCCCTTGTCGCCTATTATTATAGG
AAAGAAGAGAAGCTGCTTGTTTATGACTATGCCTCCAATGGTAGCTTGGCCAGTCACCTCCATGGTATGT
ATATGTATATTAATTAATTAGAGTATATACGTGAGTTTAAATTATTAATTACTCGGCTCTGTGTGTGTATA
TATATGTAGGCAATCACTCTAGGCTGGACTGGTCAAGCCGCTTGAAAATCATAAAAGGAGTTGCAAAGG
CGTTGGCCTACCTTCACAATGAGCTGCCAAGCCTAGCACTTCCGCATGGTCACCTCAAGTCATCAAATGT
ACTTCTGGACAAATATTCTAATCCGTTGTTGATGGATTACACATTGGTGCCCCTGGTGAATCTTTCCCAAG
TCCAACATCTTCTAGTGGCTTACAAAGCTCCCGAGTATGCACAACAAGGCCGCATCACAAGGAAGACTG
ATGTTTGGAGCCTTGGGATTCTAATACTAGAGACCCTCACCGGTAAACTCCCAACAAACTACCTTGCTCT
CAGCACTGGCTATGGCACCGAATTGGCTACCTGGGTCGACACCATCATTAAAGATAATGAATCGGCTTTT
GACAAAGAGATGGACCAGCTCACTCATCAAGGACAGATCCAAAAGCTCTTCAACATTGGAGTGGCTTGC
TGTCAAGAAGATTTGGATACAAGGTGGGATCTCAAGGAAGCTATTGAAAGTATACAAGCCTTAAATGAC
AAGGTCGACGATCAAGTTGATGTTGGATTTTAATTTTAACATCGATCGTACGTACAACAATACATATACT
CAACCCCATTAGTTAATTCCCCTCTCTATATTTATATGTGCATTACATATCATGTTTGTACACATTTTCATTG
TATAGTACTTCTCTAAATTACTCAAGGCGTAACTAGACTTGCATGCCAATCGATCGGTCTCTCACTCCATA
AGTATTCGACCCTACCATTTGCAAGTATATATGTACATCTAGAACTTTGTTTAGCTCTAAAATTT
>PGSC0003DMG400009238 LRR-RLKs
ATGAAACACAAAACATTTGTTCCTTTGTTCCCTCCCCTTATTTCACCTGAGCTCAGAGAGTATATGCAAAT
CTTGTACATAGATTTTCTTGAAACCCATTATCATTTTCTTGAAGTTTTCAGCTTTCTGACTGTCAAAGCCAA
AAACAAAAAAAAAATGAACTTAAGGTATGCTCCCCATATAGTTATTACTATATTCTTGTCTTTTTCTCTTCA
AATTGTGTTTTCTCAATTGCCCCCAAATCAAATTAATGTCATGAGAAGTGTCTATGATCTGTTTCAGAATG
ATACTGGTGCTTCTTTTGTATGGAATGGAACTGATAAAGCTTCAACCCCATGTTCTTGGAAAGGGGTTTC
TTGCAATTCTAATAATTCTTCCCTTACCAAAGTCACCTTTTCATTGTTCTCTATTTCTAGCTCTGAATTCTTG
CCTTTTATTTGTCAAATTGATACTTTGGAGTCTCTTGATGTTTCCCAAAACTTTTTGAGCTCTATCCCAAAT
GAGTTCATCACTGTTTGTGGGGGAATTAGTGGGTTGAAATTGTTGAACTTTAGTGGGAATAAATTGGGG
GGTTTTTTGCCTACTTTCACTGGTTTTGGAAAGTTGGAGTCTTTGGACTTTTCTTACAATAACATGAATGG
GAAAGTTGACTTGCAGTTGGATGGATTAGACTCACTCAAGAGTTTGAACCTTAGCTTTAACATGTTTAAT
GGTCCAGTTCCTACCAGTCTTGGAAAATTTATTCTTCTGGAGGAGCTTCATCTTTCTGCAAATTCTTTTGA

AGGTGAATTCCCCACTCAAATTGTGAACTTTGGCAACTTAACTTTGATTGACCTATCTCTAAACAGCCTAT
CTGGTGTAATTCCTGATAGATTAGGTGAACTTTCCAAATTGCAAGTTTTGATTTTGTCAGCCAATAATTTG
AGTGGCACAATCCCACAATCCCTTAGGAATATCACAACGCTGACGCGTTTTGCTGCAAATCAGAATAATT
TTGTTGGAAATATACCCTTTGGTATAACCACATACCTGAGGAATTTGGACCTCAGTTTTAACAGATTAAAT
GGTACTATTCCTCAGGACCTCTTATTCCCAATGAATTTGCAGTTTGTTGATCTCACTTCCAATAAGTTAGA
GGGACCTGTTCCTTCAAACATGTCGATAAATTTGATCAGGTTGAGATTGGGGCAAAATGCTTTGAATGG
GTCATTTCCATCTGCTTCTTTTGAAAGCCTTCAAAGTTTGACCTACTTGGAACTGGACAACAACCAGTTAA
CTGGACCAATTCCTTCTGAATTGGGAAAGTGCCAAAAGCTGGCCCTTTTGAACTTAGCCCAGAATAAGCT
GAGTGGTGTTATTCCTGTTGAGTTGGGTGATATTTCTAATCTTCAGGTACTGAGTCTTCAGTCCAATAATC
TAGTTGGAGAAATTCCAAGTAACATTTCACAGTTGAACAGATTGCAGAGGCTCAATTTCAGCTCGAATTC
ATTGACTGGTTCCATTCCAAGTTCGTTATCAAGTTTGAGAAGTCTCACAAACTTGAATTTGCAGGGGAAT
AATCTAAGTGGTCGAATTCCGGTTGACATTAGTAACTTAAATGTGCTGTTAGAACTCCAATTTGGAGGGA
ACCAACTCAGTGGGCCTATTCCTGATATGCCTTTGAGTTTACAGATTTCTTTGAATCTGAGTCACAATCTT
TTTCAAGGACCTATACCAAGTAGTTTTTCTAGATTGACTTCATTGGAAGTTTTGGATCTCTCTTACAACAG
GTTCTCGGGTCAGATTCCAGACTACCTGACGGGAATGGGAGGCTTGACTAGGTTGGTGCTTTCAAACAA
TCAACTGTCTGGAGTTGTTCCAAAGTTTGGAAGCTTTGTCAGTGTTGAGACAGATGGAAATGGGGGTCT
GATCTATCCTTCCCCAGTTGTGCCACCACAAGCTGCTGCAAAAAAGAGAAAATCAATAGTTGTTGCAGTT
GTTGTCCCAATTGCATGTGTAGCAACAATTGCCGTTTTCGTGGTGATTGCTATTTCAATCTCTAGAAGATA
TTACAGGATCAATGATGAACATTTTCACTCAGGATTAGAAATTTCTCAATCCCCTGTCGTTCAGGGAAAG
GTTTTAACTGCAAACAGCATTCACAAGTCAAATATAGACTTCACAAAGGCAATGGTAGCAGTGTCTGATC
CCTCAAATGTTGTGTTCAAGACAAGATTTTCAACCTACTATAAAGCTGTTATGCCATCAGGTACAACCTAT
TTTGTCAAGAAGCTTAATTGGAGTGACAAAATATTTCAGTTGGGAAGCCATGAACTATTTGGGGAAGAG
CTAAAAAATATTGGAAAGCTGAATAATTCAAATGTCATGATTCCACTAGGCTATCTTTTGGCTGCTGATA
GTGCTTATCTCTTCTACGAATTTGCCCCCATTGGCTCATTATATGATGTTCTTCGTGGTAGCTTGGGCTAC
TCGTTAGATTGGGCAAGTCGCTACAGCATAGCTATTGGAGTAGCTCAGGGTTTAGCTTTTCTTCATGGAT
GTGAAAAGGGTCCAATCCTCCTCCTCGACCTTTCCAGCAAGAGCATTCTCTTGAAGTCCCAGAATGAGGC
TCAGATAGGAGACATTGAACTCTACAAGGTGATGGATCCTTCGAAGAGCACAGGCAGCTTTTCTGCTGT
TGCTGGTTCTGTCGGTTATATTCCTCCAGGTAATGAACTATTACTTGGTTAATGGTTAAAGTTTAAGATCC
TTCGACATTGATTTATACTCCTTAAGGGTAATGTGACGTTTTTTGTCCTAACTGTCGTACCATGCTTTAAA

CTCATCTTTTTGTGTTTAATCTTCATATAGTTATTGTTGTTTTCGAACTTAAACACTTCATTATATCCTCCCA
CCATGAAACGGTGTTGAACTGTCTTTCGATATCTAAAGTGAAACAGTGTGTCAGTATAGCCTAGAGAGG
GAGGAGCGGGTTAAAATGTAACATGAGAAGCCTCAAAGTTGAGCATATATTAAGTGATGCATTTCCTTT
TTGTGTTACAGAATATGCATACACAATGAGAGTCACAATGGCTGGAAATGTATATAGCTTTGGGGTTGT
CCTGCTGGAATTGTTGACAGGAAGGCCTGCAGTTAGTCAGGGAACCGAGTTAGCCAAGTCAGTGCTGA
GCAACTCTGAGAAGCACAGCAAGTGGGATCACATTCTGGATTCTAGCATTTCTAAGACATCGCTCAACAT
TAGGAGCCAGATGTTAGCAGTCCTCAAATTGGCTCTAGCTTGTGTCAGTGTATCACCAGAAGGCCGGCC
AAAGATGAAGAGTGTGCTTCGAGTGCTTCTCCATGCAAGGTAATCGGATGTCAAAGGAAAGTTAAGGA
GAAAACAGCCAAGATGGTTTTCTTCTTTTTCTTTTGGGGAGGAGCTGCAAGTGGTGAAGGTGTTGTGAG
CTATGCAGAATATGCCAGTTTTGCCAGTTTATACATATTATATTAATGTAAACATATAGATATATGATGAT
AGTAGTTACATAACTTTGAATATGATGTTAGTTCTCAGTGTAACATAGTCCATTTTTCTTGTTCATGTGTA
ATATATATATATATTATATATAAAAGGTCTTTGGGAGTGAATCCAATTT
>PGSC0003DMG400009380 LRR-RLKs
AAAGCTTACACTATTTTAACAAGCTCAGCAAACACCCATTTCACCATTATTACAGAAAAATCTCTGTATCT
TCTTCCTGTGCATGAAAAACCATGCAAATGAAACTATTACTCTTCTTTCTGAGTACATTCCCTTTGATTTTT
GCTTTAAATCAAGATGGGTTGTATCTACAAAAACTGAAACACTCTTTGTCAAGCTCAGATCAAGGGGTAT
TTTCTACTTGGTATGAAAATGATCCTACCCCATGTAACTGGACAGGTGTCACCTGTAATGACGCCGGAGA
TTCTCCTTCCGTCGTCGCTGTTAACCTCTCCGGTGCTTCTCTCGCCGGAACCTTTCCGGTCTTCCTCTGCCA
TCTTACTTCGCTTTCGTCACTCTCGCTTTCGAATAATCTTATTAATTCTACTCTTCCGGTTTCTATTTCTGAA
TGCCGTAGCCTCACGTACCTTGACCTTTCTCAGAATCTCATCGGTGGAACTATCCCCGACACTATTTCCGA
TCTTCCTTACCTCAGGTAACTCAGTTTGAACCTCAATTTTGCATTTCCCCTGTTTTTTTTCCTTTCAATTTTTC
GAAAAATATCAACTAATTGGGACTTACATTTAGCCATATATGCGTATTTGGGTCTGTTTTAAGGGTGAAT
ATAAGATCTATAGTTAGTGTATATAACATGGTATGTGTAGAAAAAGACAGTCCAGTGCACAAAACATCTC
ACGTTAGCAAGCTGCGGGGAAGGGGCTGTAAGCAATGATTTAGCTGAACCAACATAACCCGTATGGAT
TTGCTTGTTGGTGTTATAGTAACTCTAAATTATTGATTGTTTGAATAAAAATGCAGATGGAACGAAATGA
ATATTATGATCTTTTATTGCAGATTTTGTTATTCTTGTTGTTTCATTAGTATATTATGATCTTTTATTGCAGG
TACCTTGATCTTAGTGGATGCTATTTTTCGGGGAATATTCCGGCAAGTTTCGGAAGATTCAGGCAACTGG
AGACTCTTATTCTGACTGAGAACATTCTTACTGGTGAAGTTCCTCCTGCATTAGGTAATGTAACGAGTCTC
AAGACACTTGAACTTGCTTACAACCCTTTTGCGCCGAGTCAGTTTCCTCCTGAACTCGGTAACTTGACGA

ATCTTGAGACATTATGGCTAAGTATGTGTAATCTTGTTGGTTCAATTCCAAAAAGTATTGAGAAATTGAG
TCGATTGACTAATTTTGATGTGTCCAATAATGGACTGGTTGGGTCAATACCAAGTGCAATTTTCCAACTTA
ATAGTATTGTTCAAATTGAGCTCTACAATAATTCCCTCACCGGAGAATTGCCTTCGGGATGGTCTAACTT
GACCAGGTTGAGAAGATTCGATGTGTCGACTAACAAGTTAAATGGGACTATTCCTGATGAGTTGTGTGA
GTTGCCACTTGAGTCACTCAATTTATTTGAGAATCAATTTGAGGGGTTTCTTCCAGAAAGTATAGCTAACT
CTCCGAATCTGTATGAGCTTAAGTTATTCTCTAACAGATTTTCTGGTTCATTGCCTAGTGAACTGGGGAA
GAACTCGGCTTTACAGTATCTTGATGTTTCATACAATACATTTTCTGGTAAAATTCCTGAAAGTTTGTGTG
AGATGGGAGCTTTAGAGGATCTTATAGTGATATATAATTCGTTCTCTGGGAGTATTCCGGCTAGTCTTGG
CAACTGCCGGAGTTTACTTAGGGTCAGGTTTCGGGATAATAAGCTATTCGGGGAAGTCCCAACTGAGTT
TTGGAGTTTGCCTCAGGTTTATCTCTTAGACCTTTTTGGCAATGCATTTTCAGGAAATATATCACACATGA
TTTCTGGTGCCAAAAATTTGTCTAACCTACAAATATCAAGAAACAAATTCTCAGGGGTTATACCTAGTGA
AGTAGGAAAGTTGAAGAACTTAGTTGAGTTTTCTGCGAGTCATAATGAGCTAACGGGAGAACTTCCAGA
CACATTAGTGCAGCTAGGGCAGTTAGGTACCCTTGATCTTAGTTTCAATGAGCTATCAGGGAAAATCCCC
TTCGGAATTCACACAATGAAGCAACTCAGTGAGCTTGACTTGGCAAACAATGGATTTTCTGGGGAAATT
CCGGAGGAAATTGGGACTTTGCCAGTGCTTAATTATCTTGATCTTTCTGGGAATTACTTCTCAGGGGAAA
TCCCACTCAGTCTGCAAAGCTTGAAGCTTAATAAGCTAAATTTGTCTAATAATCAGCTGTCAGGGATGAT
TCCTGCAGTTTTTGATAAGGGTGTTTATAGAGACAGCTTTCGAGGTAATCCAGGTTTGTGTCAAGGTGTT
GCTGGTCTTTGTCCTACCAAAGGTAGAGGACAGCATGAAGGATACTTATGGACTTTGAGAGCTATCTAT
ACAGTTGCTGGCTTCGTTTTTCTTGTCGGGATTGCTATGTTCATTTGGAAGTATCAGAAATTCAAGAAGA
TTAAGAAAGGAAACACTATGACAAAGTGGACATCATTCCATAAGCTTGGATTTAGTGAATTTGAAATACC
CGTTGGCCTAGATGAAGCTAATGTAATTGGCAATGGAGCTTCAGGAAGAGTGTACAAAGCTGTCCTAAG
CAATGGTGAGGCAGTAGCTGTCAAGAAGCTATGGGAGAGAACAGTTAAAGATGAAACCCCTTATGGTG
CTCTTGAGTCTGATAAAGACGAGTTTGAAATTGAAGTTGAAACTCTGGGTAAAATTAGGCACAAGAATA
TTGTGAGATTGTGGTGCTGTTGCGTTACTGGGGATAGCAAGCTCTTGGTATATGAGTACATGCCAAATG
GAAGTTTGGGCGATTTGCTGCACAGTTGCAAGGCCAAATTGTTGGATTGGCCGTTGAGGTTCAAGATCG
CTTTAGATGCTGCTGAGGGGCTTTCTTATTTGCACCATGGTTGTGTTCCTCCAATTGTTCACCGTGATGTT
AAGTCAAACAACATATTGCTGGATGATGAGTTTCGGGCCAAAATTTCAGATTTTGGTGTGGCAAAAATT
GTTAAAGCAGACAGCAAAGGTGACGTTGAATCCATGTCTGTAATTGCTGGTTCCTGTGGTTACATTGCAC
CAGGTATTGATTTGTCCAAACTATATCACTGGCTTTAGTCATTTTTACCGATATATTGGCACATCTAGCTT

ATATTTTTGGAAAGATTGCAATTTCATTTTAAAGTAGCCAAAACTAACATAAGTGAAATTCATTGATTGCA
GAGTATGCATATACTCTTCATGTGAATGAAAAAAGTGACATATATAGCTTTGGAGTGGTCATTTTGGAGC
TGGTGACAGGCAAAAGACCAGTCAGTCCAGAATTCGGGGAGAAAGATCTAGCTACTTGGGTACACACG
ACGTTGAACGAGAAAGGAGTTGATCAGTTGCTCGACCCAAATCTAAACTCCAGCTTCAAAGAACATATA
TGCAAGGTTCTTGATGTTGGTCTACGTTGTCTTAACCAAACTCCAGCTAATCGCCCCTCAATGCACAGAG
TGGTGAAAATGCTCCAAGAATCAGCTCCTTATAATGTGCCAGAAATGGAAAACAAGAATGGTAAACTTT
CCCCTCACTTTCCAAAGTCAGTCTAGTTAATATATTAGATCAAGTAATAGAGCAGGAGTGTTTCTTGTCAA
GAATTGGCAAGGAAGAATCTGCATAATGAAAAATTAGTAGTCTTTTTCAAGAATTAAGCATATTGTACTC
TTTGAATTCTACAACCCTTTATGAATCTATATTACGAGGATTCATCATGAATTAACAAATTTTACGCTAGC
TGTCAGCCTACCGCAACACAGATGGGGTGGGTACTGAAAGTTGTAAGTATTCTTAACTAATGCCTTTGAT
TAAGTTTGTTAAACTTAGGTCCTACTTTGCTGTCCATCATTCACTGAGCTGGGGCCTTTAGAAGTAAA
>PGSC0003DMG400009561 LRR-RLKs
AAAAATAATATTCTTTCTCCCACCTTTCTTTTGGCTTCAAAACTACATAAAAAGGTAGCATTCTTTCATTTG
TAGGAGCCCTTTGAGAGGGCGCGCACCTGCAAGTGCGCGCACCAGAAAAAAAAAAACAAAAAAGTTTT
AAAAAATAAAGAAGACTGTCTCTCTTCTCTATGCAATCATGGCATCACAAAAAAACTACAAAAACAAAAA
CAAAAACAAACATGTCCTCCTCCTTGTTATCATCATGTGTTCATTAGCATTTGTGACAGAGGGAAAATTAT
CAGAGCCTGAGGTTCTTCTCAAGTTTCGTCAATCGTTAAAGTTCGATGGAGATCCCTTCTCGACGTGGGA
TATTAATGTACCACCTTGCATCAAAGAGAACAATAAGCCTAAATGGAACAATCTATTTTGCGAAAGTGGA
AAGGTTTATGGTTTGAATCTTGAAAATCTAGGGTTAAGTGGTACCATTGATTTGGACATACTTAAAGACT
TGCCAAATTTGAGAACCATAAGTGTTTTCAAAAATAAATTTGAAGGTTCCTTACCAATATTAAATAAATTG
CCTGCATTAAAGACTGCTTATTTCTCAAATAACAAGTTTTCTGGTCCAATTGATCAAAAGATATTTGAAGG
GATGAATTCATTAAAGAAACTTCACCTAGCAAATAATGAATTTACAGGACAACTCCCTCCTATTTTTGGTG
ATATGCCAAATCTAAGGGAGTTGAATATTCAAAACAATAAGTTTGAGGGTCCAATTCCTCCTTCCTATTC
ACATCTATATTTTCCTGCTTATGATGGTAATTCCTACCTCTTTTCCTTTTTTATATTTCATTAATTATTGTGTC
CAATGAATTAACACTTTTTTTGTTTATCATCATTATGCCATGTTTTTCTTCCAATCCTAGATCATTAAAAAG
ATCATATCATTGGCTACATAATAAACAAAAGATCATTAGATTCGGGTCATCCATGTGTGTCAACTCGGAT
CCATGTAAATAATAAGGGAAGAGACACAACTATTCAGATTATTTCTGAAAAGTAGTAGTACTCCATCTCT
TTAGAATTATAAAAGAATTGTCTTAATAGAAACCAACAACACTTTTTTTTTTTTTTGAGAAAAAAAAATAAC
TTGATAGACAATCCTCTCTTCTTCTTTCAAGTTCTTGTGAAAGGATTTCATAGAACAAACTTCTGTAACAG

GTACGCGATTTTAGACTATGGCTTAATTAATATGATGTATGTAGTTCGTCTTTATATGTGATTACATCTAG
TGAAACCAAATTCAGTATTGATATATTATCTAACTAAACATAAGTATTTTTCTTCTTCTTCTTCTTATAAAA
TAGGCAATGATGGTCTTTGTGGTCCTCCTTTAGCAAAATCATGCAATAAGGAAGATGAACAAAAAAAGC
AAGAATCATCATCATCGTCATCGTCATCAGGTTGGAAGATTGCCCTTATTGTGGTTATAGTAGTTGTAGT
GATAGGCATTGTTGTGTTTGCACTAATCAATCGTCGCAAGAAGAATCACCAACAAGAAGTAGTACTTGG
AGGATCATCATTAAGTTCTTCCTCACCAACAAGTCAAGACCAAAAACTAATCCCTCAATCTCATGATCATC
TAAACAAAATGGAACAAGGCCAATCATCAGCTGCATCTACTCCTGATCGCGCGTCTAATGATGGCGGTA
AAAGAGCTGAAGTTGCTGGACAAAAACTTCTATTCTTGAAAGATGACATTGAGAAATTTGACTTACCTGA
TTTGTTGAAAGCCTCTGCTGAAGTATTGGGAAGTGGAGTGTTTGGTTCAACTTATAAAGCTGCACTTAGT
ACTGGTCCTGTCATGGTTGTTAAGAGGTTTAGACATATGAATAAAGTTGGTAAAGAAGATTTCCATGAA
CATATGAGAAGGCTTGGAAGATTGAGTCATAAGAACTTGCTTCCTGTTATTGCTTTCTATTATAGGAAAG
AGGAGAAGCTTCTTGTCTTTGAATATGTCAACAATGTCAGCCTTGCTGTTTATCTTCATGGTACCTATCTC
TACTCTCTTTTGTTTCAATTTGTTTGTCTCGTTTTAACTTGACATACAGTTTAAAAAATAAAAAAAAACCTT
TGAGACTTAAAGATACTTAAAATGTAGTAGAATATCCTTTAATTTAATTTTGTGGTATGAAAAATTTCATC
AGTAAAGTTGGAATTAAAAAGTTCCCTAAAAGAAGAAGAAGAAAGAAACATTCTTTTTGAAATCAACTA
AAAAGATAAAATAGAGTATTAATTATCCTTTCTGTCCCAATTTAAATGTCTTACTTTCACTCTTGTTCTGTT
CTAAAAAGTGTGTCGGTTTCTATATTTAGTAAGTTTTCAAATTGTGACATTCTACTTGCTTAGTTTAAGAT
CACAAAATTAAAAAAAAAAAAATCTTTATTTTTTTTAAAACCTATTTACTCACTTAGATTAAGACACTTAAAT
TGAAAGGAAGGATGTACTCCCTGCATCTATCTTTACTTGTCCAGTATATGGGATGTCCAATAATACTTGTC
CAGTTTATAAAACCAATGAATAATTTTTCATTTTATGTCTATTTTACTTTTGCTATTAAATACTATTCATTTT
TCAATGTTTAAATGACTTATATTTAAATAAGAGTGATTTAATAAAACTACCCCTATCATTTACTGCTTCATA
ATCCATGTGCCAAGTCAGTAGTGTACGACGAACAGAGGGAGAGGGAGTAATATTTTGTTCATAGTTTTG
ACCACCTTTTCTTGATTTTTCAGGTAATAGCAAATCACGTGGTAACCAAAGTCTTGATTGGCCAACTCGTT
TGAAAATTGTGAAAGGAGTAAGCAAAGGAATTTTATACCTTTACAATGAGCTACCAAGCTTAACATCACC
TCATGGTCACCTCAAGTCTTCAAATGTTCTTCTAACTGAAAACTTTGAGGCAGTTCTCACAGACTATGCCT
TACTACCAGTAGTAAATGCTGAACATGCACATGAACACATGATCTCATACAAAGCACCAGAGTTAAAAC
AATCAGGCAAAATCAATAGAAAAACTGATGTTTGGACACTTGGTATGTTAATCCTTGAGATCTTGACTGG
AAAATTCCCTTCAAATCTTTTAGGCAAAGGAACACAAGATAGCGATGATTTAGCGACATGGGTGAATAC
TACTCTTGGAGATGAATCATCAGAGAAAGAAGTGTTTGATAAAGAAATGAAAGGAACAAAAGATTGTG

AGAGTGAAATGATGAAGTTATTGAAGATTGGACTAAGTTGTTGTGAGGTTGATGTGGAGAAGAGATGG
GATATAAAAGAGGCAGTTGAAAGGATTGATGAAGTAAAAGAGAAAGGTGATTTCAGCTCTAATGTTGC
AACTAATGAAGTTGACAATATGCATACTTCAAGATAAAAAAATATTTTTGATTGATAAGCTCATGATCAT
AATATGGAAATTTTGATTATATTGGATGCATATTGCATGACTATATATACTTTGGGTATATGATTTTGTTT
TGTCATGGTGGAGGATAAATTGAACCATGATGTGGGGTTACTACAAAAGAAAAAAAGAAAAAAAAAGA
AAAAGAAAAATAAGTGATAGAAAAATGAAGTTACTTAATTTTTGTTTGCATATTGTAAATTTGTCCTTTAT
GAATCTATAGTCTAGGTGTGTTTCTTTTATTTATTTTTATTATTCATTCATCAAGCTTATTTGGGACTTGCTT
TACAAAATTGGC
>PGSC0003DMG400009843 LRR-RLKs
TTAGAGACTATTTTGACCCTACGGCCAAAATTAATGGACCATATTTGTCATTTTCTTTCTTCTTCACCCTAC
CCCTCGTTTCCCACTTCACTCTTCTCCATAGTTCCTCACTGTCTCCGAAAATCCAGTCCATTTTTCAGACCTC
CTTTCACGCACAAAATGACAACAACAAACACTAAACTTGGCCACTAGTCAACTAAACCAAATCACTCGAC
ATTTTTAGTTGCATAACTTGCAATGTCTCCATTGATGCTTCTGCTCTGTTTTCTTCTTCAACTAATCTACAGT
TTCTACTTTTGTTCTTCTTCTTCTGCTGCTGCTGCTACCTCTACCCCTTCTGCTCCATTCAACTCTTTGCTTCC
TTCCGACGCTGTTTCGTTATTGTCATTCAAGTCAAAAGCTGACCTTGACAACAAGCTTCATTACACACTTA
ACGAGCGTTTTGATTACTGTCAATGGCAAGGAGTGAAATGTGTACAGGGTCGTGTTGTGCGTTTAGTTC
TTCAAGGGTTTAGTCTCCGGGGTACTTTTCCGGCGAATAGTTTAACTCATCTTGACCAACTCCGAATACTC
AATCTTAGGAATAACTCACTTTCCGGTCCTATTCCTGATCTTTCGGGTCTTCCTAACCTGAAAACTCTGTTT
CTTGATCATAATTTTTTCTCCGGAACTTTCCCCTTTTCTGTTCTTTCTATTCACCGTCTTGTTATTCTTGATCT
TTCTCGTAACAATCTCACTGGTTCACTCCCTGTTAGGCTTACTGTTCTTGACCGGTTGAACTATCTCCGGC
TTGATTCCAACTGGTTTAACGGTTCGATTCCGCCGTTGAACCAGACCCAACTCCAAATTTTCAATGTGTCT
AATAATAACCTCACTGGTCCAGTACCTGTTACTCCAACTCTGAAGAAATTTAACATACGCTCGTTCTTACG
GAACCCTAGTCTTTGTGGTGAGGTTGTTGATAAGCCGTGCCGTTCGGCACCGTTCTTTGATTCGCCTTCCT
CTGCTGCCTCTCCGCCCACACCGTTATACCAAAACGCACAGTCGCAGGGTATTCTTATTAGTCCTCCACCT
CAGCACAAACACAAAAAAGTTGGTGTCGTTTTGGGTTTTGTAGTTGGAACATTGATTTTGATTGCAGCTG
TTCTCTGTCTTTTTGCTTTTGTGAAAAAGAGAAGAGAAGAAACTGAAACTGAATCAAAAGCAACAAAAT
GTACCATTGAGACCATCACTAATAGTGCGGCGAACGCAACGGTTTCTGAGCCAGATGATAGTAGCCAAG
AGATTAAATTAGAAAAAGAAATGAAAGTGCTTCAAGCCCCCAAACAACAGATGAAGAGTGGAAATCTG
ATATTTTGTTCAGGGGAAACAGAGTTGTATTCTTTAGAACAACTAATGAGAGCATCAGCAGAGTTGCTTG

GAAGAGGCACAATTGGAACAACGTATAAAGCTCTGATGGCTAGTCAGTTAATCGTTTCAGTAAAGCGAT
TGGATGCTGGTAAAACTTCGATTACGAGTGCAGAAGCGTTTGAGCAACACATGGAGTCAGTTGGGATGT
TGAGGCACCCAAATTTGGTTGCAGTCAGAGCATACTTTCAGGCTAAACAAGAAAGGCTTGTTATCTATG
ATTATCAGCCTAATGGCAGTCTCTTCAATCTAATTCATGGTAAAGTTTCTAACTTTTTAATATATCATTACT
AGTTCTACAAATTATTCATCTGAAATGGATCAATGTTTATTTTTCATTTTTGGTAATTCTGCTAATTGGTCA
ATGTGAAGTCAATTTGGTGGACCTGTGATGGAGAAAAAAGTGGAAGAGTGAGTTGTCTGTGGCTACTG
GCTAATCACTATCTAGCTTCTTTTTTTTTTCTTTACAGAAATTAGTTGTAGTGTTTTGGATCTTTCAGTGGT
TGCTATGGTTGTAGTAGCAGGGGACTTTTTTAGCTTTTAGTGTGGATGCCTAACATGGCTCCCACTGAGT
TCACTACCGACAATTGCAAAGTTTGTGTTTACAGGGCAGCCAAATAGTAATGTGTTTTAGTTTCAGGCTG
TCACTGACTGACCGGTATGGGTTGATAGCATTATATTCCTCAATTTTTTTTATCATGCATTGAGGCAAATG
GTCCTTGGCTCTTGCGTCTGTCTTTTTGGTATTTATGAGTATGTTTGCTTCTTCTTTAAGTTGAATAGGATG
TTGACAGTAACTTTTTGGTGTTTTCCTTTTACTTGTCCAAATGTTCTCTGTTTCTTAAATATTAAATTTACTC
AAGTGAACGTTAACGTTGTTAGATAGAAGGATTAAGGGAAAGAACTAGAACATCCTTGGGAATTCTTAA
TGTAGTGACAGAAATGGTATGAATTTGCTTAATTTACATTATTTTGAATCCTAGGTATTGCATTTTTTGTA
TCCCCTTGTGATACGCTATCAAGAAATTGGAATTTCACTTTCATAGATTACTCTGCTCAAGAAGATTGATT
CCTCTATATAATTCTGTGTGTTTAAGTGGTTGGTCTTTTCACCTAAATCCTGATAATCTGTTGTTCGTTTAC
TTATATATTGTGTTCAATTTTCTATTGTTCGTGGCAGGTTCAAGATCAACAAGGGCAAGGCCTCTTCACTG
GACATCGTGTCTAAAAATAGCAGAAGATGTGGCTCAGGGCCTTGCTTACATCCATCAAGCGTCAAAGCT
CACTCATGGCAACTTGAAATCCTCCAACGTCCTTCTAGGGTCTGATTTTGAGGCCTGTCTAACAGACTACT
CCATCATCGCGCTTGCAGATATTTCTTTGGAGGATGATCCTGATTCAGCATGCTATAAAGCACCAGAAGT
GCGCAAATCAGCACGAAGAGCCACGCCTGGGTCTGACGTATATGCCTATGGCATTCTGTTATTAGAGCT
TTTAACGGGCAAACCTCCATCTCAGCATCCACATCTCTCTCCCCCTGATGTACCGGATTGGGTTAGAGCTA
TGAGGGAGGATGATAATGAGGAGGATAGATGGCTTGCAATGCTAATTGACCTTGCTAGTATATGTAGCT
TGACATCACCAGAACAGAGACCAACAATGAGGCAAATCTTGAAAATGATACAAGATATAAAGGATAGC
GCAATGGTGGAGAATAACAAAAGAGATGCACATAATGGATACTCATAGCTTCTTCTGAATTGCAGTTAC
ACCATTAATCAGCGAGGGTACATGGTCAATTGTAAGTAAGTGTCATCTCCCCCTTGGCAAAGTGCCATTG
ATCGACAACATATCTGTAATTGAGTTTTTTCCAGATAGACCAAATGACTTATCAACTATAATGGTGCTTGT
ACCTTCACTACTCAGCAGTAAACACCTAAAACTGACAGGTTAAGGTCTATCTGCTAGAACATGGTCAACT
TAGTCACTGAAGATTATGACACATTTTGTTCCCCTCGTCGAAATCCCTCTTTTTTGGTCACACAAACATAT

GGTGCCTATATTTTTAGGGCATATTGTAGTAGGTGGTTTTGGTTGATTCTTTTTCTAAAGAACTTCAAAAT
GTATTCGCTTTACCTTAATGGCAGAAGAATATTGTATAATCTTGAATGGTAAAGCTAACCCAAATTCTGA
ACTTTCCCTTCTCTGCATGGTTGGTTAGTATCAACTTAGCAGGAGAAGAGTTGTTTAATG
>PGSC0003DMG400009941 LRR-RLKs
CTTCAAATACCCTTGAACCTATCCTAAAATTTGTACAAACCAGACTAAACTAACAGTGTAATAATGTCACT
CCCCAAAAAAATATCCCTTTTCCTCCAAATTTTCATTTTTTTTGTTTTCTCCATTAATGCAAACTCTGATCTT
GAAACCCTTTTGAAGCTCAAAGAATCCATGGTTGCTCCTGGAACTTCTGCACTTCTTGATTGGAACAACA
ACACAAATTACCCTTTTTCCCATTGTTCTTTTTCTGGTGTTACGTGTAACAATAACTCTCATGTTATATCTAT
AAACATCACTAATGTTCCTCTATTTGGTACTATTCCACCAGAAATTGGTCTTTTACTAAATCTTGAAAATCT
TATTATTTTTGGTGATAATATTACTGGTACACTCCCTTTAGAAATGTCACAACTTTCTTCTATTAAACATGT
TAATCTTTCTTACAACAATTTTTCTGGTCCTTTTCCTAGAGAAATCTTGTTGGGGTTAATAAAGCTTGAATC
TTTTGACATTTATAACAACAATTTCACTGGTGAACTTCCTACTGAGTTTGTAAAGTTGAAAAAGTTGGAA
ACTTTACATCTTGGTGGAAACTATTTTCATGGTGAAATACCAGAAGTTTATTCTCATATTGTAAGTTTAAA
GTGGTTGGGTTTGGAGGGAAATTCACTAACTGGGAAAATACCAAAGAGTTTGGCTTCGTTACCAAATCT
TGAAGAACTTAGATTGGGCTATTATAATAGTTATGAAGGGGGTATTCCATCTGAGTTTGGTAATATTAGT
ACACTTAAACTTCTTGATCTTGGAAATTGTAATCTTGATGGTGAAGTTCCTCCAAGTCTTGGAAATTTGAA
GAAGTTGCATACTTTGTTTCTACAAGTGAACAGACTTACAGGTATTCATGAACTCACTATTTGTTAATTAG
TTATACAATCAAATCTATCTTTAATAACGTCGTTTGTCTGAATATATTCTAGTTGCCATGGTTGAATATTGT
TGTAGGAAACATATTTGTCACAAATCATTTTCCAAGATGTAAGACTGAAAATCAAATTGTGTTGAAATAC
TCAATAGTAATAGTTGTTTCTTGAGTGACGTGCAGAGTGCCAGTATATAAGTAAAAAGTGAATGGAGTG
AGAATATCATAAAAAGTTTCTTTGAGAAAAACTGTATTTATAGTGAAATGATGTTAGAGTATGTGTTAAT
AGAGAGGTTTTTGTATAACATAGATGTATATGTGCAAAATATTATGTATATAAAGTTTTGAAGTCATATA
TCTGATAAATTTTAGAATCTTGAATTCGTAAAACTTAAATCTTGGATTCGCTTATGATTTACAGGTCGCAT
ACCTTCTGAACTATCTGGTTTAGAGAGTTTGATGTCGTTTGATTTGTCTTTTAATCAACTGACCGGAGAAA
TACCAGAGAGTTTTGTGAAGTTGCAGAATTTGACATTGATTAACTTGTTTAGAAACAACTTGCATGGTCC
AATTCCCCCTTTTATTGGTGACCTTCCAAATCTTGAAGTGTTGCAGATTTGGGGAAACAATTTTACTCTTG
AATTGCCCGAAAATCTTGGGCGTAACGGGAGGTTTTTGTTTCTTGATATTTCTATTAATCATTTTACTGGA
AGGATACCACCTGATTTGTGTAAAGGAGGGAAGTTAAAGACACTGATTCTAATGGAAAATTACTTCTTT
GGTCCAATTCCTGAACAACTTGGTGAGTGCAAATCGCTTGCTCGAATTCGCGTTAGGAAGAATTACTTAA

ATGGTACTATTCCAGCTGGTTTTTTCAAGTTACCTGCATTGGATATGCTTGAACTTGACAACAACTATTTC

ACTGGTGAGCTGCCAACGGAGATAAACGCGAATAATCTCACTAAACTTGTACTTTCCAACAACTGGATCA

CGGGGAACATTCCTCCATCATTAGGGAACTTGAAGAATCTAGTCACTCTATCACTTGATATGAACAGATT

ATCTGGTGAAATTCCTCAAGAAATTGCGAGTTTGAATAAACTCGTGACCATCAACTTGAGTGGTAACAAT

TTAACAGGTGAAATCCCAAGTTCAATTGCGCTTTGTTCAGAGCTAACATTGGTTGACTTGAGCAGAAACC

AACTGGTTGGTGAAGTGCCAAAAGAAATCACCAAGTTAAATAGCTTGAACGCTCTGAACTTGTCAAGAA

ACCAACTGAGTGGCGCCATTCCTGGAGAAGTCGGAGTGATGAATGGCTTGACAGTTTTGGATCTTTCTT

ACAATGATCTTTCTGGAAGGAGACCGACCAACGGACAACTAAAGTTCTTCAATGACACTTATTTTGTAGG

AAATCCAAAACTCTGTTCGCCTCATGCTACTTTTTGCCCGTCAGCTTCCAATTCACCACAAAACGCGCTCA

AAATCCATTCTGGGAAGTTCACAACTATCCAATTGGTGATTACAATAATCATCTTAGTCACTGTTGCATTG

CTGTTGGCAGTTACCGTGTTGTTCATCAAGAAGGAAAAGTTCAAGAATTCGAAACTTTGGAAGTTAACA

GCATTCCAGAAACTTGATTTCAGAGCTGAGGATGTTTTGGAGTGTTTAAAAGAGGAGAACATAATTGGG

AAAGGTGGAGCTGGCGTTGTGTACCGAGGGTCTATGTCAAATGGCATCGACGTTGCAATTAAGAAACTT

GTAGGCCGAGGAACTGGACACCATGATCACGGATTCTCAGCTGAAATCCAAACACTAGGAAGGATCAG

GCACAGAAACATCGTACGATTACTAGGATATGTCTCAAACAAAGACACAAACTTGTTGTTGTACGAATAC

ATGTCGAATGGGAGCTTAGGTGAAATGTTACATGGTGCCAAAGGAGCACATTTGAAATGGGAGACGAG

GTACCGTATTGCTGTGGAAGCTGCAAAGGGATTGTGTTATTTGCACCATGATTGTTCGCCTTCGATTATT

CATAGAGATGTCAAGTCCAATAATATTCTGCTGGATTCCGATTACGAGGCTCATGTTGCTGATTTTGGCC

TAGCCAAATTCTTGCAGGATGCTGGTGCATCAGAGTGCATGTCCTCTATTGCTGGCTCATATGGTTACAT

TGCTCCAGGTAAACTAATACGGTTACAATATTTATCATATTTATACAATTATGAATTTCCGTATTAGTTTTC

TTTTATTACTGGCTCATATGGTCATCATATTTATACAATTTGGTGGCATATAATGTAGTTACAGGTAAAAA

TTAATGATAATCTTAAAATGTGATACTAATAACTTGCTATAATAGATTAAATTATGATGATACTATGAAGT

TTGTATGCTATTGTGCATATAACCTTGAGCTATGTTGCTCAAACTCTTAAAAAATGTCAGTGGGGTACAT

GTCGGATCCTCCAAAAGTAATACTTTTTTGGAGGATTCGACACGGATGCAACATCATTTTTTGAAGAATA

CAAGCAAAATAGATCCTTAAGTCCTTTTATATACCTGTGTTATTGGTCAATATTTTCCTATTTCATGAAAG

ATACACTTCACTAACTTTTAGTACCAATTACCAAGACCTATGAACTTTTAGAATTTACTGAACTGGGGTTA

AAAATAGTAGGTATTTAAAAGCTATGCTCCAGGACAAAATCTATTTAAAACATTTGTCCAAAACATTAAA

TTGTGCAATTAATGTTTTTCTGAGGTGCATTTTTGTTTGGATATGAAACAGAGTATGCATACACATTGAAA

GTTGACCAAAAGAGTGATGTATACAGTTTTGGAGTTGTACTGTTGGAACTTATCACAGGTCACAAGCCA

GTTGGTGAATTCGGGGACGGTGTAGATATAGTCAGATGGGTAAATAAAACAATGTCCGAATTATCTCAG
CCGTCTGATGCAGCCTCAGTTTTAGCAGTCGTTGACTCGAGGCTACATAGTTACCCTCTTGCAAGTGTTAT
CAATTTGTTCAAGATTGCTATAATGTGTGTTGAAGAAGAGAGTTGTGCTAGGCCTACCATGAGGGAAGT
TGTTCACATGCTTACAAATCTTCCTCAGTCTACTACTACTACTCTCCTTGCCCTTTGAAATTGCGCGGATAT
CAAGTGTCTGGTTGAAAACTCGTGGAGTTTGAGGCCGGGAACACGAGTCTCATGAGTCTATTTGGGTAC
GGGGAACAAAAAAATTGTGCAAGTAGAGATATGTATTTGAAATGTATTGCTTGTTTTTATATTTTTGAAG
TTGTAGGCAAGTTGTTGTATAAAAAGGGGCATGAAAAAAAGAAGGAAAAAAACTCCTTTAATTACTTAT
TATGGGTATTGAGTATGAATATGTGGCCTTAGGAAATAAATTTCAAGATGGCAATTAGTCAATACTTGCT
TTTAATAACATGGCCTATGTAATGTTCTTAAGAGTTTGCAAAGTTAAAACTTG
>PGSC0003DMG400010055 LRR-RLKs
GACTGACAAGACAGTAATAAAATGATTTTCTGGCATACAAAAGCTATAGTTTTGTTCTCAATCTAAAAGA
TTATTAGAAGAAAGAAAATCCAATAGAGGGGTTTGTCTCTCTGGTATTCATTTGAGTTTCTGTTTCTTTCT
TGAAATTTAATGGAAAAGAAAAGAGAGATAAAGGTTGCATTCTTGTATGTGGCTACATTCTTGGTTCTTT
GGAGTTCTGCAGCAGGATTGCTTTCTCCTAAAGGTGTCAACTTTGAAGGTAATTAGTATTATTTTGATAA
AATTCTGCATAATGTATACTGAAAATACTAAGTTTTCATCTGAGATGTTCCTCTTTTTCTTTTTTTTTTCTCA
GTGCAAGCTTTAATGGCCATAAAAGTTGCTTTGAAGGATCCTCACGGGGTTCTTGATAATTGGGATAGT
ACTTCTGTTGATCCATGTAGTTGGGCTATGGTTACTTGTTCTTCTGAGAGTTTGGTTATTGGCTTGTAAGT
ATTTCTTGCAAAGATGAAATCTTTTTCTTTGTTTGTGTCTTTGCAATTCAAAAGTTGAATTATTTTTTTTACC
TTTCTGTATTATGCAGAGGGGCACCAAGCCAGAATCTGTCTGGTACTTTTTCTCCTAGCATTGGCAATTTG
ACAAATCTTCAGATTATGTGAGTTACTAAAATCAGTGATTTGAACCTTTCTTGATTGTTTCTGTTACTTTTT
TGAATAACCATTTTTTTTTTTCTTTTGAAACAGATTGCTGCAGAATAACAATATAACAGGACCAATCCCAAA
AGAAATTGGAAGGCTCTCAAAACTTCAGACTCTTGATCTTTCTGATAACTTCTTCACTGGTGATATTCCTG
TTTCTTTGGGACACTTGAGTGACCTCAAGTACATGTAAGAATCTTAACAGAGAGCCAGTTCTCATACTAA
ACTTCATTTTTCTATCTTCCAACAGTTTCAATATCAGTTTCCTTATATTAATTTGTTCAGGAGGCTCAACAA
TAACAGTCTATCAGGAGAAATTCCAGTCTCATTAGCCAACATGTCACAGCTTACTCTAGTGTAAATCCTTA
TTGCTTTACTTATCTTTTTCCTTTTATCCTTTTCAAAACTTGAGATTGATTTTGGTATTTAACCTTTTGATTCA
GGGACTTATCCTTCAATCACTTGAGTGGACCGGTACCTAGGTTCCCTGCTAAGAAATTCAAGTAAGCTAA
TTAACAGTTTATTCATGTCTTGTTAATAGAAAGCTGGAATGTTAAGAATAATTTATGTTGTTCTGTGATTG
TAGCATTGTTGGAAATCCATTGATCTGTGAGACGGGGTCTGAACCAGACTGCTATGGAATGCAATTGCT

GCCAATGTCAATGACATTGAATGGTTCAGAATGTATGCATTATAGAATGCCATTTTCCAAATCTAACCATT
TTTTCTTACCTGTAATTTTTGATTTTTCATTTCTTCTGTTTCACCTCACTTTATTGCTCCGTGTTTGTTATCCA
GCTTCGCCTTCTGGGAAGCAAAAAGGTCATAAAATCGCGCTTGTATTTGGCTCGACTCTTGGTTGCATTT
TTTTGCTCGTTCTTGGAATTGGACTCTTTCTATGGTCAAGGCATAGACACAATCAACAAGCCTTTTTTGAT
GTTAAAGGTTGGTCATCTGTTCTAAACTTAAGCTCAATGTTCTCATGAAAGTTATGATCATTGCTTCATAC
TCATTTGTATAATTTGCTATTATTAGATCGGCATCATGAAGAAGTTTCCCTTGGAAATTTAAGAAGATTTC
AATTCAAGGATCTCCAAATTGCAACCAACAACTTCAGCAACAAAAACATTTTAGGAAAAGGTGGTTTTGG
TAATGTTTATAAAGGCCATCTCCCAGACGGTACTCCTGTGGCTGTAAAGAGGCTAAATGATGGCAATGC
AATTGGTGGTGAGAAACAATTCCAAACAGAAGTCGAAATGATAAGCTTAGCAGTGCATCGTAACCTCCT
CAGGCTCTATGGATTTTGTATGACACAGTCGGAGAAGCTTTTGGTTTACCCTTTTATGTCCAATGGTAGT
GTAGCTTCGCGTCTCAGAGGTAAAAAAAACATATGAAATTAGCAAAGTGGCATTTCAGATATCTTATTTG
TCTTTTGCTGAAATATAGTCCATGTTACTTTCTATAGTAAAACCTGTGTTGGATTGGGGAACAAGGAAGC
GAATAGCTATAGGAGCTGCTCGAGGACTGTTATATCTTCACGAACAATGTGATCCAAAGATAATTCATA
GAGATGTTAAAGCAGCAAATATATTGCTCGATGACTTTTGTGAGGCGGTTGTGGGAGATTTTGGGCTGG
CAAAGCTTTTGGATCACCAGGATTCACATGTCACAACTGCTGTGCGCGGTACAGTAGGGCATATTGCCCC
TGAATACCTGTCAACAGGACAATCATCTGAGAAAACAGATGTGTTTGGATTTGGGATCCTTCTACTTGAG
CTGATCACAGGAATGAGAGCTATAGAATTTGGAAAAGCAGCTAACCAAAAGGGAGTAATGCTTGATTG
GGTAAGTATCAATTGACATGCCTCAAATAAGTTCAACACTTTCTATATTTGAAAACTATTTAACTTTAGAC
TTCTCATTTTACCCTTAACGACATGCTCTTATAGTCAGAGAAATGTTATGGCATGTTTCGCGACATTGTAT
ATATCCTGTTAAGTGTAACATTTGGTTTACATTATATCAGGTTAGGAAAATTCATCAAGAGAAGAAACTT
GATGTCCTTGTTGATAAAGATTTGAGAATCAACTATGATCGAATCGAGCTAGAGGAAATGGTTCAAGTT
GCTCTCCTCAGCACACAATATCTTCCAGGACACAGACCAAAAATGTCTGAAATCGTTCGAATGCTTGAAG
GTGATGGACTTGCAGAGAGATGGGAAGCATCCCAAAAATTTGATGGTAGTAACAAGTACAAAACTAAA
GAGCTATCCTCATCCGAGAGATTTTCAGATCTCACCGATGATTCATTGTTACTTGTACAAGCCATGGAGTT
GTCTGGTCCTAGGTGAGCAAGTCATTTACGTATATTTATAAAAAAAAGTAGAATTCACACTTAGAGAGGT
AATTTATTAAACTATGTGGTAATGTATAGTAATGAACCATCAAAGATTCAAAGAACTTTGATGTGTTCTG
TTATTTTTTTTGTTTCTTTATTGTATATGTTCATCTTTAAGTTAAATGATTTCAATCGAGAGTTCGATTTTTT
TTCCTGGTACAACCTGTATTTG
>PGSC0003DMG400010230 LRR-RLKs

AAAAAAGAGAAAAAAATCTTGTAGAGAGAGAAACAAGAGAGAGGAAAAAACTTTTTTTTCCCACCATTT
CCCCATATTTTCCCTCTTGTTCCTCCTCAACCATATTTCTGAGAAAAAAAATAGTTGAAAAGAAGCCCCTG
TTTTGAAATCACCCTGCAAACTGCAGTCAAGAAAAAAAAGAAAAGATATTACACTATTTTTCACAAAAAA
AAAGATTGATTTTTTCTTGTGTGATTTCAATTTTCTTCTTCAATCTCTGTTATTTCTTTAATTTTTTTCAAAAA
AGAAAAAAATAAATTTTGTCCCACCACCTGCATTTTCCTATTGCAGTTATTTGATTTTTGGCTTTTTTTCAA
GAAATGTGACGAATTGTCATTTCCAGTTCTGCAATAAAAAGCAGAAAAAGATTTCATTCTTGTCTGGTAA
GTTTTACTCTTTACATTTTACCATCTTTTGGGTTTTTGGATTTTTATTTTTCTTTTGATTGCTTGCAAATTCA
GTTATTCCCTCATCATCTTTTCTTTTTCTTTTTCTGTTTCTCTCTTTTTTTGATGGGGCTTGTGAAGTGTTCTT
AGGTTTTTCACCAACCCCTTTTCAAGAAACATATTTTTACTAATTTTTTTCTTTTGTGTTTATTCATTTTCCA
GATAAAAAATTGAATCTTGGAGATTAATCAATCGAGTTTGTGGAAGTGTTTGGTGAGGGAGATGTTGTTA
TAGTCTAAATTTTAGAAACTGGGAAAAATGAGAGACCCCAATTTTGTCCATTGTATCAATAGGGTTAATA
GTTTTTCTTTTAACTTGTAATATAGTTTTACATTGTAAAAATTGAATGATAAGCAAACAGTGGTGTATTTG
CTCTCCTTTTTTTCAACTTGTTTATATTTACGAGTGAATTCAATTGTTTGGAGAGTAATTTTGTACAAGAAA
GCTGGCGATTTTGTATAGAGTCCTCGCTGTTGACAGATATTGTTACTGCCTCAACAACTGATCCATATGG
TTGTGCAAGCTATGGGTGGTAGATGGAACACTTATGGAATCAAATTGTCGTATCTCGCTCTTTTGATTAT
CATTTTGGATATTCGCGGTTGTTGTTCTCTTAATTCGGAAGGTAATTCTTATACTTGAGTACAGTTCTATT
GGTTTTGTAGTTCTGTCTTAGGCTAGTTTTAAGTGTTTGGTATGTTTTCTTGTTTGCTAATTATTGTTGGTG
TGAGAAACAGGATTGGCACTATTGAGATTCCGCGTGAAAGTGGATTCTGATCCGTATGGCATTCTTGAA
AACTGGAATTCAGATCATTGTGACCCGTGCTTGTGGTCTGGTGTCCAGTGTATGGACGGGAGAGTGCAA
ATGTTGTAAGTCTCTTGTTAAATATACCCAAAAATTTCCTAGTGTGAATCCTTTTCTTATAAATTTATATGT
GTTGCTTTGCTATCGACAAGCCTGTTGAAATGATAATGACAGACAAAGTGCAAACACTGAGTTGTGAGT
TTTTCTGTTTGTGGTTCTTCGGATATTGTTAACTCATTTGTCTGTTCTCTATTTAGGGACCTTCATGGATAT
TCGTTGAAAGGAAAACTCGCAGCAGAACTTGGAAATCTGACTCACTTAAAATCACTGTAAGCTTCAGCTA
TACTTATATGCTTCTCATTTGTTTCTTTAGCTCATATTCCTGACCTCATCATCTGAAAAAAGATTGTTCCAA
TCATTGATGGTTTTTTCTACTTACGAAATGCTTTCATGATTCTGGTCTTAGTCTAGAAATAATATTTAGTGT
GCTATGTCATGGAACTAATACTTCCCTATCTTGGTAGACACTATCTTATCACTATACAATTTGAAATTGCA
CTAAATTTACATGTCTGGTCAGTACAGGTTTCCCTTTTTCGTTCCCATTTGTTACTTAGGAGATGTCTCAAA
ATATAAAGAGCGTCATATACATTGGGATATTTAGCATATTTGGTCATGCAAATTGCACCAAAGAAAAACT
TTACCTTTTTTTTTTCCCAAAGAAAAATGTATAAAGTGAATTGGGTAGATTTCTGGTAATCAATGTTTCAC

CTTCTGTTCTGCATCTATGTACTATGTATTAATAAACTTTCTTCCTCTTTGAATGCTTTTCTTCCACAGTGTG

CTTTCTGAAAACCGTCTTTTTGGTGCTATTCCTAAAGAATTTGGACGCCTCAGAATGCTGGAAGTGCTTG

ACATTAAGAACAACAATTTGACTGGAAGAATTCCAGCAGTAATAGGAGATTTGCAATCACTTAGAAGCT

TGTAAGTTGATAATTTGTTGTGTTTCCGTCTTATAATATATTTGTATGTATTTATGCTACAACATTGCTAAC

AAAATGTTTTTGAATTGTGCTTGATACTTCTTTTCCAGGTTGATTAGGGACAATAACTTCGAAGGGAAGA

TCCCTTTGGAGATCAGAAAGCTTCATCTGCTCTCGGAATTGCAATTTGATGATTACCTCACCTCTGGTGTT

GCTGCCGGAACTGGTTGCATAAATAGAAAATTTGGGCGTTGGTAAGACTTCCGTAGTTGTTTGGTCCTTT

TATCTTCATGTTTCAGCTGTGCTGCATTTTAAAGTCGCAGACTTGTTTCTATGTTCAATTATATTGAAAATG

CATAAAATCCATCATTTGAGGAACATATACCAATATACTACTTTCATACTAACTACCTCCTGCTATAAGTTT

GGTATTAACATTAAATTAAGTGCAAGCAATTATCCCATGGAGTTGCACTGAAGATCTTTGAGACATTTTC

TATGCATAGTGTGTCCCCACTAATCTCTCATACTTTCCTTACAATAGTGAAGCAACTATGATGGTGATTGT

CGAAAGCAATGCCATGGGGCACTTGATACTTTCCTGTCTCCAACCCTAATATTCATAGGGGATTAGAGTT

TAGTGTTTCTAAAGTTGTGATTCATCTTCTTATTCATCAAGATCTGTTTATGTGGAAATTTGACTGATCTAT

TGCATAATATTTGTTTATTTTGTCGACTTTTCTGTACATTAACCACTAAAATTGCTGGAAATGTGGTTACTC

TTTCCCATGAGTAGAAGAACACTTGGATATTATCATATTCCTGTTTCGTAGCATGAAACTTCAGCCAGCTA

TATGAATAAATTTATTCTATTCATCCCGTTGACTCTGTTTGAATTCTGGATCGAGATACTCATCTTGGACA

AGTACCTTCAGCTTTACCCTTGTAACTGATGTCAGATTACAGACTACCTCTTAATATGCTAATGTTTTATTT

CTCAATCACGCACTTTTAAGTTCCATACTCCGCTCTGAAAATTTGGTGCCTTTTTCTTGCAGCATCTGGCAT

GGCAGTTTGTGGCCATATAAGACTATAGGCTCCTTTCTCAGACCAATTAAAGGGATGCTAGCATGGTATT

TTAGTTTTTTCACACTGTAAGTAACTAAATAGGTTTTTTCCACCTTATGCTTTCTAAATAAGTTTAATGGTT

CAGAAGCAACTTTTTTGTTTCCTAAAAGGTTCCCGCGTTTTTTGGATGCTCAGGCAGACTTCTGCTCTGAC

AACCTACCAAGTGAGTGAGAATATTTTGTGACATATCTTTTATTGGATTTCAAAGAATTGTACACAAAAA

AAAAAAAAGTATTTTATTCTCTTTTGTTTTTGCAGGTTCAACTAGGCCTCACAATATCCACGATGTAGAGA

ATCAAGCAAACATTGAACGCCGTAAGTTAGCTGAGCAATCCAGTAACTTTGCTGCTGCTCCTGCTAACGG

AGGGAAACCATTGGGACCTGTTGATCCTGTTCCAAGCAGCAGAAGTAGTGGTTCATTTCGTGCTGTGCC

AAGTACTGAAGGAGCTCCTCCTCCTCCTCTGACTACACCATCACGGACCACACCTGATCATCATCCGCCTC

CAAATCCTGGGGGACACTCTAATGATGCTGTTAAACAGCCAATTGCTAGTCAAGCCCCACCTGGTGGAA

AGTCTGGAAGTACATGGAAGTATATAGGAATTGGCATTGGGGCTTTTCTGGTTGCTGTTTTTGTGTGCCT

GATAATCATCTGCAAGAGCAAAGCTGCGCAAACAATAGGCCCTTGGAAGACTGGATTGAGTGGACAGC

TGCAGAAAGCATTTATTACAGGTACTTTTGATTTAATATTGTGTCAAAGCTGATGAATCCATATGTTCTAG
TTCAACTGTTGGATTCATTTTTCTCTGGTGGATATAATCCGAATCTTCAACGCCTACAATAGTTTTTGTTTC
ATACTCCAAGAAGATGTTAAAAGCATGTATTAATACTCCTGTGCTATATTGTTTCTCATGTCTTATGCATG
AAATCAATTTTGCAGGGGTCCCAAAACTAAACAGGCCGGAGCTTGAAAATGCATGCGAGGATTTTAGCA
ACATAGTTTGCCATCAAGATGCATTTACAGTCTACAAGGGAACTCTATCGAGTGGAGTAGAGATTGCTG
TTGTGTCAACTGCTATAAATTCTTTGAAAGATTGGTCCAAGCGTTCTGAATTGGCCTTCAGGAAGAAGGT
TAGTAGTTGCCATCTCCTTCACAAATAAAAAACTGCTTCATAACTTATTACCATCTCACTCTTTCTGTTGAA
ATATCCTAGTCGTCTCGGTTTTTTTACTTATAAGATTTCTACTTATTGTGCGCTTGGGTAGATCGATTCACT
CTCAAGGATAAACCACAAGAATTTTGTTAATCTAATTGGATATTGCGAGGAAGATGAGCCTTTCACTAGG
ATGATGGTCTTCGAGTATGCAACAAATGGAACACTTTTCGAGCATCTGCATGGTAAACTAGTTAATAGTT
CAAGTTTCAAGCTTCAAGCTAATCATTTTTGTGATTTGGTTTTGATTAATGCAGTCTACATGTTTTGTTGTT
TCAGATGATGAACTTGAACCTCTTGACTGGCCTGCAAGGGTACGAGCTATAATGGGAACAGCATATTGT
CTCGAGTACATGCACAATTTGAATCCTCCTCTGTCGCATTCTGATGTGAATTCACACTCCATATTTTTTACA
GACGATTATGCTGCTAAGGTATGCTTTATTTCGTATACCACATTGAATTTAGAAACGTTTTACTTCGTGTA
ATGTCTTGAATTGAACGGATCTTTTTTTATTTACACAGATTACAGAGCTTGCTTTCTGGTCAGATATAATG
GTGAAGTCAAAGTCCTCTAGTGGTGATCTAGAGAACTCTGAGCTGCGACCACTTTCTGATCCCGAAACA
AACGTCTATAACTTTGGGATTTTGTTGCTGGAAGTAATTTCTGGAAAGTCACCTTATACGGAACGAGATT
CTCTTATGAGCTGGGTATCTATCTAACTCTATTTTATACGAAAATCCCTTATTTTCTTCGGTGTTTAATATA
GACTAGTGAAACGTTCTTCTCTGCTTTTGTTTAGGCGGAGCAATGCCTTAACGATAGACAGTTGGTTGAT
CCAAAATTGAAGTCGTTCAAGAATAATGAGCTTATGGTTATGTGTGAGGTGATGAGAGAATGTCTTGGA
GAAGATTCAAGGAAGAGACCAACAATAAAAGAAGTGATTAAAAAGCTAAGGGAAGCGATTGATATCTC
CCCTGATGCTGCAGTTCCAAGGCTATCTCCCCTGTGGTGGGCTGAACTTGAGATCCTATCCTCTGAGGCA
GCCTAATATACTGACTATTGTGTCTACTTTTGTATGTAAGTAGAGAGTTGCTTTGCTATGCATAAATGTTC
TGAATTATACTTCACTTAATGTCAACTGCAAGTCCTGTGTACCACATTTTTCTGTTCTAAATATATTAGTTC
ATTCTTTT
>PGSC0003DMG400010348 LRR-RLKs
ATGAACACCAACATTATTCCTAGTACCTCAATACAAAATTCTGAGGATATTTCAGAATTACCTATGACCAC
CACCAACATCTCTGGTTATTCATCAACGGATAAGAATTCTTCGAAAATAACTTATCCAATTACCGTTGATG
ACTATTCGTTATCTTATATATATTTCATGGGATGCAAAGATAATACTAGTGAGAAATTACAAAATCACTCC

ATCAAGTCTAAGTTGATGGTTGGTCAGCAAAATGGAATTTTGAGTTCCTTGGATAGTGAATTCCCACAAA
ATACAAGGTTTATTTTCCTTTCCCTATTTTAATTTCAAAAATACATATGTTATTGATGATATTGCCAATTATT
ATTTTTTTATTACATATTTTCAAACTTTTATTTAATCAATTCTTAGATGTAAGTAAATTTTGTTTTTGTCATTT
TGGTTTAGCAGTAGGAATGGCGGTCCTATAAGAAGGTCACCTAATAGGACAAGCTCAAGATACCAACCA
TTTGGCAAAGAAATTGATCCAAACAATCAAGGATTAGATAGGCATGTTTTCTCTCTTTTATTCTCCAAATT
ATCATCAATATATTAATTAGTAATATTGTATCTCCCATTTTCAATCACATTAGTTTACATATTATATGAGTG
TGTTCGGTACGGAGGAAACATATGGTTTTCAATTATTTTATGTTTGATTAGTGGGAATATTTTCTCATGTT
CTTTGAAAATGAGAAAAATGATAAATAGTTGCATCTATCATATATATATATAATAAAAGATCTCTTTAGAC
AATGACAACGCTCAATATAATTCATCTCATCTTGTTTATATTTTTCAGGATTCGTCAAAACAGTTCAGAGG
CGATCTTCAACACACCACACAATTTCAATGAACATACTCAAGGGTATGGCTATTCCAATGAAGCGATTAC
ATTGAGAAGTTTGAAGAGTAGGAGAGAAAATGAAGATGAAGCTCCTGCTCAGTTACAGTCTTCTTCAAT
ACCCCAACCAACTTGCCTCTATATTGAATCTGCCTAGTTCGCAAATAAGGCTCAAAATACTTGTACAATGT
GTCTTGGACGTTCAAGATATATCATTTTTAGAACAATGTTTTATTGCCTCAAACAGTATATATATATATGG
AACAAAAAATATGGAAAATAGTGTTCAGCAAATAAATATTAATCCTTTTATGTTCGATTCCTCTGTGTCTT
CATTTCTTCGTCATTAAATGCAACGTCGCCACAACTAACCAGAAGTGATCATAGGACATTTAGGTTGACA
TTTTTGGCAAACATCTCATAAAGGCAATATTAGCACTGACACTGACGAAGCAAGAAATGTGATAACTTCT
CGTTGTTGGGTTAGGATGTTGCATAATTTGTCACCTCTATATATATTGATTCTACAGTTACTTCTTCGAAC
ATTGCAAGGTGAATACTACTGGATATATGGATCGTAAGACAATTGTGTTTTACGGTTTAGTGTCGAGGT
GAGTGTCATGATGTCAGATTTTGTCAGTGGCTTACACTTACATACAAGTAAATGATGTGCTAAACCTATT
AATTATTACCAATCTCAAATAGCATTGATGAAAAAATTCTCTCAAGTTTCATAAACAAACATACAATGTTT
TTGCACTGTATTTTACTGACAATAAGAAGATACTCCTATGTGAAACCGTCATCAGCAATGAAGGCTCTGT
TGTTAAGAGAATTTGTGCTTTCCCGATCTAAGAAAAACTTGGACCAATATGTTGATTTTGCCACATCAAA
CCATAATTTATACTGTAAACACCCAAATAAAGCCACAGGAACACCAACACAAGCTGCAATGAGCTTTGGT
TCCCAACCCATATGATTACTATAGACCAAAAAGAATGTTGCTGCAAAAGCCAAGAGCATGCTAACTATCG
AGACAAACAGCATTGTGAGTCCAAACAATAACTTGGCTGGCAACGACACAAGAAAATCATCTTCAGCAT
AGTGGGATGTCACAATGGACAAGAACATAATGATGGAAACTATTGAGGTGAATAGTGCCACTGCATCT
GAATTGACAAAGATCCTAAATCCATACAATTTTAGCAATATGGGAATGCAATTATCACTGTTATTACCGC
CTGGCACAGTTAATCCAGCAGCAAAGACCATGGTGGCAATCAAAGTAGCGACAATCATACAAGAATTTG
CAGTGTCTTTCATCCACCTTTTCCCTTCTTTCAGTAATAACTTATGCTCTTCTGTGAATATTTCTCTAGGGG

TTTTCCCATCTTTGTTTTGTTAGTGCAAAGTTTTAATGATAAACAACTTATTCTTGCATCAAAAAGGAAAG
AATCAAGAATGTGTACATCCTTGACTCTGTAAAAAAACAATGGAGGACATATATGCCTAGCTTCAATGAC
TGATGATCCCTGGATTTGGCACAAGAATTTGGCCATGCAAGCATGCGACTAATAGAGAAATTAGCTAAA
AATGAGCTTGTAATTGGACACCACCACCACTTTTGTCTTTTTAGTTATTCCAAAACTATCCTTGAAAATAA
AAATAATTCAAAGTAAAAATATAAATATGAACAATTAACTCTCTTGAACTTATCACTTAGAAAAGGTAAA
TGAAAGACAAAAATGGAAGAAATTTTTAACAACTCTCTTGAACATTGAATAATTGAAAAAATGTCAAAA
GTTCAAATAGTATGGACTAGATGGAGTAAAATATATAGTTTATATTTTATTAGTCCATATATCATAAAACA
ATTTTTTTTTTTGTTTCTAGTCAATAGTAATATGAATTTTACACATCATATTAGGCCTCTTATTGTAATTTGA
AGTAATTCCGTTGACCCATCCCTGTGTAACACACTTGTGGAGATGTGTAACACATTATGAAGGGTAGAG
AGGACTATTGATAGACCCAAAAATCACGCCAAAACTAATGGCAGTATTCAAAATGCAAGGCCACAAGTA
GTGATCAGAAAGACCAAGATTCTTGCACATTTCATTGAAGGCAAAAAACATTTTCCCTGTAGGTCACTAA
TGCCATTTTCTAGTCTTCACTAAAAGAAGTTGTTGAACTCCAAATTGTCACTCTAAACCAAAAAATACTCC
AGTCATCAATTCTTGTTTTAGGAAAAAGAGAAACCTGTCAGTTATAGTTATGGGAAGAAGTTGCGATCTT
CTCTTTGCTCTTTCGGTTTTCATTCTGCTCCATACTTCACTATCTATTAGTACTGATGAAGCTGCTCTTCTTG
CCTTCAAATCTCACATTTCTTCTTCTAATCCTAACAACATCTTAGCAACCAACTGGTCTTCTTCCACCCTTGT
TTGCACCTGGATTGGAATCACTTGCACCTCCCCACACCATCGAGTCGCAGCTTTAGACATTTCTAGTATGC
AACTTCATGGTACCATTTCTCCACACCTTGGAAACCTCTCATTTCTTGTTTCCCTCAACATTAGTAACAACT
CTTTCCGTGGAGATTTGCCCAAAGAGTTGGCTCATTTGCAGAGGTTGAAATTGATTGATGTCTCAAGCAA
TAACTTCACTGGTGCCATTCCATCATTTTTAAGTTTGCTAGTAGACCTTTGCATTTTGTGCCTGTCGAGCA
ACCAATTTTCAGGAAAAATCCCATCTTCACTTTCCAATCTAACAAAGCTGGAGGTGTTGAAAATAAAGGG
AAATCTTCTTGAAGGAGAGATCCCTCGAGAACTTGGTGATCTTCATTACATGACTGCTTTAAACCTGGGA
AGTAACCACCTTACTGGCTCTATACCACCATCAATTTATAACATTACAACCATGCGAATCATTGCTCTTAC
CAACAATAATCTTAGTGGTGAGCTTCCAACAACTATATGTGACCATCTTCCAAACTTGGAAGGGCTTTGC
CTCTTAGAAAACATCCTAGGTGGCGTTATTCCACCAAACTTGGAAAAATGCAAAAACCTCAAAATTTTGT
CATTGTCTGTAAACGAGTTCACTTGGAACCATACCAAGAGAGTTAGGCAACATAACAGATCTTACAGATA
TAGCTCTTGGAATACAGCACTTGGGAGGTAGTATTGCATCTCCTAGCAAGAAATTTTGAATTTTCTTCTTT
TTCACTCTTCGACCAATTTGGTACTCATATATATTTATATTTCCCCACTTTACAGGAAAGATACCAGTGGA
GCTAGGTAATCTTAAGAAACTACAGATTCTGTCATTAAACGACTGTAAGCTTACTGGTTCCATCCCCGCA
AGCATTTTCAACATGTCAGCACTGCAGCTCCTAGGAATATCAGAAAACAGGCTTTCAGGTACTCTACCTT

CAGATTTAGGTCGTGGAATGCCCAACCTAGAAGGATTCTCTTGTTTTCAGAATAGTTTGAGTGGTTTTCT
CCCTGCTTCTATCTCAAAGTCTTCTAGACTCAGTGTACTTGAACTCTCCTATAACAGTTTTACAGGCCCAAT
TCCTGAATCAGTTAGTGACTTAGAATACATTGAGATTTTGAACTTTGGGGGGAATAATTTTTTCAGCAAT
TCAGCATTGAGCTTCTTGACATCTTTGACAAACTGTAGGAAACTGAAAGAAATCACTTTTGCCGAGAATC
CCTTGGACGGTTTTTTGCCTGCCTCCATTGGGAATTTCTCTGATTCTCTGCAAATTTTCCAAGGATGGTAT
TGTAAACTGAAAGGCTTCATTCCTGGAGAAATTGGCAATCTTACCGGTGTGATAAAGATGGATTTGTCAC
AAAATGAGTTGACTGGATATATTCCAAAAACCATCCAAGGCTTGTCAAACCTTCAAGAACTTTACTTAGG
TGGCAACATGATAAAAGGAACCATACCAGATGTTATGTGCAATTTAAATAAACTTGGAGCATTAGATTTG
TCAAAAAATCAAGCTTCTGGTTCCATACCACCATGCTTAGGGAACGTTACCAGTTTGAGGTATCTTTATCT
AGCTAACAACAGGCTGAACTGGACCTTACCTTCAAGCTTGGGGAGCCTTCAAGATCTCCTAGAATTCAAT
GTTTCGTCCAATTTATTAACTGGGGAAGTTCCTATAGAGATTGGAAATTTAAAGGTTGCAACAATAGTTG
ATCTGTCAAAAAATGATTTTAATGGTAAGATCCCTAGCACACTAGGGGGTCTGGATAGATTGATGAAAC
TTTCTCTAGCACATAATAAATTAGATGGGCCTATTCCAGATTCATTTGGCAAAATGCTGGCCTTGGAATTC
TTGGATTTGAGCATTAACAATGTTAGTGGTGAAATTCCAAAGTCATTAGAAGCTCTCGTGTATCTCAAAT
ACTTGAACTTCTCATTTAATGAACTCAGTGGAGAGATTCCCACGGGTGGTCCTTTTGCAAATGCCACAAG
TCAATCTTTCTTGAAAAATTATGCCCTTTGTGGCGACTCTAAGTTTCATGTTTCACCATGCGTCATCAAATC
TCCCAAGAGGTCAAAGAAAAAAAAGGCAATATTGGTTTTGGGAGTTGGTATGCTATTTCTTGCATTAGC
CCTCACATATGTATTTTTGAGATTGAGAAAGGAAAAAAAGAATGCAGGTCAAGCTGATGTGTCTCTGAT
AAAATGTCATGAAAGAATTTCCTATTATGAACTTGAACAAGCAACCGAAAGATTCAATGAAAGCAACTT
GCTTGGTAACGGGAGTTTCTGCAAGGTCTACAAAGGGAGACTAAAGGATGGTACTCTTTTGGCAGCAAA
GGTATTCAATGTGCAATTGGAGGGTGCATTCAAAAGTTTTGATACAGAATGTGAGATGTTGCGCAATCT
CCGCCACCGAAATCTGACCAAAGTCATCACCAGTTGCTCCAACCTTGATTTCAAAGCCTTAGTGTTGGAA
TACATGTCCAATGGGACACTTGATAAATGGCTATACTCTCACAACTTGTTCTTGGATTTATTACAGAGATT
AGCTATAATGATAGATGTTGCATCTGCAATGGTCTATCTCCACAGTGGTTGTTCAAATCCGGTGGTGCAT
TGTGACTTAAAGCCAAGCAATGTCTTGCTAGATCAAGAAATGGTTGGCCATGTCAGTGATTTTGGCATTG
CAAAATTGTTAGGTGCAGGGAAGACTTTTGTTCAAACAAGGACAATAGCAACCATTGGATATATTGCTC
CAGGTATTAAAACTTTTACATCTTTCTCATGTTTTCAGAATACCCAAAACATTAATTCTTTTCCCCGATATA
TATGATCAGTTTTAGCCTATTACTCGATAGTTAGGAAGCATTTTTGATCATTTTCAACCCATGATTGCAGA
GTATGGACAAGATGGAATGGTATCCACGAGCTGTGATGTTTATAGTTTCGGCATCCTGATGATGGAGAC

ATTTACAAGAACCAAACCAGGTGATGAAATATTTACTGGAGACTTGAGCATACGACGTTGGGTAAGTGA
TTCTTTTCCATATGAGATTCATAAGGTGGTGGATGCTAATTTGGTAGAGCTAGAGGATGAACGGATTGA
CGCAAAGATGCAGTGTTTGTTGTCTATCATAGAGTTAGCTTTGAGCTGCACTTTAGTGTCACCTGATGCA
AGAATTAGTATGGAAGATTCTCTTTCAACACTTCAAAAGATCATGCTCCAGTTGGTCAATAGTCACCGCT
AGGTGAAATTAGACCATCCTCTTATGATTGATGCTTGATTACCTATGTCAATTCTGCTATTTAAGATGAAT
TCAGTTTTGTACTTCATATTATGTACTTTTTCACTTTCTAAACATCACAGATTTCTTTCAACCATATGCTAGC
TGGAATGGTGATTTTCCAGCTACTGGAGTTCACAATTAGTCTTTCTAATCCATAATTAATTGTCTCCTGGA
TATCTTTCAGAACAAGTTTTATGACGTTGCTGATAAGAGCTTATGCCTTTTGGTGTTTTGATGATCTCCTC
ACAAATGCAGGGACCTGGTCCTCTGCAGAGTATGCTCGTCCAGTGTCGAAGTGCGGTACAGCTGTAAAA
GCTGTCTGCGCAAAGTGCGGTACAGCTGTAAAAGTTGTCTGCGTCAGAGAGTTGGTGAAAGCTGCAGA
GTACTTCACCAATCAGAAGCAAGGAGGTTGCTTGTACGTTTGGTAA
>PGSC0003DMG400010350 LRR-RLKs
ATGCAACTTCATGGTACCATTCCTCCATACCTTGGAAACCTCTCATTTCTGGTTTCCATTATAATCGACAAC
AACACTTTCCATGGAGAGTTGCCAAAAGAGTTGGCTTATTTACAGAGGTTGAAACTGATCTCGGTAAGA
AGTAATAACTTTACTGGTGCCATTCCAACATTTCTAAGTCTGTTACCAAACCTTCGCATTGTGCATCTTTCG
AGCAACCAATTTTTCGGGGAAATCCCATCTTCCCTTTCCAATATAACACAGCTGCAAGTGTTGGATATGTC
CAAAAATTTTCTCGATGGGGAGATCCCTCAAGAACTCGGTGAACTTCATCACATGACTTTGTTCAATCTG
GAAAATAACCAGCTTACTGACTCTATACCACCATCAATCTTTAACATTACAACAATGAAAAAGATTGGTCT
CACCTACAATAATCTTACGGGTAAGCTTCCAGCAACTATATGTGACCATCTTCCAAACTTGGAAGAGCTT
CACCTCTCAGCCAATCACATACATGGCGTTATTCCACCAAACATAGGAAAATGCAGAAAGCTCCAAATCT
TGTCATTATCTAGAAATGAGCTCACTGGAACTGTACCAAGAGAGATAGGCAATTTAACGGCACTTACAA
GCTTATACCTTGGAACTTTGCATCTGGAAGGTATTATGATACTTACTTTCATTTTTATCTTCTGCCAAATTG
GTACTAATCATGTATTTTCCTCACTTGACAGGAGAGATTCCAGCAAGTATTTCTAACATGTCAGAACTGC
AGAACCTAGGATTTGCAAGAAACAGGCTTTCAGGTAATTGTTGTGTAACAAAGAATCTGAAGTGTTTAT
GTAAATATATGACACGTGAGCCTAACTCAACCCTAAAAACTAGCTCATGAGGGGAGGATTCCCCAATTCC
ATATAATCTGACCACCTGTCTATTCCCCAAACAATGTGGGACTTTTAGATACTCTAACACCCCTTTCACAC
CCAGGTCCAACCGGAGCGTGGACCGGCAGCCCAAAGGGGATGGCCTGACCCAGATACCATGTAAAGAT
ACGACATCCTCTCCTCATGAACTAGCTTTTGGAGGAGAGTTAGGCCCATGTGTCATATCATACATGGTAT
TAGAGCCACGTCCATTCTCGTTTGGACTCCCAATATTCACTCCAGTTAGGCCTAGGCAAGAAGGGGTTGT

TAGAGAGGTTAAAAGTCCCACATTTGTTGGGAATGAACAGGTGGTCTGCTTATATGAACTTGGGCAATT

CTCGCCCATGAGCTAGCTTTTGGGATTGAGTTAAGCCCAGGTGCCATATCTTTACGTTCTCAAGTTCTAAC

ATGTCAACTCATATATTTCCCCCTCTTGACAGGAGAGATACCAATGGAACTAGGTTATCTTCAGAAACTA

CTGTTTCTGTCATTAGACACAAATGAGTTGACTGGTTCTATCCCTGCAAGCATTTTCAACATGTCAGCACT

GCAGATCTTAGGAATTGCAGAAAACAGGCTTTCAGGTACTCTACCTTCAGATTTAGGTCGTGGAATGCCC

AACCTAGACGGATTCTATTGTTATCAGAATAGTTTGAGTGGTTTTCTCCCTGCTTCTATCTCAAATTTTTCA

AGACTCAGAGTACTTGAACTCTCTTATAACAGTTTCACAGGCCCAATTCCTGAATCAATTAGTGACTTAG

AATACATTGAGGTTTTGAACTTAGGGGCGAATAATTTTGTCAGCAATTCAGCATTGAGCTTCTTGACATC

TTTGGCAAACTGTAGGAAACTGAAAGAAATCACTTTTGCCGAGAATCCCTTGGACGGTTTTTTGCCTGCC

TCCATTGGAAATTTCTCTGATTCTCTGCAAATTTTCCAAGGATGGTATTGTAAACTGAAAGGCTTCATTCC

CGGAGAAATTGGCAATCTTACCGGTGTGATAAAGATGGATTTGTCACAAAATGAGTTGACTGAATATAT

TCCAAAATCCATCCAAGGCTTGTCGAACCTTCAAGAACTTAACTTAGGTGGCAACATGATAAAAGGAATC

ATACCAGATGTTATCTGCAATTTATATAATCTTGGAGCATTAGATTTGTCAGGAAATCAAGTTTCTGGTTC

CATACCACCATGCTTAGGGAACATTACCAGTTTGAGGTATGTTTATCTAGCTAACAACGGGCTGAATTTG

AGCTTACCTTCAAGCTTGTGGACTCTTCAAGATCTCATAGAATTCAATATTTCGTCCAATTTATTAAGTGG

GAAAGTTTCTATGGAGATTGGAAATTTAAAGGTTGCAACACTAGTTGATCTGTCAAAAAATGATTTTAGT

GGTAAGATCCCTAGCACGCTAGGGGCTCTGGATAGATTGATGAAATTTTCTCTAGCACATAATAAATTAG

ATGGCCTATTCCAGATTCATTTGGCAAAATGCTGGCCTTAGAATTCTTGGATTTGAGCAATAACAATCTTA

GCGGTGAAATTCCAAAGTCATTAGAAGCTCTCGTGTATCTCAAATACTTGAACTTCTCATTTAATGAACTC

AGTGGAAAGATTCCCACAGGTGTTCCTTTTGCAAATGCCACAGGTCAATCCTTCTTGTCCAATTATGCACT

CTGTGGTGATTCTAAGTTTCATGTTTCACCATGCGTCATCAAATCTCCGAAGAGGTCAAAGAGAAAAAAG

ATAATCTTGGTTTTGTACATCCTTTTAGGAGTGGGTATACTATTTCTCTCATTAGCCCTCGCATATGTATTT

TTAAGATGGAGAAAGATAAAGAAGAATGCAGATCAAGCAGATGTCTTTCCGGTAAAAGGGAAATATGA

AAGAATTTCTTACTATGAACTTGAACAAGCAACAGAAGGATTCGACGAAAGCAACCTGCTTGGTAGTGG

GAGTTTCAGCAAGGTTTTCAAAGGGATACTTAAGGATGGTACTCTTTTGGCAGCAAAGGTATTCAATGT

GCAATTGGAGGGTGCATTCAAAAGTTTTGATACTGAATGTGAGATGTTGCGCAACCTCCGCCATCAAAA

TCTTACAAAAGTCATCACCAGCTGCTCCAACCCTCACTTCAAAGCCTTGGTATTAGAATATATGCCAAATG

GGACACTTGAAAAATGGCTATACAATCACAACTTTTTCTTAGACATGTTGCAGAGATTAAGTATACTGAT

AGATGTTGCATCTGCAATAGACTATCTCCACAATGGCTATTCAACACCTGTGGTGCATTGTGACTTGAAA

CCAAGCAACATCTTGCTAGATCATGAAATGGTTGGTCATGTCAGCGATTTTGGCATTGCAAAATTGTTAG
GTGCAGGGGAAGATTTTGTTCAAACAAGGACAATAGCAACCATTGGATATATTGCTCTAGGTATCGTAA
TTATAGATTAGTACTCAGTTTTAGCATGTTCCTCGGTAACTAGGAAGTTTTTTTGTGATTATCTTTTACCAT
GATTGCAGAATATGGACAAGATGGAATAGTCTCCACCAGCTGTGATGTTTATAGTTTAGGCATTGTGAT
GATGGAGACATTCACAAGAAGGAGACCAAGTGATGAAATATTTACCGGAGGAATGAATATACGATGTT
GGATTAATGATTCTTTTCCGAGTGGTATTCATAAGGTGGTGGATTCTAATTTGATACAACCATGGGATGA
ACAAATCGATGCAAAGATGCATTGTTTGTCATCTATCATGAAATTAGCTTTGAGTTGCACTGTAGTGACA
CCTGATGCAAGAATTAGCATGACAGATGCTCTTTCAACACTCGAAAAGATCAGACTCCAGTTTGTCAATA
GTCTCCGCTAG
>PGSC0003DMG400010351 LRR-RLKs
GATTAGGCAAAACACAAAACTATTCATATTGACTAATGTGCCCAGAGTTTATGTAATCAAAGTACTAAAT
ACTCGTTTAGTCTCATATTACTTGGCCTTGTTAACACTAGTAAAAGTTGACACTCTTTAAGTCTTAACCTAA
AGTATACTCCAAAAACTAATGGCAGTACTCAAAATGCAAGGCCACAAGTACTTATCAGAAAGACCAAGA
CTTAAATTTGAGGTTCAAGTTTCTTGCACATCTCATTAAACATAAAATACATTTTCCCTGTATTGTCGCCAT
TTTCTATTCTTCACTAAAAGAACTTGTCGAACTAACTCGATATTGACACTCTAAACCAAAATATACTCCAT
AGACTAATGTCAGTATATTGGTATTCGTGTGAATTTGATATCAATCATGACTATATGAAAAGTCCACATA
TGGTGAATAATGCCAAAGAAATATGACTTGAGAATTTCCATGGACTGGTTGAGGGGAGGAGACTGGAC
TGTTGACTCAGACTCACTAATACAACTTGTAGTACTAAAAAAATTTACACTCCAATGAATTTGATATCATT
AGATATACACATAATCTAAGATCATTTATTTGCCTCATCGATCAATTCTTGTTTTAGTAAAAAGAGAGATA
TCAATCTGCTATTAGTTATGGGAAGAAGTTGCAATCTTCTCTTTGCACTTGTGGTTTTCATTCTGCTCCATA
CTTCATTCTCTACTGTTCCAAATATTAGTACTGATGAAGCTGCTCTTCTTGCATTAAAATCTCACATTTCTT
TTAGTCCTAACAACATCTTAGCAACCAACTGGTCTTCTTCCACCCCTGTTTGCACCTGGATTGGAATCACT
TGCACCTCCCGACACCATCGAGTCACAGCTTTAGACATTTCTAGTATGCAACTTCATGGTACCATTCCTCC
ACACCTTGGAAACCTCTCATTTCTTGTTTCCCTCAATATTAATAACAACAGATTCCATGGGAATTTACCCC
AAGAGTTGGCTCATTTGCAGAGGTTGAAATTGATTGATGTCACAAGTAATAACTTAACTGGTGCCATTCC
AACATCTTTAGGTTTGCTAGTAGACCTTCGCATTTTGCGCCTGTCTAGCAATCAATTTTCCGGAAAAATCC
CATCTTCCCTTTCCAATCTAACAAAGCTGGAAGTGTTGAGAATAACGGGAAATTTTCTCGAAGGAGAGAT
CCCTCGAGAACTTGGTGATCTTCATTACATGACTGCTTTAAATCTGGAAAGTAACCATCTTACTGGCTCTA
TACCACCATCAATTTATAACATTACAACCATGCGAATCATTGGTCTTACCAACAATAATCTTACTGGTGAG

CTTCCAACAACTATATGTGACTATCTTCCAAACTTGGAAGGGCTTCACCTCTCAACAAACATCCTAGGTG
GCATTATTCCACCAAACTTGGAAAAATGCAAAAAGCTCAAAGTCTTGTCATTGTCTGTCAATGAGTTCAC
TGGAACTATACCGAGGGAGTTAGGCAACATAACAAATCTTGCAGAATTATACCTTGGATTGCTGCACTTC
GAAGGTAGTATTACATCTCCTAGCAAGAATATTTAATTTTTTTCTTTTTCACTTTTCCACCAATTTAGTACT
CATATATATATTTCCCCACTTAACAGGAGAGATACCAGTGGAGCTAGGTAATCTTAAGAAACTACAGATT
CTGTCATTATCCGGCTGTAAGCTTACTGGTTCCATCCCCACAAGCATTTTCAACATGTCAGCGCTGCGGG
CAGTCGTACTTGACGAAAACATGCTTTCAGGTAATCTACCAGCAGATTTAGGCAGTGGGATCCCCAGCCT
AGAAACATTGTTTTGTGCAAACAACTATCTGAGTGGATTTATATCTGCTACTATCTCAAATGCTTCAAGAC
TCAGTGAGATTGATCTTTCAATCAATAGTTTCACAGGTCCAATTCCTCAATCACTTGGTAACTTAGAAAAC
CTTGAGGTTTTGGACCTGCAGGTGAACAATTTTGTCAGCGATTCAGCATTGAGCTTCCTGACATCTTTGA
CACACTGTAGGAAACTGAGAGAACTCACCTTTGCTAAGAATCCCTTGGAAGGTTTTTCCCTGCATCAAT
TGGAAATTTCTCTGATTTTCTGCAATTTTTCGAAGGATGGACTTGTAAACTGAAAGGCTCCATTCCTGGA
GAAATTGGTAATCTTACCGGTGTGATAAGGATAAATTTTTCACAAAATGATTTGACTGGACATATTCCAA
AAACTATCCAAGGCATGCTGAACCTTCAAGAATTTTACCTAGAGAGCAACAAGATAGAAGGAGTCATAC
CAGATTCTATCTGCAATTTAAAGAATCTTGGAGCATTATTCTTGTCAGGTAATCAATTTTCTGGTCGCGTG
CCACCTTGCTTAGGAAAAGTTACCAGTATGAGATATCTTTCTCTAGCTGATAACATGCTTAATTCGAGTTT
ACCCGAAAGCTTGGGGAGCCTTCCTGATCTCATAGAGTTCAATTTTTCATCCAATTTATTGCATGGCAAA
ATTCCTATTGAGATTGGAAATTTAAAGTCTGCAACACTCATTGATCTGTCAAAAAATGATTTTTTTGGTAT
GATCCCTAGCACTCTAGAGGGTCTGGATAGATTGATTAGTCTTTCTCTAGCACATAATAAATTAGATGGG
CCTATTCCAGATTCATTTAGAAAAATACTGGCCTTGGAATACTTGGATTTGTCCAGTAACAATCTTAGTGG
TGAAATCCCAAAGTCATTAGAAGCTCTTGTGTATCTCAAATACTTGAACTTCTCATTTAATGAATTCAGTG
GAGAAATTCCCACGGGTGGTCCTTTTGCAAATGCCACAGATCAATCTTTCTTGTCCAATTATGGGCTCTGT
GGTGATTCTAAGTTTCATGTTTCACCATGCGTCATCAAATCTCCCAAGAGGTCAAAGAAAAAAAAGGCAA
TTTTGGTTTTGTACATCCTTTTGGGAGTAGGTATGCTATTTCTTGCATTAGCCGTCACATATGTATTTTTGA
GATTGAGAAAGAAAAAAAAGAATGCAGGTCAAGCTGATGTGTCTCTGATAAAATGTCATGAAAGAATTT
CCTATTATGAACTTGAACAAGCAACCGAAGGATTCAATGAAAGCAACTTGCTTGGTAACGGGAGTTTCA
GCAAGGTCTACAAAGGGATACTTAAGGATGGTACTCTTTTGGCTTCAAAGGTATTCAATGTGCAATTGG
AGGGTGCATTCAAAAGTTTTGATACAGAATGTGAGATGTTGCGCAATCTCCGCCACCGAAATCTGACCA
AAGTCATCACCAGTTGCTCCAACCTTGATTTCAAAGCCTTAGTGTTGGAATACATGTCCAATGGGACACT

TGATAAATGGCTATACTCTCACAACTTGTTCTTGGATTTATTTCACAGATTAGATATAATGATAGATGTTG
CATCTGCAATGGTCTATCTCCACAGTGGTTGTTCAAATCCTGTGGTGCATTGTGACTTAAAGCCAAGCAA
TGTCTTGCTAGATCAAGAAATGGTTGGCCATGTCAGTGATTTTGGCATTGCAAAATTGTTAGGTGCAGG
GGAGACTTTTGTTCAAACAAGGACAATAGCAACCATTGGATATATTGCTCCAGGTTTTTAAACTTTTACA
GATTCCTCATGTTTTTAGAATACCTGAAAAATATTAATTATTTCCCCATAAATATGATCAGTTTTCACATAT
TCCTCGATAATTAGGAAGCTTTTTTTTATATCATTTTCAACCATGATTGCAGAGTATGGACAAGATGGAAT
AGTATCCACGAGCTGTGATGTTTATAGTTTCGGCATCCTGATGATGGAGACATTTACAAGAATCAGACCA
GGTGATGAAAGATTTACTGGTGACTTGAGCATACGACGTTGGGTAAGCGATTCTTTTCCAGATGAGATT
CATAAGGTGGTGGATGCTAATTTGGTACAGCTAGAGGATGAACGAATTGACGCAAAGATGCAGTGTCT
GTTGTCTATCATAGAGTTAGCTTTGAGCTGCACTTTAGTGACACCTGATGCAAGAATTAGTATGGAAGAT
TCTCTTTCAACACTTCAAAAGATCAGGCTCCTGTTTGTCAATAGTCGCCGCTAGGTGGAATTAGACCATCC
CCTTATGATTGATGCTTGATTACCTATGTCAATTCCGCTATTTAAGATGAATTCAGTTTTGTACTTCATATT
ATGTACTCTTTCACTTTCAAAACATCACAGATTTCTTTCAACCATATGGTAGCTGGAATGGTGATTTTCCA
TCTACTGGAGTTTACAATTAGTCTTTCTAAATATTGACACTCTTAAGTCTTAACCAAAAAACACTCTGAAG
ACTAAACAACACTATAGGGCCTCCAGGTTGCCTGTAGCATAGCCAATACCATTTTCCAGTCCATAATAAT
GCAGTAACCTGTTCCTATCATCTACATATCCTTTCATA
>PGSC0003DMG400010400 LRR-RLKs
GGCATTTAAATCGAGCCTTCGATTGGAACCTCACCACATTTTGTCCAGAAATTGGAGCTCAGACACACAC
TTCTGCAGCTGGTATGGAGTTTCATGCTTGCACCAGAGAGTTGTAGCTTTGCAACTTCCTAATTTGGCCA
TTCAAGGCAGAGTTTCTTCAGAAATTGCCAACTTGACTCAACTTGCTCTTCTTGACCTTAGTAGCAATGAT
TTGAATGGCAATTTACCAAGTGAGTTGGGCTTCTTGCAACAACTGAGACTTTTTAATGTGACTGGGAATT
CTCTTGATGGAACTATTCCTCTGAATATAAGCCGTTGTCGTGTACTGCAAGAACTTCATTTATCAGATAAC
ATGATCAAAGGAAGCATACCTCAAGAACTTGGCCTGTTAAGTCAACTTAGAATTTTGCGTCTCAACGACA
ATAATCTGACAGGAAAGATTCTACCTTTATTAGGAAACTTATCAAAGCTGGAATATTTTTATCTGCATGA
AAATGATTTGGAAGGTGAAATACCTGATGAAATTGGTGATCTTACCAATTTGAGGCTCCTTTCCTTTCGT
GGCAATGATTTCACTGGAGCGATACCTGCTTCAATTTTCAATATTTCAAGATTGCAAATTGTGGATCTCTC
GATAAATGCCTTATCAGGGGAACTTCCAAGTGATTTGGGTAATTGTTTTCCTATCCTTGAACATCTTTTTC
TGGACAGCAACAGGATCACGGGGAAAATCCCGGTTTCTTTATCAAATGCATCAAAGATTACTCATCTCTT
CTTCACTGAAAATGATGTGCAAGGAAATATTCCAAATGAGTTTGGTAAGTTGCATGAACTAGTCTGGTTT

GAATTTGAATATAATCGAATTTCTGGTGCCATTCCATCCAGCTTATTCAATATTTCGTCTCTTGAAATCCTC
AAAGCCAGGCATAATTACTTAAATGGGCATCTTCCATATGATCTTGGTACTTGGCTACCTAACTTGCAGG
AGATTTTCCTTTGGAGATCTACCCTCTGCAATATGCAATGCTTCAAAGCTTTGAAAATCTTGAAGTTGCAA
ACAACAGTTTTACTGGACCGATTCCCATGATGTTAGGTAACCTGGTGAACCTTATAACTCTAAATCTGCA
AAATAATCTGTTGGAAAACAAGCCTGGAGCCACACAACTTGACTTCCTGAATTCATTGGTAACCAGCAG
GAATCTGGTGTACCTGATTCTTGAATCTAATCCTTTGAATGGAATTTTTCCAGAATCAATAGGAAATTTAT
CAAGCAATATCAGGGTCCTTGATGCAGCAAACTGTGGAATTAGAGGCAGTATGCCAACAAACATTGGAA
ACTTAAGTGGCTTGATTTTCCTGGGATTAGCAAGTAATAACATTGTTGGCAATGTGCCCCCCTCGTTTGTT
GGCCTGCAAAACCTGGAAAGGCTGTATCTTACTAGAAATAAATTAGAAGGGACATTTCCAGCTGAACTG
TGCAGCATAGGAAGATTAGGTCTACTGCATTTGGGAGAAAACAGACTGTCAGGAGAAATACCCTCGTGT
ATCGGAAATCTGACTGAATTAAGAGTAATGTCAATTGCTGCCAACAATTTCAGCTCAAAGTTACCTTCAA
GTTTCTGGAGATTAGTAAAGCTTGATGGTCTGAACATATCGAGAAACCTGCTGCATGGTTTCTTCCCTTCT
GATGTGGGAACTCTGAAAGCAATGAGCATCATGGACCTCTCTTTCAATAGGTTTTTTGGTGAAATACCCA
ATTCCATAGGGTCCCTTCAATATCTAGTTTCACTAGATATGTCAAGAAATGCATTCCAAGGTCCCATACCG
GACACATTTAGCGATCTAATAGTTCTTGAGGGTCTGGATTTGTCTACTAATGCTCTATCAGGTGTGATTCC
CAAATCTCTTGAGCTATTGCGAGATCTCAAGTATCTCAATGTTTCTTTCAACAACTTGCAAGGAGAAGTTC
CAAAGAAAGGGGTGTTTGCAAATGTTAATTACCGGTTCTTGATGGGAAATCCAAGACTTTGTGGAGCAA
TTGACTTATATATTCCTATATGTCCAGCTCAAGGTAGAAAGACAACAAGGAAAAAGTCCATTATTCTTAG
AACAGCAGTACCTGTTGCTGTAACATTTCTGATATTGGTCGCATTGTTTTTCACATGGATGATTTGGTCAA
GAAAAAAACATGTGATTGGAAACAATGAATCGGATTACCCTCCTAGAATTGCACATCAGAAAATCACTT
ATTATGAGCTTCTCCAAGCTACAGAAAATTTCAGTCAGTCCAAGCTAGTAGGAAGTGGAAGTTCTGGTA
CAGTCTACCAAGGTACATTTTCTGATGGTGCTGTCTTTGCAATCAAAGTTTTTGATATGCAATGGCAACG
GGATTTAAGAAATTTTGATTCAGAATGTGAAATTTTGAGCAACGTAAGACACAGAAACTTGGTCAAGAT
AGTGTCGACTTGTTCAAATTTGGATTTCACAGCAATTGTGCTGGAGTACATGCCTAATGGAAGCTTAGAC
AAAAGATTATACTCTGATGAAAATTGCTTGAGCCTGGTTGAAAGACTCAATATAATGATCGATGTAGCAC
TTGCCATGGAGTATCTTCATCATGATTATACAGTGCCCATTGTGCACTGTGATCTGAAGCCAGGTAATGT
TCTTCTTGATCAGGATCTGACTGCTCATGTAGCAGATTTTGGAATTGCAAAAATGTTGGCACAAGAAGGA
AACATGGCTCAAACCAAGACTCTCGGAACTATTGGTTACATTGCTCCAGGTATGATAATCATGTCTTGAA
ATGAAGGTGTCCTATGTTGGTTACTTTTTTCCTTTATGTCTACCATAACTTTATACTGTAACATGGATAACT

ACACATTTTAACTGGGAATTTGTTGAAACAGAATATGGTTTAGACGGGCAAATTTCCACTAGCAGTGATG
TTTATAGCTTTGGGATTTTGTTGCTTGAGACATTCACAAGAAAGAAGCCTACGGATGACTTGTTTAGCGG
AGATTTAAGCTTGCATAAATGGACATCTCTCTCCTTTCCTGATGCTGTACTGGACATTCTGGATGCTGACC
TTGTTAGCGACATTGGCTTTACAACAGATGAAAACCAAAGCGAAATCAAACAATTGCTGGTTTTGATAAT
AAATGTTGCATTCCTGTGTTTGAAAGAGTTGCCAGAGGAAAGGATAAACATGAGAGAGGTTGTGGTGC
AGCTAAACAAGATAAGAGCTGAATTAACCAAGCTTTAACTCAATCCCATTCAAAATTTCAGAAAGAGGAT
TGATGCAGCAACAAGCAAATTCAGTTTTCCACCTTCTATGACCTGTCAAAACATCTGTTATTTCTTAATATT
TTCTTTCTGTTTAGTGTAGGGGCATTTTAATATTATAAGAATGTTACATTTTAGCATTGATCGTTTGCCCCT
AAGTCAGTCATTACTCCCTCTATTCATTATTGCATTAGAATATGCATTGGTTGACTTTCATTTGTTGTAGTA
TGGTGGGAGTTAATGGAAATTCTTTTGCTGCCGTTTGGACCATTAAGTTGGTGCATTACTCCATAGAGTA
ATTGCATATGTTACTAATTCCTTGGTGCAATTGCAGCAGGTTCAGCCGTTGGTGGCTGTAAGCTTTTTTAT
CCCATTACTTCTTACTCTTTACTCCTCAATTATTC
>PGSC0003DMG400011160 LRR-RLKs
AAAAAATCTCTCTTCCCTCTCTCTGAGTCCTCTCACTTTCACTGATTTTCTTCATATCATCTCCACACCTGAT
TCTCTTACACCAAAAACACAGTACAAAAAAATTAGTAAAAAAAAAATATTTGAAAAAAAAACACAAACA
AACTCAGATAAAGCAAAAGGAGAAACAGAGTTGGTACAAATACCAACAAAATAGACAGACTGATAAAG
AACCTGTTTCTTTTCCTTTACACTCCCTCTCACTTTTTTTTTTAGTTTTTGACTGAAAAAAGACACAATTTTT
AATCATTTATATGAACCAAAATGTTAAATAAATTCATGGGGTTTGCTTAATATATGTGTTGAATAATTTGA
TTTCACTTTCTTGGGTTTTTTTTTTTTATCGTCAATGGCTTCCATTTTCTTGATTTTTGTTCTCTCTTTGCTTC
TGTTTTACCCTTTTCTTTGTTTTTCATATGAAGCTAGGAATCATGAAGGTAAACAAAAAAAAAGAATCCCA
TTTGCTTATTTTTCAGTTTTTTGTTTTCAAAATTTGATCTTGTTGTTTTGATGTATAGTGGAAGCTTTGATTG
CTATAAGGAGAAGTTTGGATGACCCAAAAAGAGCTTTAAGTAATTGGGATGAAGATTCTGTTGATCCTT
GTAGTTGGGCTATGATCAGTTGTTCTTCTGAAAATCTTGTTATTGCACTGTATGTTTAATAGTATTTTTTTT
TTTTGGAATTTTATGGTACTATATGATTATATTGGTTGTTAAAGATTGAAACTTTTACTGTTTTATCAACTA
AAAAAGTGTTTTTTTTTGTTTGTAGAGGAGCTCCAAGTCAAGGTTTATCTGGGGTTTTGTCTGGGATGAT
ATCAAATCTTACAAACCTTAAACAAGTGTAAGAGACAATTTTTTAATGTTTTTGTTGTATGTAATTTCAAA
TTTTGATGACCACATTTGGTTAATTTTCTTGTATGTTGAAAATTGTGTAGTTTATTGCAAAACAACAACAT
TTCTGGGCATATTCCAAGAGAGCTAGGGAAACTTCCTAATCTTGAAACATTGGATCTGTCTAATAATCAT
TTCTCTGGTCATGTTCCTGATTCTTTGGGTCTGTTAAACAGTCTTCAGTATCTGTAAGTGTATTAAACTTTC

AAAATTTTCATTTTTGTGTGATTATGATTATGTTTTTAGAGTTGAATGGTCTAAAACACACATGCAGTATC
ACGTTTTTGCGAGTTTGCTACGAATTATCACCTAAAACATATCTCAACGCCATCTCATAGTTGTGTTTTGA
TAAATAGTTGGTGATGGTTTAAGAAGGAAAATTGAACACATGATAGTTTAGGTAGAAAACGTGCGAAAT
CTTGATACTTCAAAGTGTGGTTTTTACCATTATCTCTTGTTTTTCCGAGTACCCTTTTTCCTTTAATTGTTGT
TTTCTCATGTAGACAATGTTTAAGTGCTATCTTATTCTTTGACTATTATGTTTTTGTTTTTTGTTGATGTCTT
GTTCTGGGGTTTGTCTTGTAATGGAGTAGAAGGTTGAACAACAACAGCTTGTCTGGTGCTATTCCTCTTT
CTTTAGCCAGGGTCCCTCAGCTTGCTTTCTTGTGAGTATTGCTTCTTTGAATCTATGACTTTTGTTTGATTT
TGTTTTATTTTTTTGTCTGATTGTCCTGAGAATTGGAATTATCTAGTCACTTTCTTGAATCAGCCTTATGTT
AAAGCTTTTTTTGTTTAAAGGAAATTGAATAGTTTGTTCTGTTTAGGGGTATTATTATATTTTAACTTATTA
TTCACTTTTATTTTATTTAGTATGACGATGATATGCAATGAGCTTAAATGAAAGTGGGGATTCATATTGTT
GACTCCAACTTGTTTGGGACTGAGGCATTGTTGTCGTCGTCGGATAGTGTTTTTTTTTTTTTTTGTATAAA
GAAATGATTAGTGTGTTCTGATTAGGGGTATTATTATATTTTAACTTATTTTTTACTTTTATTTTATTTAGT
ATGACGATGATATGCAATGAACTTAAATGAATGTGGGGATTCATTATGCGGACCCCAACTTCTTTGGAAC
TGAGGCGTTGTTGTTGTCAAATAGTGTGTCTGTTTTGTGTAAAGGAAATGAATAGTGTGTTCTGTTCTGG
GTTATTATTATATTCCTACTTATATTTCACTTTTATTTAATCTAGTGTGACGATGATATGCAGTGAGCTTAA
ATGAAAGTGGTTATTCATATTGTCGACGCCAACTTGTTTGGGACTGAGGCGTTGTTGTCATTGTTGCCGT
TGAATAGTGTGTTCTGTTTAGGGGTCTTTTTTATCTTCTAACTTATTTTTCACTTTTATTTAATCTAGTATGA
CAATGATATGCAATGAGCTTAAATGAAAGCGGGGATTCATATCGTGGACTCTAACTTGTTTGGGACTGA
GGCGTTGTTGTTTTTGTTTTCATTGTTGAATAGTGTGTTCTGATTTTATGAACCTCTTATTCTTTCAGGGAC
TTGTCTTTCAATAATCTGAGTGGACCTATTCCTAAATTTCCTACGAGAACTTTCAAGTATGGATTCTCTGC
CCAACTCTTATTATGTTACTTATAATTGTGTTCTTTTGTACATACAAATTGTCTTGTTTAAGTTCTAATTGTG
ATTTTTCTTTATCCTTGATGTTTTCGTAGTGTCGTGGGTAACCCTTTGATATGTGGAAATCACTCTGCTGA
GACTTGCTTCGGATCGGTTAACCTGATGCCCCTTTCGTTCGACATTGATTCCACCGGTAAAGTTTAGAAA
AGTTTCTCATGATTCAACAATGTTTAGACATAAATACAATTTAATTATCTTAAACTTCTTTCTGTTGTAACC
AGGTAAGCGAAACTCGAAAAGACTAGCCATTGCTATAGGACTTAGTGTCAGCTTCATCTCTCTTTTCCTCT
TGGGATTCGGATTCATAATTTGGAAAAGGAACCAAAACAGGAAACAGTCTATTCTGAACATTAATGGTG
AGTCTTAGTAGCATTTTGTGTATTTGATGACGGTTCAAAGTTATTTACGTTTCGTATTTTTGTTCCAGATAT
GCAAGAGGAGGATCTAGTTAGACTAGGGAATCTCAGAAGCTTCACATTTAAAGAACTTCAACGTGCGAC
AAACAGTTTCAGCTCTAAGAACATTCTTGGTGCTGGAGGATTTGGCAATGTTTACCGTGGAAAGTTGGG

GGATGGCTCGTTGGTCGCGGTGAAACGATTGAAAGATATTAGTGGAACTGCTGGAGAGTCACAGTTCC
GGACGGAATTGGAGCTTATAAGCTTGGCAGTACATAGAAATTTGCTCAGATTAATTGGTTATTGTGCTAC
CCCAAACGAGAGACTTCTCGTGTATCCTTTCATGTCTAATGGCAGTGTGGCATCAAGACTTAGAGGTAAA
CACCTTGTGTACTCTTTTCAGCCAATCGCATGCCTTACACATTGTACTCCTTCGTTTCTATTCGTTTGTCTG
GTTTTGATTTGACACGGAGTTTAAGAAAGTAAAGAATGATTTTTGAATCTTGAAGTCTTAAACTAAAGAT
ATGGAGAGTGTACCAATATGTCTTTTAATCTTGTGGTCTTAAACATGCCATGTGGTCTTAAAAAAATTGA
TGGAATGTACACATTCTTAATCCATTTTTCGCGATGACAGGAAAACCAGCACTAGATTGGAATACTAGGA
AGAGAATTGCAATTGGAACTGCAAGAGGTCTTCTTTACCTCCACGAGCAATGTGATCCAAAGATAATCC
ACAGAGATGTGAAGGCGGCAAATGTCCTATTAGATGACTTCTGTGAAGCTGTTGTCGGGGACTTTGGCC
TTGCAAAGCTCCTCGATCATGCTGACTCTCATGTTACCACAGCTGTTCGTGGAACAGTTGGCCATATAGC
GCCAGAGTACCTTTCCACCGGTCAATCATCCGAAAAAACGGATGTGTTTGGATTTGGTATCCTCTTATTA
GAGCTTATAACTGGGATGAGAGCTCTTGAATTGGGAAAAACAGTAAACCAGAAAGGAGCAGTACTTGA
ATGGGTATGTATGTTGCTTTATTCATCTACCAGTTATTGAATTCTATGTTGCTCGAACTCTCCAAAAATGC
ACTACTTTTGAAGGCTTCGATACGCATGTGTTTTCATTTTTGAAGAGTCCGAGCAACCAATATTTCCTTTA
AATAAAAGTGAAAAATGGAGCTCCAGTAGCATCACTTACTTAGTGATTTTTTAACCTTTGTTAGTCGGAC
TCTTCAAAATGTAGACTGGTGCATATCGAATCCTCGAAAAAACTAGTACATTTTTTGATAATCTGATACG
GATGCAACAACATTTTTGTAGAGTTCGAGCAAAGTAGGTTTTAACAAAAAGAGTCTTGCTTAGATTTTCT
CATGAACTGCTGCTTAACAACAAGTGTGGCTTGATTGCAGGTTAAGAAAATGCAGCATGAGAAGAACGT
CGAAGCTCTCATCGACAGAGAGCTTGGGAGCAACTACGACAGGATCGACGTAGGGGAGATGTTACAAG
TGGCTATACTCTGTACTCAATACCTACCGTCCCACCGTCCCAAGATGTCTGAGGTGGTCCGAATGCTCGA
AGGTGATGGCCTTGCTGAAAAATGGGCTGCATCTCATAACTATGATGGCTTTTCTCTAACCAAAAACAGT
AACAAAACTCAGACTAAACATTCTATGGTGCCTCTTGGATATGATGACAATGATCATGATCATTTGACCG
TGTTTGGGATGATGGATGATGACTATGACACACATGCCATGGAGCTTTCCGGTCCAAGATAATAGCGAA
ATATCATCGAGGTCTGGAGAGGGTACGATGTACACAAACCTTACCCCTATTTCTATGGGATAGAGATGC
TATTTCCGATGACCCTCGGCTCGTAATAGCAAAATATCCAAAAGGAAAAGAAATTGAACATAGGTACCT
AATTCTTTTCAAATTTTTATAAGTTAGTACCACAATTAACTCTATCAAAAAGCATGTTGGAGAGGAAAAA
GAAAGTAGTTCTTTTTTAAAATTTTTTTTTGTTGGTTTTCTTTAGATGGTGTGAAAATAGGAGTTTGCTAT
GTTTGATAAAAGTACACTTTTAAGGAGTTTATGGGACTAATGTAAATAACCCTCTTCATCAAAAGTATCA
ATCTTTA

>PGSC0003DMG400011205 LRR-RLKs

ATGTCTATGATTGTTTCTGATCCATTTCATGTATTAGAAAGTTGGAATTCTTCCAACATTCATGTATGTAAT
TGGACAGGAATTGTATGTGACAAGAAAATTCATAGAGTTGTTGAGCTTGATCTTAGTCATCATTCGTTAC
GTGGAACGATTTCTCCTTCTCTCTCTGGGCTCTCTTTCTTGCAGATTCTTGATTTGTCTGGTAACTTATTAG
AAGGACAAATTCCAGCTGAATTAGGCAATCTTGTTAAGCTAAATCAGCTTAGTTTGTCTTCTAATCTCCTC
GAAGGAGAAATCCCAAACGAGTTAGGATTCCTTCATGAATTGAAGTATCTTGATTTGGGAACAAACAAT
CTTTCTGGTGCAATTCCTCTGTCTTTGTTCTGTAATTGCTCTGCTTCATTACAGTATATGGACCTTTCTAAC
AATTCGCTTCGCGGTGAGATCCCTATGAATGATCATTGTGAGTTGAGTGGTTTGAAGTTTCTTCTTCTATG
GTCTAATGGACTTGTTGGTGAAGTTCCTATTGCTCTTTCGAAATCTACAAAGCTAGAATGGCTTGATCTTG
AATCGAATTCGTTGAGTGGTGAGCTTCCTTCTGATATTGTAAGTAAAATGCCAAAGTTACAGTTTTTGTAT
TTGTCTTACAACAACTTTGATAGTCATAGTGGTAATACTGACCTGACGCCTTTTTTCGCGTCTTTAGTTAA
TTCTACTAACTTGCAAGAACTTGAGTTAGCTGGGAATAATCTTGGTGGGGAGTTGCCTCCTATTATCGGT
AACATCTCCAAGAACCTTGCACAGATACATCTCGACGATAATCTTATCTATGGTCCTATCCCAACACAAAT
TTCGAGTCTTGTAAATATTACTCTATTGAACTTGTCTAGTAATCATCTGAATGGTACAATTCCTCCTGAGTT
GTGTCGAATGGGGAAACTTGAGAGGCTTTATCTGTCGAATAACTCACTCTCTGGTGTCATTCCTTCTGCT
TTTGGAAATGTTTCTCATTTAGGCCTTCTTGATCTGTCGAAAAACAACCTCTCTGGATTAATCCCAAATAC
CTTTGCTAATCTTCCGCAATTAAGGAGGCTTTTGTTGCATGATAATCACCTCTCTGGAACGATTCCCCCGA
GCTTAGGTGATTGTGTTAACTTGGAAATACTTGATCTTTCTCACAACAGAATCACGGGGACGATTCCAAG
TGCAGTTGCTGGATTGAGTAGTTTGAAGCTCTATCTGAACTTGTCTAGTAATTACTTACATGGTCCAATTC
CTTTAGAGCTCAGCAAAATGGATATGGTGTTGGCAATTGACTTGTCTTCGAATAATCTTTCGTCTAATGTC
CCTTCACAGCTTGGCAGCTGCATTGCTCTCGAATACTTGAATCTATCGCGTAATTCCTTAGAAGGACCTCT
TCCATCTTCGATAGGAAGATTGCCTTACCTTAAGGAAATCGACGTTTCATTCAATGTGTTGAGTGGGGAA
ATACCACAGACATTTCAAGCATCGTCGACTTTGCAGAACCTCAACTTCTCCTATAACCATTTCTCGGGGAA
CGTGACAGATACTGGTGCATTTTCATCACTAACCATGAGCTCATTCATGGGAAATTTTAAGCTTTGTGGC
TCAGTACAAGGGATGAAAAGTTGCCATAAAAAGAGGGGTAGACATTTCATTATCACAATCCTCCTTTCAT
TGCTTATTACTCCTATATTTTGCATAGTCGGGTACCCTCTCCTCCTAAGATCAAGATTCAGGAACCAATTC
GCGAGTTTTAATAAAAGCAAGGTAATTGAAGATGTGGAGCAAGGAGGTGGGAGGAAAGAGCAAAAGT
ACCCGAGGATATCTCGTATGCAGCTGATTGAAGCAACGGGAGGTTTTAGCAGCTCGAGTTTAATAGGAG
CAGGACGATATGGGCGTGTTTATAAAGGAGTGCTGAAGAACAACATGAGCATAGCTGTTAAAGTGATT

GACACAAAAGGAGGTGGAGAGATATCAGGGAGTTTCAAAAGGGAATGTCAAATCTTGAAAAGGACTA
GACATAGGAACTTGATAAGGATCATAACGACATGTAGCAGACCGGACTTCAAAGCTCTCGTGTTCCCTCT
GATGTCAAACGGAAGCCTAGAGAACCATCTGTACCCGAGCCATGGGCTGAGACATCAACTTGACTTAGC
TCAATTGGTTAACATTTGCAGTGATGTAGCAGAAGGAGTGTCGTATCTCCACCACTATTCGCCTGTTAAA
GTCGTGCATTGTGATCTAAAACCAAGCAATATCCTTCTTGATTACAACATGACAGCTTTGGTGACTGATTT
TGGGATTTCAACATTAGTAAAAGCTGTTGAGGAAAATACTAATCCTATCGACGAATCAGTTAATTACAAC
TCAACAGATGGATTACTTTGTGGATCAATTGGTTACATTGCTCCTGGTTAGTAACAAATAATAGTTCTATT
CTATGATTCTATCTAATTCATTTCAATTTATATGACTAAAGTTTCTGTTTCTTTGATTGTAGAATATGGAAT
GGGAAGGAGGGCTTCAACAAAAGGAGATGTATTTAGCTTTGGAGTTTTGTTACTCGAGATTGTGACAGG
TAAACGTCCCACAGACGTTCTATTTCAACAAGGCTCGAGCTTACACGAATGGGTGAAGAGTCAGTATTC
ACATAAACTCGAGGCTATGATTGATGAAGCATTACAAAGATGTACAACAGTTCAACGCGATAGCAATTT
ATGGCACGATATCGTGTTAGAGTTGATTGAACTTGGCTTAATGTGTACTCAGTACAATCCATCAACTAGG
CCAACAATGTTGGATGTTGCACTTGAAATTGGAAGGTTAAAACAGTATGTTTCAAGTTCTTCAAACAGTG
TTACTGAAGATGTCACAACAAAAAGTTGAACAGTTTTTTTTTAATATCTAACAATGTACCTTTTAACTTTA
TTCATTGTACTTCTATGTTCTTTTTTTTTTACCTTTTAATTGTTCCTTCTTGGGGTGCGGACCTTCTCTGGA
TTCTACGTGAACGTGGGATGGTTCGTGCACCAAGACTACCCTTC
>PGSC0003DMG400011509 LRR-RLKs
ATGAAAACATGGAATGCATCCACACACTTTTGCCATTGGTCTGGTGTAACTTGTGGACGTAAACATGTTA
GAGTCATTAAACTAAACGTCGAGAACCAAAAACTAGATGGCCCATTGTCATCCTTCATTGGGAACATGA
GTTTCCTCAGGTCTCTATACCTGTCGAACAATAGCTTCCGTGGTGAAATCCCTTCAGAGATTGGTCGTTTG
AGAAGACTACACCGATTGTATCTTGGTAACAACTCATTTCATGGAGAGATCCCCTCAAATTTATCAAGGT
GCTTGAATCTTGTATCCCTTGTCCTTGAAGGTAACAAGTTAGTAGGAAGCCTTCGTCCGGAGCTAGGTTC
CTTGTCCAAACTCGAGTACTTGTTACTCACTCGAAACAACTTGACAGGTGAAATCCCTTCTTCTTTTGGAA
ACTTAACATCATTGATAGGATTCTATGCACCATTGAATAATCTTCAGGGGAAAATCCCTGACTCATTTGG
GCAGCTGAAAAACTTAGAAATTATCGGAGTAGCTGCAAACCAATTGTCAGGTACTATCCCTTCTGAAATC
TTCAACATATCTTCCATCACAACATTTGATGTTGGAATGAACCAAATCCAAGGTACACTTCCTTCAAGTTT
GGGAATCACCCTCCCGAATCTTGAATTATTTATCATCGGTGGGAACAACGTTTCTGGATCGATCCCATCT
ACATTGTCCAATTCATCAAAGCTAGTGTATTTTCTTGCTGGAAGAAATCAACTTACTGGAAGTGTACCAA
GTCTTGAGAACCTGAATGAGCTTCAACAGTTAACAATTCCCGGGAATTATCTTGGAACTGGAGAATCAG

ATGATTTAAGTTTCATCGCCTCTCTGACTAACGCATCTCGTTTCAGAATTTTGGAGATTCAGTTCAATAGT
TTTGGAGGGGTTCTGCCTGCATCCTTCAGAAATCTTTCAACTGAGCTTCAGGTTGTGCAGTTGTCATACA
ACAGAATACGTGGTAACATACCGTCTGAGATTGGAAAGTTCGTCAACGTGGAAGAATTTCAGGTGAGG
GAAAACTTACTTACTGGTACTATTCCAATTAATTTTGGTAAAGTTAAGAAACTGCAGATTTTGGATTTGTC
TCAGAATAGATTTTCAGGGAATATTCCATCATCTCTAGGAAATCTTAGTGTAGTATCCATCCTTCTTCTGC
ACGATAATAATCTCACCGGTGAGATCCCAGCAAGTTTAGGAAATTGCAATTACATGATTGAGATATATGT
TGCTAAAAATAACCTGTTAGGACAGATACCTAAGGACCTTTTTGCTCTCTCTTCCTTAGTTGCTGTAGACA
TTTCTGAGAACCATTTAGATGGTTTCATTCCTCTTGAAGTTGGAAATATGATAAATCTTGAATATCTTAAT
GTTTCCGTAAATAACTTGACTGGTAAAATCCCAAGTACTATTGGTAGCTGTGTGACACTAGAAGCCTTAG
ACATGAAAGGGAACTTCTTTCAAGGGATTATTCTTCCTTCTTTCAGCTCTTTACGAGGTCTCCATGTTTTA
GACCTTTCTAGAAATAATTTGTCTGGCCAAGTACCAAAATACTTGGAAGATTTCAAGTTTCAACTCTTGAA
TTTATCATTCAATGATTTCGAAGGAGTGTTACCAAATGAAGGCATCTTTAAGAATGCAACTGCAATATCA
GTAATTGGGAATCCAAAACTTTGTGGGGGTGTGCCTGACATACATCTTCCGGAATGTGACATCAAAAGA
TCAAAAAAAATCGGTTCGAGGTTTATCCTCAAGATTGTCATCTCTGTTGTCTTTGGTATACTAGGACTTGG
TATGCTTGTCACTTTACTATTTTGCTTCTTGTTAAAGAAACCGAAAAGGGTGCCTGTTTCAAGTTCATTGG
GTGAGTCTCTCATAAATGTGTCTTACAGAAGTTTGCTTCAAGCTACTAATGGATTCTCTGAAGACAACTT
GATTGGTGCTGGGAGCTATGGATCTGTTTATAAAGGAACTCTCGATGGAGGAATGGTTGTCGCAGTTAA
AGTGCTAAACCTCTCGCGACATGGAGCTTCCAAAAGCTTTATGGCTGAGTGTGAAGTCTTGAGAAACAT
TAGACATCGGAATTTGGTTAAGGTTCTCACAGCATGTTCAGGAGTTGACTATCGAGGGAACGATTTCAA
GGCTTTGGTTTACGAGTTTATGGTTAATGGATGCCTCGAAGACTGGTTGCATCCTAGTCCAAGTGAAGAT
ACAAGTCAGGCAGCTGAAACAAAAAAGTTGAATATTCTTCAAAGGCTCAACATTGCTATAGATGTTGCTT
CTGCAATCGATTATCTTCATCTCCACTGCGAGACTCCAATAGTTCACTGTGATCTCAAGCCAAGCAACATT
CTTCTAGACAACCAATTGGTCGGGCACATTGGTGATTTTGGTTTGGCAAAGTTCCTTCAACCAACTGCAC
AAAATTCTTCTATTTCGGAAGGAAGCTCTGGTCTTGTTAGAGGAACAATTGGTTACACTGCTCCAGGTAA
CAATTAATACATAAACAAACACTCGAACGCCCTTTTTTTCTCTCATTTCTTCTATTTTTTCTTGAAAATGATA
AATTCATAGCAAAACCACACTGCTTAACCATAACTTTCTTCGACAGAGTTACTTTATCAATATCATAAACC
ATCTCTTAATTGTTACACACATACTGCCATTACTTTTATAAAATCAAGTCATACTAAGTTATGTCCCTATTA
GGAAAGTTATTGTTCCTTGTCTTTTTTTCTTTTGTGAATTTTAGAATATGGGATGGGAAGCGAGCTATCGA
CTTGTGGTGATGTGTACAGCTTCGGGATCCTTTTACTAGAAATGTTCACCGGAAAAAGGCCTACTGATGG

AATGTTTAGGGATGGTTTAGACCTTCCAAGCTTCGCTAAACATGCATTGCTTAATGGAGCTATGGAAGTC
ATAGATCCATCCCTTATTTACGGAAGTGAAGAAGACGAGAAAGGTAAAAGCACAAATATCTACCAGAAT
AAGGTGTATCTGGTTTCTGTTCTTCGAGTCGGGGTTGCTTGTTCTGCATATTCTGGAGCTGAAAGAATGA
ACATCACTGAAACTGTTTCCCAATTGTACTCTATCAAGGAAGCTCTTCTGCAATCAGAAAAAAATTCAGG
AGCACGTTGA
>PGSC0003DMG400011580 LRR-RLKs
AATAAAGGTTGAATCTTTCTCATTTTTATCCAACCCTTTTTCAAAATCTTGCATGTATATAGGAAGATCCCC
ACCATTATTGGAAAAATGTTGGAGGTTCTTGGTTTTAGATATAGTATAATCTTTGATTTTATCTTTTCCTCA
ATTCATGAGTTTTATGGTCTTTGCTCTGTCATCATCTAAAGGAATTACTTGAGAGTTCTTTGCTTTTTCTGG
GTTTTTGGTGGAATTCAGCTCTTTTTGACAAGGTGATGGGCTCAATAGCCTAGAGGTATGCTAAAGCTTA
GATCTTTAATGAGTTTCTTATTTATTCCTGAATTACTCCTTTTATTTAGTTGAGTTTCTGGGCATTTCTGAG
TTGATTGGATTCTTTGAAGTTAGTTGATTAATTGGTATGCATAGAACTTTTTCTGGTTTGTCTAGTTTATCT
AGTTAGTATTGTCTCTTCTTGATGATTGACCTTAAGGGTGTGGAGGGTAGGACTTGGATTAGGGTAGTA
GGTGGCAATGCGTTGTCTTGTTGTCCGTTCATATTAGTAGTCATACTATTGCTCATTCTGTTACTATCTATT
TGATTCCTTATACCTCGATTGTGTACTGTTTTGTTGTAGTTTTTGTTCTGCTATTCTCTATTGTTTTGCTATT
ATCTGTTGATTCTTGTACTTCCATTACTTCATTTTCTGGACTGTTTTTTCTTGAGCCGAGGGGCTACCGGA
AACAACCTCCCTACCTCTGAGATAGGGGTAAGGTTTGAGTACACTATAGTCTATACCCTCCCGAGTCCCC
ACTTTGTGTGACTACACTGGTTATGTTGTTGTTGTTGTTGTATTTTATAAGTATTGACACATAGTTGGATT
AACAGGTCAAACTGAGACTGTCTTATTGGAAGTTTGTTATGATGTCTGGAGATTTTGAATCAAGAAGTG
GTTTAGTTCTTCTAATTTGGATAAGTGCTTTGGTGGCTGCTGTACTATTGCTGGTTTCTCCTGCCGAAGGA
TTGAATCAGGAAGGAATGTACCTTCTTGAATTGAAGAAGAATTTCCAGGATTCGTTTAACCATTTGGGGA
ATTGGAATCCAAATGATGAAACTCCTTGTGGCTGGGTAGGTGTGAATTGCACTTCTGATTATAATCCAGT
TGTGCAATCTCTTTATCTGAGCTATATGAATCTTTCAGGCACGTTGAGTTCTAGCATTGGTGGTCTTGAAT
ATTTAGCTTATCTTAACCTGTCTTACAATCAATTCACCGGGAACATTCCCAAAGAGATTGGAAATTGCTCA
AAATTGCAGTCTCTCCAGCTCCATTTTAATACTTTTTACGGTCCGATTCCTGCTGAACTGTATAACCTGTCT
AATTTGAAGGATGTGAACATGAGCAGCAACATGATCTCTGGTCCTATTGCTGAGGAGTTTGGGAAACTC
TCGTCTCTAGTTACTTTCGTTGCATACACCAATAATCTTACCGGTCCAGTGCCTCGATCTATTGGAAGTTT
GAAAAATTTGACAATATTTCGAGTGGGGCAGAATGCACTTTCTGGAAGTTTACCTGCAGAGATAGGTGG
TTGTGAGAGCTTGGAATCTCTTGGTCTAACACAGAATTGCCTGGAAGGAAACATACCAAAAGAACTTGG

CATGCTTAGTAAGTTGAAAGAGCTTGTGCTTTGGGGAAATCAGTTCTCTGGTTATATTCCAAAGGAACTT
GGAAATTTAACACAAATTCAATTGCTTGCTTTATATCAGAATAACCTGATTGGAGATATTCCTGCTGAAAT
AGGTAAACTCAAAACTTTGACGAAGTTATACCTATACCGGAATGGATTAAATGGTAGCATTCCAAGGGA
GATTGGGAATCTTTCTATGGCAACAGAAATTGATTTCTCAGAAAACTTTCTGAAAGGAGAAATTCCAGTT
GAGTTTGGCCAAATAAAAAGTCTGAAATTGTTGTTTCTTTTCCAGAATCAGCTCGAAGGCGTTATACCAG
ATGAACTTACTACATTAAAGAACTTGATTAGTCTTGATTTGTCAATTAATCACCTAACAGGTCCTATTCCA
TTTGGTTTTCAGTATCAAAAGGAATTGGTTCAGTTACAGCTTTTTGAGAATTCTTTAACTGGTACTATTCC
TCAACGTCTTGGCATTTATAGTCGACTTTGGGTGCTTGATTTAAATAACAATCAACTCACAGGAAGAATT
CCTCCATTCGTTTGTCAGAATTCCAATTTGATTTTGCTGAATCTTGCATCAAATAAATTGCATGGATATATT
CCATCTGGTGTTCTTAAATGTGATTCATTGGTGCAACTTCGTCTGAACGATAACAGGCTAACAGGGACAT
TTCCTTCTGAGTTGTGCAAATTGATAAACCTGTCTGCTGTTGAACTGGGGCAGAACAAGTTCACTGGTCC
AATACCTCCTGATATTAAATATTGCCAGAAGTTGCAGAGGCTTGATTTCTCAGGCAATAGTTTCAACCAG
TTGCCAAGGGAGATAGGAAATCTTACCAGGCTTGTGACATTCAACGTCTCAGCAAATTCGCTCACTGGA
CCGATACCTCCAGAAATTCGGAACTGCAAGGCACTACAAAGACTGGATCTCAGCAAGAACAGATTTACT
GATGTTATACCTGATGACATTGGTAGCTTGTCGCAGCTAGAACGTCTTTTGCTTTCAGAGAATAAGTTAT
CTGGAAAGATACCAGCAGCATTGGGGAGCCTCTCTCATTTGACTGAGCTGCAGATGGGCAGTAATCTAT
TGTCAGGTGAAATACCATCAGAGTTGGGTAATCTTAGTGGCTTACAAATTGCAATGGATCTTAGCAACA
ACAATCTCTCTGGCAGTATACCACCCAACCTTGGTAATCTTATCCTATTAGAGTACCTATATCTCAACAAC
AATCATTTGAGTGGTGAAATACCAAGCACATTTGGAAACCTGACAAGTCTCTTGGGCATTGACTTCTCAT
ACAATGACCTGACTGGACCACTGCCGGATATACCACTCTTTCGGAACATGGATATCAGCAGCTTCATTGG
AAACAAAGGTCTTTGCGGTGGGCCTCTAGGGGAATGTAATGCATCTCCAGCTTATGATGCCAATAATCCT
CCTAGAGTCGAGAGTGCAGATTCTCCTCGAGCAAAGATTATTACTGCAGTTGCCGGTGTAATTGGTGGA
GTTTCTCTTGTTTTGATTGTGGTAGTTTTGTACTATATGAAGCAGCATCCAGTTGAGATGGTGGTAACCC
AGGATAAAGACATGTCATCTTCAGATCCAGACATTTATTTCCGTCCCAAAGAGGGTTTCACTTTCCAGGA
CTTGGTTGAGGCTACAAACAATTTTCAGGATTGTTATGTTCTTGGAAGAGGAGCCGTCGGAACAGTTTAC
AAAGCAGTCATGCAGTCTGGACAAACGATTGCAGTCAAGAAGCTTGCTTCTAACAGAGAGGGTAATAAC
ATCGACAATAGCTTCCGTGCAGAGATCTTAACTCTAGGAAAGATTAGGCATCGTAACATCGTAAAGCTAT
ATGGATTTTGCTATCATCAGGGTTCTAATTTACTTCTCTATGAGTATATGGCCAGAGGTAGCTTGGGTGA
ACTGCTTCACAGTACATCTTGCAGATTGGATTGGCCAACTCGCTTTATGGTGGCTGTTGGAGCTGCTCAA

GGACTTTCTTACTTACATCATGATTGCAAGCCCCGGATCATCCACCGTGATATAAAGTCCAATAATATTCT
GATTGATGAGAAGTTTGAAGCTCATGTTGGTGATTTTGGTTTGGCAAAAGTTGTTGATATGCCTCAATCT
AAGTCGATGTCTGCAGTTGCTGGCTCATATGGCTACATAGCCCCTGGTATGTCTTTCTGTTTTCTCCCGGA
CAACTTCTTATGTTCCCTTGTTATCTATAGAAATATGTCGAGAAATGTACTGCCATTTGATCCTTATTCCCA
CATATTTATCTGTATCTTCACTCTGAAAGTGTTACCTGTTTTTGTTGATATGAATAGGATTTTCTTGTTGAG
ATTAGATAGTTAGAATTGTATGCAACGTCTATTTCTTCGATTATCTGAAGACAACAATGAAATGCTTTTAA
CTTTTATCTGTGTATACTTCTCATTTTATATGTGTTCTATGCCAGGAGAAGCCATACAAACTGCTCTATGTA
TCTTAGGCGAACTTTGGGGACTCTTCCTGATACATATGGAGCATTCTTGTTCCCACATATTTATGTTCAGT
TTAGATATTCGACCATGTGTTGTTCACTTTGAAAATCTAATTCTTCTCTTTGTGATATGAAATGCATATCTT
GTTGAAGTTTGATAGTTAAAATTGTTTACGAACAAGTTAATTTCTTTGATGAAATGACAGAATACAAGAA
AAAATGCTTTTAACTTTTCTCTGTCTATGTAGCTTTTAATATCCCTCTCGTAATTTTCATTCTGTGTTATAAA
ACCATTTTTTCCTTTTCAATTGATGTTTTCTACCTTCTCTGTTGCAGAATATGCTTACACCATGAAAGTTAC
GGAAAAAATGTGATATCTATAGTTATGGGGTAGTCCTTTTGGAGTTGCTAACTGGAAAAGCACCCGTACA
GCCCCTAGAACAAGGCGGTGATCTTGTCTCATGGGTGAAGCATTATGTTAGGAACCATTCATTGACACC
AGGGGTACTCGATAGCCGATTGGATTTGGAAGATGTAATTACTGTTAGTCATATGCTTACAGTTCTTAAA
ATTGCTCTAATGTGCACTAGCATGTCCCCTTATGATCGTCCTTCAATGCGCGAGGTTGTTCTGATGCTGAT
TGAGTCTGATGAGCAAGAAGGGAACTTTCTCTCATCTCCAGTTTATGATCTCCCTCTGAAAGATAATTCCT
TCTAGATGCATGTAAGAAGCACATCTCAATTAACTTTGATAATTTTTTAGTACTAATTCTCCTTGTATTTTT
TTTCTTAATGGCTAAATTATAGATATTCCCATGTATATTTCTCATAGTGAAAATTTCATCAATCACTTTCTA
GAACTAGTTTGGAGGTGATTGATGTTATTTCAATAGTTCTCCATCTGATGGTTATGGAAAATAATATGCT
TTATGCTTTTCTCACTATATATTGAGGGCTACCTAGAT
>PGSC0003DMG400011833 LRR-RLKs
CTCTTCTTCTTGTTTCCCTTACTTTTCACTACTACTACTACTGATGCAAGTCCCAATGGGGAAAAAACTTTT
TCAGCCTTTGTACATCACTACTACTTTCTTGATTTTTCTATCTTTGATTCTTGTTTTCCCTGTTTTAGCTGTTG
ATCCTTTTACACAAGGACTCTTGAGCTTAAAAACTGAAATTCTTGATAATTCCAACAGTTTGAAAGATTG
GATTTTGCCTTCTGGTTCAACTGATAAAATTCATGCATGTTCTTGGTCTGGTGTGAAATGCAATGAAAATT
CTTCTTTGATTATTGGATTGGATCTTTCTGTGAAAAATCTTGGTGGGGTTTTGTCAGAGAATCAATTTAGT
GTTTTTTCAGACCTTGTTGAGCTGAATTTGAGTCACAACTCATTTTCTGAGAAACTCCCTGTTGGGATTTT
CAAGTTGAGAAATTTGAGAAGCTTGGATATTAGCAGGAACAATTTTTCTGGTCATTTCCCAAGTGGGATT

TCAAATCTTGATTCTCTTGTCATTCTTGATGCTTTTAGTAACAGCTTCTCTGGTCCTTTGCCTAAAGATGCT
TCAGAAATTGAGTCACTCAAGGTACTGTTAGGGTTTGCTTTGTTTTTTTGAGAATTATGGACAACTTTTCT
TCCACATCTGATCACATAACAACACACTAGCATTCACAAGTGTAGTCGTTAAAAGAGTCTTGTTTATAGA
TGGAGAGATAGTTCTCTCCATAGTTTCTATACTTTCTTTTAGAGTTTTTAATGGTCAAAAACAAGTTTGAA
TTATGTTTTTTCACGAGTTTCACACTCTAACTATCAATTTTAATACATAGATGGTAATAGTTCAGGTAAAA
AAAAGTGAACAACTAATAGTTGGATTGTGAAATTTCCAAAAATAGATAGTTTAGCCGCATCTTTCTTTTAT
ACTATTAGTTTTATGCCTGTTTTAGTATAGTTCTGTTTGTCTATTCATTTATCGTCGTTCAAGATTTGCTTTG
TTGGATTCTGCATGACGCGTTGTTATTTCGATCCCAACAGGTACTGAATTTTGCAGGGAGTTATTTCAGT
GGTCCAATTCCTTCTGAGTATGGCTCATTCAAGAATCTTGATTTCATTCACTTAGCTGGAAATTCTTTGAG
TGGTAAAATACCACCAGAATTAGGGATGCTGAAAACAGTAATTCATATGGAGATTGGTTATAATTTTTAT
GAAGGAACCATACCTTGGGAACTTGGGAATATGAGTAAGCTTCAGTATCTTGATATTGCTAGTGCAAAT
TTATCTGGTTCAATACCAAAAGAGCTGACTAATTTGACCAATCTTGAGTCACTTTTTTTGTTTAGAAATCA
GCTTAGTGGTAAAATCCCATGGGAGTTTGGAAAAATCATATCTTTATCAAGTTTGGATCTTTCTGATAATT
ATCTTTCAGGCCCAATCCCCGAGAGTTTCTCGGAGTTGAAAAATCTAAACTTGCTTAGTGTTATGTACAAT
GACTTAAGTGGAACTGTTCCTGAAGGAATTGCAAAGCTTCCACAGCTTGATACACTTCTTTTATGGGACA
ATTGGTTTAAAGGTTCACTCCCAAAAGACTTGGGCAAATATTCAAAGCTGAAATATGTTGATGTTTCAAC
AAACTATTTAGTTGGTAGCATTCCACCAAGTATATGTTCAGGTGGGATGTTACAAAGGTTGATCCTTTTTT
CAAATAATTTCACTGGTGGACTCTCTCCATCACTTTCCAATTGTTCCTCTCTTGTTCGGATCCGTATCGAAG
ACAATTTATTCTCAGGTGATATCTCATTGAACTTTGGCAAGTTTCCTGATTTGTCATATGTTGATATGTCTA
GAAATAGGTTCACTGGAGGGATTCCTACTGATATTGCCTTAGCTTCCAATCTTGAATACTTCAATGTGTCT
AATAATCCAAATCTTGGAGGCGTAATCTCGGAGAAGACATTGTCTTTGTATGCACTACAGAATTTCTCAG
CTACAAATTGTAGCATTTCAGGAGATTTTCCTCCTTTTGGACCTTGCAAATCCTTACTGGTTCTTGAATTG
AGTACGAACAATGTTTCCGGGATTCTCCCTCAAAGCATAAGCAACTGCCAAAATCTTGTGAGCTTAGACT
TGGCTAACAACAATTTAAGTGGTCAGATACCTGTTGGACTTGCTAGCCTTCCTGATATTAGTGTTGTAGA
CCTGTCACACAATAGTTTTAGCGGTTCTATTCCGGCCAAGTTCGGAAGTTCTTCCAGCTTGCAACTTCTTA
ATGTGTCATTCAACGATCTCTCAGGTTTGATTCCTTTTGATAAGAGTTTTAAGGTGATGGATAGTAGTGC
CTTCTGGGGTAATCCTAAGCTTTGTGGGACACAGTTGAGGCCTTGTCGTGGACCGAATGGACTGGAGTT
AGGAAGCAGAAAGACACAGAAGCTGGCATGGGTTCTCATAACCTGTGGAATCATAGTGTTGGCTATTAC
AGCTGCATTTTTCGGAGTATTTTACTTCAGAAGAAGAGGTCAAGGGCAGTGGAAAATGGTTTCTTTTAGT

GGATTTCCTCGGTTTACAGCAAATGATGTTTTGAGGAGCTTCAATTCTATTGAAGAAGCCACAGATATGG
TGCCACCCTTGGCAGGTTCTGATTGCAAAGCTGTTCTGCCAACAGGAATTACCGTCTTGGTGAAGAAGAT
CGAATGGAGACCGGAGAGAATGAATGCTATGATGGACTTGATATCAAGAATGGGCAATGCAAGGCACA
AGAATATGACCAGATTGTTGGGATTTTGCTACAACAAGTGTATGGCTTACCTGTTGTGTGACTACTTGCC
TAATGGAAATCTGGCTGAAAGGATCAGGACGAAGAGAGATTGGGCGACCAAGCATAAAATCATTGTGG
CAGTTGCTAAGGGGCTGTGTTACCTGCATCATGATTGCTACCCCGCGATACCTCATGGGGATTTGAAGAC
AAATAACATTGTGTTTGATGAAAACATGGAACCTCATTTGACTGAATTCGGTGTTAAATTCTTGATTCAAT
TAAATAATGGTCCGTCGGTAGCAAGAGTTGGAAATGAAGCAGGTATGCACCTATACTTTGATGTAGTCT
GCATTAATATTTGTTAATTAGTTAGCAATCTTAATATAAGATTAGTTATTAGACAGAATTCGGTTGCTCCA
AGGAGACGCGACTTGACTGTTGCTCCAAGGCTCCGCTTCAATTCTAATTGAAGTGTTTAATGATCTTAAT
TTGAATGACATAGCGAGAATTCCCTATTACTGGTTGTGAATCTTGTTTAATTGTGTCAAACAGGTGAAAT
CGAACGAGCTATACAGGAAGAACTTTACAGGGACATATACAACTTTGGTGAGCTCATTCTGGAAATCTT
AACCAACGGGAAGCTGTCAAATGCAGCGACGAGCATTCAGAACACCTCCAAGGAGGTTCTTTTGAGAG
AGGTTTTGGATGAGAATGATGTTGCTCCATCGAGCTCGGTCCAAGAGGAAATAAAACTGGTTCTTGAAG
TTGCTTCACTTTGCACCAGAGTTAGACCCTCAGATAGGCCATCAATGGAAGATGCACTAAAGCTTGTTAC
TGGTTTGAAGAAACAAAGCTAATGATGTTCTTGAACTAGGATGCCAATATTTAGACATGATTAGAAATTT
TGAAGCACAAAATATGTTGTAAAAGTCTTTTATTTCAGTTGTTTTTTGTTACTTCATCTGGTCAAGAAGTT
GATGCCTTTTCTTTTGTCAGGAATAGAATGTTTTGATGTAAAGTAGAGGAATGTAAGTAAGTTCTATCCT
CAGCAATATAAGGTCTGTTATTCTATTTTTTTTTTTTTTTTTTTAGTTGTTTAAAAACAAAATATGTTGTAAA
AGTCTTTTATTTCAGTTGTTTTTTGTTACTTCATCTGGTCAAGAAGTTGATGCCTTTTCTTTTGTCAGGAAT
AGAATGTTTTGATGTAAAGTAGAGGAATGTAAGTAAGTTCTATCCTCAGCAATATAAGGTCTGTTATTC
>PGSC0003DMG400011932 LRR-RLKs
ATGTCACTGCTAGCATTCAAGAATATGATCATTGATGACCCTTTCAAGATCATGGACTCCTGGAATGAAG
CTATACATTTCTGTGACTGGCCTGGGGTCTCATGTGGTCACCGTCATCGCCGAGTTACAGTTTTAAATCTT
ACTTCTCTGAAATTGAGGGGTTCTCTGTCACCAAGCATTGGTAATCTGAGCTTTCTTTATATCCTTAAGCT
CCAAAATAATAGTTTTTCAGGTGAAATCCCATCAGAGATTGGCTACTTACATAAGTTACACGTTTTACGCC
TTGATAATAACTCATTCACTGGTCATATTCCTTCAAACATTTCTGGTTGCTTCAATCTCGTTTCTGTTGGTC
TTTCGTATAATATGATGGTAGGCAAAATTCCAGCAGAATTAGGCACATTGTTGAAACTCAAACAACTTTC
TCTTGTTTCTAACTATTTTACAGGAGGAATTCCGCCTTCATTTGGTAATCTTTCTTTACTTGATACCTTTTCC

GCAAGCAAGAATAATTTGTTAGGGAAAATACCTGATGAATTATGTCAATTGCTCAACTTAAAGTACTTTG
TAGTGAATGAGAACAACTTGAGTGGTACCCTGCCTCCTTGTCTTTTCAATCTTTCCTCTATCGTGGCCATA
GATGTTGGAACAAACCATTTAGAAGGAAAATTGCCTCCATTGCTTGGTATTACTCTTCCTAGTTTAGAGTT
CCTTAGCATTTATAGAAACAATGTAACTGGAAATATTCCGGTAACATTGTCAAATGCTACGAATCTTCAG
TCTCTTATTGCTAGTAGAAATGGACTCACAGGAAAAGTACCACCCCTTGGAAATTTGCTGAAAATGCGG
AGATTTTTAGTTGCTTTCAATTATCTTGGGAAGGGGGAGGATGATGACCTTAGCTTTCTCACCACATTGG
TGAATGCCACCAGCTTGGAGCTTTTGGAGCTCAATACAAATAATTTTGGTGGAGTCTTACCTGCATCTGT
TAGTAATCTTTCGACTGAGATTATAGAGCTTTCGCTGTCTTATAATCAGATTTCTGGAGAAATCCCAAGA
GGGATATCCAATCTCAAGAAGCTTCAGGCTTTCTTTGTAGCTTACAACAGATTTCACGGTGAAATTCCGT
CTGAAATTGGTGATCTGATATATTTGCAAGAGCTGGCTTTACTTGGAAATCAGTTCTCTGGCAAAATTCC
AATCTCATTGGGAAATTTAGCTTCTTTAACCAAACTCAATTTAAGAGAAAACAATCTCCAGGGGAGAGTT
CCTTCCAGTTTAGGCAAATGCCACAATTTGGAACTGTTGGATCTTGGTTCAAATAACCTTAGTGGGTTCA
TACCATCAGAAATTCTTGAACTCTCATCCTTGTCGGAGGGCGTGGACCTATCTCAGAACCAATTAACTGG
GTTCCTTCCGATGGAAATTGGGAAGTTGAGAAATCTAGGCTACTTAAACCTGTCTGATAACAAGTTGCA
GGGCCAGATTCCTACCAGTATAGGTACTTGTGTGAAACTTGAAGCACTAGACTTGAACAACAACAACTTT
CAGGGTAGTATTCCTTCAACCATGAATAGTTTGCGAGGCCTGGAACTTTTAGTCCTTTTCTCACAACAATC
TGTCAGGTGGAATCCCGGGATTCCTGAAGGACTTTAAATTTCTGCAGATTTTAAATCTGTCTAGCAATAC
TTTGGAAGGTGCTGTCCCAACAGGAGGCATCTTTAGTAATGCTACTGTGGTTTCCATCATAGGAAATAGA
AATCTCTGTGGGGGTGTACCAGAGTTGGACCTTCCGGCTTGCATTGTAGAGGTAAAGAAAGAAAGGAA
ATCAGGTTTTCCCTTGAAAATAGTAATTCCTGTAGTTAGTGGGCTTATAGGATTGACATTCATAGTTTGTT
TTCTTGGCATACGACAGTTTAGTAGATCGAGAAAACCAACACCTACAGATATACCTGAAAACTCAACCTT
AAGAGTATCTTATCGGTGTCTACTTAGGGAAACTGATAGATTTTCTGCATCAAATTTGCTTGGAGTGGGT
GCTTTTGGGTCTGTATATAAAGGAATTTCTGAACACGATGGTACGTTTTTTGCTGTTAAGGTACTGGACC
TTTCACACCATGCAGCTTCCAGGAGTTTTTTAGCTGAATGTGAGGTTCTAAAGAACATTAGACATCGTAA
TCTTGTAAAGGTTCTAAGTGCATGCTCAGGTATTGATTATGAAGGTAATGAGTTCAAGGCCATAGTTTAT
GAATACATGGATAAGGGAAGTCTACAGGACTGGTTACATTTCACTACTCAGGAGAAATCTGAGTCACAG
GAAGAGCACAAGAAGCTCCGGTTTATTCAAAGATTAAACATTGCCATTGATGTTGCCTGTGCTCTGGATT
ATCTCCATAATGATTGCCAACCCCCAATAATTCACCGTGATCTGAAGCCAAGCAATATTCTTCTCGATGAG
AACATGACTGCACATGTTGGTGATTTTGGTTTGGCAAGATTTGTTCCACCAGCAATTCCAAACTCATCAG

CAAATTCAAAAAGCTCTACAGGTGTAGGGGGAACTATTGGTTATACACCTCCAGGTAATAATAAATCAA
GGTTTCTGCTGGCTGTCATATGTATGTCTTTTGATAAGAATATCTCTAATTCTGTAGTATTTCTTGTTGACA
GAATTAGGTATGGGAAGCGACGCCTCAATCTATGGAGATGTATACAGCTTTGGCATTCTGCTCTTGGAA
ATGTTTACCGGGAGGAAACCAACTGATGAAATGTTCAAAGACAATTTAAACCTTCATAACTATGCCAATG
CTGCTTTGCCAGACAGAGTGATGCATATTGCAGATCCCATTCTTCTCCAGGAAAGAGATGAACTTGGAAT
GAAATACAAAGTTGATGACAATACAAGCAGTGCTGGTGACATATTTCTGTCATTCTTAGTTAAGGTGATT
CAAATCGGAGTTTCCTGCTCCGTTGAATCTCCAAAAGAAAGAAAGCGGATCAGTGATGTCGTTGGAGAA
CTCAATTCATTAAGGAAGTTGTTCTTGGAGCAAGCATACCCGAAAGAAAAGGTTATAAGCTTCAACCTGC
ATTGA
>PGSC0003DMG400011989 LRR-RLKs
CACAACTTCATCACAAAACCAAAAACAATCAATCATTTAGCCTTGATGATGGTTCCCACAATATTTTGTAT
CCTTCCCTTCTTATATCTTTTCATAGTTATAACTTTTGCTTCCACTGAGGAAGCAACTGCTCTTCTTAAATG
GAAAGCAACATTCCTAAACCAGAACAATTCCTTACAGGCTTCATGGACACTAAGTAGTCCTGCTGGTACC
AAGAATTCTTCCAGAATTGAAGCGACGAGTTCTGATGCATGCAGGGGCTGGTATGGAGTTGTATGCGTT
AATGGTCGGGTTAACAGGTTGAATATTACAAATGCTAGTGTCATTGGCACACTGTATGATTTTCCATTTTC
ATCACTCCCTTTTCTTGAATATGTTGATCTTAGTATGAACCAGCTCTCTGGCACCATCCCACCTGAAATTG
GTAAGCTCGCTAATCTTGTCTACCTTGACTTGTCTGTCAATCATATTTCGGGCACAATCCCACCACAAATC
AGTTCACTAACAAAGCTTGAGACCCTCCACATCTTTGGCAACCAATTAAATGGTTCCATTCCGAGGGAAA
TAGGTCGCCTAAGGTCTCTTACTGATCTAGCTTTGAGTTCTAACTTTCTTAGTGGTTCAATTCCTGCTTCAT
TGGGGAATTTGAACAATTTGTCTTTTTTGTATCTTTATAATAATAAGCTTTCTGGCTCCATTCCTTCAGAAA
TAGGAAAACTTGTCAATCTTGTTGAAGCTTATATTAGTAGAAATCAATTAACAGGTCATATTCCTCCAGA
AATAGGTAACTTGATCAATGCAAAATTGTTCTATGCTTTCTACAATAACTTGTCTGGTCCTATTCCTGATG
AAATAGGGAAAATGAAGTCACTTGAGTACTTAAGCTTCCAGACAAACAATCTCTCCGGTCCAATTCCCAA
AACAACAGGTGACTTAACTGAGCTCAAACTCTTGCACCTTTATTCCAACGAACTTTCTGGTCCCATTCCTA
GTGAGTTGGGGAAACTGAAAAAATTGAATGATCTGCAATTATCCAATAATCAACTTACCGGTCCTATTCC
TGGTTCATTTGGCAATTTGAGAAACTTGCAATTTCTGTATCTCCGTGCCAACAAACTTTCTGGTTCTATTC
CAAAAGAACTTGCATATTTGGATAACTTGGTTATTATGCAAATGGATGAAAATCAGTTTTCAGGCCATTT
GCCTGAGCATCTTTGCCAAGGTGGGAAACTTGAGAACTTCACGGTGAATAGTAACAAACTGACAGGGCC
AATACCAAGAAGCTTTAGCAAGTGCTCGAGTTTCAAAAGAGTTCGCTTGGATAACAACAGTTTTACTGG

GAATTTATCTGAAGCTTTTGGCATCCATCCACACCTTTACTTCATTAATTTGAGTGAAAATGATTTTCATG
GTGAACTCAGCAGCAACTGGGGGAAATGCAAGAGTTTGACTGATTTGCGGGTAGCCAGAAATAACATT
AGTGGTAGCATACCACCAGAGATTGGAAACCTCAAAGGGCTTCAAGGACTTGATCTTTCATCCAATCATT
TGGTTGGACAGATACCTAGGGAATTAGGAAAATTGACCTCTCTGGTTAAGCTTTCTGTACAAAACAACA
ATATTTCTGGCAATATTCCTATAGAACTTGGGTCACTGACAAAGTTAGAGTCCCTAGATCTGTCAAACAA
TAAATTGAATGGGTCGATCCCAACGTTTATAGGAGATTACCAGCACCTATTTCACTTGAACCTGAGTTGT
AACAAGTTTGGGCAGAAAATTCCAAAGGAGATAGGGAGGATAACTCATCTTAGTGTACTTGATTTGAGC
CATAATCTTCTTGATGGAGAAATACCTGCTCAGTTAGCTAGTTTGCTGGACTTGGCGAACTTCAATCTTTC
CCACAACAGCCTTTCTGGGCGCATTCCTGAAGAATTTGAAAGTTTGACCGGTTTGCAGGATGTTATCTTG
TCATACAATGAGTTGGAAGGTCCAATCCCTAATAATAAAGCTTTCATGAATGCCTCATTAGAAGGTAATA
AAGGTCTTTGTGGTAATGTGACTGGATTCCAACCTTGCAAAAGGCCATCTTCTATTGTGAAGAAGCACTC
AATGGCGAAGGGACTTAAACTCATCCTCATCACTGTACTTCCTGTTATGGGAGCACTAGTACTACTCTGT
GCTTTCATTGGTGTTCTCTTTATGTGTAATAAAAGAAGGAGAGTTAGAGACGTTGAAAGAAGGGATTCC
AGTGATGATGTTGATGGTTTGATTTCCATATCCACATTAAATGGAAATGCATTGTACTGGGATATCATAA
AAGCCACAAAGGAGTTTGATGCAATGTTTTGCATCGGAAAAGGAGGATCTGGAAGCGTTTACAAGGTA
AAGCTTCCAACGCTTGAGAATGTAGCTGTGAAGAGACTTCATTCTTCATTTGAGGTTACCCATCGCAAAA
GCTTCATGAATGAGATAAGGTCATTGACAAGGATCAAGCATCGCAACATTGTGAAACTCTATGCCTTCTG
TTCAAATGCACAACACTCATTCTTGGTTTACGAGTACATGGAGAAGGGTAGCTTGTCTAGTATTTTGAGC
AGTGAAGTAGAGTCCAAGAAATTGGATTGGCTTACAAGGGTGAATGCCATCAAAGGTGTTGCTTATGCT
TTATCTTACATGCACCATGATTACTCACCCGCGATTGTTCATCGAGACATATCAAGCAGTAATGTTTTGCT
TGATTCCGAGTATGAAGCACGTGTTTCAGATTTTGGCATAGCTAAGATTCTTAAGCCAGACTCATCCAAT
TGTACTGCACTTGCAGGCACATATGGCTATGTAGCACCAGGTAAGATCTAACTTCTTATCCTTTTTTTAGT
TCATGAGTTATTTCTGCACAGTCTATGTTGTTTCTATCTCATTTGGAACTTTATTGTTTCAGAGCTTGCCTA
TACAATGAAGGTGACGGAAATGTGTGACGTCTATAGCTTTGGAGTATTAGCATTGGGGGTAATCAAAG
GAAAGCATCTTGGGGATTATATTACTGTGCTAGCAAATTCATCGTCTAGAGATCATGTGCAGCTTAGCGA
TTTGCTAGATGAACGCCTTCCATATCCTGAAGATAGCGTAAAGAGTTGGTTTTATCATCAAGCTAGCAAG
CTCATGTTTGCTTGAAACTCCAAAATCAAGGCCAACAATGCACTTCATCTCCAGTATGTTGTCAATGGATC
CACCTGTTTCTCAAATAACCTCTATGAAGGCGAAAAGCGTCCCCGAGATGTAAGGTGTCTATGTATGGG
GAAAAAAAGAAGCAAGTTTTGACACCAAGTGGTTGTGAAGTAAGAATAAGAAGCTATTATTACTGGGA

CTAGTATTTGTCTATTTTATTGTTAGTTGTGCTTATCTAGTTTAGTGTTTCAACCTGAAGGATTCTGAGCTC
TTGTACTAGAGGTAATACAATATCAAATAAAATGTAATATTTCAATTTTCTTGTGCCAATTAGTGCTAATA
AAAAT
>PGSC0003DMG400012218 LRR-RLKs
TAGCTATTGTGTATTTATGAGAAAATATTCGAAGTTTGATCAATCATAGCGACGCTGGTAAAGGCCCACA
CTTCCTTTATTCCGCCGCTCCTTCTATTATCTCCTTCTCCATTTTCTGCTTGATATTTTGTTCCCTTATAATTT
TAACATATAGTTTTTAACCCCAAAATTCTTCTTCTTCAGCACATAAATTCTCTGTTTCTAGACACCAAATCT
CTCTTTGAACCCAAGTTTTATTTTTTATAACTAAGTTGGTGACACTTAATAGTAGATTCCACTCAAAGGGG
TTGTTACTGATTTAGATTCTTGAATTCACTCTTAAGCTTTGTTAAGTTTTCTTTAGTGTTTATCTTTCCCCCC
TCACTTGTAAAAAAGACAACTTTATCTTTTTCCAGCTTTTTTTTTTGTTTGTTTTCATCTACTGTCTTGCGTC
TTTTCTTGTTTTCTCTTTCAATTTTGTTTGGATTATTGCTTTTTGAATCTTTTGCTTTCTGGGTTTTTCTTCTTT
TTGTTGATTGCTTAAGTTCTTGGAGACTGTTTCTTTAGATGGGTTCCTTCAGTTCTGGGGTTTTGTAGAAA
CTTAGTGTGTTCTTGTGGTGAGGAATTATTTGGGGGAAAAGATGAGACTTTGTGTTGTATTTCAAGTTTT
AGTCACTGTTGGACTAGTTCTTGGGATCTGTGATGCTTTTCCATCAAATGAAGGTGTGTATTATCAACTCA
AGTTCAGATCTTGATGTAGTTACTGCTGCAAAGATTCAATTTTTGTCTTTTAATTGAGCATTTAGTGTTGT
AATGCAGTGTTGTTTCGGAAAGATCTTGTATTTTCTTGTTTTGAGATACTTGCTGTATTTTGATGTCATAT
ATTCTTGCTACCATGGTTCAAATTGTCTATTGGAAATAGTCTCTCTACCCTGCAAAGGTAGGGGTAAGAT
CTGTGTAGTGCGTACTATGTTATTTGTTGTTGTTGTAGATAGTTGCTGTATTTTATGAGATTGTAATTCAA
AGAGTAGGTAGTTTATTGTGAGTGAATCTGATTGGGGATTCTTTGTTGATACAGTTTATGCTCTCAATAC
CTTCAAGGAAGCTATATATGAAGACCCTCTTCTGGTTTTGTCAAATTGGAATGGTTTAGATTCAGATCCTT
GTGGTTGGGCTGGAGTCTTTTGTTCTATGGCTCAAGATCATGTCATAAAAATGTGAGTTAATTGTCTTCA
ATTTTATGTGCTATACATGGATAGGTTGTTTGGTTAATGATTTTTCTCAACTAAAATGCAATCTAGTGAAT
CTTTTATGGACTCAAGTTTAGTGCAAATGAGTCTCTAATACGCATTTCCACTGGATCGTGTGACAGAAAC
ATTTCTGACGCTTCGTTGAAGGGATTTCTTTCACCCAACTTGCATTTACTCTCCTCTTTGCAAGAATTGTAA
GTTATTTCCGAATAGATTTATAAGAATTACGTAGTTCTGGTTGTTCTTTTATGTGGTTTTGTTAAAGGTTA
TGCTATGCACTGGCAATATGCAGAATTTTGCATGGAAATGTGCTGATTGGCACAATTCCTAAGGAGATT
GGCCTATTGAAAAACCTGAAGGCGTTGGATTTGGGTGCTAACCAGCTAACAGGACCAATTCCTTCCGAG
CTTGGCAATTTGACAAACATTATGAAAATGTTAGTCTAACTTGAATTTTTTAGGTTCAGTTGTACTTGCCT
TTACCTATCCTTGTTGAAGCTCCCTTACTTTTCTAGCTTGCATGTGTCTCTGCAGAAACCTACAATCTAATG

GATTAACAGGGAAATTGCCTGCTGAGCTTGGTAACTTGAAATACCTTGAGGAACTTCGGTTGGATAGGA
ATAAACTCCAAGGACTAGTGCCTGCTAATACTGGATCAGATTTTACATCTGGTGTACGTGGAATGTGAGT
TGATTAGCTACATCTTGTACTTTATCGTTTTCTTTTGAATTTTCTGGTATGTGGATAGGGATTAATCCATG
ATTCTCTGTTTACTTACAAGATATTTTTGGTTGTCCTTAGAGCGTTTTCCGTCTTGGCCCTCTAGGATTCTG
TCAGATATTTGAAGGATTGAGCGACCCAAGAATCTAATTTTCTGATCTTTATGACAGTCATTGTCGGGGA
TGATGATGACAGAGTTCAGAAAAGAGATGCTTATGGGATGATGTCTGTTCTTTCTGTCAGCATTGTTTGA
TATGTACTGCCTGCAGATTGACACTGATGGCTGTTTTACTTGAATCTAGTGTAGTACATTCAGTGTCAGC
ATAGAAAGTGGGGGTTAAACAGTGTTGTTTGATGGTGAATTGGGGTACCCCAATGGAGTGTTTTTTTGC
TATAGCCTTTAATATTTGACCAAATGAGAGCAATGTTACTGAGTTCAGAATGTATGTTTCAAAAGTTTAA
CGATGATGTTCTTGGAAATGATCTGTATTTCTATAAAGAAACAATGAGTAAACTGTTTGATGTAGCCTTA
TGTCCATCACATGTATCTTTATCTGACTGTTTTTTTCCTTTTCTGTTTTACCATTTTAGGTATGCCTCTGGTG
CCAGCACTACTGGTTTTTGTCGCACATCCCAGCTCAAAGTGGCTGATTTCTCATTCAACTTTCTTATTGGA
AGCATACCAAAGTGTTTGGGTTACCTTCCAAAGTCAGTTCTTAATTCTACGCCATAAATCTTGTTTTATCTT
ATCTTCTCTATTCCTGAATGCCTGTTAGTTTTGTTGCTGTAAATCTATATCCAAAGTAGCACTCTGATGCTC
TACTAGTGTTGTTTCTCTTACCTTTTCGGTTTCTTAATGCCTGTTATTTTGTGTGTCTATAGATCTAGCTTTC
AAGGAAATTGCCTTCAGCACAAAGACCCGAAGCAACGGTCTGCTGCTTTATGTGGTAAGTAAACAATCA
TAGTTACAATTTTCAACACTACGCATTGTTTGTGCAGCTCAGTCTAGGAGAATTGATATCTCAACCTTTTT
TCTTTAATATTTGCAGTCAAATAGATTTATTTATTTTAGTAAGAACTGAACTGGTTGTTTTGACATTGCATT
ATAGGTAGCACTCCGCCTCCCACTAGCCATGCATCGACACTCAGCAATAAGCACCAGCCTGTTGAAGAAC
CGAGACATCAGACTTCTTCCAAGCCCACTTGGCTTTTGGCTTTAGAAGTGGTAACAGGAGTTATGGTGG
GTTCTCTCTTCATTGTAGGTCTTGTGACTGGCCTTCAGAGGCTTAAGAACAAATCGTCAATCATACTTTGG
AAGAAATCAGGAAGTGAGAAAGATCATATGACAATTTATGTAGGTTAGTACTATCATGCAATTGAACTT
GAGCACTTAGTACTTTCAAAAGCATTGAGCCTAATCTTCGTTTCTGTTTACCGCTGATGTTTCTTTGTAGA
TACTGGTATGCTGAAAGATGTTGTAATATACAGCAGACAAGAACTTGAGGTAGCTTGTGAAGATTTCAG
CAACATCATTGGATCCTCACCAGATAGCATAGTTTACAAAGGAACCATGAAAGGTGGACCTGAAATTGC
TGTAATATCCCTTTGCGTCAAAGAAGAGCACTGGACAGCCTATCTAGAACTTTATTTTCAGAAAGAGGCG
AGACTCAATTCTTTTTTCATGATCGCTATTTATCTCTTCCAAAAGTTTGCATCTTAAGTTCCATGCTCTAAC
TTCTGTTAATTTACATTTAGGTGGCAGAATTGGCCAGAATAAATCACGAGAACGCTGGGAAACTTCTAG
GCTATTGCAGAGAGAGTAGTCCCTTTACGAGGATGTTGGTGTTTGAATATGCATCTAATGGGACATTATA

TGAACACCTTCATTGTGAGTAAAGTGAAATAGTTTTCGCATGCACTTCATTTTATGTTTTCCAAAGCGAAC
TCCTAACTCTTTGAATGTATCATAGATGGAGAAGGATGCCAACTATCTTGGACACGCCGGATGAAAATA
GTTATTGGCATCGCTAAGGGACTGACATATTTCCATTCAGAACTTGACCCTCCATTTACTATATCAGAACT
GAATTCAAATTCTGTATATCTCACCGAAGATTTTTCTCCCAAGGTAAACGAGAGACCATTATTTATCATTA
TCGTATAAAACAGTCCTAAACAACTCATCCCATTTTTATTCACCTTAAGCTTGTGTACCCACCCCATTATGC
TCTTAAGCAGTCTACAAAAGGCACTGGCATTTTATAATTGTCTTCTCAAAGTTTATTTCATTTGGTGCAGC
TTGTTGATTTTGAAAGCTGGAAGTCGATAATTTCCAGATCAGAAAAGAATTCAGGTGCTATCAGTAGTG
AAGGAGCAATATGTGTCCTTCCAAATACTCTCGAGAGCCGTCATCTCGATGTCCAGGGTAACATTTATGC
ATTTGGAGTACTTCTACTGGAAATAATCAGTGGGAGACCTCCATATTGCAAAGACAAAGGGTGCTTAGT
AGATTGGGTAAGCATTTCTTTTCATGTGAAATTTTTCCTACTGTAAAAAGACTTCCCTGGTAGAACCAGCT
TCTAAACGCGTTGGCAGGACCTAAAGTCGACCTTTTATGTGCAGGCTAAGGAATTTCTTGAAGTGCCGG
AGGTTTTATCCTACGTTGTGGATCCCGAGTTGAAACATTTCAAACAGGAAGACCTCAAAGTGATATGTGA
GGTGATCAACCTTTGCATCCACCCGACTACGAGTAGGAGGACATCCATGAAGGACCTGTGTGCTATTCT
GGAGAGCAACATTGACACTTCCATCACTGCAGAGCTCAAGGCGTCTTCGTTGGCATGGGCCGAGCTTGC
ACTTTCATCCTAACTGACAGAAGGCTAACACAGTATATATATCTGAAATATGTAATTGTCTAGCGCTTTCT
CCTTTTCGTTTTTTGACCTCTCGATTTCGTTCCCAATGTGTTCTGGAGTGATGGTTGAGTTCATGTAAATTT
TGAGTCTGTAGGATAAAATTAGAGTTGAAGAGAAGCTACTGTTGATTCTTTGTAAACAAAGTGGTTCTTG
TTAAAATGAATGAGTGTTCATAGTGAATGAGGCACCAACCATATTTTTTGAGTTTCAAAGAACTCGCTCG
AGTTCTCGAGTCGCAACTCAAAATATAGGTTTTGGACTTAACTCAACTCCAAAAGAAAAGTACAATACAG
ACTTTCTT
>PGSC0003DMG400012307 LRR-RLKs
TTTTTCCATGAACAAGAAGAACACAAGTGAAAAAATCAAGAAAATGTGAGAATTTTTCTACAAAAAAAA
TTCCCAGTGTCTTGGTTTTGGCATTCAAATGGAAAGTTAAAGCGCAAAGAACAAAAAGAAAAGCTTCTTC
TACTACAGATTCTGACTAGCAGCTACTTTCATTTTAAAATTACACCCTTTTGACCCTAACTTCCCCTATTTC
AAACCTTGTGAGTGACCTCCAATTTTTTTAATCACATTTTTTCAGAACCCCACATTGCTTTTTGCAGACACC
ATTCTTGGGAATTTGACTCTTTTTTCTCACACCTATATACATATATATACACATGCATCTTCATATTGTTGTT
GTCAGAGTGCATTTCTCTTTTTTGTTTGTTTCTTTCTTCTTCTTTTTTCACTTTAAGCTTGTGTTAAGCTGCT
GCTACTTCCTTTTATGAGGTTTTGAATTAAAGTTGTGAGCTTTAGATTCTTGTGGGTTGAGGGTGTTGTTG
GAGAGTTTTTGAAATAGTTGAAAAAGTTTTGAACTTTAGAATCTTGATGCTTGCTTACTTCATCTTATGTG

ACTTATAAAAGCTAAGTTCTTGAGGGGGGTTTTTTGAATTAAAGTTTTGAGCTTTAGATTTCCTTTTGTGG
ATTGAGGGTGTTTGTAGAATGTTTGAAATAGTTGAAAAGTTTTGAACTTTAGATTCTTGATGCTCTTTTCT
TTTTCTTATGTGTCTACAAAAGCTAAGTTCTTGAGGGTTTTTTTTGAATTAAAGTTTTGAGCTTTAGATTT
CCTTTTGTGGATTGAGGATGTTTGCTGAGTTTTTGAAAAGTTTTGAACTTTAGATTCTTGATGCTCTTTTT
GTTCTTCTTATGTGACTACAAAAGCTAAGTTTTTGAGGGGTTGGGGGCATTCAGAGTCTTTGAAATGGTT
AAAGTTTTGAGCTTTAGGGAGTGGTGGGGGATTGTTTCTTTGGGTTTCTTGGTGGGATTGTTTCTGTTGT
CACAAGGCACTGTTGCTCTTCTTGAAAATGATAAGCAAGCATTGTTGGATTTTGTTAATCAGTTGCCTCAT
TTGCACCCTTTGAACTGGGATGCAAATTCTTCAGTTTGCAAGAACTGGACTGGAGTGGGGTGTAATGAA
GATGGTTCTAGAGTGATAGCACTGAGGTTACCTGGGGTTGGGTTCAATGGTCCAATTCCTAATAATACA
CTGAGTCGTTTGACTGCATTGCAGATCTTGAGCCTTAGATCTAATGGTATCAATGGCACTTTCCCTATGG
ACTTTGATAACCTCAAAAATTTGTCTTATCTTTACCTTCATTACAACAATTTCTCAGGTCCTCTGCCTTTTGA
TTTCTCTGTTTGGCAAAATCTCACCAGTTTGAACTTGTCAAACAACAGATTCAATGGCACCATTCCTAGCT
CGATATCGGGTTTAAGTCATCTTACTGCCCTTAATCTTGCTAACAACTCACTTTCTGGTTCAATTCCTGATC
TGCATTTGCCCAACTTGCAGCTACTGAATTTGTCAAACAATAACCTTATAGGTACTGTGCCTAAATCACTT
CAAAAATTCCCAAAGAATGTGTTCATTGGTAATAATATGTCTTTACTTGACTATCCTGTTTCGAATTCTTCT
ATTGTTTCCCTGCCTCAGCAACCAAATCCAAAATTCAAGAATGATGGTAAATTGAGTGAGAGGGCTTTGC
TTGGGATTATAGTAGCTAGTAGTGTAATTGGGATTCTTGGATTTGGTTTCTTGATGGTTGTTTGTTGTTTT
AGAAGAAAGAAAGATGATGGTTCGTTTCCCAGCAAGATGGAGAAGGGAGATATGTCTCCTGATAAGGC
GATTTCAAGGAGTCAAGATGCTAATAATAGATTGGTTTTCTTTGAGGGTTGTAACTATGCATTTGATTTA
GAAGATTTGCTTAGAGCTTCCGCGGAGGTATTGGGGAAGGGCACGTTTGGTATGGCGTATAAAGCAAT
ATTAGAAGATGCTACCACAGTGGTAGTGAAGAGGTTGAAAGATGTAGGTGCTGGGAAGAAGGAGTTTG
AGCAGCAAATGGAGGTTGTGGGAAGTATAAAACATGAAAATGTAGTCGAGCTTCGAGCATATTACTATT
CAAAAGACGAAAAGCTTACTGTCTCTGACTATTTCAGTGAGGGTAGTGTTGCTGCAATGCTCCATGGTAT
GATCACTTTATGCACTCTCTAAATTTTATAGTTTTGAACTTTTCAACTTTGTCACATTTTGTTAGCATATTCA
TACTGCTCCGTTATTTGCTTTGAATCAGTAGATTAGTTAGTATTTTTGAGTATGATTGTTAAAACAAGTTA
TTTGTTATCCCAAACTATGTTGACTCTAACTAAGTTGCTACGACTCTTCAAAAAATGCCGCGGGGTGCAT
GTCGGATCCTCCAAATAGTGCACTTTTAGAGTATCCGACAAGGGTGTGACAACATTCTTGGAGAGTCCG
AGTTACATAGTCCCTAAGTGAGTTCTTACAATCAGACATGTTAGTTTAGTGAATTGAAAAACATAAAGCA
ATTAAAAACAACATAGAATCTTCCAAACTGAAGGCCAACTAATATAATTTGTTGTGTTATGGGTAGGGCC

AAGATGGGAATCCACATATATTATATGCTCCATATATTCTCTTTCCAGCTTAATAGTTAGTTGTACCCACA
GGATGATAATATATATATCCGTATCAAAAAAAAAAAATGTGGATGATCCTAACATGTGGTTTACGCATCT
CTGTGGGGCAGTGACTCCTTTCTGTTAACAAAATAAGCAAAATCCTATCTCTATTTTAAAATCCATACAAA
TCATTGTCACTTCTCCTTTGTCACTAGAGTCTTTCAGCTATTACACTATTTGACTGAATTATTTCATAGCAT
GTGGATAATCATTTCTGCTTTTGTTATCTGTTAGTCTTTTGTGTTTCAGTTACCGCATTATTTCGTGTCTTT
GTTGTTACTGTTCTTTTTCTTGATGTCCTGTACTGCTTTCCGTATTAATTGACATAATTTCTCCACTGCTGTT
CTTTCTTTTTTCATAACTACTTTGATTTGTTGCAATTGAACCGAGAGTCTTTTGGAAACAGCCTCTCTACCT
CCACGAGGTAGGAGTAAGGTCTGCGTACACTCTACCTTCCTCAGATCCCACTTGTGGGAGTAAGCCGGT
TATGTTGTTGTGGATAATCATTTCTGCTTTTGTTATCTGTTAGTCTTTTTGTGTTTCAGTTACCGCATTATTT
CGTGTCTTTGTTGTTACTGTTCTTTTTCTTGATGTCCTGTACTGCTTTCCGTATTAATTGACAGAATTTCTCC
ACTGCTGTTCTTTCTCTTTTCATAACTACTTTGATTTGTTGCAATTGAACCGAGAGTCTTTTGGAAACAGCC
TCTCTACCTCCACGAGGTAGGAGTAAGGTCTGCGTACACTCTACCTTCCTCAGATCCCACTTGTGGGAGT
AAGCCGGTTATGTTGTTGTGGATAATCATTTCTGCTTTTTATCTACTTTCTATAGCAAGTCAAAATAGTTC
ATGATTTTGTCGAGGTCCCAGCGTCCTTTTTCCCAATTCTTTGTCCACCAGGCAAATGCACATGATGTCAA
AGTGCATGAATGGCTAAGCTTTTCTGATAATTTATTTGGAGTTGGTTGGCATCTTTCATTACAATTTTCAC
TGGTGGGACCTGATTTAATTAACTAGGAGTTTAGGTTAAGGAATCCTAAGCTAATGTCAACCTTAATATG
CTTAGAATCTATTGAGAACATTACAACAAAATTCTTTTTGGTTATTTGCTTATTTACTCCCCTAATTTTGCT
GTTAGAGCTAGGACTTCTGTTTGTTCTTCTCAAACGAAATGAAGTGAAATGGAAACTTGTAGAAAGTGTT
ATTTCCTTTTGTATCTCAGTTATGCTAACCTAATATTTCATCCGCCGTAACTTTTGTGAACAAAAGGAAAA
GAAAAGAAGATCATTTGTGTAATGAGTAGCTTCCCACTTAGATGATAAGTCCTGCAAGGGATATTCTAA
ACTTTTAAGCACTTCGCGATTTGCTTATTCTAAGTTAAAGGATCGACCAGGAGCTACAAGGATACTGATT
ACTCTAATATTTGGCATGTTATAGAATGATAATTAAAATATGAACAAATGAGAGACAAAGGATTGAACG
CGCGATCCTTTGCTTTTGTTAAAATTGGGATGGTTACCTGTGGTATCAGAGGTCGTTTGGTAGGCAGGAT
AAGGTGAGATATTCTAGGATTGACTGTGCTACCAGTTATTCTCCTTTTCAATTCTCTACCTTTTGTTAGGC
AGTTCATATCATTGTGATCTCTGTGTTACCTGTTATCAAATATTGCTTTTGCTATTCGATCGTGTTGACACC
TATTAAAATGCAACTTATAGGTAAACGGGGAGAAAACAGGATCCCTTTAGACTGGGAAACTCGACTTCG
GATAGCAACTGGAGCAGCGAGAGGTATTGCTAGGATACATGCTGAGAATGGTGGCAAGCTAGTCCACG
GAAACGTCAAATCCTCAAATATCTTTCTGAACTCTAAACAATACGGTTGTGTATCTGATGTTGGTCTGTCA
ACAATAATGAGTTCGTTAGCTCATCCAGTTGCACGTGCTGCTGGTTTTCGCGCTCCAGAAGTAACAGACA

CACGTAAAGCCACTCAACCTTCTGATGTGTACAGCTTTGGTGTGTTGTTATTAGAGCTCTTGACAGGCAA
GTCCCCCATCCATACAACAAACGGAGATGAGGTCATACACTTAGTCCGATGGGTTCATTCAGTTGTCCGA
GAGGAGTGGACTGCTGAAGTATTCGACTTGCAACTATTGAGGTACCCGAACATAGAGGAAGAGATGGT
GGAAATGTTACAGATAGCAATGTCTTGTGTGGTAAGGATGTCAGATCAAAGACCAAAAATGTTTGAGGT
TGTTAAAATGATCGAGAACGTTCGTCCCACGAGCTTAGAGAATGAGCATTCATCTGAAGGAAAAGCAGA
AACTTCAACACCAAGAGCAGAAGCTACACCTGTGCCTGATACACAATCTGATTCTCAATGAGTTGGCCAT
TTTTATCATAGTTTACCATCTTAATTCATGTTGAAAATGCTTTACTTCACTCTCTCAGTGAAATATTGAATA
CTTTGTAACTTAATCCAATAGGCACTTTATTTTAAGGTATTCTTGAAATAGTACTTTGATTACAAATCTAA
GTTCTCTTTCTCACTCTCAAAGTACCATCTCTTCCATGTTCTTGATGCAATGATCACAAGAACATGGTTGAT
GTGGGACGAATTCAAGATTTAGAGTCAGTAAGTTCGGAGTAGGTGTGCATTATTTGTATGCATTTGTAT
CTTCTTCTTTTTTTCGATTGTAGTAGTACTTGTTTTCTTTCCTTCAACTATTGAGTTATTTGTAATGCTACTT
GTTCTCTTCTTAATTGTTGTAAACATGGTTTTTTTTCTTCG
>PGSC0003DMG400012443 LRR-RLKs
CTTTGTCTAATCTTAATAATAACTGTTCCTTTGATTTGAAATCTCAATCTCTATCAGCTTAATTAAAAACTG
TACAATCTTTTTTTTTCTTTATTAGATTCTGTCCCTCTTTCTTGAAATTAACTAAAAGAGTTTGCTTGAGTTG
AAAGTTGGGTTCTTGCTTCTCTTACAAGTGATGGGGTGTTGAAAAAATCTCATCTTTTTCATTTTTTCTTCC
TAGTTTTAGGCTTTGAGTTTTGTAATCTAGTGTATTTATTTATGATTTGGAGTTAAGGGTTTGTTAATTCAT
ACAAGTAGTTAGAGGCTTGCTTTAATTACAAGTAGTTGGTAGCCTTTTTTCTTTGAGATTTTTGAGGTGTT
TTCTTGGGATTTGTGTTAATTGAAGTTTCTGCAACTGGGGTTCTCTTCTTCCTTTACTATAATAATAAGGG
TGGTGGTGCCCTGCTGCTACCGTTAAGTGCCTTGATTTTTTCAGTCTTGGGATCTGATTTGATTTTTGGTA
CTAGGGGATCTGAGGAAGGTTCTTTCTTGGTGCTAATTTGCATCAAAGGTGGAAACTTTTCTTCATTTGG
AACTGTGAATTTGGAATACATGGTGGTGGAATCTTGATTTTGAGCTGTTTATATAAAGATTTGTGTGTTT
TCATCAATGGTGATTTGGGAGTTTCTGCCAATGAGTTTTCTGTGTTGGGTGTTTTTGGCTTATCTTTTTTG
TACATCTTTGAGTCTTGAAACCCCAGTTCAAACCTGTCATCCATATGATTTGTTGGCATTGAAAGAAATTG
CTGGCAATCTAACAGATGGGGTTATTCTATCAGCTTGGTCTAATGAACCTAATTGCTGTAAATGGGATGG
GGTTGTCTGTGGTAATGTTTCTGCTCAAAGTAGAGTGATCAGGCTAAATTTGTCAAGAAAAGGTTTGAG
AGGTGTGGTTTCACAGTCCTTGGAGAAATTGGATCAGTTGAAATTGCTCGATCTTTCGCACAATCACTTG
GAAGGTGGATTGCCTTTGGACTTGTCCAAATGGAAGCAGTTGGAAGTTCTTGATTTGAGTCATAATGTG
TTGCTTGGACCAGTGTTGAGGGTGTTTGATGGATTGGAATCAATCCATTCTCTCAATATATCAAGCAATT

TGTTCACTGGAAATTTCAGTGAGTTTGGTGAATTTCCTAACCTTGTCGCATTTAACATAAGCAACAATTCG
TTTACTGGTAGTTTCAAGTTTGAAATTTGCAGTTTTTCCAAGAAGCTTAAGGTTCTGGATATATCACTTAA
TCATCTCACTGGTGATCTTGGAGGACTGGATAATTGCAGTTCATTGCTCCAGCAGCTACATGTGGATTCT
AATGATCTCGGGGGTCACCTTCCGGACTCGTTGTATTCAATGACATCTTTGGAGCAACTTTCACTGTCTGC
CAATAATTTCTCAGGCCAGCTAAGCCCACAGCTTAGTAAGCTTTCCAAACTGAAATCCTTAGTTTTATCAG
GAAATCGCTTTCATGGTTTGCTTCCTAATGTGTTTGGTAATTTGACATTGTTAGAACAGCTAGCTGCACAT
TCTAATAGATTTTCAGGACCATTGCCCTCTACGATTTCGTATCTTTCTGTGCTTAGGGTGCTTGATCTTAG
GAATAATTCTTTGTCTGGTCCTGTTGATCTTGATTTTACTAAATTGACAAGTCTGTGCACTCTTGACCTTGC
AACTAACCATTTCAAAGGTAACCTTCCTGTATCGCTCTCTAGTCGGGAGTTGAAAATCATGAGTCTTGCC
AAAAATGAATTCACCGGGCCAATTCCTGAGAACTATGCCAACCTTTCATCCCTTGTGTTCCTCTCATTGTC
CAATAATTCCCTTTCGAATTTGTCTGGAGCTTTATCTGTTCTGCAGCACTGCAGAAATCTTTCAACTCTTAT
TCTCACCAGGAACTTCCGTGGTGAAGAGATTCCGAAAAATGTGAGTGGATTTGAGAACCTGATGATATT
TGCATTGGGGAACTGTGGTTTGGATGGGCGAATTCCGATATGGTTATACAATTGCAGTAAATTGCAAGT
GCTTGACCTGTCATGGAATCATTTGGATGGCGAAATTCCTCCTTGGATTGGTGAAATGGAAAAATTGTTC
TACTTGGATTTCTCGAATAATTCACTCACAGGTGAAATCCCAAAAAATTTAACTGATCTAAAGAGCCTCAT
TTCCCCACACAACTACGCCTCTAGTCTGAATTCTCCCACTGGTATACCGTTGTTTGTCAAGAGAAATCAGA
GCGGTAGTGGTTTGCAGTACAATCAGGCTTCAAGCTTCCCTCCGTCTATCTTATTGAGTAATAACAGACT
AAACGGGACAATCTGGCCCGAAATTGGTCGTCTGAAACAACTTCATGTCTTGGATCTCAGTAAGAACAA
CATTACGGGGACTATTCCTAGCTCGATTTCAAATATGGGGAACCTGGAAGTTTTGGATCTTTCATGTAAT
GATCTCAATGGATCAATTCCTGCTTCATTCAATAAGCTCACATTCCTTTCAAAGTTCAATGTAGCTAATAA
TCACTTGCAGGGAGCGATTCCAACAGGGGGCCAGTTCTTGAGCTTTCCCAACTCGAGCTTTGAGGGTAA
TCCTGGACTTTGTGGAAAAATCATTTCTCCTTGTGCTGCCAGCAATTTGGACCTCCGACCGGCTAGTCCTC
TTGCTTCTAGTAGCAGTAAGCTTGGCCGAGGCGGAATTATTGGAATTACAATCAGCATAGGGGTAGGAA
TTGCACTTCTTCTTGCAATAGTGCTGCTTAGAGTGTCTAGAAGAGATGCTGGTCACCAGATTGGGGACTT
CGAGGAAGATTTCAGCAGACCACCTCGATCGTCTGATACTTTTGTTCCTTCTAAGTTGGTACTTTTTCAGA
ATTCTGATTGCAAGGAACTGACTGTTGCAGACTTGCTTAAATCTACAAACAACTTCAACCAGTCGAACAT
TGTTGGATGTGGAGGATTTGGTCTTGTTTACAAGGCAGAACTTCCTAATGGCATAAAGACTGCGATCAA
GAGGCTTTCTGGGGATTGTGGTCAGATGGAGCGTGAATTCCAAGCTGAAGTGGAAGCCCTCTCGAGAG
CTCAGCACAAAAACCTTGTATCCCTTCAAGGTTACTGTCAGCACGGGAGTGATAGATTACTGATATATTC

TTACATGGAAAATGGAAGCTTGGACTATTGGCTACATGAAAGAGTTGACGGGAGCTCATTAACATGGG
ACATTAGGTTAAAGATTGCACAAGGAGCAGCTCACGGATTAGCATATTTGCATAAGGAACCAAATATAG
TTCATCGCGACATTAAAACCAGCAACATTCTTTTGAATGAGAGATTTGAAGCTCATCTAGCTGATTTTGG
ACTATCAAGGCTGTTGCATCCCTATGATACTCATGTGACGACAGATCTCGTTGGAACCTTAGGATACATT
CCTCCTGAATATAGTCAAACATTAACTGCTACTTTTCGGGGGGATGTTTACAGCTTTGGTGTTGTTCTACT
TGAGCTTCTGACAGGCAAGCGTCCCGTGGAAGTATGCAGGGGGAAAAACTGCAGGGACTTGGTATCAT
GGGTTTTTCAACTGAAATCTGAGAACAGAGTGGAGGAAATATTCGATACATCGATATGGGATACAAGTT
ACGAGAGGCAGCTTTTGGAGGTGTTAAGTATAGCTTGTCAATGCATAGTGCAAGATCCACGCCAGAGGC
CCTCGATCGATCAAGTTGTCTTATGGCTCGAGGCAATCGGAAGTGTAAAAGAAAGATGAGATTCAACTA
GTTTTTCCTTGTAGACTTGGCTTCTTTTGCTTACAAAGTGAAAAATATCAGTGTAAAGCAACAGAAAATTT
CAGTTTTATAGAGTTAGTTATAAAACTTATGAAATGCTTTGTTTGGTTCACTTTGTTTCATACACAAAAAA
TAGTGATAGTTGTGTTTGTATACATATGCTTAACTTGGGGACAAGTCCTTTACCAATATACAACTAATTAC
TCCCA
>PGSC0003DMG400012514 LRR-RLKs
AAGAAATTGGTAGCTTGCAAAATCTGATATACCTTTCTTTGAGAGACAATAAATTGCAAGGATCAATACC
TAGCTCAATGAGCAGCGTGACATCTTTGGAATTTCTAGACCTTTCTCATAACAATGTTTCAGGATTAATTC
CCACGTCTTTGGAGAAGCTTCAAAATCTCAAGTATTTCAATAAGTTAGTTGGTGAAATCCCCTCGAGCGG
TCCATTCAAGAACCTCTCCGGTCAGTCTTTCATATCCAATGAAGCATTGTGTGGTTCTCCAAGATTCCTGT
TCCCCCATGCCACACTAGTACTTCAAAGTATCTATCCAAGATGAAAAAACTACTTGCTCTAATTCTACCAG
CTGGAGTTGCACTTGTATTAGTCTCCATCGCCTTTGTGATTATATTGTATGGATAAGGTGTATAAGAGGT
AAAAGAAAGCAAGCAGTCGATTCATCGTATGTCGTCTCAACAAGAGGAAGAATTTCATACTATGAATTG
CTCCGAGCAACTGATTCACTCAGTGAGAGTAATTTGATTGGTTCTGGGAGTTTCGGCTCTGTTTACAAAG
GAATCCTCAGAGAAGGGACTTCTATTGCAGCTAAAGTTTTCAATCTGCAATTACAAGCGGCATTTAGGA
GTTTTGATACAAAATGCCAATTTTTGCGCAACCTCCGTCATAGGAATCTCACCAAAGTCATCACTAGTTGT
TCAAACCTTGAATTTAAGGCTTTGGTACTAGAGTACATGTCCAATGGGAGCCTCGATAAGTGGTTATATT
CGCACAACTACTCCTTATACATCAACCATCATGATATTTCGCTTCAAATAGGCTAGTTTTGTGTCTTTAATT
CATAACATAATAGCTTCAAGTGGTACTCTTTTAGTTTCAATAACTTGTCCTATGTCCAGAATTTCATCCAA
GTGGTACTCTTTTGGTTTCTTCATCTCTCAAGTCAATTTCACTATTATTCGATTGCTTTCTGAATTGCTCTTA
ACAATTGTTGTCCAGGTCTCAACTTTCATTAGTGATTTCATCTTGAATCTTCCTCTACTACTCTTTTAGGTTT

TGTCTTTGCTGGTTTCTTTACTCAAATTTCCCAAGTTGTTGAATTCTTTCATGTTCGTTTCGTTTTGGGCTTA
ATAAGCAAAGGTGGCTTTTTGTTATTTCTGAACTTAAAACTTATAAATGTTAAGAACTAAGCTACATTTAT
TTAGAGTAATGTGCCTGACAATGTTCGAAAATTAGGAAACTTGTATTGTTTTCAGCTTGAGTAGGGGGCT
GCACACCGCGCAAGTACAGAGTGTAGACAAGGAGGGACTTTTTCTTTTGTTGAAAGGTATTGGCCCAAA
TCTTTTAATAAATCTCATATTGTTAGGATCTTGATTAGATTCAACTGATGTGTCGAATCTCCTGAATGAAG
GATTCAGATCTTCACCATGGATGATAGAACTCCTCCCGGAAGTGAAGGCAGGAGTAAAGCCTATTGATT
CACCAGTTTGCTAATGTACTTGGTAAAGCCTATTGATTCACCAGTTTGCTAATGTACTTGGTAAAGCCTAT
TGATTCACCAGTTTGCTAACTTACTTGTTGGCTACCCGCACTTTGTAACCAGAATAGGGCTCTGGGAGGG
AGTTGTCAGTGTATTGGGAGTTGCTTTGCTATGCGTTATTCATGGTGCTAGAGAAGACTTTATTTGAAGA
CAGTAATGGTGCATATACATTCCGTACTTCTAACCCAACTCAAGCCGAAGAAACTCATATATCTTTTGACT
CTATTTATGTAGGCACCTGATATTTATGAGGTTTCACTCACCCCAGTTACCTATTTGAGGTCTAGGTCAAG
ATTTTAATAATCATGACGGTCCTTGATCCAACAGGATATTGGCTAGATGGAAAGCTTGGACCTCGAGAAT
CTGTGTACCGCCATTTTTCCTTGGACTGTGAAATCACGTGTTTCCACTCTTATTATCCTACATGGATATATT
TTTGTCTTAATTATTAAAATTTGCTCTCAATTTAATTCTTCTACTCACTCAATATTTAATACAAATATGCAGA
AACCCCAAATAGATGAGCAAATGTGTTGTGTTTGAAGTACTGATAGCATATGACATACATTGGCACGCG
TTGATTAAACATCACATATGCAATAAAGTGAATAATATTATATATACAAGTGACAAGTGAAACTAACAGC
CACCTCTTCTTGCACTGTAATTACACTGCTCAAATCTGGAATTTGTTCCTTAGCATCTCAAATCTGAATTGG
ACAATGCCAGAACGTACATCAAACGTGTTGAAGAGCTGGGTAAGGAGAGGAGGCACCAAGAGCCAGA
AGAGATGGTGGAGGTTAATACCATCATGTATATGGTGGTCAGTTTGGAAGGAAAGGAACAGTAGATGT
TTCGACAATAGATCCAATTCCATACACAAGGTCAAATGGAATTGTATAGTATCTCTACTTTTTGGTGTAAA
CAGTTGTGTATAGTAGATACAGACCAGATTGTAGATTTGATAGGAAGTTTGTAATTGACACTTTTTGGTG
GTAGCCAGCATGCCCTTAATGCTGAAGAATACAACCTTACCAGTTTCAAAAAAAAAAAAAATATTATATAT
ACACGCTTGTATACAAGGTTGATACGGTATTTACACTAAAGTGAATAATATTATATACTGGGGTATAGAA
TAATATTATACTGTTGTGGAAATGATTTAAGGTGTAAACTGAAAATATGCTTAGAGGGTGCAACGTGTTA
TGTTTGGAAGGTCACATTATTCCAGAAACGAAATACACATGCATTTGGAGCCAATGTGTTTTACGAGTGC
CTTATCTTTTTGACATATGAATTTGTATTGTTGTTTATTCAAATGTATAATGAGACAATCAATGAATGTGTT
TGAAAGGAAAATGTTTTTTTCAGAAAACAAGTTGATTTCTGACTTATTTTCTCATGTTTGGTTAGTAAGTG
AAAAATATTTTTTAGTATTTGGTTGGTAAATGAAAAATATTTTTCAAAAAATATCTTTTATTATTGGTTAG
AGCTTAGAGACTAGAAAATACTTTTTAGGAGATTAATTTCTAACCCAAAGATCCGAACCTGACAACCTGA

ACCAACACACGACCTCAATGACCGATTCGGGATCCGAACCCAATACCCAACATGTGACCTGACCTCAACC
CCAACACCCGAATTAAAACACGACCCCGATGATCAATCCGGGGATTGGACCTCAAGACCCAACCCAAGA
CTTGGCCCAGACACCCGACGCATGACCCAACCCCAACACCCAACTTCCAAATCGAGACCCGACCTCGACT
CTTGATGCGGATCCGACTTTTGAACTAGGACCCAACCTCGACACCAGATCTAGGACCCAGGACCCAACTT
CGACACTCGAACCAAGACCCAACCTCGACCTTGACTCCGGACCCAGGATACGACTTCCAAATCGAGACC
CAATTTAGAATCCCGACCTAGGACCCGATTCCTGATCATGTACCCGACCTTGACACCCAACTCGAGATAG
GAGTTCCAAATCGGGATCTGACCCCGACACAATAAATGATCCAGGATTCGACCTCGACTACCGAATCTG
ACTTCCAAATAACAACTCGACCCCAACACCCAACCCGGAATTCGACTTTCAAATTGGGACCCGCCTTTCG
AATCAGGATTTGACCCCTACATCCAACCCCAACTCCCGACTACGAAGATCAATCTGGGACCTAAAACCCA
ACACCTAACTTGGGACCCGACCTTGACTCTCAATTCGAAACACAATTTCAAAACCCAATCCCAAAATCTAA
CCTCGAATCTTGACCCAGGACCCGACACCCAAACTAGGATTCGAAACCAACCCAACCCAACCCAAAAGTT
GGGTCTCAAATTGAGGTTGGGAGTCGGGTCTCGAATCGAGAATCAGGAGTTGGATCTAGCATTAGTGA
TGAGAATCGGTACCCGAGAGTTGAGTTAGGGTGGAAAATTGAAGTGTGCTGAAAAAACTCTTCGACGC
ATCCTAATTCTCATTGAATTCAGGGCCCAAATCATTAATAAGGACTCATCCTCGACCATTTGGTTTCATAT
TAGATAAAAGGAAACTAAAAATGGGGCACTTGTAAAGCAATTGAAAAGAGACCAGAACTACGTGAAAA
GATGTTTCATATATAAGCAAACATTATTATTGTGGACCAAGGGAACTTATATACATAAAAGAAATGGCCT
AAACCCCTTTCACTCATTTAGCTCCACCCCTGCGTCCGATACTCAACACACATAACTCATTCTACAGTTCTT
TGTAAAATTGTGTATAATTAGCCTTGTAAACATTCAAAATCCTTAATATACCCTTTTCTTTTCAAAATCCCT
CTTCGTTATGAAACTAAATATCTATGGACTATATTTAGAATATTTACTTCAAAATCATCACAAAAAAATGT
AAAAGATGATTAATACTACTAACTACTATTCCAGGCTGTTATTTGCTGAAGAAAATTTTGATTTGATCAAC
AAAACGTTACTAACATTAGTCAAGTCACACGGTCCCCTTAGAGGAAAATGGAGAAAAAGACAATGAAG
ACCAAAATGGAGATCAACTGATCACTGCTATTTAAAGTCTCATAGCCCCATTGTAACTAGCCATGCCAAG
TTCTTTCTAATTTGTAAAGTACTAAAATGGAGAAAGCCACCAACACTTTTTTCTTCTTGTTTCACTTGGTTG
TGGCTAGTATAGCCATGACTCACACCAATATTACCAATGATCAATTAGCTCTTCTTTCTTTAAAATCCCAA
ATCATTTCTGACCCCTTTCACTTGTTGGATGAAAGTTGGTCTCCAACTACTTATGTCTGCTATTGGGTTGG
CATTACTTGTTCTTCCCACCATCGAGTGACGATGCTGAATATTTCCAACATGGCTCTTACAGGCAAAATTC
CTCCTGATTTCGGGAATCTCACATTTCTTGTTTCTCTTGACTTGAGTGGCAACAATTTCTATGGAAATTTG
CCTCAAGAAATGGCACGTCTACGTCGGTTAAGGTTTGTTATGCTTGGTTTTAACAACTTCAATGGGGAGG
TTCCTTCATGGTTCGGGGTCTTACACCAACTTCAAGTTCTGAATATAAGGAATAATAGTTTTAGTGGTTCC

ATTCCCTCTTCAATTTCTAATATCTCTACGCTTCAAACTTTGGATTTGACATACACCTCCTTAGGGGGCCAA
ATGCCAAAAGAAATTGGAAATCTTCAAAATCTAAGGGTTTTACGAATATCTGGTAACAATCTTATAGGTT
CTATCCCTCTGTCTCTCTCAAATGCCACAAGGTTGATGACTTTAGAACTTTCTGTCAATTACCTTGAAGGA
AACATCCCCAAAGAGATTGGAGAACTTCAAAACCTAAAAGTTTTGGCAATACAAGCTAATCAACTTATAG
GTTCTATACCATTCTCGATCTTCAACATTTTCGGAATTAAAGTCATTGGATTTTCATATAATACCTTATCAG
GAGATCTTCCTACTGATATGTGTGATCGACTTCCAATGCTGAAAGTGCTTTATCTAGGTTTCAACGAGCT
ACACGGTCATATGCCTCTAAGCTTGGCAAACTGTTCAAAACTTCAAACCTTTGTTTTATCTGATAACAAGT
TTGATGGACCCATACATAGTGAAATTGGACATTTAAGTAATTTGCAGGATTTGTCTCTCGAAAATAACCA
TTTTGAAGGTTTGGCATTGAATACTAGCTTGTGGTTATTTTTCTTCTAACTAACTACTTCCTGAAAAATTTC
AATCACATAATTTTTCTTTTGCAGGGAGAATTCCACAAGAAATCGGGAATCTTGATAATTTGGTGTCCAT
AAATCTTATGGCAAACCAGATTACCGATTGTATCCCAATCTCCATATTCAATATCTCGTCACTACAGTTTCT
CTCATTGGATGACAACAATCTCCTTGGGCCCTTGCCAGGGGAGGTTGGCAACTTGACCAAATTGCAGTA
TCTACTTCTTAATGAAAATATGCTTACAGGTAAGTTATTGCAGCTATCTTGCTACAAGTGAACTAACAAA
GTTTAAAATCATTACACGTTGTTGCTTAATTATATCTCCATACATACATTGACAGGTGAAATACCGAAAGA
GGTAAGCAATCTCATTGACTTGGCTGAAATGGGGTTGGGTTTCAACAAGTTTACTGGTTCACTTCCAATA
GAGATCTTCAACATATCAGGGATAGAAACAATTCAACTTACTTCCAATAATCTGACAGGAACCCTACCAT
TAAACATGGGTTCTACGTTACCCAACATTGAAGTTCTTTATCTAGGTCGTTTAAATCTTTTTGGGACTATA
CCTCATTCTCTCTTCAATTGTTCCAAACTCACTCATCTAGACCTTGCTGTAAACAGACTCACCGGTATGATT
CCAACCTCTCTTGAATATTTGACTCAACTCAAGTACCTATTCTTGGGGGGAAACTATTTAATAAGTGACAC
TAAGTTAAGCTTCTTGACTTCCTTATCAAATTGTAGAGATTTAGAATTTCTTTCTTTATCTTTGAACCCTCT
GAATGGAGTGATTCCAGTTTCTATAGGAAACCTTTCCAGTGCTTCTCCTCTATGGTTTAGAGCTTTTGAAT
GTGGAATTAAAGGGGAAATTCCTAAAGGAATTGGGAACTTAAGCAGCTTAGTAGACCTCGACCTTTCTG
GAAATGGCATAACTGGATCGATTCCCGCAACAATTAGAAATTTGAGATTCCTTCAGCGCTTTAAGTTGAG
TCAAAACAAACTTTCTGGATCTATTGGAGAAGATCTATGTAAATTGCAGAACTTAGGCTATTTACACTTG
ACTCAAAATAAACTTTCAGGATCTATTCCTAATTGTTTGGGGAACTTAACTTTCCTTCAAGAGATATTTCT
AGGTTCCAACAAATTGTATTCGAACATACCAGCAGGTTTAGGGAACCTCAAAAATCTCTTGAGGCTAGA
CTTATCCTCAAACAACTTGGGTGGCTCATTACCTCTGGAGATTGGAAACCTAAAGGCTGTCATATATATG
GATCTATCAATGAATGCACTCTCAAATGGCATACCCGGAGAAATTGGTGGCTTGCAGAATCTAATACACC
TTTCTTTGAGAGACAACAAGTTGCAAGGATCAATACCTGGCTCAATGACCAGCATGTCAGCTTTGGAATT

TCTAGACCTTTCTCAGAACAATGTCTCAGGATTAATTCCCAAGTCTATGGAGAAGCTACAAAATCTCAAG
TATTTCAACGTTTCATTCAACAAGTTGGTTGGTGAAATCCCCACTGGCGGTCCTTTCAAGAACCTCTCCAG
TCGGTCATTCATGTCCAATGAAGCATTGTGTGGTTCTATAAGATTTCGTGTTCCGCATTGCAACAATAGTA
CTTCAAAACATAGATCCAAGAGGAAAAAGGTACTTCTATTTCTACTAGCTGGAATACTAGTTGTATTAAT
TCCTATCGTCTTTCTGCTTGTATGGATAAGGTATATAAAAGGTAAAAGAAAGCAAGCAGTTGACTCATCC
TTTGTCGTCTCAACCAGAGAAAGAATTTCATACTATGAATTGCTCCGAGCAACTGATTCACTCAGTGAGA
GTAATTTGGTTGGTTCTGGGAGTTTCGGCTCTGTTTACAAAGGAATCCTCAGAGATGGGACTCCTATTGC
AGCTAAAGTTTTCAATCTGCAATTGCAAGCGGCATTTAAGAGCTTTGATACAGAATGTCAAGTTTTGCGC
AACCTCCGTCATAGGAATCTCACCAAAGTCATAACTAGTTGTTCCAACCTTGAATTTAAGGCTTTGGTACT
TGAGTACATGTCCAATGGGAGCCTCGATAAGTGGTTATATTCGCACAACTACTCCTTAAACATTAACCAT
CGACTAAGTATAGTGATAGATGTGGCATGTGCGTTGGAGTATCTCCACCATGGCTGCTCATTGCCCGTG
ATTCACTGTGATTTGAAGCCTAGTAACATCTTGTTGGATGAGGATATGGTTGCCCACATAAGTGATTTTG
GCATTTCAAAACTGCTTTCTGATGATGAGAGTGCTTCATACACTAAAACTCTAGCAACATTGGGCTATATT
GCACCAGGTATGTACTCTCTTAACTAATTTCTTTTGAATACTTCTCTCAAATGCTGACTTTAAAAATTGGCT
GATTGTAGAGTATGGATTGGAGGGATTGGTGTCAACAAAATGTGATGTTTATAGTTATGGCATCATGTT
GATGGAAATATTTACACGCACAAAGCCTAGTGATGAAATGTTTGATGGAGATTTTAGCTTGAAGCAATG
GGTGAGCAATTCACTCCCACAGGCAATAATGGAAGTTATCGATGCCAACTTAATAACGCCACATGATAAT
CACTTAATGAGAAAGATAGATTGTGTGGTATCCATCATGAAAGTGGCAATGGATTGTTGTGTTGAATCTC
CTAAAGGAAGAATTGACATGAAAGATGTTGGGAGGTTAAAGAAGATCAAGATTCAACTTCTTGCATGTT
GA
>PGSC0003DMG400012588 LRR-RLKs
ATGGGCTCATCTAGTCTCTTCATCAAGTACTATGTTGTCTTGCTTTTCATGATGAAATTATGTACCCTGTCC
ATAGCCGAAGATGGTTCTACGGTAGGCTATCATGGCACAGAAAGGGAGGCTCTTTGGGCGATTCGAGC
TGAATTCAACAATCCTAAATTGCTCCAAAAAAGTTGGAACAGCCTCATGTGTTACATGAATAACACTCCA
AATTGGTATGGTATTAGCTGTATCAACGGTCGAGTCACTGGACTTGTGCTAGAAAATCTTGGCCTTACTG
GGAAACTCGAGGCAAATGCACTTGTCAATCTCACTCAATTACAAACACTTAGCTTCAAGAACAATTCCAT
ATCAGGGAACTTGATGAACTTCTCCAACAACCAAAATTTGAACAACATTGATTTATCCGGTAACAGATTT
GACGGAGAAATTTCACCTTCTCTTCTAACACTTAATTCGTTAGAGTCATTGCAGGTACAGAATAACAAAT
TGAACGGTCCAATTCCCAGTTTTAATCAGTCAACTTTGAAAGTTTTCAATGTCTCAAACAACAATCTTTCT

GGTGAAATTCCAAGCACAATAGCTCTTCAAAAATTTGGTCCGTCCTCGTACTTAAGTAACCATGATTTAT
GTGGTCCTCCTTTTACTAACACAACGTGCATCACGATCAAGGATAATGAGTCATCTACACCAACTCCAAG
TGATAGTTCACATAATGAATCTTCAAATAGTTCAAAATTATCCATGTTAACTCCAATCTTGGTAGTAATTA
ACGTTGTGGGCATTGTTGTTCTTTTATATCTAGTCATTTACTACTTTAAAAAAACAAAAAAGCTTAAGAAA
ATGCTGATGAATAGTAATACAAGATTAACGGAGGTTGATAAAATGGAGACTAGCAAAGCTGAGATCAC
AACAACGCCAACAGAAACCAGGAGTGTGGAATCAAGAAGTGTGGCATCAGAGGTTGAATTAGAGAAA
GGCAAATTGATATTCTTAGGTAGTGAAATAAATTTTGAATTAGATGACTTACTGAGAGCTTCAGCAGAA
GGTTTGGGAAATGGAAATTTTGGAAATTGTTACAAGGCAATGTTGGTAGATGGACCAACTGTTGTGGTG
AAGCGTTTGAGGGTTTTGAAACCTTTAACAAATGACGAATTTGTGAGACAAGTAAGAGCTATTGGTGAT
ATAAAGCATCCAAATTTGTTGCCTATTCTTGGTTATTACTATACAAGGGATGAGAAGTTGCTTCTCTTGAA
ATCTGCACCTAACGGAAGCTTATACAACCGCATTCACGGTATCTTACTGTGATCCACTTTTCTTTGTAGTC
TATCATATAACATTTATAATTACTCAAATACATAACTGATTTTTTTTTCCGTGGTTGGTTGAACAATAGGA
GGAAAAGGGACTAGAGATAGGATACCATTTCGATGGAGCTCAAGATTATCCGTTGCTCGTGGTGTTGCT
CGAGCACTAGAGCATCTACACCTGAACGTCAGTTCCTCCCAATTCGTTGTTCCCCATGGCAACCTAAAAT
CCACCAATGTTTTACTTGACGAAAATGATGATGTTCGTGTTGCTGACTTTGGTCTCACTTCCCTTATTGCA
CTTCCCATCGCTACTCAACGAATGGTTTCCTATCGATCACCCGAATATCTGGCTTCCAAGAAAGTGTCCAA
GAAATCTGATATTTGGAGCTTTGGTTGCCTTCTCCTGGAATTATTAACAGGTATTTGTTTTTCTTGATTGA
TCAGGCAGAATCAAATATTTGCGTATATATACTACATTATTAGTTATCTAGCGATGTATATATTTAGAGCA
TACAATACATATATTTTGATGGTATATTATATTGATCACATTGTTGTATACCATTGATATATATAAGTAAAT
AGAAAATGTACCATCGGGCTGATACTCAAAACCACTTATAAATCAAATTATAATAAGAGACTTTGACACA
AGTGTGTGCGCTATAATTGTAACAGGTAGAATTTCTTCACATTCTGCTCCACCGGGGGTGACCGGTGCG
GATCTGTGCAGTTGGGTTCATAGGGCGGTCCGAGAAGAATGGACGGCTGAGATTTTCGACACGGAAAT
TGCTGTTCAGAGAAGTGCCAACTCAGGAATGTTGAAATTGTTGCAGACTGCTATAAGCTGTTGTGATAA
GTCGCCGGAAAAACGACCGGAGATTAGTGAGCTGTTGAGGGAAATGGAAAGTATTAACGGGGCGGTA
CCCGAATCGGAAGACGAAGATGATCTCTCCTTCTCAATGGATCAATCTATGACAGATGACTCCATAGCAA
CTGCAACTCCGTCTCGATAA
>PGSC0003DMG400012594 LRR-RLKs
ATGCTGTAAGCAGTAACGAAATACTATTTCCATGGACAGAATCCCTCTGCCGCAGATGTTCCCATTGACA
ATTTAAACACATTTTGAACCTCCTTTTCTTTTCTCTCAAAAGGTGTAGCTTGTGGATGCACTTATTAATTTT

TAATGTGCTACATAGCTATGTGATGACTAATGTATAGCCCCTTAATTTTTACAGATTGTGTGGTCTGTAAG
TTTTAGAACTGATTTGCTTACGACCTCTTTTTCCTAATTGCAGGGAACTTTATAGTAATAATATAAGTGGA
AGAATTCCTTATGAACTGGGGAATTTGACGGAGTTGGTGAGCTTGGATCTTTACCTGAACAAGTTAGTT
GGTCCAATCCCCGACACTTTGGGCAAGCTTCAGAAACTACGTTTCCTGTATGGGTTACACATCCTTTTTCC
TCTTCTATATGTTTGTTTTTCTTTTTCTTTTGCTTGGTTGAATAGTTTCTTTAGCCATGAGCTTTTATTTTCTG
AACATTTTGCTTAGTCAAGAAAGGCCACCTTCTATTTTAGAACATGTTAGTGGCTCTGTACTCTGTAGTAC
TATTTCTCTTGTTTAGTTATATGGAAATAGGGATAGACTTTGCTCCTTATTGGCCAAGTGGCAGACATGA
AGATAAAAACATTTCAAACACCTGGAGTATCATTGCCAATGAGACAGCATCATAATTTTCTCTGTGGCTT
AAATTATTCATAAAGAGAGAGAGAGGAAGAGGAGGCGGAAGAAGAAAACCTGAAGCAGCTCATAGAA
GTACCCAAAGTGGGTGTCTTAGGGTGTCCAAAGTGCAGGACATGCATGTCTGAAAATTCAAAAAATATA
CAGGAAGTTAGACACACACATGCTCAATGTGTGTCCAACACCAGTACTTGACAATGAAATTTGTCCATCA
TCCTTGGCAGTCATCGTTCCTATTTCCCTTATATAGGCTTGCAAAAGGCTAGGTGGATTTGAGTGGTTGA
TAAAATTGTGGTTGCTGCTCTCTATAAAGGCTCATTGGTGTGCTGCTGAACAGGATAAAAAATTTACATG
CTATTCTTGTTATTTCCCGAAGCTCTGAAAACTAGATACTTCTGATGATCATATATATGAACATAAATCTC
AACCTGCTAACAGTGTCAAGAAGCTTTCTTAGATATTGGGCATGAATTTTGTAGCATCTTCTAATACTACT
ATTTCTTCTTCATTAGTCAAGTATTTACTACAATGTACACATGGACTGATTAATTAGCCGAGTTCTGTCTGT
TTAAGCTTGATCAAACTTTTTTTTTCTTATTGTTATTGTTTTCTTTTTGCTAAATTGAAGGAGGCTTAACAA
TAACAGTTTGACTGGGCAAATTCCGATACTTTTAACCACAGTCACTTCACTTCAAGTGCTGTAAGTATAAA
GCTGGCACTTTTTTATCATGTGGTTTTTTTCGCCATCAGTATTTTCTAATTCTTAGGTACTAATGTTCATTCC
TCGCAATGCAGTGATTTGTCAAACAATAATTTAACAGGACCTATTCCAGTCAATGGTTCCTTCTCGCTTTT
TACTCCTATCAGGTCTGTGAAAAGCACTAGTTTATATTTTAAGTTCTGTCACTGGAATCATGATAATGAAA
TTCTTACATCTGAATGTCTATATTTCAGTTTTGCTAATAATCCATTGGATACTCCTCCAGCTTCTCCACCTC
CACCTATTTCTCCAACACCCACTTCTCCAGGTTTGTTAAAATAATTTATGTTTTATGGGTTAAGGCACTGC
GCCCCCCCCCCAAAGGAAGAAGGCAAAAGGGCTGACTGTTTTAGGGGAAGAGATTTGCACATTATAAT
GAGTTTTAGTAGTGTATGTGAGATAAATTTTACAGGTGATTTTGGGGTTATCTACTGTTTGATAGGTTTG
AATAAATAGATTAAACTTACAAGTGATATTGAAGAGTGGATAGAAGTGTCCTACATCAGGAAATGCAAC
TGTATGCATTGGATATTGTGACTATAAAAGGACCCAAACTTCAGAATTTATTGTCATACATTCTCTCAATT
CTTTCCCCATGTGTCTTATTTCTCACATGGTATCGGAGCTTTGGTGAAAAATTAGTTTCAGTGCATTTACC
AGCAACACGCGGGAGAAAAATCTCCTTGCTGTGCAATTCTCATTGACCATGACTGCTGTCAAAATGTATC

CTTGCCGTCTCTGACTACTGTACATAAATCAACACCACCAAAAGGCCGAATAACAAACAAGTACCTGTTG
TTATGAATAAACTGAAGGATCTTTACCTATTTCTGCATCTTTTCTATTTTGTTAGCACTTTAGCTTTACAAC
ATAGTCCTCGGTGCGAGTGTAAATGGTGAAACTACCATATCCCTTTTCTCAATTTTTTTTGAACTACTTGT
GTTAAGTTTTTGTCATCCATCAAAAAAAGGGAATTAAAACTTAAATTTTGAAATTCCTAGGTGTGGGAAA
CAGTGCAACCGGGGCGATTGCTGGCGGTGTTGCTGCAGGGGCTGCTCTTCTGTTTGCAGCTCCTGCGAT
TTTGCTTGCCTGGTGGCGCCGAAGGAAACCGGAAGACCACTTCTTTGATGTTCCAGGTGGGAAGCCTCA
ATCAAACACCTGAATTAGTTTTTTGTTTTCAAATTTAATTTTAATTTAATGAGTTCTTGCAATTTTACGTTCT
TTTGCAGCTGAGGAGGATCCAGAAGTTCATCTTGGACAACTCAAGAGGTTCTCCTTGCGCGAATTACAA
GTTGCAACTGACAGTTTTAGCAACAAAAATATACTTGGAAGAGGAGGATTTGGCAAGGTTTACAAGGGC
AGGTTGGCTGATGGCACTTTAGTTGCTGTTAAAAGATTAAAAGAGGAGCGTACTCAAGGCGGGGAGTT
ACAGTTTCAGACAGAAGTTGAAATGATTAGTATGGCCGTGCATCGAAATCTACTTCGTTTATGGGGCTTT
TGCATGACTGCAACTGAACGGTTGCTTGTTTATCCATACATGGCGAATGGAAGTGTTGCTTCACGCTTAA
GAGGTATCTCTTAGTCTTGAATTTTCCATCCTTCTTTTTCTTTGCTCCATTGAGTCGAAATACATCAGATCA
AGCACTTGAAAAAGCTGAAAAATGATAACAACGTTTTTTTTGAACTTTTAAGTTATGGTCTGGTAATATA
AGTTATCTGGAGAAGTTATTGCAGATGTGTACATCATTGTGTAAACTTGGCATTATTGGATATTGTCGTG
GTTGTATTAGGAGTTAGTTTGTTATAGTTAGAATATTCAATCCCGTAGTTAGAAGTTGTTGTTCTTTGTGA
TTTTACCCGTGGTCTATCAAGCAATTTCAAGAGCATTGCTATCCTTGATGTCCTACCAAATATTAAGATTT
CCGTTCAAGTTTTATGTTCTGAATTCATAGTGTCAGATATGGAAAGTAATCTTCATTTATGCATGAACCGC
ATATATCAATTAATTAAATTGCGTTCAACCTAAAATGGGAACTTGAACCAGTTTACTTGACATCTTGAACT
TGTCCTTACAGTAAAATTTTTTTCTTGTAGAGCGGCCAGAGTCAGATCCACCACTTGGCTGGCCAATAAG
GAAGTGTATAGCACTTGGATCTGCAAGAGGCCTTGCTTATTTGCATGATCATTGCGACCCTAAGATCATC
CACCGTGATGTCAAAGCTGCAAATATATTGTTGGATGAGGAGTATGAAGCAGTTGTTGGGGATTTTGGG
TTAGCCAAACTCATGGACTACAAGGATACTCATGTTACTACTGCTGTACGTGGTACAATTGGGCATATCG
CCCCGGAGTATTTATCCACGGGTAAATCTTCAGAGAAAACTGATGTGTTTGGCTACGGAGTTATGCTTCT
AGAGCTCATAACTGGGCAAAGGGCGTTTGATCTTGCTCGACTTGCAAATGATGATGATGTCATGTTGCT
AGATTGGGTAAGGATTTACTAACCTACATGCTCTAGTTATTCTACTTCTAGCTTATGTCTTTTTCTCTTTTC
TTTATACGTTGAAACTTTATACTACTTACAATTGCTAATTTTTATCGTGCAACTGACTTCAAACATGGAAG
TGAGCACGGGTGAGAAGATCTCACTTTCCTGCATGCATCTCCCTATAAGCAGAACTCTATCATTGCTGCA
CTCAACATGATGAAAAGCTGATTTGTAGACCCACTACAAATTAGGACTGGCGAATAAGACACTGTCATG

TCCATTCTCTTCCCCTGCGTTTTCCTTTAATATCCCCAATCCCGTTCCTGTTCTGTTATCCCTTCCCTACCCCC
GTATGTAACCCACATCCCATAATCTCGTTTTATATCCTCATCCATCAATGAGAAATCTCAGCTTCATCTCAT
CTCCCTCACTCTTTTGCACATATTACACACACTCATAGCTCTCTTTGTTCTTTACTCAAGTCTCAGGGCTGC
AGTTCTGTTTAAAGTAAAAAGTTTTATATTGACCCCCCCTCCCCCCAAAAAAAATAAATTAATAATAAAAA
AAAAATTGTTAGCTCTAGTAGGTTTTGCATTGCAGTTTGGCATGTATGATTTCTGTAATCTTTGATACTAC
ATCATATGAAACATCCAAATTTCCAAAATAATTTGATTCCTGGGACTTGTATTTGTGCACATTCTTCGTGT
AATCTTTTTGTTTCTCTTTCTAGAACTTAACCATCTTGCGAAAACATTTGTTGCATTAGCTGTATGGTCAGG
GTGGATCACTTTCATTATCGTGTAGTAGCAGATTGTTCTGGAATTGCTTTTTAGTGTTAAAATCTTTTTCCC
TTTGGTTAGCAAAAGCACTCGTATAGCATATTGCTATATCAGAAGATTTAGATACTTTTTCCATGCCATAT
TGACTGTCGCCTTCTGTGCAGATAAAACTACATCTCTTTTTTTTTCCGTTGTATGGAATTGCTAGGCTATTT
CTCTGTATTTGCATCAGAGAGTAGGTGAGATAGAGAATACACTTTACGAGAGGGGACTTCTTTGGGAGG
ATCCTCTAGAAAGGGAACCCACCCTTTAGGACTTGGGAGTGCAGAAGATGGTCAATTCTTTCACTATGGT
TGAACCGTTGAAGTATAGAAAATTGAAAGACAAGTGGCTATCTGAACTTGGAAAGTAAATAAAGTGGCT
ATCTGAACTTGGAAATTCAATATGGTTCAAATATGAGCACAGAGAATCAGGAGCCTCATGCTCTCTGATT
TTCTGTCTTTTCAGTCTCCCTATAGGGCTGCTTATCAAAAATCTCAACAACTTCTATGGTGAATGCTTTGTT
ATTTTAGTGATGAAATTAATTATTTGCTGACATGATGAGGAAAGTCCGCATGCACTGTGATTTCTTTTTAA
AAAATGGCACTCATGTGAAGAAAATGGTTATGTACCTTATTTGACCTCAAAAATTGTGTTAAAATTTCAG
GTAAAGGGACTTCTTAAGGACGAGAAGTATGAAACATTAGTAGATGCAGATCTTCAGGGTAATTACAAT
GAAGAAGAGGTGAAACAGCTTATTCAGGTAGCTCTACTCTGCACACAGAGCTCTCCTATGGAACGTCCA
AAGATGTCGGAAGTGGTCAGAATGCTTGAAGGTGATGGCCTTGCTGAGAGGTGGGAAGAATGGCAGA
AGGAAGAGATGTTCCGTCAAGACTTCAACCATGCTCACCATCCTCACACTGATTGGATTATTGCAGACTC
CACTTATAATCTACGACCCGATGAGTTATCAGGGCCAAGATGA
>PGSC0003DMG400012817 LRR-RLKs
GGCTTAATCACTTTTTCCAGAAAAAAATCCACTAATCCATTCATCTCCTTCAACAATGCATCTTCAAATCTT
GCTTATACTTCTATTACCTACATTGATTCTCTCAATAAACCAAGAATCCCTTTATTTACACACCATAAAGCT
TGGATTTGATGACCCAAATGGTGTTTTTTCAAACTGGAATCTCCATGATAACTCCTCACCCTGTAACTGGT
TTGGAGTAAAATGCGACTCTTTAACTCGTTCTGTTACATCTATTGACCTCTCCAACACCAATATCGCCGGC
CCATTTCCGGCTTCTCTTCTTTGCCGGCTCAAGTATATCAAGTACATTTCATTCTACAATAACTCCATTAAC
TCGACACTTCCGGTGGAGGAGTTATCCGCTTGTAAATCTCTTGTCCATCTCGATTTAGCTCAAAATTTGTT

AGTGGGTAGTCTTCCATCGAGTTTGGCGGAGCTTCCTGAGCTGAAATACCTTGATTTGACCGGAAATAA

CTTTACCGGCGAAATTCCGGCGCGTTTTGGGGCTTTCCGGCGACTTGAAGTTCTGGGTCTGGTTGAAAAT

TTGTTAACTGGGACTATCCCGCTGGAGATTGGAAACATTTCGAGTTTGAAGCAGCTGAATTTGTCGTACA

ACCCGTTTTCTCCGGGTCGGATCCCGCCGGAGATTGGGAACCTCACGAATCTCGAGGTGCTATGGTTAA

CTGACTGTGGGTTAATCGGTGAGGTTCCGGGTACATTAAGGGGATTAAATAAGCTTGTTAACTTGGACC

TTGCGTTAAACAACTTGTACGGTCCGATTCCGAGCTGGCTCACTGAGTTAACTAGTGTTGAGCAAATTGA

GCTGTACAATAACTCGTTTTCCGGCGAGTTTCCGGTGAATGGGTGGTCAGATATGACCTCGTTGAGGCG

GGTCGACCTGTCGATGAATCGGGTTACCGGGTCGATCCCGAGCGGGTTGTGTGAGTTGCCACTTGATTC

ACTCAATCTTTATGAGAATCAATTGTATGGTGAGTTACCTATAGCCATTGCAAATTCACCCAATTTATATG

AATTAAAGCTCTTTGGTAATAGGCTAAATGGAACTTTACCTAAAGATCTTGGTAAATTTTCGCCATTGGT

ATGGATTGATGTTTCAAACAATGAGTTTTCAGGTGAAATTCCGATGAATTTGTGCGGAAATGGGGTTTTA

GAGGAGGTTTTGATGATAGATAACTCGTTTTCCGGTGGAATTCCGGTGAGTTTAAGCCAATGCCGGAGC

TTATTACGTGTGAGATTAGCTCATAATAAGTTCTCAGGTGATGTCCCTGTGGAATTTTGGGGGCTGCCAC

GCCTTTTGCTGCTTGAGTTAACGGACAATTCATTTTCTGGTGTAATCGCGAAAACTATAGCTGGTGCATC

GAATTTATCAGCTTTGATTTTGTCAAAGAACGAATTTTCGGGTAATATTCCTGAAGAGATTGGCTTTTTGG

AAAGTTTGGTTGATTTTGTGGGAAATGATAACAAGTTTTCAGGGTCGTTACCGGTTAGTATAGTGAATCT

TGAGCAATTGGGAAGAATGGATTTTCACAACAATGAATTAAGTGGTAAGTTTCCTAGTGGGGTTCATTCT

TTGAAGAAATTGAATGAATTGAACTTCGCAAACAATGATCTTTCTGGAGAAATTCCCCGGGAAATCGGG

AGCTTGTCTGTTTTGAACTATCTTGACCTATCAGGAAACAAGTTTTCGGGGGAAATTCCAGTTGCGTTGC

AGAATTTGAAGCTCAATCAGCTGAATTTATCGAATAATGGCCTTTCGGGTGGTATTCCTCCTTCATATGCA

AAGGGAATGTACAAGAATAGCTTTCTGGGGAATCCTGGTTTATGTGGAGATATTGGAGGTTTATGTGAT

GGAAAAGATGAAGGTAAAACTGCTGGTTATGTATGGTTACTGAGATTGCTTTTCATACTTGCTGTTTTGG

TGTTTGTAGTTGGGGTAGTTTCGTTCTATTGGAAGTATAGGAATTACAAGAAAGCAAAAAGGTTGGATA

GATCGAAATGGACCTTGACGTCGTTTCATAAGTTAGGTTTCGATGAGTATGAAGTACTGGAAGCTCTAG

ATGAAGACAACTTGATTGGTAGTGGTTCTTCCGGGAAGGTTTACAAGGTCGTTTTGAGTAATGGTGAGG

CTGCTGCTGTGAAAAAACTTTCAAGAAGTTTGAAAAAAACAGATGAGAGTTGTGACATTGAGAAAGGTA

ACTATCAGGATGATGGATTTGAAGCGGAGGTTGAGACATTGGGCAAGATTCGACACAAGAACATCGTT

AGGCTATGGTGTTGTTGTACAACAAGGGGTTGCAAACTTTTGGTTTATGAGTACATGCCTAATGGAAGC

TTGGGCGATTTGCTACACAGCAGCAAAAGTGGGTTGTTGGATTGGCCTAAGAGATTTAAGATAGCTATG

GATGCTGCAGAGGGACTCTCATATTTACATCATGATTGTGCTCCTCCGATTGTTCACAGAGACTTGAAGT
CGAACAATATCTTGTTGGACGGGGAGTTTGGAGCTCGAGTTGCTGATTTTGGTGTGGCAAAGGCGATTG
ATGTCGATGACAAGGGAACCACGTCTATGTCAGTCATTGCAGGGTCTTGCGGTTATATTGCTCCAGGTTA
GAGGATTAATCCTTCACTTTGGGAGTTCTACTAACATGATTTTGTTGAATTTGGTTTATGCCTTCCATTGC
AAATTGTAGTTAAGATGTATGCCTAAATATAACATATTTCATATCTTGTTATATTATAATTAAGCGTCGAA
TAAGGTCTGCTGTACACTCTACCCTCCTCAAGCCCCACTTGTGGGATTACACGGGGTATATTGTCATTGAT
GATTGTTAAACGTCCAATATCTATAATTAGGCAGACAGCTATATATGAGTAATTTTGGTCCATTCTTGAGC
TATGTTGCTCAAATCCTTCAAAAAAATGTGGCATGTCTTTGTCGGATTCTCCCAAAGGTATTCAATTTTGG
AGGATCCAATACAGGTGTAACAACATTTTTGGAGGATCCGAGCAACATAGTACTTGAGTGCCCTTATAC
ATTAACACTCAGGTTTGCTCCAAGAACAGGTCATTCTTTGTTTACATTTGCTTTCTGGTTCTCGTAGAGCT
CCTCCGGTTGAACTAAATTCTGTTCGTCTTGCAGAATATGCCTACACACTTCAGGTGAACGAGAAGAGTG
ATATATACAGTTTTGGCGTGGTAATCCTCGAGCTAGTGACAGGGAAACTCCCTGTAGGTCCTGAATACG
GGGAAAAGGATTTGGTGAAGTGGGTTTGCGCTACTCTAGACCAGAAGGGTATAGATCATGTTATTGACC
CGAAACTCGACTCTTGTTTCAAGGAGGACATAAGCAAAGTCCTAAAAATTGGCCTCCTCTGCACTAGCCC
CCTCCCAATTAACCGACCCTCGATGAGAAAAGTCGTAAAAATGCTGCAGGAAGTTGGTGGCGGAGACCA
GCTCAAGACAGCGTTAACAGATGGCAAGTTGACCCCTTATTACCACGAAGACGCATCAGATCAAGGAAA
TGTAGCTTAAAGATAGTCAACATATCTTTGCTTAGATGTAGCGACATGGACAGTGATGATTCATATGGTC
GAGACTCGAGACCAACTTGTCTGGCATTGAGGCATAGTCGTTGTATCGTGAAAGTGAAGATGTGTGAAT
TTTTTTATTTGCTAATTGCTTAATACTTTGCTATCAATACTTGATTTTTGGTTTAATTTTGGAGAGAAGGGG
AAAACAGAGTTGAGATGAAGTGTAAAAGGATG
>PGSC0003DMG400012929 LRR-RLKs
GCTGCCTGCAGTAACACCTTGAAAACCGTACCTCATAGAAGCAAAAAACAAGAGTGTGGTATTGTTGCT
GGTTAAATGATGAATGTTGTTGTAGTTGTGATCCTTAGTTTTTTCACATTTTCAATCTCTGTTATATTTTCA
TTGAATTCAGATGGTGTTTCTCTGTTAGCTTTGAAATCTGCAATTTCTAATGACCCATCTGAGTTTCTTTCT
TCTTGGTCGGAGTTAGACTCATCAGCATGCCATTGGAATGGAATCACATGCGATGAAAATCAGAAGGTG
ACTTCTATTTCTCTGTCGTCGAAGAATTTGAGTGGTTACATTCCTTCTGAAATCGGAGCACTTTCTTCTCTA
TCAATTATTTCTCTTTCCTACAACAACTTTTCAAAACCAATCCCTATTCATCTCTTCAATGCTACTTCCCTTC
ACTCCCTTGACCTCTCTAACAATGCTTTCACAGGATTCTTACCTCAACAAATCACATCCCTTATAAACCTTA
AACACCTTGATCTTTCTGCTAATTTTCTCAATGGCTCACTTCCCAAAGAGCTAACTCATCTTACACTTCTCA

CTGGAACTTTAAATCTTTCATACAACAGATTTACCGGCCAACTTCCGGCGAGTTATGGTAAGTTTCCTGTA
ACATTGAGTTTAGACCTTCGGCATAACAATCTGACCGGAAAGATACCTTCAATCGGGTCGTTGTTGAATC
AAGGGCCTACTGCATTTTCCGGTAACCCTTTTCTTTGTGGGTTTCCATTGGAAACTCCGTGTGCAGAACCA
GAAGCTCAAAAACTACCACCGAACCCGAACCCTAATGAGGAGGAGGTGGGGTTTGTAGGAAAACGGAA
ATCGGGAAATGGGTCAATGGTGGTTTCTTTGCTCTCCGGCGTATCGGTTGTGATTGGGGTGATGTTTGTT
TCAGTGTTGGTGTTGAGGAGAAAATGGAAGTTGGATGAGGATAAAATAGGAAAAGAAAAGTTGGAGA
AGCCAGAGGAGGGGCAAAAGGGTAAATATGTAGTGTTAGACGAGGGGTTTGGGATGGAACTAGAGGA
TTTGTTAAGGGCTTCAGCTTATGTAGTAGGGAAGAGTAGGAGTGGGATAGTTTACAAGGTGGTGGCTG
GCGCTACGGTTGTTGCTGTTCGACGATTAAGCGAGGGGGATGTCACGTGGAAGTTCAAGGAGTTTGAG
ACGGAGGTGGAAGCCATTGGGAGAGTACAACATCCAAATGTGGTGAGGCTTAGGGCTTATTACTATGC
AAGTGATGAGAAATTGCTGGTAACTGATTTCATCCGCAATGGCACCTTGCACAATGCTTTACATGGTAAT
CTCACTTCATTCTTACTTGGAATTTTCTTTACTGTTAAAAGAAAATTCACTCTAAACATACTACTTACGGAG
TATTATTCTAAATCACTATAAGGATAAACTTTTAGTAGTGCTGGAGTAAGTTATCCTGCCTATGCGAGTG
AGAAAGGGTGTACTTTTTTGGAGAAAATTAGATCAAATCCTCATAAGCAGTATTATCTCTGTGTCCATGG
TACTTCAGGGGTTTTATTAATATATCAGACAACCTTGAAGGTGGCATTGTATTTGATATTTATGCAAAAA
AAGAAGTTTAATGTCTACTCCATTGAAGTAAGTGTTTACAACCTAAGATGTCCTTAAAGCTGCTCATGATT
ATTCGTTGAAGTATTCTCTAGGCACATTTAACAATTTCTGTCATGGTTTCAAGCATTCTTTGATAAATATTA
ATGATCCCAAATTATATGCTTTTAAGTCGTTAGCTGCTTACTGTCCTTATCTTCTTTTGATCAAGAATAGGC
AACCAAATGTAAATTATGATGAACAAGAACTAGTTTGGGGTAGTGGGGTGGGGAGGGGAATAATACGT
GCATGGTTCATTTGTTTTTATCTGAGCAATTGGCATTTGTGTTTTCTGCTTCCTGGAAAACATCTATGCAA
GTGTTAAGCTATTGAGACCTTTTGGGCTCCACAATTCAACATCTGTTGGTTTATTGTAATTCACTAGTTTC
TGGTCCCCATTATGGCATTTTCCCCTTGTTTTCTGCTTCCTAGGGAGTACATTTTATGTGGCCTAAGATAC
ATTGAGAGAGGCTTTAAAGTTGAAGTAAACATTCGGAAGTTACACTAAGGAAAAAAACATTAGATCTAT
CAATTGGATGTTAAGCTATTCGTTCGATTCACATTCATTAGGAATTGAACTAAGCATGGTTACACATAAA
ACGTGAATCCAAAGTCTGAAATTTTTGCAGAACAAAATATCGACTAATCATAAAATGTTGTAGTTCCTCC
TTGTAACATCTATCATTGTCAAGTTCACTTTAGGATGTTGGACTTTATGTAAAAGAGGCATTCAGCTGAC
ACCGAGAAGAAAACTATTACACCACAGGTTATTTTTGTCTTCAAATTCTTTTTTAACTAAGTGGAGAAGTC
CACGACGTTAAGCTTTCTGATAGGTAAATATAAAACAAAAAAGATAATAAGTAAGACACGAAATTTCAT
CAGATTCCAAGACTTGGTAAGTCATTCCCTGTTTCCTTTAGTCTGTTTGTCTTGTTATTCAGAGGTGTTGTT

TAATGTACCTATAATTTGTATTGTGGTTATAGAATCCCTGAATAGGCAGTTACTGGACTTGTGTTAGCCTT
GTATTTTGCTCTCTTTATTTTCAGAACCTTGCACTTCACAAAAACATTAATGTTCATTTGAACTCCAGTCCG
TTTTTCTTAACTTCAATCTTTGTGTACAAGTCTTCTCCACATAATCCTTACCTTTTTGCTCGTACTTTGGTAT
TGTCAATTGTGAACCAATCCTATATAAACACAAGTGGAAATGAAGATTGCACCTACTTTGGGGTGGCTTC
TTGAGAATGTGGTTCCTCTAGTTCATATTGATAATTTTTTGTCTCGACAAAATACCAACTGACCTATTTCTT
GTTTTCCTTGACTCAGACTTCCATTGTTTCAAGCACATATTTTTGTACCAATTTCAGGATTTGAAGTGAAA
AATGTTCTATTCGTTATTCTGAAAAGCTGTCGTCGATTTGGCATGAATCACTTTTTAATTTATCTTCCACAA
GTCCAAAAATATTTTTTGGATTTCAAAACTTGCATTTTCCGGCAAAAAATTTCTCATGGAAAAAAACACAT
ACACAACTAATTTCTCTAGAGAACTGGGTGATTTTCTTTTTATGTTGGAACATTATCCTTGTTAATCAATCT
TTTTATGTTTATTTTTTACAATTGCAGGAGGACTTGGTAATAACTTGCCACCTATGTCATGGGCAGCTAGA
CTGAAGATCGCTCAAGGCACAGCTCGGGGTCTAATGCACATTCATGAGTGTAATCCCAGGAAGTATGTT
CATGGGAACATAAATTCAACAAAAATCCTTCTTGATGATGACTTGCAAGCCTACATATCTGGTTTTGGAT
TGACTCGCCTTGTTTCAGGCTGCTCCAAGGCCATAAACAGTAGTACCAAAAAGCTAAGTACAAGTCAAAT
CATAATAAGTCCTCAAAACTCAACTTCGTCTTGTACTATGTACATGGCACCTGAGGGTCGAGTGCCTGGC
TCCAAGTTCACCCAGAAAAGCGACATCTACTCATTTGGTATGGTACTTTTGGAGATTCTGACTGGTCAGT
TGACAGATGGAGGATCGGAGGTGGAGGATGATGGGAAGGGGCTTGAGAGTCTTGTTAGAAAGGTTTT
TAGACAAGAACGACCCTTGTCTGAGATCATAGACCCTGCTCTCTTGAATGAGGTTCATGCTAAAAAGCAA
GTTGTTGCAGCATTTCATATCGCTCTCAACTGCACAGAACTGGATCCTGAGCTTCGGCCAAGGATGAGAA
CAGTTTCTGATAACCTTGATCGCATCAAACTGCAGTAAAAGAAACTTGATCAATGTAAAGGCTTTCAATG
TTTTGCTGACTGCCTAATGTAGAGCTGTTTAGTAATTGTGAATCAGAATTTTCAGTTTGTGTTGTGCCAAA
TCCTACATTTGGCGTTTCTATTGTAACTCTACTAATTCAATGTTCTATTTGTTGAATCATCAATTGAAAATT
GCTTGTGCTAGCGAATCTTACAAAGCTATTTGTGTTTGATGGTACGGACATGATCTGAATTCCAGATAAC
ATGCAAAGGAGCATGGTCAGACCCTGCCTAATCAAATGATGTAGTTCAATAGTTGGAAAGTTCTGTATG
AATTCTTGATTGCCCATCACCCTATTCAATCTTTTGAAACTACACTCCTCTATTCTCTCATTCTACCAAGTG
AAGTTGCTAC
>PGSC0003DMG400012955 LRR-RLKs
CACCCCATTCCAACAGAGAAATACAGAGAGAAAGAGCAAAAATACAAAAGAGACACCAACTCATTTGCC
ACTCTACCCCGAAATATAAAAACCCTAATTTCCCTCTGTTTTTTCTCCTTTCCCAACTCTTTCCCCACTCCCC
ACTCCCCACTCCCTCTCTCACCACCCACCTACCCTAATCTCTCTCTCTGTCTGAAGTCTGAACTCTTTCTTGA

ATTTCTATGGTGCTCTCTCCATAGTCATCCTTAAGCTTTTCTTCCTTTTACCCTTTTTCAGATTTTCCTTCTTT
ATCTCAATGCCTCATTTTTAGCACTTAAATCACATTCTCAGCCCAATGACTCTCTCTCAATCTCTCTCTTTAC
TCCTCCTTGTACTTGTGTTTCGACTCAATGGGTCTTCTGCTGCAGAAGATGAAGTTCGATCACTTTTGGAG
TTCAAAAAGGGTATTAAAAACGACCCATTAAGTAAAATCTTCAGTTCATGGAGTCAAACGGGTCTTTCAA
ATCTATCAGCATGTCCAAAGTCATTTCATGGTGTCGTTTGTGATGAAAACTCCGATTATGTTTTCTCCATT
TCTCTTGACGGGCTTGGGCTTGTGGGTGACTTGAAGTTCTCAACTCTTAGTGGGTTGAAGCAGCTCAAA
ATTCTTAGTCTTTCTGGTAACTCTTTTACGGGTAGGGTTGTTCCTGCTTTGGGTTCTATGTTAACTCTTCAG
CATTTGGATCTCTCTGGAAATCAGTTTTATGGACCAATTCCAGCACGAATTAATGAGCTTTGGGGTTTGA
ATTACCTCAATTTGTCCAATAATAATTTTACATTTGGTTACCCAAGTGGAATTAGTAATCTTCAGCAGTTG
AGAGTACTGGATTTGCATAACAATGGGCTTTGGGGTGATATCGGGGAGTTGTTTTTGGAGTTGAAGCGT
ATTGAGCATCTTGATTTGAGTAACAACTCGTTTTTCGGATCTCTTCCTACGAGCCCGGAAAATGTATCGCT
CTCTTCGACAATTCAGGTCATGAATTTGAGTCACAACAAGTTGGGTGGTGGTTTTTTTCCTGGAAAATTG
TTGGAGGCATTTGAGAATTTGATGGTTTTGGATTTGGGGAATAATGCTATAATGGGGCAGCTTCCTTCTA
CCGGGTTTATGCATAATTTGAGGGTTTTAAGGCTTGGGAATAATCAGCTTTATGGATTGATTCCCGATGA
GCTCTTGCAGGGAACAGGTCCATTGGAAGAACTGGATCTTAGTGGCAATGGATTTTCAGGTTGGGAAAT
GGATATCAGTTTTTTGGAATATTTGCATTTCTTAGATCAGTATATGTTGTTGTTGTTGAATGATCATAATC
AAATACACCTTTTATTTGTATAGTGTTTTTGTGGATGCCTTGAACTTCATGCCTCTTAATTTTTGGAGGTG
GACTAAAATTGGAAACTTTCTTGTGTTTTAGGCATTGAAACTTTTACAGTTATGCAAAGTTGCGGGGACT
CTTCACTTTTGATGCCGCACCGTGTCGGATTTTCCAAATATACACTACTTTGTAGAATCCGACACGCACCC
GTTGACATTTTTGAAGAGTCCGAGCAACATAACAGTTGTGAATGTAAAATCTCGTTCTTTTTGTTTTCTTG
TCAGGTTATAATTAGTTTTCTTGTGTGAGTAGTGGTTGACAATCTATATAACAGAGAATAGAAATCTTCA
GCTTACGCCAAATACAGAAAAGTTTGCTTTTTTGTGTAAGTAGGTTTTAAAAAAAATGTTTTAACAAAAC
CAAATTTTGAACAAACTTTTACCGCCAGCTTTTTTTGTTTGCCTCAACAGAACGTGAGCAGCGTGGACTG
CATAGACTAATTTGAATTGCAAAGCATGTTCAATAGAACTTGCCCATAATAAGTAGTCAACAAAGTGTTT
CCTAATTGTAGTAAATTTAATAAAAGCCCCTAATGGAATAGTTGCATGAATGATTGCATCATTAGTAGCC
ATAAATAATCTTTTTTGCAATGGTATAACTGTATGCTCAAAGTTCATGGCAAGCTAGCTGGAACAATATTT
AGCCCAGTGTCATCAAAGGCGCGCTTAAGCCCTGAAGCAAGGCTCAAATCGTGTTGAGCGCTTCGCTTT
GCTTAATGTGCGCTTCTGTGTCGTTATCAAGGTTCTAAGGCATACTTTTCCTTATGTTTATATATTTGTCAT
TCATGCTTAGGACTAAACATATATATTTTTATTTTTTCTCCCTTTTCGCCTTTTTCCATTAAAGCCCAATCAA

ACCTTAGAGCTTTTTTACTCTTTTTGCTTTCGATAAGACTGATTTAGCCTTTATCTTGAAAAGTGAAAATGT

TGAATTTTTTCTTCTGCTTCCAATATCCAATGCTATAGCACAATATTTGTACCTACAGTAAAATGAGTTTTA

TTTCATTAATGAGCTATTTGCTCTTAATATGCTCATCTTTCTATTTTGTTATCACTGCATTACACCGTCCAG

GGCTCCCCCCTTCCCTCCAGTGTAGGCCACTCATGATGACGTTGGGCCTTAGAATCAGAAAATAATTGAT

GACTACATTTCTTTTTTGATAAGGAATTGATGCCAACATTCCTGTCTTCATTATTATCAAATCAGTACTCAA

ATTAGAGAAAAGAATGGAGTTGGTGAGACTTGATTATTGAAGAAAAGCAGAGCAAATTTACAAGGGTG

ATAATTTGGGTCTCTGAGCTTAAAACTCCTCGGTGACAATCTGATAGGGAAGTTTGTTCTGTTTCATGAG

CTACAGAGAGGGTTCACAATTTCATATTTCTTTAGTTACACTTGGATATCTTTCATCATAGGACCAGAGCG

TGGTTCCACTTAGAGCCTAAATGTGGAGATCCTCTGTCCAGAAGTTGAATGGTGCCGTTGAGTGATGAC

TGATATTTCATTCTTCTCATCTTGCATTTATTCTTTGAGGAAGCGACTCTCAGATCAGCTAGATAGTTTTGT

ATACTTGTCTCACTTATATACTTTTTTGCCTTCTAATTTTTTTTTGGCTATATAGTATTTCTACCATATTATTT

AGCTAGAAATAGTTGCACTTAGACCTGAAAGTTAAATTGGATGTTTAATAACAATGCACATTTTTTTCAAT

AATATGAGCTCTTTTTCTGTTGGACTTGTAAACTTATTCTAAAGAGATATTTAAAGTTTAATATTTCGTTTC

TACGCATGCAGGTTCAATTCCGATTGTGAATTCAACAAAGTTGAGAGTTCTAAATATTTCATCGAACCAC

CTATTAGGTTCACTGCCATCCTCCATAGGAAATTGTGCAGTTGTGGATCTGAGCAGAAATATGCTCGTGA

ATGGTATATCAGCTATTGAGAGCTGGGAAGCCAATTTGGAAATTATTGATTTGAGTTCAAATAGGTTAAC

TGGAAATATTCCCACTATAACCTCTCAGTTTCAACTATTGACTTCACTTAATTTCGGAAACAATTCTCTTGA

GGGAACCTTACCTTCAGCACTGGACACCTTGCCAAGACTGGTTAAACTTGATCTCAGCACCAATAAACTT

GGTGGACCGATTCCATCTACATTTTTTACTTCGACGACATTGATGAACCTAAATATATCTGGAAATCAGTT

GTCAGGATCAATTCCTCTTGAAGGCTCTCATGCTAGCGAATTACTTGTTCAATCACCATACCCTGCACTGG

AGTCTCTTGATCTTTCAGAAAACACTTTAACAGGTAATTTGTCTTCTGCCATTGGTAATTTGCGGAGGCTT

CAAGTGCTAAATCTTGCTAAGAATCAGTTATCAGGCATGCTGCCTACTGAATTGGGTGACCTTAGAAGTT

TGGAATTCCTTGATATATCTAACAACAATTTTAGTGGTATGATCCCCGAAAATCTTTCATCAAATTTGAGG

GTGTTTAATGTGTCTAACAATGAATTAAGTGGTGCTATCCCTGATAACTTGAGAAACTTTAATGAAAGTT

CATTTCGTCCTGGAAACTCAAATTTGGCAATCCCAAGCAACTGGTTACATGATAATCATGGCGATCCTGA

TCAAAATAGTCAGCACCATCATAACTCGAAATCCAGTATCAGGGTGGCTATTATTCTTGCCTCAGTTGGA

GCTGCTTTAATGATTGGTGTTGTTCTCCTGGCTTACCATCGACAACGGTTCCAAGATTTCCACTTACCAAG

TGGATTTAACAGCCAATCTGCTGGAAGGGATGTTAAACTCGGGAGGTTCAGCCGGCCTGGAATTTTCAA

GTTCCATGGCTCCTCAGAACCACCACCAACTTCCTTGAGTTTCTCTAATGATCACTTGCTCACAGCGAACT

CGAGATCTCTGTCTGGGCAAATTGAGTCTGGTACAGAAATTGTTGAGCATGTTTTTCCAGAGGGAGTAA

CTGCAGTTTCAGCATCTACGCATCTTGGCACTGTGGGTAATAATCCTGCAACATCTGGCCAAAGATCCTC

TCCAGGCTCTCCAATAGCTTCCTCCCCTCGTTTCGTCGACACAGTTGAACAACCGGTGACATTGGATGTG

AATTCACCGGATCGATTGGCTGGGGAATTGTTCTTCCTGGATGGTTCACTGTCATTTACTGCTGAGGAGT

TATCTCGTGCCCCTGCTGAAGTTCTGGGTAGAAGTAGCCATGGCACATTGTATAAAGCTACTCTGAATAG

TGGGCATGTTCTTACTGTCAAGTGGTTGCGGGTTGGGTTGGTAAAAAACAAGAAAGAGTTTGCAAAGG

AAGTTAAAAAGATTCGATATATTAGGCACCCAAATGCTGTTCCTTTACGAGCATTTTACTGGGGACCTCG

GGAACAAGAAAGGCTTATTTTAGCAGACTACATACCAGGAGATAGTTTGGCGTTGCATCTCTATGGTAA

GATTTACTTATGAATAACTTCAACAATGATTTTCCATGTCTTCCATTGTTGTTTGGTTACTGTTATTAGAAA

GTTACCTTTTGCTGGTCTGGTGGGTGATTATCTTGTGTTAGGACCAATATCTAGTATCCTTCGCATAAATA

GTTTCTATAGCAGCATAGCTTATTTCTTGTGGTGGTTTTCTAATTGCTGAGATCTCGTGTTTTGTGGGATT

GTTTGATAAAAATTATGGAGGATAAAGTTGACTTGGCCCTATTATTTATTTTTGGTTGGCCTCTCTTGCAA

ATGTCCCTCTTTTACATGTCTTACAATATGCAGATAGTGATGAGATCTTATTTGGATCTGTCGTCTCTTAT

GAATCCAGTGGTAGAGTTGTTGATCAAATGGAAAAGGAAAAAAATACAACTGCAATTGTAGAATCTGAA

TGTTTTTTACAAAATTATGTTTGTTAGAATGTATGTGCAGATCTTTATGTGTCATCTGGTAACCTTCAAAG

GAACTCCAAAAAGGTACAAAAGATCTCTGGTTCACTAATTGCCGTACGCCAACAGCCACACATCAATAGC

CCATCAAAATTAGGGAGTAGACCTATTCTACGATCTCGTTAACATGACCTAGAATGTAGTGATAGTTGCT

CAACAATCTGCTTTAAATAGCATTTCCATTGTTTCTGAGTCATTCATGATGGTAAAACTTGATAAATATGG

AGAGTTGGCTTTGTCATAGTATCCCCGGTGCAGTGAGGACTGATTACTTGTAGACATGTTGGGGGCTGT

TGGAATCTTTTACTTTTTGTAATTTTTCGCAGTTTTAAATTTTTAATGAATGTCTACTTTTTACAGAGACAA

CCCCTCGGAGGTACTCCCCGTTATCCTTCAACCAAAGGTTAAAAGTTGCCATTGAAGTTGCTCGGGGTTT

GGCATATCTGCATGAGAGAGGGCTGCCTCATGGAGACTTGAAACCTACAAATATAATCTTGGTTGGTGC

TGATTACAGTGTCCGCCTCACTGATTATGGTCTCCACCGCCTGATGACTCCAGCAGGGATTGCAGAGCAG

ATACTGAACCTAGGGGCTCTAGGATACCGTGCTCCTGAACTTGCTACTGCGACCAAACCTATCCCATCAT

TTAAGGCTGATGTATATGCTCTTGGTGTGATTCTAATGGAATTGCTGACGAGAAGAAGTGCTGGAGACT

TAATCTCAGCACACTCTGCTGCAGTTGATTTAACTGATTGGGTTCGGTTGTGTGATCAAGAAGGTAGGG

GAATGGACTGTATCGATAGGGACATCGCAGGAGGAGAAGAACACTGCAAAGCGATGGATGATCTGCTA

GCTGTGTCATTAAGGTGTATTCTTTCGATAAACGAAAGGCCAAACATCAGACAAGTTGTTGAAAATCTG

GGTTCCATATCTGTGTGAAATTTTTGTACATGTTGTCTTGGAATTACAATTCTTTTTTCTTTTCCTTCACATT

TTTTATTATGTCCTATTGGTTGCCTTCTAATGTTGGTCAATCCTTACATATTCCCCATTTTCTTTTCCTTCCCC
CCTTCAATTTTTGTGTAAAGGGATGATCAGTCCATTGATTGATTGGTTGACATTTTTCTCTAATTAGAAAA
GATTGCAATTATTTATGTGTAATTTATTTTTTGC
>PGSC0003DMG400012992 LRR-RLKs
ATGAAGTCACTTGAGGTTTTACTCTTAGATAGAAACAATCTTTCTGGTCCAATTCCAACAACACTAAGTG
ACTTAACTGAGCTCAAAACTTTGTACCTTTATTCTAATCAACTTTCTAGTTTTATTCCTAGAGAGTTGGGA
AATTTGAAAAGCCTCACTTTTTTGGATTTATCAATTAATCATCTTACTGGTCCTATTCCTAGTGAGTTGGG
GAATCTGATAAATCTCACAGAGATAGATTTATCTAAAACTCAACTTACTGGTTCAATTCCGATTACTATAG
GTAACTTAACTGAGCTCAAAATTCTATACCTATTTTCTAACCAACTTTCTGGTTTTATTCCTAGAGAGTTGG
GAAATTTGAAAAGCCTCACTGATTTGGATTTATCCACTAATCATTTTACTGGTCCAATTCCTGGTGTGTTG
GGGAATCTGATAAATCTCACAAAGATAGATTTATCTAAAAGTCAACTTACTGGTTCAATTCCGATTACTAT
AGGTGACTTAACTGAGCTCAAACTTCTATACCTTTTTTCTAACCAACTTTCTGGTCCCATACCTAGTGAGTT
GGGTAATTTGAAGAACCTCAATGGTCTGTCCTTATACGAAAATCAACTTACTGGTCCAATTCCTGCTTCTT
TTGGCAATTTGAGAAGCTTGCAATTTTTGTATCTCAATGCCAACAAACTTACTGGTCCAATTCCTGCTTCC
TTTGGCAATTTGAGAAACTTGCAATTTCTGTATGTGAGTGTCAACAAACTTTCTGGTTCTATTCCAAAAGA
ACTTGCATATTTAGATAACTTGGAAGAGCTGATAATAAGTGAAAATCAGTTTTCAGGTCATTTGCCTGAG
CATCTTTGCCAAGGTGGGAAACTTGAGAACTTCACGGTGAATAGTAACAAGTTGTCTGGGCCAATACCA
AGAAGCATGTCCAAGTGCTCGAGTTTCAAAAGGGTTCGCTTTGATAACAACAGTTTTACTGGAAATTTAT
CTGAAGCTTTTGGTATCTATCCGGAGCTGCAATTTATTAATTTGAGCAAAAATGATTTTCATGGTGAACTC
AGTAGCAACTGGGGGAAATGCAAGAAGTTGACTGATTTTCGAGTAGCCAGAAATAGAATTAGTGGTCA
CATACCACCAGAGATTGGAAATCTCAAAGGTCTTCAAGGACTAGATCTTTCAGCAAATCATTTGGTTGGG
AAGATCCCTAGGGAACTTGGAAAATTGACCTCTCTAGTTAATCTTTTTCTGCAAAACAACCAGATTTCTG
GCAATATTCCTATGGAACTTGGATCAATGACAAAGTTAGAATCCCTTGATCTATCAAAAAATATGTTGAA
TGGGTCGATCCCAACGTTTATAAGAGATTACATACACTTGTTTCAGTTGAACCTGAGCAACAACAAATTT
GGCCAAAATATTCCAATGGAGATAGGGAAGATAACTCAGCTTATTGTACTTGATTTGAGCTATAATCTTC
TGGTTGGAGATATATCCCCTCAGTTAGCCAATTTGAAGGTCTTGGTAAACTTAAACCTTTCCCACAATGG
CCTATCTGGGCGCATTCCTAAAGAAGTTGAAAGTTTGACTGGTTTGCAGGATGTCGTATTGTCATACAAT
GAGTTGGAAGGTCCAATCCCCAATAATAAAGCTTTTATGAATGCTTCATTAGAAGGTAACAAAAGTCTTT
GCGGCAATGTATCAGGACTCCAGCCCTGTGAAAGACCATCTCCTGTGGTGAACAAGCACTCAATGGCGC

AAGTCATCCTCATCACTGTACTTCCTGTTATGGGAGTACTACTACTGCTATGTGTTTTCATTGGTGTTCTCT
TTATGTGTAATAAAAGAAGGAGAGTTAGAGACGTTGAAAGAAGGGATGGTGATGGTTGGCTTTCAATA
TCCATGTTAGATGGAAAGGCATTGTACAGGGACATCTTAAATGCCACAGAAGAGTTTGATGCAAAATTT
TGCATCGGCCAAGGAGGACACGGAAGCGTTTACAAGGTAAACCTTCCATCATTAGGGAATATAGCTGTG
AAGAGACTTCATTCTTCATATCAGAATACACATCCTAAAAGCTTCATGAATGAAGTAAGGGCATTGACTG
GGATTAAGCACCGAAACATTGTGAACCTCTATGGCTATTGTTCGAAAGCACAACATTCACTCTTGGTTTA
CGAGTATGCGGAGAGGGGGAGTTTGTCTAGTACTTTGAGTGATGAAGTTGAGTCCAAGAAATTGGATT
GGTTTAAAAGGGTGAATATCATCAAGGGTGTTGCTTTTGCTTTATCTTACATGCACCAGGATTGTTCATC
GAGACATATCAAGCAGTAATGTTTTGCTTGATTCAGAGTATGAAGCTCATGTTGCAGACTTTGGCATATC
AAAGCTTCTCAATCCAGACTCATCCAATTGCACAACGCTTGCAGGGACATATGGTTATGTTGCACCTGGT
AATTAAGTTTAAAATATTTCATGAACTAAAGCCAAACACTCCATTGTTTATATCTCATTTACACGTTTATAT
TGTTTTGGAAATTGTATTGTTCAGAGCTTGCATATACTATGAAGGTTACGCAAATGTGTGATGTATATAG
CTTTGGAGTATTATCTTTGGAGATAATCAAAGGAAAGCATCTTGGGGAATACATTACCGTGCTAGTAAAT
TCATCGACTATAGATCATGAGCAACTTAGTGATTTGTTGGATGAACGCCTTCCATATCCTGAAGATAGAG
TAAAAGAGGTTTTGATTTTTATCATCAAGCTAGCATGCTCTTGTTTGCTTGAAACTCCAAAATCGAGGCCC
ACAATGCACTTCATCTCTCATAAGTTATCATCGATGGATGTACGTCCATCTACTCATGTAAAGCTAGTTCA
ATTCTTTAATCCGTGA

>PGSC0003DMG400013198 LRR-RLKs

GATTCTCTCCATTTACACATCCTCACATGCTATGTTTCACTACTCTTTCTTCTACTAGTATTTATCTCTTTCTT
TTCCCTTTTCTCTTCATTTAAACTTGAAAAAAGTTGAGGGGAGGAAGAAAAAGAAGAGTTTAGCATTTCT
TTGTTTAAGTTCTTCTTCTGATTGGAGATTCTGAACTTGTTTTAAGTTTATGGATAGGTGGGATACTTCAC
TGAGTGGATTTTACAACTACCCTTTTCGATTCTTGAATTTATATTCAGAAATTAAAGATGGGTCTAGCTGG
GTTCATGACAAGAAAAAGGAAAAATGGAAGCTTTCTTCTTCTTGGCATAATACTCTGTTCTTGTTTGTTGT
GATTGTGTTTTCTGTTTTTCCTATTATATCAGCTGGGAGGAATTCAGATGGGGTTATTGTGACTCAATCTG
ATTTTCAAGCTTTAAAAGCTATTAAGCATGAATTGATTGATTTTAGGGGAATATTGAAGAGCTGGAATGA
CAGTGGTCTTGGAGCTTGTGCTGGTGGTTGGGTAGGAATTAAGTGTGTTAATGGGGAAGTAATAGCTAT
ACAGTTACCCTGGAAGGGATTGGGTGGAAGAATATCTGAAAAAATTGGGCAATTACAAGCTCTTAGAAA
GCTTAGTCTTCATGACAATGTTATTGCTGGTCCTGTTCCAACTTCTCTTAGCTTCCTACCAAATCTCAGAG
GTGTTTACCTTTTCAATAATCGGCTTTCGGGTTCAATCCCACCATCAATTGGAAGAATCCCTCTTCTACAG

ACTCTTGATCTTAGCAACAATCAGCTCAGTGGTACTATCTCTCCCAGTCTTGCAAACTCCACCAGGTTATA
CAGACTCAACTTGAGCTACAATGCACTTTCAGGGTCAATCCCAGTAAGTTTCACTCAATCTCCTTCTCTTA
CTTTTCTTGCACTTGAACATAACAATCTATCTGGATCAATTCCTGATACTTGGGGCAGTGTGGTTGTGAAC
AAGTCTTATCAACTTCAGTATCTTACACTTGATCACAATCTTTTATCTGGGAAGATTCCAGTTTCAATTAGT
AAGTTAAGCATGCTTGAGGAGATTAATCTTAGTCATAACCTTATTAATGGGACTATTCCTGATGAATTAG
GTAGTCTCTTAAGGCTTACTGTTCTTGATTTATCTAATAATACCATAAATGGAACTATTCCTGCTAGTTTCT
CAAACCTTTCAGCTCTTTCTACTTTGGATTTAAAGAGTAATCTTCTTGATAGCCAAATCCCAGATACCATG
TATAGAATGAAAAACCTTTCAGTGCTGGATTTGAGTAACAACAAATTCATTGGTCATATTCCAGCTACTA
TTGGAAATATTTCTAGGCTCACCTCACTTGATTTATCTGGAAACAATTTCACTGGTGAAATTCCAAACTCT
CTTGTTTCTTTGGCAAATCTGACTTCTTTGGATGTGTCTTATAACAATCTTTCTGGGATTGTCCCATCTCTT
CTTTCTAGAAAGTTCAATGCAAGTGCTTTTGTTGGAAATTTAGAGCTTTGTGGATATAGTCCATCAACTCC
ATGTGCTTCACCACCCCCTCAAACTCTTCCTTCTCCTGTTAGTGGGGTTGTCAAGCCTCACCGCCATCGTA
AACTTAGTACCAAAGATATCATTCTCATAGCATCAGGTGCTCTTCTAGTTGTTCTGCTTCTTTTGTGCTGC
ATGTTACTTTGCTGCTTGATTAGGAAAAAGGCAAATTCGAGAGCGAAAAATGGTAGCAAAGCCGGTGG
CCTAGCTACCACTACAGGAAGAGGTGCGAAGTCAGTTCCAGCTGTAGGAGGAGCTGAAGTTGAATCAG
GTGAAGCTGGTGGAAAGCTAGTCCATTTCGATGGACCTTTCGTGTTTGCAGCGGACGACTTGTTGTGTG
CCACTGCAGAGATCATGGGGAAGAGCACTTACGGGACAGCATATAAGGCAACATTGGAGGATGGTAAT
CAAGTTGCAGTGAAGAGGCTACGCGAGAAGATCACGAAAGGGCAGAAAGAATTTGAAGCTGAGGTTG
CTGAATTAGGAAAGATTCGACACCCAAATATTTTGGCGCTTAGAGCTTATTACTTGGGACCAAAAGGAG
AAAAGCTTCTTGTGTATGATTATATGTCTAATGGAAGTCTCTCATCCTTTCTTCATGGTAAAATTGTCTAC
ACTCACTCACTGTTTGTTTGTATATTCCTTTTGGTGTTCCTTATCATGTCGACAATCCTAACATCATACTTG
TGTTCTCTATATGCAGCTAGAGGTCCCGAGACAACGATAGACTGGCCAACAAGGATGAGGATTGCTATC
GGTATAACAAAAGGAATCTGCTTTCTGCATACCAAAGAAAACATAATTCATGGGAATCTTACATCAAGCA
ACATTTTACTTGATGAGCAAAACAATCCAAAGATTGCAGATGTTGGACTCTCCAAGCTTATGACCACTGC
TGGAAACACCAACGTGATTGCCACTGCAGGCACGCTAGGTTATCGTGCACCAGAGCTTTCTAAAATCAA
GAATGTAAGCACCAAGACTGATGTCTATAGTCTTGGAGTGATCATTTTGGAGCTCTTAACTGGAAAATCC
CCTAGCGAGGCAACGGATGGTCTCGATTTGCCTCAGTGGGTAGCTTCTATTGTGAAAGAGGAGTGGACT
AATGAAGTGTTTGATGTGGAACTTATGAGGGATGCACCTAATATTGGTGATGAGTTGCTTAACACATTG
AAACTAGCTTTGCATTGTGTTGATCCCACACCAACTGCTCGACCCGAAGCTCCGCAAGTATTACAGAAAT

TGGAGGAGATAAAACCAGAGGTGGTGTTAGCAGCCACTAGTTCTGGAGATGATGGCACAACAGTTCAA
GAAAAGAGTGATGAATAATAATCTCACAAGGCTGATTGGAAATTTTTGAGCTTTTTACTTGATATCTATTT
GTTTATTCTTTCATTTTTTTTTACAGTTAGTAGTGGAAATGCTGTTTTATACCAAGATCTACTAAAAAACTG
TATATAGGAGATTGTGATTGCTGCATAGGTTTTACTGATGAAGAAAAACATTTTATTCAATTCTTTCAATA
AAATAAACG
>PGSC0003DMG400013489 LRR-RLKs
GTTACTAACACATTCCTAGATCTCACGGTTTCCAGAATTCCAGGCTTTTTTAGTCATTGTTTTGTTTTTTTTC
CTTTCTCGAGAAAAATTCTGTCTTTATTTCCTTTTAAGCTCAAAATGTTGCCAGTTTTAGCATAATTGAGTA
AAAGAAATAAACTTGGCATTTGGGTGTTTTGAGGTTTTTCCAGTTCTTGAAAGTGCTAGTTGTGTAAAGA
CTGAAACTTTGTCATTTGGGTGTTTGAGGTTTTTTCAGTTTCTAGAAAGAGCTAGTTGTATAAAGACTGG
AACTTTATCATTTGGGTGTTTGAGTTTTTTCCAGTTCTTGAAAAGCTAGTTGTGCAAAGACTGAAACTTTA
TCATCTGAGTGTTTGAGGTTTTCAATCTTGAAATGATCTAGTTGTGTAAAGACTGAAACTTTGTCATTTGG
GTGTTTGAGGTTTTTTCAGTTTCTAGAAAGAGCTAGTTGTATAAAGACTGGAACTTTATCATTTGGGTGT
TTGAGTTTTTTCCAGTTCTTGAAAAGCTAGTTGTGCAAAGACTGAAACTTTATCATCTGAGTGTTTGAGGT
TTTCAATCTTGAAATGATCTAGTTGTGTAAAGACTGAAACTTTGTCATTTGGGTGTTTGAGATTTTCCAAT
TCTTGAAAGCTAGTTGTGTAAAGATTGAAACTTTGACATTTGGGTGTTTGAGGTTTGCAATCTTGAAATG
AACTAGTTGTGTAAATACAGAGACTTTGTCATTTGGGTGTTGAGCTTTTTCCAGTTTCTTGAAAGAGCTA
GTTATGAAAACACTGAAACTTTATCATCTGTGTGTTTGAGATTTTCAATCTTGAAATGAGCAAGTTTATCA
GGTGTTTGTGGAGGGGTTGAAAATGGATAAAAAGGGCATTTTTTTTAATGATAAATTACGAGGTTTTGT
TAGAAAAAATTACTTTGTTGAGTAGGTGAGATTGTAGAAATTTGTACTGGAAAGAATGTTGTATCTGTTTT
GGAATGTGGGGCTGCTACTGTTTTGTACATTGATGGAGAGTTCTTCTGCTACACTTTCTCCTGCTGGTGT
AAACTATGAAGGTTAGCTTCTTTCTTGAAATTTCTCACTTGTACTGAAAATTTCCTTCTATCAAATTAAGCT
TAGAGTAGAAGAACTTGTTAATTCCTCTGGATCAGGGTTAGAGCCACATGGGTGCAAGGGGTTCATCTG
AATCCCCTTCGACGGAAAATTAAACTGTATATCTAAGGTTACAAATATTTTTATGTATATATAATAGACAT
TGAATCCCCTTGTATTCGTCCTGTGTTTACTTCTTCATTATTTGAACCCCTTAGTCCTTTGCAATGTATATG
AGTATGTGACTATGTGTTCTATCTTTTTTCTTTATGATTTCCTCTCGTTATATTGAATCTTTGATGCAATGA
CAAACTGAAAAGTGAAAACAGTGGAAGTAATCTAATAGAGTGACTGTTTTTCCATATAATAATGTGCAG
TTGTAGCTTTGACGGAAATTAAGAAGGCCTTACATGACCCCTATAATGTTCTGGACAATTGGGATGTGAA
TTCTGTAGACCCTTGCAGTTGGAGAATGGTCACTTGCTCCTTGGATGGCTATGTGTCTGCTCTGTAAGTT

GTTTGGATGAAAGAGTATTTTAGCAGAAACTTAGTTGGCTCATGAAAAGGCCTGATTTAATCCAGTAAAT
TTATTGCTGAATATTTTCTCTTGCATCAGAGCACTACCTAGTCAAAGTCTAGCTGGCACCTTATCGCCTGG
AATTGGCAATCTTACTAACTTGCAATATGTGTAAGTTCTTGACTATATACATCTCTTAGTTGCTGCTACTTG
GTTCCGTAATACCTAAGGTCACTTCTTATATGATTTTCTTAGTGTTATTAAGACATTTTCTCGCAAACTGTT
CTTGACTTGTTCATGTAGATTGTTGCAAAACAATGCCATTTCTGGTCATATTCCTGATGTGATTGGGAAGT
TACAAAAGCTTCAGACACTTGATCTTTCCAACAATAAATTTGAAGGCGAAATACCCACTTCCTTCGAAGA
CCTGAAGAATTTGAATTATTTGTGAGTTCATGAATTTTCATTTTCACTTATTATTATTCACTTTCAAGACTC
CAACCATATATTTCACTCTGTCAGATTGAATACATCATGTTACCTATTGACTCAAATCTTCTGTTTCGTCCT
CATCCCTGTGATTGACTTAGCTGATCATTGCTTGCAGGGATGTCTCTTATAATAATCTAAGTGGTACCCTT
CCTAAAATATCAGCGCGAGCTTTCAAGTAAGTCAGCCTTTTCTATTGCCGTAATATACTCAGTTATTTCAT
TTTCAAGTAGTTATCTTAATATTATTGAAGTTAAACTATTGCTGTACGGCTGTACCTGTAAATCTCGATGC
TTAGTTGATAAAAAATCCATTATTAAAAATACATTCACAAATCCTTTTGGGTAAACTGGTACTTGAAAAGTA
TTTCATTAGTATTCCGCACTATAGTGAGATTGACTTCCTCTTCATGGCTTTCAGAGTGCTCGGGAATCCTT
TAATTTGTGGTCAAGGTTCTGAGAATAATTGCTCAGCAGTCTATCCAGAACCACTGTCCTTCCCACCTGAT
AGTCTGGCAGGTACATATAGCATTTTCATGCTCAAGTCCCTTCCCTTGCACCTGAAAATGGACAACTGTT
AAAACATATTATACATACTGAGGAAGTTTTGCTATTTGTGTTATTGAAAATGTGTTGCATCTATTATTTTT
GATATCACAGATCAATCTGGTGCTGCACGCAAGAGCCGTCGCGTGGTTGTTGCGTTTGTTGCAAGTTTTG
GTGCTGCTTTCTTGGTCATAGTAGCCCTGGCTTTGCTTCTTTGGTGGCGCTACAGAAATAATCAGCAGAT
CTTCTTTGATGTCAATGGTTAGTTGATTTACTTTCCCCCCTCTTCTCTATGATAATTTGAGATTTTATTAGTT
CTGCATTTCTGATTCTTTTCCTGCCACAGATATATAAGTTGAGCCAAGTGAGCTTTTCATTATTGTCTGAG
TGATTGGTGTTAGGTATCATGTGAATATTTTTTAAGCTAGGATGTTACAGAGTCACCTGCTTGTGGTTTG
AATCTTTATTTTAATCTCTAAAAAGATTTTTGTGATTGTTGATTTTAAATTCATTTGTTTTGTTTTAGAGAG
GACATGAGTGGTCAAGTAATAGGAATTGGGGATAAATTCTGTGTTTACCATAATCAATAAACCTGTTGA
ATATGAAGAACTTTCGAACCCCGTCAATTAGAAAATTATGCCCTTTGGCATACTCAGTAGTTCTGGTCAA
TTTTCCTAGTAAGGACTCAGAAAAGTTGTTTGAATTTCGTTGTTATCGGGACAAGTCCTGATCCACTACA
GGCTACCTTAAACATTTAGTTCCAAACATTGACAGAATAATTTGTTAATCACATATCTGAATAATTACACT
TGCAGTTTCTTGTGCAAGTTGAAGTAAATATTTCTGTAAAAAGGAAGATGGTGTTATGTTCACTCTCTCG
GGCCACCATGCCTGGTGAATTGCCTTGCCACATTTATTGCGTTGAAAGTTACTCTTGATAATCTTGTACTT
TGGTTGTCTGATTCATATAAGTGGATCAGTTTTGGGACCTTTTTGCCAACCTCTATTATTTTCTCCCTGTCT

CACTTCTCAAAAGATTTTCTCCATCTGTCTCTAACTAGTCCTCAAAGCTGTATCTGAGTAACGACTTCTCAA

ATGCAGATCATTGTTATCCAGAGGTATGCTTGGGTCACCTGAAAAGGTACACCCTTAAGGAACTGCGGA

CTGCCACTGACCATTTCAGCTCAAAACGTATTTTGGGAAAGGGAGGATTTGGGGTTGTGTATAAGGCCA

GCTTAAACAATGGTACTGTTGTGGCCGTCAAACGATTGAACGACTACAATGCTGTTGGTGGTGAAAATC

AATTTCAGACAGAAGTAGAATTGATTAGTTTGGCTGTCCATCGGAATCTTCTCCATCTTCTGGGGTTTTGT

TCAACTGAAAGTGAACGGCTTCTTGTTTACCCCTATATGCCAAATGGAAGTGTAGCATCACGATTAAAAG

GTTAGCTCTTGCCTCCATGTGTTAATACATAATAATCACTATTGATTTCAGATAATGAATCCAACATTTCC

ATTTATAAAAGTTAAATATTCTCACATGGTGTTTGTTTGTTACTACTCCCTTTATTCATTTTGTTTGACAGA

TAATTAATAGGATACAGAGTTTAAAATTTTAATTCCACTCATTTATTACATTGGAGGAGAAGAATTTTTTT

AAATAGTGGTTATTATACTTGTTAAAGAGAACATCATATAAATTTTAGAATGTGGATAGTATGAATTTGA

GTTCTTAAAGTTGTAAAAGGTGTACGGACAAATATAATACTAGAATAGAGCCAAATTTTGGACCATGTCT

AATTTAAAAGAAGTCATGTTAGTGAACAGTTCCTACTTCCCAATGTATTGCCATTAAACATCAGTGAGTG

CTCTGAAAACCAGCAACCGCTGTCATTGGTGAATTGAGGCCATAAGATTCTGGTTAAGTACAAGCTTAG

ATGTTACGACCCACCCTTACCTCAGTCCATCCTTGAGCCCGCGGTGGGCTGCCCACCTTAACATACTCTGT

ACTGCTGTAGTTATATGATAAGACATTATCAGTTTGCTGCTGGCTGAAGCCATTGTTGTATGTTTCTGCAT

GTTCGAAAGCCAAGCTCATTTGCTTTTCTGGAACTTCATCAGCTACTTTTTAATAGTTTCCAAGTAACTAA

ATTTTGTGTCAGTTGAAGCTGATACATATATTGGCTTGATTGAAAATGCATTGTGGTTTCTAAATTTGCAG

ATAGCATAAATGGTAAACCAGTTTTAGACTGGTCAAGGCGGAAAAAGATAGCACAAGGTACAGCGAGA

GGGCTAGTTTACTTGCATGAGCAGTGTGATCCCAAAATTATCCATCGTGATGTCAAAGCAGCAAACATTC

TGTTGGATGAAGAATTTGAGGCAGTAGTTGGTGATTTTGGGTTGGCAAAGCTCTTGGATCACCACGATT

CCCATGTGACTACTGCTGTTAGGGGCACCATTGGCCACATTGCTCCAGAATATTTGTCTACTGGCCAATC

GTCTGAAAAAACTGACGTTTTTGGGTTTGGAATCTTGCTACTTGAGCTGATTACAGGCCAGAAAGCTATG

GACTTTGGTCGAGGGGCCAACCAGAAAGGTGTTGTTATGCTTGATTGGGTGCGTCTCTCCTCTTTCTCTT

TCTCTGGATGTTGTACTGAGTACTTTTTGAAATATGTACTGTGGGTTGTGAGAGGAATAAGCTATACAGA

GGCAGGTTGTGCGTCTCTCCTCTCTCTCTCTCTCATAGCTGTTTTCTTTTCAGTTATAAGGAGTTGTAGG

TTCAAACCTCAGATATAGCTAGCAAGTATTGTGTACATCTAGTTTACAAGGCTCTTTTTCCATTAGCATAC

TTCAACGAACTCTCTCGTTTAATTACTGCTAATGATATGCAGGTGAGAAATCTGCATCAAGAGGAGAAAT

TGAACCTTATGGTAGATAGCAATTTGAAAAATAACTTTGACCAGATTGAGCTAGAAGAGATGGTTAAAG

TTGCCCTTCTATGCACCCATTTCAACCCTTCTCATCGCCCAAAAATGTCCGAAGCATTAAGGATGTTAGAA

GGCGATGGATTGGCTGAAAAATGGGAGGCATCACAAAAGCTCGAGACTCCAAGAAACCGAGCTTTTGA
ACATACACCACAAAGATATTCCAACTTCGTCGAAGACTCTTCACTAGTTGAACCAATGGAACTTTCTGGA
CCTAGATGACATCCATTTTTCGTTCTCGATTCTTATCATCCTTGAGTTGCTAAAGTTGAAGTGAGAAGTAT
TTCATTGCTGTTGAATGTATGTATTCATTTTGTAAAAGAAAAGGGAATAAGTCATGTGTAAGCTGAGTTC
ATATATGATATATTCCCTCTTGTTCTTTCAATTTTTGAAATGATATGCTATGTAATTATGATTTCATTTGTTC
TTTTTATAAGAAAGATTTTGAGATTTATGAGTAATAAAACGCCATAGGCTTGCCATTCTCATTAAGT
>PGSC0003DMG400013500 LRR-RLKs
ATGGTTGGTTCTTTACCTCTCAAAATCGGAAATTTAAAGGCTGCAACACTGATAAATCTGTCAATGAATC
AATTCTCAAATGGAATTCCTATAGAAATTGGAGGATTGCAGAATCTGGTGCACCTTTCTTTGAGGCACAA
CAAGTTACAAGGATCTATACCTGACTCATTGAGCAACATGGTAGGTTTGGAATTCCTAGACCTTTCTCAT
AAAAATATATCGGGAACCATTCCTATGTCTTTGGAGAAACTTCAAAACATGATGTATTTCAATGTTTCTGT
CAACAAGTTGCATGGTGAAATTCCCTCGGAGGGTCCGTTCAACAACCTCTCGAGTCAGTTTTTCATCAAC
AATGAAGCATTGTGTGGTTCTTCAAGGTTTAGTGTCCCGCCATGCCCCACATCATCAAAGCATAGATCAA
ATAGGAAAAAATTGCTTGTTCTATTTCTTATCCTCGGAGTTGCACTTGTATTTGTTCCTATTACCCTTTTGT
TTGTATGGATAAGGTATAGAAGAGGTAAGAAATACCCTCAACAAGATGATTCATTGTCTATTGCAACGA
GAGGTAGAATTTCATACTATGAATTGCTCCAAGCAACTGATTCTCTCAGCGAGAGTAATCTGATTGGTTC
CGGAAGTTTTGGCTCTGTCTACAAAGGCATTCTCAGAAGTGGGACTGCTATTGCAGTTAAAGTGTTTAAT
CTGCAACTGGATGTGGCATTAAAGAGTTTCGATACAGAATGTGATGGTTTGTGCAACCTTCGCCATAGG
AATCTCATAAAAGTCATCACTAGTTGTTCCAACATTGATTTTAAGGCTTTAGTGCTCGAGTATATGCCTAA
TGGGAGTCTTGAGAAGAATTTGTATTCTCACAACTACTTCTTGGACATCCAGCAAAGACTATGCATAATG
ATAGATGTGGCATGTGCATTGGAATATCTCCACCATTGTAACATCCGGCAATACGAAATAATTGAAGAG
GCTTAG
>PGSC0003DMG400013525 LRR-RLKs
GTAAACTTCCATCCAGCTGCAAGTTGTAGCTCATAATAAACCTCTAAATTATGTCATAAATTTCAATTCTC
TTAGCTTAATTCCTTCAAATTCTTCATTAATGGGGTTTTCATCGAAATGGGTTTTTGTATTTACGTTATTTT
CATTCATAAAACTTTCAATTTCTGATCCAAGAGCAACAGAAGCAGCTCTAATTTGTACAAACAGAACAGC
TGCACAATCCGATCGACAAGTTTATGTAGCAACTTTTCTAGAAGCCATGGATTCCATTACGCCATTAGTG
GGGCGAGACAAATTTGGAGCAGTAATTAACGGAACAGGGAATAATACAGTTTATGCGTTTGGTGAATG
TATGAAGGACTTATCACAAACAGATTGTAATCTCTGTTTTGCTCAGTGCAAAACTCAAATCCTACGATGCT

TACCTTTTCAAAGATTAGTTACTGGTGGGAGATTATATTATGATGGGTGTTTCCTAAGGTACGATTATTAT
AGATTTTTTAATGAAAGTTTGAGTTCTGTTGATAGGACGATCTGTGGGAACACGAGTTTTTCTGGGAATC
AGAGTTTGTTTAGTGAAAATGTTGGGAAATTGGCGAGGGATTTGGAGTTTGGGGGTTTGAGGAATGAT
GGGTTTTTGACTGCAGTTGTGAGTACTGGGAATTTGACTGTTTATGGGTTAGCTCAATGTTGGGAATTTG
TGAATGGAAGTGCTTGTCAAAGGTGTCTTTCTGATGCAGTTTTGAAGATTGGTTCTTGTACTCCTAAGGA
AGAAGGAAGGGTGTTGAATACTGGTTGTTATGTTAGGTATTCTACTCAGAGGTTCTTTAATAATTCTGCA
AGTGATACTCCTCCTGCTGGAAATGGAGGTATGTTCTTTCTTTCTTGAATGTATGAAATGAATAAATTGTT
TTCGTTTAGTTGTGCGATCATTTCACCTAAAAGTGTAAGCTATTAGAGATAGTACTTTTTCCGTGACATAT
GTCATATCTTTACATGGTATCATTGGGTCTGACCGTGAAGGGGTGTTAGAATGGTTAAAGTTTCACATTG
GTTGGGGACTGACTTGAGCAATTCTCTCGTGATGAGCTAGCTTTTGGAGTTGAGTTAGACTCAAATGTCA
TATATCTTTACGAATATCATGTTTAATTGTGTAATCATCTTATTTAACATATGCTTGCGATGTTTCTTGGTT
TCATTAGTTATTTCAATCTTATCCAATATTCAATGATCTTAAATGTTTAATTTTGTTTTTGTAGATTTGCAAT
GATACTTGCTATAAGCCTAAAACTATTGGATGTAGAGCAAGAAAAAGTTCAACTTGTTCTGCCCAATTAT
TATTTTCTAAAAAAAAGATACCTACTTTGTCATTTGGAATGATTTGAAAAACGGTGGTTGGAAATCGAGG
TGATTTTGACATTTCTTGAAAAGTTGCTCTTGTTATCTGTTTAAAATGTGGTAGTTTGTATTTCTTGGATTT
TAGTTCCATTCCAGGATTTAATAACTATCTAGACATTACTTGTGCTGATGTTTCTTTTTGTCATCTGATAAG
TTGAAATGGTTGACATATATATATACATGAAATGAATGAAGAGTGAATCAGCATTGAGGCAAATGCAGA
CAATGAATCTTTTAAAAACTGTGATAAAATAAAATGTTTATACAAAATTATGTCATTCATGTTGTCGTATT
ATGATTAATTATCAGGAGGATCGAATCGTTTGGCTGTTATTCTTGCAACAACTCTGGGTATTTCTGCTTTC
TTGCTATTTGTATCGGCTGTTTCATTCTTTGTGCGGAGGAAGGTTCTAAAACAAAAGAGAGGTACAAATT
TCTAAGTCAAATCATTTTCTTTCTTTCCGACAAAAGAAATCTCATTTAAGTCTGATTGATCAAATGGCAAT
TTGACTTGAATTTGCTTCGATGATATAGAGAAGAAGCAGCTAGGAGCTCTCATAAGAACGGTTAATAAA
TCAAAACTGAATATTTCCTATGAAACTCTTGAGAAGGCGGCAAATTACTTTAACAACTCGAATAAATTGG
GACAAGGAGGTTCTGGTTCTGTTTACAAGGTAATTTTCTTTGAACATTATATTCATTTTTCTTATACTAATG
AGATTTGCCTGACAATAGTCGTGTTTATACATGAAATAGGGGATCTTGCCAGACGGGCAGGTTGTTGCT
ATAAAGAGGCTCTTCTTCAATACGAGGCAGTGGGTTGATCATTTCTTTAATGAGGTCAATTTGATTAGCG
GCATCCATCACAAAAACTTGGTAAAGCTATTGGGTTGCAGCATTACAGGGCCGGAAAGTCTTCTAGTGT
ATGAGTACGTGCCCAATCATAGTCTTGCGGATTACGTCTTTGGTAAGTATCCAATCCACCTAAATTTATGA
AATCTTAAATTGGTGTAGTCGTCTCTCGTTATGTAAATGCCTTTGTTTTGCACTGAATCAGACACAAAGAA

CCTTCAGCCACTTACATGGAGTCAAAGATACAAAATTGTATTGGGTACTGCTGAGGGATTGGCTTATCTT
CATGAAGAATCGAAATTAAGGATCATCCACCGTGACATAAAGTTGAGCAATGTTCTTCTAGATGAAGATT
TCATGGCAAAAATAGCTGATTTTGGTCTAGCCAGGTTATTTCCTGAAGATAAGAGCCACATTAGCACAGC
TATTGCTGGCACTCTGTAAGTTCAATACCTTTTTCCGGTTACTTGTTAATTTTTCTTTAATCAATACACTAA
GAAAGTGCTGAATTAATAAGTTTTGGCATTGCAGCGGTTACATGGCTCCAGAGTACGTTGTACGTGGCA
TACTGACCGAAAAAGCTGATGTCTATAGTTTTGGAGTTCTTGTTGTCGAAGTCGTGTGTGGAAAGAAGA
ACAATTATGTTTTCAAGAACACTAATTCTCTTCTGCAGCAGGTACTTTCTTGTGATATTTTTTCTTTCCTTA
ACGGACATTAATATAGTATTTACTCAACTCATGTCATTGTGGTTTCTAGTTGTGGAACTTGTATGGGATG
GGGAAATCTTATGAAGCAGTTGACCCTTTATTAGAAGGTAACTTCAATAAAGAGGAGGCATCTAAGCTG
CTGCAAGTAGGTCTTCTTTGTGTACAAGCATCAGCAGAACTACGACCCTCCATGTCAAGTGTCGTGAAAA
TGCTCACCGAGAACCATGAAATTCCTCAGCCAACACAACCACCATTCCTCAATTCTGGTAGTTCAGAATTT
AGTCCATTTAATCTGCATGGCAAAAGATTTTTCGGTCAACCAAGCTCCTCTACACAATCTTCTGGGAATAA
ATTGACGGAGAGTTGGATTGAGCCCAGATAAACTAAGTTGATCATCTGGAATTGGTATTTGATTTTGTTC
TCAGCAAAGGCCAACTTCTTGAAGGTGAATTGTTCAGCCAACTCTAGTTTTTTCTGTGATGAACTAGTACT
CACTACTCACTCTCCATTTTCTTTACTGATATTTGTTTATAGTCTTATTTAGTCAACTTCCTTTTGTGAATAC
CCCAGTGAGCTATTTCATTTCTAATGTAAAAATTTTGTATAGTATCTGCAGGAGTTCCAATTAACTATATC
TACTGTTTTCGATTTTGGATTTTGGATTTTCGATTTAGTAATTTTCATCAAGGATTATGCTTCACAATTGTC
ATCATCTTGTCTTAGAAGTGGCTATTCAGAATCCCCTGCCTAAAGTCACAGGCATTGGTGAAACTATTCA
TTACAATTACATCTACA

>PGSC0003DMG400013682 LRR-RLKs

CTAAGCTTCTCTCTCCCTCACCTCGCTCCCATCTCTCTCCTCTTTGTGCTTCCCCATGCATGCCCACACAATC
CCATTTCCCTAGAGATGTTGAAATTCTTTTTGCATGCCCTCTAAATCATAGTATTTTCTCATTTGTAATTGC
CCTTTGTAAAGGGCGCGCACCAACAAGTGTGCGCACCCCTACAACTAGTCTATGCAATGTCGGTGGCTT
ATCGTTATAGCAACCACAATCGCCAGTATCGTCATCACCATCACCATCTCTTAATCCTCGTTGTGTTATTAT
TACTAGTCGTTGTCCCGATCAAATCAGATAGTTCTGAGGCAGAAATCCTCTTTCGATTTAGCAAGTCCTTG
CAAAAAAATGGCGCAACAGCTAATTGGAATACGAAAGTATCGCCTTGCGATAAAAATACAGATAAACCC
AATTGGGATAGTGTTATTTGCGAAAACGGATTCGTTTTCGGGTTGCAATTGGAGGGTAAAGGCTTAAGT
GGGACAATAGATGTGGATGCTCTTAAAGATTTGCCTAATTTCAGGACCATAAGTGTTATGAACAATAATT
TTGAAGGACCTTTACCGAATTTAAGTAAACTTGCGGGGTTAAAAACGGCATATTTCACAAATAACAAGTT

TTCTGGCCAAATTGACAATACTCTTTTTGAAGGGATGCATTGGTTAAAAAAACTTCATCTTGCTAATAATC
AATTAAGTGGTAAAATCCCATCTGTTTTGGGTCAATTGCCTAAGCTTACGGAATTGAGAGTTGAAAACAA
CAAATTTGAAGGCCAAATACCTGATTTTGTTCAAGAGAGACTTATGGATATGAATTTTGCTAACAACTCT
CTTGAGGGTCCAATTCCTCATGGCCTTACTAGTCTCAAACCTTCAGCATTTGAAGGTAACTTTTTTTTTCAT
GTGTAGGATAAATTCCATTTTTTGAAATTATTGTAACACTTATCTAGCTACGACGTACACAAATGTCTACA
TTTTAAAATGTAGTCGTAAAGATATGTCATGTATGCCTAACTCCACCTTAGAAGCTAGCAAGGATTGATC
AAGTTCATATCATCAGCAGATCATCAATCCATTTCCCAATCAATGTGAGACTCTTTAACACTTCTTTCACG
CCCATCTCCCAAAACGGAGATGAACCTGACTCTGACACCATATAAAGATATGACGTTTGCATCTAACTAA
ATCTCAAAAGTTAACTCACGAGGAAAAAATTGTCAAAGTCCATATTAGCAGACAATCAACACATTCCCCA
TATGGCGCTTTAACCCACATTCTAACAATAGTTCTTCGTCATATTGGCATTAGAAGAATTGTAGTTCATAA
CAATTTTTATATAGTTTTTAAATATCTAAATTTTAACTTCAAAATATTAAGTTAATCTAAACATGCTCTCATT
TTTGTTCATTTGGTACAATAATACAGGTAATGATCTTTGTGATGGTCCATTATCGAAATGCACGAGTGAA
CCAAAAGTAGCCCTCTGGACAATTATATTGGTGGTTATCGCAGTTGCAGCAGCAGTAGCAGCAATTGTT
GTTGTTATCATCATTCTTCGTCGGGGCAAGCAGACACCCGAAACAGAAACAAGACCAACGCCTACACCCT
CTGGAGCTGCAGGGGGCGCTACTAACCAAACAGGAGCGCCCTCAGCAGCAGATCTAAACAAAATGGAA
CAGGGCAGCAATCAAGCAATGGCTGCTCCTGCTCCTGACCAGTCTCCAGAGGGGACCGGTGGACTAAAT
AATAATAAGAGGCCTGAAGTACAAGCAATACAAAAACTTCTATTCTTAAAAGATGATATTGAAAAATTTG
ATTTGCCAGATCTACTAAAGGCTTCAGCTGAAATATTGGGTAGCGGTGTATTTGGTTCGACTTATAAAGC
TGCACTTAGCATGGGACGTGTTATGGTTGTCAAAAGGTTTAGGCAAATGAATAATGTAGGCAAGGAAG
ATTTTCATGAGCATATGAGAAGGCTAGGTAGGTTGAGTCATAAGAACTTACTTCCTGTTGTAGCTTTTTA
CTATAGGAAAGAGGAAAAGCTTTTGGTCTCAGAGTATGTCAACAATGTCAGCCTTGCAGTTCATCTTCAT
GGTACGTATTTCATACTTTTAAGTTTCCGCGCTAAAATTAGATCATAAAATCATGATTTGCGCTACCAGTT
TATTATATTAACTCAATCTATTTTGACCTTTTTTTTATTCAATTCATTTCAAAACAGAGTTGAGATATGCAA
CTCAAATCTTTTGAAGAGGGCTCATTTGTTGTTCTCTCTACCTACTCTACTAGCTAGAGTGATGATCTGAT
GTGTGATTATCTCATCTAAGATATTACATTTTTACTATGATGAATTCTGTGATCATCTCATTACACATTGTT
TATTTTTTTCAGGTAATAAATCGCGGGGGCAACCAAGTCTTGATTGGCCAACTCGATTGAAAATTGTAAA
GGGAGTAGCCAAAGGACTATTGTACCTCTACAACGAGCTACCAAGCTTGACAGCACCCCACGGTCACCT
CAAGTCCTCGAACGTACTTCTAAACGAATCCTACGAGCCCCTCCTCACGGACTATGCATTACTACCCGTA
GTAAACCTAGAACACGCTCAAGAACACATGATTGCTTACAAATCTCCTGAATTCAAACATAGTGGTCGTA

TCACACGTAAAAATGATGTGTGGACGCTTGGAATATTAATCCTCGAGATCATAACGGGCAAATTCCCGTC
TAATTTCTTGCAACAAGGCAAGGGGAGTGACACGGATTTGGCTACGTGGGTAAGGTCCGTGGTAAATG
AGGACATGATGGAGGTAGATGTATTCGAGAAGGAAATGAGAGGTACGACGAATTCAGAGGGGGAAAT
GATGAAGTTATTGAAGATAGCTTTGGGTTGTTGTGATCTTGATATGGAAAAGAGGTTTGATATGAAGGA
AGCAATGGAGAGGATTAAGGAAGTAAAAGAGAGAGATGGGGATGATGATTTTTACTCTACAAGAGGAT
TATCTGATGATTATACGCAAGTTTCGATGAACATTTGAAGAGTGTTGGTTATACATACTCATAGTTCGTGT
GTAACACCTTTTTATTAATTTGTTCAAAAATCATCAATGGTAAATATGGGATGGGACTTTTATTTTGTTTG
ATGCATGGTTGGGGGTCGTATATATTGGTCAATGAAACCACAAAATAAATAAATATGAAAACAAAAAAA
AGAGATAGAAAAAATGAAGTTCAGTGTTTTTTTGTTGCGATTCAATTGAAGCAAAGGAACATGTAAATT
GTCCTTGATGAATCTAGTTTAGCTAGTTGCCTTCACTTTTTTTCTTTTTTGAAACTTTATCTAGTTTATTATA
TTTGGGGTTGTAAAAATTAATCCAAAAGCATGCAAGAACAACGAATTATGGATCTTTCAATTGCAG
>PGSC0003DMG400013953 LRR-RLKs
ATGGTTGTGGCCTTGGATCTTCCTAATTTGCAACTTCAAGGTACAATTTCCCCTTCTTTGGCCAATTTGTCC
TTCCTCAATGTTCTCAATCTCCAGAACAACAGCTTCCACGGTGGCATCCCTTATGGCATTGGCCATATGCC
TCGCTTGCGAGTCATTGACGTTAAAAACAATCAGCTCAACAGAAGTATACCAACAAGTCTATTTCAAAAC
CGGAGAGTTCAAGTAATTTCATTGGATTTCAATGAACTCAGTGGTGAAATCTGGAGAGGCCCTTGGTAT
GTACCCGAACTCATAGTCTTAAATTTTAGGAACAATAGCCTCACGGGTATAATCCCTCCTTCTATTGGAAA
TGCCACAAAAATGATGATATTCAGTTTGTCTGGGAATAGAGTCAGCGGCAACATTCCAAAGGAGGTCGG
TAATCTGATCCAGCTTGCATTTTTTTCCTTGTTCAATAATCAATTAACAAGTTCCATTCCCGCAACACTGTT
TAATATCTCGTCACTACTTGTCGTAACTCTGGCAAAGAATAGCCTTTCTGGTCCTCTCTTGCTAGATGAAG
GCAATATTGTGTTGAATCTGTACTTTTTAAGTATATCTCACAACCAAATTTCTGGTTGCATTCCTTCCAACA
TATGCCAACTCACGGAGCTCCAAGTTTTGTCCTTATCTTTCAACAACATAATTGGAGACATACCCAGAAAT
ATTAGTTGTTTATCCAAACTCGAGAAGTTTTATATTGTTGATAATCTAATAAAAGGACTATTCCATTCATT
GGAAATATTCCACTCTACATACTCTTAACTGTCGAAACAATTGGATAGTGGGGGGAATTCCTCCGGAATT
AGGGAAGCTATCAAATTTGATGAAATTATGCTTTGACAATAATTCTAATCTTATTGGTCAAATTCCAGAG
GCTATTTTCAACATATCTTCTTTGGAAGTGATTGGTTTCAGTTTCAATAACCTCTCCCCAACCTTGAAGGA
CTTTACTTGGGAGTCAATCAGTTCAAAGGGAAATTCCATTGTTTATAACAAATGCTTCCAAGCTTCAGAT
ACTGGAGCTAAATAATAACTTTCTGACAGGAACTATTCCTAATAATTTGGGAAATCTTCGTCAGCTGCAA
ATGCTGCTCCTATATAATAATCAACTTACCAATGAATCAAGAGAGCGTGAGTTGTTACTCTTCAATTCTTT

GGCGGACCGTAGGATGTTGAAATATCTACAAGTGGGTAACAATCCCTTGAATGGCGTTCTGCCCAATTC
AATTGGGAATCTTTCATCTACTATTGAAAACTTTCATATAGAAGATGCACACATCAATGGCCTCATCCCCA
AAAGTATAGGCAACATGACCGGTCTTACATCCCTAAGCCTTGACGATAACAACTTGACAGGGAGTATTC
CTTCTGAGATTGGTAAGCTTAAACAACTCCAAGGGCTGTATCTATATAACAATAAATTGCAGGGACATAT
TCCAGAGGGGGTATGCCTTTTATCTAATTTAGACATATTAGCTCTAGAAGGTAATGAGCTCTCTGGATTA
ATTCCAGAATGCTTTGGAAATCTTAGCTACAACATCTTTATTTGGATTCTAATAAATTTTCATCAAAATTTC
CGTTGAGCCTTTGGAAGATGAATGGTCTTCTCTATCTAGACGTGTCACAGAATTCAATAGAGGGAGAAG
TTCCCCAGGATATTGGAGGACTGAAAGCCATTGTAGAACTACATCTTTCCAGTAACCACTTTTCAGGCAT
GATATCAACCAGATTCGAGGAATTCCAAAATCTGCAGTATCTTGACCTATCGAACAATTCATTTTTTGGCC
GAATTCCATTATCATTTGCCAACTTGATAAACTTGGAATTCTTGAATCTGTCTTTAAATGCATTGTCAGGT
ACTATTCCTAAGTCATTGGAAAAACTCTCATACCTTAAAAGCATTAATGTTTCATTTAATGATTTAGAAGG
TGAAATACCAAGTGGTGGTGTGTTTGCAAATTCCACTCTGCAATCATTTCTTNNNNNNNNNNNNNNNNN
NNNNNNNNNNNNNNNNNNNNNNNNNNNNNNNNNNNNNNNNNNNNNNNNNNNNNNNNNNNNNNNN
NNNNNGGAATGCACATATTGGAGATTCCTGCTTGTGCTATCACTAATCCTGGAAAACAATCAAAGCTTA
AGGAGGTTTTGCTAAAAATTGTTACTCCAGCGGTTACTTCATCCTTTCTGATATTCTTGTTGGTTTCAATTT
GGATAATGAAACGACAAAAGAAAGGGAAGTCCAAAGATGTGGAGAAGGTACTGGAGATCGGGACTCA
TCAATTAGTTTCTTATCACGAGATTCAACGAGCAACAAATAATTTTGATGAATCCAATTTAATTGGTGAG
GGAAGCACTGGCTCTGTGTACAAAGGCACATTATTTGGTGGAACTGCAATGGCCATAAAGGTTCTGGAT
TTGGAAAATGAGCAGGTATGCAAAAGGTTTAATACCGAATGCGAAGTGATGAGAAATGTTAGACACAA
AAATCTTGTTCCAGTGATTACTACATGTTCTAGTGACTATATAAGAGCCTTTGTTTTGCGATATATGCCCA
ATGGAAGTCTTGAGAATTGGCTGTACAAAGAAGATCGCCACTTGAACCTTCATCAAAGAGTCACTGCAA
TGCTTGATGCAGCTATGGCAGCTGAATATCTACATCATGGTCATGTCACTCCAATAGTTCATTGCGACTTA
AAGCCATCCAATGTACTTTTGGATGAAGATATAGTGGCTTATGTTGGTGAATTTGGAATCTCTAAAATTT
TATCCATAAGCAAGTCCATTGATGGTGTTCTTCCCGAGCTGCATGTTAATATGATGCTTTTTGCTTACCTG
TAG
>PGSC0003DMG400013956 LRR-RLKs
ATAAGAGCCTTTGTTCTGCAATTTATGCCCAATGGAAGTCTTGAGAATTGGCTATACAAAGAAGATCGCC
ACTTGAACCTTCATCAAAGAGTCACTGTAATGCTTGATGCAGCTATGGCAGTTGAATATCTACATCATGG
TCATGTCACTCCAATAGTTCATTGCGACTTAAAGCCAGCAAACGTACTTTTGGATGAAGATATGGTGGCT

CATGTTGGTGATTTTGGAATCTCTAAAATTTTAGCCATAAGCAAGTTCATGGCTTATAGCGAGACATTGG
GGACTCTTGGATACATTGCACCAGGTATAAAAAATCTACTCTCATTGATTTTCTCTTATCATAATAAAGCC
TCTCCAAGTTATAGAAGTAAAAAGAGAATTATTGTTGTATTTCAATTGAGTAACTTTTCTTCAATCCATTTT
TAAGAATATGGCTCAGAGGGAATAGTGTCCACTAGTGGTGATGTTTATAGCTACGGAATCATGTTGATG
GAGGTTTTGACGAAAAGAAGGCCAACAGATGAAGAGATATGCAATGGAAATCTTGACTTGAGGAAATG
GATTACACTATCATTTTCAGGTAGTATGATGGACGTTGTGGATGTCAATCTTTTTTGTGAGGAAGAACAA
ATCACTAGTAAAAGTGAAATGTGCATAGCGTCCATGATAGAATTGGGTTTAGACTGCACAAAGGAAACG
CCAGAATCAAGAGTGACCATGAAAGAAGTAGTGAAGAGGCTTAACAAAATCAAGAACGCATTTCTGGA
AACATAGAAGTGATCAGCATCTGTTCTTTACTGAGCTGCAAATCAGTTGAGAAAGTAATTATGTGAGGG
CGTTAGGCCAGCGGTAACAAGGAATGCAACTCATGCATACTATTAAACATATCTTTTTCCGCAGTCCCAT
AGATTAAGAAGTTAAGCCTGCTAATGTATTAGTGAGAACTAAAATGATATGATTAATATGTAATTCACAT
TAGATTTCTTATCTTTAACACATTTTGTGCACTCTCTGTTTAATAGATCAAAAAGTGCCATGATCCACTTTT
TGGCAGTTTTTTCCTCACTTGTTTCTGTGAGGATCAGGAACTCGGTAATTAAACTGCAGTTTATTCTCCTA
TTATATTTTATCTACAAGTTAGGCATGTGTACAGATTGTTTGCATCAGTAACAGATTGATACCGAATTCAA
AGGAAAAATATATGGTGGCTCATTTTGCAGCTGCTATATTCAGGATGGAGCCGGTAATA
>PGSC0003DMG400014281 LRR-RLKs
ATGGCTACTGGGTTTTCTTCTACTACTGCCATTGTTGTCTTTCTTCTCTGTTTCTGTGTTTCTAATGTTGTTT
TACAAAGAACAGAGGAGGACCCAGAAAGACAAATCTTGTTTTCTTTCAGGAGTTCTCTTGAAAATCCTTA
TGTTTTGTCTACATGGACCCCCACAATTTCACATTGCAAATGGGATGGTGTTTTCTGCCAAAATGGTCAA
GTTGTTTCCCTTATTCTTTCTTCTTTGTCACTCAAAGGGCCTATTTCACCCCACATTGCTTCATTGAAGAGT
TTAAGAGTGTTGGATTTGAGTAATAACCAGTTGTCTGGTGAGTTACCAATTCATCTCAGTGAACTTTCTCT
TTTAGAAACAATTAAACTTGGTTCTAACTGTTTTACTGGAGAAATATCGCCGGAGTTTGGACGGTTGACG
GAGATGAAGTTACTTGACCTTTCAGGCAATGCACTGACTGGAAAAATTCCAGCCCAACTTGGTCAGCTTA
CTAAGCTACAAGTTTTGGCACTTGGAAATAACCTTCTTTCTGGTTCTTTATCTGCAACACTTTTTACAAAGC
TTCAATCTTTAACTTCTTTTGATGTTTCTAATAACACCCTTTCTGGTATTATTCCACCTGAAATTGGGGGAT
TGAGAAGCCTTACTGATCTTTACATTGGGGAAAACAGATTCTCTGGCCATTTGCCAGCAGAAATTGGTGA
ACTTTCAAGACTCGAGATTTTCTTGGCTCCTTCATGTTTACTTGAAGGTCCATTGCCTGAATCCATCTCAA
AGTTGAAATCTTTGAAAAGATTTGACCTTTCTTATAACCCATTGAAGTGTTCAATCCCAAAAGCTATAGGT
AGTCTTGAGAATTTGACTATACTGAACCTTGCTTACTCTGAGATCAATGGTTCAATACCTTCTGAGCTTGG

AAAGTGTAGAAACTTGATGTCAGTAATGCTTTCTTTCAACTCACTCTCTGGGTCTTTGCCTGAAGAACTTG
CAGAGTTGCCTGTGCTATCTTTTTCTGCAGAAAACAATCAGCTTTCTGGTGCATTGCCTTATTGGCTTGGA
AGATGGACTCAGATGGATGCATTGTTGCTTTCGAGTAACCGGTTTTCTGGGAAAATTCCGGCTGAGATT
GGGAATTGTTCTATGTTGAGTCATATTAGTTTGAGCAATAACTTACTGACTGGTCCAATACCTAAGGAGC
TTTGCAATGCCGTGGCACTCGCGGATATTGAGCTTGGCAATAACTTTCTCACAGGCACCATTGATGACAC
ATTTGTCAAGTGTGGCAATCTGAGTCAGTTGGGATTAATGGATAATAGTATTGCTGGGATGATTCCGGA
GTATCTATCACAGCTTCCTTTGGTGGTTCTTGATTTGGATTCCAACAATTTGACAGGTCCAATTCCGGTGA
GTCTCTGGAATTCGACTTATATGTTGGCGTTTTCTGCTGCGAATAACCGGTTATGGGGTACTCTACCGGT
GGAGATTGGCAATGCTGTGTCATTGCAGAGTCTTGTTCTTAGTAACAATCAGATAACTGGTGTCATTCCT
AAGGAGATTGGTAATTTAACCTCTCTTTCAGTGTTGAACTTGAATTCCAATCTTCTTGAGGGTTACATTCC
TGATGAGTTGGGTGATTGTGTCTCTCTTACAACATTGGATCTTGGGAATAACAGGCTGCGTGGGTCGAT
ACCGGAGACACTGGGGCATCTGCCTCAGTTACAGTGTTTAGTCCTTTCACACAATGATCTTAGTGGTGCT
ATTCCTTCTAAGATTTCCAAGTATTACCGGCAAGTTAGTATCCCTGATTCGAGTTATGTGCAGCATCATGG
AGTTTATGATCTGTCTCACAACAAATTGTCTGGCTCAATACCTGAAGAGCTAGGGAGTTGTGTTGTTATA
GTGGATCTTTTGCTCAGCAATAACATGCTATCTGGAGAGATACCACGATCACTCGCCCGCCTGGTGAACC
TCACTACGTTAGACTTAACCGGGAATTTGTTAACAGGCACCATTCCAAAGGAATTTGGCAACTCACATAA
GCTGCAAGGATTTTATCTGGGGAATAACCAGCTTACAGGATCAATCCCTGAAAGCATTGGCCAAGTAAA
TAGTTTGGTGAAGCTGAATTTGACAGGGAACATGCTTTCAGGTCCAATACCCTCAAGTTTTGGGAAGTTA
AATGGGCTGACTCACTTAGATTTGAGCTCAAATATACTTGACGGTGAACTTCCTCAGTCGCTTTCCAGGA
TGGTAAACCTTGTTGGCCTTTATGTTCAGCAGAACAGGCTTTCGGGCAGTCTAGATAAGCTATTCTCAAA
CTCTGCAGCATGGAGACTTGAGATTATTAATTTGGGTACTAACTCCTTCACTGGAGACTTGCCACCATCA
TTGGGCAACTTGTCGTATTTGACGTTTCTTGATCTCCATGCAAACAGTTTAACTGGTGAAATTCCAGTTGA
GCTAGGAAATCTTGTGCAACTTGAGTATTTGGATGTCTCAGGCAACAGTCTCTCTGGCAAAATTCCCGAA
ACAATATGTGCCCTTCCCAATTTAGATATACTAAATTTTACGGATAACAAACTGAAAGGAGCTATACCGA
GGAATGGAATTTGCCAGAATCTTTCAAAAGTCTCAGTGGCTGGGAACAAAGATCTCTGTGGGGGAATCG
TGGCTCTAAAATGCCCTGCCAATAGTTTTGTTAAAAGATCATTGTTGTTTAATGTCTGGGGAATCTTATCA
GTGGTGGCTGGGACTATAATTATCACTCTTACTATTGTCATTGTGATAAGGATATGGGTTAATAGAAGCA
GCAGAAAGAGTGATCCCGAGGATAGCAAACTTGACAGTGATGATCAGCACCTTTATTTCTTAGGCAGCA
GCAAGTCGAAAGAGCCTCTTAGCATCAATGTGGCCACGTTTGAGCAGCCTCTTCTCAAGCTGACCTTGGT

TGATCTTCTTGAAGCCACCAACAACTTTTGCAAGACTAAAATTGTTGGTGATGGAGGGTTTGGAACTGTC
TATAAGGCTACTTTACCTAATGCTAAGACAGTTGCAGTTAAGAAGCTAAACCAAGCAAAAACTCAGGGT
CACCGCGAATTTTTAGCTGAAATGGAAACTCTAGGCAAAGTGAAACATCGAAATCTTGTTCCTTTGCTTG
GGTACTGTTCTTATGGTGAGGACAAAGTGCTTGTTTATGAGTATATGGTTAATGGGAGCTTGGACCACT
GGTTGAGAAACCGTACTGGAACACTTGACGTGCTGGATTGGAGCAAACGACTTAAGATTGCAGTAGGA
GCTGCTCGTGGTTTGGCATTTCTTCATCATGGATTCACACCACACATTATTCACAGGGACATTAAGCCAA
GTAACATCTTGCTCAACGAAGACTTTGAGGCCAAAGTTGCAGATTTTGGGTTGGCAAGGTTGATCAGCG
CCTGTGAGACGCATGTAAGCACTGATATTGCTGGTACATTCGGTTACATTCCTCCCGAGTATGGGCAGAC
ATGGCAGTCTACAACAAAAGGAGATGTTTATTCTTTCGGCGTGATCCTTCTTGAATTGCTGACAGGGAAA
GAGCCAACAGGACTCGATTTCAAAGATGTCGAAGGTGGAAACTTGGTTGGTTGGGTACTCCAGAAGAT
CAAGAAAGGTCATTCAGCTGATGTTCTTGATCCAACAATACTTGATGCAGATTCAAAGCAGATGATGCTT
CAGACATTACAGATTGCTACAATATGTCTCTCTGATAATCCGGCTAATCGCCCGTCTATGCTCCATGTCTT
CAAGTTCTTGAAAGGAATCAATGGGGAATAA

>PGSC0003DMG400014576 LRR-RLKs

CTTGCATTAATTGCCCCACAATTGTCACATTATTTCAACATTAATTACTATCACCACCTTTCTCTCTTCTTC
ACCTTTCACTCTGTTTTTTCTCTTCACTCTTCAATGGCTACTACTACTCTACTCATGAACTTCCATTACACTT
TTCTCTCATTCTTCTTCCTCTTCACCCTTCTTGAATCTTCCCCAAACCCAGATATTGCACCATTACTAGCTTT
CAAATCAGCTTCAGATGAAACAAACTCACTCTATAACTGGAACTCATCAACTCACCCCTGTTCTTGGACTG
GTGTATCTTGTCTTAACAATAGAGTTTCTAGACTTGTTCTTGAAGGTCTTAATCTAAAAGGGTCTTTCCAA
AATCTTGTATTTCTCAAAGAGTTAAGAGTATTGAGCTTGAAATATAACAATTTTTCCGGTTCTGTTCCGAA
TCTTTCGAACCTTACTGCTTTGAAGCTGCTGTTTCTTTCCCATAATGAGCTTTCTGGGGAGTTTTCTGAGTC
GTTTACCTCGCTTTTTAAGCTTTATAGACTTGATCTGTCGTATAATAAGTTTTCCGGTGAGATTCCGGCGA
AGGTGAATCGTTTGACCCATTTGCTTACTCTTCGTCTTGAAGGAAATGGGTTTTCCGGTGAGATTTCTGG
GGTTAATCTACCTAATCTTCAAGAGTTTAACGTTTCTGGTAACAAACTCGTTGGAGAAATACCCATTTCGC
TATCTCGTTTCCCGGAATCTGCATTTTCGAAGAACCGGGTTCTTTGTGGTTCTCCGTTGCCAAACTGTACG
GCGGCGGTTCCTCGTGAACCGTCGTCGTCGGCAGGAGCTTTAGCTTCTCCGGTTAGTCCTAAAACTACCA
TTGCATCTTCTCCGAGCTCATTGCCGGTGACCTCTGCAACTTTGAGTCCAAAAAATACCCATCATAGTAGT
GGTAAAATGAGCTCATTGGCCATAATCGCCATTATACTAGGTGATGTGTTGGTTCTATGTGTTGTGTGTA
TTTTTCTCTACTGTTTCTTCTGCATTAGGAAAGTTTCTTCTCAGAAAAATGGGTCACACATTCTTGAAGGT

GAGAAGATTGTGTACTCGTCTAGCCCGTATCCGAATACGGGCCAGACGAGCGGGTTCGAGAGAGGGAA
AATGGTGTTTTTCGAAGGGGCGAAGAGGTTTGAGCTTGAGGATTTGTTGAGAGCTTCAGCTGAAATGTT
GGGAAAAGGGGGATTTGGGACTGCTTATAAGGCTGTGTTGGATGATGGAAATGTTGTTGCTGTGAAAA
GATTGAAAGAGTTGAATGTTTGTGGGAAAAGAGAATTTGAGCAACAAATGGAAGTTTTAGGAAGACTT
AGACACCCAAATTTGGTTAGTTTGAAAGCATACTACTTTGCTAGAGATGAGAAGTTGTTGGTGTATGAGT
TCATGACTAATGGCAACTTGTTTTGGCTTCTTCATGGTAAGTTTTGTTCTTCAAGAAATTCAATAGTACTA
ACATATTTTAAAATGGAGCAACTTTGCTGGATTACACTGAGTATGTTGTTCTTGTTATTGATATGTGATTG
TGTTGTTGAACTTGTTGATTTTGAAAAGAAAAATGTGATCTTTAGGATTGTTTTGTTGAAATCGATTGATT
TGGGTGATAAAGTTTGGCAACTAGTAGCAAAAGTCACATGTGGTTGGTTTGTGTCTTGTTTTTTTTTTTTG
GATTTTAGTTGCTTTTTTTGGCTGCAACTGTCAATAAGGCCTGGTCTCTAATGCATATAAGAATAGAAACC
TAAATTTGGATAGTTTGAAAGCATATTACTTTGCTAAAGATTGAGTAGTTGTTGGTGAGTTTGGTGTATG
ATTTCATCACTTGTTTTGGCTTATTCATTTGTTTTGTGTCTTTTTTTTTTTTTTTGGATTTTAGTTGCTTTTTTT
GGCTGCAACTGTCAATAAGGCCTGGTCTCTAATGCATATAAGAATAGAAACCTAAATTTGGATAGTTTGA
AAGCATATTACTTTGCTAAAGATTGAGTAGTTGTTGGTGAGTTTGGTGTATGATTTCATCACTTGTTTTGG
CTTATTCATGGTAAATAGTATTCAACCTTTTGTTCTTTAAGAAATTCATAGTACTAAAATATTTGTGAATTA
CTTGTTTCTTAAAGAAAACTGTGATCTTTAGTTTTTTGTTGTAGAATAGATTATTGATTGACCAATAAGGA
TTATAGTGGAGTTGTAAGTACTTCGTCATCTTTAATCAGACATTTCAAGTTCGAGCCTTGAGTATGGACTT
GCCCTTGTTAGGTAACGGTTTGTCCCTATGTGGGACTTCCTAGCGCAAACCTGTATCCAGCTCCAATGCA
GTTGCCGTACACCGGATGGGTAAAAAGGAGAAGATTATTGATTGATTTGGTGGTAAAGTTTGGCAAGTA
GTATAGCATAATCACATGGGGTTTGAGTTTGGCCTTTTGTCTTTAATTGCTTTTGGTCTGCTACTGTTAAC
CAGGACTAGCCTTCTGATGCAATAGGAATAAAGATGGGATGCCTCAATACTAGACGGTATTTGCTTTAG
TTAATGTTTCTATTTACATAATTGAAACTAGCCATGTGGGGATACTGAAAATCTATGTTGCTCGGACTCTT
CAAAAATGTCGATGAGGCATTTTTGGAGGATCCGACATGGGTAAGTCCTACATAGTCTTTTCAAGCTAAA
ACTATAGAATATTGGAGTTTCTGGTCAAGGTATGTTGGTTCATTAACAATCTTTGTCACATAAGCTATTCT
TGATTTTCAAATGTAGGGAACTTAAATTATTTAACAATCTTTATCATCTTATTCTTGAAAAAGCTTTTAAAG
GAATCTAATTTACTCATGATGGTCCTAAAGTGACAGAAGATTAGAGCAAAGGACTTGTTGTACCAGATG
AATAGAATTATGTGTTCCCTCTGATTTTTGGGCCAAGCAAAAGAAAGAAATTCACACTTTTTAGGCAAGA
GATGCCCTACTGTCCCATTGTGCTCTACAGTTTTTTTGCTTGTCTTTTTGACCTCACTTAGTATTCATATCTT
GCAACTGTTTTGGTAGGTAGATAGTGAAATATAACCTCTTGTTTTTTTCTGATTCAAATGGTAAAGAATCA

ACTTCTTGTTGAATTGCACCTGTCATTCATGTAACAGGAGACAGCTGCTTCATATGTTAAATTTTCTCATTT
TAGAAATATGAACAATTATGTATTACTATTCCTACCATCAAAGTCTATTGTGTCAACTTCATACTCCTTGTC
CTCTCTGCCCATGTTTTTGTTCTTTTGTCTAGTTAAAAGTTGCCCCTTTATCTTTCTATAACAATTCTCCCTC
CGTTTCAATTTGTTTGTCTGGTTTTGATTTGGCACGAAGTTTAAGAAAAGTATAGAAGACTTTTGAATCTT
GTGGTCTTAGTTCTTAAACATGTCATGTGGCCGAAAACGGGAAGAGGCAAATAAGCGAAAATGTGCAA
GTGCTCCATTTTATAGTAGTTTGAGTAAATGTTTTGATGGGCATGAATCTTCTTGCTACAGGTAACAGAG
GGCCTGGAAGAACTCCTTTAGACTGGACAACAAGGTTAAAGATTGCAGCCGGAGCAGCTCGAGGTCTA
GCCTTCATCCACAACTCATGCAAGTCCTTGAAACTCACTCATGGCAACATCAAATCAACTAACATCCTCAT
TGACAAGAGTGGCAATGCACGTGTCTCCGACTTTGGTCTAGCCATCTTCGCCACACCATCTTCCGTCCCA
AAAACCAATGGCTACCGAGCCCCTGAAGTCGCATTGGATGGTCGTAAAATAACTCAAAAGTCCGACATC
TACTCATTCGGGGTCCTATTACTCGAGCTGCTAACCGGTAAATGCCCCTCAGTCGTGGACAATGGTAGTG
GCCTCACAACCGGCTACGGAGGGGTCGTGGACTTACCGAGATGGGTGCAATCCGTGGTGAGGGAAGA
ATGGACTGCGGAAGTGTTCGATTTGGAGTTGATGAGGTATAAGGACATTGAGGAGGAAATGGTGGGG
CTATTGCAGATAGCTATGGCATGTACTTCAACATCACCAGATCAAAGGCCTAAAATAAATTATGTTGTGA
AAATGATTGAAGAGTTGAGAGGTGTTGAAGTTTCACCTTCTCATGACACTGCTACTGATTCTGTTTCTGA
CTCTCCTGCTGTTTCTGAAGATAATACATGTGGTGCAGCTAGTCAGTAGTAAGAGTTTTTTTTTCTTCTTA
TTTTTCTAGTTTGTTATTCTGAGTTATGTAAATTAACCTCTTAGTTTTAATCCTTTTATCTATAAGATATAAG
AGAAAATTTAGGAGAAGTTTTATA

>PGSC0003DMG400014917 LRR-RLKs

GTTCATTACTACTATTTTTATTTGTATCTCAATTTTGTCAAAAATAAAAAAATCCCCAACAAATGATGTGG
AGGATATCCGTTTTTCTTGTTCTTTTACTCAATTCTCATACCGGATTTTGTCTAACTTCCGACGGACTTTCA
CTTCTATCTCTAAAATCCGCCATGGATGATGGCGGTGGTGGTACAGTTTTCTCCGATTGGAATGAAAACG
ATGATACTCCTTGTACTTGGACTGGCATTTCATGTGCTAACATTTCTGGTTCTTCAGAGCCACGTGTCGTT
GGGATTACTCTTTCCGGTAAGAATCTCCGGGGATACTTATCGTCTGAGCTGGGAACGTTACTCTATCTCC
GGCGACTGAATCTTCATGGAAATAATATTTACGGTTCTATCCCTGACCCTTTATTCAATGCGACGTCGCTT
CATAGTATTTATTTGTATGATAATAATATCTCTGGTATACTTCCTCCTTCTGTTTGTAATCTTCCTCGTTTGC
AGAATTTGGATATTTCTGATAATTCACTTTCTGGAACTTTCTCTAAGGATCTACGTAATTGTAGGCAGTTA
CAGAGGCTTATTCTAGCTCGGAATAAATTCTCCGGTGAGATTCCGGTGGGGGTGTTCCCGGAGTTGGCG
AATTTGGAACAACTTGATCTTTCGTCGAATTTGTTCAATGGTTCGATTCCTGAAGATATTGGTGAATTGAA

GTCACTATCTGGAACTTTGAATCTTTCTTTTAATCATTTTAGTGGAAGAATTCCCAAATCTGTAGGTGATT

TACCTTTAACTGTTAGTTTTGATCTAAGGAATAATAATTTATCTGGTGAAATTCCTCAGACGGGTTCTTTT

GCTAATCAAGGTCCGACGGCTTTTTTGAATAATCCCTTGTTATGTGGATTTCCTTTACAAAAAAACTGTAA

AAATAGTTCAAACAATTCAACACAAGTTCAGGGTTCGTCTGATAACGAGGGAACGAATTCGCGCAAGGG

GTTAAAACCTGGATTCATTATATTGATATGTTTGGCTGATGCATTTGGAGTGGCATTTATAGGACTAGTG

ATTATTTACTTGTATTGGAAAAAGAAAGACTCTGGTGGTTGCAGCTGTACTGGAAAAGGGAAATTTGGT

GGAAATGAGAAGAGAATGCTTTGTGGTTTCCCATGTATTAATGGATTTCCAAATAATGATTCGGAAGTT

GAGTCTGAGAAGGGAGGTGGCGGTGGGGCTAGTGGTGGTGGTGTTAGTGGTGGGGAGGGTGATCTTG

TGGCTATTGATAAAGGTTTTAGCTTTGAGCTTGATGAGCTGTTGAGGGCTTCTGCTTATGTATTGGGAAA

GAGTGGGTTGGGGATAGTGTACAAGGTTGTGCTTGGGAACGGTATCCCAGTGGCAGTGAGGAGGTTA

GGGGAAGGTGGGGAACAGAGATATAAGGAGTTTGTCGCGGAGATTCAGGCAATCGGAAGGGTTAAGC

ATCCTAATGTTGTGAAGTTAAGAGCTTATTATTGGGCTCCTGATGAGAAACTTCTCATTAGTGATTTCATT

TCCAACGGCAACTTGGCTTCTGCTCTTCACGGTGAGCTTTTCATTATAGTAAAACATCTTCAGTTTTGTTT

GTCTAGTAGATATAACCTGTTGAAAGAATCATTAACGCATTCATATTTTAGATATCGGGGTATACTATATT

CTTATCATTTAGTAGCATGATACACTGATGGATGTTTCGTACATTCCAAGATCGATATGTGTAAATTGTAA

TTCTTTTGCTGGATTGGTATTTTACTTATTGATCACTCTTTTGAGGAGTGGAAATACAGTACAAGACTTTT

GGTATTAGGTTAGTTAGGAGTTTGGGATGTTCTAGTATTTATTCACTAGTATAACTGTTTAATATATTCCC

AATGACAAAATAATGACTTTTAGCTGTTGTGTGTTCCTTGATGATTTTGAATAGCAATTATTCATATGCCA

TCTAGTGATAATTTTATATTGAATGTGTCCCATGTGCTTGCAATCCTTGAGTGAAGGATGAGTTTTGTTTG

CGACTTGATAATGTGTAGGATTTTTGCCTAATTGTATCAGCTGATAGGATAGTCTTTATTATGTATCATAG

ATGAAAAGTTATCTGCATAAAGGGTAAACCAACAACCTAACATTTTCCATCCATTTCAATGGATGTTAAA

GCACATCGTAGTATATTAACTTATAATGATCCAGCCCAAGTCAATTTGCAGACTTTAACGATTTACTGCCG

ATGCAATAATCATATGTTGACAGCATATCGAACCTGGTAGAAAGGGCATTTAGGCATGTTTTGAAGAAT

TTAGTCTCTCTTTTTTCATGTGACATATGGTTGACTGCCCCGATAAACCTTGCGGAAAATGATTTATCATC

AATTAGAAGCAAACCAATAAGTACAAGGGGCCAAGAACTGAATTATGTCATGTACTAGGTCAATTCCTT

AAGTTGTGGAGTTACGTATTCTGTCTCTCAAAAAAGTCTAGATATTACATCGTTTTATTATGTCTTTTAGA

GATGTATTACCTGATTTGCATATACTAATATTCAAGAATTTGCGAATCGCTCCGCCTGGTGCAGTTTGACT

TTACAGATGTTTAATTTTTCGAAGCAAACTGGAATCTGCAGTATCTGGTCTGGTAACTACCATCTAGCAT

GAGCGGGACTTATACTCCCTTGAGATGGATTATAGACTTCGAATTTTTAAAAATTAGTGTTCTGCAATTG

AATTTTGCACTGTTGCTGTTGGTATAGCTGCTTGAATGTTACATTTTTAAAAAGAATTAGATTTTTTCCCCT
GATACAAATTGTTGTTTACATTTATGAATATCTACTTCACTGCCTCATATACATGTCACTATGAATCTATAA
CACATTCGTTACCCAAGAGTAACTGCTTGCTTTCTGAGACAAATATATTTGATGACCTTTGGTTCTATGGT
ACCTGATGTTCCCAGTTTCTTAATTTACCTGTTTTTAAATTATGTAGCTGGAATTTGACAGTACAGTAACTT
ACCTTTTGCATTCTGAAGAAGTTGAGGAGTGTTTTGTTTCTGCATCTCTTTTTCCAAATTGTTTTGCCATGC
TAGATCTGTATGTATTGACGTAGTGACAGTAGTTTCATGCTCTAGCTGATTGAGATTAGCCGAATTAGTG
TGAACACCGATTTGCCTATTGAGCTGAAAAGTTATTACCTTGTATCTTCTTCGTCATACTGTGGTAAATGT
TGTGATGACAATCTCGTTTGGCTTTATACAGGGAGAAATGGGCAACCATCACCTAGTTTAACATGGTCAA
CGAGACTGAAAATTGCTAAGGGAACAGCGCGAGGTTTAGCCTATCTCCATGAGTGTAGCCCAAGGAAA
TTTGTCCATGGAGACGTCAAACCTTCCAATATTCTCCTTGATACTGAACTGCAGCCATATATATCTGATTT
TGGCCTCAATCGATTAATCAATATCACGGGAAACAACCCCTCCTCTTCTGGTGGATTTATGGGTGGAGCT
CTTCCTTACTTGAAGCCTGCCCAACCAGAACGTCCCAATAACTATCGAGCACCAGAAGCTCGAATCACTG
GTAACAGACCCACCCAAAAATGGGATGTGTACTCATTCGGGGTGGTCTTGCTTGAACTGCTGACTGGAA
AGTCTCCTGACCTCTCAGTTCCTACAACATCCACTTCAACGGAGGTTCCAGATCTTGTAAGATGGGTAAG
AAAGGGATTCGAGGAGCAAAATCCACTGTCGGATATGGTAGAACCAATGTTGCTTCAAGAAGTCCATGC
TAAGAAGGAAGTACTAGCTGTATTTCATATAGCTCTTGCATGCACCGAGGCAGATCCAGATATTCGACCA
AGGATGAAAACTATCTCAGAAAACATTGAGAAAGTTGGAGCTTAAACTTTTCGGTACACTCATGTCAAG
GAGTTCCTGAGTTTCATGGACTTCTTTAATCTTTTTTTTGCAGTAATTGCTCCTATTATTTTTTTTCTTTTAG
AGGTTAATCAAATCTTTAAACTTAACTACAGTACAAAATACCAGTGTGCTGCAACAAAATTCCTGTCATTT
TTGAGAAGTTAATTAGTTGCTTATCTCTTTAATCTATTTCTGTAATTCTGATGTTAAAACAACTTTTCATGG
CCAA
>PGSC0003DMG400015081 LRR-RLKs
ACACAACAAGTTGCAAGGATCTATACCTGACTCAATGAGCAACATGATAGGTTTGGAATACCTAGACATT
TCTCATAATAATATATCAGGAACCATTCCTATGTCTTTGGAGAAACTTCAAAACTTGAAGTATTTCAATGT
TTCCGTCAACAAGTTGCACGGTGAAATACCCTCAGAGGGTCCTTTCAAGAACCTCTCGAGTCAATTTTTC
ATCAACAATGAAGCATTGTGTGGTTGGTCAAGGTTTAATGTCCGGCCATGCCCCACCTCATCAACGCATA
GATCAAATAGGAAAAAATTGCTAGTTCTATTTCTTTTGCTAGGAATAGCACTTTTATGTGCTCCTACTACC
CTTGTGTTTGTATGGATAAGGTATAGAAGAGGTAAAAGATCTCCTCAACGAGCTGATTCATTGTCTATCA
CAACAACGGAAAGAATTTCATACTATGAACTGCTCCAAGCAACTGATTCTCTTAGCGAGAGTAATCTGAT

TGGTTCTGGAAGTTTTGGCTCTGTCTACAAAGGCGTTCTCAGAAGTGGGGCTGCCATTGCAGTTAAAGT
GTTCAATCTGCAACTGGAAGCGGCATTCAAGAGTTTTGATACAGAATGTGAAGTTTTGTGTAGCCTTCGC
CATAGGAATCTTGTGAAAGTCATCACCAGTTGTTCCAACCTTGATTTTAAGGCTTTAGTGCTCGAGTATAT
GCCTAATGGAAGTCTTGATAAGTATTTGTATTCCCACAACTACTTCCTAGACATCAGACAGAGACTAAGC
ATTATGATAGATGTGGCATGTGCGTTGGAATATCTCCATGATGGTTGCTCCTCTCCGGTGATTCATTGTG
ATCTGAAGCCTAGTAACGTCTTGCTGGACGAGGATATGGTTGCCCACCTAAGCGACTTTGGTATTTCAAA
ACTGCTTGGTGAAGATGAGACTGATTTATACACTAAAACCTTAGCAACATTTGGTTATATAGCACCGGGT
ACGTCTCTTTGTTTTTGCTAATTTGAACATTGATTTTTCTTCTTATTCTTTTTTTTCCAATCAAGAAAGTATA
TTGCATCTTTTAATTAATTGTTTGACTGTAGAGTATGGACTGGATGGACTGGTGTCAATAAAATGTGATG
TCTATAGTTACGGGATTATGTTGCTGGAAACATTTACTAGGAGAAAGCCTAGTGATTTTGAGGGAGATC
TTAGCTTGAAGCAATGGGTGAGTTATTCACTCCCAAAGGCAGTACTGAACGTTATGGATGCCAACTTGG
TTACACCAATGGATAATCGCTTACAGAAGGAGTTAGATATCGTGGCATCAATCATGAAAGTAGCATTAG
ATTGTTGTGCTGAATCTCCGACAAGGAGGACAAACATGAAAGATGTTGTAGGGATGCTACAGAAGATC
AAGATTCAACTTCTTGCATGCTGAGCATGTTCTCTGAATCTCTTGTTCCGAATTGATCACTTTTTTGTTTTG
TTTTAGCACTTGATTTGTGCATGATTCTGTTTTCAGAAATGCT
>PGSC0003DMG400015157 LRR-RLKs
TTCAGAACCACCAAGCAACTAAAAGAGACATCCTACTCCTCAAGCACCACCAGTGTGGTGCCTCATCCAA
CTCCCAATTCCACCACAATACTGCACCAATCCGATGGCCTTCACAGCCTCACATATACACCTTTCTTTTTTG
TCTTTTTTCACTGTGATCCTTCTTGTTCAAGCAAAACTCAACCTTTACTCACCTGATCACAGTGCTCTTTTG
CTTGTCCAAAAAGGCTTAGGCATCCCTGCTCACCGCAATGCTCTTGAGAACCCATGCAACTCCGTTGGAA
TATCATGCGAAAAACGACTCACAAACAATTCATACGTGCTAAGAGTCACAAGGGTTGTCTTCAAATCCTA
CGGGTTGAAGGGTACTCTGTCTCCTGCCATTGGGAGGCTTTCTGAGCTCAAAGAACTGTCCCTCCAAAAC
AACAAACTCTTTGACAGAATACCAACTGAAATCGTTGACTCCAGGAAATTGGAAATCTTGAACCTTCAAA
ACAACCAATTTTCTGGCAAAGTCCCACCTGAATTATCATCTCTAGTCCGCCTTCGTACCCTTGACCTTGGC
TCTAATGAGTTATCTGGGAACCTGAACTTCTTGAAATACTTTCCTAACCTTGAAAAACTCTCCCTTGCTGA
TAACATGTTTACTGGCAGAATACCTCAATCCTTGAAATCTTTCAGAAATCTCCGGCTCCTCAACATTTCAG
GCAATAGTTTCCTTGAAGGTACGGTGCCTGCCGTGAGTCAAGTTGAGCACTTATCAGCAGACTTAAATC
GAAAAGATTATGTTCCTAAGCGTTACATTCTTGCTGAGAATGAAAGAAGGCCAAGGCACACCCCTGCAA
TGGCACCAGAGTCCAAATCAGGATATGCCCAAGCTCCAGGACCTAGTACAGTTGTAGTACCAGTAAAAA

AAGACAAACACAACAAGATCAGAAGGAAGGTAGGCGCCTGGATTCTTGGATTCTTTGCTGGAGTTTTCG
CTGGGGGCTTATCGGCGTTGGTCTTCTCTGTCCTCTTCAAGGTGCTTATGTTTTTAATCAGAGGGATCAG
AAATGATTCAGGCTTAACAATTTTCAGTCCATTGATTAAGAAAGCCGAGCACTTGGCCTTTCTGGAGAAC
GAAGATGGATTGGAATCACTAGAACTCATTGGGCAAGGTGGATGTGGAAAAGTTTATAAAGCTGCGTT
ACCTGGAAGTGACGGAAAGATTATAGCCGTAAAGAAGATCATACAACCACCAAGGGATGCTGCAGAAC
TCACTGAGGAAGATAGCAAGGCTATGAGTAAGAAAATGCGCCAGATTAAATCAGAAATCAAAATTGTA
GGTCAAATCAGACACCGGAATTTGCTTCCCCTACTGGCACATATGCCAAGACCAGACTGCCATTACTTGG
TCTATGAGTACATGAAAAATGGGAGCTTACAGGATACCCTCCAGCAAGTCAGAGAGGGGACACGGGAA
CTAGATTGGTCTGCACGTCACCGAATTGCAATGGGAATAGCTGCTGGACTCGAGTATCTCCATATAAATC
ATACTCAGCGTATAATTCACAGAGATCTAAAGCCAGGCAATGTCCTCCTTGATGATGACATGGAAGCTC
GAATTGCGGATTTTGGCCTTGCAAAGGCTGTCCCAGATGCTCATACACATATTACAACTTCAAATGTGGC
AGGAACTATAGGATACATCGCTCCAGAATATCATCAGACACTGAAGTTCACTGATAAGTGTGATATATAC
AGCTTCGGGGTGCTGCTAGGCGTGCTAGTTATGGGAAAGCTTCCATCTGATGAGTTCTTCCAGAACACTT
CTGAGATGAGTTTAGTGAAATGGATGAGAAATGTCATGACTTCTGAGGATCCAAACAGAGCAATTGATC
CAAAGCTGATGGGTAATGGAAATGAGGACCAAATGCTTTTGGTTCTCAAGATTGCCTGTTTTTGTACCCT
GGAGAATCCTAAGGAGAGGCCTAATAGTAAGGATGTTAGGTGCATGTTGATGCAGATCAAGCATTAAG
TATGTAGTGGATAACTTACAGTAAATAAAAGTTTGTCATGAAACTAAGAGAATATTTCTTGTGTATGACG
GTCCTTGTGTATTCTGTTACATCTCCAATATGAGAGACTGTCCTTGTGTGTTTTGTTTCCTCTCCAAAATGA
GAAGTTTATATTTAGCTGTATGAAAGTTCTTTGCAGAAGTTTCCTCTGCTATATTATAAAGCATGGTACGA
AATGTGAATCGAATTCTTTCCACTAGA
>PGSC0003DMG400015162 LRR-RLKs
TTATGACACCTTGGTCCGCACATTCTACACTCACTCTTTCCTTCCCCTTTAAACAGTCATGCATTTACATAA
AGACAGTGACTGTTTTTTCTCTCTCTAAAATTTCATCTTCTTTCATGGCTTCTCGCCGGAAAATCAAGAATT
CCCGCCGGGAACTACATTCTTGATTCGCATTCTTCTTTAAAAGGGTCGGAACAAGAAAACAAAGGGTGT
TTATAAATGGCGGTGTTTTTAAGGTTTGTTTTTTGTGTTTTATTCTTTGCTTTACTCGGATTGAGTCGTTTC
CGGGTTTTTTCAGAGCCTACACAGGATAAGCAAGCTCTCCTTGCTTTTTTCTCTCAGATCCGACATGCGAA
TCGGGTCCAATGGAATTCTTCTGCTTCTGTTTGTACTTGGTTCGGAGTTGAATGTGACCCGAATAACTCTT
TCGTTTATTCACTGAGACTTCCTGCTGTTGGTCTCGTCGGAAAAATTCCTTCTAACAGTTTGGGCCGGCTG
AGTCAACTTCGAGTTCTTAGTCTTCACGCCAATCGTCTATCTGGTTCAATTCCTTCGGATTTTTCTAACCTT

AAGCTTCTTCGTAGTCTCTATCTTCAAAAAAATGACTTTTCCGGCGAGTTCCCGGAGAGTATTCCCGGGTT

GACCCGGTTAAACAGGCTGGATCTTTCGTCGAATAACTTCACTGGTACTATCCCTTTCTCTATCAATAATC

TGACCCATTTGACTGGACTTTTATTGCAGAACAATAGCTTTACTGGTACTCTTCCTAGCATTAATCCGCCT

GGTTTAGTGGATTTCAGTGTTTCGAATAATCAGCTTAATGGGTCAATTCCTACTGCACTTTCCAAATTCCC

AGCTTCCTCTTTTACTGGTAACATTGATTTATGTGGCGGCCCATTACCCCCATGTACCCCATTTTTTCCTTC

TCCTTCACCTTCCCCTGAAACAGAACCAAAAACCCCTCCGTCAATTAAGAAATCCAAGAAGCTTTCAACA

GCTGCCATTGTTGGAATAATTATAGGTTCAATAATTGGGGTTCTCCTACTCTTGTTACTCCTTTTCTTCTGT

TTAAAACGAAGAAAAAACGACACATCAAAGGTACAAAAACCTCCGGTAGCATCAAGAGCTATCGGGGC

AGTAACAGGGGCAGCGGCTGAGGCAGGTACATCGTCGTCTAAAGACGATTTAACAGGTGGTTCAGGTG

AAGGGGAAAGGAATAAACTTGTGTTCTTTGATGGGGGTGGGTATAGTTTTGATCTTGAAGATTTGTTAA

GAGCTTCTGCTGAGGTTCTTGGAAAAGGGAGTGTTGGAACAAGTTATAAAGCTGTTCTTGAAGAAGGTA

CTACAGTTGTTGTTAAAAGGCTTAAAGATGTCGTTGTGCCCAGAAAAGAATTCGAGCAACAATTGGAAG

TAATGGGGAAGATGAAACATGAAAATGTGCTACCTTTGAGAGCATTTTACTATTCTAAAGATGAGAAAT

TGTTGGTATCTGATTATATGCCTGCTGGAAGTTTATCAGCTCTTCTCCATGGTACGCTGCTCTTATTATTTT

TAGTTAAAAGTATGTCAAACTTGTTCGTTTTAGTGTATAATTTGCTCTATTATATGTTACTGCAATTCTGTG

TCTGTTGTTTGCTCTGAACACTTAAAAGTTCCATTTTTTCACCAATGTTTTAAAACTTTAATGGCAATAAAG

ATAGTAGCATTCAATTCTTGCTAGATTTTAGTGACTTTGTTCTGATTCTTGAAGAGTCTTGAAGGGGGTTA

GGTGGGTTATAGGTATGAAAAAGATTGAATTTTTATTTTGTTTCTTTTTTTGGAAGAATAGATGGGGCTT

CAATCTAATACACGTGCAGATCATGTACACATTGTAGCCATAGTCTTACAGCCCTTCTGAACTGGCATTG

CATTATTGTTTCACTCAATTATTGAAGTTTGGTACCACTTAATTTACTTCGTTAGTACATTTATTGTCAATT

CAACCGCAAATTGCGCATTTTGTTTCTATAATTCCTTAAGCTACAGAACATTCTTTTTTGAGAGAAATGGA

ACAATGACTTTTTTATTATGATTTATTCGAATCGAGAGCATATCGGATACAATCTCTCTATCTTAACGGAC

ACACTTGTTAGATTACACTTTTACTCTTTCTTTTTTATGAAACGAACGTTACAATGCAACAGACTAGCATG

ATTCCCTGCCTTAATAAATGATGAAATGAGTTTAAAAAGTGGAAAAAGAAGGCTTCTTTCTTGTTCAAAG

ATTTACCTTTTAAATTGCTACATTGCTTGTGTCTATGCCTTAAGGTCTTTGCTCCATAGTTCTCAATTTTTTG

CCACAATCAACAAACTACCTCTATTATTGTAGTTATATAATATATATATTTTTTTTATTATACATAGGGGAA

ATGGGGGAGGGAATAACAAAGTGTTGTGGAGTTTGGTGATATCAAATATATTAATTAAATATTAATCTTT

TGTTTTCTAATCCCACAATTTTCAAATCATGATTTAATTAGTAGTAATTGCTGTAAGTCTAAACAATACTAC

CATACTTAAAAGTTTATATTACTTTGGCTTTAAGCTATTTGGTGGTTATAACTGTCTGATTATTATTCATCT

AATCTTGATTCTTTTGCTTTACTTTATTCATCATTGTTTCTTGCTGTCCCCCATTTTTCTTCTTTTGTTGAAAT
AACGTGCTGCTTTACTCTATTGTCCATTGATTCTTGCTGTCACCCATTTTCTTCTCTTTTTTTTTGGTGAAGT
AAAGTGCAAAAGAAAAGCTAATAATACAATGTGTCCTCTTGAGTCTTGAGTAGTTCAACTGCTTTGAATT
TGAATCCAACATATTAATTTGCATTGACATTAGTTTGTTAACAACATAACCAGTGTAATCCTACGAGTGA
GGTTTGACTTTTGAGTAGGGTAGGATGTACACAGACCTTATCCTTACCTTTGGTTGTCATTGGTTTGGTT
GTCTAATTAAAATGTTTTCTTTTGGTTAATTCTTGTAGGTAGTAGAGGATCAGGGCGTACGCCACTTGAC
TGGGATAGTCGAATGAGAATAGTGTTAGGTGCAGCAAGAGGCATTGCGTACCTCCATATTTCAGGAAAA
GTTGTACATGGAAACATCAAGGCTTCGAATGTTCTCCTTAAACAAGATAACCAAGATGCATGTGTATCCG
ATTACGGATTGAACCCTCTTTTCTCTACCTCAGCACCAGTGAACCACCGTGTTGCAGGATATCGTGCACCT
GAGGTTCTTGAAACTCGAAAAGTCACATATAAATCCGATGTGTATAGTTTTGGAGTGTTAATGTTGGAGC
TCTTAACGGGGAAAGCTCCAAACCAAGCTTCACTAGGTGAAGAAGGCATTGATTTGCCTAGATGGGTCC
AAAGTGTTGTAAGAGAAGAATGGACTGCGGAGGTGTTTGATGTTGAGCTAATGCGATACCATAATGTG
GAAGAAGAGATGGTACAACTACTTCAGATAGGTATGGCATGTGTTGCTACCATGCCAGACCAACGACCT
GCCATGACCGAGGTGGTTAAGATGATCGAAGAAATGAATCATGGTGATACAGATGATGGTCTTAGACA
ATCCTCCGACGATCCCTCTAAAGGTTCTGAAGGCCAAACACCTCAGGAGTCTAGAGGTTCACCTCATGGT
GTAACACCATAAAATGAAATTAATCATAAAAATGGATGGCTTGATTTGTCAAAAATTCTCAGAAAAGCCT
AAAGTAAATTGTATTAGATTCTTAGTGGGGGGAGCCTTTTATGTGTTTTTATTTTATTTCATTTTTTTTTCT
TCTTGAAATTATTATTTTGAGAAAATTTGTGGAATGATTTTTACAGATGGTTAGTTGAGAATTTGAATATT
TCAGTGAAGTGATTGGGAAATTGAAATTTG

>PGSC0003DMG400015276 LRR-RLKs

GCTGTCTATAACTATTTTTTCAACTTTTTAATGTGTTTGACATTGTTTGAATTATACCCCCAATGTTCAATTT
TACTTCACCTACTACATACAACTGGTAAATTCTCAATCTATTAGAGTATTACTTACTTATCTATTATTCTTCT
ACGTCAAGGTTGCCACGACCAGAAATGCGTAGGACGATCCTATTTTTCGCGTTGTTTCTGGTGGTGGAAT
CAGCCGAATTTTTCCCAAATGAAAGAGATGCTTTGTTGCAGCTGAGAGATAGTGTAAATTCAAGTGTTCT
TGATTTACATTCACATTGGACAGGGCCTCCTTGTTACAACAACCAGAGTAAATGGGCTGGAATTTCGTGT
TCTAATGGGCATGTAAATCAACTTGTTTTAGAAGGAATTCAGTTAACTGGCTCACTTCCACCAACATTCTT
GTTGCACATAGCATATTTGACAAAAATCAGCTTTAGGAACAATTTATTGTATGGTCCTGTTCCAAATCTCA
CAGGACTAATCTACCTGGAGTTTGTTTTTCTGTCAGAAAATCGATTTTCGGGTTTGATTCCGTTTGGGTAC
ACTGAGTTAAAGAAACTAACAGAATTGGAGCTCCAGGGGAATGATCTTGAAGGTTCAATTCCACCATTT

GATCAACAGACTTTGATTGATTTCAATGTGTCGTCGAATAAACTGGAAGGACCTATACCTGAAACTCCTG
TCCTGAAGAGATTTTCGAAGAGTTGTTATGAGAATAACGCGAATTTGTGTGGTGAAGTTGTAGGAATAC
CTTGTTCAATATCACCTGCAAGTCCACCTAAAGCACCAAGTGAAAAGAAAAAGAATGGCCTTAAAGTAT
GGAGTATTGCTTTGATTGCAGCAGCAGTAGCAGTACTTGTTCTTATTTCAATCATGTTCATTTTGTTCCGT
TACTTTAGAAGATCTAAAAGAATGGAAACACAAGGAGAAGACAAACAAGGTAACAGTTTTATTGTATCA
TCTATGTTATCCTAGCTAGTGCCAGTTATTCTGAGGGGAAAGTTATTACTACTTAGATTGAATTAGATGAT
GAGGTTATCTGGTGTAGGGCTGTTTTGGTGATACGGAACGAGACAGGGGAGAGGAAGTTGACTGTATT
AGAGGGCTGACTGTATTTTTTTCGAAATAGTTTAACCATTTTTGTTAATCGCTAAAAGAAGGTCATTCAG
AACTCGTTGGCTCCAGAATGGGGCAGCTCCAGAGGTGCTAAAGATGGTTAATTCGTGATAGGAAGAAA
ACCAATACATGTCCATTATGAAGTCGAATTCAATCATTTCTAGCTCAATAAGATGAGAACTAGTATCTCG
CCCCTGAATTGCGGCTATACAACAACAATAACATACTAGTATAGTCCCACAAGTGAGGTCTGGGGAGAG
TGAAGTGTACGTAGACCTTACCCCTACGTTTGTGGGATAGAGAGACCGTTTCTGATAGATATTGTGGCTA
TACAACATAGAACTAGATTAGCTAATTCAATTAAACTTAAAAGATCCATGTGATTATGGAGCAGTTAATA
TTGTTGTTATTACATCTTTTAACATTTCCAATTTCCATTTTGACAGGTAATATTTCAATAGAGAATGTGGTA
AAGAGAAGTTATTGGTCCAAGAGTACAGAAGATCCAGAAAAAACATTGGACTTAATATTCTTTGAAAAG
GATATGTCAGTCTTTGACATGGATGACTTGTTAAGAGCATCAGCTGAAGTTTTAGGAAAAGGAAAGCTA
AGCACAACATATAAAGCAATCCTGGAATCTGGTTCTGTTGTTGCTGTTAAGAGACTCAAAGAAATGAATT
CTTTGAGCAAAAAGGAATTCACACAACTGGTGCAGTTGCTTGGGAAATTGAAACATGAAAACCTTGTGG
AGATGTTGTCCTTCTACTATTCCAAGGAGGAGAAGCTCATTGTTTATGAATATGTTCCACAAGGAGACTT
GTTTGGGCTCTTACATGGTTGGTTCATCAACTTTCTTTTACTTTTGGGAAAATGTCTAAAGTTAACCCCTC
CTGAACGAAATACCTTAATTTTATCTTTCATTATACTTTGAGATTATTCATATCCTTATCCTTAGTAAAACG
TCTCGTGTTATACTTCGAGGTCATTCATATTCATTTCTGATTCTAAACTATGAACTCATGATCAAATGACTT
TTGAGGTGGGCTTTCCCTTCTCTATAGTGCTACTGTGAAAATTGAAATTGCTTAGAGGTAGGTGTAAGGT
CTGCGGACACTCAACCCCTTGTGGAATTATACTGAACATGTTGTTATCGTCTTATAGTAGTAATAGAGGA
ATATCTTTTACCATTTTCCCATCTTTTTTCCCCTTAAGATTCAACCTTGTAAAAACTTATGATTCTTGATTTC
AGAAAACAGAGGAATTGGGAGACAACCACTAAACTGGACAGCAAGAATATCCCTCATAAAGGATGTAG
CAAAGGGTCTAAATTTCTTGCACCAGTCCTTACAATCACATAAGGTGCCACATGGGAACATCAGATCAAA
AAATGTTCTAATCCAGCAAGACCAAAGCAAGAACTACCATTCTAAGCTGACAGATTATGGATTCTTTCCT
TTGCTACCATCTAAAGAATCCTTAAGAAAAATAGCAGTAGGAAAATCTCCAGAGTTCTCTCAAGGGAAA

AAGTTAACAAGAAAAGCTGATATCTACTGCTTTGGTATTCTTCTGTTGGAAGTGATCACTGGAAAAGTAC
CAGGTGAATTGTTTTCACCAGAAAATGGAGTTACTAGTTATGTTGATGATGATTTGTCAGAGTGGGTAA
GGACAGTGGTGAATAATGATTGGTCAACAGACATTTTGGATATGGAAATACTTGCACAAAAAGAAGGAT
ATGATGAAATGTTGAAGCTTACTGAATTGGCTTTGCAGTGTACAGATGAGGCACCAGAAAAGAGGCCTA
AAATGAGTGAAGTGTTGAGAAGAATTGAAGAGATTGAGCTTGAACCAAAGAATTTAGTCACTGATGTG
GAAGATGATTCAGTCTCTTAGTTGAAGACTTGGAACTTTCCCTCCCAAACTGTAATCAAGCCGTAGCCGC
AACAACAACATATCCAGTATAATTTCATCAGGTGGAGCTTGAACAATATATTCAGTATAATCTCATCAAAT
GAAATCGGAGTCTGAAGAGAATTTCAAGTAGAGTCTGAACAATATATTCAATATAATCTCATCAAGTGG
AGTCTGAAAAAAATAGAGTGAATGCATACATTATCGTAACCCAATCCTATGGGAGTAAAGAAAGGGTAA
AATATGCACGTACTTTACTCTGATTCCAACCAATGGGATCTTTGAGAGAATAGAGTATGCGCATACCTTA
CCCAACAAGGATAGAGAGACTGTTTCCTAAATTGTAATCAAGAAAATAGATTAGAAATTTAGGAACAGG
CTTTTAG
>PGSC0003DMG400015391 LRR-RLKs
CAAGAATTTGGTGTCTTCTAAGTGCATGACAGCTATAATCTTTTTTATGCAGGGATATATCATACAACAAT
TTCACACAAATATCTACTTCAGTATGCGATTCCTCTACCTTGTGAGCATTGTTCCTCTTCATTTGGAACCTA
CATTCATCTACTATTTCTTTTTTTTTATAAGTGAAAATTTTGGTCCTTTAACTACATTACTTACAAACCTTCC
CTCTTTAACAGAAACTTAGCTGCCAGCTATTCAAATACAATAAACACGCTCAACAACTCGTAAGTCCTAA
ATGTTGTCTTATGCATCAACTGCTAGTTTGTTTGATGCTTTCTGTCTATTGTCTATAATTATGTGGATTAAT
CTATTTGAAATTGCAGGGATGCCTGGTGTTCTATGAAACCCCTCATTTGTCCCACAGAAACGAAATGTAA
GAGTTACAGCCTATAAACCTTTTTTTTATACTTGGAAATTCTTAACTTGACCCTTGAGAGGGCCTGAAGTA
GCAGTTTGTCAAAGGACCTCAGGGGTGGCAGCCTGTGCACTTTTAAGTACAAAAGCATAACCTTGGAGT
TGAGAGATAATTTCACAACTCTTCCTCATGTTATGCTCTTTATGTCAATTGCAGATACTTCGCTATTCATAA
ACTGTGGAGGACCTAGAACTTCATTTGAGGGAAATGATTATGAGGGGGACACGACCGACAGAGGTCCA
TCATATTTTTCTTCACCCTCCGACAGATGGGCTTTCAGCAGTTCAGGAGTGTACGTGGGTCTTCAAGCAG
CCAGTTATGTTGCAACAAATACATTTTCACTGAATGTGTCCGGTCCTGACTTTTACAATACAGCACGCCTT
GCCCCTAATTCTCTCAAATATTATGGGCTTTGCCTACGAGGAGGTAGCTACAGAGTGCGCCTTCACTTTG
CTGAAATAATGTTTTCTAATGACTCAACATATAGCAGCCTTGGAAGGCGGATATTTGATGTAGCAATACA
AGTAAGTTTGAGGCCTAACTTCTTTTGTAAATAAGGAGTATGTAATCTAAAATGTTGATTTATTTAGTTCT
GATATGATTGGCAACTTTCTGATTTAATATTGAAGGTACCAATTTGTTGGAGATAATGTGGTATATTTTG

ATTTCTTACACAGGGGAGAGTAGTTTTGAGGGATTTCAACATTATGGAGGAAGCTAACGGTGTTGGAAA
AGTCATTACCAAGGACTTTGCTGATGTTACTGTTAGTAGCACTCTGGAGATTCACTTGTATTGGACCGGG
AAGGGGACTAACGCTATTCCAGACAGAGGTGTATATGGACCGCTGATTTCAGCTATCACAGTGACACCA
AGTCCGTATATCTCTGCCTATAGACTTTACACTTATCTTCCCACAATATTATCTTAATAAAATCCCATTTTCT
TCTTGCAGACTTTGATGTTGATACAGGCAGTGGGTTACCTGTCGGAGCTGTTATTGGCATTGTACTCGCT
TCAATTGTGGTGGTTCTGCTAGTTTTATTTGCTCTGTGGAAGAAAGGCATTTTTGGAGGGAAAAATAATC
AAGAAGAATTAGGTAATACATACTTAAAAGAATTTTTTCACTCTTTCCATTTGATAAAATGGGGATTCTTC
TGTTGTGATAAATAGAATTTTTTACGAGATTTCTAGCTGCGACAGACAAATAATTAACTCCCCGTAATTG
ACCAGCATTGAGATGATTGTTCTGTTGGTGGATCGACATCATGACATATCATAGATAGTTAAACTGTCTT
CATCATACAGATGTTCATTCTTTTAAAAATTGTTGTTCATCATACAGACGTTTTAGAAGATCTATATATACT
GACGTTTGCAATATTCTTCTCATTTACAGAACTCAGAGCCCTTGATTTACAAACAGGTCATTTTAGACTTA
GACAAATTAAAGCTGCTACAAATAACTTCGATCCTGCCAACAAAATTGGTGAAGGAGGGTTTGGACCAG
TTTACAAGGTATCAATTCTCTATCCATACTGATTTTGCATAAGGACAGCGGGTTTTTGGTGATATTTCCTA
CTGGTTATCCTTGATGTCACGTTTATAGGAAAATAGAGTAGTAATAGTTGAAGTAACCCGTATCCTTCTTC
TGTCTGTAATATGATCGTTGTGAATTAATCTTGCTATCTGTAGGGAGTACTCGCCGATGGTGCCATCATA
GCTGTTAAGCAGCTGTCTTCCAAGTCGAAGCAAGGAAATCGTGAATTTGTCAATGAGATAGGCATGATT
TCAGCTCTACAACACCCAAACCTTGTCAAGCTTTACGGTTGTTGCATAGAAGGGAACCAGTTATTAGTGA
TATACGAATACATGGAAAATAACTGTCTTGCTCGAGCTCTCTTTGGTAATGCTTTGCAACAACATTTTAAG
AATCATTAATTAGGCAGTGATAGTTTATGCTTTACATTCCTCTAATTCTCGTTTCTCAGGCCGTGACGACC
AGAGGTTGAACATAGACTGGGCAACCAGAAAAAGGATATGCTCAGGAATAGCAAAAGGGTTAGCTTAC
CTTCATGAAGAATCAAGGTTGAAAATCGTACACCGAGATATAAAGTGTACCAATGTGCTTCTTGATAAG
GATCTGAACGCTAAGATTTCAGATTTTGGATTGGCTAAATTAGATGAAGAAGAGAACACTCATATTAGC
ACCCGAATTGCTGGAACAGTGTAAGCCTTCTTATCATAACTGTCTCTGTACTATCATGTGATGAGAGAGT
TACAGTTCTGTGCGTTTAAATTTGTTCATCATCAACATTACTATCGTTACTTTGAAGTATCTTACGTTTTGT
TTTCTTTTTTTGAAGAGGTTATATGGCTCCGGAGTATGCAATGAGAGGCTACTTGACAGATAAAGCTGAT
GTTTATAGCTTTGGAGTTGTTTTATTAGAGATTGTCAGTGGGAAAAGCAACACAAACTACAGGCCAAAA
GAGGAATTTGTTTACCTTCTTGACTGGGTAAACTATCTTCTTTACTTTCTTAAACTCTGTATACGTCAAAAC
TAGACAAACAAATTGAAACGAAGGGAGTATTTGCTTTGATACCTCCAAAGAAAATATTAGAATCAGAGT
TGTTTCATTAGTTCTGATTCTTTTATCCTGATATGCGGATGTCAATGTTTGTTTTCTCTTTGTCATGCAGGC

TTACGTCCTACAAGAACAGGGAAATCTGTTAGAACTAGTGGATCCACGTCTCGGTACCCATTACTCCAAA
AAAGAGGCAATGCGGATGATAAATATTTCTCTTTTGTGCACAAATCCCTCTCCTACTCTCCGACCATCCAT
GTCTTCAGTAGTGAGTATGCTCGAAGGAAAACTCCCCGTGCAAGCACCAATAATCAAGCGCACTACATC
AGACGATGAAATGAGATTTAAATCGTTTGAGAAACTTTCACATGACAGTCAAACAACACAAGTATCAAC
ATACTCTCAGGACAGTCAAGGACAAAGTGTGAATGCACCTTGGAGTGATTCCTCAGTCTCAGTGTCTGTC
CCTGGTAAAGATGGAAATGTTACTTCCACAAGTAGGCTTCTTCCAGATCTTTATAATGTAAACTTGGATT
GATTCATTGACAACTACATTTTGATTTCCATCATGTAATTATCTAACTCTATCTTCACACAGGTCCCACATA
ATTTCTTTTCGTGTATACTCCAATTTGTTCAATTGTGGTCCATGATGTTTTTGTAAAATGTATGTTACAATT
CTTATTAATTAGTTCAATTGAAAT
>PGSC0003DMG400015458 LRR-RLKs
TAAGAAAAAAAATGAAGACATAAATTTCACAACATATCCATACACCTAATATCCAAAAAAATGTTAAAGA
TTGCTCTTTTCTTCTTCTTCCTCTTCTTTTACTCAGTTTTATCTAATCAAATCTCCAATTTAGAACACAAAGC
TTTACTTGATCTTAGATCATCTTTAGGCATCAGAGCAAAAAACTGGCCAAGAAAATCCAACCCTTGTACC
AATTGGACTGGAATCCATTGCAACAATGACAAAGTTGTGGCTATTCAGTTGACAGGACTAAGAAGAATT
CACAAAGGGAACATGTCTCCACAGTTTGCTGTTGATTCTTTAGTCAATTTTACTCACTTGACATCATTCAA
CTCATCAGGTTTTGTTCTTTCAGGGCCAATACCTGATTGGTTTGGCCAAAAGTTAACTCAACTCAAACAAC
TTGATCTTAGTTCAAGTTCAATTCTTGGTTCACTTCCAACCTCATTAGGGAGTTTGAGTAATCTTGATTTTC
TGTCTTTGTTTAACAATTCAATTACAGGAACTGTTCCTTTAGAACTTGGAAAGTTGAGTTCTTTAAGGGTT
CTTAATCTTTCTTGGAATTCACTTACAGGTTCAATTCCAAGTTCTGTTTCAGATATTAAGAATCTGTCTGTT
CTTGATTTGTCTTCAAATTTCTTGTCTGGACAAATCCCAGTTGAGTTTGGTTCATTTCTTGGACTTGAGTTA
CTTAATTTGTCAAATAACAGTTTAAGTTCTTATATTCCTTCTGAAATTTCAAATCTTTCTGCATTAGTAGAA
CTTGATCTTGGCTATAATTACTTGTCAGGGTCATTGCCTGAGGGTTTATTAAGAATGAGAAATTTGAGTA
AATTGTTAGTTGGAAATAATGAGCTAGAGGGTGCATTGTCAAGTACTTTGTTTACTTCAAATGATTCTGT
GTTGGAGTATATAAATCTTAGTTGGAATCGATTCGAAGGCAAAGTTCCTGATGTTTTGTGGTCATTGTCT
GATCATTTAAGGGTTCTTGATATATCAGGGAACAATTTTACTGGTGTGGTTTCAAATAGTACCTATTTTAG
TGTTACTGGTGCTTCATATAACCTTTCTAATAATCTTTTCTATGGAAATGTGACGTTTGATGTTCGGAGTT
TTCAGTCGATTGATTTGTCTAAGAACTATTTTCAAGGACTAGCACCTGATAAATCTGGTGTTCGAGTTATG
GATAATTGCTTTGCGGGGTTATTGAATCAGAGGAGTTTAGATGATTGTAGTAAGTTTTATGCTGATAGG
GGTCTAGTTTTTGTTGATGGTGGAATCCAAACTCCGGTGCAAAGAGCCTCGAAGGGCGGGCATAAACGT

TTGTTTATCATGGTTGGAGTTTTTGGTGGAGTTGGTGTTCTTATGCTTATGGCACTAGTCATTTTATTGTT
TTGGAAGGTATGCTATAGAGGGAATTCAAACGAAAGAGTCATATCGAATGTTAGTCCTGTTGTGGAAGG
AGGCAATAATCAGGCACCAGTAAAGTTCTCTCGTGATACATCTGGTTCAATTGAATCATTTACTTATGATC
AGATACTAAAAGCCACAAGCAACTTCAATGAAACAGATCTTATCAAGAATGGTCATACTGGAGACATTTT
CCGAGGAATATTAGAAGGCGGTGTCACCGTAGTTATCAAGAAAGTTAATGTACACCATATCGAAAAAGA
AACATACAAGTTGGAGTTGGATTTGTTAAATTGGACAACACACCATAGGCTTGTCCCTTTTTTGGGACAT
TGCTTAGATCATGGGAATGAGAAGTTTTTGGTCTATAAATATATGCCAAATGGAGATTTGTTCAATTTGT
TGGCCCGGGATACGGATTTAGAAGACGAAAATGGACAGTCTTTGGATTGGATAACAAGATTAAAAATT
GCAACAGGAGCTGCAGAAGGCTTAGTTTATTTGCATCATGAGTGTAATCCTCCCCTTGTTCATAGGTATT
TTTTTCTTCCACTTTGTTGCATTCTTGTTTGATAATGTTGTACACCAAGCTAATTATATTCTGCATTTTCTGG
TTCTTGAAGAGATGTTCAAGCTAGCAGTATCCTTCTCGACGATAAATTTGAAGTAAGGCTGGGAAGTTTG
AGCAACGTGTGTGCACAGGAAGGTGATAGCCAGAATTTGATCACAAAGTTTTTCCGCACGACAAAGTAA
GTATTACTATGATCATAGTTTGATCCTAATACAAGATTTTTCAGAAATGTAATAGACATTTAGTTAGATTT
ATGTGTTTACATTTCTGTATATTGCTTTTCGTTCAATGTTCTAAGATATATTATGTTTCTTCTTGACAATAA
ACAGGAATCCTGAGGGAGAGCCTGCTGGTATGTACTATTCTTTTCGTAAAAGAACGTATAGTTGAATAG
AGTTATTTATCTGAATTTCTCATATGCTGCCTTTATACATGGACAGGATCTTCATTTACAAGTTGCAGTACT
TATGATGTATACTGTTTCGGGAAGGTCTTGCTCGAGCTTGTAACTGGTAAGGTTGGCGTAAGTCATTTAG
ATGATGCCTCAACCAAGGAGTGGTTGGACAAACTTCTTCCTTTCATCACTATACGCGAGAAAGAACTACT
GTCCAAGATAGTCGATCCATCTCTAATTATAGATGATGACTTACTTGAAGAGATGTGGGCTGTAGCAATA
GTAGCCAAAGCATGTCTTCATCCGAGGCCTTCTAGACGTCCTGAAATGAGACGCGTCCTGAAAGCTCTTG
AGAACCCGTTCAAGGTTGTTAGGGAAGGAAGTTTCAACTCTATAAGGCTACGAAATACTTCATCGAGGC
GATCATGGAGTGCTGCTTTTCTTGGTAGTTGGAATCATAGCTCACAGGATAGTGTTAATGCTAGTGGCCA
AATAAGCAAAGAAGCTGGCATGAACCTGAAACAGTCAGGAAGAGTAGGATTATCTCATTCTATTAGCAG
TGGGAACGAACATTCATCATCGTGTAAGAGATCATCGAGTGAGATTTTCCCCGAACCTCAGGACACACT
AGATATAGAGAGACAAGACATGAGCTAGCCATCCAGCACGAATCTATGCCAAATGCATTTCTGCAGGCA
GGGGTTTTGTAAGTATCAGGAGCTTTGATTTTTTATTAGATGAGCGGCAGTACTATGATCATTCTGAAGA
CATGGGGCCTACACGCTACACACATGTTATGCGGTTGGTAAACTCTTTCCTTCTGCAACAGCCGATCAAA
TCGGATCAGTATTTTTGAATATGTTGTGTTACATCATGTACATGTATTCTGTTTTCCTCTTTTCTTTTAGTGT
GTCACAAATGCTCATGTGCTTTGTAAATTTTCAAAAAGTTTACTCTTGTTCCTGTGATGATAGAACAAGTA

TATAATTGTGTCAAGAGTGTACACTATCTAATCCAGATTCACATTTCCATGATCTCATATTTTGCAATTGC
ATGAAAAAGTATGATATAAATGAATAATAAAACCTGCACTTTCTTGAGCCGAGGGTCTATCGGGAACAA
CCTCTCTATTGTAGGTAGAAGTAAGGTCTGCGTACATCCGGTCATCTCCAGACCTCATTTGTGAGATTAC
ATCAGGTATGTTGTTGTAATTGGATAAATAGGGCATCAAAACTGTTATAGTGTGTCAATCCCTTGGTGGC
CTAAATAAGGACAAGTAACTCAATACATGTAGATGCACCTGAAAGTTACAATGAGC
>PGSC0003DMG400015666 LRR-RLKs
CAAATTCCCAACTAGTAGTATTTCTTTATCTTCTCCAGCTCAGACACAGACACACAGACACACAGTAGACA
CAGTCCATTCTTCTCTCTCAAAATCAACCAACAAGAAGATGAACAACATGTTTACCACTACTTTGAACAAG
AATTGAATCAAGAAATCAAATTACCCAGAAAAAAATCCTGTTTGGGGGTTTTGGGTTTACCCAAAATCTT
CAAAAAATGAGCTCAAAAATCTTGTTTTTATTCACCATTTCTGTATCAATTTTCACTCTTTTCTTGCCGACA
AACTCCGACATTGTTTCCGATCGAGCTACCCTTTTATCTATCCGATCTGCTCTTCGTGGACGTTCACTTTTA
TGGAATATCACAAGTCCAACTTGTTCATGGCCTGGTGTTATTTGTTCACCTGATAAATCTTCTGTTTTAGA
GCTTCATCTACCTGGAATGGGACTTTTAGGCCAAATCCCACCTGGGCTTTTCTCAAATTTGACTAAATTGA
ACTTTCTCAGTCTCCGGTATAATGCACTTTCCGGTGTAATTCCGGCGGACTTGTTTACTTCACTTCATGATT
TGCGTAACTTGTACCTTCAAAATAATCTATTTTCCGGTCCAATTCCTGATTCAATCTTCTCTCTTACCAATCT
TGTCCGCCTTAATCTTGCTCATAACAATTTCTCCGGTTCAATACCGGAATCTTTCAACAATTTGACTAGTTT
GGGTACACTTTACTTGCAGGGAAATGGGTTTTCGGGTCAGATACCGGATCTGAATTTACCTGGTATGGT
TCAGTTCAATGTATCGAATAATCAGCTAAACGGGTCAATTCCTAGTAAACTTGCGGGTCAACCCAAGGAT
GCTTTTTTGGGTACTTCACTTTGTGGTAAGCCACTTGATTCATGTGATGGTAGTAGTAGTAGTAGTAGTA
GTATTGGAGAAGGTAAAAAGAAGAAACTTTCTGGTGGAGCAATTGCTGGTATAGTAATTGGTTGTGTTG
TTGGTTTGCTTTTGCTTCTTTGTTTGCTGTTTTTCTGTTGTAGAAAAAGGGGTAAAAAAGAAACAAGATCT
GCTGATGTTGGTGCTGTTAGTAAGCAGGTTGAAGTTGAAATGCCTGAAGAAAGAGGGGTTGAGAGTAA
TGGTGGAAAAGATGGATTCTTGGGTAGTGCAATTGCAGCTATTGGTGTTGGAGGGGGTAATAAGGATA
AAGGTAAAGCTGAGGCTGTTGTAAATGATGGAAAAAGTTTGGTGTTTTTTGGGAAAATGGCCAAAAATT
TCAATTTGGATGATTTGTTGAAAGCTTCTGCTGAAGTTTTGGGAAAAGGGACATTTGGTACTGCTTATAA
AGCTGCTTTGGAATCAGGGATTACATTAGTTGTGAAGAGATTGAGAGATGTTACTGTACCTGAGAAGGA
GTTTAGAGAGAAAATTGAAGATGTTGGGAAAATGAATCATGAGAATTTGGTTCCTTTGAGAGCTTATTA
TTATAGCAGAGATGAGAAGCTTCTTGTTTATGATTACATCTCAATGGGAAGCCTTTCTGCACTTTTACATG
GTGAGAAAAATAACTTTTTTTTTCTGTCATTCTTTTTCATGTCATTATATTGCTGTTATGCTGTTCCTTGAAT

CGAGCGTCTATCGAATCATGTCATTATAGTGCTGTACTGTTGTTTACCATTTACATGATCTGCAAATTTTA
GTTGTCTGATTGACACTGATACTGCTGCTTTCTATGTTGCCTAGACTCTTAAAATGTGGTGCTTGTCGGAT
CCTGGATCCTCCAAAAGTAGTGTATATTTGGAGGATCCAAACACGAGTCCGAATAACATAGACTGTTTTT
GCTGATTTTATGAGGCTAGTAAGTTTGGATGACCATTTGTTTTCTTGGATCTTGTTATCTGTTACTGAACT
GGTGCTGTTGTGTAAACGTTGCTGCATTTGGCTGATGTGTTGTAGTTGCATTATTGTGCCCAATTGTGTTC
AGCCACTAAAATTTATGTTTTTTTTTTTACATTATTGTGCTGTTGTTGCATTGTTGTTCATGTTTAATCAGAT
TTTATCGTAATCTAATGTGATGAACATTGTGTTTAGTGAATATGATATCTGACATGTTTTATATATAACTC
AACCTGGAATGGCTTTACTATGTCCAATTGTTGCTGTTCAAACTCGTGTTCATCATTTAGATTGAATGTAT
ACTTTATCCACAAGTGATCTGCACAATTTTTAGATTCCTAAGCCCGAGAGTCTATCAGAAACTACCTCTCT
ACCTACAGAAGGTAGAGGTAAGGTTTGCGTGCATCCCACCCTCCACAGACCTCACTTGTGAGATCTCATT
CGGTATGTTGTTGGAAGAGTTTAGGAAGAAGTCGTAAAATTGAATATATGTGTAGGACAGGTTCACTGC
GTTCAACAGGACGAAACAGGATCATTTAACATTTAATCTGTTTGTATGGATTTAAGCCAAATGTACCTTT
GTTAACTTATCGACTATATGTTGTGTAAAAACTATGAATTATTTGGGTGCTTTATTCGGCATATTCCACTG
CTACTATATCATGTCACAATAGTTTTGTCATTCAACTTGTCTTTTCTATAGGCAACAAAGGAGCAGGTAGG
ACACCGTTGAATTGGGAAACAAGGGCTGGCATTGCCCTTGGAGCTGCACACGGGATTGCCTACCTTCAT
GCACAAGGGCCCTCAGTATCACATGGCAACATTAAGTCATCCAATATCCTCCTCACCAAATCATACGAGG
CCCGTGTTTCAGATTTTGGCCTTGCTCAACTCGTCGGTCCATCATCAACTCCCAACCGTGTTGCAGGTTAC
AGGGCACCAGAGGTAACTGACCCTCGTAAAGTCTCCCAGAAAGCAGATGTCTACAGCTTTGGTGTCTTG
TTGTTGGAACTTCTAACAGGGAAGGCTCCCACACATTCGGTACTGAATGAGGAAGGTGTCGACCTACCA
AGATGGGTGCAATCAGTGGTACGAGAGGAATGGACAGCCGAAGTCTTTGACCTTGAGCTCCTTAGGTA
CCAAAAACGTCGAGGAGGACATGGTCCAGCTATTGCAAGTTGCAGTTGATTGCACTGCTCAGTACCCGGA
CAGACGCCCTTCAATGGCCGAGGTCACTACTCGCGTTGAAGAACTCTGTCGTATGGATTCTGGAGGTGG
TGACATAATTGACAATGATGCAGAAGTGCAAACTGCATGAAATTGAACAAGAAAAAATAGTTCCATCAC
ATTATTTCAATTTTTTTTCTTTTTCGTTTTCGATTGAGCATTTCGATTACTTTGTTCATTCTTGGAGTTCTTCA
GTGAAGATAAATTGTTCATTTTATTTTATTTTTTTGAATTTATTTTCCAGTAGCAACATAGTTTTATTAAAG
ATCTGGTTGGTAGTTGAAATGGAGCTGTTTTTTGTTTTATAGATATTGATGTATTCTTTTTTGAAGGTGTG
ATGAACTGTATAAATAATTGAGATGACCTGTAGTTACA
>PGSC0003DMG400015806 LRR-RLKs
ATTTATGAGCTTTGTGCTTGAGGGGTAGTCAACAAAGTGTCAGCTTTCCCAAAAAGAAAAACCCCAAGA

AACAAGTTCTTCCACTTCCTTCACTCATCACATTTTCCTCTTTTTTCTCTTTCATTCAATGGCACTTCAGAGG
GTTCTTCTTCTCTCCTTCTACATTGTCTTTTTCATAGCCTTCACTACTACTTCTGCTGCATCTGATGTTCAGC
TTCTGTTACAGAACATTAAACCCTCACTTCAGGGTAACACTGATAATTTGTTGTTATCTTCATGGAATATT
TCAGTTCCTCTTTGTCAATGGAGAGGTCTCAAATGGGTTTTCACCAATGGTACTTCTTTGCTCTGTAGTGA
CCTATCTTCACCTCAATGGACAAGTCTTTCTCTTTACAGAAACCCATCTTTGCATCTTGTTTCTCTACAGTT
ACCATCAGCTAATCTCTCTGGAACACTCCCTAGGGAGCTTGGTGAGCTTTCTACACTGCAAAGTCTTTATC
TTGGTGTGAATGGACTACATGGGTCCATCCCTCTTGAGCTTGGCTACAGCTCTTCTCTTTCTGATATTGAG
TTGAGTGGCAATAGTCTTACTGGGACACTTCCAACTTCAATATGGAATCTTTGTGAGAAGATGGTTTCTT
TCAAGATTCATGGTAATTCTTTGTCTGGTTCTCTTCCTAACCCTGCATTACCTGATGCTACTTGTAAGAATT
TGCAGTTTCTTGATTTTGGGCAAAACAGGTTTTCTGGGGATTTTCCTCTGTTTATAACTAGGTTTAATGGT
CTTAAAGAGCTTGATTTAGGGGATAACAATTTTTCTGGTCCTGTTCCTGAGACATTAGCTGCATTAAAGC
TGGAAAAATTGAATCTCTCACATAATAATTTTAGTGGGGTGTTGCCAAATTTTGGGGAATCTAAGTTTGG
TGTGGATGTTTTTGAGGGAAATAATCCTACTCTTTGTGGACATCCTTTGGGGCCCTGTAGTGGAAGCTCT
GGAATGAGTCCTGGTGCAATTGCTGGTATTGTTATTGGTCTAATGGCTGCATTGGTGGTTGTGGCATCAC
TATTGATTGGGTATTTTCAAGGGAAAAAGAAGAGACATGCTGATGAAGAAGAGGAGGAATTTGAGGAA
GTTGAAGATGAGGAAGCTGGTTGTGGTGAGGGGAAGCTCATCTTGTTTCAGGGAGGTGAGCATCTGAC
TCTGGAGGATGTGCTGAATGCGACGGGGCAAGTCATGGAGAAGACTAGTTATGGGACTATTTATAAGG
CAAAATTAGCTGATGGTGGAACTATAGCTTTGAGGTTGTTGAGGGAAGGCAGTTGTAAGGATGGAGGT
ACATGTTTAACTGTGATTAGACAGCTGGGAAGAGTTCGACACGAGAATTTAATTCCTTTGAGAGCCTTCT
ATCAAGGGAAGAGAGGAGAGAAGCTTCTCATATATGATTATCTCCCAAACAGGAATCTTCATGAACTCT
TACATGGTAAATTCTAGCTTCTTTATGTTCTCATGTTTGCTCTTTCAAGTTTTACTAATGAAAATCAATGCA
AGCTCAATTACGTTGACGTTATTGTATTAAGTGTTGCCTCATAGGGAAGTAGCAATAGTTCTTAGGCTCA
TTTTCAGCAGCTTTGGTTATTTGCTTGAGAGACTAAGATATGTGGGCTTCAAGTTGTTGCCTTTTCACACG
GTGGGGTTGTAATTGAAGTATTAGTGCATAAATAGATCATTTACAACAGCCTGAAAAATATAAATGTAG
CTGAACTTAGCATCTTAAGAGAAACTGAATTAGTTATTGAATGCACAATATAGAGATATTACGTTACACG
AATATAGAGAATGTTCCATTTATTCTGACCGATATCCTTTTCGCTAAGTTAAGTTGATCCAACCTATATCCT
CTCCTGTGCATCATCTTCCAATACACGAACTGCCATATGTATACCTGTTTGGTCAACAAAGAAAAAGTATC
CCTATCATATATACTTACTCATCCAAGAAAAATGGAAAAAAAAAGAACTAGCACATTATCATTAAACTGC
CGATGCAACTATCAGATGCCTTGATCAACCATTCATTTATAATTTCGCTTTAAAGAACTCTGTGGCCCTAG

ATCAACTGATGAAAGCTCATTTCCAAGCATTTGAGCTTGGTGTAGTAAGCTTCTTTAGTTCCTTTTGCTTG
ATGCAATCTATTGATGCTCTGGTTGTTTCCTGTTCCTCATTTCTTGTACTGCTAATTTAGCAGTAGTTTGGT
CATTGAGAGTAGTTTGCGCCAATAAATGTTGGAACCCCACAAATTTTTATATCCTTGTTCGTTCAACCTTA
ATAGCTCTAAGGATTAGTGAATTTGATTATGATATTTTCTGATTGTCTACAGAATCAAGAGTTGGGAAGC
CAGTGTTGAATTGGGCTAGAAGACACAAAATAGCATTGGGCATAGCCAGAGGACTAGCACACCTTCATG
GTCTGGAAACTCCCATTACTCATGGGAATGTTAGATCAAAGAATGTTCTTGTTGATGAATTCTTCGTTGCC
AGGCTTACTGAATTCGGACTTGACAAAATAATGATCCCAGCTGTAGCTGATGAAATTGTATCAGTTGCAA
AAGCTGAAGGTTACAAGGCACCAGAGCTGCAAAGGATGAAGAAGTGCAACTCAAGAAGTGATGTTTAC
GCTTTTGGTATCCTACTGTTGGAGATTCTTCTAGGAAAGAAACCTGGTAAGAATGGTAGAAACGGGGAC
AATGTTGATTTGCCTGCACTAGTTAAAGTTGCAGTTCTGGAAGAAACGACTATGGAGGTATTCGACATG
GAGCTTTTGAAGGGAATTAGGAATCCAATGGAAGAAGGTTTGGTACAGGCCTTGAGACTTGCAATGGG
ATGTTGTGCTCCTGTTGCTACTGTAAGACCGTCGATGGATGAAGTAGTAAGGCAGTTAGAGGAGAATAG
ACCTAGAAACCGGTCTGCGTTGTACAGCCCTGCTGAAACTAGGAGTGGAAGTGGTACTCCCTTTTAAGC
TCATGTTGTGACTTTAGTAATGAATATTGTCGCATATTATGCCTGTTTTTCCTCCTAAAGGAAAGAGGGCT
AAATGGCTATAGCTTTCTTACTTACTATTTCAACTATTTACCTTTGGCTTTGCATTGTTGGGGAATATCTTA
TCATAGACAAACTGACAATAGACAATGTGTCACATTAGGCATCAAATTGGATATTACAGTGTGCCTTTCT
TTTTTTCAATTTGTTCCTGCATTAGCCTACCTTCTATAAATATATATATGCAAAAGAAATTCAATG
>PGSC0003DMG400015814 LRR-RLKs
ATGCCAATGTCGAGGCGATTCTTGGACCTCCCAAATCTTGTTGTCTTTCTAACCTTTTTTCTTCTATTACAT
AACTTTGCTCTTCCTTCAAATGAAGTTGATGTCCTGTTTTCTTGGCTTCATTCTACTAATTCACCAATTCCTC
AAACATTCTCAAACTGGAATCCTTCAGATTCCACTCCTTGTAAATGGTCTCATATAGTTTGTTCTTCTAATC
TTTTCGTTACACAAATTGATATTCAGTTTATTCAGCTGGCTCTTCCTTTTCCTTCTAATCTTTCGTCTTTACA
ATCCTTGCAAAAACTGATCATTTCTGGTGCTAATCTCACTGGAACAATCCCACAGGATATTGGAGATTGT
GTTTCACTTGTAACACTTGATGTTAGTTCAAATGGTCTTGTTGGTACCATACCGAAAACTATTGGTAACCT
TATAAATCTTGAAGATTTGATTTTGAACTCTAATCAACTAACAGGGGAAATTCCAGGAGAGGTTGGCAG
TTGCATTAACCTGAAAAATCTTATTATCTTTGACAATATGTTTAGCGGAAATCTTCCTTCCGAACTTGGAA
AACTTGGAGTTTTAGAAAATATAAGAGCAGGAGGGAACAAAGATATTAGTGGGAAAATTCCGGATGAG
CTTGGGAATTGCAAGAACTTGATAGTGTTGGGACTTGCTGATACGAAAATTTCAGGTTCGCTTCCTCCAT
CGTTGGGTAACTTGGGAAAGCTTCAGGTATTATCTATTTATACTACAATGCTTTCTGGCAAAATACCTTCA

GAAATAGGCAACTGCTCAGAGCTAGTTGACTTGTATTTATACCAAAATAGTCTGTCAGGTTCACTGCCTG
CTGAGTTGGGAAAGCTTCAGAAAGTAGAAAAGATGTTGTTTTGGCAAAATAATCTTGATGGGCTAATTC
CTGATGAAATTGGGAATTGCAAAAGTTTGGTTATTCTTGATCTTTCTTTGAATTTTTTAAGCGGGAGCATT
CCGTGGTCATTTGGGAACCTTACTAATCTCCAAGAGTTGATGATTAGTAACAACAACATTTCTGGTTCAA
TCCCATCTGTTCTTTCTAATGCGACAAACTTGTTACAGTTTCAGATGGATACGAATCAGATTTCAGGCTCA
ATTCCTCTGGAAATGGGGCAGTTGAAGGAGCTAAATATATTCTTTGCTTGGCAGAACAAGCTTGAAGGA
AGCATTCCTCCTGCATTGGGTGGTTGCAGAAGCCTACAAGCTCTGGATTTATCACATAATTCTCTTACTGG
TAGCTTACCTCCTGACCTTTTTCAGTTAACTAATTTAACGAAGCTTCTGTTGATTTCCAATGATATTTCTGG
TTTTATCCCACCTGAGATAGGTAATTGTAGCTCTCTTATTCGTATACGACTTATTGGCAATAAACTTAGCG
GACAAATTCCAAGAGAGATAGGATTTCTAGACAACCTAAGTTTTCTTGATCTCTCTGAGAACCACCTTAC
AGGATCCGTTCCTGAGGAGATTGGCAATTGCAAGACACTGCAAATGCTCAATCTATCTAATAACACTCTA
AGTGGGAATTTACCTAGCTCTCTTTCTTCTCTCAGTAGGCTAGAGATTTTGGATGTTTCCTTGAATCAGTT
TAACGGACAGATTCCAGCAAGCTATGGTCAACTTGCTAACCTTAACCGGCTTGTTCTTAGTAAGAATGCC
TTCTCTGGATCGATTCCCCCAACTCTCGGCAATTGTTCAAGCCTTCAATTGCTTGATCTCAGCAGCAATGA
GTTCTCAGGGAACATGCCAGTGGAATTGTTTGACATTCAGACACTTGACATTGCCTTGAATTTAAGCTGG
AACATCTTGAGTGGGGTAGTCCCACCGCAGATATCTGCCTTAAACAAACTTTCAGTACTAGATCTTTCCC
ACAACAAGCTTGAAGGAGACCTATTGTCTCTTTCGGGGCTTGAAAATCTGGTTTCCTTGAATGTTTCCTAC
AACAATTTTACCGGCTACCTCCCTGATAATAAGTTATTCAGGCAATTATCATCAGCAGAGATGGCTGGCA
ACAAAGGTTTGTGCTCATTGGGACATGATTCTTGTTTCTTGAGCAATGTTGAAGGCGGGGGAATGATGA
GTAATAGCAATGTAAGACGGTCATGGAGGCTGAAATTGGCAATTGCACTCCTTTCTGTGGTGACTATAG
CTTTGGCACTCCTTGGAATGCTGGCAGTGTACAGAGTGAGAAAAATGAGCAGAGAAGATAATGATTCTG
AATTGGGAGGAGGGGATTCATCAGCTTGGAAGTTTACTCCATTCCAGAAGTTGAATTTTTCGGTTGAAC
AAATTTTGAGATGCCTAGTGGAATCCAATGTTATTGGAAAGGGATGCTCCGGCGTAGTTTATCGTGCAG
AACTAGAAAATGGTGAAGCAATTGCAGTCAAGAAGCTATGGCCAACCACATTGGCAACTGGATACAACT
GCCAAAATTCAAAGTCAGGAATTAGTGGATGTGTTCGCGATTCTTTCTCAACCGAGATAAAAACCCTTGG
CTCCATCCGTCACAAGAACATCGTTAAGTTCTTAGGTTGCTGCTGGAATCAAAACACTAGATTGCTCATG
TATGACTACATGCCTAATGGAAGCCTAGGTAGCCTTCTACACGAACGAAGTGATGGATGTTTGGAGTGG
GAATTGAGATACAAGATTGTCCTAGGTGCAGCTCAAGGGCTTGCTTATTTACACCACGATTGTACACCTC
CAATTGTTCATAGGGACATCAAGGCCAACAACATTCTCATTGGCCTTGACTTTGAGCCATACATTGCAGA

TTTTGGTATAGCCAAACTCGTCGATGATGGAGATTTTGCTCGATCTTCCAATACAGTAGCTGGCTCCTAC
GGATACATAGCACCAGGTTCGGCACAAGTCGTAGTACCACTTTACAATATTACACTATAATTATATACAT
TATATCTTTAATACACATGTATATATTATCTTGTGCAGAATATGGATACATGATGAAGATAACAGAGAAA
AGTGATGTTTATAGCTTTGGAGTAGTTGTGTTAGAGGTGTTAACAGGAAAGCAGCCAATTGATCCTACA
ATACCGGATGGAGTACACATTGTGGATTGGGTGAGGCAGAAAAGAGGCAGTGATGAAGTTTTAGATGT
GAGCTTATGTGCTCGTCCCGAATCAGAAGTTGATGAGATGATGCAGACTATAGGAGTAGCTATGCTGTG
TGTTAATCCATCACCAGATGATAGACCAACGATGAAAGATGTCGCAGCAATGCTGAAAGAGATAAGACT
TGAAAGAGAAGAGTACCAAAAAGTTGATATGCTCCTTAAAGATGGAGGAGACAATAAGAGTAGTAGTG
ATGGAGGGCCATCTTCAAAGATGCATAGCTTGTACTTGCAAAGCAATAATACAAGCTTTTCTGCATCTTC
ATTGTTACATTCTTCTTCCTCCAACACCAAGAATATTGGTTTCAAATAGACTTTTGTTTCTTTCTTATAGTTA
GATTTGTTTTCTCATTTTTCTTCCCGGG
>PGSC0003DMG400016015 LRR-RLKs
TTTTTTTCCAATATATATCCTTTTTCCATTTCTCTTCATAGTAGTATGTTTCAAGTGCATAGTAACATCATGG
AGAAACACATTTTCTTATTGATACTTGCTATCTTAGTTCAATTTTACTTTGTTTCTTCTATATCAGCTACTAT
TTTCTCAAATGAGACTGATCAAGAAGCTCTATTAGCTTTTCGAAATCTTGTTACGAGTGATTCTAGTCAAT
TTTTAGCCAATAATTGGACCAAAAATACTTCATTTTGCTCTTGGTTTGGTGTCACTTGTAGTCCAAAAAGG
CAAAGGGTTGTAGCCTTGACTCTTCCTAATTTGCAACTTCAAGGCACAATTTCGCCTTCTTTGGCCAATCT
ATCCTTTCTCATAGAGCTAAATCTCACGAACAACAACTTCCATGGTAACATCCCTTATGGCATTGGCCACT
TGCCTCGTTTACGAGTGATTGATATTCAGAACAACCAGCTCCAAGGTAGTATTCCAACAAGTCTATTTCA
ACACCGGAGTGTTCAAATCATTTCATTGGCTTTCAATAAACTCGGTGGTGAAATGTGGAACGGTACATG
GTATGTACCGGAACTCAGAGTCTTAAATCTCAGGAACAATACCATCACAGGTAGAATCCCTCCTTCTATT
GGAAATGCCACAAAGTTGATGAACATCAGTTTGAATTGGAATAGAATCAACGGCAACATTCCAATGGAG
ATCGGTAATCTAAGCCAACTTGTAGAGTTGTCGTTGTCTCGTAATCAATTAACAGGTTCCATTCCTTCAAC
ATTGTTTAATATCTCCTCCCTTCTCGTCGTGTCTCTGGCATACAATAGCCTTTCAGGTCCTCTGTTTCTTGA
TGATCGACGTAATGTTCTTTCATCAAACCTCGAGCATATAGGTGTATCATACAATCAAATCACTGGTCAC
ATTTCTTCCAACATCTGCCAATTCAAAGCTCTCAAAGTCTTGTCCATATCATACAACAACATAACTGGAGA
AATACCGAGAAATATTGGTTGTTTAGCCAAGCTCGAAGAGCTTTATATCGGTTATAATGCAATAGATGG
AACAATTCCTACTTCATTAGGCAATATTTCCACTCTTCAAAAACTTCATTGTGGAAACAATCACATGGAGG
GAGAACTTCCTCCGGAATTAGGAAAGCTATCAAACTTAAGACAAATCAATTTCGAAGAAAATTATAATCT

TATAGGTGAAATTCCAAATGCTATTTTCAACATATCTTCTTTGGAATTCATTGCTTTCACTTTCAACTACCT
CTCAGGTAGAATTCCAAATCTTCTTCATCTTCCAAACCTTATACAACTTCTCTTAGCAAACAATCAGCTCG
AAGGTGAAATTCCTCGGTACATCACAAATGCTACCAATCTTGAGCTATTGGAACTATCAGATAACCTTCT
CACAGGCAGTATTCCTTATGATTTAGGAAATCTTCGCGAGCTGCAAGAACTTTTCCTACATCATAATCAAC
TTACTGAGTTGGGATTCTTTGATTCTTTGGTGAAATGTAGGATGTTGAGATATGTACAAGTGGGATCGAA
TCCGTTGAATGGTGTTCTGCCAAGTAGTATTGGCAATCTTTCATCTACTGTTGAATACTTTCATATTGGAG
ATGCACAAATCAATGGATTCATTCCCACTAGTACAGGCAACATGAGTGGTCTTACAACGCTAGTTCTTCA
AGATAACAATTTGACAGGAAACATTCCTCGTGAGATCGGTAAGCTTAAACAACTCCAAGGTTTATTTCTA
GTTAACAATGAACTGCAGGGGGATATAGCAGAGGTAGTATGTGATTTATCGAATTTGGTTCGATTAGCT
CTGTCTGAAAATGAGCTCTCGGGGGTGATTCCGGAATGTCTAGGAAGTCTTACCATGCTACAACACCTTT
TTTTAGGTTCTAACAAGTTTGAATCAAAGCTTCCTTTAAGCTTTTGGAAGATGAGTAGTCTTCTCTATGTA
AACATGTCGCGTAATTCTATAGAGGGAGAAGTTCCATCAGATATCGGAGAACTTAAAGCTATTGTAGCA
ATTGAAATCTCTGGTAACCACTTTTCGGGGATGATACCAAGCAATTTGGGGGAACTTCAAAACTTGAAGT
TACTTTCCTTATCGAACAATTCGTTTTCAGGTCCAATTCCATTATCCTTTTCAAACTTGAAAAGCTTGGAAT
TCTTGGATTTGTCTTTGAATAACTTGTCAGGTACTATTCCTAAGTCTTTCGAAAAGCTTTTGTACCTTACAA
GCATCAACGTCTCGTTTAATGTTTTAGAAGGTGAAATACCTAGTGGTGGTGTGTTTGCAAACTCCACCCT
GCAATCATTTCGCGGGAACAAAGGTCTATGTGGAAGGCAAATATTGGAGGTTCCTGCTTGTGCTGTCAC
TACTCCTGAACAACAACAACCAAAATCGAAGAGGCTTGTGCTAAAAATTGTCACTCCGGTGGTTATTTCA
TTCTTTCTGATATTCTTGTTGGTTGTCTCAATTTGGATAATGAAACGAAAGAAGAAAGGAAAGTCCAAAG
ATATTGAAAAGGTTCCGGAGATGAGGACTTATCAATTGATTTCTTATCATGAGATTCAACGAGCAACAAA
CAATTTTGATGAATCCAATTTGATTGGCGTGGGAGGTTCTGGCTCTGTGTACAAAGCCACATTACCTAGT
GGAATTGTGGTTGCAATAAAGGTACTGGATTTGGAAAATGAGGAAGTATGCAAAAGGTTTGATACTGA
ATGTGAAGTGGTGAGAAATGTTAGACACAGAAATCTTGTTTCGGTGATCACTACGTGTTCTAGTGATCAC
ATAAGAGCCTTCGTTCTGCAATATATGCCCAACGGAAGTCTTGACAATTGGTTGTACAAAGAAGATCGCC
ACTTAAACCTTCGTCAAAGAGTCACCATAATGCTTGATGTAGCTATGGCAATTGAATATCTACATCATGG
TAATGACACCCCTATAGTTCATTGTGACCTCAAGCCAGCCAACGTTCTTTTGGATGAAGATATGGTGGCG
CGTGTTGGTGATTTTGGCATCTCAAAGATTTTAGCTGTAAGCAAGTCTATGGCACATACAAAGACATTAG
GCACTCTTGGATATATTGCACCAGGTATACTACTATACTAGTCTCTTTCCATTTCACTTATGTATCGAAGA
CCTTTCATTATTGTTACATCGGATGAGTAATGCTTTTTTCATTTTCATTCTTTTAAGAATATGGCTCGGAGG

GAATAGTGTCCACTCGTGGTGATGTTTACAGTTATGGCATCATGTTGATGGAGGTTTTGGCAAAAAGAA
GGCCAACAGGTGAAGAGATATTCAACGAAAATCTTGGTTTGAGGGAGTGGATAACGCGAGCATTTCCA
AGAACTATGATGGAAGTTGTGGACGCGGATATTTTTCATGATGGAGAAAAAATCACTTCCAAAAGTGAA
CTCTGCATACTTTCCATGATAGAACTGGCTTTAGATTGCACAAAGGCAACACCAGAATCAAGGATAACCA
TGAAAGATGTAGTCAAGAGGCTTAACAAAATTAAGAACACATTTTTGGAAACGTAGAAGTTAGTTAGAA
TCTCTTTTAATGTTGTTTTTTCTTACAAGTTGTAAGTTGATACTCAATCTGTTTGTCATACTTTTTAGTCCGT
TTTAAAAAGAATGCCTCTTTTCTTTTTTGGGAACTCTTAAATTCTAACTTTCTACATGTGTATGACATGTTT
AAGACCACGAGATTAAA
>PGSC0003DMG400016164 LRR-RLKs
GGGGTAACTAAGAAATACTGCTGTGAGTGACAGGAAGAGAAGACTTAAACGTAGTAATAATTGCAGTA
TGCAAAAGAGAGAACCTTTCTTGAACTTCTTCTCCATTGACTACTACTTTTTTACAGCACAACAAAGAACA
GCTTTTATTTGTGTTTTTTTCTTCAAGATTTGCTTTGTAAAGCTTATTTTTTTGCTTAATTCAAAGTAAAGTT
TCAATCTTTGGTTCAAACTCTGCTCTTTCTTTCACAGCCTAAAAAGATTGGTGCTTGCTTAAAGGTACTTA
ACTTACTCAGCTCTTGTTTTTGTTTTGTTCTGTTTTTGTTTTCAGATTCTGTCATTTACTGTTATTTGTTTGGT
GTATTGAGAATGGTGTTTTGACTTTAGATATGGTGGGTTTGTCTTTTCTGATTTGACTTGACATATGGAA
GCAAAAAAGTGAACTTTTTTCTTGGAAAAATTTGTTCTTGATGGATTTTGTGAATTTGTGGATGTGGGGT
TCTTTTCTTTTTGGGTGGAGAGGGGAGTTGCTAATTGTTTGTTTTTTGTGATACATATGTGAAATAGAAG
GAGGTCAAGTTTAAATTGTTAACCATAGGCAAGGGGCATAGTTGATTTCGGATATGGTCTCTCGACTAAT
AGTTATTATGTCAGAAAGTTCCTTTCTTTGTCGAAGAAAATTGGAGTCAAGAGGAAAGACTTTATGAGAT
AACAACGGTTAGTTGAGTGACCATGTGAGAAATCAAACCAAAAGGGACAGTGGAAAAAAGAATGAACT
TGGATTTTTGTGAGTCTGGTTAAGTTTTAGGAATTACAATGGTTGAGAAGGAGATTTGAATATGTACACA
GTCAGTGTTTTAGCTACAACAACCTTTGTTGTTTCATTTGTTGGAGTTTCTTTTGAAGAACTTGTGTCTTTC
TCCTTAATAAGTTACTGAAAGTTTTTTGTAATAGTTCATTACTCACTTGATGTATTTTGAGAAAATGGGGG
AATTATATATCAGTTTTGTTATATCTTCAAAGAATTTCTTGTGGTTATCTGATAGACATAACCAATAGACA
TTCAAAGAGTTGCTGGCGAAAACATCTGGTTTTTGTTAAAGAAAAGGATAGGTTTGTCGGTTGTTTGAA
AGTTGTGGATCAATTCTTCCTTTGGATGGTTGACATTATTGGTAGTAGCTGAAATATTGTTTGGATGGAG
AAGACGAGTGTTTAGTTGTTCTTTCAACTCCTAATAGCTTTTACGATTCTGCAATACTGCTACAGTTTGCT
TATATGGGGTTTGCAGTTCGAAGTACGTCGTTGCATGCTTTACATTATTAATAGAGGGCATTATGTAATG
CTGTATTATGCTTTTCATGTGATTGATCTATATCATCAACCACTACCATCTTCTTCTTGTTCTTTGTACTTCA

ATTATCGCACTTTCTTTGGTGTAGTTACTGGTCCTTTTCTTAGAATGCTTTGTCATACTTTCTATAGTATTTT
GCTATGATCCCTTCACTTCAGTTATTTCTTTTTCGATCTGCTTTGATATACTTTTCCTTGAGCCGAGGGTCT
TTTGGAGACTGCCTCTCTACCTCTATGAGGTAGGGCTAAGGTCTGCATACACTCTTGACTCTACCCTCTCC
TCATTTGTGGTATTACACTGGGTATGTTGTTGTTGTTCTTGCATCAAATATCTCTTTTACATATTTAATTAG
GCTTAGCTGTTCTCCTTGAGGTTATACTAATTTTGATCGTAGTAACCTTTCTAATATATTATGCAGAACTG
CCATTTGTCATTACCAAGGAAGATAGTGCTTTATGTTACAGTGATGAAGGGGAACAGGTTTACTTGATCT
ATAGAATTGAACAGCAACTGCAATATGGAACTGTTCCTTGCAATTCTGATATTAGCATGTCTCTACTCATC
TGTCTCACCTGATCAGCAGGGTACACATTTCTCCCATTTTCTACTTTAGCAAACTAACAAAGAGATGCCCT
TAGCTGTCTTTAGTCTTTTATCTATTCAAAGGTATAAATAAAAATTTAGAGGGGTTAGATAATTTTGCTTG
TGCTGACTTAATGTTGTCTGTATTCTGCTCGTAAAGTAGTGTGACATTCGTTATTTTGTAATCTCTTGATCA
GGAGATGCACTGTTTGCATTGAAGAGTTCACTAAATGCTTCAAATGATCAACTTTCTGATTGGAATCAGA
ATCAAGTTAATCCATGCACTTGGTCAAAGATAACTTGTGACGATAACAGCAATGTCTTGATGGTGTAAGT
TGAAGTTACTTTGCGAGCCATATTGATCACCATGTACATGATAAAATTATGGTCCCAAACACTTGAAAAG
TTGGTTAACTACCATTCTGAAATCCTATATTTAATGTTGTCTGTGCTTTATTTGCAGGTCGTTGTCAAATAT
GGGGTTCACTGGCACTTTAACTCCAAGATTAGGTGTTCTAGTGCATCTTAATACTCTGTACGTATAGAGA
ATTTGTGGAATTATTTTCAATTGAATGCTAATGTGTATCTATGATGTACTTATAGAGATCAGGTGTGCACT
TTAATCATAACGTCAGTAGATAAATCACTCCATTTACGAAAACTAGGTAAGTGTGATCAACTGAAACAAA
CAAAAACATACCAGTGTAATCCAACAAGTGGGATCTGGGGAGGGTAGATTTTGCTTGTGCTGACTTTAG
GTTGTCTCTGTACTGTTCGTAAAGTAGTGTGTCATTCGTTATTTTGTAATCTCTGACCTTTGATTTGGATG
ATAATTTTGAGTTTCTTGTTCTTAGGGGAGGAAATAACATTGAACTTTCAATTTGAAACCATTGTGGAAA
ATGTGATGGTGAAATAAAAATATGGTTTGTTAGGTTATGTGCTGTCAATTTTCTGCTGAATGCCTCTCAA
AGTGAGAGAGTCTTTCCATTATATAGAGAGCATAAATATTACACATCCCTATAGATCCGTCATTCCTATCC
CTAAAGATCAGCTATTCTATCTCTACAAATCTGCTATTCAATCCCTCTAAATTTGCTTTGCTATCCCTCCAA
CTCAACTATTCTATCACAATATTTATTCCTCTGATTCTACAACAAATCTATATGATAATACTAATTCATATC
ACTATATAATTACATTAATATATACAATTATCCATCATGCTAACATGTGCTACTCTAGAAGTCTATATTTAT
CGATTTCCTTCATCAAGCTGATATTAATCTACTCATGATATTGGCACATTACTAACTGCTCTTGTTTGGGG
ACTGGTGATTGCTTGTATCTTTGCCCGTGCATGGTTTGGGATCTTAGCCTTTTCCGATTGTACTACTTTCCT
ACCCACACAAGCATGTGTAGAAATCTACTCGCAATTTTGGTTCTCAATATTCCAACTAAAGAGTAGTATTC
TTTTAAGTTAATAATTAGGAAACGACATTCTTGAGTGTTTGGTCATGCTTTTGCCAGAGTGTTGGATATTT

CATCTTCATTTCTTGTCCGTTATCACATTATTCTCGTAGTGCCTCTGATAAGCATTCTAAGGTCATAACTGG
TAACATTTTCTAAGATCTCCTAAAAATAAGTTTTCATCCTTGTCTGTAGGAAGTTCTACTTTAGTGAGATCT
GTGAACTCATCAATTTTCTTGTCCTTAGTTGTGTTATGTATTGAACTGACTGGGCGGTCAATATCCTGGCA
GTTCTTTGCAAGGAAATGGAATAACTGGTAAAATTCCTGAAGCATTAGGAAATTTAACCAGCTTGACCAT
GTTGGATCTGGAAAAAAACAGTTTGTCAGGAGAAATACCAGCTTCCCTAGGCAATCTAAAAAAGCTTCA
ATTTCTGTAAGTAATATTATTTCATTGATTATTGTTTTTTTAAAACTGAGTTTTACATTTGGTGTTTGACTT
GGGAGTCTATTATAATGTTTCTTTGAATAGGTTTTTGAGTCAAAACAATCTGTCTGGGACTATCCCTGAGT
CACTTTCAGGCCTTCCCAACTTGATTAACCTGTAAGTGATTCCTTTAGCCTGATCTTTAATGTGCTTACAAC
GTGTGCCAAGTGATATTGACCTGACCCGGTGATATCTCTTTTCCAGTCAGCTTGCATTAAATGATCTTACT
GGAAAAGTTCCTGACAGTTTGTTCCAAGTCCCGAAATACAAGTATGCAGATCTCAATTCTTGCATTGTTT
AATTTGATGATAGATTGAAGCTGCTTAATAATTTGAACATTTTAAAAAGGTATAATACATAAATAGACAC
TCAAACTTAGTCTCAACTGGCAAGTAAGCGCTCCCAACTTTGAGCGAGGACATCTAGACACCTCAACTTG
GTCTCAACTGACAACTACACACTCTAACTCGTCCCCACTGTGTCTAGTGGACACCTGATGCTGACGTGGC
ATCTAAATTTTGGTGGTGTCTTGATGATCTTTTTGTAAGTTGGAGTGTTCAACTGACACAATGAAGACGA
GTTGAGGTACCTAGATATGCATACTCAGTTGGAGTGTTTACTTTCAAGCTGAAGCCAAGTTTGAATGTAT
GTGTATGTATTATGCCCTTTTTATAATAGCCCCTGCCTCTTTTGCATGGGTCAGAACTGTTGATATTAGTTT
AACTCAGTTTGCCTACTAGTTTTTCAGAGAACCATTTGAATTGTGGCTTAAACTTCAGTCGTCGTTGTGTA
TCTGACAGTGAAGGTAATTAATTGAAAGAGCAAATACCACATTTTTGAACTACAAGTATTTACCTTAGAA
GTTAGACGTTGCCGGATATGTTTGGCAGTTGGCTGGTTTATGCACCCTGGTACAATTCTAAATTCTAAAG
TTGATATTCTGGCAGGTTCTCCCAGTAAATCAAAGACTGGTCTCGTAATTGGTATCGTTGGTGGATTTCTT
GGAATTATCCTTCTTGGGGGATTAATGTTATTTTTCTGGCGGGGGAGGCACAAAGGCTACAAACGTGAA
ATCTATGTTGATGTTCCAGGTCCTTATCACCTGTTTTACACAGTATTAACTTCAATATGTGTTTGTTAGCTC
ATCAAAGAAATACTCCCTCCATTTCAATTTGTTTGTCTTGCTTGCTTTTTTAGCTCGTTTAAAAAGAATGCC
CCTTTCCCTTTTTGGCTACTCTTCAATTTCAACTTTTCACGGGGCATGTTTAGGACCACAAGATTCAAAAG
TCTAATTTACTTTTTTAAACTCCATGCCAAGTCAAAACAAAACAAACAAATTGAAACGGTGGGAGTATGT
GTTAGCTCTCCTGAAAAAAAAAGAAAGAAGAAGGCTGAAGTAGTTACCTATTGCTACATTTCTTTGTTCC
ATCTTACATTGGAGACTAGATTTTTTATGAGCATTACTTTAGTTCACTTGCAGTGTAAGATGGTATAGGTC
CATTGAGTATGTTGTGACGTTTTTCTTGTGTCTGTTCGGTGTGCCCAGGTGAGGTTGACAGACGAATTCC
ATTTGGTCAATTGAGGAGATTCGCATGGAGGGAATTGCAACTCGCTACTGATAACTTTAGTGAAAAGAA

TGTTCTTGGACAGGGAGGTTTTGGCAAGGTTTATAAAGGAGTGCTCAGTGACAGCACTAAAGTTGCTGT

GAAACGCTTAACTGACTATGAGAGTCCTGGGGGAGATGCTGCATTTCAGCGTGAAGTTGAGATGATTAG

TGTTGCTGTTCATAGAAATCTTTTGCGGCTTATAGGATTTTGCACCACACCGACTGAACGCTTGCTAGTGT

ACCCGTATATGCAGAACCTAAGTGTTGCCTATCGTCTACGAGGTACTTATTTTTCCACAATAGTGCTTAGA

TCTTGGTTTGCAGTTTTCCCAAGGCTTTCCTTGTTCATCTCAATATCAAATTTTCCTGGGATCACTTATTTCT

TTTGCTCCAAACTCAGCTGAATTTCTATAGAAGTCTGGAATTTGATGGTCAAATGCTTATGTCAATAGTCT

AACTGTCTAATAGCCTATTTGGCCAAGCTTCTAAAATCAGCTTATTTTGATTTTAGGCTATTAGACTCCTAT

CAAAGGTCTAATCACCTACTTTTTCCTAGTGATCTTTAGTAGAGTAGACAAGTTGAACAGATAATAAAGG

AGGGTATTTGCGTAGGTACTCGTATTCCTAATATTCTTAGAAGTGTGTGAGTTCTTGAAAATTTGGTTCTC

TTCAGTCTTCAAGAGCCATAGTTGGATTGTACCTCGTCACGAGGGAACTTCCATCACGTATGAATTGACC

GATATCCATAATGAGAGCTTAAATGAGTTCATGAATTCATTCTTGCGCATTCATCATTCCCCCCGAGGTG

ACTTTTTTGAAAACCTTGCATGAATTGAAGATGACACATGCAGTAGTCGGTCAAACTTTAAAATTCAGCA

TTTATCTCCCAGAGATGATGAGATTTTGGTGGCTTCTTATTTGTAGTATTTTGTTAAATGCAGAACTTAAA

CCTGGGGAGTCTGTTTTAATTTGGCCAACCAGAAAGCGTGTGGCACTGGGCACTGCACGTGGACTTGAA

TACCTGCACGAACACTGTAATCCAAAAATTATTCACCGTGATGTTAAAGCAGCCAATGTTTTACTGGATG

AAGATTTCGAGGCTGTAGTTGGTGATTTTGGCCTGGCAAAGTTAGTTGATGTTAGGAAAACCAACGTGA

CAACGCAAGTTCGGGGTACTATGGGCCATATAGCTCCTGAATACTTGTCCACTGGGAAATCATCAGAAA

AAACTGATGTTTTTGGATATGGAATCACGCTTTTGGAGATTGTAACTGGTCAACGCGCTATAGACTTCTC

ACGCCTAGAAGAAGAAGATGATGTCTTGTTACTTGACCATGTGAGTGTACATAACTCAACGACTGATATT

AACTTGTGATTCTATTGGTCTGCATATTACTTTTAACGTGTCCTCTAAAATTATTCAGTTTCTTCTAAGTTT

ATTTTTTTATCTTATACTTTCACTCTTCTCGAAGCTGCATATTGGTGGATGGCTCATTGTCCATCACAAGAC

ATTCTCATGAATTATATACGTAGTTCTTCTTTTTTATGTTGCTTTCTTGGTATATGTTGTAATTTTATATTCA

ACAGGAAGGAGGGACAAAAAAGAAGTTTCATTTTCCATATAGCACTCTTACGCAAATTCTTTTGCATGTC

AAACATAGGTCTTAATACTCAGCCTCTTTTGTCGGATACCCTTTTTAGGTGTAAAGTAAATTAGTGAAAA

AAGGTGAAATAACATTGAGAGGACGCAAAATTTCTTAATTAGGGTAGAAGGATAGTAGATAGTGCATTT

TCTCGCTATCCGTCCATTCTAGTAGTTGTAGTAGTGCTCCTTTAGTTTCTTGTCCTATGTTTTTTGTTACCAT

CTGTTGTTTCTTGTATTTCGCTTGTCGTATTATTTTGATGTAGTTATTGTTCAGCATGCTTTGCTATGCTTTC

ACATCCATTACTTTTTCTGGACTGCTTTTGTTTGTTTTTCCTTGAGCCGAGGGTCTATCGGAGACAACCTCT

CTATTCTGAGGTAGGGGTAAGGTCTCTATGTAGACTCTATCCTCCCAGATTCCACTTTGTGGGAGTACAT

TGGGTATGTTGTTGGTTACTGAATGGCTGCATGTGTCATCTTGAAGGTCAAAAAACTGCAAAGGGAGAA

AAGGTTGGATGCTATTGTCGACCGCAACCTACATAAGAACTATAACATGGATGAAGTAGAGATGATGAT

TCAAGTTGCGTTGCTGTGTACTCAAGGATCACCAGAGGATCGTCCAGCAATGTCAGAGGTAGTACGAAT

GCTTGAAGGAGAAGGACTTGCTGAGCGGTGGGAAGAGTGGCAGCATGTCGAAGTTACACGTAGGCAA

GAATACGAGAGATTACAACGACGATTTGATTGGGGCGAAGATTCAATCTATAATCAAGATGCAGTTGAA

CTGTCTGGTGGAAGATAACAAAATCTGTATCTCCAAACAAGTACATAATTTCCTGTAATAGTCTCTCCTTG

ATACATATAACTTGTCGCTAAGTGGCAATCATTCCACGTTTTCATATTGCTTGTGTCATATCTTGATTGTAT

TGTTTTTCGTATGCTTCTCTTATTTAATCGATTTTTTCCCTGTATGATTGGCCTGTGCAGCGACATAATATA

TATCGACTTTCTTTTTGTTTCAATTGTAACATCAATCTTTGTTATAGTGATTTGAGTTTTGC

>PGSC0003DMG400016654 LRR-RLKs

CTCCCACTCACACCAGAGAGAGAGAGCGCTTTAATAAATGTTTAACTGACTCGCGGTCGCCGGAAAACT

CATTCTCCCTCCGGTGGATTTTTGTCTTAATTTCATTTTTGGATAAGGACAAGTATCCGGCGCCGGTGGTT

TCTTTCCCGGAAAAAAATCTCAGCTCACATTTTGTGCTGCTTTTAGTTTGGAGGTTGAAACTTCTCTTTCTCG

CTAAATTAAACTCCAACCGAGCTGTTTTTTTCAGTGAATTCGTAGGTTTATGCGTTTTTTATTTGTCTGTTC

TTCGTTTGGGTCTTTTATAGTTTACTCTTTTTTAGCAGATTTGCACAAACTTTTTTCCAGTTTCAGCTGTATT

TTAGAATTCTAGGGTTTTGGGTTGAATTTTTCACCACTGGACCCATTGCGTGCTTATTTAGTTGTAACTGG

TCATTTCAACTCCTATTTTGGAAGTTGATTTTTTTTTGTCTTGAAAAACAAGTAGGGCCTCAACATTGTCTT

CAGCTGACCTTGTCTATGGAATTATAAGCAACATTGGTTTGGAAATGAGAAAGGTGTTTGTTACTGTGTT

TTTGTTTCTGTTGGTGGAGGCTGTGTTTGTGGAAGCTTTGAACCCATCTTTGAATGATGATGTACTGGGA

TTAATTGTGTTCAAATCGGACGTTCAAGATCCATATGGAAAGCTGAAATCTTGGAATGAAGAGGATGAT

AGTCCTTGTAACTGGGACGGAATCAACTGTAACCCCAGATCCAATAGGGTGACTGAAGTTGTTCTTGAC

GGGTTTGGTTTATCTGGAAGGATAAGCAGAGGCCTTTTACGCTTGCAGTTTCTTCACAGACTTTCACTAG

CAAAGAACAATCTTACAGGTAGCATTAGTGTTAGTCTTGCTCAACTTGCTTATCTCAAGTTTCTTGACTTG

AGTGAAAATAACTTATCTGGTAACATTCCTGGCGATTACTTTCAACAATGTGGGCCGCTTAGATCAATTT

CTTTGGCTAACAACAAAATTTCAGGGCAGATCCCAGAAAGTTTGAGCTCCTGTGTTACACTTGCGTCCCT

TAATGTATCGTCAAATCAGTTTTCAGGGTTGGTGCCTTCTGGGATTTGGTCTTTGAATGGACTAAGGTCT

CTTGATTTGTCAAACAATTTATTGGACGGCGAAATTCCAGTGGACATTCAGGGTCTGAGTAATTTGAGAG

CATTAAACTTGGGGAGAAATAATTTCAAGGGTGAAATTCCGGATGAAATTGGAGGTTGTTTGCTTTTGA

GGTCAATTGATTTGAGTGAAAATTCTCTCTCAGGAGAGCTTCCAAGCACAATGCAGAAGCTTAGCTTGTG

TAGTGAATTCATTTTGAGACGTAATGCTTTTGTGGGCATTGTTCCTAAATGGATTGGAGAAATGAAAAGC
CTTCAGATTCTTGACGTTTCTGAGAACAACTTCTCTGGTATCATTCCAACTTCAATAGGGGAACTTGAGTC
ACTGAAAGTATTAAAGGTGTCACGGAACGGGATTTCTGGAAGCTTGCCTGAGTCCTTGAGCAGTTGTGT
TAAGCTTCTGGAATTGGATGTTAGTCATAACTCTTTGGCGGGTAATCTTTCTACTTGGGAATTTAAATTGC
AATTAATTCAGGTTATGTTTTCCGTGAATAAAATTAACTGCCAATCTAAATAATGCATTGCTACACAACTC
TTATCACAATCTTGTAGCAGTGAGAGTTATCTGGTGAAATTTAATTAACTCTCTGAAACTTCTCTTGCAGG
CACCATTCCTAAGACTGTCGGCCAATTGAAATCCTTGAATATTCTTGATTTGAGTGAAAATCGCTTATATG
GTATTGTCCCTGTGGAAATTGGAGGAGCAAAATCTCTCATGGAACTTAGTCTGGAGAAAAATTCCTTGA
CTGGAGAAATTCCTAGTTCAATTGGCTATTGTTCTTCATTAGTATCCTTGTAAGTTCTCGAACCTCCTTATG
TTTCATTAATTTGTTTTTGGACATTGCCTTTCTCTGCTTGAAGATTCTTAGAGGATAGTATAGTGGTATCT
GGTGTTTAAGTTCTCTATAGTTTTCAGGCCAGGGAATATTGTGTTAGAGGCAAGTTCAAATACGAAACCA
TGTTTGATATTCTCAATCATTTTCTGCTGAAATTTAGTGGCTCTAGTAATCATGTAGAGTTTATAATTTGCA
ATGACAATAAAATGGGATTATCAGCTTCTATTATAAAAGCTAATTGCAGAGTAGTATTAGTGGTAATGTG
CCTAACTGTATCTGAATATAATTTGAGTGGTTATGACAGTAATATCTTACATGAGGCATGAAAGTAACAA
CAATAATATATTAATAGGCTCTCAATGATCTATCTATTTATATCCATTATGTTTTGGCTGGTGTTACCTGAA
TACGAATTTTTTGCTACAATTAGTTAATGTTTTACTTCAATCAACTTGGCAGGTCTTTATCACATAACGGTC
TAACTGGTCCTGTACCTGAAGCCCTAGCAAAACTGACTTATCTTCAAAGTGTTGACTTATCCTTTAACAAA
TTAACGGGAGTGTTGCCAAAGCAGCTTGGAGATCTTGGCCATCTCTCCTTATTCAACATTTCACACAACC
AGCTACAGGGCGAACTACCTTCTAATGGTTTTTTAATACTATTTCTCCTTATTCCGTGTCAGCAAACCCAT
CTCTATGTGGGGCTGCTGTCAATAGGTCTTGTTCTACAGTCATGCCCAAGCCAATTGTTCTGAATCCCAAC
TCTACTGATTCTTCTCCCAACACTGTCCCTCAAAGTATTCGCCATGAGAAGAAAATCCTAAGCATCTCTGC
CCTCATTGCTATTGGTGCAGCTGCTGTTATTTTTGTTGGTGTGATTGCCATTACTGTGCTCAATATTCGTG
TCCGGTCAGCGGCACCTCGCTCTGCTGCTGCAGCCCTTACATTTTCTGGTGGAGATGATTTTAGCCATTCT
CCATCCACGGATGCGAATTCTGGTAAGCTTGTCATGTTTTCTGGTGATCCCGACTTCAGCACAGGGGCAC
ATGCTCTGCTTAACAAAGATTGTGAGCTTGGGCGAGGTGGTTTTGGAGCAGTGTATCGCACTGTCCTTG
GGGATGGGCATCCAGTGGCCATTAAGAAGCTTACCGTTTCCAGTCTTGTTAAGTCTCAGGAAGATTTTGA
GAGGGAGGTTAGGAAATTGGGAAAAGTACGTCACCATAATCTGGTCACCCTTGAAGGTTATTATTGGAC
ACCATCTTTACAGTTACTTATATATGAATTTGTGGCTGGTGGAAATTTGTACAAGCATCTCCATGAAGGA
TCTGGTGGGTGTTTCCTCTCATGGAATGAGCGGTTTAACATCATTCTCGGGACAGCAAAAAGCTTGGCTC

ACTTGCACCAAATGAATGTAATCCACTACAACCTAAAGTCCAGCAATGTCCTAATTGATAGCTCAGGTGA
GCCTAAAGTTGCAGATTATGGCTTAGCAAGACTATTACCCATGCTTGATCGATATGTCTTGAGTAGCAAA
ATTCAGAGTGCACTCGGTTACATGGCACCTGAATTTGCATGCAAAACTGTGAAGATAACCGAGAAGTGT
GATGTGTATGGATTTGGAGTTCTTGTTCTGGAGGTAGTCACTGGAAAGAGGCCAGTTGAGTATATGGAA
GACGACGTGGTAGTACTGTGTGACATGGTACGTGGAGCATTAGAAGAAGGCAGGGTAGAAGACTGTAT
AGATGCAAGGTTGCAAGGAAAATTTCCAGCAGATGAGGTGATTCCTGTGATGAAGTTAGGATTAATCTG
CACATCACAAGTCCCATCAAACCGGCCTGATATGGGAGAAGTGGTTAACATTTTGGAGCTGATTCGATG
TCCTTCAGAAGGCCAGGATGAACTTGTATGATGTTCACTAGATAAACACAGATTGTAAGATAAATGTCTC
ATCATGGCAAAGAAGAATGCGGTTCATTTATTCAGAGAAGTTGGAATTTGTTGTTGTAGAATATTTTGCT
CTTAATTGCTATAAAAATCCCTCCTTTGAATTACATCATTTGTTATTTGCTTCTCTATGCCACTCATGGATT
CCTTTTCACCTGGTTCCAATGTTGGACTCACATGCTTATTATTACTCTTTTCTTTCTTCTCAGGGTTTAAGTC
ATCTGCCGCTCCTTAAAATTGTGCTCATGTTTCACTTAGACACA
>PGSC0003DMG400016685 LRR-RLKs
GAACATTTCTTCTTCTTCTCCACCATTTCTGCTCTGCTCACTCTTTCCCCTCCACCATTGAAGAAACATGCG
TTTTCTTCTCCTCTTCTTCCTTTCCCTTATTCTCCATTTCCATCTCCTCCACTTCACCACCGCAAAACCACCTT
ACGTGCCGGAATACCGGGCATTACTCTCCCTGAAAACTTCCATTACCGATGACCCACAATCTGCTCTTCTT
TCATGGAATATCTCAACAAGTCATTGTACATGGAGAGGTGTCACGTGCGACCGGTATCGTCACGTGACT
TCTCTCGACATCTCTGGTTTTAATCTCACCGGTACTCTCACGCCGGAAGTTGGTCATCTCCGTTTTTTGCTC
AATCTTTCTGTAGCTGTTAACCAGTTCTCTGGACCCATTCCTATAGAGCTCTCGTTTATACCAAATCTGAG
TTACCTTAACCTCTCTAACAACATTTTCAATTTGAGTTTCCCTCCCCAGCTTACCCATCTCCGGTACTTGAA
AGTTCTCGATATTTATAATAACAATATGACCGGTGACCTTCCGGTTGGGGTTTACAATTTGACTAATCTTC
GACATCTTCATTTGGGTGGCAATTTTTTTAGTGGCAGTATTCCACCGGAGTATGGTAGATTCCCATTCCTA
GAATACCTTGCAGTTTCTGGAAATGCGCTCGTCGGTATGATACCACCGGAGATCGGAAATATTACCACA
CTTCGTGAGCTTTACATTGGATACTACAACACGTTTTCCGGTGGGTTACCGGCGGAAATAGGGAACTTGT
CGGAGCTCATTCGGTTAGATGCTGCAAACTGTGGACTTTCCGGTGAGATTCCGCCGGAGATAGGGAAGC
TTCAGAAATTAGATACACTGTTCTTGCAAGTGAATGGTCTTTCTGGGTCTGTTACACCGGAATTGGGGAA
TTTAAAAAGCTTGAAATCTTTAGATCTATCAAACAATATGCTCTCCGGTGAAATACCGTTCACATTCACAG
AGCTGAAGAATCTAACCCTGCTAAATCTTTTCCGTAACAAGCTTTACGGGTCGATACCGGAGTTCATAGA
AGATTTGCCAAAACTGGAAGTATTGCAGCTTTGGGAAAACAACTTTACCGGAAGTATTCCACAAGGTTT

AGGCAAAAACAGTAAGTTAACAAACGTTGACATCAGTACCAACAAATTAACCGGAAATTTGCCCCCAAA
CATGTGTTCCGGCAACAAGTTACAGACGTTGATCACTCTCGGAAACTTCTTGTTTGGCCCAATTCCAGAA
TCTTTAGGTGAGTGTCAATCACTTAATAGGATTAGAATGGGAGAAAATTTTCTAAATGGGTCTATTCCAA
AAGGGCTATTCAGTTTGCCGAAGCTTTCACAAGTAGAACTTCAAGATAATCTTCTCACTGGTACATTTCCA
GTGACTGGTTCTGTTTCATCAAGTCTTGGACAGATTTGTCTGTCGAATAATCGTTTCACGGGGCCTTTGCC
ATCGAGCATTGGAAATTTTACTGGTGTTCAAAAGTTGCTTCTTGATGGGAACAAGTTTTCTGGTCAAATT
CCAGCTGAATTAGGGAAATTGCAGCAGCTGTCGAAAATGGATTTTAGTGGTAACAGTTTTTCAGGCCTG
ATTCCACCGGAGATAAGCCGGTGCAAGGCTTTAACTTATGTTGATCTTAGTAGGAATAAGCTATCTGGTG
AAGTTCCTACTGAGATCACTGGTATGAGGATACTGAATTACTTGAATGTATCGCGGAATCAGTTAGTTGG
GAGTATTCCTGCACCTATTGCAGCAATGCAGAGTTTAACCTCGGTTGATTTTTCGTATAACAACTTATCTG
GATTGGTTCCGGGTACTGGTCAGTTCAGTTACTTCAATTACACATCATTTATTGGTAATCCAGATCTTTGC
GGACCCTATTTGGGTCCTTGCAAAGAAGGTATTGTTGATGGGGTTAGTCGACCTCATGAGAGAGGTGCA
TTTTCGCCTTCTATGAAGCTTTTACTTGTTATCGGGTTGCTTGTTTGCTCGATTGTGTTTGCTATTGCTGCA
ATTATTAAGGCTAGATCGTTAAAGAAGGCGAGTCAGGCTCGTGCCTGGAAGCTTACTGCTTTCCAACGC
CTGGATTTCACTTGTGATGATGTATTGGAATGTTTGAAAGAGGATAACATTATTGGTAAAGGAGGTGCT
GGAATAGTATACAAGGGGGTAATGCCAAATGGTGAACTTGTTGCTGTTAAAAGGTTGCCGGTTATGAGC
CGTGGTTCTTCCCATGATCACGGGTTTAATGCCGAGATACAGACACTTGGGAGTATTCGACATAGACATA
TTGTTAGATTATTGGGATTTTGCTCAAATCATGAAACAAATCTTTTGGTTTATGAGTACATGCCTAATGGG
AGCCTTGGTGAAATGCTTCATGGAAAGAAAGGTGGTCACTTGCATTGGGATACCAGGTATAAGATAGCA
TTGGAGGCTGCAAAGGGACTTTGTTATCTTCATCACGATTGCTCGCCTTTGATCCTCCATCGTGATGTAAA
ATCAAACAACATTCTTCTGGATTCCAGCTTCGAAGCTCACGTTGCTGATTTTGGGCTTGCCAAGTTTTTGC
AAGACTCGGGAACATCAGAATGCATGTCTGCTATTGCTGGTTCTTATGGCTACATTGCACCAGGTATGCT
CTCAAATCTTTACTTCTTTGTAGTCTTGGTTTTGATGTGTGTTGTTCCTTTCAAAAGAAGTTTATTATTGTT
GTAAGTTGACAACTAAAATTGTGATTTATCAGTCTCTTCAAGTGTTTATTATGGTACTTCTTGATCCTTTAA
AGCTGGTAATGATCAGTCTCCTTGTTAAAACATGGAACAACATGACACGAATATCAGTCCTTGATTGGTA
GTTAACAAGAACAACAACAACAATGATAACTACGTGTCGAAAATGAATGCCTCTGTTCATAGATGAGCT
GCTAACTGGTGCTTTGATGAATTCAAATTTGGTTACTTGCTGTTTGGCGTTAAGTTGTTTCATTTTGACTT
ACAAAGTTTGCTTATTGCTTGAAACACAATCTCTCTTTTTTTGTATGTGCCTAATTTCGTTTTCATTTGAAC
TGTGCAGAATATGCATACACACTCAAGGTAGATGAGAAGAGTGATGTATACAGCTTTGGTGTGGTTCTG

TTAGAATTGGTGAGCGGGAAAAAGCCAGTTGGGGAATTTGGTGATGGCGTTGACATAGTCCAATGGGT

AAGGAGGATGACCGATGGGAAAAAAGAAGGAGTTCTAAAGATCCTTGATCCAAGACTCTCAACAGTTC

CCCTTCATGAGGTGATGCATGTGTTCTATGTTGCAATGCTGTGTGTCGAAGAGCAAGCTGTTGAACGCCC

CAAAATGCGTGAGGTTGTGCAAATGCTAACTGAGCTTCCCAAGCCATCTGGTCCAAAAACAGAAGATTC

AACAATCACCGAGTCGCCCCCATCATCAGGTCCTGCATTAGAGTCTCCCACTTCGACTCCCGGAGACACG

AAAGACCAGTACCACCATCAGCCATCACCTCAATCTCCTCCACCTGACCTACTCAGCATATGACCTACAAT

GTTCCCTTCTAATAGAGGATGTAGTTCTCTTCCTTTAGTTTTATTATCAGTTTTATTGGGGGTTGGTTTATT

TATTAGGCTATAAGCTTTTCCTAGGAAGTGAGTTTCATTGTACAGTAAGATTGGCTAAGGTATCTGAGTA

ACTTTATCCTTCAAATTTTCCCATTTGCTGTTTAGCTTTTTGATATTGAAAGAATC

>PGSC0003DMG400016709 LRR-RLKs

CGACACCTATAGATACACACTGCCAGTGGAGTCTTCTTGTATAAATTTTTCTCTCTCTCTTCATTAAAAGG

AATGCTTGGAATCGGAGAGTCTCTTAACGATGGTGACACCTACCGCTACTGACGATGTTACCTTCTTGTC

ATTTCCCTGTCTTTCTTTCGCCATGCTTCTTCTTCTTCCTCTTACCTCTGTCTCTGCACAACCCCTTTAATTCT

TGACTAACAAGAACCAAAACAGAAAATGAATAGAAATAGCTGTAAGCAAGACACCCTTTTCACCCTTTTG

CCAGTAGTGGTGATTGTGGTAGTGGTTTTTTACTCATCTTTTGTTGTGGAAGGAAACACTGATCCGAATG

ATTGTGAGTTTCTTCACTGGTTGAAAATCTTGGATCCGTTTTTAGAGCTTCATTAGTGATTTTGAGCTTAG

TGGTTTGGGTGTTTTTTCTGTTTTCTTGATTGTGTTGAAGGAGGAGGGTTTTTTTGTTTTTTTGTTTGTCA

CTCTGTGAATGTTGTGATATAAGAATGCCTTTCTTGTGATTGTTTGAATGCCTGATTTGGTTAATGGGGG

TAATGTATTTGTTTGCCCTTTTTCTGGGATCTTTTTCTTCCCCTTTTTGTTGGGTTTCCTAGTCAATGCAAGT

CAAAGATTGATGCGTTAGTACAAAAAATATGGATTCTTGATCTTTATACTAAAAATTATATGACTGGGAT

TTTCTGTGATTATAATGTGAGGACGCATGCAATTGAATTTGAATGCTAAAACTGGTTGATTTGGTAAGAT

AATATGTTTTCTTGCCCTTTCTTGGTGTCTTTCTTGTATTTAATTAGTTTCAGAGCCAACACTAGCTTAAGA

TTGAGGCTGTACTTGTTGAATCTTGATAAGATATATATTGAATCTTGGTTTTCTTGTTGGAGAGATGTTCT

GGCTTTGGCTTTGTCGTTGCCTGACTCTTGTAATCTGCAGTGCAAGCTCTTCAGGTCTTGTATGCTTCACT

GAATAGTCCTTCACAGCTCTCTAATTGGAAGTCGAATGGTGGCGATCCCTGTGGTGAGTCTTGGAAAGG

AATCACTTGTCAGGCCTCTTCTATTGTTTCAATGTAAGTTTGCATAGCATTAACTTTGTGCGCATCATTATA

CATTTTAGTTATGATTTGATTGATATAATGGTTGGTTCTTGCTGAAACAGTCAAATTCCTGATTTGGGACT

CAATGGAACGTTGGGATACATGCTAAATGGTCTTACCTCGTTAAAGAACTTGTAAGAGCTTGTCCTTTTA

GTGGTAAAGGGTAATATAGAGGACCTACTATCACAATAAAATTGAACTTCTGCAATCTGTTGTCTTCAGG

GATATGAGCGGCAACAACATTCATGATTCTATTCCATATCAGCTTCCACCCAATCTCACTAGCCTGTAATG
TTGACTCCTTGTTATGAGTTTGTTTTGTTTGAAAACTTCCCGGTTTATGACTGTCTCACTGGAAATATAATC
ATTGTCAATGCAGGAATCTTGCAAACAACAATATAAGTGGCAACATTCCGTATTCCTTTGCTGGCATGGT
TACACTTAGCTATCTGTGAGTGGCCAGACTCACTAGGCACTTTCCAAAACAAAAATAACATTTGCTTGGT
CTTCTCTTTATTCTATTGAATCATTTCAGGCAACTATTTAAAATAGCTTACCCTACGATTCATTTTTTCAGG
AATCTTAGCCATAACTCACTCTCTCAAACAATTGGAGAATTGTTCACCAATCACTCCGATCTGGGAGCTTT
GTAAGTGTTCTACTTAATCTTTAATTGATTGATCCTTTCTGGTAATTTTTTTTCCTTTTCTTCTTTTTTTCTAA
TAGAGTTCATTCATTGCCACACTTGAAGGGCCTCTTTTCCTACACCAGTCCAATTAGTTACTAGCTTGAAT
TCATAACACCATGGTGTCTCCGACCCACAGTAGCTGGCCCTTCATTTGTTTTGACGTGCCTGCAATGTACA
GACATGACTGCTTCCAAATATGTTCTCAAAACAAATATAGGATATAATCATATACAAATGCCTTTTTAGAA
TCATTATCTGCTTAATTTTAATGAACAATGAAGTCTGGAGGCTTGTAAAGTCCAACATTTTATTTTCCTTC
GTAGCCTTTTTATGTGGAAATTCGTTTTGATTGTATTAGGATAGGTGCCCTGCTCCTCTTTGAAGCTTTCA
GTTGCCTTTTGTTTTCATTTATTTGTTCTTTCAGAATCCCATTTAAGTAAACCTACATTAGAGCTCAGTACT
CCTGCTACAAAGAGCTTTCTGGCGATATTGATTCCTCATGTCTTTTGAACATCTGTTTCACTAGTTAATAG
TTATAAATTTTCACTGATTTGCTGCTACTGCAGGGATCTGTCCTCGAATAATTTTTCTGGAGATCTCCCCTC
TTCATTCAGTTCGTTGACAAATCTTTCCATACTGTAAGCATAATAACTTTCTACTAATAGGATTTGCTGTTC
CGTTACTTGACTTACCCATGAAATTGGCATCTCTTGTAGTCATCTGGAAAACAATCAGCTAAGTGGCCCT
CTCAATATCCTGGTTGGTCTACCGTTGACTAATTTGTAAGTTTCAATACCTCTTCTTGAATTAATGGGCTT
AGCTCGTTCGATTCAATGCCAGTAGAGTGTTTTATGGGTAAAACAAAACCTTTTTGCAGAAATGTTGCAA
AGAATCATTTCAATGGATGGATCCCGAATGAGCTTCTATCTATCCCTAACTTCATGTAAGTAATTTCACAT
CCTATATATTCTTACATGGTTTAGAATGTTACAGTTTCAAGGGGTGTGACATAACATCTAATATGACAGA
TACGATGGGAATTCCTTTGCAAATGGTCCAGCTCCTCCCCCACCACCATACACTGCACCTCCTCCTGGAA
GATCTCGCAACAATCGTAGCCATTCTCCTCCTAGTTCACGTACAAGACCAGGTTCTGATGGCCAGCCATC
TCCAGAAAATGGTGATAAAAAAAAAGGGATTGACAACTGGAGCTATCATTGGCATAGCTCTTGGATCTTC
TCTTGCAATTCTTTTTGCTCTGGTAGCACTTGTATTTTGCTTCCGGAGGGGCAAGGGCAAGGAAATTGCT
ACAAGACCCTCTACAGGAAGTCTTCCAGTTGGCACAGATAAAGGTGTGTTCCCTTGAACTTTTTTCCTTCC
TGACTCAATATTGTCATTGGTGCTAATACCCTCAGATGACTAACAGTTGTGGTGTGATGTGCCATCTTAAT
TTGGAAATTGGTACAAACTTGGTTGATTTCTCATGTATGCTGTTTAATCTTAGTGAATATGGAGGTACAA
GAGCAAAGGGCGAATCCTGTGGCTGCTGTTGCTGACTTGAAGCCCCCACCTACCGAAAAGGTGACTGTT

GAAATGATACAAGGGAAGAATGGATCTGTCAGGAGAGTGAAGTCTCCAATAACTGCATCTGCTTATACT
GTTGCTGCATTGCAAACAGCAACAAACAGCTTCAGTCAAGAAAATCTCATTGGTGAAGGATCTCTTGGG
CGTGTTTATAGGGCAGAGTTTCCTAATGGGAAGGTAATTTCAGATGTTTAACGTTGTAACTTCTTACCTTT
CCTACTTGTGATGTGCATTCAAAACTATTTGAAGCTGAACAATATTGAATAAATTCAATCTGTCTCTCATC
GACATCTTTCATTTTTGATGCTTCGGAGAATCACATACTTGTGAGTTGTATAACGTTCATCAGATACATAA
GGACATCAAAAGGGGTATATCAAGATGTTGGCCACTCCACCTATCACCCTATTGCTAAGATGCTTTAACA
TAGTATCAAGCCGTTACACGTGTGGGACAATGTTGCGTTACCTAATGTCCAACCTCGGGTAAATGAGCCT
GTAAAGATATGGTAACTCAACCCCAAAAGCTAACTCATGAGGGGAGGATTGCCCAAATCCATATAAGGA
GACCACCAGTCCATTCCCTAACCAATGTGAGACTTTTACCCACTCTAACACCCCACTTCACGCCCAGGCCC
AGGCCCGCTGGAGCATGGACCGGGAGCCCAAATGGAGATGAACCTCTGATACCATGTAAAGATATGGC
ACCTGGGCTTAACTCAACCCCAAAAGCTTAGCTCATGAGGGGAGGATTGCCCAAGTCCATATAAGGAGA
CCCCAGTCCATTCCCCACCCAATGTGAGACTTTTACCCACTCTAACAAAGGCATCCAAAGTAGTATATAA
GGTTCTTGTCCTACTATATCCATTGCCTTAACGTTATGGTTGAACCCTTAGATCCTTTCACAATATTTGTCA
AACCGGGCAAACACTAAAAGGCTACATACTTGATCTATGTGTTACTAAACCTGAGTAGAAGTTAGTAGA
CTTCTTGATACTCTGCTCGCATGGGAGTTAAAGTATTGGGTCCTCTTGCTAGTACATAATCTGTTTTGAGT
GATTCTCTTAACTGAGATTAAGTCCTTGATACTCTTTGATTCAACTTGCTCTTTTAGATCTTTCACAAAGTT
GATTGCCCACAGTAGGCTCATATTCTGATGAACACATGTCAATTGTTCACTCCTCTGGGTGGTCTGGCCA
CATCTTTGCTTTTATCTGTCTTGTGATGTTTGATGGGTGTAATTATATGGTCACTTTGATGGTCATCTCACT
TCTCTTCTCAGCCTGAATTAACTATGATACTATAACCTTGTCCTTTGGTTACATGATGCAGTCCTAAGCAA
AATTATTCTTAGCAGTTTCAAAGTTTGACATTATATATCTTCTACTTTGTGGTTTATTATTGGATGTGCTAC
TGGTTATTTCTTTTTGAATGAACCAATTGTGTTGACTATTTCTCTGCTCCAAACTTGATGCCAACTGTGTTC
CAATAAATGATTTAAGAATTTCATGTGTCAACAACATTTTTGATGAGGAAACTTTTAGTTTTTCATTCATG
TAGTTTCTACGGAAGGTGCCAACTGAAAATCTCAATTGATCTTTTGAACTGTCCTTACCAGATAATGGTC
ATCAAAAAGATCGACAGTGCTGCACTGTCGCTGCAGGAGGAAGATAACTTTCTTGAAGCTGTCTCAAAT
ATGTCAAGGCTGAGACACCCAAACATTGTTCAATTGATAGGATATTGTGCTGAGCATGGACAGCGTCTT
CTGGTTTATGACCATGTTGCAAATGGTAGCTTGCATGATATGCTGCACTTTGCTGATGAGAGGAGCGAA
TTGCTTACTTGGAACGCCCGGGTTAGAGTAGCACTTGGCACTGCTCGAGCTTTAGAGTATGCTATAGCTG
TATAGTATTTTACCTCTCTTGCCCTTTTCTTGTTGGGTTAGCTTAAAACTTGACCGTCTTCTTTTCAGGTACT
TGCATGAAGTCTGTCTACCTTCTGTTGTACATAGAAACTTCAAATCCGCAAATATTTTACTAGATGAAGA

GCTCAATCCCCATTTATCAGATTGTGGGTTAGCAGCTTTGACTCCAAACACAGAACGCCAGGTGAGTAAA

GAAGATTTAATCTACGTCGTCGTTTTAACAGAAGGTGTTGATAATATTGGGTGGTACGTGATGCATATAA

TGTTTGTGAGGTGATGTCAATCAACTTACCTAGTACTGTAGTAGGAGCAATATTAATAAGCAACTATTTC

TAGGTAGATCTTTACAGACTTAGTCATGAAATTGGAGATTCTGATTGTGGCAGCTTAGTTTTGTTTTCTAA

TTCTTCTGGAGCAATCTTGTTAGATGCTTAAACACTTGGCTTACCATAGTGCACAAACTTTAAGGAAGAC

CCCTTCCCCCTGCTTGAAATTCCATTTAGTTCTGTGAATTATTTCTGTGAAAAGAGAACCTTCTTGTACAG

AAGTTCGGGTGAAGGAAGCTAATGTGGGAAGGCTAATTGACTCTGTTATGGGCATTGCCTAATATTGTA

ATTACTTCTGAATGTTGACAGGTGTCATCCACACAAATGATTGGTTCATTCGGTTATAGCGCTCCTGAATT

TGCTTTATCTGGAATATACACGGTAAAAAGTGATGTATATAGTTTTGGTGTGGTGATGTTGGAATTGTTG

ACAGGAAGGAAGCCACTTGATAGGTAAACTAGATCTTTTTACATTGCTAGTCAACTTTTACACTATTGAA

TTCTTTTAATGAATTCATTCTTGTTAACAATCATGTAGTTCAAGGGTGAGATCGGAACAGTCCCTTGTGAG

ATGGGCTACGCCTCAACTCCATGATATAGATGCCTTGTCAAAAATGGTTGATCCTGCACTAAATGGCATG

TATCCTGCAAAGTCTTTGTCCCGTTTCGCTGATATTATTGCACTCTGTGTTCAGGTGAGCTTTACTTGCAT

GTTCTTTCATTTGGAACATAACAGTTGTTAACATGTGAAGCTTAAAAGATGAAACCACTTCTAAAATATCT

TTGATGGAGGCGTAAGTCAATGATCATAGGGAATTTACCCACTTAAGGACCAAACTTGACATTCTAACCC

GTCAAATAAATGATACACTACCAGTTTTTGCATTCTATATAAATCCAAATTCAGGGGCGTGGATAAATTG

GGACCACCTGCACTCTGAGTTTGCTCATGGCTCAGTGCTGAAGTGAATCGATGTGCTTCAGCAAATAGAT

GACCTTTCTTACACATACCCGGTGATTCTTGGAATGGCGAGAGCACCCATCTTGGTTAAGTTAGTTTGGG

AACTTCCAAGGCGTAATCCTTGCATACCATGGTCCATAGTGGCTTTGGGTGCTTAGTCTTTAAATGAGAA

ATTCTTAATTAAGCTTTTCTTGATCTAAGGTGACTTCAAGACTCACGAAGAAAAATCTGATGGACTCGAC

AAAATTATAATGAATACTTGCTCATTGACATATCTGAAGTGAATTACTTTACTTGCAATAATTTGAATGCG

ACTGTTTTCTCCTTGGTAATAACTTTTGACATCTATTTCCTGTACAGCCTGAACCAGAGTTTCGACCCCCAA

TGTCTGAAGTTGTGCAAGCACTGGTTCGGTTAATGCAAAGGGCGAGTGTGGTCAAAAGGCGATCCAGT

GATGAATCAGCTTTCACATACAAGACTCCGGATCATGAGACGGTTGACATGCCATAGATAAACATACAC

GACGAAGCAGCCTTTGCAATTTTGTAAGTAACTTCTGTGAAGGTCAAGAGCAACTATGGCAGATAAAAC

TCCGAGGTGTCAATACCAGGGACATTAAAAATGTAATTATTTTTCACATCTTCCTCGTTAAAAAAAGAAG

AAGCTTTTTTGCATCCCAATTTGAGGTACATAATTCACTGACAATGTCCTCCAGAGTATATTTTCAAACCA

TCTTGGGCTACTGGATATAATTTATTGTCAATAAGAACATTGCTACACATTATCATTCTGTGTTTGTTTATA

TTACAGTTTTTCCAATAACTTTTGGCTAGCCTAAGAGCTAACTGTAGTGTGCATCGGTCGGTTCGGTT

>PGSC0003DMG400016872 LRR-RLKs

ATGTCTGGAGTATATGACTCAAGAACTGGTCTAATTCTGATTTGGATAGTTATCTTGTTGGCTTCCGCGA
TGATGGTTTGTCCTGCAGAAGGATTGAATGCTGAAGGAATGTACCTGCTTGAGTTGAAAAAGAATTTAA
ACGATGAGTTTAACAATCTTGAAAATTGGAATCCTAGTGATGAGACGCCATGTAGATGGAAAGGTGTAA
ATTGCACTTCTGATTATAATCCAGTTGTGCAATCTCTTGATTTGAGTTTTATGAATCTTTCAGGCACTTTGA
GTTCTAGCATTGGTGGTCTCGTAAGTTTAACCATACTTGACTTGTCTTTCAATGGATTCACGGGAGAAATT
CCGAAAGAAATAGGGAACTGCTCGAAAATGCAGAGTCTTCAGCTTCATGATAATGAATTTTATGGGCAG
ATTCCTGATGAATTGTATAACCTTTCTCATTTGAAAGACTTGAATTTGTTCAATAACATGATATCTGGTCCT
ATATCAGAGGAGTTTGGGAGACTTTCTTCTTTAGTTTCTTTTGTTGCATACACCAATAATCTCACTGGTTC
ACTCCCTCGATCACTCGGGAAACTAAAAAAGTTGGAAACATTTCGAGTGGGGCAGAATCCGCTTTCTGG
AACCTTACCTGCTGAGATTGGTGATTGCAAGAGCTTACAAGTTCTTGGTCTTGCACAAAACAATATAGGA
GGAAATATACCCAAAGAGATCGGTATGCTTAGAAGATTGAAACAACTTGTACTTTGGGACAATCAACTC
TCGGGCTATATCCCGAAGGAACTTGGAAACTGTACAAAACTTGAACTGCTTGCTTTATACCAGAACAACC
TGGTTGGAGAAATTCCTGCTGCAATAGGTAAGCTCAAATCTCTCAAGAGGTTATACCTATACCGGAATG
GATTAAACGGTACAATTCCAAGGGTCATTGGGAATCTTTCGTCAGCGATAGAGATTGACTTCTCAGAGA
ACTATCTAATAGGAGACATACCAATTGAGTTCAGTCAAATAAAGGGACTGATATTGTTGTATCTGTTTCA
TAACCAGCTCAATGGTGTGATACCTCGGGAACTTTCTAGTTTAAGAAAGTTGGAACGTCTTGATCTGTCG
ATTAACGACTTATATGGGTCTATTCCATTTTCATTTCAGTATCTCACTGAATTGGTTCAGTTACAGCTCTTT
CAGAATTCTTTAAGTGGAACTATTCCCCAAGGTCTCGGGAACTATAGCCGACTTTGGGTTGTTGATTTTTC
TAACAACTACCTCACAGGAGGAATTCCTCCGAATATTTGTAGGAATTCTAATTTGATTTGGCTTAATCTTG
GTTCAAATAACTTGCATGGAGACATTCCATCTGGTGTTATTAAGTGTGACTCATTGGTACAACTTCGTCTT
GACGGTAACTGGCTACAAGGGAGTTTTCCTTCTGACTTGTGCAAACTGAGTAATCTATCTGCTCTTGAAT
TAGGACAGAACACGTTCGGTGGTCTAATTCCTCCTGAGATTGGAAACTGTCAAAAGTTGCAAAGACTTG
ATCTTTCAGGCAATTATTTCACACATGAATTGCCTCAAGAGATAGGGAATCTTGAAACACTTGTGACGTT
TAATGTCTCGTCTAATTTGCTGACAGGTCAAGTACCACAAGAAATTCTGAAATGCAAGGCGTTGCAACG
ACTTGATCTCAGTAGGAACAGCTTTAGTGGTGCTATACCTGCTGAAATTGGAAAACTAGCACAGCTTGA
ACGTCTTTTGGTTTCGGATAATAAGTTCTCTGGAAAGATACCAGTAGCATTGGGGAGGCTTTCTCGTTTG
AACGAGCTGCAGATGGGTGGAAATTCATTTTCAGGTGAAATACCATCAGAATTGGGTGATCTTACTGGT
CTACAGATCGCGATGGATCTTAGTGACAACAATCTCTCTGGTTCAATACCACCTAAGCTTGGAAATCTTA

TCCTACTAGAGTGTCTCAATCTCAACAATAATCATCTGAGTGGTGAAATACCAATCACATTCGGAAACTT
GACGAGTCTAATGAGCTGTAACTTTTCATACAATAACCTCACTGGACCATTGCCGGATATACCACTCTTTC
AGAATATGGATGTCAGCAGCTTTATTGGAAACAACGGGCTTTGTGGTGGTCGTTTAGGTGGATGTAACG
AATATCCTCCGTTCAATTCTGATCCTCCAATAAAAAATGCAGGTGCTCCAAGAGGAAAGATTGTTATTGT
TGTTGTGGCTGTTGGTAGTGGTGTTTCTCTTGTTTTGATTATGGTAATTTTGTATGTGATGAAGCGGAAA
CCAGTTGATCAGATGGTTGCATCAGTAAAAGATAAGAACGCGTCATTTCCAGCTTCGGACATATATTTCC
CTCCCGAAGAGGAGTTCACTTTCCAAGACTTAGTTGAGGCTACAAACAGTTTTCATGACAGTTATGTTGT
TGGAAGAGGAGCTGTTGGGACAGTATACAAAGCAGTCATGCAATCTGGACGAAAAATCGCTGTTAAAA
AGCTGGCTTCTAACAGAGAGGATAATAACATAGAAAAGAGTTTCCGAGCAGAGATTTCAACTCTCGGAA
AGATTAGGCATCGTAACATTGTAAAACTGTATGGCTTTTGCTATCATCAAGGTTCTAATCTGCTTCTTTAC
GAGTACATGGACAAAGGTAGCTTGGGTGAATTGCTTCACGGTGCATCTTGTAGCTTGGATTGGCCTCAA
CGCTTCATGATAGCTCTTGGGGCAGCTGAAGGACTTTCTTACTTACATCATGATTGCAAACCACAGATCA
TCCACCGCGATATAAAGTCCAATAATATTTTGCTCGATGAGAAGTTGGAAGCTCATGTTGGTGATTTTGG
TCTTGCTAAAGTTATCGACATGCCTCAAACTAAGTCGATGTCTGCAATTGCTGGATCATATGGCTACATA
GCCCCTGGTATGTACATTTCATTATTTTTTCATTAGAACTTGTGTAGTATAGCCCGTCTACTGTCTGCGAA
TAATGTCAAGAAACATATGCCTGTCTAGATTGCTCTAACAAGACTAATATATTTTAGGTAAGCAATGATT
ATAACTCTTCATCGAAAAATCTCACGTATAGTTCCTCCCATGAGCTGACTAGTGTATGTTAGTTTTCCTCA
CACTTTGACTAGCAAATAGTACTAGTGTTTCCTTGGTGAACGTTTTTGCTTCCTTTGTTTATACTCAACAAC
AACATATCCAGTGAAACCCTATAAAGTGCGATCTGGAGAGAGTAGAGTGAACACAGACCTTATACTCAT
ACCTCGTAGAGGTAGAAAGACTGTTTCCAAAAGACCCTCGACTCAAGTGAGGTGAAGAGAACAATACA
ACTAGTAGAAAAAAGTGCAGAGCATACATGCAAGGAATGATATTTGAAGTAAGGGTAATACTATTTGAT
GAACTATTTGAAGAGAGTAAGCAGACGCGTCTACTTTGTTTAACGGTTCTGCTTTTCCCATTGCAGAATA
TGCTTACACCATGAAGGTAACAGAAAAATGTGATATCTATAGTTACGGAGTAGTCCTTTTGGAGTTGCTA
ACAGGAAGAACACCGGTGCAGCCACTCGATCAAGGAGGTGATCTTGTCACTTGTGTGAGGCATTATATT
AGAGACAATTCGTTGACACCAGGGGTACTCGATATCCGATTGGATTTGACAGATAAAACTACTGTTAGT
CACATGCTTACAGTCTTGAAAATTGGTCTAGTATGTACTTGTTTGTCCCCGGCTGATCGCCCTTCAATGCG
CGAAGTTGTGTCAATGTTGATGGAGTCTGATGAGCAAGAAGGTAACTTCATCTTGTCTCAAAGTTGA
>PGSC0003DMG400017291 LRR-RLKs
TTTCTCTTCTCTATTATTATTACTATACTATAACAAAATGCCAATAAGGAAGTGACTGAAGAGCTGAAACT

CTTTGGATTTTTGTACTGTTTTTTGTACAATCTAATAGTAGCTGGAAATGAGCATAATGAAGATTTTGTTT
CTTCAATCTCTGTTATTTTCTCTTTTTCTTGTTTTCTTCACAGTTTTAGCAGCAACTTCAAAATCTAAGCTCC
CACAAGAAGAAGGTAACCCCATAGTAATCTTTTTCAGTTTCTACATGTTCCCATTTTGTTTTCTTGGAAAA
TGTCAGAAAAAGTTTCAAACTTTTTGTGTTTTGAAGTTTGCTGCTTAGTTTTTTCAATTACCCATATGTTCA
GTTTGTTGGAAAATTGAAAAAGTTTGGAACTTTCTGTTCAGTGAAGTCTGTTGTTTAGTTTTTTCAATTAC
CCATGTTTCATTTTCTTGGAAAATTTCAGAAAAAGTTTGAAACTTTCTGTTCATTGAAGTCTGCTGCTTAAT
TTTTAGTTTTTACTTTCTCAATTTCAATGTATAAAAGTAACTCTCTATTACTTTTTCAGGAACTTTTTAATTT
CTAACTTTCCAGTTTACACGTGACATGTTTAAGAGCATAAAATTAAAAAACAATTTGGTACATTTGACAA
ATCTTTTAATTGAAGGCCATTTAAAAATTGTTCTTTGCTTTTTTTAAACTTCGTGTCGAGTTAAAATAGGAC
AAGCATATTGAAATGGAGAGAGTACAAATTACTCATATGTTTCATTTTCTTGGAAAATATCAGAAAAAGT
TTGGAGCTTTCTGTTCATTGAAGAGTGCTGCTTAGTTTTTTCAATTACCCATATGTTCATTTTCTTGATGTG
CAGTGATAGCATTGAAAGTGATTGCAAAAAAGTTTGGGAAAAAAGATTGGGATTTTAACAAAGATCCTT
GTAGTGGAGAAGGAAATTGGAGTACTGCTATTACTGTTAAAGGATTTGAGAGCTATGTTGCTTGTGATT
GCTCTTTTAACAACAATTCCACCTGCCATATCACCTCCATGTATGTTTTAGTCTGTTCCCTATTAAATTGAA
ATTATGTTGTTCGGACTCTCCGAAAATGTAGCAGCACCTGTTCAACTCCTCCAAAAATACACTTCTTTTGG
AGGATCTGTCACATACTTGACTATATTTTTGAAGAGTCCGAGTAGCATAGAGGTGAAGATTTGATTTTTT
TTAAAGCAGCAACAATTAAATCTGCAGTTTTAAGTAGCTTCTTTGCAAGAGAAAAGATTAAAACTTTTCTT
TTTACAAGTTTGAAAGGTTGAGACTTTTTACAAGTTTGAACTGATTAGTGTGTGAATAGGAAATGAAATG
TGTGGACTTTAAGTACCACTTTTCTATGAGTTGACTGAAAATTGAGTCAACTTCTTATATGGCAGCCCAGC
TGTAATGAGGTGTCTGGAGTGTGGACATTATTTTTGTAAAGTATTTCAAAACTCAAAAGACATTTCACTTT
CAAAAAAATTCTACAAGATAATTTTTAAAAAATACTTGTTGTAACAAATTACATGTCTTATAATTCAGGTT
ACTAATTGAATTTTGTTATGTATAATTAATTACCTGTAATTAGTTTCTAGTTATCTTGAAAAGTACTAATTA
TCTTTTGAAACAGTTAGTGTAATGAAGTTAAAGCTCTTTCTTCAAATTACCAAACAAATCAACATTGTATA
CTTCCTGTTTATGTTAAACTTGCATGTAATGCAATGAAGTAGTCAAAAACTAACACTAAATATGCCACAG
AGCTTTGAAAGCACAGAATATTTCAGCAAATATTCCACCAGAATTCGCGCAACTTCGCCATCTTAAGCAC
TTGTAAGTCCTTGTTACAGAATTATCACTCAATAGACTGAAGCACCTGATATTACTATGTTGAAAACTTAG
GAGCTGCACTAATCCAACTACTAAATGATGATTATGCAGGGACCTCAGTCGCAATTATCTCAATGGTTCA
ATCCCATTTCAATGGGCGTCACTGCGCTTGCTTGACCTGTACATACTATTTCTCACCTAAACTATTCTTCAT
TAGTGCTTAAATTTTTCTCTTCTGGTTTAGAATAAGCTATTTGACATGAAAATAATTGTTATTTGAATGTG

CAGTTCTTTCATGGGAAACCACTTGTCTGGTCCATTTCCAAAAGTTCTCACCAGAATTACGACGCTTAGG

AACCTGTAAGATCAAATTTCTCTTCTGATGCCTACTTGTCATTTTCAGTGTTGTATACTTGCACTTAATCTG

TTTTACTATTGAATTTAAGTTTTATTTATGACTGCATAAAGTATTTACACAATCGAGTTTAAGTTCTATTCG

CTGACTATATAAGTAATTACAAGTAAAATCTCTATGATAAACACTAATTGGTAACCTGATAAAAATGTTA

ACTTAAGTCATTTACCATTTCAATATGGCGATTCATTGATTCAACATATATATTATACTGTAATTTGGTACT

GAAGTGAGAATGCGGAAAAAAGATCTCTATATGTCATGTCTGTCTGCAACAACAACATACCTAGTGAAA

TCTCGTAGGTGGTGTTTGGGGAAGCTAGAGTGTACGCAGAACTTATTTCTTCCTCGTAGAGGTAGAGAG

GCTGTTTGTGAAAGACTTCGGCTCAAGTAAAACATATCAAAACAGATTCTCTTTTCCTAAGGCATTTTATT

TCAAAAGTTAACTGCTGCTCTGACAAGAATTTTTGATCACCTGTATTTGATTCCATGAGAAATAAAATGCC

TCTTCTATAACTTTCAATACAGGAGCATTGAAGGAAATAAATTTTCAGGAACAATACCTCCAGAAATCGG

CAACTTGGTTCATATGGAAAAACTGTAATTCTCTTTTTCCTGTTTTAATTTTGTTCTGGAAATCTTTATTCG

TTTCACGTTGTATTAATGCATCTGTGCCTTAATTCTCAGCGTGCTATCGTCAAATAAATTAACCGGAGCTT

TGCCAGCCACACTAGCCAAGCTAACCAACTTAACTGATATGTGAGTTCACAGTTTTAAACTCTTTCTCATT

TGGTCATTCCTTCTGTCCCTTGTGAATTGTGATGGATGATCACATAATTAACTTTGCCGATATATTAGGAG

GATAAACGACAACAATTTTACTGGAAAGATACCAAAATTTATCAGCAGCTGGACAAAAATTGAGAAACT

GTAAGCTTTCTTTTTTCTTTACTTTTCAGACTTCAGTAGATGAAGTCTAGTTTCTAGCTTTATAACAAAAGG

GCTTCCCGGTGCACTAAGCTCCCTCTATGCGCGGGTCTAGGGAAGAGTTGGACCACAGGATCTATTGTA

CCAGCCTTAGCCTGTATTTCTATGACAGGCTGTTTCCACGGCTTGAACTCATGGAAACAGCCTCTTTCTGG

AATATTAGAAGTTTATACCCCAATTTTTCCTGCATATTTACAGTTATTGCTGTGATCTCATGAACCTCCAAT

TATACTATAATTTTTGCAGGCATATACAAGGCTGTTCTCTTGAGGGTCCCATTCCTTCCTCTATATCTTTCC

TGACAAGTTTAATTGATTTGTAAGCCAATGTGAAACTCCTTTTTGATGTCATTTGTGGTTGCTTTGAGAAG

TAAAATAATTTGTTATTTTTTTCTTTCATGAATTTCAGAAGGATCAGTGATCTAAAAAGTGGAAAATCTGG

TTTTCCACCATTAGATAACCTAGAATCCATAAAAATACTGTGAGTTTCTTTACTTTTTCTTCTTTTAATGGA

CTGCTTGAAGGACATTTCATTTCGATTTCGGAATCAATTTCTGATCAAAGATTCTGAATTCCTCTTGTGCA

ATGCAGAGTACTAAGAAATTGCTTAATTCATGGAGAAATGCCTGAATATATTGGGGAAATGAAGAAATT

GAAGACACTGTAAGTTTGAAAAAATTCAATCACTTTTTTTTGGTTCAGTTTTCCTGCATGTTGATACCTTTT

CTGTTTTCATGCTATATAGTATATACACATTCTTCAAGATTCTGAATCTTTTTTAGCTGAAATTTGTTTTTCC

ATGGAACTTACTATTTTCTGCTTTGTGATTTTAGGGACCTTAGTTTTAACAGCCTGTCTGGTGAGATTCCA

TCAACTTTTGTACACCTATCCAAAGTAGATTTTATGTAAGTGGATATTCTTCAGATATCTTTATTTCTTTGT

AGCCAGATGCTTATCAATATTAGGCTTCTGTTTTTTTCTTTTCTCTAATTGTTCTGGTACTTTGCAGATATTT
GACAGCGAACAAACTTACTGGACTTGTTCCTGGATGGATTTTAACCAGAAACAAGAACATGTGTGTGTTT
TCGCAACTTAATTCGTACTTCCCTTTTTAGTCCGTCCCAAAAAAGAATAACACAATTCCATATTTAGTAAC
AATTTTAACTTTAAAATGTTCACAAACATCGATGACTTGTTTTAGACCACAAGTTTTAAAAGTATTTCTTTC
TTTCTTAAACTCCATGCCAACTCAATCAACATCACATAAATTGAGATGGATGGAGTATAAATGTGCAAAA
AACTTGATTGCTCTTTAAAGTTTTCACTTACTGACCTACATGCTTTTATGCAAGTAGAGGCTTTAGAATGT
ATTCAATATTGAGTAATCTATTTTTGCCAAAAAAAGAATGGTGAAAAGGAACTAAAAAAATGTAAAAGA
TCATCTCTAAGCTTCAAAAAGTTATTAGTATTATGGACTTGCGAAACTTCTAGTCTAATTTTGTTGTCTATT
TTGCTTCACAGAGATGTTTCAACTAACAATTTTACATGGGAAACCTCACCTTTTGAATGTCCCCGTGGAAA
TCAGTAAGTTGTTTGATAAAATTGTTTTTCTTCATTTGACTTATTTGTTTGTGATAATTTTCTAACATCAAG
ACGATTTATTCAGAAACTTGGTGGAGAGCTATTCTGCATTGGGACAAGAATCGTAAGACTAATGTGCCT
ATAATAAATTTATCTGTTGCACTGATCTTACAGAGGAAACTATTGTTCCTTGAGTTCACCTATAACTAAGA
GTGAACTCTTACCTACTATAGAAAACTTTTGTAGCACTCTTAACAACTTTTTCTTTTGTTTTCTGTGTTGCA
GAAACAAAATTCATCCTTGCCTGAAGCAGAATTTCCCTTGTTCTGAGCCAATAGATCAGCGTAAGTAAAC
TTTTATGTGTCCATTTCTGTAATTGATAGCACAACAAAATCTGTCTCCTGTCACATAAATAGGTGTACTTG
TAGTGTGAGGTCAGCTAGTGGCAGAAAATATAAACAACAACAACATAGCCAGAGTAATCCCACAAGTG
GGGTATGGGGAGGGCGGTGTGTACGTAGTCTCACCCCTGTTAGAGTGGGTTAGAGTCCTACATTGGTT
GGGGAATGGATTGGTAGTCTGCTTATATTGACTTGGGCAATCCTCCCCTCATGAGCTAGCTTTTGGGGTT
GAGTTAGGCCCAGGTGTCATATCTGACTCCTAGTAACAACAAAAAAATACAATTTAAGAGTTTGGGACTG
ACTGCTGTCTTCTGATCTGTCCATTGTGATTTTCTGCTGAATTCTTGTTAAGAGTGCCCTTGTCTACTGGTT
TGCAGAACAATATTCTATCCACATTAATTGTGGAGGCAAAGAGGTAACCATCAAGAATGGTACAAAATA
CACAAACTATGAAGCTGATTTAGAACCCAGAGGTGCTTCTATGTACTATTCAAGACATAACTGGGCGTTT
AGCAGCACAGGAAATTTTATGGACAACGACGTTGAGTCTGATGTCTATATCAATACCAATGTCTCTGCCC
TGCAAAATGTCAAGGCCCCTGAACTTGAACTATACACTACTGCACGTATATCTCCTCTCTCCCTCACATAT
TATGGACAATGTCTAATGAATGGAAACTACACTGTCAAGCTCCATTTTGCGGAGATAATATTCACAAATG
ATACTTCATTCAACAGCCTTGGAGAGCGTATATTTGATGTCTACTTGCAGGTAAATTTCATGCTCTGGGG
GAAGGTGAAATGTGTGTGAATTTCATGTTTGCTGCCACCAGTGTATTTATTCAATCTTCTTGAGACAGGA
AAAGCTGGTGTTGAAGGATTTTAACATTGCAAAAGAGGCTGGTGGGCCTGGTAAGCCCATTGTGAAGA
CGTTCACTGTGAATGTGACAAGCCACACGCTGAAGATACATTTTTACTGGGCAGGAAGGGGGACTACAG

GAATTCCATTTAGAGGGGTTTATGGTCCTCTAATTTCAGCTATATCAGTTGTTAATAGTATGTTCCTTTAC

TCTCTTCAAGTTTATATCTGTCTTGCTTACAGTTGTATGCTCAACTCTTCTTTAATGAATGCCATAGGGATC

ATAGGACATTATTCTAATGTACTAGTCCTTAAAAAAACCTTTCCGAGACCAAATTACTCTACATACCAAGG

CTTAATAAGTTTTGTTAACCATCTTGATGTTATAAATTCTATTCCTACTGATTCATTATGCATCTAAAACCG

CTGTCAACATATTCTTTCAGGAAGTTTATGTGTATCAGAACCTAAGAAACTTCATATACCCTTAAATTTCA

TGATGCATTTCGAATACAGTGGGGAGACCACTTATAGTGTCCCCAGTTCAGTTCCTTAATCTTGTCTGGT

AGTGGAAGCATCAAGACTTATACTAAGACTTCAAAGGAAAATGTTTCAAATTGCATATGCAAGTCAATTA

AAGCTTGCTAAAATCATCACAAGTTTTGAGATGTCATATTATTTTTTCCTTTCCGTGCAGAGTTTCCCCCTC

CTCTGCCTGCTCGTCTGCCTGCTCATCTGCCTGCAGATCAAAAGAGTAAAATTAATGTTGGCATACTAGC

CGGCATAATAGCTGGATCAGTATTTCTTGTCCTTCTGATTATTGGTATCTTGTACAAGGGAGGCTGTCTA

GGCGAAAATGTATCTACTGATAGAGGTAATAACTACAAGTATTTTACTTTTGAAAGTTTAGTATCCATTTA

TTTGACCTATGAGTCAGCAATAATGTTCTCAAGTTAAATGCTAAACTACATAATTACTTTCTAGCCAGTAT

TTCAGTTCATACCAAATTATATCAGCTCAAATACTTGAAGTTATGCTTTACGTTCTATTGACTTGCTTGTTC

ATCTCATCAATCATTTATTCATTTTAGGCTTTCAAAAATCATTAATGGTCTGAAGCACTTGACCAAAGTTT

ATATGAACCTTGAAGAAACATAGTGTTATAGCCATTAGCTTCCACTGAGATACTTTTCCTCTTGAACAGA

ACTTAAAGGACTGGATCTACAAGCTGGACTATTTACTCTCAGACAGATCAAAGCCGCGACAAAGAATTTT

GACCCAGCAAACAAGATAGGGGAAGGTGGTTTTGGTTCAGTTTATAAGGTAATCATCTTTTCCTTGTCTA

TTTTAAATCAAACACATAACTATAAATGTCATTATATTAGCATGAGAAGGATCATAATGACATGGTGCAG

ATGCCTCTTAAGAGAGAAAATGAAAGACTAAAATCTTAAATTTCATAAACCTTCAAAGGAAGTGTGAAT

ATTCGCCTGTAATGAGGAGACTGTACAGGCAATGCTCTATTACCATATGTGTTTCTATAGCCCTGAGAAG

TTGGAGTAATTTACTGATCTTTTTCCATCAACTAAGATGCAGAGTTCTTATCTTCGCGACATAGGATTCAA

TCTATTATAATCTTACTTGCTGATCATAAGCTGCAGAAGTCTGAATAGAGTTTTGAATCATGACTTTTGTA

CACAGGGTCTATTATCTGATGGAACAGTAATTGCGGTGAAGCAACTTTCTGCAAAGTCAAAACAAGGAA

CTCGAGAATTTTTGAATGAAATAGGGATGATCTCTGCTGTACAACATCCAAATCTTGTAAAGCTTTATGG

ATGTTGCATTCAAGGAAACCAATTATTGCTAGTCTATGAGTACATGGAAAATAACTGTGTATCTCGTGTT

CTTTTTGGTAAATACTTCCTAACTCTGATCAATCAAAATATGTCTGCTGCTTATGCACTTGACATTGTTTAT

CTAACTGAGCTGGTATATACAACTTTTTCTTTTTGTTTGTCTCTCTCTTACAGGCAAAGGTCCAATTGGCA

AAATGAAATTAGACTGGTCTACTCGGAAAAAAATTTGCCTAGGAATTGCAAGAGGTTTAGCATATCTCCA

TGAGGAGTCGAGCCTGAAGATAGTCCACAGAGACATCAAGACCAGTAATATTTTGCTTGACAAGGACTT

CAATCCAAAAATATCTGATTTTGGCTTGGCAAAACTACATGAGGATGATACTACCCACATTAGTACCAGG
ATTGCAGGAACTGTGTAAGTATTATGAAACTAGTTCCTCTTGTTCCGATCCCATTAGCAACAACATTTGGT
TGATATGTTCCATAGAAAATGGCTCAACATTTAATCCACTCATTTATCTTGAAACACTCGTGGTACTGTTC
TCATCCGAAAGCAACTTTGCAGGTATAATCTGCCAAAAGTAAAGCAAGCCAAGAACTTATTACCTGCCTA
AAGAAACTTAACTTTTCTAGATATTAAACAGCCTTTGTAGAACAGTAACATAGAATTTTATTCGTCAATGG
TAAAACCACCTCTCTTTCCTTACCATATTTGTAACTCATTGGTTTGCTAATTATTAGCACCTCAACGTCTCA
CTTCAGTCATAATTTTACATTTTGCAATTCAACATAGCTATAGGTGAACTTTCAAGAAAATATTTTCTTGCT
ACTTCAAATTTTAAGAAATGTGAAGATTGTTTGTATCAGAATTCTCTGTAATTAGCAAGTAACTGTATTAA
TGATGAGGAAACAAAGATCTAATAAGTTGGAACATCTTTGGTATTTGGTGGTACAGGGGTTACATGGCT
CCAGAATATGCGATGCGAGGCTACTTAACTAGCAAAGCAGATATTTACAGCTATGGAGTTGTTGCATTG
GAGATTATTAGTGGGAAAAGCAATACAAATTATAGGCCAACGGAAGATTGTGTTTATCTTCTTGACTGG
GTAATTTTAATATTTCTCTCTACAACAATAAACCCAATGTAGTCCCAAAAGCGGGGAATTTCACTACTTCT
CTCTATTCGACCAAATATCTGGGCTTCCATCATCCTTAAATCACTTCCCCTTCTAGTTTCATCTCAACCAGA
GAACGTTTTATCAACATTCCGAGTAATGCTAATAGCATCTTATTAATCTTCCCCTGCATCAACTTGTACATT
TAGAGCCAAAGTACTTTAAAATTGGCTTTGGCAAATGAACATGTCATACTTTTTTAGTACATTAACTGTTC
TTCATTTTCTGTTTTGATTAGGCTTATGTTTTGCAAGAGCGCGGGAGTATATTGGAATTGGTTGATCCAG
ACTTGGGTTCTGACTACTCGTCACAAGAAGCAATTGTGTTACTGAATGTTGCCCTTTTATGCACTAATGCA
GCTCCAACACTTAGACCAATAATGTCTCAAGTGGTAAGCATGCTCCAAGGCCAAACTCTTGTTCAGGATG
TCCTTTCTGATCCAGGGATGTCAACAAGCGGTTCAGGATTCAGATCAACGAGAAGTCACTTCTGGCAAA
CTCAAAGCCTAACTGATGGTACGCTTACTGATTCCACTCTCTCAACTGGAAATGCGGAAGAAATTGGCAT
TCTTCAAAGAGTAGAGCCTAATGTATCTAACAGTTAAACAAAATGTCTGTTAACACAAGCAAGTGTATAT
ATGAACATCAAAATGTTTAATGAAGATTAAGTTTAGTCTTTACAAAACTGCTAACTGGTCCTCCTTTTATA
GTACTTCATTTGTTTATTTTCTAAGCCAACCTCATTGTTGCATAGTTCTATCAATTCCTTCTTTATTTTTC
>PGSC0003DMG400017645 LRR-RLKs
TGTCACAAAAAATTGTACAACTAGACTAGCTATAGACAAACACATCTGTACTCCATTATTTAATCTCATCAT
TTAAAAATCCCTTTTCTCTATGTATCATTACTCACTCTATTATAGATTCTTGAATTATTTCATTCCTCACTTC
TCTTTAATCACTTCAAAAAAACAAAAAAAGTGCTCACTCTTTTGTGAGCTCAAAAGAGTTTTTTGGGATTT
TGAGTCTTTTAGCTATGAAGGGTTTCTTGTTTCTTGGTTTGTTTTTGTTTTTTCTAGTTCCATTTTCATCATC
TCAGTTAGTTCCTGCAGAAAGTAGAATCCTTTTTCAAATTCAACAGTTTCTTGAAAATCCACCTGTTCTTC

AAGAATGGAATAAATGGACAAATTTTTGTTTTCTCCCACAATCTCCTTCTCTTGTTATCACTTGTTCTGGTA
ATCATATAACTGAGTTAACAATTGTTGGTAACAAAAAATCCCCTTTTGAGTCTTTAAAATCCTCTTCTCCA
CAAGCCCTTTCTGGGAAATTTTCAATTGATTCATTTTTCACTGTTGTTACTAAGCTTTCTAGTTTAAAAAAG
TTGTCTTTAGTTTCTCTTGGTTTGTGGGGTTCATTGCCTGCTAAGATTAGTAGGCTACATTCACTTGAGGT
ACTTAATATAACTTCAAATTTCATTCTTGGTGAGATTCCATCATCTATAGTGAATTTCAAGAACCTGAAAT
CTCTTGTTTTGGCTAGAAATTTGCTTAATGGAAGTGTACCAGATCTGAAAGGGTTAAAAATACTTGAAGA
GTTGGATTTGAGTGGTAATAATTTAGGACCAAAGTTTCCATCTTTAGGTGATAACAACAACAATCTTGTT
TCACTGAATTTGAGTAACAATATCTTTAGATCAGAAATACCTAATGGACTCAACAAGTTTACTCATTTGCA
AAATCTTGATTTGTCTTCTAACAAACTTGTTGGTCCAATGCCTGCTTTCCTGTTTTCACTTCCAGCAATTCA
GTCTATTAGTATTGCTAAGAATCAGCTTAGTGGTGCTCTTCCAGGAAGTGTGTCTTGTAGCAACAATCTA
AAATTTGTCGATTTTTCGAGTAATTTATTGATGGGAAAGTTGCCTGCTTGTCTTGGATCAAGTTCAAGTTC
AAGAAACAGGACAGTGATTAATGTTTGGAATTGTTTGTCAAGTACTAGTACTAAGTCTCAACATCCACAT
ACATTTTGTGAGAAACAAGCTATAGCTGTTAAGCCCCCTCCAAGAACTAGTGATGAGAAGGAACAATCC
ACAGTTAAGCTTGGTGTTGTCTTAGGACTCATTGCTGGAATTGTTGTAGTTGTGGGTGCAGTTGGTTTCC
TGATCTTTTTCATTGTCAAGAAAGTTGTGAGAAATAGAGATCAAGGATATAAAAATGACAGCTTTGCTTT
TGAGAAGAATTCAACCCTTTCCAAAACTGCTGATGGAGGTAAATTTCCTTAATGATTAGGGTCTATCGGA
AACAGTTTCTCTACGTGTCAAGGTAGGGGTAAGGTCTGTGTACACTCCCCCACTAGACCCCACACTGTGT
ATGTTGAATCTTGAATAGTGATAGGGGTCTATTGGAAACAACCTCTCTACCTCCCGAGGTAGGGGTAAG
GTCTGTGTACACTCTATCCTCTCCAAACCCCACTTGTGGAATTACGCGGGGTTTGTTGTTGAATCATGAAT
AGTGATAGTGCATCATTATGACAATTTTATTGATAACTTGATTTAATCAGGGAATGCAAGGAGGACAAT
GAGGATGGTATCATTAGGACTTCCACCATATCATGTTTTCACTTTAGAGGAAATGGAAGAAGCAACAAA
CAGTTTTGATCCAACTAATCTGGTTGGAGAAGGTTCCCAGGGTCAGGTAAAAATGTTGATTCATTATAAA
TAGCAAAAAAAATGATATTTATTACTATATCCTGTCCATATTATTCGTCATTTTGGACGCCGAACAATTCC
ATTCTAGTCAAATACATAATCTACATGAGTATACAAGTCCGTAGATACTCATATTGCTATACTTCTAGTCT
AGACCCAAAAAGTCACCCTAAAAGTCAACCTGAACAACACTTGACATTTTAGAAAAACTAAAGATAGTA
ATATGGATATGCTTTTCTTAAATCTTGGTGAATACAAACTTGTGTTTTGAACATTGGGATTGATGTTATTA
TTAATGACAGCTATATAGAGGTTGGCTTAGAGATGGATCAGTGGTCCTAGTGAAATGTCTGAAATTGAA
GCAGAAGCATTCACCTCAAATTCTGCAGCAGCACATGGAGATGATATCAAAGCTAAGGCATCGACATTT
AGTAAGTGTACTTGGACACTGTGTTGTTACTTACCAAGATCATCCAAATACAGCTAGCACTGTATTTATTG

TGCTTGAAAACGTCGTCAATGGATCACTAAAGGATCATCTTAGTGGTAAGCTATTACGAGTTGATTTCTC
TCATGGCCACTCAACTAATAGTTATTACCTCAAAAGTCACTTTCGAAAAGGTGACTTTTGAGATAATGAC
TATTAGTTGAGTTACTATCTAATAGTCATTACTTTCTTCAGTCGGAACGATGAAGGACTGAATTTTATATT
CCGGGATTCGTTCTTTGGCAGATTGGAGGAAAAGGGATGTATTGAAATGGCCACAAAGAATGGGAATA
ACGATGGGCATTGCAAAAGGAATTCAGTATTTGCACACTGGAGGAGTCACTGGAAATGATATAAAGCTT
GAGAATGTTTTGTTGGATGAAACTCTTACTGCTAGAATAAGCAGCTACAATATATCGTTGCCACCTAAGG
TAATGCGATTAAATCACTATACGTAAATCTTGCAGAAACTGTATTTTTGACCATCTAAATTTTTACAGGTT
GGTTCGGAAAGTCCTCTTGCTGGACCCGACCATTTTACCAGGTATTATTCTGCAGCTACATTCACCTTACT
TTCTCTATTTCGTGCTATCATTTCTGAATTGTCTGCTAATATAAACTTCATATTCAGCGCGAAAGAAGCAG
AAAAGGAAGACATTTATCAGTTGGGAGTTATTCTACTAGAAGTTATTATTGGTAGACCAATCAACTCTCG
AAGCGAAGCAGAGGACCTTAAGCTTCAGGTATCAATAACAATATCCTAATAACTCTCAAACGATAGAAA
TATATCATATCGTCACTCAACTAATAGTAATTATTTCAGAAAGTCACCTTTCTTCTTGATTCAGATGTTCTT
CTGAAATAATAACGATTTGTTGAGTGACCATATGAGAAATCAACTCGAGAAATAAGAGATATGCGATAA
AGAAATTAGAAGAATGCACACGAATACAAATGTCTTACATGATATGAAGCAATACACATGTTTACATATT
GCATAAGAGTCTAGTCTATTTACACAATCTATCTAACAACCTTGAACCAAATTGCCTTTTATGACATTCAG
GTGGAGACTGCCTTAGCGGAATCACCATCAAAGCTACGAGATTTAACAGATCCTTGTATACGAGGTACA
TTTGCATATGACTCATTGAAAACGACAGTCCAGATAGCAATAAACTGCCTTGAAAAAGAACCAAGCAGG
CGGCCATCAGTCGAAGATGTTCTCTGGCATATGCAATACTCAATTCAGGTTCAAGAAGGAACAACCAAC
AGTGGAAACCTCAGTGGGAACCAAAGTGGGAAACTTAGTGGAAAAAATAAGTGGAAATCTTAATAATAA
GTTGTACTGATAAGAGAGAACTGTCATTATATTGTTATATCTTGTTGTTTAGTCATTTGTATGCATCTTGT
ATAGCATAGTTCAGTAACTTACTGAATAAATGTTTTTGCGTTCTACATCACAAAAAAAATGTACCCAAGTT
TACAATACAAAGCAAATTTTCATGGAAGTTGAGAATCTCGGGGATCTCGCGAAAAACTTATCAACACCC
GGTGTTTACATCAACTCAATGAAGACATAAAATGTATCTTCATGTGCTTTGTTATGTCTAAGTCCTTCAAA
ATAAGTAACTAGCGGAAGATATAACTAAAGTAAATAACAATGCAGTCTCTACAGTCATGATATGATTCAA
TATCGTATCAGAGCAGGCAAAAATCATCGGTTCATATCTCACCACCAGAAATTTTCATGTGCGCTTGGCA
CATTATAAGACCTTTACACGTGAGGGGGCGTGTTGAGGATATAATAAAGTAAACACTAAAAGTATGTCT
CTCCAACAATTGAAACTTCTAGATGAAATGCTCACAACATACCTTTGTTGACTCAAAGGGTACTTCTTGAA
GTATAACTTCGCCTTTCCTATACCATTTTCAGCAGCCACGCCTGCCACGGTGGCTGCATGCAACAGCAGA
ATCTGCTGTTCATCGTTGGTGCTCCAAGAGCACCGACATCAATTACCAATTCCAGCCACAACAGCTGAAT

TGGCTGATCAGGCTGCTGCTTCTATCTCTGCATTCTCCAGCACCCAAAGAACAATAGCAGAGTAGTCAAG
TATTCCCAAGTATCTTTCTCTCCAGCCATTACTTTGTCGACACTACTAAAAAATCACTATTTTCTCCAAGAA
ATTTCTCACTGATATTTTGATTAAATCGGTGAGAAAAGAGTAATGTTTTCGTACAAAAAAACTAGAAAGT
TTACCACTATTTCATTGAGAATCTGTTTCCCACCATGCATATTCCCAGTGAACTGATTTGGTGGAAATGAG
TGAAAAACATGTTCTATAACACAAATTTCTCACTAATTCACCGTGGAAAAAATCTATTACTACTACTCATC
ATTAGTACTTCATCCGTTTCATCTTATGTTGAACTTGATTGATTAGGCGCGGCTAAGAGCAACATGGGAA
GTCTTAAAGTTCAATTTTTTCCAAATATAGAAAAAGACCCTTTTTATAACGTAC

>PGSC0003DMG400017690 LRR-RLKs

CACATGTTAGTTGGCATGTCTCATCTTAAGCGGCATGGTGGGCAAATTCTCAGAATAACTTGCTAATGAT
CTTTTTTTTTAATTTCAGGGAACTTTATAGTAATAACATAAGCGGAAGAATTCCAAATGAACTGGGGAAC
TTGACAGAGTTGGTTAGTTTGGATCTTTACCTGAACAACTTAAATGGTCCTATCCCTCCCTCATTGGGCAA
GCTTCAGAAACTACGCTTCCTGTACGAGTTATACATTCTATTTCCTTCCGGTTTTTTTCTTCTCTTTTTGTTT
CTTTTTCAGTTTGGTTATTTTTTTTCAATGTTTCAAATCTTTAGCATATATATCTGAAGTTTAGTTAAATCAG
AAATGATCACCTTCTATTTTTATTTTTATCAAGAAAGCAGCGTCTATGTTAAGGATCCGTTTGGTCATAGA
TTTCCCAAGTAAAATTTGGGAGAAAATTTGGCATATAGTGTTTGTCCATACAATTTGCCATTATTTGGCAA
ATAACCCAAATTCCCAATACTAGAAAAAACTAGTATTTGGGCCAAATCTCATTATTTGGGATCTTTTAAAA
ATTTAAATTCTACACCAAACTTTTATGTTTTACAAAAACACCCTCTATAGTAGTTGTTTGCGTTGTATTACA
TAATTTTTTACGTAAACACCAAACACCAAAGTAGTGATGAAATATTCAGTGAATATGAAGTTGATCATAT
GGTTATTGATGAAAATGATGAACAATTGGCTCAGGGTAACAAAGTCATGTGCTTTGTCTACTTGACGAT
GTATGGAATGATGCTTGTTGCACTCACTCTGAACTACCATATTGCTCCAATGTCATGGACACTGCTTGTTA
TTTGTTGCAACGAAGATCCAATTGACTTGTAATATAAACTTATGGTTAGTTTTGATAGTTTTTAAAACTTTT
ATAAATCATATTTCCTAAAAAAGTGAAATATGTTTCCCAAATACTATGGCCAAACACATAGTGAAATTTCA
CCCAAATTTTAACACAAATAATATTTGCCAAAAATATTTGGAAATCTATGGTCAAACGCTAGCTAAATCAT
GCAAATGGGGTTGTAGTTTCTAATTCTCTAAGCAAATCTGTATGATGTTCTGTAACTTTTCTGTTTATTAT
GTTTACATTGATGGGTTCTGTCCAAAAAAAATTGATGTGTGGAAGCATTATGAAAATCACTACTCCTGTC
AGCGAACAAACCTTTGTTGTTTGAGAGGTCAACAGGGAATATATCTGTATGATTCATTCTGTCTTTGCTCT
GTAGTGGAAAGTTAGTTGATATGCTTGGCAAACCTTGTTCTTGTTTCCCAAATACTGTATGGTTGCTGAG
TGAAGACCAGGTGACTGGACTGGACTCTTTTTTTGTACGACAAACAGAGATAAATAAGTAATAATATAA
AATGTTAAGAATAATATGAATCAACTTGAATAAAAATATATTCTAAAAGTTTCAATTATTATCTGATTAAC

AAAGTTGTGCTTGCTTGGTCTCTCTCGATATAGACTCACAGCCGTGCAGCTCAACTGAAAGTGACAATTC
TGAGATACTTCTCATCTTATCAGGAAAATGGCCTGTGGATTTAGTTCATTGCTAAAATACTTTATCTATGA
ACTGTGATTCTCAACCTGCAAACACTATCAGCAAGTTTTTTCTAGCATATTGGAATTAATCTTTCTGGAGT
ATCTAGTCCTATATATATAAATGTATTTGTCTTTGTCTACCTTTAGCACATGTACGCTACATTTAGCACATG
TACTGAGTAATGAGCTCTAGACCAGTATTCGCTTTGACGAACTTCTTTTCCTGTCTTGCTTACTCTTGATCA
ACCTTCCTTTTATCCTTTTTTTTTCTTTGCTGAATTGAAGGAGGCTCAATAATAACAGTTTGAATGAAGGT
ATTCCCGTGTCTCTAACCACCATTGTTGCACTTCAAGTACTGTAAGTATAAAGTTGTTCCTTTATTTATCAA
TGCTGTATGTTGTCTGTGTTGTCTAGTTCTTCTGGGTTACTTATTGTCATTGCCTTGCAATGCAGTGATCTC
TCAAACAACCATTTGACAGGACCAGTTCCAGTCAACGGTTCCTTTTCACTTTTTACTCCTATAAGGTGTGT
TGGAAGCATAAATTTGTAGTTCCTTCTGTTCCTGAAGTTACAATAGTGAATATCTAAAGATTGAGTACCT
ATATTTCAGTTTTGCTAATAATCAGTTGGAAGTTCCTCCAGTTTCTCCACCTCCTCCCCTTCCTCCTACGCC
CTCATCGTCATCTTCAGGTTTGTTGACATAATTTGTGTTTATGCTGTTATGAAATTGTGCTACCTCTCCTTT
TTCCCTATTCCAATTTAGTTTTCATGAAAATCCCTTAAGTTATTCACAATAAGGGGAGATGGCAATGGGA
AGAAAGGAAAGGCCTGGTTGCCTAGGGAAAGCTGAAGAGATGAGCCCCAGACTGGATTCCCTTATGAA
AGCACTTTCTTTATGAAAAAAACTACCATTACGTTAGGCACTATAAATTAGTTTATTTAGATCAATTATAC
AGGTATTGGGGTCTTGTGGTTATCCAGCATTCATGACTTTCCAAAATAAAAAATTCAAATAAGCTTGGAG
ATACTATCAACATCTTTTTCTTTGTTGGAAATTAGATTTACTGTAAACTCTGAAGTTCGAGTGATACGTGT
GTCAAAAGTACCATATATATTTTAGAATTGCCTGTATTTTAAAATTTAGGTCATACAAATAAAAAGTTAAC
TCAATCATCTTTTCAGTGGGCAACAGCGCAACTGGAGCTATCGCTGGAGGAGTTGCTGCAGGCGCTGCC
CTTCTATTTGCAGCTCCTGCAATTTTTATTGCTTGGTGGCGTCGGAGGAAACCGCAAGACCACTTCTTTGA
TGTTCCTGGTTTGGCAAAACACATTGAATCTGAGTTAATTGTACTTTCAACTTGGATGTGTTCTCACTCTT
TATAACTACTTTTGCAGCTGAGGAGGATCCAGAAGTTCATCTGGGACAACTCAAAAGGTTTTCCTTGCGT
GAACTACAAGTTGCGTCGGATAATTTTAGCAACAGAAATATACTCGGTAGAGGTGGATTTGGTAAGGTT
TATAAAGGCCGGTTAGCTGATGGCTCTTTAGTTGCAGTGAAAAGACTAAAAGAGGAACGTACTCAAGGT
GGAGAGTTACAGTTTCAGACAGAAGTAGAAATGATCAGCATGGCTGTACACCGAAACCTACTTCGTTTA
CGGGGATTTTGCATGACACCCACTGAGCGGGTGCTTGTTTATCCGTACATGGAGAATGGAAGTGTTGCA
TCACGTTTAAGAGGTATCTCTTTTTTAATTTTCATGTGCTTGCCTCTACTTTTTGCTCACCTTTAGGCAGGC
TCACAACAACCACAACAACAACATACCCACAAAGTGGCGTCTGGGGAGGGTCGAGCATATGCTGACCTT
TCCTCTACCTCAGATGTCAGGTAAAGAGGTTGTTTCCAATAGATTCTTGGCTCAAGACAAAAATAGTCTA

GAAAAAGACATAATTGAAGTACAAGAGAAAATAGATAGCAACAAAAACAACAGATAGTATCAAAACAG

TACTGCACAAAGTAATACTGTAATAGAACTACATAAAACATCATATATCAACAAAAATCGAAGAACAAG

AAACTACAAGCATAGTACTACAACTACTAGTAAGGGCAACAATATAACGCACTCCTACCTACTAGTATGG

ATGTACTTTTACCTACTAATCTTCTACCCTAATTTGTGTCCTCTACAACTTTCTATCTAAAGGTATGTCCTTG

GTAAGCTGCAATTACGCCATGCTGTGTCTAATCATCGCTCCCCAATACTTCTTCGGTCTACCTCTACCTTTC

CTAAATCCATCCATAGCCAACCTCTCTCACCTCCGCCCTTGGCATCCGTGCATCTTTTCATCACATGTCCAA

ACAATCTCAACCTCACTTTCCGCATCTTGTCTTCCACCAATGTCACTTCCACCCTGTTTTGGATATCCAAAT

CCTTAATCCTATCTCTCCTAGTAAACCCACACATCCATCGCAACATCCTTATCTCTGCCACTTTCATCTTCTA

AATGCTGGCCAACACTCTGACCTTACAACATAGCTGGTCTAACCACAGAAAAAAAGCCAATAGCTAATGT

TAATGACAAGCCTTTAGTCTTTTAACTTTTCTTTCATAAATTTGTAACAAATTTGAAAAAGTTACTGCAGA

AGTGTAAGCCAGTGGTGGAATCTTCACATCAATTTCATTGTGCTATTAGTTATTATAGTGATTGAATTTTT

TATGAGTTCTCATGATGGGTATGTATATTTAACAAGGTGTCAATGTTGTTTATGATTTTGCTTGTGTTCTA

TGGACCAATTTCACAAGTGGTGCTATCCTTGATATCTTACTGAATATAGAGACTGCAGTTCATGATAAAA

TTTATTTAATGAGCACATCTGAAAGTCAAAAAGAATTAAATGTCTTGAAAGAGAATACATATTTTTATTAT

TGTATGAAACTGGATAAGTAATGCCTCATGACTTGGTGTTTCATGATTTAGAGTGATATTGACCCAAAAA

GATCAGTTTTCTTTTTATAACTTATTCAAGCTTAAATAGGAACCTGTTTCCAGTTTGCTGAATAGCTTGAAT

TTATGCTCATGGGATAATTTACATTTTTCTAGAGAGGCCTGAATCAGAGCCCCCACTTGACTGGCCAAAA

AGGAAGCGTATTGCACTTGGATCTGCAAGAGGCCTTGCTTACTTGCATGATCATTGTGATCCTAAAATTA

TTCATCGTGACGTCAAAGCCGCAAATATCTTGTTGGATGAGGAGTTTGAAGCAGTTGTTGGGGATTTTG

GGTTAGCTAAACTCATGGACTACAAGGATACTCATGTTACCACTGCTGTACGTGGTACAATTGGGCATAT

TGCCCCTGAATATTTATCTACTGGTAAATCTTCTGAGAAAACTGATGTGTTTGGCTATGGGGTTATGCTTC

TAGAGCTCATAACTGGGCAAAGGGCTTTTGATCTTGCTCGACTTGCGAATGATGATGATGTCATGCTGCT

AGATTGGGTAAGCATTTATACTGTCCTGTATGCTTAAGTTATTTTGCTTCTGCTATCAATCAGCAATTTGG

ATTTGCTTAAAGATCAAGTCACTTCTGCTATCAGTGAACAGTCTTGGAATGTTCTGAAAAGATCAAGTCA

ACTCTCTCTTCTCTTCTATAAGTCAAAGTTTGATGTCTTTTTCTACTGTAGTGTTAGTTGTTGAACACTGAT

GTGCTCATCCTGAAGAAATGGTGTGGTACGAGACCTGCATCGTTGCTTATTATTTATTGTGGTTTTGTGG

TGTGAAAGTAACATTCGTTGCAGTTCCCTGTGAGTAATTTTTTTTAAAAAAGGCTTAGATGTTTCAGGAG

GATTTAACCATTCATTGCTAAAATTGCACGAGCATGTAGGTTATAATTAGAGTGGATTACTTTCATTATCT

TATTCTTGCAGGTGCTTCAACAGTTAGGTTGCCAAATTCGTCCCCTCAATTTGTGACTAATCTAGAAAATG

TGCATCTCCACTTCTCATTTCTGATGCTATCTGAAGATTAGAAAAATATACTTTTTTCATTTTCATTCAAAT
AAGCTAAGGTGTATCTGCAACTGCCATATTAATGTCACTTCAGTCATGTTGTTTTTGGGGGTAGATATAG
TATACTTTAGGACAGGTACTCATTCAGGAGCATCCTTGAGAAAGGCTAGTCTTATGTGGGATCGCCATCC
TGGAGAAGGAAAAGGAGATGGTCAAACTGTTAGGAAGTGACTATTAAAATTAAAAGCTTTTCTGAACCT
CTTGCGTCTCTGATGGATACAAAAACTGTAATAGCAACATGAACACTAATAGACAGCAATGCCACTAATG
TCAATCAGAAACACTTGGCTGTGTTTGAGCCTCATGCACTGATGTGCTGTTTTCTTAGTCTCCCTTCAGAT
CTTACTAGCTTTTCTAGTGACTGTGCTCTTAGAGATGGCACATATGGTTGCATGCATGGCTATTATTTGAC
TTATAACATTCCACTGGATATTCAGGTGAAGGGACTCCTGAAGGACAAGAAATATGAAACATTAGTTGA
TGCAGATCTTCAAGGTAATTACAATGAAGAAGAAGTGGAACAGCTTATTCAGGTAGCTCTACTTTGCAC
GCAGAGTACGCCTACGGAACGTCCAAAGATGTCAGAAGTTGTAAGAATGCTTGAAGGTGATGGCCTTG
CTGAGAGGTGGGAGGAATGGCAAAAGGAGGAGATGTTCCGGCAAGATTTCAACCATGTCCACCACCAC
CATACTGATTGGATAATAGCTGACTCCACTTCAAATATCCGACCGGATGAGTTGTCAGGGCCAAGATGA
TCTTTCATTTATTCTGCCATCAGCACATATAGGACCGACTCTTTTGAGGGAAAATCCTCTTTGTATCCTTGT
ATCTGTAATCGGTATATGTCATCTTCTTTTTTTTGGGTTAATTGGTATATGTCATCTTTGTGCATACCATTT
TCCTTCTTTTTTTGTTTCAGTCAGTTATATTGTATTTGATTATGTCCAAATGTGTATAGGTGATTGGGTCAT
TACAACAGGTCTGAACATGAAAATCAACTTATAGCAGGAATGGCTGTAGAAAGCATCTTTGCACCTTCTC
TTTTTCTAATGTATCAGATAGAGTGGCGTTTGTTAAAGAATATACACTTTGTAAACTTAAAGTAATCATAG
TTTCTTTCATGTTCAGTGACAGTTTGTCTCCTCCTCTTGATGGCTGAATTTTGTAGTATTATAG
>PGSC0003DMG400017713 LRR-RLKs
CAAAAACAATCTTTTAATTATTAGCAATGATGATAGTTCCCACAATTTTTAATTTCCTTCAGTTTATTACTA
TCTTATATTTTTTCACAGTTACATTTGCTACCACTGAGGAAGCAACTGCTCTTCTAAAATGGAAAGCAACT
TTCCAAAACCAGAACAATTCCTTATTGACTTCGTGGACGCTAAGCGGTCCTGCTGGTACCAACAGAATTG
GAGCGGCGAGTTCTAATGCGTGTACGGACTGGTATGGAGTTACATGTTCTAATGGTAGGGTAAACATGT
TGAATATGACAAATGCTGGTATCATTGGCACACTCTATGATTTTCCATTTTCTTCACTCCCTTTTCTTGATT
ATCTTGATCTTAGCATGAACCAACTCTCTGGCACTATCCCACCTGAAATAGGAAAGCTCACTAATCTTTTC
TATCTTGACTTGTCCATCAATCAGATTTCGGGCACAATTCCACCACAAATCAGCTCATTAACAAAGCTTGG
GACTCTCCACATCTTTGTCAACCAATTAAATGGTTCCATTCCGGAGGAAATAGGTCACCTAAGGTCTCTTA
CTGAGCTAGATTTGAGTTCTAACATTCTTAATGGTTCTATTCCTGTTTCTTTGGGGAATTTGAACAACTTG
TCTGTTTTGAGTCTGTATGAGAACCATCTTTCTGGTTCCATTCCAGCAGAAATAGGAAAACTTGTCAATCT

TGTTGAACTTTTACTTGATTCAAACAACTTAACTGGTCATATTCCTCCAGAAATCGGAGAACTTGTCAATC
TCGATAGAGTTTATCTTGATTCAAACCAATTAACTGGTCATATTCCTGCCGAAATAGGGAAGATGAAGTC
ACTTGAGGAGTTGAGCATAAACACCAACAATTTTTCTGGTCCAATTCCCAAAACTATAGGAGAATTAACT
GAGCTCCAACTCTTGTACCTTCATACCAACCAACTTTCTGGTCCCATTCCTAGTGAATTGGGGAATCTGAA
AAAACTGAATTATCTGCAGTTATCCACTAATCAACTTTCTGGTCCGATTCCCGATTCTTTCGGCAATTTGA
GAAACTTGAAAATTTTGTTTCTCCGTAACAACAAACTTTCTGGTTCTATTCCAAAAGAACTTGCATATTTG
GACAACTTGGTTGTGATGGAAATGGATGAAAATCAGTTTTCAGGCCATTTGCCTGAGAATCTTTGCCAA
GGTGGGAAACTTGTGAACTTCACGGTGAATAGTAACAAGCTAACAGGGCCAATACCAAGAAGCTTGAG
CAACTGCTCAAGTTTCGAAAGGGTTCGCTTTGATAACAACAGTTTTACTGGGAATTTATCTGAAGCTTTT
GGTATCTATCCGGAGCTGCAGTTCATTAATTTGAGCGAAAATGATTTTCATGGCGAACTAAGCAGCAACT
GGGGGAAATGCAAGAATTTGACTGATCTTCGGATAGCCAGAAATAGAATTGGTGGTCGCATACCACCA
GAGATTGGAAACCTCAAAGGGCTTCAAGGACTAGATCTTTCATCCAATCATTTGGTTGGGAAGATTCCTA
GGGAATTTGGAAAATTGACCTCTCTGGTTAATCTTCTTTTGCAAAACAACCAAATTTCTGGCAATATTCCT
ATGGAACTTGGGTCATTGACAAAGTTAGATTACCTTGATCTTTCAGACAATAGATTGAATGGTTCGATCC
CAACGTTTATAGGAGATTACCATCACCTGTTTCACTTGAACCTGAGCAACAACAAATTTGGCCAAAATAT
TCCGAAGGAGATAGGGGGTATAACTCAACTTAATGTACTTGATTTGAGCCATAATGTTCTGGTTGGAGA
AATACCCCCTCAGTTAACCAATTTGAAGGTCTTGGAAAGCTTAAATATTTCCCACAACGGCCTTTCTGGCC
ACATTCCTGAAGAATTTGAAAGTTTGACTGGTTTGCAGGATGTTGTCTTGTCATACAATGAGTTGGAAGG
TCCAATACCTAATAATAAAGCTTTCACAAATGCCTCATTGCAAGGTAATAAAGCTCTTTGTGGCAATGTA
ACAGGTTTCGTGCCATGCAACATACCATCTTCTATGGTGAAGAAGCACTCAATGGCGAAGGGACTTAAA
CTCATCCTCATCACTGTACTTCCTATTCTGGGAGCATTAGTGCTTCTTTGTGCTTTCGCTGGTGCTCTCTTT
ATGCGTGACCAAAGAAGAAGAGTTGGAGACATTGAAAGACGGGATTCCATAGACAAAGATGATGGTCT
GCTTTCAATATCCTCATTACATGGAAATTCATTATACTGGGATATCTTAAAAGCCACAGAGGAGTTTGAT
GCAACATTTTGCATTGGGAAAGGAGGGTTCGGAAGTGTTTACAAGGTAAATCTTCCATCATTGGGAAAT
GTAGCTGTGAAGCGACTTCATTCTTCATTTGAGATTAAGCATCGCAAGAGCTTCATGAATGAGGTAAGG
GCATTGACAGGGATTAAGCATCGGAACATTGTGAGACTCTATGGCTTTTGTTCGAATGCACAACACTCGT
TCTTGGTTTACGAGTATGTGGAGAGGGGGAGTTTGTCTAGTATTTTGAGCAACGAAGTAGAGTCTAAGA
AATTGGATTGGCTTACAAGGGTGAATATCATCAAAGGTATTGCTTATGCTTTATCCTACATGCACCACGA
TTGCTCACCCCCGATTGTTCATCGGGACATATCAAGCAGTAATGTTTTGCTTGACTCCGAGTTTGAAGCTC

GTGTTTCTGATTTTGGCATAGCTAAGATTCTCAAGCCAGACTCATCCAATTGTACTGCACTTGCAGGCAC
ATATGGCTATGTAGCACCAGGTAAGATCTAACTTCTTATCCTTATTTTAGTTCATGAACTATATCTGCACA
CTCTATATTGTTTCTATCTCATTTGCAACTTTATTGTTTCAGAGCTTGCCTATACATTGAAGGTGACGGAA
ATGTGTGACGTCTACAGCTTTGGAGTATTAGCATTGGAGGTAATCAAAGGAAAGCATCTTGGGGAATAC
CTTACTCTTCTAGCAAATCCATCAACAAGAGATGTGCAGCTAAGTGATTTGCTAGATGAACGCCTTCCAC
ATCCTGAGGATGAAGTAAAAGAGTTTTTGGTTTTCATCGTCAAGCTAGCAATCTCGTGTTTGCTTGAAAA
TCCAAAATCAAGGCCAACGATGCACTTCATCTCTCATATGTTATCAATGGATCCACCCAATCATCAGATTC
CATGAAAGTGAGATTCCTCGAAATGTAAGGTGCCCTGGTATGGAAAAGATTATGCTCAGCACCAAGTGG
TTGTGAAGTAACAAGTATGTTGATCAGAACTTTCACAGCAAAATATTATTAAGATTAGCCTTTCTGTATTG
TTGAGTTGCTTATATAGATTACTTATTTCAATTTGAAGGTTTCAGAGCCTTTGTACTAGAATATATACAAA
TTACAGTAATATACTGATGTTAAGTAATAAAAGCA
>PGSC0003DMG400017864 LRR-RLKs
ATGAATATGAAGAAGTTGGAAAGAAGAAATACTATTGTTATTGTTATGTTAGTTGTTGCATTTGATCACT
TTACTGCTTCTTTGGCTAACATTGCCACTGATGAAGCTGCTCTTCTTGCCTTCAAATCTCACATTTCTTCTT
CTTCTGATCCCAACAACAACAACGTAGTCGTCTTGCAAACCAACTGGTCTTCTTCAATTCCATCATCAGTT
TGCACCTGGATAGGGATCACTTGCAACGGGCGCCATAGAGTCACTGCTTTAGACATTTCTAGCATGCAA
CTTCATGGAACCATTCCTCCACACCTTGGAAATCTCTCATTTCTCTTATCCCTTGACATTAGTAACAACACT
TTCCATGGAGATCTCCCCGAAGAGTTGTCTCATTTGCGGAGGTTGAAATTGATTGATGTTACAAGAAACA
ATTTTAGTGGTACCATTCCATCATTCTTAAGTTTGTTACCTAACCTTCGATTCGTTTACCTTTCAAACAACC
AATATTCAGGGGAAATTCCATCTTCCCTTTCCAATCTAACAAATCTTCAAGAGTTAAGAATACAGAGGAA
TTTTCTTCAAGGAAAAATCCCTCCTGAAATTGGCAATCTTCGTTACTTGACTTTTTTAGACCTGCAAGGTA
ATAGACTTACTGGCTCTATACCGTCGTCAATCTTCAACATGACTTCCTTGACAACACTTGCTATAATACAC
AATCGTCTTGTCGGTAAACTTCCTGTAGATATTTGTGACAATCTACCAAATCTTGAAGTACTTTTACTCTC
AACAAACAACCTGGATGGCCTAATACCACCAAATTTACAGAAATGCTCAAAGCTACAACTGCTGACATTG
TCTGGCAATGAGTTCACTGGACCTATACCAAGAGAACTTGGGAACTTAACTATGCTCACAGTCTTGCATC
TTGGAGAAAACCATTTAGAAGGTACGTCATATGAATTACTCCCTCTGTTTCAATTTGTTTATCTAGTTTTG
ACTTGGCACTGGTTTAAGGAAGTAAAAAGGACTTTTGAATCTTATTGTCTGTAAAATGTACCAAAATACC
CAATAATCTTAGAGTCTTAAACAGGTCGGGTGAAAAGTTGAAATTAAGAAGAAAAGACGAACTGACTAA
AAAGGGAAGTAAGACAAACAAATTGAAACGGAGGAAGTATATCTTTATGCTTTTGTGTTTATTTTAAGAT

TTCTCAAAAGCTAAGCAATATCAATATGTACTAATGTTTATGCATTTTACTCTAATTGACAGGAGAGTTAC
CGGTCGAGATAGGTAACCTACAAAACCTCCAAGTACTTGGGTTAAGAAATAATAAGTTAAATGGTACTA
TTCCTGCAGAAATTTTCAACATTTTAGCATTGCAGATATTGACAATGTATGGAAATCAGTTATCAGGTAG
TTTACCTTCAGATTTAGGCGTTGGAACGCCTAGTCTTGAAGAAGTTTACCTTGGATCAAATGAACTCAGT
GGACGAATTGCTCCTACCATCTCGAATTCTTCAAAGCTAACACTTCTAGACCTTGCAGACAACAAGTTTAC
AGGTCCAATTCCTGATTCACTTGGTTCATTAGAGTTTCTTGAAGTCCTATTCTTGGGAGGTAACAATTTCA
TTAATGAGCCATCTTCTTCTGAGTTAAGATTCGTCTCGTCCCTGACTAATTGTAGATATTTAAGAGAAGTG
GTCATTGAAGATAATCCTTTGAATGGTTTTCTTCCATCCTCTATTGGAAACTTTTCTGATTCTTTTCGGATG
TTTGTTGCACGTAGAACCAAACTGAAGGGCACAATCCCGGAAGAAATTGGAAACCTGAGTAGCTTAGGT
GTGCTAGCTCTGTCTCATAATGACTTGACTGGTTCGATACCTGAGCAGTTGAGAAGCATGAAGAATCTTC
AAGAATTTTACTTAGAAAATAACAGCCTAAGTGGAACAATACCTGATGATATCTGCAGTTTACGAAATCT
TGGAGCTTTAAAATTGACTGGAAATCAAATATCTGGTTCAATTCCAGCATGTTTAGGAAATGTTTCTACTT
TAAGATATCTTCATTTAGCTTTCAACAGATTGACTTCCACCTTACCTGAAACCCTGTGGAGCCTTCAAGAT
CTCTTGGAACTCAATGCATCAGCAAATCTGTTTAGCGGGCACATACCTCCTGAGGTTGGTAATCTAAAGG
CTGTGTCACTAATTGATCTGTCAAGAAATGATTTTTCTGGTAATATTCCTAGTACTATAGGGGCTCTTGAA
AAGTTGATTAGTCTTTCCATGGCACACAATAAATTGGAAGGGCCTATTCCGAGTTCATTCGGGAAAATG
GTGGGCTTGGAATTCTTGGATTTTTCTTACAACAATCTTACTGGTGAAATTCCAAAGTCACTTGAAACACT
CTCACATCTCAATTACTTTAACATCTCTTTTAACAAATTAAGAGGAGAAATTCCTAGTAGTGGTCCATTTG
CAAACTTCACCAGTCAATCTTTCATTTCCAATTCTGCACTCTGTGGTGCTCCTCGCTTTAATGTGTCGCCAT
GCCTCATCAAATCCACCAAGAAATCAAGGAGACACAGAGTTCTAACAAGTCTGTATATCGTATTAGGTAT
TGGATCAATGATTCTCACATTGGTTCTAGGATATGTTTTGTTAAGGTGGCAAAAGAGAAGGAAGAATTC
AGGTCAAACAGATGCATCTTTGGTTAAAAGACATGAAAGAATCTCTTATTATGAACTTCAGCAAGCAACG
GAAGGATTCAGCGAGAGCAATTTGCTTGGTACCGGAAGTTTCAGTATGGTATACAAAGGGATACTTAAA
GATGGGAATCTTTTGGCAGCTAAGGTATTCAATGTGCAATTGGAGGGAGCATTCAAGAGCTTTGAAACA
GAATGTGAGATACTACGCAACCTTCGCCATAGAAATCTAACAAGAGTGATCACTAGTTGTTCCAACCCAG
ACTTCAAAGCCTTAGTATTGGAATACATGCCCAATGGAACACTTGATAAGTGGCTACACTCTCATGACTT
GTTCTTAGACATGTTGAAGAGGTTGGACATTATGATAGATGTGGCATCGGCATTAGATTATCTCCACAAC
GGGTATCCAACACCCGTGGTGCATTGTGACTTGAAGCCAAGCAACGTCTTGCTCGATCAAGACATGGTT
GGTCATGTGAGTGATTTTGGCATTTCAAAATTGCTAGGTGATGGGGAGACTTTTGTACAAACAAGGACT

ATTGCAACCATCGGATACATTGCTCCAGGTAATAGAAATTTCAAACTTGTCATCCTTTAGATTTTCACATT
TCAAACTTTATCTAACAAGATTTCTCCCATGATTGCAGAGTATGGACAGGACGGAATAGTATCCAAATGT
TGTGATGTTTATAGTTTCGGTATCATGATGATGGAAACATTTACAGGTATGAGACCAAGTGATGAAATGT
TTACGGGAGATTTGAGCCTAAGATGTTGGATTAATGATTCTTTTCCTAATGGAGTTGTGGATGGCAATTT
GTTAAGGCCAGAGGAAGAACATATTAAAGAGAAAATGCAATGTGTATTGTCTATCATGGAATTGGCTTT
GAGTTGCACTTTAGTGTCACCTGATGCAAGAGTTAACATTGAAGATGCTCTTTCAGCCCTCCAGAAAATA
AGATATCAGTTTGTCAATAGTTGTGCAAGGACTTAA
>PGSC0003DMG400017943 LRR-RLKs
AAGAATTAATAGTGGGAATTTCCACTTCGGAGGGTTGCCAATTGTGTGTGCCTTATGAATGACAGCCAA
TGAGTAGTTGCAGAAGGTGAATGTTATAGTTAAGCTAGCATAAATGGTTGAAAATGATAAGCAAGAAG
TAGTTGGAGAGGTAACCTCGAGATCTGTGCTAGGATAGCTTAGATTCCGTTTGCTTCACGAAGAAGCTC
AAACATTATTCTTTATTTCTTTTGAAAAGAAAAGTTTTTTCCTTCCTACTGCTTTGTTTTTTCCTACCGGTAC
CACCAGCTGCTGCTGCAGAGGGAAGTAAAAAGAATAATCTGATTTTGCAAAGTAAAAAAAATGCCGTAC
GTTGGAGTAGCAAGTTTAGAGGGGAATTGTAAGGCACAAGACTTCGACCTTTTTTCTCAATTCTCAACCG
TTATATATTAAGCTCTCACCCATTTTCTTCATTGCAGTAGAGTTCACCATTTTTCAAGGTATACTCTTTCCC
CTATTATTTCAACTCTCTACTACCTTTGCTTACATTAGTGTTGATACTCCATTTTTCAAGGCACACCCCTCT
GGCTCTGGCCATAGATTGTGTGTGGTAAGTGTTGGTTCATCAAATTTACTCATTATTTCAACTTCATTCAA
ACATGTTTTCACTCATGTCCTAACACAACTAAACTTTCAAATTTTGGATATGACAACCAATTTCTCAACTTA
AGACATTTAAACTACTTTCTTTGTCTAATGCTATGATGGAATGTTGAAATTAGGAAATAGAGGGTGGTAA
TGCAGAAAAATCAAATCTTTTTCATACATGTCCTGTCTGCTCGGTATGGGATGCATCTAATAAGTTCATAG
GGCTTCAGAATTTAGGATGTTTATCTCTAAACAAGGTTTTTACTCTTATTATGAATCCAACTGCTAAGCTT
CTTTTCGCCTTGGTTGACTACATATGAGAACGCCACAAAGTGTCCTTTGCTCTACGGGGAATTAATTGTCT
CTTCATTTCATACTGTGATGGGGATATTTTCCATATATCTAACTTCTCTGCCACATTTTTGTTTACCATTGTT
ACTAGGTGATGCAAATGGATGACATACAAAGTTGATATCTTATGGCAACTTGAGTTGACTTGGCAAGTC
CAGGAGTAAATAGATTTAAATGATGAAGCTACACCCAATAATAGATCTACTTCCTCTTTCTCTCTTCCTAT
TTTTGCTCCTCTTCTCAAATGTTATTGCTGACCTTAGCTCAGATAGACAAGCACTACTTGACTTTGCTTCTG
CAGTACCTCATCTTCGAAACTTCAAGTGGAACACTAACTCTTCCATTTGCACATGGCATGGCGTAAGTTG
CAGCTCAGATGGCACTCGTGTAGTTGCACTTCGGCTTCCTGGTATTGGACTTTATGGTCCTATTCCAGAC
AATACCATAGGAAGACTGGATGCACTAACAACCCTCAGCCTTCATTCCAATGCCCTCACTGGAAATCTTC

CTTCAGACATCACCTCTCTTCCGTCCCTCCGTTTCATATTTATACAACAAAACAAATTTTCTGGTGAAATAC
CTTCTTCTCTATCTCTACAGCTTAACTTCATTGATCTCTCTTTCAACTCCTTCTCAGGGGAAATTCCAACAA
CAATTCAAAATCTGACACATCTTACTGGTTTAAACCTACAAAACAACTCCCTCACAGGATCCATTCCTAAT
GTAAACCTACCAAGGCTTACGCAATTGAATATGAGTAATAATCAACTCAATGGTTCAATTCCACCATCCCT
TGCAAAGTTTTCTGCTTCTTCATTTCAAGGAAATTCTCTATTATGTGGACAACCCTTGACTCAATGCCCTTC
TTTTGCGCCTTCACCGTCTCCATTCCCTTCAATTCCACCTTCTCCTTTGAGTCTAACACCACCATCTCGTTCT
CCGAGCGTCCTGCCAGCCTCACCAACAATTCCTGAAAACCATAAAGGCAAGAAAAGCTTGAGTACACGG
GTTATCATTGGCATTGTTGCAGGAGGTGTTGGAGGGATCCTATGTCTAGCTGTGTTGATTTTCTTGTGTT
GTATGAAGCGATATTATACTAAGAGAGGTGTACAGCAAAGAAAAGACTTTAATGGAGGAGGAAGTCCG
AAGCAAACAGAGGACTTCAGTAGTGGAGTACAAGCAGCTGAAAAGAACAAATTGGTTTTCTTTGAGGG
TTGTTCTTTCAATTTTGACCTTGAAGATTTGTTGAGAGCCTCAGCTGAGGTTTTGGGTAAAGGGAGCTAT
GGAACAACCTACAAGGCCATCTTGGAGGAGGGAACGACTGTTGTTGTGAAACGGCTGAAGGAAGTTGT
TGTTGGGAAACGAGAGTTTGACCAACAGATGGAGACAATTGGCACTGTGGATCAGCATCGAAATGTTG
TTGCTCTTCGTGCTTATTACTTTTCCAAAGATGAAAAACTTCTTGTCTATGATCATGTACCGGCAGGCAGT
TTATCCACTCGAATGCATGGTAACATTTCTTCCTTCTTTTGTCCTCCCATTCTTTTAGCTGTCATACCCATCC
AGGTGCTTGGGAATATCCTTAGTTACTAAGACTGGCAAGAAAATCAGCTGTAAAAGTTGCACGAGATCA
TAGATTCCGAATGTGAAATGAAGTACCTATATTCTTTAGAAAAGCCCCTAGACAAGCATTATAAATAAAC
ACTCCTGAAATAGGCATGCAAGTTTGTCTTGAAGACTTTATAACATGTGATATTGGTTTCATGCACTCATA
GTTCTCCATATTATTTTGAAGCAAGATGCAACTTGCAAGTTGTTGAATGTCAGATTAGTTGTTCGGTGCTA
TACATAATTTTCAATCTAACTGTTTATACAAGAAAGTTCTTTTCTTTCATTGCTCCTTGTTTATTGATTATAG
GCAACAAGGACTTGGGAAGAACACTGGATTGGGAATCTAGATTGAGGATTGCCCATGGAGCTGCAAGT
GGTATTGCCCATATCCATGCTGTTTCTGGTGGCAAATTAATTCATGGAAATATCAAGTCATCCAATGTGCT
TCTCACCCACGATAATAATGGATGCATCTCAGATGTTGGTCTTACACCTTTAATGGGTTTTCCTACTATCC
CATCAAAGAGTGCAGGATATAGAGCACCCGAGGTGATAGAGACCAAGAAATGTACTCAGAAATCCGAT
GTTTACAGCTTTGGTGTTCTGCTTCTTGAGCTCCTTACTGGGAAAGCACCAGTGCAGCCACCTGGCCATG
ATGAGGTGGTAGACTTACCAAGATGGGTGCAGTCTGTTGTGAGGGAGGAATGGACTGCTGAGGTATTT
GATGTTGAGCTCATCAAGTTTCAAAATATCGAAGACGAAATGGTGCAGATGCTGCAGATTGCAATGACT
TGTGTGGCAAACGTGCCTGAAACAAGGCCTGACATGAGTCAAGTTGTCCAGATGATCGAAGATATCCAA
CAAATTGATTCTGGAAATAGGCCATCATCTGAAGATAACAAGTCTAGGAGCCCAACTAGTCCAACGCCA

TGATAACTCATGCATTTCTTACTACTCAATTCTATTACCAAATGTGTATTTGAACGTCTTTTCTTGTTTTCAA

GTATAATTCAGACCTTTTAGATACTATCATTCTTCATCACTGCCTATATCTAAAAATTCAATCCTATTATGT

CTAGCTCCACTTTTATTTT

>PGSC0003DMG400018024 LRR-RLKs

GGTTTCACTACGGACGAACAATCTCAAGGGATCCTTACCACGCGAGATTGGCAACTTAACCAAGATGCA

AATTTTAATTCTAGACGAGAATAGGTTTACTGGTACGTATACATTCAATTTAAGTGATAGCAATCTGAAC

AGTTTAAGAGGTAAACTCATTCTTTCTTCAGGTGAAATACCCAAAGAGATAAGAAATCTCGTTGAGTTGG

AGAAGCTTGGTCTTGGATCTAATAGTTTTAGTGGTTCACTTCCAATGGAGATCTTCAACATATCACGATT

GAGACATATTGCTCTTACAAACAATAATCTATCAGGAACTCTACCACCAAACATAGGTTCTACCTTACCCA

ACATTGAAGGACTTTATCTGCATGGCTTAACCAATCTTGTTGGGACTATTCCTCATTCTATCTCCAATTGTT

CAAAGCTTACTATTCTAGAGCTTTCACAAAACAAAACTCACAGGATTGATTCCCAATTCTCTTGGATATTT

GACTCATCTAAACATCTTAAACTTGCGGAGAAAACAATTTAACCAGTGATTCATTTTTAAGCTTCCTCACT

TCCTTAACCAATTGCACAAGATTAACAGTTGTTTCTCTATCTTTCAACCCTCTAAATGCCATGCTTCCGGTC

TCCGTAGGGAACTTCTCCAAATCTCTTCTAACCTTTTATGCCGATGCTTGCAACATTAACGGCAAGATTCC

AAATGAAGTTGGAAACTTAAGCAACTTATTAAACCTTGATCTTTCTGATAATAACTTGATTGGATCAATTC

CTACATCAATTCACAACTTGAGAAGCCTTCAGCGCTTCAACTTGAGTTACAACAAGCTTACGGGATTTATT

GGAGATAATTTATGTAAATTGCAACATTTGGGAGAAATTTACTTGGGACAAAATCAACTTTCAGGATCTA

TTCCTAATTGTTTAGGGAATGTTACTTCCCTTAGATTGATATATCTTACTTCCAATAAATTGAGTTCCAATA

TACCAACAAGCTTAGGAAATCTTAAAGATCTAGTGGTTCTTGACTTATCGTCAAACAACATGGTTGGTTC

TTTACCTCTGGAAATTGGAAATCTAAAGGCTGCAATATATATGGATCTGTCAATGAATCAATTCACAAAT

GGAATTCCTAGAGAAATTGGAGGATTGCAAAATCTGGTATATCTTTATTTGGAACACAACAAGTTGCAA

GGATCTATACCTGACTCAATCAGCAACATGGTAAGTTTGGAATTCCTAGACATTTCACATAATAATATATC

GGGAACTATTCCCATGTCTTTGGAGAAACTACAATACCTCAAGTATTTCAATGTTTCTGACAACAAATTGT

ATGGTGAAATACCCTTGGGGGGTCCTTTCAAGAACCTCTCGAGTCAGTTTTTCAGCGACAATGAAGCATT

GTGTGGTTCTTCAAGATTTAGTGTCCCGCGATGCCCCACTTCATCAAAGCACAGATCAAATAGGAAACAA

ATGTTTGCTCTATTTCTTATGCTGGGAATTGCACTTGTATTTGTTCCTATAGCCTTTCTGTTCATATGGATA

AGGTATAGAAGAGGTAAAAGAGCTCCTCAACAAGCTGATTCATTGTGTATTGTAACAACAGAAAGAATT

TCATACTATGAACTGCTCCAAGCAACTGATACGCTTGGCGAGAGTAATCTGATTGGTTCTGGAAGTTTTG

GCTCTGTCTACAAAGGCGTTCTCAGAAGTGGAACTGCCATTGCAGTTAAAGTGTTCAATCTGCAACTGGA

AGCAGCATTCAAGAGTTTCGATACAGAATGTGAAGTTTTGCGTAGCCTTCGCCATAGGAATCTCGTGAA
AGTCATCACTAGTTGTTCCAACCTTGATTTTAAGGCTTTAGTGCTCGAGTATATGCCTAATGGAAGTCTTG
ACAAGTATTGTATTCGCACAACTACTTCCTAGACATCAGGCAGAGACTAAGCATTATGATAGATGT
>PGSC0003DMG400018171 LRR-RLKs
TAAACAAATCCGAAAAAAACACCCATCTAAAATTCCTCCCCACTCTCTCTTACATAATGGCCTAGCTGTTC
TTGGATCCGGAGATTCTGGCAACTTTTTCAAATTTTTATATGTAACCTACATAAAATCCATTAGCCCACAA
GACATGATTTGGTTTTCCTCTCTTTGTCTCTCCTCCCTAGGCTTAAACCTCTACACCAAAACCAAGAACAA
GGCTTCCTTTCTCTGCTTAATCTTGAAGTTTGTAGCAGCAGTCACATATTTGTTCTAATCTAATGCCTGTTT
ATTCATGGACCCTCTTTTTCTTTTTCTCCTTCTTTCTCTTTACTACCTCCTCTGCTCTCAACCCTCAAGGTCA
GGCTCTTCTTTCATGGAAGACAAGCTTAAATGGATCATTAGATGTGTTGAGTAATTGGGATCCTACTGAT
GAAACTCCATGTGGTTGGTTTGGTCTTAGTTGCAACTTCAATAAGGAAGTTGTTGAATTGGAATTGAAGT
ATGTGGATTTGCTTGGGATTGTTCCTTCTAATTTTAGCTCATTGGTGTCATTGAACAAGCTTGTTTTGTCA
GGGACTAATCTTACTGGTGTAATTCCAAAAGAGATTGGGATGCTTCAGGGACTTAAGTTCTTGGACTTG
AGTGACAATGCATTGACAGGTGAAATTCCAAGTGAAATTTTTCACTTGCCAAAGTTGGAGCAACTTCATA
TCAATTCAAACCGGCTGGTCGGATCGATCCCAGAGGACATTGGTAACCTCACAAGCTTGGTGTGGCTTA
TATTCTATGATAATCAGCTGAGTGGTGGAATTCCAAGTAGTATTGGGAATTTGAAAAGGCTGGAGATTA
TTAGAGGAGGTGGAAACAAGAATCTTGAAGGTCCATTGCCTCAAGAAATTGGCAACTGTAGTAATTTGG
TTATGTTAGGATTGGCAGAAACCAGCATTTCTGGTTTTCTACCTTCTTCTCTTGGCCAGCTGAAAAGACTC
GAAACGCTTGCTGTTTACACCTCCTTGCTCTCTGGACAAATCCCACCGGAGCTCGGGGATTGCTCCAAGC
TCCAAAACATTTATCTTTATGAAAATTCACTCACTGGTTCAATCCCAGCTCGGCTTGGTAACCTCAAGAAC
CTTCAAAACCTCCTTTTGTGGCAGAATAATTTGGTTGGAACAATCCCTCCCGAGCTTGGGAACTGTCAAC
AATTACAAGTCATTGACATTTCAATGAATTCATTAACCGGAAGCATTCCGGAATCATTTGGGAGATTGAA
TTCAATGCAAGAGTTACAGCTGAGTGTTAATCAGATTTCAGGTCGGATTCCAGCACAAATTGGCAACTGC
ACTGGTCTTACTCACATTGAGCTCGACAACAATGAGATAACCGGTTCCATACCATCGGAATTCGGAAACT
TGTCGAACTTAACTTTGCTGTTCTTGTGGCAAAATCGTCTTGAAGGGAAAATCCCTTCTTCAATTTCCTCG
TGCCATAATCTCGAGGCTATTGACTTGTCGCAGAATGCATTGACTGGTTCGATTCCTAAAGAGATATTCG
ATCTTCAGAAGCTGAACAAGCTACTCCTACTGTCTAATAATCTCTCTGGTCCCATTCCACCAGAGATAGG
GAACTGTTCATCGCTGATTCGACTTCGGGCAAATGATAACAAGCTGACCGGATCACTGCCACCGGAGAT
TGGGAAATTGAAGAACTTGAATTTCCTTGATGTCGGATCCAATCACCTCACCGGGATCATTCCACCGGAA

TTATCTGGATGCCGGAATCTGACATTTCTAGACTTGCATTCCAACTCAATCAGTGGCAATTTACCGGAGA
ATTTGAATCAGCTTGGAATCCTCCAGTTCATTGATGTTTCTGATAATTTAATTGAAGGCACTTTGAGTCCG
AGTTTCGGCTCACTGACTTCACTCACCAAACTTGTTCTGGGTAAGAACAGGTTTTCTGGTCCTATTCCAAC
TCAACTCGGTTCCTGCATGAAGCTTCAGTTGATTGACTTGAGTGGTAACCAACTCTCCGGCGAGATTCCG
GCGAGTGTTGGTAAAATCCCTGGTCTAGAAATTGCACTGAACCTCAGCTGGAATCAGCTTTCCGGTGAA
ATTCCGGCAGAGTTCGCCGCATTGGACAAGCTTGGAGTGCTTGATCTTTCTCATAATCAGCTCTCGGGGG
ACCTCCATTTTCTCGCGGACCTACAAAATCTCGTGGTTCTGAATGTCTCGCACAATAATTTATCGGGGCAC
GTGCCTGACACCTCATTCTTCTCTAAGCTCCCACTCAGTGTACTTGCTGGCAATCCAGATCTATGCTTTCCC
GGCAACCAGTGCTCCGCCGACAAGGGCGGCGGTGTCCGTCGTACCAAAGCGGCGAGGGTGGCGATGG
TGGTGTTGCTTTGCGCAGCTTGCGCTCTCCTTGTGGCAGCTCTCTACATTATCCTCAGCGGCAAAATACG
GAACCGCAAGGCCCACGATTACGATCTAGATGGTGACAATGACGTTGAACTTGGCCCACCATGGGAGG
TCACAGTATACCAAAAACTCGACTTATCTATAACTGACGTCGCAAAATGTTTAACCGTTGGTAACGTCCTC
GGCCGTGGTCGGTCCGGAGTTGTGTATAAAGTGAATATTCCGTCAGGATTGACGATCGCTGTGAAAAGA
TTCCGAGCATCGGACAAGCACTCGATGTCGGCATTCTCGTCGGAGATTGCCACGCTGGCACGAATTCGA
CATCGGAACATTGTTAGATTACTTGGATGGGCAGCAAATAGAAAGACTAAGCTATTGTTCTATGATTATT
TACCAAATGGTACATTAGGTTCATTTTTACATGAAGGTTTTGGAGGGTTAATTGAGTGGGAAACCAGATT
CAAGATTGCATTAGGGGTGGCTGAGGGATTAGCTTACTTGCACCACGACTGCGTTCCACCCATCCTCCAC
CGCGATGTCAAGGCTCAGAACATTTTGTTAGGTGATCGTTACGAGCCGTGTTTAGCTGATTTTGGACTTG
CTAGACTCATGGAGGAGGAAAATTCTTCAGTTACAGCAAATCCTCAGTTTGCTGGATCTTATGGCTACTT
TGCCCCAGGTAAATATTTCTCTTCTTCCTAGTTCAAGTTCATTTCTTTCGAAAATCGACTAGTTATGATTCA
TTTATTACTTTAGTTATTAGGGGAATTCTCCTCTAATGCAATTATGTGAGGTTTCATTTATCACTTAGTTCA
AGTCCATGGTGGAGCTAGAGGGTGGAAGGGAGTTCATTTGAACCCTCTTCGTCACATAATCACACACAT
ATATAAGAACTATTTTTTCAATATATATATATATATGGAAATTGGAAGGAAGTAATCTCAAAATACGCTTGA
TATCAATACAGACTTAGGTAAAGATAGTACAAATGGAAGAAAAAGATCCATGTAGGTGATAGCTTAATG
TTAAGGATATTTTAAAGACTCGTATGAAACTTTAATCATGATTATTATTAGTATTTTATTTTTTTACTGATG
ATATGATGTGAGCTTAAATGAAATTAGGATTCATATAGCCAACCCCAAGTTTGCCTTTGGATTTTTTCCGT
TTTTTCCTTTATAGAAAAAAGAAGAACTGAATGCGAGGTAGCAGAAGAGAACAGATATAAAGCACTGCA
AACAGAGAGCAAGAAGGAGAATTCAGTTTAAGTTGCTTCCATACGGATGGAATTGTGTCACTATTTTGA
CAATATATGCATGTGAAGCCATGTGCTAGCTTTAACTATGTGCAAAACCAAGTTTGTCATGACATACTTG

GCAGCTACAGTGCTATTTCCCCATCTTTTTGCCTGTGTATGGCCCCCCACTAAAGATACTTAGGATGGGG
AATACTCTAAAAAAACCATAACCAAGTTAGTGGTCACACTTTCACAGCCAAGCATGGACACATTGCTGCT
GTCTCCATACCCCTTTGGCCATTGCTTGTTGTCCGATTGCGATATGTGAAAAATAATCGCCCAACAAATGC
CTTTGTTATGTTTCAAAAAGCATCAAGATCAAATAGGTGATAAATATCTGGCATGAAAAGGACTAAACTC
TGTTCTATTGTTTTGTCTTTTGCAGAGTACGCTTGTATGCTAAAGATTACAGAGAAGAGCGATGTTTTTAG
CTTTGGAGTTGTGTTGCTAGAAATCATTACTGGGAAAAAGCCAGCCGACCCATCCTTCCCTGATGGCCAA
CATGTTATACAATGGGTTCGCGACCACCTAAAGAGCAAGAAAGATCCAGTTGACGTAATTGATCCAAGG
TTACAAGGACATCCTGATACACAAATTCAGGAAATGCTTCAAGCCCTTGGAATAGCCTTGTTGTGTACTA
GTAACCGGGCTGAAGATCGTCCAACAATGAAAGATGTGGTGGCATTACTCAAGGAGATTATTCATGAGC
ATGCAACAGGAAATGAAGCTAAAAAGACATTGAACAACTCCTCAAAAATGTCCGATACTTCATCATTTTC
CTACTCGTCTTCATCTGTCACTCCAGCTCAGTTGCTACGCCTCCAAGGGTCTTTTAATTGCTCTCTTACTCA
CTCATCCTCCTCCGTTAGCTATAGCACCAGCAACCAATAATAATGTAGCGATAGTCATGATAGATCTGTT
ATTCTTTTGAACTAGATAGTATTAGCAACCGTAAAGAGCTAGGTAGAGGGAACCTTATTGTCAATTTAAT
TGTTAAGCAAATGAAAATGAAATCCATTGTACAGCAAAGTCAAAATTTTCTTTATCTCCC
>PGSC0003DMG400018253 LRR-RLKs
TCATTTCTGGAGCTCTTTTATTCACTCTGTTTGGTTTTTCATCATTAATGGGCAGGGTTTCTTGAATATTTG
GAGATTATAGGATGGAGGGGTGTTCTTAAATAGTTGAAAGAATTGATTTTTATGCAGTTGGTTGGTGCT
GGTGTTTGTTTTCAGTAAGATTAGCTACAAAAGATCCTAAAGAATTGATTTTTATGCAGTTGGTTGTGCTT
TTTTTCAGTATGAGGAGTCGTAAAAGATGTCAAGAATATTTTTCACTACTGGGGTGATGAAATTTTTTGT
CTTTATCATAATACTTCTGAGTTGTGGTTTTTTTGTGGTGGTGTCAAATGCAAGAACACTGCCTGAAAATG
ATCAAGTAGAGAGGTTATTAGACTTCAAGAAGTCCTCTGTTGAATCTGATCCAAATGGATTCTTGAATGA
ATGGACTTCATCTTCTTCAAGTCCTTGCACTTGGAATGGGATTTCATGTTCAAATGGTCAAGTTGTTGAGC
TTAATCTGTCTAGTGTTGGGCTTGGTGGTCCACTTCACCTCACTGATCTCATGGCTTTGCCTACTTTGCTA
AGAGTTAAGTTTAATGGCAACCATTTCTATGGCGATCTTTCCTCAATAGCTAGTTCTTGTGGCTTTGAGTT
TCTTGATTTATCAGCTAATAGTTTTAGAGCCTTTGTTGAAGTCTTGTGATAAAATTAAGTACCTAAATGTC
TCTGGGAATTCAATTCAAGGGGTTGTTCTAAAGTTTGGACCTTCACTGTTGCAGCTTGACTTATCAAGTA
ACACCATTTCAGATTTTGGCATTTTAAGTTATGCACTGTCCAATTGCCAGAATTTGAATCTGCTTAATTTTT
CCTCAAATAACTTAATTGGAGAACTCAATGATCTTGATTTTGGGACTTGTCAAAATCTCACTGTCCTAAAT
TTGTCTTTCAACAACCTTACCTCAACTGAGTTTCCAGCCTCTTTGGCAAATTGCCAGAGCCTCAATACACT

GAACATTGGCCATAATTCGATCCGAATGGAGATTCCTAGCAAGTTATTGGTGAAGTTGAAGAGCTTGAA
GCAATTGGTGCTAGCCCACAATCAGTTTTTTGATAAAATTCCATCAGAATTGGGGCAGAGTTGCAGCACA
CTTGAGGAGGTTGATCTTTCTGGAAACCGGCTTACTGGTGAACTTCCTTCAACTTTTAAATTGTGCTCCTC
ACTTTTCAGTCTCAATCTTGGTAACAATGAGCTATCGGGTGATTTCTTACATACTGTTATCAGTTCATTGA
CAAATCTTAGGTATCTTTACTTGCCATTCAATAACATAACTGGTCATGTACCAAGGTCTTTGGTTAACTGT
ACAAAACTTCAGGTGCTCGACCTCAGTTCCAACGCATTGATTGGGAATGTACCTTTTGAGTTGTGTTTAG
CAGCCTCGGGGTTTCCACTGGAGACGATGTTGTTAGCCAGTAATTATCTTACTGGAACTGTGCCTAAACA
GCTTGAACATTGTAGGAACCTCAGGAAGACTGATCTCAGCTTCAATTACCTGACTGGTTCGATCCCATTG
GAGATTTGGACCCTTCCAAATCTTTCGGAATTGGTAATGTGGGCTAATAATCTCACAGGTGAAATTCCTG
AGGGCATTTGCATCAATGGAGGAAACCTTCAAACACTCATTCTCAATAACAATTTCATCTCAGGGGCACT
TCCACAGTCCATTTCCAATTGTACCAACTTGGTTTGGGTTTCGTTGTCCAGCAATCAACTTTCTGGAGAGA
TACCACAAGGAATTGGGAATCTTGCAAATCTTGCTATCCTTCAGTTGGGTAATAACTTACTTACTGGACC
GATCCCTCGGGGATTAGGTAAGTGCAGGAACTTGATATGGCTAGATTTGAATAGCAATGCTCTAACAGG
TTTGATCCCTCTTGAACTTGCTGATCAAGCTGGCCATGTTAATCCGGGAAAGGTTTCTGAAAAACAGTTT
GCTTTTGTGAGAAATGAGGGTGGAACTGAATGCAAGAGTGCTGGTGGACTGATTGAGTTTGAGGGTAT
CCATGAAGAGAGGCTTGAGATTTTGTCTATGGTTCACTTGTGTCCATCAACTAGGATCTATTCTGGCAGG
ACGATGTACACCTTCACGAGCAATGGGAGCATGATTTACCTTGATCTTTCCTACAATTCTTTATCAGGAAC
TATTCCTGATGAACTTCCTTCAAGTTCTAAATTTGGGACACAACAATTTAACTGGAACCATTCCATTCATC
TTTGGAGGTCTAAAGATAGTTGGAATTGTTGATCTGTCACATAACAACCTACTGGGATTCATTTCACCCTC
GTTAGCAAGCCTTTCATTTCTTAGTGATCTTGATGTTTCAAACAACAACCTATCGGGAATGATTCCTTCTG
GTGGACAGTTGGCTACATTTTCAGCTTCAAGATCCGAAAACAATTTGGGCCTTTGTGGCGTCTCCCTTCCT
CCTTGTGGCCCAGGCAATGAACACCATTCATCCAGTATTTACCATAACAAGCCTACAACTATAGGAATGG
TGGTTCGTATTATGGTATCGTTTGTATCTATTACTATTATTATCCCACTTGTTATCTGAAATATAAGAATAA
CTGCACCACAAAGTCTAATTGAATTACCCATTTCGATAAGATAATATATCTTCCAAAGACCCCTAAATAAA
>PGSC0003DMG400018263 LRR-RLKs
GAGATATTATCCTCTTGCTGTTTAGAGAACAGTTTAATCCTCAAAACAAACCAAACACTTGTGGTTTCTCT
TTCTTCCCATTACTCTTCTTTTCTCTTTCTTTTTAGTACACATATTGGCCCCTAAGAAGAAACTAAGGTTGC
ATGGTGGTGGATTTAAACAATGGTCCAAACAAGAACAATCCAACTGATCATCTTTTGTCTGCTTATTATTT
CTATGCAAGTTTTGGTGCAGCTGGGAGCTTCAACAAATTCTGGTGACTGTAAGTCCTACTTATCACATCT

CTTTTGTTGTTTTTTTAACTGTATAATTATGAATTTAACTTATATACACCATATCGCGAATAAAAAATAAAG

TTATACTAGTACCTAGCACAGTTTAACATGTTATATCGTAAGTTACTTATTGTAAATAGTTCTTGTTTTAGA

TGATCTGAGTTATGAAAGCAAATTGTTTTCATTTCTTTTCTTTTTTTTTTAGTAGGGAATATATTAGTGTCC

ACTATTTTAACTCCAATTTATTTTGATCCCTTTTTCGAGTTCATTACTTAAAAGGTCATTTCAATTATTGAAC

ATAGTTATGTTTTTCGTTGTTTTGTATCAGAAAAGTCAAATATGATTTTCTTCATCAATTTCATTCCGTATG

TTCTGAATTTTTTTAAAAATAATTAGTGTATGGTATTTTTGTTCAAAAATAAAATGATTGGTGCAGATGTT

GTGTTAGAGCCACTGAAGGAAGAGTGGGAAAACATACCTCCAAGTTGGGATGGGTCTGATCCTTGTAA

CGATCCATGGGAAGGCATCGACTGCAACAATTCACGTGTTATTTCTATGTAAGCTTTTTTTTTTTGTGATT

AAATTAAAATTGTTAAAAGAGAATCATATTAATCGGAAATAATAACAATATTACCACTCTTTAAATTATTC

CTAAATTTATGAATAAAATGTTGAAATTAAGAAATAAAAACACAAGTGCACTCTGTCAAAAATTCCATTT

TTCCTTAGCTCGACAACGTCACATGTTTAATTCAATGTTCACCCACCTCATTTATGCCAGCTAAATAATATC

TTCGATTTTTAAAAAATAAAATAAAATTTAAACACTAGAATAATTAAGGAACTATTTCCAAAAAAACAGC

AAAATATTTTTTTTCTTAAAAAATCTTAATTAGTAAGAAAGTACAAAAATATGTAACTGTTACTGTATTTA

CTCTTCCATTTTGGTCAGTTCATGTGTATATATAATAATAAAACTAAAATGCTTATTATCTAATGAAACTA

GGTTAAAATTAAATCATATTAATTTTGTTAAATGACATTGCTAATTGTAATTTGAGGTACTAATTAATGAT

TGTGAATTTCAGAAAATTATCAAGCATTAATCTGAAAGGGGAGCTATCTGGAGATATCGAAGGCTTGTC

TGAATTGCAGATATTGTATGTCTTCTACTCATCATAATTGAAAGTGTTAAAATTTGGAGAAAAAAAATTG

TCTTAATTATGTTCGCTCTGTTTCAATTTATTTGTCTGATTTGAATTTGACACATAATTAATGAAAGTAAAT

AAGATTTTTGAATCTTGTGTGGAATATGACCATTTTTGTTTTTGTGCTCGCCAAAGTGTTCCTAAAGGATT

CATTACAAAAAATAATAGTTTAATGATTTTATTTGGTGCGATAAAATGAAAGTTGAGTGATCATCTGTGA

AATTAATCCTGTTCTTGAAGCACCATTTTGCTTGTGATCATGCATTGCATGCACACAAATATTTTTGACTG

TTGAAAAAGTTTTAAATTTCTGTTCTGATTATCTGTTTCCTTAATAGGGATCTATCATACAACAAAGGCTT

GACAGGTTCACTCCCTCAATCAATTGGAAATTTGAAGAGTTTATCAATTCTGTAAGGGTATTATTACATG

ATAATGATTAAATGTTGTTAAATTCACCAGCTGATTTCATTAGAGTAATTGCTCCGAGCTGCCTCGCAGG

ATCCTTGTTGGTTGTGGGTTTTCTGGACTGATACCAAACACAATCGGATCCCTTAGCCAGCTGGAATATC

TGTAAGGATATCAATTTCTTCTGTTTGCAACTTTTTATGCTTAACGAATTCAAGATATTGAGTATTTGAAG

AACTTTTAGTCCTTTATGTTTTGTATAAAATTGGAAATTTGGCATGAGATGAGTTGGATGGAGTAACTTG

GTCAGTGAGGATTTATATAGCCGACCCCAACTTGTTTGGGGACTAACGCTTAGTTATTTTTGTTGTTGTA

GTAATTCTTGTATCCCTTTACGTTGTCAAACTGTGAAGTCTTAGAGCTTCAGGTCTCTAGATATGGTATAC

TAGATTGAAATATTTTTCCTGTTTCTTCTACTGGGCTACATTTCTAAAATACTATCTGAATCATGCAGATCT
TTGAATTTAAACAACTTTATCGGACCGATACCAGCTTCTGTTGGTAATTTATCAAAGTTACATTGGCTGGA
CTTAGCTGATAATAAGCTCAGTGGACCTCTTCCAGTTTCACCTGGTCTAGACATGCTTGTTCACACAAAGC
ATTTGTGAGTAATACTGATTACTGTCAGTTTCTTAAGTAACACCTTCGTCTCTCATTTTCATCTGATATACC
ACTTACCTCGCAGTCACCTGGGGGGAAATCAGTTCTCAGGTGAGATTCCAGATCAGCTATTCAGCTCAAA
TATGACTCTCAAACATTTGTAAGTTCCACTGAACTGGAAGTGTATATATTTTTGAGAATTATTTGATGGAT
CAACTTCATTATTGTCCCACTCCAACGCTATGGTGCTAGCTCTGACACTTGTTGCAAACTACACCCGTTTG
GCTAATGGTTTCTTCTGTTTGGGTCTTGTAATTGCACAGGCTGCTTGACCAAAACCAGCTTACTGGAAAA
ATACCCCCTTCATTAGGACTCGTGCAGACTCTGGAGGTGGTGTAAGTATTTCCAAAATCCATCATGCTCTT
AAAGAATTCTAATAAGTGTCCATCTTTCTTGCTTTTGATTACCTGGTGAAGTTTTATTAACTAGATACCCAT
GTCCACTGTAGTCGTCTTGACAGGAATTCATTAGACGGATCGATTCCATCAACCCTCAAAAATCTCGCAC
TTATGAGTGAACTGTGAGTGGACAAGCATTATGCTAATATAAAAAATCCTTTTTATGAGTGTATCAGACC
ATCTATAAATTTTCCGTATCACTTGCAGCTTCTTATCAAACAATAAATTCACTGGCCCCCTGCCCAATCTTG
CTGGCATGAATGCCCTCTACTACTTGTAAGTTCCTTGAAACTCTTTTCTGCTAAGAAAATGCAGCAATCTT
GATATTGTGATAATTACAGATTATGTCTTTCGGTTACTTTAATTTGATTTTGTTGCCTATGGTCATGTTTTG
CAGAGATATGAGCAATAATACATTCAGCTCAGCTGATTTTCCGCAGTGGTTTTCAACTTTGCAGTCATTG
ACGACTTTGTATCTATCTCTCTCTCGCTTTCTCTTTTTCTCTTAACATGCATGTGTGTATCTGTCCCTGAGTT
CACGTGCCTCAGCAGAACTCACATGCGTCTGAGTGTATATGTTATCCTAATGAAGTGGTTCATAGAATTT
GTTATGCACCTCATTTGTATTTGTTCTGCTCATAGGGTAATGGAGAACACTCAACTTCAAGGAGAAATTC
CGCCAACCCTCTTCAGTCTTTTTCAGTTGCAGACTGTGTGAGTGTTCTTTTACTTTTAGTACATGGTTCATG
TCATGCAACTATTCTCATTTTCGCTGTGTGCTAGTCTTGTACAAAGTTTGAAAGGTAGTACAAAGTGACCT
AGTATATAATTCTATGTTTTGCATTTCGATTTTACGACAATGAGGTGGTTATTTATGACAGCAACTTGAGG
GGAAACAAGATCAATGGAACCCTGAATATTGCATCCAACTATAGCAGTCAATTAAAACTGATTGATTTGC
AGAACAATTCCATCGATGCATATACAGTAAGACCTGGATTTCCTTTTCAAATAATGTAAGTCCTCAAGCC
ACTTTTTCTCCACTTTAGCAATGAGAAAGAAATAATGGATCATTCATGTTAGGACATTTGATTCGTCTGAA
TACAATTTAGTCGTTTAACAATCTCAGTTTTCTACTTGGTCACTTGGCAGACTTATGCATAATCCAGTTTGT
TATGAAGAAAGATCAGAAGACTATTGTGGCATTTCCCAAACGAACTTTGTATATTCAACTCCACAAGAAA
ATTGCCTTAAAACACAATGCAGTTCTGATCAAATTCTTAGCCCTACCTGTAAATGTGCTTATCCGTACACA
GGCGACTTATTCTTCAGAGCTCCTTCCTTTTCTGACTTGACAAACACAAGCATTTATGAGTCACTTCAGAA

GTCTATGCTATCTTCTTTTAGCCCAAATCAGGTGCCAGTAGATTCAGTTTCCTTGAGTAATCCAAAAAAGA
ACTCAGAGTACTACCTTGTGTTGCACCTGCAAGTTTTTCCATTTGGCCAAGATCATTTCAACCGTACAGGG
ATTGCCACAATTGGATTTGCACTTAGCAACCAGACTTACAAACCCCCACCAAAGTTTGGACCATTCTTTTT
CAGTGGTGAAAGCTACAAATACTTCGAAGGTGATTGGTCATTTCATTTCATTGATATGTAAATGTATTTCC
TATGCACAAGTTTCATCTGATAGCCGTACTATTAGTCGTGGTATTACTCTTGTAGTTTATTGCCCTTTGAG
TTAGGTTATCGTCTATAGTTTTTTTACACTTCGATATATTGTTTTCAGTTGGATCAAGAGGAACTCACAAG
TCAATCACTAAAGGAATTATAATTGGAGCAGCTGCTGGTGGTGCTGTCCTTTTAATTTTATCAGTAATAAT
AGGAGTTTTAGTTTGCCAAAAGAAAAGAGATCAAGAAGCTATTAGAAAGAGTAACCCTTTCGGTGTGTT
CTGTTACTGTTCTTTAGTGTTGCTTCTAACTTTTTATTTCGCTGATGGTTATCGTCTTTTTTATCTGTCCAGC
TACATGGGATTCTATAAAAGACAGCGGTGGTGTTCCACAGTTAAAAGGTGTCAAATGTTTCACATTTGAA
GAGTTGAAAAAATACACCAATAATTTTTCAGAAAGCAATTACATTGGATCTGGTGGTTATGGGAAGGTT
GGTTTCTATTGACTTTTGATAATACAATGTTATATTATGATGACTGGTTAAAAAGCTGTTGCTTTAATTCT
ATCCATACTATCTGCACCATCTTTCGGGCAAGCTAAAGTTAGTTGATCACTTAAAACTTCTCGTGAACTTA
CAGGTATACAAAGGCACACTTCCAGATGGACAACTTATTGCAATTAAAAGAGCAGAACAAGCATCTAAG
CAAGGCGCCCTCGAGTTTAAAACTGAGATTGAGATTCTTTCAAGGTTTCATCACAAAAATGTTGTCAGCC
TTGTAGGATTTTGTTTCAGGCAAGGTGAACAGATGCTGGTCTATGAGTTTATACCAAATGGATCCTTGAA
AGAGAGTCTTTCAGGTATTTTGAACTCTCATCGACTGTTGTTTTGAAAATCAGCTGTAGGTTGGAATGTC
TGGGCATTGATAAGTTAGTGTTAACTTCTGCAGGAAAGTCCGGGATTAAGTTAGATTGGAAGAGGAGA
CTTCGGATAGCCCTTGGAGCAGCCAGAGGTTTGCAATATCTTCATGATCATGTTGACCCTCCTATCATCCA
CAGAGATATCAAATCGAATAACATCTTGCTGGATGAGCGCTTGACTGCAAAAGTTGCTGATTTTGGTCTT
TCCAAGACGATGTATGAGCCAGACAAGGGTTACGTCAGTACTCAGGTTAAAGGAACAATGGTAAGTCCA
GACTTTACAAATGTTGTCATATGGTAGCATTTCTCAGTTTCTATATTTCTTAAGTCACTGGAAACTCCTGTC
CTTTAATAGTTATAGACTGGTGATCGTCTTGGTTCAATTCTTAAAAGTTAACACTGAAGTTGCGTCCTGAT
CAATTACACATTGTGCTGAACTTATGTATGTAAGTTCATACAACGAAAGGAGTTCATTGGTTAAACTGCT
TAAAAAGACTAAAACAACAGTTGATTTGTAATTTTCGCAGAGTAGAAAACTTGAAACCTGAACACTTGAT
ATCAACATTGTTTGAAGAATCTGTAACAGAACATTTAATTAATACGAGCTGCTTTGTACATTTATCGGCTT
AGGGCTACATGGATCCAGAGTACTACACAACACAACAGTTGACTGAGAAGAGTGATGTCTATAGCTTCG
GAGTTCTGCTGCTAGAGCTTATAACAGCACGGAGTCCTATTGTGAGAGGAAAATACATTGTGAAGGAAT
TGAGGCAAGCGATAGACAAGTCTAAAGACATGTACAACATTGATGAGTTTGTTGATCCTGCTATCCCCTC

AAACATGACACCTATAAGCTTCAGGAAGTTTGTGGATCTAGCTTTCACATGCCTCGAAGATGCAGGAGC
TAATAGGCCAACAATGGCTGAGGTAGTGAAAGAGATTGAGAATATCATGGAAATAGATGGATTGAACA
GTTGTGCTGAATCCAGATCAACTTCTGTGAGCCACGAAGGAACCAGCGCAACCTTTGATCATCCTTACAG
TGAGGAAAGCCTAAATCGTTACAATGGGGTCACCAATTCGCGATAAACACCAACTCTTCTCCTGGAACCA
GGTACATTGTCATAGAAATGGAAAGTGATTCAAGCAACTGTTGTGCTTTTGTGTTAGCTGTGTACATTCT
GTGAACCTCTGTGTGCGGCGTAAACAGCCTTGTATGTTTCAACTGTTTGCTTCTGATAATCCAGGAGCTA
GACTCCGTGGATTCAAAATGAGTGATTTTATTGTACGTATACATTTTAATTTTGATTTATTTTTACCTATGT
ATACATAATACGAATCAAAAAGTAATGCTTCAATTGAACCTACACAGAACCACCCTAGATCAGGAA
>PGSC0003DMG400018264 LRR-RLKs
ATGGTAGTTTCTATGGCAATGATGCTGCGAATTTTCGTCAGTTTTCTGGTTGTTTTGATCCAAGTTCTGTC
AGTAGCAGCACGAACTAATCCTGTTGATTGTAAGTTGCTTTTGTTCTTCTCTATCAAATGTTTCTTAACCA
GTGTACAGAGATATTTGATTTATCTGTGAGTACTGGTGTTCAAATCTTAGTTCCTTTTTGTAATCATATGA
AGAACTTGTCTCTTAATTGCAATAATCTTGAGGGAACATTAAATATTTCCTGTTCTCTTCTAGATATGGTC
AATTAAGTGTTCTAGTGACGAACTGATTCAATATTTTATACAGCTGCTGCTTTAAAGTCTCTCAAGGATTC
TTGGTATGGTCCTTCCAATTGGACAGGAGAAGATCCCTGTGGGAGTAGTTGGGAAGGAGTTGGATGCA
GAAATTCACGAGTGATTTCTATGTAAGAATTCGGTGTGTATCCTTTTGTCAACTGCTTAAAATTACAGAA
ATCATATGGTTGATGCCATCTTTCTCAATTTCAGAACACTGTCAAGTATGGATTTGAACGGTCAGCTATCT
GGAGACGTCCAAGGATTATCTGAATTGGAGACTTTGTAGGTCTTCCTTTCACATTTCGTGTTTCTACTTCA
CTCCCTTTTTTCTTTTGACGGAGCAATACTTTGTCAGTTCTAATTATGGACTGCTCCTTATAGGGATCTATC
ATACAACAAAGATTTGACCGGATATCTTTCTCAGTCTATTGGAAAATTGACAAAGTTATCAAATTTGTAA
GTGAATGTGAATAATCATCACTCGAGTATTCACTTACCAGCTAATCTCGTGATAATATGCTGATGTTTTCT
TGATTTCATGACAGGATCCTTGTTGGTTGTGGATTTTCTGGACCAATACCAGACTCCATAGGATCTCTGA
CCCAGCTGTTTTTTCTGTAAGCATTATGTGGCACTCTTTGTACTTTGAATGTGCGACCAGTACGTGACATA
TTTTACCAGAATGCAAGGGTCATACACTATCTGCTTAAAATTGTTAACGTAATGTGTGCTTTTGGTGCTAG
CTGTACTCAAATTTTGGTCCATCTCTGGGACAGGCAATGCACTTCTAAACAGAGAAATCAGTTCATGAGG
AGTTCAATCCATACTGCTATTCTTGTGTTTTATACGTTATCAAAAGTTGAACTGTTGTGTTTACCATATCTA
TCTGTATATGATTTCTTTCAACCCTTCTTCTGGTATATGCTCTAAAACAGCCCCATTCTGTATTATGCAGAT
CTTTGAACTCTAACAAATTTACTGGAGGAATACCTGCTACCATTGGCTATCTGACAGAGCTTTATTGGCTC
GATTTATCTGACAATCAGATAGAAGGAAACATTCCAGTCTCTAGTGGGAGCAACCCTGGTCTTGATATGC

TTGTTAACACTAAACACTTGTACGCATCTCCTTTTTCTTGATGTTGTTTCGTCTCTCAACTGCATTTTCTAA
GATTATTTACATTTCTTCAGTCACTTTGGGAAGAATCAGCTATCCGGTGAAATTCCAGCTCGTCTTTTCCA
TTCTAAAATGACTCTGATACATCTGTAAGTTCCATCGAATTGGAGAATGGAGTGAATGAATAATTTTGTC
CGTAAATATTGGGGACCTCACTTGTAGTTGAGCTTGCCTTTTGATATTCTTGTTAGTGCATGTTTTTACTTC
TGAGAGTAGTCATGTGAAAGAAGCTTGATAATTATATCACAAACAACTTTCCTTTACGGTTTCCTCATGCT
TGTATATTCCAACTTGTAGGCTAGTTGAGAACAACAGGTTTACCGGGGTAATCCCAGATACATTAGGATT
TGTCCAGACGATGGAAGTGCTGTAAGTATTGCTATCTTGTGCTTTCCCTTTATCATCTAGTAAAGTTGCTT
TCTGAATTGTTTCTGGTATTGCAATAGTCGTCTTGACAGGAATTCATTCAGTGGATCTGTTCCACAAAACC
TCAACAATCTCACACATGTCAATGAACTGTAAGTAGAAAATGAAACAATTTTCTGAACCTACTGTAGGTC
CTCTTGATTTTTTGAAGTAGTATATTTTGACCATGTATTACAGCGACATATCAAACAACAATTTGAATGGC
CTTTTGCCGAATCTCACTGGCATGAATGTCCTCAACTACTTGTAAGTTGCTTATCCTAAGTTTTGTGTCTTT
ATCCCTTTGAGTTGACAATCCATTATTCTCAACATAGTACTATAAATATTTCTCCCCAATCACGATTCACGG
GCACTTTTTTTTTGCAGGGACATGAGTAGTAATACATTTAATGCATCAGATATTCCATCATGGATCCTAAG
CCTGCAATCATTAACAACATTGTATACTCTCTCTCTCTCTCTTTTTTACTAGATTTGGGAACGTGCGTTGCA
CGTTTATCCCAAAATACTTATATAAATTTTGTATTGCAAATGACATATTTTATCCCCTTCAAATCTTTGGTA
TTTACAAAGAAAAATCTATCATTGTTTCTATAGGAACTACATATATCTTCCTAATTAAAAAAAATATACCTT
TAACTATAAAATTGACTAAAAGTTAATAAAGTAAAAATAACTAACTATTGCACAAATTCAATATTCAAAT
GAGGATACTAAAACACCACATGCTAGGCTAATGTATACAAAACAACAAACCATGATAAATTCTCAATAAT
AACAACACAACAATAGAAAGTTGTACAATAACAACAAACAAGAAAAATAAATGTTAAACTAAACAAATA
AAAAGAGACAAGAGAAGAGAGCATAGAGATTTTCTCATATTTTTTTTCAATTGGTTTAAGTGTGTAAATT
ATTGTGTCAAATGTCATTATTTATAGGATAGCATTGAGGAACACAAAGAGTTGTCATAAATATGATATTA
ATCCATTTGAGATTATTGATGAGAGTAAAAACATAATGAATATAATTGGTGGGAATTACATACATATAAT
GAAGAGTTAGTGGTAATTTATGTATACTAATATGATGATTTACTATTTACTACTATTTAATTTATTAAACA
ATTTATAACATTTAAATATAATGTAGAAATAAAACCGTTATCTAAGGATAAAATATGTAAAAGAATTCAA
ATATATTAAATGTGTAGTGAAAAATCATTATGACTAATTATTAAAAAATTCATAGTAACTATGATTATGAA
AAATCATTACTATTACTACATGGAAGATGTAAAGATGGTTGTTTTTTTCTTGCTAATAATAACTTACAATT
TAATAATGAGTATTGAATTGTAAGTCTAGCTGGAAATAGGAGTGTTAACTACCATAATACGAACAATCAA
AATTCATTGAATCCCAAAATAAATATTAAATGAAAAAATAATATTTTAAATAAATTCAAGAAAATAAATT
AATAGTTAAAGGGTATTTTCGTAATTCAACTTTTGACTTAGATTCTTCCCACTTTTAATATAACTAGTTCTC

GACACGTGCGTTGCACGTGCATCCCATATTAAGTAATATAATTCATAATTAAATTGGAGGTTAATGAGCA
ACAAATTATATGGATATGCTCCATGTATGAAATTCAAAAGTCCATGTTATATGGATATGCTCCATGAAAT
GAGGAAAAGAAGTTGCATGTTATCAGAAGTCCAAAAGTCCAGGTAAGATGGATATACTCCATGTATGAA
ATTAATTAAATGAATTTAATCATTAATAACTAACGAAAATTATAATGCCATAACTACAGAATCAAAATGC
AATTGCTAGACTTCTAATTCAGTAAATAAAATATGATTATTTCATTAAATTTACTGGTTAATTAAATATTTA
ATTATATTTTAATTTAGTGTGGGGTAAATTCGTAATTCAACTTTTAAGTTATGAGCTTCCCACTGTTAATAT
AAAATATAAAATAATAATATGATTTATTGGTTAATTATATTTTAATTTAGTGTGGGGGTAAAATCGTAATT
CAACTTTTAAGTTATGAGCTTCCACTTTTAGTATTACTAGTATCTGGACACGTGCCATGTTATTTTGTTCAA
ACTTATTCATTAAGATGGAGGGAATATATAATAGTTAAATATATTGGTTTTAAAAAATGAAAGCTTATAC
TTACTAAAATAAAAAAATTACAACACATATCAATTCTCTCTTTACATATAAAGTAGAAACAACTGCTAAAA
GTATAACAAGAATATTTGAATCCTAAAATTAAAATATTACATGTTGGAATAATAATACATAAACATTGTG
CATTACAATAAATCTGTATTGAACTTTGTTTAGTGAAGTTGATGTTACTGGCTAATCGAACTCTAGACGCC
AATAGCTTTAGATACTTTCCATTCTCTCAAATTGTTGCTTTCTCTTCAAGAACAAAAAAGAAGATGAAATT
AATTAATAAATTAAAATTATGCAAGAGCCTATAAAAATGGTGAGAAAGAATTCAAGAATCCTTTCTTGCC
TTTTATAGCTATTTTTTGAGTAAGAATTCAAGAATCCTTTCTTGTTTTTTTATAGCTATATTTCAATTTTCAT
TCCTTGAACCTTTTCTTTCATTAGTCTACATTTAATTATTCAATGCAACTCTTCCTATTCTCCAAACTATTTA
GCAATGAATAACGCACACTTAAATTTGATTAATGGGTAAATCATTAAGGGCTACATTTAAATTGGACTTT
TGAATATCTACAGATTCACAAATCTCATGTAAATTTAAATACAATTACTACTATTTCAATTAGATTAGGCA
TTTTATTTTAATTAAATATAAGAGAAAAATTGTAATCTAACTTTAAACACTTGTTTTATTTTTCTCCATTTTT
ATTCATGTTTAGTACATTCACCTCAGACTAATATTCTACTTTGAACAGAAATAACACAAAGCAATAAATAT
ATAGAGATACATATTCCAAGCCATAATAAATGTCATTATTTTTTATTATGGATACATTCACCTCAGTTCTCA
CTCCATGAATTTCCGTAACATTTCATTAATTTGACTGGAAACATAATAAGTATCATTATCTTTTAGTTGGA
ATATGAATGTCAAAGAGTGCCCTCTCTTTTTTTGTGTGGATAATTTAGTGATTTTATATTTATGTATATTAA
TTTTGAAGAGATAATACATTTTTATTATTATTTATTTAGATTAGATGTTGTATCTATTACATTTTAATTAAG
TATAGGGGTAAAATTGTAATTTAACTTTGAAGATAGGTGCTTCCCACTTTTAGTAATTAGTAAAAATTACT
CCCTCTATCCCTAATTACTTGTTCACTTTTGAATTGACACACCTATTAAGAAAACAATTATTGACATAGTG
AGTGTACCATTTTACCCCTATTATTAATTATAAAGTGGATGGATTAAAAATTTAAGATTTTCAAGAAATTT
TACCTTTTTCAAAGTATTTAATTGAGGGTATAGTAGGTAAAATTATTTTGTCCTTTCTTGATTTGTCAAAAT
GAACAAGTAATTAGGGATAATTATAAAAGGAAAAGTGGACAAGTAGTTAGGGACAGAGGGAGTAGTA

AATTAGTAATATATATAATATATATATAATATAATATATGATATGATGTGTGTGTGTATGCACGACCATG
AGCAGGTGCATGTGTGTATGGGGCTCGTGTATATGCCTGCATCCATTTGAATATGGACTGATTTTATTTT
TTCTTGTTTTTATGGATTCAATTCCTAGATGTTCTTTTTAACCGTTTGACTGACCTTGTATTGTCAGGGTGT
TGGAAGACACGGGACTTCAGGGATCAGTTCCAGCTAACCTTTTCAGCCTACACCAGTTGCAGACAGTGC
AAGTGTCCCTTTTACTTTGTGTCGTTTTTGTATGAAGTATAGATTGAGTATCTGAAGTTGAATTTATCTTT
GCTGAAATTTCCGAACTCTGTATTATGCTGATAGTTGTTGACAATGTGCTCGTTGCTTACAGCATCTTGAG
GAATAACAAACTCAACGGCTCTCTTGAAACTGGAACCACATATAGCAACCAGTTGAAGCTCATTGATGTG
CAAAATAATCTTATCGAATCATTTACTCAAAGACCAGGGTATCCCTTTCAGATAAAGTACGCACTATTCCA
TGATTCTTCAAGCCATAAAATCTCCAAGTTGATAAGAAACACCATACGCGAGACAGTTTTTTGCCTAGCT
TGTTGTGTCGTGTGATTATTCTATGCTCAACTTGACTATATACTCGTTTATTTACTTTTTCCTATGCAGGCT
TGCAGGCAATCCTATCTGCAATGAAAGAGGAGAAGGGAGAAAAACTTACTGTATGGAAACTCAACAAA
CTGAGACATACTCAACCCCACCAGAGAATTGCTTGCCAACTGAGTGCAGCTCTAATCAAGTTTCTAGTCC
TACCTGTAAATGTGCCTTTCCATATACAGGAAACCTAGTCTTCAGAGCTCCTTCCTTTTCAAACTTGGGAA
ATAGAACTACTTATGAGACTCTCCAAAAGTCTTTGTTGCTTTTCTTCCAAAAACGTCAACTGCCTGTGGAA
TCAGTTTCCCTCCGTAACCCAATAAGAGATTTAAATGACCACCTTGTAATACAGCTGCAAGTTTTTCCATC
TAGCCAAGATCGTTTCACTTGGACTGGTGTTTCTGGAATAGCTTCTGTGCTTAGTAATCAGACTTTCAAG
ACCCCATCGTCATTTGGACCTTTCTTCTTCATTGGTGAAACCTATAAATACTTTGTCGGTAATAGAACTATT
TCCTTTTCCTATTTATCACTCATTTCTCATCTCCTACATATATGTGCAGCCTAATCTATAATTACAGAAAGTT
GGTGGCTGAAGAAACAATATATATTTGAAATCTTGATGATTAAGTTTGTATGTACTTTCGTTTGACAGAA
GAATTTACTGTATTTTTAAGACTTTGAACGTGAATAGCAACTATTACAGATCTACTGTTTTGATAACTATA
TCAATCCTTTCTGAAGTATTCAATTTAGAAGTACAAGACCCAAAAAAAATTGTCTAGTCATTGAATTTGA
GACAAAGATACGATAAAAAAACTAAAAATTTAGGCTACGTGCATGTGATGGATCAACTTTCGTTCATTGT
TACTAGTGATTTTTCTGTTAGAGTTTGCATAAACTTCAATGGATTTATTAATAACGTTTTAGTATAGACGC
AGGTTATTCTGATCGGTCTCCTTAGAAAAACTGGTTCATTAATTGACAGCTGAAAATTCAAATTATTGTTT
GGAACATTATGGAGCTACATCTAAACGTTTCATGTTACTGAAGTCTGAGGCTGTGCTTGCTGTTTTCTAG
GGGAATCCACAGGATCAAAAAAATCAGTTAGTACAGGCATTATTATTGGAGCTACAGTTGGTGGTTCTG
TCCTTGCAATTTTAGCACTAATCATTGGTATTTATGCTTTCCGGCAAAAGAAAAGAGCTGAAGATGCTGC
AAAGAGGAGCGATCCTTTTGGTAAGAGTTGGAACTAACTATTAGGCCATTTACCTATTACTTCATCTTAC
GGTTGTTGTTGCTATCTTCCTTCTTAATGCAGCATCCTGGGACTCAAATAAACATAGTGGTGCTGTTCCAC

AGCTGACAGGAGCTAGATTTTTCTCATTTGACGAACTAAAAAAATGGACTAATAATTTTTCAGAAACCAA
TGTTATTGGTTGTGGTGGTTATGGAAAGGTCAGCTTTTATGCTCTGATACACTAAGCATGTGTTCTGTTA
GCAGTACTGCAGCCTTCATTCGTTCTGATCTTAATCATCTTAGACTTAGTAGTTAGTACTACCAGTTTCTTT
GTCAAAGTGTCAGGCTATTTACAGACGATAATGAACTGATTGTATATTTGGATGGAGAAGTCTTGATTCT
TCCACCCAAGATAATTCACATATTTTGGTTTCGGATGTAATACAATAGCTCTAATCTATTTAATTAATAAG
ATCCGGTAAGTATGGATATTTTTTATCTTGTTTGTACCAGCTTCTCTTTCCACTGAAAAGAAAATTTCTCGA
CTACAATTGAAACTGTATTAAGTTTTTAAACTGTAGAAGTGTGGCTCTACAGGTTTACAGAGGAAGTCTT
CTGAACAGAGAATTGGTTGCAATTAAAAGAGCTCTACAAGGGTCTATGCAGGGCACACATGAGTTCAAA
ACTGAGATAGAGCTTCTCTCAAGGGTTCATCACAAGAATGTCGTTGGCCTTGCTGGCTTTTGTTTCGATC
AAGCTGAACAGATGCTTGTGTACGAGTACATTCCTAATGGCACCTTAAAAGACGGTCTTTCAGGTATATG
TTGATCCCTTCTTGTTGATTAATTGAAACTAGCTCCTTTTAACTTAACCAAAATGCAAATGGAATGAAATT
TGTACTTGTAGGTAAGACTGGGGTCAGGTTAGATTGGATGAGGAGATTGAAGATAGCCATTGGAGCAG
CCAGAGGTTTGCAGTATTTGCATGACCATGTCAATCCCCCCATTGTACACAGGGACATCAAGTCCAACAA
TATTTTGCTAGCTGATCACCTAAACGCAAAAGTTGCTGATTTTGGCCTGTCCAAGCTTCTAGGTGAAAGG
GGTCATATTACCACTCAAGTCAAAGGAACAATGGTAAGTAAAGATTCTATAAACACCTGCCTGAACTTGT
TTCGTTTTAGAATAATGTATGCTCCCTCAAATTTCTGATGTCCAGAAAGTTCAGAAATTAAATGTGTCGAT
AATTTCTAGTCCTGTAATAACCATTACAAGCACTATTTCATTGTCTAGTAAGAACACCTGAAGCTCCTCCT
TTCTCATCGACCACCCTTCCCTCCACTAAAAATGAGTAAATAAATAAGTAGGAACAGGAAAATACGAAAA
CGTTTTATTTATTTCTTGATATTCTCTCAAGGTAAGGGACTTGTAGGTAATGATGCTTAAAAACACCAAAT
TTAAGGAGAAATACTACCTTGATTAGGAAACAATGAGTTATTGCCCATAGCAGATAGAAGCTTTTTAACG
TTCATATAATGTGATCTTGCCATGTATGAAAGGAAACAAGTTGAGATGTTGCAGAAAAATGTGTCAGTA
TTTACCTTATAATCTATGTCCTTTTGATGGTTGCCTTTTATTTATTAGGGATACATGGATCCTGAATATTAC
ATGACAAACCAGTTGACAGAGAAGAGTGATGTTTTTAGCTTTGGAGTGGTGCTGCTTGAGATAGTCACA
GGAAAAGTTCCTATTGATAAAGGAAGATATATTGTCAAGGAAGTGAAGGATGCAATGGACATGACAAA
AGATTTGTATAACCTTCATGAAATTTTGGATCCTGCAGTTTGTTCGGGTGCAACTCCTAGAAGCTTAGAG
AAGTTTGTGGATCTAGCACTCAAGTGTGTTGAAGAAAAAGGAGTCAATAGACCTAAAATGAGTGAAGT
GGTGAAACAGATTGAATATATTATGGAGATCGAAGGCCTGAACCCCGATGATGAATCTGCATCTACTTC
AGCAACCTATGAAGGAGCAAACAAAGGCTTGAACCATCCTTATACTGAAAGTCTCTTTGTCTATAGTGAA
GCATAA

>PGSC0003DMG400018367 LRR-RLKs

CTCTGTTAAAACAATGCTTTGCTTGCATTTTTAGTTTTTGTTCTTTTGTTTTTCAGCTCAATATCATGATGTT
TTTTCTCAGCTTCTTCCTCATTATTTCTTCATAATTCACTTCATATTTGGTGGGTTTTTCCTTCTTGTTACTGT
ACTTCAATTTTTTACTTCTCCTTCTTCAACTGTCCAGAGTTAGTTTTTTTTTTTTGTTTCTAGATTTGTTCTAT
CCTTTTCTCAATTTTTGAATACCCATTTTGTTATCAGTTGGGTGTCATTGATTTGCTTCTTTTTGGTTGTGTT
ATGATGAATTTTGGGTTTGATTGAAGTACTGATTTTTATTTTTGGGCTCTTTCAGATTGGAGGTTTATGTA
TTGGTTATGCAGAGTGTGTTTCCAAAGAAAGAGTAGATTGAAGAAAAGGGTTACAGTTTGGATCAATGG
GTGGGAAGAGATCTGTGTTAGGGTGCTTGAATTTGGAGATCTTGGTGGGGATTTTGTTAATTTTTGCTGT
TCAGCTTTCAGATGAAACTGACCCCGGAGATGGTAAGGAACAACAAATATTAGTATTATATTTCGAGTTG
TTTTGCGTTGAAGGAGAGGAATGCTAATTTTCATGTTTTGGGGAATGCTGGTTATGAGGGACTGAATTA
TGAGCTGAGCTAGTTGTTAACTTGAACTTTATTTTGGGTAAACGTTTTCAAGTCGGGGGCGAGCTGGAG
TTATCGAAATTGGTCGGTTAAATGAATAGTCTTTATCCATTTCCGGAATTAAGTTGGGGCATGACGTTAG
ATATGAGGTTGCATAGGAAATGGATTAGGTAGAAGATTAGTAGGTAATGGAGTGTTGTCTTGCTTATCC
TTTGATGCTGGTAGTCGTAGTATTTCCCTTGGTAGTTTCTCGTCCTTGGATTTGTGTTACTATTATTGCACT
GTTTTGTTGCGGTTACCGTTTGTTTTTTTCTTCTCAGAATGCTTTGCCATGCTTTCTATAGTATTTCGCCACT
ATTTCTTCACTTCTGTTATTTCTTTTTTCGATATGCCTTTTCTTGAGCTGAGGGTCTATTAGAAACAACCTCT
CTACCTCTTAAGCTAGGGGTAAGGTCTGCGTACTCTACCCTCCCCAAACCCCACTTGTGGGATTACATTG
AGTATGTTATTGTTGTTTGAATTAATTTACAAACAACTCCATCATACACAAACAAAAAACCTTAAAAATAA
CCTAATCGTTTCACTTACATTCTCAAAAAACTTGCATGCCGCAGTTACCAATTCTACTAACAGTGGACAAG
CTGTCATTATTTCAGAAAAAAAAAAGAAAAAGAGACAGTCCCAGGAAATTGAAGGGGGAGAAGAGGA
AATGAAGTACTGACTCCTAGGTTGCTAGTCAAGTGTAGGGGGCTATGGCCTCGTCAAGTGATATGTAAA
TTCAACACATATTATTGTCCTAAATATCTACTAAATTGCCATCTTGATCGTGTAAACTTTGCTTGGTTTGAA
GTTAGCATGCTTTAAATGTTTGGAATCAAATTTTCTAGTTCATTCATGATCAATATCAACAATATTCTTATA
TGGTTTGAAATTTCTAACTATGGCATTTTCCTTTCTGCTGGTCTAGTTGCTGCAATTAATGCCTTACATGCT
TCCCTGGGATCACCTCCCCTTCCTGGCTGGGGTGTCGGTGCAGACCCATGTGATGGACAATGGCAAGGG
GTCGTATGCCAGGAGACAAATATAATATCCATGTAACTACCTCTTATTCGTTGCAGCATTTCATGGATATT
TGGAAGTCGTAATGTTAATCCTTCTAATCATCTTCTTTGCAGTCAATTAAATGCTGCTAACTTGGCAGGTG
AACTAGGGGATAACTTAGCATCATTTTCTTCTCTCAAAACGATGTACGTGTACCCCACAGATATATCTCCT
TCGACATATATTTGGTTGTGTTTTCTGTGCATGTGCTGCATATATTATGTGGATGCTTCCTTTTCTAGAGG

TTTCTTTTATTGTGTGACATGTGAACAATAATTGTGGAAACTTCTTGTGGCAGAGATCTGAGTAACAATC
ACATCGGCGGAACCTTCCCATCCAGTCTGCCAGTTACATTGCAGAATATGTATGTATTATCATTATCCTAG
AAGGGGAAACAGAGAATGAAGCTTGAACTATCACAGCTTACCTTTTGAGCAAACCATTTGATAGTCTTTA
CCTTTTGGCAGTTTTCTTTCAGATAATGATTTAGCCGGAAGTATCCCCAGTTCTTTATCTTCCCTAAGTCAA
CTGTCAGCAATGTGAGTTTCAGCTTATATTTTAGATTGGAGATTTCCCGTTACCGTAATCATGGTTCCAGT
CAGCTATACTTCTCTTTTGTTTCAGGTCTCTCAATGGAAACCAACTGACTGGAGAACTTCCTGATTCCTTTC
AAGGCCTTACAGCATTGGTTAATCTGTATGACACCAAGACAAAATCATTTCAATATTTCCATAGAGGTTTT
TAATTGTCCATAACTGGTGGATTAATTGTATACTTGCAGTGATTTGTCAAGTAACAGTTTCAGCGGAGCT
TTGCCGTCATCAGTAGGAAACTTGTCCTCCCTGACCACTCTGTGAGTGATTCTACATATTGTTGAATTGGT
TTGTAATTTATGACCAATAGAAAAGCAGCTTATTTCTTTCTCTCCAGACATGTGCAGAATAACCAGCTTTC
TGGAACTCTCGATGTTTTACAAGATCTTCCCCTCGCAGATTTGTACTCTTCGTCACCATTTTTAACTCAAAT
ATTAATTCCAGGTCCATAGAATGATATAATATCGTACTAATATCTCCCATCTTTTGTGTCCCTCCAGGAAT
GTGGAGAATAACCTGTTTTCTGGGCCCATACCTCAGAAGTTGTTATCTATTCCCAATTTCAAGTAAGTACA
TTTCACTTTCTGCTTCTAATATTGTTGTTTATCTTCCAGCATGTAATCTACAATATATTCTTGGCCACCTATT
ACATTCTTGTGCAGTTGTGCCTCTTTGTTCGACACAATCTGCTAGACAGATATTCAGAAACTATGGAGAC
AGATTCCTTGTTGAAGTTCGATTATTACATGCCAAACTGCTCTGTACTTTTTGTTAATACTTAATACCCTCT
TTCTTAAATCTGAGTGACAACCTTGATCAGCTTGTTATTCATGGACAATTAAGGCAGATAAGATCTGAAG
GGCTACAATCGTTAACATAAACAATAATTGTACTCCACCCTCCTAAAGCTGCAGCACATGTATCATCTGTA
CTCAGCTTCATATTCCCTACCCACTTCAGACCATACTAAACTTTTAGTTAACATACTGGTGAATATCAAACT
TCACTAAAGGGGAGAGTTTCTGTGCAAAATGACAAGACTTGAATAGGCTTCATCATCTCATTAAGTATAG
ATCAGCGGAAGAGTTCAGAGTGGCTTCTTTATTACACCCAAGTTTGTTGGTTGGCAAAAAAATGCAATTG
CTATTGGCAAACTTCTCAATTTTCTGAACCTATACGTTTTAGACATTTTATGTGCGTAGAAATTTGTGTGT
GAGTGTGTGTGTGTGTGTGTGTGTGTGTCTGTTTTTATGTAAGTTTGTGAATGCAAGACCTCGTTAAATTCC
TATAAGCTAGAAAAAAACCTTGATCTGAACTACATTACTCTTTCTCATAAGCAGGAAAAATATTATGAAAT
GTGTGTCGTGCTAGTCTGTATGTTAGCTGCACCATCTTTAACAATAGTATTTTAGAAAATTGTTGAGTCCC
ACATCGTGTAGCATGAGGAAGTTGACTCCTTTATATGGTCTTGGGCAATCTTCATCTCTTGAGCTAACTTT
TGGGGTTGAGTTAGGCCCAAAGTCTTTTTTATACTATCGGAGATCTGATATGTTGAGGAGTATATTTATG
TTGCAAAACCAGATTATGCTTACAGGTTTATGGCACTGACTTAGATTAGAATTGCTAGTGGGCACACTGC
ATATCAACAGCTTGAGTCCTAAATGTTAGAATAATTTCAAATTACATTAGGTAAAGTAGCCAAATTTTGA

TATCATCAGCACAATTACCTAAAAAAAGAATAATCCTAACACTATGCTGCTCAATTTAACAATTGGATAA
AAAAACATCTAGCCTACACAATAAATTGGAAAAAATGTGACGTATACAAGCTAAAGGATTATAGTAGCA
CAAAACCATATGACACCCATATATGATGTATATGTCACGCTGGACCATGGACTGCCAAGCTGGATCATG
GACTGCATTTTGGTCATATTTAAGTTCACTAGCGGGCTAACATTTATTTAAGTTTTGAACACAGGCGTGAT
TTTCATATCATGTTGTTCAATAAGATTTGTTTGCCGGGAAATTTGATTTATTGAACAATATTGGTAAGGGT
ACATATTGATCCATGCTAGGAAGATGTATCAGTTGTTTCATTATTTTTTTCAAGTGTCTTATATAGGTTTTG
ACTCTTGATTGACTTTAATTATGAGAATTGATGGCTAATGCTTTCATGTACTTACTAATTTTGTTCTTGACG
TGCAGAAACACTGGAAATCCTTTCAACAGTGTCTCTCCTTTGTCTCCACCCAACAGTAGTATTGCCCCTTT
GTCTCCACCTACTTCATTTACGAGACCCCCTCCAGCACCACCAGGACCACCCTTTTTTAAGCCTCCAACTTC
AACACAGACACCACCGACTTCAGACAGAAAACCTGGACAAAAAGCTGATGGACCATCAGCAACTGCAG
AATCTAGTTCAAAAGGAAGCAAAAAGTCTGTTAAAAGGGTAGTCTGGATATCCATTGCATCAGTTTTGTC
ATTTATAATATTGGTAATAGCAATCCTGCTCTTTCTGCCAAGATGCTTTAGGGAAAGGCAAGAAACTAAT
TGGTCCAGACGGCACGGAATAGCTCCTTATGTGGGCTCCAGGGAGAATCGTAGGGATAATGGTTTATTG
GTCCTACCTGGACATGATGTAGAGAAAGGTACTGCAAATTATTAAACCTTTCAACTTTTATATCGTGTGT
ATTGTTGCTAGTATTTGTTAGTCTGGCTTTTTTCCTTAGTTCTTACAGGGAAAACTAGTACTCTTTAATTTA
GGCTCTATTTGAATCTGCAGTCTTGACATCTAAGTATGCAGCTAAATATATAGTGGTTATCCATGTGCCCA
GGGGCGGACCCACCCTTACCCAAGGCCTTCGTCAGAAAATTACATGGTATATAGAGGTCAAATTTTGATT
TAATAAAATATATACAAGTATTGACACCTCTTGACACAAGTTAAAGGTTCAGTCTAGTGGTTAAGACATT
GCTTAATGACTACAACCATTGACACCCCTTAATGACAATGGCCGCTACAGTATTGTCAAATTCTCTATTAC
AATCATAAACGCCCTACAAAAGATTCGTGAATTTGATATCTGTTTTTGCAACATTCAGAACTAATAAGCTA
TGACTCTTAACCTCTTCTACTCTTTGATGATAAGTGGACTTCATATGTTTTGAACAGCTCCTCCTCTGGTGA
GGCCAAAGGAAGAGCAACAACCAAGGAGACCGGCCCGCACTCCCATGCCACAGCAGGAGCAGGAGTT
AAATGTGCAAAACCTGAGGGCAGTACCAAAGAAGGATACTAGTGAGATAAACCTAAGCAGAATTGATA
TTGATTCGATGTTGCCACCACCTCCACCCCCACCACCACCTCCGCCACCGCCACCACCCTTTTCTCAAGAG
AGAGTTATTGTGAAGCCAATTCCACCTGCCGACAATACTTCAATGAAGTTTCCCCACAGGCCACTTCCAC
TAACTTCTGTGAAATCTTACACAATCGCATCTCTTCAGCAATACACCAACAGCTTTTCTCAAGATAATCTG
ATAGGATCAGGAATGCTGGGAACTGTGTACAGGGCAGAGCTTCCAAAAGGGAAGGTATAAGTTATGAA
TGATATATAATGTACTGTAATTTGATACTCGAAATTACCAACTGTTTTCCTACCCTGCTGTATTCTCTTGAA
CCTAGATTGTCAGCTTTTCTCTTCTTATAATTTCCTTTTTTGGGGGTCTTTACGGTGTTCTGTGAGCACTAA

AGATGCCTCTTATCTGTTGTTGCATTACGTATCTTGCTCTTGCATTTCTATGCTTAATTGCTTAAGAATGAT

TCACGGACTGATCGAATCATATGTAAATATGGTTTTGTTGGTGGAAATCCAAAGTCATATATATTAGGGT

AAACTGTATTTCATAAATACAGATAAAAGCAAAATGACTTCCAATTGCTTCATAAGATATTTCTTGTAACT

TAAATTTTCTTTAGGACGTTTTTTGGTTCTTATCCTATATAATCTTTAAGTGGCATTTTCTTCCAAATAAAC

AACACCATTGATCAACTGTTGTCATAGAACAATCAACATTCAAGTTGCATGAGCTTATCATTCTGATTTGT

ACCTTAGTTTTGTCATTTGCTGTATAGCAGGATCTGCCAAATACTGATGCATCTTTTCTAATGAGAAACTA

TTTTATAATCAGAATATACAATGTCCCTGTTTTATTTTCAGTGTACATTTATTTATCTGGCATGCAATCTTA

ACTCTCATCCCATTGCTAACATTATTTGAATCATTTGGTGGTCACATTGTGTTACTTCTGACACTTGACCCT

TTGAATGCTTATGCAGTTACTTGCTGTCAAGAAGCTGGACAGAAGAATTTCCAATCAACAGAAGGATGA

TGAGTTTCTTGACCTAGTAAACAATATTGACAGAATTCGTCATGCTAACGTTGTCGAGTTGATGGGCTAT

TGTGCAGAGCATGGTCAAAGGCTTCTGGTTTATGAGTATTGCAGTAGTGGGACACTACAAGATGCGCTG

CACTCTGATGATGAATTCAAGCAACAACTTTCATGGGATACACGGATCCGGATGGCTCTTGGAGCTGCA

AGAGGCTTGGAGTAAGTAAAATAACCAGTCACATATACATAGAAACCACATGCAAATATACGAGTATAT

ACATATGAGCTATGGAAAATCTGATGTTGAAGAGTTATTTGGCCGTGATCTATAAGAAATTTCTGTAAGC

AGTACTAATAATAATTTAGCACTGGAAGGTAGCAAAGAACTCAAATCACAAACTAGTCCTGAACTTTCAT

GCCATGTACATACTACATGACATAAATGATAAAATACTGTTGGGAGATGGTCTTTATAAGTATATGTTAA

CATTATATTTGGTGCTGTATTTCGGTTTATACTACTTTCCATTCCTCCTCCACATGAGTTCATACAAGGGAT

GCTGAACACGATTGTTGAAAGAAAATATCCTCATAGTCATCCCACCCGTTAATTATTATTAGTGGGATTG

CTAGGTGATCTAATTCTTAGATTTGCATGACAAGCGAGTTAAAGCAAATTTTAAATCTCATGTATTAATTA

TATTTGTTCTTTTATGCAATGAAGGTATCTCCATGAGGTCTGCGAGCCACCTATTATCCACAGAAACTTCA

AATCTGTCAATCTTCTCCTTGATGAAGAGTTGGCTGTGCATATCTCTGACTGTGGTTTAGCTCCACTGATA

TCATCAGGTGCCGTGAGCCAGGTAACTTACCATCCAAATATTATGGATCAGACATTTACCTCTTGGGGTG

GAATGGTCATTTTCTGCTTGCATTTCCAGTTGAAAGAAAAGCAAGTTTCAGGTTTATGATGGAAGTGAAA

ATGTGGACTAAGTAGTAGTTGTTAAGAAAGGAGTGTAATGATGATCAAAATGCTTCCAAGTAACACACC

GGAGTAGAGCAGTAATTAACTACAAATGCAGTTTCTCGTAAATGGATAACTTATTGTTTAGAAATAGTTG

ATTTTGTAGTAGTGCCAGAAGTTATACGGTCAATTTCAGAAGGGTCAAGTCTTTTTCCCTGTAAATCTGG

AAATCAGGAAACAAATCTAGCTAGTCTGAGACTACCTGGTTGCCCAGCTAGTCTGAAGTATTTGTCATGT

TTTAGGCTTTTATAACATTTCAATGCTTCACGAGATCTAATAGACACTAGTGGAATTTGTAAGCAGGAGA

GTAGATCCTTTCCAACATATGCTGGACTTGCCTTCTCATCTTCACCCCTTGACAAAGAGATGAAATTTAGT

GATCCTTGAAACTGTTCCCAACATTCTTCAGAAGTAGTTTTGCCGCTGCACATTTAATATGATGGTGACAA
GCTCCTGTATTTTTTTCCCTGTTGGAGAATTTTGACTTCTAAAGATGTGGGAAGAACAGTTTCACTATGTT
AGTTTGACCTAGTCATACATAAAGACACTCTACAGGTGCTTTTGTTAGATGGGTTTTCAGAAACTTAAATT
CTATTTTATTTTTATGAAGTCCACATTATCTCCTCTCATTAATAAGAAACAGTAAAACACAAGATTGAACT
AAACAAGACATCACGGATATGACTGGTTTTTTCACTTGTACTTCGTATGATTATAGATCTGCTACTCTAGG
AGCATGTGGATCATCAATCTTTGCTGAAACCTTACCTTTTTTGTTTTTCTTATGCACTGTGCCGAAGAATTT
GAAATTGAATAAGTGAAGTTGTACTTGATTTTCAGTTGTCAGGACAACTTCTTACAACCTATGGCTATGG
TGCCCCAGAATTTGAGTCAGGGATTTACACTTCTCAGAGTGATGTTTACAGTTTTGGCGTGGTGATGCTG
GAACTCTTAACGGGCAGAATGTCATATGACCGGTAAGGCCATTTGCATCTCATTGAATAAATTTTGTTGT
CATGTTTTCTCCCAGCTTTATCATTACCTATATATCATATTCAGAACTCGAAGTCGAGGAGAGCAATTCTT
GGTCAGATGGGCAATCCCTCAGCTACATGATATTGATGCCTTGACAAGAATGGTTGATCCTTCTCTCAAA
GGAAAGTACCCACTTAAGTCATTATCACATTTTGCTGACATAATTTCCCGCTGCGTTTTGGTGAGTTTTTT
AATCCTTTCTTTTATTACAAGTGAGTAATTTGTCCAAATCTCCATTTTCTTCAATCCTTAATCAAACTTTGG
GTACAAATGAGGAAAAAGGTAACATGGAAAATGATTTATTTCTGTCACAGAAAATAAAATAAAAAGTAA
AAGGAAGGAGCTGTACTGTGTCAAAATAGGAACTCTACACATGGTGTCTTTTGTTTTTTATAGCCACTAG
ATTATGAATTATTTACCAGTTTAAGGACATCTGTGAATCTGTAGTATGAGATGTACCAGGTTTATAGGCA
TAGTTAAAAGTTACAGGGAGTGGAAACATCCCATGTCGATGTTCCGATATCGTTTTCTGCTCAGGTGTCT
GAATGCCTTTTCATGACTCGTTGAAGAGTGTGTGTATGTTGTAGTTGAAGCAAACAACACTAGGAAAGT
CTGGATCAATGAACAATTTACCAATAATACCCTATTCACCAAATTAAATCTTGTGGCAGAATACGCATGG
TTAACTAGCACACAATCACACACCAAACAAATAAATAATAAAAATAACACAAGGAATTTACTAGGTTTGT
CAAAACCCATTTCCTCGGGGAAGAAGCGGAGAGAATTTTCCACTATAAAAGATAAGAAAACAGTACCAA
GAGATCCCCTATTACAACCCTTTTAAATACCCAAGTGTTTGCCCAATCCAACTGCCCCAGTCTAACAATAT
AAGCAAATAATAGTGGTTCGAAGCTTTTCGGGGTTGGGTCACATTTCGGATCTGCCAGAATCCAGACAC
AAAGCTGTAGAAGCAACAGATATATTATATTTGCTTTCCTTCTTCTGCTTATTGTTCTCCTGTCTTATTGTT
TATTCGGTGGGTGTTGGATTTTTCACTAGGTAATATGCAATTTTTTGTACAACCAAATGCTTGTGTATGTG
GGGGAGAAGAAGAATGAGGCACCACTCTATTGATTGGATATTTGATTGCAACTGATATATCCATTGTATT
TATATAATGGTCAAATTTTCAATGTTTCAATGAACAGAAATCGATACTGATAAGTTAAAAGGAACCAAGT
AGTTGGTGTAACTCCAATGTGAAGCCTGTTCACAGTGTCCTCCCCATTATCATCTTTCAGTTACCCTTCTA
GCAATGACAGAGAAGAGATTAAATGCATCATATGATTTCTTAGGGACTATTTCCATTTGGCTCCACCTTC

CATATCCTATTCCCGCTATCTTATAGATTGATCGCACTCACAATTGTGTGCAGAACAACTAGTACAGAGTA
CTATATTACTGTTAATCAAGGAAGAGTGAAGTAGTTTTCGTGTGGCTGTTTTCTGAAGCCCATCTCATATC
TCTTCCTAGCATGACTAAGATTTAGCTGTTGTATAGGATTTGCCTTTGCATTTTGTTGTAACTATCTTTAAT
ATTTCCTTGCAGCCTGAGCCTGAATACAGGCCACAAATGTCAGAAGTGGTTCAAGACCTCATACAAATG
ATAAGAAGAGAATCTCCCAGTAGATCCGATGAAGAATGATTTGAACCAGACATGCATGCTATAGCAGAT
TCAGGCATGGAGTAACCAAGACTTCCAAGATTTTTTTACTTTTGCAAAGAACTTAACTCGAACTGAGATA
CTTGGAACCAAAGATTTTGAGGCTGTAGATGCAGTGCCCTGCCAGCAAAGCTTCATACCTCCAAGACAA
AGGGCTGGCTAGAAACTGCACGATTTCCACCAACACAGCTCGGGTTATCCTGATTCTTAGTACAATTTTT
TGCAATCATTCTAATGTTCATTTATAGTCCAAACAGAAACCACAATGCATAAAATGCACTAACCCCATTGT
TTTGAGAGCTTATTTATGGTTGTTAATTGGTATATCTGAAGTTGTAAATCTTTATTTGTTCTGCTGTACATT
TTCAATTGTTTGTACAAGAATATTCTTCACACTTTCAATATGGACTTTCTGTAGCACATTTTTCAGCCTTCA
GTATCATCATGGATATATTTAATAATCACTTGAAG
>PGSC0003DMG400018752 LRR-RLKs
TTGAGAAGTTGTTCTGTCTGGTTCAAATCACGAATTTCTTGAGAAAAAAAAAATCCTTGACAAGATGGAG
AAAAGCTATGTTCTTTTCCTTATCTTATTAGTATTTCAAGAATCAATTTTTTCCCTATATGCAAATTCCACA
AATGAAACAGACATGGAAACTTTGTTATCTTTTAAACAACTCATTACAAATAACTCTTCTTTCCTTTCCAA
GAATTGGACATCAAATACTTCATTTTGCTCTTGGTTTGGTATCACTTGTAATCCACAAAACCAAAGAGTCA
TAGCTTTAAATCTCCCAAACATGAATCTTCAAGGCAAAATTTCACCATCAATAGCTAACTTATCCTTTCTTA
CTAAACTTAACTTTAGTAACAACATTTTTCAAGGCACTCTTCCTTATGAACTTGGAAATTTGCCTCTTTTGG
AAGTCATTGATGTTCATAACAATCAACTAGAAGGAACTCTTCATCCTTTTGTTGGTAACATTACAAAGTTA
GAGAGATTGAGGTTAGATGGAAATAGATTTAGTGGTAAAATTCCTACTGAGATTGGGAATCTAAGCCAA
CTTGTTGAGTTAGATTTGTCACATAATCAGTTTTCAGGTTCAATCCCTGGATTGATCTTCAGCATGTCGTC
GTTACGTGCTGTGTATTTGGTTAACAATAGTCTTTCTGGTTCATTCTTGGTTGATGAAATGAAGGGTGTG
ATGAATCTTGAAGTTATAGACCTTAGTTATAATCGAATCATCGGTGAGATTCCTTCAAGACTTTGTCAATT
CAGTAAGCTAAGAACTCTTGTTCTGTCTTACAACAATTTAACAGGTCAAATTCCGAGAAATATTGGTTGTT
TATCAAGGCTTGAGAGTTTTTATATAACTCAGAATGCAATAAGTGGTACAATTCCACTTTCATTGAGTAAT
ATTTCCACATTGCAATTTCTTGGTTGTGTGAACAACCATATAAGTGGTACAATTCCTAAAGAATTAGGAA
ATTTACCGAACTTGAAGATGTTGGGATTTGATTTCAATAATCTTACTGGTGTAATACCTGAATCTATCTTC
AATATATCTTCTTTGGAGTACATTGCTTTCAGTGATAACGATCTCTCAGGGCGAATTCCAACCACTCTTGG

ACTTAAACTTCCAAACCTTAAAGGAATTTTCTTGCCTGATAATCAACTTGAAGGAGAAATCCCAATGTATA

TCACAAATGCTTCTAACCTTATTGAGCTAGAGTTGTCCTATAACTTGTTCACAGGCATAGTTCCAAGTGAT

TTGGGAAATCTACGCCAGCTCGAGTTCTTGAATCTTGGTGGTAATCAGCTTACAAATGAACCAGGACAA

CAAGAGCTTGGATTTTTGAACTCTTTGGTGGATTGTAGGAGGCTGCAGTTCCTGATATTGGCTAACAACC

CTTTGAATGGCGTCTTGCCTGATTCGGTTAGTAATCTTTCGTCTACTATTGAAATGTTTAACATAGAGAAT

GGTCAGATAAATGGACAAATTCCAAGAGGAGTAGGCAATATAAGCAGCATGTTATCGCTAGTCTTGAAC

GGTAACCAATTGACAGGAACCATTCCACCAGAGATTGGTGAGCTAAAGCAACTTCAGAGGCTTTATCTA

AGCAAAAACAAGCTACAAGGTTCAATTCCAGAGGAAATATGTGACTTGGTAAACTTGGGAGATACATTT

CTACACGAAAACGAGCTCTCTGGGGCTATTCCAAGTTGCATTGGAAAACTCACTAGGCTGCAAAGGCTT

TCATTTGGCTTCAATAAGTTCACCTCAAGCCTACCTTCAAGTCTATGGGAAATGGATAGCCTGATTTTTCT

AAACGTGACGCGAAATTCAATACAAGGGGAACTTCCTATTGACATTGGAAAATTGAAATCTATTGAAGG

TATAGATTTTTCAAGCAACCAACTTTCCGGTGTGATTCCAAGTACATTTGGAGACCTTATAGGCCTGAGG

TATCTTTCACTCTCAAACAACTCGTTACGGAGTGCAATTCCTTCATCATTTGGCAGTTTGTTAAGTTTGAA

GTTCTTGGATTTATCTTCAAATGAGTTATCAGGAAACATCCCAAAGTCCTTAGAAAAACTTCAGTTCCTTA

ATGAAATCAATCTTTCCTACAACCATTTAGAAGGTGAAATACCTTCTAGTGGTGTGTTTGCAAACTCTTCT

TCTCAATCTTTTGTTGGAAATAGAGGTTTATGTGGAAAACCTATATCAGAAGTTTCTCAATGTGCTACGA

ATAGTGCAACGAAACGATCAAAGTCTAGAAAACATGTGCTAGTAGTTGTCATTCCTGTGATTGCATCAAT

TCTCCTCATATTTGTTGTTCTGTTCGTATGGATTAAGCGAAGATCAAGAAGAACAAAACTTCAAGACCAT

GATCAAGAGTTAACAGAAATCACAACACACCAGTTGATCACATACCGTGAACTTCAACAAGCAACGGAT

AGTTTCAGTGGCTCTAACATGATTGGTTCAGGAGGTTCTGGTTCTGTTTACAAGGGCATATTGGCTAACG

GAACAATGGTCGCGATAAAGGTTCTAAATATGCAAAATGAGGAAGGATGCAAGAGGTTTGATACCGAA

TGTGAAGTGATGAGAAGCATTAAACATAGAAATCTTGTCAAGGTGATCACTACATGTTCCAATCAATACG

TTAGAGCCATCATTCTCGAGTATATGCCAAATGGAAGCCTTGAAAGTTGGTTGTATGATAAGGAACATC

AAGTCTTAGACATGTTTCAAAGAGTTAGCATAATGCTTGATGTTGCAATGGCATTGGAATATCTTCACTA

TTGTTATGATACACCTATAGTCCATTGTGACTTGAAGCCTCAAAATGTTTTATTAGATGCTGACTTGGTGG

CACATGTTGGTGATTTTGGCATTTCAAAGATTTTAGGTGAAAACAACTCCATGGCACAAACTAACACATT

GGGCACTATTGGCTACATTGCACCAGGTAAACAATTCTTCTCTGACTACTCTACCTTATATAAGGCATATT

TTTGGTCATTCTTCTCTCTGAAAAATAGAATATTGGTCAATTAAGTTGATTTCGGTGCTACTTACTATGCC

TGAATCTAACGCTAGGCTAAGTCTTGAACCTTTGAGCTGACCGTTCACTTTCTTAGTAGCAAGAGGGCTT

CTCTTTGCCCCTTTATTGGTTGTATCAACTAAACTGCCCTTCCATATAGATCGTAGCAACATGAAACTATT
ATTAATAGATTCCTCGCTTCCTATTAATCTTGAAAAGTTCTAGCTTAGTTCTTTTTTTTTTGTTAGAATTATC
TTTTTAATTAGACCGGACCCACTTTTATACTTATCCTACAAGTGCTATTGGGGACCCACTCAAAAAAAAAA
AACTTGAACTTAGAAAAGTCCACCCCTTAATAGATTAGGGTAGAATCAACTGGATTTAACTTATATCTAC
TGATAGTATAGAATATATGTTTAATTCGTTATAACATATTAATTTCCTTTCCTTTTTGTGTTTTTAGAATAT
GGCTCAGAGGGAATAGTGTCCACGAGTGGTGATGTTTATAGTTATGGTATAATGTTAATAGAGATATTG
ACAAGAAGAAGACCAACAAATGAGTTTTTCAATGAAAACATGAACTTGAGGCAATGGGTCAGTGAATCA
TTTCCAAGTTCTTTGAAGACAGTTGTTGATGAAAATATTTTATTTGGAGAAAATGAGATTTTCATATTTAG
TATGATGGAGTTGGCTTTGGAATGCACAAAAGAAAGACAAGAAGAGAGGGTAAATATGAAAGATGTAG
TGAATAGACTTGGTAAAATTAAAGAGGACCTCTTCTTGGAAACAAAGAAGAAAAAATCTTAGGAGTAAA
ATTTTGTTTATTTTTAGTTTATGGTTTAGTTATTCTTT
>PGSC0003DMG400018811 LRR-RLKs
ATAAAAGGGGCACTAGTAATTTTAGTAATTGAAAAGCAAGTGTATAGAGAGAGATAATGAGAGAGAAA
GAGTAAGTACACTACTACTGCTACTATCCCATATAGCTGTAATGTTGCAGGTCTGATTTTTGCAGTTGCA
GACCCCCTTCTTGGCACAAGCTCTTTTAACTTTTATCTTCTCAAATAATTCTCTCTCTCTCTCTCTCTTTTT
TCTCTTTTTACATTGTGAGGAAACTGAATACCCATTGTATGTATTAGTGTGAGGCCTATCTGCCACAAGG
ATGTGATGGAACACTATGCTTCCTCTGCTAAAACCCCACAACCCCAAAACTCTTTTTCACTTCACATTTAAT
CACAATTCCTCAGTGAAATTATTCTGTTGCTCTCTCTAATTTCAATTTCAATGTCGGTAAGTCCAAGAACT
GGTTTTTCAATTCAAAGGAGCTGAGTTAGTGCAAACACTTGAGGTTTTGAGTTTTGACAGAGACTTGAGT
CTCAGAGAAACTACCATGACATCATTTTTACTTCAAAGATGTAATCTTTTCTTTGAGGTTCTTCTTCTTTTG
GGGTTCTTGAATTTCTTCAGCTTTGGTTCTGTGGTGTCTGATGATGGTGAGTGAGTAGAGTAGAGTAGT
ATAACATTCTGCTTCTTATGTTCTAGTTTAATGTTTTGTTTAAATGTTGAAAAGACAAAGTGTCCTTTTTTT
AATCATTTTTTAAATGGTGTTTTTGATTAATCCCACGTTTTGTAGTTGTTATTTGTTAAAGGTTTATTTTTTT
GTCTCATTATTATAATAATAATTGGAAAATAGGTTCTGCATTGTTGGAGATTAAGAAGTCAATTAGGGAC
GTGGAGAATGTGTTATATGACTGGACTGATTCTCCTTCATCTGATTACTGTGCCTGGAGAGGTGTTACCT
GTGATAATGTCACCTTCAATGTTGTTCAACTGTAAGCCATAACTCAAAAAACACTCTTGTTACATTATTAT
CATTTGGGATTCTTTAGTATAAAGTTGTAATCTTTTGACATTATCTTGTAGTAATCTTTCGAGTTTAAATCT
TGATGGGGAGTTGTCTCCTGCAATTGGACAGCTCAAAGGCCTTATATCTATGTAATATCTTCTCCCATTAT
CCCACATTTTCCCTTTTTGTTTGATCTTTTGACTTAGTGTACATTTAGATTATGTTTGTTAATTTATTTATTTT

TCGTAGTGATGTAAGGGGAAATCGCCTTTCTGGCCAGATACCAGATGAGATTGGTGACTGTTCAGCACT
GAAAAACTTGTAAGTATAAAATGCTTCTGATTCTTGTGTTATTATTTGGAAAATAAGTAACCATTTTTTTC
CCTTAGGGACCTATCCTTCAATGAGCTTTATGGTGATATTCCCTTTTCCATATCAAAACTTAAGCAACTGG
AATATCTGTAAGTTTTGATACTCTTCTTCTTCTAGGTGTTGTATCATTTGCTTTCCAAGATTGTCAATTGAT
CATGCTTGTCTTCTTCAACTTAGGATTTTAAAGAATAATCAGTTGATTGGACCAATTCCATCTACATTGTC
ACAGATCCCCAACTTGAAGGTCTTGTAAGTATATTCTCTTCTGCTTTGTCATGATATTGGTAGATTATGAA
TAATTTCAATTTAATCCAAGAACTTCCTCCAGGGACCTAGCTCAAAATAGGTTAAGTGGAGAAATTCCTA
GGCTGATATACTGGAATGAAGTCCTGCAGTATTTGTGAGTGTTTTAATTCAGTGCTCCTCTTCTCTTGGTT
TCTAGAATACCTTAGGAGACTACCATTTTGTATATGGATGATCATATCTGTTGTATGTTTTTCATTGTATA
GGGGACTGCGTGGCAACAACTTGGGTGGATCACTTTCTCCTGATATGTGTCAGCTCACCGGCCTGTGGT
ACTTGTAAGTTTGTAATCTTTATGCTCTTCAGATCTTACTATAGCTCCTTTAGCTGATGACATTAACCATCG
TTCATTGTTTTGTGCAGTGATGTTCGGAACAATAGTTTGACTGGTTCCATTCCTCAAAATATTGGCAACTG
TACTGCTTTCCAGGTTCTGTAAGTATCTATAACAAATGAATGAAGTTGGACTATTTTCTAAATGTTTGGTT
GGCATAACACCTTGTTTTGTGCTATCAGAGATTTGTCTTATAATGACTTGACTGGAGAGATTCCTTTCAAC
ATTGGTTTCCTGCAAGTAGCGACCTTGTAAGTTTATGCTGCTTCTCTTTATCACAAAGCCATTCATTATAT
GGTTATAAGTTTGAAGTGTACTTTCATCATTCCAGGTCTTTGCAAGGTAATCGCCTTTCAGGGCAGATCC
CTTCTGTCATTGGATTGATGCAAGCTCTTGCAGTTTTGTGAGTTTTTGTGTCTTGTTATCTCAATCCAATGC
TACTGAATCATGAACGAGATTTCTTGGAAACCAAAAAATGCATCTGTTATTTAAGTTTTTCTGACCCTTTT
ACTGACAGGGACTTGAGCTGCAATATGTTGAGTGGAACAATTCCTTCAATTCTTGGGAATTTGACTTACA
CAGAAAAATTGTTAGTACTATAATAATTTAATTTAAGAGCAATTTGGATCATTTTGTGCTTCCTAAATTGT
ATACTGGATCAATTACTGTAAGTTCGCATTGTATTGCAGGTATCTACATGGGAACAAGCTATCTGGTTCC
ATTCCTCCAGAGCTGGGAAATATGACAAAGCTTCACTACTTGTATGAATGCTTTCTATTAATCATTTTTTG
TTGGCTTTGTTTTGTTCTTCCTGTTCAAACCCTTTTAAATGAATGCAAGCATTTGTTTGATTAGTGAGCTTT
TAGGCAACCACTGATTTGATTGATACAAAGCTGTTTTGAGAATTTTTTATAAGAGACTTTCCTCAATCTCA
AGGCTCAAAGATGGTAATTTGCAGAGAATTGAATGATAACCAACTTACTGGACGCATACCACCAGAACT
TGGAAAGCTGACGGAGTTGTTTGACTTGTAAATCCCGTTTCTCTTCATCTTCTACTTTGGACTTGTTAACA
TCATTATTTATTTACTTATGTGGTATGTTTCAGAAATGTTGCAAACAACCACCTTGATGGGCCCATACCTT
CCAATCTTAGCTCATGTACCAATTTGAATAGTCTGTGAGTGTTTTTCATATCCGATGTGTTTCAATTATGT
ATGATCATGCTTGTTTGGGAGTTATTGACACCTAATTTGGTTGCAGCAACGTTCATGGAAACAAATTGAA

TGGTACAATTCCACCTGCTTTTCAAAAGCTGGAAAGTATGACCTATCTGTAAGTTCTTACTTTTTGAATTTT
TCTTTTAGAGAAATATGTGCAAGGTTATCTAGTTATCATTCTACCCATTCGCGCTGTTAAGGAAGATTGTA
AACTTACTGTGCCAATTTTATATTTAAATTTTACCAACGTTGTATTATTTTGCACAGAATTCATTTTATTTAT
CTTGCTCTCAAATCGTTACTGGCTTATCCTTGCATGAAGAATCATTTCTATTGTCGAGAGCTTTCAGCTAT
GTGTGTCATATAATTTTTTTAATTATTAACTAGTTTATTTAGAGCCTCTGCATCATCTAGTTATGCGAATTT
CAATTGCTTATTCTGCATACTCTGAGCATTAACTGCATCTTTTTTTTTGTTAATTGCTCAAGTAATCTCTCCT
CCAACAACCTCAAAGGCCCAATTCCAATTGAGCTTTCTCGTATTGGGAATGTAGATACACTGTAAGTGCA
AACTTTCTCATCCACTTTCATTTCTCTCATTGCAATTATGGTTGCGGGAAAAGCACGTTTTGACAGTTTAA
GAACTCTTCAACATTTTTTTGGCTTAGGGACTTATCAAACAACAGGATCAGTGGTCGTATACCTTTGTCCC
TCGGTGATTTGGAACATCTTCTTAAACTGTGAGCATAACCGTCAGGTTGTTATGTTATCATCATATATCTG
TTGTACTTACATTCCTTTTGTCAATGCTGTAGGAACTTAAGCAAGAACGAAATAAATGGAAACTTGCCAG
CTGAATTTGGCAATTTAAGGAGCATCATGGAGATGTATGGAACCTTGCTAAATTTAGTTACTTCAAATTT
ATGGTTTGCTTGATTTTCAGCTTTTTGACCGCACTCTTAATTGTAGTGATCTGTCAAGCAATCACCTCTCTG
GTCCCTTGCCTCAGGAACTTGGTCAGCTTCCAAATCTGTACTTGCTGTAAGTACTTTAGATTTACTTCGAG
ACTCTCATCCTCTTAGCCATTGGTAATAATCTGTACAGTGAATAAGTATGAACTTCTAAACTGGGAAAGT
TAGATTTTAAATTATTTTGGATGCCATTTTCAAGAAAGTAGAGATGAAGTGGTGTTGTTGCGATTGGTTT
ATATTATCTGGCTACATATGTTAATTACTTTGGTGTTGTCTCAGTTTTTCTTTATATTGCATTATTGTACGG
GGCTCATAGGGCATCATTTCTCTATCTTCTTCTTTTCCTTGAGGCCTTATTCTACAAAATAATGAACAGGA
AACTGGAAAACAACAATTTATCAGGCGATGTGATGTCCTTAGCCAGTTGTCTCAGTCTAAATGTCCTGTG
AGTTTTCAAGCTAGTAGTATTACACCAGTACAAATGTTTTGTAAATCTAATCAACCGTTTTTTGGCAGAAA
TGTCTCATACAATAATCTGGGAGGGAATATTCCAACAGGCAATAATTTCTCTAGATTTTCACCAGACAGG
TAAGTGGAGCGATGAAGATTCCATTCATTGATCTTCCTCGTCATATGCCAAGTTCACACAAGTCACAAGC
ATTTATTGGTTTGTTTTTAATTCTTTGCTTCTAATTTTTTCCTTTTGCTATGTTTCCAATAAAGCTTCATAGG
AAATCCAGATCTGTGTGGGTATTGGCTCACTTCTCCCTGTCATGCATCTCATCCAGCAGAGCGAGGTATG
ATCAAACTGTAGCCATCATTTGACCTTTATTCTATTGCATTCTTGAAGTTTCATTTCACTTTAGACACTTGC
AACATTTATTAAGCGCGATGGACAGATATTGATTAATGAGGAATTACCCCTTGGTTGAGCAAATTTAATT
CTGTGTTAGTCTGGTAGTAGGGTGCACGATCACGGTTTCCTCTGTTCACAATCCTTGATTGTAACACTTG
GATGTGTGCAGTAACACTTGGATGTGTGCACATATTAAATTAACATGAAATAATATTCATTTGCTGGAGT
TACATTGACGTAAAGATGCCTTAGCTGTCAAATGAAACTGCATTAGTTTTATCCCCATCATCAGTACATTA

AGGGGTCATTTGGTAGAGTGTATTAGTAAAAATAATGCATGCATTAATTACATGTGTTAGTAATCCCTTG
TTTGATGCATTTTTTCATGCTATGTATAACTAATGCTATACACTCTATTGTGTATTGAGGAGTGTATTACTA
ATACCATGGATTTCTAGGTATTAGTAATGCAAAGGATTTAATACATGCATTAATATGGTTAAAGACCCAA
TTACCCCTCAAAAGTACTTCTACATCTTTTTCACCATAATTGTGAAGGATACTTTTGTAAATAAATATTTTA
TACAATGCATACTATTTTCATACACCAAACCAAACAATGCATAAGAAATAATATATGCATAACTAATACA
AGTATAATTAATACATGCATTACTAATGCTTGCATTACTAATACACTCTATTGGGTATTATTTTTATACACT
CTACCAAATGACCCCTAAGTGCATAAATATTTTAACAGTTGTCTAATGAAAAAAGATGAATTCATTGGAC
AATTGCAGTTTCAATTTCTAAAGCTGCTATACTTGGTATTGCTCTGGGTGGCTTGGTGATTCTTCTGATGA
TACTAGTAGCAGCATGCCGGCCACAGAATCCTGCACCTTTCATGGAAGGATCTATTGATAAACCAGGTA
CAATATTTTCTGGACGGTTGGATAGTGTTTGGAGACGTTCATGTCTTAAGGACAGTCATCAGAGTTAATT
GAAGTTGCCATGTATTGATTATTTAACGTTTTTGATGAACAGTTTATTACTCATCTCCAAAGCTTGTGATC
CTTCATATGAACATGGCACTTCATGTTTACGAGGACATTATGAGGATGACTGAGAACTTGAGTGAGAAG
TATATAATTGGTTGTGGAGCATCAAGTACTGTATATAAATGTGTTTTGAAAAATTGCAAACCTGTAGCTA
TCAAGAAATTGTACTCTCACAACCCGCAATACTTGAAGGAGTTTGAGACTGAACTTGAGACAGTTGGGA
GTATTAAGCATCGTAATCTTGTCTGCCTCCAAGGATATTCTCTTTCTCCATCTGGCCATCTTCTTTTCTATG
ACTACATGGAAAATGGTAGCCTTTGGGATTTGCTTCATGGTTAGTAAATCCAAAATGGTTTAGGTGATTT
GGTTCATTGAACTTGTGTTTTAAGCATTTCGTAATAATCCCTCTTATATCTTTTTTGCAGGTCCTACAACAA
AGAAGAAAAAACTTGATTGGGTTACTCGCCTTCGAATTGCATTGGGATCAGCTCAAGGGCTTGCTTATCT
TCACCATGATTGTAGCCCTCGAATTATCCACCGTGATGTTAAATCATCAAATATCTTGTTGGACAAAGACT
TTGAGGCTCATCTGACTGATTTTGGCATTGCCAAAAGCTTATGCATATCAAAGACCTATACGTCCACATA
CATTATGGGAACCATTGGTTACATTGATCCAGAGTATGCTCGCACTTCTCGCTTGACAGAGAAGTCTGAT
GTTTACAGCTATGGAATTGTTCTATTGGAATTGCTCACTGGAAGGAAAGCTGTAGATAATGAATCTAATC
TACACCATATGGTATGCTCTTGTAATTTAGTTAATATGAACTTGTGCTATGATGTATCTTCATATAATTATA
TAAAGATTCAATTGATCTCAACAGTTTTGCATATGTTACAGATTCTAACTAAGGCAGCAAACAATGCTGT
AATGGAAACAGTGGATCCTGAGATCACAGGCACATGCAAAGATCTTGCAGATGTGAAGAAGGTTTTTCA
GCTTGCCCTTCTATGTTCCAAAAGACAGCCTGCTGAGAGACCAACAATGCATGAAGTGGCAAGAGTACT
TGAAAGCCTAATACCCGTCGCTGAAACGAAACAGCCAAATCCAACCCCCTCACTTGCATTACTTCCATCT
GCTAAGGTACCTTGTTACATGGATGAATATGTCAACCTCAAGACACCCCATCTAGTGAATTGTTCATCCA
TGAGCACTTCAGATGCCCAACTTTTCCTCAAGTTTGGAGAGGTCATATCCCAGAATAGTGGCTGAAAATA

ACATGAGAGTAGATTTCTTGAGATTTTGTGTAAAAATGTAGTGCCATTATCATATTATTATTGTAGGTAGT
TGTTGTAAGATGATGCATGCAATAGTGGTCCAGTCTACTTTTTCCACTACATAGGTCTAGTGTGTGTAAA
AATATTTCACTTTTTACCATGATGAAATTGGAAGAGGTAGCACTTAGTAGAGTATTGTAATATTGGTTTTT
GGGACCACTGATGCTGAGTATGGACTATACTGTCTGTAAAATTTTTGGCACACACTTTGAGGTGGCCTTA
GCACTGCCCCTCTGTGTATAGCAATAATGGATTGCATTGTGGACCTAAGTTTGACAGTCTGCCGCTCTCT
CTTGCAGCTGCAGTACATTAGTGCATTTATTTGGTGGACTACTCAGAAACACGAAAAATAATTTTGACAT
TTTAATTATTACTCTACTTCCTCAGATTATTAAAT

>PGSC0003DMG400018849 LRR-RLKs

ACTTAACCCACTAAAATCATCATGAAACACACTCTATTTTTCCAGCACTCCTAAAAAGCATTTTTCTCTCAA
ATTTACCTCTCTTTACATACCCCATTTCTCAACCATTTCCACCATAAACAACATCACCATGAGACACTTGTT
TCCTTTCTTTTTTACTACAGTTTTCTTCTTCCTAACAAGAATTTCACTTGTTTTCTCCATTACCAATCTACCTC
TACAACTTATATCTCTTTTGACTTTAAAATCTTCTTTGCATGATCACCACAATACCTTCAATGACTGGGATC
CCACCCTCGCCTTCGCTAGACCGGGTTCTCACATTTGGTGTTCCTGGTCTGGTATCAAATGTGACAAGAA
AACTAACCAAATCACCTCTCTTGATCTGTCGAAGCGCAATCTTTCTGGTACAATTCCAGAAGATATCAGA
AACTTGGTACACCTTCATCACTTGAATTTGAGTGGAAATGCTTTGGAGGGTCCTCTTCAAACAGTCATTTT
TGAATTGCCTTTTCTCAAGACACTTGATATTAGTCACAACTTGTTTAATTCAACTTTTCCCTCTGGTGTATC
AAGGCTCAAGTCACTAACATACCTAAATGCTTATAGTAATGACTTCGTAGGCCCTTTGCCTGAAGAAGTT
GCTCAAATCCCAAATCTTGAGTACCTTAACTTTGGGGGAAACTATTTCAAGGGTGTGATTCCAAAAAGTT
ATGGTGGCTTAGCAAAACTGAAGTTTTTGCACTTGGCTGGAAATTTATTGAATGGTCCTGTTTTGCCTGA
GTTGGGTTTTTTGAAGCAGCTTGAACATGTAGAAATTGGTTATCAGAATTTCACTGGAGTTGTCCCTGCT
GAATTTTCTTCTTTGTCAAATTTGAACTATCTTGATATCTCATTAGCCAATCTTTCTGGTAATCTTCCTGTTG
GACTAGGCAACTTGACAAACCTTGAAACTCTGCTTCTTTTCAAGAACCATTTTTATGGTACAATTCCATTG
TTTTTTGCCAGACTGACATCCTTGAAATCATTGGATTTGTCTGACAACCATCTCTCCGGAACAATCCCCGA
AGGGTTTTCAGGATTCAAGGAGCTTACGGTCTTGAATTTGATGAACAATAACTTAACAGGTGAAATCCC
ACAAGGCATTGGTGAGCTTCCTAATCTTGAATTATTGGCTCTATGGAACAACTCACTCACTGGGATTTTA
CCACAGAAGTTGGGTTCAAATGCAAAGCTACAAAAACTTGATGTCTCTTCAAATTATCTCTCAGGTCCCA
TTCCACCAAATCTTTGTCTCAGCAATAGCTTAGTGAAACTTATCCTGTTTTCAAATCAGTTTATTGGTGAG
ATCCCTTCTTCCTTAGCAAATTGCACTGCATTATTCAGGTTTAGAATTCAAAACAACAGACTCAACGGGTC
AATACCATTAGGCTTCGGTATTTTGCCCAATTTAGCTTACTGGGACTTGAGCAAAAACAATTTCACTGGTC

CAATTCCAGAAGATTTAGGGAATACTGCGACATTGGCGTATTTGAACATATCGGAGAATCCATTCAACA
GTGAATTACCGGAGAGTATCTGGAGTTCACCATCTCTACAGATCTTTTCAGCTAGTTACAGTGGCCTTGT
CGGGAAAATCCCCAATTTCAAAGGGTGTAAAGCTTTCTACAGAATCGAACTGGAAGGAAACAATCTCAC
AGGAAGCATTCCATGGGATATTGAACATTGTGAGAAGCTCATTTGCATGAATTTTCGACGAAATTCGCTA
ACGGGAATCATTCCTTGGGAAATTTCTGCAATTCCTTCCTTAACAGAGGTTGATTTGTCTCACAATTTTCT
CACTGGAACAATTCCTTCGAATTTCGCCAATTCCACTACCATAGAAAACTTCAACGTATCCTACAATCAGC
TCACTGGTCCAGTTCCTTCTTCAGGCTCAATTTTCTCCAGCCTACATTCCTCTTCCTTCATCGGAAACGAAG
GCCTCTGTGGTACTGTCATTCAAAAGCCTTGCGGGACTGACGGACTCGCAGCCGGAGCAGCGGAAATCA
AGCCACAGCCGAAAAAAACAGCTGGCGCTATTGTCTGGATCATGGCAGCAGCATTCGGTATTGGTTTAT
TCGTCCTCATCGCCGGTAGCCGCTGTTTCCACGCGAAATACAGTCAGCGATTTTCAGTAGAGAGAGAAG
TTGGGCCATGGAAATTAACCGCATTTCAAAGGTTAAACTTCACGGCGGATGATGTACTTGAAAGTTTGA
CAATGACAGATAAGATCCTGGGAATGGGTTCAACAGGGACAGTGTACAAGGCGGAAATGCCAGGCGG
AGAGACCATAGCAGTAAAGAAACTATGGGGAAAACAGAAGGAAACGATCAGGAAAAGGCGAGGCGTT
TTAGCTGAGGTGGATGTGTTAGGCAATGTGAGGCACAGGAACATCGTGAGGTTGTTCGGCTGTTGCAG
CAACAACGAGTGCACCATGTTGCTGTATGAGTACATGCCTAATGGTAGCCTGGATGATCTGTTGCATGG
TAAGAATAAGGATGCTAATTTGGTGGCTGATTGGCTCACTAGGTACAAAATTGCTCTTGGGGTCGCTCAT
GGAATTTGTTATCTTCATCATGATTGTGATCCCGTCATTGTTCATCGTGATCTTAAGCCTAGTAATATTCTT
CTGGACGGTGATCTGGAGGCTAGAGTGGCTGATTTTGGTGTTGCTAAGTTGATTCAGTGTGATGAATCT
ATGTCCGTGATTGCTGGATCTTATGGCTACATCGCACCAGGTAAGTTAGTACTCCCTCCGTTTCAACTTGA
CATGAAATTTAACAAAGTAATATTCAACTTTTTATCGTGTTCTTTAATTTAATTATTAATGGCAGGAATATT
TGTCTGTTTGTTTGATGGAATTACATGATTGTTTTGGAGTTCAAGTTTTTTTCTTGTGCGGCTTATTGCTAT
CCAAAATTATTTTGTTCAGATCAGTTTAGTTCGTTCTGTAGGGTGACGAAAAAGAGTTCACTTGCGTGCT
AAAACGTGGTGTCATGTCAATAGCATGATGTATATTGTTTTTTTTTTCGTCCACTATCGTCATTAAATCTTC
TTTGCCCACACAATTGTCAAATTCCTCAACTGCTTCATACTAGTGGAATCTTCTTTTAATCATAACCAGATC
TTGGAGGCACTAATTCATTTTTTGAATTTGAGCGCCACAATATAATATAAATATCTAAATGTTGAAACTTT
TATTAGGAAGTTAAAATAATATTCTTATGAAGATTCTAATATTAGTGTAGTGAAACAGGTAATATAATTTT
TTTTCTTAATAAAGGGTATCAAGTATTTCTTTTCTCATACTTCTTTATGACAATTGGTGCAAATATTTATTC
TCTTTGTTTTTGTCCATGATTTTGTGGTGCTTAAAGAGGGCATGGCGCACTAGTTGCGCGTGAATAACTC
CATTAGCCTTTAAGCTTTTATTTTACACTTTTTGTTATTCTTTTGGACCCACCAAGATTTTGTCTTAGTTACT

TATCTGTGATTAACCCTTTGATTTATTAGTATATAATATAGTACTAGTACTACATTCCTTGACTTTAATGCT
TAAAGTCAACCATTTTTGGGCACTAGTAGACAGAGTGTTATGATAACAAATTATTTTTATATAGTTATTGT
ACTAAATATGTTTGTTGATACAGAATATGCATACACATTGCAAGTTGATGAGAAGAGTGACATCTACAGC
TACGGGGTTGTGTTACTTGAAATTTTATCAGGGAAAAGATCGGTGGAACCAGAATTTGGCGATGGAAAT
AGCATTGTGGATTGGGTTAAGTCGAAGATCAAGACTAAAAATGGCATAAATGATGTGTTGGATAAAAAT
GCTGGCGCTTCATGTCTTTCAGTTAGGGAGGAAATGATGTTGTTGCTGAGGGTGGCATTGCTGTGTACT
AGTCGTAACCCGGCTGACAGGCCATCGATGAGGGATGTTATATCTATGTTGCAAGAGGCTAAGCCCAAG
AGGAAGTTGCCTGAAACTGGTGATAATGCAATTGCTGCTATTCCTTTGGCTCAAAAAGCTAATGTGGAG
TGTTGATATTATAGGAGGAGATTTGGGGTTGTTTTGATTTTACAACTTACTGATGAATTGTGGGGGAAAT
TGGTATAGGAGAAGTTTAAATAGTTTTATTTGCTTCTTAAATTTTTGCATAGTAGGAAATTCCCGAGTGTT
GTCATTCCAATGTTGTGATGAAATGGGTATCTTTTCCAACAAATTAATCTTCGATAGCATACTTGTCTAGA
C
>PGSC0003DMG400019051 LRR-RLKs
ATGATCGGTCTTACATCCCTAAGCCTTGACAGAAACAACTTGACAGGGAGTATTTCTTCTGAGATTGGTA
AGCTTAAACAACTCCAAGGGTTGTATCTAGATAACAATAAATTGCAGGGACTTATTACAGAGGCCGTAT
GCCATTTATCTAATTTGGTTCAATTATATCTGAATGATAATGACCTCTTTGGATCAATTCTAGAATGCTTA
GGAAATCTAAGCATTCTACTACATCTTTATTTGGATTCTAATAAATTTTCATCAAAATTTCCGCTGAGCCTT
GGGAAGATGAGTCGTCTTATCTATCTAGACGTGTCACAGAATTCAACAGAGGGAGAACTTCCATCAGAT
ATTGGAGGACTAAAAGCCATTGTAGAACTATATCTTTACAATAACCACTTTTCAGGTGTGATACCAACCA
GATTCGGGGAACTCCAAAACCTGCAGTATCTTGACCTATCCAACAATTCATTTTTTGGCCAAATTCCATTA
TCCTTTGCCAACTTGATAAGCTTGGAATTCTTGAATTTGTCTTTAAATGCATTGTCAGGTAGTATTCCTAT
GTCTTTGGAAAAACACTCTTAAAAGCATTAATGTTTCATTTAATAATTTAGAAGGTAAAATACCGAGTGG
TGGCGTGTTTGCAAATTCCACTTTGCAATCATTTCTAGGGAACAAAGGTCTTTGCGGAATGCACATATTG
GAGATTCCTTCTTGTGCTATCACTAATCCTGGAAAACAATCAAAGTATAAGGAGGTTGTGCTCAAATTTG
TTACTCCAGTGGTTATTGCATCCTTTCTTATATTCTTGTTGGTTTCAATTTGGATAATGAAACGACAGAAG
AAAGGAAAGTCTAAAGATGTTGAAAAGGTACCAGAGATCGGGACTCATCAATTAGTTTCTTATCACGAG
ATTCAACGAGCAACAAATAATTTTGATGAATCCAATTTAATTGGAGAGGGAAGCTCTGGCTCTGTGTACA
AAGGCACATTGTTTGGTGGAACTGCAGTGGCCATTAAGGTTCTATATTTGGAAAATGAGCAAGTATGCA
AGAGGTTTGATACTGAATGTGAAGTGATGAGAAATGTTAGACACAGAAATCATGTTCCAGTGATTACTA

CATGTTCTAGTGACTTTATAAGAGCCTTTGTTCTGCAATTTATGCCCAATGGAAGTCTAGAAAATTGGCT
GTACAAAGAAGATCGCCACTTGAACCTTCATCAAAGGGTCACTGTAATGCTTGATGCAGCTATGGCAGT
TGAATATCTACATCATGGTCATGTCACTCCAATAGTTCATTGCGACCTAAAGCTAGCCAACATTCTTTTGG
ATGAAGATATGGTGGCTCATGTTGGTGATTTTGGAATCTCTAAAATTTTAGCCATAAGCAAGTTCATGGC
TTATACCGAGACATTGGGCACTCTTGGATACATTGCACCAGATATAAAAAAATCTACTCTCTTTGATTTTC
TCTTATATCATAATTAA
>PGSC0003DMG400019415 LRR-RLKs
ATGGCCTATTATCTTGACAAAATCTTTCTTCTCTTATTTCTCTTCACACTTAGTACTCCCTCAAAATCAGCTA
ATATGACAGATATTGAAGCACTCTTTGCTATCAAGAATGAAATAATTGATCCCTTTGAATCATTAAGTTCA
TGGAATGAGTCTGTTCCTTTATGTCAATGGCGTGGCGTTGTATGTGGAACGCGAAATCAACGAGTCATT
GAACTAAACCTTCTTGACCATAAGTTAGCCGGTGTGTTATCACCCTTTGTTGGAAATTTGAGCTTCCTTAT
ACGACTAGACGTCGGAAACAACACCATTTCTGGCAATATCCCTCCTGAATTAGGCCGTTTGACAAGATTA
CGCCATCTATATTTGCAAAACAATTCATTTTATGGTGAAATTCCTGTCAATCTATCGTTTTGTAGTAACCTC
GTTGAGTTATGGGCGTACAAGAACAATCTTGTAGGTGTTCTTCCAAGGGAGCTTGGTTTACTTACCAAAC
TCAAATATTTTGATTTCAGTTACAACAAGCTCATTGGTGAAATCCCGAAATCTTATGGTAATTTTTCAAGT
CTATTGGAAATGTACCTTTTGGATAATGATCTCGAGGGTAAAATCCCCGATGAGTTTGGGAAACTGAAG
AGTTTGGAAATATTTGACGCGGACTTTAATAGGTTATCTGGTAGAATTCCGAGCTCATTCTACAATCTTAC
CTCCTTGAAAGTCATTGATGTGTCATTTAACCAACTCGAAGGTACTCTGCCTCGGGATTTAGGGATCAAC
CTTCCTAACCTTGAGTGTCTCAGCATTAGTGAGAATCAATTCACTGGATCGCTTCCTTATTCACTTTTCAAC
CTCACAGGACTCGCGTACCTTTATGTTGGAAAAAATGACTTAGGAGGACGTGTTCCCAGGTTCGATAAG
TTGCATAATTTGTATATTTTAAGCATGGAAAATAACCAATTTGATAATGACTTGACCCTCATGTCCTCCTT
AACAAATGCAACTAACCTCAACCGTCTGTATCTGCAAAACAACGCGTTTGGAGGGACATTTCATGAGTTT
TTCAGCAACCTTTCAAGTAATCTGGCGTTTGTAGACTAGTCAAGCAATCAGTTTTCAGGAAATATCCCGA
TAGACATTGGGAAATTCAACAACTTGGAAGAGCTTAGGCTGAGCGGAAACCAACTCACAGGTATCATCC
CTTCGACTATTGGGAAGCTTTATAAGCTACGATATTTACACCTGTCCCAAAACAGAATTTCGGGTAGTAT
ACCCTTTTCCATTGCAAATCTGAGCCTTTTAACTGAGTTAAATCTTGATCATAACAATCTTAGAGGTAGAA
TACCTTCAAGTGTTGCAAAATGTCAAAGTTTGTTGTATCTTAACCTGAACCAAAACAATTTAATCGGTTCG
ATACCTAAAGAAATTTGTCTCATATCTTCCTTAGTTGTTCTAAACCTTTCTAGTAACAACTTGACTGGCGCC
CTTCCCAAAGAAATCGAAAGCCTGAAAAATCTCTTATCATTAGATGTCTCTTTGAATAATCTCAATGGTGA

AATTCCTAGTACACTTGGGAGTTGTGTGACAATTGTTAACTTAACAATGAAAAGGAATTTATTCCGCGGG
ATTATTCCTTCCACTTTCAGTTCACTAAAGAGTCTTCAAGTTTTAGATCTTTCTCAGAATAAGCTATCAGGT
ATGGTTCCAAAGTACTTGGAAGGATTGGCCTTACATTTCTTAAACTTATCGTTTAATGATTTTGAAGGTG
GCCTTCCACAACGAGGCATCTTTGAGAACGCGAGTGTAGATTCTTTCGTTGGAAATTCTAGAATCTGTGG
TGGTGTACCTGGCTTGAAACTTCCCAATTGCAACTTCAGTCATTCAAAGAAAATGAACTTTAAGCTTGTTA
CTTTAGTAATATTAGGAATTTTGGGGCTAGTTGTTATGGTTTGTGCTTTTTTCTTCTATCGATTCGGAAGG
TCCAAAAGAACAATTCCTTCATTAGATAGCAATCTAAACCAACTCATAGCTATGTCCTATCAAAGCATTCT
CAAAGTTACCAACGGGTTTTCTACGAGCAACTTGATTGGTGTTGGGAGTTATGGATATGTTTATAAGGG
AATTTTAGAAACGGATGGTAAGCACGTTGCTATAAAAGTATTGAATCTTTTACAATATGGAGCTATCAAA
AGTTTCATAGCAGAATGTGAGGCCTTGAGAAATGTTAGACATCGGAATTTAGTTAAGCTACTTACTGCAT
GTTCTTGTGTTGATTATCGTGGCAACGAATTCAAGGCATTGGTTTACGAGTTTATGGCCAATGGAAGTTT
GGAGGACTGGTTGCATCCAGATAATTCAAGACCAAATGTTCAACCAAGAAGATTAGGATTTCTACAAAG
GATGATTATTGCTATTGATGTTGCTAGTGCAATTCATTATCTTCATAATGATTGTCAAATATCAATAGTTC
ATTGTGATCTCAAACCAAGCAATATTCTCCTTGACAATGAATTGGTTGCCCATGTTGGGGATTTTGGATT
GGCAAGATTCCTACATCTTACTGATGAAGCCACTTGTAGAATTCAGACAAGCTCTTCAACTTTTAAAGGA
TCTATTGGCTACATAGCTCCAGGTAAATTTCACTCTATATAAATGTCTCTAAAGACTTTAGTGACTCTGGA
TGCAATGACATTAACTCACTTGCCGCTATATTTTCATTGCAGAGTATGGTATGGGAAGCGAGGCATCAAT
GCAAGGTGATGTGTATAGTTTTGGTATCGTCTTACTTGAAATGTTAACCGGGAAAAGACCAACAGATGA
CATGTTTGGCGGCGATTTAAGCCTTCATGATTTTGTTAGGAATGCCATGCCTGATGGAGCACTTGAGATA
GTAGATCCACTTCTTATTTTGGAAGAGGAAGAGATAAGCAGAGAGAGGTCTCAAATTCCAAGATTCATC
AGAAGGCAAAAAATGGTGGAGGGTATAATCTCCTTATTTGGAGTTGGAATTGTTTGTTCAATGTATGATT
CAAGTAAAAGAAAAAACATGAAGGAGGTTGTTAGAGAGTTGTGTTCTATAAGAGATTCTCTAGTTGGTT
GTACAATATGA

>PGSC0003DMG400019698 LRR-RLKs

TCTTCACATTCTACTACTCCTTTCCCTTTCTCTATTTGTCTGTTTAAAACTTCTACTAATCCTATCTTTCTTTT
CAGAAAAATATGGAACCCCTTCAAGAACTCAAGCTATAGATTCAAGAAAATCTCCATTTAAGCTATAAAG
TTTCAATCTTTGAAGATGAAAGCTCACAAAACTGTGTTTTACCAACACCCTTTAAGCTTAAACAAGCTTTT
CTTTGTTCTTCTTCTTATCTTCTTTCTTCCTCCAGCTTCACCAGCTTCTGTTAATGGTCTTTTTAAAGACTCCC
AACAGCTTCTTTCCTTTAAAGCTGCACTTCCACCAACCCCTACTCTGCTTCAGAACTGGTTGCCATCTACTG

ACCCTTGTAGTTTCACTGGTGTTTCATGCAAGAATTCTAGAGTTTCTTCTATAGATCTCAGTAACACTTTTT
TAAGTGTGGATTTCAGTTTGGTCACTTCTTATTTGCTTCCTCTTTCTAATTTGGAGTCTTTAGTGTTAAAGA
ATGCTAATCTTAGTGGTTCTTTAACTTCTGCTGCTAAATCCCAATGTGGGGTTTCTTTAGACTCCATAGAT
CTAGCTGAAAACACAATTTCAGGACCTATTTCTGATATCTCTAGCTTTGGTGTTTGTTCAAACCTTAAGTC
TCTTAATCTTTCTAAGAATTTCTTGGACCCTCCTGGTAAAGAAATACTTAAAGGTGCAACCTTTAGCCTCC
AAGTTCTTGATCTTTCTTACAATAATATCTCAGGTTTCAACTTGTTTCCATGGGTTTCATCTATGGGGTTTG
GTGAACTTGAGTTCTTTTCTCTCAAGGGTAACAAACTAGCTGGAAGTATTCCTGAATTAGACTTCAAGAA
TTTGTCACATTTGGATCTTTCTGCAAATAATTTCTCAACTGTTTTTCCTTCATTCAAAGATTGCTCCAATTTG
CAGCACTTGGATTTGTCATCCAACAAGTTTTATGGTGATATTGGTTCTTCACTTTCTTCATGTGGGAAGCT
CAGTTTTCTCAACCTTACCAATAACCAGTTTGTAGGTTTGGTCCCTAAACTACAAAGTGAAAGCCTACAGT
ATTTGTACTTAAGAGGGAATGATTTTCAGGGTGTGTACCCAAACCAGCTTGCTGATTTGTGCAAAACTGT
GGTGGAATTGGACTTGTCATACAATAATTTCTCAGGTATGGTTCCTGAGAGCCTTGGTGAATGTTCAAGT
TTGGAACTTGTTGATATTTCCAACAATAATTTCTCTGGTAAGTTGCCTGTTGATACTCTCTTGAAGTTGAG
TAATATGAAGACTATGGTCTTATCATTCAACAAATTTGTTGGTGTTTTGCCTGATTCTTTCTCTAATTTGCT
GAAATTGGAGACTTTGGATGTGAGTTCTAATAATCTCACAGGGGTTATTCCATCTGGGATTTGCAAAGAT
CCTATGAATAACTTAAAAGTGTTGTACCTTCAGAATAACTTGTTTGAAGGCCCTATACCTGACAGTCTAA
GCAACTGTTCACAGCTGGTGTCACTTGATCTTAGCTTTAATTACTTGACTAGGAGAATACCATCTAGTTTG
GGGTCATTGTCAAAGCTGAAAGATCTCATCCTTTGGTTGAATCAGCTTTCAGGGGAAATCCCACAGGAG
TTGATGTACTTGCAGGCTTTAGAAAATTTGATTCTTGATTTTAATGACTTAACTGGACCAATACCTGCAAG
TCTTAGCAACTGTACCAAGTTGAATTGGATTTCATTGTCAAATAACCAATTGAGTGGTGAGATACCGGCT
TCTCTTGGTCGTTTGTCGAATCTAGCCATTCTTAAGCTTGGAAACAACTCAATCTCAGGGAATATACCTGC
TGAGTTGGGTAATTGCCAGAGCTTGATATGGTTGGATCTCAATACTAATTTCCTGAGTGGCTCCATTCCA
CCGCCTTTGTTCAAGCAATCTGGAAATATTGCAGTGGCATTGCTGACCGGAAAACGATATGTGTATATTA
AGAATGATGGGAGTAAGGAGTGCCATGGAGCAGGGAATTTGCTGGAGTTTGGAGGGATTAGACAAGA
ACAGCTGGGTAGAATCTCAACAAGGCATCCTTGCAATTTCACAAGAGTTTATAGAGGTATCACTCAGCCA
ACATTTAACCACAATGGCTCTATGATATTTCTTGATTTATCTTATAATAAGTTGGAAGGTAGTATCCCAAA
GGAATTAGGGACCATGTACTATCTGTCTATATTGAATTTGGGGCATAATGATCTGTCTGGTATGATTCCT
CAAGATCTTGGAGGCTTGAAGAATGTTGCAATTCTTGATCTGTCATATAATAGGTTCAATGGCCCGATCC
CGAATTCCCTCACCAGTCTTACATTGCTTGGAGAGATTGACCTGTCAAACAATAATCTCAGTGGAATGAT

TCCTGAATCTGCACCATTTGATACATTCCCTGATTATAGGTTTGCAAATAATTCTCTCTGTGGGTATCCTCT
CCCCCTACCTTGTAGCTCGGGGCCGAAATCGGATGCAAATCAGCATCAGAAATCTCACCGCAGACAAGC
ATCGTTGGCAGGGAGTGTGGCGATGGGTTTGTTATTTTCCCTCTTTTGTATCTTTGGTTTGATTATTGTTG
CCATAGAGACGAAGAAGAGGAGGAAGAAGAAGGAGGCTGCCCTTGAAGCTTATATGGATGGTCATTCA
CATTCTGCAACTGCAAACAGTGCCTGGAAGTTTACGAGTGCTCGTGAGGCGTTAAGCATTAACCTCGCA
GCATTTGAGAAGCCTCTCAGGAAGCTCACATTTGCTGATCTTCTCGAAGCCACCAATGGTTTCCACAACG
ACAGTCTTGTAGGCTCTGGTGGTTTTGGTGATGTCTACAAAGCTCAGTTGAAGGATGGCAGTGTTGTAG
CCATTAAGAAGTTGATACATGTCAGTGGACAGGGTGATCGAGAATTCACTGCTGAAATGGAAACCATAG
GGAAGATCAAGCACAGGAACCTTGTCCCTCTTTTGGGCTACTGCAAAGTAGGGGAAGAAAGACTACTG
GTTTACGAATACATGAAGTATGGAAGTCTTGAAGATGTCCTGCACGATCGGAAGAAAATCGGGATCAA
GCTGAATTGGCCTGCAAGAAGGAAAATCGCCATTGGAGCTGCGAGAGGGTTGGCTTTCCTACACCATAA
CTGCATTCCACACATCATTCACCGGGACATGAAATCAAGTAATGTCTTGCTTGATGAAAATTTGGAAGCC
AGAGTATCTGATTTCGGAATGGCAAGGTTAATGAGTGCTATGGACACTCATTTGAGTGTCAGCACTCTTG
CAGGCACTCCCGGATACGTTCCTCCTGAATATTACCAAAGCTTTAGATGTTCAACAAAAGGAGACGTTTA
TAGTTATGGTGTCGTATTACTTGAGCTTCTAACCGGCAAACAGCCAACAGATTCAGCTGATTTTGGTGAC
AACAATCTTGTCGGATGGGTAAAGTTGCACGCTAAAGGAAAAATAACTGATGTCTTCGATCGAGAGCTA
TTGAAAGAGGATCCAAGCATTGAGATTGAACTTCTACAACACTTAAAGGTAGCTTGTGCTTGCTTAGATG
ATCGACATTGGAAACGTCCCACGATGATACAAGTTATGGCTATGTTTAAGGAGATTCAAGCAGGGTCAG
GCATGGATTCGACATCCACAATCGGAGCTGATGATGTCAATTTTAGTGCAGTTGAAGGAGGGATAGAAA
TGGGGATAAATGAAAGTATAAAAGAAGGCAATGAGCTAAGCAAACACCTTTGAATGCAGCAGCACTAA
ATCAAGAGTTTGTTGAAAGCTCACAAATTTTCCAAAATCATATGCAAAGAATTGCCAGTGTAATTTTTAG
CCAATTATTGTATGTACCACTAGTTCCCATCCATAAAATCTTGTGTTATTATACATAAAAGGAAGGTTGTT
TTTCTTTTTTAAAACATGTAACAGTGTTGTATATAAATAGTGTATGAGTTTGTTTTTGC
>PGSC0003DMG400019801 LRR-RLKs
ATGGTTTCCATGACTATTATTATAGAATTGCTAGTTTTGTTAACGTACCTCTTGGATACTAATGCATCGAA
CATTTCAACTGATGAGGCTGCTCTTTTAGCTTTCAAAGCTCATATAACTTCAGATCCAAATGGAATGTTGT
CGAAAAACTGGACCAAAGGGACTCACGTTTGCAATTGGATAGGCATATCTTGCAGTATAAGGCATCAAA
GAGTGACATCACTAGACCTCAACGGCCTCAGACTAAGAGGTTCAATTGCCACAGAAATTGGGAACCTCT
CATTTCTTAGTTTCCTGGACATTGGCAACAACAGCATCCACAGCAAAATCCCTGATGAACTCGGCTATTT

GAGGAGGCTAAAGTACTTGTCCTTGTCAATGAATAATCTCACAGGTCAAATTCCAGCAAGTCTAGGATTA
CTGAGAAGGCTCGAATCACTTGATCTTTCTGAAAATGATCTCCTTGGATCTGTTCCTTCATCTATCTTCAAT
GTCTCTTCTTTGAAGGTCATCAATTTGATTTATAATGCACTGTCTGGGACTTTGCCGAATGACATTTGCCA
TAATCTTCCGCGGCTGGAGGGATTACAAATAGCAAGAAATCAACTCTCTGGCCAACTACCAAGCAACTTA
AACAAATGCAAAGAGCTTACATACTTGTCCTTGTCATATAACCAGTTCACAGGTAAAAATACTGAACCAA
TTTCTTTTTCTGCTACTTTTGGTTCATATTGACAATAAAGATTGCAACAATTTTTAGTGAAAACAATGATAA
GCTGTTGAGAGGTATAACTTATTGTAACGTCTATTACAGGAAACTTGCCTAGAGACATGTGGAACTTGAC
AAAACTTCAACATCTATTTCTTGGATGGAATAACATAACAGGTACTTATAATGTTCTAGTTTAACTGAATC
TGCCTACAACTGCATTTATTGCTGGACTTCTCACCTCGTAGCACTAGAGATCTTTGACATTTCCTAACTTG
CTCTTCAAGTCATTTATATGAAATTAGTATTTGCATTTCAAAGGGTAGTAGCAGAAAATGTTAGTATAGC
TCTTTGTTCTTCATTTTCAAACAACAACAGCAACTACTCCATAGGTTCAATGCTAGTTTGTGATGTGAATC
CTCATTATCCATGTTGATCCATTTAGTCCCATCTTCAAGAGGACGTTAAAACAGATTGCGAGGATTGCTG
AACTATTTAAAAGGCATACAACTAACAGTTTTCTGTTATTTATAGTAACTTATTGACTGTAAAGCTGTTAT
ACAGTATTGGACATTTCAGTGTAGTAACCACACAATTCGTCATATATTTTACAGGACATATACCAAGTGA
AATTGATAACCTATCAGCAATTCGAAGATTAAGTCTACGACGGAACAATTTGGTGGGAAGTCTTCCACCA
TCTATTGGAAATTTGTCAAATCTAGAGATGATAGACCTAGGTGACAACAGTCTATATGGTGGCATTCCTC
AGGAGTTTGAACATCTTGTAAATTTGAAAGAAGTGTATCTTGGTCCAAACAGATTATCGGGTGAAGTTCC
AAGGCGTATGTACAACATTTCTGGACTAAAAAAGATTTCATTTGTAGCAAATACGCTTTCAGGAACACTT
CCCCCAAACATAGGCCATACTCTTCCAAATCTTGAAGGCCTTTACCTTGGGGAAAACCAGTTCACCGGGC
TGATTCCTACCTCCATTGTGAATTCCACCAAGCTTATCCAGCTTGATTTTGGTCGCAATCTGTTTAGTGGA
CCTGTACCTATGAATCTTGGAAAGCTACAACAGCTTCAATTCATCAACTTGCAAGTAAACCAATTGACGA
ATGATCAATCTACAGGCGAGTTAAGTTTCCTGACTTCATTATCTAATTGCAAGTATTTGAAAACCGTTCAA
ATAGGAGGTAATCAATTTAATGGAAGTCTTCCAAAATCTCTAGGTTCAGGAAACTGGTCCTTTTCCCTTG
AATATTTCATTGCCATTCAAAGTGGTATCACAGGAAAAATTCCAACTAACATCAGTAACTTTAGGAACTT
GGAGTGGTTAACATTGGGAGACAATAAGCTTATAGGATCTATTCCTCCAGATTTGGGGAACTTGAGGAA
TTTGAAAAGATTTAGCCTGGAGAAAAACAATTTAGATGGTATTATACCAACTAGCTTGTGTAACATGGAA
AACTTGTACCAAATTATCTTGGGGAAAAATCAACTTTCTGGTGAATTACCAAGTTGTTTTGGGAATCTTTC
TTCTTTAAGAGAATTCTACTTAGATTCCAATACACTAATTTCCCATATTCCATCAACTTTTTGGAGGAACA
GAGATATTTCAATTCTCACTTTGTCCTCCAATTTGCTGAATGGGTCTCTTGCAGTAGAAATGGGGAATTTA

AGGAGTTTGCGGATATTACATTTACATGGTAATCAGTTCTCTGGTCAGATTCCTAGCACAATTGGCCAAC
TACAAAATTTGGTTAGCCTTTCATTGTCAAAGAATATGTTAGATGGTTCCATACCTGAACTATTTGAAGAT
TTGGTTTCATTGGAACACTTGGATCTATCTAGCAACAATCTATCAGGCATGATCCCCAAGTCCTTGAGGA
ATCTTGAACATCTCATTTATTTCAATGTCTCGTTCAATGGGCTCATGGGTGAAATTCCAGATGAGGCCATT
CGTAAATTTTACAGCTGAATCATTCATCGGTAACCCTGCATTATGTGGATCATCACGCTTCCATGTGATGC
AATGCAGAGCCACAAGTCTTGAAAGAAAAAGAAAGAGTAGAGTCTTAACTTCTGTTCTTGCATCAGTTTC
CTCAGGAGTTGTAGTCACAACCATTTTCATCATTTGGTTTCTGAAATGCCGAAAAAGGGGTACGGAACTT
CCTCTAGTTGATACATTTGGTCAGGTACATAAGAGGATTTCGTACTATGATATTTCTCAAGGGACAAACA
ACTTTGATGAAGCAAACTTGATTGGAAGGGGTAGCCTTGGTTTGGTGTACAAAGGGACACTAGCAGAT
GGAATGGTTGTGGCAATAAAGGTATTCAATACAGAACTGCAACACGCATTTAGGAGTTTTGAAGTGGAG
TGTCAAGTTTTACGTAGCATTCGACACAGAAACCTTGTTAAGGTGATAAGTAGTTGTGCCAATTTTGATT
ATAAAGTGTTGGTGCTAGAGTACATGCCCAATGAAAACCTCGAATGTTGGCTTCACTCTACTGACAAATT
ATTGGATTTAACTCAAAGACTAAAGGTGATGATTGATGTGGCTTCTGCTGTGGAGTATTTACATGGAGG
TCATTTGTTTGTAGCCGTCCATTGTGATTTGAAACCAAGTAACATACTTTTGGATGGAGATATGGTGGCA
AAAGTGAGTGACTTTGGGATATCCAAACTTCTAACAGCTGAGACGCTAATAGCACATACCAAGACTTTG
GGTACTATCGGCTACATGGCACCAGGTAAATGTTATTATCATGTTCATTTGAAGATTTTAAATGACGTTA
ATTATTTTCACTAATGATTGCGTGATTTTTTTGCAGAGTATGGATCAGAAGGCAAAGTATCAACCATGGG
AGATGTTTATAGCTTTGGTATTTTGTTAATAGAGACTTTTACAAGAAAGAGCCCCGTGGATGATCTATTC
GTTGGAGACTTCACCTTGAAAAGATGGATATGCCAATCATTACCAAACCGAGTGGTGGATGTAGTGGAC
ATTAATCTATTTTCACTGGACGAAGAAAATCTTACTTCTAAAGAGAGATGCTTCAAATCAATCATGGAAT
TAGCTCTTGAATGCACAAACGATTTGCCTGAAGAGAGAATTTGCATGGAAGATATTACTCTGCGACTCAA
GAAGATCCTAACTCAATTTCTGCAAAATGTTGTAACAAACTAA
>PGSC0003DMG400020594 LRR-RLKs
CCCCAACACCCCCACCCCACATCTCCATTCTTCCTTATTTATGCACCCACCACCCTAACATCTCCACCACAG
CCCCTTACAATCAATGGCTTTCCACCACCACCACTACCTCCTCCTCCACCTTCTCATTTTCTTCACTTCCCTT
TCTTTCACCTTCTCAGATGATGCTGATGTTATGTCCAAGCTTCTTGCAGCTCTTTCTCCAACTCCTTCTGGT
TGGTCAACTTCTAAGCCTTTCTGTTCTTGGACTAATGTCATTTGTGACAAGTCTTCAGCTACTGTAACTTC
AATTAATCTTGATTCTCAATCTGTTTCTGGTTCTTTACCTTCTGAAATTACTCAACTTTCCAATCTTAAAACC
CTTTCTCTTCAAAAAAACAAACTTTCTGGCCCCTTACCTTCTTTTGCCAACATGTCAAAATTAGCTGAACTT

TTCTTGGACAATAACCAATTCACTTCTGTTCCTCAAGATTTCCTTTTGGGGGTTCCCAATTTGGTAACTTTA
AGCATTAGTGAAAATGGGAGACTCTCTCCTTGGCAAATCCCTATTTATTTAACTGAAAGTACAAATTTGG
GATCTTTATATGCTAGTAATGCTAGTATTGTTGGTGTCATTCCTGATTTCTTTGATGTTTTTCCTAATTTAC
AGAATTTAAGATTGTCTTATAATAATCTTACTGGTTCTTTGCCTGCTTCTTTTGGTGGTTCTGAAATTATGA
ATCTTTGGTTGAATAATCAAGTTAAAGGGTTATCAGGTAGTATTGATGTGATTGGAAGTATGACACAATT
GTCTCAAGTTTGGTTACATGCTAATTCTTTTACTGGTAGTATTCCTGATTTGTCAAAATGTGAGAATATAT
TTGATTTGCAGTTGAGGGACAATCAGTTTACTGGTATTGTTCCTGAGTCTGTGATGTCTCTTCCCAAATTG
TTGAATATTACTTTACAGAACAACAGGTTGCAGGGTCCAATACCTCAGTTTAAAGATGGGGTTGAGGTT
AAACTTGAAACTACTAATAGTTTCTGTAAGGACACTCCGGGACCGTGTGATCCACAGGTCACTACACTTC
TTGATGTGGCTGGTGGTTTTGGATATCCATTGTCTTTGGCTGAGTCTTGGAAAGGGAATGATGCTTGCAA
TAGTTGGAGTTTTATAAGTTGTGATACAACAGGGAAGAATGTAGCTGTTGTTACCTTAGGTAAGAGGGG
ATTTTCGGGTACTATTTCACCTGCTTTTGCGAATTTGACTTCATTGAGGAGCTTGTTTTTGAATGATAATA
ATCTTACTGGTACGATCCCGGAGAGGTTGACAACTTTGCCTAGTTTACAAGTTCTTGATGTGTCTAATAAT
AATTTGTCTGGACCTATACCATTGTTTCCACCTAGGGTGAAGTTTACTCATACTGGTAACTTGTTTCTCGG
GACAAATATAACTACTGGTGGTGATGGTGGGGGAAGTGGTTCGGATGGTTCTGGATCGAATTCCAGTG
GTCGAGGTGGAAGTTCATCTGGTGGTTCGAAAGGGCCTTCAGTTGGAATGATTGTTGGTGTTGTTATAG
CAGTTGTTCTTTTTGTTTTAGTAGTGTTGTTTGTGTCTTACAAATGTTACATGAAGAGACACCATAAGAGA
TTTGGAAGGGTTGAGACTCCAGAGAAAAGCAATGAAATGGTTAAGCCTAGTCTCCCAAGTGTCGTGGGT
GGTTCGAATGGATATACTGGTGGCACCAGTGAGTTACAGAGTCAGAGCAGTGGCGATCATAGTGAGAT
ACCAGTTTTTGAAAATGGAAACGTGGCCATTTCCATCCAGGTCCTTCGACAAGTGACGAACAACTTCAGT
GAAGAAAATATCTTGGGGAGAGGAGGGTTTGGAGTTGTTTATAAGGGTGAACTACACGATGGGACTAA
AATCGCGGTGAAGAGGATGGAATCTGGGGCAATGGGTACCAAAGGAATGAACGAGTTCCAAGCAGAA
ATTGCGGTCCTTACCAAAGTCAGACATAGGCATTTGGTTGCTCTACTTGGTTCATGTGTTAATGGAAATG
AGAGACTTTTGGTGTACGAGTATATGCCGCAAGGAACTTTGAGTCAGCATTTGTTTGAATGGCAAGAAT
TAGGCTACAAACCTCTTACTTGGAAGCAAAGGGTAACGATAGCATTGGATGTGGCAAGAGGGGTCGAA
TACCTTCATAGCTTGGCACAACAAAGTTTCATTCATAGAGATTTAAAACCGTTAAACATCCTTCTCGGAGA
CGACATGAGAGCCAAGGTTGCAGATTTTGGTTTGGTTAGAAATGCCCCTGATGGAAAATACTCTGTGGA
GACACGTTTGGCTGGAACTTTTGGCTATCTTGCACCTGAATACGCGGGTAAGCATTTCTACTCCTTGTATC
ATTACGTCTTTCTTTAACTGAGAACTGATTTGTATGCCCCTAGCTGTAAACTTCAGATTTTAGCAAATTTTG

AAATTATTCGATCATCTGCATTGCTAGGCATTGTTGTCTTCATTACATTTAAAGACATTTGGCATATAATG
TAGAGGCAATCTCCATGCGTAAATGCTGCTTTGCAATTTTTATCGAACTCATGAACTATATACTACTATTA
TAAAAGATAAGTTTCCATTTCGTGTGGATCAGCATCTGAATAATTATCCTATTACATCTCCTCTTTGTTACA
GCTACTGGACGAGTCACAACCAAAGTAGATGTTTACGCCTTTGGCGTTGTTTTGATGGAGATCATTACTG
GTAGAAAAGCGTTAGATGAAACAATGCCAGATGAGAGGTCTCATTTGGTTACATGGTTCCGCAGAGTCC
TTATCAACAAAGAAAACCTCCGAAAGGCTATTGACTCGACACTTGATCCTGATGATGAAACATATGAAA
GCATCTCCAAGGTTTCCGAGTTAGCTGGCCATTGCACTGCTCGGGAACCTTTTCAGCGACCTGATATGGG
ACATGCTGTTAATGTCCTTGGTCCCCTTGTAGAGCAGTGGAAGCCTACAACAAGTGAAGATGATGACGG
CTATGGCATTATTGACCTCGACATGAGCCTTCCCCAAGCCCTTCAAAGGTGGCAAGCTGATGAAGGAAC
TTCAAGAATGTTCGATGACTTCTCAATCAGTCACTCGCAGTCGAGCATACCCTCAAAACCTTCCGGATTC
GCAGATACTTTCAGTTCAACAGATTGCAGATGATATGCAAAAAATTTGACAACAGTTCAAGCCATGCCAT
GCCACACCTCGCGCGGTTTTAATCCTTTGTGGATCAACATAGAGTATGCCAATATTCATTTGATTAATTGG
TTTCTTATTGTCAATGTCATTACTTTTTATGTGATTACATATTAAGTTAATATAGTCTTGATGTAAGGTGTG
TGATAACAGTTGTAAAGTCAGGTGAATGTCACATTTTTCTTTTGCCTTTTTGGAGACAACAGAAAATTGT
AACAAATTCTTTCATATTACATTGTTAATGTAAATCAAATTTCGAGCTTCCCCCTCC
>PGSC0003DMG400020697 LRR-RLKs
ATAATGGAGAAAGTCTTCACATATTTTTACTTAATAGCACTCATGTTGCTTTACTTGGGTGTGGCTAGTTT
AGCCATGACTCAAACCAACATTACAACTGATCAATCCGCTCTTCTTTCCTTGAAATTCCAAATCATTTCGG
ATCCCTTTCACTTGTTGGATGAAAGTTGGTCTCCAGCTATTTCTGTTTGTCATTGGGTTGGAGTCACTTGT
GGTTCTCACCACAAAAGAGTGACGTCATTGAATATTTCCAACTTGGATCTTATAGGCAAAATTCCTTCTG
ATTTTGGGAATCTCACATCACTTGTTTCTCTTGACTTGAGTAGCAATAATTTCTACGGAAAATTGCCTCAA
GAAATGGCACATCTTCATAGGTTAAGGTTTGTTGAACTTAGTTTTAACAACTTTAGCGGAGAGGTTCCTT
CCTGGTTCGGGGTCTTACACCAACTTCAAGTTCTGACTCTAAGGAATAACAGTTTCAATGGTCCCATCAC
CCTCTCTTTATTTTCTAATATTTCCACACTTCAAACTTTGGATTTGACATACAATTCCTTAGAGGGCCAAAT
ACCAAAAGAAAAAGGAAATCTTGAAAACCTGAGGGTTTTAAGATTATCTGGCAACAAGTTTATAGGTTC
TATCCCTCCGTCTCTCTCGAATGCCTCAATGTTGATGACATTAGAACTATCTCACAATTTCCTTGGAGGAA
ACATTCCAGAAGAGATTGGAAAACTTCAAAACCTAAAACTTTTGTCAATAGAATCCAATCAACTTACAGG
TTCTATACCATTCTCGATCTTCAACATTTCTGGAATTGAGATCATTGCATTTACAAATAATACCTTATCAGG
AAATCTTCCTTATGATATGTGTAATTGTCTTCCAATGCTCGAAATGCTCTATCTAAGTGACAACAAGCTAT

ATGGTCATATGCCTGTAAGCTTGCCAAATTGTTCGAACCTTCAAATATTGTCTTTATCTAATAACAAGTTT
GACGGACCCATACATAGTGAAATCGGGATTCTTAATAATTTGGTGATTTTAGCACTGGATACTAACCATA
TTTCTGGTCATTTCCCAATCTCCACATTCAATATCTCCTCATTACAGGTTTTGGTACTGAATCACAACAATC
TGATTGGATCCTTGCCAAGGGAGGTTAGCAATTTGACAGAATTGCAAATTCTAGATCTTGCCGATAATAA
TCTTACAGGTATATATGTATTCAAATCATTACTAGCTAGCAACATAATTTCTTAAATCTTAGTAATTACTAA
CTATACATATACATACATTGGCAGGTGAGATACCGAAAAAGTTTAGCAATCACATTGAGCGGTTTGATCT
TAGTTTCAACAAGTTTACTGGTTCACTACCAATGGAGATTTTCAACATATCGGGTATGAGAGAGATTCAA
GTTATTTTCAATAATGTGACAGGAACTCTCCCATTAAACATAGGTTCAACGTTACCCAACATTGAAGTTAT
TCATCTGGGCAACTTAAATCTTTATGGGACTATACCTCATTCTCTCTCCAATTGTTCCAAACTCACTAGTCT
AGACCTTTCCCTCAATAGACTAACCGGTATGATTCCCAACTCTCTCGGAAGTTTGACTCATCTACAGTCCC
TAAGCTTGGCAATAAACAATTTAATGGGCGATAGTAGGTTAAGCTTCTTGACTTCCTTGTCAAATTGTAG
AGATTTAAAAATTCTTTTCCTCTCTTCGAACCCTCTATATGGTGTGCTTCCAGAATCCATAGGGAACCTTTC
CAGTACTTCTCTTCTAAGGTTTAGAGCTATTGATTGCAAAATTAAGGGGGAAATTCCAAAAGGAATTGG
GAACTTAAGCAGCTTATTAGAGCTCGATCTTTCTAGAAACGGTTTCACTGGACCAATTCCCACAACAATC
AGCAACTTGAGATCCCTTCAGAGCTTGAAGTTGAGTGTCAACAAACTTTCTGGATATATTGGAGATGATC
TATGCAAATTACAGAACTTGGGTTATTTAAACTTGACTCTGAATCAACTTTCAGGATCTCTTCCGAATTGT
TTGGGAAACTTAACTTCCCTTCGGGAGATGATTCTAGGTTCCAACAAATTGCGTTCAATCATTCCAAAAA
GCTTAGGAAACCTCATAAACCTCTTGAAGCTAGACTTATCCTCAAACAACTTGGGTGGCTCTATACCTCT
GGAAATTGGAAATCTAAAGGCTGCGATATATATGGATCTATCAATAAATACACTCTCAAATAGAATTCCT
AAAGAAATTGGTAGCTTGCAAAATCTTATATACCTTTCTTTGAGAGACAACAAATTGCAAGGATCAATTC
CTGGCTCGATGAGCAGCATTTCAGCTTTGGAATTTCTAGACCTTTCTCATAACAATGTTTCAGGATTGATT
CCCAAGTCTTTGGAGAAGCTTCTATATCTCAAGTATTTCAACATCTCATTCAACAAGTTGATTGGTGAAAT
CCCCTCGAATGGTCCTTTCGAGAACCTCTCCGGTGAGTCTTTCATGTCTAATGAAGCATTGTGTGGATCTC
CAAGATTTTGTGTTCCCCCATGCCACAGTAGCACATCAAAGCATAAATCCAAGAGGAAAAAAGTGATTG
TTCTTGTTCTACCGGGTGCACTTGTATTAGTCTCCATCGCCTTTGCCCTTTTATGGATAAGGAATACAAGA
GGTAAAAGAACCGATCCTCAGCAAGCAGTTGATTCATCCTTTGTCGTCTCAACAAAAGGAAGAATTTCAT
ACTATGAACTGCTTCGAGCAACTGATTCACTCAGTGAGATCAATTTAATTGGTTCTGGGAGTTTCGGCTC
TGTTTACAAAGGCATCCTTGGAGATGGGACTTTCATTGCAGCTAAAGTGTTCAACCCACAATTGCAAGTG
GCATTTAGGAGTTTTGATACAGAATGTCAAGTTTTGCGCAACCTTCGTCATAGGAATCTCACCAAAGTCA

TCACCAGTTGTTCCAACCTTGAATTTAAGGCTTTAATACTCGAGTACATGCCCAATGGGAGCCTCGATAA
GTGGTTGTATTCACACAACTACTTCTTAGACATCAATCATAGGCTAAGTATAATGATAGATGTAGCATGT
GCATTGGAATATCTCCATCATGGTTGCCCATTGCCCGTGATTCATTGTGATTTGAAGCCTAGTAACGTCTT
GTTGGATGAGGACATGGTTGCCCACCTAAGTGACTTTGGAATTTCAAAGCTGCTTTCTGAAGATGAGAG
TTCTTTATACACTAAAACATTAGCAACGTTTGGTTATATTGCACCAGGTACGTGATCTCTTAATCAATTGC
TTTTGATTATTTCTCTTTCCTTTTTTCCACTAGTAAATGTTGAATTTCAAAATTGATTGATTACAGAGTATG
GACTGGAGGGATTGTTGTCAATAAAATGTGATGTTTATAGTTACGGGATCATGTTGATGGAAACATTTA
CTAGGAAAAATCCTAATGATGAAATGTTCGATGGAGATTTTAGTTTGAAACAATGGGTGAGAAATTCAC
TCCCACAAGCAGCAATGGAAATTGTCGATGCCAACTTAATATCGCCACATGATAATCACTTAATGAAAAA
GCTAGATTGTGTGGTATCCATCATGAAAGTGGCAATAGATTGTTGTGTTGAATCTCCTAAAGGAAGGAT
TGACATGAAAGACGTTGTGGGAAGGTTAAAGATGATCAAGATTCAACTTCTTGCGTGTTGAGGAAGATC
TTATTAGAAACTTTTCTTCCAAATCATCTCATGTGTAACAAACATTTGCAATAATAGTTAGCTTCAAATAA
GTTATAGCGTTATGTCTTTCATAACATAGAGAATTGATGGGCATGTATACCTCTTTGGTCTTGAATTTGTA
CAGTGGTTTTGGCCTAATACCTTTACTTTTTTCCATCTTTTTAACTTTAACAATGTAAATAGAACTCTCAAT
ATTGAATAACGGAATTTCG
>PGSC0003DMG400020744 LRR-RLKs
ATGTCACTACTAGCATTCAAGAATATGATCATTGATGACCCTTTCAAGATCATGGACTCCTGGAATGAAA
CTATACATTTTTGTGACTGGCCTGGGGTTTCATGTGGTCGACGTCATCCCCGAGTTACAGTTTTAAATCTT
ACTTCTCTGAAATTGAGGGGTTCTCTGTCACCAAGCATTGGTAATCTGAGCTTTCTTTGTGTCCTTAAGCT
CCAAAATAATAGTTTTTCAGGTGAAATTCCATCAGAGATTGGCTACTTACATAAGTTAAAGTTTTTACTCC
TTGATAATAACTCGTTCACTGGTCATATTCCATCAAACATTTCAGGATGCTTCAATCTTGTTTCTGTTGTTC
TTTCATATAATATGATGGTGGGCAAAATTCCAGCAGAATTAGGCACATTGTTGAAACTCAAACAACTTTC
TCTTGTTTCTAACTATTTTACAGGAGGAATTCCGCCTTCATTTGGTAATCTTTCTTTACTTGATACCTTTTCC
GCAAGCAAGAATAATTTGTTAGGGAAAATACCTGATGAATTATGTCAATTGCTCAACTTAAAGTACTTTG
TAGTGAATGAGAACAACTTGAGTGGTACCCTGCCTCCTTGTCTTTTCAATCTTTCCTCTATCGTGGCCATA
GATGTTGGAACAAACCATTTAGAAGGAAAATTGCCTCCATTGCTTGGTATTACTCTTCCTAGTTTAGAGTT
CCTTAGCATTTATAGAAACAATGTAACTGGAAATATTCCGGTAACATTGTCAAATGCTACGAATCTTCAG
TCTCTTATTGCTAGTAGAAATGGACTCACAGGAAAAGTACCACCCCTTGGAAATTTGCTGAAAATGCGG
AGATTTTTAGTTGCTTTCAATTATCTTGGGAAGGGGGAGGATGATGACCTTAGCTTTCTCACCACATTGG

TGAATGCCACCAGCTTGGAGCTTTTGGAGCTCAATACAAATAATTTTGGTGGAGTCTTACCTGCATCTGT
TAGTAATCTTTCGACTGAGCTTATAGAGCTTTCGTTGTCTTATAATCAGATTTCTGGAAAAATCCCAAGAG
GGATATCCAATCTCAAGAAGCTTCAGGCTTTCTTTGTAGCTTACAACAGATTTCACGGTGAAATTCCGTCT
GAAATTGGTGATCTGATATATTTGCAAGAGCTGGCTTTACTTGGAAATCAGTTCTCTGGCCAAATTCCAA
TCTCATTGGGAAATTTAGCTTATTTAGCCAAACTCAGTTTAAGAGAAAACAATATCCAGGGGAGAATTCC
TTCCAGTTTAGGCAAATGCCACAATTTGGAACTGTTGGATCTTGGTTCAAATAACCTTAGTGGTTTCATAC
CATCAGAAATTCTTGAACTCTCATCCTTGTCGGAGGGCATGGACCTATCTCAGAACCACTTAACTGGTTTC
CTTTCGATGGAAATTGGGAAGTTGAGAAATCTAGGCTACTTAAACCTGTCTGATAACAAGTTGCAGGGC
CAGATTCCTACCACTATAGGTACTTGTGTGAAACTTGAAGCACTAGACATAAGCAACAACAACTTTCAGG
GTAGTATTCCTTCAACCATGAATAGTTTGNNNNNNNNNNNNNNNNNNNNNNNNNNNNNNNNNNNNNNNNN
NNNNNNNNNNNNNNNNNNNNNNNNNNNNNNNNNNNNNNNNNNNNNNNNNNNNNNNNNNNNNNNNNNNNNN
NNNNNNNNNNNNNNNNNNNNNNNNNNNNNNNNNNNNNNNNNNNNNNNNNNNNNNNNNNNNNNNNNNNNNN
NNNNNNNNNNNNNNNNNNNNNNNNNNNNNNNNNNNNNNNNNNNNNNNNNNNNNNNNNNNNNNNNNNNNNN
NNNNNNNNNNNNNNNNNNNNNNNNNNNNNNNNNNNNNNNNNNNNNNNNNNNNNNNNNNNNNNNNNNNNNN
NNNNNNNNNNNNNNNNNNNNNNNNNNNNNNNNNNNNNNNNNNNNNNNNNNNNNNNNNNNNNNNNNNNNNN
NNNNNNNNNNNNNNNNNNNNNNNNNNNNNNNNNNNNNNNNNNNNNNNNNNNNNNNNNNNNNNNNNNNNNN
NNNNNNNNNNNNNNNNNNGGGCTTATAGGATTGACATTCATAGTTTGTTTCTTGGCATACGGCAATTTAG
TAGTATCTTATCAGTGTCTACTTAGGGAAACTGATAGATTTTCTGCATCAAATTTGCTTGGAGTGGGTGC
TTTTGGGTCTGTATATAAAGGAATTTCTGAACACGATGGTACGTTTTTTGCTGTTAAGGTACTGGACCTTT
CACACCATGCAGCTTCCAGGAGTTTTTTAGCTGAATGTGAGGTTCTAAAGAACATTAGACATCGTAATCT
TGTAAAGGTTCTAAGTGCATGCTCAGGTATTGATTATGAAGGTAATGAGTTCAAGGCCATAGTTTATGA
ATACATGGATAAGGGAAGTCTACAGGACTGGTTACATTTCACTCCTCAGGAGAAATCTGAGTCACAGGA
AGAGCACAAGAAGCTCGGGTTTATTCAAAGATTAAACATTGCCATTGATGTTGCCTGTGCTCTAGATTAT
CTCCATAATGATTGCCAACCCCCAATAATTCACCGTGATCTGAAGCCAAGCAATATTCTTCTCGATGAGA
ACATGACTGCACATGTTGGTGATTTTGGTTTGGCAAGATTTGTTCCACCAGCAATTCCGAACTCATCAGC
AAATTCAAAAAGTTCTACAGGCGTAGGGGGAACTATTGGTTATACACCTCCAGGTAATAATAAATCAAG
GTTTCTGCTGGCTGTCATAAATATGTCATTTGATAAGAATATCTCTAATTCTGTAGTATTTCTTGTTGACA
GAATTAGGTATGGGAAGCGACGCCTCAATCTATGGAGATGTATACAGCTTTGGCATTCTGCTCTTGGAA
ATGTTTACCGGGAGGAAACCAACTGATGAAATGTTCAAAGACAATTTAAACCTTCATAACTATGCCAATG

CTGCTTTGCCTGACAGAGTGATGCATATTGCAGATCCCATTCTTCTCCAGGAAAGAGATGAACTTGGAAT
GAAATACAAAGTTGATGACAATACAAGCAGTGCTGTCGACATATTTCTGTCATTCTTAGTTAAGGTGATT
CAAGTCGGAGTTTCCTGCTCCGTTGAATCTCCAAAAGAAAGAAAGCAGATCAGTGATGTTGTTGGAGAA
CTCAATTCATTAAGGAAGTTGTTCTTGGAGCAAGCATACCCGAAAGAAAAGGTTATAAGCTTCAACCTGC
ATTGA

>PGSC0003DMG400020832 LRR-RLKs

ATGGACACAAGTTATACTCTCCTCTTTGCTCAACTTTCAATTTTCATTCTGCTTCTTAACCATACTTCACTTG
CTAATATTAGCACTGATGGAGCTGCTCTTCTTGCACTTAAATCACACATTTCTTCTCATCCTAACAACATCT
TAGCAAGCAACTGGTCTTCTTCAACCTCGGTTTGCAGCTGGATTGGAATCACTTGCAGCTCCCGCCACCA
TCGAGTCACTGCTTTAGACATTTCTAGCATGCAACTTCATGGTACCATTACTCCACACCTTGGAAACCTCT
CATTTCTTGTTTCCCTCAACATCAGTAATAACGCTTTTCATGGAGATTTGCCACAAGAGTTGGCTCGTTTG
CAGAGGTTGAAAGTGATTGATGTCACAAGCAATAACTTCACCGGAGAATTTCCGTCATTTTTAAGTTTGT
TACCAAATTTACACATTATGTACCTCTGGAGAAACCAATTTTCAGGTAAAATTCCATCCTCCCTTTCCAATC
TGACAAAACTGCAAGTGTTGTCAATACAGAACAATTTTCTCCAAGGAGAGATCCCTCGGGAACTCGGTC
ATCTTCGTTACCTGAGTATCCTAGACCTGCAATATAATGGACTTACAGGCTCTATACCATCATCAATCTTT
AACATTACTACAATGCAAGTAATTGCTCTTACAGGCAACAATCTTACTGGAAAGCTTCCAAAAACTATAT
GTGACCATCTTCCAAACTTGGAAGGGCTTCACCTCACAAGCAACTCCCTAGATGGAGTTATTCCACCAAA
CCTTGAGAAATGCCGAAAACTTCAAATATTGTCATTGTCTTACAATGAGTTTATTGGAATTGTACCAAGA
GAGCTGGCCAACTTAACAGCTCTTACAGAATTATATCTCGGAGATCTGCATTTGGAAGGTAGTATGATAT
TTCCTTACAAGAAAAATGAAAATTTTCTTCCTTTTCTCTCTTCCGCTAAACTGGTAACTCATATACTTCCTG
AAATTGACAGGAGAGATCCCTCGAGAAATCAGTGATCTTCGTTACTTGACTATCCTAGATCTGCAATTTA
ATCAACTTAGTGGCTCTATACCACCATCAATCTTCAACATTACCACAATGCAAGTAATTGCTCTTACGGGC
AACAATCTTACTGGAAAGCTTCCAAAAACTATATGTGACCATCTTCCAAACTTGCAAAGGCTTTACCTCTC
AAAGAACTCCTTAGATGGAGTTATTCCACCAAGCCTTGAGAAATGCCGAAAACTTCAAATATTGTCATTG
TCTTACAATGAGTTTATTGGAATTGTACCAAGAGAGCTGGCCAACTTAACAGCTCTTACAGAATTATATC
TCAGAGCCCTGCATTTGGAAGGTAGTATGATATTTCCTTACAAGATAAATGAAAATTTTCTTCCTTTTCTC
TCTTCCGCTAAACTGGTAACTCATATACTTCCTGAAATTGACAGGAGAGATCCCTCGAGAAATCAGTGAT
CTTCGTTACTTGACTATCCTAGATCTGCAATTTAATCAACTTAGTGGCTCTATACCACCATCAATCTTCAAC
ATTACCACAATGCAAATCATTGATTTTACTGGCAATAATCTTACTGGTAAGCTTCCACCAAGCCTCGAGA

AATGCAGAAAGCTTCAAGTCTTGGCATTGTCGCTCAATGAGTTTATAGGAACTATACCAAGAGAGATAG
CCAACTTAACAGTTCTTACAGAATTATATCTCAAAGCTCTGCATTTGGAAGGTAGTATGATATTTCCTTAC
AAGAAAAATGAAAATTTTCTTCCTTTTCTCTCTTCCGCTAAACTGGTAACTCATATACTTCCTGAAATTGAC
AGGAGAGATCCCTCGAGAAATCAGTGATCTTCGTTACTTGACTATCCTAGATCTGCAATTTAATCAACTT
AGTGGCTCTATACCACCATCAATCTTCAACATTACCACAATGCAAATCATTGATTTTACTGGCAATAATCT
TACTGGTAAGCTTCCACCAAGCCTCGAGAAATGCAGAAAGCTTCAAGTCTTGGCATTGTCGCTCAATGA
GTTTATAGGAACTATACCAAGAGAGATAGCCAACTTAACAGTTCTTACAGAATTATATCTCAAAGCTCTG
CATTTGGAAGGTAGTATGATATTTCCTTACAAGAAAAATGAAAATTTTCTTCCTTTTCTCTCTTCCGCTAAA
CTGGTAACTCATATACTTCCTGAAATTGACAGGAGAGATCCCTCGAGAAATCAGTGATCTTCGTTACTTG
ACTATCCTAGATCTGCAATTTAATCAACTTAGTGGCTCTATACCACCATCAATCTTCAACATTACCACAAT
GCAAATCATTGATTTTACTGGCAATAATCTTACTGGTAAGCTTCCACCAAGCCTCGAGAAATGCAGAAAG
CTTCAAGTCTTGGCATTGTCGCTCAATGAGTTTATAGGAACTATACCAAGAGAGATAGCCAACTTAACAG
TTCTTACAGAATTATATCTCAAAGCTCTGCATTTGGAAGGTAGTATGATATTTCCTTACAAGAAAAATGA
AATTTTTCTTCCTTTTCTCTCTTCCGCTAAACTGGTAACTCATATACTTCCTGAAATTGACAGGAGAGATCC
CTCGAGAAATCAGTGATCTTCGTTACTTGACTATCCTAGATCTGCAATTTAATCAACTTAGTGGCTCTATA
CCACCATCAATCTTCAACATTACCACAATGCAAATCATTGATTTTACTGGCAATAATCTTACTGGTAAGCT
TCCACCAAGCCTCGAGAAATGCAGAAAGCTTCAAGTCTTGGCATTGTCGCTCAATGAGTTTATAGGAACT
ATACCAAGAGAGATAGCCAACTTAACAGTTCTTACAGAATTATATCTCAAAGCTCTGCATTTGGAAGGTA
GTATGATATTTCCTTACAAGAAAAATGAAATTTTTCTTCCTTTTCTCTCTTCCGCTAAACTGGTAACTCATA
TACTTCCTGAAATTGACAGGAGAGATCCCTCGAGAAATCAGTGATCTTCGTTACTTGACTATCCTAGATC
TGCAATTTAATCAACTTAGTGGCTCTATACCACCATCAATCTTCAACATTACCACAATGCAAATCATTGAT
TTTACTGGCAATAATCTTACTGGTAAGCTTCCACCAAGCCTCGAGAAATGCAGAAAGCTTCAAGTCTTGG
CATTGTCGCTCAATGAGTTTATAGGAACTATACCAAGAGAGATAGCCAACTTAACAGTTCTTACAGAATT
ATATCTCAAAGCTCTGCATTTGGAAGGTAGTATGATATTTCCTTACAAGAAAAATGAAATTTTTCTTCCTT
TTCTCTCTTCCGCTAAACTGGTAACTCATATACTTCCTGAAATTGACAGGAGAGATCCCTCGAGAAATCA
GTGATCTTCGTTACTTGACTATCCTAGATCTGCAATTTAATCAACTTAGTGGCTCTATACCACCATCAATCT
TCAACATTACCACAATGCAAATCATTGATTTTACTGGCAATAATCTTACTGGTAAGCTTCCACCAAGCCTC
GAGAAATGCAGAAAGCTTCAAGTCTTGGCATTGTCGCTCAATGAGTTTATAGGAACTATACCAAGAGAG
ATAGCCAACTTAACAGTTCTTACAGAATTATATCTCAAAGCTCTGCATTTGGAAGGTAGTATGATATTTCC

TTACAAGAAAAATGAAATTTTTCTTCCTTTTCTCTCTTCCGCTAAACTGGTAACTCATATACTTCCTGAAAT
TGACAGGAGAGATCCCTCGAGAAATCAGTGATCTTCGTTACTTGACTATCCTAGATCTGCAATTTAATCA
ACTTAGTGGCTCTATACCACCATCAATCTTCAACATTACCACAATGCAAATCATTGATTTTACTGGCAATA
ATCTTACTGGTAAGCTTCCAACAACTATATGTGACCATCTTCCAAACTTGGAAGGGCTTCACCTCACAAG
CAACTCCCTAGATGGAGTTATTCCACCAAGCCTCGAGAAATGCAGAAAGCTTCAAGTCTTGGCATTGTCG
CTCAATGAGTTTATAGGAACTATACCAAGAGAGATAGCCAACTTAACAGTTCTTACAGAATTATATCTCA
AAGCTCTGCATTTGGAAGGTAGTATGATATTTCCTTACAAGAAAAATGAAATTTTTCTTCCTTTTCTCTCTT
CCGCTAAACTGGTAACTCATATACTTCCTGAAATTGACAGGAGAGATCCCTCGAGAAATCAGTGATCTTC
GTTACTTGACTATCCTAGATCTGCAATTTAATCAACTTAGTGGCTCTATACCACCATCAATCTTCAACATTA
CCACAATGCAAATCATTACTCTTACTGACAATAATCTTACTGGTAAGCTTCCACCAAGCCTCGAGAAATGC
AGAAAGCTTCAAGTCTTGGCATTGTCGCTCAATGAGTTTATAGGAACTATACCAAGAGAGATAGCCAAC
TTAACAGCTCTTACAGAATTATATCTCGAAACTCTGCATTTGGAAGGTAGTATGATATTTCTTTACAAGAA
AAATGAAAATTTTCTTCCTTTCGGAGCTCTGCATAAGGTAAGTGTAACTCATATATTTCCCAAAATTGACA
GGAGAGATACCAATGGAGCTAGGTAATCTTAAGAAACTTCAGGTACTGCAGTTAGCAGAAAATGAGCTT
ACTGGCTCTGTTCCTGACAGCATTTTCAACATGTCAGCACTGAAGATTATAGATTTTGGACAAAACAAGC
TTTCAGGTACTCTACCTTCAGATTTAGGTCGTAGAATTCCCAACCTAGAAGTATTTCTTTGTGGAGGAAAT
AATCTGAGTGGTTTTATCTCTGATTCAATCACAAATTCTTCAAGACTCAGAATGTTTGACATCTCATTCAA
CAGTTTCACAGGTCCAATTCCTAAATCATTTGGTAACTTAGAATACCTTGAGATTCTTAACTTAGAGATGA
ATAATTTTATCAGTGATTCATCATTGAGCTTCCTTACATCATTAACAAACTGTAGGAAACTAAGAGCACTC
CGATTTAATGAGAATGCATTGGATGGGGCATTACCAGCGTCTGTTGGTAATTTCTCAAACTCGATGCAAA
ATTTTCAAGGAAATGGCTGTAAGCTAAAGGGCGTCATTCCTCGAGAAATCGGTAATCTTACTGGAGTGA
TACATATGAGTCTGTTTAACAATAAGTTGGCTGGACATATTCCAAATACTGTCCAAGACATGTTGAACCT
CCAAGAATTTTATCTACATAGCAATGAGATTAAAGGTACCATACCAAATGTTATATGCAGTTTAAAGAAT
CTTGGTGCATTAGACTTGTCAGGAAATCATTTTTCTGGTTCAGTGCCCTCATGCTTAGGGAATGTAACTA
GTTTGAGGTATCTTAATCTAGCTTACAACAGGCTGAATTCAAGATTACCTGCAAACTTGGGGAGCCTTCA
AGATCTCATTGAATTAAGTGTTTCATCCAATTCATTAAGTGGTCATATTCCTCTGGAGTTGGGAAATTTAA
AGGCAGTAACACTAATTGATTTGTCAAAAAATGATTTTTCTGGTAAAATTCCTAGCACTTTAGGAGGTCT
AGCTGAATTGATTAATCTTCCTCTAGCACATAATAGATTAGAGGGGCCTATTCCAGATTCATTTGGCAAA
TTGTTGGCATTGGAATTCTTGGATTTGTCTTATAACAATCTTAGTGGTGAAATTCCAAAGTCATTAGAAGC

TCTAGTGTATCTCAAATACATGAATTTCTCATTCAATAAACTCAGTGGAGAAATTCCCATTGGTGGTCCTT
TTGTAAATGTCACTAGTCAATCCTTCTTGTCCAATGATGCACTTTGTGGTGACTCCCGGTTTAACATAAAA
CCATGTCAAACTAAATCTACAAAGAAGTCAAGAAGAAAAAGAGTGCTTATAGGTTTATATACGCTGTTA
GGGATTGGGTCGCTCTTTGTGTTGGTAGTTGGATATGTGGTGTTAAGATTGAGAAAGACAAAGAAGAAT
GCAAGTCAAGCAGATGTGTCTCTGGTAAAAGAACATGAAAGAATTTCCTATTATGAACTTGAACATGCA
ACAGAAGGATTCGACGAAAGCAACTTGCTTGGTACAGGGAGTTTTAGTGTGGTCTACAAAGGGATACTT
AAGGATGGTACTCTTTTGGCAGCAAAGGTATTCAATGTGCAATTGGAGGGTGCATTTAAAAGTTTTGAT
ACGGAATGTGAGATACTCCGGAACCTTCGCCACAGAAATCTGACCAAAGTTATTACAAGCTGTGCCAAC
CTTGATTTCAAGGCCCTGGTGTTGGAATACATGCCCAATGGGACACTTGATAAATGGTTATACTCCCACA
ACTTGTTCTTGAACTTATTACAGAGATTGGATATAATGATAGATGTTGCATCTGCAATTGACTATCTCCAC
AATGGCTATACAACGCCTGTGGTTCATTGTGACTTGAAGCCAAGTAATGTCTTGCTTGATCAGGAAATGG
TTGGTCATGTCAGTGATTTTGGCATTGCGAAATTGCTAGGTGAAGGGGAGGCTTCTGTTCAAACAAGGA
CAATTTCAACCATTGGATATATTGCTCCAGGTATATTAAAACTTCTTAAAGTTTTTCTTGTTTCTCCTTTCA
GTTTTGGCATATTCCATTTTAACTAGGAAGCTTTTATGATAATTTTTTAAAAATAATCGTAGAGTATGGAC
AAGATGGCATAGTATCCACGAGTTGTGATGTTTATAGTTTTGGCATCCTGATGATGGAGGCATTCACAA
GAAGAAGACCAAGTGATGAAACATTTACCGGTGAATTGAGCATACAGCGTTGGGTTAGTGATTCCTTTC
CGGGTGAAATTCATAAGGTGGTGGATTGCAATTTGGTACAGCCAGGGGATGAACAAATTGATGCAAAG
ATGCGATGTTTGTTATCTATCATGGAATTATCTTTGAACTGCACTTTAGTGAAACCTGATGCAAGAATTAG
CATGAAAGATGCTCTTTCAACACTCAAAAAGATCAGGCTACAGCTTGTCAATAATCGGCATTAG
>PGSC0003DMG400020848 LRR-RLKs
ATGGGAAGAAGTTGCAATCTTCTCTTTGCTCTTGCAGTTCTCATTCTACTCCATCACCATACTTTACTTGCT
ACTCTTCCTAATATTAGCACTGACGAAGCTGCTCTTCTTGCACTTAAATCACACATTTCTTCTTATCCTAAC
ATCATCTTAGCAAGCAACTGGTCTTCTTCCAGCCCGGTTTGCAGCTGGATTGGAATCACCTGCAGCTCTC
GCTACCATCGAGTCACTGCTTTAGACATTTCTAGCATGCAACTTCATGGTACCATTCCTCCACACCTTGGA
AACCTCTCATTTCTCGTCTCCCTTGATATCAGTAACAACACTTTCCATGGAGATTTGCCAGTAGAGTTGGC
TCATTTGCAGAGGTTGAAACTGATTAATGTCAGAAGTAATAACTTCACCGGAACCATTCCATCATTTTTAA
GTTTGTTACCAAACCTACGCTTTGCGTACCTCTCGAGCAACCAATTTTCGGGGAAAATTCCATCATCTATT
TCCAATCTGACAAAGCTGGAATTGTTGACAATACACACAAATTTTCTCGAAGGAGAGATTCCTAAAGAAA
TCGGTGATCTTCGTTACTTGATTGTCCTAAACATGCAAGATAATCAGCTTACTGGCTCTATACCACCCTCA

ATCTTTAACATTACTACAATGCAAGTAATTGCTCTTACTGACAACAATCTGACCGGAAATCTTCCACGAAC

TATATGTGACCGTCTTCCAAACTTGGAAGGGCTTCACCTCTCAAGCAACTACCTAGGTGGAGTTATTCCA

CCAAACCTGGAGAAATGCCGAAAACTTCAAATACTGTCTCTGTCTTACAATGAATTTACTGGAACTGTAC

CAAGAGAGCTATCCAACTTAACAGCTCTTACAGAATTATATCTTGGAATACAACACTTGGAAGGTAGTAT

GCTATTTCCTCACAAGATATTTGAAATTTTCTTCCTTTCCTCTCTTCACCAAATTTGTACTCATATATTTCCC

AAAATTGACAGGAGAGATACCAGCAGAGCTAGGTAATCTTAAGAAACTACAGCTGCTGATGTTAGACCA

GAATGAGTTTACTGGTTCTATCCCTGAAAGCATTTTCAATATATCAGCAATGCAGATCCTAGATTTTTCAA

TGAACAAGCTTTCAGGTACTCTACCTTCGGATTTAGGTCGTGGAATGCCCAATTTAGAAGAACTTTATTG

TGGAGGAAATAATCTGAGTGGTTTTATATCTGATTCAATCTCAAATTCTTCAAGACTCAGAATGGCTGAT

CTCTCAGACAATAGTTTCACAGGTGTAATTCCCGAATCACTAGGTAACTTAGAGTACCTTGAGGTTCTGA

ACTTGGAGCTGAATAATTTTATCAGCGATTCATCATTGAGCTTCCTTACATCATTAACAAACTGTAGGAAA

CTAAGAGCACTCCGGTTTAATGATAATGCATTGGATGGGGCATTACCAGCGTCTGTTGGTAATTTCTCAA

ACTCGCTGCAAAATTTTCAAGGAAATGGCTGTAAGCTAAAGGGCGTCATTCCTCGAGAAATTGGTAATC

TTACTGGAGTGATATATATGAGTTTGTATAAAAATGAGTTGACTGGACATATTCCAAATACTGTCCAAGA

CATGTTGAACCTTCAAGAATTTTATCTACAAAGCAACGAGATTGAAGGAACCATACCAAATGTTTTATGC

AGTTTAAAGGATCTTGGTGCATTAGACTTGTCAGGAAATCATATTTCTGGTTTGGTGCCATGCTTAGGGA

ATGTTACTAGTTTGAGGAAACTTAATCTAGCTTACAACAGGCTGAATTCTAGATTACCTGCAAACTTGGG

GAACCTTCAAGACCTCATAGAATTCAATGTTTCATCCAATTCGTTAAGTGGCCATATTCCTCTGGAGTTGG

GAAATTTAAAGGCAGTGACACTCATTGATTTATCAAAAAATGATTTTTCTGGTAAAATTCCTAGCACTTTA

GGGGGTCTAGCTGAATTGACGGATCTTTTTCTAACACATAATAGATTAGATGGGGCTATTCCAGATTCAT

TTGGCAAAATGTTGTCACTGGAATACTTGGATTTGTCCTATAACAATATTAGTGGTGAAATTCCAAAGTC

ATTAGAAGCTCTTGTGTATCTCAAATACATGAATTTCTCATTCAATAAACTCAGTGGAGAAATTCCCACTG

GCGGTCCTTTTAAAAATGTCACCAGTCAATCTTTCCTATCCAATGATGCACTCTGTGGTGACTCCTGGATT

AATGTAAAACCATGTCAAAGCAAGTCAACAGAGAAGCCAAGAGGAAAGAGAGTGCTTATCAGTTTATAT

ACATTGTTAGGGATTGGATCACTCCTTGTGTTGGCCGTTGGATATGTGGTGTTAAGATTGAGAAAGACA

AAGAACAATGAAAGTCAAGCAGATGTGTCTTTAGTAAAAGAGCATGAAAGGATTTCCTATTATGAACTT

GAACATGCAACAGAAAGATTCGATGAAAGCAACTTGCTTGGTACCGGGAGTTTTAGCATGGTCTACAAA

GGGATACTTAAGGATGGTACTCTTTTGGCAGTAAAGGTATTCAATGTGCAATTGGATGATGCATTCAAA

AGTTTTGACACGGAGTGTGGGATACTCCGGAACCTTCGCCACAGAAATCTGACCAAAGTCATCACCAGC

TGCTCCAATCTTGATTTCAAGGCCCTGGTGTTGGAATACATGCCCAACGGGACACTTGATAAATGGTTAT
ACTCTCACAACTTGTTCTTGAACTTATTGCAGAGATTAGATATAATGATAGATGTTGCATCTGCAATGTAC
TATCTCCACAATGGCTATTCAACGCCTGTTGTGCATTGTGACTTGAAGCCAAGCAATGTCTTGCTAGATC
AAGAAATGGTTGGTCATGTCAGTGATTTTGGCATTGCAAAATTGTTAGATGCAGGGGAGGCTTTCGTTC
AAACAAGAACAATTTCAACCATTGGATATATTGCTCCAGGTATATTAGTTAAGCAATTCTTTCCCCTAGAT
ATGGATTGATGCTTTCAGTTTTGGCATATTCCATTTTAACTAGGAGGCTTTTATGATCATTTTTTACCATGA
TTACAGAGTATGGACAAGATGGAATAGTATCCACAAGTTGTGATGTTTATAGTTTTGGCATTCTGATGAT
GGAGACATTCACAAGAAGAAGACCAAGTGATGAAATATTTACCGGAGAATTGAGCATACAACGTTGGA
TTAGTGATTCCTTCCCAAGTGGAATTCATAAGGTGGTGGATTACTCTTTGGTACAGCCAGGGGATGAAC
ATATTGATGCAAAGATGCAATGTTTGTTATCAATTATAGAAGTAGCTTTGAGTTGCACTTTAGTGACACC
TAATGCAAGAACTAGCATGAAAGATGCACTTTCAACACTCCAAAAGATTAGGCTCCAGCTTGTCAATAGT
CAACACTAG
>PGSC0003DMG400021008 LRR-RLKs
TTCCTTTCAATATGCTATCTGTCATGGTATTTGTAGTGGTTGTAATCAAGGTGTACTGGTATTGATTTCTT
GTTCTAAGTGAACCCCATTTTACCTTGTGGTGTTAGAATGAAGTTTATCAAGTTCTTGATGATTTTTGTTC
TTTATTTTGGCTCTGTCATTGGGCAGCTTCCCTCTCCGGACATATTGGCTTTGCTTGAGTTTAAGAAAGGT
GTCGAACATGATCCAACGGGGTTTCTTCTTGAATCTTGGAATGAGGAGTCTATTGATTTCAATGGATGCC
CTTCATCTTGGAATGGGATAATGTGTAATGGTGGTAATGTTGCAGGAGTTGTTCTTGACAACTTGGGTTT
AGTTGCTCATGTTGATTTGAGTGTATTTGCTAATCTTACAAAGCTTGTTAAGCTTTCAATGGCTAACAATT
CAATTGCTGGGAAAATGCCTAATAACATAGGTAGATTTAAGAGCCTTCAGTATCTAGATCTCTCAGGTAA
TCTATTCAACTCTTCTTTACCCCCAGAGGTTGGACAGTTAGGGAGGTTAAAGAATTTGTCATTAGCTGGT
AATAACTTCTCCGGTATGATACCGGAGACAATTTCAGGGCTTGTGTCTGTGCAATCTTTGGACTTGAGTC
GCAATTCTTTTTCTGGGGGTCTACCTTCATCTTTGATGAAGTTGGCTGGTTTGGTATACCTGAATCTATCT
GTTAATGGATTCACAAAGGAAATTCCTAAAGGATTTGAGCTGATGGAAAATCTTGAAGTGCTTGATTTG
CACGGAAATATGTTTGATGGCAATTTGGATGCAGAAATGTTGCTGCTTACCACTGCAACTCATGTTGACT
TGAGTGGAAATTTGCTGGTGAACACTGCTTCTCAGCAGCAGAAATTTTTGCCAGGTTTATCGGAGTCGGT
GAAGTATTTAAATCTTAGCCATAATCAGCTAAAGGGTTCACTTGTAAGTGGAAATGAAGCTCAGATATTT
GGCAACTTGAAGGTTTTGGATTTGAGCTACAATCAGCTCTCAGGGGAATTGCCTAGTTTCAATTTTGTTT
ATGATCTACAGGTGCTTAAACTTGCCAACAACTTATTTTCCGGATTTATTCCGAATGATCTTTTGAAAGGA

GATGCATTGCTTCTCACTGAGTTGGATTTAAGTGGAAACAACCTCACTGGTAAATACATGAAACCCTTTG
GTAAATAAAGTTATCTGTTTGCTAACAACTCTTTTCGCTGTTTTTCTCAATCTCTTCATTTTGTAGGTTCAA
TAAGCATGATAACATCCACAACATTGCGAATGCTTAACTTATCCTCCAATGCTCTTTCTGGTGAACTTCCC
TTTGTGACTGGAACTTCTGCGGTGCTTGATCTCTCGAAAAATCAGTTCAAAGGTAATCTAACTAGAATGC
TGAAATGGGGGAACATTGAATTTCTTGACCTTAGCCAAAACCGTTTGACTGGAAACATCCCTGAGGTTAC
GGCTCAGTTTCTGCGTTTATATCACCTCAACCTTTCGAGCAATACCTTGACAGGATCTCTCCCGAAAGTTA
TTTCACAGTTTCCTAAGATCACTGTCCTTGATCTCAGTTTCAACCAGTTGGATGGGCCTCTTCTTACTTCTC
TACTAACTTTGCCCACTATTGAAGAACTTCACCTACAAAACAATGCACTTGTTGGAAGCATTGATTTCTCT
CTTCCGGCTAGTACTCCTAAGCTTCGTGTTCTAGATCTTTCTCATAATCAGCTAGCTGGTTACTTTCCTGAT
GGATTTGGGTCATTGATTGCACTTCAAGTACTCGATATATCAGGAAATAATTTCTCTGGATCTTTGCCTAC
TTCCATGGGTAATGTTAGCTCTCTTACTTCTTTAGATGTTTCAGAGAATCATTTCTCGGGTGAACTGCCAA
AAAACCTGCCTAACAGCCTCCAAAGCTTTAATGCATCACTCAATGACTTCTCTGGTGTCGTGCCTGAGAA
TTTGAGAAAGTTTCCTTTATCATCTTTCTACCCGGGAAACTCTCAACTTCAATTTCCAAATCCTCCCTCAGG
ATCTGGTCAAGCTTCAACAGAAAAGCATAAGAGCAGGCAACTTAAAACTATTATCAAAGTGGTGATCAT
TGTTGCTTGTGTGGTTGTTCTTGTTGTTTTAATCATGCTAGCCATTTTTGTATTTTACTTGCGGGCATCGAG
GAACTCTCAGGCACATGTTACTGACAAAGACATCCGCCGTCAAGCCCAATCAAATCCTTCTGGTTTTAGC
AGTAGAGAAGGTGCAGTAGTTTCAGCACAAGATGTTACAGCTGCTTCACGGAAAGCATCATCATCAGAA
GTAATTAGTGCTGATGAGAAAACGACAGGTTTCTCCCCTTCGAAAACAAGTCATTTCACTTGGTCACCAG
AGTCAGGGGAATCATATACTGCAGAAAGCCTTGCTGATAATTTGGCTGGAGAGCTGTTCTTCCTTGATG
ATACAATCTCTTTTACAGCTGAGGAACTATCTAGGGCCCCAGCTGAAGTCTTAGGCAGAAGCAGCCATG
GGACATCTTACAGGGCAACACTAGATAACGGGTTGCTCTTGACTGTGAAATGGTTGAGAGAAGGAGTG
GCCAAACAGAGAAAGGATTTCACAAAGGAGGCTAAAAAGTTTGCCAACATTAGACATCCAAATGTGGT
GGGATTAAGAGGTTACTACTGGGGTCCCACACAACATGAGAAGCTTATTCTTTCAGATTACATATCTCCC
GGAAGTCTATCAAATTTTCTATATGGTAAACTTTATCTTCTTGCATCAACCTTGCAGTTCCTCTTATGCATA
TCGTTTGCTATAACACTTTGAACTCATAAAACACTTATAATCTTTCCTTCTTTTGATATTTTTTAGTATTTCA
TGGCAAACAATTTGTCAGTTTGATATCTTGACAGTTCTTGTGGTGCATGTATTTTCTCAGTCTGTGATACA
CGAATTAAGGATAAGAAGCCATTTCCATTTTTAGTTAGCATAGTGAGTCCAGCTAATTCATTCATTCATTA
TACAGCTTGTAATCAGTTCATTCACAATCATATATGAAACTACACTCTTTCTCTTCTGAATCTATTCTCTCA
ATATTTCTCCTTCTTTAGCTGTTCTTCTTGAGGGAACTCCTGAAACAGTCTCTTGATCCTCGAATTCCTGCA

ATCAAGTAACAGTTTGTATTATCATGAAATATAGTATCACTTTCACTGTGGAATTCTTCAACTTAAACTGC
TGAAGTCTAGCTTCTGATTTATTGTTGGAAAACTTTAAACTTGTCCAAGGCCCTATACACTACGTCCGACC
GGACGTGTAAGCAGGCCTTTACTTTCTTAATATCTCTGCACTGGCAGTAAGGTTCAGCAATTGTGGTACT
TTGCTCATAAAAAGGTCACAATGAACTGCAAATACTTCAATGCTCAAATACCTTATTTTCGATTGGTGATT
CTACCTGCTCAACTTCTCTACTATTTTGTATGCTTTTGTGTAGTTTGTATAGTGTAAGGTTTCTGTATATAC
ATTTAACGTGCGGCACCTTTGCAGATCGACCTGGAAGAAAAGGTCCACCACTAACCTGGCCCCAAAGAC
TCAAAATATCAGTTGATATTGCGCGTGGCCTGAATTATCTCCATTTTGACCGTGAGGTTCCTCATGGAAA
CCTTAAAGCAACCAACATCTTGTTGGATGGTCCTGATCTTAATGCACGAGTTGGTGATTACTGCCTTCACC
GTCTTATGACTCAAGCCGGTACAATTGAACAGATTCTTGATGCTGGAGTGTTAGGTTATCGTGCACCAGA
GTTGGCTGCATCGAAGAAACCAGTGCCTTCCTTCAAATCAGATGTATACGCTTTTGGAGTCATTTTGTTG
GAGGTCCTAACTGGGAAATGTGCAGGTGATGTTGTTTCTGGTGAAGATGGTGGTGTAGACTTGACTGAT
TGGGTAAGGTTGAAAGTTGCAGAAGGTGGAGGATTCGATTGTTTTGATAATGCATTGTCATCTGAGATG
GGAAATCAAACAACAGAGAAGCAAATGAAGGAGGTTCTTGCAATAGCTCTAAGATGCATACGTTCGGT
ATCTGAGAGGCCAGGTATCAAGACTGTATATGAGGACCTTTCATCTATTTAGTTTTCCTCATTTTGGCTTT
TTGCTTGGGAATCAAATAGCTCATTGGCTTCCCTTGAGTTGTAGAATTAGCCAGTTTAGTGTGTGAATGG
GCTAGTTTTATTAACCATTGTTTGTTGTAAATTGGGGCATATTTATAATGTATACAACTTTCACCCCCCACC
CACCCCCC
>PGSC0003DMG400021017 LRR-RLKs
ATGGAGGAGTACATTTTCTTATTGATTCTTCTCTTTCTACTTCAATTTTCTGTATCATTTGCAACCTCAAAT
GAGACAGACCAACAAGCTCTACTAGCTTTCCAAAATCTTATTACAAGTCCTAGTCATTTTTTGGCCAACAA
TTGGACCAACAATACTTATTTTTGCTCTTGGTTTGGTGTCACTTGCACTTCAAAAACCCAAAGGTTTGGGG
CCTTGACTCTTCCTAATTTGCAACTTCAAGGCACAATATCCCCGTTTTTGGCCAATTTGTCCTTCCTCGATG
TTCTCAATCTTGGGAACAACAACTTCCATGGTGGGATCCCTTATGGACTTGGCCACTTGCCTTGCTTGCG
AGTGATTGATGTTCAAAACAATCAGCTAGAAGGAAGTATTCCAACAAGTCTATTTCAACACCAGAAAGTT
CAAGTTATTTCATTGGGCTTCAATAAACTCGGTGGTGAAATGTGGAAAGGGCCATGGTGTGTACCGAAA
CTCAGAGTCTTAAATCTCAAGAACAATAGCCTCACGGGTATAATCCCTCCTTCTATTTGAAATGCCACAAA
GTTGCTTAACTTCAGTTTGTATGGAAATAGAGTTGGTGGCAACATTCCAAAGGAAATTGGTAATCTGAG
ACAACTTGAATTTTTGTCCTTGGTCGATAATCAATTAACAGGTTTCGTAACTGCAACATTGTTTAATATCT
CATCACTTCGTGTCCTATCTCTTGGAATCAAAAGACTTTCTGGTCCTCTCTTGCTTGATGAAAGGAATATT

GTGTCAAATATAGAGTTTCTAAGTATATCTAACAACCAAATTTCTGGCTGCATTCCTTCCAACATATGCCA
ACTCACAAAGCTCAAAGTCTTGTCCAGAGCTTTCAACAACATAACTGGAGAAATACCCAGAAATATTGAT
TGTTTATCCAAACTTGAACAGTTTCTTATTGGTGATAATCCAATAGAAGGGACTATCCCCACTTCATTGGG
AAATATATCCACTCTTCAATATATTTATTGTGGAAACAATCGCATAGTGGGGAAAATTCCCCAAGAATTA
GGGAAGCTATCAAATTTGAGGCAATTAAACTTTGCCTCTAATTATAATCTTATTGGTCAAATTCCAGAGG
CTATTTTTAACATATCTTCTTTGGAAATCATTGATTTCAATTTCAATAGCCTCTCAGGTAGAATTCCAGTCA
CTACAGGTCTTCATCTTCCAAACCTTAAAGTACTTGAGTTGGGAGCCAATCAGATCAACGGGGAAATTCC
ATTGTTCATAACAAATGCTTCCAAGATTGAGAAACTGGTACTAGCACGGAACTTTCTCACGGGCGCTATT
CCTAATAATTTAGGAAATCTTCGTGAGTTGCGATTACTGTTCTTGCATACTAATCAACTTACCAATGAACC
AAGAGAGCATGAATTGTGATTTTTCAATTCTTTGGCGGATTGTAGAATGTTGAAATATCTAGAAGTAGGT
TCCAATCCGTTGATTGGCATTCTTCCCAATTCTATTGGGAATCTTTCATCTACTGTTGAATACTTTGAAATA
GGATATGCACACATCAACGGCCTCATCCCCATAAGTATAGGCAACATTAGCGGCCTTACAGCCTTGTACT
TTGAAGACAACAACTTGGCAGGGAATATTCCTTCTGAGATTGGTAAGCTTAAACAACTCCAAGGGTTGT
ACCTATCTAAAAATAAATTGGAGGAACATAAGAGGCGGTATGCCATTTATCTAATTTGGTTGAATTAGGT
TTGGTTGATAATGAGCTACCTAGATTAATTCCAGAATGCTTAGGTAATCTTAGCATGCTTCAAAAGCTTTA
TTTGGGTTCTAATAAATTTTTATCAAAAATTCCCTTGAGCCTTTGGAAGATGAGTAGCCTTCTCTATCTTG
ACGTGTCACATAATTCAATAGAGGGAAAAGTTCTCGAGGATATTGGAGGACTGAAGGCCATTGTAGAA
CTACATCTTTCCGGTAACCACTTTTCAGGCATGATACCAAGCAGATTCGGGGAACTCCAAAACCTGCAGT
CTCTTGACCTGTCGAACAATTCATTTTTTGGACAAATTCCATCTTCCTTTTCCAACTTGATAAGCTTGGAAT
TCTTGAATTTGTCTTTAAATGCATTCTCAAGTACTATTTCTATGTCTTTGGAAAAACTTTCATCCCTTAAAA
GCATTAATGTTTCATTTAATGATTTAAAAGGTGAAATACCTAGTGGCGGTGTGTTTGCAAATTCCACACT
ACAATCATTTCTCGGGAACAAAGGTCTATGTGGAGTGCACATATTGGAGATTCCTGCTTGTGCTATCACT
AATCCTGGACAACAATCAAAGCTTAAGGAGGTTGTGCTAAAAATTGTTTCTCCGATGGTTATTTCATCCTT
TCTCATATTCTTGTTGGTTTCAATTTGGATAATGAAACGACAAAAGAAAGGAAAGTCCAAAGATGTGGA
GAAGGCTTTAGAGATCAAAACTCATCAATTGATTACTTATCACAAGATTCAACAAGCAACAAATAATTTT
GATGAATCCAATTTAATTGGTGTGGGAAGTTTTGGCTCTGTGTACAAAGGCACATTATCTGGGGGGAAG
TGTAGTGGCAATAAAGGTTCTAGACTTGGAAAATGAGCAAGTATGCAAGAGGTTTGATACTGAATGCG
AAGTGATGAGAAATGTTAGACATAGAAATCTTGTTCCAATGATTACTACATTTTCTAGTGACTATATAAG
AGCCTTTGTTCTGCAATTTATGCCCAATGAAAGTCTTGAGAATTGGTTGTACAAAGAAGATTGTCACTTG

AATCTTCATCAAAGAGTCACTATAATGCTTGATGTAGCTATGGCAATTGAATATCTACATTATAGTCATGT
CAATTAGATAGTTCATTGTGACCTAAAGCCAGCCAACGTTCTTTTGGATGAAGATATGGTGGCTCATATT
GGTGATTTTGGAATCTCTAAAATTTTAGCCATAAGCAAGTTCATGGCTTATACCGAGACATTAGGCACTC
TTGGATACATTGCACCAGGTATAAAGAATCTACTCCTTTGATTTTCTCTTATTATAATTATGACTCTCCAAA
TTCTAATGGTAAAAAAGGTAAGTCTTTATTTATGCAGAATTATTGTTTTATTTCAATTGAGTAACTTTTCTT
CAATCCTTTTTTTAAGAATATGGCTTGGAGGGAATAGTGTCCGCTAATGGTGATGTTTATAGTTATGGCA
TCATGTTGATGGAGGTTTTGACCAAAAGAAGGCCAATAGATGAAGAGATATGCAATGAAAATCTTGACT
TGAGAAAATGGATAACACAATCATTTTCAGGGACTATGATGGATGTTTTGGATGCTAATTTATTTTCTAA
GGAAGAACAAATCACTTCTAAAAGTGAAAGTTGCATCGCCTGCACGATGGAACTAGCTTTTGATTGCAC
AAAGGAAATGCCAGAATCAAGGATAACCATGAAAGATGTAGTCAAGAGGCTTAACAAAATCAAGAACA
CGTTTCTAGAATCATAG
>PGSC0003DMG400021018 LRR-RLKs
ATGAGTGGTCTTCTCTATCTAGACGTGTCACAGAATTCAATAGAGGGAGAAGTTCCATCAGATATTGCTG
GACTGAAAGCCATTGTAGAACTACATCTTTCCTATAACCACTTTTCAGGCATGATACCAAGCCAATTGGG
GGACCTCCAAAACTTGAAGTCTCTTGACTTGTCGAACAATTCGTTTTCAGGCTCAATTCCATTATCCTTTG
CCAACTTGATAAGCTTGGAATTCTTGAATTTGTCTTTAAATGACTTGTCAGGTACTATCCCTATGTCTTTG
GAAAAACTCTCATCCCTTAAAAGCATTAATGTTTCATTTAATGGTTTGGAAGGTGTAATACCCATTGGTG
GTGTGTTTTCAAATTCCACTCTACAATCATTTCTCGGGAACAAAGGTCTATGTGGAATGCACATATTGGA
GATTCCTGCTTGCACTATCACTAATCCAGGAAAACAATCAAAGCTTAAGGAGGTTGTGCTAAAAATTGTT
ACTCCAGTGGTTATTTCATCCTTTCTAATATTCTTGTTGGTTTCAATTTGGATAATGAAACGAAAGAAGAA
AGGAAAGTCCAAGGAAGTGGAAAAGGTACCGGAGATCAAGACTCATCAATTAGTTTCTTATCACGAGAT
TCAACGAGCAACAAATAACTTTGATAGGTCAAATTTAATTGGTGAGGGCAGCTCTGGCTCTGTGTACAA
AGGCACATTATCTAGTGGAACTGTGGTGGCAATAAAGGTTCTGGATTTGGAAAATGAGCAAGTATGCAA
GAGGTTTGATACTGAATGTGAAGTGATGAGAAATGTTAGACACAGAAATCTTGTCCCTGTGATTACTACT
TGTTCTAGTGACTATATAAGAGCCTTTCTTCTGAAATATATGTCCAACGGGAGCCTTGAGAATTGGTTGT
ACAGAGAAGAGTCCCACTTGAACCTACTCCAAAGAATCACTATAACGCTTGATGTGGCTGTGGCAATCG
AATATCTACACCATGGTCATAACACTCTGATAGTTCATTGCGACCTAAAGCCAGCCAACGTTCTTTTGGAT
GAAGATATGGTGGCTCATGTTGGTGATTTTGGAATCTCTAAAATTTTAGTCGTAAGCAAGTCCATGGCAC
ATACCGAGACATTGGGCACTCTTGGATATATTGCACCAGGTAAACGAGTCTCTTTCTATTTTCAGAAAAA

TCACAATTGAGCCCTTCCAAATTCTAGAACTAAATAAAAGCAAGTGTCTGTTAATACATAATTATTGTTAT
ATTCAAATGAGTAATTCTTTTTCATTTTTCTTCTTTTAAGAATATGGCTTAGAAGGAATAGTGTCTGCTAG
TGGTGATGTTTACAGTTACGGCATCATGTTGATGGAGGTTTTGACCAAAAGAAGGCCAACAGATGAAGT
GATATGCAATGAAAATCTTGACATGAGGAAGTGGATCACACAATCATTTTCAGGGACTATGATGGACGT
TGTGGATGCCAATATTTTCTCTGAGGAAGAACAAGTCACTTCTAAAAGTGAAATCTGCATAGCCTCCATG
ATAGAATTGGCTTTAGACTGCACAACGGAAATGCCAGAATCAAGAATTACCATGAAAGATGTAGTCAAG
AGGCTTACCAAAATCAAGAACACATGGGCCACGAGGGGTTAG
>PGSC0003DMG400021184 LRR-RLKs
TTTTTTTTTAGAGAGAGAAGCTGAGAGTTGTAAGAGAGAGAAAATATGTCTGCAAGAAGACAAATGTTG
AGTGTCACTGCCATTTCTGGGAGCTGTGAGGTTTCTCTTTCATTCATATACAAAACAAGACTAGGGTTTTA
CCATTGTGGTTTGGTTTTTTACTGAGCTCATAGAAATCTTGAACTTCATTTATCAAGATTTCTAAAGGGCT
AAATACTTTGCTGGCCCATTTCATTTTCTTGTACTTTAATGCAGATTTTTTTTGAGGCCACCAGTTGAGTGC
ATTAAAGAACTTGAGGTTCCAAGATAACCTGAAGCTGTAATCATTGCAAGAAGTTGAATTTCTTCAGTAA
ATCTTTGAAAAAGACTCTTCTTTTTTTGTTTTTCTTCTAGTTGAGCTGATGTTCTTGGTAAGGGAATGCTG
AACTTGTTTTACTATCTTGAATCTTTGAAAGTTCAGTCTGTACTTTGTTTTTGTCACTCATTTCAGCTTGTAT
ACCTAGAAAAGTTTCTATTTTGAAAAAGACTGGTGATTTTGGGTTGAAGTTGTGTACTTTTCCCCCCTGTT
GTTTTACTGCTTTGAACTGTGATGGTTGTTGTGAAAGATGGTTTTCATATGTTGAGGTTTTACTTAAAGTG
ATTCTCATACTTAAAGCTGCTCCTTTTGGATTTCTTGGAAGGTTGAAGATTTAGTTGTATTTTTGGGAGTG
AGTTTGTTGTCACTTGGGCTAGTAAAGCTTTTTCTGGTGTTGCAATGAACCTATTTAGGTTTTTGATGCTT
GTGCTTTGTTTTGGTTCTGCAATGGGGCAGCTTCCTTCCCAGGACATTTTGGCCCTGCTTGAATTTAGGA
AGGGTATCAACCATGATCCAACTGGTTATGTACTGCAGTCATGGAATGAGGAGTCCATTGATTTCAATG
GTTGCCCTTCATCTTGGAATGGTATAATGTGCAATGGTGGTAATGTTGCAGCAGTTGTCCTTGACAACAT
GGGTTTATCTGCTGTTGCAGATTTGAGTGTGTTTGCTAACCTCACAATGCTTGTGAAACTCTCTATGGCTA
ACAATTCAATTGCTGGACAAATGCCAAAGAAAATTGGTGATTTTAAGAGTCTTGAATATCTGGATATTTC
AAACAATCTATTCAACTCCTCTTTACCACCAGAGATTGGGAAAATAGGAAGCTTGAAGAATCTTTCACTT
GCTGGTAACAACTTCTCTGGCCCAATCCCTGATACTATTTCAGAGCTCATGTCAATTCAATCTTTGGATTT
GAGTCACAATTCTCTTTCTGGGCTACTTCCTTCTTCTTTAACCAAGTTAAATAATTTAGTATACCTCAATCT
TTCTCTTAATGGATTTACAAAGAAAATCCCTAAAGGTTTTGAGCTAATGGCTAACCTTGAAGTTCTTGACT
TGCATGGAAATATGCTTGATGGTACTTTGGATCCAGAGTTCTTACTGCTTACTACTGCAACTTATGTTGAC

CTAAGTGGAAATTTACTCGCGAGTGCCGCTTCTCAACATGAGAAATTTTTACCAGGCATATCATCGTCAG
TCAAGTACTTGAGTCTTAGCCATAATCAGCTTACGGGGTCACTAGTAAGTGGCGGTGAAGCTCAGGCAT
TTGGAAACTTGAAGGTTCTGGATTTGAGCTATAATCAGTTGTCCGGGGAATTACCTGGCTTCAATTTTGT
TTATGATCTTCAGGTCCTGAAACTCAGCAACAACAGATTTTCTGGTTTCGTTCCAAATGATCTTTTGAAAG
GAGATACTTTAGTTCTCGCAGAATTGGATTTAAGTGGAAACAACCTCACAGGTATATTCCAAACTGCTCT
CTGAAATTCATTTTGCAAATTAAATAGTATTTCGTTGTGTTGAACTTGAGTTACAACAAAGTAGTTAATTT
CTGGTCATTTTGTTAGTGTGCTTTACTCAGTCCCTTCATTTTACAGGGTCGATAAGCATGATAACATCAAC
AACTCTGCGCGTTCTTAACTTATCCTCCAATGCTCTTTCTGGTGAACTTCCACTGGTGACAGGTAGTACTG
CAGTTCTTGATCTCTCGAAAAACCAATTAGAGGGGAATCTAACTAGAATTCAGAAATGGGGAAATGTTG
AATTTCTTGACCTCAGCCAGAACCAATTGACTGGAAACATCCCTGAGGTTACAGCTCAGTTTCTGCGATT
AAATCGTCTAAACCTTTCTCACAATGCTCTTACTGGATCTCTCCCAAAAGTTATCACACAGTTTCCCAAGA
TCACAGTCCTTGATCTTAGTTTCAACCAGCTGAATGGACCTCTTCTCACTTCTCTGCTAACATTACCCACTA
TTGAAGAACTTCACCTTCAAAACAATGCACTTGTTGGAAATATTGATTTCGCTGCCCCCTCTGCTACACCC
AATCTCCGCGTTCTTGATCTTTCTCATAATCAGCTTGCTGGTTCTTTTCCTGAAGGGTTTGGTTTGTTGACT
GCGCTTCAAGTACTTGATATAGCTGGAAATAATTTCACTGGGTCTCTGCCTACATTGATTGGTCAAGTTG
GTTCGCTCACTTCTCTAGACATTTCACAGAATCATTTTACTGGTCCTCTGCCGATGAACCTGACTGATGGC
CTCCAAAGCTTCAATGCATCACTCAATGACTTATCTGGTGTTGTCCCTGATAATTTGAGAAAGTTTCCTTT
ATCATCTTTCTACCCTGGAAACTCTGAACTTCAATTTCCAAATCCTCCCTCAGGATCTGGCCAAGCTTCAC
CAGAAAACCAGAAAAGTCGATCACTTAAAACTATAATCAAGGTGGTAATAATAGTTTCTTGTGTAATTGC
TCTTATTATTTTGGTCTTGCTAGCCATTTTCTTTTATTATATACGAGCTTCAAGGAAGCGTCATCCTCGTGT
TGCTGAAAAAGTTGTTCATCGCCAAGCCACATCAAATCCTTCTGGCTTTAGCAGTAGGGAGGGTGCCGG
TGGTGCAGTTGTTTCAGCTGAAGATCTTATGACTTCACGGAAAGGATCATCAGAAATAATTAGTCCAGAT
GAGAAAATGGCTGCTATAACTGGTTTCTCCCCTTCAAAAGGTAGCCATTTCTCTTGGTCACCAGAGTCTG
GAGATTCGTATACTGCAGAAACTTTTGCAAGATTGGATGTGAAGTCTCCGGATCGTCTGGCTGGAGAGC
TGTACTTCCTTGATGATACAATTTCTTTTACACCTGAGGAACTTTCCAGGGCTCCGGCTGAAGTCTTAGGT
AGAAGCAGCCATGGTACATCATACAGGGCAACACTGGAGAATGGGTTGCTCCTGACTGTGAAATGGTT
GAGAGAAGGAGTAGCGAAGCAGAGGAAGGATTTCGCAAAGGAGGCTAAAAAGTTTGCCAATATTAGG
CATCCTAATGTAGTAGGATTAAGAGGTTACTACTGGGGTCCCACACAGCATGAGAAGCTCATTCTTTCGG
ATTATATATCTCCTGGAAGTCTTGCAAGTTTCCTCTATGGTAAGCTTCATGTATTCTAAACTCTCATATTAT

GTTTTGCTAATAAAGGTAAAGGCCTTGTGTACCTGTTCTTTAATCTAGTCATTTTCCTCTGACTGTGTGAT
ATATCTTACAGTTCATATGGTTTTCCCATGCACATTATATATGAGCACTTTAATCATTGCTGTGTGATCCTG
TAGTGGGAATTTGTGTGAACGACAGTATTTAGTCCTTGGTTTGATGTGGTAATACTTTAGTACATCTAGA
GCCCAATATATTATGCATGTCTTTTGTTTATGTAACTTTCACACCAAGTAGTAATACAACAATCACCATGT
GCTCTTCGTGATGGTCACTACATAGGCTGTGAGGGATTTGCAATATGGTACTTATTATTTCCAGTAGCTA
AAATTCTGTATGATTGAGTTCTTAATGGATCTGAAAATTGTGATGTTTAGCTTTTGTTTCATGATTAACTTT
GTTAGAAATCAGCTAGAATATGATACATTTTCCTTCTTTTATGAATCCTTCAAATGGACTTGCTTTCTATTC
ATTTGACTAACTAAATGTTTTCATTCAGTTGATAATTATATATGTGGTCATTCTCAATATACTTCTCTAATG
TGCTGCACGTTTGTAGATCGGCCTGGTAGAAAAGGTCCACCACTAACCTGGCCCCAAAGACTCAAAATA
TCAGTTGATGTTGCACGTGGCTTGAACTACCTCCATTTTGATCGTGAGGTTCCACACGGAAACCTTAAAG
CAACCAACATCTTGTTGGATGGGCCTGACCTTAATGCAAGGGTTGCAGATTACTGCCTTCACCGCCTCAT
GACTCAAGCCGGCACAATAGAACAGATTCTTGATGCAGGAGTGTTAGGTTATCGTGCCCCTGAGTTGGC
TGCGTCGAAGAAACCACTGCCTTCCTTCAAATCAGATGTATATGCTTTTGGAGTTATTTTGTTGGAGCTGC
TAAGTGGGAAGTGTGCAGGTGATGTTGTCTCTGGTGAAGATGGTGGAGTAGACTTGACTGACTGGGTA
AGGTTGAAGGTGGCAGAAGGTCGTAGTGCTGATTGTTTTGATAACGTGTTGTCACCTGAGTTGGGAAAT
CCAGCAATGGAGAAGCAAATGAAGGAGGTTCTTGGTATAGCCGTGCGATGCATTCGTTCTATATCTGAG
AGACCTGGTATCAAGACTATATACGAGGACCTTTCTTCTATATAGTTTGTACCAAACTCTACTTTGGGAAT
CAAATAGCTCATTGACTTCCCTTTTGAGTAGTAGAATTAGCACAGTTAGTGTGTGAATGGGGCTAGTTTT
ATTGACCATTGTTTGTTGTAAATTGGGGCATATTTAATGTGTAAACAAACTTTTCTCCCCTTTTGGCCTAT
GAGCTATCAATGTTTGTCAGTCTTTCGGAGTAAATACTTGTGACTTTATTTTCCAAGTTTTGCAGCACAGT
TTCACAGAACATTCTCAGATGGTCAATCAAACACAATGCTGACATCAACATTGCAGTGCAAACTGACCGA
AAAAGGTGTTCAAGAGAAAGTGCCAGGTTATGAACAAATGCTACTGGTTTTTCTTGTCCACTATTCAATA
AAAGTTCAAATGCAACATCATTATAAGACACCTAAGGTGGAAAAAGGAAAAGTTACAAGAACCTAGTTA
TGCCACTAGTTACTATGCAAGTGAGGAGAGGGGTGGGATAAAGGTATGAATTTAAACAACAGGAACTC
TAAATTACATGAAAATACATCATAAGATGCAACATATTTTACTGTTGCAACTTTAACAATGTATGTTCGTC
AAACCTGAATCCAGCCATCTCTGCCTCCTTGCAGATATCCTTGCACTTAGCTACCCTTCCAGATGCCATAT
AAACTTTGATCAAGCTGCAGTATATGTCAAAAGTTGGACTAAAACCTGCTTCCTTAAGTTTAGAGAAATA
GCGTACAGCCCTTGAGAGATCGTTCAAAGCAACAAAAAGCTTAATAACTACACGATAAGCTGGAAGATC
AAAAAGGCAATTTGAAGCCTCCATTTCCCATAGCATGTCTATTGCCTCTCTATAGTTGTTCGCTGAATAAC

GACAGTGAATCAGAGTAGTATACATAACTACACTTGGATTACCACCAGATTTCCTGAACCAGTCATATAG
TCCCTCCACGATCTCAAATCTTCCCAGCCTAATGCAAATCTTCATGATAGCTGTACAGTCCTGTGGTGTCA
AATTCAGATCATCTCTCACAGCAAGCTCCTCTAATAATACCAAGACAAGTGTGCTTTTATCAGGATCCTTA
CCAAGCTCTAAGATTAGCTTAGCATACACCCCAGTATCAAGCACTCTGTTAGATTCCTTAGCCATTGAAA
GTAGCTTCAAAGCAAGCTTCAGGCTTCCTCCTTTAATATAACCCCTTAACATTGCTTCATACACACCCCGA
CTTGCCAAGCCAGTGAACTTGTCCAAATCAAATGGCACTTTCAACTCATTTGACCCTGCCAAAACTTCAAT
GGTAGAACCAAGAATATGATCATCTGGGAAAAGATGGGGTTGTTTCTTCACCCAACAGAATGTTTCAAG
AGCTCTCTCCGGAAGCCCCAAGTAACCCAACTCTCGGATTGTCAACGACAATGATCCTTTCCTAAGAAAT
GGACTCCATTTACTGAGAACCTTTGATACATTTTCCTCAGTTGAAAGGCCTTGTATCTCTTTAGCCAAACC
AATCAAGAAAGTAGGATTCTTGTACACTTGCGTTGATATGGATTGCCTTGAACGATTTGTGAATCCCTTTT
GTGTAGGAAGTCCTAAAGGTCGAATCTTGTAAGGAAGGGGCAGAGGTAGAGGCCTCTCTCTATCCAATT
TTCCAGGCTTCTGAGGGATCCTCCCTTTAAAAAGAGATGAAATTGCTTCCATCTCATCCGACTCCCATATA
ATTCCCCCATCATCATCACACATCCTTCCTTTTTCTACTTCCAGCTCCAATTCTTCATCCATTCCATCAGAAC
AAGCCTCAGTTGCAACTCCCTCATCCTCCTCGTACAAGACCCTATTAAAACTATAGTCCATATCTGGTGGT
AGCTTGGGTCGTCGAGGATTTCTAAGGTTCTTTGGAAGTTTTCTATGATTAGCTCTGTTATTAGATACAG
CCATGAAAACAACTCTTCCCTTTCTTCTTCCAAAACTTAAAGGTGTTCCAGAAGCAGCTGTACTGGCACTA
CTAGACTGCGAGATAGAATATGTGGGTGAAGACCAAGTAGAGGACTCATAAGTGGCCGAGAACATGCA
ATAACTCCTGCTGAGCATTTGGTCGAGCAGGTGGTCGGCACAAATTTATAAACAAAGGAATATGAGAGA
CCTTATAGTGTGACGGAGTATAAAACCAACTGTTAAATGAAAGTTGAGCAGTATTTTTGACATATAAGTT
ACTAT
>PGSC0003DMG400021353 LRR-RLKs
AGCATTCCTCATATGAAAAATCCACTCTTTATGGTCATTTCTTGCTACTAAATCTTCACTTATAAAAAAAAA
GATTAAATTTTTTGGATTACTCTGTAAAAACTACTGTGTTGTCAGCAAGAACAACACCATAGTAGTAGCA
ACTAGCAAGCATTGTGCTTTTTCTCACTCCAATTATCACTCTCTTTCTCCTTTTTTGTTTTGGGGTTTAGTGA
TCTGAACCCCATGTTTTCCACCTCTTATTGTCTCAAGATTTGACATGGGGGTCTTCAAGAATCAACCTCAT
TAGGTGGCAAAGATTGTAGCTTTCTTGATTTTTGTTGTAGTGGGGTTTTTCTTTGTTGAATGTTAATGCAA
ATGCTCAAATGTTGTTGCTTTGTGGATCATCTATGGAGGTTTTTTTTCACCACATATGGTTTGTTGTAGCT
CTGTTTCTTGCTTTTGCTTCACTTGTTTTCACTGTCACTGACCCTAATGACTTGTCTGTTATCAATGAGTTTA
GAAAAGGGTTGGAAAATCCTGAGGTCTTGAAATGGCCTGAAAATGGTGGAGACCCTTGTGGTTCTCCA

GTTTGGCCACATATTGTGTGTAGTGGTAGTAGAATTCAACAGATTCAAGTTATGGGGTTAGGGTTAAAA
GGCCCTTTACCTCAAAACTTGAATAAGTTGTCTAGGTTGACACATTTGGGGTTGCAAAAGAATCAATTTA
GTGGAAAGTTGCCATCTTTTAGTGGTTTGTCTGAATTGAGCTTTGCTTACTTGGATTTCAATCAGTTTGAC
ACAATCCCATTAGATTTCTTTGATGGACTTGTGAATCTACAAGTTTTGGCTTTGGATGAAAATCCTCTAAA
TGCCACTAGTGGTTGGTCATTGCCAAATGGGCTTCAAGATTCAGCTCAGTTGATCAATTTAACAATGATA
AACTGCAATTTAGCTGGTCCTTTGCCTGAATTTCTTGGAACTATGTCTTCTTTAGAGGTTTTGTTGTTGTCT
ACAAATAGGCTTTCAGGGCCTATTCCAGGTACTTTCAAGGATGCAGTGCTGAAGATGCTTTGGTTGAAC
GATCAGTCGGGTGATGGTATGAGTGGTTCAATAGATGTTGTTGCAACAATGGTGTCACTTACACATCTTT
GGCTTCATGGGAACCAATTTTCAGGTAAAATCCCAGTAGAGATTGGTAATCTAACAAATCTGAAGGATC
TCAATGTGAATACAAATAACCTTGTTGGATTAATCCCTGAAAGTTTAGCAAATATGCCATTAGACAATCTT
GATTTGAATAATAATCATTTTATGGGACCAGTTCCTAAGTTCAAGGCTACTATTGTTAGTTTTATGTCCAA
TTCTTTTTGTCAAACCAAACAAGGAGCAGTATGTGCCCCTGAGGTTATGGCACTTTTAGAGTTTCTTGAT
GGTGTGAATTATCCTTCTAGGCTTGTTGAATCATGGTCTGGAAACAACCCTTGCGATGGACGTTGGTGG
GGAATAAGCTGTGACGATAACCAAAAAGTTAGTGTCATAAACTTGCCTAAGTCTAATCTTTCCGGGACCT
TGAGTCCTTCAATCGCGAACCTTGAATCCGTTACTCGCATTTATCTTGAATCAAATAATCTTTCTGGTTTTG
TTCCATCTAGTTGGACTAGTTTGAAATCTCTGTCTATTCTTGATTTGAGTAATAACAATATTTCTCCACCTC
TACCTAAATTTACCACCCCTTTGAAACTTGTTCTAAATGGAAATCCAAAGCTGACTTCTAGTCCTCCTGGA
GCAAATCCTTCACCAAACAACAACACAACGCCTGCAGCATCACCTGCGTCGTCTGTACCATCTTCACGAC
CCAACAGTTCAAGCTCTGTGATCTTTAAACCCGGTGAACAGTCACCCGAAAAAAAGGACTCAAAGTCAA
AGATAGCCATAGTTGTGGTTCCTATTGCTGGTTTTCTACTTTTGATTTTTCTTGCTATTCCACTGTACATTT
ATGTCTGTAAGAGGAGTAAAGATAACCATCAAGCTCCAACTGCTCTTGTGGTTCATCCTAGAGATCCATC
TGATTCGGATAATGTGGTCAAGATTGCGATCGCCAATCAGACTAATAGAAGTCTTTCCACAGTAAATGCA
AGTGGTTCTGCTAGCATACACAGTGGTGAATCCCATATGATTGAAGCTGGGAATTTGCTCATATCGGTTC
AAGTACTTCGGAATGTGACTAAGAATTTTTCTCCGGAAAATGAACTTGGGCGTGGTGGTTTTGGTGTGG
TTTATAAGGGAGAATTAGATGATGGGACACAAATAGCTGTCAAAAGAATGGAGGCTGGTATTGTTAGC
AACAAAGCTTTGGATGAATTTCGATCTGAAATTGATGTTCTCTCAAAAGTACGGCATCGACATTTAGTGT
CTCTACTGGGATACTCTGTTGAAGGTAGTGAAAGAATTCTGGTTTATGAGTACATGCCACAAGGTGCACT
TAGTAGACATCTTTTCCGCTGGAAAAAGTTCAAGTTGGAGCCTCTCTCTTGGAAGAAAAGGCTTAATATT
GCCTTAGATGTTGCTAGAGGTGTGGAGTATCTACACACACTAGCACATCAGAGTTTCATTCATAGAGATC

TCAAATCCTCAAATATCTTGCTGGGTGATGATTTCCGAGCAAAAGTATCCGACTTTGGACTCGTGAAACT
TGCTCCTGATAAAGAGAAATCTGTGGTCACCAGGCTGGCCGGAACTTTTGGATATCTGGCACCTGAATA
TGCTGGTAAGCAATTTTTTGCTTACTTTGTCAAAGAGAAAAGATGATTTTTTAAAATGCATGTTTATATGA
CAATTTTTACTTTTGCAGACACCTAGAGTTTGCTCAACTCAATTCTGATGTATAGCATTTTATTTTTTTGCA
TACCAATGTACTGTTTTCCGAACTAACATGAGATGTTTATTTGATCCAGTTACGGGTAAAATCACCACGA
AAGCTGATGTCTTTAGCTTTGGTGTTGTGCTAATGGAGTTGTTAACTGGGATGATGGCACTAGATGAGG
ATAGACCTGAGGAAAGCCAGTACTTGGTTTCGTGGTTCTGGAATGCCAAATCCTCTAAAGAGAAGCTTA
TGACAGTCATTGATCCAGCTCTAGACGTGAAAGATGAGATAACCAAGAGCATTTCCACCTTAGCTGAACT
TGCTGGTCATTGCACTGCAAGAGAACCTGGTCAACGGCCAGATATGGGCCACGCTGTAAACGTGCTCTC
CCCACTTGTTGAGAAATGGAAGCCTCTTGAGGATGATCCAGAGGACTATTGTGGTATCGACTACAGTCTT
CCCCTCAATCAAATGGTCAAGGGCTGGCAAGAATCGGAAGGAAAAGACTTAAGTTACGTGGACCTCGA
GGACAGTAAGGGTAGTATCCCTGCAAGACCAACTGGATTTGCAGATTCATTTACATCAGCTGATGGTAG
ATAATGGAGGTACTTCTATGTAGTAGATGTAGATATCAATTTTCTTTGTATTGTGTGAGATTTTGATCGTA
TTTTCCACGTGCCTTCGTTCATTTCTCCCCCTTCAATGTGAATGTATTAGATATTCAAACTATGTGTAGCCT
CAGTTCCTTCTGTAGATACAAAATAGTGATGAAGGAGAACTATACATCGGTTAAACTCCTCGCTCAGTCA
CTATTTTCATCTTCTATATACTATTGTGAGATTTAAGAGCATTTCTTACAACTTTGCTTATTTTCGTCGTCTA
GTTTGCTGCTTCAGAAGTTCTGTGTTGAGAAACATACTGATGTTGAGATAGCCTTGTAATAGAATATGTC
GCTTGATTCGAT
>PGSC0003DMG400021394 LRR-RLKs
AATTTGGCTGTAAAGTGCCAAACACTTGCAACAAGAACACTAGTAAATTACATTTATTATTCATTTCTCTT
TTCTCTTGTTCATTCCTCCAATCTCTAACAACGTAGATGCAATTCACAGGCTCAAGTAGTTTTCCTTTTTAT
CTAAAATTGATCATCGTTTTGTTGGGGACATTTTGTGATGCCGATCCTCGGATTTCAGAAGCAGGGCAGG
TGTGTGGCACTAACATGACATTGCCGTCCATTATCATCCCACAGTATACTAAAGAAATGCAAGTGATTAC
CCAGCTTGTGAGTGAACATGGATGGGGCAGCAGCTATGCTGTTAATTCAACAGATATTTCGATATACGC
TTTAGCAAATTGTTACCAAGATCTTCCACCTAACGACTGTGTTCAATGCTTCCTTATAAGTTGTTCTACACT
CCCTACTTGTCTCCCTGCTGTCTCTGGTCGTGTATATCTTGATGGTTGTTTCATCCGTTATGATTATTACAA
CTTCTTCGGGGAAACTACTGATTCTTTCAAAGATAAAGTGAACTGTAGCAGTTCGTTTGGAAGGGCTGTT
ACTGATTCAGATCAATTGGCTTTGGCTGCTGCAGCTGGAAATTTGATTGAAAACGTGACGAAAAAAGCA
ATTAATGAGAATGATGGATATGCAAAGGGGAAATTGAATGGAGTTTATGGATTGGCACAGTGTTGGAG

AACAGTGAGTAAACAAGGTTGCAGAGAATGTTTGGACAAGGCAAGTGAAGCTATAAAGGGATGTTTGC
CTAATAGGGATGCTAGAGCTTTAATGGCTGGTTGTTATTTGAGGTATTCCACTCACAATTTCCTCAGTCAT
CATTCTCAGGCTACTAATAAAGGTATCTATATCACTTCTCTTTTCACCATACCAACTCTATTCTTCGGTTAA
TCCATAGACTTTTGAAAATTTACTTCCTCATCAACATATCTCTTTTTATTTCTTCTTTATTAAGGGCTCGTTT
TGTAGGTGGATAAGAAATAAGTTATTTATGTTATTAATTTCTGCATAACTTATAATACTACAATTGGCAAG
TATATAGGAAATAAGGTATTCATTTATAAAATTAATAGGACATTTGAATGACAATTTAGAAACTCGCATA
AATAATATATGTATAAGTTATGAGATAATTTTTATATATATTCTACAGAATAGAATGTGGAATAAGTATTA
GCACATGTATAACTAATATCTGCATAAAGTAATACATAAATTCACTCATAACTAATCCTACATTACTAATA
CCTCTATAATTCTCACCAGCAACCAAACAACCTAAGGGTGTGTTTGTTATGGAAGAAAACATTTTCGCTA
CAAAAAACATTTTCAAGCTGTTAAGGATTATAATTTGTAAGTAGCCAACAATGACGAGAATTGCTAAGAA
AAGAAAACACTAAAAAATTTCTTCAAGGGATTCAATAATACAGAGAAAGAAAGATGAAAGAATGATAA
ACAACTTCAGATTAAGTTGTGGTACCTTTTTTTTTGTTTTTTGTTTGAGTATTTTAGATTGGCTTAATGAAA
ATAAAGAAGTATGAAAGGGAAGTGATGGATACGTACGTTCGGTGCTTTGCTGTTAAACATTATTTTTCAA
TATAGATCTAGCAGATCATAAATCTTATTTTTAGCCAAAGAAATTTCCTATTCTTTGGGTTAATACTTCATT
AAATGGTGTCTCAACATGTAATGTTTTGAAAATAATCCCCGCATCAACTCCTAGTTTTCTATTTTGGAACA
CCTCTAGTTATGGGAATTACATGTTTAATGATTGATTGGAAAAACCTTCATTGAAAAATTGATTTCAATCT
CAAAAAATTCATCTTCAGAAACTCATTGCATCTCATGAAAATGTCTTGAACTTATTATTTTCAATACAAGG
AAAAATGTCCAAACAAGATCTTAGAACGATATATATCCTGTTTGCTAGCTTTACTAAAGGCATTTTATGA
ACGTGTTTCCCCAAAAGAATTTGATATTCTCCTACCAAACACTCAAAATTATTTTTTTAAAGTATTTATTTC
AAAATATAAAATCATATATAATTTCCGTCCTGTTTGATTATCTCAAGGAAAATAAAAAATGATATTATAGG
AATATCTTGACTATGTGGGATGGTCAAACTAACGCCATATTTGCTTTAAAAAACAAGAATGGTCCAAAGT
CCGAACTTATATGAGTTCTCTAACATCCTTCCTTTTGTCTTATTCCCCTAATCCAACTATAATTCACATTGTA
CAAAAAAAAAACAGCATAAATCATTATTTTAAACGGATAGCAAAATTAAATAATTCATAACACATTAGCA
CCTAAGATTAATTAAATTGTTAAAAAAAGCTCAACTGTGGAAATAATTCTTATTTGATTTACTAAACAACG
CAAGTTAGACTCTATGTTTTTTTCAAGTACTAAAGCCAAATATATGCAAATTGGAAGGCAGAAAATTTCA
AACGTATCGATAATGACATCCATATGACCTACTCCTACAAAGATGTATTATAAATATTTCTGTTGGGTCAA
GTCTATACGGATCGACAGAGACATTTTAATGGTATGTAAAATAATTATTTTTCTTTGTTGTTATATTTTGA
TTTTGGTGAAAAAGATTGGAAGTATATATTTATATATATACAACTAGCTGAATTAATAAATGTAGAAAGT
ATAAAATATAAAACATCAATTGTGCTATTGTGGAATTGACTTGTCTTATAAGTTGAAGTAGAACCAGCTC

CTCTCCTCTCGGACTCAGGTGTAGTTCAGTTCCAATACTAAACATTTTCTTAAAAGAAAAATAGAAAATAA
ATATTCAAGAAAATAATACTCCCTCAGTCCCTTTTTATTTGTACAATTTGGACTTCTTACTTGTTAGTTTTG
ACAAATCAAGAAAAAATAATATATATTTTTTTACGTATTATACTCTCAATTTATTTACAGAGTTGATATTTA
AAAAAAAGATAAAACCTCTTTAATAAGTAAATTGATTTAGAATAGTAAACTCATTATGTTAATTATTATTT
TTCTTAATAGATGTATCAAATAAAAAGAGTATTATTGAACAAACAAAGTTTTTACTGATATACTAGACTAT
AAGGGATTGGGTTGTGAGAGTCTATAAAAACAATCTCAGAACTGACAAATTAAAATCTGCTACCTTATAC
TCTCAAGACCCCATTTTGTCAGATTACATCAATCAAATATTCTGTTGTTATTGTTGTAGTATACTATAAAAC
TTTATCTGGCTCCTCTTTGACTGCTAAACTTGAAAGCTTGTTCTTTAGAGACTTTCAACTCTGTTTTTTTTCT
TTTCTCTCAAGGACAATAATATACTTGCTTTTGACTAATTTGTAAATAAATGCTCTTGGTTTCAGGCTTAA
GCAAAGGTGTGATAATAGTTATCGTTTTTGCAATGATAGCTTTCTCCATGCTGTTTCTCTTTGTTGCGTAC
GTAGCTCGTAAAAAATCGTTAAAGCGAAAACAAGGTATCATAAAAGAAACTAACCTTAATTTGTCAATTT
AGACAATCGATCAGTCGTATATGTTATAAAATAATTACATAAATGTTTGCAATTTTTCTTGGCCACAGAAT
TCATTAATCTTGGCAAGATATCGAATTCATACAAGAGGTCCAGCTTGAACTTTAAATATGAATATCTTGA
AAAGGCAACACACTACTTTGATCCATCAACAATAGTAGGCCAAGGAGGAAATGGTTCTGTATACAAGGG
GACTCTTCCTAATGGGAAGGTTGTTGCAGTTAAGAGACTGTTTTTCAATACAAGGCACTGGGTTGATGA
GTTCTTCAATGAGGTTAACCTTATCCATGGGATTGAGCACAAAAATCTTGTGAAGTTGTTGGGTTGCAGC
ATTGAAGGCCCCGAGAGCCTCCTTGTCTATGAGTTTGTGCCTAATAAGAGCCTAGATCAATACCTTAATG
GTACGTTAATATCGATGAATTTATATCTATAAATGCAGTGACAATTAGGGACGACTTCTAATTGAGTTGT
TCAAATTTTTGTTGTGTAGATAAGAATAAGATAAAAGTTCTAAGTTGGAAAGAACGATTACACATCATAG
TCGGAACAGCAGAAGGCCTTGCGTTCCTCCATGGAGGTTCAGATATGAAAATCATCCACAGGGACATCA
AGAGCTCCAATGTACTTTTGGATGAAAATTTTGAGGCGAAAATTGCTGATTTTGGACTAGCTCGGTGTCT
TGCAGCTGATAAAACTCATCTTAGCACTGGAATTGCTGGGACATTGTAAGAATGATAAAACAGCATTCA
CAGCCTTCTTTTAACTACTTAATTATAACTAGTGATTTTATTTGTTGCGTAGAGGATATATGGCGCCTGAA
TATCTCGTAAAAGGACAACTCACAGAGATGGCTGATGTCTATAGCTATGGAGTGCTTGTTCTTGAAATTG
TTTGTGGAAGAAAAAACATTTCTTTGGCAGAGGATGACTCTGGATATCTACTGCAAACTGTAAGAATTAA
TGTTGTACATTTCTCTTAAAAGTTTGTTGGTAACAAATTGAACCTGTCATTTTTTATTTCAGGTGTGGAAA
CTGTATACAACAAATCAAGTGACTAAAGCATTAGACCCCATCTTGAAAGATGATTTTTCACCAGAAGAGG
TATCAAAAGTCCTCAAAGTAGGGCTTTTGTGCACTCAAGCTTCTGTTACATTAAGACCATCAATGTCTGAT
GTTGTCCAAATGCTAACAACATTTGGACAACCGATTCCGGAACCATGCCAACCACCATTCTTACGTTCAA

GCAGTAGCATAAAAAGATTCGTCTCCGATACACTATCCAAACTTGATGAATCTTGTATCTCCTCTGCCATG
CATAGCTCATCAGATGGACCACACATTAACTGATCAAGAATTCTTTTCAAAACCGTCTCTACTGGAAACAT
ATATACAAATTAAAAGAAGTCAAATTTCATGATGTATGTAAGTGAAAATGAAAATTTTGCACAACATTTT
TGTACCGTTCGAACTAGTAGTGATTACATGTTGCATTTTTACGATTTGATTTTACTTTAACTCTTCG
>PGSC0003DMG400021609 LRR-RLKs
CACAAAACACACAAGCCCACTACACTACCAATCTTAGTCATTATTTCTCTATCTACTATCACTCTTGTGAAT
GCAGTTACTTCATTTTCCTTTCCACCTTGTACACATGAATATCTAACAATAATCCCAAACTCAACATTCATT
CAATGAAACTTGTACTCCATTACCTAAAATTCACACCTTTTTCCCTTTTTCATTTCCATTCCCTTCTCTAACT
ACTCTGAAAAAAACATTTGTTTGCTTCTCTTATTGTAGGAACTAAAAACACCATCATAAAAATGAGATCTT
TTAGTCCCTTTTTAGTTCTAATACTTTATTTTGGCTTATTGTATGGATCTTTATTAGCTGATGATACTACTAT
GCAGCTTAATGATGATGTTTTAGGCCTCATTGTGTTCAAATCAGCTCTTCTTGATCCTTACTCAAAACTCTT
GTCATGGAGTGAAGATGATAACTCTCCTTGTGCATGGGAATACATCAAATGCAATCCAATGAATGGTAG
AGTCACTGAGCTCAACTTAAATGGCTTAAGTTTATCAGGAAAGATTGGTAGGGGGCTCGAGAAGTTGCA
ATCTTTAAAGGTACTATCTTTGTCAAACAACAACTTTACTGGTGCAATTAGCCCTGAGTTAGCCTTGTTGA
CAAATCTTGAAAATCTCAACTTTAGTCATAATGGCTTATCAGGGAATATCCCTGGATCTTTTTCAAATATG
ACATCTTTACAGTTTCTTGATCTGTCTGAGAATGCTTTATCTGGACCTGTCTCTGATACCATGTTTGATAAC
TGTGGTGATTCGCTACGTTATCTTTCTCTATCCGGGAATTTTCTTGAAGGTTCATTTCCTAAAACAGTTTCA
AAATGCACTAGTTTGAATCATCTCAATCTTTCAAGAAACCATTTTTCTGGTGACCCTGGATTTTCCGGGGG
TATCTGGGGATTGACAAGGCTTAGAACATTAGATCTTTCACATAATGAACTCTCTGGATTAGTACCAATT
GGTGTTTCAGTTTTACATCAGTTGAAAGAGTGTTTGTTACAAGGGAATCATTTCAGTGGAGATTTACCAG
CTGATATTGGTTTCTGTCCACATTTGAATAGATTAGATTTGAGTAATAATCAATTCACAGGACAAATTCCA
ATGTCACTTCAAAGGGTGAATGTTCTTTCTTTTCTCAGTTTATCAAACAATATGATAAATGGAGATTTCCC
TCAATGGATAAGTAACATGAGCAGCTTGGAGTACTTGGATCTTTCAGGCAATAGTTTAGAAGGAGCATT
GCCTGATTCAATAGGAGATTTGAAAATGTTGAAGTACTTGAGTTTATCTGGTAATAAATTGAGTGGAAA
CATTCCAAAGTCTATGGTGTATTGTACTAGTTTATCGACTATTCGGATGAAAGAAAATGCATTGACTGGT
AGCATTCCTGAAGGATTGTTTGGTATAGGATTGGAAGAAGCAGATTTTTCAAGAAATGAGTTAACTGGT
TCAATCCCTCCTGGTTCTGGTAAATTCTTTGAATCACTTCAAGTTCTTGATCTATCAGGAAACAATCTTACT
GGGAATATCCCAGCTGAAGTGGGACTTTTCTCCAAGTTGAGATACTTGAATCTTTCTTGGAACAATTTTC
AATCTAGATTGCCTCCTGAAGTTGGATACTTTCAGAATCTAACGGTGTTGGATCTTCGACACAGTGCTTT

AGTTGGATCAATTCCTGGTGATATATGTGATTCTGGTAGCTTGGGAATTCTTCAGCTTGATGGAAATTCA
TTCACTGGACCTATTCCTGATGAGATTGGAAATTGTTCATCCCTCTACTTGTTGTAAGTATATTTATTCTGA
TTTATCTCATTATAGTATGGTACAGCCTCTCTATAACAGTCATTTCACTATGAGAACCATGTTTTTTGTGGA
ACCAATCTCTCATGTTATGTTATCTATTACAACATGTTGCTATAGCAGCAAAAATAATATCGGAACAAACA
ATGTTGTTATAGAGAGGTAGCAAGGGATATGTTTATTTGAAAATGAGGTTCTGTATCACTTTTGCTTTGT
CTATTTTCCCTGTAGTTCTTGAATTGAATGTCAAATTTGTTAAATGCAGGAGTTTGTCACATAATAATTTA
AGTGGCTCAATACCAAGGTCCCTTTCAATGTTGAAGAAGCTCAAGATTTTGAAGCTAGAGTATAACCAAT
TGAGTGGTGAAATACCACAAGAACTTGGCAAGTTAGAAAATCTTCTGGCTGTTAATATATCCTACAACAG
GCTCGTTGGACGCCTTCCAATGAGTAGTATATTCCAGAATTTAGACCAGAGTTCATTGGAAGGGAATTTG
GGAATTTGTTCACCCTTGTTGAAAGGTCCCTGTAAGATGAATGTGCCAAAGCCTTTGGTTCTTGATCCTTA
TGCATATGGAAACCAGAGGGGAGGTCAAAACCGGGACGATGGAACTTCAAGAAGCAATAACAAAAGTT
TCAAGAACCATAGATTCCTCAGTGTTTCATCCATTGTTGCAATCTCTGCTGCAGCTGTGATCGCGGTTGG
AGTCATGATAATAACCTTACTCAATGCTTCTGTTCGAAGGAGGATCACATTCGTTGACAATGCCTTGGAA
AGTATGTGCTCAAGTTCTTCTAAGTCAGGGAGCCTTGCCACTGGAAAGCTTGTGTTATTGGACACCAAAT
TGTCCCCGGATTGGACTAATAGCAGTCTTGAATCAATCCTAAACAAGGCATCTCAAATCGGTGAAGGTG
TCTTCGGGACTGTTTACAAGGCTCCATTGGGAGGGGAAGGAAGAATAGTAGCTATCAAGAAGCTTGTA
ACATCAAAAATCCTCCAATATCCTGAGGACTTTGATAGAGAAGTTAGAGCTTTAGCAAAAGCAAGGCAT
CCAAATCTAATATCCTTAAAAGGGTATTACTGGACTCCTCAACTTCAGCTTCTAGTATCAGATTATGCACC
AGAAGGAAGTTTACAAACCATACTGCACGAAAGGCCTTCGTCTTCAACTTCCCTTCCCCTGTCTTGGTCCA
CTCGATTCAACATTGTGCTCGGAACAGCCAAGGGACTAGCACATTTGCACCATGCGTTTAGGCCAGCAA
TCATACACTACAACATAAAGCCTAGCAACATCCTCCTTGATGAAAACTTCAACCCTAGAATATCAGATTTC
GGGCTGGCAAGGCTAGTGACAAAACTCGATAAGCACATGATAAGCAACAGGTTCCAGAGTGCACTAGG
CTATGTAGCACCTGAATTGGCATGCCAGAGCTTAAGGGTGAACGAAAAGTGCGACGTGTATGGTTTTGG
AATGTTGATTCTTGAAATTGTCACAGGTAGAAGGCCAGTTGAGTATGGAGAGGACAATGTGTTGATACT
CAATGATCATGTGAGAGTTTTGCTTGAGCAAGGCAATGTGTTGGAATGTGTTGATCCAACTATGAAAAC
TTATCCTGAAGAGGAAGTTTTGCCTGTTTTGAAGTTGGCATTGGTGTGCACTTCTCAAATACCATCAAGT
AGGCCTTCAATGGCTGAAGTGGTTCAAATCTTGCAGGTCATCAAAACACCTGTTCCTCAAAGAATGTCAG
CATACTAAACATGTCACAAACTTGATGAATTTCCCTTTCTCTTTGGCTTTCTATCATCCTATTGTTGTTATG
TTAACCTCCTAATTTGATGTTTTTGGACTAGATAGTGATAGAGAGAGTTTTGTACTTCTCTGTCATTTGAT

GAAATATATGAGAGGTTGGTTCAATGACTTCAATCATGTTTGTTG

>PGSC0003DMG400021789 LRR-RLKs

CTTTAATGTTTTGTAGACATATTTTCATGGAAGATGGTGTCTTATGTCACAGTGATAAATTGCAACAAGG

ATACTTGACATAAAGTGCTATATCCGAAAGATGGTACTGTTCTTTGCAATTGTGATATTGGCATGTCTCC

GCTCATTTGTCTTACCTGATGGGCAAGGTACCTGGTTCTTCCAGTTTTACATCATAAAAACCAACTTTGTT

TTTATTTATCTACTCAAATGGATAAAGAATAATTTAGATGTGACCACTATATCTTGTTTACATTTTGCTTCT

GCCGGTGAAATGTTGTCTTTGTTCTTCTAATAAAGTAGGGTGACATTTGGTTTTTTATACTCTGGCCCATT

TTAGGAGATGCACTGTACACATTGAAGAACTCACTAAATGCTTCAAATGGTCAACTTGCTGATTGGAACC

AGAATCAAGTTAGTCCTTGTACTTGGTCCAAGATCACTTGTGATGATAAAGGCAATGTCATTATGGTGTA

AGTACTATTGTTAGAGCCATTAGAATCACTGTGTGCATATACTATGATGTTACTGTTTTTATGCACTAGAA

AGGTTAATCTTTGGTTGGTCAACTTCCTTTACTGGATGTAATTTTGATAATGTCTGAATCTGTATTTGCAG

GTCATTATCAAGTTTGGGATTCTCGGGCACTTTAACTCCCAGAATAGGTGTTCTAAAGAATGTGAATACA

CTGTACGTTTAAAGTACTCGGGCTGAGTTATTATTGATAGAATGATGTCTTGAATGCTGCCCACATATAA

TTTTCTAGATGCTATTTATTGCTATTTTAAAATGTTTATTTAAATTTTGTTTCTGAACATACAAGTTTGATGT

CATGGCATTGCACTAGAACAAAAATCCCTTCAAATCATCAGATGTTAACTAAAAGTTGAGAACACGATGA

TTAATATTTTCAAGGGAGCTTCACTTAGCGGAAGGGGGAAAAAGAAAAAGGAAAGTACCTACATATTGT

GGTGTTATAAATGAACAAGGAGAAATATTTGGCCATTTCACAAAGGCAAACAGCTTTAAGAGAGATGGA

AGCTGTATGAACTGATCTAGCAAATGGGAGTCTCTGTTCGGATGGCTGTCTTTTTTCTTGTGGTAACTGT

GTGGTGTCTCTACACTTGTAACTGCTGGTTCCGTTGGACTAAGAGCAACCTAAGCCATTTCACGAAGACT

AGAAATGTGAACTATACAAGATTAATCCTCAGTATCAGTGACTTTATCTTTTTTCCTGAGTGGTCATTCCG

TCACATTGGTATTTGCACTTTGTCTACTTCTTAGGGTCCTATTTTAGTATTTGACTTATATATATTTCAAATC

AATAATTTGGATGCATAACCATACGTAAGGGTACATAGTTCTTTGATGTAGCTCTTGATGAACTTTGATTT

GTTAGTATGTTGTTGAGCTCAAAATTGTAAGGTCTATGACTAAAATTTTTGGGATGATTTCTTGTCTTCTT

AGATCCATGACTAAAATTTTTGGGATGATTTCTTGTTTTCTGCTTCTAACCTAGCTGACTATGTCGCATAC

ACTCCCTGCAGGTCCTTGCAAGGAAATCGTATAACTGGTGAAATTCCCAAAGAGATTGGAAATTTAACA

AGCTTGACAATGTTGGATTTAGAAAATAACCGTCTTTCCGGAGAAATACCAGCTTCGTTGGGCAATCTTA

AAAAGCTTCAGTCTCTGTAAGTCATATTATTTCATTTAATCATTTGAGATATAACTTAGTTTACTTGGTGAC

TGGAATAGGTGTGAACTCTATTATTCCATTGAGCAGGTTTTTGAGTCGAAACAATATCACGGGGACTATC

CCTCAGTCACTTTTGGGTCTTCCAAACTTGATGAACCTGTAAGTTATTTATCTTGCCTTTTCCTTATTCCAA

GTATAAATTACTTTTCTGAATAGGAGAATAATACATTTTCTTTTGGGATGTCAACTTCAGACTTCAGTGAT
CCTTAACCTGTTACCTGTATCATTCGATATTTGACTTGATTCTCTGATTCCGCTTTGCAGTCAGCTTGGTTC
CAACGGTCTTACTGGCCAAATTCCTGAACAGTTGTTCCAAGTTCCAAAATACAAGTACGATTACAGTTCTT
ACTTTAGACAGTAGCTCAAGGCTTCTTAGTTTTTGCCTTCTCTACTAATGCATAATCTTTAATGTTAGTATA
ACTTCATTTTATTCTAGTTTTACAGGGAATCATTTGAATTGTGGCTTAAATCTCACTCATCATTGTGAATCT
GCTAATGGAGGTAAGTTCTACCAGCAAATATGTGAATTTTTGAACTGTATATGCTTCATGCTTCTCTGTAT
TCTGGAAGAATGAAAGTAATGTAGCTAAATTTTTTGAATTTTACTGCCATTATCTTGATTTTATTTGTTATT
ATCTTTTAGCTAGAAAGGAGGCTTTATCTTTTAGTTTACTTTATTAGAGAAAAAACTTTAGTATTTTCAAG
ATGTCTAGCTTGTTAGGGGTTTCATATTCTATATCTAGTATAAGTGTGTACTTTGTTTGGAACGACACTTG
ATGAATAGACTCTATTAATTTCCTTAATACAACAACAACAACATACCCAGTGAAATCCCACTAGGTGGGG
TATTAATTTCCTTAATATGTCATCTATTTTTTCTTGTTTCTTTCCGTTCCCATTCTTACTTAGTCCCTTCTCCC
TTTTCTTGGTGCTATTTCCTCCTTGTACTCAAGACATGGAACAAGATTAAAGTTCTTAGTGTTATTTAGAG
TTATACTTGCCATTGGCTGTGGGATATGTTTGTTGTGTGGTATACTGGTATCTAATTTAATGGATGGAAG
AATCAAGCACCTCATGGTGCTGCCTGCACTAAAAAGTATGAGTTTCTTTCCATTAGTACATTTAATCTTGG
AGAGGTGTAACTTGTAACACTGACTTACTTGTAGGTGCCAAGCATTCTTAACGGAGGGATAATGATGTA
GCTGAAAAATATAGCATTATTTACTACCTTTACTGTTGATCTATTGTCGTCATCTTTTAGTTAAAGTTTGAG
GTTTGATATATTTTTTTTAGAAATACTATCATATTATAGTAGGAGAGAATTTTAATAATTTTAGGAGTTGT
AATGCTTAAAAAAAAGAATTTAGTAATTCTAAGAGTTGTAATGATTCTAAAAGAAAAATTATTTTAACTCT
AAGAGTTGTAATGATTAAAAAATAGAATTTTAACAATTCTAAGAGTTATAGTTATCTAGGTTCCCTAATCT
ATAATTAGAGTCCATAATTCCTTTGCTTGGAACAACATTTGATGAGTAAATATTGTTGATCTCTTATTATA
TCTCTCGTCCCCTTTTCTTCTCGTTCTCTCCCTTAATCATCATGCTCATTCTCTCTCCTTTTGGTGCTACATCT
CAGAAAGTTTCTGAAGTTGATTACCTATCCGATGTATTACTATTCGTTTAGCAAGGAAATCGTCATTTAAC
ATATGCACCTTGTTGAACTTTCTGGACAAACCAAATTCTAAAGTTGAATTTCTTGCAGGTTCTCCTAGTAA
ACCAAAGACTGGTCTCATAGTTGGTATTGCTGTTGGATTTCTTGGACTTCTCATTCTTGGAGGATCTGTGT
TATTGTTCTGTAGGAGACGGCACAAAGGCTATAGTCGTGAAGTCTTTGTTGACGTTGTAGGTTTGTGTCA
CATATTTTTGACAATAATTTCATCAGTTTAATGTACTTGTTATTCTTCGTAAGGGAAGAAGATATTCTGGC
CATGCATTGTTGTATCTTGTAATTCCATCTTAAGTTAGTGACCTCATTCTTTATACACCAGTACTTAGTTTT
CATTTACCTTCAAGATGGTATGATTCCTTTGAGTATATTATGATATTATATTTTATGTTTTATTTGGCGCAC
TCAGGTGAGGTTGATCGACGAATTCCATTTGGTCAATTGAGGAGATTTTCATGGAGGGAATTGCAGCTT

GCTACTGATAACTTCAGTGAAAAGAATGTTCTTGGACAGGGGGGTTTCGGCAAGGTCTATAAAGGAGT
GTTCAATGACGGCATTAAAGTTGCCGTGAAACGGCTAACTGATTATGAGAGTCCTGGGGGAGATGCTGC
ATTTCAGCGAGAAGTTGAGATGATAAGTGTGGCTGTTCATAGGAATCTCTTACGGCTTATAGGATTTTGC
ACCACACCGACTGAACGCCTGCTAGTATACCCATATATGCAGAATCTAAGTGTTGCTTATCGTCTACGAG
GTATTGTTTCTTCATAGCATTGCTTTTATTCATTGTAGTTTCTTTTACATCCTGAGTATGCTATTTTCCCTTA
CTGTGCCTGTTTCATCTAATTTATTTTATCCCAATCAATAGATCCTTCATTTCATTAGTCTTACATTTCCTGT
GGCGCATATTTTGATTTTGTCAGCTTGGTTGTAAACGATCACGAGTCATTTCCCCGTTCGATCTGCGAAG
AAAGCGTGCATTTGTCATTGTACGGTCTAAATTGACTATTAAAGTACTGTAGAACTGCTGATCCGGTAAA
AGAACTTGTACATGGCATCAAGCTCGAGGGGCATGTTTATAGAGCTAATGTGTGTAGCTTAATTGGCAG
AAGCATAAACCCTTTAATTTGTGTTTTCATGCTTCAGAATCTTATTCTTTTTCCTTCTCAAAAAAGAATCTT
ATTCTTTCAACTTTGAATGAAGTGCTAGAAGTTTAATGCCTTAGGGTTGCATCACCATGAGTACAATTTGT
ATGCTTCCTTTTTTCTCCCAGAATATGGTAGGGAAAGACAAGTTCTGAGCAGATTTAAGGGTTCTGTAGA
AGTATTTTCTGATATTCATTGAAGGGGTTGAGTGTTCGGAGATTGCAATTTTCTTTGGTCAAATCAAGAG
CAATACTCAAATCATACCTCATTAATCATTAATAGATGTATTCAGTTTTTTTTTAAAATCATTAAAAGATGT
ATGTGCGTTAAGTTGGTATCACAGATCGTGATATGAAAGAGCTATGTGACTTCATAAATTGATTCTTGTG
ATCCTGGCTTGAACTAGAAGGTGTTACATGAAGTAGTTAGTGCAGCTTAAGATTTCAGCAGTTTTCTGCT
AGAGTTGATGTGTCTGTTATTAGTTTTCAGTGGTGTCTAATTAAATTTTTAATCACTTACAGAACTTAAAC
CTGGGGAGCCTGTTTTGAATTGGCCAACTAGGAAGCGTGTGGCAGTGGGTACTGCACGTGGACTTGAA
TACCTACATGAACACTGTAACCCAAAGATTATTCACCGTGATGTTAAAGCAGCTAATGTGTTACTAGATG
AAGATTTTGAAGCTGTAGTTGGTGATTTTGGCCTGGCAAAGCTAGTTGACGTTAAGAAAACCAATGTGA
CAACTCAAGTTCGTGGTACAATGGGCCATATAGCTCCTGAATACTTATCCACTGGCAAATCATCAGAAAA
AACTGATGTTTTTGGCTATGGAATCATGCTTTTGGAAATTGTAACCGGCCAACGTGCAATAGACTTCTCA
CGCCTAGAAGATGAAGATGATGTCTTGTTGCTTGACCATGTGAGTGTACTTAACTCAATGATCTTCATAT
ATTTTCAAATGGGCATCAAATTATTGCATCAAACTTTTCATATATCTTATCTCTACTGACCAATCTTTGTTT
CTCTCCATATTCTATTGGGACGACTCATTGCATGAGAGCCTCTTGGATGGATATACATTCGTTTATCTTTC
TCCTATTGGTGAAACTGCTGCAATTTTTCTATGATAATTGAAATGGTGTAAACAATTATTATTTTTCGCGT
GGCACCAGGGTACAAATGCTTTGAATATGAGCTAGGTCTTAATATTGAGCCTTTTAGGCACAAGAAATA
TGATGACTATATATATATTTGATGCTAATTTTTTCTCTCTTTCAAAAGTGTGGATATGTGGACCCTGAGCCTC
TCTTATAACTTTACATGATTATTCTTTAAGCTTCATAGGACCTTTATGTAGTAATAGTTGCTAAAAAGAGA

TCGTCTTGAAGGTCAGAAAACTGCAAAGGGATAAAAGACTGGATGCAATTGTAGACCGTAACCTGAATA
AGAACTATGAGCTGGATGAAGTGGAGATGATGATTCAGGTGGCATTGCTGTGCACTCAGGCATCACCA
GAGGACCGTCCAGCAATGTCGGACGTGGTTAGGATGCTGGAAGGAGAAGGGCTTGCTGAGAGATGGG
AAGAGTGGCAACACGTTGAAGTCAACCGTAGACAAGAATACGAGAGACTCCAGAGACGATTTGATTGG
GGAGAAGATACAATGTTTAATCATGATGCTGTTGAGTTGTCTGGTGGAAGATGACACATGAGTAATAAC
TTTGAACCACAAAAATGTTTCTTGATATATAGATGCCTGGTTGCTAAATGTTTTTTTAAAATTATTTATGG
AAAACCCCTTGTTCTAACTAGGCAGATCAACAGTGGACCTGTATTTATGTAAGTTTTAGTCATTATTTTCA
TGTTCCATTAACTACTCCTGGGAGTGGGAATTTGATTCCTCAAATTGTTACATATTTTTCCTCACTAAACTA
TTTTTCATTTCATA
>PGSC0003DMG400022000 LRR-RLKs
ATGGTTTCCAGGACTATAATTATAGCATTCCTAGTTTTGTTAATATACCTCTCAGTTACAAATGCATCGAA
CATTTCAACTGATGAGGCTTCTCTTTTAGCTTTCAAAGCACAGATAACTTCAGACCCCAATGGAATATTGT
CAAAAAACTGGACAAAAGGAACTCACATTTGCAATTGGATAGGCATATCTTGCAGTAAAAAGCATCAAA
GAGTGACATCATTAGTCCTCACAAGCTTTGGATTTAGAGGTTCAATAGCAATAGAAATTGGGAACCTCTC
CTTCCTTAACAAGTTGGATGTTGGAAACAACAGTTTCCACGGCCAAATCCCCGATGAAATCGGGAGTCT
GAGGCGTTTAAACTTCTTGTCTTTGCAAATGAATAATCTCACTGGTCAAATCCCAGAAAGCCTTGGATTTC
TCACAAGGCTTCAACTTCTTGATCTTTCTGAAAATGATCTATTTGGAAATGTTCCATTTTCCATTTCCAATG
TGTCTTCCTTAAAGATCATTGATTTGGGATTTAATCGGATTAGTGGGAATCTCCCAAGGGGATTTTGTAC
TAGGCTTCCAAATCTGCAAGGTTTATTTCTCTCAAAAAATCAATTAGCAGGCCAAATCCCAAGTGGGCTG
AATCAATGCACACAACTTATTTACCTTTCCTTGTCATATAATCAGCTCACAGGTAATTAGTAATGTTAATTC
AATTCTTGGACTATATATACTTGATTCAGAATCATTTAATCCTTTTACACTGTTATGAGGCTATCTGATTGT
GTTCATATATGTATTAACAGGAAGTTTGCCAAGAGACATGTGGAATTTGACAAAGCTTCGAGAATTATAT
CTTGGATGGAATAACATAACAGGTATCTTGGTTCTAAGTCTAATAATTTCTAGTTAATTAGATATTGTAGT
AGTAGCAAAAAATGTTCATATAGCTCCTTGTTATTCATTTTCAAACAACAATGACTACTACTACATCGGCT
CAATGCTAGTTTGTGTCAACTTTATGAATCCTCATTATCCGCGTTAATCCATTTAGTCCCCATCTCAGTTCA
AGGTTCAATAATTTAAGTTTTCTAACATTAAAATTTCTCTATATTTTCTGTTAACATACAAATATCTAACAA
TATTAAAAGCATCCTAGCGAACATTGAGTAAGTTGGAGGTACCAACATGATTAAGCTTTAATATCAACTA
GTTGGTATCACCTTTATGGATCTTACCATTCCATTTTGCTCTACTTTTCACTGAGTCAAAATGGATTCTAAC
TTATAGTTGGTCTTTCTTTCAACTTCCTCCCATGTGATTTTAGATCTACCTTGTCCTTTTTAACAAATCCAAT

CACCATATATAGTTTCATACCTGCAGACCAGTGCATCTAGAGATCCATGTAAGACTGAACTAACCATGGT
AGTTTCTAATGTCTCATCTCAAGTGACTCACATATCCATCTTAATATAGATAATTGACTGACGATCAAATA
GTTTAAGCTTCCTTTCTTCAGTTACCATATAAGAAGAGAGGAAAAGAGAATAGCAGAAGTAAATGAATA
GTTCTCATGTTGCCAATATTTAAAATTTAAATGCCCTAATTAACAGAAAACCTTTCCTAGGCTTGTTATTGT
TCGTCTTCAAGAGGACGTTGAAACAGAATACAAGGATTGCCGAACTATCCGTGATTCTAATTCTCTTACA
CAATATTGTACGTTTTAGGGTTGTAACCACACAATTTAATATATATTTTACAGGACATATCCCAAGTGAAA
TTGATAACCTATCAGCAATTCGAAGATTAAGTCTACCACGGAACAATTTGGTGGGAATTCTTCCACCATG
TATGGGAAATTTGTCAAATGTAGAGGTGATAGACCTAGGTCAGAACAGTCTACATGGTGGCATTCCCCT
AGAATTTAAACATCTTGTAAATTTGAAAGGATTGTTTCTTGGTCAAAACAGATTATCGGGTGAAGTTCCA
GGGCCTATGTACAACATTTCTGGATTAGCAATGATTTCATTTGTAGGAAATGGGCTCTCAGGAACACTTC
CCTCAAACATAGGCCATACCCTTCCAAATCTTGTCGGCCTCTATCTTGGGAACAACCAGTTTACAGGGCT
GATTCCTACCTCCATTGTGAATTCCACCAAGCTTATCCAGCTTGATTTTGGTCGCAATCTGTTTAGTGGAC
CTGTACCTATGAATCTTGAAAAGCTACAACAGCTTCAATTCATCAGCTTGCAATTTAATCAATTGACGAAT
GATCCATCTACGGACAAGTTAAGTTTTCTTACATCATTATCTAATTGCAAGTATTTGAAGACAGTTCAAAT
AGGAGGTAACCAATTTAATGGAAGTCTTCCAAAGTCCTTGGGATCAGGCAACTGGTCCTTTTCCCTTGAA
TATTTCATTGCCACCAATAGTGGTATCACAGGAAAAATACCAACTAACATCAGTAACTTTAGGAACCTGG
AGTGGTTAAGCTTGGGAGAGAATAAGCTTATAGGTTCTATTCCTCCAGATTTGGGGAACTTGAAGAATT
TGCAAAAGTTTTACCTGCAGATAAACAATTTAGATGGAATTATACCAACTAGCTTGTGTAACATGGAAAG
CTTGTACCAAGTTATCTTGGGCAAAAATCAACTTTCTGGGGAATTACCAAGTTGCTTTGGAAATATTTCTT
CTTTAAGAGAACTCTACTTAGATTCCAATGCACTAGTTTCCCATATTCCATCAACATTTTGGAGAAACAAA
GATCTTTCAGTTCTCGATTTGTCCTCCAATTTGCTGAATGGATCTCTTGCAGTAGAAATGGGAAACACAA
GGAGTTTGCGGATGTTAGATTTATCTGGCAATCAGTTCTCAGGTCAGATTCCTAGCACGATTGGCCAACT
ACAAAGCTTGGTTAGCCTTTCATTGTCAAAGAATATGTTAGATGGTCCTATACCTGAACTATTTGAAGATT
TGGTTTCATTGGAATATTTGGATCTATCTAGCAACAATCTAACAGGCATGATCCCCAAGTCCTTGAGGAA
TCTTGAACATCTCATGTATTTCAATGTCTCGTTCAATGGGCTCATGGGTGAAATTCCAGATGGAGGGCCA
TTCATAAATTTTACAGCTGAATCATTCATGGGTAACCCTGCATTATGTGGATCATCACGCTTCCATGTGAT
GCAATGCAGAGTCACAAGTCTTGAAAGCAAAAGAAAGAGTAGAGTCTTAACTTCTGTCCTTGCATCAGT
TTCCTCAGGAGTTGTAGTCACAACCATTTTCATCATTTGGTTTCTGAAATGCCGAAAAAGGAGTACGGAA
CTTCCTCTAGTTGATACATTTGGTCAGGTACATAAGAGGATTTCGTACTATGATATTTCTCAAGGGACAA

ACAACTTTGATGAAGCAAACTTGATTGGAAGGGGGAGCCTTGGTTTGGTGTACAAAGGGACACTAGCA
GATGGAATGGTTGTCGCAATAAAGGTATTCAATACAGAACTGCAAAATGCATTTAGGAGTTTTGAAGTG
GAATGTCAAGTTTTACGTAGCATTCGACACAGAAACCTTGTTAAGGTGATAAGTAGTTGTGCCAATTTTG
ATTATAAAGTGTTGGTGCTAGAGTACATGCCCAATGAAAATCTCGAATGTTGGCTTCACTCTACTGACAA
ATTTTTGGATATAACTCAAAGACTAAAGGTGATGATTGATGTGGCTTCTGCTGTGGAGTATTTACATGGA
GGTCATTTTTTTGTAGTCATCCATTGTGATTTGAAACCAAGTAACATACTTTTGGATGGAGATATGGTGG
CAAAAGTGAGTGACTTTGGGATATCCAAACTTCTAACAGCTGAGACGCTAATAGCACATACCAAGACTTT
GGGTACTATTGGCTACATGGCACCAGGTAAATGTTATTATCAAGTTCATTTGAAGATTTTAAATGACGTT
AATTATTTTCACTAATGATTGCGTGATTTTTTTTGCAGAGTATGGATCAGAAGGCAAAGTATCAACGAAG
GGAGATGTTTATAGCTTTGGTATTTTGTTAATGGAGACTTTTACAAGAAAGAGCCCCGTGGATGATCTAT
TCGTTGGAGACTTCACCTTGAAAAGATGGATATGCCAATCATTACCAAACCGATTGGTGGATGTAGTGG
ACATTAATCTATTTTCACTGGACGAAGAAAATTTTACTTCTAAAGAGAGATGCTTCAAATCAATCATGGA
ATTAGCTCTTGAATGCACAAATGATCTGCCGGAAGAGAGAATCTGCATGGAAGATATTACTCTGCGACT
CAAGAAGATCCTAACTCAATTTCTGCAGAATGCTGTGACAAACTAA
>PGSC0003DMG400022139 LRR-RLKs
GTTTTCTTGAAGATTTGGAGATTGTAGGATGGAGGAGTGTTCTTAAATAGTTGAAAGAATTGATTTTTAT
GCAGTTGGTTGGTGGTGGACTGGTGGTGTTTGTTTTCAGCAAGATGAGCTATTAAAGATTCTAAAGAAT
TGATTTTTATGCAGTTAGTGGTGTTTTTTACAGCAATATGAGTCATAAAAGATGCCAAGAATATTTTTCAC
TAAATGGGGTGTTGAAAAATGTTGTCTTTATGATAATACTTCTGAGTTGTAGTTTTTTTGTGGTGGTGTCA
AATGCAAGAAAACTAGCTGAAAATGATCAAGTAGGGAGTTTATTAGCCTTCAAGAAGTCCTCTGTTGAA
TCTGATCCAAATGGATTCTTGAATGAATGGACTTCATCTTCTTCAAGTCCTTGCACTTGGAATGGGATTTC
ATGTTCAAATGGTCAAGTTGTTGAGCTTAATCTGTCTAGTGTTGGGCTCAGTGGTCTACTTCACCTCACTG
ATCTCATGGCCTTGCCTTCTTTGCTCAGAGTTAATTTCAATGGCAACCATTTCTATGGTAATCTTTCCTCAA
TAGCTAGTTCCTGTAGCTTTGAGTTTCTTGATTTATCAGCTAATAATTTCTCTGAGGTTCTTGTTTTAGAGC
CTTTGTTGAAGTCTTGTGATAAAATTAAGTACCTAAATGGCTCTGGGAATTCAATTAAAGGGGTTGTTCT
AAAGTTTGGACCTTCACTATTGCAGCTTGACTTATCAAGTAACACCATTTCAGATTTTGGCATTTTAAGTT
ATGCACTGTCCAATTGCCAGAATTTGAATCTCCTTAATTTCTCCTCAAATAAGCTTGCTGGAAAGCTAAAA
AGCTCCATTTCTTCTTGCAAGAGTCTATCTGTTCTTGATCTTTCGCGTAATAACTTAACTGGAGAACTCAA
TGATCTTGATTTTGGGACTTGTCAAAATCTCACTGTCCTAAATTTGTCTTTCAACAACCTTACCTCAGTTGA

GTTTCCACCCTCTTTGGCAAATTGCCAGAGCCTCAATACACTGAACATTGCCCATAATTCGATCCGAATG
GAGATTCCTAGCGAGTTATTGGTGAAGTTGAAGAGCTTGAAGCGATTGGTGCTAGCCCACAATCAGTTT
TTTGATAAAATTCCATCAGAATTGGGGCAGAGTTGCAGCACACTTGAGGAGGTTGATCTTTCTGGAAAC
CGGCTTACTGGTGAACTTCCTTCAACTTTTAAATTGTGCTCCTCACTTTTCAGTCTCAATCTTGGTAACAAT
GAGCTATCGGGTGATTTCTTACATACTGTTATCAGTTCATTGACAAATCTTAGGTATCTTTACTTGCCATT
CAATAACATAACTGGTCATGTACCAAGGTCTTTGGTGAACTGTACAAAACTTCAGGTACTCGACCTTAGT
TCCAACGCGTTTATTGGGAATGTACCTTTTGAGTTGTGTTTAGCAGCCTCAGGCTTTCCACTGGAGATGA
TGTTGTTAGCCAGTAATTATCTTACTGGAACTGTGCCTAAACAGATTGGACATTGTAGGAACCTCAGGAA
GATTGATCTCAGCTTCAATTACCTGACTGGTTCGATCCCATTGGAGATTTGGACCCTTCCAAATCTTTCGG
AATTGGTAATGTGGGCTAATAATCTCACAGGTGAAATTCCTGAGGGCATTTGCATCAATGGAGGAAACC
TTCAAACACTCATTCTCAATAACAATTTCATCTCAGGGGCACTTCCACAGTCCATTTCCAATTGTACCAACT
TGGTTTGGGTTTCGTTGTCCAGCAATCGACTGTCTGGAGAGATGCCACAAGGAATTGGGAATCTTGCAA
ATCTTGCTATCCTTCAGTTAGGTAATAACTCACTTACTGGACCGATCCCTCGGGAATTAGGTTCGTGCAG
GAACTTGATATGGCTAGATTTGAATAGCAATGCTCTAACAGGTTCAATCCCTCTTGAGCTTGCTGATCAA
GCTGGCCATGTTAATCCGGGAATGGCTTCTGGAAAACAGTTTGCTTTTGTGAGAAATGAGGGTGGAACT
GAATGCAGGGGTGCTGGTGGACTGGTTGAGTTTGAGGGTATCCGTGAAGAGAGGCTTGCGATTTTACC
TATGGTTCACTTCTGTCCATCAACTAGGATCTACTCTGGCAGGACGATGTACACCTTCACGAGCAATGGA
AGCATGATTTACCTTGATCTTTCCTACAATTCTTTTTCAGGAACCATTCCTGATAATTTAGGTTCATTGAGC
TTCCTTCAAGTTCTGAATTTGGGACACAACAATTTTACCGGAACCATTCCATTCAACTTTGGAGGTCTAAA
GATAGTTGGAGTTCTTGATCTGTCACATAACAGCCTACAGGGATTCATTCCACCCTCGTTAGGAGGCCTT
TCATTTCTTAGTGATCTTGATGTTTCAAACAACAACTTATCGGGAACGATTCCTTCTGGTGGACAGCTGAC
TACATTTCCAGCTTCAAGATACGAAAACAATTCAGGCCTTTGTGGTGTCCCCCTTCCTCCTTGTGGCTCGG
GCAATGGACACCATTCATCGAGTATTTACCATCATGGGAATAAGAAGCCTACAACTATAGGAATGGTGG
TTGGTATTATGGTATCTTTTGTATGTATTATACTACTTGTTATTGCCCTTTATAAGATTAAAATGACTCAGA
ATGAGGAGGAGAAGAGAGATAAATACATTGATAGCCTTCCAACTTCTGGCAGTAGCAGCTGGAAACTAT
CAACCGTTCCTGAGCCTCTAAGCATTAATGTGGCAACATTTGAAAAACCATTGAGGAAGCTGACCTTTGG
TCATCTTATAGAAGCGACCAACGGGTTCAGCTCTGAAAGCATGATTGGCTCTGGAGGTTTTGGTGAAGT
CTACAAGGCTCAGCTGAGAGATGGCAGTACTGTTGCAATCAAGAAGCTCGTGCACGTAACAGGTCAAG
GAGACAGAGAATTCATGGCTGAAATGGAGACTATTGGAAAAATCAAACATAGAAACCTTGTCCCTTTGT

TGGGATATTGCAAGATTGGGGAGGAAAGGCTTCTCGTATACGAATACATGAAATGGGGAAGTCTTGAA
TCTGTTCTTCATGATGGGGGGAAAGCAGGGATGTTTCTTGATTGGCCAGCTAGAAAGAAAATCGTGATA
GGATCAGCAAGAGGATTAGCATTTCTTCACCACAGCTGCATGCCACACATAATTCATCGCGATATGAAGT
CCAGTAATGTACTTTTAGACGAAAACTTTGAGGCACGAGTTTCTGATTTTGGAATGGCAAGGCTGGTAA
ATGCTCTGGATACTCATTTAAGTGTGAGTACACTTGCTGGTACTCCAGGTTATGTTCCTCCAGAGTACTAC
CAAAGTTTCAGGTGCACCGCAAAAGGGGACGTGTACAGTTATGGTGTCATACTGTTGGAGCTTCTTTCA
GGGAAACGGCCAATAGATCCCCGTGTATTCGGTGATGATAACAATCTTGTTGGATGGGCAAAGCAACTT
CATAACGAGAAACGAAGTCATGAGATTCTTGATCCTGAGCTGATAACAAACCTATCTGGTGATGCTGAA
CTCTATCATTACTTGAAAGTTGCATTTGAATGCCTTGATGAGAAATCTTACAAAAGGCCAACAATGATAC
AAGTGATGACAAAGTTTAAAGAACTACAAACTGATTCAGAAAGCGATATTCTTGATGGCATTTCAGTGA
AAGGTTCTATACTCGAGGAATCGCAAGAAAGGGAACCGTAAATCAATGATATTTGATCTTTTTGTGGTTA
GAATTCTGTTCTTTTTTTCTCTTTTTTTGGATTGCCTCATGGATAGTAGTAAAAACTTTTATCTTTAATGGTT
CTTCACCATTGAGATTGATAAGAGTTTATACTTCAGATTTTGCCTACTGTGTAAATATTAGAACACGACGA
TGAATGGGAGATGTGAA
>PGSC0003DMG400022189 LRR-RLKs
ATGGACAATCTTCTCAAGTTCATTGTAAGTGTTTTACTTTCTCTTCTTTTTATCAGATTCTGTAATGGAGGC
GAGTTTATCGAATCCAAATCATTTATCAGTTTTCTTGTTGCTATTGATCCAAACAATGTACTAAACGTTGA
TCGAAATGCTTTGTTTTCTTACCCTTGTTACTACAAATTACATGGTGTTAAGTGTGATTTACGTTCCGAAG
AAATCATAGAAATAAGACTTGAGAACATGAATCTTAGTGGTGTAATTGATGCAGAGTCACTCTGTAAACT
CTCAAACTTGAGAGTTCTTAGCTTAGCTAGGAATAAGATTGAGGGAACTGTTCCTGAATCGATATCATCG
TGTAAAAGTTTAACTGTTCTTGATTTAAGCAGCAACTTTCTTAGTGGAAACTCATTAGTACTAATGCAGTC
ATTGACAAGGATCAAGAATCTTAAAAAACTTAACATTTCTAAGAACAATTTCACTTCCTCACAACGTATGA
TGAGTCTACACGCGTTGAGACGTGTAACTATTAAAGAATCTTCATCTTCAACTACAGTAAGTGACGATAA
GAAGACAAATAATCATCATGTGAGTCTGATGGTTTGGGTACTCATATCTCTTGGAACTGTGATTGTGTTA
GTAGTTTTAGTATTCTTGTATATGAGATGTGTTGGGACAGTTAAGGATAAGGAGGTTATAAAGGAAGTA
GTTGATTATCCGTCTCCTCGAAAAACTCCCTCAGATGAAGTTGTGGATGGAGTGTGTAACACTGAAGAG
AAAAGCTCAGAACTTTACTTCTTCATGGAAGATGTTGAGAAGTTTACGATGGACGATCTTCTTGAAGCAA
CTGCTAACTTAACAAAACAAGGCATTTGCAGCAGCCTTTACAAGGTTCATATCAGTAGAAGTGGTGTGTT
TGCTCTCAAGAGACTGAAGAAACTACAAGTGGGGTTCAAGGAGTTCTCGCGAACGATGAAAAAGATAG

GGAGCTTGAAGCATCAAAATGTACTTCCACTTGTTGCTTATTACTCTAGCAATGATGAAAAAATGCTCAT
CTACAAATATCAGAACAGTGGAAGTCTACTAACACTTTTCGAAAGTAAGTAGTCAAGAACTTCAAATTTC
TCATCTTGTAATTCAGAATTTTATATCATATTGTTGTTGTTTATTGTTGCAGATTATGCAGAGGGGAAATG
GAACTTCCCGTGGAACTTAAGGCTGTCGATTGCAGTTGGCATAGCAAGAGGTTTCGCGTACATATACAG
AAGTTCAAAGAAAGGCAATGTCATTCCTCATGGCAACATTAAGCCATCAAACATTCTATTAAGCGATAAC
GGGGAGCCATTGATAAGTGAGTATGGCTATTACAAATTCTTAGACCCTGACAAAAGTTGCCTCTATAAA
GACAATGGTTATACAGCCCCTGAGAAAACGTTGACAGAAGAAACTGATGTCTATAGCTTCGGGGTGATT
CTGCTGGAACTGCTAACAGGTAAAGTTGTGGAGAAGACAGGATTAGACCTTGTCAAATGGGTGAAATCT
ATAGTGAGGGAAGAATGGACTGGAGAAGTGTTTGATAGTGAAGTTGCCAACTATGAAATGTATGCTTTC
CCTCTACTAAACGTTGCACTCAAGTGTGTGGATCGTTTGCCTGATGAGCGACCTACTATGACAGAGGTCT
TGGAGATAATCGAAGAAATTGTGAATGATCAAGAAGACATTTCTCCTTCTTCTATGACTTCTTTTGAATCC
ACCCCTGGTTCAATGAAGCATGTGTATGCCCTTTAG
>PGSC0003DMG400022264 LRR-RLKs
GTTCAATAATAATAATAGAAAAATTCAAAGTGCTCTTGGATCCAGATCTTCTTCTGTTTTTCTTCTATAAAC
AAAACATGGGGTTCCCCCATTTACAATCCCAAAGTCCTCAACTTTCTCTCTTCCCTCTTAGCAGCAGCAAA
CAATGCCTGCTCCTTCAAGAAATCCCTTTCTTCTTCTTCTTTTACTTTTCCTCTCTTCTTGTTATTCACTTGAT
GTACAAACTCAAACACTTCTTGCATGGAAAAAAACATTAAACATAACTTCCAATGATGTTTTAACATCATG
GGATTCTTTAGATAAAAGTCCTTGTAATTGGTTTGGCATAAACTGCAATTCCAATGGTCATGTTGTTAGTA
TAAGTCTTAAATCAGTTGATTTACAAGGTCCATTGCCTTCAAATTTTCAGTCACTTAAATTCTTGAACACTC
TTGTTCTTTCATCTGTTAATCTTAGTGGTCCAATCCCAAAAGAGTTTGGAGATTATCTTGAACTTAAGTTC
ATTGATATTAGTGATAATTCAATCACTGGTGTCATTCCTCAAGAACTTTGTAAGCTTATAAAGTTACAAAC
TTTATCACTTAGTTCAAATTTTCTTGAAGGTGATATCCCTTCAGACATTGGGAACTTGTCTGATCTTAAGA
TGTTCTTGATTTATGACAATCAACTTAGTGGTGAAATTCCAAAGGGTATTGGAAAGTTGAAGAATCTTGA
AGAATTTAGAGCTGGAGGGAATCAAAATCTTAAAGGAGAACTCCCTTTTGAGATTGGAAACTGTTTGAA
CTTGGTTTTTTTAGGCCTTGCTGAAACAAGCATTTCAGGGAATTTGCCAGTGTCAATTGGAAACCTGAAA
AAGATTCAGACAATTGCAATTTATACAGCTCTTTTGTCTGGACCAATTCCAGAAGAGATTGGGAATTGTA
GTGAACTACAGAACTTGTATTTGTATCAGAATTCGATCTCTGGTTCGATTCCAAGGAGTATAGGGGAACT
CAGGAAGCTTCAAAGTTTGTTGCTTTGGCAGAATAGTATAGTTGGTGTGATTCCAAATGAGCTTGGGAA
TTGTAAGGCTATTACAGTTATTGACTTGTCTGAGAATCTCTTAACAGGTAGTATTCCTACAAGCTTTGGTG

AACTTTCAAGTCTTGAAGAGTTGCAGTTGAGTGTCAATAAGTTATCAGGTACAATACCTACTGAAATATC
AAATTGTACTAAATTGTCTCATTTGGAAGTTGACAATAATGGCATATCAGGTGAAATTCCTAATGAAATA
GGTAAGTTAAAGAGCTTGACTTTATTCTTTGCTTGGCAAAATAATTTAACAGGTGAAATTCCTGTTACATT
ATCTAACTGTGAGAATCTTCAGGCTATAGACCTTTCTTATAACAATCTTTTTGGTTCCATACCAAAAGAAA
TCTTTAGTTTGAAAAATCTAACAAAGTTGTTGCTTCTTTCCAATGATTTGTCTGGTTTTATACCAACTGATG
TTGGAAACTGCACAAATTTGTATAGACTTAGAGTGAATAGGAATAGGCTAGGGGGTACTGTTCCATCAG
AAATAGGAAAGTTGAAGAATTTGAATTTTCTTGACATGAGTGGCAATCACTTTATGGGAGAAATTCCTTC
AGCAATATCAGGATGTCAAAATCTTGAGTTTCTTGATCTCCATTCGAACGCGTTTACTGGTTCTTTACCAG
AGAAACTGCCAGGGAGTCTACAGTATGTGGATATTTCAGACAACAGGCTTTCGGGTTCGTTAAGTCCAA
GTGTTGGTTCTTTAACAGAATTAACAAAATTGAATCTTGGGAAGAATCAACTTTGTGGTAGAATTCCAGC
TGAGATAGTTTCTTGTAGTAAGCTTCAGCTGTTAGATCTCGGGTACAATGGCTTATCAGGCGATATACCT
AAAGAGTTGGGTCGAATACCATCCCTTGAAATCTCTCTTAACCTTAGTTGTAACCAGTTTACAGGAGTGA
TTCCAAGTGAGTTTTCAGGTCTTAGTAAATTAGGAAACCTTGATCTTTCCCACAACAAACTCACCGGGAA
TTTGGACGTTCTCACGAACCTTCAAAACCTTGTTTCACTCAATATATCATTCAATGACTTCTATGGAAAAC
TTCCCAATGCTCCATTCTTTCATAATCTTCCTTTGAGTGATCTTACTGGAAACCAAGCTCTATACATTTCTG
GAGGGGACGTGATCCAAACGGGGCCTGCTGGACATGCTAAAACCACCATGAAGCTTGCTATGTCAATCC
TAGTCAGTATAAGTGCTGTGCTGGTGCTGCTAGCCATCTACACATTGATCAGGATGCAAATGGCGGCAA
AGTATGGACCAGAAGTTGATACTTGGGAAATGACTCTTTACCAGAAGTTGGACTTTTCAATAGATGACAT
TGTTCATAATCTAACATCAGCTAATGTCATTGGCACTGGGAGCTCTGGGGTTGTCTACAGGATAATGACC
GAAAATGGGGTGACTTTAGCAGTCAAGAAAATGTGGTCATCAGAGGGATCAGGAGCATTTGGTTCAGA
AATTCAAACACTTGGTTCAATCCGGCACAAGAACATAGTACGCCTTCTCGGCTGGGCTTCAAACCAGAAC
ATGAAACTTCTGTTCTATGACTACCTTCCCAATGGGAGTTTGAGCTCATTGCTCCATGGTGTTGGCAAAG
GAGCAGCAGAATGGGAGAACAGATTTGATGTGGTCCTTGGAGTAGCTCATGCACTTGCATACTTGCATC
ATGACTGTGTACCCCCTATAATGCATGGAGATGTCAAAGCAATGAATGTATTGTTAGGCTCTAGAATGG
AGCCTTACCTGGCCGATTTCGGGTTGGCAAGGATTGTCAACACTGATGTTGATGCAGACTTGTTGAAGG
AGAGCCAAAGGCCTCATCTTGCTGGTTCCTATGGATATATGGCTCCAGGTAATTATTTTTACTGCATTTTT
ACTCATTATCTTAGATTGATGACGGAGAACGTAAAACTAGTCAATTTATGATGAATCCTCACAATACAAA
AGATATTGTTGGTGTTGTACTCATTAGTCCTTATATCAGCTTGATTAGTTGCATCAACTTTTCTTGAATTGA
CAAAGACTCTTGCTTATTATGTGTCCACAGAACATGCTTCGATGCAACGGATCACAGAAAAGAGTGATG

TATACAGCTTTGGTGTGGTCCTCTTGGAGGTACTAACAGGAAGGCATCCACTGGACCCAACGTTGCCCG
GGGGTGCACACTTAGTACAATGGATACGCGATCACTTACAAAGCAAGCGCGATCCAAATGACATTCTTG
ATCCAAAGCTTAGAGGCAGAGCTGACCCTGAGATGCATGAAATGCTGCAAACTTTAGCTGTTTCATTCCT
GTGTGTGAGCACGAAAGCTGATGATCGTCCGATGATGAGGGATGTTGTAGCCATGCTCAAGGAAATTC
GGAATGTTGATCCTGTTGTGTCCGAATCCGACTTGTTGAAGAAAAAAGCAAGTGTAACACCTCTTCCAAA
ATCACCTGGCACCAAGAATGTGGATTCTCAAATGTCTTGTAGCTGCTCTTTTGTATTTTCTGATAACTCTA
TCTCCAAATAAGTCATCAACACAGTGAACAAATTTGTGAATATAGCATGTTTTATTAGCAGTATGAGTTA
GAGCAGATTATAGATTTTAAAAGGAATATCATGTATCCAATTAATTTAGCATAAGCATTATAGAGTGAAA
>PGSC0003DMG400022425 LRR-RLKs
CTTTTGGTTGTTGGAGATTTGTCTGTTGTTTATGTTAAAGTGAAGATCTTAGTGTGTTTTGCAGAGTGTTT
TCTTTTTGCTGGAACTGTGAAGCAAAGATTTGAAGGAAAAAGGACAGAGGAAGAGGAAGCAGGAAATT
TGTGTTCTTGGAGTGTCCCTTGGTGATGATTGGTCACAAGATGTGTGGACTATATATGAAGATTTTGTTT
GTATTTGCAGTTTTTGCTGTTCAAAATTGCCAGGGGTTTACTGATCCTCGTGATGGTATTAATAAATCTCT
CTGTGGTTTGAAATTTGTGTATTATACAGTATAGAATGTCTCAATAGTTTGAATTTGTTATCTTTTCTATTA
GCTTTATTGAAATTACAGTTTGATGTTTTGTGTAAAAGAAAGGTGAAGTTTATGACTTTGTGTTCTTTTCT
TGCTCTCCAGTATTTGCCATAAATAGTTTATATACTAGTCTGGGCTACCCCTTGCTTCCTGGATGGGTGCC
ATATGGATTAGACCCTTGTGGTGATCTATGGCATGGTGTACTCTGTGTCAATTCCAATGTAACTGGAATG
TAAGATTTCCTTCTTTTTTTTTTTGCTTGTGTTTCTCACATGGTTTGTATACTTTATAATAGATTTCTTTTCTT
TTTTTGGCAGAGTACTAAATGATTCAAATTTGGGAGGTGAATTAAGTGAGGGCATAGGAAGCTTTGCTT
CAATCATACAAATGTTGGTACCATCTTTATCTTCATCATTGCATTGATTTAACTTATGTTGCTGAAATTTGC
CTTGTCATTTGGTTCTGTGCACTTTGTCCTTCCCTGCATCATTTGGCACCACTATTACATTGTTTAATTCTAT
GGCTTTAGTTAACTGCTACGTATTAAATGTCTACGCTTTGTGTTATGATCAAACCAGAGATCTCAGCAAC
AACCATATTGGAGGCAGTATACCATCCAATTTGCCTGTCACCCTCAAAACCTTGTAAGTTAATAATCTTTG
TCCTTACTGAGCTTCTTCACCACTTTATAAGTTATTTGCTCCACATACATCCACATTTTTAATCCTATTATGG
TATTTGTGTATATACAGGTCTCTCTCTGGTAACCAGCTTACTGGGAGCATTCCAGATAACATATCCTCGTT
AGGGCAATTAACAGACTTGTGAGTTATATTTGACTTATCTTGATATACTTTTTCTATGACCATGCTTTTGTT
GCCATTACATTTAATTCCTTCCTTGTAGGTCGTTGAACAACAATCATCTCAATGGAGTCATTCCAGATGCC
TTTCTGCAACTTAAGACTTTGATTAACATGTACTCATACTTCTTTTAGCTTTTTACCTTTTCGCAGTGATGA
AACTACTTTTCTTGGTGTTGTTTGTATTTATGGCTTCATGCTCTTATTTTGCAGGGACTTATCTGGGAACA

GTTTAAGTGGTCAGCTGCCTCCTTCAGTGGGGACTTTGTCATCACTTAGTACCTTGTATGTATTTCTCCTT
GTTGGTATGATAATTAGGGAAATATTAGGAAATGAAACTACTTTTCTGGCTATGAGGAATGAGTGCTTTC
CTCTCTCTAGACACTGAATAGAAGTTCAAATAAGTCCTTAATTCCTTCAGAAATTTAACATAAGTTCTGCT
CATACATAATTTAAACTGATAAATTTTGTAAAGATAAATATAGTTTTTGACATTTCATTGCATATGTAACT
ATTGTATGGTTGTGCTGTATGTTGGTTTGTCAAGATGTGTTTTCTGTGAACAAGCTAGAGGAATGATATT
TGAGGATAATACTTCACAAAGTTGATTAATTTTGTCTAGAATCTCAATTTGCAACCATTCAACTTAATATA
CTTGGATGGTAAAAGGATGTTAACATAATTTCTCAACTGAACTTGTGAATATACTTGTATTTATGGTGAA
TTGTGATACTCTAGTTTTGATTTTTGAATATATACTTTTGGCTCTATGCAGACACTTACAGAACAATCAGC
TTTCTGGGATCCTTGACGTTTTGCAAGATCTTGCTCTAACGGATTTGTAATCTCCTGGACTAATAAATTTT
CCATCATTATAGATAATGTCATCAACTATGTCATTTTCTGCTCAATGTGTGCTTAAATTTGGTCACTTTCTT
GTTCTGCAGAAACATAGAGAATAATTTGTTCTCTGGGCCTATACCACCAAAATTGTTGAGCATCCCGACC
TTCAGGTAAAAACCTAGGAATTTTTTTGCCCTCTTTAGTTTGAGTTTTCTTTTAAGCTGATGAAATCCATTT
TATTCTATCTTCAACTTCTGTAATATGAATTGGAGTATCTTATTTCTCTTTGTTTTAATTTGGCTTCATCTCT
CTGTGTACTTCCATCAATTTAAAATTGGATAAGCATTGGTCACTGTGGTAATGGGACAATGTGACTTAAA
AAATTTAAAACAAAAGGGAGTTGTCTCTTGGGCTAGAGATTGCACTATTTAGATCTAATTTAATTGCCAA
TTGTGCTTGCAATGAATATTTATGGGAAGCCAGTATAACTGTTGTTTCTAATAAACTTGACACCATTCCAT
GAGCTGACCATGAAAATGTTGGTTTTACAAGTTTTAGTTTCTATAACTTTGTCATGCATCGATCTGATAAA
TAAATCAGTACACTGGCTGATAAATGGGTAAAGTAGCAAACTTGAAGATATGGCATGTAATGATATTAC
TAAATAGTATGGTCTTTTTGATGAAGACTGCATAGTACACCTTATGAACCAATTCTTTACTCCATCAATCA
ACCATCCTCAATCCCAAATTAATTGTGGATGGCTATATGAATCCTCTTTATCCGTTCTACCTTATGCATCAT
CCTACATGTCACTCTTAACTTTATCTAAAATTCTATTTTGTGCAAGATTGATAATAGATATTTAGTTGGTGT
TTGTTGTGAAAGCTCTTTTGGAGTTCTTTTAAAGACCTTATTAAAACCATGTTTGCTTTAGTTCTTATAAGA
CATTTTTCATTTCATCAATGAAGAAGGGGGTTTGAGGGATTTTCATTAAAAAACAATTCTTCTTCCACTTA
TAAAAGTGCTCACAAATCTTCAAAAGATCAACTGTGTAAACACATTTGTTTTGCACATCTACAAAAATATT
TCTCCAGAACCATTTGATTGGTTAAAATGAAATTACAATGGATAACCAATCAACATGATGCATTTTTGTG
ATAGACTAGCCTTAGTCATGGCCTCAATTGTTTAGTTGATACTCTTTGGGGGAAGCGAACTATGGTAGTC
TTTTCTTCTTCATGAAAGGTTCATTTGTATACTTATAAATTTTGTTGCCTTACATGGTGAAATCTATGTTTTT
TATCAGACGTGCAGGGAATCCTTTTAATACTACTATCATCCCTTCACCACCCATGATATCGCCTTCTCCAG
CACCATTTGCGCTTTCTCCAGATTTAGCCCCTTCACTGCCATATATTGTACCGCCAGGACAGGAATTACAG

CCCTCTGGAAGGCAAAAGAGTAGTAATTCAACCAAACATGTCAAATTGATTGCTATAGCTGGATTAGTAT

CCCTTGTCATACTTGGATTGGGTGTTTGCCTATTGATGTCACGATTCTGCAAAAGAAGAAGAGAGACCCA

AAAAGAAACCAAGAGACATGAAACATATGATCATAGTTTGCCCAAGGCCAAGGGTAACCCTAAGCATG

ATCAATCCCTGCAAAAACCATATTATGATGCAGAAAAAGGTAAATAGTTCATATGTGTACTTTCTTCTTAT

AGATTTATGTTTATCGTCAGTTACCTGTTGTTTTTAATGGCATTATGATGACAAAATGGATATGTCCAAAA

GAAGCAGGTTTGAGGCCAGTGGCTGGAAATGGGAAAGCTCAGGGTAGGAACATGTCAATGAATACTTC

AGAGGCTGTGCAGCAGAAAGACGTGAAGAGCACTATATCATATACAGACGATGAGCTGGGCTCGGAGA

TGGAGTCAATGATATCGGATGTTCTTCCACCACCGCCACCACCTTTTCTTCAAACTCTTTCTGCTGAGAGG

ATGGTTGTAAATCCAATTGTTCCCCCAATTACCTCAGGCAAGCATGCTATGAAGAATGTAAACTCTGCAG

AGTTCTTCACCATTGCCTCTCTTCAGCAGTATACAAATAGCTTTTCTCAAGATAATCTTATTGGAGGAGGC

ATGCTTGGAACAGTCTACAGAGCTGAGTCTCCCAAAACAGTAAGCATCTTTATTTTCACGAGCTTCAAAT

CAGTAAATACTGTGTGCTTGCATGCATTGTGACTGCATCAGTACCACATCTTGATCTTCATTTCACTTTTTT

AAAGGTGGAAATTACTCGTTATTTTTTTATTAAAAGCATGGTTTTCTGTGACAGACATTATATCTATTTTA

CATTGAACTTGTAGATGTTTTGTGGGTCTGTAATGTTTGACTCGAGTTTTGCATAATAGAAGTAGTCCAA

CCTTGTAGATGGCAACAGTTTCCATTAGTAGACTGTGTAGATATACCTGACATGCCTCCTGATTCATTAAA

TTAGTTTCCTGTTTTCTCAATAACCACTTGTACTTGTCATGTTTGTATATAACCTATTTAGTTGCCGTACAA

GGCTAGAAGATGCTGAGACTTTGCTATTTCTTGATGCCTTTTGTTTTGTTAGCTATTGGCGGTCAAGAAA

TTAGACACTGCTATGACCAAGCGTCAAAGTGATCAAGAATTTGTTGAAATGGTTTCCAATATCTCTAAAC

TTCAGCATGAAAACATTGTCAAACTTGTGGGTTACTGTTCAGAGCATGGACAACGGTTGCTTGTCTACGA

GTACTGTAGAAATGGTACCCTCCACGAAGCATTACACTTAGATGATCAGATCCACAGAAAACTTTCCTGG

AGTACACGTGTACGCATTGCACTTCAAGCAGCAAGAGCATTAGAGTAAGTACGAAAAAAAAGCTAAATG

ATATTAGTTTATAAGTGCTTGTAACTTGTTTTAATGAATTGTGATGGTTAAATAAATTATAAATTGGCTGT

TAAAGTCAGCTCTGCATGTTTCCCGGGCTTAATGTTTGCTACACCACAATTAATTTTGTAGTTTTATCCTTA

GCTTGTAACCGTTTAGAGATTGAGTTGCTTCCTGTTTCACTGAAAGGTACTTGCATGAAGTTTGTCAACC

ACCTATTGTCCACCTGAACTTCAAGTCCACTAATGTTCTCCTTGATGATGAACTTGCTGTACATATGTCAG

ATTGTGGTTTGGCACCTCTGATGTCATCTGATTCCATAAGACAGGTAATCCCTTCAATCCTCAAACTAGAA

ACAAGAATAACAGGAGATTTGACTTGCAAACTGTAGTTATTCTGAATTCGTAATGGGAAAATTTATCCTT

TTGGACTGTGATCTTCATTGAACTTGTAAACACTTCCTTCTCAACTTCTAGAAACTGATTCATTATTCATTA

GTATGAAAACTAGGTGCCAATTGGCTGCTAAAAATTGGTGCATTCTGTTTAGAAATGGTTGTATGTTTCT

ATTTCGAGGGGATAAAAATGAAAGGACAAGACATGGTATTTCTGCTAATTTTGGTTTGCATCAAAAAGC
TGTCCATCTCATATTGCATTTATCGATGAATAAAGTGTTGATAACTTCAGTATAACTTTCAGTTGCAAGGA
TCCGGCTATGGTGCTCCTGAACTTGAATTGGGAAACTATACTTGTCAGAGTGATGTTTATAGCTTTGGAG
TTGTTATGCTAGAACTTCTCACAGGAAGACAATCTTACGACAGGTTAATTTCTTTTCTGACAGATATATTT
TAACACCTTGCTAGTTGTTATTGTTGCAACTACATATTTTTCTAAAATTATACGACTCCTGACTTTGAGCCC
ATTAAAATATGATTTCCAGGTCCCGGCCTAGAGGGGAGCAGTTGCTGGTTCGATGGGCTATTCCACGGT
TGCATGATATAGATGCATTATCAAGAATGGTTGATCCTTCCCTAAATGGTTGTTATCCATCCAAGTCTTTG
TCTCGGTTTGCTGACATAATTTCCTTGTGTATTCAGGTTAGTCTCTTCCTTTACAGGTTCCTCATAGCTCTT
TAATGAAGTTTAAAGTCCCACTTACAGTTTTGAGTTACCGCGTGTGAGAAACTTTACTGGTTATTCTTCAT
AAAATGAGACTTTAATCCAGTTAAATTCCAGGGCTTTGGTAAATGTCTGTTTTGGATAAACCTACTTTACA
GGCTGCATATAACTATGTCCAACAAGAAAACAAACTCACATTTGAGCTATTTAGGCTTAAGTAAATTTTA
GAATCAACTCAGTCTAGGGAGAGAAGAGGTTCTTTGTTAGTAAATTCGCCATTCTGCTATAGCTAAAAAA
CACACACATAATATTTGAACTGATGTTTTTCGAGCTGTCGCCTTTCATACTTTTCTCATGTCTTCTTACTTTT
TTGTTGGGCAGTCGGAACCTGAATTCAGGCCACCAATGTCCGAAATCGTGCAGAATCTTCTGCAGATGA
TCTAAAGATATCATCAAGATTGAGTCTTTTAGCATAGATGGCAAGGATTCAAGGAGAAATCTTTACACAA
CATTTTCATTAAGCTCAAATATTGTGTGCTAACATGAGTAATGTAATGTAAATAGCATTATTATGTTTTGC
AACATTTTCATGTGTGATTCTTTTTGACATATCTATGAGAAAGTAGAAAAAGACTCTCATTCTTTTAGTGT
AATCGAGGAGTTTGTAATATATTACCAGCCAATTCTTTATGCAAGTTTTGTGATCATTCGAAG
>PGSC0003DMG400022565 LRR-RLKs
CCAAAACTAGCTGCAACCCTGCCAATAACACTCATCTTTCATTGTCTCACTCTCACAATGATACCCTTAATC
TCCATGGCTACTTCTTCATCTTCTGTAAATTTCTTCTTCTTCTTCCTCTTTCTCTTCACCCATTCATTCTTTTG
CTTTTCTCAACAAAACCCAGAAATTCAAGCACTTCAATCCTTCAAACTTAGTATTCATGACCCACTTGGAG
CACTCACAAGTTGGGATTCTACATCTCCTTCTGCTCCATGCGATTGGCGAGGAATTTTTTGTTCAAATGAT
TCCTATGTGAGTGAAATTCGTCTTCCACATTTACAGCTTAGTGGATCTCTCACTACCCAGATTTCTGATTT
GCGTATGTTGCGTAAGATTAGTCTTCGTTCAAATTTCTTTAATGGAACTATACCTGCTTCAATCTCCAAAT
GCAAACTTCTGGATACTGTTTTCTTACAGTATAACTCTTTTTCCGGTGAAATCCCGCCGGAGATTATGAAT
TTGACTGAGCTTGAAACGTTTAATGTGGCTGGAAATCAGATGTATGGTGAAATACCCAGTGAGTTGCCG
GTGAATCTGAGGTATTTTGATGTCTCGGAGAATTTGTTTTCCGGTGGTATACCGGAGAAAATATCGGACC
TTTCTCAGGTTATTCTGCTTAATCTGTCGTACAATCGATTCTCCGGTGATATTCGGGCGAGTTTTGGTAGG

CTTCAACAACTTCAGTATCTTATGCTTGACTACAATGAGTTAGAAGGAACAGTTCCTTCTGCAATTTCGAA
CTGTTCGTCGTTGGTGCATTTGAGTGCTGAAGGGAATGGTATTACTGGTGTTATACCGGCGGGGATTGC
TGCTCTTCCGAAGATTCAGGTGATAAATTTATCGCATAATAAGCTTTCTGGATACTTAGCAACTTCATTTT
TCTGCAATGGTTCTGTGTATCCTCCTTCCCTTCAGATTGTTCAGTTGGGTTTCAATGCCTTCGCTGAGATTC
TTCACCCACAATCGTCTAGATGTTTTAGTTCATTGCGAGTTTTGGATATTCAACACAATCAGATACATGGA
GATTTCCCTTTTTTCTTGACAGACAATTCTGCATTGTCGTCCTTGGACTTGTCAGGGAATTTGTTTTCGGG
TACAATCCCGAGTTCCATTGGGAATTTGTTGAGGTTGGAGCAACTGAGAATTGGAAATAATTCATTCGA
AGGTGATATACCGGTTGGGATAACGAAATGTAGTAGTTTGAATGTTCTTGATCTTGAAGGGAACAGATT
CATAGGGGAAATTCCAGCTTTTATAGGTGATCTTAAAAGCTTGAAGATTTTGTTCATGGGAAGAAATCA
GTTTTCGGGTTCAATCCCTTCTAGTTTTAGCAATATCACTAGTCTTGAAAGTTTGAATTTGGAAGGAAACC
GTCTTACGGGAAGCTTGCCTGAGGAGTTGATGTTTTTGAGCAATTTAAGCACATTGAATCTTAGTGGGA
ACAAGTTTTCAGGTAGTATTCCCGTTGGTATTGAGAACCTTCAGCAGTTATCGGTTCTAAATCTGAGTAA
GAATGGCTTCTCAGGGACTATCCCGTCTAGCATTGGAACTTTGTATAAGCTGGTAGCTCTTGATTTGAGT
CGACAGAATTTATCAGGCGAATTGCCATCTGTTCTTGGTGGTTTGCCTAGTTTACAAGTGATTGCTTTGC
AAGAAAACAACTTGTCTGGAAATGTTCCTGAAGGTTTTAGTAGCTTAATGGGTTTGCAGTTCTTGAATCT
ATCTTCCAATTCATTTTCAGGTCATATACCGTCTACGTTTGGGTTCTTGACATCGTTAGTTGTCCTCTCGTT
GTCTAAGAATCACATATCTGGTTCAATACCTCCTGACTTGGGTAATAACACTGCTCTGAAGATTTTAAATC
TGAGGTCAAATTCATTGAGTGGCCAAATTCCTTCTGATCTTGCACGTCTCTCCCGTTTGAGTGTGTTGGAT
TTGGGTAGAAATACCCTGACAGGTGAAATTCCAGAAGTTATTTCCAATTGTTCATCCCTCACATCAGTTGT
GCTGGACACGAACCATCTTTCGGGAAACATTCCAGCATCACTCTCCAGCTTATCAAGCTTAATTACCATTG
ACCTCTCCGGAAACAACTTGAGTGGAAAAATCCCAGAAAATCTTACAGTGCTCCCAAAGTTGGTGAACTT
CAATGTATCGAACAATAAGTTGGAAGGCCAGATACCAGTGAAGCTCGGTTCTCATTTTAACGATCCATCG
GATTTCAGTGGCAACCAGGGTTTGTGTGGGGAGCCATTGAATAGAAAATGTGAGCGAACTGGTAATGG
TAAGAATAGACTGATTATGTTTATTGCAGTGTCTGCTAGTGGAGGTCTTCTCCTTGCATCATGCTGCTGTT
TTTACATATACGCCTTGCTGAGATGGCGTAGGAAGCTCAAAGAAAAAACAGCTGGAGAGAAGAAGCAT
AGCCCTGCAAGGGTTAGTTCGAGGACCAGTGGTTCTCGTGGCAGTGGCAACAATGCTGGGCCAAAACTT
GTTATGTTCAATAACAGAATTACAGTCGCTGAAACAATCGAAGCAACTAGGGAATTTGATGAGGAAAAT
GTTCTTAGCAGAACACGCCACGGAGTCCTATTCAAGGCATGTTACAGTGATGGAATGTTGCTCTCGATCT
GTAGACTACCAGATGGATCACTAGATGAAAACACATTCAGAAAAGAGGCTGAATCGCTTGGCAGAGTG

AAGCACAGAAACCTAACTGTTCTACGCGGGTACTATGCTGGCCCTCCAGACCTTAGACTACTTGCATATG
ATTACATGCCAAACGGGAACCTTGCAACACTCCTTCAAGAAGCATCTCATCAAGATGGTGGTCATGTTCT
CAATTGGCCAATGCGACACCTCGTTGCACTTGGCATTGCCCGTGGGCTTGCTTTCCTCCACGCAGCCTCT
ATTATTCACGGAGATGTTAAACCACAAAATGTACTGTTTGATGCAGACTTTGAAGCCCATCTTTCAGACTT
CGGCTTAGACAAGCTTACTGTAGCCACACCAGTCGAACCTTCCTCTTCAACATCAGTTGGGACTATAGGC
TATGTGGCGCCAGAAGCAACATTAACAGGAGAAGCCACAAGGCAATCAGATATTTATAGCTTTGGCATT
GTATTACTGGAACTGCTAACAGGGAAAAAGTCGTTAATGTTCTCGCAGGATGAGGACATTGTGAAATGG
GTAAAGAGGCAATTACAGAGAGGGCAAATTTCAGAATTGTTAGAGCCAGGATTGCTTGAACTGGACCCT
GAATCATCAGAATGGGAAGAGTTTTTATTAGGAGTAAAAGTAGGCTTGCTTTGCACAGCACCGGACCCC
CTTGATCGTCCCACCATGACCGACACTGTGTTCATGCTCGAAGGTTGTCGTGTTGATCAAATTTCACTCTC
CTGAAGAACCACGACCGGCAATGGTGACACGTAAAACCCAAGGTCTCATCCTAATTCTTTGCCCTTCCTA
TTGACTCTTTGATTTTTTGAGGAGTTGCATTGCAATGCAACAATTGCTAGCACTAGAACTTAAGTTTTTTG
TTTAAGGAAAAGAACAAAAAGAAAATATTCTCAATTTTTTTTAGCTATAGCTATTTTATTGATTTTGGTTTT
CTATTACAAAAGTGCCTCTTGAGTTGAAAACAATGAAATGAATTCTTTTTAAGTAATTCAAG
>PGSC0003DMG400022632 LRR-RLKs
CAAACCTCAGTCAAATGACAACAAGCACAGCCTCTTCATTCTTGTTTTTGCTCTTCCCCCTCTGTTCTTCTT
CTTCTTCCTAACCCCCTTCTCACTTCACTTCTCCACACTCAACCCACCCACCATGACAGCAAAATCAACTAA
CCCCATCTTCTTCTTCACTCTTTTCTTCCTCATTTTCACATTAACCCCATCTTTTTCCATTGAAAATGATGTTA
AATGTCTTGAAGGTATCAAATCTGCACTTTCTGACCCTTTAAACAAGCTCTCATCTTGGTCATTCTCCAAC
ACTTCTGTAGCTTCAATTTGTAAACTTGTTGGTGTTTCTTGCTGGAATGAGAAAGAAAACCGCCTTCTTTC
TCTTCAACTTCCTTCAATGTCTTTATCCGGTTCACTCCCACCTGCCCTTCAGTTCTGTACTTCTCTTCAATCT
CTTGATCTCTCTGGTAACTCTTTTTCCGGTCCAATCCCGGTTCAAATCTGTTCTTGGTTACCTTATCTCGTC
AATCTCGATCTCTCCAGCAACTCTTTCTCCGGTTCCATACCCCCTGAGTTTATTAATTGCAAATTCTTGAAC
ACCCTTGTTCTAAATGACAACAAACTTACTGGGTCGATCCCATTTGAGATCGGCCGGCTCGACCGGTTGA
AACGGTTCAGTGTGTCCAACAATGGCCTTTCCGGTTCAATTCCTGATGATTTAGACCGGTTTTCGAAAGA
TGATTTCGATGGGAATGATGGGCTTTGTGGGAATCCAATTGGATCTAAATGTAGTAATTTGAGTAACAA
AAATCTTGTTATTATTATAGCTGCTGGTGTTTTTGGTGCTGCTGCATCACTGATTCTTGGATTTGGGATTT
GGAGATGGTTTTTAGTTCAGCCTAGTAAGAAAGATAGAGAATTCGGTGACGGTAAAGGTGGTGGTGGT
ATCAGTGATGATTGGGTCGATAAATTGAGAGCTTACAAGCTTGTTCAGGTGACATTGTTTCAAAAACCTA

TTAATAAGATTAAGCTGAATGATTTACTAGTAGCTACAAATTCATTTGCTAGTGAAAATATTGTTATTTCA
ACTAGAACTGGTATTTCTTACAGAGCTATGTTGATTGATGGATCAGCATTGGCTATTAAAAGGTTGAGTA
GTTGTAAGTTAAGTGAAAAACAGTTTAGGTCTGAGATGAATCGATTAGGACAGCTTAGACACCCGAATT
TAGTCCCGTTATTGGGATTTTGTATTGTTGATACTGAAAGGCTTTTGGTTTATAAGCATATGCAGAATGG
AAGTTTGTATTCGCTTTTACATGGTAATCTTAGTACTGGTATTAGAAATGATAGTTCTGAGCTTGGTTGGT
TAGCAAGGGTTAGGGTTGCTGCTGGTGCAGCTAGAGGACTAGCTTGGCTTCATCATGGGTGTCAACCGC
CATACGTGCATCAATACCTTAGCTCGAATGTGATTCTTGTAGACGATGATTTTGATGCTAGGATCACGGA
TTTTGGGCTTGCAAGGCTTATTGGTTCTGCTGACTCGAATGATAGTTCGTTTGTGAATGGGGATTTGGGA
GAGTTTGGTTATGTAGCTCCTGAGTATTCGAGCACGTTGGTTGCTTCAATGAAAGGGGATGTGTATAGT
TTTGGAGTAGTGCTGCTTGAGTTGGTGACAGGGCGGAAGCCTCTTGGTGCGGGTAATGCAGAAGAGGG
ATTCAAAGGTAGTTTAGTCGATTGGGTTAATCAGCTTTCGAGCTCAGGTCATAGTAAGGACGCGATTGA
CAAAGCTTTTGCTGGAAGTGGTCAAGATGATGAAATTTTGCGAGTTCTTCAAATTGCATGTTCGTGTGTG
GTTTCAAGGCCGAAAGATAGACCTTCGATGTACACAGTGTATCAGTCCTTGAAGAGCATGGTGAAAGAA
CATTGTTTCTCTGAACACTTTGATGAGTTTCCAATCAACTTGACCAAAGAAAATCATGATCATAAAGATTA
GGTTATAACATTGAAATGATTAGACAAATGTTTATGAATTTTGAATATTCTAGTTATGGCATTTGTATTTC
TTGATAACTCTGTATAGTTTGAGAAGTAAATGCATGATCTTTGTACTTTCTTGACACATTTT
>PGSC0003DMG400022900 LRR-RLKs
AAAAATTATACTAATTCTACCTAACAAAAAAACAAGTAAATTTTTCTTGAGATAACAAATGAAAAATCCA
GAAATTTACTTCATAACGGGGACGAATATAGTAATTTTGAAGAAATAACATGGACATTTTTCGTGTTATG
ATCAAAACATGCCTTCTTTGCATCGTCTTCTTGATCAATTACCCCGTGAAAATAATTTCAGTTTCCGATACT
GATGCTTTGTTGAAGCTGAAACAATCATTTACAAATGCAGCGTCATTGGAATCCTGGAAACCTGGAACG
GATCCGTGTGACAAAAATATACGTTGGTTGGGTGTTTTTTGTCAGAAAAAAATGGTTACTGGACTTCTTC
TTGCAGAAACGAATTTATCTGGAATTATAGACGTTGAGGCATTATCACAAATGCCTGCTCTGCGTACACT
TAGTTTTCAAAGCAATTTATTTTCTGGACCAATTCCGGAATTCAATCGAATGGGTGCTTTAAAAGGTCTTT
ATTTGTCGGAAAATCAATTTTCTGGTGAAATTCCATCAAATTACTTCGAAAAAATGTTGTCGTTGAAGAA
GTTATGGCTCTCCGACAACAAATTTTCGGGTGAAATTCCGTCTTCATTAATGGAGCTGCAATATCTCATTG
AATTGCACCTGGAGAACAATCAGTTCACAGGTCCAATACCGCCAATCTCGAAGCAGTCAACATTAGAATC
AATCGATCTTTCTAATAATAACTTGAAAGGGGAAATCCCTGTTTCGTTATCAAGATTCAATGAGAGTTCG
TTTAAGGGGAATTCCGAGCTTTGTGGTGAAAAATTAGGCAAAGCTTGTAATCCAACCATAAATAGCAGC

AACGACAACAACACTAATAATAACGCAAATAATGGCACTCAACAAAGTCCGGATAGTAATTCGAATAAA
AGCATCTCAATGTGGATTATGATATCATCAGTTGTTCTTTTATTGATCATGATCATAGTAATATATCTCATA
TGTCGCTACCAACAAAATCGCCGTGCTTCAATTGAAAGTTTTGATGAGCCATCACTTGGGAGGCGAATTT
CGAGTGATAGCAAGAAGTCATTTGAACTAAGTAGACGTGGCAGTTCGATTAGAAAAGGATCGATAATG
GGAAAACGTGTAGGAGATTTGACAATGGTGAATGATGATAAAGGTGAGTTTGGTTTAGCAGATTTGAT
GAAAGCTGCTGCAGAGGTGTTAGGGAATGGGCCATTGGGTTCTTCTTATAAGGCTATGATGTCAAATGG
ATTGACAGTTGTGGTAAAGAGGATCAAAGAAATGAACAAGATCGGAAAAGATGGTTTCGATGCAGAGG
TTAAACGTTTAGGAAGATTGAGACATAAAAATATTCTAACACTTTTGGCTTACCATCATCGCAAGGAAGA
GAAGTTGTTTGTGTATGAGTACATTCCCAAAGGAAGCTTGCTGTACATATTGCACGGTAAAGTACCTACC
TCTAATTTTATCAAGTCATACTAGAAATGCATGGTAAGACTGTGTACAATAAACTCATTTTTGTTAATTTT
GTGCATGTGCTTAGAATAGGAGACCAAAAGCCAAAAACTTCTACAAGTAAGTAAGTTTACATTGTATTA
GTAGTCTAGTTGTCAAGTGTTTCATATTAGTTGAGGGAACGGAAAGTAGTCTCCTTGTATGATCTTGGCA
AACTATACGTCTATATCCATGAACTAGCCTTGAAGTTGAGTGAGGTCTAAAGGTCCATTTGATTCACAAG
TTATCAAAGCCAGACTCGTATGTACTCTTGATATCCCGATGTAGAGTTCCCATCTTACATTGGGATTATTC
GCACCTTGTGGTTTTTTTCAAGACCACTTTTTGAATTATGTTCCCGTTAGCCCAGTGGGTTAAGGGGAAC
ATAAGTTAAATAGAGACCTCAAGAAAGGTCATTTCTACAGTTGTCCTGTTATTTTCTCCATGTTAATTAGT
TGAGGAGTTGAGGGACGGATTTGAGCTATGCTTAACTTATGAACTAGTTTTCGAGGTTTAATTAGGCAT
AATATTCATTTTCTTCGCGCTAATTAGTCTGTGCATGATTACTTATAAAGATTGCTGCTGCTTTATATGAAT
GATCACAGGTGAAAGAGGGCTGCCCCATGCTGAGCTCACCTGGCCAGTGCGTCTAAATATTATACAAGG
AGTAGCTGAAGGACTAAATTATCTTCACACTGAACTTGCTTCTTCTGATTTGCCCCATGGCGATTTGAAAT
CGAGCAATATACTCATAAGCACAAACCACGAGCCAATACTCACAGGTTACGGATTCTGCACCTTAATGAA
CAACGCGCATGCAGTTCAAGCACTAATCGCGTTCAAGTCACCAGAAGCAGTACAAAATAACCAAGTTAC
ACCCAAATGCGATGTTTATTGCCTTGGAATTGTGATTCTTGAGATAATAACAGGGAAGTACCCGTCTATA
TATCTTAATACCGGTAAAGGAGGGATAGACATAGCACAATGGGCGAAATCCGCGATTGCTGAGGGAAG
AGAAACTGAATTATGTGATCCAGATATAATAGCAAGCGCGAAAGATTCAATGAGTTCAATTAAGGAACT
AATTCACATTAGTGCAGCATGTGTAGAGAGTAATCCACAACAGAGGATAAATATTAGAGAAGTCATTAG
AAGAATTGAAGAAATACAACAACAAAACGGAGTAGGCCAAACAGGACAAACAACAACAACAACGCCTC
AGTCTCAAACGGGACAAGCAATTGAGATAATGCAGTCTCCAAGAGATGGAGATGCTGAAATACAAGTG
TCGGAATCCTAACAAGGAACAGCAAATCATTAGGAATTTTAGCAGACTCCGGTAATTCAAGGCGTGGTT

AATATTGCAGCTTCTCCTATGGTCATCAGACTAATGATAGTGTTTTTTTTTCTTCATTTTTTAAGGTTCGTT
AACTAGGGATAGTACATATATTACTGACTAAGTTTTAGTTTCATTTTGTAGTCTGATGACCATTATCTCCC
CTTGTAGAACAAGAAATGAGAGGAGAAGCTGTATCCTGTGTAGAGATGAAAGAGCTTACTGCTTGCTTT
AGAATTGTATATGCAATACAATTTCCTGTTTCTACTACGTTGAGTACCAACTATTGTAGACTAAAAATTCG
TAGCGTACTTTGACCAACTCCAAGGGATACTGTCATTTTCAACCAGTAACATGTATCAGGTAACTCTGCTT
CCAAGGATAGAACACATGAGAAAGAAATTACCTAGTGTTTTTATCCATATTGATATTTGAACCTGAAACC
TCATGATTCTCGACCTACTTCATTGATCACACTCTTGCGTACCTGTATGTTAATTTTTTTCACACAATAATA
AACGGAGAAGTATGGAAGGCCCAATCAGATAACTATAAC
>PGSC0003DMG400023004 LRR-RLKs
CTACTGCCCAAAAGAAAAAAAGAAACCAACTGAGTCAGTATAGTACTTCATCTCTGAAACAAAGCAAGA
ACCTCACTCTCTCACCATTTCCAGTCAAAGGTAGGTAACAACTAACATGGGTTTTCCTCCATTATTCCTCT
GTTTTCTCTTCATACTCTTTTCACTCTCTGTAAGTGGAAATGCTGAGTTACAACCTCTAATGGACTTAAAA
GCCAGTCTTGACCCTGAAAATGCTTTACTCACTTCATGGACTGTTTCTGGTAATCCATGTGATGGTTCTTT
TGAAGGTATTGCTTGTAATGAAAAGGGTCAAGTTGCTAACATTTCTCTACAAGGTAAAGGCCTTAAAGG
GAAGTTGTCACCAGCCATTGCTGGGCTAACTCACTTGACTGGACTCTACTTGCATTATAACTCATTGTATG
GAGAGATACCAAGAGAAATTTCTAATTTGGTTGAACTCACTGATTTGTATTTGAATGTTAATGATCTCTCT
GGTGAGATCCCTCAAGAACTTGGAACCATGTCAAAATTGCAAGGTCAGTGACTAAGAAACTAACTTCTC
TGTTTTGTGCGTTATGCTTTGCTTGGTTTTTGGGCTATTTTAGATTTTGAAGTTACAAACTTGAAACTTGA
AAATTTGAATTTTTGTTACTATGATAAAAATAAGATATTTTTACATTTTCTTTGTTCTTTCTGAATATTTTGT
CCAAATGTGTTGAGCTCTAGTTGGAAAAAGTAGGGGCTTCTGCTGTTAATCATTTATGTCTCACTGAAAA
AATTGAAGTCACAAACTGGAACTTGTTCCATTTGGGAAATCATAAAGATTGTTTTTTTCCCCCAAATTGTT
TTAGAGTTCAATTTCAGTCTAGTAGTTCCATCCCTTGTATTCCTTTCATACTACTAGTTTCTTGTTCTATGAT
TTTGTATTTTGGTTATCGTATGATTTTGTTGTAGTTACTGCATAGAATGATTTGTCAGGCTTTGTATAGTAT
TTTGTCCTGACTTCTTCTCTTCTATTATTTCCTTTTCTGTACTACTTTGAACTTTTTTTTCTTGAGCCAAGGGT
TTATTGGAAGCAAACTCTCTACCTCCAAGGTAGGGTAAGGGTAAGGTCTGTGTATACTTCACCCTTCCCG
GAGCCTACTTTGTGGGATTACATTGGGTATGTTGTCGTTGTTGTAGTTCATCCCCGTATGTTCTAAAGGT
GGCTTTAAGTTTTGTTTTTGGGTGTTTATTTACAACCTCATTTTGATAATGGAGTCTGTCTCACTTTAGTTT
ATTGTCTTTTTCCGTATTGTACAGTTTTGCAACTATGCTATAACCATTTTACTGGTAGCATACCCACTCAAC
TAGGGACTCTAACAAAGCTCAATGTCCTGGCTCTGCAATCCAATCAGTTAACTGGTGCGATCCCCGCTAG

CTTGGGTGACTTGGGAGTGTTGATGAGGGTGGACTTGAGTTATAATAGTCTTTTCGGTTCAATTCCTTCA
AAACTTGCGGATGCTCCCCTACTTGAAGTCTTGGATATCCGAAACAACAAGATTTCTGGCAATGTGCCTC
TTGGTAATGAATTTTCTCGCCCTACATTGTACTCTCTCCGTTGGAGTTTCAAGAGTCAATCAAGTTTTTTT
TAGTGGCATTTCCATATATGCCTTTTAGATAATTTTAGTTGTTAATTATTGTGTTTCATAGTACTTATTACG
TAGTTTTCAAATATGAGTTTTTTTCAAAAAACTTGAAGCTTCTATGTCCGATTCATGGTCAAAGTTTAGAC
GTTTGAATCTTGAAATTCCAACCGTGCCACATAAGTTGAGGCAGAAGGAGTTATGTATATGTAGCCCCAT
AAGAGTCGTACGTATAGTTTCTTGGTAAATTATATCTTATCTAGTCTTTTGTTTTCTGTCTTGTGAAGCTTT
GAAGAGATTAGTTGGAGGTTTCCAGTATGAGAACAATCCAGGACTATGCGGTGTGGGTTTCCCATCCTT
GCGAGTTTGTACATCTTTGGATCGCTCGAATCCTAATAGACCAGAACCCTATGGAGTTGGCTCAAGTGGT
TTATCTACTAGAGATATTCCAGAAACAGCTAATCTCAATCTAAATTGCAGCGGAAATGCCTGCACAACGT
CGTCTAAAACCTCACAGGCATCTGTTGTTGTAGGGGTTATTGTGGTTACTATTATTGCATCTGCTATAGG
AATTCTTTCCTTCACACACTATAGAAGACGAAAGCAGAAACTTGGAGGTGGACTGGAAATGTGTGATAG
CCGTCTCAGTATCGATCAGGCCAAGGAAGTTAATAGGAAGAATGGTTCTCCCCTAGTCAGTCTTGAGTA
CTCGATTGGTTGGGATCCGTTGGCTGAGGGCCGGCGTTATGGTGGAGTTTCCCAAGAGATTCTGCAGAG
ATACAGATTCAATTTGGAAGAGGTGGAGTCAGCTACACAGTACTTTGCTGATAAGAATCTATTGGCTAA
GAGCAACTTTTCAACTACATATAGAGGGACTCTAAGAGATGGATCACTTGTTGCTGTTACGAGAATTGCC
AAAATTAGCTGCAAGTCAGAAGAAGCTGAGTTTTTAAGAGGACTAAACATTTTGACGTCTCTGAGACAT
GATAATTTAGTTAGGCTGAGAGGTTTCTGTTGCTCTAAAAGCCGGGGAGAATGTTTTCTCGTTTATGATT
TTGTGCCAAGAGGAAACTTGTTGCACTACCTAGATTTGAAGGAGGACGAAGCTCATGCTCTTGAATGGT
CCACGAGGGTATCTATCATTAGTGGCACTGCAAAAGGTCAGTTTTTGAATCAACTCTATCTTATAAGATC
TAAAGTACAGTAGTTTGACATTATATTACTTTTTTTGAGCTTACTGTCAACAATTCAATAATGCCTTTTTAT
CTATCTTGAGCAGGCGTTGAGTATTTGCACGGGTGCAAGGTGAATAAGCCAGCTCTAGTGCATCAGAAT
ATCTGTGCTGAAAACGTGCTCCTTGACCAACGATTCAAACCATTGCTTTCAAATTCAGGCCTGCATAAGC
TCCTCACAAATGATACCGTCTTTTCAACACTCAAGGCAAGTGCTGCCATGGGATATTTAGCTCCAGAGTA
CTCCACTACAGGCCGATTTACTGAGAGGAGTGATATTTATGCCTTTGGAGTGCTCATTTTCCAAATTTTAT
CTGGCAAGCGAAAATTCACCAGTTCAATGCGTGCTGCTGCTGAATCGTCCAAATTCCACGACTTGATGGA
CGTGAATCTCCGTGGAAGGTTCTCTGAGTCTGAAGCAACAAAACTAGCGAAAATCGCTTTGTTATGTACA
CATGAATGTCCAGAGGAGAGACCAACCATGGAAACAATCGTACGAGAAATTGGTAATTTAGCTAGTCCC
TCCTAATGCTAATGATCTTCTTCCATCTACTATTGCCTGTGGCAAATTTTCAATCCTCCTACTTATTAGCTG

GAGATAGATTCGTACTAACCTCTCACGAGTATTGGAGCTTCAAATATTTAGTCAGTCTGAGAACTTCTTC
GACTGAGGACTTGACCATTTTGTGAATGTATCTTGTATGCCATTTAACCTTAGGATTTCTCATGTTGGAG
GATTTTCCTCCGTTTATCTCATGGATTCTAATTAATCTGTTTGTAAAAAGATGAATGTTCTTCTATTTACCT
ACGATCATTGCTTCGTTTTTGTTTTGTGCCTA
>PGSC0003DMG400023057 LRR-RLKs
ATGTCGATGTTTAACAAATTATTGTTGGTTTGGTTTTTTGTTGTAACACTGGTTCTTGGATATGTGTTTTCT
GAAACAGAGCTTGAGGTTCTGTTAGAGATAAAGAAGTCATTTCTTGATGACCCTGAAAATGTTTTGTCAA
ATTGGTCTGATAAAAATCAAAATTTCTGTCAATGGAGTGGTGTTTCATGTGAAGAAGATACATTGAAAGT
TGTGAGATTAAATCTTTCTGATTGTTCAATTAGTGGTTCAATTTCACCTTCTTTTGGCTTTTTGCATGACCT
ACTCCATCTTGATCTTTCTTCTAATCTATTATCAGGTCCCATTCCACCTACTCTGTCTAATCTTTCTTCTTTAC
AATCTCTTCTTCTTTATTCTAACCAACTCACTGGCCCCATTCCCAATGAAATTGGCTTGTTGAAGAATCTCC
AAGTTCTTAGAATTGGTGATAATGTTGGCTTAACAGGGCTAATTCCAAGTAGTATTGGTGATCTTGAAAA
TTTGGTTACTCTTGGTTTGGCTTCATGTAGTTTGAGTGGTATGATTCCTCCTGAGTTGGGAAAACTCGGG
CGGGTTGAGAATATGAATCTGCAAGAAAATAATCTTGAAAATGGAATTCCTGCTGAGATTGGTAACTGC
TCTAGTCTTGTTGCGTTCAGCGTTGCAGTTAACAATCTCAATGGATCAATCCCGGAGGAGTTATCTATGC
TTAAAAACCTTCAAGTTATGAATTTAGCGAATAATAGTCTTTCAGGACAGATTCCTACTCAGCTTGGAGA
AATGAATGAGCTGCAGTATCTCAATTTGCTTGGTAATCAGTTGGAAGGTTCGATACCAAAGTCGTTGGC
AAAGTTGAGTAATGTTCGGAATTTAGACTTGTCTGGTAATAGACTTACTGGTGAAATTCCTGGAGAATTT
GGGAATATGGATCAGCTACAGTTCTTGGTTCTGACTAGTAATAATCTTTCGGGTAGCATACCGAAAACAA
TATGTAGTTCTAATCGAAACAGTAGTTTGGAACACATGATGCTATCTGAAAACCAACTTTCTGGTGAGAT
TCCTGTGGAGTTGAAAGAATGCATTTCACTAAAGCAGCTTGATTTGTCTAACAACACGCTTAATGGTTCG
ATACCAGTAGAGCTGTATGAGTTGGTTGAGTTAACTGATCTCCTACTTAACAACAACACATTGGTTGGTT
CAGTTTCTCCGTTGATTGCAAACCTAACCAATCTGCAAACATTGGCATTGTCGCATAATAATTTTCATGGC
AATATACCGAAAGAGATTGGGATGGTCGAAAATCTTGAGATTCTGTTTCTATATGAGAATCAGTTCTCTG
GAGAAATACCAATGGAGATAGGGAATTGCTCCAGATTGCAAATGATCGATTTTTATGGGAATGCATTCT
CAGGACGCATTCCCATTACAATTGGAGGGCTGAAGGAGCTGAATTTCATTGATTTTAGACAGAATGATC
TCTCTGGTGAGATTCCTGCCAGTTTGGGAAACTGTCATCAGCTGAAGATCCTTGATTTGGCGGATAATCG
TCTATCTGGTAGCGTTCCTGCTACGTTTGGTTATCTTCGAGCATTGGAGCAGCTTATGCTTTATAACAACT
CACTTGAAGGTAATCTTCCTGATGAACTGATCAATCTTTCAAACTTGACAAGAATAAATTTTTCTCATAAC

AAACTGAATGGTAGTATAGTTTCCCTGTGTAGTTCGACGTCTTTTCTTTCGTTTGATGTTACAAATAATGC
ATTTGATCATGAGGTTCCACCCCATTTAGGATATTCACCATTTCTTGAAAGGTTAAGGCTAGGAAACAAT
CGTTTCACCGGAGAAATCCCTTGGACATTGGGATTAATCCGTGAGTTATCACTGCTCGATCTCTCTGGAA
ATGAATTGATTGGTTTAATACCTCCACAGCTTTCTCTGTGTAGAAAGCTCACACATCTCGATCTCAATAAC
AACCGGCTTTATGGATCAATTCCATCTTGGCTCGGAAACTTGCCACTCTTAGGTGAGCTTAAGCTGTCCTC
AAATAAGTTCTCGGGACCTCTTCCTCGAGAATTGTTTAACTGCTCAAAGCTTTTAGTGCTATCTCTAGAAG
AGAATTCTCTCAATGGAACTCTGCCCCTTGAAATTGGTGAACTCAAGTCCCTAAATATCTTAAATTTCAAC
AAAAACCAACTCTCTGGTCCCATTCCATCTACCATTGGCAACTTAAGCAAGCTATATATACTCCGTCTCTC
GGGAAACAACTTAACCGGTGAAATACCAAGTGAGCTTGGAGAACTCAAGAATCTTCAAAGCATACTGGA
TTTGAGTTTCAACAACATCATTGGTCAAATTCCTCCTTCTGTTGGGACACTTACTAAGCTTGAAACACTTG
ATCTATCTCATAACCATCTCACCGGAGAAGTTCCTCCTCAAGTCGGTGAAATGAGCAGTTTGGGAAAGCT
TAACCTCTCCTACAACAACCTTCAAGGCAAATTGGACAAACAGTATGCACATTGGCCAGCTGATGCATTC
ACGGGGAATCCACATCTTTGTGGAAGTCCTCTACAAAATTGTGAAGTCTCTAAGTCCAACAATCGGGGTT
CAGGACTAAGTAACTCGACAGTTGTGATCATTTCAGTGATATCGACTACTGTGGCTATCATACTTATGTT
GCTTGGAGCTGCCCTCTTTTTCAAGCAAAGGCGAGAAGCCTTTAGAAGAGGAAGCGAAGTGAATAGTG
CTTACTCATCCAGCTCTTCTCAAGGACAAAAGAGACCACTTTTCGCAAGTGTAGCTGCTAAGCGTGATAT
CAGGTGGGATGACATCATGGAAGCAACAAATAATCTAAGCAACGACTTCATAATCGGATCTGGTGGTTC
AGGAACTGTCTATAAAGCTGAGCTGTTTAACGGAGAGATCGTGGCAATCAAGAGAATACCAAGCAAGG
ATGATCTCTTGCTAGACAAAAGCTTTGCAAGAGAAATCAAGACACTTTGGAGGATAAGGCACAGACACT
TGGTGAGGCTACTTGGATACTGTAACAACAGCGGAGAAGGTTCCAACGTATTGATTTATGAGTACATGG
AGAACGGAAGTGTGTGGGATTGGCTTCACAAGCAGCCAGCTAATAATAACAAGAGGAAAACATGCCTC
GATTGGGAGGCAAGGCTGAAGATAGCGGTGGGATTAGCACAAGGAGTTGAGTACCTTCATCATGATTG
TGTACCCAAGATCATTCATAGGGACATCAAATCAAGCAACATTCTACTAGACTCAAACATGGAGGCACAT
TTGGGAGATTTCGGGCTGGCTAAAGCCGTTCATGATAACTATAATTCCTACAACACTGAATCAAACTTGT
GGCTTGCTGGTTCATTTGGTTATATTGCTCCAGGTATATATCATTCCTGAATTCTCGAGTATTAGCATTCG
AAAATAGTTTTTTATTTTTTTTATACTAAGTTGCTTATTTGTTCGCAGAGTATGCTTATTCTTCGAAGGCAA
CAGAGAAGAGTGATGTTTATAGCATGGGGATTGTACTGATGGAGCTAGTGAGTGGAAGGATGCCAACT
GATGGGAGTTTCGGAGAAGACATGGACATGGTGAGGTGGGTTGAATCGTGCATCGAAATGTCTGGGAC
AGTTAGGGAAGAGCTCATTGATCCTGTTTTGAAACCATTGTTACCTAATGAAGAAAGTGCTGCACTTCAG

GTGCTGGAAATAGCACTTGAATGCACCAAAACTGCACCTGCTGAAAGGCCATCTTCGCGCAAAGTTTGT
GATCTGTTGCTACATGCATTCAACGACAAGGTGGTTCATTCTGATAAGATGAGTCCAGACAATTACGTCT
AG
>PGSC0003DMG400023192 LRR-RLKs
ATGACAACTTTGTCATATTTTTTCATTATATTCTTCTTCTTGTTAAGTTGTTTGAGTCCAAGAAGAGTGAAC
TCTCTGTCTTCTGATGGAAAAGCTTTGCTCTCTCTTCTCAAAGCAACTTATGACCCTTATGCTAAATCATCA
TCATCTTTTGTACTTTCTTCTTGGAATGCTTCAACTTCAACTCCATGTTCTTGGCAAGGCATTTCTTGTTCTC
CTCAACAAAGAGTTATTTCTGTTTCTATTCCAAATACATTCCTCAATCTTTCTTCTTTTCCTTTTGAGCTTTT
TTCACTTTCATCTCTTCAGCTTCTCAATCTTTCTTCTACTAATATATCTGGCTCAATACCTTCATCTTTTGGTT
TGTTTACACATCTTAGGCTTCTTGATCTTTCTTCTAATTCTCTTTCAGGACATGTTCCTTCAGAACTTGGTG
GACTTACCTCACTTCAGTTCCTTTTCTTGAATTCAAACAGATTGAGTGGTAGAATCCCATATCAACTTGCT
AATCTTTCTTCATTAGAAATACTCTGTCTCCAAGATAATCTCCTTAACGGGTCGATTCCGAAATATTTAGG
CTCATTGGTATCACTCCAACAGTTCAGGATTGGAGGGAATCTAGAGTTGAGTGGTGAAATACCTGCAGA
GCTAGGAATGCTCACCAATCTTACGACGTTTGGTGTTGCGGCTACTGGACTTTCAGGGGTTATTCCACAT
ACATTTGGTAATTTGATCAGTCTACAAACTCTGGCAGTATATGATACTGAAGTGTTTGGTTCAATACCTCC
TGAACTTGGGATGTGTTCAGAGCTCAGGAACTTGTATTTGCACATGAATAAGCTTACTGGTCCAATCCCT
CGTCAGTTGGGTAAGTTGCAAAAGATTACTAGCTTGCTTTTATGGGGAAATTCGCTTACTGGCCCTGTCC
CGGCTGAGCTGTCGAATTGTTCATCCCTTGTGGTTCTTGATGTCTCTGCAAATGACTTGTCTGGCGAAATT
CCGGGTGATTTAGGGAAGCTTGAAGTTCTTGAACAGCTTCACTTATCTGATAATGCACTTAGCGGGGCTA
TTCCAATGCAGTTGAGTAACTGTAGCAGCTTAACAGCTCTTCAGTTGGATAAGAACCTATTATCCGGCAC
GATTCCTGAGCAAGTTGGTGAGTTGAAGTACTTGCAGATTTTCTTGTTGTGGGGGAATTCGGTTTCAGG
AACTATCCCGGCTGCTTTCGGAAACTGTACTGAACTGTATTCACTTGACCTCTCAAGAAACAATCTCACTG
GTTCAATACCTGAAGAGATATTCAGTTTGAAGAAGCTGAGTAGGTTGTTGCTTCTTGGGAATTCGTTAAC
GGGAAGATTGTCTCCGAGTGTGGCAAAATGCCAGTCTCTGGTCAGGTTGAGGATTGGCGAAAACCAGT
TTTCTGGACCAATACCTGATGAGATAGGACAGCTGCAGAATCTTGTGTTTCTTGATTTATACATGAACCA
TTTCTCTGGTGAATTGCCTTCTGAAATTGCCAACATTACGGTTCTTGAGCTGTTGGATGTGCATAACAATT
ACCTAACCGGGGAAATACCATCATCGCTGGGAGAGCTCGTGAATTTAGAGCAGCTTGATCTCAGCAAGA
ACAGCTTCACTGGTGAGATTCCGTGGAGTTTTGGTAATCTCAGTTACTTGAACAAACTTATTCTTAGCAAT
AATCTGCTTACTGGTCCAATTCCAAAGTCATTTAGCAACTTGCAGAAGTTAACTCTACTTGATTTGAGCTC

CAATAGTTTATCTGGTGCAATTTCACCTGAGATTGGTTACATGACAAGCTTAACAATCAGTTTAGATTTGA
GCTCAAACCGATTCACAGGTGAACTCCCTGACACGTTATCCGGATTGACACTGTTGCAATCTCTTGATATT
TCTCATAATATGTTGAGTGGAAGAATCACAACTCTAAGCCTCTTGACCAGTCTCGCTACTCTAAACGTATC
GTATAATAATTTCTCAGGACCTATTCCTGTAACACCCTCCTTTAGAACTCTAACATCAAACTCTTTTCTTGA
GAATTCACTTCTTTGTGAATCAACTGATGGCTTCACTTGTTCTGCACATATAACTAGAAGGAACGGATTG
AAGTCTGCAAAAACCATAGCTTTGGCTGCTGTGATTGTGACTTCTGCATCCATAACAGTCGTGGCAACGT
GGTATCTTGTGACAAGGAAGCATAGATATGAATTTGAGAAATCACCTGGCATGTCGGTCTCTGCAATAG
GGACAGAAGATTTTTCCTATCCGTGGACTTTCATCCCTTTCCAGAAGCTCAATTGCACCGTTGACAACATC
TTGGATTGCTTGAAAGATGAAAACATTATAGGGAAAGGCTGTTCGGGAGTTGTTTATAGAGCAGAAATG
CCAAATGGCGAGTTGATTGCAGTGAAGAAGCTCTGGAAAACAAAGAAAGATGAGGAACCAATAGATTC
TTTCGCTGCTGAAATTCAGATTCTTGGTCACATTAGGCATCGAAACATAGTGAAGCTCCTCGGATATTGT
TCAAACAGGAGTGTTAAGCTTCTTCTCTACAACTACATTTCAAATGGTAATCTTCAACAGCTCTTGCAAAG
TAACAGGAACTTGGATTGGGAAATCAGGTACAAGATTGCAGTTGGATCAGCTCAAGGCCTTGCTTATCT
TCACCATGACTGTGTGCCAGCAATTCTTCATAGAGATATCAAGTGCAACAACATACTCCTCGACTCTAAA
TTCGAGGCTTATTTAGCAGATTTTGGACTTGCAAAGCTGATGAATTCTCCAAATTATCATCAAGCTATGTC
CAGAGTAGCAGGCTCTTATGGCTATATAGCTCCAGGCAAGTATCTTTCAACTTTTATTTCTCATCTTAACC
TAAATGTTGTGCAGCTATATGATGCTTTAAATCCTTGTGTTGAATCGTGTGATCATCGCATCTCTAAAAGC
TTAAGTTATTAGAAAGGATACACTTTTCTCTACTAAATCATATCTTCAACACATGCGGCTCTGATTCTTGC
CAAGCACGTGGATAATGGGTGACGCTTAGACTTCAACCTAGGATACCATGTTGAAATGTGTGACTCTCTC
ATTTAGTTTAAGTTATTAGAGATGGTACACATTTATTTACTTCATTATATCGTCAACAGTTCCCAGATATA
GGTCAAAACGAGAGTCACAACGTTATGAGCTATAGTCAAATTTGGAAGGATAAACTAAACAAGTTATGC
TTCATAGATGTTAACACTATTTTTTACCACTATGGTTGCAGAATATGGATACACAGTAAATATAACAGAA
AAGAGCGATGTCTACAGTTATGGAGTGGTGCTGCTGGAAATATTGAGTGGACGTAGTGCGATTGAGCC
TCAGATTGGTGATGGACAACACATTGTCGAGTGGGTGAAGAAGAAGATGGGAAGCTTTGAACCAGCTG
TTACAATTCTTGATTTGAAGCTACAGAGTTTACCGGATCAAATGGTGCAAGAAATGCTTCAAACACTTGG
AATAGCTATGTTCTGTGTGAATTCATCACCCACTGAAAGGCCAACTATGAAGGAAGTAGTGACACTTCTA
ATGGAAGTGAAGAGCCCAACTGAAGAATTTGGAAAAACTTCTCAGCCTTTAATCAATCAGTCAACTCAA
AGTTGA
>PGSC0003DMG400023282 LRR-RLKs

AATGAACCATCCCTCTTTCTTCATTTTCCTTTCACTCCACAATAACACTCTCTTCCTTTTAACAAAATTTTTTT

CTTCTCATTAGATTTCTGAATTCTGCTGCTCAAACATGAGACTAATTAAAGCACACATTGTTACTATTATAT

TTTAGGTAAGTAACTCTGTTTCCTCTTAACTCTTTCTTCATTATTTTTGTAATATTATGTATGCAACAAATTC

TTGAGTTATAGTATAACATGTTCTTTAATAGCAAAAGATGCATTCTACTTTTTGTTGTACTTAATGTCTCTG

TTTCTGTTAAATTTAAGAGTTAAATGATTTTAGGCACAAAAATATATGATTTTGGTTGTTAGGACTCAATT

ATGAAAGTTATATGTTTTTTTTTGTCTGTCACCACTCACCACTTTCTCTTTTTCTCTTCCTGTATGTATATGC

CATACTCATTTCCTCTTCTTTTTTTTTTTTTGATGATTTGTCATATTAGAATCTCCCAATTTGTTCATTCAATA

ATAAGTGTGTGGGTCAATTTAAGCGCATCTTGATTAATTTTATGACATATTTGCCATCTGTTATTCATGCA

TTCAGATTAGCAAATCAGAAACAATTAAGATGAAAAGAAAAAGAATTTTCTATTCACACCTTTAAAACCC

AACATCATCTCACATCAATAATAAATAGCATGTAACTCTATTTACCAAGGTTAGGACAGATGAAAAGTAA

TAACCTAGTGTTTTTTGTTTCTACTGGGATTTGAATTTATCTCATAGTTTTCACCTACTTCATTGATTAATA

GGCTGCACTGGTGGTGTAATAAGTTTAGAACTATTCGGTTCAAGATTTTAATCTTTTCTGGTGCTATGTTC

TATCCCACTCTAGTCAGATCCTTCCCCGGATATGAAGAATTTGATTGTAGGAAAGATGAAAAAACAAAG

GCCGCTCTTTAGTATCTTTCCTTATAACAAAAGACTATAAAGTCAAGCGAAACATTATATACTTGAGTCAC

TTCAAAGAAAGATGTAAATTCATTTGCAATATAGTAAACCCACAACTAAATACAAATTGACATGTTAAAC

ACATCAGCCAATCACCCAACATCCTTTGTCTGTGTATATCTGTTTAACATCTTTCATATGTGCTTTACTCAA

TTCATAAGCTATCTTATTCTTTTTAAGTGATATTTTTAATTTCTTATTATACAACAAACATTTGATTAGGTAT

ACATTGCTTTTAAAGTAAAAGGGGTTTGTTATATGTTTATTCTTGTTTCTATACTTTATTGTTTGGTTACTG

ATTGCTACTTCTCTTCTTTCAGTTACTCATTAAGAATGGGCATCCATCATATCTGTTATGGAGTTTGTCATC

CCCTTGTGAAATGACTCTTTAGCCCTTCCCATATTCAATCATGTCTAGAATTCAAACTCTCCATTCCCTCCA

AATCCTTGTTATTTTCTTGAAGTTTCTTGTTTTCTCTAATTCATTGTCTGTTGAGACAGAAGCACTTCTTGA

ATTTAAAAAGCATCTTGTTGACCCTTTGAATGTCTTGGAATCTTGGAAATATTCAGATTCCCCGTGTAAAT

TCTATGGTATTCAATGTGACAAACACACTGGCTTGGTCACTGAAATTTCACTTGACAATAAGTCCCTTTAT

GGAATTATTTCCCCTTCAATATCTGTCCTCCAAAGCCTTACTTCTCTAGTGCTTCCTTCAAATTACCTGTCT

GGAAATCTTCCAAGTGAATTGGCGGATTGCACCAATCTCAAAGTATTGAATGTTACTGACAATAACATGA

ACGGTACGATACCTGATTTATCTAGCTTGGCTAAGTTGGAGGTGTTAGACTTGTCAGATAACTGTTTTTC

TGGAAAGTTTCCAGCTTGGTTTGGAAAGTTGACTAGTTTGGTTGCATTAGGCCTTGGTGGAAATGAGTA

TGATGAAGGTAAACTTCCTGATTTATTTGGGAAGTTAAAGAAAGTTTATTGGCTTTTCTTGGCAGGGTCA

AATTTGACAGGACAAATCCCGGAATCTATTTTCGAAATGAAGGCACTAGGTACATTAGATATTTCGAAAA

ACCAGATATCCGGGAATTTTCCAAAGTCGATAAACAAGCTGCGAAATCTCTTCAAGATTGAACTTTATCA
AAACAATTTGACCGGGGAATTGCCAGTAGAACTTGTAGACCTTATACATCTGCAGGAAATTGATGTTTCT
AGAAACCAGCTGCATGGGACTTTGCCTAAGGGGATTGACAACCTGAAGAATATAACGGTTTTTCAAATA
TTCAAGAACAATTTCTCTGGGGAAATCCCTCCAGGCTTTGGAGATTTGCAGCATCTTAATGGCTTTGCTG
TGTACAACAACAGTTTTACTGGAGAAATTCCAGCTAATCTTGGCCGGTTTTCGCCACTGAATAGCATAGA
CATATCTGAGAACAAGTTTAGTGGTGCATTTCCAAAATATTTATGTCAAAACAACAACTTGCAAAATTTG
CTTGCTGTTGAAAACAGTTTCACAGGGGAGTTTCCAGGTAATTATGCTTCTTGCAAGACTTTAATGAGAT
TGAGGGTGAGTCAGAATCAACTTTCTGGGAGAATTGCTGAGGGTTTATGGGGGCTTCCAGAAGTCACA
ATGATAGATTTCAGTGATAACAACTTCACTGGAACTGTATCTCCTGGAATCGGCGCTGCGACTAAGTTGA
ATCAGTTGGTATTATCAAACAACAGATTTGCAGGTGAGCTTCCAAAAGAACTTGGAAAGCTCACACAGT
TGGAAAGGCTATATTTGGATAACAATGAATTCTCTGGCATAATACCATCTGAACTTGGCTCATTGAAGCA
AATTTCATCTTTGTATTTAGAGAAAAACTCACTTTCCGGGTCGATACCATCGGAGTTGGGTGAGTTTCCTA
GGTTGGCAAACTTGAATCTTGCTTCAAATCTTCTCACAGGCAACATACCGAATTCGTTGTCAATGATGGC
CTCGTTGAACTCTCTGAATCTTTCGAGTAACAAACTCAGTGGCTCAATTCCAACAAGCTTGGATAACCTG
AAGTTATCTTCATTGGACCTCTCTAACAATCAGCTAACTGGAAGAGTTCCTACAGATCTTTTGACGGTGG
GAGGAGAAACGGCATTTATAGGAAACAAGGGGCTTTGTGTTGATCAAAGCATCAGGAATGTCCGAAGG
AACTCAAGCATCGGCGCTTGTAGTGGAAAGGCTGCTCAGGAAGTGTTCATGAAGAGCAAACTAGTTGT
GTTTTGCATTGTATTACTTTCTTTGGCTGTTCTAATGGGTGTTTTCATGCTTGTGAGTTACTGGAAGTACA
AGTGCAATGCTGAAGCTGATTCAGAAAAGTGTCTGGGACATTCAAATGGAATGAATCCAAAATGGAAG
CTTGAAAGCTTCCAACATGTGGAACTTGATATAGATGAAATATGTGATGTTGGTGAGGACAAGCTGGTA
GGAAGTGGGGGCACAGGAAAAGTTTATCGGTTAGATTTGAAGAAAGGTTGTGGCACAGTGGCAGTAA
AGCAGCTTTGGAAAGGAAATGAGGTGAAAGTTTTGACAAGGGAAATGGACATTCTAGGAAAGATCAGG
CATCGAAATATAGTTAAACTGTATGCCAGTCTAATGAGAGAAGGTTCGAATATGCTGGTTTTCGAGTACT
TGCCAAATGGTAATTTATTCGAGGCACTACACCGGGAAGTCAAGGCTGGGAAGACAGAATTAGATTGG
TACCAGAGGTATAAAATTGCAGTTGGAACTGCAAAGGGAATCGCTTATTTGCACCATGATTGTGTCCCG
CCTATTATTCATAGAGATATCAAGTCAACAAACATTCTACTTGATGAGGAGTATGAAGCAAAAGTTTCTG
ATTTCGGGGTTGCAAAAGTTTCAGAAATTTCCTCTAGAGTCTCCGAGTTCAGCTGCTTTGCAGGCACTCA
CGGCTATTTAGCTCCTGGTATGTTTCAAAGAGGATAAACCAAATACTATGTTTCCACTTCTTTCTATTTTCA
CTCTTGCTAGTTTCCTAACATCACATTTAAATTTGTTTTATTGACAGAGATAGCATATACTTCCAGAGTGA

CAGAAAAGAGCGATGTATACAGTTTTGGAGTTGTTCTATTAGAATTAGTAACCGGAAGGAAACCAATAG
AGGAAACCTACGGAGAAGGCAAAGACCTGGTTTATTGGGCATCAACTCATCTTAATGACAAAGGAAGT
GTTCTGAATATTCTTGATCAAAAGGTGGTTTCCGAGCTTATCCAAGATGACATGATCAAAGTTCTGAGGA
TTTCTGCCCTATGTACGACTAAGCTTCCTAATTTACGCCCCAGCATGAAAGAAGTAGTAAATATGCTTGTT
GATGCTGAACCTTGAACATTTAGTCTTCAAGCAAATGCGAGAAAAATGAGAAGGATCCTTTGTAGTTTTC
TCCTTTTCCCTCTTACCATATAGTTTAAATTCCAAACTTCAGTATAGTTTTAGTTTTCTCATTATGGATATGC
CATGTAAAATTGTATCCAACTTTGTCTGTACAAGTTCATTTTGTTAAATGGAATGCATATTTCTGGTTGAG
CTTATAA

>PGSC0003DMG400023283 LRR-RLKs

ATGATTGTCTTGAACTCTAGTGAATTGTTTGGTCCATTGTCACCAGCAGTTGGTAATCTTAGTTTCCTCAG
GGTACTTTGGCTCAGCAGAAACAGTTTCACTGGCCAAATCCCTGGAGAGATAGGAAAGCTATCGAGATT
ACGGAGGTTGAACTTAGCAAACAATTCATTTTCTGGTGAAATACCAAGAAACATATCGCGGTGCTCCAAT
CTTAATTATATTCATTTAGGTAATAACAATCTGAGTGGAAGCATTCCAACAGAGTTGAGATCATTGAATA
AACTTGAGAATTTACTACTTCAGTTGAATAAATTGACTGGAGAAGTTCCAGCTTATCTAGGTAATCATTCT
TCACTCTTGGCAATTGCATTAGGAGATAATCAATTACATGGAAAGATACCTGATATTTTTGGTGGACTCA
AACATTTGGTGTTTCTTGATTTTGCTTTGAATTATCTGTCTGGAGAGATTCCTATGTCAATTTTCAACTTGT
CATCATTGCATACACTTCAGATAACTTTTAATAAACTTGAAGGGGCTCTTCCATCAGATATGGGGTTCAAT
CTTCCAAGTTTAAAGTTCTTTCTCCTTGTGGGAAATCAACTGACAGGAAAGGTTCCTACTTCAGTACTGAA
TTCTACATCACTTATGGAATTTGGTATCGACGGGAATAATTTCACTGGACAAGTACCTGCCTTTGGAAAC
CAAAAGGATTTGTACTGGCTTGGACTTGCTGAAAATCATTTTGGAAATGGAATGTTGGATGATTTGAAG
TTTATGTACTCCCTACAAAATTGTACTAGTTTAGAGCAGCTAATATTGGAAAACAACAATCTTGGAGGAG
TACTGCCAAGCTATATAGGCAACATGTCCAATCTTTTACGGCTTTCTGTAGGGGGGAATCTCATCCAAGG
AAACATTCCCACAGAGATTTTACAGCTTGAAAACCTGCAAGTACTTGGTCTCGAGAAAAATAATTTTACT
GGTATGATTCCTGAGTCTATTGGACAACTAAGCCAACTAAGTAGGTTATACATTCACCACAACAGATTTT
CAGGTGAGATTCCACATTCTCTTGGAAATTTGACGAGGTTGATTGAACTCGATTTGGGATCAAACAACTT
GCAAGGTACTGTGCCTTCAAGTCTTGGAAGCTGCAAGTTCTTATCACTCTTGTATCTGAATGGAAATCAA
CTTAGTGGTCTTATACCGAAAGAACTGTTTGAGCTCTCCCTTATAGAATTTGACCTCTCAAACAACCACTT
GACGGGGTATTTTCCTGTAGGGATTGGCTCTGGAAACTTGACTGGATTGATAAACTTGATCTACATGAAC
CATTCATACAACAATTTATCAGGTGAAATACCAAGCAGCTTTGGCACTTTGACTAGTTTGAGGGAGCTAT

ACCTTGGTAACAATGCCCTTCAGGGAGTTATTCCTGCATCCTTGAGTTCTTTGAGAAGTCTTGAATATATG
GATCTTTCTCATAACCACTTTGTAGGGAGGATCCCAAAGTTTCTGGATGAACTTGTCTCCTTGAAGTTCTT
AAATTTGTCTTATAATGATTTGGAGGGTGAGGTGCCTTTGAAAGGAGCTTTCAGAAACATGAGTGCTGT
TTCACTTGTTGGGAACAGTGAGCTATGTGGTGGTATTCCAGAGTTCAAAATGCCAAAGTGCAGCAACAA
AGTAGCGTCAAGAAGACGAAGGTTATCTCATAGATTGATAATAGTAATGCTGGTTATTGGAGGATTATT
GGCAGCAACCACAGTGGCCCTTCTTATTTTCTTGTGTGCTAGAAGAAAAAAGAAGTCCACTTCATCGGAG
AATTCATCGTTGGACGTGATCCCAAGAGTGACTTACAATAGTCTTTACAAAGAAACTAATGGCTTCTCTA
CGTCAAACATGATTGGTTCGGGTGCATTTAGCTTCGTGTACAGAGGCATTCTAGAGGAAAATGGAAAAT
TTGTTGCCATAAAGGTGCTCAAACTCCAAGTTAGGGGAGCTTCTAAGAGCTTTCTTACTGAATGTGAAGC
ACTAAGGCATATCAAGCATAGAAACCTGGTAAAACTCTTAACATCATGTTCAAGCATTGATTACCAAGGT
AATGACTTCAAGGCTCTCATTTATGAGTACATGGCCAATGGTAATTTGGCAAATTGGTTGCACAATAGAA
GTACAGATGGTGAGGAAAATCATGAGCCTAAAACTTTGAACATGCTACAGAGACTCAATGTAATAATTG
ATGTGGCATCTGCATTAGATTATCTTCATCATCAATCTGGAACGCCATTGACACATTGCGATATCAAACCA
AATAATGTGCTTTTGGATGAAGATTTTGTGGCTCATTTAGGTGACTTTGGACTTGCAAGGTTTCTGCCTG
ATGCTGCAAATCTGCTCTCCTTAAGCCAATCAGCCAGCTCTCTCAATATAAGAGGAACTATTGGATATGT
TCCACCTGGTAATTAACTCTTCTCCCTCTCTAAACATCATTTTATATTTATGAAGGTCCTCTTACTTATCAA
GTTTGTCAATTCATGTATGTGCAGAATATGCTACATGCAATACGTTTTCGACCTATGGAGATGTCTATAGT
TATGGAATTCTACTGTTGGAAACATTTACTGGACGAAGTCCAACTGATGAGATTTCCAAGGATGGTCTAA
ACCTTCATGATTTTGTTAAGAGAGCCATACCTGAGCAAGTGAAAGATGTTTCTGATCCTAAACTTGTCTAT
GATGAAATGGGCAGACTGATCAGCAACAATAAAACAATCGAGTGCCTCACATTGATTTTTCAAGTTGGG
ATTGCTTGTTCAGTGGAATCTGCAAAAGATCGCATGGACATTGCCAATGTTGTTAATGAACTGAATGTAA
TCAAAGATGCCTTCCTGAGGAACTAGCAATATTGGCTATGAAGTTTTTGAGTTTCATTATAAAGATACTA
CTTATATAAGTTATATACTCTTTATCCACTTTTCCCTTCCCCTTTTCCTGCTTCTCCTTATGTAATGGAAATT
>PGSC0003DMG400023329 LRR-RLKs
CAAAATTGTTCTATTCCTACTTTCTTGAATGCTTTGTGTGTCCCACTTTACACATTACACTAAACCCCCAAT
TGCCATTTGAATCTCACCTATTCATCATGTCATCAATTATCTACTATTACTCTTCCTTGTATAACTTAATCAT
TCATTCAACTCTTCAAAATCCATGGCTATTAGTCCAGGTCACTTTTCTTACTAACTTGTATATTTACATATT
ACTATTTGCATATATACACGTTGTGAACCTCATATAGTCGACCTCAACTTGTTAGGGACTGAGGCGTAAT
AGTTGTTATACATGTTGTGAAGCTTATATAGTTGACCTCAACTTGTCAGGGACTGAGGCGTAGTAGTTGT

TATACATGTTGTAAAGCTTATATAGTCGACCTCAACCTGTCAGGGACTAGTAGTTGTTATACATGTTGTG
AAGCGTATATAGTCGACCTCAACTTGTTATGGATTGAGGCATGGTAGTTGTTATACATGTTGTGAAGCTT
ATATAGTCGACCTCTAACTTGTTAGGGACTGAAGCATGGTAGTTGTTATACATGTTGTGAAGCTTATATA
GTCGACCTCAACTTGTTAGGGACTGAGGCGTAATAGTTGTTATACATGCTATTATATAATTTTCTTGATAT
TTTTTTGGGTATATTGAACTAAGTTAATTTTGCCTTAGCTTTTAAATGAACCTATGTTAAGGGGATTCAAC
AATCTAATATATATATGTGCCTATCATTTAACATACGTATGTCATTTAGTTTTTGTACCTTTGTCATCCCTC
AACCCCTCTCCAATCTTTTCTATCTATCATTAGAAGTAGGACGGGTAATATTCCGGTGAAGTGGATAAAA
ATTAGTTTAAAGGCTTTATGTTTTGATGATCATTTGAGAAATTAACTCTTAGGAAGACTAGTCGCTCAGA
AATAGTCATTTTGATTCTTTTATTCTTACTCAGTATGAGCATTAATTCTTATTGGTAGCACAAAAAGAAGT
GGATACTTCTTGAACTAATTGGTAATATTTTCATTGTGTTGCAGCTACAAGGCCTATTTCTTTGACATCAT
ATTCTCCGACCTAGACTTGTTTGAGATTGAGGCGTAATTATTGTTGTTGTTGTTATATAGAGTATTGGAA
ACAAGGAGAAGGATCAATTAGAAGCTCTCCTCTGGTTGATAGAGAAAATTCAATGCCTACAGCCTTTTC
GTTCCGGAATTTTCACTTGAACTTGCTGCTATTCCTACTTTTATGCAGCAATGGTCTCTCCTCAAATGATTA
TGATGTTGAAGGTATGGAGTTATAGGTTTTGGTTACTGTACCTGTGAAAATTTCATATGCTTGCAAAATA
ATGTGCTAATAAAATCGCGATAACTGTTATTCTCTATTGAAATCATATATCATATATACATCATTTCAGGT
CATGCTTTAATTGAGTTGCTGAGGGCTCTGAATGATTCACACAATAGAATCAAAGATTGGAACATTGATT
TTGTGACTCCATGTTTCAGTTGGTCTCATGTTACATGTAAAAATGGAAATGTCATATCCCTGTAAGACTAA
TTCTTTCCTTTCTGTTATAAGTAAATCAAGTAGATTCATATAGTCGACCTCAACTTGTTTAGGACTGAGTC
GTAACTATTGTTGTAATCAAACAGGAACTTAGCTTCTAATGGATTCTCAGGAACACTTTCACCGTCGATA
ACCAAATTGAAATTTTTGGTTAGCTTGTAAGTATACTGCACTTTGTATTAAAATTAGAGTTACTGGTCAAA
AACACACCTAAAGTATCCCTTTTTTCGAGTTCCCTATCACCAACTATTTATCAAAAACACACCTCAACTATT
AGTTGCTCCCTTTTTCCTACCTGAGATATCACCATCGTTCAGGCAGGAAAAATGAAATGTTGCTAATACTT
GATGTCTCGCGAACCTAGGAAAGTTTTGTGAAGTGTTGCTAAAATGTTAATGGAACTGTGCAGGGATTT
ACAGAACAATGATCTATCTGGTGCCTTACCTGATTACCTTAGCAGCATGTCAAATCTACAAAACTTGAAT
CTTGCAAACAATAATTTCAATGGCCCTATTCCACCAGCTTGGGGTCAGCTCTACCAACTTAAATATTTGTA
AGTACTTGTTTTTCTTTGTTTCTTTGTTCTCTCCGTTTTCCTAAAATATCAAGGAATGTCACTCGGACTCTTC
AAAAATGATGACAGGTTCGTGTCAGTTCCTCCAAAAGCAGTGCATTTTTGGAGGATCCAACACGAGTGT
GACAACAGTTTTGGAGAGTCCTCTCTGACCTTAACATAGCTCTTATTAAATAGTTCTTGTAACAAAGTCTT
TCTATGATATGTCCTTTTGTCCTGCATCAGGGTCATGAGGGGAAATCACATAACTGGAACTGTTCCAAAT

TCACTTACAAATATCACAGGCTTAAAAGAACTGTAAGAACTATGTTTACCTTACGGGCATAATTTACTCTT
TGTTTGAAATTTTCGGTTGGATTGAGGCCTATGTTGCTCAGACTCTTCAAAACTACTATAGTATGTGTGTC
GAACCCTTCAAAAGGTACCATTTTGGAAAGTCCGAGCAACATAGAATGAGTTGTTATTTGATGTTTTCAT
TTATTTCTGGTTGTAGTTCTTACTTGTTTTAATTCTTCACAGGGACCTGTCCTCCAATGATCTGACTGGAG
GTATCCCACTGCAGTTGTTTTCAATACCAACATACAGGTAATGTCATCATTTATTCTTCTTTCTTGCCAACT
TGTTTAGCCTTCCCATTTTGATGTTGAGCTCTCAAAAAGGGCTACTTGATGCACAAAACAGCCATGGGGG
TGTGACATAGGCAGTCTACCCTGGTGCTAGCATCAGTAGCTTATTCCACAGCTCGACCTCATGACCTATA
GGTCATATGGAGACAACTTTACGTTGCTCCAAGGCTCCCTTCAACTGATAAAAAAATGAAAATTTGTTAC
TCATGACTGCATTATTCAACTTGAAGCTATTATTATTGACTTAGTTGCTTAACTATGTTGTTTTAACAATCA
TTCTATACGTCGTAATCAGTTTTTCAGGAACTCATCTTGCTTGTGGCAATAGATTTCAGAAACCCTGTGTT
TCTAGTTCCTCTGCTCCAGGTTAATAGTCTTGTCACCAATGCATCTTTTAGTTCTTAAATCTTTGCCAACCA
AATGATTATTGCTCAAGTGATATTTTTTTCAACAGCTTCTAGCAGAAGACCAAAACTTGAGGTTGCTATTA
CTGGTGCAAGTTGTGGTGCATTCCTTCTTCTCTTAGTTGGGGCTATCGTCACATATCGTTGTAATTATAAA
CGTAAACTCAAACAAGATCAATTTTTTGATGTGGAAGGTATATGCTTTACTCCTATTTCTTCAACAGAAAG
AGTATATGGTTTTTCTGTACTGAACAAATTTCCTTAATAACTTCAAATGCCATCTACTTGTATGGCATTTTA
GGTGACGTTGAGCGCAACATTTCATTAGGCCAACTAAGAAGGTTCTCATGGCGCGAACTTCAGATTGCA
ACGGACAATTTCAGTGAAAGCAACGTTATAGGACAAGGAGGTTTTGGTAAAGTTTACAAGGGCTATCTT
TCAGACAACACAAAAGTGGCAGTGAAAAGACTCACTGATTACCACAATCCTGGTGGAGAGGCTGCATTT
CTAAGGGAAGTGCAGCTGATTAGTGTTGCAGTTCATCGAAATCTCCTACGTTTGATCGGGTTCTGTACAA
CCTCTTCTGAGAGAATTCTTGTTTATCCATTTATGCAGAATCTTAGTGTTGCATATCGGTTAAGAGGTATG
CCCCTTATCACTCAACCACTTTTTTTGACATTCTTGATACTATTCAGTCTGTTAAGTCTGTGTTTGTGTTTTT
TCCTCTCTTTTCGACAGATTTAAAACCTGGAGAGAAAGCTCTAGATTGGCCAACGAGGAGACGGATAGC
ATTTGGAGCAGCACATGGTCTAGAGTACCTACATGAGCATTGTGATCCTAAGATCATTCATAGAGATCTA
AAAGCAGCAAACATCCTCCTAGATGATAACTTTGAACCTGTTCTTGGAGATTTTGGACTAGCAAAGCTAG
TTGATACAAAACTGACTCATATTACAACTCAAGTTCGCGGAACAATGGGCCACATTGCTCCAGAATACTT
GTCTACTGGAAAATCATCTGAGAAAACAGATGTATTCGGATATGGTATTACACTTTTAGAACTTGTAACA
GGGCAACGTGCAATTGACTTTTCGCGTCTTGAAGAAGAGGAAGATGTGTTGCTACTGGATCATGTAAGT
CCCTACTTCCACACACTTTTCAATTTTTGAAGAGTTAGAGCAACATAATGGATACCTTTTTACTTCTACAG
ATCAAGAAACTACTAAGAGAGAAAAGGCTAGGTGACATTGTGGATGGAAACATGAAAACTTATGAACC

CAAAGAAGTAGAGACAATCCTTCAAGTTGCATTGCTATGCACCCAAAGTTCACCTGAGGAACGTCCGAA
AATGGCACAAGTAATCACCATGCTAAAGGGTGTAGGTTTAGCTGAAAAATGGGCAGAGTGGGAGCAAC
TTGAAGAAGTAAGAATTCAACAATTTTCGCTCATGTCGCGACAGTTTATGTGGGCTGAAGACTCAACACA
TGATCAAGAAGCTATACAATTGTCTCAAGCAAGATAAACAAAAGGAAGATGCAGAGAAATGTATGTATA
AGTACTTCTGCAATAAAATTTAAGAGTAGTTTTAGCTTCTGCTGCAGCTAGCTAGTTATGTAACATGTAA
GAGTAATTTTATCATACATCTAGCTAGTTATGTATCATGTAAGAGTAAATTTATCATACGTATATAAGGCG
CTAAGGAGTCTTTTTTTAATTTTACGATGTATATATATAGAGAGAGAGACCATCTATTACTGAATTCACTC
AATTCCTAGCTCGTTGTACTAAAGTTCCTGCTCGATACTAATACTAAAGTTCCAT
>PGSC0003DMG400023550 LRR-RLKs
ATGTTGAGATATCTAGAAGTGGGTAACAATCCGTTGAGCGGCGTTCTACCAAATTCTATTGGGAATCTCT
CATCTACTATTGAAAAAAATCATTTAGTAGATGCACACATCAATGGCCTCATCCCCACAAGTATAGGCAA
CATGAGCGGTCTTACATCCATAGACATTCAAGACAACAACTTGGCAGGGAATATTCCTTCTGAGATTGGT
AAGCTTAAACAACTCCAAGGGTTGTATCTAAGTAACAATAAATTTCAGGGACATATTCCAGAAGCGGTAT
GCCATTTATCTAATTTGGTTAAATTAACTCTGCAAGTTAATGAGCTCTCTGGATTAATTCCAGAATACTTT
GGAAATCTTAGCATGCTACAAAAGCTTTATTTGGCTTCTAATAAATTTTCATCAAAATTTCCGTTGAGCCT
TTGGAAGATTTAGTGGTCTTCTCTATCTAAGTGTGTCACAAAATTCAATAGAGGGAGAACTTCCATCAGA
TATTGGAGAACTGAAAGCCATTGTAGGACTATATCTTTACAGTAACCACTTTTCAGGTGTGATACCAACC
AGATTCGGGGAACTTCAAAACCTGCAATATCTTGACCTATCGAACAATTCATTTTTTGGCAAAAGTCCATT
ATCCTTTGCCAACTTGATAAGCTTGGTATTCTTGAATTTGTCTTTTAATGCCTTGTCTGGTACTATTCCTAT
GTCATTGGAAAAGCTCTCCTACCTTAAAAGCATTAATGTTTCATTTAATGATTTAGAAGGTGAAATACCG
AGTGGTGGCGTGTTTGCAAATTCCACTTTGCAATCATTTCGCGGGAACAAAGGTCTTTGCGGAGTGCAC
ATATTGGATATTCCTGCTTGTGATATCACTAATCCTGGAAAACAATCAAAGCTTAAGGGGGTTGTGCTAA
AAATTGTTACTCCACTGGTTATTTCATCCTTTCTGATATTCTTGTTGGTTTCAATTTGGATAATGAAACGAC
ACAAGAAAGGAAAGTCCAAAGATGTTGAAAAGGTATCAGAGATTAGGACTCACCAATTAGTTTCTTATC
ATGAGATTCAACGAGCAACAAATAATTTTGATGAATCCAATTTAATTGGTGAGGGAAGCTCTGGCTCTGT
GTACAAAGGCATTTTATCTAGTGGAACTGTAGTGGCCATAAAGGTTCTGGATTTGGAAAATGAGCAAGT
TTGCAAGAGGTTTGATACTGAATGCGAAGTGATGAGAAATGTTAGACACAGAAATCTTGTGTAACATCC
GACAATTTGAAATAGCTTTGAAGAGGCTTAGGATTGGAAATTTTCATTTTTGGAAAGGATTTAAAAATCT
GGAAGAATTTGGCTAAGTGTAGAAATCAGTGATTTTTTGGCCAACTTTGAGAAGTCATAACTTCCAGCTC

AGGATGAGTTAGGAGTAGTTCCAGTTATGGTTGTGAAGCTTGTGGAATGATCTTTCCAACGCCGCCGAG
TTTGCTCGATGACGAGTTCGTATGAGTGAGATATGCCCTTTGAAAGTTGGGAAGTTGACAAGGAAATCT
GTCCGGGAATCATTTAGGGCATTTTAGTCTTTTCCCTAGCCAATTCTTTTGGGATTATATTGTTGTATAGG
GATGATCTTTGGATCATTTTGTCCCATTTTAAAGAGAGAAAGTTAGGGTTCTTGAGAGTGAAGAAGAGA
AGAANNNNNNNNNNNNNNNNNNNNNNNNNNNNNNNNNNNNNNNNNNNNNNNNNNNNNNNNNNNNNN
NNNNNNNNNNNNNNNNNNNNNNNNNNNNNNNNNNNNNNNNNNNNNNNNNNNNNNNNNNNNNNNNNN
NNNNNNNNNNNNNNNNNNNNNNNNNNNNNNNNNNNNNNNNNNNNNNNNNNNNNNNNNNNNNNNNNN
NNNNNNNNNNNNNNNNNNNNNNNNNNNNNNNNNNNNNNNNNNNNNNNNNNNNNNNNNNNNNNNNNN
NNNNNNNNNNNNNNNNNNNNNNNNNNNNNNNNNNNNNNNNNNNNNNNNNNNNNNNNNNNNNNNNNN
NNNNNNNNNNNNNNNNNNNNNNNNNNNNNNNNNNNNNNNNNNNNNNNNNNNNNNNNNNNNNNNNNN
NNNNNNNNNNNNNNNNNNNNNNNNNNNNNNNNNNNNNNNNNNNNNNNNNNNNNNNNNNNNNNNNNN
NNNNNNNNNNNNNNNNNNNNNNNNNNNNNNNNNNNNNNNNNNNNNNNNNNNNNNNNNNNNNNNNNN
NNNNNNNNNNNNNNNNNNNNNNNNNNNNNNNNNNNNNNNNNNNNNNNNNNNNNNNNNNNNNNNNNN
NNNNNNNNNNNNNNNNNNNNNNNNNNNNNNNNNNNNNNNNNNNNNNNNNNNNNNNNNNNNNNNNNN
NNNNNNNNNNNNNNNNNNNNNNNNNNNNNNNNNNNNNNNNNNNNNNNNNNNNNNNNNNNNNNNNNN
NNNNNNNNNNNNNNNNNNNNNNNNNNNNNNNNNNNNNNNNNNNNNNNNNNNNNNNNNNNNNNNNNN
NNNNNNNNNNNNNNNNNNNNNNNNNNNNNNNNNNNNNNNNNNNNNNNNNNNNNNNNNNNNNNNNNN
NNNNNNNNNNNNNNNNNNNNNNNNNNNNNNNNNNNNNNNNNNNNNNNNNNNNNNNNNNNNNNNNNN
NNNNNNNNNNNNNNNNNNNNNNNNNNNNNNNNNNNNNNNNNNNNNNNNNNNNNNNNNNNNNNNNNN
NNNNNNNNNNNNNNNNNNNNNNNNNNNNNNNNNNNNNNNNNNNNNNNNNNNNNNNNNNNNNNNNNN
NNNNNNNNNNNNNNNNNNNNNNNNNNNNNNNNNNNNNNNNNNNNNNNNNNNNNNNNNNNNNNNNNN
NNNNNNNNNNNNNNNNNNNNNNNNNNNNNNNNNNNNNNNNNNNNNNNNNNNNNNNNNNNNNNNNNN
NNNNNNNNNNNNNNNNNNNNNNNNNNNNNNNNNNNNNNNNNNNNNNNNNNNNNNNNNNNNNNNNNN
NNNNNNNNNNNNNNNNNNNNNNNNNNNNNNNNNNNNNNNNNNNNNNNNNNNNNNNNNNNNNNNNNN
NNNNNNNNNNNNNNNNNNNNNNNNNNNNNNNNNNNNNNNNNNNNNNNNNNNNNNNNNNNNNNNNNN
NNNNNNNNNNNNNNNNNNNNNNNNNNNNNNNNNNNNNNNNNNNNNNNNNNNNNNNNNNNNNNNNNN
NNNNNNNNNNNNNNNNNNNNNNNNNNNNNNNNNNNNNNNNNNNNNNNNNNNNNNNNNNNNNNNNNN
NNNNNNNNNNNNNNNNNNNNNNNNNNNNNNNNNNNNNNNNNNNNNNNNNNNNNNNNNNNNNNNNNN
NNNNNNNNNNNNNNNNNNNNNNNNNNNNNNNNNNNNNNNNNNNNNNNNNNNNNNNNNNNNNNNNNN

NNNNNNNNNNNNNNNNNNNNNNNNNNNNNNNNNNNNNNNNNNNNNNNNNNNNNNNNNNNNTTGT
TACTCCAGTGGTTATTGCATCCTTTCTGATATTCTTGTTCGTTTCAATTTGGATAATGAAACGACAGAAGA
AAGGGAAGTCCAAAGAGGTGGAGAAGATACCGGAGATGGGGACTCATCAATTAGTTTCCTATCACGAG
ATTCAACGAGCAACAAATAATTTTGATGAATCCAATTTAATTGGTGAGGGCAGCTCTGGCACTGTGTACA
AAGGCACATTGTTTGGTGGAACTGCAGTGGCCATTAAGGTTCTGGATTTGGAAAATGAGCAAGTATGCA
AGAGGTTTGATACCGAATGTGAAGTGATGAGAAATGTTAGACACAGAAATCTTGTTCAAGTGATTACTG
CATGTTCTAGTGACTATATAAGAGCCTTTATTCTGCAATTTATGCCCAATGGAAAGTCTTGAGAATTGTCT
GTACAAAGAAGATCGCCACTTGAACCTTCATCAAAGAGTTTCTGTAATGCTTGATGCAGCTATGGCAGTT
GAATATCTACATCATGGTCATGTCACTCCAATAGTTCATTGCGACTTAAAGCCAGCCAACGTTCTTTTGGA
TGAAGATAATGGTGGCTCATGTTGGTGATTTTGGCATCTCTAAAATTTTAGCCATAAGCAAGTCCATAGC
TTATACCGAAACATTGGGCACTCTTGGATACATTGCACCAGGTATAAAAAATAATCTCTCTTTGATTTTCT
CTTATCATAGTTAAGCATTCTAAAAGTAAAAAAAGCAAGTCTTCATTTATGCAGAATTATTGTTGTATTTC
AATTGAGTAACTTTTCTTCAATCCTTTTCTAAGAATATGGCTCAGAGGGAATAGTGTCTTCTAGTGGTGAT
GTTTATAGCTATGGCATCATGTTGATGGAAGTCTTGACCAAAAGAAGGCCAACAGATGAAGAGATATGC
AATGAAAATCTTGACCTGAGGAAATGGATAATACAATCATTTTCAGGGAGTATGATGGACGTTGTCGAT
GCCAATCTTTTTTACGAGGAAGAACAAATCACTAGTAAAAGTGAAATCTGCATAGCGTCCATGATAGAAT
TGGGTTTAGATTGCACAAAGGAAATGCCAGAATCAAGAGTGACCATGAAAGATGTAGTGAAGAGACTT
AACAAAATCAAAAACGCATTTCTGGAAACATAG
>PGSC0003DMG400023554 LRR-RLKs
ATGTGGAAAGGGCCATGGTATGTACCCGAACTCAGAGTCTTGGATCTCACCAACAATAACCTCACGGGT
ATGATCCCTCCTTCTGTTGGAAATGCCACAAAAATGATGAACTTCAGTTTGTTTGGGAATAGAGTCAGCG
GCAACATTCCAAAGGAGGTCGGTAATATGAGCCAACTTGCAGACTTGTCCTTGACTGATAATCAATTAAC
AGGTTCCATCAGTGGCGAATTTAGAGCCAAAAAAAAGGGCACCCGCACCCATGGTCTTTTCGTAAAATT
AGTATATTTTATATGTATATTTTTTAAGATTTTGTATAATATTATATTCTTGCACCCGTACTACAAGAAGTC
TCATGGTTAAATGTTGAGCTTTTGACCTTGAGGATTAAGGATCAATTTTCACTTCAATACATTTTACTTGG
TGCACCCATGTTCGAAAAATCCTAGATTCGCCTCTGGGTTTCATTCCCCAGAACTGTTTAATATCTCGTCG
CTACTTGCCGTATCTCTGGCAAACAATAGCCTTTCTGGTCCTCTCTTGCTAGATGAAGGCAATATTGTGAC
GAATCTGTACTTTTTAAGTATATCTGACAACCAAATTTCTTGTTGCATTCCTTCCAACATATGCCAACTCAC
AGAGCTCAAAGTTTTGTCCGTATCTTTCAACAATATAACTGGAGACATACCCAAAAATATTGGTTGTTTAT

CCGAGCTCCATGAGTTTTATATTGGTGATAATCTAATAAAAGGGACTATTCGCACTTCATTGGGAAATAT
TTCCACTATGCATAATCTTAATTGTGGAAACTATCGCATAGTGGGGCAAATTCCTCTGGAATTAGGGAAG
CTATCAAATTTGAGGAAATTAAAGTTTGCCCATAATTCTAATCTAATTGGTGAAATTCCAGAGGCTATTTT
CAACATCTCTTCTTTGGAAGGGATCGATTTCAGTTTCAACAACCTCTCAGGGAGAATTCCAACCACTACA
GGTCTTCATCTCCCCAACCTTGAAGGACTTTACTTGGGAGTAAATCAGTTCAAAGGAGAAATTCCATTGT
TTATAACAAATGCTTCCAAGCTTCAGCTACTGGACCTAAGTTATAACTTTCTGACAGGAACTATTCCTACT
AATTTGGGAAATCTTCGTGAGCTGCAAATACTGCTCCTATATAATAATCAACTTACCAATGAATCAAGAG
AGCGTGAGTTGTTATTCTTCAATTCTTTGGCGGACTGTAGGATGTTGAGAGATCTAGAAGTGGGTTCCAA
TCCGTTAAATGGCGTTCTGCCCAATTCAATTGGGAATCTTTCATCTACTATTCATAACTTTAATATAGAAG
ATGCACACATCAATGGCCTCATCCCCAAAAGTATAGGCAACATGACCGGTCTTACAATCCTAAGCCTTAG
AGGAAACAACTTAGCGGGAAGCATTCCTTCTGAGATTGGTAAGCTTAAACAACTCCAAGGGCTGTATCT
AAGTAACAATAAATTGCAGGGACATATTCCAGAGGCGGTATATCATTTATCTAATTTGGTTGAATTAACT
CTGCAAGTTAATGAGCTCTCTGGATTAATTCCAGAATGCTTAGGAAATCTAAGCATGCTACAACATCTTTT
TTTGGATTTTAATAAATTTTCTTCAAAATTTCCGTTGAGCCTTTGGAAGATGAGTGGTCTTCTCTATCTAA
GTGTGTCACGAAATTCAATAGAGGGAGAAGTTCCATCAGATATTGGAGGACTGAAAGCCATTGTAGAA
CTATATCTTTACAATAACCACTTTTCAGGAAGGATACCAAGCACATTCGGGGAACTCCAAAACCTGCAGT
ATATTGACTTGTCGAACAATTCATTTTTTGGCAAAATTCCATTATCCTTTGCCAACTTGATAAGCTTAGAAT
GCTTGGATTTGTCTTCAAATGCGTTGTCAGGTACTATTCCTAAGTCTTTGGAAAAACTCTCATACCTTAAA
AGAATTAATGTTTCATTTAATGATTTAGAAGGGGAAATACCAAGTGGTGGCGTGTTTGCAAATTCCACTT
TGCAATCATTTCTTGGGAACAAAGGTCTTTGCGGAATGCACATACTGGAGATTCCTGCTTGTGCTATCAC
TAATCCTGGAAAACAATCAAAGCTTAAGGAGATTCTGCTAAAAATTGTTACTCCAGTGATTATTGCATCC
TTTCTGATATTCTTGTTGGTTTCAATTTGGATAATGAAACGACATAAGAAAGGGAAGTCCAAAGATGTAG
AGAAGGTACCAGAGATCGGGACTCATCAATTTGTTTCTTATCACGAGATTCGACTAGCAACAAATAATTT
TGATGAATCCAATTTAATTGGTGAGGGAAGCTCTGGCTCTGTGTACAAAGGCACATTGTTTGGTGGAAC
TGCAGTGGCCATAAAGGTTCTCGATTTGGAAAATGAGCAAGTATCCAAAAGGTTTGATACCGAATGCGA
AGTGATGAGAAATGTTAGACACAGAAATNNNNNNNNNNNNNNNNNNNNNNNNNNNNNNNNNNNNNNNN
NNNNNNNNNNNNNNNNNNNNNNNNNNNNNNNNNNNNNNNNNNNNNNNNNNNNNNNNNNNNNNNNNNNNNN
NNNNNNNNNNNNNNNNNNNNNNNNNNNNNNNNNNNNNNNNNNNNNNNNNNNNNNNNNNNNNNNNNNNNNN
NNNNNNNNNNNNNNNNNNNNNNNNNNNNNNNNNNNNNNNNNNNNNNNNNNNNNNNNNNNNNNNNNNNNNN

NNNNNNNNNNNNNNNNNNNNNNNNNNNNNNNNNNNNNNNNNNNNNCTAAAGCCAGCCAACATTCT
TTTGGATGAAGATATGGTGGCTCATGTTGGTGATTTTGGAATCTCTAAGATTTTAGCTGTCAGCAAGTCC
GTGGCACATACAGAGACATTGGGCACTCTTGGATACATTGCACCAGGTATAAAAAATCTACTCTCATTGA
TTTTCTCTTATCATAATTAAGCCTCTCCAAGTTGTAGAAGTAAAAAGAGAACTATTGTTATATTTCAATTG
AGCAACTTTTGGTCAATCATTTTCTAAGAATATGGCTCAGAGGGAATAGTGTCTGCTAGTGGTGATGTTT
ATAGCTACGGAATCATGTTGATGGAGGTTTTGACCAAAAGAAGGCCAACAGATGAAGAGATATGTAATC
AAAATCTTGACCTGAGGAAATGGATAATACAATCATTTTCAGGGAGTATGACCGACGTCGTGGATGCCA
ATATTTTTTCTGAGGAAGAACAAATTACTTGTAAAAGTAAAATGTGCATAGCCTCCATGATAGAATTGGC
TTTAGACTGCACAAAGGAAATGCCAGAATCAAGAATAACCAATGAAGGATGTAGTCAAGAGGCTTAA
>PGSC0003DMG400023587 LRR-RLKs
ATGGAGAAGCACATTTTCTTACTGATTCTTCTACTTGTAATTCAATATTACTCTGTTGCTTCCTCAAATGAA
ACTGATCAACAAGCTCTACTAGCTTTCAAAAATCTTATTACAAGTCCTAATCATTTTTTGGCCAATAATTG
GACCAAAATACTCTTTTGCTCTTGGTTTGGTGTCACTTGTAGTTCAAAAAGGCAAAGGGTTGTGGCCTTG
GCTCTTCCTAATTTGCAAATTCAAGGCACAATTTCCCCGTCTTTGGCCAATCTGTCCTTCCTCAGTGTTCTC
AATCTCGAGAACAACAGCTTCCGCGGTGGCATCCCTTATGGACTTGGCCACTTGCCTCGCTTGCGAGTGA
TTGATATTCAAAACAATCAGCTCAACGAAAGTATACCAACAAGTCTATTTCAAAACCGGAGAGTTCAAGT
AATTTCATTGGCTTTCAATGAACTCAGTGGTGAAATGTGGAGAGGGCCATGGTATGTACCCCAACTCAG
AGTCTTAAATCTCAGGAACAATAGTCTCATCGGTATAATCCCTCCTTCTGTTGGAAATGCCACAAAAATG
ATGAACTTCAGTTTGTCTGGGAATAGATTCAGCGGCAACATTCCTAAGGAGGTCGGTAATCTGAGCCAA
CTTGCAGACATGTCCTTGACTGATAATCAGTTAACAGGTTCCATTCCCGTAGAACTGTTTAATATCTCGTC
ACTACTGTCCATAAATCTGCGACACAATAGCCTTTCCGGCCCTCTCTTGCTTGGTCAAGGGAATATTGTAT
CAAATCTGGAGCATTTAATTATATCTTTCAACCAAATTTCTGGTTGCATTCTTTCCAACATATGCCAACTCA
CAGAGCTCAAAGTATTGTCCATAGTTTACAACAACATAACTGGAGAAATACCCAGAAATATTGATTGTTT
ATCCAAACTCGAGATGTTTCTTATTGGTGGTAATCCAATTAAAGGGACTATCCCCACTTCATTGGGAAAT
ATTTCCACTCTACAATATCTTTATTGTGGAGAAAATCACATAGTAGGGAAAATTCCTCAAGAATTAGGGA
AGCTATCAAAGTTGAGGGTATTAGTCATTGAGGACAATTCTAATCTTATTGGTCAAATTCCAGAGGCTAT
TTTCAACATATCTTCTTTGGAAGTGATTGCTTTCAGTTTCAATAACCTCTTGGGGAGAATTCCAACCACTA
CAGGTCTTCATCTTCCGAACCTTAAAGAACTCTACTTGGGAGACAATCAGCTTGAAGGGGAAATTCCATT
GTTCATAACAAATGCTTCCAAGCTTGAGAAATTGGAGCTAAATGATAACTTTCTGTCAGGAACTATTCCT

ACTAATTTGGGAAATCTTCATGAGCTGCGATATCTCTTCCTACATCATAATCAACTAACCAATGAACCAAG

AGAGTGTGAGTTGTTATTCTTCAATTCATTGGCGGACTGTAAGATGTTGAAATATCTACAAGTGGGTTTC

AATCTGTTGAATGGCGCTCTGCCAAATTCTATTGGGAATCTTTCATCTACTATTCAAAACTTTCATATAGG

ACATGCACACATCAACGGCCTCATCCCCACAGGTATAAGCAACATGAGCGGTCTAATGACCCTATACTTT

CAAGGAAACAACTTGACAGGGAGTATTCCTCCTGAGATTGGTAAGCTTAAACAACTCCAAGGTCTGTAT

CTACGTTACAATAAATTGCAGGGACATATTTCTGAGGCAGTATGCAATTTATCTAATTTGGTTCAATTATA

TCTGGAACATAATGAGCTCACTGGAGTGATCCCAGAATGTATAGGAGATCTAAGCTTGCTACAACATCTT

TATTTGGATTCTAATAAATTTTCATCAAAATTTCCGTTGAGTCTTTGGAAGATGAGTGGTCTTCTCTATCT

AAGTGTGTCACGAAATTCTTTAGAGGGAGAAGTTCCATCAGATATTGGAGAACTGAAAGCCATTGTAGG

ACTATATCTTTGCAGTAACCACTTTTCAGGCGTGATACCAACCAGATTCGGGGAACTCCAAAACCTGCAG

TATCTTGACCTATCCAACAATTCATTTTTTGGCCAAATTCCATTATCCTTTGCCAACTTGATAAGCTTGGAA

TTCTTGAATTTGTCTTCAAATGCATTGTCAGGTACTATTCCCAAGTCTTTGGAAAAACTCTCATACCTTAAA

AGCATTAATGTTTCATTTAATTATTTAGAAGGTGAAATACCGAGTGGTGGCGTGTTTGCAAATTCCACTC

TTCAATCATTTCTCAGGAACAAAGGTCTATGTGGAATGCACATATTGGAGATTCCTGCTTGCGCTATCAA

TAATCCTGGAAAACAATCAAAGCTTAAGGAGGTTCTGCTGAAAATTGTTACTCCAGTGGTTATATCATCC

TTGTTGATATTCTTGTTGGTTTTAATTTGGATAATGAAACAACAAAATAAAGGAAAGTTCAAAGATGTTG

AAAAGGTACGGGAGATTGGGACTCATCAGTTAGTTTCTTATCATGAGATTCAACTAGCAACAAATAATTT

TGATGAATCCAATTTAATTGGTGAGGGAAGCTCTGGTTCTGTTTACAAAGGCACATTATCTAGTGGAACT

GTAGTGGCCATTAAGGTTCTGGATTTGGAAAATGAGCAAGTATGCAAGAGGTTTGATACCGAATGCGA

AGTGATGAGAAATGTTAGACACAGAAATATTGTTCCAGTTATTACTACTTGTTCTAGTGACTATATAAGA

GCCTTTGTTCTGCGATATATGCCCAACGGGAGTCTTGAGAATTGGTTGTACAGAGAAGATTGCCACTTGA

ATCTTCATCAAAGAGTCACCATAATGCTTGACGTGGCAATGGCAATTGAATATCTACATCATGGTCATGT

CACTCCAATAGTTCATTGCGACTTAAAGCCAGCCAACGTTCTTTTGGATGAAGATATGGTGGCTCAAGTT

GGTGATTTTGGCATCTCTAAAATTTTAGCCATAAGTAAGTCCATGGCACATACCGAGACATTGGGAACTC

TTGGATACATTGCACCAGGTAGACTACTTTCTTTCTTTCTCACTTATATAATAACCTTTCTATTTGATTTTTT

TCTTATCTAAATCAAGCCCTTGCAAATACTAGAACTAAATAAAAAGCATGTCTTTGTTAGTACGCGCACTA

TTGTTATATTCAAATGAGTAATTCTTTTTCATTTTCATTTGTTTAAGAATATGGCTCAGAGGGAATAGTGT

CCACTAGTGGTGATGTTTATAGTTATGGCATCATGTTGATGGAGGTTTTGACCAAAAGAAGGCCAACAG

ATGAAGAGATATTCCATGGAGATCTTGACTTGAGGAAATGGATAACACAATTATTTTCAGGGACTATGA

TGGACGTTGTGGATGCCAATATTTTTTCCGAGGAAAAACAGATCACTTCCAAAAGTGAAATCTGCATCGT
GTCCATGATAGAATTGGCTTTAGACTGCACAAAGGAAATGCCAGAATCAAGAATAACCATGAAAGAAGT
AGTCAAAAGGCTTAACAAAATCAACAACACATTTCTGGAAACATAG
>PGSC0003DMG400023612 LRR-RLKs
ATGGAGGGGCAAATTCCTCCGGAATTAGGGAAGCTATCAAATTTGAGGAAATTAAGCTTTTCAGAGAAT
TATAATTTTATTGGTCAAATTCCAGAGGCTATTTTCAACATATCTTCTTTGGAAATGATTGATTTCAGTAAC
AATAACCTCTCGGGTAGAATTCCAACCACTACAGGTCTTCATCTTCCTAACCTTAAAGTACTTGAGTTGGG
AGTCAATCAGCTGGAAGGGGAAATTCCATTGTTCATAACAAATTCTTCCAAGCTTCAGATACTATCGCTA
ATTGATAACTTTCTCACAGGAACTATTCCTACTAATTTAGGAAATCTTCGTGAGCTGCAAGAACTGTTCCT
ACATACTAATCAACTTACCAATGAACCAAGAGAGTATGAGTTGCGATTTTTCAATTCTTTGGCGGACTGT
AGGATGTTGAAATATCTACAAGTAGGTTCCAATCTGTTGTATGGCATTCTGCCCAATTCTATTGGCAGTCT
TTCATCTACTATTCAAAAATTTGAAATAGGAGATGCACACATCAATGGAATCATCCCCACAAGTATAGGC
AACATGACCGGTCTTACATCCTTAAGCCTTGGCGGAAACAACTTGACAGGGAATATTCCTTCTGAGATTG
GTAAGCTTAAACAACTCCAAGGGTTGTATCTAGATAACAATAAATTGCAGGGACATATTCCAGAGGCAG
TATGCCATTTATCTAATTTGGTTCATTCTTATCTAGATAATAATGAGCTCTCTGGACTAATTCCAGAATGCT
TAGGTAATCTTAGCATGCTACAAAAGCTTTTTTTGAGTTCCAATAAATTATCAAAAATTCCAGGGAACCTT
TGGAAAATGAGTGGTCTTCTCTATCTAAGTGTGTCACAAAATTCTATAGAGGGAAAAATTCCATTAGATA
TTGGAGAACTGAAAGCCATTGTAGAAATACATCTTTCTGGTAACCACTTTTCAGGCATGATACCAAGCAC
ATTCGGGGCACTCCAAAACCTGCAGTCTCTTGACCTATCAAACAATTCATTCTTTGGCCAAATTCCATTAT
CCCTTGCCAACTTGATAAGCTTGGAATTCTTGAATTTGTCTTTAAATGCATTGTCAGGTAGTATTCCTATG
TCTTTGGAAAAACTCTCATACCTTAAAAGCATTAATGTTTCATTTAATGATTTAGAAGGTGTAATACCAAG
TGGTGGTGTGTTTGCACATTCCACTGTGCAATCATTTCTGGGGAACAAAGGTCTTTGCGGAATGCACATA
TTGGAGATTCCTGCTTGTGCTATCACTAATCCTGGAAAACAATCAAAGCTTAAGGAGGTTCTACTAAAAA
TTGTTACTCCTGTGGTTATTTCATCCTTGCTGATATTCTTGTTGGTTTCAATTTGGATAATGAAACGACAG
AAGAAAGAAAAGTGCAAAGATGTTGAAAAGGTACTAGAGATCAGGACTCATCAATTAGTTTCTTATTAC
GACATTCAACAAGCAACCAATAATTTTGATGAATTCAATTTAATTGGTGTGGGAAGTTCTAGCACTGTGT
TCAAAGGCACATTATCTAGTGGAACTGCAGTGGCCATTAAGGTTCTGGATTTGGAAAATGAGCAAGTAT
GCAAGAGGTTTGATACCGAATGTGAAGTCATGAGAAATGTTAGACACAGAAATCTTGTTCCAGTGATTA
CTACTTGTTCTAGTGACTATATAAGAGCCTTTGTTCTGAAATTTATGCCCAATGGAAGTCTTGAGAATTGG

TTGTACAAAGAAGATCGCCACTTGAACCTTCATCAAAGAGTTACTGTAATGCTTGATACAGCTATGGCAG
TTGAATATCTACATCATGGTCATGTAACTCCAATAGTTCATTGCGACTTAAAGCCAGCCAACGTTCTTTTG
GATGAAGATATGGTGGCTCATGTTGGTGATTTTGGTATCTCTAAAATTTTAGCCATAAGCAAGTCCATGG
CTTATACAGAGACATTGGGTACTCTTGGATACATTGCACCAGGTATAAAAAATCTACTCACTTTGATTTTC
TTTTATCATAATAAAGCCTCTCCAAATTCTACAACTATAAAAACAAGCTTTTATTTATGCAGAATTATTGTT
GTATTTCAATTGAATTAACTTTTTTCAATCCTTTTTAAGAATATGGCTCGGATGGAATAGTGTCTGCTAGT
GGCGATGTTTATAGTTACGGCATCATGTTGATGGAGGTTTTGACGAAAAGAAGGCCAACAGATGAAGA
GATATGCAATGAAAATCTTGACTTGAGGAAATGGATCACACAATCATTTTCAGGGAGTATGATGGACGT
TGTGGATGCCAATCTTTTCTCCGAGGAAGAACAGATCACTTCAGAAAGTGAAATCTGCATAGCGTCCAT
GATAGAATTGGGTTTAGACTGCACAAAGGAAATGCCAGAATCAAGAATAACCATGAAAGATGTAGTCA
CGAGGCTTAACAAAATCAACAACACATTTCTGGAAACATAGAAGTCA
>PGSC0003DMG400023614 LRR-RLKs
ATGCCTAGAAATATTGGTTGTTTATCCAAGCTCGAGAAGTTCTTGATTGGCAATAACCCAATAAAAGGGA
CTATTCCCACTTCATTGGGAAATATTTCCACTCTGCAATATATTTATTGTGGAAGCAGTCGCATAGTGGG
GCGAATTCCTCCGGAATTAGGGAAGCTATCAAATTTGAGGCAATTAAGCTTTGACAACAATTCTAATCTT
ATTGGTCAAATTCCAGAGGCTATTTTCAACATTTCTTCTTTGGAACTGATTGGATTTGCTTTCAACAACCTC
TCCGGGAGAATTCAAGCTACTACAGCTATTCATCTTCCGAACCTTAAAGAACTTTTCTTGGGAGTCAATC
AGCTGGAAGGGGAAATTCCATTGTTCATAACAAATGCTTCCAAGCTTGAGACACTGTCGCTAGCACGGA
GCTTTCTGACAGGAACTATTCCCACTAATTTGGGAAATCTTCGTGAGCTACGAAATCTGTTCCTATATGAT
AATCAACTTACCAATGAACCAACAGAGCGTGAGTTGAGATTCTTCAATTCTTTGGCGGACTGTTGGATGT
TGCGATATATATCAGTGGGTTCCAATCCGTTGAATAGCGTTCTGCCCAATTCTATTGGGAATCTTTCATCT
ACTATTGAATACATTTATATAGGAGATGCACACATCAACGGCCCCATCCCCACAAGTATAGGCAACATGA
GCGGTCTTACATCCCTAGTCTTTAATGAAAACAACTTGATGGGGAGTATTCCTCCTGAGATTGGTAAGCT
TAAACTACTCCAAGGGCTGTATCTATTAAACAATAAATTGCAGGGACATATTCCAGAGACGGTATGCCAT
TTATCTAATTTGGTTCAATTATATCTGGACGGTAATGAACTCTCTGGATTAATTCCAGAATGCTTTGGAAA
TCTTAGCATGCTACAACATCTTTATTTGGATTCTAATAAATTTTCATCAAAATTTTCGTTGAGCCTTTGGCA
CGAAATTCTTTAGAGGGAGAAGTTCCATCAGATATTGGAGGACTGAAAGCCATTGTAGAACTACATCTT
TCCAATAACCACTTTTCAGGCATGATATCAACCAGATTCGGGGAACTCCAAAATCTGCAGTATCTTGACC
TATCGAACAATTCATTTTTTGGCCAAATTCCATTATCCTTTGCCAACTTGATAAACTTGGAATTCTTGAATT

TGTCTTTAAATGCATTGTCAGGTAGTATTCCTAAGTCTTTGGAAAAACTCTACTACCTTAAAAGCATTAAT
GTTTCATTTAATGATTTAGAAGGTGAAATACCGAGTGGTGGCGTGTTTGCAAATTCCACTCTGCAATCAT
TTCTTGGGAACAAAGGTCTATGTGGAATGCACATATTGGAGATTCCTGCTTGCGCTATCAATAATCCTGG
AAAACAATCAAAGCTAAAGGAGGTTCTGCTAAAAATTGTTACTCCGGTGGTTATATCATCCTTTCTGATA
TTCTTGTTGGTTTCAATTTGGATAATGAAACGACAAAAGAAAGGGAGGTCCAAAGATGTGGAGAAGGT
ACTGGAGATCGGGACTCATCAATTAGTTTCTTATCACGAGATTCAACGAGCAACAAATAATTTTGATGAA
TCCAATTTAATTGGTGAGGGAAGCTCTGGCTCTGTGTACAAAGGCACATTGTTTGGTGGAACTGCAGTG
GCCATAAAGGTTCTCGATTTGGAAAATGAGCAAGTATCCAAAAGGTTTGATACCGAATGCGAAGTGATG
AGAAATGTTAGACACAGAAATCTTGTTTCAGTGATTACTACGTGTTCTAGTGACTATATAAGAGCCTTTG
TTCTGCGATATATGCCCAATGGAAGTCTTGAGAATTGGCTGTACGAAGAAGATCGCCACTTGAACCTTCA
TCAGAGAGTCACTGTAATGATTGATGCAGCTATGGCAGTTGAATATCTACATCATGGTCATGTCACTTCA
ATAGTTCATTGCGACTTAAAGCCAGCCAACATTCTTTTGGATGAAGCTATGGTGGTTCATGTTGGTGATT
TTGGAATCTCTAAAATTTTAGCCATAAGCAAGTCCATAGCTTATACGGAGACATTGGGCACTCTTGGATA
CATAGCACCAGGTATAAGAAAACTACTCTCTTTGATATTCTATTATCATAATTAAGCCTCTCCAAATTCTA
GAAGTAAGAAAAAAAGCAAGTCTTTATTTATGCAGAATTATTGTTGTATTTCAATTAACTTTTGTTGAATC
GGCCTTTTCTAAGAATATGGCTCAGAGGGAATAGTGTCTACTTGTGGTGATGTTTATAGCTATGGCATCA
TGTTGATGGAGGTTTTGACCAAAAGAAGGCCAACAGATGAAGAGATATGCAATGAAAATCTTGACTTGA
GGAAATGGATCACACAATCATTTTCAGGGACTATGATGGACGTTGTGGATGCCAATATGTTTTCTGAGG
AAGAACAAATCACTTGTAAAAGTGAAATATGCATAGCCTCCATGATAGAATTGGCTTTGGACTGCACTAA
GGAAACGCCAGAATCAAGAGTGACCATGAAAGAAGTAGTGAAGAGGCTTAACAAAATCAACAACACAT
TTCAAGAATCATAG

>PGSC0003DMG400023638 LRR-RLKs

ATGAAGAAGCACATTTTTATATTGACTCTTCTCTTTCTAGTTCAATTTTCTATATCAATTGCCTCCTCAAAT
GAGACGGACCAAGAAGCTCTACTAGCTTTCCAAAATCTTATTACAAGTCCGAGTCATTTTTTGGCCAATA
ATTGGACCAAAAATACTTCTTTTTGCTATTGGTTTGGTGTCACTTGCACTCGAAAAAGGCAAAGGGTTGT
GGCCTTGACTCTTCCTGATTTGCAACTTCAAGGCACAATTTCCCCGTCTTTGGCCAATTTGTCCTTTCTCAG
CGTGCTCAATCTCCGGAACAACAACTTCCATGGCGGCATCCCTTACGGACTTGGCCACTTGCCTCGCTTG
CGAGTGATTGATTTTCAAAACAATCAGCTCCAAGAAAGTATTCCAACAAGTCTATTTCAACACCAGAGAG
TTCAAATCATTTCATTGGCTTTCAATAAACTCGGTGGTGAAATGTGGAAAGGGCCATGGTATGTACCCGA

ACTCACAGTTTTAAATCTCAGGAACAATAGCCTCACAGGTATAATACCTCCTTCTATTGGAAATGCCACAA

AGTTGCTGAACTTCAGTTTGTATGGGAATAGAGTCAGCGGGAACATTCCAAAGGAAATCGGTAATCTCA

GCCAACTTGAATTTTTGTCCTTGTTCGATAATCAATTAACAGGTTCTATTCCTGCAGCACTATTTAATATCT

CCTCGCTTCTTGTCGCATCTCTAGCATTCAATAGCCTTTCCGGCCCTTTCTTGCTTGATGAAGGGAACGTT

GTGTCAAATCTAGAGTCATTAACTATATCTAACAACCAAATTTCTGGTTACATTCCTTCCAACATTTGCCA

ACTCACAGAACTCAAAGCGTTGTCCATATCTTTCAACAAGATAACTGGAGGAATACCCAAAAATATTGGT

TGTTTATCCAATCTCGAGGAGCTTTATATTGGTGACAATCCAATAAAAGGGACTATTCCCGCATCATTGG

GAAATATTTCCACTCTGCAAAATCTTTATTGTGGAAGCAATCACTTGGAGGGGCCAATTCCTCCAGAATT

GGGGAAGCTATCAAATTTGAGGCAAATTAGCTTTGTCCAAAATAATAATCTTATAGGTCATATTCCAAAG

GCTATATTTAATATATCTTCTTTGGAAATAATCGATTTCAGTTACAATAACCTCTCGGGGAGAATTCCAAC

CACTACAGGTCTTCATCTTCCAAACCTTAAAGAACTTTTCTTGGGAGTCAATAAGATCCAAGGGGAAATT

CCATTGTTCATATCAAACGCTTCCATGCTTGAGATACTGGGGCTAAACAGGAACTTCCTCACAGGCAATA

TTCCTACCAATTTAGGAAATCTTCGTGACCTGCGACGCTTATACTTAGAGGGTAATCAACTTACCAATGA

ACCAAATAATCATGAGTTGCAATTCTTTAATTCTTTAGTGGACTGTAGGAAGCTGCAATATCTAACAGTG

GGTAACAATCCATTAAATGGTATTTTGCCCGATACTATTGGAAATCTTTCATCTACTATTGAATCAATTCA

CATGGGAAATGCACAAATCAGTGGCCTCATTCCCACAGGTATAGGAAACTTAAGCGGTCTAATGTCGCT

AGCTTTTGTGGAAAACAACTTGATGGGAACCATTCCGTCTGAAGTCGGTAAGCTTGAACATCTCCAAGG

TCTGTATCTATATAGCAACAAATTGCAAGGGAATATTCCAGAGGTAGTTTGTCATTTATCTTATTTGGTTA

CATTATCTTTGCATGTTAATGAGCTCTCTGGGGTGATCCCAAAATGTTTAGAAAATCTTACCACGCTACGA

GTTCTTTCATTGAGTTCTAACAAATTTTCATCAAAGCTTCCTTTGAGTCTTTGGAAGATGAGTGGACTTCT

CTATCTTTTCATGTCACGAAATTCTATAGAGGGAGAAGTTCCACAGGACATTGGAGGATTGAAGGCCAT

TGTAAAACTAGATCTTTCTGGTAACCACTTTTCCGGCATGATACCAAGCCAATTGGGGGACCTCCAAAAC

ATGAAAGTTCTTGACCTGTCAAACAATTCATTTTCAGGCTCAATTCCCTTATCCTTTGCCAATTTGATAAGT

CTAGAATACTTGAATTTGTCTTTCAACGCATTGTCAGGTACTATTCCTAAGTCTTTGGAAAAGCTTTCATA

CCTTAAAAGCATCAATGTCTCATTTAATGATTTAGATGGTGAGATACCTAGTGGAGGTGTGTTTTCAAAT

TCCACTTTGCAATCATTTCTTGGGAACAAAGGTCTATGTGGAGTACACATATTGGAGATTCCTGCTTGTG

CTATCACTAATCCTAGAAAACAATCAAAATCTAAGGAGCTTGTGCTGAAAATTGTTATTCCAGTGGTTAC

TTCATCCTTTCTGATATTATTGGTTGTGTCAGCTTGGATAATAATGAAACAAAAGATGAAAGGAAAGTCC

AAAGACTTGGAAAAGGTACCAGAGATCAGGACTTATCAATTGGTTTCTTATCATGAAATTCAACGAGCA

ACAAATAATTTTGATGGATCCAATTTAATTGGTACGGGAGGTTCTGGCTCTGTGTACAAAGGTACATTAT
CTAGTGGAACTGTGGTGGCAATAAAGGTTCTCGATTTGCAAAATGAGGAAGTATGCAAGAGGTTTGATA
CTGAATGTGAAGTGATGAGAAATGTTAGGCACAGAAACCTTATTTCAGTGATTACTACTTGTTCTAGTGA
ATACATAAGAGCCTTTGTTCTACAGTATATGCCCAATGGAAGTCTTGATAATTGGTTGTACAAAGAAGAT
TGCCACTTGAACCTTCTTCAAAGAGTCAACATAATGCTTGATGTGGCTGTGGCAATTGAATATCTACACC
ACGGTCATGATACTCCAATAGTTCATTGCGACCTAAAGCCAGCCAACATTCTTTTGGATGAAGAAATGGT
GGCACATGTTGGTGATTTTGGCATCTCTAAAATATTAGCTGTAAGCAAGTCCATGGTACATACTGAGACA
TTAGGCACTTTTGGTTACATTGCACCAGGTACGTACACTAGTTTCGTTCTTTTTCACTTATCATTAACCCTT
TCTCTTATCAGAACTAAGCCCTTCCGAATTCTGGAACTAAACAGAAACCAAGTCTTTCATTGTACAAAACA
ATTATTATCTTCCGATTGTAATTTATTTTTCATTTTCATTCTCCTAAAGAATATGGCTTGGAAGGAGTAGTG
TCCATTAGTGGTGATGTTTACAGCTATGGCATCATGATGATGGAGGTATTGACAAAAAGAAGACCAACT
GAAGACGAGATATTCAACGAAAATCTTGGCTTGAGGCAGTGGATAAGACGAGCATTTCCCGGGACAAT
TATGGAAGTCGTAGATGCCAATCTTTTTCATGAGGAAGAATCTGTCAATTTCAAAAGTGAAATATGCATA
GCCTCCATGATGGAATTGGCTTTGGATTGCACAAAGGAAATGTCAGAGACAAGGATAACTATGAGAGA
CGTAGTCAAGAGGCTTAGCAAAATCAAAAACACATTTTTAGGAACATAG

>PGSC0003DMG400023639 LRR-RLKs

ATGGAGAAGCACATTTTTTTATTGACTCTTCTCTTTCTAGTTCCATTTTCTATATCAATTGCTTCCTCAAATG
ATACGGACCAAGAAGCTCTACTAGCTTTTCAAAATCTTATTACAAATCCGAATCATTTTTTGGCCAATAAT
TGGACCAAAAATACTTCTCTTTGCTCTTGGTTTGGTGTCACTTGCAGTTCAAAAACGCAAAGGGTTGTGG
CCTTGGCTCTTCCTAATTTGCAACTTCAAGGCACAATATCCCCGTCTTTGGCCAATTTGTCCTTTCTCAGAG
AGCTCAATCTCGAGAATAACTTATTCCATGGTGGCGTACCATACAGACTTGGCCACTTGCCTCGCTTGCG
AGTGATTATTGTTCGAAACAATCAGCTAGAAGGAAGTATTCCGACAAGTCTATTTCAACACCAGAGAGTT
CAAATCATTTCATTGGCTTACAATAAACTCAGTGGTGAAATGTGGAAAGGGCCATGGTATGTACCCGAA
CTCAGAGTCCTAAGTCTCAGGAATAATAGCCTCACAGGTATAATCCCTTCTTCTGTTGGAAATGCCACAA
AGTTGCTGAACTTCAGTTTGTCTGGGAATAGAATCAATGGTATCATTCCAACTGAGATTGGTAATCTTAG
CCAACTTATAGAGTTGCATTTGTTCAATAATCAATTAGCAGGTTCCATTCCCGCAACACTGTTTAATATTT
CGTCGCTAATTAGAGCATCTCTAGCAAGCAATAGCCTTTCTGGTCCTCTCTTACTTGATGAAGGGAATATT
GTGTCAAATCTGAAGTATCTAAGTATATCTAAGAACCAAATTTCTGGTTGCATTCCTTCTAACATATGCCA
ACTCACAGAGCTCAAAATTTTGTCCATATCTTATAACAATATGATTGGAAAAATACCCAGAAATATTGGTT

GTTTATCCAAAATCGAGGAGTTTTATATTGGAAATAATCCAATAACAGGGACTATTCCCACTTCATTGGG

TAATATTTCCACTCTGCGGAATCTTTATTGTGGAAACAGTCGCATAGTAGGGCAAATTCCAAAGGCTATT

TTTAACCTATCTTCTTCGGAAATGATTGATTGCAGTTACAGTAACCTCTCGGGTAGAATTCCAACCACATC

AGGTCTTCATGTTCAGAACCTTAAAGAACTTTTCTTGGGACACAATCGGCTCGAAGGGGAAATTCCATTG

TTCATAGCAAATGCTTCCAAGCTTGAGATATTGGGGCTAGAAAATAACTTTCTCACTGGCACTATTCCTAC

TAATTTGGGGAATCTCCGTGAGCTGCAAGAACTGTTCCTACATGATAATCAACTTACCAATGAACCAAGA

GAGCATGAGTTGCAATTCTTCAATTCTTTGGCGGACTGTAGGATGTTGCGATATCTACAAGTGGGTTCCA

ATCCGTTGAATGGCATTCTGCCCAATTCTATTGGGAATCTTTCATCTACTATTGAAAACTTTCATATAGAA

GATGCACACATCAATGGCCCCATCCCCAGGGGCTTACTCAACATGAGCGGTCTAATAGCCCTAAACCTTG

GAGAAAACAACTTGGCGGGAAGTATTCCTTCTGATGTTGTTAAGCTTGAACAACTCCAAGGTCTGTATCT

AAATAACAATAAATTGCAGGGACATATTCCAGAGGCAGTATGCCATTTATCTAATTTGGTTCAATTAAGT

CTGGGTGGTAATGAGCTCTTTGGATTAATTCCAGAATGTTTAGGAAATCTTAGCATGCTACAAGCCATTA

TGTTGAGTTCTAACAAATTTTCATCCAAGATTCCTTTGAGCATTTGGAAGATGAGTAGTCTTCTCTATCTA

ATCATGTCACAAAATTCTATAGAGGGAGAAGTTCCACAGGATATTGGAGGACTGAATGCCATTGTAGGA

CTAGATCTTTCTGGTAACCACTTTTCAGGCATGATACCAAGCCAATTGGGGGACCTCCAAAACATGAATA

CTCTTGACCTGTCAAACAATTCATTTTCAGGCTCAATTCCATTATCCTTTGCCAACTTGATAAGCTTAGAAT

ACTTGGATTTGTCTTTAAATGTGTTGTCAGGTACTATTCCCAAGTCTTTGGAAAAGCTATTATACCTTAAA

AGCATTAATGTTTCATTTAATGATTTAGAAGGTGTAATACCCAGTGGTGGTGTGTTTGCGAATTCCACTCT

GCAATCATTTATTGGGAACAAAGGTCTATGTGGAATGCACATAATGGAGATTCCTGCTTGTGCTATCACT

ACAACTGGACAACAATCAAAGTCTAAGAAGCTTGTGCTTAAAATTGTTATTCCAGTGGTTGCTGCTTCTTT

TCTGATATTTTTGTTTGCTATAGTTTGGATAATGAAACGACAGAAGAAAGCAAACTCCAAAGATGTGGA

AAAGGTACCGGAGATCAGGACTTATCAATTGGTTTCTTATCACGAGATTCAACAAGCAACAAATAATTTT

GATGGATCCAATTTAATTGGTGTGGGAGGTTCGGGCTCTGTGTACAAAGGTATATTATCTAGTGGAACT

GTGGTGGCAATAAAGGTTCTCGATTTGCAAAATGAGGAAGTATGCAAGAGGTTTGATACTGAATGTGA

AGTGATGAGAAATGTTAGGCACAGAAATCTTATTCCAGTGATTACTACTTGTTCTAGTGAATACATAAGA

GCCTTTCTTCTACAGTATATGCCCAATGGAAGTCTTGAGCGTTGGTTGTACAGAGAAGATCGCCACTTGA

ACCTTCTTCAAAGAGTCACCATAATGCTTGATGTGGCTCAGGCAATTGAATATCTACACCATGGTCATGA

AACTTTGATAGTTCATTGCGACCTAAAGCCAGCCAATGTTCTTTTGGACGAAGAAATGGTGGCACATGTC

GGTGATTTTGGGATCTCAAAAATATTAGCTGCAAGCAAGTCCATGGCACATACTGAGACATTAGGCACT

CTTGGTTACATTGCACCAGGTACGTACACTAGTCTTATCCCTTTTCACTTATCATTAACCTTTTCTCTAATC
AAAATTAAGCTCTTTCGATTTCTAGAAATATATAAAAATCAAGTCTTTGATTGTAAAAAACAATTATTATC
TTTCGACTATAATTTATATTTTTTTCATTCTTCTAAAGAATATGGCTTGGAAGGAAGAGTGTCCAGTAGTG
GTGATGTTTACAGCTATGGCATCATGATGATAGAGGTATTGACAAAAAGAAGACCAACTGATGACGAGA
TATTCAATGAAAATCTTGGCTTGAGACAGTGGATAAGACAATCATTTCCCAAGACAATTATGGAAGTTGT
GGATGTCAATTTTATTCATGAGGAAGAACATTTCAATTCAAAAAGTGAAATATGCATAGGCTCCATGATG
GAATTGGCTTTGGATTGCACAAAGGAAATGCCAGAGTCAAGGATAACAATGAGAGACGTAGTCAAGAG
GCTTGACAAAATTAAGAACACATTTTTGGGAACATAGAAATTAGCATCTTTTAAAGTGGTGTTCTTTTTTA
GTTGAAGTTAATATGTTGATCAGCTTTTTCTGTTTAAATGGTTTATATAAGGAATGAAGTTCGAGGATTA
AAATGGAGCAAGGTTGGGATGGAGTACCAAAATGAAATTTTGGTGCAAGTTAGAGGGGCTATCTATGT
ATTTGACCTTGTGTGAATGTACG
>PGSC0003DMG400023643 LRR-RLKs
ATGGAGAAGCACATTTTCTTATTGATACTTCTCTTTCTACTTCAATTTCCTATATCAATTGCTTCCTCAAAT
GATACTGACCAACAAGCCCTACTAGCTTTCCAAAATCTTATTACAAGTCCCAATCAATTTTTGGCTAATAA
TTGGACCAAGAATGCATCTTTTTGCTCTTGGTTCGGTGTCACGTGCAGTGCAAGAAGGCTAAGGCTTGT
GGCCTTGGCTCTTCCTAATTTGCAACTTCAAGGCACAATTACCGGGATTAGGGCATTGCTTGTCAGAGAG
CTCAATCTCGAGAACAACTTATTCAATGGGGGCATCCCTTACGGACTTGGCAAGTTGCCTCGCTTGAGAG
TCATTGATTTTCAAAACAATCAGCTCCAAGGAAGTATTCCAACAAGTCTATTTCAACACCAGAGAGTTCA
AATCATTTCATTGGCTTTCAATGAACTCAGTGGTGAAATGTGGAAAGGGCCATGGTATGTACCAGAACT
CAAAGTCTTAGATCTCGCGAACAATAGCCTCACGGGTATAATCCCTCCTTATGTTGGTAATGCCACAAAA
TTGATGAACTTCGATTTGTCTGGGAGTAGAATCAATGGTACCATTCCAATGGAAATTAGTAATTTGAGCC
AACTTGCATCTTTGTCCTTGGTTGATAATCAGTTAACAGGTTTTATTCCTGGATCACTGTTTAACATTTCAT
CACTACTTGGCGTAACTCTGGGATTGAATCACCTTTCTGGTCCTCTCTTGCTTGATGAAGGGAATATTGA
GTCAAATCTGAAGATACTAAGTATATCTTTCAATCAAATTTCTGGTCGCATTCCTTCTAACATATGCCAAC
TCACACAGCTCAAAATTTTGTCCATATCTTTCAATAACATAACTGGAGACATACCCAAAAATATTGATTGT
CTAGCCAAGCTCGAGATGTTCTTAATTAGTGAGAATGCAATAAGTGGAACTATTCCTACTTCATTGGGCA
ATATTTCCACTCTGCAACATCTTGATTCGGGGGACAATCGGTTGGAGGGACAAGTTCCTCAAGAATTAG
GGAAGCTATCAAATTTGAGGGTGTTAAACTTTCACAAGAATTTCAATCTTATTGGTCAAATTCCAAAGGC
TATTTTCAATATATCTTCTTTGGAAGCGATTGATTTCAGTTTCAATAACCTCTCAGGTAGAATTCCAGCCA

CTACCGATCTTCATCTTCCGAACCTTAAAGAGTTTATGTTGGCATTCAATCAGCTCGAAGGAGAAATTCCT
CAATACATAGCAAATGCTTCCAAGCTTGAGATACTGGCGCTAAATTATAACTTTCTGACAGTTGCTATTCC
TACTAATTTAGGAAATCTTCGCGAGCTGCAATCACTGTTCCTAGATCATAATCAACTTACCAATGAACCAA
GAGAGCCTAAGTTGTTATTCTTGAATTCTTTGGTGAACTGTAGGATGCTGAAATATGTAGATGTGAGTTT
CAATCCGTTGAATGGCGTTCTTCCCAATTCTATTGGGAATCTTTCATCTACTATTGAAGTATTTGATATAA
GAAATGCACACATCTATGGCCTCATTCCCACAAGTATAGGCAACATGAGCGGTCTTATATCCCTAGTCTT
TAATGAAAACAACTTGACAGGATATATTCCTCCAGAGATTGGTAAGCTTAAACAACTCCAAGGTCTGTAC
CTATTTAACAACAAATTGCAGGGACATATTCCAGAGGCGGTATGCCATTTATCTAATTTGGTCGAATTAG
AAATGGATGGTAATGAACTCTCTGGATCAATTCCAGAATGTTTAGGAAATCTTGGGATGCTACTATATCT
TTCGTTGGGTTCAAATGAATTTTCACCAAAATTTCCCTCGAGTCTTTGGAAGATGAGTGGACTTCTCTACC
TAAACATGTCACATAATTCTATAGAGGGAGAAGTTCCACCAACTATTGGAGAACTGAAAGCCATTGAAG
AACTACATCTTTCCGGTAATCACTTTTCAGGCCAGGTACCAAGCAGATTGGGAGACCTCCAAAGATTGAA
GTCTCTTGACCTATCAAACAATTCATTTTCAGGCTCAATTCCATTATCCATTACCAACTTGATGAGCTTAGA
AGACTTGGATTTGTCTTTAAATGCATTGTCAGGTACTATTCCTAAGTCTTTGGAAAAACTCTCATACCTTA
CAAGCATCAATGTTTCATTTAATGATTTAGAAGGTGAATTACCCAGTGGTGGTGTGTTTGTAAATTCCAC
TCTGCTATCATTTCTCGGGAACAAAGGTCTATGTGGAATGCACATATTGGAGCTTCCCGCTTGTCCTATCA
CTAATCCTGGACAACAATCAGTGTCTAAGAAACTTGTGCTAAAAATTGTTACTCCAGTGGTTATTTCATTC
TTTCTGATATTCTTGTTGGTTTCAATTTGGATAATGAAACGAAAGAAGAAAGGGAAGTCCAAAGATGTT
GAAAAGGTTCTGGAGATCAAAACTTATCAATTGATTTCTTATCATGAGATTCAACGAGCAACAAATAATT
TTGATGGATCCAATTTAATTGGTGAGGGAAGCTCTGGCTCTGTGTACAAAGGCACATTCTCAAGTGGAA
CTATGGTGGCCATAAAGGTTCTGGATTTGGAAAATGAGCAAGTATGCAAGAGGTTTGATACAGAATGC
GAAGTGATGAGAAATGTCAGACATAGAAATCTTGTTCCAGTGGTTACTACTTGTTCTAGTGACTATATAA
GAGCCTTTGTTCTGAAATATATGTCAAATGGGAGTCTTGAGAATTGGTTGTACAGAGAAGCTCGCCACTT
GAACCTTCTTCAAAGAGTCACTGTAATGCTTGATGCGGCTATGGCAATTGAATATCTACATCATGGCAAT
GACACTGCGATAGTTCATTGTGACATAAAGCCAGCCAACGTTCTTTTGGATGAAGATATGGTGGCGCAT
GTTGGTGATTTTGGAATCTCTAAGATTTTAGCCGTAAGCAAGTCCATGACACAAACCGAGACATTGGGC
ACTCTTGGATACATTGCACCAGGTATACTAAAATTATAACCTTTCTATTTAATTTTTCTCTTATCAAAATCA
AGCCCTTGAAAATTCTAGGACTAAATAAAAAGCAAGTCTTTGTTAGTATGAGCATTATTACTATATCCAA
ATGAGTTAGTTCTTTTTCATTTTCGTTCTTTTAAGAGTACGGCTCAGAAGGAATAGTGTCGGCTAGTGGT

GATGTTTACAGTTACGGCATCATGTTGATGGAGGTTTTGATGAAAAGAAGGCCAACAGATGAAGAGAT
ATGCAATGAAAATCTTGACTTGAGGAAATGGATAACACAATCATTTTCAGGGAGTATGATGGACGTTGT
GGATGCCAATCTTTTTTCCGAGGAAGAACAAATCACTTGTAAAAGTGAAATCTGCATAGGGTCCATGAT
AGAATTGGGTTTAGACTGCACAAAGGAAATGGCAGAATCAAGAATAAGCATGAAAGAAGTAGTGAAGA
GGCTTAACAAAATCAACAACACATTTCTCGAAACATAG
>PGSC0003DMG400023953 LRR-RLKs
TTAATTCTATTCTTTAAGATTGGAAATGAGAGATCAAGATCGAGTTTTTACTAGTTTTGAACCATACCCTT
TTCGAGTTGTTAACAAGTTTTTAACTCGAAAAGGAGGAGCTGGTAGATGTATAATTTCTCATCTTGCAAA
AAGATTCTTTCTTTTTGCTCAACTTTTCTTGTTATTTCAGTTACTGATATTTGGGATTCAGCCAACTTCAGG
TGAAGATTGGGATGGGATAATTATTACTGCAGCAGATTTTCAAGCACTTCAAGCTTTTAAACAAGAATTG
GTTGATCCAAAAGGGTTCTTGAAAAGCTGGAATGATAGTGGATTTGGAGCTTGTTCTGGTGGATGGCTT
GGTATTAAATGTGCTCAAGGACAAGTTATTGTTATTCAGCTTCCATGGAGAGGTTTAGGTGGAAGAATT
ACTGAAAGAATTGGACAATTTCAGTCTCTTAGAAAACTCAGCTTACATGATAATGTCATTGGTGGTTCAA
TTCCTTCTACTTTGGGACTCATACCTAATCTTAGAGGACTTCAACTTTTTAACAATAGGTTATCTGGTTCTA
TTCCTGCTTCTCTTGGTTTATGCCCTCTTCTTCAAACACTTGATCTCAGCAATAACTCATTCTCTGGTGCAA
TTCCAGATAGTCTTGTTAATTCAACTAAGCTTTATAGGCTTAATCTTAGCCATAATTCTTTGTCTGGTTCAA
TCCCCACAAGTCTCACACAATCTCCCTCAATCATCTTTCTTGATCTCAAGTATAACAATCTTTCAGGCTCAA
TACCAGATACTTGGGATGGAAATGGAAATGGAAAAAGACTCTTCCAACTTCAGTCTCTTACACTTGATCA
CAACTTCTTTTCTGGAAGTATGCCTGCTTCCTTAGGCAAGTTGAATGAACTTGTTGAGCTTTCACTTAGTC
ATAATCAATTGACTGGAGTTATCCCAAGTCATATTGGGGGACTCTCTAGGCTTACAACACTTGATTTGTCT
TATAATGCCATTAATGGAAGCTTGTCTGATAGCTTCTTGAATCTATCCTCACTAGTGGTCTTGAACTTGGA
AAGCAACCAACTTGATAACCAAATCCCAGCAGCCATAATAAAATTGCAGAAACTCTCAGTTCTCAACTTG
AGGAGTAACCACTTTAGTGGTGATATTCCAGTCACTATAGGAAACATCTCTACTCTTAGACAACTAGATT
TAGCTCATAATAATATAAGTGGAGAAATCCCAGCTTCTTTAGATACTTTACCAAATCTTAGTGCCTTCAAT
GTCTCATATAATAATCTGTCTGGTCCTGTTCCAACTCATCTCGCACGGGAATTCAACTCGAGTGCCTTTGT
GGGTAATCTTCAGCTATGTGGTTACAGTGCATCAACCCCATGTCCAATCTCTCCAGTTAGCCCATCCCCCG
AAACACCAAAAAAGCAACGTCGTAAGTTAAGTACTAAGGACATAATTCTCATAGCAGGAGGGGCACTTC
TCATAATCTTGGCTATGCTATGTTGCATTCTCCTATGCTGCTTGATCAGGAAAAGATCTGCAGCTGAAGC
AGGGAAGGATGGTCAAAGCACGAGTAGGGCGGCTGCAGCAGCCAGAGGTGAAAAGGGTGTTCCACCA

ACTGCTGGAGAAGTTGAAGCAGCAGGAGGAGGAGATACAGGAGGAAAGCTTGTCCATTTTGATGGACC
TATTGTGTTCACAGCAGATGATCTTCTTTGTGCAACCGCCGAAATAATGGGAAAGAGCACTTACGGTAC
GGTGTACAAGGCCACGTTAGAGGATGGCGATCAAGTTGCTGTGAAAAGATTGAGAGAGAAGATCACAA
GAGGTCAAAGGGAATTTGAGAGTGAAGTCAATATTCTTGGCAAGATCAGGCATCCAAATCTTTTGGCCC
TTAGAGCATATTACATGGGACCTAAAGGAGAGAAACTTCTTGTTTTTGACTACATGCCTAAGGGAAGTTT
GGCAACTTTTCTTCATGGTAAGCACAAAATCAGTTTTCCATTTCACTTTGTATTTGTGAGAACTTCAGATT
ATAAAAATCTTACTGTTTTTTGCAGCTCGCAGTCCTGATACACCAATTGATTGGGCAACAAGGATGAGAA
TAGCAAAGGGTACAGCAAGGGGATTGCTCTTCCTTCATACAAATGCCAACATCATTCATGGGAATCTTAC
ATCGAGCAATGTTTTACTTGATGACAACACAAATGCAAAGATTGCAGATTATGGTCTTTCACGCCTAATG
ACTGCTGCTGCAAATGCAAATGTTATTGCAACTGCTGGAGCACTTGGGTATCGGGCACCTGAACTTTCAA
AACTCAAGAAAGCAAACACAAAGACAGATGTATACAGCCTTGGTGTTATTATACTGGAACTTCTAACTG
GGAAGTCACCAGGAGAAGCAATGAATGGTGTGGATTTGCCTCAATGGGTTGCATCAATTGTGAAAGAG
GAGTGGACTAATGAGGTATTTGATTTAGAACTAATGAGGGATGCATCCGTTATCGGTGATGAATTATTG
AATACCTTGAAATTGGCTTTGCATTGTGTTGATCCCTCACCATCAGCTCGACCAGAAGTTCAGCAACTTCT
CCATCAACTAGAAGAAATTAGACCTGAAACAGCTACCAGTTCTGGGGACGACGGTGCTGCAGCTCCCTC
AGCAAGCGATGATTAGATATTAGATTTCTCATCTAAGTCATTTTTAGGCAGGTGTGTTAGGAGTTAGGTG
CCACAATTCTTTCATTCTTGAATTAGGTTGATTAAGAGAGTGATTTTTGTTCTTCTACTGTCTGTAGATACC
CACCCAAGTGTGCTATTTTGTTCTGTTATTTTGAGCAGCAATGGAATCCTCTTAATTTCTGGCCCTAGAGA
CAGAAAATACACTAAAAACTCCACACACATGTCTATTTTCAGGTCATTCTTCTATACAAC

>PGSC0003DMG400024485 LRR-RLKs

ATGTTGCTAGTACTTGCAGGTTCAGAAACTCTGGAAGTGAGACGAGCATTAGTTAAGTTTATGAACAAG
ATATCACTTGGTAACATTTCAAAAGAAGTAAACTTTGGATGGGATTTATCATCTGATCCATGTACCAACA
AATGGGAAGGGATAACTTGTGATTCAAGAAACCAACATGTAAAAAATATTGTTCTTGATCAGAAGAATC
TCACTGGAATTCTTGATGCTTCTTTTGTCTGTGAGGCTACTTCCCTTGCTGTACTGAGCTTGAACGAAAAC
GAAATTGTTGGAACATTACCTCAAGAAATATCAAACTGCAGACGTCTCACACATTTTTATCTGCGTGGCA
ACAAATTATCAAGCAACTTACCAAGCTCTCTTTCGCGGTTGAGCAATTTGAAAAGGTTTGTAATTTCAGA
CAATGCCTTCTCGGGGCAGATACCGGATATGTCAAGAATCTCAGGGCTGATAACTTTTTTGGCTGAAAG
AAATCAGCTAACAGGGCAGATACCAGAATTTGATTTTTCCAATCTTGTAGAATTCAATGTCTCATACAAT
AATTTAACTGGTCCAATACCTGATGTCAAGGACCAGTTTAGTTCCAGCAGTTTTTTGGGTAATCCTGGATT

ATGTGGAGTGCCATTGCCAAGCATATGTCCACCCTCGCCACCGCCACCTCCCCATCCAGTTGATAAGGAG
AAAAAAGTTTCATACTTTATTTATCTTGGCTATGCTATTCTTGGGCTGATTATCATACTTCTACTTGTATGG
AAGCTTTTTAAGTGCATAAGGAAGAAAAAGAGCAATAGTACTCCAATGGATCACAACAAGACTATTAGC
TCTTCTGGTGTTACAAAGACTCCTAAAAATAGGTCAGAATATTCAATAACATCTAGTCCCGAAAACTCTAT
GCTCTCAGCATCATTTGAAATCCTTTCAAGTCCTCTGGCTAATAAATTGAGATTCGAGGACTTGCTTCGAG
CTCCAGCTGAATTGATTGAGAAAGGAAAACATGGAAGTGTTTTCAAGGTCAATGTTGATGGAGTGACAT
TGGTAGTAAAGAGGATCAGTGGTTGGAATATTTCGAAATACGATTTCAAGAAGAGGATGCAGAGAATA
CATAGAATGAAGCATCCTCATGTGTTGCCCCTTGTTGCCTTCTATAGTTCCAAACAAGAGAAGCTAACAG
TTTACAAGTACCAACAGAATGGCAGTCTATTCAAGCATCTCCATAGTAAGTTTTATTAGTGCAAACTTAAT
TATTCTCACTTGAATTTCAAGCCATAGAATGATACATGAGTAATAGGCCTTCCTGTAAGAACTATCAAGA
ATTGATATCAGTAAAGGAATATGTTAGATAGAACTATAGTTCAATTTCCAGATCCAGGGTCTAGCTAAGT
CAAAATCTTTTTAAGTATTTTGTCGGTCTACGTTGCTTTGACTCCAAAATGTTGCCACACCTGTGTCAGAT
CCTCCAATAATGCATTACTTTTGGAGGATCCGACACACACCTGTATGACATTTTTGAAAAGTCCGAGCAA
CATTGTTGTCGGTTGATTAAGTAAGAAAGAAACTTTACTAATTAGTATTATTAGTTTGGGCAGCAAGATA
TAGTCATGTTTTCATTGGAATCTAACTTCTGATTCTGAACCTAGTTCAAAAGATAATGTCAACATATCAGC
TTATAGGATTTTAGGTATTTACATTGGCAATACAAAGCTAACCTATAATAGCAAGTCAATTACCTATAATT
AGTTACCATACAAGTGATCTAATCGTATAAGTATCTTTTACGCAGTCAGTGAATAGACCTTAAACTCTTAA
AAATAATCAAGTAGTAACTAGTTTGTCTATTATTCTTTGAGAATGAACATGGAAAGGTAAAGTTACTGCA
TAACTGTTCATTTCAGCATCTCAGGGCAGTAAAGTTTTCGAGTGGGCGAGCAGATTAGCTATTGCAGCCA
GTGTCGCAGAGGCCTTAGCATTCATGCATGAAGGCCTTCAGAATGACAATATTCCTCATGGAAACATGA
AATCCACCAACATTATGCTGAACGATGACATGGAAGCCTGTATCAGCGAGTATGGTCTAATGCCCAACA
ATCAAGACCAGTCATTTGTTGCTCAAAGTGATCATAGCATCAGAGAAGATGATTCAGTTGCAATTACTAC
ACGTAACACCTTCAAGATGGATGTTTATAGTTTTGGAGTGATTCTTCTTGAATTGCTCACAGGGAAGCCT
GTTCAGGCTAGTGGATATGATTTGTCTAGGTGGGTAAATTCAGTAGTTAGAGCGGAATGGACTGGTGAA
GTTTTCGACAAGTCCCTTATTACAGATGGTACAAATGAAGAAAGAATGATCAATCTGTTGCATGTAGCTC
TCAAATGCATAAATACATCACCAGATGCAAGGCCTAACATGAAGGAAGTTGCGTTCATCATCAATTCTAT
TAAGGAAGATGAAGAGAAATCCGTGTCTGCGTTCAGCTAA
>PGSC0003DMG400024661 LRR-RLKs
TTTGTATTAAAAAGTTTGGTTTGGTCCATTTTGCAGGCCCTCCCTTTCTGAAATTCAACTCCATTTTCCCAT

GTTTATTCCTTGCACAGAGATATTGTTTCTGAACTACCAAAACCCCCTTTCTTAAACATAGTAAAATACCA
TTTCTTTCTTGTTTTGCACAAATTTCTATAACAACAACAACTCATCTGTCACACAACAAAAAATAAATAAA
AAAATAGAACAAATAGTACTGGAAGAAATCAAGATTCAAGATTGAGTTATGTCAGAAGAGGAATCTGAT
ATTCTTCTTCCTGTTGTATTATTCCACTTCTTGCTTCTAATCACTACTGGTAAGTCTGAGTTTTTTTTTTTCG
ATTTTTCAAGATTGTACTCAGAATTCTGATTGTTGTTTTATGAGGTTTTTAGCTGATTTGTAATACTAAAGT
TTTGATTTTTATTTATAGTTTGTGGAGATACCCTGGAGAGTGACAGGCAAGTGTTACTGAGTTACAAGTA
TTTTCTTGAACATCAAAATCCGGTTAATAAAGGATACAGGCATACAGAATGGAATGCTTCTGATTCGTCT
CCGTGTAATTGGCGTGGGGTTTTTTGTGATGGTGGAGTTGATCGTGTTACTCGAATTGATCTCTCTGGTG
ATAGTTTGGCTGGGAATATGTTTTACAACTTCTCAGCTATGACAGAGTTGACATATATTGATTTGTCTATG
AATACAATTGGAGGGTCTATTCCTGCAGACTTAGGCCAATGTAAAAACCTGAGATTCTTGAATTTATCGC
ATAATATTATTGATGGGGAGCTTAATTTGACTGGTTTGAACAATTTGCAAGTTCTTGATTTGACCATGAA
CAGGATTCATGGTGAGATCAGTTTAACTTTCCCTGGAATTTGTGATAGTTTGGTGGTTGCTAACATTTCG
AATAACAATTTTACTGGTGAGATTGGAAGTACTTTTGATCAGTGCTGGAATCTTAGATATCTTGATTTGA
GCTACAATAACTTGACTGGAGGATTGTCATTTGGTTTTGATAAGCTTAAGGAGTTTTCGGTGTCTAAAAA
CAAGTGTAATGGCTCTCTGCCTTCGTCATTTTTCACTCAAAACTGCACCTTGCAGGTGTTGGATTTATCAG
AAAATGGATTCGTTGGAGGAGTGCCTAAAGAGATATCGAATTGTAAGAACTTGGAGGACTTGAATTTGT
CTGGAAATAACTTTTCAGGGCCAATTCCTGAGGAAATTGGATCAGTTATGAGTCTTCAAGCACTTTACTT
GGGAAGCAACAATTTTTCAAGGGATATTCCAGAGAGTCTATTAAGTTTAAGTAACTTAGTGTTTCTTGAT
CTTAGTAGAAACAACTTCAGAGGAGAAATACAAGAAATTTTCAGGCAATTTACACAGGTAAAGTTTCTTC
TGTTGCATGGTAATTCTTATACTGGAGGTATAGTTACCTCTGGAATTCCCAACTTAGTGAACCTTTCTCGA
TTGGACTTGAGCGATAACCAATTCTCCGGTCCATTGCCAGTTGAACTTTCCAAGATGAAAGGGTTGAAGT
TTCTGATTCTTGCATATAACCACTTTAATGGAAGTATACCATCAGTATACGGGGATATTCCGACACTTCAG
GCCCTTGATCTTTCATCTAATAAGTTAACTGGCTCAATACCACCAAGTTTAGGGAAGCTAAGCTCACTTTT
GTGGTTGATGCTTGCTAACAATTCACTGACCGGTGGAATCCCACCCGAGTTGGGAAATTGCAGTAGCTT
ATTGTGGTTGAATCTTGCAAACAATCAGCTTTCTGGTTCAATTCCACCTCAGTTAGCAAGAATTGGCTCAA
ATCCTATGCCTACTTTCTTGTCGAATAGGGCTAAGGATAAGGTGACTGCTGGCTCGGGGGAGTGCTTTG
CTATGAAGAGATGGATACCAGCTGATTACCCTCCATTTAGCTTTGTATATCCTCTCCTTACCAGGAAGAAT
TGTAGAAGCCTATGGGATAAATTGCTCAAAGGGTATGGTTTATTTCCAGTGTGTGAACCGGGTAGTAAT
GTTCGTTCAAATCAGATATCAGGCTATCTTCAACTTAGTATGAACAAATTTTCTGGTGGGATCCCTCCTGA

AATTGGCAGCATGCAGAATTTCAGTATGCTTCATTTGGGTGTAAATGAATTCGGTGGCACGCTCCCTTCA
GAGATTGGAAAAATGCAGCTAGTAGTCCTTAATATTTCACAAAACAGAATATCTGGTGAGATTCCAAGC
CAGATTGGCAACATTAAGTGCTTACTGAATCTTGATCTGTCCAGCAACAATTTCTCTGGTCTGTTCCCAGC
TAGTTTTAGTAACTTGACTGATCTAAGCAAGTTCAATATCTCTTACAACGCGTACATCTATGGTACTATAC
CAGAAAGTGGGCAATTAGCCACATTCGAGAAGTCATCATACCTTGGTGTTCCATTGCTGCACCTTCCACC
TTTCATTGATAACACTAGGAATAATACTATAAACAAGGGTGGAAGTTTCAAAAGGCCTACAAAGGTTGG
TACAGTTTTGGTATTCATGGCTTTGCTACTGGCTTTGCTAGTTTGTGGACTTATGACACTTGTTATCTGCCT
TGTTCTAAAATCTCCGATAGATACACCAGGATACCTACTGGAGGATTCAAAGGGCCGACACGATCTTGC
ATCGAGTTCAGGTGCATCCTCACCATGGTTGTCTAATGATGTTAAGGTTATCCGCTTGGACAAAACAAGC
TTCACACATTCTGACATATTGAAGGCCACAGGCAGATTCTCGAATGACAGAATTATAGGGAAGGGAGGA
TTTGGGACAGTTTATCGCGGAGTCTTGCCTGATGGAAGGCAAGTGGCGGTGAAAAAGCTACAAAGAGA
GGGAATTGAAGGGGAAAGAGAGTTTAGAGCTGAAATGGAAGTACTAAGTGGAAATGACTTTGGTTGG
CATCCGAATCTTGTAACACTTTACGGTTGGTGCCTTAATGGATCAGAGAAATTGTTAGTCTATGAATACA
TGGGAGGTGGAAGCTTAGATGAGATCATCACAGATAGAACCAAATTCACATGGAAGAAACGACTCAAC
GTGGCAATTGATGTTGCACGTGCTTTAGTCTTCTTACACCACGAGTGTTACCCTTGTATCGTCCACAGAG
ATGTCAAGGCTAGCAACGTGCTGCTAGACAAAGACGGAAGGGCAAGAGTCACAGATTTTGGCCTAGCT
AGGGTTATGGATGCTGGAGATAGTCATGTTAGCACAATGGTTGCTGGTACAGTCGGGTACGTTGCACCA
GAATATGGACAGACATGGCAGGCCACAACAAAAGGCGATGTCTACAGTTATGGAGTGCTGGCAATGGA
GTTAGCCACAGGGAGACGCGCTGTAGATGGAGGCGAGGAATGTCTAGTTGAATGGGCGAGACGTGTG
ATGGGAGACGGAAGGCATGGATTCACCAGAGCCATAATACCAGTTTCTCTTTTGGTATCAGGCCTAGCA
GAGGGAGCAGAGGAATTGTGTGAGTTGCTTAGAATAGGAATAAGGTGCATTGCTGATAGTCCTCATGC
TAGGCCTAACATGAAGGAAGTATTGGATTTGTTAATCGCGATTTTGCGCAGCCAAAGATCAGGATCCAG
TCGTAGCACTTCTCCTTCATTTTGATCAATTTTTGTTTCAAATTACACAGCAAAGTGTCAATAGTTTTTTTT
CTTCACATTTGTAACATACACACATTACATTCTTCTTGTAAACAGTTCTTTTTAGATTCTTTGTTCAGGCAC
TCTTGTTTGTTAGGATCATAAGAG
>PGSC0003DMG400024795 LRR-RLKs
CCTATGGCCAACCCTGTCCTACAACACTGTGTCAAGTGTCCATCTTGACAATGAATCTAATTGAAGACAG
ATATATTTGGAGGAAAATCAAGATTTCTGAAAATTCCATTTGTTTCTTTCTTTTACATAAAATTCTGCAAG
AATTTAACTGTTGTGCATTTCCAACACCAGCAAACCATGATAAAGAAATCATTGCAGATAACCTCTTATG

ACTACATTTGGTGCAAAAAAGAAAACTGAAATAGAAGTACAGTAGATCAGAGAGTACATACACACCCTC
ATTGTCATCATAATCATAATCATGTCAGGCAAACTAACAGTCATCTTATTCTTCTTGTTTCTCAACATATCT
TTTTTATTCCCAACATCCATTTCTGGTTTAAATCAAGAAGGGGTTTCTTTAATGTCATGGCTTTCCACTTTT
AACTCTTCTGCTTCTGTTGCACCTTTCTCTTCATGGAATCCAAGTCATGAAAATCCATGCAAATGGGATTA
TATCCAATGCACCAGTAATGGATTTGTTTCAGATATCAAGATCAGGTCGATCAATCTTCCCGCGATATTCC
CAAGTCAAGTTCTTTCTTTTCACTTTCTGGAAGTGCTAGTTTTATCAAATTGTAACCTCACTGGTGAAATTC
CTGCTTTTATTGGAAACTTGTCATCGTCTCTGAGGATTTTGGATCTCAGTTTCAACGCTCTAACAGGAAGT
ATTCCGCCTGAAATAGGAAGATTATCACAGCTAAAGCAACTTTTGTTGAGTTCTAATTTCTTTGAAGGTC
AGATACCAAAGGAGATAGGAAGATGTTCTGAATTGCAGCAGCTTGAGCTCTTTGACAATCAGTTTTCTG
GAAAGATACCTGAAGAGATCGGTCAGTTAACGAGTCTTGAAATCTTTCGTGCTGGTGGGAACGTGGGA
ATTCAAGGAGAAATCCCAATGCAGATATCAAGCTGCAAAGAGTTGGTCATTTTGGGTCTTGCAGATACC
GGTGTTACGGGTCAGATTCCACATAGTATAGGTGAACTCAAGAAGCTGCAGACTCTTGCTGTTTACACA
GCTAATTTGACTGGTGAAATCCCTCCAGAAATTGGGAATTGCACTTCTTTAGAAGAATTGTTTGTCTATG
AGAATCAAATTACAGGAGAAATTCCAAGTGAACTCGGTCTCTTGAAGAATCTTAAGAAGGTTTTGCTTTG
GAAGAACAATCTGACAGGGGAAATTCCAGGAAATCTTGGAAACTGTTCAAGTTTGAAAGTGATTGATTT
CTCTGTGAACTATCTATATGGTGAAATCCCTCCGTCTTTCGAGAATTTAGGCACATTGGAGGAGCTTCTAT
TATCAGAAAACAGCATTTCTGGAGAAATCCCATACTATATTGGCAACTTTTCCAGCTTGAAGCAGCTTGA
ATTGGACAACAACAACATTTCAGGTGTGATTCCACCTACCATTGGGAAACTAAAGGAGCTAAATCTGTTT
TTCGCTTGGCAGAATCAACTTCACGGGAGCATACCGACTGAGCTAGCAGATTGTCGGAAACTTCAGTCTT
TGGATCTTTCTCACAATTTCCTTACAGGGTCCATCCCAAACACTTTATTCAATCTCAGGAACTTGACTAAG
CTGCTGCTGATATCAAATGTTCTGTCCGGTGGAATTCCACCTGATATTGGAAATTGCACCAGCTTATCCA
GATTACGCCTTGGATCAAACAGGTTAGATGGTCCAATTCCTCCAGAAATAGGGCGTCTACCGAGTTTGA
GTTATCTTGAACTGTCGGAAAATCAATTCACTGGATCAATCCCTCCAGCTATTGGCAACTGTCCGCAGCT
AGAAATGGTTGATCTTCATGGAAACAATCTTCAAGGAACAGTTCCTTCCTCTTTTGTGTCCCTCACTGGTC
TTAATATATTAGACCTATCCATGAACAGAATATCAGGTAACATTCCAGAAGATATAGGGAAACTCATATT
ACTGAACAAACTCATACTGAATGGAAACAACATAGACGGAACGGTTCCTAAATCACTTGAACTCTGCCA
GGATTTGCAGTTGTTGGATTTAAGCAGCAACAGACTCGTGGGCTTAATCCCTGAAGAGATTGGTAACCT
CCAAGGACTAGATATTCTGTTCAATGTAAGTCGTAATTTTCTGACCGGACAGATTCCTGAAAGCTTCTCTA
ACCTCTCCAAGCTAGCCAACATGGATATTTCTCATAACATGTTGACTGGAAGTCTTAGAGTACTCAGTAA

CCTTGATAACCTTGTTTCTCTGAATGTTTCATACAATAACTTTTCTGGTGTTCTTCCTAACACAAAATTCTTT
CAAAGTCTCCCTCCTAGCGCTTTTATTGGTAATCAAGAGCTCTGCACTGACAGAGCTGCATGCCACTTAA
GTGGTGATCACCATGGATTAAAATCCATAAAGAAGATCACCATTGCTATTGTACTCAGCATTTTCATGAC
CATGCTAATTGTGACAGCTTCTATTGCCATCTTTATCCGAACACAGGGAGAGATATGTCAGAAGGACGA
CGAAGAAAATGGTTTGCAGTGGGAATTCACCCCATTCCGAAAGCTTAGCTTCTCTGTAACTGATGTAGTG
CCAAGACTTAGTGAATCTAACATTGTTGGAAAGGGCTGCTCAAGCTTTGTTTATCGCGTTGAGACTCCAT
CAGGACAGGTGATCGCGGTGAAGAAACTATTGGCTAAGAAAATCGGTGAGGTTCCTCAGAGGGACTTT
TTTCTGCAGAAGTTAGAACACTGGGATCAATCAGGCATAAAAACATAGTGAGGCTTCTTGGATGTTGT
AATAATGGCAAAACAAGATTGTTGTTGTTTGATTACATTAGTAACGGGAGTTTATCGGGACTTCTCCATG
AGAAGAGGGTATTTCTGGATTGGGATGCTAGGTTTAATATCATATTAGGAGCAGCTCAGGGCTTAGCTT
ATCTTCACCATGACTGTAGTCCTCCTATAGTCCATCGTGATATCAAAACCAATAACATTTTGGTAGGTCCA
CAGTTTGAAGCTTTTCTTGCAGACTTTGGACTTGCAAAGCTATTAAACACATCATCAGATACTTCAAGAG
CCTCCACCATAATAGCTGGTTCCTATGGGTATATAGCTCCGGGTGAGTACATTTCCTCAAATTTCCGCCCT
ATATATTCAAATATTTATTAAGTGCTCAACATTTTGATAACATGATTATGCTAGTACTATTGAATATTAGCT
TTAAAGAAACACTTGAAGTTCACTATATTAGTCTTGTACCCCGTGAAATTCGCTGTGCAAGACATATAGT
AGGTATATATACTATGTTTGGACAGCATTCTAAATGTTTTGGTCCTACATTGCAGAATATGGATATAGTCT
AAGAATAACTGAGAAGAGTGATGTCTATAGCTATGGCATTGTGCTTCTCGAGGTCCTAACGGGGATGGA
ACCAACTGATTCCCGGATACCAGAAGGCAGCCACATTGTCACTTGGGTCAATCAGGAGCTAAGAGTAAA
ACATAAGGAATTCACAACAATTCTTGATCAGCAATTACTTTTAAGGTCTGGTACACAAACTCAGGAGATG
CTTCAAGTTCTTGGGGTGGCACTACTTTGTGTTAACCCATGCGCGAATGAAAGACCAACAATGAAGGAT
GTAGCAGCAATGCTAATGGAAATCAGGCACGAAAATGAAGATCTTGAGAAGCCTAATCGAGGAGTGGT
ACCGAATCCTAAAGAAGCAATTAGTTGTCCTAGTTTCTCTGGTTCATCTCAACCTCTCAATAGATCCCCTC
CTCAGTAACTCTAGTTAACTTAGCCTCTCTCTAGTTCTAACTAGTTGATTCTTTCAAGTAACTAATAAGGAT
GTATAGATTATCAATATTCATTTCACAAAATAAGAACATTACTCTTTCTAGTCCCGACTATAAGTCGTTTT
GACA
>PGSC0003DMG400024890 LRR-RLKs
ACCATTTAATTTAGGCTAAAAACTAACTTTGATCTTCTATGTAATGTGTTGTTCTTCAGTGAGAGATAAAA
GGAAAAAAAGCAAAGAGAGGAAAAGGGGAGGCTTAGCTTCTTCCTTGATCTTGAAGACTCACTTTCTTT
CTTTTGAAGATGTTTGATTCTTTAAATCTATCTATACCTGAAGTTTCTCAGATTCCCTTAACTTTTTTCTCCT

TTTACATTGCCTCAAAGTCTCATAATAACTCACTTTGTTTCTTCTTCAAGTCCAAACAAATTCACTTTCCTTG
AAAAAACTTCCAAGAAAATATGTTGCCACTTATTCTTGTCACACTCTTTACTCTTGTATGCACTTCCATTTC
CTCCTCTTCCTCTTCTATTAGTACTGATTTTCATGCTTTGTTGAGTCTAAAAAAAGGATTTGACTTCTCAAA
TTCTGTTTTGAGTTCATGGGATAGTTCAAATCCTAGCTCAGTTTGTTCTTGGATGGGAATCAAATGCTTAC
AAGACCGAGTTGTATCGATAAACTTATCCAATATGGAGCTTTATGGTTCTGTTTCCCCTGCTATTTCAAGG
TTAGATAAGCTTGTTGAGCTTTCAATTGATGGAAACAACTTCACTGGTGAAATCAAGATTGAGAACATGA
GGAGTCTTGAGTCACTCAACATTTCAAACAACATGTTTAGTGGGAGTCTTGATTGGAACTACACAAGTTT
GGCTAATTTGGAGGTTCTTGATGCTTACAACAATAACTTCTCATCTTTTCTTCCTGTTGGAGTTGTTAGCTT
GGAGAAACTCAAGTACTTGGACTTAGGTGGCAATTATTTCTATGGACGAATACCGGAGAGTTATGGGGA
TTTGATTGGGTTGGAATACTTACAACTTGCTGGAAATGACCTGCATGGAAGAATCCCTAGAGCTTTGGG
GAATCTTACCAACTTGAAAGAGATTTATTTGGGATATTTCAATGTTTTTGTAGGTGGGATTCCTAAGGAA
TTTGGAAAGTTGGAGAATCTTGTTCACATGGATATTTCTAACTGTGAATTAGATGGTCCAATTCCTCCTGA
ATTAGGGAATTTGAAATTGTTAAACACACTTTTTCTACATATAAATCTTCTTTCAGGTCCAATTCCAAAGG
AACTAGGCAACCTTACTGGCTTAGTCAATCTTGATCTTTCAGCCAATGCACTTACTGGTGAAATCCCATTT
GAGCTCATTAACCTTCAGCAGCTACGCCTTTTCAACTTGTTCATGAACAAATTACATGGTTCTATACCGGA
TTTCATCGCTGATTATCCTGACCTGAAGATTCTTGGTCTCTGGATGAACAACTTCACCGGTATAATCCCGG
AGAAGTTAGGCCAAAATGAGAAGCTACAAGAGCTGGATTTGTCTTCAAATAAGCTCACTGGTACAATTC
CTAAACACTTGTGTGCTTCTAAGCAACTCATAATCTTGATTTTACTGAAGAATTTCCTCTTTGGTTCAATCC
CAGAGGATTTAGGCACATGTTTAAGTCTTGTTAGGGTGAGATTGGGACAGAACTACTTGAATGGCAGTA
TTCCAAATGGATTCATTTACATGCCTGACCTAAACTTAGTTGAGCTTCAGAATAACTACCTGTCTGGTACC
TTATCAGAAAATGGTGACACTTCTTCAAAGCCAGCCAAGTTAGGTCAATTGAATTTATCAAACAATCAAC
TTTCAGGTTCTTTGCCATTTTCACTTTCTAATTTCTCTTCCCTTCAAATCCTTTCACTTGGAGGGAACCAATT
CTCTGGTCCTATTCCTACTTCTATAGGTCAACTCTCACAAGCTTTAAAAATTGACCTCAGTCAAAATTTTCT
TTCTGGTGAAATCCCCCCTGAAATAGGCAATTGTGTCCACTTGACTTATCTAGACTTGAGCCAGAACAAC
TTCTCTGGCTCAATTCCACCTCGAGTATCCGAAATTCGAATCTTGAACTACTTGAACTTGTCAAGAAACCA
CTTGAATGAGACCATACCAAAGTCAATTGGTACCATGAGGAGTCTCACTACTGCTGATTTCTCATTCAAT
GATCTATCAGGCAAGCTGCCAGAATCAGGCCAATTTGCTTACTTCAATGCTACTTCTTTCGCGGGCAATC
CTCAACTCTGTGGTTCTTTATTGAACAACCCTTGCAATTTCACACTAATCACTGATCCACCAGGAAAGTCT
CATGGAGATTTCAAGCTGATCTTCGCCCTCGGATTGTTAATCTGTTCACTAGTCTTTGCAGCTGCAGCTAT

CATAAAGGCCAAGTCTTTCAAGAAAACAGGCGCGGACTCTTGGAAAATGACAGCATTTCAGAAGGTTGA
ATTCAGTGTTGTTAATGTTCTTGAATGTGTAAAAGATGGCAATGTGATAGGCAGAGGAGGAGCCGGGA
TTGTGTACCATGGGAAAATGCCAAATGGAGTTGAAATTGCAGTGAAAAAACTGCTTGGCTTTGGGAACA
ATAGTCATGATCATGGTTTCAAGGCGGAGATTCGGACACTAGGCAACATTCGACATAGAAACATTGTAA
GACTAGTTGCTTTTTGTTCCAACAAGGAAACGAATCTCCTTGTGTATGAGTACATGAGGAATGGTAGTTT
AGGAGAGGCGTTACACGGGAAAAAAGGCGGATTCCTTAGCTGGAATTTGAGGTATAAAATTGCTATAG
AAGCTGCTAAAGGATTGTGCTATCTTCACCACGACTGCTCGCCATTGATCGTTCACAGAGACGTCAAATC
CAACAACATTTTGCTGAATTCAAACTTTGAAGCTCATGTTGCTGATTTTGGACTAGCAAAATTCTTGGTAG
ATGGTGGTGCCTCAGAATGCATGTCTGCTGTTGCTGGCTCCTATGGTTACATTGCCCCTGGTACATTACTT
TTAACTTTTACTCACACATTCTCAATAATCAATCGTTCATATTAAGTGAAATTAATAACCTCGAAAGATAA
AATACAACTTGGTACAACATGTCAAAATACACATTGTATAGAGGGGACAATTATTGTGTAAAAATCATAT
ATGTACGTTGGTTAGTTAATTTAGTTAGGTATGTAGACATTACCTGACAAAGTGATTTTTGCCTGGTCGT
ACATACAACAACAATTAGTCTTATCAATTGGTCATAAAATCAAACTTTTAAGATAAAATATTATATTGCAT
TTTGTTTCTTTTAGTTTGGAGTTTGTTGAATTCGATAGAAATTTTGGGCTAAAGTGAAGAGGCCCAAGAT
CACCCGTGATTGAAGGCATAGAGTTTAGGGCTTTGTTGGAATTTTCTGGGTAATCGTAAATCGTATCATG
GGCCCGTTTTTCGAGAGAGCACTTAATTTCTGTGCCATAGAGAGAGTTCTTAGTGGGGGCCCAATAGAA
GATCACTCTGCTAAAGTAGGTCAAAAAACTGACTTGACTGACACACAAAGATAATTGGGCCCCCTCGATT
GTCGTGCTTGCCAACTCAACATGCACTTGTTCAAAAGGAATTCAACTAAATGGAATAGAGTATATAATAT
ATAGTCACAATATTACATATTTTACTGTATAACTCTTTAAAGTAGTCAACTTTTTTAAAATTAAAGGAGGG
AGAGAAAGCAATAGTGTGAATAGCGATGAAGTCAAGATATTCTATTTGTCACATATATTTGAGGGGTGT
TAACTGATCTCAAATATTGTTTAATGCATTTATATTAAATTTGTATATACATTGTCATAAATGTTAACCTAT
TTTTTTAAAAAAATTGTTGCAGAATATGCATATACTTTGAGAGTAGATGAAAAGAGCGATGTATATAGTT
TTGGTGTGGTTCTATTGGAGCTGATCACCGGGCGTCGTCCGGTGGGTGAATTCGGAGACGGAGTGGAC
ATAGTACAATGGAGCAAGAAAGTGACAAATTGTAAAAGGGAACAAGTAACACACATTGTTGATCCAAG
GTTAACCACTGTGCCTGAAGATGAAGCCATGCATTTGTTCTTTATTTCTATGCTATGTATTCAAGAAAATA
GCGTAGAACGTCCAACAATGAGGGAAGTCATTCAAATGTTGTCCGAATTTCCACGACAATCATCAGAAT
ATAATCAACCTTCGTCTTCAAAAGTTGTTTTACAGAAGCTAAAAAGCCTTGAGAATGACCAAACTACTTG
TCCGAAGATTCGGAAAGAACTTTTGGTCTAACCAATTTTCATTTTTATATAATATGGTCCTCTTGGCAAGT
TGTTTTAATTTTAACTTTGTATTATTCTTTATGCTTATTTAAGGTTAGGGGTTTTGTGACAAAGAGAAGTA

GGAATATAATTAGTACTATGTATTACTAAGTAGTTAATTAGTTGCTTTTGATTAGTTGTTAGGGTACTTGG
GGGTGTTTTAAGTTCTTGGCAAAGGCAGACCAGGCCCAATTAAGTTGGTTCTGAATGCTTTTTGTATCAT
ATGGTTATATACAGGTATATATTCTTCCTTTAACGGGTGAATTGATTCTCGATCCAATAATGTTTAATTGT
TTATTGTTGGTCATGTTGTTATGGATGAATTGGTTCTCG
>PGSC0003DMG400024954 LRR-RLKs
GGAAGGTTGGTTTTCGATGAGCTGTGTCTTCTTCTTCTCTCAATATTTTATAAACTTCCCTTCACATGGTCC
CCTTTTTATCATAAATCTACATTTATTCTTCTAACTTTTTTTTCTATTCAATTCCTAAAAAAAAACAGAGATG
TGGGAACATTTAGAGACACCATGAAATTCCAAACCTTTGATCTTGTTCTTCTTCTTTTTCTACTACTTGAAT
ATCAAGCTTCTGGGCTTAACAATGATGGACTTCTGTTACTGACATTCAAGTACAATATTCTGAGTGACCCT
TTAAGTGTTCTGCAGAACTGGAATTCTTGGGATGATACACCATGTTCATGGAAAGGGGTAGGTTGTGGA
AATCCAAATGCTTCAGACCCAAACCTTCGAGTGACAAATTTGTCTCTTCCTAATTCTCTGCTTCTTGGTTCT
ATTCCTTCTAGTCTTGGTATGATTCAATATCTTACAAATCTTGATCTTTCTAATAATTCCATTAATGGCTCT
ATTCCTCTTACTCTCTTTAGTGCCCCTGAGCTTCAACGCCTTGATTTTTCTAACAACAGAATATCCGGCCAG
TTGCCTGAGCTTGTCGGACATTTAAATAATCTTCAGTTTCTTAACCTCTCCGGTAATGCTTTGACGGGAAG
ATTACCGGCAAATCTTGGTAATCTAACTGTTGTTTCTTTGAAAGATAATTATTTTTTTGGTGCTGTTCCTTT
TGGGTTTGATTCAGTTCAAGTTTTAGATCTGTCTTCAAATTTGATTAATGGGTCATTGCCTCCTAATTTTG
GGGGCAGTAATCTCCGTTACTTTAATGCTTCTTTTAATAAACTTTCTGGAGATATCCCACCGGAATTTGGC
AGCAAAATCCCTCCAAATGCAACTATAGATCTCTCTTACAACAATTTCAGCGGTGCAATTCCAGAGTCAA
TTGTTTTTATCAACCAAAATAGAAAAGCTTTTTCTGGTAATCCAGAATTATGTGGTGCGCCGCTGAAAAA
TTTATGCCCAATTCCGTCAACAGTAACAACTCTGCCAAATTCCCCTGAACCAACTTCTTCCCCAGCAATTG
CAGCCATTCCGAAACCCACAGACTCAAACCCAGCCGCCGAATCTCCAAAAGAGGGCAAAAATGGGTTGA
GAACTGGCACAATTATAGGCATAATAGCAGGGGATATTGCAGCAGTAGGAGTTTTAGCACTGATTTTCA
TGTACGTATACCGAGCCAAAAAGAAGAAAAGGAACATAGAAAATAGCATTAAAAAAGAAGCTGAAACT
GCAAAAGATTTTGACTGGGCATCGTCAGCATCATCAGAAGAATACAACTGGTTAAGGTCATGGACTTGC
TTGAAAAAACAAAGGCACGGAGATGACGACGAATTATCAGAAGCTTCCCATTCAGAAAGTGAAAATTCC
CAATTGGGTCATCCTCAGCAGAATCACGTGCATACAGAGCAGAAAACAGGTGAATTAGTAACGGTTGAT
GGAGAAAGGGAACTCGAATTAGAAACACTACTAAAAGCATCTGCATACATTTTGGGAGCTAGTGGGTC
GAGTATAATGTACAAGGCTGTGCTAGAAGACGGTACTACACTGGCGGTGCGACGAATTGGTGAAAGTG
GTGTGGAACGGTTCAAAGATTTCGAGAATCAAGTAAAGGTAATTGCTAAATTGGTGCACACAAATTTGG

TTAAAATCCGTGGCTTCTATTGGGGCGCTGAAGAGAAATTGGTCATCTACGATTTCGTTCCTAATGGTAG
CCTTGCCAATGCACGTAACAGTAAGTCCACTTTTTCCCATATACATTATGTTTTACATCAAATAACGTTAC
AATTATTGAGTAGAATCGCGTCTTTTGTTATTTGTTTTACTATTTTGCTCCTGTTTTACAGTGGAGAATAAA
TTGAAACAAAATTGACTAGTCATTATGTGTGTACATTGTACCTAATCATCTCTAATTCTTAATTTGTTTCAA
TAAGTATCTTTTGAATAATTGCGAACATTTTGGAATATAAAAGTTCGGAGTAGGTTTGTACTTTTAAGAC
ATTTGGTTATGCTTAATATTCAAAAGAAAGACGTAACAAGTCACTCAAATTAAGTTCACACATGTATAAA
ATTGTGCGCAAATTAGAAAAATATTATGAATTTCTGTTAAAATGCGCGGGAAGTAAAGGAGATAGTGGA
CCTTGGGGTGGAGTAGTTGAAACTTTAGTGTTTGTTTTTAATGTGGAAAGTAGCAGTAGTTCCCTCACTG
CAAGTCAACCGTGTCTGTTTCTAAATGGGCGCCCCACAAATTCATAAATACCGCCAAACGCCCCCTCTTCT
ACTGTCACTTACATGGAAAATGACTACCTTTTAAAAAAAAAATATTACTCTGTAGGCCATAAGTATGCTCT
TTTGTTATGTCAAATTCATGCATTTCTTTTGCCCACAACACCAATAATGTTAAAAGAATCTTCACCTTATTT
ACCATTTATAGTATGTTGTTTATTTTATAGATATTATTGTGAGAATCTTAGCGCTCAGTTGATTGACTACCT
GATGTAATTATAACGTGTGCATTTTCATGGTTTCAGGAAAGGCTGGTTCTTCGCCATGTCACGTACCCTG
GGAAATCCGGCTGAAGGTTGCTAAAGGTGTGGCTCGTGGGCTCACTTACATTCATGAAAAGAAGCATGT
CCACGGCAACTTGAAGCCCAGTAACATTTTACTAGGGGAAGATATGGAGCCCAAGATTGGTGATTTTGG
AATTGAAAGGCTTGTAACAGGAGACAGTAGTCATAAGACCTATGGATCTGCCCGTAATTTTGGAAGCAA
AAGGTCCACAGCATCCCGTGAGAGCTTCCAGGACTTTACATCTGGGCCTACTCCAACCCCAAGTCCAAGT
GCATTGGGCATATCTCCTTACCATGCACCCGAATCGCTTCGTAGCCTCAAGCCCAACCCGAAATGGGATG
TATTCTCATTCGGGGTAGTCTTGCTGGAGTTGCTAACTGGAAAGGTGATTGTCTCGGATGAAATGGGAC
CCGCATCGGTCATCGGTGCTACCACATCAGCAGAGGAAGAGAAGAGTAAAGTGTTACGGTTGGCTGAT
GTGGCGATACGTGCTGATGTGGAAGGGAAAGAAGATGCTTTGTTGGCACTAATGAAAGTAGGATATAG
TTGTATATCACCAACACCTCAGAAGAGACCAGGCATGAGAGAGGTTGTTCAAGCATTGGAAAAATTTCC
AACTAATTCAACCAGTTCACCATACTATTATGGACCTTAATTAGCACTGATTAAAAGATGGACACAATTGT
AGAGACAAGAGACTTTGTATGTAATGAGACTGACATCTTTGTTATTTTATTTTTTTTCAATTTTTTTGGTTT
GTGTCTTAATATAGTATTAGCCATTGGTTTTCTTTTTTAACAGTGATTAGTGTAATTGTCCGTGCTTTGTTG
TGTTGTAGTTTTCCTAAACCTTATGGTGACTTTGGATTACCAATATATATGACAACTTATTATGTG
>PGSC0003DMG400025076 LRR-RLKs
TAATCTCTTTGCTATTTAAATGTATAGTTTCATTTGTTTGCTCTTTAGTTTCTCAAGAACTGTCACTCTCACT
CAAAATCCTCTCCGCCACCGTCAACCGCCGTCAACCACCGTCCGCCGCCGTCATGAAAAAATGCAAAATG

ACAAATGTGTTTGGTTCATGCTAGTGACCATTTATCTTCACCACCATCATTAGTAAGCTACTAGCTAGCGC
CGCCGTGTACGGAGGAGCAAGGAGCAAGGAGGAGCTGTTTTCTCCAGTAAGAGAACTTTAATAGTAGC
AGTTTAGTAAATGTTTCATTAAAGATAAACAAGAACTTTTTAGTTCACATGCATGGTAAGTAAATTTTTTG
AGTTAGTGTGAGTTTGAAGCAAACCACCATTAACACACCACATGTATGTTGTCTTGTTAGAGTGAACTTT
CAAACACTATTATCATTTTTTTCAACAACCCCATTTCTTGACTTTGATTTTCACTCAATAATGGAGAAGAAA
AGCTACCATTTTTACACTTTCCCTTCATGTTATTTCTTGTTTTTGTTGATTTCTTGGTTAAGTTTGAGTCCAA
AGATTGTATTTTTAGTGAGCTCTGTTTCTTCTGATGGACAAGCGTTGCTTTCTCTGCTTAAAGCAGCTGAA
CCTTATACTAAATCATCATCTTCTGTGCTTTCTTCTTGGAACCCTTCAAGTTTAACTCCATGTTCTTGGCAA
GGGATTACTTGTTCTCCTCAAGAAAGGGTTATTTCACTTTCTATCCCAAATACTTTCCTAAATCTCTCGTAT
TTGCCTTCTGAACTCTCTTCCCTTTCATCTTTGCAGCTTCTTAATCTTTCTTCTACTAATATTTCTGGAACAA
TCCCTCCATCTTTTGGTTCTTTTTCTCATCTTAGGCTTTTGGACCTTTCTTCTAATTCACTTTTAGGTTCTATT
CCATCAGAACTTGGTAGGCTTAGTTCACTTCAGTTCTTGTTCTTGAATTCAAATAGATTGACTGGTAAGAT
TCCACCAGAGTTGGCTAATCTTTCTTCACTAGAAATCTTTTGTCTTCAAGATAATCTCCTCAATGGGTCAA
TTCCATCACAGTTAGGGTCCTTAGTGTCACTCCAGCAGTTTAGGATTGGTGGAAATCCATATTTGAGTGG
TGAAATTCCTGCACAGTTAGGCCTGCTCACCAATCTTACCATGTTTGGTGTCGCGGCGACTGGTCTTTCG
GGTGTTATTCCACCTACGTTTGGGAACTTGATCAATCTTCAAACACTGGCAATTTATGATACTGAAGTATT
TGGTTCGATACCGCCTGAACTCGGGATGATTTCGGAGCTTAGGTACTTGTATCTGCATATGAATAAGCTC
ACTGGTTCGATACCTCCTCAGTTGGGTAAGTTGCAAAAGCTTACTAGCTTGCTTTTATGGGGAAATTCAT
TAACTGGACCTATACCAGCTGAGGTTTCGAATTGTTCGTCCCTTGTGATTCTTGATGTTTCCGCGAATGAA
TTGTCCGGTGAAATCCCTGGTGATTTGGGGAAGCTATTGGTTCTTGAACAGCTTCATTTGTCTGATAATG
CACTTACCGGTTCTATACCGTGGCAGTTAAGTAATTGCACTAGCCTGACAGCTCTTCAGCTAGATAAGAA
CCAATTATCAGGGCAAATTCCCTGGCAAGTTGGTAAGTTGAAGTACTTGCAGAGTTTTTTCTTGTGGGGG
AATTCGGTTTCAGGAACCATTCCAGCTGCTTTTGGAAACTGTACTGAGCTATATGCACTTGATCTTTCGAG
GAACAAGCTCACTGGATCGATCCCTGAGGAGATATTTGATTTGAAGCAGCTGAGTAAATTGTTGCTCCTT
GGGAACTCGTTAACTGGGCGATTGCCTCGAAGTGTTGCGAGATGCCAGTCTCTGGTGAGGTTGAGGCTT
GGTGAGAATCAGCTTTCAGGACAGATTCCTAAAGAGATTGGTCAATTGCAGAATCTCGTGTTTCTTGATT
TGTACATGAACCATTTCTCTGGAGGCTTGCCTTCTGAAATTGCCAACATTACAGTTCTTGAGCTGTTGGAT
GTGCACAACAATTACCTAACTGGGGAAATACCACACCAGATGGGGGAGCTTGTGAATTTAGAGCAACTT
GATCTTAGCCGGAACAGCTTTACCGGTGAGATCCCTTTGAGTTTTGGCAATCTCAGTTACTTGAACAAAT

TTATTCTCAGTAACAATCTGCTTACTGGTTCAATTCCAAAGTCTTTTAAGAACTTGCAGAAGTTAACTCTA

CTTGATTTGAGCTCGAATAGTCTTTCTGGCGAGATTCCATCTGAGCTTGGCTATGTCACAAGCTTAACAAT

CGGTTTGGATTTGAGCTTGAATCGTTTCACGGGTGAACTTCCTGAAACATTGTCCGGTTTGTCACAGTTG

CAATCTCTTGATATTTCTCATAATTTGTTGAGTGGAAGGATCACAATTCTAAGCTCCCTGACCAGCCTTAC

TTCCCTGAATGTTTCAGACAACAATTTCTCGGGGCCTATTCCTGTCACACCATTCTTTAGAACTCTTACTTC

AGATTCTTTTCTGGAGAATTCACTCTGTCAATCAGTTGATGGTTATAGTTGTTCTTCACATATAATGGGAA

GGAATGGGTTGAAGTCACCGAAAACTATAGCTCTGGTTGCGGTGATTCTGACATCTGTAGCCATAGCAG

TCGTGGCTACATGGATTCTTGTGACGCGAAACCACAGATATGTGTTTCAGAAGTCCCAAGGCATGTCAG

CCTCTTCGGTAGGAGCAGAAGATTTTTCCTACCCTTGGACTTTCATCCCCTTTCAAAAGTTCAATTTCACC

ATAGACAACATCTTGGATTGCTTGAAAGATGAAAACATCATTGGGAAAGGATGTTCCGGTGTTGTATAC

AAAGCAGAAATGCCTAACGGTGAGGTGATTGCAGTGAAGAAGCTTTGGAAAACAAAGAAAGACGAGG

AGCCAGTAGATTCTTTTGCAGCAGAAATTCAAATTCTCGGACATATTAGGCATCGAAACATTCTGAAGTT

ATTGGGATATTGTTCAAACAAAAGCGTTAAGCTTCTTCTCTACAACTACATTTCAAATGGCAATCTCCATC

AGCTCTTGCAAAGTAACAGAAATTTGGACTGGGAAATTAGGTACAAAATTGCAATTGGATCAGCTCAAG

GCCTTGCTTATCTTCACCATGACTGTGTTCCAGCCATTCTTCATAGAGATGTCAAGTGCAATAATATACTG

ATCGACTCCAAGTTTGACGCTTATATTGCAGACTTTGGACTTGCAAAGCTAATGAATTCGCCAAACTATC

ACCACGCTATGTCCAGTGTAGCAGGATCTTATGGATATATTGCTCCAGGTATGCATGCACTTAGAATATT

TTCAATTACTAATATTTCAACATAAAAGCCAAATCGATCATAACATCTTTTGAATATTTACTTTATGAGTCA

TAGTTCGTTGAGTGTGACACAAATATTTGTCAACGGTTGCAGAATATGGATATACAGCGAATATAACAG

AAAAGAGCGATGTCTATAGTTATGGAGTGGTGCTGCTGGAAATACTTAGTGGGCGTAGCGCTGTTGATT

CTCAGATAGGTGATGGGCTTCACATCGTTGAGTGGGTAAAGAAGAAAATGGGAAGCTTTGAACCAGCT

GTTACAGTTCTCGACACTAAGCTACAAGGTTTACCCGATCAAGTGGTCCAAGAAATGCTGCAAACGCTTG

GAATAGCGATGTTCTGTGTCAACTCATCGCCGGTTGAAAGGCCAACAATGAAGGAAGTTGTGGCACTTC

TAATGGAAGTAAAGTGTTCACCTGATCAAGAATTTGGGAAAACTTCTCAGCCTCTAATAAAACAGTCCTC

AACTCAAAGTTGATTAATTTTGTTATGTTTTTTTTTTTTTTTGCTTTTGTCTAAGAGGAGTAGGAATTAGT

TTTCTTTAGTTCCCAAGATATGATCAACTCATTGTTCCAAGCATTGGATAATGTCTTGGGGTCCATTGTGT

ACATATTTCAGAAACATTTATGTAGTTTATTTGCTTCTTCATTCTTCCTATCCATCTCTTCCATCTCTGTGCT

GAGATTTTAGCTTTCAAAATGCTTGCAGTTGCACCATATAATATTTTAGTTTACCATAGTTTCAAAAAAAG

AGAAATATAGTTTGACCTATTCAGAAAAATTGTATTGTTGGTGTATGAATGTGGTTTCAAACCTTCCGGT

GATGCTAAAAGTAGTTTCAGAAAAGAGAAATATAGAAGAAGAAATCAAAGAAGGGGAATCTTGGAGCA
ACGGTAAAATTATCTCATGGGTTCAAACTGTGGAAGCAGGCATTAATGCATTAGGTTAAATTGTCTACAT
CATATCCTTTGAAGTGTGGCCTTTCTCTGAGCCTTGCTAACACATGATGTTTTGTTCATTGAGTTGTTTGT
AGAAAGAAGAAAACAAACAACTCTTAAAACCAAATATAATACTCATGTAATGTCATTAGAGACTATTGGT
CTAGTGGTAAGAGTGTAGCGCGTAATGCGTGGGTAGTTGTGTC
>PGSC0003DMG400025330 LRR-RLKs
TTTTGAATTCTCCACTGGTGTCTGGACAACGGTTCTTCTCTCTTTTTTTTTTCTTTCTTTCTATTATACACAA
CTTTCTAGACTACTTTCAAAGTAAAGTCTTCTCTTTTTCAAAATACACACACCCCCCTCCCCTCTTCCTCTTT
TTCTCACTTCGTGCTTGAAATTTCATTATTATGGATTTTCCATCTGTAAGAACTGTAATCATACCCATTGTT
TCTATCAAAACCCCACCATTTTAGCTTTACTCTTTTTTTTTTCAAGATCTGTATTGATTAGTGAACTTGCTAC
TACTCCACATTGCACTTGATATTTATAGTCCAAAGTGAAGAAGTAAAGCTCAAATCTGCATTTTTCATCAA
GAAATCGAAATGGGTGTCTCGGATTTGATCTTTTTGGGACTATTTACGATGACCATTTTCAGTAGAAGCT
GTTTTTCACTAACCCCAGATGGTAAAGTTTACTTCTTTGTGGGTTTTATCTAGTTTTTTCAGCATTGTATGT
TGTTCTTGAGCTAAAAAGATTGTACTTTTTTAGGTTCTGCTTTGTTGGAAATGAGAACCCTTTTGAATGAT
ACAAAAAATGTTTTGAGCAACTGGAATGATTCGGATGAGTCGCCTTGTAGGTGGACAGGCATTTCTTGC
TATCCTAATGACCAAAGAGTTCAATCTATGTATGTTCAATTCACTTTTTTTTGTTTTCACTATCAGTGCTTT
ATTCACTTTGTTTAGTTCTGTTTGCTTGGATTCATTTTCTGCATTTTGTTTCTGAATTTTCAGCAACTTACCT
TATATGGAACTTGGAGGAATTATATCTCCTAGCGTTGGTAAACTCACCAGATTGCAGAGATTGTAAGTCC
CATAGTCATTGAGGCAATGCAACAAAATTGTATTAGGGCTAAAGTTTCTGTCTTGCTACTTGCCTTGATA
AACTATGTGTTCTTTCCTAAAACCAGGGCACTTCACGAAAACAGTCTGCACGGTGTGATTCCTAATGAGA
TTGGCAATTGCCCTGAACTCAGAGCTATGTAAGTAGGAGTTTCATATATAACTGAGTGATAACATGTTTT
CTTGGAGATTCCACATGTTTCTTGATGAATTTGTCACAAGCTAGGTGTTATTTATGCGTACAGATACCTCA
GGGCAAATTTTCTTCAAGGAGGCATACCATCAGATATTGGAAATCTTTCAATACTGACCATATTGTGAGA
TATATATATTTTAATTCACCATCTTATTATATAGTTAATGAGGTTGTTGTTTTAATGATTGGTACCTTTTAA
TCCTTTTTAGAGATTTATCAAGCAATTCACTTAGGGGTGCTATACCTTCTTCTCTTGGTCGTCTCAGACATC
TTCGTCTTCTGTAAGTCCAATTTCTGTTCATTGTAAATTTTTAATTTGATAACTTAATGCATTACACACTGA
TGTGTTAAACATGAAAATCAATTCAGGAACTTGTCAGCCAATTTCTTCTCGGGTGAGATCCCAGATGTTG
GAATACTGAGCACTTTTGGTAACGATTCGTAAGTTCTCCTCCATTGGAATATAATCCCACATTTGATTTTG
CCCTCTGTGTTCATCATTTTCTATATACTCTAAATAAATTTAGACAGATGGTTCAAAACTCAGAAAATACA

ATTTATCGTGAATAATGCTTCACATTATTATATGTATAGAAGTGCAGCAAAATATTCACATTCCATTTGGC
ACTACAATCACACTTATAGGAAATACAATACAAGATAAATCAGGTCTTTGCACACCAGTTTTCTGGTGAT
TAACTGCTGTTTGCTGCATAGGCTAGGTTTGTGGCAGTGTTACAATGACACCCAGCAAATACAAACTAAG
CAGAATACCCATCAAAAGATTGATGTATTAACATAACAAGTGCAGTTGAGATTTTTTACAACTGAACAAA
CCGAATCTATCAATTGATAGTCCTTTAATCATGATCCTAGAAGTTAAATACCATGTGATAATTGAGATGG
GAACTACACTAGCAAATTGATGGTGCTTCAATTTTAAAGATGGTGATTAATTTGTAAGTATACCTGACTT
AAATTTATGGTACTCCAATCATGCAGTATGTGTTGCATATGTTAAGATAGACTTTTTGGTGTTAAATTGCC
TAGTGGGTGATTGGCTGCTACATAGTTGAAACTAGGAATCTAGTCCTGTATCATGATCAGTTTTACTTGT
GTACCTTGCAAAAATTATTCTTCCCCATCATCTCAAAAGGGAAGCTTGATGCTCTCAAAGTCTCAAATAGT
TAAGCTTATAAAGTATTTTCAAATGTCACAAGGGCAAGAATGTATGTGACGGGTCAATTCACGTAGGTTT
CTTTCTGGAGTAAATTCTTAGTCATCCATTGGTAAGGGTGTTGCATCACCAACACTAGCTAGAAACAACT
TGTTTAACTGGAGTCTCGTGAACTGACCTGCCGTTTACCTTTCTTCTTCATAGTCTTGGGAGATAATATTT
CCAGTGTTAAATTTTTCTATTAATGGCTATCTTATAGCAGACTATTCTGATGAGGATAATGCTTATGCTTA
GGTTTATTGGCAATTTAGATCTCTGTGGTCAACAAGTCCGCAAGCCTTGCAGAACATCTATGGGATTCCC
TGTAGTATTGCCTCATGCTGAAAGCGATGAAGCTGCAGGTTGTTATTTGCTACTGCTTCATATCTATTTCC
TTTTAGAAGTTTACTGGCTTTTGGAACTTCCCTTTCCTCCACCAACTGAATCTATTTTTTTTCTCCTTTACTT
CTATGTAGAAAGCTTGATTTGTTGCTAAATTAATGTGATTTCTGCAGTCCCTACGAAGCGATCCTCTCACT
ATATTAGAGCAGCAGTTATTGGTGCAATATCTACATTGGGTTTTGTACTCATTGTGTTCTTTATTTTCCTCT
GGGTTTGGTTGTTATCTAAGAAGGAAAGGACAGCAAAAAAATACACAGAAGTCAAGAAACAAGTCCAC
AAAGAGCCAAGTAAGTGGGTGAATAGTACAATACCGTTTCTTTTCCTTCTCCCAATTTTTGTCTTAATTTG
TGGAGGCTTCAGGTGCCAAGCTTATTACTTTCCATGGAGATCTTCCTTATCCTTCATGCGAGCTAATAGA
GAAGATTGAATCCCTTGATGAGGAAGATGTTGTTGGGGCAGGAGGGTTTGGTACTGTATATCGGATGG
TTATGAATGATTGTGGAACATTTGCTGTTAAAAGAATAGACCGGAATCGTGAAGGCTCTGATCAAGTCTT
TGAGAGAGAGTTAGAAATCTTGGGTAGCATCAAACACATTAATCTGGTCAATCTACGAGGTTACTGCAG
GCTTCCTACTGCAAGGCTTCTCATCTATGATTATTTGGTCATGGGAAGCTTGGACAATTTCCTACATGGTA
TGTTTCCAGCTTTTCAAAATTTGTCCCCAGATCCTTCCTCCTCTCCACATCTCCTCTTAAGCTTATACAAGA
CATCAAATATGGAAAGTGTGTATATTTTTGCCATACATTTTTTATTTGGTTTATCCCGAATACAACCATTAT
TTTCAGTCATCTGTTTTTAAACTGGAGAGGGGGAAAAGCACTATTGCTTTCATTTAAACAATCTGTTTTTC
TTGGCTATTCATTGCATGCATCTTTATTGTATTTTTGCAGAACGTGTGGATGATCGGTTGTTAAACTGGAA

TGCACGTCTGAAAGTAGCTCTTGGTTCTGCAAGAGGATTGGCCTACCTACATCATGACTGTTGTCCTAAA
ATAGTTCATCGAGATATCAAATCAAGCAACATACTCCTTGATGAGAATCTAGAGCCTCGTGTTTCAGACT
TTGGCCTTGCCAAGCTTTTGGTGGATGAGGAAGCTCATGTCACGACTGTGGTTGCGGGTACTTTTGGTTA
TTTAGCACCAGGTATGCATATTCACTTAAATCAGCAAGGCATAAAGATAGATTCACACATATGTTGCCTT
TTAATTGCAAGAATGGTTTATTTTCTTATTTTTTCGCTTGGATAAACCTTAACCTATTCTTCTTTTTATAATC
ATCTATATGTAGGTTTGACTTGCTTAACCTCTATAAATTGAAATTCCAAACTCACCTCATCTTCTAATGAAA
TGTCTGACCAACGATTTCTTGTTTGTATATTGTAATATAACCAGAGTACTTGCAAAGTGGAAGAGCCACA
GAGAAATCTGATGTGTACAGCTTTGGGGTTCTCCTTTTAGAGCTTGTGACTGGAAAAAGGCCAACAGAT
CCATCCTTCGTAAACCGAGGCTTAAATGTGGTTGGCTGGGTATGCATGCTTCCCTTGAAACTCACCAAAT
TTTTGAACTTCATGAAGACTCATTAATTTTTTAAACTTCCCTTTTCTGTATTTAGCTGTACAATATCCACATA
TTGATAGTGTCACAATTTGAAAAAATGAACAGATGAATACCTTGCTGAAGGAGAATAGACTGGAAGACAT
CCTTGATGCGAGTTGCAGAGAAGCCGATGCAGAGACGGTAGAAGCAATTATAGAAATAGCTGCAAGAT
GCACTGATGCAAATCCAGATGATAGACCTACAATGCAACAGGTGCTGCAATATTTGGAGCAAGAGGTCA
TGTCACCATGCCCGAGTGATTTCTATGACGAGTCTCATTCAGATTATTCTTGATGTATGGAATAATTTCCT
GGGACCAAATATTTAGAAACAATTTGCAGCCTGATGCTGAAATGTGTAATCTGCCATTGCAGATACTCTG
TATGCTCCTTGCTTTTGGAGCCCTGCTGCTTTTCTTCCTAATGTCTTTGTGCATCACTTGTGCCTTTTTATGT
AGATACCTGCAGTTAGTTTTAAAGCTACAATTATGAGTCCTAACTAGCCATGGCATTATGTAGTATTTATA
ATCTATTTATCACTAGTGAATTTTTCCTTCACCTATAATAGAGTTTAGGTTCCTCCTAAGAAAGTTCTTTAA
GAAAATTTGATGAAATATGTGAGGACACTCAACTACTGGTGGATGTCATATCTGCAAAGCATATTTGTAT
GCCACTCAATTATCA
>PGSC0003DMG400025635 LRR-RLKs
ATGCGTCTTATATCACGTATTGGTGCTCCAATACCAACAACGACATTTCTGAGAGGTATCATCTTTTGCTT
CTTGACATTTTCCAGAATGGAAATTTTCACAGCGAGTTTTAACTTCCACACCTTTCTGGCTATACATGCTG
TGTTTCTTGTGTTCCTATTCTCATTTTCTCTCCAACATATTGTATCAGCTGCTTTTCTTGGCAATGAAACTG
ACAAACTTGCCTTACTTGGATTCAAGTCCCAAATAACTGAGGATCCAGCAAGAGTTTTCGCCTCTTGGAA
CGATTCTGTTCATTTCTGTCAGTGGACTGGAGTAAAATGTGGCCTGAGACACGGAAGAGTCATCCGTTT
GAATCTCAAAGGGCGAGGCTGGCAGGTATGATCTCAGGTAATCTTGGAAACCTCTCTTTTCTTAATTCTC
TTGACCTTGCAGAAAATGCCTTCCATAACAAAATTGAGGCTTCTTGGATTGTCAGTAAATAGCTTATCCG
GGGAGTTCCCTCCTCCGTTGTACAATTTGTCTTCCCTGGAACTAATATCACTTTCCTTTAACAACTTTTCTG

GTAATCTCAGATCCGGTTTAGGCAACTACTTCCCTAATCTCCAAATACTTTACTTGGCGAAATGTCAATTC
ATTGGTTCCATACCGTCCTCTTTGGCCAATGGTTCAAAATTGCTAGAGCTTGATTTTCCTGTAAACAATTT
CACTTGAAATATACCCAAGGGTTTTGGTAACTTGCAGATTTTCTTGTGGCTCAACGTTTGGAGTAACCAA
CTTGGATACAGTAAGCATGATGACCTTGACTTTGTAATTTTTCTTACCAACTACCAGCAGTCTACAAATGC
TCCATTTTGGAGACAACCAGTTTGTAGGTACATTACCTCATTCAACAGTCAACCTTTCTAGTCAGTTACAA
CGCCTACTCTACTAGGAAACAGAATTACTGGAAGCATACCCAGAGATATCTCAAACCTGGTGAATATGC
ATCTACTCGAGATGAGTAACAACAATCTTACAGGTAGCACTCCAGGTTCTATTGGAAGACTGACAAACTT
GGGAGGTCTCAACTTGGGTAACAGTCTCTTGACAGGGGTCACCCCTTCTTCAATAGGAAACCTCACTGA
ACTCATCTATCTTTATTTGGGACTAAACAGGTTAGAGGGTAACATACCTTCAACATTGGGAAACTGTAGC
CAACTGCTAAAATTGGGTATTTCTGAAAACAACCTAACAGGCACTATACCACAACAACTTTTTGCTCTCTC
ATCCCTCACAAATATTTATGCATCTTATAACTCTTTGACAGGTCCATTACCAGTCTATATTAGTAACTGAA
GTTATCTTACTTATCTTGATTATTCTCACAACAATTTTTCGGGGATGATTCCACGAACTCTTGGAAAATGC
TTGGCCTTGGGGGATCTATTTGAAGGGAAACTCCCTCCAAGGGACCATTCCCAATTTAGAAGATCTGCC
GGATCTCCAATCCTTGGATCTTTCCCAAAATAATCTATCAGGGCCAATTCCTCACTTCATTGCAAGTTTTA
CTTCCTTACTCTACTTGAACTTATCTTTTAACAATCTAGACAGTGAGGTTCATGTCACCGGAATATTTTCAA
ATTTGAGTGCAGATGTATTAAACTGGAACTCCGAGCATTTTGGTGGGATTCAAGAGCTGCATTTACGACC
TTGTGTTTATCAAAAGACACAGAAGAAGCATGTACTTCCCTCAAGCATGATACTTCACAGAATGAATATC
ATAATATGTGGCCTCTGCACTTCATTATCTTCACCATGAGTGCCAAACACCCATGATCCACTGTGATATAA
ACCACAGAACATTCTTCTTGATGAAGATCTCACAGCTCATTTGGATGATTTTGGTTTGGTGAGACTCATTC
TTGATGAAGATCTCACAGCTCAGTTTAGCTCACTTGGAGTTATGGGAACCATTGGCTATGCAGCTCCAGG
TGGTGTTCCTAGGTTAACAAAAACTGTGATATGAAATTTCTTCAAACAATAGTTGTTTATTTATAAGCTAA
ATTTGCTTGTTGCAGAATACGGCATGGTAAATAAGGTCTCCGCTTTAGGAGATATGTACAGTTTTGGGAT
TCTCATATTGGAGATATTCACTGGAAGAAGACCCACAGACACTTTGTTCCAAGCAAGCTCTAGTCCTCAC
CACTTTGTCGAAACAGCGTTGCCAGAGAAAGTTATGGAGATTCTAGACAAAACAACTTTTCATGGTGAG
ATGAGTAAAGCAACCAACGTCAAAGAACACTGGGGCAGTATCAAGAAAGAAGAGATGGAATGCTTGGT
TAGCATACTTGAGATTGGTGTCGCATGTTCAGCTGAATCCCCAAGAGACAGATTGACCGTGACACAAGT
TTACAGTAAGTTGACACTAATGAGAGAACAATTTCTACAAAGGGGAGGTTGAGGCCACAGGCTACAAGT
GAAAGCATTGGATTTCATCTAAAATGGTTTAAATTCTGTCTACTTGGTAAGAATGCTAACATTCTGTCCAG
CATGCAGAAGAACTAAGGTAGTGTCAGATTTCTTGTTACTTAATGGATTTAGTCTAGAAACATCAGATAT

TAGTTCTTAATGTCTACATCAGATAATACGTTTCATTCATGAATGTAAAGAATCAGATGTTTTAGGAGATC
ATAAGGCAAGATCAAATGGTCATGGTGAGAATGTTCCTAAAAGGTTGTGATTCTAGACACAGATGAGGT
ATTCTTGTCCCAAAGTTGGAGCATCATGATCAATACTTGGAGGGTTAGTGAGAACACTGTTGATCTTTGC
GTTGCTTTCTTAGCTTTGCTTGTCGTTTGTCCTTGTCCTTTATTTGAATACCAATGCCATGCCCACATGACA
TTGTTTAGCCTTCATCTTGTCAAGTCCACACCAAGTTGCCTTGCCCTCAAGTCCTTGGTCCTCACTTACCAC
TTTGGAATCAAGCCTTGGTGCTATTGTGCACACCTTAGCTTTGTCAACCGTCCGTCGCCAACTTAG
>PGSC0003DMG400025771 LRR-RLKs
CAACACTTTCTTTTTTTTGCCTTAACAATTCTCCATCTCTTGCTGCCTTTTTGGTCACCAAAACAACTTCTCC
AGTAGTTGGTTTACCTCTTCCTCTTTCTTCTTATGTATATAGAAAGTAATCATGAATAATCACTTCAAGAA
ATGATGACTATCTCAGAGCATTACTATTCTTTCTGGTCATTTGGGATCTTTTTCTTTCTTCTTTTATGTCCTT
CATTTGGTCTCAACATAGATGGAACTCTCTTGTTGTCTTTCAAATACTCTGTTCTTGATGACCCTTTATCAG
TTCTTGATAATTGGGACTATAATGATGCAACCCCATGTTTATGGAATGGGGTAACTTGTGCACCAGACAT
GTTTAGAGTCATTAGTTTACTACTCCCAAATTCTAAGCTTATTGGTTCTATTCCAGAAGAATTAGGATTTA
TTCAACATCTTCATACTATTGATCTTTCAAATAATTTCTTGAATGGAACACTTCCTCTTTCACTTCTCAATGC
TTCTGAACTTCAAGTTCTTTCACTGTCAAACAATGCTATTTCTGGTGAGCTTACTGAGTCTATTGGAGTGT
TAAAGAGCTTAAAGGTTCTAAATCTTTCTGTCAATGCATTTGTAGGAAATATCCCACAAAAGCTAAGTTC
TTTGCAGAATCTAAAAGTTGTTTCTTTGAGTAAAAACCTATTTTCTGGTGCAATACCAAGTGGATTTCAGT
TTGTTGAAGTGTTGGATCTTTCTTCTAACTTGTTGAATGGAACATTGCCAGATGATTTTGGTGGTGATAG
TTTGAAATACTTGAACTTTTCATCAAACAAGTTATCTGGTTTAGTGTCACCACAATTTGCCAAGAAAATAC
CAACAAATGCTACAATTGATCTTTCTTTCAACAGCTTTACAGGTGAAATCCCAGAGTCATTAGCTTTATCA
AACCAGAAAACAGAATTCTTTGCAGGAAATACAGATCTTTGTGGCAAACCACTCAAGAAACTCTGTACA
ATCCCTTCAACACTTTCATCTCCACCTAATATAACCACTAATCCTCCAGCAATTGCAGCCATTCCAAAAGA
AATTAACTCAACCCCATTTCAAGATTCTGATGGAACAACTCAAACTGCAGCTCAGAATCAACAACAACAT
GGGCTAAAGCCAGGTACCATAATGGGAATCATAGTTGGTGACTTAGCTGGTGTTGGTGTTCTTGCTATT
GTCTTTCTTTATGTCTACAAATTGAAGAAGAAGAATAAAGCAAGTGAGGCAACCATAGAAAGCTCAATT
GACAAAGATCAAAAATACAACAAAACACCAGAACCATCAATACTTGTTGTAAAAGAAAAAGACACCATT
TTTCCTAGTTGGCCATGCTTGACCATAAATCAAGAAAGTTCAGATACTGATGACAGCAAAAACCAAGAA
AGTGAAGACCAAATTGAGTATGAAACAGAACAAAAGAATGAGAAAAACAAGAAAACAGAAAGGTCATT
TGTAATGGTGGATGGTGAAACAGAATTAGAGCTAGAGACTTTGTTGAAAGCTTCAGCTTATATTTTAGG

GTCAAGTGGAGCTAGCATAGTTTACAAAGCTGTGTTAGAAGATGGAACTGCATTTGCTGTTAGGAGAAT
TGGAGAGAGTGGTGTTGATAAATTGAAGGATTTTGAGCAACAAATTAAAGGAATTAACAAGCTAAGGC
ATCCAAATCTTGTTCGTGTTAGAGGATTCTATTGGGGAGATGATGAGAAATTAGTCATCTATGATTATGT
TACTAATGGAAGCTTAGCCAATATTGGTTACAGTAAGTTACTTTTCTTTCATCTTCATTATCTTATCATAGT
TATAGTTTTGATAAATCTTTTCTGTCCTTATCATAATTACCCCCAAATATTCTGATTAGTCAAGTGTACTTG
ACATTTAATGTCACTGGTATATTTGCAGCAACATTCCTTGATTTGTCACTTAACAAAAACATTAAAGCCTA
TAGTTTAGCGGTAGTAATAAATATTGTACTAAGGGGTCGTTTGATAGCTGGTTAGAGTTATGCATGTATT
AATCATGTAGGAATTGGTATTATTTAATTAAGTGATTAGTTACTCCACATTCTATGTTGTATAAAGTAATA
CATAGCTTTCCTCGTAACTTATATATGTATTAGTTATAAGGGTTTTTAAATTACAAACCAGACAACTCCCA
AAGATAATACAACTTACGTCTAAACCAACTACCAAACGAGTACTTTTTTAAGTACACTGTCTGTCATTAGA
AAAACTAGTTTAGTTTTAGTGTACATATACTACTCATAGGCATGGATTTTCGTTACTAAATTTTGCAGATA
CTATACTTCATATGTGGTTGTGTTTGAGGCTCCCAATGGTCCTCCATGTGACATTGTCATAATCCGTGAAA
GTCCCCAAACTATACTACAATACATATAATACGTCAAAATACATGTGCACCCCTTCTCCTCCATATTGTAG
TACCACAAACGTTCCCGAGACTTGATTTTTTCAAACTCCACGTTTGATCAGATTATACTGAGTATAATATT
ATTGTTTAAATTTTTCAGAAAAAGTTGGTTCCTCTCCTTACCATTTATCATATGAAGTCCGGCTCAAGATA
GCAAAAGGAATTGCAAGAGGATTGACCTACATCCACGAAAAGAGACAAGTACATGGCAATATTAAACCT
AGTAACATTCTCTTAACACCAGATATGGAGCCCATAATAAGTGACATTGGGCTTCATGGGCTAATGCATG
GTAAAAACACCTCTAAACCGGATAATTCGGGCCGGCATTTCGGTAGCAAGCGGTCCACATCCTCCTCCCG
GGACGGTTTAAATGATCAACCGGTTCATGGAAGTCCTTACATTGCCCCAGCCGGGTTCGTGGTAGGGTG
CACGTCCCCCTACCACGCCCCGGAGTCGTTGGAGAGTCTCAAACCTAGTCCTAAATGGGATGTTTACTCA
TTTGGGATTGTGTTGCTTGAGCTTTTAACCGGGAAAGTATTCTCGGACCGGGAGTTGAGCCAGTGGACC
ACCGGTTCAGTATCGGATGACGTGGACCTGGTTTTAAGGATGGCTGATGTGGCGATTAGAGCTGACGTG
GAGAGCAGGGAAGAGACCACGGTGTCATTGTTTAAGTTAGGTTTTAGTTGTGCATCATTGAATCCACAA
AAAAGGCCTACAATGAAAGATGCCCTTCATGTGCTTGATAAAGTACCAGGTTATTCACATTACTAATCAT
TATGTTGGGACCAAGAAAAGCTATAAAAAGCTGTTTAGTTCCCTTTAATTTGTTCTTCTTTTGACTTGGAA
AGATTTTTGCTCCATAAAGATGAGAAGAACCGTTGTGTTGTAATTGGTGAAAGTGTTTGTAAGTTTGACA
AATTGATCAACAGTGTATTTATTTGAGTTTTTTAATAATAGTAGTTTTTTTTTTCACCCGC
>PGSC0003DMG400026004 LRR-RLKs
TTCTTCACAAAAGCCAACACTCCTTTCTTGAAATTTGTCACTAAAACCAACTTTCCCTACAAACTTTTCCTT

CCATTTTTCTTGCAAAAACCAAATATGGGTTTTCTCTCTGTTCTCTCTTCCATTTTCCTTTTCATCCTAATAT
TAACATTCCCACAACAAAGGGTTTCTTCTGAAGCCATTAATGATAGTACTAAAACTTCTTTATCTTTGCTT
ATGACTATCAAAGCTTCTTTGGACCCAAAAAGCCTCATTCTTTCATCATGGTCACCTACTTCTACTGACCC
ATGCAATGATTCTTTTGAAGGTATTGCTTGTAATGAGCTTGGTCAAGTTGTTAACATTTCTTTGCAAGGCA
AAGATCTTACTGGAAAAATCCCACCTGAGATTGGTCAGCTTCAAAGCTTGTCTGGATTGTACTTGCACTT
TAATAAGCTACATGGGGTTGTACCTAAAGAAATTGCTAACTTAACTCAGCTCTCAGATTTGTATCTTAATG
TGAATAATCTCTCTGGTGTTATTCCTCCTGAGGTTGGCAACATGTCTAGTCTTCAAGGTAGGTTCTTTTTT
GCTTTGTTGTCTTGACTAGTTATGGTAAAAAGCCTCAGTCTTGATGCTTAGTTTTGACTTAATTTGAATGA
ATGTTTGAACTTTGCCAAATAGATCTATACCTCTATTAATGAAATGGGTAGCTCAATAAAGAGTCCAATA
AGTGATAATGCTGTTAGTTATTGAGTAATTTCACAATGCCTATGGCTTTTCTTGAAAAAAGATTGGATTTT
TAGTACTCATTAGTCATTGAAAGTCTATATTAAAAAGGTTCTTGAAGTATCTTTTATGAGGTGATTTTTGT
TCCTATGTAGCTAACAGCTAGGTGCTTAGAACATGAAACACTTGAAAAAGCCTCCATCTTTGGTTTTATG
TTAAGCTTTTCTCTGTCATTTGTCTCTTTCCCCATTGATTTGAGCTTCAATATGGGCTAATGCTTGAATAAA
GTTTCAACTTTCCTGGCAAACTTGCAAAATTCCTAGTCTCTTGTTTGGGTGGCTTTTTCTGCATTTTTGGTG
GAATAAATCTCAAATAAAAGTAACCGAGCTCCATTTGTTTTCTGTTTTTACACAGTACTGCAACTATGTTA
TAACCAGTTGACTGGAAGTATACCTAATCAACTTGGAGCTGTGAAGAAACTGAGTGTTCTTGCTTTGCAA
GTCAATCAGTTAACTGGAGCAATTCCTGCAAGCTTGGGGGATTTGGAGATGTTAACCAGGCTTGACTTG
AGCTTCAATAATCTTTTCGGGTCTATTCCAGTGAAACTCGCGGATGCTCTAAAGCTAGAAGTTCTTGATAT
CAGAAATAATACCCTTTCTGGCAATGTCCCTCAAGGTAGTTCTATTTGATTTTCTTGATAGTACCTTTAATC
CTATTTCATTGCTTAATATGCTTAAGAAATAAAGAAATCTTAGGTTCTTTGATTCCCCCTAATATCAATCCA
AATGTTTACTAATATTAGACATTATAGTAGAACTAACTCTCTTTTTGGTCTTTTTGGTTTGGGCAATGGAT
GAAATGAAGCCTTGAAGAGACTGAATGAAGGATTTCAGTATGCAAACAACCCAGACTTGTGTGGAATTG
AATTTTCCTCCTTGAAACTCTGCACTGTATCTAGTCTAAACCAAAACAGACCACAACCATTTGAACCTGGT
TCGAATCGTCTTCCCACAAAAGACATTCCAGAGTCTGCAAATGTACAAACAAAACAAATGAGTCAGTCAA
GAAAATCACAAACTGCTGTGGTTGTTGGTGTTATTGCACTTTTTGTTGCAGTAGCAGTAACTGGACTTTT
CACATTTTCGTTGTACCGTCGAAGGAAACAGAAAATTGGAGGTACTCTTGATACCTCCGATAGGCGACTC
AGTACTGATGAGGTGAAGGAGATCTCTAGAAGAAGCGCGTCGCCCCTCATCAGCCTGGAATATTCCAAT
GGCTGGGATCCTCTAGGCAAAGGCCGAGGTGGAAGTGCTTTCTCTCAGGAAGTATTTGAGAGCTTTATG
TTCAATTTGGATGAAGTTGAGTCAGCTACACAGTACTTTTCAGAAGCAAACTTGTTAGGCAAGAGTAATT

TCACAGCTGTTTATAAAGGGACATTGAGGGATGGGTCTGTTGTTGCTATTAAGTGCATTTCCAAGACTAG
CTGCAAGTCAGATGAAACTGAATTTCTCAAGGGACTAAAACTCTTAACCTCATTGAAACATGAAAATCTT
CTGAGGTTGAGAGGCTTTTGTTGTTCAAAAGGTAGGGGGGAATGCTTTCTGATCTACGATTTCGTTCCAA
ATGGAAATTTGTTGCAGTATCTTGACGTGAAGGATGGAAAAGGAAAGATCCTTGATTGGTCTACCAGAT
TTTCTATAATCAAAGGCATTGCCAGAGGTGAGCTACTGTTTGATTAGTCCCATGTTTGATGTTTTCTTTAT
AGCTCTTTGACGACCAGATTTGAATAACAACATTTGTTATCTATTGTTTAACAGGTATTCATTACTTGCAT
AGAAACAAGGGAGGCAAACCTGCTCTAGTCCATCGAAATATATCAGCTGAAAAAGTACTTGTCGATCAA
CACTACAACCCATTGCTCTCGGATTCTGGTCTGCATAAACTACTAGCAGATGACATTGTCTTCTCAACACT
CAAGGAAAGTGCTGCAATGGGATACCTAGCTCCTGAGTATACAACCACTGGACGGTTCACCGAAAAGA
GTGATCTTTATGCATTTGGCATGATCATATTTCAGATTCTCTCCGGAAGACGTAAAATCACCCCTTCTAAC
CACCAAGGGGCAGAGTTGTGCAGATTTGAAGACTTCATTGATCCGAATCTTTGGGGCAACTTTGTTGAA
GCGGAGGCAGTTCAGATGGCAAAGGTAGCTCTACTTTGCACGCACGAGTCACCAAATCAGAGGCCAGA
CATTGAAATCGTCATGCAAGAACTGAATGATTTAACCTCCAGCAAGTCGTAAAAACTATTGCAAACAACA
TCCAAGTTCTCCAATGACCATTAGGAGCCTGATATTAACATCTTACTGATGCCAGGAGTAGGCACCATTT
TCCCATCCTTTTGATCCTGCATGACTTGTGCCTGAGGACGACACATGAATGCCAGGTGCCTGTTCCACAT
AGTTTATTAGTACTAATGCACCTCGACGTAGGTAGGCCTCAAGTGATCAGTTCTCTTGTATTATGTAACTT
AACCATAATTCTAAGCAAGTCTTCTCCACTTGCAAACTAGTGTACCAGAAGTGCAATTTCTATTTAGTATC
ATATGAGTTATGACCTCTTGC
>PGSC0003DMG400026072 LRR-RLKs
CTAAGACCTCATTTTTCTATCATCACTAAATGGACTAAAAACTAACAAACTCTGCATTCAGAAACTTCTTTT
TCTTTTATCTATTTCTGGTGACTGACACTGCAAATTCCCATTTTCCCTTTAGAGGGGTCTCATTCAAACTTT
TGAATTTTTTGGGTATTCAAGAAAACAAAAAGTTTGAAGCTTACTGCCTCTTTATGTGTGAATAAATAAG
AGAAACAGAAAATCATCTTACTATTTCAGGTATAACTCATGTTTTCTTTAAGCCTTATTCATTTGTTAGTCT
TTTAATTACATAAAAATCCAAACTTTATGTGTTATATGAGTTATCAGATGATTCTTAACAACTAAGCTATC
ATTTAGCCATAGATTTTAGATGATATTTTGAAAATTTATATTCCTATCTGTTTGGCCATGAAATTTGATATT
TTTGGAGTTTTGGGACTTTCCCTCTTAAAACTTCAAAATCTATAGCCAAACAGGACCTAAAGGATGCATG
TTACTTATGATTTCTTAAGAAAGTTTACTTTTTTGTTTCTATGGATATCTCTGTTTCTGTAAAATTATGGAA
GCTGTTTGTTTTCTGTCAATTAGTTGATTTCCATGTTCCTTGTTTGCCTATTGTAGTTTGCTACAACTTTCTG
GGAAAAAAAGTTGTTTTGTTATTGTTACAAATATGGAAATCCTAGATTTCAATCTTCTTGCTTATATTCCT

ATAATTCTTGCAATTATGTTTCGAGGGGTCTATTGAAAAAAGTCTCTACCTACCCCAGATTCCACACTTGT
GTTTTTGGGGTAAATGAGTATCTTCTTTCATCTGGACAGTCTGTAAAATAAGGTGAAACTATTATATTTTG
AGTCACTTTAAAGGGAGATAATTTAATTTTTATATAGTGTAATTCACAACTAAATGAGAAATTGACATCTT
AACATAAGTTTATTTCCTTCCCAACCCCACCCAACAATTCCATCTTTTATAATCTAGACTTCCTGTATCTGT
AAGTTTTCTTTTTCTTGAACTACAATTTTTATTTCATGTTGGAAAGTTATATGAAGTATATGACAAACATTT
TGTTAGGTATACATACAACACTTTAAAAAAAGACAGCTTTTTTTGAATTGGATATCAGTATTATCTTGTTT
TTTTTAATAGACCACTTTATCTTAGTGTTTGATATCTGAGTTTCCACTGTTTTTCTTGCAGCTGCTTCTTGG
ATGGACACCCATCATGGCAATAATGGAGTTCTTGATTGGATTTGTGTCCCCTTTATGAAATGACCCTTTTG
CCCTTCTCAAATTTCAAGAATGGCCAGAATCCAAAAACTTCAAAACTCTCTCTCAATTCTTGCTGTTTTCTT
GTTTTTGAATTTTTTTGTCCAGACATGTAAGTCCTTAACCTCAGAAAGTGAGGCTCTTTTGCATTTCAAAG
AGCAGCTTAATGACCCTTTGAATTACTTGGATTCTTGGAAGGACTCAGAATCTCCTTGCAAGTTCTATGG
AATTACATGTGATAAAAATACTGGTCTTGTTATCGAAATTTCCCTCGATAATAAGTCGCTTTCCGGGGTG
ATTTCACCTTCAATTTTTTCACTCCAAAGTCTTACTTCTCTTGTGCTACCTTCAAATGCACTATCAGGGAAA
CTTCCTAGTGAAGTTACCAACTGCACCAGTCTCAAAGTCTTGAATGTCACTGGAAATAACATGAACGGAA
CGATACCTGATTTGTCTAAGTTGACTAACTTAGAAGTGTTGGACTTGTCGATTAACTATTTTTCCGGGGA
ATTCCCATCTTGGGTTGGAAATATGACTGGTTTGGTTGCGCTAGGGCTTGGAGATAATGACTTTGTTGAA
GGTAAAATTCCTGAGACTCTTGGGAATTTAAAGAAAGTGTATTGGCTTTACCTGGCAGGTTCAAATTTGA
CAGGAGAAATCCCGGAATCCATTTTTGAAATGGGGGCACTAGGTACATTGGATATTTCAAGAAATCAGA
TTATTGGGAATTTCTCTAAGTCAGTAAACAAGCTGAAAAATTTATGGAAAATTGAGCTGTTTCAGAATAA
GTTGACAGGGGAACTTCCTGTAGAACTGGCAGAGCTCTCTCTTTTGCAGGAATTTGATATCTCTAGCAAC
CATATGTATGGGAAGCTGCCTCCAGAAATCGGGAACCTGAAGAAGTTAACAGTTTTTCAAGTATTCATG
AATAACTTCTCTGGTGAAATTCCTCCTGGTTTTGGAGATATGCAGCATCTTAATGCCTTTTCTGTGTACAG
GAACAATTTTTCTGGGGTATTTCCAGCAAATCTTGGTCGGTTTTCCCCCCTGAATAGCATAGACATATCTG
AAAACAAGTTTACTGGTGGATTCCCAAAGTACTTGTGCCAAAATGGGAACCTGCAGTTTTTGCTAGCTAT
TGAGAACAGTTTCTCAGGGGAGTTTCCGAGTACTTATTCATCGTGCAAGCCTTTACAGAGATTGAGGGTC
AGTAAGAATCAACTTTCTGGGCAAATTCCTAGTGGTGTATGGGGACTTCCAAATGTATTTATGATGGATT
TCAGTGATAACAAATTCAGTGGAACTATGTCCCCGGAAATTGGGGCTGCTACTAGCCTGAATCAGTTGG
TATTATCAAATAACAGATTTTCAGGTGAGCTTCCAAAAGAACTTGGGAAACTCACACAATTGGAAAGGCT
TTACTTGGATAACAATAACTTCTCGGGCGCAATACCTTCTGAACTTGGTAAACTAAAGCAAATTTCATCTT

TGCATTTGGAGAAAAACTCATTCTCGGGCACTATCCCATCAGAATTGGGTGAATTTCCTAGGCTTGCAGA
CCTGAATCTTGCTTCAAATCTTCTTACAGGTAGCATTCCCAATTCCTTATCAACAATGACCTCTTTGAACTC
TCTGAATCTTTCACACAACAGACTCACTGGTACAATACCAACTAGCTTGGACAATCTGAAGCTGTCTTCAT
TGGATCTCTCAAACAACCAGCTATCAGGAGAGGTATCATTAGATCTTTTAACACTGGGAGGAGACAAGG
CATTAGCTGGTAACAAAGGACTTTGTATCGACCAAAGCATCAGATTCTCGATCAACTCAGGCTTGGGCA
GTTGTGGTGGAAAGGCTGCTAAACATAAGTTAAACAAACTAGTTGTGTCTTGCATTGTGTTGCTTTCCTT
AGCGGTTCTAATGGGTGGGTTATTGCTCGTCAGTTACCTAAACTACAAGCATAGTCATGAAGTAGATCAT
GAAGAAAAACTGGAGGAAGCCAAGGGAACAAATGCAAAATGGAAGCTTGAGAGTTTCCACCCTGTGGA
ATTTGATGCAGATGAAGTTTGTGATTTCGATGAGGACAATTTGATCGGAAGTGGAGGTACAGGAAAAG
TTTATCGGTTAGATTTGAAGAAAGGTTGTGGAACAGTAGCAGTGAAGCAGCTGTGGAAAGGGATTGGT
GTGAAAGTTTTGACAAGGGAAATGGAAATCTTGGGAAAAATCAGGCACAGAAATATAGTTAAGTTGTAT
GCCAGTCTAATGAAAGAAGGTTCAAATATCTTGGTTTTCGAGTACATGCCAAATGGTAATCTTTTCGAGG
CACTACACCGAGAGATCAAGGCTGGCAAGCCAGAACTAGATTGGTATCAGAGATATAAAATAGCCCTTG
GAGCTGCAAAAGGAATTGCTTATCTGCATCATGATTGTTACCCTCCTATTATTCACAGAGATATCAAATCA
ACCAACATTCTACTTGATGAGTATTATGAAGCAAAAGTTTCAGATTTCGGGGTTGCAAAAGTTTCAGAAA
TTTCTTCAAGGGGATCCGAGTTCAGTTGTTTTGCAGGCACTCATGGTTACATGGCTCCTGGTAAGTTTCA
AGAAAACCTTAAATTGAGTGACCATTTTGTCTCTCCTTGTTACTTTCCATTCTTGAATTTGCATTTACTTTC
CTATATGTTTGCTTGACAGAAATGGCATACACTCTCAGAGTGACCGAAAAGAACGACATTTACAGCTTTG
GTGTCGTGCTATTGGAGCTAGTAACTGGAAGAAAACCAATAGAAGAAGCCTACGGAGAAGGAAAAGAC
CTGATCTACTGGACCTCAACTCATCTAAATGACAAGGAAAGTATTAACAAAGTTCTTGATCAAAAGGTGG
TATCTGAACTTGTTCAGGATGAAATGATCAAAGTGTTGAGAATTGCCACACTTTGTACTACTAAGCTTCCT
AATTTGCGTCCCAGCATGAAAGAGGTTGTCAATATGCTCGTTGATGCTGAACCTTTGACGTTTAGGTCTT
CAAGCAAATCTGAGAAAAAAGGGAACAGTTTTTCTGAGGTCTAGTTTCTCTTCTTTTTTTTTATTGGGTTT
TCCCTGTTGATAGTTGCTCTGTAGTATGATTCTCAAGTTAGTTTTAGGTATCTGATATACATAAATTTATA
CCATGTGATGTGATATTCCACTTTCTTTATGTAAAAGTTCGAATTGTGTAAGAAAAATGTACTTTTTTCTG
CTGTGATTGGAAACTTCAGAAGTGGGATTAATCAGAAATGTGGGTAGTTAATGTCCATGATTAAGAGTC
CCACATCGGCAAAAATACATTAGATATGTTGAGTATATAAATAAGCAGGCCTTACACTCAAATAACATGT
CTTAAAGCCGCATAGGTGTAGACTCAAAGCTAACAATATTGCCTTACACAAAAACTTGTTTTGTTGTAAA
CTTTGAATCTGATTCAATGAATATCCACATAGATAGTTGGAAGCAAAACTGATATACATAAACATTTCAT

ACATTTGTATAAGAGGGATGGCAACCAACAATGTTACTCTTTTATCTTTTGTGATCCTCCTCAGTATTACA
TGGTACATTCTGCAGATTTTTCCTGCTCTCTATGACATTACGGTTCCAAATCTTCACTCCTTTGCTTCAAGA
AGTTGAGTTAATGCAGCATCAATTTGAGCAAATACCTCCTCTTTGGAAACGCTCCCGTCCACCTGAAATT
AAAATAGGTCAGAGAAGATAGAAGTTTTAACACTGAGAAATGATAAGAACACGTTCATGCACGCGTATT
GAAAGAAAGAACATACAGTATCCTTGTTCAGTGAAGAAAAAGAGAAGGGTAAGCAAAAGGGGAGTCCT
TGATTAAGTATATTTCCCCCCAGATTGTTTGGTATGAATGTAAAACTCAAAGGATTTGCTCTTCCTTTATCT
AATTGAGGAGAGATGAATAATTTTAAGATAAAAGTGGATGTATAGCCCTTTTGCTTTGGCTTCCCTTGTT
GTTTTAACAACTGTGTGAGTATGGATCAGTCTGGATGAACTGGAACATAATTTAAATTGAACATTTTGTA
ACCTACATGTCAAACAACATAATCTTATAACCATATCATTATTCTTTCGCCAATTAGAAAACCAAACTACTT
TTTCTCCAAACCATTTCCAACCAACCCCCTCCACATTGTCTTTTTTATGGACTATATTCCTGTTAACCAAGA
CCTTGGAAACCATCAAACAACCCAACAACTATGAATCAAGCATATAAGAATGATTCCACGTTTTGAAGGT
GAGTGTTCAATCTGCATTCCCGAGTGAAGCAACTGCACCACATCTGTGGCAAAAAGAATACCTGGAATA
TAGTATCTTTGTACATTGAGAGAACTGATTCCACATTTTGATGGTGAGTGTGCAGACGCAGCTTCACCTG
TAAATGGTAAACTGAAAAAGAAATAAGACAAAAATGAAGCTGATCACAACATAAATTTATGGTGTTTTA
GAAACCTATACTTAAGTCAATTATCTACAAGGGGACTTGAGTAAGCAAGGGAAAAGAACATCAAGAATG
ATAAAG
>PGSC0003DMG400026749 LRR-RLKs
CTCAAAACCACAGGTTACTAGTACTAATATTTGCACTAATGGAGAAAGCCTTCACTATTTTTCTCTTAACA
ATACTCTTGTTGCTTCACTATGTTATGACCCAAACCAACATTACTACTGATCAATTAGCTCTTCTTTCTTTA
AAATCTCAAATCATTTCGGATCCCTTTCACTTCTTGAATGAAAGCTGGTCTCCCGCTATTTCTGTTTGTCAT
TGGGTAGGAGTCACTTGTGGTTCTCATCACCAGCGAGTGAAGTCCTTGAATCTTTCCAACATGGCTCTTA
CAGGCAGGATTCCCCGTGATTTTGGAAACCTCACATTTCTTGGTTCTCTTGACTTGGGAAGCAACAATTTC
CAGGGATATATGCCTCAAGAAATGGCATATTTGCATCGGCTTAAGTTTCTTGATTTAAGCTTCAACAACTT
TAGAGGGGAAAATCCTTCTTGGTTTGGGTTTTTACACCAACTTCAAGATCTAAATCTTGGAAATAATAGT
TTCATTGGTTCCATCCCTTCTTCATTTTCTAATATTTCCACACTTGAAACTTTGAATCTGAATTTCAATTCCA
TAGAGGGTGAAATCCCAGAAGTGATTGGAAGTCTTATAAACCTTAGAGTTTTAAGCTTGTATGGAAATA
ATCTCATAGGTTCTATTCCTCCGTCACTCTCGAATGCCTCAAGGTTGGAGGCATTAGATTTATCTCGTAAT
TTACTTCAAGGGAACATTCCAGAAGGGATTGGCAATCTACACAAGATGAAATTGTTGTCCATACAACATA
ATAAACTTACGGGTTCTATACCGTTCACAATTTTCAATATTTCTAGAATCAAATTCATTGCATTTACAGAC

AATAGCTTATCAGGAAATCTTCCCAATGGTTTATGTAATGGTCTCCCAATACTCAAAGGGCTTTATCTATC
CACAAACAAACTTGGTGGTCATATGCCTACAAGCTTGTCAAATTGTTCACAACTTCAAATATTGAATTTAT
CAGGTAATGAGTTTGACGGACGAATACATAGTGAAATTGGAAGATTGAGTAACTTGCAGATATTGTATC
TCGGATCTAACCATTTCACAGGTACGATCCTAGCTACAACATTATTAATTCTTTTATGAATACTTGTAATAT
TGAACTTGAACTGCAGGAATAATTCCACAAGAGTTAGGAAATCTTGCTAATTTGGCGGATTTAGGCATG
GAGGATAACCAGATTAGTGGCTCTATCCCGATCAACATATTCAATATCTCCTCACTGCAACGTTTTGGACT
ATGGCGGAACAATCTCAAGGGATCCTTACCACGAGAGATTGGCAACTTAACCAAGATACAGATTCTAGA
TCTTAGAGAAAATACATTTATTGGTACGTATTTGAAGTGATAGCAAAATTATAAACTTGTACTAGCTTGA
ACTAACTCATTCTTAATTTCTTTAGGTGAAATACCCAAAGAGATGAGCAATATGATGGAGTTGGAGGTAC
TTAGTCTCGGGTTAAATAGTTTCAGTGGTTCACTTCAAATAGAGATGTTCAACAGTACTTCACGCTTGAG
GATAATTTCTCTTACAAACAATAATCTATCAGGAACTCTACCATCAAACATAGATTCTGTCTTACCCAACA
TTGAAGAGCTTTATTTGGGTGCCTTAACAAATCTTGTTGGGACTATTCCTCATTCTATTTCCAATTGTTCAA
AACTTACTAATCTAGAGCTTTCAAATAATAAACTCACTGGCTTGATTCCCAATTCTCTTGGATATTTGGCT
AATCTACAGTTCCTAAACTTGGCAGCAAACAATTTAACAAGTGACTCATCGTTTAGCTTCCTCACTTCCTT
AACCAATTGTAGAAATTTAACAATTCTTTTTCTATCTTTGAACCCTCTAAATGGCATGCTTCCAGTATCTGC
AGGGAACCTTTCCACTTCTCTTACAATGTTTTACGCCAGTTCTTGCAATATTAAAGGGCGAATTCCAAATG
AAGTTGGGAACTTAAGCAACTTGTTAGAGCTTGATCTTTCTGGAAACAACCTGGTTGGATCAATTCCCAC
ATCAATTGGCAACTTGAGAAACCTTCAACGCTTCAACTTGAGTTACAACAAACTTACAGGATTTATTGGA
GATTTAATTTGTAAATTGCAGCATTTGGGTGCTATTTACTTGGGACAAAATCAACTTTCAGGATCTCTTCC
TAATTGTTTAGGGAATGTTACTTCCCTTAGATTGATACATCTTGGTTCCAATAAATTGATTTCCAATATACC
ACCAAGTTTAGGGAATCTTAAGGATTTAATGGAGCTTGACTTATCCTCAAACAACATGGTTGGTTCTTTA
CCACCAGAAATTGGAAATCTAAAAGCTGTGACACATATGGATCTGTCGATGAATCAATTCTCAAATGGA
ATTCCTAGGGAAATTGGAGGATTGCAAAATCTGGTGCATCTTTCTTTGAGACACAACAAGTTGCAAGGA
TCTATACCTGACTCAGTGAGCAACATGGTAGGTTTGGAATTCCTAGACATTTCTCATAATAATATATCAG
GAACCATTCCTATGTCTTTGGAGAAACTTCAAAACTTGAAGTATTTCAATGTTTCCGTCAACAAGTTGCAC
GGTGAAATACCCTCAGAGGGTCCTTTCAAGAACCTCTCGAGTAAGTTTTTCATCGACAATGAAGCATTGT
GTGGTTCTTCAAGTTTTAGTGTCCCACCATGCGCCACATCATCAAAGCATAGATCAAATAGGAAAAAAAT
GCTTGTTCTATTTCTTGTGCTGGGAATTGCACTTCTTATGCTGGGAATTGCACTTGTATTTGTTCCTATTAC
CTTTGTGTTTGTATGGATAAAGTATAGAAGAGGTAAAGGTGATCCTCAACAAGCTGACTCATTGTCTACC

GTAACAAGAGAAAGAATTTCATACTATGAACTGCTCCAAGCAACTGAGTCGCTTAGCGAGAGTAATCTG
ATTGGTTCTGGAAGTTTTGGCTCTGTTTACAAAGGTGTTCTCAGAAGTGGGACTGCCATTGCAGCTAAAG
TATTCAATCTGCAACTGGAAGCGGCATTCAAGAGTTTTGATACAGAATGTGAAGTTTTGCGCAGCCTTCG
CCATAGGAATCTCGTGAAAGTCATCACTAGTTGCTCCAACCTTGATTTTAAGGCTTTAGTGCTCGAGTAT
ATGCCTAATGGAAGTCTTGATAAGTATTTGTATTCGCACAACAACTTCCTAGATATCAGCCAGAGACTAA
GCATTATGATAGATGTGGCATGTGCGTTGGAATATCTCCATCATGGATGCTCCTCACCCGTGATTCATTG
TGATATAAAGCCTAGCAACGTCTTGCTGGATGAGGATATGGTTGCCCACCTAAGCGACTTTGGTATTTCA
AAACTGCTTGGTGAAGATGAGAGTGATTTATACACAAAAACTTTAGCAACATTTGGTTATATAGCACCAG
GTACGTCTCTTTGTTTTTGCTAATTTGAACATTGATTTTTCCTCTTATTCTTTTTTTTCCAATCAAGAAAGTA
TATTGCATCTTTTAACTAATTGTTTGACTGTAGAGTATGGGCTGGATGGACTGGTGTCAATAAAATGTGA
TGTCTATAGTTACGGGATCATGTTGCTGGAAACATTTACTAGGAGAAAGCCTAGTGATTTTGAGGGAGA
TCTTAGCTTGAAGCAATGGGTGAGTTATTCACTCCCAGAGGCAGTAATGGACGTTATGGATGCCAACTT
GGTTACACCAATGGATAATCGCTTACAGAAGGAGTTAGATATCGTGGCATCAATCCTGAAAGTAGCATT
AGATTGTTGTGCTGAAACTCCGACAAGAAGGACAAACATGAAAGATGTTGTAGGGATGCTACAGAAGA
TCAAGATTCAACTTCTTGCATGCTGAGCATGTTCTCTGAATCTCTTGTTCCGAATCGATCACTTTTTTTGTT
TTGTTTTAGCACTTGATTTGTGCATGATTCTGTTTTCAGAAATGCTAGTTGGTTGCAATAAATGTCACTGT
TTCCTCTTAAA
>PGSC0003DMG400026760 LRR-RLKs
CCAAATTTCAAGAAAAATAGAAAAAATAAATAAAAATTTTGTTTTTGTTTTTAGAATTGTTTGGTTGGTG
GTGGGTATTGGATCCAATGTCACCAAGTAAATGTTTTTAGTTATCCAATGTGTTTTTCTTTTTAAAATTCA
GCAAAAAAAAAAGTGTATTTTATTAGACACACAAATCATTGTTTTTAGAAGCATTTTTGGGTGTCTCTGC
AATCTCTGAGGTCTGTCATGTGAAGTGTAGGGAAAAATAATTCAAGAAACCAAAACCCCAAGTTTTGAA
TTTCTTTATTTTATTATATGGTATTTTTTTTGTAATTTTTTTGGGTTTTGGGGTTTTATGAAAAGTGAAAGT
AGTAGTAAAGAGTGAAAAAAAGGGTAGTGATGGTGATGAGGAAGTTCAATAAATTGAGTTTGTTTTGG
AGTTGTTGATTTTATAGTTTTGTGGTCAAAGTTTTTGTTTGTCAATTGATGATAATGACACTAATTGGTAG
ATCTGAAGATTCTTGTGTTGATAGTGATCTTGTTAAAACTTTTATAGACAGAAAGGAAAATGTCTTTTGG
ATCTTTTACATTTCAAGATTTAATCATCAAATAAGAAAATACTCTACTTTTGTCATATGAAAATCCAAAAA
AAAAAAAAAACTTCATTTTTAATGAACTTTCTTGTTTCCATTCATCAATGGTGACACCCCAACCAAATCAA
GAATCCATTTTTTTTCTAGGTTTCTAGTAGATCCCTTTAGTGGCAAGACTCCATTTTTAATTCTGCATATTA

AACTTTGTAGTTTGGTGACACCCAACCAAATCAAGACTCCATTTTTAGCTCTGCATTTTGAACTTTCTAGTT
TCCCATTCATCAATGGTGACACCCAACCAAATCAAGAATCCATTTTTGCAGACCCCTTTAGTCTCAAAACT
CCATTTTTAACTCTGCACTTTGGACTTTCTTGCTTCCCATTCATCCATGGTGACACCCAACCAAATCAAGAA
TCCATTTTTGCAGAGACCCCTTTAGTCTCAAGACTCCATTTTTAGCTCTGCATTAGATCCCTTTAGTGTTAA
GACTCCATTTTTAGCTCTGCAGTAGATCCCTTTAGAGTCAAGACTCCATTTTTATCTCTGTAAGTTGAGTA
TTTTAGTGTCAAGACTCCATTTTTAGCTCTGTATTGAGCATTTCAGTGCTCAAAAATTGTCAAGTTCAATG
AAAATCAAAAACCCCTTTTTCCTCTAGAATTGAACAACTTAATGCTCAATCATTCAACAATGGAAATGTCA
ATTTCAATGGAAATCAAGAACCCAGTTTTGTTCTTAAGGTACTGTTCTAGTCACAAATTCTTGTTTTTCATT
TTCTCAATATTTTCCATAGTTTTCCCACTTGTAAGATCAGATGATGCTGAAGCTCTTTTAGCATTGAAATCC
ACAATAGACCCCTCAAATTTTCTTGATTGGAAAAAAGGAACTGATTTTTGCCAATGGCAAGGTGTTAAAG
AATGCATGAAAGGTAGAGTTACTAAGCTTGTTGTTGAAAATCTCAATTTGAGTGGTTTATTGGACCAAAA
TGCTCTCAATCAACTAGATCAATTACGAGTTTTAAGCCTCAAAGAAAACTCCATTTCCGGCCAAATCCCAA
ACCTTGCCGAACTTTCCAATCTGAAATCCCTTTTCCTCAACAACAACAACTTTTCCGGTGAGTTTCCGGCT
ACCATTACCCTCCTCCACCGTATCAAAGTTGTGGTTCTCGCCGGAAACAAACTCTCCGGTGACATTCCTCC
GGCCTTAACAAATCTATCCCGGTTGTATGTTCTTTATCTAGAAGACAATCACTTCACCGGAGAAATCCCAC
CGTTGAATCAGCTCAGTTTAAGGTTCTTTAATGTGTCAAAGAATCAACTTTCTGGTGAAATCCCGAATACT
CCGGCGTTATCTCGGTTCAATGAATCATCTTTCGCCGGAAATGTCAAATTGTGCGGAGTACAACTGCATA
ATAACTGTCCTTCAACTAGTCCATCGTATCCCATCATTCCAGAAGATGTATCTCATCATAAAAAAAGCAAA
AAGCTTATCATCATCATTGTCCCAGTGGTCGCTGGAATCTTGATCCTCTGTGTTTTGATGGCGATTTTTAT
AGCATGTGTTCGAAAACGAAGAAGAAAAACTCAAGATGGAAAGAGTAAAGGAGTTGAAGCCGGTGGA
GGCGGGGGAGGGGGAGGGGAAGAAGAGGGGACTAGTCGCGGTGGCAATGGCAGTGGTGGTGGTGG
TGGCAGGGATGGTAATAATGGTGGAAAAGAAGGGGCATTTTCATGGGATCAAGGAAGTGGAGGACTA
GGGACATTGGTGTTTTGTGGACCAGGTGATCAACAAATGAATTACACCTTAGAGGATTTGTTGAAAGCA
TCAGCCGAGACATTAGGGAGGGGTATAATAGGAAGTACATATAAAGCCGTGATGGAATCCGGCTACAT
TGTGACGGTGAAGCGGTTGAGGGATTCGAGGTTTCCGAGGTTAGAGGAATTCCGGAGGCATGTGGAG
ATTATTGGTAGGCTAAGACATCCTAACTTGGTCCCACTCCGAGCATATTTTCAAGCTAAGGAAGAACGTT
TGCTCGTTTATGATTATTTCCCTAATGGCAGCCTCTTCTCCCTCGTTCATGGTATGTATTCCGTCATTCATT
TATTTATTTATAGTATATTTTGCTTTTATCCGTTTTTGTGTACTGAACTCTCGTGAAGGAGGAAAAAACAG
GTATAGTTGAACTCATGAATAGTGAAAGGAAGTTAAAAAGGGATAGATTTGGCGGAGTGAACTTGCAC

TTGAGTGGTATAAGGTGGAATAAGATAAGGGTGAGGTAACTTATTTACGTCTAGTATATGCATCGATTC

AATAGATGCATTACATGTGTTTTTTTACTAAATTAGTTATCGAATAGTTTATCCTCTCTTGAGGTTAAACTA

GTTTAGTTTTGTGCAAATACCAAGTGGGGACAAATTCTTTTTCATGAGATCACTAGTTTATAACTCAATCT

CTCGAAGATTCGGCTCCTTTATTGACATGAGTCATTTTCTTATTTTGAATTCAAATTTGTTACCTCTTTATG

CTTGGCTGTTTTGTGGTGGAGCATTGGAGGACTTTAGTGTCACTTTATTTTCCTATTTTTTTGTGTTTATAT

TTGTGTACTTTGCACATTTTCCTTGAATTGTTTATACTTTTTAATATGTTTCTTTATCTTCTTCAGTATTCTGT

CCCTTCTTATTTTGTTTTTTTGTTATTTTTGAATAATTTCCATATAATTGGTTTGGTAGTGTATCACTTTCTG

GTCTCTCCTGTGGGTTCTGTTTGGTGATGGTGGGGAAGAGAGGGCCAACAAAGGAAGCTTGTAGGAGC

CAACAAAGAATTGAGATAGCTGGCCAAGTAGGACAAATATGTAAATTTGTAAAGGAAGTTCCAAACTTG

CTTTGGTATACTGATGTGAAGATTATAGAAAAATATTGTTTAAATAATTAAGTTAAAAAAAATGAATAAT

CAAATAAGTAAACTCTTCTCATCTTGTTCTCTACGACTACGGATGCTATACGAAAAGAAGATAAGGAACA

AAAGGGGAGTATAGTTATATATTGGCCTTTTGGAATCTTACAAAGGATGATATTTCGTTCAAATGAATTG

TGAAAAAAGATGTTTAGTGTGTTTTTGCATTATTTTGTTGTTGTTACTGTTCCTTTTATGAATGCTTTGTGC

TTTCCTTATTATTTGCCATATTTCTTCAATGTCATTTTCCCTTTCATAACTACTTTGTTTAGCTGCACAAGAG

CCTAGGATCCTTCGGAAACAACCTTTCTATCTCAACGAGGTAAGGTCGCGTATACTCTACCCTCTCTAGAT

CCCACTTGTGGGATTTCACTTTGTTGTTGTTGGATTGTGAAAAAGATGGCCCTCTGGGAGGAGGAAACT

CAAATTAGGAATCAAAACAATGATGTCACCGCTACACTCGCATATTTTGGCCTTATCATTTATTGCATAGA

GCCAAAAAAGTTGTCCACCATATATGGAGATAAGGCATGATACTTTGAAATTAGAAAGACCCTGCATATT

GCTCAAATTTGCATACGTAAGAGCTTCTTTGCCTTGTGCTGATTTCAGTACTTCTCTGGTAAGGTGTTAAT

CGTTAAATTGACCAGTGAGCTTCCAAAGATACCTGGAAAAATCAACATTTTCCTTCTGATTCAAAAAGAA

CAACAACAACAACGTACCTAGTGTAGTCCCACAACTACGGTCTGGGGAGGGTGGTGTGTACGCAGAAA

AACAGAATTTTTCTGACACTAGTCACTAGCGATTAGAAGTTCCATTGCTATTTATAAAGTTTCATAATTGT

CTGTACTATTTAGCTTTTAACATCTGCTCAACATCTCTTGTTCTCTCCACATTATATCTTGTGATATAGTTTG

AGATGTTCTAAATTTGATTTGTTCCTAGTAAACTGAAGTTGCACCTACGTTGTATTCCAGAGTGTGTTCAA

TATGATATTTACTTTTCTTCTATTTAGGGAAACTAGAATGCAATACACCATTGACAAGAACAAAAAAGTTC

AATATCATATAAAACTATTGTCAACCGACATGTTGAACTCCGAGATCCCTACATGTAAGTATAGCAAATT

GGGTAATAATCAAGAATAAGGTAGTCTAGATGCTAGTCTTTTCTTCCAAGTAAAGTTGTGGTGATGCAGT

GAAAGGCATTGAAAATGAGTTGCTTTGTTAGGCCATTAGACCATTTCAGGCACTAAATCACCTATGGCG

GAATCACAGAGCAAAAGGCACACCGAAGTTTATTTTTGGAATAAGGACAACGTGGAAGGTGCTATGCA

ACAACAACAACATACTCGGTGTAATCACACAAAGTGGGGTAAAGAGGTGGTTACAATAGACCCTCGGTT
CGAGGAAAAAAAATTCAAAGCAGTTCGGATGGAAGGTGATTTATGCGTCTAGCAATTGTATACGCCGTA
TGCAAGTACCACCTCAAATAGTGTGTTACCTTTCATGTGGTCTTATCTTGTTACTAGTCATTGTAAGACCA
CATCTGATGTTTAATACATGGGGGTGCCAATCGAATATTTGAAGAGAACATATTCAAAGTTAGCCGGGA
CTCTCTTATTTTCATGTATCTGGACAACCAATATGTTTTGTTATTCAAAAAATATTCCAGATGATAACTAAG
CTATATTTTTACAATTTGCTAACTTTTTCAGGGACAAGGGCTAATAGTGGTTCAAAGCCTCTTCACTGGAC
TTCCTGTCTCAAAATTGCAGAGGATGTGGCTACTGGATTGCTTCATATCCATCAGAACCCTGGTCTGACT
CATGGAAACTTGAAATCATCAAATGTACTCTTGGGGGCCGACTTTGAGTCGTGTCTTACAGATTATGGCC
TCATGCCATTTAGGAATCTAGATTCTCCTGAGGAATCAGGTGCTTCTTCCCTCTTCTATAGAGCTCCTGAG
TGTCGTGACATTCGAAGGCCACTTACCCATCAGGCCGATGTCTATAGCTTTGGTGTCCTCCTATTGGAGC
TCCTCACGGGAAAGACTCCATTTCAAGATCTTGTTCAAGAACACGGATCAGACATACCGAGGTGGGTGA
AATCTGTTCGAGAAGAAGAAACTGAATCAGGTGATGAACCTGTTTCTAGTAATGAAGCCTCGGAAGAGA
AACTCGGTGCTCTCTTAAACGTAGCTATGGCTTGTGTTTCACTTGTACCCGAGAATCGACCCACAATGAA
GGATGTTTTGAGGATGATCCGGGATGCTAGAGCAGAAGCTCAAGGGTCCTCCAATAGCAGTGACCATTC
ACCAGGAAGATGGTCAGATACTGTCCAAAGCTTGCCAAGAGATGAACATTTGAGCATATGAGTTTGTAC
TATACTTATGGAGGCCCCGAATAACGTGTCTTGATTCTTTTGGAAGGCAGAAGTTACTTCGTAAGTTAAG
CATCATCGATGTATCTGAGTTCTCAGAGATTTGTTGATTCAGTTCTTGTAAAGTCTCAAGGGCTTTCTACT
TTTCTTGTTTTTCTAGGTTTAGTTGTTTGTATGTAGGAGATTTGCTTTTATACCACTTGGATTGACATTCAC
TAGTTGATGATACATTCCTGATTCAAAAGTTGTCATTGTGTTTTGTGAGAGATGTAATTGTAATTGAGGT
TCCAACACTTCTTTAACATAGTTTCTTATTCTAGTGGTAAGAGTGTATCACGTGATGTGTGGATTAGGAGT
ACGACAACCATTCAAACCTTGCCCTAGACAAAAATATGATTGCTACTTTCTTGACTTGGGGCATTACATTT
CGG
>PGSC0003DMG400026792 LRR-RLKs
CGGCGTTACACTCTCTCTTCACTCCAAAACTTGTCTAATTTTGACTATACAAAATCATTTGAATCCACCTCC
TCAACAAAGAACCAGCCTTGATGAACTTTTCTCTCTCACTACTCACTGTCTTTCGTCACTCTCTTCTTCTAA
AACTGTCCTCCCCCTTTTAAGTTTCTCTCTTTCTTGATTCCAACTTGTCTGCTTAAACACCCTTTTTCTTCCTC
TCCATCACACACACATTCTTGCAGAATACCCTTTTTCTTCTACTCTATCTTCAACCACTGACAATGTTTCTTT
TATTCACTCAATCTATTCTTTAACCTTTATGTTTTGTTTTCTTATATATGCAATTTACATATCTCTTCACCATA
TATTTCATTTTTGTTGGAGACAAAATGTTGTGGCATATAATAACTGTTTTATCTTTAGCTCCATTTCTGGTA

CACTCTTTAGACCCAGCATTCAATGATGATGTAATGGGTTTAATTGTTTTGAAAGCTGGATTCACTGACCC
TAAATCTAAACTTGCATCTTGGACAGAAGATGATCCTACTCCTTGTAATTGGGTTGGTATAAATTGTTATC
CTCAATCCAATAGAGTTTCTGAGATTTTACTTGATAATTTCTCTCTTTCTGGACATATTGGGAGAAGTCTC
TTGAGGTTACAATTCTTAAAGGTTTTGTCTTTGTCTAACAATAACTTTACTGGCAATATCAATCCCATTTTA
TCCCAAATACCTAGCTTGAGGGTTATTGATTTGAGTCACAACACTTTGTCAGGTTCAATCCCTGATGAATT
TTTTCAACAATGTAGCTCTTTACAATCTGTTTCTTTTGCAAACAACAATCTTACTGGTGAAATCCCTCAATC
TTTAACATCTTGTTCAACGCTCCAAAGGGTTAACTTTTCATCCAACCGCCTTTCGGGTCCGTTGCCTTCAG
CTTTATGGTCCTTAACCTCTCTTCAATCTCTTGATGTTTCTGATAATTTAATGGAGGGTGAGATTCCTAAA
GCAATTGAAGGTTTATATTCTTTGAGGTCAATTAATTTACATAAAAACAAGTTTACTGGTTGGCTGCCTG
AAAATATTGGAAACTGTGTGCAGCTCAAGTCAATTGATCTCAGTGACAATTTGTTGAATGGGGAACTTCC
TGAGTCTATGAGAAGATTGGGTTTTTGTACCAATATGGAGTTAAGATCAAATTTGCTTAATGGGCAGATT
CCAGATTGGATAGCAGAGATGAAAAGCCTCAAGGTTTTGGATCTTTCTGCGAATAATCTTTCTGGTAGG
ATTCCAACCTCTATGGGAGATGTTTCATTGCTAAAGGAACTCAATTTGTCAAACAATTACTTCGTTGGAA
GCTTGCCTCGGTCCTTGATGAAATGCAATGACCTTGTAATCTTGGACATAGGCAACAACTTCTTGACTGG
TAACCTTCCTTCTTGGACCTTTGAATTGGGTGTAGAGAGTATTTCTCTTTCGGGGAATAGGTTTACCGGG
CATATAGATTATCCGTCCATATCCATGGATGCTTCTTATCGAAGCCTTCAAGTTTTGGATTTGTCTTCCAAT
GCATTATCTGGTGAAATACCAGCTGCCATTTGGAATATCAGTAGCTTGCAGGTCTTGAATATTTCCAGGA
ACTTTTTGTCTGGTACTATTCCAGAAGCTGTAGGTAAACTAAACGCAACTCGAATTCTTGATTTAAGTCAC
AATCAACTAAACGGAAGTATTCCCAGTGAAATTGGGAGTGCTGTTTCATTGCTGGAACTGAAGCTGAGA
GAAAATCATTTGAGTGGTACAATTCCTGCAGATATAGCGAATTGCTCTGCTCTAAGTTCATTGTAAGTTTC
CTGCCACTTCCATCTCATTTTTATTCAATGTGTTGAAAACTTTTGTATTTAGCTTTTCGATCATGTTATCTTC
GTTTAAATTGCAGTTATGTGCATCATGATGTTTGCATTTGTTATGTCTGTGTGATTCTAAGAGTCATTTGT
TCTGCATTTTCGATTGTGTAGGGACTTGTCACACAACAACCTCACTGGCCCTATCCCTCCAGAGATTGCTA
AGTTGACCATTCTTGAAGTGGTAGATTTCTCCTTTAACCAATTTTCTGGAAGCTTACCTAAAGAACTAACC
AATCTTTCGCACCTTGCCACATTTAATGTCTCCCACAACCATCTCAAAGGTGAACTTCCAGTAGGTGGCTT
TTTCAACACCATTTCTCCATCATCTGTTGTTGGTAATCCATCCCTATGTGGTTCTGTTCTCAACCACTCCTG
CCCTGCTGTCCATCCAAAGCCTCTTGTACTAAACCCCAATTCATCTGACCCGAATCATGCTTCTGTTACTTC
GCTTGGTCATAAGAGAATCATGCTCAGTATATCATCTCTCATTGCCATTGGAGCAGCAGTTTTCATAGCA
CTTGGAGTAGTGGTCGTTTCTATTCTCAATTTGCATGTGCGCTCTTCTATGGCACTCTCTGCTGCAACTTTC

ACCTTATCTGGTGGCGATGACTTTAGCCATTCACATGGTACAGAAGCCAATCTAGGCAAGCTTGTTATGT
TTTCTGGTGATGCAGATTTTGTTGTTGGAACTCAAGCGCTGCTCAACAAGGATAATGAACTTGGACGTG
GTGGTTTTGGATCTGTATACAAGACAGAACTCGGAGATGGACGTTCTGTTGCCATTAAGAAGCTCAACA
TTACAAGTTTGATCAAGTCCCAAGAAGATTTTGAGAGGGAAATGAAAAGTCTCGGAAGCATCAGGCACG
AGAACCTTGTGGCACTTGAAGGTTATTATTGGACACCTTCTCTCCAGCTCCTGATTAATGAATATGTATCT
GGAGGCAGCTTGTACAAATTACTCCATGAGGAAAGTTCCAAAAGGAGTCTCTCTTGGCAGCAAAGGTTC
AACATTATTCTTGATACTGCGAAAGGCTTAGCCTATCTGCACCAGCTGAACATAATTCATTACAATATGA
AGTCAACCAACGTCCTCATAGATGATGGTTCTGCCAGCACAAAAGTTGGAGATTTTGGTTTGGCTCGTTT
ATTACCAGTCTTGGATCGTTACATATTGAGCAGCAAGATTCAGAGTGCACTTGGATACATGGCTCCTGAA
TTTGCATGCCAGACGGTGAAGATAACTGAGAAATGTGATGTCTATGGATTTGGCATCTTGATTCTTGAG
GTGGTGACAGGGAAAAGACCCATGGAGTACATGGAAGATGACGTGATTGTATTATGTGACATGGTGCG
GGGAGCACTAGAAGAAGGCAGGATAGAAGAATGCATTGACGGACGGCTCCAAGGTAACTTCCCTGTCG
AAGAGGCAATTCCAGTTGTGAAACTTGGTTTAATATGTGCATCTCAAGTTCCATCCAATCGACCAGACAT
GGAAGAGGTGATCAAAATTTTGGAGCTAATTCGATGTCCGTCAGAAAGTCCAGAGGAAATAGAGTAAT
GTTATAAGGTGTTTGTGGAAAACCAGATTATAGAGAATGAGTCATGTGGTGAACAGGAATTCAGTTTTG
TTTGTTACATTTGGAGAAGGTCACTATCCTCTTGTTATTTTGTCATATCCATACATGTTAAATCTTTATATG
TTATTTAAATAACTGGATTCCTCTTCACCAGCTCCAGTTTGGGACCCTTGTTAGATTTGGATAGACTGGCA
TTGTCTTTATATAGTATTCAGACATCCAACTTGTTGTCTTGTGCTTGTTTGATGTTGTTGTCCATACAAAGC
TGAAAAAAATACATCAGTTGTATTCTTATAGAAGAAGACCCACATGGATTCCTAATGAAATGAAAACATC
AAAGTAGCCCACCACTCA
>PGSC0003DMG400027071 LRR-RLKs
CAACCATTTTTGATTCATATAAACCACCAATACACACTTATAAAAAAATCCATCAACCACCACAGCCTGGT
TGCCTCATTCACCTCTCTCTTCCCTCCGTCCGGCCTACCGGAAAAACAAGAATCTCAATGGCTTCAAATTT
CCACTTTTTTCTCTTATACCTTGTGACCCTTTTCCTTTTTGCTCAAGCAAGACTGAATCTTTATCCACAAGAT
CATGCTGCACTTTTGCTTGTTCAAAAAGACTTAGGCATCATTGCTCTTAACAACCCCTGTACGCTCGCAGG
AATATCCTGCGAGCGTAGACCGGGTAACAGAACACAAGTTCTGAGAGTTACCCGTATTGTATTCAGATC
CAATGGATTGAAGGGAACTTTGTCTTCTGCTATTGGCAAACTCTCTGAGCTCAAAGAGCTTTCTCTTTCCG
ACAATCAACTATCTGAACAAATCCCAATTCAGATTCTTGATTGTCGGAAATTAGAGATTCTTGAACTTCAA
AGAAACAGATTTTCTGGAAAGATTCCGTATGAATTGTCATCTTTAAACCGTTTAAGGGTAGTTGACTTTTC

ATCGAATGAGTTTTCTGGGAATCTTGATTTCTTGAAATACTTTCCTAATTTGGAAAAACTGTCTCTGGCTG
ATAATATGTTCACTGGAAAAATACCCTTTTCATTGAAATCTTTCAGGAATCTTCGTTTCCTCAACATTTCAG
GGAATAGTTTCCTTGAAGGTCCAGTACCTGTCATGAGTCAAATTGAGCATTTATCAGCAGATTTGAATCG
AAACAATGCCGTTCCCAAACGTTACATTCTTGCTGAGAATTCAACAAGGTCAAATCAGATATCTGCAATG
GTGCCTGCTCCAGCTCCAGCACCGGTCAATCGTGTTGTACCGGTGGTACATAAACGTAACAAGAAGAAA
AGGAAGTTAAGATCTTGGTTTCTTGGTTTCCTAGCTGGAACTTTTGCTGGGGGTATATCTGCTGTGATCT
TTTCGTTGCTATTCAAGCTCGTAATGTTCTTCGTAAGAAGGGGAAAGAACGATAGTTCAAGTTTAACGAT
ATTTAGTCCGTTGATCAAGAAAGCGGAGGACTTGGCTTTCTTAGAGATGGAAGATGGAGTGGCATCACT
TGAAATGATTGGAAAAGGTGGATGTGGAGAAGTTTATAGAGCCGAGTTACCGGGGAGTAATGGGAAG
ATTATAGCTATAAAGAAGATTATACAATCCCCAATGGATGCTGCAGAGATCACGGAGGAAGATACTAAG
GCGTTGAACAAGAAAATGCGTCAAGTTAAATCAGAAATTCAAATTGTAGGTCAAATCAGACACCGGAAT
CTGCTTCCATTACTGGCGCATATGCCAAGGCCAGACTGTCATTACTTGGTGTATGAATATATGAAAAATG
GGAGTTTACAGGATATCCTGCAGCAAGTCACAGAAGGCACAAGAGAATTAGATTGGTTGGGACGACAC
AGAATTGCAGCGGGGGTAGCTGCTGGTCTTGAGTATCTCCATATAAACCATACTCAACGCATAATTCACA
GAGATCTAAAGCCAGCAAATATCCTACTTGATGATGACATGGAAGCTCGAATAGCTGATTTTGGGCTTG
CAAAGGCAGTTCCAGATGCTCATACACATATTACGACTTCAAATGTTGCAGGAACTATGGGATATATTGC
ACCAGAATATTATCAGACACTGAAGTTTACAGACAAGTGTGATATATACAGCTTCGGTGTGGTGCTAGCT
GTGTTGGTTATCGGAAAGGGTCCATCGGATGAATATTTCCAACATACTTCTGAGATGAGTTTAGTTAAGT
GGCTGAGAAATGTAATGACTTCTGATGATCCTAAAATAGCAATTGATCCTAAGCTGAGAGGGAATGGAT
ATGAGGAGCAAATGCTTTTGGTTCTCAAGATAGCTTGCTTTTGTACTCTCGACAATCCAAAGGAGAGGCC
TAACAGTAAGGATGTTAGGTGCATGTTAACTCAGATCAAGCATTAGCTAAGTCATACGGGGTATACGTT
GGTTATGTAACTAAGAATGTGTTCTGTATTTCAGTTTTTGTTGTAATTGTGTTATACATGTCAAATCTTGT
ATGTTTACTGCAGTGTTAATAATATTATTTCCACTTCGCTACTTAAATTACTAAAACTCTA
>PGSC0003DMG400027115 LRR-RLKs
TTCTTGTGGTGTGTGTAAAAAAAACACTGTCCCTATCCCATTTTTATACTTATCAAAAAGCAAAAAAAACA
GGGATGTCAACTGAAAAATCCCATTTTTTCTAACAAAAAAATGGAAAATCAAGACCAAGTTCAAGACTTT
ACCTGTTTTTACCAGCACCCATTAGGGGTTTTCATGTTTTTAATCCAAGAAAGAAATGTGGGGTGTGTTA
AAAAGAAGGAAAAATGGATACCATTTATCCAAATTTTGTTGTTTTTGCAGCTGATGATTTGTGGAGCTCA
GTTAGCTTTAGGTCAAGTTTGGGATGGTGTAGTTATCACTGAGGCAAATTATCAAGCACTTGAGGCGCTT

AAACATGAGTTAGTTGATCCCAGAGGGTATTTAAGTAGCTGGAATGATAGTGGTTATGGAGCTTGTTCT
GGTACTTGGGTTGGCATAAAATGTGCTCAAGGACAAGTTATTGTTATTCAGCTTCCGTTAAAAGGATTTG
GGGGAAGAATCACTGAAAGTATTGGTCAATTTCAAGCACTCAGAAAACTTAGTTTTCATGATAATGAAAT
TGGTGGTTCAGTGCCTTCATCTTTGGGTTTTTTACCCAATCTAAGAGGTGTTCAATTGTACAATAACAGGT
TTTCTGGTTCTATTCCTGGTTCACTTGGTTTATGTCCAGCTTTACAAACACTTGAACTTAGTAACAATTCTT
TATCTGGTGCAATCCCTGATAGTCTTGTGAATTCATCTAAGCTTTATAGACTTAATCTTAGTTACAATTTAT
TGTCTGGTTCCATTCCTGTTAGTATAACTCAATCGCGCTCTCTAGTCTTTATTGACTTGAAATATAACAACT
TGACAGGGTCTATTCCGGATTCTTGGGGTGGAAATGGAGATAGACAGTTTAAATTAGAGTCGTTGACGC
TTGATCATAACTCTTTGTCTGGTGGAATTCCTGTTTCTTTTGGGAAGTTGAGTGAGCTTCTTGAGATTTCG
TTGAGTCATAATCGTATCGTTGGTGTCATCCCAAATGATATTGGAAGGCTTAGTATGGTTAGGAATCTTG
ATTTTTCTTATAATGAGATTAATGGTAGTTTGCCAGAGAGTATAACCAATCTGTCCTCCCTCATGGTTCTC
AACTTGGAAAGTAACAATCTTGATGGTGAAATCCCGTTGGATGTAAAGAAGTTGCAAAAGTTGTCGTTT
CTTAACCTGAGGAATAACCGGTTTAGAGGTGATATTCCAGCTGCTATCGGGGACATTTCTGGTCTAGTCA
TGATAGATTTGTCTCGTAACAATCTAACCGGAGAAATCCCAGAGTCTGTTAGTGAGTTACCTAATCTTAG
TTCCTTTAATGTTTCATATAACTCTCTGTCTGGTCCTGTTCCTACTTATCTTGCTAAGAAATTCAATTCAAG
TGTGTTTGTGGGTAATGTTCAGTTATGTGGCTATAATACTTTGAATCCATGTCCTGTTACGCCTTCTCCAG
TTACACGAGGTAGTAAACGAAGTATTAAAGACATTATTCTCATAGTAGTAGGGTGTCTTATAATAGTCTT
GTTTCTACTCTGTTGCATTCTTCTCTGCTGTTTGATCAAGAAAAGAGGTGTGGCCAAAAAAGCAAAAGAT
GTAAAGGATGTTCCTCCAACTGCCGGAGAAGTTGAGGCGGCTGGGGATAACGGAGGAAAACTTGTCCA
TTTTGGTGGAGGTATGATGTTCAGTGCAGATGATCTTCTATGTGCTACTGCAGATATATTGGGAAAGAGT
ACTTACGGTACGGTGTATAGGGCTACATTAGAAGATGGAAGTCAAGTTGCAGTGAAAAGAATGAGAGA
AAAGATCACGAAAGTTCAGAGGGAATTCGAGAAAGAAGTCAACGTTCTTGGAAAGATAAGGCATCCGA
ATCTTCTGGCTATTAGGTCTTATTACTTAGGTACGAAAGGCGAGAAGCTTCTTATTTTCGACTACATGCCT
AAAGGAAGTCTCTCAAATTTTCTTCATGGTAAGCAGAAAACAGATTTCCATTTGACATTCTTTTGCTTCTG
TTTTTTTGAGAATCAGATACTTTCTCTGTCTCATTTTAACGTAATGAAAATTGGATTATGTATCATATCGGA
AACAACTTCTCTAACTCACACAACGTAGGTAAGGTCTACGTACACTCTACACTCCCCACTCCCCAGACCTC
ACTTGTGAGATCGCATGGGGAGGAAATTGTTATGGAGGGGGTAATATAGCTTTATTGTTTTTGTTGCAG
CTCGTGGACCGGATACACCAATTGATTGGCCAACAAGGATGAGAATAGCAAAAGGGACGACGAGGGG
ATTGCTGTACCTTCATACTGATGCCAACATCATTCATGGGAATCTTACATCAAGCAATGTTCTACTTGATG

AAAACACAGATGCAATAATTGCAGATTATGGCCTTTCACGCCTTGTAGCTGCTGCTGCAAATTCAAATGT
TATGGCCACAGCAGGGGCACTTGGTTATCGTGCACCTGAGCTTAATAAGCTCAAGAAAGCCAACGTGAA
GACGGATGTCTACAGCCTCGGAGTCATCATACTTGAACTCCTAACTGGAAAGTCTCCAGGTGAGGCAGT
GAATGGTGTAGATTTGCCTAGAAAGGTGGCGTCAATTGTGAAAGAGGAGGGAACAAAAGAGGTATTCG
ATTTGGAACTAATGAGGGATGCATCCATAATTGGTGATGAATTGTTAGTTACACTAAAATTGGCTTTGCA
CTGTGTTGATCCCTCGCCATCAGCTCGACCAGAACTTCAGCAAATTCTCCAGCAACTGGAAGACATTAGG
CGTGAAACGCCAACTGCAGGTCCCTCGACAAGTTACTAGATACTCAGTTTTTGATTTTATTAACATGATTC
TTTCATTCTTGAATTTCATTGCTAGAGTACTAGATCTGTTTCAAGTGTCTTTAAATAAAAGTGTGCAACAT
TGTGAAAAGTGTTTGAGCTTAATGGATTCCTAAATACCATCCCCTTTTGGCCTAAAGAACATAACTTGAG
ATTGAG

>PGSC0003DMG400027467 LRR-RLKs

ATGGGACTAATAGGTGAGTTTCCGCGAGGCATACAAAATTGCACTTCTTTAACTTCTCTGAATCTTTCAA
ACAACGAGCTATATGGAACCATCCCTTCCGATATATCAAAACTAATCGTATATGCTGTAATCGTTGATCTC
TCGAGCAACCTATTCTCAGGCGCGATCCCAAGTGACATTTCAAATTTCTCTTTTCTCAATTCCCTCAAATTA
GAAAACAATAAACTAGAAGGACTGATCCCACCTGAAATTCGCTTGTTACAGCGACTCAAGACGTTTAAT
GTTGCAAATAATATGTTGACAGGGCCAGTGCCAAATTTTGTCAATGCTACTTTTCCAGATGAAAGTTATG
CAAATAATCCAGGACTTTGTGGTAGTCCTCTGGAATTATGTGTTACAGAACGCGTAGTAAGCCGTCATAT
TCTGTTTGCTTGTGGTTTTGTGACGGGTTGGTCGCTTTCTACATTACTTGGTGTATATCTTTTTTTCTTTGG
TGTAAAGAAGATGCTTCTATTGATCAACAAGAGAACAAAGGTAATGGTGATTGATGGAAATGAAGTAA
ACAATGACCCAAAGGTAATTATGTCTACCTGCTCAATTATATACAAGTTTAAGTATCTATTTGTGCACATT
CAAAGTCGGAGGGCATAGATTTTAGATGAGGCCAAGTTAAAGGGAATGATTATATTATGCCAATTTGGA
AACAATTTCACTTAGTGACCAGCTTTTATAGCCAGACAGTGTTATGGCACGTTTAAACTACAAGTTTAAA
AAAACTACCTTTTTTTTTTTTGAAAATTTGGGCCCTGTCAAACTATGTTAGATAAATTGCGACGAAAGGGG
AACTACTTAATAAAAAAACTGTTGTTGTAGAGGAGAAATAAAATGTGGTAATGGTAAATATGTTTTTTTC
CTCAGATTTTAAAGCTGGAGAAGATTGTTACAAGAATGAGTTTTATGGAACTATCAAAAGCGACTTTGA
ATTTCAGCCAAGACAATGAAATCGGGAACGGAATGCTGGGGAAATTGTACAAAGCACTAGTCCCAAAT
GGATGGACTGTTGCCATAAAGAGGCTCTAAAGTTCCGAGGATTTGGAGGAAGAGTTTGTTTCGGAGATC
ACAACTCTAGGTATTTTGAGGCATCCGAATTTAGTGCCCCTAATCGGATTTTGTTATGAGAGGGACAAGC
GACTTCTGGTTTACAAATACATGCCTAATGGAAACTTACATGAGTGGCTGCATTCAACTGACGACAAGGC

AAGACTCTTGGACTTCCCTCTAAGGGTTAAAATTGTTGTTGGTGTCGCGAAAGCCCTGGCTTGGCTTCAT
GATGGTGGAAATTTCGATCCCAAGTTATCCAATTTTTGGGAGGCAACATTTGTAGAACCAAATGACATA
GGTTCAAACTTGAGTCTATCGCCAATGGTCGAGTTTTTAGACTTTACAACATACAAAAAAGATGTCTATA
AATTTGGGGTTGTGCTTCTCGAGATTTTAACGGGGAAGGAATCTTACGAATTGAGCTGCTCGAGTCTAA
ATTTGTCGAGCAGCTCTTTTGCCAGTCCTCTTGATGTGGATAAACTACTGTTAGGCCAAGGATTTGATAA
TATGGTAATGCAACTACTTGAACTTGCAAGTAACTGCATGAAATTCATCCCGGATCAAAGACCAACGATG
CAACAAGTGTATCAGATTGTTGCTGCGATTGCTCGAGTCCATGACCAAACAGGGGACTCTGAAATACAA
TTGCACTTGGACTAG
>PGSC0003DMG400027468 LRR-RLKs
TTTCAACAATGTGTAACTGCAACATTCTTTACTTAGTACTACCATTTTTCCTACTATTTTGCAGCTTTTCTCT
AAGATGCAACGCGGTGCAGAGTGATATCGATTGCTTGAAATCGATTAAAGATTCGTTGATAGACCCTTT
AAACATCTTGAATTCTACATGGAAGTTCGATAATCAGACAGAAGGTTTTATATGCAAATTTACAGGAATA
CAATGCTGGAATGCCGATGAGAACATAGTTGTATCCATTAGTCTTCCAGACATGGGACTAATAGGTGAG
TTTCCGCGAGGCATACAAAATTGCACTTCTTTAACTTCTCTGAATCTTTCAAACAACGAGCTATATGGAAC
CATCCCTTCCGATATATCAAAACTAATCGCATTTGCTGTAGACATTGATCTCTCGAGCAACCTATTCTCAG
GCGCTATCCCAAGTGACATTTCAAATTTCTCTTTTCTCAATTCCCTCAAATTAGAAAACAATAAACTAGAA
GGACTGATCCCACCTGAAATTTGCTGGTTACAGCGACTCAGGATGTTTAATGTTGCAGATAATATGTTGA
CAGGGCCAGTGCCAAATTTTGTCAATGCTGCTTTTCCAGCTGAAAGTTATGCAAATAATCCAGGACTTTG
TGGTAGTCCTCTGGAATTATGTGTTACAGAACGCGTAGTAAGCCGTCATATTCTGTTTGCTTGTGGTTTT
GTGACGGGTTGGTCGCTTTCTACATTACTTGGTGTATATTTTTTTTTTGGTGTAAAGAAGATGTTTCTCTT
GATCAACAAGAGAACAAAGGTAATGGTGATTGATGGAAATGAAGTAAACAGTGACCCAAAGGTAATTA
TGTCTATCTGTTCAATTATATACAAGTTTAAGTATCTATTTGTGCACATTTAAAGTCGGAGGGCATAGATT
TCAGATGAGGCCAAGTCAAGTTAAAGGGCATGATTATATACTATGCTAATTTGGAAACAATTTCACTTAG
TGACAAGCTTTTATAGCCAGACAAGTGTTATGGCACGTTTAAACTACAAGTTTAAAAAACTACTTTTTTTT
TCTGAAAATTTGTGCCCTGTCAAACTATGTTAGATAAATTGCGACGAAAGGGGAACTACTTAATCAAAAA
TTTTTATTGTAGAGGAGAAATAAAATGTGGTAATGGTAGATATGTTATTTTCCTCAGATTTTAAAGCTGG
AGAAGATTGTTACAAGAATGAGTTTTATGGAACTATCAAAAGCGACTTTGAATTTCAGCCAAGACAATG
AAATCGGGAACGGAATGCTGGGGAAATTGTACAAAGCACTGGTCCCAAATGGATGGACTGTTGCCATA
AAGAGGCTCCAAAGTTCCGAAGATTTGGAGGAAGAGTTTGTTTCGGAGATCACAACTCTAGGTATCTTG

AGGCATCCGAATTTAGTGCCCCTAGTCGGATTTTGTTATGAGAGGGACGAGCGACTTCTGGTTTACAAAT
ACATGCCTAATGGAAACTTACATGAGTGGCTGCATTCAACTGACGACAAGGCACAATTCTTGGACTTCCC
TCTAAGGGTAAAAATTGTTGTTAGTGTCGCGAAAGCCCTGGCTTGGCTTCATGATAGTGGAAATTTCCAT
GTCGTGCATAGTAACATTAGCACACAATGTATCTTGCTCGATGAAATCTTACATATTGAGTTGCTCGAGT
CTAAATTTGTCGAGCAGCTCTTTTGCCAGTCCTCTTGATGTGGATAAACTACTGTTAGGCCAAGGATTTG
ATAATATGGTAATGCAACTACTTGAACTTGCAAGTAACTGCATGAAATTCATTCCTGATCAAAGGCCAAC
GATGCAACAAGTGTATCAGACTGTTGCTGCGATTGCTCGAGTCCATGACCAAACAGAGGACTCTGAAAT
ACAATTGCACGTGGACTAGAAGCAGGGGCGGAGCCACCAAAGGGACGTGTAGCTTTGGCTCAAACCAT
GTATTTGTGATGAAGAACCATTTCATATGTATAAATAATCCATCTAGACTCATGTGCTACAAATTCTGGCT
CCGCCTCATACTAGAATCACCAGCTTTTGGAACCGTGTTTCCTATGGTTTCTTTGTTTAGCAAGAATGTAT
GTCTTCCTGAATTGTTGTTTTCAATATTGCAATAACTATGCTTCAGAATGATCATCATTA
>PGSC0003DMG400027482 LRR-RLKs
ATTTGGTGCCTTTAATCGGCTTTTGTTCTGAGAGGGACGAGCGAATTCTGGTTTACAAATACATGCCTAA
TGGAAACTTACATGAGTGGTTGCATTCAACCGACGACAAGGCAAGACTCTTGGACTTTCCTCTAAGGGTT
AAAATTGTTTTAGGTGTCGCGAAAGCCCTGACTTGGCTTCATGATGGTGGAAATTTCCATGTTGTGCATG
GTAACATAAGCACACAATGTATCTTGCTCGATGAAAATTTCGATCCTAAGTTATCCAATTTTTGGGAGGC
AACACTTGCAAAAACAAATGACATAGATTCAAACTTGAGTCTATTTCCAATAGTCGAGTGTTTAGACTTT
ACAACATATAAAAAAGATGTTTATCGATTTGGGGTTGTGCTTCTCGAGCTTTTAACGAGGAAAGAATCTT
ACATATTGAGCTGCTCGAGTCTAAATTTGTCGAGCAGCTCTTTTGCCAGTCCTCTTGATGTTGATAAAGTA
CTGTTAGGGCAAGGATTTGATAATATGGTAATGCAACTACTTGAACTTGCAAGTGATTGCATGAAATTTA
TTCCGGATCAAAGGCCAACGATGCAACAAGTGTATCAGACTGTTGCTGCGATTGCTCGAGTCCATGACC
AAACAGGGGGTTCTGAAATACAATTGCACTTGGACTACAAGCAGGGGCGGAGCCACCAAAGGGACGTG
TAGCTTTGGCTCAAACCAATGTATTTG
>PGSC0003DMG400027499 LRR-RLKs
GTGACTGTATTCTTTAAAAATGCATGCAGCTTCTTCAACCCCATCATTTTCTTGAAGTTTACAGCTTTAATT
CCTCAAAGAAATCAGCAAAAAAATTGCATTTGCCATGGGAATTGCAAAAACAGTGCAGGGCTCATGTTG
GCTTCTTGTATTCATGTTGTTTTTTCTGTTTAATTTATCAGATGAGTTCTTAGAACCTTCACAATCTGAAGC
TATAGTGAAAATCAAGCAGCTTTTGAACTTCCCTCAAGATTTGAGTAGTTGGAGTGACAATACAGACTTT
TGCAATAGTGAGCCAAACACTGCCTTGACACTCATGTGTTATGAAGATAACATAACACAGCTTCATATTT

CTGGCTATAACTGGTTTCCTAATTTGCCTCAGGGGTTTTCTACAGATACCCTTTTCTCTAATCTTGCTCTTT
TGCCTAATTTAAAGGTACTTTCTTTGGTTTCTTTAGGTTTGAGAGGAACTTTACCTAAAAAGATTGGATTT
TTATCTTCTTTGGAGATTATTAATATCAGTTCAAACTTCTTTTATGGTGAGATTCCAGGAGAGATTTCATA
TTTGAAGAGTCTTCAGACACTTATATTGGATGATAATAAGTTTACAGGTCAAGTTCCTGAAGGGGTTGGT
TTACTTCATTCTTTGTCTGTTTTGAGTTTCAAGAACAATTCATTTTCAGGTTCATTGCCTAATTCTTTGTCAA
ATTTACAAACCCTAAGAATCCTTTCTATTTCTGGTAATAATTTTTCTGGGGTTGTTCCAAACCTTCATAATC
TGTCAAATCTACAAGTTCTTGATTTGGAAAGTAACAATCTTGGACCTAATTTCCCTAATATTCCAACCAAG
TTGGTATCTTTGGTACTTAGGAAGAACAAGTTCAGTTTAGGGGTACCAAAAGAACTGAGTTCATGTTATC
AGTTAAAGAAACTGGATATTTCTTCTAATGAGCTTGTTGGACCCTTTTCACCAACAATTTTATCATTGCCA
TCACTAAGCTATTTGGATATTTCTGGAAATAAACTAACTGGGAAGCTTCTAAAGAATGTGACATGTAGTC
AAGACCTTTCTTTTGTGAATTTATCATCAAATTACTTGACAGGGGAGTTGCCTGATTGCTTGCACCCTAGT
TCTGGTTCCAAGATTGTATTGTTTTCTGGGAATTGTCTGTCGAATAAAGAGCAATGGCAGCATCCTTATTC
ATTTTGCCATAATGAAGCTTTGGCTGTTAGTATTGAGCCTCATAAGGGAAAGGTTAAAGGGGGAAATGG
TAAAGCTGTTCTTGCATCAAGTATGGTAGGAGGTTTTGTTGGAGTAGTAGCGATTGTCGGTTTGGCTTTC
GTGGTTGTTAGAAGAGAGTATGCCAAGCAGAAAGCTAGCAAAGCCCCTCAAACAAGATTGATACTTGAA
AAGGTCTCTCCTGCTCACACTCTTAAGCTGCTTAATGATGCAAGTAAGCTCTCTCTTTCTCTCACACAAAC
ACATTTTGTTAATGATTTCAACTTGTGAAAGTTTTTAGATCCTGATAATTGTAAATCTAATGTTCTGTAAG
GTCTATGTACACTCTACCTTCCCCTAATCCCGCCGGTGGGATTACACTGAGTATGTTGTTGTTGTTATTGT
TGTCATTGTACTTGAAAAGGCTATAAACATTTGAATTTAAGCATTATTCCACAAAAGGAGTTTCTTTATAT
ATAGATTGAAAATATCAATTTGCTTCAGGAAATTGTCCAATACTGTGTTATTGGAATAAACTCTGTTCTTG
CCTAAATTTCAAGATGTATCTTGATCTTTGATTCTTGATAGGGTATATTTCTGAAACGAGGAAGTTGGGA
TTGCTTGGGGCCCCTCCTTATCGAACGTTTGTCTTGGATGAGCTTCGAGAGGCCACTAATAACTTTGATA
TATCAAATCTAATTGGCGCAAGCTCCAGTGGACAGGTATGGATTTCCTTACTAAATACTGTAAGACTGTT
TGAACAATGTTAGTCCACATGGACTGTTTTGTAGTTCATTCCTCAATTCTCTTGCATCTATGGTGAATGCT
GATTGTGCTTTTCTATACTCTGAACAATAGATTTACAAAGGGCGTCTCACAGATGGCACTGTGGTTGCTA
TAAAAAGCATAAAAATGAGGAAGAGGCATAGTGTCCAGTCCTACACTCACCAACTCGGGCGTATTTCAA
AGATCAGATACTGCCACTTAGTTAGTACTATTGGACACTGCTTTGAGTGCTACCAAGATGACTCAAGTGT
TAGCCGAATATGTTTAGTCTTTGAATTTGTGCCAAATGTGACGCTGCGGGGGGTCATATCAGGTATATAG
TCTAGATAACTTCATCTGCTTCGCGAGTTATCATTTGGCACAACAACTGCAACAGTTTATAATTGAGCATT

TAGTTTACTTATTTGACTATTTCTACAATATTACATAAATTATACTAAGTCTAAGTAAATGACTTCTTGGTA

TGTAGACGTTATAGAAAACAAGCATCTTTGCAACAATCTGTTCCATGTTGTTTTGATATATATTACTAGAT

TGTCCTCAATCACGACAAGTACCTACTACATTGTACTTCTTGTCAAAAGTGCTGATGAATTTTAAGGAGA

CATGAAATGCTTTTAATTACTTTCTCCTAGATTATGCAACGCGTTGTTTCTTCTTTGCCCGATGCTTAAATG

TACAGGTGTTTATTGTCTTTAAGACAGTTGTTCTGAATTCTTTGCAAATGCTCGGTTTTGTAGGAGCAAAT

TCAGCTCAAAAGTTTACATGGACGCAACGGATGTCAGCTGCAATAGGGATAGCAAAGGGAATTCAGTTC

CTTCATACTGGAATAGTACCAGGGATATTCTCCAATCAATTGAAGATTACAGATGTATTACTGGATCAGA

ATTTCCACGTAAAGATTAGCAAATACAATCTGCCTTTGTTAATTGAAAATAAGAAAATGGTATGCAAACC

TCTCTTCTCTACTATATTCTCTATTAATCACTCATTTCATTCTTGAACATTCACATTTTGTTATTGCAACTGTT

CTTAGGATACTGGACCGTCTTCTAGTGGATCAAAGGGAAATGATGGGCAAAGGTAAATGATCTCATCAT

TCTCAGCTGCTACTGCTGCATTTTCGCTCTTCATTACTCCACTTTTATGGTTCGATTTCAGAAGCACCTATT

GGAAACTAATCTTGTTATACTTCTAAATGCAAATTGGTCTAAAAAGGTTTTAGAGAATCTTAAGTGTATCT

CGACCAATTCTAGACAACACCTCTCCAGCCACTGCTATTTATTGGTTATGATTCTTGAAGACAATATATAG

AGCCATAGACTTGGACCGTCTTAGGAGATTCCAAGATAGTGCTCTACCGTTTAGTAGACTACTAATGAAT

TCCTTCTTTTGTTAATATCTTCTTGAATGCAGGTTAAAATACGAGGAGAAAGATGATGTGTATGACTTTG

GAGTAATCTTACTTGAAATCATATCGGGAAGGACAATCGATACAAAAAATGATATAGATGTTTCTAAAG

ACATTGTAAGAGTCACTTATGACATTTATGAGAAATTCTTTCATGTCATCAAGTTGAAGAACTATTTTGTT

AAAACCTTTTCTTGTTGTTCTTATTGCAGCTAATAGTTAGCTTAACAGCTGACGAAATAGCTCGGAGGAA

CATCATTGATCCAGCTGTTCGGAAGGAATGCTCAGATAGTTCTCTTAGAACATTAATGGAACTATGCATA

AAATGTCTTTCAGATGAACCATCGCAACGTCCTTCTGTAGAAGACTTGATATGGAACTTGCAGTTTGCAG

CTCAGGTCCAAGACCCTTGGAATAGAGACACTTATGGCAACCAAGAGTCCCCTGGTCATGTGTAAGAAC

TTCCAATGATCAATGGACCTTTTCTTGTACAAAATGTAACATTATACCACTAGGATAGTAGTTCCAATTAT

TTAGTAGTCTCTATTGCAACACTTTTGGCTATATGAGGTTTCAGTACTCTTTGGAGGTTAAACTCTACATT

TGAGCCAAAATTTGTGAGCAAATGGCTATTTCTTGCACTTTTTTTTTAACGAACCTAAAATTTAAAAGGTG

GGCCTTTCACCGACCATCCAATGGACCATGGACTAAACGGCACCCAAAAATGTATGGGAAAATTTTCAA

AATGTCAATATTTTAGCATTTAATGGGACCTCTTATCAACATTTTCAATATTTATT

>PGSC0003DMG400027536 LRR-RLKs

CTCTGCGCACACACAACTCAGTAGAGAGAGAAAGAGAAGCAGTACAAAAATGTGAAAAGTTGCAGCAG

ATAAAAAAAAAAAAGGGGGTTCATCAGAAGAAGGAGAAGAACTAAACTGAACTGAGAAATACAAAAA

AGGGTTGTTCAGTGAAGAGTAAAAGGGGTAGCAAAATCTTGGTAAACAAGTGTAAAAATTTGATCTTTT
TTTGTTGGGTTATGAGATGAGATCGATTCTTGGGTTTTGAGCTTTGTGGGGGTGTTTATCTTGGTTCATG
GTAATGAGGGGTAGGGTTTTCTTGAATTTTTGGGTAAATGCTGGCTTTTTTGAGTTTGCTTTACTTGGATC
TACGAGATTGTTTTGTCAGCTGGCTGCTTGATTTGTAAAAAAAAAAACAGTATTTTGATGGTGAAGGTGA
TGGAGAAGGATGCTGTGGTGGTGTCACTGGTGGTATGGCTAATCTTGGTTGTACATCATCTTAAGCTCA
TTTATGCTAATATGGAAGGTTTGTGGGACCATCATCCTCATTTAGTTAATTTTTAGGTCTATATTTGTTGTT
CTATTTTGTGAGATGAGTTTCAAATGTCGAAACTTTTCGTACTAGAAATTAAATATTTGCTGAAAGTGAA
ATGAGTATGTGCAAAATTGGATTTTGAATTTGACTAATGTGTGAGTTGTTGAATTTTCCCAAAAGGGTAA
GCAAGATGTGCAGTGAGCTCCCTGCATTGTTACGGTGGCAAGCAGTTTAGTTTTAATGTCAGTTATATGT
TAAAGTTTTTAGTTATCATTGCCATTCAACCTTAGTGCATTATGTTTCCTATACAAGCTTGAATAATTTGAT
GTGATTTAATTTGGTTGTTGCCAAAATCTTGATAAACTGTTTTATTCAGTGAATAGATTAATTTTTTATGT
GCATTTTGGTGGATTAAATATCGTTTATGTTTTCCTAAATGGATACACGAACAGGTGATGCATTGCATAG
TCTACGCGTCAATTTACAAGATCCTAACAATGTGCTACAGAGCTGGGACCCTACCCTTGTTAATCCTTGCA
CATGGTTTCATGTTACCTGCAACAATGACAACAGTGTTATAAGAGTGTAAGAATCTGTTGTCTGGTTTAC
CTCCGTTTTAATGGATTTGCAATTCCACTCTCTTGTGGTGGTGAGCAAATCTAATTGCAGTTTGTGCTTCC
ATAACACTTGTTATATGATCAACCTTTCCAGTGATTTAGGAAATGCAGCTTTATCTGGTCTGTTAGTCCCA
CAGCTTGGCCTTTTGAAGAATTTGCAGTACTTGTAAGTCTCACTTCATGAACTATGTTTGGAATTATTTTA
CGAATTTAGACTTGGAAAACTGGCATTAAGTGTATTGGTTTTTCCAGATGTTGAAGATTTTTCATATTACC
TCTAGAATTTGCTGCTGGAAATACCATAGATGGCTTGCATATTAACTTTCGCATAAAACAAAGCTGTTGT
AAGTGGCAAAAGCGAGCAGAGAACACTTTCTCCTCTCCATCTCTTGTTTCACTTGGTGTTGTAGTCAATT
GCATAGTTGTTAATGTGTTCCCTGAACAACACAAATTTGAATGTATACTTCATAAAAAAAGATTTAAATTT
GTGTTTCACAATACATACTAGTAAATTAAGAAACTCGGATACTTGGATGAGACTAGCAGTGAATTAACTT
ATGTGGCAAAGTAGTGTCTGTAATTGCTTGTGAAAACATTGTAGTTGAACTTAAATTTCTGTCATACACTC
CCCTATCCAAAGTAAGCATAGATTCTAAATACCATTACTAATGCCAGTACCCTAAGCACTCTTTATTCTTTT
GGTTTCCCACCCGGTGTCCGGTACCCACATTGGAGCCTGACTAAATCCGGATTCGCGCTGGGAAGTCCC
ACATTGGGGGTAAAGCGCTCCCTAACAAAGGTGACTCCGTACCCAAGAGGGCTCGAACCCAAGACCTCT
TGGTTAAGGATGAAGGAGTACTTACCACTCCACCACAACCCTGCTTGGTACCCTAAGCACTCTTAAACAA
ACTTCCAACTCATATCAAATCCTTTATTCCCTCTTTCCTTGTCCCAGGTAGCGTCTAATCATGATTTAGGAT
GATGGAATAGGATAAAGCAAACAAGAGAAACAAGTCCATTGTAAAAGTAAAGCAAAATCTAGACTTAG

AAAGAGGAAATATGGCTTGAGGGTAGGTTGATATCTTGGAGGAGAAGATCTGAAGTTTTGTGGTCATA
TAACAGATGCTATTCTGGTTTTCACCACGAGTTTGAATCATGATTCAATTAAGCATAGATTTTGTACCTGT
GTTAGCTTAGTTTGGAGTTTGATAATATACCGTTCACTTAAATTGTCAACGTGCTATAGGCATATTCTGTC
TCAAACTGGCTTTCAAGTTCTTATTGTAACCCATGCTCTTTTGAAGATGCAACTGTATGGAAGTTCACAGA
GACAGTGAGTGCATGTGCACCTGGTGAAGCTGGGCATTACATGATGTATACTGATGCATCAGACAATGT
TTTAATGATGGGCATAATTATTACCTAGTGACTGCTTCGTTCTGCTCACACATTCATATATTCCTATGGAT
GCCTAGATTTGCTGGAGGCTTGTGTAGTATGTGTGCTAAGTATTTTCCAAGTGTATGCAAGTGACAATCA
CATCAAGAATGAAACAATATAAAAAGAACATCAAGAATGCTGATGGATTTTAACGATCACTGGGCATAC
ACACTGTTTAGATGAAGTTCGAAATAAACATAATGACAGACGTTAATTTTTTATTGATGAGCTATAAAGA
TTGTATAAAATGTTTGACGAGCTGATCCTTTGCAGCACATGGGATGACAGGTTTTTATCTAGCAAAATGC
TTGTAACCAAATATAAGAAAATTCCTCCATGACATATACATTGGTAACTAGTTATAATTGTTAGCTTGGAA
CTCCCAGACCTTTCTCATTTGTTGACATTTAACTGTAGCTTGATGCCAGTTTTGATGAATGTGTGAACTTT
GCTTCATTATCTTGGTTGTCCCATTATCCTAGGGATGTCATTGGTATGATCTGTGGAATTAATTCATTCTCT
GGTTTTCTTGTCACAGGGAGCTTTACAGTAATAACATAAGTGGTCTAATACCGAGTGATCTTGGGAATTT
GACTAATTTGGTCAGCTTGGATCTCTACTTGAACAACTTCGTCGGTCCCATCCCAGATTCCTTGGGCAAG
CTGTCGAAATTGAGATTCCTGTATGTATTTTTATTCTATATTAGATTTATGTGGATATTGTGATGCCAAAT
GTGTATGTCATCAACCAAGATGGACCTGCTTTTGCCGGCATGTTGAGAAAGGAAAGGAAACCTGAATTC
TTTTCTAGGTTTCACATAAGATTACCTCTATTAGTATTAGGTTTCACACTAAGTTTTGATGGTAGAAGGTA
TTTGAGGACTTTATGGGACTGATATCATTAATAAAACAGATCTTACATATAAGTCTTTTTATTTTCTCCCTT
TGTTCTAATCCTTATATAAAGGAAAAGAGTAGAAGCCTATGCTTTGTCTGATATTTGGGCCTGATATTGT
AAAATTAAGCAGAGTTTAGTAATTGGTGTTTTATTTTTCTCCCATTGTTGGGATCCTTTTCTAGAAGAGAA
AAGAAGTTTAGAATCCCATGCTTCATCTTACATTTTTCACTGCTGAAGTTTCTCTTGCTTAATGCTCCACAA
ACTTCTTTCTTTTGGCTTGACTGTCTAAGAAGTGTACCAAATAAGTTCTATTATGCAGACTCAGCATGAAG
CCTCATTAACTGCATCTTGGGACTTACTATAGAACTATTGCAGTACTGCCAATGTTGACTGACATGATTTA
GTCCAAAAAGTGTTTCAAGTTTCTGTTTGTCAGGGGGGAAATGTTTTGAGGCTGTTTATTGTTGTTCTGA
ACTGACAACTTCATTTCCAGCTGAAGTGCCGGTTCTGGCTTGAAGTTCGAATATGTTTTACATCCACATAT
AAATAATTTATTAAGATGATTCTACTTGTCAATAAATAAATAAATTAAGATGATTCAATTAGTAGCCTTTT
CTTCTGTTTTATTCTCCAATTATTACTTATACCAAAAGTGAAGCTCATTCCTGTACACCTTTGTGATTTTTTT
GCAAGGATAGTCGGCTCAACAATAATAGCTTAACTGGTAACATCCCTATGTCACTGACTAATATCTCATC

ACTACAAGTGTTGTAAGTACACTAGTTATTTTGTGACTTAACTTAGATAATTCTTTCTGTCTTCCATATCTT
TTCATTCATTTCCTTTTCCTTCTAGTGCATATACAGATATACTCATGCTCAATTCTTGTCTCATCTGTATATA
GGGATCTGTCAAACAACCGTCTCTCAGGTGTTGTTCCAGATAATGGTTCCTTTTCTCTATTCACGCCTATC
AGGTATATTTCATTTAAGGTGGTGCACCATCAAACTGGCTGGTGGTTTTAGTATGCTGCTATTTGCTAAT
ATATTTTGCTTGAATTTACAGTTTTGCGAATAATTTAGATCTTTGCGGGCCTGTAACTGGACGCCCTTGTC
CGGGATCTCCTCCATTCTCCCCTCCGCCTCCATTTGTTCCACCACCACCAATTTCTGCTCCAGGTGGTTCTC
TAAATTGGTGTGGATAGGTTGTTTTCCCTTTTTTTTTTTTTGCTTGTTTGAGTTTGGTGATTTTTGTTGGTC
CATCCATCTTGCCTCTCTTAGTTTTTCCTAACCTAAGTGAGAATTTGTGCTATAGAAGTACTGACAAGAAA
GGGGCAGAAGAGGAAAGCCCATCTTAGCTAGTCAGGCATTTTGGTTAAGTAGTTCTTATGGGAAACTGG
TATGCACGAGACTACATTTAGTCTCTAAGCATTCTGGTCTTTATACAAATTTAATTCAGCATTGTGGAGAT
CTTTTCTTTGGTCCCCTTGTAAATTATTATCTGTGGTATTTGAAGTCGAGTATGTCTGAATGAAATTAATC
ATATTTAAGCCAGAACTTGTGAAGATTTTCTTTGCTTTGTAAAATCTATCCTGGAAATTAGATCTGAATGA
TAGATACATATTTGTGTTCTTTCTTTTTGAAGTGATTTAAATAATGAAGGGCCAAAATTGTGTGTTAAATA
TAGTCTGGATCATATGAAGGAAATTAATCAAATTTATGGGAACTTCAGGAGGAAATGGTGCAACTGGAG
CAATTGCTGGAGGTGTAGCTGCTGGTGCTGCTCTACTATTTGCTGCTCCTGCCATTGCATTTGCCTGGTG
GCGCCGTAGAAAGCCACAAGAATATTTCTTTGATGTACCAGGTTAGCAGTATTCACAATACCCAACCATA
AGTCCATAACTCCTACTTGCTCCCTCGCGTGTTTGTGGTTTCTCTTGCATGTTTATTTTTTGGCTCCATAAT
TACCGTTTTTGCTTAAATTTATTGCAGCTGAAGAAGATCCTGAAGTTCACTTAGGTCAACTGAAAAGGTT
CTCCCTCCGAGAGCTACAAGTTGCAACCGACAGTTTTAGCAATAAAAATATTCTGGGTCGAGGTGGATTT
GGTAAGGTATACAAAGGACGCTTAGCAGATGGATCATTGGTGGCTGTTAAGCGGCTTAAGGAGGAGCG
TACTCCTGGAGGGGAGTTGCAATTTCAAACAGAAGTTGAGATGATTAGCATGGCAGTGCATAGGAATCT
TCTACGATTGCGTGGTTTCTGTATGACACCAACTGAAAGACTGCTTGTCTACCCCTACATGGCGAATGGA
AGTGTTGCATCATGCCTGAGAGGTGACGCCTTCTGAAATCTATCACTCCGTAACATATTTGGATGTTCTC
ACCTTTAATTTAGAGGGTATTATTACATAATGCAAGAACATCTTTCGCTGGTTAACGTTCTATCTGGCATA
ACATACTCTTTATAATAAAACATATTCTTGTATGTTATTCCTGTAACTTGGTAAAGGTAGAAATATAATTT
ATTTTGTATTCTCTTGACAAAATAATTTATTTTATCAGAACGACCGCCTTCTGAACCGCCACTTGATTGGC
CAACGCGAAAGCGCATTGCTTTGGGGTCTGCCAGGGGATTATCGTATTTGCATGATCATTGTGACCCTAA
GATTATCCATCGTGATGTGAAGGCTGCAAATATATTGCTAGATGAAGAATTTGAGGCTGTTGTTGGAGA
CTTTGGTTTGGCTAAACTTATGGACTACAAGGATACACATGTTACAACTGCTGTGCGTGGTACAATTGGG

CATATAGCTCCAGAATACCTGTCCACAGGGAAGTCTTCAGAAAAGACTGATGTTTTTGGGTATGGGATC

ATGCTTCTGGAGCTAATCACCGGCCAACGTGCTTTTGATCTTGCTCGGCTTGCAAATGATGACGATGTCA

TGTTGCTTGACTGGGTATGTTGTCATGCCTGCTTTACATGTAACATGACACGAGTACCATAATGTGCTCAT

TTTTTAATCTGTACAATCATAACACTAGCTGATTAATAAGTGTTTCTGCCTTTAGCAGGAATCTTTACATCT

ATGTCTGAACTTGTTGAGGTTCTTGTTTCAGGTGAAAGGACTCCTCAAAGAGAAGAAATTGGAAATGCT

GGTTGACCCTGATCTTCAGAACAAGTATGTGGAGGCTGAGGTGGAGCAACTGATCCAAGTAGCATTGCT

TTGTACACAAAGCAACCCAATGGATCGGCCTAAGATGTCGGAAGTGGTGAGAATGCTTGAAGGTGATG

GTTTGGCTGAAAGATGGGATGAGTGGCAGAAGGTAGAAGTTCTCCGGCAGGAAGTGGAACTTGCACCA

CATCCTGGTTCTGATTGGCTTGTTGACTCAACAGAGAATTTACACGCAGTGGAATTATCGGGTCCAAGGT

GACCGACCTCGTACTACCCCAAGTAAAGAAAATCAAATCATTAGGAACTTTTTATTTTTGTTGACCTTGTG

AAAGTTTTTCTTTGTGAAATTTAATGAAAATCTCCAGTTGTAAGCCCATGAAGTGTGTCTGTTACATTGTG

CATACAAATTTGTACTGACAGGAAATTCCAAATCCATGCCCTGACAAGTTGGCACATAGGCGTAGTAGTT

AAGTGCTTTAAAATTGTCCAACACAGTACACTACTGTCAAGCAGACTCTTGTTTTCAGTGACATTGAAAA

TGCTATCAATTTGACAAGGTTATTTACAAGATTTTGGATTTTACAATCAAAGAATAGTGCTTCACATTCTC

TTACGGTTTTTATCATAATCTCGTGCTACAAGATAATGGCAGATATTTCATCAATAGGGCACTTGCAGAG

TTTGTTTTGCCACTCTATGCCCAAATTTTGGGCTCTTTCTGACAATTCAGATTAGTACCAGACGCTCTGAT

ATAAGTACAAGATCTGCTCAGTGATACTTTTCGATCATTTCTGTCATTATTTTTTACTCCAAAGAAAATTTC

TTACATGCAAATTTTTTGAACTGCATACTCTTCAACATACAAGGATCCATCGAATGTACTACAGACCAAAG

GGCAATAGAATAAACAGAAGAATCTCAAAACTCACAATCAACAAACAAACCAGAGCCTGCAGAAGGTA

GAGAAATACACTTCACTTATCATGGCTGGACCTGATATTATCTTTGAATGACTGTGAACGAGAACCAGGA

TTAGAGCCATTTGTTGCATTAAACAGGTTTGGATATGAATAATATGTTTCATCGTCCATGGAAGGCAATC

TCTGTGGTTGGTGAAGACCCTGACCAGGATGATGAATATGGGAGATGGGAGACTCAACACCCACTGTT

GAATCATCGTATGAAGGAATATTCGACACCTGATGTTCCAATGGACTGCCATGAAGTGGGCTGGGGATA

CCATCAGAGTTTGACGATTGGCGTTGGAGGTCTAAGTGAGGCTTTTCTAGGATGTGAGATTCTTGTTTCT

CAGCATAAAAGGGGTTCTCACTGGGGCTTACAGGCACCTCTTCGCTCCTTTTCCTGGTAAGCTCGCTGAT

TCTAACCTGAGCAAGGCTAGCTGCTGAGCGAGCAGCAGCAGCTGCACGTTCGGCTGAATCAGCAGCTG

CTTGGGCAGCAGCCAATACATCTTGCAAATCTACATCAGTCTCAACTTTGGTCTTTGTAGTAGAGGGTGG

TTGAGTACTTTGGGTTGTGGTTCTTTCTGTTGAGGAGAATGGAGGTGATTTTGGCGGGGGAACCATGAA

TGGCAAAAACTGTTTCTCTTCTTCCGGTGCAGATAAACTATCAGCTGATCCAAAATCTTTTGAAACTACCT

TCTCTTGCAAAAGTTCATCTCGCTCCACATCTTGTTTGTGAGATTTGAGCTCACCGTCTTCAGAAGACTTA
GCTACCTCCTCATCGGAGTCAGATAGTGCAGAGGCTGGGAAAGGAAGCATTTCAGGGGCAGAAACTCC
ACCGGTGCTAGGTCGTAGTGGCTGCTTAGGGACTTCTGGAAAATCTAAGTCAAAATCAGCCTCAGATTC
CGTTTGTTTATCAAAGACTTGATCTGATGCAGAGTGCTGCACCTCATCCAACCTTTCTTTGGGAAGTGGC
ACCTTAGCCCCACTGACAAATTGTGTGGGCCCATTCTGCAAAGCAAAGGGAAGAAATCATGATGATTGA
AAAGAGGACAAAATCAAAGCCAGTACAGACTGAAATATACCAGCAAGTCCTCATGTGACTTCAACAGCT
CAGTTTCAGAAGCACTTGGATCCCAATCTAGCTGATGCTCCTCCGCAATTTCCTTTAGCAGCTTCATTTTC
ACATCAGGAGCAGGAGCTCGAATAGACAGCAACTCTATCAACTGTCAGAGACAAGAAAAAAGAGAGAA
AAATCAGAAACAAGAAACAGTTCCTCTTTCTCGTTTAGCTATGGCATAAAGGAGAACTAAGGTAACCTG
GCGATTGACTCCGCATTCTGGCATGAGCTCTGTAGCAGCAGCAATGAATTCCTTCCCATATTTACCAGCA
AACATCATCTGAACCTGCAGCAATTCCGGAAGATCTGCACATCTTGGTGCAGCAAAACAAACACTTGAA
ATAGCTTCTTTCAGATCTAGAGGGCATTCCCTGGACATTATAGAGTTTGGGGGTTCAGCTCTTTCCAAAA
ATTCAAGTTAAGAAAATTATCAACAAAATTCAACTAGAAGTCTAGAATATTAAGACTTGGAAACAAAACC
TCCACATTGCAGGACAGTCAAGTTCAAGAGTATCAGTTTGCTTTAAAACAAATATCGAAGATGAAGCGC
TTTGGGTCCATCTTGGAACTAGAGATCCACATTTATGGTCTCATGTAGATGATCAAATTATTTTAAGGTTT
CACACTCCCATAACACAAGCAAGAATGTATTGTTCATTCTATGCCCTAGATACTTCTCGGTCATATAAGCA
GAAAGTTAACGTAGTTAATCCTTGTTTGGCTAAAATTGACTTTATGCAAAAGGATCAAACATGCTGCTGC
TCGGATACATGCACATACTTCCATTTAATTGACCTACAACCTAGACATTTCAAAGTTGGTAACTTTTTTTCA
GAATGCATGAAATTTAGCAAATTAAAGGACTGTGATTCATTAATTGAACTAGAGTCGCTCCATGAAAAA
GTATCCATATCTAGGGCAGATAATTCTCGCAAACAACAGCTAGGGTGGAGTATAGTAGATCTGAAAGTT
TGGTATTGAAGGAGATATGGAACTAAATGTTAACAGCAGCATAGACAGCAAAGGCAAGAGGCAAGCAA
GGAAGACAGAGAGGAACATTTGGGACCCGTTTGGCCATAGATTTCCCAAGTAATATTTGGGGGTAAATT
TGGCAAATAGTGTTTGTCCATACAATTTGCCATTATTTGGCAAATATCCCAAATTCCCAAATACTAGTTTT
TTCTAGTATTTGAGCCAAATCTCATTATTTGGGATCTTTTAAAAATTGAAATTTTACACCAAACTTTTATCT
TTTACAAAAACTCCCTCTATAGTAGTTGTTTGCGTTGTATTACATAATTTTTTTACGTGAACCAAAGTAGT
GATGAAATATGAAAGTGATGATATGGTTGTTGATGAAAATGATGAACAAACGGTCCAGGGTACCAAAG
TCATGTGCTTTGTCTACTTCACGATGTATAGAATGATGCTTGTTGCACTCACTCCACACACCACATTGCTC
CAGTGTCATGGACACTACTTGTTATTCGTTGCAACGAAGATCCAACTGACTTATAATACAAACTTATGGTT
AGTTTTGATAGTTTTTAAAACTTATGGGTATAAATCATATTTTTTCTAAAAAAGTGAAATATGTTTCCAAA

AGATTATGGCCAAACACATGGTGAAATTTCACCCAAATAATATTTGCCAAAAATATTTGGAAATCTAT

>PGSC0003DMG400027555 LRR-RLKs

CAGCTGAAGAAGATCCTGAAGTTCACTTAGGTCAACTGAAAAGGTTCTCCCTCCGAGAGCTACAAGTTG

CAACCGACAGTTTTAGCAATAAAAATATTCTGGGTCGAGGTGGATTTGGTAAGGTATACAAAGGACGCT

TAGCAGATGGATCATTGGTGGCTGTTAAGCGGCTTAAGGAGGAGCGTACTCCTGGAGGGGAGTTGCAA

TTTCAAACAGAAGTTGAGATGATTAGCATGGCAGTGCATAGGAATCTTCTACGATTGCGTGGTTTCTGTA

TGACACCAACTGAAAGACTGCTTGTCTACCCCTACATGGCGAATGGAAGTGTTGCATCATGCCTGAGAG

GTGACGCCTTCTGAAATCTATCACTCCGTAACATATTTGGATGTTCTCACCTTTAATTTAGAGGGTATTAT

TACATAATGCAAGAACATCTTTCGCTGGTTAACGTTCTATCTGGCATAACATACTCTTTATAATAAAACAT

ATTCTTGTATGTTATTCCTGTAACTTGGTAAAGGTAGAAATATAATTTATTTTGTATTCTCTTGACAAAATA

ATTTATTTTATCAGAACGACCGCCTTCTGAACCGCCACTTGATTGGCCAACGCGAAAGCGCATTGCTTTG

GGGTCTGCCAGGGGATTATCGTATTTGCATGATCATTGTGACCCTAAGATTATCCATCGTGATGTGAAGG

CTGCAAATATATTGCTAGATGAAGAATTTGAGGCTGTTGTTGGAGACTTTGGTTTGGCTAAACTTATGGA

CTACAAGGATACACATGTTACAACTGCTGTGCGTGGTACAATTGGGCATATAGCTCCAGAATACCTGTCC

ACAGGGAAGTCTTCAGAAAAGACTGATGTTTTTGGGTATGGGATCATGCTTCTGGAGCTAATCACCGGC

CAACGTGCTTTTGATCTTGCTCGGCTTGCAAATGATGACGATGTCATGTTGCTTGACTGGGTATGTTGTC

ATGCCTGCTTTACATGTAACATGACACGAGTACCATAATGTGCTCATTTTTTAATCTGTACAATCATAACA

CTAGCTGATTAATAAGTGTTTCTGCCTTTAGCAGGAATCTTTACATCTATGTCTGAACTTGTTGAGGTTCT

TGTTTCAGGTGAAAGGACTCCTCAAAGAGAAGAAATTGGAAATGCTGGTTGACCCTGATCTTCAGAACA

AGTATGTGGAGGCTGAGGTGGAGCAACTGATCCAAGTAGCATTGCTTTGTACACAAAGCAACCCAATG

GATCGGCCTAAGATGTCGGAAGTGGTGAGAATGCTTGAAGGTGATGGTTTGGCTGAAAGATGGGATGA

GTGGCAGAAGGTAGAAGTTCTCCGGCAGGAAGTGGAACTTGCACCACATCCTGGTTCTGATTGGCTTGT

TGACTCAACAGAGAATTTACACGCAGTGGAATTATCGGGTCCAAGGTGACCGACCTCGTACTACCCCAA

GTAAAGAAAATCAAATCATTAGGAACTTTTTATTTTTGTTGACCTTGTGAAAGTTTTTCTTTGTGAAATTT

AATGAAAATCTCCAGTTGTAAGCCCATGAAGTGTGTCTGTTACATTGTGCATACAAATTTGTACTGACAG

GAAATTCCAAATCCATGCCCTGACAAGTTGGCACATAGGCGTAGTAGTTAAGTGCTTTAAAATTGTCCAA

CACAGTACACTACTGTCAAGCAGACTCTTGTTTTCAGTGACATTGAAAATGCTATCAATTTGACAAGGTT

ATTTACAAGATTTTGGATTTTACAATCAAAGAATAGTGCTTCACATTCTCTTACGGTTTTTATCATAATCTC

GTGCTACAAGATAATGGCAGATATTTCATCAATAGGGCACTTGCAGAGTTTGTTTTGCCACTCTATGCCC

AAATTTTGGGCTCTTTCTGACAATTCAGATTAGTACCAGACGCTCTGATATAAGTACAAGATCTGCTCAG
TGATACTTTTCGATCATTTCTGTCATTATTTTTTACTCCAAAGAAAATTTCTTACATGCAAATTTTTTGAACT
GCATACTCTTCAACATACAAGGATCCATCGAATGTACTACAGACCAAAGGGCAATAGAATAAACAGAAG
AATCTCAAAACTCACAATCAACAAACAAACCAGAGCCTGCAGAAGGTAGAGAAATACACTTCACTTATC
ATGGCTGGACCTGATATTATCTTTGAATGACTGTGAACGAGAACCAGGATTAGAGCCATTTGTTGCATTA
AACAGGTTTGGATATGAATAATATGTTTCATCGTCCATGGAAGGCAATCTCTGTGGTTGGTGAAGACCCT
GACCAGGATGATGAATATGGGAGATGGGAGACTCAACACCCACTGTTGAATCATCGTATGAAGGAATA
TTCGACACCTGATGTTCCAATGGACTGCCATGAAGTGGGCTGGGGATACCATCAGAGTTTGACGATTGG
CGTTGGAGGTCTAAGTGAGGCTTTTCTAGGATGTGAGATTCTTGTTTCTCAGCATAAAAGGGGTTCTCAC
TGGGGCTTACAGGCACCTCTTCGCTCCTTTTCCTGGTAAGCTCGCTGATTCTAACCTGAGCAAGGCTAGC
TGCTGAGCGAGCAGCAGCAGCTGCACGTTCGGCTGAATCAGCAGCTGCTTGGGCAGCAGCCAATACAT
CTTGCAAATCTACATCAGTCTCAACTTTGGTCTTTGTAGTAGAGGGTGGTTGAGTACTTTGGGTTGTGGT
TCTTTCTGTTGAGGAGAATGGAGGTGATTTTGGCGGGGGAACCATGAATGGCAAAAACTGTTTCTCTTC
TTCCGGTGCAGATAAACTATCAGCTGATCCAAAATCTTTTGAAACTACCTTCTCTTGCAAAAGTTCATCTC
GCTCCACATCTTGTTTGTGAGATTTGAGCTCACCGTCTTCAGAAGACTTAGCTACCTCCTCATCGGAGTCA
GATAGTGCAGAGGCTGGGAAAGGAAGCATTTCAGGGGCAGAAACTCCACCGGTGCTAGGTCGTAGTG
GCTGCTTAGGGACTTCTGGAAAATCTAAGTCAAAATCAGCCTCAGATTCCGTTTGTTTATCAAAGACTTG
ATCTGATGCAGAGTGCTGCACCTCATCCAACCTTTCTTTGGGAAGTGGCACCTTAGCCCCACTGACAAAT
TGTGTGGGCCCATTCTGCAAAGCAAAGGGAAGAAATCATGATGATTGAAAAGAGGACAAAATCAAAGC
CAGTACAGACTGAAATATACCAGCAAGTCCTCATGTGACTTCAACAGCTCAGTTTCAGAAGCACTTGGAT
CCCAATCTAGCTGATGCTCCTCCGCAATTTCCTTTAGCAGCTTCATTTTCACATCAGGAGCAGGAGCTCGA
ATAGACAGCAACTCTATCAACTGTCAGAGACAAGAAAAAAGAGAGAAAAATCAGAAACAAGAAACAGT
TCCTCTTTCTCGTTTAGCTATGGCATAAAGGAGAACTAAGGTAACCTGGCGATTGACTCCGCATTCTGGC
ATGAGCTCTGTAGCAGCAGCAATGAATTCCTTCCCATATTTACCAGCAAACATCATCTGAACCTGCAGCA
ATTCCGGAAGATCTGCACATCTTGGTGCAGCAAAACAAACACTTGAAATAGCTTCTTTCAGATCTAGAGG
GCATTCCCTGGACATTATAGAGTTTGGGGGTTCAGCTCTTTCCAAAAATTCAAGTTAAGAAAATTATCAA
CAAAATTCAACTAGAAGTCTAGAATATTAAGACTTGGAAACAAAACCTCCACATTGCAGGACAGTCAAG
TTCAAGAGTATCAGTTTGCTTTAAAACAAATATCGAAGATGAAGCGCTTTGGGTCCATCTTGGAACTAGA
GATCCACATTTATGGTCTCATGTAGATGATCAAATTATTTTAAGGTTTCACACTCCCATAACACAAGCAAG

AATGTATTGTTCATTCTATGCCCTAGATACTTCTCGGTCATATAAGCAGAAAGTTAACGTAGTTAATCCTT
GTTTGGCTAAAATTGACTTTATGCAAAAGGATCAAACATGCTGCTGCTCGGATACATGCACATACTTCCA
TTTAATTGACCTACAACCTAGACATTTCAAAGTTGGTAACTTTTTTTCAGAATGCATGAAATTTAGCAAAT
TAAAGGACTGTGATTCATTAATTGAACTAGAGTCGCTCCATGAAAAAGTATCCATATCTAGGGCAGATAA
TTCTCGCAAACAACAGCTAGGGTGGAGTATAGTAGATCTGAAAGTTTGGTATTGAAGGAGATATGGAAC
TAAATGTTAACAGCAGCATAGACAGCAAAGGCAAGAGGCAAGCAAGGAAGACAGAGAGGAACATTTG
GGACCCGTTTGGCCATAGATTTCCCAAGTAATATTTGGGGGTAAATTTGGCAAATAGTGTTTGTCCATAC
AATTTGCCATTATTTGGCAAATATCCCAAATTCCCAAATACTAGTTTTTTCTAGTATTTGAGCCAAATCTCA
TTATTTGGGATCTTTTAAAAATTGAAATTTTACACCAAACTTTTATCTTTTACAAAAACTCCCTCTATAGTA
GTTGTTTGCGTTGTATTACATAATTTTTTTACGTGAACCAAAGTAGTGATGAAATATGAAAGTGATGATA
TGGTTGTTGATGAAAATGATGAACAAACGGTCCAGGGTACCAAAGTCATGTGCTTTGTCTACTTCACGAT
GTATAGAATGATGCTTGTTGCACTCACTCCACACACCACATTGCTCCAGTGTCATGGACACTACTTGTTAT
TCGTTGCAACGAAGATCCAACTGACTTATAATACAAACTTATGGTTAGTTTTGATAGTTTTTAAAACTTAT
GGGTATAAATCATATTTTTTCTAAAAAAGTGAAATATGTTTCCAAAAGATTATGGCCAAACACATGGTGA
AATTTCACCCAAATAATATTTGCCAAAAATATTTGGAAATCTATAGCCAAACCCTAGCTTGGTATCCTATA
CTATTTTTATTAGGAAAAAAAAATGCCAAAGAACCCCCCCCCACCCCACCCCACCCCCGACTTGTAGCTTT
TTAGTTAGTGAGCACCTAAACTATCACTCGTATTAAATACCCCTGGAATTTGTTTTTTCAACCGTAGCGTA
CACCTACGACGTTTAGATCCCTCAACCGTGTATACACGCACGTGACACGGTGAAAATATGACAAATGTG
GATGTCCATGTAGATAAATAATGCCCCCACCCTCTCTAATATTTACCCTTTTTCAAAAGCTTTTTATATAAT
CCCCCAATTCACACAAATCAACCCTAATTACCCGATCTTCTCTTTCACACAAATACTCTCTAAATAATCAAA
TCCTAAGAAGTTTAACAGTCGGAGTTAAGGGTAGATTTTGAACATTTCTGGCTCTGCTATTTGAATGTTTT
CTCCTCAAATAGTACAGTCCATTTGGGTGAAGCTTTTTATTTGAGTGGATTTTTCTCCGATCTTGTCTGAA
ATTCTTATCAAGTAAGTGATTATCCATTCTTAAAATGTATTTTTGTTTGTTTTTCCTCGAATAGCCTTGGTC
AGTTACAATCCAATTACGTCCGGGGTAGTCATATGACCATGAAATTCTCACAATCGGAGGACACCCACAT
GCACAAACAAGCATCCTATTATAATTCATGGATTCATCAATTGCAACTTTAGAAAGACGGAGGAGAGAT
AAATTATAGGCAAAGAAAGAAAATGATGGCAAAGATAGGTATTTCAGAAAATTGAAAGTGATGGAGAA
GAAGGGTTTAGGGAGAATAAATAGGAAGAGAATGAAGAGAGATGCTGTGCAAAAAAGGAAAAAAATA
AAGAGAAACAAGAATTTTTAATGTTTTTCACCATTGAAGTAATATGATGCGGCAAAAAATTTACTCCCTC
ACGCACCTAGCTGAGCTTGTTGTCAGTTTCTATGCCAGGTGGACGTTGTAGGTGTATGCGACGGTTGAA

AAAACAAGTTCCGGGGGTATTTCATATGAGTGATAGTTTAGGTATTCACTGAATAAAAAGCTACAAGGT
CGGGGGGTCTCTAGCATTTCTGCCTTTTTATTATTATTAAACTGGTGTATGCGACGGTTAAAAAACAAGT
TCCAGGGATATTTCATATGAGTGATAGTTTAGGTATTCACTAAATATAAAGCTACAAGATCGGGAGGTCT
CTAGCATTTCTGCCTTTTTATAATTATTAAACCTCATTCAGCTGACTATCCTAGTCTATATCAGGTGTAACC
CAGTTTATTGACTTATTGCAGCTAGAGTAAAGTTAAATGGAGCTATTGTTTTAGAGCACAATACAGCTAT
AAAGATACATATGACCAGCATAACTACACAAGATGACAATATACTTTACTTATGAAAACGGAAGAAGAA
AAATAAGCTAAAAGGCAATTCTACAAGTAGGCCAACCTTACAGCTGGGATTTACATCTGCCAACGGTGT
CAAAAAAATCATATAACCACGAAAACCAAAAACTTGCTGTTCTATAGGCACTTCGCCAAAACATCATAGG
TGATCATCTAATGCATCATAAAGGAACCCTTTTATATTCTTTGCCATTCGCCCCATACATTCTTTATCCTAA
GAGCAACTAAAAGCATATTCAGTTGCTCATCAATAAACTGAAGAACCATCTTAGGATACTGTAAATGTTC
ACACCATATAGAATATCAAGAACATTAACACAACAGATAACTCATTACCTTTGTGCTTCAATAATTGGAA
GACGAACCGATATAAGCTCGCAGAATAGCTCAACAATCTCCTGCGCTGCCATCATTTTCTCTTCTCTTATG
ATATGCTCTACCTAAACAAGCAAACATCACAGTCAAGCTCATTACTCTGTAAAACCATCTATCAAAATACA
TAATAACCTTTCAGTATTCAAATGCGAAGAACACACAGCCTAGCACAAATTATATCAGCATGAATCGATG
AATTTAAGCAAAATAAGAGTCAGTTTATACCCGAATTCGAGCAGTAGCTTCCTGACCAGTCTCTAGTAGC
TTAGCTATTTCTTTCCTCATCTGCTTCAGCTGAAACTCCCTACGATTCCTCAGTAGTTTGATCCGAGGAATT
GTCAATTTCAACATGGTTTTACTGCAAATTGATAGGTACCATCAGCAAATTCCCATATAAAAAAGACAAC
ATTTTTCTCAAAAATTTGAAAAATTCGAAACCCCATGATTTCAACAATACAAAATTTCAAATCTGCATCAT
AAGAACCCTTAACTAAAAAGATCCAAATCATAAAATCTGACAAGAACCTTCAATAAAAGACAGGAAAGT
GGGAAATTGAGGATTACCATTTGGCAGCTTTGAAACCTTTGCTGAAGAAAGAATCCATTATAGACTTGAT
TCAGATCTTCAATATCCGTGTACCCTTTAAAATTCTTGCAGTAAAACCTTATGAATTATCACTTACCTAAAA
CCACAAAATAATTAATCAAACTTGGTAGTATTTCAGATTAAAGCAAAGAATACCTTTTTATTTTTCAAAAA
AAGAAAAGAAAAAGAGATCAAAGATCAAAACTGATGACGAAGTAGTTAAGAAAATATGTGTTTTTGTG
GAAAGCACAGAAAGTGAACCTGCTCCGCTATGCATATAAAGACAAATGGAACGCGGACAAAGCTTTCA
ATGTCTACGACAACAAGCAGCTTCCTTAAGCTCTAAGCATTATTGGATTTCAACTTTCAACGAGTATTATT
TTTGG
>PGSC0003DMG400027581 LRR-RLKs
ATGAAACTACAAGATTTACGTGCAACTATCGTCTTTCTTCTATCCTTGCTAGCAATTCTTCCTCACATTATT
GCTAATCTTGATTCAGACAAACATGCTCTACTTCAATTTGCTGCATCTGTCCCACATCTTAGAAAACTCAA

CTGGAACTCTGCTTTGTCAATCTGCAATTCTTGGATTGGGATCACTTGCAACAAAGATGGAACGCGAGTA
GTTGCAATTCATCTGCCTGGTGTTGGACTTACTGGCCATATCCCAGCAAACAGCATTGGCAAACTAGATG
CTCTCCAAGTCCTTAGCCTAAGAGCAAACAATCTAAATGGAAATCTTCCTTCTGATATTCTTTCAATCCCTT
CTCTTTACTCTATCTACCTTCAACATAATAACTTCTCTGGTGATATTCCTGTTTCCTTTTCATCAACACTTGG
TGTAGTAGATCTCTCGTTTAACTCTTTTACCGGAGAAATTCCCCCCAAGATCAAGAATTTGCCTCGACTAT
CCATGTTGAACCTAAAGTTCAATTCACTATCTGGATCAATTCCCAATCTTGATGTGTCAAGGTTGAGTTTT
CTGAATTTGAGTTACAACATGTTAAATGGCTCAGTTCCATATTCCCTCAGGAAGTTCCCACTTTCTTCTTTT
GTGGGGAACTCTAATTTATGTGGAACACCTTTGAGTAGCTGTTCTTCGAGATCTCCATCACGTAAAGGTG
ACAACTTTAAGAAACTTAGTAATGAAATTATCATTGCAATTGCAGTAGGTGGTCCTTCTGTTATAATTCTC
CTTGTTTTATTCATCTACTTCTGTTATTTTAACAAGAAGGTTGATAACAACACAAGCATGGTTGAAGAGAA
GAATGAGAAGTTAGAAGACTTTGGAAATGGGGTTCAAGATTCTGAAAAGAACGAGTTAACTTTCTTTAA
AGGGTGCTCTTATAACTTTGATCTTGAGGATCTGTTAAGTGCTTCTGCTGATTTTCTTGGTAAAGGGAGT
TATGGAACTGCTTACAGAGTTTCTTTGGATGAGGTATCGATGATAGTTGTGAAAAGGCTGAAGGAAGTA
AGGGTTGTTAAAAAGGAGTTTGAACAGCATATGGATATTGTTGGAAAGATTACAAGGCACCCAAATATA
GTGCCATTTCTTGCTTGTTATTACTCAAAGGATGAGAAACTTCTTGTTTGTGAATACATCCCTTATGGTAG
CTTGTCATCCGCCTTATATGGTATAAGCTCCTTGACTCTTCTTATATAAAACTACTTCTTAGAACAGATCAT
GCTTGCTGCACATTATGCTGTTAATAGCTATTCCAATTCCGGCTTTATGCTTACATGGTCTTAGCTTAAAT
AGAGCGACATGATTATTGGTGGTGATTCATATAGCCGAGTAGTAGTTGTTGTTTCGTACTGTTACATGAT
CTTGTTTATAACTAGCTATATGCCTACTGTTAGCTTGTCAACAGCCTTGCATGGTATACAAAATTACACTG
TTTATACATGATTAAAATTACTATTTATGTATATATAGTAGATGTTGAACCCTTATGGCTTTTGCGTCTTTT
GAACCTCCTAAGTGAAAATCTTAGCTCTGTCACTGACGGTATATGTTGCTCAACTCTGATTGAAACTGCA
GTGTCATCGATCACTTGCTACAGATTATTAATGATTCCTGTTTTGGGCTTTATTTTTACATGATATTGGTTA
TAGCAGGTAATGGAAGAACTCGACTAGACTGGGATACCAGATTGAAGGTCTCTCTTGGAGCAGCAAAA
GGAATAGCACATATCCATTCTGAAGGTGGAGTGAAATTTACTCATGGAAACATCAAAGCATCCAACATA
CTTCTTACAAGGGATCTAGATGGTTGCATTTCTGATTTTGGCTTAAGCCCTTTAATGAATTACACAGCCAT
CAAGAATAAGGCTGCTGGTTACCATGCTCCAGAAGTGATTGAAACGCGAAAAGGCACACAAAAATCAG
ATGTTTATAGCTTTGGAGTGTTGGTACTTGAGTTGCTCACTGGCAAATCTCCTCTGCCATTGCCAAGGCA
CGAGGACGTAGTCAATTTGCCTAGATGGGTACGAGCTGTTGTTAAAGAGGAATGGACAGCTGAAGTTTT
TGATGCAGAGCTGATGAAGTACCATAATATTCAAGAAGAGATGGTGCATATGCTTCAAATTGCACTTTTA

TGTGTAGCAAAGGTGCCTGACATGAGGCCTTCAATGGATGAAGTGATTAAAATGATTGAGCAAATTAAA
CATCTATAA
>PGSC0003DMG400027585 LRR-RLKs
GAGAGAGAAAGATAAAGAGAACGTTAATAAATGCTCCCACTGACCCGCCGTCCCCGGTAACACTTTACT
TTTATCTACTTTCTCCGGCTTATTCCTCCCTACCTCATCAACAACCTTCAACTTCACCGGCGCTTTACATTTT
CCGGCGAAACCCCAAATTCACTCTCATTGTCTCCTTCTTCTTCCCTTCAGATTTCCCTTTTTCTGCTGCCAA
AATTCAACTGTTTCTTCCACTCATTCATCTACATTTTCTTCTTCTCCACTTTGTTTCATTAAAAATTCACTCTT
TCCGGATTTCTAGGGTTTTTTAAGAACTGGGTGTTTTTAGAAAAAAATTCTTACCCATTTTATTGGCGGTT
ACGTTCTTCTTGTGCTAAAGATTGTATCTTTTGCTTGTTCTTGTATATAATTATGCATACCCATTTGGGGTT
GCTGATGGGATTATGAGTAACTTATATATTGCTTTGTGATTATTGAGCTGGGAGAATGGAAATGAGGAA
GGTTTTTGGTATAGTAATATTGTGTATTTTAGTATCTCCTATTTTTGTTAAAGCCTTGAACTTATCTTTTAA
TGATGATATACTGGGGTTGATGGTATTCAAGGCTGATGTTCAAGATCCACAAGGGAAGTTGGTGTCTTG
GAATGAAGAAGATGATAGCCCCTGCGGCTGGAATGGTATCCATTGCAACCCCAGATCCAATAGGGTCTC
TCAGATTGTTCTTGATGGGTTTGGACTTTCAGGTAAGATTAGTAGAGGTCTTATGAGGTTGCAATTTCTT
CGAAAGCTTTCTCTTGCGAAGAATAATTTTACAGGGAGTATAAGTAGCAGTGTTGTTCAACTTGCTTATC
TAAGGATTCTAGACTTGAGTGAGAACAACTTATTTGGGACGATTCCGGGTGATTTTTTTGAACAATGTGG
GCCATTGAGATCAATTTCTTTGGCCAAGAATAAGTTTTCAGGGAAAGTTCCAGAAAGTTTGAACTCATGT
GTGGCATTGGGGTCTTTAAACTTGTCCTCAAATCAGTTTTCAGGATTGTTACCTTCTGGAATTTGGTCTTT
GAATGGGCTAAGGTCACTTGATCTGTCAGATAATTTGCTGGATGGTGAAATTCCAGTAGGTATTGAAGG
TATGTATAATTTGAGAGCCATAAATTTGAGGAAAAATCATCTCAAAGGTGAAGTTCCTGATGGAATTGG
AAGTTGTTTGCTGTTGAGGTCAATTGATTTGAGTGAAAATTCTTTCTCTGGAGAACTTCCTAAAACAATG
CAGATGCTTAGTTTGTGCAATGAGTTGATTATGAAACACAATGCATTAGTTGGTAGTGTACCTGAATGGA
TTGGAGAAATGAAAAGCCTTGAGATGCTTGATCTTTCTGGGAACAACTTCTCTGGTCAGTTCCCGAATTC
AGTTGGGAAGCTTCAATCACTGAAATTACTGAACGTGTCGCGAAATGCTATTTCTGGGGACTTTCCCAAG
TCCATGAGCAGTTGTGTTAATCTTATGACACTGGATGTTAGTCACAACTCATTGACGGGTGATCTTCCTCC
TTGGGTATTCAAATTAGGATTGCGACATGTTTTGTTTTCTGAGAATAAATTAAGTAGGGGCTTGAAGAAT
GCTATTGCTTCTTCACTGGAAAACTCTCGACAAAAGCTTCTAGTTCTCGATATCTCTTGCAATGAGTTAGC
TGGTGAAATTCCATTTGCTATTGGTGATTTTAACAGCTTGCAGTCATTGAATTTATCTAGAAATTCACTTG
TAGGGAAAATCCCTGAGACTGTTGGCCATCTAAAGTCACTGGATGTTCTTGACTTAAGCGAGAATCAGTT

AAATGGTAGTATCCCTCTGGAGCTTGGAGGAGCCTATGCTCTCAGGGAACTGAAACTGGAGAAGAATG
CCTTGACAGGAGAAATTCCTACTTCTATTGGAAACTGTTCTGCTCTTTTGTCCTTGTAAGTTCTTGTTTCTC
TTTATCGTTCATTTATAACTTTCATTTGAGATAATCTCTCTGCTTGGAGGTTTTCATAAGGACAATGTAGT
GGTAAGTTATTGTACAGAAGAACCTGATTGATATATAGTTTGATAATTAAGGAACTAGCATTTGCATTCT
TTTTCGCAGTTTCCTCTCAGACAACGTTGTTTTGGATTGTCAAAATTCTAGTGACTAAATCAAGCACGAAT
CCTTATATTAGAGATTCTTTATCATGTTCTACTAATTAGTTAGTTCTAAGCTTCTAGCAATCATTTAGAGTT
TCCATATTGAGTTAATCAAGTAGCTTCTAGTGCATGATTTTAGATAAAATCCTTTTTTTTTTCTAGATAAGG
CAATTTCCTATACTTAATATTTTACATAAAGTATAATGTAACAACATCACATTACCTGGTTTTCGATGTTTT
TATGTGACGTAGCCTGTAAATTTATTTATGAAGTTGTCTTTGAATATCAGCTTTCCTCAAAGCTTGGTTAA
TCGGTTTTGTTTCCATCAACTTGGCAGGTCTCTGTCACATAATGGTTTAACTGGCCCTGTACCTGCAACCC
TTGCTAAACTATCTAACCTACAAAATGTTGACCTTTCCTTCAACAAATTAACCGGAATCTTACCAAAGCAG
CTGGTAAATCTTGGTCACCTTGAGTTGTTCAACATCTCACACAACCAGTTAAAGGGCGAACTGCCTTCTG
GTGGTTTTTTTAACACTATTTCTCCCTATTCAGTGTCAGCCAATCCATCTCTTTGTGGAGCTGCTGCTAATA
GGTCTTGTCCAACTGTCCTTCCCAAGCCGATTGTTCTGAATCCCAACTCCACAGAATCTATTCCTGGTACT
ATCCCTCTGACTGTGGGCCACGAAAAGAAAATCCTAAGCATCTCTGCCCTCATTGCCATTAGTGCAGCTG
CTATTATTGTTGTTGGTGTTATTGCCATTACTGTGCTAAATCTTCGCGTACGGTCTGCAACATCTCACTCT
GCTGCTGCCCTTACATTTTCTGGTGGAGATGACTATAGCCCCTCACACAGTACAGATGCAAATTCTGGTA
AGCTTGTCATGTTTTCAGGCGAACTTGACTTCAGCACAGGGTCACATGCTCTGCTTAACAAGGATTGTGA
GCTCGGACGTGGTGGTTTTGGAGCCGTTTATCGCACTGTCCTCGGAGATGGGATGCCTGTGGCCATTAA
GAAGCTTACTGTCTCAGGTCTTGTTAAGTCTCAGGTAGACTTTGAAAAGGAGGTTAAGAAATTGGGAAA
AGTTCATCACCCTAATGTTGTGGCTCTTCAAGGTTATTATTGGACTCCGTCACTACAGCTACTTATATATG
AATTCATAACTGGTGGAAATTTGTATGATCATATCCATGAAGGTTCTAGTAAAAACATGCTCTCATGGAA
TGAGCGGTTCAACGTTATTCTTGGGACAGCAAAAGGCATGGCTAACTTACATCAAATGAACATAATTCAC
TATAACCTCAAGTCCAGCAATATTTTGATTGACAGCTCTGGTGATCCTAAAGTTGCAGATTATGGGTTAG
CAAGGCTGTTACCGATGTTAGATCGATATGTTTTGAGCAGCAAAATTCAAAGTGCACTCGGTTACATGGC
ACCTGAATTTGCATGCAAGACTGTGAAGATAACTGATAAGTGTGATGTGTATGGGTTTGGAGTTCTTGTT
CTGGAGATAGTCACTGGAAAGAAACCGGTTGAGTACATGGAGGATGATGTTATTGTATTGTGTGACATG
GTACGGGGTGCATTGGAAGAAGGCAAGGTGGAAGAGTGTGTTGACAAGAGGCTGCATGGAAAATTTC
CAGCAGAGGAAGCGATTCCTGTGATGAAGTTGGGTTTAATCTGCACTTCACAAGTCCCTTCAAACAGGC

CTGATATGGCAGAAGTGGTTAACATATTGGAAATGATCAGATGTCCTTCAGAAGGCCAGGAGGAACTA
GTATGATATCCCACTAAACTGATGGAAGTTTTTTAACATATGCTATTAGTCCCGGAGAAGAAGAATCCGC
TTGATCTATTCATATTATGGTCATTTAAAGTTTCAATTTTTTTTAATTATGTTATTGGTTCTCTGTTGTTCCG
GGTTACCGGATTCCTTTTCACCGGGTTTTAGTATGTGAGGATCCTTTTTTCACGGTTCTTTTATGTAACAA
TTGATTCTAACATGTAATTCCAGTAACCTCTTGCGTTTGTTGTTCAAATGAAGGAAGGTCCAAATGTTGAT
AGTGGATGTGCCAATAAGTATATTTCATGACAAAATGATCAAACAAGC
>PGSC0003DMG400027586 LRR-RLKs
GCTCAACAAGGTATATAATTAAAAGAAACCTTGTTGACATATATAGTTATGGGCAGAAGTTGCATTGTCC
TCTTTGGTCTTGCAGCTGTTTTCATTCTGCTTCATAACCAAACTTCACTTGCTACTGTTCCTAATATTGGCA
CTGATGAAGCTGCTCTTTTTGCACTGAAATCACACATTTCTTCTCGTCCTAACAACATCTTAGAAAGCAAC
TGGTCTTCGTCCAGCCCGGTTTGCAGCTGGATTGGAATCACTTGCAGCTCCCGCCACCATCGAGTCACTG
CTTTAGACATTTCTAGCATGCAACTTCATGGTACCATTCCTCCACACATAGGAAACCTCTCATTTCTTGTTT
CCCTCAGGATTAGTTACAACGCTTTCCATGGAAATTTGCCAGCAGAGTTGGCTCATTTGAAGAGGTTGAA
ATTGATTAATGTTAGAAGCAATAACTTCACTGGAGCCATTCCATCATTTTTGAGTTTGTTACCAAACCTAC
GCACTGTGTCCCTCTGGAGCAACCAATTTTCGGGGAAAATTCCATCTTCCCTTTTCAATCTAACAAAGCTG
CAAGTGTTGAGTTTGCAGAGTAATTTTCTTGTAGGAGAGATCCCTCGAGAAATTGGTGATCTTCGTTACT
TGACTGTCCTAGACCTGCAATACAATCAGCTTACTGGCCCTATACCACCATCAATATTTAACATTACGACA
ATGCGAGTGATTGGTCTTACCAACAACAATCTTACTGGAAAGCTTCCAACAACTATATGTGACCATCTTC
CAAACTTGGAAGGGCTTTATCTGTCAAGCAACTCCTTAGATGGAGTTATTCCATCAAACCTTGAGAAATG
CAGAAAGCTTCAAAGATTGTCATTGTCGTCCAATGAGTTTATTGGAACTGTACCAAGAGAGCTAGCAAA
CTTAACGGCTCTTATAACATTATTTCTTGGACAACAAGTACACTTGGAAGGTATATAGTATGATATTTCCT
TACAACATTATATCTAAAAATTTCGATCTCTCTTCCGCCTAACTGGTAATATTTCAAACAATTGACAGGAG
AGATACCTGTGGAGCTAGGTAATCTTAAGAAACTACAGATGCTGGGATTAGCCGAGAATGAGTTTACTG
GTTCGGTCCCTACAAGCATTTTCAACATATCAGCACTGCAGTTCCTAACACTTTCACTAAACAAGCTTTCA
GGTACTCTACCTTCAGATTTAGGTCCGAACCTAGAAGAATTTTACTGTGGAGGAAATAATCTGAGTGGTT
TTATCTCTGATTCAATCTCAAATTCTTCGAGACTCAGAATGCTTGATCTCTCATTCAACAGTTTCACAGGTC
CAATTCCTAAATCACTCGGTAAATTAGAATACCTTGAGGCTCTAAACTTGGGAAACAATAGTTTTATCAG
CGATTCAACATTAAGCTTCCTTGCATCATTGAATAACTGTAGGAATCTAAGAAATCTCTGGTTCTATAATA
ATCCTTTGGATGCCATTTTTCCGGCATTTGTTGGTAATTTCTCCGACTCCTTGCACGGTTTTGAAGCAGAT

GGTTGTAAACTTAAGGGCATGATTCCCCAAGAAATTGGTAAACTTACTGGTTTGACAAAGATGAGTCTAT
CTAACAATGAGTTCACCGGATTTATTCCAAATACCATCCAAGGCATGTTGAGCCTTCAAGAACTTTACCTA
GATAAAAACAAGATAGAAGGACCCATACCAGATGATATGTGCAGTTTACAGAATCTTGGTGCATTGGAC
TTGTCACAAAATAAATTTTCTGGTTCCGTGCCACCATGCTTAGGAAAATTTACCAGTTTGAGGACACTTCA
TCTAGATTACAACATGCTGGATTCTAGATTACCAGAAAGCTTGGGGAGCCTTAAAGATCTTTTAGAATTT
AGTGTTTCATCCAATTTATTAAGTGGGCAAATTCCATTTGAAATTGGAAATTTACAGGCGGCAACAATCA
TTGATTTGTCAAAAAATGATTTTTCTGGTAAGATTCCTAGCACTCTAGGGGGTCTAGATAAATTGATTAAT
CTTTCTCTAGAACATAATAGACTAGATGGGCCTATTCCAGATTCATTTGGCAAAATGTTGGCATTGGAAT
TCTTAGATTTGTCCTATAACAATCTTGTTGGTGAAATTCCAAAGTCATTAGAAGCTCTTGTGTCTCTCAAA
TACCTCAACTTTTCATTCAATAAACTCATTGGAGAGATTCCCACTGGTGGTCCTTTTTCAAACGCCACAGG
TCAATCCTTCTTGTCTAATGATGCACTATGTGGTGATGCCAAGTTTAATGTGACAACATGTGTCATCCAAA
CACCCAAGAGGAAAAAGGCTAACTTAGTTTTGTACATCAGTTTGGGAGTGGGTATGTTACTTCTTGTATT
AGCCCTTGCATATGTATTTGTAAGATTCCGAAACACAAAAAAGAATACAGGTCAAACAAATGTGTCTCCA
TTAAAAGAGCATGAAAGAATTTCCTATTATGAAATTGAACAGGCAACAGAAGGATTCAACGAAACCAAC
TTGCTTGGTAATGGGAGTTTCAGCATGGTCTACAAAGGGATACTTAAAGATGGTACCCTTTTTGCAGCAA
AGGTATTCAATGTGCAATTGGAGCGTGCATTCAGAAGTTTTGACATAGAATGTGAGATACTCCGAAACC
TTCGCCACAGAAATTTGACCAAAGTCATCACCAGCTGCTCCAACCTTGATTTCAAAGCCCTAGTGTTGGA
ATACATGCCCAATGGGACACTTGATAAATGGTTATATTCTCATAACTTATTCTTGAACTTGTTGCAGAGAT
TAGATATAATGATAGATGTTGCATCTGCAATGGACTATCTCCACAATGGTCATTCAACAGCTGTGGTGCA
TTGTGACTTGAAGCCAAGCAATGTCCTGCTAGATCAAGAAATGGTTGGTCATGTCAGTGATTTTGGCATT
TCAAAATTGTTAGGTGTAGGGGAGGCTTTTGTTCAAACAAGGACAATTGCAACCATTGGATATATTGCTC
CAGGTATAGTACAACTTTTCAAAGTTTTCTCATATCGTTTAAATACTCAAAACAATTCTTCCTCCCCTAGAT
ATAAATGGATTTTCTTTGTTTTTTGCATATTCCATTTTAACTAGGGAACTTTTATAATTGTGATTTCAGAGT
ATGGACAAAATGGAATTGTATCCACAAGCTGTGATGTTTATAGTTTTGGCATCATGATGATGGAGATGTT
CACAAGAACAAGACCAAGTGATGAAATATTTATCGGAGGCTTGACCATACAACGTTGGGTTAGTGATTC
CTTTCCAGGTGAAATTCATAAGGTGGTGGATTCTAATCTGGTACAGCCAGGGGATGAGCAAATTGATGC
AAAGATGCAGTGCTTGTTGTCTATCATGGAATTAGCTTTGAGCTGCACTTTCGTGACACCCGATGCAAGA
ATTAGTATCGAAAATGCTCTTTCAACACTGAAAAAGATTAGGCTCCGGCTTGTCAGTAGTTGGCACTAGC
TGGTGGAATTGGACCGGCTACTCTTGTATGCTATTTGATTAACTATGCCAGTAACATTAAACTTTCAATTT

TGC

>PGSC0003DMG400027683 LRR-RLKs

GTCGTCGCCTGTCCCCGTTTAACACTCTTTTTCTTTTTTTCAATTTCTTTGCAAGTTTTCAGTTTTAGTTCTC

ACTGTTAGCCTTCTTTCTTTGACCAAAGAAAAACTGTATCATCAAAAAAATACTCTCTTTTTTTGCCCCTTT

TCTTCTTTGTAATTGACCATCAAGATCCAGCATTTGATATCCTCAACTGGGCTGTAAGTATCTTGCATCTA

TAATTTCTTGGTCTTCTTTTGGTATTTTTTATGCTCAATTTGTTTATTGGTAGAATTTCTTATTAAAAATTGT

TTCTTTATTATGTTGTTGCTTGCCTTTGACTAAGGACTTGGTAATTTGTTGAATTTTGAAAGTTGTGTAGA

AAGGGTCTGGACTTAATTTATGGAAATTGAAGTTTTTTTGGTATTTTTGCCTAGTTGCTATAAAAAGATTG

ATTTTTTTTTAATTGTGTTGGTAGCTGCTTTGAATATGGACTTTGTAAATTTGTTTAATTTTGAAAGTTGCA

TAGAAAAGCTCTGGACTTGTATTTCTGGAAATTGAAGTTGAATTTGGCCTTTTGTTGTTTGATCTTAAGAA

CCCTTTTTGGGTTTTGTGGATTGTGTGAGATGGGGTTTGTGTCTTATATCAAGTAGAGGTCTTTTTTCTTT

TTTGAACTTTCCTTTTGAAGGTCTGAACTTTTGTGCTTGTGGATGTGGAAGATCAAGTAAATTCTGTATCT

CGGAAACAATCTCTCTACCATCTCAAGGTAGGGGTAAGGTTTGTGCAGTACCCTCCCTAGACCCGACCTG

TGGGATTACACTAGGTTTGTTGTTGCCGTTGTTGTTGTTGTTGTTGACCTTAGTGATTAACTAATTATTAC

TAACCATTTCTTTAATACATTGGCTGCCTTCTTGAGTACTTTGAACAAGGACAGTGGTTTATTATTTGTTG

AAAATTTGAAGGTCATGGTCAAAAAGCTCTCAACTTCAATTAATTTTTTTTGGGATAGGAATTTGCTCTCT

TGTTGTTTGATTTTCAGAACCTTTTCTGGGTTCTATTAGACTTGCTTGTTGGGATTCCCTGTATATCAAGTA

AGGGGGCTTTTATCAGCTTCCTTCTTGGAGGTGTGAAGTTTGTGCTTGTGGCTTTGTAAGTTCAGATAAA

ACCAACTTCCATCGTGTGTTTTGTTTATATCAAGTAGGGGTGTTCTTTTTTTTCAACTTCCACGTAAAAGG

CGTGAACTTTTATGGTTGTCAATGTAGATGATAAGGTGAAATCATCTTTTGAATGAAGTTTTACTTTTGTT

GACGATAGTTTTATGGAAGTGCATCTCTTGGATTGCTGTTATTGTGCTTTTTTCTCTTCCCAAGTCTCTTTT

GCTTCCTTGTTTGCCGTTTTTCCCATTAAAATCTGTGAGGAACAGTTTTCTGCTTGTATCTGACTTGTCCTT

CAATCTTTATTTTGGTTGTCCTGTAACTGTGGGATTTGAGTACCTCTACTTCTGGAGAATCACTAGTTCGC

TTAAGAAGAAAAGTTATCTAATGTTGATATGAAATGAGTCCGAATAGAGCGGATGAAACGTAGATTCAT

ATAGTTTTTTTTTTACTAGTTTGGAAGTGAGGCAGCATTGACTGATTGATAAAACTGATTCATCATCTTAT

TTTTTTCTTGCAGCCTTACATTTTGCTTCTTAACTCGGGAGAAATTGGATTTGTTCTTGCAATGGATTTTTC

GGGGTTGCAAACTAGTCTATAGAGATCTTAAATGTTTCAGTCAACTACTTAATTAGGACCTTCGATTGTT

GCGGTTATGACTGGGCCTCTCACCAAGATGGCTTTGGATAACAGAGCTCTTACTACCTTTGCTGCTATTTT

GATCTACTTGGTCTTGAGCTGTGCTGTTTGCTCTGCTCTTCAAAGCGACATTGACTGTCTGAAATCTATAA

AAGATTCATTTGAAGATCCATTGAAGTTCTTGAACACAACATGGAAGTTCGATAATCAGACCGAAGGTTT
CATCTGCAAGTTTGCTGGGATTCAGTGCTGGCATCCTGATGAAACCAGGGTTCTGAGTATCAGTCTTCCT
GACATGGGTCTCAAGGGCAAGTTTCCACGTGGTCTTAAGAATTGCACCTCTATTACATCATTAGATCTTTC
GAGCAACAAACTCCATGGAAGTATTCCAAATGATATCTCCAAAATTATAGGTTTTATGGTAACGCTGGAT
CTCTCATCCAACAATCTATCAGGGGATATCCCAGTTAATCTTGCAAACTGCTCTTTTTTGAATAGCCTTAG
ACTTGACAACAACCAGTTTACGGGTCAAATTCCTCCAGAAATTGCCCTGCTTGGTCGGCTCAAGAATTTT
AATGTAGCAAACAATCGATTGACTGGACCAGTTCCACGTTTCATAAATTCAACTTTTCCCCCAGAGAGCT
ATGCCAATAATGCCGGACTGTGTGGACCACCATTAGCTCTTTGTGAGGGTTATGCTAAGAAATCCCCTAC
TGGAATCATTGTCGGTGCTGCTGTTGGTGGGGTGACTTTAGGCGCTGTATTATTGACCATCGGTATGTTC
TTCTATATGAGAAAGATATCCAGGAAGAGGAAAAAGGAAGATGATCCTGAAGGGAATAAATGGGCGA
AGAGTATCAAGGGTTCCAAGGCAATCCAGGTGAGAATTCTGTTCGTGCTTTGTTGATAGGTCATAAATC
AATTCTAGTGGGTTGTGGTTTGTGTTTACGGGTTCTACTGTGGCATTGCGATCATATTTAGAACCATAAA
TTCGTGTTTTAATATAAGCATTTAACTAATGAATTTTTCACCATAAGCTCGTGTGAGTTTTCTCTTGCTCAA
GCATTCTTAATTGACTTAAAGTGCCTTATTTTCAGCTTTCAATGTTTGAGAAATCTACTTCAAAAATGAGA
CTAAGTGATCTCATGAAGGCCACCAACAACTTCAGCAAGAACAATATTGTTGGGTCAGGAAGAACCGGG
ACATTCTACAAAGCAGTACTTGATGATGGCACTTCACTTATGGTTAAGAGGTTGCAGAATACTCAACACT
CAGAGAAAGAGTTTACATCCGAGATGGCTACGTTGGGAAATGTAAAGCACCGTAACCTGGTGCCTCTTT
TAGGTTTCTGCATGGCGAAAAAAGAAAGGCTGTTGGTCTACAAAGATATGCCAAATGGCACCTTGCATG
ATAGGTTGCATTCGGTGAGTGAAGGGGAAAAAACTCTTGAGTGGCCTATGAGAATGAAAATCGCTATT
GGAGCAGCCAAAGGATTTGCATGGCTTCACCACAACTGCAATCCTCGTATCATCCACAGAAATATTAGTT
CTAAATGCATCTTGCTGGATGTGGAATATGAACCTAAAATATCGGATTTTGGACTTGCTAGGCTCATGAA
TCCAGTTGACACTCATTTGAGTACCTTTGTAAATGGGGAATTCGGGGACTTTGGTTATGTTGCACCTGAG
TACGCGCGAACTCTCATGGCTACACCAAAAGGTGATGTGTACAGTTTCGGTGTTGTACTTCTCGAGTTAG
TTACAGGTGAGACACCAACTTCTGTCGCCAAAGCTCCCGAGACCTTTAAGGGGAATTTGGTGGAATGGA
TCACACAACTCTCTGGTGAATCTAAACTTCAAGATGCGATTGACCATTCATTGTCTAGTAAAGGTTATGA
CAGTGAGATCTTCCAGGTCCTTAAAGTTGCTTGTCGATGTGTGTTGGCTGCTGCTCCTAAGGAGAGGCC
AACAATGTTTGAACTGTACCAGCTTCTGAGAGCCATCGGAGAACGATATCATTTCACAACTGATGATGAC
ATTATGATGCCTGAGAGTGATAGCGGATTCCAAATGGACGAGCTCATTGTTGCTCAATAAGTAAATGAA
GCCCTTCGATAATGGTCGAGTAAGATACAATAATTACTAGATCATTTTCTATCCTGTACAAATACCACGGT

TCACTATCTATATAATATCATTCTATTTCTACTGTAAGTCCCTTATTCATAGCTACCCAACTATGTAATTTGT
GCTTCTTTTTCTGTATTTTTAATGGTGAAGAGATTTCTACAGATTGGTGTTAGTCAGTATTTTTCCTCTTTT
CAGATTTACTGCTTGAACAGTGTAGTCTCATCTTCCAGTAATTATAAAATGCAAAGTTGGAAAAAATCCA
TCACTCAAAGCTGAC
>PGSC0003DMG400027703 LRR-RLKs
TGAATCCTGATATTCAAGCTAAACATAAAAACAAAGGTGCTATCATAGCTATCGTTGTATCAGGTGTTTT
TGCTGCATTTGTTTCAGCAATTTTAACTGTGTTAATAAAAAAACGACATGCCAAATACCAAAGTCTCCTAT
CAAGGAAACGGCTATGTAAGTTATCTCATTTTGGTTATAAGCTACCTTTACTTCATAAGAATATGTTTCTT
TCAATTCTTCCCGCAAAATTGACTAGCATAAAATGTTTAATTTCATAATGCCAGCCGCAAAGCTCTCTATA
AAATTGGATGGCCTTAGAAGCTTCACTTTCAGAGAAATGACCTTGGCCACTAACAACTTTGATCACTCAA
ATCAAGTTGGTGAAGGAGGATATGGGGCTGTCTTTAAGGGAATTTTAGCTGACAAAACAGTTGTTGCTA
TCAAACGAGCAAAAGAAGGATCAGTACAAGGGCAAAAAGAATTTTTGACTGAGATATCTTTGTTATCAA
GGGTACATCACCGGAATCTAGTTTCATTGCTAGGGTATTGTGATGAAGAAGGAGAACAGGTATGATAAA
CCTCAAAACTTCAAAATTCCAAATCTTTTTGAATCCACTCATTAACATGGAATTCATCATGTTGATCCTTTC
CTCGGCTATATGAGTAGTTCAGGCTCACATTGTATGCTATAGCTTCTTTTTTTGCTCCGTAGCAATCTCTT
GTCATTCGTTCTGACATCTCTTTGGTATATCATCTATGTGAAGCAATTAGTGTGTTTCCTGTTTATGTATTT
GCAGATGTTGGTTTATGAATTCATGTGCAATGGAACTTTGCGAAACTGGCTTTCTGGTAAAGAAACTGTT
CCCTGGTTTATTCTGATCCATCATCTTCCTCTGCTTTATTTTTGGTTAGGGAAGCTATTGAGCCAGGAGAG
GAATTTGATATTTATCATTATTCTGATAATTGTGTTGTGTAAGCTGGCTTGCTTGCGCTTTGACTAATCTAT
TAAATACGTGCTACCTCCCACTAGCACAAATATCAGATAACTCCATCCTTCAAGGTTTAGGAAGATGGAT
CACCTCTGTTGGCGTTCCAACTTTAATTACCAAGTTTATTACTCCAAATCTACCACCACTAGGCCACCTTTC
TATGTTCCTTAAGCAAATAGTAGCAGTATCTTTGAACTGGTACAGTAGACTGGTAATATATGCTCTTAAA
CATTGTTTTTTAAGTCTATAGATGGAGATCAGATGTCAAGAAGCAAACTTTGATCTAATAACTGTATGTCT
ATATGGATATCACCTAATCTAGATTATCTTGTTGTAGTTACTGCTCCGTTACTATCTGCTGTTTTTGTTACT
ATGTGTTGTTTCTTGTACTTCTGTTACTTCTTTTTCTGGACTGCTTTGGACTATTTTTCCCTTGAGCCGAGG
ATCTATCAGAAACAGCCTCTCTACCTCTAAGGTAGGGGCAAGGTGTACACTCTACCCTCCCCAGACACCA
CTTTGTGGGACTACACTAGGTATGTTTTTGTAGTTGTAACACTTGATATCATTTTTTGACAGCTAATTGTA
AAGGAACCTTGTCGTTTGCTACAAGATTGGAGATTGTACTAGGTGCAGCAAAGGGCATTCTTTACCTCCA
TACAGAAGCTCATCCACCAATATTCCATAGGGACATCAAAGCCAGCAATATTCTTCTTGACTCAAAAATG

GCAGCTAAAGTTGCTGATTTCGGATTATCTAGGCTTGCACCTGTTCAGGATGATGAAGGTGTACTACCTA
ACCATGTTTCAACTATTGTGAAGGGAACACCTGTAAGTCCTTCACTACATCATTATGTTCTATTTGACCTC
ACTCGTATCATTTAAATTTGATGCATTGAGACTCTACTTGTATATGCAGGGTTATCTAGATCCCGAATACT
TCTTGACCCGTAAATTGACAGATAAAAGTGATGTTTATAGCCTTGGGGTTGTGTTTCTAGAAATCTTAAC
CGGAATGCGTCCCATCTCACATGGAAAAAACATTGTCCGCGAGGTATTACTCTCATTGTTAACTTCCCGA
TTTCCATGTCTTATCGTGTGCATATGTTTGATAGATGATTTATCCGCACACAAAGTGAAATACTGCTGACT
GATGCAAGGGTAGGAATAGAGGCAGATTCATGAATTAAACTTGTAATGCTCTTAGTTCTTACCATTGAAC
ACACTACATGTCGCACAAAATGACGCTTTTAGAATTTATATTTTATGGATACATTTTAGTGATTTTACATA
TGTATGTTATTTTTATGGTGCATCCTCCTCTGGGCTGAGAAGGGCTAGGGGAAATGTGAACATAATTTTT
ACGCAATCTTTCCTTGCATTTCTGCTATGAAGCTTCACACCTCGAATCTAGCTCCACCGTGTTTAATAGTCC
CTTGTACCATCAATGGCAGGTAAATTTGGCTCATGGCTCTGAGAAGATGTTCTCAGTTATGGACAGTACA
ATGGGATCTTATCCTTCTGAATGTGTGGAAAAGTTTGTAGCTTTGGCCCTCAAGTGTTGCGAAGACAAAC
CAGAAGACAGACCTTCAATGTTGGATGTGGTCAGGGAGCTGGAAACTATTCAGTCTATCCTCAATCTGA
TGCCAGATATTGACACTGATTCAGTAGATTCTAAAGCAAAATTTAACGAACCAAAGACATCGTCTTCATT
TTCTGATTGCACTAGCAAGGACGCGTTTTTATCGTCCTCCAATGTATCGGGAGCTTACAGTATCAGTGGT
GTCAGTCTTACTATGCCTCGCTAACAGAATAGACAATAGTTGCTCATGTTTTGTACTAGAAAAAATAGCA
TGTAGAATAGAGAGTTGTACCATTTAGCTTCTGTTTTATATATGTATATTTCAAGGATTTGTTAACAAAAA
CCTCTTTATCATAGCCTTCTGAGTTCTGACAGTATGTGTATGCTATCAAAATCCAAAACTTTGCTACAAAA
TGGACTCTGAGATAGGTACTGCTATTTGTTTGCAAGGAGTTGCACCAACCAATTCCTCAAAACAACTGAC
AACAAACAGACAATGGCAGAGCTAAAGGGGCGGAAGGGGGTTCATCGCCAGAAAATCACACTGTATAT
ATAAAAGGTCGATCTTTTTATCTATATAAATGATGAACCCTCTTGTAATCCAACATCATTACCAGAAATAG
AAATATTATCTAAGCTATGGCTACAATGAGAGTCGTACAACAAAACAGGCAAAACTCGTAGTTTTGAAG
TAATGTTAGCTTTAGATCACATAACATGTCATCGTAAACCACACAAAGGAACAGAAAACAAATCCTCAAA
TCAATATGAGGAAACTCATGTTTTGTTCTATGGAACCAAAGTTAGACATGGTTGACACACTCCAAGCTCC
TACAAGAAGTCAGAAGACTATTTCTTGGACTAAAAAGACAGCAAAGTTCATTTTTATTTATAGATCATAG
TAAGAGAGCAGCGGGTAAAATGGCTCTACCGGGAGGGAGGGAGGGAGCCGGTTTTGGATTGGTGTGT
ATTTGAAACAAATATTGATAATCACTTTGAAGCAGCATCAGCAGTATGCATACTGTCATTCTGGTCCAGC
AACCTCTCTTTCTCTCCAACTAGTGTGTCTTCAACACGAACAACCTTTCCTTCATCTGGTTTATCTGCTTTCT
GTGATCCTGTGAACTTTGCTAACATGACGAATGTCATTCCCCCAAGAATGTTATTTGCGCATCTCAAAGC

AATAACCGAGGTCTTAAATACCAAAGGAGGAAGACCTTTAGCTAGCACAAACTCAACACCGTTAAGTGT
TTGATATCTTAAATTACTACTGAGACCCATGTGTATTGCCCAAGTTGCTGCATTCAAAAGTGTAGGAGGT
GGCTTGTTTGGTGTTTCGAAATTCGGATCCATCTTCTTCCTCAACTTGATTAAACCATTAGATAATGCAGT
CCCAACAAGCCCTGCTGCAAATCCAACTGCAGCAAACTGAGTTCCTTTGTACACAAATGTACCAAGCCTA
CTCAATAAACCGTAAGAACCAGACTGAAACATGTGGCTCGGTGGACAACTGGCGAACATCAAAGGGAG
AGTTTGGATCGAAGCAGATGCCGTTGGTGCCAACAGATACATCAACACAAAATTCATAATTGAACCAAC
AACAAGTGTCGAAAACACAAAATCTAATTCATTAAGCCCAAAATTAGGCCTTGATGCCATATCTCCAAGA
ACATTAGCACTAACACCAACCAATTCCTCCATAAGAACCTTAAAAGGAAACTGTGGATCTGCAGCCACCC
TTGATCTCCAGCCATTAACAAAAGCACCAATTGGTCCAATACTATCCCAAGAAGACTTTGAATCATCTGA
ATCTCCACCTTTACTCCAATCACCACTACCCCCACCCCCACCACCCCCACTACCCCTTCCAATCCCAATACT
TCCTCCATCACCTCCACCAACACAAGGAACTTGATACCTCAGGTGATCCACTTGTTTAGACACAATATACC
CAGTTTTCAAAGGGTTAAAACAAACCCTATTATCACAAAATCTGGCTGAAACACCAGAAAGATCACGACT
TTGATTATGATTCTGATAGTAACCCGTAAGAGGAGAACAACGAAGCTGAGACATCGCCACCATTTTCTAC
CAACCAACCTAATAAAATGAGAAAATAGAAAGTAATGGCTATATTATTCATCCCATCAACAAACAAAGAT
TCAGATTCACTAAAAGATCAAGAAAGACTCTTTCTTTGACACAAATAATATATATAAATACATACACACAC
AACAAAAAAGATGCAACAGAGTTAACAATAAATCAAGAAAAGGAAAAGGGGTTACCTTTTATGAAGAG
ATGTGTTCAGGGGCTGCACATCAAGTGATCTGAGCAGAGCAAAAAAAGATGGCTGCAGCGGCTAAAGT
GACTAAAATGGGGATCTGCATGAAAGTGGGAATCTTTATAACCTAAATAAAAATACAGGCGGCAGGGC
CGAAGTTAAAGAAAAAC
>PGSC0003DMG400027710 LRR-RLKs
ATGAAGTACCTGGAAGATTGGGAGAAGGGTGATCCATGTACATCTAACTGGACAGGAATTCATTGTTTT
AACAAAGTTGGAGCCAATGGCTACTTGCATATCAAGGAACTGTATGTCATTATATTCTTCTCTCTTATTAG
TTGTTATGTTTTCTTCTTCTTTATACCTGGAATTGCTGCACTTGAGTAGAGGGTCTTTCGAAAACAGTTTCT
CTACCTCCACAAGGTAGTGGTAAGACCTGCACACACTCTACACTCTCCAAGCCCCGGGATTTCACTAAGT
ATGTTCTTGTTGTTGTAATTAGGTAGATTCAAAGCCAATTTACTCTAGCAAAACTTGGGTTGGTGTTTGAA
AAACCCGAACCAACCTTGCCTATGTACACCTCTACTTTGGGCCATAAAAATACCCTTGATGTTAGAAAATC
TTTGTATCTTTAATCGAGTTCTAGTGCGGTGGATTTCACGTGTAAGAATATATCAGGAAAAAGGTCTAAA
TTTACCCCTTTGCTTTTGGTTGTGGTTAAGAAATTACCTTTCATTATGCTTCAAGTTGGAGTTCCTCTAGCA
TTAACAGCAAAAAAAAGAGACTTGGTTCTTTCGATATCTGCATAATACTCCTGCAATCCAGTTAGCAGCA

TAACTAGTATCTCCAAGGAGGGACCTGGTTCTTTTGTAGTTGTTTGTTGATCATCAAAATCAATTTAAGCT
CATCTCAACAGTTTGCTAACGACAAGGGCATGAATAACTAAGTTAACTCATAAATTAGAGCACTTAGTTG
TAAACTCTAGCAAAAGATGCGTCTTTACTACTTTGAGTTATGACTATCTTGTATTCTTGTGTGAGGGTGAT
GCATGACGCATTAAGCATATTATATTTCCCCTTTGTGCAGCCGTATGATGGCTATGAATCTTTCTGGAAGT
TTAACACCTGAGCTTGGACAACTATCTCATCTTCAGTTTCTGTGAGTTGTTACACATATTACTACGTTGTA
CAAATATTTTGATTTTAAGATATAGTAGGGTTTATTAATTTTTCTATCTGTTTTACTTATTTTTATCTTTATA
ATTCACTTTCTGAAAGGAATTTCATGTGGAATGATTTGACTGGGAGTATACCGGAAGAAATTGGAAACA
TCAAGTCTTTGAAACTTTTGTAAGTTGAACGAAATTTGATCCGTTTTTTTTTGGTCCCATCTTCTTGATTTT
GCAGTCACCCTACATGTTTCCCCGAATGCTTTGAACCCTGAACTATCTCATCTAGGAGATCTAGATAATCA
TTTGGCTATGATGGTGGTATACTTTATGTTGGATAGAACAGGTGGATTTATGATTTTAAGTTTGATGGTT
TCAATCTTTATGTTCTTATCACGGAGTCCATTACATTTTCAAAACTAGGGGTTCAAAGTTCAATATTTTTAA
ATTTATAGTGATTTGTCATGTATGTATCTGTGCTTCCCGTCGAAAATGTTGAGTTCAGTTGAACCTATTAA
TTAGATCCTTCATCCGCCACTGGATAGAATTGAGGAACATTTTTTTTCTTGAATTTCTTATGAAAATTGTG
CTTAATGTAAGATTGTTTCAAGAAGAAGTAAAACCTAATATGCTTGGAAAGATCATGACTATGTCGATTG
GTTTAATCCCTTCGGAATTATGTCCATGCAGGCTCTTGACTGGAAACAAACTTTCAGGATCTTTATCTGAT
CAGCTTGGGTATCTTCCAAACCTGAGAATATTCCAGATAGATGAGAACCAGATTTCCGGAAGAATTCCTA
AATCCTTCTCCAATTTAAACAGTGTCCAACATATGTAAGTGCAACTCATTTGATTGTTTTAAACAGTGGAA
CAAGTCTAAGGTTCTACCTTTGTAATCTTATGTGATATATCATGTGTCTACAGCCACTTCAACAACAACAC
ACTGATTGGCCAAATCCCTCCCGAGCTTTCCAATTTGTCCACTCTGCTTCACATGTAAGTTTCTTAGTTTCT
CTTCTTCAATAGACATGCATACTCTCAGTAGAAGTTAGAGCGAAGCAAGTTTTTGTAACTTCTAAAATGA
CCTTTATCTCTGCTTATGTCTGTAAACTAACGGAGTAATGTTTTTGTGATGATACTTTCTTTTTGTCAACAG
GCTTATGGACAGCAATAATCTTTCAGGATACCTTCCTCCTGAGTTTTCAATATTACCACGTCTTCGAATAA
TGTATGTCCCTACTCACACACACATATATGATATGCATATATTGAACCTCATATGCAACTTTCATTAGCTG
TATTTTGCTCAAGAGGTATAACTAACTGTTACAAAAAAACAATGGTTATTCAGAGGCAACTTTAAACTGA
ACTTTCTGAAGTAACTAGTTATAAATTTGAAGGAATGCTGATTCCTTACTACCTATGAGTGAGACTGTCTA
TGTTTGTCTTAGTTGAAGTGGCGTTATTGGCCAGATGATCGAACAACTGAAGCTGAGAACATTTCGTTAC
AGATCTATCTCAGAACCCTCTAATTATCATCTTCAGATCTTTCTGTTGATTATAAAATTCATCGATAATCTT
TGCTTGTAATATCCAATGATAAAAAAATGATTTTATATAAGACAAATATAGTAATCCTTGTCATACAATAA
CTGCTATGCCATTTGCAGTTCCACTGAGAAGTGCATGGGTTGAAATTTATGATGAAGTTCTGAATTTTCTT

ATGCAGACAACTTGATAACAATAATTTCAGCCGTTCCGAGATTCCAGCTTCGTATGGGAACATGTCATCA
TTAGTGAAACTGTAAGTGCTCCTTGAAAATGTTCCTCTCTTTTATTCTTCCCCTATTCTCTATGACTCAGCA
TTATAAGTTATACATTTGAAGGACAACAATAGTAAATAATATGTACAAACATAAGCATATATTCATTGTG
CACAATGTATATGAAAGAGAGAATGTTCAGGACATCTTATTATGACGGTTCGGGTAGATAGAATAAGAT
GTAATAGTTGAATCGTACTGTCACCTAATCAAATGGAGATTCAATGCTTCTGATAATATTGCAGTAGTCT
CAGAAATTGCACGTTGCGAGGATCCATTCCTGACCTTAGCAGAGTACCGAGTCTACACTATCTGTAAGTT
CAAGTTTTGAAATTGTTTCTTCTGTTCCCGTTGATTTGTGACCTTTTTACTTCATGCAACATGATTATAAGA
TTTTCTAAAATGTTAACACTTCTTGCTTACTGTTTGTGCAGTGATCTCAGCTGGAATCAACTGTCTGGATC
TCTACCAAATAAGCTTTCCAACAATATGACTTCTATGTATGCTTTCTATTTCATGCTACAGATTTCTTCATA
TATCTAGCATCTTAGTTCAGCCATATTTTGTGTTAGTTCATTATCTCATGTTGTGAAGAGTTCTACATCAGC
CCTTTAAGAAACATTACTAAAGAGCTAGCCTTTGGGGTTGAGTTAGGCCCTACATCCATAACACAAGTTA
AACATAATTTTTATCATGATTGCACATTTCTCTGTGTTTTTCCTGTCATTTTCCCTAATCTAATGTGTTGTTG
TACATTCTCATGCAGTATTCTGTCACACAATCGTCTTGACGGATCTATTCCGGAGAGTTTTTCATCTCTTCC
TTTGTTGCAGAAACTGTGAGGATCTTCACTGTGTCTTGTAATACATTCTCTTTAGTACTGTTTCTTTAACAT
TTCAAAGCTCAATTTCGTATATAACAAATTGATTTGACGGACTTGTCACAGGACAATGGATAACAACCTT
TTGAATGGTTCCTTTTCTACCGACATTTGGCAAAACAAGTTTACTTCAACGTCGTCGCTGTTGATGTAAGA
CCTGAAGCTAAATTGTAACATCAAATATCTGATATGAAATGTACTATCATTTACACAGAAGTCTGGGAGT
CTTTTTAACCCTTCTATCTTCTTTGCAAAACTAGTGATCTTCGGAATAATTCACTTTCGGACATTTCCGGTA
CTCTTGAACCTCCTCTGAATGTCACTCTAAGGTAAGGTTTTTCTTTCCATATTCAACGACAAAACCTAAGA
GAAGCCATTGAATTACTAATTTTATGATCTGACTATCACTTCATATCCACATTTTCAATAATAAAAGACAT
TATTTTCAGGTTGCAGGGCAATCCTGTCTGCAGAAATGAGAATGTAAGAAACATCGTCAAGTTCTGCGG
CTCTGAAGCCGGAGCAGAACATAAGACGAATAACTCAGTGATTGTAACTGGTGCTTGTCCTATTGTTGC
ATGCCCAAAGGATAACTATTATGAATATGTTCCAAACTCACCTGTTCCCTGCACTTGTGCCTCACCTCTCA
TAGTTGGCTGGCGGCTGAAAAGTCCAAGTTTTTCTTACTTTGATCCATATGTGCATCAATTTGAGCAGTAT
ATGACTAGAGATCTTCATTTGGACCTTTACCAAATGTTGATTGAGTCAAATTCTTGGGAAGAAGGACCTC
GTCTACGAATGCAATTGAAGCTTTTCCCAGTAGTTGGGGTCAGTACATTTAATAAAAGTGAGGTCATTCG
TATCAGCGACATTCTGCAATATTGGGAAATATCTGTAGTTGACCTTTTTGGACCTTATGAGCTCCTTAGTT
TCACTCTGGAGGGACCTTATTCCTACTGTAAGTTCTCAGCGCTTCATTGTGCTTCATGGTTGATCTTTGTT
ATCATGATTAGTATCTTCCTTCCTCAGAATTTTGGTTTATCATATGATGCATGTTTTTCTGCAGTGAATCCT

GATATTCAAGCTAAACATAAAAACAAAGGTGCTATCATAGCTATCGTTGTATCAGGTGTTTTTGCTGCAT
TTGTTTCAGCAATTTTAACTGTGTTAATAAAAAAACGACATGCCAAATACCAAAGTCTCCTATCAAGGAA
ACGGCTATGTAAGTTATCTCATTTTGGTTATAAGCTACCTTTACTTCATAAGAATATGTTTCTTTCAATTCT
TCCCGCAAAATTGACTAGCATAAAATGTTTAATTTCATAATGCCAGCCGCAAAGCTCTCTATAAAATTGG
ATGGCCTTAGAAGCTTCACTTTCAGAGAAATGACCTTGGCCACTAACAACTTTGATCACTCAAATCAAGT
TGGTGAAGGAGGATATGGGGCTGTCTTTAAGGGAATTTTAGCTGACAAAACAGTTGTTGCTATCAAACG
AGCAAAAGAAGGATCAGTACAAGGGCAAAAAGAATTTTTGACTGAGATATCTTTGTTATCAAGGGTACA
TCACCGGAATCTAGTTTCATTGCTAGGGTATTGTGATGAAGAAGGAGAACAGGTATGATAAACCTCAAA
ACTTCAAAATTCCAAATCTTTTTGAATCCACTCATTAACATGGAATTCATCATGTTGATCCTTTCCTCGGCT
ATATGAGTAGTTCAGGCTCACATTGTATGCTATAGCTTCTTTTTTTGCTCCGTAGCAATCTCTTGTCATTCG
TTCTGACATCTCTTTGGTATATCATCTATGTGAAGCAATTAGTGTGTTTCCTGTTTATGTATTTGCAGATGT
TGGTTTATGAATTCATGTGCAATGGAACTTTGCGAAACTGGCTTTCTGGTAAAGAAACTGTTCCCTGGTT
TATTCTGATCCATCATCTTCCTCTGCTTTATTTTTGGTTAGGGAAGCTATTGAGCCAGGAGAGGAATTTGA
TATTTATCATTATTCTGATAATTGTGTTGTGTAAGCTGGCTTGCTTGCGCTTTGACTAATCTATTAAATACG
TGCTACCTCCCACTAGCACAAATATCAGATAACTCCATCCTTCAAGGTTTAGGAAGATGGATCACCTCTG
TTGGCGTTCCAACTTTAATTACCAAGTTTATTACTCCAAATCTACCACCACTAGGCCACCTTTCTATGTTCC
TTAAGCAAATAGTAGCAGTATCTTTGAACTGGTACAGTAGACTGGTAATATATGCTCTTAAACATTGTTTT
TTAAGTCTATAGATGGAGATCAGATGTCAAGAAGCAAACTTTGATCTAATAACTGTATGTCTATATGGAT
ATCACCTAATCTAGATTATCTTGTTGTAGTTACTGCTCCGTTACTATCTGCTGTTTTTGTTACTATGTGTTG
TTTCTTGTACTTCTGTTACTTCTTTTTCTGGACTGCTTTGGACTATTTTTCCCTTGAGCCGAGGATCTATCA
GAAACAGCCTCTCTACCTCTAAGGTAGGGGCAAGGTGTACACTCTACCCTCCCCAGACACCACTTTGTGG
GACTACACTAGGTATGTTTTTGTAGTTGTAACACTTGATATCATTTTTTGACAGCTAATTGTAAAGGAACC
TTGTCGTTTGCTACAAGATTGGAGATTGTACTAGGTGCAGCAAAGGGCATTCTTTACCTCCATACAGAAG
CTCATCCACCAATATTCCATAGGGACATCAAAGCCAGCAATATTCTTCTTGACTCAAAAATGGCAGCTAA
AGTTGCTGATTTCGGATTATCTAGGCTTGCACCTGTTCAGGATGATGAAGGTGTACTACCTAACCATGTT
TCAACTATTGTGAAGGGAACACCTGTAAGTCCTTCACTACATCATTATGTTCTATTTGACCTCACTCGTAT
CATTTAAATTTGATGCATTGAGACTCTACTTGTATATGCAGGGTTATCTAGATCCCGAATACTTCTTGACC
CGTAAATTGACAGATAAAAGTGATGTTTATAGCCTTGGGGTTGTGTTTCTAGAAATCTTAACCGGAATGC
GTCCCATCTCACATGGAAAAAACATTGTCCGCGAGGTATTACTCTCATTGTTAACTTCCCGATTTCCATGT

CTTATCGTGTGCATATGTTTGATAGATGATTTATCCGCACACAAAGTGAAATACTGCTGACTGATGCAAG
GGTAGGAATAGAGGCAGATTCATGAATTAAACTTGTAATGCTCTTAGTTCTTACCATTGAACACACTACA
TGTCGCACAAAATGACGCTTTTAGAATTTATATTTTATGGATACATTTTAGTGATTTTACATATGTATGTTA
TTTTTATGGTGCATCCTCCTCTGGGCTGAGAAGGGCTAGGGGAAATGTGAACATAATTTTTACGCAATCT
TTCCTTGCATTTCTGCTATGAAGCTTCACACCTCGAATCTAGCTCCACCGTGTTTAATAGTCCCTTGTACCA
TCAATGGCAGGTAAATTTGGCTCATGGCTCTGAGAAGATGTTCTCAGTTATGGACAGTACAATGGGATC
TTATCCTTCTGAATGTGTGGAAAAGTTTGTAGCTTTGGCCCTCAAGTGTTGCGAAGACAAACCAGAAGAC
AGACCTTCAATGTTGGATGTGGTCAGGGAGCTGGAAACTATTCAGTCTATCCTCAATCTGATGCCAGATA
TTGACACTGATTCAGTAGATTCTAAAGCAAAATTTAACGAACCAAAGACATCGTCTTCATTTTCTGATTGC
ACTAGCAAGGACGCGTTTTTATCGTCCTCCAATGTATCGGGAGCTTACAGTATCAGTGGTGTCAGTCTTA
CTATGCCTCGCTAA
>PGSC0003DMG400027967 LRR-RLKs
ATGGATCCCAAAATACTTTATTTCCTTCCTCTGGTATATCTCTTCACAATTACTTTTGCTTCCACAGAGGAA
GCAACAACTCTTCTTAAATGGAAATCAACTTTCAAAAACCAGAATAATTCCTTATTGGCTTCTTGGACACT
ACGTCATCCTGCTGCTGCCAAGAATTTTTCGAGCCATGATCCATGCAGGGACTGGTATGGAGTTAAATGC
TTTAATGGTCGAATAAACAGGTTGAATATTACAAATTCTGGCGTCATTGGTACACTCCATGATTTTCCATT
TTCATCTCTACCTTTTCTTGAATATATTGATTTTAGCATGAACAACCTTTCTGGTCCCATTCCTGCAGAAAT
AGGGAAACTTACCAACCTTGTCGAAATTGATCTTGATACAAACCAGTTAACAGGTCATATTCCTCCGGAA
ATAGGCAACTTGATCAATGCAAAATTGTTCTATGCTTACTCCAATGAATTGTCTGGTTCCATTCCTATTGA
AATAGGGAAGATGAAGTCACTTGAAAGTTTAAGCTTACAGAAAAACAATCTTTCTGGTCCAATTCCAAAA
TCTTTAGATGACTTAACTGAGCTCAAAATTTTGTACCTTTATTCTAACCAACTTTCTGGTCTCATTCCTAGT
GAGTTGGGGAATCTGAAAAACCTCAATGAGCTAGATTTATCCGATAATAAGCTTAGTGGTTCAATTCCAA
TTACTTTAGGTGACTTAACTGAGCTTGAAACTCTATACCTTCATTCTAACCAACTTTCTGGTCCCATTCCTC
GAGAATTGGGGAATTTGAAAAACCTTAACGATCTGGATATATCCGATAATAAGCTTAATGGCTCAATACC
AATTACTCTAGGTCACTTAGCTGAGCTCAAACTCTTGCATCTTTATTCCAACCAACTTTCTGGTCCCATTCC
TAGTGAGTTGGGGAATCTGAAAAAATTGAATGATCTGCAATTATCCAATAATCAACTTACCGGTCCTATT
CCTGGTTCATTTGGCAATTTGAGAAACTTGCAATTTTTGTATCTCCACACCAACAAACTTTCTGGTCCTATT
CCGAAAGAACTTGCATTTTTGGATAACTTGGTAGAGCTGATAATAAGTGAAAATCAGCTTTCAGGCCATT
TGCCTGAGCATCTTTGCCAAGGTGGGAAACTTGAGAACTTCACGGTGAATAGTAACAAGCTTACAGGGC

CAATACCAAGAAGCTTTAGCAAGTGCTCGAGTTTCAAAAGAGTTCGGTTGGATAACAACAGTTTTACTG
GGAATTTATCTGATGCTTTTGGCATCCATCCACACCTTTACTTCATTAATTTGAGCGAAAATGATTTTTATG
GTGAACTCAGCAGCAACTGGGGGAAATGCAAGAGTTTGACTGATTTGCGGGTAGCCAGAAATAACATT
AGTGGTAGCATACCACCGGAGATTGGAAACCTCAAAGGGCTTCAAGGACTTATTCTTTCATCCAATCATT
TGGTTGGACAGATACCTAGGGAATTAGGAAAATTAACCTCTCTGGTTAATCGTCTTTTGCGAAACAACCA
AATTTCTGGCAATATTCCTATGGAACTTGGATTATTGACAAAGTTAGATTACCTTGATCTATCAGACAATA
GATTGAATGGTTCGATCCCACCGTTTATAGGAGATTACCAGCACCTGTTTCACTTGAACCTAAGCAACAA
CAAATTTGGCCAAAAAATTCCAATAGAGATTGGGAAGATAACTCATCTTAATGTACTGGATTTGAGCCGT
AATCATCTTGTTGGAGAGATCCCCCCTCAGTTAGCCAATTTAAAGGTGTTGGTAAACTTAAATCTTTCACA
CAATGGCCTATCTGGGCGCATTCCTCAAGAATTTGAAAGTTCAACTGGTTTGCAGGATGTCGTATTGTCA
TACAATCTGTTGGAAGGTCCAATCCCTAATAATAAAGCTTTTATCAATGCCTCATTGGAGGGTAATAAAG
GTCTTTGCGGCAATGTAGTAGGAATCCATCCCTGCGGAATGCCGTCTTCTGTGGTGAAGAAGCACTCAA
TGGCGAAGGGACGTAAACTCATCCTCATCATTGTACTTCCTGTTATGGGAGCACTTGTTTTACTCTGTGTT
TTCATTGGTGTTCTCTTTATGTGTGATAAAAGAAGAGCTGGAGATGTTGAAAGAAGGGATGGTGATGGT
TGGCTTTCGATATCCATGTTAGATGGGAAGGNNNNNNNNNNNNNNNNNNNNNNNNNNNNNNNNNNNNNNN
NNNNNNNNNNNNNNNNNNNNNNNNNNNNNNNNNNNNNNNNNNNNNNNNNNNNNNNNNNNNNNNNNNNNNN
NNNNNNNNNNNNNNNNNNNNNNNNNNNNNNNNNNNNNNNNNNNNNNNNNNNNNNNNNNNNNNNNNNNNNN
NNNNNNNNNNNNNNNNNNNNNNNNNNNNNNNNNNNNNNNNNNNNNNNNNNNNNNNNNNNNNNNNNNNNNN
AAGGGCATTGACTGGGATTAAGCACCGGAACATTGTGAACCTCTACGGCTATTGTTCGAAAGCACAACA
CTTGCTCTTGGTTTATGAGTATGTGGAGAGGGGGAGTTTGTCTAGCATTTTGAGCAACAAGGTTGAGTC
CAAGAAATTGGATTGGTTTAAAAGAGTGAATATCATCAAGGGTGTTGCTTTTGCTTTATCTTACATGCAC
CAGGACTGTTCACCCCCGATTGTCCATCGAGACATAAGTAGCAGTAATGTTTTGCTTGATTCTGAGTATG
AAGCTCGTGTTGCAGACTTTGGCATAGCGAAGCTACTCAAGCCAGATTCATCGAATTGCACTGCACTTGC
AGGCACATATGGCTATGTTGCACCTGGTAAGTTTTAAATATTTCATGAAAGCCAAACACTATTGTTTTTAT
CTTATTTACAAGTTTAAATTGATAATATTGTTCTGGAAACTGTATTGTTCAGAGCTTGCATATACTATGAA
GGTTACACAAATGTGTGATGTCTTTAGCTTTGGAGTACTATCATTGGAGATAATAAAAGGAAAACATCTT
GGGGAATACATTACCGTGCTAGCAGATTCATCGACTATAGATCCTGAGCAGCTTAGCGATTTTCTGGATG
AACGGCTTCCATATCCTGAAGATAGAGTAAAAGAGGCTTTGGTTTTTATCATCAAGCTAGCATGCTCTTG
TTTGCTTGAAAACTCCAAAAATTCAAGGCCAACAATGCACTTCATCTCTCATAAGTTATCATCAATGGATG

CACGTCCACCTATTCATCAGAGAAACCCCCATGTAAGGCGAGCAATATAA

>PGSC0003DMG400027971 LRR-RLKs

ATGGATCCCAAAATACTTTATTTCCTTCCTCTGGTATATCTCTTCACAATTACTTTTGCTTCCACAGAGGAA

GCAACAACTCTTCTTAAATGGAAATCAACTTTCAAAAACCAGAATAATTCCTTATTGGCTTCTTGGACACT

AAGTCATCCTGCTGCTGCCAAGAATTTTTCGAGCCATGATCCATGCAGGGACTGGTATGGAGTTAAATG

CTTTAATGGTCGAATAAACAGGTTGAATATTACAAATTCTGGTGTCATTGGTACACTCCATGATTTTCCAT

TTTCATCTCTACCTTTTCTTGAATATATTGATTTTAGCATGAACAACCTTTCTGGTCCCATTCCTGCAGAAA

TAGGGAAACTTACCAACCTTGTCGAAATTGATCTTGATACAAACCAGTTAACAGGTCATATTCCTCCGGA

AATAGGCAACTTGATCAATGCAAAATTGTTCTATGCTTACTCCAATGAATTGTCTGGTTCCATTCCTATTG

AAATAGGGAAGATGAAGTCACTTGAAAGTTTAAGCTTACAGAAAAACAATCTTTCTGGTCCAATTCCAAA

ATCTTTAGATGACCTAACTGAGCTCAAAATTTTGTACCTTTATTCTAACCAACTTTCTGGTCTCATTCCTAG

TGAGTTGGGGAATCTGAAAAACCTCAATGAGCTAGATTTATCCGATAATAAGCTTAGTGGTTCAATTCCA

ATTACTTTAGGTGACTTAACTGGGCTCAAAATTTTATACCTTTATTCTAACCAACTTTCTGGTCTCATTCCT

AGTGAGTTGGGGAATCTGAAAAACCTCAATGAGCTAGATTTATCCGATAATAAGCTTAGTGGTTCAATTC

CAATTACTTTAGGTGACTTAACTGGGCTCAAAATTTTATACCTTTATTCTAACCAACTTTCTGGTCTCATTC

CTAGTGAGTTGGGGAATCTGAAAAACCTCAATGAGCTAGATTTATCCGATAATAAGCTTAGTGGTTCAAT

TCCAATTACTTTAGGTGACTTAACTGGGCTCAAAATTTTATACCTTTATTCTAACCAACTTTCTGGTCTCAT

TCCTAGTGAGTTGGGGACAGGGCCAATACCAAGAAGCTTTAGCAAGTGCTCGAGTTTCAAAAGAGTTCG

GTTGGATAACAACAGTTTTACTGGAAATTTATCTGATGCTTTTGGCATCCATCCACACCTTTACTTCATTA

ATTTGAGCGAAAATGATTTTTATGGTGAACTCAGCAGCAACTGGGGGAAATGCAAGAGTTTGACTGATT

TGCGGGTAGCCAGAAATAACATTAGTGGTAGCATACCACCGGAGATTGGAAACCTCAAAGGGCTTCAA

GGACTTATTCTTTCATCCAATCATTTGGTTGGACAGATACCTAGGGAATTAGGAAAATTAACCTCTCTGG

TTAATCTTCTTTTGCGAAACAACCAAATTTCTGGCAATATTCCTATGGAACTTGGGTTATTGACAAAGTTA

GATTACCTTGATCTATCAGACAATAGATTGAATGGTTCGATCCCACCGTTTATAGGAGATTACCAACACC

TGTTTCACTTGAACCTGAGCAACAACAAATTTGGCCAAAAAATTCCAATAGAGATTGGGAAGATAACTCA

ACTTAATGTACTGGATTTGAGCCGTAATCATCTTGTTGGAGAAATCCCCCCTCAGTTAGCCAATTTGAAG

GTGTTGGTAAACTTAAATCTTTCACACAATGGCCTATCTGGGCGCATTCCTCAAGAATTTGAAAGTTCAA

CTGGTTTGCAGGATGTCGTATTGTCATACAATCTGTTGGAAGGTCCAATCCCTAATAATAAAGCTTTTATC

AATGCCTCATTGGAGGGTAATAAAGGTCTTTGCGGCAATGTAGTAGGAATCCATCCCTGCGGAATGCCG

TCTTCTGTGGTGAAGAAGCACTCAATGGCGAAGGGACGTAAACTCATCCTCATCATTGTACTTCCTGTTA
TGGGAGCACTTGTCATCCTCATCACTGTACTTCCTGTTATGGGAGCACTTGTTCTACTCTGTGTTTTCATT
GGTGTTCTGTTTATGTGTGACAAAAGAAGTAGAGTTGGAGATGTTGAAAGACGGGATAGTGATGGTTG
GCTTTCGATATCCATGTTAGATGGGAAGGCATTGTACAGGGACATCTTAAACGCCACAGAAGAGTTTGA
TGCAAAATTTTGCATCGGGCAAGGAGGACACGGAAGCGTTTACAAGGTAAACCTTCCATCATTAGGGAA
TATAGCTGTGAAGAGACTTCATTCTTCATTTGAGAATACACCTCCCAAAAGTTTCTTAAATGAAGTAAGG
GCATTGACTGGGATTAAGCACCGGAACATTGTGAACCTCTATGGCTATTGTTCGAATGCACAACACTCGC
TCTTGGTTTATGAGTATGTGGAGAGGGGGAGTTTGTCTAGCATTTTGAGCAACAAGGTTGAGTCCAAGA
AATTGGATTGGTTTAAAAGAGTGAATATCATCAAGGGTGTTGCTTTTGCTTTATCTTACATGCACCAGGA
CTGTTCACCCCCGATTGTCCATCGAGACATAAGTAGCAGTAATGTTTTGCTTGATTCTGAGTATGAAGCT
CGTGTTGCAGACTTTGGCATAGCGAAGCTACTCAAGCCAGATTCATCGAATTGCACTGCACTTGCAGGC
ACATATGGCTATGTTGCACCTGGTAAGTTTTAAATATTTCATGAAAGCCAAACACTATTGTTTTTATCTTA
TTTACAAGTTTAAATTGATAATATTGTTCTGGAAACTGTATTGTTCAGAGCTTGCATATACAATGAAGGTT
ACACAAATGTGTGATGTCTTTAGCTTTGGAGTGCTATCATTGGAGATAATAAAAGGAAAACATCTTGGG
GAATACATTACCGTGCTAGCAGATTCATCGACTATAGATCCTGAGCAGCTTAGCAATTTTCTGGATGAAC
GCCTTCCATATCCTGAAGATAGAGTAAACGAGGCGTTGGTTTTTATCATCAAGCTAGCATGCTCTTGTTT
GCTTGAAACTCCAAAATCAAGGCCAACAATGCACTTCATCTCTCATAAGTTATCATCAATGGATGCACGT
CCACCTATTCATCAGAGAAACCCCCATGTAAGGCGAGCAATATAA
>PGSC0003DMG400028123 LRR-RLKs
CAGTTACAAGAGAGCATAATGTCTAATGCAATGTCAAGAAAGAATATGGTTGATCCAGCAATTAGAAAT
TCGTGCTCGGATGAATCATTGAAGACGATGATAGAGATATGTTGCAGGTGTTTGGAACAAGATACAGAA
GATATGCCCTCAGTTGAAGATGTAATATGGAATTTGCAGTTTGCTGCTCAAGTTGAGGATTCATGGAGA
AAAGACTCTTCAAGTAGTGATGCCTCTCCAATTTCACATCTCTACAGCTTCAAACCCAACTTGTCGCGCAA
TAGCAATAGCAATAACAATAGCAATGGCACGTCTGTGTGACAAGCTATTTCTAACGTGGATAACTAATCT
CGATACTATTATTCCATCCTGTGACAAGAATAACTAAACTAACAATTTATGTAATAATTTTCTTTGCATAG
CTTATACAAAAAAGTAAGTTTTCATACAATCTTTTTCCTAACAAGCAGATGCCATAGCATTTTCCTTGTGT
ATATACCAACCCAACCTTAATAGTACCTAGATATTACAAGAGCAACATATAGTGCAGAAACCTATAATAG
ACATGGATGATAAAAAACAAAAACAAAAGAATGCAAAGAGGGACTAGTTAATGGAAAAGGGAAGCATT
ACAAATCGATTTTTTAGACAGTAGAAACAGAATATCTATTCGTTTGCAGGAGGAGTTTCCATTTTCTGAA

CTTGAGGGGCTGTCACTGCCACTAAGCTAAGATCAGATGAGTTTGAATCTCCAGATCTTTCATGGATCTC

TCGGTACTCCTGGCATATGGCACATAAGTGGCAGAAAAAGTGTGTGGCGAAGTCTCCACATGGAGCTTC

CTGCATATATATCAGGACACACAAAATTGTTAAATGCAGAAATACTGATGTAAGCAGTTTGCATCTTTCT

ATTCAGGCATGGAACCATAGTATTTCGTAGAACAGAAAAGGGGGGCGGGAGCTAATGGGAAGAGAGG

AAGGTGAAGAATTCAGGGTAACTGGTTATAGATGCTGCAGACTCATCTGATAATAAACTCAAAACTAAG

ATTTCTAACTCGGACAACTTCTCAATGTAAACCAAACAGAAAAGGAAAGTACAGTCAGTTCTCTTTCTAC

ATGGAGTCCTGATTTTCCTGATTTTCAAGTTGTTGATAGTTTATCGAACTGAAGTCCACCACTACTAATTC

AAATGAAGTATAATCATCTCTGCCGTGGAGTCGATTACACACTGTAACTCTTTAATCTTTTGTAGCTTCTA

CGGATAACATCCAATTCAAAAGAATGTATGTGGAATTTTAAGACATTTCTGATTAATTTTTCTTTCTAATG

AAAAGGTTAACTTCAAACTATCATCATAGAAGTCTTAATATAGTCATTAATATTAATATATATCACACCAT

AATGGGGAGTTTATGCATAGACGTAAAGAAACATCCTCAAAAGGTGTGCACATCAGACTATCCTCCTAT

GCATAGCCCATGTTATAGGAGATACAACTATGAATATGGAAGGTTAGATTGAAGGTAAATTTTCAAGCA

ATCTAACTTCACTCATTTTTTCAAATGTCTTTCCTGTTGAATCAATAGAGCTACTGATAATATGCTAAACCA

CATTCTCATCCATGTGCACTATAAACAGCAAAACAACAGAGATGGCTACCTGAAGATTGTACTTTGATCT

CAATGTCTTTCGATAGCCACAGGCATAACATGCAACAAAGCACCCAGGCAAACCCAGAAGAATGCCTTC

AGTTAAGAAGCAGAAAATTGTGTTGAAAAAAGCCCACAAAATAAAGTGAGTTGTACATGATCCCATCAA

GGTTCCTGACCCCAAGAAGTCCGCATTTTTTCCAAACAGAAAGCAAGGGCAAAGAAGTCCAATAAAACC

TGTGCTCAAAATTTTGCAATTAGTGCTAACTCGAGGTTGTCAACCAACATCTAGTGTCATTCCTCTGGGAT

GACAGAAGAGCCATTGCTTAGGAACAGGTGTTGGAATTTTAAACATAAACACACATAATTAACCCTCAA

AAGAGAAGTTCCACAATCACACTTTCATCTTATTATATGTTCAGAAAGGAATGATTGATCTAGTCCCATC

GGCACTGCTGAAAACCAGTTTCGGGAAATTCCGGCAGTTGTATTATTTATAACTTCATGAATATGACAAA

CAGTGAAACCACAAGGGGGAGCAAATAGCAAATAATCTGGAAACAGAATGGAAGCAGTGTTGACTGTT

GAGAAAAGAAATGCATGTGTGTTATCCAGAACTCTATGGAATTACACTACACTGGGTATGTTGTTGTTCA

TAAGAATATATACATAAATCCTGTGGGATTACACTACACTGGGTATTATTTTGTTGTTGTTCATAAGAATA

TATACATATATCCTTATGGGTATACTTTTGTCCCAGCAAAAGAGCACCAAGAAGGTTCTGTATATGTACA

GTATACAAAAACTCAGATACCCCTTTATAAATGCAGGGCACTGAGGAACTCTAGCAAGGCACCTAAATC

ATACACACTTAAGCATGTTACACCATAAATAAAAAAAGTACAGGCACTTCTAAGTTCCTAAGTCTGGATA

GGACTAGTTTTGCAAATTTGGTAAGAAAGTGTATTTTCAAAGGCATACCAACTTCAAGCTAAATTTTTATC

TAAGAAGCATATAAGCAGAGAAAAATACTTCATTCCATTATAGAGCCCGAGGAAAACTCAATTAAATGT

ATGGCTTATAATAGCTTCCTTAGTGGTTCACTCAGAGATTTATCATTGTTTTCCATCTAATAAGATAAGAG
GGTAATTAATCATGAATCTTATTTTACTACCCAAAAATGCACCTTTCATTCACTTTTTTCCCTTCTCATAAA
GCAAGATCACAGGGACGAGAGTAGCGTACAGCTCTGTGGATCATCAAAACAAGCACAGATTCCAGAAC
TCCACTGAGCATGGTCATCTTTTTTGGTCTGTTGAATTTCTTCCGCAGTAGAAACTTGTGGTACTTCTGCA
TCCGACTGATCCAGAGGAATATAAGGTGGAGGTGAATAATTCTTGTGCCCTACCATTATTAGTCAGGTG
CAAACAGCAACCTGCAGCACAAATTCAAGAGAAAGCAAAAAATATCAATTAAGAACAAGCGAGTTACAC
ATAAACATCCAATCAGTTCTCTGATTTCATGAAGGACGGTTACTTTTTGACTAAGTTGCTCGGACTCTCCA
AAAATGTTGCCGCACCCGTGTTGGATCCTTCAAAAATACATTATTTTTGGAAGATCCGACACGCACTTGT
CGACATTTTTGTAGAGTCCGAGCAACATAGCTTGTGTGGGGGGGGGGGGGGGGGGGGGGATTTTAAA
AATCCTTAGGGGTACATGCCCCCCTCCCCCCTTAAATTCATCTCTGAATGTCTAACACTTACTAGATTCCCT
TTGCTAAACTCTACAGAGTTCTTGTTGGACATTGGAACTTAGATAGTTTGTCTATACTGTACGGGTTCAAC
AAATATATTTCAATATCTTTGTCAAATTACTCCAAATTGATGTAGTTTAATAAATATAAACGGCATCTATA
ATATTCTCTATTGGTAATTTTATAAATACATCGTAGATTTATTCTTTTTAGTTCTTCTATATGTGTATACTCC
AAATTAACACTTTCCTCTAACCGTGCTTATAAATGTCTGGTACTCATTTTCACAACTATAGTGTCTTATCAC
AGGTGAAATCTCGAATACACCCGTGTTAGTTCTGCATTAAATTGTAAAACCTTAACGTATATAAGAGAGT
TATCCTTCGTATTACTTATCAACTAAGTTGCTCGGACTCTCCAAAAATGTTGCCGCACCCGTGTTGGATCC
TTCAAAAATACATTATTTTTGGAAGATCCGACACGCACTTGTCGACATTTTTGTAGAGTCCGAGCAACAT
AGCTTTTTGATGAAGTTATAAATCATCTGCCATCGTTTCAAAAGAAAAGAAACACACACACAGATATTGA
CAGGAAAGAGAAAAAGAAAGATGCTTAACATTACAAAATCGATTCTAAGTTGTCAAACTGAAAAATGTT
TACCAACAAAAAATTAGCACTGTGTAGATTGACCACATTGCAGATTGGCTCCACTAGGACTGACGATTAT
TTTATCATGTAATGCTTACGATTAAGTATTTCCAACTTTCAATTATGGGTGTTCCTTAGAAAGGATTGCAC
CCAATTTGTGCACTTCCTTTATTTTTTGACCTTGTAATCACAAAAGGAAGCTTTCCTAGTGCTTGCCTGTTT
TACAATGTGAAAATTTCGGGCCAGCTTGCTCGCACCTCAACTAATTTAACGGGACACCTACTACCTCCCA
TCAACACAAGTACAAGATAACTCTATCCACTAATGTTTGGACATATGGGAGGAAATCATCTAATATTATT
GCACTTGGAGCCTCATGGTTCTCAAACCAATTCATTGACCATTAGCCCACAGCCTCACAACCTTGGTGCA
ACTATTCAGAATTTTTTATCAATAACTATTAACCATAGCCATTTCTGATTTAAAAAATTAGACTAGATGCA
CCTGATACCTGTTGCTACTTGTTGCTGGAGGGAGGTAACACGTATCCCGTGAAATTAGTCAAGGTGAGT
GTAAGCTGACTCAAACACGACACGATTATCAAAAGAAATTGTAATGTGGTGTAACGGTAATTAGAAGGA
TTTGACAAATGATTAGCAATTCATGACAGTATGTTTCGATTCATCTATCGGCCTTAATTTTTTTTCATTTTC

TTCAAACAATTGATAAGCAATACTTCAATGCAATTATTACTCAGATCTCAACTATAGAGCCATGACAAATA
ACGACATAGTATCGAAATTCAAGCTTTATTCATGCAGTGTAAACGAATTTAAAGCATTGAATCCAAATAT
CAAAAACAATATCGTCTATTTTACCGCTAAGAATCATTTGGGATGAGGTTTTCGTTTTTTTTTTGCTCGTA
AGAGAAAATAAAAAGGCAAAATCTGGAGAAGAAAGAGACAGACCGGAACTTACAGCTCGATGTCGCC
GGAGAGAAGGATATTGCTGAGGATTTCGCCGGAGAAAGAGATCGCCGGGCAATGAACTTCGCCGAAAA
ACAAAACAAGGAGTAGTTTCCAGAGGACAAAAAGTGGCGTTTTGTT
>PGSC0003DMG400028147 LRR-RLKs
TCTCACTCCAATTATAACACTGTATATTACTATCTCTCCATTATTCTTGGCTTTGTAGTGATCTGAATCAAC
CCATGTTTCCCACCTCTCCTTGTCTAAATTCTAACATGGGTTTGTACAAAAAACAGCCATTAGTTGGCACA
AAGAAATCAACTTTATTGATTATGTGATTCTTGTCAATACATGCTGTCTAGTGGTTCTTGGTGAAATCATC
ATCAATGGAGGCTGTTCAGCTAATGGGGTTTGCTCTTACACTCTTCATTTCACTTTTTTCAGTTGTTTACA
GCGTTACAGACCCTAATGACCTTGCCATTATCAATGAGTTCAAGAAAGGGTTAGAAAATTCTGAGCTTTT
GGAATGGCCAGTTAATGGTGATGACCCTTGTGGTCCTCCAGCTTGGCCTCATATAATTTGTACTGGAAAC
AAAATTCAACAGATTCAAGTTATGGGGTTGGGGTTAAAAGGCCCTTTGCCTCAGAACTTTAATAAGTTGT
CTAAATTGACTAATTTGGGGTTGCAAAAGAACAAATTTAGTGGAAAGTTACCATCTTTTGGTGGTTTGTC
TGAGTTGCGCTATGCTTTCTTGGATTTCAACATGTTTGATAGTATTCCATTAGATTTCTTTAATGGGCTTGT
GAGTTTAGAGGTGTTGGCATTGGATGATAATCCTTTGAATGCAACTACTGGTTGGGGTTTGCCTAATGA
GTTGCAAAGTTCAGCTCAATTGACTAACTTGACTTTGATGAACTGTAATTTGGCTGGTTCTTTGCCTGAGT
TTCTTGGAAATATGTCTTCTTTAGATGTATTGTTGTTGTCCAAGAATCGACTTTCGGGGACTATACCAGGT
ACTTTTAAGGATTCTGAGCTCAAAATGCTATGGTTGAATGATCAGTCTGGTGATGGCATGAGTGGTTCAA
TTGATGTGGTTTCAACAATGAGATCACTTACTAGTCTTTGGCTTCATGGGAACCATTTTTCAGGTAAAATT
CCAAAGGAGATTGGTAATTTGACATATCTGCAGGATCTTAATGTTAATAGCAATGATCTTGTTGGTTTAA
TTCCTGAATCTTTAGCAAATATGTCATTAGGCCATCTTGATTTGAATAATAATCATTTTATGGGGCCAATA
CCAAATTTCAAAGCTATAAATGTTAGTTATCAATCTAACTCTTTTTGTCAAGCCAAAATTTGTGCACCTGA
GGTTATGGCACTTTTAGAATTCCTTGATGAGTTGAATTATCCATCTAAACTTGTTGAATCATGGTCAGGA
GATAATCCTTGTGACGGGCCGTGGTGGGGATTAAGTTGTGACATTAACCAAAAAGTTATTGTGATAAAC
TTGCCTAAGTCCAATCTTTCTGGTACCTTGAGTCCTTCAATTGCAAAATTAGATTCTCTTACTCATATTTAT
CTTGGATCTAACAATATTTCTGGTTCTATTCCATCTAGTTGGACTAGTTTGAAACATTTGGTTCTGCTTGAT
TTGAGTAATAACCACATTTCCCTTCCTCTGCCTGAATTTACTCCCCCCTTGAAACTCGTCCTGAGTGGAAA

TTCGTTACTGAACTCTAGTCCTCTTATAGCAAGCCCTTTGCAAAAGAATAGTACATCCACTTCTGTTTCAC
CCTCTTTGCCGACCAATAAGTCAAGCTCTTCCAAGTCTAACTTAGTCATTTTTGTGGTTCCTATTGCAAGTT
TTGCACTTTTAGTTTCTTTTGCTATGCTACTATATGTTTACGTTCGAAAGAGGAGTATGGATAGGCACAAA
GGTCCAACTTCTCTAGTGGTTCATCCTAGAGATCCTTCTGATTTGGATCGGATGGTCAAGATTGCAATTTC
CGATGAAACTAAAGGAAGTCTTTCGATACTAACTGGAAGGGGTTCTTCTAGTATACACAGTGGTAAATA
CCCTGTGATGGAAGCTGGCAATTTGGTCATATCAGTTCAAGTACTTAGGGATGTAACCAAGAACTTTGCT
CCAGAAAATGAACTCGGGCGTGGTGGTTTTGGAGTGGTTTATAAAGGAGAATTAGATGATGGAACAAA
AATAGCTGTCAAAAGAATGGAGTCTGGAGTAATTAGCAGCAAAGCGTTGGATGAATTTCAATCTGAAAT
TTCTGTTCTTTCCAAAGTCAGACATAGGAATTTAGTGTCTCTGTTGGGATATTCTGTTGAAGGCAATGAA
CGGATTCTGGTTTATGAACACATGCCACAAGGTGCTCTTAGCACGCATCTTTTCAACTGGAAAAGCTTGA
ACTTAGAGCCTCTTTCGTGGAAGAGGAGGCTGAATATTGCATTAGATGTTGCTAGAGGAATGGAATATC
TACATACATTGGCTCATCAGTGCTTCATACACAGAGATCTCAAGCCCTCAAATATCTTGCTGACTGATGAT
TTCCGAGCAAAAGTATCAGACTTTGGACTCGTGAAACCCGCCCCTAATGGAGAGAAGGGCTCTGTGGTC
ACCAAGCTAGCTGGAACTTTCGGATATTTAGCACCTGAATATGCGGGTAAACATTTCATTTCCTGTTCTTT
TATTCCTAACATGACATTTTATTTTGTAAAACAGGAAACAGATGTTCTATTTGCCATTTGATGTTCTCTTTG
GTATGAGTACAGTTACATAAAACTCAAATAGTTTAGTTACTGTTGTCTGTTTTGGGGTGGATAAGTTAAG
GACTATAATAAATCTGTTCACCGATAAGATGATATTGAATATCAACTTTAGTGACTAATTCTTGTAGACAC
CTAGGATTTTCTTTAGATGATTCTGTCTTTTCCGTCTGCTGGTTTCCCAACTCATTTTGCCTGCTGTTTTAAT
ACAGTTACTGGTAAAATCACCACTAAAGCCGATGTCTTTAGTTTCGGTGTTGTCCTTATGGAGTTGTTAAC
TGGATGGATGGCACTTGATGACGACAGACCAAATGAAAGCCAATACTTAGTTGCATGGTTCTGGAACAT
CAAATCCTCTAAAGAGAAACTTATTGCAGCAATCGATCCAGCTCTGGATGTGAAACAAGAGAGTACATTT
GAGAGCATCTACACCGTTGCAGAACTTGCTGGTCACTGCACAGCAAGAGAGCCTGGCCAACGCCCTGAC
ATGTCCCATGCTGTGAATGTGCTCTCCCCGCTCGTTGAGAAATGGAAGCCTCTTGAGGAAGATTCAGAC
GACGATTGTGGTATCGATTACAGTCTTCCACTCAATCAAATGGTCAAGGGCTGGCAAGAATCAGAAGGA
AAAGACTTGAGTTGCGTCGACCTTGAAGACACTAAGGGTAGTATCCCCTCAAGGCCTACTGGATTTGCT
GAGTCCTTTACATCAGTTGATGGTAGATGAGAAAAAAGGTACTGCACTACTTCTAGTGTAGTTGATCTGA
TATTCTTATTCAGTAAATGTGGATAGCATTTTTGTTTGTTTTTGTGTGATATTTTGATCCTGTTTTTTATCTA
TGCTTTCTTTCACTTTTATTTGTAGAAAATTTTACTCCAATGTATAGTCTTTTGAAGTTATGTGGGATGTAG
ATATGGTATAGTGATGATTGATGAATGAAAACTGTTTCCA

>PGSC0003DMG400028429 LRR-RLKs

TGAATTCTAAAAAGGACTTTTTTCTGAAAGCTCTGCACCAAAATCTTTCTTTTGGGTAATTTAGCTTTTTG

GGTCCAAATGGTAACTTTGCTACTCATTTTCTCATTTTTTCTTCATTGGGTGGCCACCACTCCAGCTTCCAT

GGGTTTTCTTTCACCTAAAGGTGTCAACTATGAAGGTAAAGATTAAAACTTTACTATAATCCAATCATTTC

TTGTTTTTTTTTCCAGTGAAAAATCCAAACTTTATTGCTTAGCACTTGTTTATTTTTGTTTTCATAGTTTAGA

GTGGTAAAGATGAAAACTTTACTACAATCCAAACATTTGTTGTTGTTTTTTTTCCAGTGAAAAATCCAAAC

TTTGTTGTTTAGCACTGTTTATAAGCATTTATTTGTTTATGTTTTCATAGTTTAGAGTGGTAACTTTACTTA

AAGATTTGTTGAATTTATTGTGGGGTAAGCAGTGGCAGCATTGATGTCTATGAAGAATAGAATGAGAGA

TGAATACCATGTGTTAGATGGATGGGATGTAAATTCTGTTGATCCTTGTACTTGGTACATGGTTGGTTGT

TCTTCAGAAGGCTTTGTTATTTCTCTGTGAGTTTTCTCCCTTTCTCTTACATCTTGTCCATTTCTTTTCCCTTT

CTGTGAAAATAATTTATTATTATTCTTTGGTTATATATTCTTGGCAGTGAAATGGCAAGTATGGGATTATC

TGGCACACTTTCGCCTAGTATTGGGAATTTAACTCATCTTCGGACGATGTAAGACTAATTTGACATGAAT

TTCCCACTTTATTCTTATTTTGATGTGATGCTGCTCATATATATCTTGGAGATACTTGAAGTTGAAATCACA

TAGTACTTGTTAATAATTACTAATATTAGTAAGAGTAACATTAAGTGCTTTAATTGCAAGATATGTTGAAA

AATATCTTTTAATATGATCTGTTAAAGTCATTTTTGCATTGGTTTTTGTTGTAGGTTGTTGCAGAATAATCA

TTTATCGGGTCCAATTCCTGCTGAAATTGGACAGCTCACTGAACTGCTGACCCTTGATTTGTCTGGTAATC

AGTTTGTTGGAGGAATTCCTAGGGCGTTGGGTCGCCTGATTTATTTAAATTACCTGTGAGTGTAGGCTAA

ACTCTTGATTTTGTTCTCTCTTTATTATTCATATGTTACAGTTCCCCTTGTTGGCTGTGTATGTAATCCTCTG

GTGTGTCAAATTCTCAGGAGGCTTAGCAGGAACAGATTGTCTGGACAAATTCCTAAACCTGTGGCATAC

CTTTCAGGTCTTTCATTCTTGTATGTCTTCCATCCATGACATTTATATGTCCAATACAAGGGCTATATGAGA

GTTAAATTAACTGTGGATGGTGTTGTGTAGGGACTTATCATTCAATAACCTTAGTGGTCCAACTCCAAAA

ATTTTGGCCAAAGACTACAGGTAAGATCCAAGGAAATATTATTATATCTGTCTCAATGTTTGGTCCTCAAT

AGTCGGTGAACTGGAAGAATCCCTCGGGTCTCTAGTTTGATGAATCAAGGAATTCAGGGTTTACACTTTT

ACTATGAGGAGGATGGTATATGTCACAGTACTGTGCCAGAATCTTATTCATTAATGGAGTTTAAGATAAA

CACCTTGATCTTAAATTCAACTGCAAAATAGAAGCTTGATTCAATATTGTTGCATCTGCGACATGCCATAT

GGCGCAGTGCCACACATAATGTTTCTCTCGTAATAAAGTTATGCTTTCTATAGTTTTAGTAACTGTTATCT

TGCATTCCAGTTTGTTGTTGTGGTATGTCCCTTCACTGGAAACCAGCAGTCAGTTCTTGCAGACCTGAAA

GTAATATCTCAAATCTCATTATCAAAAAGAAGAAGAAAAGAAGAAGTAATATCTCAAATCTCCATATCAA

AAAAAGAAGAAGTAATATCTCAAATCTTATCATTGAAGTATTATCTTCTTACCTGGTCTAATGGAACTTCC

ATGATGTTTGTTTGATATAATTCCTATATTTCTAATCTGCTAATGTCAAGCAGAATATTTGCTTGAGATAA
TGTCTGATGTTATAGAGGTTTTCTATCCTACTTGCAATGCTTGTTGATTATCTTCCCCCTGGCATCATACTA
GCAACCCTTCGGGTTCTTTTTTATTCTATTTCAGCTTATATTTTGGTTGTTGCCGCCTTAGTATTGCAGGAA
ACAGATTTCTTTGCTCTGCATTGACCACACAAATTTGTGGGGGTGTGCCAAAACCAGTAAATGGTATTGT
TGAAATTTTACCAGAACTTGTCATTATATTGAGGCTGATGTTTTTAATCAAACTCTATTTTTACCATGCAG
AAACATCTTCAGACAAGAGGATTAGCAATCATCATCGATGGGTTGTTTCTGTTAGCATTGGGGTCAGTTG
CACATTCATAGTTGCAGTCACGCTTCTTGTTTGTTGGGTGCATTGGTACAGATCTCGTGTCCTCACAGGAT
ATGGTAATTACAAAATTAGAAACACATCTCCGATTTCTTGCACATTAAGACTAGATACATTATGAGTCTCT
GATCATTTGATCTGCAGTGCAACACGATTGTGAGTTCGCCACTGGCCACCTGAAGAGATTTTCATTTCGT
GAACTGCAAATTGCCACCGGAAATTTCAGCTCTAAGAACATAATAGGACAAGGTGGATTTGGAGTGGTT
TACAAAGGATACCTTCCAAATAGGACAGTAGTGGCTGTCAAAAGATTAAGAGATCCAAGTTTCACTGGA
GAAGTGCAGTTTCAAACAGAAGTTGAGATGATAGGCTTGGCTGTGCATCGGAACCTTCTACGTCTGTAT
GGATTCTGTACGACTCCCGAGGAAAGATTACTTATTTACCCTTATATGCCAAATGGGAGCGTTGCCGATT
GCTTGAGAGGTTTGCTTTGAAGTATATTTGCTTGGTGCTTTTGCTGAAGCATTCTTTTTTCTTGTCACAAT
GGATTTCGTAAACTGATCAAGTTTAACATATGCATTTCAATTATTCCTAGTGCTCTCAATGTTAATCTGAC
AAAAACTCTTGAAAGGTCATGGTTTATGGAAATCGAGGACTTTGTGCTATATTTTTACTGTACATCTGAA
AAAACCAGGGCCCTATGATGAAAACAACTTTTGATTGATTATCTTAGATAGTTAAATTGGATTTACATTG
TCAATGTTTCTTGTGAAAGTGGAATGAAATCTATTATCAACATATCAATCAACAATGCATCAAACCCAAA
CTAGTTGGGGTTGACTATATGAATATTTTGTATCCATTGCAATTAAGACTCCTGATGTAGTGCATTACCTA
AGACTCTAGCAATCTATTGCGACCTTACGTGCTATTAAGCTTAATTTTTATGTGACAAAGTTTCTATCCAT
CTTCTGAAACTCTAATGCCATTTATGGTATCGACTTACATTATCCATGAATAAAATAAATTGTTATTTGGC
AGTAAGGTAACTGTCGTGTTGTCATTAATTTTTAGCATGACCAATTCAAGTCAATTTTTAGGTATATGAAC
ACAAAATCAAGTTGGAATTATTGGAATGTTAACGCATTAGCTTGATGTAGTGACTGTGTCATCTTTTGCT
CTGTTCTCACATAATCTTTTGTTATCTACCAGACAATGGCCGGGATAAACCATTTTTGGATTGGAGAAAG
CGCATGCATATAACTCTTGGAGCTGCCAGAGGTCTTCTGTATTTGCATGAACAGTGTAATCCTAAAATAA
TTCACAGAGATGTTAAGGCTGCTAATATTCTGCTTGATGAAAGTTTTGAAGCTGTTGTTGGTGATTTTGG
TCTTGCCAAGCTTTTAGACTGTAGAGATTCTCATGTTACAACTGCAGTTCGTGGTACTATAGGTCATATAG
CACCAGAGTATTTATCAACTGGTCAATCATCTGAGAAGACAGATGTCTTTGGATATGGGATACTACTTTT
GGAACTCATTACAGGACACAAGGCATTAGATGCAGGAATTGGTCAGGGTCAGAAAGGAACTATTCTTG

ACCGGGTGTGTAGCTTCTAACCCTCCTACTGTTTGAAGCTTTATATAGTTGTGCATATAATTTCTAGACTT
TATTTGATTAACCAGTTAATAGTTAATCTTCTTGCAGGATTTTGTTTGAGTCATAATCAATATCAATATTTG
ACTTGAAATTTTTCACTAAAATTGACGCAATAACTAATGTCTAACGCAGAAGTTGCTCCACATCTTATGAG
AGAAGTACAGTTCCAAAATTCTGAATCATTGCTCGAAAACTTGTTCTTTTTACTGACTGCATAATCTGTAG
GAATTATGGACAACATATTCAAGGTTTTTGAATCACTTACTGATCAGTGCAATAAAATTATTGCTGTGTAT
TACCTTACTGTTGCATACTTAAGCCCTTAAATATAAATTATTTGGTTGATTTTTTATCACATATAATGTTAC
TCTTGCATACTTTAAGCCCTTGATTAACTTATTTGGTTGATAATTATTACATTATCATGCAAGTCTGAACAA
CCATGTATTATGCTAGAATAGTTGTGTGAAAAAAGGTACCCGTTCTATGCTTCTTCAATTATGATTTTTCT
TCCCTCTCAGGCTGTTTTATGTGGAGTAAAGATTTTCAGAACATGATAGAGCTCAGAGTTTGTTATGTCT
ATTATTCGGGCCTATTCATTCTGACAACCTATCCAATTTACAGGTCCGGAATTTATTTGAGGAAAAGAAA
GTGGAAATGCTTGCGGATAGGGATCTAAGAGGATGTTTTAATGCAGAGGAGCTAGAGAAAACAGTTGA
AGTGGCTCTACAGTGTACTCAATCTAACCCCAATAATCGGCCTAAGATGTCGGAAGTTTTGAGAATCCTG
GAAGGCGTTACAGAACAAATGGGGCATGTGGACGAATCACAAGGTGGAAGCAACACATGTCAAACAA
GGGCTTTCAGCTTCTCTAGGAATTTTAGTGATATTCATGAAGAATCCTCATTTACCTTTGAACCAATTGAG
CTTTCAGGACCCAGATAACAAAAGTAGCCTGCACTGGCCTTGTCATAATAAGTGTGCAATCTTGATATAT
CTAGTTCAGCCATACATACGAGTATGTTGTTGCTTTAAGTCAATGTCCTAGTACTCTGTAAGTTACTGTAA
TTTGTAAATTTTGTTGCAAAAGCATTCATTATACTAGTTCTTGGTGGTTCACAACCTGTGTTAGAACTAGC
TAGACTAACTTCACTTTGGAACTTCATTCTTTGGAAATGACAAACTTC
>PGSC0003DMG400028435 LRR-RLKs
ATGTTCCAACTCAAGCCGAATCTTTTCCTGCAACAACACCAGCCAGGATTTCATATATACATTGAAATTGG
AAGGGCAACTCAAGGGTTCGACCAATGCAACTTGCTAGGCTCTGGAGGTTTTGGCTCTGTTTACAAGGG
CATGTTTGAAAATGGGATGACTTTGGCAATCAAAGTGTTCAATTTACGGATTGAAGGTGCATTCAAGAG
TTTTGATATTGAATGTGAAGTTCTACACAACCTTCGCCATAGAAATCTTGCCAAAGTTATCAGCAGTTGTA
CTAACATGGATTTTAAAGCATTACTTCTAGAGTACATGCCCAATGGGAGCCTGGAGAAATGGTTACATTC
TGATGATCACTTCTTGGACATAATCCAAAGATTAGACATTAATGGTTGATGTTGCATCAGCTTTGGAGTA
TCTCCATCATGGTTATTCAACAGTCGTTGTACATAGTGACTTGAAGCCTAGTAATGTCTTGCTAGACGAA
AGATTGGTTGGACACGTGAGTGACTTTGGCCTGGCCAAGTTATTCGGAGAAGGGGAATCAGTTGCTCAT
ACTAAAACACTTGCTACAATGGGGTACATTGCACCAGGTAAATTTCTTTTAGTCCTTTTGTTACCACATAT
AAAGTGTTATTTTACATCACATTAGAACTTGGTGAAAGCATAATCAAAATGGCCAAGTTAGCTCATAGAT

TGAATCTGTATATTATTACCTTTAATCTGCCAGAAGTCATATTGTTTCAATTGATTACCTTACTCTTGTTTT
ATTCCCATTATACCTTTAATTTCTTATTTGGCTCGTCAAGAAACTAATTACTCTGGTAACATTGGATCAGA
GTATGGATCAGTAGGATTAGTTTCTACAAGATGTGATGTGTACAGCTACGGCATTATGCTCATGGAAACT
TTCACAAGAAAAAGGCCATATGATGAAATGTTTCAAGAAAATTTGAACATGAGGAGTTGGGTCTGTAAT
TCACTACCTGTCGCACCAGATGATATTATTGATGACACCTAATTTCTGTTAGGATTTTGTCCTGATTTTCTT
ACCTTCTTTGAAGTTTATTTTTTCTAAAAGAAAAGACAAAACATTACCATAAATGTTTTTACTTTTTCTTGA
AAGGAAAATATAATATTCTTTTCATATTTGGTTTATTCTTTCCTTTTAGGAAAATGTTTTTGGAGTAGCTA
GTTCTTTTCTAGTAGGAAAGGTTTTAGGACTCTAAAAATATAGGTTTGTTTCTTCTAACTCAATAACAATA
ACATCCACAATGTAGTCGTATAAGAGTCTTGTTTATGGGGAAATTTAATCTCTCAACAGTTATAATATTTT
TTTCTTTTAAATTAGTTTTCATATTAATAATATTATGTTTTAGTATCGCTTTATTTGTCATCTAATTTATCAC
CGTCTTGATTGCAATTGTAAGCTTCCGCATGACGCCCAACTCACCTTCATAACCCAACAATTTCAACCTGA
AGAGATTGATTTTGAGAAAAAGTTGTGTTTTGTGTCCTCTATTTTGGACTTGGCATTGAATTGCACAGTT
GAATCTCCTAATGAGAGGCCGAACATGAAAGATGTCCTGGCAAATATCAATAAGATCAAGCATGAATTT
CTTCACAGATGATGCACCAAGGTTTATGCAAACGGTAGGCATCCAACATAGCTCCTAGATTTAAACTTTT
CTTGTTTCTTGTGCTTTCCTCTCGTGTATATTTCAAGTTTGAGTGAGTGTGTCAAATAACTTAAGTACATA
ACATGTTTACTACAATTGGAAAGGAAGAATGCCCTGTATAATATGATTAATGAGGTTATTATGTTAATTT
CAACTACAATAGTACACAAAGAGTGGTGTATAATGTTGAACTAAAATTGAACTTTTCTTTGTTTATTGCTT
TCCTCTTGTGTATGTACCAATTTGAGTGTTTCTCTCAAATATAAGTGTAATATAGGACAAGTATATATAAG
ATGTGTACTAAAGTTGTTATAGAAGAATGCCCTGTACAATATTAATGATCGTTATTGTTTTGATGTATCTA
CTTTGCAGATGTGAATCCCTCCGCTTAAGCTTTTTAGATGATATAGTCAGACACTTCAATACTCCTTTATG
TTACACCACGCTGCTCCTTTCTCAAATAACTTGATAGAACATTCAAAAAAGACGTAATCTAACACCCTTTT
TTGGGACAGACTTTAGTAAAGTCAAGTCGACCAACTGTGGAAAATTTATAACTAGGGGTGTACAAAATC
GAACCAAACCGCAAATTAAACCAAAAAAAAAACCCGACTAGTGGTTTGGTTTGACTTGGTTTGGTATTG
GAAAAAAAACCCGACTATATTTGGGTTGGTTTGGTTTTAACTAAAAAAATCAACCCGAGGCCAAACCAA
CCCGACGTTATATATATAATTTTAAAATATTATTTTATACATAAAATAGATATAATTTTTAAATATTTCTTA
TACTTTTTCATAGTTTTTGTCTTTTAATATATTATTTCAAGTTTGTAACTTAGAATTATGAATGTTCCAATAA
AGATTATAGTCCACATATGTTGGTAATTATAATAAAGCTCAAATCAAAATCAAATTAATGCTAATGCAAA
AAGAAAATCAATTCAACACTAATAATGACAATAATATTGAATATTTGTTCTTTAGTTTTACATTGGTTTAG
ACAATTAAAATACATAATCTAATTTTAATTTCATTTAATATTTAGTCATGTAACTAATACTTATTGAACTTA

TTTTAGCATGATTTAGTACTTTTAAATTATGATCATTTTCATTATGAATTGTTAATTTGCAATTTTTGTTTTA
CGCGATTTCATTATTATTATTTTTGTTGGATATTCTAGTGTCATTAATTATATCACATTTTGTGTTATTTTTT
TAAGAAACAACTTAGATAGTTGTATTTTGATAGGACTAAAGAAATATTTGAAATACAAGTAAATTATATG
TTTGTATGAATACTTTACCAGAAAAACCCGAAAACCCGAGGTTGAAAAACCCGAGTTTTTTTGGTTTGGT
TTGGTTTATAAATTTTAAAATCCGACATAAATGGTTTGGTTTAATATTTGAAAAACCCAAACCAACCCGAC
CATGTACACCCTTATTTACAACCCCTTATCTACGGCATTGCTTGGCCCTCTTTCCTTGATAATAATTAAAAA
TCAAGGAAAAAGGTTTGCATAACATTAGAAAGGTATGCTGTACTGTCCATATGGTTTAAACAGGAAATA
ATAAGAAAACCTTTCATCATAATAAGAGCTCAGGTAAAGATCGGATATTACCGTTGACAATATAATTAAA
TAATATAAAAGTTTGTTCTCTCTAATCGCTTAAGTTTTTAGATGAGATGGTCACACAATTCAACAATTATT
AGTGGAAACTAATTTTTCTAGTACTGTTCGAACTAGATAAGTATTAGGTAAATTATATAAATTTGTATTTT
CTGCTTATTTAAAATACAAGAAAATATACACTAAAAAAATATTTTTCTTCCGACCCTACTCAATCACTGAA
TTTAATGCCTATTTGGAAGGAGAAAAAAAACTCTCTAAGAAGACCTAGTAAATCCAACTAATTTTCCATA
TACTGCTCTTTGATATAGCACATGAAGTCAAGTCACTCTGGAAAATTTATAGCCCCTTATCTACGACATTG
CTTGGCCCTCTTTTATCCCTATTTAATGACTAGCTTGGTTTGATTTTTCATTTAAAAATAACTAAATGAATT
ATATTGATGTATTAAATCTATAAATCAACCCAAATTAACAAAAAGTGTTTTTCAATTTTTTGGTTTTTTTTA
TAATTATACAATAATTGAAAAAGAGATCAAATATATTTTCACTTACATATGTGATAGGCTAAAACCTAAA
GAGATCAATTTTACTTTATATTACGAAAGAATTTTAGAAAGAAAAAAAAAAGACAAAAAAGAGAGAATT
TTGCTTTAAAACAATTGACAAATAATAAGAAGAGAGAAAATAGGAACAATAAAATAATGAACTTTTTATT
TTCAAAATATATATGATAGATTAAATATAGTCAAGAACAATGATAAAAAAATGATTAATATCATCGTGTA
CACAGGTTGAATGTAGATGCGAAGGAGAAAATTTAGGCAAAAAAAAGAATATATGCATGTATGAAAAA
ATCTTAAAGAGATAAACAAAAAAGTAAAAAAAAAAAAAAGTTGAAAAAGTTGTTATTACCAAAGAACTGT
TTAAAATAAGAAGAGAGATGAAGAAATGATAAAAAATAATTATACTTTTTCAATAAATAAAGGAAGGAA
AGAAAAAACATTTCTTGAAAAGGCAAAGAAAAAAAAACGAGTAAAAGAGAACATGGCTAATAAATGAT
AAATAGATTTTAAGAAATCTTTTTTTTGCTAAAAAGTTATAAGTGGGCTATTTATTGCCAATTTTAATTATA
AAACATGTCATGATGTGCCATTTTTTCTTTTTAATATAGTGTACAATTATTATTGGATGAAGGGATTACCA
TAGTAAAGAAAAGTTCTAAAGAAATTTGGATGTCTGATGAAATAATATATTATCTCAAAACAAAGACTTG
GGGACAAAATTTTAATAAAGTCAAGCCAACCACTGTGGAAAATGTACAAATTACAACCCCTTTATCTACG
GCATTGCTTTTGTTCCTTTTTTTTATACAAATAAGGGACCCAGTCCTTAGCAACCTTTTGTCCCTATTTAGC
TCAACACTTGTTTCCTTATCTTATGTGCAATCACATGGAGAAAGCATACTCTTTCTTTCTTTCACTACTACT

CTTGCACCTATACTTGTTAGAGACATGCATTAGCAAAAACATAGTAATTAACACAGATCAATCAACTCTA
CTTTCATTGAAAGCTAGTTTTACTCTAAACTCTTCTCATCCCTTAACTCAAAATTGGTCTTCTCAAGCTTCA
GTTTGTGATTGGATTGGAGTCACTTGCGGCTCTCGCCACCATAGGGTGAGAGCACTAAATGTATCCAACT
TGAACATTCTTGGAAACATTCCACCACAATTGGGAAACCTCTCATTTCTCGTTTCGCTTGATCTTAGTAGT
AATAATTTCCAAGGAGACCTCCCTCAAGATTTGTCTCGTTTACGTAGATTGAAGGTGATGAATCTAGCAA
ACAATAACTTCACTGGCCAGATTCCCAAAGATTTTGGTAACCTTATGAGGTTAGAGAAGTTGGTTCTAGT
CAGTAATAAACTTAGTGGCTCAATTCCATTCTCCATTTTCAACATTTCCTCGTTGGAAATTTTAGCTCTCAC
ATATAATCAATTATCAGGGAGTCTACCTGTAGATATATGTCGTCGTCTTCAAAAAATAAAGAGTATTTCA
ATCATTTCCAACCACTTGAGTGGTCAAATTCCAACAGGTTTGTCTAACTGCACAGAATTGTATGAATTGTC
ATTGTCATACAATAACTTCAGTGGAACCATTCCACCAGAAATTGTAAACTTGGAGAGGCTTCAGTTCTTA
AACCTTGGGGGAAACAACTTGCAAGGTACATTCTGCATACTTTAATTTCTCAATGTGAATATATAACATC
CTACTTTGCATATATATGTAAGCCCACAATATTTAGGGTGTGTTTGGTACCAAGGAAATTTTCCTACTTTC
CTATTTTTTTATGTTTGGTGCTTAGCATGAGGCTAAGCACCAAAGTTTGTTAATTGAGAATTAACACCTTG
AGCAACATCATAAAAATTTGCAAATTTGTTTGCCGTTTGTTGAAATTTGCAGGCACAATTCCAGCAAAGA
TTGGTAATTTAGGAAAGATGCAGCAATTACAATTGGAGAACAATCGCATCGTGGGTTCAATACCACGCA
CCATATCGAACTTATCATCACTATGGATGCTTAATTTGAACACAAATAATTTATCAGGTACCTTGGTCTTG
TTTGTCATGGAATTAATTGTTGGTTTTTATCAACTCTTATATATACTGATGGGCACAATCAAATAAAAGTA
ATTTAGATATATTCTTTCGTGTGGCAGGTATTATACCCAGAGAGATTGGAAATCTTCACAAACTGGAGAT
ACTTTATTTGCAAATTAATAAGTTAAGTGGTTCCATACCGGAAGGGCTCTTCAATATCTCTACACTAACAG
AGGTGTCAGTTTTCTTCAATAACCTTTCGGGTAGTCTTCCATCTGCTTCAGGCTACTGGCAAACAAATCTA
CAGCTTTTGAATCTTGGTCAAAACAACATTGGTGGAGTAATACCCACTTCAATCTCCAATTCATCAAATCT
AGAGATTATAGATCTTAGTATAAACAAATTCAGTGGCCAAATTCCTAACTCGTTGGGGGATTTGAGGGA
TCTTGAATATCTGGACTTGTTCGTTAATAACTTATCGTCTCCGCACCTAAGTATCTTAACTTCATTGGTGAA
CTGCAGATATTTGAAAGAAGTAATATTAGGGGAGAATCCTCTGTATGGTGTTCTTCCAGATTCCATTGGC
AATCTCTCCATTTCTATCATGGAACTTGAGTTATATAGTTCTGAAATTAGAGGCCAGATACCATTAGGAAT
TGGGAATTTAAGTAACTTAAACACTTTGTCCCTATCCGGCAATGACTTGACTGGATCAGTGCCAACAACC
TTATGTGATTTACACATTCTTCAAAGGCTAGGACTTGGTCTTAATAGGTTGAGTGGATCCTTGCCAGAGT
GCCTTTGCAAAATGTCATCGTTGGGCTTGCTTTCTTTGTACTATAATCGGATTTCTGGTCCGATACCATCT
TGCATTGGTAATGCAACCTCTTTGAGAAACGCTTATCTAAATTCAAATAGGCTCACTAACATACCCACAA

GTCTATGGAGCCTCAAATATCTTTTGGTGCTTAACTTGTCAAATAATTCTTTGATTGGTTCTATACCTCCA
GAATTAGGGAACTTGAAAGCCATAACATCCATAAATCTGTCCAGGAATCACCTTTCAGGAAGTATTCCCT
CCACAATTGGAGACTTGCAGAGCCTAGTTTATCTTTCCTTGGCTTATAATGAGTTACAAGGTTCTATTCCA
GAGTCACTTGGGAAAATGATAAGTTTAGAATCGGCGAATCTATCCAATAACATTCTTTCAGGTACCATTC
CAAAATCATTAGAGTTGCTTCGGTATCTGAAGAATTTCAATGTATCATTCAATAGATTAGAAGGTGAAAT
CCCAAGTAAAGGACCTTTTCTCAATTTCAACTCTCAATCTTTTATGGGAAATGGAGGGTTATGCGGCGGT
TTAGTTTTCCAACCTTGTATGACTAGGTCTTTTCATCATTCAAGGAGAGGCAAACTGCTTCTGATTATACT
TGTCTCACTGGGAGCTGCGGTAATGGTACTTGGCTCAATCGTTGTGTTTATGTTAAGGAGACGTTGGAA
TAGAAATATTCCAACTGAAGCTGAATCCTTTGCTGCAACAACACTAGCCAGGATTTCATACATTGAAATT
GAAAGGGCAACTCAAGGGTTTGACCAATGCAACTTGCTAGGCTCTGGAGGTTTTGGCTCTGTTTACAAG
GGCATGTTTGCAAACGGGATGACTTTGGCAATCAAAGTGTTTAATTTACAGGTTGAAGGAGCGTTAAAG
AGCTTTGATGCTGAATGTGAAGTTTTACGCAACCTTCGTCATAGAAATCTTACCAAAGTTGTCAGCAGTT
GTACTAACTTGGATTTTAAAGCATTACTTCTTGAGTATATGCCTAATGGAAGCCTAGAGCTGTGGTTACA
TTCTGATGATCGCTTCTTGAATATGATCCAAAGATTAGACATAATGATTGATGTTGCATTTGCTTTGGAAT
ATCTCCATCATGGTTATGAAACAGTTGTTGTACATAGTGATTTGAAGCCTAGCAATGTCTTGCTAGACGA
AAAGTTGGTTGGACATGTGAGTGACTTTGGCCTGACCAAGTTATTAGGAGAAGGGGAATCAATTGCTCA
TACTAATACACTTGCTACAATGGGCTACATTGCACCAGGTAACTTTCTGTAAGTCCTTTTATCATCGCCTA
TGATGTGTTATTTTACATCAAATAATTAGTTGACTGGTTCTAATATAATGCATCACATTAGAACTGGGTGA
ACGCCTAAGTGGATGGCTTATTTTAATTGGAAGTTATCACCTAGTGTTTTTTGTCACTTTGTTGGAATTTA
ATCCACTTCATTGACCATTAAGCCACAATTAGCCTTGGGTATAAATATAGATAGTATTTTGATTTCTTTGG
CTCGTCAAGAAACTAATTACTCTGGTAACATTGGATCAGAGTATGGATCAGTAGGATTAGTTTCTACAAG
ATGTGATGTGTACAGCTACGGCATTATGCTCATGGAAACTTTCACAAGAAATAGGCCATATGATGAAAT
GTTTGACGGAAATTTAAGCATGAGGAGTTGGGTCTGCAATTCAGTACCTGCAATGCCAGAGGATATTAT
TGATGTCACGTTAATGGAACCAGAAAAGACTCACTTTCAGGAAAAGTTGTGTTGTGTGTCTTCTATTTTG
GAGGTGGCGTTGCATTGCACAACTGAATCTCCCAATGAAAGGTTGAACATGATAGAAGTCCTTGCAAAT
ATCAAGAAGATAAAGCAGGAATTTCTTCGCAAATGATGTACGTAGCAGTGGTAAATATCCAACTTTTTAA
AAGTATTCTGATGTCATCTAGATATATTATTTCCTTATTCAAGAATAGAAATGATTATTGTCTTGTGAGCT
TGTCTATATCGTACAAATATATTGAATTGGATATTCTGGTTTTCCATACAAATATATCCCCTTGTATAAGA
AAAAACAAGAACTAATTGAAGCATAACTTAATTCATGATTATAAGTAGAAGAACCCAACATTTTTGCCTA

TGGAAATTTGAATTGTGATGAATGTTGAAAACTGAAGTAAACAACTCAAGTCTGTGTTACTATTAGTTTA
TTTTTGTATGTGCTGTGCCAGCTCAAGTTTAATCCTAATTGAAAATTTGTGCAGGTTGTACGTGCTGCCTT
TTCTCCTGGTTTAGCAAATTTGGGTGATACCAATTGAGATGAAAAATCAACATTTGGTCCGAGATTTGAA
CTTTTCTTGTTTCTGTTCTTGTGAATATTTCGACATTTGGTAAAATAAGTCTAATGTATAAGATGTGTACTA
CAGTTGGAATAGAAGAATGCCCTGTATAATATTGGATTGTTAATGTATAATATTGAACTAAAA
>PGSC0003DMG400028436 LRR-RLKs
CCTAAATATTTATTTCTTGAAAATTATATATACCTCTCACTTTCTTATTTGTCCCTACCTGAAAACTTAATTT
GTTCCCCAATCACTTGGAGAGATCATACTCTTTCTTGCTTTCACTACAATTATTGACACTTTACTTGTTCGA
GGCCATCGACATAAAAACAGATCAATCAGCTCTACTTGCCTTAAAAGTTAGCTTTACTCTAAACTCTTCTC
ATCCCTCAACTCAAAATTGGTCTTCTCAATCCTCCGTTTGTGATTGGATTGGAGTCACTTGCAGATCTCGT
CACCATAGGGTGAGTGCACTAAATATATCCAACATGAACATTCTTGGAAACATTCCTTCAAACTTGGGAA
ACCTCTCCTTCCTCGTTTCTCTTGATGTGAGCCAAAACAATTTACATGGAGACCTCCCTCAAGATTTGTCTC
GTTTGCGTAGACTGAAGGTGATGGATCTTGCGTTCAATAATTTCAGCGGAGAGATCCCAGTCATGTGGT
TCGGTTTCATTTCAAAGTTAGAAACTTTGAGTGTGAGAAACAATCGTCTTCAAGGCAATATCCCTAAGGA
GATTGGGAATCTTGAGTATCTAAAGGAGTTGATTTTATTTGAAAACCAATTTACTGGCTCTATCCCGCTCT
CCATTTTCAATATGTCGTTGTTGGAAATTTTAGGTCTATCACATAATCAATGAACTGGGAGTCTTCCTGTA
GATATATGTCGTCGTCTTGCAAAAATAAAGAGCATTTCAATCATTTCCAACCACTTGAGTGGTCAAATTCC
AACAGGTTTGTCTAACTGCACAGAATTGCATGATTTTTCATTGTCATACAATAACTTCAGTGGAACCATTC
CACCAGAAATTGTAAACTTGGAGAGGCATGAGCTCTTAAATCTCGGGGGAAACAACTTGCACGGTACTC
CTTTCGTCCACTTTTACTTGTTCAATTTAGAAAATAATTGTATTTATATTGCTGCTTAAGGATGGCCATCCC
ATTTGGCAAAATACATCTAACTGGTCTTTAGTTTTCATTTTGATACTTCATCTCAACCTTGTACGTTCCATT
TTAACCCTCCCAAAGCTATCATTTTATTTTCACTTTATAAAATAAGTTTATCCATATTCAAAAGCCTTCTAG
CATGAGGCTAATTTCGAAGTTTGTTAATTGAGCAACAGGATAAAAGGTTTACAAGTTTGTTTGCCGCTTG
TCAAAATAATTTGCAGGCACAATTCCAGCAAAGATTGGTAATTTACGAAAGTTGCAGCAAATACAAGTG
GAGAACAATCAAATTGTGGGTTCAATACCGCGCACCATATCCAACCTATCATCACTATGGGTGATTAATT
TGAACACAAACTATTTATCAGGTAGCATACGTGGTCTTGTTTGGTTATCCGGAGGCAATAAAATAAATTA
ATCGAGATATATGTACTCTTTCATGTGTCAGGTGTTATACCCAGAGAGATTGGGAATCTTCACAAATTGG
AGACACTTTATTTGCAATTTAATCAATTAAGTGGTAATATACCAGAAGAGCTCTTCAATATCTCTACACTT
ACAAGGGTTTCTCTCCTCTCTAACCACCTTTCGGGTAGTCTTCAATATGCTTCAAACTACTGGCCAACAAA

TCTAGAGATTTTGAATCTTGGTGTAAACAACATTGGTGGAGTTTTACCCACCTCAATCTCCAATTCCTCAT
ATCTAATGAGATTATATCTTAATGGTAACAAATTCAGTGGTCAGATTCCTAACTCATTGGGGGGTTTGAG
GCATCTTGAACATCTGGACTTGTCCAATAATAACTTATTATCTCTGCACCTAAGTATCTTAACTTCATTGAC
AAACTGCAGATCATTGAAAGAAGTTTACTTAGACCATAATCTTCTCGATGGTGTTATTCCAGATTCCATTG
GCAATCTCTCCACTACCATTATGGAATTTTATTTAGACAGTTGTGAAATAAGGAGTCAGATACCATTAGG
AATTGGGAATTTAAGTAACTTAAACACTTTGTCCCTAACCCACAATGACTTGACTGGATCAGTGCCAACA
GCATTATGTAATTTACATATTCTTCAAAGGGTAGCAATTGAGGATAATAGGTTAAGTGGACCGTTGCCCC
AGTGCCTTTGCAAATTATCATCCTTGGGCATGGTTGATTTTTCTAATAATAGGATTTCGGGTCCGGTACC
GTCTTGCATTGGTAATGCAACCTCTTTGAGAAATGTTTATCTAAATTCAAATAGGATCACTAACATACCCA
TGAGTCTATGGAGCCTCAAAGATCTTTTGGATCTTAACTTGTCAAATAATTCCTTGGTTGGTTCTATACCT
CCAGAGTTAGGAAAGTTGAAAGCCATAGCATCCATAGATCTTTCCAGGAATCACCTTTCAGGAAGTATTC
CCTCCACAATTGGAGACTTGCAGAATCTATTTTATCTTTCGTTGGCTTATAATGAGTTACAAGGATCTATT
CCGGAGTCACTCGAGAATACGATAAGTTTGGAATCAGCAAATCCATCCAATAATTTTCTTTCAGGTATGA
TTCCAAAATCATTAGAGTCACTTCGACATCTGAAGGATCTCAATGTATCATTCAATAGATTAGAAGGTGA
AATCCCAAGTAAAGGACCTTTTCTCAATTTCACCTCTCAATCTTTTATGGGAAATGAAGGGTTATGCGGC
GGTTTAGTTTTCCAACCTTGTATGACTAGGTCTTTTCATCATTCAAGGAAAAGCAAACTGCTTTTGATTAT
ACTTGTCTTCCTGGGAGCTGCAGTAGTGGTACTTGGCTCAATCGTTGTGTTTATGTTAAGGAGACGTTGG
AATAGAAATATTCCAACTCAAGCTGAATCCTTTACTGCAACAACACTAGCCAGGATTTCATACATTGAAA
TTGAAAGGGCAACTCAAGGGTTCGACCAATGCAACTTGCTAGGCACTGGAGGTTTTAGCTCTGTTTATA
AGGGCATGTTTGCAAATGGGATGACTATGGCAATCAAAGTGTTTAATTTACAGATTGAAGGTGCGTTAA
AGAGTTTCGATGCTGAATGTGAAGTTTTACGCAACCTTCGTCATAGAAATCTCACCAAAGTTATCAGCAG
TTGTACTAACTTGGATTTTAAAGCATTACTTCTTGAGTATATGCCCAATGGAAGCCTAGAGCTGTGGCTA
CATTCTGATGATCGCTTCTTGAATATGATCCAAAGACTAGACATAATGATCGATGTTGCATTTGCTTTGGA
ATATCTCCATCACGGTTATGAAACAGTTGTTGTACATAGTGACCTGAAGCCTAGCAACGTCTTGCTAGAT
GAAAAGTTGGTTATGTGAGTGACTTTGGCCTGACCAAGTTATTAGGAGAAGGGGAATCAATTGCTCATA
CTAAAACACTTGCCACAATGGGCTACATTGCACCAGGTAACTTTCTTTTAGTCCTTGAATATCGCATATGA
TGTGTTATTTACATAATAATTAGTTTACTATTACATCACATTAAAACTCGGTGAAAGCCTGAGTGGATAGC
TTAATTAAAGAAATGTAATATATCACAGATTAAGCGTGAACAAAAATGGTATAGAAAACATTGAAATAT
GACTGTCAAACTCTATATGCATGCATCTACGAATTTCTTTTTATCAAAAGTTCCCTCAAAGGATATTAAAT

AGATGAGCCAAAGGGGCATAGAGGGGGACTACGCCTGACCTCATCAATACAAATTTACGATACTAGGA
GTGAGGAAAAGAAGAAATGTGGGGGGGGGGGTTAAGGGTTGCCCCACATCAATTAAGCATACAAAAA
GATAAATTCTAGGATTTTAGAGCAAGCAGAGAAGACTCAGCCAGGATCAAAAAGAAAGCTGTTCTCTTA
ACTTAGATTCTAATGTTAGGCTTGCCAACTACATCATTCTTAAACGGAAGATTTATAGGCCTAGTCTCAGT
GAAGAAGGTAGTCATTTTGCACTATTCAGCCAAGTTAGCAAGCATGTGAGCAGGAGTGTTACCTTCTCTT
AAGGTATATGTGAAAGTGAAACGACTAAGTGAACTCATAGATTGGATCTGTCTAATATCATCTTTTATCT
GCTAGAAGTCCATATCGTTTCGATTGACTGCCTTATACTTGTTTCATTCCAATTATACTTTCAGTTTTTAAA
GTCCTTATCTACTCAAGGTAAATTCAACTATTTTCAATATCTAGTAAATATATATATATATATATATACACA
TACAGACACACACACATATATATGTATAATATTTTCATTTCTTTATCGGGCTCATCAAGAAACTAATTACT
CCAAATATTGGATCTCAGAGTATGGATCAGTTGTATTAGTTTCTAGAAGATGTGATGTGTACAGCTATGG
CATTATGCTCATGGAAACTTTCACAAGGAAAAGGCCATATGATGAAATGTTTCAAGAAAATTTGAACATG
AGGAGTTGGGTCTGTAATTCACTACCTGTAGCACCAGATGATATTATTGATGACACCTTATTGCAACCTG
AAGAGATTGATTTCAACAAAAAGTTGTGTTGTGTGACCTCTATTTTGGAGTTGGCATTGAATTGCACAGC
TGAATCTCCTAATGAGAGGCTCAACATGAAAGATGTCCTGGCAAATATCAAGAAGATCAAGCATGAATT
TCTATGCAGATGATGTAACAAGGTTTATGCAATGGTAGGTATCCAACTTTTTAAAATATTCTAATGTCATA
TGGAGAAATTATTTCCCATTTCAGGAATAGAAAGATTACTGTCTTGTCTATACCATGTAGAAATGGGTTT
TTTCCATACAAATAATAATAATAAAAATAAAAAATAAAATAATAATAATAATAATATAATAATACTTTTTT
TTTTATTAATATAATAATACTTAAATCAAGGAGAACTTCATTAGTTTTGAAATTGGTCACATAATCAAGGA
GAACTTCATTAGTGAGTAAAGTAAATCAAGAATTCATGAGGACCAACATTTGGAAATTATGATGAATGTT
GAAAAGGGAAGCAAACAACTCATGTATCCGTACAATTAGTGTATTTTTGTGTGTGCTATGAGTTTTTATC
TGCAATGACTGCCAACTCAAGTTTGATGCCATTTAAATTTTTGTACAGGTAGTGCCGCCTTTTATGCCTGT
CGTTGGCAAATTTGGGTGACACCAATTGAGTTGCCAACAAAGATTTGGTCTGAGATTCGGACTTCTCTTA
TTTCTTGTGCCTTCCTCTTGTGCATGTTTCAAGTTTGAGTATGTTTATGTAACAAATCTAATATATAAGATG
TGTACTAAAGTATAATACCGGTGATCATGAATTATTATTGAGATAGCAGCTTGAATAGTCTATGAAGA
>PGSC0003DMG400028437 LRR-RLKs
AATTTTCAAATGGTATTCCAAGAGAAATTGGAGACATGCAAAATCTGATACACCTTTCTTTGGCACAAAA
CAAGCTACAAGGATCTATACCTGACTCAATTGGTAGCATACCGAGTTTGGAATTTCTAGACCTCTCGAAT
AACAATCTATCTGGATCAATTCCGATGTCCTTAGAGAAGCTTCGGTATCTCAACTATTTCAATGTTTCCTTT
AACAGCTTGCAGGGTGAAATTCCCTTTAGTGGTCCTTTCAAGAACCTTTCCAGCCAGTCATTCATGTTCAA

TAAAGCCTTGTGTGGTGCTCCAAGATTTCGTGTCCTCCCGTGCCCCACTTCTTCAAACCATAGATCAAAGA
AGAAGAAGTTACTTCTGATAGTCTTTCCTCTACTGGGAGCAGCTGTGATAATTGTTTTTGTAACATTGGC
GTTTGTGTGGATGAGGTACAGAAGAGAAGGAAATGTTCCAATTGAAGCTGATTTGTTAGCTACAATGGA
AAGAATTTCATACTATGAAATCATACAAGCCACTAATGATTTTAGTGAGAGTAATTTCATTGGTTCGGGG
AGTTTTGGTTCTGTTTACAAAGGAATTCTCAGAGATGGGACTACCATTGCAGTTAAAGTGTTCAACCTAC
AAGCTGAAGGTGCATTCAAGAGTTTTGAAACAGAATGTGAAGTTCTTCGCAACCTTCGCCATAGGAATC
TCACCAAAGTCATTAGCAGTTGCTCCAACCTTGATTTCAAGGCTTTAGTGCTCGAGTACATGCCTAATGG
GAGCCTTGAAAAGTGGTTGTATTCACACAACTACTTCTTAGACATCCTGCATAGACTAAGCATAATGATA
GATGTGGCATGTGCATTGGAATATCTCCACAATGGTTGCTCAGCACCTGTGATTCATTGTGATCTGAAGC
CTAGCAATGTCTTGCTAGATGAAAACATGGTCGCTCATGTAAGTGACTTTGGCATTTCTAAACTACTCAG
TGAAGATGAGAGTGATTTACACACTAAAACCTTAGCAACATTCGGCTACATTGCACCAGGTAAATCAAAT
CTCTTATACTTCTTTTTTCAGTATTCTATCTTTGTTTGCCTTTTATTTCGATTATATGATATTATGATAAACC
GTAAGTGTGCTTTTATTGTAGAGTATGGAAGGGAAGGATTGGTGTCGCCAAAATGTGATGTTTATAGTT
ATGGGATCATGCTGATGGAAACATTTACGAGGAGAAGGCCTAACGATGAAATATTTGATGGAGATCTTA
GTTTAAAGAAATGGGTGAGTGATTCACTCCCAGAGGCAACAATCAAAGTTGTAGATGCGAATTTGCTAA
CGCCAGAAGATGAAAAATTCATGGACAAGATAGATTGTGTAGCATCCATCATGAAAGTGGCACTGGATT
GCTCTGCAGAATCTCCTGAAGAAAGGATCAACATGAAAGATGTTGTAGGAATACTACAAAAGATCAAGA
TTCAACTTCTTTCATGTTCTGCAAGTACATAAAAACATTTGATTCATGTCACTTGAGGGTATAGAAAGTGT
TTGTTAACTTTTTAGCTGCATACCTAGACTGTTTTCCTTTCTTGCTGCATAAGATTCTTTTCTGTAACACTA
ATGTAATATCGG
>PGSC0003DMG400028438 LRR-RLKs
ATGAAGAGAAAAATCAATTTCCTTTTCCCTCTTGTAGTTTCAGTAATATGGCATCATTTGCTGATATTATC
AGCCACTGCAGAAGTCAATCTCAAAACTGATGAAGCTGCTCTACTTGCTTCGAAATCCTATGTTACTTCTG
ATACTTACAATATCTTGACAAGCAACTGGACCTCAACTACTTCCGTTTGCAATTGGATTGGAGTCACCTGT
GGCTCTCGCCATCAAAGGATCATAACCTTGGATATTTCCAATATGGGATTAATAGGTACCATCCCTCCTC
ACTTGGGAAATCTGTCCTTTTTGGTTTCTCTCGATATCAGTAGCAACGGCTTCCAAGGAATTCTGCCACGA
GAGTTGGCTAATTTGCATAGATTGGAGTTTATAAATGTCACTTCTAATAAGTTCACAGGAGATATTCCCT
CATGGTTCTCTCTTTTGCCAGAACTCCAGCACTTGCATCTAGCTTTTAACAGTTTCACAGGTATTATTCCAC
CAGATATTTGTAACGCTTCCAAACTTGAGAGTTTAGTCTTGGGATTTAATCAGTTACAAGGGGAAATTCC

AAATGAAATTGGTAATCTTCAGAACTTGACTTGGTTGAGCTTGGGATCAAATCAGCTAACAGGCTCTGTA
CCACTTAGCCTGTACAACATTTCATCGTTACAACGTTTGGTTTTGACAAAAAATAGTTTGTCTGGAAATCT
TCCTGTCGATATATGCTTGAACCTTCCAGAACTTATGGTGCTTGCACTTTCTGACAATGAATTTGATGGTC
AAATTCCTTTGGGGATAGATAAGTGTTCAAAGCTTCAGATTTTGTCATTGTCATTCAACAAGTTCAGTGG
ATTGATACCTAAACAGATTGGGAACTTGAATGTGCTGTCTATTTTATATCTTGGTTGTAATGACTTGAAA
GGTGAGTTATACATATTTGCTTTTAGGTTCCTTCTTTGTGCATACATGAAACATATTTTTTGAGTCATGTAT
GCACTCCTTAATTGATATACTGCAGAAAGTAAACTTTCTATTTATGATTTAATTTGCAGGTGAAATTCCAG
AAGAGATTGGAAATCTTCGTAATCTAGAGATATTGGATGCACAGAACTGTAGCCTAAGTGGTCCTTTACC
ATCTTCTATAAGTAACATAACTTCACTTCAATCACTCAATCTTTATGGCAATAATCTCTCTGGCACATTGCC
CCGGTATATATGTTTGAACATGCCTGATCTCAGAGCATTTGATCTTGGGAATAATTTATTCAGTGGAAAC
ATTCCAAAAGAATTTGGAAACTGCACTTCACTTACTGACCTCTTCTTAAGAGAAAACAACTTAACAGGTA
TCATCTTGTCATTTTATTTTTCCAAGATTTGGTGTATGTTCCTATGCTTTTCATATCCTAATGAGTCGCTCA
AATGTCATCAGGTGAGTTACCAAGGGAGATTGGTAATCTTTTCAATTTGGGAAGATTAGATCTACATTAC
AATTTCTTAACAGGTCCTATTCCATCTACAATATTCAACATGTCAAACATAAGGGGCATTTCATTTTTAGG
GAACTTCTTTACTGGAAGTCTACCATCAGATATAGGACTTGGTCTTCCTAATCTTGAAGAACTTTATCTTG
GTTACAATAACCTTACTGGAGCCATCCCTAATTCTCTCTCAAATGCTTCCAATATTTTCCGGCTTGGAATT
GGATATAATGACTTTTCTGGTCATTTTCCGAGGTCGTTTGGTAATCTAAGATGTTTAGAGTACCTCAACGT
GAACGGCAATCATTTCACAAGAGAGCCTTCATCTCCAGGATTGATTTTCTTTGATTCCTTGACAAATTGCA
GACATTTGAGAAAACTGTGGATAGGTTATAATCCACTCAATGGAAATCTTCCGGCTTCTATTGGAAATCT
CTCAAGTTCTCTCGACTACATTTATGCTGTTAATAGTGAAATCAGAGGCTACATTCCCAGTGAAATAGGA
AATTTAAGTGGTTTGTCTTTCTTGTTACTTCAAGGCAATTACTTGAGTGGGTTCATTCCAAGAACAATAGG
GAATTTAAAGAATCTTCAAGCCTTGAATTTGTATGACAACAAAATGATAAGTGGACCTATCCCTGAGGAA
CTCTGCAATTTAAAGAAACTAGGATTCTTGAGCTTGGGAAATAATGAGCTTTGCTGTTCTATACCAGCAT
GTTTAGGGAACATTACATCTCTTAGATACATTTACCTAGGTTCCAACAAATTGACTTTTAGCATACCTCCC
AGCCTGTGGAACCTCAATGATCTCTTGCATCTTGATGTATCCTCAAATTCCTTGAAAAGCTCTCTACCTCC
AGAAATTGGAAATCTGAAAGTCGCAACGTTGCTGAATATATCGAAGAATCAAATCTCAGGAAGTATACC
GAGCACAATTGGAGGCATGCAAAATATGGCTGAACTTTCTTTTGCAGAAAATAGACTAGAAGGACCAAT
TCCAGAGTCAATGGGGAACATGATCGCCTTGGAATCGTTAGACTTGTCTCATAACAAGCTCTCTGGTGGC
ATTCCCAAATCATTGGTAGCTCTTTCACATCTCAACTATCTTAATGTATCTAACAACAGATTGAGTGGTGA

AATTCCAATTGGAGGTCCTTTCGTGAACTTCTCTTATGATTCATTCCTTTCAAATGAGGCATTATGTGGTG
CTGCTAGGTTGCAGATACCAGCATGTAGGTCTAATTCTCCTAGTAGGAAAAGAAAAAAGATGGTGCTTC
TTATTGTGTTGATCTTGTTGGTGGCTTCTTCAGTCATAATGCTCTCAGTCATGCTCACCATCTTTTTGGTGA
TTAGAAGTCGAAAGAGGAAGACAAACATTGCTACTCATCAGGCGGATGCCTCTCCAGCCACGGTACATG
GAAGAGTTTCATACCATGACCTTCAACAAGCAACCGACAGATTCAGTACAAGCAACTTGCTCGGTTCTGG
AAGTTATGGCTCTGTTTACAAAGCAACATTTGGGAGTACTATAGTGGCCGTTAAGGTATTCAACTTGCAA
ACAGAAGGCGCATTCAAGAGTTTTGACACAGAATGTGAAGTCTTACGCAACCTTCGTCATAGAAATCTG
ACCAAAGTCATCAATAGTTGCTCCAGCATTGATTTCAAAGCCTTGGTATTAGAGTACATGCCTAAGGGAA
GCCTGGATGATTGGCTTCATTCTGAAACTTGTTCCTTAGATATAATGAAGAGAGTAGACATAATGATTGA
TGTGGGATCAGCATTAGACTATCTTCATCAAGGCTATTTTGTGCCCGTAGTTCACTGTGATCTAAAGCCTA
GCAATGTCTTGCTCAACGAAGAAATGGTTGCACATGTGAGTGACTTTGGCCTGGCAAAGCTATTAGGGG
TAGGAGAAAGTATTGTACAAACCAAAACACTTGCAACCATTGGATACATTGCACCAGGTAATAATCATGT
CAACAAGCTTCATTTCTCCTTCAAATTTCAACCATTTTAAAGATACCATATTCTCTTGATAAATTGTTTTATT
TCAATGTAGAGTTTGGATTGGAAGGACTCGTATCTACAAGGTGCGACATTTACAGCTACGGTGTCATGTT
GATGGAAACCTTTACGAGAAAGAAACCAACGGATGGAATGTTTGCTGAAAATTTAAGCCTGAGGGAGT
GGATAAGACAATCATGGCCACACGGAATGGACGAGATAATAGACCCCGAGCTTATGATACCAGGGGAG
AAGAACAAAACAGGAAAGGTGCAGTTCTTATCATCCATCATGGAATTAGCTCTGAGGTGCACTTCAGAC
TTGCCAGAGGAAAGAATGAATATCAAACATGTTCTTGCTCAACTGAAGAACGTCAGAACTATGTTGGAA
AATGTAATAAATCACTAA
>PGSC0003DMG400028473 LRR-RLKs
ATGAATATTAGCACTGATCAATCTTCTCTTCTTGCCTTGAAATCCCACATCACTTCAGATCCTTTTCATGTT
CTTTCGACGAACTGGTCTTCTTCTACCTCTGTTTGTGATTGGATTGAAATCACTTGTAGCTCTCGCCATCA
ACGAGTGATTGCACTGAATATTTCAAACATGGGTTTATCGGGTACTATCCCGCCACAACTTGGCAACCTC
TCCTTTCTTGTTTCACTTGATCTAAGCAAAAATGATTTCTGTGGTGAACTTCCATCAGAATTTTCTCATTTA
CGGAGATTGAGAGTCATTAAACTTAGTTACATCAACTTCACCGGAGAAATTTCAATTGGAATAGCCCCTC
TTCCGAGTTTGATACGGTTTTCCATGGGATACAATAAATTACTCAATGGCTCCAATGTGCTCTCTAAATTC
AACATCTCAACCCTTGAATATTTGGATCTCAGAAATTCTGGTTTAACTGGTGATTTCCCTTCTGATTTATGT
CATCGTCTTCCTAGATTGCAAAAGCTTGCACTTGGCAACAACATGTTAAGCGGAGAGGTACCAAGAAAC
ATATCTGAATGCTCAGAGCTTCAACTCCTAATGTTGTTTCAAAATAACTTTGTTGGAACAATTCCAAGAGA

TATAGGTAACTTGCATCAGCTGCAACATTTAGATCTTGCAGATAACACGTAAAAGGTATGGCTTTACCAT
TTTTCTCGGGACATTAGATTTAACTTATTTAAAAGAAAATGTATTTTAGAGAGTTGAAGTGTTTGGACAA
TCTTTAAAAAAATGTGAGTATTTTTTTAATAGTAGCATAAGAAGATTTGTTTGTTTTCACTAATTGCTCCGT
CACTTCTCCTCTCGCTTTGTATACTTGTAGCCGGTTTGACCAATCTTCCGGCATACCCATTTGGAAGTTAA
GCTCTTGTTAAATATATGATTTTTATGTGATATTTCTGTTTAACAATATGACAGGCACAATTCCTGATGAG
ATCGGCCATCTTTATAACTTAAAGAACATATTCATGGATAAAAATTATTTAACAGGCTCAATCCCTTTAAC
CTTATTCAACATTTCATCACTTGAAATGCTATATATGAATGATAACAAGCTTGAAGGACCTTTACCAAGAC
AGGTTGGAAATTTTACTATGCTTGCCTGGTTTGATCTTTCCAATAATTACCTGGCAGGTACGGGGAAAAA
ATCTAATTCAAGTCATGTTAGAATTTTTTGTTTTTTGGGATACGTCTTCTGATCATAATTATACTGAATTTT
TCAGGTGTAATTCCACATGAAGTTGGTAACCTTCAAGAGTTGACAGACCTTCCATTGTTTTACAATGAATT
TAGTGGGTCGATCCCTATTGGTATCTTTAATATCTCATCTCTTGTAACTATTGGACTCACAGAAAACCACA
TTTCAGGTAATCTTCCATCCACTATAGGCAATGGGTTACCTAATGTTGAAAAAATATTTCTAGGTGGAAA
CAACATTAATGGTGTTTTACCTGGATCCATCTCAAATTTGTCTAAGCTCACCTTTCTCGAACTCTTTAGTAA
TGAATTTACTGGTTCAATTCCTGACTCTCTCGGAAATTTAAGACAACTTGAACTTCTCAACTTGTACGGTA
ACTCCTTTACAAGTGAATTGAGCTTCATTACTCCTTTGGCCAATTGTAAACACTTGAGAATATTGATATTG
TCTTTCAATCCTCTAAATTCAATGCTCCCGAGATCCATTGGCAACATCTCTTCTCTTCAATCATTTATTGCT
GTAGGCTGTAACCTCAAAGGCAATGTTCCAAGTGAGATCAGAAATATGAGAAATTTATCTACTTTGTCTC
TGGGAGGTAATAACCTTACTGGAATTGCCCCGACGACATTAAGTTCTTTGAAAAACCTGCAACGGCTTTT
TCTTGATGCAAACAGAATAACTGGTCCTTTCCCAATTGTTTTATGTGAGCTACCCCACTTGGGGATACTAA
GCCTTTCACAAAATAAAATTTGGGGAGATATTCCTAGTTGCTTGGGGAACATGACATCCCTAAGGGAGG
TTTATCTTGATTCCAATAACCTCACTGCTAGCATACCTTCAAGTCTATGGAACCTCAAAGACATTTTGAAG
ATGAACTTGTCTTCTAATTCCTTCATTGGATCTCTACCACTAGAAGTTTGAAACCTCAAAGCTGCACTACT
TCTGGATCTTTCCAGGAACCAAATATCAGGCAACCTTCCAAGTACATTGGGAGGTCTACAGAAATTGATT
CAACTATCTTTGGCTCATAACGGAATTGAAGGATCTATTCCTGAGACATTTGGGGAACTCCTAAGTTTGG
AAACATTAGATCTTTCATATAACAAAATGTCTGGTGTGATTCCAAAGTTATTAGAGGCACTTAAGCAGCT
AAACTCCTTTAATGTCTCATTCAATAGGTTAGATGGGGAAATTCCGAGTGGGGGACCTTTTCTTAATCTC
CCTTACCAGTCTTTCATGTCGAATAAAGGATTGTGTGGTAACCCTCAAAAGCATGTCCCAGCTTGTCCTTC
TTATTCAAAGAATTAGTCTAAGTCGAAGAAAAGAAATCTGATATGGATTATAGTTGCCTCTTCAGTTATC
TCTGTAATAGGGCTTGCTTCAGCAATAGTTTTCATGTTGATGAGACGCGGGGGTAAAACGATCAATGTT

GAAGTTGAGTGGTCTCCTGAGGTAGCACCACAAAGATTTTCTTACTATGAACTTCAAAGAGCAACTCAG
AGTTTTAATGAAAATAACTTGCTTGGTAGTGGAAGTTTTGGTTCTGTTTATAAAGGGATGTTGGCTGATG
GGATGATTATAGCTGTTAAAGTTTTCAATGTGCAGATGGAAGGAACATTTCAAACCTTTGATAGAGAGT
GTGAAATCTTGCGGAATCTTCGTCACAGAAATCTCACAAAGATCATTAGCAGCTGTTTTAACTTGGATTG
CTCCAGGTGAATTCTTTATTTAACTAGTAACAATGTTATGGTTTAATGCATTAAAAATGCTGACTTATTTC
CTTTTTTCTTTTTAGAGTATGGTCTGGAAGGCCTTATATCCAAGAGGTCTGACGTTTATAGTTTTGGCATC
ATGCTGTTGGAAACTTTTACCAAGAAGAAACCCAGTGATGAAAAGTTCGCAGGAGATTTGAATTTGAGA
TGCTGGGTGCATAATTCACTTCCTGATGAGTTGGATCAAATCATAGATGCTGACTTACTAACAGTCAATG
AACAAAACTTAAGTCAAAAGCTGCAATGTGTGTCATCCATTATGGAGTTAGCCATGAATTGCACAACTAG
TATTCCAGTTGAAAGGATGAACATGACTGATGTTGTAGAAACATTGAACAAGATCAAACAAAAGCTTTCT
TACTGTTATTGAACTACCTAACAACATGACATCAGGTGCTTTTCATATCCCTTCTGAAAAGAACGAGGTTA
TAAGTTCAGTAGCATTTGACCATCAGTAAGTGTTCCTTGTTATTGCAGGAGATTATGTGGCTATTGGAAG
TTAAAAGGATTGAAGTTCATATACTTCTTACGTCAAGAAGTTGCTATGATTTTGGGACTACGTACTATTAT
AAAAAGCCGATGATAGCTTGTGTACTTCTTCCTTTTTCTTGTGTAAACCAAGAGGGCCTTTCCATAACCTG
ATGTTCTAAGCACCCTTCCAATTTGATGATACAAGGAATGTAAGCCATTTAAATAAACTATGTTGAATCC
ATTGTATATATCTCAAGTTCTCCCAAGTATTCAACTGACTTAAAAAAATTTCTCCAAAGAATAGAGCAGCA
AGATATGCATATTCCTTGATAGTTTTCACTAGTTACTTTGACATCGGAGTTCCTTTCTGTTACTGATCTATC
CATATATATATAGTCAACTAGTCAAGGTTTTACCTTGTTATGGCATTGAACATGCATGAAGGAGAACTTT
CATACAGCCACAGATAAATGAAGGGTAAACAAGAAAATATCTTAGGCAGAAAGGGAGTTGAATTTGAA
GTTCTTGATTTGTACAGTCAAGGGTGTATGAACTGGGTGCCAAAGAGGGCAACTAGTGGGGCAAATAG
TGAGTCGAAAAAGGGTTGTAAATTTTCCAGAGTCAACTCAAATAGTTTATTTATTAATGACTGCATGAAG
CCTGGAATAAATTCATTAATTCAAATAGTTTAGGGTATTGATGCAAAGATCCAACCTAGGAAAGTTAACG
ACCTAGCTAGAGAGTCAACTCTTATTCCACTCTTTTGTATGTCTGTATACTGATAACCACAAACAAATTTT
CTATGCCAAAATCTAGATAGCTGTGTTTAGCAACATCTCACCATTAGCAAAGTTTCCTTCTCTTTAAGTAA
CAGATTAATTTAGTTCAATATATGGTGCAAAGTCAACTTCCTTAGTGGAGTAATATATTACTTAGGCCATA
AACTTATATTTTAATGTATTAGCATTTATTAGCTAGCTACCAGATCTTGAATAAATTTACTTGCACAATTAA
CGGACTAAATAGTTGCTAGAAGCATTTTACAATTCCAAGTATTATGTTGGTTTCAATAATATAGAACTAT
GTACTAGCATTATATTTTCGATTATAAATCTAATCAGTTTCAATAGGTATAGATCATTATATTGCTGCAAT
GATCAAACCAGAAAACTCATCAATGGAGAAAACATCCTCCTCCTCCTTTCCGCTTCTAGAAATTTTATTAA

TATCTTTTCATGTTATAACGACATGCTTAGCCATGAATATCACTATATCAGCACAGATCAATCTTCTCTTCT
AGCCTTGAGAGAACACATCACTTCAGATACTAATCACATTCTCTTGACAAACTGGTCTTTTTCTAACTCAG
TTTGTAACTGGATTGGAATCACTTGTAGCTCTCGTCATCACAGAGTTATTGAACTGAATATATCAGGTAT
GGATATTACTGGTACCATCCCACCGGAACTTGGCAACCTCTCTTTTCTTGTTTCACTTGATCTAAGCAAAA
ACAATTTCCATGGTGAACTTCCACCAGAGTTGCTTCGCGGAAGTTGAGAGACATTGATCTTAGTTACAAC
AACTTCACCGGAGAAATACCAAAAGGAATAGCCACTCTTCCAAATTTGAAATGGTTCATCTTGAGATGCA
ACAAATTACTCAATATCTCATCTCTTGTAGTTCTGGGAATCTTAGGAAACCACATTTCGGGTAGTATTCCT
TCCACTATAGGCAATGGCTTACCTAATATCGAAGGAATTTATCTAGTTGGAAATAACATCAATGGTGCTT
TACCTAGTTCCATCTCAAATTTGTCTAAGCTTGAATTTCTCGAACTAGGTGGAAATGAACTTACGGGTTCA
ATTCCCGAATCTCTAGGAAATTTAAGACTACTTAGAAATCTCAACTTGTACGGTAACTCCTTCACAAGTGA
ATTGAGCTTCATTACTCCTTTGGCCAATTGTGAAAACATGACAAGATTGTTATTGCCTTTCAATCCCCTAA
ATGCAATGCTTCCAAAATCTGTTGGCAATCTTTCTTCTCTTAATTATTTTTCGGCAATAAGCTGTAACCTCA
AGGGACAAATTCCAGATGAAATTGGAAATATGAGAAAGTTATCTACTTTGGAGTTGGATGACAATGACT
TAACTGGAATTATCCCGACAACGATAAATTCTTTGAAAAACCTTCAACGGCTTTCACTTGGTGCAAACAG
AATAAGTGGTCCTTTCCAAATTGTTTTATGTGAGCTACCCAACTTGGGCATGCTAAACCTTTCACAAAATC
AAATGTGGGGAAATATTCCTAGTTGCTTAGGGGATGTGACTTCTCTAAGGGAGATTTATCTTGATTCCAA
TAACTTCTCTGCTAGCATACCTTCAAGTCTATGGAACCTCAAAGACATTTTAAAGTTGAACTTGTCATCTA
ATTTCTTCAATGGTTCTCTTCCACTAGAAGTTGGAAACTTCGAGGCTGCAACTCTTCTGGATCTTTCGAGA
AACCATATCTCAGGCAACATTCCAAGTACATTGGGAGATCTACAGAAATTGACTCAACTATCTTTGGCTC
ATAACAGAATTGAAGGATCTATTCCTGACACATTTGGGGAACTCATAAATTTGGAAGCGATGGATCTCTC
GTATAACAAAATGTCTGGTGTGATTCCAAAGTCATTAGAAGAACTTAAGCAACTACGCTCCCTTAATGTC
TCATTCAACAGGTTACATGGAGAAATTCCGAGTGGAGGGCCATTTGTTAATCTCACTTACCAATCTTTCAT
GTCGAATGAAGGATTGTGTGGTAACTCTCAAGAGCATTTCCCAGCTTGTCCTTCTAATTCAAAGAATCAT
TCTGAGTCAAAGAAAAGAAGAATGATATGGATTGTAGTTGCCTTTTCAGTTATCTCTGTGATAGTGCTAA
CTTCAGCAATAGTTTTCGTGTTGATGAGACATCGGGGCATAACAGTCACTGCAGAAGACGAGGGATTGC
CTGAGGTAGTACCACAAAGAATATCTTACTATGAACTTCAAAGAGTGACTCAGGGCTTTGATGAAATTAA
CTTGCTAGGTAGTGGAAGTTTTGGTTCTGTTTACAAAGGGACATTGGCAGATGGGATGATTGTAGCTGT
TAAAGTTTTCAATGTGCAGATGGAAGGTACATTTCAAACCTTTGATAGAGAATGTGAACTCTTAAGGAAT
CTTCGTCACAGAAATCTCACAAAGATCGTTAGCAGCTGTAGTAACTTGGATTTCAAGGCATTGATACTTG

AGTACATGCCAAATGAGAGCTTAGACAAGTTGCTATATTCTAGAGATTTCTGTTCAAGTATGATGCAAAG
ATTGAATATCATGGTTGATATTGCATCTGCTCTGGAATATCTCCATCATGGTTACTCAGTAGGAGTTATTC
ATTGTGATCTGAAGCCTAGCAACGTGTTACTTGACAACGACATGGTGGGACACCTGACTGACTTTGGCA
TTGCGAAACTTTTAACCAAAGAAGAATCTATTGCTCACACTAGTTACATTGATCCAGGTGAATGCTTTATT
TAACTAGTATAACAGTGTTATGGTTTAATGTGTTAAAAATGCTGACATAATTTATTTTTTATTTTCTAGAGT
ATGGTCTGGAAGGCCTTATATCCAAGAGGTCTGATGTTTATAGTTTTGGTATCATGCTGTTGGAAACTTT
TACCAAGAAGAAACCCAGTGATGAAATGTTCGCGGGAGATTTGAATTTGAGAAGCTAG
>PGSC0003DMG400028474 LRR-RLKs
AACACATTTTCTCCCATTCACTCAGAAAAAAATGAGGATTTCATTAATGGAGAAAAATCCCTCTTTTCAGC
TTCTAACAATTTTGTTCGTTCTTCTTCATGCTCTAGCAACTTGCTTAGCCATGAATTTTAGAACTGATCAAA
CTTCTCTTCTCGCGTTGAAATCCCAAATCACTTCAGACCCTTATCAAATTCTTTCAACAAACTGGTCTTCTT
CTGCCTCTGTTTGTAACTGGATTGGTGTCACTTGTGGCTCTCGTCATCAACGAGTTACTTTACTCAATATT
TCAGACATGGGATTTTCGGGCACCATACCGTCACAACTTGGCAACCTCTCTTTTCTTGTTTCACTTGATCT
AAGCTACAACTATTTCCATGGTGAACTTCCACTTGAGTTTTCTCGTTTGCGAAAATTGAGAGCTATTAATC
TTAGTTTCAACAACTTCACCGGAGAAATCCCTAAATTCCTCGGTGATTTTCAAGACCTTCAAATATTGTCT
CTTGAAAACAATAGTTTTAGTGGGTTCATTCCTTCTTCTATCTCAAATATGAAGAATCTTGGATTCCTGAA
TCTGAGGTACAACAATTTAGAAGGAAATATTCCAGCTGGAATAGCCGCTCTTCGGAGTTTGAAATGGTT
GAGCTTTGGATTTAATAAACTCAATGGTTCCAATGTGCTCTCTATGTTCAACATCTCTACACTAGAATATT
TGGATCTCAGAAATGCTGGTTTAACTGGTGATTTTCCATCTGATTTGTGTGGTCGTCTTCCAAGATTGCAA
AAACTTGGACTTAATTTCAATATGTTAAGTGGAGAGATCCCAAGACGTATATCAGAATGCTCGCAACTTC
AAGTCTTACTGTTGATGGAAAATAACTTGATTGGAACAATTCCAGGAGAACTTGGGAAATTACAACTGC
TGCAACAATTAGCTCTCGGAAATAACAAGTTAGAAGGTATGCAGCTTCCAATCTTAACAATATCTACATA
TATAGTAATAAATCGAAGATATTGTTTTGTGCTGAGCACATGCATTCACTTTAATTTTCAGTTTCGACAAG
AGGTTAAGTGTTCAATTTTCCTCTCTTAAACAAGCATATGTATGGTTTTGACCTGGTGTGAGAATTTGTTA
CATCACCTTTGCTTATACCTGTCTGTTAAATTATTTAATGAGGTATTGAGATTGGTTCAACTTAATTCCTTA
AGACTTTCATTCGATAATTTTGCATAACAATTTGACAGGCACAATTCCCAATGAAATTGGTCATCTTCATA
ACTTGAAGCAATTGGGATTGGAACAAAATGCATTAACAGGCTCAATCCCATTAAGCATATTCAATATTTC
ATCACTTCAAGTTTTATCAATGTGGGACAACAAGCTTGAAGGGCCTCTACCAAGAGAGGTTGGAAATTT
GACTATGGTTAACGTACTTGATCTTGGAATGAATAGCTTGACGGGTATGGAATAATATTTTACCACAGGA

TTTCAGAATGCTAGTTCTTTCAATACTTCTTCTAATGGTAATCTTACTTCATTCTGCAGGCGTACTTCCAGA
TGAAATCGGTAACCTTCAAGAGTTGCTGATGCTGAAGTTGGATTTCAATAATTTTAGTGGGTCGATCCCT
ATTGGTATATTTAATATCTCAACTCTTGTATCTATTACACTCACGCAAAACCGCATTTCAGGTAATCTTCCT
AACACTATAGGCAGCGGGTCACCCAATCTTGAACGAATTTTTCTAGGTGCAAATAACATTGATGGTCTTT
TACCTAGTTCCATCTCAAATTTGTCTAAGCTTACTGTTCTCGAACTCAGTGCGAATGAACTCACGGGTTCA
ATTCCTGACTTTCTAGGAAACTTAAGACTTATTGAAATCCTCAACTTGCAAGGTAACTCCTTCACTAGTGA
CTCTTCAATGTTGAGTTTTATTACTCCTTTGGCCAATTGTAAATACTTAAGAGAACTGATATTGTCTATCAA
TCCTCTAAATGCAATACTTCCAAAATCCATAGGGAATCTTTCTTCTCTTCAAACGTTTGAGGCAATAGGTT
GTAATCTCAAAGGCCATATTCCAAATGAAATTGGAAATTTGAGAAATTTATCTTATTTGAAGCTGGATGA
AAATGACTTTACTGGAATTGTCCCTTCGACAATAAGTTCTTTGGAAAAACTTCAACAGTTTTCACTTAGTG
CAAACAGAATAAGTGGTCCTTTCCCAATTGTTTTATGTGAGCTACCCAACTTGGGCATGCTAAACCTTTCA
CAAAACCAAATGTGGGGAAGTATTCCTAGTTGCTTAGGAGATGTGACTTCTCTAAGGGAGATTTATCTTG
ATTCCAATAACTTTACTGCTAGCATACCATCAAGTCTATGGAACCTCAAAGACATTTTGAAGCTGAACTTG
TCTTCTAATTTCTTCAATGGCTCTCTACCATTAGAAGTTGGAAATCTCAAGGCTACAATACTTCTGGATCTT
TCCAGGAACCAAATCTCAGGCAACATTCCTGGTACATTGGGAGGTTTACAGAAATTGATCCAGCTATCTT
TGGCTCATAACAGAATTGAAGGATCTATTCCTGAGACATTTGGGGAACTCATAAGTTTGGAAGCATTGG
ATCTTTCAAATAACAATATTTCCGGTGTAATTCCAAAGTCATTAGAGGCACTCAAGCAACTACAGTCCTTT
AATGTCTCATTCAACAGGTTACACGGGGAAATTCCGAGTGGTGGACCTTTTCTTAATCTCCCTTACCAGTC
TTTCTTGTCGAATGAAGGATTGTGTGGTAATCCTCAAAAGCATGTCCCAGCTTGTCATTCAAATTCAAAG
AATCATTCTAATTCAAAGAAAAGAAGAATGATATGGATTGTAGTTGTCTCTTCAGTTATCTCTATAATAG
GGCTTGCTTCAGCAATAATTTTCGTGTTGATGCGACATCGGGGTAAAGTAATCAAAGGTGAAGATGAGT
GGTCGCCTGAGGTAACACCACAGAGATTTTCTTATTATGAACTTCAAAGAGCAACTCAGGGGTTTGATG
GAAATAACTTGCTAGGTAGTGGAAGTTTTGGTTCTGTTTTTAAAGGGACATTGGCAGATGGGATGATAC
TAGCTGTCAAAGTTTTTAATGTGCAGATGGAAGGTACATTTCAAACCTTTGATAGAGAATGTGAAATCTT
GAGGAATCTTCGTCACAGAAATCTCACAAAGATCATTAGCAGCTGCTGTAACTTGGATTTTAAAGCATTG
GTACTTGAGTACATGCCAAATGGGAGCTTAGACAAGTTGCTATATTCTCAAGATTATTCTCTAAATATAA
TGCAGCGATTGAATATCATGGTCGATGTTGCATCTGCTCTGGAATATCTCCATCATGGTTACTCTGTACCT
GTTATTCATTGTGATCTGAAGCCTAGCAATGTCTTACTTGACAAAGACATGGTGGGACACCTAACTGACT
TTGGCATTGCAAAACTTCTAACAAAAGAGGAATCTATTGCTCAAACTACAACCTTTGCCACAATTGGTTA

CATTGCTCCAGGTGAATCATTTATTTAACTTGTAACAAAGTTTTTGATTAACAGTTAATCAGATTTACGCC
TTTTAAGTTCAGTTGCTGACGTATTTTAATTTTTCTTTTCTAGAATATGGATTGGAAGGCCTTATATCCAA
GAGGTCTGATGTTTATAGTTATGGTATCATGTTGCTGGAAACTTTTACAAAGAAGAAACCTAATGATGAA
ATGTTTACGGGCGATTTGGATTTGAAAAGCTGGGTACATAGTTCACTTCCTGATAAGCTGGATGAAATCA
TAGATGCCGACTTACTAACAGTAGATGAACAAAAGCTAAATGAAAAGTTACAGTATGTGTCATCTATCAT
GGAGTTAGCCATGAATTGCACAGCTAAATCTCCGGCTGAAAGGATGAACATGACTGATGTTGTAGCTGC
ATTGGAAAAGATCAAGCAGCAGCTTTCTTCATGTTACTGATCGAATGGCTTAATGACATGACATCAGGTA
CTTTTTCCATATCACTTTTGGGAAGTAAAAAGGAAATATGTAAATTTCATTAGCATTTTAATCAGTAATTG
CTCTTCAAATGCTTTTGCAAGAGACTATGTGGCTATTGTAACTGTCCTTTGTTGAGATGAGTCTTCTGTAC
AATGTATTGCTAATTAAGGATCAATAAAATGCAGTGCTTCTTAATTTTAAGTGAAGTGATCCCCATCTGG
CCTTATAAATATCATCCAAAAGAGTCATTTGCCCTTGCTTTAATCTTGTATCCAGTGAAAATGATAAGTGC
TAACACTCCTAGATGTTTTGATGATCTCCTCACAAGTGCAGGGACCTGGTCCTCTGCAGAGTATGAATAC
TCGTCCAGTGGTGAAAGTGTTGAACAGCTGTAAAAGTTGTTCGTGTCAGAAAGTTGGTGAAGTTGCAGA
GTACTTCACCAATCAGAATGGGCGTAGTTGTTTGTACATTGGACAATGGCTTTTCATGGTTGATATATTC
AACACTCAAACAACAAATTCATTATTCATTCAAAAAGAGATTTTTCAAGCTTCAAAAACAGCAGGGACCT
GATCGATTGCTTAAAGTGCTTCCTGTATCAGTTGTTACTTTGTTTGCTTGTAATAGTTCTTATATGTATGAT
TCGTTTCACTTGTGAAAGAAACGTTTCAAACTTGTGTAAATCATTGTAAGAACTAGGTTAAGGGTAACTT
AGTTTCTTACTCTAAAGTCTATATTAATCAGTTAGGATTAGTATAGTGGGCAGTGTTGTATTTGCTCTGGC
TCAGCTAAATGATAGGAGATATTGCTTAGTGTGGAGATTAGCAGGCTAATCTTCATTTTGTAAACTGGCT
TTTACTTTTGCTTGGAGAAGATTAGTGGAAGCAGTTTGAAAAGTCCTATTGAGAGCAGGTCGTGGTTTTA
CTCCCTTGAGCAAGGAGGTTTCCACGTAAAGTTGTTTGTGCAATCTTTACTTTCAGCATTTATTTTACTGTT
TGTTATTGTAATTGTGCTGAGGACTTGGTCCCATCAAAATCGGTG
>PGSC0003DMG400028478 LRR-RLKs
CAATTATATTGGACTTACTTACCTCCACTTGAAATCCCCTTAATACATCTACTCTTATCAACTCTATATGGC
ATTTACATCAACTACTTCCAACTGAAAGAAACAATCACAAGTCCCACAAACAAAAAATGAAAATCTTGTT
CACACTCTTCATCTTTCTTTCTCTTTTCACATTCTCAAAATCAGAATCCCCTTGCAAAAACAACACAGATTT
AGATTTATATCTTGTTTCCAAGGCTTTCAGTTCTGTTTATGGTTTCAACATCTCTTGGTTCAACTCAAACTG
TTCTTCTCCAATAACTGAAATAAACTTATCTTCAAGAAACCTCACTGGCATAGTTTCATGGAAGTACTTAA
CAAATTTGTCTCATCTTCATACTATAGATCTATCAAATAATTCCTTAAAGGGTTCTGTTCATCCTCTGTTTT

GGTCCATTTCATCTTTGGTTCAAGTCAATTTGTCAAAGAATAAGCTAGGTGGGGCTGTTGCTGTAGCAAG
ATCATCATCATCTATTCAAAGACTTGATCTTTCTTTCAACAGGTTTACCAACTTAGGCTCTGTATTTTATGG
TTTTCCTAATCTTACTTCTCTTGACCTTTCACATAATGATATCAAGTCTTTACCTTTTTGGTTTACCAACCTA
ACTAAGCTTGAAAATTTGAGCATTTCTAGCTGTAACATTTATGGTAATCCAGAACCCCTTTCACATATCAA
GTCACTGAAGCATTTAGATGTTTCTGTCAACCATATGGATGGTAAATTTCCTAATGATTTTCCTCCTCTGT
CTAGTCTCAATTTCTTGAATATCTCATTCAATAACTTCACTGGGGAAATACCCAAAGACCAGTTTGCTAAA
TTTGGGAACTCTTCTTTCATTCATGCTGGGCACTTGCAAATCAAGAATCTCTTACCAAATCCCAAGAATTC
CTCACAGTTACACATTAAAAATCATAACTTTACTACCCCAACACACAAAAAAATGTTGCCTTCGAGACACA
AACCTATCAAACCAAACACACACAAGAAAAGGCCCAAATCAAGAAAGAAAGTTTTAACCATAGCTATTT
CCTCTGCTTCAGCATTTCTAATCTTGGCCATGGGGTTAGTCATTTTGTGTCTATACAAGAGGAGAAAAAC
ATTGGCTAGGAAGAACAAATGGCTAATTTCAAAGCCAATACAAATACCATTTAGAATGGACAAATCAGG
GCCATTTTCATTTGAAACAGAGTCAGGGAATTCATGGGTTGCTGACATAAAAGAACCAAGTTCAGCAGC
TGTAGTTATGTTTGAGAAGCCATTGATGAACCTAACATTCAAAGACCTCATAGCAGCCACAACTCACTTT
GGAAAAGAGTCATTGTTAGCTGAAGGAAGGTGTGGGCCCGTTTACAGAGCCGTACTTCCAGGTGACCTC
CACGTGGCAATCAAGGTCTTGGAACATGCTAGGGAACTAGCCCATGATGATGCTATTATCTTGTTTGAAC
AACTCTCCAGGCTTAAGCATCCTAATCTGTTGCCTATTTCTGGTTACTGCATTGCAGGTACTAATCTTCTTT
CACTTATTACAGCATCTTCTGTTAATAAGTACAATTTATGTGATACAATTCGAATTCTAGCAGTAAAAAAT
ATCTAGAGGTGAGCATATTGAATGCATTCACGTGGGAGGATGGGATGAGAAATAGGACAGACAGTAGT
AAATGAGACAGAAATCTTTGCAACCATAGAAACGTAGGCAGTAGTTATGATTACATACAACAAGATGAA
GCAGTAAATAGGAATGCGTGACTTATTTGGATGTTAGTACTATGACTACATCATTCAAATTATATGTGCT
TTTAATGCTCTTTGTAAATTGTGTCCATTTTTTACCCTACAAAGTCAAACTTTCAAATCTTATGCCAGATTG
GGATTTGAAAAATACTGAAACTGCAGTATGGTTTGGTTCGCAGAATTACGATAACCAATGATATTCTATG
AATTGAGATATTTCACGACATTTATTTATCGTGAAATTAAAATGGTAGAATAAAAAAAAATTGTGATTAA
TTAATATCTCAAACTAAACATGGAAAAAATTAATTTTTAATTTTATTTCGAGATTATTATTTGTATCACATA
TATCACCACGACATTTATGATGATGCTCTTGGTAAATTGTGTCCATTTTGCACGATAAGGTCAAATCTTTA
AAAATCTTACCAGATTGGGAGTTCTACTGTTCAGCCTTGATTTATGATTTTTGGAAGAATGTTGCACAAAT
ATTATCCATAAAGTATTATTTTATGGAGGCGTTATTGAATTAAAAAGATAAGAGGATATTTTAAAAGCAT
TAATGGTTCAGAAGAATGTCATTTTCAATTGAAAATGCTTAAAGTTGGAAAATTATTTCATGACATGCAT
TGATTGATCAATTGTAGTATCAAAGTCCTTGTCAAATATTTTTGGAAGACTTTTCTAGCTAGGAACCAAAT

TGCTCCATTTGATAAGAACAGAATCCAATTATATTTTCTTTCAATTAAGTCATATTTAAAGCTAATTTAATA
CTTCCCTTTGATGCAGGGAAAGAGAAGCTAGTCTTATACGAATTCATATCCAATGGTGACCTCCACCGAT
GGCTACACGAGCTTCCAACTGCTACCACCAACGTGGAAGATTGGACAACCGACACGTGGGAGTTACAAA
ACGGTTCTCAAATTACGTCACCCGGGAAAATGGAATGGCACACGCGCCACCGTATTGCCGTTGGCGTAG
CGCGTGGACTAGCCTACCTCCACCACGCGCAGTCGAAACCTGTGGTCCACGGTCATTTGGTGCTATCTAA
TGTCTTACTAGCCGATGACTTTGAGCCTCGGATAGCCGATTTTGGATTGAGCCAAAACCAAGCCGAGGG
ATCTAGTGAGACGGATGTGTACGATTTTGGAGTCGTATTAGTAGAGCTATTAACAGGGAAAATCGGCTC
GGATGAGACGATTAAATGGGTGAGGAGGCTAGTGAAGGATGGACTTGGAGCTGACGCGCTTGATTCG
AGACTAAGACTCGGTGGTGACTCAGTGAGTGAGATGGTGGAGTGCCTCCGAGTTGGGTACTTGTGTAC
GGCGGAGACCCCAAATAAACGGCCTAGAATGCAACAAGTTTTAGGTTTGCTAAAAGACATACAGCCCCA
CTACCAACTCGGGAAAACGCAAAATTTATAATCTTTAGGTAAACCACTCTCACTCCAGCGCGTGATTTAC
ACGACGAGGAAGATTGATTGGCAGCATTGCATTATTGTACTCCCATGTGTTTGGTTGACCATTGAAAAGT
CAAAAGTACAAGGCATCTATTCCACTTCATCAATGGTATTTAGTCACGTGCAGATCACGTGATGAAGTGC
AGTGAGAGGAACATGTTTGCTTGCTTGTCTCATTTTATTCTCTCTTTTTTTTTTTTTTTTTGTAGTTTTGTA
TAGTATTAAATTAGAAAAATGAAAAAAAATTGTGGGTAAATCTTTTTTTTTTATATATAAA
>PGSC0003DMG400028688 LRR-RLKs
TTTTGCTTCTCTATACAAACTCTCCTCATTCTTAGCTTTCCAGTTTTCTGTGGGCCCTTTTTCTCATGTGCTT
CCTTCTTCTTCCTACTCTCAACTCTGCAAAAAACCCTACCACATTGCACAATGTTTCTCTTCTAATCTTGAA
AAAAGATTGAATTTTTGCTATAAAGAACAATTGGTTTCTTGTTTTTCTTCATTGTCTTGTCAAAAAGTTTCA
ATCTTTGCAAAAGAAATGAACACCCTTCTTGTGTTAAAGCTTGTATCTTTACTTGTTCTATAAAGAACACT
TGGGTTCTTGTTTTGTCAAAAAGTTTCAATCTTTACAAAAGAAATGAGCACCCTTCTTGTGTTAAAGCTTG
TATCTTTACTTGTTCTGTAAAGAACACTTGGGTTCTTGTTTTTTATTTCATTGTGTTGTCAAAAAGTTTCAA
TCTTTACAAAAGAAAACACCCATCTTGGTTTAAAGCTTGTAGCTTTACTTGTTCTATAAAGAACACTTGGG
TTCTTGTGTTTTTTTCATTGTCTTATCAAAAAGTTTCAATCTTTACAAAAGAAATGAACACCCTTCTTGGTT
TAAAGCTTGTAGCTTTACTTGTTCTATAAAGAATACTTGGGTTCGTGTGTTTTTTTCATTGTCTTGTCAAAA
AGTTTCAATCTTTACAAAAGAAAACACCCATCTTGGTTTAAAGCTTGTAGCTTTACTTGTTCTATAAAGAA
TACTTGGGTTCGTGTGTTTTTTTCATTGTCTTGTCAAAAAGTTTCAATCTTTACAAAAGAAATGAACACCC
TTCTTGGTTTAAAGCTTGTATCTTTACTTGTTCTTGGTATTTCTGCATTTTTTTTGGGTGTTGAGTCTCAAG
ATGATGATGTTTCAGTGATGTTAGCTTTGAAAAAGAGCTTAAATCCACCTCAAGAAGTTGGATGGTCAG

ACTCGGATCCATGCAAATGGAACCATGTGGGTTGTTCAGATAAACGGGTCACCCGGATCCAAATTGGGC
GCCAGAACATTCAAGGTACTCTCCCTCCAGAAATCTCTAAACTTACTGAACTTGAACGTTTAGAGCTTCA
AGGGAACAATATTTCTGGGCCTTTGCCTAGTTTGAAAGGTTTGAGTTCATTGCAAGTGTTACTGCTTGGT
GAAAATCAGTTTAGTTCAATTCCTGCTGATTTCTTTACTGATATGTCTTCATTACTATCTGTTGATATGGAT
AAGAACCCTTTTGTTGGTTGGGAGATCCCTGAAAGCCTTAGAAATGCTTCTTCACTTAAAAACTTTTCAGC
AAATTCAGCAAATGTAATTGGGAGAATTCCTAACTTTTTTAGTCCTGATGAGTTTCCTGGATTGGTAAATT
TACATTTGGCTGGCAATAACTTGGAAGGTGAATTGCCCTCCAGTTTTTCTGGCTTGCTGCTTGAGTCTTTA
TGGTTAAATGGACAGAAGCTTAATGGAGGAATTGATGTTCTAACAAACATGACATTCCTGAAGGAGGTT
TGGTTGCATTCCAATAACTTTTCGGGGCCGTTGCCGGACTTTTCAGGATTAAAGGCTTTGGAGACATTGA
GTCTTAGAGATAATGCCTTTACTGGTCCCGTGCCAAGTTCTTTGATGAATCTTGAGTCTTTGAAGCTCGTT
AATTTGACGAATAATTTCTTTCAAGGACCAATGCCGGTGTTCAAAGGTTCTGTTGTTGTTGATTCGGCAA
AAGGTACAAATAGCTTTTGCTCGTCACAACCAGGTGATTGTGATCCTAGAGTAAATACATTGCTTTCAAT
TGCAAAAGCAATGGATTATCCAACAAATTTTGCGAAGAACTGGAAGGGAAATGATCCCTGTGCTGATTG
GTTTGGTCTTACTTGCAGTAATGGTAACATTACTGTCATAAATTTCCAGAAAATGGGGCTTTCAGGAACA
ATTTCTCCTGAATTTGCTTCACTGAAGTCGCTACAAAAAATAGTTCTTGCAGATAATAATCTGACTGGTAC
AATTCCAGAAGAGCTAACCACATTGACAGGCCTTACAGAGTTAGATGTGTCAAACAATCAAATTTATGG
AAAGGTACCAGCTTTTAGGAAAAATTTGATTCTGAAATTTAGTGGTAACCCTGATATTGGAAAGGATAA
GAGTGATGCACCTTCCCAAGGCAGTTCTCCAGGTGGTTCAACAGGCTCTGATGATGGAAATGCCCAAGC
AGCTCGTAAGAAGTCCAATCGCCGGGTTGGAATTGTGGTGTTTTCTGTAATTGGAGGTGTATTTATGCTT
TGTTTGATTGGAGTGGCAGCTTTCTGTCTGTACAAAAGCAAACAAAAGCGTTTTAGCCGAGTGCAAAGT
CCAAACACCATGGTGTTGCATCCACGTCATTCTGGTTCTGACAATGATAGTGTGAAAATCACAGTTGCAG
GATCTAGTGTCAGTGTTGGTGCAGTTACTGAAACTCATACTGTTTCTGCTAGTGAAGCCGGTGATGTTCA
AATGGTTGAAGCAGGAAATATGGTGATTTCCATCCAAGTACTGAAGAATGTGACCAACAATTTTAGTGA
AGACAACATACTAGGCCGGGGAGGCTTTGGCACTGTCTACAAAGGTGAATTGCATGATGGGACTAAAA
TTGCAGTCAAAAGAATGGAAAATGGAATCATCACTGGGAAGGGACTAGCTGAATTCAAATCCGAGATT
GCTGTTTTGACCAAGGTCAGACATAGGCATCTTGTTGGACTACTTGGGTACTGTCTTGATGGAAATGAG
AAACTTCTAGTCTATGAATACATGCCTCAAGGAACATTAAGCAGTCATCTCTTCAACTGGGCAGAAGAAG
GGCTTAAACCCATGGAATGGACAAAACGATTGACCATTGCATTAGATGTTGCTAGGGGTGTAGAGTACC
TCCATAGTTTAGCTCACCAGAGTTTCATTCACAGGGATCTGAAGCCTTCAAATATTCTTCTAGGGGATGA

TATGAGGGCTAAAGTTGCAGATTTTGGCCTTGTCAGACTTGCCCCAGAAGGGAAAGGTTCTATAGAGAC

AAGGATTGCTGGGACATTTGGATATCTGGCACCGGAATATGCAGGTATGCTCTTTGCTTACTTATATGTG

TATGCTAAATTGCTGATAAAATACTTGTATGCAATTTGTGCCTAGATAAACTTGGTCTTAAGTCCAATACC

TCTAAAGTATGAATTATGTGAACCTTAGGGGTTTGATCTCAGGATGCTTCCAGAGAATTAGCATTCATTT

GACTGATGAGAGTCAGATGATACTAACTGTACTCCAAGAAACTTAGCCTGTTTTACATTAATCAACTTTT

GCCCTATAATAGGTCTGTAACAGTATATATTAATGTTTTGTCTTTTTTCTCAGTCAATTATACTTGTGCTCA

TGGTTAAGAACCTGCCATTCATACATTGTCATATATCAAATATCCTTATTGATTCTGTCATCCCCTTTAATG

CTATGATTACTTAGTTTCTAAAAGTGGATACCCATCCTACTGGAAACACTTTTTCCTGATGCATTTGCTTA

ATGCCACCGTTTTCTACTTTGGCCTCATCACACACTTTAAACATGTTATTATTCCTATGACAGAAGATCCTC

TTGTTTTCTTTTCCCCTTAGTGGAATTTAAGGATAGCCAAATTTGTTATTTGCTCACTTTCCTGATTGTGAT

TTGTGCATAGGAGTTCTTGGAATGTAGATACATCATTATCTAAACTTGTATTTTATCTTGTTTCGTCTGCC

AGTAACTGGGAGAGTGACAACCAAGGTGGATGTGTTCAGTTTTGGTGTGATTTTGATGGAGCTCATCAC

AGGAAGAAAGGCTCTTGATGAAAGCCAGCCTGAGGAGAGTATGCATCTAGTAACATGGTTCCGAAGAA

TGCATCTGAATAAGGATACATTCAGAAAGGCAATTGACCCCGCAATTGACCTTAGTGAGGAAACACTCA

CCAGTGTCAGCACTGTAGCTGAGCTAGCTGGTCACTGTTCTGCAAGGGAGCCATATCAAAGGCCTGACA

TGGGCCATGCTGTCAATGTTCTTTCATCTCTTGTAGAGCTCTGGAAACCATCCGACGAGTGCTCAGAGGA

CATATACGGCATTGATCTCGATATGTCATTACCCCAAGCGCTTAAAAAGTGGCAGGCTTATGAAGGTACA

AGTCACATGGATTCATCCTCCTCTTCTTACCTTCCAAGCTTGGATAACACTCAAACTAGCATACCTACTCG

CCCCTACGGATTTGCCGAGTCATTTACATCTTCTGATGGTAGGTGAGAAGTTGACACTCTTTGATCTAGG

TTTGGTTGTTCATTCTCTCTGTTTTCTTCCTCATCGCGAGTCACACATCATTTTTTTCTTCTTTTTTTTTGCTT

TTAACTCTGATGCTTTTACCTTAACTCATTGTTAGTTTTGGCACTGTGGTGAATAGGGATTCTTTTTAAGA

TTTTTGATGTCTTTTGACTCTTATTGTATGATGAATCTCTCTCTCTTTTCCATGTGAATACATTGTGGTTTG

GTTGGAAATGAAAGGGATGCCATCAATTACCAGTAGATCATGGTACTTTTGTATTCTTGAATTGTTTTTTT

GTTAGAAAAAATTTGTAGGTGCAAGCTGTATTGAAATGTAAAGTGGCTTTGAAGTGTGCAAATCATAAAG

TTAGCTTTTTGCTTATGATGCTTTTTCAAGCAAAATGTAAAGTTTTTTGTTATGATTACAGATCAACTGCTG

>PGSC0003DMG400028801 LRR-RLKs

AACACATCCTAGAACTACTAAGTACACACTTTTCATCACTCCTACAAAGAACACCATTCTTTCTTCTTACTT

CTGCTACTACATTTCTCTCTGCTATGAATTTCTAGCAGTTCTTGGTTCTTGCGCCATATTCAGCTAAAACAA

GTAGGCACTGCCCATTTCAGTTGGCTAGTTCAATTTCAGTTTCACTGTCAAATTTGACAATGGTGTTGTTA

ATCATCTTCATAATTCATGTTTTCATGTTTGTAACAGCTTCGTCGGATCTTAGCTCCGACCGTGCTGCACTT
TTAGCTTTTCGCTCCGCCGTGGGTGGCCGTACATTTCTCTGGAACACCACCAGTACCTCCCCATGCAACTG
GGCTGGTGTACAATGTGAAAACAACCGTGTTACAGTTCTCCGGCTACCGGCGTCAGCTCTCTCCGGTACC
CTTCCGGTGAACACAATTTCCAACCTCACTCGTCTTCGTACACTTAGTCTTCGTCTCAATCGCCTTTCGGGT
CCTCTCCCTTCTGATCTCTCAAATTGTGTTGAGCTCCGTAACATTTATCTCCAGGGTAACTTTTTTACCGGA
GCTGTTTCTTCTTCCTTCTCTGGGCTTCACTCTTTAGTTCGGTTAAATCTCGCCGAAAACAATTTTTCTGGA
GAAATTCCATCTGGGTTTAACAGTTTGATCCGATTGAGAACATTTCTACTTGAGAAAAACCAGTTTTCTG
GGTTTATGCCTGAGCTAAAGTTTTTCCCAAATCTCGAACAGTTTAATGTGTCTTTCAATAGATTAAATGGG
TCTATTCCGAAAAGTTTGGAGGTAATGCCAGTGAGTTCTTTTACTGGGAATTCTCTATGTGGAAAGCCTA
TTAATGTTTGCCCAGGAAGTAAGACTCAGCCTGCTATAGCTACTGATGGAATCGAAATTGGGAATTCAA
ATAACAAGAAGAAGAAGCTATCTGGTGGAGCTATTTCCGGTATTGTCATTGGGTCTGTTGCTGGATTTTT
CATTCTTTTGTTGATTTTGTTTGTGTTAGGTAGGATGAAAACCGGTGATAAAACAAGGTCTCTTGATGTT
GAGACAATTAAGTCACCGGAAACAGAAGTTCCCGGGGAGAAACAAATTGAGAAACCCGATAATGGAGG
TGTAAATAATGGGAATTCAGTTGCTGTAGCAGCACCTGCTGCTGCAGTATTGAATAGTGGTGAAGAGAA
TTGGGGAGAAAATGGGGTTAGGAAAAAGCTTGTGTTTTTTGGAGATTATTATAAAGCATTTGAATTGGA
GGATTTGTTGAGAGCATCTGCTGAGGTTCTTGGAAAGGGGACATTTGGAACAGCTTATAAGGCAGTTTT
GGAGATTGGTACAATTGTTGCGGTTAAGAGATTGAAAGATGTGGCAATATCTGAAAGGGAGTGCAAGG
AGAAAATTGAAGCAGTTGGAGCAATGAATCATGAGAATTTGGTGCCTCTCAGAGCTTATTATTTCAGCA
GGGAGGAAAAACTTCTTGTCTTTGACTATATGCCAATGGGAAGCTTGTCTGCTCTTCTTCATGGTAAGTT
TTAGTACACTCTTTTACTGTCAATTCATGTTGTTCGGACTCTCTAAAAATGTTACCACATCTGTGCGAAATC
ATCAAAAAATGCACTACTTTGGAGGATTCGACACACACTCAGTGGTATGGATCCTCTAACAATACACTAC
CTTTTTGGAGGATCTCACATGCACTTAGCAGCATGGGTCCTCAAAATATGCACTACCTTTGGAGGATCCA
ACACTCAGTGGCATGGATTCTCAAAAAAAAATTCACTATTTTTGGAGGGTCCGACATGTATTGGTCGATA
TTTTTAATGAGTTCAAGCAACATAGCTACCAATATCTGGTGTTTTACTTGCTTTTCCCTTATTCAGTAGATG
CACTATATTTGTTCCAGATTTTGACATTTTGTTAGAGTAATTAGTTTGACTTGTTGCTAACAGTGTAACAT
GCAATGTCTGTAGTATTTGGCCCCTCAATCCAATGAAAATGTGATCCTAGAATTAAGCATTAATTTCTTAT
CATTACCTTAGTCTTCATTATGTAAAGCATGCTTCTAATATAAGAAAGTATGTTAAATCTAAAGTTGTTTG
TAATTAAGAGCAATAAGTAGTACTCCATTTCATTTAGTGCTTTCATTATAGCAAAAGGGTCCTTTGTTCTT
TATAGATACCCTGTCCATATGCAGGGGAAAGGTGAACAAGCACACATATAATCTTTCACTTGGCCCTACA

GTAGTAGGGTCTATCATTATATGTTCTTTTTCTATCTAGAGCATCTGTATAGGACTAGAATTTTGAATCTC
CGAAGCTAATTATTCAACTGGTGATTAGTTTCCTGGCTTGTGAATCTTGCATGAACATGTACATTTTTCGC
CTTTGGTATACTTGAAACTTGTTAAAGTGACTAAGAAGCTGTAATGAGCTGTATGCTGCAACTCTACTTTC
TATTCACTACAATTTCTTTAATTTACTAGGAAGCAAGGGGGCTGGCAGAACACCATTGAATTGGGAAATC
AGGTCCAATATTGCCCTCGGAATTGCACGTGGCATCGAATACCTTCACTCTCAAGGTCCTGATGTCTCCC
ATGGGAATATCAAATCATCCAATGTTCTTCTCACCAAATCATACGAAGCACGTGTCTCAGATTTTGGCCT
GGCTAACTTGGTTGGATCGCCATCCTCCCCTACTCGAGTTGTTGGATATCGTGCTCCAGAGGTAACTGAT
CCTCGCAAGGTTTCCCAAAAGGCTGATGTCTATAACTTTGGAGTATTACTCTTGGAGCTTCTTACTGGAA
AAGCACCTAGTCATGCCCTCTTAAATGAAGAAGGTGTTGATTTGCCAAGGTGGGTTCAATCTGTCGTTCG
TGAAGAATGGCCTTCTGAAGTTTTTGATATTGAGCTCCTCAGGTACCAGACTGCTGAAGAAGAGATGGT
CCAACTCTTGCAGCTAGCAATAAATTGTACAGCTCAATATCCTGATAAGCGACCTTCAATGGCTGAGATC
TCTAAACAGATTGAAGAGCTTCGACGCCCAACCTCACATGATAAACAAGAACTCTCTGATCTTGCCAATG
CAACAGAGTGAAGTGGTCATTATTAGTCCCCCTCGAGATGTGCAGTAATTAAGCTAAACTCAGATTCATC
ACCAATCTTTTTTAAGCATATTAAACTTGTTGTTTGTTGGGGGAGGGGTGTCTCATCTTCTTTCAGTTTGA
ATTAACTGATCATGGCCCCTTTTTCTCTATGTTTCTGTAGTAGACTAGTGGTTACCCCTTTCATTTTCCTTTA
GATGAGGGGCCAAAGCCAATATCTGTACCATTTATTCTTGTTGGATGTATAATGCTTTCTGTAATTATTAC
TGCTTCTGCTTTTACTTTTGCTTTTTCAGTTGCAACTGTCGATGGCTATTCTGTGTGTATATGAGGCATGCA
TGCATGTGGTTCTTGATATGCTGAAGCCAGCAGGGAGATTGCATTGAGAGTTTTCATAGATGCTGAGTT
GGCCAAATATTG
>PGSC0003DMG400028845 LRR-RLKs
TGTGTGGCTTTGGATTCTTGTAGCTGAATAGTGGTTTTTCTTTAGAATGGCAAAAGCATCTGACCCTTTTC
TTCTTTTAGTGTCTTTTTCTCACTCTAAAAAGAGTCTTTCTGTGTTGTTGGTGTGGGAGAGATGAGGTTGT
TGTTGCTACTGGCAGTAGTACTGTTGGTGGCCATTTTGGCGAATTTTTCAAGCTTTGTTAATGGTGATAC
AGATCCATCTGATGGTTAGCCCCTTTTGATTTATTCATCATTCTCTTTATTTATTTCTTTTTTAGTATGTGAA
GTAGTAAGTACAAGTTCCAGTTTTCTTGTTCTCAAGGAAAAATTTAGCTGAGAGTGGGCTTGAGTCTTGA
CAACTCATGAAATGAACAAACCCAACTGAAAAAACTTGTTTCTTTGAAGCCCCATGATATTGAAAAGAAA
ATAGCATTTGAAAACACTAGGTCTAGGCTTGGTAGATAGAGTTATGTGGTACATGTTGTTGGTATATATC
CTTGGAATTAGTTAGTTGCACACAAGCTGGCTCGGACACTGTGGTTATCATAAAAAAGAAATCTTTGAAA
ATGATGGATATAATAAGTCTTGCTTGGGCTGCATCAATGGTAGTCTTCACTTTCGAGTATGGGAAATACT

CATTACTTATGGCTGAACAACCCCTTAGTCTTGTCGACTAGATTGAGTCATAGTTATTGTAATTGTTGAAT
TTACTGTTGCCGATGCACTTAATAGTTACAACAACATATCCAATGAAATCTTGTAGCTGGGGTTTGGGAG
GGTAGAGTGTATGCAGACCTTACCCTACCTCGTGGAGATAGAGGGGAGACTCTTTCGGGAAGATGCACT
TAATAGCTAATAGAAAACGTCAACCTTCTCACAATAATTTTCTTTCATTAAAAATTGAACAAGTCTGGGTA
AAAGGTAAATCCTTACAGAAACTTGTGGTTTGTGAATGTGATTGGTTTTAGTTACTATTGCCAGCCTTATA
TAAGCAATTACTGGATAATGTTTTCTACTTTATGTGGTAAGGGGTGACAAACACCAATGAGTTTGAAGTT
GATCAAAACCTTAATAAAGCTATATAATTTACTTTTGGACTCCTGATGATCCAAAGAGTTTCACAGTAATG
GTTTGGTTAAGTTGCTCGCTACTTTGTACCATGCTGGTCAAGTTGCCGTAGCTGCACATTCTTTTAACAGA
TTGTCCTATGTAAAATCTGTCTCTTGAGCCGTTCTCTGTCCATATATAACTATTTCTTTATCATTAAAATTCC
TTACACAATCACATATGTCTTGATTTTAAATTAATCTAGTTGACTTCTGTTTGGCTTTCAGCATCTGTTCTG
AGAGTCCTGTATACCACCCTAAACTCACCAGGGCAGCTGACAAAATGGAATGCCAATGGCGGTGATCCT
TGTGGAGAGTCCTGGACAGGCATTACCTGCTCGGGCAATAAAGTTACTGAAATGTGGGGTTCGGTCTTC
CTTTTTGTCTTCATCTCCATGTTGTTTCAACTTTTTGGAATTATTGACTAGGTAAACTGATGATTAGTTTCT
TTTCATTTTCCGTCAGCAAGATGTCAGGTCTTGGGCTCTCAGGTTCATTGGGGTCTCAGCTAGATAGTCT
AAAATCTGTGACCAATTTGTAAGATAAATGGCCTTTGTTTGTTCCTTATTAATTGGATTTATGTTGAATTC
CTGAATGTGGTCATCTTTGTGAATGTTGCAGTGACATAAGCAATAACAATATGGGGAGCCAGTTACCTTA
CCAGCTTCCTCCAAATGTGCAAAGATTGTATGTTTGTTTATTTGCATATCTTCCTCCTTGCAGGTTCTATTT
TCTTCCTCTTATCTTTCTTAGAACTTGCCTTGCAGAAACCTCGCTTCTAGTGGTTTTAGTGGTGGCCTCCCA
TATTCCATTTCACAGATGACCTCCCTCCAGTACTTGTAAGTGAATGCTTAAATTTAATATTTGCTTAAATAC
TTGGTCCTCTTCATTCTCTTTGGGGCTCCTTATGATTCTTTGCAATGTTTATGCCTGGTTGTAGAAATGTCA
GTCATAATCAAATTCAAGGATCAGTGGTTGTCTCGTTTGACTCGCTCTCTGCACTCAACACATTGTAAGG
CCGATTCATCTCCTTCCATTACACCAGAGTTTTCATATTGCTTAATTATGATCTGAATGAGAATAGTTTGCT
GTTCTCAAATGTTATGATGAAAGTACACTTATTCTATTATTTAAGTTGTCTCGATTTGAGAAAGAGGTTAA
ACACACTTGTTCCATTTCCCACATTACTGTGCAGAAGTGAGTCATGTCTTTGGTCTGCTGCCAAAGTATTG
AATTATGCCAATTGAGCTTAGTTGCTACTTAGATTGTACTTATTCTTAAGCAAGTATTAAAAGCCTCAAAG
GTGCGCTTATGCCCTAAAACGAGGTTTAAAACGTGTTGAGCGCTTTGCCTTGCTTAATGTGTGCTTTAGT
GTCATCATCAAGGTCCTAAGGCATACTTTCCCTTGACTAAACAAGTGATATTTCACTTTATCGTAAAAAAA
TCCAATTTCTTTGTTCATATATTTGTCATGCATGCTTATAATTATTAGTCTTGGACTAAACATATATATACT
TGTATTTTTTTCTCCCTTTGCACCTTTTTTTCATTAAAGCTTACACTTTATTTGCGCTTTAAGCCCCAATGGA

CCTTAGAACTTTTTTGTGCTTTTTGCTTTTGATAACGCTGGTACCTTATCATATTTTCTTGTTTAAAACATAA
TAGTTTGAAATTTATCATTCTCGGTCACAATTTTGATTGATCTTTTTAATGCTTCAGGGATTTCTCTTTCAA
TTCTATGACGGGTAATCTTCCTCAAAGCTTCCAGTCACTAACTAGTATGAACAAAATGTAAGAAGAGTTT
TCATACTTCATCTATCTATTTGCAAACTTCCATCAGATATAGAAGTTTTGTTGTTATAATATGCAGGTATTT
GCAGAACAACCAGTTTAGTGGAACTATTGACGTCCTGGCCAATCTTCCCCTCGGAAATCTGTGAGTTGTA
TGCCACATGTGTAGATATTATGTCTTATGTCTACATCTCCTTCAGTTTCAGTTAAATCCATTGGTTGAAAA
CCATGTTCATTCTTCTATTATTTAGGAATGTTGAAAACAACCAATTTACTGGCTGGATTCCAGATCAGCTT
AAAGGGATAGTGCAGTGAGTGTCTTAGATTGCAATAATTTTCATTCTTCTTTGCAAATTCTTTGGGGAGT
TCTTTCTAATTGTTTTCTGGATTTATGAATCTTTCATAACAGATCAAATGGTAATTCATGGATCTCTGGACC
TGCACCCCCACCTCCACCTGGCACACCTCCTGCAAACAATCCCAATGGAGATCACAAATCCAGAGACAAC
AGCAGCAATTCGGGCAGTAGGAAATCAGGTATAGGGGGTGGAGGTGTTGCAGGAATAGTGATATCTAT
TCTAGTTGTTGGAGCAATAGTAGCATTCTTTATTATCAAGAAAAGATCAAGAAGGTCGTCCACAGACATA
GAAAAACTTGATAAGCAGCCACTTGCTCCTCTTACTCCACAGAAAGTAGAAGGTATTTGGCTTTCATAAT
TCTTGATATTGTTAGAAATGTCTTGAATTCTCTCTTAACTCTTTGGATATTTCTTAACGTATAATTATAATC
AATTGCGGTGTTACTATTTACTCAGTTGCTTGGGAATATGGAAAATGATGTCTAAGCCTCTATCCTTACCT
TGCTAATGATTGGTCATAAACCATGCAAGTAGTATCAAAATTTTGCAAGGGGTTGTCATTGATACAGAA
GGAAGCAGCAAAGCTGGAAAATATTTATCTTCGAAGTGGAAAAACTGTATGGATTTGAAAGATCCTAAG
TTCTGAACAAACAAAAGTTTCATACTTTATTGGTAGTCCCGTAGGTATAATCTTTAGAAATTCAAACTGAT
CTTTTGGAGGAGGTCCTAAATTACGTGGTGCTTTTAAAGCTTATGATTATTTTTTGACAAATTAACATCAG
CTACCCTTGATGGTTTAACTTCCTGGGAGACAAAAACTCTTGAGAATATAGTTGACCCGAAGAACTCTTG
CTTCAGACTGTAACTGTCAGCACAGTGAAGGAGTCATCTTCAGGTTCATATTTGGGAAGTTGCTTTCTCC
TTTTTACTCCATCAAAAGCCCACAAAACAAACCGTCTCTTACAATACGAGTTCCTTCTCATTTGCAACTTCA
TCAGTAATTTTCCAGTTCATGCTTATAACTTTCATGCCTCATTTCATTATTGACTTCAATGATGTCATGGAA
TCCGAGTTGGAGCTTGTATTTGCTTATGTCTAACTTTTTCATTGCTGTGTTGCTGTTTTTTTAAAAAGGTAA
CGTCACTGTTATATTTTGTTTGTTCCTGGAATTCCTACTCATTGTCTGTTTTTGGTTGCAGAGTTGAAACCT
TATCAAACCTTCTCCACAGTAAGCATGGGCACATTTGAAACTCCCACTGCTGTAACTCTAAGACCTCCACC
TATTAATCGCCTTAAGTCCTTTGATGAGGTTGACATGTCACCGAAACCCATTGTTCCTCCCAAGAAAATTA
ATACAGCTCAAATCAGGGCTACACAGTTTTCAATTGCAGACCTACAGATTGCTACTGATAGCTTCAATGT
TGAAAACCTTATTGGCGAGGGATCAATTGGGCGTGTTTATTGCGCTCAGTTTGATGATGGCAAAGTAAT

ACTTCTCTCTATGAGTACCCATTAAAAAAACTTTTTTTCTTTCTTATGACTATATAAGGTGATTTAGTGATG
CTAACACATGTTTAAGCAAAAATGTATTATCAGAGAAACTACATACTTATTGTGTTCCTTTCTGTTATTTG
AAATGTTGTAGGTTCTTGCTGTCAAGAAAATCAATTCTTCTGCCCTCCAAAATCATGAAGATTTTCTCTAT
ATAGTTTCTGAGATATCACAGTTGCACCATCCAAATATAATTGAGCTTGTTGGTTATTGTTCAGAGCATG
GACAGTACTTGCTAGTTCATGAATTCCATAAAAATGGTTCTCTGCATGATTTCCTGCACCTGTCAGATGAA
GACAGCAAGCCACTGGCATGGAATAGCCGAGTCAAGATTGCACTTGGTGCAGCACGAGCACTAGAGTG
AGTTTTTGCAATCTCAGAATTATTTTATCATATTTTATATTCAATTGGTTACAGTTATGCGGGACTATTTCC
TTATTGATTCATTCCAGAAAGGATGACACGTTGCTGTATTTGGAAACAATTCACTTTATATTTCCCATTTTT
ACCATTAGTGAGAAGTTTTATAGCAACACAAATGTTATGGTATGTTTAAGGCCACAAGTTTCAAAAGTTT
TATAGTCAGACAAATGCTATGCCATGTTTAAGATCGATCACAAGTTTCAATTTTTTTTTATCTCTTTCTTAG
ATTCAATGCCGATTGGAACTATGTCACATAAATTGGAAGGGGGAAGTAAAATTCATGGAGCATGCAGCA
ATGCTATGTTTTTCTCTTTATTTATTGATGGCTAATTACTAGTAGTTTACATGAATCTTCTGCTTATCTATAT
CGGAGATTCATAGAATAGATCTTCTGGGTAGAAGAAAAGGGGATTCTGTGAAAGTCAATACCAAGCACT
TAGTCGTGACTCGTGATGAACTTTACCAGAGGATCCATAGTAATATTGCATTTAGAGGTCATACTTGTTG
GACTAAAAAGACCACTATAGAGTTTAGTCTTTTTTAAACATTGATGACTAGTAAGTTAACATAAAGAAGA
GTTCATTTATTCCTTTTAGTTAGAGCCTAGTATCCTAAATATGCCGGTCTTGCCAATATAAAGCTAATCAA
AACTTCAATGCTCCCATCATTTTGTTTTATATGGTGGTGTTTGAGTGGGGACAGAGTTCAAAAAGAAAGA
AAGACTTCTGGAACTTGTGGTCTTAAACTTGCCATGTCATTTGTGTGGCTATAAAAGCATGTCATTAGGG
TAACAATGAATATTTGAAGTTAAAATTGTTTCCAAATATAGGAAGGCGTCAATCTTCTTGAAAAGGCTTA
AAAGGAAATCCGCCATATAAAATGATACAAAGGGTGTAACAAATTTCAATTTATGTGGTTTTCATATCTG
TTCATTTCCTGTTTTCCATACACAAATTTCTTCACTAGCTAATATAGGCATCCTTTGCAGGTATCTGCATGA
AGTTCATTCACCATCATTGGTTCATAAAAACATTAAATCGGCAAATATTTTACTTGATCTGGAACTCAATC
CTCATCTATCAGATTCTGGATTAGCAAGCCTTATTAATGATGCAGATCAGGTGGCTTTAAGCTCCTTACTT
TATGCATTGTTGCAAAGTGTTTCGGTGGATTCGATATAGAAGCATGCATGCACATAAGTACACATGTATT
ATATAGGCATGTTTTGACATACTATTGGCTGTGTATGTGTCCTTTTCAAGGAGATAAGAAATATCAAAAC
ACCACATGGTTGAAACGTCATTTGTTTCAGGAGAAGCTTTGTTTTGTGCACAAACCTTTGAAACATGAAA
TCATTTACAACAGTTTCTGGGGTTCATAAATTGGATTCCAAGAGAAGTTGTCAAGTAAAAAACAGAAAAT
TTTATCCAAATCTAGGGCATGGAATTATCCAGTATATTCTATAGAAGTAACACTACCATATAACTCATTTT
AATGAACTAGTCTTTCTATGGTCCCTGTTGCTTTATCTCCTGCTCTTCTCTACTTACTCAATTGGTAAAATT

GTAGCTGTTTCTTCTTCTTAAAAGATTCACTTCATTATCATCTTCTCAAGATATTTGGAAGTGATCATCTTT
GCAGGCACTAAACCATAATACAGGGTCTGGATATGGTGCACCTGAGGTAGCTATGTATGGGCAATGCAC
CATAAAAAGTGATGTCTACAGCTTTGGAGTTGTTATGTTGGAACTTCTTACCGGACGGACACCTTTTGAT
AGGTAAAGTTGCTTAGGCATCATGATATTGTGTCAAATTACTTAGAGACAAACAGGAAGAAATTCAGAA
AGTCTTAACTTTCTTGATCAATATGTATGCTCTTGATGCAGTGAGAGACCAAGATCTGAACAATCTTTAGT
TAGATGGGCAACGCCACAACTCCATGATATTGATGCCTTAGCTAAGATGGTTGATCCAGCACTCAAAGG
GCTCTATCCTGTTAAATCCATTTCCCGCTTTGCTGATGTAATTGCTCTTTGTGTCCAGGTAATATTTTGTAT
AGACGGTGTGCCTAATTCATAAATCTGCTGTGTTTATCAAGAACTTGATTCTGTTAGTTCAGTCAACACTA
TCTCATTGTCATGTATACTATTGTGGCAGTCTGAGCCTGAGTTCCGGCCACCTATGTCAGAAGTGGTTGA
AGCCTTAGTTCGGCTAGTTCAACGAGCAAATATGAGCAAGAGGACATATAGTATTGATCGGGGTTCAAG
GGGTGAAACAGATGCTCTAGACAACCAACCTTAGTTCCGATGTAATGTAGCCTTTTCTACCAGAATGTCT
CTCTTTATAGTTTGTTGTTTATGGGATCTATCAGCTTATCTGTTCAGCAAGATCTTGTTGGTTGCATTGGTT
GTACACACTGCTAGATACTAAAATCTGTTCATAGTGCATATTCAATTTTCATTTTTCCCTGTATCTGCT
>PGSC0003DMG400029146 LRR-RLKs
GGAAAATGAAGGATCATTCCTATTAGAAAAAAGTTACTCAAGCTTTATTTAGTTCCTCTTTTTCCATGCAA
CTTATGATATAGAGCTTTCATATCTAGCTAGAGAGAAAAAATGGCCAGCATAGGATCAAAATCGATCAT
GTGTTTGATCGTGTTCGTGCTATTTTACATGCAGCAAGTCAAAAGTAGCTCTGAGCTCGAGCTACTTTTAT
CGATGAAAACATCGATGAAGGATCCATTAGGATCTCTTCATGATTGGATTCCGCGGTCTCAATCTTTTTG
CCATTGGAATGGTGTTGTTTGTGATGACTTGTCACATGTTGCAAAAATTGAGCTATCAGGGAAGAACTTA
TCTGGAAAATTATCCGAGACTATTTTCAACTTTCCATACGTTGAATCGATTGATCTCTCGAATAATCAACT
CTATGGAGAAATTCCAAGCAATATCTCAACTTGTTTGGCGCTGAGATTTCTCAATCTGAGTAACAATAAC
TTCACAAGTCTACTTCCTCAAGGCTCGAGAATCCCACTTCTCGAGACATTGGATCTCTCAAACAACATGAT
TTCGGGGAAAATCCCCGAAAATATTGGTTTATTCTCAATGCTCAAAGTTCTTGATTTTGGTGGAAATGTG
TTGGTTGGAAGCATTCCAAAGTCTATTGCAAATATTTCTAATTTGGAGTTCTTGACTCTTGCTTCAAACCA
ACTAATCGGAGAAATCCCTCGAGAACTAGGTCTTTTGAAGAACTTGAAGCTTATTTATTTGGGATACAAC
AATTTCTCTGGTGGAATACCGGAGGAGATTGGTGAATTGTCTTCTTTGTATCATCTCGATCTCGTGTACA
ACAATCTCACAGGTGAAATCCCTTCATCTTTAGGTAATCTCACCAATCTTGAATACCTTTTTCTTTACATAA
ACAAGCTCACTGGCCCGATTCCAAGATCTCTATTTAATCTCAAGAAAATCATCTCGCTTGATTTAAGTGAT
AATTTCTTGTCTGGTGAAATTCCTGAGCTTATTTCCCAATTACAAAATTTGGAAGTTCTACAATTGTTCGC

GAACAATTTCACTGGTAGAATTCCAAATACTTTATCTTCCTTACCTCGACTTCAAGTTCTTCAATTATGGTC
TAATAAATTATCTGGTGAGATTCCCAAGGACCTTGGGAAACACAACAATCTCACAATTCTAGACCTTTCC
ACCAATAACCTCACGGGTAAAATACCCGAAACCATATGTTACTTCAACCATCTTTTCAAACTCATACTCTT
TTCAAATTCACTTCATGGCGAAATCCCTGTAAGCTTGAGTCATTGCAAAAGCTTACAAAGGGTCCGCCTT
CAAAACAACCACCTCACCGGTGAATTGTCCCCAGAATTCACAAAACTCCCACTTGTTTACTTCCTTGATAT
CTCGGGCAACAATCTATTTGGCTCCATTAGCGAAAGACGATGGGATATGCCATCACTTCAAATGCTAAAT
TTGGCTAAGAACAAGTTTTTTGGTACCTTGCCTGATTCTTTTGGTAGCAAGAAACTCGAAAACTTGGACT
TATCCGAAAATGATTTTAACGGTACTATTCCTAAGAATTTCGGAGAGTTATCAGAGTTAATGGAGCTCAA
ACTACGAAGCAACAAGCTTTCAGGCGAAATCCCCAATGAATTGTCTTCATGCAAGAAGATTGTGAGTCTT
GATCTGAGCCATAATCGATTTTCAGGTCAAATTCCAACTAGTCTCTCCGAAATGCAAGTTCTTAGCCTTCT
TGATTTGTCAATGAATGAATTATCAGGTGAAATCCCGCCTAATTTGGGGAAAGTTGAATCACTTGTCTTG
GTTAACATCTCTCACAACCATTTCAGTGGATATTTACCGTCCACCGGAGCTTTCCTAGCCATAAATTCAAG
TGCGGTGGTTGGAAACCAACTATGTGCCCGTGGGGACGACATTACAAGCGGCTTAACGCCTTGTAAATC
ACTCAAGAAAAGCTCAATTTGGTGGTTCTTCTTGACATTTCTTCTTGGAATACTAGTTTTGCTAGTATTCTC
GGCTTTAGTTATTGTATTTATTCAAAGAAGAAGAGAATTGAAGCTAAAGAAAGTGGAGAGTACTACTCA
AGATGGTAATAATTGGGAAATTCAATTCTTTGATTCAAAAGCCTCAAAATCCATCACCCTAGATGACATC
TTAGGGATTGGAGTTTCGTACAAAGGGTTTTATTCTGAAATAAGTAACATGCAAGTATTCGTGAAGAAG
TTGAATGTGAATATTCCAACAAGTTTTTGGACAAACATCCAAGAACTAGGAAATATTCGACATCCAAATG
TTGTGAAGATATTGGCTGCATGCAAATCGGAGAAAGGAGGTATTTTGGTTTATGAGTATGTAGAAGGG
AAAGATTTGAGTGAAGTGATTCGAGTCATGAGTTGGGAACGTAGACAAAAAGTTGCTATTGGCATATCT
AGAGCATTGAAATATTTGCATTGTTCTTGTTCACAAAGCATTTTCATCGGTGACCTTTCTACCCGGAAAGT
TATCATTGACGGAAAAGATGAACCTCGCCTGAGATTGAGTCTTCCCACTACGTCCTACGTTGGTCCAGGT
AAATTTACCATTTTACCCTTGTCATCTACGGTTACTTCTCTTGTTTTGACATTTTTTAAGAGTTTAAGTAAT
ATACAATCGTAAGCTAAAACATCATATATTCTATCAGGTAACATGTCTTATTTGCAAGATTATAAGCTTAC
TTACATACATCTTTCAACTGACTAGATCATGTAAATTAATTTATACTGATAATGCATATAACTTAATTAACT
CGTTAGTTTAATAGTTAACAACCTTTGTTATAACTTAAACAAACCACATATAGTTTCTAAAGAGTGTAACG
TAGCTATGATGACTTTAATGAAAATTGATTTTTTCAGCTTTAGCATTGATGTGATAGGAATGTCATGTTAT
GTTGAAAATTATTTGATCTAACTAAAAGAAATTTTGTATTTTTCTAATTATTAGAATAGAAATAATTCTTA
GAATTTACGTACTTGGTCTTTATTTGGTTTCTATTTTAACTTTAGTTCTCATAGTCTCTAGGAGTAATGATA

AGGTCAAGGAATTAAGCAAACAAATGTCATGGAGGCAATCATGAATGTTTTTTTCTCTTAAAATAAATGT
AGAGACATAAATCTTATCATTCCTTTCTTTACGTAGAAAACAATAAATCTAGTCTAGTGTAACAAAAGTCA
TGACCTTTTATGCCATGCACGTACAATACTGTTACTTAATTACGAGTTACAACTTTTCTCATAAATTGTAG
AATACAATGGAATATCGGAGAGGAGCGATATATATGGATTTGGGCTTGTTTTGATCGAGTTATTGACTG
GAAAAAATCGCGGTGATGCTGAATTTGGGAAGCGTGAGAGTATAGTTGATTGGGCGCGATATTGTTACT
CAGAATGTCATTTGGATACATGGATTGAACCACTATTGAAGAGTGATGCAGTGAATAATCAGAATAAAA
TGGTGGAGATGATGAATGTGGCTCTTCAATGTACTGCTAGTGAGCCAGCAGCAAGGCCTTGTGCTAGTG
ATGTTGCAAAGACATTGGACTCTTTTGTTAGATCAAATTCTTGTGGTTTAGGGTTGAAAAGGTGTGGTAG
TGTTTAGATTTTGGGATTTCTAACTTCAAGTCCAAGATTTTTGTTTTTATGTTAATGTTACTTTCTTTTTTTT
GGTTTACTTTGTATTGGGCATAAATTAATGAGTGTTGCTCAATGTTTTGGATGTTATTTGAGTGTAAAAAT
GTAGATATGAAACTAAATGCTAATATATAT
>PGSC0003DMG400029442 LRR-RLKs
TTTCAACACTCACTAAAAGCTTACTCTCTAAGCTCTCTTCTTTCATTTTTTTTTCTATCTATCTATTTTCTCTA
TTCATCATGGACAACATGAACACCAACTCTGCTATAACATTGCTCATTGTCTTGTTTTTTTCCTTGATTCTT
GCATCCGTAAACGCGGTATCTTCCTCCATCAAAACTGATGCTGAATCACTCTTGCTATTCAAGAACATGAT
ACAAAAAGACCCTTCTGGTGTTTTATCAGGATGGCAACTTAAAAACAATCCATGTTCATGGAATGGTGTA
ACTTGCAATAGTCTTGGAAGAGTAACAAATCTTGATCTTCAACAATCTGAACTTGTTGGAGAGGTTTCTT
TTTCTCCTTTCAATTCTCTAGATATGTTAACTGTCCTTAATCTATCATCGAATTCGTTTTATGTAAATGCATC
AACCTCTTTAACTCAGTTACCTTATAGTTTGAAACAACTTGAACTTTCATTCACTGGCTTAGCAGGCTATG
TACCTGAGAATTTCTTTGCAAAATGTCCAAATCTTGAATATGTTAGTCTTTCTTTCAATAACATAACAGGT
TCTTTGCCTCAGAATTTCTTGTTGCATACTGATAAGTTACAGTATCTTGCAATGGACTATAATAATCTTAC
AGGATCAATTTCTGATATCAAGATTGAGACTTGTAATTCATTGTTACGTCTCGATTTGTCAGGGAATCAG
ATAATGGATTCAATTCCTTCTGCTTTATCTAATTGTACAACACTTCAAGAATTGGTTTTGGCTGAGAATTT
CTTCTCTGGTTCAATTCCAACTTCTTTTGGTGAGCTTATAAGTTTGCAAAGATTGGATCTTTCTAAGAACC
ATTTATCCGGTTGGATCCCATCTGAATTAGGAAACTCATGTAGTTCACTTGTGGAACTCAAGTTTTCTAAT
AACAATATCACTGGTTCAATCCCGAATTCATTCTCATCATGTTCTTCTCTTCAAAATCTTGATTTGTCGAAT
AATAATCTAACCGGTCCATTCCCTGATTCCATCCTACAGAATTTAGCTTCTTTAGAGTCTTTGCAAATGAG
TAGTAACAAGATTTCTGGTTCATTTCCTGCTTCTCTTTCATATTGCAAGAAACTCCGCGTTGTAGACTTTA
GCTCCAACATGATCAATGGGATGATTCCACCAGATTTATGCTCTGGAGCTTCATCATTGGAAGAGCTGAG

AGCACCTGATAACTCACTTTATGGTCCAATTCCATCTCAATTGTCTCAATGTTCACAGCTCAAGACTATTG

ATTTTAGCTTGAACTATTTGAACGGTTCGATTCCATCAGAGCTAGGAAAACTTGAGAAACTTGAGCAGCT

TATTGCTTGGTACAATAGTTTGGAAGGGAGCATACCAGCAGAGTTGGGAAAATGCAGCAACTTGAAGA

ATCTTATACTTAATAACAACTACTTGAGTGGGAAAATTCCAGTTGAATTGTTTAATTGTGGTAATCTTGAG

TGGATTGCTCTCACAAGTAATGGACTAAGTGGTGAGATTCCGAAAGAATTTGGCCATCTTTCAAGGCTG

GCTGTTTTGCAACTAGCAAATAATAGCTTGAGTGGTCAGATTCCAAGTGAATTAGTCAATTGTAGTAGCT

TGGTTTGGTTGGATTTGAGTAGCAATAGGTTAACCGGGGAGATCCCACCGCGACTTGGTAGGCAGCAA

GGGGCAAAAGCATTGAGTGGGATTCTATCAGGTAACACTTTGGTGTTTGTTCGAAATGTAGGAAATTCG

TGCAGAGGCGTTGGAGGATTGCTTGAGTTCTATGGGATACATCCTGAAAGGCTTTTACAGGTTCCATCAT

TAAAGAGTTGTGATTTCACTAGGCTGTATTCTGGTCCAGTTCTTAGTGCTTTTACTCGGTATCAGACTATC

GAGTACCTTGATCTTTCGTACAATGAGCTTCGTGGGAAAATCCCTGATGAATTTGGTGATATGATAGCTT

TGCAAGTTCTTGTTATATCACATAACCATTTGTCAGGAGAGATTCCTTCATCACTTGGAGGGCTAAAGAA

TTTGGGAGTGTTTGATGCATCACATAACAGATTGCAGGGTCAAATTCCTGATTCATTTTCGCTTCTTTCTT

TCTTGGTGCAGATTGACTTGTCTAATAATGAATTGACAGGACAAATTCCACAAAGGGGTCAGCTCAGTA

CACTTCCTGCAAGCCAATATGCAAACAATCCAGGACTCTGTGGGGTTCCGTTGTCTGAATGCCAGTATAA

CTCACCTGCCACGAATACTGGAGACGGAGGAGGAGGAAAAAGAAGTTCAGCTGCATCATTGGCTAATA

GCATTGTTCTTGGAGTTCTCATTTCCATTGCCTCAGTATGCATTTTGATTGTTTGGGGTATTGCTATGCGT

GCAAGGCGTAGAGAGGCTGAAGGAGTGAAAATGCTTAGTAGTTTGAGTACTAACTATGCAGCTTCATCA

TGGAAGATTGACAAAGAGAAGGAGCCATTAAGCATCAATGTAGCAACTTTCCAAAGACAACTTAGGAA

GCTGAAGTTCTCACAACTTATCGAGGCCACGAATGGATTCTCAGCTGCAAGCCTCATCGGTTCTGGAGG

ATTCGGAGAAGTCTTCAAGGCAACATTGAAGGATGGATCAAGTGTTGCAATCAAGAAACTCATCAGGTT

AAGTTGCCAAGGAGACCGAGAGTTCATGGCTGAAATGGAGACATTAGGAAAGATCAAGCACAAGAACC

TTGTACCCCTCTTAGGATACTGCAAGGTTGGTGAGGAAAGACTTCTAGTTTATGAGTTCATGGAATATGG

CAGCCTAGAAGAAATGCTTCACGGGAAGACAAGAATGCCTGATAGAAGGATCCTAACATGGGAGGAAA

GGAAAAAAATAGCCAGAGGAGCAGCCAAAGGACTCTGCTTCCTTCACCACAACTGCATCCCACACATCA

TACACCGCGACATGAAGTCAAGCAACGTGCTATTAGACAACGAAATGGACGCCAGGGTGTCTGATTTTG

GGATGGCAAGGCTGATAAGCGCGCTGGACACACACCTCAGTGTGTCCACACTAGCAGGAACACCAGGT

TATGTGCCACCTGAGTACTATCAGAGTTTTAGGTGCACAGCAAAGGGAGACGTGTACTCGTTTGGAGTT

GTGCTTCTTGAACTCTTGACAGGGAAAAGGCCAACAGATAAGGAAGATTTTGGTGACACAAACTTGGTA

GGATGGGTGAAAATGAAAGTAAGAGAAGGAAAATCAATGGAAGTAATTGATCAAGAATTGTTATCAGT
GACTAAAGGGAATGATGAAGCAGAAGTAGTAGAAGTTAAAGAGATGGTAAGGTACTTAGAGATAACA
ATGCAATGTGTAGAGGATTTTGCATCAAAAAGGCCTAATATGTTACAAGTTGTGGCTATGTTGAGAGAA
CTTATGCCTGGAAGTAGTTCAAGCAACAGTGGCTAAACAGCAAACCAGAAACAAAAAAAAGGAAAAAA
AAAAACCAAAAGAATGTAAAATTCTTTCTTGTTTTGTTTGTGCAAGTATTCATCTGCATTTGTATTTAGTC
AACAACTTCTCTTATTTTTTTGCAATATTATTCCCATGTTTTAAGAATCTTGTGTATGGATATTGATAACAT
>PGSC0003DMG400029644 LRR-RLKs
AAAAAACAGAGCAAAACCCAAGAAACCAAAAAGAAGGGATTTTTACTTAGTTTTTGATATGCAATTGC
AAACCCAAGAAGAAGTTTTTGGAATTTGAGGCAAAGATGTCTTCTTCTTCAAATCCCAACAAAAAAT
ACCATTTTTTTCTTTTCTTGGTTACCCTTTTCTTCACTTCTTCTTCTTCTTCTGCTGCTGTTACTGATGTTGTT
GCACCATTTTCAAATTGGGACATTCTTGATGATACCCCATGTAAATGGTCTTTCATAAAATGCAACTTTCA
AGGTTTTATTACAGAAATCAATATTCAGTCTATTCATCTTGAACTTCCTTTGCCTACAAATCTTTCTTCATA
TAAGTACCTGAAGAAGCTTGTTATTTCTGATGCTAACATCACTGGTACAATACCCTTTAACATTGGTGACT
GTTCTTCACTTGTTACTATTGATTTGAGCTCAAATGGTCTTGTGGGTACCATTCCTTTAAGCATTGGAACC
CTTGTAAACCTTCAAGATTTGATTTTGAACTCAAATCAGCTTACTGGGAGAATCCCAGTTGAGATTGGGA
ACTGTAGAAACTTGAAAAATCTTGTTCTTTTTGATAACAGGCTTAGTGGTGGTTTACCAAGTGAAATAGG
GTTGTTGTCAAATCTTGAAGTGTTAAGAGCTGGTGGGAATAAAGATGTTACTGGTAAAATCCCAAATGA
GTTTGGGGATTGTGGGAATTTGACTGTTTTAGGCTTAGCTGATACAAGAATATCTGGTTCTTTGCCTGTT
TCTTTGGGTAAGCTTAAGAATCTTGAAACTTTGTCTATTTATACTACAATGTTGTCTGGTGAGATACCTTC
TGATTTAGGTAACTGTACTGAGCTTGTGAACTTGTATTTGTATGAAAATAGTCTTTCTGGTTCAATCCCAT
CTGAGCTAGGGAACCTTAGAAAGTTAGAAAAGTTGCTTCTTTGGCAGAATAATCTTGTTGGTGTTATTCC
AGAAGAGATTGGGAATTGTACTAAGTTGACTATGATTGATTTGTCTTTGAACTATTTGTCTGGGAGTATA
CCATTATCTTTTGGTGGTCTTGTTGTGTTACAAGAACTTATGCTTAGTAACAACAATGTTTCTGGTTCAAT
CCCTTCTGTTCTTTCACAATGTACAAGTCTTGTGCAGTTGCAGTTTGACACAAATCAGATATCAGGTTTGA
TTCCTTCTGAGTTAGGGAATTTGACTAGTCTTGTTGTGTTTTTTGCTTGGGATAATCAACTTGAAGGAAGT
GTGCCTTTAACTTTGGGTAGTTGTAGTAACCTTCAGGCATTGGACTTGTCACATAACTCACTTACTGGTAG
CATTCCTCCAGGCTTGTTTCAGTTGAAAAACCTTACTAAGCTACTGTTGATTTCTAACGATATTTCGGGTA
CTATACCTCGAGAAATTGGATATTGTAGCTCATTAGTGAGGCTAAGACTTGGAAACAACAGGATTGCTG
GTGGAATTCCGAAAGAAATTGGAGGTCTTAAGAGCTTAAACTTTCTTGATTTGTCCGGTAATCGCCTTTC

TGGACCAGTTCCTGATGAGATAAATAGCTGTACTGAGCTACAAATGGTAGACCTCAGCAGCAATACACT
GGAAGGTCCCCTGCCTAACACTCTGTCTTCACTATCAGGGATTCAAGTCTTGGACGTTTCGAATAACCGG
TTTGGAGGGCCTATATCGGCTAGTTTTGGGCGTCTTGTGTCCTTGAACAAGCTGATTCTCAGTAAGAACT
CGTTCTCAGGGTCGATACCTCCATCTATCGGCCTGTGTTCGAGTCTTCAATTGCTTGATCTTAGCAGCAAT
GAGCTCTCGGGAGGCATACCGATGCAGTTAGGAAAGATTGAGTCCCTTGAAATCACTCTCAACCTTAGT
TTCAATGAACTAACAGGTCCAATTCCTGCTGAAATTTCCTCACTAAGCAAGCTGTCGATACTTGACCTTTC
GCACAACAAGCTTGAAGGGAACTTGAATCCACTAGCGAGGCTCGATAATCTTGTCTCACTGAATGTCTCA
TACAACAACTTCACTGGTTATCTTCCGGACAATAAGCTCTTTCGACAGTTACCATCATCAGACCTAGATGG
AAATGAAGGTCTATGTTCATTCGGAAGGCCTTCGTGTTTTCTCAGCAATATCGATGGAGTAGGAGTAGCT
AAAAATGGAAATGATGAGGGAAGGTCAAAGAAGCTCAAATTAGCCATTGCATTGCTAGTCATCATGACA
ATAGCAATGGTGATCATGGGAACTATTGCTATCATACGAGCACGAAGGGCGATGAGAAGGGATGATGA
TTCAGAGATGGGAGATTCTTGGGCTTGGCAGTTTACTCCATTCCAGAAGCTCAATTTTTCAGTGGACGAA
ATACTCAGATGCCTTGTGGATACTAATGTCATTGGAAAAGGGTGCTCGGGGATGGTCTATCGTGCAGAT
ATGAACAATGGTGATGTCATAGCAGTGAAGAAGCTTTGGCCAATAACCATGACTACAACTAATGGAGGC
AATGATGAGAAGTGTGGGGTACGTGATTCGTTCTCTGCAGAAGTTAAGACGCTTGGTTCAATTCGACAC
AAGAACATTGTTCGATTCTTGGGGTGTTGTTGGAACAGAAGCACAAGATTGCTCATGTATGATTACATGC
CAAATGGGAGCTTAGGAAGTCTTCTCCATGAGAGAGGTGGTAACCCTTTGGAATGGGAATTGAGATACC
AAATATTGCTCGGGGCAGCGCAAGGGCTCGCCTACTTGCACCATGACTGTGCTCCTCCAATTGTTCACAG
AGACATCAAGGCCAACAACATTCTCATTGGTCTTGAGTTTGAGCCTTACATTGCAGACTTTGGCCTCGCA
AAACTAGTCGATGATGGTGATTTTGGTCGTTCCTCCAATACAGTTGCTGGTTCTTATGGCTACATTGCTCC
AGGTAAACATGTTTTTTTATTTCATGTCAAGTTACAATTCGTCATCATTTTCTAATTCCTAGCATATTATTC
CGAGTATACATTTTGCTTAGTTATAGTATAAGCATTGATTGCACATTGCAACAACAACAATATACCCAGC
GTAGTCCCATGCAATGCAGTCTATGAAGGGTAGAGTGTACGCAAACCTTATGCCTACCTCAGAGGTAGA
AAGGTTGTTTCCAATAGACCCTCGCTTCATGGAAAAACAAAAACAAGGCAATCTAGAAAAAGAAGTAAC
AGATGCGACAACGATTACACATTGCATTTATCACAATTTTTTCAAAGAATATCAGTAGTTCTTGATGATCA
TTGATCCAAGTGCTCCAATGTTGTTCATTTTTCATTACAGAATATGGCTATATGATGAAGATAACTGCAAA
GAGCGATGTCTACAGCTACGGAGTGGTTGTATTAGAAGTCTTGACAGGTAAGCAGCCAATCGATCCAAC
AATTCCCGAAGGAGTGCATCTAGTTGATTGGGTAAGACGGAAAAGAGGTGGAATTGAAGTCCTCGATC
CAAGTCTACACTCGAGACCAGAATCAGAAATCGAGGAAATGTTACAAGCATTAGGAGTAGCCCTGTTAT

GTGTCAACTCCACTCCTGATGAAAGGCCTACAATGAAAGATGTAGCAGCAATGCTAAAGGAAATCAAAC
ATGAAAGGGAAGAATACGCCAAAGTCGATGTTTTACTCAAAGGCTCTCCAACAACAACAACTACTACTG
ATCATAATCAAGAAAACAACAACTCATCAAAGGGTGTTTTAGCAACATCTTCATCAGGACAGAAAGCAA
GAAGTATGTATACAATGAGCAATAACACAAGCTTCTCTGCTTCATCATTGCTTTATTATTCATCATCATCA
TCAAATGCCAAAAGTCGATTCTTGGGGTGTTGTTGGAACAGAAGCACAAGATTGCTCATGTATGATTAC
ATGCCAAATGGGAGTTTAGGAAGTCTTCTTCATGAGAGGGAAGAATACGCCAAAGTCGATGTTTTACTC
AAAGGCTCTCCAACAACAACAACTACTACTGATCATAATCAAGAAAACAACAACTCATCAAAGGGTGTTT
TAGCAACATCTTCATCAGGACAGAAAGCAAGAAGTATGTATACAATGAGCAATAACACAAGCTTCTCTG
CTTCATCATTGCTTTATTATTCATCATCATCATCAAATGCCAAAAGTGGTGTTTAAGTGATTCAACAAAAC
TTATTACATTTGCATTGAATAGAAGTTTGTCATATTAGTTACTATTAGTTTTAAGTTTCAATAATCCTTTGT
TTGTTTGTTTGGAATTTTCTGTAAAGAGAATTAGAAAGGTAGATATGTGTGACAAGTTGAAACAAAACA
AGCCAACTTATGCTAATTTGGCTTTTTGAGCATTTTTAAGCTTTCATGTAGCTACTAAAGTCAACTTTATTC
CTCCTGGTTTTAAGGTTCATTTTTGAAGCTTTCAAACAAATATTTG
>PGSC0003DMG400030035 LRR-RLKs
GGCTAGGGTACCAAATGAAGTTTCCAAGTAAAGTCCTTGAATAAACAAGTGAAGATTATAACAAAGGGC
TTTGTCATATTATGATGATCTGTCGTTAGCTTTTACTCAGTCATCAAACTTTGTGAATCTTTCAATCTCTCT
CTCAAGTCTCAACCATTTTTCTACGTCATAGTTTTTTTGAATAGAAGAAGTAGGCAACTTGTATTGAAAAT
TCTATCTGAAACTCAAAAAAGAAAAATTCACGTTTTTCACAGTAAACCTTTCTTCTCCTTGTTTTTCTATTG
GCAAAGAAGACAATACCATTTTTGCCTAAAGTGAATAAAGTCAAAAAAGAGCTTCCAGTTTTTGTTTGCT
TGGTTTTGTTTGTTTTTTACGCGTTTTTCGAACAAACTCAGTCTCCTATTTGTTTTTCCCTCTTTCTTCTACTA
TTTTATTTACTGGGTTTATTCAATTTCTGGGTTTTGGAGCGGTGGACTTTTAATTAATTGTGAATTTTAGC
GATTGTTTTTCAGTTTTGGGTAAAAGATATACTAGTGGGTTTGTTTATGTTTTGTGTGAAATGAATTGGC
AATGGAAATTCAGTGAGAATAGCTTGTTGTTGGTTATTGTGCTGTTCTTGTGTTTGGTGCATCAGGAATT
GTGTTTCTGTTGGTCTCTCAATGATGAGGGTATTACTCTTCTTCTTGTTATTATTTGCTATTTAAGACTTAC
TTTGTTAGAAATGGCAATAAAAAATTTGTTATTTTGCTTAAAGTTTTAGTCTTTATTGCTGCAGGTTTGGC
ACTGTTAAGATTCAGGGAGAGAGTGGAGAGTGATCCATTTGGGGCTTTGTTGAATTGGAAAGATGGTG
ATGGAGTGGAAAATCCATGTACTTGGTTAGGAGTTGGGTGCGTTAATGGGAATGTTGTGTCTCTGTAAG
TACTAAAAATTGCTCTTTTCTCTTGTTATTTGGTTCTGCAGAAGTCTTCTTTTTCAATTTTGATCTGGTTGG
CTTATATAGATGATTCTAAATTTGGTCTCTGATTGAATTCTGAAACTTTTGTTCCATGAGTTGGCGGTGAT

TCCAGAAGGGGTATGATCAAAAGACAGATAAATGAGAAGCTTTTGGCACCAATTCCTGTTGAAGCTTTA
GTATAATGAACATCTTTTGGTGTTTTGTACCTGGAGTGTAGCTAATGTGTTCTTGTAAAGAGGGAGCTAT
TTTAAAATTTGATTTTTATTACTCAATATTTTGAGAGGCTAAGCACAATCTTATGATATGTTGGTGATCAA
GATAATTAAACATAGCCTCGAGAATGGATGTAAATAAGTTGATGTCTAAATTCAGAATGATGAAGCATC
TGACCGTGGGTCTAATGGAAGGGAGAGATTTGGAAAACCCGCCCTCCTCTAGAAAGGGCAAACATGGC
GTTAGATATAGTAACAAAGATATAAATATTATCAGAGATCAAATAAAAGGAGGCAAATTTCACATACTA
CCTTCGATGTCAACTTTCTCCAGTTTATTTATTTTTCTCAAATAAATGTAAACTAGATAATTAGAGCTACAT
GTATAACATGATCTCTTTGTTTCTTGACCTGATACATGATTCTATATTTCTATGAATTAACTGGCAGTCGA
ATTTTGTGAAACGGGTAATTATATCATAGATAGAATATATTCATCCCTAGCTAACTTTAGAAACGCATTTT
ACTGTAGTCATTTTCGTAAATGCAATAATTTTCATACACAATTATCTCCATTTATTATCATGAATATAATTT
TCAAGTCGGTAAGTAGCATAGTGTCTAAGTTGAATTGGCTATTGTAAAAATTATCCATGATACTGATGTT
CTAATCTGTGTTTTTTATCTACTCACAGGGACTTGAAAGATCTCCATCTATGTGGTACCCTAGCTCCTGAT
CTCGCGCACTTGGTTCACTTGAAGTTTATGTATGTGCACATTTAAACTAGATGTCTTCATTTTCCTAGTCG
CTGTCATTACAAACTCTTAATTCAAGTCTTTTTCTTTAAGATAATTCGCAACTCACTGGAGACTTCTTTACA
GAATTTTACATAACAATTCCTTTACTGGGTTCATACCTGATGAGATTGCAAAGCTAAAAGAATTGGAGAT
TCTGGACCTGGGATCTAATAACTTTAGCCAGCCGCTGCCTTCCGAGCTTTGCCATAATCCCTCTCTGGAAA
TAATGTGAGTTCAAACAGAGGTGGTGTCATTTTCTAATTTTACCACCTTGTTCCCTTACTTACAGAGTTAC
ACTAATGCAGCTTATCAGACAACAATGAGCTGTTTAGCAGCATATTTCCTGGAACCGATAGACTTGAAAT
GGTCCCAAATGTCCAAGAAAATAACAACCACGCTACGAGAGCCCAACCTTTGTCACTTCCCAATGGGAG
ATTGATATTAAGGTAAACATCTTTCAATCAAATGAAATGTATATTTTAAGAGCAATCATGTGTATTTGTAA
CTTTAAATGTCCAACATAGTAATAGTAAAATGACTTAAATCATTAGATTCAGTTATTGGAATACATATTCC
TGGAATAAGTATGCATCTGTAGCAATTCATTGCTTGTCCTTTTGACCCCCCTGATGCTTGCGAAGACGTTT
AGAAGTCCCATCAATTTTCCCTGTCTTCTAGTTGTCTTTCAGTTTTCTTATGGTTTGCCCTTCACATTGAAC
ATCTCTTGTTTCCCTTAATGGCCTGATAGGTGATTGTTAAGCACATTTTGATTCATGTTTGCACAAACAAT
GTTCAGTTAATTTTCTGATATTTCTGAAAAGACACTACTAATCTTTTCTTTTCATCTTGTGATAATTTTCCTC
ATTTTCAATCAAGCTTTGCACTAGCTTCTTTTATGGGTATATACTTCTATGGTAACAACTTACATAGTTATC
ATGAAATTTAAATTATTTGTAGGACTACTGAAGCACTTGGAAATGTAGCTGGAAGAAAATTACTAAGAG
TATCATCTTTTTCTGTCCTGAATAACTTCCAGAAGCAATTTCTGCCAGTCTTCCGACTTCTAGACCGTCGTG
AAGTTGCTGTTGAGAACAGCGCACCTTCATCATCACCATCTCCATCACCGAGTCCTTCTCTTTCCCCGTCT

CCTACACCGGCAATTACTGCAGAACCTCATATGGCTCATTCACCAAGATCCTCTCCTTCTCCATCTTCACTT
CCCAGTCCTGCAGAGAACAGCAGTAGAGTTGAAGATGCCAGACGAAGTAATCATCGAGTTTTAATATTG
TCTGCAGCTATAGGAGGTTCCGTTCTACTAATAATATTGATAACTGGCACTATCGTCTTTCGAAGCAACA
AAATGGCTGTTGTAAAGCCTTGGGCCACTGGATTGAGTGGACAGTTGCAGAGAGCATTCGTTACTGGTA
ATTTTCTATCTTTCGATGAATGAATAGTATTTCTATTTTTAGAACCAAAATAGTTGTCTTAAAAATCAGATC
CATAAAATAGAAGCTTATATGAACATGTGTAGCTGTAAAACCAAAATTTTTCCTTAAAAGTATTACCCAA
ATGGGAGAAGCTTAAATGGACCTTTTCGCTCCCATTATGAGCTTGTTACAAGCTTTCTTTTCTAATTGTTA
ATGATCTTTTCCCTTTTCCCTTTTTTCTTCTTGATATCTCGAAACTTTTGTTTATCAATATGAGCAAATAGTC
TTAGATAGAGAGCTGAGTTCTATCATTCTTACTGTGAACAGGTGTGCCAAAGCTTAAGAGATCAGAACTT
GAGTCAGCCTGTGAAGATTTCAGCAATGTGATCGGTTCCTCATCTGTTTGCACATTATATAAGGGCACAT
TATCCAGTGGAGTTGAGATAGCTGTGATATCTCGTACAGTGGCATCTACTAAGGATTGGTCAAAAGATA
TGGAAGCCCATTATAGGAAAAAGGTGATTTAGATTTTCCAGTTGGAATTTTACATCTCATTTTTTCCAATA
TGCGAGTTTCTAAATGCTTTTCTCCTCCTTTTACCAGATCGATACATTGTCAAAGGTCAATCACAAGAACT
TTGTCAGCCTCCTTGGGTTTTGTGAAGAAGAGGATCCATTTACCAGAATGATGGTTTTTGAGTATGCTCC
TAATGGGACCCTCTTTGAACATCTTCACAGTAAGTTTCTGATTCTAATGGTGTATCTGTTGTGATTTACTC
AATATTGTTACCCAACTCGTCTACATCTGCTTGATTGAACTGCACTAGGATATACTTTTATATAGCAGGTA
AAAACATACCTGCATTTGGTGTTTACACAAACCATATTAATTTACCATTGTTAAGTGATAAATTGACGTGA
GAATGTGTATTAATTAACTTTGGTTCAATGATACTAGTGGGTGTGAAAACACATCTTATGTATTCATTGG
GTGGTGTAAACACCAAGTGTGAATAAGTTTTTTCTCTACCTCGAGTATGATGTATACGGACGTGTCTTTG
GTCTCGTGTCGATAATTTGTCTGTAATTTGAGAAGTAACCATTTTCACTTTAGCCTCATAAGATGTGTTCA
CACCTGTACAAGACATCTTTGGTATTCACTTTACATGCTCTTTCAACAATTTATGTTTAGCTTTCCCTGTGG
TAAACAACTATTCTTATGTAATCTATTATGACCGAAAAGATCTCTAATTCTAGTGTTTTGTAGTAAGGGAA
GCTGAACACTTGGACTGGGGGATGCGAATGAGGATAACAATGGGAATGGCATACTGCCTTGAGCACAT
GCACAATTTGTCGCCATCGTTACCTCATAAAAACTTGACCTCCTCTGCCGTATACCTCACAGAAGACTCTG
CAGCCAAAATATCGGATTTTGGGTTCTGGAATGAGACAGATGCAGCTGAAATGGTGTCGAGTCCAGAA
AGTAATGTTTACAGCTTTGGTGTCATTCTGTTTGAGATGATGACTGGTAGGCTCCCTTACTCAGCAAACA
GTAGCTCTCTTGACGACTGGGCATCAGACTATCTTAGGGGAGGAGAATCCCTGAGAGAAATGGTTGATC
CAACTTTAAATTCTTTCCAAGTAGAGCAGCTAGAGAGAATAGCTGTTGTGATTAAAATGTGTGTACATCC
CGAGCCAAGACGAAGACCGACAATGAGAGAGGTTTCTTCCAGATTGAGAGAGATAACAGATATTGGAC

CAGATGGGGCAACCCCAAGACTCTCTCCTCTATGGTGGGCTGAGCTTGAGATTATATCAACTGAGGCTT
GTTAATGCATAGGTACCTCAGTTTTGCCTTTAGATGGTAATTAATATATAGGCTGTATATACAAAGATTTT
TCTTTTGTGTGGATAACAAGAGCATATCGACTGTGGGGGCTAAAGAGAAGTTGTTTGGGTACAAAAATG
GAAAATTTAGTAAGTAAAAAAAATTTCTTTTTTTATTTTATATTTTAGTATGTAAGTAATTTCTAAATCGCC
TTAATTTGCTTTCTTGGCGGCTTGGGATCAATTCTATTGCTTATGAAAACTATAAGGACCCTAGACAAAA
GGGATAAAACCTAAGGGGTCGTTTGGCTTTATCGCATTAACAAAAAC
>PGSC0003DMG400030113 LRR-RLKs
CTTCAGTCTTCATTACCATATGAAAAGAAACACAAGGGAAGCATCATTTTTAATATGCTCCAATCAAGAT
CTAACCGTTTTTTGCTTAATTTGCAAATCTTCTTCCTAATTAGCCTGTTTTTCCAAGAAACCTTACAATTTTC
GGTCAGGGAACAGTCCATATTACTAAGCTTGAAACGACACTGGAGGGATTCCGAGTTCCTCCAGTCATG
GGACTTGAATTCTTCAGCATGCACTTGGTCAGGAGTTTTGTGCATCGATGACCGGGTAGTGACTCAACTA
AATCTTGGAGAAAAGAACATTACTGGAAAAATTCCATCAACAATAATATGTCAGCTCAAGAACCTTAACT
TCATTGATCTCTCCAACAACAACATTTCAGGAACTATTCCAGTAGGCCTGAAAGATTGCTCAATGTTACA
ACATTTGGACTTGTCCAATAATTCTTTGATCGACCGGATTCCAGGTGAGTTGTTTGGGATGAAACAGTTA
CTCAGTTTGTATCTTAATGGTAACATGTTGTCTGGTGAGATGCCAAAACAAATATCATCTCAACTGGAAA
ATCTTGATCTTTCTGAGAATCACTTGAACGGTTCGATACCAGAAGATATTGGAAACTTCAAAAATCTTTTG
AAATTGGATCTGTCACATAATTCTCTCAGTGGTTCAATTACAAGCAAGTTGTTTCAGTTGCATGATTTGCG
TCACCTGTCGCTAAGTTCCAACTACTTGTCCGGTGTGATACCTGATGAAATGGACTTGTTTAGTTTATACG
ATATGGATCTTTCACATAACCAATTGACTGGTTCTATACCAAAGGGATTTCAGTATTTACCTGGACTGCAT
GCCCTGGATTTGTCTTATAATCAGTTATCAGGAGACATTTCAAAAAGTATAGAATATCTTAGGCCTAGAA
ATACTTTAAGGCTTTGTTCCAACAAATTCTCAGGAAGAATTTCTTCTGAATTTGTTAAGCTAACATATGAA
GTGAATTGCTTTGATGAGTCCAATCTTTGTTCTACATCCAACAACCGAAAACTTTTGAGATCACAACACTT
GATTATTCCTATTGTAGGAGTGGGGTTGGCTATACAGTTATCATGGATCTTTTACAGAACAAGAAAGCAT
TGGTGGAAAACAAAGAAGCAGAATGTCAAAGATGACATGAAGTTCATTTCATTTCAGAAGTTGAAGGTG
ACTACAGAAGACATTTTGTCTAGCTTGAAAGACAAAAACATAATAGGAAATGGAGGATCAGGGAAGGT
CTATCGAGTTGTGATAGACCAAACAGGCAATACTTATGCTGTTAAAAGCATAGGGCATGGAGAAAAATC
AGGTGGAAGACCCCAAAAGGAGTTCCTGGTAGAAGTTAGAACCCTTGGTAGCATTCGACACAACAACAT
TGTCAAGCTCATCTGTTGCATCTCAAGTTTAGACAGAAAGCTTCTAGTCTATGAATACTTTGAGAAACAA
AGCCTCGACAAATGGCTTCACAGAAAGAAAAGTGTAGTATCATCGGGTCAAAGTAGTACCCCGGCCCTG

GATTGGCAAAAGAGATTAAAAATAGCCACTGGTGCAGCGCAAGGACTCTGCTATATGCACCACGACTGT
ACTCGATCCATCATTCATAGAGACATTAAGTCCAGCAATATCCTTCTGGACTCAGAATTCAATGCAAAAA
TAGCAGATTTTGGACTAGCAAAAATACTATCCAGGAGGGATGATAATCCTGAGACAGTTTCTGCTATTGC
TGGAACATTTGGTTACATTGCACCAGGTATATTACAGTTTAAATATCTATTGTACCTCGAGAATTTCACAA
AAGAATTATACTCCAATATTAAATTTTAATGTTTGGTCCTCACAGAGTATGCCTCGACATTTAGAGTGAAT
ATAAAGACAGATATCTATAGCTTCGGAGTGGTGCTATTGGAATTAACAACAGGGAGGCAACCCATTCTC
AGAGAGGACCAAATGAATCTAGCACAATGGGCTCAACAGCGTTACAGAGATGGGAATTTCATTGTTGCC
CTTGATGAAGAAATCATGGAAACAAGTAATGTGGAACAAATGAGAGGTGTTTTTAAACTAGGACTAATG
TGTACCGGAGCATCACCATCTAGTAGGCCGTCAATGAAGGAGGTCTGCAACATTCTTCAAAGTCTCAGA
GACCCCATATTTTGAGAGAATAGAATGTTTCGCCTTACATAGATAAATGTAGTTGTATTTTTTTTAACAGA
ATGAGAAAGTCGTGTTGATAACGATTTCTTCCAGCAATGTATACTTTAGTAATACTGTCAGAGGACATTT
TTTATCCTCAGACAGATCAATAGCCTCATCTGGTTTAAGTGGTGTCAAACACAAGGGCTTGTAAGCTTTA
CCTTAGAATGTGATTCCTTGTTCATACTTGAGATGTCGCGAGGCAAGAGCTAAGTGTCGTGGCAATTACA
GTGTATATATATATATATATATCTTTTTTTTAATTCTTCAGAACTCTCCAAGGAAGCTTTTGGAACATACAGGT
TTGATGAACTGCAAATGCCTTCTTTCAAACATAAATCCTTCTCATGATAATCTTGTAGGAAGTCTAGTTCT
TGTGGCAGAGGTTGTAGCGGCTGGGGATTATCCTGGGGTTCCCAAAAAATAAAAAATAAAAATTCTAAC
AGTTTTACACAATTGATAAAAGCTAAAACCATTGATTAGATATTCATTAATGGTCTAGGCTAACTTATATG
ATTGGAAAAATGAAATGTTCCTATAAGCGATCAAGTTTTTCTACTCGTTAATTCTTTTGGTACTTGTTTTAA
TTAGTTTGTTAGAATTGTTCTGACCCTCAGTGATACTCCTGATGGAGCAGGGTCAAGTAGGACTAACAGA
CTTCTAGGTTCACAAGCTTCAGAAGATGAGGTGAACATGACATCTTAG
>PGSC0003DMG400030117 LRR-RLKs
ATGTTCTTCCGCATTTTTTTCGTCCTAATTAGTCCGTCATTGCAAGAAAGTTTACAATTGGAACAGTCCGT
GTTGTTAAAATTGAAACAACACTGGAGCGATTCGGAGTTCCTTCAGTCATGGGATTTGAATTCTTCAGAA
TGCACTTGGTCAGGAGTTTCGTGCATCGATGGCAGGGTAGTGACAGAACTACATCTTGGAGGAAAAGA
CATTACTGGAACAATTTCATCGACAATATGTGAGCTCAAGAACCTTACCTTCATTGATCTCTCCAACAACA
ATATTTCAGGAATCATACCAGTAAGCCTGAACGATTGCTCAATGTTACAACATTTGGACTTGTCCAATAA
TTCTTTGAATGATCGGATTCCAGGTGAGTTGTTTGGGATGAAACAAATACTCAATTTATATCTTAATGGT
AATATGTTGTCTGGTGAGATGCCAAAAGAAATATCATCATCCCAGCTGAAAAATCTTAATCTCTCTGAGA
ACTACCTGAACGGTTCGATACCAGAAGATATTGGTAACTTGAAAAATATTGTGAAGTTGGACATGTCAC

ATAATTCTTTAAGTGGTTCAATTACAAATCAGTTGTTTCAGTTGCATCATTTGCGTCACCTGTCGCTAAGT

TCCAACTATTTGTCCAGTGTGATACCTGATGAAATGGACTTGTTTAGTTTGTATGCTATGGATCTTTCTCA

TAATCAATTGACTGGTTCTATACCAAAGGGATTTCGGTATTTACCTGGATTACATGCTCTGGATTTGTCGT

ATAATCAGTTATCAGGAGATATTTCTCAAAGTATAGAACATCTTAGGCCTAGAAATACTTTAAGGCTTTG

TTCCAACAAATTCTCAGGAAGAATTTCTGCTGAATTTGTGAAGTTAACATACGAAGAGAATTGCTTTGAC

GAATCAAATCTTTGTTCTACATCCAAGAACTTATCCGTCCCTGTCCTACCAAGCTGTTCCTCTGATGATGA

GGTTCGAAAATCTTCGAGACCAAAACACTTGATTATCATTATTTCTGTTGTAGGAGTTGGGGTAGCTATA

CAGTTAACATGGATACTTTACATGGTCAGAAAGCATTGGTGGAAAACAAAGAAGCAGAATGTCAAAGAT

GACATGAAATTCATTTCATTTCAAAAGTTGAAGGTGACTACAGAAGACATTTTGTCTAGCTTGAAAGACG

AAAACATAATAGGAAAAGGAGGATCAGGGAAGGTCTATCGAGTTGTGATCGACCAAACAGGCAATACT

TATGCTGTTAAAAGCATAGGGCATGGAGAAAATTCAGGTGGAAGACACCAAAAGGAGTTCCTGGCAGA

AGTTAGAACACTTGGTAGCATTCGACACAACAACATTGTCAAGCTCATGTGTTGCATCTCAAGTTTAGAC

AGAAAGCTTCTAGTCTATGAATACTTTGAGAAACAAAGCCTCGACAAGTGGCTTCACGGAAAGAAAAGA

GCAGCATCACTAGGTCAAAGTAGTACCCCGGCCCTGGATTGGCGAAAGAGACTAAATATAGCCATTGGT

GCAGCTCAAGGACTATGCTATATGCACCATCATTGCACTCGACCAATCATTCATAGAGACATTAAGTCCA

GCAATATCCTTCTGGACTCAGAATTCAATGCAAAAATAGCAGATTTTGGACTAGCAAAAATACTATCCAG

GCGGGATGATGATCCTGAGACAGCTTCAGCTATTGCTGGAACATTTGGTTACATTGCACCAGGTACATA

ACAGTTCAAACATGTATGAATATTCAATCTTTCAAAATCCAATTCTACTATATCAAATTTTAATATTTGGTC

CTGACAGAGTATGCCTCGACATTCAAAGTGAACGTAAAGACTGATATCTATAGCTATGGAGTGGTGCTA

TTAGAATTGACAACAGGGAGAGAACCCATTCTCAGGGATGAGCAAATGAATCTAGCACAATGGGCGCT

ACAGCGTTACAGAGAGGGGAATTCCATTCTCGAGGCCCTGGATGAAGAAGTGATGGAAACAAGTAATT

TGGAACAAATGAGAAGTGTTTTTAAACTAGGAGTGATGTGTACAGGAGCATCACCATCTGGTAGGCCAT

CAATGAAGGAGGTTTGCTATGTTCTCCAAAGCTGCAGAGAGTCTAATATTTGA

>PGSC0003DMG400030119 LRR-RLKs

CTTTGCTGTTTTTTCTTGGCCACAGTCCACAGACGTCAGTGAAGTCAATTTTTGTTTCTTATCTTTTTAAAA

TTTAACAAGTCAAACTTTGTTAGCATTATACAAATTTTCGTCCATGTTGACTGACTTGTGTTGTATAATAC

ACGGTTTCTTCAAAGCTAATTCATGATCCACAAAATAAAGAGGACAATCAAACCAAAGGAGCCATGAAA

ATCAACCATATTCATGTTGTAAAATCTACATTCTCAATTCCCAGGAAACAAGTTTACCTTTTCATTGTCTCC

TTACTTGCCTTTATGCATTTCCAGGGCGGTTTACAGAGTGACGTCTACGAAAAGGAGAAGAACATTTTGT

TGGAGCTGAAGCAACTATGGGGATTGCATCAATTATGGGATTCCGATTCCGATTCCTCACACTGCAGTTG
GTATGGTATAGGCTGCAGAAATGGTTCTGTTACAAGCATCTCGATTCATGAAGGGAATCTACTTTCCGGA
ACAATTTCACTAATCATATGTGAACTCAAAAGCCTGCAATTGATAGACCTGAGTAGCAACTATATCAGAG
GAGAATTTCCTACCTCCTTGTACAATTGCTCAAAGCTGGAATATCTTGATATTTCTTGGAATCAATTCCAT
GGACCACTGCCTAGTGACCTTCATCGTTTGTCGAGGCTAATCCATTTAGATATTGCTGGAAATAACTTCA
GTAATATCCCAGAAGCCATAGGTCAGCTTTCTGAATTGCAATATCTATCTCTTAAGTTCAATAATTTCAAC
ACTTGGATATCCCGAGAAATTGGAAATTTATCAAAGCTTGAGACCTTGGATATATCCTATATTGACAGCT
TTAAGCAGGCAACAATCCCAGAGGAATTAAGAAGACTGAAGAAGTTGACAGCCCTTTTCATTGTACGGT
CAAATTTGAAAGGGGAGATTCCAGAAACATTTTCCGCTCTTTCAGGTCTCGAAGTCTTGGATCTCTCAAT
GAATTACTTAAAGGGGTCTATCCCAAGTTACTTGTTTGAATGGAAGAAGTTGACGAATCTTCATTTGGAG
TTTAACCAATTTTCCGGTAGGTTACCAATGTTGGATGCAAATATGGAATTGATGGGGAGAACTCAAGAA
ATGTTTTCTAATCTTTCTGGTCTCCAAGTCTTAGATCTCTCAGGAAATCACCTAAATGGTTCAATTCCAAG
CTACTTATTTCATTGGAAGAATTTAACATCTCTTCTTTTGGGGGATAACCAATTTTCTGGGAATTTACCTA
GTATAACTGGAAATTTGAGATTAAGAACATTGGATCTTTCATCGAATCAGCTTTCTGGACATATTCCACA
AGAGTATGACAAGAATGAATACTACTTTAGAAATAATCTGAATCTATGTAGCAAATATACCAACCATTAC
ATCCAAGTTCCACGTTGAAGTGGAAAAACAAGAGTTGTAATTGCCGTTGTGGCCCCTGTTGCATTTTTAG
TCATGATAACATTATTATGTTACACATTTAAAAAGAATTGGACTTTCTCCACAGACCAACTCATGTGGAG
GAAGAGGCATGAGAACCAAGATCCTGAGTGGATGTTCATTTCTTTCCAGCAGTTAGGATTCACAGAATT
TGAAATTCAGGTGAATATGACAGAAGAAAATTTGATTTGAAGTGGAGGATATGGGAAGGTCTATCGAG
TTGGTGTCAATCCTAATGGGAACTTTGTGGCAGTTAAGAGAATATGGAACAAGAGGAATTTAGACCACG
GGCTTGAGAAACAGTTCCTTGCAGAAGTTGAAGTATTGGGTAGCATTCGACATTCTAATATTGTGAAGTT
GTTATGTTGCATTTCTAGAGGAAACTCAAAGGTTTTGGTGTACGAGTACATGGAAAATCAAAGCTTAGA
CAAGTGGTTGCATCACAAGAGAAGAAGTGAAAAAGATATTACTGCCTCAGCACATTGTGTATTGGAATG
GCATACTAGACTGCAAATTGCAATTGGTGCTGCCCGAGGTCTATGTTACATGCATCACGAGTGCTCTACG
CCAATCATTCATCGTGACATTAAGTGCAGTAATATCCTTTTAGACCATGAACTGAATGCCAAAATTGCAG
ATTTTGGACTAGCTAAGATTTTGGCCAAGTGGGGAGAGACAGAGACTGCTTCTGCTATCGCTGGCACGT
TTGGCTACCTAGCTCCAGGTATATTTCACTGTTCTCATAATGAAATAAACAAAAACACACCAGAGTGTAC
GTATGTATCGTGTTTCTCATTGATTTTATCCTAATACCTTGTTATTCTAATTGGAATTTCAGAGTATGCATA
CACGTCGAAAGTGAATGCCAAGATTGATGTCTACAGTTTTGGTGTGGTGTTATTGGAGCTGGTTACCGG

GAGAGAACCAATTAACGGAGACGAGCATATAAACCTTGCACAAAGGGCATGGAACCACCATGAAGAAG
GCAATCCAGTGGTTGATGCTATTGATGAAGAAATCAAGGAAGCATGTTTCTTGAACGAAATGAGTTCCA
TGTTCAAGTTGGGGATTATTTGCACCAGTACTGTACCTTCTGCTAGACCTTCCATGAAGGAAGTTCTGCA
AATTCTATTACTTAGCCTTAATACTTCTTCCTAGCAATTGGCTGCGAATCCAGGATTATCGTTGCTTCTTTA
TTTGCACTGAAAAATTAAAAGTCCAAA

>PGSC0003DMG400030169 LRR-RLKs

CTTTTCTTCTTCAAGAATTTAGGGTTTCTCTTCTCCACCGACGGGAGGCGTTATTTTCCTCCTCTTCTTCAC
AATTCTTCTCGAATTTCTTCTATATATAAGTGGGGTTCTCGTTCTTGATCCTTTTGATTTCTTATTCAAGCT
GCTCGTATGGGTGAAAACGATTCAGAAAGCTCTGAAAGTGCCAAACCCAAGAATGTATCTGAAAATTCC
GACGGAGGTGATGAATTGACGGGTTTAGAGGAAGATTCGAGCAAGATGGTTTCTGGGTTTGATTCAAG
TTTTGATATTTCGGGGAAAAGTTTGGATTTTCCATTGTTGGAAGGTGTAGAAGGTGGGGTTGAAGGTTT
GTATATGTATAAGAATGTATTTAATTTGATTCCTAAGGCAATTGGTGCACTTGGGAAGGTTAAGATACTT
AAATTTTTTGGGAATGAGGTTAATTTGTTTCCAACTGGAGAGTTAAGGAATTTGGTTGAATTAGAGAGTT
TGCAAGTGAAGGTATCATTTCCTGGGATGAGTGGATTGGATTTGCAGAAGTTAAAGAATTTGAAAGAAT
TGGAGCTATGTAAAGTTCCCTCTAGACCTTCAGCTTTCCCACTTTTAAGAGATATTGCTGGTCTTAAGCGC
CTGACTAAGCTCTCTGTCTGTCATTTTTCTATCAGGTTAGATCTGTTTCTTCTTGCAATAATTCTATCTAAG
TTTATTTACGCTAAACTCATGTTTACTATAGACAAATTACCTGTAGTTGGCTGTCAGTTAACTTGATAACA
TAAAATTTCTGACTTTGTCAATGTATAAATATTAAACCAATTCGATGATGTTTCTCTGTGAACTTGTCTATA
TTGTTGCACTAAATTCATGTGCAGGTTGCAGCTCAATGTGCTTTTGGTGTTATTGGAAGATTAGGTTGCA
TCTGCACTTTTTCGCTTTTGCTGCATTCCTTAAAGTAATTTGAATTTAATAGAACAAGTTTATTGCTAAACT
ATTGTTTTTACAAGTGCTAGCTGCTGCAACTATAAGCCCCAAGTTAAATAGTATTAAGCAGGGTACATGA
TCACACAAACTTCCATTAATACAGGGGAAACTAGGTAGGTAGAGCATCCTACCAGGATGTTTTTTGTGGT
CTCTTTAAGGAGGTAAAGACAGGGAAAAAGTATTTGCAGGTTTAAATGGTTAGTTTCAGTTTTCGGGTA
CAAAATTTTCCTGTTAGTTCAAACTTGTTTTGTAGATTGACATGACCAAGCAAAACAATTTTATGCATGAT
TAGTAAAAGTTTTACAAAAACAACAGATCTTCTCAAAGAAATTTTGGATGTGGTAACACAGCTGGAGCAT
AAAAGAAAATAAAGACTTGTTTTTGCTGCTTAGATGCTTCATGAGATTGAAGTTTGTTAATTAGGACCAA
AAGATATGCACAAAATTTTGTAAGTTGTTTATCGGGAGTTTAGACTTTAGCATTCTTCAAAGGGTATCAG
GTTCTACTCTAGGACCATACTAAGTTCAACCAGGATATGATACGATATTAGGATTAGTCGATAGTACATA
GCATAACTATCCCTTGGTTAAGGGTCGTAAAGAGTGTTGTTTTTTAACTATTTATTTGTGTTTCCTTTAATT

GAAATTTTTATTTTTTGTTTATGGGATTTCTTATCCTATAGTGCATCTTAGTTAGGTTAAAATCATCTTATT
ATTTTACCTCATGTAATTTGACAGCTGAAGAGGTTATATGGTTTGCCTTTATAATGGATAAATTTATTATT
TTGAGGTTGGCATTATGCAGCCACCAGAAGACTTTTCCCTTCATCTCCGTCTTGTTGTTTCTTGCAACTAC
AACGAGTTAAAGATTTTGAGACATTAAAAATCTGGCTGGTTAATCAATTTGCAGCATATTGGTTGCTGTT
GTCTCCTGGCAATTATACCCTCTCAAGGTCCACATTTTGTTTGAGAATTATCTTTTGGTCCCTGAAGACAT
CATTCTACTTTTGATTAGCAGGTGTTATGAAAAGCCTATGCACGAGTACATCACATAATTTTAAAGTTATA
GACGTCTTAAATCTCACAGGTCTCCAATAGGAAAAAAAGAAAAGGGAAGTATATGAAGTGACTTTGTTC
GCTTGGAGTATGGAATATAGAATATGCTGCCTCAAGGAGTTTGAGGTGCAAAAGGTGTCCACACACTTC
ACACTTCAGAATTGCGAGCCTGTTGCTGTCACATCTTTCCATCACAGTAGTATGTCCTATAGCTAATAGCA
GATTAAGTGAACTTTTGCTTAGCATGTGTAATTTATCAGTTGTATATCATTGCTTAATGTTGGTAGGACTG
GAAATTAGTCGTTTTTGCAGTTGGAAGGAGGATTCAAATTGGAAATAGGTGTTAGGATTTGTATTTTATA
GTGTTCCTTTTATTGGGTGCAGTTACGCCAGAATATTGACTCTTCAGATAAAAGTCATGTTTAAGTTGTTT
GACACTTGATACATTTTGTTAGATGAGGTCTGATATCTAAAAATATCTGTACATCACATTTTATTGTTTTG
ATATCTGAAAGGCTAAAACTTCATTCTGCTACTCTTTTGGCAAGCAGTGCTTTACTTTTGGTTGCTAGAGG
TTGAAAGTTGTGAACCATAAACTTGTAGATTCAGTTCATATCTATAAACCATTTTATCTTTTTTTTATCATT
GTACTCCATTATAATGCCATTGTTCCTTGCTGTTCTTGCAGATATTTACCTCCTGAAATAGCCTGCCTCTCT
AAATTGGAGTGCCTTGATCTTTCATTCAATAAGATGAAGAATTTACCTGTTGAGATAACTCATCTTAACTC
CTTACTGTCACTGAAGGTTGCTAATAATAAACTGATTGAAGTACCTCCGGGGTTGTCTTCTCTGCAAAGA
TTAGAGAGTTTGGACTTTTCTAACAATAGATTAACATCCTTGGAGAATCTTGATCTCCTTTCAATGTATAA
TCTGCAGAGTCTCAATCTTCAGGTATGTGTTACTGAAATTACAGTTTCTTTCTAGTCTTGTTGCTTTTAGGC
TTTTTCTCTAGTTTCTTTTCCTTCTCTGATAAATGTTTATAACATGAGAGGCATCTACTCTTAATGTGGATG
AAATTCTTGCATCCCACTGGAAAAAAACCCGAAATGTATTTGTCTCAATTGTCTAGTCAAGTAACTTGATA
TATTATAATTACCTTATCCTGACCCATATATCTTCATTGAAATTTGCTGAAGCACAATAAGCTTCGTAGGT
GGTGTTCGATTCCTTCATGGGTATGCTGCAATTTGGAGGGGAATTTCATAGATTTGTCCAAGGATGACAC
CACCAGTTCGTCTTCAGAAATGGACGTGCTTGAAAGTTATGAACAGGAAACTTCTGAGAATACCCAAAA
TGGTGAGTTCCTCGGAATCTCTTGTCTCCCCTCCTTATATCTATTGCTATTTCTGCACATTCTGATTCCTTTG
AATCTTGTTTGTTGCCCTTCTAAGAAGTGTTGTTCGGAAGGGTAGTACCTTTGAGAAGTCTGATGTGTGA
ACTTTTAATTGTGTAGGAGTTTCTATCAAATTGTCAGGTCATTTATGTGGCTCATCACCCAGTCATCGATG
TTTTCGGCCTCGAAAATCCAAGAAATGGAAGCGGCAGTATTATATGCAACAGAGGGCTCGTCAAGAGC

GATTAAACAACAGCAGGAAATGTGTTGCCTGCAAACATTCTAAGCTAATTGATGATTCTCTGGTGGAGG
CCTCATCAAGCATAGTGGATGATGATACACATGATAAAGAACTGATTCCTGAAGAAGCTGAGTGCAAAG
GCTCTTTAGCTAGTGGTATTGATGAACATATCAGATTAAAGGAGGACAATTATATAGGAAGACCTTCAT
GTGTTGCTTCTGACTCCATTGAAACATGCATAGATATTCAGAATTGTAAAACATGTGATGCTTCAGTAGG
TTCTGTTTCTGATGCTGCTGATGTGGCTGAGGAAAGTTTGTCTTCAGAGGTGTCTAATAGTCCTCCAAAA
TCGAAAAGGCACCTGGATGGGGTCATTGATAATCCAAAACCATGCAAAACCCGCAGACCAACAGATCAT
TCAGAGGTATCCTGCAAATACAGCATGATGTCTTTCTGTGGTATTGATGATTATTTACCAGATGGCTTTTA
TGATGCTGGGCGAGATCGGCCATTTATGTCGCTGAGGAGTTATGAGCAGAATTTGCATCTTGATTCACG
GGAAGTCATTTTAGTTGATAGGTAACTTCCATGTTTGCATGCCATTAATAGACTAATAGTTATGGACCTC
AACTACTAACTTCAGGGAATGCTTTGCCTCTCTACTCATTTTGATTACTTATTTAATCTGTTTAGGCAAAGA
GATGAAATGCTGGATGCCATTGCTCTACGTGCTCAAGCTCTAATCTTTCATTTTAATCAGATAGATGGTCT
GTTTAAAGATAGAGAACATGTTGCTGTAGACAACTTGCAAATTGCATCACTGCTTGCCCTTTTAGTGTCG
GATCATTTTGGTGGAAGTGACAAAAGTAATATTGTACAGAAGGCAAGGAAAGATGTATCTGGGTCGAA
CTATAGCAAGCCTTTTGTCTGCACATGCCCTACTGGAAATGATGACACTACCAGCATGGTCACAAAAGAG
AGTCCTAGCATCTCAGAAGATATACTTTTCCTTAATCTTTGTGAGAAAGCACTTCACTCAATAAAATCAAG
GCAAAATTCTATTGTTGTTCCTATTGGGAGTTTGCAGTTTGGTGTATGTAGGCATAGGGCCTTGCTTATG
AAGGTATTTTGTCATCAGACATGTTTCTAATTAGATATGGTGGTTCTGAATTTAGTTTATTATTATGAACT
GATAAATTTCATTTGTCTTCTGCAGTACCTGTGTGACCGAATAGAACCTCGAATATCTTGTGAGCTTGTTA
GGGGTTATTTGGATTTTTCACCGCATGCGTGGAATGTGATCGTGGTAAAGAGGGGTGAATCATGGGTTC
GAATGATAGTTGATGCGTGTCATCCCCTTGATATCAGAGAAGAGACAGATCCAGAATACTTTTGCAGGT
CAATGATTTTCCAATTCTGTGTTACTTTCTTCATGACTAATTTCTGGTACTACAAGCATTTATATCAATACA
CGTGTATGACGATATTGCATTTTCAATTTAGCCAGTGTGATTATTGGTTATTTTGGGGTGAAATTGCCAG
CTTCTTGAGTAGTTCCTTAGTTACTAAAAATCTCTAGTTTACCTGAGTTATGTCGGAATGAAAATGGCCAG
ATCACTGAGCTTGCTGTTTCTTTCAGGTATATTCCACTGAACCGGATAAATGTTCCTGTTGTGCCAGATGC
TAGTCCTGGTCAAGTTAGTTCTTTTCCTTCTCTAACCGGTGCTGATAAAATTCATAAAGCTCCCTCCTCAAC
CCTCGTCCCGTGTAAATTGGGATCCCTTGAGACTCTGGCAAAGGTGTGGTTCCTGATTGCCTTTAAGTTC
TCCAGTTATTATTATGCAAATATACTTAAGTTCTTATTCCAAACTTCAATGACTTGAATTAAATATGTTTTA
TTCTTAGAGTGACTGAACTACATGTTTATGTGTTAACCCACAACAATTAGAAAATGATTTTTCTGGCTTTA
TTGTACTTTCCATTATTGACTGGAATTTACCAGTCTATCTGGTCTTGTGCTTTTGATTTGTTTTGGAAATTT

GAAATAGATAAGAAGCAAAGCAATCCTTTTTGACTTATTCTGCTATTCAGCTGGAATCCATTATATTGCA

GGCTTATGCTTTTCATTTTGCAAATTTTTCATTTATCTGCTGATAAAACTTGATTAATTAGTGACACGTATT

ATACCACTAGAATGTGGGAATTTTTCTCGGCTAGCATCTTTATTGACGAGGCATAGTTGATTTTTTTTTAA

ATTTATTCTGCTCAATTAACCTGAATTGAGCGGAATGAATATAAAAGAGTCATATGACTGATACCGACTA

GTTCAGGATTGAGGTTTAGATGATTGAATGAACATTTTTGCCACTGCAGGTGCGAACTCTAGAGATGTCC

AAGTCCACAGCGGATGAGATTAAGAATTTTGAGTTTAACTGTATTGGAGAAGTTAGGGTGTTGGGTGTC

TTAAACAGTTCGTGCATTGTCAAATATTATGGTCATCAAATATCTTCAAGATGGGTTCCTTCATCAGATGG

CAGTTCAGAAAGTCGTACTTTGCAGTCTGCCATTTTGATGGAGCATATTAAAGGGGGTTCACTAAAGGT

ATATGTTTTCCTAGAACTCTTCTCGTACCTGACTTTCCTCTCCTTTATTTCCTTTCCCTTTTGAGGTTGGAAG

GGTATAAATAACACCTGATTGCCTTTTGTATTCAGCTCATTTTACTTTCTTGTCCTTAACTGGCAGAAACAT

GTAGATAAGCTATCTAATGCTGGTGAGAAGCGTCTTCCCGTTGAGCTGTCGGTATTTATTGCCCGAGATG

TAGCAAGTGCGTTGACAGAGCTGCATTCTAGGCATATCATACATCGTGATATAAAAAGTGAAAACATCC

TGATAGATCTGGATAAGAAGAGAGTTGATGGTACCCCTACAGTAAAGCTATGCGACTTCGATATGGCAA

TTCCCCTTCGTTCATACTTACATACTTGCTGCATTGCCCATGCTGGAATTCCTCCACCAGATGTTTGTGTTG

GTACACCTCGTTGGATGGCTCCTGAGGTCTTTCAGGCGATGAATAAGCGCAATATCTATGGGTTGGTAA

GTCACCAATCCTTCTGACCAAAAAAAAAGTCACCAATCCTTGAAGTTAGAATCCTAACCATTGAATTCTCT

ACTAACAAGTACCAATTAAATGCCCCACCGGGTGCTCTGATCTGCTAAAACAATGTGGTTCTTTCTGATTT

GCCAATTTGTCACCCTGGGGTAAAATCAATTCAACACAATCAAGAACTTACCACCTTCAAAAGACACACA

TTTTCTACTAAAAACTGATATATCACTCATTCCTCTATTAAAGTTCAGCTTTATCTTGTCATTATTAACTTCA

AATGTTTTAAGCTAGAAAAGAAAGCATATTTTGTCTGCTCTATTGCTGCACTGCTACTGATTAAAGAACA

ATAATATGTACTCATTGACAAATTCAAATTTTCACCTTACTTTCTTGGTAATTTGTTTGTTTAAATTTCCTCA

TTTTTGATGCTTGAATCTATTTCGGAGATGAGGATTTTTGTTTTTGAGAGACTATTTTTATATGACGGCTA

ATCTCAGAGGATGTATCCTGTGGCAAAAGGGGTTGGGGAATATTATCTCTTACTTCTTTGAAGTTGAAAA

GGTCTCTAGGTTATAGATGAGTTGCTTCAAGGTAAAAAGGTTTTTCTTGCTTTGACTTTGCAGGTTCCCC

GTTTCAATAGGTAGATGAAAGCTAAATAGGACCTAAAATAAGTCCACTATGGCCAAAAAATATCAGGAG

GGGCGGGTGGATTATTTCACCCTTAACCCAAATTCAATAGATGGGTAAATTGGCTAGAATGGGTTATTG

AACAACAAAAAAGTATTTATAGGTGCTAATTTCCTGTATTATCTTTTTCCTTTTGAAGATGAACAAATGAG

TTTAATTGCTTTGAGTTGGTGACTTGAAAGATAAAGTGAGTTTTTGTCATGTCTAAACTGCAATTCTAGCT

TAATGTATTCAAAGGTTTTCCGAACTAGGTTTTTACTTGTCTTACTTTGGATGTAGGGTGCTGATATTTGG

TCGTTTGGGTGCGTCCTTCTGGAATTGTTAACACTGCAACTTCCTTACTCAGAGTCATCCGAGTTAGATAT
CCACCATTCTCTTCAGGTATTTCTTGATCTCTAACCTTCGATTATTAATGCTCTAAATACCATGATGAGTAC
CTAGTGTGCTTGAAATATCATTGTGTGATGAGGACTTCAGAAGTTCCAAAGTCCCCTGTTGGACAATCTT
TATTATGGCTGCCCTTGGTTGCTCAAAAGGCTAATGGTTCTAGCATCTTGGTTGTTGGATATTTCGGAGC
TCTCATGGTTTTCTTCCTTATATTGCTTTCGTTCATAAGAACTGCCTGCAGAAGTACACTTGTCGCATGCA
CATTTAAAAAAAAACAAGTTTTTGTTTGAATTGCTTTAAATGGTAGAAAACAATGTAATTCATGTTTAACA
CATGCAGGCGGGTAAACGGCCACAATTAACCGAAAAATTAGAGGCAATGGCTGCGTCCAAAGCAGAGC
TAGAGGATCTAGCAAAGTCTTGTTCTAGCTCGGACCTTGACAAGAAACAATCTGAATCTCGTATCCTGAA
ACTTCTCGTTTCTATTTATCGTTGGTGTACTGAGAAGGATCCAAACGACCGTCCCACAGCAGAGAACCTC
TACAACCTCTTACTTACCTGTGCTAATTCCCTCCCTTCACAACAAAGTCAAGAAGATTCTTGATCTTCCTCA
AATTTTGGTAGCCTTAGGATGTTGTAGATTAGCATTAGATTATTCAGCAGGAGTTACAGACCCAATACTT
GGGTCTGTTGACCCCTCATTGAGCAATGTAACATGTTCTTAGTCTTATTTGAAATGGAAAACCATACGGA
CGTATTCGCGTCCATAGGACATTTATGATGGTCTTGTTGTGTGTGTGTTGTGTATCAGTTTCTTTAGTC
CTTTTCCAGCTTGTAGTTGTATACATTTGTACCCATCTAATGTATTCATCTTCACTAATTCCAATTGATGTT
AATTATGAATGTATTCTGAGTTTCTAAGACTTGTTGGATTGTGC
>PGSC0003DMG400030298 LRR-RLKs
ATGTGGAGAGGGCCATGGTATGTACCCCAACTGAGCGTCTTAAATCTCAGGAATAATAGCCTCACGGGT
ATAATTCCTCCTTCTGTTGGAAATGCCACACAAATGATGAACTTCAGTTTGTATGGGAATAGAGTCAGCG
GCAACATTCCAAAGGAGGTCGGTAATCTGAGCCAGNNNNNNNNNNNNNNNNNNNNNNNNNNNNN
NNNNNNNNNNNNNNNNNNNNNNNNNNNNNNNNNNNNNNNNNNNNNNNNNNNNNNNNNNNN
NNNNNNNNNNNNNNNNNNNNNNNNNNNNNNNNNNNNNNNNNNNNNNNNNNNNNNNNNNNN
NNNNNNNNNNNNNNNNNNNNNNNNNNNNNNNNNNNNNNNNNNNNNNNNNNNNNNNNNNNN
NNNNNNNNNNNNNNNNNNNNNNNNNNNNNNNNNNNNNNNNNNNNNNNNNNNNNNNNNNNN
NNNNNNNNNNNNNNNNNNNNNNNNNNNNNNNNNNNNNNNNNNNNNNNNNNNNNNNNNNNN
NNNNNNNNNNNNNNNNNNNNNNNNNNNNNNNNNNNNNNNNNNNNNNNNNNNNNNNNNNNN
NNNNNNNNNNNNNNNNNNNNNNNNNNNNNNNNNNNNNNNNNNNNNNNNNNNNNNNNNNNN
NNNNNNNNNNNNNNNNNNNNNNNNNNNNNNNNNNNNNNNNNNNNNNNNNNNNNNNNNNNN
NNNNNNNNNNNNNNNNNNNNNNNNNNNNNNNNNNNNNNNNNNNNNNNNNNNNNNNNNNNN
NNNNNNNNNNNNNNNNNNNNNNNNNNNNNNNNNNNNNNNNNNNNNNNNNNNNNNNNNNNN

NNNNNNNNNNNNNNNNNNNNNNNNNNNNNNNNNNNNNNNNNNNNNNNNNNNNNNNNNNNN
NNNNNNNNNNNNNNNNNNNNNNNNNNNNNNNNNNNNNNNNNNNNNNNNNNNNNNNNNNNN
NNNNNNNNNNNNNNNNNNNNNNNNNNNNNNNNNNNNNNNNNNNNNNNNNNNNNNNNNNNN
NNNNNNNNNNNNNNNNNNNNNNNNNNNNNNNNNNNNNNNNNNNNNNNNNNNNNNNNNNNN
NNNNNNNNNNNNNNNNNNNNNNNNNNNNNNNNNNNNNNNNNNNNNNNNNNNNNNNNNNNN
NNNNNNNNNNNNNNNNNNNNNNNNNNNNNNNNNNNNNNNNNNNNNNNNNNNNNNNNNNNN
NNNNNNNNNNNNNNNNNNNNNNNNNNNNNNNNNNNNNNNNNNNNNNNNNNNNNNNNNNNN
NNNNNNNNNNNNNNNNNNNNNNNNNNNNNNNNNNNNNNNNNNNNNNNNNNNNNNNNNNNN
NNNNNNNNNNNNNNNNNNNNNNNNNNNNNNNNNNNNNNNNNNNNNNNNNNNNNNNNNNNN
NNNNNNNNNNNNNNNNNNNNNNNNNNNNNNNNNNNNNNNNNNNNNNNNNNNNNNNNNNNN
NNNNNNNNNNNNNNNNNNNNNNNNNNNNNNNNNNNNNNNNNNNNNNNNNNNNNNNNNNNN
NNNNNNNNNNNNNNNNNNNNNNNNNNNNNNNNNNNNNNNNNNNNNNNNNNNNNNNNNNNN
NNNNNNNNNNNNNNNNNNNNNNNNNNNNNNNNNNNNNNNNNNNNNNNNNNNNNNNNNNNN
NNNNNNNNNNNNNNNNNNNNNNNNNNNNNNNNNNNNNNNNNNNNNNNNNNNNNNNNNNNN
NNNNNNNNNNNNNNNNNNNNNNNNNNNNNNNNNNNNNNNNNNNNNNNNNNNNNNNNNNNN
NNNNNNNNNNNNNNNNNNNNNNNNNNNNNNNNNNNNNNNNNNNNNNNNNNNNNNNNNNNN
NNNNNNNNNNNNNNNNNNNNNNNNNNNNNNNNNNNNNNNNNNNNNNNNNNNNNNNNNNNN
NNNNNNNNNNNNNNNNNNNNNNNNNNNNNNNNNNNNNNNNNNNNNNNNNNNTCTTGAATTTG
TCTTTAAATGCATTGTCAGGTACAATTCCTAAGTCTTTGGAAAAACTCTCATACCTTAAAGGCATTAATGT
TTCATTTAATGATTTAGAAGGTGAAATACCGAGTGGTGGCGTGTTTGCAAATTCCACTTTGCAATCATTTC
TCGGGAACAAAGGTCTTTGCGGAATGCACATATTGGAGATTCCTGCTTGTGCTATCACTAATCTTGGAAA
ACAATCAAAGCCTAAGGAGGTTGTGCTAAAAATTGTTACTCCAGTGGCTATTGCATCCTTTATGATATTCT
TGTTCGTTTCAATTTGGATAATGAAACAACACAAGAAAGGAAAGTCTAAAGATGTTGAAAAGGTACCGG
AGATCGGGACTCATCAATTAGTTTCTTATCACGAGATTCAACGAGCAACAAATAATTTTGATGAATCCAA
TTTAATTGGTGAGGGAAGCACTGGCTCTGTGTACAAAGGCACATTATTTGGTGGAACTGCAATGGCCAT
AAAGGTTCTGGATTTGGAAAATGAGCAAGTATGCAAGAGGTTTGACACCGAATGTGAAGTGATGAGAA
ATGTTAGACACTGAAATCTTGTTTCAGTGATTACTACATGTTCTAGTGACTATATAAGAGCCTTTGTTCTG
CAATTTATGCCCAATGGTAGTCTTGAGAATTGGCTGTACAAAGAAGATCGCCACTTGAACCTTCGTCAAA
GAGTCACTGTAATGCTTGATACAGCTATGGCAGTTGAATATCTACATCATGGTAATGTCACTCCAATAGT

TCATTGCGACCTAAAGCCAGGTAACGTTCTTTTGGATGAAGATATGGTGGCTTATGTTGGTGATTTTGGA
ATCTCTAAAATTTTAGCCATAAGCATGTCCATGGCTTATACCGAGACATTGGGCACTCTTGGATACATTG
CACCAAGTATAAAAGAATCTATTCTCTTTGATTTTCTCTTATCATAATTAAGCCTCTCCAAATTCTACAAGT
AAAAAGAGCAAGTCTTTATTTTTGCAGAATGATTGTTGTATTTCAATTGAGTAACTTTTCTTCAATCCTTTT
CTAAGAATATGGCTCGGAGGGAATAGTGTCTACTAGTGGTGATGTTTATAGCTACGGCATCATGTTGAT
GGAGGTTTTGACCAAAAGAAGGCCAACAGATGAAGAAATATGCAATGAAAATCTTGACTTGAGGAAAT
GGATTACACTATCATTTGCAGGGAGTAGGGGCGGCTCAACATAATTTGTGGCCTAAAGCGAAATTTTTA
TTAGAGGCCTAAAATCTATATAAATTTTATCTATATCTATTTATTTTTCTAAATTTCTAGACATAAATTACT
ACTTAATATCTTTTATATAAATGATTTTTCAAACACTTTTTTTTTAATTATTAGTTTTAAGTAAGATTTTATC
AATTCAACATTGAAAAACATCCTTTTATTGAGAAAATTATTATATGAATTATTAGCACAATTTTATAAGTA
ATAAGTTGATAAACTTTAAAGATAAGAGTTAGAATTATAGAATTTGATTCAAAAAGTTGATAACTTGGTA
TACCTACTTTAATATGAAAATTAACAAATAAATAAAAAAAAGAATTTGTAATGCACTTTGTTCAACACTTT
TCGACTATTGATTCATATTTTTGAGAGTGTTTACTCTAACTATCAAAGATTTGAAGAAATAGAGTGCAAA
AAGAGTTACAAATTAAAGGATAAATAATGTGAGTGTTAACGAAGAAAATGTAAGAATCCAAAACAATGT
TTCCTTAATTTCTTCTATTTGAATGGGGTTAAGATACATAATTTTTTATTTATAAAAATTTGAGGCCCCCAA
AAGGCCAATGCCTAAAATTTTATAGGCAAGAGCCGGCCCTGGCAGGGAGTATGATGGACGTTGTGGAT
GTTAGTCTTTTTTCTGAGGATGAACAAATCACCAATAAAAGTGAAATCTGCATAGCGTCCATGATACAAT
TGGGTTTAGACTGCACAGAGGAAACACCAGAATCAAGAGTGACCATGAAAGAAGTAGTGAAGAGGCTT
AATAAAATCAAGAACGCATTTCTGGAAACATAGAAGTTGAGCAGCATCTCTTTTGATATGATGCTTTTTG
TTTCTTCAATAAAGTTGTTGTATAGTACTACTTGAAGTCATGTATTGTTTGGTAATGTAACATCCGACAAT
TTGAAGTGGCTTTGAGGAAGCTTGAAATTTGGAAATAGTCATTTTTGGAAAAAAAAATTTGAAATCTGGA
AATTTGGCTAAGTGTGGAAATCAGTGAGTTTTTTGGCCAACTTTGAGCAGTCATAACTCTTAGCTCAGGG
TGAGTTAGGAGTAGTTCCAGTTATGGTTGCGAAGCTCGTGGAATGACCTTTTCAACGCCACCAAATTTTC
TCGATTCCGAGTTCATATGAAGGGAAATCCGTCCGGAAATTTAAGGGCATTTTGGTCTTTTCACAAATCA
TTTCTTTTGAGCTTATATTGTTGTATTAGGCTGATCTTTGGATCATTTTTGTACCCATTTTGAAAGAGAAA
GAGTTAGGGTTCTTGAGAGTGAAGAAGAGAAGAAGAAGAGAAGAAGAGGAGAAAGGAGATCAACAA
GTTTCCGTCGTGGATTATCGTCGGGGGTGATCCCTATCAAGGTATGTGAGTTTTCGTGTGGGTTGATCCT
TTCCCTCACACACACCAAGCTTATTCTATGATTTGAGAAAAGATTGGAATTTGTTGTTGAAGTTATTGTTC
GTTGTGAGACATGATTGGGTTGTGTATTTAAGTTGAAACCTATTGTATGTTGAGGGAATTATGATTCTAA

GAGTTATGACCCTTTTGAAGTTGATTCATCATCAATTCTCAGCAAATTTTGGCTATCGTTCCCCATCTACG
GGCGTGACCTACGGACCGTAGATCGATTGACGGTCCGTACTGGTCAACCGTCAATCGGGACAGAAGTT
GGGTTTTTGGGGCATCGATCTACGGGCATGACCTACGGTCCGTAACCTGGACCTACGGACCGTAAGTCC
GGACCGTAGGTCAACACTTAGTCATTTTAAAAAAAATGAATTCTGGGGAAGTTCTGTCACGATCTACGG
ACCTGCAGGACGGACCGTAAGTCCATCTACGGTCCGTCGATGGCGTCCGTAAGTGCCATCTATGTCACC
AGAAATCTAAAGTCTCAGCCAAGTTTCCCCATTTCTTTTCCCACTTTCTCATGCATGTTCCAAAGTATTATA
CCTATATTTTACAGTTGTTTCCGACACTCCAAAGTACGTCTAAGAGTTCAAGAATCCCTCCATAACATATG
ATCGAACACCTTGAATTCATAATTCCGGTTCAAGGAAAGATAGTTCCAAGTCAAGTGAAGTTAGAAGCC
AAGTAAGAGTCTCAAGTTTCAAGTAGAGTAAGAGTCTCAGTTTCAAGTTAAGTCATGAGTTTAAAGTTG
AGTTCGTTTCTCAAAAGTTATTAGGGAACTATGTATTCCCAAAGAAGTTTTAAAAAAATGTCTTTACATTT
AAATAAGGTTGGAAACTGAGATTTCCAAAGGGGCCTTCGGGCTAGTTTTTGAGTAATTATCTCAACTAAA
GAAAAAATGTGTTTAAAGACAATTATGAGCCAAAGTATTTCTGGGAGTAGTATTGAGCACCGAATTGGG
GACACGAGTTCTGATTTACTCAACTCTCCATAAGAACCATGTGCCAACATGGGATTTCTCGGATCATACTT
TTTAGATGATCACGAAAGTTAAGCTAGTGGATCCACTAAGTAAAGTTAGCTTCCTATATCGCGGCAAGGT
ATAGGATGGTCCTTGGGCAACGCGAGGTAAATCGTTGTATCATCACTAGGGCTCATAGTGGTGGTTGTC
GGTTAAAGAACCTCCCACATAAGTTATATTACTTTTATATAAGTACAGTTGAGTTGTTATTGTTTTATCATT
AACAAGCTAAGTTGTTTATACTGTTTTACAAGCTTTATATATTGCATGCGTCTTATTGCTTTAAATATTGAG
TTTAGTTAGTCATGAGTCGTACAGAGCCGAGGTAAATGTTCTCTTGCATTTCCTTTCAAGCTTAAGTTGTG
TTTTGGCTTTCCCGCTCACATACTCGTACATTCCATGTACTGATGCCAGTTGGCCTGCATCGTTTGATGAT
GCAGATTCAGGTACCCCGGATCAGCATCCTGCACGTCGTTGATCCAGCTGAGCACTCCAGAGTCAGTGG
TGAGCCTCCTTGCATTCCAGAGGACTCAATTATTTTGGTGTTCTTAGTTTTTGTCTTATTAGGATGTTGCG
GGGTCTGTCCCAACATCCATCTCAGTATTTTAGAGGCTTCATAGACAGACAGTCAGTCAGCTAGTTTCGA
GTCTCTTATCTATATATATATGTAAATATTCTATTTTGAGATTTGGGTTGCCTTATGGCCATATTTTATAAG
TTAAGTTGTTTTTGTAATATTGCATTGCATTGAGTTATTCTGTTGAGTTAGGTTTCCGCTGAGTTAAGAAA
GCCAGGCCGAGGGTTCGCTTTGGGCCAGAAATGGTCTCCGGGTGCCGGTCCCGCCCAGGGTGTAGGCT
CGGGGTGTGACAGGTAAACTGTGTGGGAATAGACATGACACAGCCACGTTGGCGCTTTTGTCAATCAAC
AACAACGTCTCAATGTCAAACAAGTTTGGACATATTTTTGTTGATAGTTTACTTAAAATATTGACTACTAT
TTCATGACGAATTAAAATAAAACCTCTTAACAAGAATTCTTGTATCTATTATGAGTTAGTCGACTTTTATT
CCATTTTGTGAAACACAACCGAAGTAGTGTCAACTGTCAAGGAATAAATGGATAAATTCCTTTTCTTATTT

TCCTCATCCATATTAATCTGTTTTCACATTTTGGAAATTTGAGACTATTTATTTAAATTAATGAAGGTATTT
AGCTGCAACTGTTTTGCTTTTTTTTTTGCAAACTCGGAAATCTCTCTTTTCGCCTCTCTCCTTCATTTGTATT
TGCCTCAGAAGTTTTACACAGTCAATGTGTAGAGCCTAAACTCTAATGTGATTAAAAACATGAATAGATT
AAGCACCTTATCCATTTTTTTTAATCTCTTCCGGCCCGACTTGGTTTAAAAAAAGTTTTCATATGACAATTT
CCATTCATAAAAATAATTTTGTTATTTTGTTACTTTGCTTATTATTGACGAGTGTAATTCATGTATTAGAGT
ATGTTAATAGAACCAATTTAAATCAGGATAAGATGTTTAGATCGTCATAAGCAAAAGTTAAGGTTCACAA
TACGATATAGTATTATGAAACAATACGTAACAACAATAGTGAAATCAAGGTCTATTCTAGATTTTTTAAA
AAAAAAAGAGGAAGAGAAAATACAGGTAAAAATAAGATTTCGTGTACTCAAAATTGATTTTGAGATACA
TAATTCTCTACATAAGATACTTGTGTTTAAGAGACATTATATTAAGTTAATGTATTTAGATGATCACTGGA
TACATAATTTTAGTATCAAACGGATAATCAAGTTAAAGTTTAAGTATCTATCGATGTATTTTAGCAAAAAT
AATGGCATTTAAACAATGAATGTTGCCCTTTTCATTTGAATTTGATTGAATATATTATATTTTGGAATAAA
AAGATGAAAAATTTAAATAACATTTGGTACAAGATTTGTGTATCTTTGATTGTTGTAGCAAAATGAAATT
CACGAGCTGAATGATTTGCTCCAGATTTTTCTTTGTATCACTAGGAAACAAAGATAGTGCTTCACAAACT
AAATGCCCTTTTTGTTGGGTGTCTTTAATCTAAGAAGAAGATGTTACCCTTTTCTTTTGAGTTTTTTTATTT
TTGTACTTCTGTCTTTGTTAAGAGCGTAAATCATCTATTTAAAAAAGGGATTGTTTACGTTGGAATAGTTA
CAAAAATTGTTCCCTCCCTTATTTTAAATTAGCATTTATCCCTTTTACAGATAGCATTGCACCAGGTATAAA
AGATCTTCTCTCTTTTATTTTCTCTTATCATAGTAAAGCATTCTAGAAGTAAAAAAAGCAAGTCTTCATTTA
TGCAGAATTATTGTTGTATTTCAATTGAATAACTTTTCTTCAATCCTTTTCTAAGAATATGGCTCGGAGGG
AAAGTGTCTTCTAGTGGTGATGTTTATAGCTATGACATCATGTTGATGAAAGTCTTGACGAAAAGAAGG
CCAATAGATGAAGATATATGCAATGAAAATCTTGACTTGAGGAAATGGATAATACAATCATTTTCAGGG
AGTATGATGGACGATGTTGTGGATGCCAATCTTTTTTCCAAGGAAGAACAAATCACTAGTAAAAGTGAA
ATCTGCATGGCCTCCATGATAGAATTGGGTTTAGATTTTACAAAGGAAATGCCATAA
>PGSC0003DMG400030443 LRR-RLKs
ATTTGATCCATCCCTAAATTTATATTCTTTCTTTCTCTCTTTATAAACTTTGTGTGAAGTTAATTAAATTTGG
GCGAACAAGAGAGTACTAATTCAAATCGGCGAGCTTTGAAACCAGAACGCACACTACAACATGCTATCC
CTGGGATTCTCACTTCCTCCTCCTTCCAATAATAAACTAATTGTTACCAACAAAAACCAATATAATTTTCGT
ACCAATTTAGCCAAGGTTTGTTCTAATAATGTTAATGCAGCTGGGGATCAATTAGTGACACTGCCTGAGG
GCCTCAAGCATGTTGCAGTGATCATGGATGGTCATAGAAGGTGGGCAAAAAACAAAGGCTTGACCGTA
AAGCAAGGTCACCGCGCTGGCGGAGAGAAAATCCAAGTCCTCACCCGCCTTTGTAGCCAGTGGGGAGT

CAAAGTTCTCACCATTTTTGCATTCTCTACAGAAAATTGGGTTCGCCTCAAAGTAAGTCATTGTATTCCTT
CCATCCCAATTTACATGACACAGTTTGAATTTCGAGAGTAAAATAGTATAATTTTGATTGTGATTCCGGG
GAATCTCTAACTTTTGAAATAAAATTTACATATTTGAAAACTAGGTTAAAAATACTATAAGTCACCATAAT
TCATAATTCAAGATGTTTACATGAAAAAATCCGGTCAAATAAAGATTTATTTGAATCTCGAAATTTGAAAT
TAGGTGCACCATAAATTGGGGAATGAGGGAATAACTTTAATGACGATCTATCAAGCATGTTAAAATAAA
TCATCAAGAAAATAAATTAAATAAAGAATAACAATAACATATTCAGTGTAATTTTATAGGTGAGGATCTG
AAAATGATATAATGTACGCTGAACTTACCCCCACCTCGAAAAGATAATAAAAGTTGTTTTTAAAGATCAT
TGACCCAAGTAACCTGTACAAAATCAACGAATATATATATATCAGAGAAAGTGACTTTCTTTTCTAACAAT
GAAATTAATTATACAAGAAATGAATCATATGTCTGATTATATTTAACTATATATAATTATAATTGCATATG
ATTTGTTTATTTAATTTCTAATACACCAATGTTTTGTTTTATTTGTTAACAACGCAACTCTAAACTTGAGTG
CCGCAGGAGGAAGTTGATTTTCTTATGAAGTTGTTTTTAGAGCTGATAGGCTCACAAGAAAACTTGGAT
GAGTGGACAAGGTATTCTATATATGTGATAAATAAAAATATCTTGTGTGATCAGCTATCCGACTATCTAT
ATAAGAGATCGTTAATTCCACCATTTTAATGCAAAATGGTTCCCGAAATTAATTCCCCATGGTTTAGTAAA
AAAAAAGGTGGTATTTGATATTCATTTTGAAATTCAACTTATTCGAATTAAATTATATTATGTGATAGGGA
TGGTAGACGAGTATCATTTATTGGGGATAAATCAATCCTTTCCAAGTCTTTACAGAAAGCTCTTACTGTA
ATGGAGGAGAGAACAAAATGTAATTCTGGACTACATGTTATAATGGCAATCAATTACAGTGGACGACAT
GATATATTACAAGCAACCAAAAGCATTGCCAGTAAAGTGAAAAATGGACATTTAACTGTAAAAGACATT
GATCAGAGCCTATTTGAACAAGAACTGGAAACTCATTGCGCCGAATTTCCTGAACCAGATTTGTTAATTA
GAACAAGTGGAGAGAAAAGAGTTAGCAATTTCATGTTATGGCAATTAGCTTATACTGAATTCTATTTCGC
GAATAAGTTATTTCCTGACATGGAAGAAGCTGATTTTATGGAGGCTTTAATGTCGTTTAAGTCGAGACAG
AGACGTTATGGTGGACAAAATATCTAGTAATATTTTCAAGAACTTCCATTACTGAGAATTAAACCTAGCT
CGAAATATGAAGAATTATTGATTAGATCTCTTGAATTTCCTCTTGCTATTAGTACTATTAAATAATTTTATG
GTGGTCGATTGTATCTATACCAGCACTATATAAGCATGAAATATCATCATGCTAGTTATGTGGTTTGTGCT
GAGTTTTTCTTTTCTTTTCCCATCGCCCCCATTAAATTTGTATCACACACCGAAGGACTCGTGGCACTCCAA
CAGGATATTCATATTTTCATAAAAAAAGAAGAAAAAAAAAGGAGGGGGACGGGGTGGGGACAACGCC
GGAAAATGAGACTATAAAAAGTACTCAAAGTCATATGCTTCCAAGATGTCAATCACATCACCATCATTCA
TCATTTTGTTGGTTCTAAGTGATGAGTGGATATAAAATTTTCATTTTTTACATCACATTTGATTTTTGCTAG
ACTATAATATAGTAAATTAAATTATAAAATTGAGGCGAAAAATATAGTATATTAATTAACTATCAATAAT
AATATCATATATTAATTGAATTAGTATTAAATCGTAACTTGTCTTTTTAGCAAATCCTTAAATTTATTTTAG

ATGAGGGAAGTTCACTTTCTTAAGTCAAATGTCAACTCACTAGTCATTAATCATCTTATTATTCAAAGATC
CAGTGAATTTCATTACAATTCCATGGAAAATAAAAAGATTTGACAAACAAATAAGAAATTATTTGGTGTT
CTCCTTTCTCTTAAAGGGAGAGCCGTTTACCTATACAAGTCTATATTTGTCGCACTCTTAAAGTCATTGGC
CATACTTTGACTCAAGGGGTGGTATCCTGTTTGATTGGTAAATCTAGAACCTTTTTATTATTATTATAGTC
GTGGTATTCAGATCACCTTATAAGCACCTTGTTAATTTCACAAGATATGTTACCTTCCATCAACACAAGTA
CCGAGTAAGATTTGCTAAGAAAGTATCCTTTCCCTTCACATGATCTTCTTTTTTCAGTACAAGTTATATGC
ACTAATTGAGTCAAAATAACTACACAACCATGTCGCTTGTAAAATAATTCATGTAAATATGACAGTATAT
ATTATTGATAACCCGAGAAAAATAATATCTAATCTGATATAATAGGTTAAATTACACTGAAAATGGTTGT
AAAAAATTTGTACACTACCATTGTGTATAACTTTAAGCCCTTTTTCTCAAACACTTTGGTACCCCACATAA
GGAATAATGAACCCATTATAATTAGCAGAATCAATTGTGACTCAGTATTATCCTCTTTGTCCACATATCAC
CTAACCTTCCCAATCCCCAAATGGAGCTTCAAACTACTTTTGTTCATTCAAGCTCTCTTTTTTTTCTGTCATT
CCATGCTATTCTCTTCATGTTCCTATACTTATCTTTTCCAGAGTGTGCTTCAGCAGGCATACTTGGGAATC
AAACAGACAAACTTGCATTACTTGATCTTAAGTCCCAAATAACTGAAGATCCACAAGGACTCATGGATTC
TTGGAATGCCACTCTCAATGTCTGCCAATGGCCTGGTGTTACATGTGGCCATAAACATCAAAGGGTCATT
TCTTTAGATCTCAAAGACCATAGATTGGCTGGCACTATATCACCTAGCATTGGCAATCTTTCATTTCTTCG
AATTCTTGACATCTCGGATAACTCCTTTCATGGTGTAATCCCTCCAGAGCTTGGCCAATTGATCAGACTTC
AAACCATGAACTTAAGCTTCAACTTCTTGAGAGGTGAAATCCCGCTTACCCTATCTCGTTGCGTTAACGTT
GTTAACCTTATTCTAGACCATAATATCCTTGAAGGACACATTCCTACTGAACTTGGTTCATTGACAAAGCT
GGAAATGTTGTACCTCAAGAACAACAATCTCACAGGAAATGTTCCGAATTTTGTTGGAAATCTTACATCT
CTAAGAGAGCTTTACATTTCCTACAATGACTTGGAAGGTGAATTACCTGAAACAATGGCCAATATGCGA
AGCTTGATTGAGTTAGGTGTTTCAGTGAACTCTCTGTCCGGAGAATTCCCCCCTGCACTCTACAATCTTTC
ATCTCTTACACTTTTAAGCTTGTCATTTAACAAGTTCAGAGGCAGACTTAGACCAGATATTGGCCTTGCCT
TCCCAAATCTTCAGAGACTGTATTTGGCCAACAATTACTTCACAGGTTCAATACCAGCTTCATTGTCCAAT
TGTTCAGATCTTCTGCGGCTTGATATTCCTCTAAACAATTTCACAGGAAATATACCATTGAGTTTTGGAAA
CCTCAAGAATCTCTTATGGCTTAATGTTAACGATAACCAGCTTGGAGTTGGTGCACCTGATGATCTAAAT
TTCATAAATTCTTTAACCAATTGCAAAATGCTTGAGTTCCTGGACATTGCAAACAACAAATTTGGAGGTA
TGTTGCCTTACTCTATAACTAATCTCTCGACAACTTTGACAAAGCTACTGATTGGATACAACAGGATAAGT
GGAACCATACCTAGAGAGATCTCCAACCTTGTCAACTTGGATATGCTTAGCATCCAAGGAACTCTTATCA
ATGGTAGCATTCCTGATTCTATTGGAATGTTATCAACTTGAAAAATCTGCACATGGAATCGAATCAGTT

GACAGGAAATATCCCCTCTTCCTTAGGAAACATTAGAGGATTGCTGTACATTTATTTGCAAGATAATAGC
TTGGAGGGAACCATACCTTCAAGTTTAGGAAATTGTACATCTCTCCAAACCTTAGACATCGCTCAGAATA
AACTCAGCGGTAGTATACCCAAACAAGTCATTGCACTCTCCTCCCTTTCGGTACTCCTTAATATGTCTTAC
AACTCTTTGAGTGGTCCCTTGCCTGTTGAAATAGGTAACCTGACCAATCTTGCTGCACTTGACATATCGA
ACAACAAACTGTCTGGTGAAATTCCACACTCACTGGAGAGTTGTTCATCCCTTGAAATACTCTACCTGCA
AGAAAACATCTTTGAAGGAACGATTCCTCCTCTGGATGATTTGAAAAACATTCAGTATCTTGATCTTTCAC
GTAATACCTTGTCAGGCAACATTCCTAGGAGTATCACCAAGCATGTTTCATTACAGAACCTCAATTTGTCA
TTTAACCACCTTGACGGAGAGGTGCCAGTTCAAGGTGTTTTTCTGATGCAAGTAGAATTCAAGTGATGG
GAAATATGAATCTTTGTGGGGGAATTGAAGAGCTGCATTTGCATCCATGTCTCAAGCATGCAAATAAAA
GACCCCAGAAGCACATTGCCCTCATTTTGGTGCTGGCACTGGGTACTAGTGCTGCATGTTTAACTTTGTT
GCTGCTAGTTTCGTATTGCTGCGTGAAAAAGGGGAAGCATAGGCCTAGCACAGCATCTTCATTCAGGAA
AGGATACACCCAAGTTTCCTATGAAGAACTCTTAAATGCAACTGGTGGGTTCTCTTCAAATAACTTGATA
GGATCAGGAAGTTTTGGCTCCGTTTACAGAGGAAATCTTTCTCCAGAAGGAACAATTGCAGTAAAGGTA
CTAAAACTTGAAAAAAAGGGTGCTTCGAAAAGCTTTTTGGCTGAATGTGAAGCCTTGAGGAACATAAGG
CACCGGAACCTGGTGAAGATCTCAACTGTTTGCTCAAGTGTTGATTTTGATGGCAATGATTTCAAAGCTC
TCATTTATCCATTTATGGAAAATGGGAGCCTAGACGAGTGGTTGCACCCGAAGGAGGGACAGATGCTGC
AGAAGAGATTGAGTATCTTACACAGATTAAACATCACAATAGATGTGGCTTCAGCACTGCATTATCTTCA
CAGTCAATGCCATACATCTATAGTTCACTGTGATCTTAAGCCAAGCAATGTTCTACTGGACAATGATCTA
ACTGCTCTTGTTAGCGACTTTGGTTTGGCAAAGTTCCTGTCTGACTCGGGTCAAAATGCCGATGTAAATC
AGTTCAGCACAAGTGGGATTAAAGGCACAGTAGGTTATGCTGCTCCAGGTAAATATTATAGTGGGGATC
TTAGTCTAAATATTGTCTGAACTCTGAAATCCATTTTTTTTCTCCATAAGACCATAACATAATAACAATTTA
AGCAATTACATACTTCCTTGAGAGACTCCGGATGCTCCTTGTCCTAAGCCCCAGGCATCCGTTACCTTTGC
AGATCACTACCTCATGATTTTGTGCTATTGATTCTTATAACCAGAGGCAGAGGCAGCTTTAGTTTAATGG
ATTCAACCAAATCTAGTATTTTCGACACTGAATATGGATATATAAGTTTAAAAAACTACTTAAATTACAAT
AAATATCATATTTTGAACCCATAATTTCAGAAGTACAATGAGTTCAGTTCTAAGAATCTTAGACGTTGAA
CCCATCAAATTTAAATCCTGGATGTCTCTTCTCTCAAATATAATTCCTGACTAGTCATAGTTTTTCTAAGGA
CCTTAATAAGAGGTTTCAGCTTTCTAGGGCATTTTACATAACATTCTCTATTGGAAAATCGATACAAGTAA
TCTGTGTTGCTTAGGCATTTTTGACAAATGTTTAGGATCCCTAATATTACTATGAAACACCTTCTCTGATA
CATCAAATTTTTCAGAATATGGCATGGGTGGTCAGGTATCATGTCAAGGAGATGTCTATAGCTTTGGGAT

TCTTCTATTAGAGATTTTCACTGGACGAAGACCAACCAGTGAACTATTTGAGGACAATGAAACTCTTCAC
AGTTTTGTTAAGCAGGCATTGCCCGGTCAAGTGATGGATGTTGTGGATCAATCTGCTTTGTACGAGACA
GAACCAGGGGATCTTATGGATATACTCAGCTGCAGAAGTGACTTCTCGGATGAATTTGTTGAATGCTTA
GTTTCTATTCTTACTGCTGGAGTTTCTTGTTCAGAGGAAACTCCACACGCCAGAATAAGCATGGGTCAAG
TTATTTTGGATCTCATTTCCATCAGAAACAAATTGAGCAGAATTTTAGTTCATTCAGAGAAAGTGAAAAT
CTCAAGAAAAGGTGAATCATAA
>PGSC0003DMG400030474 LRR-RLKs
AAAAAACAGATTCATTACAAAAAAGCTGAAATTCCTTTTACCATTTCTCCACCTCTCACTCTTCACTTTGTA
CTTGGCACTAAACACTCCTCCTTCTTCTCCATTAACACAGATCCAAATCTCTTACTACTTCAGCTCAAGCTA
AACGGGTCGGGTCGGGTCTTATTCTGCGCAAAATGTGGTTACTGCAACTCGATGAATTCGATTTGATGTT
CATGTTTACTGTTAATTTCACCTCAGAACCTCAGTATTACTCCTAGAACTTCAAGTGCTTTCTCTATTTTAT
AGGTAATTACACTGCGATTTCATATTGTTTGTTGAAATTTATAATTTTGTATTTGTTTAGCTTGATTTCTTTT
GTGTTTTCTGCTAATTTTGGTGGCAGATCTGTTTGCTTATTTGTTTCCTATTTGTTTGGCATTGTTAGATTG
AACAGTCAATTTTTATTTATTTTGAAATATGATTTTTGAAGGTTTTCTGATAGTATTTTTTTTTTTTGTAGCT
TTAAATTTGATTTTTAAAAATTAATTAGGAAATCCGTGATGAATTCATGCAGAAAATTAGACTCATTGGA
GGGAGGAGTATTAAGTAGCGGCGGAGCTAGGATTTTCACTAAAGTGATTGAAAAATAAAGAAAGAAAT
ATAATGATAAACTATGTATGATTTTTTTCTATTTCTTTTTTTTTGAACTTGTATATATGGTATGATTTTCCAG
CTTTCACCCCTGGCTCCGGCGGTGGACTATTAAGTAGCAAGACCATGTTAGGTTCTGTATGTTGTTCAGT
CTTCAGTGTTTGCTTCTTTGATAATGTGGAAGTACAATGAATTGAGAAATTGAAAATGTTGTTAGTCATT
ATGAACTATCATTGGATTATATTTGTGTAAAGGAAGCTTAGGATTCCCTATTACTGGGACCTCATATGAG
TTGGGGTTGTTCAATAGTCTTAATTAACCCAACTCTCTTTGACATTTACTTGAAGAAGAATTATGAATGTG
GTAATAGGTAATCCCCAACCCCCAAAATAGTAATAATAATAATAATAAAAATCCCATGATCTGGTAGATT
TTTTGATGTAGTTCTTCTTCATCAACTGTTTCAAGCTCCAACCTTGAATAAGAATTATCATGACATCAGGA
TTGAAAAACTTGTAATTAAATGGGTTAGTCTAGTCCCGGGTTGACTTGATGGAGTGGTTATTTTCCTTCC
CGTTGCTTATTAGATACTATCATGTTAGAATACTTAAGTGCTAGCTATTTGTATGTTTTTTTTTTTTTCTCAA
AACATCTCTTGTTTATTAATAATATAATTACAAAAGAGGGTGACCTAAGGCCTAACACCATTTCTCACACG
TAACACCAAAAAAAAAGAGTTTCCATCCCGGAAGGTTTCTTCTTCAGTTAGAGTTTTTACCAAAACCGAG
GGTGGCTCATAATGCCAACCAGAATGGTTTTCTTTTTTTGAACCTAAAGGAGGCAAGGTTAAGTTTATCC
ATCCTCAGAAGGCTTATTTGTATGTATTAGCTCTTGCTCTTCTACTTCAAGACTCTTTTGTTCATGAAATTG

AGAATGGAATATTTGTGAACCAAAGTTTTTTCTGTTAAATGTTTCTTCTTCTACTATATTCTTTTCTAATCTC
GCTAATTCTTCTGTTTTGAGCTGTTTAATATTGTACATGGTTTGGTATATCTTAGCATCTTTTTATTTTATTT
TGGTGTAATCATTCATCCATCTGTAACATTTTCGCCCACTTCATTGTTAAGGTTTTCGTTATTTTTGGCTTA
GAAAGTGATATGGCAAGGTTTCTGGTTTTTGTTTCCAGCATCTCTTTTGCATTTTGTTTATTTCTTGGCATC
AGTATCTATGAGATTGAATAGCTTTTGAAGTAATTATAAGTTGTTAGTATGGTGGATATGATTTTTAACC
AAGTCAGCAGAGCTTTCATCATACTATGTTATTTGACTGACTGTTTTTTTCTTTTTTTTATTCTTCTTTTTATT
CCATGAGAACATGATTAGTTTTATTAATCTTCTTCTGTAGATGGTGAAAAATTAGTTAAATGTAGAACAA
TTACTATTTGGCATTCATAAGAAATTGTATAAGTCTAGTTCATTGCTAAGTGCTTTTGGAACGAATGTGCA
AAGAGAAACATTAGTTGGACACATAATAATTGTTAATTGGAATCCATAAAAAGTTATCCAGTTTTAGTTC
ATGAAAATTTAGTGCAGATTCCAAACATATTTCTTGAGGATATTCTTATATCTTTGACTGCATAGATGCTT
GCTATGTCAAAATGTCTTTGTTTATCCTTCCTCTCTCACCTTTGGTGTTTGTTTGGTTCTCAAAATGTTTATC
TACTGAATTATTCCAGGACGACCACAATACTGGCAGCATAGTGGCTTTCAATCTGTTGAAGGTTTCCTCA
TGAGAATCTACCACACCAATCAACTGCTACATTCTCTTTTTGTTGTAGCAACTCTTCTTTCGCTGTGCCGTG
GTGATCTAAACTCAGACAAACAAGCCCTTCTTGACTTTGCTGCAGCAGTCCCACATGGCTGGAAGATCAA
CTGGGATCCTGCCACTCCTATTTGCTCATCTTGGGTAGGCGTTACTTGTAGTAATGATGGAGCCCATGTG
GTGGAAGTACGACTTCCAGGAGTTGGACTCATTGGTTCACTTCCAAAAAATACTTTTGGGAAGATGGAT
AGCTTAAGGATTATCAGCCTTCGATCCAATCGTCTCAGTGGAAACCTTCCTTCCGCTATCACTTCACTTCC
TTCTCTCCAATTCCTGTTCTTACAGCAAAATAACTTATCTGGACCTATTCCTTCTTCTCTTTCAAATAAACTG
AATGTTTTGGACCTATCTCATAATTTCTTCACTGGAAAGATACCTGAAACAATTCAGAATTTGTCTCAGCT
AACAGGTTTAAGCCTCCAGAATAACTTGCTTTCTGGACCCATACCCAATATTACTCTTCCAAGGCTAAAAC
ATATTAATATAAGCCACAACAATCTTAATGGTTCTATTCCATTATCCCTCCAAAAGTTTCCAAATTCCTCCT
TTGTGGGGAACTCACTCTTGTGTGGACTACCCTTAAATCCTTGTTCACCTGTTCTACCACCATCCCCTCCTT
CCCCTTCCCCTTCCCCACCTGCACCCTCCCCAAACACTCAAGAAAAAAGTTCAAAAAAGAAACTTAAACTA
GGAGTTATTCTTGCTATTGCTGCTGGAGGGGTTGTGCTGGTCTTGCTGCTAGTTGTGATCCTCGTTTTGT
GCTGCCTGAAAAGAAAAAGGGGTGATGGCAGAGGTGTGCGGAAAGGGAAATCTCCGGGTGGAGGAA
GGAGTGAAAAACCGAGGGAAGAGTTCGGTAGTGGGGTGCAAGAACCTGACAAAAATAAGCTTGTTTTC
TTTGAAGGCAGCTCTTACAATTTCGATCTCGAGGACCTATTGAGGGCCTCAGCTGAAGTGCTAGGAAAG
GGGAGTTTTGGAACTGCTTATAAGGCTATTTTGGAGGAGTCATCAACAGTGGTGGTTAAAAGACTAAAA
GAAGTCATTGTGGGGAAGAAGGACTTTGAACAGCAGATGGAAATTATTGGTAGAGTTGGACAGCATCC

AAATGTGGTTCCTTTACGTGCTTATTATTACTCCAAGGATGAGAAGTTACTGGTGTATGGTTATTTCTCAA
TGGGCAACTTGTCAATGCTCTTGCATGGTAAGGCATCATATGCTCTCATCGTCATTTCCTTTTTCTATTTCT
AGCGTTTGGTGGAGTAACTTGATTCACTCTTTGTGATTGAATGCCTGATAGTAAGCTTCTAACACAATAC
AAAATGCACTCAAGTAATGTCCTAAACGAATCTAAACAATGTGAGTTTGAACTTTTAAACTTGTGAACTG
GTGTGAAAAGAAGCATTCTTTCTTGTAGCTTATTGTAAAGTTAGAGAAGAAAAAGTCTTCTTTAGAATAT
ATGCATCAGTTTGATCTTTATAGGTCTATTATATGAGTATAAACAGCTCTTATGTTATTCTGTATCTATTGT
TTCTGTAGCAACATATTGACACTTGCTGGCTTTCTTTTTCTTATCACTGGTAGTTTATTGCTCGCACTATCT
TGCTGTTTGTCTAACTGGAAAGCTGCTAGTGCTGAAGAATTTCATGTATGATATACCTATTATCTATCCTG
GAAGACTACACCAGAAGTGAATTCATCTTCATTCACTAACCTGCATAAACTTATCAATGTTAAGTATACTG
CCAATGATGTTACGTACTGCTGATATTTCTGTTCTCTAACCCTATCAAACCCCACTATCATTTAGGAAACA
GGACTCCGGGAAGGACTCCATTGGACTGGGAGTCGAGAGTTAAAATATCTCTTGCAGCAGCAAAGGGT
ATTGCCCACATCCATTCAATGGGTGGTCCAAAATTTACTCATGGAAATATAAAGTCCTCAAACGTCCTACT
CAAGCAAGATATGGAAGCTTGCATCTCAGATTTTGGTTTAGCCCCTATAATGAACTTCCCTGCAGCCCCA
TCTCGATATCCAGGGTACCGTGCTCCTGAGGTGATAGAAAGCCGAAAGCATAGTCATAAATCAGATGTC
TACAGCTTCGGTGTCTTACTACTCGAGATGCTTACTGGTAAGCAGCCTATGCAATCACCAGGGCGTGATG
ACATGGTTGACCTTCCTAGGTGGGTCCAATCAGTTGTCAGGGAGGAATGGACGTCAGAGGTTTTTGACG
TCGATTTGATGAGATTTCAAAACATTGAAGAAGAGATGGTACAAATGCTGCAAATTGCTATGGCATGTG
TTGTAAAGGTCCCGGACATGCGACCCAACATGGAGGAAGTTGTTAGAATGATTGAAGACGTCAGGCAA
TCTAACTCCGAAAACAGGCATTCTTCAGAAAGAGAATAGATCCTATGATTCAAATGTGCATATTCCTTGG
ATTTCTGACTTGCAATTTGTAAGTCCTGTGAAATTCATTCGATTCGATTACCCAGTGCAACAACATACATG
TTTTCTTTCCCTAAATTTTCTGATTCAATCCTATTGACAAGTGTATCTAGGAAAGTGTGGATTTGTTTTCCT
TAGGAGATATAGTAGACAAGTTAATCATCTTCCTTTTTCTTTTCGTTTAAGAACTCTTGTCATTTGTTGTAA
GATTCAATCTGCACTGCACTTATTAACATCTGATTTATGCAACATGTCGAAGCAATTCATTTGCTGTGTGT
TTGGATTTTGGTGTGAAATCATTTTTATGGCATTCAAACACCAAGTAATT

>PGSC0003DMG400030572 LRR-RLKs

CTATTTTCCCAACGTCTCTTTATTTTCTTAGCTGAAATCAACTTTACCTTTTCTCACTCTCAAAAGTCTCACC
TTTCACACTCTTTTTTCTCTCTTAAGCTCCATTCTTGGTTATCTGGTGTACAAAAGGTAAGGTCTTTGAGAC
ACCCTTTTGCTCATTTTTCATTTTTCAGCTGTCACTGTTCTTGATTTCATCATATTTGTAGATAAAAATGCG
ATCTTTTTTGTTTTTTTGCATTTTCTTGATTCTGTGTAGTTGTGTTTTTGTTACTTCTTTGAATGATGAAGGG

ATAGCTCTTTGGTCATTCAAAAAAGGTATTGGACAAGACCCTGAAGGGTCACTTAAAAATTGGAACTTTT

CTGATGAAACCCCATGTTCTTGGAATGGTGTTACATGCAAAGACCTAAAAGTTGTATCTGTTAGTATACC

TAAAAAGAAACTCACTGGGTTTCTTTCTTCTAGTCTTGGATCTCTTACTGGGCTTAGACATGTTAATTTGA

GAAGTAATTTGTTTTCTGGTAGTTTGCCTGTTGAGCTTTTTGAGGTTCAAGGTTTGCAAAGTTTGGTTCTT

TATGGGAATTCCTTTTCTGGGGTTATACCTATTGAGGTTGGGAAGCTAAATTATCTTCAAACTTTGGATTT

ATCACAAAATTTCTTGAATGGGTCTGTGCCTATAACATTACTTCAGTGTAAGAGATTGAAGTTTCTTTATT

TAAGTCATAATAACTTTACTGGTGTTGTACCAGAGGGGTTTGGGGGTAATTTGTCTGCATTGGAAGAACT

TGATCTTGGATTCAACAAATTTGATGGTAAAATTCCAAGTGATTTGGGAAATTTGTCTAATTTGCAAGGA

ACTGTTGATTTGTCTCATAATATGTTTAATGGTTCGATTCCGGCTAGTCTTGGTAATCTTCCTGAGAAGGT

GTATATTGATCTGACTTATAACAATTTGAGTGGTCCAATTCCACAAAATGGTGCTCTGATTAACAGAGGA

CCTACTGCTTTTATTGGAAATCCTGGGCTTTGTGGACCTCCGTTGAAAAACCAGTGTTCTGCACAAAGTG

ATGCAAGTTCACCGTCCTCGGAACCTTTCTTGCCTAATAACGTACCTCCCTTAGATGGTGCTGGAGGGGA

TGGGAATGGTAGAGGTCTGAGTAGAGGTGCTGTAATTGCGATAATCGTGGGGGATGTGGTTGGAATTT

GTGTTATTGGGTTGTTATTCTCGTATTGTTATTCGAGAATCTGTGGTTGTGGGAGGAAAAAGGATGAATC

CGGATTTGGTTTTCAGAAGGGGGGAGGGAAAGGAAGGAAAGAGTGTTTATGTTTTAGGAAGGATGAG

TCGGAGACGTTATCGGAAAATGTTGAACAATATGATCTAGTGGCACTAGATAATCAAGTGGCATTTGAT

CTTGATGAACTTCTCAAGGCATCTGCTTTTGTCCTTGGTAAAAGTGGTATCGGCATTGTGTACAAAGTTG

TGCTTGAAGACGGACTTAACTTGGCTGTGAGGAGATTAGGCGAGGGTGGTTCACAGAGATTTAAGGAA

TTCCAGACGGAGGTCGAAGCAATTGGAAAACTGAGACATCAAAATATTGTGACTCTTCGTGCCTATTATT

GGTCTGTCGACGAGAAGCTACTGATATATGACTTCATTCCAAATGGAAACCTTACCACCGCAATTCACGG

TATGCTTCAAACTTAGGCTGTTGCATTCTCGTTTATCGTTTTTTCTTTGATTTCCACAACTGTTTTAAGCTTT

CGTGATTATTAAGTTATTAACTTGTCTTTTTGCTTGTGTAATCAACTCTTAGGAAAACCTGGTATGGTGTC

ATTTACACCGCTTTCGTGGTCCATTCGACTGAAAATAATGAAGGGAACTGCAAAAGGTTTGGTTTATTTA

CATGAGTACAGTCCCAAGAAATATGTTCATGGTGATCTAAAGCCATCTAATATATTACTTGGGCACGACA

TGGAGCCCAAAATCTCCGACTTTGGACTTGGACGTCTTGCTAATATAGCCGGAGCATCTCCTACGTTGCA

ATCCAACCACATGACCTCTGACAAACCACAACAAAGTAAGCAAGGCAGTGCACTATCAGAGTCCGGGAC

AGTTACATCAACTACAACATCTGGTTCTTGCTACCAAGCTCCCGAGGCATTGAAAGTGGTGAAACCATCT

CAAAAATGGGACATTTACTCGTATGGAGTGATCTTGCTTGAAATGATCACGGGAAGAACCCCGATTATC

CAAGTAGGTTCTACAGAAATGGATCTAGTAAACTGGATTCACTGGTGCATTGAAGAGAAGAAGCCACTT

TCGGATGTATTGGACTCATATTTAGCTCAAGATGCTGATAAAGAAGAGGAAATGATTGCGGTTTTGAAG
ATTGCAATGGCGTGTGTTCACAGCAGCCCGGAGAGAAGACCCTCAATGAGATACATATCTGATGCATTA
GAAAGACTGCAAGCTTCCTCTGAATGAACGATCGAAACAGAACCTGAAGGCTACACATTGCAGAGTAAG
GATCACAGCATCTAACCGATAACTATGGAGCACGATCACTGCTTGGCGCGCGATCATATTGACTAACAG
TAGTAATCTCCACTTGTCTGTTTGAATATTGGCCTTCTTGCTCTCTAATTGCAAGGTTTTTGTTGTGTCTAT
TTGTTGTTTCCTTCTATTGATGAACATAAGTTATTTGTTCTCTTTATTACTACTACTATTCATCTTTGTGTGT
CACAAATGAGTTTATGTAAATAAAAATGATTGTCAAAATAATAAATAAGATAGGTGTAAGCAATAACTAT
GTCTGTTGTGAGTCTGCAAG
>PGSC0003DMG400030625 LRR-RLKs
ATTTACTCAAAACAAAAGAATATACACCAAACGGATTACCCACCCCCTCCTCACTTTTGCCTTTCTCACTCT
CACTGAGTGAAACCGCAAACCAAACAGTTGGTGGGCATTAGATTAAGGAAGGAAAAATGCGTCTTCTTT
TTCTTCTTCTTCTTGTTATGCATTTTACCGACTTTTCTGCCGGTAAACAACCACGGTTACCGGAATACCAG
GCTTTGCTTGCCCTGAAAACTGCCATTACCGATGACCCACAGTTAACACTTGCCTCATGGAACATCTCCAC
CAGTCACTGTACGTGGAATGGTGTCACGTGCGACACGCATCGTCACGTGACCTCTCTTGATATTTCTGGG
TTTAATCTTACCGGTACTCTTCCGCCGGAAGTTGGGAATCTTCGTTTCTTACAAAATCTGTCTGTTGCTGT
TAACCAGTTTACTGGACCCATTCCTGTTGAAATCTCCTTTATTCCAAATCTCGGTTACCTTAATCTTTCTAA
TAACATATTCGGGATGGAATTCCCTCCGCAGTTAACCCGTCTGCGTAACCTCCAAGTCCTTGACCTTTACA
ACAACAATATGACCGGTGAACTTCCCCTTGAGGTGTATCAGATGACTAACCTTCGACATCTACACCTCGG
CGGGAACTTTTTCGGTGGCCGCATTCCTCCGGAGTATGGAAGGTTCCCGTCTCTAGAGTACCTCGCAGTT
TCAGGCAATGCACTGGTAGGAGAGATACCACCGGAGATTGGAAACATCACTACACTTCAGCAGTTGTAT
GTAGGATACTACAATACCTTCACCGGTGGGATTCCCCCGGCAATAGGGAACTTATCGCAGCTCCTCCGGT
TTGATGCTGCTAACTGTGGACTTTCGGGGGAGATTCCACCGGAGATTGGGAAGCTTCAGAACCTTGACA
CTCTCTTCCTGCAAGTGAATTCTCTGTCTGGGTCATTAACTCCGGAGATAGGTTATCTGAAGAGCTTGAA
ATCTTTGGATCTGTCGAATAACATGTTCTCTGGCGAGATACCGCCAACATTTGCGGAGCTTAAGAATATC
ACTCTTGTTAATCTTTTTCGGAATAAGCTTTATGGGTCAATACCAGAGTTCATAGAGGACTTGCCGGAGC
TAGAGGTGTTGCAACTTTGGGAAAATAACTTTACGGGAAGCATTCCACAGGGGTTAGGCACAAAGAGC
AAGCTCAAAAATGTTGATCTCAGTTCCAATAAATTGACTGGAAATTTACCCCCAAACATGTGTTCCGGTA
ACAATCTGCAGACAATTATCACTCTAGGGAACTTCTTGTTTGGCCCAATTCCTGAATCTTTGGGTAGGTGT
GAATCACTTAATCGGATTAGAATGGGAGAGAATTATCTGAATGGGTCAATTCCAAAGGGGCTCTTAAGC

TTGCCACGTCTGTCACAAGTTGAACTTCAGAATAATATTCTCACTGGTACATTTCCTGATATTTCTTCCAAA
TCTAATAGTCTTGGGCAGATTATCCTTTCAAATAATCGCCTAACTGGACCTTTGCCGCCAAGCATTGGAA
ACTTTGCTGTAGCCCAAAAATTGCTTCTTGATGGGAACAAATTTTCGGGACGAATTCCAGCAGAAATAG
GAAAGCTTCAACAGCTATCCAAAATTGATTTCAGTCACAACAACTTTTCTGGACCCATGGCTCCGGAGAT
TAGCCAGTGCAAGTTGCTGACTTATGTTGATCTCAGCAGGAACCAACTTTCGGGTGAGATTCCTTCTGAG
ATCACAGGTATGAGGATACTCAACTACTTGAACTTATCGAGAAACCACTTAGTTGGGAGTATTCCTTCCC
CTATTTCTAGTATGCAGAGTTTAACTTCTGTTGATTTCTCATATAACAACTTTTCTGGTTTAGTTCCTGGAA
CCGGGCAATTTAGTTATTTCAACTACACCTCATTTCTGGGCAATCCAGATCTTTGCGGACCCTATTTGGGC
CCTTGCAAAGAGGGTGTTGTTGATGGGGTTAGTCAACCTCATCAACGAGGAGCCTTATCGCCTTCGATG
AAGCTTTTACTTGTTATTGGTTTGCTTGTCTGTTCTATTGTGTTTGCTGTTGCTGCAATTATAAAGGCCCGA
TCTTTAAAGAAGGCAAGTGAAGCTCGTGCCTGGAAGCTCACTGCTTTTCAGCGCCTAGATTTTACTTGTG
ATGATATTTTGGACAGCTTGAAGGAGGATAACATTATTGGAAAAGGAGGTGCTGGTATAGTCTACAAG
GGGGTAATGCCGAGCGGGGAACATGTAGCAGTTAAGAGGTTGCCAGCTATGAGCAGGGGTTCCTCTCA
TGATCATGGGTTCAATGCAGAGATACAGACTCTTGGGAGGATCCGACACAGGCACATTGTTAGATTATT
AGGATTTTGCTCGAATCATGAGACAAATCTTTTGGTTTATGAGTACATGCCTAATGGAAGTCTTGGGGAA
ATGCTTCATGGCAAGAAAGGCGGTCATCTACATTGGGATACCAGGTATAAGATAGCCGTGGAGTCTGCA
AAGGGTCTTTGCTATCTCCATCACGATTGCTCTCCTTTGATCCTCCATCGTGATGTGAAATCAAACAACAT
TCTGCTAGACTCCAGCTTTGAAGCTCATGTTGCTGATTTTGGACTTGCTAAATTCTTGCAAGATTCAGGGA
CATCAGAATGCATGTCTGCTATTGCTGGTTCTTATGGGTACATTGCTCCAGGTATTTTGTAAAAGCCTTCG
TATCTTGAAATTCTTAGTTTCTAACTGTGTTCATATTTATTTTTCTTGTCACATCATGCTATGCGAGTTTAGT
GCCGAAGTTACAAACTTACTGATGAACTAAAATTTATCAGCTGAGACTAATTATCAATCTGTTAAAATGA
AGCTTAGGTGTTCTAAACATCATAAATGTGCTATCCTGTAGTCTTGTATTTCCACTACTAATAATGTGACG
ATGAAGGACAGCATTAAGACATGGATAGTGGGGGTCCTTGACTGGTAGTAAACTAGAGGATTGGGCCT
TCTTACTTTTGAAAAGGATCTCTAACTACTTCGGTGATGGTTAGGTTGGAATTTATAAATGCTTATGTCAC
TTGATAAGTAAGGCAACTGTGTTGCATGCTCCTGAAGCAAAGCAACATTAAATAAGTGGTACTGGTACT
GATGAACTATTTACACTGTTGGAACGGGTAAGGGGATGTAAAACTCTGGTTTCTTGTTTAGTTATCTAAC
AAACCTACTGATTGCTAGCAATTATTTGTTCTGTTCTATCGTCTACTTCTCATTAGCATCTTATTTTCGTTTT
TGCAGAATATGCTTACACGCTTAAGGTTGATGAGAAAAGTGATGTATATAGCTTCGGTGTGGTGCTATT
AGAACTGGTAAGTGGCAAAAAGCCAGTTGGAGAATTTGGTGATGGTGTTGACATAGTCCAATGGGTTA

GGAAAATGACTGATGGGAAAAAGGATGGAGTTCTCAAGATCCTTGACCCAAGACTCTCAACGGTTCCCC
TTAATGAGGTGATGCATGTCTTCTATGTCGCATTGTTGTGTGTTGAAGAGCAGGCTGTGGAACGCCCCA
CCATGCGAGAGGTAGTGCAAATACTAACTGAACTTCCCAAGTCACCAGGCGCAAAATCAGATGACTCAA
CCGTCACTGATCAGTCACCCCCACCATCAGACTCTGCATTAGAGTCCCCAACCTCAATTCCCGAGGAAAC
AAAAGACCACCATCAACCAACACCTCAATCACCTCCACCTGACCTACTCAGTATCTGATTTGCAATGTTCT
TGAAATAGGAGTGTTTTATTTAGTTTGATTCTCTAGTTCTATTATGATGAATTGTGCTAGCTTTGTTCTTTT
TTTAAAAAAAATCGGGTCTTTTTAGGCTCAGGGGTTTATTCTAACTCTAAGATGGGTTTAATGCTCAGAA
GTTTTCCTCTTGTACAGTAAGATTGGTAGGGTTTTCTCAAGTGTATTAATGGAAAAATTGCCCTTCATTTG
CTA
>PGSC0003DMG400030626 LRR-RLKs
TTTTATGCCATATAATATTTTGAGTCCAAAAACCTTATCATTACTCTCTGCTTCAAGTTACTGCAACTCTTG
TCTTGTTCTTCGCCATTAGAGTCCAATACTAGCTTTTTTTTCTTCTAATGTGTGAACACTCTTCACGCTTTCA
GTACTATTTCTTCTCCTTAAACTCTGCTTCCGTTACACTTGTAATCCATGCAAAAATTCCCCCCACCTCTTTC
TAACTCTTACCTTCCCAACATTCAACCTCCATTTACAGATGTTTATGATTAATCGGTTCTTGCCTTTTTAAG
GACTTGGAGGTAGGGGAGATTCATTCAGTCATCAATGGATTACATGAAGCTTCAATTTCTGATACTCGTT
TGTTTTTTTCTCTTCATTGTTCCGGCGAGTTCATCGCCTCGGGATATCGCTATTTTACTCCGGGTTAAGTCC
GGCCAACTCGATGACCCGAATGGGTTGCTTGATGATTGGAACGGGTCTGCTCCAAATGCGCCTTGCAGC
TGGAACGGGATTAAGTGTGATCGGAAAACCGGTCAGGTTGTGTCCATTGATTTTGCAAGTTTTGGAATC
GCTGGTCGTTTTCCTGCTGATTTCTGCCGGATTTCGACTTTGCAGAAACTCAATCTGGGTGATAACAGTTT
TGGTGAGTCCATTTCCTCTGACTCATGGTCACTCTGTTCGCATCTACACTTTTTGAATATTTCTTTAAATTT
CTTTGTTGGCCGGCTGCCGGAGTTTATTACCAAGTTCGATAACTTGACCATCCTTGATGTTAATTCAAACA
ATTTCTCCGGTGAAGTTCCGGCGAGTTTAGGCCGTTTACCAAAATTACAAGTGCTGAATATAGCTAACAA
TCTCCTCAATGGTTCAATTCCTGAGTTCTTGACGAATCTTACCGAGTTGACTCGATTGGAAATTGCTGCAA
ATCCGTTTCAGCCAGGTCCATTGCCTTCCTCAATCGGCCGACTCGGTAAGCTTCGAATTTTCTATGCTCGG
TATGCGAACCTTATTGGAAATTTTCCAGATTCTATCAAAGACCTGAAATCTATTCAGAATTTTGATGTGGC
AAACAACAATCTCTCCGGAAAAAATTCCAGAAAGCTTCGGAGAACTCAAAACCATACAACAAATAGAGCT
CTTTGGAAACCATTTTTCAGGCGAATTGCCGGACATGTTTTCCGGTCTTGGTTCTCTTTCCAGGTTTGACG
CTTCTGAGAACAATCTCACCGGGAAAATACCTGAAACCCTTGCCCATTTGCCATTGGAATCTCTAAATCTC
AATGATAACCAATTAGAAGGCGAAATTTCAGAAAATTTAGCTCTTAACCCAAATCTTAGTCAGTTCAAGC

TTTTTAACAACAGATTTTCAGGTACTTTACCTCAAAATTTTGGTTTAAGTTCAGATTTAGATGAGTTTGAT

GTCTCCGGCAACAATCTAAAAGGTTCTTTACCTCCCAACCTATGTTCTAGAAAGAAACTTAGGATTTTGA

ACCTGTTTGATAATAAGTTCAATGGGCCAATCCCAGAATCCTATGGGCAGTGTTATTCACTATCATATGT

GCGTATCTATAACAATCAATTCTCTGGTGAATTACCAACTGGTTTCTGGGGATTTGATGGATACACATTTC

TTGAACTGCGAAACAACAACTTTCAAGGTTCAATTCCAGCTTCAATCTCCAATGCTCGAGGCCTGACACA

AATTCTTATCTCCGGCAACAAGTTCTCCGGGGAATTGCCAGCAGAACTATGCAATTTGGAAGAGGTTGT

GATCATGGACATTAGCAAGAATCAATTATCAGGGGAGTTGCCTTCGTGTATCACAAGATTGAAAACATT

ACAGAAGCTTGATCTTTCACAAAATAGGATCAAGGGTCAAATTCCCAAATCAGTTAGTTCTTGGAATGAC

CTGACTGAATTGAATTTAGCTAACAATCAATTGACAGGTGAAATTCCTGGTGAGCTTGGGACGTTACCG

GTCTTAACCTACTTAGACCTTGCCACAAACTTGCTTTCTGGCGAAATTCCATCTGAGCTGAGCAAACTCAA

GCTCAACAAATTCAATGTATCAAATAACAGGCTGGAAGGGAAAGTTCCACTTGGGTTTGATAACGATTTT

TTCGTCTCAGGTTTACTGGGCAATCCGGATCTTTGTAGTCCGGATCTTAAGCCTCTGCCCCAATGCCGAA

GACCCAAAAGTGTAAGCTTGTACTTGGTGTGTATTTTATCAGCTTTTGCCTTCATACTTGTCGGGTCACTT

GTTTGTGTCTTACTCAAGGCCAGTAAGCTGCTACCAATCCGTAGCAAGCGTAAAAGTGTGTGGAGAATT

ACTGCATTCCAACGCGTCGGTTTCACAGAGAGAGATGTGTTAGATGCACTGATAGAGGAAAATCTCATT

GGAGCTGGTGGGTCGGGTCGTGTGTACCGGGTCAAATTGAAAAACGGACAGATGGTAGCAGTGAAGA

AACTTTGGGCGGCTAAACGGGAAAGAGAATCCGAGGAGGTGTTCAGGTCTGAGGTGGAGACATTAGG

GAGAGTCCGGCATGGAAACATAGTCAAATTACTATACACTGGCATTGGTGATGACTTTAGGATATTGGT

GTACGAATACATGGAAAATGGAAGCTTAGGAGATGTATTACATGGGGAAAAAGGTGGCTTGTTATTGG

ATTGGCCGAGGAGATTTGCCATAGCAGTTGGAGCAGCTCACGGATTGGCCTATTTGCACCATGATTCTG

TGCCAGCAATAGTGCACAGAGATGTTAAGTCTAATAACATTTTGTTGGACGAGGATTTCAGGCCCAAAG

TGGCTGATTTTGGGCTAGCTAAGGCAATGCAACGGGATGCTGAGGAGAGTGAACAAGCCATGTCCCAC

ATTGCTGGTTCCTACGGCTACATTGCACCTGGTAACCTTAAATTCCTTATTGTTTTTCACCATTTATTTTAG

GTCCAATGACTGTTAGAAATTTTCTAGTCTTGTTAGAAATTTATTTGCCAATAAAAAATGTTGAAAAAATC

TAGTGCTAAGGAGACTATTTAAAAGAAAAAAAATATTGAACCAAGATAGGCATAAATGAAGTGGTATG

GACAGCAGGAATTCATAATGCTAACCTCTACTTGTTTGGGATCAGGGCCTAATTATTGTTGTCGTTCAAC

TTTTTTGTTAACTTGTTGTGAAAAGATATTTAATATCTTGCGTTTGGCATACCAAGGACATCCAAAAATAG

TTTTGTGGAAGTTTTGGTTAACTTCACATACTATCTTAAAATTTTGATTTGATTTGATTTGAAATTCTTTTA

TATGAAGCCAGAGAAGATTTTAGGAGATGATTCAAACTTAGAAGTAGATTGGAAACTTTAATAATATCC

ATTTAATTGTAGCATCTAGTTTCATTCCTCTAACTGGACGTTGTTTCTGAAGTCTGCATATTCTTGTTTGGC
TTGCTAATGCTTGATTATATGTTTAACAAGGGGATTTGTCAAAGTAAAATCAAAGAATGATTGGGCATCC
CATTTTTGAAGTCTGCATGACTATTTAGTTTTAACTACCTGTAATATTTAATCCTGAGTTTCGTTTGGGATT
TGTCAAAGTAAAATCAAAGCATAGTTGGGCGCTCCTTTTTCTGAAGTCGGCATGACCATTTATTCTACAC
CGGTAGTAATATTTAATTCTGAGATCACATCAAATTATTGAATGATTTTGTCACCCAATGTTTGTTAACAC
AGTCAAGATTTGTTTGAAAGAATAAAAAATAGGAGGGTCATGTGTGTCCTGTCACCTAGCATTTGTTAAC
ATATTCAAGATTTGATCTAAAAGAATAGGATTGAAAGAGACATGTGCCTAAGTATTGTTACTTATTTATC
TAATTTATCGATATCTTAAATTCTATTTACTCCCATAAGTCCTCTTTGATTGGACATACAGTTTCATCTTTTT
TGTAAAGCTGACACTATCCGGTCTGTGGCTCTGCCCATGTGCCCTTCCTTTGCAAAACTGAGAGAATACA
GAAACCAATATCGTTGATGGATATTTTTTGATGGCCTTTTGATACTAGAAAACATTCCGGTTGCAATGAC
AAACAGTTTGTTAAGTTGATCTTGGCTACTTAACGATTAGGAAGATGCTAAAGTTTGCTAAATATCTCAT
GGGATGGCACATGTGTTATCCAAGGTTTTCAGCTGCACGTTTAAACTTAACCTCGATTCCTTTGTTTCAAT
TGCAGAATATGCATACACTCTGAAGATCACTGAGAAGAGTGATGTTTATAGCTTTGGTGTGGTACTGTTG
GAACTAATAACTGGTAAAAGGCCTAATGACTCCTCTTTCGGAGAGAACAAGGACATTGTCAAGTGGGTG
TTAGAGGTTGCAACATCGTCTAAGAAAGATGAAGGAACTGGCCATATTGTTACGTGCGCAAGTGGTATT
CTTGATTTGAACCAGCTAGTTGACCAGAGAATGAATCCATCTGCAAGCAATTATTCAGAGATTAAAAATG
TTTTCGATGTGGCTTTGCTTTGCACTTCAGCATTGCCTATTAATAGGCCCTCCATGAGAAGAGTTGTTGAG
TTGCTGAAGGTTATCCCCTCTGCTCGCTCTAAAACAACCCATTAGCTTTTCACAGTTTCAACATGTCCTTGC
TCAAGTAACTTGATACTATTGTAAACTGTAAAAGAATGTGTGAAATTAGGTGTGTTTAGGTCTGCTTCAT
AGGTCGATTTAGTTTGCTTGCTGTGGTTTAACAAGTACCTTGTTAGATACTCCAGTACTACACAGAGTAG
CTTAACTAAAAGTTTGCTGACCCTGTAAAACTCATGTTGTAACGACTAAACCTTAAGAGTTTTTGTTGCAA
TATATGAACTTTACTATCATATACAAGTATTCTATTCTGTATCCATG
>PGSC0003DMG400031028 LRR-RLKs
ATGGAGGTGAGCGTGAAGATGAAATTCCCCTCACAAGCACTACTGTTGGCTCTATTGCTTGTTTTACCGA
TCGTTTTAGCTCTCACCGAAGAAGGTAACTATTTTTTTGAACACCTAGAAGCTGTTTTGGTGTTTTCTAGC
TCCTCTGTTTTTATAGCAGAAGGTAGTGCTATGTGTGCTATCTAGGAAATACGAAAGTTATTGTTCGAAT
TTAGTTTTTATTTTCAGTTTCTGGAGCAGCATTCCATGCTATTTAACTCTGATAACGAAAAATCCGTGTTAT
TTAAGATATATTTAGGCTCCAGTTTATGGCATAACTACTGGAATACTAGTTGATGAAAACTGAAAATGGT
TATGACTGCTTGCGCAGGCAAAGCATTAATGTCGATCAAGGCATCGTTTAGCAACGTGGCAAACGTGTT

GCTAGATTGGGATGATGTCCACGACGAGGATTTTTGCTCATGGCGAGGCGTGTTGTGTGGAAATTTCTC
CATTTCCGTCGTTGCCCTGTAGGTTTTCCATCCTTGTCTCCTAACTTTTACTGATACACCAGAACAAAGAA
GTAGCTGGACTCTGATGACCTGCTAGCTATTGTATAGCACTTTGTTATTTAGCTAAAACTTATACGTCTT
TTATTTAAATTTGCCTTCTCTGCTTGCGAAGGAATCTGTCTAATCTTAACTTGGGCGGGGAAATTTCACCA
GCCATTGGAGATTTGAAGAATCTAGAGACATTGTATGGTCCATGTTCTCTTTTACTGTTCTTGGTCCATTG
ACTGTTATTTTTCCGTGCTAATATTATATTCCAATTTTAATGACAGAGACCTTCAGGGAAATAAATTAACT
GGTCAAGTCCCAGATGAAATTGGCAACTGCATTTCACTGATCTATCTGTAAACTGTTTTTAACCTATGCTT
TTAATATCTATTTCTTGCGTGTCAGTAAATTTAAAGTGTTCACACTAGTTGATTTGCTATTTTCTGCAGTGA
TTTGTCTGATAACTTGCTCTATGGAGATATACCTTTCTCAATTTCTAAGCTCAAGCAGCTAGAGTTATTGT
GAGTTATTTAATCACGTTAAATTGATGTCTTCTCATATCTAGTTGATATGGTTATGATGAATTAATTCATAT
GTGGTACAGGAACCTGAAAAACAACCAGTTGTCTGGCCCAATCCCATCCACATTAACTCAAATCCCTAAT
CTAAAGACGCTGTGAGTTTCATGACTTTAGTTTATCTCACTCAAAATTTAGTCCAATATACATGGTTACTA
ATGGTTATTTGATGGTGAAGTGATCTGGCTCGAAACCAGCTCATTGGTGAGATACCAAGGTTGATCTATT
GGAATGAAGTTCTGCAATACCTGTGAGTGCATGTTCCTGGTGTTTTGGAGGTTTTCCTTTTTGTTTGTGAA
ATTTACAATGTTATAATTACCTTCTGTTTTGCAGTGGCTTAAGAGGCAACATGTTGACAGGAACATTGTCC
CCTGATATGTGCCAGTTGACTGGTTTGTGGTATTTGTATGTGCTCTGCTACAAGTTGTCTATACGGAATG
CTGTGTTGTCTGTTTGGTGTTATGTTTATTCTAACAATAGAAGTTTCATATTATTTCAGTGATGTGCGGGG
CAATAACCTCAGTGGAATAGTTCCAGATAATATTGGGAATTGTACAAGTTTTGAGATACTGTCAGTGTCC
TCTCCTCGCTCTTGTAACATTAAGCTACGGTTTTTACTCCTACTGCTGCCCAATTATAACAAAGTCTTTTTT
TTCGTGATTTCAGGGATATCTCATACAATCAGATAACTGGAGAAATTCCCTACAATATTGGATTTTTACAA
GTGGCTACCTTGTTAGTGAATTACTTTTAAATGATTTTTAGCTGTTGTCATTTTTACCTTGTCTGTTCTGTT
ATGGGGTTCATTTCTGTGAATTGTAAATGGTGATTGTGGTCCTTTCAACAGGTCTTTGCAAGGAAATAGG
CTAACTGGGAAGATCCCAGAAGTGATTGGTCTAATGCAAGCTCTTGCTGTTCTGTGAGTATTCATACTAT
TACATTTTTTTTTTAACAACTCCATTTCTTACTATTTACTACTTGCAAGTTAGAAAGTTTCATGATGTTTGTT
TCCTCTGTAGGGACTTGAGTGAAAATGAGTTGGTGGGACCAATTCCTCCAATCTTTGGCAATTTATCCTA
CACTGGGAAACTGTAAGTTTTCACCACTATTTTAATGCATATACTTTTCTACGTGTGGCTCGTTTGTCTGA
TTCACTTGTACATTCAACAGGTACCTGCACGGCAACAAACTTACAGGGCCAATACCACCGGAGCTAGGA
AATATGTCTAAACTTAGTTACTTGTAAGAGGGTTAATTCACAATTTCAGATGCATTATGCGGTCACCTTTT
TTTTTTGCTTCATGCATTGAGCTATCTTATTTACAGGCAATTAAATGACAATCAGCTAATGGGGCGAATTC

CCTCCGAACTTGGCAAACTGGACCAGTTATTTGAATTGTGAGCCTTTTATTCTTGTAGTATATAAATATGG
TTCCATTTCTCTTAGAATATTGTGTTCTTAGCTTTCCTTTACCAGGAATCTTGCAAATAACAAGTTGGAGG
GACCAATTCCTGAAAATATCAGCTCCTGCTCGGCATTGAATCAACTGTAAGATTGTTGCGTATCCTTTTGG
AACTTCTTTTTTTTTTCTTTCTCTTGCTATTTCCATTCATTCTGAGTCTAAACATATTTGTTTCTTTCTTTCAG
TAATGTTCATGGCAACAACTTAAACGGGTCCATTCCTTCAGGGTTTAAGAATCTTGAGAGCCTGACATAT
CTGTATGTTATATGCTTCTGGTTTGGCTAGTGATCATGAAAATGATTTTGAATTTCTTTCATATGGTACAA
ACTCATTGGTGATATTTTGTTGATATATACCTGGCAGAAATCTTTCAGCAAATAAATTTAAAGGTCACATA
CCCTCTCAACTAGGGCGAATCATCAACCTTGATACATTGTGAGTTCTTGACTGTCAAGGTCAATAAAATTT
ATTAGTTCAAGTGATTTCTTGTACTAAAACTTGTATTCTTTAGGGATCTCTCTGGCAATGAATTTTCTGGG
TCTATCCCTGGTTCTATTGGAGATTTGGAGCATCTCCTCACACTGTAAGATTTTAAACATTTTGATGTACA
AGGAGTTTGTATCATATTGTGACTTGAACATAATCACAATATCACTGCATATTGTAGGAATCTGAGCAGC
AATCATCTTGATGGACAAATTCCTGTAGAATTTGGCAATCTGAAAAGTATACAGACCATGTGAGTTTCTG
CTGAGAGCTTTGGTTACACTGCCTTCAAAATGTGTACTTTTTTTTAGTATACTGATTTTCTTGTTCTGAGAT
TGGTTTCTATTCTTTCAGTGATATGTCATGCAACAAGATTTCTGGTGCCATCCCAAAAGAGCTGGGACAG
CTGCAGACCATGATAACTCTGTAAGTACATTTAGTTTTTGTCAAGTAAATTGATGTAGTGTTTAATCAGTT
GTCCTTAATATCAGTCAATTCTAATGAATTTTTGATTGGCTTTGTTTCTTTATGAAGTACTTTGACAGCTAA
CGATCTTAGTGGAGCAATCCCTGACCAATTGACCAATTGTTTCAGCCTAACTAGTTTGTGAGTTCATCTAT
CTTTGCCTTAACATCATAGACATTCTAATTCTTCCTACAGTTACTGATCTTATGCCATTTTCCTTTAGGAAT
ATATCCTACAACAATTTTAGTGGTGTTGTTCCTCTTTCACGGAATTTCTCGCGGTTTGCACCTGACAGGTA
TTTTCCGTATAGAATGAAGCTTCATCATTATATATGAGCATTAGTACAAGTTAATGATCATCTTACTATGG
TCATCAGCTTTTTGGGGAACCCATTTCTTTGTGGCAACTGGAAAGGCTCAATATGTGACCCCTATGCACC
AAGGTCTAACGGTATGACTTCATTTTTGTGCATTGGTTAGTGAATCTTCTGGTATGCTAGAGTCATGTGC
ATTAAAATGACTTGTTACTTTTGCAGCCTTGTTCTCTAGAACAGCTGTTGTTTGCACAGCATTGGGTTTCA
TAGCACTCTTATCCATGGTTATAGTGGCAGTGTACAAGTCCAACCAACCACACCAGTTTCTGAAGGGGCC
TAAGACCAATCAAGGTAAAAATTAGTACATGTCCATTCTGTTCTTTTGCTTTTCAGTACTTTCAGGTATTTA
TGTTAGCTTTTATCTTGTTTCCCTCTAATTCCCTAGGTTCCCCCAAACTTGTGGTTCTTCACATGGATATGG
CCATCCATACATATGATGACATTATGAGGATTACTGAGAACTTCAATGAGAAATTCATCATAGGATATGG
TGCTTCCAGCACTGTATATAAATGTGTTTTGAAAGATTCCCGACCGATTGCCGTTAAGCGACTTTACACTA
CACATCCGCACAGCTTGCGAGAGTTTGAGACTGAACTGGAGACCATTGGAAGCATCAGGCATAGAAACC

TTGTTAGCTTGCATGGTTACTCCCTTTCCCCTCATGGGAATCTCCTTTGTTACGACTACTTGGAGAATGGT
TCACTCTGGGATCTACTTCATGGTTAGTAACCCTCTTTTCCTTGTAATCTTTTATAAAGCATCTTCATGTAA
GACAGTGTTGACTATTAGTTGATATTTGTTACTGTTTTCCCTGTCGGAGAATGGTGCTATAAGCCAGGAT
TGATTTGAAAATGGCTAATTATCAACTGAATACATGTCATAGGGCCTTCCAAAAAGGTGAAGCTTGACTG
GGAAACACGTCTGAGGATTGCTGTTGGTGCTGCTCAGGGTCTTGCTTATCTTCACCACGATTGCAACCCA
AGAATCATCCACAGAGATGTGAAATCTTCAAACATTCTTGTTGATGAAAATTTTGAGGCTCATCTTTCTGA
TTTTGGGGTTGCAAAATGCATCCCTTCTGCAAAAACTCATGCATCAACTTTGGTGTTGGGCACCATAGGT
TACATTGACCCTGAGTATGCCAGGACTTCCAGGTTAACTGAAAAATCAGACGTCTACAGCTTTGGCATTG
TTCTCCTAGAGCTTTTGACAGGAAAGAAACCGGTTGATAATGACTTGAACCTGCATCAGCTGGTATTATT
CTCCTCTTGTAGTCTACGTTGTTGCTTGTAAAAAAGATTTAACTCAGACTGGATATAGAAAAGAACAACT
TATCTCAAATTTTCCTACCTTCCTCCAGCATTTGCGATGTTATCATTCCATCAAATGTCTTTTGTCTTTTAAC
TCCTGTATTATATTTGTTGTCTTTATAGAGTACTTTGATTTGTGTTACAGATAATGTCAAAGGCGGATGAT
AACACCGTGATGGATGCTGTTGATCCTGAGGTATCTGTTACATGTATGGACTTAACACATGTGAGGAAA
ACTTTTCAGCTTGCGTTGCTGTGCACAAAAAGATTTCCATGTGAGAGGCCAACGATGCATGAGGTTGCTA
GGGTACTTGTTTCCTTGCTTCCTCCCCCGCCAACCAAACCTTGTTTAGACCCACCTCCCAAATCCATTGATT
ATACAAAGTTTGTGATTGGGAAAGGACTACCGCAAGTTCAGCAGGGTGATGATTCCTCCGAAGCACAGT
GGCTTGTTAGATTTCAAGAAGCTATATCCAAAAACTCCCTTTGACTTGGGTTCATAGACACACTTCATATC
AAATCAGAAGCGAATATGGAACATATATATGTTGGCCAAAGGTTTTTTGATGTTAGAGATTTCTCCTATA
AGATGGTTAGGTACACGGTAATAGCTAGTGTATTTCACATTTGTAGATGGAATGTTTTATGGTGGATGA
GATCCACAATAGGTTAGATCTACAACCAGTGCAGTTCAACTTAAATCTTTGTTGA
>PGSC0003DMG400031103 LRR-RLKs
CAATTTTATCATCACCATATAGTTGTATTCATTCACATATCACACTCACATGTTCTAGTACTCCTTCCATTCT
GTAAAGTTCTAGTTGACTCTCTCCCTTTGTGGTCCTTTTCCATTTTCTTGCCTTATCAATCAATCAACTTTTT
TAGCCAATTTTTTTCTCTGTTTTCGCATCCCTTTTTTATCCTCTTATTTAATTTTTAGCTAAACCCCATTTCTT
GATTTTTAGTCTTTTGCATGTCCTGAGTAGCATTATTTTTCAAGATTTCATATTATAGCTAAACCCCATTTC
TTGATTTGTATTATCTTGTCTTTTGCATGTCTTGAGTAGCATACTATCCAAGATTTCATATTATAGCTAAAC
CCCATTTCTTGATTTTGTAAAACCTTTGGTGATTTGCATGTCTTGAGTGGCATGTTTTTGTGGTCATTAAG
GTTAAAACAATTCAAGATTTCATTTTTTCCTTTGCTTCTTGAAATTGGGTTGAGTTGTATACTGAGAGTTA
TGGGCCCTGGAATCTCAAAACTTGCTTAATGGTATCTGTTTCCTGGTCTGCCATTAGCTCTGTTACAACTT

TTGGGCATTGCCATACTCATAAGTTTCCTAATTTTCATCCATAAGAAAAATATTGACATGACTTTAGTGTA
AACTAAACAGCATGTCATTCATGCTCTTTTATCCTTGGAAATGGCAAGTTATAGTAACTCTGCAATCCAAC
AGCATTTGTTTTTTGTGCTGTTGATTCTTCTTTTCTTTACTGGTTTTGCTGAATCTTTAAATGAAGAGGGAC
TTATTCTTTTGGAGTTCAAAGAATCCCTTAATGATCCTGATAACAATCTTGAAAGTTGGAATTCTTCAAAC
TTGAATCCTTGCAAATGGGATGGTGTTAAGTGTAGCAAAAATGATCAGGTGATTTCTCTCAATATCGATA
ATCGTAACTTGTCTGGTAGTTTTTCTTCAAGAATTTGTGAACTTCCTTACTTGACAGTATTGAATGTGTCA
TCAAATTTCATTTCTGGTCAAATTCCTGATGATTTTGCGTCGTGTCATAGTTTGGAGAAATTGAACCTTTG
TACCAATAGGTTCCATGGAGAGTTTCCTCTCCAATTATGCAATATTACCTCACTTAGACAGCTTTACTTAT
GTGAGAATTACATTTCTGGTGAAATCCCTCAGGATATCGGAAACTTGTCACTTCTTGAGGAGCTTGTTGT
TTATAGTAACAATCTTACTGGAAGAATACCTGTTTCAATAGGTAAATTGAAAAAACTTAGGATCATCAGG
GCTGGGCGAAACTATCTGTCTGGCCCTATTCCTGCTGAAGTTAGTGAATGTGATAGTTTACAAGTGCTTG
GTGTAGCGGAAAATAGGCTAGAAGGGTCTTTTCCAGTAGAGCTACAAAGGCTTAAGAACCTCATCAACT
TAATTCTTTGGGCTAACTCATTCTCTGGTGCAATTCCTCCTGAGGTTGGGAACTTCAGTAAGTTGGAATTA
CTCGCGTTGCATGAGAATTCATTCAGTGGACAAATTCCTAAAGAGATTGGGAAGTTAACTAATTTGAGA
AGGTTGTACATTTACACTAATCAGTTGAATGGAACAATTCCATGGCAAATGGGGAATTGTTTGAGTGCA
GTTGAGATTGATTTATCCGAAAATCAGTTGAGAGGAAGCATTCCGAAAAGTTTGGGGCAACTTTCTAAT
CTGCGGTTGCTTCACCTTTTCGAGAACAGATTACATGGGAAGATTCCAAAGGAGCTTGGAGAGTTGAAG
CTGCTTAAGAATTTTGACTTGTCTATAAACAATTTGACAGGTAGAATTCCAGCAGTGTTTCAGCATCTTGC
ATTCTTGGAGAATTTACAACTTTTTGACAATCACCTTGAAGGTCCTATTCCGCGATTTATTGGCCTCAAAA
GCAACCTCACTGTAGTGGACCTGTCCAAGAATAATCTTAAAGGAAGAATACCTTCAAATCTTTGTCAGTT
TCAGAAGTTAACATTCCTGAGCCTCGGGTCTAACAAGTTGTCTGGCAATATCCCTTATGGCCTAAAGACT
TGCAAGTCCCTCGAGCAGCTAATGCTAGGAGATAACCTGCTCACTGGGAGTTTTTCTTTTGATCTTAGCA
AGCTGGAGAACCTTTCAGCTCTTGAGCTTTTTCACAATAGATTTTCTGGATTGCTACCACCAGAGGTGGG
CAACCTTAGAAGATTAGAGAGGTTGCTCTTGTCAAATAACAACTTTTTTGGGCAAATTCCTCCTGATATTG
GAAAACTTGTGAAGCTTGTTGCTTTTAATGTTTCCTCCAACCGGCTCTCGGGTGATATTCCTCATGAATTA
GGGAATTGTTTAAGTCTCCAAAGGCTTGATCTTAGCAAGAACTCGTTCGCTGGAAATCTCCCCGATGAAC
TTGGTAGGCTAGTCAACTTGGAACTGCTTAAGCTATCTGATAATAAGTTTAATGGACAGATACCTGGTGG
CTTAGGTGGACTAGCACGACTAACGGATTTGGAAATGGGAGGCAATTTCTTCTCTGGTAGTATTCCTATT
GAGCTTGGCTACCTTGGAACTCTTCAGATTTCTCTGAATCTGAGTCACAATGCTCTCAATGGATCGATTCC

TAGCGCCCTCGGGAACTTGCAGATGCTTGAAACATTGTACTTAAATGACAACCAGCTTATTGGTGAGATT
CCAACTTCAATAGGGCAGTTGATGAGTCTGATAGTATGCAATCTTTCTAACAATAACCTTGTGGGATCAG
TACCAAACACGCCTGCCTTTAAGAGGATGGACTCGAGCAACTTTGCTGGAAATGTTGGGTTATGCACGT
CCGATTCCATCCACTGTGATCCTCCTCCGGCCCCTTGGATTGCTCCAAAGTCAAACTGGTTGAAACACGG
TTCTTCTAGGCAAAAAATAATTACTGCTGTTTCTGCGACTGTTGGGATGATCTCTTTGGTATTGATTTTAG
TTATATGTAGGATCATTAGAGGCCACAAAGCAGCTTTTGTGTCCGTGGAAAATCAAGTAAAGCCGGATG
ACTTGAATGATCATTACTTTCCACGAAAAGGGTTCACGTATCAGGACCTTGTCGATGCTACTGGAAATTT
CTCAGACAGCGCGATCATAGGAAGGGGAGCTTGTGGCACTGTCTACAGAGCTCATATGGCTGATGGTG
AGTTTGTTGCAGTTAAAAAGCTGAAGCCACAAGGAGAAACCGCGAGTGTTGACAGCAGCTTCCAAGCT
GAATTATCTACACTTGGTAAGATAAATCATAGAAATATTGTCAAGCTATATGGTTTCTGCTACCATCAAG
ATTGCAATCTTCTCTTGTATGAGTACATGGGAAACGGAAGCCTCGGGGAAGTACTTCATGGGAATAAGA
CAACTAGTTTACTAAATTGGAATAGCAGGTACAAAATTGCCTTAGGAGCTGCTGAGGGACTATGCTATTT
GCACCATGACTGTAAGCCACATATCATTCACAGGGATATAAAATCGAACAACATTTTGTTGGACGAAATG
CTTGAGGCACATGTAGGAGACTTTGGCTTGGCAAAGCTAATTGACTTCCCTTACTCAAAATCCATGTCCG
CGGTTGCTGGTTCATACGGCTACATTGCCCCTGGTAAGACCGGTGTTTAGGCACTTAAAACGATACTCTT
TATTAGCATTACTTACATACAGAAGCTAACTAAGTTCTAGTTTTTTCTTTTTTTCAGAATATGCATACACAA
TGAAAGTGACAGAGAAATGCGACATCTATAGTTATGGCGTTGTTTTGTTGGAATTGATTACTGGTAGATC
TCCAGTTCAACCCCTTGATCAGGGAGGCGATTTGGTGACTTGGGTAAGGAGATCAATTCATGAAGGAGT
GGCACTAACTGAGCTTTTTGACAAAAGACTGGATGTTAGTGTTGCAAGAACAAGAGAGGAGATGTCTCT
AGTTCTCAAGATTGCTATGTTCTGCACCAATACATCCCCTGCCAATAGGCCTACAATGCGAGAAGTCATC
GCCATGCTGATTGAAGCCCGGGAATTTGTGTGCACTTCACCACCATCACCATCCTCCGAAACTCCTCTGA
GTGAAGCTGATGCTAATAAAGGTGCATAAAAACAACTTCCTCATCTTTCAATCTTTTCACTACAGTCAAAT
ATCTCTATAACAGTCATCCTCTATAACAACATTTCACTATAACAAACGGGTTCTCTATGGAACTAATTTTC
ATGTTATGTTACATTACATGTTCTCTATAACAACATTTCACTATAACAAACGGGTTCTCTATGGAACTAAT
TTTCATGTTATGTTACATTACATGTTCTCTATAACAACATTTCACTATAACAAACGGGTTCTCTATGGAACT
AATTTTCATGTTATGTTACATTACATGTTCTCTATAACAACATTTCACTATAACAAACGGGTTCTCTATGGA
ACTAATTTTCATGTTATGTTACATTACATGTTCTCTATAACAACATTTCACTATAACAAACGGGTTCTCTAT
GGAACTAATTTTCATGTTATGTTACATTACATGTTCTCTATAACAACATTTCACTATAACAAACGGGTTCTC
TATGGAACTAATTTTCATGTTATGTTACATTACATGTTCTCTATAACAACATTTCACTATAACAAACGGGTT

CTCTATGGAACTAATTTTCATGTTATGTTACATTACATGTTCTCTATAACAACATTTCACTATAACAAACGG
GTTCTCTATGGAACTAATTTTCATGTTATGTTACATTACATGTTCTCTATAACAACATTTCACTATAACAAA
CGGGTTCTCTATGGAACTAATTTTCATGTTATGTTACATTACATGTTCTCTATAACAACATTTCGCTATAGG
AGCCAAATATTGGAACAAGTAAAACTGTTATAGAGATGTTTGATTGTATATCATTTAAACACATATCGAA
TCAATACCTGTGTTTATAGCAGCCCCAAAAGAAAGGAATTCTCATTTTTCATGTCCATAATTCTAAGTTTA
GTTAATAATAAAAGGAATTATTCAATACTGAATGGATGTTCACAAAGAAAAATCAGCTAAATCACATGCA
TGTCTGTTTACTAACAGCAACCAGTTTCTACATTTTTTTAGTAGCATTTGTGTATTCATGCTATACATGTTT
GATAATTTTGATTCAGAATGTTGCGCATACTCTCCAAAAATATCGTCAGACACTACTTTTGGAGGATCCG
ACATGCATCCAGCTGATATTTTTTTGGAGAGTTCGAGCAACATAGATTCAGATTATATCATCCACAAAAA
ACTAAAATGCATCCACTTTATGTTTCTTGCAGGTTCTAATTAAGCAACCATAGACCAAAAAACAGAAAAT
TGAAGTTGTGGCTCATCTCAAGCTCTGCCAAATTTCTAATAGAGTTAGAAGCATCTTATTATTCATGTACA
CACAACTACCTTTGATACCATACTTCCCCTCTAATAATTATTAGTTGGCATTTCTTATCTATTTATCTTCTAA
ATTATATCTACTTCTATGATCTGTCTCCACATTAACTGCC
>PGSC0003DMG400031228 LRR-RLKs
CAAACCTGCAGGAGTCTTAGGAACTGCAACTTGCAAGGAACAGTTCCTGATCTAAGCACAATACCACAC
CTTCTTTACTTGTAAGCATACTTCATCGAGAAATTCATGCGTCCAGTTAAACCACATGTTGAGGCTTATGT
TCTTGTTGTTCTAACCTCTTGGTAATGTTGATATCTGCTTAATAATTCTTATATGTCATCTGCTTAATAATTC
TTATATGCCAGATGTAGTTGAACTCATCTGCCACTCAATTTGTGCTGAATATAGAAAGTTAAATTTACAGC
CAGTGGCAATTCATCTGATATAAATTTCTCATGTCACAGAGACCTTAGCAGAAACCAACTCACGGGAAAT
ATACCTTCCAATAAGCTGTCTGATAATATCACAACCATGTAAGTTTCTGGAAGTGTTTAACTGCTGATATC
TTTCCTGGTTTGATTCACCCATCTTTAATCACACTTCCTTCCCCCCCCCCCCCAACTCTCTATCGATATCTGT
GAATAGGTGGTTGAATGGACATTAGTGTTTTAACATGCACTCTTTTCTTCTTGATGTTTGAGTAACTTGGT
CTTGGTATCAGACCCTCAATAGTGAACTAACTACTTTGATGATGATTGCAGCATTCTGTCAGGAAACATG
CTTAATGGTTCTATCCCTTCGAACTTTTCTGTCCTTCCAAATCTGCAGAGGCTGTAAGGAACTTCATCTGC
TCAATCCTTGTTTCTTGATCCTCTGTCTGTTTCAAATTTCATGTCGATACTCTAATAAATGTAGTATTGACA
CTACCTTTCCTTAAATTATTTTTCTTGTAGGTCACTCAACAATAATAGGTTAAGTGGTTTTGTGCCAACTAC
CATTTGGGAGAATAAGACATTTTCCCCAGGCGCAAAACTTAGATTGTAAGTGTATCATTTGTACATTTTCC
TCTTATCAGTAATTTGATTTTCATGTTTCTGTTGTCTGCTGCCCCACAATCTATTGGCCAGTTAAAGTTTTT
AACTGTAAGATACAGACAAAAGCTTTTTGTAGAGAATCAAGCCAAATTTGTATTCCTATTTCATTTTGGTA

GCTAGTCAGTCCACATGTAGTTTTGGATATACAAAGAAAGTATCCCGGAAAATAATAGTACAATCAAGG

AATAGTGTGTCCATTGTCAACAAGGGTTTCCTATCTCTATACAGTTAATATATAACAAGGAATAATATGTC

TGGTGTTTTGTTTCGTTCGAGGTAATGTAGCATGTATTCTTTCCTCTTACTAAACCAGGCCATGTGACTAG

AAAAGGCCCCTCAGATGTTAACATATCAGATAGCCAGGCTGATCCAGTTGTTCTCCTTTGTGCAGGAACT

TTCAACGTAATTTTCTCTCAGATATTTCGGGTATTCTTGATCCTCCTCCAAATGTCGATATTATGTAAGACC

TTGTTTCCGGTAGTCTTGTACATACATATTTGCGGACTTACTTTCAGTTCTCGGCTTTTATAATTATATGTG

GCGAATCAAGCTCCCCTAATTCAATTGCTATCTAACTTTCATTATCCTATTTTTTATAAGCTTCAAATTACA

AAGTAATTCTGTGGAGTCTTAGTTTCTATTTCAGATTTCTGTGATTCAACAGAACTACTATCATGAAGAAG

GCAGAAAAACTTGCTATAGCATATCCCCATGACAACATTTGGGATAGTTGCATAACACTGCCTTCATTTG

CTGTTTTCCTGCTAAACTCTTTCTATCAGTATAGAGATTTACTTCGAATATCCATTGATGAAGATAGAGTA

GTGAACCAACACAGTTTCAACTTCTTTTATTTTGTCATCGTTCAAAACTTCATTTCTTGTCAGAAGATGGG

TAGTTTTCTTGCTAGCAGTGCACTTTATATGGAGCTCGAAAACAATGGGGTCAAATTCATATATGTGAAT

TTCTTTCCATATGATATGGTAATATGACAAAGCTAGGGTTATGGGGGAGAAGTTGGTGGCTATGGGAGA

CTGGGGAAGCAGTGGGGATGCGACCAATATGTGGGATAGGACCGCTAGTTGCATAAGGGAAGCGGCT

AGAGAGGTCTTGGGGGTTTCAAAAGGTAACTCTAGTGGACACCGAGGAGATTTCTGGTGGAATGACAA

AGTCCACGTAAGGTAGAGGCAAAGAAGGTGGCTTATGTGAAGTTGCTGGAGAGCAAAAGGATGAGGA

GGATAAGAGGGCGACGAGAGAAGTATATAAAGTGGCAAGGAAGGAGGCGAAGTTGGCAGTTATGGA

GGCAAAAATGGCAGCTTTTGAACGCCTGTATGAGGAACTAGGGGACGAAGGCGGAGATAAGAGGTTG

TATAGGCTAGCCAAGGTGAGAGAGAGGAAGACTCGAAACCTGGACCAAATAAAGTGCATTAAAGATGA

GAAAGGCAGAGTATTGGTGGATGAGAGGCAAAGTTGGCAGTTAAGGAGGCAAAAATGGCAGCTTTCG

AATGCTTGTATGATGATCCCTGCATTATAATCTTTTTATTTTTATTACAATTCTGGCATAACTTGCCTCTAA

ACCAGACAACTAACTGGTTACATACCGAAGTAGTAACAGGCTTTCCAATTTGATGGAGAGATCACGCCT

CAGCAATACCTTTCAAGCTGTATATATTTAATTCTAAAAACAAATTGTTCAACTTCTATCATGGTTAAATA

AATTCTGGTTCACCTACTAACTTTATTTTTCTGAAGAGCAGCTTCACCTACTAACCTTATACATGTTTGTGT

GCAGTTTTGTGTGCCATATTTGCTGATTTCATGAATAACATAAATAATGAGTTTGAGTTTTGTTTTTAACC

CATTTACTGGTACTTTAACGTGCAAATATTTTCAATGGCATAGAATCCCTACACACATTTTGCTTTATCACT

CCTTTATATTTCTGATTAAGTATTCATTAGGTGTGACAAAGAACTGTAACTTTCAGGCTTTATGGAAATCC

TGTTTGTGGAAATGCAAATGAGCGCCAGATTACCCAATTTTGTAAATCAAGAGACGGAGGTGAAGAGTA

TGGAGGCTTGAACAACTCTATTCCAAGTTGTGCAGCCCAGCTGCCTTGCGATGTGGATTTTGAACATGTC

CCAGCATTAATGGATGGCTGCTTTTGTGCAGCACCTTTTGGAGTTGGTTTGCGTTTAAGAAGCCCTAGCA
TTTCAGACTTTCCACCACATTATAGTGACTTTGAACAGTGGATAACAAAAAGCGTTAATTTGAATGATTAT
CAACTGCAAATTGATTCGGTTTCATGGCAAAGCGGTCCTAGGCTTAGGTTCTTTTTGAAATTCTTCCCTCC
ACGTGCAAATGACTCTGGAACCAATGATTTTGGCAAATTTAATGACAGCGAGATAGTACGGATCGCAAA
CAAGTTTGCCTTTTTTAATCTTACTGGAAGTGATATCTTTGGCCCATATGACCTGCTCAATTTCACTGCCAT
GGCATATAATTCTGGTATGTCTCCAAATCCATTATGTTCTTGCATAAGTAGGATATAATAATACATTAACA
TGTGGACATTTTCAGTTGTAGTTCTGTAATGATTTCCTGAGGAATTTAAAGTAAAACCAATGTGAACAAT
AATAGAAACAAACGTCAATCTCGTTTGTTTTTTACAACTTAGATCATGCACGGAGAGTGGCAAAAACTCA
ATTTTTTTAGTTTAAATTTCTCTAGGGAGGCACAAGAACCCAAGGTTGAGATTAGCATGCAAGCCGAGA
GAAACACCTTTATTGTAAAACTTGTTTCTTCAGTTCTTATTTTGTGTTTAATGCCAAATATTGGATACTTTT
CAACTGGACTGGAAGTCTGGAACTCTTGTGCACCCGCATGACTAATTTTTCTTTTGTTCTTGCTGAATCTT
GAATATAATTATTATGATTATTATATTTCTTTTATCTTTACTGGTCATGCTCGGATAAAAAATTCCCTTTCC
CATTGTGCTCCATTTCGTTTCTGAAAATGTCTATTTATATCGTTTACCCTGTGATATAGAACATAGTTTTTT
CTTCTTGTCATAGAAGTATGCGAGTATGAAAATTCAATGAAATGGTTTAGATATCATGCATGCTTTGAAG
CTTAACATCCTCATGAACATGGTAACCTGACAGAGATGCTAGTAATCTCTTAGATACTCAGTAAGGGTCA
AGAGACTAAATTGACTACCTTAGTACAAGTTCTTGATTAAGTAAAATCTTAGGTGAACCATATGGTACTG
TCAAAACATGTGAATATCCCCATTCAGAAACTCAAGATGAATTGGCTACTTTTGTTTTATTTTTGGTGTAT
GAAGAATTTGTTAATGACGCAGAATCTGTGTGAAGAAGCCTTATAGAATCTTTGTAAGCAGAACAAGGA
TTTACTTTTCTTTTTTTTTCTATTTGAGATGTCTCGCAACACAGCCTTTGTCCTGAATATAAATATATCATTG
TTAAATATATGCTATCATATAAGAGAGCCACTCTGTTCTTGTCTTTCTTTCTGCTTTCTCAAGATCAAATAA
CGAATGTTTTTCCTTCTTGCAGTCCTTTTTCCTCCGTTGGGAGGAGAGAGAAGCAGAAAAAACAGAGGC
ACTGTGGTTGGAATTGTCTTAGGGTCCATATTTGCTGCAGCTGTGCTACTTATGGCCTTAATATTCGTGTT
GTTTAAAATGCGGCGGAGGTTACACGTTTCAAAAGATCAACCATGTGAGTTAATCTTCAGTAATTTTATG
TGACTTTTCCTCAGCTATGGGGAGTTTCTTCTCCATTTCTTTCATTCACCCCATTAGGACCTGTTGGTTAAG
TAGTCAATACGTTTAACTTTTCTTGGGTATTCTGTTGAATCGCCAAAAAAGAGGACATGAAACCACATTC
TTTGAGTGCTTCATGCATATAATGCAAACTTTGCACCTGATAATTTCCAATTTCCATCTTCTGAAAAACAA
AAAGATGCCAAAAACCATAAAATCTGTCTTCCTTACTGTTATTTTCTTGTTTAAGTTCTCTATTTAATCAAG
AAATAACACAGAATTGCCAGTGTCAACCATCTTTTTCAATGTGGCAAATTATTTTTCTTAGTATGTATACT
TGTAATTTAGCTATGATACTGCTTGATGTTAGTTTTTTTAAGGAGAGAGTATGGTGCTTTAATTCCTCACA

TGGACAGATAACTTTACGGTTTTATGGGAAGTTTGTGCGTATTTATGATGAGTAAACTCTCCGGCAATGC
CCCATCACCCTTAGAACAAAAAGTGGAATAGAAATAAATAGGAAGTGTGTGCTGGACAATTGGCATGTA
TATCTAAGTAATACTCTTGTTGTAGTGCTTCTGTTATTTGTACATCAATTAGACGATTATTTATGTTCTTTT
CTTAGTCAGATTAATTGATATTCTTAAAGTGAACAAAGTTAGCTGAAGCCAACTATTGTATCAAAGGTTC
ATTTGCATTAAAGTTATGCTACCATCACCTAAGCTTTTTCGTTTTCATTCTTTTTATGTCAACTTTTTATGCT
TAAGTTTTCAATGAATATCTGATTTTTAATAAATTGAATCTTGTGGCAATGATGTATTTCTTGCTTAAACA
AACTTACCCAGGACCACATGGCACTCATAGTGATATTCCTTTCATTTCTTTTGTAGTACCAAAATTTCCAAT
GCGAATAGAAGGTGTTAAGGCAATGGGTTTTAAGGAATTAGAGGCAGCAACTAACAATTTTAGCAGTA
CTACTGAAATTGGCCAAGGAGGCTATGGAAAGGTCTACAAAGGCACTTTGGCAGAGGGAACAATAGTG
GCAATTAAACGTGCACAGCAAGGCTCATTGCAGGGTGAAAGAGAGTTCTATACTGAGATAGAATTACTT
TCACGTGTGCATCATCGAAATCTGGTTTCCTTGGTGGGATACTGTAATGAAGGAAGTGAACAAGTAAGA
TTCTCCAAACTGGAAATGTAATAGACACACACACACACACACACAGAGCCAAGTCCATGTTGCTATTCCT
GCATCCAGATGGGAGAGGGGAGTATGTTCGGTGGAATAAGTTCTTTGATGTATCAGTTCAGCCACTTGT
TGAATTCTGATTGCCCAGTTCTGGTTGAATACCTAGGTCAAAATTGAATTTTTTTGCATATCCTTGATCAT
TTTGGATGCCTCATTGCCGTTGTGCAGAACCAATCAACCATAATATCAATTACCTTCTACAACTTTGAGAA
ATCCATTTGATATCACTCTAGATGCAAATGAAATTACAAGAATCACAAATGATAGTTAAATTGCAGGGCA
AAGTTCAGACTTAGCTGATTCATCTGTTAAAGTAAACTCGAGCAAAAGTCTTCTGAGATAGTATTTTCATC
ATACTTCTTTTTTTGTCACCAATTATTATCACACGTTAAGTGATTCAATTTGTGAGCATATCCAGTGTACAG
TACTTAGATCACCTAATATTAAGTTCAACCAAATTTCCATCTATGAGTAGCTCATCAGTTACATCCCTCTTA
TACTTGCGTAGTCTGGGCACATTAGTGAAATGCATGTCTTCTGAAAAAGTAATCAAATAATCAAGCAGAT
GGAAGGATAAGAACGGATATTATGTTTTATGTTTTTTTATTCTACTTTTGTAATAGTTGGTTACATGTGCA
TGCAGATGTTGGTGTATGAGTTCATGCCAAATGGTTCCTTGCATGATCTTCTAAAAGGTATGTGATGTAT
CCTTCCTTTGCCTATGTTGTCATTTGATTTTCTTTTGTTAGAATCAAGCACATCTATTCTCCTCTTATCACTT
ATTTTTTCCTGGTGTAGTACTTCTTATACGAAAAAGAGGGTGAAAGATATGTCCCTCTAGTAAAGAATAT
TGTATAGATGAAAGTAAATCAAAATTATCTAGTCACTCAGATTTTCTTTTCTGAAGTTTTTACTTCATTTTG
ACTCCTGAAAGTCACTGTTAGTTTTCCTACTTTCTTCACTTGGGTGGTCATCAACAAGTTTATCACATGGA
ACTTTATATTCTTAATTTTAGTCATGTGTCAGTTCCAATATGCATATCAAACAGGTATCTCCAAAGTACAA
TTATTAAAGATTTTTTTACTTATTTTTACAGCTAGATATGGGGAATGTCTGAGTCTTGGAACAAGGTTATA
CATTGCTTTAGGTGCTGCCAGGGGCATCCTCTACCTCCATACTGAAGCTAATCCTCCAATAATCCATCGTG

ATGTCAAAGCAAATAACATACTGTTGGACTCTAAGTTCACCGCAAAAGTTTCTGATTTTGGAATCTCAAA
GCTTGCACCATTACCTGATGCTGAAACGAGTGGGCATGTATCAACTGTTGTGAAAGGAACTCCTGTAAG
GCTTTACTCGTCTTATATGTTGAAGCCTGCAATAGAAAAGCTAGAAAATCGCAATGAGATAAGATCTGCA
ACTTGAGTAATCTAGGTAGCATTGCTACAGACCAGGAAAACTTCATTCTCT
>PGSC0003DMG400031752 LRR-RLKs
GTCAGCTTTTACTACTCCCTTTTCCCAAAAAGAAAATTGCAGTCTTTCTTCTTCTTCTTCTTTGTTCTTTCTT
GATCCCCAGAAAACCAAAAAATGAAATCTTCAATTTCAATAATGTTTCTTCAAATCTTGGTTACCCTTTTG
CTCCCAACTTTGATTTTCTCACTTAACCAAGAGGGTCTTTATTTACATAATGTGAAGCTTGGATTTGATGA
CCCTGATAATGTTCTTTCAAACTGGAATGAATATGATGATACACCATGTAACTGGTTTGGTGTTTCATGTG
ACCAATTGACTCGCACTGTTACATCATTGGACCTTTCTAATGCTAATGTTGCTGGTCCTTTTCCGACTCTG
CTTTGTCGGTTGAAGAAGCTTCGTTACATTTCGTTGTATAACAACTCTGTGAACAGTACTCTTCTTGACGA
TTTATCTGGGTGTGAAGCAGTGGAGCATCTTGATTTGGCGCAGAATTTCTTGGTGGGTACACTTCCGGC
GAGTTTATCTGAGCTCCCAAACTTGAAATATCTTGACTTGTCGGGTAATAACTTTACCGGAGATATTCCG
GCGAGTTTTGGTTCTTTTCAGCAGCTTGAAGTTCTTGGGTTAGTTGGAAACTTGCTTGACGGGAGTATAC
CGGCGTTTCTCGGGAACGTTACGACGTTGAAGCAGCTGAATCTGTCGTACAACCCGTTTACTACGGGTC
GGATCCCGCCGGAGCTGGGGAATCTGACGAATCTTGAGGTTTTGTGGCTTTCCGACTGTAATTTGATTG
GGGAAGTTCCTGACACATTGGGGAGCTTGAAGAAGATAGTGGATTTGGACCTAGCTGTTAACTACTTGG
ATGGGCCGATTCCGAGTTGGCTCACTGAGCTGACTAGTGCTGAACAAATTGAGCTGTATAACAACTCGT
TTACCGGGGAGTTTCCGGTGAATGGGTGGTCGAAAATGACGGCGTTGAGGCGAATCGACGTTTCGATG
AACCGGGTAACTGGTACGATTCCGAGGGAGTTGTGTGAGCTGCCACTTGAGTCACTCAATCTTTATGAG
AACCAGATGTTTGGTGAATTGCCACAAGGCATTGCAACTTCACCAAACTTGTATGAGTTGCGCCTTTTTC
ACAACCGTTTTAATGGGAGTTTACCTAAACATCTTGGAAAAAATTCACCTTTGTTGTGGATTGATGTGTC
AGAAAACAATTTTTCTGGTGAAATTCCGGAAAATTTGTGTGGGAAAGGGTTGTTGTTGGAGCTTTTGAT
GATAAATAACTTACTTTCCGGTGAAATTCCGGCGAGTTTGAGTGAATGCCGGAGCTTACTGCGCGTGAG
ATTGGCACACAACCAGTTATCCGGTGATGTTCCGGAGGGGTTCTGGGGTCTGCCACACCTTTCTCTGCTT
GAGCTCATGGACAATTCACTCTCCGGTGATATCGCGAAAACTATAGCCGGTGCTTCAAATTTATCAGCTT
TGATTTTGTCTAAGAACAAATTTTCAGGTTCCATCCCAGAGGAGATTGGTTCTCTGGAAAATCTTCTTGAT
TTTGTGGGCAATGATAACCAGTTTTCTGGGCCTTTACCTGCAAGCCTGGTGATTCTTGGACAATTGGGAA
GGCTGGATCTTCACAACAATGAGTTAACTGGTAAGCTTCCAAGTGGGATTCATTCTTTGAAGAAATTGAA

TGAATTGAACTTGGCAAACAATGATCTTTCTGGAGATATCCCCAAGGAGATTGGGAGCTTGTCTGTTTTG
AATTATCTTGATCTATCAGGGAACCAGTTTTCAGGGAAAATCCCAGTGGAGTTGCAGAATTTGAAGCTCA
ATCAGCTGAACTTGTCGAATAATGACCTTTCGGGTGATATTCCCCCTGTTTATGCAAAGGAAATGTATAA
GAGTAGCTTTTTGGGGAATGCTGGTTTATGTGGAGACATTGAGGGCTTGTGTGAAGGAACAGCTGAAG
GTAAAACTGCTGGTTATGTTTGGTTATTGAGGTTACTCTTCACCCTTGCTGGATTGGTGTTTGTTATTGGG
GTTGCTTGGTTCTACTGGAAGTACAAGAATTTTAAGGAAGCTAAAAGGGCTATTGATAAGTCTAAATGG
ACTTTGATGTCGTTTCATAAATTGGGTTTCAACGAGTATGAAATCTTGGATGCTCTTGATGAGGACAACT
TAATTGGCAGTGGCTCTTCTGGGAAGGTTTACAAGGTGGTTCTGAGCAAGGGTGACACTGTTGCGGTGA
AGAAGATTTTGAGAAGTGTGAAAATAGTAGATGAGAGTAGTGATATCGAGAAAGGTAGCTTTCAAGAA
GATGGATTTGAAGCAGAGGTTGAGACGTTGGGGAAGATTCGACACAAGAACATTGTTAAGCTATGGTG
TTGTTGTACAACTAGGGATTGCAAACTTCTGGTCTATGAGTATATGCCTAATGGAAGTTTGGGTGATTTG
CTACACAGCAGCAAAAGTGGCCTTCTAGACTGGCCTATGAGATCTAAGATAGCCATGGATGCAGCTGAG
GGACTCTCTTACTTGCATCATGACTGTGCTCCACCTATCGTTCACAGAGATGTTAAGTCAAATAACATCTT
GCTGGATGGTGAGTTTGGAGCTCGTGTTGCTGACTTCGGTGTTGCAAAGGCGGTCGATGCCAATGCTAA
GGCAATCAAGTCCATGTCTGTTATTGCAGGGTCTTGTGGTTACATTGCTCCAGGTTAGTGGATTAACTTT
TTCACTTTTCAGCTTCAGTTTCATGAGTTTGTGTTGAATTTGTTCATTTTGAGTTTTACTAGCAAATTGTTA
TGCCTGTAAGTTTATGGATTACTGTAGCCTTTCAAAAATGAGATCTAGTATTACTGTAGCTATGTGGCAC
CTATTGTGTAGAAGGCTAGATCTATATACTACGATATTTCCTAGATATTCCACATTTGTTAAGTAATTGGC
TCATATCTGGAGTACCCCCTAATACAGTAACAGTGTACCATTGCTCTAGATCATAGTTTGTGAACAGTTA
AGTAGGCTCCACGAGTTTCCGGCTCTTGAGCACAAAAGACTGTATTAGGACTGTAGAAAAAGATATACT
TAGCTTTCCACTTATGATGTTTGAAATCTTAACAGCTAGCTATATTGTGGGCGGCTGCTAAGTTTAATAAC
CACCCACCCATATTTTTGGTTTCATGTGGTCATTCTTTGTTGTGTTGTTTCCTTGCAACAACTTAGTTGACT
GAATTCTTTTTTGCATTCCAGAATATGCATACACACTGCGGGTGAATGAGAAGAGTGATATATACAGCTT
CGGTGTGGTCATCCTAGAGCTTGTAACGGGGAAACGCCCTGTGGATCCTGAGTTCGGGGAAAAGGATC
TGGTGAAATGGGTGTGCAGTACATTGGACCAAAAGGGCATAGATCATGTAATTGACCCTAAACTTGATA
CTTGTTTCAAGGAGGAGATATGCAAGGCCCTAAACATTGGCCTACTCTGCACTAGCCCTCTCCCAATTAA
CCGACCCTCGATGAGACGAGTCGTTAAAATGTTGCAAGAAGTGGGTGGTGGGAACTTGCCCAAGGCTG
CCTCAAAGGATGGCAAGTTGACACCCTATTACTATGAAGAAGCATCAGATCAAGGAAGTGTAGCTTAAG
AATGTCTCAACAAATTTTGTCCTTTAAAAGTGAGACTCAATTAACTTCTTTGTTCATCTGCTAACTACTCAA

TTCTTTCCCACTAGATGTTTGGGTTGAAAGAATTGAAGTTAAATGAAAAAAAGGTGAGAACAAAAGAAT
ATTAGGAAAAAAAAAATGGAGTTGGAAGAATCTTGTTTTTCATTGTAAAAATCCCATCCAACTACCAAAAG
TTATTGCAGTAAGTATTTTACTATCTGGCACTAAAAGATCTTAAATGTGCTACCTATACTCTATTTCCTTG
>PGSC0003DMG400033049 LRR-RLKs
GAGGAAAACAAGATTGTAATTAAGTGACACACTAATTATCATGTCTGAAGTGGAATCTCATGTTCTTCTT
CTCATTGTATTACTCAATTTCTTCATTCTCATAACAGGTATGTATATATCCTCATGTCATATTTGATAGCTTT
AATTTGATCAAGTTTAGTATTAGAGTCTCATTTTGTACATAATTAAAAAGAATCAATTTTTTAAACAACAT
TTATTGCAGCAAGACTAATGTTTTTTTTTACTTAGCAAAATCATAGGACCAGTCTTGTTTGTGTTACTCCA
ACACCAAGCATTAAATGTTGTTCTGTTTTGTTTTTGGGAGAATTGACTTGTATTTATTGTAGCATGATTAG
TTTATGCAAAATATTATATAGGTAGGCTTGTTTGTGGAGACTCAATTGAGACAGACAAGCAGATGCTGCT
GAACTTGAAGTCATTTCTTAAGGAACAAAATCCTGTTGACAAAGGATTCAAATATAACCATTGGAATCCT
ACAGATTTGTCACCATGTAGATGGCCTGGAATATCATGCAGTACCTCTATCAATCGTGTCACTGGAATCG
ATCTCTCGGAAAGTAATCTGGCTGGGAAATTGTTTAATAACTTCTCAGCCATGACAGAATTGAACTCTCT
TGACCTGTCCAAGAACACATTTTCAGAGTCCATTCCATCAGACTTAGGCTGGTGTAGAAATCTGAAGTTC
TTGAACTTGTCACACAATATCATTGTTGGTGAGCTCAACTTGACAGGCCTGAACAAGCTGGAAGTTCTTG
ATTTGACGATGAACAGGATCCACGGGCTAATGATCCCTGAGATTTGTGACAACTTAGCTGTTGCAAATAT
TTCTAACAACAATTTCACTAGTGGGAGTGGATTTGAATTTTCTCACTGCAAGAAACTGAAGTATCTTGAT
CTGAGCTACAATTATTTGACAGGGAATCTGTCGTTCGGGCTTGATATGCTGAATATGTTTTCAGCATCTC
ATAACAACTTAAGTGGCTCTCTGCCTTCCTGGATTTTCACTCAAAACTGCTCTTTGCAAGGTTTGGACTTA
TCAGAAAATATGTTCTTTGGTGAATTGCCTACGTCTATTGCGAACTGTAAACGATTAGTAGAGTTGAATT
TGTGGGGAAATAGCTTTTCAGGGTCAATCCCTAGAGAGATTGGATCAGTACAGAGTCTTAAAGAACTTT
GCTTGGGAAGTAACAACTTTTCAAGTGATATTCCAGACACTCTATCAGGTCTAAACAAATTGGTATTTCT
GGACCTAAGTAGAAACAACTTTGGAGGAGAAATACAAGAAATTTTCGGGAAATTGACACAGGTAAGAT
TTCTCATGCTGCACGGAAACTCATATATTGGAGGCATAGTGTCATCAGGAATTCCAAACTTGGTGAACCT
TTCGCGGTTGGACTTGAGTGATAACCACTTCTCTGGTCCATTACCAGTTGAAATTTCTGAGATGAAAGGT
TTGGAGTTTTTGATTCTTGCCCACAATCAGTTTAGTGGCAATATACCTTTAGAATATGGAGATCTTACTGC
ACTTCAGGCTGTTGATCTTTCCTCTAATAGGATCAATGGTTCAATACCACCAAGTTTCGGGAAGCTAAGG
CCACTACTGTGGTTGATGCTTGCAAACAATTCATTGAGTGGTGAAATCCCATCAGAGTTGGGGAACTGC
AGCAGCTTGTTATGGTTGAATCTCGCGAATAATCAACTTACTGGTCCAATTCCTCCTCAATTAGCAAGTAT

TGGTGCAGATCCAATGCCTACTTTTTTGTTGAATAGGGGAAAGGAGAAGCTCACTGCTAGCCCAGGGGA
TTGTTTTGCCATGAGGAGGTGGATACCAGCTGACTACCCTCCATTTAGCTTTATATATCCTCTATTGACAG
GGAAGAGTTGTAGAATCCTGGGGGATAAGTTGTTTATGGGAGATGGTTTAATGCCACTCTGTGAACCTG
GTAGTAATGTCCGAAAGAATCAGGTACCGGGCTATATTCAACTTAGTGATAACAAATTGTCTGGTGAGA
TCCCTCCTGAGATTGGCAACATGAAGAAAATGAGTATGATGCATTTGGGTGCAAATGAATTTTCTGGAA
GGCTCCCTTCAGAGATTGGACAAGCGCACCTAGTAGTCCTTAATGTTTCACAGAATAAATTTTCTGGTGA
AATCCCAAAGCAGATTGGCCATCTTAAGTGCTTGCTAAACCTTGACCTTTCATTTAACAATTTTTCTGGTC
CATTCCCAGTTAGCTTTAGTAACTTGCATGATTTGAGCAAGTTCAACATCTCTTACAACCCATACATCTAT
GGAGCTGTACCAGAAATCGGGCAACTGCTTACATTTGATAAGTCATCATTTCTTGGTAATCCGTTGTTGC
GTCTTCCATCCTTCATGCATAACTCTAACAACAACACAGAACGAAACAAGAATGACAATCACAAAAAGCC
CAAAAAGGTGGGTGCACTTTTGGTAATTGTGGTTCTGGTACTAGCTTTCCTAGTCTGTGGAGTCATGTCA
CTCCTTGTCTGCCTCCTTATCAAAGTGCCAAGGGGTTCCCCAGGAATCTTACTGGAGGATACAGAGGGC
AGACATGATTCTCCATCAAGTACTAATGCATCCTCCTCACGGTTGTGTGCATCCTCATCACGGGGTTCTGA
TGATGTTAAGGTTATCCGTTTGGACAGAACAAGCTTCACACATTCTGATATACTGAAGGCCACATGGAAC
TTCTCGAATGATAGAATTATCGGGAGGGGAGGATTTGGGATAGTCTATCGTGGAGTCTTGCCTGATGGA
AGGGAAGTAGCAGTGAAGAAGCTACAGAGGGAGGGAATCGAAGGAGAAAGAGAGTTCAGAGCTGAA
ATGGAGGGGCTCAGTGGGAATGGCTCCGGTTGGCCTCATCCCAACCTTGTAACTCTTTATGGGTGGTGC
CTTGATGGATCAGAGAAACTTCTAGTCTATGAGTACATGGAAGGTGGTACCTTAGAGGACTTCATTACA
GATAGAACAAGGTTTGCATGGAAGAGCAGAATTCAAGCAGCAATCGATGTAGCACGTGCTTTAGTCTAC
TTGCACCATGATTGCTATCCTTGCATTGTCCACAGAGATGTCAAAGCTAGCAACGTGCTTCTTGACAAGG
ACGGAAGAGCAAAAGTCACCGACTTTGGCCTTTCTAGGGTCATGATTTCTGAACATACTCATGTTAGCAC
AATGGTGGCAGGGACTATTGGTTATGTTGCACCAGAATATGGGCAGATAATGCAGGCCACTACAAAAG
GTGATGTCTACAGCTATGGGGTGCTAGCAATGGAGCTAGCAACTGGGAGACATGCTATAGATGGGGGT
GAAGAATGTCTAGTTGAATGGGCGACAAGGGTCATGGGAGACAGAAGGAAAGGGTTCACCAGAGCCA
TTATACCAGTTGCTCTATTGGTACCTGGCCTAGTAGAGGGAGCAGAGGAAATGTATGAGTTGCTTAGGA
TTGGGATAAGGTGCACAGCTGAGACGCCTCATGACCGGCCTAACATGAAGCAGGTATTAGATATGTTGA
TTAGTATTCCTAGCAGCCAAAAGGGATCCAGCCGTAGCTTTGGATCAAGTCGTAGCACTTCTCCATTATT
ATGATAGACTTTCTTGGAACCCAGAGATAAAAACGTCAATAGTTTCTTTACAAGATGTGTAACATACCTA
GCAGATTCTTCTTGTAAAACAGTTTTTCTTTTTTCACATTTGTTAGTCTAGGAAAATGTAAGTCAATTTTGT

TTGTTTGGATCACAAAATGTTCTCAAGGATATCAATTTTTGCTATGAAATTGAAGCACACAAATATTTGAC
CCACCTACAAAGTTACACTACAGTAGGTGGCAAGATTGTTCCCCTTATCAGGTACATTATTTGTCTGGTG
CAATTCAAAGTCTCTATTTCAAACAAGATTCCTCTACCAGGGACTGTGCAATAGCAACTACAAACAGGAG
TGTCTCCAAATTTCAACTTCTGGACCACATCTGATGGAAAATTTTAGTATACTTCCACTACATCAACACAC
CACCAATTGTCAACGCCACAATACTCATTTTCATATCAGGCTAGCTTCATCATCCGTGGGAATAATGTGG
ATATGCACTCAAGCATCATACTGATTTCAAGTTAAGGCTCAAAATTTTAAGTCACTCTCTGATGTATAAAC
ACTGCTAATAGAAGAGTACAAATTCTTGTCTATCTGCTTAAGAAAAAGAAACTACAGATAACGTTTAGTG
AAACTCTTAATGGTGTGAGACCTTTTAAGAAAAACATTGCGAGGTTGGATGAAAACTACCAGTATCCTAC
CATGTTAAGAATGTCTTTGGGCTAAACATAACCATGTATACCCCCATTAATCTAGAGGTCACCTATG
>PGSC0003DMG400033667 LRR-RLKs
CAATTTGGCATTTTCTTCACACCTCCAATGTTGACCAAACTTTCCAAAATCAACTCATAATTCTAACCCCAC
CTTTTTTTTTTCATGGAGTTTTCCCATTTTCCGACCGGCATCTTCGCGGGAAAACAGTTCCTCTATCTCATT
ATTCTACTAAACTTCATACCCATTTTCGTAACTTCACAATCTCCGGCCACTACTGAACGTGACACTCTACTG
AAGATTAAGCGTCAATGGGGAAACCCAGTAGCACTTGACTCATGGAACTCTACTTCTTCTCCTTGTAGCT
GACCGGAAATTGAGTGTGATGATGGGAAGGTTACCGGAATAATAATTCAAGAAAAAGATATTACTGTT
GAAATTCCAAGTTCTATTTGTGAACTTAAAAATCTTACTTTTCTTAATCTCCGGTTGAATTATCTTCCCGGA
AAATTCCCAACTTTTCTTTACAAATGTTCCAATTTGCAGCATTTGGATCTTTCTCAGAATTACTTTGTGGGT
TCCATTCCGGAGGATATTCACCGGCTTGGGAAACTCAAGTATCTTAATCTGGGTGGTAATAATTTCACCG
GAGATATCCCGTCGTCAGTCGGGAATTTGATGGAGCTGGAGACTTTGTGCATGCATTCGAACTTGTTTG
ATGGGAGTTTTCCGGCTGAAATCGGAAACTTAGCCAATCTTGAAAGTTTGGGGTTGGAGTTTAATGGGT
TTTCTCCAATGGCACTACCGCCGGAGTTTGGGAAGCTGAAGAAGATAAAGTATATTTGGATGAGAGAGA
CGAAATTAATCGGTGAAATCCCTGAAAGTTTTGGGGAATTTCAAAATCTTGAGCTTATTGATTTTGCTTAC
AACAATCTGGAAGGAGAAATCCCTTCTGGGTTGTTTCTGTTGAAGAATTTGACGATGATGTACTTGTTCA
GTAATCGGCTTTCGGGTCGAATCCCTGAAACTTTTGATTCATCAAAGTTGATGGAACTTGATGTTTCCAA
CAACAATAATTTAACTGGAACAATCCCAGAAAGTTTTGGAGGGTTTAAACATTTGGAAATTATGAATCTG
TTTGGTAACCAATTGTATGGAGCAATTCCAGAGAGCATAGCCAAGATTCCATCTTTGAAGGTATTCAAAG
TGTTTAGGAATAAACTGAATGGAAGCTTACCATCTGAAATGGGACTTCATTCGAAACTCGAATCTTTTGA
AGTTTCACTGAATTCCTTCACTGGAAACTTGCCAGAGCATTTGTGTGCTGGAGGAACTCTTTTTGGTGCT
GTAGCTTATGTTAACAATTTGTCTGGTGAAATACCAAAATCACTCGGAACTTGTTCTAGCTTACGTTCGAT

TCAGCTCTACAAGAATCAGTTTTCGGGTGAAATTCCATCTGGGGTTTGGACTTTGGTTGACATGACAAGT
TTGTTGTTGAATGATAACTCATTTTCCGGGGAGCTACCGAGCAAAATTGCATTCAATTTTACGCGGTTAG
AGATCAGTAACAACAAGTTTTCTGGTGAAATTCCTGTGGGGATATCTTCTTGGAGGAGTTTAGTGGTGCT
TTTGGCAAGTAACAATTCATTTTCAGGTCGGATTCCAGTGGAATTGACTAGTCTTTCTCAGATAACTCAGC
TAGAGCTTGATGGTAATTCGTTATCTGGAGAACTTCCAGCCGATATAATATCGTGGAAGTCGTTGACAAT
ATTGGATCTTGCCAGGAACAAACTCTCTGGCAAGATTCCAGCAACTATAGGTTTAATCCCTGATCTTGTT
GCTCTAGATTTGTCTCAAAATCAATTTTCAGGTCCAATTCCACCTCAACTAGGTGTAAAAAGGATCACTA
GCCTTAATGTATCGTCCAATCAACTTATCGGGAATATCCCTGATGCATTTGCTAACCTAGCCTTTGAAAAC
AGTTTCTTGAACAATCCCAGTCTTTGTACCACCAACTCACTTCCATATCTTCCAAGTTGTAATAATGCGAA
AGTCGCCAATTCCAAAAGATTGTCCCATAGAGTCCTAGCCCTAATTCTAGTCCTTGCATTCGCTGTTTTCC
TATTTAGTGTTGTATCGACCTTGTTCATGGTTAGAGACTACAGGAGGAAGAAGCATAAGCGCGATGTTG
CATGCTGGAAGTTGACTTCATTCCAGAGGTTGGATTTCACAGAGGCGAGCATTTTGCCAAGTTTGACGG
AAAATAACATGATTGGAAGTGGTGGATCGGGTAAGGTGTACAGAATTTCCGTTGGCAGACCAAATGAG
TATGTTGCTGTGAAGAGGATTTGGAGTGATAGGAAAGTAGACTACATTCTGGAGAGGGAATTCTTGGCT
GAGGTTCAGATATTGGGATCAATTAGACATTCCAATATAGTGAAGTTATTGTGTTGTATATCAAGTGAGG
ATTCAAAGTTGCTCGTTTACGAGTACATGGTAAATCACAGTCTTGATGGATGGCTTCATGGGAAGAAGA
GAGTTTCCTTGAGTAATAAAGTCATGGATTGGCCAAAAAGATTGGAGGTTGCCATTGGAGCTGCTCAAG
GACTTTGTTATATGCACCATGACTGCACTCCACCGATCATTCATCGCGATGTAAAAACAAGCAATATCTT
GTTGGATTCTGATTTCACAGCCAAAATAGCTGATTTTGGGTTGGCCAAGATTTTAGAGAAGAAAGGAGA
GCTTAACACCATGTCTGCTGTTGCGGGTTCCTTTGGTTACATAGCTCCAGGTAAAAGCAAAATCCAAGCT
TGAACTTCGTATTACCTCAATTTCGTTAACATTATATCAAAAGTATGACTAACTAATAATTTACTGTTTGGT
TACAGAGTATGCTTATACAACGAAAGTGAACGAGAAGATTGATATCTATAGCTTCGGGGTGGTGCTATT
GGAGCTAGTGACAGGAAGACAACCAAATTTTGGGGACGAGCATACAAGCCTGGCAGAATGGGCATGG
AAGCAGCACGGAGAAGGGAACACTGCCATCGATAATATGTTAGATACAGATATCAAAGAAACATGTTAC
TTGGAAGAAATGAAGACTGTGTTCAGACTTGGGCTTATATGCACGAGCAATTTACCTGCAAGCAGGCCT
TCCATGAAAGAGATTCTGCAGATACTTCATCGATGCAAGTCCTTTAGGTATTCTGGAGGAAAATCACCAG
GTACAGAGTATGATGTTGCTCCTCTCCTTAGCGGAAACAACAGCGAAAAATACATTGCTAGTTACAAAC
GTATTAATTCCAGTAAGGTAATAGATGATAGTAGTGATGATGGTCTAATAATTTCCAGTGTGTAATATTG
GAGATGCCAAAGTAAACTGAGTTGTTGTAAAATGATCATAGAATAGTGCCACTTAAGTAAAGTACAAGT

TTGAATTTCTAGGTACTCTTGAATTTGTTCACTGCTTGTAGACTGAGTGAAATGCATCATTTAACTTTTGC
TAGTTATGTAGATTGTAAGGAAGTGTGAAATTGGAAAATGAAAATGCCAATTTATATAATCAAAGAGAA
ATGACAGTAGAGTGTGAAATTGGAAAATAAAAATGCCAATTTCCAGGTCAACACGTAGTTCATTAAGAA
GATTTGGTTTTGACTGAAAATGACTCAATAGGAGGGTATGGAGAGGCGAT
>PGSC0003DMG400033914 LRR-RLKs
CTGGCAAGTGAGGCCAAAGTGACCACAATACATGTCCATGTCAGTGGCCACTACCTTCTAACCTGCCCG
GAGAAATTCAACTCTGCGAACTGACACACGCATAGGCATCGGTGTTTCTTTTGTTTGGCACAACTGGGG
GAAAACACCGGGCAAACCACCCCCTTTTAATTAAATCTTCTGATTACAGAAGTGGTTTAGGGTTGGGATG
GGAATCGTGTGCAGTTCTCAGCTCCACACCCCCGTGTGACAACTTAGCTTATGCTTTTGCTTTTAAATCAC
TATGTTTCCTTTTGAATATCCGATGTGGGACAAAGAGGCCATTAAAGTGATTTTGTATTGCATTATGAAG
TGAAGGCTGTATTTTGTTCTCTTTGACTGCCTCTGTTTTTATCCAACCTCCATCGAAGTGGGAGCTTACAC
ACTGTATAAAAGTAGAAAAATGGAGAGGTTCGCGCTTAACTACTTCTCCCTCCCCTTTTCACTATTTTTAA
GTATTTCCTCTGTCACCCATCAACTGAAAGAAAAGAATAACCCAACTATTATAGCACTTGTATACTTTTCA
AGAGGAAGAATCAAACCCCAGACCTTCTGATTCAGTACAACGCTGAATGATAAGCAGTGGGGTTTTGCC
TCACTTGTATTCATATTTGTATACCCCAAACCTTCACAAGTTTCAATGAAGGTTCAGCAAAAATGAGGCAC
CAACTGGGTTACCTTTCTTTCCTCCTTTTCTTGCTTTTTCATTCTACATTCCAGCAGCACGAAGATAGATTG
GCTTTACTTCAATTGAGATCGTCTTTAGGATTGAGAGCCAGAGAGTGGCCTATTAAGGGTACCCCTTGTT
CAAATTGGAGAGGTATTTCTTGCAACAATGGTAGAGTTACTGGGATTAACATTTCTGGTTTTAAGCGAAC
ACGAACTGGGAATCAAAATCCTCAATTCTTGGTTGATTCCCTTCAAAATTTGACTCTGTTGGAGTCATTTA
ATGCATCTAATTTTGCACTTCTGGGTTCTATTCCTGATTGGTTTGGCATTGGACTTGCATCTCTACAGGTT
CTTGATCTTAGCTTCTGTTCAATAACTGGTGCTATTCCTTCAACTATTGGTAATTTGAGAAGTTTAGCTGTT
TTATACATATCTAATAATGGTCTTAGTGGAATGGTGCCTTCAAGTTTGAGTCAATTGTTAAGTCTTTCAGT
TCTTGATGTTTCACACAATGCACTTGGTGGGTCAATTCCTGCATCATTTGGGTCTCTTGGGAATCTTACTT
TGCTTGATATGTCTTCAAATGTTTTGTCTGGGGCTATTCCTCCTGTAATTGGGACTTTATCTCAGTTGAAA
TTCTTGAATCTTTCTCGGAATATGTTGTATGGGAATCTGCCGCCTGTAATTGGGAGATTCAGTTTTCTTGA
TATATCAAACAACTATTTTCAAGGCAAAGTTCCTCAGTACTGGCAAAGCAATGTGACCCTTAATAGAAAT
TGCCTACAAAGATTGGAAAGTCAAAGGGATACAAGAGAATGTGCATCTTTCTATGATGAGCGGGGCTTG
ATATTTGACAATTTTGGAATGCCTAATGCTATGGAGCTTCCGGTTCTTATATCTGAAAAGAAGAGTCCCA
AAAATGTGATCATATCAGCTGCTGTTCTAGGGGTTGTCGGATTTATTGCTACTGTGATGCTCTTTGTAGT

GCTGCTGATTTGTTGCTGCAAGAGGGGCGTGACGAATCAAAGGGTCACTGGTGTAGGGCCTGTTCCTAC
TACTGCAAGTCCTCCCCCACCTCCAGGACCATCATTGAAATTTTCAAGTTTAGGAGTAGCATTTAACTACC
AGCAGATCCTTCAAACAGCCGGTCATTTCAGTGATGTAAATCTTATTAAGCATGGACATTCAGGTGATAT
TTTCCGTGGAATCCTGAAAGGTGGAGTTCATGTAGTAGTTAAAAGAATTGATTCCAGCTCAGTTAAAAA
GGAAGCATACTTGTTAGAATTGGACTTCTTCAGCAAAGTTTCTCATTCTAGAGTAGTCCCTTTTCTGGGAC
ATTGTTTAGAGAGTGAAAATGAAAAGTTTCTGGTATACAAATATATGCCAAATGGAGATCTCTCTAGTTC
TTTGTTAAGAAAAAACAATGCAGATGATGATAGTTTGCAGTCACTTGATTGGATAACGAGACTGAAAAT
CGCTATTGGAGTTGCTGAGGGTTTGTCTTATCTGCATCATGAATGTAATCCACCTCTTGTGCACAGGTATT
TTAGAGCTTGTTGTGATGCATTACTCTTACTTCCACTTATCACGCTTAATCTGACAACTTTTAAATTGCAGT
AACAAACCAAATCTATTATCTTTATACTGCCTATCTGAATCATAAGTTATCATTGTGATGGGACACTTGAT
CATGAATCTTGGAAGAGTAGTTGCATATTCCTCTACATATGTTTCTTTGGTTGTAGACTTTTGGCGAATTA
GCATAATAAATGATATGCATGTTTTATTAATACATGATATTTCAGAAAATTAAAATCATCCATTATTCATG
AAAGATGAAGTGCTTAAGTTTCCTCCATACTAATTGGCGTCACTGAAAAAGCCTCTTCTTACTGATAAAA
TTTGGCCAAGTTAATGCTGGCACCATTTTGTACTTCATGAATTTCAACTGTCTATCAGTGCCAATTAGGTC
AATACTATAAGAGACATGAGTGAAAGATATAAGCTAGAGCTGGATGGTTTGGCAATTAAATATACTCTG
AGGTGTTCTTTTTTTGGAACTAAGTGGCCACATGATACAAACCGGAGCTGGATATTTAGCAATTATATAC
AATCCAAAACTGTTCTTAGCAGTGATCTAAAAGCTTGCTAGGACACATGAATTGGGCAAATCCAATCATA
AACTATTTACAGAAAATATGAAACCAAACTTTGTCTTGAGATACCCCTGTTACAACTTACAAGAGTAAGG
GTGAATAGAATAAGTCCTTTACTCTTTTGGCCGTGTATTACTTCACAATCTTGGGCTAACTACTCCAGACG
TACTGAGTGAAACCTAATGACAAAAAAACTCCTATTGGCAACTCGGTCTAAATTTTGACAAAACTCCTAT
CACTGCCATCTTTCCTGAACAACTTGGATCACAGCCCAAATGTTAACCCTTCTTCGGAATTGTCCTTTTTA
GAAAAGATTTCTATTTAATTTTCTCATATTGTTTCATCAGAAGCTTGTAGAGAATGATAACTCAATGAACA
ACTTTGTGGATTTAATAACATGTCTGGTTGTTTGAAATTGATCCAGTATGTAGATAAATAGCAAAATATC
AGATGACAGCTGCGACAATTAGCTTCATGAAATTTTACAAAGACAAAGAAAAGGAATAGACAAGACTTC
ATCCTTGTTTTGCTCTTCATTATATGTTCTCTATGTGTTATTCACGTGCAATAAATTACAAATTTTCCATAAC
AAGACGATTTGGCCAATGAGGGGCCATGGTTATGTACACTTGTACTAGCTATTCTCTCTAGTTGCATGCA
TGTCTATTTCCACTTTAGCAAGTTCTGGTTTCTTCTAGATTCTATATCTAGTGACATGGCTTAAGGTTCACT
GTTACTAGATCTCTTACTGCTCTAGCATATTAAAATTTATTTCTGAAACTACGGCGAATTGAGGTAGCTTT
GCTTATTAATTTTTTGAAAACTTTCTAACATCGAGGTCTTCTTGTGTTTGACATGGGTGCCGCTTTTGATA

CTGAGATTTTATGAGATCGCTAAACTTGTTTAGTTGAGTACTAGCTGGTAAATATGTTAGGTAGGTCATT
TAGAAATCTCATAATGTAGAAGTGACCTTTTCTTATTTTTTCATCAAAGGATTTCTAATAGGATAGCACTT
TGACTGACTTTTTTCATTTTAAAACATCAGCATAGCTCCTTGACATATCACATACTCGTGATAAACTATGT
ACAGATTGATTGAGCTGGTATAACCGTGACTATCAAATTTATTTCTTGGATTTCCTTTGTACTTCAGTAGA
CCCATCGAGCTTCTTTCTGAAGTTAGTTTGAATATTTACTAAACTTGCAAAACAGGTTTAGATATCACCAT
GATTTATGTCCATTTCTTCCTTCCAATTTTAATCATCTTCCAATTTTAATCATGAATGTACTCTATTTCACTA
TAGGTTACTACACTTTCCTCAAGCTGTTCACCACATTTTTTCAGTTAAGTATTATTTTTTTTATGTGGCAAC
TAGAATTTCTTAAGCCAGCATGCATAAATAATTATATTCTTATCTTCTCTCTGTTTCTTTTCCACCATACCAC
GTGGTAGACAAAAGATGGTCTAAGCTGGAACTTCCCAAACGCCTCATAATAGACCATACTTAAGGTAGT
GCATGAAAAGGAGAAACCAATTGCACAAATTTCTGCATTTTCCCTGAGCTATATCAGTGAACTGTAAAAA
AGACGGATTTTATACTATTCTTAATGGTTACATTGTCTTTCTGCTGCATTGCAGATGAAAATTCACATAAT
CTCGGTTATATAATTTATGCCTTGGGGTACTATGGTGAAACACCTTTTTTGAACCTTGTCTTGCTATATTTA
CAGAGATGTCCAAGCAAGCAGCATACTTCTTGATGATAAATATGAAGTACGGTTAGGGAGTTTGAGTGA
GGTCTGTGCACAAGAAGGAGACACCCATCAAAACAAGATTTCCAGGATGTTGCGGCTGCCACAGTGAG
TAGTTGTTGCTTTTCTTTTCATTTTATTTTATATGACAATATCTTTATTTGGGATTGAGGCATAAGT
>PGSC0003DMG400038191 LRR-RLKs
ATGGGTTCCCTGCCAATGGAGATTGGAGATATAACAAACCTTGCAAGTTTTGACCTTTCTTACAACAATT
TCTCCGGCATGATTCCAAGCACCATAGGGAAATGTTTGGCCTTGGAATTACTTTACATGCAACACAATTC
ATTTGAAGGGTCCATCCCGTACATAGCTGACTTACAAAACCTACGGGAGCTAGACCTTTCAAGAAACAAC
TTATCAGGAGAAATTCCACCTTGGACTATGAATCTTTCCTCCCTACTGTACCTGAATCTTCCAGACAACAA
TTTAGAGGGTGAGGTTCCCGTCCAAGGAGTATTCTCAAATTCAACCGCATTACAAGTTGAGGGGAATCC
CAAGCTTTGTGGTGGAATCCAAGAGTTTGATTTACCACCTTGCCCGAAGCAAGAAAACAATAGAACAAA
GAGGAAGCGTCTTACACCCGTTCTGACTACAATTATCAGTATTGTTTTATTTGCTGCAATAGCATGTTTTC
TGCTTTCTATGTTTTTTATTAAAAGATCCAGAAAACGAAGAGATGCAACATCTACATCTGGGCATCTCATC
TTCCCCATGATTACATATAAGGAGCTTCATGATGTAACTAATGGTTTTTCTTCAGATAGGTTGTTAGGATC
AGGCAGTTTCGGCACTGTGTACAAAGGAATTCTTCACTCATCGAATGAAAATCCAGTTGCAGTCAAGGT
ACTCAACCTCCAAAGTCACGGTGCATCTAAAAGCTTCATGACTGAATGCCACGGCTTGAGAAACACCCG
GCATAGAAACCTGGTGAAAGTCAGAACTGCATGCTCCAGCATTGACATCAAAGGAAATGACTTTAAGGC
CATAATGTATCAGTTCATGGAAAAGGGAAGCTTGGAGGAGTGGTTACATCAAGAATACTGCAATGGTTG

CTGA

>PGSC0003DMG400039680 LRR-RLKs

ATGGGAATAGGCATTCTTGGTTCTTTTCTGCTTTTGACTGTGTTCTTTAGGCCTTTGCTTTGCCAACAGCC
AAACACTGATGGATTTTTTGTGTCTGATTTCTTCAAAAGATGGGGGTTGCTAAAGTTCACAACTTTTCAG
CTCACTTTTGTTCTTGGAAAGGGGTGGGATGTGATTCCAAGGATGAAAATGTTGTTAATTTGACAGCTAA
AAGTTTTGGTTTATCTGGTGTGATTCCTGATAACACCATTGGTAAACTCACAAAGCTGAAGTATTTGGAT
CTGAGCAACAATAAACTCACTGGTTTGCCTTCTGATATATGGAGTTTAGGTTCACTAAAGTACCTTAACCT
CTCACATAACCATATCTCTGGTGACCTTTCAAGTAACATAGGCAATTTTGGTGGTCTTGAAATCATGGACT
TTTCTGTCAACAATTTCTCTGGGAAAATCCCAGATGCCATTAGCTCCCTTTCAAGTTTGCATTCTCTTAATC
TTAGTAAGAACTTGTTTGATTCAGAAATCCCTTCTGGAATTTTGGGCTGTCATTCTTTGGAATCCCTTGAT
CTTTCAGAAAACAGACTCACTGGTTTTCCTAATGATTTTGGTTCTACATTTCACAAGCTCAAGTTCTTGAA
TCTTGCAGAAAATGAGATTCTTGGTAAAGATTCAGATTTTTCAAGAATGGGTTCAATCACTCATATCAAT
ATTTCAGGCAATCTGTTTAAGGGTTCTGTTGTTGGTCTTTTTGAGGGGCCATTGGAAGTGATTGATTTGA
GTAGAAACCAGTTTGATGGTCACATTTCTCAGGTAAACTTCAGTTCCAGCTTCAATTGGTCTCATTTGGTT
TATCTTGATTTATCTGAGAATCACCTTAGTGGAGGGATCATTAGAGAATTAAAGAATGCTCGAAATCTTC
TATACCTTAATCTTGCAGATAATAGATTTTCACATCAAGAATTTCCACAAACTGATATGTTATCTAGCTTA
GAGTATCTGAATTTGTCTGGAACTAGTTTGATTGGCCAAATTCCTCAAGAGCTCTCGTCGTTGAGTCGCT
TGAAAATCCTTGATATATCCAAAAACCATCTTAGTAAACGCATTCCTCCGCTGAGCAATAGAAATCTCCTG
GTTCTTGATGTTTCATACAACAATTTGACTGGTGATATCCCATTGCCACTTGTAGAGATACTTCCAAGGAT
GGAAAGGTTCAATTTTTCTTACAACAATCTAACTCTTTGTGCTACTGAATTCTCCCCTAAAACACTCCTTTC
GGCGTTTATCGGGTCATCAAATGGATGTCCTATTGCTGCCAACCCAGCTCTCTTCCACAAGAAAGCTCCG
AAACACAGAGGACTTAAACTTGCTTTGGCATTAACTTTCTCTATGGTTTTTCTGCTTCTTGGTTTACTGTTC
TTGGCATTTGGTTGTCGAAGGAAAACCACGATATGGGCGGTTAAACAGAACTCTTACAAGGAAGAACA
GACTATTTCTGGTCCTTTTTCATTCCAGACTGATTCAACAACTTGGGTTGCTGATGTTAAGCAAGCAAACT
CAGTGCCTGTTGTGATTTTCGAGAAGCCACTGTTGAATTTCACATTTGCAGATCTCTTATCTGCAACTTCT
AATTTCGATCGAGGCACATTGTTAGCTGAAGGGAGGTTTGGTCCTGTTTATAGAGGCTTTTTACCAGGTG
GCATTCATGTGGCAGTGAAAGTTCTTGTTCATGGTTCCACTATGACAGACCATGAAGCTGCAAGAGAGC
TCGAGTATCTTGGCCGGATAAAACATCCTAATCTTGTTCCATTGACAGGATACTGCTTGGCTGGTGAGCA
AAGAATTGCCATTTATGATTACATGGAGAACGGGAATTTGCAGAACTTGCTTCATGACTTGCCACTCGGG

GTTCGAACTACTACTGAAGACTGGAGCACGGACACGTGGGAAGAGGAGGATGATAATAACAGTATTCA
GAATGTTGGTTCTGAAGGATTGTTAACCACTTGGAGATTTAGGCACAAAATCGCGCTTGGCACTGCTAG
AGCACTTGCATTTCTACACCATGGTTGTTCACCTCCCATTATACATAGAGATGTCAAAGCCAGCAGTGTTT
ATCTTGATATGAACTTGGAGCCTAGACTATCCGATTTCGGATTGGCCAAAATCTTTGGCACGGGACCCGA
GGATGAGATCACTCGCGGTTCACCTGGATACATTCCCCCAGAATTTCTTCATCCAGAGAGCAGCTCACCT
AAATATCCTACACCAAAGTCAGATGTGTATGGATTTGGAGTCATCCTTTTCGAGCTAATTACCGGGAAAA
AACCAGTTGAAGATGATTATCCACAACACAATGATGCGAATTTGGTTGGTTGGGTTAGAGGATTAGTAA
GGAACAACGAGGGATCAAGAGTTATCGATCCAAAAATTCGTGGAACTGCATCACAGACACAAATACTAG
AAGCCTTGAAAATCGGTTATCTATGCACAGCTGAAGTTCCCGCGAAACGACCAAGTATGCATCAAGTAG
TTGGACTGCTTAAGGATATTGAGCCTACACAACAATGA
>PGSC0003DMG401000515 LRR-RLKs
TTTTATCATATAACTTAACTCAGTATATTGTCATTTTGGTTTCAGGTGTGGAAGCTGTACACAACAAATCA
GGTAACTGAAGCATTAGATCCTCTATTGAAAGGCGATTTTCCGCCAGAAGAGGCATCAAAGGTACTAAA
AGTGGGGTTGTTGTGCACTCAAGCTTCTGTCGCTTTAAGACCATCAATGAGTGAAGTTGTCCAAATGCTA
ACGTGTGAGGGCTATCAAATTCCAGAACCATGCCAACCACCATTCTTAAATTCGAACTTATTAGTTGGCG
GTTCAGTCAAATCCAGTATAAGAAGTTTAGTATCAAGTGCACTCTATAAACTTGATGAATCATCATCTTAT
GCCACCACCACTGGGTCCTCAAGTATGCAAAGCTCATCAACTGGACCACACAAAAGTGATGAATTCCTTT
TAAAAGAGTCTGAAAATAGGAAATAATTGCAAAGACAAATATAGAAATGGAAATTTTGAGAGATTTTTT
ATCAGTACATTTTTTGTACAGTAATAGAATTGCTGTAGTTGTGCATTTTTATGATTTTATTTTTGCATTTTT
GGTGAGTGATGATACAAGAAAGTGTAGGGGTGGCAATTTCAGCCCATATAAGTGAAATGAAACCATTTT
GCCCATATTAAATTAAATGGATCACTCATTTTC
>PGSC0003DMG401002925 LRR-RLKs
TGGACTTGTAGACTTGAATCAAAACCTGTCGCAAAATTTTTATCATAAGCTTGGTGAGGGGTCAAACATG
TCTATTGAAGGTTATGGAATGAGCAATGCTGGTGGTTCTGGTGCAATGTCGGTGGATAACAGCAGTGTT
GGATCAAATGATTCACACACTCATATTCTAAACCAGCAGGGTAATCACATCCACAATAACGATTCTGTTG
CTGCTAGCGTAGTCAGGGGGAGAGTTACTAGGTTGAGCGATGATGCCCTTGCTCAAGCCTTGGTTGATC
CTCAATTCCCCACTATTGGGCTTGCGATTTATCATGAGTGGACAATTGACTTGAGGAGGCTTACCATTGG
ACAAGCATTTGCTCAAGGATCTTTTGGGAAACTCTATAAGGGAACTTATAATGGTGAAGATGTTGCCATC
AAGCTTCTAGAGAGGCCAGAACATGATCTTGAGAAGGCTCACTTTATGGAGCTGCAATTTCATCATGAG

GTCAAAATGTTGGCAAATTTGAAGCATCCAAATGTAGTACGATTTATTGGTGCATGCCGTAAACCCATGG
TGTGGTGTATTGTGACTGAATATGCCAGAGGGGGATCAGTGCGTCAGTTCCTCACGAGGCGGCAAAAC
CGAGCTGTGCCATTGAGGTTAGCAGTTATGCAGGCCTTGGATGTGGCAAGGGGGATGGAGTATGTGCA
TGGCCGGAATCTGATTCACCGAGATCTGAAATCCGACAATCTACTGATTGCTGCTGACAAGTCAATCAAG
ATTGCAGACTTTGGTGTTGCTCGAATTGAGGTGCTGACTGAAGGAATGACACCAGAAACTGGCACTTAC
CGCTGGATGGCTCCGTAAGCATCTAGACTCTTTAGCAGCCTTGTCAAATCTGGTGTCTTGATCCAGTTTG
AGTTTATTGCCAAACTGTATAATAGAGCTCTTAAGCTTTTTATCCTATTCTTGTGGGCTATTTTTTCTCTGT
CTGCATTACTTTCTGTAATTTTTTTTCAAAATTGCTTTGATATGTGTTTCTTTAGCCAAGCATCTTACGGAA
ACAACCTCTCTTCTTCCACGAGGGCAGGCTAAGGTCTGCTTTCTCTCTACCCACCCCAGACCCCACTGAGT
GGGATTTCACAGGGTATGTTGTTGTTGTAGCATGCTGAATTCCCTTTATGGAATAAATTTTATTAGAACT
GAAGTGAAGAGCATGGTGGTTGATGGGTTCAAGTCCTGTATGTCCTTTTGATGCCACCTCACTGATTGTG
ATTGTATTTTCTATTGTATACCTAGTGTTTCTTCCAGTTAACTTCTTAATTTAGTTCTGTACCTGTTAATGAA
GGATAAAGAGTTGATTGACTTTACTTTCTCTTTCAATTCCTTTCCCTCCTTTTCCTTTTCCTTCTTGTCCATG
ACTTGTAACTTGATTCAAGTTAGACACACTCAAGGGTGTCACTTAGTTGTCAATGAAGTAGGAGCAAACC
TTTGGAGATTAGGGTTCAATCATCCCAGAGACAGAAAACACCAAGTGTTCTCTTCCCATATGCCTAAACC
TTAAACCTTTGTGGGTATAGTTACCCGAAATCGAGATGCATGCAAGTTGGATTGGATACCATTTTCAGAA
AGAGTAAAATTGCATATATCTGAAAATGGGTCTAGGATATTAACTACCACACTGGGTTCACTTTCAGGAT
GTGTCTGAGAGATATGAACCACAATTCTATTCTTATTGAATATGTAGATGAAAGTTCTATGGGAAGTATA
TTATCTCTTTCTCTCCTTTGTGAGTGTCGACCTTCCAGCATCATCTTTAGCATCACCTCTTTCTTCAAAGCA
GTGAGGCTATTTTGGAAATAATGTATGGGTAAGAAAGAGAATGGGGAGGTGCGATTCACCAGAGATCT
CTCTCTTTATAGGATGACATACATCCACAAGAGTCGCATTGTTTGTTGCTGCTGGAGAAACTTAAAAAGA
GCAAGGGAATGACATGTCTACCAGAAACATTTCTTGTTTAGAGTGGAAGTTGGTTGCCGAATGAAAGTT
TTTAAGTTGTTAGTAGTTGAACAGCATTCCTGTTTGCTTGGCTCGTGCTAACTTTCTCAAAGCACTGAAAT
GAATCAGTTAAAGAAGGATTGTTTAGGACATGTTATTTGGACATACACTGGTGTCTCTGGAATCTGCAG
GGCTCTTGTTAACTTTCTCAAAGCACTGAAATGAATCAGTTAAAGGAGGCCTGTTTAGGACAATTTAATT
GGACATACACTAGTGTCTCTGGAATCTGCAGGTAGATTTTAAGTTGTGCAGGTGGAAGTCATTGCTCTGT
TGGAGGATGGCAAAACAAAACATCGATTTTTTTTTATGACAAAGGAAACCCGCAGCTGCTATCCATTGG
GTGTGTACACGGTAAAACCCCCGCTCCTATGCAATAACTCACAAAATTCGGAAGAGAGGTAACCTGCAC
AAGGCAAGCTCGGTGCGACGAGCTTGACCCAGAAGACAAACCCCTTGCTTTTGCTGGTAAGGGGTTTTG

AACTTGAGACCTCCAAAATGGAAGTCCCAAGCTCAAACCACTAGGCCACCCCAAAGGGTAACTGACATA
TCTATTCTGATGTTTTTGGAAGGAATTCTAGGAGGGCCTTACAGAAACTAGACTCTACAACGATAATATG
ATTCTCCGCTGAGTTACTACATAGACTTTTTTCATCATTAGACAATCTCTACAGAAAGGTAGAAGTGGAA
TATTTGGTTCAATCTTCAATGAATCTAAATCATTTATTGGAACCTTCACTATTCTTTAGGAAGAGGCCTCTT
TAAAAGCATAAAAAGCGACAAGGTGATGTTTCTATAATCTACAACGATTATGTGGATTCTGCATTGAGTT
GCTACATGGACTTGTTCCGTCCATAAGACTATTTCAATCTCTATGGAAAGGTAGAAGTGGAATACATGGC
TTAATCTTCTCATATTAAAAGCAAAAAACGATCCATGGAGTAAAAAGCTTGGAGTGAAGCATACAAGTT
ATGGAAAAGTATAATGAAGTACTATCCACAAAGAAATTTAGTAATCTTCTCATGAACTTGAAACATTTGT
TGAAACCTCTAATTATTGAGAGTACTGTTATTAGAAGCGAGAAACACAAAAAGCGACAAAGTCCATGGA
GTTTGAAGCAAATAGCGAGAAGTGAAGCACAATGGCTATAAGAAGTAAGTAATGCCGAACATAAAAGG
ATTTAGTACAATAAAGATGCAAATAAAATAAATGGTTTTTGCATCTAGTTTACTTAATATTGATATTGATC
TTGCTCTTCAAAGTCGAGATTCAAAAAAGTAGATCCTTTCTCGTTAGGATCATTTCGCACGTCATTGTTTA
AAGCAGTGCAAGGCTTCAAGAAGTAATCCCATTCTTCAAATTGCTTTAAGCGATGGCTTTGAATAATAAT
GTGGGAGAGATGTTGTGACACATGATGAAATCAATGCCCAGTAAGAAGAATGATAAGAATGCCTTATTT
GGTATTAAGTCTTACAACACGTGTTGCTAGACCTGATTTGACAAATGCCTTATATAAGTTGAAGTAGATC
TTTTAGAAACAAAAAGAGCATGAAAACCTCATAATGATCAATCAATTAGTATAACTTGTTAAATTACGGA
GATCCTACGTCAGGCCGATTATGACTTAGTATTTCTGACATCTAACTTTTAGTGCCTCACATACTATGTTG
TATCGGTCCTTTGCATTACATACATGTTAAAATGGGTGTGTCATGGTTGCGCGTACTCTTCAAAATACACA
AATAATTTACAAAAAAATGCACGTACCTATAATGATACTTACATCTGATAAAATTCATACTCAAGGACGA
GTCCAGACAAGAAGATTTAGGATTTAAAGTTAATCGGTTCCCAATGATATCAAGTTCAAAAAGTAAATTA
TTATATGTGCCCAAGGGGAATCGAGCCTTCAATGAGGAGGGCAATAAAGCTCAAGCAAGTTCCCATTTA
TGCCGCTGGACCAAGAGCTTACTTTGTTAGTAGATCCCAACAAATAGTTTTATATATTTAATTGACTGGAT
TCCACAAAACCTACACATGATACTTTAGGTCCGCCCTTGTTCAATAGCAGCAACAACATACTCGGTGCAA
TCCCACATGTGGGGTTTGGGGAGAGTAGAGTGTATGCAAATGTTACCCTTAACTTGTTGAGATAAAAAG
GTTTGTTCTGAAAGACCCTCAACTTTAAATAAAACTGCCAAACAGTTTGCAAAAGGGAATACAAATATTC
GGAGCAATCACAATGACACATTTATGTAAAATGGCAAGTGTTACATAACAAACAAGTGAAATAATGCAA
TAATCGACACACAAAAAACTTCAAGAGTAGTGCTAATACTACTGATAAGAATACCAGTGAAAGCCTATCT
CGCAAAGAAGCAAGAGAATGCTCAATTACCAACTAACCATTTACCCTAATACACGACCTCTATACTCTCCT
ATCAAATGCCATGTCCTCGGTAAGATCTAGATGCACTCCGTCTAATCACTTCCTCCCAATACTTCTTCGGC

TCGACTTTACCTCTCTTGACGTCCTCCCTTGCCAACAACTCACACCTCCTCACTGAGGCGTCTATACTCCTC
CTCCTCCTCACGTGTCCAAATCATCTTAGTTTCACTTCCCTCATCTTGTCCAACATGGAGGCCACTCCTACC
TTGTGTCGAATATCTCCATTCTTAATCTTGCCACTCTTAGTATGCCCACACACCCACATCAATATCCTCATC
TGAACGATTTTCTTCTTCTGAACTCGAGATTTCTTAATAGGCCAACACTCCGCCTCATAAAACGTGGTCGG
TCTAACCACTACTCTGTAGAACTTACCCATCTATACTCCATATAATATGGATTTCATGTTATTTTAGTTGTG
GAAGAATAAGTATATTTGAAACATTTTGGATTTCTCCACTAAAACTCATCACCTCTAAGAATGATAGAAA
AAACGAAAGCATCAAATCTATTTAGGTGGCACGGGTGTCCACATGTGCTTGATTTGCCAAGTTTGTTAAT
ATGTACTATGAAACGCCAAATTAATTTTAGAAGTATGCATTAGGTATGTAAGAATATATTTTACTATTTGT
TTTATGTATTACGGTGCATGTATAGGCTGACTGTTGTATTGATTCATGCATTAGCGCCTGATTGTTAAACT
GTTGAAAATTTATTGACATCTATTGGAAGATCTTCTTGGTTGGCACAATTTGACCTAGAGGCTGCAAAAA
TTATGACAACTAAGAAAATTACTTCATTTTGCTCACTTTATTGTTCTACTGATACATGGGGTTTTCCTGTAT
TTAGGGGTCGTTTGGTGTGAGGGATAAAAATAAATAGTCCTGGGATAAAAAAACATGGGATTAAATTTT
GGTGTCTTGTTTGGTTGCCATGTTTGGGATAACTTATCCCACTATTTATACCATAGTGATGGGATAAGTTA
TCCCATATACATGGTGGAATAAGTAATCTCAGGATAACTAATCTCAGGATAATTAATCATGGGATAACTT
GTTCCCAACCAAATGACCCCTTAGAGTCTTATTTAAGAGTTGGTATATTTAGTGACTCTTTTGAGAAAAAA
ATTTCAAATCACGATTTTCCTTAGGATTGTGCTCTTAGAAGTTCAGTTATTTGTGTCTCAGGCATATATGG
CAGTTAGAATTTAAACCTTGAATTAGATCATCAATTCAGGTTGGTTTAGAGAATATGGTATAGAGTTAAA
TAAATCCCTTTCAATCTGGTGGAGATTGATTGCTATATCCAGTTGAGTGTAGATTACTGATGATAGAACT
GGCATTTTTTTATTTCTTTTGACCTGGACAGAAGTATCACCATCAGTTTCCGTTTGGGTTTGTACCTTACAC
AATGTTAGAGAGGAAAGCATATTCCCTATAAGCTAAGTGAGTTGGAGATTAAGCTTTTGTTTTCTTGAGA
TAATGTGATAACTGGAGAAGTTTCGATTTCTAATATGACATTTCTTTCAGACTTGATCAAAGATATGTGTT
CATGTCTGCCTGAAATATTTTCGAAATGGTTGCGATCAGAAAGGATTTAGGAGGTTGACCTGGTGAGTG
GTGGTAATTTTATAGTCTTCTCTGTGTGATAATAGGCTAGTATGCATGAATGACTTTCCTTGGGGAAATA
GACAAAAAATAATATGCTTGCTTTTGTTAATTTGATGTGATAATGAAAGATCTTGCCAAAACAGGTGTGG
ATCGAATCTCAATATCTGGTAAAACTCGAATACGTGCAGCCAGTCTGACTCTTTGTATAATTCTAGTATGC
TCAAATTTTTATTCTGATTAACTGAGAAATGAAGTTACTTTTCAAGTTTACTCTTGAGAAATTAGTTATTCA
CATTCTGCAGATCCTATCCCGTTTAACTTTGTGAATTATTGGCAAGTGTGTCTGGTTTCTTCTCTTTTCAGA
AAAAAGAAAAATGGATGTTCAAAGTTGTTAGCAATAGCATCTGTGTTTCGTTGTGGATCGCTAAGCGTTT
GACTTTGTTTTGTAGGGAGATGATCCAGCACCGGCCATACACCCAGAAAGTTGATGTTTATAGTTTTGGC

ATTGTTCTGTGGGAGCTCATAACAGGGTTGCTTCCTTACCAGAGCATGACTGCTGTGCAGGCTGCTTTTG
CTGTTGTCAACAAAGGCGTCCGTCCAACAATCCCCATTGATTGCTTGCCTGTCTTATCTGATATCATGACC
TGCTGCTGGGATTCTGACCCCGATAATCGGCCAACTTTCTCTCAGGTGGTCAAGATGCTTGAGGCGGCT
GAGACAGAAATCATGACTAATATCAGAAGGGCACGTTTCAGGTGCTCCCCTCATCCTACGGCTACAGGG
TAAGAGGAAACTGGAGGCAACTGATTTTGAATGGCAAAACTGATTTTGAGTCTGCCATGTGTATCTATC
AGGCACAATTTCTTTTCAGACAGAAAAAAGGGTTATAGTTGTGAGAAAAAACTAAACATTTCTCTGCTGT
ATTTTGTTTCTTTTCTTTAATCCGTGTATTTAAGATGGTGACTAGTTGGTAACACAGGATATCTGATGTGC
ATCTATGCATGTCAGATTATGGCTGTATTTGCTGAATTTGTTCAAAGTTATATCAGGTTCTGAATTA
>PGSC0003DMG401006401 LRR-RLKs
GTTAGCCTTCGACATGGTTCCCAGAGTATTTTATCTCCTGCCTCTCTTATATCACTTCACAATTACTTTTGC
TTCCATTGAGGAAGCAGCTGCTCTTCTAAAATGGAAAGCAACTTTCCAAAACCAGAATAATTCCTTATTC
GCTTCTTGGACACTAAGTCATCCTGCTGCTGCCAAGAATTCTTCCAGCCATGATCCATGCAGGGACTGGT
ATGGAGCTAATTGCTTTAATGGTCGGATAAACAAGTTGAATATTTCAAATTCTGGTGTCATTGGTACACT
CCATGATTTTCCATTTTCATCTCTACTTTTTCTTGAATATGTTGATCTTAGCATGAACAACCTTTCTGGTCCC
ATTCCGGCAGAAATAGGGAAACTTGTCAATCTTGTTGAAGTTGCTCTTGATACGAACCAGTTAACAGGTC
ATATTCCTCCGGAAATAGGCAACTTGATTAATGCAAAATTGTTCTATGCTTTCTCCAATGAATTGGCTGGC
TCCATTCCTATTGAAATAGGGAAGATGAAGTCACTTGAGAGTTTAAGCTTACAGAGAAACAATCTTTCTG
GTTCAATTCCAAAACATTTAGGTGAATTAACCAAGCTCAAAATTTTGTACCTTTATTCTAACCAACTTTCTG
GCCCCATTCCAACAGAAATAGGGAAACTTGTCAATCTTGTTGAAGTTGATCTTGATACAAACAAGTTAAC
AGGTCATATTCCTCCGGAAATAGGCAACTTGATTAATGCAAAAATATTCTATGCTTTTTCCAATGAATTGT
CTGGTCCCATTCCTGCAGAAATAGGGAAGATGAGGTCACTTGTGGATTTACTGTTCCAGAGAAACAATC
TTTCTGGTCCCATTCCTAGTGAGTTGGGGAATCTGAATAACCTCAATGATCTGGTTTTATCCGAAAATCAA
CTTACTGGTTCAGTTCCTACTACATTTGGCAATTTGAGAAACTTGCAGACACTGCATCTCCATACCAACAA
ACTATCTGGTTCCATTCCAAAAGAACTTGCATATTTGGATAACTTGCGAGTTCTAACAATGACTAAAAATC
AGCTTTCAGGCCATTTGCCTGAGCATCTTTGTCAAGGTGGGAAACTTGAGAGGTTCACGGTGACTAGCA
ACAAGCTGACAGGGCCAATACCAAGAAGCTTGAGCAACTGCTCGAGTTTTAAATGGGTTCGCTTTAATA
ACAACAGTTTCATTGGAAACTTATCCGAAGCTTTTGGAATCTATCCAGAGCTTCAGTTCATTGATTTGAGC
GACAATAATTTTCATGGTGAGCTCAGCAGCAACTGGGGGAAATGCGAAAATTTGATTGATCTGCGTGTA
GCCAGAAATAACATTAGTGGTAGCATACCACCAGAGATTGGAAACGTCCAAGGGCTTCTTGGACTTGAT

CTTTCATCGAATCATTTGCTTGGGCAGATTCCAAAGGAATTTGGAAGATTAACTTCTCTGGTAAGGCTTTC
TGTGCAAAACAACAATATTTCTGGCAATATATATGAGGAACTTGGGTCACTGACAAAGTTAGAGTCCCTT
GATTTATCAGACAACAGATTGAATGGATCAATCCCAACGTGGATAGGAGATTTTGTGCGGTTGTTTCAGT
TGAATCTGAGCAACAACAAGTTTGGACAGAAGATTCCAAAGGAGATAGGGAGGATAACTCATCTTACTT
TACTTGATTTGAGCCATAATCTTCTTGATGGAGAAATACCTGCTGAGCTAGCCAGTTTGCTGGACTTGTC
AAACTTGAATCTTTCCCACAATGGTCTATCTGGGCGCATTCCTAAAGAACTTGAAAGTTTGACTGGTTTG
CTGGATGTTGTATTGTCATACAATGATTTGGAAGGTCCAATCCCTAATAATAAAGCTTTTATGAATGCTTC
GTTAGAAGGTAATAAAGGTCTTTGCGGCAATGTAACAGGATTCCAGCCCTGCAAAAAGCCATCTTCTGT
GGTGAAAAAGCACTCAATAGCAAAGGGACGTAAACTCATCCTCATCACTGTACTTCCTGTTATGGGAGC
ACTAGTACTGCTCTGTGTTTTCATTGGTGTTCTCTTTATGTTTAATAAAAGAAGGAGAGTTAAAGACGTTG
AAAGAAGGGATGGTGATGGTTGGCTTTCGATATCCATGTTAGATGGAAAGGCATTGTACAGGGATATCT
TAAATGCCACTGAGGAGTTTGATGCAAAATTTTGCATTGGGCAAGGAGGACATGGAAGTGTTTACAAG
GTAAACCTTCCATTATTAGGAGATATAGCTGTGAAGAGACTTCATTCTTCATTTCAGAATACACATCCCAA
AAGCTTCATAAATGAAGTAAGGGCATTGACTGGGATTAAGCACCGGAACATTGTGAACCTCTACGGCTA
TTGTTCAAAAGCACAACACTCGCTCTTGGTTTACGAGTATGTGGAGAGGGGGAGTTTGTCGAGTATTCT
GAGCAATGAAGTTGAGTCCAAGAAATTGGATTGGCTTAAAAGGGTGAATATCATCAAAGGTGTTGCTTT
TGCTTTATCTTACATGCACCACGATTGCTCACCGCCAGTTGTTCATCGAGACATATCAAGCAGTAATGTTT
TGGTTGATTCTGAGTATGAAGCTCGTGTTTCAGATTTTGGCATAGCGAAGCTTCTCAAACCAAACTCATC
CAATTGCACTGCACTTGCAGGAACATATGGCTATGTTGCACCTGGTAAGGTCTAAATTTAATTTCTATTTT
ATTCAGACTTCGTGAACTATAGCCAAACACTCTATACTGTTTTTATCTCATTTACACATCTAAATTGTTATA
CCATTTTTGGAAATTGTATTGTTTCAGAGCTTGCCTATACAATGAAGGTTACGGAAATGTGTGATGTCTA
CAGCTTTGGAGTATTAGCATTGGAGATAATCAAAGGAAAGCATCTAGGGGAATATATTACTGTGCTAGC
AAATTCATCGACTATAGATCATGTGCAGCTTAGTGATTTGCTAGATGAACGCCTTCTATATCCTGAAGAT
GAAGTAAAGGATGTTTTGGTTTTAATCATCAAGCTAGCAAGCTCTTGTTTGGTTGAAATTCCAAAATCAA
GGCCAACAATGCACTTCATCTGTCATAGGTTATCATCAATGGATGCACGTCCACCTACTCATGTAAAGTG
ATCTTTG

>PGSC0003DMG401009161 LRR-RLKs

ATCATTTTTCATAGTAGCACAAATTATGAAGATAGCTGTTAAAAATTTCATCTTTTTTTACTGCTGCTGTTT
CTCTCTGTGGGGTTATGCTGTATATGCTCTCACTTCTGATGGCACCGCTTTACTTTCCCTTTCTAGTCACTG

GATTGGTGTACCCTCTTCTTGGAATGCTTCAGATTCCACTCCATGTTCTTGGGTTGGTGTTGAATGTGATA

ACAACCACTTTGTCACCTCTTTGAACCTCTCTGGCTATGATATCTCTGGCCAATTGGGACCTGAAATTGCT

TATTTGAAGCACTTGCTTACCATTGATTTGAGTTATAATGCTTTCTCTGCCTCCATTCCTTCTCAACTCACT

AATTGCACTCTTCTTCATTACTTGGATCTATCCTACAACACCTTTACTGGACATATTCCTTCAAACATTGGA

AATTTACACAAGTTAACCTACATAAGCCTCCTCTCTAATTCATTGACTGGTAATATTCCTGATTCCTTGTTT

TCTATCCCACATTTGAAAACTATCTACTTTAACCAAAACAGGTTAAATGGTTCCATCCCATCCAGCATTGG

TAACTTGACTCACCTTTTATCTCTCTATCTTTATCAGAATGACTTGTCCGGTCCAATTCCGTCTTCCATAGG

AAACTGTACTAACTTGCAAGAATTGTATTTGAATGATAATCATTTAGTTGGTAGTTTGCCTGAGAATTTTG

ATAAGCTACAACACCTTGTTTATTTGGACTTGAGCAATAATAGTCTACAAGGAAGTATCCCTTTCACTTTA

GGTAACTGCAAACATCTTGACACTCTGGTCTTGTCATCCAACACTTTCAATGGGGAACTTCCTCCAAGTTT

GATGAATTGTACTAACCTAAAAGTTCTTGCTGCTTTTTCTTCTGGTTTAAGTGGCCCTGTTCCTGCTTCTCT

AGGCCAGCTCACAAAGTTGGAGAAGCTTTACCTTACTGACAACAATTTCTCTGGAAAAATACCACCTGAG

CTGGGGAAGTGCCAGGCCTTGCAGGAATTACTTCTACCGGAAAACCAACTGGAAGGTGAAATTCCTAGT

GAACTAGGATCACTCAGCCAGTTGCAGTATCTCTCTCTATATTCCAACAAATTGAGTGGTGAGATTCCCC

GCACTATTTGGAAGATTCAAAGTCTTCAACATCTTCTTGTCTATCGGAACAACCTGACTGGGGAGTTACC

TCTTGAAATGACTGAGTTAAAACAATTAAAGAACATATCCTTATTTGAAAACCAGTTTACCGGAGTTATA

CCTCAAGGTTTGGGGATTAACAGCAGTCTAACGCTGCTGGATTTCACAAACAACACCTTCACAGGTCCTG

TTCCACCTAATCTTTGTTTTGGAAAGAAACTGGAGAAGCTTATTTTAGGTTATAATCATCTCGAAGGTGG

CATTCCTTCTCAATTAGGACAATGTTATACTTTGACGAGAGTAATTCTCAAAAAAAATAATCTGTCAGGT

GCCATTCCAGATTTTGTTAAGAATATAAATCCTATTTTCTTGGATCTCAGTGAGAATGGCTTCAGTGGGA

AAATATCCCCAAGTTTGGCAAATCTTGGAAATGCTACATCAATTGACTTATCAGTGAATAAGCTTTCAGG

GTTTGTACCACCAGAGCTGGCAAACCTTGTCAACCTCCAGGGTTTGAATCTTTCGTACAATGGTTTGGAA

GGTGTACTGCCATCTCAGCTTTCAAACTGGCAGAGACTGTTGAAGTTTGATGCAAGTCATAATTTGTTGA

GTGGATCGGTCCCATCCATTTTTGGAAGCTTGGAAGAGCTATCCATTTTGAGTCTGAGTGAGAATAATCT

TTCAGGAGGTATTCCAACCTCCTTGTTTGCACTCAAGAAGCTCTCCAAGCTGCAGCTTGGTGGAAATGCA

CTTGGTGGGGAAATTCATTCAGCAATTGCGACTGCATCAAGAGAAACACTGAGGTGTTTAAATCTGAGT

AGTAACAGGTTGACAGGTGAACTTCCTGCGGAGCTAGGAAAATTTACTTTCCTGGAGGAGTTAGATATA

GCTGGCAATAACATTTCTGGAACTTTAAGAGTTCTTGATGGAATGCGTTCATTGATATTCATCAATGTATC

ATACAACCTCTTTTCTGGTCCAGTACCTGCACATCTGATGAAGTTCCTCAACTTAACTCCTACCTCTTTCTC

GGGAAACTCGGGCCTTTGCGTGCACTGTGATCCAGAAGAAGGCTCAAATTGCCCTGAGAATATCACGTT
AAGGCGCTGTGATCTTCAATCAAACAATGGAAGGCACCTGAGTGTAGCAGAAACGGCAATGATTGCACT
TGGGGCACTGATATTTACCATTTCCTTACTCCTAGTAATTGCTTACATGCTTCTGTGGCGCAAAAGTTCTG
GGAAAGGAGTTGCAATCTCTGCTCAAGAAGGTGCATCGTCCCTGCTTAATAAAGTATTGGAAGCTACTG
GGAACCTAAACGATAAGTATGTCATTGGGAGGGGAGCTCATGGAGTTGTATATAAGGCCATCTTGGGTC
CAGGGAAAGTGTATGCTGTGAAAAAGCTGGTGTTTGTTGGTATAAAGGATGGAAGCAGAAGTATGGTT
AGAGAAATTCAGACAATTGGAAAGGTTAGGCACCGCAATCTTGTCAAATTAGAAGACTTCTGGCTGAGA
AAGGATTATGGGCTGATTCTTTATAATTACATGGAGAACGGGAGCCTTCATGATATTCTCCATGAGACTA
AGCCACCTGTAACATTAGAATGGAGCGTTCGGTACCGAATTGCTATTGGAGTTGCTCAAGGGTTGTCAT
ATCTCCATTTCGACTGTGATCCTGCCATTGTGCATCGAGACATCAAGCCCATGAATATCTTGTTGGACTCT
GATCTGGAGCCTCACATATCAGATTTTGGTATTGCCAAGCTTCTGGATCAGTCTGCAGCAACTTCCGCCT
CCAATGCCCTTCAGGGCACTGTTGGATACATGGCTCCAGGTATGTAATTTTCATTTTTTTCTCACACTTCCT
TTCAGTTAGCTGTCTCTTTAGAGATCTAGTCATCTGTAGAAAAATAATATTGTCTGTGTCTTGTCTGTTTTT
GTTGACAGAAACTGCATTTGCAGCTACAAAGAGCAAGGAGTCAGATGTTTATAGTTATGGTATTGTCCT
GTTAGAACTTATAACTCGAAAGAAGGTGTTGGACCGTTCACTCTATGGGGATACAGATATTATGTGTTG
GGTTAGGTCTGTTTGGACTGAAACTGAAGAAATTGAGAAGATTGTGGATCCAAGACTTTTGGACGAATT
CATAGACTCAAGTGTCATGGAACAAGTAATTGAAGTGCTTTCACTAGCTCTTAGATGTACAGAGAAGAA
AGTCAGCAAAAGACCCTCAATGAAAGAGGTAGTCAAGCTATTAACAAGGTCAAGTTCGAGTATACGAA
GCAAGTACTAGCTAGCAGGGACATGTAGTATCTAGTGTATCAGTCAGAGGGGGTTTCATATAAACGCCT
GATGTGTTCTAGGTACTCTATTTTCCAGATTAAAGTAGTCCTAGCTTCCAGCTTTCTGAATCCATGTTACA
AAATGCTCCCTGCAGGTTCAGTATCTTTTTATTGGTAGAGGGTGATAAAAATATATTTACTTCTGTAGTTT
AGAAAGAGAAGGCGTGTGCCTTTAGCTGTAAATGTTCTTTTCCATCACATGAACATGGATGAAAACTAA
AGATAACTTACTGATTTTCATCTTAGTTAATCATATTCTCAGCAAAAAGCAGTTCCTTTTGCCATTCCTAGC
AGGATTTGTTATGGTAAAGCCATATTGTTTTCTCCCTAGCTATTTGCAACTGTACAATTACTCCAATAAAA
TAAGCTTTC
>PGSC0003DMG401013731 LRR-RLKs
GCTTTGCTGTCTGGTGATGCTTATCCTGCCTGCTTCTGATAAGAATTATTTACTTGCAGGGACCTGAGCA
ACAACATGTTGAATGGTTTAATCCCAGATACATTTAGTCAGTTGCACTTTGATAACATGTAGGTACCTTG
AAGTGAAACCTTTACAAGTTGAAGCATAAATGTTGTAATGTTTTTTTTTTTCCTCAAAAGTTTTCTTTCAGT

AACAAGAAGCAAAAGTTTTATCTGTGATGCAGGTTTCTTGGTAACAACGCATTAAGCGGAGCAATTCCC

AGCTGGGTGTTTAGTAGTGATAAGAACATGTTGGTATTTGCTCAATTTGTTCCGTTTTCCTCATCTAAGTA

GTAAACTTTGGTTATTATCATGCCAGCACACTTCAAAGTTAACAGTTTTTCCCCAAAACATTTTGATTGCT

GAAAGGACGCATTTGCAAGAATTTGGTGTCTTCTAAGTGCATGGCAACTATAATTTTTTTTTTGCAGTGA

TATATCATACAACAATTTCACCCTTATATCTACTCGAGGATGCCAGTCCTCTATCATGTGAGCATTGTACT

TCTTTGTTTGGGAACCTACATCCGTGTATTATTTCTATTTTTATTAGTAAGAATTTCGGTCATTTAACTACA

TAACTTACAAACCTTCTTTCTTCAAACAGAAACTTAGCCGCCAGCTATTCAGATACAATGAACACGTTCAA

CAACACGTAAGTCCTAAATGTTGTCTTATGCATCAGGTGCTAGTTTGTTTGATGCTTTCTGTGTATTGTCT

ATAATTATGTGGCTTAATCTATTCGAAATTGCAGGGATTCCTGGTGTTCCATGAAACCCCTCATTTGTCCC

ACAGAAGCGAAATGTAAGAGTTACAGCCTATAAAAGGACCTCAGGGGTTATGTCTGGATCATTTACCAC

TGAACCAACTCTTGGAGTGGCAGCCTGTGGACTTTTTAAGTACAAAAGCATAACTTTGGAGTTGAGAGA

TAATTTCACAACCCTTACTCATGTTACGTTCTTTATGTCAATTGCAGATCATTCACTATTCATAAACTGTGG

AGGAGGTAGAATTACATATGAGGGAAATGATTATGAGGAGGACTTGACCACTAGGGGTCCATCATATTT

TTTTTCGTCCCCTGATAGATGGGCATTCAGCAGTTCGGGAGTGTACGTAGGTCTTGCAAATGTTAGTTAT

ATTGCAACAAATAGATTTTTACTGAACATATCCGGTGCCGACTTTTACAACACAGCACGCCTTGCCCCTAA

TTCTCTCAAATATTATGGGCTTTGCCTACGAGGAGGTAGCTACAGAGTGCGACTTCACTTTGCTGAAATA

ATGTTTTCTAATGACTCAACATATAGCAGCCTTGGAAGGCGGATATTTGATGTAGCAATACAAGTAAGTT

TGAGGCCCAACTTCTTTTGTAAATAATAAATATGTAATGTTGATATACTGAGTTCTGATATGATTGGCAAC

TTTCTGATTTAAGATTGAAGGTACCAATTTGTTGGAGATAACGTGGTATATTTTGATTTCTTACACAGGG

GAGAGTAGTTTTGAAGGATTTCAATATCATGGAGGAAGCTAACGGTGTTGGAGAAGTCATTATCAAGG

ACTTTGATGATATTACTGTTAGTAGCACTCTGGAGATTCACTTGTATTGGACCGGGAAGGGGACTAACG

CTATTCCAGACAGAGGTGTATATGGACCGCTGATTTCAGCTATCTCAGTGACACCAAGTCCGTATATCTC

TGCCTATAGACTTTACACTTATCTTCCCACAATCTTATCTTAAGAAAATCCCATTTTCTTCTTGCAGACTTC

AAAATAGGTCGCAGGTTACCTGTCGGAGCTGTTATTGGCATTGTACTCGCTTCAATTGTGGTGGTTCTGA

TAGTTTTATTTTCTCTGTGGAAGAAAGGCATTTTTGGAGGGAAAATTAATCAAGAAGAATTAGGTAACAC

ATACCTCAAAGAATTTATTCACTCTCTACATTTGATATGGCGATACTTCTGTTGTGATAATCAGAATTATG

TACGAGATTTCTAGCTGCGACATACAAATAATTATCATAGATGATTGTTCTGTTGGCAGATAGACATCAT

GACATATCATAGATAGTCAGCACACAACAAAATGTTCATAACATATCATACAGATGTTTATTCTTTTAAAA

ATTGTTGTTCATCATACAGATGTTTTAGAAGATCTATATATACTGACGTTGATATTATTCTCGTTTACAGA

ACTCAGAGCCCTTGATTTACAAACAGGTCATTTTAGACTTAGACAAATTAAAGCTGCTACAAATAACTTT
GATCCTGCCAATAAAATTGGTGAAGGAGGGTTTGGACCAGTTTACAAGGTATCAATTCTCTATTTATATC
TATCCGTACTGATTTTGCGTAAGGACAGCTTGAGGTTCTGTGGTGATGTTTCCTACTGGTTATGAAGGTC
TGTAATATGATCGTTGTAAATTAATCCTGCTGTCTGTAGGGTGTACTCTCCGATGGGGCAGTCATAGCTG
TTAAGCAGCTCTCTTCCAAGTCAAAGCAAGGAAATCGTGAATTTGTTAATGAGATAGGCATGATTTCAGC
TCTACAACACCCAAACCTTGTCAAGCTTTACGGTTGTTGCATAGAAGGGAACCAGTTACTGGTGATATAC
GAATACATGGAAAATAACTGTCTTGCACGAGCTCTCTTTGGTAATGCTTTGCAACAACATTTTGAAAAGC
ATTTATCATGCAGTGATAATTTTATGCTTAATACTCCTCTAATTTTCCTTTCTTAGGCCGTGACGACCAGAG
GTTGAACCTTGACTGGGCAACCAGAAAAAGGATATGCTCAGGAATAGCAAAAGGGTTAGCTTACCTTCA
TGAAGAATCAAGGTTGAAAATCGTCCACAGAGATATTAAGAGTACCAATGTGCTTCTTGATAAGGATCT
GAATGCTAAGATTTCAGATTTTGGATTGGCTAAATTAGATGAAGAAGAGAACACTCATATTAGCACCCG
AATTGCTGGAACAGTGTGAGCCTTCTTATCATAACTGTCTCTGTACTATCATGTTTTGATGAGAGAGTAA
CAGTTCTACCCATTTGAATTTGTTCGTCATACATTAACATGGTCAGTTTGAAGTATCTTACATTTCTTTTTC
CTCTTTTGAAGAGGTTATATGGCTCCGGAGTATGCAACGAGAGGCTACTTGACAGATAAAAGCTGATGT
TTATAGCTTCGGAGTTGTTGCTTTGGAGATTGTCAGTGGGAAAAGTAACACAAAACTACAGGCCAAAAG
AGGAATTTGTTTACCTTCTTGACTGGGTAAACTATCTTTTTACTCAACTGTCATTATTCCCAAATACTAAAA
TCTTTAAAAACTGCTGCAACATCATGTATTATAAGTCTTGCATGTACATGTTTAGTTTAATTCCACTCTGAA
TTATAAGCCAGAAAAAACTTCTCTTGTTAGTGTTTTGTGCAAATATATACTCCATCGGTTTCAATTTGTTTG
TCTTACTTTTATTCATAGTGAGTTTCAAAAAGGTCTCTTTTCTTTTTTGCCAGCTCTTTATTCTAACTTTCCA
CGTGACATATTTAAAACCACATGATTTAAGGGTATTTTGGTACATTCTACATATCTTTAGTTTAAATTAAA
ACCAGACAAACAAAGTGAAACGAAGGGAGTATTTGCTATGATACCTCCAAAGAAAATATCAGAATCAGA
GTTGTTTCATTAGTTCTGCTCACATTCTTATAGATGCATCTTTGCGTATTTAGTTTTTCTTTTCTCCAATTTC
ATCTTGCTTTGTATGTCAATTTTGTTTTAGGCTTACGTCCTACAAGAACAGGGAAATCTGTTAGAACTAGT
GGATCCACGT
>PGSC0003DMG401017953 LRR-RLKs
GGTCTCCGGAAAATTTTCATTTATCATAAGAGTTGTTGAGCCAGTATTGTGGTGATGGGAAAAGAAATG
GGAGTTGAAAAGCTTCTGGTTTTGTTTTTGGTACTAATCTCAATCAGATATTCAGAGCAATTACAATCTTC
TCAAGTGCAAACACTTTTGAGAATTCAACATCTTTTGAACTATCCAACAGCTTTGAGTAGTTGGAGTAAT
GAGACAGATTTCTGTAAGATTGAGCCAAGTTCAACTGTTACTGTTGTGTGCTACGACGAAAACATAACTC

AGTTGCATATAATAGGGAGAAAAGGGACTTCCCCATTACCTAGAAAGTTCTCCATTCGTTCATTTCTCAA
CGCGCTTGCCAAGCTTCCTAGCTTGAAAGTCCTTAGATTAGTTTCTCTTGGTTTATGGGGGCCGTTGCCT
GACAAACTTTCAAGACTTTCAGCTCTCGAGATACTTGACTTGAGTTCAAATTACTTTCACAGTGGCATACC
TGAAGCAGTTTCATCCTTAACGGACCTCCAAACTTTAGTGTTAGATGGAAATAGGTTGACTGGCGGAATT
CCAGATGGCCTTGGTTCACTTTCAGTTTTGGCAGTTTTGAGTTTGAAGAGCAATTCACTAGATGGACCTT
TGCCAGATACACTGCATGATTTGAATAATCTTCGTGTACTTTCACTTTCGAAGAATAATTTTAGTGGTGAT
GTCCCTGATCTTAGCAGTTTACAAAATCTTCAAGTTTTAGAATTAGAGGACAACTCATTTGGTCCAAAGTT
TCCTCAAACTGGAAGTAAAATTGAAAGTATTGCTCTAAGGAATAACAAATTCACTGCAAACATACCAGA
GAAAGTTCAGTCCTATTATCAGCTTGAACATTTTGATATTTCTTCCAACAACTTTATGGGGCCATTTCCTCC
GTCGTTGCTATCTTTGTCATCCATCACTTATCTGAATGTTGCAGGAAACAAGTTAACAGGAATGCTCTTTG
AAGATAATCCATGTAACACAGCATTAGATTTTGTTGATCTGTCTGGTAATCTGTTGACAGGATCATTACCT
AATTGCCTTCTGTCTAGTTCTAGGAACCGGATTGTGCATTTTTCCAATAACTGCTTAGCAACCGGAGATG
GTACTCAACATCCATTTTCATTTTGCCGGAATGAGGCTTTGGCTGTTGGTGTCTTACCTCAGCATCAAAGA
CAAAAACATGCTTCTAAAGTGATTCTTGCCTTGATCATATGTGGTAGCATCACCGGTGGAGTCATACTCG
TATGCCTGACCATCTTGATTGTCAAATCTTTTCTTGCAAAGAAGGCTTCTGCCCAAAATACCCCACCCAGA
TTTATTTCAGAAGATGCATCATCTTCATATACCTCCAAGTTATTCACAGATCCAAGTAAGGTTCCTCTCAA
TAACCAAATTACTGTTTAAAATAGAAGCTTAGGGCAAATATATCTGCTCTCTGCGCCCTAGATGCCTGAA
GGCGAGGGATTTCCTTCAATTCCCAAGTGCCAAGTATACTAACTTGATACCTTTGTATTTCTTGTATAAGG
ATACATTACTCAAGCAATGAAGTTAGGAGCACTTAGTCTTCCATCTTATAGAATATTCTCTCTAGAAGAAC
TTGAGGCGGCAACGAACAACTTTGATACAACAACTTTTATGGGTGAAATTCCTAATGGCCAGGTATGGT
ATATGAATTCTATTATTCTTTCCTGCTATAGCATTGAGGTCTTTGGGTGTTATACTTATGGCCATATTAGTA
ATGTAGCTCACGTGGCAGATGTGGACAAGTCATTCCCAAGAAAGGGAGTTTCAAGTATTTGGGGTAATA
ATCCATGGAAATGGAGAGATTGATGATGATGTTACACATCGTATTGGAATGAGGTGTATGAAATGGAA
GCTTGCATCTAGTGTCTTGTGTGATAATAATGTGCCACCATGTTTTAAAGGCACGTTTTATAAAGTGGTG
GTCAGACCAATCATGTTGTATAGGGCAGAGTGTTGGCCGTTCAAAAACTCGCATGTCAAGAAGATGAGA
GTAGCGGAAATGAGAATGGTGAGATGAATGTGTGGACACGCTAGGAGAGATATGATAAGGAATGAAG
CTATTCGGTACAACATGGGAGTGGCCAAGATGAGGGAGGCAATACTGAGATGGCTCGGGCAAGTGAA
GAGAAGATGCACAGATGTTCCATTGAGGAGGTGTGAGAGGTTGGCTATAGTGGTATTAAGAGAGGTAG
AGGTATGCCAAAGAAATTTTGGGGAGAGGTGATTAGACATGACATGACTTACGTGTAGCTCACCGAGG

ACATGACCCTAGATAGGAAGATATGGAGGTCGAGGATTAGGGTAGAAGGTTAGTAGGTCAAGAGTGG
TGTCTGGTTTCCCTTTTTAGTATTGTTATTACTACCCTTCTATGATCCTATTCTTCGATATTAGTTTTACTGG
TTTTTTCCTTTGCTTCAGTTATCGTATTATTTTGTTGTTGTTGCTACTAATCTTTTCTTCATTGTTTTCAGCAT
GAATTCTTCGACTATGTTTGCTTTACACACTTTTTTTCTATATGTTTTACTTGAGCCGAGTATCTTTCAAAA
CAACCTCTTTACCTTCACAAGGAAGGGGTAAGGCTGCGTACACCCCACCCCACTCTCCCCAGATCCCACT
TGGGATTATACAGAGTATGTATCAGTATGGGTGTTATATTAGAGAGGTGGTGTGGACATGTAGTAGTAA
TGCCATCTGTTTGTTCTCTATCATTTTGGCACAAAAGGATGAGTTTGACTCTTGAAGAGTAAACCTATTAA
TATTACCAGCTGTTCTCGAGGACTAAGGCTCACAAACGTTTTCTGTTTTGGATTCAGATGTACAGAGGGC
AGATGAGAGATGGTTCCTACATTACGATTAGATGCCAGATAATGAAAAGAAGCAACACTAGGCAAAATT
TTATGCATCACATAGAGTTGATATCCAAGCTCAGACATCAGCATTTGGTCAGTGCTCTTGGACACTGCTTT
CAGTTTTACTCGGATGATTCAAGTGTCAGCAGAATTTTTCTCGTCTTCGAGTATGTACCTAATGGGACCCT
AAGGAGATGGATTTCTGGTAATTACTTGACAAGCAGATATATACCCCTTCCCTATTGTTGGATGTAAATG
TGCTTCAGTAATTTGATGTTCTCTGTTGCTTAATTGCAGATAAACATGCTAGACGGAAACTAACCTGGAC
ACAACGTATAGCAGCTGCTACAGGAGTAGCGAAGGGACTACAATTCTTGCATAATGGAATTATCCCTGG
TCTCTTTTCGAATAATTTGAAGATAACAGATATTTTGCTGGATCAGACCCTTGTCGCCAAAATTAGTAGCT
ACAATCTGCCTATTCTGTCGGAGAATGTCGGGAAGGTGTGAC
>PGSC0003DMG401021514 LRR-RLKs
CAATAATTCACTACATAGCTCGCTATAAGTTCAACAGTATCCAAAACAAGCAACGTACTGAATGATGATG
GTTCATAGAATATTGCTCTTTTTTTTATTTAGAGTTTCTTTTGCTGCTTCCACTGAGGAAGCAATAGGTCTC
CTCAAATGGAAAGCAACTATCGAAAACCAGAATACTTCCTTATTTGTTTCTTGGACAATAAGTGTCAAGA
ATTCTTCCAGCCATGGAGAAGCAAATCCTGATGCATGCAGGGGCTGGTATGGAGTTAAATGTTTTAACG
GTCGGGTAAACATGTTGAATATTAATAATGCTGGTGTCATTGGTACACTCCGTGATTTTCCATTTTCATCT
CTCTCTTATCTTGAATATGTTGATCTTAGTATCAACCAGCTCTCTGGCATCATCCCTGCTGCAATAGGAAA
GCTCACTAATCTTGTCTATCTTGACTTGGGGTCTAATCAGTTTTCAGGCACAATCCCACCACAAATCGGCT
CACTAACAAAGCTTGAGACCCTCCGCATCGTTAACAATCATTTACATGGTTCCATTCCAGGGGAGTTGGG
GAATTTGCAAAACCTCACTAGACTTGCTTTGTACGATAATCAACTTACTGGTTCTATTCCTGCTTCATTTG
GCAATTTGAGAAACTTGCATATTCTGAATATACGTGCCAACAAACTTTCTGGTTCCATCCCTAAAGAGCTT
GCATATTTGGATAACTTGGTAGTGATGATAATGAGTAAAAATGAATTTTCAGGTCATTTGCCAGAGAAG
CTTTGCCAAGGTGGTAAACTTGAGAATTTTACAGTGAGCAGTAACAAACTTTCAGGGCCAATACCAAGA

AGCTTCAGCAAGTGCTCGAGTTTTAAAAGGGTTCGCTTTGATAACAACAGCTTCACTGGGAATTTATCTG
AAGCTTTTGGTATCTACACGGAGCTTCAGTTCATCGATTTGAGCGACAATGATTTCCATGGTGAACTCAG
CAGCAACTGGGGGAAATGTATGAATTTAACTAATCTGCAGGTGGCTAGAAATAACATTAGTGGTAGCAT
ACCACCAGAGATTGGAAACATCAAAGGGCTTCAAGGACTTGATCTTTCATCCAATCATTTGGTTGGGCA
GATACCTATGGAATTTGGAAAATTGACCTCTCTTGTTAATCTTTTTTTGCAAAACAACCACATTTCTGGAA
ATATACCTAGGGAACTTGGGTCACTCACAAAATTACTGTCCCTTGATCTATCAAACAATAGATTGAACGG
GTCGATCCCAACATTTATAGGAGATTACATGAACATGTTTCTCTTGAACCTAAGCAACAACAAGTTTAGC
CAAAAAATTCCAAAGGAGATAGGGAGGATAGTTCATCTTACTGCACTTGATTTGAGTCATAATCTTCTTG
ATGGAGAAATACCCCCTCAGTTAGCCAGTTTGCTGGACTTGGCAAACTTGAATCTTTCCCACAATAGCCT
CTCTGGCCGCATTCCTGAAGAATTTGAAAGTTTGACTGGTCTGCAGGATGTTGTATTGTCATACAATGAG
TTGGAAGGTCCAATACCAAATAATAAAGCTTTTACAAATGCCTCATTGGAAGGTAATAAAGGTCTTTGCG
GCAATGTAACAGGATTTGTGCAATGCAAAATGCCCTCTTCTATGATGAAGAAGCAATCAATGGCAAAGG
GACGTAAACTCATTCTCATCACTCTACTTCCTGTATTGGGAGCACTGTTACTATGTGCTTTCGTTAGCATT
CTCCTTATGCGTGTTAAAAGAAAGAGAGTACTTAGAGACGTTGATAGAGGAGATGATGGTTTGCTTTCG
ATATCAATGTTAGAAGGAAAGGCATTGTATAGGGATATCATAAACGCCACAGAGGAGTTTGATGCAACA
TTTTGCATCGGGAAAGGAGGGCATGGAAGCGTTTACAAGGCAAATCTTCCATCATTAGGGAATATAGCT
ATAAAGAGACTTCATTCTTCATTTGAGAATTCATATCACAATAGCTTCATGAATGAAATAAGGGCATTGA
TAGGGATTAAGCACCGGAACATTGTGAACCTCTACGGCTATTGTTCGAATGCACAACACTCGTTCTTGGT
TTACGAGTATGCAGAGAGGGGAAGTTTGTCTAGTATATTGAGCAACGAAGTTGAGTCCAGAAAATTGG
ATTGGCTAAAGAGGGTGAATATTATCAAAGGAGTTGCTTTTGCTTTATCTTACATGCACCATGATTGCTC
CCCACCGATTGTCCATAGAGACATATCAAGCAGTAATGTTTTGCTTGATTCCAAGTATGAAGCTCGTGTC
TCTGATTTTGGCATTGCTAAGATTCTCAAGCCAGACTCATCCAATTGCACTGCGTTGGCAGGAACATATG
GCTATGTTGCACCAGGTTAGGTCTAACTTTACATTTCCTCTTTTTTTTCAAACTTTATGAACTATAGCCAAA
GACTCCATTCTGTTTTTATTTCATTTACACATTTGAATTGTTACCTCTTTAGTTCAATTTGTTTGTCTTACTTT
CATTTTTAGTATGTTTAAAAAAAGTTGTCTCTTTCCTAATGCAACTCTTGAGTTTCAACATGTCATATAGCA
TGTTTAAGACCGTGAGATTAAAGAACATTTGGTTGCAATACATATCTTTAGTTTACAATCACAAGATTTAA
AAGTCTTCTTTTACTTCTTAAATTTTGTGGCAAGTCAAACTAAGACATACAAATTGAACTTAGGGAGTAAT
ACTTTTTCGTAAATTGTGTTGCTTCAGAGCTTGCATATACAATGAAGGTTACACAAATGTGTGATGTCTAT
AGTTTTGGAGTATTAGCATTGGAGATAATCAAAAGGAAAGCATCTTGGGGAATACATTACTGTGCTAGC

AAATTCATCGACTAGAGATAACGTACTGCAGTTTAGCGATTTGCTAGATGAACGCCTTCCATATCCTGAA
GAGAGAGTAAAAGAGGTTTTGCTTTTTATCATCAAGCTAGCAAGCTCTTGTTTGCTTGAAACTCCAAAAT
CAAGGCCAACAATGTACTTCATCTCTCATAGGTTATCATCAATAGATACGCATCCACCTATTTATGGAAG
GCGATATGAATAACCTTTGACACCAAGTGGTTGTAAAGTTCCACAAAATGTTATTACTGGGACTAGTATT
GTTGGTTTAAATCTTAGGGTGCTTAACTGGTTTATTGTACTAGAGTGTATGCAGTGTAATGTTGAAGGTA
ATTATCTAATTTCCATAGTATTTTGC
>PGSC0003DMG401023552 LRR-RLKs
ATGTTGACATATCTAGAAGTGGGTAGCAATCCGTTGAGTGGCTTTCTGCCAAATTCTATTGGGAATCTCT
CATCTACTATTGAAAAAATTAATTTAGCAGATGCACACATCAATGGCCTCATCCCCACAAGTATAGGCAA
CATGAGCGGTCCTATATCCCTATACATTGAAGAAAACAACTTGATAGGGAGTATTCCTTCTGATGTTGGT
AAGCTTAAATGACTCCAAGGACTCTATCTATCTAACAATAAATTGCAGGGACGTATTCCAGAGGCGGTAT
GCCATCTATCTAATTTGGTTCAATTATATCTGTATGGTAATGAGCTCTCTGGGTTAATTCCAGAATGCTTA
GGAAATCTTAGCATGCTTCAACAGCTTTCTTTGAATTCTAACAAATTATCTTCAAACTTTCCGTTGAGCCTT
TGGAAGATGAGTAGTCTTCTCTATCTAAGTGTGTCACAGAATTCAATAGAGGGAGAAGTTCCATCAGCT
ATTGGAGGACTGCAAGCCATTGTAGAACTACATCTTTTCAGTAACCACTTTTCAGGCGTGATACCAACCA
GATTAGGGGAACTCCAAAACCTGCAGTATATTGACCTATCAAACAATTCATTTTTTGGCCAAATTCCATTA
TCCTTTGCCAACTTGATAAACTTAGAATTCTTAGATCTGTCTTTAAATGCGTTGTCGGGTACTATTCCTAA
GTCATTGGAAAAACTCTCCTATCTTATAAGCATTAATGTTTCATTTAATGATTTAGAAGGTGAAATACCGA
GTGGTGGCGTGTTTGCAAATTCCACTCTGCAATCATTTCTCGGGAACAAAGGTCTATGCAAAATGCACAT
ATTGGAGATTCCTGATTGTGTTATCACTAATCCTGGAAAACAATCAAAGCTTAAGGAGGTTGTGCTAAAA
ATTGTTACTCCAGTGGTTACTTCATCCTTTCTGATATTCTTATTCGTTTCAATTTGGACAATGAAACGAAAA
AAGAAAGGGAAGTCCAAAGATGTGGAGAAGGTACCGGAGATCGGGACTCATCAATTAGTTTCTTATCA
TGAGATTCGAGGAGCAACAAATAATTTTGATGAATCCAATTTAATTGGTGAGGGAAGCTCTGGCTCTGT
GTACAAAGGCGCATTGTTTGGTGGAACTGCAGTGGCCATTAAGGTTCTGGATTTGGAAAATGAGCAAGT
ATGCAAGAGGTTTGATACCGAATGCGAAGTGATGAGAAATGTTAGACACGGAAATCTTGTTCCAGTGAT
TACTACATGTTCTATTGACTATATAAGAGCCTTTGTTCTGCAATTTATGCCCAATGGAAGTCTTGACAATT
GGTTGTACAATGAAGATCGCCACTTGAACCTTCATCAAAGAGTTTCTGTAATGCTTGATGCAGCTATGGC
AGTTGAATATCTACATCATGGTCATGTCACTCCAAAAGTTCATTGCGACCTAAAGCCAGACAATGTTCTTT
TGGATGAAGCTATGGTGGCTCATGTTGGTGATTTTGGAATCTCTAAAATTTTAGCCGTAAGCAAGTCCAT

GGCTTATATGGAGACATTGGGCACTCTTGGATACATTGCACCAGGTATACTAGTCTCTTTCTATATATTTT
CACAAAAATCAGAATTTAGCCCTTCCAGAATCATAAAAGCAAGTGTCTGTTAGTACATAATTATGCTATA
TTCAAATGAGTAATTCTTTTTCATTTTCATTCTTTTTTAAGAATATGGCTCGGAGGGAATAGTGTCTTCTA
GTGGTGATGTTTATAGCTATGGCATCATGGTGATGGAGGTCTTGACGAAACGAAGGCCAACAGATGAA
GAGATATGCAATGAAAACCTTGACTTGAGGAAATGGATCACACTAATCATTTTCAGGGAGTATGATGGA
CATCGTGGATGCCAATCTTTTTCGTGA
>PGSC0003DMG401027271 LRR-RLKs
GTTTTCTTCATTTCTTGATATGGCTTCTTCTTAGATAGTACTCACTACTGCTGTCTATTTCCTAAATTTAAG
GGCTTCCTAGCTTTTTGGTTATTTTACATTTTCAAGATTCCTCAAATGGAATACTCCCTCACATTTCTTGAG
CATTTCAAGACTCAATCTTTTTCACTCTAAACTCCCTTTTGTCTTCTACACCCCACCCCCACAATCTCCAATT
CTGCAGAAAGTTTCATGTGATGGATTCTTGATGGGAAAAGGAGGAAGAAGTTGAAGAAAAGGTGTTTT
CTTTGTAAACAACCTCATAACCATTTCAAAGATTCAATCTTTTTCACTGTAAACCCCTTTTTTCACCCCCCTT
AATCTCATCAATGGATTCTTGATTGTTCTATCTAGTTGTTTTGGAGGGAAAAGGAGGAAGTAATTGAAGA
AAAGGTGTTTTCTTGAAAAGAAAAAAACTCAAGAATGACACTTTTGAGGTTTCTGTTAACTGTGTTTTTTC
TAGTTGGTTTGTTATCAAGATTCCAACTTGTGAATTCCCAGTTGGTTTTTCATGATCAAGATGTAGTGGAA
GCAATTGGGAAAGAGCTATCAGTACCTGGATGGGGTGTGATGAACACAACTGATTTCTGCTCTTGGCAT
GGAGTTATTTGCAGCTCAAACAGTTCAATGGTGGAAAGACTTGATCTTTCTGGTTTTAGATTACAAGGTA
ATGCAACTCTAATATCTGAGCTCAAAGGTTTAAAGTGGCTAGACCTTTCTAATAATAACTTCCAAGGTTCT
ATTCCATTTGCATTTGGGAATTTATCTGAGCTTCAATATCTTGATTTGTCTTTTAACAAATTTGGAAACTCA
ATACCTAGTGAATTAGGTAAGCTCAGAAGCCTTAAGGCATTGAACCTTTCGAAAAACTGGCTCACAGGA
GCTATACCTGATGAGCTTGAGGGGTTAGAGAATTTGCAAGATTTTCAAATATTTACTAACAACTTGAATG
GTTCTATCCCAATGTGGATTGGTAATTTGACCAATCTTAAGGTTTTTGCTGCTTATGAGAATGAATTTAGT
GGTGGTGTTCCGGTTAACTTAGGACTGTACTCTGATCTTTCTTTGTTGAACCTTCACTCAAACCAGCTTGA
AGGTACTATTCCTGAGAGCATTTGTGCTAATGGGAATCTTGAATTTCTTGTTCTAACTCAGAATAAGTTG
ACCGGAACGATTCCTGATTCGATTGGGAATTGTAAAGGACTTTCTAGTATTAGGATTGGGAATAACAAG
TTGATTGGAGGTATCCCTAAATCAATTGGGAATATCAGTAGTCTTACTTATTTTGAAGCAGATAGTAATA
GTTTGTCTGGTGAAATTGTCACAGAGTTCGCGAAGTGCTCGAATCTCACTCTCCTTAATTTAGCTTCAAAT
GAGTTTAATGGAACTATTCCTCCTGAGTTTGGTCAGCTTAACAGTTTGCAAGAATTGATTGTTTCTGGTAA
TAACCTTTATGGAGAGATTCCAACATCAGTTCTTAAGTGCAAGAATCTCAATAAGCTTGACTTGAGCAAC

AACAAGTTCAATGGTACCATACCGGCAGATATCTGCAATACGTCCAGATTGCAGTATTTGCTGTTGGGG
GAAAACTCGATTCGAGGGGAGATACCTCATGAGATAGGGAATTGTGTAAAGTTGCTTGAGTTGGAAAT
GGGGAGTAATGAACTTACTGGAAATATCCCTCCTGAGCTTGGTCATATGAAGAACTTGCAGATTTCGTTG
AATTTGAGTCATAATCGTCTCCACGGAAAATTGCCTCAGGATTTAGGAAAACTTGACAAGTTGGTTTCTT
TAGATGTATCTGATAATCAGCTCTCTGGGAATATTCCACCGGCATTGAAGGGCATGCAGAGTTTGATAG
AGGTTGATTTTTCGAGCAACCAATTCACTGGTCCAATACCTACTTTTGCACCATTTCAGAAAAGTCCTAAT
TCAAGTTTTCTTGGAAACAAAGATCTCTGTGGTGATCCTTTGAGTGGTGGTTGTGGGGATTTAAATGGTT
ATGACCACAGCGTTTACGGTCACAAGGTCTCTTACCGAATCGTTTTGGCTGTTATCGGTTCTGGTTTGGC
AGTTTTTGTATGTGTCACAGTGGTTGTTTTGCTGTTCATGATGAGGGAGAAACAAGAGAAAGCTGCCAA
GGAAGCAGCTGGAAATACCATTGATGAAATTTCTAGCAAACCAGTGATACTGGCGGGAAACGTCTTTGT
TGACAATCTAAAGCAAGCAATTGATTTTGATGCAGTTGTAAAGGCAGTCATGAAAGACTCGAATAAGAT
TTGTGTTGGTACTTTCAGCACTGTCTATAAAGCAGACATGCCTTCCGGGATGATTTTGTCGGTGAAGAAG
CTAAAGTCAATGGACAAAACTATAATTCATCACCAGAGTAAGATGATCAGAGAGCTTGAAAAGCTTAGT
AAGCTCGTTCATGATAATCTTGCTCGTCCTATCGGATTTGGAATCTATGAAGATGTCGTTCTTCTGTTGCA
TGAATATTATGCCAACGGAACGTTGACTCAATATCTTCATAACTCTAGCCAGAAACCTGAATATAAGCCC
GACTGGCCAACAAGACTTGCCATTGCCACTGGAGTTGCAGAAGGATTGGCATTCCTTCACCATGTTGCTA
TCATCCATCTTGACATTTCTTCCGGGAATGTGCTTCTTGATTCTAATTTCAGGCCTTTGGTTAGTGAAGTT
GAAATCTCTAGGCTTCTCGATCCATCTAGAGGGACTGCAAGTATTAGTGCTGTTGCTGGTTCATTCGGAT
ATATTCCTCCAGGTACATATCATTTGAACACACATACTCCCTCTGTTTCAATTTGTTTGTCTGATTTTAACT
TGACACAAAGTTTAAGAAAGTAAAGAAAACTTTTGAATCTTAGTGTCTTAAACTAAAGATATGTAGAATG
TACCAAAATGCCCTTTAATCTTGTGGTCTTAAACATGTCACGTGAAAAGTTTGAATTTAAAAGTTGCCAA
AAAAGGAAAAAGGCATTCTTTTTTAAGTATGACAAACAAATTGAAACTATTATCTCTCTTTTCGCATTTGT
AGAGATTATAAATCTGTTATTGACTTGTTTTTTTTTTCTAAACAGAGTATGCATATACGATGCAAGTAACA
GCTCCTGGAAATGTTTACAGCTATGGGGTTGTTTTGCTTGAGATCCTAACCACCCGACTACCTGTTGATG
AAGCTTTTGGTGAAGGAATAGACTTGGTTAAGTGGGTACATGGTGCACCTACAAGGGGGGAAACACCG
GAGCAGATACTCGATGCAAAGCTCAGCACCATTTCATTTTCTTGGAGAAAGGAAATGTTAGCAGCACTA
AAGGTTGCGTTGCTATGCACTGACATGACACCAGCAAAGCGACCAAAGATGAAGAAGGTGGTAGAAAT
GCTGCAAGAAATCACGGAAAGCTAAACAAAGTTCGTATCATCAAATCAAAGTATGTCTTGATCTCTGTCT
CTTCATATGTGAATGCTCCTGTAAAAATACAAGTGTACGACATTCTTAGTCGAGAGGACAAAATGGTCG

AACTCTTAAGGGTATGATGTGTCTTACTTCAATCATATGATCACCAGTTCTTTTTCTCCAAAACATGTAGA
TGGAGATGTAGATTAATTTCTCACAAGGAGAATATGTTTACAAATAGCATTATCTTTCATTTTCCATTTCC
CCTTTCTGGTACATTGCTTCCAAATCAAGTATAGATTAAATCATAGTGAAAGATTAAGGGAATGCTACTA
TTCAAC
>PGSC0003DMG401029358 LRR-RLKs
CTCTTGTTTTATAATCCCCAAGTGTGCCATCCATTCTTATTTGTCTATTCACTTATTCCAACTTCCATATGTT
TGAAAATTCTGTGATGAGAACATCAAACCAAGAATTCAAGAATCATCGCTAAATGAAGACAAAAATTGA
AACTTTGTCATTGCTACAATATGTGTGAACCATGGAGAAAACCAATTCCAGAACAAGACCTTTCTTTTTCA
TCTGCGCATTTCTTGGCTTTCTATTGATATTACTTAGCAGAATTTGTGTCTGCAGTGCTGCAGAAAGTGAT
TTGTATTGCTTGAAATCAATAAAAGATTCATTACAAGATCCTTGTAATTCCTTGGGCTCTTGGGATTTCAG
CAATGTTACCGAAGGCTTCATCTGCCAATTTAGAGGAATTGAATGCTGGCATGATTATGAGAATAAGGT
AATGAACATTAGACTTTCAGACTCAGGACTAATAGGTGAGTTTCCACAAGGCATTCGAAATTGTACAAGT
TTGATTGGTTTAGATCTTTCAGACAACAACTTGTATGGAACTATCCCTTCAGATATTTCCGTAATAGTTGA
ACATGTTACAACACTTGATCTCTCAAGTAACAAGTTTTCTGGCAATATACCACCTGACATAGCTAATTGTG
AATACCTTAACTTTCTAATGTTGAACAACAATAATCTAGGAGGTGAAATTCCAAGTAGAATTGGTTCTCT
ATCTCGCCTTAATACATTCAGCGTAGCCAACAATAACTTGACAGGGCCAGTGCCATCATTTGTTAACATA
CAAATCACAGCTCACAGTTTTGCAAACAATTCAGAACTTTGTGGGAAGCCCTTGAAAGAATGTGAGTATT
ATGAGGATTCTTGGATTTGGAAACATGTAGATCGTGCTTCATTTATCGAGGCATTTGTGTGTGGTTGGGT
ACTTTTCTTTACATTAGTTTTAGTCCTTTGCTTCTTTAAATTTCCTGTCAAGGTTATCAACAAGATAGTCTC
ATTAAACATATGGAAGCTGAGGAAAGAGGACTTAACTTCAGGAAGTGAAGAGGATTTAAGCAGCCACC
AGAAGGTAAAGATTTTGCTAACTTTCCGTCTTTTCTCCTGCATTGTTAAGATACATATTTTTGCTGTAGCTC
TAGTTTAGTTATTAGTCCTTACAAATCCATCATGGTATATATTCTTGACTAAAGAGATGAATCGAGGTATA
AATTCTTTATTCGATGATCGAATGTGATTGAAGTAAGCTTCTCAAATTTCTGTTCTGTTCTGTGTATGTTCC
AATGATTCAACTTGCTGTTTTACAGATATTAATGTTGGAGAAGTTTGTTACAAGAATGAGCTTCACCGAG
CTGGAAAATGCAACTTCTGATTTTAGTGAAGATTATTTGGTAGGTAATGGAATGTTGGGCAAAGTGTAC
AAAGCAATTCTACCAAATGACTGGACACTTGCAATCAAGAAGCTGAATGACTCAGAGAACTTGGAGGAC
GAGTTTGTCTCGGAGATTACAACTCTTGGTGGTCTGAGGCATCGGAACCTATTGCCTCTTATAGGTTTCT
GTGTTGAAAGGGAAAAAAAACTTCTCGTTTACAAATACATGTTTAATGGAAATCTTCATGAATGGCTGCA
CTCGAACGAAGACAAGGCCAAGATTTTAGACTTCCGTCTTAGAGTTAAAATTGCACTTGGGATAGCAAA

AGGTGTAGCTTGGCTTCATCATGGTTACAAATTGCACGTTGCACACGGAAGCATAAGCACACGATGTAT
CTTGCTAGATCAAAATTTTGAGCCCAAAATATCCAACTTTTGGGAAGCAAAAATTTGGAGTAAGAATGAT
ACAGCATTAAGTTGGAGTCTTTTCCCAGTAGCTGAATATTCATGTTTAGGTTCTTACAAGAAAGACATCT
ACTGCTTTGGCGTCCTGCTTCTCGAGCTGGTTACAGGGAAGGAACCGCATGAATTGACAAGCTCGAGAA
ATCTGTTTGATCACTCGCCTTGTCTACTTGATGCAGATAAAGATTTGCTTGGGAAAGGAATCAACGATTT
GATCCTCGAATTTCTAGAGTTATCTTGTGACTGTGTGAAGTTCTTTCCTAATGAAAGGCCAACAATGCTG
AAAGTTTATGACAAACTCAAGACAATTTCTCAAGGTCGAAACGACGACTAGGTATAGAAAACTCCCTTAC
ATTCAACTGGCATTTGAAGTCTATATATAGGACTAGTACTATTGTTTATAGTTTACACTATTGGAAAGTTA
TATTGATTGCATTTGTGGTTTTTAAAAGAATCTACTGATGCTGCATTGACTAGTTAACTTCAACCCCTGTA
CCTTTAACTAGAGGTCTCGAGTTTGAGTCCCTAGGGGCTAGGAATGGAAAAAATCACGTTGAGAGCGCT
GCTCCTAGAAATGGACCCTTCAATGCCGCGAATTTGACTTTAATGGGGCCCTTGATTCCCCTGCTACTTG
ATCAGTAATATGTATTAGATGCTAACTACATTATTGAGTCTACTCAGATTGAGGCACCTAGATTGAGGCC
ACTATATGTGTGTT
>PGSC0003DMG401030114 LRR-RLKs
AAATATTTTGTTAAAGGAGAGTATCAAGTCATGGTGGATAAGTTAAAAGGGGGAGGGAGTAAATTAGT
AGCTTGACTTGGTCAAAAGGAGACAACTAGCCAATTATTATGGTTTGATTCTTCAAAGCCAAATAGTTGG
CTCTAAATGACTATTTTATTGTTGGAATAAGAGTTGACTTGACAGACTTCATTATCAAATGAAAAGAAAC
ACAAGGAAAGCATCAAATTAACATGATCCAAGCAAGCTCTAAACTTTTTTGGCTCAATATGCAAATCTTC
TTCCTAATTAGCCTGTCTTTCCAAGAAACCTTACAATTTTCAGGCACGGAACGGTCCACATTACTAAGCTT
GAAACAACACTGGAGGGATTCGGAGTTTTTCCAGTCGTGGAATTTGAATTCTTCAGAATGCAATTGGTTA
GGAGTTTCATGCATCAATGACATGGTAGAGACACAATTAAATCTTGGAGAAAAGAACATTACTGGAACA
ATTCCATCAACAATAATATGTCAGCTCAACAACCTTACCTTCATTGATCTCTCCAACAACAACATATCAGG
AACTATACCACTAAGCCTGAAAGATTGCTCAATGTTACAACATTTGGACTTGTTCAATAATTCTTTGAGCG
GCCGGATTCCAGGTGTTGTTGTTTGGGATGAAAACACTAGTCAATTTGTATCTTAATGGTAACATGTTGT
CCGGTGAAATGCCAAAGCAAATATCAGCATCCCAACTGAAAAATCTTGATCTTTCTGAGAACCACTTGAA
CGGTTCGATTCCAGAAGCTATTGGTAACTTGAAAAATCTTGTGAAATTGGATCTGTCACATAATTCTCTA
AGTGGTTCTATTACAAGCAAGTTGTTTCAATTGAACCAATTGAGTCACTTGTCGCTAAGTTACAACTACTT
ATCTGGTGTATTACCTAATGCAATGAACTTGTTTAGTTTGTATGATATGGATCTTTCTCATAACCAATTGA
CTGGTTCTATTCCAAAGGGACTTGCAAGTTCAACTGGACTGGATGCTTTGGATTTGTCGTATAATCAGTT

ATCAGGAAACATTTCAGAAAGTATAAAACATCTTAGGCCTAGAATTAGTTTAAGGCTTTGTTCCAACAAA
TTCTCAGGAAGAATTTCTTCTGAATTTGTTAAGCTAACATATGAAGAGAATTGCTTTGATGAGTCCAATCT
TTCTTCTACATCCAATAGCTTATCTATCCCTGGGCTACCAAGCTTTCTCTCTGGTGATGAGGTTCAAAAAA
TTTCGAGATCACAACACTTGATTATTCCTATTGTAGGAGTTGGGGTAGCTATACTGTTATTATGGATCTTT
CACAGAACCAGAAAGCATTGGTGGAAAACAAAGATGCAGAATGTCAAAGATAACATAAAGTTCATTTCA
TTTCAAAAGTTGAAGGTGACTACAGAAGACATATTATCTAGCTTGAAAGACGAAAACATAATAGGAAAT
GGAGGATCAGGGAAGGTCTATCGAGTTGTGATAGACCAAACAGGCAATACTTATGCTGTTAAAAGCAT
AGGGCATGGAGAAAAATCAGGTGGAAGACCCCAAAAGGAGTTCCTGGCAGAAGTTAGAACACTTGGTA
GCATTCGACACAACAACATTGTCAAGCTCATGTGTTGCATCTCAAGTTTAGACAGAAAGCTTCTAGTCTA
TGAATACTTTGAGAAACAAAGCCTAGACAAGTGGCTTCACAGAAAGAAAAGAGCAGCGTCACCCGGTC
AAAGTAGTACCCCGACCCTGGATTGGCAAAAGAGATTAAAAATAGCCACTGGTGCAGCTCAAGGACTCA
GCTATATGCACCACGACTGTACTCGACCCATCATTCATAGAGACATTAAGTCCAGCAATATACTTCTGGA
CTCAGAATTCAATGCAAAAATAGCAGATTTTGGACTAGCAAAAATACTATCCCGGAGGGATGATGATCC
TGAGACAGCTTCTGCTATTGCTGGAACATTTGGTTACATTGCACCAGGTATATTACAGTTCAAATATATAT
CGTACCTCGTGAATTCTCAAAAGAATTTATCCAACTCTAATATTTTTTAATGTCGGGTCCTCACAGAGTAT
GCCTCGACATTCAGAGTGAATATAAAGACAGATATATATAGCTTCGGAGTGGTTCTATTAGAATTAACAA
CAGGGAGACAACCCATTCTCAGAGAAGAGCAAATGAATCTAGCACAATGGGCTCAACAGCGTTACAGA
GATGGGAATTTCATTGTTGAGGCCCTTGATGAAGAAATCATGGAAACAAGTAATGTGGAACAAATGAG
AGGTGTTTTTAAACTAGGACTGATGTGTACTGGAGCAACACCATACGGTAGGCCGTCAATGAAGGAGGT
CTGCAACATTCTTCAAAGCTTCAGAGACACCATATTTTGAGAAAACAGAATGTTTAGCCTTACATGGATA
AATGTAGTTGTATTTTTTTAACAGAATGAGAAAGTCTCAATAGCCTCAGCTGGTTTAAGTGGTGTCAAAC
ACAAGGGCTTGTAAGCTTTACCTTAGAATGTTACTCCTTGTTCATAATTGAGATGTAGCGAGGCAAGAGC
TAAGTGTCTTGGCAATTACAGTGTTTGTATATATCATTCGGGAGATTAGACAGATTTTAAGCACACTGAG
CATTAAGGTCTAACAATGCTTTAGGAAGGATAATCATGATGCAGATGCTTTAGCTAAATGAGTAATGGA
GGTTAGACCCATATCTTTTTTTAATTCTTCAGACAACCTGCCTATTCCCAACCAATGTGGGACTTTTACCCA
CTCTAACACGGGAAGTAGATTTCTTTCATGAATAGAGATGCTGGTAACAGAACCATTTCTGCAGCCTATA
GCATACCAACTGCAGTGTGAGGAACTGGAATCCAATAATTGATGAAATCCCCATAGTTGCTTCAGCTCCA
ACAAAATGCTCTTCTCATTTTCGTAGACGTCACTCTGTAAACCACCCTGGAAATGCATAAAGGCAAGTAA
GGAAACAATGAAATGGTAAACTTGTTTCCTGGGAATTGAAAATGTAGATTTTACAACATGAACATGGTT

AGTTTTCATGGTTCCTTTGGTTCGATTGTACTAGCTACCTCATTCTCTACAATCTCTTGTTTTATTTTTTTCA
AGTGTTTTGATTGGATAGTTAATTAGCTTTGAAGAAACCGTGTATCATACAACACAAGTCAGTCAACATG
GACAAAAATTGTTGCTGAC
>PGSC0003DMG402002925 LRR-RLKs
GTTCACTTCTCTTTAGTTTTTACTTCGATTTTTCTCCGGAGCTCCGGTGGTCGGAAAGTTGATCGGCGTCG
GAAAACAAGTAGGATTATAAATTTATAATCACAATCGAGGTAAATTTGCTTTTTTCTCCTTAATTTTGCCC
GTTGATCATTCATTTTTTCTGAATTTTCTGTGTTTTAATTAAAGTTTAATTTTTCCTGTGATTCTGTAGTTAA
AGTTTGAAGCTTTGGGAAGTAGTTTGAGTTAGAGATGAAAATGGTGAACTTTTTTTTTACTTGGAATTGA
AACCCCACCCCCCACCCCACCCCATAATTGGAAGAAAAATGTAGGGGTGAAAGTTGAATTTGGAGTTTTT
TGTGATTTTGGAGTTGGTTTGAATGTGTATCTTTCTGGTTTTTATGTTTAAAGTTTGGAGCTTTTTTGCTTA
GGGAAGTAGTTTGAGCTGGAGATGAAAACATGTTTTGTTTAATTTGGAATTGAAACAGAAAAATTTGTC
TTGTTTGTTTGATTTTTGATAAGTCTGAAACTAATAAAATGAAAGAGGTTAGAATGATGACATTTTGTTCT
ATCATTATTGGAAGAAAGATGTAGATGTAGGGGTGAAAGTTGAATTTGGAGTTATTCATCTCTTTTATTT
TATTAGTTTTATTTTTTTTTTTGATTTTTTTTTCTGTGGTTTTGGGAAGTTCAGCATATTGGTTTATGCTTTA
AGTTTGGATTCTTTTGGCTTATGGTGATGTTTTGTTTTACTTGGAAATAAAAACCCCATATTTGGAAGAAA
AATGTAGAGGGTCAAAGTTGAATTTGGAGCTTTTTTTCCTTCTTTTGTCTTGTTAGTTTTATTTTTCGACTA
GTTCTTTTTTTTCTGTGATGGTGGTGTTGGTTGAAGGTTTATCTTGATGATTTATGTTTAGTGTTTGGAGC
TTTTTTCTCCCAAATTTTAAACATAAACCAAGAAGATAAACTTTCAACCAACTCCAAAATTACAGAGAAAA
AAAAGAAGCTAATCAAAAATAGAAATAATAAGAAAAATGAGAAAATAAAACTCCAATTTCAACTTTCAC
CACTACATTTTTTCTTCCAATTATAGGGGGTTCAATTTCAAGAAGAACAAAAGGTCACCGTTCTTATCTTT
GTGCTGGAGATAAGAATAGTAACCTTTTGTTTTACTTGAAATTGAACCCCCTATAATTGGAAGAAAAATG
TAGGGGTGACATCCATGAGGTCTCAGGTTGAATACCCCAAAGACAAAAAACACTAGGTGATTTCTTTCC
ATCTGTCCTAGCATTGTTAGATAGAGTTGTTTGGTACTTGTTGCTAGTGAAAGTGACAGGTATCTCGTGG
AATTAGTTGAGGTGCATGCAAGCTGGTCTGGACACCAAGGTTATATAAAAAGAAGAGTTGAAAGTAGC
TCACCCTGTGAGGCTACTTAGAAGAATTGATTTTTGATGTACCTAACCTTTTACATTTTTCATGTTTTGCAG
TGTCTGTGGAGGTGTGGTTTGATTCTAGAAATCCTCGTGCAAAAATAATAAGTCGGATTGGAACTTTTCT
GCTGTAGCTGCTTTGTCAGGAAAAATGATGGAGGCTCCAAAATCTTTTGGACTTACAGGCTTGAATCAA
AACCTGTCGCAGAGCTTTTACCATAAGCTTGGCGAGGGGTCAAACATGTCTACAGAGAGTTATGGGATG
AGCAATGCTGGAGGTTCTGTTGCAATGTCGGTGGATAACAGCAGTGTTGGATCAAATGATTCTCACACT

CATATTTTAAACCACCAGGACAACCACATCCACAATAACTATTCTGTTGCTGCTAGCATAGTCAGGCGGA
GAGTTTCTAGATTGAGCGATGATGCCCTCGCTCAAGCCTTGGTTGATCCTCAGTTTCCCACCATTGGGCT
TGAAATTTATGATGAATGGACAATTGACTTGAGGAGGCTTACCATTGGACAAGCTTTTGCTCAAGGATCT
TTCGGGAAACTGTATAAGGGAACTTATAATGGTGAAGATGTTGCCATCAAGCTTCTGGAGAGGCCAGAA
AATGATCTTGATAGGGCTCATCTTATGGAGCAACAATTTCAGCAGGAGGTCATGATGTTGGCAAATTTG
AAGCATCAAAATATAGTACGATTTATTGGTGCATGCCGTAAACCCATGGTGTGGTGTATTGTGACTGAAT
ATGCAAGAGGGGGATCAGTGCGTCAGTTCCTCACAAGGCGGCAAAACCGAGCTGTGCCATTGAAGTTA
GCAGTTAAGCAGGCCTTGCATGTGGCAAGGGGGATGGAGTATGTGCATGGCCTGAATCTGATTCACCG
GGATCTGAAATCCGACAATCTGCTGATTGCTGCTGACAAGTCAATCAAGATTGCAGACTTTGGTGTTGCT
CGAATTGAGGTGCTGACTGAAGGAATGACACCAGAAACTGGCACTTACCGCTGGATGGCTCCGTAAGC
ATCTAGACTCTTTAGCAGCCTTGTTAAATCTGGTGTCTTGATCCAGTTTGGGCTTATTGCCAAACCCTATA
ATAGAGCTCTCTCTGTCTGCATTACTTTCTGTATTTTATTTCTTTTTTAAAACTCCTCTGATATGTGTTACGT
GAGTCGAGCATCTTTCAAAAAAAAACCTCTCTACCTCCACGTGGGAAGGGTAAGGTCTGCTCTCAGTCTAC
CCACCCCAGACCCCACTGAGTGGGATTTCACTGGGTATGTTGTTGTTGTTGTAGCATGCTGGGTTCCCCT
TTATGGAACAAAATTTTTTAGAACTAAAGTGAAAAGCATGGAGGTTGATGGGTTCAAGTCCTGTATGTC
CTTTTGATGGCACCTCACTGACTCACTGATTGTGATTATATTTTCTATTGTATACGTGCTGTTGCTAGGAG
GGAGAAATTATTTGTTTCAGTTAGCTTTTTAACATAGTTATGTGCCTGTTAATGAAGGTTAAAGAGTTGA
GTGACTTTATTTTCTCTTTCAATTTTTTTCCCTCCTTTTCCTCTTCCTTCTTGTCCCTGATTTGTAACTTGATT
CAAGTTAGTCACATCCAAGGGTGTGGCTTAGTTGTCAATGAAGTAGGTGCAAACCGTGGAGATTAGGGT
TCAATCACACCAGAAGTGCTTTCTTCCCATATGCCTAAACCTTAAACCTTGGTGGGTATCTATTCATGTCT
GTTTATTGATCCAAATAAATGGTAAAGTGCCTTAGTTGATAACTCCCAGCACCAACCACGTCATATTCCAT
GAACTTATCATCCCAAAGTCATTATTAACAGGAAAAGAACTAATCTTAAAAAAAAAATAGTTCTTTTTCTG
TCAATAATCACTTTGAGCTGATAAGGTTCATGGAATATGACGTGGTTGGTGCTGGGAGTTATCAACTAA
GGCACTTTACCATTTATTTGGATGCATTCTCCCCGATGAAGATGATAAGTTCTACATTGTGGTGGAAATTT
TGGCCTTTTAACAGGCATCTTAGAGAAGCATGGGAGGCTTTACTTCAAATCTTAGAAGAGGCACAAAAA
CACTAGGTGATTTCACCTGATACTTGTGTTAGTGGGGTATTACAAGTACCTCATAAAATCATTGTCGGCA
TGCACTAGTGATCTTCGAGGATTTCTCGATTATCAAAAAGAAAAATCTAAATAAGTTCTATTCTTAGACAT
AATTAAGTTTCAACACTTTTATGCCTCCCAACTTATTTATGCAGTTGTTATAATTGATATATTTAAGCGACT
AATTAATGGATTCAAAAACAAAAACTGGTTACACCATAACTCAAGTATGGCTCTTTTAGTTTCTTTGCTAG

CTCTGAGTTGGAAAGAAGTTCGTTCTTCTTTGGCTGTTTCCGAAAGGAATTCCAGGAGAGGCCTTACAAA

AACTAGACTCTACAACGATAATGTGATTCTCCACTGAGTTACTACATAGACTTTTTCCATCATAAGATCAT

CTCAATCTCTACGGAAAGGTAGAAGTGGAATATTTGGCTCAATCATCCATGAATCTAAATCATTTGTTGG

AACCTTCGGTCATTCTTTACGGAGAGACCTCTTTTATAAAGCATAAAAAGCAACTAGGTGATGTTTCTAG

AATCTACAACGATTATGTGGATTCTCCATTGAGTTGCTACGTAGACTTGTTCCATCCATAAGATTATCTCA

ATCTCTACGGAAACGTAGAAGTAAAATACTTGGCTCAATCTTCTCACGACCCTAAATCATTCGTTGTAACC

TCCGAGCATTCTTTCTGGAGAGAAGTCTTATTAAAAGCAAAAGGCGATTCATGGAGTTTGAAGTGAAAA

GCTAGGAGTGAAGCATGCAAGTTTTGGGGAAGTAAAAAGAAGTACCAAACACATACAAATTTAGTCATC

TTCTCATGAACCTGAATCGTTTGTTGAAACCTCCAATTACCGAGAGTAGTGTTTTTCGAATCGAGAAATG

CAAAAAATGGCAAGGTCCATGATTTTGAAGCAAAAAGAGAGAAGTGAAGCGCACTAGCTATAAGAAGT

AAGTAATGCCAGACATAGAAGGATTTACTACTATAAAGATGCAATTAGAATAACTGATCTTTTCATCCAA

TTTACGGTAATATTGATATTAGTCTAGCTCCTCAAAGTCGAGATTCAAAAAAGTAGACCCTTTCATTAGG

ATCGTTTTACATGTCATTGTTTAAAGCCCACACTTCTCTGAAATGCATGGCTTCAAGTGGTAACCCTTACT

TCAAATTGCTTAAAGCGATGGCTTTGAATAACACTGCAGGAGAGATGTGATACATGAAGAAATCAATCT

CCGGTAAAAAGAATGATAAGAATGCCTTATTTGGTATTTAGTCTTACAACACGTGTTGCTAGACCTGATT

TAACGAATGCCTTATATAAGTTGAAGTAAGTCTTTTAGAAACAAAGGGAGCATTAAAACCCCTTAGTAAT

CAATCAACATGAACAACTTGTTAAGTCAGGGAGAGCCTACGTCAGGCCGGTTATGACTTAGTATTCCCG

ACGTCTTACTTTTTGTGCCTCACATACTATGTTATACCGGTCCTTTGCATTACACACCCGTTGAAATGGGT

GTGTCGTGGCTGTGTGTACTCTTTAAAATACACAAAAAAAATTAACAAAAAAAGAAAAATGCACGTACTT

ATAATGATACTCTCGTACAACTGATAAAATTCATACTCACAAATGAGGCCATACTTAGAAGCGGGTCTAG

AATATGAAGTTTATGGGCTCCCAACGATCTCAAATTAAAAATAAAAAATTATTATAAGTGGTCAAAGGG

AATCAGACCCTCAACGAGGAGGGCAACAAAGCATAAGCAAGTTCCATTATGCCGCTGGAGCAAGAGCT

TACTTTGTTGGTAGATCCCAACAAATAGTTTTTATATATTTAATAGATTTTTTCAATAAAAATACAAAAGTT

AGTGGATTCCCCAAAACCTACACCCGATACTCTAGGCCCACCCATGTCCAACAACAACAACAATGTACCC

AGTGCAATCCTACAAGTGGGATTTGAGGAGGGTAGAATGTACGCAAATCTTACTTTTACCTTGTAGAGA

TAGAGAGGCTTATGAAAGACCCTCGTCTTTAAATAAACATGCCAAAACAGTTCAAAAGTGGGATAAAAA

TATGGGAGCAATCAGAACGACACGTAATGCAAAATGGGAAGTGTTACATGACAAACAAGAGAAAGAAC

GGTAACGAAAACAAAATAATGCATAATCAATGCACAAGAAACTACACGAGTAGTGCTAATACTACTGGT

AAGAACACCATTGAAAGCCTATCTCGCAGGGAAGTGAGAGAATGCTTAATTACCAACTAACCTTCTACCC

TAATATGCGACCTCCATACTCTCATATCTAACACCATGTCCTCAGTAAGATCCAGATGCACTCTATCTAAT
CACTTACTCCTAATACTTCATCGGCCTGCCTTTACCTCTCCTGGCATCTTCCACTACCAATTGCTCACACCT
CCTCACTGGAGCGTCTACACTCCTCCTCCTCACATGTTCACTTAGTCTCACTTCCCTCATTTTGTCCAACAT
GGAGGCCACTCCTATCTTGTATCAGATATCTCAATTCTTAATCTTGTCTCTCTTAGTATGCCCGCACATCTA
CCTCAACATCCTCATCTCAACGACTTTCTTCTTCTGAACATGAGATTTCTTAATAGAACAACACTCAGCCCC
GTAAAACATGGTCGGTCTAACGTCTACTCTGTAGAACTTTCCCGTCCATACTCCATATAATATGGATCACA
TAGTACCTTTAGTTGCTGAAGAAGTAATATTTGAAACATTTCAAAATTTTGGATTTCTCTACTAGAATTCA
TTACCTCTAAGAATGATAGGAAAAACGAATGTATCAAATCTATTTAGGGGGAATGGGTGTCCACATGGG
CTTGATTTGCCAAGTCTGTTCATATGTATTATGGAACACCAAATTAATTTTAGAAGTAATGCATAATTAGT
ACGTAAGAATATATTTTACAATTTGTTTTATGTATTGCGGTGCATGTATAGGCTGACTATTGTATCAATTC
ATGCATTTGCGCTTGATTGTTTAACTGTTGAAAATCTTTGTTTTATTGATATCTATCAGAAGATCTCCTTGG
TTGGCTCAATCTGACATAGAAGGTGCAAAATAATGACAACGAAGATAATTATACTTCAATTTGCTTAGTT
TATTGTTCCATTGATACATGGGGTTTTCCTGTATTTAGAGTCTTATTTAAGAATGGGTATATTTAGTGAAT
CTTTCGAGAAATTTTTTCTTATCATGATTTTCCTGGAGTCTTTAGGATTCTGCTCTTGGAAGTTCAGTTATT
TGTTTCTCAGGCATATATGGCAGTTAGAATTTAAACCTTGAATTAGATCACCATTTCAGGATGAGAGAGA
GAATATGGTATAGATTGATTGCTTTATCCAGTTTATGAGTATGGATTACTGATGATAGAACTGGCATTTC
TTGATTTCCTTTGTCCCGGACAGAAGTATCACCATTAGTTTCTGTTTAGTTTTGCTCCTTACATAATGTTGG
AGACGAAAGTATATTCTTTATAAGCTAAGAGAGTTGGAGATTAAGCTTTTGATTTCTTGAGATAATGTGA
CAAACTGAGAAGTTTCCATTTCTATTAGGACATTTCTTTCAGACTTGAATCAAAGTAAGTTTTCATGTCTG
CCTGAAATATTGTTGAAATGCTTGCGATCAGAAAGGATTTAGGAGGTTGACCTTGTGAGTGGTGATAAT
TTTGGTGACCTAGTGCAGTCGTGAAACACCACATCCCTGCGATGGTGGATGATGTAGCAGTGCAATATA
GTTCTTCTCTGTGTGATAATAGGCTAGTATGCATGCTAGACTTTCCTTGGCGTAATAGACAGAAGAATCT
GCTTGCATTTGTTAATTTGATGTGATAATGAATGATCTTGCCAAAAAGAAAATGATATTGAGCGATCTTT
GCCAAAACAGGTGGGGATCGGATCTCAATATCTGGTAAAACTCGTATACGTGCAGCCAGTCTCTGACAT
TTGATTCTGATTGACTGAGAAATGAAGTTACTTTTCAAGTTCACTCTTGAGAAATTAGTTATTCACATTCT
GCAGATCCTATCTTGTTTAACATTGTGAATTTTTTGGCTAGTGTGTCTGGTTTCTTCTCTTTTCAGTAAAAG
AAAAACGATGTTCAAAGTTGTTAGCATCTGTGTTTCGTTGTGGACCGCTAAGTGTTTGACTTTGTTTTGTA
GGGAGATGATCCAGCACCGGTCATACACCCAGAAAGTTGATGTTTATAGTTTTGGCATTGTTCTGTGGG
AGCTCATAACTGGGTTGCTTCCTTTCCAGAACATGACTGCTGTGCAGGCTGCTTTTGCTGTTGTCAACAA

AGGCGTCCGTCCAACAATCCCCATTGATTGTTTGCCTGTCTTATCTGATATCATGACCTGCTGCTGGGATT
CTGACCCCGATAATAGGCCAACTTTCTCTCAGGTGGTCAAGATGCTTGAGGCAGCTGAGACAGAAATCA
TGACTAATATCAGAAAGGCACGTTTCAGGTGCTGCATTCATCCTACGGCTACAGGTTGATTCGGGAAAG
AGGAAACTGGAGGCAAAAAAATGTTGAATGGCAAAACTGATTTTGAGTCTGCCATGTGTATCTATCAGG
CACAATTTCTTTTCAGATAGAAAAAGGGTTATAGTTATGAGAAAAAACTAAACATTTCTCTGTTGTATTTT
GTTTCTTTTCTTTAAACTCTGGATTTAAGACAACGACTAGTTGTTAATACAGGATATCTGATGTGCATCTA
TGCATGTCAGATTTTGGCTCTGTGTTTGCTGAATTTGTTCAAAGTTATATCAGG
>PGSC0003DMG402004902 LRR-RLKs
TCCTATTAACTATTCCGATCCGCCATTAATCTAATTCTTCAGTCAGCTTTACTGACCATATATTTATAGATC
ATCCATCGGTATCATTGATCATATACAAGAAGATTTGCAGATCGTCCTTTTCTTTCTCCTATTAATTTGTCA
AGAGGATTACTCTCTCTTTTTTCTGATAAAGCCTTGATTTTTTTTCGAGTTCAGAGACATAGACGAATTTA
GAATTTAAATTTGATGGATCAACCTTTTAAGTTATGACTATTGAATCCATCTGATCACATTATGAGTTCGA
AATTTAATGTTACAGTACTATTTGATAAAATTCCGTATTAAAATATTGGGTTCAATTGAACGCGAAGAAG
GTGGGGGCAGGATGAGCTTGGTGGTAAAGGTGGCCCTACTCTTCTTCATTTTGCATGGCTGCAGCTTCA
TCAATGCAGACACAGATCCATCCGACGGTCAGCTTAATTCTACTGCTACGTAGCTTAATTATAAAGAAAT
TAATATATTCACGGCACTTATGATTAATTTGGAAGTTACTAACAAAGTTTCTCTGTAAATAAGAAAGAATT
TCGTTGTCAACTTATAATGTGAGCGTGGATCACTTGGTGAACAACTTTTTTTTGTCTGGTGTTCGATATCT
TGCTTTATTGGCTTGCACTAAATCGAATTTGTCTATCCTAAAACTCGTTAGAGTCCTATCTGTAATTTTTCA
TTCTCAAAACTCAAACCTGAGACTTTTTATAGCTAAGCTAGGTTGCTAATTTTCACTAAAAAGCCGGACG
ATAAGCCAAAGAGTTCACAACATGCAAAAAAAAAAGAGTTATAAAATTTAGTAACAAAATAGTGGAATT
CGATTTATATATTTTTGGAGCTGTTCTTAGTCTCTGTGTGTATATTTATATGTTCTCAATAATTTCGTTGGT
GGCGTGCAACTTTGGTTATCAGGTTCTGCCCTGAGAGTGATGTATAGCTCCCTAAATTCACCGGGGCAG
CTTACAAAATGGAGCTCATCAGACGGTGGTGATCCTTGTGGAGAATCCTGGAAAGGCATTACTTGCTCG
GGCAATAGAGTAACTGAAATGTAATTATGTTCTCTTCGCCATCCTCCTATAGTTTTTCGATATACACTTTAT
TTTTGGTAACTTCTCGTTAGTTAATTGATTTGGCAATTGCTGATTTTCAGTTTTCGTTTCATTTTCCTTCAGC
CAGATATCCGGTTTAGGACTATCCGGATCAATGGGATACCAACTAATGAGTTTAACATCCGTAACCAACT
TGTAAGATGATCAATGGTTTCAATTACTATTTCCTTACTTCGTTCTGTTATTTGTATTTACATCGTAAAATG
TTGTAATTTGCAGTGACATAAGCAACAATAATCTCGGAAACCAGATACCTTACCAGCTTCCTCCAAATGT
AAAGAGACTGTATGTGTTTTTATGCCTAATTTCTTCTTTCTTTGCTCAATTCCAGGCTCTAGTGTTTTTTAC

AAAGTTGCTTACAAAATCTTTCTGTCATTTGCCTCGTATTGCAGTAACCTTGCTGGTAATGGTTTTACCGG
TGTCCTACCTTATTCCGTTTCACAAATAACTTCCCTTCAATACTTGTAAGTGAATTTTTAAAATTAAATTTA
CATGTATAGTATTATTACACTCGTTGTTCTGATTTTCTCTCCGTTGTATGTGCCTGATTGTAGAAATGTCA
GTCATAATCAAATTCAAGGAGAACTAAATGACATGTTTGGATCACTTTCTTCTCTCAACACATTGTAAGG
CCTTGTCTCGATTTCTTCCTTTTCTTATACAGATTGATATTACCCTGTGATGATTGAGATTTTCAATGTATC
TTGTGCTATTCTCGATAGATAAATATAGTATCTCTTCTGTTTATCATGTGAACTGTTCTCACGGAGTGAAA
GTATCTTTTCTTCTTCTTGGTTGATTATATTATGTTATATCCCGTGTTTCTTACTTTGATTGTTTACATTTAT
GCTTCAGAGATATCTCTTTCAATTCAATGACCGGTAAACTTCCTCAAAGCTTTAAGTCACTGACTAGCATG
AAGAAAATGTAAGACGCGTATCTTCTGATTCATTATTGTACTGTATTGGTCCAGTGTCATTGAACTTTCTA
GTATTGTTATGTTGTGCAGCTACCTGCAGAACAACCAGTTCACTGGAAATATTGATGTCCTCGCCAATCTT
CCTCTTGACGATCTGTGAGTTATGCAACATCTTTGTGTGTGTGTGAATGTTATCGATCTCTGAATGTTCCA
TTTCAAGGCTTTCGCAGTTACTATATTGTTGATTCCTTTATGCTTTAGGAATATTGAAAACAATCAGTTTAC
TGGTGGGATTCCAGAGAAGTTTAAAGGGATAATGCAGTGAGTGTTTCAGTCTGCAAATTATTTTTCATCC
TTGAATATTTGTAGAAAATAACTTTCTAACTGAATTCGCGATTTGTGATACAATTGTAGAAACTCTAACAG
TAACACAGGGAGCTCAGGGCCTGCAGCCCCTGCGAACAAGTCCACTCAAAGTCACAAATCAGGAGGCA
ATGGCAATTCTTCAAGCGGTGGAGGTAAAAGTGATAGTGGTGAGAAATCTGGTATCGGTGGTGGAGGC
GTTGCAGGCATAGTTATATCAGTTTCAGTGGTTGGAGCAGTTGCAGTAATCTGTATTATCAAGAAAAGA
CGCAGGAAGTCGTCAACAGACATAGAAAAACTTGACGTTCAGCCATTTGTTCCTGAGTCACATGAAGTA
CAAGGTATATAATTTTATGACTTCTTTTTTGGGTCAATTGAGAACATAGCTGGTTAGTTTGACTGATACTT
CAATATCTCAACATTTATACACTCTTATGGTGGATAGGATCCTTTTCTTGCCATGTTATCTAATTTGAATAT
AGAGTTGAATTTATTGTGGTACACAAAGGGGAGCCCTGGCGTAACTAGTAAAGTTACTTCCATGTGACT
AGGTTCGAGCTGTGGAAACATCCTCTTGCAGAAATGCAGGGTAAGGTTGCATACAATAGACCATTATGG
TCCGACCCTTCCTCGTATCCCACATATAGCGGGAGCTGCACCAGGCTGTCCTATTGCAAATGTAGTTGTC
TTTATATAAACACACTTTTAACTCTGGATGCAAGTTCCAGCAAAGATACTGAATCACCTTAGGTGAATTTT
GGAAAATTAGTTTTCCTTTTGAACTCGTCGTCCTTCATTTCATGTTCAAGTGAGCATGTCTATAATGATGA
ATTATCTTTCCCTTTTGCAGCTTCATTTGTAACCTGGTATTTCATACATCTTAAGCCTTACCTCAATTGTTTT
AAGTTCTGTAGCATCATTCCATCAGAGTTCCAGCTAATATTTTCCCTTTTCTGGTTACTAGTATTATTGATT
TTTAATCATGTGTTAGTTTTGGAACCTATATTTTTCTATTCATCACCTATTTTGGCTGCAGATACGAAACTC
AGTCAAGCTCCGTCCACAATAAGCATGGAAGCTACTGAAACTCCCACTATAGTATCTCTAAGACCTCCAC

CTATTTATACTCATAAATCCATTGGTGGAGATGACATTTCAGAAAAACTCATTGTTCCACAGAAGAAAAC
TCGTACAGCTGAAAGAAAAGTTGTGCAATTTTCAATTGAAGACGTACAGATAGCTACTGACAGCTTCAG
TGTAGAAAACCTTATTGGTGACGGATCTATTGGAAGTGTATATCGAGCTCACTTTGACAATGGCAAAGT
AATATCTATTAGTTCTGTTTGTCGTTTAACTGTTTCTTGTTGCAGCTAACATTACCTATGCTGATGTAAGTG
ATTCTGATCGTTTAAGTTCCTATTCCATAGGTTCTCGCTGTCAAGAAACTTTATTCATCTGAGCTCCGGAA
TCCTGAAGATTTTCTAAAGATGATTCCTGAAATATCCCAGCTGGATCATCCAAATGTAACAGACCTAATT
GGTTATTGCTCAGAAGATGAGCAGCACCTGCTAGTTTATGAATTCCACAGAAATGGTTCTCTGCATGATT
TCCTGCATCGGATGGATGAAGACAACTCGCCACTAGCGTGGGATAGCAGAGTTCAGATTGCACTTGGCA
CAGCAAGAGCACTAGAGTGAGTTTCATAATTTACAAAATTACTTTCATTAGGCATCACATAAGATTAATG
CAGCTTGGAGAAAACTTTTGTTTGCTTGGTTTCAGATATTTTTTATGGTAAACTACCCTATCTATCATAAA
TCTTCTAGTTTTCCTTAAAACATTGATGATAGTAAGCATGTAGAAGTGTTTCAATAATATTTCGAATAGAA
AAGCAATTATATTGTTTTAAGTTGTAGAACACCGCGTATGCTGGCTTTCCACTAGATCTAATCAGAATAA
ATTTTCTATTTTGGCCGCGACCTCTCCAATTCTTATGAGTAGGTTTATTCTAGTAGCTAAATCTAAGCTTCC
TTTAATTTGCAGGTATCTACATGAAGCTTGTTCTCCATCTTTAGTTCATAAAAATATAAAATCTGAAAATA
TTTTACTTGATGCTGATCTCAATCCCCATCTATCAGACAGTGGGTTAGCAAACCTTATGGCTGATGCAGAT
CAGGTAACACTTTATAAATTTGTTGTTTTGCCTTACTCTAAAGTATAATTTCCTAAAACTCCACATCAGTAT
CTTCTTAATGGCTTCAACTTGGACCCCTTATTTTTGCTCCGTGTAGGGATTGAACCATAATATAGAGTCTG
GATATGGCGCACCGGAGGCTGCTATATCTGGGAAGTGTACCACCAAGAGTGATGTATACAGCTTTGGA
GTTGTTATGTTGGAACTTTGTACTGGTCGAAAACCTTTCGATAGGTAAATGTTAATTTAATGATATTGAAC
CCAAAATCTGTTTAAGACAAATAATAATATACGTGTGTTCTTCTCTTCTCCACTCAGCTCAAGATCAAGAT
CTGAACAGTCTTTAGTTAGATGGGCAACCCCTCAGCTTCATGATATTGATGCCCTGGAAAAGATGGTTGA
TCCAGCTCTCTCTGGATTATATTCTGTCAAATCTCTTTCCCGGTTTGCTGATGTTATTGCTCTCTGTGTTCA
GGTAATACTCTCGTTGTTTCAATTCATGTGACTAGACACAAAGTTTAAGATAGAAATGCAAACATTTGAA
ACGTGTGGTCTTAAACATGACATAACATCTCATTAGAGTAATATGAGAAGTAGTTCGTTCATTTTGTACT
GAAAGCTGCTACATGCCATGTGTTCAATTTTCAATAGAGTACTCCCCTAAATCGCGCTCACTATCACAATG
GTTTGTGTATGTTGTGTCAGCTTGAGCCTGAGTTTCGGCCACCTATGTCTGAAGTGGTTCAAGCATTAGT
CCGGCTAGTGCAACGAGCAAATATGAGCAAGAGGTTACACAATAATGATCAGGACTGAAGAGATTTGC
TCAAGACCTGCTAATTGTTACACCAAATGGTTCTCTTAAACTGTTTGCTGCTATGGGAGATCATCTGATGC
TGATCAGCCCAGCAATATCTTCTTCGGTGCAGTTGTATTTGATTAAATACCATATGCTAAAATATTTCTCC

TACATAAGCTCCTCCACTGTTGTAGCTGATTCCCCATTTGTTATGTTGAGCTAA

>PGSC0003DMG402005482 LRR-RLKs

AAATGCCAATTGAATAAATATAGTACAAAGGATAAAAAGAAGAAAAGAAAGGATTTTTCACACCAAAAA

GCACAGAATAAACCAGCTAGCTACCTCATTCTTGAATACATTGTCTGTAAGAAGTATGGTTTTTTCTCTAG

ACTTCAAGATTCCAATGCCATTCTGTTCTTTTGTTTTCTATCTAATTACCCTCCTTTCATTCATGTTCAATAT

CTCACATGCCTTGAAGTTACAGAATGGTAATGAAACTGATATCCAAGCCTTACTTGCCATCAAAGAAAAG

GTAACTCAAGATCCTCATGGTATTTTTACTTCATGGAATAATTCAGTCCATTTCTGCAGCTGGGAAGGTGT

CACTTGTGGCCATCTTCATCAAAGAGTCACTAAACTGCACTTAACATCACTTGACTTAGTAGGTACTTTGT

CACCTTTCATAGGAAATCTTACTTTTCTTACTAGTCTCAAACTTGAGCTCAACAATTTCCATGGAAAAATC

CCACCACAGGTTGGTGGTTTGTTTAGGCTTCAGCATCTTAGTTTGACTAATAACTCATTTTCTGGAGAGAT

ACCTGTAAATCTTTCTAGTTGTTTGAATCTTGTTATACTTGGTGTTGGTTGGAATCAGTTGTCTGGAAAGA

TTCCATTTGAACTTGGTTCATTGCAAAAGCTTGAGAGATTGCAAGTTCATAACAACAACCTCAATGGACC

AATCCCGGAAACCTTAGGTAATCTTTCCGCAATCAAATCACTTTCCTTATCGGTTAACAATTTGGAAGGA

ACTATTCCAAGTTCTCTTAGTCAAGTGAAGACATTGAATGTTCTTGGTTTGGGTATAAATAAGTTATCTGG

TATTGTACCTGCTGAAATTTTTAATCTTTCATCTCTTGAGATATTCACAGTTTGTTATAATCAGTTGTATGG

AACTTTGCCATCAGACTTTGGTTTAAGTCTTCTTAAGTTGAAAGTTCTTAAGATTGGTCATAACTGGTTTA

CTGGACCCCTTCCAAAGTCATTATCTAATGCTTCAAATCTAGTTGAACTTGATGCATATGGTAGCAATTTC

ACTGGCAAAGTGTCAATTGACTTCGGGGGGTTATCGGATCTTTGGTGGCTGATTCTTGCATCAAATTCCA

TTGGAACAGGAGAAGTTGATGACTTGAGTTTTTTCAATTCTTTGAGTAGATGCAGAAACCTGAAAGTTCT

GGATTTGAGTGATTGCAAATTTGGAGGTGAGCTACCTGATTCTATTGCAAATTTATCGACGACTCTTCTG

TCACTAAGATTGAGAGGTAATCAACTATTTGGTTGTATTCATTCAGGAATAGGGAATCTTGTTAACTTGA

CTGAATTGCAACTGCAAAAGAATGATTTTTCTGGTAGCATTCCTGAAGTCGTTGGCAATCTTCGTAGGCT

TCAGTTACTGGATTTGTCCGAAAACAAGTTTTCAGGAAGTATTCCACCTTCAATGTCTAACATGACTCGGT

TATATTCACTTCATTTGGAAAAGAATGAGTTAACTGGTAATATTCCCCTTACTTTTGGCAATTTCCGGTAC

TTGCAAGATTTAGACCTTTCTCAGAATCATCTAAGTGGTACCATACCAGATGGATTTATGAGTTTGTCTTC

CTTAACAAATTCTCTTAATCTTGCTGACAATCAATTGTCTGGTCCGCTGTCAGTTGAAATTGGAGCTCTCA

ATAATCTTGGAAGATTGGATATTTCTAATAACGTGTTGTCTGGGAAAATTCCTAGTAGTATAGGGAGGT

GTGTAGCTTTGGAAAGCCTTGTTTTAGCTGGCAACTTCTTTGAAGGTATAATTCCTTCATCCATTAGTTCT

TTGAAAGGTCTCGAAGAATTGGATCTTTCGCGCAACAATTTATCTGGGCAGATTCCAACATCTCTTCAGC

TCATTTCCTTAAAAAAAGTGAACTTGTCTTTCAACCAGTTTGAAGGTCAGTTGCCAACTGAAGGTGTTTTC
AGGAACGCAACTGCAATCTCCGTATCAGGGAATAGGAAACTTTGTGGGGGTATACCGGAACTGGAGTT
GCCAATATGTCCGAACGCTGACCCTGATGGTAGTGATAAGTCCAGAAGCATTAAGCTAATGATCCCTCTT
CTTTCTGGACTTGTTGCACTGGTTTTCATCATGTCTTTAGTCATAATCATTCGGTTAAGAAAGGCAAGGG
GAGAGCCTTCTTTAACATCTTCACCGGTTACTTATGAGAGCCTATATAGAGCGACAAATGGATTCTCTTCT
GCCAATTTGATTGGAAATGGTAGCTTTAGTTCTGTTTACAAAGGGGTACTTGATCCAGGTCAATGTATGG
TTGCGGTGAAGGTGATAAACATTGACCAACAAGGGGATACTTCTAAGAGCTTCATGGCTGAGTGTGAG
GCCTTGAGAAACATAAGGCATCAAAATCTTGTTAAGATCTATAATGCCTGTTCAACTAGTGATTTTGAGG
GTAATCCGTTTATAGCTTTAGTTTACGAATACATGCCTAATGGGAGCTTAGAAAGTTGGTTGCATCCTATT
CCGGGAGCTGATGCCTCAACTAATGAGGTGAGGATACTAGGTCTAGTTGAAAGACTAAGCATTTCTATT
GATGTGGCTTGTGCACTGGAATATCTTCATAACCATTGCCATAATCCAATTGTGTTCTGTGATTTGAAGCC
AGACAACATTCTTCTCGACAATGACATGACTGCCCATGTAGCTGATTTTGGATTAACAATGTTTTTCTCAG
AAACCATGAGCAAATATAGTTCAATTGGATATGCTGCACCAGGTAACGTAGTTGATAGTCCCTTGAATTT
TAGACTATCCTTGTTCAGAGCACTAACAGTCCTCCATGTTTACAGTATTGCTGCAGTTTTAGTCCGCCCTC
AAACTTGTCTCTTTTTTTATTGGGATTGAGAGGTTCATTGTGTTAGATTTCTCGTGCAACAGTACAAAATT
TTCTTTCCTTGCAACGTTATTTGTCTACAATAAACTGATATAGTTCGAAAAACAAAGGTAGATTCTTGGTT
TAGCTGCAATGATGCTTGGACTAGTTGTACAGAACGAAGAGCACATACGCTTCGTCTCACCTTCTCTCTC
ATGGTGTTATTGTTTCTACTAAACATAAAGGATGATGACTGAACATTTTATACACTAAATTATGGACAAA
ACCAATGTATTAAATAGACGGACATTTGAGGGAGGACCAAAAGTGCATGCACCAATATTGCGAACATAG
GGACTACAAGTACTCCGTGTGAAAAAAACAAAGATGACTTTTAAGTCTTAGTCCAAAGATAAGGGACTA
TTTTGGGTCTTTTCTCGTGTCTTCTTGAGAAAGTTTTCATATTTGATTTTACATGTTTTAAGTTGATTACAT
GTTTTCTTTCCTCTGTGGGCAGAGTACAGCATAGGAGGTAAGGCTTCCGAATTTGGTGACGTTTACAGCT
ATGGGATCCTTCTACTTGAGATGTTTACTGGAAAGAGACCTACTGACAGCATGTTCGAAAATGGACGGA
GTCTTCACAGTTTTGCTAAGACGGCTTTGCTTGATGAAATAGTAGACCCCATGCTTCTACCTAGCAACAG
CAGAGAAAGACAAGAGGCTGAGGAAGAAGGTGTACTTATCAATCAAGATGATACTAGTATAAAACAAG
CGCAGGAATGCTTGATCTCGATAATACAGATTGGAGTTGCATGTTCAGCTGAATCCCCGAGAGAAAGGA
TGGATATCGGGGATGTTGTCAAGGAACTTCAACTAATTAGAGACATTCTCCTTGCTTCTCATGCAATTCA
CAGCTCCACCAGCGGGTAAATTCGCTCTACTAGCTTTGTTTTCAATCGTGATATTGAGTTCCTCTGCTAGA
TATTGAGAAACTAGTTTCTAAACACATGTGTTGCACGTGTATTGTCCTGTGTAAATTCTTGTAGATCCATT

AGAACGATGAAAGTTCTATGAAGATATGCATGTGTTTACCATGTAAATTTTCTTCTTCTTGTTAATCGTTG
TCTTTCTTTCCTTGGTTTCAGGAGCTTGAGGTTCGAAGGGTCTTCCAGTCGTTCTGTTACTAGTAACTGGC
AAAATTTCACATCATTTCGTTTGCCTTGATGTTTTTAGCTCTTATATAACAAATTACTTTAGCAGATTGAAG
ATGTTAATCATCAGTTCAATCATTTGTTCAGTTGGCCTAATGCTGGTTAGTTTAGTTGAGCTTAAGCAAAT
GGAATTGGAATTGTCTATGTATATGATGTTATGACTTGGCCAAACACATACACGACCCCGTTAGCTCGTC
TCGAAATCTC
>PGSC0003DMG402008032 LRR-RLKs
AAAAAAAGTTCTCTTTCAATGCATTGCTTCATTAAAATCCCATTACTCATTTCTGGGTAGTCTTTTTTTTTT
TCTCTCTGTATTTGTTTGCCATGAAAAATGCCGTCTTTATGGAAAGCATTTTCACTATCTTCAAGTTCTCAA
CACTCTTCTTCTTCTACTACTACTGCTAGTACTACTTCTTCTCCGGCCGATTCACCGAATACCCGGCGGATT
TACGGCGGCGGAAGGAAGCTTACTAGGCAGAGGAAGTTGAGACATGTTAGTGATGATGATCTTGGTTT
GCGGAGACCTAATATTCAGGCACTGATTATTGATGAACGTTCAAAGTCTTTGCCGGGTTCTCCTGATTCT
TACGCTGATTTAGGGTCGGGTTCTAGGTCGTCTCACCACCACCTCCGTCACTGTTCTAACTCCTCTGCTGT
GCCACTGCCGTTGCCGCTCCCTGAGCTCAATTCTCTGCCTAAACAGAACTCGGTTGACTCTAATTTGCCG
GCTAGAGTTGACAGGGAGCTTTTAAGTCCTCCTCTTGCCAGGTTAGTCTTGGAAATTGAACCCATTGTTC
AGAATTTATACTCAATTTCAAATTTTAGTCTTACTTTTCGTTTTAGTCTGTTTCGAAATGAATGAGTTTTCC
ATTTTTGGCAACTCTTTAATTGTAACTTTCCACCTTACATATTTAAGAGATTAAATGATATTTTGATATATT
TTATGTATTTAGTTTAAGATCACGAGATTCAAAAGTGGTTTTTACTTTGTTAAACTTCGTGTCAAGTCAAA
ACCAGTCAAACAAATTGACATGCTGGGTATGTTTATCAAACTTTTGAGTTTTTTCTTACATGTATGTTTTAT
GCTTTAAGTAAAAAAAATGCTACCTTTCTGAGTGAAGTATTGTTTAGCCGATTCATACTGGTTTGGGATT
GAGGGGTAGTTAATTTCGGTTTGATGATGAAAGCTTTTCACCATAGGTAACTCTGTATGTAAAGGGATTT
AGTAGTAGTAGGTAGTTGGTTCGAATTTGAAGTAGTTTTTGGGGTGGATTGATTGCAGTAACACTTTATA
GGTCATTACCATAAAAGAACCATTTTTTCCTTTTTTGGGTTGTGGATTAACTGCAGTAACCTTTATAAGGT
TCACCATAAAGGTTGGTTTGGAATTGTGAAAGAGCATGTGGTTTGTAGTTGTTTCAAATGATGATTTGTA
GTGTGCTACTTTGTTTTTGGGGTAAAATGTTCGATTAGCTATGATGATTTTGTAAGAACAAAAACCGAAC
GCGGAAATGTTAACCTGTTGCGAGTTGCAGGGAAACTTTTGGTCGAACTCCAGCTGAGGTGAAAAGTTC
TCAGCATCCAAGATCATCTACTCCAACTTATCGGCGTAGGGGGTTCCCTCAGGATCTGAATGCTGAGGG
CGTTGAATTCAGGTTGAATGTTCCTGCTAGAAGTGCCCCGAGTAGTGGTTTTACAAGCCCCGTCCGAAGC
CCAAAAAGGTTTAGTACACAGGACCTTTTTCATCATCCCTTGCATCAGGCCTCATCATCACCTTCAGAGGC

TTACTCTTTCCAACTTTCACCCACAAGGATTATAAATAGCGCTGATCATTCTCCACTGAGTAGCCCAATTTT

GCCAAGTTCAGCTAGCAGAATCAGGAACAGCAGAAGTGGTGCTGTGCATTCACATCATAAATCTCTTCC

GGAAAGTTCTCTAGGTTGGAATGAAGCTAACAACAACAATGTGCATCCACTCCCCCTTCCACCAGGAGTT

CCACGACAACCAGAATCATGTACCATGCACAGCAATATGGATAAACCATGTGTATCACCAGCAAAGGGT

CAATGGCTAAAGGGGAAGCTTTTAGGTCGTGGTACATTTGGAAGTGTATATGAAGCTACTAACTGGTAA

GGGATAGAGCTTGCTTAGTAAGTAACTCGGTAGGAATGTAACATTTGCTTCTTTTGTTTTTCTTCCTTTTA

CAGTGAAACCGGTGCTTTATGTGCAATGAAAGAAGTTGATCTTACTCCTGATGATCCTAAATCTGCTGAA

TGTATAAAGCAGTTAGAACAGGTGACGTCCTCATGTCTTCTTAGGCTACATAATCAAAATTTGTATATAA

ACTGCAATTGTATGAGATACACGGTGAGAGAAAACATATTTTAAAGGCCAAATCTTTGTTAAGTGAAGT

GAACTTTAAAACAAGTACTTACTAAGCATGTTATATAATATGTCCTGGTTGTCTAACTTGCATTAGCAAAT

TAGTCAACTTAAGGTTATGTCCAACTTTGAGCTAACTATCATGTGCCTGAAGATGCAAAAATCAATATCG

TATCCATGGAGCTACTTTTATTGATCATATTCTTTGACTTCATAAGCCTCCTTCACGTTTTGCATCATTGAG

AAAATATTTTCGGAATGTTGGTAACTGACTTGGGAAAAAAGTTTCATATTGTGGAGTTTTTTATTCTACTT

GCTTATACATAGTTTGTGATGGATTGCAAAGACTATGACATGTTTTTCACTTGTTTATGCTATTCTAAATC

GATTCAGTTTCTTCCTTACTTCTGCTGTAGGAAATAAGAGTTCTCCAGCAGTTAAAGCACCAAAATATTGT

TCAATATTATGGCAGTGAAATAGTAAGAACATTTATCTTTCTCTTGCCATGTTCAGGTATTTCTTTAGAAT

ATTGAAAGATAAATGTTGTTGTTGTTGTTCAGATGGAAGACCGCTTTTGCATATATTTGGAATATGTGCA

CCCCGGTTCAATTAATAAGTATGTTCGGGAACATTGCGGAGCTATGACAGAGTCAATAGTTCGAAACTTT

ACTAGACATATTGTATCAGGGTTGGCTTACTTGCATAGCACTAAGACAATACACAGGTAGATACTGGGTT

ACATCGATGTGGTGTATGTCACTTGATTTAAAGATTCATTAACGGAACACTAAAGCGTCTGTGTTGCAAT

TCCTTGTTATAAGCAAGGAAGAAACTATTTTCTTTTGCCATTTTTTTTTAAAAAACTTTTCGTTGACAGTGA

TGCTCAGTCTAATGGTTGGTTTCAATGATTTTTTCTGAGTAGGGACATTAAGGGAGCAAATTTGCTTGTA

GATGCATCTGGTGTTGTCAAGCTTGCAGATTTTGGGTTAGCAAAACATGTAAGCTTTCAGAACTCTAATG

CCAAACTGAAAATGCTACTTTCTATGTTCGTTCTAATCTAGTATTTTTCTTGTAAACCCTGATCTGTGGTGA

AAATGAATGAGCTTGATCTTTCATCGATTATTTTCATGCATATCTTACCGCTGTAATTTATAACGTTTCTC

TTTTTCCCAGCTTTCAAGCTGTGCAACTGATCTTTCTCTGAAAGGGAGCCCTCACTGGTTGGCTCCAGAG

GTGGGCATTGTTAAGCTATTTTGCGGCTACTGCCTCTGCCTGGCTTCAGTTTTCTTTCCCCTTTATTAATTG

GCTTGTACTTATGTCTGGACTTAACTTTATATGCAGGTAATGCAGGCTGTGTTGCGCAAAGATGCCAATC

CAGAGCTTGCCTTAGCTGTTGATATATGGAGCCTGGGTTGTACTGTTATTGAGATGTTTACTGGACAGCC

CCCTTGGGGAGAACTTAGTTGGGTAAGTGTTTAAAGCATCTCATTTTGAGAACAATGTAATCGCATTACA
AGCTTAAATATATTTGTGAGCTTTTAGGTTGTCAATATAGCATCATAGCTTGTTAATATACGAGCAAGGC
TGACTGATTGGCTTAATCTCAATAAAGCCAGAGCTATGTTCTTACTTTTCATGCTTTCATGAATTGGTTTCT
GTAAGTGTCTTAATGTTCATGCTTTGGCTTTATTAGGTGCAAGCAATGTTCGGTGTCTTGAACAAATCACC
ACCTATACCAGAAAAATTATCACCAGAAGGAAAAGATTTCCTTCAGTGCTGCTTTCGGAGGAAACCTGCA
GATAGGCCATCAGCTATGATGCTACTTGAACATGCTTTTTTACGTAGTACTAGTTCCCTTGAGCATAGTAT
CAATGTTGCTGGTTGCTCAGAAGATTATCCGGGGAAGAAATTCCATGTGAGTGGTACATGCTGCTAGCC
TGCTACTGATGTGACATTAAGGGTTAACGTCACTGTATTATTTGTGAGATTCTCTAAAAAAAAAATTCCAA
ATGATTTTAAACTCTGAATTCATTACATTTCAGGATACCCTGAGTCCTAAGAACCCAATCAACCATAAAAA
GGAACAAAAACCACTGTTACCAGGAACATCTGGCAGGCATGCAAAATCACCATGTAGCAGGTAATAACT
GACTTGTGTTTGTCATGTTATATACCAAACACTTTATTCTGTGAGATTATTTTTTTCGCGTATTAGTATCTA
CAATTGCAGCCAGATACTATATCTTGTGTTGTGCCATTGACTGTCACGTCCATCTTATGCAAGTTAAACTG
TGTCTCTTCCAGTGAAACTTGCCGGCAAACTCAACCTGAAACCTGTGACTATGGAGCAGCTTCTCACCAT
TCTCCACGTTCTGCACTTGAAGTCTTTCCCTGTATTTCTTCAATGGAGCTGAACTCGAGTTCACGTGCAGC
CAGCCCATCAAGCATCCCTAGTAGCTTTCGCCTGGGACCTGAAAACAGAAGTCCTTATAGAATCATATCA
TAGGAAAGGAAATTCCAAACCTCTGTATAAGATCATAAATGAATGGAGAGGGGGAAAACTCATGTACA
GATCAGGAGAATTAGATACAGAGAACTTCAGTTGGATGATTAGTCAAAATGTTGCCATATGTGGGAGA
GATCTCAATTCCCGTATGCATCCACATCTTCTGTGTAGTATTGATGTTTGTATTTGAAGGCAAACATATCT
TTGTAACAGATTCATGAAGTACTGAAGATGATTCTGAAGTTAATAGTGTATTTTAGATTGAAAACATGAT
TTAGTGTACACAATTAAATGATTTATGCTAAATTCAATGAGTATCTTTTTCCCTCATTTTTACAACCAA
>PGSC0003DMG402014186 LRR-RLKs
GCAGCAGCAGCTGCTGCCATGACGGCTACTGGAAAGGGAGGAGAGATCGGTGGAAATGGTATTAAAA
AGCTGATCTTTTTCGGGTCTGATAGATCGTTTGATTTAGAGGATTTGTTGAGAGCTTCAGCTGAGGTTTT
AGGGAAGGGGACGTTTGGAACAGCTTACAAGGCAGTGTTGGAGATGGGCACAGTTGTAGCTGTTAAGA
GATTGAAGGATGTAACCATATCTGATATGGAGTTCCGGGAGAAAATCGATAAAGTTGGACAAATGAATC
ATGAGAATTTGGTTCCTCTTAGGGCATATTATTACAGCAGGGAGGAAAAGCTTCTTGTCTATGACTACAT
GCCAATGGGAAGCTTGTCTGCACTTCTTCATGGTGAGTCAAAGTTATACTTTTTCCTTATAAATTGATGAT
TTTTTGTTGTTGTAAATACCTGAGGGCAATCTCTATTGCTTTTCTTGCTTTAGTCTGTGGATTGGTTAGTTG
GACTTGTTAGCTTATTGTAGTACTACATGCAATGCATCTTTTTGTGGGAGAACATCTAACTGAAGTGTTG

CATTTCGTATGAATTTGAACATTTCTTTTTGATGAATTTTAAAAGCACGAGTTCAATTATTGATAATGTCCT
CTCATTTCTCTAGATTTGGTAGAGTTGTATCTGTGAAATGTTGCTTTTATAGCCTGTGAAACTCGGAAACT
TATTAAATTGCTAATAGATTGTAAGATGTTTGCTATAATTTTATTCGTGCTATTCACTACTAAGCTCTGTAA
TTTTCCAGGAAATAAGGGGGGTGGCAAGACTCCACTGGATTGGCAAGTCAGGTCAGGCATTGCCCTTG
GCACTGCTCGGGGCATTGAATACCTGCACTCTCAAGGTTCAAACGTCCATGGGAACATTAAATCATCCAA
TGTTCTTCTCACCAAATCATATGATGCACGAGTGTCAGATTTTGGCCTAGCCCAGCTGGTTGGACCTCCA
ACTTCCCCAACTCGAGTTGCTGGATATCGTGCTCCAGAGGTAACGGACCCACGCAGAGTTTCCCAAAAG
GCAGATGTATATAGCTTTGGTGTATTGCTTCTGGAGCTTCTTACTGGGAAGGCACCCACTCATGCCCTCC
TGAATGAGGAAGGTGTTGACTTGCCAAGATGGGTGCAGTCCATCGTTCAAGACCAATGGACTTCACAAG
TTTTTGATATTGAGCTCCTCAGGTACCAGAGCGTTGAGGAAGAAATGGTGCAACTCTTGCAGCTCGCAAT
AGATTGCTCGACTCAATATCCCGACCACAGACCTTCTATGTCTGCTGTCGTTGAGCGAATTCAAGAGCTC
TGTCTCTCAAGCTTGCGGGTTACTCAAGAACAGTCTGACTCTGTCAATGAATCTGATTGAGGTGGTCCTT
GGTCCTTTTACATGTGCAGTACTTGAACTAAAGAAGCACCATCACCAATTTACAGAACCCCCTTTAAGCA
TATTAAACTTTAATGCTTTCTGTTGGTTAGTAGGGCCTATCTTCTTTCATTTAAATTTTAACAATTTATGCC
CCTTTCTTTTTTCTCAGTTCTCCATTGTAGTTGAGCTAGAGCCCTTTTCATTTTCCTTTGTGTGGGGGGCCA
TTCTCTGTACCATTTGTTCTTGTTGGGTGGATAATGGTTGCTGTAATTATTACTTTCAGCCTTTACTTTGCT
TTCACTTTAACCAACTTTGTTGAGAACTTAATACCTACCTTTACTTTGTAGTACTTTCATTTAGGAGGTTTG
TTGTTTGGTAGTAGTAAATTAGTGAAGTTTTTTTTTTTGGTTTCCCACCTGGTGTCCCGTAACTACATTGG
AACTCGACTATTCAGGGGAAGCGCTCTCTACCAAGGATTGCCATGCTTTGTTTGTTGATCAGGACAAAGT
TTCTTTGGTTTCACGGATTGTGTACCCTCTTTTACTTCGTTTTTGA
>PGSC0003DMG402015976 LRR-RLKs
ATGGCAACAACGGAAAGAATTTCATACTATGCACTGCTCCAAGCAACTGATTCGCTTAGCGAGAGTAAT
CTGATTGGTTATAGAAGTTTTGGCTCTGTTTACAAAGGCGTTCTCAGGAGTGGGACTGCCATTGCAGTTA
AAGTGTTCAATCTGCAACTGGAAGCAGCATTCAAGAGTTTCGATACAGAATGTGAAGTTTTGCGTAGCC
TTCGCCATAGGAATCTCGTGAAAGTCATCACTAGTTGTTCCAACCTTGATTTTAAGGCTTTAGTGCTCGAG
TATATGCCTAATGGAAGTCTTGACAAGTATTTGTATTCGCACAACTACTTCCTAGACATCAGGCAGAGAC
TAAGCATTATGATAGATGTTGCATGTGCTTTGGAATATCTTCATCATGGATGCTCCTCTCCGGTGATTCAC
TGTGACCTGAAGCCTAGTAACGTCTTGCTGGATGAGGATATGGTTGCCCACCTAAGCGACTTTGGTATTT
CAAAACTGCTTGGTGAAGATGAGAGTGATTTATACACAAAAACCTTAGCAACATTTGGTTATATAGCACC

GGGTACGTCTCTTTGTTTTTGCTAATTTGAACATTGATTTTTCCTCTTATTCTTTTTTTTCCAATCAAGAAAG
TATATTGCATCTTTTAATTAATTGTTTGACTGTAGAGTATGGACTGGATGGACTGGTGTCAATAAAATGT
GATGTCTATAGTTACGGGATTGTGTTGCTGGAAACATTTACTAGGAGAAAGCCTAGTGAGTTTGCGGGA
GATCTTAGCTTGAAGAAATGGGTGAGTCATTCACTCCCAGAGGCAGTAATGGACGTTATGGATGCCAAT
CTGGTTACACCAATGGATAATCGCTTACAGAAGGAGCTAGATGTTGTGGCATCAATCATGAAAGTGGCA
TTAGATTGCTGTGCTGAATCTCCGGCAAGAAGGACAAACATAGAAAGATGTTGTAGGGATGCTACAGA
AGATCAAGATTCAACTTCTTGCACGCTGAGCATGTTACCGGAATCTCCTTTTCCAAATTGA
>PGSC0003DMG402020263 LRR-RLKs
CTTTAGTGGCCTAGGTTAAGACGGCCCTGGTGGCACAGTGCTTTTCTGAGACTCTGTGAAAAGAAAACT
GTCACCATTAAAGCCAGAAACTTGAATCGGTCCCCTGTTTTTTCTCCTTGACTTGCTCTGCATGCAACACT
AAATGCTTTCTCTTTTGTCAACTACCCTCAATTTTGCTCTCTGTTTTTTTATTTTCAATATTGTTCTTCTCCCA
AGAAAAAAAGAACATTCCTTTAATTTGCAATTATAGGTCAATTTATTCGCATTTTTAATGGGGTTTTATCA
AGATTGTAGCAGCATTAACTGTTTGGTAGCTCAATCTTGACTTGTCTGCACATTTAGACTTTAATGCTTTT
CATTTTCACTGAATTAGAAAGGGTCCACCCATGTAATTTTCTTATTCATGATTAGCCAAGAACGTAAAATG
GGTTGCTTCAAGATTGTTGGAAACTTGGCTCCATTTTGAAGTCACTGTTAAATGGGTTTCTTCAAGCTTTT
TGTGATAGTGTTGGTTATTTGCGCTTTCTTTATAGCAAATACTTATCAGTTACAGTCCTATGAAATTCAAG
CTCTTCTTCAGTTGAGGAAGCACTTGGAATATCCAGTTCAGTTGGATGTTTGGGAGAATTACCATGGAGA
TTTTTGCAGCTTGACTTCAACACTGCATATGAGCATTACCTGTCAAGATAACTCTGTTACTGAACTCAAAA
TCAAAGGAGATAAGTTAGTTAAGTTGAATGAATTTCATGGATTTGCAATTCCAAACCAAACATTATCTGA
GGGTTTCTCCATTGATTCTTTTGTTACCACATTGACAAGGTTAAGTAGCTTAAAGGTTCTTACTTTAGTTTC
TCTTGGTATTTGGGGACCCCTTCCTGATAAAATTCATAGGTTAGCTTCACTTGAACTTTTGGATATGAGTT
CAAATTTTCTATTTGGTTCTGTTCCTTTTCAGATGTCAAGATTGATTAAGCTTCATACTTTGACATTAGATG
CCAATTTTTTCAATGAAACTTTCCCTGATTGGTTTGATTCCTTACACAATCTTACTATATTAAGCTTGAAGA
ACAACAGGTTGAAGGGTCAGTTTCCTTCTTCAATGTCTAAAATCACAACCCTCACTGACATTGTTCTGTCA
CACAATGTGCTTTCTGGTAAATTGCCCGATTTAACGGCCTTGTCTAAGTTGCTTTTATTGGATTTAAGGGA
AAATCATTTGGATTCTGAATTGCCACCCTTGCCAAAAGGACTTACCACAGTTCTTCTTAGCAGTAACGTTT
TTACGGGTGAAATCCCAGTAGAATTTGGCACACTAAATCAACTTCAACACCTTGATCTGTCGAATAATTC
TCTCAGTGGAATGCTGCCTGCTGGCTTATTCTCTTTGTCTAGCATTAGCTACTTGAATTTAGCGTCTAATG
TTCTTAGTGGTTCACTTCCTAGCCATCTCAATTGTGGGGGTGAACTTGGTTTTGTTGATATTTCTGATAAT

AGATTAGTTGGTGGGCTTCCTTCTTGCTTGAACACTATTTCAGATAAGCGAATTGTTAAGGTTGGTGGAA
ATTGCTTGTCTCTTGACACTCAATATCAGCATTCAGAGGGCTATTGTAAACAAGCAAATTCGGATAAGAA
ACAGATCACAGGAAAACAAATAGCATTGTTGGCGGGTGTTATTGGGGGAATTGTAATTGTTGTGGTGTT
TCTGTTAGTTGTCCTTTTCATCTTCCGTAGAAAACGACATGCGCGTAACATGGTGGATAAGCACATTCCTC
CGAAAGTTGTGCAAAATACACAGCCCGGGGTTCCCTCTGAACTCCTAGCAAATGCCAGTGAGTTGATGT
TTTTCTTATTTCATTTTTAATCTGTTTAATTAATATGAAGGAAATGTTCTCCAGAAAAGCTTAATCTCAATC
TTCGAATTCTCAGGAGTCATTTCTCAAGTGGCAAACACAGGATCACAAGGTTCCCCATCATATCGAGTGT
TCTCCATGGAAGAATTGCTAGAAGCAACAGAAAATTTTGATCAGTCAGCATTACTTGGTGAAGGGTCAG
TTGGAAAAGTATGGCTTCAAAACAATACTTATGGTTCTCATTGATAATATATATCTTGATGTTTTCCATTC
AATCAATGGTCACGCTGAATTTTGGCGCAAAGTGATATACTTGCTGTAAAACTTGAATAAACGAAGCAG
GACGTAGATTTTGTTGCATAGACCATGTCCAACAATGATAATGCAATGAATGCACTAGTTCACCAAAGTA
CAAAATGGATTTAGGTGTTTTTTTTTTTTTTTAAAGTGCTTGCCTATCATATTCGCATTCTACATGTGCATC
TGTGACTTCTTTTGATGATAAAAACTGGTGTCACCAATAATGCTTTTGTGCATTTGATTTCATATCTAATG
AACACATGACAGTTCAACTAAATATATCGAATGCCCTTAAAGGCAAAATTAGATAGTCTCACACGTTTGA
GTGCCTATGCAGTGAGTTGACATCTGTGCTATTTTGATTGACATAAACAGGTGTTAGCCTTATTATGTCTT
GCTTGAATAAGACAGTTATTTTAGTTATCAGCAATTCCCTCAATATTTATCTCCATTTCAGATTTACAAGG
GAAGATTAGAGAATGGAGCTTACATAGCTGTAAGGTCACTAAATGTGTGCATAGTTCACAAAAGTTAAA
AATTGTTTTAGGTTTTTTTTTTTTTTTTAAAGTGCTTGCCTATCATATTCGCATTCTACATGTGCATCTGTGA
CTTCTTTTGATGATAAAAACTGGTGTCACCAATAATGCTTTTGTGCATTTGATTTCATATCTAATGAACAC
ATGACAGTTCAACTAAATATATCGAATGCCCTTAAAGGCAAAATTAGATAGTCTCACACGTTTGAGTGCC
TATGCAGTGAGTTGACATCTGTGCTATTTTGATTGACATAAACAGGTGTTAGCCTTATTATGTCTTGCTTG
AATAAGACAGTTATTTTAGTTATCAGCAATTCCCTCAATATTTATCTCCATTTCAGATTTACAAGGGAAGA
TTAGAGAATGGAGCTTACATAGCTGTAAGGTCACTAAATGTGTACAAAAGATACTCTAACCGGAACCTC
AAACTACGATTGGACTTACTGTCAAAGTTTCGTCACCCCCATTTAGTTAGCCTTCTGGGTCACTGCATTGA
TGGTGGAGCACCAGATGATTCAACTGTCCCCAGAATTTTTCTCATTTATGAATATATCTCTAATGGAAACT
TCCGTGCTCATCTATCAGGTCAGCTAAGTAAATCCTTTAGCATGAGCATTGCATGTGTTTTGATGCTTGGT
TGAATATGGAAATATTATATGATGCACATATGGAGTGCTGGTTGGATTCTTTTCTCCCAGAAATGATTGG
GATACCCTTGTTGATGAATCTCTATCTAGCATTGCAAGTATTTTTGCTACTTATGAGCTTGAGGCATCTTA
TTCATCATATTAATTTCACACATAGTGGTATTCAGTATGCAATTACTGTCAGTTTGATTGAGCAACAATGT

AATGATTCACTTGCACGAAGTTCTGTTGGTTGAATTTCATACTGAGTTGGACCTTTATTTTTGTTGTTATAT
ACTTTCATTTAACCCTGCATGTCCAATAAGTACAGAAAATACAAGAGACACGCCATAACGTATTCAGCTT
ATCTCAAACACTTGTACTTGCAGAAAATAGTTCAAGAAAGATCCTCAAGTGGTCAGACAGGTTGTCGGT
GCTGATTGGTGTTGCCAAGGCAGTGCACTTTCTTCACACAGGTGTAATTCCTTCTTCATTTAGCAACCGCT
TGAAGACGAGTAACATATTGCTTGATGAGCATAACATGGCAAAATTAAGTGATTTTGGGATGTCCATTCT
TATGGAAGAAAGTGAAAAGGTGGGTATTTTAATATTTTGTGTGAAAGGTGAAAATTCAAAATTTAGGAT
AGATGGTAATTTCTCATACTCAATATGTCTTTTTTCAGGCAAAAGGAGATGACGTCACCTCCTGGTATGA
AACTCTTCACTCATGTATTCCGTAACGTGCCTTACCAACTTCCTTTTACTAAACCAACTGGGATTCTATTTC
AAGAGTGTCAACTAGAGGCATATATATCATCTACTATAGCTGTTTGCAGGATATAAATCAGTGATATTTT
ATTCTTGTGTAGGCATATGACAAAGAAAGAGGACGATGTTTATAACTTTGGTTTCATATTACTGGAGTCG
CTGGTTGGCCCTTCTGTCAGTGGAAAAGGAGAAGCATTTTTGATGAATGAAATGGTATGAAACTGCTAT
ATTATTATGTATGCAACTCCTTTGTCATTGACAGTAACGTAAAGTATTCTTCAGCAATTTGTTCCCTTTAGG
ATCCTAAACGTTGTTGCAATGTTGGATAAGTAACAAAATTTTCGTTCGCTCTGCAACCTAACTTTACAATC
AGTACTAATATAGATTAACAGAACTACACACATTATTCAAGTTTTATTAGGTAAACCTTCTGATATGCAAG
TACCATAGGGAGAAAAGTTGAGCCTGATATACAAGAGTCAAAAGTCTTGATTTTTTCACTGGTGTGTTAC
ATAAATATCTCTGGATTTTCTCTTTGAATGACAGGCATCCTTTGGTAGCCAAGATGGTCGACGCAGAATT
GTGGATCCAATAGTGCTAGCCACTAGCTCCAATGAGTCATTGTCAATTGTAATATCCATCACCAACAAAT
GCATATCTACAGAGTCATCAACTCGCCCCTCGTTTGAGGATGTTCTCTGGAACCTACAATATGCTGCTCA
AGTTCAAGCTACAGCTGATGCAGATCAGAAATCTGATGCTACTTCACCGTCATAAGCTATTTGAGAAGTA
CAACTGCATCGAATCAACGATATTTAATTTGGTCAGATCATAGGAGAATGTTAGCTGATTAATTTCATGA
TGGTTGTGGGAGTACCAACCGGTAGGCTACTAGCCTTAAAGCTCAAGTTTACGCTACAGGTCCTCCCATA
TTTTCTTTATTTTGTATCAGAATTGTAAATTTTGTTGATTCTAGTAATAGGTGATGTTTTCATATAGTATAG
ACCCTCGTTTTAAATTCTATCATTGCAAGTAGGCAATTGAACTCGTAGAAGTCTAGATTCAGACAAGACA
ATACTTTAGTCATCATATATATGTTTGGTGCAACGGAAAAAAGATATGGATACCGCAAATGCAAG
>PGSC0003DMG402026747 LRR-RLKs
GACTTATCATCAAACAACATGGTTGGTTCTTTACCTCCGGAAATTGGAAATCTAAAATCTGTGATACAGA
TGGATTTGTAGATGAATCAATTCTCAAATGGAATTCCAAGAGAAATTGGAGGATTGCAAAATCTGGTGC
ATCTTTCTTTGAGACACAACAAGTTGCAAGGATCTATACCTGACTCAATGAGCAACATGGTAGGTTTGGA
ATTCCTAGACCTTTCTCATACTAATATATCAGGAATCATTCCTATGTCTTTGGAGAAACTTCAATACCTCAA

GTATTTCAATGTTTCTGACAACAAATTGCACGGCGAAATACCCTCGGCGGGTCCTTTCAAGAACGTCTCG
AGTCTTTTTTTCATCAACAATGAAGCATTGTGTGGTTCTTCAAGATTTAGCGTCCCTCCATGCCCCACTTCA
TCAAAGCATAGGTCAAATAGGAAAAAAAATCTTATGCTGGGAATTGCACTTGTATTTGTTCCTATTACCC
TTGTGTTTGTATGGATAAGGTATAGAAGAGGTAAAAGATCTCCTCAGCAAACTGATTCATTGTCTATGGC
AACAACGGAAAGAATTTCATACTATGAACTGCTCCAAGCAACTGATTCGCTTAGCGAGAGTAATCTGATT
GGTTCTGGAAGTTTTGGCTCTGTTTAAAAAGGCGTTCTCAGGAGTGGGACTGCCATTGCAGTTAAAGTG
TTCAATCTGCAACTGGAAGCGGCATTCAAGAGTTTTGATACAGAATGTGAAGTTTTACGCAGCCTTCGCC
ACAGGAATCTCGTGAAAGTCATCACTAGTTGCTCCAACCTTGATTTTAAGGCTTTAGTGCTCGAGTATAT
GCCTAATGGAAGTCTTGACAAGTATTTGTATTCGCACAACTACTTCCTAGACATCAGACAGAGACTAAGC
ATTATGATAGATGTGGCATGTGCGTTGGAATATTTTCATCATGGATGCTCCTCTCCCGTGATTCACTGTG
ATCTGAAGCCTAGTAACGTCTTGCTGGATGTGGATATGGTTGCCCACCTAAGCGACTTTGGTATTTCAAA
ACTGCTTGGTCAAGATGAGAGTGATTTATACACAAAAACCTTAGCAACATTTGGTTATATAGCACGG
>PGSC0003DMG402029358 LRR-RLKs
ATTACAAGAAAGTTGAGTTCTTAGCCTTACTAGTTTGACTGACCCCATTGCTACAAATAACTCAATAAGTT
TAAAAAAGTGATATTTTCCTTCTTGATTTGCTAGGAAGTCTCTCAAGAATATCAAAATATGGAGAAAACC
AATTTCAGAACATTTCTTGGTTTTGTATTAGTATTACTACTTAGCAGAATTTGTGTCTGTTGTGCTTCAGA
GAGTGATGTTTATTGCTTGAAATCAATAAAAGATTCATTACATGACCCTTTCAATTCTTTGGGCTTTTGGG
ATTTCAGCAATGCTACTGAAGGTTTCATATGTAAATTTTCAGAAGTTCTTTGCTGGCATCCTGATGAGAAT
AAGGTACTGAGCCTCTCACTTTCAGGCTATGGATTAATAGGTGAGTTTCCTCGTGGCATTCAAAATTGTA
CAAGCTTGACAAGTTTAGATCTTTCAGGGAACAGCTTATATGGAACTATCCCTTCTGATATTTCAGCAATA
GTTGAACATGTTACAATACTTGATCTCTCAAATAACACGTTTTCGGGTGATATACCACCTGATATAGCTAA
TTGTCAATACCTTAATGGTCTAAAGTTGGATAATAACTATCTAGAAGGTGAAATTCCAAGCAGAATAGGC
TATTTGCCTCGCCTTAAGACGTTCAGTGTAGCCAACAATTACTTGACTGGGCCAGTGCCATTGTTTTATAG
AGAAAGTATCACAGTTGAGAGTTTTGAAAACAATTCAGAGCTTTGTGGGAAGCCCTTGAAAGGATGTGA
GGATTCTTCGATATGGAAACATGTAGATCATGCTTCGTTCATCAAAGCGTTTGTGATTGGTTGGGTACTT
TTGTTTACATTGGTTTTAGTCCTTTGCTTATTTAAATTTCCTACCAAGGCTATCAACAAGATAGTCTCATTA
AACATATGGAGGCTAGGGAAAAAGGAACATTTAACTTCAGGAAGTGAAGAGGAATTAAGCAGCCAACA
TAAGGTAAAGATCTTACTAAATTTGCTGCACTGCTTGTAGTCTGTAAAGTTACCTTTTCTTCTGTAGTTCT
AATTTTGTGATTAGTCCTTGCTAGTCCATTATGTTATAAATTCTTTATTCGATCGATGATCAAACATGATA

GCAATAAGGTTCTCAAGTTTCTGTTCTGTATATGTTCAAATGATTCAACTTGTTGTTTTAATTACAGATATT
AAAGCTGGAGAAGTTTGTTACGAGAATGAACTTCACGGAGCTAGAAAATGCGACTTCTGGTTTTAGTGA
AGACTATTTAGTAGGAAATGGAATGTTGGGCAAAGTGTACAAGGCAATTCTTCCAAATGGCTGGATACT
TGCCATCAAGAAGCTCAATGACTGGGAGAATTTGGAAGACGAGTTTGTCTCGGAGATTACAACTCTTGG
TGGTCTGAGACATCGGAACCTATTGCCTCTTATAGGTTTCTGTGCTGAAAAGCAAGAAAGACTTCTTGTT
TACAAATACATGGCTAATGGAAGTCTTGATGAATGGCTGCACTCGAATGAAGTTAGCGCCAAAATTTTG
GATTTCCCTCTGAGAGCTAAAATTGCACTTGGGATAGCGAAAGGCCTGGCTTGGCTTCATCATGGTTACG
AATTGCACGTTACACATGGAAGCATAAGCACGCGATGTATCTTGCTAGATCAAAATCTTGAACCAAAAAT
ATCCAACTTTTGGGAAGCAAAATTTTGGAGTAAGAATGATTCTGCATTAAGTTGGAGTCTTTTCCCAGTA
GCTGAATATTCTGGTTTAGGCTCTTACAAGCAAGACATCTACTGTTTTGGTGTCGTGCTTCTCGAGCTGG
TAACAGGGAAGGAACCACATGAATTGACTAGCTCAAGAAATCTATTCGATCACTCGCCTTGTCTACTTGA
TGCAGATAGAAATTTTCTTGGGAAAGGAGCTGATGATTTAATCCTCCAATTTCTTGAATTGGCTTGTGAC
TGTGTGAAGTTCTTTCCTAATGAAAGGCCAACAATGCTGGAAGTGTATGACAGACTCAAGAATATTTCTC
AAGGTCGAAGGGACTGGATACAAAAGATACCATTATCAACCGATATTTCAAGTAAATATGACTAGTGCT
TATGTTTTTATGGTTACTGAGAAAAACAAATTAGGAATACACTACATTAAAGCATGAAATCCGTGCTTCG
AGTCTGTATGCTGATAGCTTCTGTTGCAACTTTAATATATATATATGAATACGTATATAATCTACAACATT
GGAAAGCCTTACTGACTGCAGTTTTCAAGTCTGGCATTAACATCTTGCAGTTTCTTTAGTTAGATAGTTTT
TATTTGCATATTGTAAAGAAGAGAAGTTGACTATGAATCTGCATATACTAATAGAACAAATCAATGGTAT
TTAATAGTTGACTTCATTGTGCTATATTGTGTAAGGTTGACAGAACAATATATAAATCATATTATTGAAAG
CTTT

>PGSC0003DMG402030114 LRR-RLKs

CCAGAAAACGATTATCAATAACATAATCAACATGTTTTTGTTTGTAATCTAAAGAACTGCCAATTATTTAT
TTGAATTATTAAAATGGGAAAACTCAATTAGCTCAAATTCAACATAATATTAATAAGAAATAGCTTTTGA
ACCTAACTCAACTGTAAAAACAAGCTAAGAATTGTCCGAAACTATATAAAGAGACCAAATCCATCATCCC
ACTACAATTTTTGTGCAAGCACAAGAAAGTGCACCTACAAATTCATTTTCACAGTCTCTTTTTCTTTCTCAC
ATGACTTCACATATTTCATAATGAGGGTATCAAGTCATCGTGGGTAAGTACAAAGGAAGGGAGGGAGT
AAATTACTAGCTTGACTTAGTCAGAAGGAGACAATTATTATGGCTTGATTTTTCAAAGCCAAATACTTGG
CTCTGGATGACTATTTAATTGTTGAAATATGATTTCTTTATTAAATCCACACTGACTTCACAGACTTCATTA
TCAAATGAAAAGAAGCACAAGGGAAGCATCAATTAACATGTTCCCAGCAAGCTCTAACCATTTTTTGCTT

AATATGCAAATTTTCTTCCTAATTAGCCTGTTTTTTCAAGAAACCTTACAATTTTCGGGCACGGAACAGTC
CATATTACTAAGCTTGAAACAACACTGGAGGGATTCGGAGTTCCTCCAGTCATGGAATTTGAATTCTTCA
GTATGCACTTGGTCAGGAGTTTCATGCCTCGATGACCGGGTAGTGACACAACTAAATCTTGGAGAAAAG
AACATTACTGGAACAGTTCCATCAACAATAATATGTGAGCTTAACAACCTTACCTTCATTGATCTCTCCAA
CAACAACATTTCAGGAACTATACCACTAAGCATGAAAGATTGCTCAATGTTACAACATTTGGACTTGTCC
AAGAATTCTTTGAGCGGACAGATTCCAGGCGAGTTGTTTGGGATGAAACAATTACTCAATTTGTATCTTA
ATGGTAACATGTTGTCTGGTGAGATGCCAAAACAAATATCATCTCAACTGGAAAATCTTGATCTTTCTGA
GAATCACTTGAACGGTTCGATACCAGAAGATATTGGAAACTTCAAAAATCTTTTGAAATTGGATCTGTCA
CATAATTCTCTCAGTGGTTCAATTACAAGCAAGTTGTTTCAGTTGCATGATTTGCGTCACCTGTCGCTAAG
TTCCAACTACTTGTCCAGTGTGATACCTGATGAAATGGACTTGTTTAGTTTGTATGATATGGATCTTTCAC
ACAACCAATTGACTGGTTCTATACCAAAGCGATTTAGGGATTTACCTGGACTGGATTCTATGGATTTGTC
TTTTAATCAGTTATCAGGAGACATTTCAAAAAGTATAGAACGTCTTAGGCCTAGAAATACTTTAAGGCTT
TGTTCCAACAAATTCTCAGGAAGAATTTCTACTGAATTTGTAAAGTTGACATACGAAGAGAATTGCTTTG
ACGAGTCCAATCTTTGTTCTACATCCAACAACTTATACATCCCCGGCCTACCAAGCTGCTCCTCTGGTGAT
GAGGTTCGAAAATCTTCGAAACCAAAACACTTGATTATTCCTATTGTAGGAGTTGGGGTAGCTACACAGT
TATCATGGATCTTTTACAGAACAAGAAAGCATTGGTGGAAAACAAAGAAGCAGAATGTCAAAGATGAC
ATGAAGTTCATTTCATTTCAAAAGTTGAAGGTGACTACAGAAGACATTTTATCTAGCTTGAAAGACGAAA
ACATAATAGGAAATGGAGGATCAGGGAAGGTCTATCGAGTTGTGATCGACCAAACAGGCAATACTTAC
GCTGTTAAAAGCATAGGGCATGGAAAAAAATCAGGTGAAAGACCCCAAAAGGAGTTCCTGGCAGAAGT
TAGAACACTTGGTAGCATTCGACACAACAACATTGTCAAGCTCATGTGTTGCATCTCAAGTGTAGACAGA
AAGCTTCTAGTCTATGAATACTTTGAGAAACAAAGCCTCGACAAGTGGCTTCACAGAAAGAAAAGAGAA
GCGTCACCCGGTCAAAGTAGTACCCCATCCCTGGATTGGCAAAAGAGATTAAAAATAGCCACTGGTGCA
GCTCAAGGACTCAGCTATATGCACCACGACTGTACTCGACCCATCATTCATAGAGACATTAAGTCCAGCA
ATATACTTCTGGACTCAGAATTCAATGCAAAAATAGCAGATTTTGGACTAGCAAAAATACTATCCAGGCG
GGATGATAATCCTGAGACAGCTTCTGCTATTGCTGGAACATTTGGTTACATTGCACCAGGTCTATTACCG
TTCAAATATATATCGTACCTCGTGAATTCTCAAAAGAATTTATCCAACTCTAATATTAAATTTTAATGTTTG
GTCCTCACAGAGTATGCCTCGACATTCAGAGTGAATATAAAGACAGATATATATAGCTTCGGAGTGGTG
CTATTAGAATTAACAACAGGGAGACAACCCATTCTCAGGGAGGAGCAAATGAATCTGGCACAATGGGCT
CAACAGCGTTACAGAGATGGAAATTTCATTGTTGAGGCCCTTGATGAAGAAATCATGGAAACAAGTAAT

GTGGAACAAATGAGAGGTGTTTTTAAACTAGGACTGATGTGTACTGGAGCAACACCATACGGTAGGCC

GTCAATGAAGGAGGTCTGCAACATTCTTCAAAGCTTCAGAGACACCATATTTTGAGAAAACAGAATGTTT

AGCCTTACATGGATAAATGTAGTTGTATTTTTTTAACAGAATGAGAAAGTCTCAATAGCCTCAGCTGGTT

TAAGTGGTGTCAAACACAAGGGCTTGTAAGCTTTACCTTAGAATGTAATTCCTTGTTCATAATTGAGATG

TCGCGAGGCAAGAGCTAAGTGTCTTGGCAATTACAGTTTATGTATATATCATTCGGGAGATTAGACAGA

TTTTAAGCACACTGAGCATTAAGGTCTAACAATGCTTTAGGAAAGTAATCATGATATCCGCATGGAGGTT

AGAATCATATCTTCTTCTTTTTTTAACTCTTCAGAACTCCCTAAGGAAGCTTTTGGAACTTACAGGTTTGAT

GAACTGCAAATGCCTTCTTTCAGATATAAATCCTTCTCATGATACATGGGGATAATCTTGTAGGAAGTCT

AGTTCTTTTAGTTAAGTTGAATGGGATCGTCCATTTTCGAAAAGGGGGCACAACTTGAAGGCATAATTG

ATTGGAAAAAAAAATAATCGAAAGATATAGGAGAGTGTTTTTTAAATGAATAAGTCCTTATTGTAGTTGA

GGAGATGATAATCAACTTCAAGAAGAACTTTCATCTCAAAATACTAAAAAAAAAGTTTTTCATACTATCAA

TGTTATTGAATTGCCTCTGCATATGAATATACTTCATAATAAATGTGGGACTTCTCACCGCGAATCTGAAT

TTAGTTAAACCTCAATGCATGTACCGGACATTGGGTGAAAACAAAAAAGGATAGTATGAAAAACTATCC

CAAGTGTTGCAAACCTATTAGCTGGAACTGTCCTCGCACTGAATGTAGAAATGCAATATTGATGGCTCCT

CTAATGAAAATCCAGACCCCAGATCGTGGACCTTTTTGTATCAGAGATATTATGGGAGAGTTAATTTATG

CTACTACTAATGTCATGAATCATGATTAACACTACTAGCCTATGTGCTGAGGCTAAAGCAATAGAAAAGG

AGTTGGAATATTCTGTGAGCCATCAATACAACCTCTTATAATAGAGACTGATTCAACCAAGTTGCGGATT

TTTTAGCTAATTATGTGTTCATTATCAATATCTACTATATGAAGCAAAGAAATTATTGAACATTGATAAGC

AACAAGTTCCCGGGTGATATTTTAAAAAAAAGGCAGGAACTAGCAACAAGAACAACAAGCAGGCTACT

CAATCAACTATCACAATAGACTATCCATACAAAATATACAAATCTTCATGAGTACAACCAGAGTAATAAC

AAAATACAGATGGTGAAATTAACTTGACAAATCCTCCAAATATTTTCTTATCAAAAGTGGTATCAATCTCA

AACAAGGTTCAATCTTTTGGGGAGGAAATATTAGAGGACTATCTCGGGGGTTTTTGACACTTTACTCGCT

TCTTGGAAACTATTTTGAGGATGGTAACATGTTACACCCCAACTTAGGGATGCAACTAGCACCTGGTGCG

ACACTTGACCCTGAGTCGATCCTTGCTGGACAACTACGCTACCAACATAACACCAGCCACAAAATCAAAA

AAACAAACAAGTATATTTCAACTTAAAAATAAGGCCTCTATCGGCAAGGACCCTGTCAACATAATCTTCA

ATAAACTCAACTACTCCCAGCAATTCATATAGAATAGAAACTAAATCATTTATTGCCCGTCAAGGTCACTG

TTATATCTAGAAACATATAAACTGAACACATATAGTCATATATGATTAAGGCCAACAAGCCACTAAGCTA

AAAGGGCAAAACAACAAGGCCAGCATAGCCAACAACACACATCTATTCAATCGAAAGTTTGCAAGCCTC

TAAGAGTAATATACTTGGTCGGGACCCTTATACTTCCTGTCCCGACCTACCTATTAGTTGAATATGACAAA

ACACTAGCAAACCTTTAAATTTGGCAACTCTAGAAGAAATAGAGCTGACCAATCTTCTGGAACTCAGCTA
CACTATTGAGGAGGCTTGTTTAGCTGTCTGTCAGGATCTGCAGGCAAAAATGCAGCACCCCTGCCCCAG
GTATGAGGCGTCATTATGAATTGATATATCGAGTATAAAAGGCAATAGAATAACTGAGCAGGTATCATA
ATGTACCAAAAGTAATCAGGTAAGGAAGTCAAGAATTAGATGAGTGTACTGACCTGTAACACCTTAGAA
TTTGGCAAGTGTGGTTTGGAAAGTGTCATGGTTTTAACGGGCAGGTTCATGGACAGTGAAGTGGTCCAC
GACCTGCCAACCCATCCGTGAACCCTACTCCCCAAGGCTCAGGAGAACTTGATCCCTCACGGACGCCTGA
ACGGTCTGTGGAAGGGTAAATGTCCAGTTGATAGGGCCGTGGTGATTGGCAATAAAATCCCATTTTTTC
TCTCTTGTTCACAATCACCTTCATGGACCGTGAAGTGATAACCGGTCCGTGAAGGCCTCCGTACAGGGGT
CCCGTTAGCTTTTAAGTCAGGGCTATTCTAGTATTTTCCTATGCGTCTAATACCTAAGCTATGTTGTTTTGA
CCCCTATTCTAAGACCTATATAAGTATTTTAATTCCTAAAACTACCCATTCTAAAATCATTCTCCTAAAATC
TCAAATTCCCTTCAAGTTCTTCCCCCAAATTCCTCTCTCTCAAGCAAAGAAAGAAAAAGCTAGGGTTTCAC
TTCAAGGTCTCCAATTCACCATAAAATTTGGGCCTTGATCATCAAGATATGTTACTATTCACCTATGGAGT
CTTTTCCTCCATTGGGTTCCCAAAGGTCCCAATTCTACAAGCTAAATTTTCAATTCAAAGTTAGGATTTTAT
TCAAATTCCATGAATTCTTCTCAATTATGATTCAATTAGTTATTTCCTAGCTTCTAATGCATGAATTGAAGT
ATTTACATGATTTTGAGTTGAATTCATATGAACTCATGCATCCATGATTTTTACTATGAACACATGATGAA
ACTTATGAACTATGCAATATGAATATATGAAGATATGCATGTTTTTGTGAAATTAATGTATGACTTGAGG
ATGCTTTAGATGAAAGTATCAATAAGATTGTGAAAAGTTTTCTCACATGCTACTATGATCATGATTGTAA
AAGGTTTTCTTACATAAGGTTGAAAGGTTTTCTCACCTAACTTGAACTAAGATTTAAGGAGCTATTCTAAA
TGACTTTAGCTTGGATTGGTAGCTTAAACGTGCCTTTCCATGGATGATGAATATGATTAATGACTATTCTG
TGGGATTTTACTTAGCACCGAGAGGATATTGAGATGGAGGCTCTCCAATTAGTAGAGGTCGGGTCTCTA
GTAGCAATCTCCTTGTCCCTAACTACGTGCCCCCGTAGGATTTCTTAGCTAGTGGATCCACGTAAACTAG
AAGTTTATGGTCCTTACTTAGCAAGTAGGACATCTCGAAATAGACACCGGATTTCATGTTTTAGAGCACA
TGTTCCATATCGGTTAATGGAAAACATCCCACAATAATGAACTAGATTATTTTCAAAGTATCTCACCAAGT
GTTTTAAATATGTTAACCTTGCATTGACCATGATTATGACTTGTGTTTCTAACACCGTATCTTATGCTTTAT
CTTGGCTATTGCATATCATGTAAAGTCTTTTTAGCATGATTTCATATTGTTTGTGCATTGTTATCATACTTA
GTATATTCCATGTACTAATGTATACTTGTGCCTATATTGTTTCCCTAATGTAGGGTCCGATGCTCCTACCTC
GCATACTTGTGGCTAGTTGATCTCTCTTGTCGATTTGAAGATTGGTGAGTCATCATGTTCCGAGGACCAA
GACACTTTTATAGCTTTTCTTTATTTCAGTTTTCAGACATTGTACGTGGCGTATGGGTTATATCCCAACCAC
TTCTTTTGATGTATTAGATGGCTTGTTGAAACTATGTTAGACTTCCGCTTTGTCAAAAAAATTTATGATAT

GAGAATATTTCTTTGAAATCTTTATTTCTATTATCTAGTATGATACATGGTAAGTGACTAGCATAGGGCCT
CTCGGGGTTCTATACGCTGTATTACACCTAGGGTATACTTTGGGTCGTGACAAACTTGGTATTCAGAGCA
CAAGGTTTAGAAAGGTCCTAGGATGGCTCACAAGCCACGTCAAGTAGAGTTTGTTCATGAGTGTGAAGC
GCACCATATTTATGAATAGGAGGTAATAGCCTATAAGATGTTTAGGAAATTCCACTTCTTTCTTTATTCTA
AAGTCGGGCGATAGAGTATAACTCTGTATGTCTCTCTCATAATCCTTACCCGATGCTTTTCGGGATATGG
CTCCTCGAAGAGCCTACGTTAGAAGGAATGCTGGAGAGAATGTGGAATAAGAAGCTCATCAATTTCTGG
CCGACCCTTTTGCCGAACAAATGACTAATGCGGAGTTCTGATTCGTTTTTCATGTTTTGCCCTCAAGTTCA
TAGGTATTGCAATCACATAAGTTTTATGTCAAACTAATTGTAATGATGTGAATATCAACAAGGAGTGATA
TGCAGCTAATTACAAAGCCATTAAGGCATCAACAACGTAATGAGCTATCAATACCCCATCTAAGCAATGA
CAATTTATCCAAGAAGATTCTTACGTCGCTTTTTAACATGTATCACCAACTAAACAAGAATAATCCCAATA
AGGAAAGCTCACATGTATACATATATATAACTCTAGCAACCAACTCCATCAACAACTATTATGACTATGA
GTACTATGGTGATAAAGAACAAGTCGCATTACCTATACGACAAAGAAACTTGCCGAAAAAAGTTAAGCT
TTAACATACCTTTTTTCATCGAATTTACTCTGTCATATTACTTCTGCCCGCACCTTATGATACTCGCTGCAG
ACACCCAAAGAACTTTCAAGAACTCAAAACACAGCCAGATCCCTACTGACACAACTTCCACAGAGCTACA
CCGCACAATCCACAGAGAAAAAATATACCAAAACAAAGAGCGCGAGACGTAGAGCAATTTCCGTTAAA
GACCCAAGACAAGATCATAAATATATATATGGATGATTTAAAGGTTGAATATCTCGC
>PGSC0003DMG400000386 LRR-RLPs
ATGGTGTTTGAGAATGATAGAATCAAGAATGCTTATATTGCTTTACAAGCATGGAAACAAGCTATCATTT
CAGATCCCTTTAATATCACTCTCAATTGGGTTGGTCCTAATGTATGCAGCTACACCGGAATCTTCTGTGCT
CCGGCTTTGGATAACCCCAAAATCCGTACAGTTGCCGGCATTGACCTTAACCATGGTGACATTGCAGGGT
ACCTCCCGGAAGAGCTTGGGCTTCTTACAGATCTTGGGATTTTCCATATCAATTCAAACCGTTTCTGTGGT
ACAATACCAAGAAAATTCAGCAAGTTGAAGATACTGTTCGAGCTGGATCTGAGTAATAACAGGTTTGCT
GGGAAGTTTCCTTATGTTGTTTTGAGTTTACCCAAGTTGATATTTTTGGATATTAGGTTCAATGAATTTGA
AGGTAATGTACCTTCACAGCTTTTTGATAAGCCATTAGACGCCATTTTTATTAATCATAATCGATTTGCGT
TTGAGTTGCCGGAGAATTTTGGGAATTCGCCGGTTTCTGTGATAGTTCTGGCGAGTAACAGTTTCCATGG
GTGTCTTCCGGCGAGTATTGGGAATATGAGTAATCTTAATGAGGCGATTTTAATGAACAATGGGTTGCG
TTCTTGTTTGCCGGCGGAGATTGGGGTGTTGAAAAATTTGACTGTATTTGATGTGAGCTTTAATCAGTTG
ATGGGTCCGTTGCCGGAGAATTTTGGTGATTTGGTGAATTTGGAGCAATTGAATGTGGCACATAATATG
CTTTCTGGGACAATTCCGAAAAGTATTTGTCAGCTTCCTAAGCTTGAGAATTTTACGTATTCTTATAATTTC

TTCACTGGTGAACCGCCGGTGTGTTTGGGGTTGCCGGAGTTTCATGATGAACGGAATTGTTTGCCCAAC
AGACCGGTGCAACGCTCTCCGGGACAGTGTAAGGCCTTTTTGTCTAAAAAAATTCATTGTAGTGCTTTCA
GGTGTCATAAATTTGTTCCTGTTTTGCCACCTCCTCCGCCACTTTCACCGCCCTTGCCTGCCCCTCCGCCAC
CTGTTTATTCGCCTCCGCCACCAGTTTATACTCCTCCTTCTCCTTCCCCACCACCTCCTCCACCTCCAGTTTA
TTCACCACCGCCACCACCACCCTCTCCACCTCCTCCATCACCTCCCCCGCCTCCACCACCAGTCTATTCACC
TCCACCCCCACCTCCATCTCCTCCTCCACCATCACCTCCACCACCACCCCCGCCATCTCCACCTCCACCACC
AGTTTATTCATCACCACCACCGCCACCATCTCCCCCTCCACCATCACCTCCACCTCCTCCCCCACCCGTTTA
TTCGCCACCACCATCTCCACCTCCTCCATCACCATTGCCTTATTGTATACGCTCACCTCCACCACCAAATTC
CCCACCTCCTAATTCTCCACCTCCACCGCTTGCTCACTCGCCTCCACCCCCTTCACCTTACTATTACAATTCA
CCAGCACCTCCGCCCCCAAATTCGCCGCCTCCACCACCAAATTCGCCTCCTCCTCCACCAGTATGCATATA
CTCATCACCTCCGCCACCAAATTCACCTCCTCCACCACCAACATACGTATATTCTTCACCTCCTCCACCTCC
TTCCCCATTGCCTTGTATAGAACCCCCTCCACCACCTCCTCCTTGCATTGAACCACCCCCACCTCCCTCCCC
TTCACCATCACCTCCTCCACCACCAGTTTATCATTACAATTCTCCGCCCCCACCATCTCCATCTCCTCCACCA
CCAGTTTATTATTACAATTCTCCACCCCCACCATCACCACCACCTACTCCAGTATATGAAGGGCCATTGCC
ACCAATAATAGGAGTTCAATATGCTTCACCACCTCCACCACCCTTTTATTAA
>PGSC0003DMG400000819 LRR-RLPs
CTCTTATTCTACCTTAGCTTTCTATAAACATTTTTAACAAGCACCTTCCTTCTTTACTTCTTTGTTCCCTCCAT
TTCCCTGTTTGATTGGATACTTTTACCATTTTCCACCACTGCCCCAATAAAACAAAAAAGTACACATTTTTT
TTTATATTTATGAAATGAAAGCATTTTAAGATACCCCCTCTTGGAGTTTCTTCAATGTTATAAGTTCTAGTT
TGGCTTTAAACACTTTTCTTGCATAGTTGACAAAGTTTATTACTTTCAAGATTTTGTCAACCCAACTCACTT
TCAAGCATTTTTTTGGGTTTTAAAGAGTGATGGGGGTCTTCAAGAACTTGTTTATGTGATAAAGTTGTTA
TCTTTGATCACTTTGTGTACTCATTTTCAAGAAATTTTAGCTTCAAAAGAGTGATGGGGGTCTTCAAGAAC
TTGTTTGTGTGACAAAGTTGTTTTCTTTTAACACTTTGTGAACTCATTGTGTGATAAAGTTGTTATCTTTGA
ACACTTTGTGTACTCATTTTCAAGAAAATTTAGCTAAGATAGGGGTCTTCAAGAATTCTAACTAGTTTGTC
TATGTGACAAAGTTCTAAACTTTCAAGAATTTGTCAACTCATTTTCAAGAAACTTTTTTGTCTACAAAAAG
TGATGAAGGGTTTCAAGAATTCAAGATTGTGTGTGTTAGTTATCTTCTTTCTCCTCCATTTTGGTGTTTGG
TGCAATGGTGATACTGAAAACATGGGAGCTCAAATGGAGAAAAATGAGATAAATTCTCTTTACTCAACC
ATTCAAGGCTTTGTTGGTAAATGGTGGAATGGTTCAGATCTTTATCCAGATCCTTGTGGTTGGACTCCAA
TACAGGTAACACAAAAAAAATTACATAATTTTTTTCACTTTTTAGTTACTTTTCTGCCTTAAGGATTCTGTT

AAGTAAAAAGGTAAAAAAAAAAAAAAAAAAAAAACTATCTTTTTTGTGCATATGGTTCTAATTGTCCTTT
TATTATTTTGTGGTATACATGACAAGATATGACTTCTCATTGTATATAGATTGTTTTAAAGCTTTAAGTGG
CAATTTTTTACTATGACATATAATCAAAGAAATTGATAGTATATTTGGAATGATAGCTAATTAGCCATAG
AAGTTTGATGAACCAAACTTCAATAATGAATATCAGTACATTCTTGGCTTGTTTTGTTTAGCAGTTTTGAC
AGACCAACATAGACAGAATATGTTTTAAAACACAATGATAATACAGGACAGGACAGAGCTTATACTAAA
TATACTCCGTTCGTTTCAATTTGTTTGTCTGGTTTTGATTTGACACGTAGTTAAAGAATGTACCATAGTTTT
CTTTAATCTTGTGGTTTTAAACATGTTATGTAGAAAGTACAAAAGATGCATTCTTTTTGGAACATACTAAA
AACGGAAGTAAGACTAACAAATTGAAACGGAGAGAGTGAGAGTGATATAGTTGATGCATTATACTCAT
ATTTCTGATTTGTTTTGTTTCTATTTCCTCTGTCCAGGGTGTATCTTGTGACCTGTTTGATGGTTTTTGGTA
TGTTACTGACTTAAACATTGGACCTATACATGATAATTCTCTAAGTTGTGCACCAAATGTGGAATTCAGCT
CCAATCTGTTCACATTAAAGCACCTAAAATCTCTCACATTTTTCAATTGTTTCGTGTCGCCTCGCCATCATC
CAACTTCAATCCCAACAGAGAGGTGGGAGTTTCTTGCTAGTAGTTTGGAGTCACTCGAGTTTCGATCAAA
TCCCGGTCTCATTGGCCAAATCCCAAGTACATTTGGTAGACTCATAAAGCTCCAATCTTTAGTACTAGTAG
AAAATGGACTCTCTGGTGAAATGCCACAAAGTCTTGGGAATTTAGTGAACTTGAAAAGACTAGTTCTTG
CTGGAAACAAACTCAACGGCGAAATCCCGGATACTTTTGGAAGGTTCACTCAACTTTTGATATGTGATTT
AAGCAGAAACAAACTATCTGGTGTCTTGCCTAAACCATTATTTGGAGGTTTGATCTCACTTTTGAAACTTG
ATTTGAGTTACAACAACTTACAAGGCAAAATCCCACAAGAGATCTCACAATTGAAGAATTTAACACTTTT
AGACCTTAGTAACAACAAGTTATCAAGTGGATTACCTAAGTCAATTCAAGAAATGACATGTTTGGAGGA
GTTAGTCTTGTCAAACAACCCAATGGGTGGATTCTTGGATGTTCTTGATTGGTATAGCATGATAAAGTTA
ACAACTTTGGATTTGTCAAACATGAATTTGACAGGTGAGATACCTAAGTCTATAGTAGAGTTAAAAAAGT
TAAGATTTTTGGGACTTAATGACAATAAACTCATTGGTAAAATCCCAAAGAATCTTGAAAATTTGCCTAA
TGTAAGTGCTATTTACCTATATGGTAACAATCTGACAGGTGAACTTCAATTCTCTCAATGGTTTTATAGGA
AAATGGGAAGGAGATTTGGGGCATGGGGGAATCAAAATTTATGTTACACTTTTGGTTTGGTGTCAAGTG
ATGTTCCATTTGGGGTAAAACAGTGTCAACAAGAACTCAAGTTTGTTATTGATGTTGAGATGAACTCAAG
TTTTGTGGATTTTCAATCTTCAATGGGATATTTGAATGATTGTGGGATTTGGAGATGTTTTGTAGTTGAAC
TATTCATGGTTATTCTACTATTGTTGAAATGAAAGGAATTTGACAATAGTGATTTACCCTTTTTTTTTTTG
ATATTTTGTTGTTGTTTTACTTGAAATGTACCACACATTTTGGTTTGTCTTTTAACATATGAATGAATGAAT
GTACATATTTTACC
>PGSC0003DMG400000888 LRR-RLPs

ATGGAAATCAATGATGTTCGGATTGTGGGGATCTGGGGAATGGGGGGAGTTGGTAAAACGACAATAGC
AAGGGCTATTTTTGATACAAACTCAAATCGATTTGGTGGTGCTTGTTTTCTTGCGGATATTAAAGAAAAC
AAATGTGGAATGCATTCTTTGCAAAATATCCTTCTCTCAGAACTATTGAGGAAAAGAGATAATTACGTGA
ATAATAAGGAGGACGGAAAGCATATGATAGCTCGTAGACTTCGGTTTAATAATAAGGAGGACGGAAAG
CATATGATATTTCGTATGTATTGAAAAAAACCAGTGCATTTGTATGTATGTCTAGTCGAAAAGCTTTTCCT
GCTGGGATATAACGAAAAGCTTTGCAAACGACAATAGCATGGGCTATAATTAACAGAAGCTGTAATTAT
GGTTTTCAGTACAACCCATGTACTGGTTTTTGTCAATACATACGAATGTTACCTTTCTCAAAAAAAAAAAG
TAGCTTTTCGACTCGACTCTAAAACCTGAGAAATTTGAGACTATTGTGTATAAGCTTTGGCATTGTATTAA
TTTATTGAGTGATAACATACGCAGGGAGTGTATTTATACACATACAAGAGTTAGACTTAACCTAAGACTA
GTTCTTCTAATACAATAGGGAGACTAACTAGTTGGATTCACTACTCTTACCTTGTCTCTGAAGAATATGGT
GGTCCTTTGCTAGAGTCTTTGTATGAATGTCAACTAGTTGATCTCTTGATCTGACAAACTGAGGATTAGTT
ACAAAGTGATAATCCAATTCTACATGTTTAGTTCTAGCATGTAGAACTGGATTTGTTGTCATACATAGTGC
ACTCATGTTATCACAAAATAGCGTAGGAGCAGACTTGATGTGTATCCCTTTATCCATGTGAGCTCAACAG
CTGTAGCTGCTAAGGCTCTATACTCAACCTCTGTACTTGACCTTGGCTAATTCTTTTAAAATTTGGATAAT
CATAATGAAATTATAGCCAATTGTCAAGTTTAAGGGGTTTAGATATTCTACCTTTTATTTATGGTAGTAAC
TCTTTTATTTAGTTTCTTTCTGTGTTTTGAATAGGAGCTTTTGCCGTCTCTACGAAAGCTAGATCTCAGCTA
CTCCAAAAGCCTGATGCAAGCACCAGATATCACAGGGATGCCAAATTTGGAGTATTTGAATTTGACGGA
TTGTATGAGTCTTGAAGAGGTTCACCATTCCCTAGGATATTGTGAAAAACTCATTGAGTTAAGTTTGACT
TGGTGTTCGAGGCTTGAAAGTTTTCCATGAGTTAACGTGGAATCTCTGCATCTAGTGGCTTGCTCTAGTT
TAGACAAATTTCCAGAAATCCTCGGAAGAGTGAAGCCTGAGTTAAAAATTTTCATGCATTTCTCTGGGAT
AAGGGAACTACCATCATCTATTGTTCAGCACAAAGCTTGTCTTCCAGAGCTAGATTTTAGTTCTAAGGAA
AATAAAGCAGCAGTTCTAAGTTGAAAGGTTTGGTGTGTCGCGTTGCTCAGAACTTGAAAGCTTGCCAGA
AGAGATAGGCTATTTAGAATACTTGGAGGTGCTTGGGCTCACAGTCTCCGCCTCCGCCTTCAATCTGTAA
TTTGTTGTTTCAGAATATGTCATCATTGCTGCACGACATCTCTGCTTCAAATTCTGTCACTAAGAGTTCTTA
CTACCAGTGGGAATATCCCAAGTTGGTTCCACCGTCGGGGAATTGGTAGAATTGTATCGGTCAATTTGCC
TGAAAATTGGTACGTTAGTTATAACTTCTTGGGATGACCCAACAATTTGCCTCATCCAACCATTCAGATTT
TGATACCAAACCTACTATTAATATTTTCTTGGTATCTTTTGCTGGCTTATGGAAAAACACCAAATGACTAT
GGACTCCTTGTGTTATGTTTATCTGGAGAAGTGAAGGAGTTTGGACTTCGTTTCTTGTATAAAGTTGAAC
AAGAGCTTGATGCCTTGTTACAAATGAGGGAAAATAATAATGAACAGATATGAGCACCATGATGAAGCC

AGTTTCTCCTCTTCGAAGAAACAAATATCTTATTTTAATATAGGTGGGAGCTGTCTTTGAGAATCTGCAGC
AACAAGTAGAGGCACCAGTTTATTCAGAAACTTTCCAGGTATTTCCCGCAAGCCCAAAATTTTGGCATAA
AAATAGGTATATACCTCATGATTTGTTTTTCCTTACTAGTTGGTAGTGAAATATAGTTTACGTATGTATTT
ATGTCAGTTAATCTAAGAAACTCCCTAAATGAAAGAATTGAATTATGAAAGATGTTTGAAGTTCTAAGGA
GTCCAGAGAAAAGGCAAAGGGTTGTTATTACTTATTCCCTAAGGTATCCACTGATCCATGGGTTTAAAAT
TTATTAAAATATCATTTTGATGCAAGGAAATCTCTGTTTTAAATACAAATATCTCTCAGGTTATGGCAGGG
AGTTTGGGAGCACAATGTGAGACAGTGGAGGAAGCTGGAGTACACGGTGAGGTTAGTAAATCACCTCA
CATCTTATGATTTGCATCATATCGTGGTGCTCCATCTGTGCAAGCCATTATTCTGCTTTTCTTAGTACCACG
TTTCAGATTTTGATGCTAAGGTTGTACTTCAGTTTTTAACTATACCTGTTCCCTTGATCATGTCTTTCTGCA
GATGTATACCCTACCAATATAAATATTTCACCGTGTCATCGTTGGTAGATGAGGATGCCTTATAAGTTCTT
CTAACAATCCACAGAGAAAAGGCAAAGTGTTGTTTCCAAAGGTAGCCACTGATCCATGACTTCAAAACT
AATTACTAGTTCAATTTGATGCCCTCTTGTTATGGAAGGGAGTTTGGGAAAAAGATAAACTCATTGAAG
GTCCAGCACAGTGTGAAACAATGGAGGAAGCTGGAGTACGAGGTTAATTTGAGGTTCGTTTCCCTCGTG
ATCTCTTTCACCTCTTAGTTTTGTTTTAAATTTTCTATTACATCAGATAAAATATGTTGCCATTATCGAAAA
CATCAGAATACTAGCTGTGATAGAGTTGGCTTATGACCTTCCATCTCTCTCTCTCTCTCTCTTTTTCGTAAA
TATTGAATGCTAGATTAGTAAGCCAAGACTGAATGCACCAATTCATGATTAGATGTTGTAATCGTTGTTG
ATTTAGGAATATGCGACCTTTTTATCTTACTGTCTTCTGTCTCGCTTGTTGGCTTTGATCTTCACAAAAGTA
ATAACAAGTAGCATCTATGTTTTGATTACTTATTTCAAGTGGTTTTAACAAGTTTCACTCTTTTGGTTTTGC
AGTGCCTGTGAAGGCACATTGTTTTCTGCGCTCGTAACAGAACAACCAGACCCTTTGCCTGAGAAAGAG
ATTCACGGAACGACAAAAGTTATCCGTTCTCTTCAAATCTTTATATATCAATTTACTGGTCTTATTAGTAA
AGGTGACACAACCCCTCTCTTGGATCAGAATACCACGTTTCATTTTGAAATAAAATTATGTCTTCACATTG
CTGATGAGTAGCCTGTCCAAAATCATATAGCAGATGAATGTGCGCGGGAAGCAAGAAAGCTATGCTAA
AAAGGGTCAATGAAAGAAACATTGGAACTACTATATCTACTACTCTGCATATATATATAGTTTCATAATCAA
TTATTGGTAATGGTAAGGAAGATTCAGAAAAGCTGTCGCAATTGTAGACACTAATATTTGTTTTAATTAA
TTTGTCAACTCTTCTCTATTGCCCTTTTCCAAAGTTAGCTGTTATCTGGTGACACAAAAATTGTTTAGATTA
TTTTTATTGATTCAGTCTGTTGAAGACTGAGCAATACTTTGCTGGATTAAAGACTTTTAGCTGTCCATTGT
GTACATATTACGATGAAGTTTTCGTGTAGGGGACTGGTCTTTGAAGGAATTTCCTAATACCACAATATTT
CTAATCAACTGGAGTAGACATTAAGAAGCTTCACAGAAATTTCCTATTACTTCTATAGTTCTATTAATATT
CAATGAAAATGTACCACATATAATTTGACTAGATCCAACAATAATAATTTATTTAACTGGAGTAAACCGA

ATGCTCATAGAAGCTTTTTGTTTCTTCTTTCCTGTATATAACTTTGTCCAAAATATCATCAATTGATCTCAT
CCATACAGTCTATTTTTCTTTTCAGACAATTAATCAAATATATGGCATCATCTTCTTCTTCTGTGAGTAATT
CACAGTACTGTACTCGATGGAAGTACAATGTCTTTCTAAGTTTTAGAGGCAAAGATACCCGGAGAAATTT
TACGAGTCACTTGTACGAAGGTTTGGACAATAGGGGAATATTCACCTTTCTAGACAATAAAAGGCTAGA
GAATGGCGATTCCATTCCACAAGAGCTCTTGAAAGCTATCGAAGAGTCTCAAGTTGCACTCATCATTTTC
TCAAAGAATTATGCTACATCCAGGTGGTGCTTGAATGAACTAGTGAAGATCATGGAATGCAAGGAGAA
GGAAAATAATGGAGAAACAGTCATACCGATCTTCTATGATGTGGATCCATCACATGTTCGAAACCAAAA
TGAGAGCTTTGCAATAGCATTTGCCGAACATGAATTAAAGTATAAGGATGATGTTGAGGGGATGCAGA
AGGTGCAAGGATGGAGGAATGCCCTAAGTGCTGCCGCAGATCTAAAAGGATATGATATCCGTCACGGG
TGAGTTAAAAACACATAAATTGTTTTTATGAAAAAGGAAATGGAATAATTTCCATCCAAATTTAGATGCT
TCTACGAAACCTAGACTTATTCTACAATACAACAGGAATCTATACCTTCAAAATGAGTTAATTTAGTTAGA
GAGAATACACAATAATCTTCAAAATGTGCAAGTTTTATACTTCAACTGTAGAGTGTTGCTGTTTCCCTGAA
CTACAATGTTTCCTATTTGATTAACAATATGAAATACTTACTACTACTTGACTTTCACATTTTCCACAATCA
ATCTTCTTATTTATGCAGGATCGAATCAGAGAATATTCACAAGATCGTCGATCACATCTCTTCCAAATTAT
GCAAGACTTCTTATTATTTATCTTCTTTGCAAGATGTTGTGGGAATAAATGCTCATTTAGAGAAACTAAAA
TCCCTACTTCAGATAGAAATAAATGATGTTCGGATTGTAGGGATTTGGGGAATAGGTGGAGTTGGTAAA
ACGACAATAGCAAAAGCCATCTTTGATACTATATCTTATCAATTTAAAGCTTCTTGTTTTCTTGCGGATGT
TAAAGAAAATGCAAATTATAATCAATTGCATTCTTTACAAAATACCCTTCTCTCTGAACTATTAAGAAAAA
AAGATGATTATGTCTATAATAAGTATGATGGAAAGTGCATGATTCAAAGTAGGCTTTCTTCTAAGAAGGT
GCTAATTGTGCTTGATGACGTAGATCATGTTGATCATTTGGAGTATTTAGCAGGTGATGTTGGTTGGTTT
GGTAATGGCAGCAGAGTTGTTGTAACAACTAGAAATAAACATTTGATAGAGAAGGATGATGCAATATAT
GAAGTATCTACACTACCTGATCATGAAGCTATGCAATTGTTCAATCGACATGCTTTCAGAAAAGAAATTC
CAAATGAATGTTTTATGAAGTTCTCATTGGAGGTAGTAAATCATGCTAAAGGCCTTCCTTTAGCCCTCAA
GGTGTGGGGTTCTTTGTTGCATAATAAAGGCCTAACTCAGTGGACAAGCACTGTAGACCAAATAAAGAA
AAATTCTAGTTTAGAAATTGTTAAAAAGCTCAAAGTAAGTTATGATGGGTTAGAGCTTGAAGAGCAAAC
AATATTTTTAGATATAGCATGTCTTTTACGAGGAAAAGAAAAAAAACTAGTCATGCAAATCCTTGAGAGC
TGTGATTTTGGAGCAGAACACACATTGGATGTCCTAATTGATAAATCTCTTGTGTTCATCTCAAAAAATG
ATAAGATTGAAATGCATGACTTAATTCAAGATATGGGTAGATATGTGGTAAAAATGCAAAAAAAATCTG
GAGAACAGAGTAGACTCTGGAATGTTGAAGATTTTGAAGAAGTGATGGTCAACAATATAGTAAGTACTA

AGTAGGCTAACAATGCAATGATATTTATTAGTCTTCATGTTTTACTTTTTATTTAAAAGAAAAAAATTGTT
GGTACTGTCTCAATTACTACTTCATTTTATCATAACATGATTTTTTTTTTTTCAATTTGACTCTTTAGAACTT
GTCAAAAATTGTAATACTAGAAATTAGAAATATAGAATTAATTGCATAAAGTATTTCTCTTTAGCAACATT
TTTCTTAAGAAAGTCCTAACTAATATTTTACCTCTTTTTTTTTTTTTTTTTTTAGTGTTGCTAGCTTTTCAAGC
GAAATAATAAATAATGCAAAATATGATGAAATTTGGCATTGCAAGGAGTTATAAAGTAGTCATGAGATT
TTAAACAAACATTTGAAGTTGTGCTTTGTAGACTAATGGATCTTTTATCAGGGGACCAACGCAATGGAAG
CAATTTGGATACATGGTGTTCAAAAACTATGCTTTAGTGAAAAAATCATGAAAAAGCTTAGATTATTATC
CATCATCAGCTTTCATACACATGATGACTCCATTGAGTATCTGCCCAACAACTTGCGTTGGTTTGTTTGTC
ATGATTATCCTTGGGAGTCATTATTAGAAAATTTTGAACCTAAAAAGCTTGTTTATCTTGATCTCCAGCAC
AGTTTGCTGCTTCAATTATGGACTGGAACAAAGGTACAATTACTGAATACAGTTTTATTTATGCTTCTTTT
TGTCAAATACATCTTGACCATTCTAATTGAAAAACTTGACATAAAAATAAAGTAAAACAATTTCTAATTTC
ACACAAAATATTGTGTTGACTCTCATAATCTGAATGTCCTTACTCATTTTTCGTGTAAGGGAGTAAAATTA
TACGATTGCCCACTAGCCTAATTCGCATTCTTTCTATGTTCTGAATTGCAGCATTTGCCGTCTCTGCGAAA
GCTAAATCTTAACCACTCCAAAAGGCTGTTGCGAACACCAGATTTCACGGGGATGCCAAATTTGGAATAT
TTGAATTTGTGTAACTGTAGTAATCTTGAAGAGGTTCACCGTTCCTTGGGATGTTCCAGAAAACTCATTT
GGTTAAAGTTGTATAATTGTGAATGTCTTAAAAGGTTTCCATGCGTTAATGTGGAATCTCTTAGATCTCTG
GATCTACGATATTGCTCTAGTTTAGAAAAATTTCCAGGAATCCTCGGAAGAATGAAGCCAGAGTTAGAG
ATCAAGATGAGACGCTCTGGGGTAAGGGAAATACCATCATCTTTTATTCAGCAACACGCATGTCGTCTTA
CAGAGTTAGATTTGAGTTTTATGAAAAACCTTGTATCTCTACCAAGCAACATTTGTCAGTTGAAAGGTTT
GGTGAAGCTAACTGTATCCTGCTGCTCAAAACTTGAAAGCTTGCCAGAAGAGATAGGTGATTTAGAAAA
CTTGGTGACGCTTAATGCCGAATGCACTCTTATTTCACGAGCCCCGTCTTCCATCACCTGGTTGAACAAGC
TTAAATACTTGAGTTTTGCATATCAAGAATCAAATTTTGGGGTGTCCTTTGTGTTCCCTGAGGTGAATGA
AGGGTTACGCTCATTGGAATCTCTGAATCTCAGTGACTGCAATTTAATAGATGGAGGACTTCCAGAAGA
CATTGGATGCTTATCCTCGTTGAAAACAATGTATCTCAATAGAAATAAATTTGAGCATTTGCCTCGAAGC
ATTGCCCAACTTGGTGCTCTTCAATCCTTGTGCTTATCAAATTGCATGGGGCTTAAAGAGTTGCCAAGTTT
CATGGGGATGCCAAACTTGGAGACTCTGAATCTCAGTAACTGCAATATGATAGATGGAAAACTTCCGGA
AGATATTGGATGTTTATCGTCTTTGAAAGAGTTGAATCTCAGTGGAAACAGTTTTGAGTATTTGCCTCGA
AGCATAGCCCAGCTTGGTGCTCTTCGATCCTTGGACTTATCAGATAGCAAGAGGCTTACACAGCTGCCAG
AATTTCCACAACAATTAGATACAATAGGTGCAGATTGGACTAATGATTCGATCTGTAATTCGTTGTTTCA

GAATATCTCATCATTGCAGCATGACATCTCTGCTTCAGATTCCTTGCCACTAAGAGTGTTCACCACTAGAG
GGAATATCCCAAGATGGTTCCACCATCGGGAAAATGGTAGAAGCATATCAGTCAAATTGCCTGAAAATT
GGTATATAAGTGATAACTTCTTGGGATTCGCTGTATGTTACTCTGGCAAATTAATTAACGCAGAAGCTCA
CTTGATTTGTTATGATGGGAGGCCAGTGACGTGTATCACCCAGAAACTTTCCTTATCCAACCATTCAAAA
TGTCCTGCAGAATCTGCTATTCATTTTTTCTTGGTACCTCTTGCTGGCTTATGGGACACATCTAAGGCAAA
TGGAAAAACACCAAATGACTATGGGCGCGTTAAGTTATTTCCCTCTGGAGAAATGAAGGAGTTTGGACT
TCGTTTGTTGTATAAAGATGAACCTAAGATTGAGGTCAGTTTCTCCTCTTCTAAGAAACAATGA
>PGSC0003DMG400001016 LRR-RLPs
ATGTTCCTAGTAATTTATGCAATTATGAAAAAAGCCAATTGCTCTTTCCTCTTGACACTCTTGTTGCTTCAC
TATGTCATGGCTACTTCAGCCATGAACCCAACAAACTTTTCTACCGATCAATCTGCTCTCCTTTCCTTAAAA
TCTCAAATCATTTTGGACCCTTTTCACTTGTTGGATGAAAGTTGGTCTCCAACTACTTCTGTTTGCCATTGG
GTCGGAGTCACTTGTGGTTCTCGCCACCAGAGAGTGAGATCACTCAATATTTCCAATATGGCATTAATCG
GAAAAATTCCTTCTCATATCGGGAATCTCACATTTCTTGTTTCCCTCGACTTGAGTCACAACAACTTGTAT
GGCAATTTGCCTCAAGAAATAGCACATCTACGTCGATTAAGGTTTCTTAATTTGGGTGTTAATATGCTTA
GCGGGGAGGTTCCTTCATGGTTTGGGAACTTACACAAACTTCAAGTTCTGAATCTTAGGAATAATAGTTT
CAATGGTTTCATCCCCTCTCTATTATCTAATATTTCCGGGCTTCAAACTTTAAATTTGAGATTCAATTCTTT
AGGTGGTCAGATACCAGAAAAGATTGGCAAACTTCAAAACTTAAAGGAATTAAATATAGAAGCTAATCG
ACTTACAGGATCTATACCATTCTCGATCTTCAACATTTCTGGAATAAAGGTCATTGCATTGACAGATAACA
CTTTATCAGGAAATCTTCCTCCTGATATGTGTGATCGACAATCAATGTTGGAAGAGCTTTATCTAAGTTTC
AACGAGCTACGTGGTCATATGCCTCTAAGCTTGCCAAACTGTTTGAAACTTCAACTATTGTCTTTATCAGT
GAACAAGTTTGATGGACCTATACATAGTGAAATTGGACATTTAAGTGACCTACGTATTTTGTATCTCGGA
CAGAACCAATTTGAAGGTTTGTTGTTGAGTGCTAGATTTTCTTCTAACTAACTATTTCCGAAAAGAAACCA
TCACACATAAATTTATATGTATGTTATTGTTTTGACAGGGACAATTCCACAAGATATTGGGATTCTTGATA
ATTTGGAGGTTTTGGGGCTAGAGAGAAACCATCTAACTGGGAGCATCCCAATCTCCATATTCAATATCTC
CTCGTTGCAAACGATGTCATTGGAAATGAACAATCTCATGGGATCCTTACCGAGGGAGATAGGCAACTT
GAACAAATTGCGGATTCTATATCTTGGTTACAATATGCTAACAGGTATGTAAGTAGTCATTGGGTTATTG
AAAACATAAATTCAAGCAACTTGAACATTAGTCACGGAAGTGGTCAAACAACTAACGTAAAAGAATTGG
CAGGGACAATACCACAGGAAATCGGGATTCTTGATAATTTGGTGGAATTAGGACTAGATAGCAACCAGA
TTACGGGTCATATCCCAATCTCCATATTCAATATCTCCTCGCTGGAGACGTTGTATTTGGAAAGGAACCA

CCTCATTGGATCCTTGCCAGGGGAGGTTGGCAACTTGACCAAATTGCAGAGTCTACGTCTTACTGAAAAC
AAGCTAACAGGTATGTACATATATAGTAAAAAAAGGTACTCCTTTCATCCCAATTTATGTGATGTAGTTT
GACTTAATACAAAATTTAAGAAAGAATGTCTTTTGAAACTTGTGGATATTTGTATGGGTACAAATAATTT
TTAAAGTTAAATTGTTACTACATATAGAAATATATTATTCTTTTTTGACGGACTAAAAAGAAAGTAAGTCG
TATAAATTAAGGCAGATGGAAACTAAAGTATCATTACTAGTTGTACCTTTACTATTTTAACAATCATACAT
TTGCAGGTAAAATACCAGACGTAAGCAATCTCGTTGAATTGGTTGAAATTTACTTGGGTCTCAACAAGTT
TACCGGTGCACTTCCAACGGAACTCTTCAACATGTCAGGGATGAGAGTGATCGAACTTGTGCGAAATAA
TCTGACAGGAAGCGTCCTCCCATTGAGCATAGGTTCTATGTTACCCAACATTGAAGGTCTTTATCTATCTA
GTTTAAATCTTTTTGGAACTATACCTCATTCTCTCTTCAATTGTTCCAAACTCATTTATCTTGACCTTTCTTA
TAACAAACTAACCGGTTTGATCCCAAACTCTTTCGGATATATGACTCATCTAAAGTACCTAGGCTTGGGG
GGGAACAATTTAATGAGTGACACTAGGTCGAGCTTCTTTTCTTCCTTATCAAATTGTAGAGAATTACAATT
TCTTGATGTATCGTTGAACCCGTTGAATGTTGTGCTTCCAGTTTCAGTTGGAAATGTCTCTCTTGTAAAGT
TAATGGTTGTTGAATGTGGACTTAAAGGGGAAATTCCGAAAGGAATTGGGAACTTAAGTAGCTTAATAG
ACCTCGATCTCTCTGGAAATGGTTTGGTTGGATCAATTCCCACAACAATTAGCAACTTGAGACTCCTTCA
GAGTCTTAAGTTGAGTGGCAATAAACTTTCTGGATTTATCGGAGACAATCTATGTAAATTGCAGAACCTG
GGGTATATACATTTGGATCAAAATCAACTTTCAGGATCTCTTCCTAATTGTTTCGGGAACTTAACTTCCCT
TCGGGAGATATTTTTAGGTACCAACAAGTTGCATTCAAACATTCCAGCTAATTTAGGAAACCTCAAAAAT
CTCCTAAACCTTGATTTATCCTCGAACAACTTGACTGGCTCTTTACCTCGAGAAATCGGAAGTCTAAAGG
CCATGATACAGATGGATCTTTCGATGAATAAACTCTCGAATGGCATACCTGAAGAAATTGGTAGCTTGCA
AAATCTGATACACCTTTCATTGAGAGACAACAAATTGCAAGGTTCAATTCCTAGCTCAATGAGCAGCATG
TCAGCTTTGGAATATCTAGACCTTTCTCATAACAATGTCTCGGGATTAATTCCCAAGTCTTTGGAGAAGCT
TCTAAATCTCAAGTATTTCAACGTTTCATTCAACAATTTGGTTGGTGAAATCCCCTCGAGCGGTCCTTTCA
AGAACCTATCCAGTCAGTCTTTCATATCCAATGAAGCATTGTGTGGTTCTTCAAGATTTCGTGTCCCCCCA
TGCCATAGTGGAACTTCAAAGCATAGATCCAAGAGAAAAAAAGTACTTGTTTTGATTCTACCGGTTGGA
ATTGCACTTGTATTAGTGTCCATCGCCTTTGCGTTTGTATGGATAAAGTATATAAGAGGAAAATCAACTG
ATCCTCAACTAGGAGTTGATCCGTCCTCTTTTGTCTCAACAAGAGGAAGAATTTCATACTATGAATTGCTC
CAAGCAACTGATTCACTCAACGAGAGCAATTTGATTGGTTCAGGGAGTTTCGGCTCTGTTTACAAAGGC
ATCCTAAGAGATGGGACTTTCATTGCAGCTAAAGTGTTCAATCTACAATTGCAAGCGGCATTTAGGAGTT
TCGATACAGAATGTGAAGTTTTGCGCAATCTTCGCCATAGGGATCTCACCAAAGTCATCACTAGTTGTTC

CAACCTTGATTTTAAGGCGTTGGTACTCGAATACATGTCCAATGGGAGCCTCGATAAGTGGTTGTATTCA
CACAACTACTTCTTAGACATCAAGCATAGGCTGAGTATAATGATTGATGTAGCATGTGCATTAGAATATC
TCCACCATGGCTGCTCATTGCCCGTGATACACTGTGATTTGAAGCCTAGTAACATCTTGTTGGACGAGGA
CATGCTTGCACACCTAAGTGACTTTGGCATTTCAAAAATGATTTCTGAAGATGAGAGTGCTTTATACACT
AAAACTTTAGCAACATTGGGTTATATCGCACCAGGTAAGTTATCTTAATTAATTGTTTTTTTTTTTGAATAT
TTTCTTTTTCCTTTTTCTGCCAAGAAATGTTGAATATAAAATTCTCATTGTAGAGTATGGATTGGAAGGAA
TGGTGTCAACCAAATGCGATGTTTATAGTTATGGGATAATGTTGATGGAAACATTTACAAGGACAAAGC
CTAGTAATGAGATGTTTGATGGAGATCTTAGCTTAAAGCAATGGGTGAGCAATTCACTCCCACATGCAG
TAATGGAAGTTATCGATCCCAACTTAATAACACCACAAGATAATCACTCAGTGAAAAAAATTGATTGTGT
GGTATCCATCATGAAAATAGCAATAAATTGTTGTGTTGAATCTCCTAAAGGAAGGATTGACATGAAAGA
TGTTGCCGCGAGGTTAAAAACCATCAAGATTCAACTTCTTGAATGTTGAACAAGTTCTCAGGTTTAACAA
ATGTTTGCTAGAACATTGATCCTTTAAATATGCTATAGATTTACATCTTTTCATCACCAACATAGAGAACA
AATGTTATGTATACCTCTTCCATCTTGAATTTTTGAAGTGGTTTTAGTCTAATCCCCCATTACTTTATTTTAT
ATTTCCTTTTTATCGTTGATAGAACTCAATACATAACTTCTAATGGCTTTCATTAAAATATGTTTCATATGT
CACTACAACAAAAAACAAATTTTAGTCGCGATAAATATACACATTAACAAAGAGTGCTAATTTTTTTACCA
GCATTAGTTAATTGTCATTGGATTCAATGTCGCTATAGGCTTTAGGGACATATGTCGTTTGAGTCTCAAA
ATAGAATGACACCAAGTAAATTGAGATAGATGGAAACATTGTTCGTGACAAGACAAAAAAACTCGTTAG
GTATGGGCAAATAACAATAATTATACAACCATCAACCTTCATCTATATACCTACTATATCCCAATACTGGC
CATATAACATGTATGCAAGACAAAATTAAACGCCACTACTTTGGTTCTCTGTTACGTATATATACGAAGA
AATAAAAACTCATTTCCAGAAGATCACTATTATATTATAACAATGGAGTCCATAATTAGATGGAGAAAAC
CGAGTCTGAAACCAATTGAAACTAAGGATAAAGCCATTGACGCATCTGAAGATCGAAGAGCCATTGAGT
CTGTCATCGAGGCTATTTCATGACCCCAACTTCAACGTGCACGTTCTAGCTATTTTGGGCATCAAAACAA
GAGCCAGCACTGAGCAAGCTCCCAGAGAGCATGTGGCTAATACTTTGCTTAAACATCCTGTTTCTCCAGC
CTGCCGTTACCAGTATAACACTTTGTTTGTCTTACTTTCCATTTTAGTAGGTTAAAAATGAATTTTCTTTTC
GTGACAACTGACAACCCTTTAATTCTAACTTTCTCATTACATGTTTAATTAGACGACAAAATAAACAACAC
TTTGGTACATTCTATGTGTATTTAGTTTAAGACCGTAAGATTTAAAAGTCTTCTTTACTTTCTTAAACTCGG
TGTCAAGTCACAAATAAACAACTTTAAACAGAAAGAACACTATTTTCTGATTAAAATCACCAAGATGAAT
AGATGTAAAAGACAATGAATTGATAACCTGCTGCTATCAAATAAAAACATACATTACACTTGATGGTAGC
CAATAAAAATTGAAACTACAAGTACTTAAGAACAAGTCTAGTGTACTTCTCCTCAGTAAGTTCAATACAT

AAATATTGTAAACTAATAAAAGGGAGAATTGTCAAAAAGCATAAAACGCTCGTATTCAAAGATCTTGTTT
TACTTTCATAGCCCTCATGGTTAATTCACTGCAGGTTATTGATGAGGGAAAAGGCGGAGAAATGAAATG
GGTGCGGACAAAGGTGGACTTAGATCAGCACATTGTGGTGCCACAAATTAATGAACAGAACCTGCAGG
AATCACCATACAAATTTGTGGAGAACTTCTCTTATTTCCCTTCTCTTGGAGTGTACACACCAAACTGCTGA
TCCAGATAAAGTTCCACCTATTCATGGAAGTAAAAAGAGGCTTATTGATTCTTCAAAGCATTCCAATAAG
GGATTGCGGCGACATGTTACAAAAATTTGGTCTTTTATGATATTGTTTGGGTACACAAAAGTGGATGTGT
TGATGTTTATGGCTACAACTATGTTCTTGAAGGACACAGCTACACCGATTAAGAGTAGACCTGGTTATGA
GTCTAATCCTAGGTGATTTGTTTACCGGACAATAAGTCTTGACGATATGAAATTGGTGAAAAATGCATAA
AATATGTTAAGCCCATACAAATTAATTCTATATTGTAGCAACATCCTTCTCTTCAATTTTGAAATGTAGAT
GTTTATAGCGTGTTGTTTGATGTTGTTGTCGGACTTTGTCATTTCTTGCACAGACAGTAACGGATGTCGCT
TTGGGAGTAACACAAGATGGTTTGTCTATCTATCTTAATAGAAGATATGGTAAGTTTCTGTTTCATGTATT
TGGACTTATTAAGCTTAGTATTAAGAAGTCCCACATCGGTAAAGGGATGGGAAATTGGTCTCCTTATATG
GACTTGGACAAACCTACCCTCATGAGCTAGGTTTTGGGTTGAGTTAGGCCCAGGTGCATATCTTTACATG
GTATCAGAACTAGACCCATCCCAAGTTGTTGTTCACCGATGTTGGCCCCCATTTAGAAGTGTTCACGCTC
CAGTTGAGTTCTGGGCGTGCGGGGAGTGTTAAGAAGTCTCACATCGATAGAGGGATGAGAAATTGGTC
TCCTTTATGGACTTAGACAAATCCCCTTCATGAACTAACTTTTGAGGTTGTGTTAGGTCCATATCTTTACA
CTTAGTTGATCGAAAATCTTACGGAAGAAATCTTTAATGCAAAATTTTGCAGGTGAGGTAAGAAAGACA
GGAGCAACAGAGAAAAAAATAATCTTCCGATGAACATTAGACTTAGATCAACTATACTTATGAACCTAA
GACCGGCAGTCGGAATTCAGGTGATGGAATCCATTCAAATTTTAATCAGCACTTAAGTTTTCTTAGATTA
TATACTTAAAGTTATTAACATTTTTTTGGGGCTTTCTTTCATGTTTAATTAATTAGCAAATAGGCTTTTGCT
GATATGATGGAGAATGACACTGAGGCAAAATGGGGGAATTGGATTGGTTTTGTTCTTTTCCCCTTAAAA
GTTGCATTACGAGATGATCCATTAGATTATATCAGAGAAGCTAAGGCAACTATTGGTCGAAAGAAGAAT
TCACTTGAAACTTTCTACACATTTTCTATTTCAGAATTAGCACTCAAGATTTTTGGGATTAAGGTAACTAA
TTTTCATTCTCAGTTCTTAATCCTACACATGAGATATCTTCATTGTAAGATGTGACTCTTTTTTCAGACATC
AAGTTCGATATCGCACAGAACTATCACCAATACAACAATGTGCTTTTCTTGTCTGCCTGGTCCTCAAGAA
GAAATTTGCTTCTGTGGGCATCCTTTGGCTTACATTGCACCCAGTTCTTATGGCTCATGTAGGTGTTTTAC
TCTTACTAATTTCAAGTTTTTGCTTCCATTCTCAGCTAGATTTTGGGGTGTGAATTAG
>PGSC0003DMG400001072 LRR-RLPs
TATCTCTTCTTCTCCACAAAACTTCATCTCTCTCATCTCACATTTTGCTTAAAGATTCCATTTTAAAACCAAA

AATAAAAAAATTCCCCAAACCCCATTTCTCAAATCCATTTTTCTTCATAGATCTTCACTTTTTTCATCTCACA
TTTGGTTAAAGATTCAATCTTTGAACAGAAAAACGCATAAAAAAACAAGCAGTCTTGATTATCAGATGAC
TCATGTGAGATGAAGGAGAAAACTCAAACACCCATTTGCTCTCTGTTTTTTCTTGTGCTCTGTTTTTTGTG
TTCAGTTTTTACTGAACACACAAATGCTGAGTTTACTGTCTCTGAAAATGGCCCTTTAACTGATTCTGAAG
CTCAGTTCATCAAACACAGGCAGCTTTTGTATTACAGAGATGAGTTTGGTGATAGAGGTGAAAATGTGA
AAATTGATCCATCTATGGTGTTTGAGAATGATAGAATCAAGAATGCTTATATTGCTTTACAAGCATGGAA
ACAAGCTATCATTTCAGATCCCTTTAATATCACTCTCAATTGGGTTGGTCCTAATGTATGCAGCTACACCG
GAATCTTCTGTGCTCCGGCTTTGGATAACCCCAAAATCCGTACAGTTGCCGGCATTGACCTTAACCATGG
TGACATTGCAGGGTACCTCCCGGAAGAGCTTGGGCTTCTTACAGATCTTGGGATTTTCCATATCAATTCA
AACCGTTTCTGTGGTACAATACCAAGAAAATTCAGCAAGTTGAAGATACTGTTCGAGCTGGATCTGAGT
AATAACAGGTTTGCTGGGAAGTTTCCTTATGTTGTTTTGAGTTTACCCAAGTTGATATTTTTGGATATTAG
GTTCAATGAATTTGAAGGTAATGTACCTTCACAGCTTTTTGATAAGCCATTAGACGCCATTTTTATTAATC
ATAATCGATTTGCGTTTGAGTTGCCGGAGAATTTTGGGAATTCGCCGGTTTCTGTGATAGTTCTGGCGAG
TAACAGTTTCCATGGGTGTCTTCCGGCGAGTATTGGGAATATGAGTAATCTTAATGAAGCGATTTTAATG
AATAATGGGTTGCGTTCTTGTTTGCCGGCGGAGATTGGGGTGTTGAAAAATTTGACTGTATTTGATGTG
AGCTTTAATCAGTTGATGGGTCCGTTGCCGGAGAATTTTGGTGATTTGGTGAATTTGGAGCAATTGAAT
GTGGCACATAATATGCTTTCTGGGACAATTCCGAAAAGTATTTGTCAGCTTCCTAAGCTTGAGAATTTTA
CGTATTCTTATAATTTCTTCACTGGTGAACCGCCGGTGTGTTTGGGGTTGCCGGAGTTTCATGATGAACG
GAATTGTTTGCCCAACAGACCGGTGCAACGCTCTCCGGGACAGTGTAAGGCCTTTTTGTCTAAAAAAATT
CATTGTAGTGCTTTCAGGTGTCATAAATTTGTTCCTGTTTTGCCACCTCCTCCGCCACTTTCACCGCCCTTG
CCTGCCCCTCCGCCACCTGTTTATTCGCCTCCGCCACCAGTTTATACTCCTCCTTCTCCTTCCCCACCACCTC
CTCCACCTCCAGTTTATTCACCACCGCCACCACCACCCTCTCCACCTCCTCCATCACCTCCCCCGCCTCCAC
CACCAGTCTATTCACCTCCACCCCCACCTCCATCTCCTCCTCCACCATCACCTCCACCACCACCCCCGCCAT
CTCCACCTCCACCACCAGTTTATTCATCACCACCACCGCCACCATCTCCCCCTCCACCATCACCTCCACCTC
CTCCCCCACCCGTTTATTCGCCACCACCATCTCCACCTCCTCCATCACCATTGCCTTATTGTATACGCTCAC
CTCCACCACCAAATTCCCCACCTCCTAATTCTCCACCTCCACCGCTTGCTCACTCGCCTCCACCCCCTTCAC
CTTACTATTACAATTCACCACCACCTCCGCCCCCAAATTCGCCTCCTCCTCCACCAGTATACATATACTCAT
CACCTCCGCCACCAAATTCACCTCCTCCTCCACCGCCAACATACGTATATTCTTCACCTCCTCCACCTCCTT
CCCCATTGCCTTGTATAGAACCCCCTCCACCACCTCCTCCTTGCATTGAACCACCCCCACCCCCCTCCCCTT

CACCATCTCCTCCTCCACCACCGGTTTATCATTACAATTCTCCGCCCCCACCATCTCCATCACCTCCACCAC

CAGTTTATTATCATTACAATTCTCCGCCCCCACCATCTCCATCACCTCCACCACCAGTTTATTATTACAATTC

TCCACCACCACCATCACCACCGCCTACTCCAGTATACGAAGGGCCATTACCACCAGTAATAGGAGTTCAA

TATGCTTCACCACCTCCACCACCCTTTTATTAATTCTTTCAGAAATTTCACTAACTCTTATCCTCAGAGCAC

AAAATAGTTCCTACAATTATTATTTCCTCAACTCTTCAAGCCATCAACAAAATCGAGCTAATATGTGATCA

CGAACCTTAAATGGTACGACAGAAAGAAAGATAGAGGAGGTAAAGAGGAGAAAGATTCAATCTCCAAT

GATTTTATTTTTGAATTGTTATCAAATAGATTGAAGTTGTGGAGAGAATAAATTGGCAAAGTAAAGATTG

AAACTTTGCTGGAAAATGTATGTATAGAGAGATCATAATCTTGAATTAGAAACCAGTTTACAGTGCTTGC

TGCAACTGGTTTTATTTAATGATCATACTTTGATTTCTTTGTAATTTTTTCTTTTTTTTTTTGGATCTTCCAAA

CTATAGTATAAGATGAGCTTTTTATTTTCTTTTAAAACTTGTATTGATTCCTCATAATTCAGATGAT

>PGSC0003DMG400001252 LRR-RLPs

ATCAATTTAGAAAATTTTAGAGAGAGAAACAAAAACAACACCCTCAGGATGGGCAGCTTCATGTACATT

GTCATCCTGCTTCTTGCCATAATCTCAACCGTTCATTCCTGCTCGCCATCAGATCGGGCAGCATTATTATCT

TTCCGAGCTGCCCTAAATGAATCTTACTTAGGAATTTTCAACTCATGGAAAGGCTACGACTGTTGCCATG

AATGGTACGGGGTGAGTTGTGATCCCACAACTCACCGCGTAGCAGACATAAACCTCCGCGGTGAATCCG

AAGATCCACTCTTCGAAAAAGCTCATCGTACCGGTTACATGACCGGTACGATCTCTCCATCTATATGCAA

ATTAGAAAGACTATCAAGTCTCACTATAGCTGATTGGAAAGGCATCACTGGACCTATACCATCATGTATT

ACATCCTTACCCTTCCTACGAATCATCGACTTAGTCGGAAACAAGCTCACCGGCTCAATCCCTTCAACTAT

AGGTCGATTAAGTCGACTCACAGTGTTAAACGTAGCTGAAAATCAACTTACTGGCTACATACCTCGATCA

TTAACCAACTTATCATCTTTAATGCATTTAGATCTCCGAAACAACTTCATCTATGGAAACATCCCAACAAA

TTTCGGAAAATTACGGATGTTGAGTCGAGCTTTACTGAGTCGAAACAAGTTAACAGGTCATATACCGTAT

TCGATTTCGTATATTTACAGGCTTTCAGATTTGGATCTGTCATTGAACAAACTTTCCGGTAGCATACCTCC

GTCTCTAGGTAAAATGCACGTACTTGCAACGTTAAATCTTGATGGTAACAATATTACTGGTACTATACCA

CCTAGTTTGATTAATTCACGTATCAACATTTTGAATTTGAGCAAAAATGCTATTGAAGGTTATATTCCTGA

TTCATTTGATGAAAGATCTTATTTTATGGTAATGGATTTATCTTACAACAAATTAAAAGGAAGGATTCCAA

AATCGATATCGTCGGCGACGTTTATCGGTCACTTTGACGTTAGTCATAACCATCTTTGTGGCCAGATTCC

GGAAGGTTCTCCGTTTGATCATCTTGAAGCTTCATCTTTTACTTACAATGATTGTCTATGTGGGAAGCCTC

TTAAACCTTGTCACTAAAGTGTGACTATTATTAGGTAAATTTAATTTTACTTTTCTTTTTTGAGATTTTTATT

AACACTACATATTGGTGAATATGTTTGTATTATTATGTGATACTGAAAAGTAAAGTGTTTTTTTTAGCATA

TATTTGTAATTTTAAATGTATAATTGTGCCTTGAGATATATGAAGGCTTTCTTA

>PGSC0003DMG400001829 LRR-RLPs

ATGGAGTACCAAAAATTGGTAATGGTTCTTTCTATACTTTTTGTCCAACAATCTCAGCTTACGTATTCTGG
GAAACATCTCTGTCCCCGCGATGAAGCTTTTTATTTGCTTCAATTTAAGCAAGAACTCACATTTCTTGATA
CAAGATCTTTTTACTTTCATAATGTCTGTGACGGCGAGACCCAAGCCAAGACTTTTTCCTGGAATGTTACA
GGAGATTGTTGCGAATGGGATGGTGTTACTTGTGATGGATTTACGGGTCATGTCATAGGCCTCGATGTT
TCTTCGAGCTGTCTCATGGGAACCATCAATGCTAACAGTAGCCTCACAAAACTTGGTCATCTCCAAAGAC
TCAACCTTGCCTTCAACTACTTGGATGACTTTCCACTTGGAAATAGTATTAGTGAATTCACTAGTTTGACG
CATCTCAATCTTTCAGATTCTGGATATATGAAAGGGAAGATGATCCCACAAGGATTATCCAACTTGTCTA
AGTTGATTTCACTTGACTTCTCCAGGAATTACATCCAAGTTGGCCGAACAACCTTCACTAGTTTGGTGCAC
AACTTAGCCAACTTAGAGGTACTGTTGTTTCACAAAGTGGACGCACCATTTGAGTTACCTAAGAGTTTTC
CTTCTTCCCTTAGGAAATTAAGTCTTGAGGGCACCAACATGTTTGGGAAAATAACTGATTCTCAGCTTTTT
CATCTACCAAACTTGCAAGTACTGAGATTGGGATGGAATCCTTCATTAACCGGCACTTTGCCAAATTTTA
ATTGGAGTTTCAGTAAAAGTATTTTAGAGTTGGACTTTTCCAATACTGGCATTTTTGGGAAGGTACCTGA
TTCTATTGGAAACCTCCATTCTCTTTGCTATTTAGATCTCTCTTACAACCATTTATCCGGCTCGATTCCAGA
ATCCATTGGCAACCTCACTGCAATCACAGAGTTGACACTTTCACGTAACAGCTTCACTGGTAATGTTCTCT
CAACTATTGCAAAATTAAACAAACTCGTTTATTTAGAACTCTATTCCAATTATTTTCAAGGCTTGATTCCA
GAATCCATTGGCAACTTCACTGCAATCAAAGAGTTGTCACTTTCAGATAACAACTTCACTGGTAATGTTCC
CTCAACTATCGGAAAGTTGAACAAACTTAACTACTTATATCTTAATTCCAATAATTTTGAAGGCTCGATTC
CAGACATCTTTGCCAACTTTTCAGAGCTATCTTTTTTAGATTTTCACGGTAACAATTTCACTGGCCCATTCC
CCTGTTCAATTGCAACCTTGACTCACCTGCAAAGGTTAGAGTTGCAAATCAATTCACTAACTGGTCCACTT
CCCTCTAATATAACCGGACTTCAAAATCTAGACATACTTGATTTGTCGTTCAATTATTTCACTGGCACAAC
ACCCTCTTGGTTATTCCATCTTCCGTCCTTGACTTCTTTATCTGTTCAAGCCAATCAATTAACTGGATTGCC
AAATGAGCTCAAGAGAACTTCCTCAAAATACTCTAGAATTGATCTTTCGCACAACAAGCTGCATGGCGAA
ATCCCTGATTGGATGTTATCCATGAGCATGAGAAGTCTGGATCTCTCTCATAACTTTCTCACAGGCTTTGA
AAAACAAGTGTGGCGCGCAGAGAACTTGTCCTACCTTAATTTGGAGAACAATTTTCTTCAAGGGCCTTTG
CATCAATCCATTTGTGACTTGATTAACCTTGGAATCCTCATTTTGGCTCAAAACAATCTCAGTGGTTCAAT
CCCAGGATGTTTAGGTAACTCCAGTAGGTTCATTTTTATTTTAGACTTGCGAATGAACAATTTTCATGGAG
AGATACCAACATTCTTACATAGAGGGTTAGAGTATCTTGGTTTATATAGCAACCAATTGAGGGGACAAG

TCCCCCGATCCTTAGTTAACTGTACAAGATTGGAAGCTCTTGATTTGGGGAACAACATGATAAATGACAC
GTTCCCCATTTATCTGCAGAAACTACAAAACCTACAAGTTTTGATTCTGAAATCAAATCTCTTTCACGGTC
CAATTGGTGACTTGGAGTCCGAATTTCCATTTCCTAAGTTACGGATCTTTGACCTTTCCTTTAATGGGTTC
ACAGGAACTTTGCCATCTAATCTTTTCAAAAGTTTTAAAGGTATGATGGATGAAGAAAAAACAGCAAGT
ACAAGCACTCGCACGGATTACCTTTACAATATTAGTTTGGTGATAAAAGGCAATGAGTATGATATGAGA
ATCACGTCAATTATGACGAGTGTTGATTTATCAAGCAACAGGTTTGAAGGAGATATTCCAAATTCCATTG
GAAATCTGAGCTCACTTGTGTTACTCAACCTGTCCCACAACAGCTTCCGTTGTCGTATTCCTGCAGAATTT
GCAAAGTTGCAGGCGCTCGAAGCATTAGATCTCTCATGGAACAGACTCATTGGAGAAATTCCAGGTCAA
TTGTCGAGTTTGACATTTCTTGAGGTCTTAAACCTTTCATACAATCATCTGGCTGGGCGCATTCCTATTGG
GAAACAGTTCAATACATTTCCAGATGATTCATATTGTGGAAATCTTGATTTATGTGGATTTCCACTATCAA
AGAAATGTGGAAACAATAATATGCGAGATGAGTCAACACTTGAACAAGATGATGATGATTCATTTTTTAT
GAGCGGGTTCACATGGGAAGCTGTTGTAATAGGTTATGGTTGTGGTATGATATTTGGATTGTTGATGGG
AGGCCTTATGTTCCTACTGGAAAAACCAAAATGGTTTTCGAACTTTGCTGAAGATATTGCTCAACAAATT
GCTGCTAAGAAGCGAACAAGGCAAAAGAAGAGACGTCAAAGACGTGGTCTAAGAATGAATTAG
>PGSC0003DMG400001831 LRR-RLPs
ATGGAGTACCAACAATTGCTAATAGCTTTCTGCTTCTATTCACTTTTTATAATTTTTTCCCAACAATCTCAG
CTTACATATGCTGGGAAACATCTCTGTGCTCGCGATGAAGCTTTTTATTTGCTTCAATTTAAGCAAGAACT
CAGAGTTGATTCAAATTATGAGTACTGTGACAACAAGGCCCGAACCAAGAATTTGTCCTGGAATGTTAC
AGGAGATTGTTGTGAATGGGATGGTGTTACTTGTGATGGATTTACGGGTCATGTCATAGGCCTCGATCT
TTCTTCGAGCTGTCTTTGGGGAACCATCCATGCTAACAGCAGCCTAAAAAAACTTGGTCATCTCCAACGA
CTCAATCTTGCCTTCAACAAGTTGGGCGAGTTTCCACTTGGAAATAACATTAGTGAACTCAGTAGTTTGA
CGCATCTCAATCTTTCGCATTCTGGAACTTTGATGCACATCCCACAAGTATTATCAAACTTGTCCAAATTG
GTTTCACTTGACCTCTCCTGGCATTTGGCTGTACTATTCCCATTTGGTCAAACAACCTTCACAAGTTTGCTT
CAAGACTTAACCAATTTGGAGGTACTGTTTCTTGACAACGTATACGCACCATTTGAGTTACCTAAGAACC
TTTCTTCTTCCCTTAGGTACTTAAGTTTTCAAGGCACCGGCATTTTTGGGAACCTAAGTGAGTCTCAGATT
TTTCATCTACCAAACTTGCAAGTGCTGCTATTGGGAGAGAATCCGTCATTAACAGGCACCTTGCCGAATT
TCAATTGGAGTTTCAGTGGAAGTATTTTAGAGTTGGACTTCTCCAATACTGATATATTTGGGAAGCTACC
TGATTCAATTGGTAATCTCCATTCTCTTTTACATTTGAACATCTGGTATTGCCATTTATCCGGCTCTATTCC
GGAATCCCTTGGCAACCTCACTACAATCACAGAGTTGAGACTTCCAGGTAACAGCTTCACTGGTAATGTT

CTCTCAACTATCTCTAAATTAAGCAAACTTGTTCATTTAGATCTCTCTCACAACCATTTTCGAGGCTCAATT
CCAGAATCCTTTGGCAACCTCACTGCAATCAGAGAGTTGCGACTTTCAGATAACAGCTTCACCGGAAATA
TTCCCTCAACTATCCAAAAGCTAAACAAACTTAGCTTCATATCTCTCTCTTCCAATAATTTTGAAGGCTCGA
TTCCAGACATCTTTGCCAACTTTTCAGAGCTAGATACTTTATATTTTGAAAGTAACAATTTTACTGGCCCAT
TCCCCGATTCAATTGCAACCTTGACTCACCTTGAAAGTTTAGACTTGCAAAACAATTCACTAACTGGTCCA
ATTCCCTCTAATATAAGCGGCTTTCAAGAGCTACAAATACTTGATTTGTCGTTCAATTATTTCACTGGCAC
AACACCCCCTTGGTTATTCCATCTTCCATCCTTGCTTTCTTTATATGTTCAAGACAATCAATTAACCGGGAA
ATTGCCAAATGAGCTCAAGAGTAATTATCTAGAATACTTTAACATTGATCTTTCATACAACAATCTGCATG
GAGATATCCCTTATGGGATGTTATCCAAGAGCATGGGTAGTCTGGATCTCTCTCATAACTTCCTCACAGG
CTTTGAAAAAAAAGTATGGCACTCAGACTCCTTACAATACCTTAATTTGGAGAACAATTTTCTTCAAGGG
ACTTTGCATCAATCCTTTTGTGACATGATTAACCTTGTATTCCTCATTTTGGCTCAGAACAATTTCAGTGGT
TCAATCCCAGACTGTTTGGGTAACTCCAATAGCTCCATTTCTATTTTAGACTTGCGAATGAACAGTTTTCA
TGGAGAGATACCAAGATTCTTACCTACAGGGTTACAGTATCTTGGTTTATATGGCAATCAATTGAGGGG
ACAAGTCCCGCGATCCTTGGTTAACTGTACAAGCTTGGAAGCTCTTGATTTGGGGAACAACAAGTTAAAT
GACACGTTCCCCATATGGCTGGAGAAACTTCCGAACCTACAAGTTTTGATTCTGAAATCAAATCTCTTTCA
CGGTTCAATTGGTGATTTGGAGTCAGAATTTCCATTTCCTGAGTTACGAATCTTTGACCTTTCCTTCAATG
GGTTCACAGGAACTTTGTCATCTAATTTTTTCAAAAGTTTCAGAGGTATGATGTATGTGGATGAAGAAAA
TACAGGAATTACTCGAGCTACTAATAGGACTCACAGAGATTACCTTTACCATGTTAGTTTGGTGATAAAA
GGTAATGAGTTTGATATGAAAATCACGTCAATTATGACGAGTGTTGATTTATCAAACAACAGGTTTGAA
GGAGATATTCCAAATTCCATTGGAAGTCTGAGCTCACTTGTGTTACTCAACCTGTCTCACAACAGCTTCTG
TGGAGTTATTCCTGCAGAAATTGCAAAGTTGCATGAGCTCGAAGCATTAGACCTTTCATGGAACAGATTC
ATTGGGGAAATTCCAGGTCAATTGTCGAGTTTGACATTTCTTGCGGTCTTAAACCTCTCCTACAATCATCT
GGCTGGGCACATTCCTACTGGGAAACAGTTCAATACATTTCCAAATGATTCGTATTGTGGAAATCCTGAT
TTATGTGGATTTCCACTGTCAAAAGAATGTGGAAACAATAATGAATCACCACTTGAACACGATGATGATG
ATGATGATGATGATCATTTTTATGAGCGGGTTCACATGGGAAGCAGTGGTAATAGGTTATGGTTGTGGT
ATGATAATTGGATTGTTGATAGGAGGCCTCATGTTTCTACTGGAAAAACCAAAATGGTATGTGAACTTTG
CTGA
>PGSC0003DMG400001832 LRR-RLPs
GTACATATAAAAGAGAAAAAGTTACAACAAAGATGTAATCAACAACTAGCGCAGGAAAATGAGAAATT

TCAAAAATATATACTCTAGAAAAATTCCTTCAAAATAATCACAGTAACTAAAAATACTATATTTCTCACAG
CTATAGAGAAGCCAACAAATAGTTGACTTCTTTGCTAAACAGGCCTCTTCAAGTGGAAGTGGTACCTTCT
ATTATTTTTTTCATCAATTTCCTAAAGAGGGGAATAGACTATTTCTACTAGATAAGGGACAACTTCCTAGC
ATAAGGAGAAGATACAACAAATGTAATTTTTTTTGTTAGTTGAACCATTGTTTTTTGCTTATAGTATGAAA
GTCTATGTCACTTTAGCCTTTCTGTAGATATTGCTGCTCAGAAGCAAAGAAGGCAAAACAAGAGACGTC
AAAGAAAAAATAAGCTTTAGACATAGCTACCTCTGCACTCTGTTTTTGGATGGATGGATGGATGTCTAAAC
AAAAATAGGCTTTAGACATAAAAACACCATTTTTCATGAAAAGGCCCTCAAAGTTTGAATTCAAAAATGA
GGATTTGATGTTGAATTCATGAAGAACTAAATGGGTAATGGAGGATTGGGGTTGAAATCACTTACCCAA
ACGTTATTTCTTAAAATCGCCTACTCCAAGCTTCCAAGGTCCAAAAATGGAAAAATGGGGAAGAAACCC
AAATTGGGAAATTTCATTAAACTGCCAAGCCATTTGGTCTTCGCAATCGCGGAGACCAGACGGAATAAC
CCTTCACGAACGCAGCCACTCTGACCCTCCACGATCGCGGCCAAGCTCCACCAACGCGAAGGAGGCACC
AGAAGACAGCAAACAGCAAATACCAAGGCATAAAAACATTCACCCAATGGACCCTTGGAATCCCGAGCA
CACAAACTACTACCCAACTAAACTCGACGTTTCGGACTCAATGGAATCATCGAAATATGGATTCGAGGTC
TCCGTGACCCGGCACCCATTAAAGGAATTAATATCTCAGGTGATCACTCAACTAATTCTTTTCTCTGAAAG
TCACTCAATTTTGATTTTTAACTCAAAAGTCACTTAACTATGAATTATTTTCTCATAAAGTCACTCAACTAT
AGATTTTTTTCTCAAAAAGTCATTCGACTTTGAATTTTAACTCAAAAGTCACTCAATTATGAGTATTTTACT
TACAAAGTCACTCAACCTATTCAAATAATTTTTTTAATTAAAATTTACTTTAATGAAATTTTTATTCCTAAG
CAAAAATAAAAAATCAATAAAAAAGTATCCATTTGTACTGCCAATTATATTATGCTTAAAAATATCCAATA
TCACCGTCAATATACTATACTTAATTATTTTTTTAAAAACTGGAATAATGTTCCTATAAATACGTGTTAAAA
GTAGTGTAGAAAATAATAATAAAACTCATGGAATTTAAAGGTTCTTAAAATAATTAATGCTAACTATTAT
ATTTGGGGGGAAAGGTAAAATAAAAGGATCACTATTATATTTTTAGTGGATCGAGTCAAGTAGGTGGGT
CATCTTTTTACAATTTTAGTTAGAAATAAAAATTTCATGTAAGTAAATTTTAATTAAAAAAATTAATTAAAT
AGGTTGAGTGACTTTCTAAAAAAAGAACGCATAGGTGAATGACTTTCTGAGAAAAGAAATCATAGTTGA
GTGACTTTTGAGTTAAAAACCAAAGTTGAGTGACTTTCTAAGAAAAGAATCAATAGTTGAGTGACTTTTG
AGTTAACAACCAAAGTTAATTGACTTTCTGAGAAAACAATCAATAGTTGAGTGACCATCTGAGATATTAC
CTACTCGTTAAAGCCACAATTTACCAACTTTCACCCAAAATATTAAAATAATATTAGTGGCAGTAAAGTTT
TGAAAAAAATATTAAAATTGTAGTAGTGGTAGTAAAGTTTTTGAAAAAAATATTAAAATTGTAGAGTGG
TAGTAAAGTTTTTGAAATTGTATTAGTGGTAGTAAATTTTTTGAAAAAAATATTAAAATAATATTAGTGG
AAGTAAAGTTTTTGAAAAAAAAATATTAAACTTGTAGTTGTGGTAGTAAAATTTTTGAAAAAAATATAAA

AATTGTATTAGTGGTAGTAAAGTTGTTTGACTAAATAAAGGAGGAAATTGTGTTAATTGAGTTGGGTGA
ACAACAATAATGCATTATCGATCACACTGTATGTATAAAAACTAGTTCTAAGAATGTGCATGTGTAACAC
GTTTGACCTCTACTGCTATATAAAGATGGAGTACAAGTTCGTAGCTAGTTAGATATAGAGAATGGAGTA
CCAACAATTGCTAATAGCTTTCTGTTTGTATGCACATTTTATACTTTTTGTCCATCAATCTCAGCTTACATA
TGCTGGGAAACATCTTTGTGCCCGTGATGAAGCTTTTTATTTGCTTGAATTTAAGCAAGGCCTCATCGTT
GGTCGAAATGATTATTACAATGGTTGTGACAGCAAAGCCCAAGCCAAGACTTTGTCCTGGAATGTTACA
GGAGATTGTTGTGAATGGGATGGTGTTACTTGCAATGGATTTACGGGTCATGTCATAGGCCTCGATCTTT
CTTGCAACTTTCTTAGTGGAACCATCTATGCTAATAATAGCCTCACAAAACTTGTTCATCTCCAAAGACTC
AACCTTACCTTCAACTACTTGAATGACTTTCCACTTGGAAATAGTATTAGTGAACTCAGTAGTTTGACGCA
TCTTTTGGCAATCTCACTAGAATTAGAGTGTTGAAACTTTCATTTAACAACTTAAATGGTAATGTTCTCTC
AACTATCTCAAAATTAAACAAACTCGATCATTTAGATCTCTCTTACAACGATTTTCAAGGCTCGATTCCAG
AATCTTTTGGCAATCTCACTAGAATTAGAAGTTTGGATCTTTCAATGAACATCTTCACCGGAAATGTTCCC
TCAACTATCGGAAAGCTCAACAAACTTCACTCCTTATCTCTTTCTTCCAATAATTTTGAAGGCTTGATTCCA
AACATATTTTCCAACTTCTCAGAGCTATCTTTTTTAGCTTTTGACACTAACAATTTCACTGGTCCACTCCCC
TATTCAATTGCAACGTTGACTCGTCTTGAAATATTATACTTGCAAAACAATTTACTAACTGGTCCACTTCC
CTCTAATATAAGTGGACTTCAAGAGCTACAACAACTTGATTTGTCCTTCAATTACTTCACTGGCGCAACAC
CCCCTTGGTTATTCCACCTTCCATCCTTACTTTCTTTATATGTTCAAGACAATCAATTAACCGGGAAATTGC
CAAATAAGCTCAAGAGTAATTATCTAGAATACTTTAACATTCGTCTTTCATACAACAAGCTGCAAGGTGA
AATTCCCGATTGGATGTTTTCTACAAGCATAGTTAGACTGGATCTCTCTCATAACTTCCTCACAGGCTTTA
AAAAACAAGTATGGCACTCAGACTCCTTACAATACCTTAATTTGGAGAACAATTTTCTTCAAGGGACTTT
GCATCAATCCTTTTGTGACATGCATAACCTTGACTTCCTCATTTTGGCTCAGAACAATTTCAGTGGTTCAA
TCCCAGACTGTTTGGGTAACTCCAATAGCTTCATTTCTATTTTAGACTTGCGAATGAACAGTTTTCATGGA
GAGATACCAAGAATCTTACCTACAACGTTACAGTATCTTGGTTTATATGGCAATCAATTGAGGGGACAA
GTCCCGCGATCCTTGGTTAACTGTACAAGCTTGGAAGCTCTTGATTTGGGGAACAACAAGATAAATGAC
ACGTTCCCCATATGGCTGGAGAAACTTCCAAACCTCCAAGTTTTGATTCTAAAATCAAATCTCTTTCACGG
TTCAATTGGTGATTTGGAGTCCGAATTTCCATTTCCTGAGTTACGAATCTTTGACCTTTCCTTCAATGGGT
TCACAGGAACTTTGTCATCTAATTTTTTCAAAAGTTTCAGAGGTATGATGGATGTGGATGAAGAAAAAAC
AGGAATTACTCGAGAAAGAACAAGCAATGACACAGATTACCTTTACCACATTAGTTTGGTGATAAAAGG
TAATGAGTTTGATATGAGAATCACGTCAATTATGACGAGTGTTGATTTATCAAGCAACAGGTTTGAAGG

AGATATTCCAAAATCCATTGGAAATCTGAGCTCACTTGTGTTACTCAACCTGTCTCACAACAACTTCCGTG
GTCATATTCCTGCAGAAATTGCAAAGGTGCAGGCACTCGAAGCATTAGATCTCTCATGGAACAGACTCA
TTGGAGAAATTCCAGGTCCATTGTCGAGTTCGACATTTCTTGAGGTCTTAAACCTTTCCTACAATCATCTG
GCCGGGCACATTCCTATTGGGAAACAGTTCAATACATTTCCAAATGACTCGTATTGTGGAAATCCTGATT
TATGTGGATTTCCACTGTCAAAAGAATGTGGAAACAATAATGAATCACCACTTGAACACGATGATGATG
ATGATTCATTTTTTATGAGCGGATTCACATGGGAAGCAGTGGTAATAGGTTATGGTTGTGGTATGATATT
TGGATTGTTGATAGGAGGCCTCATGTTCCTACTGGAAAAACCAAAATGGTATGTGAACTTTGCTGAAGA
TATACAAATTGATGCTAACAAGCAAACAAGGCAAAAGAAGAGACGTCAAAGACGCTGTTTAAGAATGA
ATTAGCAATATACCAGGTATGTATCAAAACCTTTTGTTTTGAATCAGGAACAGCACTATAGAGGTTGTTG
GATAACTATTTTTCCTAAATATTCAAAAACTCAATGTCTCTGCAGAGGTTACTGGAGAATTGGACAATTTA
ATTAGCAAGTTAGTGTTGATGTTCGTGCAAATATCAAAGGATGCATTTGTTTCTTCACTTTATGCCCATGG
TTGCCAAATACATAATAAGTTACAGTTGATTTCTATAGTTTTGTGGCAAATAATAAATTAGCTTTAGACAT
AGCAACCTCTACCCTCTGTTTGGATGGATGTCTCATAATGTATCGTATTATATTGTATTGTATTGTTA
>PGSC0003DMG400001870 LRR-RLPs
CTTGCATAAATAAAATTGTCGATCGACTGAGTCTGTGTGTGCTCTTGTTACTATTCTTGCGTTCGCTGAAG
TTATTGAAGTTTGAGATACCGCTACTTCTTTAACAGGTTAATCCGTTTTATCTTGGGAGGAATTAATCTAC
AACCTCGGGTACAGTGAGGGGATTAAATTTCTTAAGGAAACACAGTGGTTTCTGTGGACTCGGATTAAA
ATTCATTCTGTCTATTTCATCTTTTTTTGTTTCTGTATTTTTGACTAACCTAAATACAGGAGATAACACTTTC
CACTTGGAAATAGTATTAGTGAACTCAGTAGTTTGACGCATCTCAATCTTTCGGATTCTGGAATATTTAAT
GAAAGGGAGATGCAGATCCCATCAGGATTATGCAAGAGTTGTCTAAGCTGATTTCACTTGACCTCTCCTG
GAATTACATCCAATTTGGCCGAACAACCTTCACAAGTTTGCCGCACAACTTAACCAACTTAGAGATTTTGT
TGTTAGACTTCGTGAACATACCATTCAAGTTACCTAAGAATTTCTCTTCTTCCCTAAGGTACTTAAGTCTTC
AAGGCACAAATATGTTTGGGAACATGAGTGATTCTCAAATTTTTCATCTACCAAACTTGCAAGTGCTGAG
ATTGGGATCAAATCCTTTATTAACAGGCACTTTGCCGAATTTTAATTGGAGTTTCGGTGGAAGTATTTTA
GAGTTGGACTTCTCCCGTACTGGTATTTTTGGGAAGATACCTGATTCAGTTGGTAATCTCCATTCTCATTG
GCATTTGGACCTCTTGAATTGCCATTTATCTGGCTCGATTCCAGAATCCATTGGCAACCTCACTGCAATCA
GAGAGTTGATACTTTCAAGTAACAACTTCACTGGTAATGTTCCCTCAACTATCTCAAAATTAAACAAACTC
GTTAATTTAGATCTCTCTTTCAACCATTTTCGAGGCTCGATTTCGGAATCCATTGGCAACCTCACTGCAAT
TAGAGAGTTGACGCTTTCAATTAACAGCTTCACTGGAAATGTTCCCTCAACTATCGGAAAGATGAACAAT

CTTAACAACTTATTTCTCTTTTCCAATAATTTTGAAGGCTCGATTCCAGACATCTTCGCCAACTTTTCAGAG

CTATACTTTTTAGCTTTTGACACTAACAATTTCACTGGCTCATTCCCCTATTCGGTTGCAACCTTGACTTAC

CTTCGAATCTTAGAGTTGCAAAACAATTTACTAACTGGTCCACTTCCCTCTAATATAAGCGGACTTCAAGA

GCTACAACTACTTGATTTGTCATTTAATTATTTCACTGGTGCAACACCCCCTTGGTTATCTGTTCAACACAA

TCAATTAACTGGGAAATTACCAAATGAGCTCAAGAGCAATTACAAAGAATTCTTAGGTAACTCAAATGAT

GCCTCCACCACCTCAAATAAATTATTGCCAAAACAAGTATGGCGTTCAATGAATTTGGTGTCACTTAATTT

GGAGAACAATTTTTTTCAAGGGCCTTTGCATCATTCTATTTGTGACTTTATTAACCTAAAAGTTCTCATTTT

GGCTCGGAACAATTTCAGTGGTTCAATCCCAGACTGTTTGGGTAACTCCAATAGGTTTATTTCTATTTTAG

ACTTGCGAATGAACAATTTTCATGGAGAGATACCAAGATTCTTACCTACAAGGTTACAATATCTTGGTGT

GTATGGCAATCAATTGGGTGGGAGGGGGGGATCCCACGATCCTTGGTTAACTGTACAAACTTGGTAGCT

CTTGATTTGGGGAACAACACAAGATAAATGACATGTTCCCCATTTTGGATTGTTGATGGAGGCCTCATGT

TCCTACTGGAAAAACCAAAATGGTTTTCGAACTTTGCTGAAGATATTGCTCAACAAATTGCTGCTAAGAA

GCGAACAAGGCAAAAGAAGAG

>PGSC0003DMG400002118 LRR-RLPs

CAATCCTTCAGAGATGTCTGAAGATGAGGAGCGTCCTTGTCCTTCAGATCTCACCGGTGGCGCCACTGCA

AAAGCAAGGAACTGTTGCTTTAACGCCGCCGTTACCGGTGGCGGAGGGGGGATTTTTAACTTTTCGCCA

CACCCAGATCAAGTTCTTGAAAATGTGCTGGAAAATGTTCTTTGCTTCTTAACTGATCGCCGTGACCGTA

ACGCTGCCTCTCTTGTAAGCAAATCTTGGTATCGGGCTGAGGCTTTAACCAGATCCGAAGTGTTTATTGG

CAACTGTTATGCTGTTTCGCCGACCCGGGTTACGACCCGTTTCAAAAGGGTGACCTCTGTGGCCATTAAA

GGGAAACCTAGGTTTGCTGATTTCAGTTTGCTTCCTCCAGATTGGGGTGCTCACTTTACTCCTTGGGCTTC

TGTTTTGGGTGATTCTTATCGTGGGCTTGAGAAATTGTACCTCAAACGTATGTCCATATCTGATGATGAT

CTAGGTTTGTTGGCGCGTTGTTTCCCCAGTTTCAAAGAGCTTGTTCTTGTATGCTGTGATGGTTTTGGGAC

TAGTGGACTTGCTATTGTAGCTCGTGATTGCAGGTTAGTATTGTTTTGTCCGGATATAGATCTGTTTATGT

GATAACCCTTTTGGTAAAAAGTTTAGATTTTTAATTTCTAGAAGCTGAATTCTTCTTTCTGTAATGGGAAA

TTAACAGCTTAAACTAGTGTAGATATATTGTGTTATTTGAATTTGTGCTTCATGTGTGCTGGTGTTTGAAT

TAGTTATGTGATTGAGTTGTTCTTTAACTGTTTTATTTCTGCATTTGAAACTTAATGATACCTTTTGAGGAC

ACTAGCATTGAAGTTATAATATGTGGTGACTTGTTGTATTGGATTTGTGAGTTTGCAAGCTGTGAAATAT

GGTTTGGTAGTGTGATTTTCTTGTGCATAATAGAAGGTTTTTACTGCAGGCAAATTAGAGTGCTTGATCT

GATTGAGTCTGAGGTATCCGACGATGAAGTGGACTGGGTTTCCTACTTTCCTGAGAACAAAACGTGTTT

GGAGTCTTTGACCTTTGATTGTGTTGAATGCCCTATTGATTTTGAGGCATTGGAGAAGCTAGTAATCAGG
TCACCTAGTTTGAAGAGACTAAGGTTGAATCGGTTTGTTTCTATTACTCAGCTGTACCGTTTGATGATTCG
AGCTCCACAGCTTACCAATCTGGGAACAGGCTCTTTTGGCTCCTCAACGGTCATTGATGAACCAGATCCT
GATTATGCTTCAGCATTTGCTGCCTGCAAATCCATTGTCTGTCTCTCTGGTTTCAAGGAAATTGTTCCTGA
ATATCTGCCTGCAATCTATCCAGTTTGTGGCAATCTGACCTCTCTTAATTTAAGCTATGGTGCCAACATTA
ATACTGAACAATTCAAGTCTGTCATCAGTCGCTGCCATAAGCTCCAAGTATTGTGGGTATGTGGTTTGAA
TTTTCGCTAAATGTTCTTTTAAAGAAAATTGTACTCTGTAATAGCGTTATATTGAGTTCAATTTTGATGTTT
CAGGTATTTGATTCTGTATGTGACGAAGGACTTGAGGCAGTTGCTGCAACATGTAAGGATCTGCGGGAG
ATTCGAGTTTTCCCTATCGAAGCTCGGGAAGATGCAGATGCCCCAGTTTCTGAAGCAGGTCTCCTTGCGA
TTTCTGAGGGTTGCAGTAAACTTAAGTCCATCTTATATTTCTGCCAACGAATGACAAATGCGGCTGTTAT
AGCTATGTCAAAGAACTGCCCAGACCTTGTGGTATTCCGACTATGCATTATGGGTCGGCACTTGCCTGAC
CATGTTACTAATGAACCAATGGATGAAGGCTTTGGGGCTATTGTCAAGAACTGTAAGAAGCTTACTAGA
CTCGCTGTATCTGGTTTACTGACTGATAAGGCTTTCAGTTACATTGGACAATATGGAAAACTAGTCAGAA
CCTTATCAGTTGCTTTTGCTGGAAACAGTGACTTGGCTCTGAAGTATGTGCTCGAGGGCTGCCCTAAGTT
GCAGAAGTTGGAGATCAGGGATTGCCCATTTGGAGATTTGTCTTTGCGTTCTGGTGTACACCACTATTAC
AATATGAGGTTCCTTTGGCTGTCATCTTGTAGAGTAACTCTACAAGGTTGTCAGGAGATTGCTCGCCAAT
TGCCCCGTTTAGTAGTGGAAGTGATAAGTGGTGATGAGGAAGGGAGTGAGACTGGTGAGCATGTCAAT
ACCTTGTACATGTACCGATCTCTTGATGGACCGAGGGCTGATGTACCATCATTTGTGCAAATACTGTGAC
CGGCCTTTTTAGAAGATAGGAATTTTCTGCAGTAATTGATTGGGACCTTTGAGAAATGATTAACTATTTTC
TTGGGAAGAGATGCTCTCCGGAAATCTGTTGTGACCCATATGAAGGTGCAAGTTTCCTTCTTGAGAAAA
CGAATGGTGCACCACTTGCAAAGGCAGTGAGCAATCTATAATGTATTCAATGCTGGTCCCCATGGATATC
ATTGTATATGGTTCAGTACAGAGATTGTTTTTTCCAGCAAAGCAATCTAGAAGACAAATGTTCTCTGTAT
CAGTAATCTTTTCTATTCACTTTCTTCTCTTCATATTCATGGAGCCTTGAATTAGTAAGTGGAGTCATTGGT
AAGGCCACAGCAAGGGTAGATGTTCCAAGCTGCACGGTGGCTGATCAAATTGCAGCTCAATGAGATTTC
TCAAAGGCTTTCGGCAGAACCAACACTTGTGGAAACACCTGCATGCTCGGTCAGGATACTGGCTCGTCA
AGTAGTATCTTTTCATACAATTGAGGCCCTTCTTATTTTTTGTTTCATTGTTAGTTTAGAGTTGAAGTTTTT
TATTTTTAGTTGGTGATAGCCTAATGGTCAGAAGGATACCTACCGCAGTTGGATCTCTCCATCTCGCTTTC
TAGAAATATTTGTTCTTTCCAGGATGTGTTGTTTCGTAGCACCTGACTGTTGAATTTATTATTGTTATTTTT
AGAAGAAATGTGAGATGGGTAATAAATGCCAAATATGCATGGACCAGATGATGCTCTAGTGTGTGTTTG

TGTTTGTCTTTGCTTTTTCTTTATGTGGAATGATCAGCTTGATAGTGAAGACCCATAAATGATGTGTGGAA
AGCCTTGTACAAGTACAAAAATGCAGGACCCTCCTCTCCTTCACTCACTCTCTTTGTGGGATCCATTTATG
AGTCTTATCCTCTATTACCTTTCCAGCTGATGAACGGCCCTTCTCTGTCTCATCTCTTGTATCTGAAAGAGC
CAAGAGCAGGGAGTAGACTGTAGTAGTGGAAAGTATAGATGGATACTACAGCACACCCCTTAATTTTTA
GCTGATGCCAACTTTATCATTATTCTTTTGGATAACAGTCTTGAGGAGCCTGTTGTGTTATCCTGTTTACTT
TTCATATGCTGCTGCTGCCTCTATCATTGACTACAACTCAC
>PGSC0003DMG400002426 LRR-RLPs
CTTCTCAAATTTCAACAAATTAACCAAATAAATGGCATCTGCTTCTTCTTTTGCGAGTGATTCACAATACT
GTGCTCGATGGAAGTACGATGTCTTTCTAAGTTTTAGAGGTGCCGACACTCGGGCTACATTTACAAGTCA
CTTGTACGAAGGTTTGAAAAACAGGGGAATATTCACCTTTCAAGATGATAAAAGGCTAGAGCAAGGTGA
TTCCATCTCAGAAGAACTCTTAAAAGCTATCGAAGAGTCTCAAGTTGCACTCATCATTTTCTCAAAGAATT
ATGCTACATCGAGGTGGTGCTTGAATGAACTAGTGAAGATCATGGAATGCAAGGAGGAAGAAAATGGA
CAAACAGTCATACCGATCTTCTATGATGTGGATCCATCAAATGTTCGATACCAAAGTGAGAGCTTTGCAG
AAGCATTTGCCAAACATGAATCGACGTATAAGGATGATGTTGAGGGGATGCAGAAGGTGCAAGGATGG
AGGAATGCCCTAACTGCTACTGGAAATCTAAAAGGATATGATATTCGTGGCGGGTGAGTTGAAAACACA
TAATATGTTTCTATTACCCTGAACTATAATGTTTCCTATTTAATACGCTCTTGAACATTAAAAGTATGAAAA
TACTTTCTCATACTTGACTTCCTCACATTGTCAACAATCAATCTCTAATTTATGTAGGATTGATCAATCAAA
GGAAATTGAGCAGATCGTCGACCACATTTCTTCCAAATTTTGCAAGAGTGCTTGTTTTTTATCTTATTTGC
AAGATGTTGTGGGAATAAATGCTCATTTGGAGGAACTAAAATCCCTACTTCAAATAAAAATCGATGATGT
TCGAATTGTTGGGATCTGGGGAATAGGCGGAGTCGGTAAAACGACGATAGCAAAAGCCATCTTTGATA
CTTTATCTGATCAATTTAAAGCTGCTTGTTTTCTTGCCGATGTTAAAGAAAATGCAAGAAAGAATCAACTA
CATTCCTTACAAAATACCCTTCTCTCTGAATTGTTAAGAAAAAAAGATAATTACGTCAATAATAAGTATGA
TGGGAAGTGCGTGATTCCAAACAGACTTTGGTCTATGAAGGTGTTGATTGTGCTTGATGACATTGATGA
AAGAGATCATTTGGAGTATTTAGCAGGTGGTGTCGGTTGGTTTGGTAATGGCAGCAGAGTTATTGTAAC
AACTAGAAACAGACAATTGATAGAGAAGGATGCTGCAATATACGAAGTGCCTACGCTACCTGATGATGA
TGCTATGCAATTGTTCAATCAACATGCTTTCAAAAAAGAAGTTCCAGATGAGTGTTTTAAGAAGTTCTCG
TTTGAGGTAGTAAATCACGCTAAAGGGCTTCCTTTAGCCCTCAAGGTGTGGGGTTCTTTGCTGCATAAGA
AAGGCCTAACTCAGTGGACAAGAACTGTTGACCAAATAAAGAAAAAGTCTAATTTAGAAATTGTTGAAA
AACTCAAAATAAGTTATGATGGGTTGGAGCCTGAAGAGCAAAAGATATTTCTTGATATCGCATGTTTCTT

CCGTGGGCATCATAGAAAAGAAATGAAATTTTTTGATAGCTGTGATTTTGGAGCTGAATACGGATTAGA
TGTTCTAGTTGACAAATCCCTTGTGTTCATCTCTATGTATGGTAGAATTGAAATGCATGACTTGATTGAAG
ATATGGGCAAATACATTGTGAAAATGCAGAAAGATTCCGGAAAACCTAGCAGAGTATGGAATGTTGAA
GATTTCAAAGATGTGATGATGGACAATATGGTAAGTAAGCTAAACAATGCAATAGTATTCATATTTCAAA
GATATATATATACATACTCTGTTCCTCTAGCTTCATATACTCTAACTAATATTTGTACCTTAAATTTTGTTTC
GTGGTGCTAACTCTTCAAGAACTTCAAGTGTAATAATAAATATAATATAAAACATACATGACAAAAATAG
CATTTGGGGGTAGACCGGTAGAGTAAGAAAGTAATGATATTGATAAGTGAATTGGACAAGAGGTGGCT
ATATGTGAAGTTGTGATTTGTTGACTCATTACCTTTTTATAGGGGACCATGACAGTCGAAGTAATTTGGT
TGACAGATTCTGAAAAACTATGCTTTAGCAAAGAGGCAATGCAAAATATGCAAAAGCTTAGGATATTAT
GCATGTCATATCATCCATGGGTTCCTCAAAGGGCTTTCAAAGATACTCCCAAATCCTCTATTGAGTACCTT
TCAAACAACTTGCGTTGGTTTGTGTGGCATTCCTATCCTTGGAAGTTATTGCCAGAAAATTTTAATCCCAG
AAGGCTTGTTCATCTTGATCTCCGGTGGAGTTCATTGCATTATTTATGGAATGAAACAAAGGTACCATTC
TATTTTAGATACTTTCTATTTTGTCTTGTCCTAAAATTATATTTGATTCTTGTGTTTAAATGAAAATAAGTT
ATTAATTCCATAAACTCAAACTAATAGTTTCTGAGCATGTAATTTCCATAGAAAGTAGTAAAATAAATAC
GTTGAGTTATAATATAAACATGTCATTTACTATCATTCTTTCTGTGTTTTGAATTGCAGCAATTTCTGTCTC
TACGAAGGATAGATCTAAGTGGCTCTAAAAGCCTGAAGAGAACACCAGATTTCAAGGGGATGCCAAATT
TGGAGTATTTGAATCTAGAGTATTGTAGGAGTCTTGAAGAAGTTCATCATTCCCTCAAGTATAGCAAAAA
ACTCATCCACTTAGATTTGCATTATTGTAGAACACTTGAGAGGTTTCCATATGTTAATGTGGAATCTCTTG
AATCTATGAATCTAAAATTCTGCTCTAGTTTAAAGAAATTTCCAGAAATCCTTGGAATAATGAAGCAGGG
AACTGCCAGAAAGATTATGATGTCATGCTCTGGGATAAGGGAACTGCCATTATCTTTCTTTGATCACCAA
CCTCATCTTATAGAGCTACATTTGAATGGCATGAAAAACCTTGTATTTATTCCAAGCAGCATTTGTAAGTC
GAAAGGTTTGGTGAAGCTATCTGTGTCGTATTGCTCAAAACTTGAAAGCTTGCCAGAAGAGATAGGTGA
TTTAGAAAACTTGGAGGAGCTTCATGCCAGCAATACTCTAATCTCACGTCCTCCATCTTCCATCGTCCGGT
TGAACAAGCTTATAAGATTGAGTTTTGGACAACATAGATCAGAAGATAGAGTGTACTTTGTGTTCCCTCA
GGTGAATGAAGGGTTACTCTCATTGGAATATCTGGATCTCAGTGGCTGCAATATAATAGATGGAAGACT
TCCGGAAGACATTGGATGCTTGTCCTCTTTGAAAAAGTTGAATCTCAAGGGAAATAATTTTGAGCATTTG
CCTCAAAGCATATCCAAACTTGGTGCTCTTGAATACTTAAACTTATCAGATTGCAAGAGACTTACACAGC
TGCCAGAAGACATTGGATGTTTATCCTCTTTGACAAAGTTGAAGCTCAACGGAAATAATTTTGAGCATTT
GCCTCGTAGCATATCCCAGCTTGATGCTCTTGAATACTTGGACTTATCAGATTGCAAGAGACTTACTCAG

CTGCCAGAAGACATTGGATGTTTATCCTCTTTGACAAAGTTGAAACTCAACAGAAATAATTTTGAGCATT
TGCCTCAAAGCATATCCAAACTTGGTGCTCTTGAATACTTAAACTTATCAGATTGCAAGAGACTTACTCA
GCTGCCAGAAGACATTGGATGTTTATCCTCTTTGAAAAAGTTGAAGCTCAACAGAAATAATTTTGACCAT
TTGCCTCAAAGCATATCCAAACTTGGTGCTCTTGAATACTTAAACTTATCAGATTGCAAGAGACTTACAA
AGATGCCAGAAGACATTGGATGTTTATCCTCTTTGAAAAAGTTGAAGCTCAACAGAAATAATTTTGACCA
TTTGCCTCAAAGCATATCCAAACTTGGTGCTCTTGAATACTTAAACTTATCAGATTGCAAGAGACTTACAA
AGCTGCCAGAATACATTGGATGTGCATCCTCTTTGAAAGAGTTGTATCTCAAGGAAATAATTTTGAGCAT
TTGCCTCAAATCATATCCGAACTTGGTGCTCTTCGATCCTTGGACTTATCAGATTGCAAGAGGCTTACTCA
GCTGCCAGAATTTCCACAGGAATTAGATACAATATATGCAGATTGGAGCAATGATTTGATCTGTTATTCC
TTGTTTCAGAATATGTCATCATTGCAGCATGACATCTGTTCTTCAGATTCCTTGTCACTAAGAGTGTTTAA
GAGTTGGGGGGAGGGTATCCCAAGTTGGTTCAAATATCAGGGAATGGGCAAAAGTGTATCAGTCAATT
TGCCTAGGAATTGGTATGTAAGTGATAACTTCTTGGGATTTGCTGTATGTTATTCTGGCGAATATGATAT
TGATTGCATCAGAGCTCATTTGATTCCCTTATCATGTGATGATGGGATGTCGTCGATGACCCAGAAATTT
GCCTTATCCAACAATTCACAATATTCTAATGTATGGTATATTAAATTTTTGTTGGTACCTCTTGGTGGCTTA
TGGGATGCATCTAATGCAAATGAAAAAACACCAAATGACTATGGGTGCATTAAGTTAGATGTTTTTGGA
GAAAAGAAGACATTTGGAGTTCGTTTGTTGAATAAAGATGAAGCTGAGCTTCACAATGGGATAAGGAA
GAGCAGAGATGAAGAAGAGGCCACTTGCTCCTCTTCTAAGAAACAAAGGCAACTCTTTCCCACAAGCTC
GGGATTATAGATTAAATATAGGTATATACCTCATGAAATTGTGTTCTCTGTTTATATATAAATTATTTCTTC
TCATTCTTTCTTCTATTGGGAATAGGTGATGTACGTATCAGTAATCAGCAGCGAGTTTGGGGACACTGCC
TATGAAGGGACATGTACGTACAATTTTGGGTGGCAAAAATTCATTCTTTTTATATTTCATTTCGAAATATA
ATCACATTTTCCATTTCCTGACAAATAGATAAATTGCAGAAGCTTTGGAATCCAATGGAGCTCAAATAAT
TTCTCCCCAACCATCAAGTTCTACTGAAGTTGCATAGTCTCCGTTGATATCTACTGTAAAATGTTACATTCT
TATTCTATTTTAATAAGTGCAAGAAACACATTTGATTAATCATCTCTAAAATTAATGTAATCTAATTAGCA
AAGAACACTGCTATATAATTGAACCCCTCCCTATCCTCATTTCTTGTTCAATGTATTGCCAATGTCGAGCA
CGAAACGATATTTCACATCCGATTTCAAAAGACGTTCCAACGTTGTGTTCACATAGTCCATTGGCACAAC
TTCAATATTTGGTGTTATGTTATGCTTTGCCGCAAAATCCAACATTTCTTGAGTCTTCATCCCTCCTATGCA
ACTTCCAGCCACTAGCTTCCTTCCTGTTTT
>PGSC0003DMG400002478 LRR-RLPs
CTTCCACTTGATTTGAGACAATGCTTTGAGTATTGTGCGGTATTCCCAAAGGACACCGAAATGGAAAAAC

AAAACCTTATCTCTCTTTGGATGGCGCATGGTTTTCTTTTATCGAAAGGAAACATGGAGCTAGAGGATGT
GGGTAATGAAGTATGGAGAGAATTATACTCGAGGTCTTTTTTCCAAGAGATTGAAGTTAAAGATGGTCA
AACTTATTTCAAGATGCATGATCTCATCCATGATTTGGCTACATCTCTGTTTTCAGCAAACACATCAAGCA
GAAATATCCGCGAAATAAATATGATGATGTCCATTGGTTTCGCTAAATCGGTGTCTTCTTACTCTCCTTCT
CTCTTGCAAAAGTTTGTCTCGTTGAGGGTGCTTAATCTGAGTCGCATAGGAAATGAACAGTTACCATCTT
CCATTGGAGATCTAGTACATTTAAGATACTTGAACCTCTCTCGCAATAACATGTGTAGTCTTCCAAAGCA
GTTATGCAAGCTTCAAAATATGCAAACTCTTGATTTACAATATTGCACCAGACTTTGTTGTTTGCCAAGAG
AAACAAGTAAACTTGGTAGTCTCCGAAATCTTTTACTTCATGGTTGCTATAGATTGACTTGTACACCACCA
AGGATAGGATCTTTGACATGCCTTAAGACTTTAGATTGCTTTGTCGTGGGCGAGAGGAAAGGTTATCAA
CTTGGTGAACTAAGAAACCTAAATCTCTATGGCTCAATTAAAATCTGGCATCTTGAGAGAGTGAAGAAT
GATAAGGATGCAAAAGAAGCCAATTTATCTGCAAAAGGGAATCTGCATTCTTTAATCATGAGTTGGGAC
AGCGGACCACATAGATATGAATCAGAAGAAGTTAAAGTGCTTGAAGCCCTCAAACCACACCCCAATCTG
AAATATTTAGAAATCAATAGCTTCAGAGGATTCCGTCTCCCAGACTGGATGAATCACTCAGTTTTGAAAA
ATATTGTCTCTATTGTAATTTACGACTGCAGAAACTGCTCGTGCTTACCACCCTTTGGTGATCTGCCTTGT
CTAGAAAGTCTAGAGTTACAGTTGGGACCTGCGGAGTATGTTGAAGAAGTGGATATTGATATTCATTCT
GGATTCCCCACAAGAATAAGGTTTCCATCCCTGAGAAAACTTTGCATAAAATATTTTCGTAATCTAAAAG
GATTGCTGAAAAGGGGAGGAGAAGAGCAATTTCCTGTGCTTAAAGAGATGGAGATTTGCTATTGCCCTA
TTCCGACCCTTTCTTCTGATCTTAGGACTCTTACTTCCCTCAATATTAGGGATAACGAAGAAGCTACTTCA
CTCCCAGAAGAGATGTTCAAAAACCTTCTTAGGACTCTTACTACCCGCAATATTAGGGAAAGCGAAGAA
GCTACTTCACTCCCAGAAGAGATGTTCAAAAACCTTGCAAATCTCAAAGACTTGAAAATCTCTAACTTCA
AGAATCTCAGAGAGCTGCCTACCAGCCTGGCTAGTCTCAATGCTTTGAAGAGTCTGAAAATTCATATGTG
TGACGCACTAGAGAGTCTCCCAGAGGAAGGGGTGAAAGGTTTAACTTCACTCACTGAGTTGTATGTCTG
TTACTGTAAGATGCTAAAACGTTTACCTGAGGGATTGCAACACCTAACAACCCTCACAAATTTAGGAATT
TGGGGATGTCCAGAAGTGGAAAAGCGGTGCGAGAAGGGAATTGGAGAAGACTGGCACAAAATTGCAC
ACATTCCGTATCTGAAAATATATTCCTAAGTATTATTTGTAATTGTTTCTTTGTTTGTGAGTCTTTTTGGTT
CCTGCCAGTGGATGTCATTTTTTCTAGGGTTGTCTGTTTGTGAGTCTCTCTCTTTGGATGTAATTTTCTTTT
GGAAACAAATCAACAATCTATTTGTATTATACGCTTTCAGAATCTATTACTTCTTATTTGTAATGGTGAGT
ATCTCTGTTTCCT
>PGSC0003DMG400002618 LRR-RLPs

TAGTAAAGTAAAATAATGGGGAAAGGAGCAATTTTGAAATTGAAAAAAAAACAAAAACAAAGAGTGGT
GCTCTTATTCCGGAAAAGCAAGGTGGGAAATCTTAACAGGACTCGTCTCTTTTTCCTTATCAGAATCAGA
AGAAGAAAGAAGAAGATACTTTTTATTCGTCTCTCAGGTATGGTGTTCCTCTTACAATCAATCTATAATGC
TTATGTTTGTTTGTTTGAGAGGCCGATCCGCCCACCCTCATGATGGATGGGTCAAACCTGTTTTTTTCCAT
AAAAACAAACCAGACGCTCAACGTCTATCTTTTCCGTTTTCCCTTTTTGAGTTTTGACCAATTGTTAATTAG
ATGAAAATCAACAATAGGGTAAGAAAAGGGGGTTTGATTGAATTATGCTGGAATATTCTGTCTTGTTAG
CATGTGTATTTGTGAACAAGGTTCACCGCACGCGTTTATTTGACCTTTTCCGTATTATATTGGGTCTTGTC
TTCTAATTAATTAATCTCGTGCGTTTTTGTGACAGCACAAAATTTGACAGCTATATAGTCTTGCTTGTGGG
GCTGTCATTATAGTTCATCTATATTGGCAGCCCAATTTCTGAGGTGTGTCTTCTTTCTCTTCACCGGCAAA
ACAAATATATCATCATGGCTTACTTGTCTGGAATCCTCCAAAGACCTATAGTTGCAGCATCTGCAGTTGCT
ATAGCTTCTGTATCTACTGATCTTCATGAAAAATTCTGGCCATCTAAGTCATTACACAACAACAACAACAA
TTCCCAAGAAAAGGAAAAGATAACATCTTGGGTCTCTCACATATATCTTTCTAAGCTACCCCATTTCTCCT
CCTACCTCACCACAATACCTGTTCCCCTGCCTATTCTAAATAACACCAATTTTACTTCTCTTTCAACCCTTCC
TTTTCCTACTTCTTCTCTGTTGCTCAACTCTTATCAATCAGCAGCATTGCCCAACTCTGCTAATCCCTCCACA
TATACATATACACTTCCTTCTTCACCTTCCGAGGTGTTGTATACATGGCACTTACCTGAGCCAAATGCATC
ACGGAATTCTGATTCTTCACCACTAAAGTCTACCACTCTGGTAGTATTGTTGGGATGGTTAGGTGCAAAG
CAAAAGCATCTAAAGAGATATGCAGAGTGGTATGCATCAAGGGGATATCATGTCATTACATTTACTTTCC
CAATGTCTGAGATTCTAAGCTATCAAGTCGGGGGAAAGGCAGAGCAAGATATTGAAATGCTTGTGAATC
ATCTAGGTGATTGGTTGGAAGAAGAGCATGGAAAGAACTTGGTCTTCCACACTTTCAGCAATACTGGAT
GGTTAACGTGAGTTCTTGATGATACTCATTAGTCTAGGAAGAGAACAGAAACATAACTATTTACATTTTC
CTTAATGCTAGCTTGATAATTATTGATTGACTTCTTTTATTCTCTTGGCCTTACAGTTATGGTGTCATTTTG
GAAAAGTTTCAGAAACAAGATCATGCTTTAATGAGAAGGATTAAGGGTTGTATTGTTGATTCTGCCCCTG
TCGCAGCTCCCGATCCACAGGTATTGCTGACTCAAAAATTGTGATGATAAGATATCATTTTTATGTGCAA
GTGTCAGAAGAGTGTTTCTTGGAGAGTAGTCTTATTTCTGAAAGTTAATGCAGAGCACTTGTTTAGAGCT
ATACATTTCTATTGAAAACCAACCTTTTCATGCTTTTATTGGACAACTATTACAAGTGTCATATATAGTCA
GGTTTTAGCCATTCAGAATTGCACAGTTGTTTGGATTCTAAACAGGACCTGCTGTTCTCGTATTTTTATCC
TGTTGCTTTCTTTTATCTTAGGGCCTTAACCATGCAGCCTGCTTTTCGAAAACTATTCTTATGCTTGATAAC
GTGAAAGAATCTTGAGAGTTTAGCTCTCTCTGACAACTTTTTAACAGGAAGCATTCTATGCAACTTTTGTT
TCACGGTTCAAGCTTTAAGCCTACTTATTCTGGCTGAGAATAAGCCATCGAGGAATTTCCCCTTGGAGCT

ACTAAACTGCACACGTCCCTGCGACTACTGGATCTCTCCAGTAACAACTTTGGAGGAATGCTTCCATGAG
GCTTCGACAGGCTAGAAAGTCTCACATCCTTTTCAACAATAACAACTTTACTGGAAATATACTGCCTCAAA
CAGGAAACTTGACTAACTTGGAAGATTTGTACCTAATCCATAACATGCTCTCCGGAAGAATTCCAGTGGA
AATAGGAAAGCTTCAAAGGCTGTGTGAACTACCTATAGCTATGGAAACCAGTTGTCAGTAACTACCCCC
AGAGAATTGACAAATTGCTTAGTACGCATTGATTTCTTGGCTAATCAATTTTCAGGCTCTATTTCTGATGC
CATTGGTAGGCTTAAGAATCTTGTTATCTTCAGCTAACTCAGAATGAGTTGTCTGGTCCGGTCCCATCAA
GCTTAGCCTACTGCAGGAAGCATGAAAAACTGGCCTTTGCTGATAACAAACTTTCAGGATAATTGCCACC
AACTTTAAGATTTCTATCAGAGCTGGACCTGACTACTCTTTACAACAATTGTTTTGAAGGTCCACTTCCTG
AATATCTTTCCCTTCTGAAAAATCTCAGCAAAGTTAACTTTTCTCACAATAAGTTCAGTGGAAACATTTCT
CCGCTAGCTGGTTCAAATTCTCTGACAGTTGACATGACAAACAACAGCTTCTCTGGTCCTATCCCATCTGA
GCTAGTGGTGTCCAAAAATCTGACCCATCTTCGCCTTTCTAACAATTTTTTCACTGGAAAAACCCCTTCTG
AATTTGGACAACTTGAAGATCTCAGATGTCTTGACTTATTGTTCAAAAATTTGACTGGAGATCTAGCACC
TTCCCTTGCTGGCCTGAAAACCTTCGCTGTTTTCTTCTTATATCTAATCAATTTTCGGGGAAAATTTCCACA
TGGTTTGTGGAGGAATGGAAGGTCTTGGTGAGTTCGATCTTTCATTCAACAACTTCACTGGAACAGTTCC
AGCAGAACTTGGAAACTCTCCAAAATAACTCAAACTTTTTCTCAGTTACAACAGATTATTAGGTGCCATAC
CACCAGAATCAGGAAATCTCACAAGTTTAAATGTCTTGAATCTTCAATGAAACAATCTTTTTTGGCTCCAT
TCCTTCAACTCTTCAAAAATACCAGAAGCTCTATGAGCTGAGGCTCTCTGAGAATAATATAAGTGGCTCA
ATCCCTTACGAAATTGGCTCTTTATCCGAATTACAGGTGATAATAGACCTGAGCAAAAATCATTCTATTTT
ATGTGCAAGTGTCAGAATAGTGTATCTTCAAGCATAGTCTTATTTCCAAAGGTTAATGCAGTGCAATTGT
TAAGAGCCATACATTTCTATTGAAATCCAACCGTGTCATGCTTTTATTGGATAAGTATTACAAGTGTCATA
TATAGTCTGGGCTTAGCCATTTAGGAATTGCTCAGTTGCTTGTACTCTTATCAGGACCTGCCGTTCTCTTA
TTTCGATCATGTTGCTTTCTTCTATTTAGGCCTTAGACATGCAGCCTGCTTTTCAAAATATATTCTTACACT
TGATAATGTGGAAGTTGTTCATTCTTGATTTAGTCTTCAAGGGAAGGTGGATGGCCTGGGGGTCAGCTG
TGGACATTAGGAGGAGGGAGGGGAAGCTAGTACTTGTTTAGTTGCATATAATAAATGTTAATCTGTGCT
TTGGTAAGGTTAAACTAATTTTGTCAAGTTCCCTTCTTTGTTTGTATCAAGGTCTGGGCTTCTGGATTCTCT
GCTGCCTTTTTAAAGAAGAATAGTGTTGCAACAAAACGCATCATGACTTTAAACGACAGAGATGTGACA
ATAGAAGCCAAAACTTCTGTTGAAACTAAGCCTGCAGTAACTGAAGCAGCGTTGCTATTAGTACTGGAA
AAGTTCTTTGAGGTGGTTTTGAACCTTCCCACCGTAAATAGGTAAGGAGATGTCTTCCAGAATCACCTTT
TCTGCAAATGTTTATCAGCCTGCAGTATTTTTAAACAGCAGTACCAATATTAAATCTACTTTTTGTCTTTGT

ACTCTACTCAACGTGCGAAGTAGACCTTGTCGATATCATGTCCATTCACCAATGGATTGTAATTACATTTT
CAAGTCATTAGTGTGTATAGCAAGTGCCAAAAATAGTTTCGTGTTTGTACTTACATTGATGGTTGTATGA
AATTGCAGGAGGCTTTCTGACGTTCTAGATCTATTGACATCCCGGCAACCAAGTTGCCCTCAATTATACA
TATACAGCAGTGCCGACAGAGTCATTCCTGCAGTTTCGGTTGAGTCCTTTGTAGAAGAGCAACGAAGAA
TTGGTCGCGATGTTAGAGCTTGCAACTTCGTTTCTACACCTCACGTTGATCATTTCAGAAATGACCCAGA
ATTGTATACTTCACAGCTTACCCAATTTCTAGAGGACTACGTCCTAAGCTCTTGCAAACAGCCTTCTTGAA
TAATTCCTAACCACAACACATTGATTCTTTCTGCCCTTTTTTTCCCTCACTTCAACCAAATACTTGGTAAAG
CTTCTGTTTTCCAACTTTAAAGCAGATATACATTGATGTAGATTGTTACGTATACAGAGAATTAAGATTTT
TTTTTCTTTTCCATTCTTATTTAATTTAGTTGTATCTGTGGTCTCCCAGCAACAGAATTGTTGCATTGTACCC
AAAATATACTTTACTTTACTACATATATATAAAAAAGTTGGGCTTAATT
>PGSC0003DMG400002885 LRR-RLPs
ATGTGGCCTCTCTTTTTCATTTATTCACTTTTCTGTTGTGTATTTCTAAGTCTAAGTTTTTCTTCATCCTTTGA
TCATCATCTTTGCTCTCCCAGTGAAGCTTCTGCTTTGCTTCAGTTTAAGCATTCCTTTAATATATTGGACTT
CTCCCCGTGTGATACGTCTTTCCCAAAAACAGTTTCATGGAATGAGAGTAACGATTGCTGCACTTGGGAC
GGGGTCACTTGTGACATGTTAAACGGTCATGTTATCGGGTTGGATCTTAGTTGTAGTCAGCTTAATGGAA
CTATTTATCCCAACAGCAGCCTCATCCAACTTCATCATCTTCAGACACTAAACCTTGCTAAGAACTACTTTT
ATCCTTCTACGATTCCAAATGACGTTAGCCGGTTGAGGAATTTGAAGCATCTCAACCTTTCTGATGCTTAC
TTTCAAGGGGAAATCCCAACAGAAATCTCATACCTTTCCAATTTGGTTTCACTTGATCTTTCTGTTCCTTCT
TATGTGAACGGATTACAATTTGATCAGAGAGCATTTAAAGCAGTGCTTCAGAACTTGACAAATCTAGAG
GTACTTTCTCTCTTTGGTGTCAACGTCTCATCTCCGATACCTGTAAATTTGTCTTCCTCCTTAAGGTACGTC
GATCTTAAGGTAACAAATTTGGAAGGTGTTCTCACGGAGAGCGTTTTCCTTCTGCCAAAATTGGAAACGC
TCTATTTGAGTAATAACTATCTTCTCAAAGGAGTTTTACCAAAGATCCATCCAAGCAGCACATTGTTGGA
GTTGGATATTTCATACACAGGAATCTCCGGTGAGCTGCCTGATTCAATTGGCACCTTGAGTTCCTTGACT
CGCTTGTACCTCTATGGATGTCAATTCTCTGGCCGCATTCCTGATTCCATTGGCAATCTTACACAAATTAG
GTATTTGAATTTTGGTAATAATCATTTCACTGGCAATATTCCTTCCACAATCTCTAAATTGAAGCACCTCG
CAGTTTTAGCTCTTTCATCTAACTCCTTCGGAGGTGAAATTCCCAATTTTTTCTCTAACCTCCAAGAACTAC
GTTATTTAAGTCTTTCTAATTGCAGCTTCATCGGTCCGTTTCCTTCTCCACTTTTAAGCTTGACACAACTTG
AAACCTTAGACTTGTCGAGTAATTCCCTATCTGGACCACTGCCTAGAAATGCAAGCATGCTTCAAAAGCT
AGCCGAGCTGGATTTGTCTTACAACTCACTGAATGGTACCATACCATCTTGGGTGTTTAGCCTACCTTTGC

TATCTTCAGTGTCGCTCCATCATAACCGGTTAAGTGGTTCTTTTCCTCAATCACCTGTGAATCTCACAAAC
CTTTCTACCCTTGACCTTTCATCAAATAACATCACCCTTGATGCAGGAATACAAATCACTTTACCTAGCCTA
GTAGTTTTACAGTTATCATCTTGTGAACTTAGGGATTTTCCACACTTCTTGAGAAATGTAAAGACACTTAT
GGTCTTGGATATTTCTAACAATAAGATTCGTGGTCAAATCCCTAACTGGTTTAGCGGCATGAGGTGGGA
CTCCTTGTTGCACCTAAACCTTTCACATAACTCATTAACAGGCCACCTACAGCCAGTGGCGGAGGCAGCA
TGTAATGAGGGGGGGCACCCCCCTCGGCGAAAAATATTACTATTTATACATGGTTAAAATTATTTTTAG
GTATGTATAATAGATGTCGAACCCCCTTCCACTAGTTCGTGTGTTTACTTTTCAGATTTTGAACCCCCTTAT
TAAATTTTCTGGCTCCGCCACTGCCTACAGCAACTTCATTATAATAGTCTAGAGTCTCTTGATCTTAAATTT
AACTTCCTTGAGGGGGCACTACCTTCATCCATTTGTAACATGAGCAGTCTTAGCCTTCTGGATTTGTCACA
CAACTACTTCAGTGACTCGGCTCCGCATTGCTTGGGCAGCATGGTTGGGCTATCTGTGCTAGACTTGAGA
AGAAATAGTTTCACTGGGAGTCTTCCACCGTTTTGTGCGCAGAGCAATTCATTGAGAACCATTGTCTTGA
ATGGTAATCATTTTGAAGGAACTGTCCCGATGTCATTGCTCAAGTGTGATGGTCTAGAAGTCCTTGATGT
GGGGAACAATGTTATAAATGACACGTTTCCAGCTTGGCTCGGAACTCTTCAAGAGCTGCAGGTACTTAT
ATTGAAGTCTAACAAGTTCCATGGACCTATAAGTACTAGGCTGAAGTTTGGTTTCCCCGTGTTACGGATT
TTTGATCTCTCTCATAATGAGTTTGGTGGTTCACTGCCTGCAGAAGTTTTTGAAAACTTCAAAGGAATGAT
CAAAACAGATGAGAGAGACAAAGGAGAGATCGAATATATGCAACCACAGTCTTACATCGGATTTAGTA
ACGTAATGTATGAGGTTTCAGTGAGGTTGGTGATCAAAAGCCAGGAAATTCAGCTAGAAAAAATCACCA
CAATTATGACAACCATAGATCTCTCAAGCAACCATTTTGAAGGTGTCATTCCGAAAACACTAAAGGATCT
CAACTCACTTTGGCTACTCAATTTATCTCATAACAATCTCAAAGGAGATGTACCAATGGAACTGGAGAAA
TTGAATATGCTTGAAGCGTTAGATCTCTCTTGGAATCGGCTCACTGGAAAGATTCCACAGCAATTGACAA
GAATGAACTTTCTGTCATTCTTAAACCTCTCTCAAAATCTCCTTGTTGGACGCATTCCTCAAGGTTCACAAT
TCAACACATTTGAAAATGATTCATATGGTGGCAACCTTGATTTATGTGGTCCTCCTTTATCAAAGAAATGT
GGAACAAGTGATCCATCACATGTTCCTCAACCATTGGAGGAAGAAGAAGAAGATGATGAGTCATATTTT
TTTAGTGGATTTACATGGGAATCAGTAGTCATAGGCTACAGTTTTGGACTCGTTGTTGGAACTGTCCTGT
GGAGTCTCATGTTTAAATATCGTAAGCCAAAATGGTTTGTGGAATTTTTTGACGGCATCTTTCCCAAGAA
AATGAGAAGGCCAAAGAAGAGAGCTCAGAGACGACGGACATAATGAAAGATTGAAAGCAGTTCCTTTA
ACTGTTTGTATATATGCAGTATAATTGTATGTGTTGTTTTAGCATTTAGCTATTGAAAAGTGTCAATGTCC
ATTTTTTGGTCATTTTTTACTTCATTATCAAGGGATTGGGCTGAAAATTTGCA
>PGSC0003DMG400002886 LRR-RLPs

ATGTTTCAGAACTTCACAAATCTGGAGATACTTTCTCTCTCTTATGTCAACATCTTATCTCCGATACCTGTG
AATATTTCTTCTTCCTTAAGGTATGTGGATCTTGCACATACTAATCTGCGAGGTGTTCTCACAGAGAGCTT
TTTCCTTATGCCAAACTTGGAAAGACTCCAATTGAGAGGGAATGATCTTGTCAAAGGAGTTTTTCCAAAG
ATCCACCGGAGCAACACTCTGTTAATGGAGTTGGATATTTCATCCACAAGCATCTCTGGTGAGCTGCCTG
ATTCAATTAGCGCCTTCAGTTCCTTGAATATATTGTACCTCGAAGGATGTCAATTCTCTGGTTCCATTCCC
GATTCCATAGGCAACCTAACCAGAATTACGGAGTTGGCTTTGTCTTATAATCATTTGGGATAATACATAT
ATTTGACCTTAAACTTGGCTTCAAATTTTAACTTTGACCTCCAACTTTCATAGTGCACAAACAAGCACTTTA
ACTATCCAATCTTTTAATAAATAAACACGTGATTTTTGGAGCCAAGAGCGTGAAATGCAAACGCTGCCAC
GTGTATTAGCGAGCCACTCAATGGCTGCCAACTAATTAAACACTTTACACGTCCATTTTAGAACTAAAAA
AAAAAATAATTAATTAAGGGTAGAGTTGTCATTTCAGCATTTTTTTCATTGTTGTGTGCCTCAAAAATTAC
TTCTCACTCGCGCAAAATGCGTGCACATCACGCGTTTTGCCAGGTAAGACACGCGTGTTTATTTATTAAA
AGGTTGGATAGTTAAAGTGTCTATTTGTGCACTTTGAAAGTTGGAGGTCAAAGTTAAAATTTGAAGCCA
AGTTTAAGGTCNNNNNNNNNNNNNNNNNNNNNNNNNNNNNNNNNNGTCCAACTCCTTTTCAGGTGAAAT
TCCTGATGTTTTCTCTAACCTCCAAGAGTTACGTACGTTACTACTTTCTTATAACAACTTCATTGGTTTGTT
TCCCGCTTCACTTTTAAGTTTGACACATCTTGAATACTTAGGATTTACGAGTAATTCCCTATCTGGCCCACT
GCCTAATAATGTGAGCATGCTTCAAAAGCTAACCAAACTGAATTTGTCCTACAACTCAATGAATGGTACC
ATACCATCTTCTGTGTTTAGCCTCCCTTTGCTAGGTCAGTTGTGGCTCGATAACAATAGATTTAGTGGATT
ACCCGATGAGCTCAAAACAAACCCAACATTAGCACGCCTGGATTTAAGCCATAATCAACTCAGTGGTTCT
TTTCTTCAATCACTTTCGAATCTCACAAACCTTTCTATCCTTGACCTTTCATCGAATAACATCACCGTCGAT
GAGGGAATACAAATCACTTTTCCTAGCCTACAAGATTTGTGGTTATCATCTTGTGAACTGAAGGATTTTC
CAGTCAACTAAAGGATTTTCCACACTTCTTGAGAAATGTAAAGACACTTGTGTACTTGGATATTTCTAACA
ATAAGATTTGTGGTCAAATCCCTAACTGGTTTAGCGGCATGACGTGGGACTCGTTGCAGTTCCTAAACCT
TTCTCATAATTCATTAACAGGCCACCTACCACAATTTCGTTACTATAACCTACAGTATCTTGATCTGAAATT
TAACTTCCTTCAGGGGTCACTACCTTTATCTATTGGTAACATGAGAAAACTTATCTTATTAGATTTATCAC
ACAACTACTTCAGTGACTCAGTTCCACATTGCTTGGGAAGCATGGCTTCACTAGCGGTGCTGGACTTAAG
AAAGAACAATTTCACAGGGAGTCTTCCCCCATTATGTGGACACAACACTTCATTGAGTACCATTGTCGTA
AATGGTAATCGATTTGAAGGACCTGTTCCTGTATCGTTGCTCAATTGTAATGGTCTACAAGTCCTTGATGT
GGGGAACAATGCTATAAATGACACGTGTCCAGCTTGGCTCGGAAGTCTTCAAGCTCTGCAAGTCCTTAT
ATTAGAGTTGAACAAGTTCCATGGACCTATAAGTACGTGTCAGACTAAGTTTTGCTTTCCCAATTTGCGA

ATTTTTGATCTTTCTCGTAATGATTTCAGTGGCTCACTTCCTGCAAAAGTTTTTGGAAACTTCAAGGCAAT
GATCAAATTAGATGACGAAGACACAAGAGAGATCAGGTACATGGAATCTATGTTGAATTTGTCATTTGT
CAGATCGTATGAGGATTCAGTGAGTTTGGTAATCAAAGGGAAAGATATTGAGCTACAAAGAATCAGCAC
AATTACGACAACCATAGATCTCTCAAGCAACCATTTTGAAGGTGTCATTCTGAAAACGTTATAG
>PGSC0003DMG400002887 LRR-RLPs
TTTGGTTTCACTTGATCTTTCTGATAGTTATGAATTACAACTTGATGAGAGAACATTTGAAACAATGCTTC
ACAACTTTACAAATCTGGAGTTACTAGCTCTCCCTCTTGGTGACATCTCATCACCGATACCTGTAAGTATT
CATCCCAATAGCAGCCTCTTCCAGCTTCATCATCTCCACACACTAAACCTTGATAATAATTACTTGAATCCT
TCTTCTTCGATCCCACATAACATTGGCCGATTGAGGAATTTGAGGCATCTCAACCTTTCTGATGCTTNNN
NNNNNNNNNNNNNNNNNNNNNNNNNNNNNNNNNNNNNNNNNNNNNNNNNNNNNNNNNNNN
NNNNNNNNNNNNNNNNNNNNNNNNNNNNNNNNNNNNNNNNNNNNNNNNNTACAAATCTGGAG
NNNNNNNNNNNNNNNNNNNNNNNNNNNNNNNNNNNNNNNNNNNNNNNNNNNNNNNNNNNN
NNNNNNNNNNNNNNNNNNNNNNNNNNNNNNNNNNNNNNNNNNNNNNNNNNNNNNNNNNNN
NNNNNNNNNNNNNNNNNNNNNNNNNNNNNNNCAATCCCAAATGGCATTGGCCGATTGAGGAATTT
GAGGCATCTAAAACTCTCTGGCTTTGATGGGAAAATCCCAACAGAAATCTCATACCTTTCCAATTTGGTTT
CACTTCATCTTTCTGGTTATGGATTACAACTTGATGAGAGAACATTTGAAGCAATGTTTCAGAACTTGAC
AAATCTGGAGGTACTTTCTCTCTCTGAAGTCAACATCTCATCTCCGATACCTGTGAATATTTCTTCTTCCTT
AAGGTACCTGGATCTTAGTCATACTAATCTGCGAGGCATAATTCCCCATGGTTTCTCTAATCTCCAAGAG
CTAGTTGAATTGGATCTTTCTAATAACAACTTCACCGGTCCATTTCCCTCTTCAATTTTAAACTTGACAAGT
CTTCAATACTTAGATTTGTCTCACAACTCACTGAATGGCACAATACACTCTTGGGTGTTTAGCCTCCCTTC
CTTACTTGATTTGAAGCTCCATCATAACCAATTCAGTAGAGTGGCTGATGAGATCAAAACAAACCCGACA
TTAGTGACTTTGTATTTAAGTCATAATCAATTCAATGGTCCTTTTCCTCGATCACTTGTGAATCTCACAAGC
CTGGCCTTGCTTGACTTTTCATCAAATAACATCACCGGCGATGTGGGAATAAATATCACCTTTCCTAGACT
ATCAGCTTTGTTATTATCATCTTGTGAACTGAAGGATTTTCCACACTTCTTGAGAAATCTAAAGACACTTC
AGTTCTTGGATATTTCTAACAACAAGATCCGTGGTCAAATCCCTAACTGGTTTAGCGGCATGAAGTGGGA
CTCTTTGGAACAACTAAACCTTTCACGTAATTTTTTGACAGGCCATCTAGGAGAATTTCATTATTATAGTC
TTGAGTCTCTTGATCTTAAATTTAACTTCCTTCAGGGTCCACTACCTTCATCCATATGTAACCTAAGAAGCC
TTAGAATTCTGGATTTATCACGCAACAACTTCAGTAACTCAATTCCAAATTGCTTGGGAAGCATGGCTAA
GCTAACGGTGTTGGACTTGAGAAGGAACAATTTCAGTGGGAGTCTTCCACTGTTATGTACTCAAAGCAC

TTCATTGATGACCATTGTCTTGAATGGTAATCAATTCGAAGGATCTGTCCCTGCGTCATTACACAACTGTG
TTGGTTTAAAAGTTCTTGATTTGGGGAACAACGCTATAAATGACACATTTCCAGCTTGGCTTGGAACTCT
TGAAGAGCTGCAAGTCCTTATATTAAAGTCGAACAAGTTCCATGGACCTATAAGTGCTAGGAAGAAGTT
TTGCTTTCCCAGGTTGCGGATTTTTGATCTCTCTCATAATGCATTCAATGGTTCACTGCCTGCAGATATTTT
TAGAAACTTCAAGGCAATGATAAAAAATGGCACAGACAAAGGTAACATTACATATATGGAAACATCAGT
TTTCAGGTCATTGGTTGATTCATCGATCAAAGACTGGACTATGTATAAGCTAGAATTGGCCATTGATGAA
GTGTACAAGGATTCAGTGAGGTTGATGATCAAAGGAAACAATATTGAGCTAGAAAGAATCAGCACAATT
GTTACAGCCATAGATCTCTCAAGCAACCATTTTGAAGGTGACATTCCGAAATCACTAAAGGATCTCAGCT
CACTTTGGCTACTCAATTTATCCCACAATAATCTCAAAGGTGATATTCCAATGGAATTGGGGCAATTGAA
TTCACTTGAAGCCTTAGATCTCTCTTGGAATCGGCTCACTGGAAAGATTCCACAGGAATTGACAAGAATG
AACTTTCTGGCGTTCTTAAACCTCTCTCAAAACCAGCTCGTTGGACGCATTCCTCAAGGTTCACAATTCAG
CACATTTGAAAATGACTCGTATGGTGGCAACCTTGATTTATGTGGTCCTCCTTTGTCAAAGCAATGTGGA
ACGAGTGATCGCTCACATGTTCCTCAATCACTGGAGGATGAAGCAGAAGAAGATGAGTCATATTTCTTC
AGTGGATTTACGTGGGAATCAGTAGTCATAGGCTACAGTTTTGGACTAGTTGTTGGAACTGTCATGTGG
AGTCTCATGTTTAAATATCGTAAGCCAAAATGGTTTGTGGAATTTTTTGAAGGCATTTATCCCAAGAAAA
TGAGAAATAGGCTACAGTTTTGGACTAGTTGTTGGAACTGTCATGTGGAGCCTCATGTTTAATTATCGTA
AGCCAAAATGGTTTGTCGAATTTTTTGACGGCATCATGCCCCACAAAAGGAGAAGGCCAAAGAAGAGA
AGGCTGAGACGGCAGACATAATGGAAGATTGAAAGATTGAACTTTTCTGTTTCTGTTTTTTCTGTTTGTT
GTTTGTATATTCTTCATACAAACTTAACTGTATGTGTTTTTTTTTAAATTAATGTATCTTTTTCGGAGACTT
GTTTGTGGTGGAAG
>PGSC0003DMG400002906 LRR-RLPs
CGAGTGTCCAACCTTGTCAAGCCCACTATTAAAAATGGAAAAAACAACAAATAGACCAGGCCCTTTCGTA
AACCTGATATTCTAAGATAGGATTTTCGCCGCTATCTCCGCTTCTTCGCCTTAGCTCTCTTCACTGTTTCTG
TACATGAACAACAATGTTGCCTCCATGTTACTGACCTTTATGCTTTTTTCCAGTTGTGTTCAAAATTTTCTT
CCTCTTGCTTTCTTTTCTTCTTCTCTCCCTCTTTTGGTCTCTGATTCCCATCATTCTGCAAGTGATTTTGCGA
TGGAGTGCAAGGGTAGTTCAATTCCTCCATCTCCAGGTTAAAAGCTCCATCTTGACTCCAGATCCTCCCTT
TTGCTGAGATCGCTTTGTTGACTGGAATAGCAATAGCATTTTCCCTGTTTTGTTGCCTTCTAACTTCTATGT
AATTTTGTTGTTTGTGGAAAAATATATGTTGTAGCCTTCCCTTGTCCCCTATCCCCTTAAGGATGATACTCT
TAAATATGGGCATAGTAATTTTTCATTATTTATGAGTCTCCTCGCAAGTGTCTCCCTGTTAAAATTTGTGA

ATGTGCAGCTCCCTCGGTCAATAGCAAAGTTTGCCACATGATCTGCTAGCTTGTTTCCTTCCCTTAGAATA
TGTTGGAAAATAACTTGTTTATCTTGTAGACATGATTGAATCTCTGTCACCATTTCTGCAATTATCCATGG
ACATTCCCATTTCCTTTCTAATACCTTGTACAAAAGCATTGAGTCAGTCTGTATGATTATTTGATTATGTTG
TAACTTCTTGCAATGCTTGGATTCCTCCAGAATAGCTTTAGCTTCCGCGACTCTATTAGTAGTATTTTCTAT
ACTTGCTCCTTCCACGTATTCTACATCTCCTTTAGCATTCCTCAAAACGAAGGCATAGGAACTAAGACCCG
GATTTCCTCTAGAAGTTCCATCTGTATTGTATTTTACCCACCCATTTGGTGGGAATTCCCATAAGACCCTTT
TCACTTTCATTTTAGGTTTGTAGCTTGTCCATTCTATGAGCATATCAGGCCAGTTAACAGGACATGTCATA
TAGGGTTTCCTGAGTCTTACCAGCATATACATGTTCCTGGTGATATTGTGAATGACTCTTGCTACTGATGT
CTTTTTTCCTTCATGCTTCATCCTGTTTCTCTTTCTCCACAATTCCCATATTATAAAACTAGGAAGAGCTCTG
TAAAAAGGTCTCATATTTGTCCCAATTTTTGTCCCCCACCACAACATTATAACTTCTCTTAGTTGGAGACCC
TCCATGTTAAGACCTGTAAAAGAACAAAAATAGGACCAAGTCCTGTTAGCTGAATCAGATTTCAGAAAC
ACATGTTGAGGTCTTTCACAACACCAGCATTTGGATGGGCCTTCAAGACCCCATCTTCTCATTATGTCATC
AACGGGGATTTTAAATTTCCATAATCTCCACATTATGAAAGCCATTTTAAATGGTACACCCTTACTCCACA
TCCATTTATAAATTTTGTTTTCCTCCGCCTTATGTCTGATATAATGCCAAGCAGATTTAACTGAGAAACTTC
CCCTTGTCTCTAGCATCCAACAAGGTTTATCCATTGCTGTTTCTTCTTTTGGAGGATGAATATTCTGCAGA
ATATGGTTCACCAATTCCTGTGGGAGTATCTCATGTAGTACCTCTGTATCCCACTCCCCTTGTCTAGTTAA
ATCCCTAACTGTTTTATATGTATCATCACATTGAAATTCTTCTCCAGTAATAGTGTACAAATCCCCTAGGCC
CGTCCAGTTATCATGCCAGACTGATACTGATCCTTTCTCCATTTTCCATAAAATTTGGTTTTCAATGAGATC
TCTAGCTTTAAGCATTTTTTCCCATAAAATTTGGCTCGGAACTCTTCAAGAGCTACAGGTCCTTATATTAA
AGTCGAACAAGTTCCATGGACCTATAAGTATGTGTTAGACTAAGTTTTGCTTTCCCAAGTTGTGAATTTTT
GATCTTTCTCGTAATGATTTCAGTGGCTCACTTCCTGCAAACGTTTTTGGAAACTTCAAGGCAATGATCAA
ATTAGATGGTGAAGACACAGGAAATATCAAGTACATGGAATCTGTGTTGAATTCGTCATTTGTCACATCG
TATGAGGATTCAGTGAGTTTGGTAATCAAAGGGCGGGATATTGAGCTACAAAGAATCAGCACAATAAC
GACAACCATAGATCTCTCAAGCAACCATTTTGAAGGTGTCATTCCGAAAACACTGAAGGATCTCATATCA
CTTTGGCTACTCAATTTATCTCATAACAATCTAAGAGGTGATATTCCAATAAAACTAGGACAATTGAGTAT
GCTTGAAGCTTTAGATCTCTCTTGGAATCGGCTCACTGGAAAGATTCCACAAGAATTGACAAGACTGAAC
TTTCTGGCCTTCTTGAACGTCTCTCAAAATCATCTCGTCGGACCAATTCCTCACGGTCTACAATTCAACAC
ATTTGAAAATGACTCGTATGGCGGCAACCTTGATTTATGTGGTCCTCCTTTATCAAAACAATGTGGAACG
AGTGATTCATCACATGTTCCTCAACCATTGGAGTCTGAAGAAGATGAAAGCGAGTCATATTTTTTAGTGG

ATTTACATGGGAATCAGTAGTCATAGGCTACAGTTGTGGACTAGTTGTTGGAACTGTCATGTGGAGCCT
CATGTTCAAATATCGTAAGCCAAAATGGTTTGTGGAATTTTTGATGGACTCATGCCTCACGAAAGAACAA
GGCCGAAGAAGAGAGCTCAGAGACGACGAACTTAATGGAAGATTGAGAGGGGCCAAGTTGTCTTTGTT
TGATTGAACTTTTTTGGTTGTGTGTTTTAATTTTCTGTTTGTTGTGTTGTATATGTTTGATAGAAACATAAT
TATATGCATGTGTGTTATTT

>PGSC0003DMG400002908 LRR-RLPs

ATGAAACTAGGACAATTGAGTATGCTTGAAGCTTTAGATCTCTCTTGGAATCGGCTCACTGGAAAGATTC
CACAGGAATTGACAAGAATGAACTTTCTGGCCTTCTTAAACCTCTCTCAAAATCATCTCGTCGGACCAATT
CCTCAAGGTCGACAATTCAACACATTTGAAAATGACTCATATGGTGGCAACCTTGATTTATGTGGTCTTC
CTTTAACAAAGAAATGTGGAACGAGTGATTCATCGCATGTTCCTCAACAATTGGAGTCCGAAGAAGAAG
GCGAGTCATATTTTTTTAGTGGATTTACGTGGGAATCAGTAGTCATAGGCTACAGTTTTGGACTAGTTGT
TGGAACTGTCATGTGGAGCCTCATGTTTAAATATCGTAATCCAAAATGGTTTGTGGAATTTTTTGATAGA
CTCATGCCTCACAAAAGAAGAAGGCCAAAGAAGAGAGCTCAGAGACGACGGATTTAA

>PGSC0003DMG400002910 LRR-RLPs

ATGACTTCAATCCCACATAAAATCATTGGCCGATTGAGGAATTTGAGGCATCTAAAACTCTCTGGCTTTG
ATGGGAAAATCCCAACAGAAATCTCATACCTTTCTAATTTGGTTTCACTTGATCTTTCTGGTTATGGATTA
GAACTTGATGAGAGAACATTTGAAACAATGCTTCACAACTTTACAAATCTGGAGTTACTCTCTCTTGGCG
ACATCACATCACCGATACCTGTAAGTATTCATCCCAATAGCAGCCTCTTCCAGCTTCATCATCTCCACACA
CTAAACCTTGCTGGCAATTACTTTTATCCTTCTTCAATCCCACATAACATTGGCTGATTGAGGAATTTGAG
GCATCTAAAACTCTCTCACTTTCAAGGGAAAATCCCAACAGAAATCCCATACCTTTCTAATTTGGTTTCAC
TTGATCTTTCTGGTTATGGATTAGAACTTGATGAGAGAACATTTGAAACAATGCTTCACAACTTCACAAA
TCTGGAGTTACTTTCTCTCTCTTATGTCAACATCTCATCACCGATACCTGTAAGTATTCATCCCAATAGCAG
CCTCTTCCAGCTTCATCATCTCCACACACTAAACCTTGTTAACAATTTCTTTAATCCTTCTTCGATCCCATAT
GGCATTGGCCGATTGAGGAATTTGAGGAATCTGATACTCGCTGACTTTGATGGGAAAATCCCAACAGAA
ATTTCATACCTTTCCAATTTGGTTTCACTTCATCTTTCTTCTTCTTATGGTAATGAATTACAACTTGATGAG
AGAACATTTGAAGCAATGTTTCAGAACTTCACAAATCTGGAGATACTTTCTCTCTATCAAGTCAACATCTC
ATCTCCGAATATTTCTTCTTCCTTAACGGAGTTGGATATATCATACACAGGCATCTCTGGTTCCATTCCTG
ATTCAATTGGCACCTTAAAATCCTTGAATATCTTGATCCTCTATAGATGTCAATTCTCTGGTTCCATTCCTG
ATTCCATAGGAAACCTAACACAAATTACGAAGTTGATTTTATCTCATAATCATTTCACTGGCCATATTCCT

TCCACAATCTCTAAATTGAAGCACCTCACACGTTTATATCTTTCGGACAACTCCTTTTCAGGTGAAATTCCT
GATGGTTTCTCTAACCTCAAAGAGCTACGTTATTTATATCTTTCTCACAACTCCTTTTCAGGTGAAATTCCT
GATGTTTTCTCTAACCTCAAAGAGCTATATTATTTAGATCTTTCTGAAAACAGCTTCACTGGTCCATTTCCC
GATTCAATTTTAAGTTTGACACAACTTCATACCTTAGACTTGTCGAGTAATTCCCTATCTGGCCCACTGCC
TAGCAACCAAAGCATGCTTCAAAAGCTAACTTTTGTGGATTTGTCATACAACTCACTGAATGGTACCATA
CCATCTTGGGTGTTCAGCCTACCTTTGCTACATTCAGTGTCGCTCCAACATAACCGATTCAGAGGACTAG
CCGATGAAGTGATCAAAACAAACCCAACATTAAAACAACTGCATTTAAGCAATAATCAACTCAGTGGTTC
TTTTCCTCAATCACTTGTGAATCTCACAAACCTTGAAACCCTTGGAATTTCATCAAATAACATCACCATTG
ATGAGGGAATGAATCTACAGTATCTTGATCTGAAATTTAACTCCCTTCTGGGTCCACTACCTTTATCCATT
TGTAACATGAGCTCGCTTATCTTATTAGATTTATCACGCAACAACTTCAGTGACTCAGTTCCACATTGCTT
GGGAAGCATGGCTAGTCTAACGGTGCTAGACTTAAGAAGGAACAATTTCACAGGGAGTCTTCCCCCATT
ATGTGCACACAGCACTTCGTTGAGTACCATTGTCATAAATGGTAATCAATTTGAAGGACCTGTACCTGTG
TCGTTGCTCAAGTGTGGTGGTCTAGAAGTACTTGATGTGGGGAACAATGCTATAAATGACACGTTTCCAT
CTTGGCTTGAAATTCTTCAAGAGCTGCAGGTCCTTATATTAAAGTCGAACAAATTCCATGGACCTATAAG
TATATGTCAAACTAAGTTTTGCTTTTCCAAGTTGCGAATTTTTGATCTTTCTCGTAATGATTTCAGTGGCTC
ACTTCCTGCAAATGTTTTTGGAAACTTCAAGGCAATGATCAAATCAGATGGCGAAGACACAAGAGAGAT
AAAGTACATGAATTCTGTGTTGTATTCGCCATTTGTCACATCGTATGAGGATTCAGTGAGTTTGGTAATC
AAAGGGCACGATACTGAGCTAGAAAGAATCAGCACAATTACGACAACCATAGATCTCTCAAGCAACCAT
TTTGAAGGTGTCATTCCGAAAACACTAAAGGATCTCAGATCACTTTGGCTACTCAATTTATCTCATAACAA
TCTCAGAGGTCATATACCAATGGAATTGGGGCAATTGAGTATGCTTGAAGCTTTAGATTTCTCCTGGAAT
CGGCTCACTGGAAAGATTCCACAGGAATTGACAAGATTGAACTTTCTGGCCTTCTTAAACGTCTCTCAAA
ATCATCTCGTTGGACCAATTCCTCAAGTTCTACAATTCAACACATTTGAAAATGACTCATATGGTGGCAAC
CTTGATTTATGTGGTGTTCCTTTAACAAAGAAATGTGGAACGAGTGATTCATCGCATGTTCCTCAACAATT
GGAGTCCGAAGAAGAAGACGAGTCATATTTTTTTAGTGGATTTACTTGGGAATCTGTAGTCATAGGCTA
CAGCTGTGGACTAGTTGTTGGAACTGTCATGTGGAGTCTCATGTTTAAATATCGTTAA

>PGSC0003DMG400002914 LRR-RLPs

AAAAAAGAACCATTCTTTGTTTCTTCTCTCTCTAACCTCAAATCTTTCTCTCTCTATTTTTGCCCTACTTTGT
TGGGGTTCTTGAAAAAAAACTGGATTTTTGAGGGTTCTTGAAAAAAATTTGGATTTTTTCATCTGGAATGC
AAATCTTGGTTAAAGATATAATCTGGGTTGTGTTTTTCTTGGTTCAAAATCTGATCTTTTTTGCTTTACAGT

TCGGGATTTCTTCTGAAGAACAGACATGCCTACTCTTGTTAATTACAGTGGTGAGTTTCAAAAAACTTCAT
TTCTTGTTTTTATAAGTTATGGGGTTGTTCATATTTCAATGAATTTTGAAGTTCAGTTACAATTTTTTGATT
TTTCATCATGAATTACTGAGATGGAGATGGATTTGTTATGTTTAGATTCATATAAAGTTGTTTATCATGAA
TAAAGTTTATAACTTTGTATAGGTAGATTATTGTTGAGATGAATATGAGCAATAATTTATTGAGATGAAG
ATGAATTCTTTTAAGGTTTAGATCCATGAATATAGTCTATAACTATGTATAGGTGCATGATTGTTGATAAC
TTGAGATGAATATGGTTTATTAATTTACTGAAATGAAGATGATCTTTTAGGTTTTAGATCCAATCATGTTT
TTATTTAAAAAAGGAAGTTGGTTTATGAGATAATAACTAAAAGTTGAGTGACTATCTAAGAAATTAACTC
ATATAACTTTGTATAGGTGCATGATTGTTGAGGTGAATATGAGCATTAATTTACTGAAATGGATAAAAAA
GGGAAAAAAGTTATGATAAGACTAGATCCTTTGGCTTTGAAGATTAATTTCTGTGTTTGATAGATCTTTTT
TTGTCTTATGTTCTGAATCTATATTAGAGATTGGTAATATATTGAAGAAACTGTGGATTACTCTTTTGGGT
TCTGTATCTAGAATCGTGTATTTATCTGATCTGATCTGAAGATTTATGTACATAATAGGTAGATCTAAAGC
TTCAATTCACATGTGATAGTTGTTTCTCTGTTATACTTGAAGTTGAAGTGAAAATTCATAAGAAATGGGC
ATATGGATGAATACTTGTAAAATTCTTTCATTAGACAAAATTTAGTATGCTTGCTTGTTGATTTAAGGGAA
AATATAGCTGACAATGTATTTGCTCCTTGGATTTCAGGTGATGATGAGTTCTATTCTGGTGGATCGTTTTG
TTCTGCAGATTTGGGTCTCATGTTGTCTTTGGGTCATGCTGATGTTTACTGTCCTCCTAGGAAGAGGGCA
CGCATTAGTGGCCCGTTTGTTGTCGAAGACCGGTCAAAGGATCCATCTCTTGAAATTCTTCCTGATGAAT
GCCTATTTGAGATCCTCAGACGATTGCCCGGTGGCCGAGAAAGGGGTGCAGCAGCTTGTGTTTCTAAGC
GCTGGCTTACAGTGTTGAGTAGCGTGAGGAACTCTGAAATCTGCAGGAGCAAGAGCTATAACAATCTAA
ATGACGCAATAATGATCTCTAAAGATGAAGATCTTGAAGTTGAATGTGATGGATACCTTACTAGGTGTG
TGGAAGGGAAGAAAGCTACTGATGTTAGACTTGCTGCAATTGCAGTTGGGACGTCTACCCGTGGTGGA
CTAGGAAAGCTTTCTATCCGAGGGAGTAACTCAGTTCGTGGTATTACTAATGTCGGTCTATCTGCTATTG
CACATGGGTGCCCTTCTCTTAGGGTCCTATCATTGTGGAATGTTCCTTCTATTGGGGATGAAGGTCTCTT
GGAGGTTGCAAGGGAATGCCGTTCATTAGAAAAGCTAGATCTGAGCCACTGTCGTTCAATCTCCAACAA
GGGTCTTGTTGCAATAGCAGAGAATTGCCCGAGCTTGACTTCATTGACTATTGAATCCTGTCCAAAGATT
GGAAACGAAGGCTTGCAAGCTATTGGAAGATGCTGCACCAAACTACAGTCTCTTACGATTAAGGACTGC
CCACTTGTTGGAGATCAGGGAGTTGCTAGTCTTCTGTCATCAGGTGCTTCAATGTTGTCAAAAGTGAAAC
TACATGGTCTAAACATCACAGATTTCTCGCTTGCTGTCATTGGTCACTATGGCAAGCTGATTACCAATCTT
AATCTTTGTTCACTTCGTAATGTGAGTCAGAAGGGATTTTGGGTCATGGGTAATGCTCAAGGCCTCCAGT
CTCTGGTTTCTTTGACAATCACCTTATGCCAGGGAGCCACTGATGTCGGTCTTGAAGCTGTTGGAAAGGG

ATGCCCAAATCTCAAACATATGTGCATTCGCAAGTGTTGCTTTGTTTCTGACGGTGGGCTTGTTGCTTTCG
CTAAAGAGGCTGGATCTCTTGAGAGTTTGATCTTGGAGGAGTGCAACAGAATTACCCAAGTAGGTATCC
TTAATGCAGTTTCAAACTGCAGGAAGTTAAAGTCTCTTTCCCTAGTGAAGTGCATGGGAATCAAGGATCT
AGCTCTACAAACTTCCATGCTGTCTCCATGTGAATCTCTTCGGTCCTTGTCTATCCGAAGCTGTCCAGGAT
TCGGGAGCACTAGCTTAGCTATGGTCGGTAAGCTCTGCCCCAAGCTGCATCAATTAGATCTCAGTGGGC
TTTGTGGAATAACCGATGCTGGTCTTCTCCCACTCTTGGAGAACTGTGAAGGACTTGTTAAGGTGAATCT
TAGCGACTGCCTGAACTTGACAGACCAAGTGGTGCTTTCATTGGCTACGCGACATGGAGAGACCCTTGA
ATTGTTGAATCTCGATGGATGCAGGAAGGTTACCGACGCAAGTTTAGTGGCAATTGCAGATTATTGCCC
ATTACTTAATGATCTTGATGTTTCTAAGTGTGCAATAACTGATTCTGGTGTAGCTGCTTTATCCCGTGGAG
TGCAAGTGAATTTGCAGGTCCTTTCTTTATCAGGTTGCTCCATGGTATCAAACAAGAGTGTCCCTTCTCTT
AAAAAGTTAGGAGAGAATCTACTCGGCTTGAATCTCCAACATTGCTCTGTTAGTTGCAGCTCAGTCGAGC
TTCTTGTGGAGGATTTGTGGAGGTGTGACATCCTCTCCTAAACCAAAAGGGCACAAGGAAATGCATCAA
AATGGTCAAAAAGAATAAGCAGAAAGCTATAATGGTTCATTCAGCCTGGCAATTACAGAAGCAAACATG
AGTGTTCTTTTTTTAGCCCGTATATTCGTGGTTATCTAATACTTCAGTGTCTCGGTTGTTTTCCAGGAGGCT
ATGGCATCACTTTTTTCCTCCATTTTTTGCAGGCTTCGTCAAATCGAAGCCTGTTTCATCCATTTTTGGAGA
CAACTCCATCATGCCATGGTTTCCTGAGAATACAGTCACCTAGTACTGAGATTAAAATTTTCACTCATGTT
GCTCCTCGAAAGAATCAGGCCGTTTGAATCATTCAGCTACAACATCGACAGACAATATCTATCGATAGTC
TTTTAGTGTTTCATTGTAAGTATTCTACTGTGTGTCTTTTAGACTTTAGTTGCTTTGAGCAAATTTCTGTTG
TTGTTGTGCCTGTTATTCTAGGGGCTTGGCCATTACAACCTTACCTAGGTTGTTTTTTTTAACCAGTCCCTT
GTTTTTTGATAGGATTATCAATTTCCTATTTTTTACCAGGTTCCACATATTCAGATTTGTACTCTTGCAACT
ATGTTTTACATATATAATGAAGTACTAGTTTGTTTTGAA

>PGSC0003DMG400002937 LRR-RLPs

GAGTGATTTGTAAGAAGAGAACATCCATCCTTTTTTTGGCTGAATACGAAAAAGAAAGATGGGACATTT
CTTTTTCTTTTATTCATTTCTGTGTGTTGTGTTGCTAATAAGTGAATGTTTTTGTTCTTCCTTTGAGCATCAT
CTTTGCTCTCCCACTGAAGCTTCTGCTTTGCTTCAGTTTAAGCAACACTTTGAGATTATATCCTACCCTAAT
TCCCTATGTCCTATTCATTTCCCAAGAACAAAGTCTTGGAATGAGAGTAGGGACTGCTGCATTTGGAATG
GAGTCACTTGTGATATGTTAACGGGTCGTGTTATCGCGTTGGACCTTAGTTGCAGTCAGCTTTGGGGAA
GTATTCATCCCAACAGCAGCCTCTTCCAACTTCATCATCTCCACACACTAAACCTTGCTTACAATAACTTCA
ATTATTCTTCAATCCCACATAACATCGGCCAATTGACGAATTTGAGGCATCTCAACCTCTCTGAGTCTGGC

TTTGATGGGCAAATCCCAACAGAAATATCATACCTTTCGAATTTGGTTTCACTTGATCTTTCTGGGTATGA
ATTACAACTTGATGAGAGAACATTTGAAACAATGCTTCACAACTTCACAAATCTGGAGCTACTTTCTCTCT
CTCAGGTCAACATCACATCTCCGATACCTATGAATATGTCTTCCTCCTTAAGGTACGTGGATCTTGGATTC
ACTAATCTGCGAGGTGTTGTCCCAGAGAGTCTTTTCCTTCTGCCAAACTTGGAAAACCTCAAATTGGGTG
TGAATCTTCTTCTCAAAGGAGTTTTACCAAAGATCCCCCTGAGCAACACTCTGTTGGAGTTAGATATTTCA
CTCACAGGCATCTCTGGTTCCATTCCTGATTCAATTGGCACCTTAAGTTCCTTGAATATCTTGAACCTCGA
ACTATGTCAATTATCTGGTTCAATTCCTGATTCCATAGGCAACCTAACACAAATTACGGAGTTGATTTTAT
CTACTAATCATTTCACTGGCCATATTCCTTCCACAATCTCTAAATTGAAGCACCTCACACGTTTGGATCTTT
CATCTAACTCCCTTTCAGGTGAAATTCCCAACGTTTTCTCTAACCTCCAAGAGCTACGTTATTTACATCTTT
ATGATAACAGGTTCATCGGTTCATTTCCTTCTACAATTTTAAATTTGACACATCTTGAAAGCTTAGCCCTG
TCGACAAATTCCCTATCTGGCCCACTTCCAGCTAATAAAGCGAGCATTCTTCAAAAGCTAACCCAACTGG
ATTTGTCTGACAACTCACTGAATGGTACCATACCGTCTTGGGTGTTTAACCTCCCTTTAGCATCTTCATTG
TGGCTCCATAATAATCAATTTAGTGGACTACCCGATGAAATCAAAACAAACCCAACATTAGAATACCTGT
ATTTAAGCAATAATCAACTCAGTGGTTCTTTACACCAATCACTTGCGAATCTCACAAACCTTTCTACCCTT
GACCTTTCAACAAATAACATCACCGGTGATGCAGGAACAAATATCACTTTTCCTCGCCTACAAGTTTTGC
AGTTATCATGTTGTGAACTGAAGGATTTTCCATATTTCTTGAGAAATGTAAAGACACTTGAGGTCTTGGA
TATGTCTAAGAATAAGATTCGTGGTCAAATCCCTAACTGGTTTAGCAGCATGAGGTGGGACTCGTTGGA
GTTCCTAAATCTTTCACATAATTCGTTAACAGGCCACCTACCACAATTTCATTACCATAGTCTAGAGTATCT
TGATCTTAAATTTAACTCCCTTCAGGGTCCACTACCTTCATCCATTTGTAACATGAGCAGCCTTAACATTCT
AGATTTATCACGCAACAACTTCAGTAACTCGATTCCAAGTTGCTTGGGAAACATGGCTAGTCTAACAGTG
CTGGACTTAAGAAGGAACAATTTCACAGGGAGTCTTCCCTCATTATGTGCGCAGAGCACTTTATTGAGAA
CCATTGTCTTGAATGGTAATCGTTTTGAAGGAACTGTCCCGATGTCGTTGCTCAAGTGTGATGGTCTACA
AGTCCTTGACGTGGGGAACAATGCTATAAATGACACATTTCCAGCTTGGCTCGGAACTCTTCAAGAGCT
GCAGGTCCTTATATTAAAGTCGAACAAGTTCCATGGACCTATAAGTACTAGGAAGAAGTTTTACTTTCCC
AGGTTGCGGATATTTGATCTCTCTCAAAATGAGTTCAGTGGCTCACTGCCTGCAGAAGTTTTTCGAAACT
TCAAAGCGATGATCAAATTAGATGACAGAGAGGAAGGCGAGATCAAATATATGAGTCAATATTTGAAT
GGACATTCAATATATGAGGATTCAGTGAGGTTGGTGATCAAAGGCCAGTATATGGATCTAGAAAGAATC
AGCACAATTATGACAGTCATAGATCTGTCAAACAACCATTTTGAAGGTGTGATTCCGAAAACACTAAAG
GATCTCAGCTCACTTTGGCTACTCAATTTATCCCATAACAATCTCAAAGGTGATATCCCAATGGAATTGG

GGCAATTGAATATGCTTGATGCCTTAGATCTCTCCTGGAATCGGCTCACTGGAAAGATTCCGCAGGAATT
GACAGAATGAACTTTCTGGCATTCTTAAACCTCTCTCAAAATCATCTTGTCGGACCAATTCCTCAAGGTCC
ACAATTCAACACATTTGGAAATGACTCGTATGGTGGAAACCTTGATTTATGTGGTCCTCCTTTATCAAAG
AAATGTGGAACGAGTGATCCATCACATGCTCCTCAGCCATTGGAGTCTGAAGAAGATGAAGATGAGTCA
TATTTCTTAAGTGGATTTACGTGGGGATCTGTAGTCATAGGCTACAGCTTTGGACTCATTGTTGGAACTG
TCATGTGGAGCCTCATGTTTAAATATTGTAAGCCAAAATGGTTTGTGGAATTTTTTGAAGGCATTTTCCCC
AAGAAAATGAGAAGGTCAAAGAAGAGAAGGCCGAGACAGC

>PGSC0003DMG400002954 LRR-RLPs

ATGACTCAAGGTATTAACTTCTCCCTTTTTCCCTTTTAACTCTTTTCTAATAGATAGTAAAGGGTTGCTGCC
GTTGAAGATAGAATCAGTGTTCTACCAAGTAACGTTATAGATCATATCCTCGAGCTCTTGATGGTTCATG
AGGCTGCAAGAACTAGTATTTTGTCCAAAGATTGGAGATATAGTTGGGCCAAGCTTCCCAATTTGGTGCT
TGATAAGCAATTCTCCATGAAATTAGCTAAAAAATCTCACAATGTCTTCAAAGAAACAATAGATATGATT
CTCTTACAACATATTGGAGATATTGTGAAGTTTTATCTTGATGTTTCAGGAGTACATTTGTCTACATATGC
AGATATTGATAGATGGATGCTTTATGTCACCAGAAATGGTGTCAAGAAGCTAAAGCTTAACATGACAAA
AGATGGTACTTACAAACTACCTTCTTATATATTTAATTGTCCAACACTGACTCATTTGAAACTCTTCAACTG
CAACTTCAAATTACCAAATTCTTTTCTTGGCTTACAGACTCTTACCGTCCTTCGGTTGGAAAAAATAACCTT
TGTGCCTGCAATAGAGTTTTATACTATCAATGTGCCCCTTCTTGTCAAACTAACCATAGATTTATGTGATG
GTACTCAATACCTCAACATTGTGTTCTCATCTGAGTTGAAATCCTTGGTTGTTCATGAGAGTCACTATAAT
CTTGACCTAAGTAACAACTTCTTTAAGAAGTGCAAAAAATTGACAGATTTATACCTTGTGATTGATAATCC
AATACCTGCGGATAATAGATTAACTTTGGAAAAGCTTCTTTTTAGCTCGCCTACACTTGAGACACTTTGTT
TGGGTAAATTTGAACTTGAGGTAAGAATCTAACGTGCTGTTTTACTTAGATATCTTCTTTGAGTGTGATCC
TTCTTATTGTATAATGTTCCGTTAAAGCTTTTGACTGCAGGTGTAGTCCCAAAGAGGCTTCCTTGTATACT
CAACTGCTTGTGGCATCTTGATTTAGATGTAAACTTCAATAAATTGGGTCAGACTTCTTACACTCTGGAGT
TGATTAAAAGTTTCCCCAATTTTCGTGACCTTGAGATTTGGGTAAGAAATAGCAAATATATGTGGTCATA
CATTCTATATATCAATGGTCTTGTTCGTTTTTGCATTTCACGTACTTAAGAAGCTAAAATGACCTTGTATAC
CATCAGGACAAAACTACCGATGACATAGTTGAAGGAGTTATGAAATATCTAGACACACCATCATGTTTG
GACAAAACCCTCAACAAGCTTGAAGATGTTTCAATCCATTCCTTTAACTATTCTAAAGTTGTACTATATTT
CGTAAAATTATTGTTTGCTCATGCTTCATCTTTGTCAAGGATGAGCATCTATCCGGATAAAGCTTCTAATT
CCAAAGAAGAATTGAATGTTGCCACAGAGTTGATGCGTTTCCCCAGAGTATCTCCTAAGGCAGAGCTAA

TCTATCATCCAAATGAAGAGTTGGATTAA

>PGSC0003DMG400002960 LRR-RLPs

ATACACAAGAGTAGTACATTATTTGATATCTTGAGTTACATCTAAAAAATTTTTTTTGTTGTAATTCCTCTC

CATATTGACTTGATGGCAGCTTATACTGCTGTAATTTCTCTTCTTCAAACTCTTGATCAACCAAATATTTCA

GAACTCTTTCATGATCACAGTACTGAAATGTTCGATTCTCTTCGTGCTACAGCTGAATATTTTCAAGATGT

TCTTGAAAACACTAGCAATGAGAAAATCAAATCTTTGGAGGAAGATATTAGAGTTGTTGTTAGTGAGGC

AGAGGATGTTGTTGAAATGAAGATTTCTGAAATCATCAAAGAAGAAAGCTGGACATTTGGAATTTTACA

ACACCATGATATGCTACCAGTTGTTGAAAAAATGGATACCACAAAGAAACAAGTGATGGAGATTCTTTG

TCATGATGCTGATCAAATTCTTGAATTAACCGGGGATTCCTTGATTGGCGCTTCTTCTATGAGTGATCCAA

TGCTGTCAGATCAGTTGGAAGATGATATTGTGCAGGGACTTGATGGTGACTTGGAGATAATAGATAAGA

GATTGAGAGGACCAACGTCGGATCTAGACATTGTCACCATATCAGGTATGGGTGGCATTGGCAAAACAA

CACTCGCTAAAAAAACTTATGATCATCTTCCAATCAGGTATCACTTTGACATTTTTGTTGGGTTACAATAT

CTCAAGAATTTCAATATAGAAATGTATTGTTAGAAGCTTTACATTGCATTTCAAAGCAAAGAGTTATTGA

GAACACAAAAGATTATGATAAGATGAATGACAGTGAGTTAGCTGATCTGGTGCAAAAGAGTCTAAAGG

GTCCTAGATACCTTGTTGTTGTTGATGATATTTGGAGTAGGGATGTTTGGGATAGCATATCACAAATATT

TCCTAATCGCAACAATGGGAGTCGAGTCTTATTGACTACTAGGGAAACTGATGTAGCAATATATGCAAA

TACTAGTAGCCCTCATAAGATGAACCTCTTGGATTTAGATAATAGTTGGAAGTTACTCCGTGATAAGGTG

TTTGGACTAGAATACGATCATCCTCCTGAGTTGGAAGAAATTGGAAAAAAAATAGCAGAAAAATGTCAA

GGACTTCCCTTAATAATTTCAGTGATTGCAGGGCATCTCTCTAAAACGGCCAAGACATTAAAAAGTTGGG

AGTATGTTGCCAAAACCTTAAATGAAATCATTGCTAGCCATCCAAATAAATGCTTAGGAGTGCTCGGTTT

GAGTTACCATCACTTGCCTAATCGACTCAAACCATGCTTTCTTTCTATGGGTAATTTCCCAGAGGATTTTC

AAGTTGATACTTGGAGATTGATCCAATTATGGATCGCAGAAGGTTTCATAAGGGCACCTGCTGGAAGTC

GTAAAAGCTTGGAGGAAGTGGCAAAATGTTATTTGGAGGATCTTATCAGCAGGAACTTGATAATGGCTT

GAAAAAGGAGATTTAATGGTGAGGTTAAAGTATGCGGAATACATGATCTGCTGCGTGAGTTCTGTTTCA

TAGAAGCTGAAATGACTAAGTTTATGCATGTTGTGAGAACTCCATTTTCACCAACACAAAAGCCTAATGT

TCGTCGTTTCAGTATTCAAAAGGCATTTTTAGATGAGTATGCTTATAAGCTATTACCTCCTGTTGCCAGAT

CTATGTACTACTTTTCTCTATTGAATCTACCTTTTGAACCCCGTATTAAGCTTCTCGGGATATTGCCCATCT

ACCGTCATGATCCTATAATACATGGATTTTTCTCTCGTTTCAACCTTCTCAGGGTATTGGCCATCTTCAATA

CAAATGTCAGGTTCGAGTCATTTCCAGTTGTGATTACAAAGTTATTTCATTTGAGATATCTCCAAATTCGA

TGTTTTGGCAATATTCCTGAATCAATCTCAGAGCTTCAGAATTTGCAAACTCTAATTTGTGATGGTCATTC
TTTAGATATAACTTTACCTGAGAAGATATGGATGATGAAGAATTTGAGGTATATACGTTTAGGGGGAGC
CAGTTATTTACCAAGTCCTAGAATAGATAACAAGATGCCAAATCTAGAGGAATTATTCAGTATTTGTTAC
TCTAGTTGTACAAATGAAGTCTTTTCTAGCATTCCCAATCTAAAGAGATTGACCATTCGTCTACCTTTGAG
TGTCGGAGACAATATTCCCTCTCAGCTCATCGATATGTCCAGCTTGAGAAAACTCGAGTCAATCAAGTTT
AACTGGTTACTTCCTTTCAAAAACCCCATCAACATATTTGCTTTTACAACATCACTTAGGACGTTGTCTTTA
ACTGATTGTGCTAATTTTATTTGGAAAGACATATCATCAACTTTTATCATGTTACCAAATCTTGAAGAGCT
CAAACTTAAAAGTTGTCGAGCCGAAGATGATGTATGGAGATTGAGTGATAAAGACATTTTCAAAAGCTT
GAAGTTGTTGTTACTGAAATACCCAAATCTTAAGTGTTGGGAAGCTAGCAGTGATAACTTCCCAAATCTA
AAACGTCTCGTTCTCATGAATTGCAACAACCTGCAAGAAATTCCAACAGATTTTGGGGAAATTTGTACTT
TACAATCGATTGAGTTACATAATTGCAGCACTTCTGCTGATGAGTCTGCCATAAATATTGTACAAGAACA
AGAGGAAATGGGAAATAATATCCTTAAGGTCTACATCAGCCACGGTAAGTTTTAAATGATAAATAAACC
AAGAATAAGATTCCTTATTAGTTTAGGATTTGTTATCCTCTTGACCAACAAGTTTATAAATGTCAGGTACA
ATGGTGAACTTTCTGGTGGACAATGTATTGCACCTATTAAGTGAAAATGTGTTGTTAATAAAAGCCGGA
GATGTCGAATTCAGTAATTTATTGAAAGAAGTGCGATGTCTCAAACAGTTCGTTGACGTTGCATGGAAAT
ACCACAGCGAAAGCAAGCAATGGGATGAATTGGTGAGAGATATTCAACTAACATTATTTAAAGCGGAA
GATGCAATTGATGAATTTGTGATTCAATCGAAGCTGAATCGAGAAAAGAATAAAATTGGAAGAGTTTTT
GATAAAGTGAGTCATATCAGTAATGTTAGGGCTCTGGTAGCTGAGATCAAAGAGATTCATTATAAGGCG
ACAAATTTTCGACAAAACAATTAACAAGCTTTTCAGTTTGTGTGTAAATGTTGTAATTATTTTAAGTAAAA
AATCGATGATGGATGAAGAAAAATAATTTAGTTATGGACATCTCAATTAGGAGTGTACAAGTCAAATCG
AA

>PGSC0003DMG400002961 LRR-RLPs

ATGACAAAGTTTATGCATGTTGTGAGAACTCCATTTTCACCAGCACAAAAGCCTAATTTTCGTCGCTTCAG
TATTCAAAAGGCATTTTCAGATGTGTATGCTTATAAAGCATTACCCCCTGTAGCCAGATCTATGTACTGCT
TTTCATACTTGGATCTACCTTTTGAACCCCATATTAAGCGTCTCCCTAGATTGCCCATCTACCGTCATGATC
CTATAATACATGGATTTTTCTCTTGTTTCAACCATCTCAGGGTATTGGTCATCTTCCCTGCCTGGTTCGGCC
CATTTCCATTTGTGATTACAAAATTATTTCATTTGAGATATCTCCAAGTTCGATATTTTGGAAATATTCCTG
AATCAATCTCAGAGCTTCAAAATTTGCAAACTCTAATATATGATTGTTATTCTTCACATATGGCTTTACCC
GGGAAGATATGGACGATGAAGAACTTGAGGTATATACATTTAGGGAAAGCTAGTTATACACCAAATCCT

AGAATAGATAACAATCTTGTGACGGGGATGCCAAATCTAGAGGAATTTTTCAATATTTGTTACTCTAGTT
GTACAAATGAAGTCTTTTCTAGAATTCCCAATCTAAAGAGATTGAGCATTTGTGCATCTTCTATAGTCAGA
GAAAATATTCTCTATCGTCTCTTGGATATGTCCAGCTTGAGAAAACTTGAGGCATTCAAGCTTTACTGGA
GATATATTTTGGAAAAGCCCATCAAAAGATTTGTTTTTCCAACATCACTTAGGAAGTTGATTTTAGCTTGC
TGTAGTCATTTTATTTGGGAAGAGATATCATCAACTTTTATCATGTTGCCAAATCTTGAAGAGCTCAAACT
TAAATATTGTCGAGCCGAAGATGATGTATGGAGATTGACTAGCAAAGACATATTCAAAAGCTTGAAGTT
GTTACTGAGCGAGCTAAATCTTAAGCATTGGGAAGCTAGCAGTGTTAACTTCCCATGTTTAAAACGTCTT
GTTCTGAAGAATTGCCAGGACCTGCAAGAAATTCCAACAGATTTCGGGGAAATTGGTACTTTGGAATCA
ATTGAGTTACATGATTGCAACACTATTTCTGAGGAATCTGCCAGAAATATCGAACAGGAACAAGAGGAA
ATGGGAAATAATATCCTTAAGGTCTACATCCATAACAACAACAGTAAGTTATAA
>PGSC0003DMG400002980 LRR-RLPs
ATGGAGGTAGTTGGTGGGGCATTTCTCTCTTCAGCTTTGAATGTTCTCTTTGATAGGCTTGTCTCTCATGG
TGATCTGCTCAACATGTTTCGGAAGCATAAGGATCATGTTCAGCTCTTACACAAGCTGCAGGACAATTTG
CTTGGTCTTCAGATTGTGCTAAGTGATGCAGAGAAAAAGCAAGCATCAAATCGACATGTGAGCCAATGG
TTTAATAAGCTTCAGAGTGCTGTGGACGGTGCTGAAAACTTGATAGAACATGTCAACTATGAAGCTTTG
AGGCTTAAGGTAGAAGGCCAGGATCAAAATCTTGCAGAAACAAGCAACAAACAAGTAAGTGATCTCAA
CCTGTGCTTGAGTGATGATTTTTTTCTTAACATAAAGGAGAAGTTGGAAGAAACCATTGAAACATTGGA
GGTGTTGGAAAAGCAAATTGGTCGCCTTGGCTTAAAGGACCATTTTGGTTCGACTAAACAAGAAACTAG
AACACCTTCAACTTCTTTGGTTGATGATTCTGATATCTTTGGAAGGCAGAATGATATAGAGGATTTGATT
AACCGTTTATTGGCTGAAGATGCAAGTGGAAAAAAGCTGACTGTGGTTCCTATTGTTGGAATGGGCGGT
GTGGGTAAGACAACACTTGCTAAAGCCGTTTACAATGATGAGAAGGTGAAGGACCATTTTGGTTTGAAA
GCTTGGTTTTGTGTTTCTGAGGCATATGATGCTTTCAGAATAACAAAAGGTTTACTTCAAGAAATTGACT
CATTTGACTTGAAGGCTGATGACAATCTTAATCAGCTACAAGTCAAATTGAAGGAAAGCTTAAAGGGAA
AGAGGTTTCTAATTGTTTTAGATGATGTGTGGAATGACAACTACAGCGAGTGGGATGACTTGAGAAATG
TTTTTGTACAAGGAGATATAGGAAGTAAGATCATTGTGACGACACGTAAAGAGAGTGTTTCCTTGATGA
TGGGTAGTGGGGCAATCACCGTGGGGACTCTGTCTGATGAAATCTCTTGGGATCTATTCAAACGACATT
CACTAGAAAACAGGGATCCTAAAGAACATCCAGAACTTGAAGAGATTGGAAAACAAATTGCAGACAAG
TGCAAAGGGTTGCCTTTAGCTCTAAAGGCACTTGCTGGTGTTTTACGCTGCAAATCAGAGGTGGATGAG
TGGAGAGACATTTTAAGTAGTGAAATATGGGAGCTTCCAAGTTGTTCGAATGGTATATTACCAGCGCTG

ATGTTTGAGCTACAATGATCTTCCTGCACATTTGAAGCAATGTTTTGCTTATTGTGCAATATATCCCAAAG
ATTATCAATTTTGGAAAGACCAAATTATTCACCTGTGGATTGCTAATGGTCTTGTACAGCAGTTGCAATC
AGGTAACCAATACTTTCTTGAGTTGAGATCAAGATCATTGTTCGAAAGGGTCCCAGAGTCTGAATGGAG
ACAGGAGGTATTCTTAATGCATGACCTTGTCAATGATTTGGCCCAAATTGCATCTTCAAATCTTTGTATAA
GGTTGGAAGAGAACAAAGGATTGCACATCTTGGAACAATGTCGGCACATGTCCTATTCAATAGGAAAAG
ATGGTGACTTTGAGAAATTGAAATCACTCTTTAAATCAGAGCAGCTGAGGACATTACTTCCAATGGATCT
CCAGCGCCCTGATTATGGCCGAAGTAAGCTTAGCAAGAGGGTGCTGCATAACATACTGCCAAGACTAAC
ATCCTTAAGGGCATTATCATTGTCATGCTATCAGATTGTTGAGTTGCCGGATGCCTTGTTTATCAAATTAA
AGCTCCTTAGATTTTTGGACCTTTCTGGGACATTGATTAAAAAGTTGCCAGATTCCATTTGTACATTGTAT
AACTTGGAGACACTTCTCCTGTCATTTTGTGTTGATCTTGAGGAGCTACCGCTGCAGATGGAGAAGTTGA
TTAACTTGCGTCATCTTGACATACTCGATACTTGTCGCTTGAAGATGCCGCTACATCTGAGCAAGATGAA
AAGTCTCCATGGGCTAGTGGGAGCCAAGTTTCTTGTAGGTGGTCCTGGTGGTTTGAGAATGGAAGATTT
GGGTGAAGTACATAACTTATATGAATCTCTATCAGTTGTAGAGTTGCAAAATGTGGTTGATAGAAGGGA
AGCTGTGAAGGCAAAGATGTGGGAGAAGAATCATGTTGGGCAGTTATCTTTGGAGTGGAGTGAAAGTA
GTAGTGCCAACAATTCACAAACTGAAAGAGACATAGTTGATGAGCTACGCCCACATAAAAACATAAAAG
AAGTCGAAATCAGTGGATATAGAGGGCTAACATTTCCAAATTGGCTAGCTGATCTTTTGTTTCTTAAGCT
GGTGAAATTGTCTCTTAGCTACTGCAAGGTCTGTTATTCCTTGCCAGCATTAGGACAACTCCCTTGTTTGA
AAATCCTTTCCATTAAAGGGATGCATGGAATAACAGAGGTGACGGAAGAATTCTATGGCAGTTTGTCCT
CCAAAAAGCCTTTTAACTGTCTTGAGGAAATTAGTTTTGAAGATATGCCGGAGTGGAAGCAATGGCACG
TACCAGGAAATGGAGAGTTCCCTATACTTGAGAAGCTTTCCATTATAAATTGCCCTGAACTCAGTTTGGA
GACACCCATCCAACTTTCAAGTTTAAAATGGTTTGAAGTTAGTGGTTGTCCAAAGGTTGGAGTTGTTTTT
GATGATGCTCAACTACTTAGATCACAACTTGAGGGAATGAAGCAGATTGAGGTATTATATATTGGTGAT
TGTAAGTCTCTTACCTTCTTTCCTTTTAGCATACTGCCCACTACCTTGAAGAATATATGGATATCTGGTTGC
CGGAAATTGAAATTGGAGCAGCCAGTTGGTGAGATGAGTATGTTTCTGGAGAATTTGAGACTGGATGG
ATGTAATTGTATAGATGATATATCACCCGAGTTGATCCCAAGCGCACATACATTGAATGTATCTAATTGC
GACAACCTTACTAGGTTTTTGATTCCTACTGCCACTGAAAGTCTCTTCATTCGGAATTGTGAGAATGTTGA
AAAACTTTCGGTGGCATGTGGTGGGACCCAGATGACGTATTTGTATATTGACGACTGTTCAAAGCTGAA
GTGGCTGCCAGAACGTATGCAGGAACTCCTTCCATCTCTTAAGGAACTGCAACTGATTAATTGTCCAGAA
ATAGAGTCCTTTCCTCAAGGAGGATTGCCCTTCAATTTACAACAACTTGCGATCAACAATTGCACGAAAC

TGGTGAATGGCCGAAAGGAGTGGCATTTACAGAGACTCCCCTGTCTCACACAGTTAATCATCTCTCATGA
TGGCAGTGACGAAGAGATTGTTGGTGGTGAGAATTGGGAGTTGCCTTCCTCTATTCAAACACTTATCATA
AACAATCTGAAAACATTAAGCAGCCAAGTTCTCAAAAGCCTCACCTCTCTTCAATATCTATGTATTAAGG
GTAATTTACCTCAAATTCAGTCAATGCTGGAACAAGGCCAGTTTTCCTCGCTTCAAAGTCTACACATCGA
GGATATCCCTAATCTCCAATCACTTTCCGAATCAGCACTGCCCTCCTCCCTCTCTCAGCTGACCATCATCAA
TTGCCCTAATCTCCAATCACTTCCTGAATCAGCACTGCCCTCCTCCCTCTCTCAGCTGGAGATCTCCCATTG
CCCTAATCTGCAATCTCTTCCAGTAAAAGGGATGCCATCTTCACTCTCTACACTATCAATTTCCAAATGCC
CATTACTCACACCACTACTAGAATTTGTCAAGGGGAAATACTGGCCAGAAATTGCTCATTTCCCCACAAT
AGAGATCGATGAGGAATGCCTTTAA
>PGSC0003DMG400003195 LRR-RLPs
TGGAGCTTTAAATACTTCTTAATTACAAGTTCTAGAAGGAGTCAGAGAAATTTATGAGCCATCCGGGATT
GAATCCTTATCTCTCATGCACTTTATCGACCACTAGACCAAATCACTGAGGTGACGGGTGTCTAGTCATTT
ATATATAGAGTCGCAACCATCTGAATTTTTCCTGTTTGAATGTCAAAGATACTCATAGCACGATCAAACT
GTCAGTCAAACAAAAACAATAGAGTTGACATTTAACATATATGCACACAAAATTGCCTAACTATGATGCA
AACTGCATAATGATTTGACAATCAATGAGGCTGGATTTTATTACTTGTTCTAGAAGGAGTCAAGGAGAT
GTTCATGAGCAACCCAGGATTTGAATCCTGATCTCGCATGCACTTTATCGACCACTAGATCAAATCACTG
GGGTGACGGATGTCTAGTCATTTATATATAGAGTCGCAATCATCTGAATTTATCAAGTTTGAATGTCAAA
GATACTCCTAGCGCGATCAAACCGTCAGTCAAACAGAAAAAATAGAGTTGACATTTAACATATACACAC
ACAAAGTTGCCTAACTATGAATGCAAGCTGCGTAATGATTTGACGATCAATGATGCTGGATTTTGTTACT
TGTTCTAGAAGGAGTCAAGGAAGATATTTCATACCTCAAGTATATAAAATCTTGTGAATTATTCCTTCATT
CCTCAAGTACTTATTATTTTTGTGCAGATCATGGGCTGTCTATTTCTTCTTCTTCTTCTTCTTCTTGTGTTGC
AATATCCACTTGTTATCTTTTCAAGTCATCTATGTCGACGAGACGAGAGTTTGGTTTTGCAAAACCTGAA
GCTTAAACTGTCCATAGATGACTCTGCTTCTGCTGATTGTGAATCCAATGGTTACTTTCCCTATCCAAAGA
CACTTTCTTGGAATAGAACCACTGATTGTTGTACTTGGGATGGTGTAACATGTGATAAAGTTACAGGTTG
AATAACTCGTCTTGATCTTAGTTGTAGCCAACTTCATGGAACAATTGACTCCAACAGCACCCTCTTCCAAC
TTTCTCATCTTCAAAACCTTAATTTAGCTTACAATGATTTCTCACCTTCCCAAATTTCAAGCAAATTTGGAT
GGTTTCCGAGTTTGACACATCTTAATCTATCGCGTTCTGGTTTTTCAGGGACAGTACCTTTACAAGTCTCA
TATCTATCTAAGTTGATATCACTTGATCTTTCTGCAGGTATTGAGGAGTTGAGAGTTGAGCCTCACACTTT
CAAAATGCTCCTTCAAAACTTAACTCAACTAAGGGAGCTTTACCTCACCTCAACATATATATCTTCATCAC

TTCCATCAAACTTGTCCTCTTCCTTACAAGAGCTGAATCTTGTCGGAACGGAATTGTCTGGGAAAATCCCT
GATGACATTTTCCATTTACCAAGATTGCAAATGCTTAACTTAGGGAGCAACTTGTATCTCACAGGGCATT
TGCCAACAACTCAATGGAACTGCAGCAACTCTTTAAGGGAATTAGATCTTTCTTCCTCAGGCTTTTCGGG
GAATATACCAGATTCTATTGGTCATCTCAACTCTTTAAGGAAATTAGATATATCAAGCTGCTACTTCTCTG
GTAACATTCCTCCAACTATCGGTGATCTCACCGAGCTCACGAGTTTGCAACTCTTCTCCAACAACTTCAAT
GGTCAACTACCATCAACAATCTTAAAGCTGGTGCAACTTGTTGAGTTTGATATCTCATCTAACAACCTTAC
AGGCAACATTCCAAATATTTTCGGTAACTTCACCAAGCTCAAATCTTTATCCTTTTCATATAACCTCTTTAT
GGGCCTTTTTCCATCCTCGGTTACAAACCTAACAGAGCTAGAGAGTTTAGACCTCTCAAATTGTTCTATAA
CTGGTGCTATTCCTTCTAGCGTCAGCACCGGATTCCCTAACCTCATCTTGCTCTTCCTACCATATAACTCCC
TTAGCGGAGAAATACCATCTTGGATATTCAATCTTCCTTCCTTAAAATACTTAGTTTTGAGAGGCAATCAA
CTTGCTGGTCAACTTAAGGAAGTTAGATACAACTTGTTAGAAGTTGTTGATGTCAGTGACAATAAGCTGA
ATGGACCTATTCCAACATCATTCTCTAAACTAGTGAACTTGACAACACTTGATCTTTCTACAAACAACCTT
TCTGGTGGTCTAGATATTGGGTTGTTTTCAAAATGCAAACAACTTCGACGTCTTGGCCTTTCTTTTAACAA
TCTGTCTGTATTTTCCAGTCAAAAAGATGCATCCTTGCCTAGTTCTCTTGGAAGTTTGTATGCATCTTCTTG
CAACATAAGGGAGTTAAATTTCCTACGAGCTGCAAAATACATTGGTAACTTAGATCTTTCCAACAACAAC
ATGCATGGAAAAATTCCTGATTGGGCATGGTCTAATTGGCAAGTCTCAGTGTCTTATTTGAATCTTTCTA
GCAACTTTCTAACGGCTATTGACCTGCTTCATAACTTTGAGAGTCTTTACAATCTTGATATTGGATCTAAC
TTGATTCAAGGAGAATTACCTGCTCCACCTCCCTTATTGTTCCTCTTCATAGCCTCGAACAATAACTTCACC
GGGAAGTTACCTAATTCACCATTATGCAAAATGAGTTCCCTTTTGATTCTTGATTTGTCCAATAACAGCCT
GAGTGGAGTAATTCCAAAATGTTTAGTGAACATGAGCACAAGTCTCTCAGTGTTGGACCTGCACAACAA
TCATTTTAGTGGGACAATTCCAAATAACTTTGGTAAGGGAAGTAGTCTGAGAAGTCTCAATTTGCGGAA
AAATAAACTAGAAGGAATGTTACCAAGAAAGCTTACCAATTGCAGAGGGCTCGAAGTAGTTGATCTCGG
AGAAAACTTGTTGAATGATACATTTCCCAAATGGTTAGGATCACTTCCTAGGCTGCAAGTTCTCAGTTTG
AGATCCAATAAATTACATGGCCCGATTACCATTTCAAGAAACCAAGTCTTATTCTCAAAGCTGAAAATCCT
TGATCTCTCTTATAATGACTTCACTGGAAACCTGTCAGAAAGGTTTTTCAACAACTTGAAATCCATGATAA
TAGAAGATCAAACGGGAATTCCTTTAATGTATATTGGAGAAGTTCGATACCATGATTCCTTGACATTGTC
TATAAAAGGACAGCAGATTGAACTTGTGAGGATTCTTTCTATCTTCACAACCATTGATTTCTCAAATAACA
GATTTAAAGGTAATGTACCAAACTCCATTGGAAATCTTGGTTCACTTCGCGGGTTGAATTTGTCACACAA
CAGCCTTCTTGGACCTATACCTCAGTCTTTTGGAAATTTATCTGTTCTTGAGTCGTTAGACCTTTCGTGGA

ACCAACTTTCTGGGAACATTCCTCAAGAAGTTGCTTCACTGAAATCTCTTGCTGTCATGAATCTCTCACAG
AACCACCTGATGGGAAGCATACCTAGAGGTCCACAGCTTGATACATTTGAAAATGACTCATATTCAGGA
AATGCTGGGTTATGCGGATTTCCCTTGTCAAGAAACTGCGGAGATAATGAGATGCCTCCTTCTACTGTGA
CTGATATTGAAGACGAAGAAGATTCAGGTTTTATGGATTGGAGAGCTGCGATAATAGGATGTGGTTGTG
GATCAGTGTTTGGGATGTTTATAGTATATATCATTTTCTTAACAGGGAGACCAAAATGGTTTGTGAGAAT
TGTCAACCAACAGATCAACAGGATCCTTTTGAAAATTGAGGCAAAGAGAAGAATTGAGGCAAAAAAGA
GAAGACGGCAGAAAAGAAGAAGGATGAAGTTTTAGTATATACTTCATGCATTATTGTCTTGCAACATAC
ACAGTTTCAGCTTACACTTTGTTTTGGC
>PGSC0003DMG400003380 LRR-RLPs
GAGGAAGTGAAGAAATAAACTATAATACTGTGTGTTCTTCTTTCCCAGTCCACAACAGTTTAATTCTTGT
GTCACAAGGATGTATTTCAACAAAGAATTATCTAATCTGAAAGATCGACTCCTTAGCACGGTGCAAGATC
AAAATTACTCGGATGTTACAAGGGATCGAATAAATTTCTTTCTATGGGACTTAAAATTCCTCGATTGTTTT
CTCCATTTGAAAAGGTTGCCTTTTGCAAGTGAATGCGGTATGCTAAAAGTCTCACAGAAAATCATAGAAA
TTTGGAAGTTTCAGTGTAAACCCTATGATTGTCCAGATCTATTTCCGTACAAGACTCTGTATGTTTACGAT
TCATTTGGCTACTGGAAGGATGTAATTTGGAAGACTAAGCAAGAATTCAGAGCTGAATACTCTTTTCCAA
ATACATCACTTGCAGCCAACAAGGTGGATGATGATGTAGCCCCAAATTTGTGATGGAAGTCATCGATGT
TTTTGTGGAGAACCTCAATGTTCTAGTGAAGATCAATGATCCAATTTCGTGGCTTTTTGTTCCAGAACACA
AGGAACAAATAGAACAAGTGTTAAAGGAGTTGAAGTTATTGAGATTTTTTGTCTGCTTTGTTTCAAACAA
ATGTATAGAGCCTCAATACCGACATACTACTTTTTATATTCATGCTTTAATTGAGGCTAGCCACATTGCAA
TGGTTGTCTGGTTGCATTTGCCAGTCGTCTATGGAAATCAAGACGTGGCTCCAAGTGAAGTCAGTCGTTT
GCTCTCTGATTTCATGGAATTGAAGATTAAGTCCATTCAGCCAGTCATCAGCCGCAACAATATTTATATTG
ATGTCCTGCAAGCTTTGAAGTCAACCATACCACAAGCACAAAAGAAGCATGCAGCTGAGAGTAGCACTG
TGGAGATTCCAACACACAGTCTGACGGTTGGTTTGAGTGATCAAATGGCTAACCTTCAGGAGATGCTCT
GCCTTCTAAGAGATAATCTCATTCATCTGCCAATACTAGATCTGGAATTTCATCTTCAAGATATGGATTCT
GTTATTATTGATGCTGGACTTCTTATTTACTCATTATATGATATCAAGAGGGAGAAGGAAGACACAGTAT
TGGATGATATGAACCGAGCACTTGGTTTTGATCTTCCAAGAAACATTGAGCCTATCAAGGCGATGGTCTA
CCTCATCATGCAAAAGGCATTTCAATGTAACTTGCCAAGGATTCATGGACTAGGCTATGTCGATTTTCTAT
TGAAAAACCTGAAGGATTTCCAAGGCCGTTATTCAGATTCACTCGCTTTCCTCAAGAATCAAATTCAAGT
AATTCAAACGGAATTTGAGAGCTTGCAACCTTTCCTGAAGGTTGTCGTAGAAGAGCCACACAATAAGTT

CAAGAGACTGAATGAAGATTGTGCTATACAGATAATTAGGAAAGCACATGAGGTGGAATATGTAGTTG
ATGCTTGTATAAACAAAGGCATTCCTCATTGGTGCCTCGAGCGTTGGCTCCAGGATATCATAGAGGAGA
TTACTTGTATCAAAGCAAAGATTCAGGAAAATAACACTGTTGACGATACAATGAAGACTGTCATTGCTCG
TACATCATCACAACTGGCAAGGACTCCGAGGATGAATGAAGAAATTGTTGGGTTTGAGGATGTCATAGA
AAATTTAAGAAAAAAACTACTGAATGGAACCAAAGGGCAAGATGTCATTTCAATTCATGGCATGCCAGG
TTTAGGTAAGACGACTTTAGCCAACAGACTCTATTCTGACAGGTCAGTTGTTTCTCAATTTGATATTTGTG
CACAATGTTGTGTGTCTCAAGTATATTCGTATAAGGACTTGTTATTGTCCTTGATACGTGATGCTATTGGT
GAGGATTCTGATCAGCATAGAGAACTTCATGACAATGAATTAGCTGATAAGCTTCGCAAAACCTTATTGC
GCAGAAGGTACCTTATCCTTGTTGATGATGTGTGGGAAAATAGTGTTTGGGATGATTTAAGAGGTTGTT
TTCCAGATGCCAATAACAGAAGCAGAATCATTCTAACGACAAGACATCATGAAGTTGCCAAATTTGCTAG
TGTTCATAGTGATCCCCTTCATCTTCGTATGTTTGACGAAGATGAAAGTTGGAAGTTGCTTGAAAAGAAA
GTGTTTGGTGAACAAAGCTGCTCCCCTCTCCTAAGAGATGTTGGGCTAAGAATAGCAAAAATGTGTGGA
CAACTCCCTCTTTCAATTGTTTTGGTGGCTGGTATTCTGTCAGAGATGAAAAAGGAAGTAGAATGTTGGG
AGCAAGTGGCCAACAATTTGGGTACCCACATTCACAATGACTCAAGAGCCATTGTAGACCAAAGTTATC
ATGTTTTACCCTGTCATCTTAAGTCTTGCTTCCTTTATTTTGGAGCATTTTTAGAGGATAGAGTGATTGAT
ATTTCAAGGTTAATAAGGTTATGGATATCAGAATCATTTATAAAAAGTTGTGAAGGCAGGAGGTTGGAG
GATATAGCAGAAGGTTACTTGGAGAATCTTATTGAAAGAAATCTAGTAATGGTTACTCAAAGGGCCGAT
TCAGATGGTAAGGTCACAGCATGTCGCCTTCATGATGTACTACTCGACTTCTGCAAGGAAAGAGCAGCT
GAGGAGAATTTTCTACTATGGATAAAACGGTAATATGATAAGTAATGGTACTTTCAATCAATCAAGTATT
TCAAGTTATATTTGAAAATTAATGATATGATTTTGCTAATTGATATATTCACAGAGATCAGATTACCAAAG
CTGTTTACTCTCACAAGCAGCATGCTCACTTGGCGTTCACTGAAATGGATAATCTTTTAGAATGGAGTAC
GTCTGGCTCACTTGTTGGCTCTGTACTTTTTAAGAATTATGACCAATACTTTGCCGATCGTCCCTTTTTCTC
TCATGCTTTTGAAATTTCACACATTTTACCACATTTCAAGTTTCTAAAAGTACTGGATTTGGAACACCAGG
TTGTTATTGATTTTATTCCGACTGAGCTCCCTTACTTGAGGTATTTCTCTGCGCTCATTGATCAGAATTCAA
TTCCTTCAAGCATATCCAATCTTTGGAACCTTGAAACTCTTATATTAAAGCGTAGATCAGCTGCCAGATAT
AACAGGGTATTACTACCTAGTACAGTTTGGGATATGGTTAAATTGAGATATCTGTATATTCCTTACTTCAG
CCCTGAAATAAAAAGCATTACTCGAGAACTCTCCAAAACTTGATGATTTGGAAACCCTTTCCAATTCATAT
TTCACTCGTGTTGAGGATGCAGAATTGATGCTGAGAAAAACACCTAATCTTCGAAACCTGACATGTAAA
ATCGAGTGCTTGAAATACCCCCATCAGTACCATGCATTGAATTTTCCAATACGGCTTGAAATACTAAAGC

TTTATGGATCAAAAATCGTCAAAACCATCCCCTTTTACATCTCTGCACCAAATCTCAAATACCTGATACTCT
GTGGCTTTTACCTGGATTCTCAGTACTTATCAGAAACTGCTGATCATCTGAAGAACCTTGAGGTACTCAG
ACTGTCCTTCGTTATATTTGGTGGTCATATGAAATGGAAAGTGAGCAATGGCAAGTTCCCTCAACTCAAA
ATCTTGAAACTAGAATATTTGCCCATGATGAAATGGATTGTAGCTGATGATGCCTTTCCTAACCTTGAAC
AATTGGTTTTGCGTCGATGTCATGATCTTATGGAGATCCCTTCTTGTTTCATGAACATCCTTTCTCTGCAGT
ACATCGAGGTAGAAAACTGCAATGAGTCGGTTGTCAAGTCAGCCATGAATATACAAGAAACACAAGTCG
AAGATAATCAAAATACTAATTTCAAGCTCGTTCTCATCGAGGTACACTACTGAAAAAAGCTTTATTCTGCA
TGATTTTGATGACTCAGAAATAATGGCCTAAATTGTATGAACTGTTTTCTCTGTTATCTTTACCTGGTGGC
CTCGTTTTACATTTGGGTTTTTCTCTTGGTTTTCTTGCAGAAAGAGACATACCCAAGGCATTTAAACAATT
ACTTTTACTTCCAGGTACTACTTATTATTTTTTCTTTCTTTCCTTTTTTTGAGTACACCGAATAGATAGATTC
ATCTTTTTTCTCCTGTCGATATAAAAGGGATAGAATCAATTTCAACTGATATGAAAGAGAAGAAACTTAC
TGTGACCAGAGAATGTGGATGCTGATGAAGTTCAATTAGTTGTGGAGAAACTGAGAAAGCGTGGCATG
CTCTAACAAAATGCTGAACTCTGGTGAACTCAACCGTGATGGATTTCCAAATAATGACAGGGTTGTAGTC
CCAACTTGTCAACACAAATGTGCTATACTCATTTTTCTTACTGTAATACCATTTCATGACACACACACAAA
CATTAACTGTAGTAAAGTTTTGATGGATCAGCTGATATGTTAGTTCAACCCATTGTAATCCCGTTCAAATT
CAACTCAAAAAATGCCCATTGAGTTATTCTTTAACAGGGTATCCAGAGTTTGTAGCTGGAGCAATTTGGA
ATATCGCATGTAATTTCTTTATGAGTTATTTCGTTTAATAAAGATTCTGTAAAACGTCCAACGGCTGTTGC
ATTCATTGTAAACTAAATACATCTCACAATGTAACTGTTGAACAAATTTTTCATTCAAGTCCTTAAGGTTT
GATGTAAGTCATTAGATTTTAC
>PGSC0003DMG400003543 LRR-RLPs
CTTTCTTTGTTTGTTCCCTTCTTTGGACACACAATATATCCTCATGAATCCATTTAAAAACTAATCTTGAAG
GCTCTTCTTCTTCATCAATTCTCATTACGATAAAAAAAAACCACACAGAAAAAATCCAGAAAATGGGTTG
TTTTTCAATTTCTACTGTTCTGTTCTGTATAGTTCTCTATGCTCTGTTCTTGCATGCGCATTCAACTACTCAC
TGGCAAGACATTGAAGTCTTGAAGCAGCTGAAAAATAGCGTCGACCCCAATTCAGTGTCACCTGGTTCA
TGCCTGAATTCATGGGATTTCTCTGTAGACCCTTGTGATAATCTCGGGGGTGAAAAATTCACTTGTGGTC
TCCGTTGCGACCTTGTCGTTTCCTCTGTTAGCCGAGTCACTGAACTCGCTCTAGACCAGTGGAATTACTCC
GGTTCACTCACTTCTGTCTCTTGGAATTTGCCTTATCTCCAAAACTTAGACATCACTAATAATGCCTTCTCC
GGTTTAGTACCTGAGTCACTCTCGAATCTAACTCGGGTTCAACGAGTCGGTTTATCAGGTAACTCGCTAT
CCGGGTCAATTCCGAGTTCACTGGGTTCACTTTCAAACCTTGAAGAGCTTTACCTTGACAATAATTTCCTT

GAGGGAGAAATTCCCCAAAGTTTCAACGGTCTCAAGAATTTGAAACGGCTCGAATTTCAAGGCAATCAG
ATTACTGGCGAGTTTCCCGAGTTGGATCAGTTAATTAATTTGAATTTCATCGATGCTAGTGATAATGCCAT
CTCCGGCGAACTCCCGGCGAGTTATCCGGCTTCTCTCATCGAGCTTGCGATGAGAAACAACAGCATAGA
AGGTAACATTCCGGCGAGCCTCATCGGACTGAATTTCGTACAAGTTATCGATCTGAGTCACAACAAACTC
AGTGGGTCAGTGCCAGCGAGTGTCTTCACTCACCCATCCCTCGAACAGCTCACCCTATCATACAATCAAT
TCGGGTCGGTTCAGGAACCCGGGATCTCGTTTCAGAATAGTCAGCTTATTGCAGCAGACTTAAGCAACA
ATGAGATCCGTGGGTTATTACCCGGGTTCCTGGGTTTAATGCCCAGATTATCATCACTTTCACTAGAGAA
CAACAAGTTATCGGGTATGATACCGACCCAATATGCATTGAAAATGGTGTTTCCGGGTGAAGGGGTGTC
TCAATTTGAGAGGCTACTGTTAGGAGGAAACTATTTATTCGGACCCATACCGAGTCCATTGTTGGATCTG
AAAGCGGGTTCATTCACAGTGAGATTAGGAGACAACTGTTTGTACCGGTGCCCGTTGAGATTATTTTTAT
GTGAAGGTGGGGAACAGAAATCATTATCGGAGTGTCAGGCTTTTGGTCCCATCATTCCTTGAGTAATGT
GATAATGTCTTTACTTTGAGGTCCTCGTTTTCATTTTGTACTAGTAATTATTGTATCGTTTCAATTTTATCG
GAAGGGACCAGTAAGTATTGTATAAATATATTTTTTCCTGCAACCTTACCCCATGTATGGGGTTTTAGAT
ATGATCTATGGTAGAGTTGTTATTGTTGTGAAGAGTGAGACAGAAATATAAGATTTCTGACTTGATGTGT
TGAAGTAGTAATTGTGGGACAGTTGAGAAAATTTTATTAGCGGAT
>PGSC0003DMG400003544 LRR-RLPs
CAAATATGGGAATTTTGGGTAGGAAAAAATCTTTCTTGTTCATCTTATTTACATAATTTTTTCTGTTTTTG
TTCCAAGGCCATTAGAATGTTGTCATTTGGTTGATAAAACATCTTTGTTAGACTTTAAACAAAAAGTCACT
TCTGATCCTTCAAACTTATTGCAAACATGGACACTTTCAACTGATTGTTGCAAATCTTGGGAAGGTATTGC
TTGTAATTCCAATGGAAGGGTTGTTAACGTCTCTCGCCCTGGCCTTTCTTCAGGGGACGATTTCATTATCG
ATACCTCAATTTCTGGAACTCTTTCTCCTTCTCTTGCCAATCTTTCTTTTCTTGAATTGCTTGATCTTAGTAA
TCTTAAGGACTTAACAGGGCCAATTCCACCTGAATTTGGCAAGTTATCGCGCTTGAGTTACCTCTTTCTTG
ATACTAATAAACTCTCAGCATCTATACCTGTCACATTTAAATATCTATACCAACTTAAGAAGCTTTATCTCA
GTGATAATACTCTATCTGGTACTATCCCTTCGAGTATTTTCGAACGCTTCCTTTCACTCTCAGAACTTGGTT
TATCAGCAAATCAACTTTCAGGTCCAATACCATCTTCAATTGGGAATTTAGTATCATTGACCAAGCTTGAT
ATGAGTCGAAATAAGTTCTTTGGAAGCATTCCAGAGAGTATAGGAAGATTAAAAAATCTTGCATATGTT
GATTTGTCTGAGAATCAGTTATCTGGAAAGATTCCAAACTCTATTGGAAACCTTTCCCAAGTATCTCTAAT
GTATCTGAACCAAAACCAATTGAGAGGAAAAATTCCTTCATCAATATCTGGTCTTAGATCATTGGTGTTC
TGCCGTTTATCCGAAAACCAGCTGAGTGGCAGTATTCCACCTTCTATTGGCAATCTTCCAAAGATCGAAA

GGCTGATATTTGAGAACAACAAGTTAAGTGGAAAACTGCCTGCAACACTTGGACATCTTGTAACTCTCAC
TGATATGTACTTTTCCAATAATCTATTTACAGGGAAAATCCCATCAAGTTTTGGTAATCTAAAGAATCTAC
AGATACTAGATTTGTCGAAAAATAAGCTCAGTGGTGAAATCCCTCATCAGCTTGTGAAGTTAGTGAGTTT
ACAGACATTGGATCTGTCATTTAATCCTTTGGGGCTTTCAAGAATACCAAACTGGTTCAAGAAACTAAGA
CTGTTTCGTCTCCTTTTGGCGAAGACTGGGATCAGAGGGAACCTCCCAAGTTGGTTGGCTTCATCGTCAT
TATCAACACTTGATTTATCAAACAATGGTTTGACAGGAAAATTACCTACATGGATTGGTAACATGACCAA
CCTTTCACTCTTGAATTTATCAAACAACGCCTTCTATTCATCAATCCCTGATGAGTTCAAGAACCTGACACT
TTTGATGGATCTTGATCTTCACTCAAACAGATTTTCAGGCAACTTGATAGCAATTTTCTCCAAGAATTTTC
AAGACCCCCTTGGTCACTACAATTCTATTGATCTTGCATACAACATGTTCACTGGACCATTAGACGAAAA
CAGCGGAAATGAACCTGTCATGGATTCTATTTTGTCACTAAGTTTGTCCCACAATCCATTGACAGGACAC
ATACCAAAATCACTGGGAAATTTGACAACTTTGCAGGAAATTAAACTAGCTGAGAATGGGCTAACAGGT
GAAATTCCAACAGAACTTGGGAATGCCAAGGAACTGAAAACAATTTTACTTTCAAATAACAATTTGGAA
GGTGCAATACCAGCACAAGTTCTGAATTTGAAAGACCTTCGAGAATTTGATGTATCGGGAAATCGACTA
AGCGGTAGTATCCCTCCTCATAAAGCTAATATCCCAAAGTCTGCATTTTTGGGAAATTATGGCTTGTGTG
GAGCTCCACTTCCTCCTTGTAAGCACACATAGTGGAAACCTTCAAACACAATATTTCCCTTTTCTTTATTTA
TTTTTCATTTTCGACATGATTGTTCGTCATAATGTATTGTATTTCAGTTGTAATCACTCAAATGTTATTAGT
AAATACTGAACAAAGTTGATTTTTCAAAAGCAACCTTGCAGAATTCTACATCAATATCCTCAATGGTTTAA
AC

>PGSC0003DMG400003710 LRR-RLPs

ATGAAAATGCAGTTTTTTCTTCATTTTTTTTCATTTTTCATCTTCTTTGTATTATTAGCACAATGCAAAACTC
ATCCTATTGATATACAAGGACTTCAAAATCTCAAAAATGGAATAAACCCAAAATCCATTAAACCCGGTTC
ATGCTTAAACTCATGGAACTTCACTATAGACCCATGTGATCACATTTTCACCGACCGGTTCACCTGCGGTT
TCCGCTGTGATCTCATCGTCTCCGGTTTTTATCGAGTCACTGAAATCAGCCTAGACCAAGCCGGTTACTCC
GGTTCATTGCCTAATTCATTCAACCTCCCTCATCTCGAGATTTTAGATATGTCGTATAATTCCCTCTCCGGT
TCAATCCCGAATTCATTTTCAAATCTAACTCGTCTCCGGCGGCTCAGTCTTTCGAAAAACTCGTTCACCGG
TCCAGTTACTTCTTCTATCGGTTCTCTATCCCGTTTACAGGAACTATTTCTCGATAACAACAAATTCACCGG
TTCAATCCCGGAAAGTTTTAATGGTTTAATCAATCTGAATCGATTCGAGCTTCAACAAAACAATATCTCCG
GTGAGTTACCTAATTTGAATCAGCTCGGAAATCTGATCTTCTTAGATTTAAGCAATAACAGACTCGCCGG
AAATTTCTTCACGACAAATTTACCTGATTCGATGATTGAACTCTCAATACGCAACAATTATTTACACGGCG

AGTTTCCGTTGAACTTCGAAGAGTTGAAGTTCCTACAGGTGTTAGATCTGAGCCATAATTTGTTTTCCGG
GATGATTCCGGCGGTGTTATTTCATCACTCATCTCTTCAGCAGCTTACACTTTCTTACAATAACTTTACGTT
TTTGCAAGTGCCGGAAGATTTAGTGCTTCGAAGTAAGCTTATAGCTATTGATCTGAGTTACAACAATTTG
CATGGCTTATTGCCGGCGTTTATGGCATCGATGCCGGAGCTGTCGGCTCTGAATTTGGAGCATAACAAG
TTTACCGGCGTGATACCGATACAGTTTGCAGTAAAAGTTGTGGCTCCGAGGAATCATACGGCGTCGTTT
GAGAGGTTATTGCTGGGTGGAAATTATTTGTTTGGGCCGATTCCTGGGCCTTTAATGGGCCTAAAGCCT
GGTTCTGGAAATATTAGTTTGGTGGATAACTGTTTGTATATGTGTCCAGATTCGGTATACATTTGTCATG
GTGGGTATCAGAAGTCTTTATTGGATTGTAAAAAGTTTGGGCCTAGGATTCCTTGA

>PGSC0003DMG400003802 LRR-RLPs

ATGTTTGAATTCGCCCGGAAATTGCAGAAGCGAGGCGGAGAGTTGATTAGCGAGTTTTGCAGTGAAATT
GGAGTCGAAAAATGGGGTTTTAAGGTACGGTTGCACAATAGGACACCTTCAATTGATATATTGTCGGAA
TGTCTCATTCATAAAATACTTTGTTGTCTTAGTTTTAAAGAAGCAACGCGGATGAGCATTCTTTCCAAAAC
ATGGCTGCAAGCCTGGTCGACTCTTCCAAACTTGGATTTCACAGTTGATTTTTCTAAAGGCAACGATATG
AAAATAGTGGATGATATTATGGGGAGATATAGGGACGGAAAAATCCCTATAGAAAAGTTTGAATTACTA
GAGTTCGATGGTGATTCTGAAAATCTCTTCCGTATGATTGATAAGTGGCTTGACATTGTACTTACAAATG
GTGTAAAAGACCTTACCATATCATCATATTCATACCCCTTGTTTATTTTTGCAATCTTCACGGTAAAATCTG
TGAGAAAATTGGTTCTGGAATCTTGTACTCTTTTGCCTAGTGTTGTTGTGAATTGCAATTCTTTGAGAAAA
CTTTCTCTATCTTATGTAAGATTAGATGAAAACATGCTTCAGGCTCTACTTAATAGCTGTCCGTTGATTGA
ATCTTTCATATTTGAGTATTGCAAGGGGTTGGAAACAATGAATCCTCAAAAGATTAAGTCGGTTTCCCTG
AAGTTCTTGAAGATTCGGTGTTGTGGGGGAATATGGGAGATTGATGCTCCAAATTTGGTATCACTTAAG
TACACCTGGGAAGACATTCCTCAACTTAAAATTGTGAGAGAGTCAAGACAACTGAAGTACTCAAGAATC
GTTCTTCAATGCTTAGATAGTGACAATGTAAATGCAGCATGGTTTTGTAAGTTGAGGAAGTTTCTATCAA
ATTCGATCTCTTGGTCTGAAGTTTGCATTGAATCTTATAGATGCAATGACATCAACATTCAAAATTTGCAA
CTGGACCATAAAGATTCTAATCCCCACGTGGACGTTTTAAATGTCATCATTATATGGAAGAATGGGGAG
AGCTCAACATTTGTGGATGCTTTGGTATGGAGTTGTCAGCCTAGGAGACTCAACGTGGATTCAACAAGA
GAAAAGATCACATTTTTCATTGATCATTTGATGTATATGAAGAATTCGAGTCAGTCTACTTCTCATGGAA
GCACGCCTCTTCTTAGTCAATTAAAAGGAGTAAAAGCCTACAAATTTGATAGGAAGAAGGAGAGTTGGC
ATCCTGTGGAACACAAAAGTGGTGAGCTGGTAACAACAAACTCTGAAATGTGGGATTGGTGTTATTTTT
TATTAGATTGGTGA

>PGSC0003DMG400003814 LRR-RLPs

TTTTTCATGCTGTAGCCATTAAAGCTTCCACCTATCTCCACACGAGAACTCGTTTGCTTCTCACGGAAAAG
CAATACCCAAAAGTCTCTACTTTTTTCCCCTTATATAAACCCCCATTTTAATTTTACTCTCTCTTCACCTCTG
CAACAATTAATTAATCATGAGGTTTTTGTTGTTGATGTTTCTGTTTCATGTTATTGTTATTCAACAATGTGG
TGCAATGCTTCACCCCATTGATTTCTTGGCTTTACAAGCGATTCGTAAAACCCTCAATGATTTACCCGGTT
CGAATTTTTTCTCTTCTTGGGATTTTACTTCCGACCCATGTAGCTTCGCCGGAGTTTATTGTGCTGGTGAT
AAAGTGATCGCGTTGAATCTGGGTGACCCGAGAGCCGGGTCGCCGGGTCTAACCGGAAGATTAAACCC
GGCTATTGGGAAACTCTCTGCTTTAGCTGAGTTCACTGTCGTCCCCGGTAAGGTATTCGGAGTATTGCCG
GAAAGTTTTTCTCAGTTGAACAACTTAAGATTTCTCGGAGTTAGTCGAAATTTTCTATCTGGACGGATTCC
GGCGGGTTTAGGTAAACTCCGGCGACTTCAAACTCTTGATCTTAGTTTCAATCAGCTCACCGGAAACATT
CCATGGGCCATCGGAACCCTCCCGGCGCTCAACAATGTCATTCTCTGTCACAACCATTTATCCGGTTCAGT
ACCGCCTTTCGTATCCCTTAGATTAACCCGGCTCGATCTCAAACACAATGCTCTTTCTGGTTCGTTATTGCC
AATGTCACTTCCTTCTTCACTTCAATACCTCTCAATGTCATCGAACCAGTTAACCGGTCCGGTCGACCATTT
ACTTACTCGGTTAAACCGGTTAAACTATCTCGACCTTAGTATAAACCGGTTCACCGGTTGTATCCCGGGG
AACTTATTTAGATTTCCGATAGCAAATCTTCAGCTACAAAGAAACCAATTCACCAGTCGAATTTACCCGGC
CGGTTTAGTAACAATTCCGGTTGTGGACCTTAGCTTCAACCGGTTTTACGGCGAAATATCGCCATTGTTCT
CCGGTGTACAGATTTTATATATGAATAACAACCGGTTCATGGGTCAGGTTCCGGGGATCATGGTAGACC
GGTTGTTATCGGCTAGTATACATGTTTTGTATTTGCAACATAATTTTCTAACCGGAATAGCTATCAATCCG
GCCGCAGAAATTCCGGTTCGGAGTTCATTGTGTTTGCAGTATAATTGCATGGTTTTGCCGGTTCAGACCC
CTTGTCCATTGAAAGCCGGGAAGCAGAAGTCCCGGCCCACGGCTCAGTGTGGGCAATGGAAGGGGTAA
AATAGACAAATCACAAGTATGTTTTTTTTAATTATGGTTTTGGTGGATTCATTGATTAATTTTGAAATTAA
AGGGTAAAAAAAAATGTGGATAAAGAAGAAAAAATTAGAGTGAAAAAGGGTAAGAGTTTTAAGTTTAG
TTATTTGTATGTTTTTTTTTGGTATTTCTTGTAATTATAGATATATACTGATTTACCTTATTAAATATGTTGC
AAATTTCTTGTTTTTTTTTTTTTTGGTTCTTATTTTGTAAAATGTTGCTTTATGGGGTTTTCTTTTCTTTTTTAT
TCCTGTTTACAACAGCCTTATTGTTCTGATGATAAAAGTGAAGTCTTTAGGGATATAGAGTATCTTTTTCC
TGTTTTTAGGAAATTATATGTGATATACAGAGATTGTATACAGCCAAAATACAGCTGCACCAGCATATCT
CTGTTCTTTGTGGTACTAAAC

>PGSC0003DMG400003822 LRR-RLPs

TTGAAAACCAAATATTACAGATGACAACATTTCATACTACCTTAGTTTACATCTTGTATGTGCTACTTCTTT

TTTCACTTCCATTAAGTATCACATCTTCAGCAAGAACAGAAGCTGAATCTCTTGTCAAATGGAAGAGAAA
TTTACCTTCTACTTCTTTCTTGGACACATGGTCCATTTCGAATCTCGAAAACTTGTGCAATTGGACATATAT
TGTGTGTAATGATGGTGGAACAATTTCTGAGATCAATCTTTCTGATGCAGCCCTCTCAGGCACACTTGAT
CACCTTGATTTCACTTCATTTCCGAGTCTCGTGAATTTCAATCTTAATGGAAATAACTTCAGTGGATCGAT
ACCTTCGAGTATTGGCAATGCCTCATTGCTCACATTCTTGGACCTGAGTAACAATATCTTATCAGGTGTCA
TACCTGAAGAGATTGGAAAATTGAATCAACTTGAGTATCTGAGTTTTTATAACAACAATATCGAAGGTGT
GATGCCGTATCAGATTAGCAATCTGCAGAAGGTAATGCACTTGGATCTTGGATCAAATTTCTTAGAAACT
CCTGATTGGTTGAAGATGAGAAATATGCCTGTGTTGACGTATCTAAGCTTTGGTTATAATGAACTGAGGT
TAGAATTCCCTGAATTCGTACTTCGTTGTCATAATCTAACATATCTTGATTTATCGATAAATCATTTCAATG
GTTCGATTCCTGAAACAGTATTTACCAACTTGATCAACCTTGAAAGACTTAATCTTTCATCTAATTCATTTC
AAGGTTCATTGTCACCAAATTTTACTAAGTTGTCTAAGTTAAAAGAACTTCAGCTAGGTGTTAACATGTTT
TCTGGTTTAATTCCTGATGAAATTGGCTTGATTACTAGTCTTGAAGTTCTTGTACTTTTCAACAATTCATTT
GAAGGAAAGATTCCATCTTCTATAGGTAGACTCATAAATCTTCAAAAGTTGGATCTTCGAAAGAACGATT
TGAATTCAACTATTCCTTCTGAGCTTGGCTTTTGTACTAAACTCACTCTCTTAGCTCTAGCAGAAAATGAC
CTGCAAGGGTCATTGCCTTTATCATTCTCCTCTCTTGCAAAGTTATCTGATTTGGGGTTGTCTGATAATAG
TCTTTCTGGTGAAATCTCATCGAATTTCATCACCAATTGGACTGAGTTGACATCTTTGCAGCTTCAAAACA
ATATGTTTACAGGGAAAATCCCACCCGAAACCAGTCAATTGACAAACCTTGTATATCTTTACCTCTATCAT
AACAATTTCACTGGTTCCATTCCCTATCAGATTGGGAACTTGCAAAACTTGTTGGATTTAGACTTTTCTGA
TAACCAGCTTTCAGGCATAATACCTCCAACCATTGGAAATCTGACCAATCTAAAGATGTTGCAACTTTTTC
GCAACAATCTCAGTGGAACCATTCCTCCTGAGATTGGAAAATTGATATCCCTTGAAACCATCGACATCAA
TACCAACCGACTAAGTGGTGAGCTGCCGGACAGCATTTCTGACCTCAGTGAGCTGAAGTTTCTTTCTGTA
TATACTAATGATTTCTCAGGCAGTGTCCCTAAGGACTTTGGAAAGAACAGTCCTCAATTGTCAAGTGCCA
GCTTTGCAAATAACAGCTTCACTGGTGAACTACCTGCTGGATTGTGTAGCCCGAATCTTGAGGAGTTGAC
GATAAATGGGAACAAATTCAGTGGGAAGTTACCAGATTGCTTGAAGAACTGCACACTTCTAAGACGAGT
ACGGCTTGAAGGCAACAATTTATCTGGTAATCTTGCAGATGCATTTGGAGTGCACCCGAAACTTGTTTTC
CTTTCTCTTAGTGACAACCAACTTTCAGGTGAACTCTCACCTGACTGGGGGAAATGTGAAAATCTCACAA
GTCTAAGAATGGATGGAAACAAATTTTCTGGTGTAATCCCAAGTGAGCTAGGGAACCTGAGAGCGTTGC
GCGTGTTAGCTTTGGAAGGAAATGAATTGACTGGTGAAATTCCCTCTGAACTGGGAAGGTTAGACCTGC
TCTATAATCTCAGCTTGAGTAAAAACAATCTTACTGGAGGCATCCCTCAGTCTGTTGGCAATTTAACTAA

GCTCCAGTATCTTGATTTGTCGACAAACAAGTTAAGTGGTAACACACCAGTAGATCTTGGGAAGTGTGA
GAGCCTGTTGAGCTTGAATCTTGGCAACAACTCATTATCAGGTGGCATTCCATCTGACCTTGGTAATTTG
ATGCAGTTGAGTATTCTTTTGGACTTGAGTGGCAATTCACTAACGGGAACCATTCCACAAAACTTGGCCA
AACTCACCTCATTAATGCATCTCAACCTCTCACATAACAACCTCTCAGGTAGAATTCCTCCAGCATTATCTC
AGATGATCAGTCTTCAGGAGATGGATTTTTCCTACAATGAGTTTTCTGGACCAATCCCAACCGATGGAGT
ATTTCAAAGAGCACCCGCTAGATCTTTTCTTGGAAACTCTGGTTTGTGTGGAAATGTAGAGGGATTATCC
TCATGTAATTTGGATACTCCCAATGACAAGTCCAGAAATAATAATCAAAAGATCCTCATTGGGGTACTTG
TACCGGTGGTCAGCCTCATACTTTTGGCAATCCTGTTTGTTGCATGTCTCGTGTCTCGAAGGAAGGCTAA
GCAATATGATGAGGAGATCAAAGCTAGCCAGATACATGAAAATACAGAATCTCTGATTTGGGAAAGAG
AAGGGAAGTTTACATTTGGTGACATAGTGAAAGCTACTGAAGATTTCAGTGAAAAGAACTGCATTGGAA
GAGGAGGCTTTGGAAGTGTCTATAAAGCTGTTTTGCCCTCTGGGCAGATTGTTGCAGTCAAAAGACTCA
ACATGTCAGACTCAAGTGATATTCCATTGACTAATCGTCGAAGCTTTGAGAATGAGATTAGAACTTTGAC
AGAGGTGAGACACAGGAATATAATTAAGCTCTTTGGTTACTGTTCCAAGAATGGATGCATGTACTTGGT
TTATGAGTACATAGAAAGAGGTAGCCTGGGGAAAGTTCTATATGACAATGAGATGGGAATGGAACTTG
GATGGGGTACAAGAGTGAAAATTGTGCAAGGAATAGCTCATGCACTCGCTTACTTGCATCATGACTGCT
CTCCGCCCATTGTTCACCGTGATGTATCATTGAATAACATTTTGCTTGAATCAGAATTCGAGCCACGACTC
TCTGACTTTGGCACAGCAAAGTTGTTAGCTTCAGACTCATCAAATTGGACCACAGTTGCTGGTTCTTATG
GCTATATGGCACCAGGTATGTTACATACTCATTACCTAATGCATCCATATTTCTGTTGATTAAGTAGTATA
TTCATTGGAAGGATACTTCAATATTATTGTGTCTTTCTTCTTGTGCAGAGCTTGCACTTACCATGCGTGTT
ACAGAAAAGTGTGATGTTTATAGTTTTGGAGTCGTAGCGATGGAGACTATGATGGGAAGGCATCCAGG
GGAGCTTTTAACTTCATTATCAGCATCAACAACATTGTTTCCAGAAATACTTTTGAAGGATGTTCTTGACC
AAAGACTTCCACCTCCCACAGGCCACTTGGCAGAGGCAGTGGTTTTTGTGATCACGATTGCCTTGGCATG
TACACGTACCACTCCTGAATCGCGACCAACCATGCGTTCTGTAGCACAAGAATTGTCTGTTCAGACTTTG
CCTTACCTTCCACAGCCATTGGGGACAATAGAAATGAGCAAACTAACAAGTTTTCAGAAATAATGAGAA
GATAGAGCCTCATGTACAGGTGTGAAATGTACAGGCTGTAGAGTTTTAGTGTTGTAATGCAACTTCCTCA
AGATCTAGAATCTATATAGTATCAAGGATGAAACCTTGTATCTATTCAAATTACTATGTTTTGGTTGCACT
TTTCCCTAGAAAGAAAGAAATAATATCATCCTGTTAATCATGAATGTTACAAAGTTGGTATATTAATTTG
GACAAGTATGAATGTTACAAAGTTGCACAACCTTATCCTAGTTTTCACTTTATTGCTAGGCAAAACACTA
GCAAAAAGATACAATGTGTTCACTTGTAATAAAAATAGCAACCTAACAAAAATTTGGCAAAGAATACAT

CACACAGAAACCAGGCATGGTACTTAGCATTCCACTCCACACTGCTTGAGTATAGGTCCCTTCTGCTTTG
GTAGGAATGGGTCTATTCGTACCCAAACGAGTGAGAAGATTGAAGCAAGAAGGATTGACCAGAGGACC
ACAATGGTGGGAGTCCTGTTCTGCCTGCCCATCAATCCCTTTAGGAAAGGATAGAGATGCACAATAACC
CAAAAGGCAAAGAATAACTTTCCGAAGAGAGGACCCCATGAACCATAACCATTGTTGATGGCATCCGAA
ACACCAGCTACAACACCAACCATATTCAAGATGATCAGAGTGGTTGGTGGGATGAGAAGTGTGGTCCAT
TTGAAAAGGTAAAGCTCCCCAAATTCAGCGTCATCAGCTGCTTTTGATGTTACAGTGAAGTTTGTGTCTA
CACCAGCAAGAACTTTAAGAAGGCCTTGGAAAACTGCAAACAAATGTGCTGAAACACCACCGATGACCC
AAAATTGCTCGTTACGCCACCAGGCCTCAATGCTTACTCCACTCCATCGCAGCTCGAGTACACTTGTGGC
TATGATGGAGATAAAGAGAGCCATGAACCAAATGCTTGCAAAGTTGTTAAGCTACAAAACAAACATGGA
TTTTGATTTATAAAATCTAAATCAAATCTTTATATTACTGAACCAAATGACTTCAGTTCACTTACTGTGGG
GACGATAAATTTTCCAGTGAGAAGGCATACAGCTGGAAGGGTGCAGTAAGCTAGCAGAGCAATGGAAG
TGAATGGGTAAACAATAGTGTTAATGTATGCTAGCCTTTCCAACCACTTGAGTTTTCCACCCCAGGCATA
CCAAAGGGGACAATGACGACTCATGAAGATTTCGACAGAACCAAGAGCCCATCTCAGGACTTGGTGCA
ATCTATCTGATAGATTGATTGGTGCTGATCCCTTAAAAGCGGCTCTCTTCGGACAGCAATAAACCGATCT
CCATCCTCTGCAATGCATCTTGAATCCAGTCAAAATGTCCTCTGTGACAGAACCATAAATCCATCCAATCT
GTCAAAAAAAGAAAAATGAAAACCAATTTATTAGAAGTTAGCTAAGCGAACACCCATAACTCATAATTTA
TTCATGAAATATAATGCTAACCTCTTTGCCCCAGTCAGTTTTCTCTTCATACCCACAGCTAATAACATGAAT
AGCTTCTTTTATGAGTGTTGTCGGGTTTGTCCCTTCAGGAAGCCCACCATCTTCCTTAAGTGTGGAAGCTA
TGAAAACAGGGGACTGCCCGAATCGTTTCTCAAAGTTCTTTTGGGACATGAGAGACGACTTCTCGAGCT
CATCATATCCTTCAAGGCCTTCTTCAATTTCCTCAAGATCAAAGACAGGTCCAGATGGCTTCCTAGTGTAG
TTCTTTCCATTCATTGTTTTCTTTTTCTTGCTGTATAGTCCTCCAAGTCCAAGTAAGGCCTTGATGCTCTTCT
TCTTGGACTTGGACTTGGACTTTGACTTTCTTGAACCACCACAACAGCAACTGGGCCAGCAATCACATGT
CATCTTTTGACGCTTCTCTGAAACAGGTGGATCATAGCCGTATAACGCCGGCCTGTTAAAGACAGTACCT
GTTCCAACATAAACTGGTCCTTGAATTCCATCCAGGCCTTTCATGTTGATCTGCCACCATGAAGGGAAAA
AAGCATATTAATACCGGGAATAAATCCTTTGTCAACACATTGCTGTATATATAGATCAACTTACATCAAAA
AAGACAACATTACGATTGGCATATCGATCGTGGCGATCAATACCATCAAATCTCTGGGGAAATTGGACA
TAGCATAGCTTCTTTCCAATTTGTGGGTCCATTAAAAAGCACATGGCTTCTCTAACTGCCTTGCTGTTGTT
AAGGTAGTGATCACAATCCAAGTTCAACATAAATGGTGCATTCGTCAGGACTGCAGCGACTCGAACCTA
TTTAGAAAAACAGCACAAATTACATAACATTAAGAAAAGGACTTAATAGGATGTCAAGGAAGCAAGCCA

GAAACTAATATACTAATTACTAACCAAAGCATTCATGGCACCAGCTTTCTTGTGGTGTTGATAGCCCGGT

CGTTTCTCACGGGAAACATAGACAAGGCGAGGTAGCTCCTTACCTTCAATGTCAAGTGCACCTTCACTGC

CAAGATAAACTTGAATCATACCAGGATGGTCTCTGGTGTTGTTACCGGGCCACGGAGTTCCATCTTGCAT

TACCCAGCCTTCTTCTGGTTTTTTCTGAGCTTTTGCTACTAATGCATTAATTCTTACTTTGAATTCTTCATAT

TCTCTCTGCCACAAGAGAGTGAGTTTTCAATTTGCAGAAAAAATCTTATTTTAAGCCAAAGAAACTTAAG

CCCCCGATAAATGATACTCCTTCTATCCCAACTTATATGACATTCTTTACTTTGGGCACGTCCTAAAATGTT

CGACACGATTCCTTCTACAACTTCTTTTTAAACCTTGATATGATTCAGTCAACATTGTAGGCACTGCATCTC

CTCTGATATAGTCGTATGAAATGAGACGAAGGAAGTAATAGATACATACCTTCATGGCCCTGCGATCCTT

GACAAAGGTAGGCTGAACTTTATCTTTCAAGTAATCAATCTTTTCGTTGAAGTAGATATCAGGTGCTCTA

GGCTCTACACTATATTTCTTACAAAATGGCACCCATCTCCTGGCAAATTCAGCTGTTTCTGCTAATGAATC

AAACAACAGCATGGAAGCACCATCATCTGATACATAACAGCTAACCTTCTCGACAGGATAATCAACAGC

CAAGATTGACAGAACAGTGTTTGCAGTGATAATAGGAGGCTCCTTAAGAGGATCAACAGTACTCACAAA

CACATCCACTGGGGCTAATCGATTAGGCTCGCCTTCACGTTCAAACCTCAATGTCAATCGATCTAAATAA

GTTTCACGGTTTATTGGTGACCATTTGGGGAACTGATCGAGTATCCATGATAATGCAAACCATATTTCAC

ATATAACAGAGATAATCCATAATGGATAGGCATCATAGGCTGGTGTTAGGATCCGGAAATGGAAAAAG

AATCCAAGAATTACAAGCCTGAGGATAATGACTATCCGATATGGACTGATTAAACTTGATGGAAGTGGA

ACTTTTCGCCAGAGTGGTTGCCGAGCTTCAGCCAAACTGCTTGGAAATCAGTTAAGTCGTTATAATATTG

AAATATACTGCAAGTTTTATATGCTTAAACTTGATCTGTTTCAATTCATACACACTCATTTCATGTTGAAAC

ATTGTAGTATCTGATTCATTTTGTTAATAATATTATATGTAAAAGTGTTCAAATTATTTAACTATACCAGTC

AGTCACTGAGTGATACATAACTACAAGTGACACAGACGATATATAACCGAAAATCCAATATTTAAAAGTT

GTAAGGAGAATGAGGAGAAGGTGGAAACAAGACCAGATATAATTTGATCGCTATGAACAAAACCAAAT

ACCAGATTGACTGATTTGTTATAAATTTAAAGTATTTCTATAGCATGTTTTCCTTTTCCTTCTATGTTGTTTT

CTCAATGATTCCTCACATAAAATCTTCTCATATTAACGACGATATAACTTCAACACTATCAAAGAGAGGCG

GCAATTAGCTTGAATAATCAAACAAACTTATCAATATTTTCTTCCACTACTAAGAATATATGTTAAATTCC

CCTTCTAAGTCAAAGGTCTATCTAAAAAGCAAATCTAGTAGGGGTAAGGTGTGGTATATCTTACCCTCCC

AAACTTCACTTGTGGGATTACACTGGGTGTATATATCATGTTGAATTCCCCTTCTAAACCGAGCTGTTAAT

TTCCACTTCTAAACCGAGGGTCTATCAGAAACAGTAAAACTACCCTGGCCCGCAAAGGTAGTGTTAAGG

TGTGGTATATCTTACCCTCCCAAACATCACTCGTGGGATTACACCGAGGGTAAACAAGATGTAAAGTGA

AAAGAAAGAGTACATACAGAAAGTCATCATCTTCATCAGGTTGATCATTTCCACCATTATTAACAAGACC

TCTCTTTTCTTGACGATTCTTCCATTTCTCAACCCTTTCCTTCCATTCTGCATCACCATAACTTTCTTTCTGCT
CATCCTCCAATTCCTTCCCAGCAACTGTAAGTGATTTTCCATTGAAATAAAACAAAATCATGACACATATA
TATAAAGTGCAACAATTACTCATTTCGAAATTAAGTAATAGAGATCGATATAGCTTTACCGCTTCCAACA
ATTGATTGATTAAAAGCAGCAGCACCATTGGTGTTGTGATTATGCTCTCCATTCTCCTGCCAAACATACAA
CAACTTGCTTTCAGTTCAAATGAG
>PGSC0003DMG400003834 LRR-RLPs
ATGAGCATTCTCTCCAAAACATGGCTACAAGAATGGTCGAATCTTCCCAACTTGGAATTCACTATTAATT
GTTGGGAAGGCAATATAAATACAGCGAACACAGCCATGGAGAGATATAGGAAGGGGAAAATCCCTATA
CAAAAGTTTGAATTATTAGAGACCTTTGCTAATTCTCATGAAGATTTCCCTCTCATTGATAAGTGGCTCGA
TATTGCACTTCAGAATGGTGTAAAACATCTTTTTCTTAACTTTAAATCATACCCCATGCCTATTTTAAGAAT
CTTGGCAGCAAAATCTTTAAGAGAATTGGATGTGCAGGGTAGTATGCCTGATTCGTTATCTAGTGGTGTT
GTGAATTGCAAATCATTGAGAAAGCTTTCTCTATCTCATCTAAGATTAGACGAAAACATGGTCCAGACTA
TACTTAATAGTTGTCCCTTTATTGTCTCTTTAATCCTTGAGTATTGTACTGGGAAGCTTCAAAAGATCAAG
TCGGATTCCTTGAAGGTCTTGAAGATACATCATCGCGGGATAGAGGAGATCGATGCTCCAAATTTGGTA
TCACTTGACTATATGGGGAGTCAAATTCCCGAACTTAATAAAATTGCAAGAGACTCAAAAATCAGGCTTG
GATGCATCAACAATTTAAATGCAGCATGGTTTTGTAAGTTGAGGAAGTTTCTATCAAATTCGTCCTCGTG
GTCTCATGTTTCCCTTAAATTTATGAATTGCTGTGAGATCAAGATGAAAGATTTGCATATGGACCACATA
GGTTCTACCCCTCGGGTGGACGTTTTAAATGTCGATATTCAATGGATGAATCAGAATTTTGTGGATGCGT
TGCTATGGAGTTGTCATCCAAGGAGACTCAACCTACTCTCAAATATTACTCCAACTATTACACATTTCATT
GGTCGTTTAGTGTTTTTGACAAATTCCAGTCATTCTACTTCTCATAAAAGCTTACCTTGGCATAGTCAATT
AAAAGAAATAAAAGCTTTTGATGGAAAAAACCAGTCGCTACAACTCATAAATGGGGAGCTTGCAAAAA
GGACCCTTATGGAGGGGGAGAAAGTTTATTTTTTATTAGATTGGTGATGTAGTTAATTTGTTTCTATTTCA
AATTGAATGCACTTATTCTTTTCTGCATTGGTGTACAGAGGTATTAGATATTATTAAAACAGAACTTAGA
GCATAATTTTAAACTTGGATCTTATAGGCTTGCAAAAGCAAAAACAATTTTTTTAAGAAAGTAATGAGTA
TGGCAAATTTCTTAAAAGGAAAGAGACTTAATTTCTACATAATTACTAGTGTTTCAACGTAACTACAAAT
GCCTTCGATGCCATACTTTCTAGGTAGCCCGTTGATTTCTAATTTTCTCAAACTCTGTCTCGTGACATCATA
AGAGAAACATAAAGTATCCGACATTGAGTCCAGGATGAATACAATCTCTTCATCACTGGACGTGCATGA
TGATATAACTAAGGGTTTGTCCTTCCACCACATCGAAGGGAAGTCAATGATTTGAATTTTCCATTCTTCTT
CTTTCTGAGTTTGTTCTAAAATCCACAAGTTGAATTATCCATGTTGCCAATATTCATAATCCATGATCGCTA

GTTTACCTTTCACCTCTAACAACTCGTAATACCACCACGAGTTAGATGCATTCAATAAATCAATATATTTT
GAATTTTCGATTTTAACATCAAATGCATCTATAGTAAGTCTGTCCTGGAAAACAAATTTATAGATAACTCC
ATTGATGCAAACCCCGGGCTTGCACAGAATAGGAGGGGTTATGCTTTGAGTCTCTCTCCATGATTTGTCT
ACTCCTAAAGTGAAAATCCAATGTTTTATGTACCCTTTTATAATATGCTTTGTTGTGGAAAGAACTTTGTA
CTTCTTTTCTTCTGGGTCAAAACCGAATGAACAGTACCACATGGAAGAACCATCATTTAGGTCGGGAAGA
AATCTTACTTCTCTTGTACTAGGATTGAAAATTGCAGCAAAAATAATGGTTCCCAAATACAAAATAAACC
AGGTCTTGGGTCAGATCTCAGGATCGGATTTTGGAACGGTCGTTGGGGTTGTGTCCTAGTTTGGGTGTT
AGGGTCGGGTCCTACATCGATTATCGGGTTGATTTTCGAGTTAAAAATTATTCTCCTAAAGAGAATTTTCT
AGTCTCAAACAAAAAATAAAAGATTTTTTCTGGAAAATATTTTTTATTCACCAACCAAACATTAAAAAATA
TTTTTCAGAAGATATTATCTACTCATCAACCAATAAACATAAGAAAATAAGTTAGAAATCCATTTGTTTTT
TCCAAGAAAACATTTTCCTTCGTACCAAACACACCCTAAAGAGTTATAAAAAAGAAAAAAGGACAATCTT
TTTTAAATGGATTTAATTGAAAAGTAAGGCAAATATGTTAATTGAAACATATGAAGTATTTTTTTTCGACC
TTCTGGAATTTTTTTTAAAAAACAAACAAAGAAGAAAGTCTAGGGGATAATTTTATATAGTTTCTTTTTTA
CTCTTTTTGCTGACAAAGAACTTTAATATTTTGACCTTATGTAGAAGAAAATATTTTAATTTTTAAGTAGC
GCATGATGTTTTCTTCTTTTTTTAAAAAAAATCAAGCATGATGTATTAATTTTTTTGTACATTTATTTTTATT
TTTATTAATGAACCATGATACACGTGCAACACACACAATATTAAAAGCATGATTTTTTTTAGAATATAGAT
TGAACTAACTTTTTATCCTTCATTCAAAATTTTCACAATAGGCAAAACAATCATAATATTATTCGATTTTTG
CACACCTCTACTTTTCGATGCGAATCTAAATTTAATCGGAATCTAATGTATAAAAATAATGATTCATTCAA
ATATTATTTGACCGTGTGACGAGGGAGGGAGATCTTTTAGTAATATAGAGCTCCTTATTTTTTTTTTTTTT
TTTTTTTTNNCAGCAAACTCCGGCGAAGGTAAGAGCATTTTTCAGCTTCTCCGGCGAACTCATCAGCAAA
CTCCGGCGAAGGTAAGAGCATTTTTCAGCTTCTCCGGCGAACTCATCAGCAAACTCCGGCGAAGGTAAG
AGCATTTTTCAGCTTCTCCGGCGAACTCATCAGCAAACTCCGGCGAAGGTAAGAGCATTTTTCAGCTTCT
CCGGCGAACTCATCAGCAAACTCCGGCGAAGGTAAGAGCATTTTTCAGCTTCTCCGGCGAACTCATCAG
CAAACTCCGGCGAAGGTAAGAGCATTTTTCAGCTTCTCCGGCGAACTCATCTATCTTCTCCGGCGACCAG
CTCAGAAGAGAGACGGCAGTGCTACTGTTTGCCGCCGGAGATCAACCAGATCTGGCCGGAAATTACCCA
TTGATCACCGAAACCCTCCATCTTCACTGACCAGGTTCTATCTATCCTTTTCTCTCCCTTTTTCGCTGAACCT
AGTTGCTGCTGGGCTCTGTCTTGCAGACTCCTTATCCCGGAACAGTATCCCCAGTTTTTAA
>PGSC0003DMG400003835 LRR-RLPs
ATGGCTGACATATTGCCTGAATGTTTCATTCAAAAAATATTGTCTTCTCTTAGTTTTGATGAAGCAGCGCG

GATGAGCATTCTCTCCAAAACATGGCTGCAAGCCTGGTCGACTCTTCCCGACTTGGTATTTAATGTTAAG

TTTTGCAAAGGCGATATAAAGATATTGGACACAATTATGGAGAGATATGGAAACGGAAAAATCCCTATA

GAAAAGTTTGATTTATCAGAGCTCTTAGGCAATTCTCATGAAGTTTTCCCTCTGATTGATAAGTGGCTTAA

CATTGCACTTCAAAATGGCGTAAACAATCTTGTCCTTAACTATAAATCATACCCCGCGCCTATTTTAACAA

TCTTGGCAGCAAAATCTTTAAGAAAATTGGTTCTGAAGAGTTGTACTCTTTTGCCTATTTCGTTATCCAGT

GGTGTTGTGAATCGCAATTCTTTGATAAAGCTTTCTCTATCTTATGTGAAATTAGACGAAAATATGCTTCA

GACTCTACTTAATAGTTGTCCCTTGATTGTCTCTTTCATCTTTGAGTATTGTTCGGACTTGGAAACAATCG

AGCTAGTGAATCTTCAAAAGATCAAGTCGGTTTCCCTGAAGGTCTTGAAGATTCGGTTTAGTGGGAGCA

TATGGGAGATTGATGCTCCAAATTTGGTGTCATTTGAGTACATGGGGAATCAAATTCCTGAACTTCAAAT

TGTGAGAGAGTCAAGACAACTGAAGCACTCAAAAATCATTCTTCACCGCTTAGACAATATAAATGCTGAC

TGGTTTTGTAAGTTGAGGAAGTTTCTATCAAATTCAATCTCTTGGTCTCAAGTTTCCCTTTATTTTTATGAA

TGCGCGGAGATTAAAATGAAAGATCTGCAACAACACCACAGAGTTGCTACGCCCCAGGTGGACATTTTA

GATGTCAACATTCTGTGGCAGAATCGCGAATACCCTTCGTTTGTGGATGCTTTGGTATGGAGTTGTCAGC

CTAGGAGACTCAACTTGCAATTAACAAGAGAAATGGTTACAGTTTTCATTGATCGTTTGATGCATATGAA

GAATTCGATTCAGTCTACTTCTCATGGAAGCAACCCTTGGTATAGTCAATTAAAAGAAGTAAAAGTCCAC

AAATTTGATAGGAAGAAGGAGATTTGGTATCCTGTGGAACACAAACTTGGGGAGCGGGCAACAATGAA

CCTTGATAGATATTATATTTTATTAGATTGGTAA

>PGSC0003DMG400004056 LRR-RLPs

ATGTACAGAGGTGTAGCAGATGCTTCTATATCTCCAATGGAGAAACATGAGAAAGAGGCATTGTACTCT

GCTATTCAAGGTTTTGTTGGAAATGAGTGGAATGGTTCATTTCTTTATCCTGATCCTTGTGGCTGGACATC

AATTGAGGTACTAATCTTTTGCTCTAGATGTTCCTTTGCTAATTTTCAAAGCTAAATTAGATTACATTAATT

TGATATTTTAAACTAAACATTTAGATATTCAAAAACTATATAAAAAATACTATAAATTGTAATTTTTTGCAT

ATTAATATGATGAAGTTAGTCAAAGTTACTCTAAAGAAAACAGGAAAACATGACAACTAAAAAGGAACG

AAGAGTATATGATTTCATAATTTGCTAGTTGATTCTAGTCTATGTTTTTTTACCATTATTGACTTTTCTTGCT

GTAGTTCAAAATCAAGAAAACAGTAATGTCACAAAGCCATGCATGGATGTCCTCAATTTTTATTTTTATTT

TTTAATATAAAGAAATGGATCTTGGGCCTTACTCAACCGCATAAACTACATCATAGGAATTAGGATGAGT

ATTGTTCAAGACATTTGAGATCAATTACCCATCCCATATTCGACTCTTGTGGGCAATATAATATGGAAGC

ACAACATAGGTGAACAAAGAATTCGGATGAGCGGACTTTGGTAACATGATAAAAAAAATGGATCTTGG

CCTAACTCAAGACAAAAAAATATCTCATAGGGTGAGGCTTATTCAAGACCATATAGGAGATCAATTACCC

ATCTCATATCCGATGTGGGACTCTTAATAGATCAAACTGTCCTCTCAATGGTTGTTAATTAAAGTGAAAA
AGACTTTCTCAAAGTCTTACTTTTTCTGTTCTGGATTTGCAAACAGTGTTATATGAGTGTGTGTAAGAGAG
ACATCACTAGTTAGGAGCTTTCCAATTTCTAACACACCTTTTCTGGTAGTACTAGTAAATATATATGCTAG
TATTCTATTGGTAATTTGCAATATTTATTCCTAAAACCAAGTAGTTATATGTATTGAAATTATCGTTCTTGT
ATGTTAACTTGACATAACAGTGTAAAAATTCTTTACACCGTAACATAAACTCTTGTCTTAACACTTTAATCT
ACTCTGTGAACTCTTATTTTTACTATAATTCACAGGGAGTATCATGTGATTTCTCCAATGGCTTCTGGCAC
GTAACGGATTTAACTATTGGAGACCTATATGACAATTCCCTTCGTTGCTCCCAAGCTGCAGAGTTCACAG
AACACTTGTTTAACCTCAAGCATCTAAAAAGACTTTCCTTCTCAAATTGCTTCCTCTCAGATCAGCACAAA
TCAATTCCCATTTCAAATTGGGATAGTCTCGCGAACAGCCTTGAATCGTTAGAGTTCCGATCAAATCCTG
GTCTTATTGGGACAATTCCCACAAGTTTTGGCTACCTTAAGAATCTTCAATCTTTAGTACTGCTGGAAAAT
GGACTGAAAGGAGAAATACCAGAAGCTTTAGGCAATTTAACTAAACTAAAGCGCCTCGTTTTGGCAGGC
AACAATTTTATTGGTCCTATTCCAACTAGTCTAGGAAGATTGACAAATCTTTTGATACTTGACACAAGCAG
GAATTTTCTATCTGGTGCATTACCTGTGACTATTGGTGATTTATCTTCGCTCATAAAGGTCGACTTGAGCA
ACAATCGATTAGATGGAAAATTGCCTCGAGAAATTGGAGGACTAAAGAGTCTAACACTACTTGATCTCA
GCCACAACAATTTCTCTGATGGTTTGCCAGAGACATTTCAAGAAATGGATTCCTTAGAAGAACTTGTGTT
ATCTGACAATCCGATAGGCGATTATGTTACAAGAATTCAATGGAGGAACATGAAAAACTTGGAAATGTT
GGATCTTTCAAACATGGGATTGAAAGGGAATATACCAAATTCCATGACAGAAATGAAGAAACTGAGATT
CCTCAGCCTCAGTAATAACATTCTTTCAGGAATTATTCCGTCAAAATTCGAGAAAATATCAACTATTAATG
CTCTATACTTGGATGGAAATGATTTCACAGGGAAACTTGAATTTTCACAAAGGTTTTATACAAAATTAAG
GAGTCGTTTTCGAGCTTCAGGTAATGTGAAACTCTGCTTGTCCCTCGAGTTAACGTCAACAAGTCATGTT
CCAGAAGGAGTAAAACAATGTGAACAAGAAAGTACAGTAGATAGTAAAGAATCAGACTCCAATTTAAA
AAATGAAGATTTGAGTCAGAATTCCCACCATATTATAGCTTCTTTGGGGCATTCAGGACATGTTGTTAGT
CATTTTACATTTTGTTATGTCGCAAGAATAATACTTATGTCTCTTCCATGGATCGTATTTTTATAA
>PGSC0003DMG400004190 LRR-RLPs
AATCCAACGACGCGCAGTGCAAGCTAAATAGCGCGTCGTGTAAAAAAACCAATTTTGAGGAAAATTACC
AAAGTGAGGATTCATTTCACACACAGATCTTCTTCCTTCACTTCCATCATCTCTCTCAATCTCGACTATAGT
AATGTACGTATAGCTTTGCGTTTGGATTCCGGTTGATTCAGTAATTCTACTCCCTGAAGGTGATTAACCCT
ATTCAAATGGCTCGCCATGGGCATCTACCTTCAGTATTTCTCCTGATCTTCTCGTTATCCTTGATCCAAAGC
GCTCTCGTTCACTCTAAAACCCTCAAACGTGACGGTACACATTTCGATCCATTTACTTTTTAAACCTAATTT

ATTAGCAATTTAGGTGAGAAAATCAGTTGAATTTGATTGATAATAGTTTGGTTTTGTTATTTTGTAGAGTA
TGTATGCAGCTTCAAGTGAAATTAATTTTGTAGTTACAGAGAAAATGTAACGATGTTGAACTTTGTAGAT
CTTATGTATTCTAGGTGCAAGGTTATAGTTTGAGTGGAGTTTAATTGAATTTTAAACGTTCAAGTGAAAA
TAGTTAGATTTTGTAGATTATATGCAGTTTGTGGTGAAATTTTTGTAGTTTCAGCGAAAACAATATTGAAC
GTTGTACATCTTATGTAATTTCAAGGTGTAAGCTTATAGTGTGAGTGGAAGTAATTGAATTTTGTAGTTC
AAGTTGTAGTAATTGGATATTATCCAGTTTCTAGGTGAAAGCTTATAGTTTGAGTGGAATGAACCTGGAT
TTTATAGATTATGTGCAGTTTGTGGTGGATTCATGGTTTCTGCGGAAACAATTGAAAGTAAGTGGATTTT
ATGTAATTTCTAAGAGAGAGCTTATAGTTTGAGTGAAAGTAAGTGGATTTTATGTAGTCCTAGAGCAACT
TTATAGTTTGAGTTGTAGAAATTGGATTTTATAGATTTTTTCCCAGCTTCTAAGTGCAAATTTTTAGTTTGA
GTTGAAATAATTGGATTTATTCTATAAATTATATGTGGTTTCGAAGTGCACATGTATAGTTGGAAATGAT
TGGAATTTATAGATTAGATGAAGTTGTAAGTGCAAGTTTATAGTTTGAGCCAAAATAATTGGATTTTATG
CATTAGCTGCTTTCTAAGTGTATGTTTATGGCTTTCCTGGAAGTAATTGGACTTTTTAGATTTTATGCAGC
TTCTAAGCTCACGTTTATATATTTTGAGTGGAAGATTTTATGCTGTTTCTAAGTTTTACTTATTGTGTTTCA
TCTGTTCTTACTATAATTTCCTTTTTCCTGAATGTCTGTTTTCTTGGTTAAGTAATTCAAGCTTTGCAATTTG
TTGATTTTGTTGAGGAATAGCTATTCGTTTGATGTATAATCTTGATCTGTTCAATGCATTATCTATTATCTG
CAATCAATAACTAAATCAGTGCCTTGGAGTGTCATTAACTATAAATGTTTTCCCCTTTTTTTGCTCGGAGA
ATTCTATTTTCTAGTTGTTACAGGTAAATATAGCTATCCTTGGAAAGTTAAACCGATTTATCATTCAATCA
ATGCCAATTTAATATCTCTCCATGATTACATTGTATTAGTTAATTAAATGGTAGTATAAGTAAGTATATAA
TCGATCACGAGTATTTTTATGGTTGTCAGAAATAAGAATGTCCACTGTAGGTCTTGAGCCCATGATCATA
AGGTTAATAGCTTTGCTCTCTACACTTAGTGTTGTTGGAAATGTTGTGGAGTTCCAATTATATCCACTCTT
TCTCTGTTAACATTTCATTAAAATGATTTACACAGTTTAAGGCCATTGATTGCATTTATGAGAAAACCGAG
GAGTTATCGTAGTACCTGAATGTATATGTTTACATGGACTAATTTGAACTTCTGGAATATGACTGTAGTG
AAAGCTCTGAATGAAATTAAGGCATCTCTTGGATGGAGAGTGGTGTATGCATGGGTTGGGGATGATCCT
TGTGGTGATGGTGATTTACCTCCATGGTCTGGTGTTACATGTTCAACGCAGGGTGATTATAGAGTAGTTA
CTGAATTGTAAGTTTGCTTCACCAGTTCCAAGTAGCTTTTCTCTAACTAAGCTTCAAATCTGTATGAAGGT
ACTCACAGAATTGAAATATCTTTACTTGTCACTTTTTCACAATGTTCTTTTCTGTTTTACCACTTGCAGGGA
AGTTTATGCTGTTTCAATTGTTGGCCCATTTCCTACTGCTGTCACTAATTTATTAGATCTTACTAGACTGTA
AGATTCTTCTTCTCTTTGTCTTTTTACTGGAATCTAGTTTTTACAAAACTTCAGCTACTTCTTACAGATTTTT
TAACCTTGTACAAATTGAGAAGTTTCCCTAAGTTCCAATATGGATGTTTTTACACCTTCCAGTCTTAATTGT

TCATTAACCAACTTAAAGAATTGTTCTGCTTGGGTGTTGACTGTTACCTCAAAAAATGTGGGCGTTATCTA
TAATGTGCATAGTCATTTTTTTCAAATTTGATACTACTATTTAAGGCAATAGTTAGTGGACAATTGTGCCT
GTACTGTTGGATCTTTTGATTTTCAGTCTGAACTTCCTAATCCATAATATTCTTTCATATGACAGGGATCTG
CATAATAACAAGCTGACCGGTCCTCTTCCTTCTCAAATTGGGCGTTTGAAGCGTCTTAAAATATTGTATAA
TCCTTTACTTGTATACTCCTATTGAAGTATCTATATTCAGTAGCAATTATGAAGTGATATTTATGTTTAGG
GCTTATGCTCTGTCTCCGAATAGCCTGTTTCTCCAGTTTCAGGATTAACATATGTTTATCCATTAACACCTA
GCTAGATGGTGTTAATATGATTCAACTGTGTTGTACAAAGTGATTAAGTACATGGCTTTTCATCGAGATG
AAATGTGTTAATAACAAGGAAAAGCTTACCTGATTAATGATTAATCCCAAAGAAGGGAGTGAGAGGGA
GTCTGCCTGATATAATGTGCACCGAAGAAGAAAGATAAATTTTCAACTGAGAGAATATATACTCGTAGG
CTTATATTCTAAAAAATAAAAACAGAGTAGGTTTATTATCAAGGAAATGTCACTTAACTAAGAAGGCCTT
CTATACTCTTTTTAGGATTGTATTAGATGTATATCTCAACATCTACTTTCTGTTGCCTGTATCCTACCTTTA
GGTTTTCCTTTGTCCATAGGAACCTCCACGTTGTCCCAGAATGTTAACTTTAAAAATTACTTTTATTGCTTT
GTTACCCAACATACTAATATGAAATTATATCCTCTAGTTTCCTAGTGTGTTGTAATGTTGCATTATTGATTA
GTTCCTTCTCCTGATTCTTTTTACCAGAAACGTGAGGTGGAATAAGCTGCAAGATGTCATTCCACCTGAA
ATAGGTGAACTAAAGCAGTTAACACATCTGTGAGTGCTTTTTGAGCTTCTCTAGCTGTGCTCTTTATCCAT
AGATCTATTTTGCTGTTATAGACTAATAACTGATATGTATATTTTTCAGCTACCTAAGTTTTAATAATTTCA
AAGGCGAAATACCTAAGGAGCTTGCTAATCTTCCTGAACTTCGTTATCTCCATCTGCATGAAAATCATTTT
ATTGGACGAATTCCACCTGAACTGGGTTCCTTACAGTATCTTCGACACCTGTATGATACCATCTTAACTTC
TTCTCTTGTTTAACATATGGAACCTGTGTCACACATGGATCTCACTGTGATCTCATGCAATTCCAGGGATG
TTGGTAACAATCGTTTGGTTGGAACTATAAGAGAACTCATACGTATTGAAGGATGTTTTCCGGTTCTCCG
CAACCTGTAAGTTGATTATTCTTGTTTAATTGTCCTGTTGCTAGTGTTATGAATTATATGCTAATTCAAGG
ATAACACGTGCTGCAACTTGCAGATACTTAAACAATAATTATCTTACTGGAGGCATTCCAGCGCAACTTG
CAAATCTGACTAACTTGGAGATCTTGTAAGTTTCGGGAAGAAAGTTTCTCCGCGATTAGTGCTTGTCTTTT
GGACAAATAACAACTAATTTCTCCTGTACTGGTTGATAAATATTATTATGCTTAATTTGATTGCTCTGGTT
ATTACAGGTATCTATCCTACAACAAGATGACAGGAGTTATACCATATAGCATTGCTCATATTCCTAAGTTA
ACTTACTTGTAAGTCCTCCTCTATGCTCTTAGCTACTCATTTATTACACTGTATGTGGACAGTTTGGACACA
GAAAATTCAGAATTTTTGAAAGCAAATACAGAAACATTCTTAGCCTTAAGTTGAGATCTCTTTCTTTGATA
GATTTTTGGGGCAACCTGGTCTTTGTGCATGATTTAGACGCCACGTTAGATACGATCAGGGACCTGTGT
GTTATCATGCAGTAGTTTAGTGCTGTTTTCTGTTATCAATAGATTGCTTTATTAACCTATAAAAATATTAGC

ATATTGGATGTGATGTCCCAATGATTGTCTAGTGAGTGCAGTCTTTCAAGGATCGTTCTTTCTTAAAAAG

ATGAATTTTGAGGATTGTTCAACTTAAGGATGAACAAAAACAACATACTTACTACTTAGTGTAATCCTAC

AAGTGGGGTCTGGGGAGTAGTGTGTACCCAGACCGTACCCCTACCTTAAAAAGGTTGAGATGTTATTTC

CAATAGACCCTCGACTCAAAACAGATGAACTCGGGACTAGCTAATGTGGTCTTGCAAGGGTGTTTCTTTC

TTAAGGAGATAAATTTTGAGGATTGTGCAATTTATGGATGCTGTGACAAAAACATTAGTAGATGGACTT

GATGCTTGTTGGCTGACCTTATTGGACCTTATCCGTAATGATTTCCTCCGACAAGTAGTAATGTATAGAA

ATTGCCTGGAGAAATTAGATGCAAGAGTGTTCCCATCCTAGTAGGTTCTAATTTTAGCACAAAAAACACA

ATCCATTTCAATAGTAAAACATCTCATGTACTCAAGATTTCAGTTGAGCTATGTAGCAACAGCACAGTGA

TACAGGGACGCGTGAATGACATGTTCAACTTTAATTATAGAAGATTGTCTGTTAGAACATAATAGTTTTA

TTGGTATAATGTGTTAAAAAAACTGTATGTATAATTCAAAAAAATATGAACACTGATGCATGCAAATCTT

TAAGCGATTAAACAAAGCCGATTTTGCACCTGGTTGTCAATATCCATTGCATTTCCTACCCATTTTCTTCTT

TCCTCCAAAATCCATGGTCTTTCAACACTTACATTCCCCTCCATCCCCCAGATTAGAAGTCATTATTTGTAC

AAGTGAGAGGCTTGGATAATGCAGAAGGACCTGCATTTTGCTGGTCAAACAACCAAAGAATGTCAAGA

GACAAGTCTACTTGTGTCAGATATATATCCCTGCTCAAGATATGCTGTGTTGGAAATGGGAACCTTGTTT

TGAAGCTTAACCCACATACTTCCTTTGACAAAACCTTTTATCACAATATTTTCCTTTAAAGTGTTGGTGTTG

GAGCTAAACCACACAAGCTGAAACTAGGGCCTTCTCCATTTAAGTGGTTGTTAGTGTTGTCAAAGTGATG

CTTGAGCCATGACTTGAGGCTCTGCACAAGTTTGCTCCAATGAAGGTGGTAACTGGTAAGGGATATTTTC

CCAAAACCAGCCAAGCAACTTCTTTTCTGAGAATATTAGATTAATTTGTGAGGTCCATAGGTTTCTTCTTG

GATAGGGATTAAGCAACAAATATTCAATAGATAGCGAGCAAACCGATATATTAGTTGTTAGCGAGACTG

TACTAATGATACATTATTGATTAGGAGTAAGAAGCTGTAAATTTAATGCAAGAAAGCTAGCGACTAAGT

CTGAGTTGAAATTTTAAAGGTAGATGTTCCTTAAAGCTTGCCACTCAGATTCAAAATGCAAACTTGATGA

CCAATGATCCTTGTAAGTTGCAATATCCTAGATGTTCCCTTAAAGCTCAAATGGTCCTTGCAGTTGCATAT

GTGAGTGTTTTGTTCCATTTATTTATTTATTTATTTTTACGTTCTTCGCCCTTGATAAATCAGGTGTTACGC

ATCATTCCTTCACTCTTCTTGATTGTATACTGGTAGATTTTGCACCAAAAAGAAAAATAGTTCTGCATGCA

TTTGATAGTTGATGTCTGAGGCTGAATCTGATTTCAGGTATCTCGATCATAATCAATTTTCTGGGAGGAT

TCCAGATGCCTTCTACAAACACCCTTTCTTGAAGGAAATGTGAGTGTTCATTTTTTGCTACTGACCATTCTT

TGGCTTTGTGCAAATCCTAATAATATGACAAATTATGTCATAGGTACATTGAAGAGAACACGTTCCGGCC

TGGTGTAAAACCAATTGGCCTGCACAAAGTGCTAGAACTTTCGGACGCAGATTTCCTTGTTTAGAAAAA

GCCAACTCCTGTTATTTGTTCATGAGCAAAGTGTACCTAGGTTGTCGAAAGAAGCAGCCAGTCCCTGTTC

TGTCAAGTATCTATTCTTTAGTTGCCAGATGAGAAAAAAGGTTCCATCTTTTTTGTGCAAAGAAGTAATC
AACCAAACTTAGTTGCGGTTGCTGCAGTTAAATAGTACCACGTTATGAATCATTATTAAGTAAGTGCGAC
ATACGTCTCAATCAGTTCTCACTTGCCTTGAATTCTTGCATTTGGTTTTTGATTATACATGATGCGATTTCA
GAGTTAATATGAAAGGGTAGAGAATATGCTCTGATATGACAGGTTTTGGATAGGTTTTCCATTTTTTCGT
TAAATGATACAAATACATACCTAAATTATCGTATTATTTTTTTGAGTTTCACACCCTAATTATAGTTATTAT
CTATGTGCAAAAACACTTCGCTGAGTAGCTGATGGTATGCATGCTTTATTAGGTGCCAAAGTGGCAGTCC
ATGTGGATAAATATTTTCACTGGAA
>PGSC0003DMG400004195 LRR-RLPs
CTAAAACTCTTAACATAAATTGTTAAATATTTCTAACCTAAGTAGGTATTACCGAAAATCTTTAACACAGG
GTGACATCGAGCTATGGAACATAAACAATTGGTAATTGCTTCACGCTATCTATTGCTCCTTTATTCATGAA
TTCATGTATATACAATGTAATTTTCTGTTAGAGGGGTTCGAACCTTCTGAGCCCATATGGTTCTGCCCCTG
ACATCGCTTATCTAGTTAAGCTCAATAATTTGAATATGTTCTTAAAAATATAATTTTTAGAGGCTTCATTG
GTCAATTTGTTAATAAAACAAGAATATTATAGAATGGGAACAGTGATATCCTCCATAAATGAAACTCAAC
TACACTATTTGGTTAAGACTATGTTTGTGACTATAATAGTGGAGAAATAATAGTAGAAAGGCTTTCCATA
TTATTTGTCATGACATTTTTACTATTTTTTATTGCTTTAATTGTTATGAATTATTTTTCTTGAGCCGAGGGTC
TACCAGAAACAATCATTTATCTACACAGATAGGGATAATGTATGTGTACATACATTATCCTCCTCAGATCC
CACTTTATGAGATTAAACTTCAGTAGTAATATTCTAAGCAATTACAAAAATATTTCCACAAAAACGAAAA
ATATACTAAGGGGAGACATAATATTGTGTTTTATAAAATGATGTTGTAGTAATTTGTCTATTTGTTTACTT
TCTTTAAAATCAACATGGCTTGTGCAGAAACTTGACGACATGAGATGCTTAATGTATCGAGCTACCGAAT
CTATTATTGACATAATCATCCCTTTTTTGGCAGGCCAAAATGGCATCTCATCAATTTATTTGTTTCCTAGCA
ATGATATCTCTTATGATTATCAATATTAGTGCAAACAAGGCATGCCACCCTGATGATCTCAAGGGTCTCA
ATGACTTCAAAGCTAGCATACATTCGGATACTTCTGGCAGACTAAGCAAATGGATAGGCCAAGATTGTT
GCAATTGGCCAGGCATTTCTTGCAACTCTACAACTAATAGAGTTGTAGAAATCTATCTACCTGGTCACTAT
GTATCTGGTGACGATGAATCTCCAAACTTCGTGTCAAGCACAATGAGTGGTTCAATCTCTCCTTCAATTTC
ACTTCTCACTTCACTACAAGTTATTGATCTTAACAAACTAGTCGGACTAACAGGGCAAATTCCTGAGTCCA
TTGGGGTTCTCAAGAATCTCAAAGAACTCAACTTACAAACCAATCAAATCTCTAGTACAATTCCAGAGTC
AGTATTCACATTGACGTCTCTAACAACCTTGAACCTTGAAAATAACCACTTGACAGGGGAAATATCGGAG
AATATTGGCAACTTACAAGCACTTCAAAAGCTTTTCTTGTCAAACAATTCGTTCACTGGAAAAATCCCAGT
TTCAATAACAAAACTACATTCCATTTCCACAATCCACTTAGAAAAAAATCAACTTGTTGGAGAAATACAAT

TGCCTTTAACTCCTTCCCAATGGCCTTTAATTAAAACTTTGGCTCTTGACAACAATGCACTTACTGGAGCT
ATACCTGATTCAATTGGTTATTTGACAACACTTTCATCACTCTCTATATCAAATAATCAATTTACAGGACCT
ATTCCTTCTAGTTTGGGAAACATAAAGCAATTGCAAATATTGAAGGTCAATAACAACAATTTGGCTGTTG
AGATGTTGCCAACGACGATTTGTGGTCTTACTGAGCTAAGTGTTTTGTATGTTTCTCAAAACAAGATTCAT
GGACCATTACCTGGATGTCTCTATTCATTCAAGTACTTACTAGATGTCGATGTACCGTTCAAACGTACTGC
CACTCTTCCTATGGGAGTACACAAATAATAAGTAATAGTGATGGAGCCAGAAATTCTGAAGGAAGTCCA
AAAATGCCTCAATTTGAAATGTTATGTATGTCTACAACAACCAAAAAAAAAAACTTGTGTTTATTTATTATC
TCTATGTTATATGAGACTCCAACTAGCCAGAATCACTGGCTAGTATGAAAAAATTCGAATACATAAAGTA
CTTTCTTTGTTTTATTTTTG
>PGSC0003DMG400004284 LRR-RLPs
AACTTTTTTTCTTTCATTACCTACCATATTTCTTGCAAAATGAGTCTCTTGTTTCTACTTGCATCTTTGTTTCT
TTGTGAACTTGTTTTGTCCTCATTTGCAATATGCCCCAGAGATCAAAGTCTTGCCCTTGTAGAATTCAACC
AGTCTTTTGTTGTTGATGCATCTGCATCTTTTATTTGTGACAAGCAGTCTTATCCTAAAACGAGTTCATGG
AACATGAACAAAGATTGTTGTTCGTGGGATGGAATAATATGTGATGAGATGACCGGTCATGTGATTGAG
CTTGACCTCAGTTGCAGCCAACTTGTTGGGAAGATTGATTCCAATAGCAGCCTATTCCAACTCTCTCATCT
CCAAAGGCTCAATCTTTCTAATAATGACTTTGACGGTTCTCATATCTCGCCTAAATTTGGTAGGTTAGCAA
GCTTGACACATCTTGATCTTTCCCAGGCAGACTTCTCAGGTCAAATTCCTTCTGAAATCTCTCATCTCTCCA
AGTTACAGTCTGTTTTTTTATCGTCTAACAGTGAGCTTAGACTCGTAGCTTACGATTTCAAGATGTCCCTT
CAAAATTTGACTCAATTAAGAGAGCTTCATCTTAGTGGTGTGAACATCTCTTCCACCATTCCTCTGAATTT
CTCTTCTCATTTAACGACGCTGGGGCTGACAAGTACAGGAGTGTATGGGATAATACCTGAGAGTATCTTT
AACCTGCCCAACCTGGAAACACTTGACTTAAGTTCCAACGATCAGCTCAATGGTTATTTTCCAAATACCAA
ATGGAATAGCAGTGCATCTCTCATGGAGCTGAATCTCGCTGGGGTAAATTATTCTGGTAATTTCCTGCCT
GAATGTCTTGGCTATCTAACTTCACTGCAGAGATTGGTACTTAGTTCCTGCAATTTATCGGGGCAGGTTC
CGAAATCTCTTTGGAACCTCACCCATCTGGAGTATATGGACCTTGAAGATAACCGTCTTGAAGGACCGAT
ATTTCCTCAGTTTACAAGTGGACTGCAGGATCTGAACACACTTAAGCTATCAAATAACTCCCTGAATGGA
GAAATACCGTCCTGGATATTCTCCCTTCCATTACTGAGTGAACTAGACTTGAGTAATAACCACTTTTCTGG
TCAACTTAAGGAATTCAGTAACACTTCAGTACTAGTCGGTGTTGATATAAGCGAGAATGAGTTGCAAGG
ATGTCTTCCCAAATCACTTCAGAACCTTGTGAACCTAGTCTGGATTGATTTAGCAAATAACCAGTTGCAAT
GTCCTCTTCCCAAGTCACTCCAGAACCTCAGAAATCTAAAATTACTTGATCTTTTGTCTAACAATTTTAGTG

GGAGTGTAGATGTCAGTGTCTTTTCGAACCTCAAACAACTTTGGTATCTTGATCTGTCATACAATAGTATT

TCTCTGATCAATGAGAACAAAGTCAAGTCTACATTGCCCCAATCTCTTGAATACTTATATTTGGCTGCTTG

TCAAGTAAAAGAACTGGATTTTTTGAGATCAGCAAATAACTTTTATACCTTGGATCTTTCATATAACAACA

TTCAAGGAACAATTCCTGATGTGGTATTGTCTAATTGGATGCATTCCATAAAAAATCTTAATCTAGCCCAC

AACATGTTGACTAGTATTGACCACATTTCATCATTTTCCCAACTGATCTCTATTAACTTATTGTCCAATTCT

CTTCAAGGGACACTACCAATTCCACCACCCTCTATAGAATTCTTCTTCATGTCAAATAATAATGTCAGTGG

TAAGATCCCTTCTTCTATTTGCAATTTGACAACATTAAAAATCCTAGATTTGTCAAATAACAATTTGAAGG

GAGAAATTCCACAATGTTTGGGTAACATGAGTGATCAACTCGAGGTTTTGGATATGCGACGCAATAGTC

TTTCTGGGAGTCTGCAAACAACTTTTAGCTTGGGAAGTAAACTCAAAAGTTTCAACTTGCATGGCAATAA

TCTAGAGGGAAAACTCCCAAGATCCTTGGCTAATTGCAAAGAGTTGGAAGTTCTTGACTTAGGAAACAA

TCAACTCAATGACACATTCCCTATGTGGTTGGGGACTCTTCCAAATCTACAAGTTTTAAGTTTGAGACTCA

ATAATTTGCATGGACCAATTAGAACTTCAACTAGTTCGAAACTGTTTCCTCAGCTTCGGATGTTAGATCTC

TCTCGCAATGCCTTTACAGCAGAACTGCCAACAATTCTTTTCAGAAATCTGAAAGCAATGAGGAGAATTG

CCATTGATAAAACAATGAAGGCACTAGGTGATGAAGAAAAATCATATTACCAAGACTCGGTAACTGTTG

TAACAAAGGGAACGGAGTTTGAAATTGTGAGAATCTTGTCTTTGTACACCACTATGGATCTCTCAAACAA

CAAATTTGAAGGACATATTCCTAGTATGATGGGAGACCTCATTGCACTACGCGTGTTGAATTTATCTCAC

AATGAGTTGCAGGGTCATATACCACCATCATTTGGAAAGTTATCTGTAGTCGAATCATTGGACCTTTCAT

CCAACAGGCTTTCAGGAGAGATACCAAAACAACTTGTTTCTCTTACATCTCTTGCAGTTTTTAATCTCTCTC

ACAATCATCTTGAAGGATGCATTCCAAAAGGGAATCAATTTGATACATTTGAGAACAATTCATATGAAGG

AAATGATGGATTGCGTGGATTCCCAGTTTCAGGAGGTTGTGGAAGTGATCGGATACCGGATACAAACA

ACACAACATTTGTGCCAGATGAAGAAAATGATTCAACATTTCTAAGTGAGCTCAGTTGGAAAGTGGTTCT

TATGGGATATGGTACTGGATTGATTATTGGATTCTCCATAGCATATTTGATGCTTTCTTCTCAAAATCCAA

ATTGGCTTTCTGGGATAGCTGAAGATTTAGAAAACAGAATCATTATGAGAAGGCGAAAGAAGCAGCGA

GGTCAAATGCATTACAGAAGAAGAAGAAGAAATAATGGCATCTAGATGTAAGTTCAAAATTTATGAGA

GATGTAGTCATGTTAAGCTCTATACTGATTCTTTTTGTTTACTGTAACTGGTATTAAAAGAATGAGATTAG

TGTTCTGTATCTGACTCTTTCTTTTGTAACTTTTATGTAGGCAAAGGGTGTATCCAGTTGAAATATCAAGA

AGATGATGCCTGCAACAACAAATATATCTTCTTGGAGGAAATTACTTGAGCAAGAGATGCTTTTGATATT

TCCTGGAGGTTGCTACTCCTTTTTCTTCATAAAAATAAAATAAATTCTAGGATTGAAGTATGTTGCTTTAC

TATAAACAGTTGTCACAATGAAGTGTTTTTGTTTTTCCATCCAAGTGTTAGTAAAGAGAAAAAAGATATT

TGTCTGCAAAATAATAGCTTTCTTTTGACATGTGTATTGACTTGGAATATTACATTTACCTGGAATACAGT
TT
>PGSC0003DMG400004384 LRR-RLPs
CAACCCTCTTTCTCAGGGATGGTCGAAGAGAGGTTCCTCTTCTGAGATCATCCTGAACACAGCAGTCAAC
AAAAAATATTCAAAATAAAATAAAATATTTTTTTTGGGATTTTAATTCTACATTTCATAAAAAATCCCACAT
TTTCCATTGGGTTAAATTTCTTGAATTTTTTTTTCAAGAATTTGATCTCTGTTTGCTTTTTCTGGTAAATCTT
TCATCTTTACCCCCACTTTTTTAATTTTTGTTTGATTTTGCTTGAGCATATGGTTATCTTATGATGGCCTTTT
TGTTTTTCCCAAAAAAGATTCAATTTTTATTAATTCTTGTTGTTTTTATGTCTGCATTTTCAGCTAACAATTG
AATCTTGGAATTTTTGAAGACTTGGGGTTTCTGCAATGATGGAGAAAACAGAGGTAATATTGCCTATATT
ATAGAAATGTTAAGAAAATGAGAGACCCCATGAAGGGGTGGAGCCAGGTAGGGCCGAGGGGTTCAAA
ATTACGTTGTTTATACATAGTTAAAAGTTTTATATATATGTAGTACATGTTGAACCCCTTTGCCTTGTAGA
GGTGTTTACTTCTTTATATTTTGAACGTTGTTAGTGAAAATCCTGGCTCCGCAGTTGACCTCATTGTGCCT
AATATAGATAGATTTTGTTTGTAATCTTTTTTATTTTTTGTGTCTAGTGTAGAGTTGTGCAGATAAGCAAC
TGTGTGTTCGTGTGTCCGAGTGATTTTTGTTACTCGGGGGAGAGTCATTTCATATCAAAAAGCTGGCGAT
TTCGTGTGAGAATTTGTTCAGAATCCTGGCTGTTGGCAAGAAATGAGGCGTAGATGGAACATTTATGGA
ATCAAGTTATTGCTTCTCACCATTTTAATTCTTCTTTTGGAGGTTCATGAATGTTGCACTCTGAATTCTGAA
GGTAGTTACCATTCTCTAGTTTCTTTCTTTTCTTACTTCTGGAGGACCATAGTGTTCGCAATTCGGGCCAG
CTTGCATTAATTCGACTGCTCCACTGTATATCCTCTACCTCCAACCAACATAGGCATCAGTTACTATGCCC
ATCGAGGCTGAGGCATATGATGTGTTAGGCGAACCAAAGTTCTACATTGGTAGCTAAAAAGATTGGAAA
TCTACAATTCTACATAAGGTGTAGGATCTCTTAACATGGTGTGAGGCCTTTTGTGAAAACCGTGCGGGCG
TGCCCCCTAAGCGGACAATATTGCACCATGTTAAGAGTATTTTTGGGTTGACTTAGCCCAACAAGATGAA
GTTACTTACTCCCTACATCCCATTTTATGTGGTCATGTTTTGTTGGACACAAAGTTTAAGAAAGAAAGAAT
TTTGTAGGTTGTAGTCTAAAACAAGTTATAGACATTTAAGTATGGGTGTAATTTGAATATTAAGATTAAA
TTGTTTCTAAAGTCTAAACATGATGTTATTCTTTTTGTGACAAACTAAAAGGAAAATGTGCTGCGAAAATT
GAGACAGAGGGAGTCGAGTTTTTGGTCTTTGCTGGGATATGAACCTTGATCTTTCATGGTTTTTTACTCA
CTTAGGGCTGTGTCTTCTGTCTCCCTGGGCCGTGGGCTATTCTCTAGTTTCTTTAGTCTATTCATGTTATTG
GCTTATTCTTTGTTTCGCTGATTACTGTTGATTTGAGAAACAGGATTGTCGTTGTTTGAATTCAAATTGAA
TGTGTATTCTGATCCAAATGGAGTTCTTGAAAACTGGAATGCTGATCACTGTGACCCATGCATGTGGTCT
GGTGTCGAGTGCATGGATGGTAAAGTGCACATGTTGTAAGTCTCTTGTTATAGATACTCAAGTTTTCTAG

TTAAGATAGTGTCAATAAACTTTCTCTAATGGTTAGTGTTTAGTGATTTGATTCTGGAACCAGTTTGTTGA
ACTAATAATGATAAACCAAATGCAACAGGGTGAAATTGTTTGTTTCGACTCTTGTGATTCTTCTTATGTCA
TTAAATTAGTTGACCTATCTTCTTTTTTTCATTCTGTTCAGAAATCTCCATGGATTTTCTTTGGAAGGAACA
CTAGCATCAGAGCTTGGAAATCTCACTTACTTGAGATCACTGTAAGCTTTAGCTATACTAATATGCCTTTT
CTTTAGGCTCCTGCTCATTAAGTCTTAAATGGTATTTTTTACCTGAACCGTCATCCGTAGAAGGTTGTCAT
AATCTTTGAATAGTCTTCTATCTTCTTATGTAAATTCGAAAGAACGCAAGGGATCGTTTGGTGTATGATG
GACAAGCATAGATAGTCCGGGATAAAAAATTAGTAAGGGCTTATCCCTTGTTTGGTTATTAATCCTGGTA
TAAGTTATATCAGGATTAGTAATTATTATCGGTATAAGTTATCCTCATCAGAGGGTGGAATAATAGTACT
GGATTAGTTATCCTAGGATAAAATAGTGAAAATGACAAATATCCATGAAGTCCCTCAAACTCTTTTTCTA
CTAAATAAGGTGGATGGTGTTTTTGTAAAAGAACTTCTAAGAAATTTATGCAATGCATGTTATTTTTAATA
CAACAAACCAAACAATCACTAAGCAATAATACATTACCAACATAACTAATCCCAACATAACTTGTCTTCAA
ACTAAACGACCCCTAAGAGTTATGTTGGTCAAGTAAATTGGGAGGTAGAGACAAAGGTGGATAAGCTTT
GGATATCTACTAATCAGCTCTCATCATCTTGTATTCTGCATTTACATTGTTCTCCGCATAAGTTGTGTTTGA
ATCTTAACAGTATTTCTTCTTGCTGTCTGGTTGCTTTTACAGAATGCTCTCAAGGAATCATTTTTTCGGTGC
TATTCCTAAAGAATTCGGAAGACTCGATATGCTTGAAGTGCTTGACTTGAGGGATAATAATTTGAGTGG
AACAATTCCAGCAGAAATAGGAGACCTGCAATCACTAAGAATCTTGTGAGTTAATGATTTGTTGTGTGCA
CGATAAATGGTTTGTTGTGTGGATGATAAACGGTTTGTTGTGTGCAGATGTATATATACATATGCATACA
CATTCATTATGCGAAGTGATTTCAAAATGCTCATCAATCGTGTTTATTTCGTTTTTCTAGGTTGATTCATAA
CAATAACTTTGAGGAGAGTGTTCCTTTGGAAATTGGGAAGCTTCATTTAGTCACAGAATTACAATTCGAC
GAAAATCTCACTTCTGCTTTTGCTTCTGGAACTCTCTGTATAAGAAAATTTCGTCATTGGTAGGATTCTTTT
ATTTGTTTTAGTACCCTATCTTCATATTCAGCTGGTTTTTTGCATATCAGATGCTGGGTCAGATGCTGGAT
AGTATATCTTTGTGGCAACACACTATCTAATTGATCTAGCTCATTTCTCAATTTGATATTTTGCATGTCTTA
CAGCATCTGGCAAGGCAGCATGAAGCCATTCAAGACGATAACCTCCATTATTGAACCAATAAAATGTAC
ACTTTTGCATTACCTCAGTTTCTTCCAACTGTAAGTAATTAAATAGTTTTTCACATCACGAGTCATCTAGTT
AAGTTTTATTATTGAGAGACATTTTTTCTATTTCTTTAGGCCATCTCCTGGAACGGGCTTTATGGATGACC
GGAAAGACTGCTCTGACAACCTTCCAAGTGAGTAAACACGTCATATTTTTTAAAATAACTCCGAAGAACC
GTTTACAAATAATAATTCTTCATTGTTTGCAGGGTTATCCAGGCCTCACATCATCCACACTATACAGCACC
AAGAGGAGATTGCACGCCGTAAGTTGGCTGAACAATCCAGTAACCTTGCTGCTGCTCCTGCCAATATCA
ATGGACCGTTGGGTCCTGTCATTCAAATGCCAAGAAGCAGTGGGTCGTTTCGTGCGGTGCCAAATTCCG

ATGGAACACATCCTACAAACTTACCATCACCACCTCCACAGCATGAATCTCAAGACAAATTACATCCTGT
GGGACAGTCTGCAAGTGCTGTAAAACAGTCGCCTGCTGTGAAAAAGCCAACTAATAGCCAAGGCCCCCC
TGCTGGAAAATCGGAAAGTACTTGGAAGTACGTAGTTGGGATTTGCATTGGAGTAATTTTGCTCATTCTT
GCTGCTTCTGTGTTCTTAATCTGCCGAATCAGAGCGGCTAGAACAATTGGCCCTTGGAAGACTGGATTGA
GTGGACAGCTGCAAAAAGCATTTGTTACAGGTACTCACATCATATTGTGCCGAAAAGTGATACTGCTAAT
ATTCTGCTCCCCACCCTGATAGTAGTTGCATTGCTAACAATATTCTTAGCATAGCGTTACCTTATCAAAAA
AAATATTCTTAGCGTAGTGTTGTTATAACAATTTAACTGGAGTAAAGGGAGGTACTGATAATCTTCGTCT
TATGAGGAATTAGAGGACTAATATTCTCAGTGTAGCATTGTTATATACCCTTTTTCACTATATGTTTGCTA
GGACGAGAAGGTTCAAATGGAACTATTTCCTTCTTTCTCATGAGAAGTGAATTAGCTGAGACGTCAATAC
TAACGCTTGAAGGCTGGAATCCTCCCCTTATACTTCTAAAATGTCGCTGATCTTTCCTCATACATTTGCTTT
TGTCTAGCTTATATCCCCCCCTACAAAGTTGCTTTTCAACGACAACCATAGGAATGTGCAATCCTTTATGT
TAATCTATGATATTTTTCGTCTATTTTAATTCTTTTGCCCGTGAAAACATTGTTATAGGGGTTCCAAAACTG
AACAGATCCGAGCTCGAAACAGCATGCGAGGATTTTAGCAACATTATTACCAGCGGCGATTCATATATA
GTATACAAGGGAACACTGTCTAGCGGAGTGGAGATTGCCGTTATCTCAACTACTATAAGTTCTCTGGAA
GATTGGTCCATGCACTCGGAAACAGCCTTCCGGAAGAAGGTTTATCATTTCTTTGCTTTCACAAATTAACC
ACCTCCA
>PGSC0003DMG400004529 LRR-RLPs
TTTTTGGCCTACGTGGCTGAGTCCGTGACTTCACCTATGTTAGGCGCGTGGGTGCAATTTTTAGACAACA
CTGACCAATAAAAATTGACCACGTGTTAAAATAAACTTTATTGTTATTTATTGTTCTTCATCTTTAACTAAG
TTTTCTTCTTCTTTATTGTTCTTCATCTTTAGAAAAAAAAACCTCCATTATCATTATCATTCAAGTTAAATTG
CTCCTTACTTCGAAAAGGTAAAGTAAAATTTTAGTATATTCTTTATATTTACACCTTTTTATTTCGATTGTT
AGTTCTGATTTTAAATTTCTTTCACAATTTTTATGTATAAATGTTAGGGTTATGACAAAATCCGAGTTGAA
TAAGTTGTGTATGGAGCTAGGTGAAGGATGTCCCTTGCTGAGGGATATTGTGTTGTCTCACTGTCGTCA
AATAACAGATGTAGGGTTAAGTTATATTGCTAATAGGTGTACCTTGTTGGAAACTAGCCACATGGTCTAT
TGCCCTGGTATCACCGCTGCTGGAGTAGCCACGGTGATTACAAGCTGCACAAATATAAAGAAAGTTCTT
GTAAAGAAGTGGAAAGTGAGTCCAAGAACCTAAAGGAGAGCAGGATCCATCATCTCCTATCTCTGTGTT
GATCTGTAGATCT
>PGSC0003DMG400004561 LRR-RLPs
CGGAAATAGGAAAAGAAAATGAAGGAGAAATAGAGAAATTAGCAAGCAACTCATTGGTATGTTACTCT

GTCTGTTGTTTCTTGTACCTCGATTAGTATATTGTTTTGTTGTAATTTATGTTCTATTACTATGTGCTATTTT
TGTTATTATTGTCTGTTGTTTCTTGTATATTCATTAGTTCCTTTTTGGGATTGCATGCTTAAGATTGTTTTTG
TTAAGCCGAGGATCTATCAGTAACAATTTATGTACCTTTGAGGTAGGGGTAGGTTTGATGTAAATTGTTT
CTCTTGAGAAGTTGAAAGGAAGGATTTTTTTAAATTCTTTTTAACTACTTTGGCTTGATTAGTGAGAATGG
AAAGAGGAAAAGAAAATGAAGGGCAAACAAAAGGGGAAGATAACAAAATCATTGGTATGTCATACAA
AATTTAATTATTTTTTTCATTCACATAATTACATATTCCTGTAAGTATGTCTGTTTCTTTGTGTAACTTAGAT
GGTGAATATAATCTTTATAACCTATAAGACTCAATGATTTAATCTTTCTGATATAAGTTAAACTCTATGTA
CTAAATCATCTGCTATAGTTGATAAATGTAAGGGATTAAGAACTACGAAAGTAAGAGCTTTTGTTTAGAG
AGATAGCGCGTAAACAATGAGAGAGCTTAGTGCACCAGGCCGTCATTTTTTGGTTTAAGATTTGAGGTG
TAACAGGACAAACATTTTGGCTTCTCTATTAGGCTATTCTGGAGAAAGTCCTCTGCACTTGTGACAGTTG
CTTGTTGTAAAATTTAGAGCTATCACATTTTCGCGAGTTTCACACCTCAACTATATTAGTCGTTCCCTTTTT
CTATCTAAATTATCACCATCTATGTATTAACATAATTTGAAACAACATTTGCTCTTGTGGCAGGTGTCATC
CGTTGTACTTCGCGAGGACATTGTCAATCTTCTAGATTTCATAGAGAGGTTAAATAGTTGGCGTGTTCGA
ATTTCTCTTGACTTGTATCAATATAAAGAGTTGACATTGGAGCTGACGTTTGTGTCCGCATTTTGTCATCT
TTATTACTCTTTCTTTTTGGAAAGCTGTAATGACGAAATGTCCTGCATATCAAATAAGATTCATGATTTGG
TTCAGTCACTGTTTCATCCAAGTGGAGAAGACATGCTGGTTAATATAAAGAATCATGACCTCTTGGAGAA
TATCAAAAGTTATATTAACTCACATTGCTATGCTGAACCAAGTCGTGTAACCATGACCGAGGATCGTTTG
GTTGAACTTTTAGATGCCATCCTCGTGTATCTCCAATGTCTACTCAAGTGTTGTTCTGAATCTATTTTCCCA
TCAATGACTCAATGTGAGCTTCTTCAGAATGTATTTGGCAATTTAAGAGATTTCCATAGGTTGAAAGTAA
ATGGTTGCGTTGAGTATGAAACAATCGAATATGTCTTACCTCATCTTCAACTTATGGCTCTGAGAGTAGT
AAACATCTGTTTTACTCTTTTGAATTGTCAACTGGACAAATTCGATGAATCAGATGTCTCTCTAGTTAACT
CCAAGCTAGCTAATCTACTTGTGGAGGTTATTCCTGTTGAACTGGAGGTTATGCACATATGTTGTACAAA
TTTGAAAGGTTCAAAGTCGGAAGAAGTTGAACGTTTCATCAAGCAGCTCGTAGAAGCCTCTCCGGACAT
TCTTAGAGTATCTCTGATTCATCTACAAGAGCACATGGTTAATGCTGGTACTCCTAGAGCTTCTACGTGTA
ACATTCATGTCATGATAGAGTTCTTATTGATTATTCTTAGTGATGTGCTCAAGGACGTTATTCGTCATGAC
AAATTGTTTGTTTTCTTGGCACGTGTTAGAGAACTGACCAAGGAGGTATTCGTTCTTGTTCGCAACTTAG
AAGAGAATATGAATGAAACAAGTGATGCAAATCTAAACTTGCTGGAAAGTATTGAACTCCTCAAGGAAG
ATCTCAAGAATGATTTCTTGAAAGCCCGTGCAGACTCATCTCGGCTCTGCTTCCCCATGAGTGATGGACC
GCTCTTCATGACTCTTCTGCTCACAAACTTGAAAGACTTGGTCAATTCCAATGCTTCTTCAGTTTCTATGAT

AAAAGAAGAAATTAAGCAGGTGAAACAAGACCTGGAAATCATAAGATCGTTCTTTGGGTATGGTGAGC
AAGAGTTACATAGAGATCTTTGGACTCACGTTCTAGATTTGTCATATGAGGCGGAACATGCCATTAACTC
AATTCTTGCCCGAGATCATGGTCTATTACAGCTTATCTTCATACTTCCTGATACCGTAGAAAAGATCAAGC
TTGTCAAAAAAGAGGTACAAGAGAAGATCCCCAAGAGCAGCGGTATTATTGTTGCGAACGCTCCCAACA
AGCCGGTTGAAAGAAACTCATCGAGTACAGTTGGTAAAATAATTGTAGGTTTTGAGGAGGAGACAGAG
TGGATAATTAGGAAGCTCACCAGCGGACCAGCTGAGATAGATGTCATTTCCATAGTCGGCATGCCAGGG
CTTGGAAAAACTACTTTGGCATACAGAGTGTATAATGATAAGTCCATTGTTGATCATTTCGACGTTTGTG
CTTGGTGCACAATCGACCAAGAACGTAATGAGAAAAAGTTGTTGCAGAAAATTTTCAATCAAGTTATTG
GTTTGAAAGAAAGATTCAGTGAGAAGGACATAGATGATGACGTTGCTGATAAGCTGCGGAAACAACTA
TTTGGAAAACGGTACCTTATTGTCTTGGATGACATGTGGGATACTGNNNNNNNNNGATCAGACGAAAG
TTTGGAGTTATTAGAGAAAAAGGTATTCGGAGAAGAACGTTGCCCTGATGAACTAAAGGATGTTGGAG
AAAAGATAGCTCGAAAGTGTGATGGGCTTCCTTTAGTACTTGATCTAATCGGTGGAGTCATTTCAAGGA
AGGAAAAGAAAGAGGCTTTGTGGCTTGAAGTTCTCGATAATTTGAGTTCCTTTATTTTTAAGGATGAAGA
GGAAGTGATGAAGGTTATACAATTAAGTTATGACCATTTGTCAGATCACGTAAAGCCGTGTTTGGTTTAC
CTTGCAAGTTATCCAAAGGACAAAGATATAATGATCTCCGAGTTGAAAGATTTATGGATTGCTCAAGGA
CTTGAGATGAAAAATGCAGAAGAAGTAGTGGATGTGTTAATTTCCAGTAGTTTGGTAATACCTTTCAATA
ATTCTATTTGCAAAATTCATGATCTTGTGCATGACTTTTGTTATATAAAATCTAGAAAGGAAAAGTTGTTT
CACTTCATAGGAGGTTCAAACGCTCCGTCTTCTTCTTCAAATCTGATTCCGCGTGGAATTACCATTCATTA
TAATGAAGGTCTCTTTCATATGGATAGAAATTTTGTGGTGTTTAATCCAGAAAAGAAGAATCCTTATGTT
AAACACCTCCTCTCTCTGAAGGTGTGTGATGGGGTTAAGAATCGTCTTTCCTACAAGAGTCACCTAAAAC
AGTTGAGGCTTCTCAAAAGTTTGGATTTGAAGGGCATAACATTGACAGATTCTTTACTGAATGAAATCGG
TATGCTCGTTCATTTGAAGTACTTAATCATTCAGACGAAGGCTAAAGCTCTCCCTCCGTCATTTTCAAACC
TCTGCAATCTAGAAACTCTGGTGGTGGATAACCTGGAGGGATCATGCATGATACTATCGCCTTGCTTTTG
GAGTCTTGCAAAGCTACGAGATGTGCGGATGAAGTGTTGTGCTATCTTTGATCCAACTGTGTTAGACGA
GGAGTCAAGGTTAGAAAATTTTAGAACATTATGTGGTCTCTACCTTCCCGGTTTAGAAGAGACGGAGGA
TATTTTCAAAAGGTTTCCTAATCTTGAAGCCGTTAGATTCAGGATCAAACAATTACCAGATCGGTCAGGA
GAGAAGATTTGTATTCCAAGATTGGATGTCCTTAACGAACTTGAACAACTCCATTTAACTGCTTCTAGGG
ATTCATTGAGTGAATATACACATGGCTTCCCTTTGAGCTTGAAGAAGGTGATGTTGAAAGGGCTTGCTCT
GACATCCGATTCATTGTCAAGAATTGGTAGACTACCCAACCTTCAAAAACTAAGTCTAAAAGACGCAATC

ATCGAGGAGGGGAAAGAATGGAACATGGAAGATGTCACCTTCAAGAATCTCAAATCTCTAAAATTGATT
AATGTGTCATTTTCTGAATGGCAGGTTGATGCAGAGAAATCCTTTCCCGTGCTTGAGAAACTAGATATAA
CTATATGTGATATGCTTATGAACATACCAGACAGTTTTGGAGATATTGCGTCATTAAAGTTTATCCAAGT
ATGGTTCAGCCCTCAGCTTGAAGAGTCGATCTTCAAAATTAAGGAATATGTTGAAGAAATGACTGGAGA
AGACAAGCTTGAGGTAGAATTCTATGCTTGAGGTATGTTGAAGAATATGTTGAACTTTTACAAATGTTAT
GTGTTTTCCAATATATTACTTTTAACTTATATGTTCTTTGCGATCAGATCGAGCTAGCTAGCGCATACAAA
TGATGAACCGATAGATTCTCAAGACAACTTTCTCCATAGATTTGTGCCTAATTAAGAGCTCCCCTTGCTGC
AGCACCTCCCATTATGAACACATTTTTGGGGCTTGCTTTCAAAATGGAGACATGACGTGGTGGATTGGTA
AAATCCTGTTTAGCCCATTATTATGTTCAACTAAAGTAAACTAAATTCATGTCATTATTTTTCTGTTTCTCC
AATTTGCTCTCCTTGTGAAAATCTTCTGGTGTGCTTGTCCTCTCGCCATGACTGACAAACACATTTGCTTTC
TTAAGTTCCAGTTATGCTAAGCAGAAATGTCGTCTCGTTGTGAGTTGTCAATCCGACTTTGAAGTAGAGA
TGGTTAAAGGTATAGAGGAAGCAGAAGAGGATTATGTTTGTATAGAAACAACAAGGAGGCCCCTGAAT
AAAGCTTAGTGAAGAAGAGTTCATTGCATGCCTTGTATTAGTTTTCGACATTTCTGAAAAAATTGCTTCA
GACATGGCCTCATTTGCTTTGTCTCTTGTTGTCATATTATTCTTGCAATTCTGCTTCGGAAACATTAAAAAT
AACCCCATAAAGAAGGTAGTAAGGTTAGCGTTCATCCTACCTTCCCCAGATCCCACCTGTGGGATTAGAG
AGTAAAGTTCTCG
>PGSC0003DMG400004722 LRR-RLPs
ATGCATTCAGAGGAAATGACTAAGATGGATGTTGATGGCACTACGGGAATTCACTTTGAAGAAGATATA
ATCACGGAAATCCTAAGTAGGTTACCTGTCAAGTCTCTTTTTCGATTCAAATGTGTTATAAGGTCTTGGAT
GACATTGATCTCTAAACCTTACTTTGTGCAAACGCATCACAATCATTCCAAGAATAACCAAAATTCCCAAA
AAAATTGTCTTCAAGAAAATGCTTACTGTAGATACCTCATTAGTTTGGTGTAGTTACTCTTACCACACACG
AGTTCATGGAACATGAGCAGAGATTGCTGCTCATGGGATGGAGTCGTATGCAATGACATGACTGGCCAT
GTCACTGAACTTGACGTCAGTCTATTCAGGGACGAGAGCTTGAACTTGTCAAAATTTTGACCATTCACAC
CACTATTGTTCTTTCAAGTAACAGATTTGAAGGACTTATTCCAAGTATCATGAGAGAACTCATTGCTCTTC
GGGTGCTGAATTTGTCTCATAATGAATTGCAAGGTAATATACCATCATCACTTGGAAGTTTATGTGTGGT
CGAATCATTGGACCTTTCATCCAACCATCTTTCAGGAGAGATACCACAACAACTGGCTTCTCTAACGACA
CTTGAATTCTTAAATCTCTCCCACAATCATCTCGATGGATTCATCCCTCAAGGACCTCAATTCGCTACATTA
AAGAAATATTCATATGAAGGCAATAATGGATTACATGGATTTATGCATTGCCAAATTCATCATTGCATAT
GTTGGAGGAATATCAGGGGTCATAAAGTTTTATGAGCTTAAGTTGGCTCGAACTTCATGATTATTACCCG

TTGACATACTAATGACAAGGTGATATATAGAGTTGAAACGAGTTAGATATTTTTTTCTTTTTGTTTTTCAG
TAATCACCTCTTATTTGTTTTACTGTCATAAATTCAACTTTCTGATGCAATATAGTTGAAAGTAGTACCTAT
TATTTTGAAAGTACATAAATTCTGCTTATATGGATGCAGCTAAAGTATTTATGTACAACTATACTTCCTTTC
ATGATTCATAAATGTACACAACAACCTCATGACCCACTGCAATAGATTACGAAGTACCTTCACTTCAAATT
GAATGAGTTGTAGCTTTCATTTGCAAGACGCGTGACTTTTCTGTTACATAACAAATAAGGTGGCTTTCTA
AGATAATTATCAATAATTGAGTGACAATATGAGAAATCAACTCTTGATGTCAGCTATATGAAATCTCAGT
AGTCATATAGCTTTACCTAAACTCATCTCGATCTAGTACAAAGTATATTAGAGATATAATGTAGTCTAGA
ACTATTGTTCTATGACATAATTGTTGGATTGGTCCACTAATTTAAAGGTGAGATTGAGAACCAAACATGT
CCGCATCATGGCTGATAACACAACAAAACTTTTTGGCCACTTGGTTAAGTCCATAGCTGAATTACATATG
TAACCCGTGGATAAAAATAAAAAGCAAAAAGACAAGAACAGAACAACCAAAATTTGCATTGAGAATGA
GAACACTTTCTCTGATAAATATGAACAGACAAAGAAGTGTGAATAAGAGAGACAGAAGCCGAATTGGC
AACATTTGGTGGCAGTCAAACATGTTTCGCTTTGATCTTTAATATTATGCACTATATAAAATTCTACGAAC
TCTCTACAATTAGTTCATTGAAACGCAAAATATGATGATGGTTCCCAGCAAAATATCCTCTCTACTTCAGT
TTTTCACTCTTTTATATCTCTTTACAGTTACTTTTGCTTCCACTGAGGAAACAACTGCCCTCTTTAAATGGA
AAGCGACTTTCAAGAACAAAAATAATTCCTTATTGGCATCATGGACACGAAGTTCTGATGCATGCAGTG
ATTGGTATGGAGTTATATGCATTAATGGTAGGGTAAACATGTTGAATATTACAAATGCTAGTATCTTTGG
TACACTCTATGCTTTTCCGTTTTCATCCCTCCCTTTTCTCGAGTATCTTAATCTTAGCAGGAACAATTTATTT
GGCACCATCCCACCTGAGATTGGTTATCTAACTAATCTTGTCTATCTTGACTTGCATATCAATCACATTTCA
GGCTCAATCCCACCACAAATTGGCTCACTAGCCAAACTTCAGATCATCCGTTTATTTGACAATCATTTAAC
TGGTCCTATTCCAGAAACAATAGGTTACCTAAGGTCTCTTACTAAGCTATCGTTGAGTATTAACTCTCTTA
CTGGTTCTATTCCTGCTTCATTGGGAAACTTGACCAACTTGTCTTTTTTGTTTCTTCATGTTAATCACCTTTC
TGGATCCATTCCTGAAGAAATAGGTTACCTAAGGTCTCTTACTCGTCTAGATTTGAGGAATAACTTTCTTA
TTGGTTCTATTCCTACTTCCCTTGGCAACTTGAAAAATTTGCAAGTTCTGCTTCTCAATGACAACAATCTAA
TTGAGGAAATTCCTCCATCTATTTGCAATTTGACATCACTGGCAATCCTGTATTTGTCGAGAAACAACTTG
AAAGGAAAAATTCTGCAATGCTTAGGTAATATCAGTACCCTCCAGTTTGTGATGATGTCACATAATAATC
TCAGTGGAGAACTCCCTTTGTCTATTTGCAATCTAACATCACTACAAGTTCTAAATTTGGGTAGAAACAAT
CTGAATGGAGCAATTCCACAATGTTTTGGCAACATGAGTGGTCATCTTGAGATTCTTGATATGCAGCACA
ACAATCTTTCTGGTACTTTTCAAAGCACTTTTAGCATTGGAAGAGCACTCAGAAGCTTCAATTTGCATGGC
AATAAGCTAGAGGGGAAAATTCCACGATCCTTGGCAAATTGCCAACGATTGGAAGTTCTTGATCTAGGA

GCTAATCTCCTCAATGACACATTCCCGATGTGGTTGGGGACTCTGCCAGAGCTACAAGTGTTAAGCTTGA
GATCCAACAAATTGCATGGACCCATCAGAACTTCAAAGTTCATGAAGAACTTGTTTCCTAAGCTTCGAAT
CATAGACCTCGCTAGCAATGCATTTACAGCAGAGTTACCTGCAAATCTTTTTCAAAATTTCGAAGCCATG
AAGAGAGTTGATCAAACTGTGGAGGATTCAAGTAATAAAGAAGGCGGTTATTACCAAGACTCCGTAACT
GTCGTAACAAAGGGACTGGAGCTTGAAGTTGATTCAATCTTGTCTTTGTACATCACAATTGATTTTTCAA
ATAACAGATTTGAAGGACGCATTCCAAGTATTATGGGAAATCTCATTGCACTTCACATGTTGAATTTATCT
CATAATGGATTGCAAGGTCATATACCGCCATCACTCGGAAAATTATCTTCAGTTGAATCATTGGACCTAT
CAGCTAATCATCTTGAAGGAGAGATACCTAAACAACTTACATCTCTTACATTTCTTGAATTCTTGAATCTC
TCTCACAATCAGCTCCAAGGATGCATCCCAACAGGACGCCAATTTGATACATTCGAGAAGAACTCGTATG
AAGGTAATGATAGGTTACGTGGATTTCCTAATTTGGAAGGTTGTGGAAACAGCAGGTTTCCCAAGACAA
ACAACACGACACATGAGCTGGAACAAGAAAGTGATTCTACATTTCTGAGTGAACTCAACTGGAAAGTGG
TTCTTATGGGATATGTGTGTGGACTTATTATTGGATTCTCCATAACATATTTAATGCTTTCAGCTAAAAAT
CCCGATTGGCTTTCTAGGATTGCTGATGAACTAGAATACAGAATCCTTATGAGAAGGCGAAAGTAG
>PGSC0003DMG400004725 LRR-RLPs
ATGGGGTCTGGCCAATTCTCATTTCTGCTCTTTCTCTGTTTGCTTTCTTTTTCTTCATCCATACCTCATCCAT
GCCGCAAAGATCAAAGTATTTCCCTTCTAAAATTCAAGAAAACGTTTACTGTAAATTCCACAGCCTCTGAT
TCTAATGTCTGTGAAATATTTTATGGTCAGAAGCCATATCCAAAAACGAGTTCATGGAACATGAGTATAG
ATTGTTGTTCATGGGATGGAGTCATATGTGATGAGATGACTGGCCATGTCATTGAACTTGACCTCGGCT
GCAGCTGTCTCGTAGGAAAGCTTGATTCCAATAGCAGCCTTTTCCAACTCTCTCATCTCCAAAGGCTCAAC
CTTTCTGTTAATAACTTCTCTCGTGTGGATTCAATTCCTCTTCAGTCTCTAGATACTATTGATTTGCGGTCC
AATTTGCTTCAAGGTTCACTACCTATTCCACCAATTTCCACGAGATTCTTCCTCATAGCCCATAATAATCTC
ACTGAAGAAATTCCTTCACATATTTGCAATTTGAAATCATTGGTAATGCTAGATTTGGCGAGAAACAACT
TGAAGGGAGCAATTCCGCAATGTTTGGGTAATATCAGAAGCCTCAAAATTCTAGATATGCACAACAACA
AACTTTCTGGGACTCTTCCAACAACTTTCAGCAATGGAAGTTCACTTAGAAAACTCAACTTGCGTGGAAA
TAAACTAGAGGGGAAAATTCCCCGATCTTTGGCCAATTGCAAAGTGTTGCAAGTTCTTGATCTAGGAGA
TAATCATATCATTGACACATTCCCTATGTGGGTTGGAACTCTTCCAAAGCTTCATGTTTTAAGCTTGAGAT
TGAATAAGTTGCATGGACCCATTAGAACTTTAAAAAATGAAAACATGTTTCCTGATCTTCGAATTATAGA
TCTCTCTTACAATGCCTTCACAGGAAATTTACCTACGAGTCTGTTTCGACATCTGAAAACCATGAGAACAA
TTGATCCATCAAAGAAGAAACTAACATACCTTGAAGACTATTATTACCAAGATTCTATTATAGTCATAAGC

AAGGGATATGAGATTGAACTTATCAAAATTTTGACTGTTTACGCCTCTATTGATCTTTCAAGTAACAAATT
TGAAGGACATATTCCAAGTATCATGGGAGAACTTACTGCACTTAAGGCGCTGAATTTCTCTCATAATAGA
TTGCAAGGACGTATACCATCATCACTTGGAAATTTATCTTTGGTTGGATCATTGGACTTATCAGTTAACCA
GCTTTCGGGAGAGATACCAAAACAACTTGCTTCTCTTAAGTATCTTGAATACTTCTTCACATCAGAAAATA
ATCTCACTGAAGAAATTCCTTCATCTATTTGCAATTTGACATCACTGGTAATACTAGATTTGGCGAGAAAC
AATTTGAAGGGAGTAATTCCTCAATGTTTGGGTAATATTACTGGCCTCGTGGTTCTTGATATGCACAACA
ACAATCTTTCTGGGACTCTTCCAACAATTTTCAGCAATGGGAGATCACTGAGGAGCCTCAACTTGCATGG
CAATAAACTAGAGGGGAATATTCCCCGATCTTTGTCCAATTGCAAAGCGTTGCAGGTTCTTGATTTAGGA
CATAATCATCTCAACGACACATTCCCCATGTGGTTGGGAACTCTTCCAGATCTACAAGTTCTAAGCTTGA
GATCCAATAAATTGTATGGGTCCATTCCACCGTCAAGTGTTGGAAGTATGTTTCCTAAGCTTCGAATCAT
AGATCTCTCTTACAATGCCTTCTCAGGAAACCTACCTACGAGTTTGTTTCAACATCTGAAAACCATGAGG
ACAATTGATCCATCCAAGACGGCACTAATTGATGGTTATTATTACCAAGACTCAGTAGTTGTAGTTACAA
AGGGATTGGAGCTTGAAGTTGAGAGAATCTTGTTTTTGTACACCACTATGGATCTTTCAAACAACAATTT
TGAAGGACATATTCCTGATGTTATAGGAGATCTCATTGCGCTTCGGATGCTTAATTTTTCTCATAACAGAT
TGCAAGGTCATATTCCATCATCACTTGGAAATTTATTTGTAGTCGAATCATTGAACCTTTCATTTAACCAG
CTTTCGGGAGAGATACCACAACAACTCGCTTCTCTTACATCTCTTGCGGTCTTAAATCTCTCCCACAATCA
TCTACAAGGATGCATCCCAAAAGGACCTCAATTCAATACATTTGAGATCAATTCATATGAAGGTAACGAT
GGATTATGTGGATACCCTTTTCCTCAAGGTTGTGGAAGTAGCAGGATGCCAGAGACAGAAAACACAGCA
TATGTTCTGGATGAAGAAAGCAATTCTACATTTCTGAGTGAATTTTGGAATGGGATTCTCATGGGATATG
GAAGTGGACTTATTATTGGATTCTCCATAGCATCTTTCATGCTTTCGAGTCGAAATTCCAATTGGCTTTCT
AGGATTGCTGAAGAACTTGAATACAGAATCGTTATGAGAAGGCGAAAGAAGAAGCAAGGCCATCACAG
AAGATGA
>PGSC0003DMG400004727 LRR-RLPs
CCAAGTTACACTCTCTTCGTATCTCCACATTTTTTAACTTTTACACTAAGGAGCTAAGACTCACATCCCATG
ATTTTAAATCGCTTCTTCAGAACTTGACCCAATTAAGAGAGCTTGATCTTACTCAAGTAAACATCTCTTCC
ACCATTCCTCTGAATTTTTCTTCTCATTTCACGACTCTGAGGCTGTCAGAAACAGGATTGTATGGGATAAT
ACCTGAGAGTATATTTCACCTGCCCAACTTGGAAATACTTGACTTAAGTTACAATGTTCATCTCAATGGTT
ATTTTCCAAAGGCCAAATGGAACAGCAGTGCATCTCTCATAGAGTTACATCTCAGTATGGTGAATTTTTC
TGATAATTTTCCAGAGTCTATTGGCTATCATCTAACTTCGCTGCGTTATTTGTCTCTTGTAAGTTGCAACCT

TAGAGGGCCTATTCCTGAATCTCTTTCAAATCTCACCCGCATTGAGTATTTGGTCCTTTCATATAACTCCCT
GAATGGAACAATACCATCAGGGATGTTATCCCTTCCATCACTAAATACAATAGTCTTGAGTAATAATCAC
TTATCTGGTCCCCTTGAAGATTTAAAGTCCAATTCACTAATCTCGATATATTTAGAGGGCAACCAGCTGCA
GGGTCATCTTCCCCACTCAATTCAAAACCTTGTGAACCTAACAGGCCTTATCCTTTCTTTCAATAATTTTAG
TGGTCGTGTGGATGTCAGCTTCTTTTCAAACCTTAAACAGCTTCGGTATCTTAGTCTTTCATATAATAATA
TCTCATTGACCAATGACAACAACGTTACTTTGCCCGAATCTCTTGATACGTTAGAATTGGCCGCATGTGA
AGTGAAAGAGTTGGAGTTTCTAAGATCCGCAAAGCTTCTTCGGCATTTGGATCTTTCTTTCAATAATTTTA
GTGGTCGCGTGGATGTCAGCCTCTTTTCAAACCTTAAACAGCTTGTGGAACTTCGTCTTTCATATAATAAT
ATCTCATTGACCAATGACAACAACGTTACTTTGCCCGAATCTCTTGATACGTTAGAATTGGCCGCATGTG
AAGTGAAAGAGTTGGAGTTTCTAAGATCCGCAAAGAAGCTTTGGCAGTTGGATCTTTCAAATATAAGAT
TCAAGGAAGAATTCCTGATTGGGCATGGTCTAACTGGATGTTTCATTGCAAGATCTTAATATATCCCACA
ATATGCTGACGAGTGTGGATTCAATTCCTCTTCAGTTTGCAGATACTATTGATTTGCGGTCCAATTTGCTT
CAAGGATCACTACCCATTCCATCAAATTCCACTACATTCTTCTTCATATCTAATAATAGTCTCACTGGAGA
AATTCCTTCATCTATTTGCAATTTGACAACACTGGTAATGCTTGATTTGGCGACAAACAATTTGAAGGGA
GCAATTCCGCAATGTTTGGGTAATATTACTGCCCTCCAGGTTTTGGATATGCGCCACAACAATCTTTCTGG
GAATATTCCAACAACTTTCAGCAATGGAAGTTCACTGAGCAGCCTCAACTTGCATGGCAACAAACTAGA
GGGGAAAATCCCACAATCCTTGACCAATTGCAAACAACTTGAAGTTCTTGATTTAGGAGACAATCACCTC
AACGACACATTCCCCGTGTGGTTGGGGACTCTACCAAAGCTGAAAGTTCTAAGCGTGCGATTCAACAAA
TTGCATGGTCCCATCAGAACATCAAGGATTGAGAACATGTTTCCAGAGCTTCGAATCATAGATCTCTCTT
ACAATGCCTTCTCAGGAAACTTACCTTCGAGTTTGTTTCAACATCTGAAAGCCATGAGGACAATTGATCT
ATCATTGGAAGCATCAAGATATGGTGCAGATCGATATTACCAAGACTCGATAGCTGTCGTATCCAAGGG
ATATGAGCGTGAAGTTGTGAGAATCTTGTTTTTGTACACCACTATCGATCTTTCAAAGAACAAATTTGAA
GGACATATTCCAACTATTATGGGAGATCTCATTGCACTCCGTGTGCTGAATATATCTCATAATGGATTGG
AAGGTCATATACCGTCATCACTTGGAAGTTTATCTTTGGTTGAATCACTGGACCTATCAAGTAACCATCTT
GTAGGAGAGATACCAGCACAATTTGCTTCTCTTACTTCTCTTGCAGTCTTAAATCTTTCCTACAATCATCTC
GAAGGGTGCATTCCTCATGGAAATCAATTTCAGACCTTTGAGAACAATTCATATGAAGGCAATGATGGA
TTACGTGGTTTTCCAGTTTCAATAAGTTGTGGCAATGATCATGTATCAGGGAAAAATTATGCCGTAGATG
ATGAAGAAAGCAATTCTGAATTTCTGAGTGATTTTCGGAAAGCTGCTCTTATGGGGTATGGAAGTGGAC
TATGTATTGGAATATCCATAGTATATTTCATGATGTCAACTGGAAAACCGATATGGTTTGCAAGAATCAT

TGTAGAGCTGGAGCACAAAATAATGATGGGAAGGAGAAAGAAGCAGCGAAGTCAAAGGAATAACAGA
AGAAGAAATAATCACTAATCATATTACTGTTATTTCTTTAAGGAAAAGATGTAATAAGTTGTAGACGCAT
TATCGTTAAATCCTGTTTGCATAAAGTGTGTAGCATCATTTGGCCATGAAAATTGAGGGTGAG
>PGSC0003DMG400005171 LRR-RLPs
ATGAGAAAGCTAAGGCAAGTGGATATATACAATTTGTCCTTGGTATGGGAAGAGAATGATCGAGCAATT
TTTGATGAATCTCCAACAACTATGCTAGTGAACTTGAAGACATTCCGCACTTATGTTATACACGTGGATA
ATAGTACCCCAAGGTTCTGGTGGAGGTTTCCAAATATTGAAGAACTCAGCTGCCTCCGTATTGAAGCTTT
ACCTAGTTGTCCTTTGTTTCCCTCAGTGGAAGTTCATATTCGGCTTCAATCTCTTAATCTGGTTATAAGCCC
TCGGGGATTATTGGATTCAGTTGGTTGGGAGAGCTACTTTGTCTTCCCTTCAAATCTTAGGCATTTGTGC
ATTGGTGGTTGTTTCCTAACGAAAGAAATGACTTTAAACATTGCAAGATTGAAGAAGCTTGAGAGTCTC
ACATTAAAGGGAGGATTTCCTTGGGGGGAAATAAAGTACCATTGCTGGGATGTTACAAATGTGGAGTTC
CCTGCACTCAAATACTTGGCATTACACTTTATGAGAATGGAAGAATGGAAAGCTTCAGAGGAATCCTTTC
CTGTGCTTGAGAAGTTAGTTATAAGAGAATGTCACTACTTCAAGGAGATCCCTATTAGCTTTGCCGATAT
TCCAACACTGCGACTTATTGTGCTGTTTGAGTGTACTGATTCACTTGTGGCTTCAGCTGTGAACATAAAA
AATGAAATAGAAGAAAACACTGGATGTGATAGTCTCCAACTCGGGGTAACAAATTCGTATAAATAA
>PGSC0003DMG400005172 LRR-RLPs
ATGGAAGTTGTTGAGACTTGTGGAAAAGGAACTCTTTCTGCTGGAGAGCCTTCCACCAAACGTAGTATT
ACATCAATTTATGATGATGAAGAAGTAGTGGGCTTTGAGAAAGATGCAGAAATTATAATGAAGAAATTG
ATACGAGGAACAAAGGAACGGGATGTTATCTCCATCTATGGAATGCCTGGACTTGGTAAAACAACTTTG
GCAAGGAAGGTGTACAACAACCCCACTACTGTTAATTACTTTGATGTTAAAGCATGGTGTGCTGTGTCGC
AAACATATAATAGGAGGGGATTATTGGTTGAGATTTTCAAACAAGCAACAAGTAATGAGAATGAAATCA
AAGAGGATGTTGACATAGCTGACATGTTGCGCAAGAGTCTAATAGGCAGAAGATACCTCATTGTATTAG
ATGATATTTGGGATGTCGAGGCATGGGAAGATTTGGGAATATGTTTCCCTGAAGGTGAATATGGAAGC
AGAGTAATGGTCACAACACGAATTGAACAAGTGGCTAAGCATCTTCAGGTCCACAGTGTTCCTTATTCTC
TTAGCTTTCTAACGTCCGAAGAGAGTTGGGAATTATTGGAGAAGAAGGTATTTCGAGGAGAGAGTTGTC
CCCCGGATCTACTGGAAGCAGGGTTACAAGTTGCCCTACACTGTAAGGGATTGCCTCTTGTGGTTGTNN
NNNNNNNNNNNNNNNNNNNNNNNNNNNNNNNNNNNNNNNNNNNNNNNNNNNNNNNNNNNNNNNNNN
NNNNNNNNNNNNNNNNNNNNNNNNNNNNNNNNNNNNNNNNNNNNNNNNNNNNNNNNNNNNNNNNNN
NNNNNNNNNNNNNNNNNNNNNNNNNNNNNNNNNNNNNNNNNNNNNNNNNNNNNNNNNNNNNNNNNN

NNNNNNNNNNNNNNNNNNNNNNNNNNNNNNNNNNNNNNNNNNNNNNNNNNNNNNNNNNNNNNNNNNNN
NNNNNNNNNNNNNNNNNNNNNNNNNNNNNNNNNNNNNNNNNNNNNNNNNNNNNNNNNNNNNNNNNNNN
NNNNNNNNNNNNNNNNNNNNNNNNNNNNNNNNNNNNNNNNNNNNNNNNNNNNNNNNNNNNNNNNNNNN
NNNNNNNNNNNNNNNNNNNNNNNNNNNNNNNNNNNNNNNNNNNNNNNNNNNNNNNNNNNNNNNNNNNN
NNNNNNNNNNNNNNNNNNNNNNNNNNNNNNNNNNNNNNNNNNNNNNNNNNNNNNNNNNNNNNNNNNNN
NNNNNNNNNNNNNNNNNNNNNNNNNNNNNNNNNNNNNNNNNNNNNNNNNNNNNNNNNNNNNNNNNNNN
NNNNNNNNNNNNNNNNNNNNNNNNNNNNNNNNNNNNNNNNNNNNNNNNNNNNNNNNNNNNNNNNNNNN
NNNNNNNNNNNNNNNNNNNNNNNNNNNNNNNNNNNNNNNNNNNNNNNNNNNNNNNNNNNNNNNNNNNN
NNNNNNNNNNNNNNNNNNNNNNNNNNNNNNNNNNNNNNNNNNNNNNNNNNNNNNNNNNNNNNNNNNNN
NNNNNNNNNNNNNNNNNNNNNNNNNNNNNNNNNNNNNNNNNNNNNNNNNNNNNNNNNNNNNNNNNNNN
NNNNNNNNNNNNNNNNNNNNNNNNNNNNNNNNNNNNNNNNNNNNNNNNNNNNNNNNNNGATATACGCTT
GGATAATATGAATGCAAGGTTGTGGTGGAGGTTTCCGAATCTTGAAGAACTCGGCCTCCACGTTAAATA
TAAACCTAAGTTTCCTTTGTTTCCCATACCAGAAGTTCATACTCGCCTTCAGTCTGTTTCTCTGACGTTCTC
ATTAAATAGAGTCTTCAAGTCAGTTGGATGGGAGAGGAATTTTGTGTTCCCTTCAAATCTTAGGCATTTG
AACCTTCGCGGCTGTTTCTTGACGGAAGAAATGGTTTTAAACATTGCAAGATTGAAGAAGCTTGAGAGT
CTCAAATTACACATGGGATTTCCTTTTATGAGATTACAGAGGCTTCGGAACTGTTGCTGGAATGTCACAA
ATGTGGAGTTTCCTGCACTCAAATACTTGGGATTATTCTTTGTGCAGATAGAAGAATGGAAAGCTTCAGA
GGAATCCTTTCCTGTGCTTGAGAAGCTAGTTATAAGTGGATGTTCCGATTTCAAGGAGATCCCCCCAAGC
TTTGCGGATATTCCAACACTAAGACGTATTGAACTGACTAACTGCATCGACTCTCTAGGGGTTTCAGCTA
TGAATATCAGAAGAGACATTGAAGAAAACACAGGATGTGACAGTCTCCATGTTCTAATTGTAAACCGAA
TATAAATGTATTTAGAAGCAATTCAAAGTTGCTCTATTTCCACTTTTGTATTGTCACATTATCTGTTTTTTT
TTATTAGGAAACTTTTTAACTTGGAAGCTGAGATTGCAATTCAACTTTGCA
>PGSC0003DMG400005226 LRR-RLPs
CTTGATTTCTTCTCTTCCAACATAATTTTAATACTTTTAGTACAAAAATGGGTTATCAAACACTTGCATTGT
TTCTTTTATGTTCCTTTCTATGTCAACTTGTTTTCTCCTCATTCTTACCTCATTTGTGCCCGAGTGATCAAGC
CCTTGCTCTCCTACAATTCAAGCATATGTTTGCCATTAATCCTAGTGCTTCTCATGATTGTTTCGACATAAC
AGGCGAATGGATGCAGTCATACTCGAAAACATTGTCATGGAACAAGAGCACAGATTGTTGCTCATGGG
ATGGAGTTCATTGTGACGAGACAACGGGAAAGGTGATTGAGCTTAACCTCACTTGTAGCAATCTTCAAG
GCAAATTTGATTCCAACACTAGTCTCTTTCAACTCTCCAATCTCAAAAGGCTTGATTTGTCTTCGAATAATT

TTTCAGGATCGCTCATTTCACCTAAATTTGGTGAGTTTTCTAATTTGACGCATCTTGATTTGTCACATTCAG
GTTTTAGATTCGGACCTCACAATTTAGAACTTCTCCTTAAGAACTTAACCCAATTAAGAGAGCTTGACCTT
TACGGTATGAGCATCTCTTCCACCTTTCCTCTGAATTTCTCTTCTTATTTAACAACTCTACGACTTAGTGAC
ACACTATTATGGGGGATACTGCCCGAAAGTGTTTTTCACCTTTCAAACCTGGAAACACTTGATTTATCATA
CAATCCCCAACTCACAATTAGGTTTCCCACGATCAAATGGAATAGCAGTACATCTCTCATGTACTTATCTC
TACCTGAAGTGAATTTTATTGGTAAGATACCCGAATCATTTAGCTATCTAACTTCATTGCATTACTTGGAT
ATGAGATCTTGTAACCTCTCAGGTCCTATTATTAAACCTCTTTGGAATCTCTCCCACATACAAAGTTTGTAT
CTCAGAAATAACCATCTTGAAGGACCAATTTCCCAATTCTTCAAATTTGGAAACCTCAAGTTGTTATCACT
TGGAATTAACAACTTTTATGGCCAACTTGATTTTTTATCCTCCAACAGAAGTTGGACTCAACTTGAAGTGT
TCGAAGTTTCTTCCAATTCCTTAGTTGGTCCAATTCCTTCCGATGTAAGTGGTCTGCAAAGCCTACAATCA
TTGTTCTTGTCATCAAACCACTTGAATGGGACTATACCATCATGGATATTCTCCCTTCCTTCACTAGATGA
ATTATACTTGAATGATAACTATTTCAGTGGAGAAATTCAGGAGTTCAAGTCCAAAACATTGTGGGACGTT
ACTCTAAAACAAAATCAGTTGCAAGGTCCTATTCCGATGTCACTCCTAAACCAACAGAGCCTATCTTATCT
TCTCCTTTCGAAAAATAATCTTAGTGGACAGATTGCTTCAGCTGTCTGCAGTCTAAAAGCCTTGACAGTG
CTAGATCTTGGAAGTAATAATTTGAAGGGAACAATCCCACGATGTTTGGGTGAGATGAGTGAACTTTGG
GTTGTTGATCTAAGCAACAACAGTCTTAGTGGGGCAATTAATACAACATTTAGTATAGGAAACACGCTCA
GAGTCATTAAATTGCACGGGAATAAGCTAGAGGGGGAAGTCCCACGATCTTTGGTCAATTGCAAGAGTT
TGGAATGTCTAGATTTAGGTAACAATGAGTTGAGTGACACATTTCCAAAATGGTTAGGAACCCTACCGA
ATTTGAAGATTTTAAGCTTGAGATCAAATAAGTTCCACGGGCCCATTAAAACTTCAAGGGCTGGAAACTT
GTTTGTACATCTTCGAATAATTGATCTATCATCCAATGGATTTAGTGGGAATTTACCTATGAGCCTTTTTG
AGGATTTGAAAGCTATGAAGATAAATGATGAGATCACGAAAATCCCACAGTATGTGGGAGAAGACTTG
TATTATCATTTTTCTGAAACAATTACAACAAAGGGGTTGGATCTTGAATTTCGTGGAGTTTTGACTACAAA
CATAGTTATCAATCTCTCGAAGAATAGATTTGAAGGTTGTATTCCAAGTTCCATTGGAGATCTCATTGCAC
TTCGTACTTTGAATTTATCTCATAATGGCTTGGAGGGTGTCACACCAACTTCGCTGCAATACTTATCTGTA
CTTGAATCATTAGATCTCTCATTCAACAAAATAAGCGGAGAAATTCCACAACAACTTGCATCCCTCACATT
TCTTGAAGTCTTAAATCTCTCTCACAATCATCTTGTTGGATGCATCCCTAGAGGAAAACAATTTGATACGT
TTGAGAATAGTTCATACCAAGGGAATGATGGGTTACGTGGATTGCCACTGTCAAAAGATTGTGGTGGTG
ATGAAGGGGTACCACAAACAACAACTCCATTTGGGCTTGATCAAGGAGAAGAAGGAGACTCAACAATG
ATCAGTTGGCAGGCAGTTCTCATGGGGTATGGTTGCGGTCTAATTATTGGATTATCCGTAATATACATAA

TGTTATCAACTCAAAATCCAGCATGGTTTTCGAGGATGGTTGAAGAATTGGACCACAAGATTATTACGA
GAATGAAAAAATACAAGAAAAGATATTAGTGTGCAACCTAGCCAGGTATTCAACTTGATCTTTATCATTC
ATAAGATTATTTTGTATATCTCAATGTATTATATATCTACCTCCTTCATCCTTAAAATTCTTAACTTCAATTC
TTCATTTTTTAAAATTGCAGGATTCGAGCCTAATATGTTGTTGGTGATGCAAATATAAGCTTTTATTTTTC
ACATTTTGTGATGTATCTAAGTATTTATCGCCCTATGAATAAAGACATGAGCTTTCTTCCTTTGAGCTTCG
GCCAGAATTTTTAGAACGTTTTTAAATAGAGATTGTTAGCTTGAGTTCAGCCTAATAATAAGTTGAGTAA
TTAGGCTCAACCGCAAAGTCA
>PGSC0003DMG400005395 LRR-RLPs
ATGGGCTGTTGTGTGAAAGCTAATGCCTCCAAGGAGATTAGAACCCGCAATCATAGACATGTAGGACTT
GAAAAGATCTTTCGCCAAGTGCACGAACCTCTACTTTGCATTCTGGTGCCTTTGTGTATCTATTTCTAAGA
TATCAAGATAGTTACCTATATAAACCAAGTTTCCATCGAAGTTTCCGGGTGCAGGTGTATCCTCAACCTA
CTATAAACGGGCTTGCTAAATCCAGATTGAGGGAAATTCATGATTAAATGTGTCTTTTAGTAAATATTAA
CTGTGTTACTCTGTTGGAAATGCAAGGTTAAAGCAGTAACAATTTGTCCAAAATATAATGGCAAACACAA
AATTAAAGATATGCTGACAGCTTAACTTCAAAGGGAGATTTTAAGCTATCGTAGGATAAGTCTTAGGAG
GAAATAGCCCTGTGAAGGAGCTGTAATGAAAGTCAATTTTTTTGTCAACGAGGTAATATGTGGTGGGTA
AAAAGCCCAAGTGTCACACCCGAAATCCACGCTTATTGCTCAAAGTACTTCCATGCTTGTTCTAGTACGTT
TAATCACTTTTGGACTTTAGTATCCTTGCTGGTCCCCACACTTCCAACCTTGATCTGATTTTTTCAAGGTTT
GAATTTTCGGGGTCTTACACCAAGTATACTAGTGGTGTACAAGAAGTGGACCTACACCAACACATATTTC
TGGTACTCCAAAAATCTATTTAAAAAAACAAAAAATCAGTTGTAATCTGTACAAGAGTCCTCAAGACTCT
ACTGGGATGAAAATCAGTTCTACCTGGGAAAACTGAAAAGTGTCTGCTTCTCATTTATTCTTAAGGGACT
CTTCATCTGGGTTTTCACGCTGAATACTATTGATATTGACATATTGTAGGGTGTATTCTCTTAAAAAGAAA
ATTTTATAAAGAGTTTGTTATATCTGATTCTATCCAAAAAATTGAGTCTGATTATTTCTTTTCAGGTTTTGG
CTGATAACCTTATTGAGAATCTCCCGGTGAACCTGGGATTGCTTAAGTCTTTGAAAGTTGCAACACTTGA
TGGGAATCGAATCAGAGCATTGCCTGATGAATGTAATTTCCCACCGTCCTAGATCATATGTGAAGTTAAG
CAGGATTCTCTCTGCTATAGAGTATCACACTCTCTATTCTAACAACATAATGTTCTCATATTCAGTGGGCC
AGTTGGTGAAGCTTGAGCGTTTGTCTGTCTCAGCAAACTTGTTAATGAGCTTGCCTGAGACTATTGGGA
GATTGCAGAATGTGAGTGATCCATTCTGATGGCTAATACGGTGCCATCTTCCGCAATTCTAAAAAGTGTG
CCAGTTAATATATTATTTCTTAGCTTGCTTTACTGCTTTGTTATTTTTTTTTTCCGAGTATAAAGGTGCTAA
GGTGTCTAATGAAATGGTGTCAATCTTGTAGAAAATGTTCAGCATATTGATATCCATATTAATGGATCAT

TTTGCTTACAACTTTCTGTTCTTACAACTAAAAGCCCTACTTTTTTTTCTCTATTTCTATAGTGATGCCGCTT
CTTCATGAATTTCTATTATCTTCCTGGTAGTTGATGATGTCTCTTTGCTCTCTTATGTTAGTATTTTGGATTT
GGAATAGGGTCCTGCAGCTCCTGGGGGAGGGTGGTTGAATTTGGAGAATAAGTGGCTCTATTGCAGGT
TGTCTTGAGGTCCACCACCCTGAGTGCCGTCTGTTCTTCCTTTTTATTTCACTCTCTCCTTATATTGAATATT
TTTCTTCTCAAGTTTTTTTCTTCAATGTCATTGATGAACCAAGTGTGAGAAAGAAAGACTCGGAAAATACT
TTCAATATACGTAAACTACATTATTACACAAAGCATTGTTTATAGACAATACACTACAATCCTTTTCCATGT
ATGGCACCATGTACAATCCTATTTGAGCACTCCCCCTCAAGCTGGTTCATGTAAGTCATATGTACCAAGCT
TGGTACCTCCACTTGGATATATTGTGTCACTTCATTTATCACTTGGGTAAATAAAAAATGGGCTAAGAAT
CGATCCATGGTGTAACCCCATCTCTACTAGGAAGTGTTCTAAGTCTCCTCCCATTGTTCTCACTGGGTTTT
GACTCCATCGTACATGTTATTAATCCCTCTAATGTATGCCACATGTACACCTCTGGGCTCCAAGCATCTCT
ATAAAATCTTCCTAGGGACTGTCATATGCCTTTTTTAAGTCAATGAACATCATTTGTAGGTTTCTCTTCCTA
TTTCTATACTCATCCAACAATCTCATTATAAAATGAATGACTTCCATAGTTGATCGCCCTGACATGAATCT
GAATTGGTTCTCCAAAATAGACATATCCCTCTTCCTTCTCATCGTCATCTCTACCACCCTCTCCCAATCCTT
CATAGTGTGATTTAGTTGTTTAATACCACTCTATAGTTGTTGTAATTTTGAATATCATCCTTTTTCTTGTAC
AGCGGGATCATTGTCCTCCACCTCCATTCTTTGGGCAGTTAATTCATCCTAAAATGATATTAAACAGCCTA
GTTAGCCATTCAATGCCTACTTCCCCTGCGCTCTTCCAAAAATCCATAGGCATCTCATCTAGTCTGTTCGCT
CTTCCTGTTTTCATTTTACGAATTACCCCCTTGACCTCCTCAAACTTTTTACACCTACAATACCCAAAATCAC
GATGTCTCTTGGAGTGTTATAAATCACTCAACACAATAGTTCTAATTCATGCTCCCCTCTGCATTCAAGAG
TACAAGTTCCATTTGACTGTTTTTTTCTAAATTTTAATTCTCTGCGCATTGACAAACAAAGACTCAGAAAA
TATTATTAAGATACGTAAATATCACACTGAGGTCTACTTATAGATAATACACTACAATCCTTTTCCATGTA
GGACACCATGTACAACCTATTGTAACACCAAGAAGTATAGTGTTCACTTTTAGGTGTATATCTGATTCTCA
TGGTTGCTGACATGGGATTATGAATTAAAGTTTTTCAATCAAGTTAACTAGCTCTATCCTCTAGTAGTGTT
CATATCATTCTAAACACTTGCATATACTTTTACTCCAAATATGTTTTGATATTTGTGAGTTCAGTTCAATAT
GATGATGCCGTTTTTTGTCTGTCAGAGATAGTCGTTCAGCCTATACTTAAAAGTATTCTTAATTCATCTTCT
TTGGAAAATGAGGCCTTTGGAATTGACAGTAATGGTTTTTCACTGCAGTTGGTGCTGTTGGACATTTCAA
ACAATAAGTTAAAGTTTTTTCCTGAATCAATTGGAAGTTGCTTCTCTTTGGAAGAATTGCAAGCAAATGG
TACCTTCAAATTTTAGTGTCACTCATCCATTCTTGCTATTTCTCTATTTCCTTTTATTTTCCTGGCACTGGAA
CTCTGTATCAAATATCCATCGAGGAAAAAAAAACATGTTACCATCAACATAAAATTAAGTGAAATTTATG
GCCGATGATAAAATTGGAGTGTTGGGTGACCTAGAAAGCTAAAGGAAAATCTACAAATTTTCTCTGTTA

AGCTTATAAGATAATATTCTTGGCACCATCCATTTGAACTGGTACATGATTTTCACGTTCTGTTTCTTATTC

CTCAGTGTGGCATAATGAGGTTTACTGTGTTTTTTTGGCAATCTTGCCTCGTGATAACAATATTTCCTTTC

AGATAATTCCATTGAAGAGCTTCCTGCATCAGTTTGCAATCTTGTTCAACTAAAATCACTCTGCCTGAACA

ATAACAATCTCAATCAGGTTAGTGTCTAATTTTACCTGTGTGTATGTGTGTGTGACCCTTGTTATTTTGAG

GTCATATCTAGTATTCAGAGTTTACCTCGATTTGGTACTGCTCTCGTGGCCTGCACTGATACAGTGCTTTA

CCTCTACATGTGCAGTCAACTGCTGTGCATGTGTGTGTGTGTCTGTCTCTATCATCGTCACACATAAAAAG

TGCAAAGAATAGTAAACCACGTGGATCAAAAGCTTACCTGTTTGTCACACCGAAAAGCTGAACTTATTGT

TGTATTGGTGATACTTGTATGTGCTCTCTCTCTCACTTTCTATCTTAATAATACTATTAAGCAGAGTTAGG

AACTCTCTTCGCATGATCTGTAAGCCTAGTTCACTTCCCCGTGTATGAGTGTTAAAAGGAAACTCATACAT

TAACCTTAAAACGTGCATCTAAAAGCTTTTCCTTTTAATAAGACCTCATGAAGAAACGAAGATAATCAAA

CAACCAGTAGCATCTACATTGCAAACCGGCAATAAAGCCAAATACGCATAATGATCCACATTTGAGGTCA

TTGGATTGAACCTCACGTATCTTCCATTTAAAGCATCATCATCCAAGTTTTTGTACTTGTGCCTATCAAGTA

TATGTTTTTACTAGATAGTCATGCTTCCTTTTGGTAACTGGATTGTTCAGATGCCTCTAAATTTGTTAAGA

GAATGCAAGAGCTTGCAGACCATATCCCTGCATGGAAATCCGATTTCCATGGACCAATTTCAACAGGTTA

CTCACTAATATTCTACTATTTAGTTCTGCAAGTACTAAAAAAATCATTTATGAAGAATTCTATTTGCCTTTT

CTTTTTCCAGATGGATGGCTTCAAGGACTTTGAAGCCCGCCGTAAACAGAAGTTTGATAAACAAATTGAT

TCAAATGTGATAATCAGTTCTAAAGGGCTTGATGAGGGTGTTGACTTGTGATTCCTCTCCGCAGGCGTTT

TCTATAAGGCTCCGCGCAATCTTGTTAATCTCAGGTTTACTATTTTTTTGCTTCAATTCCCTGATGGAAAAT

TTCCCTTTATTTTATGTATGACCATGTAGTAGCCACACTACACAGTTGTGCTCCTTCAGTTGCTGTCAAGC

TTTTAGTATTTCTAGTGTCTGTAATGCCTAATTTGGACAATTCATTCTTGTTTACTTCATGCATGTCTCCGT

TGCTCTTAGCTCCCAGCCTGGCGTGCCTGCATAGTTGCCTCTTCTCATTTATGTTCCAGTCTTCTAGTTTGT

ATTACTCTCCATTATACCCGTGCCCCTAAGTGTATATAATTGTGGTAATTATTAGTAACACCAATTTCAAA

AGATTGAACACGAAAAAGCTTCTCATTTATGTTCCATGGAAGCAGGACCGTTTTGTCCATTTAGTTTCTG

AATTTGATAGGTAGTAGCTAATCCAGTGGCCAAATTAGTTCAAAAAGAAATTGCACTCGTTTTTCTAAAC

TTCCCAGTCAACTGGTGCTATTGATGATACAATTTTTTTTGAATAAATCATTATATCTTATTTACTGCAGGT

TAAATTTGTTCAGGTTGGATGGAATTGCGACAGTTTTGACTTTGATGAAAGGTATTGCTTCTGCTTATTTG

GCATGAGATATTTCACACCTTTCTATAGTTTGTCATTGATTGTAAGTTGTGACTTGTGCGTAATTGTATCT

ATGCTGACTTGCTGAGTATGTTTGTGATTGTAAATGACTCAACTCTAACAAATTCTGTACATTTAGCGAGC

TGTATATCACTTTAAAATCAAACTAACGATTTTTGTACCTAAAGATATTTGATTTTAATATGTTTGGATTGT

AACGATCCTTTCAACGTATCCAAGAGTAGGGCCTGGTGGCCAATAAAGTGGCTGAAAATTGTGGCCACT
TTTTATCTCCGTTCGTCGTTTTAAAATTAATTGAAACTAAGGGCGTGTTTATGATATTTTCATGTTTGGTTG
GTTAAATTTTTTGGAAAATATTTGCTCTAGTAAGTATTTCATACGCGAGCCATCATCTAAAATCAATTCAT
CGATATCAAGTCAGCAACACAAGCATTTATCTAGTGCTATATACTAGGAGTCAAGCTCACATAATCACAT
TAATCAATTCAGGAAGAAAATCTCCAATTTTTCATATTCATGGCATTGGTACTCAAAGCATTTCATATTCA
TGGCATTGGTACTCATAGAAATGCTTGTTATCTCTTTTATACCGGACCTCCATTATCCATTAACCTTTTATT
CAATTAATAAATACACTTTAACATTTCATAAAAAGTTTCAACTTGCCTTAATTGGAATTCAAAAAGTTAAT
GTGGATACCTAGGCACCCAAAGACAATGAAGGGTGTAGTAATCGACAAAATGCTTTAGGGAGTTGAAA
ATAAGCATAGATTTGGAGATTCTCGAATAAGGTAACCTTTCAAAATGCTATTGAATCCACGGGTAGGCA
AGATACAACTAACGATTTGCTCTTCTTTTATGCCTTTGAACTATTTCAATTTGGTCATAAATGTAATTTAGA
TGAGTATACAAGTTCGTAGACACACAAATTGGCACATAATTGGCTCGAAAATTGAGTGAAAAAGTCAAC
CTTGAGCCTTACTGTCAAATTTGAAAATTTGTTTACCCATAGGTGGGTCAAAAATCCGCTAAAATGGATT
GTTAATCGGTCCCCAAATCGAAAGCTACATAAATCATTCTTTGGAGAATTAAAAACCAACCAAATTCCCC
AATTCAAAAACCCCCAAATTAAATATCTATGAGCTTGTATAGCAAGGTGAGACCCTCCTCTTATTGGATTC
TAATTACTTTAAGTCACAAATAGTATTTTAGGATGAAAATTCATATCTTTCGTCGGATTGTCATAGTACTT
CTTGTTATTGATTGTTGAGTGAACTCATTGTTATAGTAATGTGGGGCTGTGGGAAAAAGTTATTGGATG
GCAAAGAGTCAATGGAAAAAGGTATGATTAGGTTACTCTCTTCTTTTAGCATGTTTTCTTAAAAGGGTAA
AGCTATATGTCAAGCGTACTTTGGATGAGCTACTCATAGCCATAAGTTTAGTGTGTTGGCTTCCTAGAGT
TGTTATGTTCGTCCCTTGTTGGGACTCCTATTATAATGATAGCGACCTCGTGCTTAGATACAACTTAGAGA
TTACTCATATAGGTCGCTTATAGTTGGTTTGCTTGCCTTAGGGCCACTTGATTGATGTTGATGCCTTTGGG
CCATATGTTGGTTGTTACTTCTTTCGGGGGGGGGGGGGGGGTTTTGTGTNNNNNNNNNNNNNNNNN
NNNNNNNNNNNNNNNNNNNNNNNNNNNNNNNNNNNNNNNNNNNNNNNNNNNNNNNNNNNN
NNNNNNNNNNNNNNNNNNNNNNNNNNNNNNNNNNNNNNNNNNNNNNNNNNNNNNNNNNNN
NNNNNNNNNNNNNNNNNNNNNNNNNNNNNNNNNNNNNNNNNNNNNNNNNNNNNNNNNNNN
NNNNNNNNNNNNNNNNNNNNNNNNNNNNNNNNNNNNNNNNNNNNNNNNNNNNNNNNNNNN
NNNNNNNNNNNNNNNNNNNNNNNNNNNNNNNNNNNNNNNNNNNNNNNNNNNNNNNNNNNN
NNNNNNNNNNNNNNNNNNNNNNNNNNNNNNNNNNNNNNNNNNNNNNNNNNNNNNNNNNNN
NNNNNNNNNNNNNNNNNNNNNNNNNNNNNNNNNNNNNNNNNNNNNNNNNNNNNNNNNNNN
NNNNNNNNNNNNNNNNNNNNNNNNNTTTGGGATGGGTGGGGGGGGGGGGGGGGGATTGTGGT
TACCTTCAGAGTTATGGTTCATGTTTTCAAGGTTATATGTAGTTTGCCCAAGGGTAATATAGAGCCTTGT

GAGCTCGGATAGATAGTCTTATGTGCTCAGACATATAGAGCCTTTTGTGCTCGATTGTATAGTTATACAA

CAAGGGTAGTATAGAGCCGAAAGAGTCCTCAACTATACAGAGCTGACGAGATCCTCAACTATACGAGCT

AGTCAATTACACAAAGCTTATGGGGCAAAATTGTTGCCAATAGGGGTATAGAGATTGATTTGTACATGT

CGAGCTTATGGGGGATAGGGCAAGTGTATTAATTGTTATTTGTACTTGTCGATTTTATAGGGACTTGGTA

AGTTGTTGTTTAATTACTGTTGTATGATCCATGTATGAATTGTTGGATTTTGCTGTGACTATTCTTACTCCC

TTGCAAGATTCCCTTTAGTATTGTATTTGTATGACATTAGTTGGCCTAGTTAGGCCGAATTGATTGTTGAG

CTGAATATGGTTTCTGAGGTTTAAGCCAAAAACATGTAAGTACGTTCTGACTATTTTTAGACTCCCTATTT

GATTACTTATAGTACTTTTATCATATCATTATTACTTATTCAATTTTCTTACATACTCGGTATTTATTTCGTA

TTAATGCTCTATTGTCTACAGGCTCTGCATTCATGCTTGCAGGTACTAACAAACTAGTAGCCTGACTTCTT

TAGTAATGTTGCCGAGTTTAGCTAATCAATAAGTTCCACTTCATTCAGAGTTGCGAGGTTTAGAGTTTGA

GAGTCTGTGATTATATATAGTTGATGGGTAGGTCGGGGCCCACTCCCGACTAGATACTATATTCTTGAGG

CTTGCAGAGCTGGGTCATGTCATAGTACATCTCAGTCGAACGTATTGGCATTACTACTGTGTTATTATTAA

GCATGTCATGTTTGTCGTCTATATCATTTTGCTATTGTAGATATTATAATGGTCTTGTCAGCCCGCATAGC

AATTGATATGCTATTATTATGAACTGTTGGGTTAGCTAAAATTGTTGTTGCTGCGGGATTCTATGATTATA

GCTTTCCCAACCTTGACTTTTA

>PGSC0003DMG400005538 LRR-RLPs

TATTCATCTATTCCACCATTCCTCTGAATTTTTCTTCTCATTTAACAACTCTGAGGCTGTCAGAAACAGGAT

TGTATGGGATAATACCTGAGAGTATATTCCACCTGCCCAACCTGGAAATACTTTTCTTAGGTTTCAATTAT

AAGCTAAGTGGTTATTTTCCAAAGACCAAATGGAACAGCAGTGCATCTCTCGTAGAGTTATATCTCAGG

GGCGTGAATTTTTCTGATAATTTGCCTGAGTCTCTTGGCTATCATCTAACTTCGGTGCGTTATTTGTCTCTT

AAAAGTTGCAACCTTAGAGGGCCTATTCCTGAATCTCTTTCAAATCTCACCCGCATTGAGTATTTGGGCCT

TCGCAATAAACTCCCTGAATGGAACAATACCACCAGGGATGTTATCCCTTCCATCACTAAATGCAATAGA

CTTGAGTTTTAATCACTTATCTGGTCCGCTTGAAGATTTCAAGTCCAATTCACTAATCTTGATAGAGTTAG

AGGGCAATCAGTTGCAGGGTCATCTTCCCCACTCAATTCAAAACCTTGTGAACCTACAATATCTCTATCTT

TCTTTCAATAATTTTAGTGGTTTTGTGGATGTCAGCTTCTTTTCAAACTTCAAACAGCTTTCGTATCTTAGT

CTTTCATATAATAATATCTCATTGACCAATGACAACAACGTTAATGCTACTTTGCCCGAATCTCTTATTGG

GTTAGAATTGGCCGCATGTGAATTGAAAGAATTCGAGTTTCTAAGATCCGCAAAGAAGCTTCAGCAGTT

GGATCTTTCAAATAATAAGATTCAAGGAAGAATTCCTAATTGGGCATGGTCGAACTGGATCTTTTCATTG

CACGATCTTAATATATCCCACAATATGCTGACGAGTGTGGATTCAATTCCTCTTCAATATCTATATACTATT

GATTTGCGGTCCAATTTGCTTCAAGGTTCACTACCTATTCCATCAAATTCCACTACATTCTTCTTCATATCC
CATAATAATCTCACTGGAGAAATTCCTTCATCTATTTGCAACTTGACATCACTGATAATGCTGGATTTGGC
GACAAACAATTTGTGTGGAGAAATTCTGCAGTGTTTTGGAAATATCACTTCCCTCCAGGTTTTGGATATG
CGCCACAACAATCTTTCTGGGAATATTCCAACAACTTTAAGCAATGGAAGTTTACTGAGAAGCCTCAACT
TGCATAGCAATAAACTAGAGGGGAAAATTCCCCGATCTTTGGCCAATTGCAAAGAGTTGCAGGTTCTTG
ATTTAGGAGATAATCATCTCATTGGCACATTCCCGATGTGGTTGGGAACTCTACCAAAGCTGCAAGTTTT
AAGCTTGCGGTTCAATGAATTGCATGGTTCCATCAGAACATCAAGGATTGAGAACATGTTTCCAGAGCTT
CGAATCATAGATCTCTCCTCCAATGGCTTCTCGGGAAACTTACCCTTGAGTTTGTTTCTACATCTGAAAGC
CATGAGGACAATTGATCCATCAATGGAAGCACCAAGTTATAGAGGAGATATATATTACCAAGATTCTATT
ACAGTTGCAACTAAGGGATTTGATCGTGAAATTGTGAGAATCCTGTATTTGTACACCGTTATCGATCTTT
CAAGTAACAGATTTGAAGGACGCATTCCAAGTATCATGGGGGATCTCATTGCAGTTCACATATTGAATTT
ATCTCATAATGGATTGCAAGGTCATATACCGCAATCATTCGGAGATTTATCTTCAGTTGAATCATTGGAC
CTATCAGGAAACCAGCTTTCGGGAGAGATACCACAACAAATGGCTTCTCTTACGTCTCTTTCATTCTTAAA
TCTCTCCCATAATCATCTCCAAGGATGCATTCCTCAAGGACCTCAATCTCTCACTTTTGGGAGCAATTCAT
ATGAAGGAAATGATGGATTACGTGGATTCCCTGTTTCAAGAAGTTGCACATATGATCGGGTATTAGATA
CAAATGATACCGTATCTGTACTAGACGATGAAGAAAGCGATTCTGAATTTCAGAGTGATTTTCGGAAAG
GTGCTCTTATGGGATATGGAAGTGGACTATGTATCGGATTATCCATAATATATTTCATGTTGTCAACTGG
AAAACCGATATGGCTTGCAAGAATCATTCTAGAGATGGAGCACAAAATTATGAGGGGAAGGAGAAAGA
AGCAGCGAAGGCAAAGGAATTACAGAAGAAGAAATAATCATTTCTAGACAAGTTATAAGTTTTGACTTC
AGGTAACTAATCGGTGCTATATACTTTTCATTTTTTTCATTTCCAATAATTTCAAAAGGGTCAGCATCAGTT
GATGTTTAACTAAAATATTTTGATTACAGCGAGGAGCTAGTGTCTATGGAAGTGGAGAAAGGGAAGAA
GAAGTTATGGCAATGAATGGAAACGAATGCCAATCAGTTTTTAAGCAATAATAATTGTCTAACTGAATAT
TTTCGTTGAACAGTCTTAGATGTGGCTTCAATTCTTACTGTGTAGTGTAAATTACAAATTTATGTAGTGTG
TTTCTGTTGATATATAAAATCTATGAAATTTCTCAGTCTTACTGCTTGTCCTAAGATGAGGCAACGCTGGG
GCACCTAAATCTCATTCCTAGTTACACTGTTAAGGCACTCATTGTAATTATGGATAATAAGATTGTTACGG
CACTTATCAATTATGTGGAAATGCAAATGATAATGCAAGTGAACCTCTGATTCATGCCTAGGTTAAAGAA
AACTCTGCTGCTACACTTTTTAGCTACTTCAGAAACAGTAAATTTGTTGTATTAAACTTCATATTATTATGA
GACGAAGAAAGTATTATTTTTGACATTTTCCTGGAAAGACTTTGTGCATTGATTTGAGAATGACAACATT
TTGTGAACTTGGAATTGATTGATGATTTGATATGGAGGCAGCAGTTCATACTTGACTTTTCCCCTTTCCTC

TCTCTTTTTGATAATAAAATTCGCAAGTAATTAATTTCAATATGAAAATTCAAAGTTAAAGGGATTTGTCT

GATAAGTAACTTTCATTGAAATTTCCGCAGCAAGACTCATTGCAATTACTTCATAATTCAAAGAACTGCAT

TAATACTTCTAGGTGCAAGAAAAAAGATAATTGGAGGGACACTCTGTGCAAAAACTTGGTGTTATTTCCC

TCTTTGATAGGCCTTACACGCCGAGAATCCAGATTATTCGGGATCCTAATCTGGATATCGAAAATCGAAT

AGAAAATCAATTTATTTTTAGAAAAAGCCATTTCTCATTATAAATATGAGGTAAACAAGTCAATATCCTGT

AATTATACCAAAAAGACACTATGGCATATGACTTAGTCGTGTCTAATATTTGAGAATGACATTTCTCTACT

TTCTTTTGAAATTTTCATAGGAAGACTTGTGCATTGACTTAAGAATAACAAACATTAATTTCCCTATTTCTT

TTCAAATTTACTATTGGTGAAGGTGGAATTTGGAAATAATTTCCCTATGCAATATGATTCTGGATTTGTTT

TTAATTTTTTTTTGTTTGTTTAAGAAAGCCTTTTCTTCTTCCTTTTGGAGTAGCTAGGAATTTGCTTTGCCAT

TTAGAGCCACTTTTATAACGAGGATATATGATATATATATATATAAGCTCTAAATGAAAAAGTTAGGGGG

TATATTAGACCCTTTTTCCTGAATAAGAAAGTAAAACAACGTACAATTATATTTTAAGAAAAAAAGATGA

TTCTCTACTCCTTTTTTAAGCTTGTTTCTTTAGAGAAATAAAACAACCCAAACTTGACTTGTGTTGAACAAT

ATAAAATCCTAAGTTAGTTCATTGAAACCCAAAATAATAAAATAAAAATATGATGATGGTTCCCAGTAAA

ATATTTTCTCTACTTCAGTTTTTCACTCTTTTATATCTCCTTACAGTTACTTTTGCTTCGATCGAGGAAGCAA

CTTCCCTACTGAAAATGGAAAGCAACTTTCAAGAACCAGAACAACTCCTTATTCGCTTCATGGACACCAA

GTTCTAACTCATGCAGGGACTGGTACGAAGTTATATGCTTAAATGGTAGGGTAAACACGTTAGAATATT

ACAAATTCTAGTGTCATTGATACCCTCTATGCTTTTCCGTTTTCATCTCTCCCTTTTCTTGAATATTTTGAAC

TTAGCATGAACAAATTTTTCAGGAATCATTCCCCCTGAGATCGGTAATCTCACTAATCTTGTCTATCTTGA

CTTGTCCATGAATCAGATTTCAGGCATAATTCCGCCACAAATCGGCTCACTAGCCAAGCTTGAGACCCTC

CACATCTTTGACAACCATTTAAGTGGTTCCATTCCACAAGAAATAGGTTACCTAAGGTCTCTAACTGAGCT

AGCTTTGTATAACAATTTTCTTAATGGTTCTATTCCTGCTTCATTGGGGAATTTGAACAACTTGTCCTATAT

GTATCTTTATGATAATCACATTTCTGGCTCTATTCCTAAAGAAATAGGAAAACTTGTCAATCTTTTTGGAG

TTTATCTTGAAACAAACCAATTAACAGGTCATATCCCTCCAGAAATAGGCAACTTGACCAATGCAAAATT

GTTCTATGCTCCCTTCAATGAATTATCTGGTCCCATTCCCGCAGAAATAGGGAAGATGAAGTCACTTCAG

GAATTAAGCTTACATACAAACAATCTTTCTGGTCTAATTCCAAGAACTACAGGTGACTTAATTGAGCTCA

AATATCTACAACTTAATGATAATCAACTCGCTGGCCTAATTCCTAGAGAGATGGGGAATCTAGAAAACCT

CAATGGTCTGGAATTATTCAATAATCAACTATTTGGCCTAATCCCTAGTGAGTTGGGGAATCTTAGTATC

CTCTATCATCTGACATTATCCAATAATCAACTCACTGGTTCAATTCCTTCTTCCTTTGGTAATTTGAGAAAC

TTGCGAGTTCTGTATCTCCATGACAACAATCTGATTGAGGAAATTCCTTCATCTATATGCAATTTGACATT

CCTCATGTTCTTGCATTTGTCGAGAAACAACTTGAAAGGAAAAATTCTGCAATGTTTGGGTAATATCAGT
ACTCTCCAGTTGGTGACACTGTCACATAATAATCTCAGTGGAGAGCTCCCTTTGTCTATTCGCAATTTAAC
ATCACTACAGATTCTAAATTTGGGCAAAAACAATATAAAGGGAGTAATTCCGCAATGTTTTGGCAACATG
AGTGGTCATCTTGAGGTTCTTGATATGCAGCCAACAATCTTTCTGTGACTCTTCCAATGACTTTTAGAACT
GGAACACTTAGAAGCTTCAACTTACGTGGCAATAAACTAAAGGGGAAAATTCCTCAATCTTTGGCCAATT
GCAAAGAGTTGCAAGTTCTTGATTTAGGAGATAACCACCTGAATGACATCTTTCCGATGTGGTTGGGAA
CTCTTCCTAAGTTGAGAGTTTTAAGCTTGAGATTGAATAAGTTGCATGGACCCATTAGAACTTTAGGAAG
TGAAAACATGTTTCTTGAGCTTCAAATATTGGATATCTCTTCCAGTGCCTTCACGGGAAACTTACCTACGG
GCCTGTTTAAACATTTGAAAGCCATGAGGACAATTGATCCATCAAAGAAGGCACCAAGTGATGGATATT
ACCAAGACACGGTAGCTGTCGTAACAAAGGGATTTGAGCGTGAAATTGCGAGTATCTTGTATTTGTACA
CTGTTATCGACCTTTCAAGTAATAAATTTGGAGGGGAAATTCCAAGTATCATGGGGGATCTCATTGCAGT
TCATACATTGAATTTATCACATAATGGATTGCAAGGTCATATACCACCATCATTTGGAGATTTATCTTCAG
TTGAATCATTGGACCTATCAGGAAACCAGCTTTCGGGAGAGATACCACAACAACTGGCTTCTCTTACGTC
TCTTTCATTCTTAAATCTCTCCCACAATCATCTCCAAGGATGCATCCCTCAAGGACCTCAATTCCACACTTT
TGAGAGCAGTTCATATGAAGGCAATGATGGATTACGTGGATTCCCTGTTTCAAAAAGTTGCAGCGATGC
TCGGGTGTTAGATACAAATGATACCGTATCTGGACTAGACGATGAAGAAAGCAATTCTGAATTTCAGAG
TGATTTTTGGAAAGGTGCTCTTATGGGCTATGGAAGTGGACTATGTATCGGATTATCCATATTATATTTC
ATGTTGTCAACTGGAAAACCGATATGGCTTGCTAGAATCATTGTAGAGATGGAGCACAAAATAATGAGG
GGAAGGAGAAAGAAGCAGCAAAGGCAAAGGAATTACAGAAGAAGAAACAATCATTTCTAGACAAGTA
ATAAGTTTTGACTTCAGGTAACTAATAGGTGCGCTATACTATTCATTTTTTTCATTTCCAATAATTTCAAAA
GGCTCAGCATCAGTTGATGTTTAAATAAGATATTTTGATTACAGCGAGGAGCTAGTGTCTATGGAAGTG
GAGAAAGGGAAGAAGAAGTTATGGCAATGAATGGAAACGAATGCCAATCAGTTTTTAAGCAATAATAA
TTGTCTAACTGAATATTTTCGCTGAACAGTTTTAGATGTGGC
>PGSC0003DMG400005539 LRR-RLPs
ACAATTTATGACTATAAGAAGCTAAGACTCACAACCCATGATTTTAAATCGCTTCTTCAGAACTTGACCCA
ATTAAGAGAGCTTCATCTTAGTGGCATAAACATCTCTTCCACCATTCCTCTGAATTTTTCTTCTCATTTAAC
AACTCTGAGGCTGACATATACAGGATTGTTTGGGATAATACCTGAGAGTATATTCCACCTGCCCAACCTG
GAAATACTTTTCTTAGGTTTCAATTATAAGCTAAGCGGTTATTTTCCAAAGACCAAATGGAACAGCAGTG
CATCTCTCGTAGAGTTATATCTCAGGGGCGTGAATTTTTCTGATAATTTGCCTGAGTCTCTTGGCTATCAT

CTAACTTCGGTGCGTTATTTGTCTCTTAAAAGTTGCAACCTTAGAGGGCCTATTCCTGAATCTCTTTCAAA
TCTCACCCGCATTTGAGTTATTTTGGGCCTTCGCAATAACTCCCTGAATGGAACAATACCACCAGGGATG
TTATCCCTTCCATCACTAAATGCAATAGACTTGAGTTTTAATCACTTATCTGGTCCCTTTGAAGATTTCAAG
TCCAATTCACTAATCTGGATATATTTAGAGGGCAACCAGCTGCAGGGTCATCTTCCCCACTCAATTCAAA
ACCTTGTGAACCTAACAGAACTTTCCCTTTCTTTCAATAATTTTAGTGGTCGTGTGGATGTCAGCTTCTTTT
CAAACTTCAAACAGCTTTCATATCTTAGTCTTTCATATAATAATATCTCATTGACCAATGACAACAACGTT
ACTTTGTCCGAATCTTTTATTGAGTTAGAATTGGCCGCATGTGAAGTGAAAGAGTTGGAGTTTCTAAGAT
CCGCAAAGCAGCTTTGGAAGTTGGATCTTTCAAATAATAAGATTCAAGGAAGAATTCCTGATTGGGCAT
GGTCTAACTGGATGTTTTCATTGCAAGATCTTAATATATCCCACAATATGCTGACGAGTGTGGATTCAATT
CCTCTTCAGTCTGTAAATACTATTGATTTGCGGTCCAATTTACTTCAAGGTTCACTACCCATTCCATCAAAT
TCCACAAGATTCATCTTCATATCCCATAATAATCTCACTGGAGAAATTCCTTCATCTATTTGCAACTTGACA
TCACTGATAATGCTGGATTTGGCGACAAACAATTTGAGTGGAGAAATTCCGCAGTGTTTCGGAAATATC
ACTTCCCTCCAGGTTTTGGATATGCGCCACAACAATCTTTCTGGGAATATTCCAACAACTTTCAGCAATGG
AAGTTCACTGAGCAGCCTCAACTTGCATGGCAATAAACTAGAGGGGAAAATCCCACAATCCTTGACCAA
TTGCAAACAACTTGAAGTTCTTGATTTAGGAGACAATCACCTCAATGACACATTCCCCGTGTGGTTGGGG
ACTCTGCCAAAGCTGAAAGTTCTAAGCTTGCGATTCAACAAATTGCATGGTCCCATCAGAACATCAAGGA
TTGAGAACATGTTTCCAGAGCTTCGAATCATAGATCTCTCTTACAATGCCTTCTCAGGAAACTTACCTTCG
AATTTGTTTCAACATCTGAAAGCCATGAGGACAACTGATCCATCAATGGAAGCATCAAAATATGGTGGA
GATTCATATTACCAAGACTCGATAGCTGTCGTATCCAAGGGATATGAGCGTGAAGTTGTGAGAATCTTG
TTTTTGTACATCACTATCGATCTTTCAAACAACACATTCGAAGGACATATTCCAACTATTATGGGAGATCT
CATTGCACTCCGTGTGCTGAATATATCTCATAATAGATTGGAAGGTCATATACCGCCATCACTTGGAAGA
TTATCTTTGGTTGAATCATTGGACCTGTCAAGTAACCATCTTGTAGGAGAGATACCAGCACAATTTGCTTC
TCTTACATCTCTTGAAGTCTTCAATCTTTCTTACAATCATCTCGAAAGGGTGCATTCCTCAAGGAAATCAA
TTTCATACCTTTGAGAACAATTCATATGAAGGCAATGATGGATTACGTGGTTTTCCTGTTTCAAAAAGTTG
TGGGACAACTTATGCCATATCTGGACAGCTAGACGGTGAAGAAAGCAATTCTGAATTTCAGAGTGATTT
TCGGAAAGCTGCTCTTATGGGGTATGGAAGTGGACTATGTATTGGAATATCCATAGTATATTTCATGATG
TCAAGTGGAAAACCGGTATGGTTTGCAAGAATCATTGTAAAGCTGGAGCACAAAATAATGATGAGAAG
GAGAAAGAAGCAGCGAAGGCAAAGGAATTGCAGAAGAAGAAATAATCACTAATAATATTACTGTTATT
TCTTTAAGGAAAAGATGTAATAAGTTGTAGACTTACTATCGTTAAATCCTGTTTGCTTAAAGTGTGTAGC

ATCATTAGGGTGAGATAATCTTTAATGATAAGTTATATGCAATATAAAATCTTATGACC

>PGSC0003DMG400005542 LRR-RLPs

ATGGGGTATGCAGTTATTATGCTCTTAATTGTGTTTTTCTGTCATGAAGTTTCTTCAATATCCATATTACCT
CACAAACATCAAACCATTTCACTTCTAAAATTCAAGAACACCTTTACTATAGATCCGTCAGCCTCTGGCGT
TTTTCACAGTAGAATCTTATTATTGTCCGAAGCCTTATCCAAAAACGAGTTCATGGAACACGAGTAGAGA
TTGCTGCTCATGGGATGGAGTCGTATGTGATGACATCACTGGGCATGTCATTGAACTTGACCTCAGTTGC
AGTAATCTCACTGGAACCATCGATTCCAATAGCAGCCTATTCCAACTCTCGCATCTCCAAAGGCTTGATCT
TTCTTGGAATAACTTCTCCAATTCTCATATCTCCCCTGAATTCAGCAAGTTTTCGAGCTTGATGCATCTTGA
TCTTTCTGGGTCCTATTTCTCAGGTCAAATTCCTTCTGAAATCTCTCATCTTTCCAAGTTACACTCTCTTCGT
ATCTCCACATTTTATTACTTTTACAGTAACAACCTTTACAATAAAAAGCTAAGACTCACAACCCATGGTTTT
AAATCGCTTCTTCAAAACTTGACCCAATTAAGAGAGCTTGATCTTAGTGGCATAAACATCTCTTCCACCAT
TCCTCTTAATTTTTCTTCTCATTTCACGACTCTGAGCCTGTCAGAAACAGGATTGTAAGGGATAATACCTG
AGAGTATATTTCACCTGCCCAACTTGGAAATACTTGACTTAGGGATCAATCATGAGCTCAATGGTAATTT
TCCGAAGACCAAATGGAACAGCAGTGCATCTCTCATTGAGTTAGATCTCCGTGGCGTGAATTTTTCTGAT
AATTTGCCCGAGTCTCTTGGCTATCATCTAACTTCGCTGCTTTATTTGTCTCTTGAAAGTTGCAACCTTAGA
GGGCCTATTCCTGAATCTCTTTCAAATCTCACCCGCATTGAGTATTTGCACCTTAATCGTAACTCCCTGAA
TGGAACAATACCACCAGGGATGTTATCCTTTCCATCACTACATAGAATAGACTTGAGTAATAATCACTTA
TCTGGTCCCTTTGAAGATTTCAAGTCCAATTTGCTTCAAGGTTCACTACCCATTCTATCAAATTCCACAGTA
CTCTTCTTCATATCCCATAATAATCTCACTGGAGAAATTCCTTCATCTATTTGCAACTTAACATCACTGATA
ATGCTGGATTTGGCGACAAACAATTTGCGTGGAGAAATTCCGCAGTGTTTCGGAAATATCACTTCCCTCC
AGGTTTTGGATATGCGCCACAACAATCTTTCTGGGAATATTCCAACAACTTTCAGTAATGGAAGTTCACT
GAGGAGCCTCTACTTGCATGGCAATAAACTAGAGGGGAAAATCCCACAATCCTTGACCAATTGCAAACA
ACTTGAAGTTCTTGATTTAGGAGACAATCACCTCAACGACACATTCCCCGTGTGGTTGGGGACTCTGCCA
AAGCTGAAAGTTCTAAGCTTGCGATTCAACAAATTGCATGGTCCCATCAGAACATCAAGGATTGAGAAC
ATATTTTCAGAGCTCCGAATCATTGATCTCTCTTACAATGCCTTCTCAGGAAACTTACCTTCGAGTTTGTTT
CAACATCTGAAAGCCATGAGGACAGCTGATCCATCAAAGGAACCATCAAAATATGGTGGAGATTCATAT
TACCAAGACTCGATAGCTTTCGTATTCAAGGGATATGAGCGTGAAGTTGTGAGAATCTTGTTTTTGTATA
TCACTATCGATCTTTCAAACAACAAATTCGAAGGACATATTCCAACTATTATGGGAGATCTCATTGCACTC
CGTGTGCTGAATATATCTCATAATGGATTGGAAGGTCCTATACCGTCATCACTTGGAAGTTTATCTTTGGT

TGAATCACTGGACCTGTCAAGTAACCATCTTGTAGGAAAGATACCAGCACAATTTGCTTCTCTTACATCTC
TTGAAGTCTTCAATCTTTCCTACAATCATCTCGAAGGGTGCATTCCTCAGGGAAATCAACTTCACACCTTT
GAGAACAATTCATATGAAGGCAATGATGGATTACGTGGATTTCCTGTTACAAAAAGTTGTGGGACAACT
TATGCCGTATCTGGACAGCAAGACGGTGAAGAAAGCAATTCTGAATTTCTGAGTGATTTTCGGAAAGCT
GCTCTTATGGGGTATGGAAGTGGACTATGCATTGGAATATCCATAGTATATTTCATGATGTCAACTGGAA
AAACCGGTATGGTTTGCAAGAATCATTGTAAAGCTGGAGCACAAAATAATGATGAGAAGGAGAAAGAA
GCAGCGAAGGCAAAGGAATTGCAGAAGAAGAAATAA
>PGSC0003DMG400005590 LRR-RLPs
ATTGATTTGAGCAATTTGGAGTATCTGGATCTCTCGTATAACAATATAGAGAGCTTTGTGACTACCAAAG
GTACACGAATCAATGCAGTTGCTCTAATGTTGAATGTTTTATTTTAACCTTAACACATCCTACAGAATAAA
ATACATCTCTACGGACTTTAGATTGAAAATTGTGGGATACAAATTAGATTTTAATAAGATCCAACTATTCA
TTCTTATTTAAAAATGTATATGATTTATTTATTCACCATTTAACAGGTAACAAAAGAATAAGTTCTCTGCG
GAATTTACAATTGGGAAGTTCCTACTCCAACTCAAGCAGAGTCATAGAATCCCTAAAATCATTCTCTTCTC
TTAAATCACTTTCTTATGAATTTAGTGATCTCACTTCTCCAACCATAATTTACGGTAAGCTCAAATTCATAT
ATACCTTAGCTTACTTCTATCGCCACTCTTTTCTTTTGATATGTACATTTAGTTTTCTTTTCATTTTTTGGACT
CTATGATATAAGCATTAAGAAATCTGAGCACGGTGGAGCACCTATATTTGGAGGGATGTTCTTTAAATG
ACAACTTTCTCCCAAACATTGGACAAATGCCTTCTCTCTTGGTGTTCAGTATAGCATTTGGTGACAATGAT
GGCACCCTCCCTAATCAAGGTACCCTATTTTTCTCTTTTTTCCCTAATGCCCGCATTTTCATGTTGGAGGTC
TGATTTTAGCATGTTCAGTTGATCAAACATTATAATAATTTTTGTTTATTACCAAACACAAGAAAATAAGT
AACACAAGCCACCTAATTTTCCTAGAAAATATTTTCCTTCGTATCGAACACATCCATAGAGACATAAAAAA
AATATAATAAGATGTCACTTGTTATAGAAATTTGTTTTAGCTAAATGTTTCTTAATATATATATATATAGGTT
GGTGTGAACTCAAACATATTCAAGAGCTGGATTTTTCAGCCAATAGTTTTCGGGGAACACTGCCTTCATG
TTTTGGAAACTTGACATCACTTCGACGATTGAGTTTTGGGGGGAATTACTTCAGAGGAAATATAGCGTCA
GATTCTATTTGGAGGAGACTCACATCACTTGAGTACATCGATATTTCATATAACCAATTTGATGTTCCTCT
GTCATTCAGCCAATTTTCCAACCATACAAAATTGATCTACTTGGATGTTAGTTCTAATACAATAATTACAG
ATACCAAATTCCAAAATCAGATCCCAAATTTCCAGTTGGAGTTTTTTGCTATCAAAGGATGTAAACACCTT
AAAAAATTGCCTTATTTCCTTCACTACCAATATGACTTGAGGATTCTTGCTATTGGAGGAAATCAATTGCC
CGGGATCTTTCCAACATGGTTGTTAGAAAATAACACCAGACTTGCAGGGATTTATAGTAGAGATAATGC
TTTCAGCGGATCATTCAAGTTGCCATCGAGTTGTCACCTCCATCTAGAGACAATTGATGTTTCTAATAACA

AATTAAATGGGCATATTCCAGATAAAATGAATTCAGCATTCCCAAAGCTTACTTATTTGAACATGTCACA
AAATTATCTTGAAGGTCCTATACCTTCCAAGATAAGTAGCATTCATTTACATAATCTAGACCTATCTCACA
ATTTCTTATTTGGAGGAGTTCCTAGTGATCTGGCAATTGGTTCTCCAGTATTAGCTTACCTTCGATTGTCA
CACAACAAGTTAAAAGGTCAAATTTTTCCAAAGGATGTCAAACCAAAGATGTTATTCTTTTTGTTCTTGAA
TGGAAATAACTTTGAGGGACCACTACCTTGCAACATGTTTATCACATCCCTTATTGTATTGGACGCTAGC
AACAATAATTTCTCTGGAGAGATTCCGAGATGCATTAGGGATAATACAAGATTGTTACAACTTGATTTAT
CCAAGAATCATCTCAAAGGTTCAATTCCAGTTGAGATTTGCAATTTAAAGCTCATTCAGGTCTTAGCTATA
TCAGAAAACAGGCTTTCAGGCTTTATTCCTTCTTGTGTGAATTCTTTACCTCTTGAACACATCCATCTACAG
AAAAATCAATTGGGTGGTGAATTGGAACATGCACTTTTCAACTTATCTTCTCTGATAACTTTGGATCTTGG
GTACAACAATTTTACAGGAAGTATCCCACACACCATAGGCTCACTTAATACTCTCAACTACCTTCTCCTTA
GCAATAATCAATTCGAAGGAGAGATTCCAACTGAGATTTGTATGCTGAACAAGCTATCTATCGTGGATTT
ATCTTTCAATAAGCTTTATGGTTCACTCCTTCCTTGTTTGAGCTACTTAACACAAGCCAAAAAGGATACAG
AGATAAGTAAGACCAATTATCCTACGACTTCTAGGCGTTCATGGATAACTTTTTTGGCCTGGATGCGTTT
GAAAAGGCACTACCACGATAGTCATGGATTTCTAAGTGACATATTTCTGATGGATGTGGAAACTCAGGT
CCAGTTTTCAACAAAAAGAAACTCATACACTTATAAGGGAAGCATTCTAAAATATATGTCAGGTATGGAT
CTCTCAAGTAACAGATTAACTGGTGAAATTCCCGTTGAGCTAGGGAAGATGAGTAATATACCTGCATTG
AATCTATCGCACAACCATCTCATCGGAAGAATACCAAATACCTTCTCCAATCTACAGAAAATTGAGAGTT
TAGATCTTTCCTACAACAACTTGAATGGGAGCATTCCTGTTGGTCTACTTGAGCTAAATTCTTTGGCAGTA
TTCAGTGTTGCATACAACAACTTATCGGGTGCAGTGCCTGATTTTAAAGCTCAATTTGGAACTTTCAACA
AAAGCAGCTACGAGGGAAATCCTTTTCTTTGCGGTTATCCATTAGACAACACGTGTGGCATTCCTAAATT
GTCATATACCTCCAAGATTAATGGAGGTGAGGAATCATCAGACTTGGAGGATATTCAGTGTTTTTACATT
GGCTTTGTTGTGTCTTATGGAGCGATCTTGTTGGGATTAGCTGCGGCACTCTGCTTCAATCGTTATTGGA
GAAAAGCATGGTTTAGGATGATAGAGGCATTGATGTTTTATGGTTACTATTTTCTCTTGGACAACGTTGT
TACACCCATCAAGAGTATATGGTACAGGAATGTTGGTTAATTCTAGGTAGTTGTTTTACAATATTGTTGT
ATGTGTATCACCATTATTAATTATATTGTCATTCTGTTTTATCAATATTCGACAAATAGGATCTGTTTGGAG
TAGTTACAACTTCATAATTTAATACCCCCTGCATAGCTTCATTAGTGTTGCTATTTATGTAGCCG
>PGSC0003DMG400005600 LRR-RLPs
CCCTGAGACTTAGTTACCATCACCTTCCATTTGATTTGAGACAATGCTTTGCATATTGTGCAGTATTCCCA
AAGGATACCGAAATGGAAAAGCAAAATCTAATCTCTCTCTGGATGGCACACGGTTTTCTTTTATCGAAAG

GAAACTTGGAGCTAGAGGATGTAGGTAATGAAGTATGGAATGAATTATACTTGAGGTCTTTCTTCCAAG
AGATTGAAGTTAAATCTGATCAAACTTATTTCAAGATGCATGATCTCATTCATGATTTGGCAACATCTCTA
TTTTCGGCAAGCACATCAAGCAGCAATATCCGTGAAATAAATGTAAAAGAATACCCACATATGATGTCG
ATTGGTTTCGCAAAAGTGGTGTCTTCGTACTCTCCTTCTCACTTGCAAAAGTTTGTCTCGTTGAGGGTGCT
TAATCTAAATAATTTAAGACTTAAGCATTTACCGTCTTCCATTGGAGATCTAGTACATTTAAGATACCTAA
ACTTGTCTGGCAATCGTAGAATTCGTAGTCTTCCAAAGCAGTTATGCAAGCTTCAAAATCTGCAGACTCT
TGATCTACATAATTGCTACTCTCTTTCTTGTTTGCCAAAAGAAACAAGTAAACTTGGTAGTCTCCGAAATC
TTTTACTTCATGGTTGCTATGAATTGAATTCTATGCCACCAAGGATAGCATCTTTGACATGCCTTAAGACT
CTTGGTTGCTTTGCAGTGGGAAGGAAGAAAAGTTCTCAAATTGGTGAATTACGAAATCTGAATCTCTAT
GGCTCAATTGAAATCACGCATCTTGAGAGAGTGAAGAATGATATGGATGCAAAAGAAGCCAATTTATCT
GCAAAAGAAAATCTGCATTCTTTAAGCATGAGATGGGATGACGTTGAACGTGCACATAGATATGAATCA
GAAGAAGTTGAAGTGATTGAAGCCCTCAAACCACACTCCAATCTGACTTGTTTAACAATCAATGGCTTTA
GAGGAATCCGTCTCCCAGACTGGATGAATCACTCAGTTTTGAAAAATGTTGTCTCTATTGAAATCAGGGG
TTGCCAAAACTGCTCATGCTTACCACCCTTTGGTGAGCTGCCTTGTCTAGAAAGTCTAGAATTATGGAGT
GTGTCTGCGGAATTGGAGTATGTTGATTCTGGATTCCCTACAAGAAGAAGGTTTCCATCTCTGAGAAAAC
TTATTATAGGTTATTTTGATAATCTGAAAGGATTGCTGAAAAAGGAAGGAGAAGAGCAATTCCCTGTGC
TTGAAGAGATGGAGATTTACAGGTGCCCTATGTTTGTTATTCCACCCTTTCTTCTATCAAGAAATTGGTAG
CTTATGGGGAGAAGTCAGATGCAATAGGTTTCAGTTCGCTATCTAATCTCAGGACTCTTACTTCCCTCCG
CATTGGCCATAACGAAGAAGATACTTCGCTCAAAGAAGACATGTTCAAAGGCCTTGCAAATCTCAAGTA
CTTGAAAATCTCTTTCTTTAAGAATCTCAAAGAGCTGCCTACCAGCCTGGCTAGTCTCAATGCTTTGAAGC
ATCTGGATATTTATCGGTGTGACGCACTAGAGAGTCTCCCCGAGGAAGGGGTGAAAGGTTTAACTTCAC
TCACACAGTTATCCATAACATACTGTCAGATGCTACAATGTTTACCGGAGGGATTGCAGCACCTAACAGC
CCTCACAAAATTATTAGTTACGTATTGTCCAACACTGGCCAAGCGATGTGAGAAGGGAATAGGAGAAGA
CTGGTACAAA
>PGSC0003DMG400005972 LRR-RLPs
AAAAATTGTATTGTTATTGAAACCAAATACTACATTAGACATGACAACTTCTCATTGTACATTCATTTACA
TTCTTCTACTTCTTTTTTCACTTCCATTCAAAGTAATCACATCATCAGCAAAAACTGAAGCTGAGGCTCTTA
TCAAATGGAAGAGTACTAACTTATCTTCTACTTCCTTATTGGATTCATGGTCCATTTCCAATCTCGAAAAC
TTGTGTAATTGGACATTCATTGCCTGCAATGCTGATGGAACAATTTCGGAGATCAATCTTTCTAATGCAA

CACTCTCAGGCTCTCTTGATCACCTTGACTTCACTTCATTTCCGAATCTCACGAGTTTCATTCTCAATGGCA
ACAACTTTAGTGGATCGATACCATCGAATATTGGCAATGCCTCTAGTCTTGAAGTTGTTGTACTTCTCAAC
AATTCATTTCAAGGGAAGATTCCATCTTCAATAGGCAAACTTAGAAATCTTCAAACACTTGATCTTCGAAA
GAATCGTTTGAACTCAACCATTCCTTCTGAGTTAGGCCTTTGTACAAACCTCATTATCTTGGCTCTAGCAG
AGAATGCTCTGCAAGGAACATTGCCTATATCTTTGTCCTCTTTGATCAAGTTATCCCAGTTGGCGTTGTCC
GATAATTTTCTTTCTGGTGAGGTCTCATCAGATCTCATTTTACCAATTGGACTGAGTTGATAGCTTTGCAG
CTTCAAAACATTTACATTACTGATTTAGGAGATAATCACTTCAACGACACATTCCTAGTGTGGTTGGGAA
ATCTGTCAGAGCTGAAAGTTTTAAGCTTGAGATCAAACAAATTCCATGGACTCATTAGAACGTCAAGGAT
TGAGAACATGTTTCCCGAGCTTCGAATCATAGATCTATCTTATAATGCCTTCTCGGGAAACTTACCTACGA
GTATGTTTCAACATCTAAAAGCCATGATGAGAGTTGATCAAACAATGAAGGCACCAATATATCTTGGAGT
GTACAGTTATTGCCATGATTCTATGACAGTTGCAACCAAGGGACGAGAGCTTGAACTTGTTAGAATTTTG
ACAACTTACACCACCATTGATCTTTCAAGTAACAGATTTGTAGGGAATATTCCAAGTATTATGGGAGAAC
TCATTGCACTTCGGGTGTTGAATTTGTCTCATAATCAATTGCAAGATCATATACCACCATCACTTGGAAAT
TTATATGTACTTGAATCATTGGACCTTTCGTTTAACCATCTTTCAGGAGAGATACCACAACAACTTGCTTC
ACTTACGTCTCTTGAATTCTTAAATCTCTCCTATAATTATCTCCAAGTATGCATTCCTCAAGGACCACAATT
CTCTACATTCCAGAACAACTCATATGAAGGTAATGTTGGATTACATGGATTCCCAATTTCAGATGGTTGT
GGCAATGATCGTGTATCTGAAACAAATTACACCGTATCTTCTCTAGAAGATCAAGAAAGCAACTCTGAAT
TTCCCAATGACTTTTGGAAAGCTGCTCTGATGGGCTATGGCAGTGGACTATGTATCGGAATGTCCATAAT
ATATTTCATGATCTCCACGGGAAATCCAAGATGGCTAGCAAGAATCATCGAAAAACTGGAACACAAATT
TATCATGCAAATGAGAAAGAAGCAACAAGGTCAAAGGAATTACAGAATAAGAAATAATCGTTTCTAGAC
AAGTTATAAGTTTTGCTATTCAATCAAGCTCTACATTGATCTTTAGTTTATTGTAATTTGTAACTATTATGA
TATCTCATTGAGTGCTTCAGCTTCATGTGATATAATAATACTGTACTATTTCCAGTGTGACTAACTGCAAC
ATGTACTTTAATTTCCTGCCTTTCTTACATTTAGACAGGAAACACAAAGGACCAAATAATAACTGGACAAT
ATATGGGCAGTAGGCAAAAGAGAAAGAAACAAACCATAAAATAGGTTACATAATGAGAAACTAAATAC
ACATTACATATGAGGTATGGTCAAAGAATCTGCCAAATTCATTCCTTGACATGAACCTTCCTCGGGTTCA
GGGGACTCGTATACGCACTCCCTACAATATAAACAAGGTACTTGATTACAATTAAGGGTAGAAAACAGA
GCAAAGAACACAATTTCAGATAAGTTTGGTCTCACTTGTTAACTAGCATCTATGGATACGAGTGATGAGT
GAGACCATGACAAGCGTATTTGGCGCCAAGAATATGGAGCTCGCTACCCAAAGAATGGTCAAAACTAG
AGTACAGGCCCTAGCTAGTATCATCATAACGGCTTAAAAATTGGATTATACAAGGATATACATGTTCTGC

GGCCTGCAGGCACCAGGAAGAGTATAAGTTGTTATGCAATAATAGAAACGGAAAAATAATAACAATGC
ACATGCAAAAGATGCCTATTTCACGGAGTTGTGAAAGTAAATAACGTATCATATTTCCTAATCTAGGTAT
ATTTAACTCTCAGGTTTACATAAGGAAACAAAAAAAACATTGAAAGGCAATTCCAATGTGACTAGGCAC
CTCTCCACTTCCAGCATCTTTCTATTCTTTATCATCCTTTTCTATACTCTGTCTGCTAATCAATCAATCAACT
AGACCTCAAATCCTCAATCCTATATCGGTAGGGTTTGGTTTTATAAATTCTCCATATCCAGTCCGGACTAT
TCAGGTTAATCTCATTCTAATACTAAATAATGTCTTTCAAAGCAAGCAAATTAAAATTCCTCTAAAACTCA
CATTTATTCCCAAATGAATAACTAGTCTAAATCCAATCAGTTAATGTCACTCATATAGCTCCTTTGTTTCCA
TTGTATTCTGTTCTTGTATTCCAAAAGAAGTTAAAAATTGTTAAAGTTTACATACCATGCGTCCTCCCACC
AAGTCATAATGAGCATAATGCTGACCATTATCATCCCCCAATACTTTGTAGGTGACCAAGTGCTCGGGGA
TAAGCTTTGCCGTTTCTGCTTGAACCAATGTGTTGCAAGAAAAGAATTGTTAGAAAACTTATCATCCTCTG
ACAGAAAAATGAGCGGAAGTACTATGAAACATACCGCACACTGCTTCTGGTGGGCAGATTATGTCTTTA
TCTCCAGCAACAGCCAACACGGGTACATTGGTTTTGTGAAGATTATCCTTGTAGAATATTTTACCACTTCT
GTCACGGAGTCCCCCTTCTCGAAAAGCTGTAGTCAACTGCAAAATAAGTTTAGCAGGAATAGTACCTGC
CAGAAGATCTATTAGAAAATCTAAGTAAGAGGTACATGTGAAATCTCAAGAGAACATAGAAAGGGCGT
GATCCCCAAAGGAATTTTTTAACAAAAGAAAGTGAACTTTCAGGATCACCGTCAGACTACAGCCCTTATA
AATCAGTTATGATAAACTATCCTTACTTTGATGGAAACACTCTTCCATTGAAGATCATCTATCAACACCGT
TCCACAGTGTCAAGAAAACAACACAGGAGTGAAACTTGTGGGAGCAAAGAGTCAATAGTTCATCAAAC
AAAGAGTCAAATAAATATATTGGTATATGCAGCTCTTTGTCAGAAGAGAAAATATCTAGCTGGCAAATA
AATATACAGCGGATGTTTACTTGATATAATTTTCTTTTTCAATCTGAAGATGTTAATAGTTCTTCGTCGCTC
ATTCTTTATGCGAACAAATAAGAATTAAGAATCAACTCTCAGTCTCTCACATAGTTTGTTTCCACTGATTA
CCTTATTGCTCAGAAGCCTATAAGGAAAGACCTTTTTAGTTTTTAGTACCGACTCCTCTTGAGGCCAAGA
AGGAGACCAACTAGACGAGACAGACCATTGTTCATACTTCATAGGTTGGTTATAAGCAAGTACAAATCA
AAATTCAATTTGGCTTGCCAAAGGACATACTTCAAACTTTGGCTGTTCTTCCTTGTATAACGCTGCATGAC
TACTGTGAAAGATGAGATATACTAATAATCATAATTAATAAGTACATTTGCCTTCCTAACTGATACCCATG
AGGATGTATCTGAAATGTAGAATATTAGTTCAACTCAGACTCAACATATCATCCAGACAGAAAGTGGAG
GGGAAAAGAATGTATTGGTGCAAAAACGCTGTAGACAGGAATGTGGGGCATAGCCAGATGGTCATTGA
ATCATTCTTCAGAACATGTAAGTTTCTCATACTGTTATCTTTAAAACTCATACTAAATATCTATGCCTATAC
AACTTGAAACCTGCCTGCTCTCCATATAAATAAATTATAAAACAAGAGGACCATAAATTTTTACAGTTCAC
AATCAATTAATACTCCCTCTGTCCCAATTTATGTGACTTATTTTCCTTTTAAGTCAGTCCCAAAAAGAATGA

CACATTTCTATATTAAGTAACAATTTAACTATAAAATGTCTATTTTACCCTTAATGAAAATGATTTACAGCC
ACACAAATTCCTATGATTCATTTTGGACCACAAGTTTTAAAAGTTTTCCTTTCTTTCTTAAACTCCGTGCCG
AGTCAAACTACCTCACATAAAATGGGACGGAGGGAGTAAGAAATACTGCTTATTGCCCATTTAAGCACT
TACAGAAGTTATTTAATACGAGCTTTTTCAGCAACTCTGGATGCATCATGTCTGCAGCTGATATTAAATCA
TTAAGCCAAGACAACACATAAGGAGGGCGAGAAGAAAGAGGATAAGCTGCTGCAAGTAATGCTCCTAA
AGGGACAACAGGTACATTGAGAGCTTGGGCAGGATCAGCCTGCAGCCAAGGTAAATAAATATAAGAAT
ATGCTGAAAGGGCTTTTTGCTGCACAACGAGAGTTGAAAGACATTTATTTTTAGCTTACAAGGGGTAGG
AGCAGTCTGAGTGCAGACTTCGAAGATGTGTAATCCAGTGATGAAGCCAAAGTTACAATAGCAGTTAAT
CCAGGCTCTCTTCCTTGCAATCCTTTCCAAGATAAAACCAGAAACATATTAAAATTATGTCATATCTGTAT
GATTCCCGGATGTAGTCAGTCTGAAAATATTTTTTTGAGACTGTGCCCACTGCACATTAACAAGGAATCA
GACAATGCTCCAAGTGACTTATCTGTTAGTTGCGAAACCTTTTTACATCTAAAACCAGGTCAAATTCCTTC
CAAATCAAATTGTCCCTTACACACTGTTAAAACGTATGGATAAAGTATAAAATCTTGCCTAGCTCCCTATA
ATCTCAATAAGGTCAAAAGATGGCTCATTTTCAGCTCTGTATGAAAGACGCTAGTAAGCAGATAAGTCCC
ACTGTTAACCAGATCCTGTTTACCTTGGTGCACCCTATCATGGAATTTTTAAGTTAATAGGAGTACTGCAC
TTGGTGGCTATTACAACTATTCTCCCAAAGTTGTTGTGAACTATAGCATCTTTCTTATTTTTCAGCAAAAA
GCTTGTTGAAGTTGAGTCTGAAGATTTTGCTCCATGAATATTAGGAATTAAAAGAACAATAAGTCAAGA
GGAGACCAGGTTAACTCAAAGTTGCACCAAGTGAAGTGGTAGCCTTTGGAATTTAATCCCAAGACTAAA
TCGTATGATACTCCAGCAGGTTCATGGAATAAATTTCTGGAATTATACTGCCTTCTTTATTTAAATAAAAT
GTTTAAATATTACTGTTTTCTTATCAACTGCAACTACAGAAAGGATTCTTTGTTACTTTTTCAAAACACTTG
CACATTAATTTATTAGTCTCTTAAGTGGCTCATAAGTAGACCCGCCAGATCACGTAAGTGTAACAACATT
AATTCCATATAAACAACGAATGAATCTTACCACACTGTGATAGCCAAGCATACAGTAATATTCCTCCCATA
GAATGTCCAATGGCAAGAAGTTTACCATCTTTTGGTGCAGTTTGGGCCTTAATGTATTCCATCTAGAATG
AAAATAAAATAATTAGACTAGTCAGAATAATAAATAAGAGTGAAAAATCAACAAATTCTGAATTTCACTT
GGCTGCGTAATCTCAAGGACAATAATATCTTTATATTCTACAACATGGGAATCTGTCACCTCACCGCAGC
AGGAACATCCTCTTCCAGGTAATGATCAAAGTCCCAATCATACTTGACAATCAAGTCAAGCTGTTTCTGG
AAATCTTCTATGGTAGTGGTTAGACGTTCATGAAGGTCAACTAACGGAGAAACAGAACGTTGGCCTTCT
TCAATGATATTTACTAGCTTTTGGCTAAGATCCTTTACTTGGTCAACAACGGCAGAGTTTTGCCCTGTCTC
CAGCAGACTTAACAACTTCCCCCTAGTCTCGTTAAAGCGTTCATAAAGAAAGGAATCCTCTAATAATTTT
GATATTTGATCAAATAGTTTAGCAGACATAATCCTTGATTGGCTTTCATTGAGGAAGCCAGAGACTCTTT

CTGACAAGCGCATAAATGTTTCCGTAAGCTCGTTTACTACTCTTGACTCATCCCACATTGTTGCAATAGCT
GTGGATTCTTCTTTGACCACAGCTGTGTCAGACTCTTCTAAGGTACTCTGGGGATCTGTTGACTGCTGTG
CTGCAGAAAGAACTTTGTCAGTAGCATTATCAGCGGCAGCTTCCATCTGTTCTGAGACTGCAATTGCAGT
CTTCTCAATGTTTGTGGAATCTGACTCCTGCACACTCAACCCAGCTCCACGAAGTTCAAGAACCCAAGTG
TCAAATCCTTCACCAGACATGTACCGGGCGAAAGATGACTGCATAATTATTTTTAAAAGTAAAGACCGTG
ATTAAATAACTTTTAGAAAGAATTTAAAAAAACCAGCCATACTATGTAAGATTTCTTTGATAAGCAGGAT
GGACATGGTTGCCTGAAGTGATAAAGAATTCTAAACCTCCACTGAAGAGTAGGGTGCACATACTTGCCT
CAAGTGATAAAAATGAACTTAAAAGGATAAGAAGATGCATCAACAGAACTTTAAACAAAAAATAGACAA
GAAATAATCCCAGTGAAATCAAGTTGACACTAGTTATTGCATTAACCAGCAGACATTTAGATTTGTCTCA
CTAAAAAAGAAAAGATAGAAATCATCACTAGCAACTAAGCAAAAAATCGCCATCACAATTCATATTTCAG
AAATGTACAATCACAGAGAGTGCTAGCCACATGACTACCATCAACACCTGATTGATGCAGCCATAAAGC
CATATAAAAAAAAGGAAGATGAAATAGATAACAAGCCAGTAATAAAAAAATCACACAAGTAAACTTTTA
TACGCTTGAAATGCATAAAGTATGAGAAAAGGACGAAGTTTTTAACCGACCCACCATTTTGATCACCGCA
CCACCATGTGTGCAGAAGGAAGTAGATGCAGATGCACTAGGAACTAGTTACCCTAGGACCGTAAACAAT
CCATTTTGTATTATCTAGGGTCGGGATGTGACCCAAACTACTTACAATGACCCACCCGTTGACACAGGTG
GGGAAACTACTAATGTTCTTGATCCCTAATTATATAATATAATATAAGTATCCTTTTGATCAAAAAAAAAA
AGTAGTAAGGATAGTACAACATCAAAACCAGGGTTTACTCTAAAAAATTTGAGCATGCCATAGAATTCTA
CCAGATTCAGAAACAAGAACCACTCCACGAAATAAAGAGTGAAACTTTAGTGGAACCTGACATATCCAC
TAAGCACTGATAATATACACATTTGACATCTGTCATTATGATCTATTAAATGTGGCTATCAATGAACGATC
CATGAAGATCCCCTAGTTCTGTTCACCTATACAGGCATAAATCTGCAAATTAGAGTGTCCCGGCTACTCTT
TCACGCAATACTGGACATACAACAAAACAGAAGAATGCACAAAGAATGGAGAGAAGGCAGACGAGTAT
CATACCCCAGGAGCAAGATCATATCCAATAGCATTAGTCCCCACACCAGACAACAGAAGCAGGGGATGA
TTTCTCTTTGGAGCCTATCATCATCAAAAATCACACACCCTTAATGCAAAGCTCAAATCCTTGAACAAAAA
AATCATGTAACAGAACTGTTAAACCTTCCTAGTATCACTCCCTGTGTTTCATTTTATATAACAGTATTTGAT
AGAACACAAAATTTAAAATATTCAATAGTTGAATTAGATAAAATGGCGTCAAATATTCTTAATGCCTTCAT
GTCTTATATATTGGGACCAATTCTATATGACCACATTTACTTCAAGCCGAGTATCTATCAGAAACGGTCTC
TCTACCTTCACAAGGTACGAATAAGGTTGCATTCGCTCCACCCTCCCCGGACCTCACCTGTGAGATTACG
GGTTGTATTCTCTATGACCACATTTAATTGATCGCAAAATTTAAAATTTGAACATATAGAAATGAAAGTG
TCGAATATATTATTTTTTTCAAATTGTCATACAGTTAAGAGTAACAGTTCAAATTAAAAAACATAAAAAAT

TGAACCTGAGGAGGAGGAATGTAGCGCCAGAGAGCAAGTTTCCAATCGGAATTATTAACAGATACGTA
GTGGAGTTCATCGGCAGTGCATAACGATGGCTTATTACAAACCTTCTGGACTGTAGTATCGCCATCATTG
GTGGTGAAAGCTCTGAGTTTAAAGGCGGAGTTCACCGTAAAGCAGCAGTTCGGGGAGTTCCGGTATGA
AATGACGGCGGGAGGAAGTCGCCGGCGGTGATTTGTGAGGTGGTGGTAATGGTTGTGGTGGCGGAAG
ATGGCGGCGATTTGAATCGCCGATCGCATATCGGATTGAATTGGATAAACCATATTTTTTGGGATTCGTG
ACAATTTGGATAAAGAAAATTAGTTTGATAAATCTTTTACTCTTTTGACTTTGGAAGGTTTGAGACGTAA
ACTGTTTTTTTTTTTTTAATAATTT
>PGSC0003DMG400006027 LRR-RLPs
TAGAGGTGTCTACTTGCTATTTAATTCAAATCAAATTCATTCATTCATTCAACAACACATTTTTACACTGTG
AAAAAAAAAACTTCATTTTCTTCTTCATAGCTTGAAAATCTCTATCAAATCCAGCAATGCGGCCTCCTAGC
CGCAGCGTATTTATCTCTTTTCTTGTTATCTCCCTCTTGTCGTACCAGATCTCGAATGTGGTAGGACAAGA
AGGTGATAACGATATCGATTTAGATGATATTAAGGCTAATGCTAGTAAGTTAAGTTTCGAAAACCCAAA
GATTCGCGATGCTTATATCGCCTTGCAATATTGGAAAACAGCAATGTTTTCTGATCCTTTTAACTTCACAG
CTAATTGGACAGGACCTGATGTTTGTTCGTATGGTGGAGTGTTCTGTGCTCCTTCGTTAAACGATGATTC
GATTAGAGTTGTAGCTGGCATTGATCTTAATCATGCTGACATTGCTGGTTCCTTAGTTGCAGAGCTTGGT
CTTCTTACAGATCTGGTTCTTTTCCATCTCAACTCGAATCGTTTTTGTGGAGTTGTACCGAAAACTTTTAGC
CATTTGAAGCTTCTTAGGGAGCTTGATTTGAGTAACAATAGGTTTGTTGGTGGGTTCCCTAAGGTAGTTC
TTTCATTACCTTCTTTGAAGTTTCTGGATCTAAGGTTTAATGATTTTGAAGGACCAGTTCCTTCCGAATTGT
TCGATAAAGATTTGGATGCGTTGTTTCTGAATGACAATAGATTCCGTTTTGGCATCCCAGAGAACTTGGG
TAACTCCCCTGTTTCTGTTCTTGTGTTTGCTAATAACGATCTTGGTGGGTGCATTCCCGCGAGTATTGGGA
AAATGGGGAAAACGTTGAATGAGTTGATATTGATGAATGATAACCTCACTGGGTGTTTGCCCATGGAGA
TTGGATTGTTGAAGAAGTTGACAGTGTTTGATGTTAGCTTCAACAAGATTCAAGGCTCATTGCCATTAAC
TGTGAGCAAAATGAGGAGTGTGGAGGAACTCAATGTGGCTCACAACAAGCTTACTGGGGTTATACCAG
CTAGCATTTGCCAACTTCCAAGGTTGCAGAACTTTACGTATTCGTTCAATTACTTCACTGGGGAGGCTCC
GGTTTGTGCTGCGACTAGATCTGGTGATGGTCAGGAGAACTGTATAGTTGGAAAGAAGAATCAAAGAT
CTGCTAAAGAATGCTCTTCGGATGATGCAAAACCGTATGATTGTAAGAAATCGAAGTGTTATAGCCCAT
ATGCTACGTCTCCTTCGACGAAACCAAAGCCAACACCAACACCAAAACCAAGAGGACCACCACCACCTA
CTTGGAAGTCTTCGGGTTCGCATAACAAGCGATCTCCGCCACCACCTAAATCAACTCCTCTTCCTCCACCA
GCACCTTACAAAAAGTCCCCAGCGTATCGACATAGAAGTCCACCACCACCTACTCACAAAATTTCACCTG

TAACTCATCATTCACCTCCTCCACCCTCACCCGTATATTACCACCCATCTCCATCGTCCCCACCACCACCAG
TTTATTATTCTCCACCACCCCCGGTGTATTACGAGCCACCTACTTACAAGCCTAAATCACCACCACCGCCA
ACACCGTCATATGAACACCCAAAAACCCCATCACCACCACCACCAACACCATCATACGAACATCCAAAAA
CCGCATCACCACCAACACCATCATACGAGCATCCTAAAACACCACCACCAACACCATCATACGAACATCC
AAAAACACCATCACCACCACCACCAACACCATCATACGAGCATCCAAAAACACCACCACCAACACCATCA
TACGAGCATCCAAAACCCCAATCACCACCACCACCATACGAACATCCAAAAACCGCATCACCACCAACAC
CATCATACGAGCATCCAAAACCCCAATCACCACCACCACCAACACCGTCATACGAGCATCCAAAACCCCA
ATCACCACCACCACCAACACCATCATACGAACACCCAACCCCACCTACCCCACCTTGTAACGAACCACCAC
CTCCACCACCTAATTCACATTGGGAACCAAAACCATCCCCACCATACACCTACTCATCACCACCACCTCCA
TCACCATCTCCTCCTCCTCCCACTTACTACTACTCATCACCACCGCCACCACCACCATCACAATCACCTCCC
CCACCCACTTACTACTACTCATCACCGCCGCCACCACCACCATTTTACGAAAATATCCCTCTCCCACCGGT
AATCGGAGTTTCCTACGCATCTCCACCACCACCAGTCATTCCATATTACTGAACAGGGGTTATCAATTAGC
CTTTGATTTTCCATTATCAAAAAAAAAAAAGAATTCATTTTTTTTTATGAATTATGTATACTTTGTTATTTAT
TTTTTGTAATATTTTTATTTTGAGTTTGATTATATTTATGAAAAATTGGGATTATGGGCGGGAGTTAGTGA
GGGGTTTAACTATCAATATTTTAATATTGTTATAT
>PGSC0003DMG400006076 LRR-RLPs
AATTCCCCTTTCAAGCAAAAAACATGGCCGCCACTGCTGACATATTCTCGGAATGTGTGATTCACAAAAT
ACTCTGTTATCTTAGCTATGGCCAAGCATCCCGGATGAGAATTCTATCTAAAACATGGCTACAAGCTTGG
TCGACTCTTCCGAACTTGGAATTCTTAGTTGAATATTTCGAAGAAACAAAAATAGTGGACGCAATTATGG
AGACATACAGGGAAAACAAAATCCCCATTGACAAGTTTGGATTTTCAAATTGTTCGAAATTCAATCGTCG
TCATCAAGTTTTCCCTCTGATTGATAAGTGGCTTGACACAGCTCTTCAAAATGGCGCAAGAGATTTAGTC
TATGTAGATCTTCTCTACTCGGTACGATTATATCCTTTTCCTATTTCTAAAGTCTTGGCTGCAAATTCTTTA
AGAGAATTGGTTCTGAAAGGTTGTAACCTTATGCATGTTTCGTCATCTAGTGGGTTCGCGAATTGTCATT
CTTTGAGAAAGCTTTCTCTATCTTATATAATTTTAAGCAAAAATTTGCTTCAGACTTTACTTAATAGTTGTC
CTTTCATCGTCTCTTTCAACCTTGATCATTGTTCGGGGTTGGTAAAGATTGAGCTTATGAATCTTCAAAAG
ATCAAGTCAGTTTCCGTATCAATAAATAGAAACCAACATGTGAAAATCCAAGCACCAACTCTTGAACACT
TGTTTTATAATGGTTATTTATCGGGAAATTTGGTTATCGTTGGATGTCAGAATCTAAAATCTTTAGAGCTA
TCATATGTGAAGATATGTAATGGATTTCTCCATAACCTTATTTCTAGATCGCAATCCCTAGAAGTATTGAA
GATTCAGTATTGTATGGGCATAAGACAGATTGAAACTTCGAATTTGGTATCACTTGAGTATATAGGATAT

CAAATTCCTGAACTTAAAATTGCGAAAGCTTCAAGTCAATTGAAGCATTCAAAAATCGTTCTTCATGGCA
ACAACAATTTAGATGCTGCATGGTTTAGTAAGTTGAGGAAGCTTCTATGGAATTCGGCCTCTTGGTCCCA
AGTTCTCCTTTGTTTTCCCGAATACAATGAGATCAAGATTAAAGATTTGCAGCAGCACAACGGAGTTGCT
TGCCCCCAGATGGACGTTTTAAATGTCAACATTACAAGGTCATATGGGGAGTGTCCAACATTTGTGGAT
GCTTTGCTATGGAGTTGTAATCCTAGGAGACTCAACCTATCATCAACGGTTGAAATGATTACATGTTTCG
TCAGTCGTTTGATGTATATGAAGAATCTGAGTCATTCTTCTTCGGAAAGAAGCAACCCCTGGCATAGTCA
ATTGAAAGAAATCAAAGTTTTTGATGTGAAAAATCAAGAGCTGCAACTCGTAAGTGGGGAACTGGCGAT
AAGCACCCTTTCGGAGGACAGGAAGGAAGTTTATTTTTTATTAGATTGGCAATGCAATTAATTTATCACT
ATTTCAAAACGAATGCACTTGTTCATTCCTAAACTTTATTAATCTGTTTTATGTCTAGCTTTAAATACTTGC
ACTTTCTCACCATTGTATAAAATTGTGTTGAAGTTGAAGCATAAGATTCTTT
>PGSC0003DMG400006095 LRR-RLPs
ATGGGGACAGCTGACATGTTGCCTGTATGCCTCATTCGCAAAATACTCTGTTATCTTAGTTATGAAGAAG
CATCTCGGATTAGAATTCTCTCCAAAACATGGCTACTAGCCTGGTTGGATCATCCCAACTTGGAATTCAC
AGTCTCGTCGGGCAAAACCATAACAATAGTGGATCATGTCATGGAGAGATATAAGAACGGAAATATTCC
TATTGACAAGTTTGAATTACACAACTCTACAAAGTCTGATCATATTTTTCCTCTGATTGATAGGTGGCTCG
GTATTGCTCTTCAAAATGGTGTAAGACATCTAGAGTACATAGATGTTGAACTTTCAACATCATACCCCTTC
CCTATTTTTAAATTCTTTGAAGCAAATTTTTTACGAGAACTGGTCCTTACGGGTTGTGATCTGATGCATGT
TTCGTTATCTAGTGCTAGTCAAGCGGTGACGTGTCATTCTTTGAGAAATCTTTCTCTATCTCTTGTACGCTT
AGACAATAACATGCTTCAGACTATACTCAGTAGTTGTCCGTTCATTGTTGATTTAATCATTGGGTATTGTA
GTAGGCTGAAAAATATTGAGATACGGAATCTTCCAAATATCAGGTCTCTTGTCATTGGTAATGACCAACC
CGTTAAAATCCAAGCACCGTCTCTTGAATACTTGTATTATAGTAGTTCTTGTTTGAATAAATTGAATATTG
TTGAACATAAGAATCTGAAAACTTTAGATATATCATGTACAGAGATATCTGATGTATATCTCAACCACCTT
ATCTCCATACCTTACTGCCTGGAGAAACTGTTATTAGCTAACATTGCTTTTGGAAGATTCGACATTTGCAG
GAGTCAATCCCTAAAGGTGTTAAAGATTCATAATTGCAAGATCATAGGAGCAATTGATGCTCCAAATTTG
GTTTTGCTTGAGTATAAAGGAAATGATATTCCTCAGCTTAAATTTGCTCAAGAATCAAGACAATTGAGGC
AATCACAAATGATTCTTTATCCCTTGTTCAATGTAGATTCAGCATGGTTTTGTCAGTTGAGGAAATCTTTA
TCAGATTCGACGATCTCTTGGTCTCAAGTTACCATTTACTTTCACAAATGCGAGGAGATCAACATGAGAG
ATTTGCTACTGCAACCTAGAGTTGCTATCCCCAAAGTGGACATTTTAGATGTAAATTTTTCAAGGCCGAA
TTGGGAGTTTCCGACATTTGTGGATGCCTTGCTGTGGAGTTGTCATCCCAGGAAACTTAACCTACAATCA

ACAACTACAATTTTTACATGTTTCATGTATCGTTTCATGCAGATGAAGAATATGCGTCATGGTCAATTAAA

AGAAGCAAAAGCCTACAAATTTGATCAGAAAAGTAAGAGTTGGCATCCTGTAGAACACAAAAGAGGAA

GAGATTTCCCAATAAGGACCAGTAGCAAGTCGAAGAAATATTTCTTTTTATTAGATTGGTGA

>PGSC0003DMG400006226 LRR-RLPs

GTACAACTCCATAATTCATTGCCCTAACCAAATGGCTACCATTATTATCTCTATTATTTTCATCCTTCAATT

ATTTTTGCAAAATTGTCTAGTCCTTGCTAGTCCAATTCTTGGCCAAAGTAGACAAGCTTTGGAGATAATCA

TCGGCGGAGGTGGTGGAGTACCTCCACCGGATAATCAAGATTGTCCACCACCTCCTCCGCCACCAGAAC

CTCCTTGCCCTCCGCCACCATCGCCACCATTGCTCCTCTTTGAAAGTAAACGTGTCGAGATTGCCTATCAT

GTCATCCAAAAGTTAAAGTCCAAAATCACATATGACCCTTTACATATTACAAAAACATGGATTGGCTCTG

ATGTTTGTAACAAGTACAAAGGTTTTCGTTGTGCCACGGTTCCTGACTTAAAGGTAAAAGCACTAGCTGC

TGTGGACTTCAATCAGTACCAATTTGATGGCCCTGACCTTACTATTGACGGCTTTCTTGATGAATTACCAG

ACATAACAATCTTCCACGCAAATACCAACAATTTTAAAGGTACAATCCCCAAGAAAATCGCGAATCTTCG

TTATCTCTATGAGCTAGACCTTAGCAACAACAAATATAATGGTGAATTCCCAAAAAACATTGTTTTTGGT

GCCAAGAAATTGTATTTCTTGGACCTTAGGTTCAATTCATTTTCCGGGTTGGTTCCCCCTGAAGTTTTCTT

GTTAAATCTCGATGTTCTGTTCATCAACAACAACAATTTAATCCAAAAACTTCCTGATAACTTGGGATCAA

CTCCAGTTCTTTACTTAACTTTAGCCAACAACAAATTCACCGGTCCAATTCCTCGTAGCATTGGTCAAGCT

TGTAAATCATTACGCGAAGTTCTGTTCTTGAACAACCAGCTCACTGGATGCTTGCCTTATGAAATTGGGC

TTTTAAGTAAAGCCACAGTTTTCGATGCGAGCAAAAATCAGTTAACAGGAAAAATACCTCATTCATTTGG

ATGTTTAGCTAAGATGGAGATACTTAACTTGGCACAAAATCAGTTCTATGGAGCTGTGCCTGAACTCGTG

TGCAAGCTCTGCAATTTGAAAAATTTGACGTTGAGTTACAATTACTTCACTGAAGTTGGACCGGAATGCA

AGAAGTTAATTCAGAAAAAGGTTCTTGATGTCAGGATGAATTGCATTCCGGGCTTGCCTATGCAAAGAT

CAGCAGAGGAATGTGCTGCGTTTTTCTGTAAGCCAAGATCATGTCCTGATGAAAAGTCAATGGGATTTG

TTCCTTGTAGCATTGGCAGTTCTGAGCAGAATCAACAGAACGCAAGTGAGACCGCGCCAGCGCCAAGAA

CTTACAGTGTTTTGAAACCGCATTCGTTGTAAATGCTCTGTTCTTTCCATGTTTGCAGATTGAACTATGGC

AGCAGGAAATGTAAATTTGCCAAGTTTAACTTCCTTATCTCTGTTGATGCATATTATGTTTGTTAATTATA

ATAAAATAATTACGTTTGGATGAGATGATCACATTGTTCAATAGAATTAGAGATGGTAGGTTGTGTAGC

ACAATGGCTCAACTAATGCATCTATGGTTTTGGATAGCA

>PGSC0003DMG400006255 LRR-RLPs

AAAAAATTTATTTCTCTCTTTCATGAATGAAGCAAGACCAAAGAGGAGGACTTCCACAAGTCAGTCTTCC

ACAGAATGAACCAAAAGGGATAACATCAAACAAGTAAAAGGAGTTAATTCAATCTGTACAAACGCGTA
GTTTTCTTTGACATTTCCCAATAACCCTTCTCCTTATTAAGTTCCTTCTAACTGCGTACTTTAGTCATATTCA
AGCTAACAAGTTGCATGAATATGACAACTCTCTACTTTCTATGGCTTTTCTTGATTCCCTTGTTTCAAATCT
TATCAGGATATCACATTTTCTTGGTTTCCTCTCAATGTTTTCACGATCAAAAGTCGTTGTTGCTACAGTTG
AAGGGCAGCCTTCAGTATGATTCTACTTTGTCAAATGAATTGGCAAAATGGAACGAGAAGACAAGTGAA
TGTTGCAATTGGGATGGAGTTAAATGTGATCTCTCTGGTCATGTGATTGCCTTGGAACTGGATAATCAGA
CTATTTCTAGTGGAATTGAGAATTCAAGTGCTCTTTTCAGTCTTCAGTATCTTGAGAGGCTAAATTTGGCT
TACAACAGGTTCAGTGTTGGCATACCAGTTGGTATAGCCAACCTCACGAACTTGAAGTACCTGAATTTAT
CCAATGCTGGTTTTGTGGGGCAAATTCGTATGATGTTATCAAGATTAACAAAGCCAGTTACTCTAGATCT
CTCAACTCTTTTCCCTTACTCTGACCAGACACTAAAACTTGAGAATTCCAATTTGAGACATTTCATTGACA
ACTCAACAGAGCTTAGAGAGCTTTACCTGGATGGGGTTGATCTTTCATCTCAGAGGACTGAGTGGTGTC
AATCTTTATCTTCATATTTGCCTTACTTGACCGTCTTGAGTTTGCATGCTTGTCAAATTTCAGGCCCTATTG
ATGAATCACTTTCTAAGCTTCAGATTCTCTCTATCATCCGTCTTGACCAGAACAATCTCTCTACCACAGTTC
CAGGATATTTTGCCAATTTCACAATCTTGACTACCTTGTCCCTCGACTCTTGTAATCTGCAAGGAGCTTTTC
CTAAAAAGATCTTTCAGGTACAAGTTTTAGAGATTTTGGACTTTCAAATAACAAGCTGCTTAGTGGTAGT
ATTCCGAGTTTTCCTCGAAATGGATCTTTGAGGACGATATCACTAAGCAACACCAACTTTTTGGGTTCATT
ACCAGAGTCCATTTCGAACCTTCAGAACCTGTCCAGGTTAGAGCTCTCCAATTGCAATTTCACTGGACAG
TACCTTCAACAATGGCAAACCTTATCAATCTTGTTCATTTGGACTTCTCTTTGAACAACTTCACTGGTTCTA
TCCCATATTTCCATAAATCCAAGAAACTCGCCCACTTAGACCTATCTCGTAATGGTTTAACTGGTTTCCTCT
CCCCGACTCATTTTGAAGGACTCTCAGAGCTTGTCTACAGATATTTAGGGAACAACTTACTCAACGGGAT
CATTCCTGAATGTATCTTTGAGCTCCCCTCCTTGCAGACGCTTTTACTTAACAGCAATCAATTTGTTGGCC
AAGTCAACGATTTTCACAATGCATCCTCCTCTCTGTATGTAATCGATTTGAGCAATAACCACCTGAATGGT
TCAATTCCCAAGTCCATGTTTGAAATAGGGATGCTTAAGGTCCTCTCATTGTCTTCCAACTTCTTTAGTGG
GACAGTTCCACCTTGACCTCATAGGGAGGCTGAGTAATCTTACAAGACTGGAGCTTTCTTACAATAACTT
GACTGTTGATGCAAGTAGCATCAACTCCACCTCTTTCATATTTCCCCAGTTGAGATTATTGAAACTAGCTT
CTTGTCGGTTGCAAAAGTTTCCTGATCTTAAAAGTCAGTCAAGGATGATCCACTTAGATCTTTCTGACAAC
CAAATACGGGGGGCAATACCAAATTGGATTTGGGGAATTGGTAGTGGAAATCTGGCTCACCTGAATCTT
TCCTTCAATCAGCTGGAGTACGCAGAACAGCCATACAATGTTTCCAGCTATCTTGTAGTCTTGGACTTTCA
TTCCAATAAGCTAAAGGGTGACATACCAATTCCACCTTCCTGTGCTGCATATTTGGACTACTCGAGCAAT

AATTTCAGCAATTCCATCCTACTAGATATTGGCAATTATCTTGGTATATTTCTCTCACTTGCAAATAATCGG
CTCGCAGGAAGAATTCCTGAATCTATATGCAATGCCAGCTACCTTCAAGTTCTTGATTTCTCTAACAATGC
CTTGAGTGGAACAATCCCACCATGTCTACTGGAATATAGTACAAATCATGGAGTGCTGAATCTAGGTAA
CAATAAACTCAATGGCGTCATACCAGATTCATTTCCAATTGGTTATGCTCTACAAACTTTAGACGTCAGTG
AGAATAAGTTACAAGGTAGGCTGCCAAAATCGCTTGTGAACTGCAAGTTGTTGGAGGTCCTGAATGTTG
GAAATAACAAACTTGTTGATCGTTTCCCATGCATGTTGAGGAACTCAAATAGTCTGAGGGTTCTAGTTTT
GCGTTCCAATCAATTCAATGGAAGTGTTCAGTGTGATGCAACCAGGAATAACTGGAAAAATCTCCAGAT
CATAGATATAGCTTCCAACAACTTCACTGGTGTGTTGAATGCAGAATTCTTTTCAAATTTGAAAGGAATG
ATGGTTGCAGATGATTACGTGGAGACAGGACGCAATCATATCCAGTACAAGTTTCTCCAACTAAGTAAC
TTATACTATCAGGACACAGTGATAATAACCATCAAAGGGCTAGAGTGGGAGCTTGTGAAAATTCTCAGG
GTCTACACAGCTATTGATTTCTCTTCAAATAGATTTCAAGGAGTGATACCAGATACTATTGGGAATCTTA
GCTCACTCTATGTTCTGAACCTATCACACAATGCCCTTGAAGGACCAATTCCAAAATTGATTGGGAAGCT
ACAAATGCTTGAATCACTAGACCTGTCAACAAACCACCTATCCGGGGAGATCCCATCAGAGCTTGCAAGT
TTCACATTCTTAGCAGCTTTGAACTTATCGTTCAACAAATTGTTTGGCAAAATCCCATCGACTAATCAGTT
TCAAACATTCTCAGCAGATTCCTTTGAAGGAAACAGTGGCCTATGCGGGCTCCCTCTCAACAACAGTTGT
CAAAGCAATGGCTCAGAGTCCCTGCCTCCACCAACTTCGCTACCAGACTCAGATGATGAATGGAAGTTC
ATTTTCGCAGCTGTTGGATACGTAGTAGGGGCAGCAAACACCATTTCACTTCTGTGGTTTTACGAACCAG
TGAAGAAATGGTTTGATAAGCATACGGAGAAATGCTTGCTTTGGTTTTCAAGAAAGTGATCCTTAAACCC
ATAAATGATGAGTTTATTTAATCCTTGCTTTGTTTAAATAAACACCAGGATCCAAATTCAAGTCAGTTTGT
TATTTCCCTTGATGAATGTATACAATTTTCTACTATCAGAAACAGCACCCACTTTTGGATTAAATAGCTTCC
CACATTGCCTGCAAC
>PGSC0003DMG400006268 LRR-RLPs
AACCAAGCACTTGTCCTATTTTTCTTTTGACTATTTCTTCCCCTCATTTTCATCTTCTTTTCTACCTTCTCCCT
TCGCTTCAATCACAAAGTGGAAAAAACAAGTTTGATGGATTATAATTCCTCCAAGACTTGAGCAATGAGA
TTTTTACACTTTCTATGGATCTTCTTGATACCCTTTTTGCAAATTTTATTAGGTAATGAGATTTTCTTAGTTT
CCTCTCAATGTCTTGATGATCAAAAGTCATTGTTGCTGCAGTTGAAGGGAAGCTTCCAATATGATTCTACT
TTGTCAAATAATTTGGCAAGATGGAACCAGAACACAAGTGAATGTTGCAATTGGAATGGGGTTACATGT
GACCTCTCTGGTCATGTGATTGCCTTGGAACTGGATAATCAGACCATTTCTAGTGGAATTGAGAATGCAA
GTGCTCTTTTCAGTCTTCAGTTTCTTGAGAGGCTAAATTTGGCTTACAACAAGTTCAATGTTAGCATACCA

GTTGGTATAGGCAACCTCACCAACTTGAAGTACCTGAATTTATCGAATGCCGGTTTTGTTGGCCAAATTC
CTATGATGTTATCAAGATTAACAAGGCTAATTACTCTTGATCTCTCAACTCTTTTCCCTGACTTTTACCAGC
CACTAAAACTTGAGAATCCCAATTTGAGTCATTTCATTGAGAACTCAACAGAGCTTAGAGAACTTTACCT
TGATGGGGTTGATCTTTCATCTCAGAGGACTGAGTGGTGTCAATCTTTATCTTCATATTTGCCTAACTTGA
CCGTCTTGAGCTTGCGTGATTGTCGAATTTCAGGCCCTATTCATGAATCACTTTCTAAGCTTCACTTTCTCT
CTTTCATCCGGCTTGACCAGAACAATCTCTCTACCACAGTTCCTGAATATTTTGCCAATTTCTCGAGCATG
ACTACCTTGAACCTCGCCTCTTGTAATTTGCAAGGAACATTTCCTGAAAGAATATTTCAGGTGTCAGTTTT
AGAGAGTTTGGACTTGTCAACTAACAAGTTGCTTCGTGGTAGTATTCCGATTTTTCTTCGAAATGGATCTT
TGAGGAGATTATCACTAAGCTACACCAACTTTTCTGGTTCATTACCAGAGTCCATTTCGAACTTTCAAAAC
CTGTCCAGGTTAGAGCTTTCCAATTGCAATTTCAATGGATCAATACCTTCCACAATGGCAAACCTTACCAA
TCTTGTTTATATAGATTTCTCCTTCAACAATTTCACTGGTTCTATTCCATATTTCCAACAGTCCAAGAAACT
CACCTACTTAGACCTTTCACGTAATGGTCTAACTGGTCTCTTGTCTAGAGCTCATTTTGAAGGACTCTCAG
AGCTTGTTAACATAAATTTAGGGAACAATTCACTCAATGGGACCCTTCCTGCATATATATTTGAGCTCCCC
TCCTTGCAGCAGCTTTTTCTTAACAACAATCAATTTGTTGGCCAAGTCCACGAATTCCGCAATGCATCCTC
CTCTCCGTTGGATACGGTTGATTTGAGTAACAACCACCTGAATGGATCGATTCCCAAGTCCACATTTGAA
ATTGGGAGGCTTAAGGTCCTCTCACTTTCTTCCAACTCCTTTAGGGGGATTGTGCCCCTCGACCTCATTGG
GAGGCTGAGCAACCTTTCAAGACTGGAGCTTTCTTACAATAACTTGACTGTTGATGCAAGTAGCAGAAA
TTCAGCCTCTTTCACATTTCCCCAGTTGAACATATTGAAACTAGCTTCTTGTAGGCTGCAAAAGTTCCCTA
ATCTCAAGAACCAGTCAAGGCTGATCCACTTAGACCTTTCAGACAACCAAATAAGGGGAGCAATACCAA
ATTGGATTTGGGGAATTGGTAGTGGAAATCTGGCTCACCTGAATCTTTCCTTCAATCAGCTGGAGTACAT
GGAACAGCCTTACAATGCTTCCAGCAATCTTGTTGTCCTTGACTTGCATTCCAACCGTTTAAAAGGTGACT
TACCAATTCCACCTTCCTCTGCTATCTATGTGGACTATTCGAGCAATAATTTAAACAATTCCATCCCACTAG
ATATTGGAAATTCTCTTGCTCTTGCCTCCTTTTTCTCGGTAGCAAACAATAACATCACTGGAATAATTCCT
GAATCCATATGCAATGTCAGCTACCTTCAAGTTCTTGATTTCTCTAACAATGCCTTGAGTGGAACAATACC
ACGATGTCTACTGAATAATAGTACAACTCTTGGAGTGCTGAATCTAGGGAACAATAGACTCCATGGTGTT
ATGCCAGATTCATTTCCAATTGGTTGTGCTCTAAAAACTTTAGACCTCAGCAGGAATATCTTTGAAGGGA
AGCTACCAAAATCGCTCGTCAACTGCACGTTGTTGGAGGTCCTGAATGTTGGAAATAACAGTCTTTTTGA
TCATTTCCCATGCATGTTGAGGAACTCAACCAGCCTGAAGGTCCTAGTCTTGCGCTCCAATAAATTCAAT
GGAAATCTTACGTGTAATATAACCAGAAATAGTTGGAAGAATCTCCAGATCATAGATATAGCTTCCAACA

ATTTTACTGGTATGTTGAATGCAGAATGCTTTTCAAATTGGAGAGGAATGATGGTTGCAGATGATTACGT
GGAGACAGGACGCAATCATATCCAGTATAAGTTCTTCCAACTAAGTAACTTGTACTATCAGGACACAGT
GACATTAACCATCAAAGGCATGGAGCTGGAGCTTGTGAAGATTCTCAGGGTCTTCACATCTATTGATTTC
TCTTCAAATAGATTTCAAGGAATGATACCAGATACGTTTGGGCACCTTAGCTCACTTTATGTTTTGAACCT
GTCACACAACGCCCTCGAGGGACCAATTCCAAAATCAATTGGGAAGCTACAAATGCTTGAATCGTTAGA
CCTGTCACGAAACCACCTGTCCGGGGAGATCCCCTCAGAGCTTTCAAGTCTCACATTCTTAGCAGCTTTG
AATTTATCATTCAACAATTTGTTTGGCAGTATCCCATTGAGTAATCAATTTCAAACATTCTCAGCAGATTC
CTATGAAGGAAACAGAGGCCTATGCGGGCTCCCTCTTAACGTCACTTGCAAAAGCGATGCTCCAGAGTT
GAAACCAGCACCAAGTTTTCAAGATGACTCTTATGATTGGCAGTTCATATTTACGGGTGTGGGATATGG
AGTAGGGGCAGCAATCTCCATTGCACCTCTGTTGTTTTACAAGCAAGGGAGCAAATACTTTGACAAACAT
TTGGAGAGAATGCTTAAACTGATGTTTCCAAGATACGGGTTCACTTACACCAGATTTGACCCTGGGAAG
GTTGTAGCTGTGGAAGAATATGAAGATGAGACCCCAGATGATACCGAAGATGACGATGATGGGGGAAA
AGAAGCATCTCTTGGGCATTATTGTGTCTTCTGTAGTAAACTTGATTTTCAGAGAAATGAAGCAATGCAT
GATCCTAAATGCACTTGTCATATGTCATCATCCCCCAATTCTTTTCCTCCTACACCATCCTCTTCTTCACCTT
TATTAGTCATATATCACAAAAAGTTTTGATTCTTAAGCTTCTTCACATCTGCACATTTGTAATCTGTATATA
GTTACAGGTAGCACAGGATTGATTCAAAGCAAATTAGTCTCTCT
>PGSC0003DMG400006279 LRR-RLPs
TTTGATCCCCCTCCATCCTGATCCCTGCCCTAATTTCAGGACCTCAAATTTCAAACAACAGGAAGCAAGAA
CAGAGGAAAATCCATGGAAATTCAGAGTCCGATGAAGCTAAAGAGATTGGTTGTGATCGGAAGGCATA
AAAAAGACCGAATCAGTGATTTACCCGATGCCATCCTTCTTCACATCCTCTCTATGGTGCCGGACGGAAA
ACAAGTTGTGCGAACCAGCGTACTGGGTAAACGATGGAGGTCTCTTTGGATGTCTGTTCCAATGTCAAT
CGATTTCAACTTCCCCCAAACTGAAAATCTAGTCGTCACACTTGATTACCTGTCGTCCACACTTACAGAAC
TTCATTACTGTAAGTCGTGCGATAAAATACCCAGATTTACCGTTGGGGGATTTAGGTACGAACACTGTTA
TGCTAAGTATGTTGATTTATGGGTTAGTTTCGCTACTAAAGTTGCTAAGGTGGAAGAATTTTTGCTTGGA
TTGTTATCTGTAAATGATCAAACTTATGACTTCCCTTCGTATGCATTTAAGAATTCGTTGCTTAAGGATTT
GGAATTGAGGAATTGCCAACTTAACCCTCGTGCTACTGTTGATTGGACTAATCTAAGCTCTCTTAAAATT
GGGTATATCGATTTGACAGATGATGCTATGGAAAAGATATTATCTGGTTCCCCGAACTTGAAAAGCTTG
GAACTAGATAATTTTTCCGGCCTACAACGTCTCGAAATTAACTCTGTCAAGTTGACACAACTGATGATAG
AATCTTATTACAATGACAATGATGAGATTTGGCTTGAAATAGTAGCTCCCCATCTTCAGCATTTAGAAGTT

TTGGAATTGTGCAGTGAGATACGCATTGGCCAGAGAAATGTGCCTTCACTTGTCACTGCAATCTTTCGTT
TAAATTTTAATTTTGAGAATGAAGAACAAGACTTGGAGATGGAATTTAGCTGTTTGAAGGAACTTCTTCA
TAGTGTTGCCCATGTCCAGAATCTTGAATTGGGTACCTGGTGCATCGAGGTTTATTCATACACTTCATACT
GCAATCTTGAATTTTTATTTACTATTGAATCATCACTATAGTAACTTTAACAGTTCACTACTTATAATCTGA
TAACTAAGTTGGTCAGTACTAAAGAGTTTGCAATGGATACAACAACATGTAAAATTTGGTTTGATTTACA
CATATCATACTTTTAGTTATGTTGATAGGTTTTTCCAAGTGTTTAAAGGTCCATCTTTGGATTTGCAAGCG
AACCATGAACACGTTGATATGTGTACAGTCATATAGTCCAAGTTGTTTTTATGTTATATCCAACAATGTGC
ATTTCATTTATTAAGTATGCCATGTTTGTTTGTTTTGACATATTTTGGTTTGTCTGTTTTTGGTTTATCAGTG
CGTGTCCATCCTAGAGTTGAAAGGGTGGCGGCCTCCACCATCAAGCCGGAAATTCTTAGAACTGAACCT
AGCCGTAATACAATTGGACTTCCCTGGAATTTACAGCTTTCTACAGAGCTCATCGGATCTTGAGACATTG
ATCATTGGTTGTTGGTACAATCACAATGAAAGAGTAAGTTTCCTTTCGACCTTTATTTATATTACTTCATGT
TATTCAAATCAAACAACTATTAGTGGAGGAATAAGCTGATATATATCAACCTATTTCTAATCTTAAGTACT
TCAGGAAAGAAAATTTATTTAAGTGTTTTTTTTAATAGCAATCTCCTCAGTCACTTTACCATCCCAACTGT
GCTTCCTTCCATGAGTTCTATTTTTGTTTTTTCCAATTCATCGTTTCCTAGGAGATATTGCAGCTTCCCTAG
CTTCATTTTTAAAATATTCATCCAGTGTGGCATCCTCCTTGCTTCTCTCAAAAATGTTTGCACCTTGAGAAC
TTCTTGGAGAAAAATCACTAGGTCTAACATGTAGTTGCTTACTAGTGTTAATAGGAGTAAGAGAATTCAC
TCTTTCAATATTCGAACTCATCAGCACATCAAGAAGCGCCATGTCTATAGTATTATTAATCTTCTGAACAT
TAGAACTGCTGCGCAGAATTCTCAGATGATGCATCTGCCAGTTTCGAAATTGTATGACATCCCTGTTTCT
GGTGCATCTGTCTAGCACTTGAACTTGCAATTTCTTTTGAACTCAACGCTCCATCTTTCATGATTTCAGGC
GATCGCTGCCCCCAAGTGACAACTTCATTCCCCTGAATATTGTCTTCTAATATCTTCTCGTTCCCAGCAGC
CTTCTTTAACACCTGATTGACTAAAATCTGTACCTCTGCTTTTGTGCCATGAATGGTCTTACACTCTTACCA
ACTTCAGAACCTGTAGCATCTGATTTCTCACCCAAATTACGAACACTCTGTGCAGCTCGTCTTGAATTAAT
AATTTCCCTAGCATTTCCCTGATGCTTAATACCTTGCATATTAATCTGCATACTTCATCTCTATGGCCTCGA
TGTTTGCATGTACGTCAATATGAAGGTTAATAATCATATGTTATTATTTGGTACACTATCAATTCATTCGA
CTTTGGCTCTAATGAGCAATCTCACACGTTCAGGCAAAGCTGCCAGTAAGTTGACTTCAACTTTAACTCTT
GCTGTGCTTGGCCTTGTTTGATCTGTGATTGCTCTATCAACAACCAAAGGTCTGCCCACTGCTGATGCTAA
CAAATATAATGCCTCTCGAACGAAAAATTGCACCAGTAAATTCTGAAACGATATCCAAGTCAAAGCAATG
GATTCTTCTTCTTTGGGATTAAACCAAGGGGTCCAACGAAAAATTCTAAATTGATAGTCTTCCTCCATGG
ATTTTCTCCTTCTCACCAATTGATGAAATAGCTATATGAATAGACAGCACCAAAATCCTCATGTGCCGTCA

ATCGAATTAAAATATGCCGTGAATCTAATGAAAATGTTTCATGCCCCTTTAACCTTTAGTTGTTGTGGAAA
ATACGCTCTCAACTCTGTCATTGCAAGTTTGCAACAAGATCATATCGCAAATTTTTCTCTTGGAAATTTAT
ACAGAGCACATTGCAAATTTTCCTCCTGAATATCAGATTATTCAAAAACTATAGTAGGTTCTCCATTGATT
ATCAAAATTGTTGCACGCCCCTCTAGCTTCTCATGCTAGTGAATTAAAGCTGAAAATGTTAACAGTATGTT
ACCTTGACTTATGATTCAACTCTTTAATCTTTTTTTGTTGTTTGGATGTACCACATTGTAACATTTGCTAAT
ATGTTCCTTGAATTTAAAAATTCTTCACCTTTTTGTTTTATCCACAATCAACTCCTTGCCTTCATGCTTTCTT
CTAAGGACAATAATTGGTTAATATAGTGTATTCTTTCCTACAGAATCTGTTGGCAAGGTACACAGATGAG
GATGAACTGAGCGAGAGGTTTGAGAAGCATAATGTTAAATGCTCATTTTCACATCTGAAGACCATCACG
ATCAATAACTTTTCTGGATCATCAAGTGAAAATAAGTTTGTGCTGCAATTGGTAAAATATTTGCTCAAGC
ATGCAGCCATGCTAAAAGAGTTTGTCATTGCTTCCACATACCAATGGATGTCTCCGGAGTATCGTAAAAT
GGCACAAAAGCTCTTAAGCTTTCCAATGTCCTCTCCGCACGCTTCAGTTGTCTTTTCTGACATATAACCTTT
GTCCCTTACAACATACCTTACTAACAATGTGTTTATGCTCTAAACAGCAAGAAAGTTTGTCGTAAATGTTT
TATTGACTTATACTCATTGTTTGTGAGAATAGTTTTTCAAGATTCAGTTATGTGTCCAAGGGCTTTGTTTTT
GTTGGCCTATGTTATTTTTAATTGGTGGAATGATTATTTCTCGACTTGGACAGTGAATGACCTTAGGAGG
GAAGCAAGGCAGTGGAACCTTGTCCCAGCATGTTTTCTGGGGTATTACAAAAGGAAAGAAATAGCATGT
GTATATCTGCAGAGTTCATATTAGATTAGTTTGTATCAGAACTTTTATGTTATTGAAGACTTTATGATAAT
CTGTATGGTAAATTGTTGAGTCCTGTACTGTTCTC
>PGSC0003DMG400006422 LRR-RLPs
ATGGTCAAAGGAAAAAAGGAAGTGGGAGATGATGATGAGTTAAATTATTCCTACATTTTTGGAGTTTTG
AGAGAGAGGGCCATAGTAACGAATAAAGGCAATAAGTACCAGTATGACACCATTTTGGCATTGTTTACA
AGTATGGATATGTCTAGCAATAAGCTTTCTGGGGATATTCCTGTAAGTTTAACAGGTCTTGTAGGATTAA
GATCGTTTAATTTTTCTAATAATAACCTAACTGGTAAGATTCCAAATGACATTGGTGACATGAAAGTCTTG
GAATCTGTTGATCTTTCAGAAAATAAATTTTACAATCAAATCCCACAAAGCTTTTCAAGTTTGTCCACTTT
GAGCTTCTTGAATCTATTTGATAATAATTCGTTATGTATGATACCATTGAGCACTCAACCTCAAAGCTTTG
ATCTCACGAGTTTTCAAGGAAACAAACTCTGCGGACTTCCACTCTTGATGAACTGCAGTTTAGATGACAA
TATTCCAAATCACGAATATGAAGATGATGAGGGTGACAAAGATGAAGGGGATTGGTTTTACATTTCATT
GGTTATAGGATTTGCGCTAAACTTTTGGGGTGTTTGTGGTTCACTGCTTTTCAAGAGATCATGGAGACAT
GCTTATTTTCGTTTTCTAGATCGTAGCTGGGAAATGTTGCTTGCAAAGATACCAATATGGTGA
>PGSC0003DMG400006458 LRR-RLPs

TTTCTCCTCTCCATTCCCTATAAGTGTTTGTGTCACCACACACAAGTAACGACCCCTCAATTTGTCAATAAA
CAAATAATAGGATTAGTTAATTAACAAAGGAAGAAGAAGATTATGGAATTTCAATGATGATTTGTGTTA
GGATTAAGAGATCATGAAGATGAGGATGGAATTAGTTGAACCACGACGACAACAACAACGACAAGAAG
AAAAAGGAGCCTTTGAGAGGAAGAGTAGTAGGGGAAAAAGTACAAGAAGAAAGATGACAATTAGTAT
TGTTGAAGAAGAAGAAGAAGAGAGCCCAAAGATTGTGGATCTAAGTGGCATGTCATTAGACTCTCTTCC
TGTAAATCCTACTATCAATTTGGGAGCTATATCCAAATTACATATTTCCAACAATAATCTTCAGGTTGGTC
AGCTTTCGTACACCTCAATTAATTTTTGTGAAATATCTGTTATCTTCTATCAGCAATAAGCAATATGTACTA
GATGACTCTGTTCATAGAGGTTAGGATAGATGAATAGATTCACTTAGTGTTTTTTTTTTGTCTTCGCTAGA
ATTAATTGAACAACCACTTCGTTGATCACTATATGCTATCATGAATTATTTATTTATGAGAATTTTATTCAT
GATGGAATATTTTTAATCTTTGATTGGAAATTAAGTGAAAAATATCTTTTTTTGAAATAAGTGGTGGCTA
AAGTATTTGATATGAAGATAAGATAATTATGAAGAAAAATGTTTTAGTCAAAACCGAGTGAAGTTATTTT
CCTCATTTTGACCGGAGGCGAACTAGTTGATTGCTTTGAATTGTATCAGTAGCACAAGGACACTATTAAA
AACACTATTGCATTTCATAAATGTACGATTAATTATATATTAGTTTATGTCGATATTAATAGGTGGACGTG
CACTACCTATATAGGTGGATCTGCCTCTAATATAATTTTGACCAAAGAATATTTTTCTAAAAATATTTTCTG
ACAAGCACTCACGACTAGTATTTTGGAAAACATTAATTTTTCAATCATACCAAATACAGTCGTTGCTTCCA
TTTTTAGTTGCTTTTGGTTCCTTGTACTTTCACAAATATTTAGTTTTGTTTAATTTCTTCCATATTTATGGAA
AGAAATATATTTATCATTTACCTTTCTTCTATTTTACGCAACAATAGTTATTATATGTTTCCTAGTTTTCAAT
TTGTTTATTCAGTGTTGAGTTGATATTGAGTTTCAAAAGGTGGATAATTTTTTTATTGTTATAGTCTTAAA
CTAAAAATATGTAAAATGTATCAAAATATTTTTTAATTTTGTGGTTTTAAACATGTCTTGTGGAAAGTTAG
AATTAAAGAGGTGTCAAAATAAGAATTTAATGTACTACTATATTTTCTTAAAATATTCCCCTTTACTTGAT
AATGAGTCGTTTATGTGAACTATACCGTATAATATTTTCAGGAATAAAAGGCAGTACTATTTTATTCTGTG
TTTAATACAAAACAAACAATATAAATTTTAACGGATCAAATTAAATCCAAATCAACCGTCCTAACGTCATA
TTTTGTCGTTCATCATTATTTTAAATTGACCCAATAGTAAGGATAAAGTCTATTAGTTTATTTTTTACTTCA
TTGATACATTTTAAAAAATATATATATAACAACTTGATAGGTTTTCTAATTATTCGTATTAAACGCAATAG
TATATTTTTCTGGTTGTTATATGTATGTGTGGATTGTGGATTTACTTAAATATAATTGACAAAATTTCACA
AAACACGATAGTACATAACGTAACAACATTTTCGTTAATTATATATCCCGTCTTTGTTTAAAGTCTAATAC
AATGTATATATCTCTTATTGTGTTTTACAATGTGTATGTGTCTCACTCATCTCTCTTCTTCTATGTATTTGTA
TTTTATAATGTATATGTGTCTCACAATTTCAATTATAAGTACCATACAAATATCTGATACACAAATACATC
GACGGTTTATATTTTTTTTTTATATCAATGTATCTATTTCATACATGTATCGATTTTAAGTAGCATGAATTG

TATCATAGATTAACACTTTATCATTTTACTCTCTCTGTCCAACAATAGTAGTCCACTATTGACTTGGCATAC
CCCTTAAGAAAGAATAAATAATAGGGGTAATATTACTATATTATCTTTTGACTTTATTAAATTTAATGTTTT
GAGAAATGTGTTAGATGATAAATAGTATTTAACAGTAAGGGTAAAATAGATCTCTTGATTTTCTTAATTG
GACAAGTATTATTGAACAACTATTTTTAGTAGGAAAAATGACAAATATACCCTCGAACTATCGTAAATGG
TTTGGAGATACCCTTCGTCATACTTTTGGGACATTGGTGCCCTGTCGTCCAAAAAGTAGAGCATATATAC
CTTTCATACTAACGGACATACACGTGTCACAATCTTATCCACTGACCCACATTTATTAAATATCGAATCGA
CAGATAAGATTGCGCCACGTATCCCTATTTAGTCTTCCGTTAGAGTGAAGGGCATATATGCTCTAGTTTTT
GGATGAAGGGGCACCAATGTCCCAAAAGTATGACGGATGATATCTGCATACCATTTACGATAGTTCGGG
GTATATTTATCCTTTTTCCCTTTTTAGTATAGTGGACAACACTATTGTTGGACAGAGGAAGTACTAGACAC
AATGATTTTAGGGTAAGTCACATTCTTATTTGGTTGGACGATTTTCAATTATCATGTATCACTTGTGTATA
TAAATACACAAACACACATCTGTATCTCTTCAAAGTTCCACTCATCTGCCATAGATACATAAATACATTGA
TACACATATATATCTCGTCAAAGCTAAGCACTCAATTATATTATATCATGGATGCACATTTACATATATAC
ACAAATACACATATATATATCTCTTAAAAATATTCCACTCGTCTTTCATGGATACACAAATACACATTCATA
GATACGCAATACACATATATATCTCCTCAAAACTACTCACTCATTTGCAATAGATGCACAAAAATATAGAT
ACACATATACACAAATATACATATGTATCTCTTCAAAGTTGCACTCTAATTTGCCATAGATGCACCAATAC
ATATAGTCCCTCTGTAAAGCGAGAGAGGGGGAGAGGGAGGAGAGTGGCGAATTTGCTACGAAAAAGG
GAAATCTCATTGAGTTATGTAATTTTTTAAAACTATAAATAGGTTGAGTAGATATATATACTAGCTATGCT
TGATTAATTATGTAATTTTTCATGTATAATTCTCACTTCATTGTTTTTGTTGTATGTTTGACAGAGTATACC
AGAGTCTTTAACAGCAAGACTACTTAACTTGGTTGAGTTAGATATGCATTCCAATCAGCTCAACTCCATTC
CCAACTCTATAGGTTGCTTATCAAAGCTCAAACTTCTCAACATTTCTGGGAATCTCCTTCTTTCGCTCCCTA
AAACTATCGAGAATTGCAGGTTAGTCATCTAAACATTTCTAATTCATAACATTATCACTACAACAAAAGA
GATATTTAGTAGCAATTGAATTACTGTCATTGCATTCAGAGTCGCTAAAGCCTTTAGAACATAAGTATAA
CCTTTTCTTCCTACTTGCAGATCATTGGAAGAGTTGAATGCCAACTTCAATATGCTTACTCATTTACCGGA
CACCATTGGATTTGAGCTCATAAATCTCAAGAAGTTATGCATAAATTCAAATAAAATAGCATACCTTCCTT
ACTCCACCTCCCACTTGACCAATCTTCGCGTGTTGGATGCTAGGCTAAACTGCCTTAGATCACTCCCCGAT
GATCTTGAGAACCTAATCAACCTTGAAATCCTTAACGTTAGTCAAAATTTCCAGTACCTCTCAAAGTTGCC
CTATTCAGTAGGATTTCTCATTTCTCTTCATGAACTAGATGTTAGTTACAACAAGATTACTGAATTACCAG
ATTCCATTGGATGCTTGAAGAAGCTCCAGAAGCTGAGTGTAGAAGGCAACCGCCTTGTTTCTCCCCCACC
GGAGGTAGTGGAGCAAGGGATACGTGCAGTTAAGCAATACTTATGTGAAAAGATAAATGGCATGCATG

ACAAGTCCCCTAAGAAGAAGTCATGGTTTGGAAAACTAGCAAGATGTAGCACATTTAGTGGTGCTAACA
TACCAAGAGATGATAGAGAGGACTTTGGTGTTCCAAGCAATCGAACTATCGATGTAATTGCCTCACCGC
GATTTATGGGAATGCTTTCTCCTCGTCGTCTTCTATCTCCAAAAACCTACTTCTCCAAATAAGGGACATTG
CAATTTGCTTGGATATCATATTGTGGTAGCTCTTAGCTTGTTTTCTTTTACGTTTGAAATCGTGCAATGTGT
GCTAGATCAAGCTGTACATTATGTTCTGTAGATTTTGTTGTCTTTGAGATTGTACAGATTTTCATAGATGA
TTAGCCTCATGAGTCATGGGTATGTTTGAGAGGTAGAGGTACAAATTTACCCGAACAGGGTGTTTATAT
CAAATCATATGAATAATTACATCGAATCAAAATTTGTTATCGTAACAATATCACTTGTACAAAAACTCATT
TTGTTCATTAATTATCAAAACTCAAAAAAAAATTATCTTACATAAACAGAACCATTAAAAAAACCCCAAAA
AAACAGAACATGACTCTTAGAAAGCAAACAGAACAAATGAAAAACTTGCCAGAATCACAAATTATGAAT
TAGTTTCTTGCACTTGCGATTCATTTCTTTGCAAATCCGATTCACTTCTCCTGTTAAACGACCATTCTCTAA
TGGTAGACAAAACTGGCTTCCATGCTCGCGATTTATTTGATCTTTCTCCAAATTTGCACGAAGTTGTGTTA
GTTGCACCACTTTTAGCTATGCAACTGAGTTTTCTAGCTATTGGAACTAAATCCATTGGAGAAACACTTG
ATTGAAATAAGGATTTAGGCAATAAATAGTAAATATGGCCTTGTTCTAACTTAGTATCAGGGTTGAGAG
GCTTTGAAGCAGCAGATAAAAGTTGGGCATGAGTGAAGACAAAGTGTTTTTGAGATTTGGCCATGAGTT
CATGTACACTAATAGGATGATCAAAATCCTCTACATGACCATTTAAATGTACAACTCTAATAGTCTTTAAT
ATATCACAAGATTTGGGAGAAAAACAACAATATGTTATTTTGATGTACATATTGTTAATTTTTTCCTTATTT
CTCTCTAGCTAGTGTGTAAAAGGGCTCCAAAGAAGATGTCCTTTTATACAAGGGGAAAGATGGTGTTGA
AACCAGGTTGTGGTGCGGTAGTGGAATTGCTCCACCCTTAATCAAAGATCTCAGTTTCGAGCTCTGGGTA
TGAAGAAAATCCTATTGGGAGTGCTATCCTCGGATGGGTCTTGCAGTGTGCAAATACAGATTTAATCTAA
GCTCCAATGCAGACTCCGAATATTGAGTGGAAAATCAAAAAAATGGTGGTGGGGAAGAGTTGTGGAGA
TAGATGATGAGAACACGCTATTTAAGTTAGAAATTTTTTTTATTTGCTTCAAGGCTTATGTGACGCTTCCA
ATAGGATTTTCTTCATATTCAAGGTTCAAAACTGAAACTTCTGATTAAAGATGGAGAAGTCTCATCA
>PGSC0003DMG400006486 LRR-RLPs
ATGGCAAAAAGAGTTTGTGTTGATGGAGAAGACAGAATATCAGAATTGCCTGTGCATATCATTCATCAC
ATCTTATGCCGCACTGACCTCGACGTCGAAGAGGCGGCGATAAGTTGTATGTTGTCCAAGAGATGGTAC
TATTGCTGGACTTCAAGACCTAATTTGATATTCAATCAATTTCAAGGCTACAAATATAGGCCCCTTGAAAA
CTATGTCAAGTTGGTTGATCAATCTCTGCGATCCCATGTCGAAAAAAATTTACATCTTGAACAATTCATCC
TTACATATAATGGTCAAGAAGTGGATCCTCACGTGGATACTTGGATTGAATTGGCGGTTAAACTCAACGT
CACAGAGCTGGGAATTCACCGTTTCGGTTTAACATCATATAACTTACCTGATGTTATTTATGATGCTAAAA

AGCTGACAACATTGCAGTTAAGCAGGTGCAATTTTGAATTTGACAAAAGATTTTCTTGTCTTGAGTCTCTT
TCTTTGAATCATGTCCATATTTCAGATGCCCAATTACAAAGAGTTATCGACAGATGCCCATCTATCAGGAC
TCTAAGGACTCGTTTGGTGTAAGGGATAATACCAAATAGTCCCGGGATTAAATTATAGTACCGCTTATTT
TGTTGTTTGGTTGGCAAGTTCGGGATAACTTATCCCGGGATTAATAATTAGTACCGGGATAAGTTATCCC
TCTCCTTGGGTGGTATAGTAATCCCGGGATAACTTATCCCGGGATAAAATAGGTAAATGACAAAAATGT
CTCTTTCAACCCTTTTGTTGTATCACTTTTTACCTTAATGAAGGGCATTTTTGTAAATAAATAAATTGTTCT
TAAAATTTATTATTTTGAATATAACAAACCAAACACTCAATAAAAATAATCCAACACAACTTATCCCATCA
TAACTAATCCCAACATAACTTATCCCATCATAACTCATTTTCAAACCAAACGACCCCTAAGGTTACAGTGT
TGTCAGGGTATAAGCAAATGTCATATTTTTGGCCTAGTACATCTCAAGTATTTCGATGCTAGTTATTGCAA
ACTCCATAGTGTCATAGTCCAAGCTCCATATCTTCAATGCTTTAGATATACAGAACCTACTGAACAGGGC
AATCTTCATCCTTGCCAAATTGCTATTTTGGATGGTTACAATACTTTACAAACACTCAAGCTCACTGGTGC
CACCATCACAGACCAACAGTTTCGGGATGTATCCTATAAGTTCCCAAACATTTCAGAATTGGATCTAGCA
AGATGCTATAAGCTGAAAAATATAGAGATCCAGAGTGAAAAACTCAAGAAATTCACCTTATTAGAAGAG
TTGAACAGTCTGGAAAAAATCACGATTCAAGCTCCAAATTTATTGAAATTTGATTTTCATGGTAGTAAAA
TGCCCTTCACATATATGGATACTTCTTCCCTAGAAAGTGCCCGGCTCAATTTCTTCTTGCCTGGCACAAAC
TTTGGATCAGTGGACAGCAGTTGGTACACGAGCCTTCAGCACTTTGTGAAAAAGTTCAACTATTCTAAAG
ATTTCATATTAATAATATTTTGCCACCAGGTAAATCAAAGTAGTTTACATTATTTTTGTGCTTATGTCATTG
ATTTATGCATTACTTGTTGAATTTGGTTTAAACTATGCCATTGACAAAATTCTTGCAGACCAGAAGCATCC
TTATTTATAAAAATCTAGGAGAAATTGTTATTCCACCAAGTCACAAAGTCGAGATATTCATTGCATCCATG
CTGCGTGTTGAATCGATCATTGAAATAATAATGTTCGAGTGTCCCTATATCATATCCATACTTCCATGTAC
AGATAGCAAAGCACTACAGGTAATTTCTTGCTTGTCTCTTGTGCTTGTATAAACTAAGACATTAATTTGAG
CTTACAATCAGAAAATTTGTTGTTAAATGTTGATTGCTCGTCTCATAAGCATTATTGTATAATATATCTTAT
GTTTTTGCTTAAATGCAGGCGATGTCCACACTAAAAGGGTGTATACAAAAGCAAAATTGTGGCAAAGAA
TGTCCATCCGATACCGTCTGGCATCGTAACTTAAAAGAAGTCATCAGTTGTAGTGGGACATCTGAGGAG
GGAATGGCTGCCTCCATGTGGAATCTCTGGTTGAAATCCACGTCTTTATTTGATCAAGTGAATAATTTCG
TGTTAGAATGGAAAGATGAAGCCTAG
>PGSC0003DMG400006487 LRR-RLPs
TATAAACCCTAACAGCAAACGGCGGCACCTACCAAAATCAGAAACCCTAATGGAAGAAAGTGTTGATGG
AGAAGACAGAATATCAGAATTGCCTGTGCATATCATTCATCACATCTTATGTCGCACTAACCTCGACCTC

AGAGAGGCGGCGATAACTTGTGTTTTATCCAAGAAATGGCAGTATTATTGGCTTTCAAGACCTTATTTGC
TGTTCCGTCAATGTAAAGGCAACAATTGTATACCCCTGGAAATTTTTGTCAAGTTGATTGATCGACCCCTA
CGAATCCATGTTGAACAAAACTTACATCTTGCAAAATTCTTCCTTGCATATCGGGATCGAGAATTGGATC
CTTATTTGGATCATTGGATTGAATCGGCTATTAAAGTCAATGTTTTGGTGCTGGTGATTCATGCTCCGGA
TATTGATTCGTCGTATTATAGCTTACCTGATGTTGTTTATGATGCGAAAAAGCTCAACACATTGTGGTTAA
GCAGGTGCAAGTTTGAATTTGATATTAGCACCACTCATATAAGATTTTGTTCTCTTGTGGCTCTTTTTTTGT
ATAATGTTCATATTTCAGATGCCCAAATGCAAAGTGTTATAGATAAATGCCCATCCATCAGGAATATAAG
AGTACTGGCTTGCAAAGGTATGAGTAAATTGTATGTTGTTGGACGGTTACATCTACACTCTTTGGCTGTA
CTATTATGCAAACTCGATAGTGTGATTGTCGAAGCTCCGAAACTTGAATCCTTTAGTTATACAGAATGTA
TTGATCAGGGAAATTTTCATCCTTGCAAAATTGATATTTTGGATGGTAACAATACTTTACAAACACTCGA
GCTCACTGGTTCCAGCATCACAGACCAACAGTTTCGAGATGTATTCCACAAGTTCCCAAACATTTCAGTG
TTGCATCTAACAAGATGTTATAAGCTGAAATATATAGAGATTCAGAGTGAAAAACTCAACAAAGTCATCT
TATCGAAGTTGGATAGTATCAAAAAAATCATGATTCAAGCCCCAAATTTATCGCAATTTGATTTTGAGGG
TGATAAAATGCCCTTCTTATCTATGGATTCTTCTTCCCTGGAAAGAGCCCGACTCAATTTCTTCTTGCCGA
GGACAGTAATATCAAACTTTGGAGATGTGGATAGCAGTTGGTTCACAAACCTTAATCACTTTGTTCAAAA
CTTCAAATATTCTAAAGGTTTGATGTTAGTAATATTTTGCCGCAAGGTACTTTCCATTCTTTTTGTGCCATT
GATTTATGCATTACTTGTTGAATTTGGTTTTAAGCTATGTCATTGGCAAAATTCTTGCAGACCAAAAACAT
CCTTATTTATGAAAATCCAAGAGAAATTCTTATTCCACCAAGTCATAATGTCGAGATACTCATTGTACCCA
TCCTGAGCATTGAATTGATCATTGGATCATTATTGCTCAACCGTCCCAGCATCATGTCCATACTTCCATGT
ACAGATAGCAAAGTACTCCAGGTAATTTCTTGCTTTACCTGGAAATCCTTTCAAAAAAACGTCCCTTGTGC
TTGTAGACACTAATGCAGTAGAGCTTAAGCATTATTGTATTCTCTATAACTTATGTTTTTGTTTACATACA
GGTGTTGCCTGCATTAGAGCGGTGTACACAAAATCAAAATTGTGGTAAAGAATGTCCATTTAGTACCAG
GTTTTTCCACAAGCATCGTATATTAGAAGAAATCATCAGTTGTACTGGGACATCTGAGGAGGAAATGGC
CTCCATTTGGTATACCTGGTTGAAATCTACGTCTCTAATTGGCAAAGTGAATAATTTCATGTTCAAATGGA
AAGAACAAGCTTAGACCGAAGAATCTTGGGGAATTCAGCATATAGTGTTTATTAGTGTGTTCAGAATAA
AGTTCAGTTAACCCGCTTGTGGCTCTGTCAAATCCATGAAAGGTTCCTTAACCTTGCTCTAGCTCTAAATA
AATTGTGCTCAAATCTGTGGTTTAATTTCATTTGTTCGATTGTACGGAATGAAGTTTAACTGTTAACAAAC
AGCATAAGAGGTCTTCATTTCATTTTTTTTTTTCTGGCTCTGCCTCTTCTTCACATTCCATTCAATTTCTCTC
ATGCTTAGACTACTTGGAGGTGATTAAAAAGAAGTGAGCACTTGAGGTAGAGAGATGTAATGATGGAT

GCATATGCCTCCTACTTGTTCTGAAATCTGGTATATATTCACTGTCATACTTGAAATGTAATATGATTTTTC
GTATAACTTTCTTTGTCA
>PGSC0003DMG400006580 LRR-RLPs
TATAGAAACTTTAATTGTGAAGAGTTGTCCTTATGTACTGTTACCAGTGTCTTTTTGGCAAATGGAAAAAT
TAAGGCATGCTCATGTTGATTGGGCTCTTTTTGGTAATCAGGGACTCTTTGAAGGATCCTCTAAATTGGA
AAATTTGAGGATATTAAAGAAAATTAGTGCATTTCCAATTGATAGGGTGGATGTGTTGTCAAGGAGGTG
TCCTAATCTTCAACAACTTCATATCGAATTTGATGTACATTCTGCAGATTCTTTTTGTCTCACATTGGAGAA
TCTTACCCAGCTTCAAATACTTCGCGTTTTCTTTCAGCGGCCCCACATCATTGTATCTGGGTTGCAATTGC
CTTCAAATTTAAACAAGTTGGTACTTAGAGGAATTCATATAGAAAGTGCTATTCCCTTCATTGCGGGACT
ACCAAGCCTGGAGTATCTTCAATTAGAAGAATGGAATTATGCAGAAGGGGAACCAATTTTTCCTCAATC
AGAAGAGTGGTGCCTTGGAGATATCACGGTCCATAACCTTAAGTTCTTGAAACTGGTGCGGTTAGGTAT
CTCAAAGTGGGATGCCTCGGAGGAATCATTTCCCCTGCTTGAAACACTTGTTATAAAAAATTGTAGTAAG
CTAGAGGAGATCCCCCTTAGCTTTACCGATATTCCATCATTGAAACAGATTAAGTTGATTGGGTCTTGGA
AAGTATCTATGGAGGCTTCAGCTGTGAGAATTAAGGAAGAAGTCGAAGAGATTGAAGGATGTGATCGT
ATACACCTCGTCAAAGAACTGGCAGATTAGTGAGGGTTTTAATAGCTAATTATTGCAGCCATGCTCATAC
TGAAGTCTAGCCCATTCTCTTTTATTGTAACACGACTACTATTTGCTATGTTTGTTTTAATTGATAAAACTC
TTTTTATGTATGATTGGAGACAAATATATATCAGTTGGTCTTTCACTGATTTCTTGTTTTT
>PGSC0003DMG400006625 LRR-RLPs
ATGAACAAATCTGGAGGAATCAAAGTACCTAAATTCATCGGTCGCCTTAAAGAACTAAGGTATCTCAATC
TATCTTCTGCGTCTTTTACTAGTTCAGTATCGTCATTCCTTGGAAACCTCTCAAATTTGTAAGTTCTTGATT
TGGGAGGTTTAGATCTTAGCAAAGCTACTAATTATTGGGTTCAAAAAATCAATGATCATATTCCTTCTCTC
TTGGAGTTACACTTGCCTCGATGTAACTTTTTAGACTTCCATCTTCTTTTCCGTCTCTACATCTATCTTCCTT
TTTGGTACTTGATCTTTCCGACAATGCTTTCAATTCAAGTACATTCCCTCAATGGATATTAAAACTTAATAA
CCTTGTGCATCTTCACTTAAGCTCCAACAATATCTTTAGTGAACTTCCTGATGAGTTTGCAAAGCTCAATT
TATTTGAATATCTTGATCTCTCTTCCATTTATGGTATCAATGGGACACTAAAGAGAAGTTTGGGAAAACT
ATGCAATTTGAAGACTTTGATCCTCTCCTACAACAACATCTTGAAAACATAATCCATTTAATCGATGCCTT
ATCTAAATGCCAAAGCAACAGCTTGGACATTGGATTTTATTTTCAATGACTTGACTGGAAATCTTCCTGAT
GAATTACGCAACTTGTGGAAGCTAAAAGATCTTCAATTGAGGTATAACTCACTGATATGCACAATAGTTG
AAACTATAGGGAACTTATCATCCTTAGAGACATTCTACCTCACATCTAACAAAATGAGTGGAAACCTCAC

CACGAATATTGGGCAGCTCATGTCATTGGTCTCATAGGACATCTTAGAGAATATGTGGGAAGGTATTGT
CAAAGAATCCCATTTACTTGATCTTTCCAACTTGCAAGAGTTTTCAATTGGCATGAAACTTGGTACAAATA
TAACCTTGGCATTCAATATCAGCCTGAATTGGAATCCTTCATTCAATCTGACATTCTTGACCATTCAGACA
CGCTAATTAGGCACTGAATTCCCACATTGGCTAAAGGATCAAAATGAGCTCAACAATATAATATTCAATA
CCACAGGGATTTCAAATGTTGTTGCAGATTGGTTTGTAGAGTTGGATTTGAAGCTTGATAATCTAGACAT
GGCTTACAACAACTTGACAGGGAAAGTTCCCAAAGCTCAAATTTCAAGCCAACGTGGATTTAAGCACCA
ATCGCTCCGAGGGACCTCGCCCACAATGGTCTTCTAGTGTTACAACATTGTATCTAAGGGATGACTTATT
TTCAGGACCTATTCTTGTTCATCCTGTATTCCTAAGATACTTAGAACAAAACTTTGAAGAATTTGGTAAGA
AACAACAAAGTTTAAAGAGTATTTTCAAAACTAGAAAATTAAAACTAGTAGAGAAAATAGAATCAGAAA
CAGATTAGAAACAATATACAAAATATTTTCTCAAAGAAGAAAATCGAGCCCACTGAATGCACAGTGTCCC
CTTAAGGAAATTATTCCCCTCAAGTACCCGAGGTTAATGAAATATATCCTCCCAGGATAGAACGATCTTA
CTCACTGGTGTATCGGTACCTACAATTACGGTGTCAGCGAGCCACTCAACGGCAATAAAGTACACTTAG
AATACTAGATTTAGTAATAGTAGAAGAAGTCCAGGAAATTGGTTCTTTCCTTGTTGAAAAATAAGAGGA
AACTCTCTATTTATAGACAACAAAGGGTAGTGTGAAAAGGTTCTTATTGTGCCTTACCGAAAGGTCACAA
CCCTTTGGAAAAGTCACAATCTTTCAAAAAGGTCACAACGTTTCATAAAAGTCACAAGTTTTCATAAAAG
TCACAACAATTCATAAAAGTCACAACTTTTCATAAAAGTCACAACTCTTCATAAAAGTCGCAACTCTTCAT
TTTCCATCCACACCTTTTAAAACCCAACAATCCCCCGCATGAATGGGGAATGACTCCAAGACAAAGAAAC
GAACAAGTGTGTGTACTTTACAAGTAAGAATTAATTGCATCTGGATAAGTAGGTTTTCCTTTGAACTTTCT
GTAGTGAACATATGTCGGATATACTCGGTCAATCGATAGATTTGGTATCTTTGAACCGTCAATCTTTGGT
GTATATGTAGACAACCATATGTCACACAATTAACTTTACCGTTTATGGTTCTTACGGTTGTGTTCGTTTTA
GCCATGAACACCTCCTGGTTTCATGAGTGTATAGAGGGATGGGCTTTTGACAATCATCATCTTTGAAGCG
GCTTACACTTCACACTCACATAGGTGATTTCTAATCGTGTTATCACGTAGGTACACTATTTGGTCAAATCT
GCCAAACTTAGCAAATCATTAAAAAACTATAAGCTTTATTAACTCATTAACAAGCCTTAATGTTATATTAT
TGTTCATAAGCATTATCTTCATCATGAGAATGGATTGAGTTGGTTGACAATGCGAAACTGTCAGTCACAA
CTTTGTTTTTCTTCTTGAACCTAGCTCTTGGGATCTCCAGTCTACTAGGTAGAGTTACCGCCATCATGACTT
GTCCTAAGCCATAAACACATTCCTTTAGATGATCTTTCAACTTTCTCTCTAGTTAGGCCTTTCTGTAAGTGG
ATCCGACACATATCCTTTGACTTTACATAGTCAATTGTGATAATTCCACTAGAGAGTAGTTTTTTAACGGT
ATTATGTCTATGTCATATATGACGAAACTTTCCATTATACATCATGCTCCATGCCCCCTACCTATTGCATCT
TGACTATTAAAGTGTATGCATACTGATGCCAAAGGTTTAGGCCAAAAAGGAATATTTATTAAGAAATTTT

GGAGTCTTTCTACTTATTCACCGGCCTTATCTAGAGCACCAAGCTCAGAGATCATTGTAGAGCTAGCGAC
ACGTCTTTTTGGAAGATTTCCAAGATATTGCTCATCCACCAAGGGTAAATACAAATCCACTCATGGATTTT
ACTTCATTTGATCCAGGTGATCCAATTTGCATCACTATATCCTTTCAATACTACTGGATATTGTTGTAATGC
AAAACATAGTTTTGAGTATGTTGTAAATACCCCAAAACTCTTTTCATTGTCATCTAATGAGTTCGATTGGG
ATCACTCATGAACCGACTCAGTTCATTAATAACACATGTTATATCTGATCATGCACACTTCATGATGTACA
TCATACTTCCCAATACTCTAACACAATCCAATTTTGAATCACTTTCACCTTCACTCTTTTGAAATGCAAAGC
TCACATTTATTGGAGTCTTGACAATATTGAAATCCAAATATTTGAACTAGTCAAGTACCTTTTCAATGCAA
TGAGACCGTGACAATGCTAAACATTATGGAGTTCTAAGAATTCTTATACCTAAGATCGCATCAGCAACTC
CAAGATCTTTCATATCAAACTTTCTTTTTAGCATACGTTTAGTAGCATTTATGTCATTAGTGTCTCTACTAT
GATCAACATGTCATCAATATACAAACAAACAATGACCTTGTGATTTTAATGTAAACACATTTATCACTTCC
ATCATTCTTAAATCTATTTGCCAACATGGTTTGGTCAAGTTTCTTATGTCATTGTTTGGGTGCTTGTTTTAG
TCCATAAAGTGACTTAATAAGTTCACACACTTTCTTTTCTTTACCAGGAACCACAAAACCCTCAGGCTGTT
CCATGTAAATTTCTTCCTCCAACATATACCGTAACTAATGTAACTAACATCCGAAGGAATGTAATTCTAGT
TACTGGTAAGTATGTGTCGACAAGATCAATACCTTGTTTTTGTCTAAAGCCTTTGACAAAAAGTCTTGCTT
TATATTTGTCAACAGTTCCATCGACTTTCATTTTCCTTTTGAAGATTCACTTTGAACCCAAAGTTTTATTTCC
TGGAGGAAGATCAATTAACTTCCAATGTGGTTGCTCAAGATTGAATCAATCTCATTAAAGACACCATCTT
TCCAAAAGGATGAGTCCACAAAAGACATAGGAAATGTTATAAAATCAGATCCAATGGTAGTATATGTCT
TTTGACATTTACTACGCGTCCGAATCTATTTATTAAGTACATTCTCTTTTTGTTATTTTCAAGGTCTTTTAGA
CATTTCGCTAGACTACTCAAGTCTAATTTTTCAGTCCACAGTTCTTAGGTCCTATTTTAATCTTTTTAGGAA
TAGGAACTTGGACTTTGGCTAGACACCCCCACACTTTGAAATATTTCAAGTTGAATTTCCTTCATTTTCATT
TCTCATATGGAATAGATTGTGTCTTGAACAAACAAAACTTTTTATTGCTTTCTTTCTGAAAAGTACAAAAC
TTTTTTGTTGTTCCCTTTTGCTGTAAGGATAGCTTCTCCACACAAGTTTTATGGTAAACCTGAACTTATAAG
TAATGTAGCCATTACTTCCTTAAAAGTTCGGTTTTTGTTTTGATTCTACAATTCCATTAGATTGAGGTGAA
TAGGGTAGTAATTTGATGGATAAATCCACTTTCCAAACATATTTCTACAAAAGGGGATTTATATTCTCCAT
CCCTATAACATCTTTTTTTCCCAACTAATTTCAACTTCAATTTTAGTATTATGCAGTTTTTCAAGATTTTCAA
GAGTCAAGATCGAAACGGAATTGATGCTACAAAATTTGTTAGGATTCATTTAAATGGCAGGCATTTTTTA
CCCTTTTTCATAATTGTTATCATTCCAATACTACAGTCTACATTCAGCCCTTGTGGGCCACTTGAATTGCCA
AGGAAGGTGGAATCTGCAGCACAGGGGGTTATCTGTTTTCTGGTCCCTGTTACACTATTGATATCCCTTC
TTCTAAGGCATGTTCTCACCTTCAGAATGCTTAATTTCCATTTTGTTTATTTTTTATGTCCATTTTCTGTTAT

CCATGAAGTAGATATTTACTTTGATGCCTTTGCACTTTTTCGATGGTCTCCTATTGTTTTCTGCGTATCAAC
AATAATCTTGTAGACTAAATCGCCTAACTATTTAGAGGTATTGATGTTATAGACCGTAAATGATTATGCA
ACTATTCATGCATTTATGTTGGAAGCATGTACATTATCTCCTCTTGCAAGTCGAGATTAGGCCAGTTTGTT
ATAATTTGGCATGATTTTTCTGCACTGAAATAGTGATTAGTTTGAACTTTGGATGTACTCGATCAAAGAT
GCCAATTGACCAAATATCAAATAAGGAGAAAGGGAATGTCTATTTCTTGTCTCCATGGCAGGAGATTAG
TTAAGTTAGTTATGTTTAGCAATTTTTTTTAAAGTACAACAACAACTATACCGTAGTCCCTGACAAGTTGG
GGTCGGCTATTTGAATCCCCACTTCTTCATTTAAGCTCATTTCTTGTATCCAACTGACTTCTCTGCTTTTGC
ATGTCCAAGGTTCTTGTTGTTCATATGTTGTTTAGGAGTTTGATTGTTTTGGCTGTTGGGATTTGTACTGC
AGGCACTTTTACTCTGCCTAGACGTTTCTATTGCTTCATCCCTACCATTCAGCAATAAACATAATTGTATCA
AATGCAATTGTCAATAAAAGTTATGAGATATTTTTTCCACCATGAGAAGATGTTGACTTCATATAAAGAA
TGTAAGCGTAAATCAAATCTAACGGATTAGAATTCCTTTCAACAGATTTATAAGAATGCTTAGCATACTT
AGATTCAACACAAACTTGACATTTTTATTTATTGCACTCAAAGGTTTTGTAATTTACATGTCCCAAGTGCT
CATGTCACAAACAGTTTGACTCAAGCAAGTAAGAACAAATAAAAATATTGAGTTTGAAAAGCTCTCAGT
GAGGTAGCTTTTTCACACAAATATTTGTTCCTATTTCTTACAATTTTGTGTGATACAAACATTGTTTAGAGT
CATCACCTTGTCGAATATCCTTTTCGGAAGGACCTTTCCAAAACTTTCAATTTGATTGTTATAGAACTACA
CATGAACAAAGTTTCATCGGGTCCAACAAAGACAATAGTCCAAATGCTTCTTTTACAAAACATGACAAAT
GACTATTCACTAATATTCATGTCTATACAAATAGTTATATTTAATAGACATATCTTTTCATGAAAAATCCCA
GCATTTTAAGTGACATTTTTTCATAAAAGAACTCAATCATTTTGAACAAGTATACATATAATTTGGTAGTT
TCATACTATACATACCGTATACTAGTAAAAGAGAAACTCAGCGGCCACAATATCAAATATACTCCAAGAA
TTTTGTGGGTCTAACATAATACAAAAGTATCATCGAATTTCATATTTTGTGCTCCAAATTTAAATTAGACA
CTAAGTCTATTTTTATCTTTGTCATAAGCAAAGAACCCCCCACATTTTAAATATGACCTCAAAAATATTTTT
CAAATAGTTGAAAATAGTTTTTCCACATATTTTCCCAGACCATGAATGGCGGCATCAAACCTCTTTTGGAA
ATATTATTCTACGAAAAAAATATAGTGCTTCAATTCTCTTTGACAATCTTTACATGGTGTCACAGTTCATTT
CATAGAAGCACCTATTACAGACACAAAATCACACACTTTAAGTCACTGGTCGTTTTTCTATCACAATTAGA
CATACCACTTAGTGTTTAGAATACTGATAATAAAGACAAAAACTTTTGTACATTTTTTTCTATTTAACAGT
GAAGTTTATAGAAAACAATAAGTACAACACTGACTTATTAGATGAAGTTTTCATATTCTTCAAACCAATTG
CATAGTCTTATTTATCCAGAGTAGAAAACTACAGAAGTTTTTACTCAACAAAAATAAGACACAATCAAAT
TCACACAGAATACAAAACTACAAAAGTTTTTTTCTCCAAACAGAATAAAACATATTCTTAGATAATCACA
TAGAAATGCTTTTATTTTTAAATAGTCCTCCAACTATAACATTTTGACATACTATTTTATCAAACATAAAAT

GTGCTCTCTCATTCTGCAAACTAAAGAATTTTAAAGGAAACATTATTTGCCAAGGAAAATCCATTCCAAA
ACATATGATTTGCAGCTATATTTCCAAACATACACATGATAAATATCAAAATAATTAACTTTTAGAAACTA
TTTTCCTCCTTAGATAAATAATAAGAAGGTTACCTGGTGTAATCTTTAACCGGAATACCACCAATTTTTCT
AGTACATTCTCAATTCGATCTTGCTAAACAAGGAACCAAATTCTGAAGTAGTAGTGCATTAATCTTCTACT
TCCATGTTTTGTTTTCCTTAATCAATAGAATACAAGAAATACCAACCGTATAATAATTTCCTTAAGATTGTT
GTTCATCATGTATTCCTAAGATACTTAGAACAAAACTTTGAAGAACATGGTAAGAAACAACAAAGTTTAA
AGAGTATTTTCAAAAATTAGAAAATTAAAACTAGTAGAGAAAATAGAATCAGAAACAGATTAGAAACAA
TATACAAAAATATTTTTTCTCAAAGAAGAAAATCGAGCCCACTGAATGCACAGTGTCCCCTTAAGGAAAT
TATTTCCCTCAAGTATCCATGGTTAATGGAATATACCCTCCCAATATAGAACGATCTTATGCACCGGTGTA
TCGGTACCTAAAACCATGGTGTCAGCGAACCACTCAACGGCAGTAAAATATACTTAGAATACTATATTTA
GTAGTAGTAGAAGAAGTCCAGGAAATTGAATCTTTCCTTCTTGAAAAATAAGAGGAAACTTTCTATTTAT
AGACAACAAAGGTTAGTGTGGAAAGGTTTTTATTGTGCCTTACCGGAAAGGTGACAACCCTTTGGAAAA
GTCATAATCTTTCGGAAAGGTCACAACCTGTCATAAAAGTCACAATTTTTCATAAAAGTCACAACTCTTCA
TAAAAGTCACAACTCTTCATAAAAGTAACAACTCTTCGTAAAAGTCGCAACTCTTCATTTTCCATTCACAC
CTTTTAAAAGTCAACACTTATTCCTCTAAACATTGTGAAGCACTTCCCAGTATAGCAGACTTGGACATCTC
TAGGAACAATCAAAATGGCACAATTTCCTTATGCATGGGAGATAGATATGAATCAGTGGACTACCTTAG
CCTTCAATAATAACCAACTTATCGGACAAGTTCCTGATTTCTAGGGTAAGCTACCATATCTTTACTAGATA
GATATGTCAGAGAATCGCCTATCCATCCAAATTCCAGACTCACTAGGCTCTCTTGGTTATCTGATGTTCTT
AAGACTCTCAGGCAATGATTTCAATTGAGAGCTGCCTTCGAGTTTGAGAAATTGCACGAAAATCATTAAC
AATTATCTTAGTAACAATCAGTTGTCAAGGCTTATCCCAGCTTGGCTAGGGGAAACAATGAGATTACTGT
TATCCTTAGTGTAAGGAATAACAGGTTTTCAGGCCACATTTCTTTTAAAGTTTGTAGCCTTTCAGGTCTTC
ACATACTAAACCTTTCAAGAAACAACTTATTTGGTTCTATCCTCTCTTGTTTTGGAAACTTGAAAGGCTTC
AAGGTTGAGCTAACTGATGTAGAAGCTAGACATTATCAAGGGACATTGAAGCTTGAGGCAAAATAAAG
AAAAAAAATTATGATAAAATCCTTTACCTAGTAAAAATAGTATCGGCCTCTTGAGCAACAGTTTGTCTAG
AGAAATCCCAGTAGAGATAACAAACTTGGAACCTTGAACTTGTCCAGGAATCATTTGAGAGGAAATATA
CCAACATATATCGGAAAGTTACGTTGGTTTGAAACGTTAGATCTTTCAATAAATCAGTTAACAGGTTTTAT
CCCACCAAGTATAGCTACTTTAGAGTTTCTGAGTCATTTGAGCTTGTCATAGAATAAGTTGACAGGAAAT
ATTCCTACTAGTACTCAGTTTCAAACCAAAGTTGATCCAACAATTTTCCAAGGAAATGTTGCACTTTGTGG
TCCTCCTTTGACTGAATGTGTTGGTGATGGAACTACAACGACATCTGAAAGTGGAAGAAATGATGACGG

AGAAACAGATGATGAAGATAAACTTGAGAAAGTATGGTTCTTTGCAGTAGTTGGTTTGGGATATTGTCA
CGACCAAGTCTACACCCTGGATGTGGCTGGCACTCAAGAACCATTGATGGTCCCCAAGTGA
>PGSC0003DMG400006652 LRR-RLPs
ATGGATCTCTCCTGGAATTGGCTCACTGGAAATATTCCAACTGAATTGACAAGACTAAAATTTCTAGCAG
TCTTAAACCTCTCTCAGAATGTTCTTGTCGGTCCAATTCCTCAAGGTCTACAATTCAACACATTTGGAAAT
GACTCGTATGGTGGCAACCTTGATTTATGTGGTCTTTCTTTATCAAAGCAATGTGGAACGAGTGGTTCAT
CACATGTTCCTCAACCATTGGAGTCGTATTTCTTTAGTGGGTTTACGTGGGAATCAGTAGTCATAGGCTA
CAGTTGTGGACTAGTTGTTGGAACTGTTGTGTGGAGCCTCATGTTTAAATATCTTAAGCCAAAATGGTTT
GTGGAATTTTTTGATGGACTCATGCCTCACAAAAGAAGGAGGCCAAATTAAGAAGAGAGCTCAGAGAC
GACGGACTTAATGGAAGATTGAGAGGGCCAACTTGTCTTTGTTTGATTTTATGGACTAATTAGTGAACTT
TTCTGGTTCTGTTTTTGTCTTTCTGTTTGTTGTTTGTATATGTTTGAAAGAAACATAATTATATGTATGTTA
TTTTAATATCAATGGTTATGTTAGTCTGTTTCTGTTTCAAGTTGACAAAAGTGATCTACACAGCCACTCAT
TAGCTCAGATTTTTCAAAAAGTTATGTATTTCTTAAAGAACCAACACCATCATATTTTAGGAACGCGGGG
TTCCTCATATATTAGCTTAGGTACAAATAACGTGAAAGTCAAATATATTCTCAAAAACAAGCTAAAAGAA
AGACATATCTGCATTTGAAGTTCGATTTTTGCCTTTTTTATATTCCGACATATTCATTCACTTTGACTTAAA
GGGCGTGTTCATCCTTGTAATGAGAATTGAGCATGAATGCTCAAGTGCCTGGACCTCGAGGAGGTGAAA
ACTTTATAATATGTATTTAACTTGTTTCTTCCATGATTTTAAACATCAAAGAATGAGTTGATTACATCTATA
AAAGCAAAAGACAGAAAACAGTGATCCTTCTATACAATTAGTCTCTCAGTTTTCTTTGCCTGTGCATCACT
ACACCTTTATCTTGAGTCGAGAGTCTATGGAAACAACACAAGAGACAACTAATCGTCTAGAGAAAATGA
GTACATAGGGGGTATTATTTTGTTAAGTCTTCTCCGTCCTCTAAAAAGTAATTTGTGGGAAACACTACCA
AAAATATGAAGTAACAAGTTGTTAGAAGTGAGTAGTACTCATCCATGGACCACAAGTTGCGTAGTTAGT
GATGTCAAGGTTGTATTATTTTCTCTCCATTTATGCCAAATTTGGACAAAATAAATATTGTATAATAGTTA
CTATCAAACAAGATTTGAAGACTTGAGTCTTTCTGGAAAATGACTCAAAACACTTTGGACAACTTTTTTGC
AATCACAAATTAGTTAGTGGTAGATGTGGGGCAAATACCCCATTTTATTTGTCTGGATATGTTTCTTCTCT
TTTTCCATTCACACCTTTTATTCATAAAATTATTCAAAACATACTAATTATGCAGGAAATTAAGACACATAT
CAAACAAAAAATTGAATAAAAAGCAAATAAAAGAATAAAAATAAAATTCCACACGGTATTATTGTTAATT
GTTTCCTCACTTGACTTAGTCAAGAAATAGTTTCTCTCATTGGCAATTTCTTAGAAGTTCTGCAGAAATTTT
TTCCACATAATGGGACCAATTTCACATCATCTTACCTAACACACAATACGGCGATACTTACTAACCTCGAA
ATGAACACAACATCTAGAGAAAATGAGGACATAAAGAGTATTATTTTGTTAGGTCTTCTCCGTCCTCCTC

TAACAAGTCTTGTGGACACTACAAAATATGAGTAACAAGTTGTTAGAAGTGACTAGTAATCATTCATGGA
CCACATGTTCTGTAGTTAGTGATGCCAACGTTGTACTATTTTCTCACCATTTATCCCGAAGTTGAAAAAAA
ACAAATATTGTATAATTGGTACTATCAAACAAGACTTGACTTAAAGACTAGAGTCTTCCTGGAAATTGAG
TTGACAACACTTGGGACAACTTCTTTTGCAACCACAAATTAATTAGTACCCCATTTTATACCTCATAGTCC
CATGCATAGACAAGTCAACAACGTTACTTTATGAAATAGTGTTTAGTTAACTGTTTACTTTATTTAATGTG
GAGGTCGTTGTAATAATTAAGAAATCGAATAGTACTTAATTAATTATAGCATCCTTTCTTAAATATATATG
TGGATAATGCTAGTTGATTTGTAAGAAGAGAACAAACATCCTGTTATTTGCTGAAAACAAAAAATGGTC
ATGAGTCTGTTTGATATTCCACACGACCTAGATTGTTTTACGTTTACCAGACTTTACTTAGGTGAACCAAG
TTGCCTAACATGTTTCAAAGACAATGTAAAGACAGGAAAGTAAGAACACAATATTTTACGTGGAAACAC
CCGGCTCAAAAGGTGTAAAAACCACGACTTGTTCCTCAACAGGATTTACCCCAACTTCACTAAATAACTA
CAAGTCTCAACCAACAAACAAGATTACAATAGTTTATCTAACCTAAAGATCAACTACTTAATCTCAATTCT
ACCACACACCTCCAAGGTATGCGTTCTCAGTGTCTTCGAGTTGCCCTCAACTTGAAGACTAAAATCCTAAT
ATCAGTTATAAATTACTGATCTAGAAAATATAAAAATCACAAATACTAAGAAAACTTCCTAGATCTGTTCT
TCTATATTCTTTGATCAGCAGCACTTCAGAAATCAATCTCTCAATTTTTTTGCTCTGTGGGTGCGTAATTAT
TTTGAACGCCTCTTCTCCTATTTATGCTTTTGTTTCCTGAAGTCTTCTACTCCTTCAGTGACTCCTCATTCGT
TCAACCACTTTATTTCATTTAGAGTTCTTCTTATACCTTTATTAATTAGAAGTCCTTCTTGAAACAGTTTCTC
ATCAACTCACTTTGCCAACGTGTTCCTTGCATTTTGTCATGTATCAAAATAATACTTATCCCAACATTTTCT
TTTATTCCTTTCTCTGTTTTGTGTTGCTAATAAGTCTATGATTTTCTTCTTCCTTTGATCATCATCTTTGCTCT
CCCACTGAAGCTTCCGCTTTGCTTCAGTTTAAGCAACCTTTCAAGTTAAATCCGACTACTCCCAGTGTGAT
ACTTCTTTCCCAAAAACAAAGTCTTTGAATGAGAGTGGAGATTGTTGTACTTGGGACGGAGTTACATGTC
ACATGTTAACCGGTCCTTAGTTGTAGTCAGATTGTTGGCAATTTTCATCCCAATAGCAGTCTCTTCGAACT
TCATCATCTCCAGACACTAAATCTTGCTTACAATGACTTTTATCCTTCTTCGATCCCACATAACATTGGCCG
ATTGACGAATTTGAGGCATCTTAACCTTTCTCGTTCTTTCTTTGATGCAAAAAATCCCAACAGAAATCTCA
TATCTTTCCAATTTGGTTTCACTTGATCTTTCTATGAATTGATTACAACAAATTGATGAGAGAACATTTGA
AGCAATGCTTCACAACTTGACAAATATGGAGGTACTATCTCTCCTTCTTGTCAACATCTCATATCCCATAC
TTGTGAATATTTCTTCCTCCTTAAGGTACCTGGATGTTGAAGCTACTAATCTGCGAGGTGTTCTCACAGAG
AGCCTTTTCCTTCTGCCAAACTTGGAAACACTCAAATTGAGTGGTAATCATCTTCTCAAAGGAGTTTTTCC
AAAGATCCACCCGAGAAACACTCTGTTAATGGAATTGGATATTTCATACACAGGAATCTCCGGTGAGCC
GCCTGATTCAATTGGCACCTTGAATTCATTGAATATCTTGAATCTCCAAGGACGTCAATTCTCTGGCCATA

TTCCTGATTCCATAGGCAACCTAACACAAATTACAGAGTTGATTTTAATCTTATAATCATTTCACTGGCCA
TATTCCTTCCACAATCTCTAAATTGAAGCACCTCACAAGTTTACTTCTTCGGTCCAACTCCTTTTCAGGTGA
AATTCCTGATGTTTTCTCTAACATCCAAGAGCTACGCTTTTTAGATCTTTTTGATAACAGCGGTTCGTTTCC
CTCTTCAATTTCAAACTTGACACATCTTCAACGGTTAGACTTGTTGAGTAATTCCTTATCTGGCCCACTTCC
TAATAATGCGAGCAATCTTCAAAAGCTAACCCAAGTGGATTTGTATGACAACTTACTGAATGGTACCATA
CCATCTTGGTTGTTTAGCCTCCATCTGCTAACACTTTTTTTGTCGCTCAGTAGAAATCAATTTAGTGGACT
ACCCGATGAGCTCAAACCAAATCCAGCATTAACAAAAGTGGATTTAAGCAATCACTTGTGAATCTCACAC
ACCTTTATAACCTTGACCTTTCATCAAATAACATCACCGGTCCATTTCCCGCTTCAATTTTAAGTTTGACAC
ATCTTGAATAGTTAAACTTGTCGAGTTATTCCCTATCTGGCCCACTGCCTAGCAATGCAAGCATGCTTCAA
AAGCTAACTTTTGTGGATTTGTCTTACAACTTGCTAAATGGTACCATACCATCTTGGAACCTCCCTTTGCT
ATCTTTGTTGTGGCTCAATCATAATCAATTTAGTGGACTAGCCTATGAGATCAAAATAAACCCAGCATTA
GAATTTTTGCATTTAAATAATAATCAACTCAGTGGTTCTTTTCCTCAATCACTTGTGAATCTCACAAACCTT
TTTGCCCTTGACCTTTCATCAAATAACATCACCATTGATGAGGGAATGAATATCATCTTTCCTAGCCTATC
ATCTTTATTCTTATCATCTTGTGGACTGAAGAATTTTCCACACATCTTGAGAAATGTAAAGACACTTCAGG
TCTTGGATAATTCTAACAATAATATTTGTGGTCAAATCCCTAACTGGTTTAGCGGCATGAGGTGGGACTT
GTTGCAGTTCCTAAACCTTTCTCATAATTCATTGACAGGCCACCTACCACAATTTCATTACTATAACGTAC
AGTATCTTGATCTGAAATTTAACTCCCTTCAGGGTCCACTACCTTCATCCATTTGTAACATGAGCAAACTT
ATCTTATTAGATTTATCACATAACTACTTCAGTGACTCAATTCCAAGTTGCTTGGGAAGCATGGCTAATTT
AAGGGTGTTGGACTTAAGAAGGAACAATCTCACAGGGAGTATTCCCATTATGTGCACACAACACTTCAT
TGAGTGCCATTGTTGTAAATGGTAATCAATTTGAAGGACCTGTACCTGTGTCGTTGCTCAAGTGTAATGG
TCTAGAAGTCCTTGATGTGGGGAACAATGCTATAAATGACACGTTTCCAGCTTGGCTCGGAACCCTTCAA
GAGCTGCAAGTCCTTATATTAAAGTCAAACAAGTTCCATGGACCTATAAGTACTTGTCAGACTAAGTTTT
GCTTTCCCAAGTTGCGAATTTTTGATCCTTCATGTAATGATTTCAGTGGCTCACTTCCTGCAAAAGTTTTT
GGAAACTTCAAGGCAATGATCAAATTATATGGCGAAGACACAAGAGAGATCAAGTACATGGAACCATCT
GAGGAGCCATCGTACGTATCGTATGAGGATTCAGTGTGTTTGGTGGTAATCAAAGGGCAGAATATTGA
GCTACAAAGAATCAGCACAATTACGACAACCATAGATCTCTCAAGCAACCATTTTGAAGGTGTGATTCCG
AAAACACTAAAGGATCTCAGCTCACTTTGGCTACTCAATTTATCCCGTAACAATCTCAGAGGCGATATTC
CAATGAAACTAGGACAATTGAGTATGCTTGAAGCTTTAGATATCTCTTGGAATCGGCTCACTGGAAAGA
TTCCACAGGAATTGACAAGAATGAACTTTCTGGCCTTCTTAAACCTCTCTCAAAATCATCTCGTCGGACCA

ATTCCTCAAGGTCCACAATTCAACACATTTGAAAATGACTCATATGGTGGCAACCTTGATTTATGTGGTCT
TCCTTTAACAAAGAAATGTGGAATGAGTGATTCATCGCATGTTCCTCAACAGTTGGAGTCCGAAGAAGA
AGGCGAGTCATATTTTTTTAGTGGATTTACGTGGGAATCAGTAGTCATAGGCTACAGTTTTGGACTAGTT
GTTGGAACTGTCATGTGGAGTCTCATGTTTAAATATCGTAAGCCAAAATGGTTTGTGGAATTTTTCTATG
GACTCATGCCTCACAAAAGAAGAAGGCCAAAGAAGAGAGCTCAGAGATGA
>PGSC0003DMG400006655 LRR-RLPs
ATGTCCAAAGAGTACTTTAGTTATTGGTCTGATGGAGATTCTTTCCCGAAAACAAAGTCTTGGAATGAGA
GTAGGGATTGCTGCAGTTGGGATGGAGTCACTTGTGACTTATTAAACGGTCATGTTATCGGGTTAGACC
TTAGTAGTAGTCTGCTTTGTGGAAGTATTCATCCCAATAGCAGCCTCTTCCAACTTCATCATCTCCAAACA
CTAAACCTTTATAACAACCTCTTCAATTATTCTTCAATCCCACATAGCATTGGCCAATTGACGAATTTGAG
GCATCTCAACCTTTCTCATTCTCACTTTGGTGGGAAAATCCCAACAGAAAACTCATACCTTTCTAATTTGG
TTTTACTTGATCTTTCTGTGTATGGATTACAACTTGATGAGAGAACATTTGAAACAATGCTTCACAACTTC
ACAAATCTGGAGGTACTAGCTCTCTCTCTTGGCGACATCTCATCACTGATACCTGTAAGTATTCATCCCAA
TAGCAGCCTCTTCCAGCTTCATCATCTCCACACACTTAACCTTGATAACAATTACTTTTATCCTTCTTCGAT
CCCAAATGGCATTGGCCGATTGAGGAATTTGAGGCATCTAAAACTCTCTGGCTTTGATGGGAAAATCCC
AACAGAAATCTCATACCTTTCCAATTTGGTTTCACTTGATCTTTCTTATAGTTATAAATTCTTACAACTTGA
TGAGATAACATTTGAAGCAATGCTTCACAACTTCACAAATCTGGAGATACTTTCTCTCTGGTATGTCAACA
TCTCATCTCCGATACCTGTGAATATTTCTTCTTCCTTAAGATATGTGGATCTTGCACATACTAATCTGCGAG
GTGTTCTCACAGAGAGCTTTTTCCTTGTGCCAAACTTGGAAAGCCTCAAATTGAGTGGGAATGATCTTCT
CAAAGGAGTTTTTCCAAAGATCCACCGGAGCAACACTCTGTTAATGGAGTTGGATATTTCATCCACAGGC
ATCTCCGGTGAGCTGCCTGATTCAATTGGCACCTTTAGTTCATTGAATATCTTGAACCTCGGAGGATGTC
AATTCTCTGGTTCCATTCCCGATTCCATAGGCAACCTAACCCAAATTACGGAGTTGGCTTTGTCTTATAAT
CATTTGGGATAATACATATATTTGACCTTAAACTTGGCTTCAAATTTTAACTTTGACCTCCAACTTTCATAG
TGCACAAACAAGCACTTTAACTATCCAATCTTTTAATAAATAAACACGTGATTTTTGGAGCCAAGAGCGT
GAAATGCAAACGCTGCCACGTGTATTAGAAATTCCTGATGTTTTCTCTAACCTCAAAGAGCTACGTACTTT
ACATCTTTCTAATAACAGCTTCATCGGTTCGTTTCCCTCTTCAATTTTAAGTTTGACACATCTTGAATACTT
AGGATTGACGAATAATTCCCTATCTGGCCCACTGCCTAGCAACCAAAGCATGCTTCAAAAGCTAACCGAA
CTGAATTTGTCATACAACTCACTGAATGGTACCATACCTTCTTGGGTGTTTAGCCTACCTTTGCTATCTTCA
GTATCGCTCCAACATAACCGATTCAGAGGACTAGCCGATGAAGTGATCAAAACAAACCCAACATTAAAA

GAACTGCATTTAAGCAATAATCACCTCAGTGGTTCTTTTCCTCAATCACTTGTGAATCTCACAAACCTTGA
AACCCTTGGAATTTCATCAAATAACATCACCATTGATGAGGGTATGAATATCACCTTTCTTAGCCTATCAT
CTTTATTCTTATCATCTTGTCAACTGAAGGATTTTCCACACTTCTTGAGAAATGTAAAGACACTTCAGGTC
TTGGATATTTCTAACAATAAGATTTGTGGTCAAATCCCTAACTGGTTTAGCGGCATGACGTGGGACTCAT
TGCAGTTCCTAAACCTTTCTCATAATTCATTAACAGGCCACCTACCACAATTTCGTTACTATAACCTACAAT
ATCTTGATCTGAAATTTAACTTCCTTCAGGGGTCACTACCTTTATCCATTTGTAACATGAGGAAACTTATC
TTATTAGATTTATCACACAACTACTTCAGTGACTCAGTTCCACATTGCTTGGGAATCATGGCTTCACTAAC
GGTGCTGAACTTAAGAAAGAACAACTTCACAGGTAGTCTTCCTCCATTATGTGCACAGAGCACTTCATTG
AGTACCATTGTCGTAAATGGCAATCGATTTGAAGGACCTGTTCCTGTGTCGTTGCTCAAGTGTGATAGAC
TAGAAGTCCTTGATGTGGGGAACAATGTTATAAATGACACATTTCCAGCTTGGCTCGGAACTCTTCAAGA
GCTGCAGGTCCTTATATTAAAGTCGAACAAGTTCCATGGACCTATAAGTACGTGTCAGACTAAGTTTTGC
TTTCCCAAGTTGCGAATTTTTGATCTTTCTCATAATGATTTTAGTGGCTCACTTCCTGCAAAAGTTTTTGGA
AACTTCAAGGCAATGATCAAATTAGATGGCGAAGATACAGGAAATATCAAGTACATGGAATCTATGTCG
AATTTGCCATTTGTCAGATCGTATGAAGATTCAGTGAGTTTGGTAATCAAAGGGCAGGATATTGAGCTA
GAAAGAATCAGCACAATTACGACAACCATCGATCTCTCAAACAACCATTTTGAAGGTGTCATTCCGAAAA
CACTAAAGGATCTCAGCTCACTTTGGCTACTCAATTTATCCCATAACAATCTCATAGGTCATATACCAATG
GAATTGGGGCAATTGAATACGCTTGAAGCTTTAGATCTCTCTTGGAATCGGCTCACTGGAAAGATTCCGC
AGGAATTGACAAGGATGAACTTTCTGGCAGTTTTTAACCTCTCTCAAAATCATCTCATCGGACCAATTCCT
CACGGTCTACAATTCAACACATTTGAAAATGACTCTTATGGCGGCAACCTTGATTTATGTGGTCCTCCTTT
ATCAAAACAATGTGGAACGAGTGATTCATCACATGTTCCTCAACCATTGGAGTCTGAAGAAGATGAAGG
TGAGACATATTTTTTTAGTGGATTTACGTGGGAATCAATAGTCATAGGCTATAGTTTTGGACTAGTTGTT
GGAACTGTCATGTGGAGTCTCATGTTTAAATATCGTAAGCCAAAATGGTTTGTGGAATTTTTTGATGGAC
TCATGCCTCACAAAAGAAGAAGGCCGAAGAAGAGAGCTCAGAGACGACGGACATAA
>PGSC0003DMG400006792 LRR-RLPs
CAACAACAGACTATACGGAAAGTTCCCCTCATGGATATCTGATGGTTTTCAAAATCTTAGAATCCTCAGA
TTAAGGTCAAATTCATTTTCCGGAGAACTTCCTCTAGGAATGTCAAAACTGAGTTCATTGCAGGTGCTTG
ATCTTGCAGAAAACAACATAACTGGCGCCATTCCAACAAGCATAGGAGACCTGAACGCCATGGTACACG
AGAAAAAAGTGAATGAATATCTGTTATATGGGAAGTATAGAGGGATTTACTATGAAGAAAGCTTGGTA
GTGAATTTGAAAAACCAGTTCCAAAAGTACACCAAGACTCTATCACTTCTTACCTCAATAGACTTGTCGA

GAAACAACCTCAACGGAGCATTTCCTGTGGAATTGACAAACTTGCATGGCCTCATAGTTCTGAACTTGTC
TGGAAACCAAATCAGTGGAAAAATACCAGAAAACATTTCAAGCTTACATCAGTTAGCATCGCTTGATCTC
TCGAGTAATACACTGTCTGGTGTCATCCCATCAAGCATGGAATCAATGTCATTCTTGAGCTATATTAACTT
CTCAAACAACAACTTATCCGGTATGGTTCCTTATAAGGGGCAGATGACAACTTTTACAGTGTCCGCGTTT
GAGGGGAATCCCCATCTTTGTGGAGCTCCACTTGTAGTACTATGTCAAAATGGAAACTCGGACAATAGA
ACAGTACTAGAGAATGACAGCAGTGATGAATTTCCTGATAAATGGTTTTATCTTAGTGTTGGATTGGGAT
TTATTACAGGTATTCTTGTTCCTTACTATATCTTGCTCGTTCGAAAGCCATGGAGACGAGCATATTTCAGT
TTCTTAGACACAGTCATCGACAGATTTGTCCCAGTTAGAAGCAACAGAACAACCAGCACCAAAAGATCA
GATACGGGCACCAGCTAAGGCAAAAGAAGTGTTCAATAGGTACTAGTCCTAGT
>PGSC0003DMG400006880 LRR-RLPs
CACAGAAGAAACACTCTCTGTAAATAGAAAATAGAGAGTAATAGTACTGCATTTTGTAATAGAGAGGAG
AAATGGGTCTCACCGGAGTAACATTGTTGGTCACTCTCATGGTGGTGGCCGCCGTCACTTTGCCGGCAA
ATGCCTGTCTGCCGGCGGACCAAGCAGCATTGATGGATATCAAAGCAGCTCTTAAGGAGCCGTATTTGG
GCATTTTCAACACATGGACCGGCAGCAACTGTTGCCAGGGCTGGCATGGTGTTAGCTGTGACCCGACTA
CTCAACGGGTTGCCGACATTGTTCTTCGGGGAGAATCCGAAGATCCGATTTACGAGAAAGCCGGCCGGT
CTGGTTACATGACCGGTTCACTCTCCCCTTCTCTCTGCAAACTCGACAAGCTCACTACACTCATCGTCGCC
GACTGGAAAGATATCTCCGGCGAAATTCCGGCATGTGTCACTTCATTACCAGATCTCCGTATCCTTGAGC
TCATCGGCAACAAAATCAGCGGTCAAATCCCGGAAAACATCGGTCAGCTGAGTAAACTCACTGTTCTAA
ACCTCGCCGACAATAAAATCTGCGGTTCAATCCCCGCCTCCATTGTCAACCTCGGAAAACTAAAGCACCT
CGAACTCAGCAACAATCAACTCACTGGCGAAATCCCGTCCGATATCGGAAAACTCGGAATGATGAGCCG
AGCACTACTCAACAAGAACAAATTCACAGGTTCAATCCCTAACTCAATCACTCAACTTCGCCGGTTAGCC
GATCTAGATCTGTCAATGAACCAAATCACCGGTTCACTCCCGGCTCAGCTCGGTTCAATGCCGGTTCTCT
CAACTCTAAACCTCGATTCCAACCAAATCTCCAGTTCAATCCCCACTAATCTACTTAGCAGCAGCGGATTA
AACATCTTAAACTTAAGCAGAAACTCATTAGAAGGAGAATTACCAGATGTGTTTTGCTCAAAAACGTACT
TCACACATCTGGATTTATCATACAATAATCTTCGAGGTTCAGTTCCAAAATCATTATCATCAGCTAAGTAC
ATTGGCCATTTGGATTTAAGCCATAATCATCTTTGTGGCCCGATTCCAAACGGGTCACCCTTCGATCATCT
AGAAGCTTCTTCTTTCTCTAACAACGATTGTTTGTGTGGATCGCCATTACGTACTTGTTAATTCTTTTTTGT
TTACTTACTAAGTTTGTAATTAATCGAAATTATCGGTTGATTACGCATAAATGGATGTGTAATTGTGTATT
ATGTTGTTATGATTAAAAAAATATTCTTATGTGTTTAGCTTAATTATTATTATTATTGCTAAGCAATATGAT

GGATTATGCGGTTCTATCATGCATGCACGTGAAAATGCGTGACAGTTTACAAGAAAAATTATTGAAGAA
ATTGTGTGCTATTGTTATTAGTAAATTGTAATATAATAAATCCCAC
>PGSC0003DMG400007033 LRR-RLPs
ATGGCCAGATCAGTTAACTCCATTTTCTTAACCTTCTTGTACATAAGTTTAATATACACACTCTCACCTTAT
GTCAATGCAACAACTTCACAATCTGATATTCAAGTTCTAGTAGCCATCAAGACGTCGATAGACCCTTTTTC
AATAACCCCGGATTCCTTCCTTAGTAGTTGGGATTTCAACTTAGACCCTTGTGAAACAACTGGTGCAAGT
TTCTTAGGCATTCTTTGCACAGTTCCTGATCAGGTAGACACTAATGCCACGAGCCGGATAATGGAGATTG
ATCTAGAAGGTGATGGACTAGAGGGGTTCTTGACATCGTCTATCGGGAACTTAACAGAACTTGTTACTCT
CAATCTAGGTAGGAACAAGTTCAGGGGACCAGTGCCAGAGAGTATTACTAATTTGAGGAAACTCACAA
GTCTTCAACTTTATGAGAATTTTTTCTCAGGTAGTCTTGTTGATGACATTGGTGTCCTTACAAAACTTGAA
ACTCTTGATGTCTCGAATAACAGGCTATCTGGCTCAATCCCTTCCTCGATTACATCACTTAGAAGTTTAAC
GCGATTAGACCTGTCTAACAATGAATTCACTGGAAAATTTCCTCAACTCAACGGATTATGGCAACTCTCTT
CATTTGACATTTCAAATAACCAAATTTATGGGAATTTACCACAATTCCCATTGAAGATCAAAACACTATCA
CTAAGCCACAACTTGTTATCAGGACACATTACCCCAGTGCATAAACTTAGACATTTAAGCACATTAGACC
TTAGTGATAACAGATTTTCAGGAGGAATAAACAAAGGGATCTTCATGTTACCTGAATTAAGTCACGTAAA
CGTTTCAGTAAACCGTTTCACAGTGTTTGAGGTGGTAGAATTCTCTGATAAAAAATCACAGCTACACACG
TTAGACGTACATGCTAATCGTCTACATGGTCATTTGCCTGTGAACTTGGTTACTTACCCAAACTTAACAGA
AATAAACCTTGGACATAACCTGTTTTCGGGTGAGATTCCATCAGAGTATTGGTCAAGACTCGGATTTTCA
TGGAGAAGTCTCAACTTGGAATATAATAACCTGGAGGGATCGGTGCCTCGAGAGCTCAACAGGACTTTA
GAAGGGGTTCAGGGAAGTTTTGAACACAACTGTCTCATTTGTCCAAAGGGATTGCAGCTATGTCATGGA
CAGAGGACAGCTTCAGAATGTCATGGAGGAGACCATAATTAG
>PGSC0003DMG400007281 LRR-RLPs
ATGCCGGCTCTACGCCGCCAAATTTCTACCCTCTCAACGGTTAACTTGAACAAAACGATCAACAGCTTCA
TTGGTAACGGAAACCTCCAAGAAGCTCGGAAGCTCTTCGATCAAAGCCCCCACTTGACAAATGTCGTATC
ATGGAATTCCCTTATCGCTGGTTACTTCAAACACAATCTCATCCAACAAGCAGAGTACCTGTTTGACAAA
ATGCCTCACAGAGATGTCGTTTCTTGGAACACCATGCTCTCCGGATATCGCAACGCCAACAACCCAGAGA
AAGTATATAGATGTTTTTAGATATGAACAGATGTGGAGAAATGAGACCCAATGAGCTCACTTTTGCAGT
GTCAATTAGCTCCTTCTTATATTTAGATTACAAGCATTTGATCCCTCAGCTTCAAGGTCTCGTGCTCTGTTT
GGGGATAAGTCTCAATGTCTTTGTAGGGTCAGCATTGATGAGGGGTTATGTTGATTTGGATGATTACAG

AGGTTTAGCTCGAGTTTTTGATGAGATTTTGGATAAAGATGTTACGCCGTGGAATGTGTTGATTTTGGGT
TATATGAAATTTGGGTGCACAAGTGAGGCGCAGAGGGCTTTTGATATGATGCCTATGAGAAATTATTTT
ACTTGGAGTACTTTGATCAATGGATATATTGAGAACAAGAAGCTTAATGAAGCTCGATTCATTTTTGATA
AGATGAGTGAGAAAGATGTGGTTTCTTGGACAGCAATGATAAGAGGGTATGCGCAGTATGGAGAGTTT
ATGAAGGCTTTGAAATTATTTAAGTTGATGTTGAACTTGGGTTCTCGTCCTAATCATTTTACGTTTTCAAC
TGTTTTAGATGCTTGCGCAGGCTACTCTGCTGTTCTGGTAGGCAATCAAGTTCATGCGTGCATCTTGAAG
TCTGGTTTCCCTCTTGATGTCATCTTGTTGACCTCCCTTCTTGACATGTACGGTAAATGTGGAGACATTGA
AGTTGCTTTCTGCATTTTTGAGTCAATCCCGGCAAGGAATTTGGTAGCCTGGAATTCGATCATTGGAGGT
TTTGCAAGACATGGGCTTGCAGAGAGGGCAATGCAGGAGTTTGAGAGGATGCTAAAGAGTGGTATCAG
GCCTGATGAAATTACTTTTATTAATTTGGTATATGCATGTGGCCATGGTGGGCTAGTCGAGGAAGGTGA
GAGAATTTTCAATTCTATGGTAACAGAGTACGGATTGAAAGCAGAGATGGAACACTATGCATGTATGGT
GGACTTGTATGGAAGGGCAGGTCAGCTTGAGAAAGCAGAAAAACTAATCGATGGGATGCCTTTTAAGC
CTGATGTGGTCGTCTGGGGGGCATTGCTAGGAGCATGTGGCTTGCACTCATGTCTAGAGCTCGGAGAG
ATTGCAGCAAATGGAATCTACACATTGGAGCACGACCATCCTGCAGTATACTCGGTGCTTTCCAAAATCT
ATGGAGACAAAGGAGTGTGCAGTGACATAACTGGTTTGGATAAGTTGATGAAGAAATGGNNNNNNNN
NNNNNNNNNNNNNNNNNNNNNNNNNNNNNNNNNNNNNNNNNNNNNNNNNNNNNNNNNNNN
NNNNNNNNNNNNNNNNNNNNNNNNNNNNNNNNNNNNNNNNNNNNNNNNNNNNNNNNNNNN
NNNNNNNNNNNNNNNNNNNNNNNNNNNNNNNNNNNNNNNNNNNNNNNNNNNNNNNNNNNN
NNNNNNNNNNNNNNNNNNNNNNNNNNNNNNNNNNNNNNNNNNNNNNNNNNNNNNNNNNNN
NNNNNNNNNNNNNNNNNNNNNNNNNNNNNNNNNNNNNNNNNNNNNNNNNNNNNNNNNNNN
NNNNNNNNNNNNNNNNNNNNNNNNNNNNNNNNNNNNNNNNNNNNNNNNNNNNNNNNNNNN
NNNNNNNNNNNNNNNNNNNNNNNNNNNNNNNNNNNNNNNNNNNNNNNNNNNNNNNNNNNN
NNNNNNNNNNNNNNNNNNNNNNNNNNNNNNNNNNNNNNNNNNNNNNNNNNNNNNNNNNNN
NNNNNNNNNNNNNNNNNNNNNNNNNNNNNNNNNNNNNNNNNNNNNNNNNNNNNNNNNNNN
NNNNNNNNNNNNNNNNNNNNNNNNNNNNNNNNNNNNNNNNNNNNNNNNNNNNNNNNNNNN
NNNNNNNNNNNNNNNNNNNNNNNNNNNNNNNNNNNNNNNNNNNNNNNNNNNNNNNNNNNN
NNNNNNNNNNNNNNNNNNNNNNNNNNNNNNNNNNNNNNNNNNNNNNNNNNNNNNNNNNNN
NNNNNNNNNNNNNNNNNNNNNNNNNNNNNNNNNNNNNNNNNNNNNNNNNNNNNNNNNNNN
NNNNNNNNNNNNNNNNNNNNNNNNNNNNNNNNNNNNNNNNNNNNNNNNNNNNNNNNNNNN

NNNNNNNNNNNNNNNNNNNNNNNNNNNNNNNNNNNNNNNNNNNNNNNNNNNNACAACCCCCCCC
CCCCCCAATATATGTGAAGGTTAATGCAATTGGTGCTTATTATGAAGAATGCACAACCATTGATTGAGAT
AAATATTACATGGCATGCCATCTCAATGTCACAATCCATTTTAGCCTCCTCTCCACTTGTTCTTCCGCTAAT
TAAACTAAGCAAAAATAACTACAATGAACTAAGCTAATTAAACTAAGCAATATTCCATAACCAACAAATC
TATAGAGAAGAGATGAGATGAGAGTCAAAATTTAGGAAGTTGTATTCTGCTCTTTGTCTGATGTTTTAAA
TCTTCTGATTTTCAGGAAATGGTGCTTTCATCCAAGGTTCATAGCCAAGTCCAAAACAATCCGCCCTGTCT
TTATCACATCGGCAAAATGGGAATGTATATTTTCCTTCTTGGAAGAGGATTTGTGTCAGTACCGCTGTTG
AAAATTTCGAGCAGGAGAAGCAGTCGTCCAATGAAATCCTTCTTGATGAATGCCTCGTTGAGGTATTCA
AACGTCTTTCCAGTGGCCAAGAGAGAAGTGCTTGTGGTTGTGTTTCCAAGCGCTGGCTTATGCTTTTAAG
CAGCATCCGCAGGGATGAAGCATTTGCCTTCAAGCCCAATCTGCCTTCAAATGCTGAAGAGGAATCTACT
TAACGTACCCAAGTTAAAATTGGTGAGATTGCAGAATCTACTGGTGTAGAAGGTGAGGGATATCTTGCC
AGGTCCCTTGCTGGCAGGGAAGCCACAGATGTCAGACTTGCTGCTATTGCCATTGGAACTGCAAACCTT
GGAGGCTTAGTAAAGCTATCTATCTAGGGAGACAACCTTTGTCGTGGTGTGACTGATACTGGTCTCAAC
GCTATCGCTCGAGGTTGCCCTACTCTCAAACATCTTTTCCTATGGGATTTGTCTTCTGTTGGTGATGAAGG
CTTATCTGAGGTTGCTCATGAGTGTCATCTGTTAGAGAAGCTTGATCTTTTCCAATGCCCAACAATTACTG
ACAAGTCTTTACTCGAAATTGCGGTGAACTGCCCCAAAATGACCTCTATAACAATGGATTCTTGTTCAAA
CATTGGGAATGAGTCTCTCAAAGCTGTCGGTCAATATTGCCTGAACCTGAAGCTTGTTGTATTTAGAAAT
TGCCCACTCTAGAATGAAGCTGTTATCAAGATGTTTGCCATACTTCTCTGAGAATGCACATCCTGGATTTT
TTTGGTCCTGGCTCCTTTGCCAGTAGTTTTTAGCTTCTAGCACTGCATTGCTGAGAAAATTGTGCTACAAT
AAAAACTTTTTTCACCATCTCTTATGTTCCTGTTTAGTTTGGAGTGAATTTATTTGAAGGTTTTTTTTTTGG
GTTGGCCCTTCTCCTGTATCTGCAATATGGAGACACTGTATGAA
>PGSC0003DMG400007606 LRR-RLPs
ATGGACCCTACACATGATAATTGGAAGAAGGTTGAGGAAAATCTGAACTCATTCTTTGGTACGGTGTCC
GAACGGTGCCAATCAATTCTGTCTCTGAGCTACAATTATTTGCCCCAGTATTTGAGGGCTTGTTTTCTCTA
TGTTGGAGGTTTTCCTGAAGATATGGAGATTGATGTTTCCAAGTTGATTAGGTTATGGATTTCAGAGCAA
TTCGTAAAGGCAAGAAGCAATAAAAGATTAGAAGTGGTGGCAGAGGAGTATCTACAAGAGTTAATTGA
TAGAAGTCTAATTTTGGCTGATAAACAATGGGTTCATGGAATGATGAGAAGTTGCAAAATTCATGATCTT
CTTCGCCAACTATGCCTAAGTGAAACTCATACTGAAAATGTTGTGCATGTCATGAATGGGAAAGTTCTCA
AAGCCATAGATGGTCAATGTCGAGTTATCCTTCTGTCAAATTATGAAGAGAAGTATGATTATTTTCCGCG

ACATAGCAGTGGTATTGTCCGTACCTTTATTTCAATGGGTGTAGCTTTTCCAAAAGAGATGTGTTCCATTA
TTTCAGAGTTCAAGTTGATTAAGGTGTTGGACGTGTTACCAGTCCTGTACGATTTCTCTAGTGTAATACCT
GAGCTTGTACATTTGAGATATGTTGCTGCAAGAATTGAGGAAGGTCTTTCACTAGCCAAATTGAGAAAT
CTACAGACCATAATTCTTCAAAGATATTTCTCCAGACCAACAACGTTGAAGCAGCCAGTAGATATCTGGA
GTATGTCAGAGATAAGACATGTGGATATTCAATTTCCACTATATATATCAAATCCTGTTGAAGCAGAACA
ACCTTTGTGCCTTCTGAATAACTTGCAAACACTTTATCCCTATAACTCTCCTTTTGTTGAGGAAATCATAAG
AAGAACTCCCAATCTAAAAAAGCTAAAGATTTTAGATATATCTGAGCATTCTGATTGGGCTGTTATTCTT
GATTCTCTCAGTATTTTACAGGATCTGGAGACACTACACATACAAAACATTGATCCGATGATTATCTCTGA
AGACATTTCTCTTCCTAATCTTAAGGAGTTGAATTTATCAAATACTTGTATTCCATGGGAAGTTGTGAATT
TGCTGGCTAATTTACCCAATCTTGAGAAGCTCTATGGGCATGCTGCATTCGATGGAACAGATTGGAAAG
TAGATGAAGATGTTGTATTTCACAAATTAAAATATCTACGTCTATATACGTGCAGAGATCTGGAAAGGTG
GGAATTAGCAGCTGGTAGTGATAATTTTCCGATGCTTGAGAATTTAATACTGTTTGAATTGCCTAAACTA
GAGGAGATTCCGGAGAGTATTGGAGAAGTAATGACACTAAAATTCATCCGAATAGTTAATTGCGGCTCT
GGTGTAAAGACAAGTGCAAAGAAAATTCAGGAAGAGCAAGAAAGCTTGGGAAATTATGAGCTTGATGT
TCAAATCTACCCCTAA

>PGSC0003DMG400007684 LRR-RLPs

ATGAAACTCATCAACTTAGCCCTTAGTGGCTTCTTGTTCATCATTTTCTTCTTCTCAAAACCCTCATTTCAA
GCTTCAATCAAATATGAACCAAACCCTAGACTTTTCGATGCTTACATAGCTCTACAAGCTTGGAAACATG
TCATGACTTCCGATCCAAGAAACTTCACAAAAGATTGGTATGATTACAATGTTTGTAACTACACCGGGGT
TTATTGTGCTCCAGCTCCAGATAACCCTAATATCACCACTGTTGCTGGTATTGACCTCAACCATGCAAATA
TCTCCGGATACTTACCTGAAAAACTTGGTTTATTGACTGATTTAGCCGTCTTTCATATAAATTCCAATAGA
TTTGAAGGCTCAATCCCGAAATCTTTTTCTAAGCTTCGAATTTTATTCGAACTTGACGTGAGTAACAATCT
TTTTTGTGGTGAGTTTCCTTCCGTTGTGTTATCTCTTCCTTCTTTAAAATTTCTTGATATTAGGTATAACCAA
TTCGAAGGAAAAGTCCCTTCAACCCTTTGGGATCGAACACTCGATGCTCTTTTCATAAACAACAACAAAT
TTCAATTTTCATGGCCTAAAAACTTAGGTAAATCACCCGTTTCTGCTTTGGTTATGGCAAATATTAATGTA
ACGGGGTGTATACCATCAAGTATAGCAAAAATGTCGAAAACATTAAATGAGATTATTCTCTCGAATTCGA
GTTTAAGTGGATGTTTACCACAAGAATTAGGGTTATTGAAAAATGTTAGGGTTCTTGACGTGAGTTTTAA
CAAGCTTGTTGGTGAGTTGCCGGAATCAATTGGTGGAATGAAGAAATTGGAACATCTTAACGTGGCACA
TAACAAGTTTTCCGGTGAGATTCCGATGAGTATTTGCTCATTACCAAAGTTGGAGAATTTCACTTACTCTT

ATAATTATTTTTGTGGTGAACCAAAGATTTGTCTCAAGTTGAAGGATAGAAATGATAAGAAGAATTGTAT
ACCATATCGACCATCGCAACGTTCAGAAAGTGAATGTAAGACATTTTATTCACGAGGTCCGGTGGATTGT
AGTTCATTTGGTTGTAGATCACCACCACCGCCACCACCGCCACCGCCACCACCANNNNNNNNNNNNNNNN
NNNNNNNNNNNNNNNNNNNNNNNNNNNNNNNNNNNNNNNNNNNNNNNNNNNNNNNNNNNNNNNNNNNNNN
NNNNNNNNNNNNNNNNNNNNNNNNNNNNNNNNNNNNNNNNNNNNNNNNNNNNNNNNNNNNNNNNNNNNNN
NNNNNNNNNNNNNNNNNNNNNNNNNNNNNNNNNNNNNNNNNNNNNNNNNNNNNNNNNNNNNNNNNNNNNN
NNNNNNNNNNNNNNNNNNNNNNNNNNNNNNNNNNNNNNNNNNNNNNNNNNNNNNNNNNNNNNNNNNNNNN
NNNNNNNNNNNNNNNNNNNNNNNNNNNNNNNNNNNNNNNNNNNNNNNNNNNNNNNNNNNNNNNNNNNNNN
NNNNNNNNNNNNNNNNNNNNNNNNNNNNNNNNNNNNNNNNNNNNNNNNNNNNNNNNNNNNNNNNNNNNNN
NNNNNNNNNNNNNNNNNNNNNNNNNNNNNNNNNNNNNNNNNNNNNNNNNNNNNNNNNNNNNNNNNNNNNN
NNNNNNNNNNNNNNNNNNNNNNNNNNNNNNNNNNNNNNNNNNNNNNNNNNNNNNNNNNNNNNNNNNNNNN
NNNNNNNNNNNNNNNNNNNNNNNNNNNNNNNNNNNNNNNNNNNNNNNNNNNNNNNNNNNNNNNNNNNNNN
NNNNNNNNNNNNNNNNNNNNNNNNNNNNNNNNNNNNNNNNNNNNNNNNNNNNNNNNNNNNNNNNNNNNNN
NNNNNNNNNNNNNNNNNNNNNNNNNNNNNNNNNNNNNNNNNNNNNNNNNNNNNNNNNNNNNNNNNNNNNN
NNNNNNNNNNNNNNNNNNNNNNNNNNNNNNNNNNNNNNNNNNNNNNNNNNNNNNNNNNNNNNNNNNNNNN
NNNNNNNNNNNNNNNNNNNNNNNNNNNNNNNNNNNNNNNNNNNNNNNNNNNNNNNNNNNNNNNNNNNNNN
NNNNNNNNNNNNNNNNNNNNNNNNNNNNNNNNNNNNNNNNNNNNNNNNNNNCACCACCGCCACCTCCACC
ACCACCGCCACCACCACCGCCACCACCGCCGCCGCCACCTCCACCACCACCAAAATCACATTATTACCATT
ATCCTTAA
>PGSC0003DMG400007876 LRR-RLPs
ATGGAAGGTGTAGCTTTAGTACTTTCACTTCTCCTTATTGTTGTTAATTTAGATGGATTTTCTTGTGTTTAT
TCAGGAAGTCATTGCATTGAGAGTGAAAAAAATGCTCTTATTAAGTTCAGCCAAGGTTTCAAGAATCCGT
CACAAAGTATGTTATCTTGGATGTTTGACGAAAACTGCTGCAGCTGGAAAGGTGTTGAATGTGACAACA
AAACCGGGCACGTGATCACTCTTGATCTACATAAGCAATTCTTGCAAGGTGAGTTCAGGACTTCTTTACT
TGGTTTGCCTCATTTAAGGCACTTAGACATGAGCCACATTGATTTCCTTGGGGAACGGATTCCTGAGTTT
ATTTCCAGTTTTAAGGACCTGGAGTATCTCAATTTGTCTAAAACGAATTTTAGGGGAATGATTCCTGAAC
ATCTTGGGAATCTGTCGCGGTTGCAGTTTCTTGATCTGAGTGGTGATTTTTCACTCCAGGTTAATAACCTT
GAATGGATTCACTCCCTCTTCTCTATGAAGATTCTTGATTTGAGTGGTGTTGACATGAGAAACGCGGAAA
ACTGGCTACATGACATCAATTTGTTAAGTTCTCTCTCTGAATTACGTTTGTCTGCTTGTCAGCTTACTACAT

TTCCTGAATTGCTTCCGACTTCTGTGAATTTCACATCCCTTCGGATTCTTGATCTTTCATTAAACTATTTTAA
TACTAGGATTCCTAGCTGGTTGTTCAACACAAGCCACAATCTTGTTTACCTTAATCTTTCGAGAAGTCAGC
TGAACGGTTCACTTCCAAATGCCTTTGGCAACATGCGTTCACTTAGAGTTCTTGATCTTTCTGGGAAGTCT
CTCCTAGGCAACAGTCCACATTCGTTTGAGAAAATGAGTTCACTTACGGGCTATTTACCACCAACCCTGC
CATCAAAGCTGAAATACTAGGATATTTCTGATAATCAAATGGAAGGTCCTTTAGCAAGAAGCATCACACA
ACTTAAGCAATTAGTTGTCCTCAATGTTGCTCGGAACTCTTTCAAGGACTCGATTACAGAACATTTCTTGA
ATATAACTCATTTGAGAGTGTTAGACTTGTCATCTAACTCATTTATTTTCAATGTAAGCGCAACTTGGATG
CCTCGTTTTCAACTTGACTCTCTAAGCCTGCAGTTTTGCGGACTTGGTGCACAGTTTCCCCAGTGGATTCA
GACACAAAAAGAGTTGTCATTTATTGATATCTCTCGCACCGATATCTCAGGTGAAGTGCCCGATTGGTTC
TGGAATTTTTCTGCCAAGGTTTATCACATAGACCTTTCAGGAAACAATTTCAGTGGAGAAATCCCTGAAT
TTACAGAAAGAGTTTGTCTTGCAAAATTGGACCTGAGTGATAACAATTTTCATGGTCCTTTGCCTCATTTT
TCACCCAAGATGATGACGTTGATTTTGGCAAGGAATTCGTTTAATGGTACCATTGCCCCTGTATGTGAAT
CACTTGTCATGAATAACTCTCTGAGTCTTCTAGACCTATCTTCAAATTTTCTATCAGGACAACTTTCAGATT
GTTGGAGATATGGGAAGAATCTGCAAGGATTGAATTTAGGCCATAATGATCTATCAGGGGAGATACCTC
ATTCGATTGGAGATCTTACTAACCTCTTCTTTTTGCAATTGCAGAACAACAGTTTCTCCAAAAATCTTCCTT
CATCATTGAAGAACATAACTGGACTAAAAATTCTTGATGTTTCTGAAAATAGTTTATCTGGGAATATACC
ATTTTGGTTAGGGGAGAATTTACATGCTCTTGAGATTCTTATATTAAGCAGAAACAACTATGACGGAATT
ATTCCCAGGGAGATTTGCCAGCTTAAATACCTATGTATTTTGGACCTTAGTTCCAACGCACTATCAGGAG
TCATTCCAAGGTGTCTTGATAATCTCCACACCATGTCCGGAGAAGAGGAAGCCCCATCCTTTACTAATGG
ACCCTATGCAAATTACAGGATTCATGGAATGCTCGTTCGTAATGGATACTTTAAGGAGTTCATTTTTAGCT
GGCCCCGGATAGCAATTGATCTAGCAAAGAACCATTTATCAGGGGAAATTCCTGTGGAAATCACCAGGC
TTACTGCACTGAGGGGCTTGAATTTATCAAGTAACAATTTCAGAGGAGCAATTCCAGGTGACATTCACAA
CTTGCAACTTTTGGAGTCTCTAGATCTTTCCAGGAATCAGCTGTCATGCTCCTTTCCTCCCCGTATGGGAC
ATCTTATGTTTCTTGAGGTCGCGAATTTTTCTTTCAATGATTTGACAGGAGAAATTCCAGTTGGAGGTCAA
TTCGCCACCTTTGAGAGTACTTCTTATGTTGGGAATGCAAACTTGTGTGGATTCCCAGTCTCACGATTCTG
CTCGGATCATTTACATGAGGACATGGTTCACTGTAGTAACAATAATCAGGAGGTTCAGGCCATTGAACA
CGAAGAAAACAACAACTGGCTCGATGAATTTTCGTTCTACATTAGTATGGGAATTGGATTCAATACAGGT
TTCTGGTTATTTTGGGTTACTTTAATGTTGAAAGAGTCATGGAGATATGCCTATATGAGGTTCCTGGAAA
ACATGGGCAATAAGATATATGTCTTTGCTGCCATCAGATTGAAAAATATTCAAGCAGCAAAGAGGCTGA

CAAATTTCCCAAATTAAGCTGCTTCTCTTTACTATTCTCTGTTTCTTTTTTTTCCTTTCTGTCTCTTCTTATAT
TTTGGTTTTG
>PGSC0003DMG400007915 LRR-RLPs
ATGAGGTGGGACTCGTTGCAGTTCCTAAACCTTTCTCATAATTCTTTAACAGGCCACCTACCACAATTTCG
TTACCATAACCTACGGTATCTTGATCTGAAATTTAACTCCCTTCAGGGTCCACTACCTTCATCCATTTGTAA
CATGGGTTCGCTTATCTTATTAGATTTATCACGCAACAACTTCAGTGACTCAGTTCCACATTGCTTGGGAA
GCATGGCTAGTCTTAAGGTGCTAGACTTAAGAAGGAACAATTTCACAGGGAGTCTTCCCCCATTATGTGC
ACACAGCACTACATTGAGTACCATTGTCATAAATGGTAATCGATTTGAAGGACATGTTCCTGTGTCGTTG
CTCAATTGTAATGGTCTACAAGTCCTTGATGTGGGGAACAATGCTATAAATGACACGTTTCCAGCTTGGT
TCGGAACTCTTCAAGAGCTGCAGGTCCTTATATTAAAGTCGAACAAATTCCATGGACCTATAAGTATATG
TCAAACTAAGTTTTGCTTTTCCAAGTTGCGAATTTTTGATCTTTCGCATAATGATTTCAGTGGCTCACTTCC
TGCAAATGTTTTTGGAAACTTCAAGGCAATGATCAAATTAGATGGCGAAGACACAGGAAATATCAAGTA
CATGGAATCTATGTTGAATTCGCTATTGGTCACATCGTATGAGGATTCAGTGAGTTTGGTAATCAAAGGG
CACGATATTGAGCTAGAAAGAATCAGCACAATTATGACAACCATAGATCTCTCACGCAACCATTTTGAAG
GTGTCATTCCGGAAACACTAAAGGATCTCAGCTCACTTTGGTTACTCAATTTATCCAGTAACAATCTCAGA
GGCGATATTCCAATGAAACTAGGACTATTGAGTATGCTTGAAGCTTTAGATCTCTCTTGGAATCGGCTCA
TTGGAAAGATTCCACAGGAATTAACAAGAATGAACTTTCTAGCCTTCTTAAACCTCTCTCAAAATCATCTC
GTCGGACCAATTCCTCAAGGTCGACAATTCAACACATTTGAAAATGACTCGTATGGTGGCAACCTTGATT
TATGTGGTCGTCCTTTAACAAAGCAATGTGAAACGAGTGATTCATCGCATGTTCCTCAACAATTGGAGTC
CGAAGAAGAAGGCGAGTCATATTTTTTTAGTGGATTTACGTGGGAATCAGTAGTCATAGGCTACAGTTT
TGGACTAGTTGTTGGAACTGTCATGTGGAGTCTTATGTATAAATATCGTAAGCCAAAATGGTTTGTGGAA
TTTTTCGATGGACTCATGCCTCACAAAAGAAGAAGGCCAAAGAAGAGAGCTCAGAGACGACAGACTTA
A
>PGSC0003DMG400007999 LRR-RLPs
CTCCAGTAGCATAATAACATCACTCAAAACTTCAACTTCTTCAAGCAGAATGTAAAATCAGTTTGAAGTCC
CCAAACATGGCGGTGACGGACTTTTTCGTCGGCGAAATCACCACCGAACTCTTAAAAAACCTTCTGCTAA
TAGTTAAAAAATCCACTTTATGCCGTTCAAGTGCCGAGAATCTCATCGACAGCATCAATGGTCTCCTTCCA
ATCATCCAAGAAATCAAACAAACCGGTGTTGAACTTCCACAGATACGTCAAACTCAGCTCGACGATTTCT
CCAAACTTCTCCGAGATGGTTACGAACTCGCCGGAAAAGTTGTCCACTCCGGCCGTTGGAACATGTACA

GGTGTCACCTTCTCATTCGACTCTAATTACAGTGAATTGAACCTCTTCCTGCTCTAATTAGGGTTATTGAA
TTGTGCGAATCTGTTAGAAATTACTTAATTTATATTATGCCTTGATAACAGGAACCTACAGTTGGCTAGG
AAAATGGAGAGGCTGGAGAAAAGAGTAGCGAGGTTCATGCAAGTTACAATGCAAGCTCATGTACTTGC
GGATGTTCATCATGTTAGGTTTAATATGGAGCAGAGATTTGACGTGCTTGAGCATAGGCTTAAAGCTAT
AAAAATCGGAGTTGACGATAAAAGTGGTGGTGGTGGTGGAGGAGGAGGGTGTTTAGGGGAAGCTGTG
AAAAGAATGGAAGAAGATGAGAAATGGTTTGAGAATAGTTTTGTAAATTTAGGTGCTGGGATTGAATT
GGGGAAGAGGAAAGTGAAGGAGATGCTGATGGGTGAACAGGATAGAGGTGTGTTTGAGATTTGTGGA
ATTGGGGGTAGTGGCAAAACTACCTTGGCTAAAGAGATTTGTAAAGATGATCAAGTTAAAAGTAAGTAA
TTAATCCCAGAAAAGTTTCTCATGTAGAGTACTTTAGCTTTTTGCGGGGTTGATTTCTCGAAAGGTTACTC
AACTAATAGTTATTATCTATCACTTTTCGTTCTTGATTCTGATGCAAAGAAAGTGACTTTATTAGATATTAA
CTATTACTTGAGTGGACATCCGTGAAATCAACTCATGTTTTGCGTTGCTGATGATCGAGTTATTAGAAGT
TAATAATCACACAAAAGTTTCTCATTTCATTTTGTTTTTACTCTTCTGTTGCGGATGAAACAGTGGATAAA
AGTTGAAAACTTTATTGGCTATTATCTCTATTTTATCAGTTGAATTCTAATAGAGATCAATTTTGCATGTAA
AGGACTCATCAAAAGTTTTGATTGGAATACAGTTTAAATTGGTCTTAAAAACCTTTTCTTTAAATAATGGT
ATGGTGTCTAAAACCGCCATGTTGTTCCAGGTTATTTCAAGGACAAGATTTTCTTTTTCACTGTTTCTCAA
TCTCCAAACGTGGAGCAATTAAGGAAAATGATTTGGGAAAAGATATCAGGGTGCAATCTCCATGGTTAT
GGACACGGGGAGATGTTACCCCAGTGGAACCTACAGTACCAATGGAATACGAAATCTGCATCCCCAGTA
CTCTTGATTCTTGATGATGTGTGGTCTGCATCTGTGCTAGAGCCACTAATTTTCAAGATCCCCGGCTGCAA
GATCCTAGTTGTATCGCGTATCAAGTTTCCTCCATCAATCATTGACTGTATTTATGATTTAGAGTTGTTAA
AGGAAGATGAAGCTATGCCCTTATTTTGCCATTTTGCATTTGGACACAATTCCTTTCCGCGTGGTTTCAGC
CAAAAGCTTGTCAAAGAGGTATTAGTGTATGTTTTTGGCAGCATATTAGTTTTTCTTTGCAAAAATTTCTT
GTTGTACTTGAGTTAAGCTAACTAAAGCTTCTTGGACTTTTTAGATTGTGGATGAATGTGAAGGGCTTCC
TTTGGCTCTTAAGGTCATTGGATCTTCATTAAAGGGAAAACCTGAGATGTTCTGGATAAGTGCAAAAAAC
AGATTATCACGATGCCAACCTGTCTGCGAGTCTCATGAACTGCAGTTGCTTGAGCGAATGAAATTGAGT
ATTGACTGTTTGCCTGAGAAGGTGAGAGAGTGTTTCCTGGACTTGGGTGCTTTCCCAGAAGACAAAAGG
ATTCCTCTTGATGTTCTAATTAACATGTGGGTGGAGCTACATGATATTGATGAGGAGGAGGCTTTTCACA
TTCTTGTTGAACTTTCAGACAAAAATCTCCTAAATCTAGTCAAAGATGCACGGTAAGCGGTGGACTCAAA
TCTCCTCAAACAATCTTTTAATTTTTGTTAGAAGGTGATTGTTTATGGAAAAAATCAATATGTTGATAGTT
TTCAATGTGGTCTTCATGTGGCAGAGCTGGAGACATGTATACAAGTTACTATGAGATATCGGTGTTTCAG

CATGATGTATTACGAGACCTAGCAATTCATATGAGCAACCGTGATGATATAAATCAGAGAAAGCGATTG

GTTATGCCACGAAGAGACGTAAACTTTCCAAGAGAATGGGAAAGAAATGTGGACAAACCTTTCCATGCA

CGAGTTATCTCTGTGCATACAGGTATTTTAGATCCTTGGTTACTGCTGATTGCTTAATAATGAAAAGTTTA

TCATGTGATATAAACAGAAATATCTCTCTTTAATGCAGATGAAATGAGAGAAATGGACTGGTTCAGAAT

GGATTGCCCGAAAGCTGAAGTACTGATTCTCAATTTTGCCTCATCTGAGTACTTCTTGCCTCCTTTCCTGG

AGAATATGCCAAAGCTAAGGGCATTGATAATCATAAACTATAGTGCTGGCAATGCAGTTCTTCATAACAT

GTCTGTATTCAGTCATTTAACCAACTTGAGAAGCCTTTGGTTTGAGAAGATATCTGTCACTCACTTATCTG

ACTCCACAAATCCTCTCAATAACTTGCGGAAGATATCTCTAGTGCTTTGTGACATGAAAAACAGCTTAGA

TGAGTCAGATGTGGACCTCCCTGGTTTGTTCCCACAGCTTTCTGAGTTCACAATGGATCATTGCATCAACT

TCAATAAGCTGCCATCAAGTATTTGCCGGTTGCATAAGCTCAACAGCCTTAGTATCACTAATTGTGATAG

TCTTTATGAACTTCCATCTGATTTAGGTGAATTACAAACTCTACAAGTTATAAGGATATATGCCTGCCCAC

ATCTGAAAAGGCTTCCCCCGGGAATTGGTCATCTGGTAAAGCTGAAGTACCTTGACATTTCACAATGTGT

TGGTTTGAGATGTCTCCCTGAAGCAATTGGTTGCTGTAGAAACTTGGAGAAGATTGATATGAGGGAGTG

CCCTCAAATTGACAGTTTGCCAAGCGCTCTATCCTTTCTTGAATCATTACGTTGTGTTATTTGTGACGAAG

AAGTTTTTTGTCAATGGAAAGATGTTGAGAAGGCTGTACCAGGTCTCTGTGTACAGGTTGCCGAGGAGT

GCTATACTCTTGATTGGCTATCTCAGTAATCAAGTTTTCAATTCCCTTATCTTGGGTTTCTTTCGTGTAAAT

GTAATAAGGCTCAGTCAATTTTCCAGTTCTGTATTTTCCCCTTTGCATGGGGTTCATTGTATCTCTTGTAAA

TAATATATAGCAATATGTAAATATGTAGATGGACTACATCTCAATAGCTGATCTTTCCTTTTCCTTTAAAA

CCATAATGCTGGAAGCAAAATTACCACAGGTAATCCAAGACTAAGGCGAGTTATCTGCTATAACATGT

>PGSC0003DMG400008036 LRR-RLPs

ATGAATAGTACTATCAAATTTCTAATGTTTTTCTTCAGTGTCACAACAGCACAACCACTAAAGGGACAGA

ACCAATTGATTTTTGCAGATCAAAGGCTTGCTATAGTATACCCCATTATACAAAAATTCAAGAATATCATC

TCTGCTGATCCTCTAGGTATTACAAAAACTTGGGTTGGTCCAAACATATGTAATTATACTGGTTTTTATTG

TGAAAGTCCTCCAGACAACAATTCAGCAATTGCTCTTGCTTCTATTGATTTCAATGGCTTTCAACTTAGCG

CGTTGACACTTGATGGCTTCCTTGATCAGCTTCCTGATATAGCTATTTTCCATGCTAATTCAAACAATTTTG

GTGGGACTCTGTCTCCTAAAATCGCGAATCTTCCTTATCTCTATGAACTTGATATTAGTAACAACCAATTT

TTCGGTCCATTTCCTAGTCCAATTTTGGGCATGAATAGCCTAAATATATTGGATATAAGATTCAATTCTTT

CACTGGATCAGTCCCACCTCAATTGTTCACTAAAGATCAATTAGACGCGCTCTTCATCAACAACAACAATT

TCATGCAAATGCTTCCTGATAACATCGTGACCAGCCATGTCACATATCTTACTTTAGCTAACAACAGATTT

TTCGGTCCTATCCCACATAGTATTTCCAAAATCTTGTCGAGTTTGTCTGAGGTTTTGCTGCTTAACAACTT
GTTAAGTGGATGTCTTCCTTACGAGATAGGATTCTTAAACGAAGCAATAGTCTTCGATGCTGGCAATAAT
AGACTCACCGGTCCACTTCCGTTCTCATTAGGGTGTTTGGAAAATGTAGAAATGTTGAATTTTGCTAGAA
ATCAGATGTATGGGATAGTGCCTGATGTGATTTGTGCCCTGGGGAATTTGGCAAATTTATCGTTGTCCGA
TAACTATTTCACAGGATTTGGGCCAATTTGCTTGAGATTGATAGAAAATGGAGTGCTTGATTTGAGGAAT
AATTGTATTCCTGGTTTTCCATTTCAAAGATCAATAGCTGAATGTGTTGCATTTTTTGCTTACCCAAGGTA
CTGTCCTCATATGGCTACATATACTTATATTCCTTGTTGGCTTTCAAATTTCAAAACACCTACTCTGGATCT
CCCTGAATTGGCTCCTTAGTAAGGGTAACACGGTGTACAAAGCATTATGCGTTAGCATGGTTCGGGAAA
AGATCATTAGAAAATTCTGGTATTTGATCATAAACATACATGTCCCATTTTCGCGATCTTTTTTTGTTTTAA
TTAAATTAAGCTTGTAATTTCCATTGAACAGAGTGTGATATCTCATAATAACTTGCGATCTCTGTTTATGG
AGTATTGCTCAGTTGTAAATTTACGTTACTTTTATGTTATGG
>PGSC0003DMG400008185 LRR-RLPs
TATATTTGGCTTTTTAAAAATATATAAATAAATGTGTGTAAGCAGTTTTGGCTTGTCCACTTATGACCTAG
TGTTTTGTTCCAATAGCTCCAACTCTATCTGCAACAATGTTTGCAGGTTTTTTGTCCCAACCTGGAAGACC
TATACATCCATGGCCTTAAAAGCATAAGTGCTCTATGCTCTCACCAACTTCCAACTGACTACTTCACCAAA
CTTCAAACATTGTTTGTATCATTTTGTGGAAAATTGAGAAACTTGATGTCTCCATCAGTGGCCACAGGTCT
TTTGAATCTCCGAATGCTATGGATACAAGACTGTGAATCAATGGAAGAAGTGATCACAAAAGAGGAACA
AGAAGGAGCAGGAATCATGACCTTATTTCCACTCTTAGAACAGCTGCATCTTATTCGTCTGCCTAAGTTG
GGGCATTTCTTTCTGACAAACCATGCCCTTGAATTTCCATATCTAAGAAATGTGGAAATTGATGACTGCC
CTGAAATGAAGACGTTTGTTCAACAGGGAATATCTGTGAGTATACCAATTCTCAAATATGATGATGAGG
TGAAATTCGTAGATGATCTCAATAAATGTATACAGCAGGAGTTCAATAACAAGGTATGTCTTATTCCACT
AGCTATATAAGTAATTGCTACATGATATTCTAATCAGGCTCTACTCCTCTCTTTCCGTTTTAGATACTTATT
TAAGTGCATCAACTTTCTTTTACTTAATAATCTAGTCATCTCTCTCTTCTTTTGCATTCTCTGTTTCCTGCAG
GAACAAGAAGCTAGTGATGACTACGAAGCTAGTGATGACGACGAAGCTGAACCTAGTCATGCCAGAAG
TTAGGAAGCTGGTAAGAAAAACTCAATGAAATAATGCAAGTAAAACCTTGAACATCACCTGATACCTAT
GCTCGTGATGGAGGTAATTAACAAGGAGAGGCAGATCTACATTATTGTAACCTATCGGTTCATGTCAAC
CTACTAGCTATTGGCTATAGATCTAGTATATGTATTAGTAATATCTAAAAAATAGTTGAT
>PGSC0003DMG400008394 LRR-RLPs
CTTCCTTTGAGTACTCTACAAACAACTACCAAAAGAAGACTTACTTTGAGAAGACTTTGGTGCAGAGGAA

GAAGACTTTGTGAAGGAGCAAACACAACAATGGCCGATCCTGTAATTGGTGCTACTGTTCAAGTTTTGCT
TGAAAAGTTGATTTCTCTCACTATCGAGGAGGTCAACAGCTCAAGGGATTTCAACAAAGATCTCGAAAT
GTTGACACAAAATGTATCCTTAATCCAAGCTTTCATTCATGATGTTGAAACACCACAAGTTGAGAAACAA
CAGTCTATCGAACAATGGCTCAACAGGCTTGAGAGAGTTGCTGAAGATGCTGAAAATGTGTTTGATCGA
TTCAGATATGAATCTCTCAAAACAAAAGTGGTGAGGAGCCCATTGAAAAAGGTCAGTGGTTTCTTTTCTC
ATACTGCTTTTAAGAGAAAAATGTCTCAAAAAATCAACAACATCAATAAAGAGTTGACGGCTATCAATAA
GGTAGCCAAAGACCTCGGTCTACAATCACTCATGGTTCCTTTTCGGAAAATACTACCAATTCGAGAAACA
GATTCCTTAGTAGTTGCTTCTGATATTGTTGGTAGAGATTTGGATGTTGCTGAGATAAAGGAGAAGATTT
TGAACATGAGAGAGGAGGATATTGTTCTGTCCACCATTCCCATAGTAGGTATGGGAGGTTTAGGGAAAA
CAACTGTGGCTAAGAGGATTTACAATGATGAACACATCCAGCAAATCTTTGAAAAGAGAATTTGGTTGT
GTCTACCTGAAATGTCTGAAACGAAGTGCTTTCTTGAACAAATCCTCGAATCATTGATAGAGAGGAGAA
TTGAGGTTGAAAGGAGAGATATAATAGTCAAGAAGCTACAAGATGAATTGAGAGGAAAAAAATATTTG
CTAGTCCTGGATGATTTGTGGTGTGTTGACTCTACATCATGGCATGAGTTCGTTGACACCTTGAGAGGAA
TAAATACATCCAGAGGAAACTGCATTCTTGTGACTACTCGTATGAAGCGGGTGGCATCCACAGTAGCAA
CAGATCTTCATATCTTGGGGAAGTTAACAGAAGATCATTGTTGGTCTATTTTCAAACAAAAAGCATTTGT
TGATGGCAGGGTTCCAGAGGAATTAGCGAGCATGGGCAACAAGATTGTTAAAATGTGCCAAGGTCTAC
CGTTGGCTGCAAGTGCGTTGGGAGGGCTCTTACACAACAAAGAAAAACATGAATGGCAAGCAATTCTTG
ATGGCAGCCTCCTTGTTGCAGGTGAAGATGATAATGGAGAAAATAGCATAAAGAAAATCCTAAAACTCA
GCTATGATTATCTACCATCTCCACATCTGAAAAAATGCTTTGCTTACTTTGCAATGTTTCCAAAAGATTAT
ATGTTTGAAAAGGACCAACTAATCCAACTCTGGATGGCAGAAGGGTTTCTTCGTCCAAGTCAAGAGATC
CCTGTGATGGAAGACGTTGGGCACAGGTTTTTTCAAATCTTGTTGCAGAATTCCTTGCTACAAGATGTTG
TGTTAGATGAACACAACACTATAACACACTATAAGATGCACGATCTTGTGCATGATTTGGCTGGAGATAT
CTTAAAATCTAGACTATTTGATCCGAAGGGAGACAATGGAGAAAAACTTTCTCAAGTTCGATACTTTGGA
TGTGAGTCACCAACGGATCAAATAGATAAGATATATGAGCCAGAACGTTTGTGCACACTGTTTTGGAGA
AGCAATTATACGTCTAAAGATATGCTGTTGAACTTTAAGTTCTTGAGAGTTTTAGATTTGTCCAGTTCAGG
AATCAAGGAGTTGTCAGCCAAAATGGGGAAGCTGATATACTTGAGATATCTTGATCTCTCGAACACTGA
GATCACAGCCTTGCCCAACTCCATTTGCGAGCTCTATAATTTGCAAACATTTAGAGTCATCAACTGCTTTT
CACTCCAGAAACTTCCATATGAGATGAGAAATATGATAAGTTTGAGACACATATATTACACTTCTGTTGA
CGAAACAAGTGGGCATTGGGGAGGATGGTGTCTTCACAATGAACATTTTCAGATTCCACTTAATATGGG

GCAATTGACTAGTCTTCAAACCCTCAAGTTTTTCAAGGTAGGTTTAGAGAAAGGTCGTCAAATAGAAGA
ATTAGGTCATTTGAAAAACCTAAGAGGTGAATTGACAATCAATGGTCTCCAATTGGTCTGTGATAAAGA
AGAGGCTCGAACAGCATATTTACACGATAAACCAAATATCTGCAAGCTGGCATATTTATGGTCCCATGAT
GAATCAGAAGGCTGTGAGATCAATGATGAGCATGTGTTGGATGGTCTTCAACCGCATCCTAACTTGAAA
ACCTTAGCAGTAGTGGACTATTTAGGGACTAAATTTCCTTCATGGTTCAGTGAAGAGTCTCTACCAAATT
TGGTCAAGTTGAAATTAAGTGGTAGCAAAAGGTGCAAAGAAATTCCATCCCTTGGCCAACTGAAATTCC
TTCGGCATCTTGAGCTGATAGGATTCCATGAGTTGGAATGCATTGGACCTGCTTTAGATGGTGTTGAGAT
TAGAAATATTGGATCAAACAGCATTATCCAAGTGTTCCCATCATTGAAAGAACTAGTATTGGAGGATATG
CGTAGCCTTATTGAGTGGAAGGGAGATGAAGTTGGAGTAAGAATGTCTCCCAGGCTTGAGAAGTTGCG
GATTACAGACTGTCCATTGTTAAAAAGTATTCCGAATCAATTTGAAATCCTCCGTCAATTAGAAATTAGA
GGAGTTGACAGTGAAATGCCATTGTTGAACTTGTGCAGCAACTTGACATCTCTCGTAAAGCTTAGTGTCT
ATGATATGAAAGAGCTCACTTGTCTTCCAGATGAGATACTACGTAACAACGTTTCTCTTCAACAGATAAC
GATTTTCAACTGCGGAGAGTTTCGTGAATTGCCACAAAGCTTGTACAATCTCCATTCTCTTAAGAGATTA
GACATTTACAACTGCACCAATTTCAGTTCTCTTCCTGTTCCCAATGGAGACAACTATTTGACTTCCCTCGA
ATTCTTTTTCTTATACAATTGTAATGGATTGATCAGTTTACCAATTGGAATGCTAGATCAATGCCGGTCTC
TAGAGTTTTTGTGTGTCAGCTGCTGTAACAACTTGGTTTCATTCCCTTTACATGTGTGGGAAATGCCTTCA
TTTTCATTTTTGGATATAACAGAATGTCCCAAATTGATTAGTGTACCCAAAGTGGGCCTTCACCATCTCAC
CGGGTTATTGAGATTGGGAATTGGTCCTTTCTCAGAGATGGTGGATTTTGATGCATTCCAATTGATTTTT
AATGGCATTCAGCAGCTGTTGTCCCTTCGTGATCTGGAGGTGTACGGACGTGGGCACTGGGATTCTCTG
CCCTATCAGATTATGCAACTATCTGACCTAAGAGAGATCACAATAGCTGATTTTGGAATTGAGGCTCTTC
CTCCTAGACTTGACAACCTTACTTCTCTTGAAAGTTTGACTCTAGTGAGGTGCAAACAGCTACAACATCTG
AACTTCTCAGATGCCATGCCCAAATTACGGCTTCTGTGTATAAGTGATTGTCCATTGTTAGAAGCTCTGTC
GGATGGGCTCGGCAACCTTGTTTCTTTGGAAGAATTATATTTACATGACTGCGAAAAACTAGAGCATCTA
CCGTCCCGAGATGCCATGCGACGCCTCACTAAATTATGGAACATGGGAATTAAAGGCTGCCCAAAGTTA
GAAGAAAGTTTCACCAACTACTCCCAGTGGTCCAAAATTTCCCATATTTCAAATATTGAATTAGGTGGGC
GGAGAAGGACAACCGTAAGTCTCGGTTTCTCTTTCACTTACTGAGTCGCTCGCATTTTTAATTAGAATATT
ATTTTCCACGAGTTCCAAAAAAGAAGTTTGGTAAGTAATGTAATAATTTTTCTTGAAATTATTTTGACATA
AAAAAAAAAAGTTAGATTAAATCAATTGCATCTGTTGCTATGCTGGATTCTGCTTGTCCACTTGAACTGA
ATTGCTGTTATTCTAAAAATTATGTTTCTTTTCCTTATAGGTAGCTGGTTCAAAACGTCTCTGTGGTAAATC

AAGCCAAATCGTATGAGCAGCAGGCAATTACGTTAGGTAAATTTTCATTTATAATTTATAGGCACTTTGT
AATCTACTTGTTTCTATCTTTCTTTTTGTTGGAAACCAATTTTCGGAGGTCTATGCTGATGAATACAACATT
ACCTTCCTTAAAAAAAAGTTTAATCATGAAAATTTATGATTTCTGCTCATAGGTTACTAGGTACAAGGAC
ATAGTAGAACGTCGTTGTGGTATATCTAGTCAAAATCCATGGGCAGAATCATGTTAGGTATTTTTATTTA
GTTGTTAGAGGAATGCTATAACAAGTTTGTAAAGTTTCTTTTTTTTACTTTCCTTTTCTCTCATGAAATAAT
TTTAATGTTTAACGGGACTTGTCGTGTTTCACAGAGGAACCACCACTGTTATTTTGTATGAAGAACAATCT
TCAACTGTAGACTCATTATGGGACACTTTCATGAGTATTTTCGGGGAACTACAGTCTACATATGAAACAT
CAAAAACTCAAAAGCGTATAGGGTGCTCAATGAGAAGCTACACGGGGTCTATTCTCCATGTAAGTATTG
ATAACTCTCAAGGAACAATACCAAGTCTCATATATGGTAGAAATCTATATAAGTTTCTTAATAGACTCATA
AAAGAATAATCTTTATGTCTTCTGCAGTTCTGGTTTTATTTTCATGCTTAAGACAGTACAACTTTTATGAAT
ATATGAAACACTTCTCGTTACTTTCACCTCACAGGACAATTTCTCCGTTCATTAAAACTTCCGCAAATGAA
GGACTTCAACAAAGATAGTTGTCATGAATATTTTTGCAAAGGGTGACCCATTGGCTCTTAAAATGTCCTT
AGCAGTATATGTTCATCTTAAACAGGTTTTCCTCTGTCCTTTTTTAGTGGTTACTTGTAATGATCCGCTTCT
CCATTCTCCAAGGAGGAAACGTGTGCCAAAACTAACTTTTTACCATTTCCCGTGTTTCAGATTCATGTTTG
AAACTCCAGCATGGACCTTCGAAATGCTACTTGTGTTAGAGATGTGTTTGAGTGTCATGATTGGCAAGG
GAAATTGCATGTGGCGGAAATTGGAGTGGAAATACTATTTACTTGTATGACTAAGATTTGACAGCAGAG
GCGCCTTTCTTTCACAGCATACGTGCCTTTTGGTACAAATAGAGTTCATTTTTCCAACAAACATTTTGAAC
TTTTATACTTAGCTGCTGCTTCATTGCCTAGTTGTTAGCTTCTGTAATGTACATCTGCTTTTCCTCTTGAAA
TTTCTCTTGGCTTTTGTTCGCAAGTATAAACAAAAAAGACAGGGCCTACCATTTGCATTGTTTAAATCCAA
ACAATATGTATTCAAAAGTCACAAGCTACGTGTGTTATTCCAATTTCGTACAGGACTGCATTTTTGTGTCT
TTGTATAACATATCGCATTGCTCATTATTCTTTCAGATAGACCAGATTGCAC
>PGSC0003DMG400008450 LRR-RLPs
ATGCATGACTTTTGTTATATAAAATCTAGAAAGGAAAAGGTGTTTGACTTCATAGGAGGTTCAAACGCTC
CGTCTTCTTCTTCTGGTGTGATGGCACGAGGAATTACCATTCGTTATGTTAAACACCTCTTCTCTCTGAAG
GTGTATGATGGGATTCATGATTGTCTTTCCTACAACAGTCACCTAAAACACTTGAGGCTTCTTAAAAGTTT
GGATTTGAAGGGCCTAACATTGACAGATTCTTTGCTGGATGAAATTGGTATGCTCGTTCATTTGAAGTAC
TTAATCATTCAGACGAAGGCAAAAGCTCTCCCTCCGTTATTTTCAAACCTCTGCAATCTAGAAACTCTATC
GGTGTATAACATTGAGAGACCATGCATGATACTATCACCTTGTTTTTGGAGTCTTGCAAAGCTACGAGAT
GTGCGGATGATGGAATGTGCTCTCTTTGAACCAACTGTGTTAGATGAGGACTCAAGGTTAGAAAATTTG

AGAACATTACAGCATCTCTCCCTTCCCGGTTCAGAAGACATGGAGGATATTTTCAAAAGGTTTCCTAATC
TTCAAATCCTTAGTGTCTCTATCATAGCAGAGAAGATTTGTTTTCCAAGATTGGATGTCCTTAACGAACTT
GAACAACTCAATTTATCTGCTTCTTGGGATTCATTCCATGAATATACACACGACTTCCCTTTGAGCTTGAA
GAAAATGAGGTTGCAAGGGCTCGCTCTGACATCCGATTCATTGTCAAGAATTGCTAGACTACCCAACCTT
CAAAAACTAAGTCTAAAAAACGCAATCATCGATGGGGGGCCAGGAGAATGGAACATGGAAGATGTCAC
CTTCAAGAATCTCAAATATCTAAAATTGGATAGTGTGGAATTTTCTGAATGGCAGGTTGATGCAGAGAA
ATCATTTCCCGTGCTCGAGATGTTAGATATAATTACATGTGAGAAGCTTATGGACATCCCAGATAGTTGT
GGAGATATTGCGTCATTAGAGCTTATCAAAGTATGGTTTAGCCCTCAGCTTAAAGAGTCAATTCTTCAAT
ATTAA
>PGSC0003DMG400008492 LRR-RLPs
CTTTTTCTTGTTACTTGCTTCATAAAATATAATGAAGCCCTCCTCCTCCTCTATACTTTCCATTCTTTCTTTTT
CTCTCTTGTTTCTTTTTCAACTCTCTTCATCTGAAAGATGCCATCCAAATGACAAAAAAGCCCTCCTAGAAT
TCAAAAAAGGTCTAGGCAATCCTGAAGACTTGGGTAATTGGGATCCGAAAACTGATTGTTGTACTGATT
GGTACGGACCTACTATCAGATGCGATGAAAAAACCAATCGTATTAACGTCATCGACTTCACCAAAATGG
ATATGGCCGGATACCTCTCCCCCGCCATAGGAGACCTTACATACCTTACAACATTCAGTATCCACAATGT
ACGTAATCTCACCGGTCCAATTCCATCAACAATTGTTAAACTGACTAATCTCGAATTCTTCAGAATTAGTC
AAACAAACATTTCAGGACCTGTCCCTGAAGTTCTTAGTAAGTTGAAAAGCTTAACGTACATTAACTTGTC
CTATAATAAGCTTGTTGGAACTATTCCACCTTCGCTATCTCAACTTCCACACTTGGAGTTTTTAGGATTGG
ATAGGAACAAACTTAGTGGACCAATCCCGGAGTCTTTTAGAAAGTTAGCACCAAATCTAACGTATCTTTA
CCTTGGACATAACCAACTTACAGGGATTGTACCAACTTCATTTGCTGGCTGGAGTTTTGCGACAATTGAC
TTGTCAAGGAACATGCTTGAAGGGGATATTTCATTTTTGTTTGGTAAAGATAAGACTACATTTGAAATGC
TTTTGGATAGGAATAAGTTTGAATTCGACTTCTCAAAGTTGACGTTTGGGAAGAGATTATGGAGGTTGG
ATTTAAATCATAACAAGATTTATGGGAGTATTCCAAAAATAATAGCCAAGAATCCGTGGCAAATCTTGAA
TGTGAGTTATAATAGACTTTGTGGCGAGATTCCAAAGGGTGAGTATATGAAGAAATTTGAGATATATGA
GTATCTTCACAACAAATGCTTGTGTGGTGCTCCATTGCCGCCCTGTAAATAATCATCACTAGTATCCTGTC
TATGTATTTCAACTTGTTCTCTTTGAATGTTGATGAATAATTAAGCGACGAAACTCAAGGCTTAAAAATGT
ACTTTAATTAATTTATGAATGAATGTTATCCTTTTTTAGTAACTTCTCGATGGATGATTATA
>PGSC0003DMG400008596 LRR-RLPs
CTTTATTTAGGTAAAGAAGACTTTGGTACAGAGGAAGAACATTTTGTGAAGGAGCAAACACAATAATGG

CCGATCCTGTAATTGGTGCTACTGCTCAAGTTTTGCTTGACAAGTTGCTTTCTCCCACTATTGAGGAGGTC
AAAAGTTTAAGGAACTGCAAGAAAAATCTCGAAATGCTGACAAAAAATGTATCCTTGATCCAAGCTTTCA
TTCATGATGCTGAAAGACGACAAGTCGAAGATCAGGCTGTGGAAAAATGGCTCAAGATGCTTGAGAGA
GTTGCTGAAAATGCTGATAATGTGTTTGACGAATTCAGGTATGAATCTCTCAAAAGACAAGTGAAGATT
CGAAACAATCCAATGAAAAAGGTCAGTGATTTCTTTTCTCATACAGCTTTTAAGAGCAAAATGTCTCAAA
AAATCAACAACATCAATGAAGAGTTGACGGCTATCAATAAGTTAGCCAAAGACCTTGGTCTCCAATCATT
GATGGTTCCTCCTCGGCAAATACTACTAATTCGAGAAACAGATTCTGTAGTGGTTGCTTTGGATGTTGTT
GGTAGAGACAAAGATGTTGCTGAAATAAAGGAGAAGATATTGACCATGAGAGAGGATGTTGTTCTGTC
AACCATTCCAATAGTGGGTATGGGAGGTTTAGGGAAAACAACTGTCGCAAAGAGAATTTACAATGATGA
ACACATCAAGCAAACCTTTGAAAAGAGAGTTTGGCTGTGTCTACCTGAAATGTCGGAGACTAAGAGCTT
TCTTGAACTGATCCTCGAATCATTGACAGAGAGGAAAGTTAAGGTCCAGAGCAGAGATATAATTGTCAT
GAAGCTCCGAGATGAACTGGCAGGAAGGAAGTATTTGCTTGTCCTGGATGATTTCTGGCGTGTTGACTC
TACATTGTGGAATGAGTTTGTGGATACCTTGAGAGGAATAAATACTTCCCGGGGAAACTTCATTCTCGTG
ACTACTCGTATGGAACAGGTGGCGTCCACAGTAGCAGCAGTAGGTCCTCATAAGTTGGAAAAACTAGCA
GAAGATCATTGTCGGTCCATTTTCAAACAAAGAGCGTTTGTTGATGGGGAAATTCCAGAGGAATTAGCA
AGCATGGGCAACAGGATTGTTAAAATGTGTCAAGGTCTACCGTTGGCTGCAAGTGTATTGGGAGGCCTC
TTACGCAGTAAGGAAAAACATGAATGGCAGGCAATTCTTGATGGAAACCCCCTTGTTGCAGGTGAAGAT
GGTAATGGAGAAAATAGCATAAAGAAAATCCTAAAACTCAGCTATGATTATCTACCATATCCACATCTGA
AAAAATGCTTTGCTTACTTTGCAATGTTTCCAAAAGACCTTGAGTTTGAAAAGGACCAACTGATCCAACT
CTGGATGGCAGAAGGCTTTCTTCATCCATGTCAAGAGACCACCGTGATGGAGGACATCGGGAACAAGTT
CTTTCAACTCTTGTTGCGGAATTCCTTGCTGCAAGATGTTAAGCTAGATGAGCATAACAATATAACACAC
TGTAAGATGCACGATCTTGTGCATGATTTGGCTGGAGATATCTTAAAATCTAAACTAATTGATCCAAAGG
GCGATGGTGGAGAAAATCTTTCTCATGCACGATACTTTGGATGGGACTCACCAAGTGATCAAATAGATA
AGATAAATGAGCCAGGACGTTTGTACACACTGTTCTGGAGAAGCAATCATATATCGGAAGATATGCTGT
TGAGCTTTAAGTTCTTGAGAGTTTTAAATTTGTCCAGTTCAGGAATCAAGGAGCTGCCAGCCAAAATCGG
CAAGCTAATATACTTGAGATATCTTGATCTCTCCAACACTAAGATCACAGCCTTGCCCAACTCCATTTGCA
AGCTCTACAATTTGCAAACATTTAGAGTCAATGACTGCTATTCACTCAAGGAACTTCCATATGAGATGGG
AAATATGATAAGTTTGAGACACATTTACTGCTATTCTCGATCTCAGTTGCCACTTAACATGGGACAATTG
ACTTGTCTTCAGACCCTGCAGTATTTCAATGTGGGGTTAGAGAAAGGGCGTCGAATAGGAGAATTAGGT

TGTTTGAAGAACCTTAGAGGTGAATTGACGATCAATGAGCTGGAATTGGTCTGTAGTATTGAAGAAGCT
CGAACAGCATATTTACAAGAGAAATCGATTATCTACAAGCTGGCATATGTGTGGTTCCACGATGAACCA
GAAGGCTGTGAAACCATTGTTGAGCATGTGTTGGATGGTCTTCAACCGCATACTAACTTGAAAACCTTAG
AAGTCAAGAACTATTTAGGTACTAGATTTCCTTCATGGTTCAGAGAAGAATCTCTACCAAATTTGGTCAA
GTTGAAATTAAGTGGTTGCAAAAGATGCAAAGAAATTCCATGGCTTGGCCAACTGAAATTGCTTCGGCA
TCTTGAGCTATTAGGGTTACATAAGGTTGAATGCATCGGAACTACTTTTTATGGTATTGATGGTAACAAC
ATTGGATCAAGCAGCAATGTCCAAGTGTTCCCATCATTGACTGAACTAGTATTGAAGGATATGTCTAGCC
TTATTGAGTGGAAGGGAGATGAAATTGGAGTAAGAATGTTTCCTCTGCTTGAGAAGTTGAGGATTAGTA
GCTGTCCATTGTTAAAAATTACTCCAAGTCAATTTGAAATCCTCCGTGAATTAAGAATTGAAAGAGTTGA
CAGTGAAATGCCATTGTTGAACTTGTGCAGTAAATTGACATCTCTCGTAAAGCTTAGTGTTTATGATGTG
AAAGAGCTCACTTGTTTTCCAGATGAGATGCTACGCAACAAGGTTTCTCTTCAACACCTATTGGTCTCAG
ACTGTGGAGAGTTTCGTGAATTGCCACAAAGCTTATACAGTCTCCGTTCTCTTAAGAGCTTAAAGATTTA
CCGCTGCACCAATTTCAGTTCCTTTCCTGTTCCCAGTGGAGAGAATTATTTGACTTCCCTCCAAAGTTTTCA
GTTATGGAACTGTGATGGATTGACCAGTTTACCAAATGGGATGCTAGAGCATTGTCGGTCTCTGGAATC
TTTGATGGTCTGCTACTGTAACAACTTAGTTTCCTTCCCTATACGTGTGGGGGAAATGCCTTTACTTTTTG
ATGTGTATCTATCAGAATGTCCCAAATTGATTAGTGTACCCGCAGGGGACCTTTACCGTCTCACTGGGTT
ACGGCATTTGGAAATTGGTCCTTTCTCAGAGATGGTGGATTTTGAGGCATTCCAATTGATTTTCAATGGC
ATTCAGCAGCTGTTGTCCCTTCGTTTTTTGTGTGTGGTAGGACATTTGCACTGGGATTCTCTGCCCTATCA
GCTTATGCAACTCTCTGACCTAACAGAGATCCAAATATATGGATTCGGAATCGAGGCTCTTCCTCATAAA
TTTGACAACCTTGCTTCTCTTGAAAGATTAACGTTAGTGAGGTGCAAACGGCTACAACATCTGGACTTCT
CAATTGCCATGCTCAAATTACGGCATCTCCAGATACAGGATTGTCCATTGTTAGAAACTCTGTTGTATGG
GCTTGGCAACCTTGTTTCTTTGCAGGAGTTAGTTTTACAGAAATGCGAGAAACTAGAGCATCTGCCATCC
GGAGATGCCATGCAACGCCTCACCAAATTACGGTACCTGAAAATTGAAGGCTGCCCAAAGTTAGAAGAA
AGTTGCACCAATCGGATTGGCTCAAACTCCCAGTGGTCCAACATTTCCCATATTCCAAAAATTAAAGTAG
GTGAAAGCATAATTCAGGACTTAAGTAAGTCTCTGTTCCTCTTTCAGTTTCTGAGTCGATTTTTTTGTTCTT
TCTTTTCCACGAGTTACTCTCCTTCTTTTTAACAAACTTTTCATTCCCCAAACTAATACATGTTATGGTACTG
AAGAATTGCAGATTTTTTTATCACTCACTTGAAACTAACTGTTCTTCTTCTTCTTCTTATCGTCATCAAAAG
AGGAATATATACATTCCCGTCCTTTAATAATGTAATATGTTCCTTCTCATAGGTAACTAGATCAAATGTCA
TGCTGGAACGTTGCTAGCTGTGGTATATCAAGTCAAAATTTATGGTTGGTAATCAGGCTGGCTAAAATTT

TCATTTAGACTTGGTAGAGGAATGCTATAACAAATTTGTCGCTTCTTTTCACTGGTCATTATTGTACTGTT

TAACTAGGTTGGCTGTGTTTTGTATGCATGCAATGTAAGCTTGGTTGCGTTTCTAGCACCATGAGGGACG

TTCATCCATTTGGTTTAAGGATTCTCTACTCTCATTGGACCAAACATCTCCATGTAAGCTTCTTCCCAGTTA

TTAATCTCCTAGATCCCATAGTTTCTTTTGTTAATCGTGCAGCAAAAGAGTATCTTTTAATCCTCAAATATT

ATAATGCTCTTCGATATTGGGTATAGTGTGTTAGCATGTTGGACAGATACTAATAGTTGTACATATCTAT

GTAATAGCTCTAAATATTTGTGTAATTACTGTGTCAATTGCTTTCCACATGTAGGAGATATCAGGGATAA

ATGTAGCATATTTTTATAGATATTACAGATATTTAGGGGTGTAAGTGGCAACTTCTAATTTAATGGAAGA

GCTCTTAATTTGAGAGCATTCATTCAGCAAATTCGACTCTGGATAAAGACAATTTCTCTTTCTCTTGAGGA

AAATGAAGTATTTGATTGACAAAACATTGGCTTATCATGGTTGACACAATCAGTTTCTTTTGCATAAGAG

AAGGTGAAACATAGAGGGGGGAAAACGACCTTCTGCAAGACAATTTTATTATCGACTATCTGGTGAAAT

AGTATAGGTAACAAAGCCATTTCATCATAAGCCTTAGATGCAAGACAGATTTCATTTTGGTAAAAAATGT

GACTTCAGTTGATAGTAGAGGCTTTTCCAAATTTTGTTGACGGTCTTCACTCTTCAATAGTATTTTCTTTAA

TTTTCATGGGACTTATCGGAACATTGTTTACATTGGTGCCTTTGGGGACGGTGATTGCACAAGCTGATTA

GGTCTTGTTTTCTCCTAAAATTTCTCTTTTTAGTGGTTTTTTTTTTTTTTTTGGTTGAAAACAGAGTAATATA

TATGTTATGTAATATGCATCGTTACATTGTTACCAGCAAGTGGCAAAACAGGACACCTACTATTGGTGTT

CTGAGGAAGGGGCATACTATTACTAGTACTATTACATGAGTTAATTACAGAGGGCATACTATTACTAGTA

CTATTACATGAGTTAATTACAGAGGGCATACTATTACTAGTACTATTACATGAGTTAATTACAGAGGGCA

TACTATTACTAGTACTATTACATGAGTTAATTACAGAGGGCATACTATTACTAGTACTATTACATGAGTTA

ATTACAGAGGGCATACTATTACTAGTACTATTACATGAGTTAATTACAGAGGGCATACTATTACTAGTAC

TATTACATGAGTTAATTACAGAGGGCATACTATTACTAGTACTATTACATGAGTTAATTACAGAGGGCAT

ACTATTACTAGTACTATTACATGAGTTAATTACAGAGGGCATACTATTACTAGTACTATTACATGAGTTAA

TTACAGAGGGCATACTATTACTAGTACTATTACATGAGTTAATTACAGAGGGCATACTATTACTAGTACT

ATTACATGAGTTAATTACAGAGGGCATACTATTACTAGTACTATTACATGAGTTAATTACAGAGGGCATA

CTATTACTAGTACTATTACATGAGTTAATTACAGAGGGCATACTATTACTAGTACTATTACATGAGTTAAT

TACAGAGGGCATACTATTACTAGTACTATTACATGAGTTGCTTGCAAAAGATATCTTAGATAGTCTAACA

AATGGAGTTCCTTCTTTGTCTGCTTCTAGTTTGCTGGATACAAACACCGGAGGAACTTCCATGCACAAAA

AAGAGTTGATTTGATCCATTTTAGCGCCTTCCTTTGCTAGAGCGTCTTTTTAGTGGTTACTTGTAATGACC

CGCGTCTCCATTTCCCAAGAAAGAACTGTGTGCCAAAACTGACTTTTTTCCCCTTGTGTTTCAGATGGTTG

AACAACGGCATGGAAAAAAGACTATGAAGTTTCATAGTCATGATTGTCAAGGAAAATTGCATTTCGTAA

ATTCATTTGCATTTGGCTGGAACTGGAATTGAGATACTATTCTGTATGTGGAAAATTCTGGACTAAGATC
TGTCAGCTGAGGACTTTCGTGCATTGTGTACATGCTACACTTGCCTTTTCTTGTATATAGAGATTTTGTCT
CCAGCAAACAGTTTTTTAATTATTTTTTTAACGTTGTGTGGGGTTAGGTTTGACCCCTACCACGTCTGTAA
CATCCAAAAAGTTGCAAAAGCAGAAGGGGACATGGACCTAACTTCCAGCAAACAGTTTATATTCGCTTA
ACTGTTGTATCATTGTCTAGTTGATAGCTTCTTTAATGTATCTCTGTTTTCCTTTCGAAATTTATCTTGGCTT
ATGTTAGTGCATACTATGATGGATTAAATATAAAACATGTGTTCCAAATCAACATTTCC
>PGSC0003DMG400008925 LRR-RLPs
ATGGATCCTAATGAGGTAATGAGAAGGTTACCTAATCTTCGGAAATTGAGATGCATATCAGTTGACTCG
GAGGACTGCTGTCATTTTCCATCACTGGAGTGTGTGAGTCGACTTGAATCACTCAAGATATTACACTATG
GTCAGTCATTTTCTTCTTGTAAGTTCAACTTCCCCTCAAATCTCAAAAGACTGACATTGCCAAAGTTTCAG
CTACCATGGACTGAAATTTCAAGAATTGGTAGCTTACCACACCTTGAGGTTCTCAAACTACTTGCCCGAG
CCTTTGAGGGTGTGCAATGGGACATGAAAGACGGAGAGTTCCTGAAACTAAGATTCTTGAAGTTAGACT
CTTTGAACATTGAACAATGGAACATCTCATCTGATTGCTTACCTTCCCTTGAGCATCTTGTTCTGCAAAGT
TGTAAGAAACTTAAGGAGTTCCCTTCATGCTTAGGCGATGTTTCAACGCTACACAGTGTTGAGCTACACT
GGTGCAGCCGTTCTTCTGTGAACTCGATTATCTCAATTCAAGAAGATGAGGGGTTCAAGGTCCATGTATA
CCCTCGAGAATTCGATTCCTGA
>PGSC0003DMG400009029 LRR-RLPs
GGTTATCCCTCGAGTAACAAGACAGTCGAATATAAAAACACTGTTCCCACAAAACACACACACATTGTTT
ACTTCACTTTTTTCAGTTGAGATTTTCTTCACTTGCACTCTCTCCGGCGGCGCGTCGAACTCACACATTCTC
CCTTGTTGTTTTCCGGTGAGTTACAACAGACACTCACTCCAACGATAATCAGAAAAATCCCAAAAAGGTT
TTTTCTTTTCTCTTGATTTCTAGGGTTTTTGCGCTTATGCTCTGTACGAGTCTACTTTTTTGGTCATGTGATT
ACACAGATCTGTGCTGGATTTTAGTAATTTTTCTTGTTTAATGTGCTTTCGTTTGTGGTTTTAGGTAGTTTT
TTTTTATGTTTATTATTTTGGGGTTTGGTAGAGTAGGTTGCAATTACTGGGAGGAGGACTACAAACTTCA
CTGTTAGGTATAAAGTTTTTATTTTTCTCGTGAGGTTTGTTTGTTTGTAGGGGTAAAAGAGGGATTTTGTT
TTGTATTACAAGTTTCTGATGTTTTAGCGGCTCTGTAAATTGTATATATGCTGCTCTGCGCTGCTCATATT
ATTCTTCGGTAATGTATTGTGGGGTATTTTTTTTGTATCCAAGAAAGTTTTTATGTGTTATAGTGTGGTGA
AATGGAATACATCTGTTGAGTATACCATTTTTGGTTTTGAGTGTTTTTTACATTGTTGGATATGAATGTGG
TGCATTGCAAGGTGGTTTATAAAAGAATTAGAAAATTCAATCTCGTTTTCGATGATGGATGATTGTAGAA
ACTTTGCCATTCTTTGGACACCTGCTTGGTGTTTTGTGGTGAAACTATTGAGTTGTCAAACAATTATATCT

GATTTCTATATGAGGAAGAATTTGAGCTCTTCAAATTTGGTTTCGATATATGTTAGTTTTTCTTACGTGGA
GTAGGTGTGCACGAACTTGTTCTTGCCTGGTACTTTGTTTTTGGTTGTTATGTTCTTAACTGCCAGAGATG
AGTCTTGTAGTGGTTATATTTCTTGAAAGATAATCACTTTTATAGAAACTGAGACATGATTCACTATATTC
CTTTGATTCTAGGTTTTCTTCCAATGGATGCCACAACAGCTAACTCTCAGCGCAGACCATTTTCAATTAAA
CTGTGGCCTCCAAGTGAGAACACAAGAAAAATGCTGGTGGAAAGAATGACGAATAATCTTTCTAGTCCA
ACCATTTTCACCCGCAAGTACCGCAGTCTATCCAAGGAAGAAGCTGCCAAAAATGCTGAAGAAATTGAA
GATGCAGCTTTTACCATTGCTAATCAACATTATGAGAAGGAGCCTGATGGTGATGGAAGTTCTGCTGTG
CAACTTTATGCCAGGGAATGCAGCAAGCTTATCCTGGAAATTCTAAAGAAGATACCCAAATCAGAGGAC
AAGGAAATTTCAATTTCTGAAGTGGTTCCTACTGTTCAGGAGACCTTTTTTGATATCTCAAAAGGTAAAA
GGGCTTTTATCGAAGCGGAAGAGGCTCAAGAACTTTTAAAGCCATTAAAAGAGCCTGGAAATTCTTACA
GCAAAATTTGTTTCAGCAATAGAAGCTTCGGTATAGATGCAGCACGTATTGCAGGCCCTATCTTGGCAGC
CTTGAAGGATCAATTGAAGGAAGTTGACTTGTCAGATTTTGTTGCCGGAAGAAATGAGGCAGAAGCACT
TGATGTCATGAATATATTCTCAGAAGCTCTGGAAGGTTCTAACTTGAAGTTTCTGAATCTCTCTGATAATG
CTCTGGGTGAGAAGGGAGTTAGGGCATTTGGAAAGCTCTTTCAGTCTCAAACCAACTTGGAAGAACTAT
TTTTGATGAATGATGGGATTTCACAGGAAGCTGCAAATGCTGTTAGCGAGCTAGTTCCTTCCACCGAGA
AGCTTAAGGTTCTTCATTTTCATAATAATATGACAGGTGATGAAGGGGCTGTTGCGATTGCGGAAATTGT
GAAGCGTTCTCCTTTATTGGAAGATTTCAGGTGCTCTTCTACCAGGGTAGGCTCTGAAGGAGGGAGTGT
CTTGTGTGAAGCACTTGGGATGTGCTCTCATTTGAAGAAGCTTGATTTGCGAGACAATATGTTTGGTCCA
GAAGTTGGTCTTGTGTTGTGTAAGGCACTCAGCAAACATGAAAATCTTACGGAAATTTACCTGAGCTACC
TTAATTTAGAGGATGAAGGAGCAATTGCAATAGCTAACGCTCTTAAAGATTCAGCCCCTTCACTTGCTGT
CTTGGAGATGGCAGGTAATGATATAACTGCTGAAGCTGCTTCAGCAATAGCTTCTTGTATAGCTGCAAA
GCAGCTTCTTGCCAAGTTAAGCTTGGGTGAGAATGAACTCAAGGATGAAGGTGCAATTCAGATTGCCAA
GGCACTGGAAGGACACAGCCATCTGATTGAAGTTGATATGAGCAGCAATGCACTAAGGAGGGCTGGGG
CTAGGGTGCTGGCTCAAACTGTGCTGCACAAAGATGAGTTTAAATTACTAAATGTTAATGGGAATTTCAT
CTCAGAAGAAGGCGTTGACGAGTTGAAAGAGATCTTCAAGAAATCTCCTGAAATGCTTGCATCCCTGGA
AGATAATGACCCGGAAGGAGAAGACGAGGATGATGAGGAGGATGAGGAGAAAGAATCTGGAGATGA
GGGCAAAGACGTTGAGGATGAACTAGAATCAAAACTCAAAAACCTGGACGTCAAGCAAGAGGCAAGTC
TTGACACACCAGATAGCAATTAGAATCCAGTACTTGACGACTTCTGCATGTTGCAGTTTTAGTTTTACCAT
TTTCTTTATGCTAAGCTGGCCATATTATGTTCCTCCAGCAAGTGCTCTAACATATAGTTCCATCAGTAATTT

AAATTTGGTGGAATTGAACTGTTGCCTTAATTGGTCAAGTTAGGTCTCTGGCTGAATTATGTAGGGTACG
AGAGAGAGAACTGCTATTTATGTTGAACCTTCTTTTGTTATGTAGACTTGATAATCTCCTCTTTTTTTCCTT
TTCTTTTTTTTTATATTTCCATGATGCATTTGACTTGTTTTATT
>PGSC0003DMG400009230 LRR-RLPs
CTTGACTGGAACTTGCACACCAAAGACCATTTCTTCATTCTTCACGCGTATTCTCTCATGAAAAACTCTATT
TAGACTAATCCTCCCACTTTGTCCTTTCATCATTTTTTCACCATGGCTAAATCTACAATATTTTTTCTCCTTT
TTCTTTCATTTTCAGTGTTACTTCACAATGTTACTGCATCAGATGATATTAGGTGTTTGCAAGGACTGAAG
GACTCATTTAAGGATCCTAATGGAAATTTGAATTCTTGGAACTTCTCAAACTACTCCATGGGGTTTATCTG
CAAGTTCATTGGTGTCGGTTGCTGGAACGACCTCGAGAATCGGGTGCTCAGCCTCTCACTTCCAAACATG
AATCTCAGTAGACAGCTCCCAGATGCCTTCAAATATTGCTCCTCTTTGACTTCTCTTGATCTCTCTGGTAAC
AAGTTCTCCGGTCCCATTCCTTCGGAGATTTGTAGCTGGACTCCTTTTTTAGTAAACCTCGACTTGTCCAA
CAACAGTTTCTCCGGCTCTATACCTGCTGAACTTGGAAACTGCACCTACCTGAACAAGTTGATGCTCAAT
AACAACAAACTCTCTGGAAATATTCCACCAGAAATCTCTCGATTAACGAGGCTTAAGGTGCTCTCCGTGG
CCAACAATAATCTTTCTGGTAAAATACCACCATTTTCAGGATTGTCTGATTTTGAATATGGAGGAAACAG
ACATCTCTGCGGTGGACCATTAGCCAAATGTGGTTGAAGATGGCAGAAAGTTAGCCAAGTTTTGGCTAA
TTGTATCTTTTATAGCCTATCTAGATTTCTGTTTCCTGTCATTTCATAATTGTATTGTATGGTGAAAATGTC
TACAATTCCATGCCTTTTAAGTTAACAAATGCAACACTGGCTTCTTCCTGTGTCCATTCCACAAGTTATTGT
ACACAACACAAAGGAACAGAAATGTTAATACTCGAACTATCATTGCCTTGCTCAAGACTGTTCTTCGCGT
ACAGAACAATTTATCAACAGAATACTAATCTACCATTTTTTTTACCCACTATACTACATCTAGGTTCCCACC
AAACCCAGAAATCTACAAACTAGGCTGAAAAATTGAGTCATAGAAATGGCCGAGAATAATCAACAGATA
TCAGACATCTCACCAAAAGAATAAAAACAGAAAACACTCTCAAGCTGAAATTGATAAAGGGGAGATGAC
CGGAAAATCCAAAATCTGGCACACCTATTTTTTTTGGACGGAAATGAACAGCCATAGAGAAGTGCCTCC
AACCAATCATTTAAGTTCTTTTGCGCTACTTGAAATGATCCTGCCTGATTATTGCAACAAACTCTGAATGC
AAACTGTCCTCTTTCTGCTTCCATATTTCTCCGTTGGCATGTATTAACCATGATATTTGAGGGGAAAGTTG
TGCGTACAAATCTGCAAGTAATATATGATATTTAAACTAATCAAAGACGTGCCCAAAAGCAACAAATTTC
TGGTTGCAAGAACTGCAACAAACAAGAATATATACATTTAGAATGTCTAGACTATGATAAGTCTTCTATT
AATGCTCTACAAAGAGAGTTATCAAGTATCACCTAAGTTTATCCACACCTATTACTACTTCATAACAACAA
AGAGGGTTATCAAGTATCACCTAAAACTTTTATCACAATAGTCAGTATGTGTAACCTTTTTGCATTGTTGC
ACAGACTAGAATGATAAGGCACATGGATTTTTCTGATCAAGCTCAGTGTAAATACCTGTACTACTAGGA

GGAGGGTCTGGATCTCTTGTACTATGAAGAACCAATGTACCAGAGAGCACAGTTAAAAAGCCACAAAGC
GCTGAAACTATGTTGCTAACACTCTGACCAGACCAATCCTGAGAGAAAGACATGACATTAGAAATGAAA
ATGAGAGAAGGCCTCCTTGCATCCCGAACATAAACTTTCAGAATACCTTAAACATTATGGCACTGGCTAA
AATTGTAAGTGATGTGAACATGGCATAATATATTGGAGAAACCACAGCAGTGTTAAATGTGTCCAATGC
CTGCAAGAGTCAAATTCAGGCATTTGTCCAATTTACAATACAAGTCTAAGCCAAGATTCAATAACATGTA
GTTTCTATAGTAATCCCTGTAAATATTAGCTCAAGCATTTGAACAAATGCCAATGAGTGTCAGTTCATAA
GTTTTCCAATTACAATTGTACTTGTAAAAAGAAATGGATCTTGGGTCTTACTCAACCTCAAAAGATAGCTC
AAGAGATCACCCATGTGGTGAGAATTGTTCAAGCCATATTAAAAAGATCTCTTCATACACTACCTCACGC
CCAAGAATGGAGATCTAGTGTATGGGCAATTTAATAAGAGGAGCGTTGAAAAATGCTCTTTTACTAATT
GTTGGTTTTTAGCAGAGAAATTTTGGAAAATAACAGAGCAAGACTGAGAGGAAAATGCTGCAATGCTAT
CAAAGGGAACTTTTTTATTCAGCTTACTCTACATTTACGGGGAACTTGAAATAAAAAACAGTAAAAATAT
GATCATTATCCTAGCCTGGAGTATCTTCATACAATCATATCTTGCCTTCACCACTATCCCAGTTAATATGG
CATACTTTACTTTTTAGTCAGTCACAAGTCACAACAAAATGACACATTTCCTTATTTGGCAAATGTTTAAT
GACATTATCAACATTTTACCCATGCTGGGTCACACTTATTTGGCAAAAGGTCCACAAAAGTGTATTCTTCT
ATTTAAAAAATTCATTTCTTTTAAGGGTAGTTTTGGAACATTGCAAAGTCTTCTCATATTGCATAAATGTTG
TGCCAAGTCAAACTAATATCACATAAACGGGAGAAACTAATTACTAGAAATTAAGGCTAGCTAGCTAAC
AAACTTTAACTAAGATTTATTCAAAAACAACACAGCTAAACAAAGTTACTGTAGTCCAAAAGCATAATTC
TAAGGGGTCGTTTGGTTTGAATATGAGTTATGATGGGATAAGTTATGTTGGGATAAGTTATGTTGGGAT
TAGTTATGATGGGATAAGTTGTCCTGGGATTATTTCTTATTGAGTGTTTGGTTTGTTATATTCAAAATAAT
ATATATTGATAAATTTTAAGAACAAGTTGTTTGTTTACAAAATTACCCTTCACTTTATTTAAACTTTTTAAA
TATTTCTTTGTGAGCTTTAGTTATTCATTTTTTAAAATAAAACTTTTATTTTATTTAACTTAAATATTTTTGTT
TATGTATTCCCATGATAAGTACCATGTGTATTTAAAAAAATAACTTTTTTTCTTATTTAGCTTAAAAATAGA
ACTTCCCTCTTAAGTTTATTAAAGAGAAGAAAATTTATGAGAAATCAAAATAAAATAAACATAAGAATTA
GTCAAAATAATTCATAAATAAAAATTATATTTATATAGTATAATTTTTTAATAGAAAAATATGTATAATTA
TCATAAAAATAAGGAGTAGTAAATATTATTATACATGGTATTAAAAAAAGTGTAAATATACATAAATGTA
AAAAATGATGTATAAAAAGGATTTAAAGGGACATATTTGTCATTTACCTATTTTATCTCGGGATAAGTTA
TTCCGGGATTACTATACCACCCATGGGGAGGGATAACTTATCCCGGTACTAATTATTAATCCCGGGATAA
GTTATCCCGGACTTGCCAACCAAACAACATAATTTTTTTTTTATCCCATCATTATTTCCTTATCCCATCACTA
TTATTTCTAATCCCTCACACCAAATGAGCCCTCTAATAGTACTCTCAGACTTCAAACATCACTGCATACCA

GTGTTTTCAAAAACGTTTTTGGGGCGAGTCTCAGGGCAGGGCGTACCAAAAACGCTCCGGAATGTAAAT
CAAAGACGTAGATCCATAAGGCGTAAGTTCTAAAATTTGAGGCATAAGCCTCGAGCATAAAAACGTAAT
TCCTGGGCGTAAAAACGTAGGCCCGGGCGTTTTTAATTTTTTTTAAACCTTTTTTGCTTTAAATCATATATT
TTATTATTGGTGTAATGATATTTCTCAAATAGTAATTATTAATACTTTTTTTCTTCATTATTGATTATTAATA
TTTATCTCAAATAAATATAATTTCTATATATTATAGGTTATTTATAAAAAAATTATTTGTAAAAATCATTGA
AAGATTTACTTTTGCTTATTTTATTAGTAATAAAACTATAAAATTGAATATACATGAGACTACGCCCCGTA
GATTTATGGGACTTACGCCCCATAGATCCATGGGACTTACGCCCCGTGTCTCGGGGCTTACGCCTCGCCC
CATACTGTATAAAGCGCCTCGCCTCACACCCCCGCCTTTTAAAACACTGCTACATACTATAAGGAGAAGT
AGAAAGTCTACCTTATTTAAGTAATTTAGTTGAGTAATTATACATGTAACAGAAACCATCACAAACACCC
AAGTTTGAAAATTTGCAGCTTGGTTTGAACCCTCCAATGTAAGCTTTATAGCTATACCGATAGCTTTGATG
CTCATAACCTACAACAGAGCAACAAACAAACTTAAAATGACTGCTACATGCAATTTTTACCAATTGTACTT
ATGAGAAGGACTAGTTCCTATATTTTCTTTACTTCTCATGAGGAATAATTTTATCATATTTCATTGGCAAC
AGAAGCACTCTCTCTAAGTCAGTTCGGCAAAGAGAACTGCAATAGTAGCTCAATTAATACAAAAAAATT
AAGAGAGGAACCAATTCATAAACTGTTATTTATCGAGTTTTTTAAGCAAAATACATGAGTATTATTCAAA
AGATAGTGAATTCGATTGATAAAGAAGTCATAAAATAACTCGACCTCGAACTAACAATGACTAGATAAA
AGCTTATTTCAACTAGCAACTTAGACTTATTTTAAATTTATGTAAAAGACAAAAACTAATAAAATAAAGTA
ATAGCACTGCAATCTTTTATTGAGTTACACACCTACTAGTCAAGGAAAAACAACAGCAACCCTGGATGCC
TTATAAATTATAACAGATCAAAGTATAGTCACTTGTACAAGTATAGTGGCAGGAAAAGACATCTACTAGC
ATGCAAACACGTTACAGCACCCTAAAGCTCTGGAGTAATATTTCAAGTTCCATACATCCATTTATACCTAA
ATGAGAAGTTTTCTTTTACTTCAGTGACAACTAGCAATGGATAAGGACCCAGGACCAGTTTGACTCTTCA
CAACTTAAAAAATATGATCCATTTAGTGGTCAAATTATGCTATTCCAACACCAAGGAGAAAGATAGAATT
TGGATAACGTTAAGTTTAAAAGATAAGGCGTCTTGCAAACTGTCAATTTAGCTTCAAGGTTAACTCAGTC
AGTTCAGAGCTACAAACTAAAGCACGGTTGTGACACATGTTTCTTCCCTTCCTTCCTTCGCTTTCTTAATTC
TTGAGTAGAAGTACAGACTCTGTAGGGATATAGATCAATACAATAGAGGCACTAACCGTCAAGGACCCA
AAGATGGAGCAAACACCAATATAGACCATAATATTTGTCCGACCATAGCGAGGCTCACAATACAAGACT
AGTACCAATGTTATTGCAACTGCTGATGCAGTGTACAAAAGAAAAGCTGCAAAACAAAGAGTGGAACAT
CAAAAAAATGATATATCAGCAAGGCAAATAAGAATGATCTTCAAGGAAATACAGCACAGTACATTAGCA
CACCTGGTTGTGTAGCTAATGCCCATATTTCATCCACTGAATTGAGATCATGTTCACGAGGTGCATGAAG
AACAATGACAACGGAACCTACTATACATAAAAGACATCCTAATACTCCCAGTTTCTTCAATTTTTCCTTCA

GTATGAAATGTGCTAGAACAGCACTGCAGCAAAGCGCCAAATTATATGGAAGAAAACCGAATAAGCCA

TTGCCCAGAAAATAATAAGGCCGAGTGGAAAATTTACCTCACAATTATACTTAAAGCTCCAAGCGGTGTC

ACAAGAACTGCTGGCGCATAAATGTAAGCCACGAAATTTGCAAATTCTCCAACAATCACTGCAAGGATT

ATGGTAAACATTGCACTCTATATTAGAATAACAAATTCAGGAACATATTCTCTAACATTTAAGAAGCAATT

CAAGACTTACTAGTTACCATCCCAATCCACCAAAGAGGCTGCCGTAGATAACCATATCCGCCCGAGCCTT

CAGCAAGAAACAAGTATCTTATTGTCAAATAGACAGAGCAACAGAGTTCATATAATCAGCTTTTAAACTA

AATTCTATTTAATGCTGCTACAGAAAAGCATCTTCTATATATAAACTTTACAAGATGTCCCCAATATATCT

ACTTTTAATGTGTAAAGTAGCTGATAAATTACAATGTCAAGGAAATTCATTCAGCTTATTACAGTGCAGA

ATGATTTAAAAAATCCACTACACTGATCATTTTCAGTTCTTAAAGTCAAGATCCATTTATTATGTCTTGAG

CTCATTTCTTCTCTGTAATACATGTATTAGTACTACCTAATACAGCAGTCTGTGTTTATTCGGAAATGATA

GATCAAATAATTTGGAATTACCAACAACAATCATTTAGTGAAGCAGAAGCATGAGTGCATATATTTAAAT

TGTTGGCCCATTCTACAAGCGGTAACTATCAAAGGTATTAAGTTTAAACAGGTGCTGCTGGAGTCGCTCT

GATTGCTAAAACAATGTCATTAGTCCTAGATAACTGCCACCGAAATCTAGGCAGCTGACTGGTCAACATT

CAGAAGAATATGAATTCTTTTGCAAATACTTCTCCAATATTATTCAAGGACTACTTACTTGCATGATAAGT

GTGAAGTGTCAATTCCAATATAAGTAACTTGATATTCATTTTCCCAGAAATGTACACCCAATACTTCCCTT

TCGGAGAGAAGTGCAAATTCCATTCCTTGAGTTCCAGAACAAGTTTTCTTTATTCCCAAACGGATAACTG

CTTTGTAAGATGCAATATCACCTTTTATAAGCAGTCATTGTGTATTAGTAATACTCATTGTGACTCTACATT

TGCTGGAAGTGTTACCCCATTGCTCTTATTTTACAGTCGGGCACCTACAACATGGTGTACCAACAGAGCA

TAACATAATCTTAAACTGTACTTAAGCAGAAAACAAATGAAGTAAGAATTTTCAGGTGTAAAACATGTGT

TTCACCCGCAAAACTAAAATGGGGACAGCAGAAAAGCAGGTACCACCATACCAGTACAAAAGCAAACC

CAACATTATCAGTCTGTTACCACTTGATATTTCAAAGTAAGAAGTAAAAGAACAATCTATTCATCAAGCAT

AATCTCCAATTCCATACATCTATAAACAGCATCTGATATATGGTAAATTGGTAATGCAGTAAAATGGTTA

GAAATATCAATCTTCAATCGACTAATTTTGACTTGATCATGTAATAGTCGGTGTCAGCTTTATGAGTCCAC

TATATTCATTCTACATATCTGGGCCCATTTCAATTTCAATACTACAGGAATTTTATATTAAGAAAAACTAG

GGGTTTTCTAGAGTTAAGAGATTCTCCAAATGTCACACTTCTTTTCTGTACACTAACATACAAGGTTTTAA

CCTTATTCCCAATTAGCTAGCGTCATCTACAATAAATCATTTACTTCAACTGTGATTCTTACCTAATTTGCC

TTAGTTGAGAGATTGTAGGGGTTTTGTTGTCATTGTTAGGATCGAAATTACCAAGTGTCATGAAGAAGC

CAATAAAGCAAACATTGGACTATGATAGTATAACAAAGAGAAATATACCAAATACATAAAAATTTAACAT

GAAGAATTCACAAAGATAAAAAAAGAGTACAAACATATAAAGAGAATAACCTCACAAAATTCACTCGTA

AAGAAGTTTACTTTTGCCCTAAACAAAATTCTCTTGAGACTACATTGTGCATGCTCTAAATTGAGAAGGA
AATCCTCAATGAATAGAAATTCAAAACATGTTCTTCCAAGAAAAGGATTAGCCAAATATGGAAGATTCAT
ACTTTCCTGATAAAATCTAGTTATCCTAAATGTTATATCCAAATATGATAAATAAATTAGGGCAAACACCT
ACAACAACCTGCGTTTGTATAATTTTAAGCAAGGTCAACCTTCCTAACCTTAGCAAGAAGTAATGCATTG
CAACACACACATATAAAGAACTGAGCATATTAAACAACTTTAGCAAAACTACAAGGTAGAAGAAAACAC
ACAAACATCGGCAAACTGAATGGGGACACAACTAAACCTCCATGAATCCACCACTAGAAGCATTGACAA
AGTTCAAAATCAACAAAAAGCAATAGATTATTGACAATTAGACCTAAAAACACTTTGCTATCAAGAAAAT
GAATTACTCACTGGCCCGAGTACCAGAGGCGCCTGCCTTCTGAAGACCCTTCTTCTTGATAATGAAACTG
GAACCAATAAAAGCGCTAGACGCCACAGCCAATCCAAAACCAGTCAAATTAGCACTGTACATGATCCCA
ATATTATATCTAAACTATTGGGCAACAATTTTGGAATTTAATTCTCATCTGAACTTTCCCAAGAACAAAAC
TTTCAACACCCAAAAGGTTCCCCTGGATATACTCAACAAGACAACACTCAAGTAAGACTAAAATAGCAAG
AAAAACGAAATGGGCAAGTTTGAAATACTTAAAACCGCTGTAGCTTTACTCAATTACCCTATAATTGTAG
AGCTGAGAATGAAAAAACAGCAAGAAAAACGAAATGGGTATATGAGAAAATTGGCGAAAACAGGTTT
GAAATGGTGAGCTAACAGTGCAAGCTCGTTATAGACAATAATGGAGAATAGTATTACAATAAGCATAAT
TTTGTCATGCTTTGATTCGTTGCTTTGGGTTGATTTGGCTATGGTATGTCTTCGACAATTGGGCATAACTA
TGAATAAATC
>PGSC0003DMG400009324 LRR-RLPs
AGGAGACCTAGTAAAAAAGAAAAGAAAAAAACATGGCCGACGAAGTTCAGCCAGTCAACCAGAGGTAC
GGAGAATTGTTGGTATATGCAGTTATCATGAAATATATAAAGTCCACACACTGCTCAGCTGAGCAAACC
AGACACTTTGAATTGGTCAATATATCTTTACTGGATTCAATACATGGCGGAAGCTTTTCTTCAAGTTCTGC
TAGAAAATCTCAATTCTTTCATAAGAGGGAAACTTGTATTGCTTTTCGGTTTCGAAAAGGAATTTGAAAA
GCTGTCCAGTGTGTTTTCCACAATTCAAGCTGTACTTCAAGATGCTCAAGAGAAGCAATTGAAGGACAA
GGCAATTGAGAATTGGTTGCAGAAACTAAATTCTGCTGCGTATGAAGTTGATGACATATTGGGCGAATG
TAAAAATGAGGCAATAAGATTCGAGCAGTCTCTATTAGGGTTTTATCATCCAGGGATTATCAGTTTCCGT
CACAAAATTGGGAAAAGGATGAAAGAGATAATGGAGAAACTAGATGCAATTGCTGAGGAAAGAAGGA
AGTTTCATTTCCTTGAAAAAATTACAGAGAGACAAGCTGCCGCTGCTACGCGTGAAACAGGTGTGAGTA
CTGAGTAATAGTAGCTTAGTTTATATTCAATTTGTTACCACATCGTGTGTTCACCGTGATCTCTACAGTAG
GATGGCAATGGGGCGGGGCGGGGTTGGAGGTGTGCAGGTGTGGGGCGCAATCCCAACTTTGAGTCTG
CACAAGTAGGTACTTAAATTTGTATAGAGTTAAACAAGTACACACGCATCCTACTTGGTGTCCTACATGG

CTCTTATGTGTATTATGTCATATAGGATGCATGTGTCTATTTGTTCAATTTTATACGAGTTTAAGTTCTACT
TGTGCACACCCAAATGAAAGTTGGAGGGCGTAGATGTCAGTTGATACCAAGTTAAAAATTCATATTAAT
GAATTATGCCTTTAAATTATGATTCAATTTTATATCAGTCTGTCCAAAAGATGTTCTAGTGAAAGTGTTAA
ACTTAGCCTGGATCTGCTATTGGAAGTGTGAATTTTTGTGGCACTAAACAATGCAATGGGTCTGGATTCA
TTTTTGCATTAACATTTGTTTAGACGATTTTCTTTATCGAATTTTACTGTCTAAAAAGGAAAAAGCAAAGA
AATAAGACGTATACAGAGGCTGACTTCTTCATAGGATTTCTCATATAAAAAAAAAACATTGATTTCTAGG
ATATGGGTTATTTTAAATTACAAATTTGTGAGTTAAAACAGTTCTGTTGGGAAGGATTTAGATACACGTG
GATAGTATCTAGAAGTTTTTTAAATAAAAAATTAGCAAATTATGTGGGCTGGGGCGGGTTGAAAACAGC
AAACTTTGCAAGGCAGGGCGGGTTGAAATCATTGCAAGTTTGTGTGGGTTTGCCCTGCACCACCCCATCT
GCCATTCCTGTCTAAATGTTTGTTTTGTCTATATAATTCTTGCTGACTCATTCTAATGAGCTCAATTGTAAC
AAATTCTTTGTGTCCACACTACTTGGAACAGGTTTTGTGTTAACTGAACCAAAAGTCTACGGAAGGGACA
AAGAGGAGGATGAGATAGTGAAAATCTTGATAAACAATGTTAATGTTGCCGAAGAACTTCCAGTCTTCC
CTATAATTGGTATGGGGGGACTAGGAAAGACGACACTTGCCCAAATGATCTTCAATGATGAGAGAGTG
ACTAAGCATTTCAATCCCAAAATATGGGTTTGTGTCTCAGATGATTTTGATGAGAAGAGGTTAATTAAGA
CAATTATAGGAAATATTGAAAGAAGTTCTCCTCGTGTCGAGGACTTGGCTTCATTTCAGAAGAAGCTCCA
AGAGTTATTGAATGGAAAACGATACTTGCTTGTCTTAGATGATGTTTGGAATGATGATCTAGAAAAGTG
GGCCAAGTTAAGAGCAGTCTTAAATGTTGGAGCAAGAGGTGCTTCTATTCTAGCTACTACTCGTCTTGAA
AAGGTTGGATCAATTATGGGAACGTCGCAGCCATATCATTTGTCAAATTTGTCTCCACATGATAGTTTAC
TGTTATTTATGCAACGCGCATTTGGGCAACAAAGAGAAGCAAATCCTAATCTAGTGGCCATTGGAAAGG
AGATTGTGAAGAAATGCGGTGGTGTGCCTTTAGCGGCCAAGACTCTTGGTGGTCTTTTACGCTTCAAGA
GAGAAGAGAGTGAATGGGAACATGTGAGAGATAATGAGATTTGGAGTCTGCCTCAAGATGAAAGTTCT
ATTTTGCCTGCTCTAAGACTGAGTTATCATCACCTTCCAGTTGATTTGAGACAATGCTTTGCGTATTGTGC
AGTATTCCCAAAGGACACCAAAATGATAAAGGAAAATCTCATCACTCTCTGGATGGCCCATGGTTTTCTT
TTATCAAAGGGAAACTTGGAGCTAGAGTATGTGGGTAATGAAGTATGGAATGAATTATACTTGAGGTCT
TTCTTCCAAGAGATTGAAGTTAAATCCGGTAATACTTATTTCAAGATACATGATCTCATCCATGATTTGGC
TACATCTCTGTATTTGGCAAGCACATCAAGCAGCAATATCCGCGAAATAAATGTCAAAGATTATAAGCAT
ATAATGTCCATTGGTTTCGCTGAAGTGGTGTCTTCTTACTCTCCTTCGCTCTTGAAAAAGTTTGTCTCGTTA
AGGGTGCTTAATCTAAGTTACTCAAAACTTGAGCAATTACCCTCTTCCATTGGAGATCTATTACATTTAAG
ATACCTGGACCTGTCTCGCAATAACTTCCGTAGTCTTCCAGAGAGGTTATGCAAGCTTCAAAATCTTCAG

ACTCTTGATGTGCATAATTGCTACTCACTTAATTGTTTGCCAAAACAAACAAGTAAACTTAGTAGTCTGCG
AAATCTTGTTCTTGATGGCTGTCCATTGACTTCTACTCCACCAAGGATAGGGTTGTTGACATGCCTTAAGA
CTCTAGGTTTCTTTATTGTGGGAAGCAAGAAAGGTTATCAACTTGGTGAACTGAAAAACCTAAATCTCTG
CGGCTCAATTTCAATCACACACCTTGAGAGAGTGAAGAACGATACGGATGCAGAAGCCAATTTATCTGC
AAAAGCAAATCTGCAATCTTTAAGCATGAGTTGGGATAACGATGGACCAAACAGATATGAATCCGAAGA
AGTTAAAGTGCTTGAAGCCCTCAAACCACACCCCAATCTGAAATATTTAGAGATCATTGCCTTCGGAGGA
TTCCGTTTTCCAAGCTGGATAAATCACTCAGTTTTGGAGAAGGTCATCTCTATTAGAATTAAAAGTTGCA
AAAACTGCTTGTGCTTACCACCCTTTGGGGAGCTGCCTTGTCTAGAAAGTCTAGAGTTACAAAACGGGTC
TGCGGAGGTGGAGTATGTTGAAGAGGATGATGTCCATTCTAGATTCTCCACAAGAAGAAGGTTTCCATC
CCTGAAAAAACTTCGTATATGGTTCTTTCGTAATTTGAAAGGGTTGGTGAAACAGGAAGGAGAAAACAA
ATTCCCCATGCTCGAAGAGATGGCGATTTTACATTGCCCTCTGTTTGTTTTTCCAATCCTTTCTTCTGTCAA
GAAATTAGAAGTTCACGGCAACACAAAAGCAAGAGGTTTGAGCTCCATATCTAATCTTAGCACTCTTACT
TCCCTCCGCATTGGTGCTAACTACAGAGCGACTTCACTCCCAGAAGAGATGTTCACAAGTCTTACAAATC
TCGAATACTTGAGTTTCTTTGACTTCAAGAATCTCAAAGAGCTGCCTACCAGCCTGACTAGTCTCAATGCT
TTGAAGCGTCTCCAAATTGAAAGTTGTGACTCACTAGAGAGTCTCCCTGAACAAGGGCTGGAAGGTTTA
ACTTCACTCACACAGTTATTCGTTAAATACTGTAAGATGCTAGAATGTTTACCCGAGGGATTGCAGCACC
TAACAGCCCTCACAAATTTTGGAGTAACCGGTTGTCCAGAAGTGGAAAAGCGCTGTGATAAGGAAATAG
GAGAAGACTGGCACAAAATTGCTCACATTCCTAATTTGAGTATATATTAGTGAGTTATTTGTAATTGTTTC
TTGGGTTATTTTTTTGTCAGTCTCTCACAGGTTACAATTCCTGCCATTGGATTATTATAGCAAAGTTTGACT
GTATGAGGAAACATTTAAAAAAAAAAAAGAAGGTAAATCGTTTAACATGACTTTTTTTTTACTGTTTAAT
TTAATATAAAAGGATTGTACTAATTTTCTATTTGAAAAC
>PGSC0003DMG400009532 LRR-RLPs
ATGGATCGAATTTCCGAATTACCAGAGCCAATTTTAGAGTACATCTTGTCTTTTCTTGTTTACAAAAATGT
TGCACAATTAAGTATGTTGTCCAAGACTTGGAACAAGGTTTGCAATTCAATCTCCTATTTGAAATTTGATG
GTGATTTTTTCTTTACGCCATCAAAATACAAACCAAAGCTGGTGGATGTTGTGCATCAAACCCTAGAAAA
TCGTCGAAAGCTGAAGATTGCCGTACACAAGTGTTGGCTACCATTAACGATTGGATCTCATGAGGTATA
CAATTGTTGGATTGATACACTTGTAGTTTGTAATATCAAAGAACTACATCTATCAATTAAATCTCATCTTT
ACAAATTGCCTGAGATGATCTTTAATGCAAAAGCCCTAAATGTGCTGAGGCTGATTGGATTTAACATTAT
TGAATTGCCTTGTAAAAGTATAAATTTGCCATCTCTGCGAGAGTTTTATCTTGACAGTGTAATTTTGGACG

ACAACTTTCTTCGAGCTATATGCACAACCTGTTCTAATTTAGTGGTTTTGTATTTGGTGGGCTGTCATGGA
TTTAACAGTTTGCAAGTTGGTGAAAATTTACCTAAACTGAAAAAATTGAAATTGGAATATTCTTATCATCA
AGTCCAATTCGTTGATATTAGTTCAACTAATCTTGAACAACTTACCATCTACGGCAATTGTGATTACCTAA
AGGCAGTTGAAATAACTGCTTCTAAAGCCCTTAAGACTCTGTACCTCCAGCGTGTGCCGATAACCCAAAA
AATTCTTTCTTGCCTTGGAAATCTAGACAAGTGTGAGTTATTTTTTTGCCACACATTGAAGACTCTAAAGA
TTGCAAGTTTCCTGCTCAAACAGCTGACGGTACATCAATGTCCTAACCTAATCGCTGTTGAATTAGACACT
CCTAATTTGATAAGGTTTTCGTACAATTGTCATTCATTGCCAACCCTGCAACTGAAAGCTTCAGCTTCAGC
TTCATTGGAAGCAAAAATTTGCTTTGTCTCAGGAACCACTGACAGTGATGTCACAAAATTTCTTGGTAAC
TTCAATCAGTCCATGGTTGTTGAATTAACATGCAAAAGTGACAAGGTAATAATCTTTTTAATTCCCACATA
TTCTTTTAGTTGTTGTTATATATTATGGATTTCTCACAAGTTGTTTACTTTTGCTGATACAGGCACTAGTTA
TACCAAAAGACATGAGAGGAAGCTTGCTTCCTCCCCTTTACGGTACTAATCGTGTTCATGTAAATATTCG
AAGTCCATTGATAAACTGTTCGATTGTCGACATTCTTGATAGTATGCTTTGGATTTCTCCTCAACTGGACA
CCCTATCTTATTTCAAACCTGACTTAAAGATCCTAATGGTAAGACATTTATCTTTCAAAGGTACTGACTTT
GTAAAAAAACGTTTTTTTAGATGCAAGGTTTCTTGCAATTCTTTTCCCTTTTAACATGCCTATTTCTTTCTTT
CTTTAGCAGTATAAGTAATTAAGTCTGCAGTTACTTGAACTAACAAGTCAATTAAGTCTAGTATAAGGAT
ACTGAGATTCTGAAATTCTTTCGCGTCCCCCCAAAAGCTGTTTATTTTAAAAAAATTGCGAGAATATATGT
GAAAATTTCGGGTTAATAATTATTGGATTGTGCTTTGTCTCTGCGGCTAGTGGATAAATTTGAAGTTATA
TGTAGCTGGTTAACTCAAAAAAATATGTTGTTAACAAACAAGACAACGAAATCAGGGTCATAAGATTGT
ATTGAAAATTGCACTTCTTAACAAGCCGTGCATGCTACATTTGCAGCAATAACTCTACTAGAAAAGAGAC
TGGTGTTTCTGATATTTGTTTTCATGACTGATTCTTTATGAAGACAGTTTTTATATTCATGCGTTTGTGTAT
TGTCAATACTTATTGACTTTTCTGGTTTTACAGTTTATATATGAAGATGCATTAGATGAAGATGAGAAGCC
TTGTTGTACATCTCAACCTTGGAAATGTTGGAGGCATAAGTTAAAGCAAGTCAAAATACAAAACATTACA
TGTATGGAACAAAAACAGGAGTTGAGAAACTACTTGTACGCAAACCCAGATATCCCGGAGATAATTGAG
GTTCCTGGCCCAGTATTCATCGATCCCACAGTATTCATCCACAGATTCGTAATTGGATAA
>PGSC0003DMG400009755 LRR-RLPs
ATGGACAAACCAAGTTATAAAGGAGATGGATATTACCAAGACTCGGTAGTTGTTGTATCAAAGGGATTG
AAGCTTGAAGTTGTGAGAATTTTGTCTTTGTACACAGTTATAGATCTTTCAAACAACAAATTTGAAGGAC
ATATTCCTGGTATTCTGGGAGATCTCATTGCGATTCGGGTACTGAATTTATCTCATAATGGATTGCAAGG
TCCTATACCACCATCACTTGGAAGCTTATCTGTAGTGGAATCACTGGACCTTTCGTTTAACCATCTTTCGG

GAGAGATACCACAACAACTTGCTTCTCTTACGTCTCTTGAATTCTTAAATCTCTCTCACAATTATCTCCAAG
GATGCATCCCTCAAGGACCTCAATTTCGTACCTTTGAGGGCAATTCATATGAAGGTAATGTGGATTACGT
GGATTCCCCATTTCAAAAGGTTGTGGCAATGATCCTGTGTCGGAGACAAATTATACAGTATCTGCGCTAG
CTGATCAAGAAAGGAATTCTGAATTTCTCAATGATTTTTGGAAAGCTGTTCTGATGGGCCATGGCAGTG
GACTATGTATTGGATTATCCATAATATATTTCATGGTCTCAACTGGAACTATGAAATGGCTTGCAAGAAT
CATTGAAGAACTGGAACACAAAATTATCATGCAAAGGAGAAAGAAGCGGCAAAGTCAAAGGAACTACA
GAAGAAGAGATAATCATTTCTAGACAAGTTACCAATAAAGAAGGATTCGATTTCAGAACTTCAGGTATTC
AAGCTAAGATCTACACTATCTTTATTAGTTTATTCTAACTACTAATATATTAGTTTACACGTTTTTTGTTAAT
AACAACAACTAATTAATGCTCAATATGAAGCATATTCGTGTTTCTTTCAACTTCAAGAAGCTAAAGATTTG
AAAAGCTTGGCAGTTGTTATAACAGTGGCTACTAAATTCAATCAGTGATCAACTCTCAAGTATTTGACTC
ACTATCTTATTTTTTCAGATGCCAAGAATAAGAAGATGTTGGCGTAATGGATTTGATTTTAGTTTCTTTTT
AGTTTAATAAGTAAGCTTCTTCTTAACTTGGAAAATGTCATATTATGAATTTTTGATGATATATAAAGTTT
ATTTAAGTTTCTAATGTTATTCAATAAATGTTGTGTGTATTGAATGAAATGTATACATTTCTCTGATCCCTA
ACTTACAATTCAAAGAATCTAGGAGGGACTTAGTCATGAATAAATTTTTTGAACAAGCTGAAGAATGTGT
TATCTTGTCCCTATTTAGCTCGAGAAAGAAGTGAAGTCAGAGTGTTGGGTGTCCCTGGATTGCTCACTGC
TGCCTCAACGTGTGCTGAGGTTTTGCAGCAGTCTAAAGAGAGAGCGTTGAGAAGAGCTAAATTAGAGG
AAGATATTTATCGTGTGATAGAGGAAAGAGCATTAACAAGGAAGAATAACGAGTTCGTGACAAGTGAT
TACATTTACTGGCCAAGGGAATTGCTTTAA
>PGSC0003DMG400009899 LRR-RLPs
ATTGTCTAATAAATTTCTCTTTGATTATTGAGCAAAATTTTGTCACCAATGGAAGGTGTAAATTTACTACT
AGTCCTTCTTCTTGTAGCTAGTAGTACTATATTAGATGGATTGTTATTATGCAGAGCTATGAATTCAGACA
TACTTTGTGATGAAAATGAAAAAAATGCTCTTATCAAGTTCAGACAGGGTTTCAGGGATCCATCACAGTC
AATGATCATGTCGTCTTGGATGGTCGAGGAAAATTGCTGCAACTGGAAAGGTGTTGAATGTGACAACAC
AACCGGATACGTGATCACTCTTGACCTGCACCAGCAATTCTTGCAAGGTGAGTTTGGGACTTACTTGCTT
GGCTTGTCTCATTTAAGACACTTAGACTTAAGCCAAAATGATTTCCTTGAGGCCTTGTTTCCTGATTTCAT
TTGCAAATTTAAGTACCTAGAGTATCTCAATTTGCACAAAACCAAGTTTAGGGGATCAATCCCTGAGTGT
CTTGGAAATCTGTCGCGGTTGCAGTTACTTGATCTTGGTGATGGTTTATCACTCCGGGTTGATAGTCTTG
AATGGATTCAGCATCTTTCCAATATGAGGATTCTTGACCTGAGTGGAGTTGACCTGAGCAATGCCAAAA
ACTGGCTACATGACATCAATTTCCTAAGGTCTCTCGTGGAATTACGTTTGTCTTCTTGTCAGCTTCCTAAA

TTGCTTCCTGTTTTTGTAAATTTCACATCCCTTCAGGTTCTTGACCTCTCGTTAAACTATCTGGATGTTCCA
TTTCCTAGCTGGTTAGTCAATACAAGCTCTCAGAGTCTTGTTTATCTTAATCTCAAAAGAAGTCTGCTGCA
TGGTTTACTTCCAAATACCGCCTTTGGAAACATGTACTCACTTAGAGTTCTTGATCTTTCTGACAACTCTAT
CCGAGGAAAGCTTCCGTCTTTTGAGAACATGACTTCAGTTACTTCTCTTGATATCTCAAGGAATTCTCTTG
TAGGCAAATAACCGCCAACCCTGCCTTCAAAGTTGAAACATTTGGATCTTTCATCCAATAATCTGGAGGG
TCCTTTAGCAAGAAGCATCGCACAACTTAGGCAATTAGTTGTCCTCAATGTTGCTTGGAACTCTTTCAATG
ACTCAATTACTGAACATTTCTTGAATTTCAGTGATTTGAGAATGTTAGACTTGTCATCCAACTCAATTATC
CTCAATATAACTGCAACTTGGATGCCTCCTTTTCAACTTGACATTATTGGCCTGATGTCTTGCCAACTTGG
TACACAGTTTCCTCAGTGGCTTCATACACAAAAGGATTACTCATTTATCGATATCTCTCATGGTAGCATCT
CAAATGAAGTGCCTGATTGGTTCTGGAGTCTCTCTGCAAAGGTACAGCACATGAACCTTTCTCTCAACTA
TTTCAGAGGGGAAGTACCTCAAGTTACATCAAGACTTGATAGTCTTGAAAAACTAGACCTGAGCCAAAA
CAACTTTTCTGGTCCTTTACCTCATTTTTCGTCCAAGATGAAGATGTTGATTTTGTCCCGGAATTCTTTTAA
TGGCGCCATTTCCCCTGTATGTGAATCACTTGTCATGAATAATTCTCTTATGTTTTTAGACCTATCGTTCAA
TAATCTATCAGGAACACTTTCAGACTGTTGGAGATATGGGAACAATCTGGCAGTATTAAAGTTAGGCTAT
AATAATCTGTCTGGTGAAATACCTCATTCTTTTGGATATCTTGAAACTCTCAGGTATCTGCAACTGAAAAA
CAACAGGCTGTCCAAAAATCTGCCTTCATCACTGAAGAACCTACAAGGACTAAAAATTCTTGATCTTTCC
GGAAATTGTTTATCTGGAAAGATACCATCTTTCTTAGGGGAGACTTCACAAAATCTATTGGTTCTTTTGTT
AAGAAAGAACAAGTTCGATGGAAACATTCCCCTACAGATTTGCCAGCTCAAAAACTTGATTATTTTGGAC
CTTAGTTCCAATTCTCTATCAGGCACCATTCCAAAGTGTGTTAAAAATTTCCTAATGATGGCTGGGGTAG
AGGAGCTCCCTTCCTTGGTTTATGGTCCCTATGAAGAATACAGGAAGGATGTGGCGTTGATGCTAAAAG
AGAGAGGCTATGATTATAGTTTTGCCTTTATGGCGGTGATTGATCTATCAGATAACCATTTATCAGGGGA
AATTCCTGAGGAAATCACCACACTTGTTCAATTGCAGGTATTGAATTTATCAAGGAATAATTTAACTGGA
GCAATTCCTTGTAAGATTAGTAAAATGCAATCTTTGGAAGCTCTTGATGTTTCCTGGAATCGTCTGTCATC
CTTCTTGCCTTCCAGCATAGTGGAGCTTTCCAGGCTCGAGATCGCGAATTTTTCGTTCAATAGTTTGACAG
GAAAAATTCCAATAGGTGGTCAATTTTTAACCTTTGAAAATAGTTCTTATATTGGGAATCCAGATTTGTGT
GGCATGCCACTTTCCAAAAGCTGCTCGGGACATTTTCTTGAGGACATTACTCACTGTAACTCTCCCAAGA
AAAAAGACGTGCAGGCCATTCATCAGCATGAGGAAGACAATTGGCTTGATGAATCATCATTCTATATCA
GCATGGTAATTGGTTTCATCACAAGTTTCTGGTTATTTTGGGCTACTCTATTAGTGAAAACATCTTGGAGA
CATTCCTATATGAGGTATCTTAATAAAATGGGCAACAATATTTATGTGTTTGTAGCCATAAGATTACCAA

ATAAGCATAAACCATCAAAGATGAAAGATTGAAGTTCTTTCAGGGATTGCTGAATAAGGTAATGAAGGT
CTTATTTCTGGTGCAACTTTCTTTTGTTGTTTTGTTTTCTAGGAGCTCTTCTGGTTTTGTTTTTGTAAAATCT
TCTAAGTACTATATTGTGTATGCCTAGTTGCATTGTTTCATCTCGTGGGATTGTACTAGGTATGTTGTTGT
TGTAGTCATAATTGTATTCTATGATATGTTTTGTGGATATCTAAGTAACTTGAGAATACTATTACTAATGT
TTTT
>PGSC0003DMG400010183 LRR-RLPs
ATGTCATTGTCAATGACCATCTTGTATTTCATTTGTGTTGTTTTGACCGAGTGAGTATAAAGGCGCTATAT
TGCAGCTGCATTTCCTCCTAAAAAGCAGTTGAGTTTAAAATACAAGACAAAGCAAAGCAAAAATAGTTA
GTCAACACTTACCTTGCATGCACCAGAGAGTCGTGACTTTTGAAATTCAATTTCAAGAACTGGATAGTTT
TTTAATGCATATTTTAAAGAAAAGCTTAGGTGGATAGAGAACCAAACTCAGAATCAATGTGCTAACAAA
ATATTTTATTGTTGATATATCATTGGCAGCTTATTTTATAATCGTGTGTCTTATACCACGTAGTGACTCTCC
ATTACCAAAAACAACATTCATCTCTTGCTCCTTGACATTTTCCAGAATGGAAATTTTCTCAATGAGTTTTAA
TTCCCACACCTTACTGGCAACGCATGCTGTGTTTCTATTCTCATCTACTCTCAAATATGCTGCAGCAGCTA
CTATATACTTGGAAACGAAACTGACGAACTAGCCTTACTTGGATTCAAGTCCCAAATAACTGAGGATCCA
TCAAGAGTTTTCGCCTCTTGGAACCAATCCGTTCATTTATGTCAGTGGACTGGAGTAAAATGTGGCCTGA
CACAGGAAAGGGTGAGTCTGGCAGGTACAATCTCAGATCATCTCGGAAATCTTTCTTTGCTCAATTCTCT
TGACCTGGCAGAAAATTCATTCCATGACGGAATTCCTCAAGAACTCAGCAGGCTGTCAAGGCATCAAAA
CCTGAATTTGAGTTTTAACTATCTAACAGGGGAAATTCCAGTTAATCTATCACATTGTGTTAACCTCAAGA
GCCTTGTGCTGGACCACAACACTCTTGTGGGACTGATTCCTTACCAAGTTGGATCTTTAACAAAGTTAGT
GAGATTGTATCTCAGAAATAACAACCTGACAGGAATCTTTCCAGTGTCTATTGGAAATCTTACATCTTTA
GAAGAGTTGTATTTATCGTACAACAGTCTAGAGGGACAAGTGCCAGCTTCTTTAGCTCGATTGACCAAGT
TGAGACTGCTTGGATTGTCAGTAAATAGCTTCTCCGGGGAGTTCCCTCCTTCCTTGTACAATTTGTCATCC
CTGGAATTAATAGCACTTTCCTTCAACCACTTTTCTGGTAATCTCAGATCTGATTTAGGCCACTACTTCCCT
AATCTCCAAAGACTTCACTTGGGGAACTGTCAATTCCATGGCTCCATACCATCCTCTTTGGCCAATGCTTC
AAAATTGCTACAGCTTGATTTCCCTGTAAACAAATTCACTGGAAATATACTAAAGGGTTTTGATAACTTG
CAGAATTTGTTGTGGCTCAATGTTGGGAGTAACCATCTTGGATACGGTAAGAATGATGACCTTGACTTTG
TAAATTCTCTTACCAACTGCAGCAGTCTACAAATGCTCCATTTTGGAGACAACCAGTTTGTAGGTACATTA
CCTCATTCAACAGTCAACCTTTCTAGTCAGTTACAACGCCTACTCTTCTTCGGGAACAGAATTGGTGGAA
GCATACCCAGAGAGATCTCAAACCTGGTGAATTTGAATCTACTCGACATGAGCAACAATAATCTTACAGG

TAGCATTCCAGATTCTATTGGAAGACTTGCAAACTTGGGAAGTCTTGACTTGCGTAACAACCTCTTGACA
GGGGTAATCCCTTCTTCAATAGGCAACCTCACTGAACTCGTCTATCTTTATTTAGGATTTAACAGGTTAGA
GGGTAACATACCTTTAAGGCTTTAA
>PGSC0003DMG400010325 LRR-RLPs
ATGGTGGAACAAGAAGCAGTGTACAATGTTCTTGAATCCATTAACTCTGATGTCTCTTGGCGTTCCCTTTT
CCCTGATGATCTCTGTTCTTCTTCCCCACATGGGGTTATTTGTGATTACTTTGTTTCCACTACAAACTCATT
TACTCCTCATGTAACTGAGATTAGTCTTGGTTATGTTTCAGATTATGCTCCTAATCCACCTTGTACTCCTAA
TAGCACACTTAATGCTTCCCTTTTTACCCCTTTTTCTTATCTAAGAAAGATCTTTGTTTACAAATGTTTTACT
GAAAATGAGGTTTCTTTGATTGACTTTGGATCTTTGCCTTCTTCTTTGGAGGAGGTGGTTTTCTTGGAGA
ATCCAGGTCTTGTTGGTTCTTTAAGTGATAAAGTTGGAAACTTGATGAGTTTGAGGAGGTTGGTTTTGAG
TGGGAGTGGTGTTTATGGGGAAATCCCAAATGGAGTTGGTGGTTTGGTCAATTTAGAGCAGCTGACTTT
ATCAAGAAACAAGATTAGTGGTGAAATTCCAGTGGGGTTGATGACATTGAAGAAACTCAAGATTCTTGA
TTTGAGTCAAAATGAGCTTGAAAGTAGTGTTCTTGAATCTATAGGGAACTTTACTCAACTTTTGAAACTT
GACTTGAGTTACAACAAGCTTACTGGGAAAATCCCTCAAAGTTTGAAGGGTTTGAAGAGTTTGGAGTTT
TTGGACTTCAGTTACAATAACTTTTCCAATTCTGGGGTTCCATTGTTTTTGGCTGAGATGTCTAGTTTGAA
GGAAGTCTACTTAAGTGGAAACTATCTTGGAGGGGAAATTCCAGAAATATGGGAGAATCTTGAGGGTA
TTGTGGGGCTAGGATTATCAAAAACAGGACTAGTTGGGAACATTCCTGTTTCTATGGGGGTGTACTTGA
AAAACATATGTTATTTGGGGCTTGATAATAACAAGCTGGAAGGGGCATTGCCTGAAGAGTTTGGGGCAT
TGGAATATGTGAATGAGTTACATTTGGAGAACAATAATTTGAGTGGTAGACTTCCATTCTCTCCAAAATT
TGTATCCAAAGTTGGAGAAAAGTTGAAGTTGGGAGGCAATCCTCAACTCTGTGTAGATGAAGGCTTGAG
AATATCTAATGTTAGTGTTAGCTTGAGGCTACTAAAAATGTGCAACAATGCTATTAGTGCCAAATTTGTC
CCATTCTCTGGGGCATCTTTAGTACTGGTGCCTGTTTCCCATGTTGTCTTTTTTCTTGGAATTTTGTTTCTC
CTGTGCTAG
>PGSC0003DMG400010328 LRR-RLPs
TACAAACATTATCTTATGTAGAACCCATGGAGAAAAGACTGCGACTTTCCAACAAATTTGCTTTAAAAAA
GGTGTTTTTCTCAACCCCATATCTCTTTTCTCCAGTCTCTTGTCATTCTTGTGGTTTGATCAATTTTTTTGTT
TCTAGTGTCAAAAGGAGGTCTGCATTGCACAAGAAGATCGTGTCAGTCAGTTACCTGATGAAATTCTGG
TATATATATTGTCTTTCTTTTCTGTTAAGGAAGCTGCAGATACAAGTCTCCTCTCAAGGCGTTGGCTAAAC
TTATGGACATATATTGATCACCTTGATTTTGATGCTACAAAATACTTGGATCAAGTGTTCGTGCAACCCAA

GCTACAAAAAGGGCATATGGACAAGTATCTGACATGGGTCAACTCTGCTTTGCAAAAGTTTAAAGGCCA

GAGACTTGACCAATTTCGTGTTTACTATGATTTAAGCAAATTTGCTCAGCACGACATTGACAAATGGCTT

GAACTTGCCTTTTCTAAGAAAGTTCAAAGACTAGAGTTAGACTTATTAAGGTGTGGATCATTCACTCGTA

ATTTTGATTCCTGTTATACTTTTCCGGAGCGGCTCCTTGGTCTTGGAGATTCTTCTTCATGCTCTAATAACC

TCCAAATACTCTCACTTATCAACCAGAATTTCAAGTCTCTAAAAGTGCTTATATTGAAATCTGTAAATGTG

ACGGGCCAAGTTCTTGAGTTCTTTCTACGCAATTGTCCATTTCTTGAAAGAATGCAAGTTCATGGATCAG

GAACATTGAAAAATTTGGAAGTTGTTGGTCCGTCCCTCAAGTTGAGACACTTGGAGATATATAGTTGCTT

CAATGTAAAATCACTTAAAATTTGTGATACAAATCTTATATCATTAAGGACTTCAGCAGGAAAAGTATTA

CTGCTTAAGAATGTCCCGATGCTGGTTGATGTAGAGATTTTGGGTATGCCATACACCCATTTATTGGATG

ACATATCCTCTCGTCTCTTGTGCGTTCTCTCTCAGCTGGAGGTCCTTACAGTAGATGCTTGTATATCATTG

GTTAGTGAACAAAGTTTGTGTCTATCTACTCTTGTTTTACTAGCCTAGCACTAACTGGTGCCTTATCTGTA

GTTTGATTATCCGCACGAAGATTTGGAGCATTACAATTTTCCTGAACTTACGAAGCTCAAGAAATTTGTTT

TTATTACATTTGGAAATTCACTAAGAGTGTCAGGCCAGAGTCTCTTAGGTTGCACGTCTATAATAAGGGC

TGCCCCACAATTGAAGGAATTTGAACTACAGGTAAGTATCATGCCTTTTCCTCTTTGGTGTACAAAAGTCT

GATTTATTCATATTAGCAATTCTGGTTCATGCAGTTATTGATGCTACTTCTTTAATAAACTTAGTTATTTAA

GTCGTTGAGCTTGAGTTGAAACAATTGTGAAGAGCTAAACTTGAATAAATGGTACTCTTGCATTTTAACT

CATCTGACTGTGCCAATATAAAAGTCCTGCCCTTACATAGGTGTCATTTTAGATAGGTAGTAGACAGCTC

TGTCGTCCCTGAGCTTTGATTTATTATCTGTAACAAAGAGCTTAAAGTCTCATCATTTCATCAAAGAGCTT

AGAGCCTCATCATTCCTTCAGTTTCTTGAATATTTTTACTGAGTTACTGCACTTGTTTAGCGTAATGGAAT

GACCTGAGTGTTGGGATTGTGGTTTCAGTTGTCGATTGTGTTGCTCCAAAATCTTAGGTTTCCACCTGTTT

GTTGCCAGTATATTATATGATGGAATTGGGAAGTACTATACAGTGATTATTATGTGTTTAATAAAACTAC

TTTTTTGAGAAAAGGTAACTTGTATTATAAACACAGGAATACTCATGACGTATTCCAGTAATACTTACAAT

CAGAAATACAGAAGTGTTGCTGTTTTCCTATAAACTCAAGAACTCTAAGATACTAGGGATATCATCATTT

TCATGAGGATATTTTTTTTTTTTTTTTTTGATAACCGAGAAATCCGTCAGTGACCCGCCCTTTGGACCAATC

ATAGCCTTCTAAACTCGGTGGATAATGGGCCCGCCCCTCTACCCTTCTCCACTTAAATACGGGGCTTCAT

GAGGATATTTATGTTTACACCAAAAACAAGAGCTAAACAATTCAGCTTCATCTTCTCCATAGAGCAGCTC

TTGTTCTCAAAACATCTTTGATTCCTTTCCAGCCAAATAGTCCACCAAATGCAGGCTGGGACAATCTTCCA

TCTCTCCCTGTGCCCTGACTTATTTCCATCCCGATTCCAACATGCCAAAGTTTCCTTTATGTTTCCTGGCAT

AGACCAACTGATGCCCTTTTTATTGATGAAAATTTGCCGAAGTTTCAATGTCTCCTTAAAGTGTAAAAAG

AGATGATTTATTGTCTCTGTCTCACACTCACAAAAAAAACATCTGGAGCACAAATCGAGGCCCTTCTTCAT

ACGGTTATCCTGTGTGAGAACAGCCTGTTTTGCTAAGAGCCAAGTAAAACATGCCACCTTGAAGGGCAC

CTTAACTTTCCAGATCATTTTCCATGGCCAGTAGCTTGATTGAGTGTGTGTCCTCTGTGAGTTTCTGTAAG

CTGTTCCAACTGAGAATCTCTCCTGTGTATCTGGAGCCCATAACAGTTTGTCTTCACTATTACTGAGTTCC

TTGTATCGTTCCAGGATATTTAGAAATTCAACCATTCTATTCACCTCCCATTCATTTAAAGGTCTTCTGAAC

CTTAAATTCCAACCCTGAGCATCTTTTATAAACCTACTTTCTTGTCATAATTATACTGGTTGGTTTCAAATG

AGAACAACCAACTAAGAAAATTGAGAAGTAAGTTGTTGCCTAGCTAGGTAGATGGGTGAAGGAGGAAT

TTAGTGGGGTAGAGGTTTCAGAGATGAGGGATTACAAAATGTTGTTAGAACCGAGGGGAACTTCCAAA

TTCTAATAGTAGGATTCGAAGGTGGCATCCTGAATCCCAATCAAATGACTCCAGGCTCAAAGAGTCAGA

ATCGAGCCTGGATGGTATTGTAGAGGAGGCTTAGGGCTTCACATGGTATCTGATGTTCTCTCTCTTGAAT

ATTTATTTTTTCAAAAAATCAAGTAACGATTATATAAGGGAAAAGAGAAAAAATACCCGAACTTGTATCG

GATTTCTTGTTACACTTATTCTTTGAAGTTGTTCTATGAATTATTTTGTAGTAGAATAATACCATAGGAAG

TGTTGATGTGGCTTTTACTTTGCACCTCCATATAGTGCATGAAAAGACAAATACAAGCTAAATGATAGAG

GCTCTAGAGTCCAAAAGATATAATTTTTCTTCTACTTTTTAGGCCCTTTTCTAAATCTCGAGCGGTGATCC

ACCACTTGTCCCACCAGGCCACCATCCATGCCCCCCACCCCCGTGTCTGAGCCAGGATTTTTTTTTTGTAG

TTGAGTAGATACACAAATTATGTTTTTCCTACTCATGAGTGGGTGCACGCACTTGGCTAAAGTTGTGTGC

ATAATATTTTCAATAAAAATCAAGCAATAAAAGTAAAAAAAATTATCTAGGGTGTGTTTTGTATGGAGAA

AAATGTTTATCTATTTTCATGATTGGTTTGTCAATATATTTTGTGAAATATTTTTTCTAGGAAAACAAGCTC

CTTAAAAATGAGGAAATTTACTTTCCTAATGGAAGTAGAAAACATAAGTTCCAAAGCAATTAAATCATAT

TAGCATTTTATAACTATAAAAAACAATCATATAAATCACTATAGACATAGAGAAGTCAATAAAATAATTTT

AGTTACTAGTTAAATTAAATAAAAAACAATACATATCACAATAAAAGTAATAAAATAATAATTAAAATAA

AAGAATTACCGCGAAACATTATAAAAAAAACTATTGATAATCTCAACCACAAAAAAACTTGAAAGGAAA

AACACGAGTACGGAGCCAGAAGGGCAAAAAAATTGACCAAAAATTCCAAAAGGGTTTGAAATCAAAAG

GGCAACATTTAAACGGTAGAGGAGATGCTGTTTAAATTAGGGTAGCGCCTTACAAGGCGCAACTCCCTA

TGCGCCTTACAAGGCGCAAGAAAACAGAGCCACCATCATTGTTTATTTTTTCCATGTCAGTACTTACGGT

GGTACTTTTTGCACTACAAAATAGTATAGGGGTCGTAGGGACATCACAAAGAATAAGTGTGTAACTGGA

AAACTGAGATAAGTCCATAGGGTTTTTATGCCTTTTCCCACTTTGTTATTTAAAAAAGCACCAAGAGGGC

TCCAACCTTTACAAGTAGAAGATTCTGTTGTTCTGTCAACCTCCCAGTCATGCAATCTCCTTCTTAATCTAA

TATTAAAAGATAGCCTTTCTCTGTATTCTTCAATCACATCTTTTATATGGATAGCTATGAGATGTAATTCTG

AATAAGTTATTAAAGCTTCTTGATCCGGCGTACATGAAACTTTATCTTCTTCTTGCAACTAAAAGCTTTCA
CAAGATTAAAAGAATCAATTCCATAAGTTCTAATGTACTTTCAAACTGTGACATCATCAGTTGTATTGATT
AGTTTTGCTTTTGAACATCTCATATCGTTGCCATGCTCTATGTTTAACAGCTTAATCCATAAATTGTTTTGC
TCTACTGTGTTTTATCTCCACAAACTAATTCGACAATAGACACTTTTTTTAATACCCAAAGTTTTTATCTAT
GAACTACTCTTCTTAGAGAATATAGCTCTATTTTTATGCTCTTTGAATCAACTTGCCACATCTTTCTCCTTG
CATTATCGAACCGTCTTCGCACCTTAAAAAATGATAGATACAGTTTCTTGCGGGTTGCCAATCTTCTCAAC
CTTATGTATCACTCTCTGCCATATTATTGCACTTCTATATCTGGCATTAGAGACTTGCTCATGTATGTCTCT
GCCTGGATTCTCAATTTAAATGCAGTCAAGTGCTGTCTCAAAGTGTAATAGTAGAGATCTGTGATGCATG
ATCTTCCGCTCCTCAAGTCCAAATAGAAATAGGGTATAGTCTGCATATCGTGTACATGAAAAATTTCCAT
ATTTTCATTTGTACAACTTTCCCCAACTTTTGACTCCTTTTATCCATACTATATTCATGGCATAATGCAGCAT
CTTACTGAATGTTTCCATTGCTTAGGAAGAGAATGGGATAACGGGGTTCTCCTATTTAACTGCATATCCT
AATTATATTTTTCCATTTGTTTGCAAACCGTGTTGATTCAAAAGCTTCAGTAATATTGCCAGTTTACATGAT
TGTTCCACCGGGTCAAATCTTTGCTTGATCCTGTGTTGTCCCTAGTCTGAGGTGATAACTTGAGAATGAA
TCATTCATCGGTGCTCTCATAGTAAAGAAGGCTTCGCTCAAGGAGTGATAGATTTGGACGGAGTCTTGG
CTTTGATGCTAAGGAGACGAGATCCATCGATCTGAATAAGTCTAGTACACCTTTCTCAAGTCCAGCTAAC
AAATAATTCTTGCTACTTTAATTCTATTTAAGGCTCCAAGAATTCATTTTCTAATAACACTGGATCTATAGT
TTGTCCTTCCCTTTACAAATACATGCATTTTTTGACCTTGATATCTACTTGCCAATACTTGTTTCATCCTTGT
TGTTAGGACCCCACCGGTAGTAATCTCATCCTTTTACCTTGTTCTTAAGAACCAATGCTGTGAATATTGCA
GTCCAGCCTCTTGTAAAAATTTCTGTCTCATGAAAGAAGTGCAATGCACTGATAATGTCCCTTTCCAAAA
AGTTCCTAGATTTCTGGTAAGAAGACATTATCTGCCCATCTGGCCCTAAAGTTGTTATCACCACTAGACCA
TCTCCATTACTTCTCCGAGAAAATTTCTTAGTAGCCAACACTCTTCCCGTGATAATAGCTCAAGGTCATTG
TAACTGGAAGGCGCCATCATTGCTCGGTTTTACTAAATAACTTCTCATAGCATTACACTCATCTGATGACC
TTTGGATTTGGTAGTTCGTCCATCTCTTACAAGTTAGTTTATATGGCTGTACTTTTTATTTGTATTGATCAA
TTTACAGTAAAACACTTAGTATTTTGTAGGGGTGTCAAAAATGAGCCCACCTATAGGTAACCCGCCCAAC
CCGCCCATAATTTTAAGGGTTGGGCTTAAGATAATTTGAATTAGGTTTAATCTCAACCCCCATTTTAAGAG
AATCCTCAATTGAGCCCAATTTAATTTTCAATTTTCAACCCATTTTAAGACTCTTTATTTGTATTGATGTAA
TGTATGTTCTTATATTGAAGGTATGAATTACTTTTGTTTTATATTTTTTAGGATTTATCTATCCATTTGTAAC
TTTTTTTGTAATTTTCTTAAGTTGAAATCTAAAATGTGGTTATAAAACTTAAATAAACAATATATTAAAACT
ATTGAGATTAAGCGGGTAAAGACCCAACCATTTTTAAGCTCATTTGAGCCCAACCCATTTGAGCCCAAAG

TAAACTTGGGCGGGTCAAGACTCAACCCAATTTCTATTTCAACCCATTTTAATATCTCCAATTTCAACCAA
CCCGCCCATTTGACACCTCTAGTATCTTGTCTCATTCATCTAACCAGAAACACCTCAATTGTTGTTTCCAGG
ATGTTTTTTAGCATTGGTTATTTGTTCCAGTTCTACCAAGTGCTTCTTTTAGGCTTTTCGTTTTTCTATATCC
GTTCACCCTTTTCTGATCATACTTACCAATCCAGCAATCTGATTATGTATAATTTTTCGACATGCATTGTTG
TTTGAACTCAAAGTCTGCCTTTTAAGTTCAGTTTCCTTTCTATTAACCATAATCAGATTGCCAACACTATCA
TAAGATTGCTATTGCTGTTCCGCTAAATCAATGGATCCCTCTATAGCTTTTTTCATTTTCTCGAATTTGCAT
TAAGATTCGCCTATTCCCAAGTCTGCACTCTGCTGGAATGGGTGTATGATTGGTTATCATCATATGCTTTG
AAAACTTGAATATCACATTCCAAAGAAATTGTATACACAAATTTCTGTGTATTCCACCTCATAAGTTTACT
GTACATGCTATTGTATTTATACAAAACTAGGTCTAAATTCTGTACCAAAATAAATGAATTAAACAATGATT
ATTTGTACACTTGGCACCTAATTATTGGACTACTTTGTAACAAACTCTAACAGCCATTTTGCTCTTCATGTG
CCTTCCTTGTGCCTAGGTTGAGAATCTTCTAGACCTTGTACAACTGGTTAGGTTGAGAATCTTCTAGACCT
TCTGATTAGGTTTCCTAGAACTTTTGTTCTTTTTGGCACAAATCTTTTGGTTCTTGAAAAGAAATGTTGGA
GAAGGTTCATTTCGGTTAGCAAGTCCGGCATGCAGCTCGAATATGTTTATATGATAAGTCACTTATGTAT
TCTTTTGTTTCCTAATTTCTCCAAGTTAAGATTTTTCTCAGAGAGAGTCTAGAACATGGATAATGAGTAGT
GGTGATTGAATATTGTTGAATTAGTCAACTTGACTAGGAGGCATCTTACATGATAATGGGAAGTTTTCAT
TCAGTCGATCAAGGAAGCCATATATCCCGAATCTTAAAATTTAGATCCACAACCGATTTCGAGCAACTTA
GTCTAAAATAAGTCGACCCAAACATGTTGAAAGAAGTTGCAATTCTCTCAGAAAATTGAGCACTCCACGT
CTGTAATATTCTTGTCCATATTCCCCACTTTCCTTCCTCCGTGTGGTAAATACTTTAGGGAATTTGTAGAA
GAACAAAGTAGTAGGAGAAGTCAAATGACATGAGCGATTGATACAATCATGGTTCATCAGTGATTGGTA
CATAGTTTTATGTTATACTGATTCCCCAAAGTTTAATTCTTTTCATTTAACTTGTGTCCCAAGAAAAAGGCC
TGTTCAAAATTTTCTTTTATTAGCTTTACTGACTTATCTTATCATTATTTTTTATTTTAATTTTAATTTGTGTT
GTATATATTTACGGCAGGTGATGTCAATCCCCTTCGAGGCAAAGAAAGAGTGTAAGAAAGCAGCCATAA
AATGCCCACTCCATCACCTCAAAGTGGTCAAATTTTCGGGGTTTTATACAGGCTTATTTCAACTTGAACTC
GTTAGGTACTTTTTGGAAAATGGTGTTGCTCTTGAGAAAATCATTATTGATCCAGGTACTCCATTTTTTTA
TCGTATCCCTCCGTGCCCTAGCGAAATCAAACACACGCAAGCTTCGAGAAATCTTGCTAAGCTCCAGCTT
GAATGTGAAGTGCCTCCGGCTATTGAGTTGGTCATACTTTAATGAAAATAATAATGTTATTAGGGTTAAA
TTTGTGATACATATCGGAGGTGAATTTTTATTTCTTAGTAGATGTTCCTCATGCTTTGTTTCATTATTGAGT
GATATTTTCACTTTTTGCGAGGTTTATATTGCTATATGAAATATGGGGGATTTTCAAAATGAAAATGTCGC
AGTTATTCCGCCA

>PGSC0003DMG400010447 LRR-RLPs

CAACTTGCACTTTCCAAGTCTAAAAAGCAGACATCACATTTAACATTTATAATTTCAGAAGCCACCAAGA
ATTTGTCATCCAAATGTATGTATATATTATGCCATTTTTTGGCACATTCTTTTCTTATAATTTCACAAAAAT
GAGAGGGCTGTACTACATAGTACTTGTCCTGTTTACTATAAAGCTTTGTTCTTGCAGTGGAATTTGCAAA
GAAAATGAGAAAACAGCTTTGCTAAGGTTAAAGAAAGAAGCAAATGATCCCACAAATGTACTATCTTCT
TGGGTTGATAAAGAAGATTGTTGCAACTGGGAAGGAGTTTTGTGCCACAATGTGACTGGTTCTGTCATT
GAGCTATCAATTAACGGATGGGATTGGTCAGAACTTAAAGGCCTAAAGATTGATGATTTCCAGTGGTTG
TCAAGTCTTGAAAATCTTGATAGTTTGGACATGTCTGGTGTGGATTTAAGTGAAGCAACAAACTGGACT
GAGGTGATTAGCATGCTTCCTTCTTTGGTTAATCTTCGTTTTTCGAATTGTAGCCTTCATTCAATACCTCCT
TTGTTTGATCATAACAGTTCTGTTCTTGAAAATCTTGATCTTTCTTTGAATAACTTTGGCTCTCCGATTCCC
GGGTGGGTTTTCAGTTTTGGTAGTCTTGTTTCTCTTGAATTCACTGGAAGTAATTTCACTGGTTCATTTCC
TGAAGGTCCTTTTAACTTGACTTCTTTCACAACATTGAGAGCTTCTAGCAACTCCTTTGGTTCTGTTTTGCC
TCAATGGTTATTTGATCTTAGTAATCTTGAATATCTTGATTTGAGTTTTAGTGGTCTTGAGGGTCCAATAC
CAAATGGAGTTGGTGATTTTACCAAGCTTACATATCTTAGTCTTGCTTCCAATAATCTGAACTCCACTATC
CCAAATGGCTCTATGGATGCAAGAATCTTGAAACACTTATCATGAAGGATAACAGTCTTGAGGGAACAG
TTTCAGACTCGATTTCAAATCTGACATCTCTAACTAGTATCGATATGTCTGAAAATTTTCTTTCTGGAAAG
TTACCGAGTGTAATTGGAAAGCTATGGAAATTAGAAGATCTCGATCTCTCAGAAAATCGATTTGAAGGA
GAAGTATATGAGGTTTTCAATACTATGAATGATTTTCCTCCAATAGGCTCAGGAAATTGTTCTTCTTTGAG
GACCTTAAGACTAAATGACAACAAACTGATTGGAAATCTTCCAAAAAGTTTTGGGCAGCTTCCAAATCTA
CAATTCTGTTTCATGTCCAATAACAGGTTGGAAGGGGTGTTAACAGAAGAACATTTCACCAACTTGACAA
ATTTGAAGTTTTTCTCTGCATCTAAGAGTAATTTGACTTTAAGGGTGAGTTCAGATTGGATTCCATCATTC
CAAGCTAGTGACATAGTAATTGGCGGTTGGCGCTTAGGCCCTGACTTCCCTTTGTGGATCCAGACTCAAC
AATCAATCATGAATTTGGACATATCAAATGCTGGTATAGTAGGTGAGGTTCCAACTTGGTTCTGGAACTT
ATCGTCTCAAATTTGTTTCCTCAACATGTCTCACAACCAGCTCATTGGTGAGATTCCAACTTTCTCAATTTC
TGATAGCTCCATGGGAAGTGGAGGTCCTTGGTTGATATACTTGAGTTCCAACAACTTCAGTGGTTTACTC
CCACATATTCCAACCATGGTAACAGAGTTAGACCTTTCAGATAACACTTTCTCGGAGGGTTTAACAAGCT
TCTTGTGTGATAACAAGAATGAATCTTACATGTTGGAGATTCTACACCTTGGGGGAAATCGTTTCTCCGA
AGAAATTCCTGATTGTTGGATGAATTGGCCAGAGTTGAGAGTTGTCAACTTGGGAGAGAATAAGCTTAC
CGGAGGAATCCCAAGATCCATTGGAGCTTTAAGCAATTTGAAATCTTTGGACTTGAAAAGAAACAAACT

TTCTGGTCCATTGCCTTCATCCTTGAACAACTGCACAAGATTGTGGAAAATAGACATGAATGAGAATGAG
TTAGATGGAAATTTACCACCTTGGTTTGGTACACTTTCAAAGCTGATAGTCCTTAGTCTTCGGTCTAATAA
ATTCGATGGTGAACTAGCTCCAGAACTTTGCAAGCTCAATGAACTTCAAATCTTGGATCTTGCCAACAAC
AATTTGTTTGGAGCTATACCTCGGTGTGTTAATAATTTCACAGCAATGGTGAATGGGAGGAAGGAAATT
AGAGATGGGGACGACGAGATGGATTATTCTTATTATGTTGGAGTTTTCAGGGAAAGTGCAAGAGTAGC
AACTAAGGGCAACATGTATCAGTATGATACCATTTTGTCATTGTTTACTAGCATGGATCTTTCTAACAATA
ATCTTTCTGGAAACATTCCTATGAGTCTCACAAGTCTTGTTGGATTGAGATCATTAAATTTCTCCCACAAT
CAGCTGACTAGTAGTATTCCCAGGGACATTGACAAGATGAAGGTATTAGAATCACTTGATATCTCGAAT
AATCAACTCTCTGGTGAAATACCACAAAGTATTTCGAGTTTGTCCTCTTTGAGCTACTTGAATGTATCTCA
CAACAACTTATCAGGGCAGATTCCTGTGAGCACTCAACTTCAAAGCTTTAGCCCTTTTAGTTTTACTGGAA
ATAAACTGTGTGGGCTTCCACTCGTGAAGCGTTGCAGTAAAAATCCTGAGGTTGAAAATGAAGAAGAA
GAAGATGATGATAATGAAGTGGAATGGTTCTACGTTTCCATGGCAATAGGATTTATTGTGGCTTTTTGG
GGTGTAAGTGCTCCTTTGCTTTTCATAAGACCATGGAGACATGCCTATTATGGGTTTTTAGATAGAAAAT
GGAAATCATGGCACACATAATTTGTAAAGATAAGACATTAGGGAGATTTCTATTTATGTATACTGGATGA
AATTTGTTTATATCTCATGAAAATCCATCACCATTCGGCGTTCTTTGAATTAAGACATTTTTATATTTGTTG
TCTCCATACAAATATGAGAAATTTGACAATTTTGATTTGGCAAGGTTTGAGTTATGGAATGAACAAAGGA
AATTAACAGTTCCT
>PGSC0003DMG400010519 LRR-RLPs
TCAGTTCAAGTCCTCGAGACATTTCAAATCAACATGTCTTGGATGACCGTCTGCAGCTAAACCATAACGA
AAATCGTATGGTGCTCCAAACAGATTTTTTCCATTTCTATAGCCTACTTCTTCCAAAGCCTTCACAAGTGG
CTCCATATATGCTGTAATTTTCCTAAACATGTTAAAAGTAAGTTACTACAAGGAATTTCTGGATGAGTTAT
TGGCTGAAACAAACTGAATTTCTTGAATCATACGTTACTAGTATGGCGTTGTAAGATAACACCATACTGG
CAACATATGACATTTTGATCAATCCAAGGCATATTTTTTTTCTTTTTTTGTAATGGTATCAGGCTAACTTGT
ATGTGCTTCAACTGGATAACAAGATTGTGCATTTTCAACACTAAGAATAATAAATAATCTAGAAGGTGAT
AGACTAGAGGGGTTCTTGACATCATCTATCGGGAACTTAACAGAACTTGTCTCTCTGAATCTAGGCAGG
AACAAGTTTAGAGGACCAGTGCCAGAGAGTATTACTTATTTAAGGAAACTTACAAGTCTTCAACTTTCTG
AGAATTTTTTCTCTAGTAGTCTTGTTAATGACATTGGTGTCCTTACAAAACTTGAAATTCTTGATGTCGCG
AATAATAAGCTATCTGGTTCAATCCCTTCATCGACTACATCACTTAGAAGTTTGACGCGATTAGACCTGTC
TAACAATGAATTGACCGGGAAAATTCCTCAACTCAACGGTTTATGGAAACTCCATTCATTTGACATTTCCA

ATAACCAAATTTAAGGGAATTTGCCACAACTTCCATCGAAGATCAAGACATTGTCACTAAGCCACAACTT
GCTATCAGGACACATTACCACAGTGCATAAACTTAGACATCTAAGAACATTAGACCTTAGTGAAAACAG
ATTTTCAGGAGGAATAAACAAAGGGATCTTCATGTTACTTGAATTAAGTCACGTAAGCATTTCAGTAAAC
CGGTTCACAGTGTTTGAGGTGGTAGAATTCTCTGATAAAGAATCACAGCTTCACACGTTAGACGTAAAT
GCCAATCGTCTACATGGTCATTTGCCTGTGAACTTGGATACTTACCCAAACTTAACAGAAATAAACCTTG
GGCATAACCTGTTTTCGGGTGAGATTCCATCGGAATATTGGTCAAGACTCAGGTTTTCATGGAGAAGTCT
CAACTTGGAATATAATAACCTGGAGGGATCGGTTCCTCGAGAGCTCAACAGGACTTTAGAAGGGGTTCA
GGGAAGTTTTGAACACAACTGTCTCATTTGTCCAAAGGGCTTTCAGCTATGTCATGGACAGAGGACAGC
TTCAGAATGTCATGGAGGAGACCATAATTAGCTGAGAATGTTTTAAAAATTACATTGCATTTTTTTTAATG
TTTTCCCAGTTTCCAATATAGTTTATAAGTTGCATTTTTTTTTTATGTTTTCGTAGTTTCCAATATAGTAGAA
GTTCCTTAAATTTGTCAGTATATTTTATTTAGATACTTGAACTACAACATGTTTCCTATTAAGCACCTGACA
TGATGAACTGTGTTAGAGTTTTGCCCTGAATGTATGATATTATCAGTTATAGCAAAGTATATTTGCATAAC
AATATTGAACAAGAGTATCAAAATTGGTTTAAGTATACGACATGAGCAAGAGTATCAAAATTAACTACT
GAAGCAACATGTAAGCCAACGTTTTAAGTGCGAGCTTGTTTTAAGTTCAACTATAAGTGAGCTTGTTTAC
TTTGTTGGTTCCAAGATTAGATAAATTCTCATAATTTATGGTGAAGCAAGGTTGCTGCAAAGAAAAGTGA
TTCACTCTCAACTACCTTACTAACATAAAGTCATTCTTTATTTTCTTAAACTCCATGTCAACTCAAAATACA
TCAAACAAATTTTAAACGGAGGAAGTACCATACTACTAGTAACTGGAAAAATCAAAACAAGCTATAATG
AAAGTTGTTTTAATTGCTAAAGAAGCTCATGAAGACAATGACATTACAACTCATTTCTGTTGCCTAGTCTA
ATAAAAACAATATTTGCCACCTAAAGATATATTTTAGCAGAATTGCATGTCTAAGAACTTAAGTACCTTTT
TCAGGGGAAGATGCTGGGAAGAGTCCTTTACAAGCATCTGTATTCCACAAACATGAAATCAAAAGAGCA
AGTCAGGCCCGAGACTACAAGCTGCTGTATTTCAAAGCATCTCAATGGATTAGAGAAAAACCTTGACCC
ACGAGTGTTAAGCTAGTAATATTATAGCTATTGACAGAAGGCTTGAAAATTGTACTACTTATGTAATGAT
ATGTGTAATTCATTGTTATTTCATACTCGTTCATGAGTTTTTGAAGTTGAGAAAGAGATTCTGAAAGTAAC
TAGTGACTGAAGATTTGAGAATAGCAATTCACACATTTTTTAAGAGCGGGAATTGTGTACAAAAATGTA
CTATTTCTTCTAAAGTAACGTCTGCATTTTGGAAGAAAATCTCACCTCCTATATGTGTCCGAGTGTTCCTTT
GCTGTCAAATGGGGGCATCCCGTACAGAAACTCAAAGCATAGGACACCAAGGCTCTAAATGTCCACACT
TGCATCATGCTCCACACTTTCCACTGCATGTTCAACAATTAACATATCAGAAAAAATGTAAAAACAGATTA
ACAAAATGTTAAAAGCTAAACTCTCATACACGCCCATGTGAAGGTAAAAAGCAATGTATAACATTCACAC
ATAGTCCGCCTTCGATTAAAGGTATGCACTGACCACCCAAAGTCTGCCGTTTTGAGTTCACCCTAAAAGC

AGAAATATTAAAGCTGTCATTTTACTGCAATGCATTGTATATACTGTTTAGCAATGCTAAGTTTGTACCTC
AATATCTTCTCTGTGTATTGTGTGATTCCCATGACAGTATATTGAAAGTTCGTAAGAATTGAGAAGAAGC
AATGCAGAAGAGGCAACGACAAGTTACCGGGTCATAAAAGTAACCATAAGTATTTCAACTTCACGACGA
AGCTGATGTTCAACCAGGGACTGTTTTAGCTGGCTCTTGAACAACACTTTTAACGCGACGATATGATTGC
TATGTTTCCAAGAAACAACTAACCTTAGTATTGGAATGTGATTGTAGAATGTTAAGTCTATCGAATAGAA
TTCGAAAAATCTTTTAAGCCATGTCCACAAGATATCATCCCCAACCTCCCACCAATCCATAGCAATTATAA
CAAATATGTTGAATGAGAGATTCTCACTCGCAGTGCATAAATCCGAGGCATTGAAAAAGTAAGTAGGTT
TATCAGCCATTCTTAACATGTAAAGGTTTAAAGGACTTACATACATGACCGAACTTTCCTCATCCAAGAG
GCTTCCCAATGTTGAAATCATTAAGTGTCCGCCTCTTTTTATTAGCCTCTGATGAAGTCTGCAGAAGTTGA
AAATTCTATAGTCCATTTAAAAGATCAGTTAATTAAAAATAACAATAAATTCTTGTTGTTGTTGTTGTGTA
CAGTATAACAGATCAGTTAATTAAAAATAACAATAACTTGTTCTTTTAATTTCATGATAACATATGGTTAT
TTCACATCACAAAGTTGTACGATCCAGCTGAGCAAGAATCAAGATGCTCTTTTGATTCCATTTAGTGCGG
GAAGAAGATATGTCAAGGAAGACAATTTGCTTCAAAAGCTCCAAGAATCAGGCTTAAATGCATTGGTAG
AAGATTATCTCAATTAACTGCACCTCTGTTCATACAGTCCTCTTAGAGGACTTCATGAACAAGCAATCAAC
CACAAGTGATCGCAACTAACATACAAGCTTAAACTCAATCAACAATGTGTACAACAAATTTTAACTAACC
TCAACATTCTTCCTTTAGCAAGGTGGTACGCTTGTGAAACTATCAAGTACAACTCAACTCAATCTACATAC
TAATTTATTATAAGCTCAAAAATAACCAAACACAGTGAGGTCAGAGCATGTTACTAGAAATTTTATTTTTC
TTTACGACAGAAACAAACATTGTTG
>PGSC0003DMG400010523 LRR-RLPs
GTTCTTTAGTGGTGACTCTCAATGAAGTACCTACTTTCAGCTTACTCTCTCTATACTCACTACTACTGCTAC
TCAGTACTGATTCCTTTCACACATACTGTGCCTGTAAACCCTGTCCAGGGACCCCCATTTCCCCTTTCCCCT
TTCCCCTTTCTCCTTCCTTCCTTAGCTATCTCTCACACAAACACTAATCTTTTTTCACCTCTACCTTACCTCCA
TGGCAGAATCAGTTCTTGAACCTTGTACAACCTCTTATTCCTTCAAAGTTTCAATCTTTATCCTATTCTTCTT
GATTTTCCCTTTCTTGAACCCATTTTCATCTGCATTTCCTCTTTCTTTTGATACTAATGCAACTGAGGCTGTC
AATCTTGAAACAGAAGAGGACATGGGTTTGCTTTTGTTCTTCAAGTTACAGTTTCGAGAAACCCCTTTAC
CAAGCTGGGATGTCAATGTTCCTCTATCAAACTGGACTGGTGTTACCCGGTCTAACCAGACCGGACGGG
TCACTGGACTTAACCTCACAAGGTTTAACTTGTCAGGACAGGTTCATCCTTGTTTGTGTAATCTTACTTTT
CTTGAAACCCTTGTGTTGTCTCATAATAGCTTTAACAATTCAATACCTTCTTGTTTATGGAAGTTGTGGAG
CCTTAAGACCTTAGATCTTAGCTATAATATGCTTACTCTTCTTATTCCTAGTACATTTGCAACAACTATGAG

TAAGTTAATTGAGCTTGACCTTAGTCATAACATGTTGAGTGATGAAATCCCAATGTGGATAGGGAATGTC
TCAATGTCACTTGAAAAACTTAACTTAGGGTTTAATAGTTTTCATGGGGATATACCTAAGAGCTTGTTGA
ATTTGATGTCTTTGAAGTATCTTGACTTGTCTCACAATAGTTTGATGGGAAATGTGGGTGATTTTAACCAA
GAATTGGTCTCACTTAATCTTGAGTCTAATTTGTTATCGGGTACTTTGCCTTGTTTATATTCGTCAAGGGA
ATCACTTACACTTCTTAATTTAGCAAACAATTCGATTCTTGGAGGTATACCAACGTGTATCTCGAGTCTTG
GGGGTTTGACACAGCTCAACTTGTCACGTAATGAATTACGATATGGTATCTCGCCTAGACTGGTTTTTTC
AGAGAGGTTATGTTTGTTGGACTTGAGTTATAATGAGCTATCAGGGAAGATTCCAAGTAGGATTGTTGA
GGCATCGGACAAGTCTGGACTTCTACTTCTTGACCTGTCTCACAATCAGTTCTCTGGTAATATTCCTGTAA
CGATAACAGAATTGAAGAGCTTGCAAGCATTGTTTCTGTCTTATAATCTTCTTGTGGGCGAAATACCAGA
AAGGATTGGTAATTTGACCTATCTACAGGTGATTGATCTCTCACATAACTTCCTCACTGGCTCGATTCCTT
TGAACATCGTAGGATGTTTCCAACTACTGGTGCTGATACTAAACAGTAATAATCTTTCTGGGGAAATTCA
GCCAGTGCTTGATGCGTTGGATAGTCTTAAGATATTTGATATAGGAAACAACAAGATTTCTGGTGAGAT
CCCACTGACATTGGCAGGCTGCAAGTCGTTGGAAGTTGTTGACTTGAGCTCTAACAATCTCTCAGGGTCT
CTAAATGATGCAATAACCAAATGGTCGAACCTCAAATTCCTCTCCCTTGCTCGGAACAAGTTCAGTGGAT
CTCTGCCAAGTTGGTTGTTTACATTTCAGGCTATTCATACTCTGGATTTTTCTGGAAACAAGTTCTCGGGA
TATATACCAGATGGTAACTTTAACACTAGTCCAAATTTCTACAACGGCGACATTAGGAAAACCATTCCTG
CAGTACCATCAATTTCAGCTCGAAGCCTGGATATCAAACTTTCACTCATTGCTGATGAAACTAGTTTGAG
CTTCAACTATAACCTGACAACCACAATTGGAATTGATCTGTCTGACAATTTGCTTCATGGTGAAATTCCAG
AGGGTCTGTTCGGATTACATGGTTTGGAGTACCTTAATTTGTCATACAATTTTCTTAATGGTCCAGTTCCA
GGGAGTTTAGGGAAGTTGCAGAAGCTAAAAGCACTTGATTTATCACATAATTCTTTATCTGGCCACATCC
CTGAAAACATTACTGTCCTCAGAAATTTGACAGTTTTAAATCTGTCTTATAATTGTTTCTCTGGTGTTATTC
CGACAAAGCGAGGTTATTGGAAATTTCCTGGAGCATTTGCTGGGAATCCAGACTTATGTATGGAATCAT
CTGGTAATGTCTGTCAAAGAACTTTGCCTGTAGAGCCAGGGAAGAAATTTGAAGAGGAAATGGAAGAG
GGACCATTATCAGTTTGGATTTTCTGTATAAGTGCTTTAGTTAGCTTCTATGTTGGCATTGTTGTTTTATTT
TGTTCATCTCGAACAAGAAGCTGTATTCTGCAAACAAAAAGTTTAGCAGGTTGAAAGTGATAAGTGATC
ACATTGTACAGCAACTTATGAATTGTATCATATAGCAAAACTGCTTCAAGCATATCTTGTTTACTAATCAG
CCCTTCATTTTTTTTAACTAGTTAACAATGCTTCACGTTG
>PGSC0003DMG400010700 LRR-RLPs
ATGGGCATTGGAAAACTTGCATTGTTTCTGCTATGCGCGTTTCTCTGTCAACTTGTTTTCTCCTCATCTTTA

CCTCATTTGTGCCTCAATGATCAAGCCCTTGCTCTTCTTCAATTCAAAAACACGTTTACTATAAATCTTGAT
GCTTCTTATAGTGGTTATTATCCTAAAACAGTTAAATGGAATAAGAGCGCAGATTGTTGTTCATGGGATG
GAGTTAAGTGTGACGAGATGACAGGACAAGTGATTCAGCTTGATCTCAGTCGCAGCCAACTTCGAGGC
GAGTTTCATTCCAACAGTAGCCTCTTTAAACTCGTTCATTTCACCTAAATTTGGTGAGCTTTCTAGTTTGAC
TCATCTTGATTTGTCATATTCAACTTTTACAGGTATAATGCCAGCAGAAATATCTCATCTTTCTAAATTGCA
CGTTCTTAGTATCGAGAGTGATCCATATGAGCTTAGATTCGAACCTCACAATTTTGAACTGCTCCTTAAGA
ACTTGACCCGATTAAGAGTGCTCCACCTTAGCTCTGTGAACATCTCTTCAACCATTCCTCTTAAATTCTCTT
CTTATTTAACAAATCTAGGACTTTCAGACACGCAGATATACGGGATATTGCCCGAAGGAGTTTTCCACCT
TTCCAACTTGGAATCTCTTGATTTATCAGACAATCCCCAGCTCACTGTTAGGTTTCCCACAACCAAATGGA
ATAGCAGTGCATCACTTATGAAGTTATATCTCAGTGGTGTGAATGCTACTGGTACGATACCAGAATCATT
TGGTCATCTAACTTCACTGCATAGATTGAGAATAGCTTCTTGTAATCTCTCAGGGTCTATTCCTAAACCTC
TCTGGAATCTCACCAACATAGAGGTTTTGGACCTTGGTTATAACCATCTTGAAGGACCAATTTCTGATTTC
TTTAGATTTGGAAAGATCAGGTCGTTATCACTTAGAAATAACAACTTTAATGGCCAACTTGAGTTTTTATC
CTTTAACAGATGGACGCAACTTGGATTATTAGATTGTTCAGCCAATTCCCTAACAGGTCCCATTCCTTCTA
ATGTAAGTGGTATGCAAAACCTATATTCACTCTTCTTGTCATCAAACCTCTTGAATGGGACTATACCTTCC
TGGATATTCTCCCTCCCTTCAATAGGGATTTTAGACTTGAGTGATAACCATTTAAGTGAAAATATTCAGG
AGTTCTATTCCGAATCATTGTATCGAGTTTCTCTAAAACAAAATCAGCTGCAAGGTCCTATTCCAAAGTCA
CTCTTAGATCTGCAGGGCCTACAATTTCTTCTCCTTTCACGAAATAATCTCAGTGGACAGATTGCTTCAAC
CATCTGCAATCTGACAAAACTGTTAGTGCTAGATTTGGGCAGCAATAATTTGGAGGGAACAATCCCACT
ATGTTTGGGTGAGATGAGTGGACTTTGGATTTTGGATTTAAGCAACAATAGTCTTAGCGGGAAAATTAA
TACAACTTTTAGTATAGGAAACCAACTTATAGTCATTAAATTTGACGGGAATAAGCTAGAGGGGAAAGT
TCCGCAATCTTTGATCAATTGCACATATTTGGAAGTTGTTGATTTAGGTAACAATGAGTTGAGTGACACA
TTTCCTAAATGGTTGGGAGCCCTACCTTATTTGCAGATTTTGATTTTGAGATCAAATAAGTTCTATGGCCC
TATAAAAGATTCAAGGACTGATAACTTGTTTGCTCAAATTCGAGTCATAGATCTCTCAGCCAATGGATTT
AGTGGAGATTTACCAGTGAGTCCTTTTTTGAGAATTTTGAAGCCATGAAAATGATTGGATGAGAGCAGT
GGAACCCGAGAGTATGTAGGAAATATATATTCTTATTATTACACGAATTCTTTGACAGTGACAACAAAGG
GACTGGATCTTGAACTTCCTCGAGTTTTGACTACAGACATGATTATGGATCTCTCAAGGAATAGATTTGA
AGGTCATATCCCAAGCATTATTGGAGATCTCGTTGGGCTTCGTACGTTAAACTTATCTCATAATGTCTTGG
AAGGTGATATACCAGCATCACTGCACCAGTTATCTGTACTTGAATCATTGGATCTCTCATCCAACAAAATC

AGTGGAGAAATTCCACAACAGCTTGCATCCCTCAAATCACTTGAAGTCTTAAATCTCTCTCACAATCATCT
TGTTGGATGCATCCCGAAAGGAAACAATTTGATACGTTTGAGAACAGTTCATACCAAGGAAATGATGGG
TTACGTGGATTTTCACTCTCAAAGGATTGTGGTGGTGATGATGGGGTAGCACAAACGACGAATCCAGTT
GAGCTAGATGAAGAAGGAGGAAGAGATTCATCAATGATCAGTTGGCAGGCGGTTCTCATGGGTTATGG
TTGTGGACTTGTTATTGGACTGTCCATAATATACATAATGTTGTCAACTCAATATCCAGCATGGTTTTCGA
GAATGGATGTAGAATTGGAACACAAAATTCTTACGAGAATGAAAAGGCATAAGAAAAGATATTAGTGT
GTTACCTCCA
>PGSC0003DMG400010940 LRR-RLPs
ATGAACTCAGTGGTGAAATGTGGAGAGGGCCATGGTATGTACCCGAACTCAGTCTTAAATCTCACGAAC
AATAGCCTCACAGGTATGATCCCTCCTTCTGTTGGAAATGCCACAAAAATGATGAGTTTCAGTTTGTCTG
GGAATAGAGTCAGCGGCAACATTCCAAAGGAGGTCAGTAATCTGAGCCAGCTTGCATATTTGCCCTTGT
TCGATAATCAATTAACAGGTTCCATTCCCGCAACACTGTTTAATATCTCGTCGCTACTTGCTGTATCTCTGT
CAATCAATAGCCTTTCTGGTCCTCTCATGCTAGATGAAGGGAATATTGTGTCCAATCTGTACTTTTTAAGT
ATATCTTACAACCAAATATCTGGTTGCATTCCTTCCAACATATGCCAACTCACAGAGCTCAAAGTTTTGTC
CATATCTTTCAACAATATAACTGGAGACATACCCAGAAATATTGGTTGTTTATCCAAGCTCGATGGGTTTT
ATATTGGCCATAATCCAATAAAAGGGACTATTCCCACTTCATTGGGAAATATTTCCACTCTGCATGATGTT
TACTGTCGAAACAATCTCATAGTGGGGGGAATTCCTCCGGAATTAGGAAAGCTATCAAATTTGAGGCAA
TTAACCTTTGACAATAATTCTAATCTTATTGGTCAAATTCCAAAGGCTATTTTCAACATATCTTCTTTGGAA
GTGATTTCTTTCCATTACAACAACCTCTTGGGGAGAATTCCAACCACTACAGGTCTTCATCTTCCGAACCT
TAAAGAACTTGACTTGGCAGTGAATCAGCTTGAAGGGGAAATTCCATTGTTCATAACAAATGCTTCCAA
GCTTGAAATGCTGGAGCTAAATAATAACTTTCTGAAAGGAACTATTCCTACTAATTTAAGAAATCTTCGT
GAGCTGCGATATCTGCTCCTACATACTAATCAACTTACCAATGAACCAAGAGACTGTGAGTTGTTATTCTT
CAATTCTTTGGCGGACGGTAAGATGTTGCGATATTTACAAGTGGGTAACAATCCGTTGAATGGCATTCTG
CCCAATTCTATTGGGAATCTTTGATCTACTATTCAAAACATTGATATAGGAGATGCTCACATCAACGGCCC
CATCCCCACAAGTATAGGCAACATGAGCGGTCTTACAGCCATAGACATTGAAGGAAACAACTTAACAGG
GAGTATTCCTTCTGAGATTGGTAAGCTTAAACAACTCCAAGGTCTGTATCTATATAATAATAAATTGCAG
GGACATATTCCAGAGGCCGTATGCCATTTATCTAATTTGGTACAACTATTTCTGAATGGTAATGAGCTCTC
TGGATTAATTCCAGAATGCTTAGGAAATCTTAGCATGCTACAAAAGCTTTATTTGAATTCTAACAAATTTT
CATCTAAATTTCCGTTGAGCCTTTGGAAGATGAGTGGTCTCCTCTATCTAAGAGTGTCACGAAATTCAATT

GAGGGAGAATTTCCATTAGATATTGGAGGACTGAAAGCCATTGTAGAACTACATCTTACCAGTAACCAC
TTTTCAGGAATGATACCAAGCACATTCGGGGAACTCCAAAACCTGCAGTCTCTTGACCTATCGAACAATT
CATTTTTTGGCCAAATTCCATTATCCTTTGCCAACTTGATAAGCTTAGAATACTTGGATTTGTCTTCAAATG
CGTTGTCAGGTACTATTCCTAAGTCTTTGGAAAAACTCTCATACCTTAAAAGCATTAATGTTTCATTTAAT
GATTTAGAAGGTCAAATACCGAGTGGTGGTGTGTTTGCAAATTCCACTTTGCAATCATTTCTCAAGAACA
AAGGTCTTTGTGGAATGCACATATTGGAGATTCCTGCTTGTGCTATCACTAATCCTGGAAAACAATCAAA
GCTTAAAGAGGTTCTGCTAAAAATTGTTACTCCAGTGGTTACTTCATCCTTTCTGATATTCTTGTTCGTTTC
AATTTGGATAATAAAACGACAGAAGAAAGGGAAGTCCAAAGATGTGGAGAAGGTACCGGAGATCGGG
ACTCATCAATTAGTTTCTTATCACGAGATTCAACGAGCAACAAATAATTTTGATGAATCCAATTTAATTGG
TGAGGGAAGCTCCAGCTCTGTGTACAAAGGCACATTGTTTGGTGGAACTGCAGTGGCCATAAAGGTTCT
CGATTTGGAAATGAGCAGGTATGCAAAAGGTTTAATACCGAATGCGAAGTGA
>PGSC0003DMG400010950 LRR-RLPs
CATTTTTCTATTAATATTTGCACTAATGGAGAAAGCCTTCACATTTTTCCTCTTGACAATACTCTTGTTGCT
TTCACTATGTTATGACCCAAACCAACATTACTACTGATCAATTAGCTCTTCTCTCTTTGAAATCCCAAATTA
GTTCAGACCTTTTCATTATTTGGATGAAAGTTGGTCTCCTGCTATTAATGTTTGTCAGTGGGTAGGAGTC
ACTTGTGACTACCGTCACCAGAGAGTGAAGTCCTTGAATCTATCCAGAATGGCTCTTACCGACAGGACTC
CTCGTGATTTTGGAAACCTCACATTTCTTGTTTCTCTTGATTTGCGAAGGAACAATTTCCATGGAAATTTG
CCTCAAGAAATGGTACGCTTACGTCGGCTTAAATTTCTTAAGTTAAGTTTCAACAACTTGAGAGGGGAAA
TTCCTTCTTGGTTTGGGTTTTTACACCAACTTCAAGTTCTAAATCTTGGAAATAATAGTTTCACTGGTTCCA
TTCCTCCGTCACTCTCAAATGCCTCAAGGTTGGAGACTTTAGAAATATCTGCCAATTTACTTGAAGGAAA
CATTCCGGAAGAGATTGGCAATCTTCAGAACCTGAAATTCTTGTCCATACAATATAATAAGCTTACGGGT
TCTATACCATTCACAATTTTCAATATTTCTAGTATCCAAGTCATTGCATTTACAAACAATAGCTTATCAGGA
AATCTTCCCAATGGTTTATGCAATGGTCTCCCGACACTCAAAAAGCTTTATCTATCAACAAACAAACTTCA
TGGTCATATGCCTACAAGCTTGTCAAATTGTTCACAACTTCAAATATTGTCTCTATCCGGAAATAATTTTG
ATGGACCAATACATAGTGAAATTGGAAGAGTGAGTAACTTGCATATATTGTATCTCGGATCCAACCATTT
CACAGGTATATATGTTTTATCTTTCTAGTTTCTGGACACGTGTGTGGTCATCTAAATTGACATAAATTGTT
TCAAATTATCATTGTATTAAAATAGATTTATAATTAAATTACATGAATATCG
>PGSC0003DMG400010952 LRR-RLPs
TTCAAAGTCTCAAAACCACAGGTTACTAGTACTAATATTTGCACTAATGGAGAAAGCCTTCACTATTTTAT

TCTTAACAATACTCTTGTTGCCTCACTATGTTATGGCCCAAACCAACATTAACACTGATCAATTAGCTCTTC
TCTCTTTTAAATCCCAAATTAGTTCCGACCCCTTTCACTTCTTGAATGAAAGTTGGTCTCCTGCTATTTCTG
TTTGCCGTTGGGTTGGAGTCATTTGTGGTTCTCGTCACCCGCGAGAGAAGACGTTGAAGCTTTCCAACTT
GGCTCTTACAGGCAGAATTCCCCGTGATTTTGGAACCCTCACATTTCTTATTTCTCTTGACTTGGGAAGCA
ACCATTTCCATGGAAATTTGCCTCAAGAAATGGCACATTTGCGTCGGCTAAAGTTTCTTGATTTAAGTTTA
AACAGCTTCAGAGGGGAAATTCCTTCTTGGTTTGGGTTTTTACACCAACTTCAAGTTGTAAAGGAGTAAC
AGTTATACTGGTTCCATTCCTCCGTCACTCTCGAACGCCTCAAGGTTGGAGACTTTACAAATACCTGCCAA
TTTACTTCAAGGAAACATCCCCGAAGAGATTGGCAATCTCCACAACCTGAATTGATTATCCATAGAAAAT
AATCAGCTTACGGGTTCTATACCATTCACAATATTCAATATTTCTAGAATTGAAGTCATTTCATTTTCATAT
AATAGCCTATCAGGAGATCTTCCCAATGGTTTATGCAGTGATCTCCCAATACTCAAAGAGCTTGATCTAT
CAATAAACAAGCTTCATGGGTCATATGCCTACAAGCTTGTCAAATTGTTCAAATTAGCAAAAACAAAGAG
ACGTACCGGGTACAATATAACCAAATGTTGCTAAGGTTTTTGTGTATAAATAAATCACTCTCATCTTCACG
GAGCAGTTTTGAAAAACCAAAGTCACTTAGGTGGGTAACCATATCCTCATCCAACAAGACGTTACTAGG
CTTTAGATCACAATGAATCACAGGTAAAGAGCACCCATGATGGAAATATTCCAACGCACATGTAACATCT
ATGATAATGCTTAGTCTCTGGCTGATATCTAGGAAGTAGTTGTGTGAATACAAATACTTTTCAAGACTTC
CATTAAGCATATACTCAAGCACTAAAGCCTTAAAATCAAGGTTGGAGCAACTAGTGATGACTTTCACGAG
ATTCCTATGGCGAAGGCTGCGCAAAACTTCACATTCTGTATCAGAACTCTTGAATGTCGCTTCCAGTTGC
AGATTGAACACTTTAGCTGCAATGGCAGTCCCACTTCAGAGAACCCCTTTGTAAACAGAGCCAAAACTTC
CAGAACCAATCAGATTACTGATTCACTTAGCGAGAGTACTAATCTGATTGGTTCTGG
>PGSC0003DMG400010964 LRR-RLPs
AGGACAGAATCTATTAAAGGCATAATGGTACCAATTGGCTCAGACCAACATATATGCAAGTGGAGCAAA
GCTTTCAGAAACATGCCTTGTCTTAGGTTACTCATTGTCAAAGGGCAGGAGGTCTGGCATCATGACCTTA
TTTGTGACCCTATTGAGTGTCTCCCCAGTAACTTGAAATGGCTTGATTGGTCATACTACAGTTTTGAATCT
TTACCAGCATATTATGAACCAGGAAACCTTGTTGGGCTCCACATGACTTTTAGTTCTCTTGTTGAAGTTTT
CAAGGAGCCAAAGGTATTATTTACTCCTTTAGAAACAAATCATATGTGAAAAAAAGAATCATTGCTAAAG
CTGAAGTTTCTATTCATTCTCTCTTTATGAACAGGCATTTGATAAGTTGACAGTTCTCAATTTAAGTTTTTC
TAGAAATTTAATCCGAACACCCAATTTTTCGGATATTCCAAACCTGCAGAGGATTATACTGAAAAGCTGT
GTAAGCTTGGTAGAAGTTCATCCATCCATTGGCCATCTCAGAAAGGTTGTCTTTTTGAATATGGAATACT
GCGAAAGTCTCAAATCTTTACCAAGCAGTATTCAAATGGAATCTCTTAAAATCTTCAATCTCTCAGGTTGT

GAAAAGTTGGAGAAATTTCCAGAAATTCAGGGAAATATGGAATTACTATCAGAGCTCCTTTTGGCACAT
ACTGCAATATGGGAACTTCCATCATCAGTTGGACTGCTCAGTGGCATCAGCTTGCTCGATTTGCATTCAT
GTAAATACCTTGTAAGACTCCCAGCCAGTGTCTCTGAGATGAGAAAACTGAAAATTTTGACTTTGAAAG
GCTGCTCAAGACTTGCACACTTCCCTGAAAATCTAGGTGATCTAAACCAGTTGGAGGAGCTCTATGCTGG
TAACACTGCCATTTGGCAACTACCAGATTCTCTTGGAAACTTAAGCAAACTTAAAGTCCTATCGTTAAGA
AGAGGCCGGAAGGTAAAGTATCAAGCTGGTGGCAGTTTAATGCTACCCCCATTCTGGGAGTTTCATGGT
TTGAGGGAATTGAAAAGCTTAGATCTCAGTGGATGCAATTTATCTGATAACCAAACTGCTGCTCTTATGA
ACTTGCCTTCTTTACTAGAACTGAATCTGAGCAGAAACAAGTTTATTTCATTACCTGATATCATCAGTCGA
CTTTCTCAGCTTCGATATCTCAACATAACACAGTGTCAGGAACTTAAGGAACTTCCCAAACTACCTCAGA
GTATAGAGGAATTATATGCAGAAGATTTTTTGGCCAAGCAAAGTATCGCAAAGCTACAAATGTACCCGA
GATTGAATTTGGTGTCATTCACAAATTACAGTTTTGATCAACAATCTTATACAAGGGAGAGCAATGGTAG
CTCAGTTTTGGATGAGATTCTCGGTTCATTTCTTTCACTCAACATGGATAATGTGGTCCAGTCCTCTCTCA
ACTCCAATTACAGGGTGACTTGTTCCATCGTCTTTCCTGAATGTGCAATTCCCACGTGGTTTAGGCACCAG
AGTGTTAAGGAAAAGATGTTGCTTGAACTTCCTATTAATTGGTATAATGATAAGTTCAAGGGCTTTGCTA
TATGCTGTGCCACTCTCATGGGAGCAGGTGTGTTGAATCCTGATTCAGGGCTATCCAAGAAGTATGATTA
TGCTTTCGTCAAAGCTAAATTGATATGCAAAGATGATTTAGAAGACCTTAAAGTGCTAGAGAAAGAATG
TAAAGTGGGAACAGCATCTAGAACGTATGGCTTGTGTGTTTGCTTTGTCTACATACCATTGTATGCTTCA
CTGCAGGTGTCCGGCACAGATGTTGGAAATATTAACCAGTATAGCCTATTTGAGGCATCTATCCATGGG
CGCATTGTGAGACAGTGGGGAGTTCATATGATCTATGAGGATGAAAGTACATTTTTTGCAGGAAAATAT
GGAAGTGTTGGTTTCACACCACTTAAGCTGAAGAGAGGCTAAATTAGCTGTATATGCTGGGATATTGAG
GGAATTACCAAGAAACCGAGTTTAAGGTAACTCAATGCCTATTATTATTGAAGTTTAATAAATTGAATTT
TGAATACAGTTAGATTAACAATTTCATTTCAATGTGGTTAAGTATTTGGTTAATAATGTTGGTAAAAATTA
GGCAAAACTAATGATTTACTTAGCATTAATGAATTAATAAGGCAAGTCAATAATTACGTTAAAATTATTA
GTAAATGGCAGTTAACAGGTTTGAAAAATATATGAGTCAATCTGATAGCTTAATAGTTATACCAATATAA
TAGTTACATTTTTATTTTTTTTTACCTGGATAGCTCTATATGAGGCATGTCTCACCCAAGACAACATAAAG
AAAAGAAAAATATAGTTCCCAAACAGATGCTATATGTGCAAAAAAGAGGCTAAAACCCCCAGACACCTT
CTTCTTCATTGTGAGGTGGCCTCAGAATTATGGAGCATTTTTTTCGTCTCTCTGGCATCAATTGGACCACA
CCCCTCACTGTTATGGATGCATATGAGAGTTGGAGTCTATGGAAAGTTGACAAAGCCATCAAGAAGATC
TGGATTATGATACCTGCTTGTATTTTTTGGTGTATTTGGTTAGAGAGGAACAAGAGATGTTTTGATGGAG

AGTCAACTGCTTTAGGCATTTTGAAGACTAGGTGCATTGAAAATTTGTTTAGTTGGTCCAACTTGTATCCT
GTTGTTAATGCAGAACAACTTCAGGATTTTACTAACTCATTAGCTCTGGCTTAGATAGTTTGTATAGTTAG
CCAGAGATTTTTTTTGTAAGCTCAAATTCCATATCTTGTAAATTTTGCATCTTCTTGATGTTGTTATTAATA
CCACTCATCTTATAAAAAAAAAATAGTTACATTTTAAATCACATTATTCTTGATGTTGTATATCCTCGATCA
TTTTTGTAGCAACCAATTCGAAATAAACTAGTTGATATACTGACCTTCAAGCGGGAACCTAATATTAGGG
ATGGTTGTGTAACAAATCTTCAAAATCTTAAGTGTAATAGTACCTCTTCCTTGTTAAATTTACTGTATAAT
ATTACAATTAAGAATTAAATTAGCAGTAAGTGTTTTAGAAAACTAAAGACTGTATTAGGCACCACCAAAG
GTTGTGATAGGGTGGTAAGTACCCTTTTATCCTAAATCACAGCTCTCGGATTTGAGCCCTGAGATTTGAT
TGGAGGCACTTTACCCCTCAAAATAGGATTTACCTGCACAAATTCATATTAGTCGGGCCCAGAGGCAGT
GTCATTTGTTTGAGCTCAAAGAGGAGACGAACATTGAGCAACCCGTCATATATATTCTGTATGTATCAGG
TGCATCCTTTTTCTTTTTCATTATTCTTAATGTTTCTCCTTGTAAATATTAGTTCTATGTTCTTTCCGATCCTT
TATATTGGGTTTGATTGCTCAATCAAGCTCTCCGGGGTTTTTGCAATTTAAAATGTCCAAGGAAAAAAAA
AGTACTTCATCTGTTTCAATTTGTTTGTCTGATCAAGAGTGTACTTTATATCTTATTCTTAATCTGTGATTT
GTAGGATGGTCGGAGCAAAAGTTGATGAGTGCACAAGTAGTGTTGTATCTAGACAGATTTGTGAGCAG
GGGAAGCACTTCTCTGTTAGTCATTGATCTGGCTAATCTGGATTCGTGCTGAATAGAGTTACTAAAGGG
GTAAAGCGTTTTCTCCAAAAGGTTCTCTTGCATTCACAAGTCTCCAACCCAAGATATCAGGAAAGCGCTA
CCATATTAATGAGGAGATTTTAAAGATGATGAACTCTCCTCCTCTGCCACAATCGTCATCTCCTCCTCCTC
CATCGTCACCACCATCACTTTCGCCGGCTCTTTCTCCGTCTTGTTTGCTTCACTTTCTATCAAAAAATTAAA
GTGTCTTTATTCATCTCCTCCAGACTATATTTTAGAATTTTTAGTGTCTTTATTGCTTGTAGCATCATATAT
GGATTTTCTTATATCGTCTTAATTCTATCCAAAAT
>PGSC0003DMG400011045 LRR-RLPs
ATGGCCGATCTTGTAATTGGTGCTACTGTTAAAGTTGTGCTTGATAAACTGCTTTCTCTCACTATTGAGGA
GGCCAAGAGTTTAAGGAACTGCAAGAAAAATCTAAGAATTCTGACAAAATATGTATCCATGATTCAAGC
TCTCATTCATGATGCTGAAAGACGACAAGTTGATGATCAGGCTGTTGAACAATGGCTCAAGATGCTTGA
GAGAGTTGCTGAAGATGCTGAAAACGTGTTTGACGAATTCAGATATGAATATCTCAAAGCACAAGTCAT
GAACATCCGAACCAAACTAATGGAAAAGGTCAGCAGCTTCTTTTCTCATACTGCTTTTAAGTACAAAATG
TCGCGAAAAATAAACAACATCAATGAAGAGTTGAAGGCTATCAATCAGTTAGCCAATGACCTCGGTCTC
AAACCACTGATGGTTCCTTCTCAGAAAATACTTCCAATTCGAGAAACAGATTCCTTAGTTGTTGCTTCGGA
TGTTGTTGGTAGAGACAAAGATGTTGCTGAAATAAAAGAGAAGATTTTGAACATGAGAAAGGATGTTG

TTCTGTGCACCATTCCCATAGTGGGTATGGCGGGTTTAGGGAAAACAACTGTGGCTAAGAGAATTTTCA
ATGATGAACACATCAAGCAGCAGTTCGAAAAGAGAGTTTGGTTGTGCCTACCTGAAATGTCAGAAACTA
AGAGCTTTCTTGAACAAATCCTTGAATCATTGACAGAGAGGAAAGTTGAGGTTCAAAGGAGAGATTTAA
TAGTTAAGAAGCTACAAGATGAATTGGAAGGAAAAATGTATTTGCTTGTCCTAGATGATATGTGGCGTG
TTGACTCTATATCGTGGCATGAGTTCATGGACACCTTGAGAGGAATAAATACATCCAGAGGAAACTGCA
TTCTTATGACTACTCGTATGAAGCAGGTGGCATCCATAGTAGCAGAAGATCTTCATATGTTGCGAAGATT
AGCAAGAGATCATTGTTGGTCCATTTTCAAACAAAGAGCGTTTGTTGATGGGGAAGTTCCGCAGGAAAT
AATGAGCATGGAGAACAAGATAGTTGAAATGTGTCAAGGTCTACCATTGGCTGCAAGTGTGTTGGGAG
GCCTCTTATGCAACAAGGAAAAACATGAATGGCGGGCAGTTCTTAATGGCAATCCCCTTGTTGCGGGGG
AAGATGATAACGGGGAAAATAGCATAAAGAAGATCCTAAAACTCAGCTATGATTATCTACCATCTCCAC
ATCTGAAAAAGTGTTTTGCTTACTTTGCCATTTTTCCAAAAGATTTTGAGTTTGAAAAGGACCAGCTTATC
CAACTCTGGATGGCAGAAGGTTTTCTCTGTCCATCTCAAGAGACCACTTTGATGGAAGACGTTGGGAAC
AAGGCTTTTCAACTTTTGTTGCAAAATTCCTTGCTGCAAGATGTTAAGCTAGATGATCTGAACAATATAAC
ACACTGTAAGATGCATGATCTTGTGCATGATTTGGCTGGAGATATTTTAAAGTCTAAACTATTTGATCAA
AAGAGTATTGGAGGAGAAAATCTTTCTCAAGTCCGATACTTTGGATGGGACTCACCAAGTGATCAAATA
GATAAGATAAACGAGTCAGGACGTTTGTGCACATTGTTCTGGAAAAGAATTATATCTGAAGAACTGTTG
TTGAGCTTTCAGTTCTTGAGAGTTTTAAATTTGTCCAGGTCAGGCATCAAGGTATTGTCAGCCAAAATCG
GCAAGCTAATATACTTGAGATATCTTGATCTCTCCAGCACTGAGATCAAAGCATTGCCCAACTCCATTTGC
AAGCTCTACAATTTGCAAACATTGAGAATCAATAGCTGTTTCTCACTTAGGAAGCTTCCAGAAGAAATGG
CAAATATGATAAGTTTGAGACACATATATTGCATCTATCATTACCCAACATATCTTTATTTTGCGGGTCGG
AGATCAAGGTTTATAGGTAATTATCAGTTTCAGATGCCACTTAATATGGGGCAATTGACTAGTCTTCAGA
CGCTACAGTTTTTCTATGTAGGTTTAGCGAAAGGTCGTCGAATAGAAGAATTAGGTCATTTGAAAAACCT
TAGAGGTGAATTGACAATCAAACATCTCCAATTAGTCGTAAATAAAGAAGAGGCTCGAACAGCATATTT
ACAGGAGAAACCAAATATCTACAAGCTGACATATTTATGGACCCATGATGAATCAGAAGGCTGTGAGAT
CAATGATGAGCATGTGTTGGATGGTCTTCAACCGCATCCGAACTTGAAAACCTTAGCAATAGTGGACTAT
TTTGGGACTAAATTTCCTTCATGGTTCAGTGAAGGATTGCTACCAAATTTGGTCAAGTTGAAATTAAGTG
GCTGCAAAAGGTGCAAAGAAATCCCATCACTTGGCCAACTGAAGTTCCTTCGGCATCTTGAACTGATAG
GATTCCTTGAATTGGAATGCATTGGATCTACATTTTATGGCGTTGATGTTAATGATAATGGATCAAGCAG
CAATAATGGCAATATCCAAGTGTTCCCATCACTGAAAGAACTAGTATTGCAGAATATGAATAGCCTTATT

GAGTGGAAGGGTGATGAAGTTGGAGTTAGAATGTTTTCTGCGCTTGAGAAGTTGAGGATCACAAAGTG
TCCACTGTTAAAAGGTACCCCACGTCAATTGGAAATCCTCCGTGAATTAAGCATTGAAGGAGTTGACAGT
GAAATGCCATTGTTGAACTTGTGTAGCAACTTGACATCTCTGGTAAAGCTTACTGTGAATGATGTGAAAG
AGCTCACTTGTCTTCCTGATGAGATGCTACGCAATAACCTTTCTCTTCAACAACTATTGGTCACACACTGC
AGAGAGTTTCGTGAATTGCCCCAAAGCCTGTACGATCTCCATTCTCTTGAGAGCTTACAAATATTCTTCTG
CCCCAATTTCAGTTCTCTTCCTGTTCCCAGTGTAGAGAATCGTTTGACTTCCCTCCAAAGTCTTGATTTATC
ACTGTGTTTTGGATTGACCAGTTTACCAAGTGGAATGCTAGAGCATTGTCGGTCTCTACAGACTTTGAAG
GTCAGCTGCTGTGACAACTTAGTTTCCTTGCCTTTACACGTATGGGAAATGCCTTCAATTTCATATTTGGG
TTTATCAAATTGTCCCAAGTTGATTAGTGTACCCACAGGGGGCCTTCACCACCTCACAGGGTTAAAGGTA
TTGGAAATTGGTCCTTTCTCAGAGATGGTGGATTTTGAGGCATTCCAATTGATTTTTAATGGCATTCAACA
GCTATCGTCCCTTCATACATTGGAGGTGTATGGATGGACGCGTTGGGATTCTCTGCCTTATCAGCTTATG
CAACTCTCTGCCCTAACAAAGATCCATATACATGATTTCGGAATTGAGGCTCTTCCTCATAGACTTGACAA
CCTTACTTCTCTTGTAACATTACATCTAATGGGGTGCAAAAGGCTACAACATGTGGACTTCTCAGATGTC
ATGCCCAAATTACGGTATCTGGAGATCCGTTATTGTCTATTGTTAGAAGGTCTGTCGGATGGGCTTGGCA
ACCTTGTTTCTTTGAAAGAATTAAGTTTACGTAACTGCGAAAAACTACAACATCTGCCATCCAGATATGCC
ATGCTGCGCCTCACTAAATTATGGAAACTAATAATTAATGGCTGCCCGCAGTTAGAAGAAAGTTGCACC
AATCGGAGTAGCCCAAACTCCCAGTGGTCCAACATTTCCCATATTCCAAAAATTGAAGTAGGCGGGAGG
ATAATTCAGAACTTACGTAAGTCTCTGTTTCTCTTTCACTTTTTGATCTGTAAAATTTTTAAATATTATTTTC
CCCGAGTTCCAGAAAAGAAGTTATGCATGTAG
>PGSC0003DMG400011108 LRR-RLPs
ATGTTTCCAAAAGATTTTGAGTTTGAGAAGGACCAACTAATCCAACTCTGGATGGCAGAAGGGTTTCTTC
GTCCATGTCAAGAGACCACTGTGATGGAAGACGCCGGGAACAAGTTTTTCCAACTCTTGTTGCGAAATT
CCTTGCTGCAAGATGTCAAGCTAGATGAGCACAACAATATAACACACTGTAAGATGCATGATCTTGTGC
ATGATTTGGCCAGAGATATATTGAAATCTAAATTATTTGATCCAAAGAGAGTTGGAGGAGAAAATCTTTC
TCAAGTCCGATACTTTGGATGGGACTCACCAAGTGATCAAATAGAGAGGATAAATGAGCCAGGACGTTT
GTGCACATTGTTCTGGAAAAGAAATCTATCTGAAGATATGTTGTTGAGCTTTCAGTTCTTGAGAGTTTTA
AATTTGTCCAGGTCAGACATCAAGGAGTTGTCAGCCTCAATCAGCAAGCTAATATACTTAAGATATCTTG
ATCTCTCCTACAATAAGATCAGAGCCTTACCCAGCTCCATTTGCAAGCTCTACAATTTGCAAACTTTTAGA
GCCAATAACTGTTTTTCACTCAGGAAGCTTCCAGTAGAAATGGCAAATATGATAAGTTTGAGACACATAT

ATTGCAAGTATCATTACCAATCATGTACAGGTAAGGATCGGTTTCAGATGCCACTTAATATGGGGCAATT
GACTAGTCTTCAGACGCTACAGTTTTTCTATGTAGGTTTAAAGAAAGGTCGTCGAATAGAAGAACTAGG
TTGTTTGAAAAAACTTAGAGGTGAATTGACGATCGAACGTCTTCAATTAGTCGGAAATAAAGAAGAGGC
TCGAACAACATATTTACAGGAGAAACCAAATATCTACAAGCTGGTGTATTCATGGTCCCGTGATGAATCA
GAAGGCTGTGAGATCAATCATGAGCATGTGTTGGATGGTCTTCAACCACATCCGAACTTGAAAACCTTA
GCAGTTGTGGACTATTTAGGGACTAAATTTCCTTCATGGTTCAGTGAAGAATTGCTACCAAATTTGGTCA
TGTTGAAATTAAGTGGTTGCAAAAGGTGCAAAGAAATTCCATCACTTGGCCAACTGAAGTTCCTTCAGCA
TCTTGAGCTGGTAGGATTCCTTGAATTGGAATGCATCGGACCTACATTTTATGGTGTTGAGATAATTGAT
AATGGATCAAGCAGCAATAATGGCAATATCCAAGCGTTCCTGTCATTGAAAGAACTAGTATTGAAGAAT
ATGGCTAGCCTTATTGAGTGGAAGGGAGTTGAAGTTGGAGTAAGAATGTTTCCTAGGCTTGAGAAGTTG
AGGATTAGTAACTGTCCCCTGTTAGAAAGTACTCCGAATCAATTTGAAATCCTATGTGAATTAGAGATTG
TTGGAGTTGACAGTGAAATGCCATTGTTGAACTTGTGCAGCAACTTGACATCTCTTGTGAATCTTGTTGT
TAGTAACGTGAAAGAGCTCATTTTTCTTCCCGATGAGATGCTACGCAACAATGTTTCTCTTCAACACCTAT
TGGTCTCACTCTGCGGAGAGTTTCGTGAATTGCCACAAAGCTTATACAATCTCCGTTCTCTTAAGAGCTTA
GAAATTACCAGCTGCACCAATTTCAGTTCTTTTCCTGTTCCCAGTGGAGAGAGCTATTTGACTTCCCTCCA
AAGTCTTGAGTTATCCAATTGTAATGGATTGACCAGTTTACCAAGTGGAATGCTAGAGCAATGCCGGTCT
CTAGAGATTTTGACGGTCAGTTACTGTAACAACTTGGTTTCCTTGCCTTTACATGTGTGGGAAATGCCTTC
ACTTTCGTATTTGTGTTTATCAGAATGTCCCAAATTGGTTAGTGTACCCGCAGGGGGACTTCACCACCTCA
CTGGGTTACAGCATTTGGAAATTGGTCCTTTCTCAGAGACGGTGAATTTTGAGGCATTCCAATTGATTTT
TAATGGCATTCAGCAGCTATCTTCCCTTCGTACATTGGAGGTATACGGACGTACGGATTGGGATTCTCTG
CCCGATCAGCTTATGCAACTCTCTGCCCTAACGGAGATCGGAATAAATGATTTCGGAATCGAGGCTCTTC
CTCATAGACTTGGCAACCTTACTTCTCTTGTAACATTAGATCTGGTGAGATGCAAACGGCTACAACATGT
GGACTTCTCAGATGCCATGCCCAAGTTACGGTATCTGGAGATCCGTGATTGTCCATTGTTAGAAGCTCTG
TCGGATGGGCTCGGCAACCTTGTTTCTTTGGAAGAGATAAGTTTATGTAACTGTGAAAAATGGAGCATC
TGCCATCCAGAGATGCCATGCAACTCCTCACTAAATTATGGAACCTGCGAATTAGTGTCTGCCCACAGTT
AG
>PGSC0003DMG400011116 LRR-RLPs
ATGTTTCCTGGGCTTGAGAAGTTGACAATTACAGAGTGTTCACTGTTAAAAAGTGCTCCAACTCAATTGG
AAACCCTGCGTGAATTAGAAATTGAAAGAGTTGACAGTGAAATGCCATTGTTGAACTTGTGCAGCAACT

TAACATGTCTTGTGGATCTCGATGTCAGTGATGTGAAAGAGCTCACTTGTCTACCTGATGAGATGCTACG
CAAGAACGTTTCTCTTCAACGCCTGTGGATCTCAGATTGCAGAAAGTTTTGTGAATTGCCACAGAGCTTG
TACAATCTCCATTCCCTTGAGAGCTTACGAATATCCTTCTGCCCCAATTTTAGTTCTTTTCCTGTTCCCAGT
GGAGAGAATTATTTAACTTCCCTCCGAAGGCTTCACTTACTTTATTGTGATGGATTGACCAGTTTACCAAA
TGGAATGCTAGAACAATGCCGGTCTCTAAAGATTTTGAGGGTCAGCAACTGTAAGAACTTGGTTTCCTTC
CCTTTACATGTGTGGGAAGTACCTTCACTTTCCATTTTGGATATGTCAAATTGTCCCAAATTGATTAGTCT
ACCCACAGGAGGCCTTCACCATCTCACTGGGTTATGGGATTTGAAAATTGGTCCTTTCTCAGAGATTGTG
GATTTTGAGGCATTCCAATTGATTTTTAATGGCATTCAGCCGCTATTGTCCCTTCATACGTTGTGGGTGTA
CGGACATGGGCACTGGGATTCTCTTCCCTATCAGCTTATGCAACTCTCTTCCCTAATTGAGATCAGATTAA
GTGATTTCGGTATCGATGATCTTCCTCATAGTCTTTGCAATCTTACTTCTCTTGAAACATTACATCTAGTGG
GGTGCAAACGGCTACAACATGTGGACTTCAGAGATGCCATGCCCAAATTACATTATCTTGAGATCTGTG
GTTGTCCATTGTTAGAAGCTCTGTCGGATGGGCTCAGCAACCCTGTTTGTTTGGAAATGTTAATTTTTTG
GAACTGCGAAATACTGGAGCGTCTGCCATCCAGAGATGTCATGCGAAGCCTCACGAAATTACAGTTCCT
GAAAATTAAAGACTGCCCACAGTTAGAAGAAAGTTGCACCAATTGGAGTAGCCCAAACTCCCAGTGGTC
CAACATTTCCCATATTCCAAAAATTAAAGTAGGTGGGAGGATATTCAGAACTTAA
>PGSC0003DMG400011209 LRR-RLPs
GCGACTACTAGGTGACCATCATACTATGCCTATAAATGACAAGAGATGTTCCTTCCTACTTCATTCACAAA
CAAGCAAATATGAAGAACAATTCACTTCAACTTATCTTTCTCATATTGTTATCTCAACTTTTCTATTTCACTT
GCATAAACCATCACTTTGTTTCCTCTACTAACACCTCTTGCATTGAAACAGAGAAAAATGCACTTCTAAAC
CTTAAAGAAAATCTCACAGATCCTTCAGACAGGCTATCTTCTTGGATTGATCAACAAGATTGTTGCCAAT
GGCTTGGTGTAACATGTAACAACAAGACCGGACATGTCGTAAAGATTGATCTTAGGAACAAATACTCAA
CAGAACATGAATTGAGAGGTGTGATTAATCCATCTTTACTTAAGTTACAACAATTGAGATACTTAGACTT
AAGCATGAACAATTTTGAAGGAATCAAAGTACCGGAATTCATCGGTCGCCTTAATGAACTAAGGTATCT
CAATCTATCTGGTGCATCTTTTACTGGTTTAGTCTCTTCATTCCTTAAAAACCTCTCAAATTTGCAAGTTCT
TGATTTAGGAGGCTTAGATCTTAGTAAAGATACTAATTCTTGGCTTCAAATAATCAATGATCATGTTCCTT
CTCTGTTGGAGTTGCGTTTGCCTCGATGTCAACTTTTAAACATTCCTTCTTCTTTTCCATTACTAAATCTTAC
TTCCCTTTTGGTACTTGATCTTTCCAACAATGCTTTTAGTTCATGTACATTCCCTCAATGGATATTTAAACT
TAGTAGCCTTGTGTATCTTGACTTAAGCTCCAACAATATGTGTAGTGAACTTGTTGATGAGTTTGGTAAG
CTCACTTTCCTTGAACATCTTGATCTGTCTTCGAACCACGGTATCAATGGGACACTCAAGAGGAGCTTGG

GGAAACTATGTAATTTGAAGAGTTTGATCCTGTCTTACAACAACATCAGTGGAGACATAATCGAGTTCAT
CGATGCGTTATCTGAATGCCAAAGCAATAGCTTAGAGACATTGGAGTTGAGTTTCAATAAATTGACAGG
AAATCTTCCTGATGCATTAGGCAACTTGAGGAAACTGAAACATCTTCAGCTGAGGTATAACTCACTGACA
GGCACAATACCTGAAAGTATAGGGAACTTATCATCCTTGGAGACATTTTACCTCACATCTAACAAAATGA
GTGGGAACATCACCACGAGTATCGGCCAGCTCATGTCGTTGGTCTCGTTGGATATCTCGGAGAATATGT
GGGATGGTATTGTCACTGAAGCTCATTTGATGTATCTTTCCAACTTGCAAGAGTTTTCAGTTGGGATGAA
ACTTAGTAAAAATATTACCTTGGCATTCGATATCAGCCCGAATTGGAATCCTTCATTCAGGCTGACATTCT
TGACTATTCAGTCATGCCAATTAGGTCCTGAATTCCCACATTGGCTGAAGGATCAGAATGAGCTCACCAG
CCTAATACTCAATACCGCGGGGATTTCTGATGCGGTACCTGATTGGTTTGTAGAGTTGGATTTGAAGCTT
GATAATCTAGACATGGCATACAACAACTTGACAGGGAAAGTTCCAAACAAATTCAAGTTCAACTTTCAAG
CCAATGTGGATTTAAGCACTAATCGCTTTGAGGGACCTCTCCCACTATGGTCTTCTAATGTTACAACATTG
TATCTGAGGGATAACTTGTTTTCAGGACCTATTCCTCTCAACATTTATGAAGCACTTCCCAATATAGGAGA
CTTGGACATCTCCAGGAACAATCTAAATGGCACAATTCCCTTATGCATAGGTGATATGAATCAGTTAACT
ACGTTAGCCCTCGATAACAACCAACTTATCGGCCAAGTTCCTGATTTCTGGGGTAAGTTACCATATCTTTA
CTGGATAGATATGTCAGAGAATCGCCTATCCGGACAAATTCCACACTCACTAGGCTCTCTTGCTAGTCTG
ATGTTCTTAAGACTCTCAGGCAACAATCTCTCTGGAGAACTGCCTTTGAGTTTGAGAAATTGCACGAAAA
TGATCAACATTGATCTTAGTAACAATCAGTTGTCAGGGCTAATCCCAGCTTGGTTAGGGGAAACAATGA
GTTCACTGTTAATCCTTAGTGTAAGGAACAACAGGTTTTTCGGTCACATTCCTTTAAAAATCTGTAGCCTT
TCAGGTCTTCACATACTAGACTTTTCAGGAAACAACTTATCCGGTTCTGTCCCGTCCTGTTTTGGAAACTT
GGAAGGGTTCAAGGTTGAGCTAACTGATGTAGAAGCTAAACAATATCAAGGTACATTGAAGCTTGAGG
CAAAAGGAAGAACACTTTCTTATGATAGAATCCTTTACCTAGTAAACAGTATCGACCTCTCGAGCAACAG
TTTGTCTGGTGAAATCCCGGAAGAGTTAACAAACTTACACAAACTTGGAACCTTGAACTTGTCTATGAAC
CATTTGACAGGACATATACCAACAGATATCGGAAAGTTACGTTGGGTTGAAACGTTAGATCTTTCAATAA
ATCAGTTATCAGGTACTATCCCACCAAGCTTAGCTACATTAGACTTTTTGAGTCATTTGAACTTGTCATAT
AATAAGTTGACAGGAAGAATTCCTACTAGTACTCAGTTTCAAACCAAAGTTGATCCAACAATTTTTCAAG
GAAATGTTGCACTTTGTGGTCCTCCTTTGGAACAATGTGTTGGTGATAGGACTACGACGACGACAACATC
ACAAAGTGGAGGAAATGATGAAGGGGAAACAGATGATGAAGATAAACTTGAGAAAGTATGGTTCTTTG
CTGTTGTTGGTTTGGGATACATAGTTGGTTTTTGGGCATTTTTTGGCACTTTGATCTTCAAGAAAAGATG
GAGAATTGCTTATTGCAGATTCATTGAGATGTGTATCTTGGAGTTCTATGAAATGTCTTGTAGTTTTGTG

AAAAAAATGATAGGAGTCTAATAAGCTATGTTGCTCAGACTCTTCAAAATTGTCGAGGGGTGCATGTGG
GATCCTCCAAATATAGTATATTTGTGAAGGATACGACAATATTTTTAGAGAGTCCGAGCAACTTAGCTAA
TAAGTGGAGCTATAGGAGTTTGTCTCTGCAATTCTGCTATATGCTATGTTTTAGTTTCAATATAGCACATA
TTACTACTTTTTCTATATGTAAAATAATGCTACCTAAGTATTGGCACTTATAGAAATGCCAGTGTTTTGGG
TAGAGTTACGACATGAGTGGTCAGTTGAACACTTTTACGTCGGTCAAAATAATACTTCTTATATATGTAT
ACATGTTCACAAGACTATTGTAGTACATATAATTAGGAAGTTGTTTAAATAGTATCTAAGACTATTCTTAT
ACTTAATTTGCATGCATCTACTTGTATA
>PGSC0003DMG400011353 LRR-RLPs
ATAATAAAACCTTATCTTCTCTGTAGAACACTCACACACACTCTCTCTCTCCTCTTTCTCTCTCTAAAATACA
GCAAATCTAAGCTCGTTAATGTCGATTCAGATCTGAGAGAAGAAGAAGAAGGAAGAAAAAGGACTACT
GTAAATCTCTCTCTCACTGTGAAATTACACTTTTGCCCTTCCCCCTCCATTACCGTACAACAATGGCGTATG
CATTCCCGGAGGAAGTACTAGAACACGTCTTCTCCTTCCTCACCACCGACAAGGACCGTAACGCAGTATC
TCTAGTCTGCAAATCATGGTACGAGATCGAACGGTGGTGTAGGAAGAGAATATTCGTTGGAAACTGCTA
TGCCGTTAGCCCTAGGATCATGATCCGACGGTTTCCGGAAGTCAGATCTGTTGAGCTTAAAGGGAAGCC
TCATTTTGCTGACTTTAATCTTGTACCGGAAGGTTGGGGAGCTTATGTTTATCCTTGGGTTTTGGCTATGT
CTAGGTCTTATCCATGGCTTGAAGAAATTAAGCTTAAGAGGATGGTAATTACTGATGAGTCGTTGGAGT
TGATTTCTAAGTCGTTTAAGAACTTTAAAGTGTTGGTTTTGTCTTCTTGTGATGGGTTTACTACTGATGGA
CTCGCTGCTATTGCTGCTAACTGCAGGTGAAATTTTTTGTCTTACTTGACATTTCTCTTCTGTTTTGCTCAC
TTTAAAGTAGTTAGTGGTGAGAGAAGTCTTAATCTGTAAAGTAATTAGATCTAATTTTGGGTATTAGTTT
GGACAATGACTTGTATTGTTGTTAAATCTTAAGCTTTTGGATGAATGCAGTAAGCATTTTGGGGGTTTGT
TATAATCCCTTGCTAAACACGAGAATGAAGAAGCACTTTTCTTGCAGCTGTTTGTTCTGTTTATAGGATAT
GTAATGTATTAGTGGAAACGAAGTATCTACTGTAGATGAATTCGTAGTGTGTGAACTTGAAGAACTGAC
ATTTCTTCAAGTGATTATGGTATTATTGGATACGTCTTGCTGCTACATTGCATCAGTCTGGTTCATCTGAC
TTTAATACTTCAAAGATTAGAGCTTTTTGTTTGTTTCTTTTAGCTTCTTTTTTTTGAGGTGAACTTCTGGGA
ATTAATTTTTCGGATTTTGATTGAATGAGCTAATGTCAATAATTTCTTGTACTTCCTTCCCCAGAATTCTAC
ATTTGCATTCTATCTTTACAGATCTCTAAGGCCTTATAACAGAAAATGCTAACTTGTGGGTTTTCTTAATT
GTTATAGGATTTAATCAGTTGGACTTCCTTTTAGCATTAATTTCTTGTGGTATACACTGTGTAAACATGCC
CTTTAGCTTTGCTTTGTTTTGAAGTTGTTGGAGATTTGAATGTTATCTTAATTATCTGGAGATTTCCATATG
TTTGTTTCTTGGTTCTCTGGTGGGATTAATTTGTTTTATCAGGAGGGAGTTTTGTAATGGAGTATAGTTGT

AGTTCAATGTTGAAATGGTTAAGTGGGTTAGGAATATGAAATGGTGATTTATTGAAATTCCAATAGGAA
TATGATTCTAAATCTTTCTTATTTATCTTGTTCATATCGTCTGTGATTTATTATCATTTGTTGTTTTCTGTTG
AGTCTGTTGATTTGCTTCAATTTATCACAGAATAATTGTGTAGATGGGTATTGTCATCTAACTCCCGTTTT
GCTGCAGGAATCTGAGAAAACTAGACTTGGGAGAGAGTGAAGTGGAGGACCTTAGTGGGCATTGGTTA
AGCCATTTCCCTGATAACTGCACGTCACTTGTTTCACTTAACATTGCCTGTTTGGCTTCTGAGGTCAGTCT
ATTAGCTTTGGAGCGCCTGGTTACTCGCTCTCCTAATTTGAGAACTCTTCGGATAAATCGTGCTGTGCCCC
TTGAGAGGCTTCCAAACTTGCTCCGCCGCACTTCCCAGTTGGTCGAATTTGGTACAGGTGTTTTCTCTGCT
GATGTGCGGTCTGATTTTTTCTCGAACCTGACAGAAGCTTTTTCGAGCTGCAAGCAACTTAAATGCCTGT
CTGGGTTTTGGGATGTTGTACCAGCTTACCTTCCAGCTTTATATCCAGTGTGCTCAAGACTCACCTCCTTG
AATTTGAGCTATGCTACATGTCAGAACCCTGAGCTTGGCAAGCTCATAAGTCAGTGTCACAATTTGCAGC
GTTTGTGGGTATGTTCTAAATGTTTTGAAGTCTTAATTGATGTTCTTGATACTGCAACTGCAAACATGTTT
TTTTTTCTTATAAAGAAGAAGAGGCAATGAGATTGTAACATCATACTTGCTGTGATGACATTTAGTATAT
ATTCTTAGTGAAGAATAAGAAAAAACCTAACTGCTACAGATCAACCAATCAACCACAACTTAATCCCAAT
TTAGTTGGTCTCAGCTTTATGGGTCCTCTTAAACCATATTGCTATATTCAGGTCATTTTCGTTCCGATGCTA
AATTATTTGTCTTTACGCAATAATTGCCAAGGATAGGAAAAAAGTAGTTCATTCTGTAGATAAACTTCAC
CTATGCTGGTTAGGAAAATTCCAGGCTACATATATTTGATATTATATTGCTAAGCAGCTCACTTTCCTTGG
AAGTTGAAGCCAATTGTTACATGCTTCATATTGTGATCTGGACGAATGGGTTTCAGTTTTTAATACTTGTG
CCTACCCAATGCACGTAAAAATTCATCTAAATGTGATGGTTCTATGCTGAAAAATGTATCTATTCTTCCCT
AGTAGCTTCCCCAAACTGGTTCTGTAATATAAACTCCAGTATCAAGTTTAAGTGCTGAATTACTTTTCTTG
TAATATATGTGACTGCAGGTGCTGGACTACATTGAAGATACTGGTCTTGAGGAACTTGCTGCCAATTGTA
AGGACCTTCAAGAGCTGAGGGTGTTTCCTTCTGACCCATTTGCTGCAGAACCAAATACAACCTTGACGGA
GCAAGGCCTTGTAGCTGTCTCAGATGGCTGCCCAAAGCTCCAGTCTGTCTTATACTTCTGCCGCCAAATG
ACTAATGCAGCTTTAGTTACTATTGCTAGGAATCGACCTAACATGATCCGTTTTCGCTTGTGTATCATTGA
GCCTCGAACTCCTGATTATTTAACCCTTGGTTCATTTGATGCTGGTTTTGGGGCCATAGTAGAGAACTGT
AAGGAATTGCGGCGACTTTCACTTGCTGGCCTCCTTACCGATCGTGTCTTCGAATACATTGGCGCCCATG
CTAAAAAGTTGGAGATGCTTTCCATAGCTTTTGCTGGTGATAGCGATTTGGGACTCCACCATGTTCTCTC
GGGTTGTGATAACCTTCGGAAGTTGGAGATCAGAGATTGTCCGTTTGGAGACAAGGCTCTTTTGGCTAA
TGCTGCAAAGCTCGAGACAATGCGATCCCTTTGGATGTCTTCTTGTTCAGTAAGTTTTGAAGCATGTAAG
ATGCTAGCTCAGAAGATGCCTAGGCTTAATGTGGAAGTCATAGATGAGAGGGGTCCTCCAGATACAAG

GCCAGAAAGCTGTCCTGTGGAGAAACTTTACATATATCGAACTGTGGCTGGACGAAGATTTGACACACC
TGGTTATGTTTGGACAATGGATGAAGATGCAGCTGTAAGGTTGACCTGAGCCATACATCAAATGGAAAA
TTCCAGCAAGACTTCACATGGAAAGATGGGCAGGTATTAGCGCTATGTTTATCCTCCTGCATTTGTTTGT
CACGCCTTATGTAGTTGCAGTGCCAGTTGAGGGTTGGAACTTCACTGCTTTGAAGTAGAGACTCCCGAG
CTCATACGCCATCAATTTTATTCTTGGCTGAGAGCTACTTTACAGCAGGTAGTTTTTAATAAAGAATTTGA
ACTCTGAGTTTCTGTGTATCATCTAGAGTAACAATTATTCGTTTTCTTTGGGTGGGATTTTGCTTGGGGGG
TTTGGGGGGCATATTTTTGCCATGTATGTGTTATTTCCAGACTTTGCTTCATTATGTAGTTCCCTTTATTTA
TTCTATTGACTTATTCTCTATGAAATGGAGTGTCT
>PGSC0003DMG400011513 LRR-RLPs
ATAAATAGCAAAACAAACACAAAATGAAAAGATAACTCCTCTTTCTTTCCAATAAGATTAGAACCTTAAA
CTTCTCCGATGGCCAGCGACGACTCTCATCTGCAAAACGACGTCGTTTCTGTCCACTGTATAAACGACAG
TCTCGGCGACGACGAGCTTCGGGCCGTACTGTCGAGGCTCGGAGACGACAAAGACAAGGAAGTTTTCG
GGCTGGTATGTAAGCGGTGGCTTCGTATTCAAAGTACTGAAAGGAAGAAGCTTTGTGCTCGGGCCGGG
CCTCACATGCTCAGAAAAATCGCGGCCAGATTCACTCGATTACATGAGCTTGATCTATCTCAATCGGTTTC
TAGATCTTTCTATCCTGGTGTTACTGATTCTGATCTTTCAGTTATTGCTACTGCTTTCTCTTGCTTACGGATT
CTCAATCTTCAGAACTGTAAAGGTTCGTTTTTGTATTTTTAAAAAGTTTTATAGTTTGAAAGTTGTTGAAT
TTGAATGTTCATTGCTGAAGTGAACCTGAGCTACTGCAGGAGAGGCCAAATGCACCAGTGAGTAGTAGT
ATGTGTAGTGATATTTCACATTTAGTACTGATTTAAAATATTGAATTTGTCTTTGTGTGGGGTGGTGGAG
CAGGGATTTTCACTTAAAGGGTTTGAAATACATAAAACATAATTTAACCACGTATAACCGGTTTGGGTGA
ACCCCTGGGCCCTTATTAGCTCCGCCCCTGCCTGTGTGGGGTGGTAGGTGTATTGTTCCCAACTTGGACA
TCAAGGAAATTTTGAGGTTTGACTAAAATCTATTGTTTGAAATGTTAAAGGTTTTGACCCGATATGGCGA
AGACTGGCTCTTCCTCTTTTCTATCTTGTAAATAATGGTTTTTATGATAAAAAGGGCGTAATGGGGTTTAT
GGAGGATTTTAGGTGAATTTATGGTTGGTTTTTTTGAAGATTTGTAGGATTTTGTGTTTCTGTGTAAAGG
TTATGTCTTTTTTGCTATGTGGTTTTTGAAGTGGTAGCAAAAGGGAGGATAGTGTAGTTATATCATATGT
ATTCCAATTTTCAAGAGAAAGTCTAACTTTGTTGCGAATCTAAGGCTCAGATGCTTCCTTGACTTAACTTG
CGCGGTTTTCTGGTCCAAATTTACAGGCATAACAGACAATGGATTGGCAGCGATTGGATCAAGTCTCTCT
TCTTTACAGTCCTTAGATGTATCCTACTGCAGAAAGATCACAGACAAGGGGCTTTCAGCTGTTGCTGAGG
GGTGTCATGACTTGAGAACGTTGCATCTTGCTGGCTGTAGATTTGTCTCAGATTCATTAATGAAAGCTCT
CTCCAAGAATTGTCATTATGTAGAAGAGCTAGGTTTGCAAGGATGCACCAACATTACCAACTCAGGTCTC

TCTGTTTTGGTTGAAGGTTGCCGGAGGATAAAGTATTTGGACATTAACAAATGCAGCAATATTGGTGAT
ATTGGGATAACTAGCGTGTCTGAAGCTTGTTCATTGACACTTAGGACATTGAAGTTGTTGGATTGCTACA
AAGTTGGTGATGACTCCATCTTATCCTTGGCCAACTACTGCAAAAATCTTGAGACACTTGTTATTGGTGG
TTGCCGTAACATATCAGATGAGCCTATGAAGTCACTGGCAGCTTCTTGTAGTAATAGTCTGAGGAAACTG
AGGATGGATTGGTGTTTGAATATTACTGACTCTTCATTGGACTGCATCATTTCCAAATGCAGAGAGCTTG
AGGTTCTCGACATTGGCTGCTGTGAGGAGTTAACTGATGCTGCATTTCAACAATTAGGAAGTGAAAACA
TTATGTTGGGGATGAAGATTTTGAAGGTCAGTAACTGTCCAAAGATAACTGTTGAAGGAATTAAGAAGC
TTATGAAGTCATGTGAATATCTTGAATATCTAGACGTACGGTCTTGTCCCCTAATCACTAAAGCAGGGTG
TGAGGAGGCTGGAATTCAGTTTCCTGAATCCTGCAAAATAAACTTCACTGGAAGCTTAGCAGAGCCAGA
TGTGTTACTTTGAGCAGTACTTGATTTAGCAATGTCTTTGAAGGACTGAAGGGGATCATGCAGTGAAATT
ATCACATTCTTTGCCTAAGACCTAAAATATGACTTTGGAAGCTCCTGATGTCAGTCCCTTGTTCCCATTAA
TTTGCCTTTTGTTAAGCATCAAGTGTTTTGTTGTAGATTATTTCCCTGCTTTTCAGTTTTTTTTTCCCCTGCT
TTTCAGTTATTTGATATCAAACTCCTGTATATCATCCTTGTAAACTACTAGTAGGCACCATTGTTTCCTGTA
GGTGTTTTCAGATTGTGAATGAAATGAGTCCATTGCTGCTAGCTTGAATGTGAACTTATACCAACAATGA
TGGTTTGATTGATTACATTTCTTGTACGAC
>PGSC0003DMG400011610 LRR-RLPs
ATGGTTAATAGTATATCCCAACAAATTGATCAAACAGCGAAGGTACCGACATATCTTGGTAAATCTGGA
GAGAGAGATTACAATGATTCTATAACAATTTCAACAAAGGGAATGGAGTATGAACTGGATAGAATTTTG
ACAATTCTCAAGACTATTGATCTTTCAAGTAACAAATTTGAAGGACATATTCCAAGTAATCTCGGAGATC
TCATTGCGCTCCGGGTGTTAAATTTGTCTCATAATAGATTGCAAGGTAATATACCGTCATCACTTGGAAG
TTTATCTTTAGTTGAATCATTGGATCTTTCATTTAACCAGCTTTCGGGAGAGATACCACAACAACTTGTTG
CTCTTACATTTCTTTCATTCTTAAATCTCTCTCACAATCATCTCCAAGGATGCATTCCTCAAGGACATCAAT
TTGATACATTTCAAAATAATTCATATGAAGGTAATGATGGATTACAAGGATTCCCTATTTCGAAAGGTTG
TGGAAGTAATTGGACACCGGAGACAAATAATAGGGATTATGAGTCAGATGATGAAGAAAGCAATTCTG
AATTTCTAAATGATTTTTGGAAAGCTGCTCTTATGGGATATGGAAGTGGGCTATGCATTGGATTATCCAT
AATATATTTCATGATCTCAACTGGAAATCTGAAATGGCTTGCAAGAATCATTGAAGAGCTAGAACACAAA
ATTATTATGCGACAGAGAAAGAAACAGCGAAGTCAAAGGCAAAACAAAAGAAGACATAATCGTGTCTA
G
>PGSC0003DMG400011611 LRR-RLPs

ATGAATAATCTCTCTGGAACTATCCCACCTGAAATTGGTAATCTCACTAATCTTGTCTATATTGACTTGCA
CACCAATCAGATTTCAGGCACAATCCCATCACAAATCGGCTCACTATCCAAGCTCCAGAACATTTACATCT
ATGACAACCTTTTAAATGGTTCCATTCCTGCCTCATTGAGCAATTTGACCAACTTGTCTATTTTGTCTCTTT
ATCAGAATAATCTTTCTGGCTCGATTCCTACAGAAATAGGTTATTTAAGGTTTCTTACTTCTCTAGATTTG
GGTACTAATTCTCTTAATGGTTCTATTCCTACTTCATTGGGAAATTTGACCAACTTATCTAGTTTGTTTCTT
AATGGGAATCACCTTTCTGGCTTTATTCCCGCTTTATTGGGAAATTTGACCAGCCTTTCTATTCTGTATCTT
CAGGAGAATGACCTTTCTGGCTCCATTCCTGAAGAAATAGGTTATTTAAGGTCTCTTAGTCAACTAGTTTT
GGCTAAGAATTCTCTAAGTAGTGCTATTCCTGCCTCATTGGGAAATTTGACTAGCTTATCTGGTTTGTATC
TTAATGAGAATCACCTTATTGGCTCTATTCCCCCTTCATTGGGAAATTTGACCAGTCTGTCTATTCTTTATC
TTAATCAGAATAACCTTTCTGGATCCATTCCTGAAGAAATAGGTTACTTAAGGTCTCTTAGTCAATTAGTT
TTGGCTAAAAATTCTCTAAGTGGTGCTATTCCTGCTTCATTGGGGAATTTGACCAGCTTGACCTCTATGTA
TCTTCGTGAGAATCATCTTTCTAGCTCCATTCCTAAAGAAATAGGTTACCTAAAGACTCTTAGTTATTTAG
ATTTTTCTACTAATTTTCTTAATGGTTCTATTCCTGCTTCTTTGGGGAATTTGAACAACTTGTATCTTTTGTC
TCTTTATGCTAACAACCTTTCTGGTTCGATTCCAAGTGAGTTGGGGAATATAGGAAGACTAGTTACTATG
TATTTAAATATTAATCAACTCATTGGTTCAATTCCGGATTCCTTTGGCAACTTGAGAAACTTGCAATGGAT
GTATCTCCACAACAACAATCTCACTGAGAAAATTCCTTCATCTTTCTGCAATTTGATGAAATTGGAAGTGG
TGTATTTGGGGAGAAACAACTTTAGGGGAGAAATTCCGCAATGTCTAGTTAATATCAGTGGACTCGAGG
TATTGAAAATAGAAGATAATAATCTCAGTGAAGATATCCCTTCGTCTATTTGCAATCTAACATCACTAAG
GATTTTAGATTTGGGTAGAAACAATTTGAAGGGAGCAATTCCACAATGTCTTGGCAACATGGGTGGTCA
TCTTGAGGTTTTGGATATCCACCAAAACAATCTTTATGGGACTCTTCCAGAAACTTTTAGCAATGGAAGT
GTACTCAGAAGCTTAGACTTGCATGACAATGAATTAGAAGGAAAAATCCCCCGATCTTTAGTCAATTGTA
AAAACTTGGAAATTCTTGATTTAGGAGACAATCACTTCAACGACACATTTCCAATATGGTTTGAAACTTT
GCCAAATCTGAAAGTTTTAAGCTTGAGATCGAACAAATTGCATGGACCAATTAGAACTTTAAGTAATGG
AAACATGTTTTCTGAGCTTCGAATCATAGATCTTTCTTATAATGCTTTCACAGGAAATTTGTCGACTAGTC
TATTTCAAAAATTGAAAGCCATGAGGACAATTGATCAAACAGCAAAGGTACCAACATATCTTGGTAAATC
TGGAGAGAGAGATTACAATGATTCTGTAACAGTTTCAACGAAGGGAATGGAGTATGAACTGGATAGAA
TTTTGACACTTTTCAAGACTATCGATCTTTCAAGTAACAAATTTGAAGGACATATTCCAAGTAGTTTGGGA
GATCTCATTGCACTTAAGGTGCTAAATTTGTCTCATAACAGATTGCAAGGTAATATACCATCATCACTTGA
AAGTTTATATTTAGTCGAATCATTGGATCTTTCATTCAACCAGCTTTCAGGAGAGATACCACAACAACTTG

CTGCTCTTACATTTCTTTCATTCTTAAATCTCTCTCACAATCATCTCCAAGGATGCATTCCTCAAGGACATC
AATTTGATACATTTCAAAATAATTCATATGAAGGTAATGATGGATTACGAGGGTTCCCAGTTTCAAAAGG
TTGTGGAAGTAATTGGATACCAGAGACAAATAATAGAGATTATGAGCCAGATGATGAAGAAAGCAATT
CTGAATTTCTAAATGATTTTTGGAAAGCTGCTCTTATGGGATATGGAAGTGGGCTATGTATTGGAGTATC
CATTATATATATCATGATCTCAACTGGAAATCTAAAATGGCTTGCAAATATTTTTGAAAAGTTAGAAGAA
AAAATTATTATGAGACAGATAAGGAAGAAGCGAAGTCAAAGGTAA
>PGSC0003DMG400011708 LRR-RLPs
GTGTTACATATGATTTTGTCTTTAAGTCAGTAAAGTTGGTTCTTTGTATATGTGGTTGGGAAAAATTTATG
TAGGAGATATTTATCTCAAGTTCACATTTCTATTTTTTTTGCGGGGCCATTGAATGGTAAGTGTTTGGATT
GGAATCTCCCTAGTTTGATTTCATCTTCTCTATCATTTGAAAAAACTCAATATGGAGGATGAGACGGAAA
CTGAAAAATCTCCGATTACTACCCGTGTGATACTTCTTTCCCAAAAACAGTCTTATGGAATACGAGTAGA
GATTGTTGTACATGGGATGGATTACAACTTGATTACAGAACATTGGACACAATACTTCATAACTTCACAA
AAACCTGTGAATTTCCCAAAAACAGTCTTATGGAATGCGAGTAGAGATTGCTGTACACTTTCTCTCCCTG
ATGTCAACATCTCATCTCCGATACCTATGAATTTGTCTTCCTCCTTAAGGTACGTGGATCTTGAATATACT
AATATACAAATTAGGTATTTGAATTTCACCGGCCATATTTCTTTCACAATCTCTAAATTGAAGCAGCTCAC
AAGTTTAGATCTTTCATCTAACTACTTTTCAGGTGAAATGCTCGACGTTTTCTCTAACCTCCAAGAGCTTA
CATCTTTCTGACAACATGAATTATGCGGTTCCTAAACCTTTCGCATAATTCATTAACAAGCCACCTAGCTA
CCACAAGTTCATTACAACAATCTAGAGTATCTTGATCTTAAATTTAACTTCTTTCAGGGTCCACTACCTTCA
TCCATTTGTAACATGAGCTAGCTTGAATTTCTAGATTTATCACACAACAACTTCAGTAACTCGATTACAAG
TTGCTTGGGAAGTATGGCTAGTTTAAGGGTGTTGGACTTAAGAAGGAACAATTTCAGAGGGAGTCTTCC
ACCATTATGTGCGCAGAGCACTTCATTGAGTACCATTTTTTTGTTTGATTTTCTTGTTTTGTTTTTAAATTTT
CTAGTTGTAGTTTGTATATGTTTGATAGCTACATAATTATGTGTGTGTTATTTTAGTATCAATGGTTATTCA
AGTTATGTTAATCTGTTTCTGTTTCAAGTTGACAGAAGTTATCTACACAGCCATGCACTCATTAGCAGGCT
CTTCAAAAAATGTCAATGGGTATGTGTTGGATCTTCAAAAGTAGTGCATTTTTAAGGAACTAACACAGATG
CAACATCATTATATAGAAGAGTCCGAGCAACATAGCTCATTAGTACAGCAAAAAGCAAAAAGCAGTAAG
CATATTGAGTTTGGTTCATGATTGAAATCACTTGATTCAATTGTTCATCATTACTCTCCATTTATCGTAATC
ACTCGTAATTGATGAATCACACTTAAAACGTATGATGATGCTTCACCTACATAAGGTAGAAAAACTAAAA
ATGTCTACTACTTTGGTTATTTTCCATCTCTTCATGTTTGAACTGTATCATATTTGTCCTTTTCCCCTTGTAG
TCTTTGTGGAAATTGGTTTAAAGACTTTGGTGGAATATTGATAAAAGTTTCCCTCGTTACCTTAATGTAAA

AAGTCTTATGGAAAATTATAGAATGTACCTAACAAGTTTGAAAACAGCACAAGAGACAACTACTTGGAA
ATTTCTCTTAAAATTTCTCAACAGGTGGAATGATCTTCACCAAGTTATTCAAAACATATTTGAGATAATTA
AAAAATTATTTGAGATAAAGGAATTGAAATAGGTAGTTGAGTCAATAATAAGTTGCTTAATATTTAAAAA
ATAGAAGTAGAGTGTCAAATGGGGCTACACGTGCAATGCGCGTGCTTCGAGACTTTCCCAAGTTGTGAA
TTTTTGATCTTTCTTGTAATGAATTCAGTGGATCACTTCCTGGAAAAGTTTTTGGAAACTTCAAGGCAATG
ATCAAATTAGATGGTGAAGACACAAGAGAGATCAAGTACATGAAACCATCTGAGAAGTCATTTTACATA
ATGTATGAGGACTCGGTGAGATTGGTGATCAAAGGCCAGGAAATCGAGCTAGAAAGAATCAACACAAT
TATGACAGCCATAGATATCTCAAGCAACCATTTTGAAGGTGTCATTCCGAAAACACTAAAGAATCTCAAC
GCTCTTTGGCTACTCAATTTATCCCGTAACAATCTCAAAGGTGATATTCCAATGGAATTAGGGCAATTGA
ATACGCTTGAAGCTTTAGAACTCTCTTGGAATTGGCTCACTGGAAAGATTCCACAGGAATTGACAAGAAT
GAACTTTCTGGCATTCTTAAACCTCTCTCAAAATCTTCTTATTGGACCAATTCCTCACGGTCAACAATTCAA
CACATTTGGAAATGACTCGTATGGTGGCAACCTTGATTTATGTGGTCTCCCTTTATCGAAGAAATGTGGA
ACAAGTGATTCATCGCATGTTCCTCAACCAATGGAATCCGAAGAAGATGAAGACGAGTCATATTTCTTAA
GTGGATTTACTTGGGAATCCGTAGTCATAGGCTACAGTTTTGGACTAGTTGTTGGAACTGTCATGTGGA
GTCTCATGTTTAAATATCGTAAACCAAAATGGTTTGTAGAATTTTTTGATGCAATCGTGCCTCACGAAAAT
AGAAGGCCAAAGAAGAAAGCTCAGAGACAACGGACTTAATGTTGGAATAATCTCATCAACATGCATTTG
ATATTTTGTTCTTTTACTTTGTCTGAATTTTCTCATCTGTTATTTGCTTCGACTACTGAATTGAAGTTATAAA
AGCAGTTTTCTTTGCTTGTGCAGCACTTTATTTT
>PGSC0003DMG400011864 LRR-RLPs
AAGCATTCTTCTTCTTCTTCTTCTTCCTCCTCCTCACTTGGTTTATTCTCAACTAGGCCAGTGTTTTTCTGTT
AAACGGTTCCTTCTCAATTTTCCATCTTCTATATGTGATCAAATTCACAAATGGACTACAAATTACTTCTCC
ACCTGCAAATTTTCCCCTAAACAATCCCACCATTACCAAGAATAACCAAATCTAAGCTGCAATTTAAGCAT
AAAGCTACAATCTTTGTCCAAAATGATCCCTAAAAGTTCAATCTTGGGAACAATGAAGAAGCAAAAGCC
AATTTCAGGAACCCATTTCAACCCCTTTGATCTACTTTCTGAAGAAATCGTCTTTTCAGTTCTTGATTGTCT
TAATGACAACCCATTGGACAAGAAATCATTTTCTCTTGTTTGCAAATCTTTTTATGCTATTGAGTCTCATCA
TAGGAAAATCTTGAAACCCTTTAGGCCAGAACACTTGACAAAGGTTCTTAGCAGATACCCACAAGTTACC
CATCTGGATCTTTCCCTTTGTCCTAGAATTACAGACGGTTCACTAACAGTTATAGCTAATTTCTGTAAGGA
AATGTTGAGGTCAATCAATCTTTCTAGGTCTAAGTTTTTTACCCATGTGGGGTTGTCGAATTTGGTGATG
AATTGTGGGAATTTGGTTGAGATTGATTTGTCAAATGCTACTGAGTTGAAGGATGTGGGTGCTGCTGCA

TTAGCTGAGGCAAAGAACTTGGAGAAGTTGTGGTTGGTGAGGTGTAAGTCTTTAACTGATATTGGACTT
GGGTGTATTGCTGTTGGCTGCAGGAAGCTCAGATTGCTTAGCCTGAGATGGTGTTTGGGTGTTGGTGAT
CTTGGTGTGGGGTTGATTGCAGTCAAGTGTAAGGAGATTAGGAGTTTGGATCTTTCTTACTTGCCGGTA
ATGGAGCTTTTCTTCCCTTATAAATTTTGTTTTCGATTATATTTTTGCTCTGTACTTTTGTGTTGGTTATGTT
TCTGTTTCTGTTTTTCATGGTAAGTTAGTTTGTTTGTCTAAGTAGTTCATTTGAACATGTTTAGACAGTATG
CTCGTCGATTGGTTGGTGTTCTGTTTACTATGATTTTCAGGGTCAAATGATGTGTGTATTCATGTTGTGAA
TCAATAATCGCTAGATAACCCTGAGGGGTAGCTCGGCTGGTAAAGGCTGAGGGGCAAAGCCCTTTGGT
CGCCAGTTCGAGCCCCCTCAGGGCCACTGGGGGCATTACAAACCGCCCGTGTCTCCAGGGGCCCGTGGA
ACTCACCGGTGTTATCCACCTTGAAACAGTGGGACCCGGAGGGTACAGCTAGTCGGGGAGTGGGTTGG
TAAAGGCTGAGGAGCATGCTCTTTGGCGCCAGCTGTAACCCCCGGAAACCACGGTTAAAAAAAAATAAA
AAAAATAAAAAAATAATCGCTAGATGTGTTGTGTGATTCTTTTTGAGTACTGCTATTTGATTTCTCTTTGC
CATACTCTGCCAAGCTTGTTTGCCAATTGATGGCTTATCAGATTATAGTCTTTCATGAATCCTTGCTTGTG
TCATGAACTCGGTTTATTGAATTGTGATGTATCATGAATGCTAAATATGCACTGTATGTTATAGAACAAA
TGTAGCTTGGTGATGATAATTTATGCGGCTTTGGGAGTGTTAACCAATGATTTAGAAGGTAATGTGCAG
ATTAAGCTGGCCAAGAAAGTATCATTATAAATAAAATGCTAAGCCGTTTAGGAATTTCTCATTGATATCC
TTAACGAAGTCTATGAGAATTGTGACATCCATGACGGAGGGCACACCAACAAGGGTGGGTCAAGACGA
GTAATGGCAGGCTTTTAGCTTGGCAAGCTTCCGAAGAAGGGGTGCACATGACACCTTAAGGGAATTCAC
ATCAGAATGGGAAAGAAGAACTTCGTGAGAATATTTTGCACAAGATTATTTTTCACTCTCTATCCTCCGT
AAGGGAGGAAAATTTCATGAGTTTGCATTGCTCAACAAGTGCTGACATGTCTTAGGTTCAAAACTAGAC
GATTTCTTCCCATCTGTATAAGCTTTAGTAGACAAAGTTACCTGGTACGTGTGCTGGTGGGAGGGAGCA
GGTCATTGGTGGGTATGCACTAGCTTGCCCAGGCACCAACGTCATAAAAAGGAAAAAAGCTAGTGCTG
ATGTGTCGTCAAACTCAATGATGATTTTTTTTAAAATTGAACGGAAAATGACATTTCACAGAACATTAAA
TATTTAATATTATTAAACATTTGCTAAGATTCTTACATTGTCTTAGTTAAATTTATCCCGTGTATTGTGCTTT
CTCTACTTGGGAGATATACAAGTTCTGAAAAATGAGTCTGCTTATGCACTTTGCACATCTCTTGCACTTGG
CATGCTTCTGACCTTGAGTTTGTTGAAAAGAAAAGATATTGCAGAGTCCAGCTTTTGAATCCTAATGAAC
ACTTCTGCTTGACATTCCGATGCATCTTCAGATGAATAATTGAATGTTCTCTTATCATACTCATGCTGAAA
TACTCTAAGTTAGTGGAGGTTTTATGCTATAGAACATCTATCCATATTCTCTCGATATCTGTGCACCACTC
AAGGATAGCATGACCAAAAAAATTGTGTTTTTAAGCACCTTGGTGCTTTGTCTGATGTCCAGGTCTCAAT
TACATTTCATGACCATCACAACATGCTCCTTTTTTCATCGTATAATATGGGAAAGTTCTTGTTCATGTGATT

CGAAGTCCTTTCTTGACTTATGAAGTTACCACGACTTGGATATGAGCTTAAATAGAGGGACATAGACAAT
GAAAATTCATATAGCTGGCCCCAACTTGCTTGAGATTAAGGTGTGGTAGTAGTTGTTGGTGCTGTAGTTT
CCCACTGCTTTGGTACAGAACTTAAATTCCAGACGTACCAAGTTTCCAGTGACTGACTGTATTTATGAAG
TTGTTACATGTAATCTGTTTCTGTGTTCTGTTATTACATTCCATCTCTTTTTCTTTCTCCGTTTTTGTTTTGTT
TTGTCTAGGGTTTCTTTATCGATCATACAATAATGTGTTGATCTTCTTATTAAACAGATCACTAATGAATG
CTTATCATCGATCTCGAAACTGCAATATCTTGAAGATTTGGTTCTAGAAGGCTGTTATGGAATTGATGAT
GACAGCCTTGCATCCCTTGAGCAAGGCTGCCAGTCACTGGAGGTATGTACTTTGGAAGACTCTGTTTAAC
AGACTTTGATTCTCATATTTGCCTGATTGCCTCTCCTTGAGATAAAATGAACAATAAAAAATGAAGCTAG
TATTGATGTATGAAATTGCCAAAAGCTCCCAGGACGTAGCTCAATTGGCGAAGGTTGATAAACTTGTGA
CCTAGGTCATTAGTTCGATCCCCATGTCAAGCGAATCTGATATTTAAGTGGTGAAGGGAGAAGTGAGCC
CATCATCCCCGAGTTTCGAAGGCTGCGGTTGGTCCTAAGGGTTGGCTCCTAATGGATTTCTCGGTCATCA
AAAAGAAATTGCCCAAAAATTTGTCAAATTGTCCTAGCTTCAAGCACTACTTTTAGATGTACATATATATG
ACTTTTTTTCCCTAATTGCCTAGTCAATAATTGGAGACCTACTGTGTGAATGCCACTATAAGTACTCTGAA
ATAGTTAAAGAACAAGGTAACTCAGATTTTTTTTTTTTGATAACCGAGAAATCCGTCTGTGACCCGCCCTT
TGGACCAATCACAGCCTTCTAAACTCAGTGGATAATGGGCCCGCCCCTCTACCCTTCTCCACTTAAATACT
GGGCTTCACTTTGCATGGTGTGGGGCATGAACCTGGGACCTAAGCCACAAATCCTCTACCTTTTGCCACT
TGAGCTAGGCCTTGGGGGCAAGGTAACTCGGATTTTATGCACCCAGAATTCTACCTAATTGTCAGATGTC
CCAAAGAAGTGGACTATTGTGCAAGTCAAGTGTTGCCTTGCATGTATCATGCCTTTTGGTCCTTTTTTTCC
TCTTAAATAACTTTTTTCTTTAGAAGGTACTATTGACTAATGTCAGTGTATGTATGTTGGCTCTTTTCAGAA
ACTTGATATGTCGAGCTGTCAGAATGTTAGTCATGTGGGTTTGTCATCTCTTACCAGCTGTGCTGGATGT
CTGCGGCAACTTGTATTGTCGTATGGTTCTCCCGTAAGGCTTAACAAACTTGAGCATCTTTCAGTAGTAT
GAGTTTTAATTTGATTGCTGATTTCAAATTACTGGTATTTCAGGTTACACCTGCTGTCGCAGATAGCTTGC
AGAAGCTTTCCAGGTTGCAGTGCGTCAAACTAGATGGTTGCCAAGTTACATGTTCTGGACTGAAGGCTA
TTGGAAATTGGTGTGTCTCACTTAAAGAACTGAGCTTAAGTAAATGTGTGGGAGTGACAGATGAAGGTC
TCTGCTCCCTCGTAACAAAGCACAAAGACTTAGGGAAGCTTGACATTACTTGTTGCCGCAAAATTACTTG
CGTATCAATTTCCCATATAACGAATTCTTGTGCCTCCCTTACTTCTCTTAGGATGGAGTCTTGTACTCTAGT
CCCAAGAGAAGCTTTTGTTCTAATTGGACAGCGTTGCCAGTACCTTGAAGAGCTTGACCTTACAGACAAC
GAAATTGATGATGAAGGTCACCCCTTTACCTTAAAGGTCTCAGGCTTCTGTTGTAATTTTGAAAGTTCCTC
CTTTATATCTGACGGTGTCCTACATATGCAGGCTTGAAGACCATTTCCAAGTGTGCCAGCCTTTCCAGCTT

AAAGCTAGGGATCTGTCTGAACATAACCGATCAGGGTCTTATCCACATTGGCATGTGCTGCTCAAATCTT
AAGGAGCTTGACTTATATAGGTATGTATTTCACAGTTCATGGGATATTATTTGAGATAGTGTAACTATAT
ATCTTGTGCAGTTTTTTTTTATATTCTTTTCTTTTTGTGATGGATTGGCTAAACTCACTTTTTAACTATGGAG
TACAAGAGATTGAGTGTGCTGATTACTGGGTGATTCTTCTTGACAGGTCTGCTGGAATTTCCGACTTGGG
AATATTAGCAATCTCTCGCGGTTGTATTGGCCTTGAAATGATCAATATTGCATATTGCAATCGTATTACTG
ATGGCTCATTCATATCTATATCAAAGTGTTCAAAGTTGAATACACTTGAAAGTCGAGGTTGTCCTCTTGTC
ACATCTTTAGGTCTTGCAGCTGTTGCTGTGGGATGTAAGCAGCTTACAACATTAGACATCAAAAATTGCC
ACAACATTGATGATGCCGGAATGATTCCACTTGCTCACTTCTCGACAAACCTCAAACAGGTAACCACAGT
TTCTCTTTCGTCGACATTGACGAACTGGAGCATTGACATCTAATATGAGCTGGCCATCTGTCAATTACACA
GATAAATTTATCCTTCACTTCAGTGACGGACGTGGGGCTGTTGTCTCTTTCCAGCATCAGCGGTCTACAG
AACATGACAATCCTGCACCTCAAGGGTTTATCTCCTGGTGGGCTAGGAGCTGCACTGTTGGCTTGTGGC
GGACTTACTAAAGTTAAGCTGCAGACATCATTTAAATCATTGCTACCACAACCTCTCCTTCAACATCTAGA
ATCCCGGGGTTGTGTTTTTCAGTGGAGAGAGAAACCATTTCAGGTATCTTTCTCATATTTATCATTACCTA
CTTTTTCTCTGAAAATATGTGAGGTACTTCTATGGTTGTCGAAATATGCCGTCGTATGAATCAATTAGAAT
TACATATTTTAGCTTAACATGACATAGTTATTACTTGATCTAGAGGGTCAATGCAGTTTCAGAGGCTTTCT
TGTTTGGCATTTAATTGAGACAATAAAATTACAGTTTTGATTATCTTTGTTGTAATAATGAAAATATCCTTT
TTGCTTGCTCTAGTCATTAATTATCTTTCTTTTATTGGTTAAACACAGGCTGAAGTAGATCCAATATACTG
GAAAATTCAGCTGGACAATTTAGACTGAAGATTTGAGCCATTTATGCGAGTTGTCCTAAGATGGATAGT
CGCCCCAGAACCTCCGGAGTCCTTACTTCATCTGTTAGCACTTCATCAGGCAAGGTGCATTGTTCATGGC
GACTGTTTTGCATGTTAAGTGTCTGCTACTTGTTCGTCTGTATCCTCTAAACACTCTGTAAATTTCAAAAAC
CGGGAAAAACTCAATGTCCATTCAGCATTGCCCCGGCGTGAAATCAATTTTGTCAACAATGTTATGAGAT
GGATTAAACAGATCCAAATCATGTTTATTGTTTAGCAATCAATATCTGGGTTTGTGAATGCTTATTCCTCT
TGACTAATGTGATTTCGCGTGAAGTTACTTCAAATTTACTCTCAATTAAATAGTTATTTG
>PGSC0003DMG400011900 LRR-RLPs
TTTTAGGGGATTTGGAGAAAGAGCTATCATCTCTTACATCCATTTTAGAGAAGGAGCTATCATCCATTTTC
AGAGATGTCGTGCACCATGAACATAAAATTCCTAAAGATCTTCAGAGACGTACCATCAATTTGGCATATG
AGGCTGAGGTTGCCATTGACTCTATTCTTGCTCAGAATAATGCTTTTTTGCATATTTTTTGCTCACTTCCTA
CAATTTTAAAAGAGATCAAGCAAATTAATGCAGAGGTGACTGAGATGTGGTCAACGGACATTCCTCTTA
ATCCTCACTACGTGGCTGCTCCATTTAAACATCTGCCAGCTCGACATAGCAATCTTGTGACTGATGAGGA

GGTAGTGGGTTTTGAGAATAAAGCAGAAAAACTAATTGCTTATCTGATTAGAGGTACAAATGAGCTAGA
CGTCATCCCAATTGTAGGCATGGGGGGACAAGGGAAAACGACAATAGCTAGAAAGTTGTACAATAATG
ACATCATTGTTTCTCGCTTTGATGTTCGAGCATGGTGCATCATTTCTCAAACATATAGCCGAAGAGAGCT
ATTAAAAGAGATTTTCAGTCAAGTTACGGGCTCCAAGGACAAGGAAGATGAGGTAGGCAAACTTGCTG
ACAGGTTGAGGAAAAGCCTAATGGGAAAGAGATATCTCATTGTATTGGATGATATGTGGGATTGTATG
GCATGGGATGACTTAAGGCTTTCTTTTCCAGATGTTGGAATGAGAAGCAGAATAGTCGTAACAACTCGA
CTTGAAGAAGTGGGTAAGCAAGTTAAGTACCATACTGATCCTTATTCTCTTCCATTCCTCACAACAGAAG
AGAGTTGCCAATTGTTGCAGAAAAAAGTGTTTCAAAAGGAAGATTGCCCGCCTGAACTACAATATGTGA
GTCAAGCAGTTGCAGAAAAATGCAAAGGACTCCCCCTAGTGGTTGTCTTGGTAGCTGGAATAATCAAAA
AAAGGAAAATGGAAGAATCTTGGTGGAATGAGGTGAAATATGCTTTATTTGACTATCTTGATAGTGAGT
GCGAAGAATATAGTCTGGCGACTATGCAGTTGAGTTTTGATAACTTACCCGATTGTTTAAAGCCTTGTCT
TCTTTATATGGGGATGTTTTCGGAGGACGCAAGAATTCCAGCATCTAAATTGATAAGTTTATGGATTGCT
GAAGGATTCGTGGAGAACACTGAATCTGGGAGATTAATGGAAGAGGAAGCTGAAGGTTACTTGATGGA
TCTCATTAGCAGTAACGTGGTAATTGTTTCAAAGAAAGGTTATAATGGTAAAATCAAATGCTGCCAGGTT
CATGATGTTGTGCATCACTTTTGCTTGGAGAAGGGTAGAGAAGAAAAGTTTATGCTTGCAGTGAAGGGT
CAATATATCCAGTCTCAACCCTTGGATTGGAAGGGAAGTCGAGTGAGCTTCAGTTTCAGTGAAGAGCTT
TCCAAGTTTGCATCTCTGGTTTCCAAAACACAGAAGCCTTTCCACCAACACTTGAGGTCTCTGATAAGGA
CAGATGGTGAAAAATCTATTGATATGATTCCCTTCTGTCAGATTAGTGAATTGCGACTTCTTAAAGTCTTG
GATTTGAGTTCTTATTATGTGGAGTTTTTGTGGTTAGCTACATTCAAACCACTTATTCATCTGAAGTACCT
CGCAGTTCGGGCAGGTGATTTCTATTTTGATCCAGGATCACATCTGCCCCATATAGAAACTTTAATTGTG
AAGAATTCTCCTTATGTATGGTTACCAGTGTCTTTTTTGCAAATGGAAAAATTAAGGCATGCTCATTTTGG
TTGGGCTGTTTTTGATAAGCAGGGACTCTTTGAAGGATCCTCTAAATTGGAAAATTTGAGGATATTAAAG
AAAATTGAGAAATTTCCAATTGATAGGGCGGATGTGTTATCAAGGAGGTGTCCTAATCTTCAACAACTTC
ATATCGAATTTGATGGACATTCTGCAGATTCTTTTTGTCTCACATTGGAGAATCTTACCCAGCTTCAAATA
CTTCGTGTTTCCTTTGAGCGGCACCACATCATTGTATCTGGGTTGCAATTGCCTTCAAATTTAAACAAGTT
GGTACTTAAAGGAATTCATATAGAAAGTGTTATTCCCTTCATTGCGGGACTACCAAGCCTGGAGTATCTC
CAATTACAAGATGTGTGTTTTCCTCAATCAGAAGAGTGGTGCCTTGGAGATATCACTTTCCATAAACTTA
AGTTGTTGAAACTGGTGCAGTTAGGTATCTCAAGGTGGGATGCCTCGGAGGAATCATTTCCCTTGCTTG
AAACACTTGTTATAAAAATGTGTGGTCACCTAGAGGAGATCCCACTTAGCTTTGCTGATATTCCATCATT

GAAACAGATTAAATTGATTGGGTCCTGGAAAGAATCTCTGGAGGATTCAGCTGTGAGAATTAAGGAAG
AAATCAAAGAGATTGAAGGATGTGATCGTATACACCTCGTCAAAGAACTGACAGATTGGGAGGCATATA
GCAATTTGTCGCAGCTGTGCTTATACTGAAGTCTATTTTGTAGCTAGCCCATTCTCTTATATTGTAACACT
ACTACTGTTTGTTATGTTTGTTTTGATTGATCAACTCTTTTTATGTATGGTTGGAGACAAATATATATCAG
ATGATCTTTCAC
>PGSC0003DMG400013644 LRR-RLPs
TTTCCACAGAACCGAACAAGACACATTTTCTGTCTTAGATAACTCATCCTTTCTCTTCCCTTCTTCTTCTTCT
CTCTGTTCTCCACATTTCACGTACACTTTCAGAAACATTGTTGATTTACTTTGGACAGAAAAGCCACTAAA
TAGTGTGAGTCAATTTCTTTCCTTAAATAATTGTTTGGAGTTTTGTTTTAATGGGAAGGTTTTTTTTTCATT
CATTTTTATGAGTGTTTTTTCCGTGTTTTTGAAAACAGTTGTTTTACATATAGGAAAATACGGCGAAAAAT
AAGCTTTACTTTGCATGCCTTCATGTGGGTAAAGGTTTGAATTTGCTGTCATCGTTGATTGGCGCTTTCTA
GAAACTTGTAGAAACAAAAAACTTAGCTGCGGTTTTCATTTTTCTTGATCTGCTGATTGGTTTAGATATAG
CGGAATTCATCTTTGTTTTTTGCTTTGAATAATACATACACAACGAGGAAAAGGGTAGTCTGAGTCTAAT
TTTTGTGGCCGGAGTTGTATTTATGTTCAATTAAATTTATCTGGGTGTTTCTTGTTTTCGAGAAATTTTCAG
AGGATTATTGAAAATGGGGTCGTCAGGAATGTCGATTGATGAGATTGTGCAAGAAATTATGAGAATTCA
TGGATCTCTACCGACAAGGCCATCGATTGATGAGGTTGAAGGAGCAAGAGGTTTAATTGGAAATCTGG
AGAAAGAAGATCAGTTGAAAATAGAAGCTATCATGAGGCAAAATAAAAGGAAAGGTGTACCAGAAGA
GCTGTTCAAAATTTTGCAAGAGATGCAGAGGAACGCTGTTCTTTTTCAGAGTAAAGAACAAAAGAGGGA
GGCGTTGAAACTTTTAGATCTCGACAATATTCACAGTGTGTTCGACGATTTGATTCAGAGAGCTACGAAA
TGTTTGCCTTCTAAATCTCACGATAATGACCCGTCTTCTTCTTTAATTGAACCGAATGATTTATCTTTGGCA
AACCAAGTTTCCTTCTCCGGCGGCGGCCTTAATTCTCCGACAACTACAACTGCGGCTGCTTCTTCGTCGA
GCTATTATAACTCAACAGAGCCTATTAAAGCGTCTGAACTGTATAGTAGAGATGACAGTTACTTGAATAA
GGCAAAAGCCACGTTTCTTGTGGATGGAATTGGTGTTGGGCTTCGATCTGGGGATGCTTTATCAGGGCC
TAAGATTGTTGATTCCACCTTAAAGCCGGCGTCAATAAGTGGTATGTTACATCTATTTGCTGCACCGTATT
TCTAGTGAATTTGATTATATAATTTGTTGTGTTGACTCAAAGTTCGAATTTTTTGATCAAGAAGACACTGG
TTTCTTTGTTTGTAGGTCAAGATGGTGAGAAATTAAGTTTAATTAAGCTTGCTAGTATGATTGAAGTGTC
ATCCAAAAAAAGGAAGTCGAGAACTTATCCTTCGGAATAAGTTGTCTGACCAAGTAGAATGGCTTCCAGA
TTCTATAGGGAAGCTATCAAGTTTGATCACTTTGGATCTGTCTGAGAATCGAATTACGGTCCTACCCACC
ACCATAGGGGGGCTTTTATCATTGCAAAAGCTAGATTTGCACTCAAACAAAATTGTTGAACTGCCTGATT

GTATTGGAGATCTTCTTAACTTGGTCTATCTTGATCTCAGTGGAAATAACTTGAAGTTGTTGCCCGCGAG
TTTTGCTAGGTTAGCTCACCTTCAAGAACTTGATTTGAGCTCTAACATGCTATCAGTACTCCCTGAGACAA
TTGGCTCCTTAGTAAGTCTTAAGAAATTGATTGTGGAAACTAATGACCTTGAGGAACTTCCTCACACTATT
GGTCAATGTACTTCGCTCAAAGAGCTTCGTGTGGACTATAATCATCTTAAAGCTCTTCCTGAAGCAGTGG
GACGAATTGAGTCTTTGGAGATTCTGACTGCGCGGTACAATAACATTAGGCTGTTGCCTACAACTATGTC
ATCCCTTACAAGTTTGAAAGAGCTGAATGTTAGTTTCAATGAAATCGAATCAGTGCCTGAGAGCTTGTGT
TTTGCTACTTCACTGGTAAAGCTGAACATAAGCAACAATTTTGCAGACCTGAGATCACTACCAAGGTCCA
TTGGTAACCTTGAGTTGCTTGAAGAATTGGACATGAGTAACAACCAGATACGCGTTCTTCCAGATTCGTT
TAGGATGCTCTCAAGTTTGCGGGTCCTTAAAACCGATGGAAATCCTTTGGAAGTGCCACCAGGAAACAT
CGTTGAGAAGGGAGCACAGGTTTAATTTTGTTTCGTTTAACTATTATGCTTTCTGAATTAGTGTAGTATTT
AATTATCTTAGCTCTTCTCTTGGATATATCAATCACTAATGTTTTATTTCCTCAGGCTGTTGTACAATATAT
GTCTGATCTGGTTGCCAATAGAGAGGTAAAGGCGCAACCCGTAAAGAAGAAGAAGAAATCATGGACTC
AAATATGCTGCTTTTCAAGTTCCAACAAACGTCAGCGCAATGGCAGTATGGACTATGTTAATGCCTGATG
TGTTCCACACAAATTTGGTATGATTATATATTTCTCCTTCTTACCAAGTTAGTCATTATGGTACTGAACTTT
CCATATTTCATAAACTTCGTGTTCAGTCAAACTACATCCCATAGACTATGGAGGGAGTTTTAAATATATAT
TGTTAATAAATGGGAATGGTGCATTATACATGGTGCTGATTAGTGTTAATTAGAATAATGAAAATGAAG
GGTTGTAAGGGTAACGGCTCACATGATGTCTGTGTTATCCGTTGTAGGGAAATGAGCTGTGATTTGGCT
GGACGAAAGCTGCAAAATTTCAGTTGAATTCGAACATTTTTGCCAGCGAGATTCAATCAACTTTCTTGTT
GTTCCAGATAAGCTCTAATCCACCACGTGGTTTTTTATTCTCATTTCATGTAATAATTCTTTTAATGCTCTTT
CCTGTATTTTCCTCGATTATGATGTAAAATATCAAATGAGATGTAAAATCTTATCTTATTTTTTTACTTTCA
ATATATTCAATGAATTCCAGAATTTCTG
>PGSC0003DMG400013766 LRR-RLPs
ATGAAGAGTGCTACAATTAGCAATTTGCCCTGTAATGTCTTGGATAAAATTCTTGGGAGCTTGCCTTTGA
AAGATGCAGTGAAGACCAGTATCTTGTCAAAAGGTTGGAGGTACAAATGGGTAACGCGTGCAGAACTT
GATTTTCGTAGTGAGTTTTTCACATCTTTCAACGACAACCAAGAAGCCAAGAAAATCATTTACCAAGTCCT
ACGACTGCATCAAGGACCAATACTAAAGTTTACTCTCCAACATCCTAATTTGATATGCTATCGTGATATTT
ATAATTGGATGCTCTTCTTGTCAAAGAAAAACGTCCAGGAACTCACCCTTCAAATATTCACAGGCAACAA
ATATCATTTACCTTCTTATCTTTTCACATTTCAGCAACTAAGACATCTGGACCTTGATATGTGCTTCTTCCA
CCCTCCACCTGATTTCAAAGGGTTTGCAAAGCTTATTAGTATTAATCTTCAACATGTCATTTTTGATCCAAC

AATATTTAGGAATCTCATAACCAAATGCCCATTGCTTGAAAGTTTGATGTTGACTAGGTGCACCGCATTT
GACGTTCTTGAAATTGATGCCCCTAATCTCAAATGCTTCGACTTTCTTGGAACATCGAAGTTTATTTGCTT
CAAGAATGTCCCTATGCTTAAAACCGTTGCTGTATGTCTCAATCAAATTTTGATGGATACGTCAACTTTTT
GTTCTAATCTTACAAAGTTCTTCCATTTCATGCCTTCCCTGGAGGAACTTGAACTAGGCGGTTCATCATTA
GAGGTAATGTACATTTGTGATAATATTACTTATGATAGTTGGTACTTATTATAATCATATGATGTGTCTTC
AACCAATTTTTTTACATGCTTCTAGTACTTGAACATGGGAGGTATACCAGAGAATCCCCCGGCTGCTCTA
AACAATGTCAAATCCCTATGTATTTCGGACATGTCTTTCAGAAGTGTTGAGGAGGTTTCTAGTGCAGTTT
ACTTGATTACAAGCTGCCCTAAATTACAAGAACTTACAATTGAATGTGTAAGCACCCTCCTACTTTCTCAT
AAAACTTATTGATATGGTTTCTTCATCTGAATCCGCCTAACTGTTACCAAATTTTTTGCAGGAAGCAGTAG
GCATTGTGGTGGAACCTGTTATACAATTCTTACGAGCCAAAGCAATCTCATGTGGTGCTATGAAGCTGCT
TAAAAGTGTGGAAATGCGTTATTTTATTGGTTTTGAGATGGAAATAGAATTTGTGAAGTTTATATTGGCA
TCTGCACCAGTACTTAAGGAAATCTTCATTTGGAGTAGTGGGCAGTTTCATCGGGGTACACAGATGATG
GATGAGATAAAACAATTCCATCGAGAATCACCCAATGTTAGATTCAAATTCGAAGAAATGTGA
>PGSC0003DMG400013898 LRR-RLPs
CCCCTTTGGCTTCAAGGCTTACAAGTCATCGTCCGCTAGTATATATATCTATGAGAACCCTTTTAATGTAA
AAGCTCAAAAAAGTTGTATTATTGTTCTTGAAACCAAGTACTCTACCTATTAGACATGACAACTTCTCACA
GTACCTTCCTTTACATTCTACTTGTGCTTCTTCTTTTTTCACTTCCATTCACAATTACATCATCAGCAACAAC
TGAAGCTGAAGCTCTTGTCAAATGGAAGATGAACTTATCTTCTGCTTCTTTCTTGGATTCATGGTCCATTT
CGAATCTCGGAAACTTGTGCAATTGGACATCTATTGTCTGCAATGCTGGTGGAACAATTTCCGATATCAA
TCTTTCTGATGCAGGCCTCTCAGGCACTCTTGATCAGCTTGACTTCACTTCATTTCCGAGTCTCACAAGTTT
TTCTCTCAATGGCAACAACTTAAGTGGATCGATACCATCAAATATTGGCAATGCTTCTATGCTCACTTTCT
TGGACCTGAGCAACAATATCTTGGAGGGTGTCATACCAGAAGAGATTGAGAAGTTAACACAACTTGAAT
ATCTCAGTTTCTATAACAACAATATCATTGATGTTATTCCCTACCAAATAAGTAATCTTCAGAAGGTATGG
CACTTGGATCTTGGATCAAATTTTTTAGAAACTCCTGATTGGTCCAAATTGAGGAATATGCCTATGTTGAC
ATATTTGAGCTTTGGTTATAATGAACTAACATTAGAATTCCCTGAATTTGTACTCCGTTGCCATAATCTGA
CTTACCTCGATTTGTCGATAAACCATTTGAATGGTTCAATACCAGAAACACTATTCACCAACTTGGATAAG
CTTGAACAACTCAATCTTTCATCTAATTCATTTCAAGGTTCATTGTCACCAAATTTTACCAAGTTGACCAAG
TTAAAAGAACTTCAGCTTGGTGTCAACATGTTTTCGGGCTTAATTCCTGATGAAATTTCTCTGATCAGCAG
TCTTGAAGTTGTTGTACTTTTCAATAATTCACTTCAAGGAAAGATTCCATCTTCTATAGGTAGACTTAGAA

ATCTTCAACAACTTGATCTTCGAAAGAATCGTTTGAGTTCAACCATTCCTTCTGAGTTAGGTCTTTGTACT
AACCTCATTTTCTTGGCTCTAGCAGAGAATATCCTGCAAGGACCATTGCCTATATCCTTGTCCGCGGTGAC
CAAGTTATCTGACTTGAACTTGTCTGATAATTTTCTTTCTGGTGAGGTTCAATCATATCTCATTACCAATTG
GACTGAGCTGACATCTTTGCAGCTTCAAAACAATTCATTTTCACGGAAAATTCCACCTGAAATCAGCCAG
TTGAAAAACCTCGTATATCTTTACCTCTATAGTAACAATTTCACTGGTTCCATTCCCTATCAGATTGGTAA
GTTGCAAAACTTGCTGGATCTAGATGTCTCTGAGAACCAGCTTTCAGGCACAATACCTCCAACCATTGGA
AACCTGACCAATCTAAGGACGTTGCAGCTTTTTCGGAACAACATCAGTGGAACCATTCCTTCTGAGATTG
GCAAATTGACATCTCTTCAAACCCTTGACCTCAACACTAACCGGTTTAGTGGTGAGCTGCCCGACAGCAT
TTCTAACCTCGGCAGTCTGAAGCTTCTTGAGTTCCTTTCTCTTAGTAACAACCAGTTTTCAGGTGTGATCC
CTTTGTCTATTTGCAATTCGACATCACTACAAACTCTAGTTTTCGCAAGAAACAATTTCAGGGGAGAGATT
CCACAATGTTTGGGTAATATCAGTGGCCTCTCCATTTTGGATATGCGCTGCAACAATCTTTCTGGGATTCT
TCCAACAAGTTTTGATATTGGATGTTCACTTAGAACCTTCAACTTTCATGGAAATGAACTACAAGGGAAA
ATCCCTCGATCTTTAGCCAATTGTAAAGAGTTGCAAGTTCTTGATTTAGGAGACAATCACCTCAATGATA
CATTCCCCATGTGGTTGGGAACTCTATCAATGTTGCAGGTTTTAAGTTTGAGATCGAATAAATTGCATGG
ACCCATTAGAACTTCAAGGATTGGAAACATGTTTCCTGGTCTTCGAATATTAGATCTCTCTCGCAATGCCT
TCACAGGAAACTTGCCGACAAGTCTATTTCAACATTTGAACGCCATGAAGACAACTAATCAAACAAAGA
ATGCACCAATTGAAGGAAGCCACTATTACCAAGACTCGGTAGTTGTTGTAATGAAGGGACGCATGTTTG
AAGTTGTGAGAATCTTGTATTTATACACGAGTATGGATCTTTCAAGTAACAAATTTGAAGGACTTATTCC
AAGTATTATGGGAGATCTCATTGCACTTCGGGTGTTGAATTTGTCTAACAATGGGTTGCAAGGTCATATA
CCGCCATCACTTGGAAATTTAGCGGTAGTAGAATCATTTGACCTTTCATTCAACCACCTTTCAGGAGAGA
TACCGGAACAACTTGCTTCTATAACAAGTCTTGCAGTTTTAAATCTCTCCTACAATCATCTTGAAGGATGC
ATTCCTCAAGGACCTCAGTTTGCTACATTTCAGAACAGTTCATATGAAGGTAATGATGGATTACATGGAT
TTCCAGTTTCAAAAGGTTGTGGTAATGATCAGGTATCAGAGACAAATGATACCGTATCTGCACCAGACG
AACAAGAAAGCAATTCGGAGTTTCTGAATGATTTTTGGAAAGCTGCTCTTATGGGCTATGGAAGTGGAC
TATGTATTGGATTATCCATAATATATTTCATGATCTCAACTCAAACTCCAAAATGGCTAGCAAGAATCATG
GAAGAGTTGGAACACAAAATTATTACGCGAAGTAGAAAGAAGCAACGAGGTAAAAGGCATTATAGAAG
AAGAAAATAATCGCTTCTAGACAAGTTACCAACTGAGTAGGATTTGATTTCAGAACTTTGGGTATTCAAG
TTAAGGTCTATGCTGATTTTTAGTTTATAGTAGCTATATGTATTGATATATATACACTTTCGTTACAACAAC
AGTTAATCGATACTTTATACTAGGTATATTTTTCTTTCCAATAATTT

>PGSC0003DMG400013955 LRR-RLPs

ATGAAGCAAGAAACTCTTGAAGATGTTCATACTGGTGATATGGTAGCTTTTATCGAAGAAATCAAAGAA

GTAACTCCTGAACATGCAGTGTTCAAAAGTATGTATGCCGATGGTGACCTTCATGAAGAAAGCATTGAG

GGACATGTATTGGAGATCGAAGTCTCTAGTCAATCATCTGTCATATCGAGGTCAGTAACTGGCTTAGATT

GAACATTGGAAACAAATGGAGAAAATGATGACCAAATAGATGAAGAAGTTGATTTTGAAGTTTCAAAG

ACAAATGATATGATTTTTGAAAGCTTTGAAGCTTCCAAAAAATCTATCGAGGAGATCATAAGAGAATATG

GCAATGGATCCTATGTTGGTGTTGAGACTTCACAAGAAATTGATAGAGTTTCTCTGGATAATATAATTGT

CGAGAAGTTAAGAGAAAGGGGGAGTTTGAGAACTCAACAAGGAGATAATTTACATGGATCAACTGTCG

GTGAAAAATCTTTATCATTTGAAGAAGGAAGAGGAGTCCGGAAGAAAGTGCTAAGATTTTCACCAAGGC

GTGGCTCAAAGCTTCGTTTGAGCTCTTCCATGACAAGTGTGGGGTAGTTTCCAAAAAATCACTTTAAGGG

GGAGTGTGAAAAATTAAAGTTGATTTTTTGGAGGTACAAAAAATATCTTAGATATTTAGTAAAACTTTCT

AGAATTTTGTAGAGTAAATATCTTTGATATTTATACTTAAGAAATATCTAGATGTTCTAGATAAGTAGATA

TTTGTAGTAAAGTATAGAAATATCTAGGTTCTTGTTACATAGTTAGAAGATAAGAATATCTAGATTTTTCT

TGTAGAAGTATCTAGAAGCATCCCTATACAAGTATAAATAGGGGTGGCATTAGGCATTTGTAAAACAAT

CTAATAACAACCCAAATAAAAGAGTGTTCTTCCATAAACAAGTTCTCCTTTCTAGCTTCCTCTTCTTTAGTT

GCATATTCCAATCTTAGTTAACGATCTTGGGCTAGCAGAAGGTTTCCCAATACTTTTCTTCTGCTATTCTCT

ACAATTCTATCATTCAGGTCTCCGGCCCAATCGCATCAGTGTACATGTAAAAATTGTTGTCTGAGTTGCG

AAGAATTCTAAGACCTGAAGTTGTTGTGGCTTTAAGATACCGTAGTAGTATCCTCTTAACCGCCTTCCAG

TGTTCATGGTAGGTTCATGCATAAACTGTGATAGTTTGTTGACAGCAGGCCGTGTGAATGATAAATCCTG

TAACTTACCAAGGGTGCGCCTGTAGAGTGTAGCATGAGCTAGCAGGCGGAGAACCATCAGCTGCCCGA

GGTAGTTTACCGGAGCACATTGGAGTCAAGACTCCTTTACAATCTTGCATATCCAATTCAGACAAAATAT

CATGAATGTATTTGGATTGAGAGAGAATAAGACCATGACCATCTGACACTTTTTTTAACTTCAACACCCA

AAAAGTAGTCTAAGTAACCAAGATCCTTCAAAGAAAACCCACTTCTATTCCATTTTGTGAAACACAACCC

AAGTAGTAGTGTAGTACTGTCAACTGGATAAATTCCTTTTCTTATTTTCCTCATACTTAATAATCTGTTTTC

ACATTAATCTTTTTGCTACTTATTTTCCTCATCCATATTAATCTGTTTTCGCATTTTGAATATATTATATTTTG

GAATTAAAAGAAAAAAAAAATAAAAAAAATAAAATAGATTTAAAAAAAAAAAGTGAAATCACACATGAC

AATGTGTGTAGTAGCGTGTGTCACGTTTGATTTTCTTCAAAAGAAAACATTAAAATTTCTTCCATTTTGTG

CTAATTAGTGAACGTATGTTGTGTAACACAGCCTTGTAAGGTTGATTTGAAAGATTGACTCCCATTGACT

TTGCTCTTAACAATTATTCTTTCTATATTATGACTTTTATTTCGTTCTGTGAAACACAACCCAATTAGTGTCT

TTTTTTTTATTTTGCTATTTATTTTCCTCCTTTTAATCTGTCTTTACATTAATCTGTTTTCACATTTTGGAAAT
TAGTCCATACTTTTCAGCATTTTATTTGTTTTGGTGCTATATATATAGTGGAGTAACTTTGATCATTGGAA
ATACCACAAACACAAATTAATTGCCTTGAAATATTCTATATCATATGGAGAAGCACATTTTCTTATTGATT
CTTCTCTTTCTAATTCAATTTTCTGTATCATTTGCTTCCTCAAATGAGAGTGACCAACAAGCTCTATTAGCT
TTCCAAAATCTTATTACAAGTCCTAATCATTTTTTGGCCACTAATTGGACCAAAAATACTTCTTTTTGCTCT
TGGTATGGTGTCACTTGTAGTCCAAAAAGGCAAAGGGTTGTGGCCTTGACTCTTCCTAATTTGCAACTTC
AAGGCACAATTTCCCCGTCTTTGGCCAATTTGTCCTTCCTCAGTGAGATCAATCTTGGGAACAATAACTTC
TGTGGTGGCATCCCTTATGGACTTGGCCACTTGCCTCGCTTGCGAGTGATTGATATTCAAAACAATCAGT
TTAACGGAAGTATACCAACAAGTCTATTTCAAAATCGGAGAGTTCAAATAATTTCATTGGCTTTCAATGA
ACTCAGTGGTGAAATGTGGAGAGGGGCCATGGTATGTACCCGAACTCAGAGTCTTAAATCTCGGGAAC
AATAGCCTAACCGGTATAGTCCCTCCTTCTGTTGGAAATGCCACAAGAATGATGAACTTCAATTTGTCTG
GGAATAGAGTCAGTGGCAACATTCCAAAGGAGGTCGGTAATCTGAGCCAGCTTGCATTTCTGTCCTTGA
TCGATAATCAATTAACTGGTTCCATTCCTACATCACTTTTTAATATCTCGTCACTTCTTGTCCTATCTCTGA
GAAATAATATCCTTTCTGGTCCTCTCTTGCTAAACGAAGGAAATATTGTTTCAAATCTGAACCTTTTAAGT
ATATCTTACAACCAAATATCTGGTTGCATTCCTTCCAACATATGCCAACTCACAGAGCTCAAAGTTTTGTC
CATATCTTTCAACAATATAGCTGGAGACATACCCAGAAATATTGGTTGTTTATCCAAACTCGAGAAATTTT
ATATGGGTGATAATCCAATAAAAGGGACTATTCCCACTTCATTGGGAAATATTTCCACTCTGCATGATCTT
TACTGTCAAAACAATCGGATAGTGGGGGGAATTCCTCCGGAATTAGGGAAGCTATCAAATTTGAGGCAA
TTAACCTTTGACAATAATTCTAATCTTATTGGTCAAATTCCAGAGGCTATTTTCAACATATCTTCTTTGGAA
GTGATTGGTTTCACTTTAAACAACCTCTCCGGGAGAATTCCAACCACTACAGGTCTTCATCTTCCGAACCT
TAAAGTACTTGAGTTGGGAGTCAATCAGCTGGAAGGGGAAATTCCATTGTTCATAGCAAATGCTTCCAA
GCTTCAGAAACTGGAGCTAAATAATAACTTTCTGACAGGAACTATTCCTAATAATTTGGGGAATCTTCGT
GAGCTGCAAGCACTGCTCCTACATACCAATCAACTTACCAATGAACCAAGAGAACGTGAGTTGTTATTCT
TCAATTCTTTGGCGGACTGTAAGATGTTGCGATATCTACAAGTGGGTAAAAATCCGTTGAATGGCATTCT
GCCCAATTCAATTGGGAATCTTTCATCTACTATTGAAAACATTTATATAGGATATGCACACATCAATGGCC
TCATCCCCACAAATGTAGGCAACATGAGCGGTCTTACAGTCATAGACATTCAAGGAAACAACTTGACAG
GGAGTATTCCTTCTGAGATTGGTAAGCTTAAACAACTCCAAGGGCTGTATCTAGATAAAAATAAATTGCA
GGGACATATTCCAGAGGCGGTATGCCATTTATCTAATTTGGTTAAACTATTTCTGAATGGTAATGAGCTC
TCTGGATTAATTCCAGAATGCTTAGGAAATCTTAGCATGCTACAAAAGCTTTATTTGTATTCTAATAAATT

TTCATCAAAATTTCCATTGAGCCTTTGGAAGATGAGTAGTCTTCTCTATCTAAGTGTGTCACTGAATTCAG

TAGAGGGAGAAGTTCCATCAGATATTGGAGGAATGAAAGCCATTGTAGAACTACATCTTTCCAGTAACC

ACTTTTCAGGAATGATACCAAGCACATTCGGGGAACTCCAAAAACCTGCAGTATCTTGACCTGTCGAACA

ATTCATTTTTTGGCAAAAATTCCATTATCCTTTGCCAACTTGATAAGCTTAGAATACTTGGATTTGTCTTCA

AATGTGTTGTCAGGTACTATTCCTAAGTCTTTGGAAAAACTCTCATACCTTAAAAGCATTAATGTTTCCTT

TAATGATTTAGAAGGTGAAATACCGAGTGGTGGTGTGTTTGCAAATTCCACTCTGCAATCATTTCTTGGG

AACAAAGGTCTATGTGGAATGCACATATTGGAGATTCCTGCTTGTGCTATCACTAATCCTGGAAAACAAT

CAAAGCTTAGGGAGGTTCTGCTAAAATTTGTTACTCCAGTGGTTATTGCATCCTTTCTGATATTCTTGTTG

GTTTCAATTTGGATAATGAAACGACAGAAGAAAGGAAAGTCCAAAGAGGTAGAGAAGGTACTGGAGAT

CGGGACTCATCAATTAGTTTCTTATCACGAGATTCAACGAGCAACAAATAATTTTGATGAATCCAATTTA

ATTGGTGAGGGAAGCTCTGGCTCTGTGTACAAAGGCACATTGTTTGGTGGAACTGCAGTGGCCATTAAG

GTTCTGCATTTGGACAATGAGCAAGTATGCAAGAGATTTGATACCAAATGCAAAGTGACGAGAAATGTT

AGACACAGAAATCTTGTTCCAGTGATTACTACATGTTCTAGTGACTATATAAGAGCCTTTGTTCTGCAATT

TATGCCCAATGGAAGTCTTGAGAATTGGNNNNNNNNNNNNNNNNNNNNNNNNNNNNNNNNNNN

NNNNNNNNNNNNNNNNNNNNNNNNNNNNNNNNNNNNNNNNNNNNNNNNNNNNNNNNNNNN

NNNNNNNNNNNNNNNNNNNNNNNNNNNNNNNNNNNNNNNNNNNNNNNNNNNNNNNNNNNN

NNNNNNNNNNNNNNNNNNNNNNNNNNNNNNNNNNNNNNNNNNNNNNNNNNNNNNNNNNNN

NNNNNNNNNNNNNNNNNNNNNNNNNNNNNNNNNNNNNNNNNNNNNNNNNNNNNNNNNNNN

NNNNNGAGACATTGGGCACTCTTGGATACATTGCACCAGGTATAATAAATCTTCTCTCTTTGATTTTCTC

TTATCATAGTTAAGCATTCTAGAAGTAAAAAAGCAAGTCTTCATTTATGCAGAATTATTGTTGTATTTCAA

TTGAGTAACTTTTCTTCAATCCGTTTTTAAGAATATGGCTCAGAGGGAATAGTGTCCACTAGTGGTGATG

TTTATAGCTATGGCATCATGTTGATGGAGGTTTTGACGAAAAGAAGGCCAACAGATGAAGAGATAGGC

AATGAAAATCTTGACTTGAGGAAATGGATCATACAATCATTTTCAGGGAGTATGATGGACGTTGTGGAT

GTCAATCTTTTTTCTGAGGAAGAAGAAATCACTAGTAAAAGTGAAATGTGCATAGCGTCCATGATAGAA

TTGGGTTTAGACTGCACAAAGGAAACGCCAGAATCAAGAGTGACCATGAAAGAAGTAGTTAAGAGGCT

TAACAAAATCAACAACACATTTCTGGAAACATAG

>PGSC0003DMG400014463 LRR-RLPs

GCTAAAGATGGGGTAATGCAATTGTGCTCACTCTCGTCTTTTTCTGTCTTGAAGTTTCTTCATCCATATTA

CCTCATCACTCTCACAAACATCAAATTCAAACCATTTCCCTTCTAAAATTCAAGAAGTCCTTTACTATAGAT

CCCTCAGCCTCTTATTTTTGTCAAGACTTCGGTAAGAAGTTTTATCCAAAAACGAGTTCATGGAACATGA

GCAGAGATTGCTGCTCATGGGATGGAGTTGTATGCGATGACATGACTGGACATGTCATTGAGCTTGACC

TCGGCTGCAGCGGTCTTGTTGGGACCATTGATTCCAATAGCAGCCTATTCCAACTCTCTCATCTCCAAAG

GCTTGATCTTTCTTTAAATAACTTCTCCAATTCTCACATCTCCCCTGAATTTGGCAGGTTTTCGAGCTTGAC

ACATCTTGATCTTTCAGGCTCCTATTTTTCAGGTCAAATTCCTTTTGAAATCTCTTATCTTTCCAAGTTACAC

TCTTTTCGTCTCTCCACAAATATTGGGTTTGTTGGCCTCCCCTCATATTTTGGGCCCATACAAAGACTCGC

GGCCCATGATTTTAAATTGCTCCTTCAGAATTTGACCCAATTAAGAGAACTTGATTTTAGTTTCATAAACA

TCTCTTCAACCATTCCTCCGAATTTTTCTTCTCATTTCACAACTCTGAGTCTGGGATTAACAGGATTGTATG

GGATAATACCTGAGCGTATTTTTCACCTTCCCAACTTGAAAAAACTTGACTTATCCAGCAGTTATGAGCTC

AGTGGTTATTTTCCAAAGACCAAATGGAACAACAACACATCTCTTAGGAAATTAGAACTCAGTGGCGTG

AATTTTTCTGATAATATACCTGAGTCTCTTGGCTATCATCTAACTTCGCTGAATTATTTTTCTCTTTCGCATT

GCAAGCTTTGGGGGCCTATTCCTAAATCTCTTTCCAATCTCACTCACATAGAGTATTTGTACCTTGATGGT

AACTTGTTGAATGGAACAATACCATTAGGGATGTTCTCCCTTCTATCACTAATTTATTTAGAATTGAGTCA

TAACCACTTTTCTGGTCAGCTTGAGGATTTCAAGTCAAATTCACTATTCTATATTGGTTTAAGCAATAATC

AGCTGGAAGGCCATCTTCCAAAGTCAATTCAAAACCTTGTGAACCTGACAACTCTTGATCTTTCATCGAA

TAATTTTAGTGGCAATGTGGATGTCAGCCTCTTTTCAAACCTCAAACAACTTTCGTATCTTATTCTTTCATC

GAATAATTTTAGTGGCAATGTGGATTTCAGCCTCTTTTCAAACCTCAAACAGCTTTCATATCTTATTCTTTC

TTCCAATAATTTTATTGGAAATGTAGATGTCAGCCTTTTTTCAGACCTCAAACAGCTTTCATATCTTAGTCT

TTCACAAAATCATTCCTCATTGACCAATGAAAACAAAGTTAAGGTTACTTTGCCAGGATCTCTTTATACGT

TACAATTGGCTGCATGTGAAGTGAAAGAATTGGAGTTTCTAAAATCCACAAATCATCTTGTTTTCTTGGA

TCTGTCAAATAATAAGCTTCAAGGAAGAATCCCAGATTGGGCATGGTCTAAATGGATGTTTTCATTGCAA

TATCTAGATCTATCCTACAACATGTTGACAAGTGTTGATTCAATTCCTCTTCAATCACTAGATACTATTGAT

TTACGGTCCAATTTGCTTCAAGGTTCACTACCTGTTCCACCAAATTCCACAATATTCTTCTTAATATCCCAA

AATAATCTCACTGGAGAAATTCCTGCATCTATTTGCAATTTGATGTCACTCCAAATTTTGGATTTGGCGAG

AAACAATTGAGAAATTCCACAATGTTTGGGAAATATGACTCCCCTCCGGGTTTTGGATATGCGCCACAAC

AATCTTTCTGGGAATATTCCACCAACTTTCAGTAATGAAAGTATATTGAGCAGCCTCAACTTGCATGGCA

ATAATCTAGAGGGGAAAATTCCCCGATCTTTGGTCAGTTGCAAAGACTTGCAGCTTCTTGATTTAGGCAA

TAATAACCTCAACGACACATTCCCTGTGTGGTTGGGAGCTCTTCCAGATCTGCTCATTTTAAGCTTGAGAT

CAAACAAATTGCATGGACCAATCAGAACATCAAGTTGGAGTGAGAACATGTTTCCTAAGCTTCGAATCAT

AGATCTTTCCTACAATGCCTTCTCGGGAAACTTACCTTCGAGTTTGTTTCAACATCTTAAAACCATGAGGA
CAATTGATCCATCAAGGGAAGCAGCAAGACATGGTGGAAATACATATTACCAAGATTCTATTATAGTTG
CAATCAAGGGATCTGAGCGTGAAATTGTCAGAATCTTGTATTTGTACACCGTTATCGATCTTTCAAGTAA
TAAATTTAGAGGGCAAATTCCAAGTATTATGGGGCATCTCAATGCAGTTCACATATTGAATTTATCTCAT
AATGCATTGCAAGGTGATATACCGACATTATTCGGAGATTTATCTTCACTTGAATCATTGGACTTGTCAG
GAAACAAACTTTCGGGAGAGATACCACAACAACTGGCTTTTCTTACTTCTCTTGCATTCTTAAATCTCTCC
CACAATCATCTCCAAGGATGCATTCCTCAAGGACCTCAGTTTTACACTTATGAGAACAATTCATATGAAG
GCAATGATGGATTACGTGGATTTCCTATTTCAAAAGGCTGTGGCGATACTCATCGTCTCTAAAGAATCCA
CGCCCTTTTTTTAGCGTTTAGGGCCGGGCGTCATGAAACAAGAATTCAGAATTTTCTAAGTGTTTCGTGT
GTATCAATTCATGAATTTTTGGAAATATGACTGATCGAATATTTTTGCCCAAATATTTATTGAAATCATCT
GTAATGACCTAGTGAAC
>PGSC0003DMG400014717 LRR-RLPs
ATGGTGAAAGTGTTTGAAGATCGTGGGGGATGGGCTGGGGCTGAGAGATACAGAAGGTAATGGGATT
TGGGGAGGGAGCAACAGAACGAAGTGAGAGAAAAACTTTTTGAATTGATAATTTATCATTAATTAATGA
GTTTAAATTTTAAGATAATTATTTAACATCATATATAAGAGATTTGTGTATCTAATTAAATATTTAAAAAA
TAAAAGTGATATTGTGCGTAATTTTTTTAAACTAGAGGTAAATTAAGTATATATGGCACTAAAGTATGTC
TAACTAGGTAGTTTTTCCAATAATGAATTCATGAAAAATGCTTTTTTCTTTTCTAGCTCCAATGGTGAAAC
AAGGTTTGATCCTTTCCTTTTCAGGAACAAGGTGGGTTAACATTGTATCACATCAAGATGTCGATTCATTT
TTTGCCCTCAATGAAACGGAGAAATTTTAACCCCGATTTTTGAGCCTTTCATCTTTCTTAATCAAGACCTA
ACAACCGCCGAGGAGCACCTCAACATATGATTGTGCACAGTAACTGAGACTCCTATTGCACCTAAGGTC
AACTTCAATTCACCAAAACTAAAGTTTATCTTTGTGTAGTCATTGTAGACTCATACAAGTAGGGGAGGAG
CCAACATGTCTTTAGGGGTTCATCCGAACCCCCTTCGAAAATATTACTATATATACATGGTTAAAATTATT
TTTTAGATGTATATAATAGATGTCGAACCCCCTTCGACTAGTTCGTATGTTTACTCCTTCGAATTTTGAACC
CCCTTATTGAAAATCCTGGCTCTGCCACTGCGTACAAGGATATTGAGTAGATAGTTGATGTATCAGACTC
GATACTTCAAAAGAATGGAGGAGAAGACGATCTACGGCTGCAAGTGTGCATGAAGACAATATATGAAA
TAACTTAGCAAACATTCTTTGAAACAAACAGTTCACAATTCATAGATCAGAAAAATAATTTCCCAGCACG
TCTAAAACAAGCAAAAATGGAAAAGAAAAACAAAACAGTTCCAAAGGTAGATAAAAAGTTCTTAAGCTT
TGCAAACCACACCATCCCTCTGCAATGTCCAACACAAACGAATCAGATCGATACATAACCATCACAACGG
TAAAAAGATTATGCAATCACACCTTGATCTTTGCCAACACGAGCTTAAACCTGTCATAATCATTGAGTGA

GGCCGTCCTCTTCTGCACGATCGACTTCCTTCCTCAGGAACTGGACTCCCACTTGGTCTTCACATATGCGA
GGACATAAAGCAAATGAGAGTTAATCAAGTTCTTACGTAACCCTGTCGTTAGCCAAATGACTCAAATTAC
CATTTCTGGATGATGAAAACCGTTTTTCCTAATTAAAAATTCAAATTTGAATACTGCAATTCAGCCAACGT
ACACGTATCTTGATCTCTAGCTCGGCCACTGTTTCCAGTGATATGTATTTTCTGAGGGGACTAATTAACAC
ATAGTCTAGTCACTTTAGTATTTAGTATTGTAACTCTAGTGCTACAAAGAGTTAAGTTTAGTAGTGACGAT
TCATGAGATTAACCTCTAAATTTTGTGATGACTGAAAATCAGAAAGTAACACAAGATGCTGTTCTATCTT
ATGAAAAAGATTAGAAAGGTGAAACTCTACTTCTTTTTAAGCTTTGTATTCTAATTGATAGAATTTATTTT
CTGTATGATAGTTTTGTTTTACTTGGAAAAAGTGGGAGTAGATGTGCAACACATGGATAAAGACAATAA
AAAGACAAAAGCACAACAACCCAAATTTGACTAAAGAATGACAACATTTTCTCTAATAAAATTGAACAGA
CAAAGTGTGAATAAGAGTTTAGAAAAAAATGGGAGTAGATGTGTAACACATGGATAAAGACAATTAAA
AGACAAAAGCACAACAACCCAAATTTGACTAAAGAATGACAATAATATTTGAGTGACTTTTCTCTTACAA
AAACAAAGAAAGGTGACTTTCTGAGATAATTATCTATAGTTGAATGACCACGTGAGAAATCAACTCTTGA
CGTCTACTACATATGAATTAGAGATAATGTAGTTTAGAAAATTATTAATTTAAGACTGAAAATTGTTGAA
ACAAATATGTCTATATCATATGCTAAGGCAGAGTGGTCATGATCAATGTGTGATAACAAAACTATTTTTG
CCACTTCGGTTAAGTCCTTGTCTGAATTAGTGTTGGTCTTTAATTATAATTTACGTACAATATAAAATCCC
ATGAACCCGAAACAATAAACTCAAAATATGATGATGGTTTCCAGCAAAATACTCTCTTCACTTCAGTGTCT
CACTCTTTTATATCTCTTTACAGTTACTTTTGCTTCGACTGAGGAAGCAACTGCCCTCTTGAAATGGAAAG
CAACTTTCAAGAACCAGAATAATTCCTTATTGGCTTCATGGACGCCAAGTTCTAATGCATGCAGGGACTG
GTATGGAGTTAAATGCTTTAATGGTAGGGTAAACACGTTGAATATTACAAATACTAGTGTCAGTGCTACA
CTCTATGCTTTTCCATTTTCATCTCTCCCTTTTCTTGAGAATCTTAATCTTAGCATGAACAATTTCTCTGGCA
TCATTCCACCTGAGATTAGTAATCTCACTAATCTTGTCTATCTTGATTTGAACATCAATCAGATTTCAGGA
ACAATTCCACCACAAATCGGATCACTAACCAAGCTTCAGATCCTCCGTATATTTGACAACCATTTAAATGA
CTCTATTCCTAAAGAAGTAGGTTACCTAAGGTCTCTTACTAAGCTATCTTTGGGTAGGAACTTTCTTAGTG
GTTCTATTCCTGCTTCATTGAGGAATTTGAACAACTTGTCCTTTTTGTTTCTTTATGAAAATCAACTTTTTG
GGCCTCTATTCCTAAGGAAATAGGTTACCTAAGGTCTCTTAGTGAACTAGATTTAAGTAGTAACTCTCTA
AATGGCTCTATTCCTGCTTCATTGGGGAATTTGAACAACTTGACTTATTTGTATCTTTCTGATAATCACATT
TCGGGCTCTATTCCTGAGGAAATAGGTTACCTAAGGTCTCATAGTGAACTAGATTTAAGTTATAACTCTC
TAAATGGTTCTATTCCTGCTTCATTGGGCAATTTGAACAACTTGTCCTTTTTGTCTCTTCGTGAAAATCAAC
TTTCTGGCTCTATTCCTCAAGAAATAGGTTACCTAAGGTCTCTTACTGATCTTCGTTTGAACAATAACTTTC

TTAATGGTTCAATTCCTCTTGAAATAAGTTACCTAAGGTCTCTTAGTTTCATAGATCTGAGTATTAACTCTC
TTAATGGTTCTATTCCTGCTTCATTGGGGAATTTGAGAAATGTGCAAAGAATGTTTCTTGATGAAAACAA
TCTCACTGAGGAAATTCCTTTATCTATGTGCAATTTAAAATCACTTGATATTTTGTTTTTGCGGAGAAACA
ACTTGAAGGGAAAAGTTCCGCAATGTTTGGGTAATATTAGCGGCCTCCAGGTTATGACGATGTCACGTA
ATAATCTTAATGGAGAGATCCCTTCATCTATTTCCAATTTAAAATCACTACAAATTCTAGATTTGGGAAGA
AATAGTCTGGAGGGAGCAATTCCACAATGTTTTGGCAATATTAATACCCTACAGGTTTTTGATATGCAGA
ACAACAAATTTTCTGGGACTCTTCCAACAAATTTTAGCATTGGAAGTTCACTTATAAGTCTCAATTTGCAT
GGCAATGAACTAGAGGGGGAAATCCCTCGATCTTTGGCCAATTGCAAAAAGTTGCAAGTTCTTGATCTA
GGAAATAATCATCTCAATGACACATTCCCCATGTGGTTGGGAACTTTGTTAGAGCTGAGAGTTTTAAGAT
TGACATCGAATAAATTGTATGGACCCATAAGATCTTCAGGGGCTGAAATCATGTTTCCTGATCTTCGAAC
CATAGATCTCTCTAACAATGCCTTCTCGAAAGACTTACCAACGAGTTTGTTTCAACATTTGAAAGGCATG
AGGACAATTGATCAAACAATGATGGTACCAAGTTATGAAGGAGATGGAAGTTACGAAGACTCGATAGT
TGTTGCATCGAAGGGATTGAAGCTTGAAGTTTTGAGAATTTTGTCTTTGTACACAGTTATCAATCTTTCAA
ACAACAAATTTGAAGGACATATTCCTAGTGTTCTAGGAGATCTCATTGCTCTTCGGGTGTTGAATATATCT
CACAATAGATTGAAAGGCCATAGACCACCATCACTTGGACGTTTATCTGTAGTGGAATCATTGGACCTTT
CATTTAACCGCCTTTCGGGAGAGATACCACAACAACTTGCTTCTCTTACGTCTCTTGAATTCTTAAATCTCT
CCCACAATTATCTCCAAGGATGCATCCCTCAAGGACCCCAATTTCATACCTTTGAGAATAGTTCATTTGAT
GGTAATGATGGATTACGTGGATTCCCCGTTTCAAAAGGTTGTGGCAATGATCCTGTGTCAGAGACAAAT
TATACAGTATCTGCGCTAGCTGATCAAGAAAGCAATTCTGAATTTCTCAATGATTTTTGGAAAGCTGCTCT
GATGGGCTATGGCAGTGGATTATGTATTGGATTATCCATAATGTATTTCATGATCTTAACTAGAAATCCG
AAATGGCTTGCTAGAATCATTGAAGAAATGGAACACAAAATTAACATGCGAAGGAGAAAGAAGCAGCG
AGGTTAA
>PGSC0003DMG400014718 LRR-RLPs
ATGATTCTCAATGATAACAATCTCATTGGGGAAATTCCTTCATTTGTGTGCAATTTGACATCACTGGAAGT
GTTGTGTATGCCGAGAACCAATTTGAAGGGAAAAGTTCCGCAATGTTTGGGTAATATAAGTGGCCTCCA
GGTTCTAGTGATGTCACATAATAATCTCAGTGGAAAGATGCCTCCATCTATTTCCAATTTAACGTCACTAC
AAATTCTAGATTTGGGCAGAAACAATCTGGAGGGAGCAATTCCACAATGTTTTGGCAATATTAGTAATCT
CCAGGTTTTTGATATGCAGAATAACAAACTTTCTGGGACTCTTCCAACAAATTTTAGCATTGGAAGTTCAC
TTATAAGTCTCAACTTGCATGACAATGAACTAGAGGGGGAAATCCCTCGGTCTTTGGCCAATTGCAAAA

AGCTGCAAGTTCTTGATCTAGGAGATAATCATCTCATTGACACATTCCCCATGTGGTTGGGAACTTTGAC
AGAGCTGAGAGTTCTAAGATTGACATCAAATAAACTGCAAGGACCTATAAGATCTTCAGGGGCTGAAAT
CATGTTTCCTGATCTTCGAATCATAGATCTCTCTAACAATGCCTTTTCAAAAGACTTACCAACGAGTCTGT
TTCAACAATTGAAAGGCAGGAGGACTGTTGATCAAACAATGAAGACACCAAGTTATCTAGGAGGTAGAT
ATAACTACCAAGACTCGGTAGTTGTTGTAACAAAGGGATTGAAGCTTGAAGTTGTGAGAATTTTGTCTTT
TTACACAGTTATCGATCTTTCAAACAACAAATTTGAAGGACATATTCCTAGTGTTCTAGGAGATCTTATTG
CGATTCGGGTACTGAATTTATCTCATAATGGATTGCAAGGCTATATATCACCATCACTTGGAAGTTTATCT
GTACTGGAATCATTGGACCTTTCATTTAACCGTCTTTCGGGAGAGATACCACAACAACTTGCTTCTCTTAC
GTCTCTTGAATTCTTAAATCTCTCTCACAATTATCTCCAAGGATGCATCCCTCAAGGACCCCAATTTCATAC
CTTTGAGAACAGTTCATTTGATGGTAATGATGGATTACGTGGATTCCCCATTTCAAAAGGTTGTGGCAAT
AATCCTGTGTCAGAGACGAACTGTACAGTGTCTGCGCTAGAAGATCAAGAAAGAAATTCTGAATTTTTC
AATGATTTTTGGAAAGCAGCTCTGATGGGCTATGGCAGTGGAATATGTATTGGATTATCCATAATATATT
TCTTGATCTCAACTGGAAATCTAAGATGGCTTGCAAGAATCATTGAAGAACTGGAACACAAAATTATCAT
GCGAAGGAGAAAGAAACAGCGAGGTCAAAAGAATTACAGAAGAAGAAATAATCGCTTCTAG
>PGSC0003DMG400014719 LRR-RLPs
ATGATGATGGTTTCTAGCAAAACATTCTCTTCGCTTCAGTTTTTCACTCTTTTGTACCTCTTTACAGTTACTT
TTGCTTCCACTGATGAAGCAACTGCCCTCTTGAAATGGAAAGCAACTTTCAAGAACCAGAATAATTCCTT
ATTGACTTCATGGATGCCAAGTTTTAATGCATGTGAGGACTGGTATGGAGTTATATGCTTTAATGGGAG
GGTAAACACGTTGAATATTACAAATTCTAGTGTCATTGGTACACTCTATGCTTTTCCGTTTTCATCCCTCCC
TTTTCTTGAAAGTCTTGATCTTAGCATGAACAATTTCTCGGGAACCATTCCACCTGAGGTTGGTAATCTCA
CTAATCTTGTCTATCTTGACTTGAACATTAATCAGATTTCAGGAACAATCCCACCACAAATCGGTTCACTG
GCCAAGCTTCAGATCCTCCGTATATTTGACAACCATTTAAATGGCTCTATTCCTGAAGAAATAGGTTACCT
AAGGTCTCTTACTAAGCTATCTTTGGGTAGGAACTTTCTTACTGGTTCTATTCCTACTTCATTGGGAAATT
TGAACAACTTGTCTTTTTTGCTTCTTTATCAAAATCAACTTTCTGGTTCTATTCCTAAAGAAATAGGTTACT
TGAGGTCTCTTACTAATCTATATTTGGGTAATAACTCTCTTAATGGATCTATTCCTGCTTCATCAGTGTGC
AATCTGACATCACTGGAAGCGTTGTTTATGTCGAGAAACAACTTGACAGGAAAAGTTCCACAATGTTTG
GGTAATATCATTCGCCTCAAGGTTATGACGATGTCACATAATAATCTCAGTGGAGAGATCCCTTCATCTA
TTTCCAATTTAACATCACTACAAATTCTAGATTTGGGCAGAAACCATCTGGAGGGAGCAATTCCACAATG
TTTTAGCAATATTAGTAGCCTCCAGGCTCTTGATATGCAGAATAACAAACTTTCTGGGACTCTTCCAACAA

ATTTTAGCATTGGAAGTTCACTTATAAGTCTCAACTTTCATTGCAATGAACTCGAGGGGGAAATCCCTCG
ATCATTGGCCAATTGCAAAAAGCTGCAAGTTCTTGACCTAGGAGATAATCATCTCAATGACACATTCCCC
ATGTGGTTGGGAACTTTGCCAGAGCTGAGAGTTTTAAGATTGACATCGAATAAATTGCATGGACCCATA
AGATCTTCAGGTGCTGAAATCATGTTTCCTGATCTTCGAATCATAGATCTCTCTAACAATGCCTTCTCGAA
AGACTTACCAACGAGTCTGTTTCAACATTTGAAAGGCATGAGGACAATTGATCAAACAATGAAGGTACC
AAGTTATGAAGGACATGGAGATTACCAAGACTCGACAATTGTTGTAACAAAGGGATTGAAGCTTGAAGT
TGTGAAAATTTTGTCGTTGTACACAGTTATCAATCTTTCAAACAACAAATTTGAAGGACATATTCCTTGTG
TTCTGGGAGATCTCATTGCGATTCGGGTATTGAATATGTCTCATAATGGATTGAAAGGTCATATACCACT
ATCACTTGGAAATTTATCTGTAGTGGAATCCTTAGACCTTTCGTTCAACCAGCTTTCGGGAGAGATACCA
CAACAACTTGCTTCTCTTACGTCTCTTGAATTCTTAAATCTCTCCCACAATTATCTCCAAGGATGCATCCCT
CAAGGACCCCAATTTCATACCTTTGAGAACAGTTCATTTGATGGTAATGATGGATTACGTGGATTCCCCG
TTTCAAAAGGTTGTGGCAATGATCCTGTGTCAGAGACAAACTTTACAGGGTCTGTGCTAGAAGATCAAG
AAAGAAATTCTGAATTGTTCAATGATTTTTGGAAAGCAGCTCTGATGGGCTATGGCAGTGGACTATGTAT
TGGATTATCCATAATATATTTCTTGATATCAACTGGAAATATGAAATGGCTTGCAAGAATCATTGAAGAA
CTGGAACACCAAATTAACACACGAAGGAGAAAGAAGCAGAGAGGTCAAAGGAATTACAGAAGAAGAA
ATAATCGCATCTACACAAGTTACCAGTAA
>PGSC0003DMG400014736 LRR-RLPs
TTTTCACTTGCTAGTCTAAAACATTACACACCATGAAAGTGTTAACAATAACACTATGGGTTCTCTTGTTT
TTTATTTTGAAAAATACCAAGTTTGTCATTTGTGTAGGGATTTGTAGAGAAAACGAGCAACGAGCATTGG
AGACCTTGAAGAAAGAAATACACGATCCATCGGATATTCCATCTTCTTGGATTGTCGGAAAAGATTGTTG
TGAATGGGAAGGGGTCGTGTGCAACAATCTATCTCGTCATGTGATTGAACTACATCTAGGTTATGTAACT
CGTCATGAAGAGATTGGGCGAGGTATGTTTTTTGATAGGCTCGAGTTTACTTATCCAAGGATAAATAGC
CTTGAGTGGCTGCCAAGTCTTTCGAGTCTTGAGAACCTACAGATGGTAAGTGTTGATCTTAGCAAAGTTA
ATGAATGGCTACAGGTTATTAACATGCTTCCTTCTCTTGTTGATCTTCGTTTATCCAGTTGTAGTCTTCATC
ATATAACACCTCTACTTGATCATCACAATTTTTCTTCACTCAAATTTCTTGATCTTTCTTTCAATTACAAATT
AAGCTCTTCTGTTCCGAAATGGGTTTTCAACCTCCCAAATCTCATTTTTCTTGACCTGAGTTACTGTAATTT
TACTAGCCCATTTCCTGATGGTCCAGTTAACTTGACTTCTCTCACAACCTTCAAGGCTTCTGGAAACTTTTT
CAATTGTCGTTTACCCAAATGGTTGTTTGATTTTAATAATCTTGAACATCTTGAGCTTTCTAGTAATATGCT
TTCTGGAAAGTTACCAAATGTAATTGGAAAGTTAGGGAAATTAGAATACCTTGATCTCTCATATAATCAA

TTTGAGGGAGATATTCCTATAAGTGTAACACGTCTTGCAGGATTAAGATCATTTAATCTTTCCAAAAATA
ACCTAACTGGTAGGATTCCAAATGACATTGGTGACATGAAAGTCTTGAAAACTGTTGATCTTTCAGAAAA
TCAACTTTATGGTCAAATCCCACAAAGCTTTTCGAGTTTGCCCACTTTGAGCTACTTGAATCTATCTGATA
ACAATTTGTCAGGTATGATACCAGTGAGCACTCAACTTCAAAGCTTTGATTCCACGAGTTTTCAAGGAAA
CAAACTCTGCGGACTTCCACTCTTGGTGAACTGCAGTTCGGATGGTAATATTCCAAATAACAAGCATGAA
GATGATGAGAGTGACAAAGATGAAGTAGATTGGTTTTACATTTCAATGGCTATAGGATTTGCACTAAGC
TTTTGGGGTGTTTGTGGTTCATTGTTTTTAAGAGGTCATGGAGACATGCTTATTTTCGTTTGTATGCTTAC
ATTTCTCATGTTAGCAATCATGTTGTTTCATTGTATATTCAAATGAATGTTTCTTAAGATGTTCAAACAATG
TTGTTTCTTTCATAGTTCCAATTTTCTTTTTTCAATCACAGAGTTGTGAG
>PGSC0003DMG400014738 LRR-RLPs
ATGGTGACGGATAAAGGCAACAAGTACCAGTATGACACCATTTTGGTGTTGTTTACAAGTACGGATATG
TCTAGAAATAACCTTTCTGGGGATATTCCTGTAAGTTTAATAGGTCTTGCAGGATTAAGATCATTTGATTT
TTCTAATAATAACCTAACTGGTAGGATTCCAAATAACATTGGTGAAATGAAAGTCTTGGAATTTGTTGAT
CTTTCAGAAAATCAATTTTACGATCAAATCCCACAAAGCTTTTCAAGTTTATCCACTTTGAGCTTATTGAAT
CTATCTGATAATAATTTGTCAGGTGTGATACCATTGAGCACTCAACTTCAAAGCTTTGATCCCACGAGTTT
TCAAGGAAACAAACTCTGCGGACTTCCACTCTTGGTGAACTGCAGTTTAGATGACAATATTCTGAATCAC
GAATATGAAGATGATGAGAGTGATAAAGATGAAGTGGATTGGTTTTACATTTCAATGGCTATAGGATTT
GCGCTAAGCTTTTGGGGTGTTTGTGGTTCACTGCTTTTCAAGAGATCATGGAGACATGCTTATTTTCATTT
CCTAGATCGTAGCTGA
>PGSC0003DMG400014739 LRR-RLPs
GCTATAAATACATATTACTTGGCCTAGCGAAAAATATAAGCAAACACATACTTTACCCAAATACTTTTCAC
TTGCAAGTCTAAAACATTACACACCATGAAAGTGTTAGCAATAACACTATGGGTTCTCTTGTTTTTTATTT
TGACAAATACCAAGTTTGTTGTTTGTGTAGGGATTTGCAGAGAAAATGAGCAACAAGCGTTGGAGACCT
TGAAGAAAGAAGTAGACGATCCCTCAAATATTCTCTCTTCTTGGGTTGTCGGAAAAGATTGTTGTGAATG
GGAAGGGGTCGTGTGCAACAATCTCACTCGTCATGTGATTGTGCTAAGCATTTATTTTGATTGGTTTGAG
TCGAGATATCTAAGGATCAATAACCTAGAGTGGTTGACAAGTCTTTCGAGTCTTGAGAACTTGGAGATG
GAAAGTGTTGATCTTAGCAAAGCTAATAATTGGCCACAGGTTATTAACATGCTTCCTTCTCTTGTTGATCT
TCGTTTATTCAATTGTAGTCTTCATCATATAGCACCTCTACTTGATCATCACAATATTTCCTCACTCAAGTC
TCTCGATCTTTCTGAGAATCGCAACTTAAATTCTTTTGTTCCCAAATGGGTTTTCTACCTCCCCAATCTTGT

TTCTCTTGACCTGAGTGATTGTAATTTTATTGGCCCGTTACCTGACGGTCCAGTTAACTTGACTTCTCTCAC
AACCTTCAAGGCTTCTGGAAACTCTTTCAATTGTCGTTTACCCAAATGGTTGTTTGATTTGAATAATCTTG
AACATCTTGACCTTTCTACTAGTGGCATTGAAGGTGTGATACAGAGTAAGAGCGGAAACATAACAAAAC
TTAAACATCTTGATCTTTCTAGCAATAACCTCAACTACACTATACCAAATTGGCTCTATCAATGCAAAGAT
CTAGAATCACTTCAACTTGGATTAAATCGTCTTGAAGGAACAGTTTCAAGTCTGATTTCAAATTTGAGCTC
AATAACTATTATTGACCTTTCTAATAATATGCTTTCTGGAAAGTTACCAAATGTAATTGGAAAGTTAGGG
AAATTAGGATACCTTGAACTCTCAGGAAATCTGTTTGAGGGAGATATATATGAATTATTCAATGTTAGGA
GTAATTTCCTTTCAGTGCCATTGCGAAATACTTCTTCTTTATCTACTTTGAGACTAGGTTGGAATAAACTC
ACAGGAGCTCTTCCGGAAAGTGTTGGCCAACTTTCAATGCTAGAAGACTTTTCCATCTATAACAATAGGT
TGGAAGGCGTTGTAACGGAAAGTCATTTCTCTAAATTAACACATTTACAATACTTTTATGCATCTAGAAAT
AATCTGACTTTAAAAGTGAGTCGGAATTGGATTCCGTCTTTTCAAGCCACAACAATGTTAATAGGCGGCT
GGAATATAGGCCCTTCGTTTCCCATGTGGCTCCGAACTCAGAACCAGATAATGTATGTCGACATATCAGA
TTGTGGAATACAAGGTGAGGTTCCAACTTGGTTCTGGAACTTGTCTTCTCAAATTCGATTACTCAATCTTT
CTCATAACCACTTTGTTGGTGAGGTTCCAATCATCTCAACAGATGAGCAGAGTGGTTCTCCGCTAATGTA
CTTGGCTTCCAACAATTTTAGTGGACCATTACCATTGATTTCAACCAATGTAAGAGAGCTAGATCTCTCAA
GCAATTCTTTTTCCAAAGGTTTATCTAACTTTTTGTGTGAAGCAAAGAATGGATCATACAAATTGAGAATC
TTAAACCTCGGGGGAAATGATTTATCAGAAGAAATTCCTGATTGTTGGATGAATTGGCCGGAGTTGAAA
GTTTTAATCTTGAGGGACAATAATTTGATTGGAAGCTTACCAAGATCTATGGAGGTTTTGAGTAATTTGC
GATCATTAGACTTACGAAGAAATAGACTTAACGGTCCGTTTCCTTCATCCTTGGAAAACTGCACAAAACT
GCATAAAATAGATTTAGCTGAGAATGAGTTTATTGGAAAATTACCATCTTGGTTGGGAATGAGGTTTCCA
ACTTTGATAGTCCTTATCCTTCGGTCCAATAAATTCGATGGTGAATTGCCTCAAGAACTTTGTCACCTCAA
AGATCTTCAAATCTTAGACCTTGCAAACAATACATTTGTTGGAATCATACCGAGGTGTATTGGCAATTTG
AGTTCGATGGTTAAAAAAAAAAAAGGAATTGGAAGATGATGAAGACTTAAATTACTCTTATTATATAGGA
GTTCTGATAGAGAGTGCAATAGTGACGACTAAAGGCAACATGTACCAGTATGACACCATTTTGGCGTTG
TTTACAAGTATGGATATGTCTAGCAATAATCTTTCTGGAGATATTCCTATAAGTGTAACACGTCTTGCAG
GATTAAGATCATTTAATCTTTCCAAAAATAATCTGACTGGTAGGATTCCAAATGACATTGGTGTCATGAA
AGTCTTGGAATCTGTTGATCTTTCAGAAAATCAACTTTATGGTCAAATCCCGCAAAGTTTTTCGAGTTTGA
CCACTTTGAGCTACTTGAATCTATCTGATAACAATTTGTCAGGTATGATACCATTGAGCACTCAACTTCAA
AGCTTTGATCCCATGAATTTTCAAGGAAACAAACTCTGCGGACTTCCACTCTTGGTGAACTGCAGTTCGA

ATGGTAATATTCCAAATCACAAGTATGAAGATGGTGAGAGTGACAAAGATGAAGTGGATTGGTTTTACA
TTTCAATGGCTATAGGATTTGCACTAAGCTTTTGGGGTGTTTGTGGTTCATTGTTTTTCAAGAGATCATGG
AGACATGCTTATTTTCGTTTTCTAGACCGTAGCTGGGAAATGTTACTTGCAAAGTTACCAATATGTTGAG
AGATATGGTTTAGGATATTTCTATCTTCAAAAGTTACTGTGCGTGCTTACATTTCTCATGTTAGAAATCAT
TTTGTATTCAATATGTAGACTTTTATGTCTTAACAACTTCCTCAAATGAATGTTTCTTAAGATGTTCAAACA
ATGGTGTTTCTTTCATAGTTCCAATTTTC
>PGSC0003DMG400014740 LRR-RLPs
GGCCTAGTGAAAAATATAAGCAAACAACTAATACACACAAAAAATACTTTTCACTTGCAAGTCTAAAACA
TTGCACACCATGAAAGTGTTAGCAATAACACTATGGGTTCTCTTGTTTTTTATTTTGACAAATACCAAGTT
TGTTGTTTGTGTAGGGATTTGCAGAGACAATGAGCAACAAGCGTTGGAGACCTTGAAGAAAGAAGTAT
ATGATCCCTCGAATCGTCTCGCTTCTTGGGTAGTCGGAAAAGATTGTTGTGAATGGGAAGGGGTCGTGT
GCAACAATCTCACTCGTCATGTGATTGAGCTAAGCATTTATAGTGATCAGATCGAGTCGAGATATCTAAG
GATCAATAGCCTAGAGTGGTTGACAAGTCTTTCGAGTCTTGAGAACCTAGAAATGTATAATGTGGATCTT
AGCAAAGCTAATGAATGGCTACAAGTTATTAACATGCTTCCTTCTCTTGTTTATCTTCGTCTATACAAATG
TAGTCTTTCAAGTCTTGAGCTCCGTTATGGTGGCATTGAAGGTGCGATACAGAGTAAGAGTGGAAACAT
AACAAAACTTAAACATCTTGATCTTTCTAGCAATAACCTTAACTCCACTATACCAAATTGGCTCTATCAAT
GTAAAGATCTAGAATCACTTTTCCTTGGATCAAATCGTCTTGAGGGAACAGTTTCAAGTCTGATTTCAAA
TTTGAGCTCAATAATTAGTATTGACCTTTTTGATAATATGCTTTCCGGAAAGTTACCAAATGTAATTGGAA
AGTTAGGGAAATTAGGATCGCTTGAACTCTCAGTAAATCAATTTGAGGGAGATATATCTGAATTATTCAA
TTTTATGAGTAATTTCCTTTCCATAGAATTTGGAAATAGTTCTTCTTTAACTGTGTTGAGTCTAGACAATAA
TAAACTCACAGGAGCTGTTCCGGAAAGTGTTGGCCATCTTTCAATGCTACAAGGCCCTTTGTTTCCCACG
TGGCTCAGAACTCAAAAAAAGATATTGAATGTCGACATATCAGATGGTGGAATACAAGGTGAGGTTCCA
ACTTGGTTTTGGAAATTGTCTCCTCAAATTCGATTACTCAATCTTTCTCACAACCAATTTGTTGGTGAGGT
TCCAATCATCTCGACAGATGAGCAAAGTGATTCTTGGCTATTAATGTACTTGGGTTCCAACAATTTTAGT
GGACCAATACCATTGATTTCAACAAATGTAAGAGAGCTAGATCTCTCAAACAATTCTTTTTCCAAAGGTTT
ATCTAACTTTTTGTGTGAAGCAAAGAATGGATCTTACAAATTGAGAATCTTAAACCTCGGGGGAAATGAT
TTATCAGAAGAAATTCCTGATTGTTGGATGAATTGGCCGGAGTTGAAAGTTTTAATCTTGAGGGACAAT
AATTTGATTGGAAGCTTACCAAGATCTATGGAGGTTTTGAGTAATTTGCGATCATTAGACTTACGAAGAA
ATAGACTTAACGGTCCGTTTCCTTCATCCTTGGAAAACTTCACAAAACTGCATAAAATAGATTTAGCTGA

GAATGACTTTTTTGGAAAATTACCATCTTGGTTGGGAATGAGGTTTCCAACCTTAATAGTTCTTATCCTTC
GGTCCAATAAATTTGATGGTGAATTGCCTAAAGAACTATGTCACCTCAAAGATCTTCAGATCTTAGACCT
TGCAAACAATACATTTGTTGGAATCATACCAAGGTGTATTGGCAATTTGAGTGCGATGGTCAAAGTAAA
AAAGAAAGTACTGGAAGATGATTTCGAGTTAAATTATTCCTCTCGTTATGGAACTTTGATAGAGAGTGCA
ATGGTGATGACTAAAGGCAACATGTACCAGTATGATACAATTTTGGTGTTGTTTACAAGTTTGGATATGT
CTAGCAATAATCTTTCTGGAGATATTCCTATAAGTGTAACACGTCTTGCAGGATTAAGATCATTTAATCTT
TCAAAAAATAATCTGATTGGTAGGATTCCAAATGACATTGGTGATATGAAAGTCTTGGAATCTGTTGATC
TTTCAGAAAATCAACTTTATGGTCAAATCCCACAAAGCTTTTCAAGTTTGACCACTTTGGACTGCTTGAAT
CTATCTGATAACAATTTGTCAGGTATGATACCATTGAGCACTCAACTTCAAAGCTTTGATCCCACGAGTTT
TCAAGGAAGTAAACTTTGCGGGCTCCCACTCTTGGTGAACTGCAGTTCGAATGGTAATGTTCCAAATCAC
GAATATGAAGATGAGGAGAGTGACAAAGATGAAGTGGATTGGTTTTACATTTCAATGGCTATAGGATTT
GCACTAAGCTTTTGGGGTGTTTGTGGTTCATTGCTTTTCAAGAGATCATGGAGATATGCTTATTTTCGTTT
TCTAGACCATGGCTGGGAAATGTTGCTTGCAAAGTTACCAGTATGTTGAGAGATATGATTAAGGATATT
GCTATCATCAAAAGTTAGTTTGTGTGCTTACATTTCTCATGTTAGAAATCATGTTGTTTCATTTTGTATTCA
AATATGTAGACTTTTATGTCTTAATAACTTCCTCAAATGAATGTTTC
>PGSC0003DMG400014753 LRR-RLPs
CAAACAACTACCACACACAAAAAATACTTTTCACTTGCTAGTCTAAAACATTGCACACCATGAAAGTGTT
AGCAATAACACTATGGGTTCTCTTGTTTTTTATTTTGACAAATACCAAGTTTGTTGTTTCTGTAGGGATTT
GTAGAGAAAACGAGCAACGAGCGTTGGAGACCTTGAAGAAAGAAGTATATGATCCCTCGAATATTCTCT
CCTCTTGGGTTGTCGGGAAAGATTGTTGTGAATGGGAAGGGGTCGTGTGCAACAATCTCACTCATCATG
TGATTGATCTAAGCATTTCTATTGATTGGCCTTATTCGAGATATCTAAGGATCAATAACCTAGAGTGGTT
GACAAGTCTTTCGAGTCTTGAGAACCTAGAAATGGAGAGCGTTGATCTTAGCAAAGCTAATGAATGGCT
ACAGGTTATTAACATGCTTCCTTCTCTTGTTGATCTTCGTTTATACAATTGTAGTCTTCATCATATAACACC
TCTACTTGATCATAAAAAAATTTCTTCACTCAAATCTCTCGATCTTTCTGGGAATTACAACTTTAATTCATC
TGTTCCAAAATGGGTTTTCAACCTCCCCAATCTTGTTTCTCTTGACCTGAGTGACTGTAATTTTACTGGCCC
GTTTCCTGACGGTCCAGTTAACTTGACTTCTCTCACAACCTTCATGGCTTCTAGAAACTCTTTCAACTGTCG
TTTACCTAAATGGTTGTTTGATTTGAATAATCTTGAACATGTTGTGCTCTATCGTAGTGGCATTGAAGGTG
CGATACAGAGTAAGAGCGGAAACATAACAAAACTTAAATATCTCGAAATTTCTTACAATAACCTCATCTC
CACTATACCAAATTGGCTCTATGGATGCAAAGATCTAGAATCACTTGTCCTTGGTGGAAATCATCTTGAG

GGAACAGTTTCAAGTCTGATTTCAAATTTGAGCTCAATAATTAATATTGACCTTTCTAATAATATGCTTTCT
GGAAAGTTACCAAATGTAATTGGAAAGTTAGGGAAATTAGGATCGCTTGACTTCTCAGGAAATCTGTTT
GAGGGAGATATATCTGAATTATTCAATGTTAGGAGTAATTTCCTTTCAGTGGGATTGCGAAATACTTCTT
CTTTATATACTTTGAGACTAGGCTGGAATAAACTCACAGGAGCTCTTCCGAAAAGTGTGGGCCAATTTTC
AATGCTAGAATACATTTCCATCTCTAACAATAGGTTGGAAGGTGTTGTAACAGAAAGTCATTTCTCTAAA
TTAACACATTTACAATCCTTCTCTGCATCTAGAAATAATCTGACTTTAAAAGTGAGTCGGACTTGGATTCC
ACCTTTTCAAGCCTTTGAAATTGAAATAGGCAGCTGGAATATAGGCCTTTCGTTTCCCATGTGGCTCCAA
ACTCAGAACCAGATAATCTATGTCGACATATCAGATTGTGGAATACAAGGTGAGGTTCCAACTTGGTTCT
GGAACTTGTCTTCTCAAATTCGATTACTCAATCTTTCTCATAACCACTTTGTTGGTGAGGTTCCATTCATCT
CGACAGATAATGAGCAGAGTGATTATCCGCCATTAATAAACTTGTCTTCCAACAATTTTAGTGGACCAAT
ACCATTGATTTCAACCAATGTAAGAGAGCTAGATCTCTCCAACAATTCTTTTTCCAAAGGTTTATCTAACT
TTTTATGTGAAGCAAAGAATGGATCTTACAAATTGAGAATCTTAAACCTCGGGGGAAATGATTTATCAG
AAGAGATTCCTGATTGTTGGATGAATTGGCCTGAGTTGAAAGTTTTAATCTTGAGGGACAATAATTTGAT
TGGAAGCTTACCAAGATCTATGGAGGTTTTGAGTAATTTGCTATCATTAGACTTGCGAAGAAATAGACTT
AACGGTCCGTTTCCTTCATCCTTGGAAAACTGCACAAAACTGTATAAAATAGATTTAGCTGAGAATGAGT
TTATCGGAAAATTACCATCTTGGTTGGGAATGAAGTTTCCAACCTTGATAGTCCTTATCCTTCGGTCCAAT
AAATTCAATGGTGAATTGCCTCAAGAACTTTGTCACCTCAAAGATCTTCAGATTTTAGACCTTGCAAACA
ATACATTTGTTGGAATCATACCAAGGTGTATTGGCAATTTGAGTGCGATGGTCAAAGTACTAGAAGATG
ATTTCGAGTTAAATTATTCCTCTTATTTTGGAACTTTGATAGAGAGTGCAATAGTGACGACTAAAGGAAA
CATGTACCAGTATGACACAATTTTGGCGTTGTTTACAAGTATGGATATGTCTAGCAATAATCTTTCTGGA
GATATTCCTATAAGTGTAACACGTCTTGCAGGATTAAGATCATTTAATCTTTCCAAAAATAACCTAACTGG
TAAGATTCCAAATGACATTGGTGACATGAAAGTCTTGGAATCTGTTGATCTTTCAGAAAATCAACTTTAT
GGTCAAATCCCACAAAGCTTTTCGAGTTTGACCACTTTGGGCTACTTGAATCTATCAGATAACAATTTGTC
AGGTATGATACCATTGAGCACTCAACTTCAAAGCTTCAATTCCACGAGTTTTCAAGGAAACAAACTCTGC
GGAGTTCCACTCTTGGTGAACTGCAGTTCAGATGACAATATTCCAAATCACGAATATGAAGTTGATGAG
AGTGACAAAGATGAAGTGGATTGGTTTTACATTTCAATGTCTATTGGATTCGCACTAAGCTTTTGGGGTG
TTTGTGGTTCATTGCTTTTCAAGAGATCATGGAGACATGCTTATTTTCGTTTTCTAGATCGTAGCTGGGAA
ATGTTACTTCCAAAGTTACCACTATGTTGAGAGATATGACTTAGGATATTTCTATCATCAAAAGTTAGTGT
GTCAGTGTGCTTACATTTCTCATGTTAGCAGTCATGTTGTTTCATTTTGTATTCAAATATGTAGACTTTTAT

GTTTTAATAACTTCCTCAAATGAATGTTTCTTAAGATGTTCAAACAATGTTGTTTCTTTC

>PGSC0003DMG400014829 LRR-RLPs

CAAATATCGAAATTTTCCTAAACTACTTTGCACGAAAAAACAAAGGGAGTAGAAAGAGGAGGAAGAAA

TAGTATTCGTAGATTACATCTGAGCTTGACAACAAATTATGCAAGATTTTGAGTGAAACAACCAGACCTT

TCATGGTGTTTCTTGTTGGCTGCTAAGAAAACAATGGCGTTTTCACCTGAAAAATCAAATTTCAGGTCAC

CATTGAAAGGGCCACCATCTCCTAGCTGGAATGATTTGTTGCTCAAGAACAAGAAAGCACTCAATCATGT

CCTTTTCACTATGCATCTTCAATCCAGGTCCAATAAGCAAACCCCATCTTCATCCCCTTCTCCACAACTTCG

CAAATCCCTAGATTTCACTCTTCCGTCACTTGTATCCGATCCAATTTCTCTTCTTTCTGATCAAATACTTCTT

TCTATTCTCTCTAAACTCCCTGATTCACAAAGAAATTGTAATTCTCTTGTATCTAAACGCTGGCTCTATCTC

CAAGGTCGTCTGGTACGGTCGATTAAGCTTCTTGATTGGGATTTCCTTGTTTCGGGTCGGTTGTTTATTCG

GTTCCCGAATCTTATCCATGTTGATTTGGTTAATGGGTCTCTGATTTCACCGGGGAATGCGGGTGTTTTTT

GCTCTAACCGGTTGCTTTCTTCTCACGTGGATTGTAATGCTGATGCGAAAGATTGGTTTCTTAAGGAAAG

ATTTGTGTTGCCTTCTGATGAGATTGATAAGGGGTTGAGAATACTAGCAAGTGGGTGTCCTAATTTGCGT

CGATTATTGGTGGTAAATGCTAGTGAATTGGGATTGTTGGGGGTAGCTGAGGAATGTCCAACTTTGCAA

GTGTTGGAATTGCACAGATGTAATGATCAGGTTCTTCGTGGTATCGCGGCCTGTCAGAACTTGCAGATAT

TGAGATTGATTGGAAATGTTGATGGGTTTTATAAATCTTCGGTTACTGATATTGGATTGACTATTTTAGCT

CAGGGGTGTAAGAGGTTGGTGAGGCTGGAGCTGAGTGGTTGTGAAGGGAGCTATGAAGGGATTAAGG

CAATAGCACAATGTTGTCAAATGCTCGAAGAGTTGATTCTTCGTGATCATAGGATGGAGGGTGGTTGGT

TGTCTGCTCTTTCCTATTGTGAAAATTTGAACACCTTGAGGTTTATGTCTTGTAAGAGTATTGATCATTGT

GGATGGTTTGATGAAGATGTAGGATCTTGCCCGACACTTGAAAGATTACATTTCGAGAAATGCCAATTA

AGAAATAAGGAGAGTTTGAGAACGTTGTTTCTACTATGTCAAGAAGTTAGAGAGGTTATTTTCCACAACT

GTTGGGGACTGGATAATGAAATGTTCAGTCTATCCACTGGTCTAAGGTATTGGTTATATTATTCTGTTCTC

GTTTACTTTGCTTGCTGTTGCTGGAAAATTTCGTGCTGGATTAGCATTTTGTTATGCATTTGACATACATG

AACTTATTGTGTTTAGGTTTTATGACCTTGAAATGTAGTTCCTTATTTTTGTTAAAAATGACTTTTTAGTGT

TGCTGTAACCCCCATCCCCCGCCCCTCGTGTATAGTAGACGACCAAATGAAAGGATAGTTTGCTTGTAAT

AGTGCATTTTATTTTGGTCATAGTATACCCTTTGATCTTATCTTCTTGGTAAAAGGATAAGTGTGATAGAT

TTCCTTAGCTATTTGTGTTGGGGATAGATTGTTTTTTTAAGGCCAGTTTGTAGATGCATTTGTAATCATGC

ACCCTCTTGGTGTTTCAGTTATTAATGTTAAAATAGCCTTACCGTTTCTCAAAGAGTATACGCTTCCATCTT

GCGTTAGAGAAAACACATAATGTTCATGGCATGACAATTATGTTTCAATTCATTATTGATGCTTGATTTAT

CAATATCATGTGTGCTATCTTGGATTATAAAGGGTCCAGTTTTGAGTTTTATACACTGAACTTGCTTCAGT
TCTGACTGGCAATTTGGCATCATATTTTGTTTTGCATTCACTTGGAGCAGCAATAAAGTAACTGTCTCCGT
GTGACCTATAGGTCACTGATTCGAGCCGTGGAAGTAGCCACTAATGCTTGCATTAGGAGGGTAGGTTGT
CTACATCACACCCCTTGTGTGAACAATGGATACTTTGTGCACCAGGCTGCCCTTTTTAGTTCACTTGAATA
AAGGGCCTGTAATTAACTAAATAACATTATGTGAGGAATTTGGGTTTCACAGCTTCATTTTGGTGTTATTT
TCAACAGGAGAGTGAAGTCTATTTGCCTAGAAAGCTGTTCACTGCTCACAACTGAAGGCCTCGAATCTG
CACTCCTTTCGTGGAAGGAGATCCAGAGCCTCAAGGTGATATCATGTGGCAATATAAAGGATAGTGAAA
TCAGTCCAGCACTTTCTACCTTGTTCTCCACGCTAAAAGATTTTCAGTGGAGACCAGACACAAAATCTCTT
CTTTCAGCTGGTCTTGTGGGAACTGGCATGCGAAAAAGAGGCAGTAAATTATTCAAGAAGACGTGCGAC
TGGAAGTCACTGCCTGGTGCATAGACTGGCTTCCTCATCATGCATTCAGACAATAAATTATTCAAGAAGA
CATGCGACTGGAAGTCACTGCCTGGTGTATAGACTGGCTTTCTCATCATGCATTCAGACGACTCTACTAG
ATTTTACCTCCTCATTTATGCATATGAGAACTCTAACACATGAAAGAGGTCAGTGCGACACTTTGCTCCAA
TACTGTCAGCAATGAGGCCTTGATGTCATTATCCTGTATTTGTAGTCGCTTAAGTATGTAACGTAGGAAC
TATAGGATCGAAGATCGGCACTTTCTCAGGGTAGAGATGGAGAAAGAGGCCTAAGTTAGTGTATAGAG
CTCGATGCTGTAACCTCATGAGCATTCAGCTAAAGCCATCTCGCTGTATCTTTGTCAAGCTTAATGGTATG
TATTTCTGTATTGGAACCCCAAAAACTCTTAAAAGGGGTTTTCTTATCTACAATGTTATAAAGAATTTTGG
CACCTTTTATGCCATGACTACTGCCTTCCACAATTGTGTGAAAGCATATTTGATATGAACCATACCAGCAA
GCATGTCATTTGTGGAGGAGACGAGAACCAGAATAATTTTCTACATCAATGGCATCTATGTC
>PGSC0003DMG400015121 LRR-RLPs
TGGAAATCAAAACCATTTAATGTTTTGCATGATTGATGTGATACGTTTGCACTTTGCAGTGAACACTTGA
GAAAAGTGATATCGTAAAGACTTGGGCAAATACCAATTGTTCGGTCAACTCAACAACATAAGTATTGCTA
TATAGTAATACATTTGAAAAGCAAATCAAGCTTAGAATCAATAGTAACAGCATGAAGAAGAACATTATCT
TATTGATTCTTCTCTTTCTAATTCAATTTTCTTTATCACTTTCTTCCTCAAATAAGACTGACCAACAAGCTCT
ACTAGCTTTCCAAAATCTTATTACAAGTCCTAATCATTTTTTGGCCAATAATTGGACCAAAAATACTTCTTT
TTGCTCTTGGTTTGGTGTCACTTGCACTCCAAAAAGGCAAAGGGTTGTGGCTTTGGCTCTTCCTAATTTGC
AACTTCAAGGCACAATTCCCGATCTTGGCCAATTTGTCCTTCCTCAGTGTTCTCAATCTCCAGAATAACAG
CTTCCGCGGTGGCATCCCTTATGGACTTGGCCACATGCCTCGCTTGCGAGTTATTGATGTTAAAAACAAT
CAGCTCAACGGAAGTATACCAACAAGTCTATTTCAAAACCGGAGAGTTCAAGTAATTTCATTGGCTTTCA
ATGAACTCAGTGGTGAAATGTGGAGAGGGCCATGGTATGTACCCGAACTCAGAGTCTTAAATCTCAGGA

ATAATAGCCTCACCGGTATAATCCCTCCTTCTATTGGAAATGCCACAACATTGATGAACTTCAGTTTGTCT
GGGAATAGAGTCAGCGGCAACATTCCAAAGGAGGTCGGTAATCTGAGCCAGCTTGCATTTTTTCTCTTG
TCCGATAATCAACTAACAGGTTCCATTCCCGCAACACTGTTTAATATCTCGTCACTACTTGCCGTATCTGT
GTTACACAATAGCCTTTCTGGTCCTCTGTTGCTAGATGAAGGCAATATTGTGTCAAATCTGTACTTTTTTA
AGTATATCTCACAACCAAATTTCTGGTTGCATTCCTTCCAACATATGCCAACTCACAGAGCTCAAAGTTTT
GTCCATATCTTACAACAATATAACTGGAGACATACCCCAGAAATATTGGTTGTTTATTCCAAACTCGAGG
AGTTTTATATTGGTGATAACCTAATAAAAGGGACTATTCCCACTTCATTGGGAAATATTTCCACTCTGCAT
AATCTTCACTGTCGAAACAATCAGATAGTAGGGGGGATTCCTCCGGAATTAGGGAAGCTATCAAATTTG
AGGGAATTAAGCTTTGACAATAATTCTAATCTTATTGGTCAAATTCCAGAGGCTATTTTCAACATATCTTC
TTTGGAAGTGATTGGTTTCAGTTTCAATAACCTCTCCGGGAGAATTCCAACCACTACAGGTCTTCATCTTC
CCAATCTTGAAGGACTTTACTTGGGAGCAAATCAGTTCAAAGGGGAAATTCCATTGTTTATAACAAATGC
TTCCAAGCTTCAGATACTGGAGCTAAATAATAACTTTCTGACAGGAACTATTCCTAATAATTTGGGAAAT
CTGCGTGAGCTGCGAGGACTGCTCCTATATAATAATCAACTTACCAATGAATTAAGAGAGCGTGAGTTG
TTATTCTTCAATTCTTTGGCGGACTGTAGGATGTTGCAATATCTACAAGTGGGTTCCAATCCGTTAAATGG
CGTTCTTCCCAATTCTATTGGAAATCTTTCATCTACTATTGAAAACTTTTATATAGTAGATGCACACATCAA
TGGCCTCATCCCCAAAAGTATAGGCAACATGACTGGTCTTACATCCCTAAGCCTTGACGGAAACAACTTG
ACAGGGAGTATTCCTTCTGAGATTGGTAAGCTTAAACAACTCCAAGGGCTGTATCTAGATAACAATAAAT
TGAAGGGACATATTCCAGAGGCCGTATGCCATTTATCTAATTTGGTTCAATTATTTCTGAATGTTAATGA
GCTCTCTGGATTAATTCCAGAATGCATAGGAAATCTTAGCATGCTACAATATCTTTATTTGGGTTCTAATA
AATTTTCATCAAAATTTACGTTGAACCTTTGGAAGATGAGTGGTCTTCTCTATCTAGACGTATCACAGAAT
TCAATGGAGGGAGAAGTTCCATCAGATATTGGAGCACTGAAAGCCATTGTAGAACTACATCTTTCCAGT
AACCACTTTTCAGGAATGATTCCAAGCACATTCGGGGAACTCCAAAACCTACAGTATCTTGACCTGTCGA
ACAATTCATTTTTTGGCAAAATTCCATTATCCTTTGCCAACTTGATAAGCTTAGAATACTTGGATTTGTCTT
CAAATGACTTGTCAGGTACTATTCCTATGTCTTTGGAAAAACTCTCCTACCTTAAAAGCATTAATGTTTCA
TTTAATGATTTAGAAGGTGAAATACCGAGTGGTGGCGTGTTTGCAAATTCCACTTTGCAATCATTTCTCG
GGAACAAAGGTCTTTGTGGAATGCACATATTGGAGATTCCTGCTTGTGCTATCACTAATCCTGGAAAACA
ATCAAAGCTTAAGGAGGTTGTGCTAACAATTGTTACTCCAGTGGTTATTGCATCCTTTCTGATATTCTTGT
TCGTTTCAATTTGGATAATGAAACGACAGAAGAAAGGGATGTCCAAAGATGTTGAAAAGGTATTGGAG
ATCGGGACTCATCAATTAGTTTCTTATCACGAGATTCAACGAGCAACAAATAATTTTTGATGAATCCAATT

TAATTGGTGAGGGAAGCTCTGGCTCTGTGTACAAAGGAACATTGTTTGGTGGAACTGCAGTGGCCATTA
AGGTTCTGGATTTGGAAAATGAGCAAGTATGCAAGAGGTTTGATACCGAATGTGAAGTGATGAGAAAT
GTTAGACACAGAAATCTTGTTTCAGTGATTACTACATGTTCTAGTGACTATATAAGAGCCTTTGTTCTGCA
ATTTATGCCCAATGGAAGTCTTGAGAATTGGCTGTACAAAGAAGATCGCCACTTGAACCTTCATCAAAGA
GTCACTGTAATGCTTGATGCTGCTATGGCAGTCGAATATCTACACCATGGTCATAACACTCCTATAGTTCA
TTGCGACCTGAAGCCAGCCAACGTTCTTTTGGATGAAGATATGGTGGCTCATGTTGGTGATTTTGGAATC
TCTAAAATTTTAGCTGTCAGCAAGTCTGTGGCACATACAGAGACATTGGGCACTCTTGGATACATTGCAC
CAGGTATAAAAAATCTACTCTCATTGATTTTCTTTTATCATAATTAAGCCTCACCAAGTTGTAGAAGTAAA
AATAGAATTATTGTTGTGTTTCAACTGAGTAAATTTTCTTCATTCCTTTTCTAAGAATATGGCTCGGAGGG
AATAGTGTCTGCTAGTGGTGATGTTTATAGCTACGGCATCATGTTGATGGAGGTTTTGACCAAAAGAAG
GCCAACAGATGAAGAGATATGCAATGGAAATCTTGACTTGAGGAAATGGATTACACTATCATTTTCGGG
GAGTATGATGGACGTTGTGGATGCCAATCTTTTTTCTGAGGAAGAACAAATCACTAGTAAAAGTGAAAT
CTGCATAGCCTCCATGATAGAATTGGGTTTAGACTGCACAAAGGAAACGCCGGAATCAAGAGTGACTAT
GAAAGAAGTAGTGAAGAGGCTTAACAAAATCAAGAACGCATTTCTGGAAACATAGAAGTCATGGTGTT
CTTTGCTGAGCTGCAAATTAATATGATGCTGTTTGTTTCTTTAATAAAGTTGTTGTATAGTACTACTTGAA
GTCTTTGAGTAGTTTTGGACCTATTTTACTCTATATATTTGTGATTATGTTTTGAATGCTTGAAAGAAACC
AGAAATAAAGGTGTTTAGCTGCACTATTTTGTTTTTTGAATGTGAT
>PGSC0003DMG400015283 LRR-RLPs
ATGCACTCCAATACCAAAACAACATTTACGAGAGGTATCGTCTTTTGCTTCTTGACATTTTCCAAAATGGA
AGTTTTCACAACGAGTTTTAATTTCCACACCTTTCTGGCTATCCGTGCTGTGTTTCTTGTGTTTCTATTGTC
ATTTTCTCTCCAACATGCTGCATCAGCTGCTTTTCTTAGCAATGAAACTGATAATCTTGCCTTACTTGGATT
CAAGTCCCAAATAACCGAGGATCCATCAAGAGTTTTCGTCTCTTGGAATGATTCTGTTCATTTCTGTCAGT
GGACTGGAGTAAAATGTGGCCTGAGACACGTAAGAGTCATCCGTTTGAATCTCAAAGGGCTGAGGCTG
GCAGGTACGATCTCAGATCATCTTGGAAATCTCTCTTTTCTCAATTCTCTTGACCTTGCAGAAAATGCCTT
CCATGAAAAAATTCCTCAACAACTCAGCAGGCTGCCAAGGCTTCAATACTTGAATTTGAGTTTTAACTAT
CTAACAGGAGAAATTCCAGCTAATCTATCACATTGTGTTAAACTCAAGAGCCTTGTCCTAGACCACAACA
CTCTAGTGGGAAAGATTCCTTACCAAGTTGGATCTTTAACGAAGTTAGTGAACTTGTACCTCAGAAATAA
CAACCTGACAGGAATCATTCCAGGTTCTATTGGAAATCTTACATCTTTAGAGAAGCTGCATTTATCGTAC
AACAATCTAGAAGGAGAAGTGCCAGTTTCTTTAGCTCGATTAACCAAGTTGAGGATTCTTGGACTGTCA

GTAAATAGCTTATCTGGGGAGTTCCCTCCTCCGTTGTACAATTTGTCATCCCTGGAACTAATATCACTTTC
CCTTAACAACTTTTCTGGTAATCTCAGATCTGAGTTAGGCAACTACTTCCCTAATCTCCAAATACTTTACTT
GGCGAACTGTCAATTCATTGGTTCCATACCATCCTCCTTGGCCAATGCTTCAAAATTGCTAGAGCTTGATT
TTCCTGTAAACAATTTCACTGGAAATATACCTAAGGGTTTTGGTAACTTGCGGAATTTGTTATGGCTCAAT
GTTTGGAATAACCATCTTGGATATGGTAAGCATGATGACCTTGACTTTGTAAATTCTCTTACCAACTGCA
GCAGTCTACAAATGCTCCATTTTGGAGGCAACCAGTTTGGAGGTACATTACCTCATTCAATAGGCAACCT
TTCTAGTCAGTTACAACGCCTACTCTTCTTCGAAAATAGAATTGGTGGAAACATACCAAGAGAGATCTCA
AACCTGGTGAATTTGAATCTACTCGACATTGGTTCCAACAATTTTATAGGTAGTATTCCAGATTCTATTGG
AAGACTTACAAACTTGGGAGCTCTCAACTTGGTTAACAATCTCTTGACAGGGGTCATCCCTTTTTCAATA
GGAAACCTCACTGAACTCGTCTATCTTTATTTGGGACTTAACAGGTTAGAGGGTAACATACCTTCAACAT
TGGGAAACTGTAACCAACTGCTAAAATTGGGATTTTCTGAAAACAACCTGACAGGAACTATACCACAAC
AACTTTTTGCTCTCTCATCCCTCACAGATATTTTTGCATCTAATAACTCTTTGACTGGTCCATTACCAGTGG
ATATTGGTAACTGGAATCATCTTACTTATCTTGATTTTTCTTACAACAATCTTTCGGGTATGATTCCACAAA
CTCTTGGAAAATGCTTGACCTTGGGGGAGATCTATATGAAGGGAAACTCCCTCCAAGGAACCATTCCCA
ATTTAGAAGATTTGCCGGATCTCCAATCCTTGGATCTTTCCCTAAACAACCTATCAGGGCCAATCCCTCAC
TTCATTGCAAATCTTACTTCCCTACACTACTTGAACTTATCTTTTAACAATCTAGAGGGTGAGGTTCCTGTC
ACCGGAATATTTTCAAATTTGAGTGCAGATGTATTAAGCGGAAACTCCAAGCTTTGTGGTGGGATTCAA
GAGCTTCATTTACAACCTTGTATTTATCAAAAGACACGGAAGAAGCATGTGCTTGCCCTCAAGTTTATTTT
GATAATTGTTTTTGCTGCTTCATTTTCAATCCTGGCCTTGTTAGTAGTTTTCCTTTGTAGGAGAAGAAATTT
GAACAATCAACCAGCACCACAAGATAGGTCTAAATCTGCGCATTTCTACCCCAATATTTCGTATGAAGAG
CTCCGCACTGCAACAGGTGGATTTTCTTCTGAAAATCTTATTGGCTCTGGCAGTTTTGGAACTGTTTATAA
GGGAACTTTTCCCTCTGATGGAACGGTCGTTGCTGTGAAGGTACTCAAACTCCAGCATGAAGGTGCTTC
AAAAAGCTTCTTAGCAGAATGCCAAGCATTGAGAAACATAAGGCACCGGAACCTTGTCAAGGTCATCAG
TGTATGCTCAAGTTCTGATTTCAAGGGAAATGATTTTAAAGCTTTAGTCTTTCAGTTCATGCCAAAAGGG
AACTTGGACGAATGGCTACATCCAGAAAAGGAGATGAATGAGAAGAGTAGCTTGACCACACTTCAGAG
AATGAATATCATAATAGATGTGGCCTCTGCACTTCATTATCTTCACCATGAGTGCCAAACACCCATGATTC
ACTGTGATATAAAACCACAGAACATTCTTCTTGATGAAGATCTCACCGCTCATTTGGGTGATTTTGGTTTG
GTGAGACTCGTTCCTGAATTCAGTAATGAATCGGATCTACATCAGTTTAGCTCACTTGGAGTTCTGGGAA
CCATTGGCTATGCAGCTCCAGGTAATTTTTATATGTAAACAAAAACTATGAATATACCATATGGTTTCTTG

TAACAATAGTGGTTTATTTATGAGATGATCAATTTGCTTGTTGCAGAATACGGCATGGGTAGTAAGGTCT
CCGTTGTAGGAGATATGTACAGTTTTGGGATCCTAATATTGGAAATATTCACTGGAAGAAGACCCACAG
ACACTTTGTTCCAAGCAAGTTCTACCCTCCACCACTTTGTAGAAACAGCATTGCCAGAGAAAGTTATGGA
GATTCTAGACAAAACAGCTTTTCATGGTGAGATGAGTAAAGCAACCTGTGGCGAAGAATACTGGGGCA
ATATCAAGAAAGAACAGATGGAATGCTTGGTTGGCATACTTGAGATCGGTGTTGCATGTTCAGCTGAAT
CCCCAAGAGACAGATTGACCATGACACAAGTTTACAGTAAGTTGACACTAATAAGAGAAAAATTCCTAC
AAAGGGGAGATGGAGGGCACAGGCTACAAGTGAAAGCATTGGATTTAATCTAA
>PGSC0003DMG400015345 LRR-RLPs
TATTTCTAACAATAAGATTCGTGGCCAAATCCCAAACTGGTTTAGCGGCATGAGGTGGGACAACTTGTTG
TACCTAAACCTTTCGCATAATTCTTTAACAGGCCACCTACAACTATTTCATTTCCATAATCTAGAGTATCTT
GATCTTAAATTTAACTTCCTTCAGGGTCCACTACCTTCATCCATTTGTAACATGAACAATCTTAAATTGCTA
GATTTATCACGCAACAACCTCAGTAACTCGATTCCAAGTTGCTTGGGAAGCATGTCTAGTCTAACAGTGT
TGGACTTAAGAAGGAACAATTTCACAGGGAGTCTTCCCCCATTATGTGTGCAGAACACTTCATTGAGAAC
CATTGTCTTGAATGGCAATCAATTTGAAGGAACTCTCCCTGTGTCGTTGCTCAACTGTAGTGGTTTAGAA
GTTCTTGATATGGGGAATAACGCTATAAATGACACATTTCCTGCTTGGATAGGAACACTTGAACAACTGC
AGGTCTTAATATTAAAGTCCAATGTGTTCCATGGACCAATTACTACTTGTCAGACTACACTTTGCTTTCCC
AAGTTGCGAATTTTTGATCTTTCGCGTAACGAATTCAGTGGCTCAATCCCTGCAAAGGTTTTTGGAAACTT
CAAGGCAATGATTAAATTAGATGGTGAAGACACAGGACAGATCGAGTACATGGAACCATCTATGTTTCA
CACATTATATGATGATTCCGTGAGTTTGGTGATCAAAGGCCAGGATATTGAGCTAGAAAGAATCAGCAA
AATTATGACAACCATAGACCTCTCGAGCAACCATTTTGAAGGTGTCATTCCGAAAACACTAAAGGATCTC
AGATCACTTTGGCTACTCAATTTATCCCATAACAATCTCATAGGTCATATTCCAATGGAATTGGGGCAATT
GAATAAGCTTGAAGCGTTAGATCTCTCTTGGAACCGGCTCACTGGAAAGATTCCGCAGGAATTGACAAG
GCTAACATTTCTGGAAATGTTAAACCTTTCTCAGAATGTTCTCGTTGGACCAATTCCTCACGTTCTACAAT
TCAACACATTTGAAAATGACTCGTATGGCGGTAACCTTGATCTATGTGGTCCTCCTTTATCAGAGCAATG
TGGAACGAGTGATCTATCCCATGTTCCTCAACCATTGGAGTCTGAAGACGAGTCATATTTTTTTAGTGGA
TTTTACGTGGGAATCAGTAGTCATTGGCTACAGCTGTGGACTAGTTGTTGGAACTGTTGTATGGAGCCTC
ATGTTTAAATATCGTAAACCAAAATGGTTTGTCGAATTTTTTGAAGGCATTTATCCCAAGAAAATGAGAA
GGTCAATGAAGAGAGCTCAGAGACGACGGACTTAGTGGAAGATTGCAAGAG
>PGSC0003DMG400015347 LRR-RLPs

GTTGGTAATTAGTGAAAGCTTTTCTTCTTCCTCTGTGATGATGCATCATGTTTGCTCTCCCACTGAAGCTTT

TGCTTTGCTTCACTTCAAACAATCCTTTCAAATCTCTGACCAACCTACATGTTGGGATGATATCCCAAAAA

CAAAGTCTTGGAATGAGAGTAGGGATTGCTGCAGTTGCTGCAGTTGCTGCAGTTGCTGCAGTTAACCGG

TTATGTTATTGGATTGGACCTTAGTTGCAGTATGCTTACTGGAACTATTCATCCCAATAGCAGCCTCTTCC

AACTTCATCATCTCCGGACACTAAACCTTGCTCACAATGCCTTCTTTTCTTCAATCCCACACAACATTGGCC

GATTGACGAATTTGAGGCATCTCAACCTTTCTCATTCATTCTTTGAAGGGGAAATTCCAACAGAAATCTC

ATACCTTTCCAATTTGGTTTCACTTGAACTTAATTGTTATGGATGTGAACTTGATGAGAGAACATTTGAAA

CAATTCTTCAAAACTTCACAAATCTGGAGGTAGTTTCTCTCTTTGGTGTCAACATCTCATCTCCGATACCTC

TGAATATATCTTCATCCTCCTTAAAGTATGTAGATCTTGGATTTACTAATCTGCGAGGTGTTCTCACAGAG

AGCTTTTTCCTTCTGCCAAAATTGGAAATGCTCTATTTGAATTACAATGATCTTCTTAAAGGAGGTTTACC

AAAGATCCACCCGAGCAACACTCTGTTGGAGTTGCATATTTCATTCACAGGAATCTCCGGTGAGCTGCCT

GATTCAATTGGCACCTTCAGTTCCTTGAATCGCTTGAACATGTATGGATGCCAATTATCTGGCCGCGTTCC

TGATTCCATTGGCAATCTTACACAAATTAGGTATTTGGCTTTCAGTTATAATCATTTCACCGGCCATATTCC

TTCCACAATATCTAAATTGAAGCACCTCAGCTATCTAAAGCTTTCGTCCAACTCCTTTTCAGGTGAAATTC

CCGACATTTTTTCTAACCTCCAAGAGCTACGTTCTTTATATCTTCATAACAACAGTTTCATTGGTTCGTTTC

CTTCTACAATTGTAAGCTTGACACATCTTCAATACTTAGACTTGTCAAGTAATTCCCTATCTGGCCCACTG

CCTAATAATTTTAGCATGCTTCAAAAGCTAACCCAACTGGATTTGTCGTACAACGCACTGAATGGGACCA

TACCATCTTCGGTGTTTAGCCTCCCTTTGCTACCTCAATTATGGCTCGATAATAATCGATTTAGTGGACTA

CCCACTGAGCTCAAAACAATCCCAACATTAGAAAGCCTAGATTTAAGCCATAATCAACTCAGTGGTTCTT

TTCCTCAATCACTTGTGAATCTCACAAGCCTTTCTACCCTCGACCTTTCATCAAATAACATCACCATCGATG

AGGGAATACAAATCACTTTTCCTAGCCTACAAGATTTGTGGTTATCATCTTGTGAACTGAAGGATATTCC

ACACTTCTTGACAAATGTAAAGAAACTTCAGGTCTTGGATATTTCTAACAATAAGATTCGTGGCCAAATC

CCAAACTGGTTTAGCGGCATGAGGTGGGACAACTTGTCCTACCTAACCCTTTCGCATAACTCTTTAACAG

GCCACCTACAACAATTTAATTTCCATAATCTAAGGTATCTTGATCTTAAATTTAACTCCCTTCAAGGTCCAA

TACCTTCATCCATTTGTAACATGAACGATCTTCAATTTCTAGATTTATCACGCAACGACTTCAGTAACTCG

ATTCCAAGTTGCTTGGGAAGCATGTCTAGTCTAACAGTGTTGGACTTAAGAAGGAACAATTTCACTGGG

AGTCTTCCATCGTTATGTGCGCAGAGCACTTCATTGAGTACCATTTACTTGAATGGTAATCAATTTGAAG

GAACTCTCCCTATGTCGTTGCTCAACTGTAGTGATTTAGAAGTTCTTGATATGGGGAATAACGCTATAAA

TGACACATTTCCAGCTTGGCTAGGAACACTTCAACAAATGCAGGTCTTAATATTAAAGTCCAATCTGTTCC

ATGGACCAATTAGTACTTGTCAGACTACATTTTGCTTTCCCAAGTTGCGAATTTTTGATCTTTCGCGTAAC
AAATTCAGTGGCTCACTCCCTGCAAAAGTTTTTGGAAACTTCAAGGCAATGATTAAATTAGACGGTGAA
GGTACAGGAGAGATCAAGTACATGGAACCATCTGAGATGTCATCTGATGATTCTGTGAGTTTGGTGATC
AAAGGGCAGGATATTGAGCTAGAAAGAATCAGCACAATTATGACAACCATAGACCTCTCGTGTAACCAT
TTTGAAGGTGTCATTCCGAAAACACTAAAGGATCTCAGCTCACTTTGGCTACTCAATTTATCCCATAACAA
TCTCATAGGTCATATTCCAATGGAATTGGGGCAATTGAATAAGCTTGAAGCGTTAGATCTCTCTTGGAAC
CGGCTCACTGGAAAGATTCCGCAGGAATTGACAAGGCTAACATTTCTGGAAAAGTTAAACGTTTCTCAG
AATGTTCTCTTCGGACCAATTCCTCTTGGTCCACAATTCAACACATTTGAAAATGACTCGTATGGCGGTAA
CCTTGATCTATGTGGTCCTCCTTTATCAAAGCAATGTGGAACGAGTGATCCATCCCATGTTCCTCAACCAT
TGGAGTCTGAAGACGAGTCATATTTTTTTAGTGGATTTACGTGGGAATCAGTAGTCATAGGCTACAGTTG
TGGACTAGTTGTTGGAACTGTTGTGTGGAGCCTCATGTTTAAATATCGTAAGCCAAAATGGTTTGTCGAA
TTTTTGAAGGCATTTATCCCAAGAAAATGAGAAGGTCAATGAAGAGAGCTCAGAGACGACGGACTTAA
>PGSC0003DMG400015348 LRR-RLPs
ATGTCGTTGCTCAACTGTAGTGGTTTAGAAGTTCTTGATATGGGGAATAACGCTATAAATGACACATTTC
CTGCTTGGATAGGAACACTTGAACAACTGCAGGTCTTAATATTAAAGTCTAATATGTTCCATGGACCAAT
TAGTACTTGTCAGACTACGTTTTGCTTTCCGAAGTTGCGAATTTTTGATCTTTCGCGTAACAAATTCAGTG
GCTCACTCCCTGCAAAAGTTTTTGGAAACTTCAAGGCAATGATTAAATTAGATGGTGAAGACACAGGAC
AGATCAAGTACATGAAAGCATTTGACACATCGTATGAGGATTCTGTGAGTTTGGTGATCAAAGGCCAGG
AAATTGCGCTAGAAAGAATCAGCACAATTATGACAACCATAGACCTCTCGAGCAACTATTTTGAAGGTGT
CATTCCGAAAACACTAAAGGATCTCAGCTCACTTTGGCTGCTCAATTTATCCCATAACAATCTCATAGGTC
ATATTCCAATGGAATTGGGGCAATTGAATATGCTTGAAGCGTTAGATCTCTCTTGGAACCGGCTCACTGG
AAAGATTCCGCAGGAATTGACAAGGCTAACATTTCTGGAAAAGTTAAACGTTTCTCAGAATGTTCTCTTC
GGACCAATTCCTCTTGGTCCACAATTCAACACATTTGAAAATGACTCGTATGGCGGTAACCTTGATCTAT
GTGGTCCTCCTTTATCAAAGCAATGTAGAACGAGTGATCCATCCCATGCTCCTCAACCATTGGAGTCTGA
AGACGAGTCATATTTTTTTAGTGGATTTACGTGGGAATCAGTAGTCATAGGTTACAGCTGTGGACTAGTT
GTTGGAACTGTTGTGTGGAGCCTCATGTTTAAATATCGTAAGCCAAAATGGTTTGTGGAATTTTTTGAAG
GCATTTATCCCAAGAAAATGAGAAGGTCAATGAAGAGAGCTCAGAGGCGACGGACTTAA
>PGSC0003DMG400015349 LRR-RLPs
ATGGGAATTCTGTTTTTCTTTTATTCACTTGTCTGTTTTTTGTTGGTAATTAGTGAAAGCTTTTCTTCTTCCT

CTGTGATGATGCATCATGTTTGCTCTCCCACTGAAGCTTTTGCTTTGCTTCACTTCAAACAATCTTTTCAAA
TCTCTAACCAACCTAGCTGTTGGGTTGAGATTAATCTGCCAGATATGACCTTTGTTGATATCCCAAAAACA
AAGTCTTGGAATGAGAGTAGGGATTGCTGCAGTTGGGACGGGGTAACTTGTGACATGTTAACCGGTCA
TGTTATCGGATTGGACCTTAGTTGCAGTCTGCTTAATGGAACTATTCATCCCAATAGCAGCCTCTTCCAAC
TCCATCATCTCCAGACACTAAACCTTGCTCGCAATGACTTCTATTCTTCTTCAATCCCACATAACATTGGCC
GATTGACGAATTTGAGGCATCTCAACCTTTCTCAATCTTTCTTTGTAGGGGAAATTCCAACAGAAATATCA
TACCTTTCCAATTTGGTTTCACTTGAACTTAATTGTTATGAATGTGAACTTGATGAGAGAACATTTGAAAC
AATTCTTCAAAACTTCACAAATCTGGAGGTAGTTTCTCTCTGTGGTGTCAACATCTCATCTTCAATACCTG
TGAATATTTCTTCTTTCTCCTTAAGGTACGTGGATCTTAGACATACTAATCTGCGAGGTTTTATCACAGAG
AGCTTTTTCCTTCTGCCAGGATTGGAAAGGCTCTATTTGAGTGACAATGATCTTCTCAAAGGAGTTTTACC
AAAGATCCACCCGAGCAACACTCTGTTGGAGCTGGATATTTCATTCACAGGAATCTCCGGTGAGCTGCCT
GATTCAATTGGCACCTTGAGTTCCTTGAATATCTTGAATTTTCTTAATAATCATTTCACCGGCTATATTCCT
TCCACAATCTCTAAATTGAAGCACCTCACATCCCTAGATCTCTCGTCCAACTCCTTTTCAGGTGAAATTCCC
GATATTTTTTCTAACCTCCAAGAGCTACGTTTTTTATATCTTCATAACAACAGCTTCATCGGTTCGTTTCCT
TCTACAATTGTAAGCTTGACACATCTTCAAGACTTAGACTTGTCGAGTAATTCCCTATCTGGCCCACTGCC
TAATAATGTGAGCATGCCTCAAGAGCTAACCAAACTGGATCTGTCGTACAACTCACTGAATGGTACCATA
CCATCTTCGGTGTTTAGCCTCCCTTTGCTATCTTCATTGTCTCTCCAACATAATCAATTTAGTGGACTAGCC
GATGAGTTCAAAACAAACTCAACATTAGATTACCTAGATTTAAGCCATAATCAACTCAGTGGTTCATTTCC
TCAATCACTTGCGAGTCTCACAAACCTTTCTACCCTCACCCTTTCATCAAATAACATCACCTTCGATGAGG
GAATACACATCACTTTTCCTAGCCTACAAGATTTGCATTTATCGTCTTGTGAACTGAAGGATTTTCCACAC
TTCTTGAGAAATGTAAAGACACTTACGGTCTTGGATATTTCTAACAATAAGATTCGTGGCCAAATCCCAA
ACTGGTTTAGTAGCATAAGGTGGGACTACTTGTACTACCTAAATCTTTCGCATAATTCTTTAACAGGCCAC
CTACAACAATTTCATTTCCATAATCTAAGGTATCTTGATCTTAAATTTAACTCCCTTCAGGGTCCACTCTCT
TCATCCATTTGTAACATGAGCGATCTTAAATTTCTAGATTTATCACTCAACAACTTCAGTAACTTGATTCCA
AGTTGCTTGGGAAATCTTCAATTTCTAGATTTATCACGCAACGACTTCAGTAACTCGATTCCAAGTTGCTT
GGGAAGCATGTCTAGTCTAACAGTGTTGGACTTAAGAAGGAACAATTTCACAGGGAGTCTTCCCCCATT
ATGTGCATAG

>PGSC0003DMG400015351 LRR-RLPs

ATGTTAACTGGTCATGTTATCGGATTGGACCTTAGTTGCAGTCTGCTTAATGGAACTATTCATCCCAATAG

CAGCCTCTTCCAACTCCATCATCTCCAGACACTAGACCTTGCTTACAATGACTTCTATTCTTCTTCAATCCC
GCATAACATTGGCCGATTGAGGAATTTGAGGCATCTCAACCTTTCTTATTCTTTCTCTGGAGGGGAAATC
CCAACAGAAATATCATACCTTTCCAATTTGGTTTCACTTGACCTTTTATGTTATGGATGTGGTCTCCAACTT
GATGAGAGAACATTTGAAACAATTCTTCAGAACTTCAGAAATCTGGAGGTACTATCTCTCTATGGTGTCA
ACATCTCATCTCCGATACCTGTGAATATTTCTTCATCCTCCTTAAGGAACATGGATCTTGGACATACTAAT
CTGCGAGGTGTTCTCCCAGAGAGTCTTTTCTTTCTGCCAAAATTGGAAACGCTCTATTTGAGTGACAATG
ATCTTCTCAAAGGAGTTTTACCAAAGATCCACCCGAGCAACACTCTGTTGATGTTGGATATTTCCTACACA
GGAATCTCTGGTGAGCTGCCTGATTCAGTTGGCACCTTCAGTTCCTTGAATCAATTGAACCTCCGAGGAT
GTCAATTCTCTGGTTCCATTCCTGATTCCATTGGCAATCTTACACAAATTTGGCGTTTGGATTTCCGTAAT
AATCATTTCACCGGCAATATTCCTTCCACAATCTCTCAATTGAAGCAGCTCACATGTCTATATCTTTCGTCC
AACTCTTTTTCAGGTGAAATTCCCGACATTTTCTCTAACCTCCAAGAGCTAAGTTGTGTATATCTTCGTAA
CAACAGCTTCATCGGTTCGTTTCCTTCTACAATTGTAAGCTTGCCACATCTTCTAGACTTAGACTTGTCGA
GAAATTCCCTATCTGGCCCACTGCCTAATAATTTTAGCATGCTTCAAAACCTAAACGAACTGCATTTGTTG
TACAACTCGCTAAATGGTACCATACCATCTTCGGTGTTTAGCCTCCCTTTACTAGTTGAGTTGCGGCTCGG
TAATAATCGATTTAGTGGACTACCCAATGAGCTCAAAACAAACCCAAAATTAGAACGCCTGGGTTTAAGC
CATAATCAACTCAGTGGTTCTTTTCCTCAATCACTTGCGAATCTCACAAACCTTTCTACCCTTGACCTTTCA
TCGAATAACATCACCGGTGATGCTGGAATAAATATCACCTTTCCTAGCCTAGAAATTTTGCAGTTATCATC
TTGTGAACTGAAGGATTTTCCACACTTCTTGACAAATGCAAAGAAACTTCATGTCTTGGATATTTCTAACA
ATAAGATTCGTGGCCAAATCCCAAACTGGTTTAGTAGCATAAGGTGGGACTACTTGTACTACCTAAATCT
TTCGCATAATTCTTTAACAGGCCACCTACAACAATTTCATTTCCATAATCTAAGGTATCTTGATCTTAAATT
TAACTCCCTTCAGGGTCCACTCTCTTCATCCATTTGTAACATGAGCGATCTTAAATTTCTAGATTTATCACT
CAACAACTTCAGTAACTTGATTCCAAGTTGCTTGGGAAGCAAGGCTAAACTAAGAGTGTTGGACTTAAG
AAGGAACAATTTCATAGGGAGTCTTCCCCCGTTATGTGCGAAGAGCACTTCATTGAGTACCATTGTCTTG
AATGACAATCACTTTGAAGGAATTCTCCCTATGTCGTTGCTCAACTGTAGTGGTTTAGAAGTTCTTGATAT
GGGGAATAATGCTATAAATGACACATTTCCAGCTTGGCTAGGAACACTTCAACAACTGCAGGTCTTAATA
TTAAAGTCTAATATGTTCCATGGACCAATTAGTACTTGTCAGACTACATTTTGCTTTCCCAAGTTGCGAAT
TTTTGATCTTTCACGTAATGAATTCAGTGGCTCTCTCCCTGCAAAAGTTTTTGGAAACTTTAAGGCAATGA
TTAAATTAGACGATGAAGTCTCAGGAGAGATCAAGTACATGAAAACATTCGGGACTGTATATTCCACAC
CGTATGAGGATTCTGTGAGTTTGGTGATCAAAGGCCAGGATATTGAGCTAGAAAGAATCAGCACAATTA

TGACAACCGTAGACCTCTCGAGCAATCATTTTGAAGGTGTCATTCCTATAACACTAAAGGATCTCAGCTC
ACTTTGGCTACTCAATTTATCCCATAACAATCTCATAGGTGATATTCCAATGGAATTGGGGCAATTGAAT
ACGCTTGAAGCGTTAGATCTGTCTTGGAATCGGCTCACTGGAAAGATTCCACAGGAATTGACAAGAATG
AATTTTCTTTCCTTCTTAAACCTCTCTCATAATCTTCTTATTGGACCAATTCCTCAAGGTCCACAGTTCAACA
CATTTGAAGATGACTCGTATGGCGGTAACCTTGATCTATGTGGTCCTCCTTTATCAAACCAATGTGGAAC
GAGTGATCCATCAGATGCTTCTCAGCCAGTATTGGACTCTGAAGAAGATGAAAACAAGTCATATTTTTTT
AGTGGATTTACGTGGGAATCAGTAGTCATAGGCTATAGCTGTGGACTAGTTGTTGGAACTGTTGTGTGG
AGCCTCATGTTTAAATATCGTAAGCCAGAATGGTTTGTCGATTTTTTTGAAGGCATTTATCCCAAGAAAAT
GAGAAGGTCAATGAAGAGAGCTCAGAGACGACGGACTTAA
>PGSC0003DMG400015352 LRR-RLPs
ATGATGGAGACTGAAGAAAACATTGTAACTGTTGGTGCTGGCATAGCTGGTCTTGCTACTTCTTTGGCCC
TTCACAGGTAATAATTGCTATTCTAAAATACTTGTTCAATAATTACATCATCTACTTCCAAAAGTTTGATTT
AGAAGGGCATATGCATTTGAACGAGCATTTATGCCCTCCACTTTTAGATGTGCATAAGTAGTAATACACT
TAGGATGCCAGGTAGATGCAGAACTGTCACGTAGGATGCCACATGGGATGGGTGTGTTTACTTAGCTTG
TTCAACTTTTGAATAATTTAACAGTCTACTTGTACCGGCTCAAAGTTGGATGACATAGATGTGGAGCAAA
GTTAGATGATATATTTATGTATTATGCCTCTCTAGAATATGATATTTATGATATAATAGCGCTATTAGTTG
AGTGACGATTTGAGAAATAACTCGATGGAAGGCGTGTCTTGTATGTAATATGTAGGTTGGGGCTGCGG
AGTGTTGTGTTGGAATCAGCAGATTCGTTGCGAGCTACTGGATTTGCATTGGCATTGTGGACTAATGCCT
GGAGAGCTTTGGATGCATTGGGCATTGGGGGCTCTCTTAGACAACGTTCCTTATCAATTACTGGGTAAG
CTTCCTCTTTATTATTAGTTAGGATTTAGGATTACTAATTGAAGTTCAATTTCGAATTTTTATGAGATTTTG
TGGTCATATGTGTTTTCCGGAGTTCTAGCTCTGGAAAAAAAAAGTTACTGATTGTCCAAAATGCATTCGA
GTTGATGTGCAGGTTTAAAAGTTTCTCAGCGGATTCAGGTGCACCTATCAAGGAGGTATCATTTGTGGG
GAATAACAGGTTAGCATTATACCAATTTTCATATTAGTTGGGGTCGGTTATATAAGTCCTTTCATCTATTC
GTGTTTATGTATGTTCATTTTGTTCCAACACTAGATAATCTAGCATGTGAGACAAATTAGGAATTCTCTAA
AATTAGAGGTTTTTTGAGTATCGCAATCTATAGAGATGTATAAATTTCTAACCATTGATATAAAGACTCCT
CACAAACGTTGAAGTAATGCAAACCTCCGATTTTCACAACATCGATCTTCGTAGAGAACAATCACATTTG
TTTACGAAGGACTTAAGTATGAATAGCATATTCAGAGGATGCTACATACCCAATTTTTTGTATTGTTCACA
AGTACAGAGTGACGTGATTTTAATGTCCAAATCCCTATGATTTCAACAATGAAATTAAGTTCATTTTGAAT
AACGTGGTTGAGTATGTCTTCCTTCCGACTATTGGATGGACTGATCATTCGTCAGCCAACATTTTTATTTT

TGAAGTCAATAGTCATCTAAAGTCAGTTATATTACGTTGCAGTGTGGAGTATGAAAGTCGTTGTGTGAG

GAGAAAAGATCTGTTGGAGACATTAGCTAATGAACTGCCTCAAGGTGCGATTAGATATTCTTCAAAGGT

CGATTCCATTGAAGAATCTGGACCATTGAAATTGGTACATCTTGCAGATGGTTCCACTATTAGAACCAAG

GTAAATCTTTCTCTGAAATCCGTAACTTGGAATCCCTATTATTGGAAACTTCAACCAACGTGATAATTCAA

ACGTGTATTTGACCATTATCTCAATTCCTTTCGTTAGTCTAGTACGTGACTAACTTTGATAAGCTTCAATTA

AGGCCTTAATTGGGTGTGATGGAGTCAATTCTGTGGTGGCAAACTGGCTTGGNNNNNNNNNNNNNNN

NNNNNNNNNNNNNNNNNNNNNNNNNNNNNNNNNNNNNNNNNNNNNNNNNNNNNNNNNNNN

NNNNNNNNNNNNNNNNNNNNNNNNNNNNNNNNNNNNNNNNNNNNNNNNNNNNNNNNNNNN

NNNNNNNNNNNNNNNNNNNNNNNNNNNNNNNNNNNNNNNNNNNNNNNNNNNNNNNNNNNN

NNNNNNNNNNNNNNNNNNNNNNNNNNNNNNNNNNNNNNNNNNNNNNNNNNNNNNNNNNNN

NNNNNNNNNNNNNNNNNNNNNNNNNNNNNNNNNNNNNNNNNNNNNNNNNNNNNNNNNNNN

NNNNNNNNNNNNNNNNNNNNNNNNNNNNNNNNNNNNNNNNNNNNNNNNNNNNNNNNNNNN

NNNNNNNNNNNNNNNNNNNNNNNNNNNNNNNNNNNNNNNNNNNNNNNNNNNNNNNNNNNN

NNNNNNNNNNNNNNNNNNNNNNNNNNNNNNNNNNNNNNNNNNNNNNNNNNNNNNNNNNNN

NNNNNNNNNNNNNNNNNNNNNNNNNNNNNNNNNNNNNNNNNNNNNNNNNNNNNNNNNNNN

NNNNNNNNNNNNNNNNNNNNNNNNNNNNNNNNNNNNNNNNNNNNNNNNNNNNNNNNNNNN

NNNNNNNNNNNNNNNNNNNNNNNNNNNNNNNNNNNNNNNNNNNNNNNNNNNNNNNNNNNN

NNNNNNNNNNNNNNNNNNNNNNNNNNNNNNNNNNNNNNNNNNNNNNNNNNNNNNNNNTCAT

CCCATGACTCCTGATCTTGGTCAAGGTGGATGTTCTGCTTTAGAGGACAGTGTTGTTATCGCAAAGTGCC

TTGGAGAAGCCTTGATAAAACCAATAACAGAACAAGAAGACGATGAATCTATGAATATTAGGAAAGGA

CTCGAGAAGTATGCTAAAGAGAGAAGATGGAGAAGTTTTACATTTATCAGTGCTGCTTATTTATCTGGTT

TCATACAAGAGAGTGATAACAAGATAATCAGTTTCTTAAGACAACATTTNNNNNNNNNNNNNNNNNN

NNNNNNNTTTTGGCTGGAGTCACTATATCAGTTACACTAAGAATAGCTAACTTCGACTGCGGAAGATTA

ACTGTTTCTTAGTAGCTCAATTGGATCCTTTAGAAGCTATGAATATCCTTTATGGACTTTGTTTAATATCTA

TCTAGCATACATGTTCTGGTTTAGTTATGTCTAAGAATTGTAAGACCGAATCGGATCCTTATTTTGCAATA

ATAAAATTGTGTCCTTCATCAAAGATATCCAATAACATCAGTTGTTTAGAAGCAAATATGGTAAAAGCTG

CATTTTATTATTATCATGGAAAAACAGTGTAAAAGGTAAATCTCTGAAGTATAGAATAAGTGTTTTCTTAT

CTGAGATCATTTCGTGCAATTAGATACTACTCCCTCTGTTGCAACTTGTCTGTCTGGTTTTGACTTGACAC

GAAGTTTAAGAAAGTAAAGAAGACTTACAATTAGAGATATGGACACGTAATATATATCCTGTATAATAA

GTAATGTTGGAATAATCTCATGAATATTTTTTGTGGTCTATGTAAGAACATTGTTATTTTTAGCATTTGCTT
CGACTACTGATTTAGTTATTTTCCATCTCATCATATTTGTCCTTTTTTTCCCCTGTAGTCTTTGAGGAAATTG
GCTTAAAGACTTGGAAGTTTTTTAAACATCTATTTCTACTACAACTACATGGATGTGAAAAGTCTTGTGG
AAAATTATAGAATATGACTCACAAGTTTGAAAAAATTCCACACGGTATCAAAAATGTTACGTTTCGTCTAA
GTAGATGTAGGAGTTAAATCATGTCTTTAATAATTGTTAATTTTTTATTTTTCTCACTCCACTTAGTCAAGC
AACAGTATCTCTCATTGGAAATTTGTTACAATTCTGCAGCAAATGTTCTACCGTGTGTCTTACATAATGGG
ACCAATTCACATTGACTTAGGTGACACACTGCAGAGATAGTCGATAACCTTGAAATGAAACAACATTTAG
AGAAAACAAATATGGAATAATTGGTACTAGCCACTAGGGGTGTACATGATCGGGTTGATTTGAATGTTC
CAAATATCAAACCAAACTATTTGTGTAAAATTTTAAATTTATAAATCAAACCAAACTAATAAAATTCAGAT
TTTTTGGGGTTTTTCAACCTCGGGTTGGTTCGCGTTTTTCACTTGTGACTTGTTAACTGGTCATGTTATCG
GATTGGACCTTAGTTGCAGTTTGCTTTTGGGTACTATTCATCCCAATAGCAGCATCTTCCAGCTCCATCAT
CTCCGGACACTAAACCTTGCTTACATGACTTCTATTCTTCTTCAATCCCACATAACATTGGCCGATTGACG
AATTTGAGGCATCTCAACCTTTCTGGTTCTTTCTTTGAAGGGGAAATCCCAACAGAAATATCATACCTTTC
CTATTTGGTTTCACTTGGACTTAATTGTTATGAATGTGAACTTGATGAGAGAACATTTGAAACAATTCAG
AACTTCACAAATCTAGAGGTACTATCTCTCCATGGTGTCAACATCTCATCTTCAATACCTGTGCATATTTCT
TCATCCTCCTTAAGGTACATGGATCTTGGACATACTAATCTGCGAGGTGTTCTCACAGAGAGCTTTTTCCT
TCTGCCAGGATTGGAAAGGCTCTATTTGAGTTCCAATGAACTTCTCAAAGGAGTTTTACCAAAGATCCAC
CCGAGCAACACTCTGTTGGAGTTGGATATTTCTAACACAGGAATCTCCGGTGAGCTGCCTGATTCAGTTG
GCACCTTGAGTTCCTTGAATATCTTGAACCTCAAAGGATGCCAATTCTCTGGTTCCATTCCTGATTCCATT
GGCAATCTTACACAAATTAAGTATTTGGATTTCAGTAATAATCATTTCACCAGCCATATTCCATCCACAAT
CTCTCAGTTGAAGCAGCTCACATATCTACATCTTTCGTCCAACTCTTTTTCAGGTGAAATTCCCGACATCTT
CTCTAACCTCCAAGAGCTAAGTTATTTATATCTTGGTAACAACAGCTTCATCGGTTCGTTTCCTTCTACAAT
TGTAAGCTTGCCACATCTACAAGACTTAGACTTGTCGAGTAATTCCCTATCTGGCCCACTGCCTAATAATG
TGAGCATGCTTCAAAAGCTAACCAAACTGAATTTGTCCTACAACTCAATGAATGGTACCATACCATCTTCT
GTGTTTAGCCTCCCTTTGCTAGGTCAGTTGTGGCTCGATAACAATAGATTTAGTGGATTACCCGATGAGC
TCAAAACAAACCCAACATTAGCACGCCTGGATTTAAGCCATAATCAACTCAGTGGTTCTTTTCTTCAATCA
CTTTCGAATCTCACAAACCTTTCTATCCTTGACCTTTCATCGAATAACATCACCGTCGATGAGGGAATACA
AATCACTTTTCCTAGCCTACAAGATTTGTGGTTATCATCTTGTGAACTGAAGGATTTTCCACACTTCTTGA
GAAATGTGAAGTCACTTTGGGTCTTGGATATTTCTAACAATAAGATTCGTGGCCAAATCCCAAACTGGTT

TAGCGGCATGAGGTGGGACTACTTGTGGTACCTAAACCTTTCGCATAATTCTTTAACAGGCCACCTACAA

CAATTTCATTTCCATAATCTATGGTATCTTGATCTTAATTTAACTCCCTTCAGGGTCCACTCTCTTCATCCAT

TTGTAACATGATCAAGCTTGAATTTCTGGATTTATCACGCAACAACTTCAGTATCTCGATTCCAAGCTGCT

TGGGAAGCATGTCTAGTCTAAAAGTGTTGGACTTAAGAAGGAGCAATTTCACTGGGAATCTTTCTCCATT

ATGTGCGAAGAGCACTTCATTGAGTACCATTGTCTTGAATGACAATCACTTTGAAGGAACTCTCCCTGTG

TCGTTGCTCAACTGTAGCGGTTTAGAAGTTCTTGATATGGGGAATAACGCTATAAATGACCTATTTCCAT

CTTGGCTAGGAACACTTCAAGAACTGCAGGTTCTAATATTAAAGTCCAATGTGTTCCATGGACCAATTAG

GACTTGTCAGACTACGTTTTGCTTTCCCAAGTTGAGAATTTTTGATCTCTCTCATAATGAATTCAGTGGCT

CACTTCCTGCAAATTTTTTTGGAAACTTCAAGGCAATGATTAAATTAGAGGGTGAAGACACAGGACAGA

TTGAGTACATGAAACCACACGAAAATACAATTTACACATCGTATGACGATTCTGTGAGTTTGGTGATCAA

AGGCCAGGATGTTGTGCTAGAAAGAATCAGCACAATTATGACAACCATAGACCTCTCGAGCAACCATTT

TGAAGGTGTCATTCAGCAAACACTAAAGGATCTCAGCTCACTTTGGCTTCTCAATTTATCCCATAACAATC

TCACAGGTGATATTCCAATGGAATTGGGGAAATTGAATACGCTTGAAGCGTTAGATCTCTCTTGGAATA

GGCTCACTGGAAAGATTCCGCAGGAATTGACAAGAATGAATTTTCTGGCCTTCTTAAACCTCTCTCATAA

TCTTCTTATTGGACCAATTCCTCAAGGTCCACAATTCAACACATTTGAAAATGACTCGTATGGCGGTAACC

TTGATTTATGTGGTCCTCCTTTATCAAAGCAATGTGGAACGAGTGATCCATCACATGCTCCTCAACCATTG

GACTCTGAAGAAGATGAAGACGAGTCATATTTTTTAAGTGGATTTACTTGGGAATCAGTAGTCATAGGC

TACAGTTTTGGACTAGTTGTTGGAACTGTTTTGTGGAGCCTCATGTTTAAATATCGTAAGCCAGAATGGT

TTGTGGAATTTTTTGAAGGCATTTTCCCCAAGAAAATGAGAAGGTCAAATAAGACAAGGCCGAGACGGC

GGATTTAA

>PGSC0003DMG400015353 LRR-RLPs

ATGCGGAAATTGAATTTTGGAAATAATCATTTCACCGGCTATAATCCTTCCACAATCTCTAAATTGAAGCA

CCTCACATCCCTAGACCTTTCGTCCAACTCCTTTTCAGGTGAAATTCCCGATATTTTTTCTAACCTCCAAGA

GCTATGTTATTTATCTCTTTTTGACAACAGCTTCATCGGTTCGTTTCCTTCTACAATTGTAAGATTGACACA

ACTTCAACACTTAGACTTGTCGCACAACTCACTGAATGGTACCATACCATCTTCGGTCTTTAGCCTCCCTTT

GCTATCTTCATTGTCGCTCCAACATAATCAATTTAGTGGACTAGCCGATGAGTTCAAAACAAACCCAACA

TTAGAACACCTAGATTTAAGCCATAATCAACTCAGTGGTTCTTTTCCTCAGTCACTTGCGAGTCTCACAAA

CCTTTCTACCCTCATCCTTTCATCAAATAACATCACCGTCAATGAGGGAATACAAATCACTTTTCCTAGCCT

AGAAGTTTTACAGTTATCGTCTTGTGAACTGAAGGATTTTCCACACTTCTTGACAAATGTGAAGTCACTTA

GGATCTTGGATATTTCTAACAATAAGGTTCGTGGCCAAATCCCAAACTGGTTTAGCGGCATGAGGTGGG
ACAACTTGTGGTACCTAAACCTTTCGCATAATTCTTTAACAGGCCACCTACAACTATTTCATTTCCATAATC
TAGAGTATCTTGATCTTAAATTTAACTCCCTTCAAGGTCCAATACCTTCATCCATTTGTAACATGAACGAT
CTTCAATTTCTACTTTTATCACACAACGACTTCAGTAACTCGATTCCAAGTTGCTTGGGAAGCATGTCTAG
TCTAACAGTGTTGGACTTAAGAAGGAACAATTTCACTGGGAGTCTTCCATCGTTATGTGCGCAGAGCACT
TCATTGAGTACCATTGTCTTGAATGGTAATCAATTTAAAGGAACTCTCCCTGTGTCGTTGCTCAACTGCAG
TGGTTTAGAAGTTCTTGATTTGGGGAATAACGCTATAAATGACACATTTCCTGCTTGGCTAGGAACACTT
CAACAACTGCAGGTCTTAATATTAAAGTCCAATGTGTTCCATGGACCAATTAGTACTTGTCAGAGTAGGT
TTTGCTTTCCCAAGTTGCGAATTTTTGATCTTTCGCATAACAAATTCAGTGGCTCTCTACCTGCAAAAGTTT
TTGGAAACTTCAAGGCAATGATTAAATTAGACGGTGAAGACACACGAGAGATCATGTACATGATGCCAT
CTATAATGCCATTTTACTCATCATCATATGAGGATTCTGTGAGTTTGGTGATCAAAGGGCAGGATATTGA
GCTAGAAAGAATCAGCACAATTATGACAACCATAGACCTCTCGTGTAACCATTTTGAAGGTGTCATTCCG
AAAACACTAAAGGATCTCAGCTCACTTTGGCTACTCAATTTATCCCATAACAATCTCATAGGTCATATTCC
AATGGAATTGGGGAAATTGAATAAGCTTGAAGCATTAGATCTCTCTTGGAATCGGCTCACTGGAAAGAT
TCCGCAGGAATTGACAAGGCTAACATTTCTGGAAAAGTTAAACCTTTCTCAGAATGTTCTCGTTGGACCA
ATTCCTCACGTTCTACAATTCAACACATTTGTAAAATAG
>PGSC0003DMG400015449 LRR-RLPs
CTCATACAACAACTATATCTCCCACAAAAAAAGTGAAAAAATCAATCTTCATTTCTTATATGATGAACATT
TCTTATCTTGAATAATTGTTATTGTGTTTAATATATAGTTCTTTTGTATATCATCTTCCTTAGGGGCAGCCT
TGGGGGTTGGTTCTTCCGAGGCAGCTTGTTAGTCGAAAATTCCCTTTGAAAAATGCAGGTGTATCGATG
TTTTTTCAGTTTGTTGCTGATCTCTGCTGTTCTGTTTTCTTTCTCTTGTGCACTCTCTGATCATGAAGCATCA
TTGCTTGCACGACGACAACTCTCAACACTTCTTGAAAATGGTAATTTACCCGATAAATATGAATTCGAGG
TGAATGTAGAATACACATTTCCTAATTCTAGGCTTAGACGTGCTTATATTGCGTTAAAGGCATGGAAAGA
AGCTATATATTCTGACCCTTCTGAATTCACTAGCAATTGGAAAGGTCCAGATGTTTGTAACTATAAAGGC
GTGTTCTGTTCGCCTGCTTTAGATGATCCTAATGTCACTGTTGTTGCTGGGATTGATCTGAATCATGCAGA
TATAGCAGGTTATTTTCCTGTAGAATTAGGATTATTGACAGATGTAGCTTTGTTTCACTTAAACTCCAATA
GATTTTGTGGAATTGTTCCTGAGAGTTTTTCGAAGCTTACGTTGATGCATGAGTTTGATGTTAGTAATAA
TCGTCTTGTTGGCCCTTTCCCTAAGGTTGTTTTGAACATGTCTAGTCTTAAGTACTTGGATTTAAGGTTTA
ACAACTTTGAGGGAGAGTTGCCTTCACAGTTGTTTGACAAAGATCTTGATGCTTTGTTCTTGAATGATAA

TCGATTCGTGTCTACCATCCCTGAGACTCTTGGTAACTCTTCAGCTTCTGTGATTGTGTTTGCTAATAACA
AGTTTCATGGTTGTATTCCCAGTAGCATTGGGAAGATGTTGAATTTGGATGAGATTGTGTTTATGAACAA
TGATCTTGGTGGATGTTTGCCTGTTGAAGTCGGGTTGTTAAAGAATGTAACAATGTTTAATGCTGCTGGG
AATTTGCTTTCTGGGATATTGCCAAAGACACTTGATGGTCTTTCACATGTTGAGGAATTGGATATTTCACA
TAATACCTTAACTGGGTTTGTTCCGGAGAATCTATGTTCATCGTTGAGTTTGAAGAAGTTTGTGTTTTCGT
TTAATTACTTTAATGGAGAGGCTAAAGGTTGTGAAGCGCGAACAAGGAAAGATATGAGTTTTGATGATA
GGAACAATTGTTTGCCTGGTAGAGCAAAACAAAAGTCTCAAAAAGAATGTCAACCTATTGTGAGTAAGC
CTATTGATTGTAGTAAGGCTAAATGTGGAGGGCCTTTAACAAAATCTCCTCCTAGTAAGAAAAATGAAG
TGCCACCAAAACCAACACCAACACCAGCACCAAAACCTCGACCTACCCCTAAGTCACCACCCACCCCAAA
GAAGGCTTCTCCTCCACCAACACAAGTCGTTTCATCGCCCCCTCTAGTACATTCTCCACCACCACCTGTTC
ACTCCCCTCCACCACCTCTAGTACATTCTCCACCACCACGTGTCCATTCTCCTCCTGTTTCCTCTCCACCACT
ACCCGTCCACTCCCCTCCATCACTACCTGTCAACTCCCCTCCACCACCTATAGTACATTCTCCACCACCACG
TGTCCATTCTCCTCCCGTGTCCTCTCCACCACCACCTCTTGTCCAATCTCCACCGCCACTACATATCAACTC
CCCTCCACTACCTATAGTACATGCTCCACCACCACATGTCCATTCTCCTCCTGTTTCCTCTCCACCACCACC
TCATGTCCAATCTCCACCGCCACCTCTAGTACATTCTCCACCACCACCTGTCCACTCTCCTTCTCCACATGT
TTCCTCTCCACCACCACCTATCCAATCTCCACCGCCACCTCCAGTACATTCTCCACCACCACCTGTCCACTC
TCCACCTCCACCTATTTCCTCTCCACCGCCACCTGTCCATTCCCCTCCACCACCACCCGTCCACTCTCCTCCT
CCACCTGTTTCCTCTCCACCACCACCTGTCTACTCCCCTCCACCACCACCCGTTCACTCTCCTCCTCCACCAC
CACCCGTCCACTCTCCTCCTCCTGTTTCTTCCCCACCACCACCTGTCCACTCTCCTCCACCTCCGGTACACTC
ACCACCACCACCTTTCGAACATGTTGTCCTTCCACCAAATATTGGTTCAATTTACGCATCGCCACCGCCAC
CAATCTTCCAAGGATATTAATTAAGCAATGAATGAAGCTTCGTCTCGTTTCCACCTTTTTAGTACGTATAA
TATTTAGTACATGCAGAATTACATAATCATTTTTCTTAATGTAAGATTATTATCTAATATATGGTGATGTTT
TTCTTTCTTTAAACTGATACAATATAATCTTGGAAAAG
>PGSC0003DMG400015603 LRR-RLPs
CAGATCATCATCACTGCAACGCCGCGCTTTATCTCCTCTTCTGGAACTCTCCCTTCGTTCCTCTCACTGTCT
AACTCACTCATTGAGTCTCTCCATGGGCTCATCTTCTACATTCTCTCTGTGCTCTCATCCAACGGTACTTTC
CTCTACTCTATTAGCTCACTCGAACTTACAATTATGACTAAATTCTCACTTTTTTTCGTAATTTTTAGGTGA
ATTTTGAATTTAATGGTAGAAAAGTCGGATACGGATTGCAGAAATCGTTGAGCAGTTCATCCAGTTATTG
TTTGATTCATATTTCGCCGATTTGTATAAGTAGTAGAAGAAAATGCTTGCAATTGAGACGTGTAATTGTT

AGAGCTGCTTCGTCGTCTAGTACTGGTCCGAGACGTTCGACGAATACTCGGAGAGTTTACAAGGAATCA

CAAGCTCAAACGCCAGCATTGCCTGTTGACAGAATTGTTTCGTTTGTTGTGCCGGCTGGTGCCTTTGTGG

TTATCTCTTTTGGTATTGACATTTTGCCTATTTTCATCTCTTTTTCTACTGATAGGCTTATAGCTTTAATTTCT

TATGCTGAATTGAGCTTTTGAGGAGGATTTTGCGTTTGAGTACATTCGGTTATCACTACTAGTGTTGGAG

CAATAAATAAAATAAGGAGTTAAAAATTACTTCTTTCAATATATGTTACACCTTTTAACTCAGACACAGAA

AAAGAAGACTTTTGAATTTATGGTCTAAAGTACTAGAGTACTTGATTATTGGTGCACCTATAAAGTTAAA

TTGTTTCCAATTATAAAAAAAGTGACATCTTTTGGACAAATTAAAAAAGGAAGTGTGCCACATAAATTAG

AACGGATTAAAAAGGGAAGTGTGCAATGTTTGCTTTCCATTATGTATCTGTAATGGTTTTAATTTTCTGTT

TTTCTTGTATTGAAATTGTTTTTGCATCCTTTTGCGTCTAGTTTAAGCTCAATTGCAATGCACCTTATAACT

GTGGCAAAAAATATGAAATTGCAAATGGTAAATTTGACATTCTTGCTCTTGTGTTTTTCACAATGCCAAG

TTAGTGGCAGGCAAGGCTTTAGTATTTGGGGAAAGGGTCTAACTTAAGTGGGTTTCCAGTTTTGGCGGA

TACTGGAAGAGGTCAGGAATCATGAGGTCTTGTGTGAAAATTCTCAACTTAATAGAAAAGTTTGAAGTT

ATTGGAGTGCTTGGCAGTATGACAGAGACCTGATATTCAACCATGAATGGAGAACTTAAGGCTCTTTAT

ATGGAACCTTTTAAGCTGTACATCCTGCAACGGATTACCCTCTTTTTGTTGAGTTATTCGTGACTAAGAAA

ATGAGTTAACACGGTAATGTTTCAGCATTTTAGAGGCTTAGATTTAAAACTTAGAAGTGCAAGGCACCTT

ACAGCCAAACAGGATTGCCATTTTCATTTTGCAGCCTTTTGATATCATTTTAGATGTTCTGCTGCCTGCAC

TCGTATGAATGTATTTTGTTTGACATTTTGACTGAGTGACACTGACCTGTTTTTTAGGTTCCCAATTGTTG

CAATCATTGTAATCAATTTAATATGCTTTAATTATCTAATATAAATTAGCCATTGAAAATTATATTTCCTCT

TTAAGAGCCATTTTCTAACAGCTTTTGAGATATAGGTTCTGCAACTTCACAAATTCATTCTCTTGAACCAA

CTTTGGTTGTAAAGCGGCTTGCTCAACACAACCATAAATCTGGTTTTCATCTTTTAGTTGCCAAATATAGT

TCTATGCTTAAGGGACACCGGACATGTAGTGTGTGGTAGTTTTTTTTTTTTAAAAAAAAAATTAGTGAGAA

GATGATGACTTGTTCCGCAACTTATGGCATCCTCATTTAACTATCTTTTGAAAATCAAGAGGATTACTAGG

AGGAATGCTCTTGTTCTTTTTAGTTGTTTTACTGGTGAAAGTTAATGGAATGTTATTAATATTGTGTAGAA

ATATTCTTAATGATTATGTTATATTGATTAAGAGCTCGTTTGGATTGACTTCAAAAAAGTAACTTTTTAAA

GTGTTGAAAGTTATTTTAGAAATAAGTAGTTATGTGTTTGGATAAAAGTGTTGCAATTGAAATTTTTTTAT

TGATGTGTCTGGCAAATAAGTGTTGTTAAACACTTTTTTTTACCAAAATGTCCTGAAATACCCTTAAAACT

GATAACATTATATAAAAAAAAAGGTTTTGAGATGAAGGTCTTCAATGAAGTTGAAGACCGTGCTCATCAA

CATTCTAAATATATGTTGGATTTTCTCTATGTGAAGATTAGTGATTGAAAGCGAAGGAGGGTTTATTGGT

AATAAATGCTTGAGGACACTTCAATGGCATCCTAATCAACAACGGAATATCCTAGAAAGAATAGTTGTG

CTGGGGATAGAAATAGAATAGCACTTATATGGATGAGGTCATGAGCCTCCCTTCAGTAACTTACTTCAGT
GTCGATCATCCTGTGAAATTTTCATGAAAAAAAAGAGAAAATTGAAATGTTGAAGAGGAACAAGAAAA
ATGTTGGTGAGAACGTTGGAGAAAACTAGGGCCACAACAACAAAAGAAAGTTGAGGGAAAGAGGAGA
TCTTAGAGTTTTATTTCACAAGGGCAAATTTGGAATTATATACATGAATTGAGGGTACAAAAGTAATATC
CTTGGTTTTAAGTTATTTTTATATTTGCCAAACACTCCTAGAAGTCAAAAACTGACTTAAGTCGATTCAAA
CAGGCCCTAAGAATCCAAAAGATTAGGAAATAATCCTTATTATTGTAGGCTAAAACTTCTATTTAGGAGT
CTTCTTGTATGGTTGTAAGCATCAATTAAAAAGAAGTTGTTATTCCCTTTTCAGATGTTATAGATCTTGCT
AATGACTTAAGTAACTTCTGCCTATCAGTGCACGGCATTATGCTACTACTTTTGGGTGGACTTCTCTTTCC
TTTTTACGTTTTACTGATCACACTTCTTTTTTCCTTCTGGTGGCACTGCAATTCTTTTTTACTTCTTAAGTTG
ACTTTACCACCAATCCGAGCTATGTGGATGATGTCCAAGAGATTTAACTTAGCATATACCTTCCGGTGGT
TGAATTATAGATTCCTTTACATGTGTCGAAAAACACTTTCTTTCATTATTCAATTACCAGTAACCTTTTAGA
AGAAAAAAATTTCAACGCTGCTTTTCTTTTCCATGATCATTCACTACTAATTAGACGATTCAGGTGACTGT
TCTTGTTCTAATTACTTTTCTAGTTATTTGACCATCTTGAATATGAAGGAGCATGCTTGACGTTAAGTGAA
TCATTAAAAATATTGTGTAAATTATTCATAATAATTATGTTTTATTAGTTAGGAGAGCGGGAATTAGTAA
ACTTTCCTTCTTAATTTAGTTCAGAATTTCTATATGAGTAGCTCCTTGAATGTGGTTAAACCTTTCATTACA
AGAGCTCATTTCTCTCTTCTTTTTTTACAATGTCAAATTCTAATAAACAGTTAATCAACCACTGGTATCGTT
GCATCTAATTTATCTTAACATTCAAAGTTCTTACATTTCCATCTCCAAAGATGTTGCTCCATACTACTATTC
TATATGATGCGATTGAATTCCTCTTTCATTGCCAATTACCTCTACTAATAACTTCTGAGCTCTTTCTGCCCA
AAAAATTGAGGAGCTACTTAACTTGCATCTGCAGAAAGAGTAAATGTCTTAGTTTTACCACAGTGTTGCT
CTGGTTCAGTCATTAACTATGGGCCCTGGCCATTTCTATTGCTGCACCCTTCCCAATGAACTCGTTGCAAG
TTATATCCTTCCAACTTTCGCCTGGCATCCTTTCTCACGGAGGTTGAATCTTGTGAGAATGTGGCTGCTAG
ACTGAAGATCTTCCTCTTATGAATTGTGACTCGAAATAGAAATCTTAAGTAGAAGGAAGATTCTCTATAT
CGTACTAATCTATGTGTTCAGTTGACAGTTTTGTGGAAGCTGGTGGAGAAAGTTCTTCTACCCAAACCAG
CCAAGACTACATCAGAAGAAAGCAAGTCTACTCAGGGATTGAAGTGGTCCATTGCCGCTGGGACAAACT
TGTTACCTGGTTTTGGTGCAAAGATTGAAAGAGAATCCAAGCTGAGGCTTAATGACTTTGCTAAAGAGC
TTAGATCTTTCAGCATTGTTGACATGTCAGGTAAAATTGCAGTCAGTTCTGGCTCAGCTGCTTCAGTATTA
AAGTTGGTGGTCGTCTATGCTAAATGTTGTCATAATGAATATCTTCCTTCTTTATACCTTTGATAAAAAAC
CTTCTATCATTTGTAGTTTTCAGAAATGTGATCACGATGTTAAAATATTTGCCTTGACTTGTTAAATTCTTG
AGTCCCACCTCTTTAGAAAGTTCAACATATAAAGATCAAAGCTTTACCGTGATTTATGATTAAAAAACATT

TTGTGCATACTGATATTGCCTGAAGGAAACTTTCCAATCTTGTTTCATTTATAAGCTTCCCCCACCAAGTG
CGCGACACACCAAAACCAAAGTTTTACTGCATCTTCCTTGTATTGTGATTCTACATTCTTTGCATACTGATT
AAGGATTAAAATTGCAAGTCACAAGAGGTATTGGATTGAATGTTTTAGGCTTTGGCAGGTCGTAACTTT
GGAGATGAGGGGCTCTTTTTCCTAGCAGAGAGTTTAGCATATAATCAGGTAATGTCTTTTGCTCTCTCTA
CCCCCCTCCCTCTTCCTTTATCTGTGTAGAGTATGTCATTATTTATCATGGTCCATCTTGTTAACCCAAGAG
GATAAAGCCATAATCCTGTAATGTAGACAAGTAACAATACAATCCTGGCTTCTTTTTTCTGGAATAATGT
TTTTGGGCAAGTTTGGTTCCTTTACCAATGTACACTGCATTTCCTTTCATGTGATTTCCAGACAGCCGAGG
AAGTAAACTTTGCTGCTAATGGAATAACTGCAGATGGATTGAAGGCCTTTGATGGCATATTGCAATCAA
ATATTGCTCTAAAGACTCTTAACCTATCTGGAAATGCTATTGGGGATGAAGGAGCCAAGGTAATGCACA
GATGATATAGGTGAAAATCCAAAGCAGTGAGTTCTTGGAATACAGATGACTAATAAGCAGATCCTAACT
TTCCCTTTGCACTTACTTAACTGTTATACCAATTTAACACGTGTATTTATGGTCATGCTTTGTATTGTATTTC
TCATCTCCGCCTTGGCACTTCAGGAGCAATCACGTCTATTTGGTGTTAATTTATGTTTGCAGTGTCTATGT
GATATACTGGCAAATAACTCTGGTATTCAAAAGCTCCAGCTGAACAGCACTGGCTTAGGAGATGAGGTC
TTGAACATGCCTTGAGCATTTAATTGAAATGCTGTTGCTAACTTGCGATGGTATTTTCTGCTTTCTGGTAA
ATTATTGCTTAAATTTCGCTGCAATGGTTTGTCTGAATTTTGCAGGGAGCCAAAGCCATTGGTGAAATGC
TGAAAAAAAATTCAACTTTGCGTGTTCTTGAACTAAACAATAATCTGATTGACTATTCTGTATGTTATTAA
GCAGAACTTGTAATTTGTATTTCCTAGTTATTAATTATCTCAATTTCTGAAGGAAAACATGTTTGATTTGG
ATATGGAAATAGGGATTTTCAGGTCTTGCTGGATCACTTCTTGAGAATAAAACTTTACAATCCTTGCATCT
GAAGTAAGCAAAATTTATTCTGCTGTCTTTCTGCGCCCATCCTCATTTGTCGTATGTCAAATGTAGCAGTT
AATTATTTTATTTTTCTTTATTGGTTTGGAGGGTGGGGGTGGGGGGAAAAGAAGTGGATTTTAATTTGTG
AGGGAAAGCATATCAATTCTTATTTTCCCTTATATCTTTTAATTTATGTGCTTTAATTAACTGTTCAGCGGC
AATTATGGTGGTGCTTTAGGGGCTGCAGCACTGGCTAAAGGCTTGGAGAGCAATAAGTCCTTGAGGGT
ACATTTCTAATCAATTCCTTCCTCCCACTGAGACCCTTCGCCTTTTGCTCTTTTTTTTGTTCTTGTAATTTGA
CTTGGCAAATAATTGAGCTTTTTTTCAGCCTTGATATCTTGCTTCTGCGTCATTGGACTGTTTCTTAGTATT
TAACTTCTGCACTCATGTTTCCCTTCTTTTTTTTTTTGGGAAACAACCTCTCTACCTCCACAAGGTATGAGT
AAGGTCTGGGTACACTCTCCCCACCCCAACCCCACTTGTGGGATCACACTGGGTATGTTGTTGTTGTTAC
TCATGTTTCCATTTGATTGTATAGAAGAGCTCTTCTTTTCAAAAGCTTCAATTTCCTCTGTGACTGCTGACA
CTGGGATTAAGTGTTAAATGCACTAATGTTATTAGATAAACTTCTTCAGGGGGTTTCTTAATGTTCCTCAC
CAACAATCCGAGGTTGAAGCTAGATCTGTTCCGCCCAAAATGGAAATGGGCTACTGTTTACATATTACTA

TATATCATTAACATACACCTGTATATTCAAAGGTGTTTGAAAAGATTCTTTATGTGCAGGAACTTTATATG
CAGGGGAACTCTGTTGGTGATGAAGGAGTACGGGCATTAATCTCGGGCCTATCTTTGCGTAAAGGTTTG
CTTATTGGATATAATTAACCATTCTCTCTAAACACATATTGGCGTTGTTTCCTGTGCATCTTTTTTGAAAGG
TCACCATCATATGTCTGCAGGGAAACTTGTCTTACTTGACCTTGCCAACAATTCAATTACTGCTAGAGGA
GCATTTCATGTTGCCGAATACGTCAAAAAAAGCAAAAGCTTACTGTGGTTGAATCTCTATATGAATGACA
TCAAAGATGAGGTAATTGAACCACTTAGAACTTTTCACAGTTTTTTTCCTTTGTAATGTAGAAGACTCTTC
TCAGCCTTGTCCTTCTCACTGCAATCAGTAGACTCTCTTGAACTATTTTATGGTTTTATTTTATGTAACAGA
AGCAAAGAATGAGAAAGCTTGAGTTTTTGTCAAGTTGAAACCACTAAAATAAGATAGAAAAGATTTTTG
GGTAAGAGGGTGAGGATTGATTGTCGAGAAAAAACAAAAAGAATTTGATTGGTTAGAGCTGAAGGTGA
TGGCTGAGTCGTTCTTGAAACCAATCATAAGTTCAGATCACATGGTTAGGAAATTTTATAATGGTACTTA
CCATTAAAGAATATAATTTTAAGACTAGGTTTAATCACTTTGGGGATTCTTGCCAGAAGAAAATTAGAAG
GGTTGCATGGTGTTGGATGAACTGAGGTCTTTTTAAATTTTGTCAGCAGTTCAGAAAGATAAAACTCAAA
TAATCCCAAATCTGTAGGGGTAGCTTTATTTTTTCCCTTCAAATACATATGCATATGGACAGACATATTAA
CTATGTAAAAATGAATACTTTTATAGTCCTGCACAACTTTAACGTTAATATTTCAACAGTGAGAAACCTAA
CTGTGCTCCTCTTGGATTATAGTAGAACTGTCTGCATCCACCATAATTAAACTGTGTTCATTGGAATCAAA
AGGAAAGCTTTGTGGTCGAAGCAGAAAGATAAAAAATTGATGATTGGAGGAAATTGGTACGGATTCAG
TAACAGAAGGTTAAATCTGTTGTGAAGAGTTCAAGGGATTTGGGCTGCAAAGAAGGATTTTTTTCAGAA
ATTTATGGGGGTGGGTTTGCATTCGACAGGGTAGTTGCTCAGAAGCACAATTAAATTTCTGCTGGAGAA
GAGCTCATGTGCTGGCAACATCAGATTGAAACATAAAGTCAAACTTTAACCATATCCTTGGTAGATTAGA
CCAAACTAGCTAATGTTGATTACCCATTCCGGAAACTTAAGGCTATGGTTTACAATGACAGAGTACAGTG
TCTACAAGCAACAAGTCCTGCACAAGAACTTGGAAAGAACTCTCTAAGTTAGCTCGCAAAAAAATTTACT
GCCTTCATTTGTGTCAATAAACTTCACATGTAATCAGTAGCCTCAAGTTTCCCACTTCTTCAATTCAACCCA
GTTTCTTTTTGTTGGTATGCGATTTCCTTGGTGTTGAAGATTAAAGGCGAAGGCTTTTTGTTTTAAAAGAG
TACACAAATCACATGCTTTTCACTTGAGAAACTACCGTTAATATTTGACGTCAAATTTGGGAAGGACCAA
AGGTGTCCATTTTTTGCAAAGACGAAGGATTTATTTTATCCACAGTTATATTTGAAGGACAAAGTGTAAC
CTGTCCCCATAAATAAGGGATCATTTGAGTTTTTACATTGCAGATCCAGACTCGATCTCTTGATATGCATT
GACTTTTCCTTCTTTTTTTTTTCCCCCATTTACATCAATATCCATCAGGGAGCTGAAAAGATTGCAGAAGCT
TTGAAGGAGAATCGCTCGATAACTAACGTTGATCTTGTAAGAGTTGTTTCCTCTTTTCTATTTTATTCAAC
ATTTCTTGTTTTGTTCATGGAGGAGGTTGGGTTTCTTAATCTTATCAGAGCATAATTTTCTATTTGCTCTGA

TGGCTGGAATTCTGCGAATTTTACAGGGGGGCAACGACATACATGCTAAGGGTATTAGTGCCATAGCAG
AAGTTCTGAAAGATAATTCTGTCATTACCTCTGTAAGTTTCATCAAAATGTTAACTTTTACTGATTTATTGG
ACAAACTTAAATACATTATACTTCAAATTTCTCACGAACAGTTAGAACTTGGTTACAACCCCATTGGACCA
GAGGGGGCAATGGCATTAGCAGAGGTACTCAAGTTTCATGGAAAAGTAAAAGATCTCATGCTTGGGTG
GTGTCAGGTAATGCATGGATTGTTTCATATATGCCTTCTTTAACTATCTCTTTTAGCATGCTCACTTAATTT
TGTTCCAGATCGGAGCCAAAGGTGCAGAATATTTTGCCGATATGTTGAAGTACAATAGTACCATATCAAC
TCTAGACTTGCGAGCCAATGGACTTAGAGACGAGGTTTCATTCAAAGTTTATAGTTCTTCTATTGTGCCA
CAATCTTTAATTTCCCTCAAAGTACATAACCATTTCCTTTTTCAGGGTGCAATCTGCCTTGCTCGGAGTTTA
AAAGTGGTCAATGAATCTTTGACGACACTCAATTTAGGGTTCAATGAAATTAGAGTATGCAACTCTTTTC
CAGTACAAGTACAACCACCTGTCTTGATAAAGGTATCATCTTGTATTAACTTAGTTTCTGTTGCACACAGG
ATGAAGGAGCTTTTGCTATCGCTCAAGCACTTAAGGCAAATGAAGATGTTAGACTCACATCTTTAAATCT
TGCCAGTAACTTCCTCACCAAATTAGGACAGGTAACATCAAATGATAATATTATTCTTTTATTTTAGCTGT
TTTACGCGATTTTTAGTTCCTTACAATATCCGGATGGATTCCAAATTTCAGACAGCTCTCACAGATGCAAG
AGACCATGTGTTTGAGATGACTGAGAAGGAGCTTGCTGTTATGTTCTAAAATACTACATTCCTTAGATCA
GATATATTTTCCTCCCTACTTGGGGCAAGGTAGATAAGTATTTTTTTTTCTATTCTGGGGGAATAGAATCG
CAGCCTACCATGATATTCTTAAGAGTCCCACCTTAGTTCCCTTTTGTGACTGAAATCATTTTGAGCGGGGA
ACAGTGGAAATTTTGATATCGAGATACACACTGATATTATAAAAAATGTTCCTTTTCTGAAACTTTTTTTTT
TGCCTGATAACGTTAGTGGCAAGTACATAGAGATTACTTCTATTCACCATTTCAGATTCATTCTCTGGTTT
GAAACTCTTCTGAGTTGTTGTTCTTTCCCTATGGCCTTTTAAGCTTATTTTGGTGTTGCAGGACAG
>PGSC0003DMG400015672 LRR-RLPs
CTTCCTTCCTCTAAAAGCTAAAGGTTCCTCAGTTTTGCTAAAATTTCACACATTGCAGCAAAAAATTCTCT
AATTTTCCCAATCAACATGACTCAAGGTATGCAAATTCTCTCTTTTGTTGTTTTTTTTGGATTATGAAAGTG
GATTTGAGTCTATAGTTGAAGAAAAATAGCATTTTTCTCTCCAGTTATTTACTAACCTGAAGTGGAAATTT
TACTATTCAGATATGAAATTTGAAGTTTTAGAATTTGATAAAGTAACTTAGATTCCTCATTTTATTGAGTT
GTGTCTTTCAATTGATCCCAAAAAAGTTTAATTTTACTCCTATTATGTAGTGGAAGAACAAGACACTAAA
GAAAGAAAAAAAATATGCTTTGAGAATTAGGTTTCAAGGCTTAAGCCCTGAAGTGAGGCTAAAAATGCAT
ACTTTTCCGAAGAGGCGCCACTAAACAAATGACAATTGATATTTCACTTTGTAGTTAAAAAAAATCAATTT
ATTTGATCATATATGTGGCATTCGTGCTTATAATTATTAGTGTTTGTATTTTTTCTTCTGTTCGCGTGTTTTC
ATTAAAGCCTATGCTTTATTTGCACGTTATGCTTAAAGCCGTTGTGCTTTTTGGTTTTTGATAACACTTGTA

GAAGCACCTTTAGTGGTCGTTTGATAGAGTGTATAAAAATAATGTATAATAGGGTGTATTAGTAATACTT
TTATTAGTTATGCTTGCATTAGTTAAACATAGAATATTTTTTATGCATTGTTTGGTTTGGTGTATTAAAAA
AAGTAAGTATTGTATAAAAATGTGTATTTACAAAAGCATCCTCCACAATTATGGTGGAAAAGATGTAAAA
GTGCTTTTGAGGGGTAATTGAGTCTTTAACCATACTAATGCATGTATTAAGTCCTTTGCATTACTAATACC
TAGAAATTTATATACATGGCATAAAAAAATGTACCAAACAAGGTATTACTAATACACAAAGCTAATGCAA
GCATTATTTTTGTTAATACACTCTACCAAACGACCCCTTAGTTTGTTCATTTTGTGCACAATAGGATGTGA
CTTAGTGGTCGATGAAGTATGAGGGAAATCGTGAGGTTAAGAAAATGCTTGGTAATTTCTATTTATCTGC
CTAGGATTTGGTGGGCAGAGACTCAGAGTTACATGATTCATGTGTTGGTGGGAGGTACTACATATTTAG
TGTAATATTCTATGTGCACGCAAGGTGGCTCGGACATCACTGTCGTTAAAGATAAGACACTACTAAACTT
AAGGGACTAATTTGATTTTTTTTTCTCCATATTCTTCTAACAGATGGAACAAGATGTGTCATTGAAGGGG
ATAGTGAAGATAGAATCAGTGGTTTACCAAGAAATGTTATCGATCGTATCCTTGAGCTCATACCAGTTGA
AGATGCAGCAAGAACTAGTATTTTGTCTCGAAACTGGAGATATATTTGGGCAACATTTCCAAATCTGGTG
CTGGATGACCGCTTCCTTCATAATTTTATAGGAAAGCTCCAATTCAACTTCACAGAAGCGGTAGATTGTA
TTCTGTTATTGCATATGGGGAACATTGTGAAGTTTGTTCTTGACACTAGAGGAGTTTTTACTGCAAATTCT
TCATATGCAATTATCGATAGATGGATTCTTTATGTCACCAGAAATGGAGTCAAGATGCTAACCCTTCACA
TTTCAAATGATGATACCTATACTCTGCCTTCTTCTATTTTTATTTGTTCAACCTTGACATATTTGAAACTCTC
CAACTGTGTCTTCAGACCACCCAATCCCTTTCTTGGTTTTCAGAATCTTATTACCCTTCGTCTGGAAGGGT
CAACCTTTGTGCCAGCCACATCATTTTGTGTTATCAAGGCTCCCCTTCTTGCCAGATTGGATTTGATCTTG
TGTCGTGGTACGCAATACCTAAACATTGTTTCACCAGGGCTGAAGTTCTTGTACATTCAGGACAGTCACT
CATATCTCGTGCTAGATTGCTTTATGAACTGCAAGAATATAAGACTGTTAAAACTTGAATTTAACAGAGT
GGTTGATGATCCAACAAATGATAAAATATCAACTTTGGAGAAGCTTCTTGTTAGCTCTCCTGCACTTGAG
GTACTCCGTTTAGATTCATTCTTTGTTGAGGTAAGATATTGACTACTTGTTTCTCTTGATATCTTATAGTGA
TGGCAATTTCGTATGATTCTAATTATTGTGTTTTCTTCAAAGCGTGCACGCATTGTTCCGAATGGGCAACC
TTCTACACTCAACTGCTTGTGGCATCTATATTTAGATGTAGACTTCAGCAAAATGGGTCAGATTTCTAATG
TTCTGCAGTTAATTAAGAGTTCCCCCAATTTGAGGAAACTCCGTATTTCGGTAAGAAAAGAAAATATATG
CACGATCTGGTTCCTTTTTGCACTTCATAATTGTGAACATGAGAATCTAAAATAGCTTGGTATTTTCTTCAT
ATACATATTAGGTCCATGCTACAAGTGACAATGCTGAAGCAATCTTGAAGTATCTTGATACGCCATCCTG
CCTGGAACAACCACTCGACAAGCTAGAGCACATTTTCATCAACTTTTTAAGAGATCAAAAACTGAACTG
CTTTTTGTAAAGCTATTGCTTTCTCGCACTCCATCTCTTTTAAAGATGTGCATTGACCAGGTTGCAGACAT

AGACGTTGATATTGCCTTAGAATTGATGCGTTTCGCCAGGGCTTCTCCGAGGGCAGAGCTATTCTATTCT
CGGATAACTCTGTGATTTGAGTGTTGGAAGGCAGACTGATGTTTATCCGTTGTCAATTAGTTTTGTTAAA
ACAAGGGATGAATTGGCAAATTTTGTAAAAAATGTGTGGTACCTTTGTGCATATGTTCTTCACTAGATGT
TTTTGATGCTCAGCATGCGTCGATGTGTTTGATATGACTAGATGTCTTTTGCTATATTTCTTTGTATTCTAC
TTCTGATTCAAGTTGCACTGGCTATTGAGAAATAGGGTGTCTGATGGTTTAGCATAAAGTGTTATTTTTTT
CACACCATTGTGTATATTGCTTGTTAAG
>PGSC0003DMG400015687 LRR-RLPs
ATGGATGGAGATGTTGTTATTGTTGTTGGAAAGAAGAAAGGACTATGTTCCATTGATCATAAGTGTTAG
TAACTATGACTATGTTCCAAAAAGGACTGAAGTAGTTATACATATCAGCAAGAATGTTAAATTCAAAATT
TAATCTATTTGCCTCGCGAAAACTTAACTGTTTAGCATGTCTGATTTTAAGTTTCTTCGTGATTTGTTTACA
GGTTTCAAGAAACCTGGACAACTAGAAATACTTGATTTGTCATTGAACTACTTGAACCGCAGCATCTTTA
AGTCTCTTAGTCAACTTTCATCTCTCAAGTCGTTGTACCTCGCGGAAAACAACATTGAATCAGGTGCTAAC
ATTTGGTTCATCTGTTTTCCCCTTTGCACTATTAGTCTTCCTCTTTTTACAGAACTGTGCTAAAAATAATCC
ATTTCCTTCTAACATTTCAGGTTCTGAGAGATTATCAGGGTTGGACAAGCTGGAAATCCTGGATTTGAGC
TACAATGCACTAAATGATGAAAATGTTCTCTCTGTTCTAGGTAATTAAACTTATAGTTTCTTTTGTGAAAA
GCCGTTTTTCCTCAGTAGCTTCCTACATAATTATTCTGAATTCCTCGACAGATCTAAATGTATCTAGAATG
ACGTTGAAGAAGCTTGATATAAGGTACAACAGATTCCGGAGTTTTATACCAAATGAAGGTATATATACA
ATTACTGAAGTTTTTCTTCCTTCAATTGATTTTATTCTACCTCCTTGTACATCTTGCTATGTAAATATATCTT
ATTGGTACCCAATCGAAATCACAAGCTTAAAAGAACTGAAAAGTCACAAAATCATTTACAAATTCCACAG
GCTCCATTTGCATTTGATGAGATGTGTATAAAATATAAATACATAGACTTACATGGAAAGATGTTATTTT
AATAACTCTCTTTGTCAACATTTTGGTTAAAAATATGACATTCCACCATTTTAGGCGTCATTTGCAAGTCG
CTATTGCCATTTTAGTTCAAACATTTACAGTTTAAAGCATACTTTTAATGTTTCTTTTGCATGTAAGAGTTG
GGAGCTTTAAGAAATATCGAGTGGTTACCGTTGGATGGAAACACATTGGATGAGAACTTTCTCAGGAGT
ACTGGTGTAATGTCATCTCTTACAGTGTTATCAGTAGCTAACTGCAACCTCAGTGGAGTCCTACCTCTTCA
AGGTAATTTCAGACATACGTAAATAGAACAATGAATAGTCGAGAACAATGAATAATTTCAGAACAATCC
ATGTTTCTAATATTTTCTGCATAACAACTATATAAGAACATGAATAACAATCATCTTCATTTTATAGGCTTG
TGTGATCTAAAATATCTTGAAGAACTAAGTCTCCGCCTAAACTACTTCATTGGAGAAGTCCCTGCATGTCT
TGGAAACTTAACACCTCTTCGGGTAATAGATCTCAGTCAAAATTACTTTATTGGAAATATTGCCTTATCTC
CACTTCGAAGTCTTGTCCCTTGAATATCTCTTGTTAGCAAACAACAAGTTTGAAATACCAATTTCATTCGA

GCCATTTGCTAACCATTCAAAACTTAAGTTTGTCATTGCTTTGTTATTCTAAAAGCTGGACTCCAAATTTCC
AATTGGAAGCCTTGTCTTTATCCAATTGCTCTCAAATGCCAAGTTTCCTTCAGTATCAACATCATTTACGG
ATTCTTTGTCTCTCGAAATGTAACATTGGTGGAAACTTCCCAAATTGGTTGTTAGAAAACAATCCCAGACT
AGGAGAAGTTTATTTGGATGGAAATGCATTCAATGGATCTCTTGAATTTCTGTTCCTTCCTAATCTGAAA
GCTTTTGATATCTCCAGCAACAAAATTCAAGGACAACTTCCTCCTAACATTGGGTCCATTTATCCGAAACT
CATGATATTCAAAATGTCCAACAATATGCTTGAAGGCTTGTTGCCTTCAAGTTTTGGTGGCATGAAAGAC
CTTCAATGCTTGGATTTGTCAGGCAATAAGCTAACAGGTGATCTACCTATTGGATTGGCTCGTAAAGGAT
CCAAGTTAAATTTTCTGAGATTATCGAATAACATGTTGAAAGGGGAAATATTTCCAGTTTTGACTAACAT
CAACAATTTTGCATATTTGTTCTTGGATGGAAATAACTTCTCAGGCCCAATTCCGCAGGCGTTATCCACTG
CCTTATTATTTTCACTGGATTTAAGTTACAATAATTTGTCAGGAAACTTACCAGTGTGGCTTGGTGACATA
TCCTCCTTAGATTCCCTCGCACTGTCCGGAAACCATCTAAGAGGTCATATTCCGCCTGATTTTTGTAGACT
TGAAGGACTTCAGGTGTTAGATCTCTCTGAAAACAACCTTGTTGGCACGATTCCATCATGTTTTAGTGCTT
TACGAGCCTTAAGACGTGTGTATTTGAGCAAAAACATGTTACAAGGGGAATTTAACGTGTTCTCGAATTC
CCATCTACTAATGTTGGATCTTGGAGATAACAACTTCAGTGGTTCAATTCCAAAATGGTTGGACGAAACT
TCTGAGTTAACTGCCTTACTCTTGAAAGGAAATCGTCTTCAAGGCACCATTCCTCCACAGTTATGTCATGC
AAGTTACTTAAGAATGTTGGATCTTTCTCACAATAATATCTCAGGACCTATACCTCATTGCTTGGGAAACA
TAATGCAGAATCCATGGGACACAGATGTATACTACAGTCCATCATTTGGATATGGAGACTTCGAGATGTT
TGGAGGAAATACCTTGATAGACGTAGATGACTCATTCGAGACAACATCAACAATTTCATTTCCAAATAGT
TTTGGCGCGTGGGTTGGAGTAGAGTTCACAACCAAATATAATACTTATTCATATGAAGGTAGCATTCTTA
GTTACATGTTTGGTGTTGATTTATCTTGCAACCAGTTAAGTGGTGAAATACCCAATGAACTCGGTAATCTT
ACTGTCATTCATGCATTAAACTTGTCACATAACCACCTTACTGGAACAATACCATCAGAATTCTCAAATCT
CCAGAATATTGAGAGTTTGGACATATCATACAACAACTTGACTGGGAGGCTTCCTACTCAACTTCTAAAG
TTGACAGCTCTAGAAGTTTTCAGCGTGGCACACAATAATTTAACAGGTATGACTCCACAGCGTACAGCGC
AGTTTGCAACTTTTGATGAAAGCAGTTATGAGGGAAATCCTGTTCTTTGTGGTCCTCCATTGCACATCAG
TTGCACAGAGGCCAAAGAGATTCCAACATACCTTCCTGCACCAGATTTCTCCGAAGATGATGCTTCTTTTT
TAGATATGGAGTGGTTCTGTATTTCATTTCTTGTAGCATATGCAAATGTGGTGGTTGCAGTGGTTGTAGT
ACTTTGCGTAAACCCCTCCTGGAGAAATGTCTGGTTTTATTATATCGAGTCTTTCATGTATTCATGTTACG
ATTACTTTAATTCCAAAAGTTCATGACTTTCATAATTATGTAACATTTCATTTATGTAAATGTATGTGTGAG
TTGTTCTTGGAATGTTGGTGCCTATCTAGTCCATTAATACTAGTGAATGTTGTGTTGATTTGCTTGTGCCT

TCTTTAAGCATTTGCCAGATTAATTAAATTATTTCGAAATATAAAAATGTGTCATATTTTTTAGAACAGAC
GAATAGCCAAAATTCTTCAGTGCAATTAATGGTTTGAAAAATATGTATACTGCTCAATGAGCCATTTCTTC
TATATGGTCCACTGAAATAAGTGCTAAAGACTTGGTAAACTCCTAAAGTAGCTGAATTTTTTTGACATTA
GCACACAAAAATTATAACTAAAAAAAAGAAAGAAGAAGAAAATGGCTAAGGAATTAATTTTTGTTGACT
AGTTAAGCACAATAATTATCAAAATTTACTCACCGTTTCAATTTGTTTGTCTTACTTTCCTTTTTAGTCTGTT
TCAAAACGAATGTCTCTTTCCTTTTTTGCAACTCTTTACATCAAACTTTCCACGTGACATGTTTAAGACCAC
ATGATTCAAAAATCTTCTTTACTTTCCTAAATTTCGTGTCAAGTCAAAATCAGAAAAACAAATTGAAACGG
AGGGAGTATATAGCAATAGAAGTGTTTGCATGAAACTTCCATACAAGTGATACTAATGGGAAATGTTTC
CTCATGAGTAAGTGTCTCAAGTCAAGTCAATGATGGTGCTAGTCAACTCAAATTGCAAGCAATGTTTTGA
TGAAAATTAGAGAAAAATATAGAGATAGAAGAACAACAAGACATATTAAAGAGTAAAATAAAGAAGTT
GAAAGGGACATAACAAGAAAAGTAGTTGCACAAAATTCCACTCTAGTCTACATCAATTATTGCACTATTC
CTTAAGATGCCTGCTACCTCTCACCAGCACAGATAACGAACAAGCTTCTTTTTCTTATATATAGTTCGGAT
ATATCTTATAGTGTTGTCAACTTGTAGGTAAAGAAGCAAAACATGGCTAATAGCAAGATAGTAGATTGG
TGGTTGTTGATTTTGTTCATGGCAAATGGATGGTTGTGTTGTTATTGTTGTTTGAATGAAGAAAAGACTG
CTCTTTTGCAACTCAAGGCAAACATAAACTACAGTACAGGCAACGATTACTTGTCATCGTGGGGGGCTA
ATGAAACCTCGGATTGTTGTCAATGGGAGGGAATTGTTTGCAACAACACCACCAGAAGAGTCGTAGAAT
TGTCCATTATTGCTAAGCAAATTAGTCAAGAAGAACAACTAAGAAATGTTACAGATAGAGATGATATGTT
GAGGAACTGGCTTTTTAATGCATCTCTATTTTTTCCCTTCAAAAATCTCAAAGCTTTGATCATACCTGGAC
ATTCATTAGCAGGCTGGGTGAAGAATGAAGGTATGTTCTCTCTATATACGCTCCCATAATATAGAACGAA
AGAGATTATACTACTCCCTCCGTTTTATATTATATGGCGATGTTTGACTAGGCTCAAAAGAACCTTAAAA
GCTTGTGGTTGTAAACATGTAATGATAATTCTATTGTGTTGTACTTTTTTTAAACGGACTAAAACAGAAA
GAATGTCATATAAAATGAAACAGATAGAGTACTACATATCTGAAAAATGGTTCTAAAGATCCATTTCTAA
ATACAAAAAGGTGTCATTCTTTTTAAGCGGGATAAAACAGAAATGTTGAAGTTTGCTTTTAGTTACTGCA
GTAACTTTTAGCAAAATAAGTTATTTAGTTTGAATAAATTTGAATTTTGAATTTTAGTTAGTTTGTTTATGT
TGTTGAGATATTTTAGGTTGTTTTAAATTTCAAATTACGCAGATTATGTGTTTTATGTTGGCTATTTAAAG
CCCCTCAATGCTTAATAAAATTATTGAAAGCTATTGAAAGTCATTTCAATCCATTGAGAGACATTTTCAAT
CTTTTTGTGCTGCCCCATTTTTAAAAAACACCAACAGTGGTATCAAGAGCTTCATTGATCTTAAGGGATCT
GTGAAATCTTCCAAAAAACTACCATATATATTTAACCCATAAGGGCTACCCATTTAACCCATCAGGGCTAC
CCATTTTAACCCTACCTTTTCAACCGGTGCTTTTTTCAATGCACAGGCTTTTCTAAATCATTTTTAACCAAA

ATAATGGTAAGCAGCAGTCTCTCTTTGAATTCCCCACAAATTTTCACAGACGAAAACTATCAAATTTGGTC
TGTGAGAATAAAACCATATTTTGAAACCTATGTGATATGTGAGGTTGTGATGGAAGAAAAACCCTTACA
ATCTCTCTCTGTGAAGTTCAGATTAAAGTACTTTTCAAAAAAACTTCTACACAAGACAATGTCATCACATG
TGTTTTTGAAGAACAAAATCATAAGAAGAAAAAGAAAAAGAAGAAATTTTGCAAGTTCAAAACTAATAG
AAAAAAATCCAGCATCAACTACCAAAGCAAAAATTGAGGAGAAGCATTTTTTTCAATTGAAGTAACTAA
AGCAAAGGAGGAGTGTTGAAGTTTGCTTTTAGTTACTGCAATAACTTTTGGCAAAATAAGTTATTTAGTT
TGAATAAATTTGAATTTTGAATTTTAGTTAGTTTGTTTATGTTGTTGAGATATTTTAGGTTGTTTTAAATTT
CAAATTATGCAGATTATGTGTTTTATGCTGGTTATTTAAAGCCCCTCAATGCTTAATAAAATTATTGAAAG
CTATTGAAAGTCATTTCAATCCATTGAGAGACATTTTCAGTCTTTTTGTGCTGTCCCATTTTTAAAAAAACA
CCAACAAGAAAGAATGTCACATAAAATGAAACGGAGGGAGTATGATCGCAATACAAACTTGAATTACA
GCTTCCAAAGGTTTAACTATATACTGGTTTAAGAATGTTAAGTTCATTGTACAATCTACTTGACTCTGTCA
CATAATTTAGCATGTCCATTTTAAGTTTCTTGTTAATTTGGTAACAGGTTTCGAGAAACTGAGACGACTGA
GGAAACTAGAAAGACTTGACTTGTCGGGGAATCAGTTCAATCGCAGCATCTTTCAGTCCCTTAGTCAGCT
TTCATCTCTCAAGTCACTGAACCTCTCAAGTAACAACAAAATTGGATCAGGTTCTGAGAGATTGTCAGGG
TTAGACAAGCTGGAAATCCTAGATTTGACCTATAACAATCTAGAGGATGAAAATGTTCTCTCTGCTCTAG
GTAAGTAAACTTATAAAAATGTTCTAAAAAAAGCTCTTCTTTTATCATCCATACCTTCCTAACATTATTATT
ATGAATTCCTGGACAGAGTTGAATAAATCTAGAACGACATTGAAGAAGCTCGATATAAGGTACAACAGA
TTCCAGAGTTTTATACCAAATGAAGGTACTTAAAGTTTCTTCTTTCAGTTGATTTTATTGTTAGAAAAACC
ATAGATTACACGCGGAGATATTATTCTAGGTGTCTCAGTATCAGTGTCTTGGTTAAAAATATGCTGTTCC
ACCATTTTAGGTGTCATTTGTTAGTTACTATTACTTCACACGTCTACGCTGCACTTTTGTAACACCTATGGC
TACATGTTGATGATTCTTTTATGTTTCTTTTGCACGAAAGAGTTGGGAGCTTTAAGAAATATAGAGTACTT
ATTGTTGGATGGAAACACATTGGATGAGAACTTTCTCAGGAGCAGTACTGGTGTAATGTCATCTCTTAAA
GTATTATCAGTAGCTCAATGTAACCTCAATGGAACTCTACCACTTCAAGGTAATTTCAGACATAGCATCG
ATCCTTTAAATTTTCGTCTTTCTAGATAGTGCTACGTAGGATTGATGTCACGTTAATCTTGTGTAAACGGG
ACAAAATTTCAGAATTTTGGTTCCATAGTAGTCTATATTTTCTGCATAACAAAATAGAACATGTATAACAA
TTATCTACTTGTTCATTTTATAGGCTTGTGTGATCTAAAATATCTTGAAGAACTAAGTCTCAGCAGCAACA
GCTTCACTGGAAAACTCCCTACATGTCTTGGAAACTTAGCGTTTCTTCGAGTAATTGATCTCACTCATAAT
CAGTTTACTGGAAACATTGCTTTATCTCCACTTTCAAGTCTCTTGTCCCTTGAATACCTCTTGATAGCAAAC
AACAACTTTGAAGTACCAATTTCATTCGAGTCATTTGCTAACCATTCAAAACTTAAGTTTGTCATTGCTGA

CTACAACTCGGTAATTGTACAAACTACGTCTAAAAGTTGGATCCCGGAGTTTCAATTAGAAGCCTTATCT
TTATTCAACTGCTCTCAAATGCCAAACTTTCTTCATTCTCAACATCACTTGAGACTTCTTCGTCTCTCGAAA
TGCAACATTGGTGGAGACTTTCCAAATTGGTTATTAGAAAACAATCCTAAACTTGGAGAAGTTTACTTGG
ATGGAAATGCATTCACTGGATCTCTTCAATTGCCATTACTTCCTAATCTGAAAACTTTCGATATCTCAAAC
AACAAGATTCGGGGGGAACTTCCTGCTAACATTGGGTCCATTTTCCCAAATCTCGTGATATCAACAATGT
CCAACAATATGCTTGAAGGCTTGTTGCCTTCAAGTTTTGCTGACATGAAAAACCTTAAATGCTTGGATTT
GTCATACAATAAGTTAAAAGGAGAGCTACCGACAAGATTGGCTCGTAAAGGATCCAAGTTATATTTACT
GAGATTGTCGAATAACATGTTGAAAGGGGAAATATTTCCAGTTTCAGCCAACATCAACAATTTCGAATAC
TTATACTTGGATGGGAACAACTTCTCCGGCCCAATTCCACAGAAGTTATCCACTGCTCCTTTACGTACATT
GGACTTAAGTTACAACAACTTGTCAGGTAACATACCGTCCTGGCTTGGTAATATCTCCTCCTTAACCTCCC
TTGCATTGTCCAAAAACCATCTAAATGGTCATATTCCACCTGATTTCTGTAGGCTTGAAGGACTTGAGGT
GTTAGATATCTCTGAAAACAACCTTGTTGGTGTGATTCCATCATGTTTTAGTAGTTTCTGGGACTTAAAAC
ATGTGTATTTGAGTAAAAACAAGCTACAAGGGGAATTCAACATGTTCTCAAACTCAGATTTAAGAGTGTT
GGATCTCGGAGATAACAACTTCAGTGGTTCCATTCCAAAATGGTTGGGACGTTCTGGTATAACCACCTTA
CTTTTGAAAGGAAATAATCTTCAAGGCACCATTCCCACAGAATTGTGTCATGCTAGTGACTTAAGAATTA
TGGATCTTTCTCACAATAATCTCTCGGGACCTATTCCTCGTTGCTTTGGGAACATAATGCAACAAGAATAT
GTCATAGAAGACAAATACAGTCCAGCATTTGGATATTCAGGATTCCAGACGTTTGGAGGAGACGCTGCG
ATAGAGGTAGAATTCTCGATCCAGTATTCATCGACAATCATGTTTCTAGATACCTATGTATGGGTTGGAG
CAGAATTTATGACCAAACACATTACATATTCCTATGAAGGTAGCATGTTTTATTATATGTCCGGAATTGAT
CTTTCTTGCAACCAGTTAAGTGGTCACATACCTAAGGAGCTCAGTAATCTTACTCAAATTCGTGCGTTGA
ACTTGTCACATAACCACATAACTGGAACAATACTATCAGCATTCTCGAAGCTTCTGAATATCGAGAGTTT
GGACTTGTCATACAACAACTTGACTGGGAATATTCCCACTCAACTTCTAGATTTGACAACTCTAGCAGTG
TTTAGTGTGGCACACAATAATTTAACAGGTATGACTCCACAGCCTATTGCACAGTTTGGAACTTTTAATG
AAAGCAGTTATGAAGGAAATCCTTTTCTTTGTGGTCCTCCACTGCTCATCAGTTGCTCGGAGCCTAAAGA
GATACCGAAATCACCTCTTGAACCAGAATGTTGTGAAGACGATACTGGTTTTCTAGATATAGAGTCATTC
TATATTTCATTTCTTGTGGCATTTGCAAATGTGGTGCTCGTAACGGTTGTAGTCCTCTGGGTGAACCCCTA
CTGGAGAAATGTTTGGTTTTATTATGTTGAGTATTACATGTATTCTTGCTATTACTTTTTTGCTTCCAAAAT
GTAA

>PGSC0003DMG400015706 LRR-RLPs

ATGAAGAGTGCTAGGAGATCTGATGTAGATACACTTAGCAACAATTTGCCTCGTGATATTCTGGATGGA
ATTCTGGGGCACTTGCCTTTGAAAGATGCAGTGAAGAGTAGTATCTTGTCGAAAGATTGGAGGTATAAA
TGGGCCACACGTCAAGAGCTTGAGTTTGATTATCACTTTTTCGAGTTGTTTGCACATTTTCAAGAAGCTAA
AACAATCATTTATCAAGTACTCTTACTCCATAAAGGACCTATACTGAAGTTTAAACTTGGAGGGTCTAATT
TGATAAGATTTCGTGATATTGATCATTGGATACTTTTCTTGTCAAAGAAAAATGTCGAGGAATTCACTCTT
CGTGTAAGATCAAACAACGATTATCATTTACCTTCTCACCTTTTTACATTCCGGAAACTAAGGCTTTTGGA
AGTTCAAAATTGCTTATTCCACCCTCCACCGGATTTCAAAGGGTTTAAGAAGCTTGTTAATCTTGGTCTTC
ATTGTGTCACTTTTCTTCCGACAGTATTAAACAATCTTATCTCTCAATGCCCGTTTCTTGAAATATTGAAGT
TGAATTGGTGCACAAACTTCGACACCCTTGAAATTGATGCTGCTAATCTCAAATGCTTTGAATTTAGAGG
AACGTCAAAGTCCATTTGCTTCAAAAATGCCCCTATGCTTAAGGAAGTTACTATATGGCTCAATTCACAA
GTTTCGACAGATCTATCAGCTCCTGTTTGTTCTAATCTTATAAACTTCTTCTATTACATGCCTTGCCTAATG
GAACTGGATATAAGTGGTGTTTCATTAAAGGTAATAGTCGAGTGAAGTTAGCTACTTATCATAACTAAAT
TATGTATTTGATTCGTATCTTGAACTAATTTTTGACATGCTTATAGGATTTAACAATGGGAGGTCTGTCAG
GGAATTCTCCGACTGTTTTGAACAATGTCAAATCTCTCAGAATTCAGAGAATGTTATTCGAAAACAGTGA
GGAGGTTTTAGGTGCTGTTTACTTGATTGCAAGCTGCCCCAAATTACAAGAGCTGACTATTGAATGTGTA
CGTACTCTCTCCTCCCTAAGGTAATTTGATAATAATTTGTCCTTTGTATCCGGCCTGACAACTTCCTGAATT
TTTTTTTAGGAAATAATGAACGATGTTGTGGGACCTGTTGCAGTTGTACAATACTTACAAGACCATCAAA
GCTTGTATGATTCTGTGAAGCTGCTTCGGAGAGTGCATATGAATACGTTTAGTGGTTTTGAGATAGAAAT
GGAATTTGTGAGGCTTATATTAGCATCTGCACCTGCACTTGAGAAAATTTTCTTTTGGAATTTTTCATGTT
TCCTTCATCAGCCGGGTAGACAACTGATGGATAAGATGAAGCAATTTCACCAAGCATCACCTAATGTTGA
ATTCACATTTGAAGAAGTTGTTGCAGAAGATCGTAGACCAATTGAACCCCAAGAAGTCATGGAAGCACC
ATATGACTTTGATTAG
[truncated: 354,882 more chars]
